# Subcellular Localization Prediction of Human Proteins Using Multifeature Selection Methods

**DOI:** 10.1155/2022/3288527

**Published:** 2022-09-12

**Authors:** Yu-Hang Zhang, ShiJian Ding, Lei Chen, Tao Huang, Yu-Dong Cai

**Affiliations:** ^1^School of Life Sciences, Shanghai University, Shanghai 200444, China; ^2^Channing Division of Network Medicine, Brigham and Women's Hospital, Harvard Medical School, Boston, MA, USA; ^3^College of Information Engineering, Shanghai Maritime University, Shanghai 201306, China; ^4^Bio-Med Big Data Center, CAS Key Laboratory of Computational Biology, Shanghai Institute of Nutrition and Health, University of Chinese Academy of Sciences, Chinese Academy of Sciences, Shanghai 200031, China; ^5^CAS Key Laboratory of Tissue Microenvironment and Tumor, Shanghai Institute of Nutrition and Health, University of Chinese Academy of Sciences, Chinese Academy of Sciences, Shanghai 200031, China

## Abstract

Subcellular localization attempts to assign proteins to one of the cell compartments that performs specific biological functions. Finding the link between proteins, biological functions, and subcellular localization is an effective way to investigate the general organization of living cells in a systematic manner. However, determining the subcellular localization of proteins by traditional experimental approaches is difficult. Here, protein–protein interaction networks, functional enrichment on gene ontology and pathway, and a set of proteins having confirmed subcellular localization were applied to build prediction models for human protein subcellular localizations. To build an effective predictive model, we employed a variety of robust machine learning algorithms, including Boruta feature selection, minimum redundancy maximum relevance, Monte Carlo feature selection, and LightGBM. Then, the incremental feature selection method with random forest and support vector machine was used to discover the essential features. Furthermore, 38 key features were determined by integrating results of different feature selection methods, which may provide critical insights into the subcellular location of proteins. Their biological functions of subcellular localizations were discussed according to recent publications. In summary, our computational framework can help advance the understanding of subcellular localization prediction techniques and provide a new perspective to investigate the patterns of protein subcellular localization and their biological importance.

## 1. Introduction

Protein subcellular localizations are connected to protein functions and associated with multiple biological and pathological conditions. Generally, the subcellular localizations in a eukaryotic cell can be divided into 12 groups: (1) chloroplast, (2) cytoplasm, (3) cytoskeleton, (4) endoplasmic reticulum, (5) extracell, (6) Golgi apparatus, (7) lysosome, (8) mitochondria, (9) nucleus, (10) peroxisome, (11) plasma membrane, and (12) vacuole [1]. Specific biological processes can only happen in certain region of the cells: for instance, the orchestrating cellular energy production mainly happens in the mitochondria and is mediated by mitochondrial region specific proteins [2]. The proteins and related biological functions of subcellular regions are regarded as a coordinated whole. Therefore, finding connections among proteins, biological functions, and subcellular localizations is an effective way to systematically study the organization of a living cell as a whole.

Traditionally, the most effective and widely used method to study subcellular localizations is labelling, either by immunolabeling or tagging with a green fluorescent protein [3, 4]. However, the method is not only expensive but also time-consuming. Therefore, with the development of computational biology, more and more statistical methods have been developed to study the subcellular localizations. Machine learning models have been applied to connect genes, Gene Ontology (GO) terms, and KEGG pathways to predict subcellular localizations. Systematic analyses on the essential genes and related biological functions (Gene Ontology and KEGG pathways) associate subcellular localization with potential biological significance [5–7]. In most previous analyses, researchers focused more on machine learning models. Multiple machine learning models such as support vector machine (SVM) [8], random forest (RF) [9], and random walk [10] have been applied to identify the key factors affecting the subcellular localization and specific biomarkers for each subcellular location.

Early in 2001, Hua and Sun from Tsinghua University have described an SVM-based pattern recognition method for subcellular localization prediction [11]. Later in 2006, integrating different SVMs, a predictor for five pre-classified subcellular clusters (i.e., secretory pathway, cytoplasm, nucleus, mitochondrion, and chloroplast) has been built, implying that machine learning models may be effective for subcellular localization prediction [12]. In 2010, a general protein localization prediction model with various new localization subcategories and capabilities for all prokaryotes was presented [13]. Further in 2017, a deep-learning-model-based subcellular localization prediction model was established using recurrent neural network architecture with optimized MCC as 0.8345 [14]. Therefore, as we have described above, computational approaches using machine learning and deep learning models have been widely reported and confirmed to be effective for subcellular localization prediction. However, the feature selection procedure is a long-neglected part for machine-learning-model-based subcellular localization analyses. The integration of feature importance evaluation and incremental feature selection (IFS) [15] can help us identify the optimized candidate features for subcellular localization prediction based on machine learning models.

Here, in this study, we applied multiple feature selection methods, including minimum redundancy maximum relevance (mRMR) [16], Monte Carlo feature selection (MCFS) [17], and light gradient boosting machine (LightGBM) [18], to optimize the candidate features for machine-learning-model-based subcellular location prediction. The results can not only help us compare and screen out the most effective feature selection methods for machine learning model application but also systematically evaluate the importance of regulatory factors associated with subcellular localization, exploring the potential biological significance of subcellular localization.

## 2. Materials and Methods

### 2.1. Dataset

The dataset employed in the present study was obtained from the Swiss-Prot [19] (release 54.0) database by searching for proteins with the annotation “subcellular location.” At first, 53,427 protein sequences were acquired. Proteins with sequence length less than 50 amino acids and lengths greater than 5000 amino acids were filtered away, and proteins that included unknown amino acid abbreviation, such as X, were also removed. In addition, protein sequences that have high similarity were excluded by CD-HIT program [20] with a cut-off threshold of 0.7. Finally, only human proteins were chosen for further investigations. After these processing operations, 4986 protein sequences were left. They were classified into 16 categories, as listed in Table 1. The number of proteins in each category is also provided in this table.

### 2.2. Feature Representation

In this study, each protein had one group of network features and two groups of functional features, where the network features were derived from a protein–protein interaction (PPI) network and functional features were about functional terms (GO terms and KEGG pathways). These features were produced through the following steps.

#### 2.2.1. Network Features

The network features were extracted from a PPI network. Such PPI network was constructed using the PPI interactions acquired from STRING (version 9.0) [21]. These interactions include verified and predicted protein interactions. 20770 proteins were treated as nodes in the PPI network, and there was an edge between two proteins once they can interact with each other. Clearly, each edge in the network indicated a PPI. In addition, each PPI in STRING is assigned a confidence score with range between 0 and 1, indicating the strength of the PPI. Such score was assigned to the corresponding edge as its weight. The adjacent matrix of this network was used to extract network features of a protein, that is, each row of the adjacent matrix was assigned to the corresponding protein as its feature vector. Accordingly, each protein was represented by 20770 network features.

#### 2.2.2. Functional Features-GO Enrichment Scores

GO term is a key annotation information for proteins, indicating the essential properties of proteins. Here, we used the enrichment theory [22] to quantify the relationship between a protein and all GO terms, called GO enrichment score. For a given protein *p*, let *P*(*p*) denote the protein set consisting of *p* and its direct neighbors in the PPI network constructed above. Its GO enrichment score to one GO term GO_*i*_ is defined as the −log10 of the *P* value on *P*(*p*) and the protein set containing proteins annotated by GO_*i*_. 20681 GO terms were used in this study, inducing 20681 GO enrichment scores for each protein.

#### 2.2.3. Functional Features-KEGG Enrichment Scores

Similar to GO enrichment score, we also applied enrichment theory [22] to KEGG pathways, inducing KEGG enrichment scores. For a protein *p*, its KEGG enrichment score to one KEGG pathway Pathway_*i*_ is defined as the −log10 of the *P* value on *P*(*p*) and the protein set containing proteins in Pathway_*i*_. 297 KEGG pathways were considered, which induced 297 KEGG enrichment scores for each protein.

By combining all above network and functional features, each protein was encoded by 41748 (20770 + 20681 + 297) features. These features were deeply analyzed in this study and essential features which can efficiently predict subcellular locations of proteins were extracted by multiple feature selection methods.

### 2.3. Boruta Feature Filtering

Lots of network and functional features were employed to represent proteins. Clearly, a small proportion of them are highly related to the prediction of protein subcellular locations. A deep analysis on these features was necessary. Considering the huge number of features, we first employed Boruta feature selection method [23] to exclude irrelevant features.

The Boruta feature selection method [23] can choose important features that are significantly relevant to target labels in a wrapper manner on the basis of the random forest (RF) algorithm. Boruta feature filtering retains important features iteratively by comparing the feature importance between the original features and the shuffled features. This method involves three steps: (i) copying and then shuffling the original data to yield shadow features; (ii) training the RF model on the dataset by appending the shuffled data to the original one, and the importance score such as z-score of each feature is calculated; and (iii) the importance score of each original feature is evaluated, and the original features achieving significantly lower importance than the shuffled ones are removed. Through repeating the above steps, the Boruta approach obtains related features.

The Boruta program that we used in this study was acquired from a public web site (https://github.com/scikit-learn-contrib/boruta.py) [24], and it was executed with the default parameters.

### 2.4. Feature Ranking Algorithm

Although irrelevant features were filtered by the Boruta method and relevant features were retained, the selected features were still too many to conduct further investigation. In view of this, we further analyzed the remaining features with the following three feature ranking algorithms: mRMR [16], MCFS [17], and LightGBM [18].

#### 2.4.1. mRMR

The mRMR approach allows ranking features according to their importance on the basis of the following assumptions. On the one hand, the mRMR considers features that have the high relevance to the labels are important. On the other hand, features that have the low redundancy to others are also important. Thus, mRMR sorts features by both considering the conditions of minimum redundancy to other features and maximum relevance to labels. The obtained feature list was called mRMR feature list in this study. Features with high ranks are crucial for discriminating the labels in subsequent model building. This study adopted the mRMR program obtained from http://home.penglab.com/proj/mRMR/. Default parameters were used.

#### 2.4.2. MCFS

The MCFS is a tree-based feature selection algorithm that randomly takes features from the original data multiple times and then trains a series of decision trees using samples consisting of these features. The importance of one feature is evaluated on the basis of the number of times the feature appears on these tree models and the classification accuracy. To qualify such importance, MCFS computes the relative importance (RI) score for each feature. Accordingly, features were ranked in the decreasing order of their RI scores. The obtained list was termed as MCFS feature list. In this study, we downloaded the MCFS program from https://home.ipipan.waw.pl/m.draminski/mcfs.html and executed it using default parameters.

#### 2.4.3. LightGBM

The LightGBM is a gradient boosting decision tree- (GBDT-) based ensemble learning algorithm that processes data with large sample size and various features using two new methods: Gradient-based one-side sampling and exclusive feature bundling, improving computing speed and ensuring model accuracy. The importance of a feature is measured by the total number of times that the feature is involved in the trees. Investigated features are ranked in a list, called LightGBM feature list in this study. The program of LightGBM was retrieved from https://lightgbm.readthedocs.io/en/latest/, which was performed using default parameters.

### 2.5. Incremental Feature Selection

Three feature lists were generated by three feature ranking algorithms, IFS method [15] was applied to each list to extract essential features from them, which is a feature selection method that can determine the optimal number of features in an iterative way. First, the IFS creates a series of subsets from a ranked feature list (e.g., the mRMR feature list). For example, when the step size is set to 1, the first feature subset is made up of the top-ranked one feature, the second feature subset contains the top-ranked two features, and so on. Then, the IFS trains a classifier on each feature subset based on one classification algorithm (e.g., RF) and tests it through 10-fold cross-validation [25]. Moreover, the feature subset on which the classifier provides the highest evaluation measurement, such as Matthews correlation coefficient (MCC) [26] (please refer to Section 2.8), is regarded as the optimal feature subset. And the classifier is called as the optimal classifier.

### 2.6. Classification Algorithm

According to the IFS method, one classification algorithm was necessary. This study tried two classic classification algorithms: RF [27] and SVM [28]. Their brief descriptions were as follows.

#### 2.6.1. Random Forest

RF [27, 29–33] is an ensemble learning algorithm that builds a classifier on the basis of a number of tree classifiers. In RF, the label of a predicted sample is decided through aggregating votes from the tree classifiers. Of note, the final consensus outcome is usually relied on the voting of all trees. With the goal of avoiding overfitting and boosting the model robustness, the diversity of decision trees is uniform in RF. To quickly implement RF, the package in Scikit-learn [34] was adopted. Default parameters were employed to execute this package, where the number of decision trees was 100.

#### 2.6.2. Support Vector Machine

SVM is based on the statistical learning theory [28, 35, 36]. It can guarantee that the solution is the global optimal solution rather than the local minimum, which determines the SVM method to have better generalization ability to unknown samples. SVM is aimed at finding a hyperplane that can locate sample points of different classes in the training set on both sides of the plane and requires the blank area on both sides to be maximum. In the present study, the tool “SMO” in Weka [37] was used, which implement the SVM mentioned above. Default parameters were used, where kernel was a polynomial function, and regularization parameter *C* was set to 1.0.

### 2.7. Synthetic Minority Oversampling Technique

As shown in Table 1, the dataset utilized in this study had unbalanced sample sizes between different classes. The classifier directly built on such dataset may produce bias. In view of this, the synthetic minority oversampling technique (SMOTE) [38] method was used to balance the sizes of categories. It iteratively generates new samples of the minor categories until the number of samples in these categories is equal to that in the major category. The well-balanced data processed by SMOTE can effectively enhance the performance of classifiers. In the present study, the tool “SMOTE” in Weka [37] was employed. Default parameters were adopted.

### 2.8. Evaluation Metrics

In this study, the predictive performance of each classifier was assessed by 10-fold cross-validation [25], and the MCC [26] was adopted as the key measurement. MCC is a widely used measurement that scales between −1 and+1; +1 is achieved when the classification model is perfect, and−1 is achieved when the model is completely wrong. Here, we used a multiclass version proposed by Gorodkin [39] as the data we analyzed consisted of 16 categories. The MCC can be calculated below:

(1) $$\begin{array}{c} \text{MCC}=\frac{\mathrm{cov}\left(X,Y\right)}{\sqrt{\mathrm{cov}\left(X,X\right)\mathrm{cov}\left(Y,Y\right)}}, \end{array}$$

where cov(·, ·) represents the covariance of two matrices, *X* is a 0–1 matrix indicating the predicted category of each sample, and *Y* is also a 0–1 matrix representing the actual categories of all samples.

In addition, the accuracy on each category and overall accuracy (ACC) were also calculated. The accuracy on one category was defined as the proportion of correctly classified samples in this category, whereas ACC was the proportion of correctly classified samples.

## 3. Results

**Figure 1:**
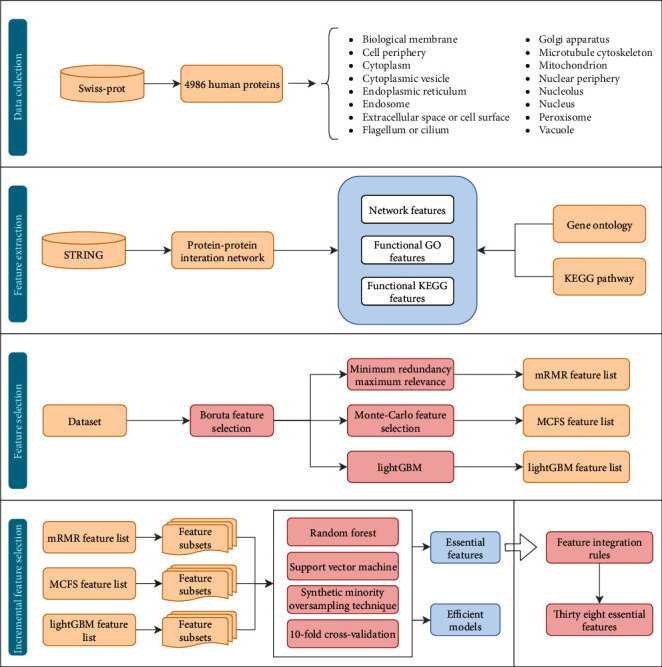
Entire procedures for constructing and evaluating protein subcellular location prediction models. Human proteins and their subcellular location information are retrieved from Swiss-Prot. Each protein is represented by three feature groups: network features, functional KEGG features, and functional GO features. All features are analyzed by Boruta and mRMR, MCFS, and LightGBM methods, resulting in three ranked feature lists. These lists are fed into the IFS method one by one, incorporating two classification algorithms, to build efficient models and extract essential features. Thirty-eight essential features are selected on the basis of the feature integration rules.

In this study, we designed a computational framework on the basis of some machine learning approaches for the identification of human protein subcellular locations. The whole procedure is shown in Figure 1. The results of feature selection and model evaluation are described in this section.

### 3.1. Results of Boruta and Feature Ranking Methods

As mentioned in Section 2.2, for each protein, it was represented by a large number of network features, functional features (KEGG enrichment scores), and functional features (GO enrichment scores). We first applied the Boruta method to discard irrelevant features. As a result, a total of 4773 features were retained, which are listed in Table S1. Within these features, the numbers of network features, functional features (KEGG enrichment scores), and functional features (GO enrichment scores) were 399, 151, and 4223, respectively. It was obvious that functional features (GO enrichment scores) dominated in the selected features (∼88%). Next, for these 4773 features, mRMR, MCFS, and LightGBM were followed to analyze their importance. Three ranked feature lists were produced, as listed in Table S1.

### 3.2. Results of the IFS Method

**Figure 2:**
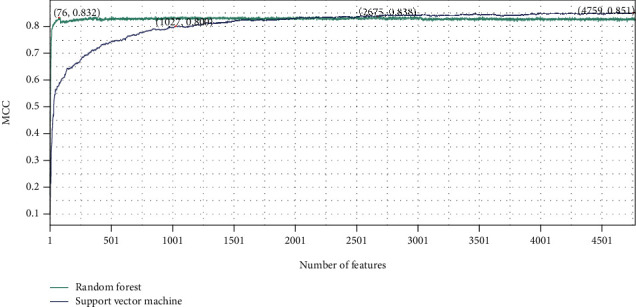
Results of the IFS method with RF and SVM in the LightGBM feature list. The highest MCC values for RF and SVM are 0.838 and 0.851, respectively. RF and SVM can provide quite high performance when much less features are used (76 for RF and 1027 for SVM).

**Figure 3:**
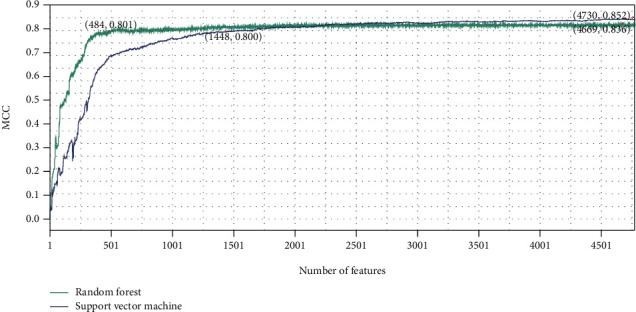
Results of the IFS method with RF and SVM in the MCFS feature list. The highest MCC values for RF and SVM are 0.836 and 0.852, respectively. RF and SVM can provide quite high performance when much less features are used (484 for RF and 1448 for SVM).

**Figure 4:**
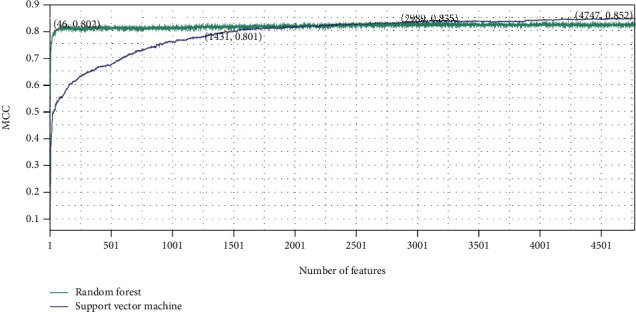
Results of the IFS method with RF and SVM in the mRMR feature list. The highest MCC values for RF and SVM are 0.835 and 0.852, respectively. RF and SVM can provide quite high performance when much less features are used (46 for RF and 1431 for SVM).

On the basis of LightGBM, mRMR, and MCFS feature lists, the IFS method was executed. A number of feature subsets were generated with a step size of 1. For each feature subset, RF and SVM classifiers were trained individually using samples composed of these feature subsets, and their performance was evaluated by 10-fold cross-validation. The evaluation results of RF and SVM classifiers with different numbers of features, including MCC, ACC, and accuracy on each category, are listed in Table S2. For easy visualization, we plotted the IFS curves for different feature lists and each classification algorithm, as shown in Figures 2–4.

**Figure 5:**
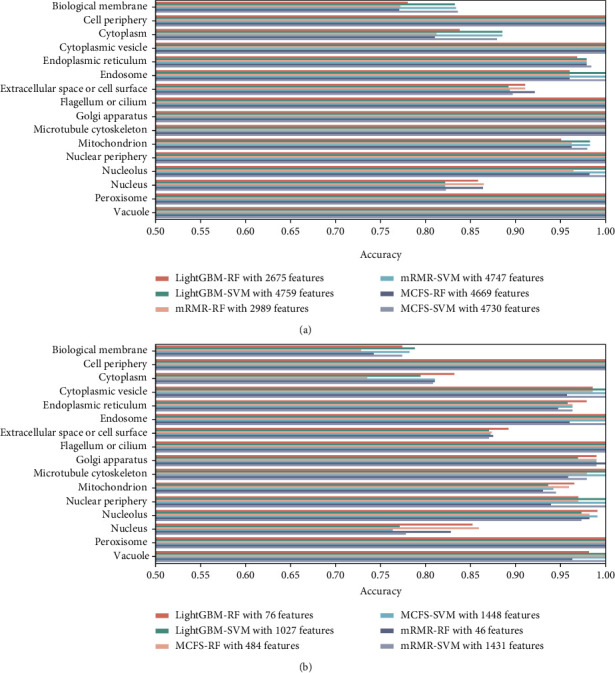
Performance of different classifiers on each category. (a) Performance of the optimal classifiers constructed from three feature lists on 16 categories. (b) Performance of the classifiers using much less features from three feature lists on 16 categories.

For the LightGBM feature list, the RF obtained the highest MCC of 0.838 when using the top 2675 features, whereas the SVM achieved the maximum MCC using the top 4759 features with MCC of 0.851 (Figure 2). For the MCFS feature list, the highest MCC of RF was 0.836 when using the top 4669 features, and the SVM reached the highest MCC of 0.852 when the top 4730 features were used (Figure 3). As for the mRMR feature list, RF and SVM generated the highest MCC of 0.835 and 0.852 when top 2989 and 4747 features were adopted, respectively (Figure 4). Thus, we can construct optimal RF and SVM classifiers from each feature list using these features. Their performance of each category is shown in Figure 5(a). The six optimal classifiers achieved an ACC of 1 in identifying cell periphery, flagellum or cilium, Golgi apparatus, microtubule cytoskeleton, nuclear periphery, peroxisome, and vacuole. In terms of performance on the three feature lists, RF performed worse than SVM in distinguishing biological membrane, but outperformed SVM in distinguishing nucleus. Moreover, the accuracies in distinguishing each category are almost higher than 0.8, indicating that the feature subsets that we identified from three feature lists were capable of distinguishing subcellular sites, and the performance of the optimal RF and SVM was extremely high and similar.

Although several optimal classifiers were built as previously discussed, their complexities were high due to the large number of features included. We thoroughly checked the performance of RF and SVM on different feature subsets for each feature list in order to develop classifiers with lower complexity. Six classifiers using fewer features can be constructed, where the RF used the top 76 (for LightGBM feature list), 484 (for MCFS feature list), and 46 (for mRMR feature list) features and the SVM adopted the top 1027 (for LightGBM feature list), 1448 (for MCFS feature list), and 1431 (for mRMR feature list) features. The MCC values yielded by these classifiers are also marked in Figures 2–4. It can be observed that although the performance of these classifiers fell short of the above-mentioned optimal classifiers, their MCC values stayed consistent at around 0.8, demonstrating that they still performed well with fewer features. The accuracies evaluated by these classifiers under each subcellular location are provided in Figure 5(b). The accuracies of six models in cell periphery, flagellum or cilium, and peroxisome are 1. There is not much of a difference from the above optimal classifiers, suggesting that the ability of these classifiers to discriminate subcellular sites using fewer features is still strong. This fact also implied that features used in these classifiers were more essential than other features in the optimal feature subsets. Furthermore, considering that the RF outperformed the SVM using a small number of features, we chose the top 76, 484, and 46 features from the LightGBM, MCFS, and mRMR feature lists for the follow-up analysis.

In summary, we identified the optimal feature subsets and constructed the optimal classifiers on different ranked features lists when using the IFS method with RF and SVM. Taking into account the computational efficiency and the number of features, the feature subsets used to construct RF classifiers with a little lower performance were applied as biomarker sets for the next step of biological analysis.

### 3.3. Results of Feature Integration

Considering the different ranks of each feature in three feature lists, we gave an integration on these ranks to further identify essential features. Here, we only considered the features used to construct RF classifiers with fewer features and a little lower performance, i.e., top 76, 484, and 46 features from the LightGBM, MCFS, and mRMR feature lists, respectively. We used the following integration rules to rank these features:

1. Number of selections for each feature by three feature selection methods
2. The highest rank of the feature selected by three feature selection methods
3. The averaged rank of the feature selected by three feature selection methods

In this step of the analysis, the top 38 key features were eventually selected, which are listed in Table 2. The biological functions of these 38 features in subcellular localization would be detailed in Section 4.

## 4. Discussion

Here, we applied three feature selection methods to optimize the candidate features for predicting protein subcellular locations. To evaluate the performance of each feature selection method, we applied RF and SVM in the IFS method. With accepted MCC value based on a small number of features, some features were selected using the performance of RF. 38 features were screened by the feature integration rules, as listed in Table 2.

Among the 38 optimized features, 15 features with six genes and nine GO terms or KEGG pathways were identified by two methods. As the first predicted gene, *PEX5* (*ENSP00000407401*) has been shown to be associated with spatiotemporal contacts between peroxisomes and lipid droplets [40], indicating that this gene contributes to the subcellular localization of peroxisomes. The next predicted gene is *SUMO2* (*ENSP00000405965*), a ubiquitin-like protein regulating covalent attachment via an isopeptide bond to its substrates [41, 42]. The subcellular location of *SUMO2* has shown to be identical for specific biological functions; on the contrary, the activation and inhibition of *SUMO2* can reflect its specific subcellular distribution patterns [41], indicating its prediction capacity. The next predicted gene *BCCIP* (*ENSP00000357748*) has been shown to be essential for p21 protein nuclear localization [43], validating our prediction. The other three genes in our list predicted by multiple methods are *DDX18* (*ENSP00000263239*), *GRK3* (*ENSP00000317578*), and *CYC1* (*ENSP00000317159*). According to recent publications, these three genes have been directly connected to nucleus cytoplasm [44], membrane structure [45], and subcellular localization of NADPH-cytochrome P450 enzyme [46].

As for the nine remaining GO or KEGG terms, all functional description terms have also been connected to specific subcellular localization. Six GO or KEGG terms were directly connected to specific subcellular locations or organelles: lysosome (*hsa04142*), nucleoplasm (*GO:0005654*), organelle membrane (*GO:0031090*), integral component of membrane (*GO:0016021*), extracellular space (*GO:0005615*), and endoplasmic reticulum membrane (*GO:0005789*). Therefore, these six biological function description terms are effective features for subcellular localization. As for the remaining three features, amino sugar and nucleotide sugar metabolism (*hsa00520*) occurs mainly in the cytosol, involving multiple *trans*-membrane transportation from the cytosol to the nucleus. Therefore, it is also reasonable to use such sugar metabolism to recognize cytosol and nucleus [47]. Similarly, glycolysis/gluconeogenesis (*hsa00010*) is another pathway associated with cytosol regions [48], validating our prediction. The remaining feature predicted by multiple method turns out to be Vibrio cholerae infection (*hsa05110*). Five core regulatory subcellular regions including the cytoplasm, periplasm, inner membrane, outer membrane, and extracellular space have been shown to be specifically associated with our predicted Vibrio cholerae infection [49], reflecting its unique association with subcellular localization.

For the remaining features selected by only one method, 11 predicted GO terms or KEGG pathways directly refer to specific subcellular localization, including intrinsic component of membrane (*GO:0031224*), intracellular organelle lumen (*GO:0070013*), envelope (*GO:0031975*), microtubule bundle formation (*GO:0001578*), integral component of plasma membrane (*GO:0005887*), nucleus (*GO:0005634*), plasma membrane (*GO:0005886*), obsolete microtubule organizing center part (*GO:0044450*), retrograde transport, endosome to Golgi (*GO:0042147*), obsolete intracellular part (*GO:0044424*), and microtubule organizing center (*GO:0005815*). Therefore, these 11 features have been confirmed to be directly associated with subcellular localization, validating our prediction.

As for the remaining features, two of them are also functional descriptors: (1) oxidoreductase activity (*GO:0016491*) describes the oxidoreductase associated biological processes. Considering that oxidoreductase activity has been shown to be connected with the mitochondrion [50, 51], it is reasonable to speculate that such feature may be connected to mitochondrion subcellular localization. (2) Aerobic respiration (*GO:0009060*) is another predicted feature only by mRMR. Considering that aerobic respiration is associated with the mitochondrion, this feature can also help us localize subcellular regions, validating our prediction.

The other nine features are all proteins predicted by MCFS. According to recent publications, such genes can be connected to specific subcellular locations with specific biological significance. For example, (1) *PES1* (*ENSP00000346725*) encodes a specific nuclear protein [52] that can be localized on telomerase [53]; (2) *SLC25A17* (*ENSP00000390722*) is a functional gene regulating the physical functions of peroxisome and located in the same organelle [54]; (3) *NOC4L* (*ENSP00000328854*), *NOP58* (*ENSP00000264279*), and *NOL10* (*ENSP00000371101*) are three nucleolar complex associated proteins with specific subcellular localization around the nucleus [55]; (4) *PUM3* (*ENSP00000380982*) is a functional DNA and mRNA binding protein that is mainly localized in the nucleus region [56], with specific subcellular location; and (5) *MPHOSPH10* (*ENSP00000244230*) regulating the pre-18S ribosomal RNA processing is observed to be mainly distributed in the nucleus and cytosol [57]. As for the remaining two Ensembl protein IDs (*ENSP00000408017*, *ENSP00000402733*), no genes can be directly annotated. However, both of them have been shown to be potential scaffold proteins, which indicate that they may be distributed in the cytosol.

As discussed above, all the top 38 features have been shown to be associated with subcellular localization, validating the reliability of our findings.

Furthermore, according to our analyses, all three methods have similar number of selected features (17 for LightGBM, 19 for MCFS, and 17 for mRMR). For each feature selection method, the average rank of the 38 selected features in their respective feature lists was calculated. The features identified by mRMR had the lowest averaged ranking (16.94) compared with the other two methods (102.05 for MCFS and 19.35 for LightGBM). Among the 15 features identified by more than one feature selection methods, all three methods identified 10 features (66.7%), reflecting the consistency of multiple feature selection methods.

Finally, considering that there are some direct subcellular location features such as nucleoplasm (*GO:0005654*) with high reliability to connect with specific subcellular location, we summarized the number of GO or KEGG terms directly describing subcellular locations and their proportions: 12/38 for LightGBM (lysosome, nucleoplasm, organelle membrane, integral component of membrane, extracellular space, endoplasmic reticulum membrane, intrinsic component of membrane, microtubule bundle formation, nucleus, obsolete microtubule organizing center part, retrograde transport-endosome to Golgi, obsolete intracellular part, and microtubule organizing center), 1/38 for MCFS (lysosome), and 9/38 for mRMR (nucleoplasm, organelle membrane, integral component of membrane, extracellular space, endoplasmic reticulum membrane, intracellular organelle lumen, envelope, integral component of plasma membrane, and plasma membrane). Therefore, according to the summary, LightGBM and mRMR were much better than MCFS.

## 5. Conclusions

In this study, we first identified a number of network features and functional annotation terms that effectively contribute to intracellular subcellular localization prediction using several feature selection methods and classification algorithms. Combining the feature ranked results obtained by mRMR, MCFS, and LightGBM, 38 key features were determined on the basis of the feature integration rules. A validation was conducted on several selected network features, functional features (KEGG pathway), and functional features (GO term) using recent literature. This study could provide a machine-learning-based investigation method for effective prediction of subcellular localization and establish a strong baseline for future experimental studies in this field.

## Figures and Tables

**Table 1:** Number of proteins in each category.

Index	Category	Number of proteins
Class 1	Biological membrane	1487
Class 2	Cell periphery	35
Class 3	Cytoplasm	506
Class 4	Cytoplasmic vesicle	70
Class 5	Endoplasmic reticulum	190
Class 6	Endosome	25
Class 7	Extracellular space or cell surface	649
Class 8	Flagellum or cilium	3
Class 9	Golgi apparatus	98
Class 10	Microtubule cytoskeleton	48
Class 11	Mitochondrion	345
Class 12	Nuclear periphery	33
Class 13	Nucleolus	112
Class 14	Nucleus	1285
Class 15	Peroxisome	46
Class 16	Vacuole	54
Total		4986

**Table 2:** Thirty-eight key features obtained by feature integration rules.

Rank	Feature name	Description
1	ENSP00000407401	PEX5 gene: peroxisomal biogenesis factor 5
2	hsa04142	Lysosome
3	GO:0005654	Nucleoplasm
4	GO:0031090	Organelle membrane
5	GO:0016021	Integral component of membrane
6	ENSP00000405965	SUMO2 gene: small ubiquitin-like modifier 2
7	ENSP00000357748	BCCIP gene: BRCA2 and CDKN1A interacting protein
8	GO:0005615	Extracellular space
9	hsa00520	Amino sugar and nucleotide sugar metabolism
10	ENSP00000263239	DDX18 gene: DEAD-box helicase 18
11	GO:0005789	Endoplasmic reticulum membrane
12	ENSP00000317578	GRK3 gene: G protein-coupled receptor kinase 3
13	ENSP00000317159	CYC1 gene: cytochrome C1
14	hsa05110	Vibrio cholerae infection
15	hsa00010	Glycolysis/gluconeogenesis
16	GO:0031224	Intrinsic component of membrane
17	GO:0070013	Intracellular organelle lumen
18	ENSP00000346725	PES1 gene: pescadillo ribosomal biogenesis factor 1
19	GO:0031975	Envelope
20	ENSP00000390722	SLC25A17 gene: solute carrier family 25 member 17
21	ENSP00000328854	NOC4L gene: nucleolar complex associated 4 homolog
22	GO:0001578	Microtubule bundle formation
23	GO:0005887	Integral component of plasma membrane
24	ENSP00000264279	NOP58 gene: NOP58 ribonucleoprotein
25	GO:0016491	Oxidoreductase activity
26	ENSP00000380982	PUM3 gene: Pumilio RNA binding family member 3
27	GO:0005634	Nucleus
28	ENSP00000371101	NOL10 gene: nucleolar protein 10
29	GO:0005886	Plasma membrane
30	GO:0044450	Obsolete microtubule organizing center part
31	ENSP00000244230	MPHOSPH10 gene: phase phosphoprotein 10
32	GO:0042147	Retrograde transport, endosome to Golgi
33	ENSP00000408017	/
34	GO:0009060	Aerobic respiration
35	GO:0044424	Obsolete intracellular part
36	ENSP00000402733	/
37	GO:0005815	Microtubule organizing center
38	GO:0044451	Obsolete nucleoplasm part

## Data Availability

The original data used to support the findings of this study are available at Swiss-Prot (http://cn.expasy.org/).

## Supplementary Materials

Supplementary Materials Table S1: three feature lists generated by the LightGBM, MCFS, and mRMR methods after the analysis of Boruta. Table S2: detailed results of IFS method with RF and SVM algorithms in the three ranked feature lists.
