# Supplementary material for: Subcellular Localization Prediction of Human Proteins Using Multifeature Selection Methods
Source: Biomed Res Int. 2022 Sep 12;2022:3288527. doi: 10.1155/2022/3288527 (PMC9484878; doi:10.1155/2022/3288527)
Supplement: Supplementary Materials — Table S1: three feature lists generated by the LightGBM, MCFS, and mRMR methods after the analysis of Boruta. Table S2: detailed results of IFS method with RF and SVM algorithms in the three ranked feature lists. [file 3288527.f1.zip › Table S1 (1).pdf]

Table S1. Three feature lists generated by the LightGBM, MCFS, and mRMR methods after the analysis of Boruta

Rank	LightGBM	MCFS	mRMR
1	GO:0031224	ENSP00000407401	GO:0070013
2	hsa04142	ENSP00000346725	GO:0031975
3	GO:0005654	ENSP00000390722	GO:0031090
4	GO:0001578	ENSP00000328854	GO:0005887
5	GO:0016021	ENSP00000264279	GO:0016491
6	GO:0005634	ENSP00000380982	GO:0005654
7	GO:0044450	ENSP00000371101	GO:0005886
8	GO:0042147	ENSP00000244230	ENSP00000405965
9	GO:0044424	ENSP00000408017	GO:0009060
10	GO:0005815	ENSP00000402733	GO:0044451
11	GO:0043296	ENSP00000223073	GO:1901564
12	GO:0005911	ENSP00000291576	GO:0043566
13	GO:0008017	ENSP00000377385	GO:0042175
14	GO:0044425	ENSP00000254605	ENSP00000357748
15	GO:0005576	ENSP00000345917	GO:0007005
16	GO:0019082	ENSP00000297990	GO:0043233
17	GO:0006664	hsa00630	GO:0008201
18	ENSP00000407401	ENSP00000362153	GO:0046128
19	GO:0044438	ENSP00000253237	GO:0031078
20	GO:0007033	ENSP00000307525	GO:0043232
21	GO:0005730	ENSP00000362105	GO:0098588
22	GO:0005615	ENSP00000268802	GO:0044459
23	GO:0003069	ENSP00000382392	GO:0045333
24	GO:0005770	ENSP00000251289	GO:0031974
25	GO:0005596	ENSP00000254940	GO:0048037
26	GO:0098742	ENSP00000327179	GO:0016021
27	GO:0005768	ENSP00000370968	GO:0016925
28	hsa00520	ENSP00000225298	GO:0016072
29	GO:0033276	ENSP00000308179	GO:0003682
30	GO:0031984	ENSP00000358563	GO:1901657
31	GO:0031090	ENSP00000247003	ENSP00000263239
32	GO:0097212	ENSP00000232888	GO:1902494
33	GO:0005783	ENSP00000320917	GO:0016604
34	GO:0032421	ENSP00000301364	GO:0045261
35	GO:0044782	ENSP00000236051	GO:0016054
36	GO:0044429	ENSP00000360828	GO:0005789
37	GO:0016197	ENSP00000311135	ENSP00000317578
38	GO:0005740	hsa00120	GO:0032991
39	GO:0031966	ENSP00000370589	GO:0046034
40	GO:0005814	ENSP00000353246	GO:0016569
41	GO:0044432	ENSP00000285814	GO:0004386
42	GO:0005789	ENSP00000259239	GO:0044743
43	GO:0005929	ENSP00000296792	GO:0044772
44	GO:0000775	ENSP00000322396	ENSP00000317159
45	GO:0032947	ENSP00000268854	GO:0005615
46	GO:0008173	ENSP00000360031	GO:0031047
47	GO:0005759	ENSP00000323858	GO:2001252
48	GO:0016327	ENSP00000258772	GO:0009161
49	GO:0016020	ENSP00000272521	ENSP00000254940

50	GO:0005262	ENSP00000328690	GO:0031224
51	GO:0007034	hsa04146	GO:0043228
52	GO:0005575	ENSP00000379760	GO:0004402
53	GO:0002250	ENSP00000308332	GO:0046129
54	GO:0043543	ENSP00000354040	GO:0004527
55	GO:0031301	ENSP00000238146	GO:0000803
56	GO:0005198	ENSP00000315674	GO:0005758
57	hsa05110	ENSP00000304151	ENSP00000355471
58	GO:0098536	ENSP00000339145	GO:0044444
59	GO:0045211	ENSP00000371169	GO:0045934
60	GO:0043687	ENSP00000314348	GO:0006353
61	GO:0043021	ENSP00000244496	GO:0070822
62	GO:0005622	ENSP00000368887	GO:0045275
63	GO:0000729	ENSP00000257829	GO:0033750
64	GO:0050684	ENSP00000367029	GO:0050662
65	GO:0005875	ENSP00000361092	GO:0034708
66	GO:0005856	ENSP00000380033	ENSP00000266544
67	GO:0005794	ENSP00000258531	GO:0006289
68	GO:0044441	ENSP00000203001	GO:0009124
69	GO:0019637	hsa00020	GO:0044454
70	GO:0016757	ENSP00000380495	ENSP00000326981
71	GO:0007041	ENSP00000366629	GO:0044798
72	GO:0005253	ENSP00000326981	GO:0051606
73	GO:0051932	ENSP00000314441	hsa00630
74	GO:0009056	ENSP00000359688	ENSP00000345917
75	GO:0005795	hsa00280	GO:0017136
76	hsa00010	ENSP00000364320	GO:1990204
77	GO:0031503	ENSP00000366519	GO:0018196
78	GO:0000715	ENSP00000319771	GO:0042254
79	GO:0034399	ENSP00000355565	GO:0098800
80	GO:0031975	ENSP00000395772	GO:0032259
81	GO:0007005	hsa04142	GO:0051186
82	GO:0004540	ENSP00000284690	ENSP00000260443
83	GO:0000242	ENSP00000229214	GO:0050911
84	GO:0000139	ENSP00000414514	GO:0009163
85	ENSP00000344818	ENSP00000250937	ENSP00000370589
86	hsa05016	ENSP00000417464	GO:0072329
87	GO:0009617	ENSP00000275820	GO:0051172
88	GO:1901137	ENSP00000358812	GO:0044452
89	GO:0090034	ENSP00000364037	GO:0009168
90	GO:0044446	ENSP00000261708	GO:0005549
91	GO:0042175	ENSP00000331815	GO:0006338
92	GO:0015631	ENSP00000260184	GO:0034660
93	GO:0005776	ENSP00000305702	GO:0051536
94	GO:0050780	ENSP00000283109	GO:0043413
95	GO:0048193	ENSP00000393101	GO:0044711
96	GO:0031967	ENSP00000202816	ENSP00000395772
97	GO:0007264	ENSP00000321320	ENSP00000269260
98	GO:0098781	ENSP00000338862	ENSP00000007516
99	GO:0031300	ENSP00000261406	GO:0005539
100	GO:0005813	ENSP00000338573	GO:0005732
101	hsa04145	ENSP00000374399	GO:0043436
102	GO:0046148	ENSP00000261015	GO:0006283

103	GO:0044455	ENSP00000355987	GO:0009205
104	GO:0043269	ENSP00000402338	GO:0045892
105	GO:0031985	ENSP00000355541	GO:0004984
106	GO:0010631	ENSP00000267425	ENSP00000229214
107	GO:0009226	ENSP00000317159	GO:0016458
108	GO:1901094	ENSP00000357748	ENSP00000322450
109	GO:0017069	ENSP00000384316	GO:0016274
110	GO:0008150	ENSP00000361232	GO:0070911
111	GO:0005975	ENSP00000337518	GO:0099537
112	ENSP00000390722	ENSP00000265245	ENSP00000301585
113	GO:0060271	ENSP00000363746	GO:0032392
114	GO:0005743	ENSP00000263774	GO:0004930
115	GO:0044766	ENSP00000266544	GO:0004532
116	GO:0043220	hsa00071	GO:0035097
117	hsa04725	ENSP00000263239	GO:0008535
118	hsa04141	ENSP00000278856	ENSP00000264279
119	GO:0046914	ENSP00000311977	GO:1901681
120	GO:0003682	hsa00640	GO:0010257
121	GO:0098589	ENSP00000297579	GO:0016363
122	GO:0071944	ENSP00000230340	GO:1901566
123	GO:0044267	ENSP00000384302	GO:0050907
124	GO:0008353	ENSP00000274849	GO:1902589
125	GO:0006892	ENSP00000260619	GO:0016651
126	GO:0004602	ENSP00000302886	GO:0010558
127	GO:0044422	ENSP00000352021	ENSP00000358563
128	GO:0034453	ENSP00000407515	GO:0045252
129	GO:0033108	ENSP00000377523	GO:0030686
130	ENSP00000405965	ENSP00000246802	GO:0006790
131	GO:0061695	ENSP00000366623	GO:0050906
132	GO:0051797	hsa00620	ENSP00000261406
133	GO:0006863	ENSP00000321449	GO:0098813
134	GO:0005788	ENSP00000348596	GO:0042775
135	GO:0004148	ENSP00000363642	GO:0043966
136	GO:0098542	hsa00190	GO:0006399
137	GO:0070661	ENSP00000386134	GO:0010389
138	GO:0046931	ENSP00000405614	ENSP00000263657
139	GO:0044743	ENSP00000298746	GO:0009593
140	GO:0043231	ENSP00000221801	GO:0045257
141	GO:0042384	ENSP00000360412	GO:0018205
142	GO:0034067	ENSP00000356548	GO:0015075
143	GO:0031623	ENSP00000367361	GO:0045239
144	GO:0016874	ENSP00000350524	GO:0045171
145	GO:0009225	ENSP00000333934	ENSP00000283109
146	GO:1902224	ENSP00000296802	hsa04146
147	GO:0016323	ENSP00000350352	hsa03008
148	GO:0014704	ENSP00000261741	hsa04740
149	GO:0010992	ENSP00000402869	GO:0000075
150	GO:0009057	ENSP00000332340	GO:0032981
151	GO:0008375	ENSP00000372695	GO:0001076
152	GO:0008270	ENSP00000361010	GO:0043603
153	GO:0005777	hsa00072	GO:0031060
154	GO:0098791	ENSP00000230640	GO:0051225
155	GO:0070925	ENSP00000263657	ENSP00000315774

156	GO:0043168	ENSP00000234170	GO:0007608
157	GO:0034708	ENSP00000348722	ENSP00000382392
158	GO:0031988	hsa03013	GO:0009062
159	GO:0002455	ENSP00000327070	GO:0006323
160	GO:0098588	ENSP00000350698	GO:0048039
161	GO:0044703	ENSP00000343081	GO:0031327
162	GO:0034260	ENSP00000301585	GO:0016591
163	GO:0019730	ENSP00000253107	ENSP00000353246
164	GO:0005774	ENSP00000264670	GO:0044710
165	GO:0005643	ENSP00000381655	GO:0007606
166	GO:0002474	ENSP00000355899	GO:0033108
167	GO:1990234	hsa05012	GO:0006325
168	GO:1900115	ENSP00000395187	ENSP00000364320
169	GO:0060456	ENSP00000260563	GO:0022857
170	GO:0044451	ENSP00000410530	GO:0045259
171	GO:0044255	ENSP00000263331	GO:0006974
172	GO:0035091	ENSP00000365806	GO:0004540
173	GO:0032580	hsa03008	GO:0005782
174	GO:0031461	ENSP00000362687	GO:0007600
175	GO:0030529	ENSP00000412483	GO:0016741
176	GO:0030054	ENSP00000233114	GO:0044839
177	GO:0000280	ENSP00000402802	GO:0090568
178	GO:2000001	ENSP00000364649	ENSP00000216254
179	GO:0099512	ENSP00000355471	ENSP00000236051
180	GO:0061726	ENSP00000225296	GO:0003713
181	GO:0051861	ENSP00000199320	GO:0006553
182	GO:0051817	ENSP00000222567	GO:0031984
183	GO:0048519	ENSP00000331111	GO:0034388
184	GO:0046037	hsa00380	GO:0044712
185	GO:0044464	ENSP00000203407	GO:0009890
186	GO:0019673	ENSP00000310668	GO:0007067
187	GO:0015874	ENSP00000374390	ENSP00000334051
188	GO:0015851	ENSP00000362873	ENSP00000327268
189	GO:0015698	ENSP00000373090	ENSP00000362105
190	GO:0007040	ENSP00000330460	GO:0006281
191	GO:0006900	ENSP00000403576	GO:0016614
192	GO:0006575	ENSP00000249269	GO:0030490
193	GO:0005623	ENSP00000317992	GO:0050877
194	GO:0002923	ENSP00000394290	GO:0005736
195	hsa04146	hsa00510	GO:0032446
196	GO:0061077	ENSP00000281701	GO:0045240
197	GO:0048524	ENSP00000399454	GO:0010501
198	GO:0042391	ENSP00000314193	GO:0044700
199	GO:0022400	ENSP00000350011	GO:0065004
200	GO:0016668	ENSP00000362036	ENSP00000387262
201	GO:0016604	ENSP00000254803	GO:1903507
202	GO:0010390	hsa00650	GO:0006575
203	GO:0009311	ENSP00000405965	GO:0016675
204	GO:0009064	ENSP00000357535	GO:0099600
205	GO:0008194	ENSP00000396454	GO:0022891
206	GO:0007017	ENSP00000399753	ENSP00000296802
207	GO:0006626	hsa03040	GO:0072524
208	GO:0005829	ENSP00000297873	GO:1902749

209	GO:0000939	ENSP00000327268	ENSP00000328854
210	hsa00510	ENSP00000225402	GO:0016407
211	GO:0051701	ENSP00000260443	ENSP00000306397
212	GO:0043015	ENSP00000356476	GO:0043270
213	GO:0042578	ENSP00000409367	GO:0038023
214	GO:0034703	ENSP00000315774	GO:0045254
215	GO:0034660	ENSP00000372199	ENSP00000225298
216	GO:0032592	ENSP00000268379	GO:0032435
217	GO:0031253	ENSP00000303515	GO:0050136
218	GO:0031023	hsa00533	GO:0032204
219	GO:0009295	ENSP00000378857	GO:0046782
220	GO:0008237	ENSP00000323424	GO:0009066
221	GO:0007126	ENSP00000215375	GO:0016577
222	GO:0007098	hsa03010	GO:0007186
223	GO:0005779	ENSP00000403536	ENSP00000380033
224	GO:0004175	ENSP00000322450	GO:0008137
225	GO:0000930	ENSP00000407436	hsa04110
226	GO:1901004	ENSP00000261637	GO:0004518
227	GO:0071383	ENSP00000411162	GO:0016676
228	GO:0048384	ENSP00000412251	GO:0004888
229	GO:0043297	ENSP00000413572	GO:0008026
230	GO:0015931	ENSP00000313953	GO:0008227
231	GO:0007276	hsa00900	GO:0097031
232	GO:0006998	hsa00310	GO:0000981
233	GO:0006396	ENSP00000328671	GO:0043144
234	GO:0005667	ENSP00000409074	GO:0051287
235	GO:0004674	ENSP00000396441	GO:0045814
236	GO:0001618	ENSP00000233190	ENSP00000234170
237	GO:0000045	ENSP00000342056	ENSP00000357535
238	GO:1901032	ENSP00000377944	GO:0006482
239	GO:0097060	hsa00410	ENSP00000223073
240	GO:0016787	ENSP00000310042	GO:0000989
241	GO:0007043	ENSP00000306397	hsa00620
242	GO:0004222	ENSP00000365837	GO:0007059
243	GO:0003954	ENSP00000345895	GO:0051537
244	GO:0050794	ENSP00000387262	GO:0022892
245	GO:0048520	hsa03020	GO:0006304
246	GO:0046903	ENSP00000377446	ENSP00000199320
247	GO:0043236	ENSP00000356972	GO:0019395
248	GO:0038023	ENSP00000309565	GO:0070034
249	GO:0036126	ENSP00000007516	ENSP00000356972
250	GO:0010604	ENSP00000360329	ENSP00000259037
251	GO:0008092	ENSP00000330737	GO:0046496
252	GO:0005581	ENSP00000320567	GO:0048285
253	GO:0002697	hsa03015	GO:0000154
254	GO:0000268	ENSP00000303983	ENSP00000314348
255	ENSP0000225298	hsa03018	GO:0006305
256	GO:1903513	ENSP00000318158	ENSP00000248572
257	GO:0070098	hsa05016	GO:0006163
258	GO:0051321	ENSP00000259037	ENSP00000362649
259	GO:0043395	ENSP00000332887	GO:0031093
260	GO:0031225	ENSP00000367934	GO:0008173
261	GO:0016607	ENSP00000262946	GO:0007154

262	GO:0009986	ENSP00000253452	GO:0007093
263	GO:0005681	ENSP00000317780	GO:0005749
264	hsa05146	ENSP00000296684	GO:0003735
265	hsa04911	hsa03022	GO:1902850
266	hsa04070	ENSP00000345793	ENSP00000370968
267	hsa00480	ENSP00000349142	GO:0060089
268	GO:1901977	ENSP00000247866	GO:0019233
269	GO:0070374	ENSP00000262607	GO:0015985
270	GO:0070201	hsa00100	GO:0006368
271	GO:0051606	hsa00010	GO:0001522
272	GO:0050962	ENSP00000258424	GO:0015002
273	GO:0044431	ENSP00000419740	hsa00071
274	GO:0043167	ENSP00000282050	ENSP00000247003
275	GO:0042748	ENSP00000216254	GO:0004872
276	GO:0042254	ENSP00000301587	GO:0070125
277	GO:0036019	ENSP00000356953	GO:0044154
278	GO:0035821	ENSP00000364699	GO:0006402
279	GO:0032922	ENSP00000358737	GO:0044283
280	GO:0019866	ENSP00000233627	ENSP00000253452
281	GO:0018149	ENSP00000346839	GO:0006303
282	GO:0016311	hsa04110	GO:0030688
283	GO:0010971	ENSP00000262030	GO:0015986
284	GO:0005874	hsa03030	GO:0050434
285	GO:0005778	ENSP00000284727	GO:0005740
286	GO:0004386	hsa00830	GO:0090305
287	GO:0003878	hsa04530	GO:0010390
288	GO:0000375	ENSP00000360492	GO:0004871
289	GO:1903900	ENSP00000268668	ENSP00000380982
290	GO:0070013	hsa00601	GO:0032434
291	GO:0051905	ENSP00000389649	GO:0055065
292	GO:0051056	ENSP00000361084	GO:0046394
293	GO:0050678	hsa05150	GO:0055085
294	GO:0045324	ENSP00000285949	GO:0008175
295	GO:0043202	hsa00670	GO:0044431
296	GO:0042470	ENSP00000308938	ENSP00000309565
297	GO:0035767	ENSP00000276585	GO:0009150
298	GO:0033692	hsa00514	ENSP00000325682
299	GO:0032993	ENSP00000276689	ENSP00000314193
300	GO:0031970	ENSP00000269260	GO:0035064
301	GO:0030173	ENSP00000361965	GO:0016866
302	GO:0022406	hsa04640	GO:0008213
303	GO:0016791	hsa00520	GO:0051168
304	GO:0016591	ENSP00000299166	GO:0007283
305	GO:0010257	hsa04740	GO:0016742
306	GO:0008277	hsa04610	ENSP00000358737
307	GO:0006997	ENSP00000362649	ENSP00000301364
308	GO:0006625	hsa05010	GO:0007165
309	GO:0005782	hsa04260	GO:0000794
310	GO:0005539	ENSP00000330937	GO:0034220
311	GO:0002360	hsa00030	GO:0044439
312	ENSP00000203407	ENSP00000254810	ENSP00000258772
313	hsa00500	hsa05203	GO:0055086
314	GO:2000785	hsa00240	GO:0044728

315	GO:1901606	ENSP00000290866	ENSP00000371377
316	GO:0090557	ENSP00000334051	GO:0034475
317	GO:0043243	ENSP00000370473	ENSP00000379760
318	GO:0043229	ENSP00000258105	GO:0006119
319	GO:0043032	hsa00260	GO:0016580
320	GO:0042276	ENSP00000317578	GO:0005741
321	GO:0031965	hsa00250	ENSP00000374399
322	GO:0030817	ENSP00000287022	hsa00640
323	GO:0030490	ENSP00000260227	GO:0000723
324	GO:0017015	ENSP00000331545	ENSP00000333934
325	GO:0008536	ENSP00000316955	GO:0006873
326	GO:0007007	ENSP00000344818	ENSP00000316955
327	GO:0006820	ENSP00000262367	GO:0043933
328	GO:0006816	ENSP00000312652	ENSP00000354040
329	GO:0006487	ENSP00000252711	GO:0051321
330	GO:0006486	hsa04670	GO:0044391
331	ENSP00000216492	ENSP00000416330	ENSP00000367029
332	hsa04744	hsa00330	ENSP00000387523
333	GO:1901879	hsa04060	GO:0001077
334	GO:0098805	ENSP00000386047	GO:0006306
335	GO:0098796	hsa00140	ENSP00000247866
336	GO:0090179	hsa00500	GO:0006753
337	GO:0071013	hsa04623	ENSP00000302886
338	GO:0045766	ENSP00000352839	GO:0004774
339	GO:0043112	ENSP00000255030	GO:1902750
340	GO:0034308	ENSP00000223095	GO:0051236
341	GO:0033233	hsa05323	ENSP00000327585
342	GO:0031935	ENSP00000290299	GO:0070545
343	GO:0031333	hsa00980	GO:0007052
344	GO:0031304	hsa00590	GO:2000756
345	GO:0030234	ENSP00000218388	ENSP00000318158
346	GO:0030027	ENSP00000307786	GO:1901361
347	GO:0018198	hsa04614	GO:0007094
348	GO:0016747	ENSP00000264498	GO:0019867
349	GO:0015296	hsa05130	ENSP00000244623
350	GO:0008037	ENSP00000306974	GO:0015992
351	GO:0005742	hsa04145	GO:0016607
352	GO:0000979	ENSP00000348170	ENSP00000328690
353	ENSP00000338573	hsa00970	GO:0007346
354	GO:1901575	hsa04062	GO:0050921
355	GO:0099568	ENSP00000209540	ENSP00000399078
356	GO:0098862	ENSP00000001146	ENSP00000339145
357	GO:0090288	hsa04921	ENSP00000258105
358	GO:0090287	ENSP00000304419	GO:0010959
359	GO:0051276	hsa03420	ENSP00000350698
360	GO:0051219	ENSP00000264563	GO:0044815
361	GO:0044798	ENSP00000258743	GO:0016042
362	GO:0044437	hsa04742	GO:0000314
363	GO:0044309	hsa00220	ENSP00000297990
364	GO:0043226	ENSP00000231751	GO:0007126
365	GO:0035267	ENSP00000264998	ENSP00000317780
366	GO:0031974	hsa04070	ENSP00000323858
367	GO:0030139	hsa04540	GO:0043565

368	GO:0022829	hsa00350	GO:0072521
369	GO:0022613	hsa04514	GO:1901575
370	GO:0019724	hsa04144	GO:0006336
371	GO:0016050	hsa04723	GO:0001676
372	GO:0015085	ENSP00000307130	ENSP00000380153
373	GO:0010739	hsa00480	GO:0008301
374	GO:0008535	hsa05110	ENSP00000254605
375	GO:0008238	ENSP00000297350	GO:0016884
376	GO:0007097	hsa05132	GO:0030141
377	GO:0006839	ENSP00000248572	GO:0000275
378	GO:0006644	hsa04810	ENSP00000254810
379	GO:0006090	ENSP00000285600	GO:0097472
380	GO:0005886	ENSP00000265171	ENSP00000357134
381	GO:0005796	hsa04512	GO:0006779
382	GO:0005775	hsa04114	GO:0005840
383	GO:0003924	hsa04080	GO:0001561
384	GO:0001824	ENSP00000350720	ENSP00000202816
385	GO:0000323	ENSP00000338548	ENSP00000332887
386	ENSP00000304133	hsa00860	GO:0044265
387	ENSP00000301585	hsa05200	GO:0043967
388	ENSP00000231751	ENSP00000304133	GO:0005102
389	GO:0065003	hsa04970	ENSP00000257829
390	GO:0060712	hsa04151	GO:0009063
391	GO:0051439	ENSP00000356671	ENSP00000308938
392	GO:0046966	hsa04330	GO:1901799
393	GO:0046488	ENSP00000357134	GO:0043527
394	GO:0046467	ENSP00000302150	ENSP00000324769
395	GO:0045454	hsa04933	GO:0030003
396	GO:0044712	ENSP00000275525	GO:0016758
397	GO:0044448	ENSP00000344453	ENSP00000362153
398	GO:0044282	hsa04911	hsa00010
399	GO:0043484	ENSP00000311038	GO:0022617
400	GO:0043228	ENSP00000325203	ENSP00000303864
401	GO:0036297	hsa05144	ENSP00000308332
402	GO:0036066	ENSP00000263253	GO:0016572
403	GO:0034622	ENSP00000295897	GO:1901606
404	GO:0033059	ENSP00000326259	ENSP00000296684
405	GO:0032467	ENSP00000355436	GO:0031903
406	GO:0031579	ENSP00000398698	ENSP00000346725
407	GO:0031047	ENSP00000220616	GO:0008528
408	GO:0030695	hsa04727	GO:0030521
409	GO:0030667	hsa05205	GO:0006446
410	GO:0030060	hsa04020	ENSP00000299166
411	GO:0016758	hsa05146	ENSP00000404102
412	GO:0009101	hsa05143	ENSP00000363746
413	GO:0007031	hsa05321	GO:0009056
414	GO:0006261	ENSP00000304188	GO:0098589
415	GO:0002251	hsa05215	ENSP00000377385
416	GO:0000805	hsa04918	GO:0045263
417	GO:0000055	ENSP00000206249	GO:0051568
418	ENSP00000264998	ENSP00000348986	GO:0015630
419	hsa05034	hsa05169	ENSP00000377799
420	hsa03013	ENSP00000368244	ENSP00000417464

421	hsa00640	hsa04730	GO:0045944
422	GO:1902305	hsa04976	GO:0016616
423	GO:1900034	ENSP00000355430	GO:0000786
424	GO:0098590	ENSP00000408153	ENSP00000355541
425	GO:0097208	ENSP00000330341	GO:0019080
426	GO:0090575	ENSP00000321246	ENSP00000355430
427	GO:0090305	hsa04932	GO:0070838
428	GO:0071875	hsa05142	GO:0044723
429	GO:0048002	hsa04728	GO:0008176
430	GO:0045933	ENSP00000306688	ENSP00000353947
431	GO:0045814	ENSP00000296099	GO:0032984
432	GO:0044248	ENSP00000325682	GO:0006370
433	GO:0043234	ENSP00000306651	ENSP00000366519
434	GO:0042393	ENSP00000355443	GO:0004739
435	GO:0038044	ENSP00000323612	GO:0031497
436	GO:0036276	hsa00983	GO:0071174
437	GO:0031577	ENSP00000353947	ENSP00000344453
438	GO:0030133	ENSP00000303076	GO:0008652
439	GO:0030036	ENSP00000325128	ENSP00000296792
440	GO:0016746	ENSP00000278407	GO:0051028
441	GO:0016605	hsa05034	GO:0043628
442	GO:0009611	ENSP00000266646	GO:0051262
443	GO:0008233	hsa04726	ENSP00000328563
444	GO:0006643	hsa05168	GO:1902600
445	GO:0006473	ENSP00000306245	ENSP00000372695
446	GO:0006391	ENSP00000295400	GO:0000724
447	GO:0005923	hsa04014	GO:0048521
448	GO:0005902	ENSP00000404102	ENSP00000364649
449	GO:0005765	hsa04120	GO:0046112
450	GO:0005739	ENSP00000322088	ENSP00000324251
451	GO:0005614	hsa04962	GO:0008324
452	GO:0003824	hsa04960	GO:0044255
453	GO:0003676	ENSP00000332504	ENSP00000360828
454	GO:0002384	hsa04744	GO:0006754
455	hsa00310	hsa04310	GO:0072503
456	GO:2000296	ENSP00000306095	ENSP00000254803
457	GO:1902115	hsa04270	ENSP00000322939
458	GO:0070372	hsa04973	GO:0002740
459	GO:0051179	hsa04141	GO:0006637
460	GO:0046541	hsa04720	ENSP00000405614
461	GO:0044430	ENSP00000409384	GO:0045277
462	GO:0043933	hsa05414	GO:0008649
463	GO:0043679	ENSP00000310337	GO:0070734
464	GO:0043209	ENSP00000399078	ENSP00000309673
465	GO:0042059	GO:0010727	GO:0010605
466	GO:0034975	GO:0010720	ENSP00000389649
467	GO:0032271	GO:0010716	GO:0061045
468	GO:0031248	GO:0010715	GO:1903900
469	GO:0016725	GO:0010714	ENSP00000362687
470	GO:0016675	GO:0010712	ENSP00000306974
471	GO:0016651	GO:0010711	GO:0008135
472	GO:0012505	GO:0010648	GO:0016602
473	GO:0007289	GO:0010647	GO:0002687

474	GO:0006901	GO:0010646	ENSP00000225296
475	GO:0002920	GO:0010644	ENSP00000306688
476	GO:0002431	GO:0010639	ENSP00000284727
477	GO:0002046	GO:0010638	GO:0008276
478	GO:0001958	GO:0010634	ENSP00000361232
479	GO:0000776	GO:0010633	GO:0016903
480	GO:0000187	GO:0010632	GO:1901565
481	hsa04080	GO:0010631	ENSP00000305207
482	hsa00240	GO:0010629	ENSP00000305702
483	GO:1901800	GO:0010628	GO:0006475
484	GO:1901616	GO:0010608	ENSP00000303834
485	GO:0060322	GO:0010605	GO:1900047
486	GO:0051310	GO:0010604	ENSP00000384316
487	GO:0051262	GO:0010596	GO:0051480
488	GO:0051251	GO:0010575	ENSP00000272521
489	GO:0050954	GO:0010574	GO:0003705
490	GO:0050657	GO:0010573	GO:0034979
491	GO:0048489	GO:0010569	hsa04080
492	GO:0045787	GO:0010564	ENSP00000361010
493	GO:0045721	GO:0010563	ENSP00000325203
494	GO:0045334	GO:0010562	GO:0005747
495	GO:0044445	hsa04725	GO:0006337
496	GO:0044273	GO:0010558	GO:0008194
497	GO:0043197	GO:0010557	GO:0043596
498	GO:0042160	GO:0010556	ENSP00000301587
499	GO:0035437	GO:0010544	ENSP00000350524
500	GO:0035064	GO:0010543	GO:0032103
501	GO:0033014	GO:0010536	ENSP00000310488
502	GO:0032401	GO:0010535	GO:0006397
503	GO:0031228	GO:0010534	GO:0034661
504	GO:0030913	GO:0010533	GO:0044236
505	GO:0030863	GO:0010522	GO:1901989
506	GO:0019886	hsa04724	GO:0000104
507	GO:0016925	GO:0010518	GO:1990837
508	GO:0010638	GO:0010517	GO:0034723
509	GO:0010256	GO:0010510	ENSP00000414514
510	GO:0008610	GO:0010506	ENSP00000369728
511	GO:0008289	GO:0010501	GO:0044724
512	GO:0007586	GO:0010498	ENSP00000412483
513	GO:0007218	GO:0010468	ENSP00000345163
514	GO:0006779	GO:0010467	ENSP00000395187
515	GO:0006621	GO:0010466	GO:0043687
516	GO:0006508	GO:0010455	hsa04911
517	GO:0006360	GO:0010453	GO:0044445
518	GO:0006352	GO:0010390	GO:0071427
519	GO:0006304	GO:0010389	ENSP00000278409
520	GO:0006081	GO:0010257	GO:0071043
521	GO:0005635	GO:0010256	GO:0015276
522	GO:0004620	GO:0010248	GO:0070933
523	GO:0004003	GO:0010243	GO:0051048
524	GO:0003713	GO:0010193	GO:0016879
525	GO:2000870	GO:0010107	ENSP00000264670
526	GO:1902494	GO:0010035	GO:0051303

527	GO:0098573	GO:0010033	GO:0061621
528	GO:0097425	GO:0009991	ENSP00000352626
529	GO:0072594	GO:0009987	ENSP00000322396
530	GO:0071559	GO:0009968	GO:0007276
531	GO:0070545	GO:0009967	GO:0009201
532	GO:0060260	GO:0009966	ENSP00000274849
533	GO:0060090	GO:0009952	GO:0019985
534	GO:0051260	GO:0009914	GO:0001696
535	GO:0051258	GO:0009896	GO:0044253
536	GO:0050854	GO:0009894	ENSP00000307598
537	GO:0050832	hsa04713	GO:0070901
538	GO:0050830	GO:0009893	GO:0006403
539	GO:0043433	GO:0009892	GO:0006766
540	GO:0043230	GO:0009891	GO:0044784
541	GO:0036498	GO:0009890	GO:1990542
542	GO:0036124	GO:0009889	ENSP00000250937
543	GO:0031256	GO:0009888	GO:0010510
544	GO:0030397	GO:0009887	ENSP00000360492
545	GO:0030140	GO:0009792	ENSP00000321506
546	GO:0030041	GO:0009790	GO:0016740
547	GO:0017124	GO:0009746	ENSP00000284690
548	GO:0016836	GO:0009743	GO:0090329
549	GO:0016589	GO:0009725	GO:0009303
550	GO:0016324	GO:0009719	GO:0009064
551	GO:0012506	GO:0009653	ENSP00000285600
552	GO:0009247	GO:0009636	ENSP00000331815
553	GO:0007063	GO:0009628	GO:0006066
554	GO:0006996	GO:0009620	GO:0016469
555	GO:0006959	GO:0009617	GO:0004591
556	GO:0006475	GO:0009612	GO:0005184
557	GO:0006006	GO:0009611	GO:0030574
558	GO:0005669	hsa04666	ENSP00000323853
559	GO:0004252	GO:0009607	ENSP00000260619
560	GO:0002385	GO:0009605	GO:0008277
561	GO:0001959	GO:0009593	GO:0044246
562	GO:0000151	GO:0009584	GO:0022613
563	hsa05010	GO:0009583	GO:0042769
564	hsa03022	GO:0009582	ENSP00000343081
565	GO:2000142	GO:0009581	GO:0048232
566	GO:1990391	GO:0009452	GO:0008380
567	GO:1901681	GO:0009451	GO:0002698
568	GO:0098687	GO:0009411	ENSP00000263331
569	GO:0097481	hsa04662	GO:0022836
570	GO:0090151	GO:0009410	GO:0006805
571	GO:0072686	GO:0009404	GO:0045841
572	GO:0072372	GO:0009396	GO:0008191
573	GO:0071840	GO:0009312	GO:0080171
574	GO:0071682	GO:0009311	GO:0006814
575	GO:0070603	GO:0009306	ENSP00000304188
576	GO:0061418	GO:0009303	GO:1903532
577	GO:0060261	GO:0009268	ENSP00000360329
578	GO:0051904	GO:0009260	GO:0044281
579	GO:0046782	GO:0009259	GO:0032436

580	GO:0045765	hsa04660	GO:0005763
581	GO:0035770	GO:0009247	GO:0002739
582	GO:0033013	GO:0009226	GO:0019226
583	GO:0032994	GO:0009225	GO:0046031
584	GO:0031386	GO:0009220	ENSP00000326259
585	GO:0031267	GO:0009218	GO:0048243
586	GO:0031227	GO:0009206	GO:0006023
587	GO:0031213	GO:0009205	ENSP00000260184
588	GO:0031010	GO:0009201	ENSP00000233627
589	GO:0030031	GO:0009199	ENSP00000285814
590	GO:0030029	GO:0009190	GO:0002690
591	GO:0019752	GO:0009187	GO:0000276
592	GO:0016903	GO:0009185	ENSP00000311977
593	GO:0009410	GO:0009179	GO:0000987
594	GO:0009081	GO:0009174	GO:0006335
595	GO:0008298	GO:0009173	ENSP00000319771
596	GO:0008144	GO:0009168	ENSP00000255030
597	GO:0006367	GO:0009167	GO:0022803
598	GO:0006139	GO:0009165	GO:0016667
599	GO:0005896	GO:0009163	GO:0009167
600	GO:0005793	GO:0009161	GO:0034655
601	GO:0005773	hsa04630	GO:0008610
602	GO:0004842	GO:0009156	hsa05144
603	GO:0003779	GO:0009152	GO:0008270
604	GO:0003723	GO:0009150	GO:0007610
605	GO:0000777	GO:0009145	GO:0015267
606	ENSP00000295897	GO:0009144	ENSP00000238146
607	GO:2000816	GO:0009142	GO:0000398
608	GO:1901992	GO:0009141	GO:0000313
609	GO:0090407	GO:0009135	ENSP00000407436
610	GO:0070822	GO:0009132	GO:0051985
611	GO:0061462	GO:0009127	GO:0007157
612	GO:0051436	GO:0009126	GO:0007213
613	GO:0051259	GO:0009124	GO:1903036
614	GO:0051015	GO:0009123	GO:0044033
615	GO:0050906	GO:0009119	ENSP00000306651
616	GO:0048609	GO:0009117	GO:0019682
617	GO:0048037	GO:0009116	ENSP00000262946
618	GO:0046605	GO:0009113	ENSP00000377944
619	GO:0046085	GO:0009112	GO:0016405
620	GO:0045932	GO:0009111	GO:0033554
621	GO:0045907	GO:0009108	GO:0004896
622	GO:0045039	GO:0009101	GO:0006614
623	GO:0043969	GO:0009100	GO:0006084
624	GO:0043966	GO:0009083	ENSP00000373090
625	GO:0043623	GO:0009081	GO:0031091
626	GO:0043568	GO:0009070	GO:0005643
627	GO:0043414	GO:0009069	GO:0035861
628	GO:0042742	GO:0009068	GO:0006479
629	GO:0035582	GO:0009066	ENSP00000411162
630	GO:0033631	GO:0009065	GO:0031304
631	GO:0033218	GO:0009064	GO:0001159
632	GO:0031491	hsa04611	GO:0019856

633	GO:0031348	GO:0009063	GO:0097529
634	GO:0030002	GO:0009062	GO:0098660
635	GO:0019789	GO:0009060	ENSP00000278856
636	GO:0019228	GO:0009059	hsa00330
637	GO:0017004	GO:0009058	GO:0006720
638	GO:0016853	GO:0009057	ENSP00000313953
639	GO:0016811	GO:0009056	GO:0030803
640	GO:0016620	GO:0008652	GO:0006154
641	GO:0016363	GO:0008610	GO:0051272
642	GO:0016328	GO:0008585	GO:0051046
643	GO:0016266	GO:0008584	GO:0070987
644	GO:0015081	GO:0008544	ENSP00000261637
645	GO:0010716	GO:0008542	ENSP00000410530
646	GO:0010467	GO:0008535	GO:0009410
647	GO:0009593	GO:0008406	GO:0052472
648	GO:0009070	GO:0008380	GO:0006775
649	GO:0008380	GO:0008361	GO:0002673
650	GO:0008023	GO:0008360	GO:0043161
651	GO:0006650	GO:0008344	GO:0050802
652	GO:0006354	GO:0008334	GO:0046132
653	GO:0005884	GO:0008300	GO:0034724
654	GO:0005096	GO:0008299	GO:0005622
655	GO:0003714	GO:0008298	GO:0043487
656	GO:0002227	GO:0008286	GO:0016278
657	GO:0001227	GO:0008285	GO:0030809
658	GO:0001205	GO:0008284	hsa05323
659	GO:0001104	GO:0008283	GO:0033558
660	GO:0001098	GO:0008277	GO:0000775
661	GO:0000779	GO:0008219	ENSP00000323424
662	GO:0000276	GO:0008217	GO:0050909
663	hsa05200	GO:0008213	GO:1902224
664	hsa05100	GO:0008211	GO:0004812
665	hsa04962	GO:0008210	GO:0030705
666	hsa04742	GO:0008209	GO:0031253
667	GO:1904018	GO:0008206	GO:0061029
668	GO:0099565	GO:0008203	GO:1903311
669	GO:0098798	GO:0008202	ENSP00000365806
670	GO:0090195	GO:0008154	GO:0044205
671	GO:0086001	GO:0008152	ENSP00000396441
672	GO:0075733	GO:0008150	GO:1902806
673	GO:0072329	GO:0008105	GO:1990351
674	GO:0070382	GO:0008104	GO:0002719
675	GO:0051668	GO:0008064	GO:0018024
676	GO:0051494	GO:0008037	GO:0050684
677	GO:0051236	GO:0008033	GO:0007077
678	GO:0050890	GO:0008016	GO:0019058
679	GO:0050856	GO:0008015	GO:1902911
680	GO:0050789	GO:0007635	ENSP00000409367
681	GO:0050673	GO:0007632	GO:0044433
682	GO:0050000	GO:0007631	GO:0030170
683	GO:0046847	GO:0007628	ENSP00000412251
684	GO:0045171	GO:0007626	GO:0002688
685	GO:0044306	GO:0007612	ENSP00000264998

686	GO:0042771	GO:0007611	ENSP00000403576
687	GO:0042054	GO:0007610	GO:0044450
688	GO:0038165	GO:0007608	GO:0090544
689	GO:0035887	GO:0007606	GO:0051339
690	GO:0034702	GO:0007603	GO:0006778
691	GO:0032991	GO:0007602	GO:0004775
692	GO:0032870	GO:0007600	GO:0090545
693	GO:0032507	hsa04510	GO:0006954
694	GO:0032480	GO:0007599	GO:0015078
695	GO:0031982	GO:0007597	GO:0007095
696	GO:0031981	GO:0007596	GO:0099513
697	GO:0031937	GO:0007588	GO:0008320
698	GO:0031513	GO:0007586	GO:0005775
699	GO:0031406	GO:0007584	GO:0006541
700	GO:0030880	GO:0007571	GO:0016324
701	GO:0022607	GO:0007568	GO:0016705
702	GO:0021545	GO:0007566	GO:0044702
703	GO:0019838	GO:0007565	GO:0006613
704	GO:0019731	hsa04360	ENSP00000253107
705	GO:0016860	GO:0007548	hsa00650
706	GO:0016741	GO:0007507	GO:0003729
707	GO:0016667	GO:0007492	GO:1903362
708	GO:0016247	GO:0007435	GO:0010466
709	GO:0015721	GO:0007431	GO:0043623
710	GO:0015030	GO:0007420	GO:0005765
711	GO:0009581	GO:0007411	GO:0051570
712	GO:0008603	GO:0007409	GO:0043632
713	GO:0008514	GO:0007399	GO:0016032
714	GO:0007213	GO:0007369	GO:0016887
715	GO:0007186	GO:0007346	GO:0009132
716	GO:0007030	GO:0007289	GO:1903531
717	GO:0007018	GO:0007283	GO:0005216
718	GO:0006612	GO:0007276	GO:0016259
719	GO:0006595	GO:0007275	GO:0005721
720	GO:0006488	GO:0007270	GO:0000459
721	GO:0006333	GO:0007269	GO:1901607
722	GO:0004722	GO:0007268	GO:0016023
723	GO:0004703	GO:0007267	GO:0016747
724	GO:0003677	GO:0007266	GO:0044057
725	GO:0001975	GO:0007265	GO:0016765
726	GO:0000118	GO:0007264	ENSP00000281701
727	ENSP00000386884	GO:0007260	GO:0031279
728	ENSP00000276585	GO:0007259	GO:0042573
729	ENSP00000253107	GO:0007229	GO:0061733
730	ENSP00000225402	GO:0007220	GO:0030595
731	hsa05166	GO:0007219	ENSP00000221801
732	hsa04120	GO:0007218	GO:0016746
733	hsa03015	GO:0007215	GO:0032967
734	GO:1904035	GO:0007213	GO:0000175
735	GO:1903902	GO:0007212	ENSP00000407401
736	GO:1903309	GO:0007210	ENSP00000297873
737	GO:1902554	GO:0007205	GO:0044764
738	GO:1901881	GO:0007204	GO:0000062

739	GO:0071987	GO:0007202	GO:0071674
740	GO:0060191	GO:0007200	GO:0051169
741	GO:0055067	GO:0007194	GO:0016709
742	GO:0055037	GO:0007193	GO:0016620
743	GO:0051270	GO:0007191	GO:0044708
744	GO:0051238	GO:0007190	ENSP00000345793
745	GO:0050435	hsa04261	GO:0006457
746	GO:0048385	GO:0007189	GO:0002701
747	GO:0046822	GO:0007188	GO:0015672
748	GO:0045176	GO:0007187	GO:0072594
749	GO:0045055	GO:0007186	GO:0042054
750	GO:0044724	GO:0007179	GO:0046906
751	GO:0044421	GO:0007178	GO:0006418
752	GO:0043410	GO:0007173	GO:0006171
753	GO:0035590	GO:0007169	GO:0016486
754	GO:0034714	GO:0007167	GO:0019941
755	GO:0034330	GO:0007166	GO:0045202
756	GO:0033365	GO:0007165	GO:0045177
757	GO:0032456	GO:0007164	GO:0005178
758	GO:0032259	GO:0007163	GO:0030808
759	GO:0031901	GO:0007162	GO:0044257
760	GO:0031826	GO:0007160	GO:0032786
761	GO:0031573	GO:0007159	GO:0031490
762	GO:0030669	GO:0007157	ENSP00000278407
763	GO:0030534	GO:0007155	GO:0004497
764	GO:0030258	GO:0007154	GO:0016875
765	GO:0030141	GO:0007131	GO:0031428
766	GO:0022618	GO:0007127	GO:0050886
767	GO:0019213	GO:0007126	GO:0000779
768	GO:0016597	GO:0007099	GO:0001046
769	GO:0015850	GO:0007098	GO:0007020
770	GO:0010533	GO:0007097	GO:0023061
771	GO:0010506	GO:0007095	GO:0044421
772	GO:0009411	GO:0007094	GO:0000781
773	GO:0009100	GO:0007093	GO:0042770
774	GO:0008171	GO:0007091	GO:0030799
775	GO:0007600	GO:0007088	GO:0007099
776	GO:0007157	GO:0007077	GO:0072677
777	GO:0007064	GO:0007067	ENSP00000246802
778	GO:0007020	GO:0007064	ENSP00000345895
779	GO:0006793	GO:0007063	GO:0098852
780	GO:0006720	GO:0007062	GO:0020037
781	GO:0006383	GO:0007059	GO:0030814
782	GO:0006364	GO:0007052	GO:0031985
783	GO:0005912	GO:0007051	ENSP00000419740
784	GO:0005905	GO:0007050	GO:0000725
785	GO:0005797	GO:0007049	GO:0016748
786	GO:0005749	GO:0007045	ENSP00000402733
787	GO:0005104	GO:0007044	GO:0016885
788	GO:0004872	GO:0007043	GO:0097530
789	GO:0002504	GO:0007041	GO:0070330
790	GO:0001649	GO:0007040	GO:0031410
791	GO:0001540	GO:0007034	ENSP00000403536

792	GO:0001510	GO:0007033	GO:0009165
793	GO:0000981	GO:0007032	GO:0022411
794	GO:0000152	GO:0007031	GO:0014910
795	hsa04730	GO:0007030	ENSP00000390722
796	hsa04728	GO:0007020	GO:0019725
797	hsa04623	GO:0007018	GO:0043425
798	hsa04062	GO:0007017	GO:0000096
799	hsa00020	GO:0007015	GO:1902847
800	GO:1903034	GO:0007010	GO:0006940
801	GO:1903008	GO:0007009	GO:0043484
802	GO:1901998	GO:0007007	GO:0005813
803	GO:1901565	GO:0007006	GO:1900046
804	GO:0098813	GO:0007005	GO:0046467
805	GO:0097502	GO:0007000	GO:0010498
806	GO:0090002	GO:0006999	GO:0009190
807	GO:0070230	GO:0006998	GO:0000380
808	GO:0061136	GO:0006997	GO:0032155
809	GO:0051537	GO:0006996	GO:0070525
810	GO:0051233	GO:0006986	GO:0098655
811	GO:0050871	GO:0006982	GO:0007050
812	GO:0050795	GO:0006979	GO:0016103
813	GO:0045453	GO:0006978	GO:0046058
814	GO:0045121	GO:0006977	GO:0003006
815	GO:0045022	GO:0006974	hsa04020
816	GO:0044702	GO:0006965	ENSP00000380495
817	GO:0044699	GO:0006963	GO:0051786
818	GO:0044440	GO:0006959	GO:0005179
819	GO:0044297	GO:0006958	ENSP00000386134
820	GO:0043596	GO:0006957	GO:0045787
821	GO:0043129	GO:0006956	ENSP00000365837
822	GO:0042599	GO:0006955	GO:0044456
823	GO:0042310	GO:0006954	GO:0032413
824	GO:0042277	GO:0006953	GO:0007155
825	GO:0042157	GO:0006952	GO:0000959
826	GO:0035412	GO:0006950	ENSP00000355565
827	GO:0034329	GO:0006940	GO:1900086
828	GO:0032420	GO:0006939	GO:1901070
829	GO:0031700	GO:0006937	GO:0051234
830	GO:0031640	GO:0006936	ENSP00000321449
831	GO:0030684	GO:0006935	GO:1901293
832	GO:0030594	GO:0006933	GO:0008584
833	GO:0030136	GO:0006931	GO:0010951
834	GO:0019884	GO:0006929	GO:1902579
835	GO:0019882	GO:0006928	GO:0045211
836	GO:0018027	GO:0006915	GO:0071478
837	GO:0018023	GO:0006914	GO:0015297
838	GO:0016887	GO:0006913	GO:0035587
839	GO:0016835	GO:0006909	GO:0010256
840	GO:0016779	GO:0006904	ENSP00000315674
841	GO:0016655	GO:0006901	GO:0031907
842	GO:0016405	GO:0006900	GO:0050880
843	GO:0016074	GO:0006898	hsa05204
844	GO:0016071	GO:0006897	GO:0070585

845	GO:0016070	GO:0006892	GO:0050818
846	GO:0016053	GO:0006888	GO:0070603
847	GO:0015108	GO:0006887	GO:0033047
848	GO:0009987	GO:0006886	GO:0035258
849	GO:0009790	GO:0006885	GO:0000451
850	GO:0009055	GO:0006884	GO:1903522
851	GO:0008757	GO:0006883	GO:0007018
852	GO:0008509	GO:0006875	GO:0030815
853	GO:0008299	GO:0006874	GO:0032501
854	GO:0007032	GO:0006873	GO:0000166
855	GO:0007006	GO:0006869	GO:1902583
856	GO:0006952	GO:0006865	hsa00601
857	GO:0006857	GO:0006863	hsa04723
858	GO:0006338	GO:0006857	GO:0090068
859	GO:0006323	GO:0006839	GO:0005793
860	GO:0006029	GO:0006836	GO:0005774
861	GO:0005840	GO:0006835	GO:0070851
862	GO:0005790	GO:0006833	GO:0035267
863	GO:0005737	GO:0006821	GO:0043230
864	GO:0005720	GO:0006820	GO:0042310
865	GO:0005719	GO:0006818	GO:0016712
866	GO:0005546	hsa04072	GO:0010569
867	GO:0003938	GO:0006816	GO:0051438
868	GO:0003735	GO:0006814	GO:0098754
869	GO:0002831	GO:0006813	GO:0007189
870	GO:0002702	GO:0006812	GO:0051179
871	GO:0002495	GO:0006811	ENSP00000344818
872	GO:0002064	GO:0006810	hsa04514
873	GO:0001650	GO:0006807	ENSP00000225402
874	GO:0000380	GO:0006805	GO:0046605
875	GO:0000138	GO:0006796	GO:0017111
876	GO:0000049	GO:0006793	GO:0015919
877	hsa04961	hsa04071	GO:1902653
878	hsa04360	GO:0006790	GO:1903052
879	hsa04260	GO:0006784	GO:0031935
880	hsa04024	GO:0006781	hsa05150
881	hsa04020	GO:0006780	GO:0006821
882	hsa04014	GO:0006779	GO:0015631
883	GO:1903793	GO:0006778	GO:0051049
884	GO:1903725	GO:0006775	GO:1903364
885	GO:1903651	GO:0006767	GO:0015291
886	GO:1902600	GO:0006766	GO:0048771
887	GO:1901605	GO:0006757	GO:0004303
888	GO:1901361	GO:0006754	GO:0046883
889	GO:0098797	GO:0006753	GO:0051298
890	GO:0098754	GO:0006749	GO:0090537
891	GO:0098631	GO:0006744	GO:0009111
892	GO:0090022	GO:0006743	GO:0042625
893	GO:0072509	GO:0006739	GO:0050830
894	GO:0072384	GO:0006735	GO:0001523
895	GO:0071320	GO:0006734	GO:0033365
896	GO:0070844	GO:0006733	GO:0042391
897	GO:0060205	GO:0006732	GO:0098869

898	GO:0052689	GO:0006725	GO:0032589
899	GO:0051567	GO:0006721	GO:0098868
900	GO:0051092	GO:0006720	hsa00380
901	GO:0050748	GO:0006706	GO:0033260
902	GO:0046872	GO:0006705	ENSP00000352839
903	GO:0046658	GO:0006704	GO:0003095
904	GO:0046579	GO:0006703	GO:0072659
905	GO:0046112	GO:0006702	GO:0044403
906	GO:0045446	GO:0006701	GO:0005773
907	GO:0045178	GO:0006700	GO:0007162
908	GO:0044463	GO:0006699	hsa04726
909	GO:0044444	GO:0006695	GO:0016050
910	GO:0043574	GO:0006694	GO:0009582
911	GO:0042995	GO:0006690	GO:0010455
912	GO:0042058	GO:0006664	GO:0072678
913	GO:0035563	GO:0006650	GO:0003684
914	GO:0034250	GO:0006644	GO:0019825
915	GO:0033116	GO:0006643	GO:1900048
916	GO:0032279	GO:0006637	GO:0016115
917	GO:0031896	GO:0006635	GO:0006096
918	GO:0031570	hsa04024	GO:0048608
919	GO:0030818	GO:0006631	GO:0046040
920	GO:0030175	GO:0006629	GO:0044419
921	GO:0030166	GO:0006626	ENSP00000258743
922	GO:0030163	GO:0006625	GO:0030659
923	GO:0022408	GO:0006621	GO:0000791
924	GO:0019901	GO:0006614	hsa00500
925	GO:0019722	GO:0006613	GO:0002548
926	GO:0018205	GO:0006612	GO:0070062
927	GO:0018200	GO:0006606	GO:0032559
928	GO:0018196	GO:0006605	GO:0070613
929	GO:0017056	hsa04022	GO:0016247
930	GO:0016628	GO:0006595	hsa00514
931	GO:0015078	GO:0006575	GO:0050679
932	GO:0010876	GO:0006554	ENSP00000413572
933	GO:0009132	GO:0006553	GO:0016860
934	GO:0008584	GO:0006551	ENSP00000223095
935	GO:0008344	GO:0006544	hsa04971
936	GO:0008154	GO:0006541	GO:0000963
937	GO:0008105	GO:0006520	GO:0061302
938	GO:0008064	GO:0006518	GO:1902554
939	GO:0007608	GO:0006516	GO:0016328
940	GO:0007268	GO:0006513	hsa03040
941	GO:0007062	GO:0006511	GO:0065010
942	GO:0007059	GO:0006509	GO:0072577
943	GO:0006457	GO:0006508	GO:0032465
944	GO:0006400	GO:0006493	GO:0051340
945	GO:0006361	GO:0006490	GO:0030425
946	GO:0006022	GO:0006488	GO:0015698
947	GO:0005903	GO:0006487	GO:0007010
948	GO:0005732	GO:0006486	GO:0051546
949	GO:0004888	GO:0006482	GO:0018685
950	GO:0004871	hsa04015	GO:0031122

951	GO:0004739	GO:0006479	GO:1901617
952	GO:0003729	GO:0006476	GO:0008023
953	GO:0002940	GO:0006475	GO:0071013
954	GO:0002070	GO:0006474	GO:0006886
955	GO:0001917	GO:0006473	GO:0051050
956	GO:0001652	GO:0006470	GO:0051538
957	GO:0001306	GO:0006468	GO:0051983
958	GO:0001078	GO:0006465	GO:0033143
959	GO:0000819	GO:0006464	GO:0030424
960	GO:0000793	GO:0006461	GO:0030194
961	GO:0000791	GO:0006457	GO:0006749
962	GO:0000785	GO:0006450	GO:0045178
963	ENSP00000009180	GO:0006446	GO:0044441
964	hsa04724	GO:0006418	GO:2000401
965	hsa04614	GO:0006417	GO:0046365
966	hsa04611	GO:0006415	GO:0008375
967	hsa04514	GO:0006414	GO:0001974
968	hsa04151	GO:0006413	GO:0072657
969	hsa00590	GO:0006412	GO:0036440
970	GO:1903649	GO:0006409	GO:0050819
971	GO:1903305	GO:0006406	hsa05034
972	GO:1901981	GO:0006405	GO:0040017
973	GO:1901661	GO:0006403	GO:0005261
974	GO:1900424	GO:0006402	GO:0009792
975	GO:0098534	GO:0006401	GO:0072522
976	GO:0090568	GO:0006400	GO:0015294
977	GO:0090101	GO:0006399	GO:0003008
978	GO:0072583	GO:0006397	GO:1901571
979	GO:0072378	GO:0006396	GO:0005244
980	GO:0072321	GO:0006391	GO:0004857
981	GO:0071804	GO:0006390	GO:0016725
982	GO:0071426	GO:0006383	hsa00350
983	GO:0071168	GO:0006370	GO:0072676
984	GO:0071103	GO:0006369	GO:0022838
985	GO:0070491	GO:0006368	GO:0006625
986	GO:0070011	GO:0006367	ENSP00000370473
987	GO:0060326	GO:0006366	GO:0000287
988	GO:0055108	GO:0006364	GO:0008187
989	GO:0051536	GO:0006363	GO:0006816
990	GO:0051348	GO:0006362	GO:0009451
991	GO:0051345	GO:0006361	GO:0008406
992	GO:0051298	GO:0006360	GO:0035242
993	GO:0051289	GO:0006357	GO:0051260
994	GO:0050714	GO:0006355	GO:0019953
995	GO:0050660	GO:0006354	GO:2000779
996	GO:0046794	GO:0006353	GO:0035725
997	GO:0046718	GO:0006352	ENSP00000218388
998	GO:0045921	GO:0006351	GO:0006006
999	GO:0045216	GO:0006344	GO:0006612
1000	GO:0043601	GO:0006342	GO:0009897
1001	GO:0043531	GO:0006338	GO:0043574
1002	GO:0043488	GO:0006337	GO:0045661
1003	GO:0043487	GO:0006336	GO:0046785

1004	GO:0042440	GO:0006335	GO:0016192
1005	GO:0040029	GO:0006334	ENSP00000343505
1006	GO:0034976	GO:0006333	GO:1902652
1007	GO:0031968	GO:0006325	GO:0015696
1008	GO:0031748	ENSP00000395046	GO:2000146
1009	GO:0031588	ENSP00000387523	GO:0005739
1010	GO:0031519	ENSP00000386884	GO:0022832
1011	GO:0031498	GO:0006323	GO:0006278
1012	GO:0030705	ENSP00000382166	GO:1903312
1013	GO:0030662	ENSP00000380153	GO:0044786
1014	GO:0023058	GO:0006310	GO:0016776
1015	GO:0017016	ENSP00000377799	GO:0001878
1016	GO:0016829	GO:0006306	GO:1902562
1017	GO:0016601	ENSP00000371377	GO:0043009
1018	GO:0016298	ENSP00000369728	GO:0031396
1019	GO:0016241	ENSP00000369647	GO:0016126
1020	GO:0015992	ENSP00000368989	GO:0051817
1021	GO:0015872	GO:0006305	GO:0051053
1022	GO:0015026	GO:0006304	hsa05321
1023	GO:0009069	ENSP00000365663	GO:0017022
1024	GO:0009060	ENSP00000362795	GO:0000138
1025	GO:0008187	GO:0006303	GO:0070977
1026	GO:0007631	GO:0006302	GO:0006813
1027	GO:0007165	GO:0006301	GO:0006364
1028	GO:0007127	GO:0006297	GO:1903707
1029	GO:0006957	ENSP00000352626	GO:0019318
1030	GO:0006936	GO:0006290	GO:0008299
1031	GO:0006931	ENSP00000350256	GO:0045137
1032	GO:0006813	ENSP00000350003	GO:0019787
1033	GO:0006766	GO:0006289	GO:0086012
1034	GO:0006605	ENSP00000345317	GO:0000307
1035	GO:0006283	ENSP00000345163	ENSP00000263341
1036	GO:0006270	GO:0006284	GO:0002943
1037	GO:0006119	ENSP00000343505	GO:0009411
1038	GO:0005924	ENSP00000334364	GO:0006140
1039	GO:0005798	GO:0006283	GO:1902807
1040	GO:0005791	ENSP00000332659	GO:0005681
1041	GO:0005758	GO:0006282	GO:0043005
1042	GO:0005747	ENSP00000330384	GO:0007492
1043	GO:0005488	ENSP00000330049	ENSP00000350003
1044	GO:0005254	ENSP00000328563	GO:0004582
1045	GO:0005109	ENSP00000327585	GO:0016101
1046	GO:0004857	GO:0006281	GO:0006885
1047	GO:0004582	ENSP00000324769	GO:0030513
1048	GO:0003727	ENSP00000324251	hsa04727
1049	GO:0003015	GO:0006278	GO:0015629
1050	GO:0002479	ENSP00000323853	GO:0016820
1051	GO:0002440	ENSP00000322939	GO:0002697
1052	GO:0002032	ENSP00000321506	GO:0045596
1053	GO:0001990	GO:0006271	ENSP00000001146
1054	GO:0001890	ENSP00000321426	GO:0070405
1055	GO:0001845	GO:0006270	GO:0015293
1056	GO:0000287	GO:0006261	GO:0005527

1057	GO:0000079	ENSP00000310488	GO:0032879
1058	GO:0000003	ENSP00000309673	GO:0001833
1059	ENSP00000382166	GO:0006260	GO:0048705
1060	hsa05130	ENSP00000308270	GO:0070129
1061	hsa04530	ENSP00000307598	GO:0045861
1062	hsa04071	GO:0006259	GO:0051208
1063	hsa03008	ENSP00000305207	GO:0006888
1064	hsa00100	ENSP00000303864	GO:0048531
1065	hsa00030	GO:0006222	GO:0071622
1066	GO:2001027	ENSP00000303834	GO:0051170
1067	GO:2000649	ENSP00000302665	GO:0006810
1068	GO:1903313	ENSP00000300773	GO:0005911
1069	GO:1903311	GO:0006189	GO:0032412
1070	GO:1903078	ENSP00000297439	GO:0051270
1071	GO:1900120	GO:0006188	GO:0010522
1072	GO:0098858	ENSP00000295453	ENSP00000350011
1073	GO:0098800	ENSP00000292301	GO:0048193
1074	GO:0097178	GO:0006177	GO:0022414
1075	GO:0090307	ENSP00000278409	GO:0046914
1076	GO:0090150	GO:0006171	GO:0035639
1077	GO:0072577	GO:0006167	GO:0044877
1078	GO:0072331	GO:0006165	GO:1990748
1079	GO:0072330	ENSP00000263341	GO:0016064
1080	GO:0071695	ENSP00000263339	GO:0000209
1081	GO:0071564	ENSP00000263321	hsa04970
1082	GO:0071535	GO:0006164	GO:0099565
1083	GO:0071384	GO:0006163	GO:1901990
1084	GO:0071322	GO:0006154	ENSP00000249075
1085	GO:0061659	GO:0006152	GO:0044437
1086	GO:0060589	ENSP00000249887	GO:0001882
1087	GO:0055119	ENSP00000249075	GO:0007160
1088	GO:0051568	ENSP00000244623	GO:0006997
1089	GO:0051461	GO:0006144	GO:0086001
1090	GO:0051220	GO:0006140	GO:0045165
1091	GO:0051050	GO:0006139	GO:1903050
1092	GO:0051001	ENSP00000216492	GO:0006290
1093	GO:0050654	GO:0006123	GO:0090002
1094	GO:0046909	ENSP00000165524	GO:0051171
1095	GO:0046907	ENSP00000009180	GO:0050900
1096	GO:0046165	GO:1990837	GO:0000460
1097	GO:0045329	GO:1990583	GO:0015849
1098	GO:0045046	GO:1904929	ENSP00000264498
1099	GO:0044802	GO:1902936	GO:0031573
1100	GO:0044763	GO:1901981	GO:1900084
1101	GO:0044710	GO:0006122	GO:0032550
1102	GO:0044085	GO:1901681	GO:0009790
1103	GO:0043923	GO:1901363	GO:0050730
1104	GO:0043902	GO:1901265	GO:0009311
1105	GO:0043235	GO:0099600	GO:2000112
1106	GO:0043169	GO:0099528	GO:0050708
1107	GO:0043152	GO:0099516	GO:0061134
1108	GO:0042623	GO:0098811	ENSP00000338548
1109	GO:0042398	GO:0098772	GO:0042383

1110	GO:0042162	GO:0098631	GO:0034654
1111	GO:0034774	GO:0097472	GO:0032553
1112	GO:0033198	GO:0006120	GO:0045807
1113	GO:0033036	GO:0097367	GO:0034705
1114	GO:0032755	GO:0097159	GO:0009952
1115	GO:0032689	GO:0097110	GO:0009620
1116	GO:0031100	GO:0072509	GO:0060986
1117	GO:0030176	GO:0071987	GO:0032774
1118	GO:0030165	GO:0071936	GO:0006643
1119	GO:0019840	GO:0071837	hsa04141
1120	GO:0019787	GO:0071535	GO:0097659
1121	GO:0019674	GO:0070851	GO:0006351
1122	GO:0018195	GO:0070742	GO:0006551
1123	GO:0017144	GO:0006119	GO:0070742
1124	GO:0017137	GO:0070615	GO:0006508
1125	GO:0016810	GO:0070612	GO:0008514
1126	GO:0016407	GO:0070577	GO:0045981
1127	GO:0016358	GO:0070491	GO:0008152
1128	GO:0016072	GO:0070405	GO:0003824
1129	GO:0016056	GO:0070330	GO:0000076
1130	GO:0009743	GO:0070181	GO:0016485
1131	GO:0009268	GO:0070119	GO:0009887
1132	GO:0009163	GO:0070061	GO:1901362
1133	GO:0008324	GO:0070035	GO:2001026
1134	GO:0008210	GO:0006112	GO:0016482
1135	GO:0007612	GO:0070034	ENSP00000264563
1136	GO:0007259	GO:0070011	GO:0018130
1137	GO:0007160	GO:0061733	GO:0040013
1138	GO:0006914	GO:0061659	GO:1902774
1139	GO:0006869	GO:0061630	GO:0042161
1140	GO:0006818	GO:0061135	GO:1901568
1141	GO:0006811	GO:0061134	GO:0042277
1142	GO:0006699	GO:0060589	GO:0006363
1143	GO:0006461	GO:0060090	GO:0051216
1144	GO:0006399	GO:0060089	GO:0002700
1145	GO:0006284	GO:0006111	GO:0006164
1146	GO:0006066	GO:0052909	GO:0051351
1147	GO:0005070	GO:0052890	GO:0044665
1148	GO:0004930	GO:0052689	GO:0031982
1149	GO:0004721	GO:0051996	GO:0015026
1150	GO:0003094	GO:0051861	GO:0051252
1151	GO:0003002	GO:0051786	GO:1901988
1152	GO:0002688	GO:0051540	GO:0030529
1153	GO:0002449	GO:0051539	GO:0008016
1154	GO:0002252	GO:0051538	GO:0030522
1155	GO:0002244	GO:0051537	GO:0006102
1156	GO:0002020	GO:0006107	GO:0010468
1157	GO:0001909	GO:0051536	GO:0008233
1158	GO:0001889	GO:0051431	GO:0030801
1159	GO:0000922	GO:0051427	GO:0042440
1160	GO:0000808	GO:0051287	GO:0007612
1161	GO:0000460	GO:0051219	GO:0043395
1162	GO:0000315	GO:0051192	GO:0046907

1163	GO:0000082	GO:0051082	GO:0010556
1164	GO:0000070	GO:0051020	GO:0003678
1165	ENSP00000377944	GO:0051015	ENSP00000285949
1166	ENSP00000275820	GO:0050840	GO:0008237
1167	ENSP00000275525	GO:0006106	GO:0016327
1168	hsa05012	GO:0050839	GO:0042787
1169	hsa04974	GO:0050780	ENSP00000302665
1170	hsa04915	GO:0050681	GO:0098552
1171	hsa03420	GO:0050662	GO:0061448
1172	hsa03030	GO:0050661	GO:1902822
1173	hsa03018	GO:0050660	GO:0071565
1174	hsa00562	GO:0050178	GO:0002702
1175	hsa00350	GO:0050136	GO:0002039
1176	hsa00190	GO:0048551	GO:0019438
1177	GO:2000779	GO:0048531	GO:0070169
1178	GO:2000021	GO:0006105	GO:0043297
1179	GO:1904950	GO:0048365	GO:0019229
1180	GO:1903509	GO:0048040	GO:0003697
1181	GO:1903019	GO:0048039	GO:0070198
1182	GO:0099600	GO:0048038	GO:0051481
1183	GO:0099516	GO:0048037	GO:0045428
1184	GO:0098900	GO:0047696	GO:0006901
1185	GO:0098802	GO:0046983	GO:0016043
1186	GO:0098644	GO:0046982	GO:1903506
1187	GO:0097305	GO:0046966	GO:0046943
1188	GO:0097164	GO:0046961	GO:0016574
1189	GO:0071822	GO:0006104	GO:0006355
1190	GO:0071104	GO:0046943	GO:0007611
1191	GO:0070977	GO:0046933	GO:0006986
1192	GO:0070932	GO:0046914	GO:0051271
1193	GO:0070830	GO:0046912	GO:0009081
1194	GO:0070734	GO:0046906	GO:1902115
1195	GO:0061630	GO:0046875	GO:0007264
1196	GO:0061517	GO:0046873	GO:0051603
1197	GO:0061135	GO:0046872	GO:0032279
1198	GO:0061134	GO:0046554	GO:0034440
1199	GO:0061097	GO:0045309	ENSP00000295897
1200	GO:0061024	GO:0006103	GO:0048523
1201	GO:0060292	GO:0045155	GO:0019219
1202	GO:0051966	GO:0044877	ENSP00000362795
1203	GO:0051650	GO:0044822	GO:0006344
1204	GO:0051287	GO:0044769	GO:0015872
1205	GO:0051240	GO:0044620	GO:0031690
1206	GO:0051123	GO:0044325	GO:0030315
1207	GO:0050886	GO:0044212	GO:0002250
1208	GO:0050806	GO:0043997	GO:0044773
1209	GO:0050727	GO:0043783	GO:0007030
1210	GO:0050680	GO:0043566	GO:0071031
1211	GO:0048840	GO:0006102	GO:0007270
1212	GO:0048545	GO:0043565	GO:0005684
1213	GO:0048387	GO:0043544	GO:0018146
1214	GO:0048246	GO:0043531	GO:0031326
1215	GO:0046983	GO:0043492	GO:0008037

1216	GO:0046785	GO:0043426	GO:0008284
1217	GO:0046710	GO:0043425	GO:0051437
1218	GO:0046427	GO:0043395	GO:2001141
1219	GO:0046034	GO:0043394	GO:0043236
1220	GO:0045815	GO:0043236	GO:0007194
1221	GO:0044822	GO:0043185	GO:0007006
1222	GO:0044819	GO:0006101	GO:0032642
1223	GO:0043618	GO:0043177	GO:0046390
1224	GO:0043588	GO:0043169	GO:1900024
1225	GO:0043113	GO:0043168	GO:0016070
1226	GO:0043044	GO:0043167	GO:0001654
1227	GO:0043043	GO:0043021	ENSP00000275525
1228	GO:0042787	GO:0043015	GO:0008064
1229	GO:0042769	GO:0042826	GO:0002443
1230	GO:0042274	GO:0042809	GO:0019731
1231	GO:0042221	GO:0042803	GO:0042698
1232	GO:0038166	GO:0042802	GO:0030810
1233	GO:0035725	GO:0006099	GO:0030139
1234	GO:0035561	GO:0042800	GO:0016811
1235	GO:0034629	GO:0042626	GO:0031571
1236	GO:0033860	GO:0042625	GO:0030282
1237	GO:0033591	GO:0042623	GO:0005451
1238	GO:0032465	GO:0042608	GO:0042508
1239	GO:0032225	GO:0042578	GO:0007548
1240	GO:0032154	GO:0042393	GO:0034446
1241	GO:0032148	GO:0042301	GO:0030968
1242	GO:0031994	GO:0042277	GO:0030673
1243	GO:0031690	GO:0042162	GO:1902824
1244	GO:0031683	GO:0006098	GO:0008289
1245	GO:0031638	GO:0042134	GO:0044271
1246	GO:0031442	GO:0042056	GO:0010574
1247	GO:0031398	GO:0042054	GO:1902582
1248	GO:0031122	GO:0042020	GO:0043231
1249	GO:0030574	GO:0042019	GO:0010833
1250	GO:0030512	GO:0042015	GO:0009889
1251	GO:0030496	GO:0038164	GO:0050673
1252	GO:0030488	GO:0038024	GO:0002460
1253	GO:0030324	GO:0038023	GO:0030332
1254	GO:0030279	GO:0038021	hsa04750
1255	GO:0030246	GO:0006096	GO:0032908
1256	GO:0030159	GO:0036442	ENSP00000295400
1257	GO:0022411	GO:0036440	GO:0030501
1258	GO:0019904	GO:0036094	GO:0090533
1259	GO:0019829	GO:0036002	GO:0016410
1260	GO:0019439	GO:0035639	GO:0005903
1261	GO:0019229	GO:0035591	GO:0090575
1262	GO:0019080	GO:0035326	GO:0023058
1263	GO:0018393	GO:0035258	GO:0016197
1264	GO:0016705	GO:0035257	GO:0035637
1265	GO:0016627	GO:0035255	GO:0019724
1266	GO:0016581	GO:0006091	GO:0015085
1267	GO:0016486	GO:0035250	GO:0007435
1268	GO:0016236	GO:0035242	GO:0005201

1269	GO:0015934	GO:0035091	GO:0046545
1270	GO:0015459	GO:0035064	GO:0032271
1271	GO:0014047	GO:0035035	GO:0086003
1272	GO:0010835	GO:0034979	GO:0050731
1273	GO:0010833	GO:0034714	GO:0003677
1274	GO:0010569	GO:0034604	GO:0030816
1275	GO:0009108	GO:0034603	GO:0005543
1276	GO:0009062	GO:0034602	GO:0031307
1277	GO:0008320	GO:0006090	GO:0045988
1278	GO:0008301	GO:0034513	GO:0031214
1279	GO:0008170	GO:0034511	GO:0003899
1280	GO:0008152	GO:0034212	GO:0005884
1281	GO:0008047	GO:0033764	GO:0045991
1282	GO:0007163	GO:0033613	GO:0030514
1283	GO:0007052	GO:0033558	GO:0090087
1284	GO:0007009	GO:0033218	GO:0048770
1285	GO:0006897	GO:0032947	ENSP00000265171
1286	GO:0006888	GO:0032561	GO:0000077
1287	GO:0006767	GO:0032559	GO:0061726
1288	GO:0006749	GO:0006086	GO:0008585
1289	GO:0006700	GO:0032555	GO:0046488
1290	GO:0006493	GO:0032553	GO:0042886
1291	GO:0006403	GO:0032550	GO:0044819
1292	GO:0006390	GO:0032549	GO:0036075
1293	GO:0006366	GO:0032451	GO:0097367
1294	GO:0006363	GO:0032403	GO:0043152
1295	GO:0006342	GO:0032041	GO:0072006
1296	GO:0006289	GO:0031995	GO:0043547
1297	GO:0006106	GO:0031994	GO:0030166
1298	GO:0005901	GO:0031896	GO:0071705
1299	GO:0005887	GO:0006085	GO:0070061
1300	GO:0005819	GO:0031859	GO:0042102
1301	GO:0005675	GO:0031826	GO:0006650
1302	GO:0005593	GO:0031821	GO:0044298
1303	GO:0005516	GO:0031762	GO:0004222
1304	GO:0005179	GO:0031748	GO:2000649
1305	GO:0003724	GO:0031701	GO:0071436
1306	GO:0002757	GO:0031700	GO:0030864
1307	GO:0002703	GO:0031692	GO:0050685
1308	GO:0001948	GO:0031691	ENSP00000292301
1309	GO:0001894	GO:0031690	GO:0021545
1310	GO:0001893	GO:0006084	GO:0006743
1311	GO:0001704	GO:0031683	ENSP00000263339
1312	GO:0001676	GO:0031625	GO:0006644
1313	GO:0001502	GO:0031492	GO:0044282
1314	GO:0001106	GO:0031491	GO:0035150
1315	GO:0000786	GO:0031490	GO:0008544
1316	GO:0000096	GO:0031489	GO:0072431
1317	ENSP00000266646	GO:0031420	hsa04972
1318	ENSP00000249887	GO:0031406	GO:0090304
1319	hsa04976	GO:0031386	GO:0042307
1320	hsa04916	GO:0031267	GO:0032580
1321	hsa04912	GO:0006082	GO:0051648

1322	hsa04713	GO:0031078	GO:0052548
1323	hsa00514	GO:0030731	GO:0071922
1324	GO:2001257	GO:0030697	GO:0005742
1325	GO:1990542	GO:0030696	GO:0007260
1326	GO:1904666	GO:0030695	GO:0060070
1327	GO:1903317	GO:0030594	GO:0010107
1328	GO:1902824	GO:0030554	GO:0031099
1329	GO:1902578	GO:0030523	GO:0034103
1330	GO:1901659	GO:0030515	GO:0010467
1331	GO:1901136	GO:0030492	GO:0071840
1332	GO:1900004	GO:0006081	ENSP00000382166
1333	GO:0099503	GO:0030414	hsa04962
1334	GO:0098794	GO:0030332	hsa04630
1335	GO:0090503	GO:0030246	GO:0030534
1336	GO:0090502	GO:0030234	GO:0042800
1337	GO:0090100	GO:0030170	GO:0034645
1338	GO:0065005	GO:0030165	GO:0009617
1339	GO:0060463	GO:0030159	GO:0043113
1340	GO:0060348	GO:0030060	GO:2000816
1341	GO:0060170	GO:0022892	GO:0008360
1342	GO:0055085	GO:0022891	GO:0030804
1343	GO:0051899	GO:0006066	GO:0072395
1344	GO:0051716	GO:0022890	GO:0075136
1345	GO:0051648	GO:0022857	GO:0090100
1346	GO:0051493	GO:0022843	GO:0003743
1347	GO:0051340	GO:0022838	GO:0061617
1348	GO:0051301	GO:0022836	GO:0051336
1349	GO:0051234	GO:0022834	hsa05130
1350	GO:0051188	GO:0022832	GO:0098609
1351	GO:0051186	GO:0022829	GO:0098760
1352	GO:0051148	GO:0022804	hsa04072
1353	GO:0051093	GO:0022803	GO:0061138
1354	GO:0051090	GO:0006029	GO:0019003
1355	GO:0050900	GO:0020037	GO:0022602
1356	GO:0050805	GO:0019981	GO:0031305
1357	GO:0048232	GO:0019969	GO:0045940
1358	GO:0046912	GO:0019962	GO:0009059
1359	GO:0046649	GO:0019961	GO:0005769
1360	GO:0045184	GO:0019955	GO:0006302
1361	GO:0045123	GO:0019904	ENSP00000302150
1362	GO:0044452	GO:0019901	GO:0043220
1363	GO:0044433	GO:0019900	GO:0002703
1364	GO:0044428	GO:0019899	GO:0038043
1365	GO:0043624	GO:0006027	hsa05166
1366	GO:0043547	GO:0019843	GO:1903524
1367	GO:0043270	GO:0019840	GO:0043401
1368	GO:0043254	GO:0019838	ENSP00000303983
1369	GO:0042249	GO:0019829	GO:0003676
1370	GO:0035270	GO:0019825	GO:0046949
1371	GO:0035235	GO:0019789	GO:0007420
1372	GO:0035150	GO:0019787	GO:0010506
1373	GO:0035067	GO:0019213	GO:0001726
1374	GO:0034502	GO:0019205	hsa04924

1375	GO:0034470	GO:0019003	GO:0016592
1376	GO:0034446	GO:0006026	GO:0060665
1377	GO:0034080	GO:0019001	GO:0042730
1378	GO:0033683	GO:0018685	GO:0086064
1379	GO:0033267	GO:0018024	GO:0098799
1380	GO:0033158	GO:0017171	GO:1902106
1381	GO:0032436	GO:0017160	GO:0060326
1382	GO:0032204	GO:0017137	ENSP00000348986
1383	GO:0032153	GO:0017136	GO:0004904
1384	GO:0031489	GO:0017124	GO:0060170
1385	GO:0031428	GO:0017111	GO:0006929
1386	GO:0031306	GO:0017076	GO:0035582
1387	GO:0031091	GO:0006024	GO:0032467
1388	GO:0030728	GO:0017069	GO:0051051
1389	GO:0030520	GO:0017056	GO:0006914
1390	GO:0030278	GO:0017048	GO:0048646
1391	GO:0030201	GO:0017025	GO:0003779
1392	GO:0030104	GO:0017022	GO:0070727
1393	GO:0022007	GO:0017016	GO:0007588
1394	GO:0019900	GO:0016907	GO:0019221
1395	GO:0019856	GO:0016903	ENSP00000266646
1396	GO:0019362	GO:0016896	GO:0060589
1397	GO:0018209	GO:0016887	GO:0008238
1398	GO:0016592	GO:0006023	GO:0048639
1399	GO:0016435	GO:0016885	GO:0060047
1400	GO:0016209	GO:0016884	ENSP00000297350
1401	GO:0015919	GO:0016879	GO:0019852
1402	GO:0015630	GO:0016878	GO:0019001
1403	GO:0015629	GO:0016875	GO:0048551
1404	GO:0015399	GO:0016874	GO:0098781
1405	GO:0015266	GO:0016866	GO:0030162
1406	GO:0010975	GO:0016863	GO:0048263
1407	GO:0010955	GO:0016860	GO:0016055
1408	GO:0010765	GO:0016853	GO:1900003
1409	GO:0009605	GO:0006022	GO:1901342
1410	GO:0009404	GO:0016836	GO:0017048
1411	GO:0009303	GO:0016835	GO:0006702
1412	GO:0008652	GO:0016832	GO:0010518
1413	GO:0008544	GO:0016830	GO:0031941
1414	GO:0008201	GO:0016829	GO:0048609
1415	GO:0008094	GO:0016820	GO:0090075
1416	GO:0008083	GO:0016818	GO:0005905
1417	GO:0007603	GO:0016817	GO:0002699
1418	GO:0007584	GO:0016811	hsa04916
1419	GO:0007369	GO:0016810	GO:0055038
1420	GO:0007077	GO:0006007	GO:0001936
1421	GO:0006986	GO:0016791	ENSP00000361084
1422	GO:0006974	GO:0016787	GO:0005875
1423	GO:0006958	GO:0016779	GO:0018149
1424	GO:0006884	GO:0016776	GO:0030203
1425	GO:0006865	GO:0016773	hsa04062
1426	GO:0006790	GO:0016772	GO:0048729
1427	GO:0006732	GO:0016765	GO:1903321

1428	GO:0006706	GO:0016758	GO:0051261
1429	GO:0006695	GO:0016757	GO:0050663
1430	GO:0006520	GO:0016751	GO:0030175
1431	GO:0006474	GO:0006006	GO:0070181
1432	GO:0006325	GO:0016748	GO:0019829
1433	GO:0005996	GO:0016747	GO:0001568
1434	GO:0005672	GO:0016746	GO:0010863
1435	GO:0005657	GO:0016742	GO:0004905
1436	GO:0005578	GO:0016741	GO:0001704
1437	GO:0005507	GO:0016740	GO:0070271
1438	GO:0005501	GO:0016725	GO:0006704
1439	GO:0005172	GO:0016712	GO:0038021
1440	GO:0004896	GO:0016709	hsa04921
1441	GO:0004866	GO:0016705	GO:0048659
1442	GO:0004527	GO:0005996	GO:0060255
1443	GO:0004108	GO:0016684	GO:0042330
1444	GO:0002922	GO:0016681	GO:0003002
1445	GO:0002181	GO:0016679	GO:0048524
1446	GO:0002090	GO:0016676	GO:0043243
1447	GO:0001887	GO:0016675	GO:1903555
1448	GO:0001664	GO:0016668	GO:0032549
1449	GO:0001091	GO:0016667	GO:0009112
1450	GO:0000790	GO:0016655	GO:0099568
1451	GO:0000723	GO:0016651	GO:0032946
1452	GO:0000209	GO:0016649	GO:0090184
1453	GO:0000123	GO:0005975	GO:0006863
1454	ENSP00000348986	GO:0016645	GO:0030324
1455	hsa05143	GO:0016635	GO:0031329
1456	hsa05032	GO:0016634	GO:2000257
1457	hsa04970	GO:0016628	GO:0003081
1458	hsa04960	GO:0016627	GO:1904063
1459	hsa04925	GO:0016624	GO:0043062
1460	hsa04918	GO:0016620	GO:0006897
1461	hsa04726	GO:0016616	GO:0005798
1462	hsa04022	GO:0016614	hsa04713
1463	hsa00900	GO:0016608	GO:1904035
1464	GO:2001236	GO:0003417	GO:0031088
1465	GO:2000060	GO:0016597	GO:0061462
1466	GO:1904029	GO:0016538	GO:0045937
1467	GO:1903510	GO:0016509	GO:0086036
1468	GO:1903506	GO:0016491	GO:0000151
1469	GO:1903052	GO:0016462	ENSP00000249887
1470	GO:1903051	GO:0016436	hsa04744
1471	GO:1902936	GO:0016435	GO:0006952
1472	GO:1902911	GO:0016433	GO:0044325
1473	GO:1901888	GO:0016429	ENSP00000220616
1474	GO:1901607	GO:0016428	GO:0071173
1475	GO:0098772	GO:0003416	GO:0001822
1476	GO:0098656	GO:0016426	GO:0034366
1477	GO:0090537	GO:0016423	GO:0005507
1478	GO:0090304	GO:0016410	GO:0050793
1479	GO:0090068	GO:0016408	GO:0032722
1480	GO:0072503	GO:0016407	GO:0048384

1481	GO:0071478	GO:0016405	ENSP00000408153
1482	GO:0070911	GO:0016301	GO:0030502
1483	GO:0070897	GO:0016300	GO:0036498
1484	GO:0070742	GO:0016298	GO:0042509
1485	GO:0070129	GO:0016279	GO:0030538
1486	GO:0061337	GO:0003095	GO:2000058
1487	GO:0060323	GO:0016278	GO:0010631
1488	GO:0060312	GO:0016274	GO:0046037
1489	GO:0060309	GO:0016273	GO:0007626
1490	GO:0060073	GO:0016247	GO:0050709
1491	GO:0055078	GO:0016229	GO:0042517
1492	GO:0055038	GO:0016209	GO:0007166
1493	GO:0051651	GO:0016150	GO:0033148
1494	GO:0051304	GO:0015631	GO:0033688
1495	GO:0051297	GO:0015491	GO:1903307
1496	GO:0051131	GO:0015464	GO:0046425
1497	GO:0051094	GO:0003094	GO:0080090
1498	GO:0051047	GO:0015459	GO:0071025
1499	GO:0051031	GO:0015405	GO:0060322
1500	GO:0051020	GO:0015399	GO:0090501
1501	GO:0050911	GO:0015370	GO:0007259
1502	GO:0050848	GO:0015298	GO:0031323
1503	GO:0050792	GO:0015297	GO:0030728
1504	GO:0050709	GO:0015296	hsa04670
1505	GO:0050434	GO:0015294	GO:0000930
1506	GO:0048640	GO:0015293	GO:0051459
1507	GO:0048598	GO:0015291	GO:0005515
1508	GO:0046599	hsa00982	GO:0044769
1509	GO:0046578	GO:0003081	GO:0050671
1510	GO:0045862	GO:0015288	GO:0016601
1511	GO:0045744	GO:0015276	GO:0005096
1512	GO:0045263	GO:0015267	GO:0071392
1513	GO:0044298	GO:0015266	ENSP00000332659
1514	GO:0044272	GO:0015108	GO:0097696
1515	GO:0044237	GO:0015103	GO:0032496
1516	GO:0043901	GO:0015085	GO:0032561
1517	GO:0043394	GO:0015081	hsa04961
1518	GO:0043268	GO:0015079	GO:0001655
1519	GO:0043241	GO:0015078	GO:0098644
1520	GO:0043233	GO:0003073	GO:0016584
1521	GO:0043227	GO:0015077	GO:0070925
1522	GO:0043114	GO:0015075	hsa04728
1523	GO:0042407	GO:0015036	GO:0001503
1524	GO:0038024	GO:0015026	GO:0002028
1525	GO:0034983	GO:0015002	GO:0002786
1526	GO:0034103	GO:0010576	GO:0072401
1527	GO:0033260	GO:0010485	GO:0030097
1528	GO:0033148	GO:0010484	GO:0005509
1529	GO:0033143	GO:0009982	GO:1903729
1530	GO:0033048	GO:0009055	GO:2000027
1531	GO:0032501	GO:0003071	GO:0008144
1532	GO:0032403	GO:0009019	GO:0048660
1533	GO:0032273	GO:0008988	GO:0071345

1534	GO:0031692	GO:0008757	GO:0006139
1535	GO:0031584	GO:0008650	GO:0006928
1536	GO:0031497	GO:0008649	GO:0071702
1537	GO:0031397	GO:0008603	GO:0002807
1538	GO:0030866	GO:0008556	GO:0032602
1539	GO:0030687	GO:0008545	GO:0016056
1540	GO:0030670	GO:0008536	GO:0050804
1541	GO:0030213	GO:0008528	GO:0045669
1542	GO:0030198	GO:0003069	GO:0002181
1543	GO:0023051	GO:0008514	GO:2000045
1544	GO:0022625	GO:0008509	GO:0033135
1545	GO:0022603	GO:0008495	GO:0005254
1546	GO:0019318	GO:0008401	GO:0001501
1547	GO:0019205	GO:0008395	GO:0003015
1548	GO:0017053	GO:0008378	GO:0004722
1549	GO:0017048	GO:0008375	GO:0002252
1550	GO:0016820	GO:0008353	GO:2000144
1551	GO:0016772	GO:0008324	GO:0061008
1552	GO:0016765	GO:0008320	GO:1904892
1553	GO:0016740	GO:0003044	ENSP00000165524
1554	GO:0016052	GO:0008301	GO:0032680
1555	GO:0015696	GO:0008289	GO:0044304
1556	GO:0015672	GO:0008276	GO:0005070
1557	GO:0015297	GO:0008270	GO:0070371
1558	GO:0014012	GO:0008238	GO:0030030
1559	GO:0010544	GO:0008237	GO:0033002
1560	GO:0010484	GO:0008236	GO:0016773
1561	GO:0010243	GO:0008233	ENSP00000332504
1562	GO:0010033	GO:0008227	ENSP00000009180
1563	GO:0009893	GO:0008201	ENSP00000259239
1564	GO:0009583	GO:0003018	ENSP00000350256
1565	GO:0009187	GO:0008194	hsa04510
1566	GO:0008649	GO:0008191	GO:1902402
1567	GO:0007632	GO:0008187	GO:0045184
1568	GO:0007566	GO:0008186	GO:0002440
1569	GO:0007204	GO:0008177	GO:0044430
1570	GO:0007179	GO:0008176	GO:0003012
1571	GO:0007164	GO:0008175	GO:0005593
1572	GO:0007088	GO:0008173	GO:0090130
1573	GO:0007015	GO:0008171	GO:0030866
1574	GO:0006977	GO:0008170	GO:0071936
1575	GO:0006955	GO:0003015	GO:0030029
1576	GO:0006954	GO:0008168	GO:0010720
1577	GO:0006904	GO:0008144	ENSP00000386884
1578	GO:0006886	GO:0008137	GO:1904062
1579	GO:0006705	GO:0008135	GO:0010575
1580	GO:0006702	GO:0008134	GO:0002815
1581	GO:0006694	GO:0008121	hsa05110
1582	GO:0006637	GO:0008094	GO:0046651
1583	GO:0006470	GO:0008092	GO:0006977
1584	GO:0006446	GO:0008083	GO:0016772
1585	GO:0006401	GO:0008080	hsa04973
1586	GO:0006297	GO:0003014	GO:0030155

1587	GO:0006260	GO:0008066	GO:2000258
1588	GO:0006112	GO:0008047	hsa04925
1589	GO:0005925	GO:0008026	GO:0031577
1590	GO:0005876	GO:0008017	GO:0034766
1591	GO:0005802	GO:0008009	hsa04151
1592	GO:0005769	GO:0005549	GO:0035255
1593	GO:0005741	GO:0005546	GO:0046483
1594	GO:0005577	GO:0005543	GO:0051258
1595	GO:0005518	GO:0005539	GO:0006725
1596	GO:0005506	GO:0005527	GO:0044853
1597	GO:0005246	GO:0003013	GO:0033043
1598	GO:0005216	GO:0005525	ENSP00000297439
1599	GO:0002526	GO:0005524	GO:0051604
1600	GO:0002063	GO:0005520	GO:0005669
1601	GO:0002029	GO:0005518	GO:0048858
1602	GO:0001974	GO:0005516	GO:1902578
1603	GO:0001935	GO:0005515	GO:0042734
1604	GO:0001775	GO:0005509	GO:0051240
1605	GO:0001179	GO:0005507	GO:0033177
1606	GO:0000932	GO:0005506	GO:0061044
1607	GO:0000806	GO:0005501	GO:0019882
1608	GO:0000788	GO:0003012	GO:0008542
1609	ENSP00000306245	GO:0005488	GO:0043087
1610	ENSP00000165524	GO:0005451	GO:0010193
1611	hsa05204	GO:0005391	GO:0005253
1612	hsa04720	GO:0005342	GO:0031347
1613	hsa04310	GO:0005267	GO:0002433
1614	hsa04261	GO:0005262	GO:0051239
1615	hsa04114	GO:0005261	GO:0032990
1616	GO:2001020	GO:0005254	GO:0001158
1617	GO:2000617	GO:0005253	GO:0042470
1618	GO:1990204	GO:0005246	GO:0034113
1619	GO:1903055	GO:0003008	GO:0036211
1620	GO:1902947	GO:0005244	GO:0046209
1621	GO:1902822	GO:0005216	GO:1901363
1622	GO:1902622	GO:0005215	GO:0031213
1623	GO:1902589	GO:0005201	GO:1903279
1624	GO:1901990	GO:0005198	GO:0001889
1625	GO:1901989	GO:0005184	GO:0006383
1626	GO:1901617	GO:0005179	GO:0010765
1627	GO:1901566	GO:0005178	ENSP00000312652
1628	GO:1901379	GO:0005172	GO:0006780
1629	GO:1900086	GO:0005138	GO:0032479
1630	GO:0097190	GO:0003006	GO:0048640
1631	GO:0097110	GO:0005126	GO:0051571
1632	GO:0090670	GO:0005125	GO:0031398
1633	GO:0090303	GO:0005109	GO:0032689
1634	GO:0090166	GO:0005104	GO:0052547
1635	GO:0090075	GO:0005102	GO:0031442
1636	GO:0086065	GO:0005096	GO:0090288
1637	GO:0080135	GO:0005070	GO:0016048
1638	GO:0072655	GO:0004993	GO:0031968
1639	GO:0072521	GO:0004984	GO:0001540

1640	GO:0072006	GO:0004930	GO:0098602
1641	GO:0071635	GO:0003002	GO:0000184
1642	GO:0071556	GO:0004924	GO:0034080
1643	GO:0071354	GO:0004923	GO:0070167
1644	GO:0070405	GO:0004917	GO:2000404
1645	GO:0070227	GO:0004915	GO:0006957
1646	GO:0061196	GO:0004914	GO:0032994
1647	GO:0060548	GO:0004913	GO:0071559
1648	GO:0060416	GO:0004912	GO:0097159
1649	GO:0060349	GO:0004906	GO:0007599
1650	GO:0060255	GO:0004905	GO:0048545
1651	GO:0060027	GO:0004904	GO:0016301
1652	GO:0055114	GO:0002943	ENSP00000262030
1653	GO:0051969	GO:0004900	GO:0050820
1654	GO:0051726	GO:0004897	GO:0097502
1655	GO:0051656	GO:0004896	GO:0042019
1656	GO:0051603	GO:0004888	GO:0016337
1657	GO:0051572	GO:0004872	GO:0033631
1658	GO:0051384	GO:0004871	GO:0048365
1659	GO:0050917	GO:0004867	ENSP00000334364
1660	GO:0050829	GO:0004866	ENSP00000306245
1661	GO:0048861	GO:0004857	GO:0004672
1662	GO:0048522	GO:0004842	GO:0022407
1663	GO:0048513	GO:0002940	GO:0097481
1664	GO:0048259	GO:0004812	GO:0007097
1665	GO:0046982	GO:0004809	GO:0030150
1666	GO:0046961	GO:0004776	GO:0051056
1667	GO:0046943	GO:0004775	GO:0042089
1668	GO:0046425	GO:0004774	GO:0018209
1669	GO:0045739	GO:0004769	GO:0030665
1670	GO:0045333	GO:0004739	GO:0019222
1671	GO:0044304	GO:0004738	GO:0002689
1672	GO:0044249	GO:0004722	GO:0002029
1673	GO:0044242	GO:0004721	GO:0005874
1674	GO:0044087	GO:0002923	GO:0001667
1675	GO:0044070	GO:0004703	GO:0031333
1676	GO:0043567	GO:0004693	GO:0006409
1677	GO:0043492	GO:0004674	GO:0005834
1678	GO:0043434	GO:0004672	GO:0050878
1679	GO:0043103	GO:0004620	GO:0038166
1680	GO:0042826	GO:0004602	GO:0044291
1681	GO:0042803	GO:0004601	GO:0048661
1682	GO:0042738	GO:0004591	GO:0061418
1683	GO:0042446	GO:0004582	GO:0090287
1684	GO:0035591	GO:0004540	GO:0006026
1685	GO:0035249	GO:0002922	GO:0008104
1686	GO:0035019	GO:0004532	GO:1901360
1687	GO:0034366	GO:0004527	GO:0019068
1688	GO:0034358	GO:0004518	GO:0070142
1689	GO:0034114	GO:0004497	GO:0045445
1690	GO:0033145	GO:0004468	GO:2000060
1691	GO:0033046	GO:0004448	GO:0032606
1692	GO:0033045	GO:0004407	ENSP00000365663

1693	GO:0032970	GO:0004402	GO:0042578
1694	GO:0032956	GO:0004386	GO:0006700
1695	GO:0032926	GO:0004385	GO:0018216
1696	GO:0032806	GO:0002920	GO:0031683
1697	GO:0032784	GO:0004362	GO:0001935
1698	GO:0032543	GO:0004333	GO:0030838
1699	GO:0032504	GO:0004311	GO:0005876
1700	GO:0032479	GO:0004310	hsa04270
1701	GO:0032392	GO:0004303	GO:0002360
1702	GO:0031526	GO:0004300	hsa05215
1703	GO:0031226	GO:0004252	GO:2000142
1704	GO:0031099	GO:0004222	GO:0046933
1705	GO:0030838	GO:0004175	GO:0048584
1706	GO:0030833	GO:0004174	GO:1900004
1707	GO:0030689	GO:0002862	GO:0045667
1708	GO:0030424	GO:0004169	GO:0043923
1709	GO:0030203	GO:0004152	GO:0051248
1710	GO:0030100	GO:0004149	GO:0010714
1711	GO:0023061	GO:0004148	GO:0030301
1712	GO:0022900	GO:0004129	hsa04912
1713	GO:0022898	GO:0004108	GO:0048514
1714	GO:0022892	GO:0004096	GO:0002009
1715	GO:0022836	GO:0004045	GO:0015918
1716	GO:0022617	GO:0004004	GO:0030168
1717	GO:0019953	GO:0004003	GO:0022007
1718	GO:0019898	GO:0002833	GO:0050870
1719	GO:0019827	GO:0003994	GO:0045862
1720	GO:0019825	GO:0003988	hsa04810
1721	GO:0019725	GO:0003963	GO:0005172
1722	GO:0019001	GO:0003954	GO:0090175
1723	GO:0018193	GO:0003938	GO:0031513
1724	GO:0016942	GO:0003924	GO:0090257
1725	GO:0016866	GO:0003899	GO:0006606
1726	GO:0016712	GO:0003878	GO:0000242
1727	GO:0016709	GO:0003857	GO:0001894
1728	GO:0016578	GO:0003854	GO:0006470
1729	GO:0016567	GO:0002831	GO:0044297
1730	GO:0016477	GO:0003824	GO:0045744
1731	GO:0016423	GO:0003779	GO:0031175
1732	GO:0016259	GO:0003746	GO:0017160
1733	GO:0015980	GO:0003743	GO:0005790
1734	GO:0015711	GO:0003735	hsa04360
1735	GO:0015298	GO:0003729	GO:0030165
1736	GO:0015291	GO:0003727	GO:0043271
1737	GO:0015075	GO:0003724	GO:0072358
1738	GO:0015036	GO:0003723	GO:0002020
1739	GO:0010812	GO:0003714	GO:0051345
1740	GO:0010715	GO:0002816	GO:0044249
1741	GO:0010596	GO:0003713	GO:0034097
1742	GO:0010498	GO:0003712	GO:0006098
1743	GO:0007626	GO:0003705	GO:0007584
1744	GO:0007492	GO:0003700	GO:0030212
1745	GO:0007270	GO:0003697	GO:0050890

1746	GO:0007188	GO:0003690	GO:0048812
1747	GO:0007162	GO:0003684	GO:0045619
1748	GO:0007154	GO:0003682	GO:0006396
1749	GO:0007095	GO:0003678	GO:0006461
1750	GO:0007051	GO:0003677	GO:0001934
1751	GO:0007010	GO:0002815	GO:0098858
1752	GO:0006814	GO:0003676	GO:0031960
1753	GO:0006807	GO:0003674	GO:0031267
1754	GO:0006805	GO:0002046	hsa03010
1755	GO:0006635	GO:0002039	GO:0005581
1756	GO:0006479	GO:0002020	GO:0010755
1757	GO:0006418	GO:0001972	GO:1902988
1758	GO:0006415	GO:0001968	GO:0045580
1759	GO:0006405	GO:0001948	GO:0090261
1760	GO:0006370	GO:0001883	GO:0080135
1761	GO:0006368	GO:0001882	GO:0000812
1762	GO:0006026	GO:0002807	GO:0051494
1763	GO:0005721	GO:0001784	GO:0048585
1764	GO:0005451	GO:0001664	GO:0048534
1765	GO:0005342	GO:0001653	GO:1901576
1766	GO:0003746	GO:0001618	GO:0005634
1767	GO:0003712	GO:0001567	GO:0071900
1768	GO:0003095	GO:0001540	GO:1903305
1769	GO:0002377	GO:0001532	GO:0032986
1770	GO:0002031	GO:0001228	GO:0032922
1771	GO:0001991	GO:0001227	GO:0097485
1772	GO:0001833	GO:0001205	GO:0010632
1773	GO:0001228	GO:0002805	GO:0009058
1774	GO:0000977	GO:0001191	GO:0043900
1775	GO:0000940	GO:0001190	GO:0031399
1776	GO:0000904	GO:0001179	GO:0048598
1777	GO:0000724	GO:0001159	GO:0030204
1778	GO:0000462	GO:0001158	GO:2000785
1779	ENSP00000278407	GO:0001106	GO:0007596
1780	ENSP00000223073	GO:0001105	GO:1901032
1781	hsa05414	GO:0001104	GO:0005912
1782	hsa05169	GO:0001103	GO:0009967
1783	hsa05132	GO:0001102	GO:0018193
1784	hsa04660	GO:0002803	GO:0006884
1785	hsa03040	GO:0001099	GO:0007167
1786	hsa00330	GO:0001098	GO:0043086
1787	hsa00072	GO:0001091	GO:0061140
1788	GO:2000045	GO:0001085	GO:0071391
1789	GO:1990351	GO:0001078	GO:0030670
1790	GO:1904951	GO:0001077	GO:0043194
1791	GO:1903524	GO:0001076	GO:1904951
1792	GO:1903364	GO:0001071	GO:0044409
1793	GO:1902953	GO:0001067	GO:0008603
1794	GO:1902042	GO:0001054	GO:0050817
1795	GO:1901799	GO:0002791	GO:0034332
1796	GO:1901796	GO:0001047	GO:0030055
1797	GO:1901571	GO:0001046	GO:0050812
1798	GO:0098801	GO:0001025	GO:0048861

1799	GO:0098799	GO:0001012	GO:0010975
1800	GO:0098793	GO:0000989	GO:0071459
1801	GO:0098576	GO:0000988	GO:0036126
1802	GO:0097435	GO:0000987	GO:0007399
1803	GO:0097431	GO:0000982	ENSP00000314441
1804	GO:0097159	GO:0000981	GO:0006112
1805	GO:0090080	GO:0000980	GO:0061337
1806	GO:0086036	GO:0002790	GO:0005925
1807	GO:0072522	GO:0000979	GO:0016538
1808	GO:0071806	GO:0000978	GO:0033591
1809	GO:0071704	GO:0000977	GO:1901299
1810	GO:0071459	GO:0000976	GO:0050867
1811	GO:0071392	GO:0000975	GO:0050810
1812	GO:0070838	GO:0000287	GO:0019897
1813	GO:0070588	GO:0000268	GO:0043234
1814	GO:0070160	GO:0000179	GO:0051668
1815	GO:0070071	GO:0000175	GO:0032637
1816	GO:0070069	GO:0000166	GO:0022604
1817	GO:0065008	GO:0002786	GO:0001909
1818	GO:0061640	GO:0000104	GO:2000105
1819	GO:0061197	GO:0000062	GO:0045662
1820	GO:0061041	GO:0000049	GO:0042327
1821	GO:0060986	GO:0000036	GO:0014068
1822	GO:0060968	GO:0000030	GO:0034641
1823	GO:0052548	GO:1990904	GO:0002251
1824	GO:0051954	GO:1990777	GO:1903557
1825	GO:0051883	GO:1990391	GO:0006029
1826	GO:0051782	GO:1990351	GO:0043931
1827	GO:0051302	GO:1990234	GO:0006703
1828	GO:0051248	GO:0002784	GO:0070897
1829	GO:0051247	GO:1990204	GO:0030073
1830	GO:0051225	GO:1904949	GO:0048167
1831	GO:0051171	GO:1903561	GO:0060712
1832	GO:0051168	GO:1902911	GO:0042107
1833	GO:0051101	GO:1902562	GO:0043227
1834	GO:0051082	GO:1902554	GO:0000060
1835	GO:0051028	GO:1902495	GO:0031100
1836	GO:0050803	GO:1902494	GO:0046872
1837	GO:0050793	GO:1902493	GO:0099572
1838	GO:0050685	GO:0099572	GO:0048011
1839	GO:0048365	GO:0002780	GO:0044272
1840	GO:0046933	GO:0099568	GO:0036094
1841	GO:0046851	GO:0099513	hsa04024
1842	GO:0046717	GO:0099512	GO:0031234
1843	GO:0046689	GO:0099503	GO:0042110
1844	GO:0045991	GO:0098862	GO:0017158
1845	GO:0045988	GO:0098858	GO:0060401
1846	GO:0045667	GO:0098857	GO:0002064
1847	GO:0044877	GO:0098852	GO:0035384
1848	GO:0044839	GO:0098805	hsa04915
1849	GO:0044815	GO:0098803	GO:0051651
1850	GO:0044723	GO:0002779	GO:0048667
1851	GO:0044456	GO:0098802	GO:0050962

1852	GO:0044427	GO:0098800	GO:0001071
1853	GO:0044291	GO:0098799	GO:0051649
1854	GO:0043486	GO:0098798	GO:0003700
1855	GO:0043406	GO:0098797	GO:0046085
1856	GO:0043401	GO:0098796	GO:0007159
1857	GO:0043207	GO:0098794	GO:0043492
1858	GO:0043185	GO:0098793	GO:0098576
1859	GO:0043170	GO:0098791	GO:0032989
1860	GO:0042448	GO:0098687	GO:0043267
1861	GO:0042383	GO:0002778	GO:0042130
1862	GO:0042339	GO:0098644	GO:1901991
1863	GO:0042176	GO:0098590	GO:0006807
1864	GO:0042168	GO:0098589	GO:1902946
1865	GO:0042134	GO:0098588	GO:0022008
1866	GO:0042056	GO:0098576	GO:0033619
1867	GO:0035966	GO:0098573	GO:0009982
1868	GO:0035588	GO:0098562	GO:0019082
1869	GO:0035411	GO:0098552	GO:0050715
1870	GO:0034663	GO:0098536	GO:0050686
1871	GO:0034602	GO:0098533	GO:0070489
1872	GO:0033613	GO:0002777	GO:0050777
1873	GO:0033262	GO:0097610	GO:0048666
1874	GO:0032989	GO:0097481	GO:1901407
1875	GO:0032925	GO:0097458	GO:1903309
1876	GO:0032880	GO:0097431	GO:0007265
1877	GO:0032787	GO:0097425	GO:0071310
1878	GO:0032620	GO:0097381	GO:0051057
1879	GO:0032435	GO:0097346	hsa04071
1880	GO:0032412	GO:0097208	GO:1904949
1881	GO:0032402	GO:0097060	GO:0003746
1882	GO:0032370	GO:0097025	GO:0050865
1883	GO:0032205	GO:0002775	GO:0043168
1884	GO:0032200	GO:0090661	GO:0033158
1885	GO:0032040	GO:0090575	GO:0006086
1886	GO:0031349	GO:0090568	GO:0030135
1887	GO:0031329	GO:0090545	GO:0051861
1888	GO:0031145	GO:0090544	GO:0009101
1889	GO:0031060	GO:0090537	GO:0051091
1890	GO:0030953	GO:0090533	GO:0043103
1891	GO:0030816	GO:0072686	GO:1904018
1892	GO:0030802	GO:0072589	GO:0022804
1893	GO:0030502	GO:0072588	GO:0008092
1894	GO:0030282	GO:0002768	GO:0032269
1895	GO:0030150	GO:0072562	GO:0008361
1896	GO:0030010	GO:0072536	GO:0006450
1897	GO:0022616	GO:0072372	GO:0046710
1898	GO:0022600	GO:0071944	GO:0007568
1899	GO:0019897	GO:0071682	GO:0032802
1900	GO:0019867	GO:0071565	ENSP00000295453
1901	GO:0019843	GO:0071564	GO:0051384
1902	GO:0019226	GO:0071556	GO:0048259
1903	GO:0019058	GO:0071339	GO:0002092
1904	GO:0016878	GO:0071062	GO:0010965

1905	GO:0016776	GO:0002764	GO:0007266
1906	GO:0016748	GO:0071013	GO:0048583
1907	GO:0016635	GO:0070993	GO:0005576
1908	GO:0016574	GO:0070822	GO:0030320
1909	GO:0016482	GO:0070761	GO:0050854
1910	GO:0016410	GO:0070603	GO:0002429
1911	GO:0016301	GO:0070545	GO:0031572
1912	GO:0016278	GO:0070469	GO:0030182
1913	GO:0016054	GO:0070461	GO:0032755
1914	GO:0016043	GO:0070382	GO:0043197
1915	GO:0016042	GO:0070195	GO:0012506
1916	GO:0015918	GO:0002760	GO:0004310
1917	GO:0014070	GO:0070161	GO:0045453
1918	GO:0010193	GO:0070160	GO:0044438
1919	GO:0009628	GO:0070110	GO:0060670
1920	GO:0009620	GO:0070069	GO:0051431
1921	GO:0009451	GO:0070062	GO:0005819
1922	GO:0009306	GO:0070013	GO:1904036
1923	GO:0009083	GO:0065010	GO:0006955
1924	GO:0008406	GO:0061695	GO:0061034
1925	GO:0008360	GO:0061617	GO:0048468
1926	GO:0008334	GO:0060205	GO:0032943
1927	GO:0008211	GO:0002759	GO:0045595
1928	GO:0008175	GO:0060170	GO:0001618
1929	GO:0008033	GO:0060076	GO:0050927
1930	GO:0008016	GO:0055038	GO:0000323
1931	GO:0007283	GO:0055037	GO:0051031
1932	GO:0007200	GO:0055029	GO:0014909
1933	GO:0006939	GO:0051233	GO:0045664
1934	GO:0006935	GO:0048770	GO:0001784
1935	GO:0006933	GO:0048471	GO:0032420
1936	GO:0006511	GO:0048188	GO:0030163
1937	GO:0006281	GO:0046658	GO:1903561
1938	GO:0006023	GO:0002757	GO:0019899
1939	GO:0005947	GO:0045335	GO:0016835
1940	GO:0005938	GO:0045334	GO:0042044
1941	GO:0005525	GO:0045283	GO:0016310
1942	GO:0005515	GO:0045281	GO:0050951
1943	GO:0005267	GO:0045277	GO:0030054
1944	GO:0005244	GO:0045275	GO:0009312
1945	GO:0005125	GO:0045273	GO:0032587
1946	GO:0004693	GO:0045271	GO:0048699
1947	GO:0004601	GO:0045267	GO:0001505
1948	GO:0004497	GO:0045263	GO:0001845
1949	GO:0004303	GO:0002753	GO:0002682
1950	GO:0003073	GO:0045261	GO:0033198
1951	GO:0002759	GO:0045259	GO:0044429
1952	GO:0002039	GO:0045257	GO:0051899
1953	GO:0001972	GO:0045254	GO:0006468
1954	GO:0001963	GO:0045252	GO:0001937
1955	GO:0001825	GO:0045240	GO:0030260
1956	GO:0001701	GO:0045239	GO:0005546
1957	GO:0001191	GO:0045211	GO:0090646

1958	GO:0001105	GO:0045202	GO:0001091
1959	GO:0001085	GO:0045178	GO:0009991
1960	GO:0000982	GO:0002740	GO:1901004
1961	GO:0000980	GO:0045177	GO:0002070
1962	GO:0000956	GO:0045171	GO:0046794
1963	GO:0000794	GO:0045121	hsa04070
1964	GO:0000784	GO:0045120	GO:1900115
1965	GO:0000381	GO:0044853	GO:0014065
1966	GO:0000178	GO:0044815	GO:0003674
1967	GO:0000175	GO:0044798	GO:0030010
1968	GO:0000077	GO:0044665	GO:0034110
1969	ENSP00000395046	GO:0044464	GO:0000054
1970	ENSP00000368244	GO:0044463	GO:0051174
1971	ENSP00000307786	GO:0002739	GO:0003723
1972	ENSP00000262367	GO:0044459	GO:0044260
1973	ENSP00000206249	GO:0044456	GO:0002753
1974	hsa05323	GO:0044455	GO:0001525
1975	hsa04144	GO:0044454	ENSP00000304133
1976	hsa04072	GO:0044452	GO:0019904
1977	hsa00860	GO:0044451	GO:0002760
1978	hsa00630	GO:0044450	GO:0031639
1979	hsa00280	GO:0044448	GO:0090279
1980	hsa00220	GO:0044446	GO:0051954
1981	hsa00071	GO:0044445	GO:0060402
1982	GO:2001252	GO:0002719	GO:0051588
1983	GO:2001251	GO:0044444	GO:0050714
1984	GO:2001026	GO:0044441	GO:1902626
1985	GO:2000736	GO:0044440	GO:0002521
1986	GO:2000144	GO:0044439	GO:0046950
1987	GO:2000105	GO:0044438	GO:0010008
1988	GO:1903909	GO:0044437	GO:0043405
1989	GO:1903532	GO:0044433	GO:0043170
1990	GO:1903523	GO:0044432	GO:0015934
1991	GO:1903362	GO:0044431	GO:0009743
1992	GO:1903335	GO:0044430	GO:0031859
1993	GO:1903321	GO:0002718	GO:0009636
1994	GO:1903050	GO:0044429	GO:0034112
1995	GO:1903035	GO:0044428	GO:0045185
1996	GO:1902495	GO:0044427	GO:0035929
1997	GO:1902430	GO:0044425	GO:1904950
1998	GO:1901987	GO:0044424	GO:0060193
1999	GO:1901700	GO:0044422	GO:0009057
2000	GO:1901343	GO:0044421	hsa05205
2001	GO:1901070	GO:0044420	GO:0070011
2002	GO:1900024	GO:0044391	GO:0072384
2003	GO:0098609	GO:0044309	GO:0036376
2004	GO:0090544	GO:0002703	GO:0009968
2005	GO:0090261	GO:0044306	GO:0006887
2006	GO:0090004	GO:0044304	GO:0030947
2007	GO:0072659	GO:0044298	GO:0050776
2008	GO:0072599	GO:0044297	GO:0006965
2009	GO:0071549	GO:0044291	GO:0001180
2010	GO:0071377	GO:0043679	GO:0002784

2011	GO:0070987	GO:0043601	GO:0001098
2012	GO:0070933	GO:0043596	GO:0050848
2013	GO:0070848	GO:0043527	GO:0031346
2014	GO:0070633	GO:0043296	GO:0031011
2015	GO:0070528	GO:0002702	GO:0099503
2016	GO:0070507	GO:0043235	GO:0034330
2017	GO:0070461	GO:0043234	GO:0031325
2018	GO:0070266	GO:0043233	GO:0008298
2019	GO:0070198	GO:0043232	GO:0071028
2020	GO:0070125	GO:0043231	GO:0000470
2021	GO:0065010	GO:0043230	GO:0000966
2022	GO:0065004	GO:0043229	GO:0031401
2023	GO:0061008	GO:0043228	GO:0050917
2024	GO:0060627	GO:0043227	GO:0019083
2025	GO:0060491	GO:0043226	GO:0050435
2026	GO:0060325	GO:0002701	GO:0030336
2027	GO:0060021	GO:0043220	GO:0051924
2028	GO:0051785	GO:0043209	GO:0007275
2029	GO:0051588	GO:0043202	GO:0023057
2030	GO:0051571	GO:0043198	GO:0034663
2031	GO:0051350	GO:0043197	GO:0048513
2032	GO:0051045	GO:0043194	GO:0001012
2033	GO:0050852	GO:0043189	GO:0050954
2034	GO:0050840	GO:0043025	GO:0033674
2035	GO:0050802	GO:0043005	GO:0018195
2036	GO:0050707	GO:0042995	GO:0014061
2037	GO:0050661	GO:0002700	GO:0046689
2038	GO:0048878	GO:0042827	GO:0050794
2039	GO:0048661	GO:0042734	GO:0006111
2040	GO:0048039	GO:0042645	GO:0050789
2041	GO:0048038	GO:0042641	GO:0050871
2042	GO:0046883	GO:0042599	GO:0065007
2043	GO:0046390	GO:0042579	GO:0070588
2044	GO:0046364	GO:0042567	GO:0006979
2045	GO:0046330	GO:0042564	GO:0002376
2046	GO:0046164	GO:0042470	GO:0010563
2047	GO:0045906	GO:0042383	GO:0008121
2048	GO:0045335	GO:0002699	GO:0099536
2049	GO:0045309	GO:0042175	GO:0060341
2050	GO:0045185	GO:0036477	GO:0006474
2051	GO:0044708	GO:0036464	GO:1901977
2052	GO:0044706	GO:0036454	GO:0006909
2053	GO:0044420	GO:0036126	GO:0007566
2054	GO:0044270	GO:0036019	GO:0072509
2055	GO:0044260	GO:0035861	GO:0010648
2056	GO:0044058	GO:0035770	GO:0050755
2057	GO:0043931	GO:0035267	GO:0051574
2058	GO:0043903	GO:0035098	GO:0006357
2059	GO:0043266	GO:0002698	GO:0048518
2060	GO:0043244	GO:0035097	GO:0034109
2061	GO:0043198	GO:0034774	GO:0032153
2062	GO:0043085	GO:0034708	GO:0071635
2063	GO:0042977	GO:0034705	GO:1901700

2064	GO:0042743	GO:0034703	GO:0004175
2065	GO:0042734	GO:0034702	GO:0010604
2066	GO:0042558	GO:0034663	GO:0002683
2067	GO:0042533	GO:0034457	GO:0048856
2068	GO:0042508	GO:0034456	GO:0048731
2069	GO:0042455	GO:0034455	GO:0044767
2070	GO:0042301	GO:0002697	GO:0043549
2071	GO:0042246	GO:0034399	GO:0032502
2072	GO:0042181	GO:0034388	GO:0001741
2073	GO:0042015	GO:0034366	GO:0001179
2074	GO:0035255	GO:0034364	GO:0044763
2075	GO:0034754	GO:0034358	GO:0044699
2076	GO:0034661	GO:0033276	GO:0002757
2077	GO:0034655	GO:0033267	GO:0010727
2078	GO:0034613	GO:0033202	GO:0050896
2079	GO:0034113	GO:0033186	GO:0048522
2080	GO:0034097	GO:0033178	GO:0043167
2081	GO:0034086	GO:0002696	GO:0001775
2082	GO:0033178	GO:0033177	GO:0030217
2083	GO:0033047	GO:0033116	GO:0007043
2084	GO:0032682	GO:0032994	GO:0048869
2085	GO:0032496	GO:0032993	GO:0030154
2086	GO:0032481	GO:0032991	GO:0032403
2087	GO:0032446	GO:0032806	GO:0009888
2088	GO:0032206	GO:0032592	GO:0009653
2089	GO:0031572	GO:0032589	GO:0090092
2090	GO:0031440	GO:0032587	GO:0009914
2091	GO:0031396	GO:0032580	GO:0042060
2092	GO:0031344	GO:0002694	GO:0042058
2093	GO:0031307	GO:0032545	GO:0009019
2094	GO:0031126	GO:0032421	GO:0034104
2095	GO:0031123	GO:0032420	GO:0043968
2096	GO:0031088	GO:0032279	GO:1903508
2097	GO:0030914	GO:0032155	GO:0051173
2098	GO:0030800	GO:0032154	GO:0045935
2099	GO:0030665	GO:0032153	GO:0045893
2100	GO:0030659	GO:0032040	GO:0042221
2101	GO:0030199	GO:0031988	GO:1902680
2102	GO:0030004	GO:0031985	GO:0051254
2103	GO:0022891	GO:0002690	GO:0010557
2104	GO:0022843	GO:0031984	GO:0010628
2105	GO:0022804	GO:0031983	GO:0009891
2106	GO:0022410	GO:0031982	GO:0031328
2107	GO:0019932	GO:0031981	GO:0006366
2108	GO:0019899	GO:0031975	GO:0000187
2109	GO:0019395	GO:0031974	GO:0071867
2110	GO:0019233	GO:0031970	GO:0031397
2111	GO:0019222	GO:0031968	GO:0030913
2112	GO:0017111	GO:0031967	GO:0060158
2113	GO:0016616	GO:0031966	GO:0051276
2114	GO:0016575	GO:0002689	GO:0007049
2115	GO:0016525	GO:0031965	ENSP00000275820
2116	GO:0016491	GO:0031941	GO:0044707

2117	GO:0016469	GO:0031907	GO:0014069
2118	GO:0016462	GO:0031904	GO:0035914
2119	GO:0016458	GO:0031903	GO:0044428
2120	GO:0016408	GO:0031901	GO:0031981
2121	GO:0016281	GO:0031588	GO:0097110
2122	GO:0015988	GO:0031526	GO:0042129
2123	GO:0015959	GO:0031519	GO:0071803
2124	GO:0015844	GO:0031515	GO:0042325
2125	GO:0015491	GO:0002688	GO:0051052
2126	GO:0015370	GO:0031513	GO:0006260
2127	GO:0014812	GO:0031461	GO:0048519
2128	GO:0010639	GO:0031428	GO:0044238
2129	GO:0010608	GO:0031410	GO:0044237
2130	GO:0010485	GO:0031307	GO:0071704
2131	GO:0010248	GO:0031306	GO:0010562
2132	GO:0010008	GO:0031305	hsa04660
2133	GO:0009152	GO:0031304	GO:0009893
2134	GO:0009142	GO:0031301	GO:0010033
2135	GO:0009123	GO:0031300	GO:0001818
2136	GO:0009058	GO:0002687	GO:0070887
2137	GO:0008542	GO:0031256	ENSP00000416330
2138	GO:0008066	GO:0031253	GO:0045121
2139	GO:0007602	GO:0031252	ENSP00000409384
2140	GO:0007588	GO:0031248	ENSP00000409074
2141	GO:0007507	GO:0031234	ENSP00000408017
2142	GO:0007275	GO:0031232	ENSP00000407515
2143	GO:0007269	GO:0031231	ENSP00000402869
2144	GO:0007267	GO:0031228	ENSP00000402802
2145	GO:0007265	GO:0031227	ENSP00000402338
2146	GO:0007220	GO:0031226	ENSP00000399753
2147	GO:0007187	GO:0002686	ENSP00000399454
2148	GO:0007099	GO:0031225	GO:0035556
2149	GO:0007049	GO:0031224	ENSP00000398698
2150	GO:0006999	GO:0031213	ENSP00000396454
2151	GO:0006979	GO:0031093	ENSP00000395046
2152	GO:0006956	GO:0031091	ENSP00000394290
2153	GO:0006909	GO:0031090	ENSP00000393101
2154	GO:0006874	GO:0031088	GO:0042306
2155	GO:0006835	GO:0031012	ENSP00000386047
2156	GO:0006734	GO:0031011	ENSP00000384302
2157	GO:0006733	GO:0031010	ENSP00000381655
2158	GO:0006704	GO:0002685	ENSP00000378857
2159	GO:0006629	GO:0030964	ENSP00000377523
2160	GO:0006606	GO:0030934	ENSP00000377446
2161	GO:0006412	GO:0030914	ENSP00000374390
2162	GO:0006344	GO:0030894	ENSP00000372199
2163	GO:0006310	GO:0030880	GO:0070848
2164	GO:0006144	GO:0030864	ENSP00000371169
2165	GO:0006120	GO:0030863	ENSP00000371101
2166	GO:0006091	GO:0030849	ENSP00000369647
2167	GO:0005852	GO:0030692	ENSP00000368989
2168	GO:0005684	GO:0030689	ENSP00000368887
2169	GO:0005662	GO:0002684	ENSP00000368244

2170	GO:0005527	GO:0030688	ENSP00000367934
2171	GO:0005520	GO:0030687	ENSP00000367361
2172	GO:0005509	GO:0030686	ENSP00000366629
2173	GO:0005215	GO:0030684	ENSP00000366623
2174	GO:0005184	GO:0030673	ENSP00000364699
2175	GO:0004900	GO:0030670	ENSP00000364037
2176	GO:0003854	GO:0030669	ENSP00000363642
2177	GO:0003697	GO:0030667	ENSP00000362873
2178	GO:0003008	GO:0030666	ENSP00000362036
2179	GO:0002862	GO:0030665	ENSP00000361965
2180	GO:0002833	GO:0002683	ENSP00000361092
2181	GO:0002700	GO:0030662	ENSP00000360412
2182	GO:0002687	GO:0030659	ENSP00000360031
2183	GO:0002478	GO:0030529	ENSP00000359688
2184	GO:0002429	GO:0030496	ENSP00000358812
2185	GO:0002062	GO:0030425	ENSP00000356953
2186	GO:0002028	GO:0030424	ENSP00000356671
2187	GO:0001816	GO:0030315	ENSP00000356548
2188	GO:0001750	GO:0030176	ENSP00000356476
2189	GO:0001738	GO:0030175	ENSP00000355987
2190	GO:0001508	GO:0030173	ENSP00000355899
2191	GO:0001077	GO:0002682	ENSP00000355443
2192	GO:0000910	GO:0030141	ENSP00000355436
2193	GO:0000792	GO:0030140	GO:0032388
2194	GO:0000447	GO:0030139	ENSP00000352021
2195	GO:0000226	GO:0030136	ENSP00000350720
2196	ENSP00000362795	GO:0030135	ENSP00000350352
2197	ENSP00000263321	GO:0030133	ENSP00000349142
2198	hsa05168	GO:0030055	ENSP00000348722
2199	hsa04670	GO:0030054	ENSP00000348596
2200	hsa04330	GO:0030027	ENSP00000348170
2201	hsa00982	GO:0022627	ENSP00000346839
2202	hsa00620	GO:0002673	ENSP00000345317
2203	hsa00601	GO:0022626	ENSP00000342056
2204	hsa00410	GO:0022625	ENSP00000338862
2205	hsa00380	GO:0019908	ENSP00000338573
2206	hsa00260	GO:0019898	ENSP00000337518
2207	hsa00140	GO:0019897	ENSP00000332340
2208	GO:2001233	GO:0019867	ENSP00000331545
2209	GO:2000377	GO:0019866	ENSP00000331111
2210	GO:2000112	GO:0017053	ENSP00000330937
2211	GO:2000058	GO:0016942	ENSP00000330737
2212	GO:1904235	GO:0016607	GO:0001932
2213	GO:1904064	GO:0002639	ENSP00000330460
2214	GO:1904063	GO:0016605	ENSP00000330384
2215	GO:1903753	GO:0016604	ENSP00000330341
2216	GO:1903729	GO:0016602	ENSP00000330049
2217	GO:1903307	GO:0016592	ENSP00000328671
2218	GO:1903169	GO:0016591	ENSP00000327179
2219	GO:1903036	GO:0016589	ENSP00000327070
2220	GO:1902004	GO:0016581	ENSP00000325128
2221	GO:1901699	GO:0016580	ENSP00000323612
2222	GO:1901615	GO:0016514	ENSP00000322088

2223	GO:1901407	GO:0016469	ENSP00000321426
2224	GO:1901135	GO:0002576	ENSP00000321320
2225	GO:1900542	GO:0016363	ENSP00000321246
2226	GO:0098552	GO:0016328	ENSP00000320917
2227	GO:0097458	GO:0016327	ENSP00000320567
2228	GO:0097367	GO:0016324	ENSP00000317992
2229	GO:0097346	GO:0016323	ENSP00000311135
2230	GO:0090197	GO:0016281	ENSP00000311038
2231	GO:0090185	GO:0016023	ENSP00000310668
2232	GO:0071902	GO:0016021	ENSP00000310337
2233	GO:0071867	GO:0016020	ENSP00000310042
2234	GO:0071565	GO:0015935	ENSP00000308270
2235	GO:0071345	GO:0002548	ENSP00000308179
2236	GO:0071260	GO:0015934	ENSP00000307786
2237	GO:0070972	GO:0015630	ENSP00000307525
2238	GO:0070851	GO:0015629	ENSP00000307130
2239	GO:0070167	GO:0015030	ENSP00000306095
2240	GO:0070161	GO:0014704	ENSP00000304419
2241	GO:0065002	GO:0014069	GO:0032869
2242	GO:0061448	GO:0012506	ENSP00000304151
2243	GO:0061028	GO:0012505	ENSP00000303515
2244	GO:0060350	GO:0010008	ENSP00000303076
2245	GO:0060070	GO:0009986	ENSP00000300773
2246	GO:0060047	GO:0002526	ENSP00000298746
2247	GO:0051984	GO:0009925	ENSP00000297579
2248	GO:0051928	GO:0009898	ENSP00000296099
2249	GO:0051569	GO:0009897	ENSP00000291576
2250	GO:0051459	GO:0009295	ENSP00000290866
2251	GO:0051427	GO:0008328	ENSP00000290299
2252	GO:0051261	GO:0008024	ENSP00000287022
2253	GO:0051223	GO:0008023	ENSP00000282050
2254	GO:0050951	GO:0005947	ENSP00000276689
2255	GO:0050804	GO:0005938	ENSP00000276585
2256	GO:0050801	GO:0005929	ENSP00000268854
2257	GO:0050776	GO:0002521	ENSP00000268802
2258	GO:0050681	GO:0005925	ENSP00000268668
2259	GO:0048871	GO:0005924	ENSP00000268379
2260	GO:0048863	GO:0005923	ENSP00000267425
2261	GO:0048667	GO:0005912	ENSP00000265245
2262	GO:0048646	GO:0005911	ENSP00000263774
2263	GO:0048568	GO:0005905	ENSP00000263321
2264	GO:0048471	GO:0005903	ENSP00000263253
2265	GO:0048260	GO:0005902	ENSP00000262607
2266	GO:0048009	GO:0005901	GO:0001817
2267	GO:0046875	GO:0005900	ENSP00000262367
2268	GO:0046873	GO:0002520	ENSP00000261741
2269	GO:0046102	GO:0005896	ENSP00000261708
2270	GO:0045926	GO:0005887	ENSP00000261015
2271	GO:0045834	GO:0005886	ENSP00000260563
2272	GO:0045778	GO:0005884	ENSP00000260227
2273	GO:0045760	GO:0005879	ENSP00000258531
2274	GO:0045582	GO:0005876	ENSP00000258424
2275	GO:0045202	GO:0005875	ENSP00000253237

2276	GO:0045187	GO:0005874	ENSP00000252711
2277	GO:0045177	GO:0005856	ENSP00000251289
2278	GO:0045005	GO:0005852	ENSP00000249269
2279	GO:0044325	GO:0002504	ENSP00000244496
2280	GO:0044243	GO:0005840	ENSP00000244230
2281	GO:0044089	GO:0005834	GO:0010976
2282	GO:0044033	GO:0005829	ENSP00000233190
2283	GO:0043974	GO:0005819	ENSP00000233114
2284	GO:0043967	GO:0005815	ENSP00000232888
2285	GO:0043921	GO:0005814	ENSP00000231751
2286	GO:0043632	GO:0005813	ENSP00000230640
2287	GO:0043628	GO:0005802	GO:0008286
2288	GO:0043627	GO:0005798	ENSP00000230340
2289	GO:0043388	GO:0005797	ENSP00000222567
2290	GO:0043267	GO:0002495	ENSP00000216492
2291	GO:0042699	GO:0005796	ENSP00000215375
2292	GO:0042635	GO:0005795	ENSP00000209540
2293	GO:0042273	GO:0005794	ENSP00000206249
2294	GO:0042130	GO:0005793	ENSP00000203407
2295	GO:0042102	GO:0005791	ENSP00000203001
2296	GO:0035988	GO:0005790	GO:1990583
2297	GO:0035929	GO:0005789	GO:1904929
2298	GO:0035567	GO:0005788	GO:1902936
2299	GO:0035250	GO:0005783	GO:1901981
2300	GO:0035097	GO:0005782	GO:1901265
2301	GO:0034765	GO:0002479	GO:0099528
2302	GO:0034705	GO:0005779	GO:0099516
2303	GO:0034248	GO:0005778	GO:0098811
2304	GO:0034116	GO:0005777	GO:0098772
2305	GO:0034112	GO:0005776	GO:0098631
2306	GO:0033993	GO:0005775	GO:0071987
2307	GO:0033617	GO:0005774	GO:0071837
2308	GO:0033139	GO:0005773	GO:0071535
2309	GO:0033044	GO:0005770	GO:0070615
2310	GO:0032946	GO:0005769	GO:0070612
2311	GO:0032587	GO:0005768	GO:0070577
2312	GO:0032388	GO:0002478	GO:0070491
2313	GO:0032098	GO:0005765	GO:0070119
2314	GO:0031995	GO:0005764	GO:0070035
2315	GO:0031859	GO:0005763	GO:0061659
2316	GO:0031667	GO:0005762	GO:0046649
2317	GO:0031625	GO:0005761	GO:0061630
2318	GO:0031410	GO:0005759	GO:0061135
2319	GO:0031347	GO:0005758	GO:0060090
2320	GO:0031279	GO:0005754	GO:0052909
2321	GO:0031252	GO:0005753	GO:0052890
2322	GO:0031234	GO:0005751	GO:0052689
2323	GO:0031012	GO:0002474	GO:0051996
2324	GO:0030865	GO:0005750	GO:0051540
2325	GO:0030864	GO:0005749	GO:0032270
2326	GO:0030855	GO:0005747	GO:0051539
2327	GO:0030492	GO:0005746	GO:0051427
2328	GO:0030414	GO:0005744	GO:0051219

2329	GO:0030301	GO:0005743	GO:0051192
2330	GO:0030260	GO:0005742	GO:0051082
2331	GO:0030219	GO:0005741	GO:0051020
2332	GO:0030204	GO:0005740	GO:0051015
2333	GO:0030195	GO:0005739	GO:0050840
2334	GO:0023052	GO:0002460	GO:0050839
2335	GO:0022409	GO:0005737	GO:0050780
2336	GO:0019852	GO:0005736	GO:0050681
2337	GO:0019682	GO:0005732	GO:0050661
2338	GO:0019083	GO:0005730	GO:0050660
2339	GO:0019003	GO:0005721	GO:0050178
2340	GO:0018171	GO:0005720	GO:0048040
2341	GO:0018024	GO:0005719	GO:0048038
2342	GO:0018022	GO:0005697	GO:0047696
2343	GO:0017171	GO:0005694	GO:0046983
2344	GO:0017025	GO:0005684	GO:0046982
2345	GO:0017022	GO:0002455	GO:0046966
2346	GO:0016863	GO:0005681	GO:0046961
2347	GO:0016742	GO:0005677	GO:0046912
2348	GO:0016634	GO:0005675	GO:0046875
2349	GO:0016573	GO:0005672	GO:0046873
2350	GO:0016572	GO:0005671	GO:0046554
2351	GO:0016310	GO:0005669	GO:0045309
2352	GO:0016075	GO:0005667	GO:0045155
2353	GO:0016064	GO:0005666	GO:0044822
2354	GO:0016032	GO:0005664	GO:0044620
2355	GO:0015718	GO:0005662	GO:0044212
2356	GO:0015293	GO:0002449	GO:0043997
2357	GO:0015288	GO:0005657	GO:0043783
2358	GO:0015103	GO:0005654	GO:0043544
2359	GO:0015079	GO:0005643	GO:0043531
2360	GO:0015077	GO:0005635	GO:0043426
2361	GO:0014821	GO:0005634	GO:0043394
2362	GO:0010976	GO:0005623	GO:0043185
2363	GO:0010959	GO:0005622	GO:0043177
2364	GO:0010828	GO:0005615	GO:0048010
2365	GO:0010727	GO:0005614	GO:0043169
2366	GO:0010634	GO:0005604	GO:0043021
2367	GO:0010563	GO:0002446	GO:0043015
2368	GO:0010501	GO:0005596	GO:0042826
2369	GO:0010455	GO:0005593	GO:0042809
2370	GO:0010453	GO:0005581	GO:0042803
2371	GO:0009925	GO:0005578	GO:0042626
2372	GO:0009746	GO:0005577	GO:0042623
2373	GO:0009612	GO:0005576	GO:0042608
2374	GO:0009607	GO:0005575	GO:0042393
2375	GO:0009119	GO:0001940	GO:0042301
2376	GO:0009063	GO:0001939	GO:0042162
2377	GO:0008378	GO:0001917	GO:0042134
2378	GO:0008328	GO:0002443	GO:0044087
2379	GO:0008286	GO:0001772	GO:0042056
2380	GO:0008177	GO:0001750	GO:0042020
2381	GO:0008024	GO:0001741	GO:0042015

2382	GO:0007635	GO:0001726	GO:0038164
2383	GO:0007611	GO:0001652	GO:0038024
2384	GO:0007606	GO:0001650	GO:0036442
2385	GO:0007597	GO:0000940	GO:0036002
2386	GO:0007565	GO:0000939	GO:0035591
2387	GO:0007411	GO:0000932	GO:0035326
2388	GO:0007205	GO:0000930	GO:0035257
2389	GO:0007191	GO:0002440	GO:0035250
2390	GO:0007189	GO:0000922	GO:0035091
2391	GO:0007173	GO:0000812	GO:0035035
2392	GO:0007155	GO:0000808	GO:0034714
2393	GO:0007094	GO:0000806	GO:0034604
2394	GO:0006913	GO:0000805	GO:0080134
2395	GO:0006885	GO:0000803	GO:0034603
2396	GO:0006833	GO:0000794	GO:0034602
2397	GO:0006812	GO:0000793	GO:0034513
2398	GO:0006775	GO:0000792	GO:0034511
2399	GO:0006744	GO:0000791	GO:0034212
2400	GO:0006518	GO:0002433	GO:0033764
2401	GO:0006417	GO:0000790	GO:0033613
2402	GO:0006413	GO:0000788	GO:0033218
2403	GO:0006397	GO:0000786	GO:0032947
2404	GO:0006302	GO:0000785	GO:0032555
2405	GO:0006301	GO:0000784	GO:0032451
2406	GO:0006222	GO:0000781	GO:0032041
2407	GO:0006163	GO:0000779	GO:0018108
2408	GO:0006154	GO:0000777	GO:0031995
2409	GO:0006123	GO:0000776	GO:0031994
2410	GO:0006082	GO:0000775	GO:0031896
2411	GO:0006007	GO:0002431	GO:0031826
2412	GO:0005879	GO:0000428	GO:0031821
2413	GO:0005697	GO:0000323	GO:0031762
2414	GO:0005694	GO:0000315	GO:0031748
2415	GO:0005102	GO:0000314	GO:0031701
2416	GO:0004924	GO:0000313	GO:0031700
2417	GO:0004867	GO:0000307	GO:0031692
2418	GO:0004769	GO:0000276	GO:0031691
2419	GO:0004672	GO:0000275	GO:0031625
2420	GO:0004468	GO:0000242	GO:0031492
2421	GO:0003994	GO:0000228	GO:0031491
2422	GO:0003899	GO:0002429	GO:0031489
2423	GO:0003857	GO:0000178	GO:0031420
2424	GO:0003705	GO:0000177	GO:0031406
2425	GO:0003012	GO:0000176	GO:0031386
2426	GO:0002753	GO:0000152	GO:0030731
2427	GO:0002683	GO:0000151	GO:0030697
2428	GO:0002460	GO:0000139	GO:0030696
2429	GO:0002443	GO:0000138	GO:0030695
2430	GO:0002433	GO:0000125	GO:0030594
2431	GO:0001937	GO:0000123	GO:0030554
2432	GO:0001883	GO:0000118	GO:0030523
2433	GO:0001840	GO:0002385	GO:0030515
2434	GO:0001653	GO:2001257	GO:0030492

2435	GO:0001501	GO:2001252	GO:0030414
2436	GO:0001067	GO:2001251	GO:0030246
2437	GO:0000975	GO:2001237	GO:0030234
2438	GO:0000959	GO:2001236	GO:0030159
2439	GO:0000803	GO:2001233	GO:0030060
2440	GO:0000377	GO:2001202	GO:0022890
2441	GO:0000228	GO:2001201	GO:0022843
2442	GO:0000062	GO:2001141	GO:0022834
2443	GO:0000054	GO:2001027	GO:0022829
2444	ENSP00000370968	GO:0002384	GO:0019981
2445	ENSP00000365663	GO:2001026	GO:0019969
2446	ENSP00000233114	GO:2001022	GO:0019962
2447	hsa05150	GO:2001020	GO:0019961
2448	hsa04972	GO:2000870	GO:0019955
2449	hsa04933	GO:2000833	GO:0019901
2450	hsa04921	GO:2000816	GO:0019900
2451	hsa04740	GO:2000785	GO:0019843
2452	hsa04727	GO:2000779	GO:0019840
2453	hsa04512	GO:2000757	GO:0019838
2454	hsa04510	GO:2000756	GO:0018212
2455	GO:2001237	GO:0002377	GO:0019789
2456	GO:2001022	GO:2000736	GO:0019213
2457	GO:2000351	GO:2000649	GO:0019205
2458	GO:2000258	GO:2000644	GO:0017171
2459	GO:1904929	GO:2000617	GO:0017137
2460	GO:1904816	GO:2000615	GO:0017124
2461	GO:1904814	GO:2000546	GO:0017076
2462	GO:1903707	GO:2000544	GO:0017069
2463	GO:1903322	GO:2000404	GO:0017056
2464	GO:1903320	GO:2000403	GO:0017025
2465	GO:1903076	GO:2000401	GO:0017016
2466	GO:1902652	GO:0002376	GO:0009612
2467	GO:1901652	GO:2000377	GO:0016907
2468	GO:1901362	GO:2000352	GO:0016896
2469	GO:1901360	GO:2000351	GO:0016878
2470	GO:1901068	GO:2000296	GO:0016874
2471	GO:1900426	GO:2000258	GO:0016863
2472	GO:0098739	GO:2000257	GO:0071417
2473	GO:0098660	GO:2000181	GO:0016853
2474	GO:0090109	GO:2000147	GO:0016836
2475	GO:0090042	GO:2000146	GO:0016832
2476	GO:0090025	GO:2000145	GO:0016830
2477	GO:0072677	GO:0002374	GO:0016829
2478	GO:0072676	GO:2000144	GO:0016818
2479	GO:0072376	GO:2000142	GO:0016817
2480	GO:0071837	GO:2000134	GO:0016810
2481	GO:0071548	GO:2000113	GO:0016791
2482	GO:0070741	GO:2000112	GO:0016787
2483	GO:0070670	GO:2000105	GO:0016779
2484	GO:0070613	GO:2000098	GO:0016757
2485	GO:0070612	GO:2000097	GO:0016751
2486	GO:0070525	GO:2000060	GO:0016684
2487	GO:0070469	GO:2000058	GO:0016681

2488	GO:0070168	GO:0002367	GO:0016679
2489	GO:0070124	GO:2000045	GO:0016668
2490	GO:0061029	GO:2000027	GO:0016655
2491	GO:0060396	GO:2000026	GO:0016649
2492	GO:0060346	GO:2000021	GO:0016645
2493	GO:0060218	GO:2000002	GO:0016635
2494	GO:0060029	GO:2000001	GO:0016634
2495	GO:0051918	GO:1990778	GO:0016628
2496	GO:0051641	GO:1990748	GO:0044093
2497	GO:0051539	GO:1990542	GO:0016627
2498	GO:0051437	GO:1990481	GO:0016624
2499	GO:0051254	GO:0002360	GO:0016608
2500	GO:0051241	GO:1990402	GO:0016597
2501	GO:0051057	GO:1990266	GO:0016509
2502	GO:0051053	GO:1904951	GO:0016462
2503	GO:0051052	GO:1904950	GO:0016436
2504	GO:0050999	GO:1904894	GO:0016435
2505	GO:0050918	GO:1904892	GO:0016433
2506	GO:0050907	GO:1904874	GO:0051130
2507	GO:0050896	GO:1904851	GO:0016429
2508	GO:0050865	GO:1904849	GO:0016428
2509	GO:0050851	GO:1904847	GO:0016426
2510	GO:0050839	GO:0002253	GO:0016423
2511	GO:0050730	GO:1904816	GO:0016408
2512	GO:0050729	GO:1904814	GO:0016300
2513	GO:0050662	GO:1904667	GO:0016298
2514	GO:0050432	GO:1904666	GO:0016279
2515	GO:0048858	GO:1904591	GO:0016273
2516	GO:0048705	GO:1904377	GO:0016229
2517	GO:0048589	GO:1904375	GO:0016209
2518	GO:0048531	GO:1904237	GO:0016150
2519	GO:0048523	GO:1904235	GO:0015491
2520	GO:0048521	GO:1904064	GO:0015464
2521	GO:0048146	GO:0002252	GO:0015459
2522	GO:0048011	GO:1904063	GO:0015405
2523	GO:0048010	GO:1904062	GO:0015399
2524	GO:0046949	GO:1904036	GO:0015370
2525	GO:0046928	GO:1904035	GO:0015298
2526	GO:0046906	GO:1904029	GO:0015296
2527	GO:0046697	GO:1904018	GO:0015288
2528	GO:0046502	GO:1903911	GO:0015266
2529	GO:0045995	GO:1903909	GO:0015108
2530	GO:0045981	GO:1903902	GO:0015103
2531	GO:0045786	GO:1903900	GO:0015081
2532	GO:0045732	GO:0002251	GO:0015079
2533	GO:0045637	GO:1903845	GO:0015077
2534	GO:0045619	GO:1903844	GO:0015036
2535	GO:0045137	GO:1903793	GO:0010576
2536	GO:0045120	GO:1903753	GO:0010485
2537	GO:0044770	GO:1903751	GO:0010484
2538	GO:0044769	GO:1903750	GO:0009055
2539	GO:0044728	GO:1903729	GO:0008988
2540	GO:0044665	GO:1903725	GO:0008757

2541	GO:0044344	GO:1903707	GO:0008650
2542	GO:0044271	GO:1903706	GO:0008556
2543	GO:0044205	GO:0002250	GO:0008545
2544	GO:0043970	GO:1903651	GO:0008536
2545	GO:0043565	GO:1903649	GO:0008509
2546	GO:0043425	GO:1903557	GO:0008495
2547	GO:0043412	GO:1903555	GO:0008401
2548	GO:0043086	GO:1903532	GO:0008395
2549	GO:0043005	GO:1903531	GO:0008378
2550	GO:0042886	GO:1903530	GO:0008353
2551	GO:0042809	GO:1903524	GO:0008236
2552	GO:0042802	GO:1903523	GO:0008186
2553	GO:0042773	GO:1903522	GO:0008177
2554	GO:0042744	GO:0002244	GO:0008171
2555	GO:0042641	GO:1903513	GO:0008170
2556	GO:0042608	GO:1903510	GO:0008168
2557	GO:0042517	GO:1903509	GO:0008134
2558	GO:0042445	GO:1903508	GO:1901699
2559	GO:0042278	GO:1903507	GO:0008094
2560	GO:0042255	GO:1903506	GO:0008083
2561	GO:0042127	GO:1903426	GO:0008080
2562	GO:0042060	GO:1903409	GO:0008066
2563	GO:0042036	GO:1903364	GO:0008047
2564	GO:0036464	GO:1903362	GO:0008017
2565	GO:0036314	GO:0002227	GO:0008009
2566	GO:0036094	GO:1903337	GO:0008283
2567	GO:0035987	GO:1903335	GO:0005525
2568	GO:0035630	GO:1903322	GO:0005524
2569	GO:0035601	GO:1903321	GO:0005520
2570	GO:0035556	GO:1903320	GO:0005518
2571	GO:0035326	GO:1903318	GO:0005516
2572	GO:0035098	GO:1903317	GO:0005506
2573	GO:0034728	GO:1903313	GO:0005501
2574	GO:0034644	GO:1903312	GO:0005488
2575	GO:0034641	GO:1903311	GO:0005391
2576	GO:0034440	GO:0002225	GO:0005342
2577	GO:0034104	GO:1903310	GO:0005267
2578	GO:0033273	GO:1903309	GO:0005262
2579	GO:0033138	GO:1903308	GO:0005246
2580	GO:0032984	GO:1903307	GO:0005215
2581	GO:0032967	GO:1903305	GO:0005198
2582	GO:0032802	GO:1903279	GO:0005138
2583	GO:0032642	GO:1903169	GO:0005126
2584	GO:0032589	GO:1903078	GO:0005125
2585	GO:0032555	GO:1903076	GO:0005109
2586	GO:0032434	GO:1903055	GO:0005104
2587	GO:0032409	GO:0002223	GO:0004993
2588	GO:0032310	GO:1903052	GO:0004924
2589	GO:0032101	GO:1903051	GO:0004923
2590	GO:0031960	GO:1903050	GO:0004917
2591	GO:0031904	GO:1903047	GO:0004915
2592	GO:0031691	GO:1903039	GO:0004914
2593	GO:0031529	GO:1903037	GO:0004913

2594	GO:0031490	GO:1903036	GO:0004912
2595	GO:0031420	GO:1903035	GO:0000302
2596	GO:0031346	GO:1903034	GO:0004906
2597	GO:0031331	GO:1903019	GO:0004900
2598	GO:0031294	GO:0002181	GO:0004897
2599	GO:0031214	GO:1903008	GO:0004867
2600	GO:0031167	GO:1902998	GO:0004866
2601	GO:0031124	GO:1902996	GO:0004842
2602	GO:0031078	GO:1902993	GO:0004809
2603	GO:0031057	GO:1902988	GO:0004776
2604	GO:0031056	GO:1902953	GO:0004769
2605	GO:0030894	GO:1902949	GO:0004738
2606	GO:0030815	GO:1902947	GO:0004721
2607	GO:0030666	GO:1902946	GO:0004703
2608	GO:0030595	GO:1902850	GO:0004693
2609	GO:0030554	GO:0002128	GO:0043491
2610	GO:0030521	GO:1902847	GO:0004674
2611	GO:0030518	GO:1902824	GO:0004620
2612	GO:0030514	GO:1902822	GO:0004602
2613	GO:0030193	GO:1902807	GO:0004601
2614	GO:0030183	GO:1902806	GO:0004468
2615	GO:0030111	GO:1902774	GO:0051129
2616	GO:0030003	GO:1902750	GO:0004448
2617	GO:0030001	GO:1902749	GO:0004407
2618	GO:0022626	GO:1902680	GO:0004385
2619	GO:0022604	GO:1902679	GO:0004362
2620	GO:0022402	GO:0002092	GO:0004333
2621	GO:0019985	GO:1902656	GO:0004311
2622	GO:0019941	GO:1902653	GO:0004300
2623	GO:0019320	GO:1902652	GO:0004252
2624	GO:0018394	GO:1902626	GO:0004174
2625	GO:0018146	GO:1902624	GO:0004169
2626	GO:0017160	GO:1902622	GO:0004152
2627	GO:0016879	GO:1902600	GO:0004149
2628	GO:0016773	GO:1902593	GO:0004148
2629	GO:0016614	GO:1902589	GO:0004129
2630	GO:0016570	GO:1902583	GO:0004108
2631	GO:0016229	GO:0002090	GO:0004096
2632	GO:0016115	GO:1902582	GO:0004045
2633	GO:0016023	GO:1902580	GO:0004004
2634	GO:0015294	GO:1902579	GO:0004003
2635	GO:0014823	GO:1902578	hsa05142
2636	GO:0010863	GO:1902572	GO:0003994
2637	GO:0010811	GO:1902571	GO:0003988
2638	GO:0010644	GO:1902533	GO:0003963
2639	GO:0010575	GO:1902531	GO:0003954
2640	GO:0010564	GO:1902476	GO:0003938
2641	GO:0010510	GO:1902430	GO:0003924
2642	GO:0009952	GO:0002070	GO:0003878
2643	GO:0009896	GO:1902403	GO:0003857
2644	GO:0009892	GO:1902402	GO:0010035
2645	GO:0009889	GO:1902400	GO:0003854
2646	GO:0009725	GO:1902305	GO:0003727

2647	GO:0009584	GO:1902275	GO:0003724
2648	GO:0009312	GO:1902224	GO:0003714
2649	GO:0009201	GO:1902115	GO:0003712
2650	GO:0009127	GO:1902106	GO:0003690
2651	GO:0009126	GO:1902105	GO:0002046
2652	GO:0009124	GO:1902100	GO:0001972
2653	GO:0009066	GO:0002064	GO:0001968
2654	GO:0008217	GO:1902099	GO:0001948
2655	GO:0008209	GO:1902042	GO:0001883
2656	GO:0008202	GO:1902004	GO:0001664
2657	GO:0008191	GO:1901998	GO:0001653
2658	GO:0008135	GO:1901992	GO:0001567
2659	GO:0008134	GO:1901991	GO:1903651
2660	GO:0008015	GO:1901990	GO:0001532
2661	GO:0007628	GO:1901989	GO:0001228
2662	GO:0007193	GO:1901988	GO:0001227
2663	GO:0007131	GO:1901987	GO:0001205
2664	GO:0007093	GO:0002063	GO:0001191
2665	GO:0007000	GO:1901983	GO:0001190
2666	GO:0006929	GO:1901977	GO:0001106
2667	GO:0006898	GO:1901888	GO:0001105
2668	GO:0006821	GO:1901881	GO:0001104
2669	GO:0006778	GO:1901879	GO:0001103
2670	GO:0006721	GO:1901800	GO:0001102
2671	GO:0006690	GO:1901799	GO:0001099
2672	GO:0006516	GO:1901796	GO:0001085
2673	GO:0006468	GO:1901701	GO:0001078
2674	GO:0006465	GO:1901700	GO:0001067
2675	GO:0006464	GO:0002062	GO:0001054
2676	GO:0006406	GO:1901699	GO:0001047
2677	GO:0006353	GO:1901698	GO:0001025
2678	GO:0006336	GO:1901675	GO:0000988
2679	GO:0006305	GO:1901674	GO:0000982
2680	GO:0006278	GO:1901663	GO:0000980
2681	GO:0006188	GO:1901661	GO:0000979
2682	GO:0006105	GO:1901659	GO:0000978
2683	GO:0006099	GO:1901657	GO:0000977
2684	GO:0005834	GO:1901652	GO:0000976
2685	GO:0005604	GO:1901617	GO:0000975
2686	GO:0005543	GO:0002032	GO:0000268
2687	GO:0004923	GO:1901616	GO:0000179
2688	GO:0004591	GO:1901615	GO:0000049
2689	GO:0004402	GO:1901607	GO:0000036
2690	GO:0004362	GO:1901606	GO:0000030
2691	GO:0004333	GO:1901605	GO:1990904
2692	GO:0004096	GO:1901576	GO:1990777
2693	GO:0003684	GO:1901575	GO:1990391
2694	GO:0003674	GO:1901571	GO:1990234
2695	GO:0003416	GO:1901568	GO:1902495
2696	GO:0003071	GO:1901566	GO:1902493
2697	GO:0003014	GO:0002031	GO:0099512
2698	GO:0003006	GO:1901565	GO:0098862
2699	GO:0002684	GO:1901564	GO:0098857

2700	GO:0002520	GO:1901522	GO:0098805
2701	GO:0002446	GO:1901407	GO:0098803
2702	GO:0002092	GO:1901379	GO:0098802
2703	GO:0001957	GO:1901362	GO:0098798
2704	GO:0001939	GO:1901361	GO:0098797
2705	GO:0001839	GO:1901360	GO:0098796
2706	GO:0001819	GO:1901343	GO:0098794
2707	GO:0001726	GO:1901342	GO:0098793
2708	GO:0001558	GO:0002029	GO:0098791
2709	GO:0001076	GO:1901299	GO:0098687
2710	GO:0000988	GO:1901293	GO:0098590
2711	GO:0000966	GO:1901185	GO:0098573
2712	GO:0000812	GO:1901164	GO:0098562
2713	GO:0000463	GO:1901137	GO:0098536
2714	GO:0000428	GO:1901136	GO:0098533
2715	GO:0000302	GO:1901135	GO:0097610
2716	GO:0000154	GO:1901094	GO:0097458
2717	GO:0000122	GO:1901070	GO:0097431
2718	GO:0000086	GO:1901068	GO:0071260
2719	GO:0000030	GO:0002028	GO:0097425
2720	GO:0000028	GO:1901032	GO:0097381
2721	ENSP00000387262	GO:1901016	GO:0097346
2722	ENSP00000361965	GO:1901006	GO:0097208
2723	ENSP00000357535	GO:1901004	GO:0097060
2724	ENSP00000350003	GO:1900864	GO:0097025
2725	ENSP00000315774	GO:1900544	GO:0090661
2726	ENSP00000310668	GO:1900542	GO:0072686
2727	ENSP00000302150	GO:1900426	GO:0072589
2728	ENSP00000007516	GO:1900424	GO:0072588
2729	hsa05160	GO:1900373	GO:0072562
2730	hsa04973	GO:0002009	GO:0072536
2731	hsa04630	GO:1900372	GO:0072372
2732	hsa04540	GO:1900371	GO:0071944
2733	hsa04270	GO:1900274	GO:0071682
2734	hsa00983	GO:1900135	GO:0071564
2735	hsa00250	GO:1900133	GO:0071556
2736	hsa00120	GO:1900122	GO:0071339
2737	GO:2000644	GO:1900120	GO:0071062
2738	GO:2000146	GO:1900116	GO:0070993
2739	GO:1904591	GO:1900115	GO:0070761
2740	GO:1904036	GO:1900086	GO:0070469
2741	GO:1903750	GO:0002002	GO:0070461
2742	GO:1903522	GO:1900084	GO:0070382
2743	GO:1903409	GO:1900048	GO:0070195
2744	GO:1903047	GO:1900047	GO:0070161
2745	GO:1902946	GO:1900046	GO:0070160
2746	GO:1902850	GO:1900034	GO:0008219
2747	GO:1902100	GO:1900024	GO:0070110
2748	GO:1901983	GO:1900004	GO:0070069
2749	GO:1901564	GO:1900003	GO:0061695
2750	GO:1901363	GO:0099565	GO:0060205
2751	GO:1901185	GO:0099537	GO:0060076
2752	GO:1901164	GO:0001999	GO:0055037

2753	GO:1900274	GO:0099536	GO:0055029
2754	GO:0098869	GO:0099531	GO:0051233
2755	GO:0098868	GO:0098900	GO:0048471
2756	GO:0098662	GO:0098869	GO:0048188
2757	GO:0098661	GO:0098868	GO:0046658
2758	GO:0098657	GO:0098813	GO:0045335
2759	GO:0098533	GO:0098801	GO:0045334
2760	GO:0097381	GO:0098781	GO:0045283
2761	GO:0090646	GO:0098771	GO:0045281
2762	GO:0090504	GO:0098761	GO:0045273
2763	GO:0090501	GO:0001991	GO:0045271
2764	GO:0090329	GO:0098760	GO:0045267
2765	GO:0090175	GO:0098756	GO:0045120
2766	GO:0090092	GO:0098754	GO:0044464
2767	GO:0086091	GO:0098743	GO:0044463
2768	GO:0086064	GO:0098742	GO:0044455
2769	GO:0072562	GO:0098739	GO:0044448
2770	GO:0072431	GO:0098732	GO:0016265
2771	GO:0072358	GO:0098727	GO:0044446
2772	GO:0072338	GO:0098662	GO:0044440
2773	GO:0072216	GO:0098661	GO:0044432
2774	GO:0071936	GO:0001990	GO:0044427
2775	GO:0071897	GO:0098660	GO:0044425
2776	GO:0071826	GO:0098657	GO:0044424
2777	GO:0071705	GO:0098656	GO:0044422
2778	GO:0071702	GO:0098655	GO:0044420
2779	GO:0070887	GO:0098609	GO:0044309
2780	GO:0070723	GO:0098602	GO:0044306
2781	GO:0070663	GO:0098542	GO:0043679
2782	GO:0070647	GO:0098534	GO:0043601
2783	GO:0070195	GO:0097696	GO:0043296
2784	GO:0070085	GO:0097659	GO:0043235
2785	GO:0070034	GO:0001975	GO:0043229
2786	GO:0061198	GO:0097581	GO:0043226
2787	GO:0061140	GO:0097553	GO:0043209
2788	GO:0061098	GO:0097531	GO:0043202
2789	GO:0061045	GO:0097530	GO:0043198
2790	GO:0060765	GO:0097529	GO:0043189
2791	GO:0060670	GO:0097502	GO:0043025
2792	GO:0060429	GO:0097485	GO:0042995
2793	GO:0060402	GO:0097480	GO:0042827
2794	GO:0060216	GO:0097435	GO:0042645
2795	GO:0060071	GO:0097306	GO:0042641
2796	GO:0055082	GO:0001974	GO:0042599
2797	GO:0055080	GO:0097305	GO:0042579
2798	GO:0052200	GO:0097212	GO:0042567
2799	GO:0051983	GO:0097190	GO:0042564
2800	GO:0051960	GO:0097178	GO:0036477
2801	GO:0051930	GO:0097164	GO:0036464
2802	GO:0051781	GO:0097094	GO:0036454
2803	GO:0051640	GO:0097034	GO:0036019
2804	GO:0051604	GO:0097031	GO:0035770
2805	GO:0051570	GO:0090672	GO:0035098

2806	GO:0051495	GO:0090671	GO:0034774
2807	GO:0051481	GO:0001963	GO:0034703
2808	GO:0051438	GO:0090670	GO:0034702
2809	GO:0051431	GO:0090659	GO:0034457
2810	GO:0051352	GO:0090646	GO:0034456
2811	GO:0051349	GO:0090557	GO:0034455
2812	GO:0051346	GO:0090505	GO:0034399
2813	GO:0051235	GO:0090504	GO:0034364
2814	GO:0051222	GO:0090503	GO:0034358
2815	GO:0051216	GO:0090502	GO:0033276
2816	GO:0051208	GO:0090501	GO:0033267
2817	GO:0051174	GO:0090407	GO:0033202
2818	GO:0051091	GO:0001959	GO:0033186
2819	GO:0051048	GO:0090387	GO:0033178
2820	GO:0051046	GO:0090386	GO:0033116
2821	GO:0050926	GO:0090329	GO:0032993
2822	GO:0050920	GO:0090316	GO:0032806
2823	GO:0050877	GO:0090307	GO:0032592
2824	GO:0050817	GO:0090305	GO:0032545
2825	GO:0050810	GO:0090304	GO:0032421
2826	GO:0050790	GO:0090303	GO:0032154
2827	GO:0050728	GO:0090288	GO:0032040
2828	GO:0050715	GO:0090287	GO:0031988
2829	GO:0050671	GO:0001958	GO:0031983
2830	GO:0048742	GO:0090280	GO:0031970
2831	GO:0048639	GO:0090279	GO:0031967
2832	GO:0048583	GO:0090276	GO:0031966
2833	GO:0048511	GO:0090261	GO:0031965
2834	GO:0048285	GO:0090257	GO:0031904
2835	GO:0048247	GO:0090197	GO:0031901
2836	GO:0048167	GO:0090196	GO:0031588
2837	GO:0046950	GO:0090195	GO:0031526
2838	GO:0046850	GO:0090185	GO:0031519
2839	GO:0046849	GO:0090184	GO:0031515
2840	GO:0046700	GO:0001957	GO:0031461
2841	GO:0046487	GO:0090179	GO:0031306
2842	GO:0046128	GO:0090178	GO:0031301
2843	GO:0046058	GO:0090177	GO:0031300
2844	GO:0045992	GO:0090175	GO:0031256
2845	GO:0045935	GO:0090166	GO:0031252
2846	GO:0045927	GO:0090151	GO:0031248
2847	GO:0045861	GO:0090150	GO:0031232
2848	GO:0045840	GO:0090132	GO:1901698
2849	GO:0045807	GO:0090130	GO:0031231
2850	GO:0045777	GO:0090109	GO:0031228
2851	GO:0045664	GO:0001944	GO:0031227
2852	GO:0045661	GO:0090101	GO:0031226
2853	GO:0045596	GO:0090100	GO:0031225
2854	GO:0045595	GO:0090092	GO:0031012
2855	GO:0045259	GO:0090087	GO:0031010
2856	GO:0045155	GO:0090080	GO:0030964
2857	GO:0044853	GO:0090075	GO:0030934
2858	GO:0044849	GO:0090068	GO:0030914

2859	GO:0044843	GO:0090066	GO:0030894
2860	GO:0044773	GO:0090042	GO:0030880
2861	GO:0044765	GO:0090035	GO:0030863
2862	GO:0044711	GO:0001938	GO:0030849
2863	GO:0044707	GO:0090034	GO:0030692
2864	GO:0044459	GO:0090025	GO:0030689
2865	GO:0044283	GO:0090022	GO:0030687
2866	GO:0044281	GO:0090004	GO:0030684
2867	GO:0044265	GO:0090003	GO:0030669
2868	GO:0044257	GO:0090002	GO:0030667
2869	GO:0044236	GO:0086091	GO:0030666
2870	GO:0043900	GO:0086065	GO:0030662
2871	GO:0043648	GO:0086064	GO:0030496
2872	GO:0043587	GO:0086036	GO:0030176
2873	GO:0043566	GO:0001937	GO:0030173
2874	GO:0043426	GO:0086012	GO:0030140
2875	GO:0043087	GO:0086003	GO:0030136
2876	GO:0043068	GO:0086001	GO:0009628
2877	GO:0042976	GO:0080171	GO:0030133
2878	GO:0042770	GO:0080135	GO:0030027
2879	GO:0042592	GO:0080134	GO:0022627
2880	GO:0042129	GO:0080090	GO:0022626
2881	GO:0042113	GO:0075733	GO:0022625
2882	GO:0040013	GO:0075136	GO:0019908
2883	GO:0036481	GO:0072678	GO:0019898
2884	GO:0036442	GO:0001936	GO:0019866
2885	GO:0035927	GO:0072677	GO:0017053
2886	GO:0035861	GO:0072676	GO:0016942
2887	GO:0035587	GO:0072659	GO:0016605
2888	GO:0035337	GO:0072657	GO:0016589
2889	GO:0035035	GO:0072655	GO:0016581
2890	GO:0034442	GO:0072599	GO:0016514
2891	GO:0034364	GO:0072594	GO:0016323
2892	GO:0033627	GO:0072593	GO:0016281
2893	GO:0033619	GO:0072583	GO:0016020
2894	GO:0033280	GO:0072577	GO:0015935
2895	GO:0033043	GO:0001935	GO:0015030
2896	GO:0032963	GO:0072524	GO:0014704
2897	GO:0032845	GO:0072522	GO:0012505
2898	GO:0032640	GO:0072521	GO:0009986
2899	GO:0032637	GO:0072511	GO:0009925
2900	GO:0032553	GO:0072507	GO:0009898
2901	GO:0032535	GO:0072503	GO:0009295
2902	GO:0032355	GO:0072431	GO:0008328
2903	GO:0032147	GO:0072422	GO:0008024
2904	GO:0032103	GO:0072413	GO:0005947
2905	GO:0031983	GO:0072401	GO:0005938
2906	GO:0031701	GO:0001934	GO:0005929
2907	GO:0031492	GO:0072395	GO:0005924
2908	GO:0031281	GO:0072384	GO:0005923
2909	GO:0031280	GO:0072378	GO:0005902
2910	GO:0031232	GO:0072376	GO:0005901
2911	GO:0031055	GO:0072359	GO:0005900

2912	GO:0031011	GO:0072358	GO:0005896
2913	GO:0030947	GO:0072350	GO:0005879
2914	GO:0030686	GO:0072344	GO:0005856
2915	GO:0030593	GO:0072338	GO:0005852
2916	GO:0030515	GO:0072331	GO:0005829
2917	GO:0030332	GO:0001933	GO:0005815
2918	GO:0030323	GO:0072330	GO:0005814
2919	GO:0030315	GO:0072329	GO:0005802
2920	GO:0030170	GO:0072321	GO:0005797
2921	GO:0030135	GO:0072216	GO:0005796
2922	GO:0030099	GO:0072006	GO:0005795
2923	GO:0023057	GO:0071929	GO:0005794
2924	GO:0022857	GO:0071922	GO:0005791
2925	GO:0022838	GO:0071921	GO:0005788
2926	GO:0019438	GO:0071902	GO:0005783
2927	GO:0019221	GO:0071900	GO:0005779
2928	GO:0019220	GO:0001932	GO:0005778
2929	GO:0018279	GO:0071897	GO:0005777
2930	GO:0018076	GO:0071880	GO:0005776
2931	GO:0017136	GO:0071875	GO:0005770
2932	GO:0016884	GO:0071867	GO:0005768
2933	GO:0016830	GO:0071840	GO:0005764
2934	GO:0016608	GO:0071826	GO:0005762
2935	GO:0016580	GO:0071824	GO:0005761
2936	GO:0016568	GO:0071822	GO:0005759
2937	GO:0016485	GO:0071806	GO:0005754
2938	GO:0016337	GO:0071805	GO:0005753
2939	GO:0016300	hsa00562	GO:0005751
2940	GO:0016192	GO:0001911	GO:0005750
2941	GO:0016101	GO:0071804	GO:0005746
2942	GO:0016073	GO:0071803	GO:0005744
2943	GO:0016048	GO:0071773	GO:0005743
2944	GO:0015935	GO:0071772	GO:0005737
2945	GO:0015837	GO:0071706	GO:0005730
2946	GO:0015276	GO:0071705	GO:0005720
2947	GO:0015267	GO:0071704	GO:0005719
2948	GO:0010965	GO:0071702	GO:0005697
2949	GO:0010948	GO:0071695	GO:0005694
2950	GO:0010711	GO:0071692	GO:0005677
2951	GO:0010633	GO:0001909	GO:0005675
2952	GO:0010628	GO:0071675	GO:0005672
2953	GO:0010557	GO:0071674	GO:0005671
2954	GO:0010522	GO:0071635	GO:0005667
2955	GO:0010466	GO:0071634	GO:0005666
2956	GO:0009888	GO:0071622	GO:0005664
2957	GO:0009452	GO:0071621	GO:0005662
2958	GO:0009396	GO:0071616	GO:0005657
2959	GO:0009144	GO:0071604	GO:0005635
2960	GO:0008361	GO:0071593	GO:0005623
2961	GO:0008285	GO:0071560	GO:0005614
2962	GO:0008121	GO:0001906	GO:0005604
2963	GO:0008080	GO:0071559	GO:0005596
2964	GO:0008026	GO:0071549	GO:0005578

2965	GO:0007346	GO:0071548	GO:0005577
2966	GO:0007169	GO:0071502	GO:0005575
2967	GO:0007167	GO:0071495	GO:0001940
2968	GO:0007159	GO:0071478	GO:0001939
2969	GO:0007091	GO:0071466	GO:0001917
2970	GO:0006940	GO:0071459	GO:0001772
2971	GO:0006937	GO:0071442	GO:0001750
2972	GO:0006928	GO:0071440	GO:0001652
2973	GO:0006887	GO:0001894	GO:0001650
2974	GO:0006873	GO:0071436	GO:0000940
2975	GO:0006810	GO:0071431	GO:0000939
2976	GO:0006781	GO:0071428	GO:0000932
2977	GO:0006743	GO:0071427	GO:0000922
2978	GO:0006725	GO:0071426	GO:0000808
2979	GO:0006513	GO:0071417	GO:0000806
2980	GO:0006337	GO:0071392	GO:0000805
2981	GO:0006334	GO:0071391	GO:0000793
2982	GO:0006290	GO:0071385	GO:0000792
2983	GO:0006177	GO:0071384	GO:0000790
2984	GO:0006103	GO:0001893	GO:0000788
2985	GO:0006084	GO:0071383	GO:0000785
2986	GO:0005751	GO:0071378	GO:0000784
2987	GO:0005736	GO:0071377	GO:0000777
2988	GO:0005261	GO:0071363	GO:0000776
2989	GO:0004407	GO:0071354	GO:0000428
2990	GO:0004004	GO:0071345	GO:0000315
2991	GO:0003044	GO:0071322	GO:0000228
2992	GO:0002764	GO:0071320	GO:0000178
2993	GO:0002699	GO:0071310	GO:0000177
2994	GO:0002698	GO:0071260	GO:0000176
2995	GO:0002694	GO:0001890	GO:0000152
2996	GO:0002682	GO:0071174	GO:0000139
2997	GO:0002576	GO:0071173	GO:0000125
2998	GO:0002367	GO:0071168	GO:0000123
2999	GO:0002253	GO:0071166	GO:0000118
3000	GO:0002002	GO:0071158	GO:2001257
3001	GO:0001938	GO:0071157	GO:2001251
3002	GO:0001885	GO:0071156	GO:2001237
3003	GO:0001837	GO:0071104	GO:2001236
3004	GO:0001822	GO:0071103	GO:2001202
3005	GO:0001818	GO:0071051	GO:2001201
3006	GO:0001817	GO:0001889	GO:2001027
3007	GO:0001772	GO:0071049	GO:2001022
3008	GO:0001763	GO:0071047	GO:2001020
3009	GO:0001696	GO:0071046	GO:2000870
3010	GO:0001654	GO:0071043	GO:2000833
3011	GO:0001561	GO:0071038	GO:2000757
3012	GO:0001542	GO:0071035	GO:2000736
3013	GO:0001158	GO:0071034	GO:2000644
3014	GO:0001103	GO:0071033	GO:2000617
3015	GO:0001071	GO:0071031	GO:2000615
3016	GO:0000978	GO:0071029	GO:2000546
3017	GO:0000781	GO:0001887	GO:2000544

3018	GO:0000726	GO:0071028	GO:2000403
3019	GO:0000466	GO:0071027	GO:2000377
3020	GO:0000459	GO:0071025	GO:2000352
3021	GO:0000307	GO:0070987	GO:2000351
3022	GO:0000278	GO:0070977	GO:2000296
3023	GO:0000184	GO:0070972	GO:2000181
3024	ENSP00000369647	GO:0070933	GO:2000147
3025	ENSP00000352021	GO:0070932	GO:2000145
3026	ENSP00000346839	GO:0070925	GO:2000134
3027	ENSP00000333934	GO:0070911	GO:2000113
3028	ENSP00000328671	GO:0001885	GO:2000098
3029	ENSP00000295453	GO:0070901	GO:2000097
3030	ENSP00000295400	GO:0070900	GO:2000026
3031	ENSP00000283109	GO:0070897	GO:2000021
3032	ENSP00000263341	GO:0070887	GO:2000002
3033	ENSP00000263331	GO:0070848	GO:2000001
3034	ENSP00000258743	GO:0070846	GO:1990778
3035	ENSP00000258424	GO:0070844	GO:1990481
3036	hsa05202	GO:0070838	GO:1990402
3037	hsa04971	GO:0070830	GO:1990266
3038	hsa04932	GO:0070816	GO:1904894
3039	hsa04666	GO:0001878	GO:1904874
3040	hsa04640	GO:0070741	GO:1904851
3041	hsa04610	GO:0070734	GO:1904849
3042	hsa04110	GO:0070727	GO:1904847
3043	hsa04060	GO:0070723	GO:1904816
3044	hsa04015	GO:0070670	GO:1904814
3045	hsa00970	GO:0070665	GO:1904667
3046	hsa00650	GO:0070663	GO:1904666
3047	GO:2000401	GO:0070661	GO:1904591
3048	GO:2000352	GO:0070647	GO:1904377
3049	GO:2000027	GO:0070633	GO:1904375
3050	GO:2000026	GO:0001869	GO:1904237
3051	GO:1990837	GO:0070613	GO:1904235
3052	GO:1990778	GO:0070588	GO:1904064
3053	GO:1990402	GO:0070585	GO:1904029
3054	GO:1904849	GO:0070528	GO:1903911
3055	GO:1904062	GO:0070525	GO:1903909
3056	GO:1903911	GO:0070509	GO:1903902
3057	GO:1903426	GO:0070507	GO:1903845
3058	GO:1902806	GO:0070489	GO:1903844
3059	GO:1902774	GO:0070486	GO:1903793
3060	GO:1902582	GO:0070374	GO:1903753
3061	GO:1902580	GO:0001845	GO:1903751
3062	GO:1902106	GO:0070372	GO:1903750
3063	GO:1901988	GO:0070371	GO:1903725
3064	GO:1901293	GO:0070278	GO:1903706
3065	GO:1900122	GO:0070272	GO:1903530
3066	GO:0098803	GO:0070271	GO:1903523
3067	GO:0098727	GO:0070266	GO:1903513
3068	GO:0097306	GO:0070230	GO:1903510
3069	GO:0097094	GO:0070229	GO:1903509
3070	GO:0090279	GO:0070228	GO:1903426

3071	GO:0090257	GO:0070227	GO:1903409
3072	GO:0090196	GO:0001840	GO:1903337
3073	GO:0090177	GO:0070201	GO:1903335
3074	GO:0080134	GO:0070198	GO:1903322
3075	GO:0080090	GO:0070169	GO:1903320
3076	GO:0072678	GO:0070168	GO:1903318
3077	GO:0072524	GO:0070167	GO:1903317
3078	GO:0072511	GO:0070142	GO:0032386
3079	GO:0072395	GO:0070129	GO:1903313
3080	GO:0071929	GO:0070126	GO:1903310
3081	GO:0071880	GO:0070125	GO:1903308
3082	GO:0071824	GO:0070124	GO:1903169
3083	GO:0071803	GO:0001839	GO:1903078
3084	GO:0071674	GO:0070120	GO:1903076
3085	GO:0071310	GO:0070102	GO:1903055
3086	GO:0071166	GO:0070098	GO:1903051
3087	GO:0071156	GO:0070085	GO:1903047
3088	GO:0070900	GO:0070076	GO:1903039
3089	GO:0070665	GO:0070071	GO:1903037
3090	GO:0070577	GO:0065009	GO:1903035
3091	GO:0070330	GO:0065008	GO:1903034
3092	GO:0070278	GO:0065007	GO:1903019
3093	GO:0070061	GO:0065005	GO:1903008
3094	GO:0061572	GO:0001837	GO:1902998
3095	GO:0061035	GO:0065004	GO:1902996
3096	GO:0060713	GO:0065003	GO:1902993
3097	GO:0060401	GO:0065002	GO:1902953
3098	GO:0060076	GO:0061732	GO:1902949
3099	GO:0055074	GO:0061726	GO:1902947
3100	GO:0052312	GO:0061718	GO:1902679
3101	GO:0051961	GO:0061647	GO:1902656
3102	GO:0051917	GO:0061641	GO:1902624
3103	GO:0051896	GO:0061640	GO:1902622
3104	GO:0051784	GO:0061621	GO:1902593
3105	GO:0051704	GO:0001833	GO:1902580
3106	GO:0051573	GO:0061620	GO:1902572
3107	GO:0051351	GO:0061615	GO:1902571
3108	GO:0051336	GO:0061572	GO:1902533
3109	GO:0051272	GO:0061564	GO:1902476
3110	GO:0051252	GO:0061518	GO:1902430
3111	GO:0051187	GO:0061517	GO:1902403
3112	GO:0051169	GO:0061462	GO:1902400
3113	GO:0051128	GO:0061458	GO:1902305
3114	GO:0051098	GO:0061448	GO:1902275
3115	GO:0050909	GO:0061430	GO:1902105
3116	GO:0050880	GO:0001825	GO:1902100
3117	GO:0050870	GO:0061418	GO:1902099
3118	GO:0050866	GO:0061337	GO:1902042
3119	GO:0050857	GO:0061302	GO:1902004
3120	GO:0050820	GO:0061198	GO:1901998
3121	GO:0050778	GO:0061197	GO:1901992
3122	GO:0050767	GO:0061196	GO:1901987
3123	GO:0050731	GO:0061140	GO:1901983

3124	GO:0050708	GO:0061138	GO:1901888
3125	GO:0050686	GO:0061136	GO:1901881
3126	GO:0050679	GO:0061098	GO:1901879
3127	GO:0048856	GO:0001824	GO:1901800
3128	GO:0048732	GO:0061097	GO:1901796
3129	GO:0048553	GO:0061078	GO:1901675
3130	GO:0048518	GO:0061077	GO:1901674
3131	GO:0048468	GO:0061061	GO:1901663
3132	GO:0048254	GO:0061045	GO:1901661
3133	GO:0048245	GO:0061044	GO:1901659
3134	GO:0048188	GO:0061041	GO:1901652
3135	GO:0048144	GO:0061035	GO:0043066
3136	GO:0046942	GO:0061034	GO:1901616
3137	GO:0046939	GO:0061029	GO:1901615
3138	GO:0046888	GO:0001822	GO:1901605
3139	GO:0046653	GO:0061028	GO:1901522
3140	GO:0046545	GO:0061024	GO:1901379
3141	GO:0046365	GO:0061008	GO:1901343
3142	GO:0046040	GO:0060986	GO:1901185
3143	GO:0045943	GO:0060968	GO:1901164
3144	GO:0045930	GO:0060765	GO:1901137
3145	GO:0045841	GO:0060713	GO:1901136
3146	GO:0045762	GO:0060712	GO:1901135
3147	GO:0045761	GO:0060670	GO:1901094
3148	GO:0045669	GO:0060669	GO:1901068
3149	GO:0045666	GO:0001819	GO:1901016
3150	GO:0045580	GO:0060665	GO:1901006
3151	GO:0045445	GO:0060664	GO:1900864
3152	GO:0045254	GO:0060627	GO:1900544
3153	GO:0045252	GO:0060591	GO:1900542
3154	GO:0045188	GO:0060548	GO:1900426
3155	GO:0045087	GO:0060491	GO:1900424
3156	GO:0044818	GO:0060463	GO:1900373
3157	GO:0044786	GO:0060462	GO:1900372
3158	GO:0044772	GO:0060456	GO:1900371
3159	GO:0044764	GO:0060429	GO:1900274
3160	GO:0044454	GO:0001818	GO:1900135
3161	GO:0044060	GO:0060416	GO:1900133
3162	GO:0043997	GO:0060402	GO:1900122
3163	GO:0043983	GO:0060401	GO:1900120
3164	GO:0043968	GO:0060396	GO:1900116
3165	GO:0043604	GO:0060351	GO:1900034
3166	GO:0043408	GO:0060350	GO:0099531
3167	GO:0043405	GO:0060349	GO:0098900
3168	GO:0043271	GO:0060348	GO:0098801
3169	GO:0043066	GO:0060346	GO:0098771
3170	GO:0043065	GO:0060341	GO:0098761
3171	GO:0042573	GO:0001817	GO:0098756
3172	GO:0042516	GO:0060326	GO:0098743
3173	GO:0042451	GO:0060325	GO:0098742
3174	GO:0042359	GO:0060323	GO:0098739
3175	GO:0042327	GO:0060322	GO:0098732
3176	GO:0042307	GO:0060312	GO:0098727

3177	GO:0042306	GO:0060311	GO:0098662
3178	GO:0042110	GO:0060310	GO:0098661
3179	GO:0042033	GO:0060309	GO:0098657
3180	GO:0040007	GO:0060292	GO:0098656
3181	GO:0039519	GO:0060284	GO:0098542
3182	GO:0038021	GO:0001816	GO:0006793
3183	GO:0036477	GO:0060271	GO:0098534
3184	GO:0036265	GO:0060261	GO:0097581
3185	GO:0036065	GO:0060260	GO:0097553
3186	GO:0035812	GO:0060255	GO:0097531
3187	GO:0035384	GO:0060218	GO:0097480
3188	GO:0035295	GO:0060216	GO:0097435
3189	GO:0035025	GO:0060193	GO:0097306
3190	GO:0034766	GO:0060191	GO:0097305
3191	GO:0034511	GO:0060158	GO:0097212
3192	GO:0034508	GO:0060135	GO:0097178
3193	GO:0034332	GO:0001775	GO:0097164
3194	GO:0034109	GO:0060083	GO:0097094
3195	GO:0033160	GO:0060073	GO:0097034
3196	GO:0032990	GO:0060071	GO:0090672
3197	GO:0032964	GO:0060070	GO:0090671
3198	GO:0032945	GO:0060047	GO:0090670
3199	GO:0032786	GO:0060029	GO:0090659
3200	GO:0032774	GO:0060027	GO:0090557
3201	GO:0032675	GO:0060021	GO:0090505
3202	GO:0032612	GO:0055119	GO:0090504
3203	GO:0032606	GO:0055114	GO:0090503
3204	GO:0032559	GO:0001763	GO:0090502
3205	GO:0032502	GO:0055108	GO:0090407
3206	GO:0032413	GO:0055086	GO:0090387
3207	GO:0032269	GO:0055085	GO:0090386
3208	GO:0032102	GO:0055082	GO:0090316
3209	GO:0031639	GO:0055080	GO:0090307
3210	GO:0031589	GO:0055078	GO:0090303
3211	GO:0031326	GO:0055075	GO:0090280
3212	GO:0031324	GO:0055074	GO:0090276
3213	GO:0031323	GO:0055067	GO:0090197
3214	GO:0031093	GO:0055065	GO:0090196
3215	GO:0031061	GO:0001738	GO:0090195
3216	GO:0030968	GO:0052652	GO:0090185
3217	GO:0030809	GO:0052548	GO:0090179
3218	GO:0030803	GO:0052547	GO:0090178
3219	GO:0030801	GO:0052472	GO:0090177
3220	GO:0030688	GO:0052312	GO:0090166
3221	GO:0030513	GO:0052200	GO:0042802
3222	GO:0030510	GO:0052192	GO:0090151
3223	GO:0030509	GO:0052126	GO:0090150
3224	GO:0030501	GO:0051985	GO:0090132
3225	GO:0030449	GO:0051984	GO:0090109
3226	GO:0030335	GO:0001731	GO:0090101
3227	GO:0030330	GO:0051983	GO:0090080
3228	GO:0030212	GO:0051969	GO:0090066
3229	GO:0030194	GO:0051966	GO:0090042

3230	GO:0030162	GO:0051962	GO:0090035
3231	GO:0030154	GO:0051961	GO:0090034
3232	GO:0030055	GO:0051960	GO:0090025
3233	GO:0030030	GO:0051954	GO:0090022
3234	GO:0022890	GO:0051952	GO:0090004
3235	GO:0019955	GO:0051937	GO:0090003
3236	GO:0019538	GO:0051932	GO:0086091
3237	GO:0019369	GO:0001706	GO:0086065
3238	GO:0019219	GO:0051931	GO:0075733
3239	GO:0019218	GO:0051930	GO:0072655
3240	GO:0018130	GO:0051928	GO:0072599
3241	GO:0018108	GO:0051924	GO:0072593
3242	GO:0017158	GO:0051918	GO:0072583
3243	GO:0017157	GO:0051917	GO:0072511
3244	GO:0016832	GO:0051905	GO:0072507
3245	GO:0016645	GO:0051904	GO:0072422
3246	GO:0016624	GO:0051899	GO:0072413
3247	GO:0016571	GO:0051897	GO:0072378
3248	GO:0016569	GO:0001704	GO:0072376
3249	GO:0016538	GO:0051896	GO:0072359
3250	GO:0016514	GO:0051883	GO:0072350
3251	GO:0016125	GO:0051828	GO:0072344
3252	GO:0015985	GO:0051817	GO:0072338
3253	GO:0015833	GO:0051806	GO:0072331
3254	GO:0015031	GO:0051798	GO:0072330
3255	GO:0014911	GO:0051797	GO:0072321
3256	GO:0014066	GO:0051785	GO:0072216
3257	GO:0010870	GO:0051784	GO:0071929
3258	GO:0010817	GO:0051783	GO:0071921
3259	GO:0010755	GO:0001701	GO:0071902
3260	GO:0010714	GO:0051782	GO:0071897
3261	GO:0010629	GO:0051781	GO:0071880
3262	GO:0010605	GO:0051726	GO:0071875
3263	GO:0010573	GO:0051716	GO:0071826
3264	GO:0010558	GO:0051707	GO:0071824
3265	GO:0010534	GO:0051704	GO:0071822
3266	GO:0010468	GO:0051701	GO:0071806
3267	GO:0009991	GO:0051674	GO:0071805
3268	GO:0009967	GO:0051668	GO:0071804
3269	GO:0009897	GO:0051656	GO:0071773
3270	GO:0009894	GO:0001696	GO:0071772
3271	GO:0009890	GO:0051651	GO:0071706
3272	GO:0009792	GO:0051650	GO:0071695
3273	GO:0009636	GO:0051649	GO:0071692
3274	GO:0009260	GO:0051648	GO:0071675
3275	GO:0009218	GO:0051641	GO:0071634
3276	GO:0009199	GO:0051640	GO:0071621
3277	GO:0009145	GO:0051606	GO:0071616
3278	GO:0009135	GO:0051604	GO:0071604
3279	GO:0008585	GO:0051603	GO:0071593
3280	GO:0008284	GO:0051588	GO:0071560
3281	GO:0008283	GO:0001676	GO:0071549
3282	GO:0008276	GO:0051574	GO:0071548

3283	GO:0008236	GO:0051573	GO:0071502
3284	GO:0008227	GO:0051572	GO:0071495
3285	GO:0008206	GO:0051571	GO:0071466
3286	GO:0008203	GO:0051570	GO:0071442
3287	GO:0008176	GO:0051569	GO:0070670
3288	GO:0008168	GO:0051568	GO:0071440
3289	GO:0008104	GO:0051567	GO:0071431
3290	GO:0007610	GO:0051549	GO:0071428
3291	GO:0007568	GO:0051547	GO:0071426
3292	GO:0007431	GO:0001667	GO:0071385
3293	GO:0007229	GO:0051546	GO:0071384
3294	GO:0007219	GO:0051495	GO:0071383
3295	GO:0007215	GO:0051494	GO:0071378
3296	GO:0007212	GO:0051493	GO:0071377
3297	GO:0007210	GO:0051481	GO:0071363
3298	GO:0007067	GO:0051480	GO:0071354
3299	GO:0007045	GO:0051461	GO:0071322
3300	GO:0006978	GO:0051459	GO:0071320
3301	GO:0006950	GO:0051444	GO:0071168
3302	GO:0006915	GO:0051443	GO:0071166
3303	GO:0006836	GO:0001655	GO:0071158
3304	GO:0006796	GO:0051439	GO:0071157
3305	GO:0006757	GO:0051438	GO:0071156
3306	GO:0006631	GO:0051437	GO:0071104
3307	GO:0006282	GO:0051436	GO:0071103
3308	GO:0006271	GO:0051384	GO:0071051
3309	GO:0006167	GO:0051352	GO:0071049
3310	GO:0006152	GO:0051351	GO:0071047
3311	GO:0006122	GO:0051350	GO:0071046
3312	GO:0006111	GO:0051349	GO:0071038
3313	GO:0006104	GO:0051348	GO:0071035
3314	GO:0006102	GO:0001654	GO:0071034
3315	GO:0006098	GO:0051347	GO:0071033
3316	GO:0005746	GO:0051346	GO:0071029
3317	GO:0005549	GO:0051345	GO:0071027
3318	GO:0005524	GO:0051340	GO:0070972
3319	GO:0005178	GO:0051339	GO:0070932
3320	GO:0005138	GO:0051338	GO:0070900
3321	GO:0005126	GO:0051336	GO:0070846
3322	GO:0004906	GO:0051321	GO:0070844
3323	GO:0004812	GO:0051310	GO:0070830
3324	GO:0004045	GO:0051306	GO:0070816
3325	GO:0003018	GO:0001649	GO:0070741
3326	GO:0003013	GO:0051304	GO:0070723
3327	GO:0002805	GO:0051303	GO:0070665
3328	GO:0002740	GO:0051302	GO:0070663
3329	GO:0002689	GO:0051301	GO:0070661
3330	GO:0002521	GO:0051298	GO:0070647
3331	GO:0002376	GO:0051297	GO:0070633
3332	GO:0002225	GO:0051292	GO:0070528
3333	GO:0001944	GO:0051289	GO:0070509
3334	GO:0001936	GO:0051283	GO:0070507
3335	GO:0001933	GO:0051282	GO:0070486

3336	GO:0001911	GO:0001578	GO:0070374
3337	GO:0001741	GO:0051276	GO:0070372
3338	GO:0001706	GO:0051272	GO:0070278
3339	GO:0001655	GO:0051271	GO:0070272
3340	GO:0001567	GO:0051270	GO:0070266
3341	GO:0001523	GO:0051262	GO:0070230
3342	GO:0001047	GO:0051261	GO:0070229
3343	GO:0001046	GO:0051260	GO:0051092
3344	GO:0001025	GO:0051259	GO:0070228
3345	GO:0000470	GO:0051258	GO:0070227
3346	GO:0000275	GO:0051254	GO:0070201
3347	GO:0000183	GO:0001568	GO:0070168
3348	GO:0000166	GO:0051253	GO:0070126
3349	GO:0000083	GO:0051252	GO:0070124
3350	GO:0000076	GO:0051251	GO:0070120
3351	GO:0000060	GO:0051250	GO:0070102
3352	ENSP00000355436	GO:0051249	GO:0070098
3353	ENSP00000343505	GO:0051248	GO:0070085
3354	ENSP00000317578	GO:0051247	GO:0070076
3355	ENSP00000314193	GO:0051246	GO:0070071
3356	ENSP00000264498	GO:0051241	GO:0065009
3357	ENSP00000260563	GO:0051240	GO:0065008
3358	ENSP00000255030	GO:0001561	GO:0065005
3359	ENSP00000249075	GO:0051239	GO:0065003
3360	hsa05215	GO:0051238	GO:0065002
3361	hsa05205	GO:0051236	GO:0061732
3362	hsa05203	GO:0051235	GO:0061718
3363	hsa05142	GO:0051234	GO:0061647
3364	hsa04810	GO:0051225	GO:0061641
3365	hsa04750	GO:0051223	GO:0061640
3366	hsa03010	GO:0051222	GO:0010942
3367	GO:2001141	GO:0051220	GO:0061620
3368	GO:2000615	GO:0051216	GO:0061615
3369	GO:2000404	GO:0001558	GO:0061572
3370	GO:2000181	GO:0051209	GO:0061564
3371	GO:2000145	GO:0051208	GO:0061518
3372	GO:2000134	GO:0051188	GO:0061517
3373	GO:1990266	GO:0051187	GO:0061458
3374	GO:1904874	GO:0051186	GO:0061430
3375	GO:1904851	GO:0051179	GO:0061198
3376	GO:1903530	GO:0051174	GO:0061197
3377	GO:1903312	GO:0051173	GO:0061196
3378	GO:1902807	GO:0051172	GO:0061136
3379	GO:1902750	GO:0051171	GO:0061098
3380	GO:1902749	GO:0001542	GO:0061097
3381	GO:1902679	GO:0051170	GO:0061078
3382	GO:1902533	GO:0051169	GO:0061077
3383	GO:1902105	GO:0051168	GO:0061061
3384	GO:1901991	GO:0051156	GO:0061041
3385	GO:1901522	GO:0051148	GO:0061035
3386	GO:1901342	GO:0051131	GO:0061028
3387	GO:1901299	GO:0051130	GO:0061024
3388	GO:1901016	GO:0051129	GO:0060968

3389	GO:1900084	GO:0051128	GO:0060765
3390	GO:1900003	GO:0051123	GO:0060713
3391	GO:0099531	GO:0001525	GO:0060669
3392	GO:0098857	GO:0051101	GO:0060664
3393	GO:0098771	GO:0051098	GO:0060627
3394	GO:0098655	GO:0051094	GO:0060591
3395	GO:0098602	GO:0051093	GO:2001233
3396	GO:0097659	GO:0051092	GO:0060491
3397	GO:0097553	GO:0051091	GO:0060463
3398	GO:0097531	GO:0051090	GO:0060462
3399	GO:0090386	GO:0051081	GO:0060456
3400	GO:0090280	GO:0051057	GO:0060429
3401	GO:0090130	GO:0051056	GO:0060416
3402	GO:0090066	GO:0001523	GO:0060396
3403	GO:0086012	GO:0051053	GO:0060351
3404	GO:0086003	GO:0051052	GO:0060350
3405	GO:0072593	GO:0051051	GO:1902531
3406	GO:0072507	GO:0051050	GO:0060349
3407	GO:0072344	GO:0051049	GO:0060348
3408	GO:0071921	GO:0051048	GO:0060346
3409	GO:0071622	GO:0051047	GO:0060325
3410	GO:0071442	GO:0051046	GO:0060323
3411	GO:0071428	GO:0051045	GO:0060312
3412	GO:0071417	GO:0051044	GO:0060311
3413	GO:0071391	GO:0001522	GO:0060310
3414	GO:0071378	GO:0051043	GO:0060309
3415	GO:0071158	GO:0051031	GO:0060292
3416	GO:0071062	GO:0051028	GO:0060284
3417	GO:0071031	GO:0051017	GO:0060271
3418	GO:0071027	GO:0051001	GO:0060261
3419	GO:0070371	GO:0050999	GO:0060260
3420	GO:0070229	GO:0050965	GO:0060218
3421	GO:0070228	GO:0050962	GO:0060216
3422	GO:0070181	GO:0050961	GO:0060191
3423	GO:0070120	GO:0050954	GO:0060135
3424	GO:0065007	GO:0001510	GO:0060083
3425	GO:0061617	GO:0050951	GO:0060073
3426	GO:0061430	GO:0050930	GO:0060071
3427	GO:0061138	GO:0050927	GO:0060029
3428	GO:0060664	GO:0050926	GO:0060027
3429	GO:0060284	GO:0050921	GO:0060021
3430	GO:0060193	GO:0050920	GO:0055119
3431	GO:0055086	GO:0050918	GO:0055114
3432	GO:0055075	GO:0050917	GO:0055108
3433	GO:0051962	GO:0050911	GO:0055082
3434	GO:0051952	GO:0050909	GO:0055080
3435	GO:0051924	GO:0001508	GO:0055078
3436	GO:0051480	GO:0050907	GO:0055075
3437	GO:0051443	GO:0050906	GO:0055074
3438	GO:0051347	GO:0050901	GO:0055067
3439	GO:0051339	GO:0050900	GO:0052652
3440	GO:0051292	GO:0050896	GO:0052312
3441	GO:0051271	GO:0050890	GO:0052200

3442	GO:0051250	GO:0050886	GO:0052192
3443	GO:0051246	GO:0050880	GO:0052126
3444	GO:0051209	GO:0050878	GO:0051984
3445	GO:0051173	GO:0050877	GO:0051969
3446	GO:0051156	GO:0001505	GO:0051966
3447	GO:0051129	GO:0050871	GO:0051962
3448	GO:0051051	GO:0050870	GO:0002253
3449	GO:0051049	GO:0050868	GO:0051961
3450	GO:0050927	GO:0050867	GO:0051960
3451	GO:0050901	GO:0050866	GO:0051952
3452	GO:0050868	GO:0050865	GO:0051937
3453	GO:0048870	GO:0050863	GO:0051932
3454	GO:0048869	GO:0050857	GO:0051931
3455	GO:0048812	GO:0050856	GO:0051930
3456	GO:0048666	GO:0050854	GO:0051928
3457	GO:0048659	GO:0001503	GO:0051918
3458	GO:0048017	GO:0050852	GO:0051917
3459	GO:0046879	GO:0050851	GO:0051905
3460	GO:0046660	GO:0050848	GO:0051904
3461	GO:0045944	GO:0050832	GO:0051897
3462	GO:0045934	GO:0050830	GO:0051896
3463	GO:0045893	GO:0050829	GO:0051883
3464	GO:0045860	GO:0050820	GO:0051828
3465	GO:0045839	GO:0050819	GO:0051806
3466	GO:0045785	GO:0050818	GO:0051798
3467	GO:0045321	GO:0050817	GO:0051797
3468	GO:0045277	GO:0001502	GO:0051785
3469	GO:0045165	GO:0050812	GO:0051784
3470	GO:0044767	GO:0050810	GO:0051783
3471	GO:0044259	GO:0050806	GO:0051782
3472	GO:0044238	GO:0050805	GO:0051781
3473	GO:0044154	GO:0050804	GO:0051726
3474	GO:0044092	GO:0050803	GO:0051716
3475	GO:0044062	GO:0050802	GO:0051707
3476	GO:0043603	GO:0050801	GO:0051701
3477	GO:0043549	GO:0050796	GO:0051674
3478	GO:0043527	GO:0050795	GO:0051656
3479	GO:0043461	GO:0001501	GO:0051650
3480	GO:0043436	GO:0050794	GO:0051641
3481	GO:0043025	GO:0050793	GO:0051640
3482	GO:0042737	GO:0050792	GO:0051573
3483	GO:0042564	GO:0050790	GO:0051572
3484	GO:0042509	GO:0050789	GO:0051569
3485	GO:0042476	GO:0050778	GO:0051567
3486	GO:0040017	GO:0050777	GO:0051549
3487	GO:0040008	GO:0050776	GO:0051547
3488	GO:0038111	GO:0050767	GO:0051495
3489	GO:0035914	GO:0050755	GO:0051493
3490	GO:0035637	GO:0001306	GO:0051461
3491	GO:0035272	GO:0050748	GO:0051444
3492	GO:0035257	GO:0050731	GO:0051443
3493	GO:0034767	GO:0050730	GO:0051439
3494	GO:0034764	GO:0050729	GO:0051436

3495	GO:0034762	GO:0050728	GO:0051352
3496	GO:0034654	GO:0050727	GO:0051350
3497	GO:0034645	GO:0050715	GO:0051349
3498	GO:0034443	GO:0050714	GO:0051348
3499	GO:0034388	GO:0050710	GO:0051346
3500	GO:0034243	GO:0050709	GO:0051338
3501	GO:0034220	GO:0001189	GO:0051310
3502	GO:0034212	GO:0050708	GO:0051306
3503	GO:0033688	GO:0050707	GO:0051304
3504	GO:0033674	GO:0050686	GO:0045087
3505	GO:0033615	GO:0050685	GO:0051302
3506	GO:0033554	GO:0050684	GO:0051301
3507	GO:0033539	GO:0050680	GO:0051297
3508	GO:0033319	GO:0050679	GO:0051292
3509	GO:0033177	GO:0050678	GO:0051289
3510	GO:0033135	GO:0050673	GO:0051283
3511	GO:0032940	GO:0050671	GO:0051282
3512	GO:0032881	GO:0001188	GO:0051259
3513	GO:0032879	GO:0050670	GO:0051253
3514	GO:0032844	GO:0050663	GO:0051251
3515	GO:0032602	GO:0050658	GO:0051250
3516	GO:0032508	GO:0050657	GO:0051249
3517	GO:0032451	GO:0050654	GO:0051247
3518	GO:0032226	GO:0050435	GO:0051246
3519	GO:0031941	GO:0050434	GO:0051241
3520	GO:0031821	GO:0050432	GO:0051238
3521	GO:0031401	GO:0050000	GO:0051235
3522	GO:0031325	GO:0048878	GO:0051223
3523	GO:0031295	GO:0001180	GO:0051222
3524	GO:0031062	GO:0048871	GO:0051220
3525	GO:0031058	GO:0048870	GO:0051209
3526	GO:0030934	GO:0048869	GO:0051188
3527	GO:0030823	GO:0048863	GO:0051187
3528	GO:0030538	GO:0048861	GO:0051156
3529	GO:0030522	GO:0048858	GO:0051148
3530	GO:0030425	GO:0048856	GO:0051131
3531	GO:0030200	GO:0048840	GO:0051128
3532	GO:0030168	GO:0048812	GO:0051123
3533	GO:0030167	GO:0048771	GO:0051101
3534	GO:0030155	GO:0001101	GO:0051098
3535	GO:0030073	GO:0048742	GO:0051094
3536	GO:0023056	GO:0048732	GO:0051093
3537	GO:0022904	GO:0048731	GO:0051090
3538	GO:0022627	GO:0048729	GO:0060548
3539	GO:0022610	GO:0048705	GO:0051081
3540	GO:0022602	GO:0048699	GO:0051047
3541	GO:0022414	GO:0048667	GO:0051045
3542	GO:0022008	GO:0048666	GO:0051044
3543	GO:0020037	GO:0048661	GO:0051043
3544	GO:0019908	GO:0048660	GO:0051017
3545	GO:0019693	GO:0000967	GO:0051001
3546	GO:0019068	GO:0048659	GO:0050999
3547	GO:0018216	GO:0048646	GO:0050965

3548	GO:0016896	GO:0048640	GO:0050961
3549	GO:0016817	GO:0048639	GO:0050930
3550	GO:0016602	GO:0048609	GO:0050926
3551	GO:0016584	GO:0048608	GO:0050920
3552	GO:0016055	GO:0048598	GO:0050918
3553	GO:0015849	GO:0048589	GO:0050901
3554	GO:0015464	GO:0048585	GO:0050868
3555	GO:0014912	GO:0048584	GO:0050866
3556	GO:0014909	GO:0000966	GO:0050863
3557	GO:0014848	GO:0048583	GO:0050857
3558	GO:0014069	GO:0048568	GO:0050856
3559	GO:0010972	GO:0048553	GO:0050852
3560	GO:0010951	GO:0048545	GO:0050851
3561	GO:0010942	GO:0048534	GO:0050832
3562	GO:0010810	GO:0048524	GO:0050829
3563	GO:0010647	GO:0048523	GO:0050806
3564	GO:0010632	GO:0048522	GO:0050805
3565	GO:0010576	GO:0048521	GO:0050803
3566	GO:0010556	GO:0048520	GO:0050801
3567	GO:0010543	GO:0000963	GO:0050796
3568	GO:0010517	GO:0048519	GO:0050795
3569	GO:0010389	GO:0048518	GO:0050792
3570	GO:0009898	GO:0048514	GO:0050790
3571	GO:0009891	GO:0048513	GO:0050778
3572	GO:0009653	GO:0048511	GO:0050767
3573	GO:0009259	GO:0048489	GO:0050748
3574	GO:0009206	GO:0048468	GO:0050729
3575	GO:0009205	GO:0048387	GO:0050728
3576	GO:0009156	GO:0048385	GO:0050727
3577	GO:0009141	GO:0048384	GO:0050710
3578	GO:0009117	GO:0000959	GO:0050707
3579	GO:0009112	GO:0048285	GO:0050680
3580	GO:0009111	GO:0048263	GO:0050678
3581	GO:0009068	GO:0048262	GO:0050670
3582	GO:0009065	GO:0048260	GO:0050658
3583	GO:0008395	GO:0048259	GO:0050657
3584	GO:0007420	GO:0048254	GO:0050654
3585	GO:0007194	GO:0048247	GO:0050432
3586	GO:0007190	GO:0048246	GO:0050000
3587	GO:0007178	GO:0048245	GO:0048878
3588	GO:0007044	GO:0048243	GO:0048871
3589	GO:0006883	GO:0000956	GO:0048870
3590	GO:0006875	GO:0048232	GO:0048863
3591	GO:0006780	GO:0048193	GO:0048840
3592	GO:0006754	GO:0048167	GO:0048742
3593	GO:0006739	GO:0048146	GO:0048732
3594	GO:0006735	GO:0048144	GO:0048589
3595	GO:0006701	GO:0048041	GO:0048568
3596	GO:0006614	GO:0048017	GO:0048553
3597	GO:0006544	GO:0048011	GO:0048520
3598	GO:0006541	GO:0048010	GO:0048511
3599	GO:0006509	GO:0048009	GO:0048489
3600	GO:0006482	GO:0000910	GO:0048387

3601	GO:0006476	GO:0048002	GO:0048385
3602	GO:0006450	GO:0046950	GO:0048262
3603	GO:0006414	GO:0046949	GO:0048260
3604	GO:0006409	GO:0046942	GO:0048254
3605	GO:0006402	GO:0046939	GO:0048247
3606	GO:0006369	GO:0046931	GO:0048246
3607	GO:0006362	GO:0046928	GO:0048245
3608	GO:0006357	GO:0046909	GO:0048146
3609	GO:0006355	GO:0046907	GO:0048144
3610	GO:0006351	GO:0046903	GO:0048041
3611	GO:0006335	GO:0000904	GO:0048017
3612	GO:0006303	GO:0046888	GO:0048009
3613	GO:0006164	GO:0046883	GO:0048002
3614	GO:0006101	GO:0046879	GO:0046942
3615	GO:0006096	GO:0046851	GO:0046939
3616	GO:0006024	GO:0046850	GO:0046931
3617	GO:0005744	GO:0046849	GO:0046928
3618	GO:0005677	GO:0046847	GO:0046909
3619	GO:0005671	GO:0046824	GO:0046903
3620	GO:0005201	GO:0046822	GO:0046888
3621	GO:0004913	GO:0046794	GO:0046879
3622	GO:0004518	GO:0000902	GO:0046851
3623	GO:0004448	GO:0046785	GO:0046850
3624	GO:0004129	GO:0046782	GO:0046849
3625	GO:0003678	GO:0046718	GO:0046847
3626	GO:0003417	GO:0046717	GO:1903649
3627	GO:0003081	GO:0046710	GO:0046824
3628	GO:0002791	GO:0046700	GO:0046822
3629	GO:0002790	GO:0046697	GO:0046718
3630	GO:0002779	GO:0046689	GO:0046717
3631	GO:0002701	GO:0046660	GO:0046700
3632	GO:0002685	GO:0046653	GO:0046697
3633	GO:0002673	GO:0000819	GO:0046660
3634	GO:0002223	GO:0046651	GO:0046653
3635	GO:0001968	GO:0046649	GO:0046599
3636	GO:0001940	GO:0046605	GO:0046579
3637	GO:0001906	GO:0046599	GO:0046578
3638	GO:0001878	GO:0046579	GO:0046546
3639	GO:0001667	GO:0046578	GO:0046541
3640	GO:0001522	GO:0046546	GO:0046502
3641	GO:0001190	GO:0046545	GO:0046487
3642	GO:0000451	GO:0046541	GO:0046440
3643	GO:0000179	GO:0046502	GO:0046427
3644	GO:0000165	GO:0000729	GO:0046395
3645	GO:0000075	GO:0046496	GO:0046364
3646	ENSP00000416330	GO:0046488	GO:0046330
3647	ENSP00000408153	GO:0046487	GO:0046165
3648	ENSP00000403536	GO:0046483	GO:0046164
3649	ENSP00000377446	GO:0046467	GO:0046160
3650	ENSP00000362105	GO:0046440	GO:0046148
3651	ENSP00000360828	GO:0046427	GO:0046103
3652	ENSP00000358812	GO:0046425	GO:0046102
3653	ENSP00000358563	GO:0046395	GO:0046049

3654	ENSP00000342056	GO:0046394	GO:0045995
3655	ENSP00000319771	GO:0000726	GO:0045992
3656	ENSP00000312652	GO:0046390	GO:0045953
3657	ENSP00000311977	GO:0046365	GO:0045943
3658	ENSP00000309565	GO:0046364	GO:0051704
3659	ENSP00000308938	GO:0046330	GO:0045933
3660	ENSP00000290866	GO:0046209	GO:0045932
3661	ENSP00000263339	GO:0046165	GO:0045930
3662	ENSP00000263239	GO:0046164	GO:0045927
3663	ENSP00000261406	GO:0046160	GO:0045926
3664	ENSP00000234170	GO:0046148	GO:0045921
3665	ENSP00000222567	GO:0046132	GO:0045907
3666	ENSP00000216254	GO:0000725	GO:0045906
3667	hsa05144	GO:0046129	GO:0045903
3668	hsa04723	GO:0046128	GO:0045860
3669	hsa03020	GO:0046112	GO:0045859
3670	hsa00980	GO:0046103	GO:0045840
3671	hsa00670	GO:0046102	GO:0045839
3672	hsa00533	GO:0046085	GO:0045834
3673	GO:2000833	GO:0046058	GO:0045815
3674	GO:2000757	GO:0046049	GO:0045786
3675	GO:2000756	GO:0046040	GO:0045785
3676	GO:2000403	GO:0046037	GO:0045778
3677	GO:2000257	GO:0000724	GO:0045777
3678	GO:2000113	GO:0046034	GO:0045766
3679	GO:1990748	GO:0046031	GO:0045765
3680	GO:1903555	GO:0045995	GO:0045762
3681	GO:1903531	GO:0045992	GO:0045761
3682	GO:1903310	GO:0045991	GO:0045760
3683	GO:1902998	GO:0045988	GO:0045739
3684	GO:1902680	GO:0045981	GO:0045732
3685	GO:1902531	GO:0045953	GO:0045721
3686	GO:1902476	GO:0045944	GO:0045666
3687	GO:1902275	GO:0045943	GO:0045637
3688	GO:1902099	GO:0000723	GO:0045621
3689	GO:1901701	GO:0045940	GO:0045597
3690	GO:1901698	GO:0045937	GO:0045582
3691	GO:1901657	GO:0045935	GO:0045454
3692	GO:1901576	GO:0045934	GO:0045446
3693	GO:0099528	GO:0045933	GO:0045329
3694	GO:0098562	GO:0045932	GO:0045324
3695	GO:0097581	GO:0045930	GO:0045321
3696	GO:0097530	GO:0045927	GO:0045216
3697	GO:0097529	GO:0045926	GO:0045188
3698	GO:0097025	GO:0045921	GO:0045187
3699	GO:0090505	GO:0000715	GO:0045176
3700	GO:0090184	GO:0045907	GO:0045123
3701	GO:0090178	GO:0045906	GO:0045055
3702	GO:0090087	GO:0045903	GO:0045047
3703	GO:0090003	GO:0045893	GO:0045046
3704	GO:0072657	GO:0045892	GO:0045039
3705	GO:0072588	GO:0045862	GO:0045022
3706	GO:0072413	GO:0045861	GO:0045005

3707	GO:0072401	GO:0045860	GO:0044851
3708	GO:0071900	GO:0045859	GO:0044849
3709	GO:0071772	GO:0045841	GO:0044843
3710	GO:0071692	GO:0000480	GO:0044818
3711	GO:0071604	GO:0045840	GO:0044802
3712	GO:0071560	GO:0045839	GO:0044783
3713	GO:0071502	GO:0045834	GO:0044782
3714	GO:0071495	GO:0045815	GO:0044774
3715	GO:0071440	GO:0045814	GO:0044770
3716	GO:0071363	GO:0045807	GO:0044766
3717	GO:0071173	GO:0045787	GO:0044765
3718	GO:0071157	GO:0045786	GO:0044706
3719	GO:0071047	GO:0045785	GO:0044703
3720	GO:0071033	GO:0045778	GO:0010941
3721	GO:0070993	GO:0000479	GO:0044364
3722	GO:0070816	GO:0045777	GO:0044344
3723	GO:0070761	GO:0045766	GO:0044273
3724	GO:0070615	GO:0045765	GO:0044270
3725	GO:0070585	GO:0045762	GO:0044267
3726	GO:0070509	GO:0045761	GO:0044259
3727	GO:0070126	GO:0045760	GO:0044248
3728	GO:0070110	GO:0045744	GO:0044243
3729	GO:0070102	GO:0045739	GO:0044242
3730	GO:0065009	GO:0045732	GO:0044092
3731	GO:0061733	GO:0045721	GO:0044089
3732	GO:0061647	GO:0000478	GO:0044085
3733	GO:0061061	GO:0045669	GO:0044070
3734	GO:0060462	GO:0045667	GO:0044062
3735	GO:0060351	GO:0045666	GO:0044060
3736	GO:0060341	GO:0045664	GO:0044058
3737	GO:0060083	GO:0045662	GO:0043983
3738	GO:0055065	GO:0045661	GO:0043974
3739	GO:0052890	GO:0045637	GO:0043970
3740	GO:0052652	GO:0045621	GO:0043969
3741	GO:0051937	GO:0045619	GO:0043932
3742	GO:0051897	GO:0045597	GO:0043921
3743	GO:0051798	GO:0000472	GO:0043903
3744	GO:0051786	GO:0045596	GO:0043902
3745	GO:0051649	GO:0045595	GO:0043901
3746	GO:0051547	GO:0045582	GO:0043648
3747	GO:0051444	GO:0045580	GO:0043634
3748	GO:0051338	GO:0045454	GO:0043633
3749	GO:0051306	GO:0045453	GO:0043627
3750	GO:0051282	GO:0045446	GO:0043624
3751	GO:0051253	GO:0045445	GO:0043618
3752	GO:0051249	GO:0045428	GO:0043604
3753	GO:0051239	GO:0045333	GO:0043588
3754	GO:0051172	GO:0000470	GO:0043587
3755	GO:0051170	GO:0045329	GO:0043568
3756	GO:0051130	GO:0045324	GO:0043567
3757	GO:0051044	GO:0045321	GO:0043543
3758	GO:0051017	GO:0045216	GO:0043488
3759	GO:0050961	GO:0045188	GO:0043486

3760	GO:0050930	GO:0045187	GO:0043461
3761	GO:0050921	GO:0045185	GO:0043434
3762	GO:0050878	GO:0045184	GO:0043433
3763	GO:0050777	GO:0045176	GO:0043414
3764	GO:0050710	GO:0045165	GO:0043412
3765	GO:0048771	GO:0000469	GO:0043408
3766	GO:0048660	GO:0045137	GO:0043406
3767	GO:0048585	GO:0045123	GO:0043388
3768	GO:0048534	GO:0045087	GO:0043269
3769	GO:0048514	GO:0045055	GO:0043268
3770	GO:0048262	GO:0045047	GO:0006950
3771	GO:0048243	GO:0045046	GO:0043266
3772	GO:0046651	GO:0045039	GO:0043254
3773	GO:0046554	GO:0045022	GO:0043244
3774	GO:0046209	GO:0045005	GO:0043241
3775	GO:0045953	GO:0044851	GO:0043207
3776	GO:0045940	GO:0000467	GO:0043129
3777	GO:0045892	GO:0044849	GO:0043114
3778	GO:0045597	GO:0044843	GO:0043112
3779	GO:0045428	GO:0044839	GO:0043085
3780	GO:0044851	GO:0044819	GO:0043069
3781	GO:0044784	GO:0044818	GO:0043065
3782	GO:0044774	GO:0044802	GO:0043044
3783	GO:0044093	GO:0044786	GO:0043043
3784	GO:0043932	GO:0044784	GO:0043032
3785	GO:0043633	GO:0044783	GO:0042981
3786	GO:0043491	GO:0044782	GO:0042977
3787	GO:0043393	GO:0000466	GO:0042976
3788	GO:0043194	GO:0044774	GO:0042776
3789	GO:0043177	GO:0044773	GO:0042773
3790	GO:0043161	GO:0044772	GO:0042772
3791	GO:0043069	GO:0044770	GO:0042771
3792	GO:0043067	GO:0044767	GO:0042748
3793	GO:0042981	GO:0044766	GO:0042744
3794	GO:0042800	GO:0044765	GO:0042743
3795	GO:0042776	GO:0044764	GO:0042742
3796	GO:0042772	GO:0044763	GO:0042738
3797	GO:0042698	GO:0044743	GO:0042737
3798	GO:0042626	GO:0000463	GO:0042699
3799	GO:0042503	GO:0044728	GO:0042635
3800	GO:0042107	GO:0044724	GO:0042592
3801	GO:0042104	GO:0044723	GO:0042558
3802	GO:0042098	GO:0044712	GO:0042534
3803	GO:0042089	GO:0044711	GO:0042533
3804	GO:0040012	GO:0044710	GO:0042531
3805	GO:0040011	GO:0044708	GO:0042516
3806	GO:0038164	GO:0044707	GO:0042503
3807	GO:0038162	GO:0044706	GO:0042493
3808	GO:0038094	GO:0044703	GO:0042476
3809	GO:0038043	GO:0000462	GO:0042455
3810	GO:0036002	GO:0044702	GO:0042451
3811	GO:0035948	GO:0044700	GO:0042448
3812	GO:0035947	GO:0044699	GO:0042446

3813	GO:0035815	GO:0044419	GO:0042445
3814	GO:0035766	GO:0044409	GO:0042407
3815	GO:0035490	GO:0044403	GO:0002684
3816	GO:0035258	GO:0044364	GO:0042398
3817	GO:0035246	GO:0044344	GO:0042384
3818	GO:0035242	GO:0044283	GO:0042363
3819	GO:0034968	GO:0044282	GO:0042359
3820	GO:0034603	GO:0000460	GO:0042339
3821	GO:0034472	GO:0044281	GO:0042278
3822	GO:0034462	GO:0044273	GO:0042276
3823	GO:0034427	GO:0044272	GO:0042274
3824	GO:0033559	GO:0044271	GO:0042273
3825	GO:0032965	GO:0044270	GO:0042255
3826	GO:0032943	GO:0044267	GO:0042249
3827	GO:0032722	GO:0044265	GO:0042246
3828	GO:0032680	GO:0044260	GO:0042181
3829	GO:0032571	GO:0044259	GO:0042176
3830	GO:0032549	GO:0044257	GO:0042168
3831	GO:0032270	GO:0000459	GO:0042160
3832	GO:0031571	GO:0044255	GO:0042157
3833	GO:0031400	GO:0044253	GO:0042147
3834	GO:0031399	GO:0044249	GO:0042127
3835	GO:0031327	GO:0044248	GO:0042113
3836	GO:0031175	GO:0044246	GO:0042104
3837	GO:0031125	GO:0044243	GO:0042098
3838	GO:0030849	GO:0044242	GO:0042059
3839	GO:0030832	GO:0044238	GO:0042036
3840	GO:0030814	GO:0044237	GO:0042035
3841	GO:0030810	GO:0044236	GO:0042033
3842	GO:0030808	GO:0000453	GO:0040029
3843	GO:0030804	GO:0044205	GO:0040012
3844	GO:0030673	GO:0044154	GO:0040011
3845	GO:0030523	GO:0044093	GO:0040008
3846	GO:0030500	GO:0044092	GO:0040007
3847	GO:0030336	GO:0044089	GO:0039519
3848	GO:0030334	GO:0044087	GO:0038165
3849	GO:0030321	GO:0044085	GO:0038162
3850	GO:0030217	GO:0044070	GO:0038156
3851	GO:0030097	GO:0044062	GO:0038155
3852	GO:0030072	GO:0044060	GO:0038127
3853	GO:0030071	GO:0000451	GO:0038114
3854	GO:0022407	GO:0044058	GO:0038111
3855	GO:0019961	GO:0044057	GO:0038096
3856	GO:0018212	GO:0044033	GO:0038094
3857	GO:0016818	GO:0043983	GO:0038044
3858	GO:0016684	GO:0043974	GO:0036481
3859	GO:0016679	GO:0043970	GO:0036314
3860	GO:0016433	GO:0043969	GO:0036297
3861	GO:0016428	GO:0043968	GO:0036276
3862	GO:0016093	GO:0043967	GO:0036265
3863	GO:0015853	GO:0043966	GO:0036260
3864	GO:0014068	GO:0000447	GO:0036124
3865	GO:0014065	GO:0043933	GO:0036123

3866	GO:0014061	GO:0043932	GO:0036066
3867	GO:0010757	GO:0043931	GO:0036065
3868	GO:0010720	GO:0043923	GO:0035988
3869	GO:0010646	GO:0043921	GO:0035987
3870	GO:0010562	GO:0043903	GO:0035966
3871	GO:0010535	GO:0043902	GO:0035948
3872	GO:0010518	GO:0043901	GO:0035947
3873	GO:0010107	GO:0043900	GO:0035927
3874	GO:0010035	GO:0043687	GO:0035887
3875	GO:0009966	GO:0000398	GO:0035821
3876	GO:0009887	GO:0043648	GO:0035815
3877	GO:0009719	GO:0043634	GO:0035812
3878	GO:0009582	GO:0043633	GO:0035768
3879	GO:0009220	GO:0043632	GO:0035767
3880	GO:0009190	GO:0043628	GO:0035766
3881	GO:0009185	GO:0043627	GO:0035630
3882	GO:0009167	GO:0043624	GO:0035601
3883	GO:0009165	GO:0043623	GO:0035590
3884	GO:0009150	GO:0043618	GO:0035588
3885	GO:0009116	GO:0043604	GO:0035567
3886	GO:0009019	GO:0000381	GO:0035563
3887	GO:0008545	GO:0043603	GO:0035561
3888	GO:0008528	GO:0043588	GO:0035491
3889	GO:0008401	GO:0043587	GO:0035490
3890	GO:0008219	GO:0043574	GO:0035437
3891	GO:0008009	GO:0043568	GO:0035412
3892	GO:0007548	GO:0043567	GO:0035411
3893	GO:0007435	GO:0043549	GO:0035383
3894	GO:0007409	GO:0043547	GO:0035337
3895	GO:0007399	GO:0043543	GO:0035295
3896	GO:0007266	GO:0043491	GO:0035272
3897	GO:0007260	GO:0000380	GO:0035270
3898	GO:0007202	GO:0043488	GO:0035249
3899	GO:0007166	GO:0043487	GO:0035247
3900	GO:0007050	GO:0043486	GO:0035246
3901	GO:0006982	GO:0043484	GO:0035235
3902	GO:0006953	GO:0043461	GO:0035067
3903	GO:0006703	GO:0043436	GO:0035065
3904	GO:0006554	GO:0043434	GO:0035025
3905	GO:0006553	GO:0043433	GO:0035019
3906	GO:0006551	GO:0043414	GO:0034983
3907	GO:0006259	GO:0043413	GO:0034976
3908	GO:0006189	GO:0000377	GO:0034975
3909	GO:0006165	GO:0043412	GO:0034968
3910	GO:0006140	GO:0043410	GO:0034767
3911	GO:0006107	GO:0043408	GO:0034765
3912	GO:0006085	GO:0043406	GO:0034764
3913	GO:0006027	GO:0043405	GO:0034762
3914	GO:0005753	GO:0043401	GO:0034754
3915	GO:0005750	GO:0043393	GO:0034728
3916	GO:0004809	GO:0043388	GO:0034653
3917	GO:0004775	GO:0043297	GO:0034644
3918	GO:0004738	GO:0043271	GO:0034629

3919	GO:0004311	GO:0000375	GO:0034622
3920	GO:0004310	GO:0043270	GO:0034613
3921	GO:0004300	GO:0043269	GO:0034508
3922	GO:0004169	GO:0043268	GO:0034502
3923	GO:0004152	GO:0043267	GO:0034476
3924	GO:0003988	GO:0043266	GO:0034473
3925	GO:0003743	GO:0043254	GO:0034472
3926	GO:0003690	GO:0043244	GO:0034471
3927	GO:0002943	GO:0043243	GO:0034470
3928	GO:0002777	GO:0043241	GO:0034462
3929	GO:0002775	GO:0043207	GO:0034453
3930	GO:0002768	GO:0000302	GO:0034443
3931	GO:0002719	GO:0043170	GO:0034442
3932	GO:0002696	GO:0043161	GO:0043067
3933	GO:0002690	GO:0043152	GO:0034427
3934	GO:0002686	GO:0043144	GO:0034329
3935	GO:0002548	GO:0043129	GO:0034308
3936	GO:0002128	GO:0043114	GO:0034260
3937	GO:0002009	GO:0043113	GO:0034250
3938	GO:0001934	GO:0043112	GO:0034248
3939	GO:0001932	GO:0043103	GO:0034243
3940	GO:0001882	GO:0043087	GO:0034116
3941	GO:0001784	GO:0000280	GO:0034114
3942	GO:0001525	GO:0043086	GO:0034086
3943	GO:0001505	GO:0043085	GO:0034067
3944	GO:0001503	GO:0043069	GO:0033993
3945	GO:0001159	GO:0043068	GO:0033860
3946	GO:0001102	GO:0043067	GO:0033753
3947	GO:0001101	GO:0043066	GO:0033692
3948	GO:0000989	GO:0043065	GO:0033683
3949	GO:0000976	GO:0043062	GO:0033627
3950	GO:0000963	GO:0043044	GO:0033617
3951	GO:0000478	GO:0043043	GO:0033615
3952	GO:0000469	GO:0000278	GO:0033559
3953	GO:0000314	GO:0043032	GO:0033539
3954	GO:0000313	GO:0043009	GO:0033512
3955	GO:0000177	GO:0042981	GO:0043410
3956	GO:0000027	GO:0042977	GO:0033320
3957	ENSP00000398698	GO:0042976	GO:0033319
3958	ENSP00000395772	GO:0042886	GO:0033280
3959	ENSP00000386134	GO:0042787	GO:0033273
3960	ENSP00000386047	GO:0042776	GO:0033262
3961	ENSP00000382392	GO:0042775	GO:0033233
3962	ENSP00000381655	GO:0042773	GO:0033160
3963	ENSP00000380033	GO:0000226	GO:0033145
3964	ENSP00000377385	GO:0042772	GO:0033141
3965	ENSP00000374399	GO:0042771	GO:0033139
3966	ENSP00000372695	GO:0042770	GO:0033138
3967	ENSP00000370589	GO:0042769	GO:0033059
3968	ENSP00000367029	GO:0042748	GO:0033048
3969	ENSP00000363746	GO:0042744	GO:0033046
3970	ENSP00000362873	GO:0042743	GO:0033045
3971	ENSP00000362649	GO:0042742	GO:0033044

3972	ENSP00000362153	GO:0042738	GO:0033036
3973	ENSP00000361092	GO:0042737	GO:0033014
3974	ENSP00000360412	GO:0000209	GO:0033013
3975	ENSP00000356953	GO:0042730	GO:0032970
3976	ENSP00000356671	GO:0042699	GO:0032965
3977	ENSP00000355987	GO:0042698	GO:0032964
3978	ENSP00000354040	GO:0042635	GO:0032963
3979	ENSP00000350720	GO:0042592	GO:0051347
3980	ENSP00000350524	GO:0042573	GO:0032956
3981	ENSP00000348170	GO:0042558	GO:0032945
3982	ENSP00000346725	GO:0042534	GO:0032944
3983	ENSP00000345917	GO:0042533	GO:0032940
3984	ENSP00000345895	GO:0042531	GO:0032926
3985	ENSP00000339145	GO:0000187	GO:0032925
3986	ENSP00000338862	GO:0042517	GO:0032905
3987	ENSP00000338548	GO:0042516	GO:0032881
3988	ENSP00000337518	GO:0042509	GO:0032880
3989	ENSP00000332659	GO:0042508	GO:0032870
3990	ENSP00000332504	GO:0042503	GO:0032845
3991	ENSP00000330460	GO:0042493	GO:0032844
3992	ENSP00000327268	GO:0042476	GO:0032803
3993	ENSP00000327070	GO:0042455	GO:0032787
3994	ENSP00000322396	GO:0042451	GO:0032784
3995	ENSP00000320567	GO:0042448	GO:0032682
3996	ENSP00000318158	GO:0000184	GO:0032675
3997	ENSP00000317992	GO:0042446	GO:0032652
3998	ENSP00000317780	GO:0042445	GO:0032640
3999	ENSP00000314348	GO:0042440	GO:0032635
4000	ENSP00000311135	GO:0042407	GO:0032620
4001	ENSP00000302665	GO:0042398	GO:0032612
4002	ENSP00000298746	GO:0042391	GO:0032571
4003	ENSP00000297579	GO:0042384	GO:0032543
4004	ENSP00000297350	GO:0042363	GO:0032535
4005	ENSP00000296792	GO:0042359	GO:0032508
4006	ENSP00000290299	GO:0042339	GO:0032507
4007	ENSP00000281701	GO:0000183	GO:0032504
4008	ENSP00000278856	GO:0042330	GO:0032481
4009	ENSP00000269260	GO:0042327	GO:0032480
4010	ENSP00000265245	GO:0042325	GO:0032456
4011	ENSP00000264279	GO:0042310	GO:0032409
4012	ENSP00000263774	GO:0042307	GO:0032402
4013	ENSP00000263253	GO:0042306	GO:0032401
4014	ENSP00000262030	GO:0042278	GO:0032370
4015	ENSP00000261637	GO:0042276	GO:0032355
4016	ENSP00000261015	GO:0042274	GO:0032310
4017	ENSP00000260619	GO:0042273	GO:0032273
4018	ENSP00000257829	GO:0000165	GO:0032268
4019	ENSP00000254810	GO:0042255	GO:0032226
4020	ENSP00000249269	GO:0042254	GO:0032225
4021	ENSP00000247866	GO:0042249	GO:0032206
4022	ENSP00000233190	GO:0042246	GO:0032205
4023	ENSP00000221801	GO:0042221	GO:0032200
4024	ENSP00000218388	GO:0042181	GO:0032148

4025	ENSP00000199320	GO:0042176	GO:0032147
4026	hsa05321	GO:0042168	GO:0032102
4027	hsa04924	GO:0042161	GO:0032101
4028	hsa04662	GO:0042160	GO:0032098
4029	hsa00830	GO:0000154	GO:0031937
4030	GO:2001202	GO:0042157	GO:0031667
4031	GO:2001201	GO:0042147	GO:0031644
4032	GO:2000546	GO:0042130	GO:0031640
4033	GO:2000544	GO:0042129	GO:0031638
4034	GO:2000147	GO:0042127	GO:0031623
4035	GO:2000098	GO:0042113	GO:0031589
4036	GO:2000097	GO:0042110	GO:0031584
4037	GO:2000002	GO:0042107	GO:0031579
4038	GO:1990904	GO:0042104	GO:0031570
4039	GO:1990777	GO:0042102	GO:0031529
4040	GO:1990583	GO:0000122	GO:0031503
4041	GO:1990481	GO:0042098	GO:0031498
4042	GO:1904949	GO:0042089	GO:0031440
4043	GO:1904894	GO:0042060	GO:0031400
4044	GO:1904892	GO:0042059	GO:0031349
4045	GO:1904847	GO:0042058	GO:0031348
4046	GO:1904667	GO:0042044	GO:0031344
4047	GO:1904377	GO:0042036	GO:0031331
4048	GO:1904375	GO:0042035	GO:0031324
4049	GO:1904237	GO:0042033	GO:0031295
4050	GO:1903845	GO:0040029	GO:0031294
4051	GO:1903844	GO:0000096	GO:0031281
4052	GO:1903751	GO:0040017	GO:0031280
4053	GO:1903706	GO:0040013	GO:0031167
4054	GO:1903561	GO:0040012	GO:0031145
4055	GO:1903557	GO:0040011	GO:0031126
4056	GO:1903508	GO:0040008	GO:0031125
4057	GO:1903507	GO:0040007	GO:0031124
4058	GO:1903337	GO:0039519	GO:0031123
4059	GO:1903318	GO:0038166	GO:0031118
4060	GO:1903308	GO:0038165	GO:0031062
4061	GO:1903279	GO:0038162	GO:0031061
4062	GO:1903039	GO:0000086	GO:0031058
4063	GO:1903037	GO:0038156	GO:0031057
4064	GO:1902996	GO:0038155	GO:0031056
4065	GO:1902993	GO:0038127	GO:0031055
4066	GO:1902988	GO:0038114	GO:0031023
4067	GO:1902949	GO:0038111	GO:0030953
4068	GO:1902847	GO:0038096	GO:0030865
4069	GO:1902656	GO:0038094	GO:0030855
4070	GO:1902653	GO:0038044	GO:0030833
4071	GO:1902626	GO:0038043	GO:0030832
4072	GO:1902624	GO:0036498	GO:0030823
4073	GO:1902593	GO:0000083	GO:0030818
4074	GO:1902583	GO:0036481	GO:0030817
4075	GO:1902579	GO:0036376	GO:0030802
4076	GO:1902572	GO:0036314	GO:0030800
4077	GO:1902571	GO:0036297	GO:0030593

4078	GO:1902562	GO:0036276	GO:0030520
4079	GO:1902493	GO:0036265	GO:0030518
4080	GO:1902403	GO:0036260	GO:0030512
4081	GO:1902402	GO:0036211	GO:0030510
4082	GO:1902400	GO:0036124	GO:0030509
4083	GO:1901675	GO:0036123	GO:0030500
4084	GO:1901674	GO:0000082	GO:0030488
4085	GO:1901663	GO:0036075	GO:0030449
4086	GO:1901568	GO:0036066	GO:0030397
4087	GO:1901265	GO:0036065	GO:0030335
4088	GO:1901006	GO:0035988	GO:0030334
4089	GO:1900864	GO:0035987	GO:0030330
4090	GO:1900544	GO:0035966	GO:0030323
4091	GO:1900373	GO:0035948	GO:0030321
4092	GO:1900372	GO:0035947	GO:0030279
4093	GO:1900371	GO:0035929	GO:0030278
4094	GO:1900135	GO:0035927	GO:0030258
4095	GO:1900133	GO:0000079	GO:0030219
4096	GO:1900116	GO:0035914	GO:0030213
4097	GO:1900048	GO:0035887	GO:0030201
4098	GO:1900047	GO:0035821	GO:0030200
4099	GO:1900046	GO:0035815	GO:0030199
4100	GO:0099572	GO:0035812	GO:0030198
4101	GO:0099537	GO:0035768	GO:0030195
4102	GO:0099536	GO:0035767	GO:0030193
4103	GO:0099513	GO:0035766	GO:0030183
4104	GO:0098852	GO:0035725	GO:0030167
4105	GO:0098811	GO:0035637	GO:0030111
4106	GO:0098761	GO:0000077	GO:0030104
4107	GO:0098760	GO:0035630	GO:0030100
4108	GO:0098756	GO:0035601	GO:0030099
4109	GO:0098743	GO:0035590	GO:0030098
4110	GO:0098732	GO:0035588	GO:0030072
4111	GO:0097696	GO:0035587	GO:0030071
4112	GO:0097610	GO:0035582	GO:0030041
4113	GO:0097485	GO:0035567	GO:0030036
4114	GO:0097480	GO:0035563	GO:0009987
4115	GO:0097472	GO:0035561	GO:0030031
4116	GO:0097034	GO:0035556	GO:0030007
4117	GO:0097031	GO:0000076	GO:0030004
4118	GO:0090672	GO:0035491	GO:0030002
4119	GO:0090671	GO:0035490	GO:0030001
4120	GO:0090661	GO:0035437	GO:0023056
4121	GO:0090659	GO:0035412	GO:0023052
4122	GO:0090545	GO:0035411	GO:0023051
4123	GO:0090533	GO:0035384	GO:0023014
4124	GO:0090387	GO:0035383	GO:0022904
4125	GO:0090316	GO:0035337	GO:0022900
4126	GO:0090276	GO:0035295	GO:0022898
4127	GO:0090132	GO:0035272	GO:0022618
4128	GO:0090035	GO:0000075	GO:0022616
4129	GO:0080171	GO:0035270	GO:0022610
4130	GO:0075136	GO:0035249	GO:0022607

4131	GO:0072589	GO:0035247	GO:0022603
4132	GO:0072536	GO:0035246	GO:0022600
4133	GO:0072422	GO:0035235	GO:0022410
4134	GO:0072359	GO:0035150	GO:0022409
4135	GO:0072350	GO:0035067	GO:0022408
4136	GO:0071922	GO:0035065	GO:0022406
4137	GO:0071805	GO:0035025	GO:0022402
4138	GO:0071773	GO:0035019	GO:0022401
4139	GO:0071706	GO:0000070	GO:0022400
4140	GO:0071675	GO:0034983	GO:0019932
4141	GO:0071634	GO:0034976	GO:0019886
4142	GO:0071621	GO:0034975	GO:0019884
4143	GO:0071616	GO:0034968	GO:0019827
4144	GO:0071593	GO:0034767	GO:0019752
4145	GO:0071466	GO:0034766	GO:0019730
4146	GO:0071436	GO:0034765	GO:0019722
4147	GO:0071431	GO:0034764	GO:0019693
4148	GO:0071427	GO:0034762	GO:0019674
4149	GO:0071385	GO:0034754	GO:0019673
4150	GO:0071339	GO:0000060	GO:0019637
4151	GO:0071174	GO:0034728	GO:0019538
4152	GO:0071051	GO:0034724	GO:0019477
4153	GO:0071049	GO:0034723	GO:0019474
4154	GO:0071046	GO:0034661	GO:0019439
4155	GO:0071043	GO:0034660	GO:0019369
4156	GO:0071038	GO:0034655	GO:0019362
4157	GO:0071035	GO:0034654	GO:0019320
4158	GO:0071034	GO:0034653	GO:0019228
4159	GO:0071029	GO:0034645	GO:0019220
4160	GO:0071028	GO:0034644	GO:0019218
4161	GO:0071025	GO:0000055	GO:0018394
4162	GO:0070901	GO:0034641	GO:0018393
4163	GO:0070846	GO:0034629	GO:0018279
4164	GO:0070727	GO:0034622	GO:0018214
4165	GO:0070489	GO:0034613	GO:0018200
4166	GO:0070486	GO:0034508	GO:0018198
4167	GO:0070272	GO:0034502	GO:0018171
4168	GO:0070271	GO:0034476	GO:0018076
4169	GO:0070169	GO:0034475	GO:0018027
4170	GO:0070142	GO:0034473	GO:0018023
4171	GO:0070119	GO:0034472	GO:0018022
4172	GO:0070076	GO:0000054	GO:0017187
4173	GO:0070062	GO:0034471	GO:0017157
4174	GO:0070035	GO:0034470	GO:0017144
4175	GO:0061732	GO:0034462	GO:0017015
4176	GO:0061718	GO:0034453	GO:0017004
4177	GO:0061641	GO:0034446	GO:0016578
4178	GO:0061621	GO:0034443	GO:0016575
4179	GO:0061620	GO:0034442	GO:0016573
4180	GO:0061615	GO:0034440	GO:0016571
4181	GO:0061564	GO:0034427	GO:0016570
4182	GO:0061518	GO:0034332	GO:0016568
4183	GO:0061458	GO:0000045	GO:0016567

4184	GO:0061302	GO:0034330	GO:0016525
4185	GO:0061078	GO:0034329	GO:0016477
4186	GO:0061044	GO:0034308	GO:0016358
4187	GO:0061034	GO:0034260	GO:0016311
4188	GO:0060669	GO:0034250	GO:0016266
4189	GO:0060665	GO:0034248	GO:0016241
4190	GO:0060591	GO:0034243	GO:0016236
4191	GO:0060311	GO:0034220	GO:0016180
4192	GO:0060310	GO:0034116	GO:0016125
4193	GO:0060158	GO:0034114	GO:0016093
4194	GO:0060135	GO:0000028	GO:0016078
4195	GO:0060089	GO:0034113	GO:0016075
4196	GO:0055029	GO:0034112	GO:0016074
4197	GO:0052909	GO:0034110	GO:0016073
4198	GO:0052547	GO:0034109	GO:0016071
4199	GO:0052472	GO:0034104	GO:0016053
4200	GO:0052192	GO:0034103	GO:0016052
4201	GO:0052126	GO:0034097	GO:0016049
4202	GO:0051996	GO:0034086	GO:0015991
4203	GO:0051985	GO:0034080	GO:0015988
4204	GO:0051931	GO:0034067	GO:0015980
4205	GO:0051828	GO:0000027	GO:0015959
4206	GO:0051806	GO:0033993	GO:0015931
4207	GO:0051783	GO:0033860	GO:0015874
4208	GO:0051707	GO:0033753	GO:0015853
4209	GO:0051674	GO:0033750	GO:0015851
4210	GO:0051574	GO:0033692	GO:0015850
4211	GO:0051549	GO:0033688	GO:0015844
4212	GO:0051546	GO:0033683	GO:0015837
4213	GO:0051540	GO:0033674	GO:0015833
4214	GO:0051538	GO:0033631	GO:0015721
4215	GO:0051303	GO:0033627	GO:0015718
4216	GO:0051283	GO:0000018	GO:0015711
4217	GO:0051192	GO:0033619	GO:0015031
4218	GO:0051081	GO:0033617	GO:0014912
4219	GO:0051043	GO:0033615	GO:0014911
4220	GO:0050965	GO:0033591	GO:0014848
4221	GO:0050867	GO:0033559	GO:0014823
4222	GO:0050863	GO:0033554	GO:0014821
4223	GO:0050819	GO:0033539	GO:0014812
4224	GO:0050818	GO:0033512	GO:0014070
4225	GO:0050812	GO:0033365	GO:0014066
4226	GO:0050796	GO:0033320	GO:0014047
4227	GO:0050755	GO:0000003	GO:0014012
4228	GO:0050670	GO:0033319	GO:0012501
4229	GO:0050663	GO:0033280	GO:0010992
4230	GO:0050658	GO:0033273	GO:0010972
4231	GO:0050178	GO:0033262	GO:0010971
4232	GO:0050136	GO:0033260	GO:0010955
4233	GO:0048770	GO:0033233	GO:0010948
4234	GO:0048731	GO:0033198	GO:0010876
4235	GO:0048729	GO:0033160	GO:0010870
4236	GO:0048699	GO:0033158	GO:0010835

4237	GO:0048608	GO:0033148	GO:0010828
4238	GO:0048584	GO:0033145	GO:0010817
4239	GO:0048551	GO:0033143	GO:0010812
4240	GO:0048263	GO:0033141	GO:0010811
4241	GO:0048041	GO:0033139	GO:0010810
4242	GO:0048040	GO:0033138	GO:0010757
4243	GO:0047696	GO:0033135	GO:0010739
4244	GO:0046824	GO:0033108	GO:0010716
4245	GO:0046546	GO:0033059	GO:0010715
4246	GO:0046496	GO:0033048	GO:0010712
4247	GO:0046483	GO:0033047	GO:0010711
4248	GO:0046440	GO:0033046	GO:0010647
4249	GO:0046395	GO:0033045	GO:0010646
4250	GO:0046394	GO:0033044	GO:0010644
4251	GO:0046160	GO:0033043	GO:0010639
4252	GO:0046132	GO:0033036	GO:0010638
4253	GO:0046129	GO:0033014	GO:0010634
4254	GO:0046103	GO:0033013	GO:0010633
4255	GO:0046049	GO:0033002	GO:0010629
4256	GO:0046031	GO:0032990	GO:0010608
4257	GO:0045937	GO:0032989	GO:0010596
4258	GO:0045903	GO:0032986	GO:0010573
4259	GO:0045859	GO:0032984	GO:0010564
4260	GO:0045662	GO:0032981	GO:0010544
4261	GO:0045621	GO:0032970	GO:0010543
4262	GO:0045283	GO:0032967	GO:0010536
4263	GO:0045281	GO:0032965	GO:0010535
4264	GO:0045275	GO:0032964	GO:0010534
4265	GO:0045273	GO:0032963	GO:0010533
4266	GO:0045271	GO:0032956	GO:0010517
4267	GO:0045267	GO:0032946	GO:0010453
4268	GO:0045261	GO:0032945	GO:0010248
4269	GO:0045257	GO:0032944	GO:0010243
4270	GO:0045240	GO:0032943	GO:0009966
4271	GO:0045239	GO:0032940	GO:0009896
4272	GO:0045047	GO:0032926	GO:0009894
4273	GO:0044783	GO:0032925	GO:0009892
4274	GO:0044700	GO:0032922	GO:0009746
4275	GO:0044620	GO:0032908	GO:0009725
4276	GO:0044439	GO:0032905	GO:0009719
4277	GO:0044419	GO:0032881	GO:0009611
4278	GO:0044409	GO:0032880	GO:0009607
4279	GO:0044403	GO:0032879	GO:0009605
4280	GO:0044391	GO:0032870	GO:0009584
4281	GO:0044364	GO:0032869	GO:0009583
4282	GO:0044253	GO:0032845	GO:0009581
4283	GO:0044246	GO:0032844	GO:0009452
4284	GO:0044212	GO:0032803	GO:0009404
4285	GO:0044057	GO:0032802	GO:0009396
4286	GO:0043783	GO:0032787	GO:0009306
4287	GO:0043634	GO:0032786	GO:0009268
4288	GO:0043544	hsa05204	GO:0009260
4289	GO:0043413	GO:0032784	GO:0009259

4290	GO:0043232	GO:0032774	GO:0009247
4291	GO:0043189	GO:0032755	GO:0009226
4292	GO:0043144	GO:0032722	GO:0009225
4293	GO:0043062	GO:0032689	GO:0009220
4294	GO:0043009	GO:0032682	GO:0009218
4295	GO:0042827	GO:0032680	GO:0009206
4296	GO:0042775	GO:0032675	GO:0009199
4297	GO:0042730	GO:0032652	GO:0009187
4298	GO:0042645	GO:0032642	GO:0009185
4299	GO:0042625	GO:0032640	GO:0009179
4300	GO:0042579	GO:0032637	GO:0009174
4301	GO:0042567	GO:0032635	GO:0009173
4302	GO:0042534	GO:0032620	GO:0009156
4303	GO:0042531	GO:0032612	GO:0009152
4304	GO:0042493	GO:0032606	GO:0009145
4305	GO:0042363	GO:0032602	GO:0009144
4306	GO:0042330	GO:0032571	GO:0009142
4307	GO:0042325	GO:0032543	GO:0009141
4308	GO:0042161	GO:0032535	GO:0009135
4309	GO:0042044	hsa05202	GO:0009127
4310	GO:0042035	GO:0032508	GO:0009126
4311	GO:0042020	GO:0032507	GO:0009123
4312	GO:0042019	GO:0032504	GO:0009119
4313	GO:0038156	GO:0032502	GO:0009117
4314	GO:0038155	GO:0032501	GO:0009116
4315	GO:0038127	GO:0032496	GO:0009113
4316	GO:0038114	GO:0032481	GO:0009108
4317	GO:0038096	GO:0032480	GO:0009100
4318	GO:0036454	GO:0032479	GO:0009083
4319	GO:0036440	GO:0032467	GO:0009070
4320	GO:0036376	GO:0032465	GO:0009069
4321	GO:0036260	GO:0032456	GO:0009068
4322	GO:0036211	GO:0032446	GO:0009065
4323	GO:0036123	GO:0032436	GO:0008344
4324	GO:0036075	GO:0032435	GO:0008334
4325	GO:0035768	GO:0032434	GO:0008300
4326	GO:0035639	GO:0032413	GO:0008285
4327	GO:0035491	GO:0032412	GO:0008217
4328	GO:0035383	GO:0032409	GO:0008211
4329	GO:0035247	GO:0032402	GO:0008210
4330	GO:0035065	GO:0032401	GO:0008209
4331	GO:0034979	GO:0032392	GO:0008206
4332	GO:0034724	GO:0032388	GO:0008203
4333	GO:0034723	GO:0032386	GO:0008202
4334	GO:0034653	GO:0032370	GO:0008154
4335	GO:0034604	GO:0032355	GO:0008150
4336	GO:0034513	GO:0032310	GO:0008105
4337	GO:0034476	GO:0032273	GO:0008033
4338	GO:0034475	GO:0032271	GO:0008015
4339	GO:0034473	GO:0032270	GO:0007635
4340	GO:0034471	GO:0032269	GO:0007632
4341	GO:0034457	GO:0032268	GO:0007631
4342	GO:0034456	GO:0032259	GO:0007628

4343	GO:0034455	GO:0032226	GO:0007603
4344	GO:0034110	GO:0032225	GO:0007602
4345	GO:0033764	GO:0032206	GO:0007597
4346	GO:0033753	GO:0032205	GO:0007586
4347	GO:0033750	GO:0032204	GO:0007571
4348	GO:0033558	GO:0032200	GO:0007565
4349	GO:0033512	GO:0032148	GO:0007507
4350	GO:0033320	hsa05166	GO:0007431
4351	GO:0033202	GO:0032147	GO:0007411
4352	GO:0033186	GO:0032103	GO:0007409
4353	GO:0033141	GO:0032102	GO:0007369
4354	GO:0033002	GO:0032101	GO:0007289
4355	GO:0032986	GO:0032098	GO:0007269
4356	GO:0032981	GO:0031960	GO:0007268
4357	GO:0032944	GO:0031937	GO:0007267
4358	GO:0032908	GO:0031935	GO:0007229
4359	GO:0032905	GO:0031667	GO:0007220
4360	GO:0032869	GO:0031644	GO:0007219
4361	GO:0032803	hsa05160	GO:0007218
4362	GO:0032652	GO:0031640	GO:0007215
4363	GO:0032635	GO:0031639	GO:0007212
4364	GO:0032561	GO:0031638	GO:0007210
4365	GO:0032550	GO:0031623	GO:0007205
4366	GO:0032545	GO:0031589	GO:0007204
4367	GO:0032386	GO:0031584	GO:0007202
4368	GO:0032268	GO:0031579	GO:0007200
4369	GO:0032155	GO:0031577	GO:0007193
4370	GO:0032041	GO:0031573	GO:0007191
4371	GO:0031907	GO:0031572	GO:0007190
4372	GO:0031903	GO:0031571	GO:0007188
4373	GO:0031762	GO:0031570	GO:0007187
4374	GO:0031644	GO:0031529	GO:0007179
4375	GO:0031515	GO:0031503	GO:0007178
4376	GO:0031328	GO:0031498	GO:0007173
4377	GO:0031305	GO:0031497	GO:0007169
4378	GO:0031231	GO:0031442	GO:0007164
4379	GO:0031118	GO:0031440	GO:0007163
4380	GO:0030964	GO:0031401	GO:0007131
4381	GO:0030799	GO:0031400	GO:0007127
4382	GO:0030731	GO:0031399	GO:0007098
4383	GO:0030697	GO:0031398	GO:0007091
4384	GO:0030696	GO:0031397	GO:0007088
4385	GO:0030692	GO:0031396	GO:0007064
4386	GO:0030320	GO:0031349	GO:0007063
4387	GO:0030182	GO:0031348	GO:0007062
4388	GO:0030098	GO:0031347	GO:0007051
4389	GO:0030007	GO:0031346	GO:0007045
4390	GO:0023014	GO:0031344	GO:0007044
4391	GO:0022834	GO:0031333	GO:0007041
4392	GO:0022832	GO:0031331	GO:0007040
4393	GO:0022803	GO:0031329	GO:0007034
4394	GO:0022401	GO:0031328	GO:0007033
4395	GO:0019981	GO:0031327	GO:0007032

4396	GO:0019969	GO:0031326	GO:0007031
4397	GO:0019962	GO:0031325	GO:0007017
4398	GO:0019477	GO:0031324	GO:0007015
4399	GO:0019474	GO:0031323	GO:0007009
4400	GO:0018685	GO:0031295	GO:0007007
4401	GO:0018214	GO:0031294	GO:0007000
4402	GO:0017187	GO:0031281	GO:0006999
4403	GO:0017076	GO:0031280	GO:0006998
4404	GO:0016907	GO:0031279	GO:0006996
4405	GO:0016885	GO:0031214	GO:0006982
4406	GO:0016875	GO:0031175	GO:0006978
4407	GO:0016751	GO:0031167	GO:0006963
4408	GO:0016681	GO:0031145	GO:0006959
4409	GO:0016676	GO:0031126	GO:0006958
4410	GO:0016649	GO:0031125	GO:0006956
4411	GO:0016577	GO:0031124	GO:0006953
4412	GO:0016509	GO:0031123	GO:0006939
4413	GO:0016436	GO:0031122	GO:0006937
4414	GO:0016429	GO:0031118	GO:0006936
4415	GO:0016426	GO:0031100	GO:0006935
4416	GO:0016279	GO:0031099	GO:0006933
4417	GO:0016274	GO:0031062	GO:0006931
4418	GO:0016273	GO:0031061	GO:0006915
4419	GO:0016265	GO:0031060	GO:0006913
4420	GO:0016180	GO:0031058	GO:0006904
4421	GO:0016150	GO:0031057	GO:0006900
4422	GO:0016126	GO:0031056	GO:0006898
4423	GO:0016103	GO:0031055	GO:0006892
4424	GO:0016078	GO:0031047	GO:0006883
4425	GO:0016049	GO:0031023	GO:0006875
4426	GO:0015991	GO:0030968	GO:0006874
4427	GO:0015986	GO:0030953	GO:0006869
4428	GO:0015405	GO:0030947	GO:0006865
4429	GO:0015002	GO:0030913	GO:0006857
4430	GO:0014910	GO:0030866	GO:0006839
4431	GO:0012501	GO:0030865	GO:0006836
4432	GO:0010941	GO:0030855	GO:0006835
4433	GO:0010712	GO:0030838	GO:0006833
4434	GO:0010648	GO:0030833	GO:0006820
4435	GO:0010574	GO:0030832	GO:0006818
4436	GO:0010536	GO:0030823	GO:0006812
4437	GO:0009982	GO:0030818	GO:0006811
4438	GO:0009968	GO:0030817	GO:0006796
4439	GO:0009914	GO:0030816	GO:0006784
4440	GO:0009179	GO:0030815	GO:0006781
4441	GO:0009174	GO:0030814	GO:0006767
4442	GO:0009173	GO:0030810	GO:0006757
4443	GO:0009168	GO:0030809	GO:0006744
4444	GO:0009161	GO:0030808	GO:0006739
4445	GO:0009113	GO:0030804	GO:0006735
4446	GO:0009059	GO:0030803	GO:0006734
4447	GO:0008988	GO:0030802	GO:0006733
4448	GO:0008650	GO:0030801	GO:0006732

4449	GO:0008556	GO:0030800	GO:0006721
4450	GO:0008495	GO:0030799	GO:0006706
4451	GO:0008300	GO:0030728	GO:0006705
4452	GO:0008213	hsa05100	GO:0006701
4453	GO:0008186	GO:0030705	GO:0006699
4454	GO:0008137	GO:0030595	GO:0006695
4455	GO:0007599	GO:0030593	GO:0006694
4456	GO:0007596	GO:0030574	GO:0006690
4457	GO:0007571	GO:0030538	GO:0006664
4458	GO:0006965	GO:0030534	GO:0006635
4459	GO:0006963	GO:0030522	GO:0006631
4460	GO:0006784	GO:0030521	GO:0006629
4461	GO:0006753	GO:0030520	GO:0006626
4462	GO:0006613	GO:0030518	GO:0006621
4463	GO:0006490	GO:0030514	GO:0006605
4464	GO:0006306	GO:0030513	GO:0006595
4465	GO:0006171	GO:0030512	GO:0006554
4466	GO:0006086	GO:0030510	GO:0006544
4467	GO:0005900	GO:0030509	GO:0006520
4468	GO:0005764	GO:0030502	GO:0006518
4469	GO:0005763	GO:0030501	GO:0006516
4470	GO:0005762	GO:0030500	GO:0006513
4471	GO:0005761	GO:0030490	GO:0006511
4472	GO:0005754	GO:0030488	GO:0006509
4473	GO:0005666	hsa05032	GO:0006493
4474	GO:0005664	GO:0030449	GO:0006490
4475	GO:0005391	GO:0030397	GO:0006488
4476	GO:0004993	GO:0030336	GO:0006487
4477	GO:0004984	GO:0030335	GO:0006486
4478	GO:0004917	GO:0030334	GO:0006476
4479	GO:0004915	GO:0030330	GO:0006473
4480	GO:0004914	GO:0030324	GO:0006465
4481	GO:0004912	GO:0030323	GO:0006464
4482	GO:0004905	GO:0030321	GO:0006417
4483	GO:0004904	GO:0030320	GO:0006415
4484	GO:0004897	GO:0030301	GO:0006414
4485	GO:0004776	GO:0030282	GO:0006413
4486	GO:0004774	GO:0030279	GO:0006412
4487	GO:0004532	GO:0030278	GO:0006406
4488	GO:0004385	GO:0030260	GO:0006405
4489	GO:0004174	GO:0030258	GO:0006401
4490	GO:0004149	GO:0030219	GO:0006400
4491	GO:0003963	GO:0030217	GO:0006391
4492	GO:0003700	GO:0030213	GO:0006390
4493	GO:0002816	GO:0030212	GO:0006369
4494	GO:0002815	GO:0030204	GO:0006367
4495	GO:0002807	GO:0030203	GO:0006362
4496	GO:0002803	GO:0030201	GO:0006361
4497	GO:0002786	GO:0030200	GO:0006360
4498	GO:0002784	GO:0030199	GO:0006354
4499	GO:0002780	GO:0030198	GO:0006352
4500	GO:0002778	GO:0030195	GO:0006342
4501	GO:0002760	GO:0030194	GO:0006334

4502	GO:0002739	GO:0030193	GO:0006333
4503	GO:0002718	GO:0030183	GO:0006310
4504	GO:0002639	GO:0030182	GO:0006301
4505	GO:0002374	GO:0030168	GO:0006297
4506	GO:0001999	GO:0030167	GO:0043393
4507	GO:0001869	GO:0030166	GO:0006284
4508	GO:0001731	GO:0030163	GO:0006282
4509	GO:0001568	GO:0030162	GO:0006271
4510	GO:0001532	GO:0030155	GO:0006270
4511	GO:0001189	GO:0030154	GO:0006261
4512	GO:0001188	GO:0030150	GO:0006259
4513	GO:0001180	GO:0030111	GO:0006222
4514	GO:0001099	GO:0030104	GO:0006189
4515	GO:0001054	GO:0030100	GO:0006188
4516	GO:0001012	GO:0030099	GO:0006177
4517	GO:0000987	GO:0030098	GO:0006167
4518	GO:0000967	GO:0030097	GO:0006165
4519	GO:0000902	GO:0030073	GO:0006152
4520	GO:0000725	GO:0030072	GO:0006144
4521	GO:0000480	GO:0030071	GO:0006123
4522	GO:0000479	GO:0030041	GO:0006122
4523	GO:0000472	GO:0030036	GO:0006120
4524	GO:0000467	hsa04974	GO:0006107
4525	GO:0000453	GO:0030031	GO:0006106
4526	GO:0000398	GO:0030030	GO:0006105
4527	GO:0000176	GO:0030029	GO:0006104
4528	GO:0000125	GO:0030010	GO:0006103
4529	GO:0000104	GO:0030007	GO:0006101
4530	GO:0000036	GO:0030004	GO:0006099
4531	GO:0000018	GO:0030003	GO:0006091
4532	ENSP00000419740	GO:0030002	GO:0006090
4533	ENSP00000417464	GO:0030001	GO:0006085
4534	ENSP00000414514	GO:0023061	GO:0006082
4535	ENSP00000413572	GO:0023058	GO:0006081
4536	ENSP00000412483	GO:0023057	GO:0006027
4537	ENSP00000412251	GO:0023056	GO:0006024
4538	ENSP00000411162	GO:0023052	GO:0006022
4539	ENSP00000410530	GO:0023051	GO:0006007
4540	ENSP00000409384	GO:0023014	GO:0005996
4541	ENSP00000409367	GO:0022904	GO:0005975
4542	ENSP00000409074	GO:0022900	GO:0003417
4543	ENSP00000408017	GO:0022898	GO:0003416
4544	ENSP00000407515	GO:0022618	GO:0003094
4545	ENSP00000407436	hsa04972	GO:0003073
4546	ENSP00000405614	GO:0022617	GO:0003071
4547	ENSP00000404102	GO:0022616	GO:0003069
4548	ENSP00000403576	GO:0022613	GO:0003044
4549	ENSP00000402869	GO:0022610	GO:0003018
4550	ENSP00000402802	GO:0022607	GO:0003014
4551	ENSP00000402733	GO:0022604	GO:0003013
4552	ENSP00000402338	GO:0022603	GO:0002940
4553	ENSP00000399753	GO:0022602	GO:0002923
4554	ENSP00000399454	GO:0022600	GO:0002922

4555	ENSP00000399078	GO:0022414	GO:0002920
4556	ENSP00000396454	hsa04971	GO:0002862
4557	ENSP00000396441	GO:0022411	GO:0002833
4558	ENSP00000395187	GO:0022410	GO:0002831
4559	ENSP00000394290	GO:0022409	GO:0002816
4560	ENSP00000393101	GO:0022408	GO:0002805
4561	ENSP00000389649	GO:0022407	GO:0002803
4562	ENSP00000387523	GO:0022406	GO:0002791
4563	ENSP00000384316	GO:0022402	GO:0002790
4564	ENSP00000384302	GO:0022401	GO:0002780
4565	ENSP00000380982	GO:0022400	GO:0002779
4566	ENSP00000380495	GO:0022008	GO:0002778
4567	ENSP00000380153	GO:0022007	GO:0002777
4568	ENSP00000379760	GO:0021545	GO:0002775
4569	ENSP00000378857	GO:0019985	GO:0002768
4570	ENSP00000377799	GO:0019953	GO:0002764
4571	ENSP00000377523	GO:0019941	GO:0002759
4572	ENSP00000374390	GO:0019932	GO:0002718
4573	ENSP00000373090	GO:0019886	GO:0002696
4574	ENSP00000372199	GO:0019884	GO:0002694
4575	ENSP00000371377	GO:0019882	GO:0002686
4576	ENSP00000371169	GO:0019856	GO:0002685
4577	ENSP00000371101	GO:0019852	GO:0002639
4578	ENSP00000370473	GO:0019827	GO:0002576
4579	ENSP00000369728	GO:0019752	GO:0002526
4580	ENSP00000368989	GO:0019731	GO:0002520
4581	ENSP00000368887	GO:0019730	GO:0002504
4582	ENSP00000367934	GO:0019725	GO:0002495
4583	ENSP00000367361	GO:0019724	GO:0002479
4584	ENSP00000366629	GO:0019722	GO:0002478
4585	ENSP00000366623	GO:0019693	GO:0002474
4586	ENSP00000366519	GO:0019682	GO:0002455
4587	ENSP00000365837	hsa04961	GO:0002449
4588	ENSP00000365806	GO:0019674	GO:0002446
4589	ENSP00000364699	GO:0019673	GO:0002431
4590	ENSP00000364649	GO:0019637	GO:0002385
4591	ENSP00000364320	GO:0019538	GO:0002384
4592	ENSP00000364037	GO:0019477	GO:0002377
4593	ENSP00000363642	GO:0019474	GO:0002374
4594	ENSP00000362687	GO:0019439	GO:0002367
4595	ENSP00000362036	GO:0019438	GO:0002244
4596	ENSP00000361232	GO:0019395	GO:0002227
4597	ENSP00000361084	GO:0019369	GO:0002225
4598	ENSP00000361010	GO:0019362	GO:0002223
4599	ENSP00000360492	GO:0019320	GO:0002128
4600	ENSP00000360329	GO:0019318	GO:0002090
4601	ENSP00000360031	GO:0019233	GO:0002063
4602	ENSP00000359688	GO:0019229	GO:0002062
4603	ENSP00000358737	GO:0019228	GO:0002032
4604	ENSP00000357748	GO:0019226	GO:0002031
4605	ENSP00000357134	GO:0019222	GO:0002002
4606	ENSP00000356972	GO:0019221	GO:0001999
4607	ENSP00000356548	GO:0019220	GO:0001991

4608	ENSP00000356476	GO:0019219	GO:0001990
4609	ENSP00000355899	GO:0019218	GO:0001975
4610	ENSP00000355565	GO:0019083	GO:0001963
4611	ENSP00000355541	GO:0019082	GO:0001959
4612	ENSP00000355471	GO:0019080	GO:0001958
4613	ENSP00000355443	GO:0019068	GO:0001957
4614	ENSP00000355430	GO:0019058	GO:0001944
4615	ENSP00000353947	GO:0018394	GO:0001938
4616	ENSP00000353246	GO:0018393	GO:0001933
4617	ENSP00000352839	GO:0018279	GO:0001911
4618	ENSP00000352626	GO:0018216	GO:0001906
4619	ENSP00000350698	GO:0018214	GO:0001893
4620	ENSP00000350352	GO:0018212	GO:0001890
4621	ENSP00000350256	GO:0018209	GO:0001887
4622	ENSP00000350011	GO:0018205	GO:0001885
4623	ENSP00000349142	GO:0018200	GO:0001869
4624	ENSP00000348722	GO:0018198	GO:0001840
4625	ENSP00000348596	GO:0018196	GO:0001839
4626	ENSP00000345793	GO:0018195	GO:0001837
4627	ENSP00000345317	GO:0018193	GO:0001825
4628	ENSP00000345163	hsa04925	GO:0001824
4629	ENSP00000344453	GO:0018171	GO:0001819
4630	ENSP00000343081	GO:0018149	GO:0001816
4631	ENSP00000334364	GO:0018146	GO:0001763
4632	ENSP00000334051	GO:0018130	GO:0001738
4633	ENSP00000332887	GO:0018108	GO:0001731
4634	ENSP00000332340	GO:0018076	GO:0001706
4635	ENSP00000331815	GO:0018027	GO:0001701
4636	ENSP00000331545	GO:0018023	GO:0001649
4637	ENSP00000331111	GO:0018022	GO:0001578
4638	ENSP00000330937	GO:0017187	GO:0001558
4639	ENSP00000330737	hsa04924	GO:0001542
4640	ENSP00000330384	GO:0017158	GO:0001510
4641	ENSP00000330341	GO:0017157	GO:0001508
4642	ENSP00000330049	GO:0017144	GO:0001502
4643	ENSP00000328854	GO:0017015	GO:0001306
4644	ENSP00000328690	GO:0017004	GO:0001189
4645	ENSP00000328563	GO:0016925	GO:0001188
4646	ENSP00000327585	GO:0016601	GO:0001101
4647	ENSP00000327179	GO:0016584	GO:0000967
4648	ENSP00000326981	GO:0016578	GO:0000956
4649	ENSP00000326259	GO:0016577	GO:0000910
4650	ENSP00000325682	GO:0016575	GO:0000904
4651	ENSP00000325203	GO:0016574	GO:0000902
4652	ENSP00000325128	GO:0016573	GO:0000819
4653	ENSP00000324769	GO:0016572	GO:0000729
4654	ENSP00000324251	GO:0016571	GO:0000726
4655	ENSP00000323858	GO:0016570	GO:0000715
4656	ENSP00000323853	GO:0016569	GO:0000480
4657	ENSP00000323612	GO:0016568	GO:0000479
4658	ENSP00000323424	GO:0016567	GO:0000478
4659	ENSP00000322939	GO:0016525	GO:0000472
4660	ENSP00000322450	GO:0016486	GO:0000469

4661	ENSP00000322088	GO:0016485	GO:0000467
4662	ENSP00000321506	GO:0016482	GO:0000466
4663	ENSP00000321449	GO:0016477	GO:0000463
4664	ENSP00000321426	GO:0016458	GO:0000462
4665	ENSP00000321320	GO:0016358	GO:0000453
4666	ENSP00000321246	GO:0016337	GO:0000447
4667	ENSP00000320917	GO:0016311	GO:0000381
4668	ENSP00000317159	GO:0016310	GO:0000377
4669	ENSP00000316955	GO:0016266	GO:0000375
4670	ENSP00000315674	hsa04916	GO:0000280
4671	ENSP00000314441	GO:0016265	GO:0000278
4672	ENSP00000313953	GO:0016259	GO:0000226
4673	ENSP00000311038	GO:0016241	GO:0000183
4674	ENSP00000310488	GO:0016236	GO:0000165
4675	ENSP00000310337	GO:0016197	GO:0000122
4676	ENSP00000310042	GO:0016192	GO:0000086
4677	ENSP00000309673	GO:0016180	GO:0000083
4678	ENSP00000308332	GO:0016126	GO:0000082
4679	ENSP00000308270	GO:0016125	GO:0000079
4680	ENSP00000308179	GO:0016115	GO:0000070
4681	ENSP00000307598	hsa04915	GO:0000055
4682	ENSP00000307525	GO:0016103	GO:0000045
4683	ENSP00000307130	GO:0016101	GO:0000028
4684	ENSP00000306974	GO:0016093	GO:0000027
4685	ENSP00000306688	GO:0016078	GO:0000018
4686	ENSP00000306651	GO:0016075	GO:0000003
4687	ENSP00000306397	GO:0016074	hsa05414
4688	ENSP00000306095	GO:0016073	hsa05203
4689	ENSP00000305702	GO:0016072	hsa05202
4690	ENSP00000305207	GO:0016071	hsa05200
4691	ENSP00000304419	GO:0016070	hsa05169
4692	ENSP00000304188	hsa04912	hsa05168
4693	ENSP00000304151	GO:0016064	hsa05160
4694	ENSP00000303983	GO:0016056	hsa05146
4695	ENSP00000303864	GO:0016055	hsa05143
4696	ENSP00000303834	GO:0016054	hsa05132
4697	ENSP00000303515	GO:0016053	hsa05100
4698	ENSP00000303076	GO:0016052	hsa05032
4699	ENSP00000302886	GO:0016050	hsa05016
4700	ENSP00000301587	GO:0016049	hsa05012
4701	ENSP00000301364	GO:0016048	hsa05010
4702	ENSP00000300773	GO:0016043	hsa04976
4703	ENSP00000299166	GO:0016042	hsa04974
4704	ENSP00000297990	GO:0016032	hsa04960
4705	ENSP00000297873	GO:0015992	hsa04933
4706	ENSP00000297439	GO:0015991	hsa04932
4707	ENSP00000296802	GO:0015988	hsa04918
4708	ENSP00000296684	GO:0015986	hsa04742
4709	ENSP00000296099	GO:0015985	hsa04730
4710	ENSP00000292301	GO:0015980	hsa04725
4711	ENSP00000291576	GO:0015959	hsa04724
4712	ENSP00000287022	GO:0015931	hsa04720
4713	ENSP00000285949	GO:0015919	hsa04666

4714	ENSP00000285814	GO:0015918	hsa04662
4715	ENSP00000285600	GO:0015874	hsa04640
4716	ENSP00000284727	GO:0015872	hsa04623
4717	ENSP00000284690	GO:0015853	hsa04614
4718	ENSP00000282050	GO:0015851	hsa04611
4719	ENSP00000278409	GO:0015850	hsa04610
4720	ENSP00000276689	GO:0015849	hsa04540
4721	ENSP00000274849	GO:0015844	hsa04530
4722	ENSP00000272521	GO:0015837	hsa04512
4723	ENSP00000268854	hsa04750	hsa04330
4724	ENSP00000268802	GO:0015833	hsa04310
4725	ENSP00000268668	GO:0015721	hsa04261
4726	ENSP00000268379	GO:0015718	hsa04260
4727	ENSP00000267425	GO:0015711	hsa04145
4728	ENSP00000266544	GO:0015698	hsa04144
4729	ENSP00000265171	GO:0015696	hsa04142
4730	ENSP00000264670	GO:0015672	hsa04120
4731	ENSP00000264563	GO:0015031	hsa04114
4732	ENSP00000263657	GO:0014912	hsa04060
4733	ENSP00000262946	GO:0014911	hsa04022
4734	ENSP00000262607	GO:0014910	hsa04015
4735	ENSP00000261741	GO:0014909	hsa04014
4736	ENSP00000261708	GO:0014848	hsa03420
4737	ENSP00000260443	GO:0014823	hsa03030
4738	ENSP00000260227	GO:0014821	hsa03022
4739	ENSP00000260184	GO:0014812	hsa03020
4740	ENSP00000259239	GO:0014070	hsa03018
4741	ENSP00000259037	GO:0014068	hsa03015
4742	ENSP00000258772	GO:0014066	hsa03013
4743	ENSP00000258531	GO:0014065	hsa00983
4744	ENSP00000258105	GO:0014061	hsa00982
4745	ENSP00000254940	GO:0014047	hsa00980
4746	ENSP00000254803	GO:0014012	hsa00970
4747	ENSP00000254605	GO:0012501	hsa00900
4748	ENSP00000253452	GO:0010992	hsa00860
4749	ENSP00000253237	GO:0010976	hsa00830
4750	ENSP00000252711	GO:0010975	hsa00670
4751	ENSP00000251289	GO:0010972	hsa00590
4752	ENSP00000250937	GO:0010971	hsa00562
4753	ENSP00000248572	GO:0010965	hsa00533
4754	ENSP00000247003	GO:0010959	hsa00520
4755	ENSP00000246802	GO:0010955	hsa00510
4756	ENSP00000244623	GO:0010951	hsa00480
4757	ENSP00000244496	GO:0010948	hsa00410
4758	ENSP00000244230	GO:0010942	hsa00310
4759	ENSP00000238146	GO:0010941	hsa00280
4760	ENSP00000236051	GO:0010876	hsa00260
4761	ENSP00000233627	GO:0010870	hsa00250
4762	ENSP00000232888	GO:0010863	hsa00240
4763	ENSP00000230640	GO:0010835	hsa00220
4764	ENSP00000230340	GO:0010833	hsa00190
4765	ENSP00000229214	GO:0010828	hsa00140
4766	ENSP00000225296	GO:0010817	hsa00120

4767	ENSP00000223095	GO:0010812	hsa00100
4768	ENSP00000220616	GO:0010811	hsa00072
4769	ENSP00000215375	GO:0010810	hsa00030
4770	ENSP00000209540	GO:0010765	hsa00020
4771	ENSP00000203001	GO:0010757	GO:0097190
4772	ENSP00000202816	GO:0010755	GO:1901701
4773	ENSP00000001146	GO:0010739	GO:0043068
