# Supplementary material for: Subcellular Localization Prediction of Human Proteins Using Multifeature Selection Methods
Source: Biomed Res Int. 2022 Sep 12;2022:3288527. doi: 10.1155/2022/3288527 (PMC9484878; doi:10.1155/2022/3288527)
Supplement: Supplementary Materials — Table S1: three feature lists generated by the LightGBM, MCFS, and mRMR methods after the analysis of Boruta. Table S2: detailed results of IFS method with RF and SVM algorithms in the three ranked feature lists. [file Table S2 (1).pdf › Table S2 (1).pdf]

**Table S2.** Detailed results of IFS method with RF and SVM algorithms in the three ranked feature lists.

| **Class index** | **Class name** |
|---|---|
| Class 1 | Biological membrane |
| Class 2 | Cell periphery |
| Class 3 | Cytoplasm |
| Class 4 | Cytoplasmic vesicle |
| Class 5 | Endoplasmic reticulum |
| Class 6 | Endosome |
| Class 7 | Extracellular space or cell surface |
| Class 8 | Flagellum or cilium |
| Class 9 | Golgi apparatus |
| Class 10 | Microtubule cytoskeleton |
| Class 11 | Mitochondrion |
| Class 12 | Nuclear periphery |
| Class 13 | Nucleolus |
| Class 14 | Nucleus |
| Class 15 | Peroxisome |
| Class 16 | Vacuole |

**1. IFS results using RF and LightGBM feature list**

| Number of | Class 1 | Class 2 | Class 3 | Class 4 | Class 5 | Class 6 | Class 7 | Class 8 | Class 9 | Class 10 | Class 11 | Class 12 | Class 13 | Class 14 | Class 15 | Class 16 | ACC | MCC |
|---|---|---|---|---|---|---|---|---|---|---|---|---|---|---|---|---|---|---|
| 1 | 0.369 | 0.286 | 0.128 | 0.129 | 0.305 | 0.320 | 0.220 | 0.333 | 0.286 | 0.063 | 0.113 | 0.091 | 0.911 | 0.015 | 0.152 | 0.222 | 0.212 | 0.172 |
| 2 | 0.467 | 0.514 | 0.283 | 0.614 | 0.621 | 0.680 | 0.330 | 0.667 | 0.602 | 0.333 | 0.357 | 0.455 | 0.670 | 0.186 | 0.565 | 0.870 | 0.371 | 0.313 |
| 3 | 0.535 | 0.771 | 0.476 | 0.771 | 0.679 | 0.760 | 0.473 | 0.333 | 0.735 | 0.813 | 0.620 | 0.697 | 0.714 | 0.531 | 0.717 | 0.852 | 0.554 | 0.492 |
| 4 | 0.579 | 0.743 | 0.555 | 0.800 | 0.742 | 0.840 | 0.544 | 1.000 | 0.755 | 0.854 | 0.681 | 0.727 | 0.750 | 0.637 | 0.717 | 0.889 | 0.622 | 0.558 |
| 5 | 0.601 | 0.800 | 0.569 | 0.800 | 0.768 | 0.800 | 0.593 | 1.000 | 0.816 | 0.875 | 0.690 | 0.727 | 0.741 | 0.642 | 0.804 | 0.870 | 0.641 | 0.580 |
| 6 | 0.639 | 0.886 | 0.567 | 0.829 | 0.789 | 0.800 | 0.618 | 0.667 | 0.837 | 0.917 | 0.780 | 0.788 | 0.839 | 0.730 | 0.761 | 0.852 | 0.689 | 0.632 |
| 7 | 0.638 | 0.886 | 0.607 | 0.857 | 0.816 | 0.840 | 0.632 | 1.000 | 0.888 | 0.938 | 0.797 | 0.879 | 0.848 | 0.750 | 0.891 | 0.889 | 0.706 | 0.653 |
| 8 | 0.662 | 0.886 | 0.619 | 0.871 | 0.784 | 0.880 | 0.687 | 1.000 | 0.908 | 0.938 | 0.800 | 0.758 | 0.848 | 0.764 | 0.891 | 0.944 | 0.725 | 0.673 |
| 9 | 0.680 | 0.971 | 0.696 | 0.957 | 0.889 | 0.960 | 0.729 | 1.000 | 0.929 | 0.979 | 0.870 | 0.879 | 0.902 | 0.795 | 0.913 | 0.981 | 0.766 | 0.721 |
| 10 | 0.695 | 0.971 | 0.725 | 0.986 | 0.874 | 1.000 | 0.740 | 1.000 | 0.929 | 0.958 | 0.884 | 0.909 | 0.938 | 0.793 | 0.913 | 0.981 | 0.776 | 0.733 |
| 11 | 0.723 | 1.000 | 0.729 | 0.986 | 0.895 | 1.000 | 0.763 | 1.000 | 0.939 | 1.000 | 0.899 | 0.939 | 0.955 | 0.806 | 0.913 | 1.000 | 0.794 | 0.755 |
| 12 | 0.708 | 0.971 | 0.741 | 0.971 | 0.879 | 0.960 | 0.773 | 1.000 | 0.980 | 0.979 | 0.893 | 0.879 | 0.973 | 0.806 | 0.957 | 1.000 | 0.792 | 0.752 |
| 13 | 0.710 | 0.971 | 0.745 | 0.971 | 0.895 | 0.960 | 0.769 | 1.000 | 0.959 | 1.000 | 0.907 | 0.909 | 0.973 | 0.817 | 1.000 | 1.000 | 0.797 | 0.758 |
| 14 | 0.724 | 0.971 | 0.721 | 0.971 | 0.905 | 0.960 | 0.781 | 1.000 | 0.969 | 1.000 | 0.901 | 0.970 | 0.955 | 0.816 | 0.978 | 0.981 | 0.800 | 0.761 |
| 15 | 0.746 | 0.971 | 0.749 | 0.943 | 0.921 | 0.960 | 0.841 | 1.000 | 0.949 | 1.000 | 0.930 | 0.879 | 0.955 | 0.839 | 0.935 | 0.981 | 0.824 | 0.789 |
| 16 | 0.750 | 0.971 | 0.747 | 0.943 | 0.916 | 0.920 | 0.861 | 1.000 | 0.939 | 1.000 | 0.930 | 0.909 | 0.902 | 0.840 | 0.935 | 0.981 | 0.826 | 0.792 |
| 17 | 0.748 | 0.971 | 0.763 | 0.957 | 0.921 | 1.000 | 0.852 | 1.000 | 0.959 | 1.000 | 0.936 | 0.909 | 0.946 | 0.835 | 0.957 | 0.981 | 0.828 | 0.794 |
| 18 | 0.742 | 0.971 | 0.767 | 0.943 | 0.911 | 1.000 | 0.847 | 1.000 | 0.969 | 1.000 | 0.922 | 0.909 | 0.938 | 0.837 | 0.978 | 0.981 | 0.825 | 0.790 |
| 19 | 0.751 | 0.971 | 0.773 | 0.943 | 0.926 | 1.000 | 0.846 | 1.000 | 0.959 | 1.000 | 0.933 | 0.909 | 0.893 | 0.835 | 0.957 | 0.981 | 0.827 | 0.793 |
| 20 | 0.748 | 0.971 | 0.777 | 0.986 | 0.937 | 0.960 | 0.854 | 1.000 | 0.959 | 1.000 | 0.942 | 0.909 | 0.929 | 0.841 | 0.935 | 0.981 | 0.831 | 0.798 |
| 21 | 0.752 | 0.971 | 0.796 | 0.986 | 0.932 | 1.000 | 0.857 | 1.000 | 0.949 | 1.000 | 0.939 | 0.970 | 0.991 | 0.833 | 0.957 | 0.981 | 0.834 | 0.802 |
| 22 | 0.741 | 0.971 | 0.767 | 0.986 | 0.932 | 0.920 | 0.880 | 1.000 | 0.959 | 1.000 | 0.933 | 0.939 | 0.982 | 0.839 | 1.000 | 0.981 | 0.832 | 0.799 |
| 23 | 0.736 | 1.000 | 0.783 | 0.986 | 0.932 | 1.000 | 0.878 | 1.000 | 0.969 | 1.000 | 0.942 | 0.939 | 0.964 | 0.837 | 1.000 | 0.981 | 0.833 | 0.800 |
| 24 | 0.744 | 1.000 | 0.791 | 1.000 | 0.942 | 1.000 | 0.881 | 1.000 | 0.969 | 1.000 | 0.933 | 0.970 | 0.982 | 0.842 | 0.957 | 0.981 | 0.838 | 0.806 |
| 25 | 0.738 | 1.000 | 0.771 | 0.971 | 0.937 | 1.000 | 0.875 | 1.000 | 0.959 | 1.000 | 0.939 | 0.970 | 0.982 | 0.840 | 0.978 | 0.981 | 0.832 | 0.800 |
| 26 | 0.750 | 0.971 | 0.775 | 0.971 | 0.942 | 1.000 | 0.878 | 1.000 | 0.959 | 1.000 | 0.942 | 0.939 | 0.991 | 0.839 | 0.935 | 0.981 | 0.836 | 0.804 |
| 27 | 0.754 | 0.971 | 0.777 | 0.971 | 0.953 | 1.000 | 0.884 | 1.000 | 0.969 | 1.000 | 0.930 | 0.909 | 0.991 | 0.844 | 0.957 | 0.981 | 0.839 | 0.808 |
| 28 | 0.749 | 0.971 | 0.781 | 0.971 | 0.932 | 1.000 | 0.877 | 1.000 | 0.969 | 1.000 | 0.936 | 0.939 | 0.991 | 0.834 | 0.978 | 0.981 | 0.835 | 0.803 |
| 29 | 0.753 | 0.971 | 0.777 | 0.971 | 0.942 | 1.000 | 0.888 | 1.000 | 0.959 | 1.000 | 0.936 | 1.000 | 0.982 | 0.844 | 0.957 | 0.981 | 0.840 | 0.809 |
| 30 | 0.748 | 0.971 | 0.785 | 0.971 | 0.942 | 1.000 | 0.874 | 1.000 | 0.980 | 1.000 | 0.948 | 0.939 | 0.991 | 0.842 | 0.957 | 0.981 | 0.838 | 0.806 |
| 31 | 0.748 | 1.000 | 0.796 | 0.986 | 0.921 | 1.000 | 0.878 | 1.000 | 0.980 | 1.000 | 0.930 | 0.939 | 0.991 | 0.846 | 0.935 | 0.981 | 0.839 | 0.807 |
| 32 | 0.760 | 1.000 | 0.796 | 0.971 | 0.942 | 1.000 | 0.864 | 1.000 | 0.969 | 1.000 | 0.939 | 0.970 | 0.991 | 0.842 | 0.957 | 0.981 | 0.841 | 0.809 |
| 33 | 0.759 | 1.000 | 0.787 | 0.986 | 0.968 | 1.000 | 0.881 | 1.000 | 0.990 | 1.000 | 0.933 | 0.909 | 0.982 | 0.844 | 0.978 | 0.981 | 0.843 | 0.812 |
| 34 | 0.759 | 0.971 | 0.785 | 0.986 | 0.968 | 1.000 | 0.891 | 1.000 | 0.969 | 1.000 | 0.930 | 0.970 | 0.991 | 0.844 | 0.935 | 0.981 | 0.844 | 0.813 |
| 35 | 0.744 | 1.000 | 0.791 | 0.986 | 0.968 | 1.000 | 0.878 | 1.000 | 0.990 | 1.000 | 0.933 | 0.939 | 0.991 | 0.840 | 0.978 | 0.981 | 0.838 | 0.807 |
| 36 | 0.757 | 1.000 | 0.808 | 1.000 | 0.968 | 1.000 | 0.880 | 1.000 | 0.969 | 1.000 | 0.957 | 0.939 | 0.991 | 0.837 | 0.978 | 0.981 | 0.844 | 0.814 |
| 37 | 0.748 | 1.000 | 0.804 | 1.000 | 0.974 | 1.000 | 0.883 | 1.000 | 0.980 | 1.000 | 0.951 | 0.970 | 0.991 | 0.838 | 0.957 | 0.981 | 0.842 | 0.812 |

| | | | | | | | | | | | | | | | | | | |
|---|---|---|---|---|---|---|---|---|---|---|---|---|---|---|---|---|---|---|
| 38 | 0.749 | 1.000 | 0.812 | 0.986 | 0.979 | 1.000 | 0.880 | 1.000 | 0.969 | 1.000 | 0.959 | 0.939 | 0.991 | 0.844 | 0.978 | 0.981 | 0.845 | 0.815 |
| 39 | 0.753 | 1.000 | 0.806 | 0.986 | 0.968 | 1.000 | 0.884 | 1.000 | 0.990 | 1.000 | 0.948 | 0.939 | 0.991 | 0.840 | 0.978 | 0.981 | 0.844 | 0.814 |
| 40 | 0.760 | 1.000 | 0.810 | 0.986 | 0.979 | 1.000 | 0.874 | 1.000 | 0.980 | 1.000 | 0.945 | 0.909 | 0.991 | 0.839 | 1.000 | 0.981 | 0.845 | 0.815 |
| 41 | 0.759 | 1.000 | 0.808 | 1.000 | 0.984 | 1.000 | 0.866 | 1.000 | 0.969 | 1.000 | 0.951 | 0.909 | 0.991 | 0.833 | 1.000 | 0.981 | 0.843 | 0.812 |
| 42 | 0.751 | 1.000 | 0.800 | 1.000 | 0.974 | 1.000 | 0.892 | 1.000 | 0.969 | 1.000 | 0.954 | 0.909 | 0.982 | 0.841 | 1.000 | 0.981 | 0.844 | 0.814 |
| 43 | 0.755 | 1.000 | 0.798 | 1.000 | 0.989 | 1.000 | 0.883 | 1.000 | 0.980 | 1.000 | 0.951 | 0.879 | 1.000 | 0.837 | 1.000 | 0.981 | 0.844 | 0.814 |
| 44 | 0.758 | 1.000 | 0.820 | 1.000 | 0.984 | 1.000 | 0.878 | 1.000 | 0.980 | 1.000 | 0.959 | 0.909 | 0.982 | 0.837 | 1.000 | 0.981 | 0.847 | 0.817 |
| 45 | 0.762 | 1.000 | 0.804 | 1.000 | 0.984 | 1.000 | 0.886 | 1.000 | 0.990 | 1.000 | 0.962 | 0.909 | 0.973 | 0.846 | 1.000 | 0.981 | 0.850 | 0.821 |
| 46 | 0.752 | 1.000 | 0.814 | 1.000 | 0.979 | 1.000 | 0.877 | 1.000 | 1.000 | 1.000 | 0.968 | 0.909 | 0.991 | 0.844 | 1.000 | 0.981 | 0.847 | 0.817 |
| 47 | 0.758 | 1.000 | 0.818 | 1.000 | 0.979 | 1.000 | 0.883 | 1.000 | 0.990 | 1.000 | 0.962 | 0.939 | 1.000 | 0.845 | 1.000 | 0.981 | 0.850 | 0.821 |
| 48 | 0.760 | 1.000 | 0.822 | 1.000 | 0.984 | 1.000 | 0.874 | 1.000 | 1.000 | 1.000 | 0.957 | 0.909 | 1.000 | 0.847 | 1.000 | 0.981 | 0.850 | 0.821 |
| 49 | 0.769 | 1.000 | 0.818 | 1.000 | 0.974 | 1.000 | 0.875 | 1.000 | 0.990 | 1.000 | 0.959 | 0.939 | 0.973 | 0.851 | 1.000 | 0.981 | 0.853 | 0.824 |
| 50 | 0.768 | 1.000 | 0.828 | 1.000 | 0.974 | 1.000 | 0.881 | 1.000 | 0.990 | 1.000 | 0.957 | 0.939 | 0.964 | 0.840 | 1.000 | 0.981 | 0.851 | 0.822 |
| 51 | 0.767 | 1.000 | 0.814 | 1.000 | 0.979 | 1.000 | 0.875 | 1.000 | 0.980 | 1.000 | 0.945 | 0.909 | 0.982 | 0.836 | 1.000 | 0.981 | 0.847 | 0.817 |
| 52 | 0.765 | 1.000 | 0.814 | 1.000 | 0.984 | 1.000 | 0.891 | 1.000 | 0.990 | 1.000 | 0.968 | 0.939 | 0.982 | 0.835 | 1.000 | 0.981 | 0.850 | 0.822 |
| 53 | 0.769 | 1.000 | 0.816 | 1.000 | 0.979 | 1.000 | 0.889 | 1.000 | 1.000 | 1.000 | 0.959 | 0.970 | 0.982 | 0.836 | 1.000 | 0.981 | 0.851 | 0.822 |
| 54 | 0.767 | 1.000 | 0.814 | 1.000 | 0.989 | 1.000 | 0.881 | 1.000 | 1.000 | 1.000 | 0.951 | 0.939 | 0.982 | 0.843 | 1.000 | 0.981 | 0.851 | 0.822 |
| 55 | 0.765 | 1.000 | 0.826 | 1.000 | 0.989 | 1.000 | 0.883 | 1.000 | 1.000 | 1.000 | 0.957 | 0.970 | 0.973 | 0.844 | 1.000 | 0.981 | 0.853 | 0.824 |
| 56 | 0.767 | 1.000 | 0.812 | 1.000 | 0.989 | 1.000 | 0.889 | 1.000 | 0.980 | 1.000 | 0.954 | 0.939 | 0.973 | 0.840 | 1.000 | 0.981 | 0.850 | 0.821 |
| 57 | 0.762 | 1.000 | 0.808 | 1.000 | 0.989 | 1.000 | 0.894 | 1.000 | 0.990 | 1.000 | 0.954 | 0.939 | 0.982 | 0.839 | 1.000 | 0.981 | 0.849 | 0.820 |
| 58 | 0.760 | 1.000 | 0.814 | 1.000 | 0.984 | 1.000 | 0.889 | 1.000 | 0.990 | 1.000 | 0.965 | 1.000 | 1.000 | 0.847 | 1.000 | 0.981 | 0.852 | 0.824 |
| 59 | 0.768 | 1.000 | 0.802 | 1.000 | 0.979 | 1.000 | 0.891 | 1.000 | 0.990 | 1.000 | 0.959 | 0.939 | 0.991 | 0.841 | 1.000 | 0.981 | 0.851 | 0.822 |
| 60 | 0.767 | 1.000 | 0.818 | 1.000 | 0.984 | 1.000 | 0.883 | 1.000 | 1.000 | 1.000 | 0.971 | 0.939 | 0.991 | 0.845 | 1.000 | 0.981 | 0.853 | 0.825 |
| 61 | 0.773 | 1.000 | 0.814 | 1.000 | 0.984 | 1.000 | 0.878 | 1.000 | 1.000 | 1.000 | 0.962 | 0.970 | 1.000 | 0.840 | 1.000 | 0.981 | 0.853 | 0.824 |
| 62 | 0.762 | 1.000 | 0.826 | 1.000 | 0.984 | 1.000 | 0.881 | 1.000 | 1.000 | 1.000 | 0.962 | 0.970 | 0.991 | 0.847 | 1.000 | 0.981 | 0.853 | 0.824 |
| 63 | 0.767 | 1.000 | 0.820 | 1.000 | 0.984 | 1.000 | 0.894 | 1.000 | 1.000 | 1.000 | 0.959 | 0.939 | 0.982 | 0.840 | 1.000 | 0.981 | 0.853 | 0.824 |
| 64 | 0.761 | 1.000 | 0.814 | 1.000 | 0.984 | 1.000 | 0.894 | 1.000 | 0.990 | 1.000 | 0.954 | 0.939 | 0.982 | 0.847 | 1.000 | 0.981 | 0.852 | 0.823 |
| 65 | 0.766 | 1.000 | 0.818 | 1.000 | 0.984 | 1.000 | 0.886 | 1.000 | 1.000 | 1.000 | 0.968 | 0.909 | 0.982 | 0.844 | 1.000 | 0.981 | 0.853 | 0.824 |
| 66 | 0.761 | 1.000 | 0.826 | 1.000 | 0.984 | 1.000 | 0.888 | 1.000 | 0.990 | 1.000 | 0.968 | 0.909 | 1.000 | 0.851 | 1.000 | 0.981 | 0.854 | 0.826 |
| 67 | 0.765 | 1.000 | 0.816 | 0.986 | 0.989 | 1.000 | 0.886 | 1.000 | 1.000 | 0.979 | 0.965 | 0.970 | 0.982 | 0.854 | 1.000 | 0.981 | 0.855 | 0.826 |
| 68 | 0.768 | 1.000 | 0.830 | 0.986 | 0.979 | 1.000 | 0.884 | 1.000 | 1.000 | 0.979 | 0.971 | 0.909 | 0.982 | 0.846 | 1.000 | 0.981 | 0.854 | 0.826 |
| 69 | 0.764 | 1.000 | 0.826 | 0.986 | 0.979 | 1.000 | 0.884 | 1.000 | 1.000 | 1.000 | 0.971 | 1.000 | 0.991 | 0.846 | 1.000 | 0.981 | 0.854 | 0.825 |
| 70 | 0.769 | 1.000 | 0.838 | 1.000 | 0.979 | 1.000 | 0.888 | 1.000 | 1.000 | 1.000 | 0.977 | 0.970 | 0.991 | 0.848 | 1.000 | 0.981 | 0.858 | 0.830 |
| 71 | 0.773 | 1.000 | 0.828 | 0.986 | 0.974 | 1.000 | 0.883 | 1.000 | 1.000 | 1.000 | 0.968 | 0.970 | 0.991 | 0.851 | 1.000 | 0.981 | 0.857 | 0.829 |
| 72 | 0.773 | 1.000 | 0.828 | 1.000 | 0.984 | 1.000 | 0.888 | 1.000 | 1.000 | 1.000 | 0.965 | 0.970 | 0.982 | 0.846 | 1.000 | 0.981 | 0.857 | 0.829 |
| 73 | 0.777 | 1.000 | 0.846 | 0.986 | 0.979 | 1.000 | 0.874 | 1.000 | 0.990 | 1.000 | 0.959 | 0.970 | 0.991 | 0.850 | 1.000 | 0.981 | 0.858 | 0.830 |
| 74 | 0.773 | 1.000 | 0.828 | 0.986 | 0.979 | 1.000 | 0.878 | 1.000 | 1.000 | 1.000 | 0.980 | 0.909 | 0.982 | 0.840 | 1.000 | 0.981 | 0.854 | 0.825 |
| 75 | 0.774 | 1.000 | 0.826 | 0.986 | 0.974 | 1.000 | 0.883 | 1.000 | 0.990 | 1.000 | 0.965 | 0.970 | 0.991 | 0.844 | 1.000 | 0.981 | 0.855 | 0.826 |
| 76 | 0.774 | 1.000 | 0.832 | 0.986 | 0.979 | 1.000 | 0.892 | 1.000 | 0.990 | 1.000 | 0.965 | 0.970 | 0.991 | 0.852 | 1.000 | 0.981 | 0.859 | 0.832 |

| | | | | | | | | | | | | | | | | | | |
|---|---|---|---|---|---|---|---|---|---|---|---|---|---|---|---|---|---|---|
| 77 | 0.767 | 1.000 | 0.830 | 0.986 | 0.968 | 1.000 | 0.877 | 1.000 | 0.990 | 1.000 | 0.962 | 1.000 | 1.000 | 0.854 | 1.000 | 0.981 | 0.855 | 0.827 |
| 78 | 0.769 | 1.000 | 0.820 | 0.986 | 0.979 | 1.000 | 0.888 | 1.000 | 0.990 | 1.000 | 0.965 | 0.939 | 1.000 | 0.849 | 1.000 | 0.981 | 0.855 | 0.827 |
| 79 | 0.770 | 1.000 | 0.816 | 0.986 | 0.968 | 1.000 | 0.891 | 1.000 | 0.990 | 1.000 | 0.965 | 0.939 | 1.000 | 0.843 | 1.000 | 0.981 | 0.853 | 0.825 |
| 80 | 0.779 | 1.000 | 0.814 | 0.986 | 0.974 | 1.000 | 0.894 | 1.000 | 0.980 | 1.000 | 0.968 | 1.000 | 0.991 | 0.849 | 1.000 | 0.981 | 0.858 | 0.830 |
| 81 | 0.773 | 1.000 | 0.826 | 0.986 | 0.979 | 1.000 | 0.897 | 1.000 | 0.990 | 1.000 | 0.968 | 0.970 | 1.000 | 0.849 | 1.000 | 0.981 | 0.858 | 0.831 |
| 82 | 0.774 | 1.000 | 0.820 | 0.986 | 0.979 | 1.000 | 0.897 | 1.000 | 0.990 | 1.000 | 0.957 | 1.000 | 0.991 | 0.843 | 1.000 | 1.000 | 0.856 | 0.828 |
| 83 | 0.767 | 1.000 | 0.830 | 0.986 | 0.979 | 1.000 | 0.898 | 1.000 | 0.990 | 1.000 | 0.965 | 0.970 | 0.991 | 0.856 | 1.000 | 0.981 | 0.859 | 0.831 |
| 84 | 0.775 | 1.000 | 0.840 | 0.986 | 0.974 | 1.000 | 0.886 | 1.000 | 0.990 | 1.000 | 0.971 | 0.970 | 0.991 | 0.854 | 1.000 | 0.981 | 0.860 | 0.832 |
| 85 | 0.763 | 1.000 | 0.806 | 0.943 | 0.942 | 1.000 | 0.889 | 1.000 | 0.939 | 0.979 | 0.951 | 1.000 | 0.964 | 0.846 | 1.000 | 0.981 | 0.847 | 0.817 |
| 86 | 0.765 | 1.000 | 0.791 | 0.943 | 0.958 | 0.960 | 0.877 | 1.000 | 0.939 | 0.979 | 0.948 | 0.970 | 0.955 | 0.842 | 1.000 | 0.981 | 0.843 | 0.812 |
| 87 | 0.763 | 1.000 | 0.792 | 0.957 | 0.958 | 0.960 | 0.898 | 1.000 | 0.949 | 0.979 | 0.954 | 0.939 | 0.946 | 0.841 | 1.000 | 0.981 | 0.845 | 0.815 |
| 88 | 0.767 | 1.000 | 0.808 | 0.943 | 0.958 | 0.960 | 0.895 | 1.000 | 0.939 | 0.979 | 0.954 | 1.000 | 0.964 | 0.844 | 1.000 | 0.981 | 0.849 | 0.820 |
| 89 | 0.757 | 1.000 | 0.802 | 0.957 | 0.963 | 0.960 | 0.888 | 1.000 | 0.939 | 0.979 | 0.954 | 0.970 | 0.955 | 0.843 | 1.000 | 0.981 | 0.844 | 0.814 |
| 90 | 0.759 | 1.000 | 0.800 | 0.957 | 0.968 | 0.960 | 0.895 | 1.000 | 0.949 | 0.979 | 0.954 | 0.970 | 0.955 | 0.847 | 1.000 | 0.981 | 0.847 | 0.817 |
| 91 | 0.764 | 1.000 | 0.800 | 0.929 | 0.963 | 1.000 | 0.894 | 1.000 | 0.939 | 0.979 | 0.957 | 0.939 | 0.955 | 0.848 | 1.000 | 0.981 | 0.848 | 0.818 |
| 92 | 0.768 | 1.000 | 0.802 | 0.929 | 0.958 | 0.960 | 0.892 | 1.000 | 0.939 | 0.979 | 0.948 | 0.879 | 0.964 | 0.850 | 1.000 | 0.981 | 0.848 | 0.818 |
| 93 | 0.770 | 1.000 | 0.791 | 0.957 | 0.958 | 0.960 | 0.891 | 1.000 | 0.939 | 0.979 | 0.948 | 0.939 | 0.964 | 0.846 | 1.000 | 0.981 | 0.847 | 0.817 |
| 94 | 0.768 | 1.000 | 0.804 | 0.943 | 0.963 | 1.000 | 0.897 | 1.000 | 0.939 | 0.979 | 0.948 | 0.879 | 0.955 | 0.840 | 1.000 | 0.981 | 0.847 | 0.817 |
| 95 | 0.767 | 1.000 | 0.798 | 0.957 | 0.968 | 0.960 | 0.892 | 1.000 | 0.939 | 0.979 | 0.951 | 0.909 | 0.955 | 0.836 | 1.000 | 0.981 | 0.845 | 0.815 |
| 96 | 0.769 | 1.000 | 0.794 | 0.957 | 0.968 | 1.000 | 0.897 | 1.000 | 0.949 | 0.979 | 0.945 | 0.879 | 0.955 | 0.840 | 1.000 | 0.981 | 0.847 | 0.817 |
| 97 | 0.758 | 1.000 | 0.808 | 0.943 | 0.963 | 0.960 | 0.884 | 1.000 | 0.949 | 0.979 | 0.942 | 0.909 | 0.955 | 0.848 | 1.000 | 0.981 | 0.845 | 0.814 |
| 98 | 0.765 | 1.000 | 0.800 | 0.971 | 0.974 | 1.000 | 0.894 | 1.000 | 0.949 | 0.979 | 0.945 | 0.970 | 0.964 | 0.845 | 1.000 | 0.981 | 0.848 | 0.819 |
| 99 | 0.762 | 1.000 | 0.806 | 0.971 | 0.968 | 0.960 | 0.886 | 1.000 | 0.949 | 0.979 | 0.945 | 0.909 | 0.955 | 0.840 | 1.000 | 0.981 | 0.845 | 0.814 |
| 100 | 0.764 | 1.000 | 0.804 | 0.971 | 0.974 | 1.000 | 0.884 | 1.000 | 0.949 | 0.979 | 0.954 | 1.000 | 0.955 | 0.838 | 1.000 | 0.981 | 0.846 | 0.816 |
| 101 | 0.769 | 1.000 | 0.796 | 0.957 | 0.963 | 0.960 | 0.889 | 1.000 | 0.949 | 0.979 | 0.959 | 0.879 | 0.964 | 0.847 | 1.000 | 0.981 | 0.849 | 0.818 |
| 102 | 0.762 | 1.000 | 0.800 | 0.957 | 0.968 | 1.000 | 0.888 | 1.000 | 0.949 | 0.979 | 0.951 | 0.909 | 0.955 | 0.840 | 1.000 | 0.981 | 0.845 | 0.814 |
| 103 | 0.773 | 1.000 | 0.796 | 0.957 | 0.958 | 0.960 | 0.888 | 1.000 | 0.949 | 0.979 | 0.962 | 0.970 | 0.964 | 0.847 | 1.000 | 0.981 | 0.850 | 0.821 |
| 104 | 0.764 | 1.000 | 0.806 | 0.957 | 0.979 | 1.000 | 0.886 | 1.000 | 0.949 | 0.979 | 0.959 | 0.970 | 0.973 | 0.850 | 1.000 | 0.981 | 0.850 | 0.821 |
| 105 | 0.765 | 1.000 | 0.800 | 0.957 | 0.958 | 0.960 | 0.884 | 1.000 | 0.949 | 0.979 | 0.957 | 0.970 | 0.955 | 0.839 | 1.000 | 0.981 | 0.845 | 0.815 |
| 106 | 0.766 | 1.000 | 0.806 | 0.957 | 0.963 | 1.000 | 0.892 | 1.000 | 0.949 | 1.000 | 0.951 | 0.970 | 0.955 | 0.843 | 1.000 | 0.981 | 0.848 | 0.818 |
| 107 | 0.763 | 1.000 | 0.791 | 0.957 | 0.974 | 1.000 | 0.891 | 1.000 | 0.949 | 0.979 | 0.957 | 0.939 | 0.964 | 0.841 | 1.000 | 0.981 | 0.846 | 0.816 |
| 108 | 0.772 | 1.000 | 0.789 | 0.943 | 0.979 | 0.960 | 0.880 | 1.000 | 0.949 | 0.979 | 0.959 | 0.939 | 0.955 | 0.847 | 1.000 | 0.981 | 0.848 | 0.817 |
| 109 | 0.765 | 1.000 | 0.812 | 0.943 | 0.974 | 1.000 | 0.898 | 1.000 | 0.949 | 0.979 | 0.959 | 0.909 | 0.964 | 0.845 | 1.000 | 0.981 | 0.850 | 0.821 |
| 110 | 0.760 | 1.000 | 0.798 | 0.943 | 0.984 | 1.000 | 0.884 | 1.000 | 0.949 | 1.000 | 0.957 | 0.939 | 0.955 | 0.847 | 1.000 | 0.981 | 0.847 | 0.816 |
| 111 | 0.764 | 1.000 | 0.792 | 0.943 | 0.968 | 1.000 | 0.892 | 1.000 | 0.949 | 0.979 | 0.959 | 0.939 | 0.964 | 0.839 | 1.000 | 1.000 | 0.846 | 0.816 |
| 112 | 0.775 | 1.000 | 0.808 | 0.943 | 0.963 | 1.000 | 0.898 | 1.000 | 0.949 | 0.979 | 0.939 | 0.939 | 0.964 | 0.839 | 1.000 | 0.981 | 0.850 | 0.820 |
| 113 | 0.767 | 1.000 | 0.785 | 0.943 | 0.968 | 0.960 | 0.892 | 1.000 | 0.949 | 0.979 | 0.954 | 0.939 | 0.964 | 0.844 | 1.000 | 0.981 | 0.846 | 0.816 |
| 114 | 0.765 | 1.000 | 0.810 | 0.943 | 0.968 | 0.960 | 0.894 | 1.000 | 0.949 | 0.979 | 0.957 | 0.939 | 0.938 | 0.843 | 1.000 | 0.981 | 0.848 | 0.818 |
| 115 | 0.764 | 1.000 | 0.796 | 0.957 | 0.979 | 1.000 | 0.895 | 1.000 | 0.949 | 1.000 | 0.948 | 0.970 | 0.955 | 0.836 | 1.000 | 0.981 | 0.846 | 0.815 |

| | | | | | | | | | | | | | | | | | |
|---|---|---|---|---|---|---|---|---|---|---|---|---|---|---|---|---|---|
| 116 | 0.765 | 1.000 | 0.794 | 0.943 | 0.979 | 1.000 | 0.892 | 1.000 | 0.949 | 0.979 | 0.954 | 0.970 | 0.955 | 0.851 | 1.000 | 0.981 | 0.849 | 0.819 |
| 117 | 0.763 | 1.000 | 0.789 | 0.943 | 0.979 | 0.960 | 0.888 | 1.000 | 0.949 | 0.979 | 0.957 | 0.909 | 0.955 | 0.844 | 1.000 | 0.981 | 0.845 | 0.815 |
| 118 | 0.768 | 1.000 | 0.800 | 0.943 | 0.979 | 1.000 | 0.894 | 1.000 | 0.949 | 0.979 | 0.954 | 0.970 | 0.964 | 0.844 | 1.000 | 0.981 | 0.849 | 0.820 |
| 119 | 0.766 | 1.000 | 0.802 | 0.957 | 0.979 | 0.960 | 0.889 | 1.000 | 0.949 | 0.979 | 0.951 | 0.970 | 0.964 | 0.845 | 1.000 | 0.981 | 0.848 | 0.818 |
| 120 | 0.751 | 1.000 | 0.789 | 0.943 | 0.968 | 1.000 | 0.888 | 1.000 | 0.949 | 1.000 | 0.945 | 0.939 | 0.964 | 0.851 | 1.000 | 0.981 | 0.843 | 0.812 |
| 121 | 0.770 | 1.000 | 0.789 | 0.943 | 0.979 | 1.000 | 0.889 | 1.000 | 0.949 | 0.979 | 0.948 | 0.939 | 0.955 | 0.840 | 1.000 | 0.981 | 0.846 | 0.816 |
| 122 | 0.761 | 1.000 | 0.779 | 0.971 | 0.979 | 1.000 | 0.886 | 1.000 | 0.949 | 0.979 | 0.954 | 0.939 | 0.964 | 0.842 | 1.000 | 0.981 | 0.844 | 0.813 |
| 123 | 0.777 | 1.000 | 0.802 | 0.957 | 0.979 | 1.000 | 0.888 | 1.000 | 0.949 | 0.979 | 0.942 | 0.939 | 0.955 | 0.841 | 1.000 | 0.981 | 0.850 | 0.820 |
| 124 | 0.765 | 1.000 | 0.783 | 0.957 | 0.984 | 0.960 | 0.891 | 1.000 | 0.949 | 0.979 | 0.945 | 0.939 | 0.964 | 0.849 | 1.000 | 0.981 | 0.847 | 0.817 |
| 125 | 0.765 | 1.000 | 0.775 | 0.957 | 0.979 | 0.960 | 0.892 | 1.000 | 0.949 | 0.979 | 0.939 | 0.939 | 0.955 | 0.849 | 1.000 | 0.981 | 0.845 | 0.814 |
| 126 | 0.770 | 1.000 | 0.798 | 0.957 | 0.989 | 0.960 | 0.888 | 1.000 | 0.949 | 0.979 | 0.951 | 0.939 | 0.955 | 0.844 | 1.000 | 0.981 | 0.849 | 0.819 |
| 127 | 0.765 | 1.000 | 0.779 | 0.957 | 0.979 | 0.960 | 0.894 | 1.000 | 0.949 | 1.000 | 0.945 | 0.970 | 0.955 | 0.846 | 1.000 | 0.981 | 0.846 | 0.815 |
| 128 | 0.775 | 1.000 | 0.787 | 0.971 | 0.974 | 0.960 | 0.886 | 1.000 | 0.959 | 0.979 | 0.939 | 0.970 | 0.973 | 0.835 | 1.000 | 0.981 | 0.846 | 0.815 |
| 129 | 0.767 | 1.000 | 0.791 | 0.957 | 0.984 | 0.960 | 0.895 | 1.000 | 0.949 | 1.000 | 0.948 | 0.970 | 0.964 | 0.850 | 1.000 | 0.981 | 0.850 | 0.820 |
| 130 | 0.766 | 1.000 | 0.785 | 0.957 | 0.979 | 0.960 | 0.895 | 1.000 | 0.949 | 1.000 | 0.942 | 0.970 | 0.955 | 0.844 | 1.000 | 0.981 | 0.846 | 0.816 |
| 131 | 0.762 | 1.000 | 0.791 | 0.943 | 0.984 | 1.000 | 0.891 | 1.000 | 0.959 | 1.000 | 0.936 | 0.970 | 0.955 | 0.846 | 1.000 | 0.981 | 0.846 | 0.816 |
| 132 | 0.763 | 1.000 | 0.787 | 0.943 | 0.974 | 0.960 | 0.881 | 1.000 | 0.949 | 1.000 | 0.948 | 0.939 | 0.964 | 0.841 | 1.000 | 0.981 | 0.843 | 0.813 |
| 133 | 0.769 | 1.000 | 0.806 | 0.957 | 0.974 | 0.960 | 0.898 | 1.000 | 0.949 | 1.000 | 0.939 | 0.970 | 0.973 | 0.849 | 1.000 | 0.981 | 0.851 | 0.822 |
| 134 | 0.777 | 1.000 | 0.791 | 0.943 | 0.984 | 0.960 | 0.884 | 1.000 | 0.949 | 1.000 | 0.942 | 0.939 | 0.964 | 0.843 | 1.000 | 0.981 | 0.849 | 0.818 |
| 135 | 0.771 | 1.000 | 0.781 | 0.943 | 0.979 | 1.000 | 0.897 | 1.000 | 0.949 | 1.000 | 0.936 | 0.970 | 0.964 | 0.840 | 1.000 | 0.981 | 0.846 | 0.816 |
| 136 | 0.767 | 1.000 | 0.779 | 0.957 | 0.984 | 1.000 | 0.892 | 1.000 | 0.939 | 1.000 | 0.936 | 0.939 | 0.982 | 0.844 | 1.000 | 0.981 | 0.846 | 0.816 |
| 137 | 0.769 | 1.000 | 0.777 | 0.957 | 0.974 | 0.960 | 0.895 | 1.000 | 0.949 | 1.000 | 0.945 | 0.939 | 0.964 | 0.847 | 1.000 | 0.981 | 0.847 | 0.817 |
| 138 | 0.773 | 1.000 | 0.791 | 0.957 | 0.979 | 0.960 | 0.892 | 1.000 | 0.949 | 1.000 | 0.951 | 0.970 | 0.964 | 0.847 | 1.000 | 0.981 | 0.851 | 0.821 |
| 139 | 0.776 | 1.000 | 0.787 | 0.943 | 0.979 | 0.960 | 0.891 | 1.000 | 0.949 | 1.000 | 0.945 | 0.939 | 0.955 | 0.851 | 1.000 | 0.981 | 0.851 | 0.821 |
| 140 | 0.767 | 1.000 | 0.794 | 0.943 | 0.968 | 0.960 | 0.897 | 1.000 | 0.939 | 0.979 | 0.936 | 0.939 | 0.973 | 0.845 | 1.000 | 0.981 | 0.847 | 0.817 |
| 141 | 0.771 | 1.000 | 0.794 | 0.957 | 0.974 | 0.960 | 0.891 | 1.000 | 0.949 | 1.000 | 0.936 | 0.939 | 0.973 | 0.846 | 1.000 | 0.981 | 0.848 | 0.818 |
| 142 | 0.775 | 1.000 | 0.802 | 0.957 | 0.968 | 0.960 | 0.891 | 1.000 | 0.949 | 1.000 | 0.945 | 0.970 | 0.955 | 0.844 | 1.000 | 0.981 | 0.850 | 0.820 |
| 143 | 0.765 | 1.000 | 0.796 | 0.957 | 0.979 | 0.960 | 0.894 | 1.000 | 0.949 | 1.000 | 0.942 | 0.939 | 0.946 | 0.840 | 1.000 | 0.981 | 0.846 | 0.815 |
| 144 | 0.767 | 1.000 | 0.792 | 0.943 | 0.974 | 0.960 | 0.892 | 1.000 | 0.949 | 1.000 | 0.936 | 0.970 | 0.955 | 0.848 | 1.000 | 0.981 | 0.848 | 0.817 |
| 145 | 0.771 | 1.000 | 0.792 | 0.943 | 0.963 | 0.960 | 0.884 | 1.000 | 0.949 | 1.000 | 0.942 | 0.970 | 0.964 | 0.841 | 1.000 | 0.981 | 0.846 | 0.815 |
| 146 | 0.778 | 1.000 | 0.792 | 0.971 | 0.974 | 1.000 | 0.888 | 1.000 | 0.949 | 1.000 | 0.942 | 0.970 | 0.955 | 0.840 | 1.000 | 0.981 | 0.849 | 0.819 |
| 147 | 0.766 | 1.000 | 0.812 | 0.929 | 0.979 | 0.960 | 0.895 | 1.000 | 0.949 | 1.000 | 0.939 | 0.939 | 0.946 | 0.847 | 1.000 | 0.981 | 0.849 | 0.820 |
| 148 | 0.775 | 1.000 | 0.789 | 0.943 | 0.974 | 0.960 | 0.891 | 1.000 | 0.949 | 1.000 | 0.939 | 0.970 | 0.938 | 0.840 | 1.000 | 0.981 | 0.847 | 0.817 |
| 149 | 0.771 | 1.000 | 0.787 | 0.943 | 0.968 | 0.960 | 0.884 | 1.000 | 0.949 | 1.000 | 0.939 | 0.939 | 0.964 | 0.850 | 1.000 | 0.981 | 0.848 | 0.817 |
| 150 | 0.757 | 1.000 | 0.804 | 0.971 | 0.979 | 0.960 | 0.901 | 1.000 | 0.949 | 1.000 | 0.933 | 0.939 | 0.964 | 0.844 | 1.000 | 0.981 | 0.846 | 0.816 |
| 151 | 0.761 | 1.000 | 0.796 | 0.943 | 0.974 | 0.960 | 0.901 | 1.000 | 0.939 | 1.000 | 0.939 | 0.939 | 0.964 | 0.847 | 1.000 | 0.981 | 0.847 | 0.817 |
| 152 | 0.769 | 1.000 | 0.785 | 0.943 | 0.974 | 0.960 | 0.904 | 1.000 | 0.949 | 1.000 | 0.945 | 0.970 | 0.964 | 0.843 | 1.000 | 0.981 | 0.848 | 0.818 |
| 153 | 0.767 | 1.000 | 0.791 | 0.943 | 0.984 | 1.000 | 0.904 | 1.000 | 0.949 | 1.000 | 0.942 | 0.970 | 0.955 | 0.846 | 1.000 | 0.981 | 0.849 | 0.820 |
| 154 | 0.771 | 1.000 | 0.792 | 0.957 | 0.979 | 0.960 | 0.892 | 1.000 | 0.949 | 1.000 | 0.930 | 0.970 | 0.964 | 0.847 | 1.000 | 0.981 | 0.849 | 0.819 |

| | | | | | | | | | | | | | | | | | |
|---|---|---|---|---|---|---|---|---|---|---|---|---|---|---|---|---|---|
| 155 | 0.764 | 1.000 | 0.800 | 0.943 | 0.968 | 0.960 | 0.897 | 1.000 | 0.959 | 1.000 | 0.948 | 0.970 | 0.964 | 0.847 | 1.000 | 0.981 | 0.849 | 0.819 |
| 156 | 0.769 | 1.000 | 0.798 | 0.957 | 0.979 | 1.000 | 0.894 | 1.000 | 0.949 | 1.000 | 0.933 | 0.970 | 0.973 | 0.852 | 1.000 | 0.981 | 0.851 | 0.821 |
| 157 | 0.769 | 1.000 | 0.806 | 0.943 | 0.979 | 0.960 | 0.889 | 1.000 | 0.959 | 1.000 | 0.957 | 0.970 | 0.982 | 0.849 | 1.000 | 0.981 | 0.852 | 0.822 |
| 158 | 0.764 | 1.000 | 0.804 | 0.957 | 0.984 | 0.960 | 0.900 | 1.000 | 0.949 | 1.000 | 0.948 | 0.939 | 0.991 | 0.846 | 1.000 | 0.981 | 0.850 | 0.821 |
| 159 | 0.766 | 1.000 | 0.792 | 0.957 | 0.984 | 0.960 | 0.898 | 1.000 | 0.939 | 1.000 | 0.933 | 0.970 | 0.973 | 0.850 | 1.000 | 0.981 | 0.849 | 0.819 |
| 160 | 0.773 | 1.000 | 0.800 | 0.957 | 0.979 | 0.960 | 0.900 | 1.000 | 0.949 | 1.000 | 0.939 | 0.970 | 0.964 | 0.852 | 1.000 | 0.981 | 0.853 | 0.824 |
| 161 | 0.769 | 1.000 | 0.789 | 0.943 | 0.979 | 1.000 | 0.900 | 1.000 | 0.949 | 1.000 | 0.933 | 0.970 | 0.982 | 0.854 | 1.000 | 0.981 | 0.851 | 0.822 |
| 162 | 0.769 | 1.000 | 0.804 | 0.971 | 0.974 | 1.000 | 0.891 | 1.000 | 0.949 | 1.000 | 0.945 | 0.970 | 0.964 | 0.848 | 1.000 | 0.981 | 0.850 | 0.821 |
| 163 | 0.771 | 1.000 | 0.806 | 0.986 | 0.984 | 1.000 | 0.898 | 1.000 | 0.949 | 1.000 | 0.942 | 0.939 | 0.982 | 0.848 | 1.000 | 0.981 | 0.853 | 0.824 |
| 164 | 0.766 | 1.000 | 0.804 | 0.957 | 0.974 | 0.960 | 0.898 | 1.000 | 0.949 | 1.000 | 0.942 | 0.970 | 0.991 | 0.846 | 1.000 | 0.981 | 0.850 | 0.821 |
| 165 | 0.775 | 1.000 | 0.794 | 0.971 | 0.974 | 1.000 | 0.903 | 1.000 | 0.949 | 1.000 | 0.939 | 0.970 | 0.964 | 0.842 | 1.000 | 0.963 | 0.851 | 0.821 |
| 166 | 0.768 | 1.000 | 0.796 | 0.957 | 0.974 | 1.000 | 0.904 | 1.000 | 0.949 | 1.000 | 0.948 | 0.970 | 0.973 | 0.853 | 1.000 | 0.981 | 0.853 | 0.823 |
| 167 | 0.771 | 1.000 | 0.802 | 0.943 | 0.979 | 0.960 | 0.911 | 1.000 | 0.949 | 1.000 | 0.942 | 0.970 | 0.982 | 0.845 | 1.000 | 0.963 | 0.852 | 0.823 |
| 168 | 0.770 | 1.000 | 0.802 | 0.957 | 0.979 | 0.960 | 0.903 | 1.000 | 0.949 | 1.000 | 0.942 | 0.970 | 0.973 | 0.844 | 1.000 | 0.981 | 0.851 | 0.822 |
| 169 | 0.769 | 1.000 | 0.808 | 0.943 | 0.979 | 0.960 | 0.898 | 1.000 | 0.949 | 1.000 | 0.954 | 0.909 | 0.955 | 0.848 | 1.000 | 0.981 | 0.851 | 0.822 |
| 170 | 0.773 | 1.000 | 0.787 | 0.957 | 0.984 | 1.000 | 0.895 | 1.000 | 0.949 | 1.000 | 0.942 | 0.939 | 0.982 | 0.842 | 1.000 | 0.981 | 0.849 | 0.819 |
| 171 | 0.773 | 1.000 | 0.806 | 0.957 | 0.979 | 1.000 | 0.897 | 1.000 | 0.949 | 1.000 | 0.939 | 0.939 | 0.982 | 0.850 | 1.000 | 0.981 | 0.853 | 0.824 |
| 172 | 0.769 | 1.000 | 0.791 | 0.971 | 0.984 | 1.000 | 0.891 | 1.000 | 0.959 | 1.000 | 0.948 | 0.970 | 0.973 | 0.845 | 1.000 | 0.981 | 0.849 | 0.820 |
| 173 | 0.766 | 1.000 | 0.791 | 0.943 | 0.984 | 0.960 | 0.904 | 1.000 | 0.949 | 1.000 | 0.936 | 0.909 | 0.973 | 0.847 | 1.000 | 0.981 | 0.849 | 0.819 |
| 174 | 0.763 | 1.000 | 0.796 | 0.971 | 0.979 | 0.960 | 0.898 | 1.000 | 0.949 | 1.000 | 0.945 | 0.970 | 0.982 | 0.841 | 1.000 | 0.981 | 0.847 | 0.817 |
| 175 | 0.772 | 1.000 | 0.806 | 0.943 | 0.984 | 1.000 | 0.891 | 1.000 | 0.949 | 1.000 | 0.945 | 0.970 | 0.973 | 0.851 | 1.000 | 0.981 | 0.852 | 0.823 |
| 176 | 0.775 | 1.000 | 0.800 | 0.957 | 0.984 | 0.960 | 0.886 | 1.000 | 0.949 | 1.000 | 0.945 | 0.939 | 0.982 | 0.847 | 1.000 | 0.981 | 0.851 | 0.821 |
| 177 | 0.766 | 1.000 | 0.798 | 0.943 | 0.984 | 1.000 | 0.894 | 1.000 | 0.949 | 1.000 | 0.951 | 0.970 | 0.964 | 0.840 | 1.000 | 0.981 | 0.848 | 0.818 |
| 178 | 0.767 | 1.000 | 0.802 | 0.943 | 0.979 | 0.960 | 0.904 | 1.000 | 0.949 | 1.000 | 0.942 | 0.970 | 0.973 | 0.854 | 1.000 | 0.981 | 0.853 | 0.824 |
| 179 | 0.769 | 1.000 | 0.800 | 0.971 | 0.979 | 1.000 | 0.903 | 1.000 | 0.959 | 1.000 | 0.936 | 0.970 | 0.973 | 0.845 | 1.000 | 0.981 | 0.851 | 0.822 |
| 180 | 0.772 | 1.000 | 0.804 | 0.943 | 0.989 | 0.960 | 0.897 | 1.000 | 0.959 | 1.000 | 0.954 | 0.970 | 0.964 | 0.845 | 1.000 | 0.981 | 0.852 | 0.823 |
| 181 | 0.766 | 1.000 | 0.802 | 0.971 | 0.984 | 0.960 | 0.895 | 1.000 | 0.949 | 1.000 | 0.945 | 0.970 | 0.964 | 0.850 | 1.000 | 0.981 | 0.851 | 0.821 |
| 182 | 0.770 | 1.000 | 0.802 | 0.943 | 0.984 | 1.000 | 0.898 | 1.000 | 0.949 | 1.000 | 0.936 | 0.939 | 0.955 | 0.846 | 1.000 | 0.981 | 0.850 | 0.820 |
| 183 | 0.769 | 1.000 | 0.806 | 0.971 | 0.989 | 0.960 | 0.900 | 1.000 | 0.959 | 1.000 | 0.945 | 0.939 | 0.973 | 0.847 | 1.000 | 0.981 | 0.852 | 0.823 |
| 184 | 0.768 | 1.000 | 0.792 | 0.943 | 0.979 | 1.000 | 0.880 | 1.000 | 0.959 | 1.000 | 0.948 | 0.939 | 0.982 | 0.848 | 1.000 | 0.981 | 0.848 | 0.818 |
| 185 | 0.776 | 1.000 | 0.798 | 0.943 | 0.989 | 1.000 | 0.895 | 1.000 | 0.959 | 1.000 | 0.951 | 0.939 | 0.973 | 0.847 | 1.000 | 0.981 | 0.853 | 0.824 |
| 186 | 0.770 | 1.000 | 0.804 | 0.957 | 0.989 | 0.960 | 0.894 | 1.000 | 0.959 | 1.000 | 0.948 | 0.970 | 0.982 | 0.837 | 1.000 | 0.981 | 0.849 | 0.820 |
| 187 | 0.780 | 1.000 | 0.796 | 0.943 | 0.989 | 0.960 | 0.892 | 1.000 | 0.959 | 1.000 | 0.936 | 0.970 | 0.973 | 0.849 | 1.000 | 0.981 | 0.853 | 0.824 |
| 188 | 0.770 | 1.000 | 0.798 | 0.957 | 0.979 | 0.960 | 0.897 | 1.000 | 0.959 | 1.000 | 0.939 | 0.939 | 0.982 | 0.847 | 1.000 | 0.981 | 0.851 | 0.821 |
| 189 | 0.767 | 1.000 | 0.796 | 0.957 | 0.984 | 0.960 | 0.888 | 1.000 | 0.959 | 1.000 | 0.948 | 0.970 | 0.973 | 0.850 | 1.000 | 0.981 | 0.850 | 0.820 |
| 190 | 0.767 | 1.000 | 0.798 | 0.957 | 0.984 | 1.000 | 0.901 | 1.000 | 0.959 | 1.000 | 0.948 | 0.970 | 0.973 | 0.842 | 1.000 | 0.981 | 0.850 | 0.820 |
| 191 | 0.773 | 1.000 | 0.785 | 0.957 | 0.989 | 1.000 | 0.895 | 1.000 | 0.959 | 1.000 | 0.942 | 0.970 | 0.991 | 0.849 | 1.000 | 0.981 | 0.852 | 0.822 |
| 192 | 0.777 | 1.000 | 0.810 | 0.943 | 0.984 | 1.000 | 0.898 | 1.000 | 0.959 | 1.000 | 0.945 | 0.939 | 0.973 | 0.855 | 1.000 | 0.981 | 0.857 | 0.828 |
| 193 | 0.765 | 1.000 | 0.808 | 0.943 | 0.984 | 0.960 | 0.897 | 1.000 | 0.959 | 1.000 | 0.957 | 0.939 | 0.955 | 0.853 | 1.000 | 0.981 | 0.852 | 0.823 |

| | | | | | | | | | | | | | | | | | | |
|---|---|---|---|---|---|---|---|---|---|---|---|---|---|---|---|---|---|---|
| 194 | 0.771 | 1.000 | 0.798 | 0.957 | 0.979 | 1.000 | 0.898 | 1.000 | 0.959 | 0.979 | 0.951 | 0.970 | 0.973 | 0.852 | 1.000 | 0.981 | 0.853 | 0.824 |
| 195 | 0.765 | 1.000 | 0.794 | 0.971 | 0.984 | 1.000 | 0.898 | 1.000 | 0.959 | 1.000 | 0.954 | 0.909 | 0.982 | 0.845 | 1.000 | 0.981 | 0.850 | 0.820 |
| 196 | 0.781 | 1.000 | 0.804 | 0.957 | 0.989 | 1.000 | 0.906 | 1.000 | 0.959 | 1.000 | 0.945 | 0.970 | 0.964 | 0.848 | 1.000 | 0.981 | 0.857 | 0.828 |
| 197 | 0.767 | 1.000 | 0.781 | 0.957 | 0.979 | 1.000 | 0.904 | 1.000 | 0.959 | 1.000 | 0.942 | 0.970 | 0.973 | 0.849 | 1.000 | 0.981 | 0.850 | 0.820 |
| 198 | 0.779 | 1.000 | 0.806 | 0.957 | 0.989 | 0.960 | 0.900 | 1.000 | 0.959 | 1.000 | 0.954 | 0.939 | 0.973 | 0.844 | 1.000 | 0.981 | 0.855 | 0.826 |
| 199 | 0.773 | 1.000 | 0.792 | 0.957 | 0.989 | 1.000 | 0.900 | 1.000 | 0.959 | 1.000 | 0.954 | 0.970 | 0.973 | 0.851 | 1.000 | 0.981 | 0.854 | 0.825 |
| 200 | 0.761 | 1.000 | 0.792 | 0.971 | 0.989 | 1.000 | 0.903 | 1.000 | 0.959 | 1.000 | 0.951 | 0.939 | 0.982 | 0.845 | 1.000 | 0.981 | 0.849 | 0.820 |
| 201 | 0.773 | 1.000 | 0.800 | 0.943 | 0.989 | 1.000 | 0.894 | 1.000 | 0.959 | 1.000 | 0.948 | 0.939 | 0.973 | 0.856 | 1.000 | 0.981 | 0.854 | 0.825 |
| 202 | 0.773 | 1.000 | 0.800 | 0.943 | 0.989 | 1.000 | 0.895 | 1.000 | 0.959 | 1.000 | 0.945 | 0.970 | 0.982 | 0.849 | 1.000 | 0.981 | 0.853 | 0.824 |
| 203 | 0.762 | 1.000 | 0.792 | 0.986 | 0.984 | 1.000 | 0.897 | 1.000 | 0.959 | 1.000 | 0.951 | 0.939 | 0.991 | 0.847 | 1.000 | 0.981 | 0.849 | 0.820 |
| 204 | 0.777 | 1.000 | 0.804 | 0.971 | 0.989 | 0.960 | 0.898 | 1.000 | 0.959 | 1.000 | 0.945 | 0.970 | 0.973 | 0.848 | 1.000 | 0.981 | 0.855 | 0.826 |
| 205 | 0.775 | 1.000 | 0.796 | 0.957 | 0.984 | 0.960 | 0.900 | 1.000 | 0.959 | 1.000 | 0.954 | 0.970 | 0.964 | 0.855 | 1.000 | 0.981 | 0.855 | 0.826 |
| 206 | 0.771 | 1.000 | 0.785 | 0.957 | 0.984 | 1.000 | 0.898 | 1.000 | 0.959 | 1.000 | 0.945 | 0.970 | 0.973 | 0.846 | 1.000 | 0.981 | 0.850 | 0.820 |
| 207 | 0.773 | 1.000 | 0.802 | 0.957 | 0.989 | 1.000 | 0.908 | 1.000 | 0.959 | 1.000 | 0.945 | 0.970 | 0.973 | 0.851 | 1.000 | 0.981 | 0.855 | 0.827 |
| 208 | 0.768 | 1.000 | 0.796 | 0.957 | 0.989 | 1.000 | 0.904 | 1.000 | 0.959 | 1.000 | 0.951 | 0.970 | 0.964 | 0.840 | 1.000 | 0.981 | 0.850 | 0.821 |
| 209 | 0.772 | 1.000 | 0.791 | 0.957 | 0.989 | 1.000 | 0.904 | 1.000 | 0.959 | 1.000 | 0.939 | 0.939 | 0.991 | 0.847 | 1.000 | 0.981 | 0.852 | 0.823 |
| 210 | 0.771 | 1.000 | 0.810 | 0.957 | 0.989 | 0.960 | 0.898 | 1.000 | 0.949 | 1.000 | 0.945 | 0.970 | 0.973 | 0.851 | 1.000 | 0.981 | 0.854 | 0.825 |
| 211 | 0.769 | 1.000 | 0.800 | 0.971 | 0.989 | 1.000 | 0.903 | 1.000 | 0.959 | 1.000 | 0.948 | 0.939 | 0.973 | 0.847 | 1.000 | 0.981 | 0.852 | 0.823 |
| 212 | 0.773 | 1.000 | 0.789 | 0.957 | 0.989 | 0.960 | 0.892 | 1.000 | 0.959 | 1.000 | 0.948 | 0.970 | 0.973 | 0.846 | 1.000 | 0.981 | 0.851 | 0.821 |
| 213 | 0.769 | 1.000 | 0.802 | 0.957 | 0.984 | 0.960 | 0.898 | 1.000 | 0.959 | 1.000 | 0.942 | 0.970 | 0.964 | 0.853 | 1.000 | 0.981 | 0.852 | 0.823 |
| 214 | 0.779 | 1.000 | 0.798 | 0.957 | 0.995 | 0.960 | 0.900 | 1.000 | 0.959 | 1.000 | 0.954 | 0.970 | 0.973 | 0.844 | 1.000 | 0.981 | 0.855 | 0.826 |
| 215 | 0.770 | 1.000 | 0.808 | 0.957 | 0.989 | 1.000 | 0.900 | 1.000 | 0.949 | 1.000 | 0.954 | 0.939 | 0.991 | 0.844 | 1.000 | 0.981 | 0.853 | 0.824 |
| 216 | 0.777 | 1.000 | 0.806 | 0.957 | 0.989 | 1.000 | 0.901 | 1.000 | 0.959 | 1.000 | 0.945 | 0.970 | 0.991 | 0.849 | 1.000 | 0.981 | 0.856 | 0.828 |
| 217 | 0.771 | 1.000 | 0.804 | 0.957 | 0.995 | 1.000 | 0.895 | 1.000 | 0.959 | 1.000 | 0.939 | 0.939 | 0.991 | 0.854 | 1.000 | 0.981 | 0.854 | 0.825 |
| 218 | 0.769 | 1.000 | 0.808 | 0.957 | 0.995 | 1.000 | 0.903 | 1.000 | 0.959 | 1.000 | 0.948 | 0.970 | 0.982 | 0.844 | 1.000 | 0.981 | 0.853 | 0.824 |
| 219 | 0.769 | 1.000 | 0.794 | 0.957 | 0.989 | 1.000 | 0.904 | 1.000 | 0.959 | 1.000 | 0.959 | 0.970 | 0.982 | 0.847 | 1.000 | 0.981 | 0.853 | 0.824 |
| 220 | 0.772 | 1.000 | 0.814 | 0.957 | 0.995 | 0.960 | 0.894 | 1.000 | 0.959 | 1.000 | 0.951 | 0.970 | 0.991 | 0.858 | 1.000 | 0.981 | 0.857 | 0.828 |
| 221 | 0.765 | 1.000 | 0.812 | 0.957 | 0.989 | 0.960 | 0.904 | 1.000 | 0.949 | 1.000 | 0.951 | 0.939 | 0.991 | 0.839 | 1.000 | 0.981 | 0.850 | 0.821 |
| 222 | 0.775 | 1.000 | 0.798 | 0.957 | 0.989 | 0.960 | 0.892 | 1.000 | 0.959 | 1.000 | 0.959 | 0.939 | 0.973 | 0.847 | 1.000 | 0.981 | 0.853 | 0.824 |
| 223 | 0.771 | 1.000 | 0.804 | 0.957 | 0.995 | 0.960 | 0.897 | 1.000 | 0.959 | 1.000 | 0.936 | 0.970 | 0.982 | 0.854 | 1.000 | 0.981 | 0.854 | 0.825 |
| 224 | 0.766 | 1.000 | 0.796 | 0.971 | 0.984 | 1.000 | 0.906 | 1.000 | 0.949 | 1.000 | 0.939 | 0.970 | 1.000 | 0.847 | 1.000 | 0.981 | 0.851 | 0.822 |
| 225 | 0.771 | 1.000 | 0.800 | 0.943 | 0.989 | 1.000 | 0.901 | 1.000 | 0.959 | 1.000 | 0.939 | 0.970 | 0.991 | 0.851 | 1.000 | 0.981 | 0.853 | 0.825 |
| 226 | 0.772 | 1.000 | 0.792 | 0.957 | 0.984 | 0.960 | 0.908 | 1.000 | 0.959 | 1.000 | 0.942 | 0.970 | 0.982 | 0.850 | 1.000 | 0.981 | 0.853 | 0.824 |
| 227 | 0.771 | 1.000 | 0.818 | 0.971 | 0.979 | 0.960 | 0.903 | 1.000 | 0.959 | 1.000 | 0.936 | 0.970 | 0.991 | 0.854 | 1.000 | 0.981 | 0.856 | 0.828 |
| 228 | 0.771 | 1.000 | 0.783 | 0.943 | 0.989 | 1.000 | 0.898 | 1.000 | 0.959 | 1.000 | 0.948 | 0.939 | 1.000 | 0.860 | 1.000 | 0.981 | 0.854 | 0.825 |
| 229 | 0.777 | 1.000 | 0.808 | 0.943 | 0.989 | 1.000 | 0.892 | 1.000 | 0.959 | 1.000 | 0.951 | 0.909 | 0.973 | 0.843 | 1.000 | 0.981 | 0.853 | 0.823 |
| 230 | 0.767 | 1.000 | 0.804 | 0.943 | 0.989 | 0.960 | 0.897 | 1.000 | 0.949 | 1.000 | 0.948 | 0.909 | 0.982 | 0.843 | 1.000 | 0.981 | 0.850 | 0.820 |
| 231 | 0.759 | 1.000 | 0.808 | 0.943 | 0.989 | 0.960 | 0.900 | 1.000 | 0.959 | 1.000 | 0.945 | 0.939 | 0.991 | 0.847 | 1.000 | 0.981 | 0.850 | 0.820 |
| 232 | 0.771 | 1.000 | 0.802 | 0.957 | 0.989 | 0.960 | 0.891 | 1.000 | 0.959 | 1.000 | 0.942 | 0.939 | 0.982 | 0.841 | 1.000 | 0.981 | 0.850 | 0.820 |

| | | | | | | | | | | | | | | | | | | |
|---|---|---|---|---|---|---|---|---|---|---|---|---|---|---|---|---|---|---|
| 233 | 0.763 | 1.000 | 0.808 | 0.986 | 0.984 | 1.000 | 0.898 | 1.000 | 0.959 | 1.000 | 0.948 | 0.970 | 1.000 | 0.852 | 1.000 | 0.981 | 0.853 | 0.824 |
| 234 | 0.771 | 1.000 | 0.808 | 0.943 | 0.984 | 0.960 | 0.895 | 1.000 | 0.959 | 1.000 | 0.948 | 0.970 | 0.991 | 0.851 | 1.000 | 0.981 | 0.853 | 0.824 |
| 235 | 0.773 | 1.000 | 0.808 | 0.957 | 0.979 | 1.000 | 0.909 | 1.000 | 0.959 | 1.000 | 0.942 | 0.879 | 0.982 | 0.849 | 1.000 | 0.981 | 0.855 | 0.826 |
| 236 | 0.772 | 1.000 | 0.808 | 0.957 | 0.979 | 1.000 | 0.901 | 1.000 | 0.959 | 1.000 | 0.951 | 0.970 | 0.991 | 0.849 | 1.000 | 0.981 | 0.855 | 0.826 |
| 237 | 0.774 | 1.000 | 0.808 | 0.943 | 0.989 | 1.000 | 0.898 | 1.000 | 0.959 | 1.000 | 0.951 | 0.970 | 0.982 | 0.846 | 1.000 | 0.981 | 0.854 | 0.825 |
| 238 | 0.774 | 1.000 | 0.812 | 0.971 | 0.989 | 1.000 | 0.904 | 1.000 | 0.959 | 1.000 | 0.954 | 0.970 | 0.982 | 0.843 | 1.000 | 0.981 | 0.855 | 0.827 |
| 239 | 0.775 | 1.000 | 0.810 | 0.971 | 0.979 | 1.000 | 0.897 | 1.000 | 0.959 | 1.000 | 0.951 | 0.970 | 0.982 | 0.844 | 1.000 | 0.981 | 0.854 | 0.825 |
| 240 | 0.779 | 1.000 | 0.812 | 0.986 | 0.984 | 0.960 | 0.897 | 1.000 | 0.959 | 1.000 | 0.945 | 0.909 | 0.973 | 0.854 | 1.000 | 0.981 | 0.857 | 0.828 |
| 241 | 0.779 | 1.000 | 0.818 | 0.971 | 0.979 | 0.960 | 0.895 | 1.000 | 0.959 | 1.000 | 0.951 | 0.939 | 0.991 | 0.848 | 1.000 | 0.981 | 0.857 | 0.828 |
| 242 | 0.771 | 1.000 | 0.794 | 0.943 | 0.989 | 0.960 | 0.903 | 1.000 | 0.949 | 1.000 | 0.951 | 0.939 | 0.973 | 0.849 | 1.000 | 0.981 | 0.852 | 0.823 |
| 243 | 0.769 | 1.000 | 0.822 | 0.957 | 0.984 | 1.000 | 0.895 | 1.000 | 0.959 | 1.000 | 0.948 | 0.970 | 0.991 | 0.843 | 1.000 | 0.981 | 0.853 | 0.824 |
| 244 | 0.781 | 1.000 | 0.808 | 0.957 | 0.984 | 1.000 | 0.894 | 1.000 | 0.959 | 1.000 | 0.948 | 0.970 | 0.973 | 0.848 | 1.000 | 0.981 | 0.856 | 0.827 |
| 245 | 0.774 | 1.000 | 0.806 | 0.957 | 0.984 | 1.000 | 0.897 | 1.000 | 0.959 | 1.000 | 0.951 | 0.970 | 0.991 | 0.846 | 1.000 | 0.981 | 0.854 | 0.825 |
| 246 | 0.779 | 1.000 | 0.794 | 0.943 | 0.984 | 1.000 | 0.895 | 1.000 | 0.969 | 1.000 | 0.951 | 0.939 | 0.991 | 0.846 | 1.000 | 0.981 | 0.854 | 0.825 |
| 247 | 0.773 | 1.000 | 0.816 | 0.957 | 0.984 | 1.000 | 0.903 | 1.000 | 0.959 | 1.000 | 0.945 | 0.939 | 0.991 | 0.851 | 1.000 | 0.981 | 0.856 | 0.827 |
| 248 | 0.782 | 1.000 | 0.791 | 0.943 | 0.989 | 1.000 | 0.895 | 1.000 | 0.959 | 1.000 | 0.957 | 0.970 | 0.982 | 0.843 | 1.000 | 0.981 | 0.854 | 0.825 |
| 249 | 0.774 | 1.000 | 0.785 | 0.943 | 0.984 | 1.000 | 0.906 | 1.000 | 0.949 | 1.000 | 0.951 | 0.970 | 0.991 | 0.847 | 1.000 | 0.981 | 0.853 | 0.823 |
| 250 | 0.773 | 1.000 | 0.796 | 0.971 | 0.989 | 1.000 | 0.900 | 1.000 | 0.949 | 1.000 | 0.948 | 0.970 | 0.991 | 0.844 | 1.000 | 0.981 | 0.853 | 0.824 |
| 251 | 0.767 | 1.000 | 0.796 | 0.943 | 0.989 | 0.960 | 0.909 | 1.000 | 0.949 | 1.000 | 0.954 | 0.939 | 1.000 | 0.844 | 1.000 | 0.981 | 0.852 | 0.823 |
| 252 | 0.776 | 1.000 | 0.802 | 0.957 | 0.989 | 1.000 | 0.894 | 1.000 | 0.969 | 1.000 | 0.948 | 0.970 | 1.000 | 0.852 | 1.000 | 0.981 | 0.856 | 0.827 |
| 253 | 0.773 | 1.000 | 0.806 | 0.943 | 0.989 | 1.000 | 0.898 | 1.000 | 0.949 | 1.000 | 0.954 | 0.970 | 0.991 | 0.853 | 1.000 | 0.981 | 0.855 | 0.827 |
| 254 | 0.769 | 1.000 | 0.796 | 0.943 | 0.989 | 1.000 | 0.904 | 1.000 | 0.959 | 1.000 | 0.954 | 0.909 | 0.982 | 0.844 | 1.000 | 0.981 | 0.851 | 0.822 |
| 255 | 0.773 | 1.000 | 0.794 | 0.957 | 0.984 | 1.000 | 0.901 | 1.000 | 0.959 | 1.000 | 0.951 | 0.970 | 0.982 | 0.848 | 1.000 | 0.981 | 0.853 | 0.825 |
| 256 | 0.768 | 1.000 | 0.792 | 0.971 | 0.984 | 1.000 | 0.898 | 1.000 | 0.959 | 1.000 | 0.951 | 0.939 | 0.982 | 0.851 | 1.000 | 1.000 | 0.852 | 0.823 |
| 257 | 0.777 | 1.000 | 0.796 | 0.957 | 0.984 | 1.000 | 0.909 | 1.000 | 0.959 | 1.000 | 0.951 | 0.970 | 1.000 | 0.852 | 1.000 | 0.981 | 0.857 | 0.829 |
| 258 | 0.777 | 1.000 | 0.810 | 0.943 | 0.979 | 1.000 | 0.908 | 1.000 | 0.959 | 1.000 | 0.954 | 0.939 | 0.991 | 0.837 | 1.000 | 0.981 | 0.854 | 0.825 |
| 259 | 0.773 | 1.000 | 0.800 | 0.943 | 0.979 | 1.000 | 0.901 | 1.000 | 0.959 | 1.000 | 0.942 | 0.939 | 1.000 | 0.853 | 1.000 | 1.000 | 0.855 | 0.826 |
| 260 | 0.771 | 1.000 | 0.798 | 0.957 | 0.984 | 1.000 | 0.897 | 1.000 | 0.969 | 1.000 | 0.948 | 0.970 | 0.991 | 0.845 | 1.000 | 0.981 | 0.852 | 0.822 |
| 261 | 0.767 | 1.000 | 0.798 | 0.986 | 0.984 | 0.960 | 0.906 | 1.000 | 0.949 | 1.000 | 0.948 | 0.970 | 0.991 | 0.849 | 1.000 | 0.981 | 0.853 | 0.824 |
| 262 | 0.774 | 1.000 | 0.794 | 0.957 | 0.979 | 1.000 | 0.908 | 1.000 | 0.949 | 1.000 | 0.954 | 0.970 | 0.991 | 0.852 | 1.000 | 0.981 | 0.855 | 0.827 |
| 263 | 0.780 | 1.000 | 0.800 | 0.943 | 0.984 | 1.000 | 0.908 | 1.000 | 0.959 | 1.000 | 0.939 | 0.970 | 1.000 | 0.837 | 1.000 | 0.981 | 0.853 | 0.824 |
| 264 | 0.779 | 1.000 | 0.802 | 0.957 | 0.984 | 0.960 | 0.908 | 1.000 | 0.959 | 1.000 | 0.954 | 0.970 | 0.991 | 0.848 | 1.000 | 1.000 | 0.857 | 0.829 |
| 265 | 0.783 | 1.000 | 0.804 | 0.943 | 0.984 | 1.000 | 0.911 | 1.000 | 0.959 | 1.000 | 0.951 | 0.939 | 0.991 | 0.845 | 1.000 | 0.981 | 0.857 | 0.829 |
| 266 | 0.769 | 1.000 | 0.810 | 0.943 | 0.984 | 1.000 | 0.898 | 1.000 | 0.959 | 1.000 | 0.945 | 0.939 | 0.991 | 0.843 | 1.000 | 0.981 | 0.851 | 0.822 |
| 267 | 0.769 | 1.000 | 0.800 | 0.957 | 0.979 | 0.960 | 0.906 | 1.000 | 0.959 | 1.000 | 0.945 | 0.939 | 0.991 | 0.847 | 1.000 | 0.981 | 0.852 | 0.823 |
| 268 | 0.775 | 1.000 | 0.798 | 0.957 | 0.984 | 0.960 | 0.895 | 1.000 | 0.949 | 1.000 | 0.939 | 0.939 | 0.991 | 0.851 | 1.000 | 0.981 | 0.853 | 0.824 |
| 269 | 0.769 | 1.000 | 0.814 | 0.943 | 0.984 | 0.960 | 0.906 | 1.000 | 0.959 | 1.000 | 0.951 | 0.909 | 0.991 | 0.852 | 1.000 | 0.981 | 0.855 | 0.827 |
| 270 | 0.777 | 1.000 | 0.798 | 0.957 | 0.979 | 1.000 | 0.900 | 1.000 | 0.959 | 1.000 | 0.939 | 0.939 | 0.991 | 0.851 | 1.000 | 0.981 | 0.855 | 0.826 |
| 271 | 0.778 | 1.000 | 0.810 | 0.943 | 0.979 | 0.960 | 0.900 | 1.000 | 0.959 | 1.000 | 0.957 | 0.909 | 1.000 | 0.844 | 1.000 | 0.981 | 0.855 | 0.826 |

| | | | | | | | | | | | | | | | | | | |
|---|---|---|---|---|---|---|---|---|---|---|---|---|---|---|---|---|---|---|
| 272 | 0.773 | 1.000 | 0.806 | 0.943 | 0.979 | 0.960 | 0.904 | 1.000 | 0.949 | 1.000 | 0.945 | 0.939 | 1.000 | 0.861 | 1.000 | 0.981 | 0.857 | 0.829 |
| 273 | 0.778 | 1.000 | 0.796 | 0.957 | 0.984 | 0.960 | 0.904 | 1.000 | 0.949 | 1.000 | 0.945 | 0.939 | 0.991 | 0.855 | 1.000 | 0.981 | 0.856 | 0.828 |
| 274 | 0.775 | 1.000 | 0.810 | 0.957 | 0.979 | 0.960 | 0.909 | 1.000 | 0.980 | 0.979 | 0.945 | 0.970 | 0.991 | 0.855 | 1.000 | 0.981 | 0.858 | 0.830 |
| 275 | 0.770 | 1.000 | 0.802 | 0.943 | 0.979 | 1.000 | 0.914 | 1.000 | 0.969 | 0.979 | 0.945 | 0.939 | 1.000 | 0.852 | 1.000 | 0.981 | 0.855 | 0.827 |
| 276 | 0.774 | 1.000 | 0.820 | 0.929 | 0.984 | 0.960 | 0.901 | 1.000 | 0.969 | 0.979 | 0.945 | 0.939 | 0.991 | 0.857 | 1.000 | 0.981 | 0.857 | 0.829 |
| 277 | 0.767 | 1.000 | 0.798 | 0.943 | 0.979 | 1.000 | 0.909 | 1.000 | 0.969 | 0.979 | 0.945 | 0.939 | 0.991 | 0.847 | 1.000 | 0.981 | 0.852 | 0.823 |
| 278 | 0.769 | 1.000 | 0.804 | 0.957 | 0.984 | 0.960 | 0.915 | 1.000 | 0.969 | 0.979 | 0.942 | 0.970 | 1.000 | 0.850 | 1.000 | 0.981 | 0.855 | 0.826 |
| 279 | 0.773 | 1.000 | 0.822 | 0.943 | 0.984 | 1.000 | 0.900 | 1.000 | 0.980 | 1.000 | 0.942 | 0.939 | 1.000 | 0.854 | 1.000 | 0.981 | 0.857 | 0.829 |
| 280 | 0.771 | 1.000 | 0.812 | 0.943 | 0.984 | 1.000 | 0.898 | 1.000 | 0.980 | 1.000 | 0.942 | 0.939 | 0.991 | 0.852 | 1.000 | 0.981 | 0.855 | 0.826 |
| 281 | 0.771 | 1.000 | 0.818 | 0.943 | 0.984 | 1.000 | 0.903 | 1.000 | 0.980 | 1.000 | 0.942 | 0.939 | 1.000 | 0.852 | 1.000 | 1.000 | 0.856 | 0.828 |
| 282 | 0.774 | 1.000 | 0.812 | 0.943 | 0.979 | 0.960 | 0.901 | 1.000 | 0.969 | 0.979 | 0.945 | 0.939 | 0.991 | 0.855 | 1.000 | 0.981 | 0.856 | 0.828 |
| 283 | 0.773 | 1.000 | 0.806 | 0.943 | 0.989 | 1.000 | 0.906 | 1.000 | 0.969 | 0.979 | 0.951 | 0.939 | 0.982 | 0.853 | 1.000 | 1.000 | 0.856 | 0.828 |
| 284 | 0.775 | 1.000 | 0.818 | 0.943 | 0.984 | 0.960 | 0.911 | 1.000 | 0.980 | 0.979 | 0.945 | 0.939 | 1.000 | 0.842 | 1.000 | 1.000 | 0.856 | 0.827 |
| 285 | 0.774 | 1.000 | 0.802 | 0.957 | 0.979 | 1.000 | 0.906 | 1.000 | 0.990 | 0.979 | 0.939 | 0.939 | 1.000 | 0.850 | 1.000 | 0.981 | 0.855 | 0.827 |
| 286 | 0.776 | 1.000 | 0.804 | 0.957 | 0.974 | 1.000 | 0.912 | 1.000 | 0.980 | 0.979 | 0.942 | 0.939 | 1.000 | 0.852 | 1.000 | 0.981 | 0.857 | 0.829 |
| 287 | 0.771 | 1.000 | 0.812 | 0.957 | 0.984 | 0.960 | 0.920 | 1.000 | 0.969 | 1.000 | 0.948 | 0.939 | 0.991 | 0.851 | 1.000 | 1.000 | 0.858 | 0.830 |
| 288 | 0.781 | 1.000 | 0.810 | 0.957 | 0.979 | 0.960 | 0.906 | 1.000 | 0.969 | 1.000 | 0.945 | 0.939 | 1.000 | 0.855 | 1.000 | 0.981 | 0.859 | 0.832 |
| 289 | 0.764 | 1.000 | 0.816 | 0.957 | 0.984 | 1.000 | 0.900 | 1.000 | 0.969 | 1.000 | 0.951 | 0.939 | 0.991 | 0.856 | 1.000 | 0.981 | 0.855 | 0.826 |
| 290 | 0.783 | 1.000 | 0.804 | 0.971 | 0.984 | 1.000 | 0.898 | 1.000 | 0.980 | 1.000 | 0.945 | 0.939 | 0.991 | 0.859 | 1.000 | 1.000 | 0.860 | 0.832 |
| 291 | 0.767 | 1.000 | 0.810 | 0.943 | 0.979 | 1.000 | 0.903 | 1.000 | 0.980 | 0.979 | 0.945 | 0.939 | 0.991 | 0.859 | 1.000 | 0.981 | 0.855 | 0.827 |
| 292 | 0.770 | 1.000 | 0.806 | 0.943 | 0.989 | 1.000 | 0.918 | 1.000 | 0.980 | 0.979 | 0.942 | 0.909 | 0.991 | 0.853 | 1.000 | 0.981 | 0.856 | 0.828 |
| 293 | 0.773 | 1.000 | 0.806 | 0.943 | 0.984 | 0.960 | 0.909 | 1.000 | 0.980 | 0.979 | 0.945 | 0.939 | 0.991 | 0.848 | 1.000 | 0.981 | 0.855 | 0.826 |
| 294 | 0.770 | 1.000 | 0.806 | 0.943 | 0.989 | 0.960 | 0.897 | 1.000 | 0.969 | 0.979 | 0.951 | 0.939 | 1.000 | 0.850 | 1.000 | 0.981 | 0.853 | 0.825 |
| 295 | 0.775 | 1.000 | 0.804 | 0.943 | 0.984 | 1.000 | 0.901 | 1.000 | 0.980 | 0.979 | 0.948 | 0.939 | 1.000 | 0.845 | 1.000 | 0.981 | 0.854 | 0.826 |
| 296 | 0.777 | 1.000 | 0.808 | 0.957 | 0.984 | 0.960 | 0.901 | 1.000 | 0.980 | 1.000 | 0.945 | 0.939 | 1.000 | 0.847 | 1.000 | 0.981 | 0.856 | 0.827 |
| 297 | 0.781 | 1.000 | 0.816 | 0.943 | 0.979 | 1.000 | 0.904 | 1.000 | 0.980 | 0.979 | 0.939 | 0.939 | 0.991 | 0.854 | 1.000 | 0.981 | 0.859 | 0.831 |
| 298 | 0.776 | 1.000 | 0.810 | 0.957 | 0.984 | 0.960 | 0.901 | 1.000 | 0.980 | 1.000 | 0.945 | 0.939 | 1.000 | 0.848 | 1.000 | 0.981 | 0.856 | 0.827 |
| 299 | 0.768 | 1.000 | 0.810 | 0.943 | 0.979 | 0.960 | 0.906 | 1.000 | 0.969 | 1.000 | 0.948 | 0.939 | 0.991 | 0.851 | 1.000 | 1.000 | 0.854 | 0.826 |
| 300 | 0.781 | 1.000 | 0.798 | 0.943 | 0.984 | 1.000 | 0.895 | 1.000 | 0.969 | 1.000 | 0.948 | 0.939 | 0.991 | 0.846 | 1.000 | 0.981 | 0.854 | 0.825 |
| 301 | 0.776 | 1.000 | 0.794 | 0.971 | 0.984 | 1.000 | 0.906 | 1.000 | 0.969 | 1.000 | 0.939 | 0.939 | 1.000 | 0.851 | 1.000 | 1.000 | 0.855 | 0.827 |
| 302 | 0.775 | 1.000 | 0.804 | 0.957 | 0.984 | 0.960 | 0.901 | 1.000 | 0.980 | 0.979 | 0.942 | 0.939 | 1.000 | 0.849 | 1.000 | 0.981 | 0.855 | 0.826 |
| 303 | 0.775 | 1.000 | 0.800 | 0.957 | 0.979 | 1.000 | 0.909 | 1.000 | 0.969 | 1.000 | 0.930 | 0.939 | 1.000 | 0.852 | 1.000 | 0.981 | 0.855 | 0.827 |
| 304 | 0.781 | 1.000 | 0.804 | 0.957 | 0.989 | 0.960 | 0.895 | 1.000 | 0.969 | 0.979 | 0.942 | 0.939 | 0.991 | 0.854 | 1.000 | 0.981 | 0.857 | 0.828 |
| 305 | 0.774 | 1.000 | 0.806 | 0.957 | 0.984 | 1.000 | 0.898 | 1.000 | 0.969 | 1.000 | 0.945 | 0.939 | 1.000 | 0.847 | 1.000 | 1.000 | 0.854 | 0.826 |
| 306 | 0.767 | 1.000 | 0.798 | 0.957 | 0.984 | 0.960 | 0.895 | 1.000 | 0.969 | 1.000 | 0.945 | 0.939 | 1.000 | 0.855 | 1.000 | 0.981 | 0.853 | 0.824 |
| 307 | 0.775 | 1.000 | 0.794 | 0.943 | 0.979 | 1.000 | 0.917 | 1.000 | 0.980 | 1.000 | 0.948 | 0.939 | 0.991 | 0.846 | 1.000 | 1.000 | 0.855 | 0.827 |
| 308 | 0.766 | 1.000 | 0.800 | 0.957 | 0.984 | 0.960 | 0.904 | 1.000 | 0.980 | 0.979 | 0.951 | 0.939 | 1.000 | 0.851 | 1.000 | 1.000 | 0.853 | 0.825 |
| 309 | 0.769 | 1.000 | 0.818 | 0.943 | 0.984 | 1.000 | 0.904 | 1.000 | 0.980 | 0.979 | 0.945 | 0.939 | 0.991 | 0.848 | 1.000 | 0.981 | 0.855 | 0.826 |
| 310 | 0.774 | 1.000 | 0.796 | 0.943 | 0.984 | 1.000 | 0.901 | 1.000 | 0.980 | 1.000 | 0.945 | 0.939 | 1.000 | 0.854 | 1.000 | 1.000 | 0.855 | 0.827 |

| | | | | | | | | | | | | | | | | | | |
|---|---|---|---|---|---|---|---|---|---|---|---|---|---|---|---|---|---|---|
| 311 | 0.781 | 1.000 | 0.806 | 0.943 | 0.984 | 0.960 | 0.903 | 1.000 | 0.980 | 0.979 | 0.942 | 0.939 | 0.991 | 0.853 | 1.000 | 0.981 | 0.857 | 0.829 |
| 312 | 0.778 | 1.000 | 0.792 | 0.957 | 0.974 | 0.960 | 0.912 | 1.000 | 0.980 | 0.979 | 0.945 | 0.939 | 0.991 | 0.855 | 1.000 | 0.981 | 0.857 | 0.829 |
| 313 | 0.780 | 1.000 | 0.810 | 0.943 | 0.984 | 1.000 | 0.906 | 1.000 | 0.980 | 1.000 | 0.936 | 0.939 | 1.000 | 0.852 | 1.000 | 1.000 | 0.858 | 0.830 |
| 314 | 0.779 | 1.000 | 0.812 | 0.957 | 0.984 | 0.960 | 0.906 | 1.000 | 0.980 | 1.000 | 0.945 | 0.939 | 1.000 | 0.857 | 1.000 | 1.000 | 0.860 | 0.832 |
| 315 | 0.775 | 1.000 | 0.802 | 0.971 | 0.984 | 0.960 | 0.895 | 1.000 | 0.980 | 0.979 | 0.948 | 0.939 | 0.991 | 0.854 | 1.000 | 0.981 | 0.855 | 0.826 |
| 316 | 0.779 | 1.000 | 0.814 | 0.971 | 0.984 | 1.000 | 0.892 | 1.000 | 0.980 | 0.979 | 0.942 | 0.939 | 0.982 | 0.858 | 1.000 | 0.981 | 0.858 | 0.830 |
| 317 | 0.775 | 1.000 | 0.824 | 0.943 | 0.979 | 0.960 | 0.904 | 1.000 | 0.980 | 1.000 | 0.948 | 0.939 | 1.000 | 0.856 | 1.000 | 1.000 | 0.859 | 0.832 |
| 318 | 0.771 | 1.000 | 0.806 | 0.943 | 0.989 | 0.960 | 0.895 | 1.000 | 0.980 | 0.979 | 0.948 | 0.909 | 0.982 | 0.858 | 1.000 | 0.981 | 0.855 | 0.826 |
| 319 | 0.775 | 1.000 | 0.808 | 0.943 | 0.984 | 0.960 | 0.909 | 1.000 | 0.969 | 0.979 | 0.954 | 0.939 | 1.000 | 0.855 | 1.000 | 1.000 | 0.858 | 0.830 |
| 320 | 0.778 | 1.000 | 0.783 | 0.957 | 0.984 | 1.000 | 0.904 | 1.000 | 0.980 | 0.979 | 0.951 | 0.939 | 1.000 | 0.851 | 1.000 | 1.000 | 0.855 | 0.827 |
| 321 | 0.775 | 1.000 | 0.810 | 0.957 | 0.979 | 1.000 | 0.901 | 1.000 | 0.969 | 0.979 | 0.954 | 0.939 | 1.000 | 0.853 | 1.000 | 0.981 | 0.857 | 0.829 |
| 322 | 0.779 | 1.000 | 0.810 | 0.957 | 0.989 | 1.000 | 0.911 | 1.000 | 0.969 | 0.979 | 0.948 | 0.939 | 1.000 | 0.860 | 1.000 | 1.000 | 0.861 | 0.834 |
| 323 | 0.787 | 1.000 | 0.806 | 0.971 | 0.984 | 0.960 | 0.914 | 1.000 | 0.969 | 1.000 | 0.945 | 0.939 | 0.973 | 0.854 | 1.000 | 0.981 | 0.861 | 0.833 |
| 324 | 0.778 | 1.000 | 0.800 | 0.943 | 0.984 | 0.960 | 0.908 | 1.000 | 0.969 | 0.979 | 0.939 | 0.939 | 0.991 | 0.856 | 1.000 | 0.981 | 0.857 | 0.829 |
| 325 | 0.776 | 1.000 | 0.798 | 0.943 | 0.984 | 1.000 | 0.911 | 1.000 | 0.969 | 1.000 | 0.942 | 0.939 | 0.982 | 0.854 | 1.000 | 0.981 | 0.857 | 0.828 |
| 326 | 0.780 | 1.000 | 0.810 | 0.943 | 0.984 | 0.960 | 0.904 | 1.000 | 0.969 | 0.979 | 0.936 | 0.939 | 0.991 | 0.851 | 1.000 | 0.981 | 0.857 | 0.828 |
| 327 | 0.778 | 1.000 | 0.800 | 0.957 | 0.984 | 0.960 | 0.909 | 1.000 | 0.969 | 0.979 | 0.945 | 0.939 | 0.991 | 0.860 | 1.000 | 0.981 | 0.859 | 0.831 |
| 328 | 0.782 | 1.000 | 0.808 | 0.943 | 0.989 | 1.000 | 0.920 | 1.000 | 0.980 | 1.000 | 0.945 | 0.939 | 1.000 | 0.856 | 1.000 | 0.981 | 0.862 | 0.835 |
| 329 | 0.774 | 1.000 | 0.808 | 0.957 | 0.979 | 0.960 | 0.908 | 1.000 | 0.980 | 0.979 | 0.948 | 0.939 | 0.982 | 0.853 | 1.000 | 0.981 | 0.856 | 0.828 |
| 330 | 0.778 | 1.000 | 0.802 | 0.957 | 0.984 | 0.960 | 0.898 | 1.000 | 0.980 | 1.000 | 0.945 | 0.939 | 1.000 | 0.856 | 1.000 | 0.981 | 0.857 | 0.829 |
| 331 | 0.773 | 1.000 | 0.808 | 0.957 | 0.984 | 0.960 | 0.901 | 1.000 | 0.969 | 1.000 | 0.945 | 0.939 | 0.991 | 0.857 | 1.000 | 0.981 | 0.857 | 0.828 |
| 332 | 0.773 | 1.000 | 0.802 | 0.986 | 0.984 | 0.960 | 0.904 | 1.000 | 0.980 | 1.000 | 0.945 | 0.939 | 0.991 | 0.856 | 1.000 | 1.000 | 0.857 | 0.829 |
| 333 | 0.777 | 1.000 | 0.796 | 0.971 | 0.979 | 0.960 | 0.904 | 1.000 | 0.980 | 0.979 | 0.942 | 0.939 | 1.000 | 0.851 | 1.000 | 1.000 | 0.855 | 0.827 |
| 334 | 0.780 | 1.000 | 0.816 | 0.957 | 0.979 | 0.960 | 0.909 | 1.000 | 0.980 | 1.000 | 0.945 | 0.939 | 0.991 | 0.858 | 1.000 | 0.981 | 0.861 | 0.833 |
| 335 | 0.782 | 1.000 | 0.798 | 0.957 | 0.979 | 0.960 | 0.911 | 1.000 | 0.969 | 1.000 | 0.942 | 0.939 | 1.000 | 0.861 | 1.000 | 0.981 | 0.860 | 0.832 |
| 336 | 0.776 | 1.000 | 0.794 | 0.957 | 0.979 | 0.960 | 0.906 | 1.000 | 0.980 | 1.000 | 0.936 | 0.939 | 1.000 | 0.853 | 1.000 | 1.000 | 0.855 | 0.827 |
| 337 | 0.774 | 1.000 | 0.800 | 0.929 | 0.984 | 0.960 | 0.914 | 1.000 | 0.980 | 0.979 | 0.945 | 0.939 | 1.000 | 0.854 | 1.000 | 1.000 | 0.857 | 0.829 |
| 338 | 0.778 | 1.000 | 0.802 | 0.971 | 0.984 | 1.000 | 0.895 | 1.000 | 0.980 | 1.000 | 0.939 | 0.939 | 0.982 | 0.850 | 1.000 | 0.981 | 0.855 | 0.826 |
| 339 | 0.771 | 1.000 | 0.812 | 0.971 | 0.979 | 1.000 | 0.911 | 1.000 | 0.969 | 1.000 | 0.939 | 0.939 | 1.000 | 0.854 | 1.000 | 0.981 | 0.857 | 0.829 |
| 340 | 0.770 | 1.000 | 0.791 | 0.957 | 0.984 | 1.000 | 0.912 | 1.000 | 0.980 | 1.000 | 0.945 | 0.939 | 1.000 | 0.851 | 1.000 | 1.000 | 0.854 | 0.826 |
| 341 | 0.778 | 1.000 | 0.800 | 0.957 | 0.989 | 0.960 | 0.904 | 1.000 | 0.980 | 0.979 | 0.936 | 0.939 | 0.991 | 0.860 | 1.000 | 0.981 | 0.858 | 0.830 |
| 342 | 0.770 | 1.000 | 0.794 | 0.943 | 0.984 | 0.960 | 0.904 | 1.000 | 0.980 | 1.000 | 0.939 | 0.939 | 1.000 | 0.857 | 1.000 | 0.981 | 0.854 | 0.826 |
| 343 | 0.777 | 1.000 | 0.804 | 0.957 | 0.984 | 0.960 | 0.903 | 1.000 | 0.980 | 0.979 | 0.945 | 0.939 | 0.991 | 0.849 | 1.000 | 0.981 | 0.856 | 0.827 |
| 344 | 0.771 | 1.000 | 0.808 | 0.929 | 0.984 | 0.960 | 0.911 | 1.000 | 0.969 | 0.979 | 0.945 | 0.939 | 1.000 | 0.856 | 1.000 | 0.981 | 0.857 | 0.828 |
| 345 | 0.772 | 1.000 | 0.808 | 0.957 | 0.984 | 0.960 | 0.915 | 1.000 | 0.980 | 0.979 | 0.945 | 0.939 | 0.982 | 0.856 | 1.000 | 0.981 | 0.858 | 0.830 |
| 346 | 0.771 | 1.000 | 0.787 | 0.943 | 0.979 | 0.960 | 0.917 | 1.000 | 0.980 | 1.000 | 0.945 | 0.939 | 0.982 | 0.854 | 1.000 | 0.981 | 0.855 | 0.826 |
| 347 | 0.773 | 1.000 | 0.798 | 0.929 | 0.984 | 0.960 | 0.909 | 1.000 | 0.980 | 1.000 | 0.942 | 0.939 | 0.991 | 0.853 | 1.000 | 0.981 | 0.855 | 0.827 |
| 348 | 0.769 | 1.000 | 0.789 | 0.943 | 0.984 | 0.960 | 0.906 | 1.000 | 0.980 | 0.979 | 0.942 | 0.939 | 0.991 | 0.851 | 1.000 | 1.000 | 0.852 | 0.823 |
| 349 | 0.782 | 1.000 | 0.812 | 0.957 | 0.984 | 0.960 | 0.911 | 1.000 | 0.980 | 0.979 | 0.951 | 0.909 | 0.991 | 0.858 | 1.000 | 0.981 | 0.861 | 0.834 |

| | | | | | | | | | | | | | | | | | | |
|---|---|---|---|---|---|---|---|---|---|---|---|---|---|---|---|---|---|---|
| 350 | 0.774 | 1.000 | 0.810 | 0.943 | 0.984 | 0.960 | 0.912 | 1.000 | 0.980 | 0.979 | 0.939 | 0.939 | 0.991 | 0.861 | 1.000 | 0.981 | 0.859 | 0.831 |
| 351 | 0.778 | 1.000 | 0.818 | 0.929 | 0.984 | 0.960 | 0.900 | 1.000 | 0.969 | 1.000 | 0.939 | 0.939 | 0.991 | 0.848 | 1.000 | 0.981 | 0.856 | 0.827 |
| 352 | 0.775 | 1.000 | 0.806 | 0.971 | 0.979 | 0.960 | 0.904 | 1.000 | 0.969 | 1.000 | 0.936 | 0.939 | 1.000 | 0.859 | 1.000 | 1.000 | 0.858 | 0.830 |
| 353 | 0.772 | 1.000 | 0.789 | 0.943 | 0.989 | 1.000 | 0.906 | 1.000 | 0.980 | 1.000 | 0.942 | 0.939 | 0.991 | 0.845 | 1.000 | 0.981 | 0.852 | 0.823 |
| 354 | 0.770 | 1.000 | 0.808 | 0.957 | 0.979 | 0.960 | 0.914 | 1.000 | 0.980 | 0.979 | 0.948 | 0.939 | 0.991 | 0.858 | 1.000 | 0.981 | 0.858 | 0.830 |
| 355 | 0.773 | 1.000 | 0.800 | 0.971 | 0.979 | 1.000 | 0.917 | 1.000 | 0.969 | 0.979 | 0.948 | 0.939 | 0.991 | 0.858 | 1.000 | 1.000 | 0.859 | 0.831 |
| 356 | 0.778 | 1.000 | 0.814 | 0.943 | 0.989 | 0.960 | 0.908 | 1.000 | 0.969 | 1.000 | 0.948 | 0.939 | 0.982 | 0.861 | 1.000 | 1.000 | 0.861 | 0.833 |
| 357 | 0.772 | 1.000 | 0.822 | 0.986 | 0.984 | 0.960 | 0.904 | 1.000 | 0.969 | 0.979 | 0.951 | 0.939 | 1.000 | 0.850 | 1.000 | 1.000 | 0.857 | 0.829 |
| 358 | 0.769 | 1.000 | 0.796 | 0.971 | 0.979 | 0.960 | 0.915 | 1.000 | 0.969 | 1.000 | 0.945 | 0.939 | 1.000 | 0.850 | 1.000 | 1.000 | 0.855 | 0.826 |
| 359 | 0.780 | 1.000 | 0.812 | 0.971 | 0.974 | 0.960 | 0.909 | 1.000 | 0.980 | 1.000 | 0.951 | 0.939 | 1.000 | 0.860 | 1.000 | 1.000 | 0.862 | 0.834 |
| 360 | 0.771 | 1.000 | 0.804 | 0.971 | 0.979 | 0.960 | 0.906 | 1.000 | 0.969 | 1.000 | 0.948 | 0.939 | 0.991 | 0.862 | 1.000 | 0.981 | 0.858 | 0.830 |
| 361 | 0.779 | 1.000 | 0.796 | 0.957 | 0.984 | 0.960 | 0.908 | 1.000 | 0.980 | 1.000 | 0.936 | 0.939 | 0.991 | 0.853 | 1.000 | 1.000 | 0.857 | 0.828 |
| 362 | 0.777 | 1.000 | 0.796 | 0.957 | 0.979 | 0.960 | 0.904 | 1.000 | 0.980 | 1.000 | 0.942 | 0.939 | 1.000 | 0.856 | 1.000 | 1.000 | 0.857 | 0.829 |
| 363 | 0.773 | 1.000 | 0.810 | 0.971 | 0.979 | 0.960 | 0.908 | 1.000 | 0.980 | 0.979 | 0.951 | 0.939 | 1.000 | 0.858 | 1.000 | 0.981 | 0.858 | 0.830 |
| 364 | 0.776 | 1.000 | 0.806 | 0.943 | 0.989 | 0.960 | 0.904 | 1.000 | 0.980 | 1.000 | 0.945 | 0.939 | 1.000 | 0.858 | 1.000 | 1.000 | 0.858 | 0.830 |
| 365 | 0.776 | 1.000 | 0.796 | 0.971 | 0.979 | 1.000 | 0.909 | 1.000 | 0.980 | 1.000 | 0.951 | 0.939 | 0.991 | 0.861 | 1.000 | 1.000 | 0.859 | 0.831 |
| 366 | 0.781 | 1.000 | 0.798 | 0.986 | 0.984 | 0.960 | 0.912 | 1.000 | 0.980 | 1.000 | 0.945 | 0.939 | 1.000 | 0.857 | 1.000 | 1.000 | 0.860 | 0.833 |
| 367 | 0.777 | 1.000 | 0.824 | 0.986 | 0.989 | 0.960 | 0.908 | 1.000 | 0.980 | 1.000 | 0.951 | 0.939 | 0.991 | 0.861 | 1.000 | 1.000 | 0.863 | 0.835 |
| 368 | 0.781 | 1.000 | 0.804 | 0.971 | 0.989 | 0.960 | 0.912 | 1.000 | 0.990 | 0.979 | 0.951 | 0.939 | 1.000 | 0.858 | 1.000 | 1.000 | 0.862 | 0.834 |
| 369 | 0.779 | 1.000 | 0.812 | 0.986 | 0.984 | 0.960 | 0.918 | 1.000 | 0.980 | 1.000 | 0.948 | 0.939 | 1.000 | 0.856 | 1.000 | 1.000 | 0.862 | 0.834 |
| 370 | 0.777 | 1.000 | 0.802 | 0.986 | 0.974 | 0.960 | 0.912 | 1.000 | 0.990 | 1.000 | 0.942 | 0.939 | 0.991 | 0.855 | 1.000 | 1.000 | 0.858 | 0.830 |
| 371 | 0.769 | 1.000 | 0.808 | 0.971 | 0.974 | 0.960 | 0.909 | 1.000 | 0.990 | 1.000 | 0.942 | 0.939 | 1.000 | 0.854 | 1.000 | 1.000 | 0.856 | 0.828 |
| 372 | 0.774 | 1.000 | 0.808 | 0.971 | 0.979 | 0.960 | 0.911 | 1.000 | 0.980 | 1.000 | 0.948 | 0.939 | 1.000 | 0.854 | 1.000 | 1.000 | 0.858 | 0.830 |
| 373 | 0.775 | 1.000 | 0.806 | 0.971 | 0.984 | 1.000 | 0.904 | 1.000 | 0.980 | 0.979 | 0.954 | 0.939 | 1.000 | 0.860 | 1.000 | 1.000 | 0.859 | 0.832 |
| 374 | 0.777 | 1.000 | 0.806 | 0.943 | 0.989 | 1.000 | 0.904 | 1.000 | 0.969 | 0.979 | 0.948 | 0.939 | 0.991 | 0.857 | 1.000 | 1.000 | 0.858 | 0.830 |
| 375 | 0.776 | 1.000 | 0.802 | 0.971 | 0.984 | 0.960 | 0.908 | 1.000 | 0.969 | 1.000 | 0.942 | 0.939 | 1.000 | 0.859 | 1.000 | 1.000 | 0.859 | 0.831 |
| 376 | 0.781 | 1.000 | 0.814 | 0.943 | 0.974 | 0.960 | 0.912 | 1.000 | 0.980 | 1.000 | 0.939 | 0.939 | 1.000 | 0.858 | 1.000 | 1.000 | 0.861 | 0.833 |
| 377 | 0.775 | 1.000 | 0.808 | 0.943 | 0.979 | 0.960 | 0.912 | 1.000 | 0.980 | 1.000 | 0.942 | 0.939 | 1.000 | 0.856 | 1.000 | 0.981 | 0.858 | 0.830 |
| 378 | 0.780 | 1.000 | 0.802 | 0.957 | 0.974 | 0.960 | 0.914 | 1.000 | 0.990 | 1.000 | 0.939 | 0.909 | 1.000 | 0.858 | 1.000 | 1.000 | 0.860 | 0.832 |
| 379 | 0.771 | 1.000 | 0.802 | 0.957 | 0.984 | 0.960 | 0.901 | 1.000 | 0.969 | 0.979 | 0.948 | 0.939 | 1.000 | 0.851 | 1.000 | 1.000 | 0.854 | 0.825 |
| 380 | 0.775 | 1.000 | 0.783 | 0.943 | 0.974 | 0.960 | 0.900 | 1.000 | 0.980 | 0.979 | 0.942 | 0.909 | 1.000 | 0.852 | 1.000 | 1.000 | 0.852 | 0.823 |
| 381 | 0.790 | 1.000 | 0.800 | 0.986 | 0.974 | 1.000 | 0.903 | 1.000 | 0.969 | 0.979 | 0.933 | 0.939 | 1.000 | 0.861 | 1.000 | 1.000 | 0.862 | 0.834 |
| 382 | 0.779 | 1.000 | 0.798 | 0.971 | 0.979 | 1.000 | 0.909 | 1.000 | 0.980 | 1.000 | 0.951 | 0.939 | 1.000 | 0.859 | 1.000 | 1.000 | 0.860 | 0.832 |
| 383 | 0.779 | 1.000 | 0.802 | 0.957 | 0.979 | 0.960 | 0.898 | 1.000 | 0.969 | 1.000 | 0.951 | 0.939 | 1.000 | 0.865 | 1.000 | 0.981 | 0.860 | 0.832 |
| 384 | 0.772 | 1.000 | 0.808 | 0.943 | 0.974 | 1.000 | 0.909 | 1.000 | 0.990 | 1.000 | 0.939 | 0.939 | 1.000 | 0.854 | 1.000 | 0.981 | 0.856 | 0.828 |
| 385 | 0.779 | 1.000 | 0.794 | 0.957 | 0.984 | 0.960 | 0.904 | 1.000 | 0.980 | 0.979 | 0.948 | 0.939 | 1.000 | 0.862 | 1.000 | 1.000 | 0.859 | 0.831 |
| 386 | 0.771 | 1.000 | 0.804 | 0.986 | 0.984 | 0.960 | 0.901 | 1.000 | 0.980 | 1.000 | 0.951 | 0.939 | 1.000 | 0.857 | 1.000 | 1.000 | 0.857 | 0.829 |
| 387 | 0.773 | 1.000 | 0.792 | 0.957 | 0.984 | 0.960 | 0.906 | 1.000 | 0.969 | 0.979 | 0.939 | 0.939 | 1.000 | 0.851 | 1.000 | 1.000 | 0.854 | 0.825 |
| 388 | 0.775 | 1.000 | 0.787 | 0.971 | 0.984 | 0.960 | 0.906 | 1.000 | 0.990 | 1.000 | 0.936 | 0.909 | 1.000 | 0.853 | 1.000 | 0.981 | 0.855 | 0.826 |

| | | | | | | | | | | | | | | | | | | |
|---|---|---|---|---|---|---|---|---|---|---|---|---|---|---|---|---|---|---|
| 389 | 0.777 | 1.000 | 0.802 | 0.986 | 0.984 | 0.960 | 0.901 | 1.000 | 0.980 | 1.000 | 0.936 | 0.939 | 1.000 | 0.854 | 1.000 | 1.000 | 0.857 | 0.829 |
| 390 | 0.773 | 1.000 | 0.802 | 0.971 | 0.984 | 0.960 | 0.901 | 1.000 | 0.969 | 1.000 | 0.945 | 0.939 | 1.000 | 0.856 | 1.000 | 1.000 | 0.856 | 0.828 |
| 391 | 0.773 | 1.000 | 0.791 | 0.943 | 0.989 | 0.960 | 0.897 | 1.000 | 0.969 | 1.000 | 0.939 | 0.939 | 0.991 | 0.849 | 1.000 | 1.000 | 0.852 | 0.822 |
| 392 | 0.767 | 1.000 | 0.791 | 0.957 | 0.979 | 0.960 | 0.895 | 1.000 | 0.990 | 1.000 | 0.948 | 0.970 | 1.000 | 0.857 | 1.000 | 1.000 | 0.853 | 0.824 |
| 393 | 0.775 | 1.000 | 0.785 | 0.957 | 0.979 | 0.960 | 0.914 | 1.000 | 0.980 | 0.979 | 0.945 | 0.939 | 1.000 | 0.850 | 1.000 | 1.000 | 0.855 | 0.826 |
| 394 | 0.765 | 1.000 | 0.806 | 0.971 | 0.989 | 0.960 | 0.914 | 1.000 | 0.980 | 1.000 | 0.942 | 0.939 | 1.000 | 0.847 | 1.000 | 1.000 | 0.854 | 0.825 |
| 395 | 0.774 | 1.000 | 0.787 | 0.971 | 0.974 | 0.960 | 0.906 | 1.000 | 0.969 | 1.000 | 0.933 | 0.939 | 1.000 | 0.850 | 1.000 | 1.000 | 0.853 | 0.824 |
| 396 | 0.772 | 1.000 | 0.794 | 0.957 | 0.989 | 0.960 | 0.903 | 1.000 | 0.980 | 0.979 | 0.945 | 0.909 | 1.000 | 0.855 | 1.000 | 1.000 | 0.855 | 0.826 |
| 397 | 0.777 | 1.000 | 0.792 | 0.971 | 0.979 | 0.960 | 0.903 | 1.000 | 0.990 | 1.000 | 0.945 | 0.939 | 1.000 | 0.847 | 1.000 | 1.000 | 0.855 | 0.826 |
| 398 | 0.768 | 1.000 | 0.800 | 0.957 | 0.984 | 0.960 | 0.901 | 1.000 | 0.990 | 1.000 | 0.942 | 0.939 | 0.991 | 0.856 | 1.000 | 1.000 | 0.854 | 0.826 |
| 399 | 0.775 | 1.000 | 0.802 | 0.957 | 0.979 | 0.960 | 0.900 | 1.000 | 0.980 | 0.979 | 0.948 | 0.909 | 0.991 | 0.854 | 1.000 | 1.000 | 0.856 | 0.827 |
| 400 | 0.777 | 1.000 | 0.794 | 0.957 | 0.989 | 0.960 | 0.901 | 1.000 | 0.980 | 1.000 | 0.942 | 0.909 | 1.000 | 0.855 | 1.000 | 1.000 | 0.856 | 0.828 |
| 401 | 0.772 | 1.000 | 0.792 | 0.943 | 0.989 | 1.000 | 0.906 | 1.000 | 0.980 | 1.000 | 0.948 | 0.939 | 1.000 | 0.854 | 1.000 | 0.981 | 0.855 | 0.826 |
| 402 | 0.781 | 1.000 | 0.818 | 0.957 | 0.979 | 1.000 | 0.900 | 1.000 | 0.990 | 1.000 | 0.939 | 0.939 | 1.000 | 0.858 | 1.000 | 1.000 | 0.860 | 0.832 |
| 403 | 0.771 | 1.000 | 0.789 | 0.971 | 0.984 | 0.960 | 0.909 | 1.000 | 0.969 | 1.000 | 0.951 | 0.939 | 1.000 | 0.848 | 1.000 | 1.000 | 0.854 | 0.825 |
| 404 | 0.771 | 1.000 | 0.791 | 0.971 | 0.979 | 0.960 | 0.903 | 1.000 | 0.969 | 1.000 | 0.942 | 0.939 | 1.000 | 0.854 | 1.000 | 1.000 | 0.854 | 0.825 |
| 405 | 0.772 | 1.000 | 0.792 | 0.971 | 0.984 | 1.000 | 0.909 | 1.000 | 0.980 | 1.000 | 0.936 | 0.939 | 1.000 | 0.853 | 1.000 | 1.000 | 0.855 | 0.826 |
| 406 | 0.773 | 1.000 | 0.787 | 0.957 | 0.984 | 0.960 | 0.908 | 1.000 | 0.980 | 1.000 | 0.945 | 0.939 | 1.000 | 0.851 | 1.000 | 1.000 | 0.854 | 0.826 |
| 407 | 0.765 | 1.000 | 0.796 | 0.957 | 0.989 | 0.960 | 0.897 | 1.000 | 0.969 | 1.000 | 0.939 | 0.939 | 1.000 | 0.851 | 1.000 | 0.981 | 0.851 | 0.821 |
| 408 | 0.768 | 1.000 | 0.791 | 0.971 | 0.984 | 0.960 | 0.904 | 1.000 | 0.980 | 1.000 | 0.933 | 0.939 | 1.000 | 0.851 | 1.000 | 1.000 | 0.852 | 0.823 |
| 409 | 0.781 | 1.000 | 0.804 | 0.971 | 0.989 | 0.960 | 0.909 | 1.000 | 0.980 | 1.000 | 0.942 | 0.909 | 1.000 | 0.854 | 1.000 | 1.000 | 0.859 | 0.832 |
| 410 | 0.772 | 1.000 | 0.798 | 0.986 | 0.984 | 0.960 | 0.901 | 1.000 | 0.990 | 1.000 | 0.948 | 0.970 | 1.000 | 0.855 | 1.000 | 1.000 | 0.856 | 0.828 |
| 411 | 0.772 | 1.000 | 0.785 | 0.957 | 0.979 | 0.960 | 0.911 | 1.000 | 0.980 | 1.000 | 0.939 | 0.939 | 1.000 | 0.858 | 1.000 | 0.981 | 0.855 | 0.826 |
| 412 | 0.783 | 1.000 | 0.798 | 0.957 | 0.989 | 1.000 | 0.911 | 1.000 | 0.990 | 0.979 | 0.948 | 0.939 | 1.000 | 0.850 | 1.000 | 0.981 | 0.859 | 0.831 |
| 413 | 0.760 | 1.000 | 0.785 | 0.986 | 0.989 | 0.960 | 0.901 | 1.000 | 0.980 | 1.000 | 0.942 | 0.939 | 1.000 | 0.851 | 1.000 | 0.981 | 0.849 | 0.820 |
| 414 | 0.773 | 1.000 | 0.789 | 0.957 | 0.984 | 0.960 | 0.901 | 1.000 | 0.990 | 1.000 | 0.936 | 0.939 | 1.000 | 0.854 | 1.000 | 1.000 | 0.854 | 0.825 |
| 415 | 0.768 | 1.000 | 0.808 | 0.957 | 0.984 | 1.000 | 0.903 | 1.000 | 0.980 | 0.979 | 0.945 | 0.939 | 1.000 | 0.855 | 1.000 | 1.000 | 0.855 | 0.827 |
| 416 | 0.779 | 1.000 | 0.796 | 0.957 | 0.984 | 0.960 | 0.911 | 1.000 | 0.980 | 1.000 | 0.942 | 0.939 | 1.000 | 0.847 | 1.000 | 0.981 | 0.856 | 0.827 |
| 417 | 0.783 | 1.000 | 0.802 | 0.971 | 0.989 | 1.000 | 0.903 | 1.000 | 0.980 | 1.000 | 0.945 | 0.909 | 1.000 | 0.849 | 1.000 | 1.000 | 0.858 | 0.830 |
| 418 | 0.775 | 1.000 | 0.785 | 0.943 | 0.979 | 1.000 | 0.901 | 1.000 | 1.000 | 1.000 | 0.939 | 0.939 | 1.000 | 0.856 | 1.000 | 0.981 | 0.855 | 0.826 |
| 419 | 0.773 | 1.000 | 0.783 | 0.971 | 0.984 | 1.000 | 0.894 | 1.000 | 1.000 | 1.000 | 0.948 | 0.939 | 1.000 | 0.847 | 1.000 | 0.981 | 0.852 | 0.822 |
| 420 | 0.776 | 1.000 | 0.798 | 0.971 | 0.989 | 0.960 | 0.897 | 1.000 | 0.990 | 1.000 | 0.939 | 0.909 | 1.000 | 0.858 | 1.000 | 0.963 | 0.856 | 0.827 |
| 421 | 0.771 | 1.000 | 0.792 | 0.971 | 0.979 | 0.960 | 0.906 | 1.000 | 0.990 | 1.000 | 0.948 | 0.939 | 1.000 | 0.854 | 1.000 | 0.981 | 0.855 | 0.826 |
| 422 | 0.769 | 1.000 | 0.796 | 0.943 | 0.968 | 0.960 | 0.897 | 1.000 | 0.990 | 1.000 | 0.942 | 0.939 | 1.000 | 0.863 | 1.000 | 0.981 | 0.855 | 0.826 |
| 423 | 0.771 | 1.000 | 0.791 | 0.943 | 0.984 | 0.960 | 0.886 | 1.000 | 1.000 | 1.000 | 0.945 | 0.939 | 1.000 | 0.844 | 1.000 | 0.981 | 0.850 | 0.820 |
| 424 | 0.773 | 1.000 | 0.787 | 0.957 | 0.979 | 0.960 | 0.903 | 1.000 | 0.990 | 1.000 | 0.954 | 0.970 | 1.000 | 0.850 | 1.000 | 0.981 | 0.854 | 0.825 |
| 425 | 0.772 | 1.000 | 0.783 | 0.971 | 0.989 | 1.000 | 0.889 | 1.000 | 0.990 | 1.000 | 0.945 | 0.939 | 1.000 | 0.857 | 1.000 | 0.981 | 0.853 | 0.824 |
| 426 | 0.780 | 1.000 | 0.783 | 0.957 | 0.984 | 0.960 | 0.878 | 1.000 | 0.980 | 1.000 | 0.945 | 0.939 | 1.000 | 0.850 | 1.000 | 0.981 | 0.852 | 0.822 |
| 427 | 0.771 | 1.000 | 0.789 | 0.957 | 0.974 | 0.960 | 0.898 | 1.000 | 0.990 | 1.000 | 0.942 | 0.909 | 1.000 | 0.849 | 1.000 | 0.981 | 0.851 | 0.822 |

| | | | | | | | | | | | | | | | | | |
|---|---|---|---|---|---|---|---|---|---|---|---|---|---|---|---|---|---|
| 428 | 0.778 | 1.000 | 0.785 | 0.971 | 0.979 | 0.960 | 0.904 | 1.000 | 1.000 | 1.000 | 0.945 | 0.909 | 1.000 | 0.856 | 1.000 | 1.000 | 0.857 | 0.828 |
| 429 | 0.770 | 1.000 | 0.802 | 0.957 | 0.979 | 0.960 | 0.897 | 1.000 | 0.980 | 1.000 | 0.948 | 0.939 | 1.000 | 0.858 | 1.000 | 0.981 | 0.855 | 0.827 |
| 430 | 0.773 | 1.000 | 0.791 | 0.957 | 0.968 | 1.000 | 0.891 | 1.000 | 0.990 | 1.000 | 0.951 | 0.939 | 1.000 | 0.851 | 1.000 | 0.981 | 0.852 | 0.823 |
| 431 | 0.774 | 1.000 | 0.785 | 0.957 | 0.984 | 0.960 | 0.895 | 1.000 | 1.000 | 1.000 | 0.951 | 0.939 | 0.991 | 0.849 | 1.000 | 0.981 | 0.853 | 0.823 |
| 432 | 0.776 | 1.000 | 0.789 | 0.971 | 0.979 | 1.000 | 0.898 | 1.000 | 0.980 | 1.000 | 0.954 | 0.939 | 1.000 | 0.850 | 1.000 | 0.981 | 0.854 | 0.825 |
| 433 | 0.777 | 1.000 | 0.783 | 0.957 | 0.984 | 0.960 | 0.898 | 1.000 | 0.990 | 1.000 | 0.948 | 0.939 | 1.000 | 0.860 | 1.000 | 0.981 | 0.856 | 0.828 |
| 434 | 0.780 | 1.000 | 0.802 | 0.957 | 0.979 | 0.960 | 0.889 | 1.000 | 0.990 | 1.000 | 0.948 | 0.879 | 1.000 | 0.852 | 1.000 | 0.963 | 0.855 | 0.826 |
| 435 | 0.767 | 1.000 | 0.779 | 0.957 | 0.989 | 0.960 | 0.903 | 1.000 | 0.990 | 1.000 | 0.951 | 0.909 | 1.000 | 0.857 | 1.000 | 0.981 | 0.853 | 0.824 |
| 436 | 0.775 | 1.000 | 0.791 | 0.957 | 0.989 | 0.960 | 0.894 | 1.000 | 0.990 | 1.000 | 0.945 | 0.909 | 1.000 | 0.847 | 1.000 | 0.981 | 0.852 | 0.823 |
| 437 | 0.776 | 1.000 | 0.792 | 0.957 | 0.984 | 0.960 | 0.898 | 1.000 | 0.980 | 1.000 | 0.954 | 0.909 | 1.000 | 0.851 | 1.000 | 0.981 | 0.855 | 0.826 |
| 438 | 0.767 | 1.000 | 0.798 | 0.971 | 0.984 | 0.960 | 0.891 | 1.000 | 0.990 | 1.000 | 0.945 | 0.909 | 0.991 | 0.854 | 1.000 | 0.981 | 0.852 | 0.822 |
| 439 | 0.774 | 1.000 | 0.798 | 0.929 | 0.979 | 1.000 | 0.900 | 1.000 | 0.990 | 1.000 | 0.948 | 0.909 | 1.000 | 0.851 | 1.000 | 0.981 | 0.854 | 0.825 |
| 440 | 0.772 | 1.000 | 0.816 | 0.971 | 0.984 | 0.960 | 0.889 | 1.000 | 1.000 | 1.000 | 0.936 | 0.939 | 1.000 | 0.857 | 1.000 | 0.981 | 0.856 | 0.827 |
| 441 | 0.779 | 1.000 | 0.804 | 0.943 | 0.979 | 0.960 | 0.894 | 1.000 | 1.000 | 1.000 | 0.945 | 0.909 | 1.000 | 0.846 | 1.000 | 0.981 | 0.854 | 0.825 |
| 442 | 0.774 | 1.000 | 0.789 | 0.957 | 0.979 | 1.000 | 0.897 | 1.000 | 0.990 | 1.000 | 0.942 | 0.970 | 1.000 | 0.854 | 1.000 | 0.981 | 0.854 | 0.825 |
| 443 | 0.772 | 1.000 | 0.796 | 0.971 | 0.979 | 0.960 | 0.900 | 1.000 | 0.990 | 1.000 | 0.945 | 0.879 | 1.000 | 0.847 | 1.000 | 1.000 | 0.853 | 0.824 |
| 444 | 0.777 | 1.000 | 0.806 | 0.971 | 0.974 | 0.960 | 0.901 | 1.000 | 0.990 | 1.000 | 0.945 | 0.939 | 1.000 | 0.847 | 1.000 | 0.981 | 0.855 | 0.826 |
| 445 | 0.775 | 1.000 | 0.789 | 0.957 | 0.979 | 0.960 | 0.908 | 1.000 | 0.990 | 1.000 | 0.945 | 0.939 | 1.000 | 0.854 | 1.000 | 0.981 | 0.855 | 0.827 |
| 446 | 0.771 | 1.000 | 0.798 | 0.957 | 0.984 | 0.960 | 0.908 | 1.000 | 1.000 | 1.000 | 0.959 | 0.909 | 1.000 | 0.858 | 1.000 | 0.981 | 0.858 | 0.830 |
| 447 | 0.782 | 1.000 | 0.787 | 0.957 | 0.984 | 0.960 | 0.895 | 1.000 | 1.000 | 1.000 | 0.945 | 0.909 | 1.000 | 0.848 | 1.000 | 0.981 | 0.855 | 0.825 |
| 448 | 0.774 | 1.000 | 0.804 | 0.971 | 0.989 | 1.000 | 0.889 | 1.000 | 0.990 | 1.000 | 0.948 | 0.939 | 1.000 | 0.848 | 1.000 | 0.981 | 0.854 | 0.825 |
| 449 | 0.777 | 1.000 | 0.779 | 0.971 | 0.984 | 1.000 | 0.898 | 1.000 | 0.980 | 1.000 | 0.942 | 0.939 | 1.000 | 0.851 | 1.000 | 0.981 | 0.853 | 0.824 |
| 450 | 0.778 | 1.000 | 0.810 | 0.957 | 0.979 | 1.000 | 0.901 | 1.000 | 1.000 | 1.000 | 0.948 | 0.879 | 1.000 | 0.864 | 1.000 | 0.981 | 0.861 | 0.833 |
| 451 | 0.776 | 1.000 | 0.796 | 0.957 | 0.974 | 1.000 | 0.901 | 1.000 | 0.990 | 1.000 | 0.945 | 0.939 | 1.000 | 0.850 | 1.000 | 0.981 | 0.855 | 0.826 |
| 452 | 0.778 | 1.000 | 0.818 | 0.971 | 0.989 | 0.960 | 0.897 | 1.000 | 0.990 | 1.000 | 0.948 | 0.909 | 1.000 | 0.848 | 1.000 | 0.981 | 0.857 | 0.829 |
| 453 | 0.780 | 1.000 | 0.794 | 0.957 | 0.984 | 1.000 | 0.897 | 1.000 | 0.990 | 1.000 | 0.957 | 0.879 | 1.000 | 0.851 | 1.000 | 0.981 | 0.856 | 0.828 |
| 454 | 0.774 | 1.000 | 0.800 | 0.971 | 0.979 | 0.960 | 0.892 | 1.000 | 0.990 | 1.000 | 0.957 | 0.909 | 1.000 | 0.854 | 1.000 | 0.981 | 0.855 | 0.826 |
| 455 | 0.778 | 1.000 | 0.789 | 0.971 | 0.984 | 0.960 | 0.903 | 1.000 | 0.990 | 1.000 | 0.954 | 0.909 | 0.991 | 0.852 | 1.000 | 0.981 | 0.856 | 0.827 |
| 456 | 0.788 | 1.000 | 0.792 | 0.943 | 0.989 | 0.960 | 0.900 | 1.000 | 0.990 | 1.000 | 0.959 | 0.909 | 1.000 | 0.858 | 1.000 | 0.981 | 0.861 | 0.833 |
| 457 | 0.771 | 1.000 | 0.802 | 0.971 | 0.989 | 0.960 | 0.895 | 1.000 | 0.990 | 1.000 | 0.951 | 0.939 | 0.991 | 0.857 | 1.000 | 0.981 | 0.856 | 0.827 |
| 458 | 0.782 | 1.000 | 0.781 | 0.957 | 0.989 | 1.000 | 0.903 | 1.000 | 0.969 | 1.000 | 0.951 | 0.939 | 1.000 | 0.848 | 1.000 | 0.981 | 0.855 | 0.827 |
| 459 | 0.774 | 1.000 | 0.798 | 0.943 | 0.989 | 0.960 | 0.888 | 1.000 | 1.000 | 1.000 | 0.951 | 0.939 | 1.000 | 0.858 | 1.000 | 0.981 | 0.856 | 0.827 |
| 460 | 0.774 | 1.000 | 0.792 | 0.971 | 0.979 | 1.000 | 0.908 | 1.000 | 0.980 | 1.000 | 0.962 | 0.939 | 1.000 | 0.849 | 1.000 | 0.981 | 0.856 | 0.828 |
| 461 | 0.777 | 1.000 | 0.791 | 0.957 | 0.979 | 0.960 | 0.897 | 1.000 | 0.990 | 1.000 | 0.951 | 0.879 | 1.000 | 0.854 | 1.000 | 0.981 | 0.855 | 0.826 |
| 462 | 0.789 | 1.000 | 0.794 | 0.943 | 0.984 | 1.000 | 0.891 | 1.000 | 0.990 | 1.000 | 0.951 | 0.939 | 1.000 | 0.849 | 1.000 | 0.981 | 0.857 | 0.829 |
| 463 | 0.771 | 1.000 | 0.792 | 0.986 | 0.989 | 0.960 | 0.900 | 1.000 | 0.990 | 1.000 | 0.951 | 0.970 | 1.000 | 0.851 | 1.000 | 0.981 | 0.854 | 0.826 |
| 464 | 0.772 | 1.000 | 0.787 | 0.971 | 0.989 | 1.000 | 0.895 | 1.000 | 0.990 | 1.000 | 0.951 | 0.909 | 1.000 | 0.858 | 1.000 | 0.981 | 0.855 | 0.826 |
| 465 | 0.771 | 1.000 | 0.802 | 0.957 | 0.984 | 1.000 | 0.886 | 1.000 | 0.990 | 1.000 | 0.959 | 0.909 | 1.000 | 0.857 | 1.000 | 0.963 | 0.855 | 0.826 |
| 466 | 0.773 | 1.000 | 0.787 | 0.957 | 0.989 | 1.000 | 0.897 | 1.000 | 1.000 | 1.000 | 0.948 | 0.939 | 1.000 | 0.854 | 1.000 | 0.981 | 0.854 | 0.825 |

| | | | | | | | | | | | | | | | | | |
|---|---|---|---|---|---|---|---|---|---|---|---|---|---|---|---|---|---|
| 467 | 0.775 | 1.000 | 0.796 | 0.957 | 0.989 | 0.960 | 0.894 | 1.000 | 1.000 | 1.000 | 0.945 | 0.939 | 1.000 | 0.855 | 1.000 | 0.981 | 0.855 | 0.827 |
| 468 | 0.779 | 1.000 | 0.777 | 0.957 | 0.984 | 0.960 | 0.897 | 1.000 | 1.000 | 1.000 | 0.948 | 0.909 | 0.991 | 0.854 | 1.000 | 0.981 | 0.854 | 0.825 |
| 469 | 0.784 | 1.000 | 0.785 | 0.971 | 0.974 | 0.960 | 0.898 | 1.000 | 0.980 | 1.000 | 0.951 | 0.909 | 1.000 | 0.850 | 1.000 | 0.981 | 0.856 | 0.827 |
| 470 | 0.772 | 1.000 | 0.798 | 0.971 | 0.979 | 0.960 | 0.895 | 1.000 | 0.990 | 1.000 | 0.951 | 0.909 | 1.000 | 0.864 | 1.000 | 0.981 | 0.857 | 0.829 |
| 471 | 0.782 | 1.000 | 0.783 | 0.971 | 0.989 | 1.000 | 0.897 | 1.000 | 0.990 | 1.000 | 0.945 | 0.939 | 1.000 | 0.858 | 1.000 | 0.981 | 0.858 | 0.829 |
| 472 | 0.769 | 1.000 | 0.789 | 0.971 | 0.979 | 0.960 | 0.884 | 1.000 | 0.990 | 1.000 | 0.948 | 0.879 | 1.000 | 0.853 | 1.000 | 0.981 | 0.850 | 0.820 |
| 473 | 0.773 | 1.000 | 0.787 | 0.971 | 0.984 | 0.960 | 0.909 | 1.000 | 0.990 | 1.000 | 0.951 | 0.879 | 0.991 | 0.854 | 1.000 | 0.981 | 0.855 | 0.827 |
| 474 | 0.778 | 1.000 | 0.791 | 0.971 | 0.984 | 0.960 | 0.897 | 1.000 | 0.990 | 1.000 | 0.954 | 0.970 | 1.000 | 0.854 | 1.000 | 0.981 | 0.857 | 0.828 |
| 475 | 0.776 | 1.000 | 0.789 | 0.986 | 0.984 | 0.960 | 0.892 | 1.000 | 0.990 | 1.000 | 0.951 | 0.909 | 0.982 | 0.854 | 1.000 | 0.981 | 0.854 | 0.825 |
| 476 | 0.775 | 1.000 | 0.783 | 0.971 | 0.984 | 0.960 | 0.898 | 1.000 | 0.990 | 1.000 | 0.939 | 0.939 | 0.991 | 0.851 | 1.000 | 0.981 | 0.853 | 0.824 |
| 477 | 0.775 | 1.000 | 0.789 | 0.971 | 0.984 | 0.960 | 0.894 | 1.000 | 1.000 | 1.000 | 0.951 | 0.848 | 1.000 | 0.854 | 1.000 | 1.000 | 0.854 | 0.825 |
| 478 | 0.768 | 1.000 | 0.798 | 0.986 | 0.989 | 0.960 | 0.894 | 1.000 | 0.990 | 1.000 | 0.954 | 0.909 | 0.991 | 0.864 | 1.000 | 0.981 | 0.856 | 0.828 |
| 479 | 0.773 | 1.000 | 0.812 | 0.986 | 0.984 | 1.000 | 0.889 | 1.000 | 1.000 | 1.000 | 0.959 | 0.939 | 1.000 | 0.857 | 1.000 | 1.000 | 0.858 | 0.830 |
| 480 | 0.779 | 1.000 | 0.800 | 0.971 | 0.984 | 0.960 | 0.889 | 1.000 | 1.000 | 1.000 | 0.954 | 0.909 | 0.991 | 0.852 | 1.000 | 0.981 | 0.856 | 0.827 |
| 481 | 0.771 | 1.000 | 0.787 | 0.986 | 0.974 | 0.960 | 0.891 | 1.000 | 0.990 | 1.000 | 0.945 | 0.939 | 1.000 | 0.858 | 1.000 | 0.981 | 0.853 | 0.824 |
| 482 | 0.769 | 1.000 | 0.796 | 0.986 | 0.984 | 1.000 | 0.897 | 1.000 | 0.980 | 1.000 | 0.945 | 0.909 | 1.000 | 0.858 | 1.000 | 0.981 | 0.854 | 0.826 |
| 483 | 0.787 | 1.000 | 0.804 | 0.971 | 0.974 | 0.960 | 0.884 | 1.000 | 0.990 | 1.000 | 0.957 | 0.939 | 1.000 | 0.848 | 1.000 | 0.981 | 0.857 | 0.828 |
| 484 | 0.775 | 1.000 | 0.796 | 0.971 | 0.989 | 0.960 | 0.895 | 1.000 | 0.980 | 1.000 | 0.948 | 0.939 | 1.000 | 0.858 | 1.000 | 0.963 | 0.856 | 0.827 |
| 485 | 0.780 | 1.000 | 0.792 | 0.986 | 0.989 | 1.000 | 0.894 | 1.000 | 0.980 | 1.000 | 0.957 | 0.939 | 0.982 | 0.858 | 1.000 | 0.981 | 0.858 | 0.829 |
| 486 | 0.777 | 1.000 | 0.791 | 0.986 | 0.984 | 0.960 | 0.881 | 1.000 | 0.990 | 1.000 | 0.954 | 0.909 | 1.000 | 0.852 | 1.000 | 0.981 | 0.854 | 0.824 |
| 487 | 0.781 | 1.000 | 0.814 | 0.986 | 0.979 | 0.960 | 0.891 | 1.000 | 0.990 | 1.000 | 0.942 | 0.909 | 1.000 | 0.854 | 1.000 | 0.981 | 0.858 | 0.829 |
| 488 | 0.773 | 1.000 | 0.781 | 0.971 | 0.979 | 0.960 | 0.898 | 1.000 | 0.990 | 1.000 | 0.942 | 0.909 | 1.000 | 0.857 | 1.000 | 0.981 | 0.854 | 0.825 |
| 489 | 0.782 | 1.000 | 0.798 | 0.971 | 0.979 | 1.000 | 0.898 | 1.000 | 0.990 | 1.000 | 0.957 | 0.909 | 1.000 | 0.863 | 1.000 | 0.981 | 0.861 | 0.833 |
| 490 | 0.782 | 1.000 | 0.792 | 0.971 | 0.989 | 0.960 | 0.892 | 1.000 | 0.969 | 1.000 | 0.951 | 0.939 | 1.000 | 0.856 | 1.000 | 0.981 | 0.857 | 0.828 |
| 491 | 0.784 | 1.000 | 0.791 | 1.000 | 0.989 | 1.000 | 0.884 | 1.000 | 0.990 | 1.000 | 0.954 | 0.909 | 1.000 | 0.853 | 1.000 | 0.981 | 0.857 | 0.828 |
| 492 | 0.774 | 1.000 | 0.785 | 0.986 | 0.989 | 0.960 | 0.901 | 1.000 | 0.990 | 1.000 | 0.951 | 0.909 | 1.000 | 0.858 | 1.000 | 0.981 | 0.856 | 0.828 |
| 493 | 0.779 | 1.000 | 0.791 | 0.971 | 0.984 | 0.960 | 0.894 | 1.000 | 0.990 | 1.000 | 0.948 | 0.909 | 0.991 | 0.859 | 1.000 | 0.963 | 0.856 | 0.827 |
| 494 | 0.779 | 1.000 | 0.792 | 0.971 | 0.984 | 1.000 | 0.889 | 1.000 | 0.990 | 1.000 | 0.948 | 0.909 | 0.991 | 0.860 | 1.000 | 0.981 | 0.857 | 0.828 |
| 495 | 0.782 | 1.000 | 0.769 | 0.986 | 0.989 | 0.960 | 0.894 | 1.000 | 0.990 | 1.000 | 0.942 | 0.939 | 1.000 | 0.860 | 1.000 | 0.981 | 0.856 | 0.827 |
| 496 | 0.777 | 1.000 | 0.794 | 0.971 | 0.984 | 0.960 | 0.892 | 1.000 | 0.990 | 1.000 | 0.945 | 0.939 | 1.000 | 0.858 | 1.000 | 0.981 | 0.856 | 0.827 |
| 497 | 0.775 | 1.000 | 0.787 | 0.986 | 0.984 | 1.000 | 0.888 | 1.000 | 0.990 | 1.000 | 0.942 | 0.939 | 1.000 | 0.856 | 1.000 | 0.981 | 0.854 | 0.825 |
| 498 | 0.783 | 1.000 | 0.796 | 0.971 | 0.989 | 0.960 | 0.889 | 1.000 | 0.990 | 1.000 | 0.954 | 0.909 | 1.000 | 0.860 | 1.000 | 0.981 | 0.859 | 0.830 |
| 499 | 0.774 | 1.000 | 0.798 | 0.971 | 0.984 | 0.960 | 0.894 | 1.000 | 0.990 | 1.000 | 0.945 | 0.939 | 1.000 | 0.860 | 1.000 | 0.981 | 0.856 | 0.828 |
| 500 | 0.781 | 1.000 | 0.798 | 0.986 | 0.979 | 0.960 | 0.895 | 1.000 | 0.980 | 1.000 | 0.951 | 0.939 | 1.000 | 0.851 | 1.000 | 0.981 | 0.857 | 0.828 |
| 501 | 0.781 | 1.000 | 0.796 | 0.986 | 0.984 | 0.960 | 0.894 | 1.000 | 0.990 | 1.000 | 0.945 | 0.909 | 0.991 | 0.859 | 1.000 | 0.981 | 0.858 | 0.829 |
| 502 | 0.765 | 1.000 | 0.792 | 0.986 | 0.979 | 0.960 | 0.897 | 1.000 | 1.000 | 1.000 | 0.942 | 0.909 | 1.000 | 0.854 | 1.000 | 0.981 | 0.852 | 0.822 |
| 503 | 0.786 | 1.000 | 0.816 | 0.971 | 0.989 | 0.960 | 0.901 | 1.000 | 0.990 | 1.000 | 0.951 | 0.939 | 1.000 | 0.863 | 1.000 | 0.981 | 0.864 | 0.837 |
| 504 | 0.773 | 1.000 | 0.794 | 0.971 | 0.979 | 0.960 | 0.892 | 1.000 | 0.990 | 1.000 | 0.948 | 0.939 | 1.000 | 0.861 | 1.000 | 0.981 | 0.856 | 0.827 |
| 505 | 0.781 | 1.000 | 0.796 | 0.986 | 0.974 | 1.000 | 0.891 | 1.000 | 1.000 | 1.000 | 0.948 | 0.909 | 1.000 | 0.854 | 1.000 | 0.981 | 0.857 | 0.828 |

| | | | | | | | | | | | | | | | | | | |
|---|---|---|---|---|---|---|---|---|---|---|---|---|---|---|---|---|---|---|
| 506 | 0.773 | 1.000 | 0.810 | 0.971 | 0.984 | 0.960 | 0.894 | 1.000 | 1.000 | 1.000 | 0.951 | 0.909 | 0.991 | 0.857 | 1.000 | 0.963 | 0.856 | 0.828 |
| 507 | 0.780 | 1.000 | 0.804 | 0.957 | 0.984 | 0.960 | 0.888 | 1.000 | 0.980 | 1.000 | 0.945 | 0.909 | 1.000 | 0.861 | 1.000 | 0.981 | 0.858 | 0.829 |
| 508 | 0.784 | 1.000 | 0.792 | 0.971 | 0.989 | 0.960 | 0.898 | 1.000 | 0.990 | 1.000 | 0.945 | 0.909 | 0.991 | 0.858 | 1.000 | 0.981 | 0.859 | 0.830 |
| 509 | 0.782 | 1.000 | 0.798 | 0.986 | 0.979 | 0.960 | 0.901 | 1.000 | 0.980 | 1.000 | 0.954 | 0.939 | 1.000 | 0.851 | 1.000 | 0.981 | 0.858 | 0.830 |
| 510 | 0.776 | 1.000 | 0.785 | 0.971 | 0.974 | 0.960 | 0.895 | 1.000 | 0.990 | 1.000 | 0.939 | 0.939 | 1.000 | 0.858 | 1.000 | 0.981 | 0.855 | 0.825 |
| 511 | 0.779 | 1.000 | 0.806 | 0.986 | 0.974 | 1.000 | 0.892 | 1.000 | 0.990 | 1.000 | 0.951 | 0.939 | 1.000 | 0.853 | 1.000 | 0.963 | 0.857 | 0.828 |
| 512 | 0.777 | 1.000 | 0.789 | 0.986 | 0.984 | 0.960 | 0.900 | 1.000 | 0.990 | 1.000 | 0.939 | 0.939 | 1.000 | 0.858 | 1.000 | 0.963 | 0.856 | 0.827 |
| 513 | 0.783 | 1.000 | 0.800 | 0.986 | 0.984 | 0.960 | 0.909 | 1.000 | 1.000 | 1.000 | 0.951 | 0.939 | 0.991 | 0.862 | 1.000 | 0.981 | 0.863 | 0.835 |
| 514 | 0.777 | 1.000 | 0.794 | 0.986 | 0.984 | 0.960 | 0.894 | 1.000 | 0.990 | 1.000 | 0.954 | 0.970 | 0.991 | 0.861 | 1.000 | 0.981 | 0.858 | 0.830 |
| 515 | 0.782 | 1.000 | 0.804 | 0.986 | 0.989 | 1.000 | 0.889 | 1.000 | 0.980 | 1.000 | 0.959 | 0.939 | 1.000 | 0.857 | 1.000 | 0.963 | 0.859 | 0.831 |
| 516 | 0.781 | 1.000 | 0.812 | 0.971 | 0.984 | 0.960 | 0.894 | 1.000 | 0.990 | 1.000 | 0.942 | 0.939 | 1.000 | 0.859 | 1.000 | 1.000 | 0.860 | 0.832 |
| 517 | 0.788 | 1.000 | 0.796 | 0.971 | 0.979 | 0.960 | 0.895 | 1.000 | 0.990 | 1.000 | 0.945 | 0.939 | 1.000 | 0.861 | 1.000 | 0.981 | 0.861 | 0.832 |
| 518 | 0.773 | 1.000 | 0.775 | 0.986 | 0.979 | 0.960 | 0.900 | 1.000 | 0.990 | 1.000 | 0.954 | 0.909 | 0.991 | 0.854 | 1.000 | 0.981 | 0.853 | 0.824 |
| 519 | 0.775 | 1.000 | 0.802 | 0.986 | 0.974 | 0.960 | 0.886 | 1.000 | 0.980 | 1.000 | 0.951 | 0.909 | 1.000 | 0.850 | 1.000 | 0.981 | 0.853 | 0.824 |
| 520 | 0.779 | 1.000 | 0.794 | 0.971 | 0.989 | 0.960 | 0.906 | 1.000 | 0.980 | 1.000 | 0.942 | 0.970 | 1.000 | 0.858 | 1.000 | 0.981 | 0.859 | 0.831 |
| 521 | 0.779 | 1.000 | 0.781 | 0.986 | 0.979 | 0.960 | 0.892 | 1.000 | 0.990 | 1.000 | 0.954 | 0.909 | 1.000 | 0.851 | 1.000 | 0.981 | 0.854 | 0.824 |
| 522 | 0.779 | 1.000 | 0.777 | 0.986 | 0.984 | 0.960 | 0.898 | 1.000 | 0.990 | 1.000 | 0.939 | 0.939 | 0.991 | 0.860 | 1.000 | 0.981 | 0.856 | 0.827 |
| 523 | 0.770 | 1.000 | 0.800 | 0.986 | 0.984 | 0.960 | 0.900 | 1.000 | 0.990 | 1.000 | 0.945 | 0.879 | 1.000 | 0.853 | 1.000 | 0.981 | 0.854 | 0.826 |
| 524 | 0.779 | 1.000 | 0.792 | 0.986 | 0.974 | 0.960 | 0.895 | 1.000 | 0.990 | 1.000 | 0.954 | 0.939 | 1.000 | 0.853 | 1.000 | 0.963 | 0.856 | 0.827 |
| 525 | 0.772 | 1.000 | 0.792 | 0.986 | 0.979 | 0.960 | 0.900 | 1.000 | 0.990 | 1.000 | 0.945 | 0.909 | 0.991 | 0.857 | 1.000 | 0.981 | 0.855 | 0.826 |
| 526 | 0.776 | 1.000 | 0.794 | 0.971 | 0.984 | 1.000 | 0.904 | 1.000 | 0.990 | 1.000 | 0.951 | 0.939 | 1.000 | 0.863 | 1.000 | 0.981 | 0.859 | 0.831 |
| 527 | 0.769 | 1.000 | 0.791 | 0.971 | 0.989 | 1.000 | 0.897 | 1.000 | 0.990 | 1.000 | 0.954 | 0.939 | 0.991 | 0.858 | 1.000 | 0.981 | 0.855 | 0.826 |
| 528 | 0.776 | 1.000 | 0.789 | 0.986 | 0.984 | 0.960 | 0.903 | 1.000 | 0.980 | 1.000 | 0.948 | 0.939 | 0.991 | 0.861 | 1.000 | 0.981 | 0.857 | 0.829 |
| 529 | 0.785 | 1.000 | 0.800 | 0.957 | 0.979 | 1.000 | 0.895 | 1.000 | 0.969 | 1.000 | 0.951 | 0.970 | 0.991 | 0.849 | 1.000 | 0.981 | 0.857 | 0.828 |
| 530 | 0.780 | 1.000 | 0.802 | 0.986 | 0.979 | 0.960 | 0.895 | 1.000 | 1.000 | 1.000 | 0.954 | 0.970 | 1.000 | 0.854 | 1.000 | 0.963 | 0.858 | 0.829 |
| 531 | 0.771 | 1.000 | 0.775 | 0.986 | 0.984 | 1.000 | 0.895 | 1.000 | 0.990 | 1.000 | 0.951 | 0.939 | 0.991 | 0.853 | 1.000 | 1.000 | 0.852 | 0.823 |
| 532 | 0.779 | 1.000 | 0.798 | 0.986 | 0.984 | 1.000 | 0.891 | 1.000 | 0.990 | 1.000 | 0.951 | 0.939 | 1.000 | 0.858 | 1.000 | 0.981 | 0.858 | 0.829 |
| 533 | 0.774 | 1.000 | 0.802 | 0.986 | 0.989 | 0.960 | 0.894 | 1.000 | 1.000 | 1.000 | 0.957 | 0.939 | 1.000 | 0.861 | 1.000 | 0.963 | 0.858 | 0.830 |
| 534 | 0.782 | 1.000 | 0.798 | 0.986 | 0.984 | 0.960 | 0.900 | 1.000 | 0.980 | 1.000 | 0.942 | 0.939 | 0.991 | 0.856 | 1.000 | 0.963 | 0.858 | 0.830 |
| 535 | 0.782 | 1.000 | 0.806 | 0.986 | 0.984 | 0.960 | 0.895 | 1.000 | 0.990 | 1.000 | 0.951 | 0.970 | 0.991 | 0.854 | 1.000 | 0.981 | 0.859 | 0.831 |
| 536 | 0.776 | 1.000 | 0.816 | 0.971 | 0.979 | 1.000 | 0.908 | 1.000 | 0.990 | 1.000 | 0.948 | 0.939 | 1.000 | 0.858 | 1.000 | 0.981 | 0.860 | 0.833 |
| 537 | 0.783 | 1.000 | 0.798 | 0.986 | 0.984 | 0.960 | 0.900 | 1.000 | 0.980 | 1.000 | 0.954 | 0.939 | 1.000 | 0.857 | 1.000 | 0.981 | 0.860 | 0.831 |
| 538 | 0.771 | 1.000 | 0.808 | 0.986 | 0.979 | 0.960 | 0.901 | 1.000 | 0.990 | 1.000 | 0.948 | 0.909 | 1.000 | 0.857 | 1.000 | 0.981 | 0.857 | 0.828 |
| 539 | 0.775 | 1.000 | 0.804 | 0.986 | 0.979 | 1.000 | 0.894 | 1.000 | 1.000 | 1.000 | 0.945 | 0.939 | 1.000 | 0.858 | 1.000 | 0.981 | 0.857 | 0.829 |
| 540 | 0.776 | 1.000 | 0.810 | 0.986 | 0.984 | 0.960 | 0.888 | 1.000 | 0.990 | 1.000 | 0.954 | 0.909 | 1.000 | 0.855 | 1.000 | 0.981 | 0.857 | 0.828 |
| 541 | 0.772 | 1.000 | 0.810 | 0.986 | 0.979 | 0.960 | 0.897 | 1.000 | 0.990 | 1.000 | 0.951 | 0.939 | 1.000 | 0.850 | 1.000 | 0.981 | 0.855 | 0.827 |
| 542 | 0.775 | 1.000 | 0.804 | 1.000 | 0.974 | 0.960 | 0.898 | 1.000 | 0.990 | 1.000 | 0.948 | 0.939 | 1.000 | 0.852 | 1.000 | 0.981 | 0.856 | 0.828 |
| 543 | 0.773 | 1.000 | 0.796 | 0.971 | 0.974 | 0.960 | 0.900 | 1.000 | 0.980 | 1.000 | 0.954 | 0.909 | 1.000 | 0.861 | 1.000 | 0.981 | 0.857 | 0.828 |
| 544 | 0.775 | 1.000 | 0.804 | 0.986 | 0.984 | 1.000 | 0.898 | 1.000 | 0.990 | 1.000 | 0.948 | 0.879 | 1.000 | 0.851 | 1.000 | 0.981 | 0.856 | 0.827 |

| | | | | | | | | | | | | | | | | | | |
|---|---|---|---|---|---|---|---|---|---|---|---|---|---|---|---|---|---|---|
| 545 | 0.776 | 1.000 | 0.796 | 1.000 | 0.979 | 0.960 | 0.900 | 1.000 | 0.990 | 1.000 | 0.959 | 0.909 | 1.000 | 0.860 | 1.000 | 0.963 | 0.858 | 0.830 |
| 546 | 0.779 | 1.000 | 0.796 | 1.000 | 0.984 | 0.960 | 0.898 | 1.000 | 0.990 | 1.000 | 0.945 | 0.909 | 1.000 | 0.851 | 1.000 | 0.981 | 0.856 | 0.827 |
| 547 | 0.775 | 1.000 | 0.806 | 1.000 | 0.979 | 0.960 | 0.897 | 1.000 | 0.990 | 1.000 | 0.945 | 0.939 | 1.000 | 0.858 | 1.000 | 0.981 | 0.858 | 0.830 |
| 548 | 0.774 | 1.000 | 0.794 | 1.000 | 0.979 | 0.960 | 0.895 | 1.000 | 0.990 | 1.000 | 0.954 | 0.939 | 1.000 | 0.854 | 1.000 | 0.981 | 0.855 | 0.827 |
| 549 | 0.779 | 1.000 | 0.789 | 0.986 | 0.984 | 0.960 | 0.904 | 1.000 | 1.000 | 1.000 | 0.945 | 0.939 | 0.991 | 0.854 | 1.000 | 0.981 | 0.857 | 0.829 |
| 550 | 0.774 | 1.000 | 0.816 | 1.000 | 0.989 | 0.960 | 0.898 | 1.000 | 0.990 | 1.000 | 0.945 | 0.909 | 0.991 | 0.854 | 1.000 | 0.981 | 0.858 | 0.830 |
| 551 | 0.780 | 1.000 | 0.785 | 1.000 | 0.968 | 1.000 | 0.898 | 1.000 | 0.990 | 1.000 | 0.948 | 0.909 | 1.000 | 0.851 | 1.000 | 1.000 | 0.855 | 0.826 |
| 552 | 0.775 | 1.000 | 0.791 | 1.000 | 0.979 | 1.000 | 0.900 | 1.000 | 0.990 | 1.000 | 0.945 | 0.909 | 1.000 | 0.863 | 1.000 | 0.981 | 0.858 | 0.829 |
| 553 | 0.775 | 1.000 | 0.792 | 1.000 | 0.974 | 1.000 | 0.903 | 1.000 | 0.980 | 1.000 | 0.945 | 0.939 | 1.000 | 0.854 | 1.000 | 0.981 | 0.856 | 0.827 |
| 554 | 0.773 | 1.000 | 0.791 | 0.986 | 0.984 | 0.960 | 0.904 | 1.000 | 0.990 | 1.000 | 0.951 | 0.879 | 1.000 | 0.855 | 1.000 | 0.981 | 0.856 | 0.827 |
| 555 | 0.779 | 1.000 | 0.800 | 1.000 | 0.984 | 1.000 | 0.906 | 1.000 | 0.990 | 1.000 | 0.948 | 0.909 | 1.000 | 0.855 | 1.000 | 0.981 | 0.859 | 0.831 |
| 556 | 0.779 | 1.000 | 0.810 | 1.000 | 0.984 | 1.000 | 0.892 | 1.000 | 0.980 | 1.000 | 0.951 | 0.939 | 1.000 | 0.851 | 1.000 | 0.963 | 0.857 | 0.829 |
| 557 | 0.771 | 1.000 | 0.804 | 1.000 | 0.984 | 0.960 | 0.897 | 1.000 | 0.980 | 1.000 | 0.959 | 0.939 | 1.000 | 0.854 | 1.000 | 0.981 | 0.856 | 0.828 |
| 558 | 0.783 | 1.000 | 0.800 | 0.986 | 0.984 | 0.960 | 0.888 | 1.000 | 0.990 | 1.000 | 0.948 | 0.939 | 1.000 | 0.851 | 1.000 | 0.981 | 0.857 | 0.828 |
| 559 | 0.777 | 1.000 | 0.796 | 0.986 | 0.984 | 0.960 | 0.888 | 1.000 | 0.990 | 1.000 | 0.948 | 0.939 | 1.000 | 0.862 | 1.000 | 0.981 | 0.857 | 0.829 |
| 560 | 0.775 | 1.000 | 0.796 | 1.000 | 0.984 | 0.960 | 0.892 | 1.000 | 0.990 | 1.000 | 0.948 | 0.939 | 1.000 | 0.854 | 1.000 | 0.963 | 0.855 | 0.826 |
| 561 | 0.777 | 1.000 | 0.783 | 1.000 | 0.984 | 0.960 | 0.898 | 1.000 | 0.990 | 1.000 | 0.951 | 0.939 | 1.000 | 0.856 | 1.000 | 0.981 | 0.856 | 0.827 |
| 562 | 0.774 | 1.000 | 0.794 | 1.000 | 0.984 | 1.000 | 0.904 | 1.000 | 0.990 | 1.000 | 0.948 | 0.909 | 1.000 | 0.864 | 1.000 | 0.981 | 0.859 | 0.831 |
| 563 | 0.775 | 1.000 | 0.806 | 1.000 | 0.984 | 0.960 | 0.895 | 1.000 | 0.990 | 1.000 | 0.948 | 0.939 | 1.000 | 0.863 | 1.000 | 0.981 | 0.859 | 0.831 |
| 564 | 0.777 | 1.000 | 0.794 | 1.000 | 0.989 | 1.000 | 0.897 | 1.000 | 0.990 | 1.000 | 0.954 | 0.909 | 1.000 | 0.854 | 1.000 | 0.981 | 0.857 | 0.829 |
| 565 | 0.779 | 1.000 | 0.802 | 1.000 | 0.979 | 1.000 | 0.894 | 1.000 | 0.980 | 1.000 | 0.954 | 0.939 | 1.000 | 0.857 | 1.000 | 1.000 | 0.858 | 0.830 |
| 566 | 0.773 | 1.000 | 0.798 | 1.000 | 0.984 | 0.960 | 0.901 | 1.000 | 0.990 | 1.000 | 0.945 | 0.939 | 1.000 | 0.853 | 1.000 | 0.981 | 0.856 | 0.827 |
| 567 | 0.776 | 1.000 | 0.791 | 1.000 | 0.979 | 0.960 | 0.895 | 1.000 | 0.990 | 1.000 | 0.948 | 0.939 | 0.991 | 0.853 | 1.000 | 1.000 | 0.855 | 0.826 |
| 568 | 0.780 | 1.000 | 0.802 | 0.986 | 0.979 | 0.960 | 0.891 | 1.000 | 0.990 | 1.000 | 0.948 | 0.909 | 0.991 | 0.850 | 1.000 | 0.981 | 0.855 | 0.826 |
| 569 | 0.781 | 1.000 | 0.775 | 1.000 | 0.979 | 1.000 | 0.889 | 1.000 | 0.990 | 1.000 | 0.948 | 0.939 | 0.982 | 0.855 | 1.000 | 0.981 | 0.854 | 0.825 |
| 570 | 0.770 | 1.000 | 0.800 | 0.986 | 0.984 | 1.000 | 0.894 | 1.000 | 0.990 | 1.000 | 0.945 | 0.939 | 0.991 | 0.861 | 1.000 | 0.981 | 0.856 | 0.827 |
| 571 | 0.771 | 1.000 | 0.804 | 0.986 | 0.984 | 1.000 | 0.894 | 1.000 | 0.980 | 1.000 | 0.942 | 0.939 | 0.991 | 0.851 | 1.000 | 0.981 | 0.854 | 0.825 |
| 572 | 0.771 | 1.000 | 0.814 | 0.957 | 0.984 | 1.000 | 0.883 | 1.000 | 0.990 | 1.000 | 0.948 | 0.939 | 0.991 | 0.850 | 1.000 | 0.981 | 0.853 | 0.824 |
| 573 | 0.785 | 1.000 | 0.812 | 1.000 | 0.984 | 0.960 | 0.889 | 1.000 | 0.990 | 1.000 | 0.951 | 0.939 | 0.991 | 0.854 | 1.000 | 0.981 | 0.860 | 0.832 |
| 574 | 0.771 | 1.000 | 0.802 | 1.000 | 0.974 | 1.000 | 0.894 | 1.000 | 0.990 | 1.000 | 0.948 | 0.939 | 0.991 | 0.850 | 1.000 | 0.981 | 0.854 | 0.825 |
| 575 | 0.777 | 1.000 | 0.800 | 1.000 | 0.989 | 0.960 | 0.898 | 1.000 | 1.000 | 1.000 | 0.951 | 0.909 | 1.000 | 0.847 | 1.000 | 1.000 | 0.856 | 0.827 |
| 576 | 0.779 | 1.000 | 0.804 | 1.000 | 0.984 | 0.960 | 0.892 | 1.000 | 0.990 | 1.000 | 0.954 | 0.939 | 0.991 | 0.854 | 1.000 | 0.981 | 0.858 | 0.829 |
| 577 | 0.785 | 1.000 | 0.802 | 0.986 | 0.989 | 0.960 | 0.884 | 1.000 | 0.980 | 1.000 | 0.951 | 0.909 | 0.991 | 0.851 | 1.000 | 0.981 | 0.857 | 0.828 |
| 578 | 0.778 | 1.000 | 0.798 | 1.000 | 0.979 | 0.960 | 0.901 | 1.000 | 0.980 | 1.000 | 0.948 | 0.939 | 0.991 | 0.863 | 1.000 | 0.981 | 0.859 | 0.831 |
| 579 | 0.779 | 1.000 | 0.796 | 1.000 | 0.984 | 1.000 | 0.898 | 1.000 | 1.000 | 1.000 | 0.954 | 0.909 | 0.982 | 0.857 | 1.000 | 0.963 | 0.858 | 0.830 |
| 580 | 0.780 | 1.000 | 0.796 | 1.000 | 0.989 | 0.960 | 0.892 | 1.000 | 1.000 | 1.000 | 0.951 | 0.909 | 0.991 | 0.858 | 1.000 | 0.981 | 0.858 | 0.830 |
| 581 | 0.780 | 1.000 | 0.796 | 1.000 | 0.989 | 1.000 | 0.901 | 1.000 | 0.980 | 1.000 | 0.951 | 0.939 | 0.982 | 0.857 | 1.000 | 0.981 | 0.859 | 0.830 |
| 582 | 0.774 | 1.000 | 0.804 | 1.000 | 0.984 | 0.960 | 0.901 | 1.000 | 0.980 | 1.000 | 0.954 | 0.939 | 0.991 | 0.859 | 1.000 | 0.981 | 0.858 | 0.830 |
| 583 | 0.771 | 1.000 | 0.792 | 1.000 | 0.984 | 1.000 | 0.891 | 1.000 | 0.990 | 1.000 | 0.942 | 0.909 | 0.991 | 0.865 | 1.000 | 0.981 | 0.856 | 0.827 |

| | | | | | | | | | | | | | | | | | |
|---|---|---|---|---|---|---|---|---|---|---|---|---|---|---|---|---|---|
| 584 | 0.775 | 1.000 | 0.812 | 1.000 | 0.984 | 0.960 | 0.897 | 1.000 | 0.990 | 1.000 | 0.948 | 0.909 | 0.991 | 0.855 | 1.000 | 0.981 | 0.857 | 0.829 |
| 585 | 0.769 | 1.000 | 0.802 | 1.000 | 0.984 | 0.960 | 0.900 | 1.000 | 0.990 | 1.000 | 0.954 | 0.939 | 0.991 | 0.853 | 1.000 | 0.981 | 0.855 | 0.826 |
| 586 | 0.775 | 1.000 | 0.804 | 1.000 | 0.984 | 0.960 | 0.904 | 1.000 | 0.990 | 1.000 | 0.951 | 0.939 | 0.991 | 0.858 | 1.000 | 0.981 | 0.859 | 0.831 |
| 587 | 0.781 | 1.000 | 0.810 | 0.986 | 0.989 | 0.960 | 0.901 | 1.000 | 0.990 | 1.000 | 0.951 | 0.939 | 0.991 | 0.852 | 1.000 | 0.981 | 0.859 | 0.831 |
| 588 | 0.764 | 1.000 | 0.812 | 0.986 | 0.984 | 0.960 | 0.894 | 1.000 | 0.980 | 1.000 | 0.954 | 0.909 | 0.991 | 0.853 | 1.000 | 0.981 | 0.853 | 0.824 |
| 589 | 0.773 | 1.000 | 0.796 | 1.000 | 0.989 | 0.960 | 0.901 | 1.000 | 1.000 | 1.000 | 0.957 | 0.939 | 0.991 | 0.853 | 1.000 | 0.981 | 0.856 | 0.828 |
| 590 | 0.779 | 1.000 | 0.798 | 1.000 | 0.979 | 0.960 | 0.895 | 1.000 | 0.990 | 1.000 | 0.951 | 0.939 | 0.991 | 0.853 | 1.000 | 0.981 | 0.857 | 0.828 |
| 591 | 0.778 | 1.000 | 0.816 | 1.000 | 0.989 | 0.960 | 0.898 | 1.000 | 0.980 | 1.000 | 0.957 | 0.909 | 0.991 | 0.858 | 1.000 | 1.000 | 0.861 | 0.833 |
| 592 | 0.777 | 1.000 | 0.808 | 1.000 | 0.979 | 0.960 | 0.903 | 1.000 | 0.990 | 1.000 | 0.954 | 0.939 | 0.991 | 0.851 | 1.000 | 0.981 | 0.858 | 0.830 |
| 593 | 0.773 | 1.000 | 0.804 | 1.000 | 0.984 | 1.000 | 0.891 | 1.000 | 0.990 | 1.000 | 0.948 | 0.909 | 0.991 | 0.849 | 1.000 | 0.981 | 0.854 | 0.825 |
| 594 | 0.779 | 1.000 | 0.810 | 0.986 | 0.979 | 0.960 | 0.895 | 1.000 | 0.980 | 1.000 | 0.954 | 0.909 | 0.991 | 0.851 | 1.000 | 0.981 | 0.857 | 0.828 |
| 595 | 0.777 | 1.000 | 0.791 | 1.000 | 0.989 | 0.960 | 0.904 | 1.000 | 0.990 | 1.000 | 0.948 | 0.879 | 0.991 | 0.861 | 1.000 | 0.981 | 0.859 | 0.830 |
| 596 | 0.780 | 1.000 | 0.796 | 1.000 | 0.979 | 0.960 | 0.889 | 1.000 | 0.969 | 1.000 | 0.957 | 0.909 | 0.982 | 0.857 | 1.000 | 0.981 | 0.857 | 0.828 |
| 597 | 0.770 | 1.000 | 0.812 | 0.986 | 0.989 | 1.000 | 0.895 | 1.000 | 0.980 | 1.000 | 0.951 | 0.909 | 0.991 | 0.859 | 1.000 | 0.981 | 0.857 | 0.829 |
| 598 | 0.777 | 1.000 | 0.794 | 1.000 | 0.979 | 0.960 | 0.906 | 1.000 | 1.000 | 1.000 | 0.948 | 0.939 | 0.991 | 0.864 | 1.000 | 0.981 | 0.860 | 0.832 |
| 599 | 0.775 | 1.000 | 0.804 | 1.000 | 0.984 | 1.000 | 0.892 | 1.000 | 0.980 | 1.000 | 0.948 | 0.939 | 1.000 | 0.851 | 1.000 | 0.981 | 0.855 | 0.827 |
| 600 | 0.781 | 1.000 | 0.812 | 0.986 | 0.989 | 0.960 | 0.898 | 1.000 | 1.000 | 1.000 | 0.954 | 0.939 | 0.991 | 0.857 | 1.000 | 1.000 | 0.861 | 0.833 |
| 601 | 0.778 | 1.000 | 0.798 | 1.000 | 0.984 | 0.960 | 0.897 | 1.000 | 0.990 | 1.000 | 0.948 | 0.939 | 1.000 | 0.846 | 1.000 | 0.981 | 0.855 | 0.826 |
| 602 | 0.773 | 1.000 | 0.806 | 1.000 | 0.984 | 0.960 | 0.903 | 1.000 | 0.990 | 1.000 | 0.957 | 0.879 | 1.000 | 0.860 | 1.000 | 0.981 | 0.859 | 0.831 |
| 603 | 0.771 | 1.000 | 0.814 | 1.000 | 0.984 | 1.000 | 0.898 | 1.000 | 0.990 | 1.000 | 0.957 | 0.848 | 1.000 | 0.853 | 1.000 | 0.981 | 0.857 | 0.828 |
| 604 | 0.777 | 1.000 | 0.800 | 1.000 | 0.989 | 1.000 | 0.901 | 1.000 | 1.000 | 1.000 | 0.951 | 0.879 | 1.000 | 0.861 | 1.000 | 0.981 | 0.860 | 0.832 |
| 605 | 0.781 | 1.000 | 0.794 | 1.000 | 0.984 | 0.960 | 0.897 | 1.000 | 0.990 | 1.000 | 0.945 | 0.879 | 1.000 | 0.865 | 1.000 | 1.000 | 0.860 | 0.832 |
| 606 | 0.766 | 1.000 | 0.802 | 1.000 | 0.979 | 0.960 | 0.886 | 1.000 | 0.969 | 1.000 | 0.942 | 0.970 | 1.000 | 0.861 | 1.000 | 0.981 | 0.853 | 0.824 |
| 607 | 0.779 | 1.000 | 0.806 | 1.000 | 0.984 | 1.000 | 0.884 | 1.000 | 0.969 | 1.000 | 0.945 | 0.909 | 1.000 | 0.864 | 1.000 | 1.000 | 0.859 | 0.830 |
| 608 | 0.777 | 1.000 | 0.798 | 1.000 | 0.984 | 1.000 | 0.883 | 1.000 | 0.969 | 1.000 | 0.948 | 0.970 | 1.000 | 0.850 | 1.000 | 1.000 | 0.854 | 0.825 |
| 609 | 0.773 | 1.000 | 0.796 | 1.000 | 0.979 | 1.000 | 0.892 | 1.000 | 0.980 | 1.000 | 0.948 | 0.970 | 0.991 | 0.857 | 1.000 | 0.981 | 0.855 | 0.826 |
| 610 | 0.774 | 1.000 | 0.794 | 1.000 | 0.968 | 0.960 | 0.884 | 1.000 | 0.969 | 1.000 | 0.954 | 0.939 | 1.000 | 0.860 | 1.000 | 0.981 | 0.855 | 0.825 |
| 611 | 0.785 | 1.000 | 0.792 | 1.000 | 0.984 | 0.960 | 0.886 | 1.000 | 0.980 | 1.000 | 0.939 | 0.939 | 1.000 | 0.855 | 1.000 | 1.000 | 0.857 | 0.828 |
| 612 | 0.777 | 1.000 | 0.804 | 1.000 | 0.984 | 0.960 | 0.889 | 1.000 | 0.980 | 1.000 | 0.957 | 0.970 | 0.991 | 0.862 | 1.000 | 1.000 | 0.859 | 0.831 |
| 613 | 0.774 | 1.000 | 0.822 | 1.000 | 0.974 | 0.960 | 0.892 | 1.000 | 0.980 | 1.000 | 0.957 | 0.939 | 1.000 | 0.858 | 1.000 | 1.000 | 0.859 | 0.831 |
| 614 | 0.769 | 1.000 | 0.808 | 1.000 | 0.979 | 1.000 | 0.898 | 1.000 | 0.980 | 1.000 | 0.951 | 0.970 | 1.000 | 0.863 | 1.000 | 1.000 | 0.858 | 0.830 |
| 615 | 0.765 | 1.000 | 0.808 | 1.000 | 0.979 | 0.960 | 0.889 | 1.000 | 0.980 | 1.000 | 0.942 | 0.939 | 1.000 | 0.857 | 1.000 | 0.981 | 0.853 | 0.824 |
| 616 | 0.777 | 1.000 | 0.808 | 1.000 | 0.979 | 0.960 | 0.895 | 1.000 | 0.980 | 1.000 | 0.948 | 0.939 | 1.000 | 0.857 | 1.000 | 1.000 | 0.858 | 0.830 |
| 617 | 0.778 | 1.000 | 0.798 | 1.000 | 0.979 | 1.000 | 0.892 | 1.000 | 0.969 | 1.000 | 0.954 | 0.939 | 1.000 | 0.847 | 1.000 | 1.000 | 0.855 | 0.826 |
| 618 | 0.781 | 1.000 | 0.800 | 1.000 | 0.979 | 1.000 | 0.891 | 1.000 | 0.980 | 1.000 | 0.959 | 0.970 | 0.991 | 0.857 | 1.000 | 1.000 | 0.859 | 0.831 |
| 619 | 0.776 | 1.000 | 0.808 | 1.000 | 0.974 | 0.960 | 0.891 | 1.000 | 0.980 | 1.000 | 0.945 | 0.909 | 1.000 | 0.861 | 1.000 | 1.000 | 0.858 | 0.829 |
| 620 | 0.777 | 1.000 | 0.792 | 1.000 | 0.979 | 0.960 | 0.889 | 1.000 | 0.969 | 1.000 | 0.954 | 0.970 | 0.991 | 0.854 | 1.000 | 0.981 | 0.855 | 0.826 |
| 621 | 0.771 | 1.000 | 0.812 | 1.000 | 0.979 | 0.960 | 0.888 | 1.000 | 0.980 | 1.000 | 0.957 | 0.939 | 1.000 | 0.849 | 1.000 | 1.000 | 0.854 | 0.825 |
| 622 | 0.773 | 1.000 | 0.796 | 1.000 | 0.974 | 0.960 | 0.883 | 1.000 | 0.980 | 1.000 | 0.954 | 0.970 | 1.000 | 0.854 | 1.000 | 1.000 | 0.854 | 0.824 |

| | | | | | | | | | | | | | | | | | |
|---|---|---|---|---|---|---|---|---|---|---|---|---|---|---|---|---|---|
| 623 | 0.777 | 1.000 | 0.806 | 1.000 | 0.979 | 0.960 | 0.894 | 1.000 | 0.980 | 1.000 | 0.951 | 0.939 | 1.000 | 0.854 | 1.000 | 0.981 | 0.857 | 0.828 |
| 624 | 0.780 | 1.000 | 0.816 | 1.000 | 0.984 | 0.960 | 0.895 | 1.000 | 0.980 | 1.000 | 0.951 | 0.909 | 1.000 | 0.858 | 1.000 | 1.000 | 0.860 | 0.832 |
| 625 | 0.782 | 1.000 | 0.804 | 1.000 | 0.984 | 0.960 | 0.892 | 1.000 | 0.980 | 1.000 | 0.954 | 0.909 | 1.000 | 0.853 | 1.000 | 1.000 | 0.858 | 0.830 |
| 626 | 0.785 | 1.000 | 0.812 | 1.000 | 0.984 | 0.960 | 0.895 | 1.000 | 0.980 | 1.000 | 0.954 | 0.939 | 1.000 | 0.858 | 1.000 | 1.000 | 0.862 | 0.834 |
| 627 | 0.787 | 1.000 | 0.792 | 1.000 | 0.984 | 0.960 | 0.894 | 1.000 | 0.980 | 1.000 | 0.954 | 0.970 | 1.000 | 0.856 | 1.000 | 1.000 | 0.860 | 0.832 |
| 628 | 0.765 | 1.000 | 0.806 | 1.000 | 0.979 | 1.000 | 0.891 | 1.000 | 0.980 | 1.000 | 0.948 | 0.939 | 1.000 | 0.847 | 1.000 | 1.000 | 0.851 | 0.822 |
| 629 | 0.779 | 1.000 | 0.812 | 1.000 | 0.979 | 1.000 | 0.889 | 1.000 | 0.980 | 1.000 | 0.954 | 0.939 | 1.000 | 0.847 | 1.000 | 1.000 | 0.856 | 0.828 |
| 630 | 0.772 | 1.000 | 0.828 | 1.000 | 0.979 | 0.960 | 0.888 | 1.000 | 0.980 | 1.000 | 0.957 | 0.939 | 0.991 | 0.858 | 1.000 | 1.000 | 0.858 | 0.830 |
| 631 | 0.783 | 1.000 | 0.800 | 1.000 | 0.984 | 1.000 | 0.891 | 1.000 | 0.980 | 1.000 | 0.957 | 0.970 | 1.000 | 0.865 | 1.000 | 1.000 | 0.862 | 0.834 |
| 632 | 0.763 | 1.000 | 0.798 | 1.000 | 0.984 | 1.000 | 0.904 | 1.000 | 0.980 | 1.000 | 0.951 | 1.000 | 1.000 | 0.856 | 1.000 | 1.000 | 0.855 | 0.827 |
| 633 | 0.775 | 1.000 | 0.812 | 0.986 | 0.979 | 1.000 | 0.891 | 1.000 | 0.980 | 1.000 | 0.945 | 0.970 | 1.000 | 0.855 | 1.000 | 1.000 | 0.857 | 0.828 |
| 634 | 0.779 | 1.000 | 0.816 | 1.000 | 0.974 | 0.960 | 0.881 | 1.000 | 0.980 | 1.000 | 0.954 | 0.939 | 1.000 | 0.860 | 1.000 | 0.981 | 0.859 | 0.830 |
| 635 | 0.774 | 1.000 | 0.798 | 1.000 | 0.984 | 0.960 | 0.877 | 1.000 | 0.969 | 1.000 | 0.951 | 0.970 | 1.000 | 0.859 | 1.000 | 1.000 | 0.855 | 0.825 |
| 636 | 0.775 | 1.000 | 0.796 | 1.000 | 0.979 | 0.960 | 0.894 | 1.000 | 0.980 | 1.000 | 0.948 | 0.939 | 1.000 | 0.854 | 1.000 | 1.000 | 0.855 | 0.826 |
| 637 | 0.767 | 1.000 | 0.810 | 1.000 | 0.984 | 1.000 | 0.894 | 1.000 | 0.980 | 1.000 | 0.951 | 0.909 | 1.000 | 0.851 | 1.000 | 1.000 | 0.854 | 0.826 |
| 638 | 0.779 | 1.000 | 0.802 | 1.000 | 0.984 | 0.960 | 0.889 | 1.000 | 0.969 | 1.000 | 0.962 | 0.970 | 1.000 | 0.860 | 1.000 | 0.981 | 0.859 | 0.831 |
| 639 | 0.781 | 1.000 | 0.810 | 1.000 | 0.968 | 1.000 | 0.888 | 1.000 | 0.980 | 1.000 | 0.959 | 0.939 | 1.000 | 0.854 | 1.000 | 1.000 | 0.858 | 0.830 |
| 640 | 0.773 | 1.000 | 0.810 | 1.000 | 0.979 | 1.000 | 0.881 | 1.000 | 0.980 | 1.000 | 0.951 | 0.970 | 1.000 | 0.860 | 1.000 | 0.981 | 0.856 | 0.827 |
| 641 | 0.781 | 1.000 | 0.816 | 1.000 | 0.974 | 1.000 | 0.884 | 1.000 | 0.980 | 1.000 | 0.945 | 0.970 | 1.000 | 0.859 | 1.000 | 1.000 | 0.859 | 0.831 |
| 642 | 0.778 | 1.000 | 0.808 | 1.000 | 0.979 | 0.960 | 0.891 | 1.000 | 0.980 | 1.000 | 0.948 | 0.970 | 1.000 | 0.857 | 1.000 | 1.000 | 0.858 | 0.829 |
| 643 | 0.775 | 1.000 | 0.806 | 1.000 | 0.979 | 0.960 | 0.897 | 1.000 | 0.980 | 1.000 | 0.948 | 0.909 | 1.000 | 0.856 | 1.000 | 1.000 | 0.857 | 0.829 |
| 644 | 0.783 | 1.000 | 0.806 | 1.000 | 0.974 | 0.960 | 0.886 | 1.000 | 0.969 | 1.000 | 0.954 | 0.939 | 1.000 | 0.853 | 1.000 | 1.000 | 0.857 | 0.829 |
| 645 | 0.771 | 1.000 | 0.804 | 1.000 | 0.974 | 0.960 | 0.897 | 1.000 | 0.980 | 1.000 | 0.951 | 0.939 | 1.000 | 0.860 | 1.000 | 0.981 | 0.857 | 0.828 |
| 646 | 0.781 | 1.000 | 0.808 | 1.000 | 0.984 | 0.960 | 0.888 | 1.000 | 0.980 | 1.000 | 0.945 | 0.970 | 1.000 | 0.862 | 1.000 | 1.000 | 0.860 | 0.832 |
| 647 | 0.774 | 1.000 | 0.806 | 1.000 | 0.984 | 1.000 | 0.889 | 1.000 | 0.980 | 1.000 | 0.951 | 0.909 | 1.000 | 0.860 | 1.000 | 1.000 | 0.857 | 0.829 |
| 648 | 0.781 | 1.000 | 0.792 | 0.986 | 0.979 | 1.000 | 0.888 | 1.000 | 0.980 | 1.000 | 0.951 | 0.939 | 1.000 | 0.857 | 1.000 | 1.000 | 0.857 | 0.828 |
| 649 | 0.770 | 1.000 | 0.818 | 1.000 | 0.963 | 1.000 | 0.884 | 1.000 | 0.980 | 1.000 | 0.951 | 0.939 | 1.000 | 0.854 | 1.000 | 1.000 | 0.855 | 0.826 |
| 650 | 0.777 | 1.000 | 0.804 | 1.000 | 0.984 | 0.960 | 0.897 | 1.000 | 0.990 | 1.000 | 0.954 | 0.970 | 1.000 | 0.857 | 1.000 | 1.000 | 0.859 | 0.831 |
| 651 | 0.779 | 1.000 | 0.802 | 1.000 | 0.979 | 1.000 | 0.895 | 1.000 | 0.980 | 1.000 | 0.954 | 1.000 | 1.000 | 0.853 | 1.000 | 1.000 | 0.858 | 0.830 |
| 652 | 0.767 | 1.000 | 0.808 | 1.000 | 0.984 | 1.000 | 0.900 | 1.000 | 0.980 | 1.000 | 0.948 | 0.939 | 1.000 | 0.856 | 1.000 | 1.000 | 0.856 | 0.828 |
| 653 | 0.778 | 1.000 | 0.796 | 1.000 | 0.979 | 1.000 | 0.892 | 1.000 | 0.969 | 1.000 | 0.945 | 0.939 | 1.000 | 0.855 | 1.000 | 1.000 | 0.856 | 0.827 |
| 654 | 0.779 | 1.000 | 0.800 | 1.000 | 0.984 | 0.960 | 0.898 | 1.000 | 0.980 | 1.000 | 0.957 | 0.970 | 1.000 | 0.856 | 1.000 | 1.000 | 0.859 | 0.831 |
| 655 | 0.783 | 1.000 | 0.787 | 1.000 | 0.979 | 0.960 | 0.891 | 1.000 | 0.980 | 1.000 | 0.948 | 0.970 | 1.000 | 0.858 | 1.000 | 1.000 | 0.857 | 0.829 |
| 656 | 0.775 | 1.000 | 0.792 | 1.000 | 0.979 | 1.000 | 0.886 | 1.000 | 0.980 | 1.000 | 0.948 | 0.970 | 1.000 | 0.847 | 1.000 | 1.000 | 0.853 | 0.823 |
| 657 | 0.779 | 1.000 | 0.804 | 1.000 | 0.979 | 0.960 | 0.888 | 1.000 | 0.969 | 1.000 | 0.939 | 0.970 | 1.000 | 0.859 | 1.000 | 0.981 | 0.857 | 0.828 |
| 658 | 0.781 | 1.000 | 0.820 | 1.000 | 0.974 | 0.960 | 0.886 | 1.000 | 0.980 | 1.000 | 0.957 | 0.939 | 1.000 | 0.852 | 1.000 | 0.981 | 0.858 | 0.830 |
| 659 | 0.780 | 1.000 | 0.806 | 1.000 | 0.974 | 1.000 | 0.892 | 1.000 | 0.980 | 1.000 | 0.951 | 0.970 | 1.000 | 0.852 | 1.000 | 1.000 | 0.858 | 0.829 |
| 660 | 0.777 | 1.000 | 0.804 | 1.000 | 0.979 | 1.000 | 0.894 | 1.000 | 0.980 | 1.000 | 0.948 | 0.970 | 1.000 | 0.856 | 1.000 | 0.981 | 0.858 | 0.829 |
| 661 | 0.775 | 1.000 | 0.789 | 1.000 | 0.984 | 0.960 | 0.894 | 1.000 | 0.980 | 1.000 | 0.951 | 0.970 | 1.000 | 0.854 | 1.000 | 1.000 | 0.855 | 0.826 |

| | | | | | | | | | | | | | | | | | | |
|---|---|---|---|---|---|---|---|---|---|---|---|---|---|---|---|---|---|---|
| 662 | 0.775 | 1.000 | 0.804 | 1.000 | 0.984 | 0.960 | 0.891 | 1.000 | 0.969 | 1.000 | 0.945 | 1.000 | 1.000 | 0.857 | 1.000 | 1.000 | 0.857 | 0.828 |
| 663 | 0.772 | 1.000 | 0.808 | 1.000 | 0.984 | 0.960 | 0.880 | 1.000 | 0.980 | 1.000 | 0.959 | 0.970 | 1.000 | 0.850 | 1.000 | 1.000 | 0.854 | 0.825 |
| 664 | 0.775 | 1.000 | 0.804 | 1.000 | 0.979 | 1.000 | 0.889 | 1.000 | 0.980 | 1.000 | 0.959 | 0.970 | 1.000 | 0.860 | 1.000 | 1.000 | 0.858 | 0.830 |
| 665 | 0.774 | 1.000 | 0.796 | 1.000 | 0.984 | 1.000 | 0.881 | 1.000 | 0.980 | 1.000 | 0.957 | 0.970 | 0.991 | 0.861 | 1.000 | 1.000 | 0.856 | 0.827 |
| 666 | 0.782 | 1.000 | 0.808 | 1.000 | 0.979 | 1.000 | 0.884 | 1.000 | 0.980 | 1.000 | 0.959 | 0.939 | 1.000 | 0.858 | 1.000 | 1.000 | 0.860 | 0.831 |
| 667 | 0.772 | 1.000 | 0.812 | 1.000 | 0.979 | 0.960 | 0.886 | 1.000 | 0.969 | 1.000 | 0.954 | 0.939 | 1.000 | 0.851 | 1.000 | 1.000 | 0.855 | 0.826 |
| 668 | 0.777 | 1.000 | 0.798 | 1.000 | 0.979 | 1.000 | 0.891 | 1.000 | 0.980 | 1.000 | 0.945 | 0.970 | 1.000 | 0.858 | 1.000 | 0.981 | 0.857 | 0.828 |
| 669 | 0.774 | 1.000 | 0.798 | 1.000 | 0.979 | 1.000 | 0.883 | 1.000 | 0.980 | 1.000 | 0.954 | 1.000 | 1.000 | 0.853 | 1.000 | 1.000 | 0.855 | 0.826 |
| 670 | 0.779 | 1.000 | 0.812 | 1.000 | 0.979 | 0.960 | 0.886 | 1.000 | 0.980 | 1.000 | 0.951 | 0.909 | 1.000 | 0.860 | 1.000 | 1.000 | 0.859 | 0.830 |
| 671 | 0.778 | 1.000 | 0.814 | 1.000 | 0.974 | 0.960 | 0.883 | 1.000 | 0.980 | 1.000 | 0.959 | 0.939 | 1.000 | 0.860 | 1.000 | 1.000 | 0.859 | 0.830 |
| 672 | 0.776 | 1.000 | 0.804 | 1.000 | 0.979 | 1.000 | 0.891 | 1.000 | 0.980 | 1.000 | 0.962 | 0.970 | 0.991 | 0.852 | 1.000 | 1.000 | 0.857 | 0.828 |
| 673 | 0.782 | 1.000 | 0.808 | 1.000 | 0.979 | 0.960 | 0.894 | 1.000 | 0.980 | 1.000 | 0.957 | 0.970 | 1.000 | 0.854 | 1.000 | 1.000 | 0.860 | 0.832 |
| 674 | 0.781 | 1.000 | 0.814 | 1.000 | 0.984 | 0.960 | 0.892 | 1.000 | 0.990 | 1.000 | 0.951 | 0.939 | 1.000 | 0.851 | 1.000 | 1.000 | 0.859 | 0.830 |
| 675 | 0.776 | 1.000 | 0.806 | 1.000 | 0.979 | 0.960 | 0.894 | 1.000 | 0.969 | 1.000 | 0.948 | 0.970 | 1.000 | 0.858 | 1.000 | 1.000 | 0.858 | 0.829 |
| 676 | 0.777 | 1.000 | 0.798 | 1.000 | 0.974 | 0.960 | 0.889 | 1.000 | 0.990 | 0.979 | 0.948 | 0.970 | 1.000 | 0.856 | 1.000 | 1.000 | 0.856 | 0.827 |
| 677 | 0.778 | 1.000 | 0.804 | 1.000 | 0.979 | 0.960 | 0.891 | 1.000 | 0.980 | 1.000 | 0.959 | 0.879 | 1.000 | 0.857 | 1.000 | 1.000 | 0.858 | 0.829 |
| 678 | 0.779 | 1.000 | 0.802 | 1.000 | 0.979 | 1.000 | 0.880 | 1.000 | 0.980 | 1.000 | 0.954 | 0.939 | 1.000 | 0.852 | 1.000 | 1.000 | 0.856 | 0.826 |
| 679 | 0.772 | 1.000 | 0.804 | 1.000 | 0.984 | 0.960 | 0.884 | 1.000 | 0.980 | 1.000 | 0.962 | 0.939 | 1.000 | 0.858 | 1.000 | 1.000 | 0.856 | 0.827 |
| 680 | 0.785 | 1.000 | 0.808 | 1.000 | 0.974 | 1.000 | 0.886 | 1.000 | 0.980 | 1.000 | 0.959 | 0.939 | 1.000 | 0.849 | 1.000 | 1.000 | 0.858 | 0.829 |
| 681 | 0.769 | 1.000 | 0.798 | 1.000 | 0.984 | 0.960 | 0.889 | 1.000 | 0.980 | 1.000 | 0.951 | 0.970 | 1.000 | 0.851 | 1.000 | 1.000 | 0.853 | 0.823 |
| 682 | 0.773 | 1.000 | 0.802 | 1.000 | 0.979 | 1.000 | 0.891 | 1.000 | 0.980 | 1.000 | 0.965 | 0.939 | 1.000 | 0.859 | 1.000 | 1.000 | 0.858 | 0.829 |
| 683 | 0.770 | 1.000 | 0.808 | 1.000 | 0.979 | 0.960 | 0.891 | 1.000 | 0.980 | 1.000 | 0.948 | 0.939 | 1.000 | 0.855 | 1.000 | 0.981 | 0.855 | 0.826 |
| 684 | 0.777 | 1.000 | 0.808 | 1.000 | 0.979 | 0.960 | 0.884 | 1.000 | 0.980 | 1.000 | 0.954 | 0.970 | 1.000 | 0.865 | 1.000 | 1.000 | 0.859 | 0.831 |
| 685 | 0.775 | 1.000 | 0.816 | 1.000 | 0.984 | 0.960 | 0.889 | 1.000 | 0.980 | 1.000 | 0.957 | 0.970 | 1.000 | 0.851 | 1.000 | 1.000 | 0.857 | 0.828 |
| 686 | 0.775 | 1.000 | 0.804 | 1.000 | 0.984 | 1.000 | 0.883 | 1.000 | 0.980 | 1.000 | 0.962 | 0.939 | 1.000 | 0.851 | 1.000 | 1.000 | 0.855 | 0.826 |
| 687 | 0.777 | 1.000 | 0.804 | 1.000 | 0.984 | 1.000 | 0.886 | 1.000 | 0.980 | 1.000 | 0.951 | 0.970 | 1.000 | 0.858 | 1.000 | 1.000 | 0.858 | 0.829 |
| 688 | 0.767 | 1.000 | 0.816 | 1.000 | 0.984 | 0.960 | 0.891 | 1.000 | 0.980 | 1.000 | 0.957 | 0.970 | 1.000 | 0.857 | 1.000 | 0.981 | 0.856 | 0.828 |
| 689 | 0.776 | 1.000 | 0.789 | 1.000 | 0.984 | 1.000 | 0.904 | 1.000 | 0.980 | 1.000 | 0.957 | 0.939 | 1.000 | 0.851 | 1.000 | 0.981 | 0.856 | 0.828 |
| 690 | 0.773 | 1.000 | 0.791 | 1.000 | 0.984 | 1.000 | 0.892 | 1.000 | 0.980 | 1.000 | 0.954 | 0.909 | 1.000 | 0.861 | 1.000 | 1.000 | 0.856 | 0.827 |
| 691 | 0.773 | 1.000 | 0.820 | 1.000 | 0.984 | 1.000 | 0.894 | 1.000 | 0.980 | 1.000 | 0.957 | 0.970 | 1.000 | 0.854 | 1.000 | 1.000 | 0.858 | 0.830 |
| 692 | 0.773 | 1.000 | 0.794 | 1.000 | 0.974 | 0.960 | 0.900 | 1.000 | 0.980 | 1.000 | 0.959 | 0.939 | 0.991 | 0.861 | 1.000 | 1.000 | 0.857 | 0.829 |
| 693 | 0.779 | 1.000 | 0.791 | 1.000 | 0.984 | 0.960 | 0.886 | 1.000 | 0.980 | 1.000 | 0.954 | 1.000 | 1.000 | 0.855 | 1.000 | 1.000 | 0.856 | 0.827 |
| 694 | 0.780 | 1.000 | 0.802 | 1.000 | 0.984 | 1.000 | 0.889 | 1.000 | 0.980 | 1.000 | 0.948 | 0.970 | 1.000 | 0.854 | 1.000 | 1.000 | 0.857 | 0.829 |
| 695 | 0.773 | 1.000 | 0.798 | 1.000 | 0.984 | 0.960 | 0.889 | 1.000 | 0.980 | 1.000 | 0.954 | 0.909 | 1.000 | 0.857 | 1.000 | 1.000 | 0.856 | 0.826 |
| 696 | 0.778 | 1.000 | 0.820 | 1.000 | 0.984 | 1.000 | 0.889 | 1.000 | 0.980 | 1.000 | 0.962 | 0.970 | 1.000 | 0.857 | 1.000 | 0.981 | 0.860 | 0.832 |
| 697 | 0.782 | 1.000 | 0.798 | 1.000 | 0.979 | 1.000 | 0.891 | 1.000 | 0.980 | 1.000 | 0.951 | 0.939 | 1.000 | 0.856 | 1.000 | 1.000 | 0.858 | 0.830 |
| 698 | 0.784 | 1.000 | 0.812 | 1.000 | 0.979 | 0.960 | 0.892 | 1.000 | 0.980 | 1.000 | 0.954 | 0.939 | 1.000 | 0.851 | 1.000 | 1.000 | 0.859 | 0.831 |
| 699 | 0.774 | 1.000 | 0.791 | 1.000 | 0.984 | 0.960 | 0.884 | 1.000 | 0.980 | 1.000 | 0.948 | 0.939 | 1.000 | 0.854 | 1.000 | 1.000 | 0.854 | 0.824 |
| 700 | 0.771 | 1.000 | 0.806 | 1.000 | 0.979 | 1.000 | 0.889 | 1.000 | 0.980 | 1.000 | 0.948 | 0.939 | 1.000 | 0.854 | 1.000 | 1.000 | 0.855 | 0.826 |

| | | | | | | | | | | | | | | | | | |
|---|---|---|---|---|---|---|---|---|---|---|---|---|---|---|---|---|---|
| 701 | 0.777 | 1.000 | 0.806 | 1.000 | 0.984 | 0.960 | 0.889 | 1.000 | 0.980 | 1.000 | 0.957 | 0.970 | 1.000 | 0.861 | 1.000 | 1.000 | 0.859 | 0.831 |
| 702 | 0.773 | 1.000 | 0.791 | 1.000 | 0.979 | 0.960 | 0.888 | 1.000 | 0.980 | 1.000 | 0.954 | 0.909 | 0.991 | 0.857 | 1.000 | 1.000 | 0.854 | 0.825 |
| 703 | 0.777 | 1.000 | 0.806 | 1.000 | 0.984 | 0.960 | 0.891 | 1.000 | 0.980 | 1.000 | 0.948 | 0.970 | 1.000 | 0.854 | 1.000 | 1.000 | 0.857 | 0.828 |
| 704 | 0.773 | 1.000 | 0.792 | 1.000 | 0.979 | 0.960 | 0.892 | 1.000 | 0.980 | 1.000 | 0.959 | 0.970 | 1.000 | 0.860 | 1.000 | 0.981 | 0.856 | 0.828 |
| 705 | 0.777 | 1.000 | 0.798 | 1.000 | 0.974 | 0.960 | 0.886 | 1.000 | 0.980 | 1.000 | 0.959 | 0.939 | 1.000 | 0.854 | 1.000 | 1.000 | 0.856 | 0.827 |
| 706 | 0.777 | 1.000 | 0.812 | 1.000 | 0.974 | 1.000 | 0.891 | 1.000 | 0.980 | 1.000 | 0.957 | 0.939 | 1.000 | 0.852 | 1.000 | 1.000 | 0.857 | 0.829 |
| 707 | 0.769 | 1.000 | 0.806 | 1.000 | 0.979 | 1.000 | 0.904 | 1.000 | 0.980 | 1.000 | 0.959 | 0.939 | 1.000 | 0.856 | 1.000 | 1.000 | 0.858 | 0.829 |
| 708 | 0.785 | 1.000 | 0.806 | 1.000 | 0.984 | 0.960 | 0.889 | 1.000 | 0.980 | 1.000 | 0.951 | 0.970 | 1.000 | 0.851 | 1.000 | 0.981 | 0.858 | 0.830 |
| 709 | 0.780 | 1.000 | 0.802 | 1.000 | 0.984 | 1.000 | 0.898 | 1.000 | 0.980 | 1.000 | 0.951 | 0.939 | 1.000 | 0.852 | 1.000 | 1.000 | 0.858 | 0.830 |
| 710 | 0.775 | 1.000 | 0.800 | 0.986 | 0.984 | 0.960 | 0.892 | 1.000 | 0.980 | 1.000 | 0.962 | 0.909 | 1.000 | 0.855 | 1.000 | 0.981 | 0.856 | 0.828 |
| 711 | 0.774 | 1.000 | 0.792 | 1.000 | 0.984 | 0.960 | 0.888 | 1.000 | 0.980 | 1.000 | 0.954 | 1.000 | 1.000 | 0.856 | 1.000 | 1.000 | 0.855 | 0.826 |
| 712 | 0.781 | 1.000 | 0.810 | 1.000 | 0.984 | 0.960 | 0.892 | 1.000 | 0.980 | 1.000 | 0.962 | 0.939 | 1.000 | 0.854 | 1.000 | 1.000 | 0.859 | 0.831 |
| 713 | 0.778 | 1.000 | 0.794 | 1.000 | 0.979 | 1.000 | 0.901 | 1.000 | 0.980 | 1.000 | 0.962 | 0.909 | 1.000 | 0.858 | 1.000 | 1.000 | 0.859 | 0.831 |
| 714 | 0.769 | 1.000 | 0.802 | 1.000 | 0.984 | 0.960 | 0.889 | 1.000 | 0.980 | 1.000 | 0.954 | 0.939 | 1.000 | 0.853 | 1.000 | 1.000 | 0.854 | 0.825 |
| 715 | 0.783 | 1.000 | 0.792 | 1.000 | 0.979 | 0.960 | 0.889 | 1.000 | 0.980 | 1.000 | 0.957 | 0.939 | 1.000 | 0.859 | 1.000 | 1.000 | 0.859 | 0.830 |
| 716 | 0.775 | 1.000 | 0.800 | 1.000 | 0.979 | 0.960 | 0.881 | 1.000 | 0.980 | 1.000 | 0.957 | 0.939 | 1.000 | 0.854 | 1.000 | 1.000 | 0.855 | 0.826 |
| 717 | 0.770 | 1.000 | 0.806 | 1.000 | 0.979 | 0.960 | 0.884 | 1.000 | 0.969 | 1.000 | 0.957 | 0.970 | 1.000 | 0.852 | 1.000 | 1.000 | 0.854 | 0.825 |
| 718 | 0.784 | 1.000 | 0.810 | 1.000 | 0.974 | 1.000 | 0.883 | 1.000 | 0.980 | 1.000 | 0.957 | 0.970 | 1.000 | 0.851 | 1.000 | 1.000 | 0.858 | 0.830 |
| 719 | 0.772 | 1.000 | 0.806 | 1.000 | 0.974 | 1.000 | 0.883 | 1.000 | 0.980 | 1.000 | 0.959 | 0.970 | 1.000 | 0.854 | 1.000 | 1.000 | 0.855 | 0.826 |
| 720 | 0.781 | 1.000 | 0.800 | 1.000 | 0.979 | 1.000 | 0.886 | 1.000 | 0.980 | 1.000 | 0.957 | 0.879 | 1.000 | 0.855 | 1.000 | 1.000 | 0.857 | 0.828 |
| 721 | 0.778 | 1.000 | 0.794 | 1.000 | 0.984 | 1.000 | 0.886 | 1.000 | 0.980 | 1.000 | 0.951 | 0.939 | 1.000 | 0.859 | 1.000 | 0.981 | 0.857 | 0.828 |
| 722 | 0.769 | 1.000 | 0.816 | 1.000 | 0.974 | 0.960 | 0.903 | 1.000 | 0.980 | 1.000 | 0.954 | 0.970 | 1.000 | 0.853 | 1.000 | 1.000 | 0.857 | 0.828 |
| 723 | 0.779 | 1.000 | 0.812 | 1.000 | 0.979 | 1.000 | 0.898 | 1.000 | 0.980 | 1.000 | 0.954 | 0.939 | 1.000 | 0.854 | 1.000 | 1.000 | 0.859 | 0.831 |
| 724 | 0.776 | 1.000 | 0.814 | 1.000 | 0.984 | 0.960 | 0.889 | 1.000 | 0.990 | 1.000 | 0.954 | 0.909 | 1.000 | 0.862 | 1.000 | 1.000 | 0.860 | 0.831 |
| 725 | 0.778 | 1.000 | 0.802 | 1.000 | 0.979 | 0.960 | 0.892 | 1.000 | 0.969 | 1.000 | 0.951 | 0.970 | 1.000 | 0.854 | 1.000 | 1.000 | 0.857 | 0.828 |
| 726 | 0.781 | 1.000 | 0.810 | 1.000 | 0.974 | 0.960 | 0.886 | 1.000 | 0.980 | 1.000 | 0.962 | 0.939 | 1.000 | 0.851 | 1.000 | 1.000 | 0.858 | 0.829 |
| 727 | 0.787 | 1.000 | 0.810 | 1.000 | 0.979 | 0.960 | 0.883 | 1.000 | 0.980 | 1.000 | 0.951 | 0.970 | 1.000 | 0.858 | 1.000 | 0.981 | 0.860 | 0.832 |
| 728 | 0.779 | 1.000 | 0.804 | 1.000 | 0.979 | 0.960 | 0.894 | 1.000 | 0.969 | 1.000 | 0.957 | 0.970 | 1.000 | 0.862 | 1.000 | 0.981 | 0.860 | 0.832 |
| 729 | 0.779 | 1.000 | 0.812 | 1.000 | 0.974 | 0.960 | 0.895 | 1.000 | 0.980 | 1.000 | 0.959 | 0.939 | 1.000 | 0.847 | 1.000 | 0.981 | 0.857 | 0.829 |
| 730 | 0.771 | 1.000 | 0.818 | 1.000 | 0.968 | 0.960 | 0.901 | 1.000 | 0.980 | 1.000 | 0.957 | 0.939 | 1.000 | 0.859 | 1.000 | 0.981 | 0.859 | 0.831 |
| 731 | 0.779 | 1.000 | 0.818 | 1.000 | 0.968 | 0.960 | 0.900 | 1.000 | 0.980 | 1.000 | 0.957 | 0.939 | 1.000 | 0.848 | 1.000 | 0.981 | 0.858 | 0.830 |
| 732 | 0.772 | 1.000 | 0.828 | 1.000 | 0.968 | 0.960 | 0.888 | 1.000 | 0.980 | 1.000 | 0.951 | 0.939 | 1.000 | 0.862 | 1.000 | 1.000 | 0.859 | 0.831 |
| 733 | 0.780 | 1.000 | 0.800 | 1.000 | 0.974 | 0.960 | 0.892 | 1.000 | 0.990 | 1.000 | 0.954 | 0.939 | 1.000 | 0.857 | 1.000 | 1.000 | 0.858 | 0.830 |
| 734 | 0.774 | 1.000 | 0.796 | 1.000 | 0.968 | 0.960 | 0.891 | 1.000 | 0.980 | 1.000 | 0.957 | 0.939 | 1.000 | 0.851 | 1.000 | 0.981 | 0.854 | 0.825 |
| 735 | 0.779 | 1.000 | 0.804 | 1.000 | 0.979 | 0.960 | 0.888 | 1.000 | 0.980 | 1.000 | 0.959 | 0.939 | 1.000 | 0.856 | 1.000 | 0.981 | 0.858 | 0.829 |
| 736 | 0.774 | 1.000 | 0.820 | 1.000 | 0.974 | 0.960 | 0.888 | 1.000 | 0.990 | 1.000 | 0.954 | 0.970 | 1.000 | 0.856 | 1.000 | 0.981 | 0.858 | 0.829 |
| 737 | 0.778 | 1.000 | 0.804 | 1.000 | 0.968 | 0.960 | 0.895 | 1.000 | 0.990 | 1.000 | 0.951 | 1.000 | 1.000 | 0.859 | 1.000 | 0.981 | 0.859 | 0.831 |
| 738 | 0.779 | 1.000 | 0.800 | 1.000 | 0.974 | 0.960 | 0.886 | 1.000 | 0.980 | 1.000 | 0.945 | 0.970 | 1.000 | 0.859 | 1.000 | 1.000 | 0.857 | 0.828 |
| 739 | 0.774 | 1.000 | 0.804 | 1.000 | 0.974 | 0.960 | 0.892 | 1.000 | 0.969 | 1.000 | 0.954 | 0.970 | 1.000 | 0.853 | 1.000 | 0.981 | 0.855 | 0.827 |

| | | | | | | | | | | | | | | | | | |
|---|---|---|---|---|---|---|---|---|---|---|---|---|---|---|---|---|---|
| 740 | 0.775 | 1.000 | 0.804 | 1.000 | 0.974 | 0.960 | 0.888 | 1.000 | 0.980 | 1.000 | 0.954 | 0.939 | 1.000 | 0.858 | 1.000 | 0.981 | 0.856 | 0.827 |
| 741 | 0.768 | 1.000 | 0.798 | 1.000 | 0.968 | 0.960 | 0.892 | 1.000 | 0.969 | 1.000 | 0.954 | 0.909 | 1.000 | 0.860 | 1.000 | 0.981 | 0.854 | 0.825 |
| 742 | 0.783 | 1.000 | 0.808 | 1.000 | 0.974 | 0.960 | 0.888 | 1.000 | 0.980 | 1.000 | 0.951 | 0.909 | 1.000 | 0.854 | 1.000 | 0.981 | 0.858 | 0.829 |
| 743 | 0.775 | 1.000 | 0.816 | 1.000 | 0.968 | 0.960 | 0.883 | 1.000 | 0.980 | 1.000 | 0.951 | 0.970 | 1.000 | 0.856 | 1.000 | 0.981 | 0.856 | 0.828 |
| 744 | 0.781 | 1.000 | 0.812 | 1.000 | 0.974 | 0.960 | 0.894 | 1.000 | 0.980 | 1.000 | 0.957 | 0.970 | 1.000 | 0.856 | 1.000 | 1.000 | 0.860 | 0.832 |
| 745 | 0.780 | 1.000 | 0.814 | 1.000 | 0.963 | 0.960 | 0.883 | 1.000 | 0.990 | 1.000 | 0.945 | 0.970 | 1.000 | 0.858 | 1.000 | 0.981 | 0.858 | 0.829 |
| 746 | 0.781 | 1.000 | 0.810 | 1.000 | 0.968 | 0.960 | 0.892 | 1.000 | 0.980 | 1.000 | 0.954 | 0.939 | 1.000 | 0.854 | 1.000 | 0.981 | 0.858 | 0.829 |
| 747 | 0.773 | 1.000 | 0.820 | 1.000 | 0.968 | 0.960 | 0.888 | 1.000 | 0.980 | 1.000 | 0.957 | 0.879 | 1.000 | 0.860 | 1.000 | 1.000 | 0.858 | 0.829 |
| 748 | 0.773 | 1.000 | 0.810 | 1.000 | 0.979 | 0.960 | 0.895 | 1.000 | 0.980 | 1.000 | 0.951 | 0.970 | 1.000 | 0.855 | 1.000 | 0.981 | 0.857 | 0.829 |
| 749 | 0.778 | 1.000 | 0.816 | 1.000 | 0.963 | 0.960 | 0.883 | 1.000 | 0.980 | 1.000 | 0.948 | 0.939 | 1.000 | 0.847 | 1.000 | 0.981 | 0.854 | 0.825 |
| 750 | 0.774 | 1.000 | 0.812 | 1.000 | 0.968 | 0.960 | 0.891 | 1.000 | 0.980 | 1.000 | 0.959 | 0.939 | 1.000 | 0.856 | 1.000 | 1.000 | 0.857 | 0.829 |
| 751 | 0.781 | 1.000 | 0.816 | 1.000 | 0.974 | 0.960 | 0.895 | 1.000 | 0.980 | 1.000 | 0.948 | 0.939 | 1.000 | 0.857 | 1.000 | 0.981 | 0.860 | 0.832 |
| 752 | 0.773 | 1.000 | 0.814 | 1.000 | 0.979 | 0.960 | 0.883 | 1.000 | 0.980 | 1.000 | 0.957 | 0.909 | 1.000 | 0.852 | 1.000 | 0.981 | 0.855 | 0.826 |
| 753 | 0.775 | 1.000 | 0.826 | 1.000 | 0.968 | 0.960 | 0.895 | 1.000 | 0.980 | 1.000 | 0.945 | 0.909 | 1.000 | 0.859 | 1.000 | 0.981 | 0.859 | 0.831 |
| 754 | 0.765 | 1.000 | 0.810 | 1.000 | 0.974 | 0.960 | 0.884 | 1.000 | 0.980 | 1.000 | 0.951 | 0.970 | 1.000 | 0.856 | 1.000 | 1.000 | 0.853 | 0.824 |
| 755 | 0.777 | 1.000 | 0.810 | 1.000 | 0.963 | 1.000 | 0.891 | 1.000 | 0.980 | 1.000 | 0.948 | 0.970 | 1.000 | 0.859 | 1.000 | 1.000 | 0.858 | 0.830 |
| 756 | 0.780 | 1.000 | 0.791 | 1.000 | 0.968 | 0.960 | 0.898 | 1.000 | 0.980 | 1.000 | 0.957 | 0.939 | 1.000 | 0.859 | 1.000 | 0.981 | 0.858 | 0.830 |
| 757 | 0.780 | 1.000 | 0.814 | 1.000 | 0.968 | 0.960 | 0.889 | 1.000 | 0.980 | 1.000 | 0.957 | 0.939 | 1.000 | 0.851 | 1.000 | 0.981 | 0.857 | 0.829 |
| 758 | 0.776 | 1.000 | 0.822 | 1.000 | 0.974 | 0.960 | 0.886 | 1.000 | 0.969 | 1.000 | 0.959 | 0.939 | 1.000 | 0.861 | 1.000 | 0.981 | 0.859 | 0.831 |
| 759 | 0.773 | 1.000 | 0.804 | 1.000 | 0.963 | 0.960 | 0.883 | 1.000 | 0.980 | 1.000 | 0.959 | 0.970 | 1.000 | 0.859 | 1.000 | 0.981 | 0.856 | 0.826 |
| 760 | 0.769 | 1.000 | 0.820 | 1.000 | 0.968 | 0.960 | 0.892 | 1.000 | 0.990 | 1.000 | 0.954 | 0.939 | 1.000 | 0.854 | 1.000 | 1.000 | 0.856 | 0.827 |
| 761 | 0.765 | 1.000 | 0.806 | 1.000 | 0.958 | 0.960 | 0.891 | 1.000 | 0.980 | 1.000 | 0.954 | 1.000 | 1.000 | 0.847 | 1.000 | 1.000 | 0.851 | 0.822 |
| 762 | 0.771 | 1.000 | 0.808 | 1.000 | 0.974 | 0.960 | 0.884 | 1.000 | 0.980 | 1.000 | 0.951 | 0.970 | 1.000 | 0.852 | 1.000 | 1.000 | 0.854 | 0.825 |
| 763 | 0.775 | 1.000 | 0.802 | 1.000 | 0.974 | 0.960 | 0.883 | 1.000 | 0.980 | 1.000 | 0.957 | 0.970 | 1.000 | 0.864 | 1.000 | 0.963 | 0.857 | 0.828 |
| 764 | 0.779 | 1.000 | 0.804 | 1.000 | 0.974 | 0.960 | 0.884 | 1.000 | 0.969 | 1.000 | 0.957 | 0.939 | 1.000 | 0.858 | 1.000 | 0.981 | 0.857 | 0.828 |
| 765 | 0.774 | 1.000 | 0.814 | 1.000 | 0.963 | 0.960 | 0.886 | 1.000 | 0.980 | 1.000 | 0.948 | 0.939 | 1.000 | 0.853 | 1.000 | 0.981 | 0.855 | 0.826 |
| 766 | 0.773 | 1.000 | 0.810 | 1.000 | 0.968 | 0.960 | 0.895 | 1.000 | 0.980 | 1.000 | 0.954 | 0.939 | 1.000 | 0.855 | 1.000 | 0.981 | 0.856 | 0.828 |
| 767 | 0.779 | 1.000 | 0.804 | 1.000 | 0.968 | 0.960 | 0.892 | 1.000 | 0.980 | 1.000 | 0.948 | 1.000 | 1.000 | 0.857 | 1.000 | 0.981 | 0.858 | 0.829 |
| 768 | 0.778 | 1.000 | 0.804 | 1.000 | 0.968 | 0.960 | 0.886 | 1.000 | 0.990 | 1.000 | 0.954 | 0.939 | 1.000 | 0.853 | 1.000 | 0.981 | 0.856 | 0.827 |
| 769 | 0.780 | 1.000 | 0.800 | 1.000 | 0.968 | 0.960 | 0.889 | 1.000 | 0.980 | 1.000 | 0.954 | 0.939 | 1.000 | 0.854 | 1.000 | 0.981 | 0.856 | 0.827 |
| 770 | 0.784 | 1.000 | 0.818 | 1.000 | 0.974 | 0.960 | 0.889 | 1.000 | 0.980 | 1.000 | 0.957 | 0.939 | 1.000 | 0.854 | 1.000 | 1.000 | 0.860 | 0.832 |
| 771 | 0.774 | 1.000 | 0.806 | 1.000 | 0.968 | 0.960 | 0.880 | 1.000 | 0.969 | 1.000 | 0.951 | 0.970 | 1.000 | 0.859 | 1.000 | 0.981 | 0.855 | 0.826 |
| 772 | 0.778 | 1.000 | 0.808 | 1.000 | 0.963 | 1.000 | 0.886 | 1.000 | 0.990 | 1.000 | 0.951 | 0.970 | 1.000 | 0.856 | 1.000 | 1.000 | 0.857 | 0.828 |
| 773 | 0.775 | 1.000 | 0.828 | 1.000 | 0.968 | 0.960 | 0.895 | 1.000 | 0.990 | 1.000 | 0.954 | 0.970 | 1.000 | 0.857 | 1.000 | 1.000 | 0.860 | 0.832 |
| 774 | 0.772 | 1.000 | 0.804 | 1.000 | 0.974 | 0.960 | 0.892 | 1.000 | 0.980 | 1.000 | 0.942 | 0.939 | 1.000 | 0.859 | 1.000 | 1.000 | 0.856 | 0.827 |
| 775 | 0.772 | 1.000 | 0.822 | 1.000 | 0.974 | 0.960 | 0.883 | 1.000 | 0.980 | 1.000 | 0.948 | 0.970 | 1.000 | 0.856 | 1.000 | 1.000 | 0.856 | 0.828 |
| 776 | 0.777 | 1.000 | 0.814 | 1.000 | 0.963 | 0.960 | 0.897 | 1.000 | 0.980 | 1.000 | 0.951 | 1.000 | 1.000 | 0.851 | 1.000 | 0.981 | 0.857 | 0.829 |
| 777 | 0.777 | 1.000 | 0.808 | 1.000 | 0.974 | 0.960 | 0.898 | 1.000 | 0.980 | 1.000 | 0.957 | 0.939 | 1.000 | 0.858 | 1.000 | 0.981 | 0.859 | 0.831 |
| 778 | 0.769 | 1.000 | 0.804 | 1.000 | 0.958 | 0.960 | 0.889 | 1.000 | 0.980 | 1.000 | 0.957 | 0.909 | 1.000 | 0.857 | 1.000 | 0.981 | 0.854 | 0.825 |

| | | | | | | | | | | | | | | | | | | |
|---|---|---|---|---|---|---|---|---|---|---|---|---|---|---|---|---|---|---|
| 779 | 0.779 | 1.000 | 0.802 | 1.000 | 0.968 | 0.960 | 0.892 | 1.000 | 0.969 | 1.000 | 0.954 | 0.970 | 1.000 | 0.861 | 1.000 | 0.963 | 0.858 | 0.830 |
| 780 | 0.768 | 1.000 | 0.812 | 1.000 | 0.968 | 0.960 | 0.897 | 1.000 | 0.990 | 1.000 | 0.951 | 0.879 | 1.000 | 0.858 | 1.000 | 0.981 | 0.856 | 0.827 |
| 781 | 0.775 | 1.000 | 0.820 | 1.000 | 0.984 | 0.960 | 0.900 | 1.000 | 0.980 | 1.000 | 0.948 | 0.909 | 1.000 | 0.863 | 1.000 | 0.981 | 0.861 | 0.833 |
| 782 | 0.778 | 1.000 | 0.814 | 1.000 | 0.974 | 0.960 | 0.881 | 1.000 | 0.980 | 1.000 | 0.951 | 0.970 | 1.000 | 0.854 | 1.000 | 0.981 | 0.856 | 0.828 |
| 783 | 0.776 | 1.000 | 0.812 | 1.000 | 0.968 | 0.960 | 0.894 | 1.000 | 0.980 | 1.000 | 0.948 | 0.970 | 1.000 | 0.854 | 1.000 | 0.981 | 0.857 | 0.828 |
| 784 | 0.773 | 1.000 | 0.802 | 1.000 | 0.968 | 0.960 | 0.891 | 1.000 | 0.990 | 1.000 | 0.954 | 0.970 | 1.000 | 0.858 | 1.000 | 0.981 | 0.856 | 0.827 |
| 785 | 0.772 | 1.000 | 0.820 | 1.000 | 0.968 | 0.960 | 0.901 | 1.000 | 0.980 | 1.000 | 0.951 | 0.970 | 1.000 | 0.858 | 1.000 | 0.981 | 0.859 | 0.831 |
| 786 | 0.776 | 1.000 | 0.820 | 1.000 | 0.968 | 0.960 | 0.895 | 1.000 | 0.980 | 1.000 | 0.948 | 0.939 | 1.000 | 0.858 | 1.000 | 0.981 | 0.859 | 0.830 |
| 787 | 0.774 | 1.000 | 0.814 | 1.000 | 0.968 | 0.960 | 0.900 | 1.000 | 0.980 | 0.979 | 0.954 | 0.939 | 1.000 | 0.863 | 1.000 | 0.981 | 0.860 | 0.832 |
| 788 | 0.785 | 1.000 | 0.820 | 1.000 | 0.974 | 0.960 | 0.894 | 1.000 | 0.990 | 1.000 | 0.951 | 0.939 | 1.000 | 0.858 | 1.000 | 0.981 | 0.862 | 0.834 |
| 789 | 0.778 | 1.000 | 0.814 | 1.000 | 0.953 | 0.960 | 0.897 | 1.000 | 0.980 | 1.000 | 0.951 | 0.970 | 1.000 | 0.856 | 1.000 | 1.000 | 0.858 | 0.830 |
| 790 | 0.781 | 1.000 | 0.802 | 1.000 | 0.968 | 0.960 | 0.898 | 1.000 | 0.990 | 1.000 | 0.948 | 0.970 | 1.000 | 0.860 | 1.000 | 0.981 | 0.860 | 0.832 |
| 791 | 0.785 | 1.000 | 0.808 | 1.000 | 0.968 | 0.960 | 0.895 | 1.000 | 0.980 | 1.000 | 0.957 | 0.939 | 1.000 | 0.854 | 1.000 | 0.981 | 0.860 | 0.832 |
| 792 | 0.772 | 1.000 | 0.810 | 1.000 | 0.968 | 0.960 | 0.895 | 1.000 | 0.980 | 1.000 | 0.962 | 0.939 | 1.000 | 0.855 | 1.000 | 0.981 | 0.857 | 0.828 |
| 793 | 0.776 | 1.000 | 0.810 | 1.000 | 0.979 | 0.960 | 0.891 | 1.000 | 0.990 | 1.000 | 0.957 | 0.970 | 1.000 | 0.863 | 1.000 | 0.981 | 0.860 | 0.832 |
| 794 | 0.770 | 1.000 | 0.816 | 1.000 | 0.968 | 0.960 | 0.898 | 1.000 | 0.990 | 1.000 | 0.948 | 0.909 | 1.000 | 0.862 | 1.000 | 1.000 | 0.858 | 0.830 |
| 795 | 0.773 | 1.000 | 0.824 | 1.000 | 0.968 | 0.960 | 0.895 | 1.000 | 0.980 | 1.000 | 0.957 | 0.970 | 0.991 | 0.846 | 1.000 | 0.981 | 0.856 | 0.827 |
| 796 | 0.781 | 1.000 | 0.810 | 1.000 | 0.974 | 0.960 | 0.898 | 1.000 | 0.980 | 1.000 | 0.959 | 0.939 | 1.000 | 0.849 | 1.000 | 1.000 | 0.858 | 0.830 |
| 797 | 0.769 | 1.000 | 0.816 | 1.000 | 0.963 | 0.960 | 0.898 | 1.000 | 0.980 | 1.000 | 0.948 | 0.970 | 1.000 | 0.859 | 1.000 | 1.000 | 0.857 | 0.829 |
| 798 | 0.779 | 1.000 | 0.812 | 1.000 | 0.974 | 0.960 | 0.878 | 1.000 | 0.980 | 1.000 | 0.957 | 0.970 | 1.000 | 0.853 | 1.000 | 0.981 | 0.856 | 0.827 |
| 799 | 0.776 | 1.000 | 0.824 | 1.000 | 0.963 | 0.960 | 0.891 | 1.000 | 0.980 | 1.000 | 0.945 | 0.939 | 1.000 | 0.855 | 1.000 | 0.981 | 0.857 | 0.829 |
| 800 | 0.773 | 1.000 | 0.808 | 1.000 | 0.968 | 0.960 | 0.881 | 1.000 | 0.980 | 1.000 | 0.951 | 0.939 | 1.000 | 0.854 | 1.000 | 0.981 | 0.854 | 0.825 |
| 801 | 0.768 | 1.000 | 0.828 | 1.000 | 0.974 | 0.960 | 0.895 | 1.000 | 0.980 | 1.000 | 0.945 | 0.970 | 1.000 | 0.850 | 1.000 | 0.981 | 0.855 | 0.827 |
| 802 | 0.771 | 1.000 | 0.830 | 1.000 | 0.974 | 0.960 | 0.889 | 1.000 | 0.980 | 1.000 | 0.939 | 0.970 | 1.000 | 0.846 | 1.000 | 1.000 | 0.854 | 0.826 |
| 803 | 0.776 | 1.000 | 0.804 | 1.000 | 0.963 | 0.960 | 0.894 | 1.000 | 0.990 | 1.000 | 0.945 | 0.970 | 1.000 | 0.854 | 1.000 | 1.000 | 0.856 | 0.827 |
| 804 | 0.780 | 1.000 | 0.816 | 1.000 | 0.968 | 0.960 | 0.898 | 1.000 | 0.980 | 1.000 | 0.957 | 0.970 | 1.000 | 0.857 | 1.000 | 0.981 | 0.860 | 0.833 |
| 805 | 0.779 | 1.000 | 0.816 | 1.000 | 0.963 | 0.960 | 0.894 | 1.000 | 0.980 | 1.000 | 0.948 | 0.970 | 1.000 | 0.855 | 1.000 | 1.000 | 0.858 | 0.830 |
| 806 | 0.774 | 1.000 | 0.810 | 1.000 | 0.968 | 0.960 | 0.897 | 1.000 | 0.980 | 1.000 | 0.951 | 0.970 | 1.000 | 0.861 | 1.000 | 1.000 | 0.859 | 0.831 |
| 807 | 0.775 | 1.000 | 0.806 | 1.000 | 0.958 | 0.960 | 0.892 | 1.000 | 0.969 | 1.000 | 0.948 | 0.970 | 1.000 | 0.854 | 1.000 | 0.981 | 0.855 | 0.826 |
| 808 | 0.775 | 1.000 | 0.798 | 1.000 | 0.979 | 0.960 | 0.894 | 1.000 | 0.980 | 1.000 | 0.954 | 0.939 | 1.000 | 0.857 | 1.000 | 1.000 | 0.857 | 0.828 |
| 809 | 0.777 | 1.000 | 0.826 | 1.000 | 0.968 | 0.960 | 0.889 | 1.000 | 0.980 | 1.000 | 0.954 | 0.970 | 1.000 | 0.859 | 1.000 | 1.000 | 0.860 | 0.832 |
| 810 | 0.782 | 1.000 | 0.802 | 1.000 | 0.974 | 0.960 | 0.891 | 1.000 | 0.990 | 1.000 | 0.939 | 0.939 | 1.000 | 0.854 | 1.000 | 0.981 | 0.857 | 0.828 |
| 811 | 0.769 | 1.000 | 0.806 | 1.000 | 0.968 | 0.960 | 0.895 | 1.000 | 0.990 | 1.000 | 0.945 | 0.970 | 1.000 | 0.860 | 1.000 | 0.963 | 0.856 | 0.827 |
| 812 | 0.775 | 1.000 | 0.816 | 1.000 | 0.974 | 0.960 | 0.901 | 1.000 | 0.980 | 1.000 | 0.942 | 0.939 | 1.000 | 0.851 | 1.000 | 0.981 | 0.857 | 0.829 |
| 813 | 0.777 | 1.000 | 0.816 | 1.000 | 0.958 | 0.960 | 0.891 | 1.000 | 0.980 | 1.000 | 0.957 | 0.970 | 1.000 | 0.856 | 1.000 | 1.000 | 0.858 | 0.830 |
| 814 | 0.773 | 1.000 | 0.808 | 1.000 | 0.974 | 0.960 | 0.889 | 1.000 | 0.980 | 1.000 | 0.945 | 0.939 | 1.000 | 0.854 | 1.000 | 0.981 | 0.855 | 0.826 |
| 815 | 0.775 | 1.000 | 0.804 | 1.000 | 0.974 | 0.960 | 0.884 | 1.000 | 0.990 | 1.000 | 0.951 | 0.970 | 1.000 | 0.851 | 1.000 | 0.981 | 0.855 | 0.825 |
| 816 | 0.771 | 1.000 | 0.796 | 1.000 | 0.968 | 0.960 | 0.897 | 1.000 | 0.980 | 1.000 | 0.942 | 0.970 | 1.000 | 0.858 | 1.000 | 1.000 | 0.855 | 0.826 |
| 817 | 0.781 | 1.000 | 0.814 | 1.000 | 0.974 | 0.960 | 0.894 | 1.000 | 0.990 | 1.000 | 0.954 | 0.970 | 1.000 | 0.858 | 1.000 | 0.981 | 0.860 | 0.833 |

| | | | | | | | | | | | | | | | | | |
|---|---|---|---|---|---|---|---|---|---|---|---|---|---|---|---|---|---|
| 818 | 0.783 | 1.000 | 0.816 | 1.000 | 0.974 | 0.960 | 0.900 | 1.000 | 0.980 | 1.000 | 0.942 | 0.939 | 1.000 | 0.854 | 1.000 | 0.981 | 0.860 | 0.832 |
| 819 | 0.773 | 1.000 | 0.798 | 1.000 | 0.968 | 0.960 | 0.908 | 1.000 | 0.980 | 1.000 | 0.951 | 0.939 | 1.000 | 0.851 | 1.000 | 0.963 | 0.855 | 0.827 |
| 820 | 0.774 | 1.000 | 0.826 | 1.000 | 0.974 | 0.960 | 0.884 | 1.000 | 0.980 | 1.000 | 0.954 | 0.970 | 1.000 | 0.854 | 1.000 | 0.981 | 0.857 | 0.829 |
| 821 | 0.773 | 1.000 | 0.812 | 1.000 | 0.974 | 0.960 | 0.892 | 1.000 | 0.990 | 1.000 | 0.945 | 0.970 | 1.000 | 0.862 | 1.000 | 0.963 | 0.858 | 0.829 |
| 822 | 0.775 | 1.000 | 0.814 | 1.000 | 0.974 | 0.960 | 0.892 | 1.000 | 0.969 | 1.000 | 0.951 | 0.970 | 1.000 | 0.861 | 1.000 | 0.981 | 0.858 | 0.830 |
| 823 | 0.773 | 1.000 | 0.810 | 1.000 | 0.974 | 0.960 | 0.889 | 1.000 | 0.980 | 1.000 | 0.954 | 0.970 | 1.000 | 0.861 | 1.000 | 0.981 | 0.858 | 0.829 |
| 824 | 0.777 | 1.000 | 0.814 | 1.000 | 0.968 | 0.960 | 0.895 | 1.000 | 0.990 | 1.000 | 0.945 | 0.939 | 1.000 | 0.855 | 1.000 | 0.981 | 0.858 | 0.829 |
| 825 | 0.776 | 1.000 | 0.816 | 1.000 | 0.968 | 0.960 | 0.900 | 1.000 | 0.980 | 1.000 | 0.962 | 0.939 | 1.000 | 0.856 | 1.000 | 1.000 | 0.860 | 0.832 |
| 826 | 0.769 | 1.000 | 0.820 | 1.000 | 0.968 | 1.000 | 0.894 | 1.000 | 0.990 | 1.000 | 0.948 | 0.970 | 1.000 | 0.854 | 1.000 | 1.000 | 0.856 | 0.828 |
| 827 | 0.778 | 1.000 | 0.810 | 1.000 | 0.974 | 0.960 | 0.886 | 1.000 | 0.990 | 1.000 | 0.951 | 1.000 | 1.000 | 0.854 | 1.000 | 0.981 | 0.857 | 0.828 |
| 828 | 0.783 | 1.000 | 0.816 | 1.000 | 0.979 | 0.960 | 0.906 | 1.000 | 0.980 | 1.000 | 0.951 | 0.970 | 1.000 | 0.855 | 1.000 | 0.963 | 0.862 | 0.834 |
| 829 | 0.771 | 1.000 | 0.822 | 1.000 | 0.968 | 0.960 | 0.894 | 1.000 | 0.980 | 1.000 | 0.954 | 1.000 | 1.000 | 0.858 | 1.000 | 1.000 | 0.858 | 0.830 |
| 830 | 0.771 | 1.000 | 0.808 | 1.000 | 0.974 | 0.960 | 0.904 | 1.000 | 0.990 | 1.000 | 0.945 | 0.970 | 1.000 | 0.854 | 1.000 | 0.981 | 0.856 | 0.828 |
| 831 | 0.775 | 1.000 | 0.816 | 1.000 | 0.974 | 0.960 | 0.894 | 1.000 | 0.980 | 1.000 | 0.951 | 0.970 | 1.000 | 0.858 | 1.000 | 0.981 | 0.858 | 0.830 |
| 832 | 0.775 | 1.000 | 0.812 | 1.000 | 0.968 | 0.960 | 0.895 | 1.000 | 0.980 | 1.000 | 0.948 | 0.970 | 1.000 | 0.861 | 1.000 | 1.000 | 0.859 | 0.830 |
| 833 | 0.781 | 1.000 | 0.814 | 1.000 | 0.968 | 0.960 | 0.897 | 1.000 | 0.980 | 1.000 | 0.951 | 0.939 | 1.000 | 0.853 | 1.000 | 0.981 | 0.859 | 0.830 |
| 834 | 0.773 | 1.000 | 0.814 | 1.000 | 0.974 | 0.960 | 0.895 | 1.000 | 0.980 | 1.000 | 0.942 | 0.970 | 1.000 | 0.857 | 1.000 | 1.000 | 0.857 | 0.828 |
| 835 | 0.780 | 1.000 | 0.810 | 1.000 | 0.974 | 0.960 | 0.891 | 1.000 | 0.980 | 1.000 | 0.939 | 0.970 | 1.000 | 0.853 | 1.000 | 0.981 | 0.857 | 0.828 |
| 836 | 0.777 | 1.000 | 0.806 | 1.000 | 0.968 | 0.960 | 0.891 | 1.000 | 0.980 | 1.000 | 0.951 | 0.970 | 1.000 | 0.854 | 1.000 | 1.000 | 0.857 | 0.828 |
| 837 | 0.774 | 1.000 | 0.818 | 1.000 | 0.974 | 0.960 | 0.900 | 1.000 | 0.980 | 1.000 | 0.954 | 0.970 | 1.000 | 0.854 | 1.000 | 1.000 | 0.858 | 0.830 |
| 838 | 0.779 | 1.000 | 0.808 | 1.000 | 0.979 | 0.960 | 0.898 | 1.000 | 0.980 | 1.000 | 0.951 | 0.939 | 1.000 | 0.858 | 1.000 | 1.000 | 0.860 | 0.832 |
| 839 | 0.773 | 1.000 | 0.812 | 1.000 | 0.974 | 0.960 | 0.898 | 1.000 | 0.980 | 1.000 | 0.957 | 0.970 | 1.000 | 0.859 | 1.000 | 0.981 | 0.859 | 0.830 |
| 840 | 0.767 | 1.000 | 0.818 | 1.000 | 0.968 | 0.960 | 0.895 | 1.000 | 0.980 | 1.000 | 0.948 | 0.970 | 1.000 | 0.853 | 1.000 | 1.000 | 0.855 | 0.826 |
| 841 | 0.777 | 1.000 | 0.822 | 1.000 | 0.968 | 0.960 | 0.889 | 1.000 | 0.980 | 1.000 | 0.957 | 0.970 | 1.000 | 0.854 | 1.000 | 1.000 | 0.859 | 0.830 |
| 842 | 0.773 | 1.000 | 0.808 | 1.000 | 0.974 | 0.960 | 0.903 | 1.000 | 0.980 | 1.000 | 0.939 | 0.939 | 1.000 | 0.858 | 1.000 | 1.000 | 0.857 | 0.829 |
| 843 | 0.767 | 1.000 | 0.818 | 1.000 | 0.979 | 0.960 | 0.895 | 1.000 | 0.980 | 1.000 | 0.945 | 0.909 | 1.000 | 0.858 | 1.000 | 0.981 | 0.856 | 0.827 |
| 844 | 0.776 | 1.000 | 0.808 | 1.000 | 0.974 | 0.960 | 0.900 | 1.000 | 0.980 | 1.000 | 0.945 | 0.909 | 1.000 | 0.854 | 1.000 | 0.981 | 0.857 | 0.828 |
| 845 | 0.769 | 1.000 | 0.806 | 1.000 | 0.974 | 0.960 | 0.886 | 1.000 | 0.980 | 1.000 | 0.948 | 0.970 | 1.000 | 0.861 | 1.000 | 0.981 | 0.855 | 0.826 |
| 846 | 0.772 | 1.000 | 0.814 | 1.000 | 0.968 | 0.960 | 0.892 | 1.000 | 0.980 | 1.000 | 0.957 | 0.939 | 1.000 | 0.854 | 1.000 | 0.981 | 0.856 | 0.828 |
| 847 | 0.773 | 1.000 | 0.818 | 1.000 | 0.974 | 0.960 | 0.892 | 1.000 | 0.980 | 1.000 | 0.942 | 0.970 | 1.000 | 0.856 | 1.000 | 0.981 | 0.857 | 0.828 |
| 848 | 0.768 | 1.000 | 0.798 | 1.000 | 0.974 | 0.960 | 0.897 | 1.000 | 0.980 | 1.000 | 0.954 | 0.970 | 1.000 | 0.856 | 1.000 | 0.981 | 0.855 | 0.826 |
| 849 | 0.777 | 1.000 | 0.820 | 1.000 | 0.979 | 0.960 | 0.897 | 1.000 | 0.980 | 1.000 | 0.948 | 0.939 | 1.000 | 0.853 | 1.000 | 1.000 | 0.858 | 0.830 |
| 850 | 0.776 | 1.000 | 0.824 | 1.000 | 0.974 | 0.960 | 0.894 | 1.000 | 0.980 | 1.000 | 0.957 | 0.939 | 1.000 | 0.859 | 1.000 | 1.000 | 0.860 | 0.832 |
| 851 | 0.781 | 1.000 | 0.802 | 1.000 | 0.974 | 0.960 | 0.883 | 1.000 | 0.980 | 1.000 | 0.936 | 0.939 | 1.000 | 0.860 | 1.000 | 1.000 | 0.857 | 0.828 |
| 852 | 0.777 | 1.000 | 0.820 | 1.000 | 0.968 | 0.960 | 0.889 | 1.000 | 0.980 | 1.000 | 0.951 | 0.970 | 1.000 | 0.857 | 1.000 | 1.000 | 0.859 | 0.830 |
| 853 | 0.781 | 1.000 | 0.822 | 1.000 | 0.974 | 0.960 | 0.886 | 1.000 | 0.969 | 1.000 | 0.945 | 0.939 | 1.000 | 0.858 | 1.000 | 0.981 | 0.859 | 0.831 |
| 854 | 0.768 | 1.000 | 0.812 | 1.000 | 0.963 | 0.960 | 0.897 | 1.000 | 0.980 | 1.000 | 0.957 | 0.939 | 1.000 | 0.856 | 1.000 | 0.981 | 0.856 | 0.827 |
| 855 | 0.767 | 1.000 | 0.810 | 1.000 | 0.974 | 0.960 | 0.892 | 1.000 | 0.990 | 1.000 | 0.957 | 0.939 | 1.000 | 0.856 | 1.000 | 0.981 | 0.855 | 0.826 |
| 856 | 0.772 | 1.000 | 0.812 | 1.000 | 0.974 | 0.960 | 0.894 | 1.000 | 0.980 | 1.000 | 0.951 | 0.970 | 1.000 | 0.855 | 1.000 | 0.981 | 0.856 | 0.828 |

| | | | | | | | | | | | | | | | | | | |
|---|---|---|---|---|---|---|---|---|---|---|---|---|---|---|---|---|---|---|
| 857 | 0.775 | 1.000 | 0.824 | 1.000 | 0.968 | 0.960 | 0.894 | 1.000 | 0.980 | 1.000 | 0.945 | 0.970 | 1.000 | 0.850 | 1.000 | 1.000 | 0.857 | 0.828 |
| 858 | 0.771 | 1.000 | 0.816 | 1.000 | 0.974 | 0.960 | 0.892 | 1.000 | 0.980 | 1.000 | 0.954 | 0.939 | 1.000 | 0.848 | 1.000 | 1.000 | 0.855 | 0.826 |
| 859 | 0.770 | 1.000 | 0.808 | 1.000 | 0.958 | 0.960 | 0.892 | 1.000 | 0.980 | 1.000 | 0.951 | 0.939 | 1.000 | 0.858 | 1.000 | 0.981 | 0.855 | 0.826 |
| 860 | 0.792 | 1.000 | 0.816 | 1.000 | 0.979 | 0.960 | 0.900 | 1.000 | 0.980 | 1.000 | 0.954 | 0.970 | 1.000 | 0.857 | 1.000 | 0.981 | 0.864 | 0.837 |
| 861 | 0.777 | 1.000 | 0.808 | 1.000 | 0.974 | 0.960 | 0.897 | 1.000 | 0.980 | 1.000 | 0.951 | 0.970 | 1.000 | 0.854 | 1.000 | 1.000 | 0.858 | 0.829 |
| 862 | 0.777 | 1.000 | 0.830 | 1.000 | 0.979 | 0.960 | 0.888 | 1.000 | 0.980 | 1.000 | 0.945 | 0.970 | 1.000 | 0.861 | 1.000 | 0.981 | 0.860 | 0.832 |
| 863 | 0.767 | 1.000 | 0.806 | 1.000 | 0.968 | 0.960 | 0.897 | 1.000 | 0.980 | 1.000 | 0.948 | 0.939 | 1.000 | 0.848 | 1.000 | 0.981 | 0.852 | 0.823 |
| 864 | 0.770 | 1.000 | 0.800 | 1.000 | 0.968 | 0.960 | 0.903 | 1.000 | 0.959 | 1.000 | 0.957 | 0.939 | 1.000 | 0.860 | 1.000 | 0.981 | 0.857 | 0.828 |
| 865 | 0.771 | 1.000 | 0.802 | 1.000 | 0.963 | 0.960 | 0.883 | 1.000 | 0.969 | 1.000 | 0.948 | 1.000 | 1.000 | 0.847 | 1.000 | 0.981 | 0.851 | 0.822 |
| 866 | 0.774 | 1.000 | 0.800 | 1.000 | 0.984 | 0.960 | 0.895 | 1.000 | 0.990 | 1.000 | 0.959 | 0.939 | 1.000 | 0.856 | 1.000 | 0.981 | 0.857 | 0.829 |
| 867 | 0.767 | 1.000 | 0.806 | 1.000 | 0.979 | 0.960 | 0.894 | 1.000 | 0.969 | 1.000 | 0.951 | 0.970 | 1.000 | 0.855 | 1.000 | 1.000 | 0.855 | 0.826 |
| 868 | 0.780 | 1.000 | 0.812 | 1.000 | 0.974 | 0.960 | 0.889 | 1.000 | 0.980 | 1.000 | 0.954 | 0.970 | 1.000 | 0.851 | 1.000 | 0.981 | 0.857 | 0.829 |
| 869 | 0.779 | 1.000 | 0.818 | 1.000 | 0.979 | 0.960 | 0.892 | 1.000 | 0.980 | 1.000 | 0.959 | 0.939 | 1.000 | 0.864 | 1.000 | 0.981 | 0.862 | 0.834 |
| 870 | 0.773 | 1.000 | 0.822 | 1.000 | 0.974 | 0.960 | 0.889 | 1.000 | 0.980 | 1.000 | 0.945 | 0.970 | 1.000 | 0.853 | 1.000 | 0.981 | 0.856 | 0.828 |
| 871 | 0.773 | 1.000 | 0.826 | 1.000 | 0.974 | 0.960 | 0.897 | 1.000 | 0.980 | 1.000 | 0.951 | 0.970 | 1.000 | 0.860 | 1.000 | 1.000 | 0.860 | 0.832 |
| 872 | 0.775 | 1.000 | 0.814 | 1.000 | 0.979 | 0.960 | 0.898 | 1.000 | 0.980 | 1.000 | 0.951 | 0.970 | 1.000 | 0.861 | 1.000 | 0.981 | 0.860 | 0.832 |
| 873 | 0.770 | 1.000 | 0.802 | 1.000 | 0.979 | 0.960 | 0.891 | 1.000 | 0.980 | 1.000 | 0.945 | 0.939 | 1.000 | 0.853 | 1.000 | 0.981 | 0.853 | 0.824 |
| 874 | 0.771 | 1.000 | 0.810 | 1.000 | 0.968 | 0.960 | 0.898 | 1.000 | 0.980 | 1.000 | 0.954 | 0.939 | 1.000 | 0.854 | 1.000 | 1.000 | 0.856 | 0.828 |
| 875 | 0.776 | 1.000 | 0.816 | 1.000 | 0.968 | 0.960 | 0.894 | 1.000 | 0.980 | 1.000 | 0.951 | 0.939 | 1.000 | 0.855 | 1.000 | 1.000 | 0.858 | 0.830 |
| 876 | 0.781 | 1.000 | 0.804 | 1.000 | 0.979 | 0.960 | 0.892 | 1.000 | 0.980 | 1.000 | 0.945 | 0.939 | 1.000 | 0.852 | 1.000 | 0.981 | 0.857 | 0.828 |
| 877 | 0.760 | 1.000 | 0.796 | 1.000 | 0.974 | 0.960 | 0.898 | 1.000 | 0.980 | 1.000 | 0.957 | 0.939 | 1.000 | 0.857 | 1.000 | 0.981 | 0.852 | 0.823 |
| 878 | 0.775 | 1.000 | 0.802 | 1.000 | 0.974 | 0.960 | 0.898 | 1.000 | 0.980 | 1.000 | 0.951 | 0.970 | 1.000 | 0.858 | 1.000 | 0.981 | 0.857 | 0.829 |
| 879 | 0.779 | 1.000 | 0.810 | 1.000 | 0.974 | 0.960 | 0.901 | 1.000 | 0.980 | 1.000 | 0.948 | 0.970 | 1.000 | 0.853 | 1.000 | 0.981 | 0.858 | 0.830 |
| 880 | 0.777 | 1.000 | 0.818 | 1.000 | 0.968 | 0.960 | 0.891 | 1.000 | 0.980 | 1.000 | 0.948 | 0.939 | 1.000 | 0.855 | 1.000 | 0.981 | 0.857 | 0.829 |
| 881 | 0.772 | 1.000 | 0.800 | 1.000 | 0.974 | 0.960 | 0.897 | 1.000 | 0.980 | 1.000 | 0.945 | 0.939 | 1.000 | 0.854 | 1.000 | 0.981 | 0.855 | 0.826 |
| 882 | 0.778 | 1.000 | 0.816 | 1.000 | 0.979 | 0.960 | 0.889 | 1.000 | 0.980 | 1.000 | 0.951 | 0.970 | 1.000 | 0.861 | 1.000 | 0.981 | 0.860 | 0.831 |
| 883 | 0.778 | 1.000 | 0.818 | 1.000 | 0.979 | 0.960 | 0.891 | 1.000 | 0.980 | 1.000 | 0.942 | 0.909 | 1.000 | 0.858 | 1.000 | 1.000 | 0.859 | 0.830 |
| 884 | 0.771 | 1.000 | 0.820 | 1.000 | 0.974 | 0.960 | 0.898 | 1.000 | 0.980 | 1.000 | 0.954 | 0.970 | 1.000 | 0.865 | 1.000 | 0.981 | 0.860 | 0.832 |
| 885 | 0.773 | 1.000 | 0.816 | 1.000 | 0.979 | 0.960 | 0.897 | 1.000 | 0.980 | 1.000 | 0.954 | 0.970 | 1.000 | 0.858 | 1.000 | 0.981 | 0.859 | 0.830 |
| 886 | 0.773 | 1.000 | 0.812 | 1.000 | 0.974 | 0.960 | 0.895 | 1.000 | 0.980 | 1.000 | 0.942 | 0.970 | 1.000 | 0.856 | 1.000 | 1.000 | 0.857 | 0.828 |
| 887 | 0.771 | 1.000 | 0.810 | 1.000 | 0.968 | 0.960 | 0.894 | 1.000 | 0.980 | 1.000 | 0.954 | 1.000 | 1.000 | 0.858 | 1.000 | 0.981 | 0.857 | 0.828 |
| 888 | 0.769 | 1.000 | 0.812 | 1.000 | 0.974 | 0.960 | 0.892 | 1.000 | 0.980 | 1.000 | 0.951 | 0.939 | 1.000 | 0.853 | 1.000 | 0.981 | 0.854 | 0.826 |
| 889 | 0.775 | 1.000 | 0.824 | 1.000 | 0.974 | 0.960 | 0.892 | 1.000 | 0.980 | 1.000 | 0.948 | 0.970 | 1.000 | 0.854 | 1.000 | 1.000 | 0.858 | 0.830 |
| 890 | 0.766 | 1.000 | 0.802 | 1.000 | 0.974 | 0.960 | 0.909 | 1.000 | 0.980 | 1.000 | 0.954 | 0.970 | 1.000 | 0.859 | 1.000 | 0.981 | 0.857 | 0.829 |
| 891 | 0.780 | 1.000 | 0.818 | 1.000 | 0.979 | 0.960 | 0.895 | 1.000 | 0.980 | 1.000 | 0.945 | 0.939 | 1.000 | 0.856 | 1.000 | 0.981 | 0.859 | 0.831 |
| 892 | 0.775 | 1.000 | 0.810 | 1.000 | 0.979 | 0.960 | 0.891 | 1.000 | 0.980 | 1.000 | 0.951 | 1.000 | 1.000 | 0.846 | 1.000 | 0.981 | 0.855 | 0.826 |
| 893 | 0.777 | 1.000 | 0.820 | 1.000 | 0.984 | 0.960 | 0.888 | 1.000 | 0.980 | 1.000 | 0.948 | 0.970 | 1.000 | 0.853 | 1.000 | 0.981 | 0.858 | 0.829 |
| 894 | 0.779 | 1.000 | 0.804 | 1.000 | 0.979 | 0.960 | 0.895 | 1.000 | 0.990 | 1.000 | 0.959 | 0.939 | 1.000 | 0.854 | 1.000 | 0.981 | 0.858 | 0.830 |
| 895 | 0.770 | 1.000 | 0.818 | 1.000 | 0.974 | 0.960 | 0.898 | 1.000 | 0.990 | 1.000 | 0.942 | 0.939 | 1.000 | 0.851 | 1.000 | 0.981 | 0.855 | 0.827 |

| | | | | | | | | | | | | | | | | | |
|---|---|---|---|---|---|---|---|---|---|---|---|---|---|---|---|---|---|
| 896 | 0.773 | 1.000 | 0.814 | 1.000 | 0.979 | 0.960 | 0.891 | 1.000 | 0.980 | 1.000 | 0.948 | 0.970 | 1.000 | 0.853 | 1.000 | 0.981 | 0.856 | 0.827 |
| 897 | 0.775 | 1.000 | 0.808 | 1.000 | 0.974 | 0.960 | 0.900 | 1.000 | 0.980 | 1.000 | 0.945 | 0.939 | 1.000 | 0.858 | 1.000 | 0.963 | 0.858 | 0.829 |
| 898 | 0.769 | 1.000 | 0.824 | 1.000 | 0.979 | 0.960 | 0.897 | 1.000 | 0.969 | 1.000 | 0.951 | 0.970 | 1.000 | 0.859 | 1.000 | 1.000 | 0.858 | 0.830 |
| 899 | 0.776 | 1.000 | 0.796 | 1.000 | 0.974 | 0.960 | 0.895 | 1.000 | 0.980 | 1.000 | 0.942 | 0.939 | 1.000 | 0.862 | 1.000 | 0.981 | 0.857 | 0.828 |
| 900 | 0.766 | 1.000 | 0.810 | 1.000 | 0.974 | 0.960 | 0.897 | 1.000 | 0.980 | 1.000 | 0.948 | 1.000 | 1.000 | 0.850 | 1.000 | 0.981 | 0.853 | 0.825 |
| 901 | 0.772 | 1.000 | 0.806 | 1.000 | 0.984 | 0.960 | 0.895 | 1.000 | 0.980 | 1.000 | 0.948 | 0.939 | 1.000 | 0.854 | 1.000 | 0.981 | 0.856 | 0.827 |
| 902 | 0.782 | 1.000 | 0.800 | 1.000 | 0.974 | 0.960 | 0.895 | 1.000 | 0.990 | 1.000 | 0.951 | 0.939 | 1.000 | 0.855 | 1.000 | 0.981 | 0.858 | 0.830 |
| 903 | 0.765 | 1.000 | 0.808 | 1.000 | 0.979 | 0.960 | 0.901 | 1.000 | 0.980 | 1.000 | 0.951 | 0.970 | 1.000 | 0.861 | 1.000 | 0.981 | 0.857 | 0.829 |
| 904 | 0.779 | 1.000 | 0.806 | 1.000 | 0.979 | 1.000 | 0.888 | 1.000 | 0.990 | 1.000 | 0.942 | 0.970 | 1.000 | 0.858 | 1.000 | 0.963 | 0.857 | 0.829 |
| 905 | 0.777 | 1.000 | 0.810 | 1.000 | 0.979 | 0.960 | 0.904 | 1.000 | 0.980 | 1.000 | 0.948 | 0.939 | 1.000 | 0.861 | 1.000 | 0.981 | 0.860 | 0.833 |
| 906 | 0.769 | 1.000 | 0.800 | 1.000 | 0.979 | 0.960 | 0.894 | 1.000 | 0.990 | 1.000 | 0.945 | 0.939 | 1.000 | 0.860 | 1.000 | 0.981 | 0.855 | 0.826 |
| 907 | 0.765 | 1.000 | 0.826 | 1.000 | 0.974 | 0.960 | 0.897 | 1.000 | 0.990 | 1.000 | 0.957 | 0.970 | 1.000 | 0.857 | 1.000 | 0.981 | 0.857 | 0.829 |
| 908 | 0.782 | 1.000 | 0.816 | 1.000 | 0.974 | 0.960 | 0.894 | 1.000 | 0.980 | 1.000 | 0.951 | 0.970 | 1.000 | 0.851 | 1.000 | 1.000 | 0.859 | 0.831 |
| 909 | 0.772 | 1.000 | 0.816 | 1.000 | 0.974 | 0.960 | 0.892 | 1.000 | 0.990 | 1.000 | 0.957 | 0.970 | 1.000 | 0.863 | 1.000 | 0.981 | 0.859 | 0.831 |
| 910 | 0.771 | 1.000 | 0.818 | 1.000 | 0.974 | 0.960 | 0.889 | 1.000 | 0.990 | 1.000 | 0.945 | 0.970 | 1.000 | 0.849 | 1.000 | 1.000 | 0.854 | 0.825 |
| 911 | 0.781 | 1.000 | 0.830 | 1.000 | 0.979 | 0.960 | 0.883 | 1.000 | 0.990 | 1.000 | 0.945 | 0.970 | 1.000 | 0.861 | 1.000 | 0.981 | 0.861 | 0.833 |
| 912 | 0.773 | 1.000 | 0.820 | 1.000 | 0.979 | 0.960 | 0.880 | 1.000 | 0.990 | 1.000 | 0.951 | 0.909 | 1.000 | 0.858 | 1.000 | 0.981 | 0.856 | 0.828 |
| 913 | 0.782 | 1.000 | 0.808 | 1.000 | 0.979 | 0.960 | 0.891 | 1.000 | 0.990 | 1.000 | 0.948 | 0.939 | 1.000 | 0.855 | 1.000 | 0.981 | 0.859 | 0.830 |
| 914 | 0.776 | 1.000 | 0.814 | 1.000 | 0.979 | 0.960 | 0.892 | 1.000 | 0.990 | 1.000 | 0.954 | 0.939 | 1.000 | 0.847 | 1.000 | 1.000 | 0.856 | 0.828 |
| 915 | 0.781 | 1.000 | 0.810 | 1.000 | 0.974 | 0.960 | 0.895 | 1.000 | 0.990 | 1.000 | 0.945 | 0.939 | 1.000 | 0.857 | 1.000 | 0.981 | 0.859 | 0.831 |
| 916 | 0.772 | 1.000 | 0.804 | 1.000 | 0.974 | 0.960 | 0.898 | 1.000 | 0.990 | 1.000 | 0.951 | 0.970 | 1.000 | 0.856 | 1.000 | 0.981 | 0.857 | 0.828 |
| 917 | 0.773 | 1.000 | 0.826 | 1.000 | 0.979 | 0.960 | 0.894 | 1.000 | 0.980 | 1.000 | 0.954 | 0.939 | 1.000 | 0.858 | 1.000 | 1.000 | 0.859 | 0.832 |
| 918 | 0.767 | 1.000 | 0.814 | 1.000 | 0.968 | 0.960 | 0.892 | 1.000 | 0.990 | 1.000 | 0.948 | 0.939 | 1.000 | 0.853 | 1.000 | 1.000 | 0.854 | 0.825 |
| 919 | 0.769 | 1.000 | 0.812 | 1.000 | 0.974 | 0.960 | 0.898 | 1.000 | 0.990 | 1.000 | 0.954 | 0.939 | 1.000 | 0.853 | 1.000 | 0.981 | 0.856 | 0.827 |
| 920 | 0.767 | 1.000 | 0.800 | 1.000 | 0.979 | 0.960 | 0.903 | 1.000 | 0.980 | 1.000 | 0.951 | 0.939 | 1.000 | 0.860 | 1.000 | 0.981 | 0.856 | 0.828 |
| 921 | 0.780 | 1.000 | 0.816 | 1.000 | 0.974 | 0.960 | 0.898 | 1.000 | 0.980 | 1.000 | 0.951 | 0.939 | 1.000 | 0.851 | 1.000 | 0.981 | 0.858 | 0.830 |
| 922 | 0.766 | 1.000 | 0.808 | 1.000 | 0.979 | 0.960 | 0.898 | 1.000 | 0.990 | 1.000 | 0.948 | 0.970 | 1.000 | 0.855 | 1.000 | 0.981 | 0.855 | 0.826 |
| 923 | 0.778 | 1.000 | 0.820 | 1.000 | 0.974 | 0.960 | 0.895 | 1.000 | 0.980 | 1.000 | 0.948 | 0.909 | 1.000 | 0.850 | 1.000 | 0.981 | 0.857 | 0.829 |
| 924 | 0.768 | 1.000 | 0.822 | 1.000 | 0.979 | 0.960 | 0.891 | 1.000 | 0.990 | 1.000 | 0.942 | 0.970 | 1.000 | 0.856 | 1.000 | 0.981 | 0.856 | 0.827 |
| 925 | 0.770 | 1.000 | 0.814 | 1.000 | 0.979 | 0.960 | 0.898 | 1.000 | 0.990 | 1.000 | 0.948 | 0.939 | 1.000 | 0.856 | 1.000 | 0.981 | 0.857 | 0.828 |
| 926 | 0.774 | 1.000 | 0.834 | 1.000 | 0.968 | 0.960 | 0.903 | 1.000 | 0.990 | 1.000 | 0.945 | 0.909 | 1.000 | 0.853 | 1.000 | 0.981 | 0.859 | 0.831 |
| 927 | 0.784 | 1.000 | 0.818 | 1.000 | 0.974 | 0.960 | 0.894 | 1.000 | 0.990 | 1.000 | 0.942 | 0.939 | 1.000 | 0.862 | 1.000 | 0.981 | 0.862 | 0.834 |
| 928 | 0.785 | 1.000 | 0.818 | 1.000 | 0.979 | 0.960 | 0.897 | 1.000 | 0.990 | 1.000 | 0.939 | 0.909 | 1.000 | 0.859 | 1.000 | 0.981 | 0.862 | 0.834 |
| 929 | 0.774 | 1.000 | 0.810 | 1.000 | 0.984 | 0.960 | 0.892 | 1.000 | 0.990 | 1.000 | 0.948 | 0.970 | 1.000 | 0.853 | 1.000 | 0.981 | 0.856 | 0.828 |
| 930 | 0.775 | 1.000 | 0.808 | 1.000 | 0.979 | 0.960 | 0.892 | 1.000 | 0.990 | 1.000 | 0.951 | 1.000 | 1.000 | 0.852 | 1.000 | 1.000 | 0.857 | 0.828 |
| 931 | 0.776 | 1.000 | 0.826 | 1.000 | 0.974 | 0.960 | 0.901 | 1.000 | 0.990 | 1.000 | 0.959 | 0.970 | 1.000 | 0.856 | 1.000 | 0.981 | 0.861 | 0.833 |
| 932 | 0.771 | 1.000 | 0.814 | 1.000 | 0.974 | 0.960 | 0.897 | 1.000 | 0.980 | 1.000 | 0.951 | 0.970 | 1.000 | 0.856 | 1.000 | 0.981 | 0.857 | 0.828 |
| 933 | 0.779 | 1.000 | 0.814 | 1.000 | 0.979 | 0.960 | 0.897 | 1.000 | 0.990 | 1.000 | 0.951 | 0.909 | 1.000 | 0.854 | 1.000 | 0.981 | 0.859 | 0.831 |
| 934 | 0.777 | 1.000 | 0.808 | 1.000 | 0.979 | 0.960 | 0.888 | 1.000 | 0.980 | 1.000 | 0.939 | 0.970 | 1.000 | 0.853 | 1.000 | 0.981 | 0.856 | 0.826 |

| | | | | | | | | | | | | | | | | | | |
|---|---|---|---|---|---|---|---|---|---|---|---|---|---|---|---|---|---|---|
| 935 | 0.773 | 1.000 | 0.806 | 1.000 | 0.984 | 0.960 | 0.898 | 1.000 | 0.990 | 1.000 | 0.954 | 0.970 | 1.000 | 0.846 | 1.000 | 0.981 | 0.855 | 0.826 |
| 936 | 0.778 | 1.000 | 0.810 | 1.000 | 0.968 | 0.960 | 0.898 | 1.000 | 0.990 | 1.000 | 0.959 | 0.939 | 1.000 | 0.857 | 1.000 | 1.000 | 0.860 | 0.832 |
| 937 | 0.776 | 1.000 | 0.820 | 1.000 | 0.979 | 0.960 | 0.909 | 1.000 | 0.980 | 1.000 | 0.959 | 0.970 | 1.000 | 0.851 | 1.000 | 0.981 | 0.860 | 0.832 |
| 938 | 0.783 | 1.000 | 0.796 | 1.000 | 0.974 | 0.960 | 0.906 | 1.000 | 0.990 | 1.000 | 0.954 | 0.939 | 1.000 | 0.855 | 1.000 | 0.981 | 0.860 | 0.832 |
| 939 | 0.783 | 1.000 | 0.814 | 1.000 | 0.979 | 0.960 | 0.894 | 1.000 | 0.990 | 1.000 | 0.954 | 0.970 | 1.000 | 0.862 | 1.000 | 0.981 | 0.862 | 0.834 |
| 940 | 0.782 | 1.000 | 0.806 | 1.000 | 0.974 | 0.960 | 0.892 | 1.000 | 0.990 | 1.000 | 0.948 | 0.970 | 1.000 | 0.851 | 1.000 | 0.981 | 0.858 | 0.829 |
| 941 | 0.777 | 1.000 | 0.804 | 1.000 | 0.974 | 0.960 | 0.901 | 1.000 | 0.990 | 1.000 | 0.957 | 0.909 | 1.000 | 0.861 | 1.000 | 0.981 | 0.860 | 0.831 |
| 942 | 0.783 | 1.000 | 0.802 | 1.000 | 0.974 | 0.960 | 0.889 | 1.000 | 0.990 | 1.000 | 0.954 | 0.970 | 1.000 | 0.864 | 1.000 | 0.981 | 0.861 | 0.832 |
| 943 | 0.786 | 1.000 | 0.812 | 1.000 | 0.979 | 0.960 | 0.895 | 1.000 | 0.990 | 1.000 | 0.951 | 0.909 | 1.000 | 0.864 | 1.000 | 0.981 | 0.863 | 0.835 |
| 944 | 0.783 | 1.000 | 0.804 | 1.000 | 0.974 | 0.960 | 0.883 | 1.000 | 0.990 | 1.000 | 0.951 | 0.909 | 1.000 | 0.861 | 1.000 | 1.000 | 0.859 | 0.830 |
| 945 | 0.772 | 1.000 | 0.814 | 1.000 | 0.979 | 0.960 | 0.903 | 1.000 | 0.990 | 1.000 | 0.948 | 0.939 | 1.000 | 0.851 | 1.000 | 0.981 | 0.857 | 0.829 |
| 946 | 0.772 | 1.000 | 0.820 | 1.000 | 0.974 | 0.960 | 0.898 | 1.000 | 0.990 | 1.000 | 0.951 | 0.909 | 1.000 | 0.855 | 1.000 | 0.981 | 0.858 | 0.829 |
| 947 | 0.770 | 1.000 | 0.832 | 1.000 | 0.963 | 0.960 | 0.897 | 1.000 | 0.990 | 1.000 | 0.954 | 0.909 | 1.000 | 0.860 | 1.000 | 1.000 | 0.859 | 0.831 |
| 948 | 0.768 | 1.000 | 0.816 | 1.000 | 0.984 | 0.960 | 0.891 | 1.000 | 0.980 | 1.000 | 0.951 | 0.970 | 1.000 | 0.854 | 1.000 | 1.000 | 0.856 | 0.827 |
| 949 | 0.774 | 1.000 | 0.822 | 1.000 | 0.979 | 0.960 | 0.891 | 1.000 | 0.990 | 1.000 | 0.951 | 0.939 | 1.000 | 0.852 | 1.000 | 1.000 | 0.857 | 0.829 |
| 950 | 0.779 | 1.000 | 0.826 | 1.000 | 0.974 | 0.960 | 0.903 | 1.000 | 0.990 | 1.000 | 0.957 | 0.970 | 1.000 | 0.851 | 1.000 | 0.981 | 0.861 | 0.833 |
| 951 | 0.779 | 1.000 | 0.814 | 1.000 | 0.979 | 0.960 | 0.888 | 1.000 | 0.990 | 1.000 | 0.951 | 0.970 | 1.000 | 0.858 | 1.000 | 0.981 | 0.859 | 0.830 |
| 952 | 0.775 | 1.000 | 0.824 | 1.000 | 0.979 | 0.960 | 0.883 | 1.000 | 0.990 | 1.000 | 0.951 | 0.970 | 1.000 | 0.858 | 1.000 | 0.981 | 0.858 | 0.830 |
| 953 | 0.776 | 1.000 | 0.822 | 1.000 | 0.979 | 0.960 | 0.888 | 1.000 | 0.990 | 1.000 | 0.951 | 0.909 | 1.000 | 0.854 | 1.000 | 0.981 | 0.858 | 0.829 |
| 954 | 0.777 | 1.000 | 0.818 | 1.000 | 0.979 | 0.960 | 0.888 | 1.000 | 0.969 | 1.000 | 0.948 | 0.970 | 1.000 | 0.851 | 1.000 | 0.981 | 0.857 | 0.828 |
| 955 | 0.768 | 1.000 | 0.814 | 1.000 | 0.979 | 0.960 | 0.901 | 1.000 | 0.980 | 1.000 | 0.948 | 0.939 | 1.000 | 0.851 | 1.000 | 1.000 | 0.855 | 0.827 |
| 956 | 0.775 | 1.000 | 0.812 | 1.000 | 0.979 | 0.960 | 0.904 | 1.000 | 0.990 | 1.000 | 0.942 | 0.970 | 1.000 | 0.856 | 1.000 | 1.000 | 0.859 | 0.831 |
| 957 | 0.775 | 1.000 | 0.822 | 1.000 | 0.974 | 0.960 | 0.904 | 1.000 | 0.990 | 1.000 | 0.945 | 0.970 | 1.000 | 0.856 | 1.000 | 0.981 | 0.860 | 0.832 |
| 958 | 0.774 | 1.000 | 0.800 | 1.000 | 0.979 | 0.960 | 0.898 | 1.000 | 0.980 | 1.000 | 0.951 | 0.970 | 1.000 | 0.849 | 1.000 | 0.981 | 0.855 | 0.826 |
| 959 | 0.780 | 1.000 | 0.808 | 1.000 | 0.979 | 0.960 | 0.903 | 1.000 | 0.990 | 1.000 | 0.951 | 0.939 | 1.000 | 0.858 | 1.000 | 1.000 | 0.861 | 0.833 |
| 960 | 0.775 | 1.000 | 0.818 | 1.000 | 0.979 | 0.960 | 0.883 | 1.000 | 0.990 | 1.000 | 0.948 | 0.970 | 1.000 | 0.855 | 1.000 | 1.000 | 0.857 | 0.828 |
| 961 | 0.769 | 1.000 | 0.826 | 1.000 | 0.979 | 0.960 | 0.889 | 1.000 | 0.990 | 1.000 | 0.959 | 1.000 | 1.000 | 0.854 | 1.000 | 0.981 | 0.857 | 0.829 |
| 962 | 0.776 | 1.000 | 0.820 | 1.000 | 0.979 | 0.960 | 0.892 | 1.000 | 0.990 | 1.000 | 0.951 | 0.970 | 1.000 | 0.859 | 1.000 | 0.981 | 0.860 | 0.831 |
| 963 | 0.782 | 1.000 | 0.830 | 1.000 | 0.974 | 0.960 | 0.889 | 1.000 | 0.990 | 1.000 | 0.959 | 0.939 | 1.000 | 0.855 | 1.000 | 1.000 | 0.861 | 0.834 |
| 964 | 0.769 | 1.000 | 0.814 | 1.000 | 0.979 | 0.960 | 0.895 | 1.000 | 0.980 | 1.000 | 0.951 | 0.909 | 1.000 | 0.855 | 1.000 | 0.981 | 0.856 | 0.827 |
| 965 | 0.784 | 1.000 | 0.808 | 1.000 | 0.974 | 0.960 | 0.897 | 1.000 | 0.980 | 1.000 | 0.951 | 0.939 | 1.000 | 0.851 | 1.000 | 0.981 | 0.859 | 0.830 |
| 966 | 0.787 | 1.000 | 0.816 | 1.000 | 0.979 | 0.960 | 0.891 | 1.000 | 0.990 | 1.000 | 0.942 | 0.970 | 1.000 | 0.858 | 1.000 | 0.981 | 0.861 | 0.833 |
| 967 | 0.777 | 1.000 | 0.800 | 1.000 | 0.974 | 0.960 | 0.898 | 1.000 | 0.990 | 1.000 | 0.948 | 0.939 | 1.000 | 0.865 | 1.000 | 0.981 | 0.860 | 0.832 |
| 968 | 0.781 | 1.000 | 0.822 | 1.000 | 0.974 | 0.960 | 0.904 | 1.000 | 0.990 | 1.000 | 0.954 | 0.939 | 1.000 | 0.851 | 1.000 | 0.981 | 0.861 | 0.833 |
| 969 | 0.777 | 1.000 | 0.804 | 1.000 | 0.968 | 0.960 | 0.891 | 1.000 | 0.990 | 1.000 | 0.951 | 0.970 | 1.000 | 0.855 | 1.000 | 1.000 | 0.857 | 0.828 |
| 970 | 0.773 | 1.000 | 0.828 | 1.000 | 0.974 | 0.960 | 0.903 | 1.000 | 0.990 | 1.000 | 0.954 | 0.939 | 1.000 | 0.856 | 1.000 | 0.981 | 0.860 | 0.832 |
| 971 | 0.767 | 1.000 | 0.804 | 1.000 | 0.974 | 0.960 | 0.895 | 1.000 | 0.980 | 1.000 | 0.954 | 0.970 | 1.000 | 0.861 | 1.000 | 0.981 | 0.856 | 0.827 |
| 972 | 0.778 | 1.000 | 0.808 | 1.000 | 0.968 | 0.960 | 0.898 | 1.000 | 0.990 | 1.000 | 0.957 | 0.939 | 1.000 | 0.857 | 1.000 | 0.981 | 0.859 | 0.831 |
| 973 | 0.780 | 1.000 | 0.802 | 1.000 | 0.968 | 0.960 | 0.904 | 1.000 | 0.990 | 1.000 | 0.954 | 0.939 | 1.000 | 0.858 | 1.000 | 0.981 | 0.860 | 0.832 |

| | | | | | | | | | | | | | | | | | | |
|---|---|---|---|---|---|---|---|---|---|---|---|---|---|---|---|---|---|---|
| 974 | 0.770 | 1.000 | 0.814 | 1.000 | 0.974 | 0.960 | 0.889 | 1.000 | 0.990 | 1.000 | 0.954 | 0.939 | 1.000 | 0.851 | 1.000 | 0.981 | 0.855 | 0.826 |
| 975 | 0.780 | 1.000 | 0.806 | 1.000 | 0.974 | 0.960 | 0.892 | 1.000 | 0.990 | 1.000 | 0.957 | 0.970 | 1.000 | 0.849 | 1.000 | 0.981 | 0.857 | 0.828 |
| 976 | 0.771 | 1.000 | 0.812 | 1.000 | 0.979 | 0.960 | 0.894 | 1.000 | 0.990 | 1.000 | 0.959 | 0.939 | 1.000 | 0.861 | 1.000 | 1.000 | 0.859 | 0.830 |
| 977 | 0.773 | 1.000 | 0.816 | 1.000 | 0.968 | 0.960 | 0.897 | 1.000 | 0.980 | 1.000 | 0.951 | 0.909 | 1.000 | 0.854 | 1.000 | 0.981 | 0.857 | 0.828 |
| 978 | 0.769 | 1.000 | 0.826 | 1.000 | 0.968 | 0.960 | 0.894 | 1.000 | 0.990 | 1.000 | 0.948 | 0.970 | 1.000 | 0.852 | 1.000 | 0.981 | 0.856 | 0.827 |
| 979 | 0.790 | 1.000 | 0.808 | 1.000 | 0.974 | 1.000 | 0.888 | 1.000 | 0.990 | 1.000 | 0.957 | 0.970 | 1.000 | 0.861 | 1.000 | 1.000 | 0.863 | 0.835 |
| 980 | 0.775 | 1.000 | 0.806 | 1.000 | 0.974 | 0.960 | 0.894 | 1.000 | 0.990 | 1.000 | 0.951 | 0.970 | 1.000 | 0.856 | 1.000 | 0.981 | 0.857 | 0.828 |
| 981 | 0.789 | 1.000 | 0.802 | 1.000 | 0.974 | 0.960 | 0.886 | 1.000 | 0.990 | 1.000 | 0.957 | 0.939 | 1.000 | 0.854 | 1.000 | 0.981 | 0.860 | 0.831 |
| 982 | 0.775 | 1.000 | 0.808 | 1.000 | 0.974 | 0.960 | 0.895 | 1.000 | 0.990 | 1.000 | 0.945 | 0.970 | 1.000 | 0.865 | 1.000 | 0.981 | 0.860 | 0.832 |
| 983 | 0.776 | 1.000 | 0.810 | 1.000 | 0.974 | 0.960 | 0.889 | 1.000 | 0.990 | 1.000 | 0.954 | 0.909 | 1.000 | 0.858 | 1.000 | 0.981 | 0.858 | 0.829 |
| 984 | 0.777 | 1.000 | 0.826 | 1.000 | 0.968 | 0.960 | 0.897 | 1.000 | 0.990 | 1.000 | 0.954 | 0.970 | 1.000 | 0.854 | 1.000 | 0.981 | 0.860 | 0.832 |
| 985 | 0.769 | 1.000 | 0.830 | 1.000 | 0.974 | 0.960 | 0.894 | 1.000 | 0.990 | 1.000 | 0.951 | 0.939 | 1.000 | 0.858 | 1.000 | 1.000 | 0.858 | 0.830 |
| 986 | 0.777 | 1.000 | 0.816 | 1.000 | 0.968 | 0.960 | 0.898 | 1.000 | 0.990 | 1.000 | 0.948 | 0.939 | 1.000 | 0.853 | 1.000 | 0.981 | 0.858 | 0.830 |
| 987 | 0.777 | 1.000 | 0.822 | 1.000 | 0.974 | 0.960 | 0.894 | 1.000 | 0.990 | 1.000 | 0.954 | 0.909 | 1.000 | 0.855 | 1.000 | 0.981 | 0.859 | 0.831 |
| 988 | 0.784 | 1.000 | 0.806 | 1.000 | 0.963 | 0.960 | 0.900 | 1.000 | 0.990 | 1.000 | 0.948 | 0.970 | 1.000 | 0.855 | 1.000 | 0.981 | 0.860 | 0.832 |
| 989 | 0.784 | 1.000 | 0.828 | 1.000 | 0.968 | 0.960 | 0.897 | 1.000 | 0.990 | 1.000 | 0.954 | 0.970 | 1.000 | 0.861 | 1.000 | 0.981 | 0.864 | 0.836 |
| 990 | 0.782 | 1.000 | 0.816 | 1.000 | 0.963 | 0.960 | 0.897 | 1.000 | 0.990 | 1.000 | 0.942 | 0.970 | 1.000 | 0.866 | 1.000 | 0.981 | 0.862 | 0.834 |
| 991 | 0.776 | 1.000 | 0.822 | 1.000 | 0.974 | 0.960 | 0.906 | 1.000 | 0.990 | 1.000 | 0.945 | 0.909 | 1.000 | 0.855 | 1.000 | 0.981 | 0.860 | 0.832 |
| 992 | 0.773 | 1.000 | 0.810 | 1.000 | 0.974 | 0.960 | 0.895 | 1.000 | 0.990 | 1.000 | 0.957 | 0.939 | 1.000 | 0.852 | 1.000 | 0.981 | 0.856 | 0.828 |
| 993 | 0.780 | 1.000 | 0.828 | 1.000 | 0.968 | 0.960 | 0.886 | 1.000 | 0.990 | 1.000 | 0.948 | 0.939 | 1.000 | 0.860 | 1.000 | 0.981 | 0.860 | 0.832 |
| 994 | 0.777 | 1.000 | 0.804 | 1.000 | 0.968 | 0.960 | 0.891 | 1.000 | 0.990 | 1.000 | 0.951 | 0.970 | 1.000 | 0.855 | 1.000 | 1.000 | 0.857 | 0.828 |
| 995 | 0.777 | 1.000 | 0.804 | 1.000 | 0.974 | 0.960 | 0.898 | 1.000 | 0.990 | 0.979 | 0.959 | 0.939 | 1.000 | 0.861 | 1.000 | 0.981 | 0.860 | 0.831 |
| 996 | 0.784 | 1.000 | 0.822 | 1.000 | 0.974 | 0.960 | 0.897 | 1.000 | 0.990 | 1.000 | 0.954 | 0.970 | 1.000 | 0.854 | 1.000 | 0.981 | 0.862 | 0.834 |
| 997 | 0.772 | 1.000 | 0.792 | 1.000 | 0.968 | 0.960 | 0.888 | 1.000 | 0.990 | 1.000 | 0.951 | 0.970 | 1.000 | 0.859 | 1.000 | 0.981 | 0.855 | 0.825 |
| 998 | 0.779 | 1.000 | 0.806 | 1.000 | 0.974 | 0.960 | 0.908 | 1.000 | 0.980 | 1.000 | 0.962 | 0.939 | 1.000 | 0.857 | 1.000 | 0.981 | 0.861 | 0.833 |
| 999 | 0.775 | 1.000 | 0.818 | 1.000 | 0.979 | 0.960 | 0.894 | 1.000 | 0.990 | 1.000 | 0.957 | 0.970 | 1.000 | 0.857 | 1.000 | 0.981 | 0.859 | 0.831 |
| 1000 | 0.771 | 1.000 | 0.806 | 1.000 | 0.974 | 0.960 | 0.884 | 1.000 | 0.990 | 1.000 | 0.954 | 0.939 | 1.000 | 0.858 | 1.000 | 1.000 | 0.855 | 0.827 |
| 1001 | 0.781 | 1.000 | 0.816 | 1.000 | 0.979 | 0.960 | 0.894 | 1.000 | 0.990 | 1.000 | 0.954 | 0.970 | 1.000 | 0.854 | 1.000 | 0.981 | 0.860 | 0.832 |
| 1002 | 0.775 | 1.000 | 0.832 | 1.000 | 0.968 | 0.960 | 0.903 | 1.000 | 0.990 | 1.000 | 0.957 | 0.879 | 1.000 | 0.850 | 1.000 | 0.963 | 0.859 | 0.831 |
| 1003 | 0.783 | 1.000 | 0.814 | 1.000 | 0.974 | 0.960 | 0.901 | 1.000 | 0.990 | 1.000 | 0.957 | 0.970 | 1.000 | 0.854 | 1.000 | 1.000 | 0.861 | 0.834 |
| 1004 | 0.779 | 1.000 | 0.816 | 1.000 | 0.968 | 0.960 | 0.894 | 1.000 | 0.990 | 1.000 | 0.957 | 0.970 | 1.000 | 0.851 | 1.000 | 0.981 | 0.858 | 0.830 |
| 1005 | 0.767 | 1.000 | 0.814 | 1.000 | 0.974 | 0.960 | 0.894 | 1.000 | 0.990 | 1.000 | 0.954 | 0.970 | 1.000 | 0.848 | 1.000 | 0.981 | 0.854 | 0.825 |
| 1006 | 0.780 | 1.000 | 0.814 | 1.000 | 0.968 | 0.960 | 0.898 | 1.000 | 0.990 | 1.000 | 0.957 | 0.909 | 1.000 | 0.858 | 1.000 | 0.981 | 0.860 | 0.832 |
| 1007 | 0.782 | 1.000 | 0.818 | 1.000 | 0.979 | 0.960 | 0.892 | 1.000 | 0.980 | 1.000 | 0.957 | 0.970 | 1.000 | 0.860 | 1.000 | 0.981 | 0.862 | 0.834 |
| 1008 | 0.776 | 1.000 | 0.838 | 1.000 | 0.968 | 0.960 | 0.900 | 1.000 | 0.990 | 1.000 | 0.954 | 0.939 | 1.000 | 0.861 | 1.000 | 1.000 | 0.863 | 0.835 |
| 1009 | 0.789 | 1.000 | 0.810 | 1.000 | 0.974 | 0.960 | 0.894 | 1.000 | 0.990 | 1.000 | 0.959 | 0.939 | 1.000 | 0.857 | 1.000 | 0.981 | 0.862 | 0.834 |
| 1010 | 0.781 | 1.000 | 0.808 | 1.000 | 0.968 | 0.960 | 0.903 | 1.000 | 0.990 | 1.000 | 0.959 | 0.939 | 1.000 | 0.856 | 1.000 | 0.981 | 0.861 | 0.833 |
| 1011 | 0.785 | 1.000 | 0.822 | 1.000 | 0.974 | 0.960 | 0.891 | 1.000 | 0.990 | 1.000 | 0.945 | 0.909 | 1.000 | 0.853 | 1.000 | 0.981 | 0.860 | 0.832 |
| 1012 | 0.785 | 1.000 | 0.826 | 1.000 | 0.974 | 0.960 | 0.894 | 1.000 | 0.980 | 1.000 | 0.948 | 0.970 | 1.000 | 0.853 | 1.000 | 0.981 | 0.861 | 0.833 |

| | | | | | | | | | | | | | | | | | |
|---|---|---|---|---|---|---|---|---|---|---|---|---|---|---|---|---|---|
| 1013 | 0.775 | 1.000 | 0.812 | 1.000 | 0.968 | 0.960 | 0.891 | 1.000 | 0.990 | 1.000 | 0.959 | 1.000 | 1.000 | 0.859 | 1.000 | 1.000 | 0.859 | 0.831 |
| 1014 | 0.779 | 1.000 | 0.816 | 1.000 | 0.974 | 0.960 | 0.892 | 1.000 | 0.990 | 1.000 | 0.954 | 0.939 | 1.000 | 0.861 | 1.000 | 0.981 | 0.860 | 0.832 |
| 1015 | 0.788 | 1.000 | 0.814 | 1.000 | 0.974 | 0.960 | 0.897 | 1.000 | 0.990 | 1.000 | 0.957 | 0.879 | 1.000 | 0.860 | 1.000 | 0.981 | 0.863 | 0.835 |
| 1016 | 0.779 | 1.000 | 0.802 | 1.000 | 0.968 | 0.960 | 0.897 | 1.000 | 0.990 | 1.000 | 0.951 | 0.970 | 1.000 | 0.855 | 1.000 | 1.000 | 0.858 | 0.830 |
| 1017 | 0.775 | 1.000 | 0.816 | 1.000 | 0.968 | 0.960 | 0.901 | 1.000 | 0.990 | 1.000 | 0.957 | 0.970 | 1.000 | 0.853 | 1.000 | 0.981 | 0.858 | 0.830 |
| 1018 | 0.780 | 1.000 | 0.818 | 1.000 | 0.968 | 0.960 | 0.898 | 1.000 | 0.980 | 1.000 | 0.948 | 0.970 | 1.000 | 0.849 | 1.000 | 0.981 | 0.858 | 0.830 |
| 1019 | 0.785 | 1.000 | 0.808 | 1.000 | 0.968 | 0.960 | 0.891 | 1.000 | 0.990 | 1.000 | 0.951 | 0.939 | 1.000 | 0.856 | 1.000 | 0.981 | 0.859 | 0.831 |
| 1020 | 0.776 | 1.000 | 0.812 | 1.000 | 0.974 | 0.960 | 0.898 | 1.000 | 0.980 | 1.000 | 0.954 | 0.970 | 1.000 | 0.861 | 1.000 | 0.981 | 0.860 | 0.832 |
| 1021 | 0.780 | 1.000 | 0.820 | 1.000 | 0.968 | 0.960 | 0.892 | 1.000 | 0.990 | 1.000 | 0.945 | 0.970 | 1.000 | 0.854 | 1.000 | 0.981 | 0.859 | 0.831 |
| 1022 | 0.775 | 1.000 | 0.806 | 1.000 | 0.974 | 0.960 | 0.895 | 1.000 | 0.990 | 1.000 | 0.959 | 0.970 | 1.000 | 0.863 | 1.000 | 0.981 | 0.860 | 0.832 |
| 1023 | 0.781 | 1.000 | 0.812 | 1.000 | 0.963 | 0.960 | 0.892 | 1.000 | 0.990 | 1.000 | 0.948 | 0.939 | 1.000 | 0.858 | 1.000 | 0.981 | 0.859 | 0.831 |
| 1024 | 0.775 | 1.000 | 0.814 | 1.000 | 0.963 | 0.960 | 0.891 | 1.000 | 0.990 | 1.000 | 0.954 | 0.939 | 1.000 | 0.859 | 1.000 | 0.981 | 0.858 | 0.830 |
| 1025 | 0.783 | 1.000 | 0.812 | 1.000 | 0.968 | 0.960 | 0.892 | 1.000 | 0.990 | 1.000 | 0.959 | 0.970 | 1.000 | 0.858 | 1.000 | 0.981 | 0.861 | 0.833 |
| 1026 | 0.785 | 1.000 | 0.810 | 1.000 | 0.974 | 0.960 | 0.901 | 1.000 | 0.990 | 1.000 | 0.951 | 0.970 | 1.000 | 0.854 | 1.000 | 0.981 | 0.861 | 0.833 |
| 1027 | 0.787 | 1.000 | 0.810 | 1.000 | 0.963 | 0.960 | 0.894 | 1.000 | 0.990 | 1.000 | 0.948 | 0.909 | 1.000 | 0.858 | 1.000 | 0.981 | 0.861 | 0.833 |
| 1028 | 0.775 | 1.000 | 0.806 | 1.000 | 0.968 | 0.960 | 0.898 | 1.000 | 0.990 | 1.000 | 0.948 | 0.939 | 1.000 | 0.853 | 1.000 | 1.000 | 0.856 | 0.828 |
| 1029 | 0.783 | 1.000 | 0.812 | 1.000 | 0.963 | 0.960 | 0.897 | 1.000 | 0.980 | 1.000 | 0.951 | 0.970 | 1.000 | 0.848 | 1.000 | 0.981 | 0.858 | 0.829 |
| 1030 | 0.766 | 1.000 | 0.810 | 1.000 | 0.963 | 1.000 | 0.892 | 1.000 | 0.980 | 1.000 | 0.962 | 0.970 | 1.000 | 0.858 | 1.000 | 0.981 | 0.855 | 0.827 |
| 1031 | 0.769 | 1.000 | 0.794 | 1.000 | 0.968 | 0.960 | 0.897 | 1.000 | 0.990 | 1.000 | 0.959 | 0.939 | 1.000 | 0.858 | 1.000 | 0.981 | 0.855 | 0.826 |
| 1032 | 0.765 | 1.000 | 0.804 | 1.000 | 0.968 | 0.960 | 0.900 | 1.000 | 0.990 | 1.000 | 0.948 | 0.939 | 1.000 | 0.860 | 1.000 | 0.981 | 0.855 | 0.826 |
| 1033 | 0.781 | 1.000 | 0.806 | 1.000 | 0.974 | 0.960 | 0.888 | 1.000 | 0.990 | 1.000 | 0.957 | 0.909 | 1.000 | 0.860 | 1.000 | 0.981 | 0.859 | 0.831 |
| 1034 | 0.771 | 1.000 | 0.812 | 1.000 | 0.974 | 0.960 | 0.901 | 1.000 | 0.990 | 1.000 | 0.959 | 0.909 | 1.000 | 0.851 | 1.000 | 1.000 | 0.857 | 0.829 |
| 1035 | 0.783 | 1.000 | 0.820 | 1.000 | 0.968 | 0.960 | 0.894 | 1.000 | 0.990 | 1.000 | 0.954 | 0.939 | 1.000 | 0.855 | 1.000 | 0.981 | 0.860 | 0.832 |
| 1036 | 0.778 | 1.000 | 0.810 | 1.000 | 0.974 | 0.960 | 0.900 | 1.000 | 0.990 | 1.000 | 0.957 | 1.000 | 1.000 | 0.858 | 1.000 | 1.000 | 0.860 | 0.832 |
| 1037 | 0.778 | 1.000 | 0.800 | 1.000 | 0.968 | 0.960 | 0.895 | 1.000 | 0.980 | 1.000 | 0.945 | 0.970 | 1.000 | 0.851 | 1.000 | 1.000 | 0.856 | 0.827 |
| 1038 | 0.781 | 1.000 | 0.814 | 1.000 | 0.968 | 0.960 | 0.889 | 1.000 | 0.990 | 1.000 | 0.951 | 0.970 | 1.000 | 0.847 | 1.000 | 0.981 | 0.857 | 0.828 |
| 1039 | 0.782 | 1.000 | 0.818 | 1.000 | 0.968 | 0.960 | 0.883 | 1.000 | 0.990 | 1.000 | 0.954 | 0.970 | 1.000 | 0.858 | 1.000 | 0.981 | 0.860 | 0.831 |
| 1040 | 0.779 | 1.000 | 0.820 | 1.000 | 0.968 | 0.960 | 0.900 | 1.000 | 0.990 | 1.000 | 0.948 | 0.970 | 0.991 | 0.862 | 1.000 | 0.981 | 0.861 | 0.834 |
| 1041 | 0.781 | 1.000 | 0.800 | 1.000 | 0.968 | 0.960 | 0.901 | 1.000 | 0.990 | 1.000 | 0.942 | 0.939 | 1.000 | 0.859 | 1.000 | 0.981 | 0.859 | 0.831 |
| 1042 | 0.776 | 1.000 | 0.812 | 1.000 | 0.963 | 0.960 | 0.892 | 1.000 | 0.990 | 1.000 | 0.957 | 1.000 | 1.000 | 0.855 | 1.000 | 0.981 | 0.858 | 0.829 |
| 1043 | 0.787 | 1.000 | 0.818 | 1.000 | 0.963 | 0.960 | 0.897 | 1.000 | 0.990 | 1.000 | 0.951 | 0.909 | 1.000 | 0.857 | 1.000 | 0.981 | 0.862 | 0.834 |
| 1044 | 0.779 | 1.000 | 0.824 | 1.000 | 0.968 | 0.960 | 0.904 | 1.000 | 0.990 | 1.000 | 0.954 | 0.939 | 1.000 | 0.854 | 1.000 | 0.981 | 0.861 | 0.833 |
| 1045 | 0.781 | 1.000 | 0.814 | 1.000 | 0.963 | 0.960 | 0.900 | 1.000 | 0.990 | 1.000 | 0.957 | 0.939 | 1.000 | 0.861 | 1.000 | 0.981 | 0.862 | 0.834 |
| 1046 | 0.785 | 1.000 | 0.808 | 1.000 | 0.963 | 0.960 | 0.901 | 1.000 | 0.990 | 1.000 | 0.951 | 0.939 | 1.000 | 0.851 | 1.000 | 0.981 | 0.859 | 0.831 |
| 1047 | 0.777 | 1.000 | 0.814 | 1.000 | 0.968 | 0.960 | 0.898 | 1.000 | 0.990 | 1.000 | 0.948 | 0.970 | 1.000 | 0.851 | 1.000 | 0.981 | 0.858 | 0.829 |
| 1048 | 0.780 | 1.000 | 0.804 | 1.000 | 0.968 | 0.960 | 0.904 | 1.000 | 0.990 | 1.000 | 0.948 | 0.970 | 1.000 | 0.859 | 1.000 | 1.000 | 0.860 | 0.832 |
| 1049 | 0.771 | 1.000 | 0.814 | 1.000 | 0.968 | 0.960 | 0.889 | 1.000 | 0.990 | 1.000 | 0.951 | 0.970 | 1.000 | 0.852 | 1.000 | 0.981 | 0.855 | 0.826 |
| 1050 | 0.775 | 1.000 | 0.812 | 1.000 | 0.963 | 0.960 | 0.886 | 1.000 | 0.990 | 1.000 | 0.951 | 0.939 | 1.000 | 0.855 | 1.000 | 0.981 | 0.856 | 0.827 |
| 1051 | 0.783 | 1.000 | 0.808 | 1.000 | 0.974 | 0.960 | 0.903 | 1.000 | 0.990 | 1.000 | 0.945 | 0.939 | 1.000 | 0.856 | 1.000 | 1.000 | 0.860 | 0.832 |

| | | | | | | | | | | | | | | | | | | |
|---|---|---|---|---|---|---|---|---|---|---|---|---|---|---|---|---|---|---|
| 1052 | 0.780 | 1.000 | 0.810 | 1.000 | 0.968 | 0.960 | 0.881 | 1.000 | 0.990 | 1.000 | 0.945 | 0.939 | 1.000 | 0.850 | 1.000 | 0.981 | 0.855 | 0.826 |
| 1053 | 0.765 | 1.000 | 0.814 | 1.000 | 0.968 | 0.960 | 0.898 | 1.000 | 0.990 | 1.000 | 0.945 | 0.939 | 1.000 | 0.862 | 1.000 | 0.981 | 0.856 | 0.828 |
| 1054 | 0.778 | 1.000 | 0.808 | 1.000 | 0.974 | 0.960 | 0.888 | 1.000 | 0.990 | 1.000 | 0.951 | 0.970 | 1.000 | 0.859 | 1.000 | 0.981 | 0.858 | 0.830 |
| 1055 | 0.781 | 1.000 | 0.820 | 1.000 | 0.979 | 0.960 | 0.897 | 1.000 | 0.990 | 1.000 | 0.954 | 0.970 | 1.000 | 0.860 | 1.000 | 0.981 | 0.862 | 0.834 |
| 1056 | 0.773 | 1.000 | 0.812 | 1.000 | 0.968 | 0.960 | 0.903 | 1.000 | 0.990 | 1.000 | 0.951 | 0.939 | 1.000 | 0.854 | 1.000 | 0.981 | 0.857 | 0.829 |
| 1057 | 0.775 | 1.000 | 0.800 | 1.000 | 0.968 | 0.960 | 0.908 | 1.000 | 0.980 | 1.000 | 0.954 | 0.939 | 1.000 | 0.858 | 1.000 | 1.000 | 0.858 | 0.830 |
| 1058 | 0.776 | 1.000 | 0.812 | 1.000 | 0.974 | 0.960 | 0.894 | 1.000 | 0.990 | 1.000 | 0.951 | 0.970 | 1.000 | 0.863 | 1.000 | 0.981 | 0.860 | 0.832 |
| 1059 | 0.780 | 1.000 | 0.798 | 1.000 | 0.968 | 0.960 | 0.884 | 1.000 | 0.990 | 1.000 | 0.954 | 0.939 | 1.000 | 0.858 | 1.000 | 1.000 | 0.857 | 0.828 |
| 1060 | 0.775 | 1.000 | 0.826 | 1.000 | 0.968 | 0.960 | 0.908 | 1.000 | 0.990 | 1.000 | 0.945 | 0.970 | 1.000 | 0.849 | 1.000 | 0.981 | 0.858 | 0.831 |
| 1061 | 0.776 | 1.000 | 0.822 | 1.000 | 0.968 | 0.960 | 0.900 | 1.000 | 0.990 | 1.000 | 0.954 | 0.970 | 1.000 | 0.848 | 1.000 | 1.000 | 0.858 | 0.830 |
| 1062 | 0.773 | 1.000 | 0.828 | 1.000 | 0.968 | 0.960 | 0.897 | 1.000 | 0.990 | 1.000 | 0.954 | 1.000 | 1.000 | 0.861 | 1.000 | 0.981 | 0.861 | 0.833 |
| 1063 | 0.771 | 1.000 | 0.812 | 1.000 | 0.968 | 0.960 | 0.894 | 1.000 | 0.990 | 1.000 | 0.948 | 0.939 | 1.000 | 0.851 | 1.000 | 0.981 | 0.855 | 0.825 |
| 1064 | 0.778 | 1.000 | 0.798 | 1.000 | 0.968 | 0.960 | 0.892 | 1.000 | 0.990 | 1.000 | 0.957 | 0.939 | 1.000 | 0.856 | 1.000 | 0.981 | 0.857 | 0.828 |
| 1065 | 0.773 | 1.000 | 0.820 | 1.000 | 0.968 | 0.960 | 0.892 | 1.000 | 0.990 | 1.000 | 0.957 | 0.939 | 1.000 | 0.859 | 1.000 | 1.000 | 0.859 | 0.831 |
| 1066 | 0.779 | 1.000 | 0.804 | 1.000 | 0.974 | 0.960 | 0.895 | 1.000 | 0.980 | 1.000 | 0.945 | 0.939 | 1.000 | 0.851 | 1.000 | 1.000 | 0.856 | 0.827 |
| 1067 | 0.775 | 1.000 | 0.822 | 1.000 | 0.968 | 0.960 | 0.889 | 1.000 | 0.990 | 1.000 | 0.948 | 0.970 | 1.000 | 0.863 | 1.000 | 0.981 | 0.860 | 0.831 |
| 1068 | 0.781 | 1.000 | 0.836 | 1.000 | 0.968 | 0.960 | 0.903 | 1.000 | 0.990 | 1.000 | 0.951 | 0.939 | 1.000 | 0.858 | 1.000 | 0.981 | 0.863 | 0.836 |
| 1069 | 0.776 | 1.000 | 0.822 | 1.000 | 0.968 | 0.960 | 0.892 | 1.000 | 0.990 | 1.000 | 0.954 | 0.970 | 1.000 | 0.851 | 1.000 | 0.981 | 0.858 | 0.829 |
| 1070 | 0.777 | 1.000 | 0.824 | 1.000 | 0.968 | 0.960 | 0.886 | 1.000 | 0.980 | 1.000 | 0.954 | 0.970 | 1.000 | 0.852 | 1.000 | 0.981 | 0.857 | 0.829 |
| 1071 | 0.773 | 1.000 | 0.826 | 1.000 | 0.968 | 0.960 | 0.891 | 1.000 | 0.990 | 1.000 | 0.942 | 0.970 | 1.000 | 0.859 | 1.000 | 0.981 | 0.858 | 0.830 |
| 1072 | 0.781 | 1.000 | 0.836 | 1.000 | 0.968 | 0.960 | 0.898 | 1.000 | 0.990 | 1.000 | 0.942 | 0.909 | 1.000 | 0.860 | 1.000 | 0.981 | 0.862 | 0.835 |
| 1073 | 0.777 | 1.000 | 0.810 | 1.000 | 0.968 | 0.960 | 0.892 | 1.000 | 0.990 | 1.000 | 0.957 | 0.939 | 1.000 | 0.855 | 1.000 | 0.981 | 0.858 | 0.829 |
| 1074 | 0.776 | 1.000 | 0.826 | 1.000 | 0.968 | 0.960 | 0.895 | 1.000 | 0.990 | 1.000 | 0.957 | 0.909 | 1.000 | 0.857 | 1.000 | 0.981 | 0.860 | 0.832 |
| 1075 | 0.777 | 1.000 | 0.822 | 1.000 | 0.968 | 0.960 | 0.894 | 1.000 | 0.990 | 1.000 | 0.957 | 0.970 | 1.000 | 0.862 | 1.000 | 0.981 | 0.861 | 0.834 |
| 1076 | 0.788 | 1.000 | 0.816 | 1.000 | 0.974 | 0.960 | 0.898 | 1.000 | 0.990 | 1.000 | 0.954 | 0.970 | 1.000 | 0.851 | 1.000 | 0.981 | 0.861 | 0.834 |
| 1077 | 0.786 | 1.000 | 0.826 | 1.000 | 0.968 | 0.960 | 0.895 | 1.000 | 0.990 | 1.000 | 0.951 | 0.909 | 1.000 | 0.853 | 1.000 | 0.981 | 0.861 | 0.833 |
| 1078 | 0.773 | 1.000 | 0.808 | 1.000 | 0.974 | 0.960 | 0.894 | 1.000 | 0.990 | 1.000 | 0.948 | 0.909 | 1.000 | 0.864 | 1.000 | 0.981 | 0.858 | 0.830 |
| 1079 | 0.773 | 1.000 | 0.822 | 1.000 | 0.974 | 0.960 | 0.898 | 1.000 | 0.990 | 1.000 | 0.948 | 0.939 | 1.000 | 0.858 | 1.000 | 0.981 | 0.859 | 0.831 |
| 1080 | 0.779 | 1.000 | 0.834 | 1.000 | 0.974 | 0.960 | 0.889 | 1.000 | 0.990 | 1.000 | 0.957 | 0.909 | 1.000 | 0.860 | 1.000 | 0.981 | 0.862 | 0.834 |
| 1081 | 0.767 | 1.000 | 0.810 | 1.000 | 0.968 | 0.960 | 0.888 | 1.000 | 0.990 | 1.000 | 0.954 | 1.000 | 1.000 | 0.854 | 1.000 | 1.000 | 0.854 | 0.826 |
| 1082 | 0.790 | 1.000 | 0.810 | 1.000 | 0.974 | 0.960 | 0.903 | 1.000 | 0.990 | 1.000 | 0.948 | 0.970 | 1.000 | 0.852 | 1.000 | 0.981 | 0.862 | 0.834 |
| 1083 | 0.779 | 1.000 | 0.830 | 1.000 | 0.968 | 0.960 | 0.914 | 1.000 | 0.990 | 1.000 | 0.951 | 0.970 | 1.000 | 0.858 | 1.000 | 0.981 | 0.864 | 0.837 |
| 1084 | 0.775 | 1.000 | 0.818 | 1.000 | 0.968 | 0.960 | 0.892 | 1.000 | 0.990 | 1.000 | 0.954 | 0.970 | 1.000 | 0.861 | 1.000 | 0.963 | 0.859 | 0.831 |
| 1085 | 0.775 | 1.000 | 0.812 | 1.000 | 0.963 | 0.960 | 0.891 | 1.000 | 0.980 | 1.000 | 0.954 | 0.970 | 1.000 | 0.854 | 1.000 | 1.000 | 0.857 | 0.828 |
| 1086 | 0.767 | 1.000 | 0.826 | 1.000 | 0.968 | 0.960 | 0.894 | 1.000 | 0.990 | 1.000 | 0.962 | 1.000 | 1.000 | 0.860 | 1.000 | 1.000 | 0.859 | 0.831 |
| 1087 | 0.767 | 1.000 | 0.820 | 1.000 | 0.968 | 0.960 | 0.894 | 1.000 | 0.980 | 1.000 | 0.959 | 0.970 | 1.000 | 0.861 | 1.000 | 0.981 | 0.857 | 0.829 |
| 1088 | 0.780 | 1.000 | 0.816 | 1.000 | 0.968 | 0.960 | 0.901 | 1.000 | 0.980 | 1.000 | 0.951 | 0.939 | 1.000 | 0.854 | 1.000 | 0.981 | 0.859 | 0.831 |
| 1089 | 0.772 | 1.000 | 0.826 | 1.000 | 0.974 | 0.960 | 0.894 | 1.000 | 0.990 | 1.000 | 0.957 | 0.939 | 1.000 | 0.860 | 1.000 | 0.981 | 0.859 | 0.831 |
| 1090 | 0.781 | 1.000 | 0.806 | 1.000 | 0.968 | 0.960 | 0.900 | 1.000 | 0.990 | 1.000 | 0.945 | 0.939 | 1.000 | 0.857 | 1.000 | 0.981 | 0.859 | 0.831 |

| | | | | | | | | | | | | | | | | | | |
|---|---|---|---|---|---|---|---|---|---|---|---|---|---|---|---|---|---|---|
| 1091 | 0.785 | 1.000 | 0.810 | 1.000 | 0.968 | 0.960 | 0.891 | 1.000 | 0.990 | 1.000 | 0.951 | 0.970 | 1.000 | 0.854 | 1.000 | 0.981 | 0.859 | 0.831 |
| 1092 | 0.776 | 1.000 | 0.824 | 1.000 | 0.974 | 0.960 | 0.898 | 1.000 | 0.990 | 1.000 | 0.954 | 0.970 | 1.000 | 0.856 | 1.000 | 1.000 | 0.860 | 0.833 |
| 1093 | 0.767 | 1.000 | 0.828 | 1.000 | 0.963 | 0.960 | 0.900 | 1.000 | 0.990 | 1.000 | 0.957 | 0.939 | 1.000 | 0.856 | 1.000 | 1.000 | 0.858 | 0.830 |
| 1094 | 0.781 | 1.000 | 0.840 | 1.000 | 0.968 | 0.960 | 0.881 | 1.000 | 0.990 | 1.000 | 0.954 | 0.970 | 1.000 | 0.860 | 1.000 | 0.981 | 0.862 | 0.834 |
| 1095 | 0.783 | 1.000 | 0.832 | 1.000 | 0.974 | 0.960 | 0.889 | 1.000 | 0.990 | 1.000 | 0.954 | 0.970 | 1.000 | 0.855 | 1.000 | 1.000 | 0.862 | 0.834 |
| 1096 | 0.777 | 1.000 | 0.832 | 1.000 | 0.974 | 0.960 | 0.900 | 1.000 | 0.990 | 1.000 | 0.954 | 0.909 | 1.000 | 0.860 | 1.000 | 1.000 | 0.862 | 0.835 |
| 1097 | 0.783 | 1.000 | 0.826 | 1.000 | 0.968 | 1.000 | 0.895 | 1.000 | 0.980 | 1.000 | 0.954 | 0.970 | 1.000 | 0.854 | 1.000 | 0.981 | 0.861 | 0.833 |
| 1098 | 0.777 | 1.000 | 0.830 | 1.000 | 0.968 | 0.960 | 0.897 | 1.000 | 0.990 | 1.000 | 0.951 | 1.000 | 1.000 | 0.855 | 1.000 | 0.981 | 0.860 | 0.832 |
| 1099 | 0.772 | 1.000 | 0.818 | 1.000 | 0.968 | 0.960 | 0.903 | 1.000 | 0.990 | 1.000 | 0.954 | 0.970 | 1.000 | 0.857 | 1.000 | 0.981 | 0.859 | 0.831 |
| 1100 | 0.774 | 1.000 | 0.826 | 1.000 | 0.968 | 0.960 | 0.897 | 1.000 | 0.990 | 1.000 | 0.942 | 0.939 | 1.000 | 0.857 | 1.000 | 0.981 | 0.858 | 0.830 |
| 1101 | 0.777 | 1.000 | 0.828 | 1.000 | 0.968 | 0.960 | 0.903 | 1.000 | 0.990 | 1.000 | 0.951 | 0.970 | 1.000 | 0.854 | 1.000 | 0.981 | 0.860 | 0.833 |
| 1102 | 0.775 | 1.000 | 0.816 | 1.000 | 0.968 | 0.960 | 0.894 | 1.000 | 0.990 | 1.000 | 0.951 | 0.970 | 1.000 | 0.850 | 1.000 | 1.000 | 0.857 | 0.828 |
| 1103 | 0.775 | 1.000 | 0.832 | 1.000 | 0.968 | 0.960 | 0.900 | 1.000 | 0.990 | 1.000 | 0.951 | 0.939 | 1.000 | 0.854 | 1.000 | 0.981 | 0.859 | 0.832 |
| 1104 | 0.767 | 1.000 | 0.824 | 1.000 | 0.968 | 0.960 | 0.895 | 1.000 | 0.980 | 1.000 | 0.948 | 0.939 | 1.000 | 0.849 | 1.000 | 0.981 | 0.854 | 0.825 |
| 1105 | 0.775 | 1.000 | 0.830 | 1.000 | 0.968 | 0.960 | 0.888 | 1.000 | 0.990 | 1.000 | 0.948 | 0.939 | 1.000 | 0.859 | 1.000 | 1.000 | 0.859 | 0.831 |
| 1106 | 0.779 | 1.000 | 0.830 | 1.000 | 0.968 | 0.960 | 0.886 | 1.000 | 0.990 | 1.000 | 0.957 | 0.939 | 1.000 | 0.855 | 1.000 | 1.000 | 0.860 | 0.832 |
| 1107 | 0.779 | 1.000 | 0.820 | 1.000 | 0.968 | 0.960 | 0.894 | 1.000 | 0.990 | 1.000 | 0.954 | 0.939 | 1.000 | 0.861 | 1.000 | 0.981 | 0.861 | 0.833 |
| 1108 | 0.786 | 1.000 | 0.836 | 1.000 | 0.968 | 0.960 | 0.895 | 1.000 | 0.980 | 1.000 | 0.945 | 0.970 | 1.000 | 0.858 | 1.000 | 0.981 | 0.863 | 0.836 |
| 1109 | 0.775 | 1.000 | 0.826 | 1.000 | 0.968 | 0.960 | 0.892 | 1.000 | 0.980 | 1.000 | 0.951 | 0.909 | 1.000 | 0.851 | 1.000 | 0.981 | 0.857 | 0.829 |
| 1110 | 0.773 | 1.000 | 0.836 | 1.000 | 0.974 | 0.960 | 0.894 | 1.000 | 0.990 | 1.000 | 0.954 | 0.939 | 1.000 | 0.859 | 1.000 | 0.981 | 0.860 | 0.833 |
| 1111 | 0.775 | 1.000 | 0.836 | 1.000 | 0.968 | 0.960 | 0.895 | 1.000 | 0.990 | 1.000 | 0.957 | 0.970 | 1.000 | 0.853 | 1.000 | 0.981 | 0.860 | 0.832 |
| 1112 | 0.777 | 1.000 | 0.842 | 1.000 | 0.968 | 0.960 | 0.892 | 1.000 | 0.990 | 1.000 | 0.954 | 0.909 | 1.000 | 0.856 | 1.000 | 1.000 | 0.861 | 0.833 |
| 1113 | 0.777 | 1.000 | 0.832 | 1.000 | 0.968 | 0.960 | 0.891 | 1.000 | 0.990 | 1.000 | 0.951 | 0.939 | 1.000 | 0.859 | 1.000 | 0.981 | 0.860 | 0.833 |
| 1114 | 0.767 | 1.000 | 0.808 | 1.000 | 0.968 | 0.960 | 0.878 | 1.000 | 0.990 | 1.000 | 0.951 | 0.939 | 1.000 | 0.865 | 1.000 | 0.981 | 0.855 | 0.826 |
| 1115 | 0.767 | 1.000 | 0.822 | 1.000 | 0.968 | 0.960 | 0.897 | 1.000 | 0.990 | 1.000 | 0.951 | 0.909 | 1.000 | 0.859 | 1.000 | 0.981 | 0.857 | 0.829 |
| 1116 | 0.777 | 1.000 | 0.822 | 1.000 | 0.968 | 0.960 | 0.891 | 1.000 | 0.990 | 1.000 | 0.954 | 0.939 | 1.000 | 0.858 | 1.000 | 0.981 | 0.859 | 0.831 |
| 1117 | 0.776 | 1.000 | 0.826 | 1.000 | 0.963 | 0.960 | 0.900 | 1.000 | 0.990 | 1.000 | 0.945 | 0.909 | 1.000 | 0.850 | 1.000 | 1.000 | 0.858 | 0.829 |
| 1118 | 0.773 | 1.000 | 0.834 | 1.000 | 0.974 | 0.960 | 0.900 | 1.000 | 0.980 | 1.000 | 0.948 | 0.970 | 1.000 | 0.858 | 1.000 | 0.981 | 0.860 | 0.832 |
| 1119 | 0.777 | 1.000 | 0.822 | 1.000 | 0.968 | 0.960 | 0.900 | 1.000 | 0.990 | 1.000 | 0.959 | 0.970 | 1.000 | 0.852 | 1.000 | 0.981 | 0.860 | 0.832 |
| 1120 | 0.762 | 1.000 | 0.822 | 1.000 | 0.968 | 0.960 | 0.898 | 1.000 | 0.990 | 1.000 | 0.951 | 0.939 | 1.000 | 0.854 | 1.000 | 0.981 | 0.854 | 0.826 |
| 1121 | 0.781 | 1.000 | 0.816 | 1.000 | 0.974 | 0.960 | 0.897 | 1.000 | 0.990 | 1.000 | 0.951 | 0.939 | 1.000 | 0.857 | 1.000 | 0.981 | 0.860 | 0.832 |
| 1122 | 0.787 | 1.000 | 0.820 | 1.000 | 0.974 | 0.960 | 0.892 | 1.000 | 0.990 | 1.000 | 0.948 | 0.939 | 1.000 | 0.853 | 1.000 | 0.981 | 0.861 | 0.833 |
| 1123 | 0.775 | 1.000 | 0.834 | 1.000 | 0.968 | 0.960 | 0.901 | 1.000 | 0.990 | 1.000 | 0.945 | 0.939 | 1.000 | 0.858 | 1.000 | 1.000 | 0.861 | 0.833 |
| 1124 | 0.774 | 1.000 | 0.824 | 1.000 | 0.974 | 0.960 | 0.889 | 1.000 | 0.980 | 1.000 | 0.951 | 0.939 | 1.000 | 0.852 | 1.000 | 0.981 | 0.857 | 0.828 |
| 1125 | 0.781 | 1.000 | 0.828 | 1.000 | 0.963 | 0.960 | 0.897 | 1.000 | 0.990 | 1.000 | 0.951 | 0.939 | 1.000 | 0.851 | 1.000 | 0.981 | 0.860 | 0.832 |
| 1126 | 0.775 | 1.000 | 0.830 | 1.000 | 0.968 | 0.960 | 0.895 | 1.000 | 0.990 | 1.000 | 0.951 | 0.939 | 1.000 | 0.858 | 1.000 | 0.981 | 0.860 | 0.832 |
| 1127 | 0.783 | 1.000 | 0.824 | 1.000 | 0.974 | 0.960 | 0.892 | 1.000 | 0.990 | 1.000 | 0.948 | 0.939 | 1.000 | 0.849 | 1.000 | 0.981 | 0.859 | 0.831 |
| 1128 | 0.781 | 1.000 | 0.836 | 1.000 | 0.968 | 0.960 | 0.892 | 1.000 | 0.990 | 1.000 | 0.957 | 0.939 | 1.000 | 0.853 | 1.000 | 0.981 | 0.861 | 0.833 |
| 1129 | 0.777 | 1.000 | 0.820 | 1.000 | 0.963 | 0.960 | 0.894 | 1.000 | 0.990 | 1.000 | 0.951 | 0.939 | 1.000 | 0.849 | 1.000 | 0.981 | 0.857 | 0.828 |

| | | | | | | | | | | | | | | | | | | |
|---|---|---|---|---|---|---|---|---|---|---|---|---|---|---|---|---|---|---|
| 1130 | 0.774 | 1.000 | 0.824 | 1.000 | 0.974 | 0.960 | 0.895 | 1.000 | 0.990 | 1.000 | 0.948 | 1.000 | 1.000 | 0.854 | 1.000 | 0.981 | 0.858 | 0.830 |
| 1131 | 0.769 | 1.000 | 0.818 | 1.000 | 0.968 | 0.960 | 0.894 | 1.000 | 0.990 | 1.000 | 0.954 | 0.939 | 1.000 | 0.854 | 1.000 | 0.981 | 0.856 | 0.827 |
| 1132 | 0.775 | 1.000 | 0.820 | 1.000 | 0.963 | 0.960 | 0.895 | 1.000 | 0.990 | 1.000 | 0.957 | 0.970 | 1.000 | 0.858 | 1.000 | 1.000 | 0.859 | 0.831 |
| 1133 | 0.768 | 1.000 | 0.830 | 1.000 | 0.968 | 0.960 | 0.892 | 1.000 | 0.980 | 1.000 | 0.948 | 0.939 | 1.000 | 0.860 | 1.000 | 1.000 | 0.858 | 0.829 |
| 1134 | 0.781 | 1.000 | 0.814 | 1.000 | 0.974 | 0.960 | 0.894 | 1.000 | 0.990 | 1.000 | 0.954 | 0.939 | 1.000 | 0.855 | 1.000 | 0.981 | 0.859 | 0.831 |
| 1135 | 0.775 | 1.000 | 0.828 | 1.000 | 0.979 | 0.960 | 0.897 | 1.000 | 0.990 | 1.000 | 0.954 | 0.970 | 1.000 | 0.849 | 1.000 | 1.000 | 0.858 | 0.830 |
| 1136 | 0.773 | 1.000 | 0.834 | 1.000 | 0.974 | 0.960 | 0.891 | 1.000 | 0.990 | 1.000 | 0.948 | 0.939 | 1.000 | 0.856 | 1.000 | 1.000 | 0.859 | 0.830 |
| 1137 | 0.773 | 1.000 | 0.814 | 1.000 | 0.968 | 0.960 | 0.898 | 1.000 | 0.990 | 1.000 | 0.957 | 0.970 | 1.000 | 0.867 | 1.000 | 0.981 | 0.861 | 0.833 |
| 1138 | 0.772 | 1.000 | 0.806 | 1.000 | 0.968 | 0.960 | 0.892 | 1.000 | 0.990 | 1.000 | 0.951 | 0.939 | 1.000 | 0.854 | 1.000 | 0.981 | 0.855 | 0.826 |
| 1139 | 0.777 | 1.000 | 0.812 | 1.000 | 0.968 | 0.960 | 0.903 | 1.000 | 0.990 | 1.000 | 0.948 | 0.939 | 1.000 | 0.862 | 1.000 | 0.981 | 0.860 | 0.833 |
| 1140 | 0.778 | 1.000 | 0.820 | 1.000 | 0.968 | 1.000 | 0.894 | 1.000 | 0.980 | 1.000 | 0.951 | 0.939 | 1.000 | 0.857 | 1.000 | 0.981 | 0.859 | 0.831 |
| 1141 | 0.785 | 1.000 | 0.822 | 1.000 | 0.963 | 0.960 | 0.884 | 1.000 | 0.990 | 1.000 | 0.957 | 0.939 | 1.000 | 0.857 | 1.000 | 0.981 | 0.861 | 0.832 |
| 1142 | 0.780 | 1.000 | 0.810 | 1.000 | 0.974 | 0.960 | 0.892 | 1.000 | 0.990 | 1.000 | 0.957 | 0.970 | 1.000 | 0.854 | 1.000 | 0.981 | 0.859 | 0.830 |
| 1143 | 0.769 | 1.000 | 0.830 | 1.000 | 0.968 | 0.960 | 0.892 | 1.000 | 0.990 | 1.000 | 0.948 | 0.909 | 1.000 | 0.863 | 1.000 | 0.981 | 0.858 | 0.831 |
| 1144 | 0.776 | 1.000 | 0.816 | 1.000 | 0.974 | 0.960 | 0.892 | 1.000 | 0.990 | 1.000 | 0.951 | 0.939 | 1.000 | 0.853 | 1.000 | 1.000 | 0.857 | 0.829 |
| 1145 | 0.783 | 1.000 | 0.814 | 1.000 | 0.968 | 0.960 | 0.895 | 1.000 | 0.990 | 1.000 | 0.954 | 0.970 | 1.000 | 0.854 | 1.000 | 0.981 | 0.860 | 0.832 |
| 1146 | 0.767 | 1.000 | 0.802 | 1.000 | 0.974 | 0.960 | 0.892 | 1.000 | 0.990 | 1.000 | 0.951 | 0.939 | 1.000 | 0.854 | 1.000 | 0.981 | 0.853 | 0.824 |
| 1147 | 0.780 | 1.000 | 0.824 | 1.000 | 0.968 | 0.960 | 0.898 | 1.000 | 0.980 | 1.000 | 0.954 | 0.939 | 1.000 | 0.856 | 1.000 | 0.981 | 0.861 | 0.833 |
| 1148 | 0.777 | 1.000 | 0.804 | 1.000 | 0.974 | 0.960 | 0.892 | 1.000 | 0.990 | 1.000 | 0.951 | 0.939 | 1.000 | 0.854 | 1.000 | 1.000 | 0.857 | 0.828 |
| 1149 | 0.763 | 1.000 | 0.812 | 1.000 | 0.974 | 0.960 | 0.903 | 1.000 | 0.990 | 1.000 | 0.948 | 0.939 | 1.000 | 0.861 | 1.000 | 0.981 | 0.856 | 0.828 |
| 1150 | 0.778 | 1.000 | 0.814 | 1.000 | 0.974 | 0.960 | 0.897 | 1.000 | 0.990 | 1.000 | 0.951 | 0.939 | 1.000 | 0.861 | 1.000 | 0.981 | 0.860 | 0.832 |
| 1151 | 0.779 | 1.000 | 0.826 | 1.000 | 0.974 | 0.960 | 0.898 | 1.000 | 0.990 | 1.000 | 0.954 | 0.939 | 1.000 | 0.864 | 1.000 | 1.000 | 0.863 | 0.836 |
| 1152 | 0.780 | 1.000 | 0.816 | 1.000 | 0.968 | 0.960 | 0.886 | 1.000 | 0.990 | 1.000 | 0.951 | 0.939 | 1.000 | 0.853 | 1.000 | 0.981 | 0.857 | 0.829 |
| 1153 | 0.777 | 1.000 | 0.826 | 1.000 | 0.968 | 0.960 | 0.888 | 1.000 | 0.980 | 1.000 | 0.951 | 0.909 | 1.000 | 0.859 | 1.000 | 0.981 | 0.859 | 0.830 |
| 1154 | 0.779 | 1.000 | 0.814 | 1.000 | 0.968 | 0.960 | 0.894 | 1.000 | 0.990 | 1.000 | 0.957 | 0.970 | 1.000 | 0.858 | 1.000 | 0.981 | 0.860 | 0.832 |
| 1155 | 0.775 | 1.000 | 0.814 | 1.000 | 0.968 | 0.960 | 0.892 | 1.000 | 0.990 | 1.000 | 0.948 | 0.939 | 1.000 | 0.862 | 1.000 | 1.000 | 0.859 | 0.830 |
| 1156 | 0.773 | 1.000 | 0.816 | 1.000 | 0.968 | 0.960 | 0.892 | 1.000 | 0.990 | 1.000 | 0.945 | 0.909 | 1.000 | 0.854 | 1.000 | 1.000 | 0.856 | 0.828 |
| 1157 | 0.779 | 1.000 | 0.822 | 1.000 | 0.974 | 0.960 | 0.901 | 1.000 | 0.980 | 1.000 | 0.945 | 0.939 | 1.000 | 0.852 | 1.000 | 1.000 | 0.859 | 0.831 |
| 1158 | 0.778 | 1.000 | 0.836 | 1.000 | 0.968 | 0.960 | 0.892 | 1.000 | 0.990 | 1.000 | 0.954 | 0.939 | 1.000 | 0.854 | 1.000 | 0.981 | 0.860 | 0.832 |
| 1159 | 0.774 | 1.000 | 0.832 | 1.000 | 0.974 | 0.960 | 0.889 | 1.000 | 0.990 | 1.000 | 0.948 | 0.970 | 1.000 | 0.854 | 1.000 | 0.981 | 0.858 | 0.830 |
| 1160 | 0.781 | 1.000 | 0.798 | 1.000 | 0.968 | 0.960 | 0.891 | 1.000 | 0.990 | 1.000 | 0.954 | 0.970 | 1.000 | 0.851 | 1.000 | 0.981 | 0.856 | 0.827 |
| 1161 | 0.774 | 1.000 | 0.812 | 1.000 | 0.974 | 0.960 | 0.881 | 1.000 | 0.990 | 1.000 | 0.957 | 0.939 | 1.000 | 0.857 | 1.000 | 0.981 | 0.856 | 0.827 |
| 1162 | 0.791 | 1.000 | 0.826 | 1.000 | 0.968 | 0.960 | 0.892 | 1.000 | 0.990 | 1.000 | 0.954 | 0.970 | 1.000 | 0.851 | 1.000 | 1.000 | 0.863 | 0.835 |
| 1163 | 0.774 | 1.000 | 0.814 | 1.000 | 0.974 | 0.960 | 0.895 | 1.000 | 0.990 | 1.000 | 0.962 | 0.939 | 1.000 | 0.858 | 1.000 | 0.981 | 0.859 | 0.831 |
| 1164 | 0.773 | 1.000 | 0.826 | 1.000 | 0.968 | 0.960 | 0.889 | 1.000 | 0.990 | 1.000 | 0.945 | 0.909 | 1.000 | 0.852 | 1.000 | 0.981 | 0.856 | 0.827 |
| 1165 | 0.777 | 1.000 | 0.832 | 1.000 | 0.968 | 0.960 | 0.886 | 1.000 | 0.990 | 1.000 | 0.948 | 0.970 | 1.000 | 0.856 | 1.000 | 0.981 | 0.859 | 0.831 |
| 1166 | 0.780 | 1.000 | 0.820 | 1.000 | 0.968 | 0.960 | 0.900 | 1.000 | 0.990 | 1.000 | 0.954 | 0.939 | 1.000 | 0.857 | 1.000 | 0.981 | 0.861 | 0.833 |
| 1167 | 0.774 | 1.000 | 0.812 | 1.000 | 0.968 | 0.960 | 0.908 | 1.000 | 0.990 | 1.000 | 0.962 | 0.939 | 1.000 | 0.851 | 1.000 | 0.981 | 0.858 | 0.830 |
| 1168 | 0.776 | 1.000 | 0.842 | 1.000 | 0.974 | 0.960 | 0.903 | 1.000 | 0.980 | 1.000 | 0.959 | 0.970 | 1.000 | 0.852 | 1.000 | 1.000 | 0.862 | 0.835 |

| | | | | | | | | | | | | | | | | | |
|---|---|---|---|---|---|---|---|---|---|---|---|---|---|---|---|---|---|
| 1169 | 0.779 | 1.000 | 0.828 | 1.000 | 0.979 | 0.960 | 0.898 | 1.000 | 0.990 | 1.000 | 0.954 | 0.939 | 1.000 | 0.848 | 1.000 | 1.000 | 0.860 | 0.832 |
| 1170 | 0.773 | 1.000 | 0.814 | 0.986 | 0.979 | 0.960 | 0.886 | 1.000 | 0.990 | 1.000 | 0.962 | 0.939 | 1.000 | 0.857 | 1.000 | 0.981 | 0.857 | 0.828 |
| 1171 | 0.782 | 1.000 | 0.814 | 1.000 | 0.974 | 0.960 | 0.894 | 1.000 | 0.990 | 1.000 | 0.951 | 0.970 | 1.000 | 0.853 | 1.000 | 0.981 | 0.859 | 0.831 |
| 1172 | 0.779 | 1.000 | 0.812 | 1.000 | 0.979 | 0.960 | 0.897 | 1.000 | 0.990 | 1.000 | 0.951 | 0.939 | 1.000 | 0.855 | 1.000 | 1.000 | 0.859 | 0.831 |
| 1173 | 0.781 | 1.000 | 0.812 | 1.000 | 0.968 | 0.960 | 0.897 | 1.000 | 0.980 | 1.000 | 0.954 | 0.939 | 1.000 | 0.853 | 1.000 | 0.981 | 0.859 | 0.830 |
| 1174 | 0.771 | 1.000 | 0.814 | 1.000 | 0.968 | 0.960 | 0.895 | 1.000 | 0.980 | 1.000 | 0.954 | 0.939 | 1.000 | 0.855 | 1.000 | 1.000 | 0.857 | 0.828 |
| 1175 | 0.779 | 1.000 | 0.820 | 1.000 | 0.968 | 0.960 | 0.892 | 1.000 | 0.980 | 1.000 | 0.954 | 0.909 | 1.000 | 0.847 | 1.000 | 1.000 | 0.857 | 0.828 |
| 1176 | 0.777 | 1.000 | 0.822 | 1.000 | 0.968 | 0.960 | 0.898 | 1.000 | 0.990 | 1.000 | 0.957 | 0.939 | 1.000 | 0.855 | 1.000 | 1.000 | 0.860 | 0.832 |
| 1177 | 0.781 | 1.000 | 0.820 | 1.000 | 0.974 | 0.960 | 0.892 | 1.000 | 0.980 | 1.000 | 0.948 | 0.939 | 1.000 | 0.853 | 1.000 | 0.981 | 0.859 | 0.830 |
| 1178 | 0.782 | 1.000 | 0.818 | 1.000 | 0.974 | 0.960 | 0.892 | 1.000 | 0.990 | 1.000 | 0.954 | 0.939 | 1.000 | 0.857 | 1.000 | 1.000 | 0.861 | 0.833 |
| 1179 | 0.785 | 1.000 | 0.802 | 1.000 | 0.974 | 0.960 | 0.903 | 1.000 | 0.990 | 1.000 | 0.957 | 0.970 | 1.000 | 0.847 | 1.000 | 0.981 | 0.859 | 0.830 |
| 1180 | 0.777 | 1.000 | 0.812 | 1.000 | 0.968 | 0.960 | 0.906 | 1.000 | 0.969 | 1.000 | 0.954 | 0.909 | 1.000 | 0.862 | 1.000 | 0.981 | 0.861 | 0.833 |
| 1181 | 0.782 | 1.000 | 0.840 | 1.000 | 0.968 | 0.960 | 0.909 | 1.000 | 0.990 | 1.000 | 0.959 | 0.939 | 1.000 | 0.852 | 1.000 | 1.000 | 0.864 | 0.837 |
| 1182 | 0.766 | 1.000 | 0.826 | 1.000 | 0.968 | 0.960 | 0.895 | 1.000 | 0.980 | 1.000 | 0.954 | 0.939 | 1.000 | 0.855 | 1.000 | 0.981 | 0.856 | 0.827 |
| 1183 | 0.779 | 1.000 | 0.812 | 1.000 | 0.968 | 0.960 | 0.904 | 1.000 | 0.990 | 1.000 | 0.957 | 1.000 | 1.000 | 0.855 | 1.000 | 0.981 | 0.860 | 0.833 |
| 1184 | 0.785 | 1.000 | 0.826 | 1.000 | 0.958 | 0.960 | 0.895 | 1.000 | 0.990 | 1.000 | 0.951 | 1.000 | 1.000 | 0.861 | 1.000 | 1.000 | 0.863 | 0.836 |
| 1185 | 0.771 | 1.000 | 0.826 | 1.000 | 0.968 | 0.960 | 0.900 | 1.000 | 0.980 | 1.000 | 0.957 | 1.000 | 1.000 | 0.858 | 1.000 | 0.981 | 0.860 | 0.832 |
| 1186 | 0.772 | 1.000 | 0.830 | 1.000 | 0.968 | 0.960 | 0.904 | 1.000 | 0.980 | 1.000 | 0.957 | 0.939 | 1.000 | 0.854 | 1.000 | 0.981 | 0.859 | 0.831 |
| 1187 | 0.781 | 1.000 | 0.824 | 1.000 | 0.968 | 0.960 | 0.898 | 1.000 | 0.990 | 1.000 | 0.957 | 0.970 | 1.000 | 0.854 | 1.000 | 0.981 | 0.861 | 0.833 |
| 1188 | 0.787 | 1.000 | 0.822 | 1.000 | 0.968 | 0.960 | 0.898 | 1.000 | 0.990 | 1.000 | 0.962 | 0.939 | 1.000 | 0.851 | 1.000 | 0.981 | 0.862 | 0.834 |
| 1189 | 0.781 | 1.000 | 0.826 | 0.986 | 0.968 | 0.960 | 0.901 | 1.000 | 0.990 | 1.000 | 0.948 | 0.939 | 1.000 | 0.857 | 1.000 | 0.981 | 0.861 | 0.834 |
| 1190 | 0.773 | 1.000 | 0.822 | 1.000 | 0.968 | 0.960 | 0.892 | 1.000 | 0.990 | 1.000 | 0.954 | 0.909 | 1.000 | 0.853 | 1.000 | 0.981 | 0.857 | 0.828 |
| 1191 | 0.781 | 1.000 | 0.822 | 1.000 | 0.979 | 0.960 | 0.904 | 1.000 | 0.990 | 1.000 | 0.954 | 0.939 | 1.000 | 0.850 | 1.000 | 0.981 | 0.861 | 0.833 |
| 1192 | 0.777 | 1.000 | 0.810 | 1.000 | 0.979 | 0.960 | 0.901 | 1.000 | 0.980 | 1.000 | 0.951 | 0.909 | 1.000 | 0.861 | 1.000 | 1.000 | 0.860 | 0.832 |
| 1193 | 0.774 | 1.000 | 0.822 | 1.000 | 0.974 | 0.960 | 0.895 | 1.000 | 0.990 | 1.000 | 0.957 | 0.939 | 1.000 | 0.854 | 1.000 | 1.000 | 0.858 | 0.830 |
| 1194 | 0.771 | 1.000 | 0.816 | 1.000 | 0.968 | 0.960 | 0.912 | 1.000 | 0.980 | 1.000 | 0.957 | 0.970 | 1.000 | 0.851 | 1.000 | 0.981 | 0.858 | 0.830 |
| 1195 | 0.783 | 1.000 | 0.824 | 1.000 | 0.968 | 0.960 | 0.895 | 1.000 | 0.990 | 1.000 | 0.962 | 0.970 | 1.000 | 0.852 | 1.000 | 0.981 | 0.861 | 0.833 |
| 1196 | 0.775 | 1.000 | 0.808 | 1.000 | 0.974 | 0.960 | 0.897 | 1.000 | 0.990 | 1.000 | 0.954 | 0.970 | 1.000 | 0.857 | 1.000 | 0.981 | 0.858 | 0.829 |
| 1197 | 0.779 | 1.000 | 0.820 | 1.000 | 0.974 | 0.960 | 0.901 | 1.000 | 0.990 | 1.000 | 0.954 | 1.000 | 1.000 | 0.854 | 1.000 | 0.981 | 0.860 | 0.833 |
| 1198 | 0.771 | 1.000 | 0.804 | 1.000 | 0.979 | 0.960 | 0.894 | 1.000 | 0.980 | 1.000 | 0.954 | 0.909 | 1.000 | 0.853 | 1.000 | 0.981 | 0.855 | 0.825 |
| 1199 | 0.769 | 1.000 | 0.824 | 1.000 | 0.974 | 0.960 | 0.904 | 1.000 | 0.980 | 1.000 | 0.954 | 0.939 | 1.000 | 0.858 | 1.000 | 0.981 | 0.859 | 0.831 |
| 1200 | 0.775 | 1.000 | 0.812 | 1.000 | 0.968 | 0.960 | 0.895 | 1.000 | 0.990 | 1.000 | 0.954 | 0.970 | 1.000 | 0.854 | 1.000 | 0.981 | 0.857 | 0.829 |
| 1201 | 0.784 | 1.000 | 0.826 | 1.000 | 0.974 | 0.960 | 0.901 | 1.000 | 0.990 | 1.000 | 0.951 | 0.939 | 1.000 | 0.854 | 1.000 | 0.981 | 0.862 | 0.835 |
| 1202 | 0.777 | 1.000 | 0.816 | 1.000 | 0.968 | 0.960 | 0.898 | 1.000 | 0.990 | 1.000 | 0.957 | 0.939 | 1.000 | 0.850 | 1.000 | 1.000 | 0.858 | 0.829 |
| 1203 | 0.771 | 1.000 | 0.812 | 1.000 | 0.963 | 0.960 | 0.889 | 1.000 | 0.980 | 1.000 | 0.962 | 0.939 | 1.000 | 0.845 | 1.000 | 1.000 | 0.853 | 0.824 |
| 1204 | 0.778 | 1.000 | 0.810 | 1.000 | 0.968 | 0.960 | 0.892 | 1.000 | 0.990 | 1.000 | 0.954 | 0.939 | 1.000 | 0.851 | 1.000 | 1.000 | 0.857 | 0.828 |
| 1205 | 0.767 | 1.000 | 0.826 | 1.000 | 0.968 | 0.960 | 0.900 | 1.000 | 0.980 | 1.000 | 0.948 | 1.000 | 1.000 | 0.851 | 1.000 | 0.981 | 0.856 | 0.828 |
| 1206 | 0.783 | 1.000 | 0.820 | 1.000 | 0.968 | 0.960 | 0.894 | 1.000 | 0.980 | 1.000 | 0.957 | 0.939 | 1.000 | 0.854 | 1.000 | 1.000 | 0.860 | 0.832 |
| 1207 | 0.773 | 1.000 | 0.818 | 1.000 | 0.968 | 0.960 | 0.894 | 1.000 | 0.990 | 1.000 | 0.951 | 0.939 | 1.000 | 0.850 | 1.000 | 0.981 | 0.856 | 0.827 |

| | | | | | | | | | | | | | | | | | |
|---|---|---|---|---|---|---|---|---|---|---|---|---|---|---|---|---|---|
| 1208 | 0.779 | 1.000 | 0.822 | 1.000 | 0.968 | 0.960 | 0.900 | 1.000 | 0.980 | 1.000 | 0.962 | 0.970 | 1.000 | 0.854 | 1.000 | 0.981 | 0.861 | 0.833 |
| 1209 | 0.775 | 1.000 | 0.798 | 1.000 | 0.968 | 0.960 | 0.903 | 1.000 | 0.980 | 1.000 | 0.957 | 0.970 | 1.000 | 0.854 | 1.000 | 0.981 | 0.857 | 0.829 |
| 1210 | 0.775 | 1.000 | 0.840 | 1.000 | 0.968 | 0.960 | 0.898 | 1.000 | 0.980 | 1.000 | 0.954 | 0.970 | 1.000 | 0.859 | 1.000 | 0.981 | 0.862 | 0.834 |
| 1211 | 0.775 | 1.000 | 0.826 | 1.000 | 0.968 | 0.960 | 0.898 | 1.000 | 0.980 | 1.000 | 0.957 | 0.970 | 1.000 | 0.848 | 1.000 | 1.000 | 0.858 | 0.830 |
| 1212 | 0.769 | 1.000 | 0.804 | 1.000 | 0.974 | 0.960 | 0.889 | 1.000 | 0.990 | 1.000 | 0.945 | 0.970 | 1.000 | 0.852 | 1.000 | 0.981 | 0.853 | 0.824 |
| 1213 | 0.779 | 1.000 | 0.816 | 1.000 | 0.968 | 0.960 | 0.895 | 1.000 | 0.980 | 1.000 | 0.954 | 0.939 | 1.000 | 0.850 | 1.000 | 0.981 | 0.857 | 0.829 |
| 1214 | 0.771 | 1.000 | 0.808 | 1.000 | 0.974 | 0.960 | 0.895 | 1.000 | 0.990 | 1.000 | 0.957 | 0.939 | 1.000 | 0.855 | 1.000 | 0.981 | 0.856 | 0.828 |
| 1215 | 0.770 | 1.000 | 0.812 | 1.000 | 0.968 | 0.960 | 0.897 | 1.000 | 0.990 | 1.000 | 0.962 | 0.970 | 1.000 | 0.858 | 1.000 | 0.981 | 0.858 | 0.829 |
| 1216 | 0.777 | 1.000 | 0.830 | 1.000 | 0.974 | 0.960 | 0.891 | 1.000 | 0.990 | 1.000 | 0.954 | 0.939 | 1.000 | 0.849 | 1.000 | 0.981 | 0.858 | 0.829 |
| 1217 | 0.775 | 1.000 | 0.828 | 1.000 | 0.968 | 0.960 | 0.901 | 1.000 | 0.990 | 1.000 | 0.957 | 0.970 | 1.000 | 0.851 | 1.000 | 1.000 | 0.860 | 0.832 |
| 1218 | 0.773 | 1.000 | 0.808 | 1.000 | 0.974 | 0.960 | 0.891 | 1.000 | 0.990 | 1.000 | 0.959 | 0.939 | 1.000 | 0.854 | 1.000 | 0.981 | 0.856 | 0.827 |
| 1219 | 0.773 | 1.000 | 0.800 | 1.000 | 0.968 | 0.960 | 0.884 | 1.000 | 0.990 | 1.000 | 0.959 | 0.939 | 1.000 | 0.851 | 1.000 | 0.981 | 0.854 | 0.824 |
| 1220 | 0.779 | 1.000 | 0.812 | 1.000 | 0.963 | 0.960 | 0.894 | 1.000 | 0.969 | 1.000 | 0.951 | 0.939 | 1.000 | 0.851 | 1.000 | 0.981 | 0.857 | 0.828 |
| 1221 | 0.783 | 1.000 | 0.820 | 1.000 | 0.968 | 0.960 | 0.898 | 1.000 | 0.980 | 1.000 | 0.959 | 0.939 | 1.000 | 0.857 | 1.000 | 0.981 | 0.862 | 0.834 |
| 1222 | 0.773 | 1.000 | 0.800 | 1.000 | 0.974 | 0.960 | 0.894 | 1.000 | 0.980 | 1.000 | 0.957 | 0.939 | 1.000 | 0.861 | 1.000 | 0.981 | 0.857 | 0.828 |
| 1223 | 0.771 | 1.000 | 0.824 | 1.000 | 0.974 | 0.960 | 0.891 | 1.000 | 0.990 | 1.000 | 0.962 | 0.909 | 1.000 | 0.853 | 1.000 | 1.000 | 0.857 | 0.829 |
| 1224 | 0.779 | 1.000 | 0.828 | 1.000 | 0.968 | 0.960 | 0.901 | 1.000 | 0.990 | 1.000 | 0.957 | 0.970 | 1.000 | 0.854 | 1.000 | 0.981 | 0.861 | 0.834 |
| 1225 | 0.782 | 1.000 | 0.810 | 1.000 | 0.974 | 0.960 | 0.900 | 1.000 | 0.980 | 1.000 | 0.957 | 0.970 | 1.000 | 0.851 | 1.000 | 0.981 | 0.859 | 0.831 |
| 1226 | 0.783 | 1.000 | 0.826 | 1.000 | 0.968 | 0.960 | 0.894 | 1.000 | 0.990 | 1.000 | 0.957 | 0.970 | 1.000 | 0.848 | 1.000 | 0.981 | 0.860 | 0.832 |
| 1227 | 0.777 | 1.000 | 0.818 | 1.000 | 0.968 | 0.960 | 0.898 | 1.000 | 0.990 | 1.000 | 0.959 | 0.909 | 1.000 | 0.847 | 1.000 | 0.981 | 0.857 | 0.829 |
| 1228 | 0.779 | 1.000 | 0.812 | 1.000 | 0.974 | 0.960 | 0.895 | 1.000 | 0.980 | 1.000 | 0.954 | 0.939 | 1.000 | 0.859 | 1.000 | 1.000 | 0.860 | 0.832 |
| 1229 | 0.775 | 1.000 | 0.818 | 1.000 | 0.968 | 0.960 | 0.903 | 1.000 | 0.990 | 1.000 | 0.954 | 0.909 | 1.000 | 0.853 | 1.000 | 1.000 | 0.859 | 0.830 |
| 1230 | 0.775 | 1.000 | 0.824 | 1.000 | 0.968 | 0.960 | 0.906 | 1.000 | 0.990 | 1.000 | 0.959 | 0.939 | 1.000 | 0.857 | 1.000 | 0.981 | 0.861 | 0.833 |
| 1231 | 0.777 | 1.000 | 0.830 | 1.000 | 0.979 | 0.960 | 0.892 | 1.000 | 0.980 | 1.000 | 0.957 | 0.909 | 1.000 | 0.856 | 1.000 | 0.981 | 0.860 | 0.832 |
| 1232 | 0.783 | 1.000 | 0.818 | 1.000 | 0.968 | 0.960 | 0.891 | 1.000 | 0.990 | 1.000 | 0.957 | 0.909 | 1.000 | 0.856 | 1.000 | 0.981 | 0.860 | 0.832 |
| 1233 | 0.771 | 1.000 | 0.830 | 1.000 | 0.974 | 0.960 | 0.892 | 1.000 | 0.990 | 1.000 | 0.959 | 0.909 | 1.000 | 0.858 | 1.000 | 0.981 | 0.859 | 0.831 |
| 1234 | 0.776 | 1.000 | 0.806 | 1.000 | 0.963 | 0.960 | 0.897 | 1.000 | 0.990 | 1.000 | 0.951 | 0.970 | 1.000 | 0.855 | 1.000 | 1.000 | 0.857 | 0.829 |
| 1235 | 0.779 | 1.000 | 0.804 | 1.000 | 0.968 | 0.960 | 0.888 | 1.000 | 0.990 | 1.000 | 0.957 | 1.000 | 1.000 | 0.856 | 1.000 | 0.981 | 0.858 | 0.829 |
| 1236 | 0.779 | 1.000 | 0.814 | 1.000 | 0.979 | 0.960 | 0.883 | 1.000 | 0.990 | 1.000 | 0.951 | 0.970 | 1.000 | 0.849 | 1.000 | 1.000 | 0.856 | 0.828 |
| 1237 | 0.775 | 1.000 | 0.810 | 1.000 | 0.974 | 0.960 | 0.901 | 1.000 | 0.980 | 1.000 | 0.959 | 0.970 | 1.000 | 0.855 | 1.000 | 1.000 | 0.859 | 0.831 |
| 1238 | 0.775 | 1.000 | 0.820 | 1.000 | 0.974 | 0.960 | 0.900 | 1.000 | 0.990 | 1.000 | 0.957 | 0.970 | 1.000 | 0.854 | 1.000 | 0.981 | 0.859 | 0.831 |
| 1239 | 0.779 | 1.000 | 0.822 | 1.000 | 0.974 | 0.960 | 0.889 | 1.000 | 0.980 | 1.000 | 0.957 | 1.000 | 1.000 | 0.858 | 1.000 | 0.981 | 0.860 | 0.832 |
| 1240 | 0.772 | 1.000 | 0.806 | 1.000 | 0.974 | 0.960 | 0.898 | 1.000 | 0.990 | 1.000 | 0.954 | 0.970 | 1.000 | 0.854 | 1.000 | 1.000 | 0.857 | 0.828 |
| 1241 | 0.783 | 1.000 | 0.802 | 1.000 | 0.974 | 0.960 | 0.897 | 1.000 | 0.990 | 1.000 | 0.954 | 1.000 | 1.000 | 0.854 | 1.000 | 1.000 | 0.859 | 0.831 |
| 1242 | 0.783 | 1.000 | 0.806 | 1.000 | 0.974 | 0.960 | 0.892 | 1.000 | 0.980 | 1.000 | 0.957 | 0.939 | 1.000 | 0.853 | 1.000 | 1.000 | 0.859 | 0.830 |
| 1243 | 0.772 | 1.000 | 0.818 | 1.000 | 0.968 | 0.960 | 0.906 | 1.000 | 0.980 | 1.000 | 0.962 | 0.939 | 1.000 | 0.857 | 1.000 | 0.981 | 0.859 | 0.832 |
| 1244 | 0.773 | 1.000 | 0.810 | 1.000 | 0.968 | 0.960 | 0.894 | 1.000 | 0.990 | 1.000 | 0.951 | 0.970 | 1.000 | 0.854 | 1.000 | 0.981 | 0.856 | 0.827 |
| 1245 | 0.773 | 1.000 | 0.814 | 1.000 | 0.968 | 0.960 | 0.900 | 1.000 | 0.980 | 1.000 | 0.954 | 0.939 | 1.000 | 0.851 | 1.000 | 0.981 | 0.856 | 0.828 |
| 1246 | 0.779 | 1.000 | 0.822 | 1.000 | 0.974 | 0.960 | 0.901 | 1.000 | 0.969 | 1.000 | 0.957 | 0.939 | 1.000 | 0.854 | 1.000 | 1.000 | 0.860 | 0.833 |

| | | | | | | | | | | | | | | | | | |
|---|---|---|---|---|---|---|---|---|---|---|---|---|---|---|---|---|---|
| 1247 | 0.773 | 1.000 | 0.824 | 1.000 | 0.968 | 0.960 | 0.898 | 1.000 | 0.980 | 1.000 | 0.954 | 1.000 | 1.000 | 0.849 | 1.000 | 0.981 | 0.857 | 0.829 |
| 1248 | 0.770 | 1.000 | 0.810 | 1.000 | 0.968 | 0.960 | 0.894 | 1.000 | 0.990 | 1.000 | 0.945 | 0.939 | 1.000 | 0.851 | 1.000 | 1.000 | 0.854 | 0.825 |
| 1249 | 0.782 | 1.000 | 0.804 | 1.000 | 0.979 | 0.960 | 0.889 | 1.000 | 0.990 | 1.000 | 0.951 | 0.939 | 1.000 | 0.849 | 1.000 | 1.000 | 0.857 | 0.828 |
| 1250 | 0.772 | 1.000 | 0.808 | 1.000 | 0.974 | 0.960 | 0.897 | 1.000 | 0.969 | 1.000 | 0.951 | 0.939 | 1.000 | 0.851 | 1.000 | 0.981 | 0.855 | 0.826 |
| 1251 | 0.773 | 1.000 | 0.814 | 0.986 | 0.968 | 0.960 | 0.904 | 1.000 | 0.980 | 1.000 | 0.954 | 0.970 | 1.000 | 0.854 | 1.000 | 0.981 | 0.858 | 0.830 |
| 1252 | 0.778 | 1.000 | 0.806 | 1.000 | 0.974 | 0.960 | 0.897 | 1.000 | 0.990 | 1.000 | 0.957 | 0.939 | 1.000 | 0.852 | 1.000 | 1.000 | 0.858 | 0.829 |
| 1253 | 0.771 | 1.000 | 0.820 | 1.000 | 0.974 | 0.960 | 0.898 | 1.000 | 0.990 | 1.000 | 0.962 | 0.939 | 1.000 | 0.851 | 1.000 | 0.981 | 0.857 | 0.829 |
| 1254 | 0.775 | 1.000 | 0.810 | 1.000 | 0.968 | 0.960 | 0.894 | 1.000 | 0.990 | 1.000 | 0.959 | 0.970 | 1.000 | 0.854 | 1.000 | 1.000 | 0.858 | 0.829 |
| 1255 | 0.778 | 1.000 | 0.800 | 1.000 | 0.979 | 0.960 | 0.898 | 1.000 | 0.990 | 1.000 | 0.959 | 0.939 | 1.000 | 0.854 | 1.000 | 0.981 | 0.858 | 0.829 |
| 1256 | 0.775 | 1.000 | 0.808 | 1.000 | 0.968 | 0.960 | 0.881 | 1.000 | 0.990 | 1.000 | 0.957 | 0.970 | 1.000 | 0.854 | 1.000 | 0.981 | 0.855 | 0.826 |
| 1257 | 0.775 | 1.000 | 0.806 | 1.000 | 0.974 | 0.960 | 0.900 | 1.000 | 0.990 | 1.000 | 0.962 | 0.939 | 1.000 | 0.858 | 1.000 | 0.981 | 0.859 | 0.831 |
| 1258 | 0.773 | 1.000 | 0.814 | 1.000 | 0.974 | 0.960 | 0.895 | 1.000 | 0.980 | 1.000 | 0.959 | 0.939 | 1.000 | 0.855 | 1.000 | 0.981 | 0.857 | 0.829 |
| 1259 | 0.771 | 1.000 | 0.820 | 1.000 | 0.979 | 0.960 | 0.897 | 1.000 | 0.980 | 1.000 | 0.962 | 0.939 | 1.000 | 0.854 | 1.000 | 1.000 | 0.858 | 0.830 |
| 1260 | 0.775 | 1.000 | 0.822 | 1.000 | 0.968 | 0.960 | 0.900 | 1.000 | 0.990 | 1.000 | 0.962 | 0.909 | 1.000 | 0.853 | 1.000 | 1.000 | 0.859 | 0.831 |
| 1261 | 0.779 | 1.000 | 0.798 | 1.000 | 0.974 | 0.960 | 0.886 | 1.000 | 0.990 | 1.000 | 0.951 | 0.970 | 1.000 | 0.860 | 1.000 | 1.000 | 0.858 | 0.829 |
| 1262 | 0.778 | 1.000 | 0.822 | 1.000 | 0.974 | 0.960 | 0.888 | 1.000 | 0.990 | 1.000 | 0.957 | 0.970 | 1.000 | 0.854 | 1.000 | 0.981 | 0.859 | 0.830 |
| 1263 | 0.773 | 1.000 | 0.808 | 1.000 | 0.968 | 0.960 | 0.888 | 1.000 | 1.000 | 1.000 | 0.957 | 0.939 | 1.000 | 0.850 | 1.000 | 0.981 | 0.854 | 0.825 |
| 1264 | 0.779 | 1.000 | 0.826 | 1.000 | 0.968 | 0.960 | 0.889 | 1.000 | 0.990 | 1.000 | 0.954 | 0.939 | 1.000 | 0.854 | 1.000 | 0.981 | 0.859 | 0.831 |
| 1265 | 0.777 | 1.000 | 0.806 | 1.000 | 0.974 | 0.960 | 0.881 | 1.000 | 0.990 | 1.000 | 0.957 | 0.939 | 1.000 | 0.853 | 1.000 | 1.000 | 0.856 | 0.827 |
| 1266 | 0.777 | 1.000 | 0.812 | 1.000 | 0.968 | 0.960 | 0.901 | 1.000 | 0.990 | 1.000 | 0.957 | 0.939 | 1.000 | 0.858 | 1.000 | 1.000 | 0.860 | 0.832 |
| 1267 | 0.776 | 1.000 | 0.810 | 1.000 | 0.963 | 0.960 | 0.891 | 1.000 | 0.990 | 1.000 | 0.948 | 0.909 | 1.000 | 0.856 | 1.000 | 1.000 | 0.857 | 0.828 |
| 1268 | 0.779 | 1.000 | 0.814 | 1.000 | 0.979 | 0.960 | 0.895 | 1.000 | 0.990 | 1.000 | 0.962 | 0.939 | 1.000 | 0.857 | 1.000 | 1.000 | 0.860 | 0.832 |
| 1269 | 0.771 | 1.000 | 0.804 | 1.000 | 0.968 | 0.960 | 0.898 | 1.000 | 0.980 | 1.000 | 0.954 | 0.939 | 1.000 | 0.854 | 1.000 | 1.000 | 0.855 | 0.827 |
| 1270 | 0.776 | 1.000 | 0.822 | 1.000 | 0.968 | 0.960 | 0.895 | 1.000 | 0.990 | 1.000 | 0.959 | 0.939 | 1.000 | 0.841 | 1.000 | 1.000 | 0.856 | 0.827 |
| 1271 | 0.773 | 1.000 | 0.820 | 1.000 | 0.968 | 0.960 | 0.909 | 1.000 | 0.990 | 1.000 | 0.945 | 0.970 | 1.000 | 0.854 | 1.000 | 0.981 | 0.859 | 0.831 |
| 1272 | 0.779 | 1.000 | 0.824 | 1.000 | 0.974 | 0.960 | 0.897 | 1.000 | 0.980 | 1.000 | 0.959 | 0.909 | 1.000 | 0.851 | 1.000 | 0.981 | 0.859 | 0.831 |
| 1273 | 0.775 | 1.000 | 0.824 | 1.000 | 0.974 | 0.960 | 0.898 | 1.000 | 0.990 | 1.000 | 0.957 | 0.939 | 1.000 | 0.859 | 1.000 | 0.981 | 0.861 | 0.833 |
| 1274 | 0.769 | 1.000 | 0.814 | 0.986 | 0.974 | 0.960 | 0.900 | 1.000 | 0.969 | 1.000 | 0.959 | 0.939 | 1.000 | 0.854 | 1.000 | 0.981 | 0.856 | 0.828 |
| 1275 | 0.781 | 1.000 | 0.820 | 0.986 | 0.974 | 0.960 | 0.901 | 1.000 | 0.990 | 1.000 | 0.954 | 0.939 | 1.000 | 0.851 | 1.000 | 0.981 | 0.860 | 0.832 |
| 1276 | 0.781 | 1.000 | 0.820 | 1.000 | 0.974 | 1.000 | 0.894 | 1.000 | 0.980 | 1.000 | 0.954 | 0.970 | 1.000 | 0.850 | 1.000 | 0.981 | 0.859 | 0.831 |
| 1277 | 0.773 | 1.000 | 0.822 | 1.000 | 0.974 | 0.960 | 0.911 | 1.000 | 0.980 | 1.000 | 0.957 | 0.970 | 0.991 | 0.847 | 1.000 | 0.981 | 0.858 | 0.830 |
| 1278 | 0.776 | 1.000 | 0.826 | 1.000 | 0.968 | 0.960 | 0.897 | 1.000 | 0.990 | 1.000 | 0.954 | 0.939 | 1.000 | 0.857 | 1.000 | 0.981 | 0.860 | 0.832 |
| 1279 | 0.782 | 1.000 | 0.820 | 1.000 | 0.968 | 0.960 | 0.894 | 1.000 | 0.980 | 1.000 | 0.954 | 0.970 | 1.000 | 0.852 | 1.000 | 0.981 | 0.859 | 0.831 |
| 1280 | 0.781 | 1.000 | 0.806 | 1.000 | 0.974 | 0.960 | 0.892 | 1.000 | 0.990 | 1.000 | 0.962 | 0.939 | 1.000 | 0.851 | 1.000 | 0.981 | 0.858 | 0.829 |
| 1281 | 0.776 | 1.000 | 0.812 | 1.000 | 0.974 | 0.960 | 0.904 | 1.000 | 0.990 | 1.000 | 0.959 | 0.970 | 1.000 | 0.849 | 1.000 | 0.981 | 0.858 | 0.830 |
| 1282 | 0.773 | 1.000 | 0.828 | 1.000 | 0.974 | 0.960 | 0.901 | 1.000 | 0.990 | 1.000 | 0.959 | 0.939 | 1.000 | 0.849 | 1.000 | 1.000 | 0.859 | 0.831 |
| 1283 | 0.785 | 1.000 | 0.822 | 1.000 | 0.979 | 0.960 | 0.892 | 1.000 | 0.990 | 1.000 | 0.959 | 0.939 | 1.000 | 0.851 | 1.000 | 1.000 | 0.861 | 0.833 |
| 1284 | 0.778 | 1.000 | 0.818 | 1.000 | 0.968 | 0.960 | 0.898 | 1.000 | 0.990 | 1.000 | 0.951 | 0.970 | 1.000 | 0.851 | 1.000 | 0.981 | 0.858 | 0.830 |
| 1285 | 0.779 | 1.000 | 0.816 | 1.000 | 0.974 | 0.960 | 0.895 | 1.000 | 0.980 | 1.000 | 0.962 | 0.909 | 1.000 | 0.846 | 1.000 | 1.000 | 0.857 | 0.829 |

| | | | | | | | | | | | | | | | | | | |
|---|---|---|---|---|---|---|---|---|---|---|---|---|---|---|---|---|---|---|
| 1286 | 0.785 | 1.000 | 0.824 | 1.000 | 0.968 | 0.960 | 0.891 | 1.000 | 0.980 | 1.000 | 0.962 | 0.939 | 1.000 | 0.858 | 1.000 | 0.981 | 0.862 | 0.834 |
| 1287 | 0.783 | 1.000 | 0.822 | 1.000 | 0.968 | 0.960 | 0.898 | 1.000 | 0.990 | 1.000 | 0.951 | 0.939 | 1.000 | 0.846 | 1.000 | 0.981 | 0.859 | 0.830 |
| 1288 | 0.777 | 1.000 | 0.810 | 1.000 | 0.968 | 0.960 | 0.894 | 1.000 | 0.990 | 1.000 | 0.959 | 0.939 | 1.000 | 0.851 | 1.000 | 0.981 | 0.857 | 0.829 |
| 1289 | 0.779 | 1.000 | 0.812 | 1.000 | 0.968 | 0.960 | 0.897 | 1.000 | 0.980 | 1.000 | 0.951 | 0.909 | 1.000 | 0.856 | 1.000 | 1.000 | 0.859 | 0.830 |
| 1290 | 0.775 | 1.000 | 0.818 | 1.000 | 0.968 | 0.960 | 0.898 | 1.000 | 0.990 | 1.000 | 0.962 | 0.970 | 1.000 | 0.847 | 1.000 | 0.981 | 0.857 | 0.829 |
| 1291 | 0.787 | 1.000 | 0.818 | 0.986 | 0.963 | 0.960 | 0.897 | 1.000 | 0.990 | 1.000 | 0.957 | 0.939 | 1.000 | 0.858 | 1.000 | 0.981 | 0.862 | 0.835 |
| 1292 | 0.774 | 1.000 | 0.824 | 1.000 | 0.974 | 0.960 | 0.889 | 1.000 | 0.980 | 1.000 | 0.959 | 0.939 | 1.000 | 0.852 | 1.000 | 0.981 | 0.857 | 0.829 |
| 1293 | 0.785 | 1.000 | 0.808 | 1.000 | 0.963 | 0.960 | 0.900 | 1.000 | 0.980 | 1.000 | 0.962 | 0.970 | 1.000 | 0.850 | 1.000 | 1.000 | 0.860 | 0.832 |
| 1294 | 0.777 | 1.000 | 0.818 | 1.000 | 0.958 | 0.960 | 0.900 | 1.000 | 0.990 | 1.000 | 0.957 | 0.939 | 1.000 | 0.854 | 1.000 | 1.000 | 0.859 | 0.831 |
| 1295 | 0.781 | 1.000 | 0.826 | 1.000 | 0.974 | 0.960 | 0.897 | 1.000 | 0.980 | 1.000 | 0.959 | 0.939 | 1.000 | 0.853 | 1.000 | 1.000 | 0.861 | 0.833 |
| 1296 | 0.771 | 1.000 | 0.808 | 1.000 | 0.974 | 0.960 | 0.898 | 1.000 | 0.990 | 1.000 | 0.959 | 1.000 | 1.000 | 0.845 | 1.000 | 0.981 | 0.855 | 0.826 |
| 1297 | 0.768 | 1.000 | 0.820 | 1.000 | 0.968 | 0.960 | 0.897 | 1.000 | 0.990 | 1.000 | 0.957 | 0.970 | 1.000 | 0.854 | 1.000 | 0.981 | 0.857 | 0.828 |
| 1298 | 0.767 | 1.000 | 0.802 | 1.000 | 0.968 | 0.960 | 0.895 | 1.000 | 0.980 | 1.000 | 0.951 | 0.939 | 1.000 | 0.857 | 1.000 | 0.981 | 0.854 | 0.825 |
| 1299 | 0.783 | 1.000 | 0.810 | 1.000 | 0.974 | 0.960 | 0.888 | 1.000 | 0.990 | 1.000 | 0.959 | 0.970 | 1.000 | 0.850 | 1.000 | 0.981 | 0.858 | 0.830 |
| 1300 | 0.779 | 1.000 | 0.826 | 1.000 | 0.974 | 0.960 | 0.892 | 1.000 | 0.990 | 1.000 | 0.954 | 1.000 | 1.000 | 0.849 | 1.000 | 0.981 | 0.859 | 0.830 |
| 1301 | 0.783 | 1.000 | 0.832 | 1.000 | 0.963 | 0.960 | 0.901 | 1.000 | 0.990 | 1.000 | 0.962 | 0.970 | 1.000 | 0.848 | 1.000 | 1.000 | 0.862 | 0.834 |
| 1302 | 0.775 | 1.000 | 0.812 | 1.000 | 0.974 | 0.960 | 0.901 | 1.000 | 0.980 | 1.000 | 0.962 | 0.970 | 1.000 | 0.850 | 1.000 | 0.981 | 0.858 | 0.830 |
| 1303 | 0.771 | 1.000 | 0.814 | 1.000 | 0.974 | 0.960 | 0.898 | 1.000 | 0.980 | 1.000 | 0.959 | 0.939 | 1.000 | 0.847 | 1.000 | 0.981 | 0.855 | 0.827 |
| 1304 | 0.770 | 1.000 | 0.814 | 1.000 | 0.974 | 0.960 | 0.891 | 1.000 | 0.980 | 1.000 | 0.962 | 0.939 | 1.000 | 0.854 | 1.000 | 1.000 | 0.856 | 0.828 |
| 1305 | 0.772 | 1.000 | 0.830 | 1.000 | 0.968 | 0.960 | 0.901 | 1.000 | 0.990 | 1.000 | 0.954 | 0.939 | 1.000 | 0.849 | 1.000 | 0.981 | 0.858 | 0.829 |
| 1306 | 0.771 | 1.000 | 0.810 | 1.000 | 0.963 | 0.960 | 0.895 | 1.000 | 0.980 | 1.000 | 0.965 | 0.939 | 1.000 | 0.854 | 1.000 | 0.981 | 0.856 | 0.827 |
| 1307 | 0.784 | 1.000 | 0.820 | 1.000 | 0.974 | 0.960 | 0.894 | 1.000 | 0.980 | 1.000 | 0.957 | 0.939 | 1.000 | 0.847 | 1.000 | 0.981 | 0.859 | 0.831 |
| 1308 | 0.771 | 1.000 | 0.812 | 1.000 | 0.974 | 0.960 | 0.906 | 1.000 | 0.990 | 1.000 | 0.959 | 0.970 | 1.000 | 0.852 | 1.000 | 0.981 | 0.858 | 0.830 |
| 1309 | 0.783 | 1.000 | 0.828 | 1.000 | 0.968 | 0.960 | 0.897 | 1.000 | 0.990 | 1.000 | 0.959 | 0.970 | 1.000 | 0.854 | 1.000 | 0.981 | 0.862 | 0.834 |
| 1310 | 0.773 | 1.000 | 0.822 | 0.986 | 0.968 | 0.960 | 0.904 | 1.000 | 0.980 | 1.000 | 0.954 | 0.939 | 1.000 | 0.854 | 1.000 | 0.981 | 0.858 | 0.830 |
| 1311 | 0.782 | 1.000 | 0.810 | 1.000 | 0.974 | 0.960 | 0.900 | 1.000 | 0.980 | 1.000 | 0.959 | 0.939 | 1.000 | 0.851 | 1.000 | 0.981 | 0.859 | 0.831 |
| 1312 | 0.787 | 1.000 | 0.820 | 1.000 | 0.968 | 0.960 | 0.900 | 1.000 | 0.990 | 1.000 | 0.959 | 0.970 | 1.000 | 0.852 | 1.000 | 1.000 | 0.863 | 0.835 |
| 1313 | 0.777 | 1.000 | 0.810 | 1.000 | 0.968 | 0.960 | 0.901 | 1.000 | 0.990 | 1.000 | 0.951 | 0.970 | 1.000 | 0.850 | 1.000 | 1.000 | 0.857 | 0.829 |
| 1314 | 0.775 | 1.000 | 0.828 | 1.000 | 0.968 | 0.960 | 0.892 | 1.000 | 0.990 | 1.000 | 0.959 | 0.939 | 1.000 | 0.849 | 1.000 | 1.000 | 0.858 | 0.830 |
| 1315 | 0.775 | 1.000 | 0.818 | 1.000 | 0.968 | 0.960 | 0.891 | 1.000 | 0.980 | 1.000 | 0.951 | 0.970 | 1.000 | 0.851 | 1.000 | 1.000 | 0.857 | 0.828 |
| 1316 | 0.773 | 1.000 | 0.792 | 1.000 | 0.968 | 0.960 | 0.911 | 1.000 | 0.980 | 1.000 | 0.962 | 1.000 | 1.000 | 0.845 | 1.000 | 0.981 | 0.855 | 0.826 |
| 1317 | 0.769 | 1.000 | 0.808 | 1.000 | 0.974 | 0.960 | 0.895 | 1.000 | 0.980 | 1.000 | 0.959 | 0.939 | 1.000 | 0.852 | 1.000 | 0.981 | 0.855 | 0.826 |
| 1318 | 0.778 | 1.000 | 0.810 | 1.000 | 0.968 | 0.960 | 0.892 | 1.000 | 0.990 | 1.000 | 0.962 | 0.970 | 1.000 | 0.858 | 1.000 | 1.000 | 0.859 | 0.831 |
| 1319 | 0.775 | 1.000 | 0.808 | 1.000 | 0.968 | 0.960 | 0.898 | 1.000 | 0.990 | 1.000 | 0.957 | 0.970 | 1.000 | 0.850 | 1.000 | 1.000 | 0.857 | 0.828 |
| 1320 | 0.777 | 1.000 | 0.814 | 1.000 | 0.979 | 0.960 | 0.901 | 1.000 | 0.980 | 1.000 | 0.965 | 0.939 | 1.000 | 0.855 | 1.000 | 1.000 | 0.860 | 0.832 |
| 1321 | 0.772 | 1.000 | 0.808 | 1.000 | 0.974 | 0.960 | 0.898 | 1.000 | 0.990 | 1.000 | 0.957 | 0.939 | 1.000 | 0.851 | 1.000 | 1.000 | 0.856 | 0.828 |
| 1322 | 0.775 | 1.000 | 0.832 | 1.000 | 0.979 | 0.960 | 0.903 | 1.000 | 0.980 | 1.000 | 0.959 | 0.939 | 1.000 | 0.852 | 1.000 | 1.000 | 0.860 | 0.833 |
| 1323 | 0.777 | 1.000 | 0.810 | 1.000 | 0.974 | 0.960 | 0.901 | 1.000 | 0.990 | 1.000 | 0.954 | 0.970 | 1.000 | 0.851 | 1.000 | 0.981 | 0.858 | 0.830 |
| 1324 | 0.778 | 1.000 | 0.816 | 1.000 | 0.968 | 0.960 | 0.903 | 1.000 | 0.980 | 1.000 | 0.962 | 0.970 | 1.000 | 0.848 | 1.000 | 0.981 | 0.859 | 0.830 |

| | | | | | | | | | | | | | | | | | | |
|---|---|---|---|---|---|---|---|---|---|---|---|---|---|---|---|---|---|---|
| 1325 | 0.769 | 1.000 | 0.816 | 1.000 | 0.974 | 0.960 | 0.906 | 1.000 | 0.990 | 1.000 | 0.959 | 1.000 | 1.000 | 0.852 | 1.000 | 0.981 | 0.858 | 0.830 |
| 1326 | 0.777 | 1.000 | 0.816 | 1.000 | 0.968 | 0.960 | 0.897 | 1.000 | 0.980 | 1.000 | 0.959 | 0.939 | 1.000 | 0.852 | 1.000 | 0.981 | 0.858 | 0.830 |
| 1327 | 0.777 | 1.000 | 0.820 | 1.000 | 0.974 | 0.960 | 0.901 | 1.000 | 0.990 | 1.000 | 0.959 | 0.939 | 1.000 | 0.852 | 1.000 | 0.981 | 0.860 | 0.832 |
| 1328 | 0.775 | 1.000 | 0.812 | 1.000 | 0.974 | 0.960 | 0.901 | 1.000 | 0.990 | 1.000 | 0.962 | 0.970 | 1.000 | 0.848 | 1.000 | 0.981 | 0.858 | 0.829 |
| 1329 | 0.782 | 1.000 | 0.824 | 1.000 | 0.974 | 0.960 | 0.895 | 1.000 | 0.990 | 1.000 | 0.965 | 0.939 | 1.000 | 0.845 | 1.000 | 0.981 | 0.859 | 0.831 |
| 1330 | 0.783 | 1.000 | 0.814 | 1.000 | 0.974 | 0.960 | 0.900 | 1.000 | 0.990 | 1.000 | 0.954 | 0.970 | 1.000 | 0.850 | 1.000 | 1.000 | 0.860 | 0.832 |
| 1331 | 0.770 | 1.000 | 0.806 | 1.000 | 0.968 | 0.960 | 0.901 | 1.000 | 0.990 | 1.000 | 0.962 | 0.970 | 1.000 | 0.855 | 1.000 | 0.981 | 0.857 | 0.829 |
| 1332 | 0.775 | 1.000 | 0.814 | 1.000 | 0.968 | 0.960 | 0.897 | 1.000 | 0.980 | 1.000 | 0.959 | 1.000 | 1.000 | 0.851 | 1.000 | 0.981 | 0.858 | 0.829 |
| 1333 | 0.773 | 1.000 | 0.814 | 1.000 | 0.974 | 0.960 | 0.908 | 1.000 | 0.990 | 1.000 | 0.959 | 0.939 | 1.000 | 0.846 | 1.000 | 1.000 | 0.857 | 0.829 |
| 1334 | 0.777 | 1.000 | 0.810 | 1.000 | 0.979 | 0.960 | 0.901 | 1.000 | 0.990 | 1.000 | 0.957 | 0.939 | 1.000 | 0.863 | 1.000 | 0.981 | 0.861 | 0.834 |
| 1335 | 0.774 | 1.000 | 0.830 | 1.000 | 0.974 | 0.960 | 0.901 | 1.000 | 0.980 | 1.000 | 0.959 | 0.939 | 1.000 | 0.852 | 1.000 | 0.981 | 0.859 | 0.832 |
| 1336 | 0.781 | 1.000 | 0.824 | 1.000 | 0.968 | 0.960 | 0.908 | 1.000 | 0.990 | 1.000 | 0.954 | 0.939 | 1.000 | 0.847 | 1.000 | 0.981 | 0.860 | 0.832 |
| 1337 | 0.778 | 1.000 | 0.812 | 1.000 | 0.979 | 0.960 | 0.901 | 1.000 | 0.990 | 1.000 | 0.959 | 0.939 | 1.000 | 0.849 | 1.000 | 1.000 | 0.859 | 0.830 |
| 1338 | 0.785 | 1.000 | 0.822 | 1.000 | 0.968 | 0.960 | 0.897 | 1.000 | 0.990 | 1.000 | 0.965 | 0.939 | 1.000 | 0.847 | 1.000 | 1.000 | 0.861 | 0.832 |
| 1339 | 0.777 | 1.000 | 0.820 | 1.000 | 0.963 | 0.960 | 0.897 | 1.000 | 0.980 | 1.000 | 0.957 | 0.939 | 1.000 | 0.854 | 1.000 | 1.000 | 0.859 | 0.830 |
| 1340 | 0.765 | 1.000 | 0.836 | 1.000 | 0.968 | 0.960 | 0.906 | 1.000 | 0.990 | 1.000 | 0.957 | 0.970 | 1.000 | 0.857 | 1.000 | 1.000 | 0.859 | 0.832 |
| 1341 | 0.779 | 1.000 | 0.814 | 1.000 | 0.968 | 0.960 | 0.901 | 1.000 | 0.980 | 1.000 | 0.959 | 0.939 | 1.000 | 0.850 | 1.000 | 1.000 | 0.859 | 0.831 |
| 1342 | 0.775 | 1.000 | 0.814 | 1.000 | 0.974 | 0.960 | 0.900 | 1.000 | 0.980 | 1.000 | 0.965 | 0.970 | 1.000 | 0.853 | 1.000 | 1.000 | 0.859 | 0.831 |
| 1343 | 0.782 | 1.000 | 0.812 | 1.000 | 0.974 | 0.960 | 0.901 | 1.000 | 0.969 | 1.000 | 0.959 | 0.939 | 1.000 | 0.859 | 1.000 | 0.981 | 0.862 | 0.834 |
| 1344 | 0.770 | 1.000 | 0.824 | 1.000 | 0.968 | 0.960 | 0.903 | 1.000 | 0.990 | 1.000 | 0.962 | 0.970 | 1.000 | 0.851 | 1.000 | 1.000 | 0.858 | 0.830 |
| 1345 | 0.787 | 1.000 | 0.816 | 1.000 | 0.968 | 0.960 | 0.903 | 1.000 | 0.990 | 1.000 | 0.957 | 0.939 | 1.000 | 0.848 | 1.000 | 1.000 | 0.861 | 0.833 |
| 1346 | 0.775 | 1.000 | 0.812 | 1.000 | 0.974 | 0.960 | 0.906 | 1.000 | 0.980 | 1.000 | 0.965 | 0.939 | 1.000 | 0.856 | 1.000 | 1.000 | 0.860 | 0.832 |
| 1347 | 0.779 | 1.000 | 0.834 | 1.000 | 0.968 | 0.960 | 0.894 | 1.000 | 0.990 | 1.000 | 0.957 | 1.000 | 1.000 | 0.845 | 1.000 | 0.981 | 0.859 | 0.831 |
| 1348 | 0.773 | 1.000 | 0.838 | 1.000 | 0.968 | 0.960 | 0.898 | 1.000 | 0.980 | 1.000 | 0.959 | 0.939 | 1.000 | 0.853 | 1.000 | 0.981 | 0.859 | 0.832 |
| 1349 | 0.771 | 1.000 | 0.830 | 1.000 | 0.968 | 0.960 | 0.904 | 1.000 | 0.980 | 1.000 | 0.968 | 0.939 | 1.000 | 0.850 | 1.000 | 1.000 | 0.859 | 0.831 |
| 1350 | 0.777 | 1.000 | 0.792 | 1.000 | 0.979 | 0.960 | 0.906 | 1.000 | 0.990 | 1.000 | 0.965 | 0.970 | 1.000 | 0.851 | 1.000 | 0.981 | 0.858 | 0.829 |
| 1351 | 0.777 | 1.000 | 0.822 | 1.000 | 0.968 | 0.960 | 0.908 | 1.000 | 0.980 | 1.000 | 0.962 | 0.939 | 1.000 | 0.851 | 1.000 | 0.981 | 0.860 | 0.832 |
| 1352 | 0.778 | 1.000 | 0.812 | 1.000 | 0.968 | 0.960 | 0.892 | 1.000 | 0.990 | 1.000 | 0.954 | 0.970 | 1.000 | 0.846 | 1.000 | 1.000 | 0.856 | 0.827 |
| 1353 | 0.775 | 1.000 | 0.820 | 1.000 | 0.968 | 0.960 | 0.894 | 1.000 | 0.980 | 1.000 | 0.962 | 0.970 | 1.000 | 0.854 | 1.000 | 0.981 | 0.858 | 0.830 |
| 1354 | 0.781 | 1.000 | 0.828 | 1.000 | 0.968 | 0.960 | 0.904 | 1.000 | 0.980 | 1.000 | 0.962 | 0.939 | 1.000 | 0.848 | 1.000 | 0.981 | 0.861 | 0.833 |
| 1355 | 0.774 | 1.000 | 0.816 | 1.000 | 0.968 | 0.960 | 0.906 | 1.000 | 0.980 | 1.000 | 0.957 | 0.970 | 1.000 | 0.853 | 1.000 | 1.000 | 0.859 | 0.831 |
| 1356 | 0.772 | 1.000 | 0.814 | 1.000 | 0.979 | 0.960 | 0.901 | 1.000 | 0.980 | 1.000 | 0.954 | 0.970 | 1.000 | 0.852 | 1.000 | 1.000 | 0.857 | 0.829 |
| 1357 | 0.771 | 1.000 | 0.838 | 1.000 | 0.974 | 0.960 | 0.904 | 1.000 | 0.980 | 1.000 | 0.965 | 0.970 | 1.000 | 0.849 | 1.000 | 0.981 | 0.859 | 0.832 |
| 1358 | 0.769 | 1.000 | 0.820 | 1.000 | 0.968 | 0.960 | 0.895 | 1.000 | 0.980 | 1.000 | 0.962 | 0.970 | 1.000 | 0.858 | 1.000 | 1.000 | 0.858 | 0.830 |
| 1359 | 0.781 | 1.000 | 0.818 | 1.000 | 0.968 | 0.960 | 0.898 | 1.000 | 0.980 | 1.000 | 0.959 | 0.909 | 1.000 | 0.854 | 1.000 | 1.000 | 0.860 | 0.832 |
| 1360 | 0.781 | 1.000 | 0.810 | 1.000 | 0.974 | 0.960 | 0.906 | 1.000 | 0.990 | 1.000 | 0.945 | 0.970 | 1.000 | 0.849 | 1.000 | 0.981 | 0.859 | 0.830 |
| 1361 | 0.776 | 1.000 | 0.814 | 1.000 | 0.974 | 0.960 | 0.881 | 1.000 | 0.990 | 1.000 | 0.959 | 0.970 | 1.000 | 0.847 | 1.000 | 1.000 | 0.855 | 0.826 |
| 1362 | 0.775 | 1.000 | 0.818 | 1.000 | 0.974 | 0.960 | 0.904 | 1.000 | 0.990 | 1.000 | 0.959 | 1.000 | 0.991 | 0.852 | 1.000 | 0.981 | 0.859 | 0.831 |
| 1363 | 0.777 | 1.000 | 0.836 | 1.000 | 0.979 | 0.960 | 0.897 | 1.000 | 0.990 | 1.000 | 0.951 | 0.970 | 1.000 | 0.849 | 1.000 | 1.000 | 0.860 | 0.832 |

| | | | | | | | | | | | | | | | | | | |
|---|---|---|---|---|---|---|---|---|---|---|---|---|---|---|---|---|---|---|
| 1364 | 0.775 | 1.000 | 0.818 | 1.000 | 0.974 | 0.960 | 0.901 | 1.000 | 0.980 | 1.000 | 0.962 | 0.939 | 1.000 | 0.856 | 1.000 | 0.981 | 0.860 | 0.832 |
| 1365 | 0.785 | 1.000 | 0.804 | 1.000 | 0.974 | 0.960 | 0.906 | 1.000 | 0.990 | 1.000 | 0.954 | 0.939 | 1.000 | 0.851 | 1.000 | 0.981 | 0.860 | 0.832 |
| 1366 | 0.771 | 1.000 | 0.834 | 0.986 | 0.974 | 0.960 | 0.901 | 1.000 | 0.980 | 1.000 | 0.957 | 0.939 | 1.000 | 0.851 | 1.000 | 1.000 | 0.858 | 0.830 |
| 1367 | 0.779 | 1.000 | 0.826 | 1.000 | 0.974 | 0.960 | 0.898 | 1.000 | 0.990 | 1.000 | 0.959 | 0.970 | 1.000 | 0.854 | 1.000 | 0.981 | 0.861 | 0.833 |
| 1368 | 0.780 | 1.000 | 0.812 | 1.000 | 0.968 | 0.960 | 0.901 | 1.000 | 0.990 | 1.000 | 0.954 | 0.939 | 1.000 | 0.856 | 1.000 | 0.981 | 0.860 | 0.832 |
| 1369 | 0.781 | 1.000 | 0.814 | 1.000 | 0.974 | 0.960 | 0.904 | 1.000 | 0.990 | 1.000 | 0.968 | 0.970 | 1.000 | 0.850 | 1.000 | 0.981 | 0.861 | 0.833 |
| 1370 | 0.775 | 1.000 | 0.820 | 1.000 | 0.968 | 0.960 | 0.889 | 1.000 | 0.980 | 1.000 | 0.962 | 1.000 | 1.000 | 0.854 | 1.000 | 0.981 | 0.858 | 0.829 |
| 1371 | 0.776 | 1.000 | 0.830 | 1.000 | 0.963 | 0.960 | 0.906 | 1.000 | 0.990 | 1.000 | 0.954 | 0.970 | 1.000 | 0.854 | 1.000 | 0.981 | 0.861 | 0.833 |
| 1372 | 0.777 | 1.000 | 0.820 | 1.000 | 0.968 | 0.960 | 0.897 | 1.000 | 0.990 | 1.000 | 0.959 | 0.939 | 1.000 | 0.854 | 1.000 | 1.000 | 0.859 | 0.831 |
| 1373 | 0.771 | 1.000 | 0.806 | 1.000 | 0.968 | 0.960 | 0.906 | 1.000 | 0.990 | 1.000 | 0.962 | 0.939 | 1.000 | 0.852 | 1.000 | 1.000 | 0.857 | 0.829 |
| 1374 | 0.771 | 1.000 | 0.816 | 1.000 | 0.968 | 0.960 | 0.897 | 1.000 | 0.990 | 1.000 | 0.962 | 0.970 | 1.000 | 0.858 | 1.000 | 1.000 | 0.859 | 0.831 |
| 1375 | 0.778 | 1.000 | 0.824 | 1.000 | 0.974 | 0.960 | 0.906 | 1.000 | 0.990 | 1.000 | 0.954 | 0.970 | 1.000 | 0.849 | 1.000 | 0.981 | 0.860 | 0.832 |
| 1376 | 0.783 | 1.000 | 0.820 | 1.000 | 0.968 | 0.960 | 0.892 | 1.000 | 0.990 | 1.000 | 0.959 | 0.970 | 1.000 | 0.856 | 1.000 | 1.000 | 0.861 | 0.833 |
| 1377 | 0.779 | 1.000 | 0.826 | 1.000 | 0.974 | 0.960 | 0.901 | 1.000 | 0.980 | 1.000 | 0.962 | 0.939 | 1.000 | 0.852 | 1.000 | 0.981 | 0.861 | 0.833 |
| 1378 | 0.781 | 1.000 | 0.816 | 1.000 | 0.968 | 0.960 | 0.900 | 1.000 | 0.990 | 1.000 | 0.965 | 0.939 | 1.000 | 0.856 | 1.000 | 0.981 | 0.861 | 0.834 |
| 1379 | 0.775 | 1.000 | 0.820 | 1.000 | 0.968 | 0.960 | 0.900 | 1.000 | 0.990 | 1.000 | 0.959 | 0.939 | 1.000 | 0.854 | 1.000 | 1.000 | 0.859 | 0.832 |
| 1380 | 0.783 | 1.000 | 0.814 | 1.000 | 0.974 | 0.960 | 0.900 | 1.000 | 0.990 | 1.000 | 0.959 | 0.970 | 1.000 | 0.855 | 1.000 | 0.981 | 0.862 | 0.834 |
| 1381 | 0.775 | 1.000 | 0.826 | 1.000 | 0.974 | 0.960 | 0.892 | 1.000 | 0.990 | 1.000 | 0.959 | 0.970 | 1.000 | 0.850 | 1.000 | 0.981 | 0.858 | 0.829 |
| 1382 | 0.779 | 1.000 | 0.832 | 0.986 | 0.974 | 0.960 | 0.903 | 1.000 | 0.990 | 1.000 | 0.959 | 0.970 | 1.000 | 0.855 | 1.000 | 1.000 | 0.862 | 0.835 |
| 1383 | 0.773 | 1.000 | 0.810 | 1.000 | 0.974 | 0.960 | 0.904 | 1.000 | 0.980 | 1.000 | 0.962 | 0.970 | 1.000 | 0.849 | 1.000 | 0.981 | 0.857 | 0.829 |
| 1384 | 0.771 | 1.000 | 0.826 | 1.000 | 0.974 | 0.960 | 0.891 | 1.000 | 0.990 | 1.000 | 0.957 | 0.939 | 0.991 | 0.852 | 1.000 | 1.000 | 0.857 | 0.828 |
| 1385 | 0.767 | 1.000 | 0.806 | 1.000 | 0.979 | 1.000 | 0.903 | 1.000 | 0.980 | 1.000 | 0.957 | 0.939 | 1.000 | 0.854 | 1.000 | 0.981 | 0.856 | 0.827 |
| 1386 | 0.778 | 1.000 | 0.812 | 1.000 | 0.968 | 0.960 | 0.898 | 1.000 | 0.980 | 1.000 | 0.962 | 1.000 | 1.000 | 0.854 | 1.000 | 0.981 | 0.859 | 0.831 |
| 1387 | 0.775 | 1.000 | 0.818 | 1.000 | 0.968 | 0.960 | 0.911 | 1.000 | 0.980 | 1.000 | 0.959 | 1.000 | 1.000 | 0.851 | 1.000 | 0.981 | 0.860 | 0.832 |
| 1388 | 0.778 | 1.000 | 0.822 | 1.000 | 0.979 | 0.960 | 0.892 | 1.000 | 0.980 | 1.000 | 0.957 | 0.970 | 1.000 | 0.852 | 1.000 | 1.000 | 0.859 | 0.831 |
| 1389 | 0.781 | 1.000 | 0.824 | 1.000 | 0.979 | 0.960 | 0.901 | 1.000 | 0.990 | 1.000 | 0.962 | 0.939 | 1.000 | 0.852 | 1.000 | 0.981 | 0.862 | 0.834 |
| 1390 | 0.774 | 1.000 | 0.832 | 1.000 | 0.968 | 0.960 | 0.898 | 1.000 | 0.980 | 1.000 | 0.957 | 0.939 | 1.000 | 0.850 | 1.000 | 0.981 | 0.858 | 0.830 |
| 1391 | 0.775 | 1.000 | 0.826 | 1.000 | 0.968 | 0.960 | 0.895 | 1.000 | 0.980 | 1.000 | 0.957 | 0.939 | 1.000 | 0.856 | 1.000 | 1.000 | 0.859 | 0.831 |
| 1392 | 0.771 | 1.000 | 0.810 | 1.000 | 0.974 | 0.960 | 0.900 | 1.000 | 0.990 | 1.000 | 0.959 | 0.939 | 1.000 | 0.851 | 1.000 | 1.000 | 0.856 | 0.828 |
| 1393 | 0.777 | 1.000 | 0.824 | 1.000 | 0.979 | 0.960 | 0.898 | 1.000 | 0.990 | 1.000 | 0.965 | 0.939 | 1.000 | 0.846 | 1.000 | 0.981 | 0.858 | 0.830 |
| 1394 | 0.773 | 1.000 | 0.826 | 1.000 | 0.974 | 0.960 | 0.911 | 1.000 | 0.990 | 1.000 | 0.962 | 0.939 | 1.000 | 0.848 | 1.000 | 0.981 | 0.859 | 0.832 |
| 1395 | 0.777 | 1.000 | 0.804 | 1.000 | 0.974 | 0.960 | 0.900 | 1.000 | 0.990 | 1.000 | 0.959 | 0.970 | 1.000 | 0.856 | 1.000 | 0.981 | 0.859 | 0.831 |
| 1396 | 0.782 | 1.000 | 0.824 | 1.000 | 0.968 | 0.960 | 0.895 | 1.000 | 0.990 | 1.000 | 0.954 | 0.970 | 1.000 | 0.857 | 1.000 | 0.981 | 0.861 | 0.834 |
| 1397 | 0.778 | 1.000 | 0.830 | 1.000 | 0.968 | 0.960 | 0.903 | 1.000 | 0.990 | 1.000 | 0.954 | 0.939 | 1.000 | 0.852 | 1.000 | 1.000 | 0.861 | 0.833 |
| 1398 | 0.777 | 1.000 | 0.818 | 1.000 | 0.968 | 0.960 | 0.901 | 1.000 | 0.990 | 1.000 | 0.951 | 0.939 | 1.000 | 0.861 | 1.000 | 1.000 | 0.861 | 0.833 |
| 1399 | 0.777 | 1.000 | 0.816 | 1.000 | 0.968 | 0.960 | 0.897 | 1.000 | 0.990 | 1.000 | 0.962 | 0.939 | 1.000 | 0.854 | 1.000 | 0.981 | 0.859 | 0.831 |
| 1400 | 0.786 | 1.000 | 0.800 | 1.000 | 0.968 | 0.960 | 0.895 | 1.000 | 0.980 | 1.000 | 0.957 | 0.939 | 1.000 | 0.851 | 1.000 | 1.000 | 0.859 | 0.830 |
| 1401 | 0.770 | 1.000 | 0.830 | 1.000 | 0.974 | 1.000 | 0.897 | 1.000 | 0.980 | 1.000 | 0.962 | 1.000 | 1.000 | 0.849 | 1.000 | 0.981 | 0.858 | 0.829 |
| 1402 | 0.776 | 1.000 | 0.818 | 1.000 | 0.974 | 0.960 | 0.897 | 1.000 | 0.980 | 1.000 | 0.954 | 0.970 | 1.000 | 0.855 | 1.000 | 1.000 | 0.859 | 0.831 |

| | | | | | | | | | | | | | | | | | |
|---|---|---|---|---|---|---|---|---|---|---|---|---|---|---|---|---|---|
| 1403 | 0.776 | 1.000 | 0.818 | 0.986 | 0.974 | 0.960 | 0.909 | 1.000 | 0.980 | 1.000 | 0.954 | 0.939 | 1.000 | 0.851 | 1.000 | 0.981 | 0.859 | 0.831 |
| 1404 | 0.770 | 1.000 | 0.816 | 1.000 | 0.968 | 0.960 | 0.908 | 1.000 | 0.980 | 1.000 | 0.954 | 0.970 | 1.000 | 0.854 | 1.000 | 0.981 | 0.858 | 0.829 |
| 1405 | 0.780 | 1.000 | 0.806 | 1.000 | 0.968 | 0.960 | 0.903 | 1.000 | 0.980 | 1.000 | 0.959 | 0.939 | 1.000 | 0.847 | 1.000 | 1.000 | 0.858 | 0.829 |
| 1406 | 0.783 | 1.000 | 0.824 | 1.000 | 0.974 | 0.960 | 0.904 | 1.000 | 0.980 | 1.000 | 0.954 | 0.970 | 1.000 | 0.854 | 1.000 | 1.000 | 0.862 | 0.835 |
| 1407 | 0.784 | 1.000 | 0.804 | 0.986 | 0.963 | 0.960 | 0.906 | 1.000 | 0.990 | 1.000 | 0.957 | 0.939 | 1.000 | 0.846 | 1.000 | 1.000 | 0.858 | 0.830 |
| 1408 | 0.774 | 1.000 | 0.820 | 1.000 | 0.968 | 0.960 | 0.900 | 1.000 | 0.980 | 1.000 | 0.962 | 0.970 | 1.000 | 0.847 | 1.000 | 1.000 | 0.857 | 0.829 |
| 1409 | 0.776 | 1.000 | 0.828 | 0.986 | 0.968 | 0.960 | 0.897 | 1.000 | 0.990 | 1.000 | 0.957 | 0.939 | 1.000 | 0.858 | 1.000 | 0.981 | 0.860 | 0.832 |
| 1410 | 0.769 | 1.000 | 0.810 | 1.000 | 0.968 | 0.960 | 0.892 | 1.000 | 0.980 | 1.000 | 0.959 | 0.970 | 1.000 | 0.853 | 1.000 | 1.000 | 0.855 | 0.826 |
| 1411 | 0.773 | 1.000 | 0.814 | 0.986 | 0.968 | 0.960 | 0.897 | 1.000 | 0.990 | 1.000 | 0.957 | 0.970 | 1.000 | 0.854 | 1.000 | 0.981 | 0.857 | 0.829 |
| 1412 | 0.781 | 1.000 | 0.816 | 1.000 | 0.968 | 0.960 | 0.897 | 1.000 | 0.980 | 1.000 | 0.951 | 0.970 | 1.000 | 0.858 | 1.000 | 1.000 | 0.861 | 0.833 |
| 1413 | 0.784 | 1.000 | 0.820 | 1.000 | 0.968 | 0.960 | 0.888 | 1.000 | 0.990 | 1.000 | 0.948 | 0.939 | 1.000 | 0.851 | 1.000 | 1.000 | 0.859 | 0.830 |
| 1414 | 0.771 | 1.000 | 0.822 | 1.000 | 0.968 | 0.960 | 0.906 | 1.000 | 0.990 | 1.000 | 0.959 | 0.970 | 1.000 | 0.854 | 1.000 | 1.000 | 0.859 | 0.831 |
| 1415 | 0.775 | 1.000 | 0.814 | 1.000 | 0.974 | 0.960 | 0.903 | 1.000 | 0.990 | 1.000 | 0.951 | 0.970 | 1.000 | 0.851 | 1.000 | 1.000 | 0.858 | 0.830 |
| 1416 | 0.785 | 1.000 | 0.834 | 1.000 | 0.968 | 0.960 | 0.901 | 1.000 | 0.980 | 1.000 | 0.948 | 0.939 | 1.000 | 0.856 | 1.000 | 1.000 | 0.863 | 0.836 |
| 1417 | 0.779 | 1.000 | 0.822 | 1.000 | 0.974 | 0.960 | 0.892 | 1.000 | 0.990 | 1.000 | 0.957 | 0.970 | 1.000 | 0.856 | 1.000 | 1.000 | 0.860 | 0.833 |
| 1418 | 0.784 | 1.000 | 0.808 | 1.000 | 0.974 | 0.960 | 0.895 | 1.000 | 0.969 | 1.000 | 0.954 | 0.939 | 1.000 | 0.858 | 1.000 | 1.000 | 0.860 | 0.832 |
| 1419 | 0.777 | 1.000 | 0.822 | 1.000 | 0.963 | 0.960 | 0.903 | 1.000 | 0.990 | 1.000 | 0.954 | 0.970 | 1.000 | 0.852 | 1.000 | 0.981 | 0.859 | 0.832 |
| 1420 | 0.776 | 1.000 | 0.820 | 1.000 | 0.968 | 0.960 | 0.895 | 1.000 | 0.980 | 1.000 | 0.957 | 0.970 | 1.000 | 0.856 | 1.000 | 0.981 | 0.859 | 0.831 |
| 1421 | 0.782 | 1.000 | 0.822 | 1.000 | 0.968 | 0.960 | 0.901 | 1.000 | 0.990 | 1.000 | 0.957 | 1.000 | 1.000 | 0.850 | 1.000 | 1.000 | 0.861 | 0.833 |
| 1422 | 0.776 | 1.000 | 0.826 | 1.000 | 0.974 | 0.960 | 0.895 | 1.000 | 0.990 | 1.000 | 0.965 | 0.970 | 1.000 | 0.858 | 1.000 | 1.000 | 0.861 | 0.834 |
| 1423 | 0.765 | 1.000 | 0.828 | 1.000 | 0.974 | 0.960 | 0.889 | 1.000 | 0.980 | 1.000 | 0.959 | 1.000 | 1.000 | 0.856 | 1.000 | 0.981 | 0.856 | 0.828 |
| 1424 | 0.776 | 1.000 | 0.822 | 1.000 | 0.963 | 0.960 | 0.898 | 1.000 | 0.980 | 1.000 | 0.951 | 0.970 | 1.000 | 0.848 | 1.000 | 0.981 | 0.857 | 0.828 |
| 1425 | 0.780 | 1.000 | 0.814 | 0.986 | 0.968 | 0.960 | 0.892 | 1.000 | 0.990 | 1.000 | 0.965 | 0.970 | 1.000 | 0.858 | 1.000 | 0.981 | 0.860 | 0.832 |
| 1426 | 0.778 | 1.000 | 0.834 | 1.000 | 0.968 | 0.960 | 0.897 | 1.000 | 0.990 | 1.000 | 0.962 | 0.939 | 1.000 | 0.854 | 1.000 | 1.000 | 0.861 | 0.833 |
| 1427 | 0.765 | 1.000 | 0.822 | 1.000 | 0.974 | 0.960 | 0.895 | 1.000 | 0.990 | 1.000 | 0.954 | 1.000 | 1.000 | 0.854 | 1.000 | 1.000 | 0.856 | 0.828 |
| 1428 | 0.778 | 1.000 | 0.830 | 1.000 | 0.974 | 0.960 | 0.897 | 1.000 | 0.990 | 1.000 | 0.957 | 0.939 | 1.000 | 0.855 | 1.000 | 0.981 | 0.861 | 0.833 |
| 1429 | 0.783 | 1.000 | 0.816 | 1.000 | 0.974 | 0.960 | 0.895 | 1.000 | 0.980 | 1.000 | 0.962 | 0.970 | 1.000 | 0.858 | 1.000 | 1.000 | 0.862 | 0.834 |
| 1430 | 0.774 | 1.000 | 0.804 | 1.000 | 0.968 | 0.960 | 0.900 | 1.000 | 0.990 | 1.000 | 0.959 | 0.939 | 1.000 | 0.856 | 1.000 | 1.000 | 0.858 | 0.829 |
| 1431 | 0.773 | 1.000 | 0.828 | 1.000 | 0.974 | 0.960 | 0.894 | 1.000 | 0.990 | 1.000 | 0.957 | 0.939 | 1.000 | 0.858 | 1.000 | 0.981 | 0.859 | 0.831 |
| 1432 | 0.777 | 1.000 | 0.818 | 1.000 | 0.974 | 0.960 | 0.897 | 1.000 | 0.980 | 1.000 | 0.959 | 0.909 | 1.000 | 0.857 | 1.000 | 1.000 | 0.860 | 0.832 |
| 1433 | 0.777 | 1.000 | 0.822 | 1.000 | 0.968 | 0.960 | 0.903 | 1.000 | 0.980 | 1.000 | 0.959 | 0.970 | 1.000 | 0.851 | 1.000 | 1.000 | 0.859 | 0.832 |
| 1434 | 0.771 | 1.000 | 0.816 | 1.000 | 0.968 | 0.960 | 0.900 | 1.000 | 0.990 | 1.000 | 0.959 | 1.000 | 1.000 | 0.854 | 1.000 | 0.981 | 0.858 | 0.830 |
| 1435 | 0.785 | 1.000 | 0.816 | 1.000 | 0.968 | 0.960 | 0.897 | 1.000 | 0.990 | 1.000 | 0.957 | 0.970 | 1.000 | 0.850 | 1.000 | 0.981 | 0.860 | 0.832 |
| 1436 | 0.776 | 1.000 | 0.814 | 1.000 | 0.968 | 0.960 | 0.904 | 1.000 | 0.980 | 1.000 | 0.962 | 0.939 | 1.000 | 0.849 | 1.000 | 0.981 | 0.858 | 0.830 |
| 1437 | 0.770 | 1.000 | 0.830 | 1.000 | 0.968 | 0.960 | 0.904 | 1.000 | 0.990 | 1.000 | 0.959 | 0.909 | 1.000 | 0.856 | 1.000 | 1.000 | 0.860 | 0.832 |
| 1438 | 0.781 | 1.000 | 0.802 | 1.000 | 0.968 | 0.960 | 0.908 | 1.000 | 0.980 | 1.000 | 0.962 | 0.939 | 1.000 | 0.851 | 1.000 | 1.000 | 0.859 | 0.831 |
| 1439 | 0.774 | 1.000 | 0.824 | 1.000 | 0.968 | 0.960 | 0.900 | 1.000 | 0.980 | 1.000 | 0.959 | 0.939 | 1.000 | 0.846 | 1.000 | 1.000 | 0.857 | 0.829 |
| 1440 | 0.779 | 1.000 | 0.814 | 1.000 | 0.974 | 0.960 | 0.901 | 1.000 | 0.990 | 1.000 | 0.962 | 0.939 | 1.000 | 0.851 | 1.000 | 1.000 | 0.860 | 0.832 |
| 1441 | 0.781 | 1.000 | 0.822 | 1.000 | 0.968 | 0.960 | 0.903 | 1.000 | 0.990 | 1.000 | 0.957 | 0.939 | 1.000 | 0.852 | 1.000 | 1.000 | 0.861 | 0.833 |

| | | | | | | | | | | | | | | | | | |
|---|---|---|---|---|---|---|---|---|---|---|---|---|---|---|---|---|---|
| 1442 | 0.778 | 1.000 | 0.816 | 1.000 | 0.963 | 0.960 | 0.900 | 1.000 | 0.990 | 1.000 | 0.962 | 0.939 | 1.000 | 0.861 | 1.000 | 0.981 | 0.861 | 0.833 |
| 1443 | 0.781 | 1.000 | 0.816 | 1.000 | 0.979 | 0.960 | 0.894 | 1.000 | 0.980 | 1.000 | 0.954 | 0.970 | 1.000 | 0.857 | 1.000 | 0.981 | 0.860 | 0.832 |
| 1444 | 0.771 | 1.000 | 0.820 | 1.000 | 0.968 | 0.960 | 0.898 | 1.000 | 0.990 | 1.000 | 0.954 | 1.000 | 1.000 | 0.855 | 1.000 | 0.981 | 0.858 | 0.830 |
| 1445 | 0.773 | 1.000 | 0.834 | 1.000 | 0.974 | 0.960 | 0.895 | 1.000 | 0.990 | 1.000 | 0.962 | 0.939 | 1.000 | 0.855 | 1.000 | 0.981 | 0.860 | 0.832 |
| 1446 | 0.776 | 1.000 | 0.820 | 0.986 | 0.968 | 0.960 | 0.889 | 1.000 | 0.980 | 1.000 | 0.959 | 1.000 | 1.000 | 0.857 | 1.000 | 1.000 | 0.859 | 0.830 |
| 1447 | 0.773 | 1.000 | 0.800 | 1.000 | 0.968 | 0.960 | 0.901 | 1.000 | 0.990 | 1.000 | 0.965 | 0.970 | 1.000 | 0.851 | 1.000 | 0.981 | 0.857 | 0.828 |
| 1448 | 0.774 | 1.000 | 0.820 | 1.000 | 0.968 | 0.960 | 0.884 | 1.000 | 0.980 | 1.000 | 0.959 | 0.939 | 1.000 | 0.859 | 1.000 | 1.000 | 0.858 | 0.830 |
| 1449 | 0.779 | 1.000 | 0.820 | 1.000 | 0.968 | 0.960 | 0.903 | 1.000 | 0.990 | 1.000 | 0.957 | 1.000 | 1.000 | 0.854 | 1.000 | 1.000 | 0.861 | 0.834 |
| 1450 | 0.774 | 1.000 | 0.808 | 1.000 | 0.974 | 0.960 | 0.894 | 1.000 | 0.980 | 1.000 | 0.957 | 0.939 | 1.000 | 0.858 | 1.000 | 0.981 | 0.857 | 0.829 |
| 1451 | 0.773 | 1.000 | 0.826 | 1.000 | 0.968 | 0.960 | 0.898 | 1.000 | 0.990 | 1.000 | 0.962 | 0.970 | 1.000 | 0.854 | 1.000 | 0.981 | 0.859 | 0.831 |
| 1452 | 0.770 | 1.000 | 0.816 | 1.000 | 0.974 | 0.960 | 0.897 | 1.000 | 0.990 | 1.000 | 0.957 | 0.909 | 1.000 | 0.854 | 1.000 | 1.000 | 0.857 | 0.828 |
| 1453 | 0.779 | 1.000 | 0.798 | 1.000 | 0.979 | 0.960 | 0.895 | 1.000 | 0.980 | 1.000 | 0.962 | 0.970 | 1.000 | 0.850 | 1.000 | 1.000 | 0.857 | 0.828 |
| 1454 | 0.769 | 1.000 | 0.810 | 1.000 | 0.974 | 0.960 | 0.904 | 1.000 | 0.980 | 1.000 | 0.954 | 0.939 | 1.000 | 0.851 | 1.000 | 0.981 | 0.856 | 0.827 |
| 1455 | 0.770 | 1.000 | 0.810 | 1.000 | 0.974 | 0.960 | 0.895 | 1.000 | 0.990 | 1.000 | 0.954 | 1.000 | 1.000 | 0.858 | 1.000 | 1.000 | 0.857 | 0.829 |
| 1456 | 0.777 | 1.000 | 0.830 | 0.986 | 0.968 | 0.960 | 0.898 | 1.000 | 0.980 | 1.000 | 0.959 | 0.970 | 1.000 | 0.852 | 1.000 | 1.000 | 0.860 | 0.832 |
| 1457 | 0.771 | 1.000 | 0.812 | 1.000 | 0.968 | 0.960 | 0.898 | 1.000 | 0.980 | 1.000 | 0.957 | 0.939 | 1.000 | 0.848 | 1.000 | 0.981 | 0.855 | 0.826 |
| 1458 | 0.776 | 1.000 | 0.816 | 1.000 | 0.979 | 0.960 | 0.895 | 1.000 | 0.980 | 1.000 | 0.959 | 0.939 | 1.000 | 0.856 | 1.000 | 0.981 | 0.859 | 0.831 |
| 1459 | 0.773 | 1.000 | 0.818 | 1.000 | 0.979 | 0.960 | 0.894 | 1.000 | 0.969 | 1.000 | 0.954 | 0.939 | 1.000 | 0.858 | 1.000 | 1.000 | 0.858 | 0.830 |
| 1460 | 0.769 | 1.000 | 0.822 | 1.000 | 0.968 | 0.960 | 0.891 | 1.000 | 0.980 | 1.000 | 0.957 | 0.970 | 1.000 | 0.850 | 1.000 | 1.000 | 0.855 | 0.826 |
| 1461 | 0.769 | 1.000 | 0.814 | 1.000 | 0.968 | 0.960 | 0.904 | 1.000 | 0.969 | 1.000 | 0.954 | 0.970 | 1.000 | 0.858 | 1.000 | 0.981 | 0.858 | 0.829 |
| 1462 | 0.765 | 1.000 | 0.818 | 1.000 | 0.984 | 0.960 | 0.889 | 1.000 | 0.990 | 1.000 | 0.957 | 0.970 | 1.000 | 0.848 | 1.000 | 1.000 | 0.854 | 0.825 |
| 1463 | 0.777 | 1.000 | 0.808 | 1.000 | 0.974 | 0.960 | 0.895 | 1.000 | 0.990 | 1.000 | 0.959 | 0.970 | 1.000 | 0.857 | 1.000 | 1.000 | 0.859 | 0.831 |
| 1464 | 0.775 | 1.000 | 0.810 | 1.000 | 0.968 | 0.960 | 0.901 | 1.000 | 0.980 | 1.000 | 0.957 | 0.939 | 1.000 | 0.858 | 1.000 | 1.000 | 0.859 | 0.831 |
| 1465 | 0.773 | 1.000 | 0.822 | 1.000 | 0.974 | 0.960 | 0.897 | 1.000 | 0.980 | 1.000 | 0.959 | 0.970 | 1.000 | 0.855 | 1.000 | 0.981 | 0.859 | 0.831 |
| 1466 | 0.776 | 1.000 | 0.808 | 1.000 | 0.974 | 0.960 | 0.898 | 1.000 | 0.990 | 1.000 | 0.965 | 0.970 | 1.000 | 0.854 | 1.000 | 1.000 | 0.859 | 0.831 |
| 1467 | 0.777 | 1.000 | 0.808 | 1.000 | 0.974 | 0.960 | 0.894 | 1.000 | 0.980 | 1.000 | 0.957 | 1.000 | 1.000 | 0.859 | 1.000 | 0.981 | 0.859 | 0.831 |
| 1468 | 0.780 | 1.000 | 0.818 | 1.000 | 0.979 | 0.960 | 0.897 | 1.000 | 0.969 | 1.000 | 0.959 | 0.970 | 0.991 | 0.858 | 1.000 | 1.000 | 0.861 | 0.833 |
| 1469 | 0.779 | 1.000 | 0.820 | 1.000 | 0.968 | 0.960 | 0.903 | 1.000 | 0.969 | 1.000 | 0.962 | 0.970 | 1.000 | 0.851 | 1.000 | 0.981 | 0.860 | 0.832 |
| 1470 | 0.781 | 1.000 | 0.812 | 0.986 | 0.963 | 0.960 | 0.898 | 1.000 | 0.980 | 1.000 | 0.959 | 0.970 | 0.991 | 0.856 | 1.000 | 0.981 | 0.860 | 0.831 |
| 1471 | 0.781 | 1.000 | 0.812 | 1.000 | 0.979 | 0.960 | 0.906 | 1.000 | 0.990 | 1.000 | 0.954 | 0.970 | 1.000 | 0.855 | 1.000 | 0.981 | 0.861 | 0.833 |
| 1472 | 0.776 | 1.000 | 0.814 | 1.000 | 0.963 | 0.960 | 0.897 | 1.000 | 0.980 | 1.000 | 0.959 | 0.970 | 1.000 | 0.851 | 1.000 | 0.981 | 0.857 | 0.829 |
| 1473 | 0.777 | 1.000 | 0.828 | 1.000 | 0.968 | 0.960 | 0.898 | 1.000 | 0.969 | 1.000 | 0.954 | 0.970 | 1.000 | 0.849 | 1.000 | 0.981 | 0.858 | 0.830 |
| 1474 | 0.784 | 1.000 | 0.826 | 1.000 | 0.979 | 0.960 | 0.892 | 1.000 | 0.990 | 1.000 | 0.957 | 0.970 | 1.000 | 0.854 | 1.000 | 1.000 | 0.862 | 0.834 |
| 1475 | 0.765 | 1.000 | 0.834 | 1.000 | 0.979 | 0.960 | 0.901 | 1.000 | 0.969 | 1.000 | 0.957 | 0.970 | 1.000 | 0.854 | 1.000 | 1.000 | 0.858 | 0.830 |
| 1476 | 0.782 | 1.000 | 0.818 | 1.000 | 0.979 | 0.960 | 0.898 | 1.000 | 0.969 | 1.000 | 0.962 | 0.970 | 0.991 | 0.856 | 1.000 | 0.981 | 0.861 | 0.833 |
| 1477 | 0.778 | 1.000 | 0.810 | 1.000 | 0.968 | 0.960 | 0.898 | 1.000 | 0.980 | 1.000 | 0.957 | 1.000 | 1.000 | 0.847 | 1.000 | 0.981 | 0.857 | 0.828 |
| 1478 | 0.777 | 1.000 | 0.814 | 1.000 | 0.968 | 0.960 | 0.898 | 1.000 | 0.980 | 1.000 | 0.957 | 0.970 | 1.000 | 0.861 | 1.000 | 1.000 | 0.860 | 0.833 |
| 1479 | 0.775 | 1.000 | 0.818 | 1.000 | 0.974 | 0.960 | 0.898 | 1.000 | 0.990 | 1.000 | 0.957 | 1.000 | 1.000 | 0.854 | 1.000 | 1.000 | 0.859 | 0.831 |
| 1480 | 0.769 | 1.000 | 0.810 | 1.000 | 0.974 | 0.960 | 0.906 | 1.000 | 0.980 | 1.000 | 0.962 | 1.000 | 0.991 | 0.850 | 1.000 | 0.981 | 0.856 | 0.828 |

| | | | | | | | | | | | | | | | | | |
|---|---|---|---|---|---|---|---|---|---|---|---|---|---|---|---|---|---|
| 1481 | 0.772 | 1.000 | 0.822 | 1.000 | 0.968 | 0.960 | 0.891 | 1.000 | 0.980 | 1.000 | 0.957 | 0.970 | 1.000 | 0.858 | 1.000 | 1.000 | 0.858 | 0.830 |
| 1482 | 0.781 | 1.000 | 0.808 | 1.000 | 0.974 | 0.960 | 0.895 | 1.000 | 0.980 | 1.000 | 0.957 | 0.970 | 1.000 | 0.858 | 1.000 | 0.981 | 0.860 | 0.832 |
| 1483 | 0.777 | 1.000 | 0.816 | 0.986 | 0.974 | 0.960 | 0.898 | 1.000 | 0.990 | 1.000 | 0.957 | 1.000 | 1.000 | 0.846 | 1.000 | 0.981 | 0.857 | 0.829 |
| 1484 | 0.780 | 1.000 | 0.818 | 1.000 | 0.979 | 0.960 | 0.892 | 1.000 | 0.969 | 1.000 | 0.957 | 0.939 | 1.000 | 0.854 | 1.000 | 0.981 | 0.859 | 0.831 |
| 1485 | 0.768 | 1.000 | 0.828 | 1.000 | 0.968 | 0.960 | 0.892 | 1.000 | 0.990 | 1.000 | 0.959 | 0.970 | 1.000 | 0.849 | 1.000 | 1.000 | 0.856 | 0.827 |
| 1486 | 0.776 | 1.000 | 0.816 | 0.986 | 0.974 | 1.000 | 0.892 | 1.000 | 0.969 | 1.000 | 0.959 | 0.970 | 1.000 | 0.844 | 1.000 | 0.981 | 0.855 | 0.826 |
| 1487 | 0.774 | 1.000 | 0.826 | 1.000 | 0.974 | 0.960 | 0.900 | 1.000 | 0.980 | 1.000 | 0.957 | 0.970 | 1.000 | 0.859 | 1.000 | 0.981 | 0.861 | 0.833 |
| 1488 | 0.768 | 1.000 | 0.822 | 1.000 | 0.974 | 0.960 | 0.897 | 1.000 | 0.980 | 1.000 | 0.959 | 0.970 | 1.000 | 0.857 | 1.000 | 1.000 | 0.858 | 0.830 |
| 1489 | 0.774 | 1.000 | 0.820 | 1.000 | 0.974 | 0.960 | 0.901 | 1.000 | 0.990 | 1.000 | 0.962 | 0.939 | 1.000 | 0.851 | 1.000 | 1.000 | 0.859 | 0.830 |
| 1490 | 0.780 | 1.000 | 0.828 | 1.000 | 0.974 | 0.960 | 0.894 | 1.000 | 0.980 | 1.000 | 0.962 | 0.970 | 1.000 | 0.844 | 1.000 | 1.000 | 0.859 | 0.830 |
| 1491 | 0.781 | 1.000 | 0.826 | 0.986 | 0.974 | 0.960 | 0.898 | 1.000 | 0.980 | 1.000 | 0.951 | 0.970 | 1.000 | 0.852 | 1.000 | 0.981 | 0.860 | 0.832 |
| 1492 | 0.779 | 1.000 | 0.812 | 1.000 | 0.968 | 0.960 | 0.901 | 1.000 | 0.980 | 1.000 | 0.962 | 0.970 | 1.000 | 0.857 | 1.000 | 1.000 | 0.861 | 0.833 |
| 1493 | 0.778 | 1.000 | 0.812 | 0.986 | 0.968 | 0.960 | 0.895 | 1.000 | 0.980 | 1.000 | 0.954 | 0.970 | 1.000 | 0.852 | 1.000 | 1.000 | 0.858 | 0.829 |
| 1494 | 0.785 | 1.000 | 0.814 | 0.986 | 0.968 | 0.960 | 0.900 | 1.000 | 0.980 | 1.000 | 0.962 | 0.970 | 1.000 | 0.854 | 1.000 | 1.000 | 0.861 | 0.834 |
| 1495 | 0.776 | 1.000 | 0.832 | 1.000 | 0.968 | 0.960 | 0.900 | 1.000 | 0.980 | 1.000 | 0.959 | 0.970 | 0.991 | 0.853 | 1.000 | 0.981 | 0.860 | 0.832 |
| 1496 | 0.773 | 1.000 | 0.812 | 1.000 | 0.974 | 0.960 | 0.889 | 1.000 | 0.969 | 1.000 | 0.951 | 0.970 | 1.000 | 0.855 | 1.000 | 1.000 | 0.856 | 0.827 |
| 1497 | 0.772 | 1.000 | 0.822 | 1.000 | 0.968 | 0.960 | 0.897 | 1.000 | 0.969 | 1.000 | 0.962 | 0.970 | 1.000 | 0.851 | 1.000 | 0.981 | 0.857 | 0.829 |
| 1498 | 0.776 | 1.000 | 0.816 | 1.000 | 0.974 | 0.960 | 0.894 | 1.000 | 0.990 | 1.000 | 0.957 | 1.000 | 1.000 | 0.854 | 1.000 | 1.000 | 0.859 | 0.830 |
| 1499 | 0.774 | 1.000 | 0.814 | 1.000 | 0.974 | 0.960 | 0.895 | 1.000 | 0.990 | 1.000 | 0.962 | 1.000 | 1.000 | 0.852 | 1.000 | 0.981 | 0.858 | 0.830 |
| 1500 | 0.777 | 1.000 | 0.810 | 0.986 | 0.968 | 0.960 | 0.903 | 1.000 | 0.990 | 1.000 | 0.951 | 0.970 | 1.000 | 0.854 | 1.000 | 1.000 | 0.858 | 0.830 |
| 1501 | 0.771 | 1.000 | 0.818 | 1.000 | 0.974 | 0.960 | 0.903 | 1.000 | 0.980 | 1.000 | 0.962 | 0.970 | 1.000 | 0.859 | 1.000 | 1.000 | 0.860 | 0.832 |
| 1502 | 0.774 | 1.000 | 0.828 | 1.000 | 0.968 | 0.960 | 0.906 | 1.000 | 0.980 | 1.000 | 0.959 | 0.970 | 1.000 | 0.852 | 1.000 | 1.000 | 0.860 | 0.832 |
| 1503 | 0.775 | 1.000 | 0.814 | 1.000 | 0.974 | 0.960 | 0.886 | 1.000 | 0.980 | 1.000 | 0.959 | 0.970 | 1.000 | 0.863 | 1.000 | 0.981 | 0.859 | 0.831 |
| 1504 | 0.770 | 1.000 | 0.810 | 1.000 | 0.979 | 0.960 | 0.891 | 1.000 | 0.990 | 1.000 | 0.954 | 1.000 | 1.000 | 0.859 | 1.000 | 1.000 | 0.857 | 0.829 |
| 1505 | 0.771 | 1.000 | 0.802 | 0.986 | 0.979 | 0.960 | 0.892 | 1.000 | 0.969 | 1.000 | 0.959 | 0.970 | 0.991 | 0.851 | 1.000 | 1.000 | 0.854 | 0.825 |
| 1506 | 0.780 | 1.000 | 0.832 | 1.000 | 0.968 | 0.960 | 0.903 | 1.000 | 0.969 | 1.000 | 0.957 | 0.970 | 1.000 | 0.852 | 1.000 | 0.981 | 0.861 | 0.833 |
| 1507 | 0.777 | 1.000 | 0.812 | 0.986 | 0.963 | 0.960 | 0.897 | 1.000 | 0.969 | 1.000 | 0.957 | 0.970 | 1.000 | 0.847 | 1.000 | 1.000 | 0.856 | 0.827 |
| 1508 | 0.770 | 1.000 | 0.808 | 1.000 | 0.974 | 0.960 | 0.901 | 1.000 | 0.980 | 1.000 | 0.954 | 0.970 | 1.000 | 0.849 | 1.000 | 1.000 | 0.855 | 0.827 |
| 1509 | 0.773 | 1.000 | 0.834 | 1.000 | 0.979 | 0.960 | 0.903 | 1.000 | 0.980 | 1.000 | 0.959 | 0.970 | 1.000 | 0.851 | 1.000 | 1.000 | 0.860 | 0.833 |
| 1510 | 0.778 | 1.000 | 0.824 | 0.986 | 0.974 | 0.960 | 0.908 | 1.000 | 0.980 | 1.000 | 0.957 | 0.970 | 1.000 | 0.847 | 1.000 | 1.000 | 0.860 | 0.832 |
| 1511 | 0.767 | 1.000 | 0.806 | 0.986 | 0.974 | 0.960 | 0.904 | 1.000 | 0.969 | 1.000 | 0.957 | 0.939 | 0.991 | 0.854 | 1.000 | 1.000 | 0.855 | 0.826 |
| 1512 | 0.771 | 1.000 | 0.808 | 0.986 | 0.968 | 0.960 | 0.904 | 1.000 | 0.980 | 1.000 | 0.962 | 1.000 | 1.000 | 0.857 | 1.000 | 0.981 | 0.858 | 0.830 |
| 1513 | 0.782 | 1.000 | 0.832 | 1.000 | 0.968 | 0.960 | 0.895 | 1.000 | 0.959 | 1.000 | 0.957 | 0.970 | 1.000 | 0.844 | 1.000 | 1.000 | 0.859 | 0.831 |
| 1514 | 0.769 | 1.000 | 0.826 | 1.000 | 0.963 | 0.960 | 0.901 | 1.000 | 0.980 | 1.000 | 0.954 | 0.970 | 1.000 | 0.850 | 1.000 | 1.000 | 0.857 | 0.828 |
| 1515 | 0.771 | 1.000 | 0.826 | 1.000 | 0.968 | 0.960 | 0.888 | 1.000 | 0.990 | 1.000 | 0.954 | 1.000 | 1.000 | 0.859 | 1.000 | 0.981 | 0.858 | 0.830 |
| 1516 | 0.780 | 1.000 | 0.822 | 1.000 | 0.974 | 0.960 | 0.897 | 1.000 | 0.969 | 1.000 | 0.954 | 0.970 | 1.000 | 0.858 | 1.000 | 0.981 | 0.861 | 0.833 |
| 1517 | 0.768 | 1.000 | 0.824 | 1.000 | 0.968 | 0.960 | 0.895 | 1.000 | 0.980 | 1.000 | 0.957 | 0.970 | 1.000 | 0.846 | 1.000 | 1.000 | 0.855 | 0.826 |
| 1518 | 0.785 | 1.000 | 0.832 | 1.000 | 0.963 | 0.960 | 0.894 | 1.000 | 0.980 | 1.000 | 0.954 | 0.970 | 1.000 | 0.851 | 1.000 | 0.981 | 0.861 | 0.833 |
| 1519 | 0.777 | 1.000 | 0.808 | 1.000 | 0.974 | 0.960 | 0.903 | 1.000 | 0.969 | 1.000 | 0.959 | 0.970 | 1.000 | 0.854 | 1.000 | 0.981 | 0.859 | 0.831 |

| | | | | | | | | | | | | | | | | | |
|---|---|---|---|---|---|---|---|---|---|---|---|---|---|---|---|---|---|
| 1520 | 0.775 | 1.000 | 0.822 | 1.000 | 0.974 | 0.960 | 0.903 | 1.000 | 0.969 | 1.000 | 0.957 | 0.970 | 1.000 | 0.848 | 1.000 | 1.000 | 0.858 | 0.830 |
| 1521 | 0.782 | 1.000 | 0.810 | 1.000 | 0.974 | 1.000 | 0.897 | 1.000 | 0.980 | 1.000 | 0.962 | 0.970 | 1.000 | 0.849 | 1.000 | 0.981 | 0.859 | 0.831 |
| 1522 | 0.788 | 1.000 | 0.804 | 1.000 | 0.963 | 0.960 | 0.895 | 1.000 | 0.990 | 1.000 | 0.959 | 0.970 | 1.000 | 0.847 | 1.000 | 1.000 | 0.859 | 0.831 |
| 1523 | 0.766 | 1.000 | 0.830 | 1.000 | 0.979 | 0.960 | 0.900 | 1.000 | 0.990 | 1.000 | 0.954 | 0.970 | 1.000 | 0.851 | 1.000 | 1.000 | 0.857 | 0.829 |
| 1524 | 0.777 | 1.000 | 0.830 | 1.000 | 0.974 | 0.960 | 0.901 | 1.000 | 0.990 | 1.000 | 0.951 | 0.970 | 1.000 | 0.853 | 1.000 | 0.981 | 0.860 | 0.833 |
| 1525 | 0.771 | 1.000 | 0.816 | 1.000 | 0.974 | 0.960 | 0.894 | 1.000 | 0.990 | 1.000 | 0.959 | 0.970 | 1.000 | 0.850 | 1.000 | 1.000 | 0.856 | 0.828 |
| 1526 | 0.767 | 1.000 | 0.830 | 0.986 | 0.968 | 0.960 | 0.898 | 1.000 | 0.980 | 1.000 | 0.959 | 1.000 | 1.000 | 0.862 | 1.000 | 1.000 | 0.860 | 0.832 |
| 1527 | 0.772 | 1.000 | 0.834 | 1.000 | 0.968 | 0.960 | 0.894 | 1.000 | 0.990 | 1.000 | 0.959 | 0.970 | 0.991 | 0.854 | 1.000 | 1.000 | 0.859 | 0.831 |
| 1528 | 0.772 | 1.000 | 0.830 | 0.986 | 0.974 | 0.960 | 0.892 | 1.000 | 0.969 | 1.000 | 0.959 | 1.000 | 1.000 | 0.852 | 1.000 | 0.981 | 0.858 | 0.829 |
| 1529 | 0.773 | 1.000 | 0.834 | 1.000 | 0.968 | 0.960 | 0.895 | 1.000 | 0.980 | 1.000 | 0.962 | 1.000 | 0.991 | 0.854 | 1.000 | 1.000 | 0.860 | 0.832 |
| 1530 | 0.771 | 1.000 | 0.834 | 1.000 | 0.974 | 0.960 | 0.903 | 1.000 | 0.980 | 1.000 | 0.957 | 0.970 | 1.000 | 0.854 | 1.000 | 0.981 | 0.860 | 0.832 |
| 1531 | 0.776 | 1.000 | 0.812 | 1.000 | 0.968 | 0.960 | 0.894 | 1.000 | 0.990 | 1.000 | 0.959 | 0.970 | 1.000 | 0.850 | 1.000 | 0.981 | 0.857 | 0.828 |
| 1532 | 0.769 | 1.000 | 0.830 | 1.000 | 0.974 | 0.960 | 0.891 | 1.000 | 0.990 | 1.000 | 0.954 | 0.970 | 1.000 | 0.857 | 1.000 | 0.981 | 0.858 | 0.830 |
| 1533 | 0.783 | 1.000 | 0.812 | 1.000 | 0.979 | 0.960 | 0.901 | 1.000 | 0.969 | 1.000 | 0.959 | 1.000 | 1.000 | 0.860 | 1.000 | 0.981 | 0.863 | 0.835 |
| 1534 | 0.769 | 1.000 | 0.798 | 0.986 | 0.968 | 0.960 | 0.897 | 1.000 | 0.959 | 1.000 | 0.959 | 0.970 | 1.000 | 0.857 | 1.000 | 1.000 | 0.855 | 0.826 |
| 1535 | 0.781 | 1.000 | 0.824 | 1.000 | 0.979 | 0.960 | 0.900 | 1.000 | 0.980 | 1.000 | 0.962 | 0.970 | 1.000 | 0.853 | 1.000 | 0.981 | 0.861 | 0.834 |
| 1536 | 0.781 | 1.000 | 0.810 | 1.000 | 0.974 | 0.960 | 0.903 | 1.000 | 0.980 | 1.000 | 0.959 | 0.970 | 1.000 | 0.850 | 1.000 | 1.000 | 0.860 | 0.832 |
| 1537 | 0.778 | 1.000 | 0.836 | 1.000 | 0.974 | 0.960 | 0.900 | 1.000 | 0.969 | 1.000 | 0.951 | 0.970 | 1.000 | 0.858 | 1.000 | 1.000 | 0.862 | 0.835 |
| 1538 | 0.777 | 1.000 | 0.806 | 1.000 | 0.974 | 1.000 | 0.895 | 1.000 | 0.990 | 1.000 | 0.957 | 0.970 | 1.000 | 0.855 | 1.000 | 0.981 | 0.858 | 0.830 |
| 1539 | 0.779 | 1.000 | 0.826 | 1.000 | 0.968 | 0.960 | 0.895 | 1.000 | 0.990 | 1.000 | 0.959 | 1.000 | 1.000 | 0.855 | 1.000 | 1.000 | 0.861 | 0.833 |
| 1540 | 0.783 | 1.000 | 0.814 | 1.000 | 0.974 | 0.960 | 0.904 | 1.000 | 0.980 | 1.000 | 0.959 | 0.970 | 1.000 | 0.853 | 1.000 | 0.981 | 0.861 | 0.834 |
| 1541 | 0.765 | 1.000 | 0.830 | 1.000 | 0.968 | 0.960 | 0.898 | 1.000 | 0.969 | 1.000 | 0.957 | 0.970 | 1.000 | 0.858 | 1.000 | 0.981 | 0.857 | 0.829 |
| 1542 | 0.778 | 1.000 | 0.826 | 1.000 | 0.974 | 0.960 | 0.888 | 1.000 | 0.969 | 1.000 | 0.959 | 1.000 | 1.000 | 0.844 | 1.000 | 1.000 | 0.857 | 0.828 |
| 1543 | 0.767 | 1.000 | 0.828 | 1.000 | 0.974 | 0.960 | 0.908 | 1.000 | 0.990 | 1.000 | 0.954 | 0.970 | 1.000 | 0.851 | 1.000 | 1.000 | 0.858 | 0.830 |
| 1544 | 0.773 | 1.000 | 0.806 | 1.000 | 0.968 | 1.000 | 0.906 | 1.000 | 0.990 | 1.000 | 0.957 | 1.000 | 1.000 | 0.851 | 1.000 | 1.000 | 0.858 | 0.829 |
| 1545 | 0.778 | 1.000 | 0.834 | 1.000 | 0.974 | 0.960 | 0.906 | 1.000 | 0.980 | 1.000 | 0.959 | 1.000 | 1.000 | 0.850 | 1.000 | 0.981 | 0.861 | 0.834 |
| 1546 | 0.778 | 1.000 | 0.836 | 1.000 | 0.968 | 0.960 | 0.894 | 1.000 | 0.969 | 1.000 | 0.957 | 0.970 | 1.000 | 0.851 | 1.000 | 0.981 | 0.860 | 0.832 |
| 1547 | 0.777 | 1.000 | 0.820 | 1.000 | 0.968 | 0.960 | 0.900 | 1.000 | 0.990 | 1.000 | 0.959 | 1.000 | 1.000 | 0.853 | 1.000 | 0.981 | 0.860 | 0.832 |
| 1548 | 0.771 | 1.000 | 0.828 | 1.000 | 0.968 | 0.960 | 0.892 | 1.000 | 0.969 | 1.000 | 0.948 | 1.000 | 1.000 | 0.854 | 1.000 | 1.000 | 0.857 | 0.829 |
| 1549 | 0.776 | 1.000 | 0.826 | 1.000 | 0.968 | 0.960 | 0.889 | 1.000 | 0.990 | 1.000 | 0.957 | 1.000 | 1.000 | 0.850 | 1.000 | 1.000 | 0.858 | 0.829 |
| 1550 | 0.769 | 1.000 | 0.822 | 0.986 | 0.968 | 0.960 | 0.886 | 1.000 | 0.990 | 1.000 | 0.948 | 1.000 | 1.000 | 0.844 | 1.000 | 0.981 | 0.853 | 0.823 |
| 1551 | 0.771 | 1.000 | 0.826 | 1.000 | 0.974 | 0.960 | 0.900 | 1.000 | 0.980 | 1.000 | 0.957 | 1.000 | 1.000 | 0.854 | 1.000 | 0.981 | 0.858 | 0.830 |
| 1552 | 0.776 | 1.000 | 0.824 | 1.000 | 0.968 | 0.960 | 0.909 | 1.000 | 0.990 | 1.000 | 0.951 | 0.970 | 1.000 | 0.858 | 1.000 | 0.981 | 0.861 | 0.834 |
| 1553 | 0.771 | 1.000 | 0.826 | 1.000 | 0.963 | 0.960 | 0.891 | 1.000 | 0.980 | 1.000 | 0.957 | 0.970 | 1.000 | 0.858 | 1.000 | 0.981 | 0.858 | 0.829 |
| 1554 | 0.774 | 1.000 | 0.820 | 0.986 | 0.979 | 0.960 | 0.904 | 1.000 | 0.980 | 1.000 | 0.959 | 0.970 | 1.000 | 0.853 | 1.000 | 0.981 | 0.859 | 0.831 |
| 1555 | 0.771 | 1.000 | 0.816 | 1.000 | 0.968 | 0.960 | 0.897 | 1.000 | 0.990 | 1.000 | 0.957 | 0.970 | 0.991 | 0.856 | 1.000 | 1.000 | 0.858 | 0.829 |
| 1556 | 0.769 | 1.000 | 0.828 | 0.986 | 0.968 | 0.960 | 0.897 | 1.000 | 0.980 | 1.000 | 0.957 | 0.970 | 1.000 | 0.851 | 1.000 | 1.000 | 0.856 | 0.828 |
| 1557 | 0.764 | 1.000 | 0.820 | 1.000 | 0.974 | 0.960 | 0.906 | 1.000 | 0.959 | 1.000 | 0.959 | 0.970 | 0.991 | 0.852 | 1.000 | 1.000 | 0.856 | 0.827 |
| 1558 | 0.776 | 1.000 | 0.816 | 1.000 | 0.963 | 0.960 | 0.906 | 1.000 | 0.980 | 1.000 | 0.959 | 0.970 | 0.991 | 0.861 | 1.000 | 1.000 | 0.861 | 0.834 |

| | | | | | | | | | | | | | | | | | | |
|---|---|---|---|---|---|---|---|---|---|---|---|---|---|---|---|---|---|---|
| 1559 | 0.774 | 1.000 | 0.826 | 1.000 | 0.968 | 0.960 | 0.895 | 1.000 | 0.990 | 1.000 | 0.959 | 0.970 | 1.000 | 0.858 | 1.000 | 1.000 | 0.860 | 0.832 |
| 1560 | 0.774 | 1.000 | 0.818 | 1.000 | 0.974 | 0.960 | 0.900 | 1.000 | 0.980 | 1.000 | 0.962 | 1.000 | 1.000 | 0.851 | 1.000 | 0.981 | 0.858 | 0.830 |
| 1561 | 0.771 | 1.000 | 0.830 | 1.000 | 0.968 | 0.960 | 0.897 | 1.000 | 0.969 | 1.000 | 0.957 | 0.970 | 0.991 | 0.850 | 1.000 | 1.000 | 0.857 | 0.829 |
| 1562 | 0.777 | 1.000 | 0.814 | 0.986 | 0.968 | 0.960 | 0.904 | 1.000 | 0.990 | 1.000 | 0.965 | 1.000 | 1.000 | 0.854 | 1.000 | 1.000 | 0.860 | 0.832 |
| 1563 | 0.769 | 1.000 | 0.832 | 1.000 | 0.968 | 0.960 | 0.901 | 1.000 | 0.990 | 1.000 | 0.957 | 0.970 | 1.000 | 0.850 | 1.000 | 0.981 | 0.858 | 0.830 |
| 1564 | 0.783 | 1.000 | 0.812 | 0.986 | 0.974 | 0.960 | 0.900 | 1.000 | 0.980 | 1.000 | 0.957 | 0.970 | 1.000 | 0.847 | 1.000 | 0.981 | 0.859 | 0.830 |
| 1565 | 0.763 | 1.000 | 0.824 | 1.000 | 0.963 | 0.960 | 0.886 | 1.000 | 0.990 | 1.000 | 0.957 | 0.939 | 0.991 | 0.853 | 1.000 | 0.981 | 0.853 | 0.824 |
| 1566 | 0.779 | 1.000 | 0.816 | 1.000 | 0.963 | 0.960 | 0.900 | 1.000 | 0.990 | 1.000 | 0.965 | 0.970 | 1.000 | 0.858 | 1.000 | 1.000 | 0.861 | 0.833 |
| 1567 | 0.779 | 1.000 | 0.832 | 1.000 | 0.963 | 0.960 | 0.909 | 1.000 | 0.990 | 1.000 | 0.951 | 0.970 | 1.000 | 0.860 | 1.000 | 0.981 | 0.864 | 0.836 |
| 1568 | 0.774 | 1.000 | 0.836 | 0.986 | 0.968 | 0.960 | 0.891 | 1.000 | 0.990 | 1.000 | 0.962 | 0.970 | 1.000 | 0.857 | 1.000 | 1.000 | 0.860 | 0.832 |
| 1569 | 0.770 | 1.000 | 0.808 | 1.000 | 0.974 | 0.960 | 0.881 | 1.000 | 0.990 | 1.000 | 0.959 | 0.970 | 1.000 | 0.853 | 1.000 | 1.000 | 0.854 | 0.825 |
| 1570 | 0.779 | 1.000 | 0.822 | 1.000 | 0.974 | 0.960 | 0.891 | 1.000 | 0.980 | 1.000 | 0.954 | 1.000 | 1.000 | 0.855 | 1.000 | 1.000 | 0.860 | 0.832 |
| 1571 | 0.771 | 1.000 | 0.826 | 1.000 | 0.979 | 0.960 | 0.897 | 1.000 | 0.969 | 1.000 | 0.962 | 0.970 | 0.991 | 0.854 | 1.000 | 1.000 | 0.858 | 0.830 |
| 1572 | 0.769 | 1.000 | 0.812 | 1.000 | 0.968 | 0.960 | 0.894 | 1.000 | 0.990 | 1.000 | 0.954 | 0.970 | 1.000 | 0.853 | 1.000 | 1.000 | 0.855 | 0.827 |
| 1573 | 0.777 | 1.000 | 0.816 | 1.000 | 0.968 | 0.960 | 0.889 | 1.000 | 0.980 | 1.000 | 0.957 | 1.000 | 1.000 | 0.851 | 1.000 | 1.000 | 0.857 | 0.829 |
| 1574 | 0.777 | 1.000 | 0.818 | 1.000 | 0.968 | 0.960 | 0.892 | 1.000 | 0.969 | 1.000 | 0.959 | 1.000 | 1.000 | 0.854 | 1.000 | 1.000 | 0.859 | 0.831 |
| 1575 | 0.772 | 1.000 | 0.812 | 1.000 | 0.968 | 0.960 | 0.897 | 1.000 | 0.980 | 1.000 | 0.957 | 0.970 | 1.000 | 0.851 | 1.000 | 0.981 | 0.856 | 0.828 |
| 1576 | 0.767 | 1.000 | 0.822 | 1.000 | 0.968 | 0.960 | 0.908 | 1.000 | 0.980 | 1.000 | 0.957 | 0.970 | 1.000 | 0.849 | 1.000 | 1.000 | 0.856 | 0.828 |
| 1577 | 0.780 | 1.000 | 0.828 | 0.986 | 0.968 | 0.960 | 0.888 | 1.000 | 0.969 | 1.000 | 0.954 | 0.970 | 1.000 | 0.849 | 1.000 | 1.000 | 0.858 | 0.829 |
| 1578 | 0.777 | 1.000 | 0.822 | 1.000 | 0.968 | 0.960 | 0.901 | 1.000 | 0.980 | 1.000 | 0.959 | 0.970 | 1.000 | 0.856 | 1.000 | 0.981 | 0.860 | 0.833 |
| 1579 | 0.775 | 1.000 | 0.818 | 1.000 | 0.968 | 0.960 | 0.891 | 1.000 | 0.980 | 1.000 | 0.957 | 1.000 | 1.000 | 0.850 | 1.000 | 0.981 | 0.856 | 0.828 |
| 1580 | 0.777 | 1.000 | 0.834 | 0.986 | 0.963 | 0.960 | 0.891 | 1.000 | 0.980 | 1.000 | 0.959 | 0.970 | 1.000 | 0.860 | 1.000 | 1.000 | 0.861 | 0.833 |
| 1581 | 0.785 | 1.000 | 0.822 | 1.000 | 0.968 | 0.960 | 0.897 | 1.000 | 0.980 | 1.000 | 0.959 | 0.970 | 1.000 | 0.854 | 1.000 | 0.981 | 0.862 | 0.834 |
| 1582 | 0.778 | 1.000 | 0.816 | 1.000 | 0.968 | 0.960 | 0.894 | 1.000 | 0.980 | 1.000 | 0.962 | 1.000 | 1.000 | 0.851 | 1.000 | 0.981 | 0.858 | 0.830 |
| 1583 | 0.774 | 1.000 | 0.824 | 1.000 | 0.968 | 0.960 | 0.894 | 1.000 | 0.980 | 1.000 | 0.957 | 0.970 | 1.000 | 0.859 | 1.000 | 1.000 | 0.860 | 0.832 |
| 1584 | 0.772 | 1.000 | 0.830 | 0.986 | 0.968 | 0.960 | 0.897 | 1.000 | 0.980 | 1.000 | 0.959 | 0.970 | 1.000 | 0.852 | 1.000 | 1.000 | 0.858 | 0.830 |
| 1585 | 0.784 | 1.000 | 0.816 | 1.000 | 0.963 | 0.960 | 0.891 | 1.000 | 0.980 | 1.000 | 0.954 | 0.939 | 1.000 | 0.856 | 1.000 | 0.981 | 0.860 | 0.831 |
| 1586 | 0.777 | 1.000 | 0.812 | 1.000 | 0.968 | 0.960 | 0.894 | 1.000 | 0.990 | 1.000 | 0.959 | 0.970 | 1.000 | 0.850 | 1.000 | 1.000 | 0.857 | 0.829 |
| 1587 | 0.767 | 1.000 | 0.816 | 1.000 | 0.968 | 0.960 | 0.906 | 1.000 | 0.980 | 1.000 | 0.959 | 1.000 | 1.000 | 0.856 | 1.000 | 1.000 | 0.858 | 0.830 |
| 1588 | 0.773 | 1.000 | 0.828 | 0.986 | 0.968 | 0.960 | 0.903 | 1.000 | 0.980 | 1.000 | 0.965 | 1.000 | 1.000 | 0.850 | 1.000 | 1.000 | 0.859 | 0.831 |
| 1589 | 0.787 | 1.000 | 0.822 | 1.000 | 0.968 | 0.960 | 0.900 | 1.000 | 0.980 | 1.000 | 0.957 | 0.970 | 1.000 | 0.854 | 1.000 | 1.000 | 0.863 | 0.835 |
| 1590 | 0.783 | 1.000 | 0.810 | 1.000 | 0.968 | 0.960 | 0.903 | 1.000 | 0.990 | 1.000 | 0.965 | 0.970 | 1.000 | 0.854 | 1.000 | 0.981 | 0.862 | 0.834 |
| 1591 | 0.778 | 1.000 | 0.818 | 1.000 | 0.968 | 0.960 | 0.898 | 1.000 | 0.990 | 1.000 | 0.959 | 0.939 | 1.000 | 0.854 | 1.000 | 1.000 | 0.860 | 0.832 |
| 1592 | 0.767 | 1.000 | 0.804 | 1.000 | 0.968 | 0.960 | 0.883 | 1.000 | 0.990 | 1.000 | 0.962 | 0.970 | 0.991 | 0.854 | 1.000 | 1.000 | 0.853 | 0.823 |
| 1593 | 0.775 | 1.000 | 0.804 | 1.000 | 0.963 | 0.960 | 0.897 | 1.000 | 0.990 | 1.000 | 0.957 | 0.970 | 1.000 | 0.858 | 1.000 | 1.000 | 0.858 | 0.830 |
| 1594 | 0.781 | 1.000 | 0.812 | 0.986 | 0.968 | 0.960 | 0.898 | 1.000 | 0.969 | 1.000 | 0.954 | 1.000 | 1.000 | 0.865 | 1.000 | 0.981 | 0.862 | 0.834 |
| 1595 | 0.780 | 1.000 | 0.816 | 1.000 | 0.968 | 0.960 | 0.894 | 1.000 | 0.990 | 1.000 | 0.957 | 0.970 | 1.000 | 0.848 | 1.000 | 1.000 | 0.858 | 0.830 |
| 1596 | 0.771 | 1.000 | 0.808 | 1.000 | 0.968 | 0.960 | 0.897 | 1.000 | 0.990 | 1.000 | 0.957 | 1.000 | 1.000 | 0.847 | 1.000 | 1.000 | 0.855 | 0.826 |
| 1597 | 0.779 | 1.000 | 0.818 | 1.000 | 0.968 | 0.960 | 0.892 | 1.000 | 0.990 | 1.000 | 0.962 | 0.970 | 1.000 | 0.852 | 1.000 | 1.000 | 0.859 | 0.831 |

| | | | | | | | | | | | | | | | | | | |
|---|---|---|---|---|---|---|---|---|---|---|---|---|---|---|---|---|---|---|
| 1598 | 0.767 | 1.000 | 0.836 | 1.000 | 0.963 | 0.960 | 0.891 | 1.000 | 0.990 | 1.000 | 0.959 | 0.970 | 1.000 | 0.859 | 1.000 | 1.000 | 0.858 | 0.830 |
| 1599 | 0.770 | 1.000 | 0.820 | 0.986 | 0.963 | 0.960 | 0.892 | 1.000 | 0.980 | 1.000 | 0.957 | 0.970 | 0.991 | 0.856 | 1.000 | 0.981 | 0.856 | 0.828 |
| 1600 | 0.780 | 1.000 | 0.826 | 1.000 | 0.974 | 0.960 | 0.894 | 1.000 | 0.969 | 1.000 | 0.962 | 0.939 | 0.991 | 0.851 | 1.000 | 1.000 | 0.859 | 0.831 |
| 1601 | 0.779 | 1.000 | 0.808 | 1.000 | 0.979 | 0.960 | 0.895 | 1.000 | 0.990 | 1.000 | 0.959 | 0.970 | 0.991 | 0.854 | 1.000 | 0.981 | 0.859 | 0.830 |
| 1602 | 0.771 | 1.000 | 0.818 | 1.000 | 0.974 | 0.960 | 0.903 | 1.000 | 0.990 | 1.000 | 0.951 | 1.000 | 1.000 | 0.848 | 1.000 | 1.000 | 0.857 | 0.828 |
| 1603 | 0.779 | 1.000 | 0.814 | 1.000 | 0.974 | 0.960 | 0.897 | 1.000 | 0.969 | 1.000 | 0.959 | 0.970 | 1.000 | 0.851 | 1.000 | 1.000 | 0.859 | 0.831 |
| 1604 | 0.783 | 1.000 | 0.820 | 1.000 | 0.968 | 0.960 | 0.888 | 1.000 | 0.980 | 1.000 | 0.957 | 1.000 | 1.000 | 0.854 | 1.000 | 1.000 | 0.860 | 0.832 |
| 1605 | 0.778 | 1.000 | 0.814 | 1.000 | 0.968 | 0.960 | 0.897 | 1.000 | 0.990 | 1.000 | 0.959 | 0.970 | 1.000 | 0.846 | 1.000 | 1.000 | 0.857 | 0.829 |
| 1606 | 0.778 | 1.000 | 0.818 | 1.000 | 0.968 | 0.960 | 0.898 | 1.000 | 0.990 | 1.000 | 0.954 | 0.970 | 1.000 | 0.848 | 1.000 | 0.963 | 0.858 | 0.829 |
| 1607 | 0.774 | 1.000 | 0.810 | 1.000 | 0.968 | 0.960 | 0.900 | 1.000 | 0.990 | 1.000 | 0.954 | 0.970 | 1.000 | 0.850 | 1.000 | 1.000 | 0.857 | 0.828 |
| 1608 | 0.775 | 1.000 | 0.824 | 0.986 | 0.963 | 0.960 | 0.904 | 1.000 | 0.980 | 1.000 | 0.954 | 1.000 | 1.000 | 0.847 | 1.000 | 0.981 | 0.858 | 0.830 |
| 1609 | 0.777 | 1.000 | 0.820 | 1.000 | 0.968 | 0.960 | 0.895 | 1.000 | 0.980 | 1.000 | 0.954 | 1.000 | 1.000 | 0.853 | 1.000 | 1.000 | 0.859 | 0.831 |
| 1610 | 0.776 | 1.000 | 0.816 | 1.000 | 0.963 | 0.960 | 0.894 | 1.000 | 0.969 | 1.000 | 0.948 | 0.970 | 1.000 | 0.857 | 1.000 | 1.000 | 0.858 | 0.829 |
| 1611 | 0.772 | 1.000 | 0.810 | 1.000 | 0.963 | 0.960 | 0.884 | 1.000 | 0.990 | 1.000 | 0.959 | 0.970 | 1.000 | 0.849 | 1.000 | 1.000 | 0.854 | 0.825 |
| 1612 | 0.779 | 1.000 | 0.810 | 1.000 | 0.968 | 0.960 | 0.894 | 1.000 | 0.990 | 1.000 | 0.959 | 0.970 | 1.000 | 0.845 | 1.000 | 1.000 | 0.856 | 0.828 |
| 1613 | 0.771 | 1.000 | 0.802 | 1.000 | 0.963 | 0.960 | 0.895 | 1.000 | 0.990 | 1.000 | 0.948 | 0.939 | 1.000 | 0.854 | 1.000 | 1.000 | 0.855 | 0.826 |
| 1614 | 0.779 | 1.000 | 0.812 | 1.000 | 0.963 | 0.960 | 0.888 | 1.000 | 0.969 | 1.000 | 0.957 | 0.970 | 0.991 | 0.855 | 1.000 | 1.000 | 0.858 | 0.829 |
| 1615 | 0.765 | 1.000 | 0.810 | 1.000 | 0.968 | 0.960 | 0.894 | 1.000 | 0.969 | 1.000 | 0.951 | 0.970 | 1.000 | 0.854 | 1.000 | 0.981 | 0.853 | 0.824 |
| 1616 | 0.772 | 1.000 | 0.818 | 1.000 | 0.963 | 0.960 | 0.892 | 1.000 | 0.990 | 1.000 | 0.951 | 0.970 | 1.000 | 0.847 | 1.000 | 0.981 | 0.855 | 0.826 |
| 1617 | 0.771 | 1.000 | 0.830 | 1.000 | 0.963 | 0.960 | 0.901 | 1.000 | 0.990 | 1.000 | 0.954 | 0.939 | 1.000 | 0.859 | 1.000 | 1.000 | 0.860 | 0.832 |
| 1618 | 0.777 | 1.000 | 0.800 | 1.000 | 0.963 | 0.960 | 0.900 | 1.000 | 0.980 | 1.000 | 0.957 | 0.970 | 1.000 | 0.854 | 1.000 | 1.000 | 0.858 | 0.829 |
| 1619 | 0.779 | 1.000 | 0.820 | 0.986 | 0.963 | 0.960 | 0.891 | 1.000 | 0.969 | 1.000 | 0.948 | 0.970 | 1.000 | 0.851 | 1.000 | 1.000 | 0.857 | 0.828 |
| 1620 | 0.763 | 1.000 | 0.818 | 1.000 | 0.963 | 0.960 | 0.894 | 1.000 | 0.969 | 1.000 | 0.951 | 0.970 | 1.000 | 0.856 | 1.000 | 1.000 | 0.854 | 0.825 |
| 1621 | 0.781 | 1.000 | 0.806 | 0.986 | 0.963 | 0.960 | 0.892 | 1.000 | 0.969 | 1.000 | 0.945 | 0.970 | 1.000 | 0.851 | 1.000 | 0.981 | 0.856 | 0.827 |
| 1622 | 0.769 | 1.000 | 0.818 | 0.986 | 0.958 | 0.960 | 0.888 | 1.000 | 0.990 | 1.000 | 0.959 | 0.970 | 1.000 | 0.851 | 1.000 | 0.981 | 0.854 | 0.825 |
| 1623 | 0.781 | 1.000 | 0.830 | 1.000 | 0.963 | 0.960 | 0.895 | 1.000 | 0.990 | 1.000 | 0.954 | 0.970 | 1.000 | 0.854 | 1.000 | 1.000 | 0.861 | 0.833 |
| 1624 | 0.773 | 1.000 | 0.822 | 1.000 | 0.963 | 0.960 | 0.892 | 1.000 | 0.980 | 1.000 | 0.959 | 0.970 | 1.000 | 0.854 | 1.000 | 1.000 | 0.858 | 0.829 |
| 1625 | 0.777 | 1.000 | 0.804 | 1.000 | 0.963 | 0.960 | 0.894 | 1.000 | 0.990 | 1.000 | 0.957 | 0.970 | 1.000 | 0.858 | 1.000 | 0.981 | 0.858 | 0.830 |
| 1626 | 0.765 | 1.000 | 0.818 | 1.000 | 0.963 | 0.960 | 0.891 | 1.000 | 0.990 | 1.000 | 0.951 | 1.000 | 1.000 | 0.851 | 1.000 | 1.000 | 0.854 | 0.825 |
| 1627 | 0.776 | 1.000 | 0.816 | 1.000 | 0.958 | 0.960 | 0.886 | 1.000 | 0.980 | 1.000 | 0.951 | 0.970 | 0.991 | 0.853 | 1.000 | 1.000 | 0.856 | 0.827 |
| 1628 | 0.773 | 1.000 | 0.800 | 1.000 | 0.963 | 0.960 | 0.895 | 1.000 | 0.990 | 1.000 | 0.942 | 0.939 | 1.000 | 0.854 | 1.000 | 0.981 | 0.854 | 0.825 |
| 1629 | 0.777 | 1.000 | 0.816 | 1.000 | 0.963 | 0.960 | 0.895 | 1.000 | 0.980 | 1.000 | 0.951 | 0.970 | 1.000 | 0.849 | 1.000 | 1.000 | 0.857 | 0.828 |
| 1630 | 0.775 | 1.000 | 0.818 | 1.000 | 0.963 | 0.960 | 0.886 | 1.000 | 0.990 | 1.000 | 0.957 | 0.970 | 0.991 | 0.858 | 1.000 | 0.981 | 0.858 | 0.829 |
| 1631 | 0.764 | 1.000 | 0.814 | 0.986 | 0.963 | 0.960 | 0.892 | 1.000 | 0.980 | 1.000 | 0.962 | 0.970 | 0.991 | 0.851 | 1.000 | 1.000 | 0.853 | 0.824 |
| 1632 | 0.775 | 1.000 | 0.804 | 0.986 | 0.963 | 0.960 | 0.889 | 1.000 | 0.980 | 1.000 | 0.957 | 0.970 | 1.000 | 0.857 | 1.000 | 1.000 | 0.856 | 0.827 |
| 1633 | 0.773 | 1.000 | 0.828 | 1.000 | 0.963 | 0.960 | 0.889 | 1.000 | 0.980 | 1.000 | 0.965 | 1.000 | 0.991 | 0.853 | 1.000 | 1.000 | 0.858 | 0.829 |
| 1634 | 0.779 | 1.000 | 0.806 | 1.000 | 0.963 | 0.960 | 0.895 | 1.000 | 0.990 | 1.000 | 0.954 | 0.970 | 1.000 | 0.855 | 1.000 | 0.981 | 0.858 | 0.830 |
| 1635 | 0.777 | 1.000 | 0.816 | 1.000 | 0.963 | 0.960 | 0.891 | 1.000 | 0.990 | 1.000 | 0.959 | 1.000 | 1.000 | 0.858 | 1.000 | 0.981 | 0.859 | 0.831 |
| 1636 | 0.775 | 1.000 | 0.820 | 1.000 | 0.968 | 0.960 | 0.886 | 1.000 | 0.980 | 1.000 | 0.948 | 1.000 | 1.000 | 0.848 | 1.000 | 1.000 | 0.855 | 0.826 |

| | | | | | | | | | | | | | | | | | |
|---|---|---|---|---|---|---|---|---|---|---|---|---|---|---|---|---|---|
| 1637 | 0.773 | 1.000 | 0.798 | 0.986 | 0.963 | 0.960 | 0.900 | 1.000 | 0.980 | 1.000 | 0.957 | 0.970 | 1.000 | 0.854 | 1.000 | 1.000 | 0.856 | 0.827 |
| 1638 | 0.773 | 1.000 | 0.820 | 1.000 | 0.963 | 0.960 | 0.891 | 1.000 | 0.980 | 1.000 | 0.957 | 0.970 | 1.000 | 0.854 | 1.000 | 0.981 | 0.857 | 0.828 |
| 1639 | 0.774 | 1.000 | 0.818 | 1.000 | 0.963 | 0.960 | 0.892 | 1.000 | 0.990 | 1.000 | 0.962 | 0.970 | 0.991 | 0.849 | 1.000 | 1.000 | 0.856 | 0.828 |
| 1640 | 0.767 | 1.000 | 0.810 | 0.986 | 0.963 | 0.960 | 0.906 | 1.000 | 0.990 | 1.000 | 0.957 | 0.970 | 1.000 | 0.854 | 1.000 | 1.000 | 0.856 | 0.828 |
| 1641 | 0.771 | 1.000 | 0.816 | 1.000 | 0.963 | 0.960 | 0.900 | 1.000 | 0.990 | 1.000 | 0.948 | 1.000 | 1.000 | 0.857 | 1.000 | 1.000 | 0.858 | 0.829 |
| 1642 | 0.785 | 1.000 | 0.828 | 1.000 | 0.963 | 0.960 | 0.895 | 1.000 | 0.980 | 1.000 | 0.959 | 0.970 | 0.991 | 0.850 | 1.000 | 1.000 | 0.861 | 0.833 |
| 1643 | 0.772 | 1.000 | 0.824 | 1.000 | 0.963 | 0.960 | 0.897 | 1.000 | 0.990 | 1.000 | 0.957 | 0.970 | 1.000 | 0.854 | 1.000 | 0.981 | 0.858 | 0.830 |
| 1644 | 0.774 | 1.000 | 0.820 | 1.000 | 0.963 | 0.960 | 0.908 | 1.000 | 0.990 | 1.000 | 0.962 | 1.000 | 1.000 | 0.855 | 1.000 | 1.000 | 0.861 | 0.833 |
| 1645 | 0.777 | 1.000 | 0.822 | 1.000 | 0.963 | 0.960 | 0.897 | 1.000 | 0.969 | 1.000 | 0.965 | 0.970 | 1.000 | 0.853 | 1.000 | 1.000 | 0.859 | 0.831 |
| 1646 | 0.777 | 1.000 | 0.820 | 1.000 | 0.963 | 0.960 | 0.898 | 1.000 | 0.969 | 1.000 | 0.951 | 1.000 | 1.000 | 0.854 | 1.000 | 1.000 | 0.859 | 0.830 |
| 1647 | 0.766 | 1.000 | 0.826 | 1.000 | 0.963 | 0.960 | 0.888 | 1.000 | 0.990 | 1.000 | 0.951 | 0.970 | 0.991 | 0.862 | 1.000 | 1.000 | 0.857 | 0.828 |
| 1648 | 0.767 | 1.000 | 0.818 | 1.000 | 0.963 | 0.960 | 0.889 | 1.000 | 0.969 | 1.000 | 0.948 | 1.000 | 1.000 | 0.854 | 1.000 | 1.000 | 0.854 | 0.825 |
| 1649 | 0.781 | 1.000 | 0.804 | 1.000 | 0.963 | 0.960 | 0.897 | 1.000 | 0.980 | 1.000 | 0.954 | 0.970 | 1.000 | 0.853 | 1.000 | 1.000 | 0.858 | 0.829 |
| 1650 | 0.777 | 1.000 | 0.814 | 1.000 | 0.968 | 0.960 | 0.901 | 1.000 | 0.980 | 1.000 | 0.942 | 1.000 | 1.000 | 0.844 | 1.000 | 1.000 | 0.856 | 0.828 |
| 1651 | 0.768 | 1.000 | 0.818 | 0.986 | 0.963 | 0.960 | 0.895 | 1.000 | 0.980 | 1.000 | 0.959 | 0.970 | 0.991 | 0.851 | 1.000 | 0.981 | 0.855 | 0.826 |
| 1652 | 0.775 | 1.000 | 0.808 | 1.000 | 0.963 | 0.960 | 0.886 | 1.000 | 0.969 | 1.000 | 0.945 | 0.939 | 0.991 | 0.847 | 1.000 | 1.000 | 0.853 | 0.823 |
| 1653 | 0.780 | 1.000 | 0.822 | 1.000 | 0.963 | 0.960 | 0.897 | 1.000 | 0.980 | 1.000 | 0.959 | 0.970 | 1.000 | 0.858 | 1.000 | 1.000 | 0.861 | 0.833 |
| 1654 | 0.770 | 1.000 | 0.810 | 0.986 | 0.963 | 0.960 | 0.898 | 1.000 | 0.990 | 1.000 | 0.959 | 0.909 | 1.000 | 0.850 | 1.000 | 1.000 | 0.855 | 0.826 |
| 1655 | 0.780 | 1.000 | 0.798 | 1.000 | 0.963 | 0.960 | 0.898 | 1.000 | 0.980 | 1.000 | 0.954 | 0.970 | 1.000 | 0.859 | 1.000 | 1.000 | 0.859 | 0.830 |
| 1656 | 0.775 | 1.000 | 0.806 | 1.000 | 0.963 | 0.960 | 0.904 | 1.000 | 0.980 | 1.000 | 0.954 | 0.970 | 1.000 | 0.857 | 1.000 | 0.981 | 0.858 | 0.830 |
| 1657 | 0.777 | 1.000 | 0.820 | 1.000 | 0.963 | 0.960 | 0.901 | 1.000 | 0.980 | 1.000 | 0.957 | 0.970 | 1.000 | 0.856 | 1.000 | 1.000 | 0.860 | 0.832 |
| 1658 | 0.767 | 1.000 | 0.814 | 1.000 | 0.968 | 0.960 | 0.889 | 1.000 | 0.980 | 1.000 | 0.965 | 0.970 | 1.000 | 0.847 | 1.000 | 1.000 | 0.854 | 0.825 |
| 1659 | 0.776 | 1.000 | 0.820 | 1.000 | 0.963 | 0.960 | 0.891 | 1.000 | 0.990 | 1.000 | 0.951 | 1.000 | 1.000 | 0.848 | 1.000 | 1.000 | 0.856 | 0.828 |
| 1660 | 0.772 | 1.000 | 0.814 | 1.000 | 0.963 | 0.960 | 0.901 | 1.000 | 0.990 | 1.000 | 0.951 | 0.970 | 0.991 | 0.858 | 1.000 | 1.000 | 0.858 | 0.830 |
| 1661 | 0.783 | 1.000 | 0.806 | 1.000 | 0.968 | 0.960 | 0.894 | 1.000 | 0.990 | 1.000 | 0.959 | 0.970 | 1.000 | 0.860 | 1.000 | 1.000 | 0.861 | 0.833 |
| 1662 | 0.774 | 1.000 | 0.818 | 1.000 | 0.963 | 0.960 | 0.898 | 1.000 | 0.990 | 1.000 | 0.962 | 0.970 | 1.000 | 0.860 | 1.000 | 0.981 | 0.860 | 0.832 |
| 1663 | 0.774 | 1.000 | 0.822 | 1.000 | 0.963 | 0.960 | 0.894 | 1.000 | 0.980 | 1.000 | 0.951 | 0.970 | 1.000 | 0.848 | 1.000 | 1.000 | 0.856 | 0.827 |
| 1664 | 0.777 | 1.000 | 0.802 | 1.000 | 0.958 | 0.960 | 0.889 | 1.000 | 0.990 | 1.000 | 0.957 | 0.970 | 1.000 | 0.852 | 1.000 | 0.981 | 0.855 | 0.826 |
| 1665 | 0.775 | 1.000 | 0.822 | 1.000 | 0.963 | 0.960 | 0.904 | 1.000 | 0.980 | 1.000 | 0.957 | 0.970 | 1.000 | 0.856 | 1.000 | 1.000 | 0.860 | 0.832 |
| 1666 | 0.778 | 1.000 | 0.814 | 1.000 | 0.963 | 0.960 | 0.898 | 1.000 | 0.969 | 1.000 | 0.954 | 1.000 | 0.991 | 0.850 | 1.000 | 1.000 | 0.857 | 0.829 |
| 1667 | 0.767 | 1.000 | 0.794 | 1.000 | 0.963 | 0.960 | 0.894 | 1.000 | 0.990 | 1.000 | 0.957 | 0.970 | 1.000 | 0.858 | 1.000 | 1.000 | 0.854 | 0.825 |
| 1668 | 0.782 | 1.000 | 0.792 | 0.986 | 0.968 | 0.960 | 0.895 | 1.000 | 0.980 | 1.000 | 0.959 | 0.970 | 1.000 | 0.854 | 1.000 | 1.000 | 0.858 | 0.829 |
| 1669 | 0.772 | 1.000 | 0.812 | 1.000 | 0.963 | 0.960 | 0.886 | 1.000 | 0.990 | 1.000 | 0.962 | 0.970 | 0.991 | 0.851 | 1.000 | 0.981 | 0.855 | 0.826 |
| 1670 | 0.776 | 1.000 | 0.812 | 1.000 | 0.958 | 0.960 | 0.900 | 1.000 | 0.990 | 1.000 | 0.954 | 0.970 | 1.000 | 0.851 | 1.000 | 1.000 | 0.857 | 0.829 |
| 1671 | 0.771 | 1.000 | 0.802 | 0.986 | 0.963 | 0.960 | 0.897 | 1.000 | 0.980 | 1.000 | 0.954 | 1.000 | 1.000 | 0.854 | 1.000 | 0.981 | 0.855 | 0.826 |
| 1672 | 0.771 | 1.000 | 0.830 | 0.986 | 0.963 | 0.960 | 0.894 | 1.000 | 0.990 | 1.000 | 0.945 | 0.939 | 1.000 | 0.858 | 1.000 | 1.000 | 0.858 | 0.829 |
| 1673 | 0.774 | 1.000 | 0.824 | 1.000 | 0.963 | 0.960 | 0.898 | 1.000 | 0.980 | 1.000 | 0.948 | 0.970 | 1.000 | 0.858 | 1.000 | 0.981 | 0.859 | 0.831 |
| 1674 | 0.772 | 1.000 | 0.810 | 1.000 | 0.963 | 0.960 | 0.898 | 1.000 | 0.969 | 1.000 | 0.951 | 0.970 | 1.000 | 0.855 | 1.000 | 1.000 | 0.856 | 0.828 |
| 1675 | 0.770 | 1.000 | 0.816 | 0.986 | 0.968 | 0.960 | 0.895 | 1.000 | 0.990 | 1.000 | 0.945 | 0.970 | 0.991 | 0.857 | 1.000 | 1.000 | 0.856 | 0.827 |

| | | | | | | | | | | | | | | | | | | |
|---|---|---|---|---|---|---|---|---|---|---|---|---|---|---|---|---|---|---|
| 1676 | 0.777 | 1.000 | 0.814 | 1.000 | 0.963 | 0.960 | 0.891 | 1.000 | 0.980 | 1.000 | 0.951 | 0.970 | 1.000 | 0.847 | 1.000 | 1.000 | 0.855 | 0.826 |
| 1677 | 0.771 | 1.000 | 0.800 | 1.000 | 0.968 | 0.960 | 0.903 | 1.000 | 0.990 | 1.000 | 0.951 | 0.939 | 1.000 | 0.847 | 1.000 | 1.000 | 0.854 | 0.825 |
| 1678 | 0.773 | 1.000 | 0.804 | 1.000 | 0.963 | 0.960 | 0.897 | 1.000 | 0.980 | 1.000 | 0.959 | 0.970 | 1.000 | 0.855 | 1.000 | 1.000 | 0.857 | 0.828 |
| 1679 | 0.777 | 1.000 | 0.816 | 1.000 | 0.968 | 1.000 | 0.900 | 1.000 | 0.969 | 1.000 | 0.962 | 1.000 | 1.000 | 0.857 | 1.000 | 1.000 | 0.860 | 0.833 |
| 1680 | 0.769 | 1.000 | 0.816 | 1.000 | 0.963 | 0.960 | 0.900 | 1.000 | 0.980 | 1.000 | 0.951 | 0.970 | 1.000 | 0.855 | 1.000 | 1.000 | 0.856 | 0.828 |
| 1681 | 0.781 | 1.000 | 0.814 | 1.000 | 0.963 | 0.960 | 0.897 | 1.000 | 0.969 | 1.000 | 0.948 | 0.970 | 1.000 | 0.851 | 1.000 | 1.000 | 0.858 | 0.829 |
| 1682 | 0.770 | 1.000 | 0.820 | 0.986 | 0.963 | 0.960 | 0.912 | 1.000 | 0.990 | 1.000 | 0.951 | 0.970 | 1.000 | 0.853 | 1.000 | 0.981 | 0.858 | 0.830 |
| 1683 | 0.775 | 1.000 | 0.820 | 1.000 | 0.963 | 0.960 | 0.898 | 1.000 | 0.969 | 1.000 | 0.954 | 0.970 | 1.000 | 0.852 | 1.000 | 1.000 | 0.858 | 0.829 |
| 1684 | 0.778 | 1.000 | 0.824 | 0.986 | 0.963 | 0.960 | 0.904 | 1.000 | 0.990 | 1.000 | 0.959 | 0.970 | 1.000 | 0.854 | 1.000 | 1.000 | 0.861 | 0.833 |
| 1685 | 0.777 | 1.000 | 0.804 | 1.000 | 0.963 | 0.960 | 0.904 | 1.000 | 0.980 | 1.000 | 0.957 | 0.970 | 0.991 | 0.861 | 1.000 | 1.000 | 0.860 | 0.832 |
| 1686 | 0.777 | 1.000 | 0.816 | 0.986 | 0.963 | 0.960 | 0.901 | 1.000 | 0.980 | 1.000 | 0.954 | 0.970 | 1.000 | 0.848 | 1.000 | 0.981 | 0.857 | 0.829 |
| 1687 | 0.770 | 1.000 | 0.814 | 1.000 | 0.963 | 0.960 | 0.901 | 1.000 | 0.980 | 1.000 | 0.945 | 1.000 | 1.000 | 0.858 | 1.000 | 1.000 | 0.857 | 0.829 |
| 1688 | 0.765 | 1.000 | 0.818 | 1.000 | 0.968 | 0.960 | 0.903 | 1.000 | 0.980 | 1.000 | 0.965 | 1.000 | 1.000 | 0.854 | 1.000 | 0.981 | 0.857 | 0.828 |
| 1689 | 0.773 | 1.000 | 0.824 | 1.000 | 0.963 | 1.000 | 0.901 | 1.000 | 0.969 | 1.000 | 0.951 | 0.970 | 1.000 | 0.854 | 1.000 | 1.000 | 0.858 | 0.830 |
| 1690 | 0.779 | 1.000 | 0.820 | 1.000 | 0.968 | 0.960 | 0.891 | 1.000 | 0.980 | 1.000 | 0.957 | 0.970 | 1.000 | 0.847 | 1.000 | 1.000 | 0.857 | 0.828 |
| 1691 | 0.780 | 1.000 | 0.828 | 1.000 | 0.963 | 1.000 | 0.901 | 1.000 | 0.990 | 1.000 | 0.959 | 0.970 | 1.000 | 0.848 | 1.000 | 1.000 | 0.860 | 0.833 |
| 1692 | 0.780 | 1.000 | 0.822 | 0.986 | 0.963 | 0.960 | 0.903 | 1.000 | 0.980 | 1.000 | 0.951 | 0.970 | 1.000 | 0.856 | 1.000 | 1.000 | 0.861 | 0.833 |
| 1693 | 0.771 | 1.000 | 0.808 | 1.000 | 0.968 | 0.960 | 0.904 | 1.000 | 0.969 | 1.000 | 0.948 | 0.970 | 1.000 | 0.854 | 1.000 | 0.981 | 0.856 | 0.828 |
| 1694 | 0.774 | 1.000 | 0.810 | 0.986 | 0.963 | 0.960 | 0.901 | 1.000 | 0.969 | 1.000 | 0.959 | 0.970 | 1.000 | 0.859 | 1.000 | 1.000 | 0.859 | 0.831 |
| 1695 | 0.769 | 1.000 | 0.796 | 1.000 | 0.963 | 1.000 | 0.901 | 1.000 | 0.980 | 1.000 | 0.957 | 0.970 | 1.000 | 0.849 | 1.000 | 0.981 | 0.853 | 0.824 |
| 1696 | 0.775 | 1.000 | 0.826 | 1.000 | 0.963 | 0.960 | 0.897 | 1.000 | 0.990 | 1.000 | 0.957 | 0.970 | 1.000 | 0.857 | 1.000 | 1.000 | 0.860 | 0.832 |
| 1697 | 0.775 | 1.000 | 0.810 | 1.000 | 0.963 | 0.960 | 0.904 | 1.000 | 0.990 | 1.000 | 0.957 | 0.970 | 1.000 | 0.850 | 1.000 | 1.000 | 0.857 | 0.829 |
| 1698 | 0.777 | 1.000 | 0.826 | 1.000 | 0.963 | 0.960 | 0.898 | 1.000 | 0.990 | 1.000 | 0.962 | 0.939 | 1.000 | 0.851 | 1.000 | 1.000 | 0.859 | 0.831 |
| 1699 | 0.775 | 1.000 | 0.830 | 1.000 | 0.958 | 0.960 | 0.904 | 1.000 | 0.980 | 1.000 | 0.962 | 1.000 | 1.000 | 0.861 | 1.000 | 1.000 | 0.863 | 0.836 |
| 1700 | 0.777 | 1.000 | 0.810 | 0.986 | 0.963 | 0.960 | 0.897 | 1.000 | 0.980 | 1.000 | 0.957 | 1.000 | 1.000 | 0.851 | 1.000 | 1.000 | 0.857 | 0.829 |
| 1701 | 0.773 | 1.000 | 0.828 | 1.000 | 0.963 | 0.960 | 0.892 | 1.000 | 0.980 | 1.000 | 0.959 | 0.970 | 1.000 | 0.852 | 1.000 | 0.981 | 0.858 | 0.829 |
| 1702 | 0.782 | 1.000 | 0.824 | 1.000 | 0.968 | 0.960 | 0.898 | 1.000 | 0.980 | 1.000 | 0.957 | 0.970 | 1.000 | 0.850 | 1.000 | 0.981 | 0.860 | 0.832 |
| 1703 | 0.764 | 1.000 | 0.826 | 1.000 | 0.963 | 0.960 | 0.898 | 1.000 | 0.980 | 1.000 | 0.957 | 0.970 | 1.000 | 0.850 | 1.000 | 1.000 | 0.855 | 0.826 |
| 1704 | 0.781 | 1.000 | 0.816 | 1.000 | 0.963 | 0.960 | 0.892 | 1.000 | 0.980 | 1.000 | 0.962 | 0.970 | 1.000 | 0.862 | 1.000 | 1.000 | 0.862 | 0.834 |
| 1705 | 0.774 | 1.000 | 0.826 | 1.000 | 0.963 | 0.960 | 0.906 | 1.000 | 0.980 | 1.000 | 0.957 | 0.970 | 1.000 | 0.848 | 1.000 | 1.000 | 0.858 | 0.830 |
| 1706 | 0.773 | 1.000 | 0.830 | 0.986 | 0.968 | 0.960 | 0.904 | 1.000 | 0.980 | 1.000 | 0.954 | 1.000 | 0.991 | 0.850 | 1.000 | 0.981 | 0.858 | 0.831 |
| 1707 | 0.775 | 1.000 | 0.814 | 1.000 | 0.963 | 0.960 | 0.886 | 1.000 | 0.990 | 1.000 | 0.954 | 1.000 | 0.991 | 0.847 | 1.000 | 1.000 | 0.855 | 0.825 |
| 1708 | 0.773 | 1.000 | 0.814 | 1.000 | 0.963 | 0.960 | 0.886 | 1.000 | 0.980 | 1.000 | 0.954 | 1.000 | 1.000 | 0.853 | 1.000 | 1.000 | 0.856 | 0.827 |
| 1709 | 0.775 | 1.000 | 0.820 | 1.000 | 0.963 | 0.960 | 0.900 | 1.000 | 0.990 | 1.000 | 0.959 | 0.970 | 1.000 | 0.859 | 1.000 | 1.000 | 0.860 | 0.833 |
| 1710 | 0.770 | 1.000 | 0.814 | 1.000 | 0.968 | 0.960 | 0.909 | 1.000 | 0.980 | 1.000 | 0.948 | 0.970 | 1.000 | 0.854 | 1.000 | 0.981 | 0.857 | 0.829 |
| 1711 | 0.767 | 1.000 | 0.818 | 0.986 | 0.963 | 0.960 | 0.903 | 1.000 | 0.990 | 1.000 | 0.945 | 0.970 | 1.000 | 0.846 | 1.000 | 0.981 | 0.854 | 0.825 |
| 1712 | 0.783 | 1.000 | 0.824 | 1.000 | 0.968 | 0.960 | 0.898 | 1.000 | 0.990 | 1.000 | 0.954 | 1.000 | 1.000 | 0.855 | 1.000 | 1.000 | 0.862 | 0.834 |
| 1713 | 0.771 | 1.000 | 0.820 | 1.000 | 0.963 | 0.960 | 0.897 | 1.000 | 0.990 | 1.000 | 0.957 | 0.970 | 1.000 | 0.851 | 1.000 | 0.981 | 0.856 | 0.828 |
| 1714 | 0.771 | 1.000 | 0.824 | 1.000 | 0.963 | 0.960 | 0.898 | 1.000 | 0.990 | 1.000 | 0.959 | 1.000 | 1.000 | 0.854 | 1.000 | 1.000 | 0.858 | 0.830 |

| | | | | | | | | | | | | | | | | | |
|---|---|---|---|---|---|---|---|---|---|---|---|---|---|---|---|---|---|
| 1715 | 0.775 | 1.000 | 0.806 | 1.000 | 0.963 | 0.960 | 0.897 | 1.000 | 0.980 | 1.000 | 0.954 | 0.970 | 0.991 | 0.846 | 1.000 | 0.981 | 0.854 | 0.825 |
| 1716 | 0.769 | 1.000 | 0.810 | 1.000 | 0.963 | 0.960 | 0.895 | 1.000 | 0.980 | 1.000 | 0.948 | 1.000 | 1.000 | 0.857 | 1.000 | 1.000 | 0.856 | 0.827 |
| 1717 | 0.767 | 1.000 | 0.828 | 1.000 | 0.963 | 0.960 | 0.891 | 1.000 | 0.980 | 1.000 | 0.957 | 0.970 | 1.000 | 0.861 | 1.000 | 1.000 | 0.858 | 0.829 |
| 1718 | 0.775 | 1.000 | 0.820 | 1.000 | 0.963 | 0.960 | 0.897 | 1.000 | 0.969 | 1.000 | 0.948 | 0.970 | 1.000 | 0.856 | 1.000 | 1.000 | 0.858 | 0.830 |
| 1719 | 0.777 | 1.000 | 0.818 | 1.000 | 0.963 | 0.960 | 0.892 | 1.000 | 0.980 | 1.000 | 0.951 | 0.970 | 0.991 | 0.852 | 1.000 | 1.000 | 0.857 | 0.829 |
| 1720 | 0.780 | 1.000 | 0.812 | 1.000 | 0.968 | 0.960 | 0.888 | 1.000 | 0.969 | 1.000 | 0.957 | 0.970 | 1.000 | 0.855 | 1.000 | 0.963 | 0.858 | 0.829 |
| 1721 | 0.770 | 1.000 | 0.816 | 1.000 | 0.963 | 0.960 | 0.892 | 1.000 | 0.980 | 1.000 | 0.957 | 0.939 | 0.991 | 0.851 | 1.000 | 1.000 | 0.855 | 0.826 |
| 1722 | 0.775 | 1.000 | 0.812 | 1.000 | 0.963 | 0.960 | 0.894 | 1.000 | 0.990 | 1.000 | 0.954 | 1.000 | 1.000 | 0.852 | 1.000 | 1.000 | 0.857 | 0.828 |
| 1723 | 0.771 | 1.000 | 0.810 | 1.000 | 0.958 | 0.960 | 0.900 | 1.000 | 0.980 | 1.000 | 0.954 | 1.000 | 1.000 | 0.854 | 1.000 | 1.000 | 0.856 | 0.828 |
| 1724 | 0.779 | 1.000 | 0.818 | 1.000 | 0.963 | 0.960 | 0.898 | 1.000 | 0.980 | 1.000 | 0.957 | 1.000 | 1.000 | 0.851 | 1.000 | 0.981 | 0.859 | 0.831 |
| 1725 | 0.771 | 1.000 | 0.828 | 1.000 | 0.963 | 0.960 | 0.889 | 1.000 | 0.980 | 1.000 | 0.957 | 1.000 | 1.000 | 0.850 | 1.000 | 1.000 | 0.856 | 0.828 |
| 1726 | 0.774 | 1.000 | 0.822 | 1.000 | 0.974 | 0.960 | 0.889 | 1.000 | 0.990 | 1.000 | 0.959 | 0.970 | 1.000 | 0.848 | 1.000 | 1.000 | 0.857 | 0.828 |
| 1727 | 0.771 | 1.000 | 0.822 | 1.000 | 0.958 | 0.960 | 0.894 | 1.000 | 0.980 | 1.000 | 0.957 | 1.000 | 1.000 | 0.848 | 1.000 | 1.000 | 0.855 | 0.827 |
| 1728 | 0.777 | 1.000 | 0.812 | 0.986 | 0.963 | 0.960 | 0.894 | 1.000 | 0.990 | 1.000 | 0.965 | 1.000 | 1.000 | 0.854 | 1.000 | 0.981 | 0.858 | 0.830 |
| 1729 | 0.773 | 1.000 | 0.818 | 1.000 | 0.958 | 0.960 | 0.895 | 1.000 | 0.969 | 1.000 | 0.948 | 0.970 | 1.000 | 0.856 | 1.000 | 1.000 | 0.857 | 0.828 |
| 1730 | 0.760 | 1.000 | 0.818 | 0.986 | 0.958 | 0.960 | 0.898 | 1.000 | 0.980 | 1.000 | 0.959 | 0.970 | 1.000 | 0.856 | 1.000 | 1.000 | 0.854 | 0.826 |
| 1731 | 0.770 | 1.000 | 0.812 | 1.000 | 0.958 | 0.960 | 0.889 | 1.000 | 0.980 | 1.000 | 0.954 | 0.970 | 1.000 | 0.858 | 1.000 | 1.000 | 0.856 | 0.827 |
| 1732 | 0.771 | 1.000 | 0.814 | 1.000 | 0.963 | 0.960 | 0.898 | 1.000 | 0.980 | 1.000 | 0.945 | 1.000 | 1.000 | 0.854 | 1.000 | 0.981 | 0.856 | 0.828 |
| 1733 | 0.773 | 1.000 | 0.818 | 0.986 | 0.968 | 0.960 | 0.901 | 1.000 | 0.980 | 1.000 | 0.945 | 0.970 | 1.000 | 0.855 | 1.000 | 1.000 | 0.858 | 0.830 |
| 1734 | 0.775 | 1.000 | 0.826 | 1.000 | 0.963 | 0.960 | 0.894 | 1.000 | 0.980 | 1.000 | 0.959 | 0.970 | 1.000 | 0.853 | 1.000 | 1.000 | 0.858 | 0.830 |
| 1735 | 0.770 | 1.000 | 0.816 | 1.000 | 0.963 | 0.960 | 0.904 | 1.000 | 0.990 | 1.000 | 0.959 | 0.970 | 1.000 | 0.858 | 1.000 | 1.000 | 0.859 | 0.831 |
| 1736 | 0.782 | 1.000 | 0.816 | 1.000 | 0.963 | 1.000 | 0.891 | 1.000 | 0.990 | 1.000 | 0.954 | 1.000 | 1.000 | 0.853 | 1.000 | 1.000 | 0.859 | 0.831 |
| 1737 | 0.769 | 1.000 | 0.824 | 1.000 | 0.963 | 0.960 | 0.894 | 1.000 | 0.990 | 1.000 | 0.962 | 0.970 | 1.000 | 0.852 | 1.000 | 1.000 | 0.857 | 0.828 |
| 1738 | 0.771 | 1.000 | 0.816 | 0.986 | 0.963 | 0.960 | 0.897 | 1.000 | 0.980 | 1.000 | 0.948 | 0.939 | 1.000 | 0.860 | 1.000 | 1.000 | 0.857 | 0.829 |
| 1739 | 0.773 | 1.000 | 0.814 | 1.000 | 0.963 | 0.960 | 0.892 | 1.000 | 0.990 | 1.000 | 0.948 | 0.970 | 1.000 | 0.850 | 1.000 | 1.000 | 0.855 | 0.826 |
| 1740 | 0.765 | 1.000 | 0.812 | 1.000 | 0.963 | 0.960 | 0.883 | 1.000 | 0.990 | 1.000 | 0.951 | 0.970 | 1.000 | 0.851 | 1.000 | 1.000 | 0.852 | 0.822 |
| 1741 | 0.767 | 1.000 | 0.808 | 1.000 | 0.958 | 0.960 | 0.888 | 1.000 | 0.969 | 1.000 | 0.948 | 0.970 | 1.000 | 0.849 | 1.000 | 1.000 | 0.851 | 0.822 |
| 1742 | 0.773 | 1.000 | 0.808 | 1.000 | 0.968 | 0.960 | 0.888 | 1.000 | 0.980 | 1.000 | 0.948 | 1.000 | 1.000 | 0.855 | 1.000 | 1.000 | 0.855 | 0.826 |
| 1743 | 0.771 | 1.000 | 0.802 | 1.000 | 0.968 | 0.960 | 0.900 | 1.000 | 0.990 | 1.000 | 0.951 | 0.970 | 1.000 | 0.861 | 1.000 | 1.000 | 0.858 | 0.829 |
| 1744 | 0.772 | 1.000 | 0.806 | 1.000 | 0.958 | 0.960 | 0.894 | 1.000 | 0.990 | 1.000 | 0.954 | 0.970 | 1.000 | 0.854 | 1.000 | 1.000 | 0.856 | 0.827 |
| 1745 | 0.776 | 1.000 | 0.814 | 1.000 | 0.963 | 0.960 | 0.894 | 1.000 | 0.980 | 1.000 | 0.945 | 0.970 | 1.000 | 0.851 | 1.000 | 1.000 | 0.856 | 0.827 |
| 1746 | 0.775 | 1.000 | 0.820 | 1.000 | 0.963 | 0.960 | 0.897 | 1.000 | 0.990 | 1.000 | 0.948 | 1.000 | 1.000 | 0.853 | 1.000 | 1.000 | 0.858 | 0.830 |
| 1747 | 0.767 | 1.000 | 0.828 | 1.000 | 0.968 | 0.960 | 0.895 | 1.000 | 0.990 | 1.000 | 0.948 | 0.970 | 1.000 | 0.860 | 1.000 | 1.000 | 0.858 | 0.830 |
| 1748 | 0.768 | 1.000 | 0.802 | 1.000 | 0.963 | 0.960 | 0.895 | 1.000 | 0.990 | 1.000 | 0.948 | 0.970 | 1.000 | 0.858 | 1.000 | 1.000 | 0.855 | 0.826 |
| 1749 | 0.776 | 1.000 | 0.826 | 1.000 | 0.958 | 0.960 | 0.900 | 1.000 | 0.980 | 1.000 | 0.957 | 0.970 | 1.000 | 0.844 | 1.000 | 0.981 | 0.857 | 0.828 |
| 1750 | 0.769 | 1.000 | 0.832 | 1.000 | 0.958 | 0.960 | 0.891 | 1.000 | 0.980 | 1.000 | 0.962 | 0.970 | 1.000 | 0.852 | 1.000 | 1.000 | 0.857 | 0.828 |
| 1751 | 0.761 | 1.000 | 0.820 | 1.000 | 0.968 | 0.960 | 0.904 | 1.000 | 0.990 | 1.000 | 0.948 | 0.970 | 1.000 | 0.857 | 1.000 | 1.000 | 0.856 | 0.827 |
| 1752 | 0.781 | 1.000 | 0.802 | 1.000 | 0.963 | 0.960 | 0.903 | 1.000 | 0.980 | 1.000 | 0.954 | 0.970 | 1.000 | 0.854 | 1.000 | 1.000 | 0.859 | 0.831 |
| 1753 | 0.772 | 1.000 | 0.808 | 1.000 | 0.963 | 0.960 | 0.888 | 1.000 | 0.990 | 1.000 | 0.945 | 1.000 | 1.000 | 0.851 | 1.000 | 0.981 | 0.854 | 0.825 |

| | | | | | | | | | | | | | | | | | |
|---|---|---|---|---|---|---|---|---|---|---|---|---|---|---|---|---|---|
| 1754 | 0.780 | 1.000 | 0.830 | 1.000 | 0.963 | 0.960 | 0.898 | 1.000 | 0.980 | 1.000 | 0.957 | 1.000 | 1.000 | 0.854 | 1.000 | 1.000 | 0.861 | 0.834 |
| 1755 | 0.763 | 1.000 | 0.806 | 1.000 | 0.968 | 0.960 | 0.894 | 1.000 | 0.990 | 1.000 | 0.954 | 1.000 | 1.000 | 0.855 | 1.000 | 1.000 | 0.854 | 0.825 |
| 1756 | 0.773 | 1.000 | 0.816 | 1.000 | 0.963 | 0.960 | 0.892 | 1.000 | 0.980 | 1.000 | 0.951 | 1.000 | 1.000 | 0.849 | 1.000 | 0.981 | 0.855 | 0.826 |
| 1757 | 0.780 | 1.000 | 0.824 | 1.000 | 0.963 | 0.960 | 0.889 | 1.000 | 0.990 | 1.000 | 0.959 | 0.970 | 1.000 | 0.852 | 1.000 | 1.000 | 0.859 | 0.831 |
| 1758 | 0.783 | 1.000 | 0.826 | 0.986 | 0.968 | 0.960 | 0.897 | 1.000 | 0.990 | 1.000 | 0.957 | 0.939 | 1.000 | 0.852 | 1.000 | 1.000 | 0.861 | 0.833 |
| 1759 | 0.773 | 1.000 | 0.816 | 1.000 | 0.963 | 0.960 | 0.900 | 1.000 | 0.980 | 1.000 | 0.962 | 1.000 | 1.000 | 0.854 | 1.000 | 1.000 | 0.859 | 0.831 |
| 1760 | 0.769 | 1.000 | 0.826 | 1.000 | 0.963 | 0.960 | 0.894 | 1.000 | 0.990 | 1.000 | 0.957 | 0.970 | 1.000 | 0.851 | 1.000 | 1.000 | 0.856 | 0.828 |
| 1761 | 0.774 | 1.000 | 0.820 | 1.000 | 0.958 | 0.960 | 0.898 | 1.000 | 0.980 | 1.000 | 0.959 | 0.970 | 1.000 | 0.854 | 1.000 | 1.000 | 0.858 | 0.830 |
| 1762 | 0.779 | 1.000 | 0.824 | 1.000 | 0.963 | 0.960 | 0.891 | 1.000 | 0.990 | 1.000 | 0.951 | 0.970 | 1.000 | 0.847 | 1.000 | 1.000 | 0.857 | 0.829 |
| 1763 | 0.775 | 1.000 | 0.816 | 1.000 | 0.963 | 0.960 | 0.897 | 1.000 | 0.990 | 1.000 | 0.957 | 1.000 | 1.000 | 0.847 | 1.000 | 0.981 | 0.857 | 0.828 |
| 1764 | 0.766 | 1.000 | 0.838 | 1.000 | 0.963 | 0.960 | 0.900 | 1.000 | 0.990 | 1.000 | 0.957 | 1.000 | 1.000 | 0.858 | 1.000 | 1.000 | 0.859 | 0.831 |
| 1765 | 0.778 | 1.000 | 0.808 | 1.000 | 0.963 | 0.960 | 0.891 | 1.000 | 0.980 | 1.000 | 0.962 | 0.970 | 1.000 | 0.861 | 1.000 | 1.000 | 0.859 | 0.831 |
| 1766 | 0.775 | 1.000 | 0.814 | 1.000 | 0.963 | 0.960 | 0.892 | 1.000 | 0.980 | 1.000 | 0.962 | 0.970 | 1.000 | 0.854 | 1.000 | 1.000 | 0.858 | 0.829 |
| 1767 | 0.774 | 1.000 | 0.832 | 1.000 | 0.958 | 0.960 | 0.895 | 1.000 | 0.990 | 1.000 | 0.959 | 0.970 | 1.000 | 0.858 | 1.000 | 1.000 | 0.860 | 0.833 |
| 1768 | 0.769 | 1.000 | 0.824 | 1.000 | 0.958 | 0.960 | 0.894 | 1.000 | 1.000 | 1.000 | 0.951 | 0.970 | 1.000 | 0.858 | 1.000 | 0.981 | 0.857 | 0.829 |
| 1769 | 0.770 | 1.000 | 0.818 | 1.000 | 0.963 | 0.960 | 0.894 | 1.000 | 0.980 | 1.000 | 0.959 | 0.970 | 1.000 | 0.854 | 1.000 | 1.000 | 0.857 | 0.828 |
| 1770 | 0.767 | 1.000 | 0.826 | 1.000 | 0.958 | 0.960 | 0.894 | 1.000 | 0.980 | 1.000 | 0.954 | 1.000 | 1.000 | 0.853 | 1.000 | 1.000 | 0.856 | 0.827 |
| 1771 | 0.772 | 1.000 | 0.822 | 1.000 | 0.958 | 0.960 | 0.900 | 1.000 | 0.980 | 1.000 | 0.948 | 1.000 | 1.000 | 0.849 | 1.000 | 1.000 | 0.856 | 0.828 |
| 1772 | 0.781 | 1.000 | 0.810 | 1.000 | 0.958 | 0.960 | 0.889 | 1.000 | 0.990 | 1.000 | 0.951 | 0.970 | 1.000 | 0.859 | 1.000 | 0.981 | 0.859 | 0.830 |
| 1773 | 0.775 | 1.000 | 0.804 | 1.000 | 0.968 | 0.960 | 0.895 | 1.000 | 0.990 | 1.000 | 0.957 | 0.970 | 0.991 | 0.852 | 1.000 | 1.000 | 0.856 | 0.828 |
| 1774 | 0.773 | 1.000 | 0.822 | 1.000 | 0.968 | 0.960 | 0.894 | 1.000 | 0.990 | 1.000 | 0.954 | 0.970 | 1.000 | 0.849 | 1.000 | 1.000 | 0.857 | 0.828 |
| 1775 | 0.782 | 1.000 | 0.822 | 1.000 | 0.968 | 0.960 | 0.888 | 1.000 | 0.990 | 1.000 | 0.957 | 0.970 | 1.000 | 0.859 | 1.000 | 1.000 | 0.861 | 0.833 |
| 1776 | 0.778 | 1.000 | 0.818 | 1.000 | 0.958 | 0.960 | 0.901 | 1.000 | 0.980 | 1.000 | 0.948 | 0.970 | 1.000 | 0.848 | 1.000 | 1.000 | 0.857 | 0.829 |
| 1777 | 0.771 | 1.000 | 0.804 | 0.986 | 0.958 | 0.960 | 0.901 | 1.000 | 0.980 | 1.000 | 0.954 | 1.000 | 1.000 | 0.854 | 1.000 | 1.000 | 0.856 | 0.827 |
| 1778 | 0.777 | 1.000 | 0.834 | 0.986 | 0.963 | 0.960 | 0.894 | 1.000 | 0.990 | 1.000 | 0.951 | 1.000 | 1.000 | 0.852 | 1.000 | 1.000 | 0.859 | 0.831 |
| 1779 | 0.782 | 1.000 | 0.826 | 0.986 | 0.968 | 0.960 | 0.908 | 1.000 | 0.990 | 1.000 | 0.945 | 1.000 | 0.991 | 0.853 | 1.000 | 1.000 | 0.862 | 0.834 |
| 1780 | 0.769 | 1.000 | 0.824 | 1.000 | 0.963 | 0.960 | 0.900 | 1.000 | 0.969 | 1.000 | 0.948 | 1.000 | 1.000 | 0.843 | 1.000 | 1.000 | 0.854 | 0.825 |
| 1781 | 0.776 | 1.000 | 0.816 | 1.000 | 0.963 | 0.960 | 0.900 | 1.000 | 0.980 | 1.000 | 0.951 | 0.970 | 1.000 | 0.851 | 1.000 | 1.000 | 0.857 | 0.829 |
| 1782 | 0.777 | 1.000 | 0.818 | 1.000 | 0.963 | 0.960 | 0.900 | 1.000 | 0.990 | 1.000 | 0.959 | 1.000 | 1.000 | 0.852 | 1.000 | 1.000 | 0.859 | 0.831 |
| 1783 | 0.773 | 1.000 | 0.822 | 0.986 | 0.958 | 0.960 | 0.903 | 1.000 | 0.980 | 1.000 | 0.954 | 0.970 | 1.000 | 0.858 | 1.000 | 1.000 | 0.859 | 0.831 |
| 1784 | 0.773 | 1.000 | 0.824 | 1.000 | 0.968 | 0.960 | 0.901 | 1.000 | 0.980 | 1.000 | 0.959 | 1.000 | 1.000 | 0.853 | 1.000 | 1.000 | 0.859 | 0.831 |
| 1785 | 0.769 | 1.000 | 0.812 | 1.000 | 0.963 | 0.960 | 0.901 | 1.000 | 0.990 | 1.000 | 0.954 | 0.970 | 1.000 | 0.852 | 1.000 | 1.000 | 0.856 | 0.827 |
| 1786 | 0.779 | 1.000 | 0.810 | 0.986 | 0.963 | 0.960 | 0.903 | 1.000 | 0.959 | 1.000 | 0.951 | 0.970 | 1.000 | 0.854 | 1.000 | 1.000 | 0.858 | 0.830 |
| 1787 | 0.772 | 1.000 | 0.822 | 0.986 | 0.963 | 0.960 | 0.904 | 1.000 | 0.980 | 1.000 | 0.959 | 1.000 | 1.000 | 0.850 | 1.000 | 1.000 | 0.858 | 0.830 |
| 1788 | 0.767 | 1.000 | 0.812 | 1.000 | 0.963 | 0.960 | 0.894 | 1.000 | 0.990 | 1.000 | 0.948 | 1.000 | 1.000 | 0.856 | 1.000 | 1.000 | 0.855 | 0.826 |
| 1789 | 0.775 | 1.000 | 0.814 | 1.000 | 0.963 | 0.960 | 0.903 | 1.000 | 0.980 | 1.000 | 0.951 | 0.970 | 1.000 | 0.858 | 1.000 | 1.000 | 0.859 | 0.831 |
| 1790 | 0.779 | 1.000 | 0.810 | 1.000 | 0.958 | 0.960 | 0.892 | 1.000 | 0.990 | 1.000 | 0.954 | 0.970 | 1.000 | 0.857 | 1.000 | 1.000 | 0.858 | 0.830 |
| 1791 | 0.773 | 1.000 | 0.816 | 1.000 | 0.963 | 0.960 | 0.897 | 1.000 | 0.990 | 1.000 | 0.954 | 1.000 | 1.000 | 0.851 | 1.000 | 1.000 | 0.857 | 0.828 |
| 1792 | 0.773 | 1.000 | 0.806 | 1.000 | 0.963 | 0.960 | 0.900 | 1.000 | 0.990 | 1.000 | 0.945 | 1.000 | 0.991 | 0.859 | 1.000 | 1.000 | 0.858 | 0.829 |

| | | | | | | | | | | | | | | | | | | |
|---|---|---|---|---|---|---|---|---|---|---|---|---|---|---|---|---|---|---|
| 1793 | 0.765 | 1.000 | 0.822 | 1.000 | 0.958 | 0.960 | 0.898 | 1.000 | 0.980 | 1.000 | 0.957 | 1.000 | 1.000 | 0.849 | 1.000 | 0.981 | 0.854 | 0.826 |
| 1794 | 0.774 | 1.000 | 0.824 | 1.000 | 0.963 | 0.960 | 0.903 | 1.000 | 0.980 | 1.000 | 0.954 | 1.000 | 0.991 | 0.856 | 1.000 | 1.000 | 0.860 | 0.832 |
| 1795 | 0.769 | 1.000 | 0.824 | 1.000 | 0.958 | 0.960 | 0.897 | 1.000 | 0.990 | 1.000 | 0.957 | 0.970 | 1.000 | 0.850 | 1.000 | 1.000 | 0.856 | 0.828 |
| 1796 | 0.769 | 1.000 | 0.816 | 1.000 | 0.958 | 0.960 | 0.901 | 1.000 | 0.990 | 1.000 | 0.962 | 1.000 | 1.000 | 0.849 | 1.000 | 1.000 | 0.856 | 0.828 |
| 1797 | 0.777 | 1.000 | 0.820 | 1.000 | 0.968 | 0.960 | 0.904 | 1.000 | 0.980 | 1.000 | 0.951 | 0.970 | 1.000 | 0.846 | 1.000 | 1.000 | 0.858 | 0.830 |
| 1798 | 0.772 | 1.000 | 0.820 | 1.000 | 0.963 | 0.960 | 0.900 | 1.000 | 0.990 | 1.000 | 0.959 | 1.000 | 1.000 | 0.854 | 1.000 | 1.000 | 0.859 | 0.831 |
| 1799 | 0.773 | 1.000 | 0.826 | 1.000 | 0.963 | 0.960 | 0.897 | 1.000 | 0.990 | 1.000 | 0.951 | 1.000 | 1.000 | 0.858 | 1.000 | 1.000 | 0.860 | 0.832 |
| 1800 | 0.769 | 1.000 | 0.812 | 1.000 | 0.963 | 0.960 | 0.901 | 1.000 | 0.990 | 1.000 | 0.957 | 1.000 | 0.991 | 0.854 | 1.000 | 1.000 | 0.856 | 0.828 |
| 1801 | 0.781 | 1.000 | 0.830 | 0.986 | 0.963 | 0.960 | 0.895 | 1.000 | 0.980 | 1.000 | 0.957 | 1.000 | 1.000 | 0.853 | 1.000 | 1.000 | 0.861 | 0.833 |
| 1802 | 0.773 | 1.000 | 0.830 | 1.000 | 0.963 | 0.960 | 0.906 | 1.000 | 0.990 | 1.000 | 0.954 | 0.970 | 1.000 | 0.850 | 1.000 | 1.000 | 0.859 | 0.831 |
| 1803 | 0.771 | 1.000 | 0.822 | 1.000 | 0.963 | 0.960 | 0.889 | 1.000 | 0.990 | 1.000 | 0.959 | 0.970 | 1.000 | 0.854 | 1.000 | 1.000 | 0.857 | 0.828 |
| 1804 | 0.780 | 1.000 | 0.800 | 1.000 | 0.963 | 0.960 | 0.898 | 1.000 | 0.990 | 1.000 | 0.957 | 1.000 | 1.000 | 0.857 | 1.000 | 0.981 | 0.859 | 0.831 |
| 1805 | 0.768 | 1.000 | 0.814 | 1.000 | 0.963 | 0.960 | 0.895 | 1.000 | 0.980 | 1.000 | 0.951 | 1.000 | 1.000 | 0.849 | 1.000 | 0.981 | 0.854 | 0.825 |
| 1806 | 0.783 | 1.000 | 0.822 | 1.000 | 0.963 | 0.960 | 0.903 | 1.000 | 0.990 | 1.000 | 0.957 | 0.970 | 1.000 | 0.859 | 1.000 | 0.981 | 0.863 | 0.835 |
| 1807 | 0.771 | 1.000 | 0.820 | 1.000 | 0.958 | 0.960 | 0.897 | 1.000 | 0.990 | 1.000 | 0.957 | 1.000 | 1.000 | 0.865 | 1.000 | 0.981 | 0.860 | 0.832 |
| 1808 | 0.771 | 1.000 | 0.818 | 1.000 | 0.958 | 0.960 | 0.897 | 1.000 | 0.980 | 1.000 | 0.951 | 1.000 | 0.991 | 0.854 | 1.000 | 1.000 | 0.856 | 0.828 |
| 1809 | 0.776 | 1.000 | 0.800 | 1.000 | 0.968 | 0.960 | 0.911 | 1.000 | 0.980 | 1.000 | 0.957 | 0.970 | 1.000 | 0.850 | 1.000 | 1.000 | 0.858 | 0.829 |
| 1810 | 0.773 | 1.000 | 0.824 | 1.000 | 0.958 | 0.960 | 0.897 | 1.000 | 0.980 | 1.000 | 0.951 | 0.970 | 1.000 | 0.852 | 1.000 | 1.000 | 0.857 | 0.829 |
| 1811 | 0.777 | 1.000 | 0.810 | 1.000 | 0.958 | 0.960 | 0.900 | 1.000 | 0.990 | 1.000 | 0.945 | 0.970 | 1.000 | 0.854 | 1.000 | 1.000 | 0.858 | 0.829 |
| 1812 | 0.777 | 1.000 | 0.816 | 1.000 | 0.963 | 0.960 | 0.891 | 1.000 | 0.990 | 1.000 | 0.954 | 0.970 | 1.000 | 0.852 | 1.000 | 1.000 | 0.857 | 0.828 |
| 1813 | 0.777 | 1.000 | 0.822 | 1.000 | 0.963 | 0.960 | 0.904 | 1.000 | 0.990 | 1.000 | 0.954 | 1.000 | 1.000 | 0.858 | 1.000 | 1.000 | 0.861 | 0.834 |
| 1814 | 0.767 | 1.000 | 0.810 | 1.000 | 0.963 | 0.960 | 0.908 | 1.000 | 0.980 | 1.000 | 0.954 | 0.970 | 1.000 | 0.843 | 1.000 | 1.000 | 0.853 | 0.825 |
| 1815 | 0.771 | 1.000 | 0.824 | 1.000 | 0.963 | 0.960 | 0.904 | 1.000 | 0.990 | 1.000 | 0.957 | 1.000 | 0.991 | 0.856 | 1.000 | 1.000 | 0.859 | 0.832 |
| 1816 | 0.768 | 1.000 | 0.830 | 1.000 | 0.963 | 0.960 | 0.891 | 1.000 | 0.980 | 1.000 | 0.957 | 1.000 | 1.000 | 0.850 | 1.000 | 1.000 | 0.856 | 0.827 |
| 1817 | 0.764 | 1.000 | 0.820 | 1.000 | 0.958 | 0.960 | 0.895 | 1.000 | 0.990 | 1.000 | 0.957 | 0.970 | 1.000 | 0.854 | 1.000 | 0.981 | 0.855 | 0.826 |
| 1818 | 0.774 | 1.000 | 0.816 | 1.000 | 0.963 | 0.960 | 0.900 | 1.000 | 0.990 | 1.000 | 0.962 | 1.000 | 1.000 | 0.846 | 1.000 | 1.000 | 0.857 | 0.829 |
| 1819 | 0.777 | 1.000 | 0.818 | 1.000 | 0.958 | 0.960 | 0.892 | 1.000 | 0.990 | 1.000 | 0.965 | 1.000 | 1.000 | 0.851 | 1.000 | 0.981 | 0.858 | 0.830 |
| 1820 | 0.772 | 1.000 | 0.810 | 0.986 | 0.963 | 0.960 | 0.894 | 1.000 | 0.990 | 1.000 | 0.954 | 0.970 | 1.000 | 0.852 | 1.000 | 1.000 | 0.855 | 0.827 |
| 1821 | 0.775 | 1.000 | 0.824 | 1.000 | 0.963 | 0.960 | 0.914 | 1.000 | 0.990 | 1.000 | 0.954 | 1.000 | 1.000 | 0.855 | 1.000 | 1.000 | 0.862 | 0.834 |
| 1822 | 0.769 | 1.000 | 0.808 | 1.000 | 0.968 | 0.960 | 0.904 | 1.000 | 0.969 | 1.000 | 0.957 | 1.000 | 1.000 | 0.848 | 1.000 | 1.000 | 0.855 | 0.826 |
| 1823 | 0.769 | 1.000 | 0.820 | 1.000 | 0.963 | 0.960 | 0.903 | 1.000 | 0.980 | 1.000 | 0.959 | 1.000 | 1.000 | 0.854 | 1.000 | 1.000 | 0.858 | 0.830 |
| 1824 | 0.770 | 1.000 | 0.824 | 1.000 | 0.958 | 0.960 | 0.908 | 1.000 | 0.980 | 1.000 | 0.954 | 1.000 | 1.000 | 0.851 | 1.000 | 1.000 | 0.858 | 0.830 |
| 1825 | 0.775 | 1.000 | 0.820 | 1.000 | 0.963 | 0.960 | 0.898 | 1.000 | 0.990 | 1.000 | 0.959 | 1.000 | 0.991 | 0.851 | 1.000 | 1.000 | 0.858 | 0.830 |
| 1826 | 0.773 | 1.000 | 0.820 | 1.000 | 0.963 | 0.960 | 0.903 | 1.000 | 0.990 | 1.000 | 0.951 | 0.970 | 1.000 | 0.854 | 1.000 | 1.000 | 0.859 | 0.831 |
| 1827 | 0.773 | 1.000 | 0.820 | 1.000 | 0.963 | 0.960 | 0.904 | 1.000 | 0.980 | 1.000 | 0.954 | 1.000 | 0.991 | 0.851 | 1.000 | 0.981 | 0.858 | 0.829 |
| 1828 | 0.765 | 1.000 | 0.804 | 0.986 | 0.958 | 0.960 | 0.908 | 1.000 | 0.980 | 1.000 | 0.951 | 0.970 | 1.000 | 0.849 | 1.000 | 1.000 | 0.853 | 0.824 |
| 1829 | 0.767 | 1.000 | 0.836 | 1.000 | 0.958 | 0.960 | 0.892 | 1.000 | 0.990 | 1.000 | 0.948 | 0.970 | 0.991 | 0.854 | 1.000 | 1.000 | 0.856 | 0.828 |
| 1830 | 0.767 | 1.000 | 0.816 | 1.000 | 0.963 | 0.960 | 0.903 | 1.000 | 0.980 | 1.000 | 0.959 | 0.970 | 1.000 | 0.851 | 1.000 | 1.000 | 0.856 | 0.827 |
| 1831 | 0.775 | 1.000 | 0.822 | 0.986 | 0.963 | 0.960 | 0.903 | 1.000 | 0.990 | 1.000 | 0.954 | 1.000 | 0.991 | 0.858 | 1.000 | 1.000 | 0.860 | 0.832 |

| | | | | | | | | | | | | | | | | | |
|---|---|---|---|---|---|---|---|---|---|---|---|---|---|---|---|---|---|
| 1832 | 0.773 | 1.000 | 0.830 | 1.000 | 0.958 | 0.960 | 0.903 | 1.000 | 0.980 | 1.000 | 0.957 | 0.970 | 1.000 | 0.854 | 1.000 | 0.981 | 0.859 | 0.831 |
| 1833 | 0.777 | 1.000 | 0.814 | 1.000 | 0.958 | 1.000 | 0.903 | 1.000 | 0.980 | 1.000 | 0.959 | 1.000 | 1.000 | 0.849 | 1.000 | 0.981 | 0.858 | 0.830 |
| 1834 | 0.777 | 1.000 | 0.826 | 1.000 | 0.968 | 0.960 | 0.898 | 1.000 | 0.990 | 1.000 | 0.959 | 0.970 | 1.000 | 0.852 | 1.000 | 1.000 | 0.860 | 0.832 |
| 1835 | 0.774 | 1.000 | 0.810 | 1.000 | 0.968 | 0.960 | 0.895 | 1.000 | 0.990 | 1.000 | 0.959 | 1.000 | 1.000 | 0.847 | 1.000 | 0.981 | 0.856 | 0.827 |
| 1836 | 0.774 | 1.000 | 0.818 | 0.986 | 0.963 | 0.960 | 0.898 | 1.000 | 0.990 | 1.000 | 0.962 | 1.000 | 0.991 | 0.845 | 1.000 | 1.000 | 0.856 | 0.828 |
| 1837 | 0.771 | 1.000 | 0.818 | 1.000 | 0.963 | 0.960 | 0.906 | 1.000 | 0.980 | 1.000 | 0.948 | 1.000 | 1.000 | 0.855 | 1.000 | 1.000 | 0.858 | 0.830 |
| 1838 | 0.767 | 1.000 | 0.834 | 1.000 | 0.963 | 0.960 | 0.892 | 1.000 | 0.980 | 1.000 | 0.954 | 1.000 | 1.000 | 0.851 | 1.000 | 1.000 | 0.856 | 0.828 |
| 1839 | 0.768 | 1.000 | 0.818 | 1.000 | 0.968 | 1.000 | 0.900 | 1.000 | 0.990 | 1.000 | 0.962 | 0.970 | 1.000 | 0.849 | 1.000 | 1.000 | 0.856 | 0.828 |
| 1840 | 0.769 | 1.000 | 0.824 | 1.000 | 0.963 | 0.960 | 0.897 | 1.000 | 0.990 | 1.000 | 0.959 | 1.000 | 1.000 | 0.848 | 1.000 | 1.000 | 0.856 | 0.828 |
| 1841 | 0.776 | 1.000 | 0.816 | 1.000 | 0.963 | 0.960 | 0.901 | 1.000 | 0.990 | 1.000 | 0.948 | 0.970 | 1.000 | 0.854 | 1.000 | 1.000 | 0.858 | 0.830 |
| 1842 | 0.769 | 1.000 | 0.818 | 1.000 | 0.963 | 0.960 | 0.904 | 1.000 | 0.980 | 1.000 | 0.959 | 1.000 | 1.000 | 0.852 | 1.000 | 1.000 | 0.857 | 0.829 |
| 1843 | 0.765 | 1.000 | 0.818 | 1.000 | 0.963 | 0.960 | 0.900 | 1.000 | 0.980 | 1.000 | 0.962 | 1.000 | 0.991 | 0.851 | 1.000 | 0.981 | 0.855 | 0.827 |
| 1844 | 0.763 | 1.000 | 0.806 | 0.986 | 0.958 | 0.960 | 0.901 | 1.000 | 0.980 | 1.000 | 0.951 | 1.000 | 1.000 | 0.851 | 1.000 | 1.000 | 0.852 | 0.823 |
| 1845 | 0.773 | 1.000 | 0.814 | 1.000 | 0.963 | 0.960 | 0.918 | 1.000 | 0.980 | 1.000 | 0.959 | 0.970 | 1.000 | 0.851 | 1.000 | 1.000 | 0.859 | 0.832 |
| 1846 | 0.774 | 1.000 | 0.838 | 1.000 | 0.963 | 0.960 | 0.901 | 1.000 | 0.990 | 1.000 | 0.959 | 1.000 | 0.991 | 0.855 | 1.000 | 1.000 | 0.861 | 0.834 |
| 1847 | 0.786 | 1.000 | 0.838 | 1.000 | 0.963 | 0.960 | 0.900 | 1.000 | 0.980 | 1.000 | 0.962 | 0.970 | 1.000 | 0.851 | 1.000 | 1.000 | 0.864 | 0.836 |
| 1848 | 0.771 | 1.000 | 0.836 | 1.000 | 0.963 | 0.960 | 0.894 | 1.000 | 0.990 | 1.000 | 0.957 | 0.970 | 0.982 | 0.856 | 1.000 | 1.000 | 0.859 | 0.831 |
| 1849 | 0.775 | 1.000 | 0.826 | 0.986 | 0.963 | 0.960 | 0.906 | 1.000 | 0.990 | 1.000 | 0.957 | 0.970 | 1.000 | 0.844 | 1.000 | 0.981 | 0.857 | 0.829 |
| 1850 | 0.767 | 1.000 | 0.826 | 1.000 | 0.963 | 0.960 | 0.901 | 1.000 | 0.980 | 1.000 | 0.954 | 1.000 | 0.991 | 0.856 | 1.000 | 0.981 | 0.857 | 0.829 |
| 1851 | 0.774 | 1.000 | 0.840 | 1.000 | 0.963 | 1.000 | 0.897 | 1.000 | 0.969 | 1.000 | 0.954 | 1.000 | 1.000 | 0.843 | 1.000 | 1.000 | 0.857 | 0.829 |
| 1852 | 0.769 | 1.000 | 0.826 | 1.000 | 0.963 | 0.960 | 0.901 | 1.000 | 0.990 | 1.000 | 0.959 | 0.970 | 1.000 | 0.855 | 1.000 | 1.000 | 0.859 | 0.831 |
| 1853 | 0.771 | 1.000 | 0.818 | 1.000 | 0.958 | 0.960 | 0.900 | 1.000 | 0.980 | 1.000 | 0.962 | 0.970 | 1.000 | 0.852 | 1.000 | 0.981 | 0.857 | 0.828 |
| 1854 | 0.778 | 1.000 | 0.824 | 1.000 | 0.963 | 0.960 | 0.897 | 1.000 | 0.990 | 1.000 | 0.959 | 1.000 | 1.000 | 0.848 | 1.000 | 1.000 | 0.859 | 0.831 |
| 1855 | 0.779 | 1.000 | 0.824 | 1.000 | 0.963 | 0.960 | 0.894 | 1.000 | 0.990 | 1.000 | 0.954 | 1.000 | 1.000 | 0.854 | 1.000 | 1.000 | 0.860 | 0.832 |
| 1856 | 0.782 | 1.000 | 0.820 | 1.000 | 0.958 | 0.960 | 0.900 | 1.000 | 0.990 | 1.000 | 0.951 | 1.000 | 1.000 | 0.850 | 1.000 | 1.000 | 0.860 | 0.832 |
| 1857 | 0.777 | 1.000 | 0.814 | 1.000 | 0.968 | 0.960 | 0.898 | 1.000 | 0.990 | 1.000 | 0.959 | 0.970 | 0.991 | 0.859 | 1.000 | 1.000 | 0.860 | 0.832 |
| 1858 | 0.769 | 1.000 | 0.812 | 1.000 | 0.958 | 0.960 | 0.895 | 1.000 | 0.990 | 1.000 | 0.957 | 1.000 | 1.000 | 0.850 | 1.000 | 1.000 | 0.855 | 0.826 |
| 1859 | 0.775 | 1.000 | 0.808 | 0.986 | 0.963 | 0.960 | 0.906 | 1.000 | 0.990 | 1.000 | 0.957 | 0.970 | 0.991 | 0.857 | 1.000 | 1.000 | 0.859 | 0.831 |
| 1860 | 0.765 | 1.000 | 0.828 | 0.986 | 0.963 | 0.960 | 0.894 | 1.000 | 0.990 | 1.000 | 0.962 | 1.000 | 1.000 | 0.858 | 1.000 | 1.000 | 0.857 | 0.829 |
| 1861 | 0.767 | 1.000 | 0.804 | 1.000 | 0.963 | 0.960 | 0.898 | 1.000 | 0.990 | 1.000 | 0.957 | 1.000 | 0.991 | 0.858 | 1.000 | 0.981 | 0.856 | 0.827 |
| 1862 | 0.780 | 1.000 | 0.810 | 1.000 | 0.963 | 0.960 | 0.900 | 1.000 | 0.990 | 1.000 | 0.962 | 0.970 | 1.000 | 0.859 | 1.000 | 1.000 | 0.861 | 0.834 |
| 1863 | 0.770 | 1.000 | 0.822 | 1.000 | 0.963 | 0.960 | 0.903 | 1.000 | 0.990 | 1.000 | 0.954 | 0.970 | 1.000 | 0.859 | 1.000 | 1.000 | 0.859 | 0.831 |
| 1864 | 0.771 | 1.000 | 0.822 | 1.000 | 0.963 | 0.960 | 0.903 | 1.000 | 0.980 | 1.000 | 0.954 | 1.000 | 0.991 | 0.851 | 1.000 | 1.000 | 0.857 | 0.829 |
| 1865 | 0.761 | 1.000 | 0.816 | 0.986 | 0.958 | 0.960 | 0.886 | 1.000 | 0.990 | 1.000 | 0.959 | 1.000 | 0.991 | 0.855 | 1.000 | 1.000 | 0.853 | 0.824 |
| 1866 | 0.771 | 1.000 | 0.832 | 1.000 | 0.963 | 0.960 | 0.909 | 1.000 | 0.980 | 1.000 | 0.954 | 1.000 | 1.000 | 0.848 | 1.000 | 1.000 | 0.859 | 0.831 |
| 1867 | 0.776 | 1.000 | 0.812 | 1.000 | 0.963 | 0.960 | 0.903 | 1.000 | 0.990 | 1.000 | 0.962 | 0.970 | 1.000 | 0.854 | 1.000 | 0.981 | 0.859 | 0.831 |
| 1868 | 0.763 | 1.000 | 0.832 | 1.000 | 0.968 | 0.960 | 0.895 | 1.000 | 1.000 | 1.000 | 0.965 | 1.000 | 0.991 | 0.851 | 1.000 | 0.981 | 0.856 | 0.828 |
| 1869 | 0.775 | 1.000 | 0.814 | 0.986 | 0.968 | 0.960 | 0.897 | 1.000 | 0.990 | 1.000 | 0.951 | 1.000 | 1.000 | 0.855 | 1.000 | 1.000 | 0.858 | 0.830 |
| 1870 | 0.773 | 1.000 | 0.812 | 1.000 | 0.963 | 0.960 | 0.898 | 1.000 | 0.980 | 1.000 | 0.954 | 1.000 | 1.000 | 0.847 | 1.000 | 1.000 | 0.856 | 0.827 |

| | | | | | | | | | | | | | | | | | | |
|---|---|---|---|---|---|---|---|---|---|---|---|---|---|---|---|---|---|---|
| 1871 | 0.768 | 1.000 | 0.826 | 1.000 | 0.968 | 0.960 | 0.901 | 1.000 | 0.990 | 1.000 | 0.957 | 1.000 | 0.991 | 0.853 | 1.000 | 0.981 | 0.857 | 0.829 |
| 1872 | 0.771 | 1.000 | 0.824 | 1.000 | 0.963 | 0.960 | 0.897 | 1.000 | 0.969 | 1.000 | 0.951 | 0.970 | 1.000 | 0.851 | 1.000 | 1.000 | 0.856 | 0.828 |
| 1873 | 0.770 | 1.000 | 0.826 | 1.000 | 0.963 | 0.960 | 0.900 | 1.000 | 0.980 | 1.000 | 0.951 | 1.000 | 0.991 | 0.850 | 1.000 | 1.000 | 0.856 | 0.828 |
| 1874 | 0.780 | 1.000 | 0.826 | 1.000 | 0.963 | 0.960 | 0.903 | 1.000 | 0.980 | 1.000 | 0.954 | 1.000 | 0.991 | 0.852 | 1.000 | 1.000 | 0.861 | 0.833 |
| 1875 | 0.771 | 1.000 | 0.826 | 1.000 | 0.963 | 0.960 | 0.906 | 1.000 | 0.990 | 1.000 | 0.951 | 0.970 | 1.000 | 0.851 | 1.000 | 1.000 | 0.858 | 0.830 |
| 1876 | 0.774 | 1.000 | 0.820 | 1.000 | 0.958 | 0.960 | 0.892 | 1.000 | 0.990 | 1.000 | 0.957 | 0.970 | 1.000 | 0.856 | 1.000 | 1.000 | 0.858 | 0.830 |
| 1877 | 0.763 | 1.000 | 0.824 | 1.000 | 0.968 | 0.960 | 0.901 | 1.000 | 0.980 | 1.000 | 0.959 | 0.970 | 0.991 | 0.850 | 1.000 | 1.000 | 0.855 | 0.826 |
| 1878 | 0.764 | 1.000 | 0.820 | 1.000 | 0.968 | 0.960 | 0.906 | 1.000 | 0.980 | 1.000 | 0.951 | 1.000 | 1.000 | 0.851 | 1.000 | 1.000 | 0.856 | 0.827 |
| 1879 | 0.768 | 1.000 | 0.820 | 1.000 | 0.968 | 0.960 | 0.906 | 1.000 | 0.990 | 1.000 | 0.957 | 0.970 | 1.000 | 0.850 | 1.000 | 1.000 | 0.857 | 0.829 |
| 1880 | 0.778 | 1.000 | 0.814 | 1.000 | 0.963 | 0.960 | 0.898 | 1.000 | 0.980 | 1.000 | 0.951 | 0.970 | 1.000 | 0.851 | 1.000 | 1.000 | 0.858 | 0.829 |
| 1881 | 0.775 | 1.000 | 0.820 | 1.000 | 0.968 | 0.960 | 0.906 | 1.000 | 0.969 | 1.000 | 0.954 | 0.970 | 0.991 | 0.857 | 1.000 | 1.000 | 0.860 | 0.832 |
| 1882 | 0.773 | 1.000 | 0.820 | 1.000 | 0.974 | 0.960 | 0.897 | 1.000 | 0.990 | 1.000 | 0.957 | 1.000 | 1.000 | 0.853 | 1.000 | 1.000 | 0.858 | 0.830 |
| 1883 | 0.772 | 1.000 | 0.818 | 1.000 | 0.963 | 0.960 | 0.903 | 1.000 | 1.000 | 1.000 | 0.951 | 1.000 | 1.000 | 0.854 | 1.000 | 0.981 | 0.858 | 0.830 |
| 1884 | 0.770 | 1.000 | 0.816 | 1.000 | 0.963 | 0.960 | 0.901 | 1.000 | 0.990 | 1.000 | 0.951 | 1.000 | 1.000 | 0.854 | 1.000 | 1.000 | 0.857 | 0.829 |
| 1885 | 0.765 | 1.000 | 0.830 | 1.000 | 0.974 | 0.960 | 0.904 | 1.000 | 0.990 | 1.000 | 0.965 | 1.000 | 1.000 | 0.845 | 1.000 | 0.981 | 0.856 | 0.828 |
| 1886 | 0.769 | 1.000 | 0.812 | 1.000 | 0.968 | 0.960 | 0.897 | 1.000 | 0.980 | 1.000 | 0.962 | 1.000 | 0.991 | 0.854 | 1.000 | 1.000 | 0.856 | 0.828 |
| 1887 | 0.771 | 1.000 | 0.830 | 1.000 | 0.974 | 0.960 | 0.897 | 1.000 | 0.990 | 1.000 | 0.951 | 1.000 | 1.000 | 0.851 | 1.000 | 1.000 | 0.858 | 0.829 |
| 1888 | 0.775 | 1.000 | 0.822 | 1.000 | 0.968 | 0.960 | 0.904 | 1.000 | 0.990 | 1.000 | 0.942 | 1.000 | 1.000 | 0.851 | 1.000 | 1.000 | 0.859 | 0.831 |
| 1889 | 0.773 | 1.000 | 0.820 | 1.000 | 0.963 | 0.960 | 0.911 | 1.000 | 0.990 | 1.000 | 0.957 | 0.970 | 1.000 | 0.854 | 1.000 | 1.000 | 0.860 | 0.832 |
| 1890 | 0.773 | 1.000 | 0.814 | 0.986 | 0.974 | 0.960 | 0.900 | 1.000 | 0.990 | 1.000 | 0.945 | 0.970 | 0.991 | 0.851 | 1.000 | 1.000 | 0.856 | 0.828 |
| 1891 | 0.777 | 1.000 | 0.814 | 1.000 | 0.963 | 0.960 | 0.903 | 1.000 | 0.980 | 1.000 | 0.959 | 1.000 | 0.991 | 0.854 | 1.000 | 0.981 | 0.859 | 0.831 |
| 1892 | 0.775 | 1.000 | 0.810 | 1.000 | 0.968 | 0.960 | 0.891 | 1.000 | 0.990 | 1.000 | 0.954 | 1.000 | 1.000 | 0.855 | 1.000 | 0.981 | 0.857 | 0.828 |
| 1893 | 0.765 | 1.000 | 0.822 | 1.000 | 0.968 | 0.960 | 0.900 | 1.000 | 0.990 | 1.000 | 0.959 | 0.970 | 1.000 | 0.858 | 1.000 | 0.981 | 0.858 | 0.830 |
| 1894 | 0.768 | 1.000 | 0.826 | 1.000 | 0.968 | 0.960 | 0.903 | 1.000 | 0.990 | 1.000 | 0.954 | 1.000 | 0.991 | 0.846 | 1.000 | 1.000 | 0.856 | 0.828 |
| 1895 | 0.766 | 1.000 | 0.826 | 1.000 | 0.968 | 0.960 | 0.903 | 1.000 | 0.980 | 1.000 | 0.948 | 0.970 | 0.982 | 0.853 | 1.000 | 0.981 | 0.856 | 0.828 |
| 1896 | 0.781 | 1.000 | 0.830 | 1.000 | 0.968 | 0.960 | 0.906 | 1.000 | 1.000 | 1.000 | 0.957 | 0.970 | 0.991 | 0.844 | 1.000 | 1.000 | 0.860 | 0.832 |
| 1897 | 0.766 | 1.000 | 0.824 | 0.986 | 0.974 | 0.960 | 0.898 | 1.000 | 0.980 | 1.000 | 0.954 | 0.970 | 1.000 | 0.864 | 1.000 | 1.000 | 0.859 | 0.831 |
| 1898 | 0.779 | 1.000 | 0.816 | 1.000 | 0.963 | 0.960 | 0.908 | 1.000 | 0.990 | 1.000 | 0.954 | 0.970 | 1.000 | 0.854 | 1.000 | 1.000 | 0.860 | 0.833 |
| 1899 | 0.770 | 1.000 | 0.828 | 1.000 | 0.963 | 0.960 | 0.897 | 1.000 | 0.990 | 1.000 | 0.959 | 0.970 | 1.000 | 0.850 | 1.000 | 1.000 | 0.857 | 0.829 |
| 1900 | 0.767 | 1.000 | 0.816 | 1.000 | 0.963 | 0.960 | 0.909 | 1.000 | 0.980 | 1.000 | 0.954 | 1.000 | 0.991 | 0.853 | 1.000 | 1.000 | 0.857 | 0.829 |
| 1901 | 0.769 | 1.000 | 0.820 | 1.000 | 0.968 | 0.960 | 0.909 | 1.000 | 0.990 | 1.000 | 0.959 | 0.970 | 1.000 | 0.846 | 1.000 | 0.981 | 0.857 | 0.828 |
| 1902 | 0.773 | 1.000 | 0.816 | 1.000 | 0.968 | 0.960 | 0.898 | 1.000 | 0.990 | 1.000 | 0.951 | 0.970 | 1.000 | 0.853 | 1.000 | 1.000 | 0.857 | 0.829 |
| 1903 | 0.775 | 1.000 | 0.826 | 0.986 | 0.968 | 0.960 | 0.898 | 1.000 | 0.990 | 1.000 | 0.951 | 0.970 | 1.000 | 0.844 | 1.000 | 1.000 | 0.857 | 0.828 |
| 1904 | 0.781 | 1.000 | 0.818 | 1.000 | 0.968 | 0.960 | 0.906 | 1.000 | 0.980 | 1.000 | 0.959 | 0.970 | 1.000 | 0.852 | 1.000 | 1.000 | 0.861 | 0.834 |
| 1905 | 0.777 | 1.000 | 0.820 | 1.000 | 0.963 | 0.960 | 0.901 | 1.000 | 0.980 | 1.000 | 0.959 | 1.000 | 1.000 | 0.847 | 1.000 | 1.000 | 0.858 | 0.830 |
| 1906 | 0.779 | 1.000 | 0.818 | 1.000 | 0.968 | 0.960 | 0.898 | 1.000 | 0.990 | 1.000 | 0.962 | 0.970 | 0.991 | 0.854 | 1.000 | 1.000 | 0.860 | 0.832 |
| 1907 | 0.775 | 1.000 | 0.828 | 1.000 | 0.963 | 0.960 | 0.908 | 1.000 | 0.980 | 1.000 | 0.954 | 1.000 | 0.991 | 0.855 | 1.000 | 0.981 | 0.861 | 0.833 |
| 1908 | 0.763 | 1.000 | 0.820 | 1.000 | 0.968 | 0.960 | 0.911 | 1.000 | 0.990 | 1.000 | 0.942 | 1.000 | 0.991 | 0.854 | 1.000 | 1.000 | 0.856 | 0.828 |
| 1909 | 0.769 | 1.000 | 0.818 | 1.000 | 0.974 | 0.960 | 0.900 | 1.000 | 0.980 | 1.000 | 0.959 | 1.000 | 1.000 | 0.854 | 1.000 | 1.000 | 0.857 | 0.829 |

| | | | | | | | | | | | | | | | | | |
|---|---|---|---|---|---|---|---|---|---|---|---|---|---|---|---|---|---|
| 1910 | 0.773 | 1.000 | 0.828 | 0.986 | 0.968 | 0.960 | 0.903 | 1.000 | 0.980 | 1.000 | 0.951 | 0.970 | 1.000 | 0.850 | 1.000 | 0.981 | 0.858 | 0.830 |
| 1911 | 0.769 | 1.000 | 0.826 | 1.000 | 0.968 | 0.960 | 0.906 | 1.000 | 1.000 | 1.000 | 0.957 | 1.000 | 1.000 | 0.857 | 1.000 | 1.000 | 0.860 | 0.832 |
| 1912 | 0.773 | 1.000 | 0.816 | 0.986 | 0.963 | 0.960 | 0.901 | 1.000 | 0.990 | 1.000 | 0.951 | 0.970 | 1.000 | 0.855 | 1.000 | 1.000 | 0.858 | 0.830 |
| 1913 | 0.777 | 1.000 | 0.816 | 1.000 | 0.963 | 0.960 | 0.898 | 1.000 | 0.990 | 1.000 | 0.954 | 1.000 | 1.000 | 0.855 | 1.000 | 1.000 | 0.859 | 0.832 |
| 1914 | 0.773 | 1.000 | 0.826 | 1.000 | 0.958 | 0.960 | 0.904 | 1.000 | 0.990 | 1.000 | 0.957 | 0.970 | 1.000 | 0.853 | 1.000 | 1.000 | 0.859 | 0.831 |
| 1915 | 0.770 | 1.000 | 0.822 | 1.000 | 0.963 | 0.960 | 0.903 | 1.000 | 0.980 | 1.000 | 0.954 | 0.970 | 1.000 | 0.854 | 1.000 | 1.000 | 0.858 | 0.830 |
| 1916 | 0.772 | 1.000 | 0.814 | 1.000 | 0.958 | 0.960 | 0.898 | 1.000 | 0.990 | 1.000 | 0.959 | 0.970 | 1.000 | 0.846 | 1.000 | 1.000 | 0.855 | 0.826 |
| 1917 | 0.772 | 1.000 | 0.824 | 1.000 | 0.963 | 0.960 | 0.909 | 1.000 | 0.980 | 1.000 | 0.948 | 1.000 | 1.000 | 0.853 | 1.000 | 0.981 | 0.859 | 0.831 |
| 1918 | 0.771 | 1.000 | 0.818 | 1.000 | 0.958 | 1.000 | 0.909 | 1.000 | 0.990 | 1.000 | 0.954 | 1.000 | 1.000 | 0.846 | 1.000 | 1.000 | 0.857 | 0.829 |
| 1919 | 0.771 | 1.000 | 0.806 | 1.000 | 0.958 | 0.960 | 0.906 | 1.000 | 0.990 | 1.000 | 0.954 | 1.000 | 1.000 | 0.851 | 1.000 | 1.000 | 0.856 | 0.828 |
| 1920 | 0.771 | 1.000 | 0.826 | 1.000 | 0.968 | 0.960 | 0.903 | 1.000 | 0.990 | 1.000 | 0.962 | 1.000 | 0.991 | 0.859 | 1.000 | 1.000 | 0.861 | 0.833 |
| 1921 | 0.768 | 1.000 | 0.832 | 0.986 | 0.963 | 0.960 | 0.901 | 1.000 | 0.990 | 1.000 | 0.965 | 1.000 | 1.000 | 0.848 | 1.000 | 1.000 | 0.857 | 0.830 |
| 1922 | 0.768 | 1.000 | 0.816 | 1.000 | 0.958 | 0.960 | 0.900 | 1.000 | 0.969 | 1.000 | 0.951 | 1.000 | 1.000 | 0.850 | 1.000 | 1.000 | 0.855 | 0.826 |
| 1923 | 0.779 | 1.000 | 0.828 | 1.000 | 0.958 | 0.960 | 0.898 | 1.000 | 0.990 | 1.000 | 0.957 | 0.970 | 1.000 | 0.849 | 1.000 | 0.981 | 0.859 | 0.831 |
| 1924 | 0.772 | 1.000 | 0.826 | 1.000 | 0.963 | 0.960 | 0.904 | 1.000 | 0.990 | 1.000 | 0.948 | 1.000 | 0.991 | 0.857 | 1.000 | 1.000 | 0.859 | 0.832 |
| 1925 | 0.775 | 1.000 | 0.828 | 1.000 | 0.963 | 0.960 | 0.909 | 1.000 | 0.990 | 1.000 | 0.957 | 1.000 | 0.991 | 0.849 | 1.000 | 0.981 | 0.859 | 0.832 |
| 1926 | 0.773 | 1.000 | 0.838 | 1.000 | 0.963 | 0.960 | 0.900 | 1.000 | 0.990 | 1.000 | 0.959 | 0.970 | 0.991 | 0.844 | 1.000 | 1.000 | 0.858 | 0.830 |
| 1927 | 0.782 | 1.000 | 0.822 | 1.000 | 0.968 | 0.960 | 0.900 | 1.000 | 0.980 | 1.000 | 0.951 | 1.000 | 0.991 | 0.847 | 1.000 | 1.000 | 0.859 | 0.831 |
| 1928 | 0.769 | 1.000 | 0.826 | 1.000 | 0.963 | 0.960 | 0.900 | 1.000 | 0.990 | 1.000 | 0.965 | 0.970 | 1.000 | 0.858 | 1.000 | 1.000 | 0.859 | 0.831 |
| 1929 | 0.771 | 1.000 | 0.814 | 1.000 | 0.963 | 0.960 | 0.900 | 1.000 | 0.980 | 1.000 | 0.948 | 0.970 | 0.991 | 0.857 | 1.000 | 0.981 | 0.857 | 0.828 |
| 1930 | 0.771 | 1.000 | 0.822 | 1.000 | 0.963 | 0.960 | 0.901 | 1.000 | 0.980 | 1.000 | 0.948 | 1.000 | 1.000 | 0.854 | 1.000 | 1.000 | 0.857 | 0.829 |
| 1931 | 0.783 | 1.000 | 0.812 | 1.000 | 0.963 | 0.960 | 0.906 | 1.000 | 0.990 | 1.000 | 0.957 | 0.970 | 1.000 | 0.849 | 1.000 | 1.000 | 0.860 | 0.832 |
| 1932 | 0.767 | 1.000 | 0.830 | 1.000 | 0.968 | 0.960 | 0.901 | 1.000 | 0.980 | 1.000 | 0.954 | 0.970 | 1.000 | 0.847 | 1.000 | 1.000 | 0.856 | 0.828 |
| 1933 | 0.777 | 1.000 | 0.812 | 0.986 | 0.963 | 0.960 | 0.900 | 1.000 | 0.980 | 1.000 | 0.959 | 1.000 | 1.000 | 0.861 | 1.000 | 0.981 | 0.860 | 0.832 |
| 1934 | 0.767 | 1.000 | 0.818 | 1.000 | 0.963 | 0.960 | 0.909 | 1.000 | 0.969 | 1.000 | 0.962 | 1.000 | 1.000 | 0.851 | 1.000 | 1.000 | 0.857 | 0.829 |
| 1935 | 0.769 | 1.000 | 0.820 | 1.000 | 0.963 | 0.960 | 0.901 | 1.000 | 0.990 | 1.000 | 0.948 | 0.970 | 0.991 | 0.856 | 1.000 | 1.000 | 0.857 | 0.829 |
| 1936 | 0.777 | 1.000 | 0.822 | 1.000 | 0.958 | 0.960 | 0.903 | 1.000 | 0.990 | 1.000 | 0.962 | 1.000 | 1.000 | 0.857 | 1.000 | 1.000 | 0.861 | 0.834 |
| 1937 | 0.774 | 1.000 | 0.830 | 1.000 | 0.958 | 0.960 | 0.909 | 1.000 | 0.990 | 1.000 | 0.954 | 1.000 | 1.000 | 0.852 | 1.000 | 1.000 | 0.860 | 0.833 |
| 1938 | 0.774 | 1.000 | 0.808 | 1.000 | 0.963 | 0.960 | 0.906 | 1.000 | 0.980 | 1.000 | 0.954 | 0.970 | 0.991 | 0.852 | 1.000 | 1.000 | 0.857 | 0.829 |
| 1939 | 0.771 | 1.000 | 0.814 | 1.000 | 0.963 | 0.960 | 0.912 | 1.000 | 0.980 | 1.000 | 0.957 | 0.970 | 1.000 | 0.848 | 1.000 | 1.000 | 0.857 | 0.829 |
| 1940 | 0.767 | 1.000 | 0.824 | 1.000 | 0.963 | 0.960 | 0.903 | 1.000 | 0.980 | 1.000 | 0.954 | 0.970 | 1.000 | 0.854 | 1.000 | 1.000 | 0.857 | 0.829 |
| 1941 | 0.773 | 1.000 | 0.818 | 1.000 | 0.968 | 0.960 | 0.914 | 1.000 | 0.990 | 1.000 | 0.951 | 1.000 | 1.000 | 0.858 | 1.000 | 0.981 | 0.861 | 0.833 |
| 1942 | 0.777 | 1.000 | 0.824 | 1.000 | 0.958 | 0.960 | 0.901 | 1.000 | 0.990 | 1.000 | 0.957 | 0.970 | 1.000 | 0.858 | 1.000 | 0.981 | 0.861 | 0.833 |
| 1943 | 0.785 | 1.000 | 0.808 | 1.000 | 0.963 | 0.960 | 0.903 | 1.000 | 0.969 | 1.000 | 0.959 | 0.970 | 0.982 | 0.851 | 1.000 | 1.000 | 0.860 | 0.832 |
| 1944 | 0.770 | 1.000 | 0.820 | 1.000 | 0.963 | 0.960 | 0.904 | 1.000 | 0.980 | 1.000 | 0.954 | 1.000 | 1.000 | 0.860 | 1.000 | 0.981 | 0.859 | 0.831 |
| 1945 | 0.763 | 1.000 | 0.824 | 1.000 | 0.968 | 0.960 | 0.898 | 1.000 | 0.980 | 1.000 | 0.948 | 1.000 | 0.991 | 0.847 | 1.000 | 1.000 | 0.853 | 0.825 |
| 1946 | 0.774 | 1.000 | 0.832 | 1.000 | 0.963 | 0.960 | 0.912 | 1.000 | 0.990 | 1.000 | 0.948 | 0.970 | 1.000 | 0.858 | 1.000 | 0.981 | 0.862 | 0.835 |
| 1947 | 0.775 | 1.000 | 0.832 | 1.000 | 0.968 | 0.960 | 0.897 | 1.000 | 0.990 | 1.000 | 0.954 | 1.000 | 1.000 | 0.851 | 1.000 | 1.000 | 0.859 | 0.831 |
| 1948 | 0.770 | 1.000 | 0.828 | 1.000 | 0.968 | 0.960 | 0.909 | 1.000 | 0.990 | 1.000 | 0.957 | 0.970 | 1.000 | 0.857 | 1.000 | 1.000 | 0.860 | 0.833 |

| | | | | | | | | | | | | | | | | | |
|---|---|---|---|---|---|---|---|---|---|---|---|---|---|---|---|---|---|
| 1949 | 0.763 | 1.000 | 0.824 | 1.000 | 0.963 | 0.960 | 0.904 | 1.000 | 0.969 | 1.000 | 0.954 | 1.000 | 1.000 | 0.861 | 1.000 | 1.000 | 0.858 | 0.830 |
| 1950 | 0.769 | 1.000 | 0.816 | 1.000 | 0.963 | 0.960 | 0.911 | 1.000 | 0.990 | 1.000 | 0.957 | 0.970 | 1.000 | 0.853 | 1.000 | 1.000 | 0.858 | 0.830 |
| 1951 | 0.767 | 1.000 | 0.826 | 1.000 | 0.968 | 0.960 | 0.903 | 1.000 | 0.990 | 1.000 | 0.957 | 0.970 | 0.991 | 0.850 | 1.000 | 1.000 | 0.856 | 0.828 |
| 1952 | 0.775 | 1.000 | 0.822 | 1.000 | 0.968 | 0.960 | 0.908 | 1.000 | 0.980 | 1.000 | 0.954 | 1.000 | 0.991 | 0.857 | 1.000 | 1.000 | 0.861 | 0.833 |
| 1953 | 0.782 | 1.000 | 0.826 | 1.000 | 0.968 | 0.960 | 0.901 | 1.000 | 0.990 | 1.000 | 0.959 | 0.970 | 1.000 | 0.851 | 1.000 | 1.000 | 0.861 | 0.834 |
| 1954 | 0.769 | 1.000 | 0.808 | 1.000 | 0.968 | 0.960 | 0.909 | 1.000 | 0.990 | 1.000 | 0.954 | 1.000 | 1.000 | 0.853 | 1.000 | 0.981 | 0.857 | 0.829 |
| 1955 | 0.773 | 1.000 | 0.830 | 1.000 | 0.968 | 0.960 | 0.908 | 1.000 | 0.980 | 1.000 | 0.959 | 1.000 | 1.000 | 0.855 | 1.000 | 1.000 | 0.861 | 0.834 |
| 1956 | 0.776 | 1.000 | 0.820 | 1.000 | 0.968 | 0.960 | 0.912 | 1.000 | 0.990 | 1.000 | 0.954 | 1.000 | 1.000 | 0.853 | 1.000 | 1.000 | 0.861 | 0.833 |
| 1957 | 0.770 | 1.000 | 0.816 | 1.000 | 0.968 | 0.960 | 0.892 | 1.000 | 0.990 | 1.000 | 0.951 | 0.970 | 1.000 | 0.852 | 1.000 | 1.000 | 0.855 | 0.827 |
| 1958 | 0.772 | 1.000 | 0.818 | 1.000 | 0.974 | 0.960 | 0.909 | 1.000 | 0.990 | 1.000 | 0.951 | 0.970 | 1.000 | 0.851 | 1.000 | 1.000 | 0.858 | 0.831 |
| 1959 | 0.785 | 1.000 | 0.824 | 1.000 | 0.974 | 0.960 | 0.904 | 1.000 | 0.990 | 1.000 | 0.951 | 1.000 | 0.991 | 0.850 | 1.000 | 0.981 | 0.862 | 0.834 |
| 1960 | 0.773 | 1.000 | 0.826 | 1.000 | 0.963 | 0.960 | 0.900 | 1.000 | 0.980 | 1.000 | 0.957 | 0.970 | 0.991 | 0.845 | 1.000 | 0.981 | 0.856 | 0.828 |
| 1961 | 0.786 | 1.000 | 0.818 | 1.000 | 0.963 | 0.960 | 0.903 | 1.000 | 0.969 | 1.000 | 0.951 | 0.970 | 1.000 | 0.846 | 1.000 | 1.000 | 0.860 | 0.832 |
| 1962 | 0.774 | 1.000 | 0.828 | 1.000 | 0.963 | 0.960 | 0.901 | 1.000 | 0.990 | 1.000 | 0.959 | 1.000 | 0.991 | 0.851 | 1.000 | 0.981 | 0.859 | 0.831 |
| 1963 | 0.773 | 1.000 | 0.834 | 1.000 | 0.974 | 0.960 | 0.903 | 1.000 | 0.990 | 1.000 | 0.948 | 0.970 | 1.000 | 0.853 | 1.000 | 1.000 | 0.860 | 0.832 |
| 1964 | 0.773 | 1.000 | 0.820 | 0.986 | 0.963 | 0.960 | 0.904 | 1.000 | 0.990 | 1.000 | 0.962 | 0.970 | 0.991 | 0.856 | 1.000 | 1.000 | 0.860 | 0.832 |
| 1965 | 0.776 | 1.000 | 0.828 | 1.000 | 0.968 | 0.960 | 0.906 | 1.000 | 0.980 | 1.000 | 0.954 | 0.970 | 0.991 | 0.853 | 1.000 | 1.000 | 0.860 | 0.833 |
| 1966 | 0.769 | 1.000 | 0.830 | 1.000 | 0.968 | 1.000 | 0.904 | 1.000 | 0.980 | 1.000 | 0.959 | 0.970 | 0.982 | 0.853 | 1.000 | 1.000 | 0.859 | 0.831 |
| 1967 | 0.769 | 1.000 | 0.828 | 1.000 | 0.963 | 0.960 | 0.895 | 1.000 | 0.990 | 1.000 | 0.945 | 0.970 | 1.000 | 0.851 | 1.000 | 1.000 | 0.856 | 0.827 |
| 1968 | 0.781 | 1.000 | 0.814 | 1.000 | 0.963 | 0.960 | 0.900 | 1.000 | 0.980 | 1.000 | 0.948 | 0.970 | 1.000 | 0.847 | 1.000 | 1.000 | 0.857 | 0.829 |
| 1969 | 0.765 | 1.000 | 0.822 | 1.000 | 0.968 | 0.960 | 0.906 | 1.000 | 0.980 | 1.000 | 0.959 | 1.000 | 1.000 | 0.845 | 1.000 | 1.000 | 0.855 | 0.827 |
| 1970 | 0.771 | 1.000 | 0.810 | 1.000 | 0.974 | 0.960 | 0.909 | 1.000 | 0.980 | 1.000 | 0.962 | 1.000 | 1.000 | 0.856 | 1.000 | 0.981 | 0.859 | 0.832 |
| 1971 | 0.768 | 1.000 | 0.828 | 0.986 | 0.963 | 0.960 | 0.904 | 1.000 | 0.980 | 1.000 | 0.962 | 0.970 | 0.991 | 0.853 | 1.000 | 1.000 | 0.858 | 0.830 |
| 1972 | 0.773 | 1.000 | 0.834 | 1.000 | 0.958 | 0.960 | 0.901 | 1.000 | 0.980 | 1.000 | 0.962 | 0.970 | 0.991 | 0.854 | 1.000 | 1.000 | 0.860 | 0.832 |
| 1973 | 0.769 | 1.000 | 0.832 | 1.000 | 0.968 | 0.960 | 0.895 | 1.000 | 0.990 | 1.000 | 0.968 | 1.000 | 1.000 | 0.852 | 1.000 | 0.981 | 0.858 | 0.830 |
| 1974 | 0.779 | 1.000 | 0.822 | 1.000 | 0.968 | 0.960 | 0.898 | 1.000 | 0.990 | 1.000 | 0.959 | 0.970 | 0.973 | 0.851 | 1.000 | 1.000 | 0.859 | 0.831 |
| 1975 | 0.767 | 1.000 | 0.822 | 1.000 | 0.963 | 0.960 | 0.906 | 1.000 | 0.980 | 1.000 | 0.959 | 1.000 | 0.991 | 0.845 | 1.000 | 0.981 | 0.855 | 0.827 |
| 1976 | 0.776 | 1.000 | 0.830 | 1.000 | 0.968 | 0.960 | 0.894 | 1.000 | 0.969 | 1.000 | 0.962 | 1.000 | 1.000 | 0.851 | 1.000 | 1.000 | 0.859 | 0.831 |
| 1977 | 0.771 | 1.000 | 0.802 | 1.000 | 0.968 | 0.960 | 0.903 | 1.000 | 0.980 | 1.000 | 0.965 | 1.000 | 1.000 | 0.847 | 1.000 | 0.981 | 0.855 | 0.827 |
| 1978 | 0.769 | 1.000 | 0.826 | 1.000 | 0.958 | 0.960 | 0.909 | 1.000 | 0.980 | 1.000 | 0.962 | 1.000 | 0.991 | 0.852 | 1.000 | 0.981 | 0.858 | 0.830 |
| 1979 | 0.777 | 1.000 | 0.820 | 1.000 | 0.958 | 0.960 | 0.886 | 1.000 | 0.980 | 1.000 | 0.957 | 1.000 | 1.000 | 0.853 | 1.000 | 1.000 | 0.857 | 0.828 |
| 1980 | 0.767 | 1.000 | 0.832 | 1.000 | 0.963 | 0.960 | 0.888 | 1.000 | 0.980 | 1.000 | 0.957 | 1.000 | 0.982 | 0.858 | 1.000 | 0.981 | 0.856 | 0.828 |
| 1981 | 0.775 | 1.000 | 0.820 | 1.000 | 0.963 | 0.960 | 0.900 | 1.000 | 0.980 | 1.000 | 0.959 | 0.970 | 0.991 | 0.844 | 1.000 | 0.981 | 0.856 | 0.827 |
| 1982 | 0.767 | 1.000 | 0.826 | 0.986 | 0.963 | 0.960 | 0.892 | 1.000 | 0.980 | 1.000 | 0.959 | 1.000 | 1.000 | 0.854 | 1.000 | 0.981 | 0.856 | 0.828 |
| 1983 | 0.775 | 1.000 | 0.820 | 1.000 | 0.963 | 0.960 | 0.892 | 1.000 | 0.990 | 1.000 | 0.959 | 0.970 | 1.000 | 0.851 | 1.000 | 1.000 | 0.857 | 0.829 |
| 1984 | 0.778 | 1.000 | 0.824 | 1.000 | 0.968 | 0.960 | 0.909 | 1.000 | 0.969 | 1.000 | 0.959 | 1.000 | 1.000 | 0.860 | 1.000 | 1.000 | 0.863 | 0.836 |
| 1985 | 0.776 | 1.000 | 0.814 | 0.986 | 0.963 | 0.960 | 0.898 | 1.000 | 0.990 | 1.000 | 0.962 | 1.000 | 0.991 | 0.852 | 1.000 | 0.981 | 0.858 | 0.830 |
| 1986 | 0.767 | 1.000 | 0.844 | 1.000 | 0.958 | 0.960 | 0.901 | 1.000 | 0.990 | 1.000 | 0.959 | 1.000 | 0.991 | 0.852 | 1.000 | 1.000 | 0.859 | 0.831 |
| 1987 | 0.777 | 1.000 | 0.824 | 1.000 | 0.968 | 0.960 | 0.892 | 1.000 | 0.969 | 1.000 | 0.945 | 1.000 | 1.000 | 0.856 | 1.000 | 1.000 | 0.859 | 0.831 |

| | | | | | | | | | | | | | | | | | |
|---|---|---|---|---|---|---|---|---|---|---|---|---|---|---|---|---|---|
| 1988 | 0.773 | 1.000 | 0.816 | 1.000 | 0.958 | 0.960 | 0.888 | 1.000 | 0.969 | 1.000 | 0.962 | 0.970 | 1.000 | 0.856 | 1.000 | 0.981 | 0.856 | 0.828 |
| 1989 | 0.774 | 1.000 | 0.816 | 1.000 | 0.963 | 0.960 | 0.898 | 1.000 | 1.000 | 1.000 | 0.965 | 1.000 | 1.000 | 0.857 | 1.000 | 0.981 | 0.860 | 0.832 |
| 1990 | 0.775 | 1.000 | 0.826 | 1.000 | 0.958 | 0.960 | 0.892 | 1.000 | 0.990 | 1.000 | 0.957 | 1.000 | 0.991 | 0.847 | 1.000 | 1.000 | 0.856 | 0.828 |
| 1991 | 0.777 | 1.000 | 0.824 | 1.000 | 0.958 | 1.000 | 0.892 | 1.000 | 0.990 | 1.000 | 0.962 | 1.000 | 0.991 | 0.852 | 1.000 | 0.981 | 0.859 | 0.830 |
| 1992 | 0.766 | 1.000 | 0.828 | 1.000 | 0.963 | 0.960 | 0.898 | 1.000 | 0.990 | 1.000 | 0.954 | 1.000 | 0.991 | 0.852 | 1.000 | 1.000 | 0.856 | 0.828 |
| 1993 | 0.767 | 1.000 | 0.830 | 1.000 | 0.968 | 0.960 | 0.909 | 1.000 | 0.980 | 1.000 | 0.968 | 1.000 | 0.991 | 0.853 | 1.000 | 0.981 | 0.859 | 0.832 |
| 1994 | 0.769 | 1.000 | 0.828 | 1.000 | 0.963 | 0.960 | 0.901 | 1.000 | 0.969 | 1.000 | 0.954 | 1.000 | 0.991 | 0.840 | 1.000 | 0.981 | 0.854 | 0.825 |
| 1995 | 0.761 | 1.000 | 0.828 | 0.986 | 0.963 | 0.960 | 0.894 | 1.000 | 0.980 | 1.000 | 0.959 | 1.000 | 1.000 | 0.850 | 1.000 | 1.000 | 0.854 | 0.825 |
| 1996 | 0.769 | 1.000 | 0.824 | 1.000 | 0.958 | 0.960 | 0.906 | 1.000 | 0.980 | 1.000 | 0.962 | 0.970 | 1.000 | 0.849 | 1.000 | 0.981 | 0.857 | 0.829 |
| 1997 | 0.769 | 1.000 | 0.828 | 1.000 | 0.963 | 0.960 | 0.906 | 1.000 | 0.990 | 1.000 | 0.965 | 1.000 | 0.991 | 0.853 | 1.000 | 0.981 | 0.859 | 0.831 |
| 1998 | 0.771 | 1.000 | 0.834 | 1.000 | 0.958 | 0.960 | 0.898 | 1.000 | 0.990 | 1.000 | 0.951 | 1.000 | 1.000 | 0.857 | 1.000 | 0.981 | 0.859 | 0.831 |
| 1999 | 0.769 | 1.000 | 0.824 | 0.986 | 0.963 | 0.960 | 0.895 | 1.000 | 0.980 | 1.000 | 0.959 | 0.970 | 1.000 | 0.854 | 1.000 | 0.981 | 0.857 | 0.828 |
| 2000 | 0.776 | 1.000 | 0.818 | 1.000 | 0.963 | 0.960 | 0.901 | 1.000 | 0.990 | 1.000 | 0.962 | 0.970 | 0.991 | 0.851 | 1.000 | 0.981 | 0.858 | 0.830 |
| 2001 | 0.764 | 1.000 | 0.812 | 1.000 | 0.963 | 0.960 | 0.897 | 1.000 | 0.980 | 1.000 | 0.959 | 0.970 | 0.991 | 0.854 | 1.000 | 1.000 | 0.854 | 0.825 |
| 2002 | 0.778 | 1.000 | 0.816 | 1.000 | 0.963 | 0.960 | 0.894 | 1.000 | 0.990 | 1.000 | 0.962 | 0.970 | 0.991 | 0.855 | 1.000 | 0.981 | 0.859 | 0.831 |
| 2003 | 0.779 | 1.000 | 0.828 | 1.000 | 0.963 | 0.960 | 0.898 | 1.000 | 0.980 | 1.000 | 0.957 | 1.000 | 0.982 | 0.858 | 1.000 | 0.981 | 0.861 | 0.834 |
| 2004 | 0.771 | 1.000 | 0.818 | 1.000 | 0.958 | 0.960 | 0.903 | 1.000 | 0.990 | 1.000 | 0.959 | 0.939 | 0.982 | 0.858 | 1.000 | 0.981 | 0.858 | 0.830 |
| 2005 | 0.775 | 1.000 | 0.822 | 1.000 | 0.963 | 0.960 | 0.886 | 1.000 | 0.980 | 1.000 | 0.954 | 0.970 | 0.982 | 0.844 | 1.000 | 1.000 | 0.854 | 0.825 |
| 2006 | 0.780 | 1.000 | 0.814 | 1.000 | 0.963 | 0.960 | 0.897 | 1.000 | 0.990 | 1.000 | 0.965 | 1.000 | 1.000 | 0.847 | 1.000 | 1.000 | 0.858 | 0.830 |
| 2007 | 0.777 | 1.000 | 0.826 | 0.986 | 0.968 | 0.960 | 0.895 | 1.000 | 0.990 | 1.000 | 0.957 | 0.970 | 0.982 | 0.859 | 1.000 | 1.000 | 0.860 | 0.833 |
| 2008 | 0.771 | 1.000 | 0.832 | 1.000 | 0.963 | 0.960 | 0.903 | 1.000 | 0.990 | 1.000 | 0.962 | 1.000 | 0.982 | 0.854 | 1.000 | 0.981 | 0.859 | 0.831 |
| 2009 | 0.779 | 1.000 | 0.824 | 1.000 | 0.963 | 0.960 | 0.897 | 1.000 | 0.980 | 1.000 | 0.962 | 0.970 | 0.991 | 0.854 | 1.000 | 1.000 | 0.860 | 0.832 |
| 2010 | 0.770 | 1.000 | 0.810 | 1.000 | 0.963 | 0.960 | 0.897 | 1.000 | 0.980 | 1.000 | 0.951 | 0.970 | 0.991 | 0.851 | 1.000 | 1.000 | 0.854 | 0.825 |
| 2011 | 0.782 | 1.000 | 0.826 | 1.000 | 0.963 | 0.960 | 0.898 | 1.000 | 0.980 | 1.000 | 0.962 | 1.000 | 1.000 | 0.856 | 1.000 | 0.981 | 0.862 | 0.835 |
| 2012 | 0.777 | 1.000 | 0.834 | 0.986 | 0.958 | 0.960 | 0.901 | 1.000 | 0.980 | 1.000 | 0.962 | 1.000 | 0.991 | 0.847 | 1.000 | 1.000 | 0.859 | 0.831 |
| 2013 | 0.771 | 1.000 | 0.816 | 1.000 | 0.958 | 1.000 | 0.894 | 1.000 | 0.980 | 1.000 | 0.957 | 1.000 | 0.991 | 0.855 | 1.000 | 0.981 | 0.856 | 0.828 |
| 2014 | 0.773 | 1.000 | 0.824 | 0.986 | 0.963 | 0.960 | 0.894 | 1.000 | 0.990 | 1.000 | 0.951 | 0.970 | 0.982 | 0.846 | 1.000 | 1.000 | 0.855 | 0.826 |
| 2015 | 0.781 | 1.000 | 0.828 | 1.000 | 0.963 | 0.960 | 0.898 | 1.000 | 0.990 | 1.000 | 0.957 | 1.000 | 0.991 | 0.855 | 1.000 | 0.981 | 0.861 | 0.834 |
| 2016 | 0.785 | 1.000 | 0.820 | 1.000 | 0.958 | 0.960 | 0.904 | 1.000 | 0.990 | 1.000 | 0.959 | 1.000 | 0.982 | 0.858 | 1.000 | 1.000 | 0.863 | 0.836 |
| 2017 | 0.771 | 1.000 | 0.828 | 1.000 | 0.958 | 0.960 | 0.894 | 1.000 | 0.990 | 1.000 | 0.957 | 1.000 | 0.991 | 0.847 | 1.000 | 1.000 | 0.856 | 0.827 |
| 2018 | 0.777 | 1.000 | 0.830 | 1.000 | 0.958 | 0.960 | 0.898 | 1.000 | 0.990 | 1.000 | 0.962 | 0.970 | 0.991 | 0.858 | 1.000 | 1.000 | 0.861 | 0.834 |
| 2019 | 0.770 | 1.000 | 0.830 | 1.000 | 0.963 | 0.960 | 0.898 | 1.000 | 0.990 | 1.000 | 0.957 | 0.970 | 1.000 | 0.851 | 1.000 | 1.000 | 0.857 | 0.830 |
| 2020 | 0.777 | 1.000 | 0.816 | 1.000 | 0.963 | 0.960 | 0.892 | 1.000 | 0.990 | 1.000 | 0.959 | 1.000 | 0.991 | 0.855 | 1.000 | 1.000 | 0.859 | 0.830 |
| 2021 | 0.779 | 1.000 | 0.828 | 1.000 | 0.958 | 0.960 | 0.894 | 1.000 | 0.969 | 1.000 | 0.965 | 0.970 | 0.991 | 0.848 | 1.000 | 1.000 | 0.859 | 0.830 |
| 2022 | 0.774 | 1.000 | 0.834 | 1.000 | 0.963 | 0.960 | 0.897 | 1.000 | 0.990 | 1.000 | 0.962 | 0.970 | 1.000 | 0.851 | 1.000 | 0.981 | 0.859 | 0.831 |
| 2023 | 0.774 | 1.000 | 0.832 | 1.000 | 0.963 | 0.960 | 0.895 | 1.000 | 0.990 | 1.000 | 0.962 | 0.970 | 0.991 | 0.853 | 1.000 | 1.000 | 0.859 | 0.831 |
| 2024 | 0.777 | 1.000 | 0.828 | 1.000 | 0.963 | 0.960 | 0.901 | 1.000 | 0.980 | 1.000 | 0.962 | 1.000 | 1.000 | 0.853 | 1.000 | 1.000 | 0.861 | 0.833 |
| 2025 | 0.773 | 1.000 | 0.830 | 1.000 | 0.958 | 0.960 | 0.900 | 1.000 | 0.990 | 1.000 | 0.954 | 0.970 | 0.991 | 0.851 | 1.000 | 1.000 | 0.858 | 0.830 |
| 2026 | 0.777 | 1.000 | 0.828 | 1.000 | 0.963 | 0.960 | 0.891 | 1.000 | 0.990 | 1.000 | 0.962 | 1.000 | 0.982 | 0.857 | 1.000 | 1.000 | 0.860 | 0.832 |

| | | | | | | | | | | | | | | | | | | |
|---|---|---|---|---|---|---|---|---|---|---|---|---|---|---|---|---|---|---|
| 2027 | 0.772 | 1.000 | 0.812 | 1.000 | 0.958 | 0.960 | 0.903 | 1.000 | 0.990 | 1.000 | 0.959 | 0.970 | 0.991 | 0.855 | 1.000 | 1.000 | 0.858 | 0.830 |
| 2028 | 0.767 | 1.000 | 0.832 | 1.000 | 0.958 | 0.960 | 0.894 | 1.000 | 0.990 | 1.000 | 0.962 | 1.000 | 0.991 | 0.846 | 1.000 | 1.000 | 0.855 | 0.827 |
| 2029 | 0.776 | 1.000 | 0.832 | 0.986 | 0.963 | 0.960 | 0.908 | 1.000 | 0.980 | 1.000 | 0.957 | 1.000 | 0.991 | 0.850 | 1.000 | 1.000 | 0.860 | 0.833 |
| 2030 | 0.773 | 1.000 | 0.844 | 1.000 | 0.963 | 0.960 | 0.894 | 1.000 | 0.990 | 1.000 | 0.965 | 1.000 | 0.991 | 0.851 | 1.000 | 0.981 | 0.860 | 0.832 |
| 2031 | 0.778 | 1.000 | 0.822 | 1.000 | 0.963 | 0.960 | 0.901 | 1.000 | 0.990 | 1.000 | 0.962 | 1.000 | 0.991 | 0.854 | 1.000 | 1.000 | 0.861 | 0.833 |
| 2032 | 0.779 | 1.000 | 0.836 | 1.000 | 0.963 | 0.960 | 0.901 | 1.000 | 0.969 | 1.000 | 0.959 | 1.000 | 0.991 | 0.850 | 1.000 | 1.000 | 0.861 | 0.833 |
| 2033 | 0.774 | 1.000 | 0.836 | 1.000 | 0.958 | 0.960 | 0.903 | 1.000 | 1.000 | 1.000 | 0.959 | 1.000 | 1.000 | 0.851 | 1.000 | 0.981 | 0.860 | 0.833 |
| 2034 | 0.780 | 1.000 | 0.846 | 1.000 | 0.963 | 0.960 | 0.900 | 1.000 | 0.990 | 1.000 | 0.971 | 0.970 | 0.991 | 0.855 | 1.000 | 0.981 | 0.864 | 0.837 |
| 2035 | 0.764 | 1.000 | 0.826 | 1.000 | 0.958 | 0.960 | 0.901 | 1.000 | 0.980 | 1.000 | 0.962 | 0.970 | 1.000 | 0.850 | 1.000 | 0.981 | 0.855 | 0.827 |
| 2036 | 0.774 | 1.000 | 0.806 | 1.000 | 0.963 | 0.960 | 0.889 | 1.000 | 0.980 | 1.000 | 0.962 | 1.000 | 0.982 | 0.851 | 1.000 | 1.000 | 0.855 | 0.826 |
| 2037 | 0.764 | 1.000 | 0.822 | 1.000 | 0.958 | 0.960 | 0.904 | 1.000 | 0.990 | 1.000 | 0.959 | 1.000 | 0.991 | 0.851 | 1.000 | 0.981 | 0.855 | 0.827 |
| 2038 | 0.774 | 1.000 | 0.818 | 1.000 | 0.963 | 0.960 | 0.906 | 1.000 | 0.990 | 1.000 | 0.965 | 0.970 | 0.991 | 0.856 | 1.000 | 1.000 | 0.860 | 0.833 |
| 2039 | 0.765 | 1.000 | 0.832 | 1.000 | 0.963 | 0.960 | 0.903 | 1.000 | 0.969 | 1.000 | 0.965 | 1.000 | 1.000 | 0.850 | 1.000 | 0.981 | 0.857 | 0.829 |
| 2040 | 0.774 | 1.000 | 0.830 | 0.986 | 0.958 | 0.960 | 0.908 | 1.000 | 0.990 | 1.000 | 0.959 | 1.000 | 0.982 | 0.862 | 1.000 | 1.000 | 0.862 | 0.835 |
| 2041 | 0.774 | 1.000 | 0.834 | 1.000 | 0.963 | 0.960 | 0.898 | 1.000 | 0.990 | 1.000 | 0.962 | 1.000 | 0.982 | 0.859 | 1.000 | 0.981 | 0.861 | 0.834 |
| 2042 | 0.769 | 1.000 | 0.824 | 1.000 | 0.963 | 0.960 | 0.903 | 1.000 | 0.980 | 1.000 | 0.965 | 0.970 | 1.000 | 0.851 | 1.000 | 0.981 | 0.858 | 0.830 |
| 2043 | 0.773 | 1.000 | 0.840 | 1.000 | 0.963 | 0.960 | 0.891 | 1.000 | 0.990 | 1.000 | 0.962 | 1.000 | 0.991 | 0.852 | 1.000 | 0.981 | 0.859 | 0.831 |
| 2044 | 0.771 | 1.000 | 0.832 | 0.986 | 0.958 | 0.960 | 0.901 | 1.000 | 0.990 | 1.000 | 0.959 | 1.000 | 0.991 | 0.847 | 1.000 | 0.981 | 0.857 | 0.829 |
| 2045 | 0.773 | 1.000 | 0.822 | 0.986 | 0.963 | 0.960 | 0.878 | 1.000 | 0.990 | 1.000 | 0.962 | 0.970 | 1.000 | 0.850 | 1.000 | 1.000 | 0.855 | 0.826 |
| 2046 | 0.775 | 1.000 | 0.818 | 1.000 | 0.963 | 0.960 | 0.900 | 1.000 | 0.980 | 1.000 | 0.959 | 1.000 | 1.000 | 0.852 | 1.000 | 0.981 | 0.858 | 0.830 |
| 2047 | 0.765 | 1.000 | 0.824 | 1.000 | 0.963 | 0.960 | 0.897 | 1.000 | 0.980 | 1.000 | 0.954 | 1.000 | 0.991 | 0.850 | 1.000 | 1.000 | 0.855 | 0.826 |
| 2048 | 0.783 | 1.000 | 0.814 | 1.000 | 0.963 | 0.960 | 0.898 | 1.000 | 0.990 | 1.000 | 0.962 | 0.970 | 1.000 | 0.847 | 1.000 | 0.981 | 0.859 | 0.831 |
| 2049 | 0.774 | 1.000 | 0.834 | 1.000 | 0.963 | 0.960 | 0.892 | 1.000 | 0.990 | 1.000 | 0.954 | 0.970 | 1.000 | 0.858 | 1.000 | 1.000 | 0.860 | 0.832 |
| 2050 | 0.774 | 1.000 | 0.804 | 1.000 | 0.958 | 0.960 | 0.908 | 1.000 | 0.990 | 1.000 | 0.965 | 1.000 | 0.991 | 0.852 | 1.000 | 0.981 | 0.858 | 0.830 |
| 2051 | 0.772 | 1.000 | 0.844 | 1.000 | 0.963 | 0.960 | 0.900 | 1.000 | 0.969 | 1.000 | 0.957 | 1.000 | 0.991 | 0.858 | 1.000 | 1.000 | 0.861 | 0.834 |
| 2052 | 0.778 | 1.000 | 0.814 | 1.000 | 0.963 | 0.960 | 0.903 | 1.000 | 0.980 | 1.000 | 0.959 | 0.970 | 0.991 | 0.844 | 1.000 | 1.000 | 0.857 | 0.828 |
| 2053 | 0.775 | 1.000 | 0.826 | 1.000 | 0.963 | 0.960 | 0.906 | 1.000 | 0.980 | 1.000 | 0.965 | 1.000 | 0.991 | 0.844 | 1.000 | 0.981 | 0.858 | 0.830 |
| 2054 | 0.770 | 1.000 | 0.824 | 1.000 | 0.963 | 0.960 | 0.903 | 1.000 | 0.990 | 1.000 | 0.962 | 1.000 | 0.982 | 0.849 | 1.000 | 1.000 | 0.857 | 0.829 |
| 2055 | 0.771 | 1.000 | 0.826 | 0.986 | 0.958 | 0.960 | 0.895 | 1.000 | 0.990 | 1.000 | 0.962 | 1.000 | 1.000 | 0.854 | 1.000 | 0.981 | 0.858 | 0.830 |
| 2056 | 0.777 | 1.000 | 0.808 | 1.000 | 0.963 | 0.960 | 0.898 | 1.000 | 0.990 | 1.000 | 0.965 | 0.970 | 1.000 | 0.851 | 1.000 | 1.000 | 0.858 | 0.830 |
| 2057 | 0.764 | 1.000 | 0.832 | 1.000 | 0.968 | 0.960 | 0.903 | 1.000 | 0.990 | 1.000 | 0.962 | 0.970 | 0.991 | 0.857 | 1.000 | 0.981 | 0.858 | 0.830 |
| 2058 | 0.769 | 1.000 | 0.836 | 1.000 | 0.963 | 0.960 | 0.904 | 1.000 | 0.980 | 1.000 | 0.965 | 1.000 | 1.000 | 0.854 | 1.000 | 1.000 | 0.860 | 0.833 |
| 2059 | 0.775 | 1.000 | 0.820 | 1.000 | 0.958 | 0.960 | 0.906 | 1.000 | 0.980 | 1.000 | 0.965 | 0.970 | 0.991 | 0.854 | 1.000 | 0.981 | 0.860 | 0.832 |
| 2060 | 0.766 | 1.000 | 0.828 | 1.000 | 0.968 | 0.960 | 0.895 | 1.000 | 0.990 | 1.000 | 0.965 | 1.000 | 0.991 | 0.842 | 1.000 | 0.981 | 0.854 | 0.825 |
| 2061 | 0.771 | 1.000 | 0.820 | 1.000 | 0.968 | 0.960 | 0.912 | 1.000 | 0.990 | 1.000 | 0.968 | 1.000 | 0.991 | 0.855 | 1.000 | 0.981 | 0.860 | 0.833 |
| 2062 | 0.777 | 1.000 | 0.830 | 1.000 | 0.963 | 0.960 | 0.900 | 1.000 | 0.980 | 1.000 | 0.957 | 1.000 | 1.000 | 0.846 | 1.000 | 0.981 | 0.858 | 0.830 |
| 2063 | 0.765 | 1.000 | 0.814 | 1.000 | 0.958 | 0.960 | 0.897 | 1.000 | 0.980 | 1.000 | 0.962 | 1.000 | 0.991 | 0.853 | 1.000 | 1.000 | 0.855 | 0.826 |
| 2064 | 0.763 | 1.000 | 0.822 | 0.986 | 0.953 | 0.960 | 0.891 | 1.000 | 0.990 | 1.000 | 0.957 | 0.970 | 0.991 | 0.851 | 1.000 | 1.000 | 0.853 | 0.824 |
| 2065 | 0.765 | 1.000 | 0.832 | 1.000 | 0.958 | 0.960 | 0.895 | 1.000 | 0.990 | 1.000 | 0.954 | 0.970 | 0.991 | 0.849 | 1.000 | 0.981 | 0.855 | 0.826 |

| | | | | | | | | | | | | | | | | | | |
|---|---|---|---|---|---|---|---|---|---|---|---|---|---|---|---|---|---|---|
| 2066 | 0.769 | 1.000 | 0.826 | 1.000 | 0.963 | 0.960 | 0.898 | 1.000 | 0.980 | 1.000 | 0.962 | 1.000 | 1.000 | 0.854 | 1.000 | 1.000 | 0.858 | 0.830 |
| 2067 | 0.772 | 1.000 | 0.810 | 0.986 | 0.963 | 0.960 | 0.894 | 1.000 | 0.980 | 1.000 | 0.965 | 1.000 | 1.000 | 0.852 | 1.000 | 0.981 | 0.856 | 0.827 |
| 2068 | 0.773 | 1.000 | 0.832 | 1.000 | 0.963 | 0.960 | 0.900 | 1.000 | 0.990 | 1.000 | 0.959 | 1.000 | 0.991 | 0.861 | 1.000 | 1.000 | 0.862 | 0.834 |
| 2069 | 0.773 | 1.000 | 0.814 | 1.000 | 0.963 | 1.000 | 0.901 | 1.000 | 0.990 | 1.000 | 0.957 | 1.000 | 0.991 | 0.849 | 1.000 | 0.981 | 0.857 | 0.829 |
| 2070 | 0.773 | 1.000 | 0.844 | 1.000 | 0.968 | 0.960 | 0.901 | 1.000 | 0.990 | 1.000 | 0.965 | 1.000 | 0.991 | 0.851 | 1.000 | 0.981 | 0.861 | 0.834 |
| 2071 | 0.779 | 1.000 | 0.828 | 1.000 | 0.963 | 0.960 | 0.895 | 1.000 | 0.990 | 1.000 | 0.957 | 1.000 | 1.000 | 0.852 | 1.000 | 1.000 | 0.860 | 0.832 |
| 2072 | 0.770 | 1.000 | 0.826 | 1.000 | 0.963 | 0.960 | 0.897 | 1.000 | 0.980 | 1.000 | 0.951 | 1.000 | 1.000 | 0.855 | 1.000 | 0.981 | 0.857 | 0.829 |
| 2073 | 0.767 | 1.000 | 0.828 | 1.000 | 0.963 | 0.960 | 0.901 | 1.000 | 0.980 | 1.000 | 0.962 | 0.970 | 0.991 | 0.851 | 1.000 | 0.981 | 0.857 | 0.828 |
| 2074 | 0.775 | 1.000 | 0.822 | 0.986 | 0.958 | 0.960 | 0.895 | 1.000 | 0.990 | 1.000 | 0.957 | 0.970 | 0.982 | 0.851 | 1.000 | 1.000 | 0.857 | 0.829 |
| 2075 | 0.770 | 1.000 | 0.824 | 1.000 | 0.963 | 1.000 | 0.904 | 1.000 | 0.990 | 1.000 | 0.965 | 0.970 | 0.991 | 0.852 | 1.000 | 1.000 | 0.859 | 0.831 |
| 2076 | 0.773 | 1.000 | 0.820 | 1.000 | 0.963 | 0.960 | 0.903 | 1.000 | 0.990 | 1.000 | 0.959 | 0.970 | 0.991 | 0.852 | 1.000 | 1.000 | 0.858 | 0.830 |
| 2077 | 0.767 | 1.000 | 0.834 | 1.000 | 0.963 | 0.960 | 0.901 | 1.000 | 0.990 | 1.000 | 0.959 | 1.000 | 0.991 | 0.845 | 1.000 | 1.000 | 0.856 | 0.828 |
| 2078 | 0.783 | 1.000 | 0.820 | 1.000 | 0.963 | 0.960 | 0.897 | 1.000 | 0.969 | 1.000 | 0.957 | 1.000 | 1.000 | 0.850 | 1.000 | 0.981 | 0.859 | 0.831 |
| 2079 | 0.777 | 1.000 | 0.844 | 1.000 | 0.968 | 0.960 | 0.903 | 1.000 | 0.980 | 1.000 | 0.962 | 1.000 | 0.991 | 0.847 | 1.000 | 1.000 | 0.861 | 0.834 |
| 2080 | 0.767 | 1.000 | 0.818 | 1.000 | 0.963 | 0.960 | 0.897 | 1.000 | 0.980 | 1.000 | 0.962 | 1.000 | 0.991 | 0.848 | 1.000 | 1.000 | 0.855 | 0.826 |
| 2081 | 0.770 | 1.000 | 0.838 | 1.000 | 0.968 | 0.960 | 0.898 | 1.000 | 0.980 | 1.000 | 0.965 | 1.000 | 0.991 | 0.847 | 1.000 | 1.000 | 0.858 | 0.830 |
| 2082 | 0.759 | 1.000 | 0.834 | 1.000 | 0.963 | 0.960 | 0.898 | 1.000 | 0.990 | 1.000 | 0.968 | 1.000 | 0.991 | 0.847 | 1.000 | 0.981 | 0.854 | 0.826 |
| 2083 | 0.775 | 1.000 | 0.818 | 1.000 | 0.958 | 0.960 | 0.901 | 1.000 | 0.969 | 1.000 | 0.962 | 1.000 | 1.000 | 0.851 | 1.000 | 1.000 | 0.858 | 0.830 |
| 2084 | 0.779 | 1.000 | 0.834 | 1.000 | 0.958 | 0.960 | 0.898 | 1.000 | 0.990 | 1.000 | 0.957 | 1.000 | 1.000 | 0.851 | 1.000 | 1.000 | 0.861 | 0.833 |
| 2085 | 0.771 | 1.000 | 0.828 | 1.000 | 0.963 | 0.960 | 0.906 | 1.000 | 0.990 | 1.000 | 0.962 | 1.000 | 1.000 | 0.851 | 1.000 | 0.981 | 0.859 | 0.831 |
| 2086 | 0.771 | 1.000 | 0.826 | 1.000 | 0.963 | 0.960 | 0.909 | 1.000 | 0.990 | 1.000 | 0.962 | 0.970 | 0.991 | 0.851 | 1.000 | 1.000 | 0.859 | 0.831 |
| 2087 | 0.777 | 1.000 | 0.844 | 1.000 | 0.963 | 0.960 | 0.898 | 1.000 | 0.980 | 1.000 | 0.962 | 0.970 | 0.991 | 0.855 | 1.000 | 1.000 | 0.862 | 0.835 |
| 2088 | 0.771 | 1.000 | 0.840 | 1.000 | 0.968 | 0.960 | 0.897 | 1.000 | 0.990 | 1.000 | 0.959 | 1.000 | 1.000 | 0.846 | 1.000 | 0.981 | 0.858 | 0.830 |
| 2089 | 0.779 | 1.000 | 0.840 | 1.000 | 0.963 | 0.960 | 0.897 | 1.000 | 0.969 | 1.000 | 0.959 | 1.000 | 0.991 | 0.852 | 1.000 | 1.000 | 0.861 | 0.834 |
| 2090 | 0.771 | 1.000 | 0.832 | 1.000 | 0.963 | 0.960 | 0.906 | 1.000 | 0.990 | 1.000 | 0.965 | 1.000 | 1.000 | 0.847 | 1.000 | 1.000 | 0.859 | 0.831 |
| 2091 | 0.775 | 1.000 | 0.826 | 1.000 | 0.968 | 0.960 | 0.897 | 1.000 | 0.990 | 1.000 | 0.962 | 1.000 | 0.991 | 0.851 | 1.000 | 0.981 | 0.859 | 0.831 |
| 2092 | 0.767 | 1.000 | 0.820 | 1.000 | 0.958 | 0.960 | 0.900 | 1.000 | 1.000 | 1.000 | 0.948 | 0.970 | 0.991 | 0.847 | 1.000 | 1.000 | 0.854 | 0.825 |
| 2093 | 0.763 | 1.000 | 0.830 | 1.000 | 0.963 | 0.960 | 0.895 | 1.000 | 0.990 | 1.000 | 0.962 | 1.000 | 0.991 | 0.858 | 1.000 | 1.000 | 0.857 | 0.829 |
| 2094 | 0.785 | 1.000 | 0.812 | 1.000 | 0.958 | 0.960 | 0.901 | 1.000 | 0.980 | 1.000 | 0.968 | 0.970 | 0.991 | 0.851 | 1.000 | 1.000 | 0.861 | 0.833 |
| 2095 | 0.771 | 1.000 | 0.832 | 1.000 | 0.963 | 0.960 | 0.906 | 1.000 | 0.980 | 1.000 | 0.959 | 1.000 | 0.991 | 0.849 | 1.000 | 1.000 | 0.859 | 0.831 |
| 2096 | 0.769 | 1.000 | 0.826 | 1.000 | 0.963 | 0.960 | 0.898 | 1.000 | 0.990 | 1.000 | 0.965 | 0.970 | 1.000 | 0.843 | 1.000 | 1.000 | 0.855 | 0.827 |
| 2097 | 0.776 | 1.000 | 0.826 | 1.000 | 0.963 | 0.960 | 0.897 | 1.000 | 0.990 | 1.000 | 0.962 | 0.970 | 0.991 | 0.847 | 1.000 | 1.000 | 0.858 | 0.830 |
| 2098 | 0.764 | 1.000 | 0.828 | 1.000 | 0.963 | 0.960 | 0.903 | 1.000 | 0.990 | 1.000 | 0.959 | 1.000 | 1.000 | 0.846 | 1.000 | 0.981 | 0.855 | 0.827 |
| 2099 | 0.772 | 1.000 | 0.832 | 1.000 | 0.963 | 0.960 | 0.900 | 1.000 | 0.990 | 1.000 | 0.959 | 1.000 | 1.000 | 0.857 | 1.000 | 1.000 | 0.860 | 0.833 |
| 2100 | 0.775 | 1.000 | 0.820 | 1.000 | 0.968 | 0.960 | 0.898 | 1.000 | 0.980 | 1.000 | 0.954 | 1.000 | 0.982 | 0.856 | 1.000 | 1.000 | 0.859 | 0.831 |
| 2101 | 0.779 | 1.000 | 0.822 | 1.000 | 0.963 | 0.960 | 0.900 | 1.000 | 0.990 | 1.000 | 0.959 | 0.970 | 0.991 | 0.861 | 1.000 | 1.000 | 0.862 | 0.835 |
| 2102 | 0.773 | 1.000 | 0.818 | 1.000 | 0.963 | 0.960 | 0.908 | 1.000 | 0.980 | 1.000 | 0.968 | 0.970 | 1.000 | 0.853 | 1.000 | 1.000 | 0.859 | 0.831 |
| 2103 | 0.777 | 1.000 | 0.820 | 1.000 | 0.958 | 0.960 | 0.903 | 1.000 | 0.990 | 1.000 | 0.965 | 1.000 | 0.991 | 0.853 | 1.000 | 0.981 | 0.860 | 0.832 |
| 2104 | 0.767 | 1.000 | 0.844 | 1.000 | 0.963 | 0.960 | 0.904 | 1.000 | 0.990 | 1.000 | 0.957 | 1.000 | 0.991 | 0.846 | 1.000 | 1.000 | 0.857 | 0.830 |

| | | | | | | | | | | | | | | | | | |
|---|---|---|---|---|---|---|---|---|---|---|---|---|---|---|---|---|---|
| 2105 | 0.773 | 1.000 | 0.826 | 1.000 | 0.958 | 0.960 | 0.904 | 1.000 | 0.990 | 1.000 | 0.954 | 1.000 | 0.991 | 0.851 | 1.000 | 0.981 | 0.858 | 0.830 |
| 2106 | 0.774 | 1.000 | 0.814 | 1.000 | 0.958 | 0.960 | 0.903 | 1.000 | 0.990 | 1.000 | 0.968 | 0.970 | 1.000 | 0.854 | 1.000 | 1.000 | 0.859 | 0.831 |
| 2107 | 0.761 | 1.000 | 0.830 | 0.986 | 0.958 | 0.960 | 0.898 | 1.000 | 1.000 | 1.000 | 0.959 | 1.000 | 1.000 | 0.847 | 1.000 | 0.981 | 0.854 | 0.826 |
| 2108 | 0.779 | 1.000 | 0.824 | 1.000 | 0.963 | 0.960 | 0.895 | 1.000 | 0.969 | 1.000 | 0.962 | 1.000 | 1.000 | 0.844 | 1.000 | 0.981 | 0.858 | 0.829 |
| 2109 | 0.775 | 1.000 | 0.824 | 1.000 | 0.963 | 0.960 | 0.894 | 1.000 | 0.990 | 1.000 | 0.954 | 1.000 | 0.991 | 0.851 | 1.000 | 0.981 | 0.857 | 0.829 |
| 2110 | 0.765 | 1.000 | 0.820 | 1.000 | 0.963 | 0.960 | 0.914 | 1.000 | 1.000 | 1.000 | 0.965 | 0.970 | 0.991 | 0.856 | 1.000 | 0.981 | 0.859 | 0.831 |
| 2111 | 0.775 | 1.000 | 0.838 | 1.000 | 0.963 | 0.960 | 0.911 | 1.000 | 0.980 | 1.000 | 0.962 | 1.000 | 1.000 | 0.854 | 1.000 | 1.000 | 0.862 | 0.835 |
| 2112 | 0.767 | 1.000 | 0.824 | 1.000 | 0.963 | 0.960 | 0.897 | 1.000 | 0.990 | 1.000 | 0.962 | 0.970 | 1.000 | 0.855 | 1.000 | 1.000 | 0.857 | 0.829 |
| 2113 | 0.769 | 1.000 | 0.822 | 0.986 | 0.963 | 0.960 | 0.900 | 1.000 | 0.980 | 1.000 | 0.962 | 0.970 | 1.000 | 0.854 | 1.000 | 1.000 | 0.858 | 0.829 |
| 2114 | 0.786 | 1.000 | 0.822 | 1.000 | 0.963 | 0.960 | 0.906 | 1.000 | 0.980 | 1.000 | 0.968 | 0.970 | 0.991 | 0.844 | 1.000 | 1.000 | 0.861 | 0.834 |
| 2115 | 0.779 | 1.000 | 0.824 | 1.000 | 0.958 | 0.960 | 0.894 | 1.000 | 0.990 | 1.000 | 0.959 | 0.939 | 0.991 | 0.851 | 1.000 | 1.000 | 0.859 | 0.830 |
| 2116 | 0.773 | 1.000 | 0.830 | 1.000 | 0.963 | 0.960 | 0.903 | 1.000 | 0.990 | 1.000 | 0.962 | 1.000 | 0.991 | 0.859 | 1.000 | 0.981 | 0.861 | 0.834 |
| 2117 | 0.777 | 1.000 | 0.828 | 1.000 | 0.968 | 0.960 | 0.895 | 1.000 | 0.990 | 1.000 | 0.965 | 1.000 | 0.991 | 0.854 | 1.000 | 0.981 | 0.860 | 0.832 |
| 2118 | 0.777 | 1.000 | 0.830 | 0.986 | 0.968 | 0.960 | 0.895 | 1.000 | 0.980 | 1.000 | 0.959 | 0.970 | 1.000 | 0.852 | 1.000 | 1.000 | 0.860 | 0.832 |
| 2119 | 0.762 | 1.000 | 0.822 | 1.000 | 0.974 | 0.960 | 0.897 | 1.000 | 0.990 | 1.000 | 0.957 | 0.970 | 0.991 | 0.854 | 1.000 | 0.981 | 0.855 | 0.826 |
| 2120 | 0.767 | 1.000 | 0.820 | 1.000 | 0.963 | 0.960 | 0.895 | 1.000 | 0.980 | 1.000 | 0.957 | 1.000 | 0.991 | 0.852 | 1.000 | 1.000 | 0.855 | 0.827 |
| 2121 | 0.769 | 1.000 | 0.828 | 1.000 | 0.968 | 0.960 | 0.914 | 1.000 | 0.990 | 1.000 | 0.959 | 1.000 | 0.991 | 0.852 | 1.000 | 1.000 | 0.860 | 0.833 |
| 2122 | 0.767 | 1.000 | 0.828 | 1.000 | 0.968 | 0.960 | 0.903 | 1.000 | 0.990 | 1.000 | 0.957 | 0.970 | 1.000 | 0.842 | 1.000 | 1.000 | 0.855 | 0.826 |
| 2123 | 0.774 | 1.000 | 0.834 | 1.000 | 0.968 | 0.960 | 0.894 | 1.000 | 0.990 | 1.000 | 0.962 | 1.000 | 1.000 | 0.852 | 1.000 | 0.981 | 0.859 | 0.832 |
| 2124 | 0.768 | 1.000 | 0.824 | 1.000 | 0.968 | 0.960 | 0.901 | 1.000 | 0.980 | 1.000 | 0.957 | 1.000 | 1.000 | 0.854 | 1.000 | 1.000 | 0.858 | 0.830 |
| 2125 | 0.776 | 1.000 | 0.828 | 1.000 | 0.963 | 0.960 | 0.906 | 1.000 | 0.980 | 1.000 | 0.957 | 1.000 | 0.991 | 0.849 | 1.000 | 1.000 | 0.859 | 0.831 |
| 2126 | 0.757 | 1.000 | 0.828 | 1.000 | 0.963 | 0.960 | 0.904 | 1.000 | 0.980 | 1.000 | 0.962 | 0.970 | 0.991 | 0.855 | 1.000 | 1.000 | 0.855 | 0.827 |
| 2127 | 0.768 | 1.000 | 0.814 | 1.000 | 0.974 | 0.960 | 0.906 | 1.000 | 0.990 | 1.000 | 0.957 | 0.970 | 0.991 | 0.856 | 1.000 | 0.981 | 0.858 | 0.830 |
| 2128 | 0.783 | 1.000 | 0.836 | 1.000 | 0.968 | 0.960 | 0.900 | 1.000 | 0.990 | 1.000 | 0.962 | 0.970 | 0.991 | 0.844 | 1.000 | 1.000 | 0.861 | 0.833 |
| 2129 | 0.769 | 1.000 | 0.828 | 1.000 | 0.968 | 0.960 | 0.894 | 1.000 | 0.990 | 1.000 | 0.962 | 0.970 | 1.000 | 0.847 | 1.000 | 1.000 | 0.856 | 0.828 |
| 2130 | 0.770 | 1.000 | 0.840 | 1.000 | 0.963 | 1.000 | 0.903 | 1.000 | 0.980 | 1.000 | 0.965 | 1.000 | 1.000 | 0.855 | 1.000 | 0.981 | 0.861 | 0.834 |
| 2131 | 0.783 | 1.000 | 0.822 | 1.000 | 0.968 | 0.960 | 0.901 | 1.000 | 0.980 | 1.000 | 0.968 | 0.970 | 0.991 | 0.850 | 1.000 | 1.000 | 0.861 | 0.833 |
| 2132 | 0.775 | 1.000 | 0.834 | 1.000 | 0.968 | 0.960 | 0.903 | 1.000 | 0.990 | 1.000 | 0.968 | 0.970 | 0.991 | 0.846 | 1.000 | 1.000 | 0.860 | 0.832 |
| 2133 | 0.766 | 1.000 | 0.822 | 1.000 | 0.963 | 0.960 | 0.894 | 1.000 | 0.990 | 1.000 | 0.954 | 0.970 | 0.982 | 0.858 | 1.000 | 1.000 | 0.856 | 0.828 |
| 2134 | 0.773 | 1.000 | 0.816 | 1.000 | 0.968 | 0.960 | 0.894 | 1.000 | 0.990 | 1.000 | 0.954 | 1.000 | 1.000 | 0.856 | 1.000 | 1.000 | 0.858 | 0.830 |
| 2135 | 0.777 | 1.000 | 0.820 | 1.000 | 0.968 | 0.960 | 0.903 | 1.000 | 0.990 | 1.000 | 0.962 | 0.970 | 0.991 | 0.852 | 1.000 | 0.981 | 0.860 | 0.832 |
| 2136 | 0.777 | 1.000 | 0.836 | 1.000 | 0.968 | 0.960 | 0.900 | 1.000 | 0.990 | 1.000 | 0.962 | 1.000 | 0.991 | 0.854 | 1.000 | 1.000 | 0.862 | 0.834 |
| 2137 | 0.774 | 1.000 | 0.832 | 1.000 | 0.974 | 0.960 | 0.901 | 1.000 | 0.990 | 1.000 | 0.959 | 0.970 | 0.982 | 0.847 | 1.000 | 1.000 | 0.859 | 0.831 |
| 2138 | 0.772 | 1.000 | 0.824 | 1.000 | 0.968 | 0.960 | 0.901 | 1.000 | 0.990 | 1.000 | 0.962 | 1.000 | 0.991 | 0.851 | 1.000 | 1.000 | 0.859 | 0.831 |
| 2139 | 0.774 | 1.000 | 0.838 | 1.000 | 0.963 | 0.960 | 0.909 | 1.000 | 0.990 | 1.000 | 0.959 | 1.000 | 0.991 | 0.854 | 1.000 | 1.000 | 0.862 | 0.835 |
| 2140 | 0.769 | 1.000 | 0.828 | 1.000 | 0.963 | 0.960 | 0.891 | 1.000 | 0.990 | 1.000 | 0.962 | 1.000 | 0.991 | 0.850 | 1.000 | 1.000 | 0.856 | 0.828 |
| 2141 | 0.772 | 1.000 | 0.818 | 0.986 | 0.968 | 0.960 | 0.906 | 1.000 | 0.969 | 1.000 | 0.962 | 1.000 | 0.991 | 0.851 | 1.000 | 1.000 | 0.858 | 0.830 |
| 2142 | 0.779 | 1.000 | 0.838 | 1.000 | 0.974 | 0.960 | 0.897 | 1.000 | 0.990 | 1.000 | 0.959 | 0.939 | 0.982 | 0.854 | 1.000 | 0.981 | 0.862 | 0.834 |
| 2143 | 0.779 | 1.000 | 0.814 | 1.000 | 0.968 | 0.960 | 0.901 | 1.000 | 0.980 | 1.000 | 0.959 | 1.000 | 0.991 | 0.845 | 1.000 | 1.000 | 0.858 | 0.829 |

| | | | | | | | | | | | | | | | | | |
|---|---|---|---|---|---|---|---|---|---|---|---|---|---|---|---|---|---|
| 2144 | 0.776 | 1.000 | 0.830 | 1.000 | 0.968 | 0.960 | 0.909 | 1.000 | 0.990 | 1.000 | 0.962 | 1.000 | 1.000 | 0.841 | 1.000 | 1.000 | 0.859 | 0.831 |
| 2145 | 0.768 | 1.000 | 0.832 | 1.000 | 0.968 | 0.960 | 0.903 | 1.000 | 0.990 | 1.000 | 0.965 | 1.000 | 0.991 | 0.851 | 1.000 | 0.981 | 0.858 | 0.830 |
| 2146 | 0.771 | 1.000 | 0.840 | 1.000 | 0.968 | 0.960 | 0.901 | 1.000 | 0.990 | 1.000 | 0.962 | 1.000 | 1.000 | 0.854 | 1.000 | 0.981 | 0.861 | 0.833 |
| 2147 | 0.778 | 1.000 | 0.822 | 0.986 | 0.963 | 0.960 | 0.892 | 1.000 | 0.980 | 1.000 | 0.962 | 0.939 | 1.000 | 0.853 | 1.000 | 1.000 | 0.859 | 0.830 |
| 2148 | 0.771 | 1.000 | 0.822 | 1.000 | 0.968 | 0.960 | 0.894 | 1.000 | 0.990 | 1.000 | 0.959 | 0.970 | 0.982 | 0.852 | 1.000 | 1.000 | 0.857 | 0.828 |
| 2149 | 0.771 | 1.000 | 0.838 | 0.986 | 0.968 | 1.000 | 0.909 | 1.000 | 0.990 | 1.000 | 0.959 | 0.970 | 0.991 | 0.845 | 1.000 | 0.981 | 0.859 | 0.831 |
| 2150 | 0.755 | 1.000 | 0.838 | 1.000 | 0.963 | 0.960 | 0.904 | 1.000 | 0.990 | 1.000 | 0.965 | 0.970 | 0.991 | 0.847 | 1.000 | 0.981 | 0.854 | 0.826 |
| 2151 | 0.771 | 1.000 | 0.820 | 1.000 | 0.968 | 0.960 | 0.914 | 1.000 | 0.969 | 1.000 | 0.954 | 0.970 | 0.991 | 0.852 | 1.000 | 1.000 | 0.858 | 0.831 |
| 2152 | 0.781 | 1.000 | 0.840 | 1.000 | 0.968 | 0.960 | 0.895 | 1.000 | 0.969 | 1.000 | 0.962 | 0.970 | 0.991 | 0.859 | 1.000 | 0.981 | 0.863 | 0.836 |
| 2153 | 0.770 | 1.000 | 0.836 | 1.000 | 0.968 | 0.960 | 0.901 | 1.000 | 0.980 | 1.000 | 0.965 | 1.000 | 0.991 | 0.855 | 1.000 | 0.981 | 0.860 | 0.832 |
| 2154 | 0.767 | 1.000 | 0.834 | 1.000 | 0.968 | 0.960 | 0.909 | 1.000 | 0.980 | 1.000 | 0.957 | 1.000 | 1.000 | 0.851 | 1.000 | 0.981 | 0.858 | 0.831 |
| 2155 | 0.768 | 1.000 | 0.834 | 1.000 | 0.963 | 0.960 | 0.901 | 1.000 | 0.990 | 1.000 | 0.962 | 1.000 | 1.000 | 0.849 | 1.000 | 1.000 | 0.858 | 0.830 |
| 2156 | 0.769 | 1.000 | 0.836 | 1.000 | 0.968 | 0.960 | 0.908 | 1.000 | 0.980 | 1.000 | 0.965 | 1.000 | 0.991 | 0.852 | 1.000 | 1.000 | 0.860 | 0.832 |
| 2157 | 0.774 | 1.000 | 0.824 | 1.000 | 0.968 | 0.960 | 0.906 | 1.000 | 0.980 | 1.000 | 0.965 | 1.000 | 1.000 | 0.847 | 1.000 | 1.000 | 0.859 | 0.831 |
| 2158 | 0.765 | 1.000 | 0.836 | 1.000 | 0.963 | 0.960 | 0.894 | 1.000 | 0.990 | 1.000 | 0.962 | 0.970 | 1.000 | 0.851 | 1.000 | 1.000 | 0.856 | 0.828 |
| 2159 | 0.778 | 1.000 | 0.814 | 1.000 | 0.968 | 0.960 | 0.895 | 1.000 | 0.990 | 1.000 | 0.962 | 0.970 | 1.000 | 0.854 | 1.000 | 0.981 | 0.859 | 0.831 |
| 2160 | 0.775 | 1.000 | 0.808 | 1.000 | 0.963 | 0.960 | 0.898 | 1.000 | 0.980 | 1.000 | 0.959 | 0.970 | 1.000 | 0.856 | 1.000 | 0.981 | 0.858 | 0.829 |
| 2161 | 0.771 | 1.000 | 0.830 | 1.000 | 0.963 | 0.960 | 0.903 | 1.000 | 0.990 | 1.000 | 0.957 | 1.000 | 0.982 | 0.849 | 1.000 | 1.000 | 0.858 | 0.830 |
| 2162 | 0.787 | 1.000 | 0.822 | 0.986 | 0.963 | 0.960 | 0.897 | 1.000 | 0.990 | 1.000 | 0.962 | 1.000 | 1.000 | 0.847 | 1.000 | 0.981 | 0.861 | 0.833 |
| 2163 | 0.774 | 1.000 | 0.830 | 1.000 | 0.968 | 0.960 | 0.903 | 1.000 | 0.990 | 1.000 | 0.959 | 0.970 | 0.991 | 0.854 | 1.000 | 0.981 | 0.860 | 0.832 |
| 2164 | 0.777 | 1.000 | 0.848 | 1.000 | 0.968 | 0.960 | 0.901 | 1.000 | 0.990 | 1.000 | 0.959 | 0.970 | 1.000 | 0.852 | 1.000 | 0.981 | 0.862 | 0.835 |
| 2165 | 0.777 | 1.000 | 0.838 | 1.000 | 0.968 | 0.960 | 0.897 | 1.000 | 0.990 | 1.000 | 0.959 | 0.970 | 1.000 | 0.855 | 1.000 | 0.981 | 0.862 | 0.834 |
| 2166 | 0.771 | 1.000 | 0.830 | 1.000 | 0.968 | 0.960 | 0.904 | 1.000 | 0.990 | 1.000 | 0.957 | 0.970 | 1.000 | 0.854 | 1.000 | 1.000 | 0.860 | 0.832 |
| 2167 | 0.774 | 1.000 | 0.836 | 1.000 | 0.974 | 0.960 | 0.908 | 1.000 | 0.980 | 1.000 | 0.959 | 1.000 | 1.000 | 0.856 | 1.000 | 1.000 | 0.862 | 0.835 |
| 2168 | 0.773 | 1.000 | 0.826 | 1.000 | 0.974 | 0.960 | 0.900 | 1.000 | 0.980 | 1.000 | 0.959 | 0.970 | 1.000 | 0.859 | 1.000 | 1.000 | 0.861 | 0.833 |
| 2169 | 0.779 | 1.000 | 0.818 | 1.000 | 0.968 | 0.960 | 0.897 | 1.000 | 0.990 | 1.000 | 0.959 | 1.000 | 1.000 | 0.851 | 1.000 | 1.000 | 0.860 | 0.832 |
| 2170 | 0.776 | 1.000 | 0.838 | 1.000 | 0.968 | 0.960 | 0.898 | 1.000 | 0.980 | 1.000 | 0.957 | 0.970 | 0.991 | 0.856 | 1.000 | 1.000 | 0.861 | 0.834 |
| 2171 | 0.779 | 1.000 | 0.828 | 1.000 | 0.968 | 0.960 | 0.908 | 1.000 | 0.990 | 1.000 | 0.965 | 1.000 | 1.000 | 0.856 | 1.000 | 1.000 | 0.863 | 0.836 |
| 2172 | 0.779 | 1.000 | 0.840 | 1.000 | 0.968 | 0.960 | 0.903 | 1.000 | 0.969 | 1.000 | 0.957 | 0.970 | 1.000 | 0.847 | 1.000 | 1.000 | 0.861 | 0.833 |
| 2173 | 0.771 | 1.000 | 0.820 | 1.000 | 0.963 | 0.960 | 0.900 | 1.000 | 0.990 | 1.000 | 0.959 | 1.000 | 1.000 | 0.855 | 1.000 | 1.000 | 0.859 | 0.831 |
| 2174 | 0.774 | 1.000 | 0.826 | 1.000 | 0.963 | 0.960 | 0.900 | 1.000 | 0.990 | 1.000 | 0.957 | 0.909 | 0.991 | 0.853 | 1.000 | 1.000 | 0.858 | 0.830 |
| 2175 | 0.773 | 1.000 | 0.828 | 1.000 | 0.963 | 0.960 | 0.906 | 1.000 | 0.980 | 1.000 | 0.965 | 1.000 | 0.991 | 0.846 | 1.000 | 1.000 | 0.858 | 0.830 |
| 2176 | 0.762 | 1.000 | 0.828 | 1.000 | 0.963 | 0.960 | 0.897 | 1.000 | 1.000 | 1.000 | 0.959 | 0.970 | 1.000 | 0.841 | 1.000 | 0.981 | 0.852 | 0.823 |
| 2177 | 0.768 | 1.000 | 0.824 | 1.000 | 0.968 | 0.960 | 0.911 | 1.000 | 0.980 | 1.000 | 0.951 | 0.939 | 0.982 | 0.854 | 1.000 | 1.000 | 0.858 | 0.830 |
| 2178 | 0.779 | 1.000 | 0.830 | 1.000 | 0.968 | 0.960 | 0.908 | 1.000 | 0.990 | 1.000 | 0.951 | 1.000 | 0.991 | 0.854 | 1.000 | 1.000 | 0.862 | 0.835 |
| 2179 | 0.775 | 1.000 | 0.818 | 1.000 | 0.968 | 0.960 | 0.900 | 1.000 | 0.990 | 1.000 | 0.959 | 0.970 | 1.000 | 0.851 | 1.000 | 1.000 | 0.859 | 0.831 |
| 2180 | 0.773 | 1.000 | 0.828 | 1.000 | 0.968 | 0.960 | 0.901 | 1.000 | 0.990 | 1.000 | 0.954 | 0.970 | 1.000 | 0.845 | 1.000 | 1.000 | 0.857 | 0.829 |
| 2181 | 0.773 | 1.000 | 0.826 | 1.000 | 0.968 | 0.960 | 0.901 | 1.000 | 0.990 | 1.000 | 0.965 | 1.000 | 0.982 | 0.863 | 1.000 | 1.000 | 0.862 | 0.835 |
| 2182 | 0.765 | 1.000 | 0.822 | 1.000 | 0.968 | 0.960 | 0.900 | 1.000 | 0.980 | 1.000 | 0.957 | 0.970 | 0.991 | 0.855 | 1.000 | 1.000 | 0.856 | 0.828 |

| | | | | | | | | | | | | | | | | | |
|---|---|---|---|---|---|---|---|---|---|---|---|---|---|---|---|---|---|
| 2183 | 0.781 | 1.000 | 0.812 | 1.000 | 0.968 | 1.000 | 0.898 | 1.000 | 0.990 | 1.000 | 0.965 | 1.000 | 0.991 | 0.847 | 1.000 | 0.981 | 0.859 | 0.831 |
| 2184 | 0.771 | 1.000 | 0.816 | 1.000 | 0.968 | 0.960 | 0.908 | 1.000 | 0.990 | 1.000 | 0.954 | 1.000 | 1.000 | 0.850 | 1.000 | 0.981 | 0.857 | 0.829 |
| 2185 | 0.771 | 1.000 | 0.822 | 1.000 | 0.968 | 1.000 | 0.894 | 1.000 | 0.990 | 1.000 | 0.962 | 0.970 | 0.982 | 0.857 | 1.000 | 0.981 | 0.858 | 0.830 |
| 2186 | 0.774 | 1.000 | 0.832 | 1.000 | 0.974 | 0.960 | 0.892 | 1.000 | 0.980 | 1.000 | 0.962 | 1.000 | 1.000 | 0.851 | 1.000 | 1.000 | 0.859 | 0.831 |
| 2187 | 0.767 | 1.000 | 0.824 | 1.000 | 0.968 | 0.960 | 0.901 | 1.000 | 0.990 | 1.000 | 0.959 | 1.000 | 1.000 | 0.854 | 1.000 | 1.000 | 0.858 | 0.830 |
| 2188 | 0.777 | 1.000 | 0.826 | 1.000 | 0.968 | 0.960 | 0.908 | 1.000 | 0.990 | 1.000 | 0.959 | 0.970 | 0.982 | 0.859 | 1.000 | 1.000 | 0.862 | 0.835 |
| 2189 | 0.767 | 1.000 | 0.834 | 1.000 | 0.968 | 0.960 | 0.900 | 1.000 | 0.980 | 1.000 | 0.954 | 0.970 | 0.991 | 0.851 | 1.000 | 1.000 | 0.857 | 0.829 |
| 2190 | 0.783 | 1.000 | 0.832 | 1.000 | 0.958 | 0.960 | 0.897 | 1.000 | 0.990 | 1.000 | 0.965 | 0.970 | 0.991 | 0.854 | 1.000 | 0.981 | 0.862 | 0.835 |
| 2191 | 0.765 | 1.000 | 0.842 | 1.000 | 0.963 | 0.960 | 0.911 | 1.000 | 0.990 | 1.000 | 0.965 | 0.970 | 0.991 | 0.849 | 1.000 | 0.981 | 0.859 | 0.831 |
| 2192 | 0.769 | 1.000 | 0.818 | 1.000 | 0.963 | 0.960 | 0.892 | 1.000 | 1.000 | 1.000 | 0.951 | 1.000 | 0.991 | 0.853 | 1.000 | 1.000 | 0.856 | 0.827 |
| 2193 | 0.775 | 1.000 | 0.838 | 1.000 | 0.968 | 0.960 | 0.914 | 1.000 | 0.990 | 1.000 | 0.962 | 1.000 | 0.991 | 0.858 | 1.000 | 0.981 | 0.864 | 0.838 |
| 2194 | 0.771 | 1.000 | 0.822 | 0.986 | 0.968 | 0.960 | 0.909 | 1.000 | 0.990 | 1.000 | 0.962 | 0.970 | 0.991 | 0.844 | 1.000 | 1.000 | 0.857 | 0.829 |
| 2195 | 0.767 | 1.000 | 0.832 | 1.000 | 0.974 | 0.960 | 0.895 | 1.000 | 0.990 | 1.000 | 0.954 | 0.939 | 1.000 | 0.851 | 1.000 | 1.000 | 0.857 | 0.828 |
| 2196 | 0.770 | 1.000 | 0.814 | 1.000 | 0.968 | 0.960 | 0.895 | 1.000 | 0.969 | 1.000 | 0.962 | 0.970 | 1.000 | 0.855 | 1.000 | 1.000 | 0.857 | 0.828 |
| 2197 | 0.767 | 1.000 | 0.818 | 1.000 | 0.974 | 0.960 | 0.895 | 1.000 | 0.990 | 1.000 | 0.954 | 0.970 | 0.991 | 0.853 | 1.000 | 1.000 | 0.855 | 0.827 |
| 2198 | 0.768 | 1.000 | 0.826 | 1.000 | 0.974 | 1.000 | 0.908 | 1.000 | 0.990 | 1.000 | 0.957 | 0.970 | 0.991 | 0.851 | 1.000 | 1.000 | 0.858 | 0.830 |
| 2199 | 0.773 | 1.000 | 0.814 | 1.000 | 0.968 | 0.960 | 0.904 | 1.000 | 0.990 | 1.000 | 0.957 | 1.000 | 1.000 | 0.852 | 1.000 | 1.000 | 0.858 | 0.830 |
| 2200 | 0.772 | 1.000 | 0.822 | 1.000 | 0.963 | 0.960 | 0.891 | 1.000 | 0.990 | 1.000 | 0.959 | 1.000 | 0.991 | 0.853 | 1.000 | 1.000 | 0.857 | 0.828 |
| 2201 | 0.777 | 1.000 | 0.832 | 1.000 | 0.974 | 0.960 | 0.901 | 1.000 | 0.990 | 1.000 | 0.948 | 0.970 | 1.000 | 0.853 | 1.000 | 1.000 | 0.860 | 0.833 |
| 2202 | 0.775 | 1.000 | 0.808 | 1.000 | 0.974 | 0.960 | 0.900 | 1.000 | 0.990 | 1.000 | 0.959 | 0.970 | 0.991 | 0.855 | 1.000 | 1.000 | 0.859 | 0.831 |
| 2203 | 0.763 | 1.000 | 0.834 | 1.000 | 0.968 | 0.960 | 0.903 | 1.000 | 0.990 | 1.000 | 0.951 | 0.970 | 0.991 | 0.848 | 1.000 | 1.000 | 0.855 | 0.827 |
| 2204 | 0.767 | 1.000 | 0.830 | 1.000 | 0.968 | 0.960 | 0.909 | 1.000 | 0.990 | 1.000 | 0.954 | 0.970 | 1.000 | 0.855 | 1.000 | 1.000 | 0.859 | 0.832 |
| 2205 | 0.769 | 1.000 | 0.824 | 1.000 | 0.968 | 0.960 | 0.908 | 1.000 | 0.990 | 1.000 | 0.959 | 0.970 | 0.991 | 0.855 | 1.000 | 1.000 | 0.859 | 0.832 |
| 2206 | 0.773 | 1.000 | 0.826 | 1.000 | 0.974 | 0.960 | 0.903 | 1.000 | 0.980 | 1.000 | 0.962 | 0.970 | 1.000 | 0.844 | 1.000 | 1.000 | 0.858 | 0.829 |
| 2207 | 0.771 | 1.000 | 0.814 | 1.000 | 0.963 | 0.960 | 0.906 | 1.000 | 0.980 | 1.000 | 0.951 | 1.000 | 1.000 | 0.851 | 1.000 | 1.000 | 0.857 | 0.829 |
| 2208 | 0.775 | 1.000 | 0.832 | 1.000 | 0.963 | 0.960 | 0.908 | 1.000 | 1.000 | 1.000 | 0.959 | 1.000 | 0.991 | 0.849 | 1.000 | 1.000 | 0.860 | 0.833 |
| 2209 | 0.765 | 1.000 | 0.820 | 0.986 | 0.963 | 0.960 | 0.898 | 1.000 | 0.980 | 1.000 | 0.951 | 0.970 | 1.000 | 0.854 | 1.000 | 1.000 | 0.855 | 0.827 |
| 2210 | 0.779 | 1.000 | 0.828 | 0.986 | 0.968 | 0.960 | 0.897 | 1.000 | 0.990 | 1.000 | 0.965 | 1.000 | 0.991 | 0.848 | 1.000 | 1.000 | 0.860 | 0.832 |
| 2211 | 0.777 | 1.000 | 0.832 | 1.000 | 0.963 | 0.960 | 0.901 | 1.000 | 0.990 | 1.000 | 0.959 | 0.970 | 0.991 | 0.843 | 1.000 | 1.000 | 0.858 | 0.830 |
| 2212 | 0.769 | 1.000 | 0.810 | 1.000 | 0.968 | 0.960 | 0.903 | 1.000 | 0.980 | 1.000 | 0.951 | 1.000 | 1.000 | 0.851 | 1.000 | 1.000 | 0.856 | 0.827 |
| 2213 | 0.770 | 1.000 | 0.824 | 1.000 | 0.968 | 0.960 | 0.900 | 1.000 | 0.990 | 1.000 | 0.954 | 1.000 | 0.991 | 0.855 | 1.000 | 1.000 | 0.858 | 0.830 |
| 2214 | 0.767 | 1.000 | 0.812 | 1.000 | 0.963 | 0.960 | 0.906 | 1.000 | 0.980 | 1.000 | 0.954 | 0.970 | 1.000 | 0.857 | 1.000 | 1.000 | 0.857 | 0.829 |
| 2215 | 0.771 | 1.000 | 0.830 | 1.000 | 0.974 | 0.960 | 0.903 | 1.000 | 0.990 | 1.000 | 0.948 | 1.000 | 1.000 | 0.846 | 1.000 | 1.000 | 0.857 | 0.829 |
| 2216 | 0.775 | 1.000 | 0.832 | 1.000 | 0.963 | 0.960 | 0.906 | 1.000 | 0.980 | 1.000 | 0.957 | 0.970 | 1.000 | 0.853 | 1.000 | 1.000 | 0.861 | 0.833 |
| 2217 | 0.767 | 1.000 | 0.816 | 1.000 | 0.968 | 0.960 | 0.909 | 1.000 | 0.990 | 1.000 | 0.959 | 0.970 | 0.991 | 0.862 | 1.000 | 1.000 | 0.860 | 0.832 |
| 2218 | 0.767 | 1.000 | 0.826 | 1.000 | 0.974 | 1.000 | 0.908 | 1.000 | 0.980 | 1.000 | 0.957 | 1.000 | 0.991 | 0.845 | 1.000 | 1.000 | 0.856 | 0.828 |
| 2219 | 0.775 | 1.000 | 0.810 | 1.000 | 0.958 | 0.960 | 0.900 | 1.000 | 0.990 | 1.000 | 0.959 | 0.970 | 1.000 | 0.848 | 1.000 | 1.000 | 0.856 | 0.828 |
| 2220 | 0.777 | 1.000 | 0.816 | 1.000 | 0.968 | 0.960 | 0.895 | 1.000 | 0.990 | 1.000 | 0.951 | 0.970 | 1.000 | 0.854 | 1.000 | 1.000 | 0.858 | 0.830 |
| 2221 | 0.764 | 1.000 | 0.818 | 1.000 | 0.968 | 0.960 | 0.894 | 1.000 | 0.990 | 1.000 | 0.951 | 0.970 | 1.000 | 0.845 | 1.000 | 1.000 | 0.852 | 0.823 |

| | | | | | | | | | | | | | | | | | |
|---|---|---|---|---|---|---|---|---|---|---|---|---|---|---|---|---|---|
| 2222 | 0.770 | 1.000 | 0.814 | 1.000 | 0.968 | 0.960 | 0.903 | 1.000 | 0.990 | 1.000 | 0.948 | 0.970 | 0.991 | 0.847 | 1.000 | 1.000 | 0.855 | 0.826 |
| 2223 | 0.762 | 1.000 | 0.808 | 1.000 | 0.963 | 0.960 | 0.900 | 1.000 | 0.990 | 1.000 | 0.957 | 1.000 | 0.991 | 0.854 | 1.000 | 1.000 | 0.854 | 0.825 |
| 2224 | 0.767 | 1.000 | 0.818 | 1.000 | 0.968 | 0.960 | 0.906 | 1.000 | 0.990 | 1.000 | 0.957 | 0.970 | 0.991 | 0.850 | 1.000 | 1.000 | 0.856 | 0.828 |
| 2225 | 0.779 | 1.000 | 0.818 | 1.000 | 0.968 | 0.960 | 0.906 | 1.000 | 0.980 | 1.000 | 0.959 | 0.970 | 1.000 | 0.844 | 1.000 | 1.000 | 0.858 | 0.830 |
| 2226 | 0.767 | 1.000 | 0.818 | 1.000 | 0.974 | 0.960 | 0.897 | 1.000 | 0.980 | 1.000 | 0.954 | 0.970 | 1.000 | 0.854 | 1.000 | 1.000 | 0.856 | 0.828 |
| 2227 | 0.763 | 1.000 | 0.818 | 1.000 | 0.968 | 0.960 | 0.903 | 1.000 | 0.990 | 1.000 | 0.951 | 0.970 | 1.000 | 0.847 | 1.000 | 1.000 | 0.854 | 0.825 |
| 2228 | 0.769 | 1.000 | 0.830 | 1.000 | 0.963 | 0.960 | 0.901 | 1.000 | 1.000 | 1.000 | 0.954 | 0.970 | 0.991 | 0.856 | 1.000 | 1.000 | 0.859 | 0.831 |
| 2229 | 0.774 | 1.000 | 0.822 | 1.000 | 0.963 | 0.960 | 0.898 | 1.000 | 1.000 | 1.000 | 0.948 | 0.970 | 0.991 | 0.847 | 1.000 | 1.000 | 0.856 | 0.828 |
| 2230 | 0.775 | 1.000 | 0.812 | 1.000 | 0.963 | 0.960 | 0.894 | 1.000 | 1.000 | 1.000 | 0.962 | 1.000 | 0.991 | 0.849 | 1.000 | 1.000 | 0.857 | 0.828 |
| 2231 | 0.767 | 1.000 | 0.820 | 1.000 | 0.968 | 0.960 | 0.894 | 1.000 | 1.000 | 1.000 | 0.959 | 1.000 | 1.000 | 0.853 | 1.000 | 1.000 | 0.856 | 0.828 |
| 2232 | 0.775 | 1.000 | 0.822 | 1.000 | 0.968 | 0.960 | 0.895 | 1.000 | 1.000 | 1.000 | 0.959 | 1.000 | 1.000 | 0.858 | 1.000 | 1.000 | 0.861 | 0.833 |
| 2233 | 0.773 | 1.000 | 0.826 | 1.000 | 0.968 | 0.960 | 0.900 | 1.000 | 1.000 | 1.000 | 0.954 | 0.970 | 1.000 | 0.847 | 1.000 | 1.000 | 0.858 | 0.829 |
| 2234 | 0.773 | 1.000 | 0.822 | 1.000 | 0.968 | 0.960 | 0.895 | 1.000 | 1.000 | 1.000 | 0.957 | 1.000 | 0.982 | 0.847 | 1.000 | 1.000 | 0.856 | 0.828 |
| 2235 | 0.773 | 1.000 | 0.834 | 1.000 | 0.968 | 0.960 | 0.906 | 1.000 | 1.000 | 1.000 | 0.954 | 0.970 | 0.982 | 0.846 | 1.000 | 1.000 | 0.858 | 0.830 |
| 2236 | 0.762 | 1.000 | 0.824 | 1.000 | 0.963 | 0.960 | 0.894 | 1.000 | 1.000 | 1.000 | 0.951 | 1.000 | 1.000 | 0.851 | 1.000 | 1.000 | 0.854 | 0.825 |
| 2237 | 0.781 | 1.000 | 0.810 | 1.000 | 0.963 | 0.960 | 0.894 | 1.000 | 1.000 | 1.000 | 0.959 | 0.970 | 0.991 | 0.854 | 1.000 | 1.000 | 0.859 | 0.831 |
| 2238 | 0.769 | 1.000 | 0.816 | 1.000 | 0.979 | 0.960 | 0.906 | 1.000 | 1.000 | 1.000 | 0.954 | 1.000 | 0.991 | 0.855 | 1.000 | 1.000 | 0.858 | 0.830 |
| 2239 | 0.773 | 1.000 | 0.820 | 1.000 | 0.968 | 1.000 | 0.904 | 1.000 | 1.000 | 1.000 | 0.957 | 0.939 | 0.991 | 0.851 | 1.000 | 1.000 | 0.859 | 0.831 |
| 2240 | 0.779 | 1.000 | 0.812 | 1.000 | 0.963 | 0.960 | 0.911 | 1.000 | 1.000 | 1.000 | 0.951 | 0.970 | 1.000 | 0.847 | 1.000 | 1.000 | 0.859 | 0.831 |
| 2241 | 0.765 | 1.000 | 0.828 | 1.000 | 0.968 | 0.960 | 0.906 | 1.000 | 0.990 | 1.000 | 0.948 | 1.000 | 1.000 | 0.844 | 1.000 | 1.000 | 0.855 | 0.826 |
| 2242 | 0.773 | 1.000 | 0.818 | 1.000 | 0.963 | 0.960 | 0.900 | 1.000 | 1.000 | 1.000 | 0.957 | 1.000 | 0.991 | 0.858 | 1.000 | 1.000 | 0.860 | 0.832 |
| 2243 | 0.768 | 1.000 | 0.812 | 0.986 | 0.963 | 0.960 | 0.904 | 1.000 | 1.000 | 1.000 | 0.962 | 1.000 | 0.991 | 0.849 | 1.000 | 1.000 | 0.856 | 0.827 |
| 2244 | 0.769 | 1.000 | 0.824 | 1.000 | 0.968 | 0.960 | 0.901 | 1.000 | 1.000 | 1.000 | 0.957 | 0.970 | 0.991 | 0.844 | 1.000 | 1.000 | 0.856 | 0.827 |
| 2245 | 0.761 | 1.000 | 0.808 | 1.000 | 0.968 | 0.960 | 0.898 | 1.000 | 1.000 | 1.000 | 0.962 | 0.970 | 1.000 | 0.858 | 1.000 | 1.000 | 0.855 | 0.826 |
| 2246 | 0.763 | 1.000 | 0.822 | 1.000 | 0.968 | 0.960 | 0.906 | 1.000 | 0.990 | 1.000 | 0.965 | 1.000 | 1.000 | 0.858 | 1.000 | 1.000 | 0.858 | 0.831 |
| 2247 | 0.778 | 1.000 | 0.824 | 1.000 | 0.968 | 0.960 | 0.903 | 1.000 | 1.000 | 1.000 | 0.959 | 1.000 | 0.991 | 0.851 | 1.000 | 1.000 | 0.861 | 0.833 |
| 2248 | 0.765 | 1.000 | 0.814 | 1.000 | 0.968 | 0.960 | 0.898 | 1.000 | 1.000 | 1.000 | 0.951 | 1.000 | 1.000 | 0.856 | 1.000 | 1.000 | 0.856 | 0.828 |
| 2249 | 0.765 | 1.000 | 0.822 | 1.000 | 0.968 | 0.960 | 0.895 | 1.000 | 1.000 | 1.000 | 0.957 | 0.970 | 1.000 | 0.856 | 1.000 | 1.000 | 0.856 | 0.828 |
| 2250 | 0.767 | 1.000 | 0.830 | 1.000 | 0.968 | 0.960 | 0.898 | 1.000 | 1.000 | 1.000 | 0.957 | 0.970 | 1.000 | 0.851 | 1.000 | 1.000 | 0.857 | 0.829 |
| 2251 | 0.769 | 1.000 | 0.820 | 1.000 | 0.974 | 0.960 | 0.900 | 1.000 | 1.000 | 1.000 | 0.959 | 1.000 | 1.000 | 0.852 | 1.000 | 1.000 | 0.858 | 0.830 |
| 2252 | 0.765 | 1.000 | 0.818 | 1.000 | 0.968 | 0.960 | 0.908 | 1.000 | 1.000 | 1.000 | 0.951 | 0.970 | 0.991 | 0.847 | 1.000 | 1.000 | 0.855 | 0.826 |
| 2253 | 0.757 | 1.000 | 0.816 | 1.000 | 0.968 | 0.960 | 0.904 | 1.000 | 1.000 | 1.000 | 0.957 | 0.970 | 0.982 | 0.850 | 1.000 | 1.000 | 0.853 | 0.824 |
| 2254 | 0.779 | 1.000 | 0.820 | 1.000 | 0.968 | 0.960 | 0.904 | 1.000 | 1.000 | 1.000 | 0.954 | 0.970 | 0.991 | 0.848 | 1.000 | 1.000 | 0.859 | 0.831 |
| 2255 | 0.765 | 1.000 | 0.808 | 1.000 | 0.968 | 0.960 | 0.901 | 1.000 | 1.000 | 1.000 | 0.954 | 0.970 | 1.000 | 0.854 | 1.000 | 1.000 | 0.855 | 0.827 |
| 2256 | 0.777 | 1.000 | 0.818 | 1.000 | 0.963 | 0.960 | 0.904 | 1.000 | 1.000 | 1.000 | 0.957 | 0.970 | 0.991 | 0.855 | 1.000 | 1.000 | 0.860 | 0.832 |
| 2257 | 0.764 | 1.000 | 0.820 | 1.000 | 0.968 | 0.960 | 0.900 | 1.000 | 1.000 | 1.000 | 0.954 | 0.970 | 0.991 | 0.849 | 1.000 | 1.000 | 0.854 | 0.826 |
| 2258 | 0.768 | 1.000 | 0.810 | 1.000 | 0.968 | 0.960 | 0.904 | 1.000 | 1.000 | 1.000 | 0.957 | 1.000 | 1.000 | 0.851 | 1.000 | 1.000 | 0.856 | 0.828 |
| 2259 | 0.767 | 1.000 | 0.828 | 1.000 | 0.968 | 0.960 | 0.901 | 1.000 | 1.000 | 1.000 | 0.959 | 1.000 | 1.000 | 0.850 | 1.000 | 1.000 | 0.857 | 0.829 |
| 2260 | 0.772 | 1.000 | 0.804 | 1.000 | 0.963 | 0.960 | 0.903 | 1.000 | 1.000 | 1.000 | 0.959 | 0.970 | 0.991 | 0.851 | 1.000 | 1.000 | 0.856 | 0.828 |

| | | | | | | | | | | | | | | | | | | |
|---|---|---|---|---|---|---|---|---|---|---|---|---|---|---|---|---|---|---|
| 2261 | 0.764 | 1.000 | 0.808 | 1.000 | 0.963 | 0.960 | 0.908 | 1.000 | 1.000 | 1.000 | 0.957 | 0.970 | 1.000 | 0.851 | 1.000 | 1.000 | 0.855 | 0.827 |
| 2262 | 0.773 | 1.000 | 0.826 | 1.000 | 0.968 | 0.960 | 0.908 | 1.000 | 1.000 | 1.000 | 0.951 | 1.000 | 0.991 | 0.848 | 1.000 | 1.000 | 0.859 | 0.831 |
| 2263 | 0.777 | 1.000 | 0.826 | 0.986 | 0.963 | 0.960 | 0.906 | 1.000 | 1.000 | 1.000 | 0.951 | 1.000 | 1.000 | 0.851 | 1.000 | 1.000 | 0.860 | 0.832 |
| 2264 | 0.767 | 1.000 | 0.810 | 1.000 | 0.968 | 0.960 | 0.912 | 1.000 | 1.000 | 1.000 | 0.957 | 0.970 | 0.991 | 0.851 | 1.000 | 1.000 | 0.857 | 0.829 |
| 2265 | 0.771 | 1.000 | 0.826 | 1.000 | 0.963 | 0.960 | 0.908 | 1.000 | 1.000 | 1.000 | 0.948 | 1.000 | 0.982 | 0.853 | 1.000 | 1.000 | 0.858 | 0.831 |
| 2266 | 0.767 | 1.000 | 0.826 | 1.000 | 0.974 | 0.960 | 0.906 | 1.000 | 1.000 | 1.000 | 0.951 | 1.000 | 0.991 | 0.858 | 1.000 | 1.000 | 0.859 | 0.832 |
| 2267 | 0.772 | 1.000 | 0.820 | 0.986 | 0.963 | 0.960 | 0.898 | 1.000 | 1.000 | 1.000 | 0.957 | 0.970 | 1.000 | 0.854 | 1.000 | 1.000 | 0.858 | 0.830 |
| 2268 | 0.764 | 1.000 | 0.820 | 1.000 | 0.963 | 0.960 | 0.904 | 1.000 | 1.000 | 1.000 | 0.945 | 1.000 | 1.000 | 0.855 | 1.000 | 1.000 | 0.856 | 0.828 |
| 2269 | 0.771 | 1.000 | 0.824 | 1.000 | 0.963 | 0.960 | 0.904 | 1.000 | 1.000 | 1.000 | 0.948 | 1.000 | 0.991 | 0.845 | 1.000 | 1.000 | 0.856 | 0.828 |
| 2270 | 0.770 | 1.000 | 0.814 | 1.000 | 0.968 | 0.960 | 0.900 | 1.000 | 1.000 | 1.000 | 0.957 | 1.000 | 1.000 | 0.856 | 1.000 | 1.000 | 0.858 | 0.830 |
| 2271 | 0.762 | 1.000 | 0.822 | 1.000 | 0.968 | 0.960 | 0.908 | 1.000 | 1.000 | 1.000 | 0.951 | 1.000 | 1.000 | 0.854 | 1.000 | 1.000 | 0.856 | 0.828 |
| 2272 | 0.767 | 1.000 | 0.816 | 1.000 | 0.968 | 0.960 | 0.908 | 1.000 | 1.000 | 1.000 | 0.954 | 1.000 | 0.991 | 0.851 | 1.000 | 1.000 | 0.856 | 0.828 |
| 2273 | 0.766 | 1.000 | 0.822 | 1.000 | 0.968 | 0.960 | 0.895 | 1.000 | 1.000 | 1.000 | 0.951 | 0.970 | 1.000 | 0.851 | 1.000 | 1.000 | 0.855 | 0.826 |
| 2274 | 0.778 | 1.000 | 0.824 | 1.000 | 0.968 | 0.960 | 0.915 | 1.000 | 1.000 | 1.000 | 0.951 | 0.970 | 0.991 | 0.850 | 1.000 | 1.000 | 0.861 | 0.834 |
| 2275 | 0.774 | 1.000 | 0.822 | 1.000 | 0.963 | 0.960 | 0.908 | 1.000 | 1.000 | 1.000 | 0.948 | 1.000 | 0.991 | 0.850 | 1.000 | 1.000 | 0.858 | 0.831 |
| 2276 | 0.767 | 1.000 | 0.824 | 1.000 | 0.968 | 0.960 | 0.909 | 1.000 | 1.000 | 1.000 | 0.957 | 1.000 | 0.991 | 0.854 | 1.000 | 1.000 | 0.859 | 0.831 |
| 2277 | 0.777 | 1.000 | 0.822 | 1.000 | 0.963 | 0.960 | 0.908 | 1.000 | 1.000 | 1.000 | 0.951 | 1.000 | 0.982 | 0.850 | 1.000 | 1.000 | 0.859 | 0.831 |
| 2278 | 0.769 | 1.000 | 0.824 | 1.000 | 0.968 | 0.960 | 0.908 | 1.000 | 1.000 | 1.000 | 0.962 | 0.970 | 0.991 | 0.854 | 1.000 | 1.000 | 0.859 | 0.831 |
| 2279 | 0.766 | 1.000 | 0.826 | 0.986 | 0.974 | 0.960 | 0.912 | 1.000 | 0.990 | 1.000 | 0.957 | 0.970 | 0.991 | 0.854 | 1.000 | 1.000 | 0.859 | 0.831 |
| 2280 | 0.773 | 1.000 | 0.820 | 1.000 | 0.963 | 0.960 | 0.900 | 1.000 | 1.000 | 1.000 | 0.957 | 0.970 | 1.000 | 0.853 | 1.000 | 1.000 | 0.858 | 0.830 |
| 2281 | 0.771 | 1.000 | 0.812 | 1.000 | 0.963 | 0.960 | 0.906 | 1.000 | 1.000 | 1.000 | 0.957 | 0.970 | 0.991 | 0.851 | 1.000 | 1.000 | 0.857 | 0.829 |
| 2282 | 0.767 | 1.000 | 0.810 | 1.000 | 0.958 | 0.960 | 0.894 | 1.000 | 1.000 | 1.000 | 0.951 | 0.970 | 0.991 | 0.851 | 1.000 | 1.000 | 0.853 | 0.824 |
| 2283 | 0.773 | 1.000 | 0.822 | 0.986 | 0.963 | 0.960 | 0.903 | 1.000 | 0.990 | 1.000 | 0.962 | 1.000 | 1.000 | 0.851 | 1.000 | 1.000 | 0.858 | 0.830 |
| 2284 | 0.773 | 1.000 | 0.814 | 1.000 | 0.968 | 0.960 | 0.904 | 1.000 | 0.990 | 1.000 | 0.945 | 1.000 | 0.991 | 0.851 | 1.000 | 1.000 | 0.857 | 0.828 |
| 2285 | 0.762 | 1.000 | 0.816 | 1.000 | 0.958 | 0.960 | 0.906 | 1.000 | 1.000 | 1.000 | 0.957 | 1.000 | 1.000 | 0.854 | 1.000 | 1.000 | 0.856 | 0.827 |
| 2286 | 0.771 | 1.000 | 0.830 | 1.000 | 0.968 | 0.960 | 0.900 | 1.000 | 1.000 | 1.000 | 0.954 | 1.000 | 0.991 | 0.859 | 1.000 | 1.000 | 0.860 | 0.833 |
| 2287 | 0.777 | 1.000 | 0.820 | 1.000 | 0.958 | 0.960 | 0.909 | 1.000 | 1.000 | 1.000 | 0.957 | 1.000 | 0.991 | 0.851 | 1.000 | 1.000 | 0.860 | 0.832 |
| 2288 | 0.777 | 1.000 | 0.826 | 1.000 | 0.968 | 0.960 | 0.903 | 1.000 | 1.000 | 1.000 | 0.948 | 1.000 | 0.991 | 0.857 | 1.000 | 1.000 | 0.861 | 0.834 |
| 2289 | 0.773 | 1.000 | 0.826 | 0.986 | 0.963 | 0.960 | 0.901 | 1.000 | 1.000 | 1.000 | 0.959 | 1.000 | 1.000 | 0.854 | 1.000 | 1.000 | 0.859 | 0.832 |
| 2290 | 0.773 | 1.000 | 0.826 | 1.000 | 0.963 | 0.960 | 0.900 | 1.000 | 1.000 | 1.000 | 0.959 | 1.000 | 0.991 | 0.850 | 1.000 | 1.000 | 0.858 | 0.830 |
| 2291 | 0.778 | 1.000 | 0.830 | 1.000 | 0.963 | 0.960 | 0.903 | 1.000 | 0.990 | 1.000 | 0.965 | 1.000 | 1.000 | 0.847 | 1.000 | 1.000 | 0.860 | 0.833 |
| 2292 | 0.775 | 1.000 | 0.816 | 0.986 | 0.968 | 0.960 | 0.886 | 1.000 | 1.000 | 1.000 | 0.957 | 1.000 | 0.991 | 0.857 | 1.000 | 1.000 | 0.858 | 0.829 |
| 2293 | 0.766 | 1.000 | 0.816 | 1.000 | 0.963 | 0.960 | 0.909 | 1.000 | 0.990 | 1.000 | 0.948 | 1.000 | 0.991 | 0.858 | 1.000 | 1.000 | 0.857 | 0.830 |
| 2294 | 0.775 | 1.000 | 0.814 | 1.000 | 0.963 | 0.960 | 0.901 | 1.000 | 0.990 | 1.000 | 0.948 | 1.000 | 0.991 | 0.850 | 1.000 | 1.000 | 0.857 | 0.829 |
| 2295 | 0.773 | 1.000 | 0.808 | 1.000 | 0.958 | 0.960 | 0.903 | 1.000 | 0.990 | 1.000 | 0.948 | 1.000 | 1.000 | 0.849 | 1.000 | 1.000 | 0.856 | 0.827 |
| 2296 | 0.768 | 1.000 | 0.818 | 1.000 | 0.963 | 0.960 | 0.906 | 1.000 | 0.990 | 1.000 | 0.951 | 1.000 | 1.000 | 0.847 | 1.000 | 1.000 | 0.855 | 0.827 |
| 2297 | 0.772 | 1.000 | 0.822 | 1.000 | 0.968 | 0.960 | 0.904 | 1.000 | 1.000 | 1.000 | 0.957 | 1.000 | 1.000 | 0.854 | 1.000 | 1.000 | 0.859 | 0.832 |
| 2298 | 0.777 | 1.000 | 0.812 | 1.000 | 0.958 | 0.960 | 0.901 | 1.000 | 1.000 | 1.000 | 0.962 | 0.970 | 0.991 | 0.853 | 1.000 | 1.000 | 0.859 | 0.831 |
| 2299 | 0.761 | 1.000 | 0.816 | 1.000 | 0.958 | 0.960 | 0.904 | 1.000 | 1.000 | 1.000 | 0.951 | 0.970 | 1.000 | 0.849 | 1.000 | 1.000 | 0.853 | 0.825 |

| | | | | | | | | | | | | | | | | | | |
|---|---|---|---|---|---|---|---|---|---|---|---|---|---|---|---|---|---|---|
| 2300 | 0.769 | 1.000 | 0.828 | 1.000 | 0.968 | 0.960 | 0.906 | 1.000 | 1.000 | 1.000 | 0.959 | 0.970 | 1.000 | 0.848 | 1.000 | 1.000 | 0.858 | 0.830 |
| 2301 | 0.773 | 1.000 | 0.828 | 1.000 | 0.963 | 0.960 | 0.901 | 1.000 | 1.000 | 1.000 | 0.951 | 1.000 | 0.991 | 0.849 | 1.000 | 1.000 | 0.858 | 0.830 |
| 2302 | 0.769 | 1.000 | 0.808 | 1.000 | 0.968 | 0.960 | 0.908 | 1.000 | 1.000 | 1.000 | 0.954 | 1.000 | 0.991 | 0.850 | 1.000 | 1.000 | 0.856 | 0.828 |
| 2303 | 0.773 | 1.000 | 0.814 | 1.000 | 0.963 | 0.960 | 0.891 | 1.000 | 1.000 | 1.000 | 0.957 | 1.000 | 1.000 | 0.851 | 1.000 | 1.000 | 0.856 | 0.828 |
| 2304 | 0.775 | 1.000 | 0.818 | 1.000 | 0.968 | 0.960 | 0.906 | 1.000 | 1.000 | 1.000 | 0.957 | 0.970 | 0.991 | 0.854 | 1.000 | 1.000 | 0.860 | 0.832 |
| 2305 | 0.779 | 1.000 | 0.812 | 1.000 | 0.958 | 1.000 | 0.895 | 1.000 | 1.000 | 1.000 | 0.957 | 0.970 | 1.000 | 0.853 | 1.000 | 1.000 | 0.859 | 0.830 |
| 2306 | 0.763 | 1.000 | 0.808 | 1.000 | 0.963 | 0.960 | 0.908 | 1.000 | 1.000 | 1.000 | 0.948 | 0.970 | 1.000 | 0.847 | 1.000 | 1.000 | 0.853 | 0.824 |
| 2307 | 0.773 | 1.000 | 0.816 | 1.000 | 0.968 | 0.960 | 0.895 | 1.000 | 1.000 | 1.000 | 0.954 | 1.000 | 1.000 | 0.854 | 1.000 | 1.000 | 0.858 | 0.830 |
| 2308 | 0.768 | 1.000 | 0.818 | 1.000 | 0.963 | 0.960 | 0.906 | 1.000 | 1.000 | 1.000 | 0.951 | 1.000 | 1.000 | 0.850 | 1.000 | 1.000 | 0.856 | 0.828 |
| 2309 | 0.769 | 1.000 | 0.844 | 1.000 | 0.968 | 0.960 | 0.903 | 1.000 | 1.000 | 1.000 | 0.957 | 0.970 | 1.000 | 0.853 | 1.000 | 1.000 | 0.860 | 0.833 |
| 2310 | 0.763 | 1.000 | 0.812 | 1.000 | 0.963 | 0.960 | 0.904 | 1.000 | 1.000 | 1.000 | 0.957 | 0.970 | 0.991 | 0.841 | 1.000 | 1.000 | 0.852 | 0.823 |
| 2311 | 0.770 | 1.000 | 0.812 | 1.000 | 0.968 | 0.960 | 0.897 | 1.000 | 1.000 | 1.000 | 0.959 | 0.970 | 0.991 | 0.852 | 1.000 | 1.000 | 0.856 | 0.828 |
| 2312 | 0.760 | 1.000 | 0.820 | 1.000 | 0.968 | 0.960 | 0.909 | 1.000 | 1.000 | 1.000 | 0.948 | 0.970 | 1.000 | 0.849 | 1.000 | 1.000 | 0.854 | 0.826 |
| 2313 | 0.771 | 1.000 | 0.806 | 1.000 | 0.968 | 0.960 | 0.900 | 1.000 | 1.000 | 1.000 | 0.948 | 0.939 | 1.000 | 0.847 | 1.000 | 1.000 | 0.854 | 0.825 |
| 2314 | 0.769 | 1.000 | 0.812 | 1.000 | 0.968 | 0.960 | 0.897 | 1.000 | 1.000 | 1.000 | 0.954 | 1.000 | 1.000 | 0.847 | 1.000 | 1.000 | 0.855 | 0.826 |
| 2315 | 0.765 | 1.000 | 0.814 | 1.000 | 0.968 | 0.960 | 0.911 | 1.000 | 1.000 | 1.000 | 0.957 | 0.970 | 0.991 | 0.854 | 1.000 | 1.000 | 0.857 | 0.829 |
| 2316 | 0.769 | 1.000 | 0.816 | 1.000 | 0.968 | 0.960 | 0.903 | 1.000 | 1.000 | 1.000 | 0.951 | 1.000 | 1.000 | 0.861 | 1.000 | 1.000 | 0.859 | 0.831 |
| 2317 | 0.774 | 1.000 | 0.824 | 1.000 | 0.963 | 0.960 | 0.898 | 1.000 | 1.000 | 1.000 | 0.957 | 1.000 | 0.991 | 0.854 | 1.000 | 1.000 | 0.859 | 0.831 |
| 2318 | 0.771 | 1.000 | 0.826 | 1.000 | 0.963 | 0.960 | 0.897 | 1.000 | 1.000 | 1.000 | 0.954 | 0.970 | 0.982 | 0.843 | 1.000 | 1.000 | 0.855 | 0.826 |
| 2319 | 0.779 | 1.000 | 0.808 | 1.000 | 0.963 | 0.960 | 0.901 | 1.000 | 1.000 | 1.000 | 0.957 | 1.000 | 1.000 | 0.847 | 1.000 | 1.000 | 0.858 | 0.830 |
| 2320 | 0.777 | 1.000 | 0.822 | 1.000 | 0.963 | 0.960 | 0.901 | 1.000 | 0.990 | 1.000 | 0.951 | 1.000 | 1.000 | 0.848 | 1.000 | 1.000 | 0.858 | 0.830 |
| 2321 | 0.771 | 1.000 | 0.810 | 1.000 | 0.968 | 0.960 | 0.904 | 1.000 | 1.000 | 1.000 | 0.948 | 0.970 | 0.991 | 0.850 | 1.000 | 1.000 | 0.856 | 0.828 |
| 2322 | 0.775 | 1.000 | 0.810 | 1.000 | 0.968 | 0.960 | 0.904 | 1.000 | 1.000 | 1.000 | 0.959 | 1.000 | 0.991 | 0.854 | 1.000 | 1.000 | 0.859 | 0.832 |
| 2323 | 0.771 | 1.000 | 0.820 | 1.000 | 0.968 | 0.960 | 0.898 | 1.000 | 0.990 | 1.000 | 0.957 | 1.000 | 0.982 | 0.850 | 1.000 | 1.000 | 0.856 | 0.828 |
| 2324 | 0.772 | 1.000 | 0.820 | 1.000 | 0.963 | 0.960 | 0.901 | 1.000 | 0.990 | 1.000 | 0.957 | 0.970 | 0.991 | 0.851 | 1.000 | 1.000 | 0.857 | 0.829 |
| 2325 | 0.765 | 1.000 | 0.820 | 1.000 | 0.974 | 0.960 | 0.903 | 1.000 | 1.000 | 1.000 | 0.954 | 0.939 | 1.000 | 0.850 | 1.000 | 1.000 | 0.856 | 0.827 |
| 2326 | 0.768 | 1.000 | 0.814 | 1.000 | 0.968 | 0.960 | 0.904 | 1.000 | 0.990 | 1.000 | 0.957 | 1.000 | 1.000 | 0.851 | 1.000 | 1.000 | 0.857 | 0.828 |
| 2327 | 0.775 | 1.000 | 0.818 | 1.000 | 0.963 | 0.960 | 0.906 | 1.000 | 0.990 | 1.000 | 0.957 | 0.970 | 1.000 | 0.853 | 1.000 | 1.000 | 0.859 | 0.831 |
| 2328 | 0.765 | 1.000 | 0.818 | 1.000 | 0.963 | 0.960 | 0.904 | 1.000 | 0.990 | 1.000 | 0.957 | 1.000 | 1.000 | 0.847 | 1.000 | 1.000 | 0.855 | 0.826 |
| 2329 | 0.778 | 1.000 | 0.824 | 1.000 | 0.968 | 0.960 | 0.908 | 1.000 | 1.000 | 1.000 | 0.954 | 1.000 | 1.000 | 0.855 | 1.000 | 1.000 | 0.862 | 0.835 |
| 2330 | 0.774 | 1.000 | 0.830 | 0.986 | 0.968 | 0.960 | 0.900 | 1.000 | 1.000 | 1.000 | 0.954 | 1.000 | 0.982 | 0.839 | 1.000 | 1.000 | 0.856 | 0.827 |
| 2331 | 0.767 | 1.000 | 0.808 | 1.000 | 0.968 | 0.960 | 0.900 | 1.000 | 1.000 | 1.000 | 0.959 | 0.970 | 0.991 | 0.847 | 1.000 | 1.000 | 0.854 | 0.825 |
| 2332 | 0.771 | 1.000 | 0.818 | 1.000 | 0.963 | 0.960 | 0.903 | 1.000 | 1.000 | 1.000 | 0.957 | 0.970 | 1.000 | 0.848 | 1.000 | 1.000 | 0.857 | 0.829 |
| 2333 | 0.768 | 1.000 | 0.820 | 1.000 | 0.963 | 0.960 | 0.903 | 1.000 | 1.000 | 1.000 | 0.957 | 0.970 | 1.000 | 0.857 | 1.000 | 1.000 | 0.858 | 0.830 |
| 2334 | 0.774 | 1.000 | 0.804 | 1.000 | 0.968 | 0.960 | 0.901 | 1.000 | 1.000 | 1.000 | 0.951 | 1.000 | 0.991 | 0.858 | 1.000 | 1.000 | 0.858 | 0.830 |
| 2335 | 0.773 | 1.000 | 0.826 | 1.000 | 0.968 | 0.960 | 0.912 | 1.000 | 1.000 | 1.000 | 0.957 | 0.970 | 0.991 | 0.843 | 1.000 | 1.000 | 0.858 | 0.830 |
| 2336 | 0.767 | 1.000 | 0.810 | 1.000 | 0.963 | 0.960 | 0.895 | 1.000 | 1.000 | 1.000 | 0.959 | 0.970 | 0.982 | 0.848 | 1.000 | 1.000 | 0.853 | 0.824 |
| 2337 | 0.774 | 1.000 | 0.812 | 1.000 | 0.968 | 0.960 | 0.888 | 1.000 | 1.000 | 1.000 | 0.957 | 0.970 | 1.000 | 0.851 | 1.000 | 1.000 | 0.856 | 0.827 |
| 2338 | 0.765 | 1.000 | 0.810 | 1.000 | 0.968 | 0.960 | 0.897 | 1.000 | 1.000 | 1.000 | 0.954 | 0.939 | 1.000 | 0.855 | 1.000 | 1.000 | 0.855 | 0.826 |

| | | | | | | | | | | | | | | | | | |
|---|---|---|---|---|---|---|---|---|---|---|---|---|---|---|---|---|---|
| 2339 | 0.778 | 1.000 | 0.804 | 1.000 | 0.963 | 0.960 | 0.900 | 1.000 | 1.000 | 1.000 | 0.957 | 1.000 | 0.991 | 0.848 | 1.000 | 1.000 | 0.857 | 0.829 |
| 2340 | 0.770 | 1.000 | 0.806 | 1.000 | 0.963 | 0.960 | 0.898 | 1.000 | 1.000 | 1.000 | 0.959 | 1.000 | 1.000 | 0.847 | 1.000 | 1.000 | 0.855 | 0.826 |
| 2341 | 0.771 | 1.000 | 0.804 | 1.000 | 0.958 | 0.960 | 0.908 | 1.000 | 1.000 | 1.000 | 0.954 | 1.000 | 0.991 | 0.851 | 1.000 | 1.000 | 0.856 | 0.828 |
| 2342 | 0.768 | 1.000 | 0.814 | 1.000 | 0.963 | 0.960 | 0.892 | 1.000 | 1.000 | 1.000 | 0.945 | 0.970 | 0.991 | 0.849 | 1.000 | 1.000 | 0.853 | 0.824 |
| 2343 | 0.774 | 1.000 | 0.798 | 1.000 | 0.958 | 0.960 | 0.897 | 1.000 | 1.000 | 1.000 | 0.962 | 0.970 | 0.991 | 0.846 | 1.000 | 1.000 | 0.854 | 0.825 |
| 2344 | 0.774 | 1.000 | 0.824 | 1.000 | 0.968 | 0.960 | 0.909 | 1.000 | 1.000 | 1.000 | 0.951 | 1.000 | 0.991 | 0.845 | 1.000 | 1.000 | 0.858 | 0.830 |
| 2345 | 0.777 | 1.000 | 0.804 | 1.000 | 0.968 | 0.960 | 0.900 | 1.000 | 1.000 | 1.000 | 0.959 | 0.970 | 0.991 | 0.858 | 1.000 | 1.000 | 0.859 | 0.831 |
| 2346 | 0.772 | 1.000 | 0.816 | 1.000 | 0.963 | 0.960 | 0.904 | 1.000 | 1.000 | 1.000 | 0.954 | 1.000 | 1.000 | 0.851 | 1.000 | 1.000 | 0.858 | 0.830 |
| 2347 | 0.770 | 1.000 | 0.802 | 1.000 | 0.963 | 0.960 | 0.904 | 1.000 | 0.990 | 1.000 | 0.962 | 1.000 | 0.991 | 0.860 | 1.000 | 1.000 | 0.858 | 0.830 |
| 2348 | 0.769 | 1.000 | 0.818 | 1.000 | 0.968 | 0.960 | 0.903 | 1.000 | 0.990 | 1.000 | 0.954 | 1.000 | 0.991 | 0.851 | 1.000 | 1.000 | 0.856 | 0.828 |
| 2349 | 0.771 | 1.000 | 0.806 | 1.000 | 0.963 | 0.960 | 0.891 | 1.000 | 1.000 | 1.000 | 0.951 | 0.939 | 0.991 | 0.847 | 1.000 | 1.000 | 0.853 | 0.824 |
| 2350 | 0.780 | 1.000 | 0.800 | 1.000 | 0.963 | 0.960 | 0.906 | 1.000 | 1.000 | 1.000 | 0.951 | 1.000 | 1.000 | 0.856 | 1.000 | 1.000 | 0.860 | 0.832 |
| 2351 | 0.773 | 1.000 | 0.806 | 1.000 | 0.963 | 0.960 | 0.909 | 1.000 | 1.000 | 1.000 | 0.954 | 1.000 | 0.991 | 0.852 | 1.000 | 1.000 | 0.858 | 0.830 |
| 2352 | 0.775 | 1.000 | 0.814 | 1.000 | 0.958 | 0.960 | 0.906 | 1.000 | 1.000 | 1.000 | 0.962 | 0.970 | 0.982 | 0.848 | 1.000 | 1.000 | 0.858 | 0.830 |
| 2353 | 0.775 | 1.000 | 0.818 | 1.000 | 0.974 | 0.960 | 0.903 | 1.000 | 1.000 | 1.000 | 0.959 | 0.970 | 1.000 | 0.848 | 1.000 | 1.000 | 0.858 | 0.830 |
| 2354 | 0.775 | 1.000 | 0.820 | 1.000 | 0.968 | 0.960 | 0.901 | 1.000 | 1.000 | 1.000 | 0.954 | 0.970 | 1.000 | 0.854 | 1.000 | 1.000 | 0.859 | 0.832 |
| 2355 | 0.769 | 1.000 | 0.820 | 1.000 | 0.968 | 0.960 | 0.904 | 1.000 | 0.990 | 1.000 | 0.957 | 0.970 | 0.991 | 0.851 | 1.000 | 1.000 | 0.857 | 0.829 |
| 2356 | 0.763 | 1.000 | 0.828 | 1.000 | 0.958 | 0.960 | 0.911 | 1.000 | 1.000 | 1.000 | 0.942 | 1.000 | 0.991 | 0.846 | 1.000 | 1.000 | 0.855 | 0.826 |
| 2357 | 0.770 | 1.000 | 0.818 | 1.000 | 0.974 | 0.960 | 0.908 | 1.000 | 1.000 | 1.000 | 0.951 | 1.000 | 1.000 | 0.853 | 1.000 | 1.000 | 0.858 | 0.831 |
| 2358 | 0.778 | 1.000 | 0.822 | 1.000 | 0.968 | 0.960 | 0.906 | 1.000 | 1.000 | 1.000 | 0.957 | 0.970 | 0.982 | 0.851 | 1.000 | 1.000 | 0.860 | 0.832 |
| 2359 | 0.763 | 1.000 | 0.824 | 1.000 | 0.968 | 0.960 | 0.901 | 1.000 | 0.990 | 1.000 | 0.957 | 0.970 | 0.991 | 0.851 | 1.000 | 1.000 | 0.855 | 0.827 |
| 2360 | 0.762 | 1.000 | 0.832 | 1.000 | 0.968 | 0.960 | 0.904 | 1.000 | 0.990 | 1.000 | 0.951 | 1.000 | 1.000 | 0.849 | 1.000 | 1.000 | 0.856 | 0.828 |
| 2361 | 0.769 | 1.000 | 0.806 | 1.000 | 0.963 | 0.960 | 0.903 | 1.000 | 1.000 | 1.000 | 0.962 | 1.000 | 0.982 | 0.845 | 1.000 | 1.000 | 0.854 | 0.826 |
| 2362 | 0.766 | 1.000 | 0.814 | 0.986 | 0.963 | 0.960 | 0.897 | 1.000 | 1.000 | 1.000 | 0.948 | 1.000 | 1.000 | 0.851 | 1.000 | 1.000 | 0.854 | 0.825 |
| 2363 | 0.772 | 1.000 | 0.812 | 1.000 | 0.958 | 0.960 | 0.908 | 1.000 | 1.000 | 1.000 | 0.948 | 0.970 | 1.000 | 0.846 | 1.000 | 1.000 | 0.856 | 0.827 |
| 2364 | 0.776 | 1.000 | 0.824 | 1.000 | 0.963 | 0.960 | 0.903 | 1.000 | 1.000 | 1.000 | 0.957 | 0.970 | 1.000 | 0.850 | 1.000 | 1.000 | 0.859 | 0.831 |
| 2365 | 0.776 | 1.000 | 0.816 | 1.000 | 0.963 | 0.960 | 0.903 | 1.000 | 1.000 | 1.000 | 0.957 | 1.000 | 1.000 | 0.854 | 1.000 | 1.000 | 0.860 | 0.832 |
| 2366 | 0.767 | 1.000 | 0.824 | 1.000 | 0.963 | 0.960 | 0.895 | 1.000 | 1.000 | 1.000 | 0.951 | 1.000 | 1.000 | 0.856 | 1.000 | 1.000 | 0.857 | 0.829 |
| 2367 | 0.766 | 1.000 | 0.820 | 1.000 | 0.968 | 0.960 | 0.906 | 1.000 | 1.000 | 1.000 | 0.959 | 0.970 | 0.991 | 0.845 | 1.000 | 1.000 | 0.855 | 0.827 |
| 2368 | 0.770 | 1.000 | 0.828 | 1.000 | 0.968 | 0.960 | 0.898 | 1.000 | 1.000 | 1.000 | 0.957 | 1.000 | 0.991 | 0.852 | 1.000 | 1.000 | 0.858 | 0.830 |
| 2369 | 0.766 | 1.000 | 0.810 | 1.000 | 0.963 | 0.960 | 0.892 | 1.000 | 0.990 | 1.000 | 0.962 | 1.000 | 0.991 | 0.852 | 1.000 | 1.000 | 0.854 | 0.825 |
| 2370 | 0.767 | 1.000 | 0.806 | 1.000 | 0.968 | 0.960 | 0.901 | 1.000 | 1.000 | 1.000 | 0.954 | 1.000 | 0.991 | 0.849 | 1.000 | 1.000 | 0.854 | 0.826 |
| 2371 | 0.773 | 1.000 | 0.806 | 1.000 | 0.963 | 0.960 | 0.911 | 1.000 | 1.000 | 1.000 | 0.957 | 1.000 | 0.982 | 0.848 | 1.000 | 1.000 | 0.857 | 0.829 |
| 2372 | 0.769 | 1.000 | 0.822 | 1.000 | 0.968 | 0.960 | 0.904 | 1.000 | 1.000 | 1.000 | 0.957 | 1.000 | 1.000 | 0.854 | 1.000 | 1.000 | 0.859 | 0.831 |
| 2373 | 0.778 | 1.000 | 0.812 | 1.000 | 0.963 | 0.960 | 0.897 | 1.000 | 1.000 | 1.000 | 0.951 | 0.970 | 0.991 | 0.851 | 1.000 | 1.000 | 0.857 | 0.829 |
| 2374 | 0.775 | 1.000 | 0.806 | 1.000 | 0.963 | 0.960 | 0.903 | 1.000 | 1.000 | 1.000 | 0.957 | 1.000 | 0.991 | 0.858 | 1.000 | 1.000 | 0.859 | 0.831 |
| 2375 | 0.768 | 1.000 | 0.808 | 1.000 | 0.963 | 0.960 | 0.895 | 1.000 | 1.000 | 1.000 | 0.957 | 1.000 | 1.000 | 0.854 | 1.000 | 1.000 | 0.855 | 0.827 |
| 2376 | 0.779 | 1.000 | 0.816 | 1.000 | 0.968 | 0.960 | 0.898 | 1.000 | 1.000 | 1.000 | 0.948 | 0.970 | 1.000 | 0.843 | 1.000 | 1.000 | 0.856 | 0.828 |
| 2377 | 0.771 | 1.000 | 0.818 | 0.986 | 0.974 | 0.960 | 0.898 | 1.000 | 1.000 | 1.000 | 0.957 | 0.970 | 0.991 | 0.845 | 1.000 | 1.000 | 0.855 | 0.827 |

| | | | | | | | | | | | | | | | | | | |
|---|---|---|---|---|---|---|---|---|---|---|---|---|---|---|---|---|---|---|
| 2378 | 0.769 | 1.000 | 0.816 | 1.000 | 0.963 | 0.960 | 0.909 | 1.000 | 1.000 | 1.000 | 0.962 | 1.000 | 0.991 | 0.847 | 1.000 | 1.000 | 0.857 | 0.828 |
| 2379 | 0.769 | 1.000 | 0.796 | 1.000 | 0.968 | 0.960 | 0.900 | 1.000 | 0.990 | 1.000 | 0.957 | 0.970 | 0.991 | 0.857 | 1.000 | 1.000 | 0.855 | 0.827 |
| 2380 | 0.773 | 1.000 | 0.806 | 1.000 | 0.963 | 0.960 | 0.908 | 1.000 | 1.000 | 1.000 | 0.957 | 1.000 | 0.991 | 0.851 | 1.000 | 1.000 | 0.857 | 0.829 |
| 2381 | 0.768 | 1.000 | 0.808 | 1.000 | 0.968 | 0.960 | 0.904 | 1.000 | 1.000 | 1.000 | 0.957 | 1.000 | 0.991 | 0.850 | 1.000 | 1.000 | 0.856 | 0.827 |
| 2382 | 0.780 | 1.000 | 0.816 | 1.000 | 0.963 | 0.960 | 0.903 | 1.000 | 1.000 | 1.000 | 0.954 | 1.000 | 0.982 | 0.846 | 1.000 | 0.981 | 0.858 | 0.830 |
| 2383 | 0.774 | 1.000 | 0.818 | 1.000 | 0.958 | 0.960 | 0.914 | 1.000 | 1.000 | 1.000 | 0.957 | 0.970 | 0.991 | 0.854 | 1.000 | 1.000 | 0.860 | 0.833 |
| 2384 | 0.781 | 1.000 | 0.826 | 1.000 | 0.963 | 0.960 | 0.908 | 1.000 | 1.000 | 1.000 | 0.957 | 1.000 | 1.000 | 0.854 | 1.000 | 1.000 | 0.863 | 0.836 |
| 2385 | 0.769 | 1.000 | 0.810 | 1.000 | 0.968 | 0.960 | 0.904 | 1.000 | 1.000 | 1.000 | 0.948 | 0.970 | 0.991 | 0.851 | 1.000 | 1.000 | 0.856 | 0.827 |
| 2386 | 0.775 | 1.000 | 0.816 | 1.000 | 0.958 | 0.960 | 0.903 | 1.000 | 1.000 | 1.000 | 0.954 | 1.000 | 1.000 | 0.854 | 1.000 | 1.000 | 0.859 | 0.831 |
| 2387 | 0.775 | 1.000 | 0.820 | 1.000 | 0.968 | 0.960 | 0.903 | 1.000 | 1.000 | 1.000 | 0.951 | 0.970 | 0.991 | 0.851 | 1.000 | 1.000 | 0.859 | 0.831 |
| 2388 | 0.770 | 1.000 | 0.824 | 1.000 | 0.963 | 0.960 | 0.914 | 1.000 | 1.000 | 1.000 | 0.939 | 0.970 | 0.991 | 0.851 | 1.000 | 1.000 | 0.858 | 0.830 |
| 2389 | 0.769 | 1.000 | 0.822 | 1.000 | 0.963 | 0.960 | 0.909 | 1.000 | 1.000 | 1.000 | 0.951 | 0.970 | 0.991 | 0.848 | 1.000 | 1.000 | 0.857 | 0.829 |
| 2390 | 0.775 | 1.000 | 0.818 | 1.000 | 0.963 | 0.960 | 0.911 | 1.000 | 1.000 | 1.000 | 0.957 | 0.970 | 1.000 | 0.847 | 1.000 | 1.000 | 0.859 | 0.831 |
| 2391 | 0.779 | 1.000 | 0.820 | 1.000 | 0.968 | 0.960 | 0.897 | 1.000 | 0.990 | 1.000 | 0.959 | 0.970 | 1.000 | 0.845 | 1.000 | 1.000 | 0.858 | 0.829 |
| 2392 | 0.774 | 1.000 | 0.804 | 1.000 | 0.968 | 0.960 | 0.889 | 1.000 | 0.990 | 1.000 | 0.954 | 0.970 | 1.000 | 0.857 | 1.000 | 1.000 | 0.856 | 0.828 |
| 2393 | 0.767 | 1.000 | 0.828 | 1.000 | 0.963 | 0.960 | 0.906 | 1.000 | 1.000 | 1.000 | 0.957 | 1.000 | 0.991 | 0.856 | 1.000 | 1.000 | 0.859 | 0.831 |
| 2394 | 0.778 | 1.000 | 0.820 | 1.000 | 0.968 | 0.960 | 0.909 | 1.000 | 1.000 | 1.000 | 0.954 | 1.000 | 0.991 | 0.851 | 1.000 | 1.000 | 0.860 | 0.833 |
| 2395 | 0.778 | 1.000 | 0.828 | 1.000 | 0.963 | 0.960 | 0.914 | 1.000 | 1.000 | 1.000 | 0.951 | 0.970 | 1.000 | 0.848 | 1.000 | 1.000 | 0.861 | 0.833 |
| 2396 | 0.762 | 1.000 | 0.806 | 1.000 | 0.968 | 0.960 | 0.906 | 1.000 | 1.000 | 1.000 | 0.959 | 1.000 | 1.000 | 0.851 | 1.000 | 1.000 | 0.854 | 0.826 |
| 2397 | 0.765 | 1.000 | 0.836 | 1.000 | 0.963 | 0.960 | 0.909 | 1.000 | 1.000 | 1.000 | 0.948 | 1.000 | 1.000 | 0.847 | 1.000 | 1.000 | 0.857 | 0.829 |
| 2398 | 0.776 | 1.000 | 0.816 | 1.000 | 0.963 | 0.960 | 0.901 | 1.000 | 1.000 | 1.000 | 0.945 | 0.970 | 1.000 | 0.855 | 1.000 | 1.000 | 0.859 | 0.831 |
| 2399 | 0.775 | 1.000 | 0.796 | 1.000 | 0.968 | 0.960 | 0.900 | 1.000 | 1.000 | 1.000 | 0.959 | 1.000 | 1.000 | 0.847 | 1.000 | 1.000 | 0.856 | 0.827 |
| 2400 | 0.773 | 1.000 | 0.806 | 1.000 | 0.968 | 0.960 | 0.908 | 1.000 | 1.000 | 1.000 | 0.959 | 1.000 | 0.991 | 0.854 | 1.000 | 1.000 | 0.858 | 0.830 |
| 2401 | 0.767 | 1.000 | 0.822 | 1.000 | 0.968 | 0.960 | 0.906 | 1.000 | 1.000 | 1.000 | 0.948 | 1.000 | 1.000 | 0.849 | 1.000 | 1.000 | 0.856 | 0.828 |
| 2402 | 0.776 | 1.000 | 0.812 | 1.000 | 0.968 | 0.960 | 0.912 | 1.000 | 1.000 | 1.000 | 0.959 | 1.000 | 1.000 | 0.848 | 1.000 | 1.000 | 0.859 | 0.832 |
| 2403 | 0.768 | 1.000 | 0.826 | 1.000 | 0.968 | 0.960 | 0.897 | 1.000 | 1.000 | 1.000 | 0.957 | 1.000 | 1.000 | 0.846 | 1.000 | 1.000 | 0.856 | 0.827 |
| 2404 | 0.775 | 1.000 | 0.806 | 1.000 | 0.963 | 0.960 | 0.908 | 1.000 | 1.000 | 1.000 | 0.957 | 1.000 | 1.000 | 0.851 | 1.000 | 1.000 | 0.858 | 0.830 |
| 2405 | 0.769 | 1.000 | 0.800 | 1.000 | 0.963 | 0.960 | 0.903 | 1.000 | 1.000 | 1.000 | 0.948 | 0.970 | 1.000 | 0.850 | 1.000 | 1.000 | 0.854 | 0.825 |
| 2406 | 0.770 | 1.000 | 0.802 | 1.000 | 0.968 | 0.960 | 0.898 | 1.000 | 0.990 | 1.000 | 0.962 | 0.970 | 0.991 | 0.847 | 1.000 | 1.000 | 0.854 | 0.825 |
| 2407 | 0.768 | 1.000 | 0.834 | 1.000 | 0.963 | 0.960 | 0.908 | 1.000 | 0.990 | 1.000 | 0.965 | 0.970 | 0.991 | 0.845 | 1.000 | 1.000 | 0.857 | 0.830 |
| 2408 | 0.775 | 1.000 | 0.804 | 1.000 | 0.968 | 0.960 | 0.903 | 1.000 | 1.000 | 1.000 | 0.948 | 1.000 | 1.000 | 0.853 | 1.000 | 1.000 | 0.858 | 0.829 |
| 2409 | 0.774 | 1.000 | 0.822 | 1.000 | 0.968 | 0.960 | 0.898 | 1.000 | 1.000 | 1.000 | 0.962 | 1.000 | 0.982 | 0.860 | 1.000 | 1.000 | 0.861 | 0.833 |
| 2410 | 0.773 | 1.000 | 0.802 | 1.000 | 0.974 | 0.960 | 0.903 | 1.000 | 1.000 | 1.000 | 0.957 | 0.970 | 0.991 | 0.855 | 1.000 | 1.000 | 0.858 | 0.829 |
| 2411 | 0.770 | 1.000 | 0.810 | 1.000 | 0.968 | 0.960 | 0.901 | 1.000 | 0.990 | 1.000 | 0.954 | 1.000 | 0.991 | 0.855 | 1.000 | 1.000 | 0.857 | 0.829 |
| 2412 | 0.766 | 1.000 | 0.814 | 0.986 | 0.968 | 0.960 | 0.909 | 1.000 | 1.000 | 1.000 | 0.948 | 0.970 | 0.991 | 0.849 | 1.000 | 1.000 | 0.855 | 0.827 |
| 2413 | 0.767 | 1.000 | 0.810 | 1.000 | 0.963 | 0.960 | 0.895 | 1.000 | 1.000 | 1.000 | 0.957 | 0.970 | 1.000 | 0.851 | 1.000 | 1.000 | 0.855 | 0.826 |
| 2414 | 0.773 | 1.000 | 0.802 | 1.000 | 0.963 | 0.960 | 0.897 | 1.000 | 0.990 | 1.000 | 0.959 | 1.000 | 0.991 | 0.856 | 1.000 | 1.000 | 0.857 | 0.828 |
| 2415 | 0.765 | 1.000 | 0.816 | 1.000 | 0.968 | 0.960 | 0.903 | 1.000 | 1.000 | 1.000 | 0.954 | 1.000 | 1.000 | 0.851 | 1.000 | 1.000 | 0.856 | 0.828 |
| 2416 | 0.769 | 1.000 | 0.798 | 1.000 | 0.968 | 0.960 | 0.912 | 1.000 | 1.000 | 1.000 | 0.948 | 0.970 | 1.000 | 0.847 | 1.000 | 1.000 | 0.855 | 0.826 |

| | | | | | | | | | | | | | | | | | |
|---|---|---|---|---|---|---|---|---|---|---|---|---|---|---|---|---|---|
| 2417 | 0.769 | 1.000 | 0.826 | 1.000 | 0.968 | 0.960 | 0.903 | 1.000 | 1.000 | 1.000 | 0.954 | 1.000 | 1.000 | 0.847 | 1.000 | 1.000 | 0.857 | 0.829 |
| 2418 | 0.773 | 1.000 | 0.810 | 1.000 | 0.958 | 0.960 | 0.901 | 1.000 | 0.990 | 1.000 | 0.959 | 0.970 | 0.982 | 0.857 | 1.000 | 1.000 | 0.858 | 0.830 |
| 2419 | 0.770 | 1.000 | 0.814 | 1.000 | 0.968 | 0.960 | 0.921 | 1.000 | 1.000 | 1.000 | 0.951 | 1.000 | 0.991 | 0.851 | 1.000 | 1.000 | 0.859 | 0.832 |
| 2420 | 0.766 | 1.000 | 0.832 | 1.000 | 0.968 | 0.960 | 0.904 | 1.000 | 1.000 | 1.000 | 0.948 | 1.000 | 0.991 | 0.854 | 1.000 | 1.000 | 0.858 | 0.830 |
| 2421 | 0.767 | 1.000 | 0.814 | 1.000 | 0.963 | 0.960 | 0.911 | 1.000 | 1.000 | 1.000 | 0.954 | 0.970 | 1.000 | 0.849 | 1.000 | 1.000 | 0.856 | 0.828 |
| 2422 | 0.767 | 1.000 | 0.814 | 1.000 | 0.963 | 0.960 | 0.904 | 1.000 | 1.000 | 1.000 | 0.962 | 0.939 | 0.991 | 0.851 | 1.000 | 1.000 | 0.856 | 0.828 |
| 2423 | 0.769 | 1.000 | 0.820 | 0.986 | 0.958 | 0.960 | 0.911 | 1.000 | 0.990 | 1.000 | 0.957 | 0.970 | 1.000 | 0.854 | 1.000 | 1.000 | 0.858 | 0.830 |
| 2424 | 0.772 | 1.000 | 0.818 | 1.000 | 0.974 | 0.960 | 0.900 | 1.000 | 0.990 | 1.000 | 0.959 | 1.000 | 0.991 | 0.858 | 1.000 | 1.000 | 0.859 | 0.831 |
| 2425 | 0.773 | 1.000 | 0.808 | 1.000 | 0.974 | 0.960 | 0.898 | 1.000 | 1.000 | 1.000 | 0.957 | 1.000 | 0.991 | 0.852 | 1.000 | 1.000 | 0.857 | 0.828 |
| 2426 | 0.778 | 1.000 | 0.802 | 1.000 | 0.968 | 0.960 | 0.897 | 1.000 | 1.000 | 1.000 | 0.959 | 1.000 | 1.000 | 0.849 | 1.000 | 1.000 | 0.857 | 0.829 |
| 2427 | 0.779 | 1.000 | 0.816 | 0.986 | 0.963 | 0.960 | 0.904 | 1.000 | 0.990 | 1.000 | 0.954 | 0.970 | 1.000 | 0.851 | 1.000 | 1.000 | 0.859 | 0.831 |
| 2428 | 0.775 | 1.000 | 0.822 | 1.000 | 0.974 | 0.960 | 0.901 | 1.000 | 1.000 | 1.000 | 0.954 | 1.000 | 1.000 | 0.849 | 1.000 | 1.000 | 0.859 | 0.831 |
| 2429 | 0.767 | 1.000 | 0.822 | 1.000 | 0.963 | 0.960 | 0.897 | 1.000 | 1.000 | 1.000 | 0.959 | 1.000 | 0.982 | 0.849 | 1.000 | 1.000 | 0.855 | 0.827 |
| 2430 | 0.776 | 1.000 | 0.818 | 1.000 | 0.963 | 0.960 | 0.900 | 1.000 | 0.990 | 1.000 | 0.948 | 0.970 | 0.991 | 0.851 | 1.000 | 1.000 | 0.857 | 0.829 |
| 2431 | 0.779 | 1.000 | 0.810 | 1.000 | 0.968 | 0.960 | 0.904 | 1.000 | 1.000 | 1.000 | 0.965 | 1.000 | 1.000 | 0.858 | 1.000 | 1.000 | 0.862 | 0.834 |
| 2432 | 0.769 | 1.000 | 0.828 | 1.000 | 0.968 | 0.960 | 0.901 | 1.000 | 1.000 | 1.000 | 0.957 | 0.970 | 0.982 | 0.856 | 1.000 | 1.000 | 0.859 | 0.831 |
| 2433 | 0.778 | 1.000 | 0.820 | 1.000 | 0.968 | 0.960 | 0.906 | 1.000 | 1.000 | 1.000 | 0.957 | 1.000 | 1.000 | 0.848 | 1.000 | 1.000 | 0.860 | 0.832 |
| 2434 | 0.763 | 1.000 | 0.812 | 1.000 | 0.968 | 0.960 | 0.909 | 1.000 | 1.000 | 1.000 | 0.951 | 0.970 | 0.991 | 0.853 | 1.000 | 1.000 | 0.855 | 0.827 |
| 2435 | 0.769 | 1.000 | 0.816 | 1.000 | 0.968 | 0.960 | 0.897 | 1.000 | 1.000 | 1.000 | 0.954 | 1.000 | 0.991 | 0.844 | 1.000 | 1.000 | 0.854 | 0.825 |
| 2436 | 0.777 | 1.000 | 0.834 | 1.000 | 0.963 | 0.960 | 0.900 | 1.000 | 1.000 | 1.000 | 0.954 | 0.970 | 1.000 | 0.851 | 1.000 | 1.000 | 0.860 | 0.832 |
| 2437 | 0.770 | 1.000 | 0.820 | 1.000 | 0.968 | 0.960 | 0.898 | 1.000 | 1.000 | 1.000 | 0.957 | 1.000 | 0.991 | 0.851 | 1.000 | 1.000 | 0.857 | 0.828 |
| 2438 | 0.768 | 1.000 | 0.812 | 1.000 | 0.974 | 0.960 | 0.908 | 1.000 | 1.000 | 1.000 | 0.962 | 1.000 | 0.991 | 0.859 | 1.000 | 1.000 | 0.859 | 0.832 |
| 2439 | 0.772 | 1.000 | 0.810 | 0.986 | 0.963 | 0.960 | 0.898 | 1.000 | 1.000 | 1.000 | 0.957 | 0.970 | 0.991 | 0.855 | 1.000 | 1.000 | 0.857 | 0.828 |
| 2440 | 0.776 | 1.000 | 0.812 | 1.000 | 0.963 | 0.960 | 0.901 | 1.000 | 1.000 | 1.000 | 0.959 | 0.970 | 1.000 | 0.860 | 1.000 | 1.000 | 0.861 | 0.833 |
| 2441 | 0.769 | 1.000 | 0.838 | 1.000 | 0.963 | 0.960 | 0.898 | 1.000 | 1.000 | 1.000 | 0.959 | 1.000 | 1.000 | 0.851 | 1.000 | 1.000 | 0.859 | 0.831 |
| 2442 | 0.773 | 1.000 | 0.820 | 0.986 | 0.968 | 0.960 | 0.895 | 1.000 | 1.000 | 1.000 | 0.954 | 0.970 | 1.000 | 0.848 | 1.000 | 1.000 | 0.856 | 0.828 |
| 2443 | 0.774 | 1.000 | 0.810 | 1.000 | 0.963 | 0.960 | 0.904 | 1.000 | 1.000 | 1.000 | 0.954 | 1.000 | 0.991 | 0.847 | 1.000 | 1.000 | 0.857 | 0.828 |
| 2444 | 0.768 | 1.000 | 0.830 | 1.000 | 0.963 | 0.960 | 0.898 | 1.000 | 1.000 | 1.000 | 0.959 | 1.000 | 1.000 | 0.847 | 1.000 | 1.000 | 0.857 | 0.829 |
| 2445 | 0.763 | 1.000 | 0.834 | 1.000 | 0.968 | 0.960 | 0.915 | 1.000 | 1.000 | 1.000 | 0.959 | 1.000 | 1.000 | 0.851 | 1.000 | 1.000 | 0.859 | 0.832 |
| 2446 | 0.782 | 1.000 | 0.806 | 1.000 | 0.968 | 0.960 | 0.906 | 1.000 | 1.000 | 1.000 | 0.957 | 1.000 | 0.991 | 0.851 | 1.000 | 1.000 | 0.860 | 0.832 |
| 2447 | 0.768 | 1.000 | 0.808 | 1.000 | 0.968 | 0.960 | 0.908 | 1.000 | 1.000 | 1.000 | 0.954 | 0.970 | 0.982 | 0.860 | 1.000 | 1.000 | 0.858 | 0.830 |
| 2448 | 0.780 | 1.000 | 0.816 | 1.000 | 0.963 | 0.960 | 0.900 | 1.000 | 1.000 | 1.000 | 0.954 | 1.000 | 0.991 | 0.853 | 1.000 | 1.000 | 0.860 | 0.832 |
| 2449 | 0.772 | 1.000 | 0.820 | 1.000 | 0.974 | 0.960 | 0.906 | 1.000 | 1.000 | 1.000 | 0.957 | 1.000 | 0.991 | 0.850 | 1.000 | 1.000 | 0.858 | 0.831 |
| 2450 | 0.775 | 1.000 | 0.830 | 1.000 | 0.963 | 0.960 | 0.912 | 1.000 | 1.000 | 1.000 | 0.959 | 0.970 | 1.000 | 0.857 | 1.000 | 1.000 | 0.863 | 0.836 |
| 2451 | 0.775 | 1.000 | 0.810 | 1.000 | 0.968 | 0.960 | 0.906 | 1.000 | 1.000 | 1.000 | 0.962 | 1.000 | 1.000 | 0.851 | 1.000 | 1.000 | 0.859 | 0.831 |
| 2452 | 0.773 | 1.000 | 0.818 | 1.000 | 0.968 | 0.960 | 0.918 | 1.000 | 1.000 | 1.000 | 0.959 | 0.970 | 0.991 | 0.849 | 1.000 | 1.000 | 0.860 | 0.832 |
| 2453 | 0.771 | 1.000 | 0.828 | 1.000 | 0.968 | 0.960 | 0.906 | 1.000 | 1.000 | 1.000 | 0.959 | 1.000 | 0.991 | 0.856 | 1.000 | 1.000 | 0.861 | 0.833 |
| 2454 | 0.768 | 1.000 | 0.836 | 1.000 | 0.968 | 0.960 | 0.901 | 1.000 | 1.000 | 1.000 | 0.954 | 1.000 | 0.991 | 0.851 | 1.000 | 1.000 | 0.858 | 0.831 |
| 2455 | 0.766 | 1.000 | 0.816 | 1.000 | 0.968 | 0.960 | 0.911 | 1.000 | 1.000 | 1.000 | 0.962 | 0.970 | 1.000 | 0.845 | 1.000 | 1.000 | 0.856 | 0.828 |

| | | | | | | | | | | | | | | | | | |
|---|---|---|---|---|---|---|---|---|---|---|---|---|---|---|---|---|---|
| 2456 | 0.773 | 1.000 | 0.834 | 0.986 | 0.968 | 0.960 | 0.904 | 1.000 | 1.000 | 1.000 | 0.962 | 0.970 | 0.991 | 0.847 | 1.000 | 1.000 | 0.859 | 0.831 |
| 2457 | 0.773 | 1.000 | 0.822 | 1.000 | 0.968 | 0.960 | 0.906 | 1.000 | 1.000 | 1.000 | 0.959 | 0.970 | 1.000 | 0.850 | 1.000 | 1.000 | 0.859 | 0.831 |
| 2458 | 0.776 | 1.000 | 0.800 | 0.986 | 0.963 | 0.960 | 0.912 | 1.000 | 1.000 | 1.000 | 0.957 | 0.939 | 1.000 | 0.848 | 1.000 | 1.000 | 0.857 | 0.829 |
| 2459 | 0.772 | 1.000 | 0.832 | 1.000 | 0.968 | 0.960 | 0.911 | 1.000 | 1.000 | 1.000 | 0.959 | 1.000 | 0.991 | 0.847 | 1.000 | 1.000 | 0.860 | 0.832 |
| 2460 | 0.769 | 1.000 | 0.818 | 1.000 | 0.968 | 0.960 | 0.898 | 1.000 | 1.000 | 1.000 | 0.968 | 1.000 | 1.000 | 0.846 | 1.000 | 1.000 | 0.856 | 0.828 |
| 2461 | 0.765 | 1.000 | 0.830 | 1.000 | 0.974 | 0.960 | 0.904 | 1.000 | 1.000 | 1.000 | 0.954 | 1.000 | 1.000 | 0.851 | 1.000 | 1.000 | 0.857 | 0.830 |
| 2462 | 0.778 | 1.000 | 0.834 | 1.000 | 0.968 | 0.960 | 0.909 | 1.000 | 1.000 | 1.000 | 0.959 | 1.000 | 1.000 | 0.858 | 1.000 | 1.000 | 0.864 | 0.837 |
| 2463 | 0.767 | 1.000 | 0.832 | 1.000 | 0.968 | 0.960 | 0.901 | 1.000 | 1.000 | 1.000 | 0.962 | 0.970 | 0.991 | 0.856 | 1.000 | 1.000 | 0.859 | 0.832 |
| 2464 | 0.769 | 1.000 | 0.824 | 1.000 | 0.963 | 0.960 | 0.908 | 1.000 | 1.000 | 1.000 | 0.957 | 1.000 | 0.991 | 0.856 | 1.000 | 1.000 | 0.859 | 0.832 |
| 2465 | 0.773 | 1.000 | 0.810 | 1.000 | 0.968 | 0.960 | 0.901 | 1.000 | 1.000 | 1.000 | 0.957 | 1.000 | 0.991 | 0.853 | 1.000 | 1.000 | 0.858 | 0.829 |
| 2466 | 0.779 | 1.000 | 0.826 | 1.000 | 0.968 | 0.960 | 0.900 | 1.000 | 1.000 | 1.000 | 0.954 | 1.000 | 1.000 | 0.854 | 1.000 | 1.000 | 0.861 | 0.834 |
| 2467 | 0.772 | 1.000 | 0.832 | 0.986 | 0.963 | 0.960 | 0.906 | 1.000 | 1.000 | 1.000 | 0.962 | 0.970 | 0.991 | 0.848 | 1.000 | 1.000 | 0.859 | 0.831 |
| 2468 | 0.774 | 1.000 | 0.822 | 1.000 | 0.974 | 0.960 | 0.909 | 1.000 | 1.000 | 1.000 | 0.959 | 0.970 | 0.991 | 0.850 | 1.000 | 1.000 | 0.860 | 0.832 |
| 2469 | 0.768 | 1.000 | 0.832 | 1.000 | 0.968 | 0.960 | 0.897 | 1.000 | 1.000 | 1.000 | 0.962 | 0.939 | 1.000 | 0.860 | 1.000 | 1.000 | 0.860 | 0.832 |
| 2470 | 0.775 | 1.000 | 0.822 | 1.000 | 0.974 | 0.960 | 0.908 | 1.000 | 1.000 | 1.000 | 0.957 | 1.000 | 1.000 | 0.854 | 1.000 | 1.000 | 0.861 | 0.833 |
| 2471 | 0.773 | 1.000 | 0.818 | 1.000 | 0.968 | 0.960 | 0.904 | 1.000 | 1.000 | 1.000 | 0.957 | 0.970 | 1.000 | 0.847 | 1.000 | 1.000 | 0.858 | 0.830 |
| 2472 | 0.775 | 1.000 | 0.814 | 1.000 | 0.968 | 0.960 | 0.914 | 1.000 | 1.000 | 1.000 | 0.962 | 1.000 | 0.991 | 0.847 | 1.000 | 1.000 | 0.859 | 0.831 |
| 2473 | 0.776 | 1.000 | 0.824 | 1.000 | 0.963 | 0.960 | 0.904 | 1.000 | 1.000 | 1.000 | 0.965 | 0.970 | 0.982 | 0.847 | 1.000 | 1.000 | 0.859 | 0.831 |
| 2474 | 0.768 | 1.000 | 0.816 | 1.000 | 0.968 | 0.960 | 0.908 | 1.000 | 1.000 | 1.000 | 0.959 | 0.970 | 0.991 | 0.842 | 1.000 | 1.000 | 0.855 | 0.827 |
| 2475 | 0.768 | 1.000 | 0.820 | 1.000 | 0.968 | 0.960 | 0.911 | 1.000 | 1.000 | 1.000 | 0.959 | 0.970 | 0.991 | 0.854 | 1.000 | 1.000 | 0.859 | 0.831 |
| 2476 | 0.773 | 1.000 | 0.816 | 1.000 | 0.974 | 0.960 | 0.909 | 1.000 | 1.000 | 1.000 | 0.954 | 1.000 | 1.000 | 0.849 | 1.000 | 1.000 | 0.859 | 0.831 |
| 2477 | 0.771 | 1.000 | 0.816 | 1.000 | 0.974 | 0.960 | 0.901 | 1.000 | 1.000 | 1.000 | 0.959 | 1.000 | 0.991 | 0.846 | 1.000 | 1.000 | 0.856 | 0.828 |
| 2478 | 0.771 | 1.000 | 0.812 | 1.000 | 0.968 | 0.960 | 0.901 | 1.000 | 1.000 | 1.000 | 0.965 | 0.970 | 1.000 | 0.851 | 1.000 | 1.000 | 0.857 | 0.829 |
| 2479 | 0.767 | 1.000 | 0.820 | 1.000 | 0.974 | 0.960 | 0.906 | 1.000 | 1.000 | 1.000 | 0.962 | 0.970 | 0.991 | 0.846 | 1.000 | 1.000 | 0.856 | 0.828 |
| 2480 | 0.773 | 1.000 | 0.824 | 1.000 | 0.963 | 0.960 | 0.914 | 1.000 | 1.000 | 1.000 | 0.962 | 1.000 | 0.991 | 0.848 | 1.000 | 1.000 | 0.860 | 0.832 |
| 2481 | 0.768 | 1.000 | 0.814 | 1.000 | 0.968 | 0.960 | 0.908 | 1.000 | 1.000 | 1.000 | 0.959 | 0.970 | 1.000 | 0.850 | 1.000 | 1.000 | 0.857 | 0.829 |
| 2482 | 0.775 | 1.000 | 0.812 | 1.000 | 0.968 | 0.960 | 0.909 | 1.000 | 1.000 | 1.000 | 0.962 | 1.000 | 0.991 | 0.858 | 1.000 | 1.000 | 0.861 | 0.834 |
| 2483 | 0.765 | 1.000 | 0.814 | 1.000 | 0.968 | 0.960 | 0.911 | 1.000 | 1.000 | 1.000 | 0.959 | 1.000 | 1.000 | 0.849 | 1.000 | 1.000 | 0.856 | 0.829 |
| 2484 | 0.777 | 1.000 | 0.824 | 1.000 | 0.979 | 0.960 | 0.901 | 1.000 | 1.000 | 1.000 | 0.962 | 0.970 | 0.991 | 0.845 | 1.000 | 1.000 | 0.859 | 0.831 |
| 2485 | 0.772 | 1.000 | 0.830 | 1.000 | 0.968 | 0.960 | 0.898 | 1.000 | 1.000 | 1.000 | 0.965 | 0.970 | 0.991 | 0.844 | 1.000 | 1.000 | 0.857 | 0.829 |
| 2486 | 0.772 | 1.000 | 0.824 | 1.000 | 0.968 | 0.960 | 0.906 | 1.000 | 1.000 | 1.000 | 0.954 | 1.000 | 0.991 | 0.851 | 1.000 | 1.000 | 0.859 | 0.831 |
| 2487 | 0.779 | 1.000 | 0.814 | 1.000 | 0.968 | 0.960 | 0.908 | 1.000 | 1.000 | 1.000 | 0.965 | 1.000 | 0.991 | 0.848 | 1.000 | 1.000 | 0.860 | 0.833 |
| 2488 | 0.770 | 1.000 | 0.818 | 1.000 | 0.968 | 0.960 | 0.900 | 1.000 | 1.000 | 1.000 | 0.965 | 0.970 | 1.000 | 0.859 | 1.000 | 1.000 | 0.860 | 0.832 |
| 2489 | 0.775 | 1.000 | 0.834 | 1.000 | 0.963 | 0.960 | 0.891 | 1.000 | 1.000 | 1.000 | 0.965 | 0.970 | 0.991 | 0.849 | 1.000 | 1.000 | 0.859 | 0.831 |
| 2490 | 0.776 | 1.000 | 0.826 | 1.000 | 0.968 | 0.960 | 0.900 | 1.000 | 1.000 | 1.000 | 0.951 | 1.000 | 1.000 | 0.847 | 1.000 | 1.000 | 0.858 | 0.830 |
| 2491 | 0.780 | 1.000 | 0.816 | 1.000 | 0.968 | 0.960 | 0.904 | 1.000 | 1.000 | 1.000 | 0.951 | 1.000 | 0.991 | 0.849 | 1.000 | 1.000 | 0.859 | 0.832 |
| 2492 | 0.765 | 1.000 | 0.832 | 1.000 | 0.968 | 0.960 | 0.911 | 1.000 | 1.000 | 1.000 | 0.962 | 0.970 | 1.000 | 0.855 | 1.000 | 1.000 | 0.860 | 0.832 |
| 2493 | 0.775 | 1.000 | 0.826 | 1.000 | 0.974 | 0.960 | 0.903 | 1.000 | 1.000 | 1.000 | 0.959 | 1.000 | 1.000 | 0.854 | 1.000 | 1.000 | 0.861 | 0.834 |
| 2494 | 0.777 | 1.000 | 0.818 | 1.000 | 0.968 | 0.960 | 0.901 | 1.000 | 1.000 | 1.000 | 0.954 | 0.970 | 1.000 | 0.851 | 1.000 | 1.000 | 0.859 | 0.831 |

| | | | | | | | | | | | | | | | | | | |
|---|---|---|---|---|---|---|---|---|---|---|---|---|---|---|---|---|---|---|
| 2495 | 0.783 | 1.000 | 0.806 | 1.000 | 0.963 | 0.960 | 0.900 | 1.000 | 1.000 | 1.000 | 0.957 | 1.000 | 1.000 | 0.845 | 1.000 | 1.000 | 0.858 | 0.830 |
| 2496 | 0.777 | 1.000 | 0.804 | 1.000 | 0.968 | 0.960 | 0.909 | 1.000 | 1.000 | 1.000 | 0.957 | 0.970 | 1.000 | 0.854 | 1.000 | 1.000 | 0.860 | 0.832 |
| 2497 | 0.771 | 1.000 | 0.822 | 1.000 | 0.968 | 0.960 | 0.908 | 1.000 | 1.000 | 1.000 | 0.965 | 1.000 | 1.000 | 0.852 | 1.000 | 1.000 | 0.860 | 0.832 |
| 2498 | 0.766 | 1.000 | 0.840 | 1.000 | 0.963 | 0.960 | 0.906 | 1.000 | 1.000 | 1.000 | 0.959 | 1.000 | 0.991 | 0.854 | 1.000 | 1.000 | 0.859 | 0.832 |
| 2499 | 0.765 | 1.000 | 0.824 | 1.000 | 0.974 | 0.960 | 0.911 | 1.000 | 1.000 | 1.000 | 0.957 | 1.000 | 0.991 | 0.851 | 1.000 | 1.000 | 0.858 | 0.830 |
| 2500 | 0.771 | 1.000 | 0.814 | 1.000 | 0.968 | 0.960 | 0.903 | 1.000 | 1.000 | 1.000 | 0.957 | 0.970 | 1.000 | 0.854 | 1.000 | 1.000 | 0.858 | 0.830 |
| 2501 | 0.771 | 1.000 | 0.822 | 1.000 | 0.968 | 0.960 | 0.903 | 1.000 | 1.000 | 1.000 | 0.959 | 1.000 | 1.000 | 0.853 | 1.000 | 1.000 | 0.859 | 0.831 |
| 2502 | 0.772 | 1.000 | 0.818 | 1.000 | 0.974 | 0.960 | 0.912 | 1.000 | 1.000 | 1.000 | 0.962 | 0.970 | 1.000 | 0.846 | 1.000 | 1.000 | 0.858 | 0.831 |
| 2503 | 0.783 | 1.000 | 0.818 | 1.000 | 0.968 | 0.960 | 0.906 | 1.000 | 0.990 | 1.000 | 0.965 | 1.000 | 1.000 | 0.861 | 1.000 | 1.000 | 0.865 | 0.838 |
| 2504 | 0.775 | 1.000 | 0.800 | 1.000 | 0.968 | 0.960 | 0.898 | 1.000 | 1.000 | 1.000 | 0.954 | 1.000 | 1.000 | 0.850 | 1.000 | 1.000 | 0.856 | 0.828 |
| 2505 | 0.773 | 1.000 | 0.828 | 1.000 | 0.979 | 0.960 | 0.906 | 1.000 | 1.000 | 1.000 | 0.965 | 1.000 | 0.991 | 0.847 | 1.000 | 1.000 | 0.860 | 0.832 |
| 2506 | 0.770 | 1.000 | 0.814 | 1.000 | 0.974 | 0.960 | 0.911 | 1.000 | 1.000 | 1.000 | 0.968 | 1.000 | 1.000 | 0.852 | 1.000 | 1.000 | 0.859 | 0.832 |
| 2507 | 0.767 | 1.000 | 0.830 | 0.986 | 0.968 | 0.960 | 0.903 | 1.000 | 1.000 | 1.000 | 0.957 | 1.000 | 0.982 | 0.854 | 1.000 | 1.000 | 0.858 | 0.830 |
| 2508 | 0.771 | 1.000 | 0.828 | 1.000 | 0.963 | 0.960 | 0.904 | 1.000 | 1.000 | 1.000 | 0.959 | 1.000 | 1.000 | 0.847 | 1.000 | 1.000 | 0.858 | 0.830 |
| 2509 | 0.767 | 1.000 | 0.822 | 1.000 | 0.968 | 0.960 | 0.901 | 1.000 | 1.000 | 1.000 | 0.962 | 1.000 | 1.000 | 0.856 | 1.000 | 1.000 | 0.858 | 0.830 |
| 2510 | 0.774 | 1.000 | 0.820 | 1.000 | 0.968 | 0.960 | 0.911 | 1.000 | 1.000 | 1.000 | 0.965 | 1.000 | 0.982 | 0.852 | 1.000 | 1.000 | 0.860 | 0.833 |
| 2511 | 0.768 | 1.000 | 0.814 | 1.000 | 0.968 | 0.960 | 0.909 | 1.000 | 1.000 | 1.000 | 0.951 | 1.000 | 1.000 | 0.853 | 1.000 | 1.000 | 0.857 | 0.829 |
| 2512 | 0.763 | 1.000 | 0.806 | 1.000 | 0.968 | 0.960 | 0.904 | 1.000 | 1.000 | 1.000 | 0.957 | 1.000 | 0.991 | 0.856 | 1.000 | 1.000 | 0.856 | 0.827 |
| 2513 | 0.775 | 1.000 | 0.828 | 1.000 | 0.963 | 0.960 | 0.911 | 1.000 | 1.000 | 1.000 | 0.951 | 1.000 | 1.000 | 0.852 | 1.000 | 1.000 | 0.861 | 0.833 |
| 2514 | 0.778 | 1.000 | 0.826 | 1.000 | 0.968 | 0.960 | 0.917 | 1.000 | 1.000 | 1.000 | 0.968 | 1.000 | 0.991 | 0.854 | 1.000 | 1.000 | 0.864 | 0.837 |
| 2515 | 0.767 | 1.000 | 0.814 | 0.986 | 0.968 | 0.960 | 0.897 | 1.000 | 1.000 | 1.000 | 0.968 | 0.970 | 1.000 | 0.845 | 1.000 | 1.000 | 0.854 | 0.826 |
| 2516 | 0.777 | 1.000 | 0.830 | 1.000 | 0.968 | 0.960 | 0.901 | 1.000 | 1.000 | 1.000 | 0.962 | 1.000 | 1.000 | 0.851 | 1.000 | 1.000 | 0.861 | 0.834 |
| 2517 | 0.773 | 1.000 | 0.846 | 1.000 | 0.968 | 0.960 | 0.903 | 1.000 | 1.000 | 1.000 | 0.957 | 1.000 | 0.991 | 0.846 | 1.000 | 1.000 | 0.860 | 0.832 |
| 2518 | 0.776 | 1.000 | 0.814 | 1.000 | 0.963 | 0.960 | 0.909 | 1.000 | 0.990 | 1.000 | 0.959 | 0.970 | 0.991 | 0.854 | 1.000 | 1.000 | 0.860 | 0.832 |
| 2519 | 0.775 | 1.000 | 0.838 | 1.000 | 0.968 | 0.960 | 0.900 | 1.000 | 1.000 | 1.000 | 0.959 | 1.000 | 1.000 | 0.851 | 1.000 | 1.000 | 0.861 | 0.833 |
| 2520 | 0.770 | 1.000 | 0.812 | 1.000 | 0.963 | 0.960 | 0.901 | 1.000 | 1.000 | 1.000 | 0.957 | 1.000 | 0.991 | 0.851 | 1.000 | 1.000 | 0.856 | 0.828 |
| 2521 | 0.770 | 1.000 | 0.826 | 1.000 | 0.963 | 0.960 | 0.904 | 1.000 | 1.000 | 1.000 | 0.957 | 0.970 | 0.991 | 0.847 | 1.000 | 1.000 | 0.857 | 0.829 |
| 2522 | 0.762 | 1.000 | 0.822 | 1.000 | 0.963 | 0.960 | 0.906 | 1.000 | 1.000 | 1.000 | 0.954 | 0.970 | 0.991 | 0.847 | 1.000 | 1.000 | 0.854 | 0.826 |
| 2523 | 0.771 | 1.000 | 0.814 | 1.000 | 0.968 | 0.960 | 0.903 | 1.000 | 1.000 | 1.000 | 0.968 | 1.000 | 1.000 | 0.849 | 1.000 | 1.000 | 0.858 | 0.830 |
| 2524 | 0.761 | 1.000 | 0.824 | 1.000 | 0.968 | 0.960 | 0.900 | 1.000 | 1.000 | 1.000 | 0.959 | 0.970 | 1.000 | 0.850 | 1.000 | 1.000 | 0.855 | 0.827 |
| 2525 | 0.774 | 1.000 | 0.828 | 1.000 | 0.968 | 0.960 | 0.904 | 1.000 | 1.000 | 1.000 | 0.962 | 0.970 | 1.000 | 0.850 | 1.000 | 1.000 | 0.860 | 0.832 |
| 2526 | 0.769 | 1.000 | 0.822 | 1.000 | 0.968 | 0.960 | 0.908 | 1.000 | 1.000 | 1.000 | 0.957 | 1.000 | 0.991 | 0.857 | 1.000 | 1.000 | 0.860 | 0.832 |
| 2527 | 0.769 | 1.000 | 0.826 | 1.000 | 0.968 | 0.960 | 0.897 | 1.000 | 1.000 | 1.000 | 0.959 | 0.970 | 1.000 | 0.851 | 1.000 | 1.000 | 0.857 | 0.829 |
| 2528 | 0.769 | 1.000 | 0.812 | 1.000 | 0.968 | 0.960 | 0.904 | 1.000 | 1.000 | 1.000 | 0.962 | 1.000 | 0.991 | 0.850 | 1.000 | 1.000 | 0.857 | 0.829 |
| 2529 | 0.775 | 1.000 | 0.816 | 1.000 | 0.968 | 0.960 | 0.901 | 1.000 | 1.000 | 1.000 | 0.954 | 0.970 | 1.000 | 0.847 | 1.000 | 1.000 | 0.857 | 0.829 |
| 2530 | 0.766 | 1.000 | 0.848 | 1.000 | 0.968 | 0.960 | 0.901 | 1.000 | 1.000 | 1.000 | 0.957 | 1.000 | 0.991 | 0.847 | 1.000 | 1.000 | 0.858 | 0.830 |
| 2531 | 0.771 | 1.000 | 0.832 | 1.000 | 0.963 | 0.960 | 0.911 | 1.000 | 1.000 | 1.000 | 0.959 | 0.970 | 1.000 | 0.847 | 1.000 | 1.000 | 0.859 | 0.832 |
| 2532 | 0.772 | 1.000 | 0.810 | 1.000 | 0.968 | 0.960 | 0.909 | 1.000 | 1.000 | 1.000 | 0.962 | 0.970 | 0.991 | 0.852 | 1.000 | 1.000 | 0.858 | 0.831 |
| 2533 | 0.768 | 1.000 | 0.820 | 1.000 | 0.968 | 0.960 | 0.904 | 1.000 | 1.000 | 1.000 | 0.954 | 0.970 | 0.973 | 0.843 | 1.000 | 1.000 | 0.854 | 0.826 |

| | | | | | | | | | | | | | | | | | |
|---|---|---|---|---|---|---|---|---|---|---|---|---|---|---|---|---|---|
| 2534 | 0.769 | 1.000 | 0.816 | 1.000 | 0.963 | 0.960 | 0.897 | 1.000 | 1.000 | 1.000 | 0.957 | 1.000 | 0.982 | 0.852 | 1.000 | 1.000 | 0.856 | 0.827 |
| 2535 | 0.777 | 1.000 | 0.812 | 1.000 | 0.974 | 0.960 | 0.904 | 1.000 | 1.000 | 1.000 | 0.959 | 1.000 | 1.000 | 0.854 | 1.000 | 1.000 | 0.860 | 0.833 |
| 2536 | 0.773 | 1.000 | 0.818 | 1.000 | 0.963 | 0.960 | 0.901 | 1.000 | 1.000 | 1.000 | 0.959 | 1.000 | 1.000 | 0.851 | 1.000 | 1.000 | 0.858 | 0.830 |
| 2537 | 0.757 | 1.000 | 0.816 | 1.000 | 0.968 | 0.960 | 0.909 | 1.000 | 1.000 | 1.000 | 0.957 | 0.970 | 1.000 | 0.847 | 1.000 | 1.000 | 0.853 | 0.825 |
| 2538 | 0.767 | 1.000 | 0.822 | 1.000 | 0.963 | 0.960 | 0.912 | 1.000 | 0.990 | 1.000 | 0.957 | 0.970 | 1.000 | 0.848 | 1.000 | 1.000 | 0.857 | 0.829 |
| 2539 | 0.771 | 1.000 | 0.830 | 1.000 | 0.968 | 0.960 | 0.912 | 1.000 | 1.000 | 1.000 | 0.957 | 1.000 | 1.000 | 0.848 | 1.000 | 1.000 | 0.859 | 0.832 |
| 2540 | 0.767 | 1.000 | 0.822 | 1.000 | 0.968 | 0.960 | 0.908 | 1.000 | 1.000 | 1.000 | 0.965 | 1.000 | 0.991 | 0.852 | 1.000 | 1.000 | 0.858 | 0.831 |
| 2541 | 0.771 | 1.000 | 0.826 | 1.000 | 0.974 | 0.960 | 0.909 | 1.000 | 1.000 | 1.000 | 0.957 | 0.970 | 0.991 | 0.858 | 1.000 | 1.000 | 0.861 | 0.834 |
| 2542 | 0.773 | 1.000 | 0.836 | 1.000 | 0.968 | 0.960 | 0.909 | 1.000 | 1.000 | 1.000 | 0.962 | 1.000 | 0.991 | 0.840 | 1.000 | 1.000 | 0.858 | 0.831 |
| 2543 | 0.770 | 1.000 | 0.828 | 1.000 | 0.963 | 0.960 | 0.895 | 1.000 | 1.000 | 1.000 | 0.959 | 0.970 | 1.000 | 0.858 | 1.000 | 1.000 | 0.859 | 0.831 |
| 2544 | 0.771 | 1.000 | 0.818 | 1.000 | 0.963 | 0.960 | 0.904 | 1.000 | 1.000 | 1.000 | 0.959 | 1.000 | 0.991 | 0.853 | 1.000 | 1.000 | 0.858 | 0.830 |
| 2545 | 0.763 | 1.000 | 0.820 | 1.000 | 0.968 | 0.960 | 0.900 | 1.000 | 1.000 | 1.000 | 0.959 | 0.970 | 1.000 | 0.851 | 1.000 | 1.000 | 0.855 | 0.827 |
| 2546 | 0.773 | 1.000 | 0.824 | 0.986 | 0.958 | 0.960 | 0.903 | 1.000 | 1.000 | 1.000 | 0.957 | 1.000 | 1.000 | 0.853 | 1.000 | 1.000 | 0.859 | 0.831 |
| 2547 | 0.774 | 1.000 | 0.834 | 1.000 | 0.968 | 0.960 | 0.908 | 1.000 | 0.990 | 1.000 | 0.957 | 1.000 | 0.991 | 0.844 | 1.000 | 1.000 | 0.859 | 0.831 |
| 2548 | 0.772 | 1.000 | 0.826 | 1.000 | 0.968 | 0.960 | 0.903 | 1.000 | 1.000 | 1.000 | 0.959 | 0.970 | 0.991 | 0.850 | 1.000 | 1.000 | 0.858 | 0.831 |
| 2549 | 0.772 | 1.000 | 0.832 | 1.000 | 0.968 | 0.960 | 0.904 | 1.000 | 1.000 | 1.000 | 0.957 | 0.970 | 0.991 | 0.848 | 1.000 | 1.000 | 0.859 | 0.831 |
| 2550 | 0.774 | 1.000 | 0.830 | 1.000 | 0.979 | 0.960 | 0.912 | 1.000 | 1.000 | 1.000 | 0.959 | 0.970 | 0.991 | 0.845 | 1.000 | 1.000 | 0.860 | 0.833 |
| 2551 | 0.773 | 1.000 | 0.814 | 1.000 | 0.968 | 0.960 | 0.903 | 1.000 | 1.000 | 1.000 | 0.959 | 1.000 | 0.991 | 0.854 | 1.000 | 1.000 | 0.859 | 0.831 |
| 2552 | 0.765 | 1.000 | 0.828 | 0.986 | 0.963 | 0.960 | 0.897 | 1.000 | 1.000 | 1.000 | 0.965 | 1.000 | 0.991 | 0.852 | 1.000 | 1.000 | 0.857 | 0.829 |
| 2553 | 0.766 | 1.000 | 0.826 | 1.000 | 0.974 | 0.960 | 0.904 | 1.000 | 0.990 | 1.000 | 0.962 | 1.000 | 1.000 | 0.849 | 1.000 | 1.000 | 0.857 | 0.829 |
| 2554 | 0.769 | 1.000 | 0.828 | 1.000 | 0.968 | 0.960 | 0.906 | 1.000 | 1.000 | 1.000 | 0.962 | 0.970 | 1.000 | 0.849 | 1.000 | 1.000 | 0.858 | 0.831 |
| 2555 | 0.773 | 1.000 | 0.826 | 1.000 | 0.968 | 0.960 | 0.901 | 1.000 | 1.000 | 1.000 | 0.965 | 1.000 | 1.000 | 0.845 | 1.000 | 1.000 | 0.858 | 0.831 |
| 2556 | 0.767 | 1.000 | 0.822 | 1.000 | 0.968 | 0.960 | 0.901 | 1.000 | 0.990 | 1.000 | 0.948 | 1.000 | 1.000 | 0.846 | 1.000 | 1.000 | 0.855 | 0.826 |
| 2557 | 0.767 | 1.000 | 0.830 | 1.000 | 0.963 | 0.960 | 0.906 | 1.000 | 1.000 | 1.000 | 0.959 | 0.970 | 0.991 | 0.851 | 1.000 | 1.000 | 0.858 | 0.830 |
| 2558 | 0.773 | 1.000 | 0.824 | 1.000 | 0.968 | 0.960 | 0.904 | 1.000 | 1.000 | 1.000 | 0.959 | 1.000 | 1.000 | 0.847 | 1.000 | 1.000 | 0.858 | 0.830 |
| 2559 | 0.773 | 1.000 | 0.834 | 1.000 | 0.968 | 0.960 | 0.903 | 1.000 | 1.000 | 1.000 | 0.962 | 1.000 | 1.000 | 0.848 | 1.000 | 1.000 | 0.860 | 0.832 |
| 2560 | 0.774 | 1.000 | 0.822 | 1.000 | 0.963 | 0.960 | 0.900 | 1.000 | 1.000 | 1.000 | 0.957 | 0.970 | 1.000 | 0.858 | 1.000 | 1.000 | 0.860 | 0.833 |
| 2561 | 0.775 | 1.000 | 0.830 | 1.000 | 0.968 | 0.960 | 0.908 | 1.000 | 1.000 | 1.000 | 0.954 | 0.970 | 0.991 | 0.846 | 1.000 | 1.000 | 0.859 | 0.831 |
| 2562 | 0.771 | 1.000 | 0.824 | 1.000 | 0.958 | 0.960 | 0.894 | 1.000 | 1.000 | 1.000 | 0.959 | 1.000 | 0.991 | 0.851 | 1.000 | 1.000 | 0.857 | 0.828 |
| 2563 | 0.765 | 1.000 | 0.804 | 1.000 | 0.968 | 0.960 | 0.904 | 1.000 | 1.000 | 1.000 | 0.957 | 0.970 | 0.991 | 0.853 | 1.000 | 1.000 | 0.855 | 0.826 |
| 2564 | 0.772 | 1.000 | 0.810 | 1.000 | 0.968 | 0.960 | 0.906 | 1.000 | 1.000 | 1.000 | 0.957 | 1.000 | 0.991 | 0.855 | 1.000 | 1.000 | 0.859 | 0.831 |
| 2565 | 0.773 | 1.000 | 0.824 | 1.000 | 0.963 | 0.960 | 0.909 | 1.000 | 1.000 | 1.000 | 0.954 | 0.970 | 0.991 | 0.855 | 1.000 | 1.000 | 0.860 | 0.833 |
| 2566 | 0.779 | 1.000 | 0.816 | 1.000 | 0.968 | 0.960 | 0.909 | 1.000 | 1.000 | 1.000 | 0.959 | 1.000 | 1.000 | 0.849 | 1.000 | 1.000 | 0.861 | 0.833 |
| 2567 | 0.757 | 1.000 | 0.830 | 1.000 | 0.968 | 0.960 | 0.894 | 1.000 | 1.000 | 1.000 | 0.959 | 1.000 | 0.991 | 0.849 | 1.000 | 1.000 | 0.853 | 0.825 |
| 2568 | 0.771 | 1.000 | 0.818 | 1.000 | 0.968 | 0.960 | 0.906 | 1.000 | 1.000 | 1.000 | 0.954 | 1.000 | 0.991 | 0.853 | 1.000 | 1.000 | 0.858 | 0.830 |
| 2569 | 0.778 | 1.000 | 0.820 | 1.000 | 0.974 | 0.960 | 0.897 | 1.000 | 1.000 | 1.000 | 0.954 | 1.000 | 0.991 | 0.847 | 1.000 | 1.000 | 0.858 | 0.830 |
| 2570 | 0.770 | 1.000 | 0.838 | 1.000 | 0.968 | 0.960 | 0.898 | 1.000 | 1.000 | 1.000 | 0.965 | 1.000 | 1.000 | 0.861 | 1.000 | 1.000 | 0.862 | 0.835 |
| 2571 | 0.770 | 1.000 | 0.822 | 1.000 | 0.968 | 0.960 | 0.904 | 1.000 | 0.990 | 1.000 | 0.951 | 0.970 | 1.000 | 0.862 | 1.000 | 1.000 | 0.860 | 0.833 |
| 2572 | 0.765 | 1.000 | 0.830 | 1.000 | 0.963 | 0.960 | 0.920 | 1.000 | 1.000 | 1.000 | 0.962 | 0.970 | 0.991 | 0.851 | 1.000 | 1.000 | 0.859 | 0.832 |

| | | | | | | | | | | | | | | | | | |
|---|---|---|---|---|---|---|---|---|---|---|---|---|---|---|---|---|---|
| 2573 | 0.775 | 1.000 | 0.834 | 1.000 | 0.968 | 0.960 | 0.894 | 1.000 | 1.000 | 1.000 | 0.962 | 1.000 | 1.000 | 0.849 | 1.000 | 1.000 | 0.859 | 0.831 |
| 2574 | 0.774 | 1.000 | 0.826 | 1.000 | 0.968 | 0.960 | 0.898 | 1.000 | 1.000 | 1.000 | 0.957 | 1.000 | 0.991 | 0.851 | 1.000 | 1.000 | 0.859 | 0.831 |
| 2575 | 0.779 | 1.000 | 0.842 | 1.000 | 0.974 | 0.960 | 0.901 | 1.000 | 1.000 | 1.000 | 0.948 | 0.970 | 1.000 | 0.849 | 1.000 | 1.000 | 0.861 | 0.834 |
| 2576 | 0.768 | 1.000 | 0.822 | 1.000 | 0.968 | 0.960 | 0.904 | 1.000 | 1.000 | 1.000 | 0.959 | 0.970 | 0.991 | 0.847 | 1.000 | 1.000 | 0.856 | 0.828 |
| 2577 | 0.769 | 1.000 | 0.832 | 1.000 | 0.974 | 0.960 | 0.904 | 1.000 | 1.000 | 1.000 | 0.957 | 0.970 | 0.991 | 0.845 | 1.000 | 1.000 | 0.857 | 0.829 |
| 2578 | 0.773 | 1.000 | 0.824 | 1.000 | 0.968 | 0.960 | 0.897 | 1.000 | 1.000 | 1.000 | 0.957 | 1.000 | 0.991 | 0.850 | 1.000 | 1.000 | 0.858 | 0.829 |
| 2579 | 0.771 | 1.000 | 0.818 | 1.000 | 0.968 | 0.960 | 0.914 | 1.000 | 1.000 | 1.000 | 0.959 | 0.970 | 1.000 | 0.851 | 1.000 | 1.000 | 0.859 | 0.832 |
| 2580 | 0.769 | 1.000 | 0.832 | 1.000 | 0.963 | 0.960 | 0.898 | 1.000 | 1.000 | 1.000 | 0.959 | 0.970 | 0.982 | 0.849 | 1.000 | 1.000 | 0.857 | 0.829 |
| 2581 | 0.773 | 1.000 | 0.828 | 1.000 | 0.968 | 0.960 | 0.900 | 1.000 | 1.000 | 1.000 | 0.962 | 1.000 | 0.991 | 0.854 | 1.000 | 1.000 | 0.860 | 0.833 |
| 2582 | 0.767 | 1.000 | 0.834 | 1.000 | 0.968 | 0.960 | 0.903 | 1.000 | 1.000 | 1.000 | 0.959 | 0.970 | 0.991 | 0.851 | 1.000 | 1.000 | 0.858 | 0.830 |
| 2583 | 0.772 | 1.000 | 0.836 | 1.000 | 0.974 | 0.960 | 0.900 | 1.000 | 1.000 | 1.000 | 0.957 | 0.970 | 0.991 | 0.855 | 1.000 | 1.000 | 0.860 | 0.833 |
| 2584 | 0.775 | 1.000 | 0.830 | 1.000 | 0.968 | 0.960 | 0.903 | 1.000 | 1.000 | 1.000 | 0.957 | 1.000 | 0.991 | 0.852 | 1.000 | 1.000 | 0.860 | 0.833 |
| 2585 | 0.761 | 1.000 | 0.836 | 1.000 | 0.968 | 0.960 | 0.908 | 1.000 | 1.000 | 1.000 | 0.951 | 1.000 | 1.000 | 0.855 | 1.000 | 1.000 | 0.858 | 0.831 |
| 2586 | 0.767 | 1.000 | 0.814 | 1.000 | 0.963 | 0.960 | 0.894 | 1.000 | 1.000 | 1.000 | 0.959 | 1.000 | 1.000 | 0.855 | 1.000 | 1.000 | 0.856 | 0.828 |
| 2587 | 0.772 | 1.000 | 0.824 | 1.000 | 0.968 | 0.960 | 0.904 | 1.000 | 1.000 | 1.000 | 0.959 | 1.000 | 0.991 | 0.855 | 1.000 | 1.000 | 0.860 | 0.832 |
| 2588 | 0.765 | 1.000 | 0.832 | 1.000 | 0.968 | 0.960 | 0.900 | 1.000 | 1.000 | 1.000 | 0.959 | 1.000 | 1.000 | 0.854 | 1.000 | 1.000 | 0.858 | 0.831 |
| 2589 | 0.766 | 1.000 | 0.826 | 1.000 | 0.963 | 0.960 | 0.915 | 1.000 | 1.000 | 1.000 | 0.959 | 0.970 | 0.982 | 0.853 | 1.000 | 1.000 | 0.859 | 0.831 |
| 2590 | 0.775 | 1.000 | 0.814 | 1.000 | 0.974 | 0.960 | 0.898 | 1.000 | 1.000 | 1.000 | 0.957 | 0.970 | 0.991 | 0.854 | 1.000 | 1.000 | 0.859 | 0.831 |
| 2591 | 0.771 | 1.000 | 0.834 | 1.000 | 0.968 | 0.960 | 0.897 | 1.000 | 1.000 | 1.000 | 0.965 | 1.000 | 1.000 | 0.851 | 1.000 | 1.000 | 0.859 | 0.832 |
| 2592 | 0.765 | 1.000 | 0.826 | 1.000 | 0.968 | 0.960 | 0.904 | 1.000 | 1.000 | 1.000 | 0.959 | 0.970 | 0.991 | 0.854 | 1.000 | 1.000 | 0.857 | 0.830 |
| 2593 | 0.761 | 1.000 | 0.818 | 1.000 | 0.963 | 0.960 | 0.911 | 1.000 | 1.000 | 1.000 | 0.957 | 0.970 | 0.982 | 0.848 | 1.000 | 1.000 | 0.854 | 0.826 |
| 2594 | 0.769 | 1.000 | 0.840 | 1.000 | 0.974 | 0.960 | 0.898 | 1.000 | 1.000 | 1.000 | 0.959 | 0.970 | 0.991 | 0.843 | 1.000 | 1.000 | 0.857 | 0.829 |
| 2595 | 0.767 | 1.000 | 0.830 | 1.000 | 0.968 | 0.960 | 0.904 | 1.000 | 1.000 | 1.000 | 0.962 | 0.970 | 0.982 | 0.857 | 1.000 | 1.000 | 0.859 | 0.832 |
| 2596 | 0.767 | 1.000 | 0.842 | 1.000 | 0.974 | 0.960 | 0.912 | 1.000 | 1.000 | 1.000 | 0.959 | 1.000 | 1.000 | 0.847 | 1.000 | 1.000 | 0.860 | 0.833 |
| 2597 | 0.774 | 1.000 | 0.840 | 0.986 | 0.968 | 0.960 | 0.908 | 1.000 | 1.000 | 1.000 | 0.959 | 0.970 | 0.991 | 0.854 | 1.000 | 1.000 | 0.862 | 0.835 |
| 2598 | 0.778 | 1.000 | 0.828 | 1.000 | 0.968 | 0.960 | 0.903 | 1.000 | 1.000 | 1.000 | 0.968 | 0.970 | 1.000 | 0.849 | 1.000 | 1.000 | 0.861 | 0.834 |
| 2599 | 0.777 | 1.000 | 0.836 | 1.000 | 0.968 | 0.960 | 0.911 | 1.000 | 1.000 | 1.000 | 0.959 | 0.970 | 1.000 | 0.853 | 1.000 | 1.000 | 0.863 | 0.836 |
| 2600 | 0.771 | 1.000 | 0.832 | 1.000 | 0.968 | 0.960 | 0.895 | 1.000 | 1.000 | 1.000 | 0.962 | 0.970 | 1.000 | 0.855 | 1.000 | 1.000 | 0.859 | 0.832 |
| 2601 | 0.768 | 1.000 | 0.832 | 1.000 | 0.974 | 0.960 | 0.901 | 1.000 | 1.000 | 1.000 | 0.957 | 1.000 | 0.991 | 0.849 | 1.000 | 1.000 | 0.858 | 0.830 |
| 2602 | 0.775 | 1.000 | 0.840 | 1.000 | 0.968 | 0.960 | 0.909 | 1.000 | 1.000 | 1.000 | 0.959 | 0.970 | 1.000 | 0.849 | 1.000 | 1.000 | 0.862 | 0.834 |
| 2603 | 0.765 | 1.000 | 0.832 | 1.000 | 0.968 | 0.960 | 0.898 | 1.000 | 1.000 | 1.000 | 0.957 | 0.939 | 0.991 | 0.851 | 1.000 | 1.000 | 0.856 | 0.828 |
| 2604 | 0.777 | 1.000 | 0.840 | 0.986 | 0.968 | 0.960 | 0.900 | 1.000 | 1.000 | 1.000 | 0.962 | 1.000 | 0.991 | 0.847 | 1.000 | 1.000 | 0.860 | 0.833 |
| 2605 | 0.764 | 1.000 | 0.830 | 1.000 | 0.968 | 0.960 | 0.903 | 1.000 | 1.000 | 1.000 | 0.959 | 1.000 | 1.000 | 0.849 | 1.000 | 1.000 | 0.857 | 0.829 |
| 2606 | 0.767 | 1.000 | 0.828 | 1.000 | 0.968 | 0.960 | 0.904 | 1.000 | 1.000 | 1.000 | 0.962 | 0.939 | 1.000 | 0.854 | 1.000 | 1.000 | 0.858 | 0.831 |
| 2607 | 0.773 | 1.000 | 0.840 | 1.000 | 0.974 | 0.960 | 0.914 | 1.000 | 1.000 | 1.000 | 0.959 | 0.970 | 0.982 | 0.845 | 1.000 | 1.000 | 0.860 | 0.833 |
| 2608 | 0.779 | 1.000 | 0.828 | 1.000 | 0.968 | 0.960 | 0.909 | 1.000 | 1.000 | 1.000 | 0.954 | 1.000 | 0.991 | 0.853 | 1.000 | 1.000 | 0.862 | 0.835 |
| 2609 | 0.772 | 1.000 | 0.832 | 1.000 | 0.968 | 0.960 | 0.897 | 1.000 | 1.000 | 1.000 | 0.959 | 0.939 | 1.000 | 0.853 | 1.000 | 1.000 | 0.859 | 0.831 |
| 2610 | 0.766 | 1.000 | 0.836 | 0.986 | 0.968 | 0.960 | 0.904 | 1.000 | 1.000 | 1.000 | 0.948 | 0.970 | 0.991 | 0.851 | 1.000 | 1.000 | 0.857 | 0.829 |
| 2611 | 0.777 | 1.000 | 0.826 | 1.000 | 0.963 | 0.960 | 0.904 | 1.000 | 1.000 | 1.000 | 0.957 | 1.000 | 1.000 | 0.847 | 1.000 | 1.000 | 0.860 | 0.832 |

| | | | | | | | | | | | | | | | | | | |
|---|---|---|---|---|---|---|---|---|---|---|---|---|---|---|---|---|---|---|
| 2612 | 0.772 | 1.000 | 0.838 | 1.000 | 0.968 | 0.960 | 0.904 | 1.000 | 1.000 | 1.000 | 0.948 | 1.000 | 1.000 | 0.849 | 1.000 | 1.000 | 0.859 | 0.832 |
| 2613 | 0.777 | 1.000 | 0.838 | 1.000 | 0.968 | 0.960 | 0.894 | 1.000 | 1.000 | 1.000 | 0.962 | 1.000 | 0.991 | 0.849 | 1.000 | 1.000 | 0.860 | 0.833 |
| 2614 | 0.763 | 1.000 | 0.848 | 1.000 | 0.968 | 0.960 | 0.894 | 1.000 | 1.000 | 1.000 | 0.948 | 1.000 | 1.000 | 0.855 | 1.000 | 1.000 | 0.858 | 0.830 |
| 2615 | 0.763 | 1.000 | 0.830 | 1.000 | 0.968 | 0.960 | 0.901 | 1.000 | 1.000 | 1.000 | 0.957 | 1.000 | 1.000 | 0.852 | 1.000 | 1.000 | 0.857 | 0.829 |
| 2616 | 0.764 | 1.000 | 0.836 | 1.000 | 0.968 | 0.960 | 0.911 | 1.000 | 1.000 | 1.000 | 0.957 | 1.000 | 0.991 | 0.853 | 1.000 | 1.000 | 0.859 | 0.832 |
| 2617 | 0.777 | 1.000 | 0.840 | 1.000 | 0.963 | 0.960 | 0.904 | 1.000 | 1.000 | 1.000 | 0.951 | 0.970 | 1.000 | 0.846 | 1.000 | 1.000 | 0.860 | 0.832 |
| 2618 | 0.772 | 1.000 | 0.822 | 1.000 | 0.963 | 0.960 | 0.908 | 1.000 | 1.000 | 1.000 | 0.957 | 1.000 | 0.982 | 0.852 | 1.000 | 1.000 | 0.859 | 0.831 |
| 2619 | 0.772 | 1.000 | 0.808 | 1.000 | 0.968 | 0.960 | 0.906 | 1.000 | 1.000 | 1.000 | 0.954 | 0.970 | 1.000 | 0.847 | 1.000 | 1.000 | 0.856 | 0.828 |
| 2620 | 0.775 | 1.000 | 0.836 | 1.000 | 0.968 | 0.960 | 0.894 | 1.000 | 1.000 | 1.000 | 0.957 | 0.939 | 1.000 | 0.852 | 1.000 | 1.000 | 0.860 | 0.832 |
| 2621 | 0.770 | 1.000 | 0.820 | 1.000 | 0.968 | 0.960 | 0.895 | 1.000 | 1.000 | 1.000 | 0.954 | 0.970 | 0.991 | 0.850 | 1.000 | 1.000 | 0.856 | 0.827 |
| 2622 | 0.769 | 1.000 | 0.846 | 1.000 | 0.963 | 0.960 | 0.903 | 1.000 | 1.000 | 1.000 | 0.959 | 0.970 | 1.000 | 0.850 | 1.000 | 1.000 | 0.860 | 0.832 |
| 2623 | 0.773 | 1.000 | 0.830 | 1.000 | 0.963 | 0.960 | 0.901 | 1.000 | 1.000 | 1.000 | 0.957 | 1.000 | 1.000 | 0.854 | 1.000 | 1.000 | 0.860 | 0.833 |
| 2624 | 0.777 | 1.000 | 0.838 | 1.000 | 0.963 | 0.960 | 0.912 | 1.000 | 1.000 | 1.000 | 0.954 | 1.000 | 1.000 | 0.858 | 1.000 | 1.000 | 0.864 | 0.837 |
| 2625 | 0.771 | 1.000 | 0.834 | 1.000 | 0.963 | 0.960 | 0.906 | 1.000 | 1.000 | 1.000 | 0.965 | 1.000 | 0.991 | 0.852 | 1.000 | 1.000 | 0.860 | 0.833 |
| 2626 | 0.773 | 1.000 | 0.834 | 0.986 | 0.968 | 0.960 | 0.906 | 1.000 | 1.000 | 1.000 | 0.962 | 1.000 | 0.991 | 0.852 | 1.000 | 1.000 | 0.861 | 0.833 |
| 2627 | 0.769 | 1.000 | 0.816 | 1.000 | 0.968 | 0.960 | 0.897 | 1.000 | 1.000 | 1.000 | 0.951 | 1.000 | 0.991 | 0.854 | 1.000 | 1.000 | 0.856 | 0.828 |
| 2628 | 0.777 | 1.000 | 0.840 | 1.000 | 0.968 | 0.960 | 0.891 | 1.000 | 1.000 | 1.000 | 0.951 | 0.970 | 1.000 | 0.850 | 1.000 | 1.000 | 0.859 | 0.832 |
| 2629 | 0.769 | 1.000 | 0.828 | 0.986 | 0.974 | 0.960 | 0.900 | 1.000 | 1.000 | 1.000 | 0.945 | 1.000 | 0.991 | 0.853 | 1.000 | 1.000 | 0.857 | 0.829 |
| 2630 | 0.767 | 1.000 | 0.820 | 1.000 | 0.968 | 0.960 | 0.906 | 1.000 | 1.000 | 1.000 | 0.962 | 0.970 | 0.991 | 0.847 | 1.000 | 1.000 | 0.856 | 0.828 |
| 2631 | 0.763 | 1.000 | 0.826 | 1.000 | 0.968 | 1.000 | 0.908 | 1.000 | 1.000 | 1.000 | 0.951 | 1.000 | 0.991 | 0.850 | 1.000 | 1.000 | 0.856 | 0.828 |
| 2632 | 0.777 | 1.000 | 0.820 | 1.000 | 0.968 | 0.960 | 0.903 | 1.000 | 1.000 | 1.000 | 0.954 | 1.000 | 1.000 | 0.850 | 1.000 | 1.000 | 0.859 | 0.831 |
| 2633 | 0.772 | 1.000 | 0.828 | 1.000 | 0.968 | 0.960 | 0.904 | 1.000 | 1.000 | 1.000 | 0.959 | 1.000 | 0.991 | 0.854 | 1.000 | 1.000 | 0.860 | 0.833 |
| 2634 | 0.769 | 1.000 | 0.826 | 0.986 | 0.968 | 0.960 | 0.904 | 1.000 | 1.000 | 1.000 | 0.954 | 1.000 | 1.000 | 0.850 | 1.000 | 1.000 | 0.858 | 0.830 |
| 2635 | 0.771 | 1.000 | 0.830 | 1.000 | 0.968 | 0.960 | 0.900 | 1.000 | 1.000 | 1.000 | 0.957 | 1.000 | 1.000 | 0.852 | 1.000 | 1.000 | 0.859 | 0.831 |
| 2636 | 0.773 | 1.000 | 0.826 | 1.000 | 0.968 | 0.960 | 0.901 | 1.000 | 1.000 | 1.000 | 0.962 | 1.000 | 1.000 | 0.847 | 1.000 | 1.000 | 0.859 | 0.831 |
| 2637 | 0.777 | 1.000 | 0.830 | 1.000 | 0.974 | 0.960 | 0.898 | 1.000 | 1.000 | 1.000 | 0.957 | 0.970 | 0.991 | 0.851 | 1.000 | 1.000 | 0.860 | 0.832 |
| 2638 | 0.774 | 1.000 | 0.832 | 0.986 | 0.968 | 0.960 | 0.906 | 1.000 | 1.000 | 1.000 | 0.957 | 1.000 | 0.991 | 0.854 | 1.000 | 1.000 | 0.861 | 0.834 |
| 2639 | 0.772 | 1.000 | 0.830 | 1.000 | 0.963 | 0.960 | 0.909 | 1.000 | 1.000 | 1.000 | 0.962 | 1.000 | 1.000 | 0.851 | 1.000 | 1.000 | 0.860 | 0.833 |
| 2640 | 0.769 | 1.000 | 0.830 | 0.986 | 0.974 | 0.960 | 0.911 | 1.000 | 1.000 | 1.000 | 0.948 | 0.970 | 1.000 | 0.853 | 1.000 | 1.000 | 0.859 | 0.832 |
| 2641 | 0.776 | 1.000 | 0.830 | 1.000 | 0.958 | 0.960 | 0.908 | 1.000 | 1.000 | 1.000 | 0.962 | 1.000 | 1.000 | 0.854 | 1.000 | 1.000 | 0.862 | 0.835 |
| 2642 | 0.773 | 1.000 | 0.828 | 1.000 | 0.968 | 0.960 | 0.909 | 1.000 | 1.000 | 1.000 | 0.959 | 0.970 | 1.000 | 0.850 | 1.000 | 1.000 | 0.860 | 0.832 |
| 2643 | 0.775 | 1.000 | 0.830 | 1.000 | 0.968 | 0.960 | 0.894 | 1.000 | 1.000 | 1.000 | 0.945 | 0.970 | 1.000 | 0.854 | 1.000 | 1.000 | 0.859 | 0.831 |
| 2644 | 0.773 | 1.000 | 0.820 | 0.986 | 0.958 | 0.960 | 0.909 | 1.000 | 1.000 | 1.000 | 0.954 | 1.000 | 1.000 | 0.859 | 1.000 | 1.000 | 0.861 | 0.834 |
| 2645 | 0.771 | 1.000 | 0.826 | 0.986 | 0.963 | 0.960 | 0.906 | 1.000 | 1.000 | 1.000 | 0.959 | 1.000 | 1.000 | 0.854 | 1.000 | 1.000 | 0.859 | 0.832 |
| 2646 | 0.769 | 1.000 | 0.828 | 1.000 | 0.968 | 0.960 | 0.900 | 1.000 | 1.000 | 1.000 | 0.954 | 1.000 | 1.000 | 0.851 | 1.000 | 1.000 | 0.857 | 0.830 |
| 2647 | 0.767 | 1.000 | 0.844 | 0.986 | 0.968 | 0.960 | 0.908 | 1.000 | 1.000 | 1.000 | 0.954 | 1.000 | 1.000 | 0.845 | 1.000 | 1.000 | 0.858 | 0.831 |
| 2648 | 0.767 | 1.000 | 0.828 | 1.000 | 0.963 | 0.960 | 0.906 | 1.000 | 1.000 | 1.000 | 0.957 | 1.000 | 0.991 | 0.857 | 1.000 | 1.000 | 0.859 | 0.831 |
| 2649 | 0.780 | 1.000 | 0.840 | 1.000 | 0.963 | 0.960 | 0.904 | 1.000 | 1.000 | 1.000 | 0.959 | 1.000 | 1.000 | 0.852 | 1.000 | 1.000 | 0.863 | 0.836 |
| 2650 | 0.766 | 1.000 | 0.830 | 1.000 | 0.968 | 0.960 | 0.903 | 1.000 | 1.000 | 1.000 | 0.954 | 0.970 | 1.000 | 0.848 | 1.000 | 1.000 | 0.856 | 0.828 |

| | | | | | | | | | | | | | | | | | | |
|---|---|---|---|---|---|---|---|---|---|---|---|---|---|---|---|---|---|---|
| 2651 | 0.769 | 1.000 | 0.822 | 1.000 | 0.968 | 0.960 | 0.911 | 1.000 | 1.000 | 1.000 | 0.957 | 1.000 | 1.000 | 0.852 | 1.000 | 1.000 | 0.859 | 0.831 |
| 2652 | 0.771 | 1.000 | 0.826 | 1.000 | 0.968 | 0.960 | 0.895 | 1.000 | 1.000 | 1.000 | 0.954 | 1.000 | 0.991 | 0.850 | 1.000 | 1.000 | 0.857 | 0.829 |
| 2653 | 0.773 | 1.000 | 0.826 | 0.986 | 0.968 | 0.960 | 0.911 | 1.000 | 1.000 | 1.000 | 0.959 | 1.000 | 0.991 | 0.845 | 1.000 | 1.000 | 0.859 | 0.831 |
| 2654 | 0.771 | 1.000 | 0.836 | 1.000 | 0.958 | 0.960 | 0.903 | 1.000 | 1.000 | 1.000 | 0.957 | 1.000 | 1.000 | 0.851 | 1.000 | 1.000 | 0.859 | 0.832 |
| 2655 | 0.772 | 1.000 | 0.828 | 0.986 | 0.974 | 0.960 | 0.906 | 1.000 | 1.000 | 1.000 | 0.965 | 1.000 | 1.000 | 0.854 | 1.000 | 1.000 | 0.861 | 0.834 |
| 2656 | 0.769 | 1.000 | 0.824 | 0.986 | 0.968 | 0.960 | 0.903 | 1.000 | 1.000 | 1.000 | 0.962 | 1.000 | 1.000 | 0.841 | 1.000 | 1.000 | 0.855 | 0.827 |
| 2657 | 0.767 | 1.000 | 0.836 | 1.000 | 0.974 | 0.960 | 0.909 | 1.000 | 1.000 | 1.000 | 0.957 | 1.000 | 1.000 | 0.841 | 1.000 | 1.000 | 0.857 | 0.829 |
| 2658 | 0.765 | 1.000 | 0.822 | 1.000 | 0.974 | 0.960 | 0.906 | 1.000 | 1.000 | 1.000 | 0.954 | 1.000 | 1.000 | 0.852 | 1.000 | 1.000 | 0.857 | 0.829 |
| 2659 | 0.763 | 1.000 | 0.832 | 1.000 | 0.968 | 0.960 | 0.904 | 1.000 | 1.000 | 1.000 | 0.962 | 0.970 | 0.991 | 0.844 | 1.000 | 1.000 | 0.855 | 0.827 |
| 2660 | 0.770 | 1.000 | 0.828 | 0.986 | 0.968 | 0.960 | 0.892 | 1.000 | 1.000 | 1.000 | 0.954 | 1.000 | 0.991 | 0.851 | 1.000 | 1.000 | 0.857 | 0.828 |
| 2661 | 0.773 | 1.000 | 0.832 | 0.986 | 0.968 | 0.960 | 0.904 | 1.000 | 1.000 | 1.000 | 0.959 | 0.970 | 0.991 | 0.852 | 1.000 | 1.000 | 0.860 | 0.832 |
| 2662 | 0.775 | 1.000 | 0.820 | 1.000 | 0.963 | 0.960 | 0.909 | 1.000 | 1.000 | 1.000 | 0.959 | 0.970 | 1.000 | 0.847 | 1.000 | 1.000 | 0.859 | 0.831 |
| 2663 | 0.774 | 1.000 | 0.836 | 1.000 | 0.974 | 0.960 | 0.906 | 1.000 | 1.000 | 1.000 | 0.954 | 0.970 | 1.000 | 0.858 | 1.000 | 1.000 | 0.862 | 0.835 |
| 2664 | 0.775 | 1.000 | 0.818 | 0.986 | 0.974 | 0.960 | 0.908 | 1.000 | 1.000 | 1.000 | 0.957 | 1.000 | 0.991 | 0.861 | 1.000 | 1.000 | 0.862 | 0.835 |
| 2665 | 0.779 | 1.000 | 0.828 | 1.000 | 0.968 | 0.960 | 0.897 | 1.000 | 1.000 | 1.000 | 0.965 | 1.000 | 1.000 | 0.852 | 1.000 | 1.000 | 0.861 | 0.834 |
| 2666 | 0.769 | 1.000 | 0.836 | 0.986 | 0.958 | 0.960 | 0.906 | 1.000 | 1.000 | 1.000 | 0.951 | 1.000 | 1.000 | 0.854 | 1.000 | 1.000 | 0.859 | 0.832 |
| 2667 | 0.771 | 1.000 | 0.834 | 1.000 | 0.968 | 0.960 | 0.901 | 1.000 | 1.000 | 1.000 | 0.962 | 1.000 | 0.991 | 0.855 | 1.000 | 1.000 | 0.861 | 0.833 |
| 2668 | 0.771 | 1.000 | 0.818 | 0.986 | 0.958 | 0.960 | 0.908 | 1.000 | 1.000 | 1.000 | 0.962 | 1.000 | 0.982 | 0.847 | 1.000 | 1.000 | 0.857 | 0.829 |
| 2669 | 0.781 | 1.000 | 0.818 | 0.986 | 0.963 | 0.960 | 0.909 | 1.000 | 1.000 | 1.000 | 0.954 | 0.970 | 0.991 | 0.846 | 1.000 | 1.000 | 0.859 | 0.832 |
| 2670 | 0.770 | 1.000 | 0.812 | 1.000 | 0.968 | 0.960 | 0.906 | 1.000 | 1.000 | 1.000 | 0.954 | 1.000 | 1.000 | 0.851 | 1.000 | 1.000 | 0.857 | 0.829 |
| 2671 | 0.773 | 1.000 | 0.832 | 1.000 | 0.963 | 0.960 | 0.912 | 1.000 | 1.000 | 1.000 | 0.948 | 0.970 | 0.982 | 0.858 | 1.000 | 1.000 | 0.861 | 0.834 |
| 2672 | 0.775 | 1.000 | 0.824 | 1.000 | 0.968 | 0.960 | 0.908 | 1.000 | 1.000 | 1.000 | 0.954 | 1.000 | 1.000 | 0.855 | 1.000 | 1.000 | 0.861 | 0.834 |
| 2673 | 0.766 | 1.000 | 0.832 | 0.986 | 0.963 | 0.960 | 0.897 | 1.000 | 1.000 | 1.000 | 0.968 | 1.000 | 1.000 | 0.854 | 1.000 | 1.000 | 0.858 | 0.830 |
| 2674 | 0.775 | 1.000 | 0.816 | 0.986 | 0.968 | 0.960 | 0.906 | 1.000 | 1.000 | 1.000 | 0.957 | 1.000 | 0.991 | 0.859 | 1.000 | 1.000 | 0.861 | 0.833 |
| 2675 | 0.780 | 1.000 | 0.838 | 1.000 | 0.968 | 0.960 | 0.911 | 1.000 | 1.000 | 1.000 | 0.951 | 1.000 | 1.000 | 0.858 | 1.000 | 1.000 | 0.865 | 0.838 |
| 2676 | 0.763 | 1.000 | 0.830 | 1.000 | 0.963 | 0.960 | 0.901 | 1.000 | 1.000 | 1.000 | 0.954 | 1.000 | 0.991 | 0.856 | 1.000 | 1.000 | 0.857 | 0.829 |
| 2677 | 0.773 | 1.000 | 0.802 | 1.000 | 0.968 | 0.960 | 0.904 | 1.000 | 1.000 | 1.000 | 0.968 | 1.000 | 1.000 | 0.851 | 1.000 | 1.000 | 0.858 | 0.830 |
| 2678 | 0.769 | 1.000 | 0.828 | 1.000 | 0.968 | 0.960 | 0.897 | 1.000 | 1.000 | 1.000 | 0.962 | 1.000 | 0.991 | 0.854 | 1.000 | 1.000 | 0.859 | 0.831 |
| 2679 | 0.777 | 1.000 | 0.824 | 1.000 | 0.963 | 0.960 | 0.909 | 1.000 | 1.000 | 1.000 | 0.957 | 0.970 | 1.000 | 0.849 | 1.000 | 1.000 | 0.860 | 0.833 |
| 2680 | 0.776 | 1.000 | 0.836 | 1.000 | 0.963 | 0.960 | 0.900 | 1.000 | 1.000 | 1.000 | 0.954 | 0.939 | 1.000 | 0.849 | 1.000 | 1.000 | 0.859 | 0.832 |
| 2681 | 0.772 | 1.000 | 0.832 | 1.000 | 0.968 | 0.960 | 0.909 | 1.000 | 1.000 | 1.000 | 0.965 | 0.970 | 0.991 | 0.851 | 1.000 | 1.000 | 0.860 | 0.833 |
| 2682 | 0.775 | 1.000 | 0.822 | 1.000 | 0.974 | 0.960 | 0.906 | 1.000 | 1.000 | 1.000 | 0.957 | 1.000 | 1.000 | 0.858 | 1.000 | 1.000 | 0.862 | 0.834 |
| 2683 | 0.767 | 1.000 | 0.830 | 1.000 | 0.968 | 0.960 | 0.900 | 1.000 | 1.000 | 1.000 | 0.954 | 1.000 | 1.000 | 0.856 | 1.000 | 1.000 | 0.859 | 0.831 |
| 2684 | 0.770 | 1.000 | 0.828 | 0.986 | 0.968 | 0.960 | 0.903 | 1.000 | 1.000 | 1.000 | 0.965 | 1.000 | 0.991 | 0.847 | 1.000 | 1.000 | 0.858 | 0.830 |
| 2685 | 0.773 | 1.000 | 0.826 | 1.000 | 0.963 | 0.960 | 0.894 | 1.000 | 1.000 | 1.000 | 0.959 | 1.000 | 1.000 | 0.853 | 1.000 | 1.000 | 0.859 | 0.831 |
| 2686 | 0.773 | 1.000 | 0.828 | 0.986 | 0.968 | 0.960 | 0.909 | 1.000 | 1.000 | 1.000 | 0.965 | 0.970 | 1.000 | 0.847 | 1.000 | 1.000 | 0.859 | 0.832 |
| 2687 | 0.768 | 1.000 | 0.834 | 0.986 | 0.958 | 0.960 | 0.909 | 1.000 | 0.990 | 1.000 | 0.959 | 1.000 | 1.000 | 0.851 | 1.000 | 1.000 | 0.859 | 0.831 |
| 2688 | 0.762 | 1.000 | 0.822 | 1.000 | 0.958 | 0.960 | 0.900 | 1.000 | 1.000 | 1.000 | 0.951 | 0.970 | 1.000 | 0.853 | 1.000 | 1.000 | 0.855 | 0.826 |
| 2689 | 0.769 | 1.000 | 0.830 | 1.000 | 0.963 | 0.960 | 0.908 | 1.000 | 1.000 | 1.000 | 0.954 | 1.000 | 1.000 | 0.849 | 1.000 | 1.000 | 0.858 | 0.831 |

| | | | | | | | | | | | | | | | | | |
|---|---|---|---|---|---|---|---|---|---|---|---|---|---|---|---|---|---|
| 2690 | 0.774 | 1.000 | 0.834 | 0.986 | 0.968 | 0.960 | 0.897 | 1.000 | 1.000 | 1.000 | 0.962 | 1.000 | 0.991 | 0.851 | 1.000 | 1.000 | 0.859 | 0.832 |
| 2691 | 0.778 | 1.000 | 0.832 | 0.986 | 0.974 | 0.960 | 0.906 | 1.000 | 1.000 | 1.000 | 0.965 | 0.970 | 0.991 | 0.854 | 1.000 | 1.000 | 0.863 | 0.836 |
| 2692 | 0.772 | 1.000 | 0.832 | 1.000 | 0.974 | 0.960 | 0.901 | 1.000 | 1.000 | 1.000 | 0.954 | 1.000 | 1.000 | 0.857 | 1.000 | 1.000 | 0.861 | 0.833 |
| 2693 | 0.770 | 1.000 | 0.816 | 0.986 | 0.963 | 0.960 | 0.901 | 1.000 | 1.000 | 1.000 | 0.965 | 1.000 | 0.982 | 0.851 | 1.000 | 1.000 | 0.857 | 0.829 |
| 2694 | 0.770 | 1.000 | 0.820 | 0.986 | 0.968 | 0.960 | 0.900 | 1.000 | 0.990 | 1.000 | 0.957 | 1.000 | 0.991 | 0.857 | 1.000 | 1.000 | 0.858 | 0.830 |
| 2695 | 0.773 | 1.000 | 0.814 | 1.000 | 0.968 | 0.960 | 0.900 | 1.000 | 0.990 | 1.000 | 0.954 | 1.000 | 0.991 | 0.858 | 1.000 | 1.000 | 0.859 | 0.831 |
| 2696 | 0.775 | 1.000 | 0.828 | 1.000 | 0.963 | 0.960 | 0.892 | 1.000 | 1.000 | 1.000 | 0.962 | 0.970 | 1.000 | 0.851 | 1.000 | 1.000 | 0.859 | 0.831 |
| 2697 | 0.767 | 1.000 | 0.822 | 1.000 | 0.963 | 0.960 | 0.895 | 1.000 | 1.000 | 1.000 | 0.957 | 0.939 | 0.991 | 0.855 | 1.000 | 1.000 | 0.856 | 0.828 |
| 2698 | 0.770 | 1.000 | 0.836 | 1.000 | 0.968 | 0.960 | 0.900 | 1.000 | 1.000 | 1.000 | 0.959 | 1.000 | 0.991 | 0.847 | 1.000 | 1.000 | 0.858 | 0.830 |
| 2699 | 0.766 | 1.000 | 0.836 | 0.986 | 0.968 | 0.960 | 0.898 | 1.000 | 1.000 | 1.000 | 0.957 | 0.970 | 0.991 | 0.844 | 1.000 | 1.000 | 0.855 | 0.827 |
| 2700 | 0.777 | 1.000 | 0.828 | 1.000 | 0.968 | 0.960 | 0.904 | 1.000 | 1.000 | 1.000 | 0.957 | 0.970 | 1.000 | 0.854 | 1.000 | 1.000 | 0.861 | 0.834 |
| 2701 | 0.773 | 1.000 | 0.832 | 1.000 | 0.963 | 0.960 | 0.898 | 1.000 | 1.000 | 1.000 | 0.959 | 1.000 | 1.000 | 0.851 | 1.000 | 1.000 | 0.859 | 0.832 |
| 2702 | 0.779 | 1.000 | 0.820 | 1.000 | 0.968 | 0.960 | 0.915 | 1.000 | 1.000 | 1.000 | 0.957 | 1.000 | 1.000 | 0.847 | 1.000 | 1.000 | 0.861 | 0.834 |
| 2703 | 0.771 | 1.000 | 0.844 | 0.986 | 0.968 | 0.960 | 0.906 | 1.000 | 1.000 | 1.000 | 0.965 | 1.000 | 0.991 | 0.850 | 1.000 | 1.000 | 0.861 | 0.834 |
| 2704 | 0.773 | 1.000 | 0.824 | 1.000 | 0.963 | 0.960 | 0.904 | 1.000 | 1.000 | 1.000 | 0.954 | 0.970 | 1.000 | 0.856 | 1.000 | 1.000 | 0.860 | 0.832 |
| 2705 | 0.771 | 1.000 | 0.824 | 1.000 | 0.963 | 0.960 | 0.903 | 1.000 | 1.000 | 1.000 | 0.962 | 1.000 | 0.991 | 0.851 | 1.000 | 1.000 | 0.858 | 0.830 |
| 2706 | 0.775 | 1.000 | 0.826 | 1.000 | 0.968 | 0.960 | 0.903 | 1.000 | 1.000 | 1.000 | 0.959 | 0.970 | 1.000 | 0.854 | 1.000 | 1.000 | 0.861 | 0.833 |
| 2707 | 0.764 | 1.000 | 0.828 | 1.000 | 0.968 | 0.960 | 0.904 | 1.000 | 1.000 | 1.000 | 0.954 | 1.000 | 0.991 | 0.851 | 1.000 | 1.000 | 0.856 | 0.829 |
| 2708 | 0.767 | 1.000 | 0.822 | 0.986 | 0.968 | 0.960 | 0.909 | 1.000 | 1.000 | 1.000 | 0.948 | 0.970 | 0.991 | 0.851 | 1.000 | 1.000 | 0.857 | 0.829 |
| 2709 | 0.771 | 1.000 | 0.820 | 1.000 | 0.968 | 0.960 | 0.904 | 1.000 | 1.000 | 1.000 | 0.954 | 0.970 | 1.000 | 0.844 | 1.000 | 1.000 | 0.856 | 0.827 |
| 2710 | 0.771 | 1.000 | 0.834 | 1.000 | 0.968 | 0.960 | 0.903 | 1.000 | 1.000 | 1.000 | 0.957 | 0.970 | 0.982 | 0.852 | 1.000 | 1.000 | 0.859 | 0.831 |
| 2711 | 0.773 | 1.000 | 0.836 | 1.000 | 0.974 | 0.960 | 0.891 | 1.000 | 1.000 | 1.000 | 0.957 | 0.970 | 0.991 | 0.847 | 1.000 | 1.000 | 0.858 | 0.830 |
| 2712 | 0.777 | 1.000 | 0.804 | 0.986 | 0.974 | 0.960 | 0.904 | 1.000 | 1.000 | 1.000 | 0.962 | 1.000 | 1.000 | 0.844 | 1.000 | 1.000 | 0.857 | 0.829 |
| 2713 | 0.769 | 1.000 | 0.832 | 1.000 | 0.968 | 0.960 | 0.904 | 1.000 | 1.000 | 1.000 | 0.948 | 1.000 | 0.991 | 0.844 | 1.000 | 1.000 | 0.856 | 0.828 |
| 2714 | 0.767 | 1.000 | 0.824 | 0.986 | 0.974 | 0.960 | 0.911 | 1.000 | 1.000 | 1.000 | 0.951 | 0.970 | 0.991 | 0.855 | 1.000 | 1.000 | 0.858 | 0.831 |
| 2715 | 0.762 | 1.000 | 0.816 | 1.000 | 0.968 | 0.960 | 0.900 | 1.000 | 1.000 | 1.000 | 0.959 | 0.970 | 0.991 | 0.848 | 1.000 | 1.000 | 0.854 | 0.825 |
| 2716 | 0.772 | 1.000 | 0.804 | 1.000 | 0.968 | 0.960 | 0.908 | 1.000 | 1.000 | 1.000 | 0.959 | 0.970 | 0.991 | 0.846 | 1.000 | 1.000 | 0.856 | 0.827 |
| 2717 | 0.774 | 1.000 | 0.822 | 1.000 | 0.968 | 0.960 | 0.911 | 1.000 | 1.000 | 1.000 | 0.965 | 0.970 | 0.991 | 0.853 | 1.000 | 1.000 | 0.861 | 0.833 |
| 2718 | 0.775 | 1.000 | 0.828 | 0.986 | 0.974 | 0.960 | 0.895 | 1.000 | 1.000 | 1.000 | 0.959 | 0.939 | 0.991 | 0.849 | 1.000 | 1.000 | 0.858 | 0.830 |
| 2719 | 0.771 | 1.000 | 0.838 | 0.986 | 0.963 | 0.960 | 0.903 | 1.000 | 1.000 | 1.000 | 0.954 | 0.970 | 0.991 | 0.854 | 1.000 | 1.000 | 0.859 | 0.832 |
| 2720 | 0.771 | 1.000 | 0.830 | 0.986 | 0.968 | 0.960 | 0.904 | 1.000 | 1.000 | 1.000 | 0.957 | 1.000 | 0.991 | 0.851 | 1.000 | 1.000 | 0.859 | 0.831 |
| 2721 | 0.775 | 1.000 | 0.838 | 1.000 | 0.968 | 0.960 | 0.903 | 1.000 | 1.000 | 1.000 | 0.954 | 1.000 | 1.000 | 0.854 | 1.000 | 1.000 | 0.862 | 0.835 |
| 2722 | 0.771 | 1.000 | 0.820 | 1.000 | 0.968 | 0.960 | 0.908 | 1.000 | 1.000 | 1.000 | 0.954 | 1.000 | 1.000 | 0.854 | 1.000 | 1.000 | 0.859 | 0.831 |
| 2723 | 0.779 | 1.000 | 0.816 | 1.000 | 0.963 | 0.960 | 0.908 | 1.000 | 1.000 | 1.000 | 0.954 | 0.939 | 1.000 | 0.855 | 1.000 | 1.000 | 0.861 | 0.833 |
| 2724 | 0.767 | 1.000 | 0.814 | 1.000 | 0.963 | 0.960 | 0.904 | 1.000 | 1.000 | 1.000 | 0.957 | 1.000 | 0.991 | 0.854 | 1.000 | 1.000 | 0.857 | 0.829 |
| 2725 | 0.770 | 1.000 | 0.832 | 1.000 | 0.963 | 0.960 | 0.895 | 1.000 | 1.000 | 1.000 | 0.954 | 1.000 | 0.982 | 0.847 | 1.000 | 1.000 | 0.856 | 0.828 |
| 2726 | 0.775 | 1.000 | 0.814 | 1.000 | 0.963 | 0.960 | 0.903 | 1.000 | 0.990 | 1.000 | 0.957 | 1.000 | 0.991 | 0.860 | 1.000 | 1.000 | 0.860 | 0.832 |
| 2727 | 0.765 | 1.000 | 0.824 | 1.000 | 0.968 | 0.960 | 0.901 | 1.000 | 0.990 | 1.000 | 0.951 | 0.970 | 1.000 | 0.858 | 1.000 | 1.000 | 0.857 | 0.829 |
| 2728 | 0.767 | 1.000 | 0.824 | 1.000 | 0.963 | 0.960 | 0.914 | 1.000 | 1.000 | 1.000 | 0.965 | 1.000 | 0.991 | 0.849 | 1.000 | 1.000 | 0.858 | 0.831 |

| | | | | | | | | | | | | | | | | | |
|---|---|---|---|---|---|---|---|---|---|---|---|---|---|---|---|---|---|
| 2729 | 0.767 | 1.000 | 0.824 | 1.000 | 0.968 | 0.960 | 0.901 | 1.000 | 1.000 | 1.000 | 0.959 | 0.970 | 0.973 | 0.847 | 1.000 | 1.000 | 0.856 | 0.827 |
| 2730 | 0.771 | 1.000 | 0.828 | 1.000 | 0.968 | 0.960 | 0.908 | 1.000 | 1.000 | 1.000 | 0.942 | 0.970 | 0.991 | 0.861 | 1.000 | 1.000 | 0.861 | 0.833 |
| 2731 | 0.761 | 1.000 | 0.808 | 1.000 | 0.968 | 0.960 | 0.906 | 1.000 | 1.000 | 1.000 | 0.945 | 0.939 | 0.991 | 0.853 | 1.000 | 1.000 | 0.853 | 0.825 |
| 2732 | 0.772 | 1.000 | 0.818 | 1.000 | 0.968 | 0.960 | 0.906 | 1.000 | 0.990 | 1.000 | 0.957 | 0.970 | 0.982 | 0.858 | 1.000 | 1.000 | 0.859 | 0.831 |
| 2733 | 0.775 | 1.000 | 0.818 | 1.000 | 0.968 | 0.960 | 0.908 | 1.000 | 0.990 | 1.000 | 0.945 | 0.970 | 1.000 | 0.858 | 1.000 | 1.000 | 0.860 | 0.833 |
| 2734 | 0.773 | 1.000 | 0.828 | 1.000 | 0.974 | 0.960 | 0.892 | 1.000 | 1.000 | 1.000 | 0.948 | 0.970 | 0.991 | 0.856 | 1.000 | 1.000 | 0.859 | 0.830 |
| 2735 | 0.771 | 1.000 | 0.812 | 1.000 | 0.963 | 0.960 | 0.903 | 1.000 | 0.990 | 1.000 | 0.948 | 1.000 | 0.991 | 0.854 | 1.000 | 1.000 | 0.857 | 0.829 |
| 2736 | 0.771 | 1.000 | 0.814 | 1.000 | 0.968 | 0.960 | 0.904 | 1.000 | 1.000 | 1.000 | 0.957 | 1.000 | 1.000 | 0.852 | 1.000 | 1.000 | 0.858 | 0.830 |
| 2737 | 0.761 | 1.000 | 0.794 | 1.000 | 0.963 | 0.960 | 0.906 | 1.000 | 1.000 | 1.000 | 0.962 | 0.970 | 0.982 | 0.853 | 1.000 | 1.000 | 0.853 | 0.824 |
| 2738 | 0.763 | 1.000 | 0.816 | 1.000 | 0.968 | 0.960 | 0.909 | 1.000 | 1.000 | 1.000 | 0.962 | 0.970 | 0.991 | 0.852 | 1.000 | 1.000 | 0.856 | 0.828 |
| 2739 | 0.765 | 1.000 | 0.814 | 1.000 | 0.968 | 0.960 | 0.915 | 1.000 | 1.000 | 1.000 | 0.959 | 0.970 | 0.991 | 0.850 | 1.000 | 1.000 | 0.857 | 0.829 |
| 2740 | 0.778 | 1.000 | 0.820 | 1.000 | 0.968 | 0.960 | 0.901 | 1.000 | 0.990 | 1.000 | 0.951 | 0.970 | 1.000 | 0.849 | 1.000 | 1.000 | 0.859 | 0.831 |
| 2741 | 0.761 | 1.000 | 0.812 | 1.000 | 0.968 | 0.960 | 0.898 | 1.000 | 0.990 | 1.000 | 0.951 | 0.970 | 1.000 | 0.854 | 1.000 | 1.000 | 0.854 | 0.825 |
| 2742 | 0.772 | 1.000 | 0.810 | 1.000 | 0.968 | 0.960 | 0.903 | 1.000 | 0.990 | 1.000 | 0.951 | 1.000 | 0.991 | 0.854 | 1.000 | 1.000 | 0.857 | 0.829 |
| 2743 | 0.771 | 1.000 | 0.820 | 1.000 | 0.968 | 0.960 | 0.898 | 1.000 | 0.990 | 1.000 | 0.957 | 1.000 | 1.000 | 0.861 | 1.000 | 1.000 | 0.860 | 0.832 |
| 2744 | 0.767 | 1.000 | 0.812 | 1.000 | 0.968 | 0.960 | 0.901 | 1.000 | 1.000 | 1.000 | 0.954 | 1.000 | 1.000 | 0.850 | 1.000 | 1.000 | 0.855 | 0.827 |
| 2745 | 0.759 | 1.000 | 0.806 | 1.000 | 0.958 | 0.960 | 0.903 | 1.000 | 1.000 | 1.000 | 0.957 | 0.970 | 1.000 | 0.851 | 1.000 | 1.000 | 0.852 | 0.823 |
| 2746 | 0.763 | 1.000 | 0.820 | 1.000 | 0.968 | 0.960 | 0.906 | 1.000 | 1.000 | 1.000 | 0.954 | 1.000 | 0.973 | 0.852 | 1.000 | 1.000 | 0.856 | 0.828 |
| 2747 | 0.769 | 1.000 | 0.828 | 1.000 | 0.958 | 0.960 | 0.906 | 1.000 | 1.000 | 1.000 | 0.945 | 0.970 | 0.982 | 0.851 | 1.000 | 1.000 | 0.857 | 0.829 |
| 2748 | 0.772 | 1.000 | 0.828 | 1.000 | 0.968 | 0.960 | 0.901 | 1.000 | 0.990 | 1.000 | 0.948 | 0.970 | 0.991 | 0.844 | 1.000 | 1.000 | 0.856 | 0.828 |
| 2749 | 0.771 | 1.000 | 0.820 | 1.000 | 0.968 | 0.960 | 0.904 | 1.000 | 1.000 | 1.000 | 0.951 | 0.970 | 1.000 | 0.857 | 1.000 | 1.000 | 0.859 | 0.832 |
| 2750 | 0.776 | 1.000 | 0.808 | 1.000 | 0.963 | 0.960 | 0.909 | 1.000 | 0.990 | 1.000 | 0.957 | 0.970 | 0.982 | 0.847 | 1.000 | 1.000 | 0.857 | 0.829 |
| 2751 | 0.764 | 1.000 | 0.814 | 0.986 | 0.968 | 0.960 | 0.915 | 1.000 | 0.990 | 1.000 | 0.945 | 0.970 | 0.991 | 0.857 | 1.000 | 1.000 | 0.857 | 0.829 |
| 2752 | 0.761 | 1.000 | 0.812 | 1.000 | 0.963 | 0.960 | 0.912 | 1.000 | 0.990 | 1.000 | 0.954 | 0.970 | 0.982 | 0.850 | 1.000 | 1.000 | 0.854 | 0.825 |
| 2753 | 0.764 | 1.000 | 0.822 | 1.000 | 0.968 | 0.960 | 0.914 | 1.000 | 1.000 | 1.000 | 0.945 | 0.970 | 0.991 | 0.862 | 1.000 | 1.000 | 0.859 | 0.832 |
| 2754 | 0.767 | 1.000 | 0.816 | 1.000 | 0.968 | 0.960 | 0.903 | 1.000 | 0.990 | 1.000 | 0.951 | 0.970 | 1.000 | 0.860 | 1.000 | 1.000 | 0.858 | 0.830 |
| 2755 | 0.763 | 1.000 | 0.812 | 1.000 | 0.963 | 0.960 | 0.898 | 1.000 | 0.990 | 1.000 | 0.959 | 0.970 | 0.991 | 0.850 | 1.000 | 1.000 | 0.853 | 0.824 |
| 2756 | 0.775 | 1.000 | 0.806 | 1.000 | 0.968 | 0.960 | 0.901 | 1.000 | 1.000 | 1.000 | 0.962 | 0.970 | 0.991 | 0.853 | 1.000 | 1.000 | 0.858 | 0.830 |
| 2757 | 0.772 | 1.000 | 0.806 | 0.986 | 0.963 | 0.960 | 0.897 | 1.000 | 0.990 | 1.000 | 0.948 | 0.939 | 0.991 | 0.856 | 1.000 | 1.000 | 0.856 | 0.827 |
| 2758 | 0.763 | 1.000 | 0.820 | 1.000 | 0.968 | 0.960 | 0.906 | 1.000 | 0.990 | 1.000 | 0.951 | 1.000 | 0.982 | 0.856 | 1.000 | 1.000 | 0.856 | 0.828 |
| 2759 | 0.773 | 1.000 | 0.806 | 0.986 | 0.968 | 0.960 | 0.912 | 1.000 | 1.000 | 1.000 | 0.957 | 1.000 | 0.991 | 0.851 | 1.000 | 1.000 | 0.858 | 0.830 |
| 2760 | 0.768 | 1.000 | 0.810 | 1.000 | 0.968 | 0.960 | 0.898 | 1.000 | 1.000 | 1.000 | 0.954 | 0.970 | 0.991 | 0.856 | 1.000 | 1.000 | 0.856 | 0.828 |
| 2761 | 0.773 | 1.000 | 0.810 | 0.986 | 0.974 | 0.960 | 0.900 | 1.000 | 1.000 | 1.000 | 0.951 | 1.000 | 0.991 | 0.854 | 1.000 | 1.000 | 0.857 | 0.829 |
| 2762 | 0.769 | 1.000 | 0.824 | 0.986 | 0.968 | 0.960 | 0.911 | 1.000 | 1.000 | 1.000 | 0.959 | 0.970 | 1.000 | 0.853 | 1.000 | 1.000 | 0.859 | 0.832 |
| 2763 | 0.773 | 1.000 | 0.832 | 1.000 | 0.963 | 0.960 | 0.908 | 1.000 | 1.000 | 1.000 | 0.959 | 0.970 | 0.991 | 0.860 | 1.000 | 1.000 | 0.862 | 0.835 |
| 2764 | 0.773 | 1.000 | 0.816 | 1.000 | 0.968 | 0.960 | 0.903 | 1.000 | 0.990 | 1.000 | 0.962 | 1.000 | 0.991 | 0.859 | 1.000 | 1.000 | 0.860 | 0.833 |
| 2765 | 0.766 | 1.000 | 0.806 | 1.000 | 0.963 | 0.960 | 0.901 | 1.000 | 1.000 | 1.000 | 0.948 | 0.970 | 1.000 | 0.852 | 1.000 | 1.000 | 0.854 | 0.826 |
| 2766 | 0.767 | 1.000 | 0.814 | 1.000 | 0.968 | 0.960 | 0.900 | 1.000 | 1.000 | 1.000 | 0.954 | 1.000 | 0.991 | 0.854 | 1.000 | 1.000 | 0.856 | 0.828 |
| 2767 | 0.772 | 1.000 | 0.816 | 1.000 | 0.963 | 0.960 | 0.914 | 1.000 | 1.000 | 1.000 | 0.957 | 0.970 | 0.991 | 0.847 | 1.000 | 1.000 | 0.858 | 0.830 |

| | | | | | | | | | | | | | | | | | |
|---|---|---|---|---|---|---|---|---|---|---|---|---|---|---|---|---|---|
| 2768 | 0.775 | 1.000 | 0.828 | 1.000 | 0.968 | 0.960 | 0.906 | 1.000 | 1.000 | 1.000 | 0.959 | 0.970 | 0.991 | 0.847 | 1.000 | 1.000 | 0.859 | 0.832 |
| 2769 | 0.768 | 1.000 | 0.802 | 1.000 | 0.968 | 0.960 | 0.904 | 1.000 | 0.990 | 1.000 | 0.957 | 1.000 | 0.991 | 0.854 | 1.000 | 1.000 | 0.856 | 0.827 |
| 2770 | 0.776 | 1.000 | 0.828 | 0.986 | 0.963 | 0.960 | 0.906 | 1.000 | 1.000 | 1.000 | 0.959 | 1.000 | 1.000 | 0.860 | 1.000 | 1.000 | 0.863 | 0.836 |
| 2771 | 0.776 | 1.000 | 0.818 | 1.000 | 0.968 | 0.960 | 0.898 | 1.000 | 1.000 | 1.000 | 0.962 | 0.939 | 1.000 | 0.849 | 1.000 | 1.000 | 0.858 | 0.830 |
| 2772 | 0.768 | 1.000 | 0.818 | 1.000 | 0.963 | 0.960 | 0.892 | 1.000 | 1.000 | 1.000 | 0.957 | 0.970 | 0.991 | 0.853 | 1.000 | 1.000 | 0.855 | 0.827 |
| 2773 | 0.762 | 1.000 | 0.826 | 1.000 | 0.968 | 0.960 | 0.911 | 1.000 | 1.000 | 1.000 | 0.957 | 0.970 | 0.991 | 0.857 | 1.000 | 1.000 | 0.858 | 0.830 |
| 2774 | 0.772 | 1.000 | 0.818 | 1.000 | 0.968 | 0.960 | 0.901 | 1.000 | 1.000 | 1.000 | 0.954 | 1.000 | 0.991 | 0.860 | 1.000 | 1.000 | 0.860 | 0.832 |
| 2775 | 0.768 | 1.000 | 0.826 | 1.000 | 0.974 | 0.960 | 0.906 | 1.000 | 0.990 | 1.000 | 0.945 | 0.970 | 1.000 | 0.853 | 1.000 | 1.000 | 0.858 | 0.830 |
| 2776 | 0.768 | 1.000 | 0.830 | 1.000 | 0.968 | 0.960 | 0.904 | 1.000 | 1.000 | 1.000 | 0.951 | 0.970 | 0.991 | 0.850 | 1.000 | 1.000 | 0.857 | 0.830 |
| 2777 | 0.782 | 1.000 | 0.812 | 0.986 | 0.968 | 0.960 | 0.909 | 1.000 | 0.990 | 1.000 | 0.957 | 0.970 | 1.000 | 0.853 | 1.000 | 1.000 | 0.861 | 0.834 |
| 2778 | 0.779 | 1.000 | 0.832 | 1.000 | 0.968 | 0.960 | 0.904 | 1.000 | 1.000 | 1.000 | 0.957 | 0.970 | 1.000 | 0.855 | 1.000 | 1.000 | 0.863 | 0.836 |
| 2779 | 0.771 | 1.000 | 0.810 | 0.986 | 0.963 | 0.960 | 0.900 | 1.000 | 0.990 | 1.000 | 0.959 | 0.970 | 0.982 | 0.854 | 1.000 | 1.000 | 0.856 | 0.828 |
| 2780 | 0.763 | 1.000 | 0.828 | 1.000 | 0.963 | 0.960 | 0.908 | 1.000 | 1.000 | 1.000 | 0.951 | 0.970 | 0.991 | 0.851 | 1.000 | 1.000 | 0.856 | 0.828 |
| 2781 | 0.766 | 1.000 | 0.824 | 1.000 | 0.968 | 0.960 | 0.901 | 1.000 | 0.990 | 1.000 | 0.957 | 0.970 | 0.991 | 0.858 | 1.000 | 1.000 | 0.858 | 0.830 |
| 2782 | 0.765 | 1.000 | 0.802 | 1.000 | 0.968 | 0.960 | 0.904 | 1.000 | 1.000 | 1.000 | 0.957 | 1.000 | 1.000 | 0.858 | 1.000 | 1.000 | 0.856 | 0.828 |
| 2783 | 0.772 | 1.000 | 0.816 | 1.000 | 0.968 | 0.960 | 0.908 | 1.000 | 1.000 | 1.000 | 0.965 | 0.970 | 0.991 | 0.855 | 1.000 | 1.000 | 0.860 | 0.832 |
| 2784 | 0.775 | 1.000 | 0.804 | 0.986 | 0.968 | 0.960 | 0.895 | 1.000 | 0.990 | 1.000 | 0.957 | 0.970 | 0.982 | 0.844 | 1.000 | 1.000 | 0.854 | 0.825 |
| 2785 | 0.775 | 1.000 | 0.846 | 1.000 | 0.968 | 0.960 | 0.911 | 1.000 | 0.980 | 1.000 | 0.954 | 0.970 | 1.000 | 0.843 | 1.000 | 1.000 | 0.860 | 0.833 |
| 2786 | 0.767 | 1.000 | 0.820 | 1.000 | 0.968 | 0.960 | 0.901 | 1.000 | 0.990 | 1.000 | 0.957 | 0.970 | 0.982 | 0.854 | 1.000 | 1.000 | 0.857 | 0.829 |
| 2787 | 0.761 | 1.000 | 0.816 | 1.000 | 0.963 | 0.960 | 0.904 | 1.000 | 1.000 | 1.000 | 0.962 | 0.970 | 1.000 | 0.858 | 1.000 | 1.000 | 0.857 | 0.829 |
| 2788 | 0.768 | 1.000 | 0.798 | 0.986 | 0.968 | 0.960 | 0.901 | 1.000 | 1.000 | 1.000 | 0.948 | 1.000 | 0.991 | 0.850 | 1.000 | 1.000 | 0.853 | 0.825 |
| 2789 | 0.769 | 1.000 | 0.822 | 1.000 | 0.963 | 0.960 | 0.900 | 1.000 | 0.990 | 1.000 | 0.957 | 1.000 | 0.982 | 0.849 | 1.000 | 1.000 | 0.856 | 0.828 |
| 2790 | 0.762 | 1.000 | 0.826 | 1.000 | 0.963 | 0.960 | 0.906 | 1.000 | 1.000 | 1.000 | 0.951 | 0.970 | 1.000 | 0.860 | 1.000 | 1.000 | 0.858 | 0.830 |
| 2791 | 0.769 | 1.000 | 0.830 | 1.000 | 0.968 | 0.960 | 0.901 | 1.000 | 0.990 | 1.000 | 0.951 | 1.000 | 0.991 | 0.854 | 1.000 | 1.000 | 0.858 | 0.831 |
| 2792 | 0.772 | 1.000 | 0.824 | 0.986 | 0.963 | 0.960 | 0.903 | 1.000 | 1.000 | 1.000 | 0.962 | 0.970 | 1.000 | 0.861 | 1.000 | 1.000 | 0.861 | 0.834 |
| 2793 | 0.766 | 1.000 | 0.816 | 0.986 | 0.963 | 0.960 | 0.904 | 1.000 | 1.000 | 1.000 | 0.959 | 0.970 | 0.991 | 0.845 | 1.000 | 1.000 | 0.854 | 0.826 |
| 2794 | 0.761 | 1.000 | 0.818 | 0.986 | 0.963 | 0.960 | 0.911 | 1.000 | 1.000 | 1.000 | 0.951 | 0.970 | 0.991 | 0.862 | 1.000 | 1.000 | 0.858 | 0.830 |
| 2795 | 0.766 | 1.000 | 0.830 | 1.000 | 0.974 | 0.960 | 0.895 | 1.000 | 1.000 | 1.000 | 0.959 | 0.970 | 1.000 | 0.846 | 1.000 | 1.000 | 0.855 | 0.827 |
| 2796 | 0.772 | 1.000 | 0.814 | 1.000 | 0.968 | 0.960 | 0.908 | 1.000 | 0.990 | 1.000 | 0.959 | 0.970 | 0.982 | 0.854 | 1.000 | 1.000 | 0.859 | 0.830 |
| 2797 | 0.770 | 1.000 | 0.796 | 1.000 | 0.968 | 0.960 | 0.901 | 1.000 | 1.000 | 1.000 | 0.951 | 0.970 | 0.991 | 0.854 | 1.000 | 1.000 | 0.855 | 0.827 |
| 2798 | 0.773 | 1.000 | 0.830 | 1.000 | 0.963 | 0.960 | 0.918 | 1.000 | 0.990 | 1.000 | 0.957 | 0.970 | 1.000 | 0.851 | 1.000 | 1.000 | 0.861 | 0.834 |
| 2799 | 0.772 | 1.000 | 0.822 | 1.000 | 0.968 | 0.960 | 0.908 | 1.000 | 1.000 | 1.000 | 0.951 | 0.970 | 1.000 | 0.847 | 1.000 | 1.000 | 0.858 | 0.830 |
| 2800 | 0.775 | 1.000 | 0.830 | 1.000 | 0.968 | 0.960 | 0.909 | 1.000 | 0.990 | 1.000 | 0.962 | 1.000 | 1.000 | 0.852 | 1.000 | 1.000 | 0.862 | 0.835 |
| 2801 | 0.769 | 1.000 | 0.818 | 1.000 | 0.968 | 0.960 | 0.912 | 1.000 | 1.000 | 1.000 | 0.968 | 0.970 | 1.000 | 0.861 | 1.000 | 1.000 | 0.862 | 0.835 |
| 2802 | 0.765 | 1.000 | 0.816 | 1.000 | 0.968 | 0.960 | 0.901 | 1.000 | 1.000 | 1.000 | 0.957 | 0.970 | 0.991 | 0.856 | 1.000 | 1.000 | 0.856 | 0.828 |
| 2803 | 0.765 | 1.000 | 0.824 | 1.000 | 0.968 | 0.960 | 0.909 | 1.000 | 1.000 | 1.000 | 0.962 | 1.000 | 0.991 | 0.854 | 1.000 | 1.000 | 0.858 | 0.831 |
| 2804 | 0.781 | 1.000 | 0.818 | 1.000 | 0.963 | 0.960 | 0.900 | 1.000 | 1.000 | 1.000 | 0.954 | 0.970 | 1.000 | 0.848 | 1.000 | 1.000 | 0.859 | 0.831 |
| 2805 | 0.772 | 1.000 | 0.828 | 1.000 | 0.968 | 0.960 | 0.904 | 1.000 | 1.000 | 1.000 | 0.951 | 0.970 | 0.991 | 0.854 | 1.000 | 1.000 | 0.859 | 0.832 |
| 2806 | 0.765 | 1.000 | 0.822 | 0.986 | 0.963 | 0.960 | 0.906 | 1.000 | 1.000 | 1.000 | 0.951 | 0.970 | 0.991 | 0.855 | 1.000 | 1.000 | 0.857 | 0.829 |

| | | | | | | | | | | | | | | | | | |
|---|---|---|---|---|---|---|---|---|---|---|---|---|---|---|---|---|---|
| 2807 | 0.767 | 1.000 | 0.836 | 1.000 | 0.963 | 0.960 | 0.909 | 1.000 | 0.990 | 1.000 | 0.951 | 0.970 | 0.991 | 0.853 | 1.000 | 1.000 | 0.858 | 0.831 |
| 2808 | 0.766 | 1.000 | 0.820 | 1.000 | 0.968 | 0.960 | 0.909 | 1.000 | 1.000 | 1.000 | 0.959 | 0.970 | 1.000 | 0.851 | 1.000 | 1.000 | 0.857 | 0.829 |
| 2809 | 0.770 | 1.000 | 0.806 | 1.000 | 0.968 | 0.960 | 0.901 | 1.000 | 1.000 | 1.000 | 0.957 | 1.000 | 0.991 | 0.849 | 1.000 | 1.000 | 0.855 | 0.827 |
| 2810 | 0.769 | 1.000 | 0.832 | 0.986 | 0.968 | 0.960 | 0.911 | 1.000 | 1.000 | 1.000 | 0.957 | 0.970 | 0.982 | 0.851 | 1.000 | 1.000 | 0.859 | 0.831 |
| 2811 | 0.764 | 1.000 | 0.800 | 1.000 | 0.963 | 0.960 | 0.912 | 1.000 | 1.000 | 1.000 | 0.957 | 0.970 | 1.000 | 0.858 | 1.000 | 1.000 | 0.856 | 0.828 |
| 2812 | 0.757 | 1.000 | 0.820 | 0.986 | 0.963 | 0.960 | 0.911 | 1.000 | 0.990 | 1.000 | 0.959 | 0.970 | 0.991 | 0.850 | 1.000 | 1.000 | 0.854 | 0.826 |
| 2813 | 0.779 | 1.000 | 0.810 | 0.986 | 0.968 | 0.960 | 0.917 | 1.000 | 0.990 | 1.000 | 0.962 | 0.970 | 0.991 | 0.858 | 1.000 | 1.000 | 0.863 | 0.836 |
| 2814 | 0.771 | 1.000 | 0.816 | 1.000 | 0.968 | 0.960 | 0.912 | 1.000 | 1.000 | 1.000 | 0.962 | 1.000 | 0.991 | 0.854 | 1.000 | 1.000 | 0.860 | 0.832 |
| 2815 | 0.768 | 1.000 | 0.828 | 1.000 | 0.963 | 0.960 | 0.901 | 1.000 | 1.000 | 1.000 | 0.959 | 0.970 | 0.991 | 0.854 | 1.000 | 1.000 | 0.858 | 0.830 |
| 2816 | 0.773 | 1.000 | 0.818 | 1.000 | 0.963 | 0.960 | 0.903 | 1.000 | 1.000 | 1.000 | 0.957 | 0.970 | 0.991 | 0.860 | 1.000 | 1.000 | 0.860 | 0.832 |
| 2817 | 0.771 | 1.000 | 0.818 | 1.000 | 0.968 | 0.960 | 0.908 | 1.000 | 0.990 | 1.000 | 0.959 | 0.970 | 1.000 | 0.852 | 1.000 | 1.000 | 0.858 | 0.831 |
| 2818 | 0.765 | 1.000 | 0.822 | 1.000 | 0.968 | 0.960 | 0.894 | 1.000 | 1.000 | 1.000 | 0.957 | 1.000 | 0.991 | 0.851 | 1.000 | 1.000 | 0.855 | 0.827 |
| 2819 | 0.762 | 1.000 | 0.824 | 1.000 | 0.963 | 0.960 | 0.911 | 1.000 | 0.990 | 1.000 | 0.948 | 0.970 | 1.000 | 0.849 | 1.000 | 1.000 | 0.855 | 0.827 |
| 2820 | 0.770 | 1.000 | 0.812 | 1.000 | 0.968 | 0.960 | 0.897 | 1.000 | 0.990 | 1.000 | 0.957 | 0.970 | 0.982 | 0.856 | 1.000 | 1.000 | 0.857 | 0.828 |
| 2821 | 0.777 | 1.000 | 0.826 | 1.000 | 0.968 | 0.960 | 0.901 | 1.000 | 0.990 | 1.000 | 0.959 | 0.970 | 0.991 | 0.854 | 1.000 | 1.000 | 0.861 | 0.833 |
| 2822 | 0.771 | 1.000 | 0.820 | 1.000 | 0.968 | 0.960 | 0.906 | 1.000 | 1.000 | 1.000 | 0.954 | 0.970 | 0.991 | 0.854 | 1.000 | 1.000 | 0.859 | 0.831 |
| 2823 | 0.772 | 1.000 | 0.806 | 1.000 | 0.963 | 0.960 | 0.909 | 1.000 | 0.990 | 1.000 | 0.954 | 1.000 | 0.991 | 0.856 | 1.000 | 1.000 | 0.858 | 0.830 |
| 2824 | 0.767 | 1.000 | 0.810 | 0.986 | 0.968 | 0.960 | 0.903 | 1.000 | 0.990 | 1.000 | 0.945 | 0.970 | 1.000 | 0.862 | 1.000 | 1.000 | 0.857 | 0.829 |
| 2825 | 0.777 | 1.000 | 0.830 | 0.986 | 0.963 | 0.960 | 0.908 | 1.000 | 1.000 | 1.000 | 0.957 | 0.939 | 1.000 | 0.856 | 1.000 | 1.000 | 0.862 | 0.834 |
| 2826 | 0.767 | 1.000 | 0.810 | 1.000 | 0.974 | 0.960 | 0.908 | 1.000 | 0.990 | 1.000 | 0.957 | 0.970 | 0.991 | 0.844 | 1.000 | 1.000 | 0.854 | 0.825 |
| 2827 | 0.767 | 1.000 | 0.820 | 0.986 | 0.968 | 0.960 | 0.901 | 1.000 | 0.990 | 1.000 | 0.959 | 0.970 | 0.991 | 0.854 | 1.000 | 1.000 | 0.857 | 0.829 |
| 2828 | 0.773 | 1.000 | 0.816 | 0.986 | 0.968 | 0.960 | 0.894 | 1.000 | 1.000 | 1.000 | 0.957 | 1.000 | 0.982 | 0.857 | 1.000 | 1.000 | 0.858 | 0.830 |
| 2829 | 0.772 | 1.000 | 0.826 | 1.000 | 0.968 | 0.960 | 0.901 | 1.000 | 0.990 | 1.000 | 0.954 | 1.000 | 1.000 | 0.849 | 1.000 | 1.000 | 0.858 | 0.830 |
| 2830 | 0.759 | 1.000 | 0.818 | 1.000 | 0.974 | 0.960 | 0.912 | 1.000 | 1.000 | 1.000 | 0.962 | 0.970 | 0.982 | 0.849 | 1.000 | 1.000 | 0.855 | 0.827 |
| 2831 | 0.775 | 1.000 | 0.822 | 1.000 | 0.968 | 0.960 | 0.903 | 1.000 | 0.990 | 1.000 | 0.957 | 0.970 | 1.000 | 0.849 | 1.000 | 1.000 | 0.858 | 0.831 |
| 2832 | 0.767 | 1.000 | 0.834 | 1.000 | 0.968 | 0.960 | 0.900 | 1.000 | 1.000 | 1.000 | 0.948 | 0.970 | 1.000 | 0.854 | 1.000 | 1.000 | 0.858 | 0.830 |
| 2833 | 0.771 | 1.000 | 0.816 | 1.000 | 0.963 | 0.960 | 0.903 | 1.000 | 1.000 | 1.000 | 0.957 | 0.970 | 0.991 | 0.850 | 1.000 | 1.000 | 0.857 | 0.829 |
| 2834 | 0.771 | 1.000 | 0.816 | 1.000 | 0.968 | 0.960 | 0.901 | 1.000 | 1.000 | 1.000 | 0.957 | 0.970 | 0.991 | 0.853 | 1.000 | 1.000 | 0.858 | 0.830 |
| 2835 | 0.771 | 1.000 | 0.820 | 1.000 | 0.963 | 0.960 | 0.903 | 1.000 | 1.000 | 1.000 | 0.954 | 1.000 | 0.991 | 0.851 | 1.000 | 1.000 | 0.857 | 0.829 |
| 2836 | 0.765 | 1.000 | 0.796 | 1.000 | 0.963 | 0.960 | 0.920 | 1.000 | 1.000 | 1.000 | 0.957 | 0.970 | 1.000 | 0.856 | 1.000 | 1.000 | 0.857 | 0.829 |
| 2837 | 0.776 | 1.000 | 0.810 | 1.000 | 0.963 | 0.960 | 0.908 | 1.000 | 1.000 | 1.000 | 0.959 | 0.970 | 0.991 | 0.858 | 1.000 | 1.000 | 0.860 | 0.833 |
| 2838 | 0.769 | 1.000 | 0.836 | 0.986 | 0.968 | 0.960 | 0.908 | 1.000 | 1.000 | 1.000 | 0.942 | 1.000 | 0.991 | 0.862 | 1.000 | 1.000 | 0.861 | 0.834 |
| 2839 | 0.767 | 1.000 | 0.804 | 1.000 | 0.968 | 0.960 | 0.901 | 1.000 | 0.990 | 1.000 | 0.954 | 0.970 | 1.000 | 0.848 | 1.000 | 1.000 | 0.854 | 0.825 |
| 2840 | 0.769 | 1.000 | 0.822 | 1.000 | 0.968 | 0.960 | 0.908 | 1.000 | 0.990 | 1.000 | 0.954 | 1.000 | 0.991 | 0.862 | 1.000 | 1.000 | 0.860 | 0.833 |
| 2841 | 0.769 | 1.000 | 0.820 | 1.000 | 0.963 | 0.960 | 0.906 | 1.000 | 0.980 | 1.000 | 0.957 | 0.970 | 0.982 | 0.856 | 1.000 | 1.000 | 0.858 | 0.830 |
| 2842 | 0.771 | 1.000 | 0.824 | 1.000 | 0.968 | 0.960 | 0.904 | 1.000 | 1.000 | 1.000 | 0.954 | 0.970 | 1.000 | 0.859 | 1.000 | 1.000 | 0.860 | 0.833 |
| 2843 | 0.768 | 1.000 | 0.826 | 1.000 | 0.963 | 0.960 | 0.912 | 1.000 | 1.000 | 1.000 | 0.951 | 1.000 | 0.991 | 0.856 | 1.000 | 1.000 | 0.859 | 0.832 |
| 2844 | 0.763 | 1.000 | 0.830 | 1.000 | 0.968 | 0.960 | 0.904 | 1.000 | 0.990 | 1.000 | 0.957 | 0.970 | 0.991 | 0.855 | 1.000 | 1.000 | 0.857 | 0.829 |
| 2845 | 0.768 | 1.000 | 0.816 | 0.986 | 0.968 | 0.960 | 0.903 | 1.000 | 1.000 | 1.000 | 0.959 | 0.970 | 0.991 | 0.851 | 1.000 | 1.000 | 0.856 | 0.829 |

| | | | | | | | | | | | | | | | | | |
|---|---|---|---|---|---|---|---|---|---|---|---|---|---|---|---|---|---|
| 2846 | 0.774 | 1.000 | 0.818 | 0.986 | 0.963 | 0.960 | 0.900 | 1.000 | 1.000 | 1.000 | 0.959 | 0.970 | 0.991 | 0.857 | 1.000 | 1.000 | 0.859 | 0.831 |
| 2847 | 0.774 | 1.000 | 0.818 | 1.000 | 0.974 | 0.960 | 0.915 | 1.000 | 0.990 | 1.000 | 0.954 | 0.970 | 0.982 | 0.847 | 1.000 | 1.000 | 0.859 | 0.831 |
| 2848 | 0.773 | 1.000 | 0.806 | 1.000 | 0.963 | 0.960 | 0.908 | 1.000 | 1.000 | 1.000 | 0.959 | 0.970 | 1.000 | 0.853 | 1.000 | 1.000 | 0.858 | 0.830 |
| 2849 | 0.778 | 1.000 | 0.828 | 0.986 | 0.968 | 0.960 | 0.906 | 1.000 | 1.000 | 1.000 | 0.957 | 1.000 | 0.991 | 0.854 | 1.000 | 1.000 | 0.862 | 0.835 |
| 2850 | 0.777 | 1.000 | 0.810 | 0.986 | 0.968 | 0.960 | 0.917 | 1.000 | 1.000 | 1.000 | 0.957 | 0.970 | 0.982 | 0.854 | 1.000 | 1.000 | 0.861 | 0.833 |
| 2851 | 0.777 | 1.000 | 0.812 | 0.986 | 0.968 | 0.960 | 0.898 | 1.000 | 1.000 | 1.000 | 0.954 | 0.970 | 1.000 | 0.847 | 1.000 | 1.000 | 0.857 | 0.829 |
| 2852 | 0.775 | 1.000 | 0.808 | 1.000 | 0.968 | 0.960 | 0.911 | 1.000 | 0.990 | 1.000 | 0.954 | 0.970 | 1.000 | 0.854 | 1.000 | 1.000 | 0.859 | 0.832 |
| 2853 | 0.769 | 1.000 | 0.824 | 1.000 | 0.968 | 0.960 | 0.903 | 1.000 | 0.990 | 1.000 | 0.948 | 0.970 | 0.991 | 0.854 | 1.000 | 1.000 | 0.857 | 0.829 |
| 2854 | 0.785 | 1.000 | 0.816 | 1.000 | 0.968 | 0.960 | 0.911 | 1.000 | 0.990 | 1.000 | 0.957 | 1.000 | 1.000 | 0.852 | 1.000 | 1.000 | 0.863 | 0.836 |
| 2855 | 0.761 | 1.000 | 0.822 | 0.986 | 0.974 | 0.960 | 0.906 | 1.000 | 1.000 | 1.000 | 0.959 | 0.970 | 1.000 | 0.848 | 1.000 | 1.000 | 0.855 | 0.827 |
| 2856 | 0.763 | 1.000 | 0.808 | 1.000 | 0.953 | 0.960 | 0.908 | 1.000 | 1.000 | 1.000 | 0.954 | 0.970 | 0.991 | 0.850 | 1.000 | 1.000 | 0.853 | 0.825 |
| 2857 | 0.769 | 1.000 | 0.808 | 1.000 | 0.963 | 0.960 | 0.897 | 1.000 | 1.000 | 1.000 | 0.959 | 0.970 | 0.982 | 0.853 | 1.000 | 1.000 | 0.855 | 0.827 |
| 2858 | 0.769 | 1.000 | 0.822 | 1.000 | 0.963 | 0.960 | 0.897 | 1.000 | 1.000 | 1.000 | 0.954 | 0.970 | 1.000 | 0.858 | 1.000 | 1.000 | 0.858 | 0.830 |
| 2859 | 0.779 | 1.000 | 0.836 | 1.000 | 0.968 | 0.960 | 0.900 | 1.000 | 0.990 | 1.000 | 0.959 | 0.970 | 0.991 | 0.852 | 1.000 | 1.000 | 0.861 | 0.834 |
| 2860 | 0.771 | 1.000 | 0.836 | 0.986 | 0.963 | 0.960 | 0.901 | 1.000 | 1.000 | 1.000 | 0.957 | 0.970 | 0.991 | 0.854 | 1.000 | 1.000 | 0.859 | 0.832 |
| 2861 | 0.759 | 1.000 | 0.820 | 0.986 | 0.968 | 0.960 | 0.898 | 1.000 | 0.990 | 1.000 | 0.954 | 0.939 | 1.000 | 0.851 | 1.000 | 1.000 | 0.853 | 0.825 |
| 2862 | 0.770 | 1.000 | 0.806 | 1.000 | 0.968 | 0.960 | 0.909 | 1.000 | 0.990 | 1.000 | 0.957 | 0.970 | 0.991 | 0.850 | 1.000 | 1.000 | 0.856 | 0.828 |
| 2863 | 0.769 | 1.000 | 0.806 | 1.000 | 0.963 | 0.960 | 0.912 | 1.000 | 1.000 | 1.000 | 0.954 | 1.000 | 0.982 | 0.851 | 1.000 | 1.000 | 0.856 | 0.828 |
| 2864 | 0.773 | 1.000 | 0.808 | 1.000 | 0.968 | 0.960 | 0.903 | 1.000 | 1.000 | 1.000 | 0.957 | 0.970 | 0.982 | 0.849 | 1.000 | 1.000 | 0.856 | 0.828 |
| 2865 | 0.763 | 1.000 | 0.818 | 1.000 | 0.968 | 1.000 | 0.901 | 1.000 | 0.990 | 1.000 | 0.954 | 0.970 | 0.991 | 0.851 | 1.000 | 1.000 | 0.855 | 0.827 |
| 2866 | 0.768 | 1.000 | 0.796 | 0.986 | 0.968 | 0.960 | 0.906 | 1.000 | 1.000 | 1.000 | 0.957 | 0.970 | 0.991 | 0.852 | 1.000 | 1.000 | 0.855 | 0.826 |
| 2867 | 0.763 | 1.000 | 0.808 | 0.986 | 0.963 | 0.960 | 0.918 | 1.000 | 1.000 | 1.000 | 0.962 | 0.970 | 1.000 | 0.853 | 1.000 | 1.000 | 0.857 | 0.829 |
| 2868 | 0.768 | 1.000 | 0.830 | 0.986 | 0.974 | 0.960 | 0.903 | 1.000 | 0.990 | 1.000 | 0.954 | 0.970 | 0.991 | 0.854 | 1.000 | 1.000 | 0.858 | 0.831 |
| 2869 | 0.773 | 1.000 | 0.828 | 0.986 | 0.968 | 0.960 | 0.903 | 1.000 | 1.000 | 1.000 | 0.959 | 0.970 | 0.991 | 0.856 | 1.000 | 1.000 | 0.860 | 0.833 |
| 2870 | 0.771 | 1.000 | 0.834 | 0.986 | 0.963 | 0.960 | 0.908 | 1.000 | 0.990 | 1.000 | 0.954 | 0.970 | 1.000 | 0.858 | 1.000 | 1.000 | 0.861 | 0.834 |
| 2871 | 0.768 | 1.000 | 0.822 | 0.986 | 0.968 | 0.960 | 0.901 | 1.000 | 1.000 | 1.000 | 0.951 | 0.970 | 1.000 | 0.859 | 1.000 | 1.000 | 0.858 | 0.830 |
| 2872 | 0.776 | 1.000 | 0.800 | 1.000 | 0.968 | 0.960 | 0.911 | 1.000 | 1.000 | 1.000 | 0.957 | 0.970 | 1.000 | 0.856 | 1.000 | 1.000 | 0.860 | 0.832 |
| 2873 | 0.763 | 1.000 | 0.830 | 1.000 | 0.968 | 0.960 | 0.900 | 1.000 | 1.000 | 1.000 | 0.954 | 1.000 | 1.000 | 0.856 | 1.000 | 1.000 | 0.857 | 0.830 |
| 2874 | 0.771 | 1.000 | 0.814 | 0.986 | 0.968 | 0.960 | 0.917 | 1.000 | 1.000 | 1.000 | 0.954 | 0.970 | 1.000 | 0.858 | 1.000 | 1.000 | 0.860 | 0.833 |
| 2875 | 0.778 | 1.000 | 0.820 | 0.986 | 0.963 | 0.960 | 0.901 | 1.000 | 1.000 | 1.000 | 0.954 | 0.970 | 1.000 | 0.852 | 1.000 | 1.000 | 0.859 | 0.832 |
| 2876 | 0.779 | 1.000 | 0.818 | 0.986 | 0.974 | 0.960 | 0.904 | 1.000 | 1.000 | 1.000 | 0.954 | 0.970 | 0.991 | 0.858 | 1.000 | 1.000 | 0.862 | 0.834 |
| 2877 | 0.778 | 1.000 | 0.810 | 1.000 | 0.958 | 0.960 | 0.898 | 1.000 | 1.000 | 1.000 | 0.957 | 0.970 | 0.991 | 0.859 | 1.000 | 1.000 | 0.860 | 0.832 |
| 2878 | 0.777 | 1.000 | 0.816 | 1.000 | 0.968 | 0.960 | 0.911 | 1.000 | 0.990 | 1.000 | 0.957 | 0.970 | 1.000 | 0.861 | 1.000 | 1.000 | 0.862 | 0.835 |
| 2879 | 0.761 | 1.000 | 0.822 | 1.000 | 0.968 | 0.960 | 0.895 | 1.000 | 0.990 | 1.000 | 0.959 | 1.000 | 0.982 | 0.847 | 1.000 | 1.000 | 0.853 | 0.824 |
| 2880 | 0.775 | 1.000 | 0.818 | 0.986 | 0.958 | 0.960 | 0.901 | 1.000 | 0.990 | 1.000 | 0.957 | 0.970 | 0.982 | 0.851 | 1.000 | 1.000 | 0.857 | 0.829 |
| 2881 | 0.777 | 1.000 | 0.812 | 1.000 | 0.963 | 0.960 | 0.909 | 1.000 | 1.000 | 1.000 | 0.948 | 0.970 | 0.991 | 0.856 | 1.000 | 1.000 | 0.860 | 0.832 |
| 2882 | 0.767 | 1.000 | 0.828 | 1.000 | 0.968 | 0.960 | 0.912 | 1.000 | 1.000 | 1.000 | 0.957 | 0.970 | 1.000 | 0.849 | 1.000 | 1.000 | 0.858 | 0.831 |
| 2883 | 0.772 | 1.000 | 0.812 | 1.000 | 0.968 | 0.960 | 0.908 | 1.000 | 0.990 | 1.000 | 0.957 | 0.970 | 1.000 | 0.852 | 1.000 | 1.000 | 0.858 | 0.830 |
| 2884 | 0.769 | 1.000 | 0.828 | 1.000 | 0.963 | 0.960 | 0.904 | 1.000 | 0.990 | 1.000 | 0.957 | 0.970 | 0.991 | 0.851 | 1.000 | 1.000 | 0.857 | 0.829 |

| | | | | | | | | | | | | | | | | | |
|---|---|---|---|---|---|---|---|---|---|---|---|---|---|---|---|---|---|
| 2885 | 0.769 | 1.000 | 0.820 | 1.000 | 0.968 | 0.960 | 0.912 | 1.000 | 0.990 | 1.000 | 0.959 | 0.970 | 1.000 | 0.857 | 1.000 | 1.000 | 0.860 | 0.833 |
| 2886 | 0.770 | 1.000 | 0.834 | 1.000 | 0.963 | 0.960 | 0.897 | 1.000 | 1.000 | 1.000 | 0.957 | 0.970 | 0.991 | 0.856 | 1.000 | 1.000 | 0.859 | 0.831 |
| 2887 | 0.776 | 1.000 | 0.804 | 0.986 | 0.963 | 0.960 | 0.906 | 1.000 | 1.000 | 1.000 | 0.957 | 0.970 | 1.000 | 0.854 | 1.000 | 1.000 | 0.858 | 0.830 |
| 2888 | 0.771 | 1.000 | 0.816 | 1.000 | 0.958 | 0.960 | 0.906 | 1.000 | 1.000 | 1.000 | 0.959 | 0.970 | 0.991 | 0.852 | 1.000 | 1.000 | 0.858 | 0.830 |
| 2889 | 0.769 | 1.000 | 0.822 | 1.000 | 0.968 | 0.960 | 0.908 | 1.000 | 1.000 | 1.000 | 0.959 | 0.970 | 0.982 | 0.852 | 1.000 | 1.000 | 0.858 | 0.830 |
| 2890 | 0.770 | 1.000 | 0.826 | 1.000 | 0.963 | 0.960 | 0.904 | 1.000 | 1.000 | 1.000 | 0.959 | 0.970 | 1.000 | 0.850 | 1.000 | 1.000 | 0.858 | 0.830 |
| 2891 | 0.763 | 1.000 | 0.808 | 1.000 | 0.968 | 0.960 | 0.912 | 1.000 | 0.990 | 1.000 | 0.951 | 0.970 | 0.982 | 0.850 | 1.000 | 1.000 | 0.854 | 0.826 |
| 2892 | 0.774 | 1.000 | 0.802 | 0.986 | 0.963 | 0.960 | 0.900 | 1.000 | 0.990 | 1.000 | 0.962 | 1.000 | 0.991 | 0.854 | 0.978 | 1.000 | 0.857 | 0.829 |
| 2893 | 0.768 | 1.000 | 0.820 | 1.000 | 0.968 | 0.960 | 0.901 | 1.000 | 0.990 | 1.000 | 0.962 | 0.970 | 1.000 | 0.851 | 1.000 | 1.000 | 0.857 | 0.829 |
| 2894 | 0.777 | 1.000 | 0.824 | 1.000 | 0.968 | 0.960 | 0.906 | 1.000 | 1.000 | 1.000 | 0.959 | 0.970 | 0.982 | 0.860 | 1.000 | 1.000 | 0.862 | 0.835 |
| 2895 | 0.773 | 1.000 | 0.812 | 1.000 | 0.963 | 0.960 | 0.914 | 1.000 | 1.000 | 1.000 | 0.957 | 0.970 | 0.982 | 0.851 | 1.000 | 1.000 | 0.858 | 0.830 |
| 2896 | 0.763 | 1.000 | 0.828 | 1.000 | 0.968 | 0.960 | 0.901 | 1.000 | 1.000 | 1.000 | 0.965 | 0.970 | 1.000 | 0.851 | 1.000 | 1.000 | 0.856 | 0.829 |
| 2897 | 0.772 | 1.000 | 0.822 | 1.000 | 0.963 | 0.960 | 0.904 | 1.000 | 0.990 | 1.000 | 0.954 | 0.970 | 1.000 | 0.859 | 1.000 | 1.000 | 0.860 | 0.832 |
| 2898 | 0.772 | 1.000 | 0.802 | 1.000 | 0.968 | 0.960 | 0.911 | 1.000 | 1.000 | 1.000 | 0.959 | 0.970 | 1.000 | 0.859 | 1.000 | 1.000 | 0.860 | 0.832 |
| 2899 | 0.768 | 1.000 | 0.814 | 1.000 | 0.968 | 0.960 | 0.906 | 1.000 | 1.000 | 1.000 | 0.957 | 0.970 | 1.000 | 0.847 | 1.000 | 1.000 | 0.856 | 0.828 |
| 2900 | 0.777 | 1.000 | 0.820 | 1.000 | 0.968 | 0.960 | 0.895 | 1.000 | 0.990 | 1.000 | 0.957 | 0.970 | 0.991 | 0.858 | 1.000 | 1.000 | 0.860 | 0.832 |
| 2901 | 0.776 | 1.000 | 0.830 | 1.000 | 0.968 | 0.960 | 0.904 | 1.000 | 1.000 | 1.000 | 0.957 | 1.000 | 0.991 | 0.860 | 1.000 | 1.000 | 0.863 | 0.836 |
| 2902 | 0.767 | 1.000 | 0.824 | 0.986 | 0.968 | 0.960 | 0.912 | 1.000 | 1.000 | 1.000 | 0.959 | 0.970 | 0.991 | 0.854 | 1.000 | 1.000 | 0.859 | 0.831 |
| 2903 | 0.771 | 1.000 | 0.820 | 0.986 | 0.968 | 0.960 | 0.904 | 1.000 | 1.000 | 1.000 | 0.962 | 0.970 | 1.000 | 0.854 | 1.000 | 1.000 | 0.859 | 0.831 |
| 2904 | 0.760 | 1.000 | 0.826 | 0.986 | 0.968 | 0.960 | 0.906 | 1.000 | 0.990 | 1.000 | 0.959 | 1.000 | 1.000 | 0.846 | 1.000 | 1.000 | 0.854 | 0.826 |
| 2905 | 0.775 | 1.000 | 0.828 | 0.986 | 0.963 | 0.960 | 0.901 | 1.000 | 0.990 | 1.000 | 0.951 | 1.000 | 1.000 | 0.851 | 1.000 | 1.000 | 0.859 | 0.831 |
| 2906 | 0.772 | 1.000 | 0.816 | 1.000 | 0.963 | 0.960 | 0.900 | 1.000 | 1.000 | 1.000 | 0.965 | 0.939 | 1.000 | 0.860 | 1.000 | 1.000 | 0.860 | 0.832 |
| 2907 | 0.773 | 1.000 | 0.816 | 0.986 | 0.963 | 0.960 | 0.918 | 1.000 | 1.000 | 1.000 | 0.957 | 0.970 | 0.991 | 0.852 | 1.000 | 1.000 | 0.860 | 0.832 |
| 2908 | 0.766 | 1.000 | 0.808 | 1.000 | 0.968 | 0.960 | 0.897 | 1.000 | 0.990 | 1.000 | 0.954 | 1.000 | 1.000 | 0.862 | 1.000 | 1.000 | 0.857 | 0.829 |
| 2909 | 0.767 | 1.000 | 0.826 | 1.000 | 0.968 | 0.960 | 0.904 | 1.000 | 1.000 | 1.000 | 0.959 | 0.970 | 0.991 | 0.859 | 1.000 | 1.000 | 0.860 | 0.832 |
| 2910 | 0.773 | 1.000 | 0.808 | 1.000 | 0.968 | 0.960 | 0.908 | 1.000 | 1.000 | 1.000 | 0.959 | 0.970 | 1.000 | 0.852 | 1.000 | 1.000 | 0.858 | 0.831 |
| 2911 | 0.777 | 1.000 | 0.822 | 1.000 | 0.968 | 0.960 | 0.906 | 1.000 | 0.990 | 1.000 | 0.957 | 0.970 | 0.982 | 0.854 | 1.000 | 1.000 | 0.861 | 0.833 |
| 2912 | 0.773 | 1.000 | 0.822 | 0.986 | 0.968 | 0.960 | 0.904 | 1.000 | 1.000 | 1.000 | 0.951 | 0.970 | 0.991 | 0.850 | 1.000 | 1.000 | 0.858 | 0.830 |
| 2913 | 0.768 | 1.000 | 0.822 | 1.000 | 0.968 | 0.960 | 0.908 | 1.000 | 1.000 | 1.000 | 0.962 | 0.970 | 1.000 | 0.857 | 1.000 | 1.000 | 0.860 | 0.832 |
| 2914 | 0.771 | 1.000 | 0.826 | 0.986 | 0.963 | 0.960 | 0.909 | 1.000 | 1.000 | 1.000 | 0.954 | 0.970 | 1.000 | 0.858 | 1.000 | 1.000 | 0.860 | 0.833 |
| 2915 | 0.769 | 1.000 | 0.822 | 1.000 | 0.968 | 0.960 | 0.914 | 1.000 | 1.000 | 1.000 | 0.959 | 1.000 | 1.000 | 0.856 | 1.000 | 1.000 | 0.861 | 0.833 |
| 2916 | 0.768 | 1.000 | 0.828 | 1.000 | 0.963 | 0.960 | 0.906 | 1.000 | 1.000 | 1.000 | 0.954 | 0.970 | 0.991 | 0.862 | 1.000 | 1.000 | 0.860 | 0.833 |
| 2917 | 0.769 | 1.000 | 0.826 | 0.986 | 0.968 | 0.960 | 0.904 | 1.000 | 0.990 | 1.000 | 0.954 | 0.970 | 0.991 | 0.859 | 1.000 | 1.000 | 0.859 | 0.832 |
| 2918 | 0.776 | 1.000 | 0.828 | 1.000 | 0.963 | 0.960 | 0.908 | 1.000 | 0.990 | 1.000 | 0.957 | 0.970 | 1.000 | 0.852 | 1.000 | 1.000 | 0.861 | 0.833 |
| 2919 | 0.771 | 1.000 | 0.810 | 1.000 | 0.968 | 0.960 | 0.906 | 1.000 | 1.000 | 1.000 | 0.951 | 0.970 | 0.991 | 0.847 | 1.000 | 1.000 | 0.856 | 0.827 |
| 2920 | 0.770 | 1.000 | 0.830 | 1.000 | 0.968 | 0.960 | 0.911 | 1.000 | 1.000 | 1.000 | 0.959 | 1.000 | 1.000 | 0.854 | 1.000 | 1.000 | 0.861 | 0.834 |
| 2921 | 0.782 | 1.000 | 0.818 | 1.000 | 0.968 | 0.960 | 0.904 | 1.000 | 1.000 | 1.000 | 0.959 | 0.970 | 1.000 | 0.850 | 1.000 | 1.000 | 0.861 | 0.834 |
| 2922 | 0.768 | 1.000 | 0.828 | 1.000 | 0.968 | 0.960 | 0.912 | 1.000 | 1.000 | 1.000 | 0.957 | 1.000 | 1.000 | 0.859 | 1.000 | 1.000 | 0.861 | 0.834 |
| 2923 | 0.774 | 1.000 | 0.826 | 0.986 | 0.963 | 0.960 | 0.903 | 1.000 | 1.000 | 1.000 | 0.957 | 0.970 | 0.991 | 0.860 | 1.000 | 1.000 | 0.861 | 0.833 |

| | | | | | | | | | | | | | | | | | |
|---|---|---|---|---|---|---|---|---|---|---|---|---|---|---|---|---|---|
| 2924 | 0.773 | 1.000 | 0.834 | 1.000 | 0.963 | 0.960 | 0.901 | 1.000 | 0.990 | 1.000 | 0.954 | 0.970 | 1.000 | 0.851 | 1.000 | 1.000 | 0.859 | 0.831 |
| 2925 | 0.766 | 1.000 | 0.820 | 0.986 | 0.974 | 0.960 | 0.906 | 1.000 | 0.990 | 1.000 | 0.957 | 0.970 | 1.000 | 0.854 | 1.000 | 1.000 | 0.857 | 0.829 |
| 2926 | 0.776 | 1.000 | 0.812 | 1.000 | 0.968 | 0.960 | 0.900 | 1.000 | 1.000 | 1.000 | 0.951 | 0.970 | 0.991 | 0.858 | 1.000 | 1.000 | 0.859 | 0.831 |
| 2927 | 0.773 | 1.000 | 0.812 | 0.986 | 0.963 | 0.960 | 0.911 | 1.000 | 0.990 | 1.000 | 0.959 | 1.000 | 1.000 | 0.857 | 1.000 | 1.000 | 0.860 | 0.832 |
| 2928 | 0.769 | 1.000 | 0.824 | 1.000 | 0.968 | 0.960 | 0.906 | 1.000 | 1.000 | 1.000 | 0.957 | 1.000 | 0.991 | 0.853 | 1.000 | 1.000 | 0.858 | 0.831 |
| 2929 | 0.771 | 1.000 | 0.806 | 1.000 | 0.968 | 0.960 | 0.901 | 1.000 | 0.990 | 1.000 | 0.951 | 0.970 | 0.991 | 0.856 | 1.000 | 1.000 | 0.857 | 0.829 |
| 2930 | 0.769 | 1.000 | 0.814 | 0.986 | 0.968 | 0.960 | 0.906 | 1.000 | 0.990 | 1.000 | 0.957 | 0.970 | 0.991 | 0.858 | 1.000 | 1.000 | 0.858 | 0.830 |
| 2931 | 0.775 | 1.000 | 0.826 | 0.986 | 0.963 | 0.960 | 0.909 | 1.000 | 1.000 | 1.000 | 0.954 | 0.970 | 0.991 | 0.851 | 1.000 | 1.000 | 0.860 | 0.832 |
| 2932 | 0.775 | 1.000 | 0.818 | 1.000 | 0.968 | 0.960 | 0.900 | 1.000 | 1.000 | 1.000 | 0.954 | 1.000 | 0.991 | 0.856 | 1.000 | 1.000 | 0.860 | 0.832 |
| 2933 | 0.769 | 1.000 | 0.820 | 1.000 | 0.963 | 0.960 | 0.903 | 1.000 | 1.000 | 1.000 | 0.957 | 1.000 | 1.000 | 0.854 | 1.000 | 1.000 | 0.858 | 0.830 |
| 2934 | 0.773 | 1.000 | 0.814 | 1.000 | 0.968 | 0.960 | 0.909 | 1.000 | 1.000 | 1.000 | 0.959 | 1.000 | 1.000 | 0.852 | 1.000 | 1.000 | 0.859 | 0.832 |
| 2935 | 0.770 | 1.000 | 0.820 | 1.000 | 0.968 | 0.960 | 0.908 | 1.000 | 0.990 | 1.000 | 0.959 | 0.970 | 1.000 | 0.851 | 1.000 | 1.000 | 0.858 | 0.830 |
| 2936 | 0.772 | 1.000 | 0.810 | 0.986 | 0.968 | 0.960 | 0.915 | 1.000 | 0.990 | 1.000 | 0.965 | 0.970 | 1.000 | 0.850 | 1.000 | 1.000 | 0.859 | 0.831 |
| 2937 | 0.776 | 1.000 | 0.818 | 1.000 | 0.968 | 0.960 | 0.901 | 1.000 | 1.000 | 1.000 | 0.957 | 0.970 | 1.000 | 0.858 | 1.000 | 1.000 | 0.861 | 0.833 |
| 2938 | 0.771 | 1.000 | 0.818 | 1.000 | 0.968 | 0.960 | 0.904 | 1.000 | 0.990 | 1.000 | 0.962 | 1.000 | 0.991 | 0.853 | 1.000 | 1.000 | 0.859 | 0.831 |
| 2939 | 0.765 | 1.000 | 0.818 | 1.000 | 0.963 | 0.960 | 0.903 | 1.000 | 0.990 | 1.000 | 0.959 | 0.970 | 0.982 | 0.860 | 1.000 | 1.000 | 0.858 | 0.830 |
| 2940 | 0.775 | 1.000 | 0.822 | 0.986 | 0.963 | 0.960 | 0.903 | 1.000 | 0.990 | 1.000 | 0.962 | 0.970 | 1.000 | 0.854 | 1.000 | 1.000 | 0.860 | 0.832 |
| 2941 | 0.767 | 1.000 | 0.828 | 0.986 | 0.968 | 0.960 | 0.908 | 1.000 | 1.000 | 1.000 | 0.954 | 0.970 | 0.991 | 0.854 | 1.000 | 1.000 | 0.858 | 0.830 |
| 2942 | 0.777 | 1.000 | 0.828 | 1.000 | 0.968 | 0.960 | 0.909 | 1.000 | 0.990 | 1.000 | 0.959 | 1.000 | 1.000 | 0.858 | 1.000 | 1.000 | 0.863 | 0.836 |
| 2943 | 0.775 | 1.000 | 0.830 | 1.000 | 0.963 | 0.960 | 0.912 | 1.000 | 1.000 | 1.000 | 0.959 | 0.970 | 1.000 | 0.849 | 1.000 | 1.000 | 0.861 | 0.834 |
| 2944 | 0.769 | 1.000 | 0.804 | 0.986 | 0.968 | 0.960 | 0.908 | 1.000 | 0.990 | 1.000 | 0.957 | 0.970 | 1.000 | 0.843 | 1.000 | 1.000 | 0.854 | 0.825 |
| 2945 | 0.773 | 1.000 | 0.820 | 1.000 | 0.963 | 0.960 | 0.904 | 1.000 | 1.000 | 1.000 | 0.954 | 0.970 | 0.982 | 0.851 | 1.000 | 1.000 | 0.858 | 0.830 |
| 2946 | 0.769 | 1.000 | 0.812 | 1.000 | 0.968 | 0.960 | 0.898 | 1.000 | 0.990 | 1.000 | 0.954 | 0.970 | 1.000 | 0.856 | 1.000 | 1.000 | 0.857 | 0.828 |
| 2947 | 0.768 | 1.000 | 0.822 | 0.986 | 0.968 | 0.960 | 0.912 | 1.000 | 1.000 | 1.000 | 0.959 | 1.000 | 0.991 | 0.848 | 1.000 | 1.000 | 0.858 | 0.830 |
| 2948 | 0.767 | 1.000 | 0.806 | 1.000 | 0.968 | 0.960 | 0.909 | 1.000 | 0.990 | 1.000 | 0.954 | 0.970 | 1.000 | 0.861 | 1.000 | 1.000 | 0.858 | 0.830 |
| 2949 | 0.766 | 1.000 | 0.824 | 0.986 | 0.963 | 0.960 | 0.909 | 1.000 | 0.990 | 1.000 | 0.962 | 1.000 | 1.000 | 0.850 | 1.000 | 1.000 | 0.857 | 0.829 |
| 2950 | 0.777 | 1.000 | 0.820 | 1.000 | 0.968 | 0.960 | 0.909 | 1.000 | 0.990 | 1.000 | 0.959 | 0.970 | 1.000 | 0.848 | 1.000 | 1.000 | 0.860 | 0.832 |
| 2951 | 0.776 | 1.000 | 0.832 | 1.000 | 0.968 | 0.960 | 0.909 | 1.000 | 0.990 | 1.000 | 0.959 | 0.970 | 0.991 | 0.855 | 1.000 | 1.000 | 0.862 | 0.835 |
| 2952 | 0.762 | 1.000 | 0.818 | 1.000 | 0.968 | 0.960 | 0.894 | 1.000 | 0.990 | 1.000 | 0.954 | 0.970 | 0.991 | 0.853 | 1.000 | 1.000 | 0.854 | 0.825 |
| 2953 | 0.763 | 1.000 | 0.812 | 1.000 | 0.968 | 0.960 | 0.911 | 1.000 | 1.000 | 1.000 | 0.957 | 0.970 | 0.991 | 0.856 | 1.000 | 1.000 | 0.857 | 0.829 |
| 2954 | 0.775 | 1.000 | 0.806 | 1.000 | 0.963 | 0.960 | 0.901 | 1.000 | 1.000 | 1.000 | 0.959 | 0.970 | 0.982 | 0.862 | 1.000 | 1.000 | 0.860 | 0.832 |
| 2955 | 0.781 | 1.000 | 0.810 | 0.986 | 0.974 | 0.960 | 0.908 | 1.000 | 0.990 | 1.000 | 0.959 | 0.970 | 0.991 | 0.852 | 1.000 | 1.000 | 0.861 | 0.833 |
| 2956 | 0.783 | 1.000 | 0.822 | 0.986 | 0.963 | 0.960 | 0.912 | 1.000 | 1.000 | 1.000 | 0.957 | 0.970 | 0.991 | 0.853 | 1.000 | 1.000 | 0.863 | 0.836 |
| 2957 | 0.772 | 1.000 | 0.808 | 1.000 | 0.968 | 0.960 | 0.897 | 1.000 | 1.000 | 1.000 | 0.954 | 0.970 | 1.000 | 0.858 | 1.000 | 1.000 | 0.858 | 0.830 |
| 2958 | 0.769 | 1.000 | 0.824 | 0.986 | 0.968 | 0.960 | 0.900 | 1.000 | 1.000 | 1.000 | 0.957 | 0.970 | 1.000 | 0.856 | 1.000 | 1.000 | 0.858 | 0.830 |
| 2959 | 0.771 | 1.000 | 0.808 | 1.000 | 0.974 | 0.960 | 0.914 | 1.000 | 1.000 | 1.000 | 0.954 | 0.970 | 0.991 | 0.857 | 1.000 | 1.000 | 0.859 | 0.832 |
| 2960 | 0.768 | 1.000 | 0.822 | 1.000 | 0.963 | 0.960 | 0.901 | 1.000 | 0.990 | 1.000 | 0.957 | 0.970 | 1.000 | 0.851 | 1.000 | 1.000 | 0.857 | 0.829 |
| 2961 | 0.765 | 1.000 | 0.830 | 1.000 | 0.963 | 0.960 | 0.906 | 1.000 | 0.990 | 1.000 | 0.957 | 1.000 | 1.000 | 0.852 | 1.000 | 1.000 | 0.857 | 0.829 |
| 2962 | 0.762 | 1.000 | 0.826 | 0.986 | 0.958 | 0.960 | 0.908 | 1.000 | 1.000 | 1.000 | 0.948 | 0.970 | 0.991 | 0.855 | 1.000 | 1.000 | 0.856 | 0.828 |

| | | | | | | | | | | | | | | | | | |
|---|---|---|---|---|---|---|---|---|---|---|---|---|---|---|---|---|---|
| 2963 | 0.767 | 1.000 | 0.820 | 1.000 | 0.958 | 0.960 | 0.911 | 1.000 | 0.990 | 1.000 | 0.959 | 1.000 | 0.991 | 0.859 | 1.000 | 1.000 | 0.859 | 0.832 |
| 2964 | 0.773 | 1.000 | 0.816 | 1.000 | 0.968 | 0.960 | 0.912 | 1.000 | 0.990 | 1.000 | 0.957 | 0.970 | 1.000 | 0.850 | 1.000 | 1.000 | 0.859 | 0.831 |
| 2965 | 0.755 | 1.000 | 0.814 | 1.000 | 0.963 | 0.960 | 0.900 | 1.000 | 0.990 | 1.000 | 0.957 | 0.970 | 1.000 | 0.860 | 1.000 | 1.000 | 0.854 | 0.825 |
| 2966 | 0.761 | 1.000 | 0.828 | 1.000 | 0.968 | 0.960 | 0.912 | 1.000 | 0.990 | 1.000 | 0.962 | 0.970 | 1.000 | 0.856 | 1.000 | 1.000 | 0.858 | 0.831 |
| 2967 | 0.770 | 1.000 | 0.810 | 1.000 | 0.963 | 0.960 | 0.904 | 1.000 | 1.000 | 1.000 | 0.951 | 0.970 | 1.000 | 0.846 | 1.000 | 1.000 | 0.855 | 0.827 |
| 2968 | 0.772 | 1.000 | 0.816 | 1.000 | 0.968 | 0.960 | 0.909 | 1.000 | 1.000 | 1.000 | 0.957 | 0.970 | 0.991 | 0.855 | 1.000 | 1.000 | 0.859 | 0.832 |
| 2969 | 0.775 | 1.000 | 0.836 | 0.986 | 0.968 | 0.960 | 0.909 | 1.000 | 1.000 | 1.000 | 0.954 | 0.970 | 0.991 | 0.855 | 1.000 | 1.000 | 0.862 | 0.835 |
| 2970 | 0.766 | 1.000 | 0.822 | 1.000 | 0.968 | 0.960 | 0.901 | 1.000 | 0.990 | 1.000 | 0.951 | 0.970 | 0.991 | 0.854 | 1.000 | 1.000 | 0.856 | 0.828 |
| 2971 | 0.780 | 1.000 | 0.820 | 0.986 | 0.968 | 0.960 | 0.904 | 1.000 | 1.000 | 1.000 | 0.954 | 0.970 | 0.991 | 0.860 | 1.000 | 1.000 | 0.862 | 0.835 |
| 2972 | 0.774 | 1.000 | 0.816 | 0.986 | 0.963 | 0.960 | 0.897 | 1.000 | 1.000 | 1.000 | 0.957 | 1.000 | 0.991 | 0.859 | 1.000 | 1.000 | 0.859 | 0.831 |
| 2973 | 0.775 | 1.000 | 0.804 | 0.986 | 0.968 | 0.960 | 0.908 | 1.000 | 0.990 | 1.000 | 0.945 | 0.970 | 0.991 | 0.851 | 1.000 | 1.000 | 0.857 | 0.828 |
| 2974 | 0.780 | 1.000 | 0.820 | 0.986 | 0.968 | 0.960 | 0.901 | 1.000 | 0.990 | 1.000 | 0.954 | 0.970 | 0.991 | 0.858 | 1.000 | 1.000 | 0.861 | 0.834 |
| 2975 | 0.767 | 1.000 | 0.824 | 1.000 | 0.974 | 0.960 | 0.908 | 1.000 | 1.000 | 1.000 | 0.962 | 0.970 | 0.991 | 0.852 | 1.000 | 1.000 | 0.858 | 0.831 |
| 2976 | 0.771 | 1.000 | 0.806 | 1.000 | 0.968 | 0.960 | 0.908 | 1.000 | 1.000 | 1.000 | 0.954 | 0.970 | 0.991 | 0.849 | 1.000 | 1.000 | 0.856 | 0.828 |
| 2977 | 0.765 | 1.000 | 0.822 | 1.000 | 0.968 | 0.960 | 0.906 | 1.000 | 1.000 | 1.000 | 0.959 | 0.970 | 1.000 | 0.851 | 1.000 | 1.000 | 0.857 | 0.829 |
| 2978 | 0.775 | 1.000 | 0.842 | 1.000 | 0.968 | 0.960 | 0.903 | 1.000 | 1.000 | 1.000 | 0.957 | 0.970 | 1.000 | 0.851 | 1.000 | 1.000 | 0.861 | 0.834 |
| 2979 | 0.772 | 1.000 | 0.810 | 1.000 | 0.963 | 0.960 | 0.900 | 1.000 | 1.000 | 1.000 | 0.954 | 0.970 | 1.000 | 0.848 | 1.000 | 1.000 | 0.856 | 0.827 |
| 2980 | 0.772 | 1.000 | 0.820 | 1.000 | 0.963 | 0.960 | 0.908 | 1.000 | 1.000 | 1.000 | 0.954 | 0.970 | 1.000 | 0.851 | 1.000 | 1.000 | 0.858 | 0.830 |
| 2981 | 0.769 | 1.000 | 0.838 | 1.000 | 0.979 | 0.960 | 0.911 | 1.000 | 1.000 | 1.000 | 0.959 | 0.970 | 1.000 | 0.854 | 1.000 | 1.000 | 0.861 | 0.834 |
| 2982 | 0.775 | 1.000 | 0.816 | 0.986 | 0.963 | 0.960 | 0.908 | 1.000 | 1.000 | 1.000 | 0.957 | 0.970 | 0.991 | 0.849 | 1.000 | 1.000 | 0.858 | 0.830 |
| 2983 | 0.766 | 1.000 | 0.826 | 0.986 | 0.963 | 0.960 | 0.915 | 1.000 | 1.000 | 1.000 | 0.954 | 1.000 | 0.991 | 0.856 | 1.000 | 1.000 | 0.859 | 0.832 |
| 2984 | 0.765 | 1.000 | 0.826 | 1.000 | 0.958 | 0.960 | 0.914 | 1.000 | 1.000 | 1.000 | 0.957 | 0.970 | 0.991 | 0.849 | 1.000 | 1.000 | 0.857 | 0.829 |
| 2985 | 0.778 | 1.000 | 0.820 | 1.000 | 0.968 | 0.960 | 0.912 | 1.000 | 0.990 | 1.000 | 0.951 | 0.970 | 1.000 | 0.858 | 1.000 | 1.000 | 0.862 | 0.835 |
| 2986 | 0.771 | 1.000 | 0.810 | 1.000 | 0.968 | 0.960 | 0.909 | 1.000 | 1.000 | 1.000 | 0.951 | 0.970 | 0.991 | 0.852 | 1.000 | 1.000 | 0.857 | 0.830 |
| 2987 | 0.773 | 1.000 | 0.826 | 1.000 | 0.963 | 0.960 | 0.904 | 1.000 | 1.000 | 1.000 | 0.968 | 0.970 | 1.000 | 0.861 | 1.000 | 1.000 | 0.862 | 0.835 |
| 2988 | 0.771 | 1.000 | 0.816 | 0.986 | 0.963 | 0.960 | 0.903 | 1.000 | 1.000 | 1.000 | 0.951 | 1.000 | 0.991 | 0.853 | 1.000 | 1.000 | 0.857 | 0.829 |
| 2989 | 0.771 | 1.000 | 0.820 | 1.000 | 0.963 | 0.960 | 0.901 | 1.000 | 0.990 | 1.000 | 0.954 | 0.970 | 1.000 | 0.860 | 1.000 | 1.000 | 0.859 | 0.831 |
| 2990 | 0.777 | 1.000 | 0.814 | 1.000 | 0.958 | 0.960 | 0.906 | 1.000 | 1.000 | 1.000 | 0.951 | 0.970 | 1.000 | 0.857 | 1.000 | 1.000 | 0.860 | 0.832 |
| 2991 | 0.767 | 1.000 | 0.828 | 0.986 | 0.968 | 0.960 | 0.906 | 1.000 | 1.000 | 1.000 | 0.959 | 0.970 | 1.000 | 0.863 | 1.000 | 1.000 | 0.861 | 0.834 |
| 2992 | 0.757 | 1.000 | 0.824 | 0.986 | 0.963 | 0.960 | 0.898 | 1.000 | 1.000 | 1.000 | 0.957 | 0.970 | 1.000 | 0.852 | 1.000 | 1.000 | 0.853 | 0.825 |
| 2993 | 0.779 | 1.000 | 0.826 | 1.000 | 0.963 | 0.960 | 0.900 | 1.000 | 0.990 | 1.000 | 0.951 | 0.970 | 0.991 | 0.854 | 1.000 | 1.000 | 0.860 | 0.832 |
| 2994 | 0.769 | 1.000 | 0.814 | 1.000 | 0.963 | 0.960 | 0.900 | 1.000 | 0.990 | 1.000 | 0.954 | 0.970 | 1.000 | 0.858 | 1.000 | 1.000 | 0.858 | 0.829 |
| 2995 | 0.773 | 1.000 | 0.828 | 0.986 | 0.968 | 0.960 | 0.908 | 1.000 | 0.990 | 1.000 | 0.957 | 0.970 | 1.000 | 0.857 | 1.000 | 1.000 | 0.861 | 0.834 |
| 2996 | 0.773 | 1.000 | 0.826 | 0.986 | 0.968 | 0.960 | 0.915 | 1.000 | 1.000 | 1.000 | 0.954 | 0.970 | 1.000 | 0.850 | 1.000 | 1.000 | 0.860 | 0.833 |
| 2997 | 0.767 | 1.000 | 0.816 | 1.000 | 0.963 | 0.960 | 0.917 | 1.000 | 1.000 | 1.000 | 0.954 | 1.000 | 1.000 | 0.849 | 1.000 | 1.000 | 0.857 | 0.829 |
| 2998 | 0.781 | 1.000 | 0.818 | 1.000 | 0.968 | 0.960 | 0.904 | 1.000 | 0.990 | 1.000 | 0.962 | 0.970 | 1.000 | 0.854 | 1.000 | 1.000 | 0.862 | 0.834 |
| 2999 | 0.765 | 1.000 | 0.820 | 1.000 | 0.968 | 0.960 | 0.911 | 1.000 | 0.990 | 1.000 | 0.962 | 0.970 | 0.982 | 0.848 | 1.000 | 1.000 | 0.856 | 0.828 |
| 3000 | 0.764 | 1.000 | 0.822 | 1.000 | 0.968 | 0.960 | 0.901 | 1.000 | 1.000 | 1.000 | 0.951 | 1.000 | 0.991 | 0.855 | 1.000 | 1.000 | 0.856 | 0.828 |
| 3001 | 0.769 | 1.000 | 0.824 | 1.000 | 0.968 | 0.960 | 0.895 | 1.000 | 1.000 | 1.000 | 0.968 | 0.970 | 0.991 | 0.858 | 1.000 | 1.000 | 0.859 | 0.831 |

| | | | | | | | | | | | | | | | | | |
|---|---|---|---|---|---|---|---|---|---|---|---|---|---|---|---|---|---|
| 3002 | 0.771 | 1.000 | 0.822 | 1.000 | 0.968 | 0.960 | 0.904 | 1.000 | 1.000 | 1.000 | 0.957 | 1.000 | 1.000 | 0.855 | 1.000 | 1.000 | 0.860 | 0.832 |
| 3003 | 0.765 | 1.000 | 0.830 | 0.986 | 0.974 | 0.960 | 0.906 | 1.000 | 1.000 | 1.000 | 0.954 | 0.970 | 0.982 | 0.856 | 1.000 | 1.000 | 0.858 | 0.831 |
| 3004 | 0.773 | 1.000 | 0.832 | 0.986 | 0.968 | 0.960 | 0.904 | 1.000 | 0.990 | 1.000 | 0.957 | 0.970 | 0.991 | 0.854 | 1.000 | 1.000 | 0.860 | 0.832 |
| 3005 | 0.777 | 1.000 | 0.808 | 0.986 | 0.958 | 0.960 | 0.892 | 1.000 | 1.000 | 1.000 | 0.954 | 0.939 | 0.991 | 0.854 | 1.000 | 1.000 | 0.857 | 0.828 |
| 3006 | 0.761 | 1.000 | 0.814 | 0.986 | 0.968 | 0.960 | 0.901 | 1.000 | 1.000 | 1.000 | 0.957 | 1.000 | 0.991 | 0.847 | 1.000 | 1.000 | 0.853 | 0.824 |
| 3007 | 0.767 | 1.000 | 0.828 | 0.986 | 0.968 | 0.960 | 0.908 | 1.000 | 1.000 | 1.000 | 0.954 | 0.970 | 0.991 | 0.849 | 1.000 | 1.000 | 0.857 | 0.829 |
| 3008 | 0.774 | 1.000 | 0.826 | 1.000 | 0.974 | 0.960 | 0.914 | 1.000 | 1.000 | 1.000 | 0.954 | 0.970 | 0.991 | 0.850 | 1.000 | 1.000 | 0.860 | 0.833 |
| 3009 | 0.769 | 1.000 | 0.818 | 1.000 | 0.968 | 0.960 | 0.906 | 1.000 | 1.000 | 1.000 | 0.951 | 1.000 | 1.000 | 0.851 | 1.000 | 1.000 | 0.857 | 0.829 |
| 3010 | 0.771 | 1.000 | 0.814 | 0.986 | 0.963 | 0.960 | 0.900 | 1.000 | 1.000 | 1.000 | 0.945 | 0.939 | 0.991 | 0.851 | 1.000 | 1.000 | 0.855 | 0.827 |
| 3011 | 0.771 | 1.000 | 0.822 | 1.000 | 0.968 | 0.960 | 0.911 | 1.000 | 0.990 | 1.000 | 0.954 | 0.970 | 1.000 | 0.859 | 1.000 | 1.000 | 0.861 | 0.833 |
| 3012 | 0.763 | 1.000 | 0.828 | 1.000 | 0.974 | 0.960 | 0.906 | 1.000 | 0.990 | 1.000 | 0.951 | 0.970 | 0.991 | 0.861 | 1.000 | 1.000 | 0.859 | 0.831 |
| 3013 | 0.767 | 1.000 | 0.812 | 1.000 | 0.963 | 0.960 | 0.911 | 1.000 | 1.000 | 1.000 | 0.951 | 0.970 | 1.000 | 0.861 | 1.000 | 1.000 | 0.859 | 0.831 |
| 3014 | 0.771 | 1.000 | 0.826 | 1.000 | 0.968 | 0.960 | 0.897 | 1.000 | 1.000 | 1.000 | 0.957 | 0.970 | 0.991 | 0.850 | 1.000 | 1.000 | 0.857 | 0.829 |
| 3015 | 0.773 | 1.000 | 0.824 | 1.000 | 0.968 | 0.960 | 0.909 | 1.000 | 1.000 | 1.000 | 0.954 | 0.970 | 1.000 | 0.860 | 1.000 | 1.000 | 0.862 | 0.835 |
| 3016 | 0.772 | 1.000 | 0.820 | 1.000 | 0.968 | 0.960 | 0.895 | 1.000 | 1.000 | 1.000 | 0.957 | 0.939 | 0.982 | 0.857 | 1.000 | 1.000 | 0.858 | 0.830 |
| 3017 | 0.784 | 1.000 | 0.820 | 1.000 | 0.974 | 0.960 | 0.909 | 1.000 | 1.000 | 1.000 | 0.951 | 0.970 | 1.000 | 0.856 | 1.000 | 1.000 | 0.864 | 0.837 |
| 3018 | 0.766 | 1.000 | 0.830 | 1.000 | 0.974 | 0.960 | 0.909 | 1.000 | 0.990 | 1.000 | 0.954 | 0.970 | 0.991 | 0.851 | 1.000 | 1.000 | 0.858 | 0.830 |
| 3019 | 0.772 | 1.000 | 0.824 | 0.986 | 0.968 | 0.960 | 0.900 | 1.000 | 1.000 | 1.000 | 0.957 | 1.000 | 0.991 | 0.858 | 1.000 | 1.000 | 0.860 | 0.832 |
| 3020 | 0.777 | 1.000 | 0.816 | 0.986 | 0.963 | 0.960 | 0.903 | 1.000 | 1.000 | 1.000 | 0.957 | 0.970 | 1.000 | 0.854 | 1.000 | 1.000 | 0.860 | 0.832 |
| 3021 | 0.774 | 1.000 | 0.824 | 1.000 | 0.968 | 0.960 | 0.912 | 1.000 | 0.990 | 1.000 | 0.959 | 0.970 | 0.991 | 0.854 | 1.000 | 1.000 | 0.861 | 0.833 |
| 3022 | 0.775 | 1.000 | 0.826 | 0.986 | 0.968 | 0.960 | 0.908 | 1.000 | 1.000 | 1.000 | 0.957 | 0.970 | 0.991 | 0.845 | 1.000 | 1.000 | 0.858 | 0.831 |
| 3023 | 0.767 | 1.000 | 0.820 | 1.000 | 0.968 | 0.960 | 0.920 | 1.000 | 0.990 | 1.000 | 0.954 | 0.970 | 1.000 | 0.851 | 1.000 | 1.000 | 0.859 | 0.831 |
| 3024 | 0.773 | 1.000 | 0.818 | 1.000 | 0.963 | 0.960 | 0.917 | 1.000 | 1.000 | 1.000 | 0.962 | 0.970 | 0.982 | 0.856 | 1.000 | 1.000 | 0.861 | 0.834 |
| 3025 | 0.760 | 1.000 | 0.822 | 1.000 | 0.963 | 0.960 | 0.914 | 1.000 | 1.000 | 1.000 | 0.951 | 0.970 | 1.000 | 0.861 | 1.000 | 1.000 | 0.858 | 0.830 |
| 3026 | 0.765 | 1.000 | 0.818 | 1.000 | 0.963 | 0.960 | 0.906 | 1.000 | 1.000 | 1.000 | 0.959 | 0.939 | 0.991 | 0.850 | 1.000 | 1.000 | 0.855 | 0.827 |
| 3027 | 0.774 | 1.000 | 0.816 | 1.000 | 0.963 | 0.960 | 0.912 | 1.000 | 0.990 | 1.000 | 0.965 | 0.970 | 0.991 | 0.857 | 1.000 | 1.000 | 0.861 | 0.834 |
| 3028 | 0.772 | 1.000 | 0.812 | 0.986 | 0.958 | 0.960 | 0.897 | 1.000 | 1.000 | 1.000 | 0.957 | 1.000 | 1.000 | 0.853 | 1.000 | 1.000 | 0.857 | 0.828 |
| 3029 | 0.767 | 1.000 | 0.796 | 1.000 | 0.963 | 0.960 | 0.900 | 1.000 | 1.000 | 1.000 | 0.959 | 1.000 | 0.982 | 0.856 | 1.000 | 1.000 | 0.855 | 0.826 |
| 3030 | 0.779 | 1.000 | 0.808 | 1.000 | 0.963 | 0.960 | 0.892 | 1.000 | 0.990 | 1.000 | 0.957 | 0.970 | 0.991 | 0.850 | 1.000 | 1.000 | 0.857 | 0.828 |
| 3031 | 0.783 | 1.000 | 0.820 | 1.000 | 0.963 | 0.960 | 0.894 | 1.000 | 0.990 | 1.000 | 0.957 | 1.000 | 1.000 | 0.851 | 1.000 | 1.000 | 0.860 | 0.832 |
| 3032 | 0.775 | 1.000 | 0.802 | 1.000 | 0.963 | 0.960 | 0.895 | 1.000 | 1.000 | 1.000 | 0.948 | 1.000 | 0.991 | 0.854 | 1.000 | 1.000 | 0.856 | 0.828 |
| 3033 | 0.777 | 1.000 | 0.808 | 1.000 | 0.963 | 0.960 | 0.891 | 1.000 | 0.990 | 1.000 | 0.968 | 1.000 | 0.991 | 0.858 | 1.000 | 1.000 | 0.859 | 0.831 |
| 3034 | 0.762 | 1.000 | 0.810 | 1.000 | 0.947 | 0.960 | 0.897 | 1.000 | 1.000 | 1.000 | 0.959 | 1.000 | 0.991 | 0.855 | 1.000 | 1.000 | 0.854 | 0.825 |
| 3035 | 0.768 | 1.000 | 0.810 | 1.000 | 0.953 | 0.960 | 0.898 | 1.000 | 1.000 | 1.000 | 0.959 | 1.000 | 1.000 | 0.856 | 1.000 | 1.000 | 0.856 | 0.828 |
| 3036 | 0.775 | 1.000 | 0.816 | 1.000 | 0.958 | 0.960 | 0.908 | 1.000 | 0.980 | 1.000 | 0.962 | 0.970 | 0.991 | 0.852 | 1.000 | 1.000 | 0.859 | 0.831 |
| 3037 | 0.779 | 1.000 | 0.810 | 0.971 | 0.953 | 0.960 | 0.894 | 1.000 | 1.000 | 1.000 | 0.954 | 1.000 | 1.000 | 0.857 | 1.000 | 1.000 | 0.858 | 0.830 |
| 3038 | 0.780 | 1.000 | 0.814 | 1.000 | 0.953 | 0.960 | 0.880 | 1.000 | 0.990 | 1.000 | 0.965 | 1.000 | 1.000 | 0.851 | 1.000 | 1.000 | 0.857 | 0.828 |
| 3039 | 0.764 | 1.000 | 0.818 | 0.986 | 0.953 | 0.960 | 0.878 | 1.000 | 1.000 | 1.000 | 0.965 | 1.000 | 1.000 | 0.855 | 1.000 | 1.000 | 0.853 | 0.824 |
| 3040 | 0.761 | 1.000 | 0.810 | 0.986 | 0.958 | 0.960 | 0.903 | 1.000 | 1.000 | 1.000 | 0.962 | 0.970 | 1.000 | 0.854 | 1.000 | 1.000 | 0.854 | 0.826 |

| | | | | | | | | | | | | | | | | | |
|---|---|---|---|---|---|---|---|---|---|---|---|---|---|---|---|---|---|
| 3041 | 0.764 | 1.000 | 0.800 | 1.000 | 0.953 | 0.960 | 0.895 | 1.000 | 0.990 | 1.000 | 0.962 | 1.000 | 0.991 | 0.849 | 1.000 | 1.000 | 0.852 | 0.823 |
| 3042 | 0.769 | 1.000 | 0.810 | 0.986 | 0.958 | 0.960 | 0.900 | 1.000 | 0.990 | 1.000 | 0.959 | 0.970 | 0.991 | 0.854 | 1.000 | 1.000 | 0.856 | 0.827 |
| 3043 | 0.761 | 1.000 | 0.785 | 1.000 | 0.953 | 0.960 | 0.891 | 1.000 | 0.990 | 1.000 | 0.962 | 1.000 | 1.000 | 0.854 | 1.000 | 1.000 | 0.850 | 0.820 |
| 3044 | 0.773 | 1.000 | 0.820 | 1.000 | 0.953 | 0.960 | 0.883 | 1.000 | 0.990 | 1.000 | 0.962 | 0.970 | 1.000 | 0.859 | 1.000 | 1.000 | 0.858 | 0.829 |
| 3045 | 0.763 | 1.000 | 0.798 | 1.000 | 0.953 | 0.960 | 0.898 | 1.000 | 0.980 | 1.000 | 0.962 | 0.970 | 1.000 | 0.851 | 1.000 | 1.000 | 0.852 | 0.823 |
| 3046 | 0.779 | 1.000 | 0.802 | 1.000 | 0.958 | 0.960 | 0.892 | 1.000 | 0.990 | 1.000 | 0.959 | 0.970 | 0.991 | 0.847 | 1.000 | 1.000 | 0.855 | 0.826 |
| 3047 | 0.770 | 1.000 | 0.804 | 1.000 | 0.958 | 0.960 | 0.889 | 1.000 | 1.000 | 1.000 | 0.965 | 1.000 | 1.000 | 0.856 | 1.000 | 1.000 | 0.856 | 0.827 |
| 3048 | 0.772 | 1.000 | 0.804 | 1.000 | 0.958 | 0.960 | 0.891 | 1.000 | 0.990 | 1.000 | 0.962 | 1.000 | 0.991 | 0.851 | 1.000 | 1.000 | 0.855 | 0.826 |
| 3049 | 0.772 | 1.000 | 0.808 | 1.000 | 0.958 | 0.960 | 0.892 | 1.000 | 1.000 | 1.000 | 0.968 | 1.000 | 1.000 | 0.851 | 1.000 | 1.000 | 0.856 | 0.827 |
| 3050 | 0.758 | 1.000 | 0.820 | 1.000 | 0.947 | 0.960 | 0.897 | 1.000 | 0.990 | 1.000 | 0.959 | 0.970 | 0.991 | 0.854 | 1.000 | 1.000 | 0.853 | 0.824 |
| 3051 | 0.773 | 1.000 | 0.808 | 1.000 | 0.953 | 0.960 | 0.892 | 1.000 | 1.000 | 1.000 | 0.962 | 0.970 | 1.000 | 0.854 | 1.000 | 1.000 | 0.856 | 0.828 |
| 3052 | 0.771 | 1.000 | 0.794 | 1.000 | 0.953 | 0.960 | 0.897 | 1.000 | 1.000 | 1.000 | 0.954 | 1.000 | 0.991 | 0.855 | 1.000 | 1.000 | 0.855 | 0.826 |
| 3053 | 0.767 | 1.000 | 0.789 | 1.000 | 0.958 | 0.960 | 0.892 | 1.000 | 0.990 | 1.000 | 0.968 | 0.970 | 1.000 | 0.851 | 1.000 | 1.000 | 0.852 | 0.823 |
| 3054 | 0.775 | 1.000 | 0.824 | 1.000 | 0.947 | 0.960 | 0.889 | 1.000 | 0.990 | 1.000 | 0.962 | 1.000 | 1.000 | 0.850 | 1.000 | 1.000 | 0.857 | 0.828 |
| 3055 | 0.773 | 1.000 | 0.808 | 0.986 | 0.958 | 0.960 | 0.897 | 1.000 | 0.990 | 1.000 | 0.962 | 1.000 | 1.000 | 0.851 | 1.000 | 1.000 | 0.856 | 0.828 |
| 3056 | 0.778 | 1.000 | 0.822 | 1.000 | 0.947 | 0.960 | 0.897 | 1.000 | 1.000 | 1.000 | 0.962 | 0.970 | 1.000 | 0.846 | 1.000 | 1.000 | 0.858 | 0.829 |
| 3057 | 0.772 | 1.000 | 0.812 | 0.986 | 0.953 | 0.960 | 0.891 | 1.000 | 1.000 | 1.000 | 0.957 | 0.970 | 0.991 | 0.848 | 1.000 | 1.000 | 0.854 | 0.825 |
| 3058 | 0.776 | 1.000 | 0.824 | 0.986 | 0.958 | 0.960 | 0.897 | 1.000 | 0.990 | 1.000 | 0.954 | 0.939 | 1.000 | 0.858 | 1.000 | 1.000 | 0.859 | 0.831 |
| 3059 | 0.771 | 1.000 | 0.822 | 1.000 | 0.953 | 0.960 | 0.884 | 1.000 | 0.990 | 1.000 | 0.965 | 0.970 | 1.000 | 0.847 | 1.000 | 1.000 | 0.854 | 0.825 |
| 3060 | 0.772 | 1.000 | 0.810 | 1.000 | 0.958 | 0.960 | 0.888 | 1.000 | 0.980 | 1.000 | 0.959 | 0.970 | 1.000 | 0.858 | 1.000 | 1.000 | 0.856 | 0.828 |
| 3061 | 0.767 | 1.000 | 0.808 | 1.000 | 0.958 | 0.960 | 0.888 | 1.000 | 0.990 | 1.000 | 0.957 | 0.970 | 0.991 | 0.853 | 1.000 | 1.000 | 0.853 | 0.824 |
| 3062 | 0.776 | 1.000 | 0.810 | 1.000 | 0.953 | 0.960 | 0.892 | 1.000 | 0.990 | 1.000 | 0.959 | 1.000 | 0.991 | 0.852 | 1.000 | 1.000 | 0.857 | 0.828 |
| 3063 | 0.770 | 1.000 | 0.810 | 1.000 | 0.958 | 0.960 | 0.891 | 1.000 | 1.000 | 1.000 | 0.959 | 0.970 | 1.000 | 0.861 | 1.000 | 1.000 | 0.857 | 0.829 |
| 3064 | 0.768 | 1.000 | 0.812 | 1.000 | 0.947 | 0.960 | 0.894 | 1.000 | 1.000 | 1.000 | 0.962 | 0.970 | 1.000 | 0.851 | 1.000 | 1.000 | 0.854 | 0.826 |
| 3065 | 0.771 | 1.000 | 0.828 | 1.000 | 0.942 | 0.960 | 0.903 | 1.000 | 1.000 | 1.000 | 0.959 | 0.970 | 0.991 | 0.858 | 1.000 | 1.000 | 0.859 | 0.832 |
| 3066 | 0.764 | 1.000 | 0.822 | 1.000 | 0.953 | 0.960 | 0.898 | 1.000 | 0.990 | 1.000 | 0.962 | 0.970 | 0.991 | 0.854 | 1.000 | 1.000 | 0.855 | 0.827 |
| 3067 | 0.775 | 1.000 | 0.810 | 1.000 | 0.958 | 0.960 | 0.894 | 1.000 | 0.990 | 1.000 | 0.954 | 0.970 | 1.000 | 0.857 | 1.000 | 1.000 | 0.857 | 0.829 |
| 3068 | 0.770 | 1.000 | 0.826 | 0.986 | 0.942 | 0.960 | 0.901 | 1.000 | 1.000 | 1.000 | 0.959 | 1.000 | 1.000 | 0.854 | 1.000 | 1.000 | 0.858 | 0.830 |
| 3069 | 0.773 | 1.000 | 0.818 | 1.000 | 0.958 | 0.960 | 0.901 | 1.000 | 1.000 | 1.000 | 0.959 | 1.000 | 0.991 | 0.849 | 1.000 | 1.000 | 0.857 | 0.829 |
| 3070 | 0.773 | 1.000 | 0.804 | 0.986 | 0.947 | 0.960 | 0.889 | 1.000 | 0.990 | 1.000 | 0.959 | 1.000 | 1.000 | 0.857 | 1.000 | 1.000 | 0.856 | 0.827 |
| 3071 | 0.782 | 1.000 | 0.800 | 0.986 | 0.958 | 0.960 | 0.888 | 1.000 | 1.000 | 1.000 | 0.962 | 0.970 | 1.000 | 0.849 | 1.000 | 1.000 | 0.856 | 0.828 |
| 3072 | 0.765 | 1.000 | 0.808 | 0.986 | 0.953 | 0.960 | 0.892 | 1.000 | 1.000 | 1.000 | 0.965 | 0.970 | 0.991 | 0.856 | 1.000 | 1.000 | 0.854 | 0.825 |
| 3073 | 0.765 | 1.000 | 0.800 | 1.000 | 0.953 | 0.960 | 0.884 | 1.000 | 0.990 | 1.000 | 0.959 | 1.000 | 0.991 | 0.850 | 1.000 | 1.000 | 0.851 | 0.821 |
| 3074 | 0.772 | 1.000 | 0.810 | 1.000 | 0.958 | 0.960 | 0.889 | 1.000 | 0.990 | 1.000 | 0.962 | 0.970 | 0.991 | 0.858 | 1.000 | 1.000 | 0.857 | 0.828 |
| 3075 | 0.765 | 1.000 | 0.812 | 0.986 | 0.958 | 0.960 | 0.897 | 1.000 | 0.990 | 1.000 | 0.957 | 0.970 | 0.991 | 0.845 | 1.000 | 1.000 | 0.852 | 0.823 |
| 3076 | 0.767 | 1.000 | 0.806 | 1.000 | 0.953 | 0.960 | 0.892 | 1.000 | 1.000 | 1.000 | 0.959 | 0.970 | 1.000 | 0.850 | 1.000 | 1.000 | 0.853 | 0.824 |
| 3077 | 0.771 | 1.000 | 0.808 | 1.000 | 0.958 | 0.960 | 0.888 | 1.000 | 0.990 | 1.000 | 0.959 | 0.970 | 0.991 | 0.856 | 1.000 | 1.000 | 0.855 | 0.827 |
| 3078 | 0.779 | 1.000 | 0.808 | 1.000 | 0.953 | 0.960 | 0.875 | 1.000 | 1.000 | 1.000 | 0.957 | 0.970 | 1.000 | 0.851 | 1.000 | 1.000 | 0.855 | 0.826 |
| 3079 | 0.778 | 1.000 | 0.806 | 1.000 | 0.953 | 0.960 | 0.881 | 1.000 | 1.000 | 1.000 | 0.962 | 0.970 | 1.000 | 0.847 | 1.000 | 1.000 | 0.855 | 0.825 |

| | | | | | | | | | | | | | | | | | |
|---|---|---|---|---|---|---|---|---|---|---|---|---|---|---|---|---|---|
| 3080 | 0.771 | 1.000 | 0.806 | 1.000 | 0.953 | 0.960 | 0.897 | 1.000 | 1.000 | 1.000 | 0.954 | 0.970 | 1.000 | 0.845 | 1.000 | 1.000 | 0.853 | 0.824 |
| 3081 | 0.765 | 1.000 | 0.814 | 1.000 | 0.958 | 0.960 | 0.895 | 1.000 | 1.000 | 1.000 | 0.962 | 1.000 | 0.991 | 0.849 | 1.000 | 1.000 | 0.854 | 0.825 |
| 3082 | 0.763 | 1.000 | 0.804 | 1.000 | 0.947 | 0.960 | 0.897 | 1.000 | 0.990 | 1.000 | 0.959 | 0.970 | 1.000 | 0.858 | 1.000 | 1.000 | 0.854 | 0.825 |
| 3083 | 0.779 | 1.000 | 0.808 | 0.986 | 0.953 | 0.960 | 0.891 | 1.000 | 1.000 | 1.000 | 0.959 | 0.970 | 0.991 | 0.852 | 1.000 | 1.000 | 0.857 | 0.828 |
| 3084 | 0.775 | 1.000 | 0.816 | 1.000 | 0.958 | 0.960 | 0.894 | 1.000 | 1.000 | 1.000 | 0.962 | 0.970 | 1.000 | 0.856 | 1.000 | 1.000 | 0.859 | 0.831 |
| 3085 | 0.773 | 1.000 | 0.818 | 1.000 | 0.953 | 0.960 | 0.895 | 1.000 | 1.000 | 1.000 | 0.959 | 1.000 | 1.000 | 0.853 | 1.000 | 1.000 | 0.857 | 0.829 |
| 3086 | 0.769 | 1.000 | 0.816 | 1.000 | 0.958 | 0.960 | 0.889 | 1.000 | 0.990 | 1.000 | 0.965 | 1.000 | 1.000 | 0.854 | 1.000 | 1.000 | 0.856 | 0.828 |
| 3087 | 0.773 | 1.000 | 0.816 | 1.000 | 0.942 | 0.960 | 0.898 | 1.000 | 1.000 | 1.000 | 0.954 | 1.000 | 1.000 | 0.851 | 1.000 | 1.000 | 0.856 | 0.828 |
| 3088 | 0.772 | 1.000 | 0.810 | 0.986 | 0.958 | 0.960 | 0.888 | 1.000 | 0.990 | 1.000 | 0.962 | 0.970 | 0.991 | 0.851 | 1.000 | 1.000 | 0.855 | 0.826 |
| 3089 | 0.768 | 1.000 | 0.806 | 0.986 | 0.953 | 0.960 | 0.894 | 1.000 | 0.990 | 1.000 | 0.957 | 0.970 | 1.000 | 0.845 | 1.000 | 1.000 | 0.852 | 0.823 |
| 3090 | 0.773 | 1.000 | 0.814 | 0.986 | 0.958 | 0.960 | 0.894 | 1.000 | 0.990 | 1.000 | 0.962 | 1.000 | 1.000 | 0.854 | 1.000 | 1.000 | 0.857 | 0.829 |
| 3091 | 0.767 | 1.000 | 0.796 | 0.986 | 0.958 | 0.960 | 0.892 | 1.000 | 0.990 | 1.000 | 0.957 | 0.970 | 1.000 | 0.844 | 1.000 | 1.000 | 0.850 | 0.820 |
| 3092 | 0.778 | 1.000 | 0.816 | 0.986 | 0.947 | 0.960 | 0.888 | 1.000 | 1.000 | 1.000 | 0.962 | 1.000 | 1.000 | 0.854 | 1.000 | 1.000 | 0.858 | 0.830 |
| 3093 | 0.777 | 1.000 | 0.814 | 0.986 | 0.947 | 0.960 | 0.898 | 1.000 | 1.000 | 1.000 | 0.957 | 0.970 | 1.000 | 0.850 | 1.000 | 1.000 | 0.857 | 0.829 |
| 3094 | 0.765 | 1.000 | 0.818 | 0.986 | 0.958 | 0.960 | 0.901 | 1.000 | 0.990 | 1.000 | 0.957 | 1.000 | 0.991 | 0.847 | 1.000 | 1.000 | 0.854 | 0.825 |
| 3095 | 0.776 | 1.000 | 0.818 | 1.000 | 0.958 | 0.960 | 0.900 | 1.000 | 0.990 | 1.000 | 0.968 | 1.000 | 1.000 | 0.855 | 1.000 | 1.000 | 0.860 | 0.832 |
| 3096 | 0.775 | 1.000 | 0.816 | 0.986 | 0.947 | 0.960 | 0.891 | 1.000 | 1.000 | 1.000 | 0.962 | 0.970 | 0.991 | 0.858 | 1.000 | 1.000 | 0.858 | 0.829 |
| 3097 | 0.773 | 1.000 | 0.789 | 0.986 | 0.953 | 0.960 | 0.883 | 1.000 | 1.000 | 1.000 | 0.968 | 1.000 | 1.000 | 0.854 | 1.000 | 1.000 | 0.854 | 0.824 |
| 3098 | 0.772 | 1.000 | 0.816 | 0.986 | 0.947 | 0.960 | 0.904 | 1.000 | 1.000 | 1.000 | 0.959 | 1.000 | 1.000 | 0.852 | 1.000 | 1.000 | 0.858 | 0.829 |
| 3099 | 0.771 | 1.000 | 0.804 | 1.000 | 0.958 | 0.960 | 0.889 | 1.000 | 0.990 | 1.000 | 0.959 | 1.000 | 0.991 | 0.858 | 1.000 | 1.000 | 0.856 | 0.827 |
| 3100 | 0.782 | 1.000 | 0.808 | 1.000 | 0.958 | 0.960 | 0.901 | 1.000 | 0.990 | 1.000 | 0.965 | 0.970 | 0.991 | 0.850 | 1.000 | 1.000 | 0.859 | 0.831 |
| 3101 | 0.775 | 1.000 | 0.800 | 1.000 | 0.958 | 0.960 | 0.891 | 1.000 | 1.000 | 1.000 | 0.959 | 0.970 | 0.991 | 0.847 | 1.000 | 1.000 | 0.854 | 0.825 |
| 3102 | 0.771 | 1.000 | 0.802 | 0.986 | 0.958 | 0.960 | 0.881 | 1.000 | 0.990 | 1.000 | 0.968 | 0.970 | 1.000 | 0.854 | 1.000 | 1.000 | 0.854 | 0.825 |
| 3103 | 0.775 | 1.000 | 0.808 | 1.000 | 0.958 | 0.960 | 0.895 | 1.000 | 0.990 | 1.000 | 0.959 | 1.000 | 0.991 | 0.854 | 1.000 | 1.000 | 0.857 | 0.829 |
| 3104 | 0.773 | 1.000 | 0.808 | 1.000 | 0.958 | 0.960 | 0.900 | 1.000 | 0.990 | 1.000 | 0.959 | 0.970 | 1.000 | 0.853 | 1.000 | 1.000 | 0.857 | 0.829 |
| 3105 | 0.775 | 1.000 | 0.802 | 1.000 | 0.958 | 0.960 | 0.891 | 1.000 | 0.990 | 1.000 | 0.965 | 0.970 | 1.000 | 0.851 | 1.000 | 1.000 | 0.856 | 0.827 |
| 3106 | 0.768 | 1.000 | 0.822 | 1.000 | 0.953 | 0.960 | 0.891 | 1.000 | 0.990 | 1.000 | 0.959 | 1.000 | 1.000 | 0.855 | 1.000 | 1.000 | 0.856 | 0.828 |
| 3107 | 0.779 | 1.000 | 0.802 | 1.000 | 0.958 | 0.960 | 0.898 | 1.000 | 0.990 | 1.000 | 0.959 | 1.000 | 1.000 | 0.854 | 1.000 | 1.000 | 0.858 | 0.830 |
| 3108 | 0.783 | 1.000 | 0.806 | 0.986 | 0.953 | 0.960 | 0.895 | 1.000 | 0.990 | 1.000 | 0.965 | 0.970 | 1.000 | 0.855 | 1.000 | 1.000 | 0.860 | 0.832 |
| 3109 | 0.767 | 1.000 | 0.789 | 0.986 | 0.958 | 0.960 | 0.889 | 1.000 | 0.990 | 1.000 | 0.968 | 0.970 | 0.991 | 0.856 | 1.000 | 1.000 | 0.853 | 0.823 |
| 3110 | 0.773 | 1.000 | 0.818 | 0.986 | 0.958 | 0.960 | 0.884 | 1.000 | 0.990 | 1.000 | 0.965 | 1.000 | 1.000 | 0.851 | 1.000 | 1.000 | 0.856 | 0.827 |
| 3111 | 0.768 | 1.000 | 0.832 | 1.000 | 0.953 | 0.960 | 0.906 | 1.000 | 1.000 | 1.000 | 0.965 | 0.970 | 0.991 | 0.848 | 1.000 | 1.000 | 0.858 | 0.830 |
| 3112 | 0.771 | 1.000 | 0.798 | 0.986 | 0.958 | 0.960 | 0.878 | 1.000 | 0.990 | 1.000 | 0.965 | 0.970 | 0.991 | 0.852 | 1.000 | 1.000 | 0.852 | 0.823 |
| 3113 | 0.767 | 1.000 | 0.792 | 1.000 | 0.953 | 0.960 | 0.897 | 1.000 | 0.990 | 1.000 | 0.959 | 1.000 | 1.000 | 0.849 | 1.000 | 1.000 | 0.852 | 0.823 |
| 3114 | 0.776 | 1.000 | 0.796 | 1.000 | 0.953 | 0.960 | 0.889 | 1.000 | 1.000 | 1.000 | 0.962 | 0.970 | 0.991 | 0.851 | 1.000 | 1.000 | 0.855 | 0.825 |
| 3115 | 0.763 | 1.000 | 0.810 | 1.000 | 0.953 | 0.960 | 0.897 | 1.000 | 0.990 | 1.000 | 0.954 | 1.000 | 1.000 | 0.848 | 1.000 | 1.000 | 0.852 | 0.823 |
| 3116 | 0.772 | 1.000 | 0.802 | 1.000 | 0.958 | 0.960 | 0.894 | 1.000 | 1.000 | 1.000 | 0.968 | 0.970 | 1.000 | 0.852 | 1.000 | 1.000 | 0.856 | 0.827 |
| 3117 | 0.757 | 1.000 | 0.794 | 0.986 | 0.947 | 0.960 | 0.889 | 1.000 | 1.000 | 1.000 | 0.954 | 1.000 | 1.000 | 0.854 | 1.000 | 1.000 | 0.849 | 0.819 |
| 3118 | 0.769 | 1.000 | 0.812 | 1.000 | 0.953 | 0.960 | 0.898 | 1.000 | 1.000 | 1.000 | 0.968 | 1.000 | 0.991 | 0.849 | 1.000 | 1.000 | 0.855 | 0.827 |

| | | | | | | | | | | | | | | | | | |
|---|---|---|---|---|---|---|---|---|---|---|---|---|---|---|---|---|---|
| 3119 | 0.760 | 1.000 | 0.798 | 1.000 | 0.953 | 0.960 | 0.897 | 1.000 | 1.000 | 1.000 | 0.965 | 1.000 | 1.000 | 0.852 | 1.000 | 1.000 | 0.852 | 0.823 |
| 3120 | 0.777 | 1.000 | 0.802 | 1.000 | 0.953 | 0.960 | 0.884 | 1.000 | 1.000 | 1.000 | 0.965 | 1.000 | 1.000 | 0.848 | 1.000 | 1.000 | 0.855 | 0.826 |
| 3121 | 0.769 | 1.000 | 0.812 | 0.971 | 0.958 | 0.960 | 0.891 | 1.000 | 0.990 | 1.000 | 0.962 | 1.000 | 0.991 | 0.849 | 1.000 | 1.000 | 0.854 | 0.825 |
| 3122 | 0.769 | 1.000 | 0.812 | 0.986 | 0.958 | 0.960 | 0.901 | 1.000 | 0.990 | 1.000 | 0.959 | 0.970 | 0.991 | 0.852 | 1.000 | 1.000 | 0.856 | 0.827 |
| 3123 | 0.783 | 1.000 | 0.818 | 0.986 | 0.958 | 0.960 | 0.889 | 1.000 | 1.000 | 1.000 | 0.965 | 1.000 | 1.000 | 0.851 | 1.000 | 1.000 | 0.860 | 0.832 |
| 3124 | 0.773 | 1.000 | 0.816 | 0.986 | 0.958 | 0.960 | 0.891 | 1.000 | 1.000 | 1.000 | 0.962 | 0.970 | 1.000 | 0.847 | 1.000 | 1.000 | 0.855 | 0.826 |
| 3125 | 0.763 | 1.000 | 0.804 | 1.000 | 0.947 | 0.960 | 0.891 | 1.000 | 0.990 | 1.000 | 0.965 | 1.000 | 1.000 | 0.850 | 1.000 | 1.000 | 0.852 | 0.823 |
| 3126 | 0.769 | 1.000 | 0.802 | 0.986 | 0.958 | 0.960 | 0.888 | 1.000 | 1.000 | 1.000 | 0.971 | 1.000 | 0.991 | 0.852 | 1.000 | 1.000 | 0.854 | 0.825 |
| 3127 | 0.761 | 1.000 | 0.810 | 0.986 | 0.958 | 0.960 | 0.889 | 1.000 | 0.990 | 1.000 | 0.962 | 0.970 | 0.991 | 0.865 | 1.000 | 1.000 | 0.855 | 0.826 |
| 3128 | 0.769 | 1.000 | 0.806 | 0.986 | 0.958 | 0.960 | 0.895 | 1.000 | 0.990 | 1.000 | 0.965 | 0.970 | 0.991 | 0.856 | 1.000 | 1.000 | 0.856 | 0.827 |
| 3129 | 0.770 | 1.000 | 0.818 | 0.986 | 0.958 | 0.960 | 0.889 | 1.000 | 1.000 | 1.000 | 0.962 | 1.000 | 1.000 | 0.851 | 1.000 | 1.000 | 0.856 | 0.827 |
| 3130 | 0.781 | 1.000 | 0.818 | 0.986 | 0.953 | 0.960 | 0.892 | 1.000 | 0.990 | 1.000 | 0.962 | 0.970 | 1.000 | 0.853 | 1.000 | 1.000 | 0.859 | 0.831 |
| 3131 | 0.774 | 1.000 | 0.800 | 1.000 | 0.953 | 0.960 | 0.883 | 1.000 | 0.990 | 1.000 | 0.962 | 1.000 | 1.000 | 0.845 | 1.000 | 1.000 | 0.852 | 0.823 |
| 3132 | 0.775 | 1.000 | 0.796 | 1.000 | 0.953 | 0.960 | 0.891 | 1.000 | 0.990 | 1.000 | 0.965 | 1.000 | 0.991 | 0.852 | 1.000 | 1.000 | 0.855 | 0.826 |
| 3133 | 0.765 | 1.000 | 0.816 | 0.986 | 0.953 | 0.960 | 0.900 | 1.000 | 1.000 | 1.000 | 0.971 | 0.970 | 1.000 | 0.857 | 1.000 | 1.000 | 0.857 | 0.829 |
| 3134 | 0.775 | 1.000 | 0.810 | 0.986 | 0.947 | 0.960 | 0.892 | 1.000 | 0.990 | 1.000 | 0.968 | 0.970 | 0.991 | 0.848 | 1.000 | 1.000 | 0.855 | 0.826 |
| 3135 | 0.781 | 1.000 | 0.806 | 0.986 | 0.953 | 0.960 | 0.894 | 1.000 | 0.990 | 1.000 | 0.959 | 1.000 | 0.991 | 0.844 | 1.000 | 1.000 | 0.856 | 0.827 |
| 3136 | 0.767 | 1.000 | 0.800 | 1.000 | 0.947 | 0.960 | 0.892 | 1.000 | 1.000 | 1.000 | 0.962 | 0.970 | 1.000 | 0.855 | 1.000 | 1.000 | 0.854 | 0.825 |
| 3137 | 0.769 | 1.000 | 0.806 | 1.000 | 0.953 | 0.960 | 0.900 | 1.000 | 0.990 | 1.000 | 0.962 | 0.970 | 0.991 | 0.854 | 1.000 | 1.000 | 0.856 | 0.827 |
| 3138 | 0.769 | 1.000 | 0.812 | 0.986 | 0.958 | 0.960 | 0.892 | 1.000 | 1.000 | 1.000 | 0.962 | 1.000 | 0.991 | 0.849 | 1.000 | 1.000 | 0.854 | 0.826 |
| 3139 | 0.769 | 1.000 | 0.812 | 1.000 | 0.953 | 0.960 | 0.892 | 1.000 | 1.000 | 1.000 | 0.965 | 0.970 | 0.991 | 0.849 | 1.000 | 1.000 | 0.854 | 0.825 |
| 3140 | 0.774 | 1.000 | 0.800 | 0.986 | 0.953 | 0.960 | 0.889 | 1.000 | 1.000 | 1.000 | 0.959 | 0.970 | 1.000 | 0.852 | 1.000 | 1.000 | 0.855 | 0.826 |
| 3141 | 0.772 | 1.000 | 0.810 | 1.000 | 0.958 | 0.960 | 0.904 | 1.000 | 1.000 | 1.000 | 0.965 | 1.000 | 1.000 | 0.853 | 1.000 | 1.000 | 0.858 | 0.830 |
| 3142 | 0.775 | 1.000 | 0.824 | 1.000 | 0.953 | 0.960 | 0.900 | 1.000 | 0.990 | 1.000 | 0.968 | 0.970 | 1.000 | 0.855 | 1.000 | 1.000 | 0.860 | 0.832 |
| 3143 | 0.766 | 1.000 | 0.812 | 0.986 | 0.947 | 0.960 | 0.892 | 1.000 | 1.000 | 1.000 | 0.959 | 0.970 | 0.991 | 0.861 | 1.000 | 1.000 | 0.856 | 0.827 |
| 3144 | 0.772 | 1.000 | 0.816 | 1.000 | 0.958 | 0.960 | 0.895 | 1.000 | 0.990 | 1.000 | 0.962 | 0.970 | 1.000 | 0.859 | 1.000 | 1.000 | 0.859 | 0.830 |
| 3145 | 0.761 | 1.000 | 0.806 | 1.000 | 0.958 | 0.960 | 0.894 | 1.000 | 1.000 | 1.000 | 0.965 | 0.970 | 0.991 | 0.854 | 1.000 | 1.000 | 0.853 | 0.824 |
| 3146 | 0.763 | 1.000 | 0.800 | 1.000 | 0.953 | 0.960 | 0.894 | 1.000 | 0.990 | 1.000 | 0.962 | 1.000 | 0.991 | 0.851 | 1.000 | 1.000 | 0.852 | 0.823 |
| 3147 | 0.775 | 1.000 | 0.810 | 0.986 | 0.958 | 0.960 | 0.892 | 1.000 | 0.990 | 1.000 | 0.959 | 0.970 | 0.991 | 0.847 | 1.000 | 1.000 | 0.855 | 0.826 |
| 3148 | 0.782 | 1.000 | 0.800 | 0.986 | 0.953 | 0.960 | 0.898 | 1.000 | 0.990 | 1.000 | 0.965 | 0.970 | 0.991 | 0.851 | 1.000 | 1.000 | 0.858 | 0.830 |
| 3149 | 0.775 | 1.000 | 0.806 | 0.986 | 0.958 | 0.960 | 0.901 | 1.000 | 0.990 | 1.000 | 0.962 | 1.000 | 0.991 | 0.859 | 1.000 | 1.000 | 0.859 | 0.831 |
| 3150 | 0.770 | 1.000 | 0.796 | 1.000 | 0.958 | 0.960 | 0.891 | 1.000 | 0.990 | 1.000 | 0.968 | 0.970 | 0.991 | 0.856 | 1.000 | 1.000 | 0.855 | 0.826 |
| 3151 | 0.770 | 1.000 | 0.820 | 0.986 | 0.958 | 0.960 | 0.897 | 1.000 | 1.000 | 1.000 | 0.962 | 0.970 | 0.991 | 0.847 | 1.000 | 1.000 | 0.855 | 0.827 |
| 3152 | 0.769 | 1.000 | 0.810 | 1.000 | 0.947 | 0.960 | 0.892 | 1.000 | 0.990 | 1.000 | 0.962 | 0.970 | 0.991 | 0.850 | 1.000 | 1.000 | 0.854 | 0.825 |
| 3153 | 0.766 | 1.000 | 0.820 | 1.000 | 0.958 | 0.960 | 0.900 | 1.000 | 0.990 | 1.000 | 0.962 | 0.970 | 0.991 | 0.855 | 1.000 | 1.000 | 0.857 | 0.828 |
| 3154 | 0.773 | 1.000 | 0.820 | 1.000 | 0.953 | 0.960 | 0.897 | 1.000 | 0.990 | 1.000 | 0.959 | 1.000 | 0.991 | 0.857 | 1.000 | 1.000 | 0.859 | 0.831 |
| 3155 | 0.771 | 1.000 | 0.808 | 0.986 | 0.947 | 0.960 | 0.891 | 1.000 | 0.990 | 1.000 | 0.959 | 0.970 | 0.991 | 0.860 | 1.000 | 1.000 | 0.856 | 0.828 |
| 3156 | 0.771 | 1.000 | 0.818 | 0.986 | 0.958 | 0.960 | 0.903 | 1.000 | 0.990 | 1.000 | 0.968 | 1.000 | 0.991 | 0.847 | 1.000 | 1.000 | 0.856 | 0.828 |
| 3157 | 0.772 | 1.000 | 0.806 | 0.986 | 0.958 | 0.960 | 0.881 | 1.000 | 1.000 | 1.000 | 0.959 | 1.000 | 1.000 | 0.850 | 1.000 | 1.000 | 0.853 | 0.824 |

| | | | | | | | | | | | | | | | | | |
|---|---|---|---|---|---|---|---|---|---|---|---|---|---|---|---|---|---|
| 3158 | 0.777 | 1.000 | 0.808 | 0.986 | 0.953 | 0.960 | 0.894 | 1.000 | 0.990 | 1.000 | 0.962 | 0.970 | 0.982 | 0.857 | 1.000 | 1.000 | 0.858 | 0.829 |
| 3159 | 0.772 | 1.000 | 0.806 | 0.986 | 0.958 | 0.960 | 0.888 | 1.000 | 0.990 | 1.000 | 0.959 | 0.970 | 0.991 | 0.848 | 1.000 | 1.000 | 0.853 | 0.824 |
| 3160 | 0.774 | 1.000 | 0.816 | 1.000 | 0.958 | 0.960 | 0.883 | 1.000 | 0.980 | 1.000 | 0.965 | 1.000 | 0.991 | 0.848 | 1.000 | 1.000 | 0.855 | 0.826 |
| 3161 | 0.772 | 1.000 | 0.820 | 0.986 | 0.942 | 0.960 | 0.892 | 1.000 | 0.990 | 1.000 | 0.959 | 0.970 | 0.991 | 0.857 | 1.000 | 1.000 | 0.857 | 0.828 |
| 3162 | 0.767 | 1.000 | 0.818 | 1.000 | 0.953 | 0.960 | 0.894 | 1.000 | 0.990 | 1.000 | 0.965 | 0.970 | 1.000 | 0.856 | 1.000 | 1.000 | 0.856 | 0.828 |
| 3163 | 0.781 | 1.000 | 0.800 | 1.000 | 0.958 | 0.960 | 0.894 | 1.000 | 0.990 | 1.000 | 0.971 | 0.970 | 0.991 | 0.858 | 1.000 | 1.000 | 0.860 | 0.831 |
| 3164 | 0.770 | 1.000 | 0.812 | 1.000 | 0.953 | 0.960 | 0.895 | 1.000 | 0.990 | 1.000 | 0.965 | 1.000 | 1.000 | 0.853 | 1.000 | 1.000 | 0.856 | 0.828 |
| 3165 | 0.776 | 1.000 | 0.820 | 1.000 | 0.937 | 0.960 | 0.897 | 1.000 | 0.980 | 1.000 | 0.965 | 0.970 | 0.991 | 0.847 | 1.000 | 1.000 | 0.856 | 0.828 |
| 3166 | 0.768 | 1.000 | 0.814 | 0.986 | 0.947 | 0.960 | 0.901 | 1.000 | 0.990 | 1.000 | 0.962 | 1.000 | 0.991 | 0.852 | 1.000 | 1.000 | 0.856 | 0.827 |
| 3167 | 0.772 | 1.000 | 0.818 | 1.000 | 0.958 | 0.960 | 0.898 | 1.000 | 1.000 | 1.000 | 0.962 | 0.970 | 1.000 | 0.851 | 1.000 | 1.000 | 0.857 | 0.829 |
| 3168 | 0.777 | 1.000 | 0.798 | 0.986 | 0.958 | 0.960 | 0.894 | 1.000 | 1.000 | 1.000 | 0.954 | 0.970 | 1.000 | 0.846 | 1.000 | 1.000 | 0.854 | 0.825 |
| 3169 | 0.767 | 1.000 | 0.812 | 1.000 | 0.953 | 0.960 | 0.892 | 1.000 | 0.990 | 1.000 | 0.962 | 0.970 | 0.991 | 0.857 | 1.000 | 1.000 | 0.855 | 0.827 |
| 3170 | 0.775 | 1.000 | 0.798 | 0.971 | 0.953 | 0.960 | 0.889 | 1.000 | 0.990 | 1.000 | 0.959 | 0.970 | 1.000 | 0.854 | 1.000 | 1.000 | 0.855 | 0.826 |
| 3171 | 0.771 | 1.000 | 0.806 | 0.986 | 0.953 | 0.960 | 0.886 | 1.000 | 1.000 | 1.000 | 0.968 | 0.970 | 1.000 | 0.857 | 1.000 | 1.000 | 0.856 | 0.827 |
| 3172 | 0.778 | 1.000 | 0.810 | 1.000 | 0.958 | 0.960 | 0.886 | 1.000 | 0.990 | 1.000 | 0.962 | 1.000 | 1.000 | 0.853 | 1.000 | 1.000 | 0.857 | 0.829 |
| 3173 | 0.771 | 1.000 | 0.804 | 1.000 | 0.947 | 0.960 | 0.897 | 1.000 | 1.000 | 1.000 | 0.962 | 1.000 | 0.991 | 0.856 | 1.000 | 1.000 | 0.856 | 0.828 |
| 3174 | 0.771 | 1.000 | 0.820 | 1.000 | 0.942 | 0.960 | 0.897 | 1.000 | 0.990 | 1.000 | 0.962 | 1.000 | 0.991 | 0.852 | 1.000 | 1.000 | 0.857 | 0.828 |
| 3175 | 0.774 | 1.000 | 0.814 | 1.000 | 0.958 | 0.960 | 0.891 | 1.000 | 0.990 | 1.000 | 0.965 | 0.970 | 1.000 | 0.851 | 1.000 | 1.000 | 0.856 | 0.828 |
| 3176 | 0.773 | 1.000 | 0.810 | 0.986 | 0.953 | 0.960 | 0.897 | 1.000 | 0.990 | 1.000 | 0.971 | 1.000 | 1.000 | 0.852 | 1.000 | 1.000 | 0.857 | 0.829 |
| 3177 | 0.779 | 1.000 | 0.808 | 1.000 | 0.953 | 0.960 | 0.895 | 1.000 | 1.000 | 1.000 | 0.968 | 0.970 | 0.991 | 0.851 | 1.000 | 1.000 | 0.858 | 0.829 |
| 3178 | 0.766 | 1.000 | 0.804 | 0.986 | 0.953 | 0.960 | 0.901 | 1.000 | 1.000 | 1.000 | 0.962 | 0.970 | 0.991 | 0.850 | 1.000 | 1.000 | 0.854 | 0.825 |
| 3179 | 0.777 | 1.000 | 0.810 | 0.986 | 0.953 | 0.960 | 0.895 | 1.000 | 0.990 | 1.000 | 0.968 | 0.970 | 0.991 | 0.852 | 1.000 | 1.000 | 0.858 | 0.829 |
| 3180 | 0.773 | 1.000 | 0.802 | 0.986 | 0.947 | 0.960 | 0.889 | 1.000 | 0.990 | 1.000 | 0.959 | 0.970 | 0.991 | 0.852 | 1.000 | 1.000 | 0.854 | 0.825 |
| 3181 | 0.767 | 1.000 | 0.808 | 1.000 | 0.958 | 0.960 | 0.891 | 1.000 | 1.000 | 1.000 | 0.962 | 0.970 | 1.000 | 0.851 | 1.000 | 1.000 | 0.854 | 0.825 |
| 3182 | 0.761 | 1.000 | 0.800 | 0.986 | 0.953 | 0.960 | 0.895 | 1.000 | 0.990 | 1.000 | 0.965 | 0.970 | 0.991 | 0.853 | 1.000 | 1.000 | 0.852 | 0.823 |
| 3183 | 0.767 | 1.000 | 0.810 | 1.000 | 0.947 | 0.960 | 0.895 | 1.000 | 0.990 | 1.000 | 0.959 | 1.000 | 0.991 | 0.852 | 1.000 | 1.000 | 0.854 | 0.825 |
| 3184 | 0.774 | 1.000 | 0.806 | 1.000 | 0.958 | 0.960 | 0.894 | 1.000 | 1.000 | 1.000 | 0.962 | 0.970 | 0.991 | 0.849 | 1.000 | 1.000 | 0.855 | 0.827 |
| 3185 | 0.767 | 1.000 | 0.810 | 1.000 | 0.947 | 0.960 | 0.897 | 1.000 | 1.000 | 1.000 | 0.954 | 0.970 | 1.000 | 0.851 | 1.000 | 1.000 | 0.854 | 0.825 |
| 3186 | 0.765 | 1.000 | 0.822 | 0.971 | 0.953 | 0.960 | 0.898 | 1.000 | 0.990 | 1.000 | 0.957 | 0.970 | 1.000 | 0.851 | 1.000 | 1.000 | 0.854 | 0.826 |
| 3187 | 0.769 | 1.000 | 0.806 | 0.986 | 0.953 | 0.960 | 0.897 | 1.000 | 1.000 | 1.000 | 0.965 | 1.000 | 0.991 | 0.856 | 1.000 | 1.000 | 0.856 | 0.827 |
| 3188 | 0.767 | 1.000 | 0.810 | 1.000 | 0.953 | 0.960 | 0.892 | 1.000 | 0.990 | 1.000 | 0.968 | 0.970 | 1.000 | 0.857 | 1.000 | 1.000 | 0.856 | 0.828 |
| 3189 | 0.774 | 1.000 | 0.814 | 1.000 | 0.958 | 0.960 | 0.888 | 1.000 | 1.000 | 1.000 | 0.971 | 0.970 | 1.000 | 0.855 | 1.000 | 1.000 | 0.858 | 0.829 |
| 3190 | 0.768 | 1.000 | 0.802 | 1.000 | 0.953 | 0.960 | 0.891 | 1.000 | 1.000 | 1.000 | 0.962 | 1.000 | 0.991 | 0.851 | 1.000 | 1.000 | 0.853 | 0.824 |
| 3191 | 0.771 | 1.000 | 0.808 | 1.000 | 0.958 | 0.960 | 0.888 | 1.000 | 0.990 | 1.000 | 0.965 | 1.000 | 1.000 | 0.856 | 1.000 | 1.000 | 0.856 | 0.827 |
| 3192 | 0.761 | 1.000 | 0.802 | 1.000 | 0.953 | 0.960 | 0.898 | 1.000 | 1.000 | 1.000 | 0.962 | 1.000 | 1.000 | 0.852 | 1.000 | 1.000 | 0.853 | 0.824 |
| 3193 | 0.772 | 1.000 | 0.800 | 0.986 | 0.953 | 0.960 | 0.889 | 1.000 | 0.990 | 1.000 | 0.959 | 0.970 | 1.000 | 0.847 | 1.000 | 1.000 | 0.853 | 0.823 |
| 3194 | 0.769 | 1.000 | 0.810 | 1.000 | 0.953 | 0.960 | 0.895 | 1.000 | 1.000 | 1.000 | 0.971 | 1.000 | 0.991 | 0.857 | 1.000 | 1.000 | 0.857 | 0.829 |
| 3195 | 0.766 | 1.000 | 0.820 | 1.000 | 0.953 | 0.960 | 0.901 | 1.000 | 0.990 | 1.000 | 0.965 | 1.000 | 0.991 | 0.844 | 1.000 | 1.000 | 0.854 | 0.825 |
| 3196 | 0.769 | 1.000 | 0.812 | 0.986 | 0.947 | 0.960 | 0.892 | 1.000 | 1.000 | 1.000 | 0.962 | 0.970 | 1.000 | 0.856 | 1.000 | 1.000 | 0.856 | 0.827 |

| | | | | | | | | | | | | | | | | | | |
|---|---|---|---|---|---|---|---|---|---|---|---|---|---|---|---|---|---|---|
| 3197 | 0.769 | 1.000 | 0.804 | 1.000 | 0.958 | 0.960 | 0.886 | 1.000 | 0.990 | 1.000 | 0.965 | 0.970 | 0.991 | 0.854 | 1.000 | 1.000 | 0.854 | 0.825 |
| 3198 | 0.777 | 1.000 | 0.804 | 1.000 | 0.953 | 0.960 | 0.886 | 1.000 | 0.990 | 1.000 | 0.968 | 1.000 | 1.000 | 0.854 | 1.000 | 1.000 | 0.857 | 0.828 |
| 3199 | 0.766 | 1.000 | 0.802 | 0.986 | 0.953 | 0.960 | 0.892 | 1.000 | 0.990 | 1.000 | 0.971 | 0.970 | 1.000 | 0.855 | 1.000 | 1.000 | 0.854 | 0.825 |
| 3200 | 0.769 | 1.000 | 0.812 | 0.986 | 0.953 | 0.960 | 0.889 | 1.000 | 0.990 | 1.000 | 0.965 | 1.000 | 0.991 | 0.846 | 1.000 | 1.000 | 0.853 | 0.824 |
| 3201 | 0.770 | 1.000 | 0.802 | 0.986 | 0.947 | 0.960 | 0.886 | 1.000 | 1.000 | 1.000 | 0.971 | 1.000 | 1.000 | 0.858 | 1.000 | 1.000 | 0.856 | 0.827 |
| 3202 | 0.763 | 1.000 | 0.818 | 0.986 | 0.953 | 0.960 | 0.901 | 1.000 | 1.000 | 1.000 | 0.957 | 0.970 | 1.000 | 0.855 | 1.000 | 1.000 | 0.855 | 0.827 |
| 3203 | 0.773 | 1.000 | 0.802 | 1.000 | 0.947 | 0.960 | 0.901 | 1.000 | 1.000 | 1.000 | 0.957 | 0.970 | 1.000 | 0.851 | 1.000 | 1.000 | 0.856 | 0.827 |
| 3204 | 0.767 | 1.000 | 0.810 | 1.000 | 0.958 | 0.960 | 0.901 | 1.000 | 0.990 | 1.000 | 0.959 | 1.000 | 0.991 | 0.852 | 1.000 | 1.000 | 0.855 | 0.827 |
| 3205 | 0.771 | 1.000 | 0.818 | 1.000 | 0.953 | 0.960 | 0.889 | 1.000 | 1.000 | 1.000 | 0.965 | 1.000 | 0.991 | 0.850 | 1.000 | 1.000 | 0.856 | 0.827 |
| 3206 | 0.775 | 1.000 | 0.816 | 1.000 | 0.947 | 0.960 | 0.892 | 1.000 | 1.000 | 1.000 | 0.968 | 0.970 | 1.000 | 0.847 | 1.000 | 1.000 | 0.856 | 0.828 |
| 3207 | 0.768 | 1.000 | 0.794 | 0.986 | 0.958 | 0.960 | 0.894 | 1.000 | 1.000 | 1.000 | 0.959 | 0.970 | 0.991 | 0.851 | 1.000 | 1.000 | 0.853 | 0.823 |
| 3208 | 0.772 | 1.000 | 0.806 | 1.000 | 0.963 | 0.960 | 0.894 | 1.000 | 1.000 | 1.000 | 0.957 | 0.970 | 1.000 | 0.849 | 1.000 | 1.000 | 0.855 | 0.826 |
| 3209 | 0.765 | 1.000 | 0.796 | 0.986 | 0.953 | 0.960 | 0.894 | 1.000 | 0.990 | 1.000 | 0.965 | 0.970 | 0.991 | 0.859 | 1.000 | 1.000 | 0.854 | 0.825 |
| 3210 | 0.772 | 1.000 | 0.810 | 0.986 | 0.953 | 0.960 | 0.901 | 1.000 | 1.000 | 1.000 | 0.968 | 0.970 | 0.991 | 0.852 | 1.000 | 1.000 | 0.857 | 0.829 |
| 3211 | 0.760 | 1.000 | 0.806 | 1.000 | 0.958 | 0.960 | 0.894 | 1.000 | 1.000 | 1.000 | 0.965 | 1.000 | 1.000 | 0.849 | 1.000 | 1.000 | 0.852 | 0.823 |
| 3212 | 0.781 | 1.000 | 0.785 | 0.986 | 0.953 | 0.960 | 0.886 | 1.000 | 0.990 | 1.000 | 0.959 | 1.000 | 0.991 | 0.855 | 1.000 | 1.000 | 0.855 | 0.826 |
| 3213 | 0.772 | 1.000 | 0.808 | 1.000 | 0.958 | 0.960 | 0.895 | 1.000 | 0.990 | 1.000 | 0.968 | 1.000 | 1.000 | 0.855 | 1.000 | 1.000 | 0.857 | 0.829 |
| 3214 | 0.767 | 1.000 | 0.802 | 1.000 | 0.947 | 0.960 | 0.901 | 1.000 | 0.990 | 1.000 | 0.965 | 0.970 | 0.991 | 0.853 | 1.000 | 1.000 | 0.855 | 0.826 |
| 3215 | 0.774 | 1.000 | 0.806 | 0.986 | 0.947 | 0.960 | 0.898 | 1.000 | 0.990 | 1.000 | 0.965 | 0.970 | 1.000 | 0.857 | 1.000 | 1.000 | 0.858 | 0.829 |
| 3216 | 0.772 | 1.000 | 0.828 | 1.000 | 0.947 | 0.960 | 0.889 | 1.000 | 1.000 | 1.000 | 0.965 | 0.970 | 1.000 | 0.850 | 1.000 | 1.000 | 0.857 | 0.828 |
| 3217 | 0.779 | 1.000 | 0.802 | 0.986 | 0.953 | 0.960 | 0.892 | 1.000 | 0.990 | 1.000 | 0.959 | 0.970 | 1.000 | 0.858 | 1.000 | 1.000 | 0.858 | 0.829 |
| 3218 | 0.767 | 1.000 | 0.814 | 1.000 | 0.953 | 0.960 | 0.891 | 1.000 | 0.990 | 1.000 | 0.965 | 0.970 | 1.000 | 0.854 | 1.000 | 1.000 | 0.855 | 0.826 |
| 3219 | 0.779 | 1.000 | 0.810 | 1.000 | 0.953 | 0.960 | 0.894 | 1.000 | 1.000 | 1.000 | 0.965 | 1.000 | 1.000 | 0.850 | 1.000 | 1.000 | 0.858 | 0.829 |
| 3220 | 0.770 | 1.000 | 0.814 | 1.000 | 0.958 | 0.960 | 0.898 | 1.000 | 1.000 | 1.000 | 0.971 | 0.970 | 0.991 | 0.856 | 1.000 | 1.000 | 0.858 | 0.830 |
| 3221 | 0.758 | 1.000 | 0.818 | 0.986 | 0.963 | 0.960 | 0.895 | 1.000 | 1.000 | 1.000 | 0.959 | 1.000 | 1.000 | 0.856 | 1.000 | 1.000 | 0.854 | 0.825 |
| 3222 | 0.771 | 1.000 | 0.802 | 1.000 | 0.958 | 0.960 | 0.892 | 1.000 | 1.000 | 1.000 | 0.962 | 0.970 | 0.991 | 0.851 | 1.000 | 1.000 | 0.854 | 0.826 |
| 3223 | 0.768 | 1.000 | 0.814 | 1.000 | 0.953 | 0.960 | 0.888 | 1.000 | 1.000 | 1.000 | 0.968 | 1.000 | 1.000 | 0.852 | 1.000 | 1.000 | 0.855 | 0.826 |
| 3224 | 0.777 | 1.000 | 0.798 | 1.000 | 0.958 | 0.960 | 0.900 | 1.000 | 0.990 | 1.000 | 0.968 | 0.970 | 1.000 | 0.855 | 1.000 | 1.000 | 0.858 | 0.830 |
| 3225 | 0.773 | 1.000 | 0.816 | 1.000 | 0.953 | 0.960 | 0.897 | 1.000 | 1.000 | 1.000 | 0.962 | 0.970 | 1.000 | 0.854 | 1.000 | 1.000 | 0.858 | 0.830 |
| 3226 | 0.770 | 1.000 | 0.818 | 1.000 | 0.958 | 0.960 | 0.900 | 1.000 | 0.990 | 1.000 | 0.959 | 0.970 | 1.000 | 0.851 | 1.000 | 1.000 | 0.857 | 0.828 |
| 3227 | 0.773 | 1.000 | 0.822 | 1.000 | 0.953 | 0.960 | 0.894 | 1.000 | 1.000 | 1.000 | 0.968 | 0.970 | 1.000 | 0.847 | 1.000 | 1.000 | 0.857 | 0.828 |
| 3228 | 0.780 | 1.000 | 0.814 | 1.000 | 0.953 | 0.960 | 0.891 | 1.000 | 0.990 | 1.000 | 0.962 | 0.970 | 0.991 | 0.857 | 1.000 | 1.000 | 0.859 | 0.831 |
| 3229 | 0.781 | 1.000 | 0.804 | 1.000 | 0.958 | 0.960 | 0.889 | 1.000 | 0.990 | 1.000 | 0.968 | 0.970 | 1.000 | 0.851 | 1.000 | 1.000 | 0.858 | 0.829 |
| 3230 | 0.775 | 1.000 | 0.804 | 0.986 | 0.958 | 0.960 | 0.884 | 1.000 | 1.000 | 1.000 | 0.965 | 1.000 | 0.991 | 0.851 | 1.000 | 1.000 | 0.855 | 0.826 |
| 3231 | 0.779 | 1.000 | 0.816 | 1.000 | 0.953 | 0.960 | 0.897 | 1.000 | 0.990 | 1.000 | 0.968 | 1.000 | 1.000 | 0.852 | 1.000 | 1.000 | 0.860 | 0.832 |
| 3232 | 0.765 | 1.000 | 0.816 | 0.986 | 0.953 | 0.960 | 0.889 | 1.000 | 1.000 | 1.000 | 0.959 | 0.970 | 1.000 | 0.853 | 1.000 | 1.000 | 0.854 | 0.825 |
| 3233 | 0.765 | 1.000 | 0.806 | 1.000 | 0.947 | 0.960 | 0.903 | 1.000 | 0.990 | 1.000 | 0.968 | 1.000 | 0.991 | 0.852 | 1.000 | 1.000 | 0.855 | 0.826 |
| 3234 | 0.776 | 1.000 | 0.824 | 1.000 | 0.953 | 0.960 | 0.900 | 1.000 | 1.000 | 1.000 | 0.962 | 1.000 | 0.991 | 0.857 | 1.000 | 1.000 | 0.861 | 0.833 |
| 3235 | 0.768 | 1.000 | 0.806 | 1.000 | 0.953 | 0.960 | 0.897 | 1.000 | 0.990 | 1.000 | 0.971 | 1.000 | 0.991 | 0.855 | 1.000 | 1.000 | 0.856 | 0.827 |

| | | | | | | | | | | | | | | | | | | |
|---|---|---|---|---|---|---|---|---|---|---|---|---|---|---|---|---|---|---|
| 3236 | 0.768 | 1.000 | 0.816 | 1.000 | 0.958 | 0.960 | 0.900 | 1.000 | 0.990 | 1.000 | 0.965 | 0.970 | 1.000 | 0.854 | 1.000 | 1.000 | 0.857 | 0.829 |
| 3237 | 0.766 | 1.000 | 0.816 | 1.000 | 0.958 | 0.960 | 0.886 | 1.000 | 0.980 | 1.000 | 0.962 | 0.970 | 0.991 | 0.853 | 1.000 | 1.000 | 0.854 | 0.825 |
| 3238 | 0.771 | 1.000 | 0.816 | 1.000 | 0.953 | 0.960 | 0.898 | 1.000 | 0.980 | 1.000 | 0.965 | 1.000 | 1.000 | 0.847 | 1.000 | 1.000 | 0.856 | 0.827 |
| 3239 | 0.768 | 1.000 | 0.824 | 0.986 | 0.958 | 0.960 | 0.894 | 1.000 | 0.990 | 1.000 | 0.968 | 0.970 | 1.000 | 0.854 | 1.000 | 1.000 | 0.857 | 0.829 |
| 3240 | 0.773 | 1.000 | 0.800 | 1.000 | 0.953 | 0.960 | 0.892 | 1.000 | 0.990 | 1.000 | 0.965 | 0.970 | 1.000 | 0.855 | 1.000 | 1.000 | 0.856 | 0.827 |
| 3241 | 0.775 | 1.000 | 0.810 | 1.000 | 0.953 | 0.960 | 0.894 | 1.000 | 1.000 | 1.000 | 0.962 | 0.970 | 1.000 | 0.852 | 1.000 | 1.000 | 0.857 | 0.829 |
| 3242 | 0.762 | 1.000 | 0.818 | 1.000 | 0.953 | 0.960 | 0.891 | 1.000 | 0.990 | 1.000 | 0.968 | 0.970 | 1.000 | 0.853 | 1.000 | 1.000 | 0.854 | 0.825 |
| 3243 | 0.775 | 1.000 | 0.812 | 1.000 | 0.953 | 0.960 | 0.892 | 1.000 | 1.000 | 1.000 | 0.962 | 1.000 | 0.991 | 0.851 | 1.000 | 1.000 | 0.857 | 0.828 |
| 3244 | 0.775 | 1.000 | 0.824 | 1.000 | 0.958 | 0.960 | 0.891 | 1.000 | 1.000 | 1.000 | 0.965 | 1.000 | 1.000 | 0.844 | 1.000 | 1.000 | 0.857 | 0.828 |
| 3245 | 0.778 | 1.000 | 0.834 | 0.986 | 0.958 | 0.960 | 0.895 | 1.000 | 1.000 | 1.000 | 0.959 | 1.000 | 0.991 | 0.855 | 1.000 | 1.000 | 0.861 | 0.833 |
| 3246 | 0.773 | 1.000 | 0.816 | 1.000 | 0.958 | 0.960 | 0.900 | 1.000 | 0.990 | 1.000 | 0.959 | 1.000 | 1.000 | 0.851 | 1.000 | 1.000 | 0.858 | 0.830 |
| 3247 | 0.773 | 1.000 | 0.820 | 1.000 | 0.953 | 0.960 | 0.898 | 1.000 | 0.990 | 1.000 | 0.971 | 1.000 | 0.991 | 0.851 | 1.000 | 1.000 | 0.858 | 0.830 |
| 3248 | 0.780 | 1.000 | 0.818 | 1.000 | 0.953 | 0.960 | 0.898 | 1.000 | 0.990 | 1.000 | 0.959 | 0.970 | 1.000 | 0.851 | 1.000 | 1.000 | 0.859 | 0.831 |
| 3249 | 0.773 | 1.000 | 0.818 | 1.000 | 0.953 | 0.960 | 0.891 | 1.000 | 1.000 | 1.000 | 0.965 | 1.000 | 0.991 | 0.853 | 1.000 | 1.000 | 0.857 | 0.829 |
| 3250 | 0.767 | 1.000 | 0.828 | 1.000 | 0.958 | 0.960 | 0.895 | 1.000 | 0.990 | 1.000 | 0.965 | 0.939 | 0.991 | 0.857 | 1.000 | 1.000 | 0.858 | 0.830 |
| 3251 | 0.773 | 1.000 | 0.818 | 1.000 | 0.958 | 0.960 | 0.888 | 1.000 | 0.980 | 1.000 | 0.962 | 1.000 | 1.000 | 0.858 | 1.000 | 1.000 | 0.858 | 0.830 |
| 3252 | 0.762 | 1.000 | 0.814 | 1.000 | 0.947 | 0.960 | 0.891 | 1.000 | 0.990 | 1.000 | 0.965 | 0.970 | 0.991 | 0.848 | 1.000 | 1.000 | 0.852 | 0.823 |
| 3253 | 0.768 | 1.000 | 0.820 | 1.000 | 0.958 | 0.960 | 0.898 | 1.000 | 1.000 | 1.000 | 0.965 | 1.000 | 0.991 | 0.855 | 1.000 | 1.000 | 0.858 | 0.830 |
| 3254 | 0.762 | 1.000 | 0.814 | 1.000 | 0.953 | 0.960 | 0.901 | 1.000 | 1.000 | 1.000 | 0.968 | 1.000 | 0.991 | 0.847 | 1.000 | 1.000 | 0.854 | 0.825 |
| 3255 | 0.773 | 1.000 | 0.806 | 0.986 | 0.953 | 0.960 | 0.891 | 1.000 | 0.990 | 1.000 | 0.971 | 0.970 | 1.000 | 0.855 | 1.000 | 1.000 | 0.857 | 0.828 |
| 3256 | 0.766 | 1.000 | 0.818 | 1.000 | 0.947 | 0.960 | 0.889 | 1.000 | 0.990 | 1.000 | 0.968 | 1.000 | 0.991 | 0.857 | 1.000 | 1.000 | 0.856 | 0.827 |
| 3257 | 0.771 | 1.000 | 0.810 | 0.986 | 0.958 | 0.960 | 0.892 | 1.000 | 1.000 | 1.000 | 0.959 | 1.000 | 1.000 | 0.850 | 1.000 | 1.000 | 0.855 | 0.826 |
| 3258 | 0.765 | 1.000 | 0.826 | 0.986 | 0.953 | 0.960 | 0.891 | 1.000 | 0.990 | 1.000 | 0.968 | 0.970 | 0.991 | 0.857 | 1.000 | 1.000 | 0.856 | 0.828 |
| 3259 | 0.770 | 1.000 | 0.828 | 1.000 | 0.958 | 0.960 | 0.891 | 1.000 | 0.990 | 1.000 | 0.962 | 0.970 | 1.000 | 0.854 | 1.000 | 1.000 | 0.857 | 0.829 |
| 3260 | 0.777 | 1.000 | 0.816 | 1.000 | 0.953 | 0.960 | 0.895 | 1.000 | 1.000 | 1.000 | 0.965 | 1.000 | 1.000 | 0.849 | 1.000 | 1.000 | 0.858 | 0.829 |
| 3261 | 0.778 | 1.000 | 0.814 | 1.000 | 0.953 | 0.960 | 0.888 | 1.000 | 1.000 | 1.000 | 0.962 | 0.970 | 0.991 | 0.857 | 1.000 | 1.000 | 0.858 | 0.830 |
| 3262 | 0.773 | 1.000 | 0.810 | 1.000 | 0.947 | 0.960 | 0.901 | 1.000 | 1.000 | 1.000 | 0.957 | 0.970 | 0.991 | 0.857 | 1.000 | 1.000 | 0.858 | 0.829 |
| 3263 | 0.765 | 1.000 | 0.814 | 1.000 | 0.953 | 0.960 | 0.897 | 1.000 | 0.990 | 1.000 | 0.965 | 0.970 | 0.991 | 0.846 | 1.000 | 1.000 | 0.853 | 0.824 |
| 3264 | 0.770 | 1.000 | 0.820 | 0.986 | 0.953 | 0.960 | 0.894 | 1.000 | 0.990 | 1.000 | 0.965 | 0.970 | 0.991 | 0.850 | 1.000 | 1.000 | 0.855 | 0.827 |
| 3265 | 0.773 | 1.000 | 0.810 | 1.000 | 0.953 | 0.960 | 0.892 | 1.000 | 0.990 | 1.000 | 0.959 | 0.970 | 0.991 | 0.851 | 1.000 | 1.000 | 0.855 | 0.826 |
| 3266 | 0.761 | 1.000 | 0.816 | 1.000 | 0.953 | 0.960 | 0.898 | 1.000 | 0.990 | 1.000 | 0.965 | 0.970 | 0.991 | 0.854 | 1.000 | 1.000 | 0.854 | 0.826 |
| 3267 | 0.769 | 1.000 | 0.808 | 0.986 | 0.942 | 0.960 | 0.892 | 1.000 | 1.000 | 1.000 | 0.962 | 1.000 | 1.000 | 0.854 | 1.000 | 1.000 | 0.855 | 0.826 |
| 3268 | 0.784 | 1.000 | 0.816 | 0.986 | 0.953 | 0.960 | 0.894 | 1.000 | 0.990 | 1.000 | 0.962 | 1.000 | 0.991 | 0.851 | 1.000 | 1.000 | 0.859 | 0.831 |
| 3269 | 0.771 | 1.000 | 0.818 | 1.000 | 0.953 | 0.960 | 0.892 | 1.000 | 0.990 | 1.000 | 0.971 | 0.970 | 1.000 | 0.855 | 1.000 | 1.000 | 0.857 | 0.829 |
| 3270 | 0.775 | 1.000 | 0.804 | 0.986 | 0.958 | 0.960 | 0.889 | 1.000 | 1.000 | 1.000 | 0.965 | 1.000 | 0.991 | 0.853 | 1.000 | 1.000 | 0.856 | 0.827 |
| 3271 | 0.768 | 1.000 | 0.816 | 0.986 | 0.953 | 0.960 | 0.886 | 1.000 | 1.000 | 1.000 | 0.962 | 0.970 | 1.000 | 0.842 | 1.000 | 1.000 | 0.852 | 0.822 |
| 3272 | 0.771 | 1.000 | 0.806 | 1.000 | 0.958 | 0.960 | 0.881 | 1.000 | 0.990 | 1.000 | 0.959 | 0.970 | 0.991 | 0.853 | 1.000 | 1.000 | 0.853 | 0.824 |
| 3273 | 0.759 | 1.000 | 0.828 | 1.000 | 0.958 | 0.960 | 0.892 | 1.000 | 0.990 | 1.000 | 0.965 | 0.970 | 1.000 | 0.854 | 1.000 | 1.000 | 0.855 | 0.826 |
| 3274 | 0.774 | 1.000 | 0.810 | 1.000 | 0.947 | 0.960 | 0.892 | 1.000 | 1.000 | 1.000 | 0.965 | 1.000 | 1.000 | 0.854 | 1.000 | 1.000 | 0.857 | 0.829 |

| | | | | | | | | | | | | | | | | | |
|---|---|---|---|---|---|---|---|---|---|---|---|---|---|---|---|---|---|
| 3275 | 0.763 | 1.000 | 0.806 | 1.000 | 0.958 | 0.960 | 0.889 | 1.000 | 1.000 | 1.000 | 0.962 | 0.970 | 1.000 | 0.849 | 1.000 | 1.000 | 0.852 | 0.822 |
| 3276 | 0.771 | 1.000 | 0.816 | 1.000 | 0.958 | 0.960 | 0.891 | 1.000 | 0.980 | 1.000 | 0.968 | 1.000 | 1.000 | 0.850 | 1.000 | 1.000 | 0.856 | 0.827 |
| 3277 | 0.777 | 1.000 | 0.810 | 0.986 | 0.958 | 0.960 | 0.895 | 1.000 | 0.990 | 1.000 | 0.962 | 0.970 | 1.000 | 0.851 | 1.000 | 1.000 | 0.857 | 0.829 |
| 3278 | 0.771 | 1.000 | 0.818 | 1.000 | 0.953 | 0.960 | 0.895 | 1.000 | 0.990 | 1.000 | 0.959 | 1.000 | 1.000 | 0.842 | 1.000 | 1.000 | 0.854 | 0.825 |
| 3279 | 0.764 | 1.000 | 0.798 | 0.986 | 0.953 | 0.960 | 0.894 | 1.000 | 0.990 | 1.000 | 0.965 | 0.970 | 0.991 | 0.848 | 1.000 | 1.000 | 0.851 | 0.822 |
| 3280 | 0.780 | 1.000 | 0.820 | 1.000 | 0.953 | 0.960 | 0.898 | 1.000 | 1.000 | 1.000 | 0.965 | 0.970 | 0.991 | 0.858 | 1.000 | 1.000 | 0.861 | 0.834 |
| 3281 | 0.763 | 1.000 | 0.818 | 0.986 | 0.953 | 0.960 | 0.894 | 1.000 | 1.000 | 1.000 | 0.965 | 1.000 | 1.000 | 0.851 | 1.000 | 1.000 | 0.854 | 0.826 |
| 3282 | 0.771 | 1.000 | 0.810 | 0.986 | 0.953 | 0.960 | 0.897 | 1.000 | 0.990 | 1.000 | 0.962 | 0.970 | 1.000 | 0.846 | 1.000 | 1.000 | 0.854 | 0.825 |
| 3283 | 0.765 | 1.000 | 0.816 | 1.000 | 0.953 | 0.960 | 0.895 | 1.000 | 1.000 | 1.000 | 0.962 | 1.000 | 1.000 | 0.849 | 1.000 | 1.000 | 0.854 | 0.826 |
| 3284 | 0.769 | 1.000 | 0.810 | 0.986 | 0.958 | 0.960 | 0.897 | 1.000 | 0.990 | 1.000 | 0.962 | 1.000 | 1.000 | 0.852 | 1.000 | 1.000 | 0.855 | 0.827 |
| 3285 | 0.769 | 1.000 | 0.816 | 1.000 | 0.958 | 0.960 | 0.898 | 1.000 | 0.990 | 1.000 | 0.962 | 1.000 | 0.991 | 0.851 | 1.000 | 1.000 | 0.856 | 0.828 |
| 3286 | 0.773 | 1.000 | 0.826 | 0.986 | 0.953 | 0.960 | 0.898 | 1.000 | 1.000 | 1.000 | 0.962 | 1.000 | 0.991 | 0.851 | 1.000 | 1.000 | 0.858 | 0.830 |
| 3287 | 0.767 | 1.000 | 0.830 | 0.986 | 0.958 | 0.960 | 0.888 | 1.000 | 1.000 | 1.000 | 0.962 | 0.970 | 0.991 | 0.854 | 1.000 | 1.000 | 0.856 | 0.827 |
| 3288 | 0.772 | 1.000 | 0.814 | 1.000 | 0.953 | 0.960 | 0.881 | 1.000 | 0.980 | 1.000 | 0.959 | 0.970 | 1.000 | 0.851 | 1.000 | 1.000 | 0.854 | 0.825 |
| 3289 | 0.765 | 1.000 | 0.808 | 0.986 | 0.963 | 0.960 | 0.884 | 1.000 | 0.990 | 1.000 | 0.968 | 0.970 | 0.991 | 0.850 | 1.000 | 1.000 | 0.852 | 0.823 |
| 3290 | 0.781 | 1.000 | 0.806 | 1.000 | 0.958 | 0.960 | 0.895 | 1.000 | 0.990 | 1.000 | 0.959 | 1.000 | 1.000 | 0.854 | 1.000 | 1.000 | 0.859 | 0.831 |
| 3291 | 0.768 | 1.000 | 0.830 | 1.000 | 0.958 | 0.960 | 0.894 | 1.000 | 0.990 | 1.000 | 0.962 | 1.000 | 1.000 | 0.852 | 1.000 | 1.000 | 0.857 | 0.829 |
| 3292 | 0.773 | 1.000 | 0.818 | 1.000 | 0.958 | 0.960 | 0.897 | 1.000 | 1.000 | 1.000 | 0.965 | 0.970 | 0.991 | 0.851 | 1.000 | 1.000 | 0.857 | 0.829 |
| 3293 | 0.768 | 1.000 | 0.806 | 1.000 | 0.953 | 0.960 | 0.875 | 1.000 | 1.000 | 1.000 | 0.962 | 0.970 | 1.000 | 0.851 | 1.000 | 1.000 | 0.852 | 0.822 |
| 3294 | 0.762 | 1.000 | 0.820 | 1.000 | 0.953 | 0.960 | 0.908 | 1.000 | 1.000 | 1.000 | 0.959 | 0.970 | 1.000 | 0.854 | 1.000 | 1.000 | 0.856 | 0.828 |
| 3295 | 0.774 | 1.000 | 0.804 | 1.000 | 0.953 | 0.960 | 0.892 | 1.000 | 0.990 | 1.000 | 0.968 | 0.970 | 0.991 | 0.859 | 1.000 | 1.000 | 0.858 | 0.829 |
| 3296 | 0.763 | 1.000 | 0.828 | 1.000 | 0.953 | 0.960 | 0.897 | 1.000 | 0.980 | 1.000 | 0.965 | 1.000 | 0.991 | 0.849 | 1.000 | 1.000 | 0.854 | 0.826 |
| 3297 | 0.772 | 1.000 | 0.818 | 1.000 | 0.958 | 0.960 | 0.892 | 1.000 | 0.990 | 1.000 | 0.965 | 1.000 | 0.991 | 0.854 | 1.000 | 1.000 | 0.857 | 0.829 |
| 3298 | 0.767 | 1.000 | 0.826 | 0.986 | 0.953 | 0.960 | 0.908 | 1.000 | 1.000 | 1.000 | 0.965 | 1.000 | 1.000 | 0.851 | 1.000 | 1.000 | 0.858 | 0.830 |
| 3299 | 0.778 | 1.000 | 0.806 | 1.000 | 0.953 | 0.960 | 0.894 | 1.000 | 1.000 | 1.000 | 0.965 | 1.000 | 1.000 | 0.846 | 1.000 | 1.000 | 0.856 | 0.828 |
| 3300 | 0.768 | 1.000 | 0.818 | 1.000 | 0.953 | 0.960 | 0.892 | 1.000 | 0.990 | 1.000 | 0.962 | 0.970 | 1.000 | 0.851 | 1.000 | 1.000 | 0.855 | 0.826 |
| 3301 | 0.781 | 1.000 | 0.822 | 1.000 | 0.958 | 0.960 | 0.894 | 1.000 | 0.990 | 1.000 | 0.965 | 0.970 | 0.991 | 0.854 | 1.000 | 1.000 | 0.861 | 0.833 |
| 3302 | 0.779 | 1.000 | 0.824 | 1.000 | 0.958 | 0.960 | 0.900 | 1.000 | 0.990 | 1.000 | 0.965 | 1.000 | 1.000 | 0.851 | 1.000 | 1.000 | 0.860 | 0.833 |
| 3303 | 0.777 | 1.000 | 0.818 | 0.986 | 0.958 | 0.960 | 0.904 | 1.000 | 1.000 | 1.000 | 0.962 | 1.000 | 0.991 | 0.852 | 1.000 | 1.000 | 0.860 | 0.832 |
| 3304 | 0.771 | 1.000 | 0.820 | 0.986 | 0.947 | 0.960 | 0.908 | 1.000 | 0.990 | 1.000 | 0.968 | 1.000 | 0.991 | 0.853 | 1.000 | 1.000 | 0.858 | 0.830 |
| 3305 | 0.775 | 1.000 | 0.816 | 0.986 | 0.958 | 0.960 | 0.889 | 1.000 | 0.990 | 1.000 | 0.959 | 1.000 | 0.991 | 0.856 | 1.000 | 1.000 | 0.857 | 0.829 |
| 3306 | 0.765 | 1.000 | 0.818 | 0.986 | 0.953 | 0.960 | 0.898 | 1.000 | 0.990 | 1.000 | 0.971 | 0.970 | 1.000 | 0.854 | 1.000 | 1.000 | 0.856 | 0.828 |
| 3307 | 0.771 | 1.000 | 0.818 | 1.000 | 0.958 | 0.960 | 0.892 | 1.000 | 0.990 | 1.000 | 0.959 | 0.970 | 0.991 | 0.850 | 1.000 | 1.000 | 0.855 | 0.826 |
| 3308 | 0.773 | 1.000 | 0.830 | 1.000 | 0.953 | 0.960 | 0.888 | 1.000 | 1.000 | 1.000 | 0.965 | 1.000 | 0.991 | 0.853 | 1.000 | 1.000 | 0.858 | 0.830 |
| 3309 | 0.775 | 1.000 | 0.816 | 1.000 | 0.958 | 0.960 | 0.884 | 1.000 | 0.990 | 1.000 | 0.962 | 0.970 | 0.991 | 0.851 | 1.000 | 1.000 | 0.856 | 0.827 |
| 3310 | 0.773 | 1.000 | 0.818 | 1.000 | 0.958 | 0.960 | 0.898 | 1.000 | 0.990 | 1.000 | 0.962 | 0.970 | 0.991 | 0.854 | 1.000 | 1.000 | 0.858 | 0.830 |
| 3311 | 0.766 | 1.000 | 0.820 | 0.986 | 0.958 | 0.960 | 0.891 | 1.000 | 0.990 | 1.000 | 0.965 | 0.970 | 0.991 | 0.849 | 1.000 | 1.000 | 0.854 | 0.825 |
| 3312 | 0.762 | 1.000 | 0.818 | 1.000 | 0.953 | 0.960 | 0.906 | 1.000 | 0.980 | 1.000 | 0.959 | 0.970 | 1.000 | 0.853 | 1.000 | 1.000 | 0.855 | 0.827 |
| 3313 | 0.776 | 1.000 | 0.820 | 0.986 | 0.953 | 0.960 | 0.884 | 1.000 | 1.000 | 1.000 | 0.962 | 0.970 | 1.000 | 0.861 | 1.000 | 1.000 | 0.859 | 0.831 |

| | | | | | | | | | | | | | | | | | |
|---|---|---|---|---|---|---|---|---|---|---|---|---|---|---|---|---|---|
| 3314 | 0.773 | 1.000 | 0.812 | 1.000 | 0.958 | 0.960 | 0.892 | 1.000 | 0.990 | 1.000 | 0.965 | 1.000 | 0.991 | 0.854 | 1.000 | 1.000 | 0.857 | 0.828 |
| 3315 | 0.771 | 1.000 | 0.810 | 1.000 | 0.947 | 0.960 | 0.891 | 1.000 | 0.990 | 1.000 | 0.962 | 1.000 | 0.991 | 0.853 | 1.000 | 1.000 | 0.855 | 0.827 |
| 3316 | 0.777 | 1.000 | 0.822 | 1.000 | 0.963 | 0.960 | 0.895 | 1.000 | 0.990 | 1.000 | 0.959 | 0.970 | 0.991 | 0.856 | 1.000 | 1.000 | 0.860 | 0.832 |
| 3317 | 0.771 | 1.000 | 0.814 | 0.986 | 0.958 | 0.960 | 0.891 | 1.000 | 0.990 | 1.000 | 0.965 | 0.970 | 1.000 | 0.848 | 1.000 | 1.000 | 0.855 | 0.826 |
| 3318 | 0.769 | 1.000 | 0.814 | 1.000 | 0.958 | 0.960 | 0.898 | 1.000 | 1.000 | 1.000 | 0.957 | 0.970 | 0.991 | 0.850 | 1.000 | 1.000 | 0.855 | 0.827 |
| 3319 | 0.772 | 1.000 | 0.824 | 1.000 | 0.958 | 0.960 | 0.892 | 1.000 | 0.990 | 1.000 | 0.959 | 0.970 | 1.000 | 0.853 | 1.000 | 1.000 | 0.857 | 0.829 |
| 3320 | 0.773 | 1.000 | 0.794 | 1.000 | 0.958 | 0.960 | 0.901 | 1.000 | 0.990 | 1.000 | 0.959 | 1.000 | 0.991 | 0.848 | 1.000 | 1.000 | 0.854 | 0.825 |
| 3321 | 0.766 | 1.000 | 0.816 | 1.000 | 0.953 | 0.960 | 0.897 | 1.000 | 1.000 | 1.000 | 0.968 | 0.970 | 0.991 | 0.851 | 1.000 | 1.000 | 0.855 | 0.827 |
| 3322 | 0.774 | 1.000 | 0.816 | 1.000 | 0.953 | 0.960 | 0.895 | 1.000 | 1.000 | 1.000 | 0.965 | 1.000 | 0.991 | 0.859 | 1.000 | 1.000 | 0.859 | 0.831 |
| 3323 | 0.761 | 1.000 | 0.830 | 0.986 | 0.953 | 0.960 | 0.877 | 1.000 | 0.990 | 1.000 | 0.957 | 0.970 | 0.991 | 0.853 | 1.000 | 1.000 | 0.852 | 0.823 |
| 3324 | 0.758 | 1.000 | 0.796 | 1.000 | 0.958 | 0.960 | 0.892 | 1.000 | 1.000 | 1.000 | 0.959 | 0.970 | 1.000 | 0.854 | 1.000 | 1.000 | 0.851 | 0.821 |
| 3325 | 0.775 | 1.000 | 0.824 | 1.000 | 0.958 | 0.960 | 0.895 | 1.000 | 0.990 | 1.000 | 0.971 | 0.970 | 1.000 | 0.847 | 1.000 | 1.000 | 0.858 | 0.830 |
| 3326 | 0.770 | 1.000 | 0.822 | 1.000 | 0.953 | 0.960 | 0.889 | 1.000 | 1.000 | 1.000 | 0.968 | 0.970 | 1.000 | 0.847 | 1.000 | 1.000 | 0.855 | 0.826 |
| 3327 | 0.774 | 1.000 | 0.816 | 0.971 | 0.953 | 0.960 | 0.894 | 1.000 | 1.000 | 1.000 | 0.962 | 1.000 | 0.991 | 0.848 | 1.000 | 1.000 | 0.856 | 0.827 |
| 3328 | 0.769 | 1.000 | 0.820 | 1.000 | 0.953 | 0.960 | 0.892 | 1.000 | 0.990 | 1.000 | 0.965 | 1.000 | 0.991 | 0.853 | 1.000 | 1.000 | 0.856 | 0.828 |
| 3329 | 0.766 | 1.000 | 0.822 | 1.000 | 0.953 | 0.960 | 0.898 | 1.000 | 0.990 | 1.000 | 0.962 | 1.000 | 1.000 | 0.852 | 1.000 | 1.000 | 0.856 | 0.828 |
| 3330 | 0.779 | 1.000 | 0.808 | 1.000 | 0.958 | 0.960 | 0.895 | 1.000 | 0.990 | 1.000 | 0.971 | 1.000 | 0.991 | 0.851 | 1.000 | 1.000 | 0.859 | 0.830 |
| 3331 | 0.771 | 1.000 | 0.812 | 0.986 | 0.958 | 0.960 | 0.894 | 1.000 | 1.000 | 1.000 | 0.959 | 0.970 | 1.000 | 0.853 | 1.000 | 1.000 | 0.856 | 0.827 |
| 3332 | 0.775 | 1.000 | 0.818 | 1.000 | 0.953 | 0.960 | 0.897 | 1.000 | 0.990 | 1.000 | 0.959 | 1.000 | 0.991 | 0.855 | 1.000 | 1.000 | 0.859 | 0.831 |
| 3333 | 0.775 | 1.000 | 0.822 | 0.986 | 0.958 | 0.960 | 0.898 | 1.000 | 1.000 | 1.000 | 0.962 | 0.970 | 0.991 | 0.851 | 1.000 | 1.000 | 0.858 | 0.830 |
| 3334 | 0.767 | 1.000 | 0.812 | 1.000 | 0.947 | 0.960 | 0.880 | 1.000 | 0.990 | 1.000 | 0.959 | 1.000 | 1.000 | 0.855 | 1.000 | 1.000 | 0.853 | 0.824 |
| 3335 | 0.763 | 1.000 | 0.812 | 1.000 | 0.953 | 0.960 | 0.894 | 1.000 | 0.990 | 1.000 | 0.968 | 1.000 | 1.000 | 0.852 | 1.000 | 1.000 | 0.854 | 0.825 |
| 3336 | 0.770 | 1.000 | 0.820 | 1.000 | 0.958 | 0.960 | 0.891 | 1.000 | 1.000 | 1.000 | 0.957 | 0.970 | 1.000 | 0.845 | 1.000 | 1.000 | 0.854 | 0.825 |
| 3337 | 0.776 | 1.000 | 0.824 | 0.986 | 0.953 | 0.960 | 0.898 | 1.000 | 0.990 | 1.000 | 0.965 | 0.970 | 1.000 | 0.850 | 1.000 | 1.000 | 0.858 | 0.831 |
| 3338 | 0.776 | 1.000 | 0.818 | 1.000 | 0.953 | 0.960 | 0.894 | 1.000 | 1.000 | 1.000 | 0.965 | 1.000 | 1.000 | 0.850 | 1.000 | 1.000 | 0.858 | 0.830 |
| 3339 | 0.771 | 1.000 | 0.810 | 1.000 | 0.958 | 0.960 | 0.898 | 1.000 | 0.990 | 1.000 | 0.962 | 0.970 | 0.991 | 0.851 | 1.000 | 1.000 | 0.856 | 0.827 |
| 3340 | 0.760 | 1.000 | 0.810 | 1.000 | 0.953 | 0.960 | 0.895 | 1.000 | 1.000 | 1.000 | 0.957 | 0.970 | 0.991 | 0.853 | 1.000 | 1.000 | 0.852 | 0.823 |
| 3341 | 0.765 | 1.000 | 0.810 | 1.000 | 0.958 | 0.960 | 0.900 | 1.000 | 0.990 | 1.000 | 0.965 | 1.000 | 0.991 | 0.862 | 1.000 | 1.000 | 0.857 | 0.829 |
| 3342 | 0.765 | 1.000 | 0.804 | 1.000 | 0.953 | 0.960 | 0.894 | 1.000 | 1.000 | 1.000 | 0.965 | 0.970 | 1.000 | 0.848 | 1.000 | 1.000 | 0.852 | 0.823 |
| 3343 | 0.765 | 1.000 | 0.802 | 1.000 | 0.953 | 0.960 | 0.888 | 1.000 | 1.000 | 1.000 | 0.959 | 1.000 | 1.000 | 0.849 | 1.000 | 1.000 | 0.852 | 0.822 |
| 3344 | 0.771 | 1.000 | 0.818 | 1.000 | 0.953 | 0.960 | 0.894 | 1.000 | 0.990 | 1.000 | 0.971 | 0.970 | 1.000 | 0.849 | 1.000 | 1.000 | 0.856 | 0.827 |
| 3345 | 0.767 | 1.000 | 0.812 | 0.986 | 0.947 | 0.960 | 0.888 | 1.000 | 0.990 | 1.000 | 0.962 | 1.000 | 1.000 | 0.854 | 1.000 | 1.000 | 0.854 | 0.825 |
| 3346 | 0.787 | 1.000 | 0.810 | 1.000 | 0.953 | 0.960 | 0.889 | 1.000 | 0.990 | 1.000 | 0.962 | 0.970 | 0.991 | 0.854 | 1.000 | 1.000 | 0.860 | 0.832 |
| 3347 | 0.777 | 1.000 | 0.812 | 0.986 | 0.953 | 0.960 | 0.889 | 1.000 | 1.000 | 1.000 | 0.959 | 0.970 | 0.991 | 0.866 | 1.000 | 1.000 | 0.860 | 0.832 |
| 3348 | 0.771 | 1.000 | 0.824 | 1.000 | 0.953 | 0.960 | 0.894 | 1.000 | 0.990 | 1.000 | 0.965 | 1.000 | 1.000 | 0.847 | 1.000 | 1.000 | 0.856 | 0.828 |
| 3349 | 0.772 | 1.000 | 0.802 | 1.000 | 0.958 | 0.960 | 0.894 | 1.000 | 0.990 | 1.000 | 0.965 | 0.970 | 1.000 | 0.851 | 1.000 | 1.000 | 0.855 | 0.826 |
| 3350 | 0.779 | 1.000 | 0.804 | 1.000 | 0.953 | 0.960 | 0.900 | 1.000 | 1.000 | 1.000 | 0.962 | 0.970 | 0.991 | 0.848 | 1.000 | 1.000 | 0.857 | 0.829 |
| 3351 | 0.765 | 1.000 | 0.816 | 1.000 | 0.958 | 0.960 | 0.888 | 1.000 | 1.000 | 1.000 | 0.971 | 1.000 | 1.000 | 0.853 | 1.000 | 1.000 | 0.855 | 0.826 |
| 3352 | 0.774 | 1.000 | 0.808 | 0.986 | 0.958 | 0.960 | 0.889 | 1.000 | 0.990 | 1.000 | 0.968 | 0.970 | 0.991 | 0.858 | 1.000 | 1.000 | 0.857 | 0.829 |

| | | | | | | | | | | | | | | | | | | |
|---|---|---|---|---|---|---|---|---|---|---|---|---|---|---|---|---|---|---|
| 3353 | 0.779 | 1.000 | 0.806 | 0.986 | 0.958 | 0.960 | 0.901 | 1.000 | 0.990 | 1.000 | 0.968 | 0.970 | 0.991 | 0.858 | 1.000 | 1.000 | 0.860 | 0.832 |
| 3354 | 0.763 | 1.000 | 0.806 | 1.000 | 0.953 | 0.960 | 0.892 | 1.000 | 0.990 | 1.000 | 0.965 | 0.970 | 0.991 | 0.849 | 1.000 | 1.000 | 0.852 | 0.822 |
| 3355 | 0.772 | 1.000 | 0.824 | 0.986 | 0.958 | 0.960 | 0.903 | 1.000 | 0.969 | 1.000 | 0.965 | 0.970 | 1.000 | 0.853 | 1.000 | 1.000 | 0.858 | 0.831 |
| 3356 | 0.765 | 1.000 | 0.808 | 1.000 | 0.953 | 0.960 | 0.891 | 1.000 | 0.980 | 1.000 | 0.965 | 1.000 | 0.991 | 0.859 | 1.000 | 1.000 | 0.855 | 0.826 |
| 3357 | 0.763 | 1.000 | 0.822 | 1.000 | 0.958 | 0.960 | 0.908 | 1.000 | 0.990 | 1.000 | 0.965 | 0.970 | 0.991 | 0.853 | 1.000 | 1.000 | 0.856 | 0.828 |
| 3358 | 0.775 | 1.000 | 0.806 | 1.000 | 0.958 | 0.960 | 0.895 | 1.000 | 0.980 | 1.000 | 0.968 | 1.000 | 1.000 | 0.851 | 1.000 | 1.000 | 0.857 | 0.828 |
| 3359 | 0.769 | 1.000 | 0.812 | 1.000 | 0.958 | 0.960 | 0.911 | 1.000 | 1.000 | 1.000 | 0.965 | 1.000 | 1.000 | 0.851 | 1.000 | 1.000 | 0.858 | 0.830 |
| 3360 | 0.775 | 1.000 | 0.818 | 1.000 | 0.953 | 0.960 | 0.903 | 1.000 | 0.990 | 1.000 | 0.965 | 0.970 | 0.991 | 0.851 | 1.000 | 1.000 | 0.858 | 0.830 |
| 3361 | 0.768 | 1.000 | 0.810 | 1.000 | 0.953 | 0.960 | 0.903 | 1.000 | 0.990 | 1.000 | 0.965 | 0.970 | 0.991 | 0.851 | 1.000 | 1.000 | 0.855 | 0.827 |
| 3362 | 0.773 | 1.000 | 0.830 | 1.000 | 0.947 | 1.000 | 0.897 | 1.000 | 0.990 | 1.000 | 0.968 | 1.000 | 1.000 | 0.858 | 1.000 | 1.000 | 0.861 | 0.833 |
| 3363 | 0.767 | 1.000 | 0.816 | 0.971 | 0.958 | 0.960 | 0.906 | 1.000 | 1.000 | 1.000 | 0.968 | 0.970 | 0.991 | 0.848 | 1.000 | 1.000 | 0.856 | 0.828 |
| 3364 | 0.775 | 1.000 | 0.806 | 1.000 | 0.953 | 0.960 | 0.894 | 1.000 | 0.990 | 1.000 | 0.957 | 1.000 | 0.991 | 0.853 | 1.000 | 1.000 | 0.856 | 0.828 |
| 3365 | 0.768 | 1.000 | 0.816 | 1.000 | 0.953 | 0.960 | 0.897 | 1.000 | 0.980 | 1.000 | 0.968 | 1.000 | 1.000 | 0.853 | 1.000 | 1.000 | 0.856 | 0.828 |
| 3366 | 0.771 | 1.000 | 0.816 | 1.000 | 0.953 | 0.960 | 0.892 | 1.000 | 0.980 | 1.000 | 0.968 | 0.970 | 1.000 | 0.852 | 1.000 | 1.000 | 0.856 | 0.827 |
| 3367 | 0.771 | 1.000 | 0.812 | 1.000 | 0.953 | 0.960 | 0.897 | 1.000 | 0.990 | 1.000 | 0.965 | 1.000 | 1.000 | 0.847 | 1.000 | 1.000 | 0.855 | 0.827 |
| 3368 | 0.767 | 1.000 | 0.806 | 0.986 | 0.958 | 0.960 | 0.900 | 1.000 | 0.990 | 1.000 | 0.965 | 0.970 | 0.991 | 0.851 | 1.000 | 1.000 | 0.854 | 0.826 |
| 3369 | 0.767 | 1.000 | 0.828 | 1.000 | 0.958 | 0.960 | 0.906 | 1.000 | 1.000 | 1.000 | 0.965 | 0.970 | 1.000 | 0.851 | 1.000 | 1.000 | 0.858 | 0.830 |
| 3370 | 0.769 | 1.000 | 0.812 | 0.986 | 0.963 | 0.960 | 0.897 | 1.000 | 0.990 | 1.000 | 0.971 | 0.970 | 1.000 | 0.855 | 1.000 | 1.000 | 0.857 | 0.829 |
| 3371 | 0.771 | 1.000 | 0.820 | 0.986 | 0.958 | 0.960 | 0.897 | 1.000 | 1.000 | 1.000 | 0.957 | 0.970 | 1.000 | 0.859 | 1.000 | 1.000 | 0.858 | 0.830 |
| 3372 | 0.779 | 1.000 | 0.800 | 1.000 | 0.947 | 0.960 | 0.900 | 1.000 | 0.990 | 1.000 | 0.959 | 1.000 | 1.000 | 0.852 | 1.000 | 1.000 | 0.857 | 0.829 |
| 3373 | 0.774 | 1.000 | 0.806 | 1.000 | 0.958 | 1.000 | 0.895 | 1.000 | 1.000 | 1.000 | 0.968 | 1.000 | 0.991 | 0.858 | 1.000 | 1.000 | 0.859 | 0.831 |
| 3374 | 0.773 | 1.000 | 0.798 | 1.000 | 0.953 | 0.960 | 0.898 | 1.000 | 0.980 | 1.000 | 0.968 | 0.970 | 0.991 | 0.854 | 1.000 | 1.000 | 0.856 | 0.827 |
| 3375 | 0.776 | 1.000 | 0.806 | 0.986 | 0.953 | 0.960 | 0.900 | 1.000 | 0.980 | 1.000 | 0.965 | 1.000 | 1.000 | 0.850 | 0.978 | 1.000 | 0.857 | 0.828 |
| 3376 | 0.773 | 1.000 | 0.802 | 1.000 | 0.958 | 0.960 | 0.894 | 1.000 | 0.969 | 1.000 | 0.962 | 1.000 | 1.000 | 0.853 | 1.000 | 1.000 | 0.856 | 0.827 |
| 3377 | 0.771 | 1.000 | 0.810 | 1.000 | 0.947 | 0.960 | 0.901 | 1.000 | 0.990 | 1.000 | 0.971 | 1.000 | 1.000 | 0.853 | 1.000 | 1.000 | 0.857 | 0.829 |
| 3378 | 0.763 | 1.000 | 0.824 | 0.986 | 0.958 | 0.960 | 0.906 | 1.000 | 0.980 | 1.000 | 0.971 | 0.970 | 1.000 | 0.859 | 1.000 | 1.000 | 0.858 | 0.831 |
| 3379 | 0.763 | 1.000 | 0.826 | 1.000 | 0.958 | 0.960 | 0.903 | 1.000 | 0.980 | 1.000 | 0.965 | 1.000 | 1.000 | 0.844 | 1.000 | 1.000 | 0.854 | 0.826 |
| 3380 | 0.767 | 1.000 | 0.806 | 1.000 | 0.958 | 0.960 | 0.898 | 1.000 | 1.000 | 1.000 | 0.968 | 1.000 | 1.000 | 0.853 | 1.000 | 1.000 | 0.856 | 0.827 |
| 3381 | 0.777 | 1.000 | 0.810 | 1.000 | 0.953 | 0.960 | 0.904 | 1.000 | 0.980 | 1.000 | 0.965 | 1.000 | 1.000 | 0.858 | 1.000 | 1.000 | 0.860 | 0.833 |
| 3382 | 0.769 | 1.000 | 0.814 | 1.000 | 0.953 | 0.960 | 0.906 | 1.000 | 0.980 | 1.000 | 0.959 | 1.000 | 0.991 | 0.857 | 1.000 | 1.000 | 0.858 | 0.830 |
| 3383 | 0.763 | 1.000 | 0.810 | 0.971 | 0.958 | 0.960 | 0.894 | 1.000 | 0.990 | 1.000 | 0.968 | 0.970 | 0.991 | 0.849 | 1.000 | 1.000 | 0.852 | 0.823 |
| 3384 | 0.756 | 1.000 | 0.812 | 0.986 | 0.947 | 0.960 | 0.891 | 1.000 | 0.990 | 1.000 | 0.968 | 0.970 | 0.991 | 0.859 | 1.000 | 1.000 | 0.852 | 0.823 |
| 3385 | 0.775 | 1.000 | 0.810 | 1.000 | 0.963 | 0.960 | 0.895 | 1.000 | 0.990 | 1.000 | 0.965 | 0.970 | 1.000 | 0.856 | 1.000 | 1.000 | 0.858 | 0.830 |
| 3386 | 0.775 | 1.000 | 0.808 | 1.000 | 0.958 | 0.960 | 0.900 | 1.000 | 0.980 | 1.000 | 0.962 | 0.970 | 1.000 | 0.854 | 1.000 | 1.000 | 0.858 | 0.830 |
| 3387 | 0.768 | 1.000 | 0.824 | 0.986 | 0.953 | 0.960 | 0.900 | 1.000 | 0.990 | 1.000 | 0.959 | 0.970 | 1.000 | 0.858 | 1.000 | 1.000 | 0.858 | 0.830 |
| 3388 | 0.775 | 1.000 | 0.806 | 1.000 | 0.958 | 0.960 | 0.906 | 1.000 | 0.969 | 1.000 | 0.959 | 1.000 | 1.000 | 0.855 | 1.000 | 1.000 | 0.859 | 0.831 |
| 3389 | 0.769 | 1.000 | 0.822 | 0.986 | 0.968 | 0.960 | 0.898 | 1.000 | 0.980 | 1.000 | 0.968 | 0.970 | 1.000 | 0.854 | 1.000 | 1.000 | 0.858 | 0.830 |
| 3390 | 0.771 | 1.000 | 0.812 | 1.000 | 0.958 | 0.960 | 0.906 | 1.000 | 0.980 | 1.000 | 0.965 | 0.970 | 0.991 | 0.860 | 1.000 | 1.000 | 0.859 | 0.831 |
| 3391 | 0.770 | 1.000 | 0.822 | 0.986 | 0.947 | 0.960 | 0.904 | 1.000 | 1.000 | 1.000 | 0.965 | 0.970 | 1.000 | 0.849 | 1.000 | 1.000 | 0.857 | 0.829 |

| | | | | | | | | | | | | | | | | | | |
|---|---|---|---|---|---|---|---|---|---|---|---|---|---|---|---|---|---|---|
| 3392 | 0.770 | 1.000 | 0.814 | 1.000 | 0.947 | 0.960 | 0.901 | 1.000 | 0.990 | 1.000 | 0.965 | 1.000 | 1.000 | 0.855 | 1.000 | 1.000 | 0.858 | 0.830 |
| 3393 | 0.775 | 1.000 | 0.818 | 1.000 | 0.947 | 0.960 | 0.903 | 1.000 | 1.000 | 1.000 | 0.965 | 0.970 | 0.991 | 0.858 | 1.000 | 1.000 | 0.860 | 0.832 |
| 3394 | 0.775 | 1.000 | 0.822 | 1.000 | 0.958 | 0.960 | 0.900 | 1.000 | 1.000 | 1.000 | 0.962 | 0.970 | 1.000 | 0.849 | 1.000 | 1.000 | 0.858 | 0.831 |
| 3395 | 0.775 | 1.000 | 0.824 | 1.000 | 0.953 | 0.960 | 0.895 | 1.000 | 0.969 | 1.000 | 0.965 | 0.970 | 0.991 | 0.858 | 1.000 | 1.000 | 0.859 | 0.831 |
| 3396 | 0.771 | 1.000 | 0.816 | 1.000 | 0.958 | 0.960 | 0.898 | 1.000 | 0.990 | 1.000 | 0.968 | 0.970 | 1.000 | 0.854 | 1.000 | 1.000 | 0.858 | 0.830 |
| 3397 | 0.772 | 1.000 | 0.808 | 1.000 | 0.958 | 0.960 | 0.897 | 1.000 | 1.000 | 1.000 | 0.962 | 0.970 | 0.982 | 0.859 | 1.000 | 1.000 | 0.858 | 0.830 |
| 3398 | 0.779 | 1.000 | 0.810 | 1.000 | 0.953 | 0.960 | 0.894 | 1.000 | 0.980 | 1.000 | 0.962 | 1.000 | 1.000 | 0.851 | 1.000 | 1.000 | 0.858 | 0.829 |
| 3399 | 0.775 | 1.000 | 0.812 | 0.986 | 0.968 | 0.960 | 0.901 | 1.000 | 0.990 | 1.000 | 0.959 | 0.970 | 1.000 | 0.856 | 1.000 | 1.000 | 0.859 | 0.831 |
| 3400 | 0.766 | 1.000 | 0.806 | 1.000 | 0.947 | 0.960 | 0.901 | 1.000 | 1.000 | 1.000 | 0.968 | 0.970 | 1.000 | 0.848 | 1.000 | 1.000 | 0.854 | 0.825 |
| 3401 | 0.780 | 1.000 | 0.818 | 1.000 | 0.953 | 0.960 | 0.898 | 1.000 | 0.980 | 1.000 | 0.968 | 0.970 | 0.991 | 0.852 | 1.000 | 1.000 | 0.860 | 0.832 |
| 3402 | 0.764 | 1.000 | 0.802 | 1.000 | 0.953 | 0.960 | 0.903 | 1.000 | 0.990 | 1.000 | 0.962 | 0.970 | 1.000 | 0.855 | 1.000 | 1.000 | 0.855 | 0.826 |
| 3403 | 0.774 | 1.000 | 0.818 | 1.000 | 0.958 | 0.960 | 0.898 | 1.000 | 0.990 | 1.000 | 0.965 | 0.970 | 0.982 | 0.854 | 1.000 | 1.000 | 0.858 | 0.830 |
| 3404 | 0.769 | 1.000 | 0.810 | 1.000 | 0.958 | 0.960 | 0.901 | 1.000 | 1.000 | 1.000 | 0.965 | 0.970 | 1.000 | 0.855 | 1.000 | 1.000 | 0.857 | 0.829 |
| 3405 | 0.775 | 1.000 | 0.806 | 0.986 | 0.958 | 0.960 | 0.892 | 1.000 | 0.980 | 1.000 | 0.965 | 0.970 | 1.000 | 0.855 | 1.000 | 1.000 | 0.857 | 0.828 |
| 3406 | 0.763 | 1.000 | 0.804 | 1.000 | 0.953 | 0.960 | 0.903 | 1.000 | 0.980 | 1.000 | 0.959 | 0.970 | 1.000 | 0.853 | 1.000 | 1.000 | 0.854 | 0.825 |
| 3407 | 0.766 | 1.000 | 0.806 | 1.000 | 0.963 | 0.960 | 0.898 | 1.000 | 0.980 | 1.000 | 0.965 | 0.970 | 1.000 | 0.854 | 1.000 | 1.000 | 0.855 | 0.827 |
| 3408 | 0.764 | 1.000 | 0.820 | 1.000 | 0.953 | 0.960 | 0.895 | 1.000 | 0.980 | 1.000 | 0.959 | 0.970 | 1.000 | 0.856 | 1.000 | 1.000 | 0.855 | 0.827 |
| 3409 | 0.765 | 1.000 | 0.822 | 0.986 | 0.953 | 0.960 | 0.897 | 1.000 | 0.980 | 1.000 | 0.962 | 1.000 | 1.000 | 0.848 | 1.000 | 1.000 | 0.854 | 0.826 |
| 3410 | 0.765 | 1.000 | 0.816 | 1.000 | 0.953 | 0.960 | 0.900 | 1.000 | 0.980 | 1.000 | 0.962 | 0.970 | 1.000 | 0.850 | 1.000 | 1.000 | 0.854 | 0.826 |
| 3411 | 0.767 | 1.000 | 0.816 | 0.986 | 0.953 | 0.960 | 0.898 | 1.000 | 0.990 | 1.000 | 0.968 | 0.970 | 1.000 | 0.854 | 1.000 | 1.000 | 0.856 | 0.828 |
| 3412 | 0.763 | 1.000 | 0.802 | 1.000 | 0.958 | 0.960 | 0.898 | 1.000 | 0.990 | 1.000 | 0.965 | 0.970 | 1.000 | 0.849 | 1.000 | 1.000 | 0.853 | 0.823 |
| 3413 | 0.770 | 1.000 | 0.806 | 0.986 | 0.953 | 0.960 | 0.903 | 1.000 | 0.980 | 1.000 | 0.965 | 0.970 | 1.000 | 0.848 | 1.000 | 1.000 | 0.855 | 0.826 |
| 3414 | 0.768 | 1.000 | 0.792 | 0.986 | 0.953 | 0.960 | 0.903 | 1.000 | 0.990 | 1.000 | 0.962 | 0.970 | 1.000 | 0.850 | 1.000 | 1.000 | 0.853 | 0.824 |
| 3415 | 0.767 | 1.000 | 0.798 | 1.000 | 0.963 | 0.960 | 0.911 | 1.000 | 0.980 | 1.000 | 0.965 | 0.970 | 1.000 | 0.851 | 1.000 | 1.000 | 0.855 | 0.827 |
| 3416 | 0.778 | 1.000 | 0.794 | 0.986 | 0.958 | 0.960 | 0.904 | 1.000 | 0.990 | 1.000 | 0.965 | 0.970 | 1.000 | 0.853 | 1.000 | 1.000 | 0.858 | 0.829 |
| 3417 | 0.771 | 1.000 | 0.816 | 0.986 | 0.953 | 0.960 | 0.900 | 1.000 | 0.990 | 1.000 | 0.959 | 1.000 | 1.000 | 0.851 | 1.000 | 1.000 | 0.856 | 0.828 |
| 3418 | 0.779 | 1.000 | 0.808 | 1.000 | 0.963 | 0.960 | 0.891 | 1.000 | 0.990 | 1.000 | 0.965 | 0.970 | 0.991 | 0.847 | 1.000 | 1.000 | 0.857 | 0.828 |
| 3419 | 0.769 | 1.000 | 0.816 | 0.986 | 0.958 | 0.960 | 0.901 | 1.000 | 0.990 | 1.000 | 0.962 | 0.970 | 1.000 | 0.852 | 1.000 | 1.000 | 0.856 | 0.828 |
| 3420 | 0.776 | 1.000 | 0.810 | 1.000 | 0.953 | 0.960 | 0.901 | 1.000 | 0.969 | 1.000 | 0.965 | 1.000 | 1.000 | 0.858 | 1.000 | 1.000 | 0.860 | 0.832 |
| 3421 | 0.762 | 1.000 | 0.816 | 1.000 | 0.953 | 0.960 | 0.903 | 1.000 | 0.980 | 1.000 | 0.968 | 0.970 | 0.991 | 0.847 | 1.000 | 1.000 | 0.853 | 0.825 |
| 3422 | 0.775 | 1.000 | 0.810 | 1.000 | 0.953 | 0.960 | 0.906 | 1.000 | 1.000 | 1.000 | 0.962 | 0.970 | 1.000 | 0.858 | 1.000 | 1.000 | 0.860 | 0.832 |
| 3423 | 0.767 | 1.000 | 0.800 | 1.000 | 0.947 | 0.960 | 0.903 | 1.000 | 0.990 | 1.000 | 0.959 | 0.970 | 0.991 | 0.845 | 1.000 | 1.000 | 0.852 | 0.823 |
| 3424 | 0.764 | 1.000 | 0.814 | 1.000 | 0.958 | 0.960 | 0.892 | 1.000 | 0.990 | 1.000 | 0.965 | 1.000 | 1.000 | 0.856 | 1.000 | 1.000 | 0.855 | 0.827 |
| 3425 | 0.765 | 1.000 | 0.812 | 0.986 | 0.958 | 0.960 | 0.900 | 1.000 | 0.980 | 1.000 | 0.957 | 1.000 | 1.000 | 0.861 | 1.000 | 1.000 | 0.857 | 0.828 |
| 3426 | 0.767 | 1.000 | 0.804 | 1.000 | 0.953 | 0.960 | 0.897 | 1.000 | 1.000 | 1.000 | 0.965 | 0.970 | 1.000 | 0.854 | 1.000 | 1.000 | 0.855 | 0.826 |
| 3427 | 0.772 | 1.000 | 0.808 | 1.000 | 0.958 | 0.960 | 0.900 | 1.000 | 0.980 | 1.000 | 0.971 | 0.970 | 0.991 | 0.851 | 1.000 | 1.000 | 0.856 | 0.828 |
| 3428 | 0.771 | 1.000 | 0.808 | 1.000 | 0.953 | 0.960 | 0.897 | 1.000 | 0.969 | 1.000 | 0.965 | 0.970 | 0.991 | 0.852 | 1.000 | 1.000 | 0.855 | 0.827 |
| 3429 | 0.769 | 1.000 | 0.818 | 1.000 | 0.963 | 0.960 | 0.898 | 1.000 | 1.000 | 1.000 | 0.965 | 0.970 | 0.991 | 0.857 | 1.000 | 1.000 | 0.858 | 0.830 |
| 3430 | 0.762 | 1.000 | 0.818 | 1.000 | 0.953 | 0.960 | 0.895 | 1.000 | 0.990 | 1.000 | 0.965 | 0.970 | 1.000 | 0.854 | 1.000 | 1.000 | 0.854 | 0.826 |

| | | | | | | | | | | | | | | | | | |
|---|---|---|---|---|---|---|---|---|---|---|---|---|---|---|---|---|---|
| 3431 | 0.761 | 1.000 | 0.806 | 0.986 | 0.953 | 0.960 | 0.895 | 1.000 | 0.980 | 1.000 | 0.965 | 1.000 | 1.000 | 0.855 | 1.000 | 1.000 | 0.853 | 0.824 |
| 3432 | 0.767 | 1.000 | 0.816 | 0.986 | 0.953 | 0.960 | 0.898 | 1.000 | 1.000 | 1.000 | 0.968 | 0.970 | 0.991 | 0.855 | 1.000 | 1.000 | 0.856 | 0.828 |
| 3433 | 0.763 | 1.000 | 0.800 | 1.000 | 0.958 | 0.960 | 0.894 | 1.000 | 1.000 | 1.000 | 0.962 | 0.970 | 0.991 | 0.858 | 1.000 | 1.000 | 0.854 | 0.825 |
| 3434 | 0.768 | 1.000 | 0.796 | 1.000 | 0.953 | 0.960 | 0.901 | 1.000 | 0.990 | 1.000 | 0.962 | 0.970 | 1.000 | 0.861 | 1.000 | 1.000 | 0.856 | 0.828 |
| 3435 | 0.772 | 1.000 | 0.820 | 1.000 | 0.958 | 0.960 | 0.897 | 1.000 | 0.990 | 1.000 | 0.959 | 1.000 | 0.991 | 0.842 | 1.000 | 1.000 | 0.855 | 0.826 |
| 3436 | 0.771 | 1.000 | 0.818 | 1.000 | 0.953 | 0.960 | 0.895 | 1.000 | 0.990 | 1.000 | 0.962 | 0.970 | 1.000 | 0.856 | 1.000 | 1.000 | 0.858 | 0.829 |
| 3437 | 0.763 | 1.000 | 0.802 | 0.986 | 0.958 | 0.960 | 0.894 | 1.000 | 1.000 | 1.000 | 0.968 | 1.000 | 0.991 | 0.851 | 1.000 | 1.000 | 0.853 | 0.824 |
| 3438 | 0.769 | 1.000 | 0.812 | 1.000 | 0.953 | 0.960 | 0.895 | 1.000 | 0.990 | 1.000 | 0.962 | 1.000 | 1.000 | 0.845 | 1.000 | 1.000 | 0.854 | 0.825 |
| 3439 | 0.771 | 1.000 | 0.818 | 1.000 | 0.953 | 0.960 | 0.894 | 1.000 | 0.990 | 1.000 | 0.962 | 0.970 | 0.991 | 0.851 | 1.000 | 1.000 | 0.856 | 0.827 |
| 3440 | 0.769 | 1.000 | 0.810 | 1.000 | 0.958 | 0.960 | 0.906 | 1.000 | 0.990 | 1.000 | 0.968 | 1.000 | 0.991 | 0.851 | 1.000 | 1.000 | 0.857 | 0.829 |
| 3441 | 0.771 | 1.000 | 0.816 | 1.000 | 0.953 | 0.960 | 0.889 | 1.000 | 1.000 | 1.000 | 0.968 | 1.000 | 0.991 | 0.858 | 1.000 | 1.000 | 0.857 | 0.829 |
| 3442 | 0.775 | 1.000 | 0.806 | 1.000 | 0.953 | 0.960 | 0.891 | 1.000 | 0.980 | 1.000 | 0.968 | 1.000 | 1.000 | 0.856 | 1.000 | 1.000 | 0.857 | 0.829 |
| 3443 | 0.767 | 1.000 | 0.798 | 0.986 | 0.958 | 0.960 | 0.909 | 1.000 | 0.990 | 1.000 | 0.965 | 0.970 | 0.991 | 0.851 | 1.000 | 1.000 | 0.855 | 0.826 |
| 3444 | 0.776 | 1.000 | 0.806 | 1.000 | 0.947 | 0.960 | 0.900 | 1.000 | 0.969 | 1.000 | 0.965 | 1.000 | 1.000 | 0.856 | 1.000 | 1.000 | 0.858 | 0.830 |
| 3445 | 0.764 | 1.000 | 0.812 | 1.000 | 0.953 | 0.960 | 0.895 | 1.000 | 1.000 | 1.000 | 0.957 | 0.970 | 1.000 | 0.851 | 1.000 | 1.000 | 0.853 | 0.825 |
| 3446 | 0.773 | 1.000 | 0.802 | 1.000 | 0.953 | 0.960 | 0.895 | 1.000 | 0.980 | 1.000 | 0.962 | 0.970 | 1.000 | 0.853 | 1.000 | 1.000 | 0.855 | 0.827 |
| 3447 | 0.770 | 1.000 | 0.806 | 0.986 | 0.963 | 0.960 | 0.897 | 1.000 | 1.000 | 1.000 | 0.965 | 0.970 | 1.000 | 0.854 | 1.000 | 1.000 | 0.856 | 0.828 |
| 3448 | 0.767 | 1.000 | 0.812 | 0.986 | 0.958 | 0.960 | 0.894 | 1.000 | 0.990 | 1.000 | 0.965 | 0.970 | 0.991 | 0.848 | 1.000 | 1.000 | 0.854 | 0.825 |
| 3449 | 0.770 | 1.000 | 0.818 | 1.000 | 0.958 | 0.960 | 0.897 | 1.000 | 1.000 | 1.000 | 0.965 | 0.970 | 1.000 | 0.856 | 1.000 | 1.000 | 0.858 | 0.830 |
| 3450 | 0.768 | 1.000 | 0.808 | 0.986 | 0.958 | 0.960 | 0.895 | 1.000 | 0.980 | 1.000 | 0.965 | 1.000 | 0.991 | 0.861 | 1.000 | 1.000 | 0.857 | 0.828 |
| 3451 | 0.772 | 1.000 | 0.812 | 1.000 | 0.958 | 0.960 | 0.888 | 1.000 | 0.980 | 1.000 | 0.959 | 1.000 | 0.991 | 0.854 | 1.000 | 1.000 | 0.855 | 0.826 |
| 3452 | 0.771 | 1.000 | 0.796 | 0.986 | 0.958 | 0.960 | 0.903 | 1.000 | 0.980 | 1.000 | 0.965 | 0.970 | 0.991 | 0.854 | 1.000 | 1.000 | 0.856 | 0.827 |
| 3453 | 0.763 | 1.000 | 0.808 | 0.986 | 0.953 | 0.960 | 0.897 | 1.000 | 0.990 | 1.000 | 0.959 | 1.000 | 1.000 | 0.851 | 1.000 | 1.000 | 0.853 | 0.824 |
| 3454 | 0.769 | 1.000 | 0.822 | 1.000 | 0.963 | 0.960 | 0.900 | 1.000 | 0.969 | 1.000 | 0.959 | 1.000 | 0.991 | 0.858 | 1.000 | 1.000 | 0.858 | 0.830 |
| 3455 | 0.764 | 1.000 | 0.816 | 1.000 | 0.958 | 0.960 | 0.906 | 1.000 | 0.980 | 1.000 | 0.962 | 0.970 | 0.991 | 0.848 | 1.000 | 1.000 | 0.854 | 0.826 |
| 3456 | 0.775 | 1.000 | 0.802 | 1.000 | 0.953 | 0.960 | 0.897 | 1.000 | 1.000 | 1.000 | 0.965 | 0.970 | 1.000 | 0.854 | 1.000 | 1.000 | 0.857 | 0.829 |
| 3457 | 0.772 | 1.000 | 0.812 | 1.000 | 0.942 | 0.960 | 0.909 | 1.000 | 0.990 | 1.000 | 0.971 | 0.970 | 0.991 | 0.853 | 1.000 | 1.000 | 0.858 | 0.830 |
| 3458 | 0.777 | 1.000 | 0.824 | 1.000 | 0.958 | 0.960 | 0.888 | 1.000 | 0.969 | 1.000 | 0.965 | 0.970 | 0.991 | 0.849 | 1.000 | 1.000 | 0.857 | 0.828 |
| 3459 | 0.777 | 1.000 | 0.808 | 1.000 | 0.958 | 0.960 | 0.904 | 1.000 | 0.969 | 1.000 | 0.965 | 0.970 | 1.000 | 0.853 | 1.000 | 1.000 | 0.859 | 0.831 |
| 3460 | 0.771 | 1.000 | 0.808 | 1.000 | 0.953 | 0.960 | 0.903 | 1.000 | 0.980 | 1.000 | 0.965 | 0.970 | 0.991 | 0.857 | 1.000 | 1.000 | 0.858 | 0.829 |
| 3461 | 0.765 | 1.000 | 0.812 | 1.000 | 0.953 | 0.960 | 0.900 | 1.000 | 0.969 | 1.000 | 0.968 | 0.970 | 1.000 | 0.854 | 1.000 | 1.000 | 0.855 | 0.827 |
| 3462 | 0.760 | 1.000 | 0.794 | 1.000 | 0.953 | 0.960 | 0.888 | 1.000 | 0.980 | 1.000 | 0.965 | 1.000 | 0.991 | 0.850 | 1.000 | 1.000 | 0.849 | 0.819 |
| 3463 | 0.771 | 1.000 | 0.808 | 1.000 | 0.958 | 0.960 | 0.906 | 1.000 | 0.990 | 1.000 | 0.965 | 0.970 | 1.000 | 0.859 | 1.000 | 1.000 | 0.859 | 0.831 |
| 3464 | 0.767 | 1.000 | 0.814 | 0.986 | 0.958 | 0.960 | 0.900 | 1.000 | 1.000 | 1.000 | 0.959 | 0.970 | 1.000 | 0.855 | 1.000 | 1.000 | 0.856 | 0.828 |
| 3465 | 0.775 | 1.000 | 0.820 | 0.986 | 0.953 | 0.960 | 0.894 | 1.000 | 1.000 | 1.000 | 0.965 | 0.970 | 1.000 | 0.850 | 1.000 | 1.000 | 0.857 | 0.829 |
| 3466 | 0.770 | 1.000 | 0.824 | 1.000 | 0.953 | 0.960 | 0.895 | 1.000 | 0.980 | 1.000 | 0.965 | 1.000 | 1.000 | 0.852 | 1.000 | 1.000 | 0.857 | 0.829 |
| 3467 | 0.763 | 1.000 | 0.814 | 0.986 | 0.953 | 0.960 | 0.897 | 1.000 | 1.000 | 1.000 | 0.965 | 1.000 | 0.982 | 0.857 | 1.000 | 1.000 | 0.855 | 0.827 |
| 3468 | 0.767 | 1.000 | 0.796 | 1.000 | 0.947 | 0.960 | 0.886 | 1.000 | 0.980 | 1.000 | 0.968 | 1.000 | 1.000 | 0.854 | 1.000 | 1.000 | 0.852 | 0.823 |
| 3469 | 0.765 | 1.000 | 0.812 | 0.986 | 0.958 | 0.960 | 0.906 | 1.000 | 0.990 | 1.000 | 0.968 | 0.970 | 1.000 | 0.856 | 1.000 | 1.000 | 0.857 | 0.829 |

| | | | | | | | | | | | | | | | | | | |
|---|---|---|---|---|---|---|---|---|---|---|---|---|---|---|---|---|---|---|
| 3470 | 0.763 | 1.000 | 0.812 | 1.000 | 0.958 | 0.960 | 0.898 | 1.000 | 0.980 | 1.000 | 0.974 | 0.970 | 1.000 | 0.852 | 1.000 | 1.000 | 0.855 | 0.826 |
| 3471 | 0.769 | 1.000 | 0.820 | 0.986 | 0.958 | 0.960 | 0.888 | 1.000 | 0.990 | 1.000 | 0.971 | 1.000 | 0.991 | 0.860 | 1.000 | 1.000 | 0.858 | 0.830 |
| 3472 | 0.776 | 1.000 | 0.810 | 1.000 | 0.958 | 0.960 | 0.895 | 1.000 | 0.980 | 1.000 | 0.962 | 0.970 | 0.991 | 0.850 | 1.000 | 1.000 | 0.856 | 0.828 |
| 3473 | 0.769 | 1.000 | 0.802 | 1.000 | 0.953 | 0.960 | 0.898 | 1.000 | 0.990 | 1.000 | 0.962 | 0.970 | 1.000 | 0.863 | 1.000 | 1.000 | 0.857 | 0.829 |
| 3474 | 0.770 | 1.000 | 0.810 | 1.000 | 0.953 | 0.960 | 0.901 | 1.000 | 0.990 | 1.000 | 0.968 | 0.970 | 0.991 | 0.856 | 1.000 | 1.000 | 0.857 | 0.829 |
| 3475 | 0.776 | 1.000 | 0.800 | 1.000 | 0.958 | 0.960 | 0.903 | 1.000 | 0.990 | 1.000 | 0.962 | 1.000 | 0.991 | 0.852 | 1.000 | 1.000 | 0.857 | 0.829 |
| 3476 | 0.766 | 1.000 | 0.806 | 1.000 | 0.953 | 0.960 | 0.898 | 1.000 | 0.969 | 1.000 | 0.959 | 0.970 | 1.000 | 0.851 | 1.000 | 1.000 | 0.853 | 0.824 |
| 3477 | 0.766 | 1.000 | 0.812 | 1.000 | 0.953 | 0.960 | 0.906 | 1.000 | 0.990 | 1.000 | 0.957 | 0.970 | 0.991 | 0.850 | 1.000 | 1.000 | 0.855 | 0.826 |
| 3478 | 0.757 | 1.000 | 0.806 | 1.000 | 0.958 | 0.960 | 0.898 | 1.000 | 0.980 | 1.000 | 0.971 | 0.970 | 1.000 | 0.850 | 1.000 | 1.000 | 0.852 | 0.823 |
| 3479 | 0.772 | 1.000 | 0.818 | 0.986 | 0.958 | 0.960 | 0.895 | 1.000 | 0.990 | 1.000 | 0.971 | 1.000 | 1.000 | 0.858 | 1.000 | 1.000 | 0.859 | 0.831 |
| 3480 | 0.763 | 1.000 | 0.812 | 0.986 | 0.953 | 0.960 | 0.901 | 1.000 | 0.990 | 1.000 | 0.965 | 1.000 | 1.000 | 0.847 | 1.000 | 1.000 | 0.853 | 0.825 |
| 3481 | 0.765 | 1.000 | 0.810 | 1.000 | 0.958 | 0.960 | 0.903 | 1.000 | 0.990 | 1.000 | 0.965 | 0.970 | 1.000 | 0.851 | 1.000 | 1.000 | 0.855 | 0.827 |
| 3482 | 0.771 | 1.000 | 0.820 | 1.000 | 0.958 | 0.960 | 0.898 | 1.000 | 1.000 | 1.000 | 0.962 | 0.970 | 1.000 | 0.857 | 1.000 | 1.000 | 0.859 | 0.831 |
| 3483 | 0.772 | 1.000 | 0.810 | 1.000 | 0.963 | 0.960 | 0.898 | 1.000 | 1.000 | 1.000 | 0.971 | 1.000 | 1.000 | 0.852 | 1.000 | 1.000 | 0.858 | 0.830 |
| 3484 | 0.769 | 1.000 | 0.818 | 1.000 | 0.953 | 0.960 | 0.900 | 1.000 | 0.990 | 1.000 | 0.959 | 0.970 | 1.000 | 0.854 | 1.000 | 1.000 | 0.857 | 0.829 |
| 3485 | 0.765 | 1.000 | 0.814 | 0.986 | 0.953 | 0.960 | 0.903 | 1.000 | 0.990 | 1.000 | 0.968 | 0.970 | 1.000 | 0.851 | 1.000 | 1.000 | 0.855 | 0.827 |
| 3486 | 0.767 | 1.000 | 0.802 | 1.000 | 0.953 | 0.960 | 0.894 | 1.000 | 0.980 | 1.000 | 0.971 | 1.000 | 1.000 | 0.859 | 1.000 | 1.000 | 0.856 | 0.827 |
| 3487 | 0.771 | 1.000 | 0.820 | 1.000 | 0.958 | 0.960 | 0.897 | 1.000 | 0.980 | 1.000 | 0.965 | 0.970 | 1.000 | 0.851 | 1.000 | 1.000 | 0.857 | 0.828 |
| 3488 | 0.765 | 1.000 | 0.822 | 1.000 | 0.953 | 0.960 | 0.900 | 1.000 | 0.990 | 1.000 | 0.968 | 1.000 | 0.991 | 0.860 | 1.000 | 1.000 | 0.858 | 0.830 |
| 3489 | 0.770 | 1.000 | 0.808 | 0.986 | 0.958 | 0.960 | 0.892 | 1.000 | 0.969 | 1.000 | 0.962 | 1.000 | 0.991 | 0.849 | 1.000 | 1.000 | 0.854 | 0.825 |
| 3490 | 0.768 | 1.000 | 0.808 | 1.000 | 0.958 | 0.960 | 0.889 | 1.000 | 0.990 | 1.000 | 0.965 | 0.970 | 1.000 | 0.847 | 1.000 | 1.000 | 0.853 | 0.824 |
| 3491 | 0.772 | 1.000 | 0.812 | 1.000 | 0.953 | 0.960 | 0.911 | 1.000 | 0.990 | 1.000 | 0.962 | 0.970 | 0.991 | 0.855 | 1.000 | 1.000 | 0.859 | 0.831 |
| 3492 | 0.772 | 1.000 | 0.816 | 0.986 | 0.953 | 0.960 | 0.897 | 1.000 | 0.969 | 1.000 | 0.965 | 1.000 | 1.000 | 0.851 | 1.000 | 1.000 | 0.856 | 0.828 |
| 3493 | 0.769 | 1.000 | 0.804 | 0.986 | 0.953 | 0.960 | 0.889 | 1.000 | 0.980 | 1.000 | 0.957 | 1.000 | 1.000 | 0.846 | 1.000 | 1.000 | 0.852 | 0.822 |
| 3494 | 0.760 | 1.000 | 0.830 | 1.000 | 0.937 | 0.960 | 0.898 | 1.000 | 0.990 | 1.000 | 0.965 | 0.970 | 0.991 | 0.851 | 1.000 | 1.000 | 0.854 | 0.825 |
| 3495 | 0.771 | 1.000 | 0.820 | 0.986 | 0.958 | 0.960 | 0.900 | 1.000 | 1.000 | 1.000 | 0.965 | 0.970 | 1.000 | 0.857 | 1.000 | 1.000 | 0.859 | 0.831 |
| 3496 | 0.763 | 1.000 | 0.802 | 1.000 | 0.953 | 0.960 | 0.900 | 1.000 | 1.000 | 1.000 | 0.965 | 1.000 | 0.991 | 0.851 | 1.000 | 1.000 | 0.853 | 0.824 |
| 3497 | 0.773 | 1.000 | 0.818 | 1.000 | 0.953 | 0.960 | 0.897 | 1.000 | 1.000 | 1.000 | 0.965 | 1.000 | 1.000 | 0.858 | 1.000 | 1.000 | 0.859 | 0.832 |
| 3498 | 0.782 | 1.000 | 0.824 | 1.000 | 0.958 | 0.960 | 0.894 | 1.000 | 0.980 | 1.000 | 0.965 | 1.000 | 1.000 | 0.856 | 1.000 | 1.000 | 0.862 | 0.834 |
| 3499 | 0.771 | 1.000 | 0.830 | 0.986 | 0.953 | 0.960 | 0.904 | 1.000 | 0.980 | 1.000 | 0.965 | 0.970 | 1.000 | 0.855 | 1.000 | 1.000 | 0.859 | 0.832 |
| 3500 | 0.765 | 1.000 | 0.814 | 0.986 | 0.953 | 0.960 | 0.892 | 1.000 | 0.969 | 1.000 | 0.968 | 1.000 | 0.991 | 0.851 | 1.000 | 1.000 | 0.853 | 0.824 |
| 3501 | 0.770 | 1.000 | 0.824 | 0.986 | 0.947 | 0.960 | 0.898 | 1.000 | 1.000 | 1.000 | 0.959 | 1.000 | 0.991 | 0.860 | 1.000 | 1.000 | 0.859 | 0.831 |
| 3502 | 0.769 | 1.000 | 0.814 | 1.000 | 0.953 | 0.960 | 0.901 | 1.000 | 0.990 | 1.000 | 0.959 | 1.000 | 1.000 | 0.854 | 1.000 | 1.000 | 0.857 | 0.828 |
| 3503 | 0.769 | 1.000 | 0.810 | 1.000 | 0.958 | 0.960 | 0.906 | 1.000 | 0.990 | 1.000 | 0.968 | 0.970 | 1.000 | 0.851 | 1.000 | 1.000 | 0.857 | 0.829 |
| 3504 | 0.765 | 1.000 | 0.830 | 0.986 | 0.958 | 0.960 | 0.904 | 1.000 | 0.980 | 1.000 | 0.968 | 0.970 | 1.000 | 0.863 | 1.000 | 1.000 | 0.860 | 0.833 |
| 3505 | 0.769 | 1.000 | 0.836 | 0.986 | 0.963 | 0.960 | 0.895 | 1.000 | 0.969 | 1.000 | 0.962 | 1.000 | 0.991 | 0.851 | 1.000 | 1.000 | 0.857 | 0.829 |
| 3506 | 0.764 | 1.000 | 0.810 | 0.986 | 0.953 | 0.960 | 0.897 | 1.000 | 0.980 | 1.000 | 0.965 | 1.000 | 1.000 | 0.853 | 1.000 | 1.000 | 0.854 | 0.825 |
| 3507 | 0.765 | 1.000 | 0.804 | 0.986 | 0.958 | 0.960 | 0.908 | 1.000 | 0.980 | 1.000 | 0.965 | 0.970 | 0.991 | 0.851 | 1.000 | 1.000 | 0.854 | 0.826 |
| 3508 | 0.777 | 1.000 | 0.824 | 1.000 | 0.953 | 0.960 | 0.903 | 1.000 | 0.980 | 1.000 | 0.968 | 0.970 | 0.991 | 0.858 | 1.000 | 1.000 | 0.861 | 0.834 |

| | | | | | | | | | | | | | | | | | |
|---|---|---|---|---|---|---|---|---|---|---|---|---|---|---|---|---|---|
| 3509 | 0.769 | 1.000 | 0.804 | 0.986 | 0.953 | 0.960 | 0.888 | 1.000 | 0.980 | 1.000 | 0.965 | 0.970 | 0.991 | 0.857 | 1.000 | 1.000 | 0.854 | 0.825 |
| 3510 | 0.770 | 1.000 | 0.808 | 1.000 | 0.947 | 0.960 | 0.895 | 1.000 | 1.000 | 1.000 | 0.965 | 1.000 | 0.991 | 0.852 | 1.000 | 1.000 | 0.855 | 0.827 |
| 3511 | 0.767 | 1.000 | 0.824 | 1.000 | 0.947 | 0.960 | 0.897 | 1.000 | 0.980 | 1.000 | 0.971 | 0.970 | 1.000 | 0.850 | 1.000 | 1.000 | 0.856 | 0.827 |
| 3512 | 0.775 | 1.000 | 0.810 | 1.000 | 0.953 | 0.960 | 0.895 | 1.000 | 0.980 | 1.000 | 0.965 | 0.970 | 1.000 | 0.855 | 1.000 | 1.000 | 0.858 | 0.829 |
| 3513 | 0.771 | 1.000 | 0.816 | 1.000 | 0.953 | 0.960 | 0.898 | 1.000 | 0.990 | 1.000 | 0.968 | 0.970 | 1.000 | 0.847 | 1.000 | 1.000 | 0.856 | 0.828 |
| 3514 | 0.774 | 1.000 | 0.834 | 1.000 | 0.963 | 0.960 | 0.892 | 1.000 | 0.990 | 1.000 | 0.968 | 0.970 | 0.991 | 0.854 | 1.000 | 1.000 | 0.860 | 0.832 |
| 3515 | 0.771 | 1.000 | 0.804 | 1.000 | 0.947 | 0.960 | 0.895 | 1.000 | 0.990 | 1.000 | 0.965 | 0.970 | 1.000 | 0.854 | 1.000 | 1.000 | 0.855 | 0.827 |
| 3516 | 0.771 | 1.000 | 0.808 | 0.986 | 0.958 | 0.960 | 0.898 | 1.000 | 0.990 | 1.000 | 0.968 | 0.970 | 1.000 | 0.856 | 1.000 | 1.000 | 0.857 | 0.829 |
| 3517 | 0.774 | 1.000 | 0.804 | 0.986 | 0.953 | 0.960 | 0.897 | 1.000 | 0.980 | 1.000 | 0.965 | 1.000 | 1.000 | 0.854 | 1.000 | 1.000 | 0.857 | 0.828 |
| 3518 | 0.771 | 1.000 | 0.802 | 1.000 | 0.958 | 0.960 | 0.903 | 1.000 | 0.980 | 1.000 | 0.965 | 1.000 | 0.991 | 0.849 | 1.000 | 1.000 | 0.855 | 0.827 |
| 3519 | 0.770 | 1.000 | 0.820 | 0.986 | 0.947 | 0.960 | 0.898 | 1.000 | 0.990 | 1.000 | 0.974 | 1.000 | 0.991 | 0.854 | 1.000 | 1.000 | 0.858 | 0.830 |
| 3520 | 0.779 | 1.000 | 0.812 | 0.986 | 0.958 | 0.960 | 0.900 | 1.000 | 0.959 | 1.000 | 0.971 | 1.000 | 1.000 | 0.850 | 1.000 | 1.000 | 0.858 | 0.830 |
| 3521 | 0.765 | 1.000 | 0.808 | 0.986 | 0.958 | 0.960 | 0.897 | 1.000 | 0.980 | 1.000 | 0.965 | 1.000 | 1.000 | 0.856 | 1.000 | 1.000 | 0.855 | 0.827 |
| 3522 | 0.767 | 1.000 | 0.824 | 1.000 | 0.953 | 0.960 | 0.906 | 1.000 | 1.000 | 1.000 | 0.965 | 1.000 | 1.000 | 0.858 | 1.000 | 1.000 | 0.860 | 0.832 |
| 3523 | 0.769 | 1.000 | 0.818 | 1.000 | 0.958 | 0.960 | 0.903 | 1.000 | 0.969 | 1.000 | 0.971 | 1.000 | 1.000 | 0.856 | 1.000 | 1.000 | 0.859 | 0.831 |
| 3524 | 0.772 | 1.000 | 0.810 | 1.000 | 0.953 | 0.960 | 0.900 | 1.000 | 0.990 | 1.000 | 0.959 | 0.970 | 1.000 | 0.854 | 1.000 | 1.000 | 0.857 | 0.828 |
| 3525 | 0.764 | 1.000 | 0.804 | 1.000 | 0.947 | 0.960 | 0.886 | 1.000 | 1.000 | 1.000 | 0.965 | 0.970 | 0.991 | 0.858 | 1.000 | 1.000 | 0.853 | 0.824 |
| 3526 | 0.769 | 1.000 | 0.808 | 0.986 | 0.953 | 0.960 | 0.903 | 1.000 | 0.969 | 1.000 | 0.959 | 0.970 | 1.000 | 0.847 | 1.000 | 1.000 | 0.854 | 0.825 |
| 3527 | 0.773 | 1.000 | 0.830 | 1.000 | 0.963 | 0.960 | 0.904 | 1.000 | 1.000 | 1.000 | 0.968 | 1.000 | 1.000 | 0.861 | 1.000 | 1.000 | 0.863 | 0.836 |
| 3528 | 0.758 | 1.000 | 0.820 | 1.000 | 0.947 | 0.960 | 0.900 | 1.000 | 0.990 | 1.000 | 0.965 | 0.970 | 1.000 | 0.855 | 1.000 | 1.000 | 0.854 | 0.826 |
| 3529 | 0.781 | 1.000 | 0.806 | 1.000 | 0.958 | 0.960 | 0.908 | 1.000 | 0.990 | 1.000 | 0.965 | 0.970 | 1.000 | 0.862 | 1.000 | 1.000 | 0.863 | 0.835 |
| 3530 | 0.762 | 1.000 | 0.824 | 1.000 | 0.953 | 0.960 | 0.898 | 1.000 | 0.990 | 1.000 | 0.965 | 0.970 | 1.000 | 0.858 | 1.000 | 1.000 | 0.856 | 0.828 |
| 3531 | 0.771 | 1.000 | 0.820 | 1.000 | 0.947 | 0.960 | 0.906 | 1.000 | 0.980 | 1.000 | 0.959 | 0.970 | 1.000 | 0.851 | 1.000 | 1.000 | 0.857 | 0.829 |
| 3532 | 0.771 | 1.000 | 0.816 | 1.000 | 0.953 | 0.960 | 0.894 | 1.000 | 0.980 | 1.000 | 0.962 | 1.000 | 1.000 | 0.855 | 1.000 | 1.000 | 0.857 | 0.829 |
| 3533 | 0.769 | 1.000 | 0.804 | 1.000 | 0.958 | 0.960 | 0.884 | 1.000 | 0.980 | 1.000 | 0.962 | 0.970 | 1.000 | 0.851 | 1.000 | 1.000 | 0.853 | 0.824 |
| 3534 | 0.770 | 1.000 | 0.816 | 0.986 | 0.953 | 0.960 | 0.891 | 1.000 | 1.000 | 1.000 | 0.971 | 0.970 | 0.991 | 0.848 | 1.000 | 1.000 | 0.855 | 0.826 |
| 3535 | 0.777 | 1.000 | 0.814 | 1.000 | 0.963 | 0.960 | 0.901 | 1.000 | 0.990 | 1.000 | 0.965 | 0.970 | 1.000 | 0.849 | 1.000 | 1.000 | 0.858 | 0.830 |
| 3536 | 0.767 | 1.000 | 0.828 | 1.000 | 0.947 | 0.960 | 0.895 | 1.000 | 0.969 | 1.000 | 0.971 | 0.970 | 1.000 | 0.844 | 1.000 | 1.000 | 0.854 | 0.826 |
| 3537 | 0.769 | 1.000 | 0.822 | 1.000 | 0.958 | 0.960 | 0.904 | 1.000 | 0.990 | 1.000 | 0.959 | 1.000 | 1.000 | 0.855 | 1.000 | 1.000 | 0.859 | 0.831 |
| 3538 | 0.773 | 1.000 | 0.804 | 0.971 | 0.963 | 0.960 | 0.895 | 1.000 | 1.000 | 1.000 | 0.962 | 0.970 | 0.991 | 0.844 | 1.000 | 1.000 | 0.853 | 0.824 |
| 3539 | 0.773 | 1.000 | 0.810 | 1.000 | 0.963 | 0.960 | 0.909 | 1.000 | 1.000 | 1.000 | 0.965 | 0.970 | 0.991 | 0.860 | 1.000 | 1.000 | 0.861 | 0.833 |
| 3540 | 0.771 | 1.000 | 0.822 | 0.986 | 0.953 | 0.960 | 0.900 | 1.000 | 0.969 | 1.000 | 0.959 | 1.000 | 1.000 | 0.847 | 1.000 | 1.000 | 0.856 | 0.827 |
| 3541 | 0.773 | 1.000 | 0.814 | 1.000 | 0.958 | 0.960 | 0.891 | 1.000 | 1.000 | 1.000 | 0.968 | 0.970 | 1.000 | 0.851 | 1.000 | 1.000 | 0.857 | 0.828 |
| 3542 | 0.770 | 1.000 | 0.824 | 1.000 | 0.947 | 0.960 | 0.895 | 1.000 | 0.990 | 1.000 | 0.968 | 0.970 | 1.000 | 0.852 | 1.000 | 1.000 | 0.857 | 0.829 |
| 3543 | 0.777 | 1.000 | 0.818 | 0.986 | 0.958 | 0.960 | 0.897 | 1.000 | 0.969 | 1.000 | 0.962 | 1.000 | 1.000 | 0.859 | 1.000 | 1.000 | 0.860 | 0.832 |
| 3544 | 0.765 | 1.000 | 0.800 | 1.000 | 0.953 | 0.960 | 0.898 | 1.000 | 0.990 | 1.000 | 0.968 | 0.970 | 1.000 | 0.852 | 1.000 | 1.000 | 0.854 | 0.825 |
| 3545 | 0.765 | 1.000 | 0.802 | 0.986 | 0.953 | 0.960 | 0.903 | 1.000 | 0.980 | 1.000 | 0.965 | 0.970 | 0.991 | 0.849 | 1.000 | 1.000 | 0.853 | 0.824 |
| 3546 | 0.767 | 1.000 | 0.800 | 0.986 | 0.963 | 0.960 | 0.898 | 1.000 | 0.990 | 1.000 | 0.962 | 1.000 | 1.000 | 0.851 | 1.000 | 1.000 | 0.854 | 0.825 |
| 3547 | 0.769 | 1.000 | 0.816 | 1.000 | 0.963 | 0.960 | 0.908 | 1.000 | 0.969 | 1.000 | 0.959 | 1.000 | 0.991 | 0.854 | 1.000 | 1.000 | 0.858 | 0.830 |

| | | | | | | | | | | | | | | | | | | |
|---|---|---|---|---|---|---|---|---|---|---|---|---|---|---|---|---|---|---|
| 3548 | 0.772 | 1.000 | 0.796 | 1.000 | 0.958 | 0.960 | 0.892 | 1.000 | 0.990 | 1.000 | 0.962 | 0.970 | 1.000 | 0.860 | 1.000 | 1.000 | 0.856 | 0.828 |
| 3549 | 0.761 | 1.000 | 0.816 | 0.986 | 0.963 | 0.960 | 0.901 | 1.000 | 0.990 | 1.000 | 0.962 | 0.970 | 0.991 | 0.846 | 1.000 | 1.000 | 0.853 | 0.824 |
| 3550 | 0.771 | 1.000 | 0.806 | 1.000 | 0.953 | 0.960 | 0.892 | 1.000 | 0.990 | 1.000 | 0.959 | 1.000 | 0.991 | 0.853 | 1.000 | 1.000 | 0.855 | 0.826 |
| 3551 | 0.773 | 1.000 | 0.802 | 0.986 | 0.953 | 0.960 | 0.906 | 1.000 | 0.990 | 1.000 | 0.965 | 0.970 | 1.000 | 0.854 | 1.000 | 1.000 | 0.857 | 0.829 |
| 3552 | 0.769 | 1.000 | 0.818 | 0.986 | 0.963 | 0.960 | 0.900 | 1.000 | 0.980 | 1.000 | 0.962 | 0.970 | 1.000 | 0.850 | 1.000 | 1.000 | 0.856 | 0.828 |
| 3553 | 0.764 | 1.000 | 0.800 | 1.000 | 0.947 | 0.960 | 0.898 | 1.000 | 1.000 | 1.000 | 0.971 | 0.970 | 0.991 | 0.847 | 1.000 | 1.000 | 0.852 | 0.823 |
| 3554 | 0.768 | 1.000 | 0.812 | 1.000 | 0.958 | 0.960 | 0.888 | 1.000 | 0.969 | 1.000 | 0.959 | 0.970 | 1.000 | 0.858 | 1.000 | 1.000 | 0.855 | 0.826 |
| 3555 | 0.774 | 1.000 | 0.818 | 1.000 | 0.953 | 0.960 | 0.894 | 1.000 | 0.990 | 1.000 | 0.965 | 1.000 | 1.000 | 0.856 | 1.000 | 1.000 | 0.859 | 0.831 |
| 3556 | 0.774 | 1.000 | 0.814 | 1.000 | 0.953 | 0.960 | 0.901 | 1.000 | 1.000 | 1.000 | 0.957 | 1.000 | 0.991 | 0.850 | 1.000 | 1.000 | 0.857 | 0.829 |
| 3557 | 0.775 | 1.000 | 0.800 | 1.000 | 0.958 | 0.960 | 0.889 | 1.000 | 0.990 | 1.000 | 0.965 | 1.000 | 1.000 | 0.851 | 1.000 | 1.000 | 0.855 | 0.827 |
| 3558 | 0.769 | 1.000 | 0.804 | 1.000 | 0.958 | 0.960 | 0.895 | 1.000 | 1.000 | 1.000 | 0.962 | 1.000 | 0.991 | 0.851 | 1.000 | 1.000 | 0.854 | 0.826 |
| 3559 | 0.773 | 1.000 | 0.828 | 1.000 | 0.958 | 0.960 | 0.898 | 1.000 | 1.000 | 1.000 | 0.962 | 0.970 | 1.000 | 0.852 | 1.000 | 1.000 | 0.859 | 0.831 |
| 3560 | 0.765 | 1.000 | 0.824 | 1.000 | 0.963 | 0.960 | 0.894 | 1.000 | 0.990 | 1.000 | 0.968 | 1.000 | 1.000 | 0.854 | 1.000 | 1.000 | 0.857 | 0.829 |
| 3561 | 0.769 | 1.000 | 0.806 | 0.986 | 0.958 | 0.960 | 0.900 | 1.000 | 0.980 | 1.000 | 0.962 | 1.000 | 0.991 | 0.855 | 1.000 | 1.000 | 0.856 | 0.827 |
| 3562 | 0.765 | 1.000 | 0.838 | 1.000 | 0.953 | 0.960 | 0.897 | 1.000 | 0.990 | 1.000 | 0.962 | 0.970 | 1.000 | 0.849 | 1.000 | 1.000 | 0.856 | 0.828 |
| 3563 | 0.777 | 1.000 | 0.816 | 1.000 | 0.958 | 0.960 | 0.901 | 1.000 | 0.990 | 1.000 | 0.968 | 1.000 | 0.991 | 0.854 | 1.000 | 1.000 | 0.860 | 0.832 |
| 3564 | 0.777 | 1.000 | 0.816 | 1.000 | 0.947 | 0.960 | 0.911 | 1.000 | 0.969 | 1.000 | 0.965 | 1.000 | 0.991 | 0.849 | 1.000 | 1.000 | 0.859 | 0.831 |
| 3565 | 0.764 | 1.000 | 0.814 | 0.986 | 0.947 | 0.960 | 0.904 | 1.000 | 1.000 | 1.000 | 0.962 | 0.970 | 0.991 | 0.859 | 1.000 | 1.000 | 0.857 | 0.828 |
| 3566 | 0.773 | 1.000 | 0.800 | 1.000 | 0.947 | 0.960 | 0.903 | 1.000 | 0.990 | 1.000 | 0.965 | 1.000 | 1.000 | 0.854 | 1.000 | 1.000 | 0.857 | 0.829 |
| 3567 | 0.769 | 1.000 | 0.816 | 1.000 | 0.953 | 0.960 | 0.906 | 1.000 | 0.980 | 1.000 | 0.962 | 1.000 | 1.000 | 0.859 | 1.000 | 1.000 | 0.859 | 0.831 |
| 3568 | 0.776 | 1.000 | 0.802 | 0.986 | 0.953 | 0.960 | 0.903 | 1.000 | 0.980 | 1.000 | 0.957 | 0.970 | 0.991 | 0.856 | 1.000 | 1.000 | 0.857 | 0.829 |
| 3569 | 0.765 | 1.000 | 0.808 | 0.971 | 0.958 | 0.960 | 0.901 | 1.000 | 0.980 | 1.000 | 0.965 | 1.000 | 0.991 | 0.849 | 1.000 | 1.000 | 0.854 | 0.825 |
| 3570 | 0.777 | 1.000 | 0.806 | 1.000 | 0.953 | 0.960 | 0.904 | 1.000 | 1.000 | 1.000 | 0.957 | 1.000 | 0.991 | 0.854 | 1.000 | 1.000 | 0.859 | 0.830 |
| 3571 | 0.772 | 1.000 | 0.816 | 1.000 | 0.953 | 0.960 | 0.897 | 1.000 | 0.990 | 1.000 | 0.957 | 0.970 | 1.000 | 0.853 | 1.000 | 1.000 | 0.857 | 0.828 |
| 3572 | 0.772 | 1.000 | 0.808 | 1.000 | 0.963 | 0.960 | 0.897 | 1.000 | 0.990 | 1.000 | 0.965 | 1.000 | 1.000 | 0.847 | 1.000 | 1.000 | 0.856 | 0.827 |
| 3573 | 0.765 | 1.000 | 0.789 | 0.986 | 0.953 | 0.960 | 0.903 | 1.000 | 0.990 | 1.000 | 0.968 | 1.000 | 1.000 | 0.854 | 1.000 | 1.000 | 0.854 | 0.825 |
| 3574 | 0.771 | 1.000 | 0.796 | 0.986 | 0.958 | 0.960 | 0.909 | 1.000 | 0.959 | 1.000 | 0.965 | 0.970 | 1.000 | 0.851 | 1.000 | 1.000 | 0.855 | 0.827 |
| 3575 | 0.774 | 1.000 | 0.794 | 1.000 | 0.953 | 0.960 | 0.892 | 1.000 | 0.990 | 1.000 | 0.965 | 1.000 | 1.000 | 0.854 | 1.000 | 1.000 | 0.856 | 0.826 |
| 3576 | 0.761 | 1.000 | 0.822 | 1.000 | 0.953 | 0.960 | 0.909 | 1.000 | 0.980 | 1.000 | 0.965 | 1.000 | 0.991 | 0.855 | 1.000 | 1.000 | 0.857 | 0.829 |
| 3577 | 0.762 | 1.000 | 0.800 | 1.000 | 0.963 | 0.960 | 0.904 | 1.000 | 0.990 | 1.000 | 0.968 | 1.000 | 1.000 | 0.850 | 1.000 | 1.000 | 0.854 | 0.825 |
| 3578 | 0.771 | 1.000 | 0.808 | 1.000 | 0.953 | 0.960 | 0.895 | 1.000 | 1.000 | 1.000 | 0.968 | 0.970 | 0.991 | 0.849 | 1.000 | 1.000 | 0.855 | 0.826 |
| 3579 | 0.773 | 1.000 | 0.814 | 1.000 | 0.953 | 0.960 | 0.894 | 1.000 | 1.000 | 1.000 | 0.965 | 0.970 | 0.991 | 0.856 | 1.000 | 1.000 | 0.858 | 0.829 |
| 3580 | 0.773 | 1.000 | 0.806 | 1.000 | 0.958 | 0.960 | 0.909 | 1.000 | 0.980 | 1.000 | 0.962 | 0.970 | 1.000 | 0.854 | 1.000 | 1.000 | 0.858 | 0.830 |
| 3581 | 0.762 | 1.000 | 0.804 | 1.000 | 0.953 | 0.960 | 0.898 | 1.000 | 0.990 | 1.000 | 0.959 | 0.970 | 0.991 | 0.855 | 1.000 | 1.000 | 0.853 | 0.824 |
| 3582 | 0.772 | 1.000 | 0.810 | 1.000 | 0.958 | 0.960 | 0.898 | 1.000 | 0.980 | 1.000 | 0.965 | 0.970 | 1.000 | 0.858 | 1.000 | 1.000 | 0.858 | 0.830 |
| 3583 | 0.783 | 1.000 | 0.816 | 1.000 | 0.953 | 0.960 | 0.886 | 1.000 | 0.990 | 1.000 | 0.968 | 1.000 | 1.000 | 0.851 | 1.000 | 1.000 | 0.859 | 0.831 |
| 3584 | 0.774 | 1.000 | 0.814 | 1.000 | 0.958 | 0.960 | 0.898 | 1.000 | 0.980 | 1.000 | 0.968 | 0.970 | 1.000 | 0.858 | 1.000 | 1.000 | 0.859 | 0.831 |
| 3585 | 0.763 | 1.000 | 0.812 | 0.986 | 0.953 | 0.960 | 0.895 | 1.000 | 0.980 | 1.000 | 0.968 | 1.000 | 0.991 | 0.849 | 1.000 | 1.000 | 0.853 | 0.824 |
| 3586 | 0.767 | 1.000 | 0.806 | 1.000 | 0.953 | 0.960 | 0.901 | 1.000 | 0.990 | 1.000 | 0.962 | 1.000 | 0.991 | 0.852 | 1.000 | 1.000 | 0.855 | 0.826 |

| | | | | | | | | | | | | | | | | | | |
|---|---|---|---|---|---|---|---|---|---|---|---|---|---|---|---|---|---|---|
| 3587 | 0.770 | 1.000 | 0.812 | 1.000 | 0.963 | 0.960 | 0.900 | 1.000 | 0.980 | 1.000 | 0.965 | 0.970 | 1.000 | 0.857 | 1.000 | 1.000 | 0.858 | 0.830 |
| 3588 | 0.769 | 1.000 | 0.816 | 1.000 | 0.953 | 0.960 | 0.901 | 1.000 | 0.980 | 1.000 | 0.971 | 1.000 | 0.991 | 0.854 | 1.000 | 1.000 | 0.857 | 0.829 |
| 3589 | 0.765 | 1.000 | 0.824 | 1.000 | 0.953 | 0.960 | 0.900 | 1.000 | 0.969 | 1.000 | 0.962 | 0.970 | 1.000 | 0.849 | 1.000 | 1.000 | 0.855 | 0.826 |
| 3590 | 0.773 | 1.000 | 0.812 | 1.000 | 0.963 | 0.960 | 0.891 | 1.000 | 0.990 | 1.000 | 0.957 | 0.970 | 1.000 | 0.857 | 1.000 | 1.000 | 0.857 | 0.829 |
| 3591 | 0.769 | 1.000 | 0.804 | 1.000 | 0.953 | 0.960 | 0.889 | 1.000 | 0.980 | 1.000 | 0.962 | 0.970 | 1.000 | 0.860 | 1.000 | 1.000 | 0.856 | 0.827 |
| 3592 | 0.766 | 1.000 | 0.816 | 0.986 | 0.958 | 0.960 | 0.897 | 1.000 | 0.990 | 1.000 | 0.968 | 1.000 | 1.000 | 0.857 | 1.000 | 1.000 | 0.857 | 0.829 |
| 3593 | 0.769 | 1.000 | 0.808 | 0.986 | 0.963 | 0.960 | 0.909 | 1.000 | 0.990 | 1.000 | 0.965 | 0.970 | 0.991 | 0.862 | 1.000 | 1.000 | 0.859 | 0.832 |
| 3594 | 0.773 | 1.000 | 0.802 | 1.000 | 0.947 | 0.960 | 0.900 | 1.000 | 0.980 | 1.000 | 0.971 | 0.970 | 0.991 | 0.855 | 1.000 | 1.000 | 0.857 | 0.828 |
| 3595 | 0.767 | 1.000 | 0.802 | 0.986 | 0.963 | 0.960 | 0.900 | 1.000 | 1.000 | 1.000 | 0.962 | 1.000 | 1.000 | 0.854 | 1.000 | 1.000 | 0.856 | 0.827 |
| 3596 | 0.775 | 1.000 | 0.818 | 1.000 | 0.942 | 0.960 | 0.897 | 1.000 | 0.990 | 1.000 | 0.959 | 0.970 | 1.000 | 0.849 | 1.000 | 1.000 | 0.857 | 0.828 |
| 3597 | 0.766 | 1.000 | 0.826 | 1.000 | 0.958 | 0.960 | 0.904 | 1.000 | 1.000 | 1.000 | 0.971 | 0.970 | 1.000 | 0.854 | 1.000 | 1.000 | 0.858 | 0.831 |
| 3598 | 0.761 | 1.000 | 0.812 | 0.986 | 0.947 | 0.960 | 0.903 | 1.000 | 0.980 | 1.000 | 0.962 | 1.000 | 0.991 | 0.859 | 1.000 | 1.000 | 0.855 | 0.827 |
| 3599 | 0.771 | 1.000 | 0.808 | 0.986 | 0.953 | 0.960 | 0.903 | 1.000 | 0.990 | 1.000 | 0.962 | 0.970 | 0.991 | 0.852 | 1.000 | 1.000 | 0.856 | 0.828 |
| 3600 | 0.770 | 1.000 | 0.794 | 1.000 | 0.947 | 0.960 | 0.901 | 1.000 | 1.000 | 1.000 | 0.962 | 0.970 | 1.000 | 0.854 | 1.000 | 1.000 | 0.855 | 0.826 |
| 3601 | 0.780 | 1.000 | 0.822 | 1.000 | 0.947 | 0.960 | 0.894 | 1.000 | 1.000 | 1.000 | 0.968 | 0.970 | 1.000 | 0.854 | 1.000 | 1.000 | 0.860 | 0.832 |
| 3602 | 0.769 | 1.000 | 0.791 | 1.000 | 0.958 | 0.960 | 0.900 | 1.000 | 0.980 | 1.000 | 0.959 | 1.000 | 1.000 | 0.850 | 1.000 | 1.000 | 0.853 | 0.824 |
| 3603 | 0.767 | 1.000 | 0.804 | 1.000 | 0.947 | 0.960 | 0.895 | 1.000 | 0.990 | 1.000 | 0.962 | 1.000 | 0.991 | 0.854 | 1.000 | 1.000 | 0.854 | 0.825 |
| 3604 | 0.768 | 1.000 | 0.816 | 1.000 | 0.958 | 0.960 | 0.895 | 1.000 | 0.990 | 1.000 | 0.962 | 0.970 | 1.000 | 0.852 | 1.000 | 1.000 | 0.856 | 0.827 |
| 3605 | 0.775 | 1.000 | 0.802 | 1.000 | 0.958 | 0.960 | 0.886 | 1.000 | 1.000 | 1.000 | 0.962 | 0.970 | 0.991 | 0.848 | 1.000 | 1.000 | 0.854 | 0.825 |
| 3606 | 0.776 | 1.000 | 0.810 | 1.000 | 0.953 | 0.960 | 0.901 | 1.000 | 1.000 | 1.000 | 0.968 | 1.000 | 0.991 | 0.858 | 1.000 | 1.000 | 0.860 | 0.832 |
| 3607 | 0.777 | 1.000 | 0.818 | 1.000 | 0.958 | 0.960 | 0.898 | 1.000 | 0.980 | 1.000 | 0.965 | 0.970 | 0.991 | 0.849 | 1.000 | 1.000 | 0.858 | 0.829 |
| 3608 | 0.764 | 1.000 | 0.812 | 1.000 | 0.963 | 0.960 | 0.895 | 1.000 | 0.990 | 1.000 | 0.965 | 1.000 | 1.000 | 0.844 | 1.000 | 1.000 | 0.853 | 0.824 |
| 3609 | 0.769 | 1.000 | 0.792 | 1.000 | 0.953 | 0.960 | 0.903 | 1.000 | 0.980 | 1.000 | 0.962 | 0.970 | 0.991 | 0.850 | 1.000 | 1.000 | 0.853 | 0.824 |
| 3610 | 0.768 | 1.000 | 0.814 | 1.000 | 0.953 | 0.960 | 0.895 | 1.000 | 0.980 | 1.000 | 0.962 | 1.000 | 1.000 | 0.854 | 1.000 | 1.000 | 0.856 | 0.827 |
| 3611 | 0.772 | 1.000 | 0.814 | 1.000 | 0.958 | 0.960 | 0.903 | 1.000 | 0.980 | 1.000 | 0.965 | 0.970 | 0.991 | 0.853 | 1.000 | 1.000 | 0.858 | 0.829 |
| 3612 | 0.767 | 1.000 | 0.810 | 1.000 | 0.958 | 0.960 | 0.894 | 1.000 | 0.980 | 1.000 | 0.962 | 0.970 | 1.000 | 0.853 | 1.000 | 1.000 | 0.854 | 0.826 |
| 3613 | 0.763 | 1.000 | 0.814 | 0.986 | 0.958 | 0.960 | 0.898 | 1.000 | 1.000 | 1.000 | 0.965 | 1.000 | 1.000 | 0.853 | 1.000 | 1.000 | 0.855 | 0.826 |
| 3614 | 0.771 | 1.000 | 0.808 | 1.000 | 0.958 | 0.960 | 0.892 | 1.000 | 0.990 | 1.000 | 0.965 | 0.970 | 0.991 | 0.854 | 1.000 | 1.000 | 0.856 | 0.827 |
| 3615 | 0.765 | 1.000 | 0.818 | 1.000 | 0.953 | 0.960 | 0.904 | 1.000 | 1.000 | 1.000 | 0.965 | 0.970 | 1.000 | 0.850 | 1.000 | 1.000 | 0.856 | 0.827 |
| 3616 | 0.774 | 1.000 | 0.808 | 1.000 | 0.953 | 0.960 | 0.897 | 1.000 | 0.990 | 1.000 | 0.962 | 0.970 | 1.000 | 0.859 | 1.000 | 1.000 | 0.858 | 0.830 |
| 3617 | 0.765 | 1.000 | 0.820 | 0.986 | 0.953 | 0.960 | 0.900 | 1.000 | 0.990 | 1.000 | 0.968 | 0.970 | 0.991 | 0.858 | 1.000 | 1.000 | 0.857 | 0.829 |
| 3618 | 0.766 | 1.000 | 0.818 | 0.986 | 0.958 | 0.960 | 0.898 | 1.000 | 0.990 | 1.000 | 0.968 | 0.970 | 0.991 | 0.853 | 1.000 | 1.000 | 0.856 | 0.828 |
| 3619 | 0.773 | 1.000 | 0.802 | 1.000 | 0.958 | 0.960 | 0.895 | 1.000 | 0.980 | 1.000 | 0.968 | 0.970 | 1.000 | 0.853 | 1.000 | 1.000 | 0.856 | 0.827 |
| 3620 | 0.772 | 1.000 | 0.818 | 0.986 | 0.953 | 0.960 | 0.906 | 1.000 | 0.980 | 1.000 | 0.962 | 0.970 | 1.000 | 0.855 | 1.000 | 1.000 | 0.859 | 0.831 |
| 3621 | 0.777 | 1.000 | 0.828 | 1.000 | 0.958 | 0.960 | 0.892 | 1.000 | 0.990 | 1.000 | 0.959 | 0.970 | 1.000 | 0.858 | 1.000 | 1.000 | 0.860 | 0.833 |
| 3622 | 0.768 | 1.000 | 0.802 | 1.000 | 0.953 | 0.960 | 0.901 | 1.000 | 0.990 | 1.000 | 0.957 | 1.000 | 1.000 | 0.850 | 1.000 | 1.000 | 0.854 | 0.825 |
| 3623 | 0.771 | 1.000 | 0.830 | 1.000 | 0.958 | 0.960 | 0.898 | 1.000 | 0.990 | 1.000 | 0.957 | 1.000 | 1.000 | 0.851 | 1.000 | 1.000 | 0.858 | 0.830 |
| 3624 | 0.767 | 1.000 | 0.810 | 1.000 | 0.953 | 0.960 | 0.889 | 1.000 | 0.969 | 1.000 | 0.965 | 0.970 | 1.000 | 0.858 | 1.000 | 1.000 | 0.855 | 0.826 |
| 3625 | 0.782 | 1.000 | 0.810 | 0.986 | 0.947 | 0.960 | 0.894 | 1.000 | 0.990 | 1.000 | 0.965 | 0.970 | 1.000 | 0.854 | 1.000 | 1.000 | 0.859 | 0.831 |

| | | | | | | | | | | | | | | | | | | |
|---|---|---|---|---|---|---|---|---|---|---|---|---|---|---|---|---|---|---|
| 3626 | 0.770 | 1.000 | 0.804 | 1.000 | 0.953 | 0.960 | 0.903 | 1.000 | 0.980 | 1.000 | 0.968 | 0.970 | 1.000 | 0.856 | 1.000 | 1.000 | 0.857 | 0.829 |
| 3627 | 0.771 | 1.000 | 0.818 | 1.000 | 0.953 | 0.960 | 0.901 | 1.000 | 0.980 | 1.000 | 0.959 | 1.000 | 1.000 | 0.856 | 1.000 | 1.000 | 0.858 | 0.830 |
| 3628 | 0.761 | 1.000 | 0.798 | 1.000 | 0.958 | 0.960 | 0.889 | 1.000 | 0.980 | 1.000 | 0.962 | 1.000 | 1.000 | 0.863 | 1.000 | 1.000 | 0.854 | 0.824 |
| 3629 | 0.780 | 1.000 | 0.810 | 0.986 | 0.953 | 0.960 | 0.898 | 1.000 | 0.969 | 1.000 | 0.965 | 0.970 | 1.000 | 0.850 | 1.000 | 1.000 | 0.858 | 0.829 |
| 3630 | 0.763 | 1.000 | 0.810 | 1.000 | 0.947 | 0.960 | 0.895 | 1.000 | 0.980 | 1.000 | 0.965 | 0.970 | 0.991 | 0.858 | 1.000 | 1.000 | 0.854 | 0.826 |
| 3631 | 0.775 | 1.000 | 0.820 | 1.000 | 0.953 | 0.960 | 0.898 | 1.000 | 0.969 | 1.000 | 0.965 | 1.000 | 0.991 | 0.854 | 1.000 | 1.000 | 0.859 | 0.830 |
| 3632 | 0.769 | 1.000 | 0.812 | 0.986 | 0.963 | 0.960 | 0.894 | 1.000 | 0.990 | 1.000 | 0.965 | 0.970 | 1.000 | 0.855 | 1.000 | 1.000 | 0.856 | 0.828 |
| 3633 | 0.773 | 1.000 | 0.810 | 1.000 | 0.958 | 0.960 | 0.895 | 1.000 | 1.000 | 1.000 | 0.968 | 1.000 | 1.000 | 0.850 | 1.000 | 1.000 | 0.857 | 0.828 |
| 3634 | 0.763 | 1.000 | 0.804 | 0.986 | 0.953 | 0.960 | 0.900 | 1.000 | 0.969 | 1.000 | 0.962 | 0.970 | 1.000 | 0.854 | 1.000 | 1.000 | 0.853 | 0.824 |
| 3635 | 0.769 | 1.000 | 0.808 | 1.000 | 0.958 | 0.960 | 0.895 | 1.000 | 0.990 | 1.000 | 0.968 | 0.970 | 1.000 | 0.854 | 1.000 | 1.000 | 0.856 | 0.828 |
| 3636 | 0.773 | 1.000 | 0.808 | 1.000 | 0.953 | 0.960 | 0.908 | 1.000 | 0.980 | 1.000 | 0.968 | 1.000 | 1.000 | 0.853 | 1.000 | 1.000 | 0.858 | 0.830 |
| 3637 | 0.773 | 1.000 | 0.824 | 1.000 | 0.953 | 0.960 | 0.892 | 1.000 | 0.990 | 1.000 | 0.965 | 1.000 | 1.000 | 0.861 | 1.000 | 1.000 | 0.860 | 0.832 |
| 3638 | 0.767 | 1.000 | 0.802 | 1.000 | 0.958 | 0.960 | 0.897 | 1.000 | 0.969 | 1.000 | 0.962 | 1.000 | 0.991 | 0.846 | 1.000 | 1.000 | 0.852 | 0.823 |
| 3639 | 0.768 | 1.000 | 0.810 | 1.000 | 0.953 | 0.960 | 0.898 | 1.000 | 0.980 | 1.000 | 0.962 | 0.970 | 1.000 | 0.851 | 1.000 | 1.000 | 0.855 | 0.826 |
| 3640 | 0.775 | 1.000 | 0.792 | 0.986 | 0.953 | 0.960 | 0.894 | 1.000 | 0.990 | 1.000 | 0.957 | 1.000 | 1.000 | 0.847 | 1.000 | 1.000 | 0.853 | 0.824 |
| 3641 | 0.769 | 1.000 | 0.818 | 1.000 | 0.953 | 0.960 | 0.908 | 1.000 | 0.980 | 1.000 | 0.965 | 1.000 | 1.000 | 0.851 | 1.000 | 1.000 | 0.857 | 0.829 |
| 3642 | 0.777 | 1.000 | 0.816 | 1.000 | 0.953 | 0.960 | 0.898 | 1.000 | 1.000 | 1.000 | 0.962 | 0.970 | 0.991 | 0.854 | 1.000 | 1.000 | 0.859 | 0.831 |
| 3643 | 0.771 | 1.000 | 0.812 | 1.000 | 0.958 | 0.960 | 0.904 | 1.000 | 1.000 | 1.000 | 0.965 | 1.000 | 1.000 | 0.857 | 1.000 | 1.000 | 0.859 | 0.831 |
| 3644 | 0.761 | 1.000 | 0.808 | 1.000 | 0.958 | 0.960 | 0.903 | 1.000 | 0.980 | 1.000 | 0.965 | 0.970 | 0.991 | 0.850 | 1.000 | 1.000 | 0.853 | 0.824 |
| 3645 | 0.769 | 1.000 | 0.808 | 1.000 | 0.953 | 0.960 | 0.889 | 1.000 | 0.990 | 1.000 | 0.971 | 1.000 | 1.000 | 0.855 | 1.000 | 1.000 | 0.856 | 0.827 |
| 3646 | 0.765 | 1.000 | 0.800 | 1.000 | 0.963 | 0.960 | 0.900 | 1.000 | 0.990 | 1.000 | 0.971 | 1.000 | 0.991 | 0.857 | 1.000 | 1.000 | 0.856 | 0.827 |
| 3647 | 0.771 | 1.000 | 0.804 | 1.000 | 0.953 | 0.960 | 0.897 | 1.000 | 0.990 | 1.000 | 0.965 | 1.000 | 1.000 | 0.858 | 1.000 | 1.000 | 0.857 | 0.829 |
| 3648 | 0.759 | 1.000 | 0.820 | 0.986 | 0.953 | 0.960 | 0.897 | 1.000 | 1.000 | 1.000 | 0.962 | 1.000 | 1.000 | 0.858 | 1.000 | 1.000 | 0.855 | 0.827 |
| 3649 | 0.773 | 1.000 | 0.802 | 0.986 | 0.947 | 0.960 | 0.911 | 1.000 | 0.990 | 1.000 | 0.962 | 1.000 | 1.000 | 0.853 | 1.000 | 1.000 | 0.857 | 0.829 |
| 3650 | 0.773 | 1.000 | 0.806 | 1.000 | 0.947 | 0.960 | 0.904 | 1.000 | 0.990 | 1.000 | 0.971 | 1.000 | 1.000 | 0.854 | 1.000 | 1.000 | 0.858 | 0.830 |
| 3651 | 0.767 | 1.000 | 0.814 | 1.000 | 0.947 | 0.960 | 0.901 | 1.000 | 0.990 | 1.000 | 0.962 | 1.000 | 0.991 | 0.854 | 1.000 | 1.000 | 0.856 | 0.828 |
| 3652 | 0.773 | 1.000 | 0.806 | 0.986 | 0.953 | 0.960 | 0.897 | 1.000 | 1.000 | 1.000 | 0.962 | 1.000 | 1.000 | 0.852 | 1.000 | 1.000 | 0.856 | 0.828 |
| 3653 | 0.769 | 1.000 | 0.781 | 1.000 | 0.958 | 0.960 | 0.891 | 1.000 | 0.959 | 1.000 | 0.959 | 1.000 | 0.982 | 0.854 | 1.000 | 1.000 | 0.851 | 0.822 |
| 3654 | 0.767 | 1.000 | 0.800 | 1.000 | 0.953 | 0.960 | 0.898 | 1.000 | 0.990 | 1.000 | 0.968 | 1.000 | 0.991 | 0.854 | 1.000 | 1.000 | 0.855 | 0.826 |
| 3655 | 0.766 | 1.000 | 0.804 | 1.000 | 0.963 | 0.960 | 0.904 | 1.000 | 0.990 | 1.000 | 0.962 | 1.000 | 1.000 | 0.858 | 1.000 | 1.000 | 0.857 | 0.829 |
| 3656 | 0.765 | 1.000 | 0.806 | 1.000 | 0.958 | 0.960 | 0.894 | 1.000 | 0.980 | 1.000 | 0.962 | 1.000 | 0.982 | 0.858 | 1.000 | 1.000 | 0.854 | 0.826 |
| 3657 | 0.757 | 1.000 | 0.808 | 1.000 | 0.968 | 0.960 | 0.898 | 1.000 | 0.980 | 1.000 | 0.954 | 1.000 | 0.991 | 0.858 | 1.000 | 1.000 | 0.853 | 0.824 |
| 3658 | 0.761 | 1.000 | 0.814 | 1.000 | 0.968 | 0.960 | 0.903 | 1.000 | 0.969 | 1.000 | 0.965 | 1.000 | 0.991 | 0.851 | 1.000 | 1.000 | 0.854 | 0.826 |
| 3659 | 0.765 | 1.000 | 0.802 | 1.000 | 0.963 | 0.960 | 0.895 | 1.000 | 0.980 | 1.000 | 0.957 | 1.000 | 0.991 | 0.857 | 1.000 | 1.000 | 0.854 | 0.825 |
| 3660 | 0.768 | 1.000 | 0.794 | 1.000 | 0.963 | 0.960 | 0.904 | 1.000 | 1.000 | 1.000 | 0.954 | 1.000 | 0.991 | 0.849 | 1.000 | 1.000 | 0.854 | 0.825 |
| 3661 | 0.777 | 1.000 | 0.802 | 1.000 | 0.963 | 0.960 | 0.904 | 1.000 | 0.980 | 1.000 | 0.959 | 1.000 | 0.982 | 0.853 | 1.000 | 1.000 | 0.858 | 0.829 |
| 3662 | 0.772 | 1.000 | 0.812 | 0.986 | 0.963 | 0.960 | 0.897 | 1.000 | 0.990 | 1.000 | 0.959 | 1.000 | 0.991 | 0.853 | 1.000 | 1.000 | 0.857 | 0.828 |
| 3663 | 0.770 | 1.000 | 0.806 | 0.986 | 0.963 | 0.960 | 0.901 | 1.000 | 1.000 | 1.000 | 0.951 | 1.000 | 0.991 | 0.851 | 1.000 | 1.000 | 0.855 | 0.826 |
| 3664 | 0.775 | 1.000 | 0.820 | 1.000 | 0.963 | 0.960 | 0.898 | 1.000 | 1.000 | 1.000 | 0.962 | 0.970 | 1.000 | 0.853 | 1.000 | 1.000 | 0.859 | 0.831 |

| | | | | | | | | | | | | | | | | | |
|---|---|---|---|---|---|---|---|---|---|---|---|---|---|---|---|---|---|
| 3665 | 0.771 | 1.000 | 0.808 | 1.000 | 0.963 | 0.960 | 0.898 | 1.000 | 0.980 | 1.000 | 0.951 | 1.000 | 0.991 | 0.854 | 1.000 | 1.000 | 0.856 | 0.827 |
| 3666 | 0.770 | 1.000 | 0.798 | 1.000 | 0.963 | 0.960 | 0.900 | 1.000 | 0.990 | 1.000 | 0.957 | 1.000 | 1.000 | 0.853 | 1.000 | 1.000 | 0.855 | 0.826 |
| 3667 | 0.770 | 1.000 | 0.802 | 1.000 | 0.968 | 0.960 | 0.906 | 1.000 | 0.990 | 1.000 | 0.959 | 1.000 | 0.991 | 0.852 | 1.000 | 1.000 | 0.856 | 0.828 |
| 3668 | 0.778 | 1.000 | 0.804 | 1.000 | 0.963 | 0.960 | 0.900 | 1.000 | 1.000 | 1.000 | 0.959 | 0.970 | 0.982 | 0.856 | 1.000 | 1.000 | 0.859 | 0.831 |
| 3669 | 0.763 | 1.000 | 0.798 | 0.986 | 0.963 | 0.960 | 0.901 | 1.000 | 1.000 | 1.000 | 0.954 | 1.000 | 0.991 | 0.850 | 1.000 | 1.000 | 0.852 | 0.823 |
| 3670 | 0.765 | 1.000 | 0.806 | 1.000 | 0.963 | 0.960 | 0.901 | 1.000 | 0.980 | 1.000 | 0.959 | 1.000 | 0.991 | 0.854 | 1.000 | 1.000 | 0.855 | 0.826 |
| 3671 | 0.768 | 1.000 | 0.802 | 0.986 | 0.958 | 0.960 | 0.906 | 1.000 | 0.990 | 1.000 | 0.971 | 0.970 | 1.000 | 0.843 | 1.000 | 1.000 | 0.854 | 0.825 |
| 3672 | 0.778 | 1.000 | 0.808 | 0.986 | 0.968 | 0.960 | 0.901 | 1.000 | 0.980 | 1.000 | 0.951 | 1.000 | 0.982 | 0.854 | 1.000 | 1.000 | 0.858 | 0.830 |
| 3673 | 0.763 | 1.000 | 0.814 | 0.986 | 0.963 | 0.960 | 0.898 | 1.000 | 0.969 | 1.000 | 0.957 | 1.000 | 0.991 | 0.854 | 1.000 | 1.000 | 0.854 | 0.825 |
| 3674 | 0.773 | 1.000 | 0.808 | 0.986 | 0.958 | 0.960 | 0.891 | 1.000 | 0.980 | 1.000 | 0.965 | 1.000 | 0.982 | 0.854 | 1.000 | 1.000 | 0.856 | 0.827 |
| 3675 | 0.768 | 1.000 | 0.806 | 0.986 | 0.958 | 0.960 | 0.891 | 1.000 | 0.990 | 1.000 | 0.968 | 0.970 | 1.000 | 0.854 | 1.000 | 1.000 | 0.855 | 0.826 |
| 3676 | 0.769 | 1.000 | 0.804 | 0.986 | 0.958 | 0.960 | 0.904 | 1.000 | 1.000 | 1.000 | 0.962 | 1.000 | 1.000 | 0.858 | 1.000 | 1.000 | 0.858 | 0.829 |
| 3677 | 0.767 | 1.000 | 0.802 | 1.000 | 0.968 | 0.960 | 0.895 | 1.000 | 0.980 | 1.000 | 0.957 | 1.000 | 0.982 | 0.855 | 1.000 | 1.000 | 0.854 | 0.825 |
| 3678 | 0.776 | 1.000 | 0.812 | 0.986 | 0.958 | 0.960 | 0.906 | 1.000 | 0.990 | 1.000 | 0.957 | 1.000 | 0.991 | 0.860 | 1.000 | 1.000 | 0.860 | 0.833 |
| 3679 | 0.773 | 1.000 | 0.822 | 1.000 | 0.968 | 0.960 | 0.891 | 1.000 | 0.969 | 1.000 | 0.962 | 1.000 | 0.991 | 0.847 | 1.000 | 1.000 | 0.856 | 0.827 |
| 3680 | 0.773 | 1.000 | 0.806 | 0.986 | 0.963 | 0.960 | 0.888 | 1.000 | 0.990 | 1.000 | 0.965 | 1.000 | 0.991 | 0.857 | 1.000 | 1.000 | 0.856 | 0.828 |
| 3681 | 0.775 | 1.000 | 0.820 | 1.000 | 0.963 | 0.960 | 0.897 | 1.000 | 0.980 | 1.000 | 0.959 | 1.000 | 0.991 | 0.850 | 1.000 | 1.000 | 0.857 | 0.829 |
| 3682 | 0.775 | 1.000 | 0.804 | 1.000 | 0.963 | 0.960 | 0.904 | 1.000 | 0.980 | 1.000 | 0.954 | 1.000 | 1.000 | 0.851 | 1.000 | 1.000 | 0.857 | 0.829 |
| 3683 | 0.773 | 1.000 | 0.810 | 0.986 | 0.963 | 0.960 | 0.900 | 1.000 | 0.980 | 1.000 | 0.959 | 1.000 | 0.991 | 0.844 | 1.000 | 1.000 | 0.855 | 0.826 |
| 3684 | 0.756 | 1.000 | 0.804 | 0.986 | 0.958 | 0.960 | 0.897 | 1.000 | 1.000 | 1.000 | 0.965 | 1.000 | 0.991 | 0.848 | 1.000 | 1.000 | 0.850 | 0.821 |
| 3685 | 0.776 | 1.000 | 0.806 | 0.986 | 0.963 | 0.960 | 0.889 | 1.000 | 0.980 | 1.000 | 0.962 | 0.970 | 0.991 | 0.853 | 1.000 | 1.000 | 0.856 | 0.827 |
| 3686 | 0.765 | 1.000 | 0.802 | 0.986 | 0.958 | 0.960 | 0.904 | 1.000 | 0.990 | 1.000 | 0.959 | 1.000 | 1.000 | 0.847 | 1.000 | 1.000 | 0.853 | 0.824 |
| 3687 | 0.771 | 0.971 | 0.806 | 0.986 | 0.968 | 0.960 | 0.897 | 1.000 | 0.990 | 1.000 | 0.965 | 1.000 | 1.000 | 0.857 | 1.000 | 1.000 | 0.857 | 0.829 |
| 3688 | 0.769 | 1.000 | 0.796 | 1.000 | 0.958 | 0.960 | 0.903 | 1.000 | 1.000 | 1.000 | 0.959 | 1.000 | 0.991 | 0.852 | 1.000 | 1.000 | 0.855 | 0.826 |
| 3689 | 0.759 | 1.000 | 0.806 | 1.000 | 0.968 | 0.960 | 0.898 | 1.000 | 0.990 | 1.000 | 0.957 | 0.970 | 0.991 | 0.856 | 1.000 | 1.000 | 0.853 | 0.824 |
| 3690 | 0.768 | 1.000 | 0.804 | 0.986 | 0.968 | 0.960 | 0.892 | 1.000 | 0.990 | 1.000 | 0.962 | 1.000 | 0.982 | 0.851 | 1.000 | 1.000 | 0.854 | 0.825 |
| 3691 | 0.763 | 1.000 | 0.791 | 0.986 | 0.958 | 0.960 | 0.898 | 1.000 | 0.990 | 1.000 | 0.965 | 1.000 | 1.000 | 0.853 | 1.000 | 1.000 | 0.852 | 0.823 |
| 3692 | 0.765 | 1.000 | 0.804 | 0.986 | 0.963 | 0.960 | 0.900 | 1.000 | 1.000 | 1.000 | 0.962 | 1.000 | 0.991 | 0.862 | 1.000 | 1.000 | 0.857 | 0.829 |
| 3693 | 0.771 | 1.000 | 0.792 | 1.000 | 0.958 | 0.960 | 0.888 | 1.000 | 0.980 | 1.000 | 0.959 | 0.970 | 1.000 | 0.853 | 1.000 | 1.000 | 0.853 | 0.823 |
| 3694 | 0.762 | 1.000 | 0.812 | 0.986 | 0.963 | 0.960 | 0.891 | 1.000 | 0.969 | 1.000 | 0.965 | 1.000 | 0.991 | 0.858 | 1.000 | 1.000 | 0.854 | 0.825 |
| 3695 | 0.767 | 1.000 | 0.800 | 1.000 | 0.963 | 0.960 | 0.895 | 1.000 | 0.990 | 1.000 | 0.959 | 1.000 | 0.991 | 0.852 | 1.000 | 1.000 | 0.854 | 0.825 |
| 3696 | 0.767 | 1.000 | 0.808 | 1.000 | 0.968 | 0.960 | 0.900 | 1.000 | 0.990 | 1.000 | 0.957 | 1.000 | 0.991 | 0.854 | 1.000 | 1.000 | 0.856 | 0.827 |
| 3697 | 0.780 | 1.000 | 0.816 | 1.000 | 0.963 | 0.960 | 0.889 | 1.000 | 1.000 | 1.000 | 0.962 | 1.000 | 1.000 | 0.850 | 1.000 | 1.000 | 0.858 | 0.830 |
| 3698 | 0.772 | 1.000 | 0.814 | 0.986 | 0.968 | 0.960 | 0.891 | 1.000 | 1.000 | 1.000 | 0.959 | 1.000 | 1.000 | 0.844 | 1.000 | 1.000 | 0.854 | 0.825 |
| 3699 | 0.776 | 1.000 | 0.789 | 0.986 | 0.963 | 0.960 | 0.895 | 1.000 | 1.000 | 1.000 | 0.951 | 0.970 | 0.991 | 0.855 | 1.000 | 1.000 | 0.855 | 0.826 |
| 3700 | 0.775 | 1.000 | 0.812 | 0.986 | 0.963 | 0.960 | 0.895 | 1.000 | 0.990 | 1.000 | 0.959 | 1.000 | 0.991 | 0.857 | 1.000 | 1.000 | 0.858 | 0.830 |
| 3701 | 0.769 | 1.000 | 0.798 | 1.000 | 0.963 | 0.960 | 0.903 | 1.000 | 0.980 | 1.000 | 0.968 | 1.000 | 1.000 | 0.850 | 1.000 | 1.000 | 0.855 | 0.827 |
| 3702 | 0.761 | 1.000 | 0.820 | 0.986 | 0.968 | 0.960 | 0.897 | 1.000 | 0.980 | 1.000 | 0.954 | 1.000 | 1.000 | 0.855 | 1.000 | 1.000 | 0.854 | 0.826 |
| 3703 | 0.767 | 1.000 | 0.787 | 1.000 | 0.979 | 0.960 | 0.897 | 1.000 | 0.990 | 1.000 | 0.959 | 1.000 | 0.982 | 0.851 | 1.000 | 1.000 | 0.853 | 0.823 |

| | | | | | | | | | | | | | | | | | |
|---|---|---|---|---|---|---|---|---|---|---|---|---|---|---|---|---|---|
| 3704 | 0.770 | 1.000 | 0.798 | 0.986 | 0.963 | 0.960 | 0.897 | 1.000 | 0.990 | 1.000 | 0.962 | 1.000 | 0.991 | 0.851 | 1.000 | 1.000 | 0.854 | 0.825 |
| 3705 | 0.764 | 1.000 | 0.812 | 0.986 | 0.963 | 0.960 | 0.901 | 1.000 | 0.980 | 1.000 | 0.957 | 0.970 | 1.000 | 0.856 | 1.000 | 1.000 | 0.855 | 0.827 |
| 3706 | 0.773 | 1.000 | 0.808 | 0.986 | 0.958 | 0.960 | 0.894 | 1.000 | 0.980 | 1.000 | 0.957 | 0.970 | 0.991 | 0.855 | 1.000 | 1.000 | 0.856 | 0.827 |
| 3707 | 0.777 | 1.000 | 0.804 | 1.000 | 0.968 | 0.960 | 0.900 | 1.000 | 0.990 | 1.000 | 0.965 | 1.000 | 0.982 | 0.848 | 1.000 | 1.000 | 0.857 | 0.829 |
| 3708 | 0.771 | 1.000 | 0.810 | 0.986 | 0.958 | 0.960 | 0.897 | 1.000 | 0.980 | 1.000 | 0.965 | 1.000 | 1.000 | 0.853 | 1.000 | 1.000 | 0.856 | 0.828 |
| 3709 | 0.769 | 1.000 | 0.812 | 1.000 | 0.963 | 0.960 | 0.891 | 1.000 | 0.990 | 1.000 | 0.965 | 1.000 | 0.982 | 0.858 | 1.000 | 1.000 | 0.857 | 0.828 |
| 3710 | 0.769 | 1.000 | 0.804 | 1.000 | 0.963 | 0.960 | 0.892 | 1.000 | 0.980 | 1.000 | 0.962 | 1.000 | 0.991 | 0.858 | 1.000 | 1.000 | 0.856 | 0.827 |
| 3711 | 0.777 | 1.000 | 0.794 | 1.000 | 0.968 | 0.960 | 0.898 | 1.000 | 0.990 | 1.000 | 0.959 | 1.000 | 0.991 | 0.857 | 1.000 | 1.000 | 0.858 | 0.830 |
| 3712 | 0.769 | 1.000 | 0.796 | 1.000 | 0.968 | 0.960 | 0.891 | 1.000 | 1.000 | 1.000 | 0.954 | 1.000 | 0.991 | 0.849 | 1.000 | 1.000 | 0.852 | 0.823 |
| 3713 | 0.759 | 1.000 | 0.814 | 1.000 | 0.953 | 0.960 | 0.898 | 1.000 | 0.980 | 1.000 | 0.962 | 1.000 | 0.991 | 0.861 | 1.000 | 1.000 | 0.855 | 0.826 |
| 3714 | 0.767 | 1.000 | 0.802 | 0.986 | 0.958 | 0.960 | 0.900 | 1.000 | 0.980 | 1.000 | 0.959 | 1.000 | 1.000 | 0.854 | 1.000 | 1.000 | 0.855 | 0.826 |
| 3715 | 0.768 | 1.000 | 0.804 | 1.000 | 0.963 | 0.960 | 0.901 | 1.000 | 0.990 | 1.000 | 0.959 | 1.000 | 0.991 | 0.858 | 1.000 | 1.000 | 0.857 | 0.828 |
| 3716 | 0.763 | 1.000 | 0.808 | 0.986 | 0.963 | 0.960 | 0.897 | 1.000 | 1.000 | 1.000 | 0.968 | 1.000 | 0.991 | 0.854 | 1.000 | 1.000 | 0.854 | 0.826 |
| 3717 | 0.773 | 1.000 | 0.808 | 1.000 | 0.963 | 0.960 | 0.900 | 1.000 | 1.000 | 1.000 | 0.965 | 1.000 | 1.000 | 0.858 | 1.000 | 1.000 | 0.859 | 0.831 |
| 3718 | 0.767 | 1.000 | 0.804 | 0.986 | 0.963 | 0.960 | 0.912 | 1.000 | 0.990 | 1.000 | 0.962 | 1.000 | 0.982 | 0.855 | 1.000 | 1.000 | 0.857 | 0.829 |
| 3719 | 0.761 | 1.000 | 0.812 | 0.986 | 0.963 | 0.960 | 0.900 | 1.000 | 0.990 | 1.000 | 0.965 | 1.000 | 1.000 | 0.857 | 1.000 | 1.000 | 0.855 | 0.827 |
| 3720 | 0.773 | 1.000 | 0.806 | 1.000 | 0.963 | 0.960 | 0.906 | 1.000 | 0.980 | 1.000 | 0.968 | 1.000 | 0.991 | 0.849 | 1.000 | 1.000 | 0.857 | 0.829 |
| 3721 | 0.775 | 1.000 | 0.814 | 1.000 | 0.958 | 0.960 | 0.900 | 1.000 | 1.000 | 1.000 | 0.954 | 1.000 | 1.000 | 0.846 | 1.000 | 1.000 | 0.856 | 0.828 |
| 3722 | 0.775 | 1.000 | 0.818 | 1.000 | 0.974 | 0.960 | 0.903 | 1.000 | 0.990 | 1.000 | 0.965 | 1.000 | 0.991 | 0.849 | 1.000 | 1.000 | 0.859 | 0.831 |
| 3723 | 0.761 | 1.000 | 0.798 | 1.000 | 0.968 | 0.960 | 0.891 | 1.000 | 0.980 | 1.000 | 0.951 | 1.000 | 0.991 | 0.852 | 1.000 | 1.000 | 0.851 | 0.821 |
| 3724 | 0.760 | 1.000 | 0.798 | 1.000 | 0.968 | 0.960 | 0.908 | 1.000 | 0.980 | 1.000 | 0.954 | 1.000 | 0.991 | 0.852 | 1.000 | 1.000 | 0.853 | 0.824 |
| 3725 | 0.772 | 1.000 | 0.796 | 1.000 | 0.968 | 0.960 | 0.901 | 1.000 | 0.990 | 1.000 | 0.962 | 1.000 | 0.991 | 0.850 | 1.000 | 1.000 | 0.855 | 0.827 |
| 3726 | 0.760 | 1.000 | 0.812 | 1.000 | 0.958 | 0.960 | 0.886 | 1.000 | 1.000 | 1.000 | 0.954 | 1.000 | 0.991 | 0.859 | 1.000 | 1.000 | 0.853 | 0.824 |
| 3727 | 0.777 | 1.000 | 0.808 | 0.986 | 0.968 | 0.960 | 0.904 | 1.000 | 0.990 | 1.000 | 0.962 | 1.000 | 0.982 | 0.855 | 1.000 | 1.000 | 0.860 | 0.832 |
| 3728 | 0.769 | 1.000 | 0.816 | 0.986 | 0.958 | 0.960 | 0.895 | 1.000 | 1.000 | 1.000 | 0.971 | 1.000 | 0.991 | 0.854 | 1.000 | 1.000 | 0.857 | 0.829 |
| 3729 | 0.763 | 1.000 | 0.810 | 1.000 | 0.974 | 0.960 | 0.906 | 1.000 | 0.990 | 1.000 | 0.971 | 1.000 | 0.982 | 0.851 | 1.000 | 1.000 | 0.855 | 0.827 |
| 3730 | 0.773 | 1.000 | 0.824 | 1.000 | 0.958 | 0.960 | 0.901 | 1.000 | 1.000 | 1.000 | 0.968 | 0.970 | 0.991 | 0.848 | 1.000 | 1.000 | 0.858 | 0.830 |
| 3731 | 0.763 | 1.000 | 0.814 | 1.000 | 0.963 | 0.960 | 0.908 | 1.000 | 1.000 | 1.000 | 0.957 | 1.000 | 0.991 | 0.854 | 1.000 | 1.000 | 0.856 | 0.828 |
| 3732 | 0.777 | 1.000 | 0.798 | 0.986 | 0.963 | 0.960 | 0.883 | 1.000 | 0.990 | 1.000 | 0.962 | 0.970 | 0.991 | 0.860 | 1.000 | 1.000 | 0.857 | 0.828 |
| 3733 | 0.769 | 1.000 | 0.798 | 0.986 | 0.963 | 0.960 | 0.894 | 1.000 | 1.000 | 1.000 | 0.965 | 0.970 | 1.000 | 0.848 | 1.000 | 1.000 | 0.853 | 0.824 |
| 3734 | 0.759 | 1.000 | 0.796 | 1.000 | 0.947 | 0.960 | 0.895 | 1.000 | 0.990 | 1.000 | 0.959 | 1.000 | 1.000 | 0.852 | 1.000 | 1.000 | 0.851 | 0.821 |
| 3735 | 0.762 | 1.000 | 0.808 | 1.000 | 0.963 | 0.960 | 0.900 | 1.000 | 1.000 | 1.000 | 0.951 | 1.000 | 0.991 | 0.849 | 1.000 | 1.000 | 0.852 | 0.823 |
| 3736 | 0.771 | 1.000 | 0.791 | 1.000 | 0.968 | 0.960 | 0.901 | 1.000 | 0.969 | 1.000 | 0.965 | 1.000 | 0.991 | 0.848 | 1.000 | 1.000 | 0.854 | 0.825 |
| 3737 | 0.770 | 1.000 | 0.814 | 1.000 | 0.963 | 0.960 | 0.895 | 1.000 | 0.990 | 1.000 | 0.971 | 1.000 | 0.991 | 0.859 | 1.000 | 1.000 | 0.859 | 0.831 |
| 3738 | 0.773 | 1.000 | 0.794 | 1.000 | 0.963 | 0.960 | 0.895 | 1.000 | 0.969 | 1.000 | 0.962 | 1.000 | 0.982 | 0.846 | 1.000 | 1.000 | 0.853 | 0.824 |
| 3739 | 0.773 | 1.000 | 0.800 | 1.000 | 0.968 | 0.960 | 0.908 | 1.000 | 0.980 | 1.000 | 0.959 | 0.970 | 1.000 | 0.853 | 1.000 | 1.000 | 0.857 | 0.829 |
| 3740 | 0.767 | 1.000 | 0.816 | 0.986 | 0.953 | 0.960 | 0.895 | 1.000 | 0.990 | 1.000 | 0.962 | 1.000 | 1.000 | 0.854 | 1.000 | 1.000 | 0.856 | 0.827 |
| 3741 | 0.773 | 1.000 | 0.812 | 1.000 | 0.963 | 0.960 | 0.895 | 1.000 | 0.980 | 1.000 | 0.959 | 1.000 | 0.991 | 0.851 | 1.000 | 1.000 | 0.856 | 0.828 |
| 3742 | 0.773 | 1.000 | 0.798 | 1.000 | 0.963 | 0.960 | 0.892 | 1.000 | 0.980 | 1.000 | 0.965 | 1.000 | 0.991 | 0.849 | 1.000 | 1.000 | 0.854 | 0.825 |

| | | | | | | | | | | | | | | | | | |
|---|---|---|---|---|---|---|---|---|---|---|---|---|---|---|---|---|---|
| 3743 | 0.774 | 1.000 | 0.816 | 1.000 | 0.958 | 0.960 | 0.897 | 1.000 | 0.990 | 1.000 | 0.957 | 1.000 | 0.991 | 0.855 | 1.000 | 1.000 | 0.858 | 0.830 |
| 3744 | 0.772 | 1.000 | 0.787 | 0.986 | 0.963 | 0.960 | 0.886 | 1.000 | 0.980 | 1.000 | 0.965 | 1.000 | 1.000 | 0.849 | 1.000 | 1.000 | 0.852 | 0.822 |
| 3745 | 0.780 | 1.000 | 0.802 | 1.000 | 0.963 | 0.960 | 0.906 | 1.000 | 0.969 | 1.000 | 0.951 | 1.000 | 1.000 | 0.856 | 1.000 | 1.000 | 0.859 | 0.831 |
| 3746 | 0.773 | 1.000 | 0.818 | 1.000 | 0.958 | 0.960 | 0.894 | 1.000 | 0.969 | 1.000 | 0.957 | 1.000 | 1.000 | 0.860 | 1.000 | 1.000 | 0.859 | 0.830 |
| 3747 | 0.771 | 1.000 | 0.818 | 1.000 | 0.958 | 0.960 | 0.911 | 1.000 | 0.990 | 1.000 | 0.959 | 1.000 | 0.991 | 0.854 | 1.000 | 1.000 | 0.859 | 0.832 |
| 3748 | 0.767 | 1.000 | 0.828 | 1.000 | 0.963 | 0.960 | 0.891 | 1.000 | 1.000 | 1.000 | 0.959 | 1.000 | 0.991 | 0.852 | 1.000 | 1.000 | 0.856 | 0.828 |
| 3749 | 0.777 | 1.000 | 0.792 | 1.000 | 0.958 | 0.960 | 0.901 | 1.000 | 0.990 | 1.000 | 0.962 | 0.970 | 0.991 | 0.844 | 1.000 | 1.000 | 0.855 | 0.826 |
| 3750 | 0.781 | 1.000 | 0.814 | 1.000 | 0.963 | 0.960 | 0.898 | 1.000 | 1.000 | 1.000 | 0.962 | 1.000 | 0.982 | 0.848 | 1.000 | 1.000 | 0.859 | 0.831 |
| 3751 | 0.763 | 1.000 | 0.791 | 1.000 | 0.963 | 0.960 | 0.889 | 1.000 | 1.000 | 1.000 | 0.965 | 0.970 | 1.000 | 0.847 | 1.000 | 1.000 | 0.850 | 0.820 |
| 3752 | 0.773 | 1.000 | 0.794 | 1.000 | 0.958 | 0.960 | 0.903 | 1.000 | 0.980 | 1.000 | 0.954 | 1.000 | 0.991 | 0.850 | 1.000 | 1.000 | 0.854 | 0.825 |
| 3753 | 0.771 | 1.000 | 0.796 | 1.000 | 0.963 | 0.960 | 0.900 | 1.000 | 0.969 | 1.000 | 0.962 | 1.000 | 1.000 | 0.860 | 1.000 | 1.000 | 0.857 | 0.829 |
| 3754 | 0.759 | 1.000 | 0.804 | 0.986 | 0.968 | 0.960 | 0.892 | 1.000 | 0.990 | 1.000 | 0.968 | 1.000 | 0.991 | 0.859 | 1.000 | 1.000 | 0.854 | 0.825 |
| 3755 | 0.775 | 1.000 | 0.806 | 1.000 | 0.968 | 0.960 | 0.894 | 1.000 | 0.990 | 1.000 | 0.968 | 1.000 | 1.000 | 0.849 | 1.000 | 1.000 | 0.857 | 0.828 |
| 3756 | 0.770 | 1.000 | 0.816 | 1.000 | 0.953 | 0.960 | 0.906 | 1.000 | 0.990 | 1.000 | 0.965 | 1.000 | 1.000 | 0.858 | 1.000 | 1.000 | 0.859 | 0.832 |
| 3757 | 0.771 | 1.000 | 0.791 | 0.986 | 0.963 | 0.960 | 0.901 | 1.000 | 0.980 | 1.000 | 0.957 | 1.000 | 1.000 | 0.853 | 1.000 | 1.000 | 0.854 | 0.825 |
| 3758 | 0.769 | 1.000 | 0.798 | 1.000 | 0.958 | 0.960 | 0.891 | 1.000 | 1.000 | 1.000 | 0.968 | 1.000 | 1.000 | 0.854 | 1.000 | 1.000 | 0.855 | 0.826 |
| 3759 | 0.773 | 1.000 | 0.789 | 1.000 | 0.958 | 0.960 | 0.900 | 1.000 | 0.990 | 1.000 | 0.962 | 1.000 | 0.982 | 0.837 | 1.000 | 1.000 | 0.851 | 0.821 |
| 3760 | 0.771 | 1.000 | 0.822 | 0.986 | 0.963 | 0.960 | 0.906 | 1.000 | 0.990 | 1.000 | 0.962 | 0.970 | 1.000 | 0.852 | 1.000 | 1.000 | 0.859 | 0.831 |
| 3761 | 0.765 | 1.000 | 0.808 | 1.000 | 0.963 | 0.960 | 0.903 | 1.000 | 0.990 | 1.000 | 0.968 | 1.000 | 0.991 | 0.851 | 1.000 | 1.000 | 0.855 | 0.827 |
| 3762 | 0.765 | 1.000 | 0.802 | 1.000 | 0.963 | 1.000 | 0.901 | 1.000 | 0.980 | 1.000 | 0.971 | 1.000 | 1.000 | 0.851 | 1.000 | 1.000 | 0.855 | 0.826 |
| 3763 | 0.768 | 1.000 | 0.796 | 0.986 | 0.958 | 0.960 | 0.894 | 1.000 | 0.990 | 1.000 | 0.962 | 1.000 | 0.982 | 0.854 | 1.000 | 1.000 | 0.854 | 0.825 |
| 3764 | 0.777 | 1.000 | 0.812 | 0.986 | 0.968 | 0.960 | 0.894 | 1.000 | 1.000 | 1.000 | 0.954 | 1.000 | 0.991 | 0.851 | 1.000 | 1.000 | 0.857 | 0.829 |
| 3765 | 0.765 | 1.000 | 0.814 | 1.000 | 0.958 | 0.960 | 0.895 | 1.000 | 1.000 | 1.000 | 0.962 | 0.970 | 1.000 | 0.864 | 1.000 | 1.000 | 0.858 | 0.830 |
| 3766 | 0.770 | 1.000 | 0.826 | 1.000 | 0.958 | 0.960 | 0.900 | 1.000 | 0.990 | 1.000 | 0.959 | 1.000 | 1.000 | 0.844 | 1.000 | 1.000 | 0.856 | 0.827 |
| 3767 | 0.763 | 1.000 | 0.804 | 1.000 | 0.963 | 0.960 | 0.888 | 1.000 | 0.990 | 1.000 | 0.968 | 1.000 | 1.000 | 0.851 | 1.000 | 1.000 | 0.852 | 0.823 |
| 3768 | 0.769 | 1.000 | 0.820 | 0.986 | 0.968 | 0.960 | 0.901 | 1.000 | 0.990 | 1.000 | 0.962 | 1.000 | 1.000 | 0.850 | 1.000 | 1.000 | 0.857 | 0.829 |
| 3769 | 0.763 | 1.000 | 0.792 | 0.986 | 0.963 | 0.960 | 0.900 | 1.000 | 1.000 | 1.000 | 0.965 | 1.000 | 1.000 | 0.847 | 1.000 | 1.000 | 0.852 | 0.822 |
| 3770 | 0.768 | 1.000 | 0.818 | 0.986 | 0.958 | 0.960 | 0.901 | 1.000 | 1.000 | 1.000 | 0.965 | 1.000 | 1.000 | 0.849 | 1.000 | 1.000 | 0.856 | 0.828 |
| 3771 | 0.768 | 1.000 | 0.798 | 1.000 | 0.958 | 0.960 | 0.895 | 1.000 | 0.990 | 1.000 | 0.968 | 1.000 | 0.982 | 0.859 | 1.000 | 1.000 | 0.856 | 0.827 |
| 3772 | 0.773 | 1.000 | 0.802 | 0.971 | 0.963 | 0.960 | 0.884 | 1.000 | 1.000 | 1.000 | 0.959 | 1.000 | 0.991 | 0.857 | 1.000 | 1.000 | 0.855 | 0.826 |
| 3773 | 0.769 | 1.000 | 0.816 | 1.000 | 0.963 | 0.960 | 0.903 | 1.000 | 0.990 | 1.000 | 0.968 | 1.000 | 0.982 | 0.851 | 1.000 | 1.000 | 0.857 | 0.829 |
| 3774 | 0.774 | 1.000 | 0.804 | 1.000 | 0.958 | 0.960 | 0.903 | 1.000 | 0.990 | 1.000 | 0.971 | 1.000 | 1.000 | 0.857 | 1.000 | 1.000 | 0.859 | 0.831 |
| 3775 | 0.774 | 1.000 | 0.808 | 0.986 | 0.968 | 0.960 | 0.894 | 1.000 | 0.990 | 1.000 | 0.974 | 1.000 | 0.991 | 0.851 | 1.000 | 1.000 | 0.857 | 0.829 |
| 3776 | 0.777 | 1.000 | 0.796 | 1.000 | 0.968 | 0.960 | 0.901 | 1.000 | 0.990 | 1.000 | 0.965 | 1.000 | 0.982 | 0.856 | 1.000 | 1.000 | 0.859 | 0.830 |
| 3777 | 0.759 | 1.000 | 0.796 | 1.000 | 0.963 | 0.960 | 0.903 | 1.000 | 0.990 | 1.000 | 0.962 | 0.970 | 0.991 | 0.854 | 1.000 | 1.000 | 0.853 | 0.824 |
| 3778 | 0.767 | 1.000 | 0.796 | 1.000 | 0.963 | 0.960 | 0.895 | 1.000 | 1.000 | 1.000 | 0.957 | 1.000 | 1.000 | 0.856 | 1.000 | 1.000 | 0.854 | 0.825 |
| 3779 | 0.773 | 1.000 | 0.802 | 0.986 | 0.953 | 0.960 | 0.895 | 1.000 | 0.980 | 1.000 | 0.959 | 1.000 | 1.000 | 0.851 | 1.000 | 1.000 | 0.855 | 0.826 |
| 3780 | 0.772 | 1.000 | 0.818 | 1.000 | 0.968 | 0.960 | 0.894 | 1.000 | 1.000 | 1.000 | 0.962 | 1.000 | 0.991 | 0.858 | 1.000 | 1.000 | 0.859 | 0.831 |
| 3781 | 0.774 | 1.000 | 0.822 | 0.986 | 0.953 | 0.960 | 0.895 | 1.000 | 0.980 | 1.000 | 0.957 | 1.000 | 0.982 | 0.854 | 1.000 | 1.000 | 0.857 | 0.829 |

| | | | | | | | | | | | | | | | | | |
|---|---|---|---|---|---|---|---|---|---|---|---|---|---|---|---|---|---|
| 3782 | 0.773 | 1.000 | 0.802 | 0.986 | 0.958 | 0.960 | 0.892 | 1.000 | 0.980 | 1.000 | 0.965 | 1.000 | 0.991 | 0.850 | 1.000 | 1.000 | 0.855 | 0.826 |
| 3783 | 0.774 | 1.000 | 0.800 | 1.000 | 0.968 | 0.960 | 0.897 | 1.000 | 0.990 | 1.000 | 0.957 | 1.000 | 0.982 | 0.851 | 1.000 | 1.000 | 0.855 | 0.827 |
| 3784 | 0.761 | 1.000 | 0.792 | 0.971 | 0.963 | 0.960 | 0.889 | 1.000 | 1.000 | 1.000 | 0.962 | 1.000 | 0.982 | 0.846 | 1.000 | 1.000 | 0.849 | 0.819 |
| 3785 | 0.757 | 1.000 | 0.794 | 1.000 | 0.963 | 0.960 | 0.904 | 1.000 | 1.000 | 1.000 | 0.965 | 0.970 | 1.000 | 0.854 | 1.000 | 1.000 | 0.853 | 0.824 |
| 3786 | 0.769 | 1.000 | 0.808 | 0.986 | 0.958 | 0.960 | 0.903 | 1.000 | 0.980 | 1.000 | 0.959 | 1.000 | 0.991 | 0.857 | 1.000 | 1.000 | 0.857 | 0.828 |
| 3787 | 0.767 | 1.000 | 0.794 | 1.000 | 0.968 | 0.960 | 0.889 | 1.000 | 0.990 | 1.000 | 0.948 | 1.000 | 1.000 | 0.850 | 1.000 | 1.000 | 0.851 | 0.822 |
| 3788 | 0.768 | 1.000 | 0.826 | 1.000 | 0.968 | 0.960 | 0.895 | 1.000 | 0.990 | 1.000 | 0.968 | 1.000 | 1.000 | 0.850 | 1.000 | 1.000 | 0.857 | 0.829 |
| 3789 | 0.772 | 1.000 | 0.794 | 1.000 | 0.963 | 0.960 | 0.897 | 1.000 | 0.980 | 1.000 | 0.965 | 1.000 | 1.000 | 0.848 | 1.000 | 1.000 | 0.854 | 0.825 |
| 3790 | 0.762 | 1.000 | 0.806 | 1.000 | 0.963 | 0.960 | 0.892 | 1.000 | 0.990 | 1.000 | 0.957 | 1.000 | 0.991 | 0.845 | 1.000 | 1.000 | 0.850 | 0.821 |
| 3791 | 0.774 | 1.000 | 0.800 | 0.971 | 0.953 | 0.960 | 0.891 | 1.000 | 0.990 | 1.000 | 0.965 | 1.000 | 0.991 | 0.853 | 1.000 | 1.000 | 0.855 | 0.826 |
| 3792 | 0.767 | 1.000 | 0.802 | 1.000 | 0.963 | 0.960 | 0.895 | 1.000 | 0.980 | 1.000 | 0.959 | 1.000 | 0.982 | 0.852 | 1.000 | 1.000 | 0.853 | 0.824 |
| 3793 | 0.769 | 1.000 | 0.822 | 0.986 | 0.963 | 0.960 | 0.898 | 1.000 | 1.000 | 1.000 | 0.965 | 1.000 | 0.982 | 0.849 | 1.000 | 1.000 | 0.856 | 0.828 |
| 3794 | 0.767 | 1.000 | 0.792 | 1.000 | 0.963 | 0.960 | 0.904 | 1.000 | 1.000 | 1.000 | 0.968 | 1.000 | 0.991 | 0.847 | 1.000 | 1.000 | 0.854 | 0.825 |
| 3795 | 0.757 | 1.000 | 0.806 | 1.000 | 0.968 | 0.960 | 0.894 | 1.000 | 0.980 | 1.000 | 0.965 | 1.000 | 0.991 | 0.858 | 1.000 | 1.000 | 0.853 | 0.824 |
| 3796 | 0.767 | 1.000 | 0.810 | 0.986 | 0.963 | 0.960 | 0.895 | 1.000 | 0.990 | 1.000 | 0.959 | 1.000 | 0.991 | 0.850 | 1.000 | 1.000 | 0.854 | 0.825 |
| 3797 | 0.763 | 1.000 | 0.818 | 1.000 | 0.963 | 0.960 | 0.900 | 1.000 | 0.990 | 1.000 | 0.959 | 1.000 | 0.991 | 0.852 | 1.000 | 1.000 | 0.855 | 0.827 |
| 3798 | 0.770 | 1.000 | 0.796 | 1.000 | 0.958 | 0.960 | 0.903 | 1.000 | 0.990 | 1.000 | 0.968 | 1.000 | 0.991 | 0.851 | 1.000 | 1.000 | 0.855 | 0.827 |
| 3799 | 0.771 | 1.000 | 0.810 | 1.000 | 0.968 | 0.960 | 0.894 | 1.000 | 0.980 | 1.000 | 0.965 | 0.970 | 0.991 | 0.858 | 1.000 | 1.000 | 0.857 | 0.829 |
| 3800 | 0.766 | 1.000 | 0.810 | 0.986 | 0.963 | 0.960 | 0.906 | 1.000 | 0.980 | 1.000 | 0.954 | 1.000 | 1.000 | 0.840 | 1.000 | 1.000 | 0.852 | 0.823 |
| 3801 | 0.773 | 1.000 | 0.806 | 0.971 | 0.968 | 0.960 | 0.898 | 1.000 | 1.000 | 1.000 | 0.962 | 1.000 | 0.991 | 0.848 | 1.000 | 1.000 | 0.856 | 0.827 |
| 3802 | 0.767 | 1.000 | 0.808 | 0.986 | 0.968 | 0.960 | 0.891 | 1.000 | 0.980 | 1.000 | 0.959 | 1.000 | 0.991 | 0.844 | 1.000 | 1.000 | 0.852 | 0.822 |
| 3803 | 0.769 | 1.000 | 0.810 | 1.000 | 0.963 | 0.960 | 0.894 | 1.000 | 0.990 | 1.000 | 0.962 | 1.000 | 1.000 | 0.858 | 1.000 | 1.000 | 0.857 | 0.829 |
| 3804 | 0.769 | 1.000 | 0.806 | 1.000 | 0.963 | 0.960 | 0.901 | 1.000 | 0.969 | 1.000 | 0.957 | 1.000 | 1.000 | 0.854 | 1.000 | 1.000 | 0.856 | 0.827 |
| 3805 | 0.765 | 1.000 | 0.800 | 1.000 | 0.958 | 0.960 | 0.903 | 1.000 | 1.000 | 1.000 | 0.962 | 1.000 | 0.991 | 0.849 | 1.000 | 1.000 | 0.854 | 0.825 |
| 3806 | 0.761 | 1.000 | 0.802 | 0.986 | 0.963 | 0.960 | 0.895 | 1.000 | 0.980 | 1.000 | 0.959 | 1.000 | 0.991 | 0.851 | 1.000 | 1.000 | 0.852 | 0.822 |
| 3807 | 0.778 | 1.000 | 0.796 | 1.000 | 0.963 | 0.960 | 0.894 | 1.000 | 0.969 | 1.000 | 0.959 | 1.000 | 1.000 | 0.857 | 1.000 | 1.000 | 0.857 | 0.829 |
| 3808 | 0.767 | 1.000 | 0.796 | 1.000 | 0.958 | 0.960 | 0.900 | 1.000 | 0.980 | 1.000 | 0.957 | 1.000 | 0.991 | 0.853 | 1.000 | 1.000 | 0.853 | 0.824 |
| 3809 | 0.765 | 1.000 | 0.810 | 1.000 | 0.968 | 0.960 | 0.891 | 1.000 | 0.980 | 1.000 | 0.968 | 1.000 | 0.991 | 0.858 | 1.000 | 1.000 | 0.856 | 0.827 |
| 3810 | 0.773 | 1.000 | 0.810 | 1.000 | 0.963 | 0.960 | 0.889 | 1.000 | 1.000 | 1.000 | 0.974 | 1.000 | 0.982 | 0.860 | 1.000 | 1.000 | 0.859 | 0.830 |
| 3811 | 0.762 | 1.000 | 0.804 | 0.986 | 0.968 | 0.960 | 0.881 | 1.000 | 0.980 | 1.000 | 0.959 | 0.970 | 0.982 | 0.844 | 1.000 | 1.000 | 0.848 | 0.818 |
| 3812 | 0.771 | 1.000 | 0.814 | 1.000 | 0.963 | 0.960 | 0.900 | 1.000 | 0.990 | 1.000 | 0.965 | 1.000 | 0.982 | 0.865 | 1.000 | 1.000 | 0.861 | 0.833 |
| 3813 | 0.769 | 1.000 | 0.804 | 1.000 | 0.963 | 0.960 | 0.898 | 1.000 | 0.990 | 1.000 | 0.959 | 1.000 | 1.000 | 0.847 | 1.000 | 1.000 | 0.854 | 0.825 |
| 3814 | 0.767 | 1.000 | 0.802 | 1.000 | 0.963 | 0.960 | 0.889 | 1.000 | 0.990 | 1.000 | 0.954 | 0.970 | 0.991 | 0.855 | 1.000 | 1.000 | 0.853 | 0.824 |
| 3815 | 0.778 | 1.000 | 0.808 | 1.000 | 0.968 | 0.960 | 0.895 | 1.000 | 0.990 | 1.000 | 0.971 | 1.000 | 1.000 | 0.854 | 1.000 | 1.000 | 0.859 | 0.832 |
| 3816 | 0.774 | 1.000 | 0.812 | 1.000 | 0.963 | 0.960 | 0.900 | 1.000 | 0.990 | 1.000 | 0.957 | 1.000 | 0.991 | 0.848 | 1.000 | 1.000 | 0.856 | 0.828 |
| 3817 | 0.779 | 1.000 | 0.796 | 0.986 | 0.963 | 0.960 | 0.892 | 1.000 | 0.990 | 1.000 | 0.968 | 1.000 | 1.000 | 0.849 | 1.000 | 1.000 | 0.856 | 0.827 |
| 3818 | 0.772 | 1.000 | 0.794 | 1.000 | 0.968 | 0.960 | 0.903 | 1.000 | 1.000 | 1.000 | 0.971 | 1.000 | 1.000 | 0.847 | 1.000 | 1.000 | 0.856 | 0.827 |
| 3819 | 0.768 | 1.000 | 0.808 | 1.000 | 0.963 | 0.960 | 0.892 | 1.000 | 0.990 | 1.000 | 0.968 | 1.000 | 0.991 | 0.851 | 1.000 | 1.000 | 0.855 | 0.826 |
| 3820 | 0.767 | 1.000 | 0.818 | 1.000 | 0.963 | 0.960 | 0.889 | 1.000 | 1.000 | 1.000 | 0.968 | 1.000 | 0.982 | 0.861 | 1.000 | 1.000 | 0.857 | 0.829 |

| | | | | | | | | | | | | | | | | | |
|---|---|---|---|---|---|---|---|---|---|---|---|---|---|---|---|---|---|
| 3821 | 0.767 | 1.000 | 0.806 | 1.000 | 0.968 | 0.960 | 0.889 | 1.000 | 0.980 | 1.000 | 0.957 | 1.000 | 0.991 | 0.854 | 1.000 | 1.000 | 0.854 | 0.824 |
| 3822 | 0.766 | 1.000 | 0.798 | 1.000 | 0.963 | 0.960 | 0.911 | 1.000 | 1.000 | 1.000 | 0.959 | 1.000 | 1.000 | 0.848 | 1.000 | 1.000 | 0.855 | 0.826 |
| 3823 | 0.767 | 1.000 | 0.794 | 0.986 | 0.963 | 0.960 | 0.888 | 1.000 | 0.990 | 1.000 | 0.959 | 1.000 | 1.000 | 0.852 | 1.000 | 1.000 | 0.852 | 0.823 |
| 3824 | 0.771 | 1.000 | 0.796 | 1.000 | 0.968 | 0.960 | 0.901 | 1.000 | 0.990 | 1.000 | 0.962 | 0.970 | 0.991 | 0.852 | 1.000 | 1.000 | 0.856 | 0.827 |
| 3825 | 0.769 | 1.000 | 0.806 | 1.000 | 0.968 | 0.960 | 0.903 | 1.000 | 1.000 | 1.000 | 0.959 | 1.000 | 0.991 | 0.849 | 1.000 | 1.000 | 0.855 | 0.827 |
| 3826 | 0.770 | 1.000 | 0.800 | 1.000 | 0.958 | 0.960 | 0.901 | 1.000 | 0.969 | 1.000 | 0.965 | 1.000 | 1.000 | 0.851 | 1.000 | 1.000 | 0.855 | 0.826 |
| 3827 | 0.769 | 1.000 | 0.800 | 1.000 | 0.968 | 0.960 | 0.900 | 1.000 | 0.980 | 1.000 | 0.962 | 0.970 | 1.000 | 0.854 | 1.000 | 1.000 | 0.856 | 0.827 |
| 3828 | 0.772 | 1.000 | 0.820 | 1.000 | 0.963 | 0.960 | 0.901 | 1.000 | 1.000 | 1.000 | 0.971 | 1.000 | 0.991 | 0.846 | 1.000 | 1.000 | 0.857 | 0.829 |
| 3829 | 0.777 | 1.000 | 0.787 | 0.986 | 0.963 | 0.960 | 0.906 | 1.000 | 0.990 | 1.000 | 0.965 | 1.000 | 1.000 | 0.847 | 1.000 | 1.000 | 0.856 | 0.827 |
| 3830 | 0.766 | 1.000 | 0.791 | 0.986 | 0.963 | 0.960 | 0.903 | 1.000 | 1.000 | 1.000 | 0.962 | 1.000 | 0.991 | 0.855 | 1.000 | 1.000 | 0.854 | 0.825 |
| 3831 | 0.769 | 1.000 | 0.806 | 1.000 | 0.963 | 0.960 | 0.897 | 1.000 | 1.000 | 1.000 | 0.962 | 1.000 | 0.991 | 0.851 | 1.000 | 1.000 | 0.855 | 0.827 |
| 3832 | 0.769 | 1.000 | 0.804 | 1.000 | 0.958 | 0.960 | 0.906 | 1.000 | 0.980 | 1.000 | 0.959 | 1.000 | 0.991 | 0.854 | 1.000 | 1.000 | 0.856 | 0.828 |
| 3833 | 0.767 | 1.000 | 0.798 | 1.000 | 0.958 | 0.960 | 0.898 | 1.000 | 0.969 | 1.000 | 0.957 | 1.000 | 1.000 | 0.849 | 1.000 | 1.000 | 0.853 | 0.824 |
| 3834 | 0.775 | 1.000 | 0.818 | 1.000 | 0.963 | 0.960 | 0.898 | 1.000 | 0.980 | 1.000 | 0.965 | 1.000 | 1.000 | 0.851 | 1.000 | 1.000 | 0.859 | 0.831 |
| 3835 | 0.767 | 1.000 | 0.806 | 0.971 | 0.963 | 0.960 | 0.883 | 1.000 | 1.000 | 1.000 | 0.965 | 1.000 | 1.000 | 0.852 | 1.000 | 1.000 | 0.853 | 0.824 |
| 3836 | 0.764 | 1.000 | 0.810 | 1.000 | 0.963 | 0.960 | 0.892 | 1.000 | 0.980 | 1.000 | 0.968 | 1.000 | 0.991 | 0.851 | 1.000 | 1.000 | 0.853 | 0.824 |
| 3837 | 0.773 | 1.000 | 0.808 | 0.986 | 0.963 | 0.960 | 0.894 | 1.000 | 1.000 | 1.000 | 0.959 | 1.000 | 1.000 | 0.850 | 1.000 | 1.000 | 0.856 | 0.827 |
| 3838 | 0.775 | 1.000 | 0.804 | 0.986 | 0.974 | 0.960 | 0.897 | 1.000 | 1.000 | 1.000 | 0.962 | 1.000 | 0.991 | 0.847 | 1.000 | 1.000 | 0.856 | 0.828 |
| 3839 | 0.767 | 1.000 | 0.830 | 1.000 | 0.963 | 0.960 | 0.900 | 1.000 | 0.990 | 1.000 | 0.965 | 0.970 | 1.000 | 0.847 | 1.000 | 1.000 | 0.856 | 0.828 |
| 3840 | 0.773 | 1.000 | 0.808 | 1.000 | 0.963 | 0.960 | 0.897 | 1.000 | 0.990 | 1.000 | 0.962 | 1.000 | 0.991 | 0.853 | 1.000 | 1.000 | 0.857 | 0.828 |
| 3841 | 0.779 | 1.000 | 0.808 | 1.000 | 0.958 | 0.960 | 0.884 | 1.000 | 0.990 | 1.000 | 0.962 | 1.000 | 1.000 | 0.857 | 1.000 | 1.000 | 0.858 | 0.829 |
| 3842 | 0.761 | 1.000 | 0.810 | 0.986 | 0.963 | 0.960 | 0.897 | 1.000 | 0.980 | 1.000 | 0.959 | 1.000 | 0.991 | 0.849 | 1.000 | 1.000 | 0.852 | 0.823 |
| 3843 | 0.771 | 1.000 | 0.814 | 1.000 | 0.963 | 0.960 | 0.900 | 1.000 | 0.980 | 1.000 | 0.974 | 0.970 | 1.000 | 0.847 | 1.000 | 1.000 | 0.856 | 0.828 |
| 3844 | 0.775 | 1.000 | 0.800 | 1.000 | 0.958 | 0.960 | 0.897 | 1.000 | 0.980 | 1.000 | 0.965 | 1.000 | 0.982 | 0.857 | 1.000 | 1.000 | 0.857 | 0.829 |
| 3845 | 0.771 | 1.000 | 0.800 | 0.986 | 0.963 | 0.960 | 0.903 | 1.000 | 1.000 | 1.000 | 0.965 | 0.970 | 0.991 | 0.853 | 1.000 | 1.000 | 0.856 | 0.828 |
| 3846 | 0.759 | 1.000 | 0.810 | 1.000 | 0.963 | 0.960 | 0.888 | 1.000 | 0.980 | 1.000 | 0.971 | 1.000 | 0.991 | 0.847 | 1.000 | 1.000 | 0.850 | 0.821 |
| 3847 | 0.771 | 1.000 | 0.794 | 0.971 | 0.958 | 0.960 | 0.900 | 1.000 | 0.990 | 1.000 | 0.957 | 0.970 | 0.991 | 0.858 | 1.000 | 1.000 | 0.855 | 0.826 |
| 3848 | 0.761 | 1.000 | 0.808 | 1.000 | 0.968 | 0.960 | 0.898 | 1.000 | 0.990 | 1.000 | 0.965 | 1.000 | 1.000 | 0.848 | 1.000 | 1.000 | 0.853 | 0.824 |
| 3849 | 0.775 | 0.971 | 0.804 | 1.000 | 0.963 | 0.960 | 0.898 | 1.000 | 0.980 | 1.000 | 0.962 | 1.000 | 0.991 | 0.844 | 1.000 | 1.000 | 0.855 | 0.826 |
| 3850 | 0.772 | 1.000 | 0.804 | 0.986 | 0.958 | 0.960 | 0.901 | 1.000 | 0.980 | 1.000 | 0.962 | 1.000 | 0.982 | 0.854 | 1.000 | 1.000 | 0.856 | 0.828 |
| 3851 | 0.765 | 1.000 | 0.798 | 1.000 | 0.958 | 0.960 | 0.906 | 1.000 | 1.000 | 1.000 | 0.951 | 1.000 | 0.982 | 0.855 | 1.000 | 1.000 | 0.854 | 0.826 |
| 3852 | 0.781 | 1.000 | 0.804 | 1.000 | 0.963 | 0.960 | 0.891 | 1.000 | 0.990 | 1.000 | 0.965 | 1.000 | 0.991 | 0.849 | 1.000 | 1.000 | 0.857 | 0.829 |
| 3853 | 0.774 | 1.000 | 0.810 | 0.986 | 0.963 | 0.960 | 0.898 | 1.000 | 1.000 | 1.000 | 0.965 | 1.000 | 0.982 | 0.853 | 1.000 | 1.000 | 0.858 | 0.829 |
| 3854 | 0.777 | 1.000 | 0.808 | 1.000 | 0.958 | 0.960 | 0.898 | 1.000 | 0.990 | 1.000 | 0.957 | 1.000 | 0.982 | 0.851 | 1.000 | 1.000 | 0.857 | 0.828 |
| 3855 | 0.771 | 1.000 | 0.810 | 0.986 | 0.963 | 0.960 | 0.901 | 1.000 | 0.990 | 1.000 | 0.965 | 1.000 | 0.991 | 0.856 | 1.000 | 1.000 | 0.858 | 0.830 |
| 3856 | 0.767 | 1.000 | 0.785 | 1.000 | 0.953 | 0.960 | 0.897 | 1.000 | 1.000 | 1.000 | 0.968 | 1.000 | 0.991 | 0.854 | 1.000 | 1.000 | 0.853 | 0.824 |
| 3857 | 0.764 | 1.000 | 0.785 | 1.000 | 0.963 | 0.960 | 0.901 | 1.000 | 0.980 | 1.000 | 0.962 | 1.000 | 0.982 | 0.845 | 1.000 | 1.000 | 0.850 | 0.820 |
| 3858 | 0.765 | 1.000 | 0.802 | 1.000 | 0.968 | 0.960 | 0.897 | 1.000 | 0.980 | 1.000 | 0.959 | 1.000 | 0.982 | 0.853 | 1.000 | 1.000 | 0.853 | 0.824 |
| 3859 | 0.765 | 1.000 | 0.785 | 1.000 | 0.968 | 0.960 | 0.894 | 1.000 | 1.000 | 1.000 | 0.965 | 1.000 | 0.982 | 0.854 | 1.000 | 1.000 | 0.852 | 0.823 |

| | | | | | | | | | | | | | | | | | |
|---|---|---|---|---|---|---|---|---|---|---|---|---|---|---|---|---|---|
| 3860 | 0.770 | 1.000 | 0.808 | 1.000 | 0.953 | 0.960 | 0.895 | 1.000 | 0.980 | 1.000 | 0.962 | 1.000 | 0.982 | 0.849 | 1.000 | 1.000 | 0.854 | 0.825 |
| 3861 | 0.773 | 1.000 | 0.806 | 1.000 | 0.963 | 0.960 | 0.889 | 1.000 | 0.990 | 1.000 | 0.959 | 1.000 | 0.991 | 0.846 | 1.000 | 1.000 | 0.854 | 0.824 |
| 3862 | 0.777 | 1.000 | 0.804 | 0.986 | 0.953 | 0.960 | 0.906 | 1.000 | 0.980 | 1.000 | 0.951 | 1.000 | 0.991 | 0.850 | 1.000 | 1.000 | 0.856 | 0.828 |
| 3863 | 0.768 | 1.000 | 0.804 | 1.000 | 0.968 | 0.960 | 0.900 | 1.000 | 0.969 | 1.000 | 0.957 | 1.000 | 0.991 | 0.857 | 1.000 | 1.000 | 0.856 | 0.827 |
| 3864 | 0.766 | 1.000 | 0.808 | 1.000 | 0.968 | 0.960 | 0.898 | 1.000 | 0.990 | 1.000 | 0.965 | 1.000 | 0.991 | 0.845 | 1.000 | 1.000 | 0.853 | 0.825 |
| 3865 | 0.772 | 1.000 | 0.802 | 1.000 | 0.958 | 0.960 | 0.898 | 1.000 | 0.990 | 1.000 | 0.957 | 1.000 | 0.991 | 0.840 | 1.000 | 1.000 | 0.852 | 0.823 |
| 3866 | 0.774 | 1.000 | 0.810 | 1.000 | 0.968 | 0.960 | 0.892 | 1.000 | 0.980 | 1.000 | 0.965 | 1.000 | 0.982 | 0.852 | 1.000 | 1.000 | 0.857 | 0.828 |
| 3867 | 0.775 | 1.000 | 0.814 | 1.000 | 0.963 | 0.960 | 0.901 | 1.000 | 0.980 | 1.000 | 0.959 | 1.000 | 0.991 | 0.852 | 1.000 | 1.000 | 0.858 | 0.830 |
| 3868 | 0.761 | 1.000 | 0.810 | 0.986 | 0.963 | 0.960 | 0.898 | 1.000 | 0.990 | 1.000 | 0.959 | 1.000 | 0.991 | 0.849 | 1.000 | 1.000 | 0.852 | 0.823 |
| 3869 | 0.762 | 1.000 | 0.804 | 0.986 | 0.958 | 0.960 | 0.901 | 1.000 | 0.990 | 1.000 | 0.965 | 0.970 | 0.991 | 0.852 | 1.000 | 1.000 | 0.853 | 0.825 |
| 3870 | 0.767 | 1.000 | 0.796 | 1.000 | 0.968 | 0.960 | 0.880 | 1.000 | 0.990 | 1.000 | 0.965 | 1.000 | 1.000 | 0.847 | 1.000 | 1.000 | 0.851 | 0.821 |
| 3871 | 0.767 | 1.000 | 0.812 | 1.000 | 0.958 | 0.960 | 0.897 | 1.000 | 0.980 | 1.000 | 0.962 | 0.970 | 1.000 | 0.849 | 1.000 | 1.000 | 0.854 | 0.826 |
| 3872 | 0.771 | 1.000 | 0.802 | 1.000 | 0.963 | 0.960 | 0.900 | 1.000 | 0.990 | 1.000 | 0.959 | 1.000 | 1.000 | 0.851 | 1.000 | 1.000 | 0.856 | 0.827 |
| 3873 | 0.769 | 1.000 | 0.802 | 1.000 | 0.963 | 0.960 | 0.894 | 1.000 | 1.000 | 1.000 | 0.962 | 1.000 | 1.000 | 0.851 | 1.000 | 1.000 | 0.855 | 0.826 |
| 3874 | 0.768 | 1.000 | 0.806 | 1.000 | 0.958 | 0.960 | 0.900 | 1.000 | 0.980 | 1.000 | 0.957 | 1.000 | 0.991 | 0.854 | 1.000 | 1.000 | 0.855 | 0.826 |
| 3875 | 0.771 | 1.000 | 0.792 | 1.000 | 0.968 | 0.960 | 0.900 | 1.000 | 1.000 | 1.000 | 0.957 | 0.970 | 0.991 | 0.854 | 1.000 | 1.000 | 0.855 | 0.826 |
| 3876 | 0.770 | 1.000 | 0.794 | 1.000 | 0.958 | 0.960 | 0.903 | 1.000 | 0.990 | 1.000 | 0.968 | 0.970 | 1.000 | 0.849 | 1.000 | 1.000 | 0.855 | 0.826 |
| 3877 | 0.770 | 1.000 | 0.802 | 0.986 | 0.963 | 0.960 | 0.906 | 1.000 | 0.980 | 1.000 | 0.962 | 1.000 | 0.991 | 0.849 | 1.000 | 1.000 | 0.855 | 0.827 |
| 3878 | 0.765 | 1.000 | 0.798 | 1.000 | 0.953 | 0.960 | 0.891 | 1.000 | 0.990 | 1.000 | 0.968 | 1.000 | 1.000 | 0.851 | 1.000 | 1.000 | 0.853 | 0.823 |
| 3879 | 0.765 | 0.971 | 0.796 | 1.000 | 0.963 | 0.960 | 0.903 | 1.000 | 0.990 | 1.000 | 0.962 | 1.000 | 0.991 | 0.858 | 1.000 | 1.000 | 0.855 | 0.827 |
| 3880 | 0.775 | 1.000 | 0.806 | 1.000 | 0.963 | 0.960 | 0.892 | 1.000 | 0.990 | 1.000 | 0.965 | 0.970 | 0.991 | 0.851 | 1.000 | 1.000 | 0.856 | 0.828 |
| 3881 | 0.769 | 1.000 | 0.802 | 1.000 | 0.963 | 0.960 | 0.894 | 1.000 | 0.990 | 1.000 | 0.965 | 1.000 | 1.000 | 0.857 | 1.000 | 1.000 | 0.856 | 0.827 |
| 3882 | 0.766 | 1.000 | 0.798 | 1.000 | 0.963 | 0.960 | 0.906 | 1.000 | 0.990 | 1.000 | 0.957 | 1.000 | 0.982 | 0.854 | 1.000 | 1.000 | 0.855 | 0.826 |
| 3883 | 0.777 | 1.000 | 0.808 | 0.986 | 0.968 | 0.960 | 0.900 | 1.000 | 0.980 | 1.000 | 0.962 | 1.000 | 0.991 | 0.855 | 1.000 | 1.000 | 0.859 | 0.830 |
| 3884 | 0.771 | 1.000 | 0.804 | 1.000 | 0.968 | 0.960 | 0.898 | 1.000 | 0.990 | 1.000 | 0.959 | 0.970 | 0.982 | 0.855 | 1.000 | 1.000 | 0.856 | 0.827 |
| 3885 | 0.768 | 1.000 | 0.812 | 1.000 | 0.963 | 0.960 | 0.903 | 1.000 | 0.990 | 1.000 | 0.959 | 1.000 | 0.991 | 0.853 | 1.000 | 1.000 | 0.856 | 0.828 |
| 3886 | 0.768 | 1.000 | 0.808 | 1.000 | 0.968 | 0.960 | 0.904 | 1.000 | 1.000 | 1.000 | 0.965 | 1.000 | 1.000 | 0.854 | 1.000 | 1.000 | 0.857 | 0.829 |
| 3887 | 0.775 | 1.000 | 0.798 | 1.000 | 0.963 | 0.960 | 0.900 | 1.000 | 0.980 | 1.000 | 0.957 | 1.000 | 0.991 | 0.851 | 1.000 | 1.000 | 0.856 | 0.827 |
| 3888 | 0.773 | 1.000 | 0.816 | 0.986 | 0.958 | 0.960 | 0.897 | 1.000 | 0.969 | 1.000 | 0.962 | 1.000 | 1.000 | 0.861 | 1.000 | 1.000 | 0.859 | 0.831 |
| 3889 | 0.779 | 1.000 | 0.800 | 1.000 | 0.963 | 0.960 | 0.898 | 1.000 | 0.980 | 1.000 | 0.965 | 1.000 | 0.982 | 0.848 | 1.000 | 1.000 | 0.857 | 0.828 |
| 3890 | 0.775 | 1.000 | 0.787 | 0.986 | 0.963 | 0.960 | 0.898 | 1.000 | 0.980 | 1.000 | 0.965 | 1.000 | 0.991 | 0.854 | 1.000 | 1.000 | 0.855 | 0.827 |
| 3891 | 0.767 | 1.000 | 0.789 | 1.000 | 0.963 | 0.960 | 0.895 | 1.000 | 1.000 | 1.000 | 0.968 | 1.000 | 0.991 | 0.849 | 1.000 | 1.000 | 0.852 | 0.823 |
| 3892 | 0.771 | 1.000 | 0.798 | 1.000 | 0.963 | 0.960 | 0.897 | 1.000 | 0.990 | 1.000 | 0.965 | 1.000 | 0.991 | 0.854 | 1.000 | 1.000 | 0.856 | 0.827 |
| 3893 | 0.762 | 1.000 | 0.791 | 0.986 | 0.963 | 0.960 | 0.900 | 1.000 | 0.980 | 1.000 | 0.962 | 1.000 | 0.991 | 0.854 | 1.000 | 1.000 | 0.852 | 0.823 |
| 3894 | 0.769 | 1.000 | 0.791 | 1.000 | 0.963 | 0.960 | 0.891 | 1.000 | 0.990 | 1.000 | 0.968 | 1.000 | 0.991 | 0.854 | 1.000 | 1.000 | 0.854 | 0.825 |
| 3895 | 0.766 | 1.000 | 0.791 | 1.000 | 0.958 | 0.960 | 0.901 | 1.000 | 0.980 | 1.000 | 0.959 | 1.000 | 0.991 | 0.857 | 1.000 | 1.000 | 0.854 | 0.825 |
| 3896 | 0.763 | 0.971 | 0.808 | 1.000 | 0.963 | 0.960 | 0.900 | 1.000 | 0.990 | 1.000 | 0.965 | 1.000 | 0.991 | 0.854 | 1.000 | 1.000 | 0.855 | 0.826 |
| 3897 | 0.766 | 1.000 | 0.796 | 1.000 | 0.963 | 0.960 | 0.886 | 1.000 | 0.969 | 1.000 | 0.965 | 1.000 | 0.991 | 0.851 | 1.000 | 1.000 | 0.851 | 0.822 |
| 3898 | 0.775 | 1.000 | 0.812 | 1.000 | 0.968 | 0.960 | 0.900 | 1.000 | 0.980 | 1.000 | 0.962 | 1.000 | 0.991 | 0.854 | 1.000 | 1.000 | 0.859 | 0.831 |

| | | | | | | | | | | | | | | | | | |
|---|---|---|---|---|---|---|---|---|---|---|---|---|---|---|---|---|---|
| 3899 | 0.773 | 1.000 | 0.810 | 1.000 | 0.953 | 0.960 | 0.900 | 1.000 | 0.980 | 1.000 | 0.968 | 1.000 | 0.982 | 0.851 | 1.000 | 1.000 | 0.857 | 0.828 |
| 3900 | 0.771 | 1.000 | 0.796 | 1.000 | 0.963 | 0.960 | 0.903 | 1.000 | 0.980 | 1.000 | 0.962 | 1.000 | 0.982 | 0.856 | 1.000 | 1.000 | 0.856 | 0.828 |
| 3901 | 0.776 | 1.000 | 0.806 | 1.000 | 0.963 | 0.960 | 0.894 | 1.000 | 1.000 | 1.000 | 0.957 | 1.000 | 1.000 | 0.845 | 1.000 | 1.000 | 0.855 | 0.826 |
| 3902 | 0.768 | 1.000 | 0.808 | 1.000 | 0.968 | 0.960 | 0.898 | 1.000 | 1.000 | 1.000 | 0.968 | 1.000 | 1.000 | 0.852 | 1.000 | 1.000 | 0.856 | 0.828 |
| 3903 | 0.765 | 1.000 | 0.802 | 1.000 | 0.963 | 0.960 | 0.908 | 1.000 | 1.000 | 1.000 | 0.959 | 1.000 | 1.000 | 0.849 | 1.000 | 1.000 | 0.855 | 0.826 |
| 3904 | 0.764 | 1.000 | 0.812 | 1.000 | 0.963 | 0.960 | 0.900 | 1.000 | 1.000 | 1.000 | 0.959 | 1.000 | 0.991 | 0.847 | 1.000 | 1.000 | 0.854 | 0.825 |
| 3905 | 0.768 | 1.000 | 0.808 | 1.000 | 0.968 | 0.960 | 0.895 | 1.000 | 0.990 | 1.000 | 0.965 | 1.000 | 1.000 | 0.854 | 1.000 | 1.000 | 0.856 | 0.828 |
| 3906 | 0.781 | 1.000 | 0.808 | 1.000 | 0.963 | 0.960 | 0.901 | 1.000 | 0.990 | 1.000 | 0.971 | 1.000 | 1.000 | 0.851 | 1.000 | 1.000 | 0.860 | 0.832 |
| 3907 | 0.775 | 1.000 | 0.796 | 1.000 | 0.963 | 0.960 | 0.894 | 1.000 | 0.990 | 1.000 | 0.962 | 1.000 | 1.000 | 0.852 | 1.000 | 1.000 | 0.856 | 0.827 |
| 3908 | 0.769 | 1.000 | 0.794 | 0.986 | 0.963 | 0.960 | 0.909 | 1.000 | 0.990 | 1.000 | 0.965 | 1.000 | 1.000 | 0.851 | 1.000 | 1.000 | 0.856 | 0.827 |
| 3909 | 0.763 | 1.000 | 0.796 | 1.000 | 0.958 | 0.960 | 0.894 | 1.000 | 0.990 | 1.000 | 0.962 | 0.970 | 0.991 | 0.850 | 1.000 | 1.000 | 0.851 | 0.822 |
| 3910 | 0.761 | 1.000 | 0.810 | 1.000 | 0.968 | 0.960 | 0.901 | 1.000 | 1.000 | 1.000 | 0.971 | 0.970 | 0.991 | 0.854 | 1.000 | 1.000 | 0.855 | 0.827 |
| 3911 | 0.768 | 1.000 | 0.804 | 0.986 | 0.968 | 0.960 | 0.901 | 1.000 | 1.000 | 1.000 | 0.965 | 0.970 | 0.991 | 0.853 | 1.000 | 1.000 | 0.856 | 0.827 |
| 3912 | 0.767 | 1.000 | 0.806 | 1.000 | 0.968 | 0.960 | 0.901 | 1.000 | 0.980 | 1.000 | 0.971 | 1.000 | 0.991 | 0.856 | 1.000 | 1.000 | 0.857 | 0.829 |
| 3913 | 0.769 | 1.000 | 0.806 | 1.000 | 0.958 | 0.960 | 0.901 | 1.000 | 1.000 | 1.000 | 0.959 | 1.000 | 0.991 | 0.853 | 1.000 | 1.000 | 0.856 | 0.827 |
| 3914 | 0.767 | 1.000 | 0.824 | 1.000 | 0.958 | 0.960 | 0.889 | 1.000 | 0.980 | 1.000 | 0.965 | 1.000 | 0.991 | 0.857 | 1.000 | 1.000 | 0.857 | 0.828 |
| 3915 | 0.771 | 1.000 | 0.808 | 1.000 | 0.963 | 0.960 | 0.894 | 1.000 | 0.990 | 1.000 | 0.968 | 1.000 | 0.991 | 0.851 | 1.000 | 1.000 | 0.856 | 0.827 |
| 3916 | 0.759 | 1.000 | 0.808 | 1.000 | 0.963 | 0.960 | 0.911 | 1.000 | 0.990 | 1.000 | 0.977 | 1.000 | 0.982 | 0.855 | 1.000 | 1.000 | 0.856 | 0.828 |
| 3917 | 0.772 | 1.000 | 0.800 | 1.000 | 0.963 | 0.960 | 0.897 | 1.000 | 0.990 | 1.000 | 0.965 | 1.000 | 1.000 | 0.859 | 1.000 | 1.000 | 0.858 | 0.829 |
| 3918 | 0.773 | 1.000 | 0.808 | 0.986 | 0.968 | 0.960 | 0.898 | 1.000 | 0.969 | 1.000 | 0.962 | 1.000 | 1.000 | 0.853 | 1.000 | 1.000 | 0.857 | 0.829 |
| 3919 | 0.767 | 1.000 | 0.824 | 0.986 | 0.963 | 0.960 | 0.903 | 1.000 | 0.980 | 1.000 | 0.959 | 1.000 | 0.991 | 0.848 | 1.000 | 1.000 | 0.856 | 0.827 |
| 3920 | 0.767 | 1.000 | 0.808 | 0.986 | 0.963 | 0.960 | 0.898 | 1.000 | 0.969 | 1.000 | 0.968 | 1.000 | 0.991 | 0.852 | 1.000 | 1.000 | 0.855 | 0.826 |
| 3921 | 0.775 | 1.000 | 0.808 | 0.986 | 0.958 | 0.960 | 0.892 | 1.000 | 0.990 | 1.000 | 0.968 | 1.000 | 0.982 | 0.855 | 1.000 | 1.000 | 0.857 | 0.829 |
| 3922 | 0.768 | 1.000 | 0.814 | 1.000 | 0.968 | 0.960 | 0.897 | 1.000 | 0.990 | 1.000 | 0.962 | 1.000 | 1.000 | 0.858 | 1.000 | 1.000 | 0.858 | 0.830 |
| 3923 | 0.770 | 1.000 | 0.798 | 1.000 | 0.958 | 0.960 | 0.900 | 1.000 | 0.969 | 1.000 | 0.962 | 1.000 | 0.991 | 0.851 | 1.000 | 1.000 | 0.854 | 0.825 |
| 3924 | 0.768 | 1.000 | 0.798 | 1.000 | 0.963 | 0.960 | 0.901 | 1.000 | 0.980 | 1.000 | 0.957 | 0.970 | 0.991 | 0.850 | 1.000 | 1.000 | 0.853 | 0.824 |
| 3925 | 0.764 | 1.000 | 0.812 | 1.000 | 0.958 | 0.960 | 0.892 | 1.000 | 1.000 | 1.000 | 0.962 | 0.970 | 0.982 | 0.848 | 1.000 | 1.000 | 0.852 | 0.823 |
| 3926 | 0.767 | 1.000 | 0.808 | 1.000 | 0.958 | 0.960 | 0.904 | 1.000 | 1.000 | 1.000 | 0.965 | 1.000 | 0.982 | 0.849 | 1.000 | 1.000 | 0.855 | 0.827 |
| 3927 | 0.764 | 1.000 | 0.806 | 1.000 | 0.963 | 0.960 | 0.898 | 1.000 | 0.969 | 1.000 | 0.968 | 0.970 | 0.982 | 0.848 | 1.000 | 1.000 | 0.853 | 0.824 |
| 3928 | 0.773 | 1.000 | 0.820 | 1.000 | 0.958 | 0.960 | 0.903 | 1.000 | 0.980 | 1.000 | 0.971 | 1.000 | 1.000 | 0.848 | 1.000 | 1.000 | 0.858 | 0.830 |
| 3929 | 0.776 | 1.000 | 0.800 | 1.000 | 0.963 | 0.960 | 0.891 | 1.000 | 0.990 | 1.000 | 0.962 | 1.000 | 0.991 | 0.853 | 1.000 | 1.000 | 0.856 | 0.827 |
| 3930 | 0.764 | 1.000 | 0.791 | 1.000 | 0.963 | 0.960 | 0.904 | 1.000 | 0.990 | 1.000 | 0.957 | 1.000 | 1.000 | 0.847 | 1.000 | 1.000 | 0.852 | 0.823 |
| 3931 | 0.775 | 1.000 | 0.802 | 1.000 | 0.968 | 0.960 | 0.897 | 1.000 | 1.000 | 1.000 | 0.962 | 1.000 | 0.982 | 0.850 | 1.000 | 1.000 | 0.856 | 0.828 |
| 3932 | 0.772 | 1.000 | 0.812 | 1.000 | 0.963 | 0.960 | 0.892 | 1.000 | 0.990 | 1.000 | 0.965 | 1.000 | 1.000 | 0.851 | 1.000 | 1.000 | 0.856 | 0.828 |
| 3933 | 0.780 | 1.000 | 0.792 | 1.000 | 0.958 | 0.960 | 0.901 | 1.000 | 0.990 | 1.000 | 0.974 | 1.000 | 0.982 | 0.858 | 1.000 | 1.000 | 0.860 | 0.831 |
| 3934 | 0.775 | 1.000 | 0.804 | 0.971 | 0.963 | 0.960 | 0.904 | 1.000 | 1.000 | 1.000 | 0.959 | 0.970 | 0.982 | 0.845 | 1.000 | 1.000 | 0.855 | 0.827 |
| 3935 | 0.762 | 1.000 | 0.810 | 0.986 | 0.958 | 0.960 | 0.898 | 1.000 | 0.990 | 1.000 | 0.971 | 1.000 | 0.982 | 0.849 | 1.000 | 1.000 | 0.853 | 0.824 |
| 3936 | 0.761 | 1.000 | 0.806 | 1.000 | 0.963 | 0.960 | 0.895 | 1.000 | 0.980 | 1.000 | 0.968 | 1.000 | 0.991 | 0.847 | 1.000 | 1.000 | 0.852 | 0.823 |
| 3937 | 0.761 | 1.000 | 0.808 | 1.000 | 0.963 | 0.960 | 0.901 | 1.000 | 0.990 | 1.000 | 0.959 | 1.000 | 0.982 | 0.850 | 1.000 | 1.000 | 0.853 | 0.824 |

| | | | | | | | | | | | | | | | | | | |
|---|---|---|---|---|---|---|---|---|---|---|---|---|---|---|---|---|---|---|
| 3938 | 0.770 | 1.000 | 0.802 | 1.000 | 0.953 | 0.960 | 0.891 | 1.000 | 0.990 | 1.000 | 0.968 | 1.000 | 0.991 | 0.851 | 1.000 | 1.000 | 0.854 | 0.825 |
| 3939 | 0.776 | 1.000 | 0.804 | 1.000 | 0.963 | 0.960 | 0.903 | 1.000 | 0.969 | 1.000 | 0.962 | 1.000 | 1.000 | 0.851 | 1.000 | 1.000 | 0.857 | 0.829 |
| 3940 | 0.763 | 1.000 | 0.814 | 0.986 | 0.953 | 0.960 | 0.891 | 1.000 | 0.980 | 1.000 | 0.954 | 1.000 | 0.982 | 0.859 | 1.000 | 1.000 | 0.854 | 0.825 |
| 3941 | 0.764 | 1.000 | 0.798 | 1.000 | 0.963 | 0.960 | 0.891 | 1.000 | 0.990 | 1.000 | 0.965 | 1.000 | 1.000 | 0.854 | 1.000 | 1.000 | 0.853 | 0.824 |
| 3942 | 0.771 | 1.000 | 0.806 | 1.000 | 0.963 | 0.960 | 0.895 | 1.000 | 0.969 | 1.000 | 0.962 | 1.000 | 0.982 | 0.849 | 1.000 | 1.000 | 0.854 | 0.825 |
| 3943 | 0.767 | 1.000 | 0.798 | 1.000 | 0.963 | 0.960 | 0.895 | 1.000 | 1.000 | 1.000 | 0.962 | 1.000 | 0.991 | 0.852 | 1.000 | 1.000 | 0.854 | 0.825 |
| 3944 | 0.770 | 1.000 | 0.800 | 1.000 | 0.958 | 0.960 | 0.891 | 1.000 | 0.980 | 1.000 | 0.962 | 1.000 | 0.991 | 0.857 | 1.000 | 1.000 | 0.855 | 0.826 |
| 3945 | 0.773 | 1.000 | 0.818 | 1.000 | 0.968 | 0.960 | 0.897 | 1.000 | 0.980 | 1.000 | 0.968 | 1.000 | 1.000 | 0.848 | 1.000 | 1.000 | 0.857 | 0.829 |
| 3946 | 0.773 | 1.000 | 0.792 | 0.986 | 0.963 | 0.960 | 0.903 | 1.000 | 1.000 | 1.000 | 0.965 | 1.000 | 0.991 | 0.851 | 1.000 | 1.000 | 0.856 | 0.827 |
| 3947 | 0.765 | 1.000 | 0.791 | 0.986 | 0.963 | 0.960 | 0.901 | 1.000 | 0.990 | 1.000 | 0.954 | 1.000 | 1.000 | 0.850 | 1.000 | 1.000 | 0.852 | 0.823 |
| 3948 | 0.770 | 1.000 | 0.798 | 0.971 | 0.968 | 0.960 | 0.892 | 1.000 | 0.990 | 1.000 | 0.959 | 1.000 | 0.991 | 0.851 | 1.000 | 1.000 | 0.853 | 0.824 |
| 3949 | 0.773 | 1.000 | 0.798 | 1.000 | 0.963 | 0.960 | 0.897 | 1.000 | 1.000 | 1.000 | 0.962 | 1.000 | 0.991 | 0.847 | 1.000 | 1.000 | 0.855 | 0.826 |
| 3950 | 0.764 | 1.000 | 0.796 | 1.000 | 0.963 | 0.960 | 0.900 | 1.000 | 1.000 | 1.000 | 0.962 | 1.000 | 0.991 | 0.853 | 1.000 | 1.000 | 0.854 | 0.825 |
| 3951 | 0.771 | 1.000 | 0.810 | 1.000 | 0.958 | 0.960 | 0.895 | 1.000 | 0.990 | 1.000 | 0.965 | 1.000 | 0.982 | 0.857 | 1.000 | 1.000 | 0.857 | 0.829 |
| 3952 | 0.779 | 1.000 | 0.804 | 0.986 | 0.963 | 0.960 | 0.888 | 1.000 | 1.000 | 1.000 | 0.959 | 1.000 | 1.000 | 0.852 | 1.000 | 1.000 | 0.857 | 0.828 |
| 3953 | 0.765 | 1.000 | 0.794 | 1.000 | 0.968 | 0.960 | 0.898 | 1.000 | 0.980 | 1.000 | 0.965 | 0.970 | 0.991 | 0.854 | 1.000 | 1.000 | 0.854 | 0.825 |
| 3954 | 0.764 | 1.000 | 0.794 | 1.000 | 0.963 | 0.960 | 0.897 | 1.000 | 0.990 | 1.000 | 0.959 | 1.000 | 0.991 | 0.851 | 1.000 | 1.000 | 0.852 | 0.823 |
| 3955 | 0.765 | 1.000 | 0.810 | 1.000 | 0.963 | 0.960 | 0.903 | 1.000 | 0.969 | 1.000 | 0.968 | 1.000 | 0.991 | 0.848 | 1.000 | 1.000 | 0.854 | 0.826 |
| 3956 | 0.767 | 1.000 | 0.806 | 1.000 | 0.963 | 0.960 | 0.908 | 1.000 | 1.000 | 1.000 | 0.962 | 1.000 | 0.991 | 0.850 | 1.000 | 1.000 | 0.856 | 0.827 |
| 3957 | 0.764 | 1.000 | 0.808 | 0.986 | 0.968 | 0.960 | 0.901 | 1.000 | 0.990 | 1.000 | 0.957 | 0.970 | 0.991 | 0.851 | 1.000 | 1.000 | 0.854 | 0.825 |
| 3958 | 0.767 | 1.000 | 0.796 | 1.000 | 0.968 | 0.960 | 0.906 | 1.000 | 0.990 | 1.000 | 0.962 | 1.000 | 0.991 | 0.852 | 1.000 | 1.000 | 0.855 | 0.827 |
| 3959 | 0.769 | 1.000 | 0.802 | 1.000 | 0.974 | 0.960 | 0.904 | 1.000 | 0.990 | 1.000 | 0.957 | 1.000 | 1.000 | 0.848 | 1.000 | 1.000 | 0.855 | 0.827 |
| 3960 | 0.773 | 1.000 | 0.787 | 1.000 | 0.968 | 0.960 | 0.904 | 1.000 | 0.990 | 1.000 | 0.959 | 1.000 | 1.000 | 0.851 | 1.000 | 1.000 | 0.855 | 0.826 |
| 3961 | 0.769 | 1.000 | 0.812 | 1.000 | 0.974 | 0.960 | 0.903 | 1.000 | 1.000 | 1.000 | 0.965 | 0.970 | 0.982 | 0.858 | 1.000 | 1.000 | 0.859 | 0.831 |
| 3962 | 0.765 | 1.000 | 0.810 | 1.000 | 0.974 | 0.960 | 0.904 | 1.000 | 1.000 | 1.000 | 0.962 | 1.000 | 0.991 | 0.851 | 1.000 | 1.000 | 0.856 | 0.828 |
| 3963 | 0.769 | 1.000 | 0.804 | 0.986 | 0.968 | 0.960 | 0.894 | 1.000 | 0.990 | 1.000 | 0.974 | 1.000 | 0.982 | 0.854 | 1.000 | 1.000 | 0.856 | 0.827 |
| 3964 | 0.773 | 1.000 | 0.800 | 0.986 | 0.968 | 0.960 | 0.900 | 1.000 | 1.000 | 1.000 | 0.957 | 1.000 | 0.982 | 0.848 | 1.000 | 1.000 | 0.855 | 0.826 |
| 3965 | 0.772 | 1.000 | 0.814 | 1.000 | 0.968 | 0.960 | 0.911 | 1.000 | 1.000 | 1.000 | 0.962 | 1.000 | 1.000 | 0.858 | 1.000 | 1.000 | 0.861 | 0.833 |
| 3966 | 0.768 | 1.000 | 0.816 | 1.000 | 0.968 | 0.960 | 0.894 | 1.000 | 1.000 | 1.000 | 0.962 | 1.000 | 1.000 | 0.844 | 1.000 | 1.000 | 0.854 | 0.825 |
| 3967 | 0.769 | 1.000 | 0.834 | 0.986 | 0.974 | 0.960 | 0.906 | 1.000 | 1.000 | 1.000 | 0.962 | 1.000 | 0.982 | 0.858 | 1.000 | 1.000 | 0.861 | 0.834 |
| 3968 | 0.772 | 1.000 | 0.830 | 0.986 | 0.974 | 0.960 | 0.903 | 1.000 | 0.980 | 1.000 | 0.965 | 1.000 | 1.000 | 0.857 | 1.000 | 1.000 | 0.861 | 0.833 |
| 3969 | 0.765 | 1.000 | 0.802 | 1.000 | 0.974 | 0.960 | 0.897 | 1.000 | 0.990 | 1.000 | 0.959 | 1.000 | 0.991 | 0.854 | 1.000 | 1.000 | 0.854 | 0.826 |
| 3970 | 0.765 | 1.000 | 0.812 | 1.000 | 0.958 | 0.960 | 0.900 | 1.000 | 0.990 | 1.000 | 0.965 | 1.000 | 1.000 | 0.858 | 1.000 | 1.000 | 0.857 | 0.828 |
| 3971 | 0.765 | 1.000 | 0.806 | 1.000 | 0.974 | 0.960 | 0.900 | 1.000 | 0.980 | 1.000 | 0.971 | 1.000 | 0.982 | 0.853 | 1.000 | 1.000 | 0.855 | 0.827 |
| 3972 | 0.779 | 1.000 | 0.812 | 1.000 | 0.974 | 0.960 | 0.900 | 1.000 | 0.990 | 1.000 | 0.962 | 1.000 | 0.991 | 0.849 | 1.000 | 1.000 | 0.859 | 0.831 |
| 3973 | 0.773 | 1.000 | 0.802 | 1.000 | 0.968 | 0.960 | 0.897 | 1.000 | 0.990 | 1.000 | 0.974 | 0.970 | 0.991 | 0.856 | 1.000 | 1.000 | 0.858 | 0.830 |
| 3974 | 0.782 | 0.971 | 0.794 | 1.000 | 0.958 | 0.960 | 0.897 | 1.000 | 0.990 | 1.000 | 0.965 | 1.000 | 0.991 | 0.843 | 1.000 | 1.000 | 0.855 | 0.826 |
| 3975 | 0.775 | 1.000 | 0.812 | 1.000 | 0.963 | 0.960 | 0.895 | 1.000 | 0.980 | 1.000 | 0.968 | 0.970 | 0.982 | 0.852 | 1.000 | 1.000 | 0.857 | 0.828 |
| 3976 | 0.777 | 1.000 | 0.796 | 1.000 | 0.979 | 0.960 | 0.909 | 1.000 | 0.990 | 1.000 | 0.968 | 1.000 | 1.000 | 0.858 | 1.000 | 1.000 | 0.861 | 0.833 |

| | | | | | | | | | | | | | | | | | |
|---|---|---|---|---|---|---|---|---|---|---|---|---|---|---|---|---|---|
| 3977 | 0.767 | 1.000 | 0.804 | 1.000 | 0.958 | 0.960 | 0.903 | 1.000 | 0.990 | 1.000 | 0.957 | 1.000 | 0.982 | 0.846 | 1.000 | 1.000 | 0.853 | 0.824 |
| 3978 | 0.769 | 1.000 | 0.806 | 0.986 | 0.963 | 0.960 | 0.897 | 1.000 | 0.980 | 1.000 | 0.965 | 1.000 | 1.000 | 0.861 | 1.000 | 1.000 | 0.858 | 0.829 |
| 3979 | 0.775 | 1.000 | 0.820 | 1.000 | 0.963 | 0.960 | 0.900 | 1.000 | 0.990 | 1.000 | 0.971 | 1.000 | 1.000 | 0.858 | 1.000 | 1.000 | 0.861 | 0.833 |
| 3980 | 0.771 | 1.000 | 0.812 | 1.000 | 0.963 | 0.960 | 0.894 | 1.000 | 0.990 | 1.000 | 0.957 | 0.970 | 1.000 | 0.854 | 1.000 | 1.000 | 0.856 | 0.827 |
| 3981 | 0.775 | 1.000 | 0.808 | 1.000 | 0.974 | 0.960 | 0.892 | 1.000 | 0.980 | 1.000 | 0.957 | 0.970 | 0.991 | 0.851 | 1.000 | 1.000 | 0.856 | 0.827 |
| 3982 | 0.772 | 1.000 | 0.818 | 1.000 | 0.963 | 0.960 | 0.892 | 1.000 | 0.980 | 1.000 | 0.962 | 1.000 | 0.991 | 0.854 | 1.000 | 1.000 | 0.857 | 0.829 |
| 3983 | 0.779 | 1.000 | 0.810 | 1.000 | 0.968 | 0.960 | 0.891 | 1.000 | 1.000 | 1.000 | 0.965 | 1.000 | 1.000 | 0.855 | 1.000 | 1.000 | 0.860 | 0.831 |
| 3984 | 0.767 | 1.000 | 0.818 | 1.000 | 0.963 | 0.960 | 0.904 | 1.000 | 0.980 | 1.000 | 0.951 | 1.000 | 0.991 | 0.854 | 1.000 | 1.000 | 0.856 | 0.828 |
| 3985 | 0.771 | 1.000 | 0.812 | 1.000 | 0.968 | 0.960 | 0.904 | 1.000 | 0.990 | 1.000 | 0.962 | 1.000 | 0.991 | 0.855 | 1.000 | 1.000 | 0.859 | 0.830 |
| 3986 | 0.777 | 1.000 | 0.804 | 1.000 | 0.968 | 0.960 | 0.891 | 1.000 | 0.990 | 1.000 | 0.959 | 0.970 | 0.991 | 0.853 | 1.000 | 1.000 | 0.857 | 0.828 |
| 3987 | 0.780 | 1.000 | 0.796 | 1.000 | 0.979 | 0.960 | 0.895 | 1.000 | 0.980 | 1.000 | 0.959 | 1.000 | 0.991 | 0.860 | 1.000 | 1.000 | 0.860 | 0.831 |
| 3988 | 0.769 | 1.000 | 0.796 | 0.986 | 0.968 | 0.960 | 0.894 | 1.000 | 0.990 | 1.000 | 0.962 | 1.000 | 1.000 | 0.858 | 1.000 | 1.000 | 0.855 | 0.827 |
| 3989 | 0.765 | 1.000 | 0.802 | 0.986 | 0.963 | 0.960 | 0.906 | 1.000 | 0.969 | 1.000 | 0.957 | 1.000 | 1.000 | 0.853 | 1.000 | 1.000 | 0.854 | 0.825 |
| 3990 | 0.774 | 1.000 | 0.812 | 1.000 | 0.968 | 0.960 | 0.897 | 1.000 | 0.990 | 1.000 | 0.954 | 1.000 | 1.000 | 0.858 | 1.000 | 1.000 | 0.859 | 0.831 |
| 3991 | 0.770 | 1.000 | 0.812 | 1.000 | 0.963 | 0.960 | 0.900 | 1.000 | 0.990 | 1.000 | 0.951 | 1.000 | 0.982 | 0.854 | 1.000 | 1.000 | 0.856 | 0.828 |
| 3992 | 0.771 | 1.000 | 0.808 | 0.986 | 0.963 | 0.960 | 0.892 | 1.000 | 0.990 | 1.000 | 0.962 | 1.000 | 1.000 | 0.861 | 1.000 | 1.000 | 0.858 | 0.829 |
| 3993 | 0.771 | 1.000 | 0.822 | 1.000 | 0.968 | 0.960 | 0.895 | 1.000 | 0.990 | 1.000 | 0.948 | 1.000 | 0.991 | 0.857 | 1.000 | 1.000 | 0.858 | 0.830 |
| 3994 | 0.769 | 1.000 | 0.802 | 0.986 | 0.963 | 0.960 | 0.894 | 1.000 | 0.980 | 1.000 | 0.965 | 1.000 | 0.991 | 0.849 | 1.000 | 1.000 | 0.853 | 0.824 |
| 3995 | 0.765 | 1.000 | 0.826 | 1.000 | 0.958 | 0.960 | 0.897 | 1.000 | 0.990 | 1.000 | 0.957 | 1.000 | 0.982 | 0.848 | 1.000 | 1.000 | 0.854 | 0.826 |
| 3996 | 0.763 | 1.000 | 0.812 | 1.000 | 0.968 | 0.960 | 0.901 | 1.000 | 1.000 | 1.000 | 0.951 | 1.000 | 0.991 | 0.861 | 1.000 | 1.000 | 0.857 | 0.828 |
| 3997 | 0.768 | 1.000 | 0.816 | 1.000 | 0.974 | 0.960 | 0.904 | 1.000 | 0.980 | 1.000 | 0.959 | 0.970 | 0.991 | 0.861 | 1.000 | 1.000 | 0.859 | 0.831 |
| 3998 | 0.778 | 1.000 | 0.824 | 1.000 | 0.979 | 0.960 | 0.908 | 1.000 | 1.000 | 1.000 | 0.959 | 1.000 | 0.991 | 0.859 | 1.000 | 1.000 | 0.864 | 0.837 |
| 3999 | 0.762 | 1.000 | 0.808 | 1.000 | 0.974 | 0.960 | 0.892 | 1.000 | 1.000 | 1.000 | 0.945 | 1.000 | 0.991 | 0.847 | 1.000 | 1.000 | 0.851 | 0.821 |
| 4000 | 0.773 | 1.000 | 0.789 | 1.000 | 0.979 | 0.960 | 0.892 | 1.000 | 0.990 | 1.000 | 0.945 | 1.000 | 0.991 | 0.857 | 1.000 | 1.000 | 0.855 | 0.826 |
| 4001 | 0.765 | 1.000 | 0.812 | 0.986 | 0.979 | 0.960 | 0.892 | 1.000 | 0.980 | 1.000 | 0.951 | 1.000 | 1.000 | 0.858 | 1.000 | 1.000 | 0.855 | 0.826 |
| 4002 | 0.765 | 1.000 | 0.796 | 0.986 | 0.979 | 0.960 | 0.897 | 1.000 | 0.980 | 1.000 | 0.957 | 1.000 | 0.991 | 0.853 | 1.000 | 1.000 | 0.853 | 0.824 |
| 4003 | 0.771 | 1.000 | 0.802 | 1.000 | 0.974 | 0.960 | 0.900 | 1.000 | 0.990 | 1.000 | 0.962 | 1.000 | 0.991 | 0.861 | 1.000 | 1.000 | 0.859 | 0.831 |
| 4004 | 0.767 | 1.000 | 0.812 | 1.000 | 0.974 | 0.960 | 0.909 | 1.000 | 0.980 | 1.000 | 0.959 | 1.000 | 0.991 | 0.857 | 1.000 | 1.000 | 0.858 | 0.830 |
| 4005 | 0.773 | 1.000 | 0.804 | 0.986 | 0.963 | 0.960 | 0.884 | 1.000 | 0.990 | 1.000 | 0.957 | 1.000 | 0.991 | 0.850 | 1.000 | 1.000 | 0.853 | 0.824 |
| 4006 | 0.771 | 1.000 | 0.804 | 1.000 | 0.974 | 0.960 | 0.895 | 1.000 | 0.980 | 1.000 | 0.954 | 1.000 | 0.991 | 0.854 | 1.000 | 1.000 | 0.856 | 0.827 |
| 4007 | 0.762 | 1.000 | 0.812 | 1.000 | 0.974 | 0.960 | 0.904 | 1.000 | 0.980 | 1.000 | 0.965 | 1.000 | 0.991 | 0.851 | 1.000 | 1.000 | 0.855 | 0.827 |
| 4008 | 0.765 | 1.000 | 0.810 | 1.000 | 0.974 | 0.960 | 0.886 | 1.000 | 0.990 | 1.000 | 0.962 | 1.000 | 0.991 | 0.855 | 1.000 | 1.000 | 0.854 | 0.825 |
| 4009 | 0.771 | 1.000 | 0.802 | 0.986 | 0.974 | 0.960 | 0.892 | 1.000 | 0.990 | 1.000 | 0.954 | 0.970 | 0.991 | 0.856 | 1.000 | 1.000 | 0.855 | 0.826 |
| 4010 | 0.765 | 1.000 | 0.802 | 1.000 | 0.968 | 0.960 | 0.903 | 1.000 | 0.990 | 1.000 | 0.948 | 1.000 | 1.000 | 0.858 | 1.000 | 1.000 | 0.856 | 0.827 |
| 4011 | 0.777 | 1.000 | 0.812 | 1.000 | 0.974 | 0.960 | 0.895 | 1.000 | 0.990 | 1.000 | 0.957 | 1.000 | 0.991 | 0.861 | 1.000 | 1.000 | 0.860 | 0.832 |
| 4012 | 0.769 | 1.000 | 0.798 | 1.000 | 0.979 | 0.960 | 0.891 | 1.000 | 0.990 | 1.000 | 0.962 | 1.000 | 0.991 | 0.854 | 1.000 | 1.000 | 0.855 | 0.826 |
| 4013 | 0.770 | 1.000 | 0.783 | 1.000 | 0.974 | 0.960 | 0.901 | 1.000 | 0.990 | 1.000 | 0.962 | 1.000 | 1.000 | 0.860 | 1.000 | 1.000 | 0.856 | 0.828 |
| 4014 | 0.771 | 1.000 | 0.808 | 0.986 | 0.968 | 0.960 | 0.897 | 1.000 | 0.990 | 1.000 | 0.959 | 1.000 | 0.991 | 0.849 | 1.000 | 1.000 | 0.855 | 0.826 |
| 4015 | 0.776 | 1.000 | 0.806 | 1.000 | 0.979 | 0.960 | 0.898 | 1.000 | 0.990 | 1.000 | 0.965 | 1.000 | 0.991 | 0.844 | 1.000 | 1.000 | 0.856 | 0.828 |

| | | | | | | | | | | | | | | | | | |
|---|---|---|---|---|---|---|---|---|---|---|---|---|---|---|---|---|---|
| 4016 | 0.766 | 1.000 | 0.808 | 1.000 | 0.968 | 0.960 | 0.889 | 1.000 | 0.990 | 1.000 | 0.945 | 1.000 | 1.000 | 0.854 | 1.000 | 1.000 | 0.853 | 0.824 |
| 4017 | 0.772 | 1.000 | 0.812 | 1.000 | 0.979 | 0.960 | 0.888 | 1.000 | 0.990 | 1.000 | 0.957 | 1.000 | 0.991 | 0.851 | 1.000 | 1.000 | 0.856 | 0.827 |
| 4018 | 0.772 | 1.000 | 0.808 | 1.000 | 0.974 | 0.960 | 0.903 | 1.000 | 1.000 | 1.000 | 0.959 | 1.000 | 1.000 | 0.860 | 1.000 | 1.000 | 0.860 | 0.832 |
| 4019 | 0.769 | 1.000 | 0.796 | 1.000 | 0.968 | 0.960 | 0.903 | 1.000 | 1.000 | 1.000 | 0.954 | 1.000 | 0.991 | 0.851 | 1.000 | 1.000 | 0.855 | 0.826 |
| 4020 | 0.770 | 1.000 | 0.804 | 1.000 | 0.979 | 0.960 | 0.895 | 1.000 | 0.990 | 1.000 | 0.959 | 1.000 | 0.982 | 0.849 | 1.000 | 1.000 | 0.855 | 0.826 |
| 4021 | 0.773 | 1.000 | 0.792 | 1.000 | 0.979 | 0.960 | 0.894 | 1.000 | 0.980 | 1.000 | 0.959 | 1.000 | 0.991 | 0.855 | 1.000 | 1.000 | 0.856 | 0.827 |
| 4022 | 0.765 | 1.000 | 0.798 | 1.000 | 0.979 | 0.960 | 0.903 | 1.000 | 0.990 | 1.000 | 0.965 | 1.000 | 0.991 | 0.855 | 1.000 | 1.000 | 0.856 | 0.827 |
| 4023 | 0.760 | 1.000 | 0.791 | 1.000 | 0.984 | 0.960 | 0.891 | 1.000 | 1.000 | 1.000 | 0.948 | 1.000 | 0.991 | 0.850 | 1.000 | 1.000 | 0.850 | 0.820 |
| 4024 | 0.767 | 1.000 | 0.800 | 1.000 | 0.974 | 0.960 | 0.900 | 1.000 | 0.990 | 1.000 | 0.957 | 1.000 | 1.000 | 0.847 | 1.000 | 1.000 | 0.853 | 0.824 |
| 4025 | 0.773 | 1.000 | 0.798 | 0.986 | 0.974 | 0.960 | 0.901 | 1.000 | 0.990 | 1.000 | 0.959 | 1.000 | 0.991 | 0.854 | 1.000 | 1.000 | 0.857 | 0.828 |
| 4026 | 0.758 | 1.000 | 0.820 | 1.000 | 0.984 | 0.960 | 0.891 | 1.000 | 0.980 | 1.000 | 0.962 | 1.000 | 0.991 | 0.849 | 1.000 | 1.000 | 0.852 | 0.824 |
| 4027 | 0.771 | 1.000 | 0.794 | 1.000 | 0.974 | 0.960 | 0.891 | 1.000 | 0.990 | 1.000 | 0.957 | 1.000 | 1.000 | 0.854 | 1.000 | 1.000 | 0.854 | 0.826 |
| 4028 | 0.765 | 1.000 | 0.804 | 1.000 | 0.974 | 0.960 | 0.889 | 1.000 | 0.980 | 1.000 | 0.954 | 1.000 | 0.991 | 0.850 | 1.000 | 1.000 | 0.852 | 0.822 |
| 4029 | 0.769 | 1.000 | 0.798 | 1.000 | 0.979 | 0.960 | 0.900 | 1.000 | 0.980 | 1.000 | 0.959 | 1.000 | 0.991 | 0.862 | 1.000 | 1.000 | 0.858 | 0.829 |
| 4030 | 0.771 | 1.000 | 0.806 | 1.000 | 0.979 | 0.960 | 0.901 | 1.000 | 1.000 | 1.000 | 0.957 | 1.000 | 1.000 | 0.858 | 1.000 | 1.000 | 0.858 | 0.830 |
| 4031 | 0.761 | 1.000 | 0.800 | 1.000 | 0.968 | 0.960 | 0.901 | 1.000 | 0.990 | 1.000 | 0.965 | 1.000 | 0.991 | 0.861 | 1.000 | 1.000 | 0.855 | 0.827 |
| 4032 | 0.762 | 1.000 | 0.794 | 1.000 | 0.974 | 0.960 | 0.898 | 1.000 | 1.000 | 1.000 | 0.954 | 1.000 | 0.991 | 0.851 | 1.000 | 1.000 | 0.852 | 0.823 |
| 4033 | 0.770 | 1.000 | 0.787 | 1.000 | 0.979 | 0.960 | 0.904 | 1.000 | 1.000 | 1.000 | 0.959 | 1.000 | 1.000 | 0.853 | 1.000 | 1.000 | 0.856 | 0.827 |
| 4034 | 0.764 | 1.000 | 0.804 | 1.000 | 0.979 | 0.960 | 0.909 | 1.000 | 0.990 | 1.000 | 0.957 | 1.000 | 1.000 | 0.850 | 1.000 | 1.000 | 0.855 | 0.827 |
| 4035 | 0.771 | 1.000 | 0.796 | 1.000 | 0.974 | 0.960 | 0.900 | 1.000 | 0.969 | 1.000 | 0.954 | 1.000 | 0.982 | 0.858 | 1.000 | 1.000 | 0.856 | 0.827 |
| 4036 | 0.769 | 1.000 | 0.802 | 1.000 | 0.963 | 0.960 | 0.904 | 1.000 | 0.990 | 1.000 | 0.962 | 1.000 | 0.991 | 0.854 | 1.000 | 1.000 | 0.856 | 0.828 |
| 4037 | 0.768 | 1.000 | 0.800 | 1.000 | 0.968 | 0.960 | 0.901 | 1.000 | 1.000 | 1.000 | 0.957 | 1.000 | 1.000 | 0.852 | 1.000 | 1.000 | 0.855 | 0.827 |
| 4038 | 0.769 | 1.000 | 0.783 | 1.000 | 0.979 | 0.960 | 0.895 | 1.000 | 0.990 | 1.000 | 0.962 | 1.000 | 0.991 | 0.855 | 1.000 | 1.000 | 0.854 | 0.825 |
| 4039 | 0.768 | 1.000 | 0.800 | 1.000 | 0.979 | 0.960 | 0.889 | 1.000 | 0.990 | 1.000 | 0.954 | 1.000 | 0.991 | 0.861 | 1.000 | 1.000 | 0.856 | 0.827 |
| 4040 | 0.768 | 1.000 | 0.802 | 1.000 | 0.968 | 0.960 | 0.900 | 1.000 | 0.990 | 1.000 | 0.957 | 1.000 | 0.991 | 0.858 | 1.000 | 1.000 | 0.856 | 0.827 |
| 4041 | 0.770 | 1.000 | 0.802 | 1.000 | 0.979 | 0.960 | 0.903 | 1.000 | 0.980 | 1.000 | 0.962 | 1.000 | 0.991 | 0.849 | 1.000 | 1.000 | 0.856 | 0.827 |
| 4042 | 0.769 | 1.000 | 0.822 | 1.000 | 0.979 | 1.000 | 0.897 | 1.000 | 1.000 | 1.000 | 0.962 | 1.000 | 1.000 | 0.850 | 1.000 | 1.000 | 0.857 | 0.829 |
| 4043 | 0.771 | 1.000 | 0.808 | 1.000 | 0.968 | 0.960 | 0.898 | 1.000 | 1.000 | 1.000 | 0.959 | 1.000 | 1.000 | 0.855 | 1.000 | 1.000 | 0.857 | 0.829 |
| 4044 | 0.763 | 1.000 | 0.796 | 1.000 | 0.974 | 0.960 | 0.909 | 1.000 | 0.990 | 1.000 | 0.965 | 1.000 | 0.991 | 0.858 | 1.000 | 1.000 | 0.856 | 0.828 |
| 4045 | 0.767 | 1.000 | 0.798 | 1.000 | 0.968 | 0.960 | 0.900 | 1.000 | 1.000 | 1.000 | 0.965 | 1.000 | 1.000 | 0.856 | 1.000 | 1.000 | 0.856 | 0.828 |
| 4046 | 0.771 | 1.000 | 0.812 | 1.000 | 0.974 | 0.960 | 0.898 | 1.000 | 0.990 | 1.000 | 0.959 | 1.000 | 1.000 | 0.851 | 1.000 | 1.000 | 0.857 | 0.828 |
| 4047 | 0.777 | 1.000 | 0.792 | 1.000 | 0.974 | 0.960 | 0.901 | 1.000 | 1.000 | 1.000 | 0.959 | 1.000 | 0.991 | 0.849 | 1.000 | 1.000 | 0.856 | 0.828 |
| 4048 | 0.776 | 1.000 | 0.792 | 1.000 | 0.979 | 0.960 | 0.895 | 1.000 | 0.990 | 1.000 | 0.965 | 1.000 | 0.991 | 0.850 | 1.000 | 1.000 | 0.856 | 0.827 |
| 4049 | 0.768 | 1.000 | 0.792 | 1.000 | 0.984 | 0.960 | 0.909 | 1.000 | 0.990 | 1.000 | 0.962 | 1.000 | 0.991 | 0.854 | 1.000 | 1.000 | 0.856 | 0.828 |
| 4050 | 0.771 | 1.000 | 0.794 | 1.000 | 0.984 | 0.960 | 0.895 | 1.000 | 0.990 | 1.000 | 0.951 | 1.000 | 0.982 | 0.853 | 1.000 | 1.000 | 0.854 | 0.825 |
| 4051 | 0.762 | 1.000 | 0.804 | 1.000 | 0.979 | 0.960 | 0.892 | 1.000 | 0.980 | 1.000 | 0.957 | 1.000 | 1.000 | 0.848 | 1.000 | 1.000 | 0.852 | 0.822 |
| 4052 | 0.767 | 1.000 | 0.802 | 1.000 | 0.968 | 0.960 | 0.904 | 1.000 | 1.000 | 1.000 | 0.965 | 1.000 | 0.991 | 0.852 | 1.000 | 1.000 | 0.856 | 0.828 |
| 4053 | 0.763 | 1.000 | 0.789 | 1.000 | 0.979 | 0.960 | 0.901 | 1.000 | 0.980 | 1.000 | 0.962 | 1.000 | 1.000 | 0.846 | 1.000 | 1.000 | 0.851 | 0.822 |
| 4054 | 0.767 | 1.000 | 0.800 | 1.000 | 0.974 | 0.960 | 0.897 | 1.000 | 0.990 | 1.000 | 0.965 | 1.000 | 1.000 | 0.848 | 1.000 | 1.000 | 0.854 | 0.825 |

| | | | | | | | | | | | | | | | | | |
|---|---|---|---|---|---|---|---|---|---|---|---|---|---|---|---|---|---|
| 4055 | 0.759 | 1.000 | 0.794 | 1.000 | 0.968 | 0.960 | 0.901 | 1.000 | 1.000 | 1.000 | 0.965 | 1.000 | 0.982 | 0.854 | 1.000 | 1.000 | 0.852 | 0.823 |
| 4056 | 0.774 | 1.000 | 0.802 | 1.000 | 0.974 | 0.960 | 0.901 | 1.000 | 1.000 | 1.000 | 0.959 | 1.000 | 1.000 | 0.851 | 1.000 | 1.000 | 0.857 | 0.829 |
| 4057 | 0.777 | 1.000 | 0.789 | 1.000 | 0.974 | 0.960 | 0.895 | 1.000 | 0.990 | 1.000 | 0.962 | 1.000 | 0.991 | 0.853 | 1.000 | 1.000 | 0.856 | 0.827 |
| 4058 | 0.771 | 1.000 | 0.789 | 1.000 | 0.963 | 0.960 | 0.900 | 1.000 | 0.990 | 1.000 | 0.962 | 1.000 | 0.982 | 0.849 | 1.000 | 1.000 | 0.853 | 0.824 |
| 4059 | 0.771 | 1.000 | 0.794 | 1.000 | 0.974 | 0.960 | 0.898 | 1.000 | 1.000 | 1.000 | 0.957 | 1.000 | 0.991 | 0.847 | 1.000 | 1.000 | 0.854 | 0.825 |
| 4060 | 0.780 | 1.000 | 0.789 | 1.000 | 0.979 | 0.960 | 0.903 | 1.000 | 0.980 | 1.000 | 0.959 | 1.000 | 1.000 | 0.848 | 1.000 | 1.000 | 0.857 | 0.828 |
| 4061 | 0.772 | 1.000 | 0.796 | 1.000 | 0.974 | 0.960 | 0.895 | 1.000 | 0.990 | 1.000 | 0.962 | 1.000 | 0.991 | 0.861 | 1.000 | 1.000 | 0.858 | 0.829 |
| 4062 | 0.775 | 1.000 | 0.798 | 1.000 | 0.968 | 0.960 | 0.897 | 1.000 | 0.990 | 1.000 | 0.959 | 1.000 | 1.000 | 0.849 | 1.000 | 1.000 | 0.856 | 0.827 |
| 4063 | 0.775 | 1.000 | 0.794 | 1.000 | 0.979 | 0.960 | 0.903 | 1.000 | 0.990 | 1.000 | 0.962 | 0.970 | 0.982 | 0.851 | 1.000 | 1.000 | 0.856 | 0.828 |
| 4064 | 0.765 | 1.000 | 0.796 | 1.000 | 0.974 | 0.960 | 0.903 | 1.000 | 0.990 | 1.000 | 0.957 | 1.000 | 0.982 | 0.847 | 1.000 | 1.000 | 0.852 | 0.823 |
| 4065 | 0.777 | 1.000 | 0.791 | 1.000 | 0.974 | 0.960 | 0.903 | 1.000 | 0.969 | 1.000 | 0.951 | 1.000 | 1.000 | 0.852 | 1.000 | 1.000 | 0.856 | 0.827 |
| 4066 | 0.769 | 1.000 | 0.812 | 1.000 | 0.979 | 0.960 | 0.892 | 1.000 | 1.000 | 1.000 | 0.959 | 1.000 | 1.000 | 0.853 | 1.000 | 1.000 | 0.856 | 0.828 |
| 4067 | 0.772 | 1.000 | 0.794 | 1.000 | 0.974 | 0.960 | 0.897 | 1.000 | 0.980 | 1.000 | 0.959 | 1.000 | 0.991 | 0.851 | 1.000 | 1.000 | 0.855 | 0.826 |
| 4068 | 0.773 | 1.000 | 0.806 | 1.000 | 0.979 | 0.960 | 0.898 | 1.000 | 0.990 | 1.000 | 0.965 | 1.000 | 1.000 | 0.849 | 1.000 | 1.000 | 0.857 | 0.829 |
| 4069 | 0.768 | 1.000 | 0.798 | 1.000 | 0.968 | 0.960 | 0.894 | 1.000 | 0.990 | 1.000 | 0.962 | 1.000 | 1.000 | 0.851 | 1.000 | 1.000 | 0.854 | 0.825 |
| 4070 | 0.770 | 1.000 | 0.802 | 1.000 | 0.968 | 0.960 | 0.892 | 1.000 | 0.980 | 1.000 | 0.959 | 1.000 | 0.991 | 0.854 | 1.000 | 1.000 | 0.855 | 0.826 |
| 4071 | 0.767 | 1.000 | 0.812 | 1.000 | 0.974 | 0.960 | 0.895 | 1.000 | 0.990 | 1.000 | 0.954 | 1.000 | 1.000 | 0.857 | 1.000 | 1.000 | 0.856 | 0.828 |
| 4072 | 0.765 | 1.000 | 0.798 | 1.000 | 0.974 | 0.960 | 0.898 | 1.000 | 0.990 | 1.000 | 0.959 | 1.000 | 1.000 | 0.859 | 1.000 | 1.000 | 0.856 | 0.827 |
| 4073 | 0.774 | 1.000 | 0.806 | 1.000 | 0.979 | 0.960 | 0.901 | 1.000 | 0.990 | 1.000 | 0.954 | 1.000 | 0.991 | 0.854 | 1.000 | 1.000 | 0.858 | 0.830 |
| 4074 | 0.768 | 1.000 | 0.800 | 1.000 | 0.963 | 0.960 | 0.908 | 1.000 | 0.990 | 1.000 | 0.959 | 1.000 | 1.000 | 0.858 | 1.000 | 1.000 | 0.857 | 0.829 |
| 4075 | 0.769 | 1.000 | 0.792 | 0.986 | 0.974 | 0.960 | 0.892 | 1.000 | 1.000 | 1.000 | 0.968 | 1.000 | 1.000 | 0.858 | 1.000 | 1.000 | 0.856 | 0.827 |
| 4076 | 0.769 | 1.000 | 0.804 | 1.000 | 0.984 | 0.960 | 0.908 | 1.000 | 1.000 | 1.000 | 0.959 | 1.000 | 1.000 | 0.851 | 1.000 | 1.000 | 0.857 | 0.829 |
| 4077 | 0.768 | 1.000 | 0.798 | 1.000 | 0.979 | 0.960 | 0.895 | 1.000 | 0.990 | 1.000 | 0.965 | 1.000 | 0.991 | 0.851 | 1.000 | 1.000 | 0.854 | 0.825 |
| 4078 | 0.777 | 1.000 | 0.800 | 1.000 | 0.968 | 0.960 | 0.891 | 1.000 | 0.990 | 1.000 | 0.954 | 1.000 | 0.982 | 0.854 | 1.000 | 1.000 | 0.856 | 0.827 |
| 4079 | 0.761 | 1.000 | 0.783 | 1.000 | 0.963 | 0.960 | 0.892 | 1.000 | 0.990 | 1.000 | 0.957 | 1.000 | 1.000 | 0.851 | 1.000 | 1.000 | 0.850 | 0.820 |
| 4080 | 0.774 | 1.000 | 0.792 | 1.000 | 0.974 | 0.960 | 0.895 | 1.000 | 1.000 | 1.000 | 0.959 | 1.000 | 1.000 | 0.853 | 1.000 | 1.000 | 0.856 | 0.827 |
| 4081 | 0.775 | 1.000 | 0.789 | 1.000 | 0.974 | 0.960 | 0.904 | 1.000 | 0.990 | 1.000 | 0.965 | 1.000 | 0.991 | 0.854 | 1.000 | 1.000 | 0.857 | 0.829 |
| 4082 | 0.767 | 1.000 | 0.800 | 1.000 | 0.979 | 0.960 | 0.898 | 1.000 | 0.990 | 1.000 | 0.951 | 1.000 | 0.991 | 0.849 | 1.000 | 1.000 | 0.853 | 0.824 |
| 4083 | 0.767 | 1.000 | 0.806 | 1.000 | 0.974 | 0.960 | 0.903 | 1.000 | 0.990 | 1.000 | 0.965 | 1.000 | 0.991 | 0.849 | 1.000 | 1.000 | 0.855 | 0.827 |
| 4084 | 0.771 | 1.000 | 0.812 | 1.000 | 0.979 | 0.960 | 0.897 | 1.000 | 0.990 | 1.000 | 0.948 | 1.000 | 0.991 | 0.857 | 1.000 | 1.000 | 0.857 | 0.829 |
| 4085 | 0.775 | 1.000 | 0.798 | 1.000 | 0.974 | 0.960 | 0.892 | 1.000 | 0.990 | 1.000 | 0.959 | 1.000 | 1.000 | 0.851 | 1.000 | 1.000 | 0.856 | 0.827 |
| 4086 | 0.760 | 1.000 | 0.806 | 1.000 | 0.979 | 0.960 | 0.908 | 1.000 | 0.990 | 1.000 | 0.951 | 0.970 | 0.982 | 0.858 | 1.000 | 1.000 | 0.855 | 0.826 |
| 4087 | 0.778 | 1.000 | 0.806 | 1.000 | 0.968 | 0.960 | 0.903 | 1.000 | 0.990 | 1.000 | 0.965 | 1.000 | 1.000 | 0.847 | 1.000 | 1.000 | 0.858 | 0.830 |
| 4088 | 0.777 | 1.000 | 0.796 | 1.000 | 0.974 | 0.960 | 0.891 | 1.000 | 1.000 | 1.000 | 0.959 | 1.000 | 0.982 | 0.854 | 1.000 | 1.000 | 0.856 | 0.828 |
| 4089 | 0.771 | 1.000 | 0.792 | 1.000 | 0.979 | 0.960 | 0.904 | 1.000 | 0.990 | 1.000 | 0.957 | 1.000 | 1.000 | 0.860 | 1.000 | 1.000 | 0.858 | 0.829 |
| 4090 | 0.776 | 1.000 | 0.796 | 1.000 | 0.979 | 0.960 | 0.898 | 1.000 | 0.990 | 1.000 | 0.962 | 1.000 | 0.991 | 0.855 | 1.000 | 1.000 | 0.858 | 0.830 |
| 4091 | 0.768 | 1.000 | 0.777 | 1.000 | 0.979 | 0.960 | 0.892 | 1.000 | 0.990 | 1.000 | 0.957 | 1.000 | 1.000 | 0.855 | 1.000 | 1.000 | 0.853 | 0.823 |
| 4092 | 0.783 | 1.000 | 0.800 | 0.986 | 0.968 | 0.960 | 0.894 | 1.000 | 0.980 | 1.000 | 0.962 | 1.000 | 1.000 | 0.845 | 1.000 | 1.000 | 0.857 | 0.828 |
| 4093 | 0.768 | 1.000 | 0.800 | 1.000 | 0.968 | 0.960 | 0.898 | 1.000 | 0.990 | 1.000 | 0.957 | 1.000 | 0.991 | 0.856 | 1.000 | 1.000 | 0.855 | 0.827 |

| | | | | | | | | | | | | | | | | | | |
|---|---|---|---|---|---|---|---|---|---|---|---|---|---|---|---|---|---|---|
| 4094 | 0.776 | 1.000 | 0.798 | 1.000 | 0.974 | 0.960 | 0.898 | 1.000 | 0.990 | 1.000 | 0.962 | 1.000 | 0.991 | 0.859 | 1.000 | 1.000 | 0.859 | 0.831 |
| 4095 | 0.773 | 1.000 | 0.781 | 1.000 | 0.974 | 0.960 | 0.901 | 1.000 | 0.990 | 1.000 | 0.954 | 1.000 | 0.991 | 0.856 | 1.000 | 1.000 | 0.855 | 0.826 |
| 4096 | 0.760 | 1.000 | 0.792 | 1.000 | 0.968 | 0.960 | 0.895 | 1.000 | 0.990 | 1.000 | 0.959 | 1.000 | 0.982 | 0.854 | 1.000 | 1.000 | 0.851 | 0.822 |
| 4097 | 0.771 | 1.000 | 0.808 | 1.000 | 0.979 | 0.960 | 0.894 | 1.000 | 0.990 | 1.000 | 0.965 | 1.000 | 1.000 | 0.851 | 1.000 | 1.000 | 0.856 | 0.828 |
| 4098 | 0.764 | 1.000 | 0.785 | 1.000 | 0.979 | 0.960 | 0.904 | 1.000 | 0.990 | 1.000 | 0.959 | 1.000 | 1.000 | 0.856 | 1.000 | 1.000 | 0.854 | 0.825 |
| 4099 | 0.763 | 1.000 | 0.804 | 1.000 | 0.974 | 0.960 | 0.898 | 1.000 | 1.000 | 1.000 | 0.962 | 1.000 | 0.991 | 0.850 | 1.000 | 1.000 | 0.854 | 0.825 |
| 4100 | 0.763 | 1.000 | 0.808 | 1.000 | 0.974 | 0.960 | 0.906 | 1.000 | 0.990 | 1.000 | 0.957 | 1.000 | 0.991 | 0.851 | 1.000 | 1.000 | 0.855 | 0.826 |
| 4101 | 0.765 | 1.000 | 0.800 | 1.000 | 0.963 | 0.960 | 0.900 | 1.000 | 1.000 | 1.000 | 0.965 | 1.000 | 0.991 | 0.851 | 1.000 | 1.000 | 0.854 | 0.825 |
| 4102 | 0.774 | 1.000 | 0.794 | 1.000 | 0.968 | 0.960 | 0.903 | 1.000 | 0.990 | 1.000 | 0.968 | 1.000 | 0.991 | 0.854 | 1.000 | 1.000 | 0.857 | 0.829 |
| 4103 | 0.774 | 1.000 | 0.810 | 1.000 | 0.968 | 0.960 | 0.895 | 1.000 | 0.990 | 1.000 | 0.959 | 0.970 | 1.000 | 0.856 | 1.000 | 1.000 | 0.858 | 0.830 |
| 4104 | 0.765 | 1.000 | 0.798 | 1.000 | 0.979 | 0.960 | 0.903 | 1.000 | 0.990 | 1.000 | 0.962 | 0.970 | 1.000 | 0.856 | 1.000 | 1.000 | 0.856 | 0.827 |
| 4105 | 0.761 | 1.000 | 0.798 | 1.000 | 0.974 | 0.960 | 0.895 | 1.000 | 0.990 | 1.000 | 0.962 | 1.000 | 0.982 | 0.856 | 1.000 | 1.000 | 0.853 | 0.824 |
| 4106 | 0.757 | 1.000 | 0.802 | 1.000 | 0.974 | 0.960 | 0.898 | 1.000 | 0.990 | 1.000 | 0.957 | 1.000 | 0.991 | 0.862 | 1.000 | 1.000 | 0.854 | 0.825 |
| 4107 | 0.777 | 1.000 | 0.802 | 1.000 | 0.968 | 0.960 | 0.903 | 1.000 | 0.990 | 1.000 | 0.968 | 1.000 | 1.000 | 0.849 | 1.000 | 1.000 | 0.858 | 0.830 |
| 4108 | 0.769 | 1.000 | 0.812 | 1.000 | 0.968 | 0.960 | 0.897 | 1.000 | 0.980 | 1.000 | 0.954 | 1.000 | 0.991 | 0.857 | 1.000 | 1.000 | 0.856 | 0.828 |
| 4109 | 0.767 | 1.000 | 0.796 | 1.000 | 0.979 | 0.960 | 0.897 | 1.000 | 1.000 | 1.000 | 0.959 | 1.000 | 0.991 | 0.849 | 1.000 | 1.000 | 0.854 | 0.824 |
| 4110 | 0.774 | 1.000 | 0.806 | 1.000 | 0.974 | 0.960 | 0.906 | 1.000 | 0.990 | 1.000 | 0.959 | 1.000 | 1.000 | 0.854 | 1.000 | 1.000 | 0.859 | 0.830 |
| 4111 | 0.771 | 1.000 | 0.806 | 1.000 | 0.979 | 0.960 | 0.895 | 1.000 | 0.990 | 1.000 | 0.965 | 1.000 | 0.991 | 0.857 | 1.000 | 1.000 | 0.858 | 0.829 |
| 4112 | 0.774 | 1.000 | 0.785 | 1.000 | 0.974 | 0.960 | 0.897 | 1.000 | 0.990 | 1.000 | 0.962 | 0.970 | 0.991 | 0.857 | 1.000 | 1.000 | 0.856 | 0.827 |
| 4113 | 0.766 | 1.000 | 0.816 | 1.000 | 0.974 | 0.960 | 0.881 | 1.000 | 0.980 | 1.000 | 0.965 | 0.970 | 0.982 | 0.858 | 1.000 | 1.000 | 0.855 | 0.826 |
| 4114 | 0.770 | 1.000 | 0.798 | 1.000 | 0.979 | 0.960 | 0.897 | 1.000 | 0.980 | 1.000 | 0.959 | 1.000 | 1.000 | 0.858 | 1.000 | 1.000 | 0.857 | 0.828 |
| 4115 | 0.774 | 1.000 | 0.794 | 1.000 | 0.974 | 0.960 | 0.900 | 1.000 | 0.990 | 1.000 | 0.957 | 1.000 | 0.991 | 0.852 | 1.000 | 1.000 | 0.856 | 0.827 |
| 4116 | 0.755 | 1.000 | 0.794 | 1.000 | 0.979 | 0.960 | 0.900 | 1.000 | 0.990 | 1.000 | 0.954 | 1.000 | 1.000 | 0.852 | 1.000 | 1.000 | 0.850 | 0.821 |
| 4117 | 0.770 | 1.000 | 0.812 | 1.000 | 0.968 | 0.960 | 0.894 | 1.000 | 0.990 | 1.000 | 0.954 | 1.000 | 0.991 | 0.854 | 1.000 | 1.000 | 0.856 | 0.827 |
| 4118 | 0.765 | 1.000 | 0.812 | 1.000 | 0.979 | 0.960 | 0.898 | 1.000 | 0.990 | 1.000 | 0.962 | 1.000 | 0.982 | 0.850 | 1.000 | 1.000 | 0.855 | 0.826 |
| 4119 | 0.771 | 1.000 | 0.800 | 1.000 | 0.979 | 0.960 | 0.900 | 1.000 | 0.990 | 1.000 | 0.957 | 1.000 | 1.000 | 0.856 | 1.000 | 1.000 | 0.857 | 0.829 |
| 4120 | 0.770 | 1.000 | 0.806 | 1.000 | 0.979 | 0.960 | 0.901 | 1.000 | 1.000 | 1.000 | 0.954 | 1.000 | 0.991 | 0.849 | 1.000 | 1.000 | 0.856 | 0.827 |
| 4121 | 0.771 | 1.000 | 0.796 | 1.000 | 0.968 | 0.960 | 0.898 | 1.000 | 0.980 | 1.000 | 0.951 | 1.000 | 0.982 | 0.853 | 1.000 | 1.000 | 0.854 | 0.825 |
| 4122 | 0.771 | 1.000 | 0.802 | 1.000 | 0.979 | 0.960 | 0.900 | 1.000 | 0.990 | 1.000 | 0.968 | 1.000 | 0.991 | 0.858 | 1.000 | 1.000 | 0.858 | 0.830 |
| 4123 | 0.765 | 1.000 | 0.800 | 1.000 | 0.974 | 0.960 | 0.901 | 1.000 | 0.990 | 1.000 | 0.959 | 1.000 | 1.000 | 0.855 | 1.000 | 1.000 | 0.855 | 0.827 |
| 4124 | 0.773 | 1.000 | 0.810 | 1.000 | 0.963 | 0.960 | 0.894 | 1.000 | 0.990 | 1.000 | 0.951 | 1.000 | 1.000 | 0.843 | 1.000 | 1.000 | 0.854 | 0.825 |
| 4125 | 0.765 | 1.000 | 0.816 | 1.000 | 0.979 | 0.960 | 0.894 | 1.000 | 0.990 | 1.000 | 0.962 | 1.000 | 0.982 | 0.847 | 1.000 | 1.000 | 0.854 | 0.825 |
| 4126 | 0.776 | 1.000 | 0.808 | 1.000 | 0.979 | 0.960 | 0.903 | 1.000 | 0.990 | 1.000 | 0.957 | 1.000 | 1.000 | 0.850 | 1.000 | 1.000 | 0.858 | 0.830 |
| 4127 | 0.763 | 1.000 | 0.810 | 1.000 | 0.974 | 0.960 | 0.894 | 1.000 | 1.000 | 1.000 | 0.968 | 1.000 | 0.991 | 0.851 | 1.000 | 1.000 | 0.854 | 0.825 |
| 4128 | 0.773 | 1.000 | 0.800 | 1.000 | 0.974 | 0.960 | 0.904 | 1.000 | 1.000 | 1.000 | 0.962 | 1.000 | 1.000 | 0.849 | 1.000 | 1.000 | 0.857 | 0.828 |
| 4129 | 0.767 | 1.000 | 0.791 | 1.000 | 0.974 | 0.960 | 0.898 | 1.000 | 0.990 | 1.000 | 0.962 | 1.000 | 1.000 | 0.858 | 1.000 | 1.000 | 0.855 | 0.826 |
| 4130 | 0.765 | 1.000 | 0.794 | 1.000 | 0.979 | 0.960 | 0.909 | 1.000 | 0.980 | 1.000 | 0.968 | 1.000 | 0.991 | 0.860 | 1.000 | 1.000 | 0.857 | 0.829 |
| 4131 | 0.771 | 1.000 | 0.794 | 1.000 | 0.974 | 0.960 | 0.911 | 1.000 | 1.000 | 1.000 | 0.959 | 0.970 | 1.000 | 0.852 | 1.000 | 1.000 | 0.857 | 0.828 |
| 4132 | 0.769 | 1.000 | 0.804 | 1.000 | 0.984 | 0.960 | 0.904 | 1.000 | 1.000 | 1.000 | 0.954 | 1.000 | 0.991 | 0.854 | 1.000 | 1.000 | 0.857 | 0.829 |

| | | | | | | | | | | | | | | | | | |
|---|---|---|---|---|---|---|---|---|---|---|---|---|---|---|---|---|---|
| 4133 | 0.774 | 1.000 | 0.783 | 1.000 | 0.979 | 0.960 | 0.906 | 1.000 | 1.000 | 1.000 | 0.959 | 1.000 | 1.000 | 0.854 | 1.000 | 1.000 | 0.857 | 0.828 |
| 4134 | 0.774 | 1.000 | 0.791 | 1.000 | 0.979 | 0.960 | 0.903 | 1.000 | 1.000 | 1.000 | 0.951 | 1.000 | 0.991 | 0.855 | 1.000 | 1.000 | 0.857 | 0.828 |
| 4135 | 0.768 | 1.000 | 0.812 | 1.000 | 0.974 | 0.960 | 0.901 | 1.000 | 1.000 | 1.000 | 0.959 | 1.000 | 1.000 | 0.851 | 1.000 | 1.000 | 0.857 | 0.828 |
| 4136 | 0.767 | 1.000 | 0.802 | 1.000 | 0.984 | 0.960 | 0.900 | 1.000 | 1.000 | 1.000 | 0.959 | 0.970 | 1.000 | 0.853 | 1.000 | 1.000 | 0.856 | 0.827 |
| 4137 | 0.770 | 1.000 | 0.796 | 1.000 | 0.974 | 0.960 | 0.900 | 1.000 | 0.990 | 1.000 | 0.957 | 1.000 | 0.991 | 0.857 | 1.000 | 1.000 | 0.856 | 0.828 |
| 4138 | 0.767 | 1.000 | 0.810 | 1.000 | 0.968 | 0.960 | 0.901 | 1.000 | 0.990 | 1.000 | 0.957 | 1.000 | 0.991 | 0.855 | 1.000 | 1.000 | 0.856 | 0.828 |
| 4139 | 0.768 | 1.000 | 0.800 | 1.000 | 0.979 | 0.960 | 0.888 | 1.000 | 0.990 | 1.000 | 0.962 | 1.000 | 0.991 | 0.851 | 1.000 | 1.000 | 0.854 | 0.824 |
| 4140 | 0.764 | 1.000 | 0.800 | 1.000 | 0.974 | 0.960 | 0.897 | 1.000 | 0.980 | 1.000 | 0.968 | 1.000 | 0.991 | 0.849 | 1.000 | 1.000 | 0.853 | 0.824 |
| 4141 | 0.761 | 1.000 | 0.808 | 1.000 | 0.979 | 0.960 | 0.901 | 1.000 | 0.990 | 1.000 | 0.962 | 1.000 | 1.000 | 0.852 | 1.000 | 1.000 | 0.854 | 0.826 |
| 4142 | 0.772 | 1.000 | 0.791 | 1.000 | 0.968 | 0.960 | 0.897 | 1.000 | 0.990 | 1.000 | 0.965 | 1.000 | 0.991 | 0.850 | 1.000 | 1.000 | 0.854 | 0.825 |
| 4143 | 0.775 | 1.000 | 0.798 | 1.000 | 0.968 | 0.960 | 0.894 | 1.000 | 0.990 | 1.000 | 0.962 | 1.000 | 0.982 | 0.853 | 1.000 | 1.000 | 0.856 | 0.827 |
| 4144 | 0.763 | 1.000 | 0.789 | 1.000 | 0.974 | 0.960 | 0.894 | 1.000 | 0.990 | 1.000 | 0.965 | 1.000 | 1.000 | 0.854 | 1.000 | 1.000 | 0.852 | 0.823 |
| 4145 | 0.767 | 1.000 | 0.794 | 1.000 | 0.974 | 0.960 | 0.908 | 1.000 | 1.000 | 1.000 | 0.962 | 1.000 | 1.000 | 0.846 | 1.000 | 1.000 | 0.854 | 0.825 |
| 4146 | 0.773 | 1.000 | 0.808 | 1.000 | 0.974 | 0.960 | 0.894 | 1.000 | 0.990 | 1.000 | 0.965 | 1.000 | 0.991 | 0.852 | 1.000 | 1.000 | 0.857 | 0.828 |
| 4147 | 0.773 | 1.000 | 0.806 | 1.000 | 0.979 | 0.960 | 0.914 | 1.000 | 0.990 | 1.000 | 0.965 | 1.000 | 1.000 | 0.858 | 1.000 | 1.000 | 0.861 | 0.834 |
| 4148 | 0.767 | 1.000 | 0.816 | 0.986 | 0.968 | 0.960 | 0.909 | 1.000 | 0.990 | 1.000 | 0.962 | 0.970 | 0.991 | 0.850 | 1.000 | 1.000 | 0.856 | 0.828 |
| 4149 | 0.765 | 1.000 | 0.798 | 1.000 | 0.974 | 0.960 | 0.897 | 1.000 | 0.980 | 1.000 | 0.957 | 1.000 | 0.991 | 0.857 | 1.000 | 1.000 | 0.854 | 0.825 |
| 4150 | 0.769 | 1.000 | 0.798 | 1.000 | 0.974 | 0.960 | 0.900 | 1.000 | 0.990 | 1.000 | 0.965 | 1.000 | 0.991 | 0.853 | 1.000 | 1.000 | 0.856 | 0.827 |
| 4151 | 0.767 | 1.000 | 0.804 | 1.000 | 0.974 | 0.960 | 0.894 | 1.000 | 1.000 | 1.000 | 0.959 | 1.000 | 1.000 | 0.855 | 1.000 | 1.000 | 0.856 | 0.827 |
| 4152 | 0.777 | 1.000 | 0.810 | 1.000 | 0.974 | 0.960 | 0.897 | 1.000 | 0.990 | 1.000 | 0.959 | 1.000 | 1.000 | 0.842 | 1.000 | 1.000 | 0.856 | 0.827 |
| 4153 | 0.769 | 1.000 | 0.796 | 1.000 | 0.979 | 0.960 | 0.891 | 1.000 | 0.990 | 1.000 | 0.962 | 1.000 | 1.000 | 0.857 | 1.000 | 1.000 | 0.856 | 0.827 |
| 4154 | 0.769 | 1.000 | 0.792 | 1.000 | 0.974 | 0.960 | 0.894 | 1.000 | 1.000 | 1.000 | 0.962 | 0.970 | 0.982 | 0.858 | 1.000 | 1.000 | 0.855 | 0.826 |
| 4155 | 0.764 | 1.000 | 0.804 | 1.000 | 0.979 | 0.960 | 0.908 | 1.000 | 1.000 | 1.000 | 0.959 | 1.000 | 1.000 | 0.858 | 1.000 | 1.000 | 0.857 | 0.829 |
| 4156 | 0.777 | 1.000 | 0.804 | 1.000 | 0.979 | 0.960 | 0.895 | 1.000 | 0.990 | 1.000 | 0.971 | 1.000 | 0.991 | 0.851 | 1.000 | 1.000 | 0.858 | 0.830 |
| 4157 | 0.769 | 1.000 | 0.804 | 1.000 | 0.968 | 0.960 | 0.894 | 1.000 | 0.980 | 1.000 | 0.959 | 1.000 | 1.000 | 0.844 | 1.000 | 1.000 | 0.852 | 0.823 |
| 4158 | 0.767 | 1.000 | 0.820 | 1.000 | 0.979 | 0.960 | 0.898 | 1.000 | 0.990 | 1.000 | 0.962 | 1.000 | 1.000 | 0.849 | 1.000 | 1.000 | 0.856 | 0.828 |
| 4159 | 0.766 | 1.000 | 0.800 | 1.000 | 0.979 | 0.960 | 0.901 | 1.000 | 1.000 | 1.000 | 0.954 | 1.000 | 0.982 | 0.847 | 1.000 | 1.000 | 0.853 | 0.824 |
| 4160 | 0.765 | 1.000 | 0.796 | 1.000 | 0.974 | 0.960 | 0.898 | 1.000 | 0.990 | 1.000 | 0.954 | 1.000 | 1.000 | 0.863 | 1.000 | 1.000 | 0.856 | 0.827 |
| 4161 | 0.767 | 1.000 | 0.814 | 1.000 | 0.974 | 0.960 | 0.906 | 1.000 | 0.990 | 1.000 | 0.957 | 1.000 | 0.973 | 0.847 | 1.000 | 1.000 | 0.855 | 0.826 |
| 4162 | 0.771 | 1.000 | 0.796 | 1.000 | 0.974 | 0.960 | 0.906 | 1.000 | 0.990 | 1.000 | 0.965 | 1.000 | 1.000 | 0.855 | 1.000 | 1.000 | 0.858 | 0.829 |
| 4163 | 0.774 | 1.000 | 0.806 | 1.000 | 0.974 | 0.960 | 0.901 | 1.000 | 0.990 | 1.000 | 0.948 | 1.000 | 1.000 | 0.851 | 1.000 | 1.000 | 0.857 | 0.828 |
| 4164 | 0.769 | 1.000 | 0.800 | 1.000 | 0.984 | 0.960 | 0.895 | 1.000 | 1.000 | 1.000 | 0.948 | 1.000 | 0.991 | 0.859 | 1.000 | 1.000 | 0.856 | 0.828 |
| 4165 | 0.769 | 1.000 | 0.796 | 1.000 | 0.979 | 0.960 | 0.897 | 1.000 | 1.000 | 1.000 | 0.959 | 1.000 | 0.991 | 0.855 | 1.000 | 1.000 | 0.856 | 0.827 |
| 4166 | 0.773 | 1.000 | 0.792 | 1.000 | 0.979 | 0.960 | 0.898 | 1.000 | 0.980 | 1.000 | 0.962 | 1.000 | 1.000 | 0.861 | 1.000 | 1.000 | 0.858 | 0.830 |
| 4167 | 0.769 | 1.000 | 0.791 | 1.000 | 0.974 | 0.960 | 0.897 | 1.000 | 1.000 | 1.000 | 0.954 | 1.000 | 1.000 | 0.861 | 1.000 | 1.000 | 0.856 | 0.827 |
| 4168 | 0.766 | 1.000 | 0.802 | 1.000 | 0.974 | 0.960 | 0.908 | 1.000 | 1.000 | 1.000 | 0.959 | 1.000 | 0.991 | 0.858 | 1.000 | 1.000 | 0.857 | 0.829 |
| 4169 | 0.767 | 1.000 | 0.792 | 1.000 | 0.963 | 0.960 | 0.897 | 1.000 | 1.000 | 1.000 | 0.954 | 1.000 | 1.000 | 0.851 | 1.000 | 1.000 | 0.853 | 0.823 |
| 4170 | 0.765 | 1.000 | 0.791 | 1.000 | 0.979 | 0.960 | 0.897 | 1.000 | 0.980 | 1.000 | 0.968 | 0.970 | 1.000 | 0.855 | 1.000 | 1.000 | 0.854 | 0.825 |
| 4171 | 0.770 | 1.000 | 0.789 | 1.000 | 0.974 | 0.960 | 0.901 | 1.000 | 0.980 | 1.000 | 0.959 | 1.000 | 0.982 | 0.847 | 1.000 | 1.000 | 0.853 | 0.824 |

| | | | | | | | | | | | | | | | | | |
|---|---|---|---|---|---|---|---|---|---|---|---|---|---|---|---|---|---|
| 4172 | 0.773 | 1.000 | 0.798 | 1.000 | 0.974 | 0.960 | 0.894 | 1.000 | 0.990 | 1.000 | 0.962 | 1.000 | 0.982 | 0.856 | 1.000 | 1.000 | 0.856 | 0.828 |
| 4173 | 0.765 | 1.000 | 0.798 | 1.000 | 0.968 | 0.960 | 0.900 | 1.000 | 0.990 | 1.000 | 0.951 | 1.000 | 1.000 | 0.851 | 1.000 | 1.000 | 0.853 | 0.824 |
| 4174 | 0.767 | 1.000 | 0.802 | 1.000 | 0.963 | 0.960 | 0.901 | 1.000 | 0.980 | 1.000 | 0.962 | 1.000 | 0.991 | 0.855 | 1.000 | 1.000 | 0.856 | 0.827 |
| 4175 | 0.778 | 1.000 | 0.808 | 1.000 | 0.974 | 0.960 | 0.888 | 1.000 | 0.990 | 1.000 | 0.962 | 1.000 | 0.991 | 0.859 | 1.000 | 1.000 | 0.859 | 0.831 |
| 4176 | 0.773 | 1.000 | 0.800 | 1.000 | 0.974 | 0.960 | 0.901 | 1.000 | 0.990 | 1.000 | 0.962 | 1.000 | 0.991 | 0.860 | 1.000 | 1.000 | 0.859 | 0.831 |
| 4177 | 0.771 | 1.000 | 0.800 | 1.000 | 0.974 | 0.960 | 0.898 | 1.000 | 0.990 | 1.000 | 0.959 | 1.000 | 0.991 | 0.858 | 1.000 | 1.000 | 0.857 | 0.829 |
| 4178 | 0.761 | 1.000 | 0.802 | 1.000 | 0.979 | 0.960 | 0.901 | 1.000 | 0.990 | 1.000 | 0.965 | 0.970 | 0.991 | 0.854 | 1.000 | 1.000 | 0.854 | 0.825 |
| 4179 | 0.766 | 1.000 | 0.802 | 1.000 | 0.974 | 0.960 | 0.897 | 1.000 | 0.990 | 1.000 | 0.957 | 1.000 | 1.000 | 0.851 | 1.000 | 1.000 | 0.854 | 0.825 |
| 4180 | 0.769 | 1.000 | 0.802 | 1.000 | 0.968 | 0.960 | 0.906 | 1.000 | 0.990 | 1.000 | 0.957 | 1.000 | 1.000 | 0.863 | 1.000 | 1.000 | 0.859 | 0.831 |
| 4181 | 0.765 | 1.000 | 0.798 | 1.000 | 0.979 | 0.960 | 0.901 | 1.000 | 0.990 | 1.000 | 0.959 | 1.000 | 0.991 | 0.861 | 1.000 | 1.000 | 0.857 | 0.828 |
| 4182 | 0.767 | 1.000 | 0.802 | 1.000 | 0.974 | 0.960 | 0.903 | 1.000 | 0.990 | 1.000 | 0.954 | 1.000 | 0.991 | 0.848 | 1.000 | 1.000 | 0.854 | 0.825 |
| 4183 | 0.776 | 1.000 | 0.798 | 1.000 | 0.968 | 0.960 | 0.898 | 1.000 | 0.990 | 1.000 | 0.965 | 1.000 | 0.991 | 0.856 | 1.000 | 1.000 | 0.858 | 0.830 |
| 4184 | 0.769 | 1.000 | 0.812 | 1.000 | 0.974 | 0.960 | 0.897 | 1.000 | 0.980 | 1.000 | 0.968 | 1.000 | 0.991 | 0.849 | 1.000 | 1.000 | 0.856 | 0.827 |
| 4185 | 0.769 | 1.000 | 0.798 | 1.000 | 0.974 | 0.960 | 0.904 | 1.000 | 0.980 | 1.000 | 0.954 | 1.000 | 0.991 | 0.857 | 1.000 | 1.000 | 0.856 | 0.828 |
| 4186 | 0.767 | 1.000 | 0.814 | 1.000 | 0.974 | 0.960 | 0.900 | 1.000 | 0.980 | 1.000 | 0.957 | 1.000 | 0.991 | 0.852 | 1.000 | 1.000 | 0.856 | 0.827 |
| 4187 | 0.765 | 1.000 | 0.791 | 1.000 | 0.979 | 0.960 | 0.903 | 1.000 | 1.000 | 1.000 | 0.959 | 1.000 | 1.000 | 0.865 | 1.000 | 1.000 | 0.857 | 0.829 |
| 4188 | 0.774 | 1.000 | 0.798 | 1.000 | 0.974 | 0.960 | 0.886 | 1.000 | 0.990 | 1.000 | 0.962 | 1.000 | 1.000 | 0.854 | 1.000 | 1.000 | 0.856 | 0.827 |
| 4189 | 0.763 | 1.000 | 0.796 | 1.000 | 0.974 | 0.960 | 0.897 | 1.000 | 0.990 | 1.000 | 0.962 | 1.000 | 0.991 | 0.862 | 1.000 | 1.000 | 0.855 | 0.827 |
| 4190 | 0.772 | 1.000 | 0.796 | 1.000 | 0.974 | 0.960 | 0.900 | 1.000 | 0.990 | 1.000 | 0.957 | 1.000 | 0.982 | 0.855 | 1.000 | 1.000 | 0.856 | 0.827 |
| 4191 | 0.773 | 1.000 | 0.802 | 1.000 | 0.979 | 0.960 | 0.906 | 1.000 | 0.980 | 1.000 | 0.959 | 1.000 | 1.000 | 0.855 | 1.000 | 1.000 | 0.858 | 0.830 |
| 4192 | 0.767 | 1.000 | 0.791 | 1.000 | 0.979 | 0.960 | 0.897 | 1.000 | 0.980 | 1.000 | 0.962 | 1.000 | 0.991 | 0.857 | 1.000 | 1.000 | 0.855 | 0.826 |
| 4193 | 0.780 | 1.000 | 0.791 | 1.000 | 0.979 | 0.960 | 0.888 | 1.000 | 1.000 | 1.000 | 0.965 | 1.000 | 1.000 | 0.857 | 1.000 | 1.000 | 0.858 | 0.829 |
| 4194 | 0.767 | 1.000 | 0.802 | 0.986 | 0.974 | 0.960 | 0.898 | 1.000 | 1.000 | 1.000 | 0.957 | 1.000 | 1.000 | 0.856 | 1.000 | 1.000 | 0.856 | 0.827 |
| 4195 | 0.761 | 1.000 | 0.806 | 1.000 | 0.979 | 0.960 | 0.892 | 1.000 | 1.000 | 1.000 | 0.954 | 1.000 | 0.991 | 0.851 | 1.000 | 1.000 | 0.852 | 0.823 |
| 4196 | 0.765 | 1.000 | 0.806 | 1.000 | 0.974 | 0.960 | 0.897 | 1.000 | 0.980 | 1.000 | 0.962 | 1.000 | 0.991 | 0.854 | 1.000 | 1.000 | 0.855 | 0.826 |
| 4197 | 0.773 | 1.000 | 0.791 | 0.986 | 0.984 | 0.960 | 0.897 | 1.000 | 0.990 | 1.000 | 0.965 | 1.000 | 1.000 | 0.857 | 1.000 | 1.000 | 0.857 | 0.828 |
| 4198 | 0.763 | 1.000 | 0.802 | 1.000 | 0.974 | 0.960 | 0.901 | 1.000 | 0.990 | 1.000 | 0.965 | 1.000 | 0.991 | 0.854 | 1.000 | 1.000 | 0.855 | 0.826 |
| 4199 | 0.770 | 1.000 | 0.800 | 1.000 | 0.979 | 0.960 | 0.886 | 1.000 | 0.990 | 1.000 | 0.965 | 1.000 | 0.991 | 0.844 | 1.000 | 1.000 | 0.852 | 0.823 |
| 4200 | 0.774 | 1.000 | 0.800 | 1.000 | 0.979 | 0.960 | 0.898 | 1.000 | 1.000 | 1.000 | 0.954 | 1.000 | 0.991 | 0.859 | 1.000 | 1.000 | 0.858 | 0.830 |
| 4201 | 0.764 | 1.000 | 0.794 | 1.000 | 0.968 | 0.960 | 0.898 | 1.000 | 0.980 | 1.000 | 0.951 | 1.000 | 0.991 | 0.857 | 1.000 | 1.000 | 0.853 | 0.824 |
| 4202 | 0.767 | 1.000 | 0.792 | 1.000 | 0.974 | 0.960 | 0.898 | 1.000 | 0.990 | 1.000 | 0.965 | 1.000 | 0.982 | 0.858 | 1.000 | 1.000 | 0.855 | 0.827 |
| 4203 | 0.765 | 1.000 | 0.810 | 0.986 | 0.974 | 0.960 | 0.900 | 1.000 | 0.990 | 1.000 | 0.957 | 1.000 | 1.000 | 0.849 | 1.000 | 1.000 | 0.854 | 0.825 |
| 4204 | 0.777 | 1.000 | 0.791 | 1.000 | 0.974 | 0.960 | 0.898 | 1.000 | 1.000 | 1.000 | 0.957 | 1.000 | 1.000 | 0.853 | 1.000 | 1.000 | 0.857 | 0.828 |
| 4205 | 0.779 | 1.000 | 0.812 | 1.000 | 0.979 | 0.960 | 0.897 | 1.000 | 0.990 | 1.000 | 0.965 | 1.000 | 0.991 | 0.855 | 1.000 | 1.000 | 0.861 | 0.833 |
| 4206 | 0.761 | 1.000 | 0.796 | 1.000 | 0.984 | 0.960 | 0.895 | 1.000 | 0.990 | 1.000 | 0.962 | 1.000 | 1.000 | 0.861 | 1.000 | 1.000 | 0.855 | 0.826 |
| 4207 | 0.765 | 1.000 | 0.804 | 1.000 | 0.974 | 0.960 | 0.897 | 1.000 | 0.990 | 1.000 | 0.959 | 1.000 | 1.000 | 0.857 | 1.000 | 1.000 | 0.855 | 0.827 |
| 4208 | 0.767 | 1.000 | 0.806 | 1.000 | 0.974 | 0.960 | 0.894 | 1.000 | 0.990 | 1.000 | 0.957 | 1.000 | 1.000 | 0.849 | 1.000 | 1.000 | 0.854 | 0.825 |
| 4209 | 0.765 | 1.000 | 0.794 | 1.000 | 0.974 | 0.960 | 0.906 | 1.000 | 1.000 | 1.000 | 0.954 | 1.000 | 0.991 | 0.857 | 1.000 | 1.000 | 0.855 | 0.826 |
| 4210 | 0.771 | 1.000 | 0.792 | 1.000 | 0.968 | 0.960 | 0.904 | 1.000 | 0.990 | 1.000 | 0.959 | 0.970 | 0.991 | 0.850 | 1.000 | 1.000 | 0.855 | 0.826 |

| | | | | | | | | | | | | | | | | | | |
|---|---|---|---|---|---|---|---|---|---|---|---|---|---|---|---|---|---|---|
| 4211 | 0.763 | 1.000 | 0.812 | 1.000 | 0.974 | 0.960 | 0.900 | 1.000 | 1.000 | 1.000 | 0.959 | 1.000 | 1.000 | 0.848 | 1.000 | 1.000 | 0.854 | 0.826 |
| 4212 | 0.767 | 1.000 | 0.810 | 1.000 | 0.974 | 0.960 | 0.912 | 1.000 | 0.980 | 1.000 | 0.962 | 1.000 | 0.991 | 0.854 | 1.000 | 1.000 | 0.858 | 0.830 |
| 4213 | 0.768 | 1.000 | 0.800 | 0.986 | 0.968 | 0.960 | 0.903 | 1.000 | 0.990 | 1.000 | 0.962 | 1.000 | 1.000 | 0.849 | 1.000 | 1.000 | 0.855 | 0.826 |
| 4214 | 0.774 | 1.000 | 0.802 | 1.000 | 0.974 | 0.960 | 0.897 | 1.000 | 1.000 | 1.000 | 0.959 | 1.000 | 0.991 | 0.857 | 1.000 | 1.000 | 0.858 | 0.830 |
| 4215 | 0.771 | 1.000 | 0.802 | 1.000 | 0.968 | 0.960 | 0.904 | 1.000 | 1.000 | 1.000 | 0.957 | 0.970 | 0.991 | 0.858 | 1.000 | 1.000 | 0.858 | 0.830 |
| 4216 | 0.768 | 1.000 | 0.802 | 1.000 | 0.979 | 0.960 | 0.897 | 1.000 | 1.000 | 1.000 | 0.959 | 1.000 | 0.982 | 0.840 | 1.000 | 1.000 | 0.852 | 0.823 |
| 4217 | 0.767 | 1.000 | 0.812 | 1.000 | 0.963 | 0.960 | 0.897 | 1.000 | 0.990 | 1.000 | 0.959 | 1.000 | 0.991 | 0.852 | 1.000 | 1.000 | 0.855 | 0.826 |
| 4218 | 0.771 | 1.000 | 0.808 | 1.000 | 0.974 | 0.960 | 0.894 | 1.000 | 0.990 | 1.000 | 0.965 | 1.000 | 0.991 | 0.848 | 1.000 | 1.000 | 0.855 | 0.827 |
| 4219 | 0.771 | 1.000 | 0.794 | 1.000 | 0.974 | 0.960 | 0.900 | 1.000 | 0.990 | 1.000 | 0.957 | 1.000 | 1.000 | 0.852 | 1.000 | 1.000 | 0.855 | 0.827 |
| 4220 | 0.773 | 1.000 | 0.804 | 1.000 | 0.984 | 0.960 | 0.897 | 1.000 | 0.990 | 1.000 | 0.962 | 1.000 | 0.991 | 0.856 | 1.000 | 1.000 | 0.858 | 0.830 |
| 4221 | 0.772 | 1.000 | 0.800 | 1.000 | 0.984 | 0.960 | 0.903 | 1.000 | 1.000 | 1.000 | 0.962 | 1.000 | 0.991 | 0.857 | 1.000 | 1.000 | 0.859 | 0.830 |
| 4222 | 0.775 | 1.000 | 0.824 | 1.000 | 0.974 | 0.960 | 0.900 | 1.000 | 0.990 | 1.000 | 0.962 | 1.000 | 0.991 | 0.851 | 1.000 | 1.000 | 0.859 | 0.831 |
| 4223 | 0.767 | 1.000 | 0.798 | 0.986 | 0.974 | 0.960 | 0.898 | 1.000 | 0.990 | 1.000 | 0.957 | 1.000 | 0.991 | 0.856 | 1.000 | 1.000 | 0.855 | 0.826 |
| 4224 | 0.781 | 1.000 | 0.802 | 1.000 | 0.968 | 0.960 | 0.904 | 1.000 | 0.980 | 1.000 | 0.957 | 1.000 | 0.982 | 0.851 | 1.000 | 1.000 | 0.858 | 0.830 |
| 4225 | 0.768 | 1.000 | 0.810 | 1.000 | 0.979 | 0.960 | 0.900 | 1.000 | 0.990 | 1.000 | 0.957 | 1.000 | 0.991 | 0.851 | 1.000 | 1.000 | 0.856 | 0.827 |
| 4226 | 0.772 | 1.000 | 0.787 | 1.000 | 0.968 | 0.960 | 0.904 | 1.000 | 1.000 | 1.000 | 0.957 | 1.000 | 1.000 | 0.853 | 1.000 | 1.000 | 0.856 | 0.827 |
| 4227 | 0.767 | 1.000 | 0.800 | 1.000 | 0.979 | 0.960 | 0.903 | 1.000 | 0.990 | 1.000 | 0.959 | 1.000 | 1.000 | 0.848 | 1.000 | 1.000 | 0.855 | 0.826 |
| 4228 | 0.769 | 1.000 | 0.808 | 1.000 | 0.979 | 0.960 | 0.901 | 1.000 | 1.000 | 1.000 | 0.959 | 1.000 | 0.991 | 0.853 | 1.000 | 1.000 | 0.857 | 0.829 |
| 4229 | 0.759 | 1.000 | 0.800 | 1.000 | 0.968 | 0.960 | 0.900 | 1.000 | 0.990 | 1.000 | 0.962 | 1.000 | 0.991 | 0.854 | 1.000 | 1.000 | 0.853 | 0.824 |
| 4230 | 0.763 | 1.000 | 0.787 | 1.000 | 0.974 | 0.960 | 0.889 | 1.000 | 0.980 | 1.000 | 0.959 | 1.000 | 0.991 | 0.851 | 1.000 | 1.000 | 0.850 | 0.820 |
| 4231 | 0.771 | 1.000 | 0.787 | 1.000 | 0.974 | 0.960 | 0.906 | 1.000 | 0.990 | 1.000 | 0.959 | 1.000 | 1.000 | 0.847 | 1.000 | 1.000 | 0.854 | 0.825 |
| 4232 | 0.765 | 1.000 | 0.794 | 1.000 | 0.968 | 0.960 | 0.898 | 1.000 | 0.990 | 1.000 | 0.959 | 0.970 | 0.991 | 0.854 | 1.000 | 1.000 | 0.853 | 0.824 |
| 4233 | 0.777 | 1.000 | 0.794 | 1.000 | 0.974 | 0.960 | 0.891 | 1.000 | 1.000 | 1.000 | 0.959 | 1.000 | 1.000 | 0.853 | 1.000 | 1.000 | 0.856 | 0.828 |
| 4234 | 0.776 | 1.000 | 0.800 | 1.000 | 0.974 | 0.960 | 0.891 | 1.000 | 0.990 | 1.000 | 0.962 | 1.000 | 0.982 | 0.854 | 1.000 | 1.000 | 0.857 | 0.828 |
| 4235 | 0.771 | 1.000 | 0.789 | 1.000 | 0.974 | 0.960 | 0.901 | 1.000 | 0.980 | 1.000 | 0.959 | 1.000 | 1.000 | 0.852 | 1.000 | 1.000 | 0.855 | 0.826 |
| 4236 | 0.774 | 1.000 | 0.812 | 1.000 | 0.968 | 0.960 | 0.901 | 1.000 | 0.990 | 1.000 | 0.957 | 1.000 | 0.991 | 0.858 | 1.000 | 1.000 | 0.859 | 0.831 |
| 4237 | 0.764 | 1.000 | 0.808 | 1.000 | 0.974 | 0.960 | 0.903 | 1.000 | 0.990 | 1.000 | 0.959 | 1.000 | 0.982 | 0.849 | 1.000 | 1.000 | 0.854 | 0.825 |
| 4238 | 0.771 | 1.000 | 0.816 | 1.000 | 0.979 | 0.960 | 0.884 | 1.000 | 0.990 | 1.000 | 0.957 | 0.970 | 1.000 | 0.854 | 1.000 | 1.000 | 0.856 | 0.828 |
| 4239 | 0.783 | 1.000 | 0.800 | 1.000 | 0.974 | 0.960 | 0.903 | 1.000 | 0.990 | 1.000 | 0.965 | 1.000 | 1.000 | 0.857 | 1.000 | 1.000 | 0.862 | 0.834 |
| 4240 | 0.768 | 1.000 | 0.804 | 1.000 | 0.974 | 0.960 | 0.901 | 1.000 | 1.000 | 1.000 | 0.957 | 1.000 | 0.991 | 0.847 | 1.000 | 1.000 | 0.854 | 0.826 |
| 4241 | 0.776 | 1.000 | 0.812 | 1.000 | 0.979 | 0.960 | 0.900 | 1.000 | 0.990 | 1.000 | 0.957 | 1.000 | 0.991 | 0.843 | 1.000 | 1.000 | 0.856 | 0.828 |
| 4242 | 0.774 | 1.000 | 0.796 | 1.000 | 0.979 | 0.960 | 0.900 | 1.000 | 0.980 | 1.000 | 0.959 | 0.970 | 1.000 | 0.846 | 1.000 | 1.000 | 0.855 | 0.826 |
| 4243 | 0.762 | 1.000 | 0.800 | 1.000 | 0.974 | 0.960 | 0.900 | 1.000 | 1.000 | 1.000 | 0.962 | 1.000 | 0.982 | 0.854 | 1.000 | 1.000 | 0.854 | 0.825 |
| 4244 | 0.766 | 1.000 | 0.791 | 1.000 | 0.968 | 0.960 | 0.889 | 1.000 | 0.990 | 1.000 | 0.965 | 1.000 | 0.982 | 0.851 | 1.000 | 1.000 | 0.852 | 0.822 |
| 4245 | 0.761 | 1.000 | 0.802 | 1.000 | 0.974 | 0.960 | 0.900 | 1.000 | 0.990 | 1.000 | 0.957 | 1.000 | 0.991 | 0.865 | 1.000 | 1.000 | 0.856 | 0.827 |
| 4246 | 0.779 | 1.000 | 0.812 | 1.000 | 0.979 | 0.960 | 0.891 | 1.000 | 0.990 | 1.000 | 0.962 | 0.970 | 1.000 | 0.846 | 1.000 | 1.000 | 0.857 | 0.828 |
| 4247 | 0.768 | 1.000 | 0.794 | 1.000 | 0.979 | 0.960 | 0.892 | 1.000 | 0.990 | 1.000 | 0.962 | 1.000 | 1.000 | 0.847 | 1.000 | 1.000 | 0.853 | 0.823 |
| 4248 | 0.765 | 1.000 | 0.798 | 1.000 | 0.979 | 0.960 | 0.901 | 1.000 | 0.990 | 1.000 | 0.962 | 1.000 | 0.991 | 0.852 | 1.000 | 1.000 | 0.854 | 0.826 |
| 4249 | 0.768 | 1.000 | 0.792 | 1.000 | 0.974 | 0.960 | 0.895 | 1.000 | 0.990 | 1.000 | 0.957 | 0.970 | 1.000 | 0.856 | 1.000 | 1.000 | 0.854 | 0.825 |

| | | | | | | | | | | | | | | | | | | |
|---|---|---|---|---|---|---|---|---|---|---|---|---|---|---|---|---|---|---|
| 4250 | 0.767 | 1.000 | 0.781 | 1.000 | 0.968 | 0.960 | 0.908 | 1.000 | 0.990 | 1.000 | 0.957 | 1.000 | 0.982 | 0.851 | 1.000 | 1.000 | 0.853 | 0.824 |
| 4251 | 0.770 | 1.000 | 0.798 | 1.000 | 0.974 | 0.960 | 0.889 | 1.000 | 0.990 | 1.000 | 0.962 | 1.000 | 0.991 | 0.857 | 1.000 | 1.000 | 0.855 | 0.826 |
| 4252 | 0.777 | 1.000 | 0.794 | 1.000 | 0.974 | 0.960 | 0.895 | 1.000 | 0.990 | 1.000 | 0.965 | 1.000 | 0.991 | 0.847 | 1.000 | 1.000 | 0.856 | 0.827 |
| 4253 | 0.761 | 1.000 | 0.802 | 1.000 | 0.979 | 0.960 | 0.903 | 1.000 | 0.990 | 1.000 | 0.971 | 1.000 | 0.982 | 0.855 | 1.000 | 1.000 | 0.855 | 0.827 |
| 4254 | 0.762 | 1.000 | 0.798 | 1.000 | 0.979 | 0.960 | 0.901 | 1.000 | 0.990 | 1.000 | 0.954 | 1.000 | 1.000 | 0.845 | 1.000 | 1.000 | 0.851 | 0.822 |
| 4255 | 0.773 | 1.000 | 0.794 | 1.000 | 0.979 | 0.960 | 0.901 | 1.000 | 0.990 | 1.000 | 0.957 | 0.970 | 0.991 | 0.852 | 1.000 | 1.000 | 0.856 | 0.827 |
| 4256 | 0.767 | 1.000 | 0.806 | 1.000 | 0.979 | 0.960 | 0.900 | 1.000 | 0.969 | 1.000 | 0.951 | 1.000 | 1.000 | 0.854 | 1.000 | 1.000 | 0.855 | 0.827 |
| 4257 | 0.765 | 1.000 | 0.800 | 1.000 | 0.984 | 0.960 | 0.895 | 1.000 | 1.000 | 1.000 | 0.959 | 1.000 | 0.991 | 0.854 | 1.000 | 1.000 | 0.855 | 0.826 |
| 4258 | 0.770 | 1.000 | 0.800 | 1.000 | 0.979 | 0.960 | 0.892 | 1.000 | 1.000 | 1.000 | 0.968 | 1.000 | 1.000 | 0.854 | 1.000 | 1.000 | 0.856 | 0.828 |
| 4259 | 0.771 | 1.000 | 0.812 | 1.000 | 0.968 | 0.960 | 0.906 | 1.000 | 0.990 | 1.000 | 0.971 | 1.000 | 0.991 | 0.862 | 1.000 | 1.000 | 0.861 | 0.834 |
| 4260 | 0.764 | 1.000 | 0.806 | 1.000 | 0.974 | 0.960 | 0.901 | 1.000 | 0.980 | 1.000 | 0.959 | 1.000 | 0.991 | 0.848 | 1.000 | 1.000 | 0.853 | 0.824 |
| 4261 | 0.770 | 1.000 | 0.804 | 1.000 | 0.979 | 0.960 | 0.906 | 1.000 | 1.000 | 1.000 | 0.959 | 1.000 | 1.000 | 0.847 | 1.000 | 1.000 | 0.856 | 0.828 |
| 4262 | 0.779 | 1.000 | 0.796 | 1.000 | 0.968 | 0.960 | 0.903 | 1.000 | 0.990 | 1.000 | 0.954 | 1.000 | 0.991 | 0.850 | 1.000 | 1.000 | 0.857 | 0.828 |
| 4263 | 0.775 | 1.000 | 0.791 | 1.000 | 0.968 | 0.960 | 0.904 | 1.000 | 0.990 | 1.000 | 0.959 | 1.000 | 1.000 | 0.854 | 1.000 | 1.000 | 0.857 | 0.828 |
| 4264 | 0.772 | 1.000 | 0.794 | 1.000 | 0.979 | 0.960 | 0.912 | 1.000 | 0.990 | 1.000 | 0.965 | 1.000 | 0.991 | 0.854 | 1.000 | 1.000 | 0.858 | 0.830 |
| 4265 | 0.772 | 1.000 | 0.789 | 1.000 | 0.979 | 0.960 | 0.903 | 1.000 | 0.990 | 1.000 | 0.962 | 1.000 | 1.000 | 0.851 | 1.000 | 1.000 | 0.856 | 0.827 |
| 4266 | 0.774 | 1.000 | 0.785 | 1.000 | 0.974 | 0.960 | 0.892 | 1.000 | 1.000 | 1.000 | 0.959 | 1.000 | 1.000 | 0.854 | 1.000 | 1.000 | 0.855 | 0.826 |
| 4267 | 0.768 | 1.000 | 0.808 | 1.000 | 0.963 | 0.960 | 0.894 | 1.000 | 0.990 | 1.000 | 0.957 | 1.000 | 1.000 | 0.854 | 1.000 | 1.000 | 0.855 | 0.826 |
| 4268 | 0.765 | 1.000 | 0.792 | 1.000 | 0.968 | 0.960 | 0.904 | 1.000 | 0.990 | 1.000 | 0.957 | 1.000 | 0.991 | 0.861 | 1.000 | 1.000 | 0.856 | 0.827 |
| 4269 | 0.770 | 1.000 | 0.800 | 1.000 | 0.979 | 0.960 | 0.889 | 1.000 | 0.980 | 1.000 | 0.959 | 1.000 | 0.991 | 0.853 | 1.000 | 1.000 | 0.854 | 0.826 |
| 4270 | 0.769 | 1.000 | 0.800 | 0.986 | 0.963 | 0.960 | 0.901 | 1.000 | 0.990 | 1.000 | 0.951 | 1.000 | 0.991 | 0.858 | 1.000 | 1.000 | 0.856 | 0.827 |
| 4271 | 0.774 | 1.000 | 0.804 | 0.986 | 0.974 | 0.960 | 0.906 | 1.000 | 0.990 | 1.000 | 0.959 | 1.000 | 0.991 | 0.851 | 1.000 | 1.000 | 0.857 | 0.829 |
| 4272 | 0.769 | 1.000 | 0.806 | 1.000 | 0.974 | 0.960 | 0.900 | 1.000 | 0.980 | 1.000 | 0.959 | 1.000 | 0.991 | 0.858 | 1.000 | 1.000 | 0.857 | 0.829 |
| 4273 | 0.765 | 1.000 | 0.791 | 1.000 | 0.979 | 0.960 | 0.901 | 1.000 | 0.990 | 1.000 | 0.962 | 0.970 | 0.991 | 0.854 | 1.000 | 1.000 | 0.854 | 0.825 |
| 4274 | 0.767 | 1.000 | 0.800 | 1.000 | 0.958 | 0.960 | 0.898 | 1.000 | 1.000 | 1.000 | 0.965 | 1.000 | 0.982 | 0.848 | 1.000 | 1.000 | 0.853 | 0.824 |
| 4275 | 0.764 | 1.000 | 0.791 | 1.000 | 0.968 | 0.960 | 0.901 | 1.000 | 0.990 | 1.000 | 0.954 | 1.000 | 0.991 | 0.851 | 1.000 | 1.000 | 0.852 | 0.823 |
| 4276 | 0.774 | 1.000 | 0.794 | 1.000 | 0.974 | 0.960 | 0.895 | 1.000 | 0.990 | 1.000 | 0.965 | 1.000 | 1.000 | 0.849 | 1.000 | 1.000 | 0.855 | 0.827 |
| 4277 | 0.771 | 1.000 | 0.804 | 1.000 | 0.974 | 0.960 | 0.904 | 1.000 | 1.000 | 1.000 | 0.962 | 1.000 | 0.982 | 0.863 | 1.000 | 1.000 | 0.860 | 0.832 |
| 4278 | 0.755 | 1.000 | 0.818 | 1.000 | 0.984 | 0.960 | 0.897 | 1.000 | 0.990 | 1.000 | 0.945 | 1.000 | 0.991 | 0.851 | 1.000 | 1.000 | 0.852 | 0.823 |
| 4279 | 0.775 | 1.000 | 0.800 | 1.000 | 0.968 | 0.960 | 0.900 | 1.000 | 0.990 | 1.000 | 0.957 | 1.000 | 1.000 | 0.854 | 1.000 | 1.000 | 0.857 | 0.829 |
| 4280 | 0.769 | 1.000 | 0.810 | 1.000 | 0.974 | 0.960 | 0.892 | 1.000 | 0.990 | 1.000 | 0.957 | 1.000 | 1.000 | 0.853 | 1.000 | 1.000 | 0.856 | 0.827 |
| 4281 | 0.769 | 1.000 | 0.796 | 1.000 | 0.974 | 0.960 | 0.906 | 1.000 | 0.990 | 1.000 | 0.959 | 1.000 | 1.000 | 0.851 | 1.000 | 1.000 | 0.856 | 0.827 |
| 4282 | 0.771 | 1.000 | 0.798 | 1.000 | 0.979 | 0.960 | 0.892 | 1.000 | 0.980 | 1.000 | 0.962 | 1.000 | 1.000 | 0.854 | 1.000 | 1.000 | 0.855 | 0.827 |
| 4283 | 0.767 | 1.000 | 0.804 | 1.000 | 0.974 | 0.960 | 0.904 | 1.000 | 1.000 | 1.000 | 0.957 | 1.000 | 0.991 | 0.845 | 1.000 | 1.000 | 0.854 | 0.825 |
| 4284 | 0.768 | 1.000 | 0.810 | 1.000 | 0.979 | 0.960 | 0.914 | 1.000 | 1.000 | 1.000 | 0.965 | 1.000 | 1.000 | 0.855 | 1.000 | 1.000 | 0.860 | 0.832 |
| 4285 | 0.778 | 1.000 | 0.802 | 1.000 | 0.979 | 0.960 | 0.894 | 1.000 | 0.990 | 1.000 | 0.954 | 1.000 | 1.000 | 0.849 | 1.000 | 1.000 | 0.857 | 0.828 |
| 4286 | 0.765 | 1.000 | 0.804 | 0.986 | 0.984 | 0.960 | 0.904 | 1.000 | 0.990 | 1.000 | 0.959 | 1.000 | 1.000 | 0.851 | 1.000 | 1.000 | 0.855 | 0.827 |
| 4287 | 0.767 | 1.000 | 0.804 | 1.000 | 0.979 | 0.960 | 0.897 | 1.000 | 1.000 | 1.000 | 0.968 | 1.000 | 0.991 | 0.847 | 1.000 | 1.000 | 0.854 | 0.825 |
| 4288 | 0.767 | 1.000 | 0.804 | 1.000 | 0.974 | 0.960 | 0.895 | 1.000 | 0.990 | 1.000 | 0.965 | 1.000 | 0.991 | 0.856 | 1.000 | 1.000 | 0.856 | 0.827 |

| | | | | | | | | | | | | | | | | | |
|---|---|---|---|---|---|---|---|---|---|---|---|---|---|---|---|---|---|
| 4289 | 0.771 | 1.000 | 0.800 | 1.000 | 0.979 | 0.960 | 0.898 | 1.000 | 1.000 | 1.000 | 0.954 | 1.000 | 0.991 | 0.855 | 1.000 | 1.000 | 0.857 | 0.828 |
| 4290 | 0.765 | 1.000 | 0.798 | 1.000 | 0.974 | 0.960 | 0.897 | 1.000 | 0.990 | 1.000 | 0.962 | 1.000 | 0.991 | 0.858 | 1.000 | 1.000 | 0.855 | 0.827 |
| 4291 | 0.771 | 1.000 | 0.800 | 1.000 | 0.974 | 0.960 | 0.900 | 1.000 | 0.990 | 1.000 | 0.957 | 1.000 | 0.991 | 0.854 | 1.000 | 1.000 | 0.856 | 0.827 |
| 4292 | 0.766 | 1.000 | 0.792 | 1.000 | 0.974 | 0.960 | 0.904 | 1.000 | 0.990 | 1.000 | 0.954 | 0.970 | 1.000 | 0.856 | 1.000 | 1.000 | 0.855 | 0.826 |
| 4293 | 0.766 | 1.000 | 0.822 | 1.000 | 0.979 | 0.960 | 0.898 | 1.000 | 0.990 | 1.000 | 0.959 | 1.000 | 1.000 | 0.854 | 1.000 | 1.000 | 0.857 | 0.829 |
| 4294 | 0.775 | 1.000 | 0.806 | 1.000 | 0.968 | 0.960 | 0.895 | 1.000 | 0.990 | 1.000 | 0.951 | 1.000 | 0.991 | 0.847 | 1.000 | 1.000 | 0.855 | 0.826 |
| 4295 | 0.769 | 1.000 | 0.794 | 1.000 | 0.979 | 0.960 | 0.901 | 1.000 | 1.000 | 1.000 | 0.962 | 0.970 | 0.991 | 0.848 | 1.000 | 1.000 | 0.854 | 0.826 |
| 4296 | 0.769 | 1.000 | 0.792 | 1.000 | 0.979 | 0.960 | 0.894 | 1.000 | 0.980 | 1.000 | 0.957 | 1.000 | 1.000 | 0.853 | 1.000 | 1.000 | 0.854 | 0.825 |
| 4297 | 0.765 | 1.000 | 0.789 | 0.986 | 0.979 | 0.960 | 0.903 | 1.000 | 0.990 | 1.000 | 0.962 | 1.000 | 0.991 | 0.860 | 1.000 | 1.000 | 0.856 | 0.827 |
| 4298 | 0.766 | 1.000 | 0.800 | 1.000 | 0.984 | 0.960 | 0.888 | 1.000 | 1.000 | 1.000 | 0.962 | 1.000 | 1.000 | 0.847 | 1.000 | 1.000 | 0.852 | 0.823 |
| 4299 | 0.764 | 1.000 | 0.802 | 1.000 | 0.974 | 0.960 | 0.897 | 1.000 | 0.990 | 1.000 | 0.951 | 1.000 | 0.991 | 0.857 | 1.000 | 1.000 | 0.854 | 0.825 |
| 4300 | 0.771 | 1.000 | 0.800 | 1.000 | 0.974 | 0.960 | 0.895 | 1.000 | 1.000 | 1.000 | 0.965 | 1.000 | 1.000 | 0.851 | 1.000 | 1.000 | 0.856 | 0.827 |
| 4301 | 0.766 | 1.000 | 0.798 | 0.986 | 0.974 | 0.960 | 0.901 | 1.000 | 1.000 | 1.000 | 0.957 | 1.000 | 1.000 | 0.858 | 1.000 | 1.000 | 0.856 | 0.827 |
| 4302 | 0.772 | 1.000 | 0.791 | 1.000 | 0.979 | 0.960 | 0.897 | 1.000 | 1.000 | 1.000 | 0.959 | 0.970 | 0.991 | 0.845 | 1.000 | 1.000 | 0.853 | 0.824 |
| 4303 | 0.769 | 1.000 | 0.785 | 1.000 | 0.974 | 0.960 | 0.895 | 1.000 | 1.000 | 1.000 | 0.971 | 1.000 | 0.982 | 0.858 | 1.000 | 1.000 | 0.855 | 0.826 |
| 4304 | 0.763 | 1.000 | 0.816 | 1.000 | 0.979 | 0.960 | 0.892 | 1.000 | 1.000 | 1.000 | 0.965 | 1.000 | 1.000 | 0.854 | 1.000 | 1.000 | 0.856 | 0.827 |
| 4305 | 0.767 | 1.000 | 0.800 | 1.000 | 0.974 | 0.960 | 0.904 | 1.000 | 0.980 | 1.000 | 0.965 | 1.000 | 0.991 | 0.856 | 1.000 | 1.000 | 0.857 | 0.828 |
| 4306 | 0.770 | 1.000 | 0.792 | 1.000 | 0.968 | 0.960 | 0.903 | 1.000 | 0.980 | 1.000 | 0.962 | 1.000 | 0.991 | 0.850 | 1.000 | 1.000 | 0.854 | 0.825 |
| 4307 | 0.767 | 1.000 | 0.798 | 1.000 | 0.974 | 0.960 | 0.901 | 1.000 | 0.990 | 1.000 | 0.965 | 1.000 | 0.991 | 0.854 | 1.000 | 1.000 | 0.856 | 0.827 |
| 4308 | 0.773 | 1.000 | 0.802 | 1.000 | 0.974 | 0.960 | 0.908 | 1.000 | 0.980 | 1.000 | 0.959 | 1.000 | 1.000 | 0.848 | 1.000 | 1.000 | 0.857 | 0.829 |
| 4309 | 0.763 | 1.000 | 0.802 | 1.000 | 0.979 | 0.960 | 0.904 | 1.000 | 0.980 | 1.000 | 0.962 | 1.000 | 0.991 | 0.854 | 1.000 | 1.000 | 0.855 | 0.827 |
| 4310 | 0.767 | 1.000 | 0.794 | 1.000 | 0.968 | 0.960 | 0.891 | 1.000 | 0.990 | 1.000 | 0.957 | 1.000 | 1.000 | 0.862 | 1.000 | 1.000 | 0.855 | 0.826 |
| 4311 | 0.777 | 1.000 | 0.792 | 1.000 | 0.974 | 0.960 | 0.895 | 1.000 | 0.990 | 1.000 | 0.957 | 1.000 | 1.000 | 0.852 | 1.000 | 1.000 | 0.856 | 0.827 |
| 4312 | 0.769 | 1.000 | 0.806 | 1.000 | 0.979 | 0.960 | 0.892 | 1.000 | 0.990 | 1.000 | 0.951 | 1.000 | 1.000 | 0.848 | 1.000 | 1.000 | 0.854 | 0.825 |
| 4313 | 0.769 | 1.000 | 0.814 | 1.000 | 0.984 | 0.960 | 0.895 | 1.000 | 1.000 | 1.000 | 0.954 | 0.970 | 0.991 | 0.851 | 1.000 | 1.000 | 0.856 | 0.827 |
| 4314 | 0.771 | 1.000 | 0.785 | 1.000 | 0.968 | 0.960 | 0.891 | 1.000 | 0.990 | 1.000 | 0.957 | 1.000 | 0.982 | 0.861 | 1.000 | 1.000 | 0.855 | 0.825 |
| 4315 | 0.765 | 1.000 | 0.798 | 0.986 | 0.974 | 0.960 | 0.901 | 1.000 | 0.990 | 1.000 | 0.962 | 0.970 | 0.982 | 0.855 | 1.000 | 1.000 | 0.855 | 0.826 |
| 4316 | 0.774 | 1.000 | 0.798 | 0.986 | 0.979 | 0.960 | 0.900 | 1.000 | 0.990 | 1.000 | 0.971 | 1.000 | 1.000 | 0.847 | 1.000 | 1.000 | 0.856 | 0.828 |
| 4317 | 0.763 | 1.000 | 0.789 | 1.000 | 0.974 | 0.960 | 0.897 | 1.000 | 1.000 | 1.000 | 0.959 | 1.000 | 0.982 | 0.854 | 1.000 | 1.000 | 0.852 | 0.823 |
| 4318 | 0.767 | 1.000 | 0.796 | 1.000 | 0.974 | 0.960 | 0.901 | 1.000 | 0.990 | 1.000 | 0.968 | 1.000 | 0.982 | 0.851 | 1.000 | 1.000 | 0.854 | 0.826 |
| 4319 | 0.765 | 1.000 | 0.802 | 1.000 | 0.979 | 0.960 | 0.894 | 1.000 | 0.990 | 1.000 | 0.954 | 1.000 | 1.000 | 0.852 | 1.000 | 1.000 | 0.853 | 0.824 |
| 4320 | 0.776 | 1.000 | 0.791 | 1.000 | 0.968 | 0.960 | 0.898 | 1.000 | 0.980 | 1.000 | 0.968 | 1.000 | 1.000 | 0.851 | 1.000 | 1.000 | 0.856 | 0.828 |
| 4321 | 0.772 | 1.000 | 0.798 | 1.000 | 0.974 | 0.960 | 0.904 | 1.000 | 1.000 | 1.000 | 0.957 | 1.000 | 0.982 | 0.852 | 1.000 | 1.000 | 0.856 | 0.828 |
| 4322 | 0.769 | 1.000 | 0.800 | 0.986 | 0.979 | 0.960 | 0.906 | 1.000 | 0.990 | 1.000 | 0.951 | 0.970 | 0.982 | 0.853 | 1.000 | 1.000 | 0.855 | 0.827 |
| 4323 | 0.777 | 1.000 | 0.822 | 1.000 | 0.979 | 0.960 | 0.892 | 1.000 | 0.990 | 1.000 | 0.962 | 1.000 | 0.982 | 0.853 | 1.000 | 1.000 | 0.859 | 0.832 |
| 4324 | 0.770 | 1.000 | 0.796 | 0.986 | 0.979 | 0.960 | 0.894 | 1.000 | 0.990 | 1.000 | 0.954 | 1.000 | 1.000 | 0.857 | 1.000 | 1.000 | 0.855 | 0.826 |
| 4325 | 0.765 | 1.000 | 0.791 | 1.000 | 0.974 | 0.960 | 0.898 | 1.000 | 0.969 | 1.000 | 0.959 | 1.000 | 0.991 | 0.859 | 1.000 | 1.000 | 0.854 | 0.825 |
| 4326 | 0.776 | 1.000 | 0.804 | 1.000 | 0.979 | 0.960 | 0.897 | 1.000 | 0.990 | 1.000 | 0.951 | 1.000 | 1.000 | 0.853 | 1.000 | 1.000 | 0.857 | 0.829 |
| 4327 | 0.764 | 1.000 | 0.804 | 1.000 | 0.979 | 0.960 | 0.895 | 1.000 | 1.000 | 1.000 | 0.962 | 0.970 | 1.000 | 0.860 | 1.000 | 1.000 | 0.856 | 0.828 |

| | | | | | | | | | | | | | | | | | |
|---|---|---|---|---|---|---|---|---|---|---|---|---|---|---|---|---|---|
| 4328 | 0.760 | 1.000 | 0.789 | 1.000 | 0.968 | 0.960 | 0.897 | 1.000 | 0.990 | 1.000 | 0.959 | 1.000 | 0.982 | 0.847 | 1.000 | 1.000 | 0.849 | 0.820 |
| 4329 | 0.763 | 1.000 | 0.800 | 1.000 | 0.974 | 0.960 | 0.892 | 1.000 | 0.990 | 1.000 | 0.959 | 0.970 | 0.991 | 0.855 | 1.000 | 1.000 | 0.853 | 0.824 |
| 4330 | 0.767 | 1.000 | 0.787 | 1.000 | 0.968 | 0.960 | 0.892 | 1.000 | 1.000 | 1.000 | 0.959 | 1.000 | 0.982 | 0.851 | 1.000 | 1.000 | 0.852 | 0.822 |
| 4331 | 0.771 | 1.000 | 0.812 | 1.000 | 0.974 | 0.960 | 0.900 | 1.000 | 0.990 | 1.000 | 0.965 | 1.000 | 1.000 | 0.850 | 1.000 | 1.000 | 0.857 | 0.829 |
| 4332 | 0.767 | 1.000 | 0.792 | 1.000 | 0.979 | 0.960 | 0.895 | 1.000 | 0.990 | 1.000 | 0.962 | 1.000 | 1.000 | 0.849 | 1.000 | 1.000 | 0.853 | 0.824 |
| 4333 | 0.765 | 1.000 | 0.792 | 1.000 | 0.979 | 0.960 | 0.904 | 1.000 | 0.980 | 1.000 | 0.962 | 1.000 | 1.000 | 0.858 | 1.000 | 1.000 | 0.856 | 0.827 |
| 4334 | 0.773 | 1.000 | 0.806 | 1.000 | 0.984 | 0.960 | 0.898 | 1.000 | 0.969 | 1.000 | 0.957 | 1.000 | 1.000 | 0.851 | 1.000 | 1.000 | 0.856 | 0.828 |
| 4335 | 0.767 | 1.000 | 0.814 | 1.000 | 0.974 | 0.960 | 0.897 | 1.000 | 0.990 | 1.000 | 0.962 | 0.970 | 1.000 | 0.857 | 1.000 | 1.000 | 0.857 | 0.829 |
| 4336 | 0.775 | 1.000 | 0.802 | 1.000 | 0.974 | 0.960 | 0.894 | 1.000 | 1.000 | 1.000 | 0.957 | 1.000 | 0.973 | 0.854 | 1.000 | 1.000 | 0.857 | 0.828 |
| 4337 | 0.768 | 1.000 | 0.806 | 1.000 | 0.968 | 0.960 | 0.897 | 1.000 | 0.980 | 1.000 | 0.968 | 1.000 | 0.982 | 0.854 | 1.000 | 1.000 | 0.856 | 0.827 |
| 4338 | 0.769 | 1.000 | 0.787 | 1.000 | 0.974 | 0.960 | 0.888 | 1.000 | 0.969 | 1.000 | 0.959 | 1.000 | 1.000 | 0.851 | 1.000 | 1.000 | 0.852 | 0.822 |
| 4339 | 0.770 | 1.000 | 0.802 | 1.000 | 0.974 | 0.960 | 0.891 | 1.000 | 0.990 | 1.000 | 0.959 | 1.000 | 0.991 | 0.852 | 1.000 | 1.000 | 0.855 | 0.825 |
| 4340 | 0.777 | 1.000 | 0.808 | 1.000 | 0.974 | 0.960 | 0.894 | 1.000 | 1.000 | 1.000 | 0.957 | 1.000 | 1.000 | 0.851 | 1.000 | 1.000 | 0.857 | 0.829 |
| 4341 | 0.763 | 1.000 | 0.798 | 1.000 | 0.979 | 0.960 | 0.892 | 1.000 | 0.990 | 1.000 | 0.962 | 1.000 | 1.000 | 0.852 | 1.000 | 1.000 | 0.853 | 0.824 |
| 4342 | 0.767 | 1.000 | 0.804 | 1.000 | 0.979 | 0.960 | 0.900 | 1.000 | 1.000 | 1.000 | 0.962 | 1.000 | 0.991 | 0.853 | 1.000 | 1.000 | 0.856 | 0.827 |
| 4343 | 0.770 | 1.000 | 0.800 | 1.000 | 0.968 | 0.960 | 0.908 | 1.000 | 1.000 | 1.000 | 0.954 | 1.000 | 0.991 | 0.851 | 1.000 | 1.000 | 0.856 | 0.827 |
| 4344 | 0.769 | 1.000 | 0.789 | 1.000 | 0.984 | 0.960 | 0.897 | 1.000 | 0.990 | 1.000 | 0.954 | 1.000 | 0.982 | 0.855 | 1.000 | 1.000 | 0.854 | 0.825 |
| 4345 | 0.768 | 1.000 | 0.798 | 1.000 | 0.979 | 0.960 | 0.904 | 1.000 | 0.980 | 1.000 | 0.954 | 1.000 | 1.000 | 0.857 | 1.000 | 1.000 | 0.856 | 0.828 |
| 4346 | 0.764 | 1.000 | 0.802 | 1.000 | 0.974 | 0.960 | 0.900 | 1.000 | 1.000 | 1.000 | 0.965 | 1.000 | 0.991 | 0.861 | 1.000 | 1.000 | 0.857 | 0.829 |
| 4347 | 0.773 | 1.000 | 0.814 | 1.000 | 0.984 | 0.960 | 0.894 | 1.000 | 0.990 | 1.000 | 0.957 | 1.000 | 1.000 | 0.856 | 1.000 | 1.000 | 0.859 | 0.831 |
| 4348 | 0.767 | 1.000 | 0.804 | 1.000 | 0.979 | 0.960 | 0.895 | 1.000 | 0.990 | 1.000 | 0.959 | 1.000 | 0.991 | 0.854 | 1.000 | 1.000 | 0.855 | 0.827 |
| 4349 | 0.771 | 1.000 | 0.798 | 1.000 | 0.979 | 0.960 | 0.898 | 1.000 | 1.000 | 1.000 | 0.965 | 1.000 | 1.000 | 0.857 | 1.000 | 1.000 | 0.858 | 0.829 |
| 4350 | 0.770 | 1.000 | 0.792 | 1.000 | 0.979 | 0.960 | 0.897 | 1.000 | 1.000 | 1.000 | 0.968 | 1.000 | 0.982 | 0.851 | 1.000 | 1.000 | 0.855 | 0.826 |
| 4351 | 0.773 | 1.000 | 0.806 | 1.000 | 0.968 | 0.960 | 0.891 | 1.000 | 0.990 | 1.000 | 0.959 | 1.000 | 1.000 | 0.855 | 1.000 | 1.000 | 0.857 | 0.828 |
| 4352 | 0.772 | 1.000 | 0.798 | 1.000 | 0.984 | 0.960 | 0.895 | 1.000 | 0.990 | 1.000 | 0.959 | 1.000 | 0.991 | 0.858 | 1.000 | 1.000 | 0.857 | 0.829 |
| 4353 | 0.772 | 1.000 | 0.800 | 1.000 | 0.974 | 0.960 | 0.897 | 1.000 | 1.000 | 1.000 | 0.957 | 1.000 | 0.991 | 0.851 | 1.000 | 1.000 | 0.855 | 0.827 |
| 4354 | 0.773 | 1.000 | 0.802 | 1.000 | 0.979 | 0.960 | 0.891 | 1.000 | 1.000 | 1.000 | 0.965 | 1.000 | 0.991 | 0.850 | 1.000 | 1.000 | 0.856 | 0.827 |
| 4355 | 0.771 | 1.000 | 0.808 | 1.000 | 0.968 | 0.960 | 0.898 | 1.000 | 0.990 | 1.000 | 0.965 | 1.000 | 0.991 | 0.853 | 1.000 | 1.000 | 0.857 | 0.828 |
| 4356 | 0.766 | 1.000 | 0.794 | 1.000 | 0.974 | 0.960 | 0.894 | 1.000 | 0.990 | 1.000 | 0.968 | 0.970 | 1.000 | 0.847 | 1.000 | 1.000 | 0.852 | 0.823 |
| 4357 | 0.768 | 1.000 | 0.798 | 0.986 | 0.974 | 0.960 | 0.894 | 1.000 | 0.980 | 1.000 | 0.959 | 1.000 | 0.991 | 0.857 | 1.000 | 1.000 | 0.855 | 0.826 |
| 4358 | 0.763 | 1.000 | 0.792 | 1.000 | 0.968 | 0.960 | 0.891 | 1.000 | 0.990 | 1.000 | 0.954 | 1.000 | 0.991 | 0.851 | 1.000 | 1.000 | 0.850 | 0.821 |
| 4359 | 0.776 | 1.000 | 0.800 | 1.000 | 0.979 | 0.960 | 0.900 | 1.000 | 1.000 | 1.000 | 0.959 | 1.000 | 0.991 | 0.850 | 1.000 | 1.000 | 0.857 | 0.829 |
| 4360 | 0.762 | 1.000 | 0.791 | 1.000 | 0.974 | 0.960 | 0.892 | 1.000 | 0.969 | 1.000 | 0.968 | 1.000 | 0.982 | 0.867 | 1.000 | 1.000 | 0.855 | 0.826 |
| 4361 | 0.775 | 1.000 | 0.800 | 1.000 | 0.968 | 0.960 | 0.895 | 1.000 | 0.980 | 1.000 | 0.954 | 1.000 | 0.991 | 0.855 | 1.000 | 1.000 | 0.856 | 0.828 |
| 4362 | 0.771 | 1.000 | 0.814 | 0.986 | 0.974 | 0.960 | 0.903 | 1.000 | 0.969 | 1.000 | 0.954 | 1.000 | 1.000 | 0.850 | 1.000 | 1.000 | 0.856 | 0.828 |
| 4363 | 0.769 | 1.000 | 0.800 | 1.000 | 0.968 | 0.960 | 0.900 | 1.000 | 0.990 | 1.000 | 0.957 | 0.970 | 1.000 | 0.858 | 1.000 | 1.000 | 0.856 | 0.828 |
| 4364 | 0.759 | 1.000 | 0.798 | 1.000 | 0.968 | 0.960 | 0.892 | 1.000 | 0.990 | 1.000 | 0.954 | 1.000 | 0.991 | 0.851 | 1.000 | 1.000 | 0.850 | 0.820 |
| 4365 | 0.767 | 1.000 | 0.792 | 1.000 | 0.974 | 0.960 | 0.906 | 1.000 | 0.990 | 1.000 | 0.962 | 0.970 | 1.000 | 0.861 | 1.000 | 1.000 | 0.857 | 0.829 |
| 4366 | 0.778 | 1.000 | 0.781 | 1.000 | 0.968 | 0.960 | 0.897 | 1.000 | 1.000 | 1.000 | 0.957 | 1.000 | 0.982 | 0.855 | 1.000 | 1.000 | 0.856 | 0.827 |

| | | | | | | | | | | | | | | | | | | |
|---|---|---|---|---|---|---|---|---|---|---|---|---|---|---|---|---|---|---|
| 4367 | 0.773 | 1.000 | 0.806 | 1.000 | 0.974 | 0.960 | 0.891 | 1.000 | 0.990 | 1.000 | 0.959 | 1.000 | 0.991 | 0.849 | 1.000 | 1.000 | 0.855 | 0.826 |
| 4368 | 0.767 | 1.000 | 0.806 | 1.000 | 0.968 | 0.960 | 0.897 | 1.000 | 0.990 | 1.000 | 0.959 | 1.000 | 0.991 | 0.847 | 1.000 | 1.000 | 0.853 | 0.824 |
| 4369 | 0.772 | 1.000 | 0.800 | 0.986 | 0.974 | 0.960 | 0.895 | 1.000 | 1.000 | 1.000 | 0.959 | 1.000 | 1.000 | 0.863 | 1.000 | 1.000 | 0.859 | 0.830 |
| 4370 | 0.764 | 1.000 | 0.806 | 1.000 | 0.974 | 0.960 | 0.898 | 1.000 | 0.980 | 1.000 | 0.962 | 1.000 | 1.000 | 0.847 | 1.000 | 1.000 | 0.853 | 0.824 |
| 4371 | 0.775 | 1.000 | 0.812 | 1.000 | 0.968 | 0.960 | 0.897 | 1.000 | 0.990 | 1.000 | 0.954 | 1.000 | 0.991 | 0.854 | 1.000 | 1.000 | 0.858 | 0.829 |
| 4372 | 0.768 | 1.000 | 0.808 | 1.000 | 0.963 | 0.960 | 0.892 | 1.000 | 1.000 | 1.000 | 0.959 | 1.000 | 0.991 | 0.856 | 1.000 | 1.000 | 0.856 | 0.827 |
| 4373 | 0.765 | 1.000 | 0.800 | 0.986 | 0.958 | 0.960 | 0.898 | 1.000 | 0.990 | 1.000 | 0.962 | 1.000 | 0.991 | 0.858 | 1.000 | 1.000 | 0.855 | 0.826 |
| 4374 | 0.775 | 1.000 | 0.800 | 1.000 | 0.979 | 0.960 | 0.901 | 1.000 | 0.980 | 1.000 | 0.959 | 1.000 | 0.991 | 0.844 | 1.000 | 1.000 | 0.855 | 0.826 |
| 4375 | 0.771 | 1.000 | 0.798 | 1.000 | 0.968 | 0.960 | 0.900 | 1.000 | 0.990 | 1.000 | 0.959 | 1.000 | 1.000 | 0.848 | 1.000 | 1.000 | 0.855 | 0.826 |
| 4376 | 0.771 | 1.000 | 0.816 | 1.000 | 0.979 | 0.960 | 0.895 | 1.000 | 0.990 | 1.000 | 0.959 | 1.000 | 1.000 | 0.849 | 1.000 | 1.000 | 0.856 | 0.828 |
| 4377 | 0.776 | 1.000 | 0.812 | 1.000 | 0.979 | 0.960 | 0.904 | 1.000 | 0.990 | 1.000 | 0.954 | 1.000 | 0.991 | 0.858 | 1.000 | 1.000 | 0.860 | 0.833 |
| 4378 | 0.767 | 1.000 | 0.798 | 1.000 | 0.984 | 0.960 | 0.897 | 1.000 | 1.000 | 1.000 | 0.959 | 1.000 | 1.000 | 0.849 | 1.000 | 1.000 | 0.854 | 0.825 |
| 4379 | 0.775 | 1.000 | 0.791 | 1.000 | 0.968 | 0.960 | 0.892 | 1.000 | 0.990 | 1.000 | 0.959 | 0.970 | 0.991 | 0.853 | 1.000 | 1.000 | 0.855 | 0.826 |
| 4380 | 0.766 | 1.000 | 0.806 | 1.000 | 0.974 | 0.960 | 0.909 | 1.000 | 1.000 | 1.000 | 0.959 | 1.000 | 0.982 | 0.852 | 1.000 | 1.000 | 0.856 | 0.828 |
| 4381 | 0.769 | 1.000 | 0.798 | 1.000 | 0.979 | 0.960 | 0.900 | 1.000 | 0.990 | 1.000 | 0.959 | 0.970 | 0.982 | 0.861 | 1.000 | 1.000 | 0.857 | 0.828 |
| 4382 | 0.769 | 1.000 | 0.789 | 1.000 | 0.974 | 0.960 | 0.900 | 1.000 | 0.990 | 1.000 | 0.962 | 1.000 | 0.991 | 0.854 | 1.000 | 1.000 | 0.855 | 0.826 |
| 4383 | 0.763 | 1.000 | 0.806 | 1.000 | 0.974 | 0.960 | 0.908 | 1.000 | 0.980 | 1.000 | 0.959 | 1.000 | 0.991 | 0.854 | 1.000 | 1.000 | 0.855 | 0.827 |
| 4384 | 0.768 | 1.000 | 0.791 | 1.000 | 0.974 | 0.960 | 0.895 | 1.000 | 1.000 | 1.000 | 0.959 | 1.000 | 0.991 | 0.851 | 1.000 | 1.000 | 0.853 | 0.824 |
| 4385 | 0.768 | 1.000 | 0.800 | 1.000 | 0.979 | 0.960 | 0.895 | 1.000 | 0.990 | 1.000 | 0.959 | 1.000 | 0.991 | 0.849 | 1.000 | 1.000 | 0.854 | 0.825 |
| 4386 | 0.763 | 1.000 | 0.791 | 1.000 | 0.974 | 0.960 | 0.894 | 1.000 | 0.990 | 1.000 | 0.965 | 1.000 | 0.991 | 0.855 | 1.000 | 1.000 | 0.853 | 0.824 |
| 4387 | 0.776 | 1.000 | 0.804 | 1.000 | 0.974 | 0.960 | 0.908 | 1.000 | 0.990 | 1.000 | 0.957 | 1.000 | 0.991 | 0.854 | 1.000 | 1.000 | 0.859 | 0.831 |
| 4388 | 0.763 | 1.000 | 0.816 | 1.000 | 0.963 | 0.960 | 0.903 | 1.000 | 1.000 | 1.000 | 0.962 | 1.000 | 0.991 | 0.851 | 1.000 | 1.000 | 0.855 | 0.827 |
| 4389 | 0.769 | 1.000 | 0.808 | 1.000 | 0.968 | 0.960 | 0.909 | 1.000 | 0.990 | 1.000 | 0.962 | 1.000 | 0.991 | 0.858 | 1.000 | 1.000 | 0.859 | 0.831 |
| 4390 | 0.766 | 1.000 | 0.781 | 1.000 | 0.968 | 0.960 | 0.906 | 1.000 | 1.000 | 1.000 | 0.957 | 1.000 | 0.982 | 0.851 | 1.000 | 1.000 | 0.853 | 0.823 |
| 4391 | 0.771 | 1.000 | 0.802 | 1.000 | 0.974 | 0.960 | 0.906 | 1.000 | 0.980 | 1.000 | 0.962 | 1.000 | 1.000 | 0.847 | 1.000 | 1.000 | 0.856 | 0.827 |
| 4392 | 0.771 | 1.000 | 0.806 | 1.000 | 0.979 | 0.960 | 0.908 | 1.000 | 1.000 | 1.000 | 0.957 | 1.000 | 1.000 | 0.851 | 1.000 | 1.000 | 0.857 | 0.829 |
| 4393 | 0.761 | 1.000 | 0.800 | 1.000 | 0.974 | 0.960 | 0.906 | 1.000 | 1.000 | 1.000 | 0.951 | 0.970 | 1.000 | 0.852 | 1.000 | 1.000 | 0.853 | 0.824 |
| 4394 | 0.772 | 1.000 | 0.810 | 1.000 | 0.974 | 0.960 | 0.894 | 1.000 | 0.990 | 1.000 | 0.962 | 1.000 | 1.000 | 0.853 | 1.000 | 1.000 | 0.857 | 0.828 |
| 4395 | 0.776 | 1.000 | 0.802 | 0.986 | 0.974 | 0.960 | 0.895 | 1.000 | 1.000 | 1.000 | 0.965 | 1.000 | 1.000 | 0.860 | 1.000 | 1.000 | 0.860 | 0.831 |
| 4396 | 0.769 | 1.000 | 0.794 | 1.000 | 0.979 | 0.960 | 0.903 | 1.000 | 0.990 | 1.000 | 0.962 | 1.000 | 1.000 | 0.857 | 1.000 | 1.000 | 0.857 | 0.828 |
| 4397 | 0.763 | 1.000 | 0.800 | 1.000 | 0.979 | 0.960 | 0.903 | 1.000 | 0.980 | 1.000 | 0.962 | 1.000 | 0.991 | 0.864 | 1.000 | 1.000 | 0.857 | 0.829 |
| 4398 | 0.770 | 1.000 | 0.800 | 1.000 | 0.968 | 0.960 | 0.897 | 1.000 | 0.990 | 1.000 | 0.954 | 1.000 | 0.982 | 0.847 | 1.000 | 1.000 | 0.853 | 0.824 |
| 4399 | 0.769 | 1.000 | 0.792 | 1.000 | 0.974 | 0.960 | 0.904 | 1.000 | 0.990 | 1.000 | 0.965 | 0.970 | 0.991 | 0.849 | 1.000 | 1.000 | 0.854 | 0.826 |
| 4400 | 0.775 | 1.000 | 0.822 | 1.000 | 0.974 | 0.960 | 0.904 | 1.000 | 0.990 | 1.000 | 0.959 | 1.000 | 1.000 | 0.852 | 1.000 | 1.000 | 0.860 | 0.833 |
| 4401 | 0.766 | 1.000 | 0.800 | 1.000 | 0.979 | 0.960 | 0.901 | 1.000 | 1.000 | 1.000 | 0.954 | 1.000 | 0.991 | 0.859 | 1.000 | 1.000 | 0.856 | 0.828 |
| 4402 | 0.771 | 1.000 | 0.787 | 1.000 | 0.979 | 0.960 | 0.894 | 1.000 | 0.990 | 1.000 | 0.965 | 1.000 | 1.000 | 0.853 | 1.000 | 1.000 | 0.855 | 0.826 |
| 4403 | 0.771 | 1.000 | 0.800 | 1.000 | 0.984 | 0.960 | 0.901 | 1.000 | 0.990 | 1.000 | 0.959 | 1.000 | 0.991 | 0.856 | 1.000 | 1.000 | 0.857 | 0.829 |
| 4404 | 0.765 | 1.000 | 0.792 | 1.000 | 0.979 | 0.960 | 0.903 | 1.000 | 0.990 | 1.000 | 0.965 | 1.000 | 0.991 | 0.856 | 1.000 | 1.000 | 0.855 | 0.827 |
| 4405 | 0.771 | 1.000 | 0.791 | 1.000 | 0.974 | 0.960 | 0.884 | 1.000 | 1.000 | 1.000 | 0.968 | 1.000 | 0.991 | 0.854 | 1.000 | 1.000 | 0.854 | 0.825 |

| | | | | | | | | | | | | | | | | | |
|---|---|---|---|---|---|---|---|---|---|---|---|---|---|---|---|---|---|
| 4406 | 0.761 | 1.000 | 0.802 | 1.000 | 0.974 | 0.960 | 0.892 | 1.000 | 1.000 | 1.000 | 0.962 | 1.000 | 0.991 | 0.854 | 1.000 | 1.000 | 0.853 | 0.824 |
| 4407 | 0.768 | 1.000 | 0.804 | 1.000 | 0.974 | 0.960 | 0.895 | 1.000 | 0.990 | 1.000 | 0.959 | 1.000 | 1.000 | 0.854 | 1.000 | 1.000 | 0.856 | 0.827 |
| 4408 | 0.776 | 1.000 | 0.800 | 1.000 | 0.974 | 0.960 | 0.900 | 1.000 | 0.980 | 1.000 | 0.959 | 1.000 | 0.991 | 0.856 | 1.000 | 1.000 | 0.858 | 0.830 |
| 4409 | 0.771 | 1.000 | 0.796 | 1.000 | 0.979 | 0.960 | 0.898 | 1.000 | 0.990 | 1.000 | 0.954 | 1.000 | 1.000 | 0.854 | 1.000 | 1.000 | 0.856 | 0.827 |
| 4410 | 0.770 | 1.000 | 0.791 | 1.000 | 0.974 | 0.960 | 0.892 | 1.000 | 0.990 | 1.000 | 0.962 | 1.000 | 1.000 | 0.847 | 1.000 | 1.000 | 0.853 | 0.823 |
| 4411 | 0.775 | 1.000 | 0.779 | 1.000 | 0.974 | 0.960 | 0.909 | 1.000 | 0.990 | 1.000 | 0.962 | 1.000 | 1.000 | 0.853 | 1.000 | 1.000 | 0.857 | 0.828 |
| 4412 | 0.771 | 1.000 | 0.791 | 1.000 | 0.979 | 0.960 | 0.895 | 1.000 | 0.990 | 1.000 | 0.962 | 1.000 | 0.991 | 0.855 | 1.000 | 1.000 | 0.856 | 0.827 |
| 4413 | 0.773 | 1.000 | 0.800 | 1.000 | 0.968 | 0.960 | 0.894 | 1.000 | 1.000 | 1.000 | 0.957 | 1.000 | 1.000 | 0.854 | 1.000 | 1.000 | 0.856 | 0.828 |
| 4414 | 0.766 | 1.000 | 0.792 | 1.000 | 0.979 | 0.960 | 0.897 | 1.000 | 1.000 | 1.000 | 0.959 | 1.000 | 0.991 | 0.861 | 1.000 | 1.000 | 0.856 | 0.827 |
| 4415 | 0.766 | 1.000 | 0.791 | 1.000 | 0.979 | 0.960 | 0.903 | 1.000 | 0.990 | 1.000 | 0.962 | 1.000 | 1.000 | 0.854 | 1.000 | 1.000 | 0.855 | 0.826 |
| 4416 | 0.764 | 1.000 | 0.802 | 0.986 | 0.979 | 0.960 | 0.891 | 1.000 | 0.990 | 1.000 | 0.965 | 1.000 | 0.991 | 0.851 | 1.000 | 1.000 | 0.853 | 0.824 |
| 4417 | 0.767 | 1.000 | 0.804 | 1.000 | 0.979 | 0.960 | 0.900 | 1.000 | 0.990 | 1.000 | 0.962 | 1.000 | 0.982 | 0.858 | 1.000 | 1.000 | 0.857 | 0.828 |
| 4418 | 0.775 | 1.000 | 0.816 | 1.000 | 0.979 | 0.960 | 0.895 | 1.000 | 0.980 | 1.000 | 0.959 | 1.000 | 1.000 | 0.848 | 1.000 | 1.000 | 0.857 | 0.829 |
| 4419 | 0.769 | 1.000 | 0.800 | 1.000 | 0.979 | 0.960 | 0.908 | 1.000 | 1.000 | 1.000 | 0.959 | 0.970 | 0.982 | 0.855 | 1.000 | 1.000 | 0.857 | 0.829 |
| 4420 | 0.765 | 1.000 | 0.800 | 1.000 | 0.974 | 0.960 | 0.912 | 1.000 | 0.990 | 1.000 | 0.951 | 1.000 | 0.991 | 0.858 | 1.000 | 1.000 | 0.857 | 0.828 |
| 4421 | 0.769 | 1.000 | 0.785 | 1.000 | 0.968 | 0.960 | 0.900 | 1.000 | 1.000 | 1.000 | 0.957 | 1.000 | 1.000 | 0.858 | 1.000 | 1.000 | 0.855 | 0.826 |
| 4422 | 0.767 | 1.000 | 0.808 | 1.000 | 0.974 | 0.960 | 0.895 | 1.000 | 1.000 | 1.000 | 0.959 | 1.000 | 0.991 | 0.851 | 1.000 | 1.000 | 0.855 | 0.826 |
| 4423 | 0.773 | 1.000 | 0.816 | 1.000 | 0.968 | 0.960 | 0.891 | 1.000 | 0.990 | 1.000 | 0.957 | 1.000 | 1.000 | 0.860 | 1.000 | 1.000 | 0.859 | 0.831 |
| 4424 | 0.773 | 1.000 | 0.787 | 1.000 | 0.974 | 0.960 | 0.904 | 1.000 | 0.980 | 1.000 | 0.959 | 1.000 | 0.991 | 0.856 | 1.000 | 1.000 | 0.857 | 0.828 |
| 4425 | 0.762 | 1.000 | 0.812 | 1.000 | 0.968 | 0.960 | 0.897 | 1.000 | 1.000 | 1.000 | 0.962 | 1.000 | 0.982 | 0.858 | 1.000 | 1.000 | 0.855 | 0.827 |
| 4426 | 0.760 | 1.000 | 0.798 | 1.000 | 0.974 | 0.960 | 0.894 | 1.000 | 0.990 | 1.000 | 0.965 | 1.000 | 0.991 | 0.858 | 1.000 | 1.000 | 0.853 | 0.824 |
| 4427 | 0.769 | 1.000 | 0.804 | 1.000 | 0.974 | 0.960 | 0.895 | 1.000 | 0.990 | 1.000 | 0.959 | 1.000 | 0.991 | 0.858 | 1.000 | 1.000 | 0.857 | 0.828 |
| 4428 | 0.768 | 1.000 | 0.804 | 0.986 | 0.979 | 0.960 | 0.906 | 1.000 | 0.990 | 1.000 | 0.951 | 1.000 | 1.000 | 0.852 | 1.000 | 1.000 | 0.856 | 0.827 |
| 4429 | 0.774 | 1.000 | 0.796 | 1.000 | 0.984 | 0.960 | 0.897 | 1.000 | 0.990 | 1.000 | 0.959 | 1.000 | 0.991 | 0.858 | 1.000 | 1.000 | 0.858 | 0.830 |
| 4430 | 0.763 | 1.000 | 0.800 | 1.000 | 0.979 | 0.960 | 0.892 | 1.000 | 0.990 | 1.000 | 0.962 | 1.000 | 0.991 | 0.847 | 1.000 | 1.000 | 0.852 | 0.822 |
| 4431 | 0.772 | 1.000 | 0.800 | 0.986 | 0.979 | 0.960 | 0.898 | 1.000 | 0.980 | 1.000 | 0.965 | 1.000 | 1.000 | 0.851 | 1.000 | 1.000 | 0.856 | 0.827 |
| 4432 | 0.773 | 1.000 | 0.796 | 1.000 | 0.979 | 0.960 | 0.906 | 1.000 | 1.000 | 1.000 | 0.968 | 1.000 | 1.000 | 0.857 | 1.000 | 1.000 | 0.859 | 0.831 |
| 4433 | 0.766 | 1.000 | 0.802 | 1.000 | 0.974 | 0.960 | 0.908 | 1.000 | 0.980 | 1.000 | 0.962 | 1.000 | 1.000 | 0.854 | 1.000 | 1.000 | 0.856 | 0.828 |
| 4434 | 0.772 | 1.000 | 0.818 | 1.000 | 0.974 | 0.960 | 0.898 | 1.000 | 0.980 | 1.000 | 0.957 | 0.970 | 1.000 | 0.851 | 1.000 | 1.000 | 0.857 | 0.829 |
| 4435 | 0.765 | 1.000 | 0.796 | 1.000 | 0.979 | 0.960 | 0.904 | 1.000 | 1.000 | 1.000 | 0.948 | 1.000 | 0.991 | 0.856 | 1.000 | 1.000 | 0.855 | 0.827 |
| 4436 | 0.765 | 1.000 | 0.791 | 1.000 | 0.979 | 0.960 | 0.895 | 1.000 | 0.990 | 1.000 | 0.962 | 1.000 | 0.982 | 0.846 | 1.000 | 1.000 | 0.851 | 0.822 |
| 4437 | 0.767 | 1.000 | 0.810 | 1.000 | 0.979 | 0.960 | 0.904 | 1.000 | 0.990 | 1.000 | 0.965 | 1.000 | 1.000 | 0.858 | 1.000 | 1.000 | 0.858 | 0.830 |
| 4438 | 0.773 | 1.000 | 0.804 | 1.000 | 0.979 | 0.960 | 0.904 | 1.000 | 0.990 | 1.000 | 0.954 | 1.000 | 0.991 | 0.850 | 1.000 | 1.000 | 0.857 | 0.828 |
| 4439 | 0.765 | 1.000 | 0.814 | 1.000 | 0.968 | 0.960 | 0.904 | 1.000 | 0.990 | 1.000 | 0.965 | 1.000 | 0.991 | 0.845 | 1.000 | 1.000 | 0.855 | 0.826 |
| 4440 | 0.774 | 1.000 | 0.800 | 1.000 | 0.974 | 0.960 | 0.903 | 1.000 | 0.990 | 1.000 | 0.957 | 1.000 | 0.982 | 0.856 | 1.000 | 1.000 | 0.858 | 0.830 |
| 4441 | 0.772 | 1.000 | 0.808 | 1.000 | 0.979 | 0.960 | 0.903 | 1.000 | 0.990 | 1.000 | 0.965 | 1.000 | 0.982 | 0.852 | 1.000 | 1.000 | 0.858 | 0.830 |
| 4442 | 0.766 | 1.000 | 0.789 | 1.000 | 0.958 | 0.960 | 0.912 | 1.000 | 0.990 | 1.000 | 0.959 | 1.000 | 0.991 | 0.854 | 1.000 | 1.000 | 0.855 | 0.826 |
| 4443 | 0.770 | 1.000 | 0.810 | 1.000 | 0.974 | 0.960 | 0.894 | 1.000 | 0.980 | 1.000 | 0.957 | 1.000 | 1.000 | 0.848 | 1.000 | 1.000 | 0.855 | 0.826 |
| 4444 | 0.769 | 1.000 | 0.806 | 1.000 | 0.968 | 0.960 | 0.906 | 1.000 | 0.990 | 1.000 | 0.954 | 1.000 | 1.000 | 0.844 | 1.000 | 1.000 | 0.854 | 0.825 |

| | | | | | | | | | | | | | | | | | | |
|---|---|---|---|---|---|---|---|---|---|---|---|---|---|---|---|---|---|---|
| 4445 | 0.777 | 1.000 | 0.806 | 1.000 | 0.968 | 0.960 | 0.889 | 1.000 | 0.990 | 1.000 | 0.957 | 1.000 | 0.991 | 0.844 | 1.000 | 1.000 | 0.855 | 0.826 |
| 4446 | 0.776 | 1.000 | 0.796 | 0.986 | 0.979 | 0.960 | 0.895 | 1.000 | 0.990 | 1.000 | 0.965 | 1.000 | 0.991 | 0.855 | 1.000 | 1.000 | 0.858 | 0.829 |
| 4447 | 0.763 | 1.000 | 0.794 | 1.000 | 0.974 | 0.960 | 0.901 | 1.000 | 0.990 | 1.000 | 0.965 | 1.000 | 0.982 | 0.851 | 1.000 | 1.000 | 0.853 | 0.824 |
| 4448 | 0.773 | 1.000 | 0.796 | 1.000 | 0.968 | 0.960 | 0.912 | 1.000 | 0.980 | 1.000 | 0.962 | 0.970 | 0.991 | 0.851 | 1.000 | 1.000 | 0.857 | 0.828 |
| 4449 | 0.776 | 1.000 | 0.806 | 1.000 | 0.974 | 0.960 | 0.904 | 1.000 | 0.990 | 1.000 | 0.957 | 1.000 | 0.991 | 0.854 | 1.000 | 1.000 | 0.859 | 0.831 |
| 4450 | 0.763 | 1.000 | 0.796 | 1.000 | 0.974 | 0.960 | 0.888 | 1.000 | 0.990 | 1.000 | 0.957 | 1.000 | 0.982 | 0.862 | 1.000 | 1.000 | 0.854 | 0.825 |
| 4451 | 0.766 | 1.000 | 0.808 | 1.000 | 0.979 | 0.960 | 0.909 | 1.000 | 0.990 | 1.000 | 0.957 | 1.000 | 0.991 | 0.848 | 1.000 | 1.000 | 0.855 | 0.827 |
| 4452 | 0.765 | 1.000 | 0.794 | 1.000 | 0.979 | 0.960 | 0.895 | 1.000 | 0.990 | 1.000 | 0.957 | 1.000 | 1.000 | 0.852 | 1.000 | 1.000 | 0.853 | 0.824 |
| 4453 | 0.757 | 1.000 | 0.798 | 1.000 | 0.974 | 0.960 | 0.904 | 1.000 | 0.990 | 1.000 | 0.957 | 0.970 | 1.000 | 0.848 | 1.000 | 1.000 | 0.851 | 0.822 |
| 4454 | 0.765 | 1.000 | 0.810 | 1.000 | 0.974 | 0.960 | 0.900 | 1.000 | 0.980 | 1.000 | 0.962 | 1.000 | 0.991 | 0.847 | 1.000 | 1.000 | 0.854 | 0.825 |
| 4455 | 0.767 | 1.000 | 0.804 | 1.000 | 0.968 | 0.960 | 0.894 | 1.000 | 0.990 | 1.000 | 0.965 | 1.000 | 0.991 | 0.853 | 1.000 | 1.000 | 0.855 | 0.826 |
| 4456 | 0.772 | 1.000 | 0.808 | 1.000 | 0.979 | 0.960 | 0.897 | 1.000 | 1.000 | 1.000 | 0.957 | 1.000 | 0.991 | 0.854 | 1.000 | 1.000 | 0.857 | 0.829 |
| 4457 | 0.763 | 1.000 | 0.789 | 1.000 | 0.984 | 0.960 | 0.904 | 1.000 | 0.990 | 1.000 | 0.965 | 0.970 | 0.991 | 0.852 | 1.000 | 1.000 | 0.854 | 0.825 |
| 4458 | 0.769 | 1.000 | 0.816 | 0.986 | 0.974 | 0.960 | 0.883 | 1.000 | 1.000 | 1.000 | 0.959 | 1.000 | 0.982 | 0.850 | 1.000 | 1.000 | 0.854 | 0.825 |
| 4459 | 0.770 | 1.000 | 0.822 | 1.000 | 0.974 | 0.960 | 0.898 | 1.000 | 0.990 | 1.000 | 0.959 | 1.000 | 1.000 | 0.861 | 1.000 | 1.000 | 0.860 | 0.832 |
| 4460 | 0.776 | 1.000 | 0.785 | 1.000 | 0.974 | 0.960 | 0.904 | 1.000 | 1.000 | 1.000 | 0.957 | 0.970 | 0.973 | 0.856 | 1.000 | 1.000 | 0.857 | 0.828 |
| 4461 | 0.769 | 1.000 | 0.808 | 1.000 | 0.968 | 0.960 | 0.903 | 1.000 | 0.980 | 1.000 | 0.962 | 1.000 | 0.991 | 0.853 | 1.000 | 1.000 | 0.856 | 0.828 |
| 4462 | 0.768 | 1.000 | 0.794 | 1.000 | 0.968 | 0.960 | 0.897 | 1.000 | 0.990 | 1.000 | 0.957 | 1.000 | 0.991 | 0.857 | 1.000 | 1.000 | 0.855 | 0.826 |
| 4463 | 0.774 | 1.000 | 0.812 | 0.986 | 0.974 | 0.960 | 0.898 | 1.000 | 0.990 | 1.000 | 0.959 | 1.000 | 1.000 | 0.854 | 1.000 | 1.000 | 0.858 | 0.830 |
| 4464 | 0.767 | 1.000 | 0.806 | 1.000 | 0.979 | 0.960 | 0.909 | 1.000 | 1.000 | 1.000 | 0.962 | 1.000 | 0.982 | 0.862 | 1.000 | 1.000 | 0.859 | 0.832 |
| 4465 | 0.764 | 1.000 | 0.787 | 1.000 | 0.979 | 0.960 | 0.901 | 1.000 | 0.990 | 1.000 | 0.968 | 1.000 | 1.000 | 0.857 | 1.000 | 1.000 | 0.855 | 0.826 |
| 4466 | 0.767 | 1.000 | 0.792 | 1.000 | 0.984 | 0.960 | 0.897 | 1.000 | 0.990 | 1.000 | 0.959 | 1.000 | 0.991 | 0.854 | 1.000 | 1.000 | 0.854 | 0.826 |
| 4467 | 0.763 | 1.000 | 0.808 | 1.000 | 0.974 | 0.960 | 0.895 | 1.000 | 0.990 | 1.000 | 0.962 | 1.000 | 0.991 | 0.854 | 1.000 | 1.000 | 0.854 | 0.825 |
| 4468 | 0.767 | 1.000 | 0.816 | 1.000 | 0.968 | 0.960 | 0.897 | 1.000 | 0.990 | 1.000 | 0.957 | 0.970 | 0.982 | 0.851 | 1.000 | 1.000 | 0.855 | 0.827 |
| 4469 | 0.776 | 1.000 | 0.796 | 1.000 | 0.979 | 0.960 | 0.906 | 1.000 | 0.980 | 1.000 | 0.962 | 1.000 | 1.000 | 0.861 | 1.000 | 1.000 | 0.860 | 0.832 |
| 4470 | 0.763 | 1.000 | 0.773 | 1.000 | 0.974 | 0.960 | 0.900 | 1.000 | 1.000 | 1.000 | 0.951 | 1.000 | 1.000 | 0.841 | 1.000 | 1.000 | 0.848 | 0.818 |
| 4471 | 0.779 | 1.000 | 0.802 | 1.000 | 0.979 | 0.960 | 0.906 | 1.000 | 0.990 | 1.000 | 0.962 | 1.000 | 0.973 | 0.854 | 1.000 | 1.000 | 0.860 | 0.832 |
| 4472 | 0.775 | 1.000 | 0.810 | 1.000 | 0.984 | 0.960 | 0.898 | 1.000 | 0.990 | 1.000 | 0.959 | 1.000 | 0.982 | 0.847 | 1.000 | 1.000 | 0.857 | 0.828 |
| 4473 | 0.769 | 1.000 | 0.802 | 0.986 | 0.974 | 0.960 | 0.888 | 1.000 | 1.000 | 1.000 | 0.957 | 1.000 | 1.000 | 0.846 | 1.000 | 1.000 | 0.852 | 0.823 |
| 4474 | 0.768 | 1.000 | 0.789 | 1.000 | 0.979 | 0.960 | 0.892 | 1.000 | 1.000 | 1.000 | 0.962 | 1.000 | 0.991 | 0.848 | 1.000 | 1.000 | 0.852 | 0.823 |
| 4475 | 0.771 | 1.000 | 0.792 | 1.000 | 0.974 | 0.960 | 0.908 | 1.000 | 1.000 | 1.000 | 0.965 | 1.000 | 1.000 | 0.847 | 1.000 | 1.000 | 0.855 | 0.827 |
| 4476 | 0.775 | 1.000 | 0.783 | 1.000 | 0.979 | 0.960 | 0.897 | 1.000 | 0.980 | 1.000 | 0.959 | 1.000 | 0.991 | 0.852 | 1.000 | 1.000 | 0.855 | 0.826 |
| 4477 | 0.767 | 1.000 | 0.798 | 1.000 | 0.974 | 0.960 | 0.911 | 1.000 | 0.990 | 1.000 | 0.957 | 1.000 | 0.991 | 0.853 | 1.000 | 1.000 | 0.856 | 0.828 |
| 4478 | 0.773 | 1.000 | 0.800 | 1.000 | 0.979 | 0.960 | 0.906 | 1.000 | 0.990 | 1.000 | 0.959 | 1.000 | 0.991 | 0.854 | 1.000 | 1.000 | 0.858 | 0.830 |
| 4479 | 0.777 | 1.000 | 0.800 | 1.000 | 0.979 | 0.960 | 0.892 | 1.000 | 0.990 | 1.000 | 0.959 | 1.000 | 0.991 | 0.846 | 1.000 | 1.000 | 0.855 | 0.826 |
| 4480 | 0.775 | 1.000 | 0.792 | 1.000 | 0.974 | 0.960 | 0.901 | 1.000 | 0.990 | 1.000 | 0.965 | 1.000 | 0.991 | 0.856 | 1.000 | 1.000 | 0.858 | 0.829 |
| 4481 | 0.765 | 1.000 | 0.802 | 1.000 | 0.974 | 0.960 | 0.895 | 1.000 | 0.990 | 1.000 | 0.965 | 0.970 | 0.991 | 0.844 | 1.000 | 1.000 | 0.852 | 0.822 |
| 4482 | 0.769 | 1.000 | 0.796 | 1.000 | 0.968 | 0.960 | 0.908 | 1.000 | 0.990 | 1.000 | 0.959 | 1.000 | 0.982 | 0.852 | 1.000 | 1.000 | 0.855 | 0.827 |
| 4483 | 0.757 | 1.000 | 0.808 | 1.000 | 0.968 | 0.960 | 0.900 | 1.000 | 0.990 | 1.000 | 0.957 | 1.000 | 0.991 | 0.856 | 1.000 | 1.000 | 0.853 | 0.824 |

| | | | | | | | | | | | | | | | | | | |
|---|---|---|---|---|---|---|---|---|---|---|---|---|---|---|---|---|---|---|
| 4484 | 0.765 | 1.000 | 0.796 | 1.000 | 0.974 | 0.960 | 0.901 | 1.000 | 0.990 | 1.000 | 0.965 | 1.000 | 0.982 | 0.856 | 1.000 | 1.000 | 0.855 | 0.826 |
| 4485 | 0.773 | 1.000 | 0.787 | 1.000 | 0.979 | 0.960 | 0.898 | 1.000 | 1.000 | 1.000 | 0.959 | 0.970 | 0.991 | 0.858 | 1.000 | 1.000 | 0.857 | 0.828 |
| 4486 | 0.761 | 1.000 | 0.800 | 1.000 | 0.974 | 0.960 | 0.900 | 1.000 | 0.969 | 1.000 | 0.957 | 0.970 | 0.991 | 0.855 | 1.000 | 1.000 | 0.853 | 0.824 |
| 4487 | 0.767 | 1.000 | 0.806 | 1.000 | 0.968 | 0.960 | 0.897 | 1.000 | 0.990 | 1.000 | 0.962 | 1.000 | 0.982 | 0.853 | 1.000 | 1.000 | 0.855 | 0.826 |
| 4488 | 0.776 | 1.000 | 0.792 | 1.000 | 0.979 | 0.960 | 0.897 | 1.000 | 0.990 | 1.000 | 0.965 | 1.000 | 1.000 | 0.848 | 1.000 | 1.000 | 0.856 | 0.827 |
| 4489 | 0.764 | 1.000 | 0.804 | 1.000 | 0.974 | 0.960 | 0.894 | 1.000 | 0.990 | 1.000 | 0.965 | 0.970 | 0.991 | 0.858 | 1.000 | 1.000 | 0.855 | 0.826 |
| 4490 | 0.777 | 1.000 | 0.796 | 1.000 | 0.974 | 0.960 | 0.895 | 1.000 | 1.000 | 1.000 | 0.965 | 1.000 | 1.000 | 0.863 | 1.000 | 1.000 | 0.860 | 0.832 |
| 4491 | 0.760 | 1.000 | 0.798 | 1.000 | 0.968 | 0.960 | 0.897 | 1.000 | 1.000 | 1.000 | 0.957 | 1.000 | 0.991 | 0.847 | 1.000 | 1.000 | 0.850 | 0.821 |
| 4492 | 0.765 | 1.000 | 0.810 | 1.000 | 0.974 | 0.960 | 0.901 | 1.000 | 0.990 | 1.000 | 0.954 | 1.000 | 0.991 | 0.857 | 1.000 | 1.000 | 0.856 | 0.828 |
| 4493 | 0.760 | 1.000 | 0.800 | 1.000 | 0.974 | 0.960 | 0.904 | 1.000 | 0.990 | 1.000 | 0.962 | 0.970 | 0.991 | 0.854 | 1.000 | 1.000 | 0.854 | 0.825 |
| 4494 | 0.759 | 1.000 | 0.798 | 1.000 | 0.984 | 0.960 | 0.898 | 1.000 | 0.980 | 1.000 | 0.951 | 1.000 | 0.991 | 0.853 | 1.000 | 1.000 | 0.852 | 0.822 |
| 4495 | 0.774 | 1.000 | 0.798 | 0.986 | 0.984 | 0.960 | 0.904 | 1.000 | 0.980 | 1.000 | 0.957 | 1.000 | 0.991 | 0.856 | 1.000 | 1.000 | 0.858 | 0.830 |
| 4496 | 0.771 | 1.000 | 0.798 | 1.000 | 0.974 | 0.960 | 0.892 | 1.000 | 0.990 | 1.000 | 0.957 | 1.000 | 1.000 | 0.850 | 1.000 | 1.000 | 0.854 | 0.825 |
| 4497 | 0.767 | 1.000 | 0.791 | 1.000 | 0.979 | 0.960 | 0.895 | 1.000 | 0.980 | 1.000 | 0.959 | 0.970 | 1.000 | 0.853 | 1.000 | 1.000 | 0.853 | 0.824 |
| 4498 | 0.775 | 1.000 | 0.802 | 1.000 | 0.979 | 0.960 | 0.888 | 1.000 | 1.000 | 1.000 | 0.968 | 1.000 | 1.000 | 0.860 | 1.000 | 1.000 | 0.859 | 0.831 |
| 4499 | 0.760 | 1.000 | 0.806 | 1.000 | 0.974 | 0.960 | 0.911 | 1.000 | 0.990 | 1.000 | 0.957 | 1.000 | 0.982 | 0.852 | 1.000 | 1.000 | 0.854 | 0.826 |
| 4500 | 0.766 | 1.000 | 0.796 | 1.000 | 0.979 | 0.960 | 0.897 | 1.000 | 0.990 | 1.000 | 0.962 | 1.000 | 0.982 | 0.853 | 1.000 | 1.000 | 0.854 | 0.825 |
| 4501 | 0.766 | 1.000 | 0.814 | 1.000 | 0.974 | 0.960 | 0.911 | 1.000 | 0.990 | 1.000 | 0.959 | 1.000 | 0.991 | 0.858 | 1.000 | 1.000 | 0.859 | 0.831 |
| 4502 | 0.766 | 1.000 | 0.802 | 1.000 | 0.974 | 0.960 | 0.903 | 1.000 | 0.980 | 1.000 | 0.959 | 1.000 | 0.982 | 0.859 | 1.000 | 1.000 | 0.856 | 0.828 |
| 4503 | 0.771 | 1.000 | 0.785 | 1.000 | 0.968 | 0.960 | 0.901 | 1.000 | 1.000 | 1.000 | 0.959 | 1.000 | 0.991 | 0.850 | 1.000 | 1.000 | 0.854 | 0.825 |
| 4504 | 0.775 | 1.000 | 0.796 | 1.000 | 0.974 | 0.960 | 0.911 | 1.000 | 0.990 | 1.000 | 0.954 | 1.000 | 1.000 | 0.852 | 1.000 | 1.000 | 0.858 | 0.830 |
| 4505 | 0.764 | 1.000 | 0.802 | 1.000 | 0.974 | 0.960 | 0.898 | 1.000 | 0.980 | 1.000 | 0.959 | 1.000 | 0.991 | 0.844 | 1.000 | 1.000 | 0.852 | 0.822 |
| 4506 | 0.765 | 1.000 | 0.794 | 1.000 | 0.968 | 0.960 | 0.895 | 1.000 | 0.990 | 1.000 | 0.965 | 1.000 | 0.991 | 0.857 | 1.000 | 1.000 | 0.854 | 0.825 |
| 4507 | 0.771 | 1.000 | 0.794 | 1.000 | 0.979 | 0.960 | 0.895 | 1.000 | 1.000 | 1.000 | 0.968 | 1.000 | 0.991 | 0.860 | 1.000 | 1.000 | 0.858 | 0.829 |
| 4508 | 0.763 | 1.000 | 0.806 | 1.000 | 0.979 | 0.960 | 0.901 | 1.000 | 0.990 | 1.000 | 0.957 | 1.000 | 1.000 | 0.858 | 1.000 | 1.000 | 0.856 | 0.828 |
| 4509 | 0.758 | 1.000 | 0.808 | 1.000 | 0.974 | 0.960 | 0.900 | 1.000 | 1.000 | 1.000 | 0.962 | 1.000 | 1.000 | 0.857 | 1.000 | 1.000 | 0.855 | 0.826 |
| 4510 | 0.765 | 1.000 | 0.792 | 1.000 | 0.974 | 0.960 | 0.897 | 1.000 | 0.969 | 1.000 | 0.965 | 1.000 | 0.991 | 0.858 | 1.000 | 1.000 | 0.854 | 0.825 |
| 4511 | 0.773 | 1.000 | 0.779 | 1.000 | 0.979 | 0.960 | 0.900 | 1.000 | 0.990 | 1.000 | 0.959 | 0.970 | 1.000 | 0.850 | 1.000 | 1.000 | 0.854 | 0.824 |
| 4512 | 0.765 | 1.000 | 0.800 | 1.000 | 0.974 | 0.960 | 0.901 | 1.000 | 0.990 | 1.000 | 0.971 | 0.970 | 0.982 | 0.854 | 1.000 | 1.000 | 0.855 | 0.826 |
| 4513 | 0.757 | 1.000 | 0.798 | 1.000 | 0.979 | 0.960 | 0.891 | 1.000 | 0.980 | 1.000 | 0.957 | 0.970 | 0.991 | 0.857 | 1.000 | 1.000 | 0.851 | 0.821 |
| 4514 | 0.776 | 1.000 | 0.802 | 1.000 | 0.974 | 0.960 | 0.897 | 1.000 | 0.990 | 1.000 | 0.959 | 0.970 | 1.000 | 0.847 | 1.000 | 1.000 | 0.856 | 0.827 |
| 4515 | 0.774 | 1.000 | 0.802 | 1.000 | 0.979 | 0.960 | 0.894 | 1.000 | 0.980 | 1.000 | 0.962 | 1.000 | 0.991 | 0.851 | 1.000 | 1.000 | 0.856 | 0.828 |
| 4516 | 0.774 | 1.000 | 0.816 | 1.000 | 0.979 | 0.960 | 0.904 | 1.000 | 0.990 | 1.000 | 0.968 | 1.000 | 1.000 | 0.846 | 1.000 | 1.000 | 0.858 | 0.830 |
| 4517 | 0.763 | 1.000 | 0.777 | 1.000 | 0.974 | 0.960 | 0.898 | 1.000 | 0.990 | 1.000 | 0.971 | 1.000 | 0.991 | 0.861 | 1.000 | 1.000 | 0.854 | 0.825 |
| 4518 | 0.770 | 1.000 | 0.806 | 1.000 | 0.974 | 0.960 | 0.898 | 1.000 | 0.980 | 1.000 | 0.962 | 1.000 | 1.000 | 0.851 | 1.000 | 1.000 | 0.856 | 0.827 |
| 4519 | 0.772 | 1.000 | 0.791 | 1.000 | 0.979 | 0.960 | 0.898 | 1.000 | 0.990 | 1.000 | 0.962 | 1.000 | 1.000 | 0.857 | 1.000 | 1.000 | 0.857 | 0.828 |
| 4520 | 0.765 | 1.000 | 0.804 | 1.000 | 0.974 | 0.960 | 0.891 | 1.000 | 0.990 | 1.000 | 0.968 | 1.000 | 0.991 | 0.857 | 1.000 | 1.000 | 0.855 | 0.826 |
| 4521 | 0.767 | 1.000 | 0.789 | 1.000 | 0.979 | 0.960 | 0.901 | 1.000 | 1.000 | 1.000 | 0.957 | 1.000 | 1.000 | 0.862 | 1.000 | 1.000 | 0.857 | 0.828 |
| 4522 | 0.763 | 1.000 | 0.791 | 1.000 | 0.968 | 0.960 | 0.895 | 1.000 | 1.000 | 1.000 | 0.962 | 0.970 | 1.000 | 0.855 | 1.000 | 1.000 | 0.853 | 0.824 |

| | | | | | | | | | | | | | | | | | | |
|---|---|---|---|---|---|---|---|---|---|---|---|---|---|---|---|---|---|---|
| 4523 | 0.769 | 1.000 | 0.802 | 1.000 | 0.974 | 0.960 | 0.894 | 1.000 | 1.000 | 1.000 | 0.957 | 1.000 | 1.000 | 0.852 | 1.000 | 1.000 | 0.855 | 0.826 |
| 4524 | 0.771 | 1.000 | 0.792 | 1.000 | 0.979 | 0.960 | 0.900 | 1.000 | 0.980 | 1.000 | 0.959 | 1.000 | 0.982 | 0.854 | 1.000 | 1.000 | 0.856 | 0.827 |
| 4525 | 0.763 | 1.000 | 0.794 | 1.000 | 0.974 | 0.960 | 0.909 | 1.000 | 0.990 | 1.000 | 0.959 | 1.000 | 0.991 | 0.854 | 1.000 | 1.000 | 0.854 | 0.826 |
| 4526 | 0.776 | 1.000 | 0.792 | 1.000 | 0.968 | 0.960 | 0.908 | 1.000 | 1.000 | 1.000 | 0.957 | 1.000 | 1.000 | 0.856 | 1.000 | 1.000 | 0.859 | 0.830 |
| 4527 | 0.769 | 1.000 | 0.800 | 1.000 | 0.979 | 0.960 | 0.894 | 1.000 | 1.000 | 1.000 | 0.959 | 1.000 | 1.000 | 0.853 | 1.000 | 1.000 | 0.855 | 0.827 |
| 4528 | 0.769 | 1.000 | 0.785 | 1.000 | 0.979 | 0.960 | 0.895 | 1.000 | 0.990 | 1.000 | 0.968 | 1.000 | 1.000 | 0.855 | 1.000 | 1.000 | 0.855 | 0.826 |
| 4529 | 0.763 | 1.000 | 0.800 | 1.000 | 0.974 | 0.960 | 0.898 | 1.000 | 0.980 | 1.000 | 0.962 | 1.000 | 0.991 | 0.850 | 1.000 | 1.000 | 0.853 | 0.824 |
| 4530 | 0.769 | 1.000 | 0.800 | 1.000 | 0.974 | 0.960 | 0.892 | 1.000 | 0.990 | 1.000 | 0.957 | 1.000 | 0.991 | 0.851 | 1.000 | 1.000 | 0.854 | 0.825 |
| 4531 | 0.771 | 1.000 | 0.796 | 1.000 | 0.968 | 0.960 | 0.897 | 1.000 | 0.990 | 1.000 | 0.957 | 1.000 | 1.000 | 0.844 | 1.000 | 1.000 | 0.853 | 0.823 |
| 4532 | 0.777 | 1.000 | 0.804 | 1.000 | 0.979 | 0.960 | 0.891 | 1.000 | 1.000 | 1.000 | 0.962 | 1.000 | 1.000 | 0.848 | 1.000 | 1.000 | 0.857 | 0.828 |
| 4533 | 0.782 | 1.000 | 0.794 | 1.000 | 0.974 | 0.960 | 0.906 | 1.000 | 0.980 | 1.000 | 0.954 | 1.000 | 0.991 | 0.850 | 1.000 | 1.000 | 0.858 | 0.830 |
| 4534 | 0.775 | 1.000 | 0.816 | 1.000 | 0.974 | 0.960 | 0.889 | 1.000 | 1.000 | 1.000 | 0.957 | 1.000 | 0.991 | 0.856 | 1.000 | 1.000 | 0.858 | 0.830 |
| 4535 | 0.771 | 1.000 | 0.804 | 1.000 | 0.974 | 0.960 | 0.891 | 1.000 | 0.990 | 1.000 | 0.954 | 1.000 | 0.982 | 0.858 | 1.000 | 1.000 | 0.856 | 0.827 |
| 4536 | 0.765 | 1.000 | 0.802 | 1.000 | 0.979 | 0.960 | 0.900 | 1.000 | 1.000 | 1.000 | 0.965 | 1.000 | 0.991 | 0.856 | 1.000 | 1.000 | 0.856 | 0.828 |
| 4537 | 0.770 | 1.000 | 0.775 | 1.000 | 0.974 | 0.960 | 0.900 | 1.000 | 0.990 | 1.000 | 0.965 | 1.000 | 1.000 | 0.843 | 1.000 | 1.000 | 0.851 | 0.821 |
| 4538 | 0.777 | 1.000 | 0.804 | 1.000 | 0.974 | 1.000 | 0.892 | 1.000 | 1.000 | 1.000 | 0.968 | 1.000 | 0.991 | 0.853 | 1.000 | 1.000 | 0.858 | 0.830 |
| 4539 | 0.759 | 1.000 | 0.802 | 1.000 | 0.979 | 0.960 | 0.895 | 1.000 | 0.990 | 1.000 | 0.962 | 1.000 | 0.991 | 0.850 | 1.000 | 1.000 | 0.852 | 0.822 |
| 4540 | 0.771 | 1.000 | 0.783 | 1.000 | 0.979 | 0.960 | 0.894 | 1.000 | 1.000 | 1.000 | 0.957 | 1.000 | 0.973 | 0.856 | 1.000 | 1.000 | 0.854 | 0.825 |
| 4541 | 0.777 | 1.000 | 0.796 | 1.000 | 0.968 | 0.960 | 0.895 | 1.000 | 1.000 | 1.000 | 0.957 | 1.000 | 0.991 | 0.850 | 1.000 | 1.000 | 0.856 | 0.827 |
| 4542 | 0.759 | 1.000 | 0.796 | 1.000 | 0.974 | 0.960 | 0.894 | 1.000 | 1.000 | 1.000 | 0.962 | 0.970 | 1.000 | 0.856 | 1.000 | 1.000 | 0.853 | 0.824 |
| 4543 | 0.770 | 1.000 | 0.800 | 1.000 | 0.974 | 0.960 | 0.895 | 1.000 | 0.990 | 1.000 | 0.948 | 1.000 | 0.991 | 0.850 | 1.000 | 1.000 | 0.854 | 0.824 |
| 4544 | 0.764 | 1.000 | 0.796 | 1.000 | 0.974 | 0.960 | 0.911 | 1.000 | 0.990 | 1.000 | 0.957 | 1.000 | 0.991 | 0.856 | 1.000 | 1.000 | 0.856 | 0.827 |
| 4545 | 0.766 | 1.000 | 0.806 | 1.000 | 0.974 | 0.960 | 0.894 | 1.000 | 0.980 | 1.000 | 0.962 | 1.000 | 0.982 | 0.854 | 1.000 | 1.000 | 0.854 | 0.826 |
| 4546 | 0.775 | 1.000 | 0.802 | 1.000 | 0.974 | 0.960 | 0.898 | 1.000 | 1.000 | 1.000 | 0.959 | 1.000 | 0.991 | 0.845 | 1.000 | 1.000 | 0.855 | 0.827 |
| 4547 | 0.769 | 1.000 | 0.796 | 1.000 | 0.979 | 0.960 | 0.897 | 1.000 | 1.000 | 1.000 | 0.959 | 1.000 | 0.982 | 0.859 | 1.000 | 1.000 | 0.856 | 0.828 |
| 4548 | 0.770 | 1.000 | 0.812 | 1.000 | 0.979 | 0.960 | 0.894 | 1.000 | 0.990 | 1.000 | 0.959 | 1.000 | 0.982 | 0.854 | 1.000 | 1.000 | 0.857 | 0.828 |
| 4549 | 0.770 | 1.000 | 0.791 | 0.986 | 0.979 | 0.960 | 0.901 | 1.000 | 0.990 | 1.000 | 0.968 | 1.000 | 0.991 | 0.853 | 1.000 | 1.000 | 0.856 | 0.827 |
| 4550 | 0.766 | 1.000 | 0.804 | 1.000 | 0.979 | 0.960 | 0.895 | 1.000 | 1.000 | 1.000 | 0.959 | 1.000 | 0.991 | 0.851 | 1.000 | 1.000 | 0.854 | 0.825 |
| 4551 | 0.771 | 1.000 | 0.792 | 1.000 | 0.974 | 0.960 | 0.904 | 1.000 | 0.990 | 1.000 | 0.962 | 1.000 | 0.991 | 0.853 | 1.000 | 1.000 | 0.856 | 0.828 |
| 4552 | 0.775 | 1.000 | 0.794 | 1.000 | 0.974 | 0.960 | 0.897 | 1.000 | 0.990 | 1.000 | 0.965 | 0.970 | 1.000 | 0.851 | 1.000 | 1.000 | 0.856 | 0.828 |
| 4553 | 0.765 | 1.000 | 0.800 | 1.000 | 0.984 | 0.960 | 0.897 | 1.000 | 0.990 | 1.000 | 0.965 | 1.000 | 0.991 | 0.850 | 1.000 | 1.000 | 0.854 | 0.825 |
| 4554 | 0.770 | 1.000 | 0.802 | 1.000 | 0.979 | 0.960 | 0.903 | 1.000 | 0.990 | 1.000 | 0.951 | 1.000 | 1.000 | 0.851 | 1.000 | 1.000 | 0.856 | 0.827 |
| 4555 | 0.767 | 1.000 | 0.800 | 1.000 | 0.974 | 0.960 | 0.895 | 1.000 | 0.980 | 1.000 | 0.962 | 1.000 | 0.991 | 0.851 | 1.000 | 1.000 | 0.854 | 0.825 |
| 4556 | 0.762 | 1.000 | 0.791 | 1.000 | 0.968 | 0.960 | 0.901 | 1.000 | 0.980 | 1.000 | 0.957 | 1.000 | 0.991 | 0.856 | 1.000 | 1.000 | 0.853 | 0.824 |
| 4557 | 0.763 | 1.000 | 0.812 | 1.000 | 0.974 | 0.960 | 0.898 | 1.000 | 1.000 | 1.000 | 0.959 | 1.000 | 0.991 | 0.855 | 1.000 | 1.000 | 0.855 | 0.827 |
| 4558 | 0.773 | 1.000 | 0.792 | 1.000 | 0.979 | 0.960 | 0.898 | 1.000 | 0.990 | 1.000 | 0.957 | 1.000 | 1.000 | 0.853 | 1.000 | 1.000 | 0.856 | 0.827 |
| 4559 | 0.779 | 1.000 | 0.802 | 1.000 | 0.968 | 0.960 | 0.891 | 1.000 | 0.990 | 1.000 | 0.968 | 1.000 | 0.991 | 0.858 | 1.000 | 1.000 | 0.859 | 0.831 |
| 4560 | 0.765 | 1.000 | 0.802 | 1.000 | 0.974 | 0.960 | 0.895 | 1.000 | 0.990 | 1.000 | 0.954 | 1.000 | 1.000 | 0.846 | 1.000 | 1.000 | 0.852 | 0.823 |
| 4561 | 0.772 | 1.000 | 0.798 | 1.000 | 0.979 | 0.960 | 0.908 | 1.000 | 0.990 | 1.000 | 0.968 | 1.000 | 0.991 | 0.844 | 1.000 | 1.000 | 0.856 | 0.827 |

| | | | | | | | | | | | | | | | | | |
|---|---|---|---|---|---|---|---|---|---|---|---|---|---|---|---|---|---|
| 4562 | 0.771 | 1.000 | 0.791 | 1.000 | 0.974 | 0.960 | 0.900 | 1.000 | 1.000 | 1.000 | 0.959 | 1.000 | 0.991 | 0.851 | 1.000 | 1.000 | 0.855 | 0.826 |
| 4563 | 0.771 | 1.000 | 0.796 | 1.000 | 0.979 | 0.960 | 0.894 | 1.000 | 0.980 | 1.000 | 0.959 | 1.000 | 0.991 | 0.846 | 1.000 | 1.000 | 0.853 | 0.824 |
| 4564 | 0.769 | 1.000 | 0.791 | 1.000 | 0.974 | 0.960 | 0.891 | 1.000 | 0.990 | 1.000 | 0.962 | 1.000 | 0.982 | 0.854 | 1.000 | 1.000 | 0.853 | 0.824 |
| 4565 | 0.771 | 1.000 | 0.816 | 1.000 | 0.974 | 0.960 | 0.901 | 1.000 | 0.990 | 1.000 | 0.948 | 1.000 | 1.000 | 0.853 | 1.000 | 1.000 | 0.857 | 0.829 |
| 4566 | 0.771 | 1.000 | 0.800 | 1.000 | 0.974 | 0.960 | 0.894 | 1.000 | 1.000 | 1.000 | 0.965 | 1.000 | 1.000 | 0.851 | 1.000 | 1.000 | 0.855 | 0.827 |
| 4567 | 0.773 | 1.000 | 0.792 | 1.000 | 0.968 | 0.960 | 0.888 | 1.000 | 0.990 | 1.000 | 0.959 | 1.000 | 0.991 | 0.852 | 1.000 | 1.000 | 0.854 | 0.824 |
| 4568 | 0.778 | 1.000 | 0.804 | 1.000 | 0.974 | 0.960 | 0.895 | 1.000 | 0.990 | 1.000 | 0.954 | 0.970 | 0.973 | 0.857 | 1.000 | 1.000 | 0.858 | 0.830 |
| 4569 | 0.765 | 1.000 | 0.820 | 1.000 | 0.979 | 0.960 | 0.900 | 1.000 | 0.990 | 1.000 | 0.962 | 1.000 | 0.991 | 0.858 | 1.000 | 1.000 | 0.858 | 0.830 |
| 4570 | 0.777 | 1.000 | 0.802 | 1.000 | 0.968 | 0.960 | 0.901 | 1.000 | 0.990 | 1.000 | 0.962 | 1.000 | 0.982 | 0.859 | 1.000 | 1.000 | 0.860 | 0.831 |
| 4571 | 0.767 | 1.000 | 0.796 | 1.000 | 0.974 | 0.960 | 0.892 | 1.000 | 1.000 | 1.000 | 0.962 | 1.000 | 0.991 | 0.854 | 1.000 | 1.000 | 0.854 | 0.825 |
| 4572 | 0.771 | 1.000 | 0.806 | 1.000 | 0.974 | 0.960 | 0.889 | 1.000 | 0.990 | 1.000 | 0.962 | 0.970 | 0.991 | 0.854 | 1.000 | 1.000 | 0.855 | 0.826 |
| 4573 | 0.768 | 1.000 | 0.814 | 1.000 | 0.974 | 0.960 | 0.900 | 1.000 | 1.000 | 1.000 | 0.954 | 1.000 | 0.991 | 0.848 | 1.000 | 1.000 | 0.855 | 0.827 |
| 4574 | 0.767 | 1.000 | 0.796 | 1.000 | 0.979 | 0.960 | 0.908 | 1.000 | 1.000 | 1.000 | 0.957 | 1.000 | 0.991 | 0.855 | 1.000 | 1.000 | 0.856 | 0.828 |
| 4575 | 0.761 | 1.000 | 0.802 | 1.000 | 0.974 | 0.960 | 0.908 | 1.000 | 0.990 | 1.000 | 0.962 | 1.000 | 0.982 | 0.863 | 1.000 | 1.000 | 0.857 | 0.829 |
| 4576 | 0.763 | 1.000 | 0.792 | 1.000 | 0.968 | 0.960 | 0.901 | 1.000 | 0.990 | 1.000 | 0.965 | 0.970 | 1.000 | 0.854 | 1.000 | 1.000 | 0.854 | 0.825 |
| 4577 | 0.767 | 1.000 | 0.808 | 1.000 | 0.974 | 0.960 | 0.891 | 1.000 | 1.000 | 1.000 | 0.962 | 1.000 | 0.982 | 0.855 | 1.000 | 1.000 | 0.855 | 0.826 |
| 4578 | 0.765 | 1.000 | 0.796 | 0.986 | 0.984 | 0.960 | 0.909 | 1.000 | 0.990 | 1.000 | 0.959 | 1.000 | 1.000 | 0.852 | 1.000 | 1.000 | 0.855 | 0.827 |
| 4579 | 0.767 | 1.000 | 0.808 | 1.000 | 0.979 | 0.960 | 0.904 | 1.000 | 1.000 | 1.000 | 0.959 | 1.000 | 0.991 | 0.858 | 1.000 | 1.000 | 0.858 | 0.830 |
| 4580 | 0.769 | 1.000 | 0.796 | 1.000 | 0.968 | 0.960 | 0.897 | 1.000 | 0.990 | 1.000 | 0.959 | 1.000 | 1.000 | 0.860 | 1.000 | 1.000 | 0.857 | 0.828 |
| 4581 | 0.769 | 1.000 | 0.812 | 1.000 | 0.979 | 0.960 | 0.891 | 1.000 | 0.990 | 1.000 | 0.962 | 0.970 | 0.982 | 0.851 | 1.000 | 1.000 | 0.855 | 0.826 |
| 4582 | 0.765 | 1.000 | 0.812 | 1.000 | 0.963 | 0.960 | 0.891 | 1.000 | 0.990 | 1.000 | 0.945 | 1.000 | 0.982 | 0.857 | 1.000 | 1.000 | 0.854 | 0.824 |
| 4583 | 0.760 | 1.000 | 0.800 | 1.000 | 0.984 | 0.960 | 0.888 | 1.000 | 0.990 | 1.000 | 0.954 | 1.000 | 0.991 | 0.858 | 1.000 | 1.000 | 0.853 | 0.823 |
| 4584 | 0.767 | 1.000 | 0.798 | 1.000 | 0.974 | 0.960 | 0.894 | 1.000 | 0.990 | 1.000 | 0.957 | 1.000 | 0.991 | 0.853 | 1.000 | 1.000 | 0.854 | 0.825 |
| 4585 | 0.773 | 1.000 | 0.812 | 0.986 | 0.974 | 0.960 | 0.892 | 1.000 | 0.980 | 1.000 | 0.962 | 1.000 | 0.982 | 0.851 | 1.000 | 1.000 | 0.856 | 0.828 |
| 4586 | 0.771 | 1.000 | 0.792 | 1.000 | 0.979 | 0.960 | 0.894 | 1.000 | 0.990 | 1.000 | 0.968 | 1.000 | 0.991 | 0.851 | 1.000 | 1.000 | 0.855 | 0.826 |
| 4587 | 0.775 | 1.000 | 0.800 | 1.000 | 0.968 | 0.960 | 0.900 | 1.000 | 0.980 | 1.000 | 0.951 | 1.000 | 1.000 | 0.851 | 1.000 | 1.000 | 0.856 | 0.827 |
| 4588 | 0.774 | 1.000 | 0.808 | 1.000 | 0.979 | 0.960 | 0.888 | 1.000 | 0.990 | 1.000 | 0.962 | 1.000 | 0.982 | 0.861 | 1.000 | 1.000 | 0.858 | 0.830 |
| 4589 | 0.770 | 1.000 | 0.798 | 1.000 | 0.974 | 0.960 | 0.900 | 1.000 | 1.000 | 1.000 | 0.959 | 1.000 | 0.991 | 0.860 | 1.000 | 1.000 | 0.858 | 0.829 |
| 4590 | 0.766 | 1.000 | 0.796 | 1.000 | 0.968 | 0.960 | 0.904 | 1.000 | 0.990 | 1.000 | 0.959 | 1.000 | 1.000 | 0.854 | 1.000 | 1.000 | 0.855 | 0.827 |
| 4591 | 0.775 | 1.000 | 0.800 | 1.000 | 0.958 | 0.960 | 0.898 | 1.000 | 0.980 | 1.000 | 0.962 | 1.000 | 0.991 | 0.847 | 1.000 | 1.000 | 0.855 | 0.826 |
| 4592 | 0.768 | 1.000 | 0.814 | 1.000 | 0.974 | 0.960 | 0.901 | 1.000 | 0.990 | 1.000 | 0.965 | 1.000 | 1.000 | 0.853 | 1.000 | 1.000 | 0.857 | 0.829 |
| 4593 | 0.761 | 1.000 | 0.798 | 1.000 | 0.979 | 0.960 | 0.900 | 1.000 | 0.990 | 1.000 | 0.962 | 0.970 | 1.000 | 0.847 | 1.000 | 1.000 | 0.852 | 0.823 |
| 4594 | 0.771 | 1.000 | 0.820 | 1.000 | 0.979 | 0.960 | 0.904 | 1.000 | 1.000 | 1.000 | 0.959 | 1.000 | 0.991 | 0.861 | 1.000 | 1.000 | 0.861 | 0.834 |
| 4595 | 0.769 | 1.000 | 0.791 | 1.000 | 0.979 | 0.960 | 0.895 | 1.000 | 0.980 | 1.000 | 0.965 | 1.000 | 0.991 | 0.854 | 1.000 | 1.000 | 0.854 | 0.825 |
| 4596 | 0.769 | 1.000 | 0.808 | 1.000 | 0.979 | 0.960 | 0.888 | 1.000 | 0.990 | 1.000 | 0.959 | 1.000 | 1.000 | 0.854 | 1.000 | 1.000 | 0.856 | 0.827 |
| 4597 | 0.771 | 1.000 | 0.794 | 1.000 | 0.974 | 0.960 | 0.895 | 1.000 | 0.990 | 1.000 | 0.968 | 1.000 | 0.991 | 0.855 | 1.000 | 1.000 | 0.856 | 0.828 |
| 4598 | 0.770 | 1.000 | 0.806 | 1.000 | 0.974 | 0.960 | 0.903 | 1.000 | 1.000 | 1.000 | 0.962 | 1.000 | 0.991 | 0.858 | 1.000 | 1.000 | 0.858 | 0.830 |
| 4599 | 0.767 | 1.000 | 0.791 | 0.986 | 0.979 | 0.960 | 0.900 | 1.000 | 0.990 | 1.000 | 0.957 | 1.000 | 1.000 | 0.851 | 1.000 | 1.000 | 0.853 | 0.824 |
| 4600 | 0.764 | 1.000 | 0.798 | 1.000 | 0.974 | 0.960 | 0.888 | 1.000 | 0.990 | 1.000 | 0.959 | 1.000 | 0.982 | 0.853 | 1.000 | 1.000 | 0.852 | 0.823 |

| | | | | | | | | | | | | | | | | | | |
|---|---|---|---|---|---|---|---|---|---|---|---|---|---|---|---|---|---|---|
| 4601 | 0.763 | 1.000 | 0.802 | 1.000 | 0.979 | 0.960 | 0.883 | 1.000 | 1.000 | 1.000 | 0.968 | 1.000 | 1.000 | 0.850 | 1.000 | 1.000 | 0.852 | 0.823 |
| 4602 | 0.766 | 1.000 | 0.796 | 1.000 | 0.974 | 0.960 | 0.898 | 1.000 | 0.980 | 1.000 | 0.968 | 1.000 | 0.982 | 0.851 | 1.000 | 1.000 | 0.854 | 0.825 |
| 4603 | 0.765 | 1.000 | 0.802 | 1.000 | 0.974 | 0.960 | 0.901 | 1.000 | 0.980 | 1.000 | 0.959 | 1.000 | 1.000 | 0.858 | 1.000 | 1.000 | 0.856 | 0.828 |
| 4604 | 0.769 | 1.000 | 0.810 | 1.000 | 0.979 | 0.960 | 0.889 | 1.000 | 0.980 | 1.000 | 0.962 | 1.000 | 1.000 | 0.852 | 1.000 | 1.000 | 0.855 | 0.827 |
| 4605 | 0.764 | 1.000 | 0.791 | 0.986 | 0.963 | 0.960 | 0.892 | 1.000 | 0.990 | 1.000 | 0.957 | 1.000 | 0.991 | 0.854 | 1.000 | 1.000 | 0.851 | 0.822 |
| 4606 | 0.771 | 1.000 | 0.794 | 1.000 | 0.979 | 0.960 | 0.903 | 1.000 | 0.980 | 1.000 | 0.959 | 1.000 | 0.982 | 0.854 | 1.000 | 1.000 | 0.856 | 0.827 |
| 4607 | 0.771 | 1.000 | 0.798 | 0.986 | 0.979 | 0.960 | 0.883 | 1.000 | 0.990 | 1.000 | 0.959 | 1.000 | 0.982 | 0.852 | 1.000 | 1.000 | 0.853 | 0.824 |
| 4608 | 0.773 | 1.000 | 0.836 | 1.000 | 0.963 | 0.960 | 0.900 | 1.000 | 0.990 | 1.000 | 0.959 | 1.000 | 1.000 | 0.854 | 1.000 | 1.000 | 0.860 | 0.833 |
| 4609 | 0.775 | 1.000 | 0.804 | 1.000 | 0.974 | 0.960 | 0.891 | 1.000 | 0.990 | 1.000 | 0.948 | 1.000 | 0.991 | 0.852 | 1.000 | 1.000 | 0.855 | 0.826 |
| 4610 | 0.773 | 1.000 | 0.789 | 1.000 | 0.979 | 0.960 | 0.901 | 1.000 | 1.000 | 1.000 | 0.957 | 0.970 | 1.000 | 0.857 | 1.000 | 1.000 | 0.857 | 0.828 |
| 4611 | 0.769 | 1.000 | 0.824 | 1.000 | 0.974 | 0.960 | 0.889 | 1.000 | 0.990 | 1.000 | 0.954 | 1.000 | 0.991 | 0.851 | 1.000 | 1.000 | 0.856 | 0.827 |
| 4612 | 0.768 | 1.000 | 0.789 | 1.000 | 0.974 | 0.960 | 0.898 | 1.000 | 0.990 | 1.000 | 0.959 | 1.000 | 0.982 | 0.855 | 1.000 | 1.000 | 0.854 | 0.825 |
| 4613 | 0.775 | 1.000 | 0.800 | 1.000 | 0.974 | 0.960 | 0.901 | 1.000 | 0.990 | 1.000 | 0.962 | 1.000 | 1.000 | 0.860 | 1.000 | 1.000 | 0.860 | 0.832 |
| 4614 | 0.765 | 1.000 | 0.798 | 1.000 | 0.974 | 0.960 | 0.891 | 1.000 | 0.980 | 1.000 | 0.962 | 1.000 | 0.991 | 0.857 | 1.000 | 1.000 | 0.854 | 0.825 |
| 4615 | 0.766 | 1.000 | 0.791 | 1.000 | 0.979 | 0.960 | 0.900 | 1.000 | 0.990 | 1.000 | 0.962 | 1.000 | 0.991 | 0.857 | 1.000 | 1.000 | 0.855 | 0.826 |
| 4616 | 0.769 | 1.000 | 0.796 | 0.986 | 0.979 | 0.960 | 0.903 | 1.000 | 0.980 | 1.000 | 0.959 | 1.000 | 1.000 | 0.856 | 1.000 | 1.000 | 0.856 | 0.828 |
| 4617 | 0.771 | 1.000 | 0.800 | 1.000 | 0.974 | 0.960 | 0.888 | 1.000 | 1.000 | 1.000 | 0.957 | 1.000 | 0.982 | 0.861 | 1.000 | 1.000 | 0.856 | 0.828 |
| 4618 | 0.768 | 1.000 | 0.810 | 1.000 | 0.974 | 0.960 | 0.880 | 1.000 | 1.000 | 1.000 | 0.962 | 1.000 | 0.991 | 0.857 | 1.000 | 1.000 | 0.855 | 0.826 |
| 4619 | 0.777 | 1.000 | 0.812 | 1.000 | 0.968 | 0.960 | 0.901 | 1.000 | 0.980 | 1.000 | 0.957 | 1.000 | 0.991 | 0.851 | 1.000 | 1.000 | 0.858 | 0.830 |
| 4620 | 0.764 | 1.000 | 0.792 | 1.000 | 0.968 | 0.960 | 0.895 | 1.000 | 0.990 | 1.000 | 0.962 | 1.000 | 1.000 | 0.859 | 1.000 | 1.000 | 0.854 | 0.825 |
| 4621 | 0.776 | 1.000 | 0.789 | 1.000 | 0.974 | 0.960 | 0.889 | 1.000 | 0.990 | 1.000 | 0.962 | 1.000 | 1.000 | 0.850 | 1.000 | 1.000 | 0.855 | 0.825 |
| 4622 | 0.776 | 1.000 | 0.818 | 1.000 | 0.974 | 0.960 | 0.894 | 1.000 | 1.000 | 1.000 | 0.957 | 0.970 | 1.000 | 0.847 | 1.000 | 1.000 | 0.857 | 0.829 |
| 4623 | 0.770 | 1.000 | 0.806 | 1.000 | 0.974 | 0.960 | 0.894 | 1.000 | 0.990 | 1.000 | 0.959 | 1.000 | 0.991 | 0.854 | 1.000 | 1.000 | 0.856 | 0.827 |
| 4624 | 0.773 | 1.000 | 0.802 | 1.000 | 0.979 | 0.960 | 0.895 | 1.000 | 0.980 | 1.000 | 0.962 | 1.000 | 1.000 | 0.854 | 1.000 | 1.000 | 0.857 | 0.828 |
| 4625 | 0.756 | 1.000 | 0.810 | 0.986 | 0.974 | 0.960 | 0.883 | 1.000 | 0.990 | 1.000 | 0.957 | 1.000 | 0.982 | 0.847 | 1.000 | 1.000 | 0.848 | 0.819 |
| 4626 | 0.765 | 1.000 | 0.796 | 1.000 | 0.974 | 0.960 | 0.889 | 1.000 | 0.990 | 1.000 | 0.954 | 1.000 | 1.000 | 0.851 | 1.000 | 1.000 | 0.852 | 0.823 |
| 4627 | 0.763 | 1.000 | 0.796 | 1.000 | 0.974 | 0.960 | 0.895 | 1.000 | 0.980 | 1.000 | 0.965 | 1.000 | 1.000 | 0.862 | 1.000 | 1.000 | 0.856 | 0.827 |
| 4628 | 0.781 | 1.000 | 0.796 | 1.000 | 0.974 | 0.960 | 0.895 | 1.000 | 1.000 | 1.000 | 0.968 | 1.000 | 1.000 | 0.859 | 1.000 | 1.000 | 0.861 | 0.833 |
| 4629 | 0.768 | 1.000 | 0.794 | 0.986 | 0.979 | 0.960 | 0.898 | 1.000 | 0.990 | 1.000 | 0.968 | 1.000 | 0.991 | 0.860 | 1.000 | 1.000 | 0.857 | 0.828 |
| 4630 | 0.769 | 1.000 | 0.818 | 1.000 | 0.968 | 0.960 | 0.888 | 1.000 | 0.980 | 1.000 | 0.957 | 1.000 | 1.000 | 0.846 | 1.000 | 1.000 | 0.853 | 0.824 |
| 4631 | 0.763 | 1.000 | 0.798 | 1.000 | 0.963 | 0.960 | 0.892 | 1.000 | 1.000 | 1.000 | 0.962 | 1.000 | 0.982 | 0.854 | 1.000 | 1.000 | 0.853 | 0.823 |
| 4632 | 0.775 | 1.000 | 0.810 | 1.000 | 0.974 | 0.960 | 0.891 | 1.000 | 0.990 | 1.000 | 0.954 | 1.000 | 0.982 | 0.858 | 1.000 | 1.000 | 0.858 | 0.830 |
| 4633 | 0.767 | 1.000 | 0.792 | 1.000 | 0.979 | 0.960 | 0.898 | 1.000 | 0.990 | 1.000 | 0.959 | 1.000 | 0.991 | 0.858 | 1.000 | 1.000 | 0.855 | 0.827 |
| 4634 | 0.766 | 1.000 | 0.789 | 1.000 | 0.968 | 0.960 | 0.897 | 1.000 | 0.980 | 1.000 | 0.959 | 0.970 | 0.973 | 0.851 | 1.000 | 1.000 | 0.851 | 0.822 |
| 4635 | 0.772 | 1.000 | 0.796 | 1.000 | 0.979 | 0.960 | 0.895 | 1.000 | 0.990 | 1.000 | 0.959 | 1.000 | 0.982 | 0.865 | 1.000 | 1.000 | 0.858 | 0.830 |
| 4636 | 0.765 | 1.000 | 0.798 | 1.000 | 0.979 | 0.960 | 0.891 | 1.000 | 1.000 | 1.000 | 0.954 | 1.000 | 0.991 | 0.851 | 1.000 | 1.000 | 0.853 | 0.823 |
| 4637 | 0.770 | 1.000 | 0.810 | 1.000 | 0.974 | 0.960 | 0.886 | 1.000 | 0.990 | 1.000 | 0.957 | 1.000 | 0.982 | 0.849 | 1.000 | 1.000 | 0.854 | 0.825 |
| 4638 | 0.771 | 1.000 | 0.792 | 1.000 | 0.979 | 0.960 | 0.891 | 1.000 | 0.980 | 1.000 | 0.954 | 1.000 | 0.991 | 0.853 | 1.000 | 1.000 | 0.854 | 0.824 |
| 4639 | 0.772 | 1.000 | 0.810 | 1.000 | 0.968 | 0.960 | 0.891 | 1.000 | 0.980 | 1.000 | 0.962 | 1.000 | 0.991 | 0.848 | 1.000 | 1.000 | 0.855 | 0.826 |

| | | | | | | | | | | | | | | | | | | |
|---|---|---|---|---|---|---|---|---|---|---|---|---|---|---|---|---|---|---|
| 4640 | 0.779 | 1.000 | 0.804 | 1.000 | 0.968 | 0.960 | 0.891 | 1.000 | 0.990 | 1.000 | 0.959 | 1.000 | 0.973 | 0.857 | 1.000 | 1.000 | 0.858 | 0.830 |
| 4641 | 0.765 | 1.000 | 0.792 | 1.000 | 0.974 | 0.960 | 0.898 | 1.000 | 0.990 | 1.000 | 0.968 | 1.000 | 1.000 | 0.859 | 1.000 | 1.000 | 0.856 | 0.827 |
| 4642 | 0.755 | 1.000 | 0.785 | 1.000 | 0.974 | 0.960 | 0.894 | 1.000 | 0.990 | 1.000 | 0.959 | 1.000 | 0.982 | 0.854 | 1.000 | 1.000 | 0.849 | 0.819 |
| 4643 | 0.770 | 1.000 | 0.794 | 1.000 | 0.979 | 0.960 | 0.901 | 1.000 | 0.990 | 1.000 | 0.962 | 1.000 | 0.991 | 0.857 | 1.000 | 1.000 | 0.857 | 0.828 |
| 4644 | 0.772 | 1.000 | 0.792 | 1.000 | 0.979 | 0.960 | 0.892 | 1.000 | 0.990 | 1.000 | 0.968 | 1.000 | 1.000 | 0.853 | 1.000 | 1.000 | 0.856 | 0.827 |
| 4645 | 0.768 | 1.000 | 0.777 | 0.986 | 0.963 | 0.960 | 0.892 | 1.000 | 0.980 | 1.000 | 0.957 | 1.000 | 0.982 | 0.850 | 1.000 | 1.000 | 0.850 | 0.820 |
| 4646 | 0.771 | 1.000 | 0.804 | 1.000 | 0.979 | 0.960 | 0.897 | 1.000 | 1.000 | 1.000 | 0.959 | 1.000 | 0.991 | 0.852 | 1.000 | 1.000 | 0.856 | 0.828 |
| 4647 | 0.771 | 1.000 | 0.785 | 1.000 | 0.979 | 0.960 | 0.892 | 1.000 | 0.990 | 1.000 | 0.965 | 0.970 | 1.000 | 0.846 | 1.000 | 1.000 | 0.852 | 0.823 |
| 4648 | 0.768 | 1.000 | 0.789 | 1.000 | 0.968 | 0.960 | 0.884 | 1.000 | 0.990 | 1.000 | 0.965 | 1.000 | 1.000 | 0.853 | 1.000 | 1.000 | 0.852 | 0.823 |
| 4649 | 0.773 | 1.000 | 0.787 | 1.000 | 0.963 | 0.960 | 0.897 | 1.000 | 1.000 | 1.000 | 0.971 | 1.000 | 0.991 | 0.856 | 1.000 | 1.000 | 0.856 | 0.828 |
| 4650 | 0.777 | 1.000 | 0.775 | 1.000 | 0.968 | 0.960 | 0.901 | 1.000 | 0.990 | 1.000 | 0.962 | 1.000 | 1.000 | 0.853 | 1.000 | 1.000 | 0.856 | 0.827 |
| 4651 | 0.779 | 1.000 | 0.789 | 1.000 | 0.974 | 0.960 | 0.901 | 1.000 | 0.990 | 1.000 | 0.957 | 1.000 | 1.000 | 0.856 | 1.000 | 1.000 | 0.858 | 0.830 |
| 4652 | 0.766 | 1.000 | 0.792 | 1.000 | 0.968 | 0.960 | 0.903 | 1.000 | 0.990 | 1.000 | 0.962 | 1.000 | 0.991 | 0.850 | 1.000 | 1.000 | 0.853 | 0.825 |
| 4653 | 0.781 | 1.000 | 0.808 | 1.000 | 0.984 | 0.960 | 0.891 | 1.000 | 0.980 | 1.000 | 0.965 | 1.000 | 1.000 | 0.856 | 1.000 | 1.000 | 0.860 | 0.832 |
| 4654 | 0.773 | 1.000 | 0.794 | 1.000 | 0.974 | 0.960 | 0.901 | 1.000 | 0.990 | 1.000 | 0.962 | 1.000 | 0.982 | 0.859 | 1.000 | 1.000 | 0.858 | 0.829 |
| 4655 | 0.764 | 1.000 | 0.798 | 1.000 | 0.974 | 0.960 | 0.900 | 1.000 | 0.990 | 1.000 | 0.968 | 1.000 | 0.991 | 0.853 | 1.000 | 1.000 | 0.854 | 0.826 |
| 4656 | 0.771 | 1.000 | 0.787 | 1.000 | 0.968 | 0.960 | 0.900 | 1.000 | 1.000 | 1.000 | 0.965 | 1.000 | 0.982 | 0.857 | 1.000 | 1.000 | 0.856 | 0.827 |
| 4657 | 0.767 | 1.000 | 0.796 | 1.000 | 0.968 | 0.960 | 0.897 | 1.000 | 1.000 | 1.000 | 0.962 | 1.000 | 0.991 | 0.854 | 1.000 | 1.000 | 0.855 | 0.826 |
| 4658 | 0.764 | 1.000 | 0.804 | 1.000 | 0.974 | 0.960 | 0.897 | 1.000 | 0.990 | 1.000 | 0.959 | 1.000 | 0.991 | 0.854 | 1.000 | 1.000 | 0.854 | 0.826 |
| 4659 | 0.769 | 1.000 | 0.791 | 1.000 | 0.979 | 0.960 | 0.884 | 1.000 | 0.980 | 1.000 | 0.959 | 1.000 | 0.973 | 0.856 | 1.000 | 1.000 | 0.853 | 0.824 |
| 4660 | 0.763 | 1.000 | 0.783 | 1.000 | 0.974 | 0.960 | 0.888 | 1.000 | 0.990 | 1.000 | 0.959 | 1.000 | 0.982 | 0.856 | 1.000 | 1.000 | 0.851 | 0.821 |
| 4661 | 0.767 | 1.000 | 0.800 | 1.000 | 0.974 | 0.960 | 0.900 | 1.000 | 1.000 | 1.000 | 0.957 | 1.000 | 0.991 | 0.846 | 1.000 | 1.000 | 0.853 | 0.824 |
| 4662 | 0.766 | 1.000 | 0.787 | 0.986 | 0.974 | 0.960 | 0.895 | 1.000 | 0.990 | 1.000 | 0.959 | 0.970 | 1.000 | 0.855 | 1.000 | 1.000 | 0.853 | 0.823 |
| 4663 | 0.771 | 1.000 | 0.800 | 1.000 | 0.979 | 0.960 | 0.904 | 1.000 | 1.000 | 1.000 | 0.957 | 0.970 | 0.991 | 0.850 | 1.000 | 1.000 | 0.856 | 0.827 |
| 4664 | 0.772 | 1.000 | 0.796 | 1.000 | 0.974 | 0.960 | 0.892 | 1.000 | 0.990 | 1.000 | 0.954 | 1.000 | 0.991 | 0.851 | 1.000 | 1.000 | 0.854 | 0.825 |
| 4665 | 0.770 | 1.000 | 0.792 | 0.986 | 0.979 | 0.960 | 0.908 | 1.000 | 0.990 | 1.000 | 0.968 | 1.000 | 0.991 | 0.858 | 1.000 | 1.000 | 0.858 | 0.830 |
| 4666 | 0.771 | 1.000 | 0.794 | 1.000 | 0.979 | 0.960 | 0.894 | 1.000 | 0.990 | 1.000 | 0.965 | 1.000 | 0.991 | 0.853 | 1.000 | 1.000 | 0.855 | 0.827 |
| 4667 | 0.761 | 1.000 | 0.810 | 1.000 | 0.968 | 0.960 | 0.889 | 1.000 | 1.000 | 1.000 | 0.959 | 1.000 | 1.000 | 0.846 | 1.000 | 1.000 | 0.851 | 0.822 |
| 4668 | 0.763 | 1.000 | 0.792 | 1.000 | 0.974 | 0.960 | 0.894 | 1.000 | 0.990 | 1.000 | 0.959 | 1.000 | 1.000 | 0.856 | 1.000 | 1.000 | 0.853 | 0.824 |
| 4669 | 0.769 | 1.000 | 0.792 | 1.000 | 0.974 | 0.960 | 0.898 | 1.000 | 0.969 | 1.000 | 0.962 | 1.000 | 0.991 | 0.857 | 1.000 | 1.000 | 0.855 | 0.826 |
| 4670 | 0.768 | 1.000 | 0.787 | 1.000 | 0.974 | 0.960 | 0.894 | 1.000 | 0.980 | 1.000 | 0.965 | 1.000 | 0.982 | 0.851 | 1.000 | 1.000 | 0.853 | 0.823 |
| 4671 | 0.772 | 1.000 | 0.794 | 1.000 | 0.974 | 0.960 | 0.904 | 1.000 | 1.000 | 1.000 | 0.959 | 1.000 | 0.982 | 0.858 | 1.000 | 1.000 | 0.858 | 0.829 |
| 4672 | 0.764 | 1.000 | 0.796 | 1.000 | 0.958 | 0.960 | 0.894 | 1.000 | 0.980 | 1.000 | 0.965 | 1.000 | 0.982 | 0.853 | 1.000 | 1.000 | 0.852 | 0.823 |
| 4673 | 0.767 | 1.000 | 0.792 | 1.000 | 0.968 | 0.960 | 0.901 | 1.000 | 0.990 | 1.000 | 0.965 | 1.000 | 0.991 | 0.858 | 1.000 | 1.000 | 0.856 | 0.827 |
| 4674 | 0.766 | 1.000 | 0.798 | 1.000 | 0.974 | 0.960 | 0.900 | 1.000 | 0.990 | 1.000 | 0.965 | 1.000 | 0.982 | 0.854 | 1.000 | 1.000 | 0.855 | 0.826 |
| 4675 | 0.770 | 1.000 | 0.798 | 1.000 | 0.974 | 0.960 | 0.889 | 1.000 | 0.980 | 1.000 | 0.962 | 1.000 | 0.991 | 0.860 | 1.000 | 1.000 | 0.856 | 0.827 |
| 4676 | 0.773 | 1.000 | 0.800 | 1.000 | 0.984 | 0.960 | 0.895 | 1.000 | 1.000 | 1.000 | 0.959 | 1.000 | 0.991 | 0.852 | 1.000 | 1.000 | 0.856 | 0.828 |
| 4677 | 0.769 | 1.000 | 0.806 | 0.986 | 0.968 | 0.960 | 0.898 | 1.000 | 0.990 | 1.000 | 0.959 | 1.000 | 0.991 | 0.858 | 1.000 | 1.000 | 0.857 | 0.828 |
| 4678 | 0.767 | 1.000 | 0.783 | 1.000 | 0.979 | 0.960 | 0.898 | 1.000 | 0.990 | 1.000 | 0.965 | 1.000 | 0.991 | 0.853 | 1.000 | 1.000 | 0.854 | 0.825 |

| | | | | | | | | | | | | | | | | | | |
|---|---|---|---|---|---|---|---|---|---|---|---|---|---|---|---|---|---|---|
| 4679 | 0.775 | 1.000 | 0.789 | 1.000 | 0.979 | 0.960 | 0.894 | 1.000 | 0.980 | 1.000 | 0.968 | 1.000 | 0.991 | 0.853 | 1.000 | 1.000 | 0.856 | 0.827 |
| 4680 | 0.769 | 1.000 | 0.779 | 1.000 | 0.979 | 0.960 | 0.906 | 1.000 | 1.000 | 1.000 | 0.957 | 1.000 | 0.991 | 0.856 | 1.000 | 1.000 | 0.855 | 0.827 |
| 4681 | 0.763 | 1.000 | 0.794 | 1.000 | 0.974 | 0.960 | 0.901 | 1.000 | 0.980 | 1.000 | 0.957 | 1.000 | 0.991 | 0.852 | 1.000 | 1.000 | 0.853 | 0.824 |
| 4682 | 0.774 | 1.000 | 0.781 | 1.000 | 0.974 | 0.960 | 0.900 | 1.000 | 0.990 | 1.000 | 0.959 | 1.000 | 0.991 | 0.856 | 1.000 | 1.000 | 0.856 | 0.827 |
| 4683 | 0.769 | 1.000 | 0.791 | 1.000 | 0.984 | 0.960 | 0.901 | 1.000 | 0.990 | 1.000 | 0.957 | 1.000 | 0.982 | 0.851 | 1.000 | 1.000 | 0.854 | 0.825 |
| 4684 | 0.763 | 1.000 | 0.794 | 1.000 | 0.974 | 0.960 | 0.895 | 1.000 | 0.980 | 1.000 | 0.962 | 1.000 | 0.964 | 0.853 | 1.000 | 1.000 | 0.852 | 0.823 |
| 4685 | 0.771 | 1.000 | 0.796 | 1.000 | 0.968 | 0.960 | 0.897 | 1.000 | 0.990 | 1.000 | 0.959 | 1.000 | 0.991 | 0.862 | 1.000 | 1.000 | 0.857 | 0.829 |
| 4686 | 0.761 | 1.000 | 0.804 | 1.000 | 0.979 | 0.960 | 0.900 | 1.000 | 0.980 | 1.000 | 0.954 | 1.000 | 0.964 | 0.854 | 1.000 | 1.000 | 0.853 | 0.824 |
| 4687 | 0.765 | 1.000 | 0.800 | 1.000 | 0.968 | 0.960 | 0.897 | 1.000 | 0.980 | 1.000 | 0.957 | 0.970 | 0.991 | 0.851 | 1.000 | 1.000 | 0.852 | 0.823 |
| 4688 | 0.772 | 1.000 | 0.791 | 1.000 | 0.974 | 0.960 | 0.897 | 1.000 | 1.000 | 1.000 | 0.959 | 1.000 | 0.982 | 0.848 | 1.000 | 1.000 | 0.854 | 0.825 |
| 4689 | 0.765 | 1.000 | 0.792 | 1.000 | 0.974 | 0.960 | 0.897 | 1.000 | 0.980 | 1.000 | 0.962 | 1.000 | 0.982 | 0.854 | 1.000 | 1.000 | 0.853 | 0.824 |
| 4690 | 0.762 | 1.000 | 0.804 | 1.000 | 0.979 | 0.960 | 0.900 | 1.000 | 1.000 | 1.000 | 0.957 | 1.000 | 0.982 | 0.859 | 1.000 | 1.000 | 0.855 | 0.827 |
| 4691 | 0.764 | 1.000 | 0.802 | 1.000 | 0.979 | 0.960 | 0.895 | 1.000 | 1.000 | 1.000 | 0.957 | 1.000 | 0.991 | 0.852 | 1.000 | 1.000 | 0.854 | 0.825 |
| 4692 | 0.754 | 1.000 | 0.804 | 1.000 | 0.968 | 0.960 | 0.909 | 1.000 | 0.980 | 1.000 | 0.957 | 0.970 | 0.982 | 0.857 | 1.000 | 1.000 | 0.853 | 0.824 |
| 4693 | 0.768 | 1.000 | 0.800 | 1.000 | 0.974 | 0.960 | 0.903 | 1.000 | 0.980 | 1.000 | 0.957 | 1.000 | 0.973 | 0.858 | 1.000 | 1.000 | 0.856 | 0.828 |
| 4694 | 0.771 | 1.000 | 0.787 | 1.000 | 0.974 | 0.960 | 0.908 | 1.000 | 0.980 | 1.000 | 0.954 | 1.000 | 0.982 | 0.852 | 1.000 | 1.000 | 0.855 | 0.826 |
| 4695 | 0.775 | 1.000 | 0.779 | 1.000 | 0.974 | 0.960 | 0.898 | 1.000 | 1.000 | 1.000 | 0.968 | 1.000 | 0.982 | 0.858 | 1.000 | 1.000 | 0.857 | 0.828 |
| 4696 | 0.773 | 1.000 | 0.787 | 1.000 | 0.984 | 0.960 | 0.898 | 1.000 | 0.990 | 1.000 | 0.962 | 1.000 | 0.991 | 0.853 | 1.000 | 1.000 | 0.856 | 0.827 |
| 4697 | 0.766 | 1.000 | 0.792 | 1.000 | 0.974 | 0.960 | 0.892 | 1.000 | 0.990 | 1.000 | 0.951 | 1.000 | 0.982 | 0.858 | 1.000 | 1.000 | 0.853 | 0.824 |
| 4698 | 0.767 | 1.000 | 0.798 | 1.000 | 0.974 | 0.960 | 0.898 | 1.000 | 0.990 | 1.000 | 0.959 | 1.000 | 0.982 | 0.861 | 1.000 | 1.000 | 0.856 | 0.828 |
| 4699 | 0.765 | 1.000 | 0.796 | 0.986 | 0.974 | 0.960 | 0.904 | 1.000 | 0.990 | 1.000 | 0.968 | 1.000 | 0.991 | 0.858 | 1.000 | 1.000 | 0.856 | 0.828 |
| 4700 | 0.776 | 1.000 | 0.802 | 1.000 | 0.974 | 0.960 | 0.895 | 1.000 | 0.990 | 1.000 | 0.954 | 1.000 | 0.982 | 0.856 | 1.000 | 1.000 | 0.857 | 0.829 |
| 4701 | 0.767 | 1.000 | 0.796 | 1.000 | 0.968 | 0.960 | 0.898 | 1.000 | 1.000 | 1.000 | 0.962 | 1.000 | 0.973 | 0.847 | 1.000 | 1.000 | 0.853 | 0.824 |
| 4702 | 0.773 | 1.000 | 0.792 | 1.000 | 0.979 | 0.960 | 0.898 | 1.000 | 0.990 | 1.000 | 0.962 | 1.000 | 0.991 | 0.855 | 1.000 | 1.000 | 0.857 | 0.828 |
| 4703 | 0.777 | 1.000 | 0.800 | 1.000 | 0.974 | 0.960 | 0.908 | 1.000 | 1.000 | 1.000 | 0.971 | 1.000 | 1.000 | 0.852 | 1.000 | 1.000 | 0.860 | 0.832 |
| 4704 | 0.772 | 1.000 | 0.808 | 1.000 | 0.979 | 0.960 | 0.901 | 1.000 | 0.980 | 1.000 | 0.962 | 1.000 | 0.982 | 0.861 | 1.000 | 1.000 | 0.859 | 0.831 |
| 4705 | 0.764 | 1.000 | 0.789 | 1.000 | 0.979 | 0.960 | 0.904 | 1.000 | 0.990 | 1.000 | 0.959 | 1.000 | 0.991 | 0.853 | 1.000 | 1.000 | 0.854 | 0.825 |
| 4706 | 0.761 | 1.000 | 0.785 | 1.000 | 0.968 | 0.960 | 0.886 | 1.000 | 0.969 | 1.000 | 0.951 | 1.000 | 0.964 | 0.851 | 1.000 | 1.000 | 0.847 | 0.817 |
| 4707 | 0.762 | 1.000 | 0.789 | 1.000 | 0.979 | 0.960 | 0.908 | 1.000 | 1.000 | 1.000 | 0.965 | 1.000 | 0.982 | 0.858 | 1.000 | 1.000 | 0.855 | 0.826 |
| 4708 | 0.767 | 1.000 | 0.810 | 1.000 | 0.968 | 0.960 | 0.892 | 1.000 | 0.990 | 1.000 | 0.959 | 1.000 | 0.982 | 0.861 | 1.000 | 1.000 | 0.857 | 0.828 |
| 4709 | 0.775 | 1.000 | 0.802 | 1.000 | 0.979 | 0.960 | 0.895 | 1.000 | 1.000 | 1.000 | 0.957 | 1.000 | 0.973 | 0.852 | 1.000 | 1.000 | 0.856 | 0.828 |
| 4710 | 0.773 | 1.000 | 0.791 | 1.000 | 0.984 | 0.960 | 0.900 | 1.000 | 0.990 | 1.000 | 0.962 | 1.000 | 0.982 | 0.853 | 1.000 | 1.000 | 0.856 | 0.827 |
| 4711 | 0.761 | 1.000 | 0.789 | 1.000 | 0.979 | 0.960 | 0.895 | 1.000 | 0.980 | 1.000 | 0.959 | 1.000 | 0.973 | 0.855 | 1.000 | 1.000 | 0.851 | 0.822 |
| 4712 | 0.767 | 1.000 | 0.783 | 1.000 | 0.974 | 0.960 | 0.904 | 1.000 | 0.990 | 1.000 | 0.959 | 1.000 | 0.982 | 0.860 | 1.000 | 1.000 | 0.855 | 0.827 |
| 4713 | 0.767 | 1.000 | 0.783 | 1.000 | 0.984 | 0.960 | 0.898 | 1.000 | 1.000 | 1.000 | 0.959 | 1.000 | 0.973 | 0.861 | 1.000 | 1.000 | 0.855 | 0.826 |
| 4714 | 0.773 | 1.000 | 0.792 | 1.000 | 0.984 | 0.960 | 0.892 | 1.000 | 0.990 | 1.000 | 0.957 | 1.000 | 0.991 | 0.855 | 1.000 | 1.000 | 0.856 | 0.827 |
| 4715 | 0.761 | 1.000 | 0.789 | 1.000 | 0.979 | 0.960 | 0.898 | 1.000 | 0.990 | 1.000 | 0.971 | 1.000 | 0.982 | 0.854 | 1.000 | 1.000 | 0.853 | 0.824 |
| 4716 | 0.779 | 1.000 | 0.798 | 1.000 | 0.974 | 0.960 | 0.901 | 1.000 | 1.000 | 1.000 | 0.959 | 1.000 | 0.973 | 0.854 | 1.000 | 1.000 | 0.859 | 0.830 |
| 4717 | 0.771 | 1.000 | 0.789 | 1.000 | 0.979 | 0.960 | 0.895 | 1.000 | 1.000 | 1.000 | 0.962 | 1.000 | 0.991 | 0.850 | 1.000 | 1.000 | 0.854 | 0.825 |

| | | | | | | | | | | | | | | | | | |
|---|---|---|---|---|---|---|---|---|---|---|---|---|---|---|---|---|---|
| 4718 | 0.775 | 1.000 | 0.802 | 1.000 | 0.968 | 0.960 | 0.898 | 1.000 | 0.980 | 1.000 | 0.965 | 1.000 | 0.964 | 0.859 | 1.000 | 1.000 | 0.858 | 0.830 |
| 4719 | 0.768 | 1.000 | 0.800 | 1.000 | 0.984 | 0.960 | 0.892 | 1.000 | 1.000 | 1.000 | 0.962 | 1.000 | 0.982 | 0.848 | 1.000 | 1.000 | 0.854 | 0.825 |
| 4720 | 0.761 | 1.000 | 0.802 | 1.000 | 0.974 | 0.960 | 0.900 | 1.000 | 1.000 | 1.000 | 0.957 | 1.000 | 0.982 | 0.856 | 1.000 | 1.000 | 0.854 | 0.825 |
| 4721 | 0.770 | 1.000 | 0.800 | 1.000 | 0.974 | 0.960 | 0.892 | 1.000 | 0.990 | 1.000 | 0.957 | 0.970 | 1.000 | 0.856 | 1.000 | 1.000 | 0.855 | 0.827 |
| 4722 | 0.768 | 1.000 | 0.777 | 1.000 | 0.979 | 0.960 | 0.908 | 1.000 | 0.990 | 1.000 | 0.962 | 1.000 | 0.991 | 0.862 | 1.000 | 1.000 | 0.857 | 0.828 |
| 4723 | 0.769 | 1.000 | 0.785 | 0.986 | 0.974 | 0.960 | 0.895 | 1.000 | 0.980 | 1.000 | 0.962 | 0.970 | 0.982 | 0.861 | 1.000 | 1.000 | 0.855 | 0.826 |
| 4724 | 0.773 | 1.000 | 0.781 | 1.000 | 0.979 | 0.960 | 0.900 | 1.000 | 0.990 | 1.000 | 0.957 | 1.000 | 0.964 | 0.851 | 1.000 | 1.000 | 0.854 | 0.825 |
| 4725 | 0.777 | 1.000 | 0.792 | 1.000 | 0.968 | 0.960 | 0.895 | 1.000 | 1.000 | 1.000 | 0.957 | 1.000 | 0.991 | 0.858 | 1.000 | 1.000 | 0.857 | 0.829 |
| 4726 | 0.769 | 1.000 | 0.785 | 0.986 | 0.974 | 0.960 | 0.892 | 1.000 | 0.990 | 1.000 | 0.962 | 1.000 | 0.982 | 0.854 | 1.000 | 1.000 | 0.853 | 0.824 |
| 4727 | 0.766 | 1.000 | 0.794 | 1.000 | 0.974 | 0.960 | 0.892 | 1.000 | 0.990 | 1.000 | 0.957 | 1.000 | 0.982 | 0.860 | 1.000 | 1.000 | 0.854 | 0.825 |
| 4728 | 0.774 | 1.000 | 0.804 | 1.000 | 0.979 | 0.960 | 0.898 | 1.000 | 0.980 | 1.000 | 0.962 | 1.000 | 0.973 | 0.858 | 1.000 | 1.000 | 0.858 | 0.830 |
| 4729 | 0.771 | 1.000 | 0.791 | 1.000 | 0.979 | 0.960 | 0.898 | 1.000 | 1.000 | 1.000 | 0.962 | 1.000 | 0.991 | 0.862 | 1.000 | 1.000 | 0.858 | 0.830 |
| 4730 | 0.763 | 1.000 | 0.804 | 1.000 | 0.963 | 0.960 | 0.901 | 1.000 | 0.990 | 1.000 | 0.965 | 1.000 | 0.973 | 0.855 | 1.000 | 1.000 | 0.854 | 0.826 |
| 4731 | 0.767 | 1.000 | 0.791 | 1.000 | 0.979 | 0.960 | 0.903 | 1.000 | 0.980 | 1.000 | 0.959 | 1.000 | 0.991 | 0.850 | 1.000 | 1.000 | 0.853 | 0.824 |
| 4732 | 0.765 | 1.000 | 0.794 | 1.000 | 0.979 | 0.960 | 0.908 | 1.000 | 1.000 | 1.000 | 0.965 | 1.000 | 0.964 | 0.857 | 1.000 | 1.000 | 0.856 | 0.827 |
| 4733 | 0.769 | 1.000 | 0.791 | 0.986 | 0.979 | 0.960 | 0.891 | 1.000 | 0.990 | 1.000 | 0.962 | 0.970 | 0.982 | 0.858 | 1.000 | 1.000 | 0.855 | 0.826 |
| 4734 | 0.765 | 1.000 | 0.794 | 1.000 | 0.974 | 0.960 | 0.904 | 1.000 | 0.990 | 1.000 | 0.957 | 1.000 | 0.991 | 0.860 | 1.000 | 1.000 | 0.856 | 0.827 |
| 4735 | 0.765 | 1.000 | 0.791 | 1.000 | 0.984 | 0.960 | 0.897 | 1.000 | 0.990 | 1.000 | 0.962 | 1.000 | 0.964 | 0.856 | 1.000 | 1.000 | 0.854 | 0.825 |
| 4736 | 0.763 | 1.000 | 0.806 | 1.000 | 0.974 | 0.960 | 0.901 | 1.000 | 1.000 | 1.000 | 0.965 | 1.000 | 0.982 | 0.856 | 1.000 | 1.000 | 0.856 | 0.827 |
| 4737 | 0.778 | 1.000 | 0.792 | 1.000 | 0.984 | 0.960 | 0.897 | 1.000 | 0.980 | 1.000 | 0.962 | 1.000 | 0.982 | 0.860 | 1.000 | 1.000 | 0.859 | 0.831 |
| 4738 | 0.774 | 1.000 | 0.785 | 1.000 | 0.968 | 0.960 | 0.909 | 1.000 | 1.000 | 1.000 | 0.959 | 1.000 | 0.991 | 0.861 | 1.000 | 1.000 | 0.859 | 0.830 |
| 4739 | 0.769 | 1.000 | 0.804 | 1.000 | 0.979 | 0.960 | 0.908 | 1.000 | 1.000 | 1.000 | 0.954 | 1.000 | 0.982 | 0.856 | 1.000 | 1.000 | 0.857 | 0.829 |
| 4740 | 0.775 | 1.000 | 0.798 | 1.000 | 0.984 | 0.960 | 0.903 | 1.000 | 1.000 | 1.000 | 0.965 | 1.000 | 0.982 | 0.853 | 1.000 | 1.000 | 0.858 | 0.830 |
| 4741 | 0.765 | 1.000 | 0.789 | 1.000 | 0.984 | 0.960 | 0.911 | 1.000 | 1.000 | 1.000 | 0.954 | 1.000 | 0.991 | 0.857 | 1.000 | 1.000 | 0.856 | 0.827 |
| 4742 | 0.761 | 1.000 | 0.796 | 1.000 | 0.974 | 0.960 | 0.897 | 1.000 | 0.980 | 1.000 | 0.959 | 1.000 | 0.982 | 0.858 | 1.000 | 1.000 | 0.853 | 0.824 |
| 4743 | 0.769 | 1.000 | 0.794 | 1.000 | 0.974 | 0.960 | 0.911 | 1.000 | 0.980 | 1.000 | 0.965 | 0.970 | 0.973 | 0.855 | 1.000 | 1.000 | 0.856 | 0.828 |
| 4744 | 0.778 | 1.000 | 0.810 | 1.000 | 0.974 | 0.960 | 0.901 | 1.000 | 0.990 | 1.000 | 0.957 | 0.970 | 0.991 | 0.858 | 1.000 | 1.000 | 0.860 | 0.833 |
| 4745 | 0.765 | 1.000 | 0.789 | 1.000 | 0.974 | 0.960 | 0.898 | 1.000 | 0.980 | 1.000 | 0.959 | 1.000 | 0.973 | 0.858 | 1.000 | 1.000 | 0.854 | 0.825 |
| 4746 | 0.765 | 1.000 | 0.791 | 1.000 | 0.979 | 0.960 | 0.903 | 1.000 | 0.980 | 1.000 | 0.962 | 1.000 | 0.982 | 0.854 | 1.000 | 1.000 | 0.854 | 0.825 |
| 4747 | 0.771 | 1.000 | 0.796 | 1.000 | 0.974 | 0.960 | 0.914 | 1.000 | 1.000 | 1.000 | 0.959 | 1.000 | 0.982 | 0.861 | 1.000 | 1.000 | 0.859 | 0.832 |
| 4748 | 0.769 | 1.000 | 0.792 | 1.000 | 0.974 | 0.960 | 0.903 | 1.000 | 0.990 | 1.000 | 0.962 | 1.000 | 0.991 | 0.854 | 1.000 | 1.000 | 0.856 | 0.827 |
| 4749 | 0.769 | 1.000 | 0.806 | 1.000 | 0.984 | 0.960 | 0.911 | 1.000 | 0.980 | 1.000 | 0.974 | 1.000 | 0.991 | 0.863 | 1.000 | 1.000 | 0.861 | 0.834 |
| 4750 | 0.765 | 1.000 | 0.792 | 1.000 | 0.979 | 0.960 | 0.898 | 1.000 | 1.000 | 1.000 | 0.965 | 1.000 | 0.973 | 0.859 | 1.000 | 1.000 | 0.855 | 0.826 |
| 4751 | 0.768 | 1.000 | 0.806 | 1.000 | 0.974 | 0.960 | 0.900 | 1.000 | 0.990 | 1.000 | 0.962 | 1.000 | 0.973 | 0.856 | 1.000 | 1.000 | 0.856 | 0.828 |
| 4752 | 0.771 | 1.000 | 0.806 | 1.000 | 0.968 | 0.960 | 0.903 | 1.000 | 0.990 | 1.000 | 0.959 | 1.000 | 0.973 | 0.858 | 1.000 | 1.000 | 0.858 | 0.829 |
| 4753 | 0.766 | 1.000 | 0.789 | 1.000 | 0.979 | 0.960 | 0.908 | 1.000 | 1.000 | 1.000 | 0.962 | 1.000 | 0.991 | 0.860 | 1.000 | 1.000 | 0.857 | 0.829 |
| 4754 | 0.762 | 1.000 | 0.792 | 1.000 | 0.974 | 0.960 | 0.901 | 1.000 | 0.969 | 1.000 | 0.954 | 1.000 | 0.991 | 0.856 | 1.000 | 1.000 | 0.853 | 0.824 |
| 4755 | 0.769 | 1.000 | 0.787 | 0.986 | 0.968 | 0.960 | 0.906 | 1.000 | 1.000 | 1.000 | 0.965 | 1.000 | 0.982 | 0.852 | 1.000 | 1.000 | 0.855 | 0.826 |
| 4756 | 0.755 | 1.000 | 0.791 | 1.000 | 0.984 | 0.960 | 0.900 | 1.000 | 0.990 | 1.000 | 0.965 | 0.970 | 0.991 | 0.857 | 1.000 | 1.000 | 0.852 | 0.823 |

| | | | | | | | | | | | | | | | | | |
|---|---|---|---|---|---|---|---|---|---|---|---|---|---|---|---|---|---|
| 4757 | 0.771 | 1.000 | 0.783 | 1.000 | 0.974 | 0.960 | 0.906 | 1.000 | 0.990 | 1.000 | 0.959 | 1.000 | 0.982 | 0.858 | 1.000 | 1.000 | 0.856 | 0.827 |
| 4758 | 0.763 | 1.000 | 0.796 | 0.986 | 0.979 | 0.960 | 0.898 | 1.000 | 1.000 | 1.000 | 0.962 | 1.000 | 0.973 | 0.861 | 1.000 | 1.000 | 0.855 | 0.826 |
| 4759 | 0.765 | 1.000 | 0.800 | 1.000 | 0.974 | 0.960 | 0.921 | 1.000 | 0.990 | 1.000 | 0.954 | 1.000 | 0.991 | 0.855 | 1.000 | 1.000 | 0.857 | 0.829 |
| 4760 | 0.765 | 1.000 | 0.792 | 1.000 | 0.974 | 0.960 | 0.906 | 1.000 | 0.990 | 1.000 | 0.965 | 1.000 | 0.955 | 0.855 | 1.000 | 1.000 | 0.855 | 0.826 |
| 4761 | 0.771 | 1.000 | 0.800 | 1.000 | 0.979 | 0.960 | 0.908 | 1.000 | 0.990 | 1.000 | 0.954 | 1.000 | 0.982 | 0.858 | 1.000 | 1.000 | 0.858 | 0.830 |
| 4762 | 0.773 | 1.000 | 0.802 | 1.000 | 0.974 | 0.960 | 0.903 | 1.000 | 0.990 | 1.000 | 0.965 | 1.000 | 0.955 | 0.865 | 1.000 | 1.000 | 0.860 | 0.832 |
| 4763 | 0.770 | 1.000 | 0.794 | 0.986 | 0.974 | 0.960 | 0.908 | 1.000 | 0.980 | 1.000 | 0.959 | 1.000 | 0.964 | 0.855 | 1.000 | 1.000 | 0.856 | 0.827 |
| 4764 | 0.775 | 1.000 | 0.792 | 1.000 | 0.974 | 0.960 | 0.904 | 1.000 | 1.000 | 1.000 | 0.962 | 1.000 | 0.973 | 0.865 | 1.000 | 1.000 | 0.860 | 0.832 |
| 4765 | 0.765 | 1.000 | 0.789 | 1.000 | 0.968 | 0.960 | 0.903 | 1.000 | 1.000 | 1.000 | 0.965 | 1.000 | 0.964 | 0.848 | 1.000 | 1.000 | 0.852 | 0.823 |
| 4766 | 0.771 | 1.000 | 0.777 | 1.000 | 0.979 | 0.960 | 0.906 | 1.000 | 0.990 | 1.000 | 0.962 | 1.000 | 0.973 | 0.861 | 1.000 | 1.000 | 0.856 | 0.828 |
| 4767 | 0.773 | 1.000 | 0.794 | 1.000 | 0.974 | 0.960 | 0.898 | 1.000 | 0.990 | 1.000 | 0.951 | 1.000 | 0.973 | 0.856 | 1.000 | 1.000 | 0.856 | 0.827 |
| 4768 | 0.761 | 1.000 | 0.800 | 1.000 | 0.974 | 0.960 | 0.904 | 1.000 | 0.990 | 1.000 | 0.968 | 1.000 | 0.964 | 0.864 | 1.000 | 1.000 | 0.856 | 0.828 |
| 4769 | 0.768 | 1.000 | 0.791 | 1.000 | 0.974 | 0.960 | 0.895 | 1.000 | 0.980 | 1.000 | 0.971 | 1.000 | 0.982 | 0.848 | 1.000 | 1.000 | 0.853 | 0.824 |
| 4770 | 0.772 | 1.000 | 0.785 | 1.000 | 0.974 | 0.960 | 0.901 | 1.000 | 0.990 | 1.000 | 0.959 | 0.970 | 0.964 | 0.851 | 1.000 | 1.000 | 0.854 | 0.824 |
| 4771 | 0.771 | 1.000 | 0.787 | 0.986 | 0.974 | 0.960 | 0.901 | 1.000 | 0.969 | 1.000 | 0.965 | 1.000 | 0.964 | 0.848 | 1.000 | 1.000 | 0.853 | 0.824 |
| 4772 | 0.763 | 1.000 | 0.802 | 1.000 | 0.968 | 0.960 | 0.901 | 1.000 | 0.990 | 1.000 | 0.957 | 1.000 | 0.973 | 0.859 | 1.000 | 1.000 | 0.855 | 0.826 |
| 4773 | 0.757 | 1.000 | 0.792 | 1.000 | 0.974 | 0.960 | 0.904 | 1.000 | 1.000 | 1.000 | 0.957 | 1.000 | 0.982 | 0.852 | 1.000 | 1.000 | 0.851 | 0.822 |

**2. IFS results using SVM and LightGBM feature list**

| Number of features | Class 1 | Class 2 | Class 3 | Class 4 | Class 5 | Class 6 | Class 7 | Class 8 | Class 9 | Class 10 | Class 11 | Class 12 | Class 13 | Class 14 | Class 15 | Class 16 | ACC | MCC |
|---|---|---|---|---|---|---|---|---|---|---|---|---|---|---|---|---|---|---|
| 1 | 0.289 | 0.171 | 0.107 | 0.000 | 0.000 | 0.040 | 0.000 | 0.333 | 0.071 | 0.021 | 0.000 | 0.000 | 0.286 | 0.292 | 0.000 | 0.000 | 0.182 | 0.097 |
| 2 | 0.369 | 0.171 | 0.000 | 0.057 | 0.000 | 0.200 | 0.000 | 0.333 | 0.082 | 0.167 | 0.287 | 0.000 | 0.000 | 0.101 | 0.065 | 0.722 | 0.171 | 0.120 |
| 3 | 0.367 | 0.171 | 0.002 | 0.057 | 0.005 | 0.200 | 0.000 | 0.667 | 0.092 | 0.000 | 0.000 | 0.333 | 0.429 | 0.252 | 0.587 | 0.722 | 0.205 | 0.177 |
| 4 | 0.373 | 0.171 | 0.004 | 0.071 | 0.005 | 0.200 | 0.000 | 0.667 | 0.082 | 0.563 | 0.009 | 0.212 | 0.580 | 0.302 | 0.870 | 0.722 | 0.231 | 0.212 |
| 5 | 0.387 | 0.171 | 0.004 | 0.071 | 0.011 | 0.200 | 0.000 | 0.667 | 0.112 | 0.563 | 0.009 | 0.212 | 0.571 | 0.308 | 0.848 | 0.722 | 0.237 | 0.216 |
| 6 | 0.386 | 0.171 | 0.004 | 0.071 | 0.011 | 0.200 | 0.000 | 0.667 | 0.102 | 0.563 | 0.009 | 0.212 | 0.563 | 0.299 | 0.848 | 0.722 | 0.234 | 0.213 |
| 7 | 0.387 | 0.171 | 0.010 | 0.071 | 0.011 | 0.200 | 0.000 | 1.000 | 0.092 | 0.688 | 0.009 | 0.273 | 0.598 | 0.294 | 0.848 | 0.722 | 0.236 | 0.216 |
| 8 | 0.416 | 0.171 | 0.028 | 0.029 | 0.047 | 0.480 | 0.015 | 1.000 | 0.031 | 0.688 | 0.009 | 0.242 | 0.607 | 0.295 | 0.891 | 0.741 | 0.251 | 0.229 |
| 9 | 0.413 | 0.171 | 0.038 | 0.029 | 0.100 | 0.400 | 0.741 | 1.000 | 0.041 | 0.750 | 0.412 | 0.364 | 0.527 | 0.264 | 0.391 | 0.704 | 0.361 | 0.300 |
| 10 | 0.412 | 0.200 | 0.071 | 0.029 | 0.105 | 0.400 | 0.744 | 1.000 | 0.041 | 0.813 | 0.412 | 0.394 | 0.589 | 0.289 | 0.391 | 0.704 | 0.374 | 0.312 |
| 11 | 0.467 | 0.629 | 0.065 | 0.029 | 0.142 | 0.360 | 0.784 | 1.000 | 0.041 | 0.813 | 0.412 | 0.394 | 0.589 | 0.293 | 0.391 | 0.704 | 0.400 | 0.336 |
| 12 | 0.465 | 0.743 | 0.077 | 0.029 | 0.168 | 0.360 | 0.784 | 1.000 | 0.061 | 0.813 | 0.414 | 0.394 | 0.589 | 0.299 | 0.457 | 0.704 | 0.405 | 0.341 |
| 13 | 0.467 | 0.743 | 0.087 | 0.029 | 0.163 | 0.400 | 0.787 | 1.000 | 0.092 | 0.813 | 0.412 | 0.394 | 0.589 | 0.305 | 0.457 | 0.704 | 0.409 | 0.346 |
| 14 | 0.477 | 0.743 | 0.097 | 0.029 | 0.253 | 0.480 | 0.775 | 1.000 | 0.163 | 0.813 | 0.423 | 0.394 | 0.598 | 0.311 | 0.500 | 0.704 | 0.420 | 0.357 |
| 15 | 0.454 | 0.743 | 0.132 | 0.043 | 0.347 | 0.480 | 0.663 | 1.000 | 0.173 | 0.833 | 0.412 | 0.394 | 0.661 | 0.318 | 0.739 | 0.704 | 0.411 | 0.364 |
| 16 | 0.457 | 0.771 | 0.140 | 0.100 | 0.474 | 0.480 | 0.737 | 1.000 | 0.214 | 0.833 | 0.429 | 0.394 | 0.607 | 0.313 | 0.587 | 0.704 | 0.426 | 0.377 |
| 17 | 0.492 | 0.771 | 0.136 | 0.100 | 0.389 | 0.440 | 0.740 | 1.000 | 0.378 | 0.833 | 0.438 | 0.394 | 0.616 | 0.323 | 0.674 | 0.685 | 0.440 | 0.391 |
| 18 | 0.488 | 0.771 | 0.294 | 0.100 | 0.489 | 0.480 | 0.781 | 1.000 | 0.367 | 0.833 | 0.623 | 0.333 | 0.643 | 0.324 | 0.804 | 0.685 | 0.479 | 0.421 |
| 19 | 0.489 | 0.743 | 0.306 | 0.129 | 0.479 | 0.480 | 0.777 | 1.000 | 0.367 | 0.833 | 0.571 | 0.364 | 0.661 | 0.325 | 0.913 | 0.685 | 0.478 | 0.420 |
| 20 | 0.490 | 0.771 | 0.312 | 0.143 | 0.479 | 0.560 | 0.787 | 1.000 | 0.439 | 0.833 | 0.568 | 0.364 | 0.679 | 0.324 | 0.913 | 0.704 | 0.483 | 0.426 |
| 21 | 0.490 | 0.771 | 0.334 | 0.171 | 0.495 | 0.560 | 0.790 | 1.000 | 0.439 | 0.833 | 0.542 | 0.455 | 0.732 | 0.401 | 0.913 | 0.704 | 0.506 | 0.447 |
| 22 | 0.505 | 0.743 | 0.383 | 0.157 | 0.532 | 0.560 | 0.752 | 1.000 | 0.429 | 0.833 | 0.577 | 0.455 | 0.741 | 0.378 | 0.913 | 0.704 | 0.508 | 0.451 |
| 23 | 0.504 | 0.771 | 0.411 | 0.157 | 0.553 | 0.600 | 0.778 | 1.000 | 0.439 | 0.833 | 0.449 | 0.485 | 0.759 | 0.392 | 0.913 | 0.685 | 0.510 | 0.453 |
| 24 | 0.506 | 0.771 | 0.413 | 0.186 | 0.584 | 0.680 | 0.783 | 1.000 | 0.480 | 0.833 | 0.452 | 0.485 | 0.759 | 0.391 | 0.913 | 0.722 | 0.515 | 0.459 |
| 25 | 0.507 | 0.771 | 0.413 | 0.214 | 0.542 | 0.680 | 0.764 | 1.000 | 0.480 | 0.833 | 0.449 | 0.455 | 0.759 | 0.430 | 0.913 | 0.704 | 0.521 | 0.463 |
| 26 | 0.514 | 0.800 | 0.427 | 0.229 | 0.547 | 0.720 | 0.763 | 1.000 | 0.480 | 0.833 | 0.449 | 0.455 | 0.759 | 0.434 | 0.913 | 0.704 | 0.526 | 0.468 |
| 27 | 0.514 | 0.800 | 0.423 | 0.214 | 0.553 | 0.720 | 0.757 | 1.000 | 0.520 | 0.833 | 0.449 | 0.455 | 0.759 | 0.431 | 0.913 | 0.704 | 0.525 | 0.467 |
| 28 | 0.518 | 0.771 | 0.433 | 0.243 | 0.553 | 0.720 | 0.760 | 1.000 | 0.510 | 0.833 | 0.452 | 0.455 | 0.759 | 0.444 | 0.913 | 0.704 | 0.531 | 0.473 |
| 29 | 0.517 | 0.771 | 0.431 | 0.243 | 0.547 | 0.720 | 0.761 | 1.000 | 0.520 | 0.833 | 0.452 | 0.485 | 0.759 | 0.408 | 0.913 | 0.704 | 0.522 | 0.465 |
| 30 | 0.516 | 0.771 | 0.433 | 0.229 | 0.589 | 0.760 | 0.761 | 1.000 | 0.571 | 0.833 | 0.452 | 0.485 | 0.759 | 0.410 | 0.913 | 0.741 | 0.525 | 0.469 |
| 31 | 0.520 | 0.743 | 0.496 | 0.257 | 0.605 | 0.720 | 0.781 | 1.000 | 0.592 | 0.833 | 0.536 | 0.485 | 0.759 | 0.498 | 0.891 | 0.778 | 0.565 | 0.510 |
| 32 | 0.519 | 0.714 | 0.494 | 0.300 | 0.632 | 0.680 | 0.769 | 1.000 | 0.602 | 0.854 | 0.548 | 0.485 | 0.759 | 0.503 | 0.891 | 0.778 | 0.567 | 0.512 |
| 33 | 0.518 | 0.714 | 0.470 | 0.314 | 0.732 | 0.640 | 0.778 | 1.000 | 0.602 | 0.854 | 0.690 | 0.545 | 0.759 | 0.507 | 0.891 | 0.796 | 0.580 | 0.528 |
| 34 | 0.518 | 0.743 | 0.476 | 0.314 | 0.737 | 0.640 | 0.783 | 1.000 | 0.602 | 0.854 | 0.690 | 0.545 | 0.768 | 0.507 | 0.891 | 0.796 | 0.582 | 0.530 |
| 35 | 0.517 | 0.771 | 0.474 | 0.314 | 0.737 | 0.640 | 0.783 | 1.000 | 0.602 | 0.854 | 0.690 | 0.545 | 0.768 | 0.508 | 0.891 | 0.796 | 0.582 | 0.530 |
| 36 | 0.519 | 0.771 | 0.512 | 0.300 | 0.732 | 0.680 | 0.783 | 1.000 | 0.602 | 0.854 | 0.759 | 0.545 | 0.768 | 0.521 | 0.913 | 0.796 | 0.595 | 0.544 |
| 37 | 0.519 | 0.743 | 0.520 | 0.343 | 0.732 | 0.680 | 0.784 | 1.000 | 0.602 | 0.854 | 0.759 | 0.545 | 0.768 | 0.517 | 0.913 | 0.796 | 0.595 | 0.545 |

| | | | | | | | | | | | | | | | | | | |
|---|---|---|---|---|---|---|---|---|---|---|---|---|---|---|---|---|---|---|
| 38 | 0.519 | 0.743 | 0.518 | 0.343 | 0.726 | 0.720 | 0.783 | 1.000 | 0.592 | 0.854 | 0.780 | 0.545 | 0.768 | 0.516 | 0.913 | 0.796 | 0.596 | 0.545 |
| 39 | 0.518 | 0.743 | 0.520 | 0.343 | 0.726 | 0.760 | 0.783 | 1.000 | 0.592 | 0.854 | 0.777 | 0.545 | 0.768 | 0.514 | 0.913 | 0.796 | 0.595 | 0.545 |
| 40 | 0.518 | 0.743 | 0.522 | 0.343 | 0.726 | 0.760 | 0.781 | 1.000 | 0.592 | 0.854 | 0.777 | 0.545 | 0.768 | 0.513 | 0.913 | 0.796 | 0.595 | 0.544 |
| 41 | 0.519 | 0.771 | 0.528 | 0.343 | 0.716 | 0.760 | 0.777 | 1.000 | 0.582 | 0.854 | 0.777 | 0.545 | 0.768 | 0.514 | 0.913 | 0.815 | 0.595 | 0.545 |
| 42 | 0.520 | 0.771 | 0.526 | 0.343 | 0.716 | 0.760 | 0.775 | 1.000 | 0.582 | 0.854 | 0.780 | 0.545 | 0.768 | 0.519 | 0.913 | 0.815 | 0.597 | 0.546 |
| 43 | 0.519 | 0.771 | 0.538 | 0.357 | 0.721 | 0.760 | 0.778 | 1.000 | 0.592 | 0.854 | 0.780 | 0.576 | 0.768 | 0.520 | 0.913 | 0.815 | 0.599 | 0.549 |
| 44 | 0.519 | 0.771 | 0.538 | 0.357 | 0.721 | 0.760 | 0.778 | 1.000 | 0.602 | 0.833 | 0.780 | 0.455 | 0.768 | 0.593 | 0.913 | 0.815 | 0.617 | 0.565 |
| 45 | 0.514 | 0.714 | 0.502 | 0.371 | 0.726 | 0.720 | 0.792 | 1.000 | 0.602 | 0.854 | 0.780 | 0.455 | 0.768 | 0.589 | 0.913 | 0.815 | 0.613 | 0.561 |
| 46 | 0.514 | 0.714 | 0.488 | 0.371 | 0.721 | 0.720 | 0.800 | 1.000 | 0.602 | 0.854 | 0.777 | 0.424 | 0.768 | 0.588 | 0.935 | 0.815 | 0.612 | 0.559 |
| 47 | 0.514 | 0.714 | 0.482 | 0.371 | 0.716 | 0.680 | 0.800 | 1.000 | 0.602 | 0.854 | 0.774 | 0.455 | 0.759 | 0.591 | 0.935 | 0.815 | 0.611 | 0.559 |
| 48 | 0.514 | 0.743 | 0.496 | 0.357 | 0.716 | 0.680 | 0.795 | 1.000 | 0.602 | 0.854 | 0.777 | 0.455 | 0.759 | 0.594 | 0.935 | 0.815 | 0.613 | 0.561 |
| 49 | 0.527 | 0.743 | 0.488 | 0.343 | 0.711 | 0.680 | 0.787 | 1.000 | 0.612 | 0.854 | 0.774 | 0.455 | 0.759 | 0.602 | 0.935 | 0.815 | 0.617 | 0.563 |
| 50 | 0.526 | 0.743 | 0.498 | 0.329 | 0.716 | 0.680 | 0.787 | 1.000 | 0.622 | 0.854 | 0.774 | 0.455 | 0.759 | 0.605 | 0.935 | 0.815 | 0.618 | 0.565 |
| 51 | 0.527 | 0.743 | 0.500 | 0.371 | 0.711 | 0.680 | 0.787 | 1.000 | 0.622 | 0.854 | 0.774 | 0.485 | 0.759 | 0.603 | 0.935 | 0.815 | 0.619 | 0.566 |
| 52 | 0.531 | 0.743 | 0.492 | 0.329 | 0.716 | 0.720 | 0.787 | 1.000 | 0.622 | 0.854 | 0.780 | 0.455 | 0.759 | 0.607 | 0.935 | 0.815 | 0.620 | 0.567 |
| 54 | 0.531 | 0.743 | 0.508 | 0.357 | 0.716 | 0.720 | 0.787 | 1.000 | 0.612 | 0.833 | 0.786 | 0.515 | 0.759 | 0.579 | 0.935 | 0.815 | 0.616 | 0.563 |
| 55 | 0.528 | 0.743 | 0.504 | 0.386 | 0.721 | 0.760 | 0.789 | 1.000 | 0.633 | 0.833 | 0.780 | 0.545 | 0.768 | 0.583 | 0.935 | 0.815 | 0.617 | 0.564 |
| 56 | 0.533 | 0.743 | 0.504 | 0.386 | 0.726 | 0.760 | 0.787 | 1.000 | 0.622 | 0.833 | 0.783 | 0.576 | 0.759 | 0.592 | 0.935 | 0.815 | 0.621 | 0.568 |
| 57 | 0.533 | 0.800 | 0.506 | 0.386 | 0.726 | 0.800 | 0.792 | 1.000 | 0.622 | 0.833 | 0.783 | 0.545 | 0.750 | 0.594 | 0.957 | 0.815 | 0.622 | 0.570 |
| 58 | 0.534 | 0.829 | 0.506 | 0.400 | 0.732 | 0.800 | 0.792 | 1.000 | 0.633 | 0.833 | 0.783 | 0.576 | 0.759 | 0.602 | 0.957 | 0.815 | 0.626 | 0.573 |
| 59 | 0.534 | 0.829 | 0.518 | 0.400 | 0.737 | 0.840 | 0.792 | 1.000 | 0.673 | 0.833 | 0.783 | 0.576 | 0.759 | 0.602 | 0.957 | 0.815 | 0.628 | 0.577 |
| 60 | 0.533 | 0.829 | 0.520 | 0.414 | 0.747 | 0.840 | 0.792 | 1.000 | 0.663 | 0.854 | 0.786 | 0.606 | 0.759 | 0.595 | 0.957 | 0.815 | 0.627 | 0.576 |
| 61 | 0.539 | 0.829 | 0.500 | 0.443 | 0.737 | 0.800 | 0.795 | 1.000 | 0.663 | 0.854 | 0.786 | 0.636 | 0.750 | 0.594 | 0.957 | 0.815 | 0.627 | 0.575 |
| 62 | 0.533 | 0.829 | 0.514 | 0.443 | 0.732 | 0.800 | 0.803 | 1.000 | 0.663 | 0.854 | 0.780 | 0.636 | 0.750 | 0.596 | 0.957 | 0.815 | 0.628 | 0.576 |
| 63 | 0.533 | 0.829 | 0.508 | 0.429 | 0.732 | 0.800 | 0.801 | 1.000 | 0.663 | 0.854 | 0.783 | 0.636 | 0.750 | 0.598 | 0.957 | 0.815 | 0.627 | 0.575 |
| 64 | 0.533 | 0.829 | 0.510 | 0.429 | 0.737 | 0.800 | 0.800 | 1.000 | 0.663 | 0.854 | 0.780 | 0.636 | 0.750 | 0.600 | 0.957 | 0.815 | 0.628 | 0.576 |
| 65 | 0.533 | 0.829 | 0.512 | 0.457 | 0.742 | 0.800 | 0.800 | 1.000 | 0.663 | 0.854 | 0.780 | 0.636 | 0.750 | 0.602 | 0.957 | 0.815 | 0.629 | 0.577 |
| 66 | 0.536 | 0.829 | 0.486 | 0.457 | 0.747 | 0.800 | 0.803 | 1.000 | 0.663 | 0.854 | 0.777 | 0.606 | 0.750 | 0.605 | 0.957 | 0.815 | 0.628 | 0.576 |
| 67 | 0.537 | 0.829 | 0.490 | 0.471 | 0.737 | 0.840 | 0.804 | 1.000 | 0.663 | 0.854 | 0.777 | 0.606 | 0.750 | 0.605 | 0.957 | 0.815 | 0.630 | 0.577 |
| 68 | 0.537 | 0.829 | 0.492 | 0.471 | 0.737 | 0.840 | 0.806 | 1.000 | 0.663 | 0.854 | 0.777 | 0.606 | 0.750 | 0.605 | 0.957 | 0.796 | 0.630 | 0.577 |
| 69 | 0.533 | 0.829 | 0.480 | 0.443 | 0.747 | 0.840 | 0.812 | 1.000 | 0.653 | 0.854 | 0.780 | 0.606 | 0.750 | 0.616 | 0.935 | 0.796 | 0.631 | 0.578 |
| 70 | 0.533 | 0.829 | 0.484 | 0.443 | 0.747 | 0.840 | 0.809 | 1.000 | 0.663 | 0.854 | 0.780 | 0.606 | 0.750 | 0.616 | 0.957 | 0.796 | 0.631 | 0.579 |
| 71 | 0.538 | 0.829 | 0.484 | 0.471 | 0.742 | 0.800 | 0.812 | 1.000 | 0.643 | 0.854 | 0.780 | 0.606 | 0.750 | 0.615 | 0.957 | 0.796 | 0.632 | 0.579 |
| 72 | 0.536 | 0.829 | 0.486 | 0.457 | 0.742 | 0.800 | 0.810 | 1.000 | 0.643 | 0.854 | 0.780 | 0.606 | 0.750 | 0.616 | 0.957 | 0.796 | 0.632 | 0.579 |
| 73 | 0.537 | 0.829 | 0.478 | 0.486 | 0.742 | 0.800 | 0.815 | 1.000 | 0.653 | 0.854 | 0.780 | 0.636 | 0.741 | 0.616 | 0.957 | 0.796 | 0.632 | 0.580 |
| 74 | 0.541 | 0.829 | 0.504 | 0.486 | 0.742 | 0.880 | 0.823 | 1.000 | 0.663 | 0.854 | 0.791 | 0.636 | 0.759 | 0.609 | 0.935 | 0.815 | 0.637 | 0.586 |
| 75 | 0.540 | 0.829 | 0.508 | 0.500 | 0.747 | 0.880 | 0.821 | 1.000 | 0.673 | 0.854 | 0.788 | 0.636 | 0.759 | 0.609 | 0.935 | 0.796 | 0.637 | 0.586 |
| 76 | 0.541 | 0.857 | 0.516 | 0.514 | 0.747 | 0.880 | 0.821 | 1.000 | 0.673 | 0.854 | 0.791 | 0.636 | 0.759 | 0.608 | 0.935 | 0.796 | 0.639 | 0.588 |
| 77 | 0.543 | 0.857 | 0.514 | 0.486 | 0.753 | 0.840 | 0.820 | 1.000 | 0.684 | 0.854 | 0.788 | 0.636 | 0.759 | 0.616 | 0.957 | 0.796 | 0.641 | 0.590 |

| | | | | | | | | | | | | | | | | | | |
|---|---|---|---|---|---|---|---|---|---|---|---|---|---|---|---|---|---|---|
| 78 | 0.548 | 0.857 | 0.512 | 0.486 | 0.747 | 0.840 | 0.821 | 1.000 | 0.694 | 0.854 | 0.786 | 0.636 | 0.759 | 0.619 | 0.957 | 0.796 | 0.643 | 0.592 |
| 79 | 0.547 | 0.857 | 0.504 | 0.471 | 0.753 | 0.880 | 0.810 | 1.000 | 0.684 | 0.854 | 0.791 | 0.727 | 0.759 | 0.602 | 0.957 | 0.796 | 0.637 | 0.586 |
| 80 | 0.547 | 0.857 | 0.504 | 0.471 | 0.753 | 0.880 | 0.812 | 1.000 | 0.684 | 0.854 | 0.794 | 0.727 | 0.759 | 0.609 | 0.957 | 0.796 | 0.639 | 0.587 |
| 81 | 0.545 | 0.857 | 0.506 | 0.471 | 0.758 | 0.880 | 0.812 | 1.000 | 0.684 | 0.854 | 0.794 | 0.727 | 0.759 | 0.609 | 0.957 | 0.796 | 0.639 | 0.588 |
| 82 | 0.548 | 0.829 | 0.520 | 0.457 | 0.763 | 0.880 | 0.807 | 1.000 | 0.684 | 0.854 | 0.788 | 0.727 | 0.786 | 0.609 | 0.957 | 0.796 | 0.641 | 0.589 |
| 83 | 0.549 | 0.857 | 0.512 | 0.457 | 0.758 | 0.840 | 0.807 | 1.000 | 0.673 | 0.833 | 0.794 | 0.758 | 0.786 | 0.613 | 0.957 | 0.796 | 0.641 | 0.590 |
| 84 | 0.552 | 0.857 | 0.514 | 0.457 | 0.753 | 0.840 | 0.807 | 1.000 | 0.673 | 0.833 | 0.791 | 0.758 | 0.786 | 0.615 | 0.957 | 0.796 | 0.642 | 0.591 |
| 85 | 0.561 | 0.857 | 0.502 | 0.443 | 0.758 | 0.840 | 0.814 | 1.000 | 0.684 | 0.833 | 0.791 | 0.758 | 0.795 | 0.616 | 0.935 | 0.796 | 0.645 | 0.594 |
| 86 | 0.562 | 0.857 | 0.498 | 0.443 | 0.758 | 0.840 | 0.812 | 1.000 | 0.673 | 0.833 | 0.788 | 0.758 | 0.795 | 0.618 | 0.935 | 0.796 | 0.645 | 0.593 |
| 87 | 0.567 | 0.857 | 0.500 | 0.457 | 0.758 | 0.840 | 0.812 | 1.000 | 0.684 | 0.833 | 0.788 | 0.758 | 0.795 | 0.616 | 0.935 | 0.796 | 0.646 | 0.595 |
| 88 | 0.566 | 0.857 | 0.504 | 0.457 | 0.758 | 0.840 | 0.812 | 1.000 | 0.684 | 0.833 | 0.788 | 0.758 | 0.795 | 0.618 | 0.935 | 0.796 | 0.647 | 0.596 |
| 89 | 0.567 | 0.857 | 0.496 | 0.471 | 0.753 | 0.880 | 0.812 | 1.000 | 0.694 | 0.854 | 0.791 | 0.758 | 0.795 | 0.621 | 0.935 | 0.796 | 0.648 | 0.597 |
| 90 | 0.569 | 0.857 | 0.496 | 0.500 | 0.753 | 0.880 | 0.812 | 1.000 | 0.694 | 0.854 | 0.797 | 0.758 | 0.795 | 0.616 | 0.957 | 0.796 | 0.649 | 0.597 |
| 91 | 0.570 | 0.857 | 0.492 | 0.500 | 0.753 | 0.880 | 0.810 | 1.000 | 0.694 | 0.854 | 0.797 | 0.758 | 0.795 | 0.618 | 0.957 | 0.796 | 0.649 | 0.597 |
| 92 | 0.569 | 0.857 | 0.486 | 0.500 | 0.753 | 0.880 | 0.812 | 1.000 | 0.694 | 0.854 | 0.797 | 0.758 | 0.795 | 0.618 | 0.957 | 0.796 | 0.648 | 0.597 |
| 93 | 0.568 | 0.857 | 0.496 | 0.514 | 0.753 | 0.880 | 0.810 | 1.000 | 0.694 | 0.854 | 0.797 | 0.758 | 0.795 | 0.620 | 0.957 | 0.796 | 0.649 | 0.598 |
| 94 | 0.572 | 0.857 | 0.494 | 0.529 | 0.753 | 0.920 | 0.823 | 1.000 | 0.694 | 0.875 | 0.797 | 0.758 | 0.795 | 0.626 | 0.957 | 0.815 | 0.654 | 0.603 |
| 95 | 0.572 | 0.829 | 0.500 | 0.529 | 0.753 | 0.920 | 0.821 | 1.000 | 0.704 | 0.875 | 0.794 | 0.758 | 0.795 | 0.626 | 0.957 | 0.815 | 0.655 | 0.604 |
| 96 | 0.572 | 0.829 | 0.496 | 0.514 | 0.758 | 0.920 | 0.824 | 1.000 | 0.704 | 0.875 | 0.791 | 0.758 | 0.795 | 0.627 | 0.957 | 0.815 | 0.655 | 0.604 |
| 97 | 0.574 | 0.829 | 0.506 | 0.514 | 0.758 | 0.960 | 0.826 | 1.000 | 0.694 | 0.875 | 0.791 | 0.758 | 0.795 | 0.625 | 0.957 | 0.833 | 0.656 | 0.605 |
| 98 | 0.575 | 0.857 | 0.502 | 0.514 | 0.758 | 0.960 | 0.829 | 1.000 | 0.694 | 0.854 | 0.791 | 0.758 | 0.795 | 0.626 | 0.957 | 0.833 | 0.656 | 0.605 |
| 99 | 0.574 | 0.857 | 0.506 | 0.514 | 0.758 | 0.920 | 0.826 | 1.000 | 0.704 | 0.854 | 0.794 | 0.758 | 0.795 | 0.625 | 0.957 | 0.833 | 0.656 | 0.605 |
| 100 | 0.576 | 0.857 | 0.504 | 0.514 | 0.758 | 0.920 | 0.826 | 1.000 | 0.694 | 0.875 | 0.791 | 0.758 | 0.795 | 0.624 | 0.957 | 0.833 | 0.656 | 0.605 |
| 101 | 0.578 | 0.829 | 0.518 | 0.529 | 0.758 | 0.920 | 0.824 | 1.000 | 0.704 | 0.875 | 0.797 | 0.758 | 0.777 | 0.619 | 0.957 | 0.833 | 0.657 | 0.606 |
| 102 | 0.585 | 0.829 | 0.518 | 0.543 | 0.763 | 0.920 | 0.823 | 1.000 | 0.704 | 0.875 | 0.794 | 0.758 | 0.786 | 0.615 | 0.978 | 0.815 | 0.658 | 0.607 |
| 103 | 0.584 | 0.829 | 0.516 | 0.543 | 0.763 | 0.920 | 0.823 | 1.000 | 0.714 | 0.875 | 0.794 | 0.758 | 0.786 | 0.615 | 0.978 | 0.815 | 0.658 | 0.607 |
| 104 | 0.583 | 0.829 | 0.520 | 0.543 | 0.763 | 0.920 | 0.824 | 1.000 | 0.704 | 0.875 | 0.791 | 0.758 | 0.786 | 0.616 | 0.978 | 0.815 | 0.658 | 0.607 |
| 105 | 0.582 | 0.829 | 0.522 | 0.543 | 0.763 | 0.920 | 0.824 | 1.000 | 0.714 | 0.875 | 0.791 | 0.758 | 0.786 | 0.616 | 0.978 | 0.815 | 0.658 | 0.607 |
| 106 | 0.587 | 0.886 | 0.512 | 0.543 | 0.774 | 0.920 | 0.821 | 1.000 | 0.714 | 0.875 | 0.794 | 0.758 | 0.786 | 0.613 | 0.978 | 0.833 | 0.659 | 0.608 |
| 107 | 0.588 | 0.886 | 0.508 | 0.543 | 0.774 | 0.920 | 0.823 | 1.000 | 0.714 | 0.875 | 0.797 | 0.758 | 0.795 | 0.614 | 0.978 | 0.815 | 0.659 | 0.608 |
| 108 | 0.585 | 0.886 | 0.500 | 0.557 | 0.784 | 0.920 | 0.824 | 1.000 | 0.714 | 0.875 | 0.797 | 0.758 | 0.795 | 0.616 | 1.000 | 0.815 | 0.659 | 0.608 |
| 109 | 0.588 | 0.914 | 0.506 | 0.557 | 0.784 | 0.920 | 0.823 | 1.000 | 0.714 | 0.875 | 0.800 | 0.788 | 0.795 | 0.615 | 1.000 | 0.815 | 0.660 | 0.609 |
| 110 | 0.589 | 0.886 | 0.508 | 0.557 | 0.779 | 0.920 | 0.823 | 1.000 | 0.735 | 0.875 | 0.800 | 0.788 | 0.795 | 0.612 | 1.000 | 0.796 | 0.660 | 0.609 |
| 111 | 0.588 | 0.886 | 0.508 | 0.543 | 0.779 | 0.920 | 0.823 | 1.000 | 0.735 | 0.875 | 0.803 | 0.788 | 0.795 | 0.612 | 1.000 | 0.796 | 0.660 | 0.609 |
| 112 | 0.588 | 0.886 | 0.510 | 0.557 | 0.779 | 0.920 | 0.823 | 1.000 | 0.735 | 0.875 | 0.803 | 0.788 | 0.795 | 0.612 | 1.000 | 0.815 | 0.661 | 0.610 |
| 113 | 0.592 | 0.886 | 0.508 | 0.557 | 0.779 | 0.920 | 0.821 | 1.000 | 0.724 | 0.875 | 0.803 | 0.788 | 0.795 | 0.612 | 1.000 | 0.796 | 0.661 | 0.610 |
| 114 | 0.592 | 0.886 | 0.510 | 0.543 | 0.779 | 0.920 | 0.821 | 1.000 | 0.724 | 0.875 | 0.797 | 0.788 | 0.795 | 0.612 | 1.000 | 0.796 | 0.660 | 0.609 |
| 115 | 0.594 | 0.914 | 0.500 | 0.571 | 0.784 | 0.960 | 0.824 | 1.000 | 0.714 | 0.875 | 0.800 | 0.788 | 0.804 | 0.614 | 1.000 | 0.796 | 0.662 | 0.611 |
| 116 | 0.597 | 0.914 | 0.486 | 0.571 | 0.779 | 0.960 | 0.826 | 1.000 | 0.704 | 0.854 | 0.803 | 0.788 | 0.804 | 0.612 | 1.000 | 0.796 | 0.661 | 0.609 |

| | | | | | | | | | | | | | | | | | | |
|---|---|---|---|---|---|---|---|---|---|---|---|---|---|---|---|---|---|---|
| 117 | 0.590 | 0.914 | 0.492 | 0.586 | 0.774 | 0.960 | 0.831 | 1.000 | 0.704 | 0.854 | 0.806 | 0.788 | 0.804 | 0.614 | 1.000 | 0.796 | 0.661 | 0.610 |
| 118 | 0.590 | 0.914 | 0.492 | 0.586 | 0.774 | 0.960 | 0.829 | 1.000 | 0.704 | 0.854 | 0.806 | 0.788 | 0.804 | 0.616 | 1.000 | 0.796 | 0.661 | 0.610 |
| 119 | 0.593 | 0.914 | 0.498 | 0.600 | 0.774 | 0.960 | 0.829 | 1.000 | 0.704 | 0.854 | 0.806 | 0.818 | 0.804 | 0.626 | 1.000 | 0.796 | 0.666 | 0.615 |
| 120 | 0.599 | 0.914 | 0.496 | 0.614 | 0.774 | 0.960 | 0.829 | 1.000 | 0.704 | 0.875 | 0.806 | 0.788 | 0.813 | 0.637 | 1.000 | 0.796 | 0.670 | 0.620 |
| 121 | 0.594 | 0.886 | 0.498 | 0.629 | 0.768 | 0.960 | 0.827 | 1.000 | 0.704 | 0.875 | 0.803 | 0.818 | 0.813 | 0.640 | 1.000 | 0.833 | 0.670 | 0.620 |
| 122 | 0.601 | 0.886 | 0.496 | 0.629 | 0.774 | 0.960 | 0.826 | 1.000 | 0.704 | 0.875 | 0.803 | 0.818 | 0.813 | 0.639 | 1.000 | 0.833 | 0.671 | 0.621 |
| 123 | 0.597 | 0.886 | 0.490 | 0.629 | 0.774 | 0.960 | 0.827 | 1.000 | 0.704 | 0.875 | 0.800 | 0.818 | 0.813 | 0.637 | 1.000 | 0.852 | 0.669 | 0.619 |
| 124 | 0.601 | 0.886 | 0.498 | 0.629 | 0.774 | 0.960 | 0.824 | 1.000 | 0.704 | 0.875 | 0.797 | 0.818 | 0.813 | 0.628 | 1.000 | 0.852 | 0.668 | 0.618 |
| 125 | 0.603 | 0.886 | 0.496 | 0.600 | 0.768 | 0.960 | 0.826 | 1.000 | 0.704 | 0.875 | 0.797 | 0.818 | 0.813 | 0.629 | 1.000 | 0.852 | 0.669 | 0.618 |
| 126 | 0.603 | 0.886 | 0.502 | 0.643 | 0.779 | 0.960 | 0.829 | 1.000 | 0.704 | 0.875 | 0.797 | 0.818 | 0.813 | 0.632 | 1.000 | 0.852 | 0.671 | 0.622 |
| 127 | 0.604 | 0.886 | 0.504 | 0.643 | 0.779 | 0.960 | 0.827 | 1.000 | 0.704 | 0.875 | 0.800 | 0.818 | 0.813 | 0.632 | 1.000 | 0.852 | 0.672 | 0.622 |
| 128 | 0.604 | 0.886 | 0.508 | 0.657 | 0.779 | 0.960 | 0.829 | 1.000 | 0.704 | 0.875 | 0.803 | 0.818 | 0.813 | 0.637 | 1.000 | 0.852 | 0.674 | 0.625 |
| 129 | 0.604 | 0.886 | 0.506 | 0.657 | 0.779 | 0.960 | 0.829 | 1.000 | 0.704 | 0.875 | 0.806 | 0.818 | 0.813 | 0.636 | 1.000 | 0.852 | 0.674 | 0.624 |
| 130 | 0.604 | 0.886 | 0.522 | 0.643 | 0.779 | 0.960 | 0.831 | 1.000 | 0.704 | 0.875 | 0.803 | 0.788 | 0.804 | 0.651 | 1.000 | 0.852 | 0.679 | 0.630 |
| 131 | 0.605 | 0.886 | 0.526 | 0.657 | 0.779 | 0.960 | 0.832 | 1.000 | 0.704 | 0.875 | 0.803 | 0.788 | 0.795 | 0.650 | 1.000 | 0.852 | 0.679 | 0.630 |
| 132 | 0.606 | 0.886 | 0.532 | 0.686 | 0.774 | 0.960 | 0.827 | 1.000 | 0.704 | 0.875 | 0.809 | 0.788 | 0.804 | 0.651 | 1.000 | 0.833 | 0.681 | 0.632 |
| 133 | 0.610 | 0.886 | 0.534 | 0.686 | 0.779 | 0.960 | 0.827 | 1.000 | 0.714 | 0.875 | 0.806 | 0.788 | 0.821 | 0.655 | 1.000 | 0.852 | 0.684 | 0.635 |
| 134 | 0.607 | 0.886 | 0.542 | 0.671 | 0.784 | 0.960 | 0.827 | 1.000 | 0.714 | 0.875 | 0.806 | 0.788 | 0.830 | 0.654 | 1.000 | 0.852 | 0.684 | 0.635 |
| 135 | 0.607 | 0.886 | 0.542 | 0.671 | 0.789 | 0.960 | 0.829 | 1.000 | 0.724 | 0.875 | 0.812 | 0.788 | 0.804 | 0.653 | 1.000 | 0.889 | 0.684 | 0.636 |
| 136 | 0.614 | 0.886 | 0.543 | 0.686 | 0.789 | 0.960 | 0.826 | 1.000 | 0.735 | 0.875 | 0.812 | 0.788 | 0.804 | 0.653 | 1.000 | 0.889 | 0.687 | 0.638 |
| 137 | 0.617 | 0.886 | 0.547 | 0.686 | 0.789 | 0.960 | 0.824 | 1.000 | 0.735 | 0.875 | 0.812 | 0.788 | 0.804 | 0.654 | 1.000 | 0.889 | 0.688 | 0.639 |
| 138 | 0.615 | 0.886 | 0.549 | 0.714 | 0.784 | 0.960 | 0.829 | 1.000 | 0.735 | 0.875 | 0.809 | 0.788 | 0.821 | 0.660 | 1.000 | 0.889 | 0.690 | 0.642 |
| 139 | 0.613 | 0.886 | 0.553 | 0.714 | 0.784 | 0.960 | 0.827 | 1.000 | 0.735 | 0.875 | 0.806 | 0.788 | 0.821 | 0.662 | 1.000 | 0.907 | 0.690 | 0.642 |
| 140 | 0.614 | 0.886 | 0.553 | 0.729 | 0.784 | 0.960 | 0.829 | 1.000 | 0.735 | 0.875 | 0.812 | 0.788 | 0.821 | 0.666 | 1.000 | 0.889 | 0.692 | 0.644 |
| 141 | 0.615 | 0.886 | 0.553 | 0.729 | 0.784 | 0.960 | 0.829 | 1.000 | 0.735 | 0.875 | 0.812 | 0.788 | 0.821 | 0.664 | 1.000 | 0.889 | 0.692 | 0.644 |
| 142 | 0.613 | 0.886 | 0.543 | 0.714 | 0.784 | 0.960 | 0.829 | 1.000 | 0.745 | 0.875 | 0.809 | 0.788 | 0.821 | 0.664 | 1.000 | 0.870 | 0.690 | 0.641 |
| 143 | 0.616 | 0.886 | 0.545 | 0.729 | 0.789 | 0.960 | 0.827 | 1.000 | 0.745 | 0.875 | 0.809 | 0.788 | 0.830 | 0.664 | 1.000 | 0.889 | 0.692 | 0.643 |
| 144 | 0.615 | 0.886 | 0.545 | 0.729 | 0.789 | 0.960 | 0.829 | 1.000 | 0.745 | 0.875 | 0.803 | 0.788 | 0.821 | 0.662 | 1.000 | 0.870 | 0.690 | 0.642 |
| 145 | 0.617 | 0.886 | 0.545 | 0.729 | 0.789 | 0.960 | 0.831 | 1.000 | 0.745 | 0.875 | 0.809 | 0.788 | 0.821 | 0.661 | 1.000 | 0.907 | 0.691 | 0.643 |
| 146 | 0.616 | 0.886 | 0.545 | 0.714 | 0.789 | 0.960 | 0.829 | 1.000 | 0.745 | 0.875 | 0.814 | 0.788 | 0.821 | 0.664 | 1.000 | 0.889 | 0.692 | 0.644 |
| 147 | 0.619 | 0.886 | 0.542 | 0.714 | 0.789 | 0.960 | 0.829 | 1.000 | 0.745 | 0.875 | 0.817 | 0.788 | 0.821 | 0.663 | 1.000 | 0.852 | 0.692 | 0.644 |
| 148 | 0.619 | 0.914 | 0.540 | 0.729 | 0.795 | 0.960 | 0.826 | 1.000 | 0.745 | 0.875 | 0.812 | 0.788 | 0.821 | 0.662 | 1.000 | 0.852 | 0.691 | 0.643 |
| 149 | 0.613 | 0.914 | 0.543 | 0.671 | 0.784 | 0.960 | 0.824 | 1.000 | 0.765 | 0.875 | 0.809 | 0.788 | 0.830 | 0.666 | 1.000 | 0.852 | 0.690 | 0.641 |
| 150 | 0.613 | 0.914 | 0.534 | 0.671 | 0.784 | 0.960 | 0.824 | 1.000 | 0.765 | 0.875 | 0.809 | 0.788 | 0.830 | 0.668 | 1.000 | 0.852 | 0.690 | 0.641 |
| 151 | 0.613 | 0.914 | 0.534 | 0.671 | 0.784 | 0.960 | 0.824 | 1.000 | 0.765 | 0.875 | 0.809 | 0.788 | 0.830 | 0.667 | 1.000 | 0.852 | 0.689 | 0.640 |
| 152 | 0.610 | 0.914 | 0.538 | 0.686 | 0.789 | 0.960 | 0.826 | 1.000 | 0.765 | 0.875 | 0.803 | 0.788 | 0.830 | 0.666 | 1.000 | 0.852 | 0.689 | 0.640 |
| 153 | 0.609 | 0.914 | 0.540 | 0.686 | 0.789 | 0.960 | 0.824 | 1.000 | 0.765 | 0.875 | 0.806 | 0.818 | 0.830 | 0.666 | 1.000 | 0.852 | 0.689 | 0.640 |
| 154 | 0.610 | 0.914 | 0.540 | 0.686 | 0.789 | 0.960 | 0.824 | 1.000 | 0.765 | 0.875 | 0.806 | 0.818 | 0.830 | 0.666 | 1.000 | 0.852 | 0.689 | 0.640 |
| 155 | 0.610 | 0.914 | 0.538 | 0.686 | 0.789 | 0.960 | 0.827 | 1.000 | 0.765 | 0.875 | 0.806 | 0.818 | 0.830 | 0.667 | 1.000 | 0.852 | 0.689 | 0.641 |

| | | | | | | | | | | | | | | | | | | |
|---|---|---|---|---|---|---|---|---|---|---|---|---|---|---|---|---|---|---|
| 156 | 0.617 | 0.943 | 0.545 | 0.714 | 0.784 | 0.960 | 0.829 | 1.000 | 0.765 | 0.875 | 0.803 | 0.818 | 0.830 | 0.666 | 1.000 | 0.889 | 0.693 | 0.644 |
| 157 | 0.616 | 0.943 | 0.545 | 0.714 | 0.784 | 1.000 | 0.829 | 1.000 | 0.765 | 0.875 | 0.803 | 0.818 | 0.830 | 0.661 | 1.000 | 0.889 | 0.691 | 0.643 |
| 158 | 0.615 | 0.914 | 0.547 | 0.743 | 0.779 | 1.000 | 0.829 | 1.000 | 0.765 | 0.875 | 0.800 | 0.818 | 0.830 | 0.662 | 1.000 | 0.870 | 0.691 | 0.643 |
| 159 | 0.618 | 0.914 | 0.545 | 0.743 | 0.779 | 1.000 | 0.826 | 1.000 | 0.765 | 0.875 | 0.800 | 0.818 | 0.830 | 0.661 | 1.000 | 0.889 | 0.692 | 0.643 |
| 160 | 0.617 | 0.914 | 0.543 | 0.743 | 0.784 | 1.000 | 0.829 | 1.000 | 0.765 | 0.875 | 0.803 | 0.818 | 0.830 | 0.661 | 1.000 | 0.889 | 0.692 | 0.644 |
| 161 | 0.620 | 0.914 | 0.538 | 0.714 | 0.779 | 1.000 | 0.824 | 1.000 | 0.765 | 0.875 | 0.812 | 0.818 | 0.821 | 0.661 | 1.000 | 0.889 | 0.691 | 0.643 |
| 162 | 0.615 | 0.943 | 0.536 | 0.771 | 0.784 | 1.000 | 0.820 | 1.000 | 0.765 | 0.875 | 0.817 | 0.879 | 0.821 | 0.660 | 1.000 | 0.907 | 0.691 | 0.642 |
| 163 | 0.617 | 0.943 | 0.540 | 0.757 | 0.779 | 1.000 | 0.826 | 1.000 | 0.765 | 0.875 | 0.820 | 0.879 | 0.821 | 0.658 | 1.000 | 0.926 | 0.692 | 0.644 |
| 164 | 0.617 | 0.943 | 0.542 | 0.757 | 0.779 | 1.000 | 0.827 | 1.000 | 0.755 | 0.875 | 0.820 | 0.879 | 0.821 | 0.658 | 1.000 | 0.926 | 0.692 | 0.644 |
| 165 | 0.617 | 0.943 | 0.538 | 0.757 | 0.779 | 1.000 | 0.827 | 1.000 | 0.755 | 0.875 | 0.820 | 0.909 | 0.821 | 0.655 | 1.000 | 0.926 | 0.692 | 0.643 |
| 166 | 0.619 | 0.943 | 0.545 | 0.757 | 0.784 | 1.000 | 0.823 | 1.000 | 0.765 | 0.875 | 0.823 | 0.879 | 0.821 | 0.651 | 1.000 | 0.926 | 0.692 | 0.644 |
| 167 | 0.620 | 0.943 | 0.545 | 0.757 | 0.789 | 1.000 | 0.823 | 1.000 | 0.765 | 0.875 | 0.823 | 0.879 | 0.821 | 0.654 | 1.000 | 0.926 | 0.693 | 0.645 |
| 168 | 0.621 | 0.943 | 0.547 | 0.743 | 0.779 | 1.000 | 0.826 | 1.000 | 0.755 | 0.875 | 0.820 | 0.909 | 0.821 | 0.650 | 1.000 | 0.907 | 0.692 | 0.644 |
| 169 | 0.621 | 0.943 | 0.551 | 0.757 | 0.779 | 1.000 | 0.824 | 1.000 | 0.755 | 0.875 | 0.817 | 0.909 | 0.821 | 0.650 | 1.000 | 0.926 | 0.692 | 0.644 |
| 170 | 0.621 | 0.943 | 0.553 | 0.757 | 0.779 | 1.000 | 0.824 | 1.000 | 0.755 | 0.875 | 0.817 | 0.909 | 0.821 | 0.649 | 1.000 | 0.926 | 0.692 | 0.644 |
| 171 | 0.622 | 0.943 | 0.567 | 0.757 | 0.784 | 1.000 | 0.826 | 1.000 | 0.755 | 0.875 | 0.820 | 0.909 | 0.821 | 0.653 | 1.000 | 0.926 | 0.695 | 0.648 |
| 172 | 0.623 | 0.943 | 0.567 | 0.757 | 0.784 | 1.000 | 0.826 | 1.000 | 0.755 | 0.875 | 0.820 | 0.909 | 0.821 | 0.652 | 1.000 | 0.926 | 0.696 | 0.648 |
| 173 | 0.623 | 0.943 | 0.569 | 0.757 | 0.784 | 1.000 | 0.826 | 1.000 | 0.755 | 0.875 | 0.820 | 0.909 | 0.821 | 0.651 | 1.000 | 0.926 | 0.695 | 0.648 |
| 174 | 0.623 | 0.943 | 0.567 | 0.757 | 0.784 | 1.000 | 0.826 | 1.000 | 0.755 | 0.875 | 0.820 | 0.909 | 0.821 | 0.656 | 1.000 | 0.926 | 0.696 | 0.649 |
| 175 | 0.622 | 0.943 | 0.561 | 0.743 | 0.784 | 1.000 | 0.826 | 1.000 | 0.755 | 0.875 | 0.812 | 0.909 | 0.821 | 0.654 | 1.000 | 0.926 | 0.694 | 0.647 |
| 176 | 0.623 | 0.943 | 0.557 | 0.757 | 0.784 | 1.000 | 0.827 | 1.000 | 0.755 | 0.875 | 0.812 | 0.909 | 0.821 | 0.654 | 1.000 | 0.926 | 0.694 | 0.647 |
| 177 | 0.624 | 0.943 | 0.553 | 0.757 | 0.784 | 1.000 | 0.829 | 1.000 | 0.755 | 0.875 | 0.812 | 0.909 | 0.821 | 0.653 | 1.000 | 0.926 | 0.694 | 0.647 |
| 178 | 0.622 | 0.943 | 0.569 | 0.757 | 0.789 | 1.000 | 0.821 | 1.000 | 0.755 | 0.875 | 0.812 | 0.909 | 0.821 | 0.647 | 1.000 | 0.926 | 0.693 | 0.645 |
| 179 | 0.621 | 0.943 | 0.569 | 0.757 | 0.789 | 1.000 | 0.821 | 1.000 | 0.755 | 0.875 | 0.812 | 0.909 | 0.821 | 0.647 | 1.000 | 0.926 | 0.693 | 0.645 |
| 180 | 0.619 | 0.943 | 0.565 | 0.757 | 0.789 | 1.000 | 0.821 | 1.000 | 0.755 | 0.875 | 0.823 | 0.909 | 0.821 | 0.645 | 1.000 | 0.926 | 0.692 | 0.644 |
| 181 | 0.621 | 0.943 | 0.563 | 0.757 | 0.789 | 1.000 | 0.820 | 1.000 | 0.755 | 0.875 | 0.826 | 0.909 | 0.821 | 0.647 | 1.000 | 0.963 | 0.693 | 0.646 |
| 182 | 0.626 | 0.943 | 0.573 | 0.757 | 0.789 | 1.000 | 0.820 | 1.000 | 0.755 | 0.875 | 0.826 | 0.939 | 0.821 | 0.651 | 1.000 | 0.963 | 0.697 | 0.650 |
| 183 | 0.627 | 0.943 | 0.573 | 0.757 | 0.789 | 1.000 | 0.820 | 1.000 | 0.755 | 0.875 | 0.826 | 0.939 | 0.821 | 0.651 | 1.000 | 0.963 | 0.697 | 0.650 |
| 184 | 0.625 | 0.943 | 0.581 | 0.757 | 0.795 | 1.000 | 0.821 | 1.000 | 0.755 | 0.875 | 0.829 | 0.939 | 0.821 | 0.652 | 1.000 | 0.963 | 0.699 | 0.651 |
| 185 | 0.626 | 0.943 | 0.579 | 0.757 | 0.795 | 1.000 | 0.821 | 1.000 | 0.755 | 0.875 | 0.832 | 0.939 | 0.821 | 0.654 | 1.000 | 0.963 | 0.699 | 0.652 |
| 186 | 0.627 | 0.943 | 0.585 | 0.757 | 0.795 | 1.000 | 0.821 | 1.000 | 0.755 | 0.875 | 0.838 | 0.939 | 0.821 | 0.652 | 1.000 | 0.963 | 0.700 | 0.653 |
| 187 | 0.625 | 0.943 | 0.581 | 0.771 | 0.795 | 1.000 | 0.814 | 1.000 | 0.765 | 0.875 | 0.829 | 0.939 | 0.821 | 0.653 | 1.000 | 0.963 | 0.698 | 0.651 |
| 188 | 0.626 | 0.943 | 0.579 | 0.757 | 0.800 | 1.000 | 0.818 | 1.000 | 0.765 | 0.875 | 0.826 | 0.939 | 0.830 | 0.653 | 1.000 | 0.963 | 0.699 | 0.652 |
| 189 | 0.627 | 0.943 | 0.579 | 0.757 | 0.800 | 1.000 | 0.818 | 1.000 | 0.765 | 0.875 | 0.826 | 0.939 | 0.830 | 0.653 | 1.000 | 0.963 | 0.699 | 0.652 |
| 190 | 0.628 | 0.943 | 0.577 | 0.771 | 0.800 | 1.000 | 0.818 | 1.000 | 0.755 | 0.875 | 0.826 | 0.939 | 0.821 | 0.654 | 1.000 | 0.963 | 0.699 | 0.652 |
| 191 | 0.628 | 0.943 | 0.579 | 0.771 | 0.800 | 1.000 | 0.817 | 1.000 | 0.765 | 0.875 | 0.826 | 0.939 | 0.821 | 0.655 | 1.000 | 0.963 | 0.700 | 0.653 |
| 192 | 0.629 | 0.943 | 0.587 | 0.771 | 0.800 | 1.000 | 0.817 | 1.000 | 0.765 | 0.875 | 0.823 | 0.939 | 0.821 | 0.654 | 1.000 | 0.963 | 0.700 | 0.653 |
| 193 | 0.631 | 0.943 | 0.589 | 0.771 | 0.800 | 1.000 | 0.821 | 1.000 | 0.765 | 0.875 | 0.823 | 0.939 | 0.821 | 0.654 | 1.000 | 0.963 | 0.702 | 0.655 |
| 194 | 0.636 | 0.943 | 0.587 | 0.771 | 0.800 | 1.000 | 0.818 | 1.000 | 0.755 | 0.875 | 0.832 | 0.939 | 0.830 | 0.654 | 1.000 | 0.963 | 0.703 | 0.656 |

| | | | | | | | | | | | | | | | | | | |
|---|---|---|---|---|---|---|---|---|---|---|---|---|---|---|---|---|---|---|
| 195 | 0.634 | 0.943 | 0.591 | 0.771 | 0.800 | 1.000 | 0.820 | 1.000 | 0.755 | 0.875 | 0.826 | 0.939 | 0.839 | 0.654 | 1.000 | 0.963 | 0.703 | 0.656 |
| 196 | 0.636 | 0.943 | 0.589 | 0.786 | 0.805 | 1.000 | 0.821 | 1.000 | 0.755 | 0.875 | 0.832 | 0.939 | 0.830 | 0.651 | 1.000 | 0.963 | 0.704 | 0.657 |
| 197 | 0.637 | 0.943 | 0.589 | 0.786 | 0.805 | 1.000 | 0.817 | 1.000 | 0.755 | 0.875 | 0.832 | 0.939 | 0.830 | 0.650 | 1.000 | 0.963 | 0.703 | 0.656 |
| 198 | 0.637 | 0.943 | 0.589 | 0.800 | 0.805 | 1.000 | 0.815 | 1.000 | 0.755 | 0.875 | 0.832 | 0.939 | 0.830 | 0.649 | 1.000 | 0.963 | 0.703 | 0.655 |
| 199 | 0.638 | 0.943 | 0.589 | 0.800 | 0.811 | 1.000 | 0.814 | 1.000 | 0.755 | 0.875 | 0.835 | 0.939 | 0.830 | 0.653 | 1.000 | 0.963 | 0.704 | 0.657 |
| 200 | 0.638 | 0.943 | 0.587 | 0.800 | 0.811 | 1.000 | 0.815 | 1.000 | 0.755 | 0.875 | 0.832 | 0.939 | 0.830 | 0.651 | 1.000 | 0.963 | 0.703 | 0.656 |
| 201 | 0.638 | 0.943 | 0.589 | 0.800 | 0.811 | 1.000 | 0.817 | 1.000 | 0.755 | 0.875 | 0.832 | 0.939 | 0.839 | 0.651 | 1.000 | 0.963 | 0.704 | 0.657 |
| 202 | 0.638 | 0.943 | 0.593 | 0.800 | 0.811 | 1.000 | 0.818 | 1.000 | 0.745 | 0.875 | 0.832 | 0.939 | 0.839 | 0.654 | 1.000 | 0.963 | 0.705 | 0.658 |
| 203 | 0.638 | 0.943 | 0.593 | 0.786 | 0.805 | 1.000 | 0.818 | 1.000 | 0.745 | 0.875 | 0.832 | 0.939 | 0.839 | 0.654 | 1.000 | 0.963 | 0.705 | 0.658 |
| 204 | 0.639 | 0.943 | 0.599 | 0.786 | 0.805 | 1.000 | 0.818 | 1.000 | 0.755 | 0.875 | 0.832 | 0.939 | 0.839 | 0.659 | 1.000 | 0.963 | 0.707 | 0.661 |
| 205 | 0.638 | 0.943 | 0.599 | 0.786 | 0.805 | 1.000 | 0.818 | 1.000 | 0.755 | 0.875 | 0.832 | 0.939 | 0.839 | 0.659 | 1.000 | 0.963 | 0.707 | 0.660 |
| 206 | 0.638 | 0.943 | 0.597 | 0.786 | 0.805 | 1.000 | 0.818 | 1.000 | 0.755 | 0.875 | 0.835 | 0.939 | 0.839 | 0.661 | 1.000 | 0.963 | 0.708 | 0.661 |
| 207 | 0.640 | 0.943 | 0.595 | 0.786 | 0.805 | 1.000 | 0.818 | 1.000 | 0.755 | 0.875 | 0.841 | 0.939 | 0.839 | 0.661 | 1.000 | 0.963 | 0.708 | 0.662 |
| 208 | 0.641 | 0.943 | 0.591 | 0.786 | 0.805 | 1.000 | 0.818 | 1.000 | 0.755 | 0.875 | 0.843 | 0.939 | 0.830 | 0.660 | 1.000 | 0.963 | 0.708 | 0.661 |
| 209 | 0.644 | 0.943 | 0.591 | 0.800 | 0.805 | 1.000 | 0.823 | 1.000 | 0.755 | 0.875 | 0.846 | 0.939 | 0.830 | 0.653 | 1.000 | 0.963 | 0.708 | 0.661 |
| 210 | 0.645 | 0.943 | 0.593 | 0.800 | 0.805 | 1.000 | 0.823 | 1.000 | 0.755 | 0.875 | 0.846 | 0.939 | 0.830 | 0.654 | 1.000 | 0.963 | 0.709 | 0.662 |
| 211 | 0.644 | 0.943 | 0.593 | 0.800 | 0.805 | 1.000 | 0.821 | 1.000 | 0.755 | 0.875 | 0.849 | 0.939 | 0.830 | 0.647 | 1.000 | 0.963 | 0.707 | 0.660 |
| 212 | 0.642 | 0.943 | 0.593 | 0.814 | 0.805 | 1.000 | 0.824 | 1.000 | 0.765 | 0.896 | 0.846 | 0.970 | 0.821 | 0.657 | 1.000 | 0.963 | 0.709 | 0.663 |
| 213 | 0.639 | 0.943 | 0.593 | 0.814 | 0.805 | 1.000 | 0.824 | 1.000 | 0.755 | 0.896 | 0.846 | 0.939 | 0.821 | 0.651 | 1.000 | 0.963 | 0.707 | 0.660 |
| 214 | 0.642 | 0.943 | 0.593 | 0.814 | 0.805 | 1.000 | 0.826 | 1.000 | 0.755 | 0.896 | 0.846 | 0.939 | 0.821 | 0.653 | 1.000 | 0.963 | 0.708 | 0.662 |
| 215 | 0.642 | 0.943 | 0.587 | 0.814 | 0.805 | 1.000 | 0.826 | 1.000 | 0.755 | 0.896 | 0.849 | 0.970 | 0.821 | 0.653 | 1.000 | 0.963 | 0.708 | 0.661 |
| 216 | 0.642 | 0.943 | 0.587 | 0.814 | 0.805 | 1.000 | 0.824 | 1.000 | 0.765 | 0.896 | 0.846 | 0.970 | 0.821 | 0.651 | 1.000 | 0.963 | 0.707 | 0.661 |
| 217 | 0.644 | 0.943 | 0.593 | 0.814 | 0.805 | 1.000 | 0.831 | 1.000 | 0.776 | 0.896 | 0.843 | 0.970 | 0.821 | 0.650 | 1.000 | 0.963 | 0.709 | 0.663 |
| 218 | 0.644 | 0.943 | 0.595 | 0.814 | 0.805 | 1.000 | 0.831 | 1.000 | 0.776 | 0.896 | 0.843 | 0.970 | 0.821 | 0.652 | 1.000 | 0.963 | 0.710 | 0.664 |
| 219 | 0.646 | 0.943 | 0.591 | 0.814 | 0.805 | 1.000 | 0.827 | 1.000 | 0.776 | 0.896 | 0.861 | 0.970 | 0.830 | 0.651 | 1.000 | 0.963 | 0.711 | 0.665 |
| 220 | 0.645 | 0.943 | 0.589 | 0.814 | 0.800 | 1.000 | 0.826 | 1.000 | 0.776 | 0.896 | 0.861 | 0.970 | 0.830 | 0.654 | 1.000 | 0.963 | 0.711 | 0.664 |
| 221 | 0.644 | 0.943 | 0.589 | 0.814 | 0.800 | 1.000 | 0.826 | 1.000 | 0.776 | 0.875 | 0.861 | 0.970 | 0.830 | 0.649 | 1.000 | 0.963 | 0.709 | 0.663 |
| 222 | 0.643 | 0.943 | 0.593 | 0.814 | 0.800 | 1.000 | 0.824 | 1.000 | 0.776 | 0.875 | 0.861 | 0.970 | 0.830 | 0.649 | 1.000 | 0.963 | 0.709 | 0.662 |
| 223 | 0.643 | 0.943 | 0.591 | 0.814 | 0.800 | 1.000 | 0.826 | 1.000 | 0.776 | 0.875 | 0.861 | 0.970 | 0.830 | 0.650 | 1.000 | 0.963 | 0.709 | 0.663 |
| 224 | 0.646 | 0.943 | 0.591 | 0.814 | 0.800 | 1.000 | 0.814 | 1.000 | 0.776 | 0.875 | 0.858 | 0.970 | 0.830 | 0.649 | 1.000 | 0.963 | 0.708 | 0.661 |
| 225 | 0.644 | 0.943 | 0.593 | 0.814 | 0.800 | 1.000 | 0.817 | 1.000 | 0.776 | 0.896 | 0.858 | 0.970 | 0.830 | 0.654 | 1.000 | 0.963 | 0.709 | 0.663 |
| 226 | 0.644 | 0.943 | 0.589 | 0.814 | 0.800 | 1.000 | 0.817 | 1.000 | 0.776 | 0.896 | 0.864 | 0.970 | 0.830 | 0.654 | 1.000 | 0.963 | 0.709 | 0.663 |
| 227 | 0.646 | 0.943 | 0.601 | 0.814 | 0.795 | 1.000 | 0.818 | 1.000 | 0.776 | 0.896 | 0.870 | 0.970 | 0.830 | 0.651 | 1.000 | 0.963 | 0.711 | 0.665 |
| 228 | 0.646 | 0.943 | 0.601 | 0.814 | 0.800 | 1.000 | 0.817 | 1.000 | 0.776 | 0.896 | 0.870 | 0.970 | 0.830 | 0.654 | 1.000 | 0.963 | 0.711 | 0.666 |
| 229 | 0.644 | 0.943 | 0.597 | 0.829 | 0.800 | 1.000 | 0.815 | 1.000 | 0.776 | 0.896 | 0.870 | 0.970 | 0.830 | 0.655 | 1.000 | 0.963 | 0.711 | 0.665 |
| 230 | 0.645 | 0.943 | 0.595 | 0.829 | 0.800 | 1.000 | 0.817 | 1.000 | 0.776 | 0.896 | 0.870 | 0.970 | 0.830 | 0.655 | 1.000 | 0.963 | 0.711 | 0.665 |
| 231 | 0.644 | 0.943 | 0.595 | 0.843 | 0.800 | 1.000 | 0.814 | 1.000 | 0.776 | 0.896 | 0.872 | 0.970 | 0.830 | 0.657 | 1.000 | 0.963 | 0.711 | 0.665 |
| 232 | 0.644 | 0.943 | 0.595 | 0.843 | 0.805 | 1.000 | 0.815 | 1.000 | 0.776 | 0.896 | 0.872 | 0.970 | 0.830 | 0.657 | 1.000 | 0.963 | 0.711 | 0.665 |
| 233 | 0.644 | 0.943 | 0.591 | 0.843 | 0.805 | 1.000 | 0.815 | 1.000 | 0.776 | 0.896 | 0.872 | 0.970 | 0.830 | 0.658 | 1.000 | 0.963 | 0.711 | 0.665 |

| | | | | | | | | | | | | | | | | | |
|---|---|---|---|---|---|---|---|---|---|---|---|---|---|---|---|---|---|
| 234 | 0.644 | 0.943 | 0.595 | 0.843 | 0.805 | 1.000 | 0.815 | 1.000 | 0.776 | 0.896 | 0.870 | 0.970 | 0.830 | 0.655 | 1.000 | 0.963 | 0.711 | 0.665 |
| 235 | 0.644 | 0.943 | 0.589 | 0.843 | 0.805 | 1.000 | 0.814 | 1.000 | 0.776 | 0.896 | 0.867 | 0.970 | 0.839 | 0.654 | 1.000 | 0.963 | 0.710 | 0.664 |
| 236 | 0.647 | 0.943 | 0.597 | 0.829 | 0.811 | 1.000 | 0.809 | 1.000 | 0.776 | 0.896 | 0.872 | 0.970 | 0.839 | 0.655 | 1.000 | 0.963 | 0.712 | 0.666 |
| 237 | 0.648 | 0.943 | 0.599 | 0.829 | 0.811 | 1.000 | 0.809 | 1.000 | 0.776 | 0.896 | 0.870 | 0.970 | 0.839 | 0.655 | 1.000 | 0.963 | 0.712 | 0.666 |
| 238 | 0.649 | 0.943 | 0.597 | 0.814 | 0.805 | 1.000 | 0.807 | 1.000 | 0.776 | 0.896 | 0.870 | 1.000 | 0.857 | 0.657 | 1.000 | 0.963 | 0.712 | 0.666 |
| 239 | 0.649 | 0.943 | 0.597 | 0.800 | 0.805 | 1.000 | 0.809 | 1.000 | 0.776 | 0.896 | 0.872 | 1.000 | 0.857 | 0.656 | 1.000 | 0.963 | 0.712 | 0.666 |
| 240 | 0.649 | 0.943 | 0.599 | 0.814 | 0.805 | 1.000 | 0.810 | 1.000 | 0.776 | 0.896 | 0.872 | 1.000 | 0.866 | 0.659 | 1.000 | 0.963 | 0.714 | 0.668 |
| 241 | 0.651 | 0.943 | 0.597 | 0.814 | 0.805 | 1.000 | 0.814 | 1.000 | 0.776 | 0.896 | 0.872 | 1.000 | 0.866 | 0.658 | 1.000 | 0.963 | 0.715 | 0.669 |
| 242 | 0.650 | 0.943 | 0.595 | 0.829 | 0.805 | 1.000 | 0.815 | 1.000 | 0.776 | 0.896 | 0.872 | 1.000 | 0.866 | 0.660 | 1.000 | 0.963 | 0.715 | 0.669 |
| 243 | 0.650 | 0.943 | 0.595 | 0.829 | 0.805 | 1.000 | 0.815 | 1.000 | 0.776 | 0.896 | 0.870 | 1.000 | 0.866 | 0.658 | 1.000 | 0.963 | 0.714 | 0.669 |
| 244 | 0.652 | 0.943 | 0.595 | 0.829 | 0.805 | 1.000 | 0.815 | 1.000 | 0.755 | 0.896 | 0.872 | 1.000 | 0.866 | 0.658 | 1.000 | 0.963 | 0.715 | 0.669 |
| 245 | 0.656 | 0.943 | 0.593 | 0.814 | 0.805 | 1.000 | 0.824 | 1.000 | 0.755 | 0.896 | 0.867 | 1.000 | 0.866 | 0.658 | 1.000 | 0.963 | 0.716 | 0.671 |
| 246 | 0.660 | 0.943 | 0.593 | 0.829 | 0.805 | 1.000 | 0.832 | 1.000 | 0.755 | 0.896 | 0.870 | 1.000 | 0.866 | 0.659 | 1.000 | 0.963 | 0.719 | 0.674 |
| 247 | 0.660 | 0.943 | 0.605 | 0.814 | 0.811 | 1.000 | 0.827 | 1.000 | 0.755 | 0.896 | 0.872 | 1.000 | 0.866 | 0.659 | 1.000 | 0.963 | 0.720 | 0.675 |
| 248 | 0.660 | 0.943 | 0.605 | 0.814 | 0.811 | 1.000 | 0.827 | 1.000 | 0.755 | 0.896 | 0.872 | 1.000 | 0.866 | 0.659 | 1.000 | 0.963 | 0.720 | 0.675 |
| 249 | 0.660 | 0.943 | 0.603 | 0.814 | 0.811 | 1.000 | 0.824 | 1.000 | 0.755 | 0.896 | 0.870 | 1.000 | 0.866 | 0.664 | 1.000 | 0.963 | 0.720 | 0.675 |
| 250 | 0.660 | 0.943 | 0.603 | 0.814 | 0.811 | 1.000 | 0.824 | 1.000 | 0.765 | 0.896 | 0.870 | 1.000 | 0.866 | 0.664 | 1.000 | 0.963 | 0.720 | 0.675 |
| 251 | 0.662 | 0.943 | 0.603 | 0.814 | 0.811 | 1.000 | 0.824 | 1.000 | 0.765 | 0.896 | 0.870 | 1.000 | 0.866 | 0.666 | 1.000 | 0.963 | 0.722 | 0.676 |
| 252 | 0.659 | 0.943 | 0.613 | 0.814 | 0.811 | 1.000 | 0.826 | 1.000 | 0.765 | 0.896 | 0.867 | 1.000 | 0.866 | 0.666 | 1.000 | 0.963 | 0.722 | 0.677 |
| 253 | 0.660 | 0.943 | 0.617 | 0.814 | 0.811 | 1.000 | 0.826 | 1.000 | 0.765 | 0.896 | 0.867 | 1.000 | 0.866 | 0.665 | 1.000 | 0.963 | 0.722 | 0.678 |
| 254 | 0.659 | 0.943 | 0.623 | 0.829 | 0.811 | 1.000 | 0.829 | 1.000 | 0.776 | 0.896 | 0.867 | 1.000 | 0.866 | 0.662 | 1.000 | 0.944 | 0.722 | 0.678 |
| 255 | 0.658 | 0.943 | 0.621 | 0.829 | 0.811 | 1.000 | 0.829 | 1.000 | 0.776 | 0.896 | 0.870 | 1.000 | 0.848 | 0.665 | 1.000 | 0.944 | 0.722 | 0.678 |
| 256 | 0.659 | 0.943 | 0.617 | 0.829 | 0.816 | 1.000 | 0.829 | 1.000 | 0.755 | 0.896 | 0.870 | 1.000 | 0.857 | 0.665 | 1.000 | 0.944 | 0.722 | 0.677 |
| 257 | 0.666 | 0.943 | 0.621 | 0.829 | 0.816 | 1.000 | 0.829 | 1.000 | 0.755 | 0.896 | 0.870 | 1.000 | 0.857 | 0.665 | 1.000 | 0.944 | 0.725 | 0.680 |
| 258 | 0.667 | 0.943 | 0.619 | 0.829 | 0.821 | 1.000 | 0.827 | 1.000 | 0.765 | 0.896 | 0.870 | 1.000 | 0.875 | 0.665 | 1.000 | 0.944 | 0.725 | 0.681 |
| 259 | 0.667 | 0.943 | 0.619 | 0.829 | 0.821 | 1.000 | 0.824 | 1.000 | 0.755 | 0.896 | 0.872 | 1.000 | 0.875 | 0.664 | 1.000 | 0.944 | 0.725 | 0.680 |
| 260 | 0.671 | 0.943 | 0.628 | 0.829 | 0.816 | 1.000 | 0.823 | 1.000 | 0.776 | 0.896 | 0.881 | 1.000 | 0.875 | 0.667 | 1.000 | 0.944 | 0.728 | 0.684 |
| 261 | 0.671 | 0.943 | 0.628 | 0.829 | 0.816 | 1.000 | 0.823 | 1.000 | 0.776 | 0.896 | 0.881 | 1.000 | 0.875 | 0.665 | 1.000 | 0.944 | 0.728 | 0.683 |
| 262 | 0.670 | 0.943 | 0.630 | 0.829 | 0.821 | 1.000 | 0.824 | 1.000 | 0.776 | 0.896 | 0.878 | 1.000 | 0.875 | 0.663 | 1.000 | 0.944 | 0.727 | 0.683 |
| 263 | 0.669 | 0.943 | 0.630 | 0.829 | 0.821 | 1.000 | 0.824 | 1.000 | 0.776 | 0.896 | 0.878 | 1.000 | 0.866 | 0.665 | 1.000 | 0.944 | 0.728 | 0.683 |
| 264 | 0.677 | 0.943 | 0.628 | 0.829 | 0.821 | 1.000 | 0.823 | 1.000 | 0.776 | 0.896 | 0.884 | 1.000 | 0.866 | 0.666 | 1.000 | 0.944 | 0.730 | 0.686 |
| 265 | 0.677 | 0.943 | 0.630 | 0.829 | 0.816 | 1.000 | 0.818 | 1.000 | 0.776 | 0.896 | 0.881 | 1.000 | 0.866 | 0.666 | 1.000 | 0.981 | 0.730 | 0.685 |
| 266 | 0.677 | 0.943 | 0.628 | 0.829 | 0.816 | 1.000 | 0.818 | 1.000 | 0.796 | 0.896 | 0.881 | 1.000 | 0.866 | 0.668 | 1.000 | 0.981 | 0.730 | 0.686 |
| 267 | 0.677 | 0.943 | 0.625 | 0.829 | 0.816 | 1.000 | 0.815 | 1.000 | 0.796 | 0.896 | 0.881 | 1.000 | 0.866 | 0.668 | 1.000 | 0.981 | 0.730 | 0.685 |
| 268 | 0.677 | 0.943 | 0.623 | 0.829 | 0.811 | 1.000 | 0.817 | 1.000 | 0.806 | 0.896 | 0.884 | 1.000 | 0.866 | 0.665 | 1.000 | 0.981 | 0.729 | 0.684 |
| 269 | 0.679 | 0.943 | 0.625 | 0.829 | 0.811 | 1.000 | 0.812 | 1.000 | 0.806 | 0.896 | 0.881 | 1.000 | 0.866 | 0.667 | 1.000 | 0.981 | 0.730 | 0.685 |
| 270 | 0.679 | 0.943 | 0.628 | 0.829 | 0.811 | 1.000 | 0.817 | 1.000 | 0.806 | 0.896 | 0.884 | 1.000 | 0.866 | 0.668 | 1.000 | 0.981 | 0.731 | 0.686 |
| 271 | 0.679 | 0.943 | 0.628 | 0.829 | 0.811 | 1.000 | 0.817 | 1.000 | 0.806 | 0.896 | 0.884 | 1.000 | 0.866 | 0.668 | 1.000 | 0.981 | 0.731 | 0.686 |
| 272 | 0.677 | 0.943 | 0.626 | 0.829 | 0.811 | 1.000 | 0.815 | 1.000 | 0.796 | 0.896 | 0.884 | 1.000 | 0.866 | 0.667 | 1.000 | 0.981 | 0.729 | 0.685 |

| | | | | | | | | | | | | | | | | | | |
|---|---|---|---|---|---|---|---|---|---|---|---|---|---|---|---|---|---|---|
| 273 | 0.678 | 0.943 | 0.628 | 0.829 | 0.811 | 1.000 | 0.815 | 1.000 | 0.796 | 0.896 | 0.884 | 1.000 | 0.866 | 0.667 | 1.000 | 0.981 | 0.730 | 0.685 |
| 274 | 0.679 | 0.943 | 0.628 | 0.829 | 0.811 | 1.000 | 0.814 | 1.000 | 0.796 | 0.896 | 0.884 | 1.000 | 0.866 | 0.666 | 1.000 | 0.981 | 0.730 | 0.685 |
| 275 | 0.679 | 0.943 | 0.634 | 0.829 | 0.816 | 1.000 | 0.814 | 1.000 | 0.796 | 0.896 | 0.884 | 1.000 | 0.875 | 0.669 | 1.000 | 0.981 | 0.732 | 0.687 |
| 276 | 0.679 | 0.943 | 0.634 | 0.829 | 0.816 | 1.000 | 0.814 | 1.000 | 0.796 | 0.896 | 0.884 | 1.000 | 0.875 | 0.668 | 1.000 | 0.981 | 0.732 | 0.687 |
| 277 | 0.683 | 0.943 | 0.640 | 0.829 | 0.826 | 1.000 | 0.809 | 1.000 | 0.796 | 0.896 | 0.884 | 1.000 | 0.875 | 0.669 | 1.000 | 0.981 | 0.733 | 0.689 |
| 278 | 0.682 | 0.943 | 0.640 | 0.829 | 0.826 | 1.000 | 0.814 | 1.000 | 0.796 | 0.896 | 0.884 | 1.000 | 0.875 | 0.670 | 1.000 | 0.981 | 0.734 | 0.690 |
| 279 | 0.682 | 0.943 | 0.644 | 0.829 | 0.821 | 1.000 | 0.815 | 1.000 | 0.796 | 0.896 | 0.881 | 1.000 | 0.884 | 0.670 | 1.000 | 0.981 | 0.734 | 0.690 |
| 280 | 0.682 | 0.943 | 0.644 | 0.829 | 0.816 | 1.000 | 0.814 | 1.000 | 0.796 | 0.896 | 0.881 | 1.000 | 0.884 | 0.670 | 1.000 | 0.981 | 0.734 | 0.690 |
| 281 | 0.681 | 0.943 | 0.628 | 0.829 | 0.821 | 1.000 | 0.814 | 1.000 | 0.806 | 0.896 | 0.878 | 1.000 | 0.875 | 0.675 | 1.000 | 0.981 | 0.733 | 0.689 |
| 282 | 0.683 | 0.943 | 0.625 | 0.829 | 0.821 | 1.000 | 0.815 | 1.000 | 0.806 | 0.896 | 0.878 | 1.000 | 0.884 | 0.674 | 1.000 | 0.981 | 0.734 | 0.689 |
| 283 | 0.684 | 0.943 | 0.644 | 0.843 | 0.826 | 1.000 | 0.815 | 1.000 | 0.806 | 0.896 | 0.881 | 1.000 | 0.875 | 0.679 | 1.000 | 0.981 | 0.737 | 0.693 |
| 284 | 0.684 | 0.943 | 0.642 | 0.843 | 0.826 | 1.000 | 0.815 | 1.000 | 0.806 | 0.896 | 0.881 | 1.000 | 0.875 | 0.678 | 1.000 | 0.981 | 0.737 | 0.693 |
| 285 | 0.685 | 0.943 | 0.642 | 0.843 | 0.826 | 1.000 | 0.814 | 1.000 | 0.806 | 0.896 | 0.878 | 1.000 | 0.884 | 0.679 | 1.000 | 0.981 | 0.737 | 0.693 |
| 286 | 0.685 | 0.943 | 0.648 | 0.843 | 0.826 | 1.000 | 0.815 | 1.000 | 0.806 | 0.896 | 0.878 | 1.000 | 0.866 | 0.676 | 1.000 | 0.981 | 0.737 | 0.693 |
| 287 | 0.683 | 0.943 | 0.644 | 0.843 | 0.832 | 1.000 | 0.812 | 1.000 | 0.806 | 0.896 | 0.875 | 1.000 | 0.866 | 0.675 | 1.000 | 0.981 | 0.735 | 0.691 |
| 288 | 0.683 | 0.943 | 0.644 | 0.843 | 0.832 | 1.000 | 0.814 | 1.000 | 0.806 | 0.896 | 0.875 | 1.000 | 0.866 | 0.674 | 1.000 | 0.981 | 0.735 | 0.691 |
| 289 | 0.683 | 0.943 | 0.644 | 0.843 | 0.832 | 1.000 | 0.814 | 1.000 | 0.806 | 0.896 | 0.875 | 1.000 | 0.866 | 0.679 | 1.000 | 0.981 | 0.737 | 0.692 |
| 290 | 0.683 | 0.943 | 0.644 | 0.843 | 0.832 | 1.000 | 0.814 | 1.000 | 0.806 | 0.896 | 0.875 | 1.000 | 0.866 | 0.677 | 1.000 | 0.981 | 0.736 | 0.692 |
| 291 | 0.682 | 0.943 | 0.646 | 0.843 | 0.837 | 1.000 | 0.814 | 1.000 | 0.806 | 0.896 | 0.875 | 1.000 | 0.866 | 0.675 | 1.000 | 0.981 | 0.736 | 0.691 |
| 292 | 0.686 | 0.943 | 0.650 | 0.857 | 0.837 | 1.000 | 0.814 | 1.000 | 0.816 | 0.896 | 0.872 | 1.000 | 0.866 | 0.676 | 1.000 | 0.981 | 0.738 | 0.694 |
| 293 | 0.685 | 0.943 | 0.652 | 0.857 | 0.837 | 1.000 | 0.814 | 1.000 | 0.816 | 0.896 | 0.872 | 1.000 | 0.866 | 0.674 | 1.000 | 0.981 | 0.737 | 0.693 |
| 294 | 0.683 | 0.943 | 0.650 | 0.857 | 0.832 | 1.000 | 0.812 | 1.000 | 0.816 | 0.896 | 0.875 | 1.000 | 0.866 | 0.676 | 1.000 | 0.981 | 0.737 | 0.693 |
| 295 | 0.683 | 0.943 | 0.650 | 0.857 | 0.832 | 1.000 | 0.810 | 1.000 | 0.816 | 0.896 | 0.875 | 1.000 | 0.875 | 0.675 | 1.000 | 0.981 | 0.736 | 0.692 |
| 296 | 0.683 | 0.943 | 0.654 | 0.857 | 0.826 | 1.000 | 0.812 | 1.000 | 0.816 | 0.896 | 0.872 | 1.000 | 0.866 | 0.675 | 1.000 | 0.981 | 0.736 | 0.692 |
| 297 | 0.688 | 0.943 | 0.650 | 0.871 | 0.826 | 1.000 | 0.810 | 1.000 | 0.816 | 0.896 | 0.872 | 1.000 | 0.875 | 0.675 | 1.000 | 0.981 | 0.738 | 0.694 |
| 298 | 0.689 | 0.943 | 0.654 | 0.871 | 0.826 | 1.000 | 0.801 | 1.000 | 0.816 | 0.896 | 0.878 | 1.000 | 0.875 | 0.677 | 1.000 | 0.981 | 0.738 | 0.694 |
| 299 | 0.689 | 0.943 | 0.660 | 0.871 | 0.826 | 1.000 | 0.801 | 1.000 | 0.816 | 0.896 | 0.878 | 1.000 | 0.875 | 0.675 | 1.000 | 0.981 | 0.738 | 0.694 |
| 300 | 0.689 | 0.943 | 0.658 | 0.886 | 0.826 | 1.000 | 0.804 | 1.000 | 0.816 | 0.896 | 0.875 | 1.000 | 0.875 | 0.675 | 1.000 | 0.981 | 0.739 | 0.695 |
| 301 | 0.687 | 0.943 | 0.656 | 0.871 | 0.832 | 1.000 | 0.806 | 1.000 | 0.816 | 0.896 | 0.875 | 1.000 | 0.875 | 0.675 | 1.000 | 0.981 | 0.738 | 0.694 |
| 302 | 0.684 | 0.943 | 0.658 | 0.886 | 0.832 | 1.000 | 0.810 | 1.000 | 0.816 | 0.896 | 0.875 | 1.000 | 0.875 | 0.675 | 1.000 | 0.981 | 0.738 | 0.694 |
| 303 | 0.685 | 0.943 | 0.656 | 0.886 | 0.832 | 1.000 | 0.809 | 1.000 | 0.816 | 0.896 | 0.875 | 1.000 | 0.875 | 0.673 | 1.000 | 0.981 | 0.737 | 0.693 |
| 304 | 0.685 | 0.943 | 0.656 | 0.886 | 0.832 | 1.000 | 0.809 | 1.000 | 0.816 | 0.896 | 0.875 | 1.000 | 0.884 | 0.676 | 1.000 | 0.981 | 0.738 | 0.694 |
| 305 | 0.685 | 0.943 | 0.658 | 0.886 | 0.832 | 1.000 | 0.809 | 1.000 | 0.816 | 0.896 | 0.875 | 1.000 | 0.884 | 0.676 | 1.000 | 0.981 | 0.738 | 0.694 |
| 306 | 0.683 | 0.943 | 0.660 | 0.886 | 0.837 | 1.000 | 0.807 | 1.000 | 0.816 | 0.896 | 0.875 | 1.000 | 0.884 | 0.675 | 1.000 | 0.981 | 0.738 | 0.694 |
| 307 | 0.683 | 0.943 | 0.662 | 0.886 | 0.832 | 1.000 | 0.807 | 1.000 | 0.816 | 0.896 | 0.875 | 1.000 | 0.884 | 0.672 | 1.000 | 0.981 | 0.737 | 0.693 |
| 308 | 0.683 | 0.943 | 0.660 | 0.886 | 0.832 | 1.000 | 0.807 | 1.000 | 0.816 | 0.896 | 0.875 | 1.000 | 0.884 | 0.672 | 1.000 | 0.981 | 0.737 | 0.693 |
| 309 | 0.687 | 0.943 | 0.658 | 0.886 | 0.837 | 1.000 | 0.807 | 1.000 | 0.816 | 0.896 | 0.872 | 1.000 | 0.884 | 0.672 | 1.000 | 0.981 | 0.738 | 0.694 |
| 310 | 0.685 | 0.943 | 0.660 | 0.886 | 0.837 | 1.000 | 0.812 | 1.000 | 0.816 | 0.896 | 0.872 | 1.000 | 0.884 | 0.672 | 1.000 | 0.981 | 0.738 | 0.694 |
| 311 | 0.685 | 0.943 | 0.658 | 0.886 | 0.832 | 1.000 | 0.814 | 1.000 | 0.827 | 0.896 | 0.875 | 1.000 | 0.884 | 0.675 | 1.000 | 0.981 | 0.739 | 0.695 |

| | | | | | | | | | | | | | | | | | | |
|---|---|---|---|---|---|---|---|---|---|---|---|---|---|---|---|---|---|---|
| 312 | 0.686 | 0.943 | 0.666 | 0.886 | 0.832 | 1.000 | 0.815 | 1.000 | 0.827 | 0.896 | 0.875 | 1.000 | 0.884 | 0.672 | 1.000 | 0.981 | 0.740 | 0.696 |
| 313 | 0.692 | 0.943 | 0.668 | 0.886 | 0.837 | 1.000 | 0.810 | 1.000 | 0.827 | 0.896 | 0.875 | 1.000 | 0.884 | 0.673 | 1.000 | 0.981 | 0.741 | 0.698 |
| 314 | 0.693 | 0.943 | 0.666 | 0.886 | 0.842 | 1.000 | 0.809 | 1.000 | 0.827 | 0.896 | 0.872 | 1.000 | 0.884 | 0.674 | 1.000 | 0.981 | 0.742 | 0.698 |
| 315 | 0.693 | 0.943 | 0.662 | 0.886 | 0.847 | 1.000 | 0.812 | 1.000 | 0.827 | 0.896 | 0.878 | 1.000 | 0.884 | 0.676 | 1.000 | 0.981 | 0.743 | 0.699 |
| 316 | 0.695 | 0.943 | 0.666 | 0.886 | 0.842 | 1.000 | 0.809 | 1.000 | 0.847 | 0.896 | 0.878 | 1.000 | 0.884 | 0.677 | 1.000 | 0.981 | 0.744 | 0.700 |
| 317 | 0.691 | 0.943 | 0.662 | 0.886 | 0.847 | 1.000 | 0.804 | 1.000 | 0.847 | 0.896 | 0.875 | 1.000 | 0.884 | 0.677 | 1.000 | 0.981 | 0.742 | 0.698 |
| 318 | 0.692 | 0.943 | 0.664 | 0.886 | 0.847 | 1.000 | 0.804 | 1.000 | 0.847 | 0.896 | 0.875 | 1.000 | 0.884 | 0.677 | 1.000 | 0.981 | 0.742 | 0.698 |
| 319 | 0.693 | 0.943 | 0.658 | 0.871 | 0.842 | 1.000 | 0.806 | 1.000 | 0.847 | 0.896 | 0.875 | 1.000 | 0.884 | 0.683 | 1.000 | 0.981 | 0.743 | 0.699 |
| 320 | 0.693 | 0.943 | 0.668 | 0.900 | 0.842 | 1.000 | 0.807 | 1.000 | 0.847 | 0.896 | 0.875 | 1.000 | 0.884 | 0.682 | 1.000 | 0.981 | 0.744 | 0.701 |
| 321 | 0.695 | 0.943 | 0.668 | 0.900 | 0.847 | 1.000 | 0.807 | 1.000 | 0.847 | 0.896 | 0.875 | 1.000 | 0.893 | 0.682 | 1.000 | 0.981 | 0.745 | 0.702 |
| 322 | 0.694 | 0.943 | 0.668 | 0.900 | 0.847 | 1.000 | 0.812 | 1.000 | 0.847 | 0.896 | 0.875 | 1.000 | 0.893 | 0.682 | 1.000 | 0.981 | 0.746 | 0.702 |
| 323 | 0.695 | 0.943 | 0.668 | 0.900 | 0.847 | 1.000 | 0.810 | 1.000 | 0.847 | 0.917 | 0.878 | 1.000 | 0.884 | 0.686 | 1.000 | 0.981 | 0.747 | 0.704 |
| 324 | 0.696 | 0.943 | 0.664 | 0.900 | 0.853 | 1.000 | 0.810 | 1.000 | 0.847 | 0.917 | 0.881 | 1.000 | 0.884 | 0.686 | 1.000 | 0.981 | 0.747 | 0.704 |
| 325 | 0.697 | 0.943 | 0.662 | 0.886 | 0.853 | 1.000 | 0.810 | 1.000 | 0.857 | 0.917 | 0.881 | 1.000 | 0.884 | 0.682 | 1.000 | 0.981 | 0.746 | 0.703 |
| 326 | 0.695 | 0.943 | 0.664 | 0.900 | 0.853 | 1.000 | 0.814 | 1.000 | 0.857 | 0.917 | 0.878 | 1.000 | 0.884 | 0.681 | 1.000 | 0.981 | 0.746 | 0.703 |
| 327 | 0.694 | 0.943 | 0.674 | 0.886 | 0.853 | 1.000 | 0.810 | 1.000 | 0.857 | 0.917 | 0.875 | 1.000 | 0.884 | 0.681 | 1.000 | 0.981 | 0.746 | 0.703 |
| 328 | 0.696 | 0.943 | 0.674 | 0.886 | 0.847 | 1.000 | 0.810 | 1.000 | 0.857 | 0.917 | 0.875 | 1.000 | 0.884 | 0.681 | 1.000 | 0.981 | 0.746 | 0.703 |
| 329 | 0.695 | 0.943 | 0.672 | 0.886 | 0.847 | 1.000 | 0.812 | 1.000 | 0.857 | 0.917 | 0.875 | 1.000 | 0.884 | 0.681 | 1.000 | 0.981 | 0.746 | 0.703 |
| 330 | 0.695 | 0.943 | 0.672 | 0.886 | 0.847 | 1.000 | 0.812 | 1.000 | 0.857 | 0.917 | 0.875 | 1.000 | 0.884 | 0.682 | 1.000 | 0.981 | 0.746 | 0.703 |
| 331 | 0.698 | 0.943 | 0.670 | 0.914 | 0.847 | 1.000 | 0.815 | 1.000 | 0.857 | 0.917 | 0.878 | 1.000 | 0.884 | 0.683 | 1.000 | 0.981 | 0.748 | 0.706 |
| 332 | 0.698 | 0.943 | 0.668 | 0.900 | 0.853 | 1.000 | 0.814 | 1.000 | 0.857 | 0.917 | 0.881 | 1.000 | 0.884 | 0.684 | 1.000 | 0.981 | 0.748 | 0.706 |
| 333 | 0.697 | 0.943 | 0.668 | 0.914 | 0.853 | 1.000 | 0.812 | 1.000 | 0.857 | 0.917 | 0.881 | 1.000 | 0.884 | 0.684 | 1.000 | 0.981 | 0.748 | 0.705 |
| 334 | 0.697 | 0.943 | 0.666 | 0.914 | 0.853 | 1.000 | 0.815 | 1.000 | 0.857 | 0.917 | 0.881 | 1.000 | 0.884 | 0.684 | 1.000 | 0.981 | 0.748 | 0.705 |
| 335 | 0.694 | 0.943 | 0.662 | 0.914 | 0.853 | 1.000 | 0.815 | 1.000 | 0.857 | 0.917 | 0.878 | 1.000 | 0.884 | 0.683 | 1.000 | 0.981 | 0.747 | 0.703 |
| 336 | 0.695 | 0.943 | 0.668 | 0.929 | 0.853 | 1.000 | 0.812 | 1.000 | 0.857 | 0.917 | 0.881 | 1.000 | 0.884 | 0.690 | 1.000 | 0.981 | 0.749 | 0.706 |
| 337 | 0.698 | 0.943 | 0.666 | 0.929 | 0.853 | 1.000 | 0.812 | 1.000 | 0.857 | 0.917 | 0.881 | 1.000 | 0.884 | 0.690 | 1.000 | 0.981 | 0.750 | 0.707 |
| 338 | 0.701 | 0.943 | 0.670 | 0.929 | 0.853 | 1.000 | 0.812 | 1.000 | 0.857 | 0.917 | 0.881 | 1.000 | 0.884 | 0.693 | 1.000 | 0.981 | 0.752 | 0.709 |
| 339 | 0.699 | 0.943 | 0.676 | 0.929 | 0.853 | 1.000 | 0.810 | 1.000 | 0.867 | 0.917 | 0.881 | 1.000 | 0.884 | 0.693 | 1.000 | 0.981 | 0.752 | 0.710 |
| 340 | 0.703 | 0.943 | 0.672 | 0.929 | 0.858 | 1.000 | 0.814 | 1.000 | 0.867 | 0.917 | 0.887 | 1.000 | 0.884 | 0.694 | 1.000 | 0.981 | 0.754 | 0.712 |
| 341 | 0.701 | 0.943 | 0.670 | 0.929 | 0.858 | 1.000 | 0.814 | 1.000 | 0.867 | 0.917 | 0.896 | 1.000 | 0.875 | 0.690 | 1.000 | 1.000 | 0.753 | 0.710 |
| 342 | 0.703 | 0.943 | 0.674 | 0.929 | 0.858 | 1.000 | 0.814 | 1.000 | 0.867 | 0.896 | 0.893 | 1.000 | 0.884 | 0.689 | 1.000 | 1.000 | 0.754 | 0.711 |
| 343 | 0.702 | 0.943 | 0.680 | 0.929 | 0.858 | 1.000 | 0.814 | 1.000 | 0.878 | 0.896 | 0.890 | 1.000 | 0.875 | 0.691 | 1.000 | 1.000 | 0.754 | 0.712 |
| 344 | 0.703 | 0.943 | 0.676 | 0.929 | 0.858 | 1.000 | 0.812 | 1.000 | 0.878 | 0.896 | 0.884 | 1.000 | 0.875 | 0.692 | 1.000 | 1.000 | 0.754 | 0.711 |
| 345 | 0.701 | 0.943 | 0.676 | 0.914 | 0.858 | 1.000 | 0.814 | 1.000 | 0.878 | 0.896 | 0.884 | 1.000 | 0.875 | 0.694 | 1.000 | 1.000 | 0.754 | 0.711 |
| 346 | 0.699 | 0.943 | 0.676 | 0.914 | 0.858 | 1.000 | 0.812 | 1.000 | 0.878 | 0.896 | 0.884 | 1.000 | 0.875 | 0.694 | 1.000 | 1.000 | 0.753 | 0.710 |
| 347 | 0.697 | 0.943 | 0.680 | 0.914 | 0.858 | 1.000 | 0.812 | 1.000 | 0.878 | 0.917 | 0.887 | 1.000 | 0.884 | 0.699 | 1.000 | 1.000 | 0.754 | 0.712 |
| 348 | 0.696 | 0.943 | 0.678 | 0.914 | 0.858 | 1.000 | 0.812 | 1.000 | 0.878 | 0.917 | 0.881 | 1.000 | 0.875 | 0.696 | 1.000 | 1.000 | 0.753 | 0.710 |
| 349 | 0.696 | 0.943 | 0.676 | 0.914 | 0.858 | 1.000 | 0.810 | 1.000 | 0.888 | 0.917 | 0.884 | 1.000 | 0.875 | 0.697 | 1.000 | 1.000 | 0.753 | 0.710 |
| 350 | 0.702 | 0.943 | 0.682 | 0.914 | 0.858 | 1.000 | 0.815 | 1.000 | 0.888 | 0.917 | 0.884 | 1.000 | 0.875 | 0.697 | 1.000 | 1.000 | 0.756 | 0.713 |

| | | | | | | | | | | | | | | | | | | |
|---|---|---|---|---|---|---|---|---|---|---|---|---|---|---|---|---|---|---|
| 351 | 0.701 | 0.943 | 0.678 | 0.914 | 0.858 | 1.000 | 0.814 | 1.000 | 0.878 | 0.917 | 0.890 | 1.000 | 0.875 | 0.698 | 1.000 | 1.000 | 0.755 | 0.713 |
| 352 | 0.701 | 0.943 | 0.680 | 0.914 | 0.858 | 1.000 | 0.810 | 1.000 | 0.878 | 0.917 | 0.887 | 1.000 | 0.866 | 0.695 | 1.000 | 1.000 | 0.754 | 0.711 |
| 353 | 0.699 | 0.943 | 0.672 | 0.914 | 0.858 | 1.000 | 0.810 | 1.000 | 0.878 | 0.917 | 0.887 | 1.000 | 0.875 | 0.693 | 1.000 | 1.000 | 0.752 | 0.709 |
| 354 | 0.699 | 0.943 | 0.670 | 0.914 | 0.858 | 1.000 | 0.810 | 1.000 | 0.878 | 0.917 | 0.887 | 1.000 | 0.875 | 0.692 | 1.000 | 1.000 | 0.752 | 0.708 |
| 355 | 0.700 | 0.943 | 0.672 | 0.914 | 0.858 | 1.000 | 0.810 | 1.000 | 0.878 | 0.917 | 0.884 | 1.000 | 0.875 | 0.693 | 1.000 | 1.000 | 0.752 | 0.709 |
| 356 | 0.699 | 0.943 | 0.674 | 0.914 | 0.858 | 1.000 | 0.814 | 1.000 | 0.878 | 0.917 | 0.884 | 1.000 | 0.875 | 0.693 | 1.000 | 1.000 | 0.753 | 0.710 |
| 357 | 0.703 | 0.943 | 0.676 | 0.914 | 0.858 | 1.000 | 0.810 | 1.000 | 0.878 | 0.917 | 0.884 | 1.000 | 0.875 | 0.693 | 1.000 | 1.000 | 0.754 | 0.711 |
| 358 | 0.701 | 0.943 | 0.680 | 0.914 | 0.858 | 1.000 | 0.810 | 1.000 | 0.878 | 0.917 | 0.887 | 1.000 | 0.875 | 0.693 | 1.000 | 1.000 | 0.754 | 0.711 |
| 359 | 0.701 | 0.943 | 0.680 | 0.914 | 0.858 | 1.000 | 0.812 | 1.000 | 0.878 | 0.917 | 0.887 | 1.000 | 0.875 | 0.692 | 1.000 | 1.000 | 0.754 | 0.711 |
| 360 | 0.704 | 0.943 | 0.672 | 0.914 | 0.853 | 1.000 | 0.823 | 1.000 | 0.888 | 0.938 | 0.887 | 1.000 | 0.866 | 0.688 | 1.000 | 1.000 | 0.754 | 0.711 |
| 361 | 0.705 | 0.943 | 0.676 | 0.914 | 0.853 | 1.000 | 0.823 | 1.000 | 0.888 | 0.938 | 0.887 | 1.000 | 0.866 | 0.692 | 1.000 | 1.000 | 0.756 | 0.713 |
| 362 | 0.701 | 0.943 | 0.678 | 0.914 | 0.853 | 1.000 | 0.821 | 1.000 | 0.888 | 0.938 | 0.887 | 1.000 | 0.866 | 0.693 | 1.000 | 1.000 | 0.755 | 0.712 |
| 363 | 0.702 | 0.943 | 0.678 | 0.914 | 0.853 | 1.000 | 0.823 | 1.000 | 0.888 | 0.938 | 0.887 | 1.000 | 0.866 | 0.690 | 1.000 | 1.000 | 0.755 | 0.712 |
| 364 | 0.701 | 0.943 | 0.674 | 0.914 | 0.853 | 1.000 | 0.823 | 1.000 | 0.888 | 0.938 | 0.887 | 1.000 | 0.866 | 0.689 | 1.000 | 1.000 | 0.754 | 0.711 |
| 365 | 0.701 | 0.943 | 0.676 | 0.914 | 0.853 | 1.000 | 0.823 | 1.000 | 0.888 | 0.938 | 0.887 | 1.000 | 0.857 | 0.692 | 1.000 | 1.000 | 0.754 | 0.711 |
| 366 | 0.701 | 0.943 | 0.676 | 0.914 | 0.853 | 1.000 | 0.823 | 1.000 | 0.888 | 0.938 | 0.887 | 1.000 | 0.857 | 0.692 | 1.000 | 1.000 | 0.754 | 0.711 |
| 367 | 0.701 | 0.943 | 0.676 | 0.914 | 0.853 | 1.000 | 0.824 | 1.000 | 0.888 | 0.938 | 0.887 | 1.000 | 0.857 | 0.693 | 1.000 | 1.000 | 0.755 | 0.712 |
| 368 | 0.707 | 0.943 | 0.680 | 0.914 | 0.853 | 1.000 | 0.821 | 1.000 | 0.888 | 0.938 | 0.887 | 1.000 | 0.848 | 0.689 | 1.000 | 1.000 | 0.756 | 0.713 |
| 369 | 0.705 | 0.943 | 0.682 | 0.914 | 0.853 | 1.000 | 0.824 | 1.000 | 0.888 | 0.938 | 0.887 | 1.000 | 0.848 | 0.690 | 1.000 | 1.000 | 0.756 | 0.713 |
| 370 | 0.707 | 0.943 | 0.682 | 0.929 | 0.853 | 1.000 | 0.821 | 1.000 | 0.888 | 0.938 | 0.887 | 1.000 | 0.848 | 0.694 | 1.000 | 1.000 | 0.757 | 0.715 |
| 371 | 0.705 | 0.943 | 0.684 | 0.929 | 0.853 | 1.000 | 0.820 | 1.000 | 0.888 | 0.938 | 0.887 | 1.000 | 0.848 | 0.694 | 1.000 | 1.000 | 0.757 | 0.714 |
| 372 | 0.706 | 0.943 | 0.682 | 0.929 | 0.853 | 1.000 | 0.820 | 1.000 | 0.888 | 0.938 | 0.890 | 1.000 | 0.848 | 0.694 | 1.000 | 1.000 | 0.757 | 0.714 |
| 373 | 0.706 | 0.943 | 0.674 | 0.929 | 0.853 | 1.000 | 0.812 | 1.000 | 0.888 | 0.958 | 0.881 | 1.000 | 0.848 | 0.692 | 1.000 | 1.000 | 0.754 | 0.711 |
| 374 | 0.707 | 0.943 | 0.674 | 0.929 | 0.858 | 1.000 | 0.814 | 1.000 | 0.888 | 0.958 | 0.884 | 1.000 | 0.857 | 0.691 | 1.000 | 1.000 | 0.755 | 0.712 |
| 375 | 0.707 | 0.943 | 0.674 | 0.929 | 0.858 | 1.000 | 0.814 | 1.000 | 0.888 | 0.958 | 0.890 | 1.000 | 0.857 | 0.690 | 1.000 | 1.000 | 0.755 | 0.712 |
| 376 | 0.705 | 0.943 | 0.678 | 0.929 | 0.858 | 1.000 | 0.818 | 1.000 | 0.888 | 0.958 | 0.887 | 1.000 | 0.875 | 0.693 | 1.000 | 1.000 | 0.757 | 0.714 |
| 377 | 0.705 | 0.943 | 0.678 | 0.929 | 0.858 | 1.000 | 0.818 | 1.000 | 0.888 | 0.958 | 0.896 | 1.000 | 0.875 | 0.693 | 1.000 | 1.000 | 0.757 | 0.714 |
| 378 | 0.711 | 0.943 | 0.680 | 0.929 | 0.863 | 1.000 | 0.820 | 1.000 | 0.888 | 0.958 | 0.893 | 1.000 | 0.875 | 0.693 | 1.000 | 1.000 | 0.759 | 0.717 |
| 379 | 0.708 | 0.943 | 0.682 | 0.929 | 0.863 | 1.000 | 0.818 | 1.000 | 0.888 | 0.958 | 0.896 | 1.000 | 0.884 | 0.695 | 1.000 | 1.000 | 0.759 | 0.717 |
| 380 | 0.710 | 0.943 | 0.680 | 0.929 | 0.858 | 1.000 | 0.820 | 1.000 | 0.888 | 0.958 | 0.893 | 1.000 | 0.884 | 0.694 | 1.000 | 1.000 | 0.759 | 0.717 |
| 381 | 0.707 | 0.943 | 0.684 | 0.929 | 0.858 | 1.000 | 0.823 | 1.000 | 0.888 | 0.958 | 0.893 | 1.000 | 0.884 | 0.695 | 1.000 | 1.000 | 0.760 | 0.718 |
| 382 | 0.708 | 0.943 | 0.682 | 0.929 | 0.858 | 1.000 | 0.826 | 1.000 | 0.888 | 0.958 | 0.893 | 1.000 | 0.884 | 0.694 | 1.000 | 1.000 | 0.760 | 0.718 |
| 383 | 0.708 | 0.943 | 0.686 | 0.929 | 0.858 | 1.000 | 0.824 | 1.000 | 0.888 | 0.958 | 0.899 | 1.000 | 0.875 | 0.695 | 1.000 | 1.000 | 0.760 | 0.718 |
| 384 | 0.707 | 0.943 | 0.696 | 0.929 | 0.858 | 1.000 | 0.820 | 1.000 | 0.888 | 0.958 | 0.899 | 1.000 | 0.884 | 0.697 | 1.000 | 1.000 | 0.761 | 0.720 |
| 385 | 0.709 | 0.943 | 0.694 | 0.943 | 0.858 | 1.000 | 0.823 | 1.000 | 0.888 | 0.958 | 0.899 | 1.000 | 0.884 | 0.697 | 1.000 | 1.000 | 0.762 | 0.721 |
| 386 | 0.704 | 0.943 | 0.686 | 0.943 | 0.858 | 1.000 | 0.824 | 1.000 | 0.888 | 0.958 | 0.899 | 1.000 | 0.884 | 0.698 | 1.000 | 1.000 | 0.760 | 0.718 |
| 387 | 0.703 | 0.943 | 0.694 | 0.943 | 0.858 | 1.000 | 0.820 | 1.000 | 0.888 | 0.958 | 0.901 | 1.000 | 0.884 | 0.698 | 1.000 | 1.000 | 0.761 | 0.719 |
| 388 | 0.704 | 0.943 | 0.686 | 0.943 | 0.858 | 1.000 | 0.812 | 1.000 | 0.888 | 0.958 | 0.901 | 1.000 | 0.884 | 0.699 | 1.000 | 1.000 | 0.759 | 0.717 |
| 389 | 0.706 | 0.943 | 0.696 | 0.943 | 0.858 | 1.000 | 0.817 | 1.000 | 0.898 | 0.958 | 0.901 | 1.000 | 0.884 | 0.696 | 1.000 | 1.000 | 0.761 | 0.719 |

| | | | | | | | | | | | | | | | | | | |
|---|---|---|---|---|---|---|---|---|---|---|---|---|---|---|---|---|---|---|
| 390 | 0.707 | 0.943 | 0.684 | 0.943 | 0.853 | 1.000 | 0.817 | 1.000 | 0.898 | 0.958 | 0.901 | 1.000 | 0.893 | 0.701 | 1.000 | 1.000 | 0.761 | 0.719 |
| 391 | 0.708 | 0.943 | 0.686 | 0.943 | 0.853 | 1.000 | 0.818 | 1.000 | 0.898 | 0.958 | 0.901 | 1.000 | 0.911 | 0.700 | 1.000 | 1.000 | 0.762 | 0.720 |
| 392 | 0.709 | 0.943 | 0.686 | 0.943 | 0.853 | 1.000 | 0.818 | 1.000 | 0.898 | 0.958 | 0.901 | 1.000 | 0.911 | 0.701 | 1.000 | 1.000 | 0.763 | 0.721 |
| 393 | 0.709 | 0.943 | 0.688 | 0.943 | 0.853 | 1.000 | 0.818 | 1.000 | 0.898 | 0.958 | 0.901 | 1.000 | 0.911 | 0.700 | 1.000 | 1.000 | 0.763 | 0.721 |
| 394 | 0.707 | 0.943 | 0.680 | 0.943 | 0.853 | 1.000 | 0.817 | 1.000 | 0.898 | 0.958 | 0.901 | 1.000 | 0.911 | 0.701 | 1.000 | 1.000 | 0.761 | 0.719 |
| 395 | 0.709 | 0.943 | 0.690 | 0.943 | 0.853 | 1.000 | 0.815 | 1.000 | 0.898 | 0.958 | 0.899 | 1.000 | 0.911 | 0.700 | 1.000 | 1.000 | 0.762 | 0.720 |
| 396 | 0.707 | 0.943 | 0.690 | 0.943 | 0.853 | 1.000 | 0.815 | 1.000 | 0.898 | 0.958 | 0.901 | 1.000 | 0.911 | 0.700 | 1.000 | 1.000 | 0.762 | 0.720 |
| 397 | 0.706 | 0.943 | 0.696 | 0.943 | 0.853 | 1.000 | 0.818 | 1.000 | 0.898 | 0.958 | 0.901 | 1.000 | 0.911 | 0.701 | 1.000 | 1.000 | 0.763 | 0.721 |
| 398 | 0.706 | 0.943 | 0.694 | 0.943 | 0.853 | 1.000 | 0.820 | 1.000 | 0.888 | 0.958 | 0.899 | 1.000 | 0.911 | 0.700 | 1.000 | 1.000 | 0.762 | 0.720 |
| 399 | 0.705 | 0.943 | 0.688 | 0.943 | 0.853 | 1.000 | 0.817 | 1.000 | 0.888 | 0.958 | 0.899 | 1.000 | 0.911 | 0.703 | 1.000 | 1.000 | 0.762 | 0.719 |
| 400 | 0.706 | 0.943 | 0.684 | 0.943 | 0.853 | 1.000 | 0.818 | 1.000 | 0.898 | 0.958 | 0.899 | 1.000 | 0.920 | 0.703 | 1.000 | 1.000 | 0.762 | 0.720 |
| 401 | 0.707 | 0.943 | 0.684 | 0.943 | 0.853 | 1.000 | 0.818 | 1.000 | 0.898 | 0.958 | 0.901 | 1.000 | 0.911 | 0.702 | 1.000 | 1.000 | 0.762 | 0.720 |
| 402 | 0.705 | 0.943 | 0.686 | 0.943 | 0.853 | 1.000 | 0.817 | 1.000 | 0.898 | 0.958 | 0.899 | 1.000 | 0.911 | 0.701 | 1.000 | 1.000 | 0.761 | 0.719 |
| 403 | 0.705 | 0.943 | 0.684 | 0.943 | 0.853 | 1.000 | 0.817 | 1.000 | 0.898 | 0.958 | 0.899 | 1.000 | 0.920 | 0.704 | 1.000 | 1.000 | 0.762 | 0.720 |
| 404 | 0.708 | 0.943 | 0.686 | 0.943 | 0.853 | 1.000 | 0.818 | 1.000 | 0.898 | 0.958 | 0.899 | 1.000 | 0.920 | 0.704 | 1.000 | 1.000 | 0.763 | 0.721 |
| 405 | 0.711 | 0.943 | 0.684 | 0.943 | 0.842 | 1.000 | 0.821 | 1.000 | 0.898 | 0.958 | 0.899 | 1.000 | 0.902 | 0.708 | 1.000 | 1.000 | 0.764 | 0.722 |
| 406 | 0.716 | 0.943 | 0.680 | 0.943 | 0.853 | 1.000 | 0.831 | 1.000 | 0.888 | 0.958 | 0.901 | 1.000 | 0.902 | 0.704 | 1.000 | 1.000 | 0.766 | 0.724 |
| 407 | 0.714 | 0.943 | 0.682 | 0.943 | 0.853 | 1.000 | 0.829 | 1.000 | 0.888 | 0.958 | 0.899 | 1.000 | 0.893 | 0.704 | 1.000 | 1.000 | 0.765 | 0.723 |
| 408 | 0.716 | 0.943 | 0.682 | 0.943 | 0.853 | 1.000 | 0.829 | 1.000 | 0.888 | 0.958 | 0.899 | 1.000 | 0.893 | 0.705 | 1.000 | 1.000 | 0.766 | 0.724 |
| 409 | 0.715 | 0.943 | 0.684 | 0.943 | 0.853 | 1.000 | 0.829 | 1.000 | 0.898 | 0.958 | 0.899 | 1.000 | 0.893 | 0.707 | 1.000 | 1.000 | 0.767 | 0.725 |
| 410 | 0.716 | 0.943 | 0.694 | 0.943 | 0.853 | 1.000 | 0.826 | 1.000 | 0.898 | 0.958 | 0.896 | 1.000 | 0.893 | 0.707 | 1.000 | 1.000 | 0.767 | 0.726 |
| 411 | 0.717 | 0.943 | 0.694 | 0.943 | 0.853 | 1.000 | 0.826 | 1.000 | 0.898 | 0.958 | 0.896 | 1.000 | 0.893 | 0.707 | 1.000 | 1.000 | 0.768 | 0.726 |
| 412 | 0.716 | 0.943 | 0.694 | 0.943 | 0.853 | 1.000 | 0.824 | 1.000 | 0.898 | 0.958 | 0.896 | 1.000 | 0.893 | 0.707 | 1.000 | 1.000 | 0.767 | 0.726 |
| 413 | 0.716 | 0.943 | 0.692 | 0.943 | 0.853 | 1.000 | 0.824 | 1.000 | 0.898 | 0.958 | 0.896 | 1.000 | 0.893 | 0.707 | 1.000 | 1.000 | 0.767 | 0.725 |
| 414 | 0.716 | 0.943 | 0.694 | 0.943 | 0.853 | 1.000 | 0.823 | 1.000 | 0.898 | 0.958 | 0.896 | 1.000 | 0.893 | 0.708 | 1.000 | 1.000 | 0.767 | 0.726 |
| 415 | 0.716 | 0.943 | 0.686 | 0.943 | 0.853 | 1.000 | 0.826 | 1.000 | 0.898 | 0.958 | 0.899 | 1.000 | 0.902 | 0.714 | 1.000 | 1.000 | 0.769 | 0.727 |
| 416 | 0.718 | 0.943 | 0.684 | 0.943 | 0.853 | 1.000 | 0.824 | 1.000 | 0.898 | 0.958 | 0.907 | 1.000 | 0.893 | 0.715 | 1.000 | 1.000 | 0.770 | 0.728 |
| 417 | 0.715 | 0.943 | 0.688 | 0.943 | 0.858 | 1.000 | 0.821 | 1.000 | 0.898 | 0.958 | 0.910 | 1.000 | 0.893 | 0.717 | 1.000 | 1.000 | 0.769 | 0.728 |
| 418 | 0.723 | 0.943 | 0.696 | 0.957 | 0.858 | 1.000 | 0.824 | 1.000 | 0.898 | 0.958 | 0.910 | 1.000 | 0.893 | 0.712 | 1.000 | 1.000 | 0.772 | 0.731 |
| 419 | 0.725 | 0.943 | 0.690 | 0.957 | 0.858 | 1.000 | 0.823 | 1.000 | 0.898 | 0.958 | 0.910 | 1.000 | 0.893 | 0.711 | 1.000 | 1.000 | 0.772 | 0.730 |
| 420 | 0.725 | 0.943 | 0.692 | 0.957 | 0.858 | 1.000 | 0.821 | 1.000 | 0.898 | 0.958 | 0.910 | 1.000 | 0.902 | 0.714 | 1.000 | 1.000 | 0.773 | 0.731 |
| 421 | 0.726 | 0.943 | 0.692 | 0.957 | 0.858 | 1.000 | 0.820 | 1.000 | 0.898 | 0.958 | 0.910 | 1.000 | 0.902 | 0.716 | 1.000 | 1.000 | 0.773 | 0.732 |
| 422 | 0.724 | 0.943 | 0.692 | 0.957 | 0.858 | 1.000 | 0.823 | 1.000 | 0.898 | 0.958 | 0.910 | 1.000 | 0.902 | 0.717 | 1.000 | 1.000 | 0.773 | 0.732 |
| 423 | 0.726 | 0.943 | 0.688 | 0.957 | 0.853 | 1.000 | 0.820 | 1.000 | 0.898 | 0.958 | 0.910 | 1.000 | 0.902 | 0.718 | 1.000 | 1.000 | 0.773 | 0.732 |
| 424 | 0.725 | 0.943 | 0.690 | 0.957 | 0.853 | 1.000 | 0.820 | 1.000 | 0.898 | 0.958 | 0.910 | 1.000 | 0.902 | 0.718 | 1.000 | 1.000 | 0.773 | 0.732 |
| 425 | 0.726 | 0.943 | 0.698 | 0.957 | 0.853 | 1.000 | 0.814 | 1.000 | 0.898 | 0.958 | 0.910 | 1.000 | 0.911 | 0.718 | 1.000 | 1.000 | 0.773 | 0.732 |
| 426 | 0.727 | 0.943 | 0.698 | 0.957 | 0.853 | 1.000 | 0.814 | 1.000 | 0.898 | 0.958 | 0.910 | 1.000 | 0.911 | 0.718 | 1.000 | 1.000 | 0.774 | 0.733 |
| 427 | 0.727 | 0.943 | 0.696 | 0.957 | 0.853 | 1.000 | 0.814 | 1.000 | 0.898 | 0.958 | 0.910 | 1.000 | 0.920 | 0.719 | 1.000 | 1.000 | 0.774 | 0.733 |
| 428 | 0.728 | 0.943 | 0.698 | 0.957 | 0.853 | 1.000 | 0.814 | 1.000 | 0.898 | 0.958 | 0.907 | 1.000 | 0.911 | 0.717 | 1.000 | 1.000 | 0.773 | 0.732 |

| | | | | | | | | | | | | | | | | | | |
|---|---|---|---|---|---|---|---|---|---|---|---|---|---|---|---|---|---|---|
| 429 | 0.727 | 0.943 | 0.698 | 0.957 | 0.858 | 1.000 | 0.815 | 1.000 | 0.898 | 0.958 | 0.907 | 1.000 | 0.911 | 0.717 | 1.000 | 1.000 | 0.774 | 0.732 |
| 430 | 0.726 | 0.943 | 0.696 | 0.957 | 0.858 | 1.000 | 0.815 | 1.000 | 0.898 | 0.958 | 0.907 | 1.000 | 0.911 | 0.717 | 1.000 | 1.000 | 0.773 | 0.732 |
| 431 | 0.727 | 0.943 | 0.700 | 0.957 | 0.858 | 1.000 | 0.815 | 1.000 | 0.898 | 0.958 | 0.907 | 1.000 | 0.911 | 0.718 | 1.000 | 1.000 | 0.774 | 0.733 |
| 432 | 0.726 | 0.943 | 0.698 | 0.957 | 0.858 | 1.000 | 0.815 | 1.000 | 0.898 | 0.958 | 0.907 | 1.000 | 0.911 | 0.718 | 1.000 | 1.000 | 0.774 | 0.732 |
| 433 | 0.728 | 0.943 | 0.696 | 0.957 | 0.858 | 1.000 | 0.815 | 1.000 | 0.898 | 0.958 | 0.907 | 1.000 | 0.902 | 0.716 | 1.000 | 1.000 | 0.773 | 0.732 |
| 434 | 0.728 | 0.943 | 0.694 | 0.957 | 0.858 | 1.000 | 0.815 | 1.000 | 0.898 | 0.958 | 0.904 | 1.000 | 0.902 | 0.714 | 1.000 | 1.000 | 0.772 | 0.731 |
| 435 | 0.731 | 0.943 | 0.690 | 0.957 | 0.863 | 1.000 | 0.818 | 1.000 | 0.898 | 0.958 | 0.901 | 1.000 | 0.893 | 0.715 | 1.000 | 1.000 | 0.773 | 0.732 |
| 436 | 0.728 | 0.943 | 0.700 | 0.971 | 0.863 | 1.000 | 0.827 | 1.000 | 0.898 | 0.958 | 0.901 | 1.000 | 0.884 | 0.718 | 1.000 | 1.000 | 0.775 | 0.735 |
| 437 | 0.725 | 0.943 | 0.696 | 0.971 | 0.863 | 1.000 | 0.829 | 1.000 | 0.898 | 0.958 | 0.901 | 1.000 | 0.902 | 0.714 | 1.000 | 1.000 | 0.774 | 0.733 |
| 438 | 0.724 | 0.943 | 0.698 | 0.971 | 0.863 | 1.000 | 0.829 | 1.000 | 0.898 | 0.958 | 0.904 | 1.000 | 0.902 | 0.716 | 1.000 | 1.000 | 0.774 | 0.734 |
| 439 | 0.722 | 0.943 | 0.700 | 0.971 | 0.863 | 1.000 | 0.831 | 1.000 | 0.898 | 0.958 | 0.901 | 1.000 | 0.902 | 0.716 | 1.000 | 1.000 | 0.774 | 0.733 |
| 440 | 0.722 | 0.943 | 0.702 | 0.971 | 0.863 | 1.000 | 0.826 | 1.000 | 0.898 | 0.958 | 0.904 | 1.000 | 0.902 | 0.717 | 1.000 | 1.000 | 0.774 | 0.733 |
| 441 | 0.723 | 0.943 | 0.706 | 0.971 | 0.863 | 1.000 | 0.831 | 1.000 | 0.898 | 0.958 | 0.904 | 1.000 | 0.902 | 0.718 | 1.000 | 1.000 | 0.775 | 0.735 |
| 442 | 0.722 | 0.943 | 0.708 | 0.971 | 0.863 | 1.000 | 0.831 | 1.000 | 0.898 | 0.958 | 0.904 | 1.000 | 0.902 | 0.718 | 1.000 | 1.000 | 0.776 | 0.735 |
| 443 | 0.720 | 0.943 | 0.711 | 0.957 | 0.863 | 1.000 | 0.832 | 1.000 | 0.898 | 0.958 | 0.904 | 1.000 | 0.902 | 0.718 | 1.000 | 1.000 | 0.775 | 0.735 |
| 444 | 0.722 | 0.943 | 0.706 | 0.957 | 0.863 | 1.000 | 0.831 | 1.000 | 0.898 | 0.958 | 0.904 | 1.000 | 0.902 | 0.718 | 1.000 | 1.000 | 0.775 | 0.734 |
| 445 | 0.721 | 0.943 | 0.706 | 0.957 | 0.863 | 1.000 | 0.832 | 1.000 | 0.898 | 0.958 | 0.904 | 1.000 | 0.902 | 0.717 | 1.000 | 1.000 | 0.775 | 0.734 |
| 446 | 0.721 | 0.943 | 0.704 | 0.957 | 0.863 | 1.000 | 0.831 | 1.000 | 0.898 | 0.958 | 0.901 | 1.000 | 0.902 | 0.716 | 1.000 | 1.000 | 0.774 | 0.733 |
| 447 | 0.722 | 0.943 | 0.702 | 0.957 | 0.863 | 1.000 | 0.832 | 1.000 | 0.898 | 0.958 | 0.901 | 1.000 | 0.902 | 0.716 | 1.000 | 1.000 | 0.774 | 0.733 |
| 448 | 0.721 | 0.943 | 0.696 | 0.957 | 0.868 | 1.000 | 0.826 | 1.000 | 0.898 | 0.958 | 0.901 | 1.000 | 0.893 | 0.716 | 1.000 | 1.000 | 0.772 | 0.731 |
| 449 | 0.722 | 0.943 | 0.696 | 0.957 | 0.874 | 1.000 | 0.824 | 1.000 | 0.898 | 0.958 | 0.901 | 1.000 | 0.893 | 0.717 | 1.000 | 1.000 | 0.773 | 0.732 |
| 450 | 0.724 | 0.943 | 0.690 | 0.957 | 0.874 | 1.000 | 0.824 | 1.000 | 0.898 | 0.958 | 0.901 | 1.000 | 0.893 | 0.715 | 1.000 | 1.000 | 0.773 | 0.732 |
| 451 | 0.727 | 0.943 | 0.704 | 0.957 | 0.874 | 1.000 | 0.820 | 1.000 | 0.898 | 0.958 | 0.901 | 1.000 | 0.893 | 0.718 | 1.000 | 1.000 | 0.775 | 0.734 |
| 452 | 0.726 | 0.943 | 0.704 | 0.957 | 0.868 | 1.000 | 0.818 | 1.000 | 0.898 | 0.958 | 0.904 | 1.000 | 0.893 | 0.717 | 1.000 | 1.000 | 0.774 | 0.733 |
| 453 | 0.728 | 0.943 | 0.706 | 0.957 | 0.874 | 1.000 | 0.818 | 1.000 | 0.898 | 0.958 | 0.904 | 1.000 | 0.911 | 0.716 | 1.000 | 1.000 | 0.775 | 0.735 |
| 454 | 0.727 | 0.943 | 0.702 | 0.957 | 0.879 | 1.000 | 0.826 | 1.000 | 0.898 | 0.979 | 0.904 | 1.000 | 0.893 | 0.721 | 1.000 | 1.000 | 0.777 | 0.737 |
| 455 | 0.728 | 0.943 | 0.708 | 0.957 | 0.879 | 1.000 | 0.826 | 1.000 | 0.898 | 0.979 | 0.901 | 1.000 | 0.884 | 0.721 | 1.000 | 1.000 | 0.777 | 0.737 |
| 456 | 0.728 | 0.943 | 0.706 | 0.957 | 0.868 | 1.000 | 0.823 | 1.000 | 0.898 | 0.979 | 0.901 | 1.000 | 0.875 | 0.721 | 1.000 | 1.000 | 0.776 | 0.736 |
| 457 | 0.730 | 0.943 | 0.702 | 0.957 | 0.868 | 1.000 | 0.827 | 1.000 | 0.898 | 0.979 | 0.901 | 1.000 | 0.893 | 0.723 | 1.000 | 1.000 | 0.778 | 0.738 |
| 458 | 0.728 | 0.943 | 0.702 | 0.957 | 0.868 | 1.000 | 0.824 | 1.000 | 0.898 | 0.979 | 0.901 | 1.000 | 0.893 | 0.721 | 1.000 | 1.000 | 0.777 | 0.736 |
| 459 | 0.728 | 0.943 | 0.702 | 0.957 | 0.868 | 1.000 | 0.824 | 1.000 | 0.898 | 0.979 | 0.899 | 1.000 | 0.893 | 0.720 | 1.000 | 1.000 | 0.776 | 0.735 |
| 460 | 0.728 | 0.943 | 0.709 | 0.957 | 0.874 | 1.000 | 0.826 | 1.000 | 0.898 | 0.979 | 0.899 | 1.000 | 0.884 | 0.721 | 1.000 | 1.000 | 0.777 | 0.737 |
| 461 | 0.727 | 0.943 | 0.709 | 0.957 | 0.874 | 1.000 | 0.826 | 1.000 | 0.898 | 0.979 | 0.899 | 1.000 | 0.884 | 0.721 | 1.000 | 1.000 | 0.777 | 0.737 |
| 462 | 0.726 | 0.943 | 0.709 | 0.957 | 0.874 | 1.000 | 0.827 | 1.000 | 0.898 | 0.979 | 0.899 | 1.000 | 0.902 | 0.718 | 1.000 | 1.000 | 0.777 | 0.737 |
| 463 | 0.724 | 0.943 | 0.706 | 0.957 | 0.874 | 1.000 | 0.824 | 1.000 | 0.898 | 0.979 | 0.899 | 1.000 | 0.902 | 0.720 | 1.000 | 1.000 | 0.776 | 0.735 |
| 464 | 0.725 | 0.943 | 0.706 | 0.957 | 0.868 | 1.000 | 0.826 | 1.000 | 0.898 | 0.979 | 0.899 | 1.000 | 0.902 | 0.718 | 1.000 | 1.000 | 0.775 | 0.735 |
| 465 | 0.727 | 0.943 | 0.711 | 0.971 | 0.868 | 1.000 | 0.829 | 1.000 | 0.898 | 0.979 | 0.899 | 1.000 | 0.893 | 0.721 | 1.000 | 1.000 | 0.778 | 0.738 |
| 466 | 0.727 | 0.943 | 0.702 | 0.971 | 0.879 | 1.000 | 0.824 | 1.000 | 0.908 | 0.979 | 0.899 | 1.000 | 0.902 | 0.720 | 1.000 | 1.000 | 0.777 | 0.736 |
| 467 | 0.725 | 0.943 | 0.704 | 0.971 | 0.879 | 1.000 | 0.824 | 1.000 | 0.908 | 0.979 | 0.899 | 1.000 | 0.902 | 0.721 | 1.000 | 1.000 | 0.776 | 0.736 |

| | | | | | | | | | | | | | | | | | | |
|---|---|---|---|---|---|---|---|---|---|---|---|---|---|---|---|---|---|---|
| 468 | 0.725 | 0.943 | 0.704 | 0.971 | 0.879 | 1.000 | 0.824 | 1.000 | 0.908 | 0.979 | 0.899 | 1.000 | 0.902 | 0.719 | 1.000 | 1.000 | 0.776 | 0.736 |
| 469 | 0.726 | 0.943 | 0.700 | 0.971 | 0.874 | 1.000 | 0.827 | 1.000 | 0.908 | 0.979 | 0.901 | 1.000 | 0.893 | 0.718 | 1.000 | 1.000 | 0.776 | 0.735 |
| 470 | 0.725 | 0.943 | 0.700 | 0.971 | 0.868 | 1.000 | 0.827 | 1.000 | 0.908 | 0.979 | 0.904 | 1.000 | 0.902 | 0.718 | 1.000 | 1.000 | 0.776 | 0.735 |
| 471 | 0.725 | 0.943 | 0.700 | 0.971 | 0.868 | 1.000 | 0.827 | 1.000 | 0.908 | 0.979 | 0.901 | 1.000 | 0.893 | 0.717 | 1.000 | 1.000 | 0.775 | 0.735 |
| 472 | 0.725 | 0.943 | 0.700 | 0.971 | 0.868 | 1.000 | 0.827 | 1.000 | 0.908 | 0.979 | 0.901 | 1.000 | 0.893 | 0.718 | 1.000 | 1.000 | 0.775 | 0.735 |
| 473 | 0.721 | 0.943 | 0.694 | 0.971 | 0.874 | 1.000 | 0.827 | 1.000 | 0.908 | 0.979 | 0.901 | 1.000 | 0.902 | 0.720 | 1.000 | 1.000 | 0.774 | 0.734 |
| 474 | 0.721 | 0.943 | 0.700 | 0.971 | 0.879 | 1.000 | 0.827 | 1.000 | 0.908 | 0.979 | 0.901 | 1.000 | 0.902 | 0.720 | 1.000 | 1.000 | 0.775 | 0.735 |
| 475 | 0.722 | 0.943 | 0.698 | 0.971 | 0.879 | 1.000 | 0.826 | 1.000 | 0.908 | 0.979 | 0.901 | 1.000 | 0.902 | 0.721 | 1.000 | 1.000 | 0.775 | 0.735 |
| 476 | 0.724 | 0.943 | 0.696 | 0.971 | 0.879 | 1.000 | 0.826 | 1.000 | 0.908 | 0.979 | 0.901 | 1.000 | 0.902 | 0.721 | 1.000 | 1.000 | 0.776 | 0.736 |
| 477 | 0.728 | 0.943 | 0.704 | 0.971 | 0.884 | 1.000 | 0.821 | 1.000 | 0.908 | 0.979 | 0.901 | 1.000 | 0.902 | 0.722 | 1.000 | 1.000 | 0.778 | 0.737 |
| 478 | 0.726 | 0.943 | 0.706 | 0.971 | 0.879 | 1.000 | 0.823 | 1.000 | 0.908 | 0.979 | 0.901 | 1.000 | 0.902 | 0.724 | 1.000 | 1.000 | 0.778 | 0.738 |
| 479 | 0.726 | 0.943 | 0.706 | 0.971 | 0.879 | 1.000 | 0.823 | 1.000 | 0.908 | 0.979 | 0.901 | 1.000 | 0.902 | 0.723 | 1.000 | 1.000 | 0.777 | 0.737 |
| 480 | 0.726 | 0.943 | 0.704 | 0.971 | 0.884 | 1.000 | 0.827 | 1.000 | 0.908 | 0.979 | 0.901 | 1.000 | 0.902 | 0.725 | 1.000 | 1.000 | 0.779 | 0.739 |
| 481 | 0.726 | 0.943 | 0.702 | 0.971 | 0.884 | 1.000 | 0.829 | 1.000 | 0.908 | 0.979 | 0.901 | 1.000 | 0.902 | 0.726 | 1.000 | 1.000 | 0.779 | 0.739 |
| 482 | 0.728 | 0.943 | 0.698 | 0.971 | 0.884 | 1.000 | 0.827 | 1.000 | 0.908 | 0.979 | 0.904 | 1.000 | 0.902 | 0.726 | 1.000 | 1.000 | 0.779 | 0.739 |
| 483 | 0.728 | 0.943 | 0.702 | 0.971 | 0.884 | 1.000 | 0.831 | 1.000 | 0.908 | 0.979 | 0.901 | 1.000 | 0.902 | 0.722 | 1.000 | 1.000 | 0.779 | 0.739 |
| 484 | 0.730 | 0.943 | 0.708 | 0.971 | 0.889 | 1.000 | 0.827 | 1.000 | 0.908 | 0.979 | 0.901 | 1.000 | 0.902 | 0.725 | 1.000 | 1.000 | 0.780 | 0.741 |
| 485 | 0.731 | 0.943 | 0.711 | 0.971 | 0.889 | 1.000 | 0.829 | 1.000 | 0.908 | 0.979 | 0.899 | 1.000 | 0.902 | 0.723 | 1.000 | 1.000 | 0.781 | 0.741 |
| 486 | 0.735 | 0.943 | 0.709 | 0.971 | 0.889 | 1.000 | 0.831 | 1.000 | 0.908 | 0.979 | 0.899 | 1.000 | 0.902 | 0.722 | 1.000 | 1.000 | 0.782 | 0.742 |
| 487 | 0.732 | 0.943 | 0.704 | 0.971 | 0.889 | 1.000 | 0.831 | 1.000 | 0.898 | 0.979 | 0.901 | 1.000 | 0.902 | 0.723 | 1.000 | 1.000 | 0.780 | 0.740 |
| 488 | 0.730 | 0.943 | 0.708 | 0.971 | 0.889 | 1.000 | 0.831 | 1.000 | 0.898 | 0.979 | 0.901 | 1.000 | 0.902 | 0.724 | 1.000 | 1.000 | 0.780 | 0.740 |
| 489 | 0.731 | 0.943 | 0.704 | 0.971 | 0.889 | 1.000 | 0.831 | 1.000 | 0.898 | 0.979 | 0.901 | 1.000 | 0.902 | 0.724 | 1.000 | 1.000 | 0.780 | 0.740 |
| 490 | 0.731 | 0.943 | 0.706 | 0.971 | 0.889 | 1.000 | 0.831 | 1.000 | 0.898 | 0.979 | 0.901 | 1.000 | 0.902 | 0.724 | 1.000 | 1.000 | 0.780 | 0.741 |
| 491 | 0.730 | 0.943 | 0.704 | 0.971 | 0.889 | 1.000 | 0.832 | 1.000 | 0.898 | 0.979 | 0.901 | 1.000 | 0.902 | 0.724 | 1.000 | 1.000 | 0.780 | 0.740 |
| 492 | 0.732 | 0.943 | 0.706 | 0.971 | 0.889 | 1.000 | 0.832 | 1.000 | 0.898 | 0.979 | 0.901 | 1.000 | 0.902 | 0.722 | 1.000 | 1.000 | 0.780 | 0.741 |
| 493 | 0.731 | 0.943 | 0.708 | 0.971 | 0.895 | 1.000 | 0.829 | 1.000 | 0.898 | 0.979 | 0.899 | 1.000 | 0.902 | 0.721 | 1.000 | 1.000 | 0.780 | 0.740 |
| 494 | 0.730 | 0.943 | 0.709 | 0.971 | 0.895 | 1.000 | 0.831 | 1.000 | 0.898 | 0.979 | 0.899 | 1.000 | 0.902 | 0.721 | 1.000 | 1.000 | 0.780 | 0.740 |
| 495 | 0.731 | 0.943 | 0.709 | 0.971 | 0.889 | 1.000 | 0.831 | 1.000 | 0.898 | 0.979 | 0.899 | 1.000 | 0.893 | 0.724 | 1.000 | 1.000 | 0.780 | 0.741 |
| 496 | 0.732 | 0.943 | 0.711 | 0.971 | 0.895 | 1.000 | 0.826 | 1.000 | 0.898 | 0.979 | 0.904 | 1.000 | 0.893 | 0.723 | 1.000 | 1.000 | 0.781 | 0.741 |
| 497 | 0.731 | 0.943 | 0.713 | 0.971 | 0.895 | 1.000 | 0.829 | 1.000 | 0.898 | 0.979 | 0.904 | 1.000 | 0.893 | 0.721 | 1.000 | 1.000 | 0.781 | 0.741 |
| 498 | 0.733 | 0.971 | 0.719 | 0.971 | 0.895 | 1.000 | 0.829 | 1.000 | 0.898 | 0.979 | 0.904 | 1.000 | 0.893 | 0.723 | 1.000 | 1.000 | 0.782 | 0.743 |
| 499 | 0.730 | 0.971 | 0.729 | 0.971 | 0.895 | 1.000 | 0.834 | 1.000 | 0.898 | 0.979 | 0.907 | 1.000 | 0.902 | 0.727 | 1.000 | 1.000 | 0.784 | 0.745 |
| 500 | 0.729 | 0.971 | 0.729 | 0.971 | 0.895 | 1.000 | 0.831 | 1.000 | 0.898 | 0.979 | 0.904 | 1.000 | 0.893 | 0.728 | 1.000 | 1.000 | 0.784 | 0.744 |
| 501 | 0.729 | 0.971 | 0.733 | 0.971 | 0.895 | 1.000 | 0.829 | 1.000 | 0.898 | 0.979 | 0.901 | 1.000 | 0.893 | 0.728 | 1.000 | 1.000 | 0.784 | 0.744 |
| 502 | 0.730 | 0.971 | 0.731 | 0.971 | 0.895 | 1.000 | 0.831 | 1.000 | 0.898 | 0.979 | 0.901 | 1.000 | 0.893 | 0.729 | 1.000 | 1.000 | 0.784 | 0.745 |
| 503 | 0.729 | 0.971 | 0.729 | 0.971 | 0.895 | 1.000 | 0.832 | 1.000 | 0.898 | 0.979 | 0.901 | 1.000 | 0.893 | 0.729 | 1.000 | 1.000 | 0.784 | 0.745 |
| 504 | 0.729 | 0.971 | 0.727 | 0.986 | 0.900 | 1.000 | 0.834 | 1.000 | 0.898 | 0.979 | 0.901 | 1.000 | 0.893 | 0.728 | 1.000 | 1.000 | 0.784 | 0.745 |
| 505 | 0.728 | 0.971 | 0.721 | 0.986 | 0.905 | 1.000 | 0.835 | 1.000 | 0.898 | 0.979 | 0.901 | 1.000 | 0.893 | 0.726 | 1.000 | 1.000 | 0.783 | 0.744 |
| 506 | 0.728 | 0.971 | 0.721 | 0.986 | 0.911 | 1.000 | 0.835 | 1.000 | 0.898 | 0.979 | 0.901 | 1.000 | 0.893 | 0.729 | 1.000 | 1.000 | 0.784 | 0.745 |

| | | | | | | | | | | | | | | | | | | |
|---|---|---|---|---|---|---|---|---|---|---|---|---|---|---|---|---|---|---|
| 507 | 0.728 | 0.971 | 0.721 | 0.986 | 0.905 | 1.000 | 0.835 | 1.000 | 0.898 | 0.979 | 0.901 | 1.000 | 0.893 | 0.728 | 1.000 | 1.000 | 0.784 | 0.744 |
| 508 | 0.728 | 0.971 | 0.719 | 0.986 | 0.911 | 1.000 | 0.835 | 1.000 | 0.898 | 0.979 | 0.901 | 1.000 | 0.884 | 0.728 | 1.000 | 1.000 | 0.783 | 0.744 |
| 509 | 0.728 | 0.971 | 0.719 | 0.986 | 0.905 | 1.000 | 0.835 | 1.000 | 0.898 | 0.979 | 0.901 | 1.000 | 0.884 | 0.728 | 1.000 | 1.000 | 0.783 | 0.744 |
| 510 | 0.726 | 0.971 | 0.719 | 0.986 | 0.900 | 1.000 | 0.835 | 1.000 | 0.898 | 0.979 | 0.904 | 1.000 | 0.884 | 0.727 | 1.000 | 1.000 | 0.782 | 0.743 |
| 511 | 0.728 | 0.971 | 0.715 | 0.986 | 0.900 | 1.000 | 0.834 | 1.000 | 0.898 | 0.979 | 0.904 | 1.000 | 0.884 | 0.727 | 1.000 | 1.000 | 0.782 | 0.743 |
| 512 | 0.729 | 0.971 | 0.717 | 0.986 | 0.895 | 1.000 | 0.834 | 1.000 | 0.898 | 0.979 | 0.904 | 1.000 | 0.884 | 0.728 | 1.000 | 1.000 | 0.783 | 0.743 |
| 513 | 0.728 | 0.971 | 0.717 | 0.986 | 0.895 | 1.000 | 0.837 | 1.000 | 0.898 | 0.979 | 0.904 | 1.000 | 0.884 | 0.728 | 1.000 | 1.000 | 0.783 | 0.743 |
| 514 | 0.728 | 0.971 | 0.717 | 0.986 | 0.895 | 1.000 | 0.838 | 1.000 | 0.898 | 0.979 | 0.904 | 1.000 | 0.884 | 0.728 | 1.000 | 1.000 | 0.783 | 0.744 |
| 515 | 0.729 | 0.971 | 0.721 | 0.986 | 0.895 | 1.000 | 0.838 | 1.000 | 0.908 | 0.979 | 0.904 | 1.000 | 0.884 | 0.728 | 1.000 | 1.000 | 0.784 | 0.745 |
| 516 | 0.728 | 0.971 | 0.717 | 0.986 | 0.895 | 1.000 | 0.838 | 1.000 | 0.908 | 0.979 | 0.904 | 1.000 | 0.884 | 0.728 | 1.000 | 1.000 | 0.784 | 0.744 |
| 517 | 0.730 | 0.971 | 0.713 | 0.986 | 0.895 | 1.000 | 0.840 | 1.000 | 0.908 | 0.979 | 0.904 | 1.000 | 0.884 | 0.729 | 1.000 | 1.000 | 0.784 | 0.744 |
| 518 | 0.728 | 0.971 | 0.715 | 0.986 | 0.895 | 1.000 | 0.840 | 1.000 | 0.908 | 0.979 | 0.904 | 1.000 | 0.884 | 0.731 | 1.000 | 1.000 | 0.784 | 0.744 |
| 519 | 0.727 | 0.971 | 0.721 | 0.986 | 0.895 | 1.000 | 0.840 | 1.000 | 0.898 | 0.979 | 0.904 | 1.000 | 0.875 | 0.730 | 1.000 | 1.000 | 0.784 | 0.744 |
| 520 | 0.728 | 0.971 | 0.721 | 0.986 | 0.889 | 1.000 | 0.840 | 1.000 | 0.908 | 0.979 | 0.904 | 1.000 | 0.875 | 0.730 | 1.000 | 1.000 | 0.784 | 0.745 |
| 521 | 0.728 | 0.971 | 0.719 | 0.986 | 0.889 | 1.000 | 0.840 | 1.000 | 0.908 | 0.979 | 0.904 | 1.000 | 0.875 | 0.731 | 1.000 | 1.000 | 0.784 | 0.745 |
| 522 | 0.726 | 0.971 | 0.709 | 0.986 | 0.889 | 1.000 | 0.841 | 1.000 | 0.898 | 0.979 | 0.904 | 1.000 | 0.884 | 0.733 | 1.000 | 1.000 | 0.783 | 0.744 |
| 523 | 0.727 | 0.971 | 0.713 | 0.986 | 0.889 | 1.000 | 0.840 | 1.000 | 0.908 | 0.979 | 0.904 | 1.000 | 0.875 | 0.733 | 1.000 | 1.000 | 0.784 | 0.744 |
| 524 | 0.726 | 0.971 | 0.711 | 0.986 | 0.889 | 1.000 | 0.840 | 1.000 | 0.908 | 0.979 | 0.904 | 1.000 | 0.875 | 0.733 | 1.000 | 1.000 | 0.783 | 0.743 |
| 525 | 0.728 | 0.971 | 0.727 | 0.986 | 0.900 | 1.000 | 0.840 | 1.000 | 0.908 | 0.979 | 0.904 | 1.000 | 0.875 | 0.733 | 1.000 | 1.000 | 0.786 | 0.746 |
| 526 | 0.728 | 0.971 | 0.727 | 0.986 | 0.900 | 1.000 | 0.840 | 1.000 | 0.908 | 0.979 | 0.904 | 1.000 | 0.884 | 0.735 | 1.000 | 1.000 | 0.787 | 0.747 |
| 527 | 0.730 | 0.971 | 0.723 | 0.986 | 0.905 | 1.000 | 0.838 | 1.000 | 0.908 | 0.979 | 0.904 | 1.000 | 0.884 | 0.735 | 1.000 | 1.000 | 0.787 | 0.747 |
| 528 | 0.726 | 0.971 | 0.721 | 0.986 | 0.889 | 1.000 | 0.841 | 1.000 | 0.908 | 0.979 | 0.907 | 1.000 | 0.884 | 0.735 | 1.000 | 1.000 | 0.785 | 0.746 |
| 529 | 0.726 | 0.971 | 0.721 | 0.986 | 0.889 | 1.000 | 0.844 | 1.000 | 0.908 | 0.979 | 0.907 | 1.000 | 0.893 | 0.736 | 1.000 | 1.000 | 0.786 | 0.747 |
| 530 | 0.728 | 0.971 | 0.721 | 0.986 | 0.889 | 1.000 | 0.844 | 1.000 | 0.908 | 0.979 | 0.907 | 1.000 | 0.893 | 0.736 | 1.000 | 1.000 | 0.787 | 0.748 |
| 531 | 0.724 | 0.971 | 0.723 | 0.986 | 0.889 | 1.000 | 0.843 | 1.000 | 0.918 | 0.979 | 0.907 | 1.000 | 0.902 | 0.732 | 1.000 | 1.000 | 0.785 | 0.746 |
| 532 | 0.727 | 0.971 | 0.721 | 0.986 | 0.889 | 1.000 | 0.843 | 1.000 | 0.918 | 0.979 | 0.907 | 1.000 | 0.911 | 0.732 | 1.000 | 1.000 | 0.786 | 0.747 |
| 533 | 0.725 | 0.971 | 0.721 | 0.986 | 0.889 | 1.000 | 0.843 | 1.000 | 0.918 | 0.979 | 0.907 | 1.000 | 0.920 | 0.733 | 1.000 | 1.000 | 0.786 | 0.747 |
| 534 | 0.727 | 0.971 | 0.723 | 0.986 | 0.884 | 1.000 | 0.843 | 1.000 | 0.918 | 0.979 | 0.904 | 1.000 | 0.920 | 0.735 | 1.000 | 1.000 | 0.787 | 0.747 |
| 535 | 0.727 | 0.971 | 0.725 | 0.986 | 0.884 | 1.000 | 0.843 | 1.000 | 0.918 | 0.979 | 0.904 | 1.000 | 0.911 | 0.735 | 1.000 | 1.000 | 0.787 | 0.747 |
| 536 | 0.728 | 1.000 | 0.721 | 0.986 | 0.884 | 1.000 | 0.843 | 1.000 | 0.918 | 0.979 | 0.901 | 1.000 | 0.911 | 0.735 | 1.000 | 1.000 | 0.787 | 0.748 |
| 537 | 0.730 | 1.000 | 0.721 | 0.986 | 0.884 | 1.000 | 0.838 | 1.000 | 0.929 | 0.979 | 0.901 | 1.000 | 0.911 | 0.735 | 1.000 | 1.000 | 0.787 | 0.748 |
| 538 | 0.726 | 1.000 | 0.725 | 0.986 | 0.879 | 1.000 | 0.840 | 1.000 | 0.929 | 0.979 | 0.901 | 1.000 | 0.911 | 0.735 | 1.000 | 1.000 | 0.786 | 0.747 |
| 539 | 0.730 | 1.000 | 0.727 | 0.986 | 0.879 | 1.000 | 0.840 | 1.000 | 0.929 | 0.979 | 0.904 | 1.000 | 0.911 | 0.735 | 1.000 | 1.000 | 0.787 | 0.748 |
| 540 | 0.730 | 1.000 | 0.721 | 0.986 | 0.884 | 1.000 | 0.838 | 1.000 | 0.929 | 0.979 | 0.904 | 1.000 | 0.911 | 0.734 | 1.000 | 1.000 | 0.787 | 0.747 |
| 541 | 0.730 | 1.000 | 0.725 | 0.986 | 0.889 | 1.000 | 0.838 | 1.000 | 0.929 | 0.979 | 0.904 | 1.000 | 0.911 | 0.734 | 1.000 | 1.000 | 0.787 | 0.748 |
| 542 | 0.730 | 1.000 | 0.721 | 0.986 | 0.889 | 1.000 | 0.835 | 1.000 | 0.929 | 0.979 | 0.904 | 1.000 | 0.920 | 0.735 | 1.000 | 1.000 | 0.787 | 0.748 |
| 543 | 0.728 | 0.971 | 0.721 | 0.986 | 0.889 | 1.000 | 0.838 | 1.000 | 0.929 | 0.979 | 0.907 | 1.000 | 0.920 | 0.736 | 1.000 | 1.000 | 0.787 | 0.748 |
| 544 | 0.727 | 0.971 | 0.721 | 0.986 | 0.889 | 1.000 | 0.838 | 1.000 | 0.929 | 0.979 | 0.907 | 1.000 | 0.920 | 0.735 | 1.000 | 1.000 | 0.787 | 0.748 |
| 545 | 0.727 | 1.000 | 0.725 | 0.986 | 0.889 | 1.000 | 0.838 | 1.000 | 0.929 | 0.979 | 0.907 | 1.000 | 0.920 | 0.734 | 1.000 | 1.000 | 0.787 | 0.748 |

| | | | | | | | | | | | | | | | | | | |
|---|---|---|---|---|---|---|---|---|---|---|---|---|---|---|---|---|---|---|
| 546 | 0.726 | 1.000 | 0.727 | 0.986 | 0.889 | 1.000 | 0.840 | 1.000 | 0.929 | 0.979 | 0.907 | 1.000 | 0.920 | 0.735 | 1.000 | 1.000 | 0.787 | 0.748 |
| 547 | 0.726 | 1.000 | 0.725 | 0.986 | 0.889 | 1.000 | 0.843 | 1.000 | 0.929 | 0.979 | 0.907 | 1.000 | 0.920 | 0.735 | 1.000 | 1.000 | 0.787 | 0.749 |
| 548 | 0.727 | 1.000 | 0.727 | 0.986 | 0.889 | 1.000 | 0.841 | 1.000 | 0.939 | 0.979 | 0.899 | 1.000 | 0.911 | 0.733 | 1.000 | 1.000 | 0.787 | 0.748 |
| 549 | 0.726 | 1.000 | 0.727 | 0.986 | 0.895 | 1.000 | 0.841 | 1.000 | 0.939 | 0.979 | 0.899 | 1.000 | 0.920 | 0.729 | 1.000 | 1.000 | 0.786 | 0.747 |
| 550 | 0.727 | 1.000 | 0.727 | 0.986 | 0.895 | 1.000 | 0.841 | 1.000 | 0.939 | 0.979 | 0.896 | 1.000 | 0.920 | 0.729 | 1.000 | 1.000 | 0.786 | 0.747 |
| 551 | 0.725 | 1.000 | 0.725 | 0.986 | 0.895 | 1.000 | 0.841 | 1.000 | 0.939 | 0.979 | 0.896 | 1.000 | 0.920 | 0.729 | 1.000 | 1.000 | 0.785 | 0.746 |
| 552 | 0.726 | 1.000 | 0.727 | 0.986 | 0.895 | 1.000 | 0.841 | 1.000 | 0.929 | 0.979 | 0.899 | 1.000 | 0.920 | 0.729 | 1.000 | 1.000 | 0.785 | 0.746 |
| 553 | 0.726 | 1.000 | 0.719 | 0.986 | 0.895 | 1.000 | 0.843 | 1.000 | 0.949 | 0.979 | 0.899 | 1.000 | 0.920 | 0.728 | 1.000 | 1.000 | 0.785 | 0.746 |
| 554 | 0.726 | 1.000 | 0.723 | 0.986 | 0.895 | 1.000 | 0.843 | 1.000 | 0.949 | 0.979 | 0.899 | 1.000 | 0.920 | 0.728 | 1.000 | 1.000 | 0.786 | 0.747 |
| 555 | 0.727 | 1.000 | 0.725 | 0.986 | 0.900 | 1.000 | 0.841 | 1.000 | 0.939 | 0.979 | 0.899 | 1.000 | 0.920 | 0.729 | 1.000 | 1.000 | 0.786 | 0.747 |
| 556 | 0.727 | 1.000 | 0.725 | 0.986 | 0.900 | 1.000 | 0.840 | 1.000 | 0.949 | 0.979 | 0.896 | 1.000 | 0.920 | 0.729 | 1.000 | 1.000 | 0.786 | 0.747 |
| 557 | 0.728 | 1.000 | 0.725 | 0.986 | 0.900 | 1.000 | 0.840 | 1.000 | 0.939 | 0.979 | 0.896 | 1.000 | 0.920 | 0.729 | 1.000 | 1.000 | 0.786 | 0.747 |
| 558 | 0.728 | 1.000 | 0.721 | 0.986 | 0.900 | 1.000 | 0.838 | 1.000 | 0.939 | 0.979 | 0.896 | 1.000 | 0.920 | 0.728 | 1.000 | 1.000 | 0.785 | 0.746 |
| 559 | 0.729 | 1.000 | 0.727 | 0.986 | 0.900 | 1.000 | 0.835 | 1.000 | 0.939 | 0.979 | 0.896 | 1.000 | 0.920 | 0.728 | 1.000 | 1.000 | 0.786 | 0.747 |
| 560 | 0.726 | 1.000 | 0.723 | 0.986 | 0.900 | 1.000 | 0.837 | 1.000 | 0.939 | 0.979 | 0.899 | 1.000 | 0.920 | 0.731 | 1.000 | 1.000 | 0.785 | 0.746 |
| 561 | 0.724 | 1.000 | 0.723 | 0.986 | 0.900 | 1.000 | 0.835 | 1.000 | 0.939 | 0.979 | 0.899 | 1.000 | 0.920 | 0.732 | 1.000 | 1.000 | 0.785 | 0.745 |
| 562 | 0.724 | 1.000 | 0.723 | 0.986 | 0.900 | 1.000 | 0.835 | 1.000 | 0.939 | 0.979 | 0.899 | 1.000 | 0.920 | 0.732 | 1.000 | 1.000 | 0.785 | 0.745 |
| 563 | 0.724 | 1.000 | 0.723 | 0.986 | 0.900 | 1.000 | 0.835 | 1.000 | 0.939 | 0.979 | 0.899 | 1.000 | 0.920 | 0.733 | 1.000 | 1.000 | 0.785 | 0.746 |
| 564 | 0.724 | 1.000 | 0.721 | 0.986 | 0.900 | 1.000 | 0.835 | 1.000 | 0.939 | 0.979 | 0.899 | 1.000 | 0.920 | 0.732 | 1.000 | 1.000 | 0.785 | 0.745 |
| 565 | 0.728 | 1.000 | 0.719 | 0.986 | 0.895 | 1.000 | 0.838 | 1.000 | 0.949 | 0.979 | 0.899 | 1.000 | 0.920 | 0.739 | 1.000 | 1.000 | 0.788 | 0.749 |
| 566 | 0.728 | 1.000 | 0.719 | 0.986 | 0.895 | 1.000 | 0.840 | 1.000 | 0.949 | 0.979 | 0.899 | 1.000 | 0.920 | 0.739 | 1.000 | 1.000 | 0.788 | 0.749 |
| 567 | 0.729 | 1.000 | 0.719 | 0.986 | 0.895 | 1.000 | 0.840 | 1.000 | 0.959 | 0.979 | 0.899 | 1.000 | 0.920 | 0.738 | 1.000 | 1.000 | 0.788 | 0.749 |
| 568 | 0.728 | 1.000 | 0.721 | 0.986 | 0.895 | 1.000 | 0.840 | 1.000 | 0.959 | 0.979 | 0.899 | 1.000 | 0.920 | 0.738 | 1.000 | 1.000 | 0.788 | 0.749 |
| 569 | 0.728 | 1.000 | 0.727 | 0.986 | 0.895 | 1.000 | 0.838 | 1.000 | 0.959 | 0.979 | 0.899 | 1.000 | 0.920 | 0.735 | 1.000 | 1.000 | 0.788 | 0.749 |
| 570 | 0.730 | 1.000 | 0.727 | 0.986 | 0.895 | 1.000 | 0.838 | 1.000 | 0.959 | 0.979 | 0.899 | 1.000 | 0.920 | 0.738 | 1.000 | 1.000 | 0.789 | 0.750 |
| 571 | 0.728 | 1.000 | 0.725 | 0.986 | 0.895 | 1.000 | 0.841 | 1.000 | 0.959 | 0.979 | 0.899 | 1.000 | 0.920 | 0.737 | 1.000 | 1.000 | 0.789 | 0.750 |
| 572 | 0.731 | 1.000 | 0.725 | 0.986 | 0.895 | 1.000 | 0.841 | 1.000 | 0.959 | 0.979 | 0.899 | 1.000 | 0.920 | 0.736 | 1.000 | 1.000 | 0.789 | 0.751 |
| 573 | 0.730 | 1.000 | 0.727 | 0.986 | 0.895 | 1.000 | 0.843 | 1.000 | 0.959 | 0.979 | 0.899 | 1.000 | 0.920 | 0.738 | 1.000 | 1.000 | 0.790 | 0.751 |
| 574 | 0.731 | 1.000 | 0.723 | 0.986 | 0.895 | 1.000 | 0.834 | 1.000 | 0.959 | 0.979 | 0.896 | 1.000 | 0.920 | 0.737 | 1.000 | 1.000 | 0.788 | 0.749 |
| 575 | 0.730 | 1.000 | 0.725 | 0.986 | 0.895 | 1.000 | 0.834 | 1.000 | 0.959 | 0.979 | 0.899 | 1.000 | 0.920 | 0.735 | 1.000 | 1.000 | 0.788 | 0.748 |
| 576 | 0.731 | 1.000 | 0.731 | 0.986 | 0.895 | 1.000 | 0.832 | 1.000 | 0.959 | 0.979 | 0.901 | 1.000 | 0.920 | 0.737 | 1.000 | 1.000 | 0.789 | 0.750 |
| 577 | 0.729 | 1.000 | 0.733 | 0.986 | 0.895 | 1.000 | 0.832 | 1.000 | 0.959 | 0.979 | 0.896 | 1.000 | 0.920 | 0.736 | 1.000 | 1.000 | 0.788 | 0.749 |
| 578 | 0.731 | 1.000 | 0.733 | 0.986 | 0.895 | 1.000 | 0.832 | 1.000 | 0.959 | 0.979 | 0.896 | 1.000 | 0.920 | 0.736 | 1.000 | 1.000 | 0.789 | 0.750 |
| 579 | 0.730 | 1.000 | 0.733 | 0.986 | 0.895 | 1.000 | 0.832 | 1.000 | 0.959 | 0.979 | 0.896 | 1.000 | 0.920 | 0.735 | 1.000 | 1.000 | 0.788 | 0.749 |
| 580 | 0.727 | 1.000 | 0.729 | 0.986 | 0.895 | 1.000 | 0.832 | 1.000 | 0.959 | 0.979 | 0.896 | 1.000 | 0.920 | 0.737 | 1.000 | 1.000 | 0.787 | 0.748 |
| 581 | 0.728 | 1.000 | 0.731 | 0.986 | 0.895 | 1.000 | 0.831 | 1.000 | 0.959 | 0.979 | 0.896 | 1.000 | 0.920 | 0.735 | 1.000 | 1.000 | 0.787 | 0.748 |
| 582 | 0.729 | 1.000 | 0.733 | 0.986 | 0.895 | 1.000 | 0.834 | 1.000 | 0.959 | 0.979 | 0.896 | 1.000 | 0.920 | 0.735 | 1.000 | 1.000 | 0.788 | 0.749 |
| 583 | 0.729 | 1.000 | 0.735 | 0.986 | 0.895 | 1.000 | 0.832 | 1.000 | 0.959 | 0.979 | 0.896 | 1.000 | 0.920 | 0.735 | 1.000 | 1.000 | 0.788 | 0.749 |
| 584 | 0.728 | 1.000 | 0.721 | 0.986 | 0.889 | 1.000 | 0.832 | 1.000 | 0.949 | 0.979 | 0.904 | 1.000 | 0.920 | 0.736 | 1.000 | 1.000 | 0.787 | 0.747 |

| | | | | | | | | | | | | | | | | | | |
|---|---|---|---|---|---|---|---|---|---|---|---|---|---|---|---|---|---|---|
| 585 | 0.727 | 1.000 | 0.715 | 0.986 | 0.889 | 1.000 | 0.834 | 1.000 | 0.949 | 0.979 | 0.904 | 1.000 | 0.920 | 0.736 | 1.000 | 1.000 | 0.786 | 0.746 |
| 586 | 0.727 | 1.000 | 0.723 | 0.986 | 0.889 | 1.000 | 0.832 | 1.000 | 0.959 | 0.979 | 0.907 | 1.000 | 0.920 | 0.738 | 1.000 | 1.000 | 0.787 | 0.748 |
| 587 | 0.727 | 1.000 | 0.725 | 0.986 | 0.889 | 1.000 | 0.832 | 1.000 | 0.959 | 0.979 | 0.901 | 1.000 | 0.920 | 0.738 | 1.000 | 1.000 | 0.787 | 0.748 |
| 588 | 0.726 | 1.000 | 0.729 | 0.986 | 0.889 | 1.000 | 0.832 | 1.000 | 0.959 | 0.979 | 0.901 | 1.000 | 0.920 | 0.738 | 1.000 | 1.000 | 0.787 | 0.748 |
| 589 | 0.727 | 1.000 | 0.725 | 0.986 | 0.889 | 1.000 | 0.832 | 1.000 | 0.959 | 0.979 | 0.904 | 1.000 | 0.920 | 0.739 | 1.000 | 1.000 | 0.788 | 0.748 |
| 590 | 0.726 | 1.000 | 0.725 | 0.986 | 0.889 | 1.000 | 0.832 | 1.000 | 0.959 | 0.979 | 0.904 | 1.000 | 0.920 | 0.740 | 1.000 | 1.000 | 0.788 | 0.749 |
| 591 | 0.728 | 1.000 | 0.719 | 0.986 | 0.889 | 1.000 | 0.834 | 1.000 | 0.959 | 0.979 | 0.904 | 1.000 | 0.920 | 0.741 | 1.000 | 1.000 | 0.788 | 0.749 |
| 592 | 0.728 | 1.000 | 0.731 | 0.986 | 0.889 | 1.000 | 0.834 | 1.000 | 0.959 | 0.979 | 0.907 | 1.000 | 0.920 | 0.742 | 1.000 | 1.000 | 0.790 | 0.751 |
| 593 | 0.730 | 1.000 | 0.733 | 0.986 | 0.889 | 1.000 | 0.834 | 1.000 | 0.959 | 0.979 | 0.907 | 1.000 | 0.920 | 0.742 | 1.000 | 1.000 | 0.791 | 0.752 |
| 594 | 0.730 | 1.000 | 0.733 | 0.986 | 0.889 | 1.000 | 0.837 | 1.000 | 0.959 | 0.979 | 0.907 | 1.000 | 0.920 | 0.741 | 1.000 | 1.000 | 0.791 | 0.752 |
| 595 | 0.730 | 1.000 | 0.729 | 0.986 | 0.889 | 1.000 | 0.832 | 1.000 | 0.949 | 0.979 | 0.907 | 1.000 | 0.938 | 0.742 | 1.000 | 1.000 | 0.790 | 0.751 |
| 596 | 0.730 | 1.000 | 0.729 | 0.986 | 0.889 | 1.000 | 0.840 | 1.000 | 0.959 | 0.979 | 0.910 | 1.000 | 0.946 | 0.742 | 1.000 | 1.000 | 0.792 | 0.753 |
| 597 | 0.730 | 1.000 | 0.729 | 0.986 | 0.889 | 1.000 | 0.840 | 1.000 | 0.949 | 0.979 | 0.910 | 1.000 | 0.946 | 0.742 | 1.000 | 1.000 | 0.792 | 0.753 |
| 598 | 0.730 | 1.000 | 0.731 | 0.986 | 0.889 | 1.000 | 0.840 | 1.000 | 0.949 | 0.979 | 0.910 | 1.000 | 0.946 | 0.743 | 1.000 | 1.000 | 0.792 | 0.754 |
| 599 | 0.731 | 1.000 | 0.729 | 0.986 | 0.889 | 1.000 | 0.841 | 1.000 | 0.949 | 0.979 | 0.910 | 1.000 | 0.946 | 0.742 | 1.000 | 1.000 | 0.792 | 0.754 |
| 600 | 0.732 | 1.000 | 0.729 | 0.986 | 0.889 | 1.000 | 0.841 | 1.000 | 0.949 | 0.979 | 0.907 | 1.000 | 0.946 | 0.742 | 1.000 | 1.000 | 0.792 | 0.754 |
| 601 | 0.732 | 1.000 | 0.731 | 0.986 | 0.889 | 1.000 | 0.841 | 1.000 | 0.949 | 0.979 | 0.907 | 1.000 | 0.946 | 0.742 | 1.000 | 1.000 | 0.792 | 0.754 |
| 602 | 0.734 | 1.000 | 0.723 | 0.986 | 0.889 | 1.000 | 0.841 | 1.000 | 0.949 | 0.979 | 0.907 | 1.000 | 0.946 | 0.743 | 1.000 | 1.000 | 0.793 | 0.754 |
| 603 | 0.733 | 1.000 | 0.721 | 0.986 | 0.889 | 1.000 | 0.841 | 1.000 | 0.949 | 0.979 | 0.907 | 1.000 | 0.946 | 0.742 | 1.000 | 1.000 | 0.792 | 0.753 |
| 604 | 0.733 | 1.000 | 0.721 | 0.986 | 0.889 | 1.000 | 0.841 | 1.000 | 0.949 | 0.979 | 0.907 | 1.000 | 0.946 | 0.742 | 1.000 | 1.000 | 0.792 | 0.753 |
| 605 | 0.732 | 1.000 | 0.721 | 0.986 | 0.889 | 1.000 | 0.841 | 1.000 | 0.949 | 0.979 | 0.907 | 1.000 | 0.946 | 0.742 | 1.000 | 1.000 | 0.792 | 0.753 |
| 606 | 0.728 | 1.000 | 0.729 | 0.986 | 0.884 | 1.000 | 0.837 | 1.000 | 0.959 | 0.979 | 0.901 | 1.000 | 0.946 | 0.746 | 1.000 | 1.000 | 0.791 | 0.752 |
| 607 | 0.728 | 1.000 | 0.733 | 0.986 | 0.884 | 1.000 | 0.835 | 1.000 | 0.959 | 0.979 | 0.904 | 1.000 | 0.946 | 0.746 | 1.000 | 1.000 | 0.791 | 0.753 |
| 608 | 0.728 | 1.000 | 0.735 | 0.986 | 0.889 | 1.000 | 0.837 | 1.000 | 0.959 | 0.979 | 0.901 | 1.000 | 0.946 | 0.746 | 1.000 | 1.000 | 0.792 | 0.754 |
| 609 | 0.730 | 1.000 | 0.739 | 0.986 | 0.889 | 1.000 | 0.835 | 1.000 | 0.959 | 0.979 | 0.904 | 1.000 | 0.946 | 0.746 | 1.000 | 1.000 | 0.793 | 0.754 |
| 610 | 0.730 | 1.000 | 0.739 | 0.986 | 0.889 | 1.000 | 0.835 | 1.000 | 0.959 | 0.979 | 0.907 | 1.000 | 0.946 | 0.743 | 1.000 | 1.000 | 0.792 | 0.754 |
| 611 | 0.736 | 1.000 | 0.733 | 0.986 | 0.900 | 1.000 | 0.835 | 1.000 | 0.959 | 0.979 | 0.904 | 1.000 | 0.946 | 0.742 | 1.000 | 1.000 | 0.793 | 0.755 |
| 612 | 0.736 | 1.000 | 0.733 | 0.986 | 0.900 | 1.000 | 0.835 | 1.000 | 0.959 | 0.979 | 0.904 | 1.000 | 0.946 | 0.741 | 1.000 | 1.000 | 0.793 | 0.755 |
| 613 | 0.732 | 1.000 | 0.735 | 0.986 | 0.900 | 1.000 | 0.837 | 1.000 | 0.959 | 0.979 | 0.904 | 1.000 | 0.946 | 0.741 | 1.000 | 1.000 | 0.792 | 0.754 |
| 614 | 0.730 | 1.000 | 0.735 | 0.986 | 0.900 | 1.000 | 0.837 | 1.000 | 0.959 | 0.979 | 0.904 | 1.000 | 0.946 | 0.740 | 1.000 | 1.000 | 0.792 | 0.753 |
| 615 | 0.731 | 1.000 | 0.735 | 0.986 | 0.900 | 1.000 | 0.837 | 1.000 | 0.959 | 0.979 | 0.904 | 1.000 | 0.946 | 0.740 | 1.000 | 1.000 | 0.792 | 0.753 |
| 616 | 0.734 | 1.000 | 0.735 | 0.986 | 0.900 | 1.000 | 0.838 | 1.000 | 0.959 | 0.979 | 0.904 | 1.000 | 0.946 | 0.741 | 1.000 | 1.000 | 0.793 | 0.755 |
| 617 | 0.733 | 1.000 | 0.737 | 0.986 | 0.900 | 1.000 | 0.838 | 1.000 | 0.959 | 0.979 | 0.901 | 1.000 | 0.946 | 0.740 | 1.000 | 1.000 | 0.793 | 0.754 |
| 618 | 0.733 | 1.000 | 0.737 | 0.986 | 0.900 | 1.000 | 0.838 | 1.000 | 0.959 | 0.979 | 0.901 | 1.000 | 0.938 | 0.742 | 1.000 | 1.000 | 0.793 | 0.755 |
| 619 | 0.734 | 1.000 | 0.737 | 0.986 | 0.900 | 1.000 | 0.840 | 1.000 | 0.959 | 0.979 | 0.899 | 1.000 | 0.946 | 0.743 | 1.000 | 1.000 | 0.794 | 0.756 |
| 620 | 0.737 | 1.000 | 0.739 | 0.986 | 0.900 | 1.000 | 0.841 | 1.000 | 0.959 | 0.979 | 0.901 | 1.000 | 0.946 | 0.742 | 1.000 | 1.000 | 0.795 | 0.757 |
| 621 | 0.736 | 1.000 | 0.741 | 0.986 | 0.900 | 1.000 | 0.840 | 1.000 | 0.959 | 0.979 | 0.901 | 1.000 | 0.946 | 0.745 | 1.000 | 1.000 | 0.795 | 0.757 |
| 622 | 0.735 | 1.000 | 0.741 | 0.986 | 0.900 | 1.000 | 0.840 | 1.000 | 0.959 | 0.979 | 0.910 | 1.000 | 0.938 | 0.746 | 1.000 | 1.000 | 0.796 | 0.758 |
| 623 | 0.732 | 1.000 | 0.739 | 0.986 | 0.905 | 1.000 | 0.837 | 1.000 | 0.959 | 0.979 | 0.913 | 1.000 | 0.946 | 0.750 | 1.000 | 1.000 | 0.796 | 0.758 |

| | | | | | | | | | | | | | | | | | | |
|---|---|---|---|---|---|---|---|---|---|---|---|---|---|---|---|---|---|---|
| 624 | 0.733 | 1.000 | 0.739 | 0.986 | 0.905 | 1.000 | 0.837 | 1.000 | 0.959 | 0.979 | 0.916 | 1.000 | 0.946 | 0.752 | 1.000 | 1.000 | 0.797 | 0.759 |
| 625 | 0.734 | 1.000 | 0.739 | 0.986 | 0.905 | 1.000 | 0.837 | 1.000 | 0.959 | 0.979 | 0.913 | 1.000 | 0.938 | 0.752 | 1.000 | 1.000 | 0.797 | 0.759 |
| 626 | 0.733 | 1.000 | 0.737 | 0.986 | 0.900 | 1.000 | 0.838 | 1.000 | 0.959 | 0.979 | 0.910 | 1.000 | 0.938 | 0.751 | 1.000 | 1.000 | 0.796 | 0.758 |
| 627 | 0.734 | 1.000 | 0.741 | 0.986 | 0.900 | 1.000 | 0.838 | 1.000 | 0.959 | 0.979 | 0.910 | 1.000 | 0.938 | 0.752 | 1.000 | 1.000 | 0.797 | 0.759 |
| 628 | 0.736 | 1.000 | 0.743 | 0.986 | 0.900 | 1.000 | 0.837 | 1.000 | 0.959 | 0.979 | 0.913 | 1.000 | 0.938 | 0.752 | 1.000 | 1.000 | 0.797 | 0.760 |
| 629 | 0.736 | 1.000 | 0.745 | 0.986 | 0.905 | 1.000 | 0.841 | 1.000 | 0.959 | 0.979 | 0.916 | 1.000 | 0.938 | 0.750 | 1.000 | 1.000 | 0.798 | 0.761 |
| 630 | 0.736 | 1.000 | 0.749 | 0.986 | 0.905 | 1.000 | 0.843 | 1.000 | 0.959 | 0.979 | 0.913 | 1.000 | 0.938 | 0.751 | 1.000 | 1.000 | 0.799 | 0.761 |
| 631 | 0.735 | 1.000 | 0.753 | 0.986 | 0.900 | 1.000 | 0.844 | 1.000 | 0.959 | 1.000 | 0.907 | 1.000 | 0.938 | 0.753 | 1.000 | 1.000 | 0.799 | 0.762 |
| 632 | 0.736 | 1.000 | 0.753 | 0.986 | 0.900 | 1.000 | 0.843 | 1.000 | 0.959 | 1.000 | 0.910 | 1.000 | 0.938 | 0.753 | 1.000 | 1.000 | 0.800 | 0.763 |
| 633 | 0.737 | 1.000 | 0.755 | 0.986 | 0.905 | 1.000 | 0.844 | 1.000 | 0.959 | 1.000 | 0.907 | 1.000 | 0.938 | 0.753 | 1.000 | 1.000 | 0.800 | 0.763 |
| 634 | 0.735 | 1.000 | 0.751 | 0.986 | 0.905 | 1.000 | 0.846 | 1.000 | 0.959 | 1.000 | 0.910 | 1.000 | 0.938 | 0.756 | 1.000 | 1.000 | 0.801 | 0.764 |
| 635 | 0.735 | 1.000 | 0.755 | 0.986 | 0.905 | 1.000 | 0.844 | 1.000 | 0.959 | 1.000 | 0.919 | 1.000 | 0.938 | 0.757 | 1.000 | 1.000 | 0.802 | 0.765 |
| 636 | 0.736 | 1.000 | 0.755 | 0.986 | 0.905 | 1.000 | 0.843 | 1.000 | 0.959 | 1.000 | 0.919 | 1.000 | 0.938 | 0.758 | 1.000 | 1.000 | 0.802 | 0.765 |
| 637 | 0.736 | 1.000 | 0.751 | 0.986 | 0.911 | 1.000 | 0.843 | 1.000 | 0.959 | 1.000 | 0.919 | 1.000 | 0.938 | 0.758 | 1.000 | 1.000 | 0.802 | 0.765 |
| 638 | 0.734 | 1.000 | 0.755 | 0.986 | 0.911 | 1.000 | 0.843 | 1.000 | 0.959 | 1.000 | 0.913 | 1.000 | 0.938 | 0.758 | 1.000 | 1.000 | 0.801 | 0.764 |
| 639 | 0.734 | 1.000 | 0.751 | 0.986 | 0.916 | 1.000 | 0.843 | 1.000 | 0.959 | 1.000 | 0.913 | 1.000 | 0.938 | 0.756 | 1.000 | 1.000 | 0.800 | 0.763 |
| 640 | 0.735 | 1.000 | 0.749 | 0.986 | 0.916 | 1.000 | 0.844 | 1.000 | 0.959 | 1.000 | 0.913 | 1.000 | 0.938 | 0.757 | 1.000 | 1.000 | 0.801 | 0.764 |
| 641 | 0.736 | 1.000 | 0.749 | 0.986 | 0.916 | 1.000 | 0.844 | 1.000 | 0.959 | 1.000 | 0.913 | 1.000 | 0.929 | 0.756 | 1.000 | 1.000 | 0.801 | 0.764 |
| 642 | 0.734 | 1.000 | 0.749 | 0.986 | 0.916 | 1.000 | 0.844 | 1.000 | 0.959 | 1.000 | 0.913 | 1.000 | 0.938 | 0.754 | 1.000 | 1.000 | 0.800 | 0.763 |
| 643 | 0.734 | 1.000 | 0.749 | 0.986 | 0.916 | 1.000 | 0.844 | 1.000 | 0.959 | 1.000 | 0.913 | 1.000 | 0.938 | 0.753 | 1.000 | 1.000 | 0.800 | 0.763 |
| 644 | 0.737 | 1.000 | 0.749 | 0.986 | 0.921 | 1.000 | 0.844 | 1.000 | 0.959 | 1.000 | 0.913 | 1.000 | 0.938 | 0.753 | 1.000 | 1.000 | 0.801 | 0.764 |
| 645 | 0.739 | 1.000 | 0.751 | 0.986 | 0.921 | 1.000 | 0.838 | 1.000 | 0.959 | 1.000 | 0.913 | 1.000 | 0.938 | 0.754 | 1.000 | 1.000 | 0.801 | 0.764 |
| 646 | 0.739 | 1.000 | 0.751 | 0.986 | 0.921 | 1.000 | 0.838 | 1.000 | 0.959 | 1.000 | 0.913 | 1.000 | 0.938 | 0.751 | 1.000 | 1.000 | 0.800 | 0.763 |
| 647 | 0.739 | 1.000 | 0.751 | 0.986 | 0.921 | 1.000 | 0.838 | 1.000 | 0.959 | 1.000 | 0.916 | 1.000 | 0.938 | 0.751 | 1.000 | 1.000 | 0.800 | 0.763 |
| 648 | 0.741 | 1.000 | 0.755 | 0.986 | 0.926 | 1.000 | 0.840 | 1.000 | 0.959 | 1.000 | 0.916 | 1.000 | 0.938 | 0.752 | 1.000 | 1.000 | 0.802 | 0.765 |
| 649 | 0.740 | 1.000 | 0.755 | 0.986 | 0.926 | 1.000 | 0.840 | 1.000 | 0.959 | 1.000 | 0.916 | 1.000 | 0.938 | 0.752 | 1.000 | 1.000 | 0.802 | 0.765 |
| 650 | 0.741 | 1.000 | 0.755 | 0.986 | 0.926 | 1.000 | 0.840 | 1.000 | 0.959 | 1.000 | 0.916 | 1.000 | 0.938 | 0.753 | 1.000 | 1.000 | 0.802 | 0.765 |
| 651 | 0.739 | 1.000 | 0.755 | 0.986 | 0.926 | 1.000 | 0.840 | 1.000 | 0.959 | 1.000 | 0.919 | 1.000 | 0.938 | 0.752 | 1.000 | 1.000 | 0.802 | 0.765 |
| 652 | 0.738 | 1.000 | 0.755 | 0.986 | 0.926 | 1.000 | 0.840 | 1.000 | 0.959 | 1.000 | 0.919 | 1.000 | 0.938 | 0.753 | 1.000 | 1.000 | 0.802 | 0.765 |
| 653 | 0.738 | 1.000 | 0.753 | 0.986 | 0.926 | 1.000 | 0.840 | 1.000 | 0.959 | 1.000 | 0.919 | 1.000 | 0.938 | 0.749 | 1.000 | 1.000 | 0.801 | 0.764 |
| 654 | 0.739 | 1.000 | 0.755 | 0.986 | 0.926 | 1.000 | 0.841 | 1.000 | 0.959 | 1.000 | 0.919 | 1.000 | 0.938 | 0.749 | 1.000 | 1.000 | 0.801 | 0.764 |
| 655 | 0.740 | 1.000 | 0.749 | 0.986 | 0.926 | 1.000 | 0.841 | 1.000 | 0.969 | 1.000 | 0.919 | 1.000 | 0.938 | 0.752 | 1.000 | 1.000 | 0.802 | 0.765 |
| 656 | 0.738 | 1.000 | 0.747 | 0.986 | 0.926 | 1.000 | 0.843 | 1.000 | 0.969 | 1.000 | 0.919 | 1.000 | 0.929 | 0.753 | 1.000 | 1.000 | 0.801 | 0.764 |
| 657 | 0.741 | 1.000 | 0.747 | 0.986 | 0.926 | 1.000 | 0.843 | 1.000 | 0.959 | 1.000 | 0.919 | 1.000 | 0.938 | 0.754 | 1.000 | 1.000 | 0.802 | 0.765 |
| 658 | 0.740 | 1.000 | 0.753 | 0.986 | 0.926 | 1.000 | 0.843 | 1.000 | 0.959 | 1.000 | 0.916 | 1.000 | 0.938 | 0.752 | 1.000 | 1.000 | 0.802 | 0.765 |
| 659 | 0.739 | 1.000 | 0.753 | 0.986 | 0.926 | 1.000 | 0.841 | 1.000 | 0.959 | 1.000 | 0.916 | 1.000 | 0.938 | 0.751 | 1.000 | 1.000 | 0.801 | 0.764 |
| 660 | 0.739 | 1.000 | 0.753 | 0.986 | 0.926 | 1.000 | 0.844 | 1.000 | 0.959 | 1.000 | 0.919 | 1.000 | 0.938 | 0.751 | 1.000 | 1.000 | 0.802 | 0.765 |
| 661 | 0.740 | 1.000 | 0.753 | 0.986 | 0.926 | 1.000 | 0.844 | 1.000 | 0.959 | 1.000 | 0.919 | 1.000 | 0.938 | 0.752 | 1.000 | 1.000 | 0.802 | 0.766 |
| 662 | 0.739 | 1.000 | 0.753 | 0.986 | 0.926 | 1.000 | 0.844 | 1.000 | 0.959 | 1.000 | 0.916 | 1.000 | 0.938 | 0.751 | 1.000 | 1.000 | 0.802 | 0.765 |

| | | | | | | | | | | | | | | | | | | |
|---|---|---|---|---|---|---|---|---|---|---|---|---|---|---|---|---|---|---|
| 663 | 0.742 | 1.000 | 0.749 | 0.986 | 0.926 | 1.000 | 0.846 | 1.000 | 0.959 | 1.000 | 0.922 | 1.000 | 0.938 | 0.752 | 1.000 | 1.000 | 0.803 | 0.766 |
| 664 | 0.740 | 1.000 | 0.747 | 0.986 | 0.926 | 1.000 | 0.846 | 1.000 | 0.959 | 1.000 | 0.919 | 1.000 | 0.938 | 0.753 | 1.000 | 1.000 | 0.802 | 0.765 |
| 665 | 0.742 | 1.000 | 0.751 | 0.986 | 0.916 | 1.000 | 0.844 | 1.000 | 0.959 | 1.000 | 0.922 | 1.000 | 0.938 | 0.753 | 1.000 | 1.000 | 0.803 | 0.766 |
| 666 | 0.742 | 1.000 | 0.751 | 0.986 | 0.916 | 1.000 | 0.843 | 1.000 | 0.959 | 1.000 | 0.922 | 1.000 | 0.938 | 0.753 | 1.000 | 1.000 | 0.803 | 0.766 |
| 667 | 0.743 | 1.000 | 0.751 | 0.986 | 0.916 | 1.000 | 0.843 | 1.000 | 0.959 | 1.000 | 0.922 | 1.000 | 0.938 | 0.753 | 1.000 | 1.000 | 0.803 | 0.766 |
| 668 | 0.743 | 1.000 | 0.751 | 0.986 | 0.921 | 1.000 | 0.843 | 1.000 | 0.959 | 1.000 | 0.922 | 1.000 | 0.938 | 0.753 | 1.000 | 1.000 | 0.803 | 0.766 |
| 669 | 0.743 | 1.000 | 0.753 | 0.986 | 0.921 | 1.000 | 0.843 | 1.000 | 0.959 | 1.000 | 0.922 | 1.000 | 0.938 | 0.753 | 1.000 | 1.000 | 0.803 | 0.767 |
| 670 | 0.748 | 1.000 | 0.749 | 0.986 | 0.921 | 1.000 | 0.846 | 1.000 | 0.969 | 1.000 | 0.922 | 1.000 | 0.938 | 0.753 | 1.000 | 1.000 | 0.805 | 0.768 |
| 671 | 0.748 | 1.000 | 0.749 | 0.986 | 0.921 | 1.000 | 0.846 | 1.000 | 0.969 | 1.000 | 0.922 | 1.000 | 0.938 | 0.753 | 1.000 | 1.000 | 0.805 | 0.768 |
| 672 | 0.747 | 1.000 | 0.749 | 0.986 | 0.921 | 1.000 | 0.846 | 1.000 | 0.969 | 1.000 | 0.919 | 1.000 | 0.938 | 0.752 | 1.000 | 1.000 | 0.804 | 0.768 |
| 673 | 0.746 | 1.000 | 0.747 | 0.986 | 0.921 | 1.000 | 0.846 | 1.000 | 0.969 | 1.000 | 0.919 | 1.000 | 0.938 | 0.752 | 1.000 | 1.000 | 0.804 | 0.767 |
| 674 | 0.746 | 1.000 | 0.745 | 0.986 | 0.921 | 1.000 | 0.847 | 1.000 | 0.969 | 1.000 | 0.919 | 1.000 | 0.938 | 0.750 | 1.000 | 1.000 | 0.803 | 0.767 |
| 675 | 0.747 | 1.000 | 0.745 | 0.986 | 0.921 | 1.000 | 0.847 | 1.000 | 0.969 | 1.000 | 0.919 | 1.000 | 0.938 | 0.750 | 1.000 | 1.000 | 0.804 | 0.767 |
| 676 | 0.747 | 1.000 | 0.743 | 0.986 | 0.921 | 1.000 | 0.844 | 1.000 | 0.969 | 1.000 | 0.919 | 1.000 | 0.938 | 0.752 | 1.000 | 1.000 | 0.803 | 0.767 |
| 677 | 0.746 | 1.000 | 0.743 | 0.986 | 0.921 | 1.000 | 0.844 | 1.000 | 0.969 | 1.000 | 0.919 | 1.000 | 0.938 | 0.753 | 1.000 | 1.000 | 0.803 | 0.767 |
| 678 | 0.751 | 1.000 | 0.745 | 0.986 | 0.921 | 1.000 | 0.844 | 1.000 | 0.969 | 1.000 | 0.919 | 1.000 | 0.938 | 0.752 | 1.000 | 1.000 | 0.805 | 0.768 |
| 679 | 0.752 | 1.000 | 0.741 | 0.986 | 0.921 | 1.000 | 0.849 | 1.000 | 0.969 | 1.000 | 0.919 | 1.000 | 0.938 | 0.754 | 1.000 | 1.000 | 0.806 | 0.769 |
| 680 | 0.753 | 1.000 | 0.741 | 0.986 | 0.921 | 1.000 | 0.849 | 1.000 | 0.969 | 1.000 | 0.919 | 1.000 | 0.938 | 0.755 | 1.000 | 1.000 | 0.806 | 0.770 |
| 681 | 0.752 | 1.000 | 0.743 | 0.986 | 0.921 | 1.000 | 0.849 | 1.000 | 0.969 | 1.000 | 0.919 | 1.000 | 0.938 | 0.754 | 1.000 | 1.000 | 0.806 | 0.770 |
| 682 | 0.752 | 1.000 | 0.743 | 0.986 | 0.921 | 1.000 | 0.849 | 1.000 | 0.969 | 1.000 | 0.916 | 1.000 | 0.938 | 0.755 | 1.000 | 1.000 | 0.806 | 0.770 |
| 683 | 0.753 | 1.000 | 0.743 | 0.986 | 0.921 | 1.000 | 0.851 | 1.000 | 0.969 | 1.000 | 0.916 | 1.000 | 0.938 | 0.756 | 1.000 | 1.000 | 0.807 | 0.770 |
| 684 | 0.749 | 1.000 | 0.745 | 0.986 | 0.921 | 1.000 | 0.852 | 1.000 | 0.969 | 1.000 | 0.916 | 1.000 | 0.938 | 0.756 | 1.000 | 1.000 | 0.806 | 0.770 |
| 685 | 0.748 | 1.000 | 0.745 | 0.986 | 0.921 | 1.000 | 0.851 | 1.000 | 0.969 | 1.000 | 0.916 | 1.000 | 0.938 | 0.757 | 1.000 | 1.000 | 0.806 | 0.769 |
| 686 | 0.752 | 1.000 | 0.737 | 0.986 | 0.921 | 1.000 | 0.849 | 1.000 | 0.959 | 1.000 | 0.916 | 1.000 | 0.938 | 0.756 | 1.000 | 1.000 | 0.806 | 0.769 |
| 687 | 0.753 | 1.000 | 0.741 | 0.986 | 0.921 | 1.000 | 0.849 | 1.000 | 0.959 | 1.000 | 0.916 | 1.000 | 0.938 | 0.756 | 1.000 | 1.000 | 0.806 | 0.770 |
| 688 | 0.749 | 1.000 | 0.743 | 0.986 | 0.916 | 1.000 | 0.841 | 1.000 | 0.959 | 1.000 | 0.916 | 1.000 | 0.938 | 0.755 | 1.000 | 1.000 | 0.804 | 0.767 |
| 689 | 0.750 | 1.000 | 0.749 | 0.986 | 0.921 | 1.000 | 0.844 | 1.000 | 0.959 | 1.000 | 0.916 | 1.000 | 0.938 | 0.756 | 1.000 | 1.000 | 0.805 | 0.769 |
| 690 | 0.748 | 1.000 | 0.749 | 0.986 | 0.921 | 1.000 | 0.844 | 1.000 | 0.959 | 1.000 | 0.916 | 1.000 | 0.938 | 0.756 | 1.000 | 1.000 | 0.805 | 0.768 |
| 691 | 0.749 | 1.000 | 0.751 | 0.986 | 0.921 | 1.000 | 0.844 | 1.000 | 0.959 | 1.000 | 0.916 | 1.000 | 0.938 | 0.755 | 1.000 | 1.000 | 0.805 | 0.769 |
| 692 | 0.748 | 1.000 | 0.751 | 0.986 | 0.921 | 1.000 | 0.844 | 1.000 | 0.959 | 1.000 | 0.919 | 1.000 | 0.938 | 0.754 | 1.000 | 1.000 | 0.805 | 0.769 |
| 693 | 0.750 | 1.000 | 0.739 | 0.986 | 0.921 | 1.000 | 0.846 | 1.000 | 0.959 | 1.000 | 0.922 | 1.000 | 0.938 | 0.754 | 1.000 | 1.000 | 0.805 | 0.768 |
| 694 | 0.748 | 1.000 | 0.737 | 0.986 | 0.921 | 1.000 | 0.846 | 1.000 | 0.959 | 1.000 | 0.922 | 1.000 | 0.938 | 0.753 | 1.000 | 1.000 | 0.804 | 0.767 |
| 695 | 0.748 | 1.000 | 0.739 | 0.986 | 0.921 | 1.000 | 0.846 | 1.000 | 0.969 | 1.000 | 0.919 | 1.000 | 0.938 | 0.753 | 1.000 | 1.000 | 0.804 | 0.767 |
| 696 | 0.748 | 1.000 | 0.739 | 0.986 | 0.921 | 1.000 | 0.846 | 1.000 | 0.959 | 1.000 | 0.919 | 1.000 | 0.938 | 0.753 | 1.000 | 1.000 | 0.804 | 0.767 |
| 697 | 0.751 | 1.000 | 0.739 | 0.986 | 0.926 | 1.000 | 0.847 | 1.000 | 0.959 | 1.000 | 0.919 | 1.000 | 0.938 | 0.752 | 1.000 | 1.000 | 0.804 | 0.768 |
| 698 | 0.752 | 1.000 | 0.739 | 0.986 | 0.926 | 1.000 | 0.847 | 1.000 | 0.959 | 1.000 | 0.919 | 1.000 | 0.938 | 0.752 | 1.000 | 1.000 | 0.805 | 0.768 |
| 699 | 0.749 | 1.000 | 0.737 | 0.986 | 0.926 | 1.000 | 0.847 | 1.000 | 0.959 | 1.000 | 0.919 | 1.000 | 0.938 | 0.755 | 1.000 | 1.000 | 0.805 | 0.768 |
| 700 | 0.749 | 1.000 | 0.739 | 0.986 | 0.926 | 1.000 | 0.846 | 1.000 | 0.959 | 1.000 | 0.919 | 1.000 | 0.938 | 0.753 | 1.000 | 1.000 | 0.804 | 0.767 |
| 701 | 0.747 | 1.000 | 0.737 | 0.986 | 0.926 | 1.000 | 0.844 | 1.000 | 0.959 | 1.000 | 0.919 | 1.000 | 0.938 | 0.753 | 1.000 | 1.000 | 0.803 | 0.766 |

| | | | | | | | | | | | | | | | | | |
|---|---|---|---|---|---|---|---|---|---|---|---|---|---|---|---|---|---|
| 702 | 0.747 | 1.000 | 0.741 | 0.986 | 0.926 | 1.000 | 0.846 | 1.000 | 0.959 | 1.000 | 0.919 | 1.000 | 0.938 | 0.756 | 1.000 | 1.000 | 0.805 | 0.768 |
| 703 | 0.745 | 1.000 | 0.737 | 0.986 | 0.926 | 1.000 | 0.844 | 1.000 | 0.959 | 1.000 | 0.919 | 1.000 | 0.938 | 0.756 | 1.000 | 1.000 | 0.803 | 0.766 |
| 704 | 0.748 | 1.000 | 0.741 | 0.986 | 0.926 | 1.000 | 0.843 | 1.000 | 0.959 | 1.000 | 0.916 | 1.000 | 0.938 | 0.758 | 1.000 | 1.000 | 0.805 | 0.768 |
| 705 | 0.746 | 1.000 | 0.743 | 0.986 | 0.932 | 1.000 | 0.847 | 1.000 | 0.959 | 1.000 | 0.919 | 1.000 | 0.938 | 0.759 | 1.000 | 1.000 | 0.806 | 0.769 |
| 706 | 0.748 | 1.000 | 0.741 | 0.986 | 0.932 | 1.000 | 0.847 | 1.000 | 0.959 | 1.000 | 0.919 | 1.000 | 0.938 | 0.757 | 1.000 | 1.000 | 0.805 | 0.769 |
| 707 | 0.748 | 1.000 | 0.741 | 0.986 | 0.932 | 1.000 | 0.849 | 1.000 | 0.969 | 1.000 | 0.919 | 1.000 | 0.938 | 0.757 | 1.000 | 1.000 | 0.806 | 0.769 |
| 708 | 0.748 | 1.000 | 0.741 | 0.986 | 0.932 | 1.000 | 0.849 | 1.000 | 0.969 | 1.000 | 0.919 | 1.000 | 0.938 | 0.757 | 1.000 | 1.000 | 0.806 | 0.769 |
| 709 | 0.751 | 1.000 | 0.743 | 0.986 | 0.932 | 1.000 | 0.846 | 1.000 | 0.959 | 1.000 | 0.919 | 1.000 | 0.938 | 0.758 | 1.000 | 1.000 | 0.807 | 0.770 |
| 710 | 0.753 | 1.000 | 0.749 | 0.986 | 0.932 | 1.000 | 0.846 | 1.000 | 0.949 | 1.000 | 0.919 | 1.000 | 0.938 | 0.760 | 1.000 | 1.000 | 0.808 | 0.771 |
| 711 | 0.752 | 1.000 | 0.749 | 0.986 | 0.932 | 1.000 | 0.846 | 1.000 | 0.949 | 1.000 | 0.919 | 1.000 | 0.938 | 0.759 | 1.000 | 1.000 | 0.807 | 0.771 |
| 712 | 0.753 | 1.000 | 0.749 | 0.986 | 0.932 | 1.000 | 0.846 | 1.000 | 0.959 | 1.000 | 0.922 | 1.000 | 0.938 | 0.758 | 1.000 | 1.000 | 0.808 | 0.772 |
| 713 | 0.752 | 1.000 | 0.749 | 0.986 | 0.932 | 1.000 | 0.847 | 1.000 | 0.959 | 1.000 | 0.922 | 1.000 | 0.938 | 0.757 | 1.000 | 1.000 | 0.808 | 0.771 |
| 714 | 0.752 | 1.000 | 0.749 | 0.986 | 0.932 | 1.000 | 0.851 | 1.000 | 0.959 | 1.000 | 0.922 | 1.000 | 0.938 | 0.756 | 1.000 | 1.000 | 0.808 | 0.771 |
| 715 | 0.753 | 1.000 | 0.749 | 0.986 | 0.932 | 1.000 | 0.849 | 1.000 | 0.959 | 1.000 | 0.922 | 1.000 | 0.938 | 0.756 | 1.000 | 1.000 | 0.808 | 0.772 |
| 716 | 0.752 | 1.000 | 0.751 | 0.986 | 0.932 | 1.000 | 0.849 | 1.000 | 0.959 | 1.000 | 0.922 | 1.000 | 0.938 | 0.757 | 1.000 | 1.000 | 0.808 | 0.772 |
| 717 | 0.752 | 1.000 | 0.749 | 0.986 | 0.932 | 1.000 | 0.851 | 1.000 | 0.959 | 1.000 | 0.922 | 1.000 | 0.938 | 0.757 | 1.000 | 1.000 | 0.808 | 0.772 |
| 718 | 0.752 | 1.000 | 0.749 | 0.986 | 0.932 | 1.000 | 0.849 | 1.000 | 0.959 | 1.000 | 0.922 | 1.000 | 0.938 | 0.756 | 1.000 | 1.000 | 0.808 | 0.771 |
| 719 | 0.751 | 1.000 | 0.745 | 0.986 | 0.932 | 1.000 | 0.851 | 1.000 | 0.959 | 1.000 | 0.919 | 1.000 | 0.938 | 0.756 | 1.000 | 1.000 | 0.807 | 0.771 |
| 720 | 0.751 | 1.000 | 0.743 | 0.986 | 0.932 | 1.000 | 0.851 | 1.000 | 0.959 | 1.000 | 0.919 | 1.000 | 0.938 | 0.757 | 1.000 | 1.000 | 0.807 | 0.771 |
| 721 | 0.751 | 1.000 | 0.743 | 0.986 | 0.932 | 1.000 | 0.851 | 1.000 | 0.959 | 1.000 | 0.919 | 1.000 | 0.938 | 0.757 | 1.000 | 1.000 | 0.807 | 0.771 |
| 722 | 0.753 | 1.000 | 0.751 | 0.986 | 0.926 | 1.000 | 0.849 | 1.000 | 0.959 | 1.000 | 0.922 | 1.000 | 0.938 | 0.758 | 1.000 | 1.000 | 0.808 | 0.772 |
| 723 | 0.752 | 1.000 | 0.751 | 0.986 | 0.926 | 1.000 | 0.851 | 1.000 | 0.969 | 1.000 | 0.922 | 1.000 | 0.938 | 0.757 | 1.000 | 1.000 | 0.808 | 0.772 |
| 724 | 0.751 | 1.000 | 0.757 | 0.986 | 0.926 | 1.000 | 0.851 | 1.000 | 0.959 | 1.000 | 0.919 | 1.000 | 0.938 | 0.756 | 1.000 | 1.000 | 0.808 | 0.772 |
| 725 | 0.749 | 1.000 | 0.755 | 0.986 | 0.926 | 1.000 | 0.851 | 1.000 | 0.959 | 1.000 | 0.919 | 1.000 | 0.938 | 0.755 | 1.000 | 1.000 | 0.807 | 0.771 |
| 726 | 0.750 | 1.000 | 0.755 | 0.986 | 0.926 | 1.000 | 0.851 | 1.000 | 0.959 | 1.000 | 0.919 | 1.000 | 0.938 | 0.756 | 1.000 | 1.000 | 0.807 | 0.771 |
| 727 | 0.759 | 1.000 | 0.755 | 0.986 | 0.926 | 1.000 | 0.849 | 1.000 | 0.959 | 1.000 | 0.916 | 1.000 | 0.938 | 0.756 | 1.000 | 1.000 | 0.810 | 0.774 |
| 728 | 0.759 | 1.000 | 0.753 | 0.986 | 0.926 | 1.000 | 0.849 | 1.000 | 0.969 | 1.000 | 0.916 | 1.000 | 0.938 | 0.755 | 1.000 | 1.000 | 0.809 | 0.773 |
| 729 | 0.755 | 1.000 | 0.767 | 0.986 | 0.926 | 1.000 | 0.852 | 1.000 | 0.969 | 1.000 | 0.916 | 1.000 | 0.946 | 0.754 | 1.000 | 1.000 | 0.810 | 0.774 |
| 730 | 0.764 | 1.000 | 0.765 | 1.000 | 0.921 | 1.000 | 0.851 | 1.000 | 0.969 | 1.000 | 0.913 | 1.000 | 0.946 | 0.753 | 1.000 | 1.000 | 0.812 | 0.776 |
| 731 | 0.765 | 1.000 | 0.767 | 1.000 | 0.921 | 1.000 | 0.854 | 1.000 | 0.969 | 1.000 | 0.913 | 1.000 | 0.946 | 0.753 | 1.000 | 1.000 | 0.812 | 0.777 |
| 732 | 0.766 | 1.000 | 0.767 | 1.000 | 0.921 | 1.000 | 0.855 | 1.000 | 0.969 | 1.000 | 0.913 | 1.000 | 0.946 | 0.753 | 1.000 | 1.000 | 0.813 | 0.778 |
| 733 | 0.764 | 1.000 | 0.767 | 1.000 | 0.921 | 1.000 | 0.855 | 1.000 | 0.969 | 1.000 | 0.913 | 1.000 | 0.946 | 0.753 | 1.000 | 1.000 | 0.813 | 0.777 |
| 734 | 0.762 | 1.000 | 0.769 | 1.000 | 0.926 | 1.000 | 0.854 | 1.000 | 0.969 | 1.000 | 0.913 | 1.000 | 0.946 | 0.753 | 1.000 | 1.000 | 0.812 | 0.777 |
| 735 | 0.764 | 1.000 | 0.769 | 1.000 | 0.926 | 1.000 | 0.854 | 1.000 | 0.969 | 1.000 | 0.913 | 1.000 | 0.946 | 0.753 | 1.000 | 1.000 | 0.813 | 0.777 |
| 736 | 0.767 | 1.000 | 0.767 | 1.000 | 0.932 | 1.000 | 0.852 | 1.000 | 0.969 | 1.000 | 0.913 | 1.000 | 0.946 | 0.753 | 1.000 | 1.000 | 0.814 | 0.778 |
| 737 | 0.765 | 1.000 | 0.767 | 1.000 | 0.932 | 1.000 | 0.852 | 1.000 | 0.969 | 1.000 | 0.913 | 1.000 | 0.946 | 0.752 | 1.000 | 1.000 | 0.813 | 0.777 |
| 738 | 0.769 | 1.000 | 0.765 | 1.000 | 0.932 | 1.000 | 0.847 | 1.000 | 0.969 | 1.000 | 0.913 | 1.000 | 0.946 | 0.751 | 1.000 | 1.000 | 0.813 | 0.777 |
| 739 | 0.769 | 1.000 | 0.757 | 1.000 | 0.947 | 1.000 | 0.846 | 1.000 | 0.969 | 1.000 | 0.916 | 1.000 | 0.955 | 0.751 | 1.000 | 1.000 | 0.813 | 0.777 |
| 740 | 0.770 | 1.000 | 0.763 | 1.000 | 0.947 | 1.000 | 0.847 | 1.000 | 0.969 | 1.000 | 0.916 | 1.000 | 0.955 | 0.751 | 1.000 | 1.000 | 0.814 | 0.779 |

| | | | | | | | | | | | | | | | | | | |
|---|---|---|---|---|---|---|---|---|---|---|---|---|---|---|---|---|---|---|
| 741 | 0.768 | 1.000 | 0.761 | 1.000 | 0.947 | 1.000 | 0.844 | 1.000 | 0.969 | 1.000 | 0.916 | 1.000 | 0.955 | 0.750 | 1.000 | 1.000 | 0.812 | 0.777 |
| 742 | 0.768 | 1.000 | 0.763 | 1.000 | 0.947 | 1.000 | 0.843 | 1.000 | 0.969 | 1.000 | 0.916 | 1.000 | 0.955 | 0.751 | 1.000 | 1.000 | 0.813 | 0.777 |
| 743 | 0.768 | 1.000 | 0.761 | 1.000 | 0.947 | 1.000 | 0.841 | 1.000 | 0.969 | 1.000 | 0.916 | 1.000 | 0.955 | 0.750 | 1.000 | 1.000 | 0.812 | 0.776 |
| 744 | 0.766 | 1.000 | 0.759 | 1.000 | 0.947 | 1.000 | 0.843 | 1.000 | 0.969 | 1.000 | 0.916 | 1.000 | 0.955 | 0.752 | 1.000 | 1.000 | 0.812 | 0.776 |
| 745 | 0.769 | 1.000 | 0.761 | 0.986 | 0.947 | 1.000 | 0.844 | 1.000 | 0.969 | 1.000 | 0.913 | 1.000 | 0.964 | 0.751 | 1.000 | 1.000 | 0.813 | 0.777 |
| 746 | 0.769 | 1.000 | 0.759 | 0.986 | 0.947 | 1.000 | 0.843 | 1.000 | 0.969 | 1.000 | 0.913 | 1.000 | 0.964 | 0.750 | 1.000 | 1.000 | 0.812 | 0.777 |
| 747 | 0.766 | 1.000 | 0.763 | 0.986 | 0.947 | 1.000 | 0.847 | 1.000 | 0.969 | 1.000 | 0.913 | 1.000 | 0.964 | 0.751 | 1.000 | 1.000 | 0.812 | 0.777 |
| 748 | 0.769 | 1.000 | 0.757 | 0.986 | 0.942 | 1.000 | 0.846 | 1.000 | 0.969 | 1.000 | 0.913 | 1.000 | 0.964 | 0.749 | 1.000 | 1.000 | 0.812 | 0.776 |
| 749 | 0.769 | 1.000 | 0.753 | 0.986 | 0.942 | 1.000 | 0.844 | 1.000 | 0.969 | 1.000 | 0.913 | 1.000 | 0.964 | 0.749 | 1.000 | 1.000 | 0.811 | 0.775 |
| 750 | 0.770 | 1.000 | 0.753 | 0.986 | 0.942 | 1.000 | 0.846 | 1.000 | 0.969 | 1.000 | 0.913 | 1.000 | 0.964 | 0.749 | 1.000 | 1.000 | 0.812 | 0.776 |
| 751 | 0.771 | 1.000 | 0.755 | 0.986 | 0.942 | 1.000 | 0.844 | 1.000 | 0.969 | 1.000 | 0.913 | 1.000 | 0.964 | 0.749 | 1.000 | 1.000 | 0.812 | 0.776 |
| 752 | 0.771 | 1.000 | 0.749 | 0.986 | 0.937 | 1.000 | 0.844 | 1.000 | 0.969 | 1.000 | 0.916 | 1.000 | 0.964 | 0.750 | 1.000 | 1.000 | 0.812 | 0.776 |
| 753 | 0.773 | 1.000 | 0.747 | 0.986 | 0.942 | 1.000 | 0.844 | 1.000 | 0.969 | 1.000 | 0.916 | 1.000 | 0.964 | 0.750 | 1.000 | 1.000 | 0.812 | 0.776 |
| 754 | 0.770 | 1.000 | 0.761 | 0.986 | 0.947 | 1.000 | 0.847 | 1.000 | 0.969 | 1.000 | 0.916 | 1.000 | 0.964 | 0.750 | 1.000 | 1.000 | 0.813 | 0.778 |
| 755 | 0.771 | 1.000 | 0.763 | 0.986 | 0.947 | 1.000 | 0.847 | 1.000 | 0.969 | 1.000 | 0.913 | 1.000 | 0.964 | 0.749 | 1.000 | 1.000 | 0.814 | 0.778 |
| 756 | 0.771 | 1.000 | 0.757 | 0.986 | 0.947 | 1.000 | 0.847 | 1.000 | 0.969 | 1.000 | 0.916 | 1.000 | 0.964 | 0.748 | 1.000 | 1.000 | 0.813 | 0.777 |
| 757 | 0.771 | 1.000 | 0.761 | 0.986 | 0.947 | 1.000 | 0.847 | 1.000 | 0.969 | 1.000 | 0.916 | 1.000 | 0.964 | 0.748 | 1.000 | 1.000 | 0.813 | 0.778 |
| 758 | 0.771 | 1.000 | 0.763 | 0.986 | 0.947 | 1.000 | 0.846 | 1.000 | 0.969 | 1.000 | 0.916 | 1.000 | 0.964 | 0.749 | 1.000 | 1.000 | 0.813 | 0.778 |
| 759 | 0.771 | 1.000 | 0.763 | 0.986 | 0.947 | 1.000 | 0.847 | 1.000 | 0.969 | 1.000 | 0.916 | 1.000 | 0.964 | 0.749 | 1.000 | 1.000 | 0.814 | 0.778 |
| 760 | 0.773 | 1.000 | 0.765 | 0.986 | 0.947 | 1.000 | 0.851 | 1.000 | 0.969 | 1.000 | 0.916 | 1.000 | 0.946 | 0.753 | 1.000 | 1.000 | 0.815 | 0.780 |
| 761 | 0.773 | 1.000 | 0.765 | 0.986 | 0.947 | 1.000 | 0.847 | 1.000 | 0.969 | 1.000 | 0.916 | 1.000 | 0.946 | 0.755 | 1.000 | 1.000 | 0.816 | 0.780 |
| 762 | 0.775 | 1.000 | 0.765 | 0.986 | 0.947 | 1.000 | 0.847 | 1.000 | 0.969 | 1.000 | 0.916 | 1.000 | 0.946 | 0.754 | 1.000 | 1.000 | 0.816 | 0.780 |
| 763 | 0.772 | 1.000 | 0.763 | 0.986 | 0.947 | 1.000 | 0.846 | 1.000 | 0.969 | 1.000 | 0.916 | 1.000 | 0.946 | 0.752 | 1.000 | 1.000 | 0.814 | 0.778 |
| 764 | 0.770 | 1.000 | 0.763 | 0.986 | 0.947 | 1.000 | 0.847 | 1.000 | 0.969 | 1.000 | 0.916 | 1.000 | 0.946 | 0.753 | 1.000 | 1.000 | 0.814 | 0.778 |
| 765 | 0.770 | 1.000 | 0.765 | 1.000 | 0.947 | 1.000 | 0.847 | 1.000 | 0.969 | 1.000 | 0.916 | 1.000 | 0.946 | 0.753 | 1.000 | 1.000 | 0.814 | 0.779 |
| 766 | 0.773 | 1.000 | 0.765 | 1.000 | 0.947 | 1.000 | 0.847 | 1.000 | 0.969 | 1.000 | 0.916 | 1.000 | 0.946 | 0.753 | 1.000 | 1.000 | 0.815 | 0.780 |
| 767 | 0.771 | 1.000 | 0.763 | 1.000 | 0.947 | 1.000 | 0.847 | 1.000 | 0.969 | 1.000 | 0.916 | 1.000 | 0.946 | 0.752 | 1.000 | 1.000 | 0.814 | 0.779 |
| 768 | 0.773 | 1.000 | 0.759 | 1.000 | 0.947 | 1.000 | 0.849 | 1.000 | 0.969 | 1.000 | 0.916 | 1.000 | 0.946 | 0.752 | 1.000 | 1.000 | 0.814 | 0.779 |
| 769 | 0.773 | 1.000 | 0.759 | 1.000 | 0.947 | 1.000 | 0.849 | 1.000 | 0.969 | 1.000 | 0.916 | 1.000 | 0.946 | 0.752 | 1.000 | 1.000 | 0.814 | 0.779 |
| 770 | 0.772 | 1.000 | 0.757 | 1.000 | 0.947 | 1.000 | 0.849 | 1.000 | 0.969 | 1.000 | 0.916 | 1.000 | 0.946 | 0.753 | 1.000 | 1.000 | 0.814 | 0.779 |
| 771 | 0.770 | 1.000 | 0.757 | 1.000 | 0.947 | 1.000 | 0.854 | 1.000 | 0.969 | 1.000 | 0.916 | 1.000 | 0.946 | 0.753 | 1.000 | 1.000 | 0.814 | 0.779 |
| 772 | 0.772 | 1.000 | 0.759 | 1.000 | 0.947 | 1.000 | 0.851 | 1.000 | 0.969 | 1.000 | 0.916 | 1.000 | 0.955 | 0.754 | 1.000 | 1.000 | 0.815 | 0.780 |
| 773 | 0.771 | 1.000 | 0.759 | 1.000 | 0.947 | 1.000 | 0.852 | 1.000 | 0.969 | 1.000 | 0.916 | 1.000 | 0.955 | 0.755 | 1.000 | 1.000 | 0.815 | 0.780 |
| 774 | 0.772 | 1.000 | 0.759 | 1.000 | 0.953 | 1.000 | 0.852 | 1.000 | 0.969 | 1.000 | 0.916 | 1.000 | 0.955 | 0.756 | 1.000 | 1.000 | 0.816 | 0.781 |
| 776 | 0.776 | 1.000 | 0.765 | 1.000 | 0.953 | 1.000 | 0.847 | 1.000 | 0.969 | 1.000 | 0.916 | 1.000 | 0.955 | 0.756 | 1.000 | 1.000 | 0.817 | 0.782 |
| 777 | 0.777 | 1.000 | 0.765 | 1.000 | 0.953 | 1.000 | 0.846 | 1.000 | 0.969 | 1.000 | 0.916 | 1.000 | 0.955 | 0.755 | 1.000 | 1.000 | 0.817 | 0.782 |
| 778 | 0.775 | 1.000 | 0.763 | 1.000 | 0.947 | 1.000 | 0.846 | 1.000 | 0.969 | 1.000 | 0.916 | 1.000 | 0.955 | 0.756 | 1.000 | 1.000 | 0.817 | 0.781 |
| 779 | 0.777 | 1.000 | 0.763 | 1.000 | 0.947 | 1.000 | 0.846 | 1.000 | 0.969 | 1.000 | 0.916 | 1.000 | 0.955 | 0.756 | 1.000 | 1.000 | 0.817 | 0.782 |
| 780 | 0.776 | 1.000 | 0.763 | 1.000 | 0.947 | 1.000 | 0.847 | 1.000 | 0.969 | 1.000 | 0.916 | 1.000 | 0.955 | 0.756 | 1.000 | 1.000 | 0.817 | 0.782 |

| | | | | | | | | | | | | | | | | | | |
|---|---|---|---|---|---|---|---|---|---|---|---|---|---|---|---|---|---|---|
| 781 | 0.776 | 1.000 | 0.767 | 1.000 | 0.947 | 1.000 | 0.846 | 1.000 | 0.969 | 1.000 | 0.916 | 1.000 | 0.955 | 0.756 | 1.000 | 1.000 | 0.817 | 0.782 |
| 782 | 0.776 | 1.000 | 0.765 | 1.000 | 0.947 | 1.000 | 0.847 | 1.000 | 0.969 | 1.000 | 0.916 | 1.000 | 0.955 | 0.756 | 1.000 | 1.000 | 0.817 | 0.782 |
| 783 | 0.775 | 1.000 | 0.765 | 1.000 | 0.947 | 1.000 | 0.846 | 1.000 | 0.969 | 1.000 | 0.916 | 1.000 | 0.955 | 0.756 | 1.000 | 1.000 | 0.817 | 0.781 |
| 784 | 0.776 | 1.000 | 0.767 | 1.000 | 0.947 | 1.000 | 0.847 | 1.000 | 0.969 | 1.000 | 0.916 | 1.000 | 0.955 | 0.757 | 1.000 | 1.000 | 0.818 | 0.782 |
| 785 | 0.775 | 1.000 | 0.767 | 1.000 | 0.947 | 1.000 | 0.849 | 1.000 | 0.969 | 1.000 | 0.916 | 1.000 | 0.955 | 0.758 | 1.000 | 1.000 | 0.818 | 0.783 |
| 786 | 0.775 | 1.000 | 0.767 | 1.000 | 0.947 | 1.000 | 0.849 | 1.000 | 0.969 | 1.000 | 0.919 | 1.000 | 0.955 | 0.760 | 1.000 | 1.000 | 0.818 | 0.783 |
| 787 | 0.774 | 1.000 | 0.771 | 1.000 | 0.947 | 1.000 | 0.849 | 1.000 | 0.969 | 1.000 | 0.922 | 1.000 | 0.955 | 0.758 | 1.000 | 1.000 | 0.818 | 0.783 |
| 788 | 0.774 | 1.000 | 0.769 | 1.000 | 0.947 | 1.000 | 0.847 | 1.000 | 0.969 | 1.000 | 0.922 | 1.000 | 0.955 | 0.757 | 1.000 | 1.000 | 0.818 | 0.782 |
| 789 | 0.773 | 1.000 | 0.767 | 1.000 | 0.947 | 1.000 | 0.849 | 1.000 | 0.969 | 1.000 | 0.922 | 1.000 | 0.955 | 0.758 | 1.000 | 1.000 | 0.818 | 0.782 |
| 790 | 0.773 | 1.000 | 0.767 | 1.000 | 0.947 | 1.000 | 0.847 | 1.000 | 0.969 | 1.000 | 0.922 | 1.000 | 0.955 | 0.757 | 1.000 | 1.000 | 0.817 | 0.782 |
| 791 | 0.774 | 1.000 | 0.769 | 1.000 | 0.953 | 1.000 | 0.851 | 1.000 | 0.969 | 1.000 | 0.922 | 1.000 | 0.955 | 0.760 | 1.000 | 1.000 | 0.819 | 0.784 |
| 792 | 0.774 | 1.000 | 0.765 | 1.000 | 0.953 | 1.000 | 0.851 | 1.000 | 0.969 | 1.000 | 0.922 | 1.000 | 0.955 | 0.759 | 1.000 | 1.000 | 0.818 | 0.783 |
| 793 | 0.774 | 1.000 | 0.765 | 1.000 | 0.953 | 1.000 | 0.851 | 1.000 | 0.969 | 1.000 | 0.922 | 1.000 | 0.955 | 0.756 | 1.000 | 1.000 | 0.817 | 0.782 |
| 794 | 0.775 | 1.000 | 0.765 | 1.000 | 0.953 | 1.000 | 0.851 | 1.000 | 0.969 | 1.000 | 0.922 | 1.000 | 0.955 | 0.756 | 1.000 | 1.000 | 0.818 | 0.782 |
| 795 | 0.776 | 1.000 | 0.765 | 1.000 | 0.953 | 1.000 | 0.852 | 1.000 | 0.969 | 1.000 | 0.922 | 1.000 | 0.955 | 0.757 | 1.000 | 1.000 | 0.819 | 0.784 |
| 796 | 0.775 | 1.000 | 0.765 | 1.000 | 0.953 | 1.000 | 0.852 | 1.000 | 0.969 | 1.000 | 0.922 | 1.000 | 0.955 | 0.758 | 1.000 | 1.000 | 0.818 | 0.783 |
| 797 | 0.775 | 1.000 | 0.769 | 1.000 | 0.953 | 1.000 | 0.852 | 1.000 | 0.969 | 1.000 | 0.922 | 1.000 | 0.955 | 0.756 | 1.000 | 1.000 | 0.818 | 0.783 |
| 798 | 0.775 | 1.000 | 0.769 | 1.000 | 0.953 | 1.000 | 0.852 | 1.000 | 0.969 | 1.000 | 0.922 | 1.000 | 0.955 | 0.756 | 1.000 | 1.000 | 0.818 | 0.783 |
| 799 | 0.775 | 1.000 | 0.769 | 1.000 | 0.953 | 1.000 | 0.852 | 1.000 | 0.969 | 1.000 | 0.919 | 1.000 | 0.955 | 0.755 | 1.000 | 1.000 | 0.818 | 0.783 |
| 800 | 0.777 | 1.000 | 0.769 | 1.000 | 0.953 | 1.000 | 0.851 | 1.000 | 0.969 | 1.000 | 0.919 | 1.000 | 0.955 | 0.755 | 1.000 | 1.000 | 0.818 | 0.783 |
| 801 | 0.776 | 1.000 | 0.767 | 1.000 | 0.947 | 1.000 | 0.852 | 1.000 | 0.969 | 1.000 | 0.922 | 1.000 | 0.955 | 0.756 | 1.000 | 1.000 | 0.818 | 0.783 |
| 802 | 0.775 | 1.000 | 0.773 | 1.000 | 0.947 | 1.000 | 0.854 | 1.000 | 0.969 | 1.000 | 0.922 | 1.000 | 0.955 | 0.753 | 1.000 | 1.000 | 0.818 | 0.783 |
| 803 | 0.775 | 1.000 | 0.775 | 1.000 | 0.953 | 1.000 | 0.854 | 1.000 | 0.969 | 1.000 | 0.928 | 1.000 | 0.955 | 0.754 | 1.000 | 1.000 | 0.819 | 0.784 |
| 804 | 0.775 | 1.000 | 0.775 | 1.000 | 0.953 | 1.000 | 0.854 | 1.000 | 0.969 | 1.000 | 0.928 | 1.000 | 0.955 | 0.753 | 1.000 | 1.000 | 0.819 | 0.784 |
| 805 | 0.773 | 1.000 | 0.775 | 1.000 | 0.953 | 1.000 | 0.854 | 1.000 | 0.969 | 1.000 | 0.928 | 1.000 | 0.955 | 0.754 | 1.000 | 1.000 | 0.819 | 0.784 |
| 806 | 0.773 | 1.000 | 0.769 | 1.000 | 0.953 | 1.000 | 0.857 | 1.000 | 0.969 | 1.000 | 0.928 | 1.000 | 0.955 | 0.753 | 1.000 | 1.000 | 0.818 | 0.783 |
| 807 | 0.775 | 1.000 | 0.771 | 1.000 | 0.953 | 1.000 | 0.858 | 1.000 | 0.969 | 1.000 | 0.922 | 1.000 | 0.955 | 0.755 | 1.000 | 1.000 | 0.819 | 0.784 |
| 808 | 0.775 | 1.000 | 0.773 | 1.000 | 0.953 | 1.000 | 0.858 | 1.000 | 0.969 | 1.000 | 0.922 | 1.000 | 0.955 | 0.754 | 1.000 | 1.000 | 0.819 | 0.784 |
| 809 | 0.774 | 1.000 | 0.777 | 1.000 | 0.953 | 1.000 | 0.858 | 1.000 | 0.969 | 1.000 | 0.925 | 1.000 | 0.955 | 0.754 | 1.000 | 1.000 | 0.819 | 0.785 |
| 810 | 0.774 | 1.000 | 0.775 | 1.000 | 0.953 | 1.000 | 0.858 | 1.000 | 0.969 | 1.000 | 0.925 | 1.000 | 0.955 | 0.756 | 1.000 | 1.000 | 0.820 | 0.785 |
| 811 | 0.774 | 1.000 | 0.779 | 1.000 | 0.953 | 1.000 | 0.860 | 1.000 | 0.969 | 1.000 | 0.925 | 1.000 | 0.955 | 0.756 | 1.000 | 1.000 | 0.820 | 0.786 |
| 812 | 0.775 | 1.000 | 0.775 | 1.000 | 0.953 | 1.000 | 0.855 | 1.000 | 0.969 | 1.000 | 0.925 | 1.000 | 0.955 | 0.756 | 1.000 | 1.000 | 0.820 | 0.785 |
| 813 | 0.774 | 1.000 | 0.771 | 1.000 | 0.953 | 1.000 | 0.855 | 1.000 | 0.969 | 1.000 | 0.925 | 1.000 | 0.955 | 0.760 | 1.000 | 1.000 | 0.820 | 0.785 |
| 814 | 0.774 | 1.000 | 0.769 | 1.000 | 0.953 | 1.000 | 0.855 | 1.000 | 0.969 | 1.000 | 0.925 | 1.000 | 0.955 | 0.760 | 1.000 | 1.000 | 0.820 | 0.785 |
| 815 | 0.772 | 1.000 | 0.769 | 1.000 | 0.953 | 1.000 | 0.858 | 1.000 | 0.969 | 1.000 | 0.925 | 1.000 | 0.955 | 0.760 | 1.000 | 1.000 | 0.820 | 0.785 |
| 816 | 0.771 | 1.000 | 0.769 | 1.000 | 0.953 | 1.000 | 0.857 | 1.000 | 0.969 | 1.000 | 0.925 | 1.000 | 0.955 | 0.761 | 1.000 | 1.000 | 0.819 | 0.785 |
| 817 | 0.771 | 1.000 | 0.769 | 1.000 | 0.953 | 1.000 | 0.857 | 1.000 | 0.969 | 1.000 | 0.922 | 1.000 | 0.955 | 0.760 | 1.000 | 1.000 | 0.819 | 0.784 |
| 818 | 0.771 | 1.000 | 0.769 | 1.000 | 0.953 | 1.000 | 0.855 | 1.000 | 0.969 | 1.000 | 0.922 | 1.000 | 0.955 | 0.760 | 1.000 | 1.000 | 0.819 | 0.784 |
| 819 | 0.771 | 1.000 | 0.769 | 1.000 | 0.953 | 1.000 | 0.857 | 1.000 | 0.969 | 1.000 | 0.922 | 1.000 | 0.955 | 0.760 | 1.000 | 1.000 | 0.819 | 0.784 |

| | | | | | | | | | | | | | | | | | | |
|---|---|---|---|---|---|---|---|---|---|---|---|---|---|---|---|---|---|---|
| 820 | 0.771 | 1.000 | 0.771 | 1.000 | 0.953 | 1.000 | 0.857 | 1.000 | 0.969 | 1.000 | 0.922 | 1.000 | 0.955 | 0.760 | 1.000 | 1.000 | 0.819 | 0.784 |
| 821 | 0.777 | 1.000 | 0.771 | 1.000 | 0.953 | 1.000 | 0.858 | 1.000 | 0.969 | 1.000 | 0.922 | 1.000 | 0.955 | 0.760 | 1.000 | 1.000 | 0.821 | 0.786 |
| 822 | 0.776 | 1.000 | 0.771 | 1.000 | 0.953 | 1.000 | 0.857 | 1.000 | 0.969 | 1.000 | 0.922 | 1.000 | 0.955 | 0.760 | 1.000 | 1.000 | 0.820 | 0.786 |
| 823 | 0.777 | 1.000 | 0.771 | 1.000 | 0.953 | 1.000 | 0.857 | 1.000 | 0.969 | 1.000 | 0.922 | 1.000 | 0.955 | 0.760 | 1.000 | 1.000 | 0.821 | 0.786 |
| 824 | 0.777 | 1.000 | 0.773 | 1.000 | 0.953 | 1.000 | 0.857 | 1.000 | 0.969 | 1.000 | 0.925 | 1.000 | 0.955 | 0.757 | 1.000 | 1.000 | 0.821 | 0.786 |
| 825 | 0.778 | 1.000 | 0.773 | 1.000 | 0.953 | 1.000 | 0.855 | 1.000 | 0.969 | 1.000 | 0.925 | 1.000 | 0.955 | 0.758 | 1.000 | 1.000 | 0.821 | 0.786 |
| 826 | 0.774 | 1.000 | 0.771 | 1.000 | 0.953 | 1.000 | 0.857 | 1.000 | 0.969 | 1.000 | 0.925 | 1.000 | 0.955 | 0.758 | 1.000 | 1.000 | 0.820 | 0.785 |
| 827 | 0.774 | 1.000 | 0.775 | 1.000 | 0.953 | 1.000 | 0.858 | 1.000 | 0.969 | 1.000 | 0.925 | 1.000 | 0.955 | 0.759 | 1.000 | 1.000 | 0.820 | 0.786 |
| 828 | 0.775 | 1.000 | 0.775 | 1.000 | 0.953 | 1.000 | 0.858 | 1.000 | 0.969 | 1.000 | 0.925 | 1.000 | 0.955 | 0.760 | 1.000 | 1.000 | 0.821 | 0.786 |
| 829 | 0.774 | 1.000 | 0.779 | 1.000 | 0.953 | 1.000 | 0.858 | 1.000 | 0.969 | 1.000 | 0.925 | 1.000 | 0.955 | 0.759 | 1.000 | 1.000 | 0.821 | 0.786 |
| 830 | 0.775 | 1.000 | 0.779 | 1.000 | 0.953 | 1.000 | 0.861 | 1.000 | 0.969 | 1.000 | 0.925 | 1.000 | 0.955 | 0.759 | 1.000 | 1.000 | 0.822 | 0.787 |
| 831 | 0.775 | 1.000 | 0.779 | 1.000 | 0.953 | 1.000 | 0.860 | 1.000 | 0.969 | 1.000 | 0.925 | 1.000 | 0.955 | 0.759 | 1.000 | 1.000 | 0.822 | 0.787 |
| 832 | 0.778 | 1.000 | 0.783 | 1.000 | 0.953 | 1.000 | 0.858 | 1.000 | 0.969 | 1.000 | 0.925 | 1.000 | 0.955 | 0.759 | 1.000 | 1.000 | 0.823 | 0.788 |
| 833 | 0.778 | 1.000 | 0.781 | 1.000 | 0.953 | 1.000 | 0.858 | 1.000 | 0.969 | 1.000 | 0.925 | 1.000 | 0.955 | 0.759 | 1.000 | 1.000 | 0.822 | 0.788 |
| 834 | 0.775 | 1.000 | 0.781 | 1.000 | 0.953 | 1.000 | 0.858 | 1.000 | 0.969 | 1.000 | 0.925 | 1.000 | 0.955 | 0.759 | 1.000 | 1.000 | 0.821 | 0.787 |
| 835 | 0.776 | 1.000 | 0.779 | 1.000 | 0.953 | 1.000 | 0.858 | 1.000 | 0.969 | 1.000 | 0.925 | 1.000 | 0.955 | 0.759 | 1.000 | 1.000 | 0.822 | 0.787 |
| 836 | 0.777 | 1.000 | 0.781 | 1.000 | 0.958 | 1.000 | 0.858 | 1.000 | 0.969 | 1.000 | 0.925 | 1.000 | 0.955 | 0.760 | 1.000 | 1.000 | 0.823 | 0.789 |
| 837 | 0.777 | 1.000 | 0.783 | 1.000 | 0.958 | 1.000 | 0.858 | 1.000 | 0.969 | 1.000 | 0.925 | 1.000 | 0.955 | 0.760 | 1.000 | 1.000 | 0.823 | 0.789 |
| 838 | 0.780 | 1.000 | 0.783 | 1.000 | 0.958 | 1.000 | 0.858 | 1.000 | 0.969 | 1.000 | 0.922 | 1.000 | 0.955 | 0.760 | 1.000 | 1.000 | 0.824 | 0.789 |
| 839 | 0.779 | 1.000 | 0.791 | 1.000 | 0.958 | 1.000 | 0.858 | 1.000 | 0.969 | 1.000 | 0.925 | 1.000 | 0.955 | 0.762 | 1.000 | 1.000 | 0.825 | 0.791 |
| 840 | 0.780 | 1.000 | 0.785 | 1.000 | 0.958 | 1.000 | 0.857 | 1.000 | 0.969 | 1.000 | 0.925 | 1.000 | 0.955 | 0.762 | 1.000 | 1.000 | 0.824 | 0.790 |
| 841 | 0.780 | 1.000 | 0.787 | 1.000 | 0.958 | 1.000 | 0.857 | 1.000 | 0.969 | 1.000 | 0.925 | 1.000 | 0.955 | 0.762 | 1.000 | 1.000 | 0.824 | 0.790 |
| 842 | 0.781 | 1.000 | 0.783 | 1.000 | 0.953 | 1.000 | 0.858 | 1.000 | 0.969 | 1.000 | 0.936 | 1.000 | 0.955 | 0.763 | 1.000 | 1.000 | 0.825 | 0.791 |
| 843 | 0.779 | 1.000 | 0.787 | 1.000 | 0.953 | 1.000 | 0.857 | 1.000 | 0.969 | 1.000 | 0.936 | 1.000 | 0.964 | 0.761 | 1.000 | 1.000 | 0.825 | 0.791 |
| 844 | 0.779 | 1.000 | 0.787 | 1.000 | 0.953 | 1.000 | 0.857 | 1.000 | 0.969 | 1.000 | 0.936 | 1.000 | 0.964 | 0.762 | 1.000 | 1.000 | 0.825 | 0.791 |
| 845 | 0.779 | 1.000 | 0.787 | 1.000 | 0.958 | 1.000 | 0.857 | 1.000 | 0.969 | 1.000 | 0.936 | 1.000 | 0.964 | 0.761 | 1.000 | 1.000 | 0.825 | 0.791 |
| 846 | 0.778 | 1.000 | 0.781 | 1.000 | 0.953 | 1.000 | 0.858 | 1.000 | 0.969 | 1.000 | 0.936 | 1.000 | 0.964 | 0.762 | 1.000 | 1.000 | 0.824 | 0.790 |
| 847 | 0.778 | 1.000 | 0.781 | 1.000 | 0.958 | 1.000 | 0.858 | 1.000 | 0.969 | 1.000 | 0.936 | 1.000 | 0.964 | 0.762 | 1.000 | 1.000 | 0.824 | 0.791 |
| 848 | 0.779 | 1.000 | 0.781 | 1.000 | 0.958 | 1.000 | 0.858 | 1.000 | 0.969 | 1.000 | 0.936 | 1.000 | 0.964 | 0.762 | 1.000 | 1.000 | 0.825 | 0.791 |
| 849 | 0.779 | 1.000 | 0.781 | 1.000 | 0.958 | 1.000 | 0.858 | 1.000 | 0.969 | 1.000 | 0.936 | 1.000 | 0.964 | 0.761 | 1.000 | 1.000 | 0.824 | 0.791 |
| 850 | 0.778 | 1.000 | 0.783 | 1.000 | 0.958 | 1.000 | 0.858 | 1.000 | 0.969 | 1.000 | 0.936 | 1.000 | 0.964 | 0.761 | 1.000 | 1.000 | 0.824 | 0.791 |
| 851 | 0.778 | 1.000 | 0.783 | 1.000 | 0.958 | 1.000 | 0.858 | 1.000 | 0.969 | 1.000 | 0.936 | 1.000 | 0.964 | 0.761 | 1.000 | 1.000 | 0.824 | 0.791 |
| 852 | 0.778 | 1.000 | 0.783 | 1.000 | 0.958 | 1.000 | 0.857 | 1.000 | 0.969 | 1.000 | 0.936 | 1.000 | 0.964 | 0.761 | 1.000 | 1.000 | 0.824 | 0.790 |
| 853 | 0.779 | 1.000 | 0.785 | 1.000 | 0.958 | 1.000 | 0.857 | 1.000 | 0.969 | 1.000 | 0.936 | 1.000 | 0.964 | 0.762 | 1.000 | 1.000 | 0.825 | 0.791 |
| 854 | 0.779 | 1.000 | 0.785 | 1.000 | 0.958 | 1.000 | 0.857 | 1.000 | 0.969 | 1.000 | 0.936 | 1.000 | 0.964 | 0.761 | 1.000 | 1.000 | 0.825 | 0.791 |
| 855 | 0.778 | 1.000 | 0.785 | 1.000 | 0.958 | 1.000 | 0.857 | 1.000 | 0.969 | 1.000 | 0.936 | 1.000 | 0.964 | 0.760 | 1.000 | 1.000 | 0.824 | 0.790 |
| 856 | 0.778 | 1.000 | 0.783 | 1.000 | 0.958 | 1.000 | 0.858 | 1.000 | 0.969 | 1.000 | 0.936 | 1.000 | 0.964 | 0.761 | 1.000 | 1.000 | 0.824 | 0.790 |
| 857 | 0.779 | 1.000 | 0.775 | 1.000 | 0.958 | 1.000 | 0.855 | 1.000 | 0.969 | 1.000 | 0.936 | 1.000 | 0.964 | 0.761 | 1.000 | 1.000 | 0.824 | 0.789 |
| 858 | 0.779 | 1.000 | 0.775 | 1.000 | 0.958 | 1.000 | 0.855 | 1.000 | 0.969 | 1.000 | 0.936 | 1.000 | 0.964 | 0.760 | 1.000 | 1.000 | 0.823 | 0.789 |

| | | | | | | | | | | | | | | | | | | |
|---|---|---|---|---|---|---|---|---|---|---|---|---|---|---|---|---|---|---|
| 859 | 0.779 | 1.000 | 0.775 | 1.000 | 0.958 | 1.000 | 0.855 | 1.000 | 0.969 | 1.000 | 0.936 | 1.000 | 0.964 | 0.760 | 1.000 | 1.000 | 0.823 | 0.789 |
| 860 | 0.779 | 1.000 | 0.777 | 1.000 | 0.958 | 1.000 | 0.855 | 1.000 | 0.969 | 1.000 | 0.936 | 1.000 | 0.964 | 0.761 | 1.000 | 1.000 | 0.824 | 0.789 |
| 861 | 0.779 | 1.000 | 0.773 | 1.000 | 0.958 | 1.000 | 0.855 | 1.000 | 0.969 | 1.000 | 0.936 | 1.000 | 0.964 | 0.761 | 1.000 | 1.000 | 0.823 | 0.789 |
| 862 | 0.776 | 1.000 | 0.771 | 1.000 | 0.953 | 1.000 | 0.854 | 1.000 | 0.969 | 1.000 | 0.936 | 1.000 | 0.964 | 0.760 | 1.000 | 1.000 | 0.821 | 0.787 |
| 863 | 0.776 | 1.000 | 0.771 | 1.000 | 0.953 | 1.000 | 0.854 | 1.000 | 0.969 | 1.000 | 0.936 | 1.000 | 0.964 | 0.759 | 1.000 | 1.000 | 0.821 | 0.787 |
| 864 | 0.777 | 1.000 | 0.773 | 1.000 | 0.953 | 1.000 | 0.854 | 1.000 | 0.969 | 1.000 | 0.936 | 1.000 | 0.964 | 0.760 | 1.000 | 1.000 | 0.822 | 0.787 |
| 865 | 0.777 | 1.000 | 0.765 | 1.000 | 0.958 | 1.000 | 0.854 | 1.000 | 0.969 | 1.000 | 0.936 | 1.000 | 0.964 | 0.759 | 1.000 | 1.000 | 0.821 | 0.786 |
| 866 | 0.778 | 1.000 | 0.777 | 1.000 | 0.958 | 1.000 | 0.852 | 1.000 | 0.969 | 1.000 | 0.930 | 1.000 | 0.964 | 0.760 | 1.000 | 1.000 | 0.822 | 0.788 |
| 867 | 0.779 | 1.000 | 0.779 | 1.000 | 0.958 | 1.000 | 0.852 | 1.000 | 0.969 | 1.000 | 0.928 | 1.000 | 0.964 | 0.760 | 1.000 | 1.000 | 0.822 | 0.788 |
| 868 | 0.778 | 1.000 | 0.777 | 1.000 | 0.958 | 1.000 | 0.852 | 1.000 | 0.969 | 1.000 | 0.928 | 1.000 | 0.964 | 0.759 | 1.000 | 1.000 | 0.822 | 0.787 |
| 869 | 0.779 | 1.000 | 0.775 | 1.000 | 0.958 | 1.000 | 0.851 | 1.000 | 0.969 | 1.000 | 0.928 | 1.000 | 0.964 | 0.760 | 1.000 | 1.000 | 0.822 | 0.787 |
| 870 | 0.781 | 1.000 | 0.775 | 1.000 | 0.958 | 1.000 | 0.854 | 1.000 | 0.969 | 1.000 | 0.928 | 1.000 | 0.964 | 0.760 | 1.000 | 1.000 | 0.823 | 0.789 |
| 871 | 0.780 | 1.000 | 0.775 | 1.000 | 0.958 | 1.000 | 0.851 | 1.000 | 0.969 | 1.000 | 0.928 | 1.000 | 0.964 | 0.760 | 1.000 | 1.000 | 0.822 | 0.788 |
| 872 | 0.781 | 1.000 | 0.771 | 1.000 | 0.958 | 1.000 | 0.854 | 1.000 | 0.969 | 1.000 | 0.930 | 1.000 | 0.964 | 0.760 | 1.000 | 1.000 | 0.823 | 0.788 |
| 873 | 0.779 | 1.000 | 0.773 | 1.000 | 0.958 | 1.000 | 0.855 | 1.000 | 0.969 | 1.000 | 0.930 | 1.000 | 0.964 | 0.761 | 1.000 | 1.000 | 0.823 | 0.789 |
| 874 | 0.779 | 1.000 | 0.775 | 1.000 | 0.958 | 1.000 | 0.855 | 1.000 | 0.969 | 1.000 | 0.930 | 1.000 | 0.964 | 0.760 | 1.000 | 1.000 | 0.823 | 0.788 |
| 875 | 0.779 | 1.000 | 0.777 | 1.000 | 0.958 | 1.000 | 0.851 | 1.000 | 0.969 | 1.000 | 0.930 | 1.000 | 0.964 | 0.761 | 1.000 | 1.000 | 0.823 | 0.788 |
| 876 | 0.777 | 1.000 | 0.779 | 1.000 | 0.958 | 1.000 | 0.852 | 1.000 | 0.969 | 1.000 | 0.928 | 1.000 | 0.964 | 0.760 | 1.000 | 1.000 | 0.822 | 0.788 |
| 877 | 0.779 | 1.000 | 0.773 | 1.000 | 0.958 | 1.000 | 0.855 | 1.000 | 0.969 | 1.000 | 0.928 | 1.000 | 0.964 | 0.762 | 1.000 | 1.000 | 0.823 | 0.788 |
| 878 | 0.777 | 1.000 | 0.777 | 1.000 | 0.958 | 1.000 | 0.854 | 1.000 | 0.969 | 1.000 | 0.928 | 1.000 | 0.964 | 0.763 | 1.000 | 1.000 | 0.823 | 0.788 |
| 879 | 0.777 | 1.000 | 0.777 | 1.000 | 0.958 | 1.000 | 0.854 | 1.000 | 0.980 | 1.000 | 0.928 | 1.000 | 0.964 | 0.763 | 1.000 | 1.000 | 0.823 | 0.788 |
| 880 | 0.778 | 1.000 | 0.775 | 1.000 | 0.958 | 1.000 | 0.854 | 1.000 | 0.980 | 1.000 | 0.928 | 1.000 | 0.964 | 0.763 | 1.000 | 1.000 | 0.823 | 0.789 |
| 881 | 0.777 | 1.000 | 0.775 | 1.000 | 0.958 | 1.000 | 0.854 | 1.000 | 0.980 | 1.000 | 0.928 | 1.000 | 0.964 | 0.763 | 1.000 | 1.000 | 0.823 | 0.788 |
| 882 | 0.779 | 1.000 | 0.777 | 1.000 | 0.958 | 1.000 | 0.854 | 1.000 | 0.980 | 1.000 | 0.930 | 1.000 | 0.964 | 0.762 | 1.000 | 1.000 | 0.823 | 0.789 |
| 883 | 0.779 | 1.000 | 0.775 | 1.000 | 0.958 | 1.000 | 0.852 | 1.000 | 0.969 | 1.000 | 0.928 | 1.000 | 0.964 | 0.762 | 1.000 | 1.000 | 0.823 | 0.788 |
| 884 | 0.778 | 1.000 | 0.777 | 1.000 | 0.958 | 1.000 | 0.852 | 1.000 | 0.969 | 1.000 | 0.930 | 1.000 | 0.964 | 0.761 | 1.000 | 1.000 | 0.823 | 0.788 |
| 885 | 0.776 | 1.000 | 0.779 | 1.000 | 0.958 | 1.000 | 0.852 | 1.000 | 0.969 | 1.000 | 0.930 | 1.000 | 0.964 | 0.763 | 1.000 | 1.000 | 0.823 | 0.788 |
| 886 | 0.775 | 1.000 | 0.781 | 1.000 | 0.958 | 1.000 | 0.852 | 1.000 | 0.969 | 1.000 | 0.930 | 1.000 | 0.964 | 0.763 | 1.000 | 1.000 | 0.823 | 0.788 |
| 887 | 0.777 | 1.000 | 0.783 | 1.000 | 0.958 | 1.000 | 0.852 | 1.000 | 0.969 | 1.000 | 0.930 | 1.000 | 0.964 | 0.762 | 1.000 | 1.000 | 0.823 | 0.789 |
| 888 | 0.777 | 1.000 | 0.779 | 1.000 | 0.958 | 1.000 | 0.852 | 1.000 | 0.969 | 1.000 | 0.930 | 1.000 | 0.964 | 0.761 | 1.000 | 1.000 | 0.822 | 0.788 |
| 889 | 0.778 | 1.000 | 0.779 | 1.000 | 0.958 | 1.000 | 0.852 | 1.000 | 0.969 | 1.000 | 0.930 | 1.000 | 0.964 | 0.761 | 1.000 | 1.000 | 0.823 | 0.788 |
| 890 | 0.777 | 1.000 | 0.783 | 1.000 | 0.958 | 1.000 | 0.854 | 1.000 | 0.969 | 1.000 | 0.930 | 1.000 | 0.964 | 0.762 | 1.000 | 1.000 | 0.823 | 0.789 |
| 891 | 0.776 | 1.000 | 0.787 | 1.000 | 0.958 | 1.000 | 0.851 | 1.000 | 0.969 | 1.000 | 0.930 | 1.000 | 0.964 | 0.762 | 1.000 | 1.000 | 0.823 | 0.789 |
| 892 | 0.777 | 1.000 | 0.787 | 1.000 | 0.958 | 1.000 | 0.851 | 1.000 | 0.969 | 1.000 | 0.930 | 1.000 | 0.964 | 0.762 | 1.000 | 1.000 | 0.823 | 0.789 |
| 893 | 0.775 | 1.000 | 0.783 | 1.000 | 0.958 | 1.000 | 0.849 | 1.000 | 0.969 | 1.000 | 0.930 | 1.000 | 0.964 | 0.762 | 1.000 | 1.000 | 0.822 | 0.788 |
| 894 | 0.775 | 1.000 | 0.779 | 1.000 | 0.958 | 1.000 | 0.849 | 1.000 | 0.969 | 1.000 | 0.933 | 1.000 | 0.964 | 0.760 | 1.000 | 1.000 | 0.822 | 0.787 |
| 895 | 0.777 | 1.000 | 0.781 | 1.000 | 0.958 | 1.000 | 0.847 | 1.000 | 0.969 | 1.000 | 0.936 | 1.000 | 0.964 | 0.759 | 1.000 | 1.000 | 0.822 | 0.787 |
| 896 | 0.775 | 1.000 | 0.787 | 1.000 | 0.958 | 1.000 | 0.847 | 1.000 | 0.969 | 1.000 | 0.930 | 1.000 | 0.964 | 0.762 | 1.000 | 1.000 | 0.822 | 0.788 |
| 897 | 0.775 | 1.000 | 0.783 | 1.000 | 0.958 | 1.000 | 0.847 | 1.000 | 0.969 | 1.000 | 0.930 | 1.000 | 0.964 | 0.761 | 1.000 | 1.000 | 0.822 | 0.787 |

| | | | | | | | | | | | | | | | | | | |
|---|---|---|---|---|---|---|---|---|---|---|---|---|---|---|---|---|---|---|
| 898 | 0.774 | 1.000 | 0.783 | 1.000 | 0.958 | 1.000 | 0.851 | 1.000 | 0.969 | 1.000 | 0.928 | 1.000 | 0.964 | 0.762 | 1.000 | 1.000 | 0.822 | 0.787 |
| 899 | 0.775 | 1.000 | 0.785 | 1.000 | 0.958 | 1.000 | 0.849 | 1.000 | 0.969 | 1.000 | 0.928 | 1.000 | 0.964 | 0.761 | 1.000 | 1.000 | 0.822 | 0.787 |
| 900 | 0.776 | 1.000 | 0.787 | 1.000 | 0.958 | 1.000 | 0.847 | 1.000 | 0.969 | 1.000 | 0.925 | 1.000 | 0.964 | 0.762 | 1.000 | 1.000 | 0.822 | 0.788 |
| 901 | 0.772 | 1.000 | 0.785 | 1.000 | 0.958 | 1.000 | 0.847 | 1.000 | 0.969 | 1.000 | 0.928 | 1.000 | 0.964 | 0.761 | 1.000 | 1.000 | 0.821 | 0.786 |
| 902 | 0.773 | 1.000 | 0.783 | 1.000 | 0.958 | 1.000 | 0.849 | 1.000 | 0.969 | 1.000 | 0.928 | 1.000 | 0.964 | 0.763 | 1.000 | 1.000 | 0.821 | 0.787 |
| 903 | 0.774 | 1.000 | 0.787 | 1.000 | 0.958 | 1.000 | 0.849 | 1.000 | 0.969 | 1.000 | 0.930 | 1.000 | 0.964 | 0.760 | 1.000 | 1.000 | 0.822 | 0.787 |
| 904 | 0.775 | 1.000 | 0.785 | 1.000 | 0.958 | 1.000 | 0.847 | 1.000 | 0.969 | 1.000 | 0.930 | 1.000 | 0.964 | 0.761 | 1.000 | 1.000 | 0.822 | 0.787 |
| 905 | 0.775 | 1.000 | 0.785 | 1.000 | 0.958 | 1.000 | 0.847 | 1.000 | 0.969 | 1.000 | 0.928 | 1.000 | 0.964 | 0.763 | 1.000 | 1.000 | 0.822 | 0.788 |
| 906 | 0.775 | 1.000 | 0.785 | 1.000 | 0.958 | 1.000 | 0.846 | 1.000 | 0.969 | 1.000 | 0.928 | 1.000 | 0.964 | 0.764 | 1.000 | 1.000 | 0.822 | 0.788 |
| 907 | 0.772 | 1.000 | 0.789 | 1.000 | 0.958 | 1.000 | 0.844 | 1.000 | 0.969 | 1.000 | 0.933 | 1.000 | 0.964 | 0.763 | 1.000 | 1.000 | 0.822 | 0.788 |
| 908 | 0.773 | 1.000 | 0.787 | 1.000 | 0.958 | 1.000 | 0.844 | 1.000 | 0.969 | 1.000 | 0.933 | 1.000 | 0.964 | 0.764 | 1.000 | 1.000 | 0.822 | 0.788 |
| 909 | 0.774 | 1.000 | 0.787 | 1.000 | 0.958 | 1.000 | 0.844 | 1.000 | 0.969 | 1.000 | 0.930 | 1.000 | 0.964 | 0.763 | 1.000 | 1.000 | 0.822 | 0.788 |
| 910 | 0.773 | 1.000 | 0.787 | 1.000 | 0.958 | 1.000 | 0.844 | 1.000 | 0.969 | 1.000 | 0.930 | 1.000 | 0.964 | 0.763 | 1.000 | 1.000 | 0.822 | 0.787 |
| 911 | 0.774 | 1.000 | 0.787 | 1.000 | 0.958 | 1.000 | 0.844 | 1.000 | 0.969 | 1.000 | 0.933 | 1.000 | 0.964 | 0.764 | 1.000 | 1.000 | 0.822 | 0.788 |
| 912 | 0.773 | 1.000 | 0.785 | 1.000 | 0.958 | 1.000 | 0.847 | 1.000 | 0.969 | 1.000 | 0.933 | 1.000 | 0.964 | 0.765 | 1.000 | 1.000 | 0.823 | 0.788 |
| 913 | 0.775 | 1.000 | 0.792 | 1.000 | 0.958 | 1.000 | 0.844 | 1.000 | 0.969 | 1.000 | 0.936 | 1.000 | 0.964 | 0.760 | 1.000 | 1.000 | 0.822 | 0.788 |
| 914 | 0.775 | 1.000 | 0.789 | 1.000 | 0.958 | 1.000 | 0.844 | 1.000 | 0.969 | 1.000 | 0.930 | 1.000 | 0.964 | 0.763 | 1.000 | 1.000 | 0.822 | 0.788 |
| 915 | 0.775 | 1.000 | 0.789 | 1.000 | 0.958 | 1.000 | 0.844 | 1.000 | 0.969 | 1.000 | 0.930 | 1.000 | 0.964 | 0.763 | 1.000 | 1.000 | 0.822 | 0.788 |
| 916 | 0.774 | 1.000 | 0.789 | 1.000 | 0.958 | 1.000 | 0.846 | 1.000 | 0.969 | 1.000 | 0.933 | 1.000 | 0.964 | 0.762 | 1.000 | 1.000 | 0.822 | 0.788 |
| 917 | 0.775 | 1.000 | 0.796 | 1.000 | 0.958 | 1.000 | 0.844 | 1.000 | 0.969 | 1.000 | 0.933 | 1.000 | 0.964 | 0.761 | 1.000 | 1.000 | 0.823 | 0.789 |
| 918 | 0.773 | 1.000 | 0.796 | 1.000 | 0.958 | 1.000 | 0.844 | 1.000 | 0.969 | 1.000 | 0.933 | 1.000 | 0.964 | 0.760 | 1.000 | 1.000 | 0.822 | 0.788 |
| 919 | 0.777 | 1.000 | 0.796 | 1.000 | 0.958 | 1.000 | 0.843 | 1.000 | 0.969 | 1.000 | 0.933 | 1.000 | 0.964 | 0.760 | 1.000 | 1.000 | 0.823 | 0.789 |
| 920 | 0.778 | 1.000 | 0.791 | 1.000 | 0.958 | 1.000 | 0.844 | 1.000 | 0.969 | 1.000 | 0.933 | 1.000 | 0.964 | 0.758 | 1.000 | 1.000 | 0.822 | 0.788 |
| 921 | 0.778 | 1.000 | 0.792 | 1.000 | 0.958 | 1.000 | 0.844 | 1.000 | 0.969 | 1.000 | 0.933 | 1.000 | 0.964 | 0.759 | 1.000 | 1.000 | 0.823 | 0.789 |
| 922 | 0.778 | 1.000 | 0.792 | 1.000 | 0.958 | 1.000 | 0.844 | 1.000 | 0.969 | 1.000 | 0.933 | 1.000 | 0.964 | 0.759 | 1.000 | 1.000 | 0.823 | 0.789 |
| 923 | 0.777 | 1.000 | 0.792 | 1.000 | 0.958 | 1.000 | 0.838 | 1.000 | 0.969 | 1.000 | 0.936 | 1.000 | 0.964 | 0.759 | 1.000 | 1.000 | 0.822 | 0.787 |
| 924 | 0.776 | 1.000 | 0.794 | 1.000 | 0.958 | 1.000 | 0.838 | 1.000 | 0.969 | 1.000 | 0.933 | 1.000 | 0.964 | 0.759 | 1.000 | 1.000 | 0.822 | 0.787 |
| 925 | 0.776 | 1.000 | 0.792 | 1.000 | 0.958 | 1.000 | 0.840 | 1.000 | 0.969 | 1.000 | 0.933 | 1.000 | 0.964 | 0.763 | 1.000 | 1.000 | 0.823 | 0.789 |
| 926 | 0.776 | 1.000 | 0.792 | 1.000 | 0.958 | 1.000 | 0.840 | 1.000 | 0.969 | 1.000 | 0.933 | 1.000 | 0.964 | 0.762 | 1.000 | 1.000 | 0.822 | 0.788 |
| 927 | 0.775 | 1.000 | 0.792 | 1.000 | 0.958 | 1.000 | 0.840 | 1.000 | 0.969 | 1.000 | 0.933 | 1.000 | 0.964 | 0.763 | 1.000 | 1.000 | 0.822 | 0.788 |
| 928 | 0.775 | 1.000 | 0.792 | 1.000 | 0.958 | 1.000 | 0.841 | 1.000 | 0.969 | 1.000 | 0.933 | 1.000 | 0.964 | 0.763 | 1.000 | 1.000 | 0.823 | 0.789 |
| 929 | 0.775 | 1.000 | 0.792 | 1.000 | 0.958 | 1.000 | 0.841 | 1.000 | 0.969 | 1.000 | 0.933 | 1.000 | 0.964 | 0.761 | 1.000 | 1.000 | 0.822 | 0.788 |
| 930 | 0.775 | 1.000 | 0.794 | 1.000 | 0.958 | 1.000 | 0.844 | 1.000 | 0.969 | 1.000 | 0.930 | 1.000 | 0.964 | 0.761 | 1.000 | 1.000 | 0.823 | 0.788 |
| 931 | 0.775 | 1.000 | 0.794 | 1.000 | 0.958 | 1.000 | 0.844 | 1.000 | 0.969 | 1.000 | 0.930 | 1.000 | 0.964 | 0.762 | 1.000 | 1.000 | 0.823 | 0.789 |
| 932 | 0.775 | 1.000 | 0.792 | 1.000 | 0.958 | 1.000 | 0.843 | 1.000 | 0.969 | 1.000 | 0.930 | 1.000 | 0.964 | 0.761 | 1.000 | 1.000 | 0.822 | 0.788 |
| 933 | 0.774 | 1.000 | 0.789 | 1.000 | 0.963 | 1.000 | 0.841 | 1.000 | 0.969 | 1.000 | 0.930 | 1.000 | 0.964 | 0.761 | 1.000 | 1.000 | 0.821 | 0.787 |
| 934 | 0.773 | 1.000 | 0.787 | 1.000 | 0.963 | 1.000 | 0.844 | 1.000 | 0.969 | 1.000 | 0.930 | 1.000 | 0.964 | 0.760 | 1.000 | 1.000 | 0.821 | 0.787 |
| 935 | 0.774 | 1.000 | 0.794 | 1.000 | 0.963 | 1.000 | 0.844 | 1.000 | 0.969 | 1.000 | 0.933 | 1.000 | 0.964 | 0.763 | 1.000 | 1.000 | 0.823 | 0.789 |
| 936 | 0.773 | 1.000 | 0.794 | 1.000 | 0.958 | 1.000 | 0.844 | 1.000 | 0.969 | 1.000 | 0.933 | 1.000 | 0.964 | 0.761 | 1.000 | 1.000 | 0.822 | 0.788 |

| | | | | | | | | | | | | | | | | | | |
|---|---|---|---|---|---|---|---|---|---|---|---|---|---|---|---|---|---|---|
| 937 | 0.773 | 1.000 | 0.792 | 1.000 | 0.958 | 1.000 | 0.847 | 1.000 | 0.969 | 1.000 | 0.930 | 1.000 | 0.964 | 0.764 | 1.000 | 1.000 | 0.823 | 0.789 |
| 938 | 0.774 | 1.000 | 0.792 | 1.000 | 0.958 | 1.000 | 0.847 | 1.000 | 0.969 | 1.000 | 0.930 | 1.000 | 0.964 | 0.765 | 1.000 | 1.000 | 0.823 | 0.789 |
| 939 | 0.773 | 1.000 | 0.792 | 1.000 | 0.958 | 1.000 | 0.847 | 1.000 | 0.969 | 1.000 | 0.930 | 1.000 | 0.964 | 0.765 | 1.000 | 1.000 | 0.823 | 0.789 |
| 940 | 0.773 | 1.000 | 0.791 | 1.000 | 0.958 | 1.000 | 0.849 | 1.000 | 0.969 | 1.000 | 0.930 | 1.000 | 0.964 | 0.765 | 1.000 | 1.000 | 0.823 | 0.789 |
| 941 | 0.775 | 1.000 | 0.791 | 1.000 | 0.958 | 1.000 | 0.847 | 1.000 | 0.969 | 1.000 | 0.930 | 1.000 | 0.964 | 0.765 | 1.000 | 1.000 | 0.824 | 0.790 |
| 942 | 0.775 | 1.000 | 0.791 | 1.000 | 0.963 | 1.000 | 0.847 | 1.000 | 0.969 | 1.000 | 0.930 | 1.000 | 0.964 | 0.765 | 1.000 | 1.000 | 0.824 | 0.790 |
| 943 | 0.773 | 1.000 | 0.791 | 1.000 | 0.958 | 1.000 | 0.847 | 1.000 | 0.969 | 1.000 | 0.928 | 1.000 | 0.964 | 0.763 | 1.000 | 1.000 | 0.822 | 0.788 |
| 944 | 0.773 | 1.000 | 0.792 | 1.000 | 0.958 | 1.000 | 0.847 | 1.000 | 0.969 | 1.000 | 0.928 | 1.000 | 0.964 | 0.763 | 1.000 | 1.000 | 0.823 | 0.789 |
| 945 | 0.774 | 1.000 | 0.794 | 1.000 | 0.958 | 1.000 | 0.847 | 1.000 | 0.969 | 1.000 | 0.928 | 1.000 | 0.964 | 0.764 | 1.000 | 1.000 | 0.823 | 0.789 |
| 946 | 0.775 | 1.000 | 0.794 | 1.000 | 0.958 | 1.000 | 0.847 | 1.000 | 0.969 | 1.000 | 0.928 | 1.000 | 0.964 | 0.764 | 1.000 | 1.000 | 0.824 | 0.790 |
| 947 | 0.774 | 1.000 | 0.796 | 1.000 | 0.958 | 1.000 | 0.849 | 1.000 | 0.969 | 1.000 | 0.928 | 1.000 | 0.964 | 0.764 | 1.000 | 1.000 | 0.824 | 0.790 |
| 948 | 0.775 | 1.000 | 0.796 | 1.000 | 0.963 | 1.000 | 0.849 | 1.000 | 0.969 | 1.000 | 0.928 | 1.000 | 0.964 | 0.764 | 1.000 | 1.000 | 0.824 | 0.790 |
| 949 | 0.775 | 1.000 | 0.794 | 1.000 | 0.963 | 1.000 | 0.849 | 1.000 | 0.969 | 1.000 | 0.928 | 1.000 | 0.964 | 0.764 | 1.000 | 1.000 | 0.824 | 0.790 |
| 950 | 0.775 | 1.000 | 0.794 | 1.000 | 0.963 | 1.000 | 0.849 | 1.000 | 0.969 | 1.000 | 0.928 | 1.000 | 0.964 | 0.764 | 1.000 | 1.000 | 0.824 | 0.790 |
| 951 | 0.775 | 1.000 | 0.791 | 1.000 | 0.963 | 1.000 | 0.849 | 1.000 | 0.969 | 1.000 | 0.933 | 1.000 | 0.964 | 0.764 | 1.000 | 1.000 | 0.824 | 0.790 |
| 952 | 0.774 | 1.000 | 0.791 | 1.000 | 0.963 | 1.000 | 0.849 | 1.000 | 0.969 | 1.000 | 0.933 | 1.000 | 0.964 | 0.764 | 1.000 | 1.000 | 0.824 | 0.790 |
| 953 | 0.775 | 1.000 | 0.794 | 1.000 | 0.963 | 1.000 | 0.851 | 1.000 | 0.969 | 1.000 | 0.933 | 1.000 | 0.964 | 0.764 | 1.000 | 1.000 | 0.825 | 0.791 |
| 954 | 0.777 | 1.000 | 0.792 | 1.000 | 0.963 | 1.000 | 0.854 | 1.000 | 0.969 | 1.000 | 0.933 | 1.000 | 0.964 | 0.763 | 1.000 | 1.000 | 0.825 | 0.792 |
| 955 | 0.777 | 1.000 | 0.792 | 1.000 | 0.968 | 1.000 | 0.854 | 1.000 | 0.969 | 1.000 | 0.933 | 1.000 | 0.964 | 0.764 | 1.000 | 1.000 | 0.825 | 0.792 |
| 956 | 0.779 | 1.000 | 0.791 | 1.000 | 0.968 | 1.000 | 0.852 | 1.000 | 0.969 | 1.000 | 0.936 | 1.000 | 0.964 | 0.767 | 1.000 | 1.000 | 0.827 | 0.793 |
| 957 | 0.779 | 1.000 | 0.787 | 1.000 | 0.968 | 1.000 | 0.851 | 1.000 | 0.969 | 1.000 | 0.939 | 1.000 | 0.964 | 0.767 | 1.000 | 1.000 | 0.826 | 0.793 |
| 958 | 0.779 | 1.000 | 0.787 | 1.000 | 0.968 | 1.000 | 0.852 | 1.000 | 0.969 | 1.000 | 0.939 | 1.000 | 0.964 | 0.766 | 1.000 | 1.000 | 0.826 | 0.793 |
| 959 | 0.779 | 1.000 | 0.787 | 1.000 | 0.968 | 1.000 | 0.852 | 1.000 | 0.969 | 1.000 | 0.939 | 1.000 | 0.964 | 0.767 | 1.000 | 1.000 | 0.826 | 0.793 |
| 960 | 0.780 | 1.000 | 0.787 | 1.000 | 0.968 | 1.000 | 0.852 | 1.000 | 0.969 | 1.000 | 0.939 | 1.000 | 0.964 | 0.767 | 1.000 | 1.000 | 0.827 | 0.793 |
| 961 | 0.778 | 1.000 | 0.785 | 1.000 | 0.968 | 1.000 | 0.851 | 1.000 | 0.969 | 1.000 | 0.939 | 1.000 | 0.964 | 0.767 | 1.000 | 1.000 | 0.826 | 0.792 |
| 962 | 0.778 | 1.000 | 0.781 | 1.000 | 0.968 | 1.000 | 0.849 | 1.000 | 0.969 | 1.000 | 0.939 | 1.000 | 0.964 | 0.767 | 1.000 | 1.000 | 0.825 | 0.791 |
| 963 | 0.787 | 1.000 | 0.785 | 1.000 | 0.968 | 1.000 | 0.858 | 1.000 | 0.969 | 1.000 | 0.939 | 1.000 | 0.964 | 0.767 | 1.000 | 1.000 | 0.829 | 0.796 |
| 964 | 0.790 | 1.000 | 0.783 | 1.000 | 0.968 | 1.000 | 0.858 | 1.000 | 0.969 | 1.000 | 0.939 | 1.000 | 0.964 | 0.767 | 1.000 | 1.000 | 0.830 | 0.797 |
| 965 | 0.787 | 1.000 | 0.781 | 1.000 | 0.968 | 1.000 | 0.860 | 1.000 | 0.969 | 1.000 | 0.936 | 1.000 | 0.964 | 0.766 | 1.000 | 1.000 | 0.829 | 0.796 |
| 966 | 0.787 | 1.000 | 0.783 | 1.000 | 0.968 | 1.000 | 0.860 | 1.000 | 0.969 | 1.000 | 0.936 | 1.000 | 0.964 | 0.766 | 1.000 | 1.000 | 0.829 | 0.796 |
| 967 | 0.789 | 1.000 | 0.785 | 1.000 | 0.968 | 1.000 | 0.858 | 1.000 | 0.969 | 1.000 | 0.936 | 1.000 | 0.964 | 0.767 | 1.000 | 1.000 | 0.830 | 0.797 |
| 968 | 0.787 | 1.000 | 0.787 | 1.000 | 0.968 | 1.000 | 0.858 | 1.000 | 0.969 | 1.000 | 0.936 | 1.000 | 0.964 | 0.767 | 1.000 | 1.000 | 0.830 | 0.797 |
| 969 | 0.786 | 1.000 | 0.785 | 1.000 | 0.958 | 1.000 | 0.861 | 1.000 | 0.969 | 1.000 | 0.936 | 1.000 | 0.964 | 0.767 | 1.000 | 1.000 | 0.829 | 0.796 |
| 970 | 0.785 | 1.000 | 0.785 | 1.000 | 0.958 | 1.000 | 0.860 | 1.000 | 0.969 | 1.000 | 0.936 | 1.000 | 0.964 | 0.767 | 1.000 | 1.000 | 0.829 | 0.795 |
| 971 | 0.787 | 1.000 | 0.781 | 1.000 | 0.958 | 1.000 | 0.861 | 1.000 | 0.969 | 1.000 | 0.936 | 1.000 | 0.964 | 0.768 | 1.000 | 1.000 | 0.829 | 0.796 |
| 972 | 0.787 | 1.000 | 0.781 | 1.000 | 0.958 | 1.000 | 0.860 | 1.000 | 0.969 | 1.000 | 0.936 | 1.000 | 0.964 | 0.767 | 1.000 | 1.000 | 0.828 | 0.795 |
| 973 | 0.782 | 1.000 | 0.783 | 1.000 | 0.958 | 1.000 | 0.861 | 1.000 | 0.969 | 1.000 | 0.939 | 1.000 | 0.973 | 0.766 | 1.000 | 1.000 | 0.828 | 0.794 |
| 974 | 0.785 | 1.000 | 0.785 | 1.000 | 0.958 | 1.000 | 0.860 | 1.000 | 0.969 | 1.000 | 0.936 | 1.000 | 0.973 | 0.766 | 1.000 | 1.000 | 0.828 | 0.795 |
| 975 | 0.785 | 1.000 | 0.787 | 1.000 | 0.958 | 1.000 | 0.858 | 1.000 | 0.969 | 1.000 | 0.936 | 1.000 | 0.973 | 0.766 | 1.000 | 1.000 | 0.828 | 0.795 |

| | | | | | | | | | | | | | | | | | | |
|---|---|---|---|---|---|---|---|---|---|---|---|---|---|---|---|---|---|---|
| 976 | 0.785 | 1.000 | 0.791 | 1.000 | 0.958 | 1.000 | 0.858 | 1.000 | 0.969 | 1.000 | 0.936 | 1.000 | 0.973 | 0.768 | 1.000 | 1.000 | 0.829 | 0.796 |
| 977 | 0.785 | 1.000 | 0.791 | 1.000 | 0.958 | 1.000 | 0.858 | 1.000 | 0.969 | 1.000 | 0.933 | 1.000 | 0.973 | 0.768 | 1.000 | 1.000 | 0.829 | 0.796 |
| 978 | 0.786 | 1.000 | 0.791 | 1.000 | 0.958 | 1.000 | 0.858 | 1.000 | 0.969 | 1.000 | 0.936 | 1.000 | 0.973 | 0.767 | 1.000 | 1.000 | 0.829 | 0.796 |
| 979 | 0.786 | 1.000 | 0.791 | 1.000 | 0.958 | 1.000 | 0.860 | 1.000 | 0.969 | 1.000 | 0.936 | 1.000 | 0.973 | 0.767 | 1.000 | 1.000 | 0.830 | 0.796 |
| 980 | 0.786 | 1.000 | 0.792 | 1.000 | 0.958 | 1.000 | 0.861 | 1.000 | 0.969 | 1.000 | 0.936 | 1.000 | 0.973 | 0.769 | 1.000 | 1.000 | 0.830 | 0.797 |
| 981 | 0.786 | 1.000 | 0.792 | 1.000 | 0.958 | 1.000 | 0.861 | 1.000 | 0.969 | 1.000 | 0.936 | 1.000 | 0.973 | 0.769 | 1.000 | 1.000 | 0.830 | 0.797 |
| 982 | 0.786 | 1.000 | 0.792 | 1.000 | 0.958 | 1.000 | 0.861 | 1.000 | 0.969 | 1.000 | 0.936 | 1.000 | 0.973 | 0.771 | 1.000 | 1.000 | 0.831 | 0.798 |
| 983 | 0.786 | 1.000 | 0.791 | 1.000 | 0.958 | 1.000 | 0.861 | 1.000 | 0.969 | 1.000 | 0.936 | 1.000 | 0.973 | 0.769 | 1.000 | 1.000 | 0.830 | 0.797 |
| 984 | 0.786 | 1.000 | 0.789 | 1.000 | 0.958 | 1.000 | 0.861 | 1.000 | 0.969 | 1.000 | 0.936 | 1.000 | 0.973 | 0.769 | 1.000 | 1.000 | 0.830 | 0.797 |
| 985 | 0.787 | 1.000 | 0.789 | 1.000 | 0.958 | 1.000 | 0.864 | 1.000 | 0.969 | 1.000 | 0.936 | 1.000 | 0.973 | 0.765 | 1.000 | 1.000 | 0.830 | 0.796 |
| 986 | 0.787 | 1.000 | 0.789 | 1.000 | 0.958 | 1.000 | 0.864 | 1.000 | 0.969 | 1.000 | 0.936 | 1.000 | 0.973 | 0.767 | 1.000 | 1.000 | 0.830 | 0.797 |
| 987 | 0.787 | 1.000 | 0.789 | 1.000 | 0.958 | 1.000 | 0.863 | 1.000 | 0.969 | 1.000 | 0.936 | 1.000 | 0.973 | 0.767 | 1.000 | 1.000 | 0.830 | 0.797 |
| 988 | 0.782 | 1.000 | 0.791 | 1.000 | 0.958 | 1.000 | 0.863 | 1.000 | 0.969 | 1.000 | 0.939 | 1.000 | 0.973 | 0.767 | 1.000 | 1.000 | 0.829 | 0.796 |
| 989 | 0.782 | 1.000 | 0.787 | 1.000 | 0.958 | 1.000 | 0.863 | 1.000 | 0.969 | 1.000 | 0.939 | 1.000 | 0.973 | 0.766 | 1.000 | 1.000 | 0.828 | 0.795 |
| 990 | 0.781 | 1.000 | 0.789 | 1.000 | 0.958 | 1.000 | 0.863 | 1.000 | 0.969 | 1.000 | 0.939 | 1.000 | 0.973 | 0.766 | 1.000 | 1.000 | 0.828 | 0.795 |
| 991 | 0.781 | 1.000 | 0.789 | 1.000 | 0.958 | 1.000 | 0.863 | 1.000 | 0.969 | 1.000 | 0.939 | 1.000 | 0.973 | 0.766 | 1.000 | 1.000 | 0.828 | 0.795 |
| 992 | 0.782 | 1.000 | 0.789 | 1.000 | 0.958 | 1.000 | 0.864 | 1.000 | 0.969 | 1.000 | 0.939 | 1.000 | 0.973 | 0.766 | 1.000 | 1.000 | 0.829 | 0.795 |
| 993 | 0.784 | 1.000 | 0.787 | 1.000 | 0.958 | 1.000 | 0.864 | 1.000 | 0.969 | 1.000 | 0.939 | 1.000 | 0.973 | 0.767 | 1.000 | 1.000 | 0.829 | 0.796 |
| 994 | 0.784 | 1.000 | 0.787 | 1.000 | 0.958 | 1.000 | 0.863 | 1.000 | 0.969 | 1.000 | 0.939 | 1.000 | 0.973 | 0.767 | 1.000 | 1.000 | 0.829 | 0.796 |
| 995 | 0.784 | 1.000 | 0.787 | 1.000 | 0.958 | 1.000 | 0.864 | 1.000 | 0.969 | 1.000 | 0.939 | 1.000 | 0.973 | 0.766 | 1.000 | 1.000 | 0.829 | 0.796 |
| 996 | 0.787 | 1.000 | 0.787 | 1.000 | 0.958 | 1.000 | 0.863 | 1.000 | 0.969 | 1.000 | 0.939 | 1.000 | 0.973 | 0.767 | 1.000 | 1.000 | 0.830 | 0.797 |
| 997 | 0.786 | 1.000 | 0.783 | 1.000 | 0.958 | 1.000 | 0.861 | 1.000 | 0.969 | 1.000 | 0.939 | 1.000 | 0.973 | 0.766 | 1.000 | 1.000 | 0.829 | 0.795 |
| 998 | 0.785 | 1.000 | 0.779 | 1.000 | 0.958 | 1.000 | 0.866 | 1.000 | 0.969 | 1.000 | 0.939 | 1.000 | 0.973 | 0.767 | 1.000 | 1.000 | 0.829 | 0.796 |
| 999 | 0.785 | 1.000 | 0.779 | 1.000 | 0.958 | 1.000 | 0.866 | 1.000 | 0.969 | 1.000 | 0.939 | 1.000 | 0.973 | 0.767 | 1.000 | 1.000 | 0.829 | 0.796 |
| 1000 | 0.784 | 1.000 | 0.781 | 1.000 | 0.958 | 1.000 | 0.866 | 1.000 | 0.969 | 1.000 | 0.939 | 1.000 | 0.973 | 0.766 | 1.000 | 1.000 | 0.829 | 0.795 |
| 1001 | 0.785 | 1.000 | 0.785 | 1.000 | 0.963 | 1.000 | 0.866 | 1.000 | 0.969 | 1.000 | 0.936 | 1.000 | 0.973 | 0.767 | 1.000 | 1.000 | 0.830 | 0.797 |
| 1002 | 0.785 | 1.000 | 0.785 | 1.000 | 0.963 | 1.000 | 0.866 | 1.000 | 0.969 | 1.000 | 0.936 | 1.000 | 0.973 | 0.767 | 1.000 | 1.000 | 0.830 | 0.797 |
| 1003 | 0.786 | 1.000 | 0.785 | 1.000 | 0.963 | 1.000 | 0.863 | 1.000 | 0.969 | 1.000 | 0.936 | 1.000 | 0.973 | 0.767 | 1.000 | 1.000 | 0.829 | 0.796 |
| 1004 | 0.785 | 1.000 | 0.779 | 1.000 | 0.958 | 1.000 | 0.867 | 1.000 | 0.969 | 1.000 | 0.936 | 1.000 | 0.973 | 0.766 | 1.000 | 1.000 | 0.829 | 0.795 |
| 1005 | 0.785 | 1.000 | 0.779 | 1.000 | 0.958 | 1.000 | 0.867 | 1.000 | 0.969 | 1.000 | 0.939 | 1.000 | 0.973 | 0.766 | 1.000 | 1.000 | 0.829 | 0.795 |
| 1006 | 0.785 | 1.000 | 0.781 | 1.000 | 0.958 | 1.000 | 0.866 | 1.000 | 0.969 | 1.000 | 0.936 | 1.000 | 0.973 | 0.764 | 1.000 | 1.000 | 0.828 | 0.795 |
| 1007 | 0.784 | 1.000 | 0.781 | 1.000 | 0.958 | 1.000 | 0.864 | 1.000 | 0.969 | 1.000 | 0.936 | 1.000 | 0.973 | 0.765 | 1.000 | 1.000 | 0.828 | 0.794 |
| 1008 | 0.786 | 1.000 | 0.785 | 1.000 | 0.958 | 1.000 | 0.864 | 1.000 | 0.969 | 1.000 | 0.936 | 1.000 | 0.973 | 0.767 | 1.000 | 1.000 | 0.829 | 0.796 |
| 1009 | 0.787 | 1.000 | 0.785 | 1.000 | 0.958 | 1.000 | 0.863 | 1.000 | 0.969 | 1.000 | 0.939 | 1.000 | 0.973 | 0.767 | 1.000 | 1.000 | 0.830 | 0.796 |
| 1010 | 0.786 | 1.000 | 0.783 | 1.000 | 0.958 | 1.000 | 0.864 | 1.000 | 0.969 | 1.000 | 0.939 | 1.000 | 0.973 | 0.767 | 1.000 | 1.000 | 0.829 | 0.796 |
| 1011 | 0.787 | 1.000 | 0.785 | 1.000 | 0.958 | 1.000 | 0.866 | 1.000 | 0.969 | 1.000 | 0.936 | 1.000 | 0.973 | 0.766 | 1.000 | 1.000 | 0.830 | 0.797 |
| 1012 | 0.787 | 1.000 | 0.785 | 1.000 | 0.958 | 1.000 | 0.866 | 1.000 | 0.969 | 1.000 | 0.936 | 1.000 | 0.973 | 0.766 | 1.000 | 1.000 | 0.830 | 0.797 |
| 1013 | 0.790 | 1.000 | 0.785 | 1.000 | 0.958 | 1.000 | 0.866 | 1.000 | 0.969 | 1.000 | 0.936 | 1.000 | 0.973 | 0.766 | 1.000 | 1.000 | 0.830 | 0.797 |
| 1014 | 0.787 | 1.000 | 0.789 | 1.000 | 0.958 | 1.000 | 0.867 | 1.000 | 0.969 | 1.000 | 0.939 | 1.000 | 0.973 | 0.767 | 1.000 | 1.000 | 0.831 | 0.798 |

| | | | | | | | | | | | | | | | | | |
|---|---|---|---|---|---|---|---|---|---|---|---|---|---|---|---|---|---|
| 1015 | 0.792 | 1.000 | 0.787 | 1.000 | 0.958 | 1.000 | 0.867 | 1.000 | 0.969 | 1.000 | 0.939 | 1.000 | 0.973 | 0.767 | 1.000 | 1.000 | 0.832 | 0.799 |
| 1016 | 0.791 | 1.000 | 0.792 | 1.000 | 0.958 | 1.000 | 0.867 | 1.000 | 0.969 | 1.000 | 0.939 | 1.000 | 0.973 | 0.768 | 1.000 | 1.000 | 0.833 | 0.800 |
| 1017 | 0.789 | 1.000 | 0.792 | 1.000 | 0.958 | 1.000 | 0.869 | 1.000 | 0.969 | 1.000 | 0.939 | 1.000 | 0.973 | 0.767 | 1.000 | 1.000 | 0.832 | 0.799 |
| 1018 | 0.788 | 1.000 | 0.792 | 1.000 | 0.958 | 1.000 | 0.871 | 1.000 | 0.969 | 1.000 | 0.939 | 1.000 | 0.973 | 0.767 | 1.000 | 1.000 | 0.832 | 0.799 |
| 1019 | 0.789 | 1.000 | 0.789 | 1.000 | 0.958 | 1.000 | 0.871 | 1.000 | 0.969 | 1.000 | 0.939 | 1.000 | 0.973 | 0.767 | 1.000 | 1.000 | 0.832 | 0.799 |
| 1020 | 0.789 | 1.000 | 0.791 | 1.000 | 0.958 | 1.000 | 0.871 | 1.000 | 0.969 | 1.000 | 0.939 | 1.000 | 0.973 | 0.768 | 1.000 | 1.000 | 0.832 | 0.800 |
| 1021 | 0.790 | 1.000 | 0.791 | 1.000 | 0.958 | 1.000 | 0.871 | 1.000 | 0.969 | 1.000 | 0.936 | 1.000 | 0.973 | 0.768 | 1.000 | 1.000 | 0.832 | 0.800 |
| 1022 | 0.787 | 1.000 | 0.789 | 1.000 | 0.958 | 1.000 | 0.869 | 1.000 | 0.969 | 1.000 | 0.939 | 1.000 | 0.973 | 0.772 | 1.000 | 1.000 | 0.832 | 0.800 |
| 1023 | 0.787 | 1.000 | 0.789 | 1.000 | 0.958 | 1.000 | 0.869 | 1.000 | 0.969 | 1.000 | 0.939 | 1.000 | 0.973 | 0.773 | 1.000 | 1.000 | 0.833 | 0.800 |
| 1024 | 0.787 | 1.000 | 0.792 | 1.000 | 0.958 | 1.000 | 0.869 | 1.000 | 0.969 | 1.000 | 0.939 | 1.000 | 0.973 | 0.772 | 1.000 | 1.000 | 0.833 | 0.800 |
| 1025 | 0.787 | 1.000 | 0.792 | 1.000 | 0.958 | 1.000 | 0.867 | 1.000 | 0.969 | 1.000 | 0.936 | 1.000 | 0.973 | 0.770 | 1.000 | 1.000 | 0.832 | 0.799 |
| 1026 | 0.788 | 1.000 | 0.792 | 1.000 | 0.958 | 1.000 | 0.869 | 1.000 | 0.969 | 1.000 | 0.936 | 1.000 | 0.973 | 0.770 | 1.000 | 1.000 | 0.832 | 0.799 |
| 1027 | 0.788 | 1.000 | 0.794 | 1.000 | 0.958 | 1.000 | 0.871 | 1.000 | 0.969 | 1.000 | 0.936 | 1.000 | 0.973 | 0.771 | 1.000 | 1.000 | 0.833 | 0.800 |
| 1028 | 0.786 | 1.000 | 0.792 | 1.000 | 0.958 | 1.000 | 0.871 | 1.000 | 0.969 | 1.000 | 0.939 | 1.000 | 0.973 | 0.773 | 1.000 | 1.000 | 0.833 | 0.800 |
| 1029 | 0.786 | 1.000 | 0.791 | 1.000 | 0.958 | 1.000 | 0.872 | 1.000 | 0.969 | 1.000 | 0.939 | 1.000 | 0.973 | 0.772 | 1.000 | 1.000 | 0.833 | 0.800 |
| 1030 | 0.787 | 1.000 | 0.791 | 1.000 | 0.958 | 1.000 | 0.871 | 1.000 | 0.969 | 1.000 | 0.936 | 1.000 | 0.973 | 0.773 | 1.000 | 1.000 | 0.833 | 0.800 |
| 1031 | 0.786 | 1.000 | 0.791 | 1.000 | 0.958 | 1.000 | 0.867 | 1.000 | 0.969 | 1.000 | 0.936 | 1.000 | 0.973 | 0.772 | 1.000 | 1.000 | 0.832 | 0.799 |
| 1032 | 0.786 | 1.000 | 0.791 | 1.000 | 0.958 | 1.000 | 0.867 | 1.000 | 0.969 | 1.000 | 0.936 | 1.000 | 0.973 | 0.772 | 1.000 | 1.000 | 0.832 | 0.799 |
| 1033 | 0.786 | 1.000 | 0.794 | 1.000 | 0.958 | 1.000 | 0.869 | 1.000 | 0.969 | 1.000 | 0.939 | 1.000 | 0.973 | 0.773 | 1.000 | 1.000 | 0.833 | 0.800 |
| 1034 | 0.786 | 1.000 | 0.791 | 1.000 | 0.958 | 1.000 | 0.869 | 1.000 | 0.969 | 1.000 | 0.939 | 1.000 | 0.973 | 0.771 | 1.000 | 1.000 | 0.832 | 0.799 |
| 1035 | 0.786 | 1.000 | 0.791 | 1.000 | 0.958 | 1.000 | 0.869 | 1.000 | 0.969 | 1.000 | 0.939 | 1.000 | 0.973 | 0.772 | 1.000 | 1.000 | 0.832 | 0.799 |
| 1036 | 0.787 | 1.000 | 0.789 | 1.000 | 0.958 | 1.000 | 0.869 | 1.000 | 0.969 | 1.000 | 0.939 | 1.000 | 0.973 | 0.771 | 1.000 | 1.000 | 0.832 | 0.799 |
| 1037 | 0.787 | 1.000 | 0.789 | 1.000 | 0.958 | 1.000 | 0.869 | 1.000 | 0.969 | 1.000 | 0.939 | 1.000 | 0.973 | 0.771 | 1.000 | 1.000 | 0.832 | 0.799 |
| 1038 | 0.786 | 1.000 | 0.789 | 1.000 | 0.958 | 1.000 | 0.869 | 1.000 | 0.969 | 1.000 | 0.939 | 1.000 | 0.973 | 0.771 | 1.000 | 1.000 | 0.832 | 0.799 |
| 1039 | 0.786 | 1.000 | 0.789 | 1.000 | 0.958 | 1.000 | 0.867 | 1.000 | 0.969 | 1.000 | 0.939 | 1.000 | 0.973 | 0.772 | 1.000 | 1.000 | 0.832 | 0.799 |
| 1040 | 0.785 | 1.000 | 0.800 | 1.000 | 0.958 | 1.000 | 0.869 | 1.000 | 0.969 | 1.000 | 0.942 | 1.000 | 0.964 | 0.771 | 1.000 | 1.000 | 0.833 | 0.800 |
| 1041 | 0.785 | 1.000 | 0.798 | 1.000 | 0.958 | 1.000 | 0.869 | 1.000 | 0.969 | 1.000 | 0.942 | 1.000 | 0.964 | 0.771 | 1.000 | 1.000 | 0.833 | 0.800 |
| 1042 | 0.785 | 1.000 | 0.798 | 1.000 | 0.958 | 1.000 | 0.869 | 1.000 | 0.969 | 1.000 | 0.942 | 1.000 | 0.964 | 0.771 | 1.000 | 1.000 | 0.833 | 0.800 |
| 1043 | 0.786 | 1.000 | 0.796 | 1.000 | 0.958 | 1.000 | 0.871 | 1.000 | 0.969 | 1.000 | 0.942 | 1.000 | 0.964 | 0.772 | 1.000 | 1.000 | 0.833 | 0.800 |
| 1044 | 0.785 | 1.000 | 0.796 | 1.000 | 0.958 | 1.000 | 0.871 | 1.000 | 0.969 | 1.000 | 0.942 | 1.000 | 0.964 | 0.772 | 1.000 | 1.000 | 0.833 | 0.800 |
| 1045 | 0.785 | 1.000 | 0.798 | 1.000 | 0.958 | 1.000 | 0.872 | 1.000 | 0.969 | 1.000 | 0.942 | 1.000 | 0.964 | 0.770 | 1.000 | 1.000 | 0.833 | 0.800 |
| 1046 | 0.785 | 1.000 | 0.798 | 1.000 | 0.958 | 1.000 | 0.872 | 1.000 | 0.969 | 1.000 | 0.942 | 1.000 | 0.964 | 0.770 | 1.000 | 1.000 | 0.833 | 0.800 |
| 1047 | 0.785 | 1.000 | 0.800 | 1.000 | 0.958 | 1.000 | 0.872 | 1.000 | 0.969 | 1.000 | 0.942 | 1.000 | 0.964 | 0.770 | 1.000 | 1.000 | 0.833 | 0.800 |
| 1048 | 0.787 | 1.000 | 0.804 | 1.000 | 0.958 | 1.000 | 0.872 | 1.000 | 0.969 | 1.000 | 0.942 | 1.000 | 0.964 | 0.770 | 1.000 | 1.000 | 0.834 | 0.801 |
| 1049 | 0.787 | 1.000 | 0.804 | 1.000 | 0.958 | 1.000 | 0.872 | 1.000 | 0.969 | 1.000 | 0.942 | 1.000 | 0.964 | 0.770 | 1.000 | 1.000 | 0.834 | 0.801 |
| 1050 | 0.787 | 1.000 | 0.804 | 1.000 | 0.958 | 1.000 | 0.872 | 1.000 | 0.969 | 1.000 | 0.942 | 1.000 | 0.964 | 0.770 | 1.000 | 1.000 | 0.834 | 0.801 |
| 1051 | 0.788 | 1.000 | 0.804 | 1.000 | 0.958 | 1.000 | 0.872 | 1.000 | 0.969 | 1.000 | 0.942 | 1.000 | 0.964 | 0.770 | 1.000 | 1.000 | 0.834 | 0.802 |
| 1052 | 0.790 | 1.000 | 0.808 | 1.000 | 0.958 | 1.000 | 0.872 | 1.000 | 0.969 | 1.000 | 0.942 | 1.000 | 0.964 | 0.771 | 1.000 | 1.000 | 0.835 | 0.803 |
| 1053 | 0.788 | 1.000 | 0.808 | 1.000 | 0.958 | 1.000 | 0.871 | 1.000 | 0.969 | 1.000 | 0.939 | 1.000 | 0.964 | 0.771 | 1.000 | 1.000 | 0.834 | 0.802 |

| | | | | | | | | | | | | | | | | | | |
|---|---|---|---|---|---|---|---|---|---|---|---|---|---|---|---|---|---|---|
| 1054 | 0.788 | 1.000 | 0.810 | 1.000 | 0.958 | 1.000 | 0.871 | 1.000 | 0.969 | 1.000 | 0.939 | 1.000 | 0.964 | 0.769 | 1.000 | 1.000 | 0.834 | 0.802 |
| 1055 | 0.787 | 1.000 | 0.802 | 1.000 | 0.958 | 1.000 | 0.871 | 1.000 | 0.969 | 1.000 | 0.939 | 1.000 | 0.964 | 0.767 | 1.000 | 1.000 | 0.832 | 0.800 |
| 1056 | 0.785 | 1.000 | 0.800 | 1.000 | 0.958 | 1.000 | 0.872 | 1.000 | 0.969 | 1.000 | 0.936 | 1.000 | 0.964 | 0.767 | 1.000 | 1.000 | 0.831 | 0.799 |
| 1057 | 0.785 | 1.000 | 0.800 | 1.000 | 0.958 | 1.000 | 0.872 | 1.000 | 0.969 | 1.000 | 0.936 | 1.000 | 0.964 | 0.767 | 1.000 | 1.000 | 0.832 | 0.799 |
| 1058 | 0.785 | 1.000 | 0.800 | 1.000 | 0.958 | 1.000 | 0.872 | 1.000 | 0.969 | 1.000 | 0.936 | 1.000 | 0.964 | 0.767 | 1.000 | 1.000 | 0.832 | 0.799 |
| 1059 | 0.785 | 1.000 | 0.800 | 1.000 | 0.958 | 1.000 | 0.871 | 1.000 | 0.969 | 1.000 | 0.936 | 1.000 | 0.964 | 0.767 | 1.000 | 1.000 | 0.831 | 0.799 |
| 1060 | 0.785 | 1.000 | 0.798 | 1.000 | 0.958 | 1.000 | 0.872 | 1.000 | 0.969 | 1.000 | 0.936 | 1.000 | 0.964 | 0.767 | 1.000 | 1.000 | 0.831 | 0.799 |
| 1061 | 0.785 | 1.000 | 0.800 | 1.000 | 0.958 | 1.000 | 0.874 | 1.000 | 0.969 | 1.000 | 0.936 | 1.000 | 0.964 | 0.766 | 1.000 | 1.000 | 0.832 | 0.799 |
| 1062 | 0.787 | 1.000 | 0.800 | 1.000 | 0.958 | 1.000 | 0.872 | 1.000 | 0.969 | 1.000 | 0.939 | 1.000 | 0.964 | 0.767 | 1.000 | 1.000 | 0.832 | 0.800 |
| 1063 | 0.787 | 1.000 | 0.798 | 1.000 | 0.958 | 1.000 | 0.872 | 1.000 | 0.969 | 1.000 | 0.939 | 1.000 | 0.964 | 0.767 | 1.000 | 1.000 | 0.832 | 0.800 |
| 1064 | 0.787 | 1.000 | 0.800 | 1.000 | 0.958 | 1.000 | 0.872 | 1.000 | 0.969 | 1.000 | 0.939 | 1.000 | 0.964 | 0.767 | 1.000 | 1.000 | 0.833 | 0.800 |
| 1065 | 0.787 | 1.000 | 0.800 | 1.000 | 0.958 | 1.000 | 0.871 | 1.000 | 0.969 | 1.000 | 0.933 | 1.000 | 0.964 | 0.768 | 1.000 | 1.000 | 0.832 | 0.800 |
| 1066 | 0.786 | 1.000 | 0.800 | 1.000 | 0.958 | 1.000 | 0.869 | 1.000 | 0.969 | 1.000 | 0.936 | 1.000 | 0.964 | 0.768 | 1.000 | 1.000 | 0.832 | 0.799 |
| 1067 | 0.785 | 1.000 | 0.800 | 1.000 | 0.958 | 1.000 | 0.871 | 1.000 | 0.969 | 1.000 | 0.936 | 1.000 | 0.964 | 0.768 | 1.000 | 1.000 | 0.832 | 0.799 |
| 1068 | 0.786 | 1.000 | 0.800 | 1.000 | 0.958 | 1.000 | 0.871 | 1.000 | 0.969 | 1.000 | 0.936 | 1.000 | 0.964 | 0.772 | 1.000 | 1.000 | 0.833 | 0.800 |
| 1069 | 0.786 | 1.000 | 0.800 | 1.000 | 0.958 | 1.000 | 0.871 | 1.000 | 0.969 | 1.000 | 0.936 | 1.000 | 0.964 | 0.772 | 1.000 | 1.000 | 0.833 | 0.801 |
| 1070 | 0.785 | 1.000 | 0.804 | 1.000 | 0.958 | 1.000 | 0.869 | 1.000 | 0.969 | 1.000 | 0.936 | 1.000 | 0.964 | 0.770 | 1.000 | 1.000 | 0.832 | 0.800 |
| 1071 | 0.787 | 1.000 | 0.796 | 1.000 | 0.958 | 1.000 | 0.869 | 1.000 | 0.969 | 1.000 | 0.936 | 1.000 | 0.964 | 0.771 | 1.000 | 1.000 | 0.832 | 0.800 |
| 1072 | 0.786 | 1.000 | 0.798 | 1.000 | 0.958 | 1.000 | 0.869 | 1.000 | 0.969 | 1.000 | 0.936 | 1.000 | 0.964 | 0.772 | 1.000 | 1.000 | 0.833 | 0.800 |
| 1073 | 0.786 | 1.000 | 0.798 | 1.000 | 0.958 | 1.000 | 0.869 | 1.000 | 0.969 | 1.000 | 0.936 | 1.000 | 0.964 | 0.772 | 1.000 | 1.000 | 0.833 | 0.800 |
| 1074 | 0.785 | 1.000 | 0.792 | 1.000 | 0.958 | 1.000 | 0.867 | 1.000 | 0.969 | 1.000 | 0.936 | 1.000 | 0.964 | 0.773 | 1.000 | 1.000 | 0.832 | 0.799 |
| 1075 | 0.785 | 1.000 | 0.792 | 1.000 | 0.958 | 1.000 | 0.869 | 1.000 | 0.969 | 1.000 | 0.936 | 1.000 | 0.964 | 0.774 | 1.000 | 1.000 | 0.832 | 0.799 |
| 1076 | 0.785 | 1.000 | 0.792 | 1.000 | 0.958 | 1.000 | 0.869 | 1.000 | 0.969 | 1.000 | 0.936 | 1.000 | 0.964 | 0.774 | 1.000 | 1.000 | 0.832 | 0.799 |
| 1077 | 0.786 | 1.000 | 0.792 | 1.000 | 0.958 | 1.000 | 0.872 | 1.000 | 0.969 | 1.000 | 0.936 | 1.000 | 0.964 | 0.773 | 1.000 | 1.000 | 0.833 | 0.800 |
| 1078 | 0.786 | 1.000 | 0.792 | 1.000 | 0.958 | 1.000 | 0.872 | 1.000 | 0.969 | 1.000 | 0.936 | 1.000 | 0.964 | 0.773 | 1.000 | 1.000 | 0.833 | 0.800 |
| 1079 | 0.787 | 1.000 | 0.792 | 1.000 | 0.958 | 1.000 | 0.872 | 1.000 | 0.969 | 1.000 | 0.936 | 1.000 | 0.964 | 0.773 | 1.000 | 1.000 | 0.833 | 0.800 |
| 1080 | 0.787 | 1.000 | 0.800 | 1.000 | 0.958 | 1.000 | 0.864 | 1.000 | 0.969 | 1.000 | 0.939 | 1.000 | 0.964 | 0.773 | 1.000 | 1.000 | 0.833 | 0.800 |
| 1081 | 0.788 | 1.000 | 0.798 | 1.000 | 0.958 | 1.000 | 0.867 | 1.000 | 0.969 | 1.000 | 0.939 | 1.000 | 0.964 | 0.772 | 1.000 | 1.000 | 0.833 | 0.801 |
| 1082 | 0.788 | 1.000 | 0.796 | 1.000 | 0.958 | 1.000 | 0.869 | 1.000 | 0.969 | 1.000 | 0.939 | 1.000 | 0.964 | 0.767 | 1.000 | 1.000 | 0.832 | 0.799 |
| 1083 | 0.789 | 1.000 | 0.798 | 1.000 | 0.958 | 1.000 | 0.864 | 1.000 | 0.969 | 1.000 | 0.939 | 1.000 | 0.964 | 0.768 | 1.000 | 1.000 | 0.832 | 0.799 |
| 1084 | 0.788 | 1.000 | 0.794 | 1.000 | 0.958 | 1.000 | 0.863 | 1.000 | 0.969 | 1.000 | 0.939 | 1.000 | 0.964 | 0.769 | 1.000 | 1.000 | 0.831 | 0.798 |
| 1085 | 0.788 | 1.000 | 0.796 | 1.000 | 0.958 | 1.000 | 0.863 | 1.000 | 0.969 | 1.000 | 0.939 | 1.000 | 0.964 | 0.769 | 1.000 | 1.000 | 0.832 | 0.799 |
| 1086 | 0.787 | 1.000 | 0.798 | 1.000 | 0.958 | 1.000 | 0.863 | 1.000 | 0.969 | 1.000 | 0.939 | 1.000 | 0.964 | 0.770 | 1.000 | 1.000 | 0.832 | 0.799 |
| 1087 | 0.790 | 1.000 | 0.800 | 1.000 | 0.958 | 1.000 | 0.866 | 1.000 | 0.969 | 1.000 | 0.939 | 1.000 | 0.964 | 0.769 | 1.000 | 1.000 | 0.833 | 0.800 |
| 1088 | 0.788 | 1.000 | 0.798 | 1.000 | 0.958 | 1.000 | 0.866 | 1.000 | 0.969 | 1.000 | 0.939 | 1.000 | 0.964 | 0.768 | 1.000 | 1.000 | 0.832 | 0.799 |
| 1089 | 0.787 | 1.000 | 0.798 | 1.000 | 0.958 | 1.000 | 0.861 | 1.000 | 0.969 | 1.000 | 0.939 | 1.000 | 0.964 | 0.766 | 1.000 | 1.000 | 0.830 | 0.797 |
| 1090 | 0.785 | 1.000 | 0.796 | 1.000 | 0.958 | 1.000 | 0.864 | 1.000 | 0.969 | 1.000 | 0.939 | 1.000 | 0.964 | 0.767 | 1.000 | 1.000 | 0.830 | 0.797 |
| 1091 | 0.785 | 1.000 | 0.798 | 1.000 | 0.953 | 1.000 | 0.864 | 1.000 | 0.969 | 1.000 | 0.939 | 1.000 | 0.964 | 0.767 | 1.000 | 1.000 | 0.830 | 0.797 |
| 1092 | 0.787 | 1.000 | 0.800 | 1.000 | 0.953 | 1.000 | 0.863 | 1.000 | 0.969 | 1.000 | 0.942 | 1.000 | 0.964 | 0.767 | 1.000 | 1.000 | 0.831 | 0.798 |

| | | | | | | | | | | | | | | | | | |
|---|---|---|---|---|---|---|---|---|---|---|---|---|---|---|---|---|---|
| 1093 | 0.787 | 1.000 | 0.800 | 1.000 | 0.953 | 1.000 | 0.863 | 1.000 | 0.969 | 1.000 | 0.942 | 1.000 | 0.964 | 0.767 | 1.000 | 1.000 | 0.831 | 0.798 |
| 1094 | 0.787 | 1.000 | 0.796 | 1.000 | 0.953 | 1.000 | 0.861 | 1.000 | 0.969 | 1.000 | 0.945 | 1.000 | 0.964 | 0.766 | 1.000 | 1.000 | 0.831 | 0.798 |
| 1095 | 0.787 | 1.000 | 0.798 | 1.000 | 0.953 | 1.000 | 0.861 | 1.000 | 0.969 | 1.000 | 0.945 | 1.000 | 0.964 | 0.765 | 1.000 | 1.000 | 0.830 | 0.797 |
| 1096 | 0.787 | 1.000 | 0.798 | 1.000 | 0.953 | 1.000 | 0.864 | 1.000 | 0.969 | 1.000 | 0.948 | 1.000 | 0.964 | 0.765 | 1.000 | 1.000 | 0.831 | 0.798 |
| 1097 | 0.788 | 1.000 | 0.789 | 1.000 | 0.953 | 1.000 | 0.864 | 1.000 | 0.969 | 1.000 | 0.945 | 1.000 | 0.964 | 0.767 | 1.000 | 1.000 | 0.831 | 0.797 |
| 1098 | 0.789 | 1.000 | 0.792 | 1.000 | 0.953 | 1.000 | 0.864 | 1.000 | 0.969 | 1.000 | 0.945 | 1.000 | 0.964 | 0.767 | 1.000 | 1.000 | 0.831 | 0.798 |
| 1099 | 0.787 | 1.000 | 0.789 | 1.000 | 0.953 | 1.000 | 0.863 | 1.000 | 0.969 | 1.000 | 0.945 | 1.000 | 0.964 | 0.768 | 1.000 | 1.000 | 0.831 | 0.797 |
| 1100 | 0.787 | 1.000 | 0.789 | 1.000 | 0.953 | 1.000 | 0.864 | 1.000 | 0.969 | 1.000 | 0.945 | 1.000 | 0.964 | 0.768 | 1.000 | 1.000 | 0.831 | 0.798 |
| 1101 | 0.786 | 1.000 | 0.787 | 1.000 | 0.953 | 1.000 | 0.863 | 1.000 | 0.969 | 1.000 | 0.945 | 1.000 | 0.964 | 0.768 | 1.000 | 1.000 | 0.830 | 0.797 |
| 1102 | 0.786 | 1.000 | 0.789 | 1.000 | 0.953 | 1.000 | 0.863 | 1.000 | 0.969 | 1.000 | 0.945 | 1.000 | 0.964 | 0.767 | 1.000 | 1.000 | 0.830 | 0.797 |
| 1103 | 0.788 | 1.000 | 0.792 | 1.000 | 0.953 | 1.000 | 0.860 | 1.000 | 0.969 | 1.000 | 0.945 | 1.000 | 0.964 | 0.766 | 1.000 | 1.000 | 0.830 | 0.797 |
| 1104 | 0.789 | 1.000 | 0.794 | 1.000 | 0.958 | 1.000 | 0.860 | 1.000 | 0.969 | 1.000 | 0.945 | 1.000 | 0.964 | 0.764 | 1.000 | 1.000 | 0.830 | 0.797 |
| 1105 | 0.787 | 1.000 | 0.792 | 1.000 | 0.958 | 1.000 | 0.860 | 1.000 | 0.969 | 1.000 | 0.948 | 1.000 | 0.964 | 0.764 | 1.000 | 1.000 | 0.830 | 0.797 |
| 1106 | 0.787 | 1.000 | 0.791 | 1.000 | 0.958 | 1.000 | 0.860 | 1.000 | 0.969 | 1.000 | 0.948 | 1.000 | 0.964 | 0.765 | 1.000 | 1.000 | 0.830 | 0.796 |
| 1107 | 0.785 | 1.000 | 0.796 | 1.000 | 0.958 | 1.000 | 0.858 | 1.000 | 0.969 | 1.000 | 0.948 | 1.000 | 0.964 | 0.767 | 1.000 | 1.000 | 0.830 | 0.797 |
| 1108 | 0.787 | 1.000 | 0.796 | 1.000 | 0.958 | 1.000 | 0.858 | 1.000 | 0.969 | 1.000 | 0.945 | 1.000 | 0.964 | 0.769 | 1.000 | 1.000 | 0.831 | 0.798 |
| 1109 | 0.786 | 1.000 | 0.796 | 1.000 | 0.953 | 1.000 | 0.860 | 1.000 | 0.969 | 1.000 | 0.948 | 1.000 | 0.964 | 0.769 | 1.000 | 1.000 | 0.831 | 0.798 |
| 1110 | 0.787 | 1.000 | 0.798 | 1.000 | 0.953 | 1.000 | 0.860 | 1.000 | 0.969 | 1.000 | 0.948 | 1.000 | 0.964 | 0.770 | 1.000 | 1.000 | 0.832 | 0.799 |
| 1111 | 0.787 | 1.000 | 0.796 | 1.000 | 0.953 | 1.000 | 0.860 | 1.000 | 0.969 | 1.000 | 0.948 | 1.000 | 0.964 | 0.770 | 1.000 | 1.000 | 0.832 | 0.799 |
| 1112 | 0.787 | 1.000 | 0.794 | 1.000 | 0.953 | 1.000 | 0.861 | 1.000 | 0.969 | 1.000 | 0.948 | 1.000 | 0.964 | 0.770 | 1.000 | 1.000 | 0.832 | 0.798 |
| 1113 | 0.787 | 1.000 | 0.796 | 1.000 | 0.953 | 1.000 | 0.861 | 1.000 | 0.969 | 1.000 | 0.948 | 1.000 | 0.964 | 0.771 | 1.000 | 1.000 | 0.832 | 0.799 |
| 1114 | 0.788 | 1.000 | 0.796 | 1.000 | 0.953 | 1.000 | 0.861 | 1.000 | 0.969 | 1.000 | 0.948 | 1.000 | 0.964 | 0.771 | 1.000 | 1.000 | 0.832 | 0.799 |
| 1115 | 0.788 | 1.000 | 0.798 | 1.000 | 0.953 | 1.000 | 0.861 | 1.000 | 0.969 | 1.000 | 0.948 | 1.000 | 0.964 | 0.770 | 1.000 | 1.000 | 0.832 | 0.799 |
| 1116 | 0.787 | 1.000 | 0.796 | 1.000 | 0.953 | 1.000 | 0.861 | 1.000 | 0.969 | 1.000 | 0.948 | 1.000 | 0.964 | 0.770 | 1.000 | 1.000 | 0.832 | 0.799 |
| 1117 | 0.788 | 1.000 | 0.794 | 1.000 | 0.953 | 1.000 | 0.860 | 1.000 | 0.969 | 1.000 | 0.948 | 1.000 | 0.964 | 0.770 | 1.000 | 1.000 | 0.832 | 0.798 |
| 1118 | 0.787 | 1.000 | 0.796 | 1.000 | 0.953 | 1.000 | 0.860 | 1.000 | 0.969 | 1.000 | 0.948 | 1.000 | 0.964 | 0.770 | 1.000 | 1.000 | 0.832 | 0.798 |
| 1119 | 0.783 | 1.000 | 0.796 | 1.000 | 0.953 | 1.000 | 0.860 | 1.000 | 0.969 | 1.000 | 0.951 | 1.000 | 0.964 | 0.769 | 1.000 | 1.000 | 0.830 | 0.797 |
| 1120 | 0.783 | 1.000 | 0.796 | 1.000 | 0.953 | 1.000 | 0.860 | 1.000 | 0.969 | 1.000 | 0.951 | 1.000 | 0.964 | 0.769 | 1.000 | 1.000 | 0.830 | 0.797 |
| 1121 | 0.782 | 1.000 | 0.794 | 1.000 | 0.958 | 1.000 | 0.860 | 1.000 | 0.969 | 1.000 | 0.948 | 1.000 | 0.964 | 0.769 | 1.000 | 1.000 | 0.830 | 0.796 |
| 1122 | 0.785 | 1.000 | 0.798 | 1.000 | 0.958 | 1.000 | 0.860 | 1.000 | 0.969 | 1.000 | 0.948 | 1.000 | 0.973 | 0.770 | 1.000 | 1.000 | 0.831 | 0.798 |
| 1123 | 0.783 | 1.000 | 0.796 | 1.000 | 0.958 | 1.000 | 0.858 | 1.000 | 0.969 | 1.000 | 0.948 | 1.000 | 0.973 | 0.770 | 1.000 | 1.000 | 0.831 | 0.797 |
| 1124 | 0.784 | 1.000 | 0.796 | 1.000 | 0.958 | 1.000 | 0.858 | 1.000 | 0.969 | 1.000 | 0.948 | 1.000 | 0.973 | 0.768 | 1.000 | 1.000 | 0.830 | 0.797 |
| 1125 | 0.785 | 1.000 | 0.798 | 1.000 | 0.958 | 1.000 | 0.860 | 1.000 | 0.969 | 1.000 | 0.948 | 1.000 | 0.973 | 0.769 | 1.000 | 1.000 | 0.831 | 0.798 |
| 1126 | 0.785 | 1.000 | 0.796 | 1.000 | 0.958 | 1.000 | 0.860 | 1.000 | 0.969 | 1.000 | 0.948 | 1.000 | 0.973 | 0.768 | 1.000 | 1.000 | 0.831 | 0.798 |
| 1127 | 0.783 | 1.000 | 0.798 | 1.000 | 0.958 | 1.000 | 0.861 | 1.000 | 0.969 | 1.000 | 0.948 | 1.000 | 0.973 | 0.767 | 1.000 | 1.000 | 0.831 | 0.797 |
| 1128 | 0.783 | 1.000 | 0.796 | 1.000 | 0.958 | 1.000 | 0.861 | 1.000 | 0.969 | 1.000 | 0.948 | 1.000 | 0.973 | 0.767 | 1.000 | 1.000 | 0.830 | 0.797 |
| 1129 | 0.785 | 1.000 | 0.798 | 1.000 | 0.958 | 1.000 | 0.861 | 1.000 | 0.969 | 1.000 | 0.948 | 1.000 | 0.973 | 0.769 | 1.000 | 1.000 | 0.831 | 0.798 |
| 1130 | 0.786 | 1.000 | 0.798 | 1.000 | 0.958 | 1.000 | 0.861 | 1.000 | 0.969 | 1.000 | 0.948 | 1.000 | 0.973 | 0.769 | 1.000 | 1.000 | 0.832 | 0.799 |
| 1131 | 0.790 | 1.000 | 0.800 | 1.000 | 0.958 | 1.000 | 0.861 | 1.000 | 0.969 | 1.000 | 0.951 | 1.000 | 0.973 | 0.768 | 1.000 | 1.000 | 0.833 | 0.800 |

| | | | | | | | | | | | | | | | | | | |
|---|---|---|---|---|---|---|---|---|---|---|---|---|---|---|---|---|---|---|
| 1132 | 0.790 | 1.000 | 0.796 | 1.000 | 0.958 | 1.000 | 0.861 | 1.000 | 0.969 | 1.000 | 0.948 | 1.000 | 0.973 | 0.767 | 1.000 | 1.000 | 0.832 | 0.799 |
| 1133 | 0.788 | 1.000 | 0.796 | 1.000 | 0.958 | 1.000 | 0.863 | 1.000 | 0.969 | 1.000 | 0.948 | 1.000 | 0.973 | 0.767 | 1.000 | 1.000 | 0.832 | 0.799 |
| 1134 | 0.790 | 1.000 | 0.796 | 1.000 | 0.958 | 1.000 | 0.860 | 1.000 | 0.969 | 1.000 | 0.948 | 1.000 | 0.973 | 0.767 | 1.000 | 1.000 | 0.832 | 0.799 |
| 1135 | 0.790 | 1.000 | 0.796 | 1.000 | 0.958 | 1.000 | 0.860 | 1.000 | 0.969 | 1.000 | 0.948 | 1.000 | 0.973 | 0.767 | 1.000 | 1.000 | 0.832 | 0.799 |
| 1136 | 0.792 | 1.000 | 0.798 | 1.000 | 0.958 | 1.000 | 0.855 | 1.000 | 0.969 | 1.000 | 0.948 | 1.000 | 0.973 | 0.767 | 1.000 | 1.000 | 0.832 | 0.799 |
| 1137 | 0.794 | 1.000 | 0.796 | 1.000 | 0.958 | 1.000 | 0.857 | 1.000 | 0.969 | 1.000 | 0.948 | 1.000 | 0.973 | 0.767 | 1.000 | 1.000 | 0.833 | 0.800 |
| 1138 | 0.794 | 1.000 | 0.798 | 1.000 | 0.958 | 1.000 | 0.857 | 1.000 | 0.969 | 1.000 | 0.948 | 1.000 | 0.973 | 0.767 | 1.000 | 1.000 | 0.833 | 0.800 |
| 1139 | 0.794 | 1.000 | 0.798 | 1.000 | 0.958 | 1.000 | 0.855 | 1.000 | 0.969 | 1.000 | 0.948 | 1.000 | 0.973 | 0.767 | 1.000 | 1.000 | 0.833 | 0.800 |
| 1140 | 0.794 | 1.000 | 0.798 | 1.000 | 0.958 | 1.000 | 0.857 | 1.000 | 0.969 | 1.000 | 0.948 | 1.000 | 0.973 | 0.767 | 1.000 | 1.000 | 0.833 | 0.800 |
| 1141 | 0.795 | 1.000 | 0.798 | 1.000 | 0.958 | 1.000 | 0.858 | 1.000 | 0.969 | 1.000 | 0.948 | 1.000 | 0.973 | 0.767 | 1.000 | 1.000 | 0.833 | 0.800 |
| 1142 | 0.796 | 1.000 | 0.802 | 1.000 | 0.958 | 1.000 | 0.860 | 1.000 | 0.969 | 1.000 | 0.948 | 1.000 | 0.973 | 0.767 | 1.000 | 1.000 | 0.834 | 0.802 |
| 1143 | 0.794 | 1.000 | 0.802 | 1.000 | 0.958 | 1.000 | 0.860 | 1.000 | 0.969 | 1.000 | 0.948 | 1.000 | 0.973 | 0.767 | 1.000 | 1.000 | 0.834 | 0.801 |
| 1144 | 0.794 | 1.000 | 0.802 | 1.000 | 0.958 | 1.000 | 0.858 | 1.000 | 0.969 | 1.000 | 0.948 | 1.000 | 0.973 | 0.767 | 1.000 | 1.000 | 0.834 | 0.801 |
| 1145 | 0.794 | 1.000 | 0.800 | 1.000 | 0.958 | 1.000 | 0.858 | 1.000 | 0.969 | 1.000 | 0.948 | 1.000 | 0.973 | 0.767 | 1.000 | 1.000 | 0.833 | 0.801 |
| 1146 | 0.794 | 1.000 | 0.802 | 1.000 | 0.958 | 1.000 | 0.858 | 1.000 | 0.969 | 1.000 | 0.948 | 1.000 | 0.973 | 0.766 | 1.000 | 1.000 | 0.833 | 0.801 |
| 1147 | 0.794 | 1.000 | 0.802 | 1.000 | 0.958 | 1.000 | 0.860 | 1.000 | 0.969 | 1.000 | 0.948 | 1.000 | 0.973 | 0.766 | 1.000 | 1.000 | 0.833 | 0.801 |
| 1148 | 0.793 | 1.000 | 0.802 | 1.000 | 0.958 | 1.000 | 0.860 | 1.000 | 0.969 | 1.000 | 0.948 | 1.000 | 0.973 | 0.766 | 1.000 | 1.000 | 0.833 | 0.800 |
| 1149 | 0.794 | 1.000 | 0.802 | 1.000 | 0.958 | 1.000 | 0.860 | 1.000 | 0.969 | 1.000 | 0.948 | 1.000 | 0.973 | 0.766 | 1.000 | 1.000 | 0.833 | 0.801 |
| 1150 | 0.796 | 1.000 | 0.800 | 1.000 | 0.958 | 1.000 | 0.863 | 1.000 | 0.969 | 1.000 | 0.948 | 1.000 | 0.973 | 0.767 | 1.000 | 1.000 | 0.834 | 0.802 |
| 1151 | 0.796 | 1.000 | 0.800 | 1.000 | 0.958 | 1.000 | 0.861 | 1.000 | 0.969 | 1.000 | 0.948 | 1.000 | 0.973 | 0.767 | 1.000 | 1.000 | 0.834 | 0.802 |
| 1152 | 0.796 | 1.000 | 0.800 | 1.000 | 0.958 | 1.000 | 0.861 | 1.000 | 0.969 | 1.000 | 0.948 | 1.000 | 0.973 | 0.767 | 1.000 | 1.000 | 0.834 | 0.802 |
| 1153 | 0.796 | 1.000 | 0.802 | 1.000 | 0.958 | 1.000 | 0.861 | 1.000 | 0.969 | 1.000 | 0.948 | 1.000 | 0.973 | 0.767 | 1.000 | 1.000 | 0.834 | 0.802 |
| 1154 | 0.796 | 1.000 | 0.802 | 1.000 | 0.958 | 1.000 | 0.860 | 1.000 | 0.969 | 1.000 | 0.948 | 1.000 | 0.973 | 0.767 | 1.000 | 1.000 | 0.834 | 0.801 |
| 1155 | 0.796 | 1.000 | 0.802 | 1.000 | 0.958 | 1.000 | 0.861 | 1.000 | 0.969 | 1.000 | 0.948 | 1.000 | 0.973 | 0.765 | 1.000 | 1.000 | 0.834 | 0.801 |
| 1156 | 0.794 | 1.000 | 0.802 | 1.000 | 0.958 | 1.000 | 0.861 | 1.000 | 0.969 | 1.000 | 0.948 | 1.000 | 0.973 | 0.764 | 1.000 | 1.000 | 0.833 | 0.801 |
| 1157 | 0.796 | 1.000 | 0.802 | 1.000 | 0.958 | 1.000 | 0.861 | 1.000 | 0.969 | 1.000 | 0.948 | 1.000 | 0.973 | 0.765 | 1.000 | 1.000 | 0.834 | 0.801 |
| 1158 | 0.792 | 1.000 | 0.802 | 1.000 | 0.958 | 1.000 | 0.861 | 1.000 | 0.969 | 1.000 | 0.948 | 1.000 | 0.973 | 0.767 | 1.000 | 1.000 | 0.833 | 0.800 |
| 1159 | 0.792 | 1.000 | 0.802 | 1.000 | 0.958 | 1.000 | 0.861 | 1.000 | 0.969 | 1.000 | 0.948 | 1.000 | 0.973 | 0.767 | 1.000 | 1.000 | 0.833 | 0.801 |
| 1160 | 0.792 | 1.000 | 0.802 | 1.000 | 0.958 | 1.000 | 0.861 | 1.000 | 0.969 | 1.000 | 0.948 | 1.000 | 0.973 | 0.767 | 1.000 | 1.000 | 0.833 | 0.800 |
| 1161 | 0.792 | 1.000 | 0.802 | 1.000 | 0.958 | 1.000 | 0.861 | 1.000 | 0.969 | 1.000 | 0.948 | 1.000 | 0.973 | 0.767 | 1.000 | 1.000 | 0.833 | 0.801 |
| 1162 | 0.792 | 1.000 | 0.800 | 1.000 | 0.958 | 1.000 | 0.861 | 1.000 | 0.969 | 1.000 | 0.945 | 1.000 | 0.973 | 0.766 | 1.000 | 1.000 | 0.833 | 0.800 |
| 1163 | 0.792 | 1.000 | 0.800 | 1.000 | 0.958 | 1.000 | 0.861 | 1.000 | 0.969 | 1.000 | 0.948 | 1.000 | 0.973 | 0.766 | 1.000 | 1.000 | 0.833 | 0.800 |
| 1164 | 0.792 | 1.000 | 0.802 | 1.000 | 0.958 | 1.000 | 0.861 | 1.000 | 0.969 | 1.000 | 0.948 | 1.000 | 0.973 | 0.765 | 1.000 | 1.000 | 0.833 | 0.800 |
| 1165 | 0.794 | 1.000 | 0.796 | 1.000 | 0.958 | 1.000 | 0.863 | 1.000 | 0.969 | 1.000 | 0.948 | 1.000 | 0.973 | 0.769 | 1.000 | 1.000 | 0.834 | 0.801 |
| 1166 | 0.794 | 1.000 | 0.798 | 1.000 | 0.958 | 1.000 | 0.863 | 1.000 | 0.969 | 1.000 | 0.951 | 1.000 | 0.964 | 0.768 | 1.000 | 1.000 | 0.834 | 0.801 |
| 1167 | 0.796 | 1.000 | 0.798 | 1.000 | 0.958 | 1.000 | 0.864 | 1.000 | 0.969 | 1.000 | 0.957 | 1.000 | 0.964 | 0.771 | 1.000 | 1.000 | 0.836 | 0.803 |
| 1168 | 0.796 | 1.000 | 0.798 | 1.000 | 0.958 | 1.000 | 0.864 | 1.000 | 0.969 | 1.000 | 0.957 | 1.000 | 0.964 | 0.771 | 1.000 | 1.000 | 0.836 | 0.803 |
| 1169 | 0.794 | 1.000 | 0.798 | 1.000 | 0.958 | 1.000 | 0.864 | 1.000 | 0.969 | 1.000 | 0.957 | 1.000 | 0.964 | 0.770 | 1.000 | 1.000 | 0.835 | 0.803 |
| 1170 | 0.794 | 1.000 | 0.796 | 1.000 | 0.958 | 1.000 | 0.863 | 1.000 | 0.969 | 1.000 | 0.957 | 1.000 | 0.964 | 0.770 | 1.000 | 1.000 | 0.835 | 0.802 |

| | | | | | | | | | | | | | | | | | | |
|---|---|---|---|---|---|---|---|---|---|---|---|---|---|---|---|---|---|---|
| 1171 | 0.794 | 1.000 | 0.794 | 1.000 | 0.958 | 1.000 | 0.863 | 1.000 | 0.969 | 1.000 | 0.957 | 1.000 | 0.964 | 0.771 | 1.000 | 1.000 | 0.835 | 0.802 |
| 1172 | 0.794 | 1.000 | 0.796 | 1.000 | 0.958 | 1.000 | 0.863 | 1.000 | 0.969 | 1.000 | 0.957 | 1.000 | 0.964 | 0.771 | 1.000 | 1.000 | 0.835 | 0.802 |
| 1173 | 0.794 | 1.000 | 0.794 | 1.000 | 0.958 | 1.000 | 0.863 | 1.000 | 0.969 | 1.000 | 0.957 | 1.000 | 0.964 | 0.772 | 1.000 | 1.000 | 0.835 | 0.802 |
| 1174 | 0.795 | 1.000 | 0.794 | 1.000 | 0.958 | 1.000 | 0.863 | 1.000 | 0.969 | 1.000 | 0.957 | 1.000 | 0.964 | 0.775 | 1.000 | 1.000 | 0.836 | 0.804 |
| 1175 | 0.796 | 1.000 | 0.791 | 1.000 | 0.958 | 1.000 | 0.864 | 1.000 | 0.969 | 1.000 | 0.957 | 1.000 | 0.964 | 0.777 | 1.000 | 1.000 | 0.837 | 0.804 |
| 1176 | 0.795 | 1.000 | 0.791 | 1.000 | 0.958 | 1.000 | 0.864 | 1.000 | 0.969 | 1.000 | 0.957 | 1.000 | 0.964 | 0.775 | 1.000 | 1.000 | 0.836 | 0.803 |
| 1177 | 0.798 | 1.000 | 0.792 | 1.000 | 0.958 | 1.000 | 0.864 | 1.000 | 0.969 | 1.000 | 0.957 | 1.000 | 0.964 | 0.773 | 1.000 | 1.000 | 0.836 | 0.804 |
| 1178 | 0.798 | 1.000 | 0.794 | 1.000 | 0.958 | 1.000 | 0.866 | 1.000 | 0.969 | 1.000 | 0.957 | 1.000 | 0.964 | 0.773 | 1.000 | 1.000 | 0.837 | 0.805 |
| 1179 | 0.795 | 1.000 | 0.791 | 1.000 | 0.958 | 1.000 | 0.867 | 1.000 | 0.969 | 1.000 | 0.957 | 1.000 | 0.964 | 0.770 | 1.000 | 1.000 | 0.835 | 0.802 |
| 1180 | 0.796 | 1.000 | 0.791 | 1.000 | 0.958 | 1.000 | 0.869 | 1.000 | 0.969 | 1.000 | 0.957 | 1.000 | 0.964 | 0.770 | 1.000 | 1.000 | 0.836 | 0.803 |
| 1181 | 0.801 | 1.000 | 0.787 | 1.000 | 0.958 | 1.000 | 0.869 | 1.000 | 0.969 | 1.000 | 0.951 | 1.000 | 0.964 | 0.770 | 1.000 | 1.000 | 0.836 | 0.804 |
| 1182 | 0.802 | 1.000 | 0.787 | 1.000 | 0.958 | 1.000 | 0.869 | 1.000 | 0.969 | 1.000 | 0.951 | 1.000 | 0.964 | 0.770 | 1.000 | 1.000 | 0.836 | 0.804 |
| 1183 | 0.801 | 1.000 | 0.787 | 1.000 | 0.958 | 1.000 | 0.869 | 1.000 | 0.969 | 1.000 | 0.951 | 1.000 | 0.964 | 0.771 | 1.000 | 1.000 | 0.837 | 0.804 |
| 1184 | 0.802 | 1.000 | 0.785 | 1.000 | 0.958 | 1.000 | 0.869 | 1.000 | 0.969 | 1.000 | 0.954 | 1.000 | 0.964 | 0.773 | 1.000 | 1.000 | 0.837 | 0.805 |
| 1185 | 0.802 | 1.000 | 0.787 | 1.000 | 0.958 | 1.000 | 0.869 | 1.000 | 0.969 | 1.000 | 0.954 | 1.000 | 0.964 | 0.773 | 1.000 | 1.000 | 0.837 | 0.805 |
| 1186 | 0.802 | 1.000 | 0.787 | 1.000 | 0.958 | 1.000 | 0.871 | 1.000 | 0.969 | 1.000 | 0.954 | 1.000 | 0.964 | 0.774 | 1.000 | 1.000 | 0.838 | 0.806 |
| 1187 | 0.801 | 1.000 | 0.787 | 1.000 | 0.958 | 1.000 | 0.871 | 1.000 | 0.969 | 1.000 | 0.954 | 1.000 | 0.964 | 0.774 | 1.000 | 1.000 | 0.838 | 0.805 |
| 1188 | 0.802 | 1.000 | 0.785 | 1.000 | 0.958 | 1.000 | 0.871 | 1.000 | 0.969 | 1.000 | 0.951 | 1.000 | 0.964 | 0.772 | 1.000 | 1.000 | 0.837 | 0.804 |
| 1189 | 0.802 | 1.000 | 0.785 | 1.000 | 0.958 | 1.000 | 0.871 | 1.000 | 0.969 | 1.000 | 0.951 | 1.000 | 0.964 | 0.772 | 1.000 | 1.000 | 0.837 | 0.804 |
| 1190 | 0.802 | 1.000 | 0.785 | 1.000 | 0.963 | 1.000 | 0.863 | 1.000 | 0.969 | 1.000 | 0.951 | 1.000 | 0.964 | 0.770 | 1.000 | 1.000 | 0.836 | 0.803 |
| 1191 | 0.802 | 1.000 | 0.785 | 1.000 | 0.958 | 1.000 | 0.861 | 1.000 | 0.969 | 1.000 | 0.954 | 1.000 | 0.964 | 0.777 | 1.000 | 1.000 | 0.837 | 0.805 |
| 1192 | 0.804 | 1.000 | 0.785 | 1.000 | 0.958 | 1.000 | 0.861 | 1.000 | 0.969 | 1.000 | 0.951 | 1.000 | 0.964 | 0.777 | 1.000 | 1.000 | 0.838 | 0.805 |
| 1193 | 0.803 | 1.000 | 0.785 | 1.000 | 0.958 | 1.000 | 0.860 | 1.000 | 0.969 | 1.000 | 0.951 | 1.000 | 0.964 | 0.777 | 1.000 | 1.000 | 0.837 | 0.804 |
| 1194 | 0.803 | 1.000 | 0.785 | 1.000 | 0.958 | 1.000 | 0.861 | 1.000 | 0.969 | 1.000 | 0.951 | 1.000 | 0.964 | 0.777 | 1.000 | 1.000 | 0.838 | 0.805 |
| 1195 | 0.804 | 1.000 | 0.789 | 1.000 | 0.958 | 1.000 | 0.861 | 1.000 | 0.969 | 1.000 | 0.951 | 1.000 | 0.964 | 0.778 | 1.000 | 1.000 | 0.838 | 0.806 |
| 1196 | 0.802 | 1.000 | 0.792 | 1.000 | 0.963 | 1.000 | 0.863 | 1.000 | 0.969 | 1.000 | 0.959 | 1.000 | 0.964 | 0.777 | 1.000 | 1.000 | 0.839 | 0.807 |
| 1197 | 0.803 | 1.000 | 0.792 | 1.000 | 0.963 | 1.000 | 0.863 | 1.000 | 0.969 | 1.000 | 0.959 | 1.000 | 0.964 | 0.777 | 1.000 | 1.000 | 0.839 | 0.807 |
| 1198 | 0.803 | 1.000 | 0.792 | 1.000 | 0.958 | 1.000 | 0.866 | 1.000 | 0.969 | 1.000 | 0.959 | 1.000 | 0.964 | 0.776 | 1.000 | 1.000 | 0.839 | 0.807 |
| 1200 | 0.804 | 1.000 | 0.796 | 1.000 | 0.958 | 1.000 | 0.864 | 1.000 | 0.969 | 1.000 | 0.959 | 1.000 | 0.964 | 0.775 | 1.000 | 1.000 | 0.840 | 0.808 |
| 1201 | 0.804 | 1.000 | 0.800 | 1.000 | 0.958 | 1.000 | 0.863 | 1.000 | 0.969 | 1.000 | 0.957 | 1.000 | 0.964 | 0.776 | 1.000 | 1.000 | 0.840 | 0.808 |
| 1202 | 0.803 | 1.000 | 0.804 | 1.000 | 0.958 | 1.000 | 0.860 | 1.000 | 0.969 | 1.000 | 0.957 | 1.000 | 0.964 | 0.774 | 1.000 | 1.000 | 0.839 | 0.807 |
| 1203 | 0.803 | 1.000 | 0.804 | 1.000 | 0.958 | 1.000 | 0.860 | 1.000 | 0.969 | 1.000 | 0.957 | 1.000 | 0.964 | 0.774 | 1.000 | 1.000 | 0.839 | 0.807 |
| 1204 | 0.803 | 1.000 | 0.802 | 1.000 | 0.958 | 1.000 | 0.860 | 1.000 | 0.969 | 1.000 | 0.957 | 1.000 | 0.964 | 0.774 | 1.000 | 1.000 | 0.839 | 0.806 |
| 1205 | 0.803 | 1.000 | 0.804 | 1.000 | 0.958 | 1.000 | 0.860 | 1.000 | 0.969 | 1.000 | 0.957 | 1.000 | 0.964 | 0.774 | 1.000 | 1.000 | 0.839 | 0.807 |
| 1206 | 0.803 | 1.000 | 0.802 | 1.000 | 0.958 | 1.000 | 0.861 | 1.000 | 0.969 | 1.000 | 0.957 | 1.000 | 0.964 | 0.774 | 1.000 | 1.000 | 0.839 | 0.807 |
| 1207 | 0.802 | 1.000 | 0.800 | 1.000 | 0.958 | 1.000 | 0.863 | 1.000 | 0.969 | 1.000 | 0.957 | 1.000 | 0.964 | 0.774 | 1.000 | 1.000 | 0.839 | 0.807 |
| 1208 | 0.802 | 1.000 | 0.804 | 1.000 | 0.958 | 1.000 | 0.863 | 1.000 | 0.969 | 1.000 | 0.957 | 1.000 | 0.964 | 0.774 | 1.000 | 1.000 | 0.839 | 0.807 |
| 1209 | 0.802 | 1.000 | 0.800 | 1.000 | 0.958 | 1.000 | 0.863 | 1.000 | 0.969 | 1.000 | 0.957 | 1.000 | 0.964 | 0.774 | 1.000 | 1.000 | 0.839 | 0.807 |
| 1210 | 0.802 | 1.000 | 0.802 | 1.000 | 0.958 | 1.000 | 0.863 | 1.000 | 0.969 | 1.000 | 0.957 | 1.000 | 0.964 | 0.775 | 1.000 | 1.000 | 0.839 | 0.807 |

| | | | | | | | | | | | | | | | | | | |
|---|---|---|---|---|---|---|---|---|---|---|---|---|---|---|---|---|---|---|
| 1211 | 0.800 | 1.000 | 0.802 | 1.000 | 0.958 | 1.000 | 0.858 | 1.000 | 0.969 | 1.000 | 0.957 | 1.000 | 0.964 | 0.772 | 1.000 | 1.000 | 0.837 | 0.805 |
| 1212 | 0.801 | 1.000 | 0.802 | 1.000 | 0.958 | 1.000 | 0.858 | 1.000 | 0.969 | 1.000 | 0.957 | 1.000 | 0.964 | 0.772 | 1.000 | 1.000 | 0.837 | 0.805 |
| 1213 | 0.801 | 1.000 | 0.798 | 1.000 | 0.958 | 1.000 | 0.858 | 1.000 | 0.969 | 1.000 | 0.957 | 1.000 | 0.964 | 0.773 | 1.000 | 1.000 | 0.837 | 0.805 |
| 1214 | 0.802 | 1.000 | 0.798 | 1.000 | 0.958 | 1.000 | 0.857 | 1.000 | 0.969 | 1.000 | 0.957 | 1.000 | 0.964 | 0.773 | 1.000 | 1.000 | 0.837 | 0.805 |
| 1215 | 0.804 | 1.000 | 0.798 | 1.000 | 0.958 | 1.000 | 0.857 | 1.000 | 0.969 | 1.000 | 0.957 | 1.000 | 0.964 | 0.773 | 1.000 | 1.000 | 0.838 | 0.805 |
| 1216 | 0.802 | 1.000 | 0.800 | 1.000 | 0.958 | 1.000 | 0.858 | 1.000 | 0.969 | 1.000 | 0.957 | 1.000 | 0.964 | 0.776 | 1.000 | 1.000 | 0.839 | 0.806 |
| 1217 | 0.800 | 1.000 | 0.800 | 1.000 | 0.958 | 1.000 | 0.855 | 1.000 | 0.969 | 1.000 | 0.957 | 1.000 | 0.964 | 0.776 | 1.000 | 1.000 | 0.838 | 0.805 |
| 1218 | 0.801 | 1.000 | 0.800 | 1.000 | 0.963 | 1.000 | 0.857 | 1.000 | 0.969 | 1.000 | 0.957 | 1.000 | 0.964 | 0.777 | 1.000 | 1.000 | 0.838 | 0.806 |
| 1219 | 0.801 | 1.000 | 0.800 | 1.000 | 0.963 | 1.000 | 0.857 | 1.000 | 0.969 | 1.000 | 0.957 | 1.000 | 0.964 | 0.777 | 1.000 | 1.000 | 0.838 | 0.806 |
| 1220 | 0.802 | 1.000 | 0.802 | 1.000 | 0.963 | 1.000 | 0.857 | 1.000 | 0.969 | 1.000 | 0.957 | 1.000 | 0.964 | 0.776 | 1.000 | 1.000 | 0.839 | 0.806 |
| 1221 | 0.802 | 1.000 | 0.800 | 1.000 | 0.963 | 1.000 | 0.857 | 1.000 | 0.969 | 1.000 | 0.957 | 1.000 | 0.964 | 0.775 | 1.000 | 1.000 | 0.838 | 0.806 |
| 1222 | 0.801 | 1.000 | 0.800 | 1.000 | 0.963 | 1.000 | 0.857 | 1.000 | 0.969 | 1.000 | 0.957 | 1.000 | 0.964 | 0.774 | 1.000 | 1.000 | 0.838 | 0.805 |
| 1223 | 0.801 | 1.000 | 0.802 | 1.000 | 0.958 | 1.000 | 0.858 | 1.000 | 0.969 | 1.000 | 0.957 | 1.000 | 0.964 | 0.773 | 1.000 | 1.000 | 0.838 | 0.805 |
| 1224 | 0.801 | 1.000 | 0.804 | 1.000 | 0.958 | 1.000 | 0.858 | 1.000 | 0.969 | 1.000 | 0.957 | 1.000 | 0.964 | 0.772 | 1.000 | 1.000 | 0.838 | 0.805 |
| 1225 | 0.799 | 1.000 | 0.798 | 1.000 | 0.958 | 1.000 | 0.857 | 1.000 | 0.969 | 1.000 | 0.957 | 1.000 | 0.964 | 0.770 | 1.000 | 1.000 | 0.836 | 0.803 |
| 1226 | 0.799 | 1.000 | 0.802 | 1.000 | 0.958 | 1.000 | 0.857 | 1.000 | 0.969 | 1.000 | 0.957 | 1.000 | 0.964 | 0.773 | 1.000 | 1.000 | 0.837 | 0.804 |
| 1227 | 0.798 | 1.000 | 0.802 | 1.000 | 0.958 | 1.000 | 0.857 | 1.000 | 0.969 | 1.000 | 0.957 | 1.000 | 0.964 | 0.773 | 1.000 | 1.000 | 0.836 | 0.804 |
| 1228 | 0.798 | 1.000 | 0.806 | 1.000 | 0.963 | 1.000 | 0.857 | 1.000 | 0.969 | 1.000 | 0.957 | 1.000 | 0.964 | 0.772 | 1.000 | 1.000 | 0.837 | 0.805 |
| 1229 | 0.801 | 1.000 | 0.806 | 1.000 | 0.958 | 1.000 | 0.857 | 1.000 | 0.969 | 1.000 | 0.957 | 1.000 | 0.964 | 0.772 | 1.000 | 1.000 | 0.838 | 0.805 |
| 1230 | 0.801 | 1.000 | 0.804 | 1.000 | 0.958 | 1.000 | 0.857 | 1.000 | 0.969 | 1.000 | 0.957 | 1.000 | 0.964 | 0.772 | 1.000 | 1.000 | 0.837 | 0.805 |
| 1231 | 0.801 | 1.000 | 0.804 | 1.000 | 0.963 | 1.000 | 0.857 | 1.000 | 0.969 | 1.000 | 0.957 | 1.000 | 0.964 | 0.772 | 1.000 | 1.000 | 0.838 | 0.805 |
| 1232 | 0.802 | 1.000 | 0.804 | 1.000 | 0.963 | 1.000 | 0.860 | 1.000 | 0.969 | 1.000 | 0.957 | 1.000 | 0.964 | 0.773 | 1.000 | 1.000 | 0.838 | 0.806 |
| 1233 | 0.802 | 1.000 | 0.806 | 1.000 | 0.963 | 1.000 | 0.860 | 1.000 | 0.969 | 1.000 | 0.957 | 1.000 | 0.964 | 0.774 | 1.000 | 1.000 | 0.839 | 0.807 |
| 1234 | 0.801 | 1.000 | 0.806 | 1.000 | 0.968 | 1.000 | 0.861 | 1.000 | 0.969 | 1.000 | 0.954 | 1.000 | 0.973 | 0.778 | 1.000 | 1.000 | 0.840 | 0.808 |
| 1235 | 0.800 | 1.000 | 0.804 | 1.000 | 0.963 | 1.000 | 0.863 | 1.000 | 0.969 | 1.000 | 0.957 | 1.000 | 0.973 | 0.781 | 1.000 | 1.000 | 0.841 | 0.809 |
| 1236 | 0.800 | 1.000 | 0.804 | 1.000 | 0.958 | 1.000 | 0.861 | 1.000 | 0.969 | 1.000 | 0.957 | 1.000 | 0.973 | 0.779 | 1.000 | 1.000 | 0.840 | 0.808 |
| 1237 | 0.799 | 1.000 | 0.798 | 1.000 | 0.963 | 1.000 | 0.864 | 1.000 | 0.969 | 1.000 | 0.957 | 1.000 | 0.973 | 0.777 | 1.000 | 1.000 | 0.839 | 0.807 |
| 1238 | 0.800 | 1.000 | 0.798 | 1.000 | 0.963 | 1.000 | 0.864 | 1.000 | 0.969 | 1.000 | 0.959 | 1.000 | 0.973 | 0.778 | 1.000 | 1.000 | 0.840 | 0.808 |
| 1239 | 0.801 | 1.000 | 0.796 | 1.000 | 0.963 | 1.000 | 0.864 | 1.000 | 0.969 | 1.000 | 0.957 | 1.000 | 0.973 | 0.777 | 1.000 | 1.000 | 0.839 | 0.807 |
| 1240 | 0.802 | 1.000 | 0.800 | 1.000 | 0.963 | 1.000 | 0.864 | 1.000 | 0.969 | 1.000 | 0.957 | 1.000 | 0.973 | 0.778 | 1.000 | 1.000 | 0.840 | 0.808 |
| 1241 | 0.802 | 1.000 | 0.798 | 1.000 | 0.963 | 1.000 | 0.861 | 1.000 | 0.969 | 1.000 | 0.957 | 1.000 | 0.973 | 0.778 | 1.000 | 1.000 | 0.840 | 0.807 |
| 1242 | 0.802 | 1.000 | 0.798 | 1.000 | 0.963 | 1.000 | 0.861 | 1.000 | 0.969 | 1.000 | 0.957 | 1.000 | 0.973 | 0.778 | 1.000 | 1.000 | 0.840 | 0.808 |
| 1243 | 0.801 | 1.000 | 0.802 | 1.000 | 0.963 | 1.000 | 0.863 | 1.000 | 0.969 | 1.000 | 0.957 | 1.000 | 0.973 | 0.777 | 1.000 | 1.000 | 0.840 | 0.808 |
| 1244 | 0.802 | 1.000 | 0.800 | 1.000 | 0.963 | 1.000 | 0.861 | 1.000 | 0.969 | 1.000 | 0.957 | 1.000 | 0.973 | 0.780 | 1.000 | 1.000 | 0.840 | 0.808 |
| 1245 | 0.801 | 1.000 | 0.800 | 1.000 | 0.963 | 1.000 | 0.861 | 1.000 | 0.969 | 1.000 | 0.957 | 1.000 | 0.973 | 0.778 | 1.000 | 1.000 | 0.840 | 0.808 |
| 1246 | 0.804 | 1.000 | 0.800 | 1.000 | 0.958 | 1.000 | 0.863 | 1.000 | 0.969 | 1.000 | 0.957 | 1.000 | 0.973 | 0.780 | 1.000 | 1.000 | 0.841 | 0.809 |
| 1247 | 0.804 | 1.000 | 0.798 | 1.000 | 0.958 | 1.000 | 0.863 | 1.000 | 0.969 | 1.000 | 0.957 | 1.000 | 0.973 | 0.780 | 1.000 | 1.000 | 0.841 | 0.809 |
| 1248 | 0.803 | 1.000 | 0.800 | 1.000 | 0.958 | 1.000 | 0.863 | 1.000 | 0.969 | 1.000 | 0.957 | 1.000 | 0.973 | 0.781 | 1.000 | 1.000 | 0.841 | 0.809 |
| 1249 | 0.802 | 1.000 | 0.800 | 1.000 | 0.958 | 1.000 | 0.863 | 1.000 | 0.969 | 1.000 | 0.957 | 1.000 | 0.973 | 0.781 | 1.000 | 1.000 | 0.841 | 0.809 |

| | | | | | | | | | | | | | | | | | |
|---|---|---|---|---|---|---|---|---|---|---|---|---|---|---|---|---|---|
| 1250 | 0.802 | 1.000 | 0.802 | 1.000 | 0.958 | 1.000 | 0.861 | 1.000 | 0.969 | 1.000 | 0.957 | 1.000 | 0.973 | 0.781 | 1.000 | 1.000 | 0.841 | 0.809 |
| 1251 | 0.803 | 1.000 | 0.800 | 1.000 | 0.958 | 1.000 | 0.860 | 1.000 | 0.969 | 1.000 | 0.957 | 1.000 | 0.973 | 0.780 | 1.000 | 1.000 | 0.840 | 0.808 |
| 1252 | 0.803 | 1.000 | 0.804 | 1.000 | 0.958 | 1.000 | 0.860 | 1.000 | 0.969 | 1.000 | 0.957 | 1.000 | 0.973 | 0.781 | 1.000 | 1.000 | 0.841 | 0.809 |
| 1253 | 0.804 | 1.000 | 0.804 | 1.000 | 0.958 | 1.000 | 0.860 | 1.000 | 0.969 | 1.000 | 0.957 | 1.000 | 0.973 | 0.780 | 1.000 | 1.000 | 0.841 | 0.809 |
| 1254 | 0.808 | 1.000 | 0.808 | 1.000 | 0.958 | 1.000 | 0.863 | 1.000 | 0.969 | 1.000 | 0.957 | 1.000 | 0.973 | 0.781 | 1.000 | 1.000 | 0.843 | 0.812 |
| 1255 | 0.804 | 1.000 | 0.806 | 1.000 | 0.958 | 1.000 | 0.864 | 1.000 | 0.969 | 1.000 | 0.957 | 1.000 | 0.973 | 0.780 | 1.000 | 1.000 | 0.842 | 0.810 |
| 1256 | 0.805 | 1.000 | 0.806 | 1.000 | 0.958 | 1.000 | 0.866 | 1.000 | 0.969 | 1.000 | 0.957 | 1.000 | 0.973 | 0.781 | 1.000 | 1.000 | 0.842 | 0.811 |
| 1257 | 0.806 | 1.000 | 0.804 | 1.000 | 0.958 | 1.000 | 0.866 | 1.000 | 0.969 | 1.000 | 0.957 | 1.000 | 0.973 | 0.780 | 1.000 | 1.000 | 0.842 | 0.811 |
| 1258 | 0.805 | 1.000 | 0.804 | 1.000 | 0.958 | 1.000 | 0.863 | 1.000 | 0.969 | 1.000 | 0.957 | 1.000 | 0.973 | 0.780 | 1.000 | 1.000 | 0.842 | 0.810 |
| 1259 | 0.805 | 1.000 | 0.800 | 1.000 | 0.958 | 1.000 | 0.863 | 1.000 | 0.969 | 1.000 | 0.957 | 1.000 | 0.973 | 0.781 | 1.000 | 1.000 | 0.841 | 0.809 |
| 1260 | 0.804 | 1.000 | 0.802 | 1.000 | 0.958 | 1.000 | 0.863 | 1.000 | 0.969 | 1.000 | 0.957 | 1.000 | 0.973 | 0.782 | 1.000 | 1.000 | 0.842 | 0.810 |
| 1261 | 0.804 | 1.000 | 0.804 | 1.000 | 0.958 | 1.000 | 0.863 | 1.000 | 0.969 | 1.000 | 0.957 | 1.000 | 0.973 | 0.780 | 1.000 | 1.000 | 0.841 | 0.809 |
| 1262 | 0.804 | 1.000 | 0.804 | 1.000 | 0.958 | 1.000 | 0.863 | 1.000 | 0.969 | 1.000 | 0.957 | 1.000 | 0.973 | 0.779 | 1.000 | 1.000 | 0.841 | 0.809 |
| 1263 | 0.803 | 1.000 | 0.804 | 1.000 | 0.958 | 1.000 | 0.863 | 1.000 | 0.969 | 1.000 | 0.957 | 1.000 | 0.973 | 0.780 | 1.000 | 1.000 | 0.841 | 0.809 |
| 1264 | 0.803 | 1.000 | 0.804 | 1.000 | 0.958 | 1.000 | 0.861 | 1.000 | 0.969 | 1.000 | 0.957 | 1.000 | 0.973 | 0.780 | 1.000 | 1.000 | 0.841 | 0.809 |
| 1265 | 0.802 | 1.000 | 0.806 | 1.000 | 0.958 | 1.000 | 0.861 | 1.000 | 0.969 | 1.000 | 0.959 | 1.000 | 0.973 | 0.781 | 1.000 | 1.000 | 0.841 | 0.809 |
| 1266 | 0.803 | 1.000 | 0.802 | 1.000 | 0.963 | 1.000 | 0.863 | 1.000 | 0.969 | 1.000 | 0.959 | 1.000 | 0.973 | 0.781 | 1.000 | 1.000 | 0.841 | 0.810 |
| 1267 | 0.805 | 1.000 | 0.804 | 1.000 | 0.968 | 1.000 | 0.863 | 1.000 | 0.969 | 1.000 | 0.959 | 1.000 | 0.973 | 0.785 | 1.000 | 1.000 | 0.844 | 0.812 |
| 1268 | 0.806 | 1.000 | 0.806 | 1.000 | 0.968 | 1.000 | 0.863 | 1.000 | 0.969 | 1.000 | 0.959 | 1.000 | 0.973 | 0.785 | 1.000 | 1.000 | 0.844 | 0.813 |
| 1269 | 0.805 | 1.000 | 0.804 | 1.000 | 0.968 | 1.000 | 0.863 | 1.000 | 0.969 | 1.000 | 0.959 | 1.000 | 0.973 | 0.785 | 1.000 | 1.000 | 0.844 | 0.812 |
| 1270 | 0.805 | 1.000 | 0.804 | 1.000 | 0.968 | 1.000 | 0.863 | 1.000 | 0.969 | 1.000 | 0.959 | 1.000 | 0.973 | 0.786 | 1.000 | 1.000 | 0.844 | 0.812 |
| 1271 | 0.804 | 1.000 | 0.800 | 1.000 | 0.968 | 1.000 | 0.864 | 1.000 | 0.969 | 1.000 | 0.959 | 1.000 | 0.973 | 0.785 | 1.000 | 1.000 | 0.843 | 0.812 |
| 1272 | 0.805 | 1.000 | 0.814 | 1.000 | 0.968 | 1.000 | 0.866 | 1.000 | 0.969 | 1.000 | 0.959 | 1.000 | 0.973 | 0.787 | 1.000 | 1.000 | 0.845 | 0.814 |
| 1273 | 0.806 | 1.000 | 0.814 | 1.000 | 0.968 | 1.000 | 0.866 | 1.000 | 0.969 | 1.000 | 0.959 | 1.000 | 0.973 | 0.787 | 1.000 | 1.000 | 0.846 | 0.815 |
| 1274 | 0.806 | 1.000 | 0.806 | 1.000 | 0.963 | 1.000 | 0.866 | 1.000 | 0.969 | 1.000 | 0.959 | 1.000 | 0.973 | 0.785 | 1.000 | 1.000 | 0.844 | 0.813 |
| 1275 | 0.807 | 1.000 | 0.806 | 1.000 | 0.968 | 1.000 | 0.863 | 1.000 | 0.969 | 1.000 | 0.959 | 1.000 | 0.973 | 0.786 | 1.000 | 1.000 | 0.845 | 0.813 |
| 1276 | 0.807 | 1.000 | 0.806 | 1.000 | 0.968 | 1.000 | 0.860 | 1.000 | 0.969 | 1.000 | 0.959 | 1.000 | 0.973 | 0.785 | 1.000 | 1.000 | 0.844 | 0.813 |
| 1277 | 0.808 | 1.000 | 0.806 | 1.000 | 0.968 | 1.000 | 0.861 | 1.000 | 0.969 | 1.000 | 0.959 | 1.000 | 0.973 | 0.786 | 1.000 | 1.000 | 0.845 | 0.813 |
| 1278 | 0.806 | 1.000 | 0.816 | 1.000 | 0.968 | 1.000 | 0.861 | 1.000 | 0.969 | 1.000 | 0.959 | 1.000 | 0.973 | 0.787 | 1.000 | 1.000 | 0.845 | 0.814 |
| 1279 | 0.806 | 1.000 | 0.814 | 1.000 | 0.968 | 1.000 | 0.861 | 1.000 | 0.969 | 1.000 | 0.959 | 1.000 | 0.973 | 0.787 | 1.000 | 1.000 | 0.845 | 0.814 |
| 1280 | 0.805 | 1.000 | 0.814 | 1.000 | 0.968 | 1.000 | 0.861 | 1.000 | 0.969 | 1.000 | 0.959 | 1.000 | 0.973 | 0.788 | 1.000 | 1.000 | 0.845 | 0.814 |
| 1281 | 0.806 | 1.000 | 0.810 | 1.000 | 0.968 | 1.000 | 0.861 | 1.000 | 0.969 | 1.000 | 0.959 | 1.000 | 0.973 | 0.788 | 1.000 | 1.000 | 0.845 | 0.814 |
| 1282 | 0.808 | 1.000 | 0.808 | 1.000 | 0.968 | 1.000 | 0.860 | 1.000 | 0.969 | 1.000 | 0.959 | 1.000 | 0.973 | 0.788 | 1.000 | 1.000 | 0.845 | 0.814 |
| 1283 | 0.808 | 1.000 | 0.812 | 1.000 | 0.968 | 1.000 | 0.860 | 1.000 | 0.969 | 1.000 | 0.959 | 1.000 | 0.973 | 0.788 | 1.000 | 1.000 | 0.845 | 0.814 |
| 1284 | 0.808 | 1.000 | 0.812 | 1.000 | 0.968 | 1.000 | 0.860 | 1.000 | 0.969 | 1.000 | 0.959 | 1.000 | 0.973 | 0.786 | 1.000 | 1.000 | 0.845 | 0.814 |
| 1285 | 0.806 | 1.000 | 0.808 | 1.000 | 0.968 | 1.000 | 0.858 | 1.000 | 0.969 | 1.000 | 0.959 | 1.000 | 0.973 | 0.785 | 1.000 | 1.000 | 0.844 | 0.812 |
| 1286 | 0.806 | 1.000 | 0.808 | 1.000 | 0.968 | 1.000 | 0.860 | 1.000 | 0.969 | 1.000 | 0.959 | 1.000 | 0.973 | 0.786 | 1.000 | 1.000 | 0.844 | 0.813 |
| 1287 | 0.806 | 1.000 | 0.806 | 1.000 | 0.963 | 1.000 | 0.858 | 1.000 | 0.969 | 1.000 | 0.959 | 1.000 | 0.973 | 0.786 | 1.000 | 1.000 | 0.844 | 0.812 |
| 1288 | 0.807 | 1.000 | 0.808 | 1.000 | 0.963 | 1.000 | 0.858 | 1.000 | 0.969 | 1.000 | 0.959 | 1.000 | 0.973 | 0.786 | 1.000 | 1.000 | 0.844 | 0.813 |

| | | | | | | | | | | | | | | | | | | |
|---|---|---|---|---|---|---|---|---|---|---|---|---|---|---|---|---|---|---|
| 1289 | 0.806 | 1.000 | 0.810 | 1.000 | 0.963 | 1.000 | 0.860 | 1.000 | 0.969 | 1.000 | 0.959 | 1.000 | 0.973 | 0.784 | 1.000 | 1.000 | 0.844 | 0.812 |
| 1290 | 0.806 | 1.000 | 0.810 | 1.000 | 0.963 | 1.000 | 0.860 | 1.000 | 0.969 | 1.000 | 0.959 | 1.000 | 0.973 | 0.784 | 1.000 | 1.000 | 0.844 | 0.812 |
| 1291 | 0.806 | 1.000 | 0.806 | 1.000 | 0.963 | 1.000 | 0.860 | 1.000 | 0.969 | 1.000 | 0.959 | 1.000 | 0.973 | 0.784 | 1.000 | 1.000 | 0.843 | 0.812 |
| 1292 | 0.806 | 1.000 | 0.804 | 1.000 | 0.963 | 1.000 | 0.860 | 1.000 | 0.969 | 1.000 | 0.959 | 1.000 | 0.973 | 0.784 | 1.000 | 1.000 | 0.843 | 0.811 |
| 1293 | 0.806 | 1.000 | 0.804 | 1.000 | 0.963 | 1.000 | 0.860 | 1.000 | 0.969 | 1.000 | 0.959 | 1.000 | 0.973 | 0.784 | 1.000 | 1.000 | 0.843 | 0.811 |
| 1294 | 0.806 | 1.000 | 0.806 | 1.000 | 0.963 | 1.000 | 0.860 | 1.000 | 0.969 | 1.000 | 0.959 | 1.000 | 0.973 | 0.785 | 1.000 | 1.000 | 0.843 | 0.812 |
| 1295 | 0.806 | 1.000 | 0.806 | 1.000 | 0.963 | 1.000 | 0.860 | 1.000 | 0.969 | 1.000 | 0.959 | 1.000 | 0.973 | 0.787 | 1.000 | 1.000 | 0.844 | 0.812 |
| 1297 | 0.805 | 1.000 | 0.802 | 1.000 | 0.963 | 1.000 | 0.860 | 1.000 | 0.969 | 1.000 | 0.959 | 1.000 | 0.973 | 0.787 | 1.000 | 1.000 | 0.843 | 0.811 |
| 1298 | 0.806 | 1.000 | 0.804 | 1.000 | 0.963 | 1.000 | 0.860 | 1.000 | 0.969 | 1.000 | 0.959 | 1.000 | 0.973 | 0.787 | 1.000 | 1.000 | 0.844 | 0.812 |
| 1299 | 0.807 | 1.000 | 0.806 | 1.000 | 0.963 | 1.000 | 0.858 | 1.000 | 0.969 | 1.000 | 0.959 | 1.000 | 0.973 | 0.788 | 1.000 | 1.000 | 0.844 | 0.813 |
| 1300 | 0.807 | 1.000 | 0.806 | 1.000 | 0.963 | 1.000 | 0.858 | 1.000 | 0.969 | 1.000 | 0.959 | 1.000 | 0.973 | 0.788 | 1.000 | 1.000 | 0.844 | 0.813 |
| 1301 | 0.807 | 1.000 | 0.806 | 1.000 | 0.963 | 1.000 | 0.858 | 1.000 | 0.969 | 1.000 | 0.959 | 1.000 | 0.973 | 0.785 | 1.000 | 1.000 | 0.844 | 0.812 |
| 1302 | 0.808 | 1.000 | 0.804 | 1.000 | 0.963 | 1.000 | 0.861 | 1.000 | 0.969 | 1.000 | 0.959 | 1.000 | 0.973 | 0.784 | 1.000 | 1.000 | 0.844 | 0.812 |
| 1303 | 0.807 | 1.000 | 0.804 | 1.000 | 0.963 | 1.000 | 0.863 | 1.000 | 0.969 | 1.000 | 0.959 | 1.000 | 0.973 | 0.784 | 1.000 | 1.000 | 0.844 | 0.812 |
| 1304 | 0.806 | 1.000 | 0.806 | 1.000 | 0.963 | 1.000 | 0.861 | 1.000 | 0.969 | 1.000 | 0.959 | 1.000 | 0.973 | 0.786 | 1.000 | 1.000 | 0.844 | 0.813 |
| 1305 | 0.806 | 1.000 | 0.806 | 1.000 | 0.963 | 1.000 | 0.861 | 1.000 | 0.969 | 1.000 | 0.959 | 1.000 | 0.973 | 0.785 | 1.000 | 1.000 | 0.844 | 0.812 |
| 1306 | 0.808 | 1.000 | 0.804 | 1.000 | 0.963 | 1.000 | 0.861 | 1.000 | 0.969 | 1.000 | 0.959 | 1.000 | 0.973 | 0.787 | 1.000 | 1.000 | 0.845 | 0.813 |
| 1307 | 0.809 | 1.000 | 0.804 | 1.000 | 0.963 | 1.000 | 0.861 | 1.000 | 0.969 | 1.000 | 0.959 | 1.000 | 0.973 | 0.784 | 1.000 | 1.000 | 0.844 | 0.813 |
| 1308 | 0.808 | 1.000 | 0.804 | 1.000 | 0.963 | 1.000 | 0.861 | 1.000 | 0.969 | 1.000 | 0.959 | 1.000 | 0.973 | 0.783 | 1.000 | 1.000 | 0.844 | 0.812 |
| 1309 | 0.809 | 1.000 | 0.808 | 1.000 | 0.963 | 1.000 | 0.861 | 1.000 | 0.969 | 1.000 | 0.959 | 1.000 | 0.973 | 0.784 | 1.000 | 1.000 | 0.845 | 0.813 |
| 1310 | 0.808 | 1.000 | 0.808 | 1.000 | 0.958 | 1.000 | 0.860 | 1.000 | 0.969 | 1.000 | 0.959 | 1.000 | 0.973 | 0.784 | 1.000 | 1.000 | 0.844 | 0.812 |
| 1311 | 0.808 | 1.000 | 0.808 | 1.000 | 0.963 | 1.000 | 0.860 | 1.000 | 0.969 | 1.000 | 0.959 | 1.000 | 0.973 | 0.784 | 1.000 | 1.000 | 0.844 | 0.813 |
| 1312 | 0.810 | 1.000 | 0.808 | 1.000 | 0.963 | 1.000 | 0.860 | 1.000 | 0.969 | 1.000 | 0.959 | 1.000 | 0.973 | 0.786 | 1.000 | 1.000 | 0.845 | 0.814 |
| 1313 | 0.810 | 1.000 | 0.810 | 1.000 | 0.963 | 1.000 | 0.861 | 1.000 | 0.969 | 1.000 | 0.957 | 1.000 | 0.973 | 0.787 | 1.000 | 1.000 | 0.846 | 0.814 |
| 1314 | 0.808 | 1.000 | 0.810 | 1.000 | 0.963 | 1.000 | 0.860 | 1.000 | 0.969 | 1.000 | 0.957 | 1.000 | 0.973 | 0.786 | 1.000 | 1.000 | 0.845 | 0.813 |
| 1315 | 0.809 | 1.000 | 0.810 | 1.000 | 0.963 | 1.000 | 0.860 | 1.000 | 0.969 | 1.000 | 0.957 | 1.000 | 0.973 | 0.786 | 1.000 | 1.000 | 0.845 | 0.813 |
| 1316 | 0.808 | 1.000 | 0.806 | 1.000 | 0.963 | 1.000 | 0.858 | 1.000 | 0.969 | 1.000 | 0.959 | 1.000 | 0.973 | 0.786 | 1.000 | 1.000 | 0.844 | 0.812 |
| 1317 | 0.808 | 1.000 | 0.804 | 1.000 | 0.963 | 1.000 | 0.857 | 1.000 | 0.969 | 1.000 | 0.959 | 1.000 | 0.973 | 0.786 | 1.000 | 1.000 | 0.844 | 0.812 |
| 1318 | 0.806 | 1.000 | 0.804 | 1.000 | 0.963 | 1.000 | 0.864 | 1.000 | 0.969 | 1.000 | 0.959 | 1.000 | 0.964 | 0.784 | 1.000 | 1.000 | 0.844 | 0.812 |
| 1319 | 0.806 | 1.000 | 0.804 | 1.000 | 0.958 | 1.000 | 0.863 | 1.000 | 0.969 | 1.000 | 0.959 | 1.000 | 0.964 | 0.784 | 1.000 | 1.000 | 0.843 | 0.812 |
| 1320 | 0.806 | 1.000 | 0.804 | 1.000 | 0.963 | 1.000 | 0.864 | 1.000 | 0.969 | 1.000 | 0.957 | 1.000 | 0.964 | 0.784 | 1.000 | 1.000 | 0.843 | 0.812 |
| 1321 | 0.807 | 1.000 | 0.802 | 1.000 | 0.963 | 1.000 | 0.863 | 1.000 | 0.969 | 1.000 | 0.957 | 1.000 | 0.964 | 0.785 | 1.000 | 1.000 | 0.843 | 0.812 |
| 1322 | 0.808 | 1.000 | 0.802 | 1.000 | 0.963 | 1.000 | 0.861 | 1.000 | 0.969 | 1.000 | 0.957 | 1.000 | 0.964 | 0.784 | 1.000 | 1.000 | 0.843 | 0.811 |
| 1323 | 0.806 | 1.000 | 0.802 | 1.000 | 0.958 | 1.000 | 0.861 | 1.000 | 0.969 | 1.000 | 0.957 | 1.000 | 0.964 | 0.784 | 1.000 | 1.000 | 0.843 | 0.811 |
| 1324 | 0.805 | 1.000 | 0.802 | 1.000 | 0.958 | 1.000 | 0.861 | 1.000 | 0.969 | 1.000 | 0.957 | 1.000 | 0.964 | 0.784 | 1.000 | 1.000 | 0.842 | 0.810 |
| 1325 | 0.806 | 1.000 | 0.802 | 1.000 | 0.958 | 1.000 | 0.861 | 1.000 | 0.969 | 1.000 | 0.959 | 1.000 | 0.964 | 0.784 | 1.000 | 1.000 | 0.843 | 0.811 |
| 1326 | 0.807 | 1.000 | 0.802 | 1.000 | 0.958 | 1.000 | 0.863 | 1.000 | 0.969 | 1.000 | 0.959 | 1.000 | 0.964 | 0.783 | 1.000 | 1.000 | 0.843 | 0.811 |
| 1327 | 0.807 | 1.000 | 0.802 | 1.000 | 0.958 | 1.000 | 0.867 | 1.000 | 0.969 | 1.000 | 0.959 | 1.000 | 0.964 | 0.783 | 1.000 | 1.000 | 0.843 | 0.812 |
| 1328 | 0.808 | 1.000 | 0.804 | 1.000 | 0.958 | 1.000 | 0.864 | 1.000 | 0.969 | 1.000 | 0.957 | 1.000 | 0.964 | 0.784 | 1.000 | 1.000 | 0.843 | 0.812 |

| | | | | | | | | | | | | | | | | | | |
|---|---|---|---|---|---|---|---|---|---|---|---|---|---|---|---|---|---|---|
| 1329 | 0.808 | 1.000 | 0.804 | 1.000 | 0.958 | 1.000 | 0.864 | 1.000 | 0.969 | 1.000 | 0.957 | 1.000 | 0.964 | 0.784 | 1.000 | 1.000 | 0.843 | 0.812 |
| 1330 | 0.808 | 1.000 | 0.804 | 1.000 | 0.958 | 1.000 | 0.864 | 1.000 | 0.969 | 1.000 | 0.957 | 1.000 | 0.964 | 0.784 | 1.000 | 1.000 | 0.843 | 0.812 |
| 1331 | 0.810 | 1.000 | 0.798 | 1.000 | 0.958 | 1.000 | 0.864 | 1.000 | 0.969 | 1.000 | 0.957 | 1.000 | 0.964 | 0.784 | 1.000 | 1.000 | 0.844 | 0.812 |
| 1332 | 0.808 | 1.000 | 0.804 | 1.000 | 0.958 | 1.000 | 0.864 | 1.000 | 0.969 | 1.000 | 0.957 | 1.000 | 0.964 | 0.784 | 1.000 | 1.000 | 0.844 | 0.812 |
| 1333 | 0.808 | 1.000 | 0.804 | 1.000 | 0.958 | 1.000 | 0.864 | 1.000 | 0.969 | 1.000 | 0.957 | 1.000 | 0.964 | 0.784 | 1.000 | 1.000 | 0.844 | 0.812 |
| 1334 | 0.808 | 1.000 | 0.804 | 1.000 | 0.958 | 1.000 | 0.864 | 1.000 | 0.969 | 1.000 | 0.957 | 1.000 | 0.964 | 0.784 | 1.000 | 1.000 | 0.844 | 0.812 |
| 1335 | 0.808 | 1.000 | 0.802 | 1.000 | 0.958 | 1.000 | 0.864 | 1.000 | 0.969 | 1.000 | 0.957 | 1.000 | 0.964 | 0.784 | 1.000 | 1.000 | 0.843 | 0.812 |
| 1336 | 0.808 | 1.000 | 0.800 | 1.000 | 0.963 | 1.000 | 0.864 | 1.000 | 0.969 | 1.000 | 0.957 | 1.000 | 0.964 | 0.784 | 1.000 | 1.000 | 0.843 | 0.812 |
| 1337 | 0.807 | 1.000 | 0.802 | 1.000 | 0.963 | 1.000 | 0.866 | 1.000 | 0.969 | 1.000 | 0.957 | 1.000 | 0.964 | 0.784 | 1.000 | 1.000 | 0.843 | 0.812 |
| 1338 | 0.810 | 1.000 | 0.804 | 1.000 | 0.963 | 1.000 | 0.866 | 1.000 | 0.969 | 1.000 | 0.954 | 1.000 | 0.964 | 0.784 | 1.000 | 1.000 | 0.844 | 0.813 |
| 1339 | 0.805 | 1.000 | 0.812 | 1.000 | 0.963 | 1.000 | 0.871 | 1.000 | 0.969 | 1.000 | 0.954 | 1.000 | 0.964 | 0.787 | 1.000 | 1.000 | 0.845 | 0.814 |
| 1340 | 0.804 | 1.000 | 0.812 | 1.000 | 0.963 | 1.000 | 0.866 | 1.000 | 0.969 | 1.000 | 0.954 | 1.000 | 0.964 | 0.786 | 1.000 | 1.000 | 0.844 | 0.813 |
| 1341 | 0.806 | 1.000 | 0.812 | 1.000 | 0.963 | 1.000 | 0.866 | 1.000 | 0.969 | 1.000 | 0.954 | 1.000 | 0.964 | 0.786 | 1.000 | 1.000 | 0.844 | 0.813 |
| 1342 | 0.806 | 1.000 | 0.812 | 1.000 | 0.963 | 1.000 | 0.866 | 1.000 | 0.969 | 1.000 | 0.954 | 1.000 | 0.964 | 0.786 | 1.000 | 1.000 | 0.844 | 0.813 |
| 1343 | 0.806 | 1.000 | 0.812 | 1.000 | 0.963 | 1.000 | 0.866 | 1.000 | 0.969 | 1.000 | 0.954 | 1.000 | 0.964 | 0.785 | 1.000 | 1.000 | 0.844 | 0.813 |
| 1344 | 0.806 | 1.000 | 0.812 | 1.000 | 0.963 | 1.000 | 0.866 | 1.000 | 0.969 | 1.000 | 0.954 | 1.000 | 0.964 | 0.785 | 1.000 | 1.000 | 0.844 | 0.813 |
| 1345 | 0.805 | 1.000 | 0.812 | 1.000 | 0.963 | 1.000 | 0.866 | 1.000 | 0.969 | 1.000 | 0.954 | 1.000 | 0.964 | 0.785 | 1.000 | 1.000 | 0.844 | 0.813 |
| 1346 | 0.804 | 1.000 | 0.814 | 1.000 | 0.963 | 1.000 | 0.866 | 1.000 | 0.969 | 1.000 | 0.954 | 1.000 | 0.964 | 0.785 | 1.000 | 1.000 | 0.844 | 0.813 |
| 1347 | 0.805 | 1.000 | 0.814 | 1.000 | 0.963 | 1.000 | 0.866 | 1.000 | 0.969 | 1.000 | 0.954 | 1.000 | 0.964 | 0.785 | 1.000 | 1.000 | 0.844 | 0.813 |
| 1348 | 0.804 | 1.000 | 0.814 | 1.000 | 0.963 | 1.000 | 0.866 | 1.000 | 0.969 | 1.000 | 0.954 | 1.000 | 0.964 | 0.785 | 1.000 | 1.000 | 0.844 | 0.813 |
| 1349 | 0.804 | 1.000 | 0.812 | 1.000 | 0.963 | 1.000 | 0.866 | 1.000 | 0.969 | 1.000 | 0.954 | 1.000 | 0.964 | 0.785 | 1.000 | 1.000 | 0.844 | 0.812 |
| 1350 | 0.806 | 1.000 | 0.812 | 1.000 | 0.963 | 1.000 | 0.866 | 1.000 | 0.969 | 1.000 | 0.954 | 1.000 | 0.964 | 0.785 | 1.000 | 1.000 | 0.844 | 0.813 |
| 1351 | 0.806 | 1.000 | 0.812 | 1.000 | 0.963 | 1.000 | 0.866 | 1.000 | 0.969 | 1.000 | 0.957 | 1.000 | 0.964 | 0.785 | 1.000 | 1.000 | 0.844 | 0.813 |
| 1352 | 0.806 | 1.000 | 0.812 | 1.000 | 0.963 | 1.000 | 0.869 | 1.000 | 0.969 | 1.000 | 0.957 | 1.000 | 0.964 | 0.785 | 1.000 | 1.000 | 0.845 | 0.814 |
| 1353 | 0.805 | 1.000 | 0.810 | 1.000 | 0.963 | 1.000 | 0.869 | 1.000 | 0.969 | 1.000 | 0.957 | 1.000 | 0.964 | 0.785 | 1.000 | 1.000 | 0.844 | 0.813 |
| 1354 | 0.806 | 1.000 | 0.810 | 1.000 | 0.963 | 1.000 | 0.871 | 1.000 | 0.969 | 1.000 | 0.957 | 1.000 | 0.964 | 0.784 | 1.000 | 1.000 | 0.845 | 0.813 |
| 1355 | 0.806 | 1.000 | 0.810 | 1.000 | 0.963 | 1.000 | 0.869 | 1.000 | 0.969 | 1.000 | 0.957 | 1.000 | 0.964 | 0.784 | 1.000 | 1.000 | 0.844 | 0.813 |
| 1356 | 0.805 | 1.000 | 0.812 | 1.000 | 0.963 | 1.000 | 0.867 | 1.000 | 0.969 | 1.000 | 0.957 | 1.000 | 0.964 | 0.784 | 1.000 | 1.000 | 0.844 | 0.813 |
| 1357 | 0.805 | 1.000 | 0.812 | 1.000 | 0.963 | 1.000 | 0.867 | 1.000 | 0.969 | 1.000 | 0.957 | 1.000 | 0.964 | 0.784 | 1.000 | 1.000 | 0.844 | 0.813 |
| 1358 | 0.806 | 1.000 | 0.814 | 1.000 | 0.963 | 1.000 | 0.867 | 1.000 | 0.969 | 1.000 | 0.957 | 1.000 | 0.964 | 0.784 | 1.000 | 1.000 | 0.845 | 0.814 |
| 1359 | 0.807 | 1.000 | 0.814 | 1.000 | 0.963 | 1.000 | 0.867 | 1.000 | 0.969 | 1.000 | 0.957 | 1.000 | 0.964 | 0.785 | 1.000 | 1.000 | 0.845 | 0.814 |
| 1360 | 0.807 | 1.000 | 0.814 | 1.000 | 0.963 | 1.000 | 0.867 | 1.000 | 0.969 | 1.000 | 0.957 | 1.000 | 0.964 | 0.785 | 1.000 | 1.000 | 0.845 | 0.814 |
| 1361 | 0.806 | 1.000 | 0.814 | 1.000 | 0.963 | 1.000 | 0.864 | 1.000 | 0.969 | 1.000 | 0.957 | 1.000 | 0.964 | 0.786 | 1.000 | 1.000 | 0.845 | 0.813 |
| 1362 | 0.806 | 1.000 | 0.814 | 1.000 | 0.963 | 1.000 | 0.864 | 1.000 | 0.969 | 1.000 | 0.957 | 1.000 | 0.964 | 0.786 | 1.000 | 1.000 | 0.845 | 0.813 |
| 1363 | 0.806 | 1.000 | 0.812 | 1.000 | 0.963 | 1.000 | 0.864 | 1.000 | 0.969 | 1.000 | 0.957 | 1.000 | 0.964 | 0.786 | 1.000 | 1.000 | 0.844 | 0.813 |
| 1364 | 0.806 | 1.000 | 0.812 | 1.000 | 0.963 | 1.000 | 0.864 | 1.000 | 0.969 | 1.000 | 0.957 | 1.000 | 0.964 | 0.786 | 1.000 | 1.000 | 0.844 | 0.813 |
| 1365 | 0.806 | 1.000 | 0.812 | 1.000 | 0.963 | 1.000 | 0.864 | 1.000 | 0.969 | 1.000 | 0.957 | 1.000 | 0.964 | 0.786 | 1.000 | 1.000 | 0.845 | 0.813 |
| 1366 | 0.806 | 1.000 | 0.812 | 1.000 | 0.963 | 1.000 | 0.864 | 1.000 | 0.969 | 1.000 | 0.957 | 1.000 | 0.964 | 0.786 | 1.000 | 1.000 | 0.845 | 0.813 |
| 1367 | 0.806 | 1.000 | 0.812 | 1.000 | 0.963 | 1.000 | 0.863 | 1.000 | 0.969 | 1.000 | 0.957 | 1.000 | 0.964 | 0.786 | 1.000 | 1.000 | 0.844 | 0.813 |

| | | | | | | | | | | | | | | | | | | |
|---|---|---|---|---|---|---|---|---|---|---|---|---|---|---|---|---|---|---|
| 1368 | 0.806 | 1.000 | 0.814 | 1.000 | 0.963 | 1.000 | 0.863 | 1.000 | 0.969 | 1.000 | 0.957 | 1.000 | 0.964 | 0.786 | 1.000 | 1.000 | 0.844 | 0.813 |
| 1369 | 0.806 | 1.000 | 0.814 | 1.000 | 0.963 | 1.000 | 0.863 | 1.000 | 0.969 | 1.000 | 0.957 | 1.000 | 0.964 | 0.786 | 1.000 | 1.000 | 0.845 | 0.813 |
| 1370 | 0.806 | 1.000 | 0.814 | 1.000 | 0.963 | 1.000 | 0.861 | 1.000 | 0.969 | 1.000 | 0.957 | 1.000 | 0.964 | 0.786 | 1.000 | 1.000 | 0.844 | 0.813 |
| 1371 | 0.806 | 1.000 | 0.816 | 1.000 | 0.963 | 1.000 | 0.864 | 1.000 | 0.969 | 1.000 | 0.957 | 1.000 | 0.964 | 0.787 | 1.000 | 1.000 | 0.845 | 0.814 |
| 1372 | 0.805 | 1.000 | 0.814 | 1.000 | 0.963 | 1.000 | 0.863 | 1.000 | 0.969 | 1.000 | 0.957 | 1.000 | 0.964 | 0.787 | 1.000 | 1.000 | 0.844 | 0.813 |
| 1373 | 0.808 | 1.000 | 0.814 | 1.000 | 0.963 | 1.000 | 0.866 | 1.000 | 0.969 | 1.000 | 0.957 | 1.000 | 0.964 | 0.784 | 1.000 | 1.000 | 0.845 | 0.814 |
| 1374 | 0.808 | 1.000 | 0.816 | 1.000 | 0.963 | 1.000 | 0.864 | 1.000 | 0.969 | 1.000 | 0.957 | 1.000 | 0.964 | 0.785 | 1.000 | 1.000 | 0.845 | 0.814 |
| 1375 | 0.808 | 1.000 | 0.816 | 1.000 | 0.963 | 1.000 | 0.864 | 1.000 | 0.969 | 1.000 | 0.957 | 1.000 | 0.964 | 0.785 | 1.000 | 1.000 | 0.845 | 0.814 |
| 1376 | 0.808 | 1.000 | 0.816 | 1.000 | 0.963 | 1.000 | 0.863 | 1.000 | 0.969 | 1.000 | 0.957 | 1.000 | 0.964 | 0.785 | 1.000 | 1.000 | 0.845 | 0.814 |
| 1377 | 0.808 | 1.000 | 0.814 | 1.000 | 0.963 | 1.000 | 0.863 | 1.000 | 0.969 | 1.000 | 0.957 | 1.000 | 0.964 | 0.784 | 1.000 | 1.000 | 0.845 | 0.813 |
| 1378 | 0.808 | 1.000 | 0.814 | 1.000 | 0.963 | 1.000 | 0.864 | 1.000 | 0.969 | 1.000 | 0.957 | 1.000 | 0.964 | 0.785 | 1.000 | 1.000 | 0.845 | 0.814 |
| 1379 | 0.806 | 1.000 | 0.814 | 1.000 | 0.963 | 1.000 | 0.864 | 1.000 | 0.969 | 1.000 | 0.957 | 1.000 | 0.964 | 0.785 | 1.000 | 1.000 | 0.845 | 0.813 |
| 1380 | 0.810 | 1.000 | 0.812 | 1.000 | 0.963 | 1.000 | 0.864 | 1.000 | 0.969 | 1.000 | 0.957 | 1.000 | 0.973 | 0.788 | 1.000 | 1.000 | 0.846 | 0.815 |
| 1381 | 0.810 | 1.000 | 0.810 | 1.000 | 0.963 | 1.000 | 0.866 | 1.000 | 0.969 | 1.000 | 0.957 | 1.000 | 0.973 | 0.786 | 1.000 | 1.000 | 0.846 | 0.815 |
| 1382 | 0.810 | 1.000 | 0.810 | 1.000 | 0.963 | 1.000 | 0.867 | 1.000 | 0.969 | 1.000 | 0.957 | 1.000 | 0.973 | 0.786 | 1.000 | 1.000 | 0.846 | 0.815 |
| 1383 | 0.808 | 1.000 | 0.810 | 1.000 | 0.963 | 1.000 | 0.867 | 1.000 | 0.969 | 1.000 | 0.957 | 1.000 | 0.973 | 0.786 | 1.000 | 1.000 | 0.846 | 0.814 |
| 1384 | 0.808 | 1.000 | 0.814 | 1.000 | 0.963 | 1.000 | 0.867 | 1.000 | 0.969 | 1.000 | 0.957 | 1.000 | 0.973 | 0.784 | 1.000 | 1.000 | 0.846 | 0.814 |
| 1385 | 0.807 | 1.000 | 0.812 | 1.000 | 0.963 | 1.000 | 0.867 | 1.000 | 0.969 | 1.000 | 0.957 | 1.000 | 0.964 | 0.783 | 1.000 | 1.000 | 0.844 | 0.813 |
| 1386 | 0.807 | 1.000 | 0.812 | 1.000 | 0.963 | 1.000 | 0.864 | 1.000 | 0.969 | 1.000 | 0.957 | 1.000 | 0.964 | 0.784 | 1.000 | 1.000 | 0.844 | 0.813 |
| 1387 | 0.807 | 1.000 | 0.812 | 1.000 | 0.963 | 1.000 | 0.864 | 1.000 | 0.969 | 1.000 | 0.957 | 1.000 | 0.964 | 0.784 | 1.000 | 1.000 | 0.844 | 0.813 |
| 1388 | 0.807 | 1.000 | 0.812 | 1.000 | 0.963 | 1.000 | 0.867 | 1.000 | 0.969 | 1.000 | 0.957 | 1.000 | 0.964 | 0.783 | 1.000 | 1.000 | 0.844 | 0.813 |
| 1389 | 0.809 | 1.000 | 0.810 | 1.000 | 0.963 | 1.000 | 0.863 | 1.000 | 0.969 | 1.000 | 0.957 | 1.000 | 0.973 | 0.787 | 1.000 | 1.000 | 0.845 | 0.814 |
| 1390 | 0.808 | 1.000 | 0.808 | 1.000 | 0.963 | 1.000 | 0.863 | 1.000 | 0.969 | 1.000 | 0.957 | 1.000 | 0.973 | 0.788 | 1.000 | 1.000 | 0.845 | 0.814 |
| 1391 | 0.808 | 1.000 | 0.810 | 1.000 | 0.963 | 1.000 | 0.863 | 1.000 | 0.969 | 1.000 | 0.957 | 1.000 | 0.973 | 0.788 | 1.000 | 1.000 | 0.845 | 0.814 |
| 1392 | 0.807 | 1.000 | 0.810 | 1.000 | 0.968 | 1.000 | 0.864 | 1.000 | 0.969 | 1.000 | 0.957 | 1.000 | 0.973 | 0.786 | 1.000 | 1.000 | 0.845 | 0.814 |
| 1393 | 0.808 | 1.000 | 0.808 | 1.000 | 0.968 | 1.000 | 0.866 | 1.000 | 0.969 | 1.000 | 0.957 | 1.000 | 0.973 | 0.787 | 1.000 | 1.000 | 0.846 | 0.814 |
| 1394 | 0.808 | 1.000 | 0.810 | 1.000 | 0.968 | 1.000 | 0.866 | 1.000 | 0.969 | 1.000 | 0.957 | 1.000 | 0.973 | 0.787 | 1.000 | 1.000 | 0.846 | 0.815 |
| 1395 | 0.812 | 1.000 | 0.806 | 1.000 | 0.968 | 1.000 | 0.864 | 1.000 | 0.969 | 1.000 | 0.954 | 1.000 | 0.973 | 0.788 | 1.000 | 1.000 | 0.846 | 0.815 |
| 1396 | 0.812 | 1.000 | 0.804 | 1.000 | 0.968 | 1.000 | 0.866 | 1.000 | 0.969 | 1.000 | 0.954 | 1.000 | 0.973 | 0.788 | 1.000 | 1.000 | 0.846 | 0.815 |
| 1397 | 0.812 | 1.000 | 0.804 | 1.000 | 0.968 | 1.000 | 0.866 | 1.000 | 0.969 | 1.000 | 0.951 | 1.000 | 0.973 | 0.787 | 1.000 | 1.000 | 0.846 | 0.814 |
| 1398 | 0.812 | 1.000 | 0.802 | 1.000 | 0.968 | 1.000 | 0.866 | 1.000 | 0.969 | 1.000 | 0.951 | 1.000 | 0.973 | 0.787 | 1.000 | 1.000 | 0.846 | 0.814 |
| 1399 | 0.808 | 1.000 | 0.810 | 1.000 | 0.974 | 1.000 | 0.864 | 1.000 | 0.969 | 1.000 | 0.954 | 1.000 | 0.973 | 0.788 | 1.000 | 1.000 | 0.846 | 0.814 |
| 1400 | 0.808 | 1.000 | 0.808 | 1.000 | 0.974 | 1.000 | 0.864 | 1.000 | 0.969 | 1.000 | 0.954 | 1.000 | 0.973 | 0.788 | 1.000 | 1.000 | 0.846 | 0.814 |
| 1401 | 0.808 | 1.000 | 0.808 | 1.000 | 0.974 | 1.000 | 0.864 | 1.000 | 0.969 | 1.000 | 0.954 | 1.000 | 0.973 | 0.788 | 1.000 | 1.000 | 0.846 | 0.814 |
| 1402 | 0.808 | 1.000 | 0.808 | 1.000 | 0.974 | 1.000 | 0.864 | 1.000 | 0.969 | 1.000 | 0.954 | 1.000 | 0.973 | 0.788 | 1.000 | 1.000 | 0.846 | 0.814 |
| 1403 | 0.808 | 1.000 | 0.808 | 1.000 | 0.974 | 1.000 | 0.864 | 1.000 | 0.969 | 1.000 | 0.954 | 1.000 | 0.973 | 0.788 | 1.000 | 1.000 | 0.846 | 0.814 |
| 1404 | 0.810 | 1.000 | 0.806 | 1.000 | 0.974 | 1.000 | 0.864 | 1.000 | 0.969 | 1.000 | 0.954 | 1.000 | 0.973 | 0.788 | 1.000 | 1.000 | 0.846 | 0.815 |
| 1405 | 0.810 | 1.000 | 0.808 | 1.000 | 0.974 | 1.000 | 0.864 | 1.000 | 0.969 | 1.000 | 0.957 | 1.000 | 0.973 | 0.788 | 1.000 | 1.000 | 0.846 | 0.815 |
| 1406 | 0.810 | 1.000 | 0.810 | 1.000 | 0.974 | 1.000 | 0.864 | 1.000 | 0.969 | 1.000 | 0.957 | 1.000 | 0.973 | 0.788 | 1.000 | 1.000 | 0.847 | 0.816 |

| | | | | | | | | | | | | | | | | | | |
|---|---|---|---|---|---|---|---|---|---|---|---|---|---|---|---|---|---|---|
| 1407 | 0.808 | 1.000 | 0.808 | 1.000 | 0.974 | 1.000 | 0.869 | 1.000 | 0.969 | 1.000 | 0.957 | 1.000 | 0.973 | 0.787 | 1.000 | 1.000 | 0.846 | 0.815 |
| 1408 | 0.808 | 1.000 | 0.812 | 1.000 | 0.974 | 1.000 | 0.869 | 1.000 | 0.969 | 1.000 | 0.957 | 1.000 | 0.973 | 0.787 | 1.000 | 1.000 | 0.847 | 0.816 |
| 1409 | 0.808 | 1.000 | 0.812 | 1.000 | 0.974 | 1.000 | 0.867 | 1.000 | 0.969 | 1.000 | 0.957 | 1.000 | 0.973 | 0.786 | 1.000 | 1.000 | 0.846 | 0.815 |
| 1410 | 0.806 | 1.000 | 0.808 | 1.000 | 0.974 | 1.000 | 0.869 | 1.000 | 0.969 | 1.000 | 0.954 | 1.000 | 0.973 | 0.788 | 1.000 | 1.000 | 0.846 | 0.814 |
| 1411 | 0.808 | 1.000 | 0.808 | 1.000 | 0.974 | 1.000 | 0.869 | 1.000 | 0.969 | 1.000 | 0.954 | 1.000 | 0.973 | 0.785 | 1.000 | 1.000 | 0.845 | 0.814 |
| 1412 | 0.808 | 1.000 | 0.808 | 1.000 | 0.974 | 1.000 | 0.869 | 1.000 | 0.969 | 1.000 | 0.954 | 1.000 | 0.973 | 0.785 | 1.000 | 1.000 | 0.845 | 0.814 |
| 1413 | 0.808 | 1.000 | 0.808 | 1.000 | 0.974 | 1.000 | 0.871 | 1.000 | 0.969 | 1.000 | 0.954 | 1.000 | 0.973 | 0.786 | 1.000 | 1.000 | 0.846 | 0.815 |
| 1414 | 0.808 | 1.000 | 0.810 | 1.000 | 0.974 | 1.000 | 0.866 | 1.000 | 0.969 | 1.000 | 0.954 | 1.000 | 0.973 | 0.786 | 1.000 | 1.000 | 0.846 | 0.814 |
| 1415 | 0.808 | 1.000 | 0.810 | 1.000 | 0.974 | 1.000 | 0.866 | 1.000 | 0.969 | 1.000 | 0.954 | 1.000 | 0.973 | 0.787 | 1.000 | 1.000 | 0.846 | 0.815 |
| 1416 | 0.809 | 1.000 | 0.810 | 1.000 | 0.974 | 1.000 | 0.863 | 1.000 | 0.969 | 1.000 | 0.954 | 1.000 | 0.973 | 0.786 | 1.000 | 1.000 | 0.845 | 0.814 |
| 1417 | 0.810 | 1.000 | 0.812 | 1.000 | 0.974 | 1.000 | 0.864 | 1.000 | 0.969 | 1.000 | 0.954 | 1.000 | 0.973 | 0.787 | 1.000 | 1.000 | 0.846 | 0.815 |
| 1418 | 0.810 | 1.000 | 0.810 | 1.000 | 0.974 | 1.000 | 0.864 | 1.000 | 0.969 | 1.000 | 0.954 | 1.000 | 0.973 | 0.787 | 1.000 | 1.000 | 0.846 | 0.815 |
| 1419 | 0.810 | 1.000 | 0.810 | 1.000 | 0.974 | 1.000 | 0.864 | 1.000 | 0.969 | 1.000 | 0.954 | 1.000 | 0.973 | 0.787 | 1.000 | 1.000 | 0.846 | 0.815 |
| 1420 | 0.810 | 1.000 | 0.810 | 1.000 | 0.974 | 1.000 | 0.864 | 1.000 | 0.969 | 1.000 | 0.954 | 1.000 | 0.973 | 0.785 | 1.000 | 1.000 | 0.846 | 0.814 |
| 1421 | 0.809 | 1.000 | 0.810 | 1.000 | 0.974 | 1.000 | 0.864 | 1.000 | 0.969 | 1.000 | 0.954 | 1.000 | 0.973 | 0.786 | 1.000 | 1.000 | 0.846 | 0.814 |
| 1422 | 0.809 | 1.000 | 0.810 | 1.000 | 0.974 | 1.000 | 0.864 | 1.000 | 0.969 | 1.000 | 0.954 | 1.000 | 0.973 | 0.787 | 1.000 | 1.000 | 0.846 | 0.815 |
| 1423 | 0.809 | 1.000 | 0.810 | 1.000 | 0.974 | 1.000 | 0.864 | 1.000 | 0.969 | 1.000 | 0.954 | 1.000 | 0.973 | 0.788 | 1.000 | 1.000 | 0.846 | 0.815 |
| 1424 | 0.812 | 1.000 | 0.810 | 1.000 | 0.974 | 1.000 | 0.863 | 1.000 | 0.969 | 1.000 | 0.954 | 1.000 | 0.973 | 0.788 | 1.000 | 1.000 | 0.847 | 0.816 |
| 1425 | 0.811 | 1.000 | 0.808 | 1.000 | 0.974 | 1.000 | 0.863 | 1.000 | 0.969 | 1.000 | 0.954 | 1.000 | 0.973 | 0.788 | 1.000 | 1.000 | 0.846 | 0.815 |
| 1426 | 0.812 | 1.000 | 0.808 | 1.000 | 0.974 | 1.000 | 0.864 | 1.000 | 0.969 | 1.000 | 0.954 | 1.000 | 0.973 | 0.788 | 1.000 | 1.000 | 0.847 | 0.815 |
| 1427 | 0.812 | 1.000 | 0.810 | 1.000 | 0.974 | 1.000 | 0.864 | 1.000 | 0.969 | 1.000 | 0.957 | 1.000 | 0.973 | 0.788 | 1.000 | 1.000 | 0.847 | 0.816 |
| 1428 | 0.810 | 1.000 | 0.808 | 1.000 | 0.974 | 1.000 | 0.864 | 1.000 | 0.969 | 1.000 | 0.957 | 1.000 | 0.973 | 0.787 | 1.000 | 1.000 | 0.846 | 0.815 |
| 1429 | 0.810 | 1.000 | 0.808 | 1.000 | 0.974 | 1.000 | 0.864 | 1.000 | 0.969 | 1.000 | 0.957 | 1.000 | 0.973 | 0.788 | 1.000 | 1.000 | 0.846 | 0.815 |
| 1430 | 0.811 | 1.000 | 0.808 | 1.000 | 0.974 | 1.000 | 0.866 | 1.000 | 0.969 | 1.000 | 0.957 | 1.000 | 0.973 | 0.788 | 1.000 | 1.000 | 0.847 | 0.816 |
| 1431 | 0.810 | 1.000 | 0.808 | 1.000 | 0.974 | 1.000 | 0.863 | 1.000 | 0.969 | 1.000 | 0.957 | 1.000 | 0.973 | 0.788 | 1.000 | 1.000 | 0.846 | 0.815 |
| 1432 | 0.810 | 1.000 | 0.808 | 1.000 | 0.974 | 1.000 | 0.864 | 1.000 | 0.969 | 1.000 | 0.957 | 1.000 | 0.973 | 0.788 | 1.000 | 1.000 | 0.846 | 0.815 |
| 1433 | 0.810 | 1.000 | 0.810 | 1.000 | 0.974 | 1.000 | 0.864 | 1.000 | 0.969 | 1.000 | 0.957 | 1.000 | 0.973 | 0.788 | 1.000 | 1.000 | 0.847 | 0.816 |
| 1434 | 0.811 | 1.000 | 0.808 | 1.000 | 0.974 | 1.000 | 0.866 | 1.000 | 0.969 | 1.000 | 0.957 | 1.000 | 0.973 | 0.789 | 1.000 | 1.000 | 0.847 | 0.816 |
| 1435 | 0.811 | 1.000 | 0.808 | 1.000 | 0.974 | 1.000 | 0.864 | 1.000 | 0.969 | 1.000 | 0.957 | 1.000 | 0.973 | 0.789 | 1.000 | 1.000 | 0.847 | 0.816 |
| 1436 | 0.812 | 1.000 | 0.808 | 1.000 | 0.974 | 1.000 | 0.861 | 1.000 | 0.969 | 1.000 | 0.957 | 1.000 | 0.973 | 0.789 | 1.000 | 1.000 | 0.847 | 0.816 |
| 1437 | 0.812 | 1.000 | 0.808 | 1.000 | 0.974 | 1.000 | 0.864 | 1.000 | 0.969 | 1.000 | 0.957 | 1.000 | 0.973 | 0.788 | 1.000 | 1.000 | 0.847 | 0.816 |
| 1438 | 0.813 | 1.000 | 0.808 | 1.000 | 0.974 | 1.000 | 0.864 | 1.000 | 0.969 | 1.000 | 0.957 | 1.000 | 0.973 | 0.789 | 1.000 | 1.000 | 0.848 | 0.817 |
| 1439 | 0.814 | 1.000 | 0.808 | 1.000 | 0.968 | 1.000 | 0.866 | 1.000 | 0.969 | 1.000 | 0.957 | 1.000 | 0.973 | 0.788 | 1.000 | 1.000 | 0.847 | 0.816 |
| 1440 | 0.811 | 1.000 | 0.808 | 1.000 | 0.968 | 1.000 | 0.864 | 1.000 | 0.969 | 1.000 | 0.957 | 1.000 | 0.973 | 0.788 | 1.000 | 1.000 | 0.847 | 0.815 |
| 1441 | 0.813 | 1.000 | 0.806 | 1.000 | 0.968 | 1.000 | 0.866 | 1.000 | 0.969 | 1.000 | 0.957 | 1.000 | 0.973 | 0.789 | 1.000 | 1.000 | 0.847 | 0.816 |
| 1442 | 0.812 | 1.000 | 0.808 | 1.000 | 0.968 | 1.000 | 0.864 | 1.000 | 0.969 | 1.000 | 0.957 | 1.000 | 0.973 | 0.788 | 1.000 | 1.000 | 0.847 | 0.816 |
| 1443 | 0.816 | 1.000 | 0.814 | 1.000 | 0.968 | 1.000 | 0.867 | 1.000 | 0.969 | 1.000 | 0.954 | 1.000 | 0.973 | 0.787 | 1.000 | 1.000 | 0.848 | 0.818 |
| 1444 | 0.814 | 1.000 | 0.818 | 1.000 | 0.974 | 1.000 | 0.861 | 1.000 | 0.969 | 1.000 | 0.954 | 1.000 | 0.973 | 0.787 | 1.000 | 1.000 | 0.848 | 0.817 |
| 1445 | 0.814 | 1.000 | 0.818 | 1.000 | 0.974 | 1.000 | 0.861 | 1.000 | 0.969 | 1.000 | 0.954 | 1.000 | 0.973 | 0.788 | 1.000 | 1.000 | 0.848 | 0.817 |

| | | | | | | | | | | | | | | | | | |
|---|---|---|---|---|---|---|---|---|---|---|---|---|---|---|---|---|---|
| 1446 | 0.814 | 1.000 | 0.818 | 1.000 | 0.979 | 1.000 | 0.863 | 1.000 | 0.969 | 1.000 | 0.954 | 1.000 | 0.973 | 0.785 | 1.000 | 1.000 | 0.848 | 0.817 |
| 1447 | 0.814 | 1.000 | 0.818 | 1.000 | 0.979 | 1.000 | 0.863 | 1.000 | 0.969 | 1.000 | 0.951 | 1.000 | 0.973 | 0.786 | 1.000 | 1.000 | 0.848 | 0.817 |
| 1448 | 0.813 | 1.000 | 0.818 | 1.000 | 0.979 | 1.000 | 0.860 | 1.000 | 0.969 | 1.000 | 0.951 | 1.000 | 0.973 | 0.785 | 1.000 | 1.000 | 0.847 | 0.816 |
| 1449 | 0.810 | 1.000 | 0.814 | 1.000 | 0.974 | 1.000 | 0.866 | 1.000 | 0.969 | 1.000 | 0.951 | 1.000 | 0.973 | 0.784 | 1.000 | 1.000 | 0.846 | 0.815 |
| 1450 | 0.810 | 1.000 | 0.816 | 1.000 | 0.974 | 1.000 | 0.866 | 1.000 | 0.969 | 1.000 | 0.951 | 1.000 | 0.973 | 0.783 | 1.000 | 1.000 | 0.846 | 0.814 |
| 1451 | 0.810 | 1.000 | 0.816 | 1.000 | 0.974 | 1.000 | 0.866 | 1.000 | 0.969 | 1.000 | 0.951 | 1.000 | 0.973 | 0.785 | 1.000 | 1.000 | 0.846 | 0.815 |
| 1452 | 0.810 | 1.000 | 0.816 | 1.000 | 0.974 | 1.000 | 0.866 | 1.000 | 0.969 | 1.000 | 0.954 | 1.000 | 0.973 | 0.784 | 1.000 | 1.000 | 0.846 | 0.815 |
| 1453 | 0.810 | 1.000 | 0.816 | 1.000 | 0.974 | 1.000 | 0.866 | 1.000 | 0.969 | 1.000 | 0.954 | 1.000 | 0.973 | 0.784 | 1.000 | 1.000 | 0.846 | 0.815 |
| 1454 | 0.815 | 1.000 | 0.814 | 1.000 | 0.979 | 1.000 | 0.869 | 1.000 | 0.969 | 1.000 | 0.957 | 1.000 | 0.973 | 0.789 | 1.000 | 1.000 | 0.850 | 0.819 |
| 1455 | 0.813 | 1.000 | 0.812 | 1.000 | 0.979 | 1.000 | 0.871 | 1.000 | 0.969 | 1.000 | 0.954 | 1.000 | 0.973 | 0.788 | 1.000 | 1.000 | 0.849 | 0.818 |
| 1456 | 0.812 | 1.000 | 0.812 | 1.000 | 0.979 | 1.000 | 0.872 | 1.000 | 0.969 | 1.000 | 0.954 | 1.000 | 0.973 | 0.788 | 1.000 | 1.000 | 0.849 | 0.818 |
| 1457 | 0.813 | 1.000 | 0.812 | 1.000 | 0.979 | 1.000 | 0.871 | 1.000 | 0.969 | 1.000 | 0.954 | 1.000 | 0.973 | 0.788 | 1.000 | 1.000 | 0.849 | 0.818 |
| 1458 | 0.811 | 1.000 | 0.812 | 1.000 | 0.974 | 1.000 | 0.867 | 1.000 | 0.969 | 1.000 | 0.957 | 1.000 | 0.973 | 0.789 | 1.000 | 1.000 | 0.848 | 0.817 |
| 1459 | 0.812 | 1.000 | 0.812 | 1.000 | 0.974 | 1.000 | 0.867 | 1.000 | 0.969 | 1.000 | 0.957 | 1.000 | 0.973 | 0.790 | 1.000 | 1.000 | 0.848 | 0.817 |
| 1460 | 0.814 | 1.000 | 0.816 | 1.000 | 0.974 | 1.000 | 0.867 | 1.000 | 0.969 | 1.000 | 0.959 | 1.000 | 0.973 | 0.788 | 1.000 | 1.000 | 0.849 | 0.819 |
| 1461 | 0.814 | 1.000 | 0.816 | 1.000 | 0.974 | 1.000 | 0.867 | 1.000 | 0.969 | 1.000 | 0.959 | 1.000 | 0.973 | 0.788 | 1.000 | 1.000 | 0.849 | 0.818 |
| 1462 | 0.813 | 1.000 | 0.816 | 1.000 | 0.974 | 1.000 | 0.867 | 1.000 | 0.969 | 1.000 | 0.957 | 1.000 | 0.973 | 0.788 | 1.000 | 1.000 | 0.849 | 0.818 |
| 1463 | 0.815 | 1.000 | 0.814 | 1.000 | 0.974 | 1.000 | 0.867 | 1.000 | 0.969 | 1.000 | 0.957 | 1.000 | 0.973 | 0.789 | 1.000 | 1.000 | 0.849 | 0.819 |
| 1464 | 0.814 | 1.000 | 0.814 | 1.000 | 0.974 | 1.000 | 0.864 | 1.000 | 0.969 | 1.000 | 0.962 | 1.000 | 0.973 | 0.790 | 1.000 | 1.000 | 0.849 | 0.819 |
| 1465 | 0.815 | 1.000 | 0.814 | 1.000 | 0.974 | 1.000 | 0.864 | 1.000 | 0.969 | 1.000 | 0.962 | 1.000 | 0.973 | 0.790 | 1.000 | 1.000 | 0.849 | 0.819 |
| 1466 | 0.812 | 1.000 | 0.814 | 1.000 | 0.974 | 1.000 | 0.864 | 1.000 | 0.969 | 1.000 | 0.962 | 1.000 | 0.973 | 0.787 | 1.000 | 1.000 | 0.848 | 0.817 |
| 1467 | 0.812 | 1.000 | 0.814 | 1.000 | 0.974 | 1.000 | 0.864 | 1.000 | 0.969 | 1.000 | 0.962 | 1.000 | 0.973 | 0.787 | 1.000 | 1.000 | 0.848 | 0.817 |
| 1468 | 0.812 | 1.000 | 0.814 | 1.000 | 0.974 | 1.000 | 0.866 | 1.000 | 0.969 | 1.000 | 0.962 | 1.000 | 0.973 | 0.788 | 1.000 | 1.000 | 0.848 | 0.817 |
| 1470 | 0.813 | 1.000 | 0.814 | 1.000 | 0.974 | 1.000 | 0.866 | 1.000 | 0.969 | 1.000 | 0.962 | 1.000 | 0.973 | 0.785 | 1.000 | 1.000 | 0.848 | 0.817 |
| 1471 | 0.813 | 1.000 | 0.814 | 1.000 | 0.974 | 1.000 | 0.867 | 1.000 | 0.969 | 1.000 | 0.959 | 1.000 | 0.973 | 0.785 | 1.000 | 1.000 | 0.848 | 0.817 |
| 1472 | 0.813 | 1.000 | 0.814 | 1.000 | 0.974 | 1.000 | 0.864 | 1.000 | 0.969 | 1.000 | 0.959 | 1.000 | 0.973 | 0.786 | 1.000 | 1.000 | 0.848 | 0.817 |
| 1473 | 0.813 | 1.000 | 0.814 | 1.000 | 0.974 | 1.000 | 0.864 | 1.000 | 0.969 | 1.000 | 0.959 | 1.000 | 0.973 | 0.786 | 1.000 | 1.000 | 0.848 | 0.817 |
| 1474 | 0.813 | 1.000 | 0.814 | 1.000 | 0.974 | 1.000 | 0.866 | 1.000 | 0.969 | 1.000 | 0.959 | 1.000 | 0.973 | 0.787 | 1.000 | 1.000 | 0.848 | 0.817 |
| 1475 | 0.813 | 1.000 | 0.814 | 1.000 | 0.974 | 1.000 | 0.867 | 1.000 | 0.969 | 1.000 | 0.959 | 1.000 | 0.973 | 0.787 | 1.000 | 1.000 | 0.848 | 0.817 |
| 1476 | 0.813 | 1.000 | 0.814 | 1.000 | 0.974 | 1.000 | 0.867 | 1.000 | 0.969 | 1.000 | 0.957 | 1.000 | 0.973 | 0.787 | 1.000 | 1.000 | 0.848 | 0.817 |
| 1477 | 0.812 | 1.000 | 0.812 | 1.000 | 0.974 | 1.000 | 0.866 | 1.000 | 0.969 | 1.000 | 0.957 | 1.000 | 0.964 | 0.789 | 1.000 | 1.000 | 0.848 | 0.817 |
| 1478 | 0.812 | 1.000 | 0.812 | 1.000 | 0.974 | 1.000 | 0.864 | 1.000 | 0.969 | 1.000 | 0.957 | 1.000 | 0.964 | 0.788 | 1.000 | 1.000 | 0.847 | 0.816 |
| 1479 | 0.812 | 1.000 | 0.810 | 1.000 | 0.974 | 1.000 | 0.863 | 1.000 | 0.969 | 1.000 | 0.957 | 1.000 | 0.964 | 0.788 | 1.000 | 1.000 | 0.847 | 0.815 |
| 1480 | 0.812 | 1.000 | 0.810 | 1.000 | 0.974 | 1.000 | 0.863 | 1.000 | 0.969 | 1.000 | 0.957 | 1.000 | 0.964 | 0.788 | 1.000 | 1.000 | 0.847 | 0.816 |
| 1481 | 0.812 | 1.000 | 0.812 | 1.000 | 0.974 | 1.000 | 0.863 | 1.000 | 0.969 | 1.000 | 0.959 | 1.000 | 0.964 | 0.788 | 1.000 | 1.000 | 0.847 | 0.816 |
| 1482 | 0.812 | 1.000 | 0.812 | 1.000 | 0.974 | 1.000 | 0.863 | 1.000 | 0.969 | 1.000 | 0.959 | 1.000 | 0.964 | 0.788 | 1.000 | 1.000 | 0.847 | 0.816 |
| 1483 | 0.812 | 1.000 | 0.812 | 1.000 | 0.974 | 1.000 | 0.864 | 1.000 | 0.969 | 1.000 | 0.959 | 1.000 | 0.964 | 0.788 | 1.000 | 1.000 | 0.848 | 0.817 |
| 1484 | 0.812 | 1.000 | 0.810 | 1.000 | 0.974 | 1.000 | 0.866 | 1.000 | 0.969 | 1.000 | 0.959 | 1.000 | 0.964 | 0.790 | 1.000 | 1.000 | 0.848 | 0.817 |
| 1485 | 0.812 | 1.000 | 0.814 | 1.000 | 0.974 | 1.000 | 0.864 | 1.000 | 0.969 | 1.000 | 0.962 | 1.000 | 0.964 | 0.791 | 1.000 | 1.000 | 0.849 | 0.818 |

| | | | | | | | | | | | | | | | | | |
|---|---|---|---|---|---|---|---|---|---|---|---|---|---|---|---|---|---|
| 1486 | 0.812 | 1.000 | 0.814 | 1.000 | 0.974 | 1.000 | 0.866 | 1.000 | 0.969 | 1.000 | 0.962 | 1.000 | 0.964 | 0.791 | 1.000 | 1.000 | 0.849 | 0.818 |
| 1487 | 0.811 | 1.000 | 0.814 | 1.000 | 0.974 | 1.000 | 0.867 | 1.000 | 0.969 | 1.000 | 0.962 | 1.000 | 0.964 | 0.791 | 1.000 | 1.000 | 0.849 | 0.818 |
| 1488 | 0.812 | 1.000 | 0.812 | 1.000 | 0.974 | 1.000 | 0.872 | 1.000 | 0.969 | 1.000 | 0.965 | 1.000 | 0.964 | 0.790 | 1.000 | 1.000 | 0.849 | 0.819 |
| 1489 | 0.812 | 1.000 | 0.812 | 1.000 | 0.974 | 1.000 | 0.874 | 1.000 | 0.969 | 1.000 | 0.965 | 1.000 | 0.964 | 0.791 | 1.000 | 1.000 | 0.850 | 0.819 |
| 1490 | 0.814 | 1.000 | 0.812 | 1.000 | 0.974 | 1.000 | 0.872 | 1.000 | 0.969 | 1.000 | 0.962 | 1.000 | 0.964 | 0.792 | 1.000 | 1.000 | 0.850 | 0.820 |
| 1491 | 0.814 | 1.000 | 0.812 | 1.000 | 0.974 | 1.000 | 0.872 | 1.000 | 0.969 | 1.000 | 0.962 | 1.000 | 0.964 | 0.791 | 1.000 | 1.000 | 0.850 | 0.820 |
| 1492 | 0.814 | 1.000 | 0.812 | 1.000 | 0.974 | 1.000 | 0.869 | 1.000 | 0.969 | 1.000 | 0.965 | 1.000 | 0.964 | 0.790 | 1.000 | 1.000 | 0.850 | 0.819 |
| 1493 | 0.814 | 1.000 | 0.812 | 1.000 | 0.974 | 1.000 | 0.869 | 1.000 | 0.969 | 1.000 | 0.965 | 1.000 | 0.964 | 0.788 | 1.000 | 1.000 | 0.849 | 0.818 |
| 1494 | 0.814 | 1.000 | 0.812 | 1.000 | 0.974 | 1.000 | 0.869 | 1.000 | 0.969 | 1.000 | 0.965 | 1.000 | 0.964 | 0.787 | 1.000 | 1.000 | 0.849 | 0.818 |
| 1495 | 0.814 | 1.000 | 0.812 | 1.000 | 0.974 | 1.000 | 0.869 | 1.000 | 0.969 | 1.000 | 0.965 | 1.000 | 0.964 | 0.786 | 1.000 | 1.000 | 0.848 | 0.818 |
| 1496 | 0.817 | 1.000 | 0.812 | 1.000 | 0.974 | 1.000 | 0.871 | 1.000 | 0.969 | 1.000 | 0.965 | 1.000 | 0.964 | 0.788 | 1.000 | 1.000 | 0.850 | 0.820 |
| 1497 | 0.816 | 1.000 | 0.812 | 1.000 | 0.974 | 1.000 | 0.871 | 1.000 | 0.969 | 1.000 | 0.965 | 1.000 | 0.964 | 0.788 | 1.000 | 1.000 | 0.850 | 0.819 |
| 1498 | 0.815 | 1.000 | 0.812 | 1.000 | 0.974 | 1.000 | 0.871 | 1.000 | 0.969 | 1.000 | 0.965 | 1.000 | 0.964 | 0.788 | 1.000 | 1.000 | 0.849 | 0.819 |
| 1499 | 0.813 | 1.000 | 0.814 | 1.000 | 0.974 | 1.000 | 0.871 | 1.000 | 0.969 | 1.000 | 0.959 | 1.000 | 0.964 | 0.791 | 1.000 | 1.000 | 0.850 | 0.819 |
| 1500 | 0.813 | 1.000 | 0.814 | 1.000 | 0.974 | 1.000 | 0.871 | 1.000 | 0.969 | 1.000 | 0.959 | 1.000 | 0.964 | 0.791 | 1.000 | 1.000 | 0.850 | 0.819 |
| 1501 | 0.813 | 1.000 | 0.812 | 1.000 | 0.974 | 1.000 | 0.871 | 1.000 | 0.969 | 1.000 | 0.959 | 1.000 | 0.964 | 0.791 | 1.000 | 1.000 | 0.849 | 0.819 |
| 1502 | 0.814 | 1.000 | 0.810 | 1.000 | 0.974 | 1.000 | 0.871 | 1.000 | 0.969 | 1.000 | 0.957 | 1.000 | 0.964 | 0.794 | 1.000 | 1.000 | 0.850 | 0.819 |
| 1503 | 0.814 | 1.000 | 0.812 | 1.000 | 0.974 | 1.000 | 0.871 | 1.000 | 0.969 | 1.000 | 0.957 | 1.000 | 0.964 | 0.793 | 1.000 | 1.000 | 0.850 | 0.819 |
| 1504 | 0.815 | 1.000 | 0.810 | 1.000 | 0.974 | 1.000 | 0.871 | 1.000 | 0.969 | 1.000 | 0.954 | 1.000 | 0.964 | 0.792 | 1.000 | 1.000 | 0.850 | 0.819 |
| 1505 | 0.814 | 1.000 | 0.810 | 1.000 | 0.974 | 1.000 | 0.871 | 1.000 | 0.969 | 1.000 | 0.959 | 1.000 | 0.964 | 0.791 | 1.000 | 1.000 | 0.850 | 0.819 |
| 1506 | 0.814 | 1.000 | 0.814 | 1.000 | 0.974 | 1.000 | 0.871 | 1.000 | 0.969 | 1.000 | 0.957 | 1.000 | 0.964 | 0.792 | 1.000 | 1.000 | 0.850 | 0.819 |
| 1507 | 0.814 | 1.000 | 0.814 | 1.000 | 0.974 | 1.000 | 0.871 | 1.000 | 0.969 | 1.000 | 0.957 | 1.000 | 0.964 | 0.792 | 1.000 | 1.000 | 0.850 | 0.819 |
| 1508 | 0.814 | 1.000 | 0.820 | 1.000 | 0.974 | 1.000 | 0.871 | 1.000 | 0.969 | 1.000 | 0.957 | 1.000 | 0.973 | 0.792 | 1.000 | 1.000 | 0.851 | 0.820 |
| 1509 | 0.814 | 1.000 | 0.820 | 1.000 | 0.974 | 1.000 | 0.871 | 1.000 | 0.980 | 1.000 | 0.957 | 1.000 | 0.973 | 0.792 | 1.000 | 1.000 | 0.851 | 0.821 |
| 1510 | 0.814 | 1.000 | 0.820 | 1.000 | 0.974 | 1.000 | 0.871 | 1.000 | 0.980 | 1.000 | 0.957 | 1.000 | 0.973 | 0.792 | 1.000 | 1.000 | 0.851 | 0.821 |
| 1511 | 0.814 | 1.000 | 0.820 | 1.000 | 0.974 | 1.000 | 0.869 | 1.000 | 0.980 | 1.000 | 0.957 | 1.000 | 0.973 | 0.792 | 1.000 | 1.000 | 0.851 | 0.820 |
| 1512 | 0.814 | 1.000 | 0.820 | 1.000 | 0.974 | 1.000 | 0.869 | 1.000 | 0.980 | 1.000 | 0.957 | 1.000 | 0.973 | 0.793 | 1.000 | 1.000 | 0.851 | 0.821 |
| 1513 | 0.815 | 1.000 | 0.820 | 1.000 | 0.974 | 1.000 | 0.869 | 1.000 | 0.980 | 1.000 | 0.957 | 1.000 | 0.973 | 0.794 | 1.000 | 1.000 | 0.851 | 0.821 |
| 1514 | 0.815 | 1.000 | 0.820 | 1.000 | 0.974 | 1.000 | 0.869 | 1.000 | 0.980 | 1.000 | 0.957 | 1.000 | 0.973 | 0.794 | 1.000 | 1.000 | 0.851 | 0.821 |
| 1515 | 0.815 | 1.000 | 0.820 | 1.000 | 0.974 | 1.000 | 0.869 | 1.000 | 0.980 | 1.000 | 0.957 | 1.000 | 0.973 | 0.793 | 1.000 | 1.000 | 0.851 | 0.821 |
| 1516 | 0.816 | 1.000 | 0.820 | 1.000 | 0.974 | 1.000 | 0.869 | 1.000 | 0.980 | 1.000 | 0.957 | 1.000 | 0.973 | 0.792 | 1.000 | 1.000 | 0.851 | 0.821 |
| 1517 | 0.816 | 1.000 | 0.820 | 1.000 | 0.974 | 1.000 | 0.869 | 1.000 | 0.980 | 1.000 | 0.957 | 1.000 | 0.973 | 0.792 | 1.000 | 1.000 | 0.851 | 0.821 |
| 1518 | 0.817 | 1.000 | 0.820 | 1.000 | 0.974 | 1.000 | 0.867 | 1.000 | 0.980 | 1.000 | 0.957 | 1.000 | 0.973 | 0.792 | 1.000 | 1.000 | 0.851 | 0.821 |
| 1519 | 0.816 | 1.000 | 0.820 | 1.000 | 0.974 | 1.000 | 0.867 | 1.000 | 0.980 | 1.000 | 0.957 | 1.000 | 0.973 | 0.792 | 1.000 | 1.000 | 0.851 | 0.821 |
| 1520 | 0.817 | 1.000 | 0.820 | 1.000 | 0.974 | 1.000 | 0.867 | 1.000 | 0.980 | 1.000 | 0.957 | 1.000 | 0.973 | 0.793 | 1.000 | 1.000 | 0.852 | 0.821 |
| 1521 | 0.816 | 1.000 | 0.820 | 1.000 | 0.974 | 1.000 | 0.866 | 1.000 | 0.980 | 1.000 | 0.957 | 1.000 | 0.973 | 0.793 | 1.000 | 1.000 | 0.851 | 0.821 |
| 1522 | 0.819 | 1.000 | 0.822 | 1.000 | 0.974 | 1.000 | 0.867 | 1.000 | 0.980 | 1.000 | 0.957 | 1.000 | 0.973 | 0.791 | 1.000 | 1.000 | 0.852 | 0.822 |
| 1523 | 0.818 | 1.000 | 0.822 | 1.000 | 0.974 | 1.000 | 0.867 | 1.000 | 0.980 | 1.000 | 0.957 | 1.000 | 0.973 | 0.791 | 1.000 | 1.000 | 0.852 | 0.821 |
| 1524 | 0.819 | 1.000 | 0.820 | 1.000 | 0.979 | 1.000 | 0.867 | 1.000 | 0.980 | 1.000 | 0.957 | 1.000 | 0.973 | 0.791 | 1.000 | 1.000 | 0.852 | 0.822 |

| | | | | | | | | | | | | | | | | | | |
|---|---|---|---|---|---|---|---|---|---|---|---|---|---|---|---|---|---|---|
| 1525 | 0.820 | 1.000 | 0.822 | 1.000 | 0.974 | 1.000 | 0.867 | 1.000 | 0.980 | 1.000 | 0.954 | 1.000 | 0.973 | 0.792 | 1.000 | 1.000 | 0.852 | 0.822 |
| 1526 | 0.819 | 1.000 | 0.822 | 1.000 | 0.979 | 1.000 | 0.867 | 1.000 | 0.980 | 1.000 | 0.954 | 1.000 | 0.973 | 0.791 | 1.000 | 1.000 | 0.852 | 0.822 |
| 1527 | 0.819 | 1.000 | 0.820 | 1.000 | 0.979 | 1.000 | 0.867 | 1.000 | 0.980 | 1.000 | 0.954 | 1.000 | 0.973 | 0.791 | 1.000 | 1.000 | 0.852 | 0.821 |
| 1528 | 0.820 | 1.000 | 0.818 | 1.000 | 0.979 | 1.000 | 0.871 | 1.000 | 0.980 | 1.000 | 0.954 | 1.000 | 0.973 | 0.787 | 1.000 | 1.000 | 0.851 | 0.821 |
| 1529 | 0.820 | 1.000 | 0.818 | 1.000 | 0.979 | 1.000 | 0.869 | 1.000 | 0.980 | 1.000 | 0.954 | 1.000 | 0.973 | 0.788 | 1.000 | 1.000 | 0.851 | 0.821 |
| 1530 | 0.821 | 1.000 | 0.818 | 1.000 | 0.979 | 1.000 | 0.867 | 1.000 | 0.980 | 1.000 | 0.954 | 1.000 | 0.973 | 0.788 | 1.000 | 1.000 | 0.851 | 0.821 |
| 1531 | 0.822 | 1.000 | 0.818 | 1.000 | 0.979 | 1.000 | 0.867 | 1.000 | 0.980 | 1.000 | 0.954 | 1.000 | 0.973 | 0.788 | 1.000 | 1.000 | 0.852 | 0.821 |
| 1532 | 0.821 | 1.000 | 0.820 | 1.000 | 0.979 | 1.000 | 0.867 | 1.000 | 0.980 | 1.000 | 0.954 | 1.000 | 0.973 | 0.788 | 1.000 | 1.000 | 0.852 | 0.821 |
| 1533 | 0.822 | 1.000 | 0.820 | 1.000 | 0.979 | 1.000 | 0.871 | 1.000 | 0.980 | 1.000 | 0.954 | 1.000 | 0.973 | 0.788 | 1.000 | 1.000 | 0.852 | 0.822 |
| 1534 | 0.819 | 1.000 | 0.818 | 1.000 | 0.979 | 1.000 | 0.869 | 1.000 | 0.980 | 1.000 | 0.957 | 1.000 | 0.973 | 0.787 | 1.000 | 1.000 | 0.851 | 0.820 |
| 1535 | 0.819 | 1.000 | 0.818 | 1.000 | 0.979 | 1.000 | 0.869 | 1.000 | 0.969 | 1.000 | 0.957 | 1.000 | 0.973 | 0.788 | 1.000 | 1.000 | 0.851 | 0.820 |
| 1536 | 0.819 | 1.000 | 0.820 | 1.000 | 0.979 | 1.000 | 0.869 | 1.000 | 0.969 | 1.000 | 0.957 | 1.000 | 0.973 | 0.788 | 1.000 | 1.000 | 0.851 | 0.821 |
| 1537 | 0.819 | 1.000 | 0.820 | 1.000 | 0.979 | 1.000 | 0.869 | 1.000 | 0.969 | 1.000 | 0.957 | 1.000 | 0.973 | 0.788 | 1.000 | 1.000 | 0.851 | 0.821 |
| 1538 | 0.818 | 1.000 | 0.820 | 1.000 | 0.979 | 1.000 | 0.869 | 1.000 | 0.969 | 1.000 | 0.957 | 1.000 | 0.973 | 0.789 | 1.000 | 1.000 | 0.851 | 0.821 |
| 1539 | 0.819 | 1.000 | 0.824 | 1.000 | 0.979 | 1.000 | 0.869 | 1.000 | 0.969 | 1.000 | 0.954 | 1.000 | 0.973 | 0.789 | 1.000 | 1.000 | 0.852 | 0.821 |
| 1540 | 0.818 | 1.000 | 0.824 | 1.000 | 0.979 | 1.000 | 0.869 | 1.000 | 0.969 | 1.000 | 0.954 | 1.000 | 0.973 | 0.789 | 1.000 | 1.000 | 0.851 | 0.821 |
| 1541 | 0.823 | 1.000 | 0.828 | 1.000 | 0.979 | 1.000 | 0.867 | 1.000 | 0.969 | 1.000 | 0.954 | 1.000 | 0.973 | 0.788 | 1.000 | 1.000 | 0.853 | 0.823 |
| 1542 | 0.823 | 1.000 | 0.828 | 1.000 | 0.979 | 1.000 | 0.867 | 1.000 | 0.969 | 1.000 | 0.954 | 1.000 | 0.973 | 0.788 | 1.000 | 1.000 | 0.853 | 0.823 |
| 1543 | 0.824 | 1.000 | 0.828 | 1.000 | 0.979 | 1.000 | 0.867 | 1.000 | 0.969 | 1.000 | 0.954 | 1.000 | 0.973 | 0.788 | 1.000 | 1.000 | 0.853 | 0.823 |
| 1544 | 0.824 | 1.000 | 0.828 | 1.000 | 0.979 | 1.000 | 0.867 | 1.000 | 0.969 | 1.000 | 0.954 | 1.000 | 0.973 | 0.788 | 1.000 | 1.000 | 0.853 | 0.823 |
| 1545 | 0.823 | 1.000 | 0.828 | 1.000 | 0.979 | 1.000 | 0.869 | 1.000 | 0.969 | 1.000 | 0.954 | 1.000 | 0.973 | 0.788 | 1.000 | 1.000 | 0.853 | 0.823 |
| 1546 | 0.823 | 1.000 | 0.828 | 1.000 | 0.979 | 1.000 | 0.869 | 1.000 | 0.969 | 1.000 | 0.954 | 1.000 | 0.973 | 0.788 | 1.000 | 1.000 | 0.853 | 0.823 |
| 1547 | 0.823 | 1.000 | 0.828 | 1.000 | 0.979 | 1.000 | 0.869 | 1.000 | 0.969 | 1.000 | 0.954 | 1.000 | 0.973 | 0.788 | 1.000 | 1.000 | 0.853 | 0.823 |
| 1548 | 0.823 | 1.000 | 0.828 | 1.000 | 0.979 | 1.000 | 0.867 | 1.000 | 0.969 | 1.000 | 0.954 | 1.000 | 0.973 | 0.787 | 1.000 | 1.000 | 0.852 | 0.822 |
| 1549 | 0.823 | 1.000 | 0.828 | 1.000 | 0.979 | 1.000 | 0.867 | 1.000 | 0.969 | 1.000 | 0.954 | 1.000 | 0.973 | 0.787 | 1.000 | 1.000 | 0.852 | 0.822 |
| 1550 | 0.824 | 1.000 | 0.828 | 1.000 | 0.984 | 1.000 | 0.867 | 1.000 | 0.969 | 1.000 | 0.954 | 1.000 | 0.973 | 0.788 | 1.000 | 1.000 | 0.853 | 0.823 |
| 1551 | 0.822 | 1.000 | 0.828 | 1.000 | 0.984 | 1.000 | 0.867 | 1.000 | 0.969 | 1.000 | 0.957 | 1.000 | 0.973 | 0.787 | 1.000 | 1.000 | 0.852 | 0.822 |
| 1552 | 0.822 | 1.000 | 0.830 | 1.000 | 0.984 | 1.000 | 0.867 | 1.000 | 0.969 | 1.000 | 0.957 | 1.000 | 0.973 | 0.787 | 1.000 | 1.000 | 0.853 | 0.823 |
| 1553 | 0.822 | 1.000 | 0.830 | 1.000 | 0.984 | 1.000 | 0.867 | 1.000 | 0.969 | 1.000 | 0.957 | 1.000 | 0.973 | 0.788 | 1.000 | 1.000 | 0.853 | 0.823 |
| 1554 | 0.823 | 1.000 | 0.830 | 1.000 | 0.984 | 1.000 | 0.867 | 1.000 | 0.969 | 1.000 | 0.957 | 1.000 | 0.973 | 0.788 | 1.000 | 1.000 | 0.853 | 0.823 |
| 1555 | 0.822 | 1.000 | 0.828 | 1.000 | 0.984 | 1.000 | 0.867 | 1.000 | 0.969 | 1.000 | 0.957 | 1.000 | 0.973 | 0.788 | 1.000 | 1.000 | 0.853 | 0.823 |
| 1556 | 0.822 | 1.000 | 0.828 | 1.000 | 0.984 | 1.000 | 0.867 | 1.000 | 0.969 | 1.000 | 0.957 | 1.000 | 0.973 | 0.788 | 1.000 | 1.000 | 0.853 | 0.823 |
| 1557 | 0.822 | 1.000 | 0.826 | 1.000 | 0.984 | 1.000 | 0.867 | 1.000 | 0.969 | 1.000 | 0.957 | 1.000 | 0.973 | 0.788 | 1.000 | 1.000 | 0.853 | 0.823 |
| 1558 | 0.824 | 1.000 | 0.818 | 1.000 | 0.979 | 1.000 | 0.867 | 1.000 | 0.969 | 1.000 | 0.962 | 1.000 | 0.973 | 0.790 | 1.000 | 1.000 | 0.853 | 0.823 |
| 1559 | 0.824 | 1.000 | 0.822 | 1.000 | 0.979 | 1.000 | 0.867 | 1.000 | 0.969 | 1.000 | 0.962 | 1.000 | 0.973 | 0.789 | 1.000 | 1.000 | 0.853 | 0.823 |
| 1560 | 0.824 | 1.000 | 0.822 | 1.000 | 0.979 | 1.000 | 0.867 | 1.000 | 0.980 | 1.000 | 0.962 | 1.000 | 0.973 | 0.791 | 1.000 | 1.000 | 0.854 | 0.824 |
| 1561 | 0.824 | 1.000 | 0.822 | 1.000 | 0.979 | 1.000 | 0.867 | 1.000 | 0.980 | 1.000 | 0.962 | 1.000 | 0.973 | 0.791 | 1.000 | 1.000 | 0.854 | 0.824 |
| 1562 | 0.824 | 1.000 | 0.822 | 1.000 | 0.979 | 1.000 | 0.867 | 1.000 | 0.980 | 1.000 | 0.962 | 1.000 | 0.973 | 0.791 | 1.000 | 1.000 | 0.854 | 0.824 |
| 1563 | 0.824 | 1.000 | 0.822 | 1.000 | 0.979 | 1.000 | 0.867 | 1.000 | 0.980 | 1.000 | 0.962 | 1.000 | 0.973 | 0.790 | 1.000 | 1.000 | 0.854 | 0.824 |

| | | | | | | | | | | | | | | | | | | |
|---|---|---|---|---|---|---|---|---|---|---|---|---|---|---|---|---|---|---|
| 1564 | 0.823 | 1.000 | 0.822 | 1.000 | 0.979 | 1.000 | 0.867 | 1.000 | 0.980 | 1.000 | 0.962 | 1.000 | 0.973 | 0.790 | 1.000 | 1.000 | 0.853 | 0.823 |
| 1565 | 0.824 | 1.000 | 0.822 | 1.000 | 0.979 | 1.000 | 0.869 | 1.000 | 0.980 | 1.000 | 0.962 | 1.000 | 0.973 | 0.791 | 1.000 | 1.000 | 0.854 | 0.824 |
| 1566 | 0.824 | 1.000 | 0.822 | 1.000 | 0.979 | 1.000 | 0.869 | 1.000 | 0.980 | 1.000 | 0.962 | 1.000 | 0.973 | 0.789 | 1.000 | 1.000 | 0.854 | 0.824 |
| 1567 | 0.825 | 1.000 | 0.822 | 1.000 | 0.979 | 1.000 | 0.867 | 1.000 | 0.980 | 1.000 | 0.962 | 1.000 | 0.973 | 0.791 | 1.000 | 1.000 | 0.854 | 0.824 |
| 1568 | 0.822 | 1.000 | 0.820 | 1.000 | 0.979 | 1.000 | 0.869 | 1.000 | 0.980 | 1.000 | 0.962 | 1.000 | 0.973 | 0.792 | 1.000 | 1.000 | 0.854 | 0.824 |
| 1569 | 0.822 | 1.000 | 0.820 | 1.000 | 0.979 | 1.000 | 0.867 | 1.000 | 0.980 | 1.000 | 0.962 | 1.000 | 0.973 | 0.792 | 1.000 | 1.000 | 0.853 | 0.823 |
| 1570 | 0.822 | 1.000 | 0.820 | 1.000 | 0.979 | 1.000 | 0.869 | 1.000 | 0.980 | 1.000 | 0.962 | 1.000 | 0.973 | 0.792 | 1.000 | 1.000 | 0.854 | 0.824 |
| 1571 | 0.822 | 1.000 | 0.822 | 1.000 | 0.979 | 1.000 | 0.869 | 1.000 | 0.980 | 1.000 | 0.962 | 1.000 | 0.973 | 0.792 | 1.000 | 1.000 | 0.854 | 0.824 |
| 1572 | 0.823 | 1.000 | 0.818 | 1.000 | 0.979 | 1.000 | 0.869 | 1.000 | 0.980 | 1.000 | 0.962 | 1.000 | 0.973 | 0.792 | 1.000 | 1.000 | 0.854 | 0.824 |
| 1573 | 0.822 | 1.000 | 0.820 | 1.000 | 0.979 | 1.000 | 0.869 | 1.000 | 0.980 | 1.000 | 0.962 | 1.000 | 0.973 | 0.792 | 1.000 | 1.000 | 0.854 | 0.824 |
| 1574 | 0.822 | 1.000 | 0.822 | 1.000 | 0.979 | 1.000 | 0.869 | 1.000 | 0.980 | 1.000 | 0.962 | 1.000 | 0.973 | 0.792 | 1.000 | 1.000 | 0.854 | 0.824 |
| 1575 | 0.823 | 1.000 | 0.822 | 1.000 | 0.979 | 1.000 | 0.869 | 1.000 | 0.980 | 1.000 | 0.962 | 1.000 | 0.973 | 0.792 | 1.000 | 1.000 | 0.854 | 0.824 |
| 1576 | 0.823 | 1.000 | 0.822 | 1.000 | 0.979 | 1.000 | 0.871 | 1.000 | 0.980 | 1.000 | 0.962 | 1.000 | 0.973 | 0.792 | 1.000 | 1.000 | 0.854 | 0.825 |
| 1577 | 0.823 | 1.000 | 0.818 | 1.000 | 0.979 | 1.000 | 0.871 | 1.000 | 0.980 | 1.000 | 0.962 | 1.000 | 0.973 | 0.792 | 1.000 | 1.000 | 0.854 | 0.824 |
| 1578 | 0.824 | 1.000 | 0.820 | 1.000 | 0.979 | 1.000 | 0.871 | 1.000 | 0.980 | 1.000 | 0.962 | 1.000 | 0.973 | 0.792 | 1.000 | 1.000 | 0.854 | 0.825 |
| 1579 | 0.821 | 1.000 | 0.820 | 1.000 | 0.979 | 1.000 | 0.869 | 1.000 | 0.980 | 1.000 | 0.962 | 1.000 | 0.973 | 0.792 | 1.000 | 1.000 | 0.853 | 0.823 |
| 1580 | 0.820 | 1.000 | 0.820 | 1.000 | 0.984 | 1.000 | 0.867 | 1.000 | 0.980 | 1.000 | 0.962 | 1.000 | 0.973 | 0.792 | 1.000 | 1.000 | 0.853 | 0.823 |
| 1581 | 0.820 | 1.000 | 0.818 | 1.000 | 0.984 | 1.000 | 0.867 | 1.000 | 0.980 | 1.000 | 0.962 | 1.000 | 0.973 | 0.792 | 1.000 | 1.000 | 0.853 | 0.823 |
| 1582 | 0.822 | 1.000 | 0.822 | 1.000 | 0.984 | 1.000 | 0.867 | 1.000 | 0.980 | 1.000 | 0.962 | 1.000 | 0.973 | 0.792 | 1.000 | 1.000 | 0.854 | 0.824 |
| 1583 | 0.820 | 1.000 | 0.824 | 1.000 | 0.984 | 1.000 | 0.867 | 1.000 | 0.980 | 1.000 | 0.962 | 1.000 | 0.973 | 0.792 | 1.000 | 1.000 | 0.854 | 0.824 |
| 1584 | 0.821 | 1.000 | 0.826 | 1.000 | 0.984 | 1.000 | 0.867 | 1.000 | 0.980 | 1.000 | 0.962 | 1.000 | 0.973 | 0.792 | 1.000 | 1.000 | 0.854 | 0.824 |
| 1585 | 0.820 | 1.000 | 0.826 | 1.000 | 0.984 | 1.000 | 0.867 | 1.000 | 0.980 | 1.000 | 0.962 | 1.000 | 0.973 | 0.793 | 1.000 | 1.000 | 0.854 | 0.824 |
| 1586 | 0.821 | 1.000 | 0.824 | 1.000 | 0.984 | 1.000 | 0.867 | 1.000 | 0.980 | 1.000 | 0.962 | 1.000 | 0.973 | 0.793 | 1.000 | 1.000 | 0.854 | 0.824 |
| 1587 | 0.821 | 1.000 | 0.826 | 1.000 | 0.984 | 1.000 | 0.867 | 1.000 | 0.980 | 1.000 | 0.962 | 1.000 | 0.973 | 0.793 | 1.000 | 1.000 | 0.854 | 0.824 |
| 1588 | 0.822 | 1.000 | 0.826 | 1.000 | 0.984 | 1.000 | 0.867 | 1.000 | 0.980 | 1.000 | 0.962 | 1.000 | 0.973 | 0.793 | 1.000 | 1.000 | 0.855 | 0.825 |
| 1589 | 0.822 | 1.000 | 0.826 | 1.000 | 0.984 | 1.000 | 0.867 | 1.000 | 0.980 | 1.000 | 0.962 | 1.000 | 0.973 | 0.792 | 1.000 | 1.000 | 0.854 | 0.825 |
| 1590 | 0.822 | 1.000 | 0.826 | 1.000 | 0.984 | 1.000 | 0.867 | 1.000 | 0.980 | 1.000 | 0.962 | 1.000 | 0.973 | 0.793 | 1.000 | 1.000 | 0.855 | 0.825 |
| 1591 | 0.823 | 1.000 | 0.826 | 1.000 | 0.984 | 1.000 | 0.867 | 1.000 | 0.980 | 1.000 | 0.962 | 1.000 | 0.973 | 0.794 | 1.000 | 1.000 | 0.855 | 0.825 |
| 1592 | 0.822 | 1.000 | 0.826 | 1.000 | 0.984 | 1.000 | 0.867 | 1.000 | 0.980 | 1.000 | 0.962 | 1.000 | 0.973 | 0.794 | 1.000 | 1.000 | 0.855 | 0.825 |
| 1593 | 0.823 | 1.000 | 0.826 | 1.000 | 0.984 | 1.000 | 0.867 | 1.000 | 0.980 | 1.000 | 0.962 | 1.000 | 0.973 | 0.794 | 1.000 | 1.000 | 0.855 | 0.825 |
| 1594 | 0.822 | 1.000 | 0.824 | 1.000 | 0.984 | 1.000 | 0.867 | 1.000 | 0.980 | 1.000 | 0.962 | 1.000 | 0.973 | 0.794 | 1.000 | 1.000 | 0.854 | 0.825 |
| 1595 | 0.822 | 1.000 | 0.824 | 1.000 | 0.984 | 1.000 | 0.872 | 1.000 | 0.980 | 1.000 | 0.962 | 1.000 | 0.973 | 0.794 | 1.000 | 1.000 | 0.855 | 0.826 |
| 1596 | 0.824 | 1.000 | 0.826 | 1.000 | 0.984 | 1.000 | 0.872 | 1.000 | 0.980 | 1.000 | 0.962 | 1.000 | 0.973 | 0.794 | 1.000 | 1.000 | 0.856 | 0.826 |
| 1597 | 0.821 | 1.000 | 0.822 | 1.000 | 0.984 | 1.000 | 0.872 | 1.000 | 0.980 | 1.000 | 0.962 | 1.000 | 0.973 | 0.795 | 1.000 | 1.000 | 0.855 | 0.825 |
| 1598 | 0.821 | 1.000 | 0.822 | 1.000 | 0.984 | 1.000 | 0.872 | 1.000 | 0.980 | 1.000 | 0.962 | 1.000 | 0.973 | 0.795 | 1.000 | 1.000 | 0.855 | 0.825 |
| 1599 | 0.821 | 1.000 | 0.822 | 1.000 | 0.984 | 1.000 | 0.872 | 1.000 | 0.980 | 1.000 | 0.962 | 1.000 | 0.973 | 0.795 | 1.000 | 1.000 | 0.855 | 0.825 |
| 1600 | 0.819 | 1.000 | 0.826 | 1.000 | 0.984 | 1.000 | 0.874 | 1.000 | 0.980 | 1.000 | 0.962 | 1.000 | 0.973 | 0.794 | 1.000 | 1.000 | 0.855 | 0.825 |
| 1601 | 0.818 | 1.000 | 0.826 | 1.000 | 0.984 | 1.000 | 0.871 | 1.000 | 0.980 | 1.000 | 0.962 | 1.000 | 0.973 | 0.793 | 1.000 | 1.000 | 0.854 | 0.824 |
| 1602 | 0.820 | 1.000 | 0.826 | 1.000 | 0.984 | 1.000 | 0.872 | 1.000 | 0.980 | 1.000 | 0.962 | 1.000 | 0.973 | 0.793 | 1.000 | 1.000 | 0.855 | 0.825 |

| | | | | | | | | | | | | | | | | | |
|---|---|---|---|---|---|---|---|---|---|---|---|---|---|---|---|---|---|
| 1603 | 0.820 | 1.000 | 0.824 | 1.000 | 0.984 | 1.000 | 0.872 | 1.000 | 0.980 | 1.000 | 0.962 | 1.000 | 0.973 | 0.794 | 1.000 | 1.000 | 0.855 | 0.825 |
| 1604 | 0.819 | 1.000 | 0.824 | 1.000 | 0.984 | 1.000 | 0.872 | 1.000 | 0.980 | 1.000 | 0.962 | 1.000 | 0.973 | 0.793 | 1.000 | 1.000 | 0.854 | 0.824 |
| 1605 | 0.817 | 1.000 | 0.824 | 1.000 | 0.984 | 1.000 | 0.874 | 1.000 | 0.980 | 1.000 | 0.962 | 1.000 | 0.973 | 0.794 | 1.000 | 1.000 | 0.854 | 0.824 |
| 1606 | 0.817 | 1.000 | 0.822 | 1.000 | 0.984 | 1.000 | 0.874 | 1.000 | 0.980 | 1.000 | 0.962 | 1.000 | 0.973 | 0.793 | 1.000 | 1.000 | 0.853 | 0.824 |
| 1607 | 0.820 | 1.000 | 0.824 | 1.000 | 0.984 | 1.000 | 0.875 | 1.000 | 0.980 | 1.000 | 0.959 | 1.000 | 0.973 | 0.789 | 1.000 | 1.000 | 0.854 | 0.824 |
| 1608 | 0.820 | 1.000 | 0.824 | 1.000 | 0.984 | 1.000 | 0.875 | 1.000 | 0.980 | 1.000 | 0.959 | 1.000 | 0.973 | 0.791 | 1.000 | 1.000 | 0.854 | 0.824 |
| 1609 | 0.818 | 1.000 | 0.832 | 1.000 | 0.984 | 1.000 | 0.874 | 1.000 | 0.980 | 1.000 | 0.965 | 1.000 | 0.973 | 0.788 | 1.000 | 1.000 | 0.854 | 0.824 |
| 1610 | 0.818 | 1.000 | 0.832 | 1.000 | 0.984 | 1.000 | 0.869 | 1.000 | 0.980 | 1.000 | 0.965 | 1.000 | 0.973 | 0.788 | 1.000 | 1.000 | 0.853 | 0.823 |
| 1611 | 0.818 | 1.000 | 0.832 | 1.000 | 0.984 | 1.000 | 0.871 | 1.000 | 0.980 | 1.000 | 0.965 | 1.000 | 0.973 | 0.788 | 1.000 | 1.000 | 0.853 | 0.824 |
| 1612 | 0.818 | 1.000 | 0.832 | 1.000 | 0.984 | 1.000 | 0.869 | 1.000 | 0.980 | 1.000 | 0.965 | 1.000 | 0.973 | 0.788 | 1.000 | 1.000 | 0.853 | 0.823 |
| 1613 | 0.818 | 1.000 | 0.830 | 1.000 | 0.984 | 1.000 | 0.869 | 1.000 | 0.980 | 1.000 | 0.965 | 1.000 | 0.973 | 0.788 | 1.000 | 1.000 | 0.853 | 0.823 |
| 1614 | 0.817 | 1.000 | 0.830 | 1.000 | 0.984 | 1.000 | 0.867 | 1.000 | 0.980 | 1.000 | 0.965 | 1.000 | 0.973 | 0.788 | 1.000 | 1.000 | 0.852 | 0.823 |
| 1615 | 0.819 | 1.000 | 0.830 | 1.000 | 0.984 | 1.000 | 0.871 | 1.000 | 0.980 | 1.000 | 0.965 | 1.000 | 0.973 | 0.792 | 1.000 | 1.000 | 0.854 | 0.825 |
| 1616 | 0.819 | 1.000 | 0.830 | 1.000 | 0.984 | 1.000 | 0.869 | 1.000 | 0.980 | 1.000 | 0.965 | 1.000 | 0.973 | 0.792 | 1.000 | 1.000 | 0.854 | 0.825 |
| 1617 | 0.822 | 1.000 | 0.834 | 1.000 | 0.984 | 1.000 | 0.867 | 1.000 | 0.980 | 1.000 | 0.968 | 1.000 | 0.973 | 0.789 | 1.000 | 1.000 | 0.855 | 0.825 |
| 1618 | 0.823 | 1.000 | 0.834 | 1.000 | 0.984 | 1.000 | 0.867 | 1.000 | 0.980 | 1.000 | 0.968 | 1.000 | 0.973 | 0.789 | 1.000 | 1.000 | 0.855 | 0.826 |
| 1619 | 0.819 | 1.000 | 0.838 | 1.000 | 0.984 | 1.000 | 0.867 | 1.000 | 0.980 | 1.000 | 0.968 | 1.000 | 0.973 | 0.789 | 1.000 | 1.000 | 0.854 | 0.825 |
| 1620 | 0.820 | 1.000 | 0.834 | 1.000 | 0.984 | 1.000 | 0.867 | 1.000 | 0.980 | 1.000 | 0.965 | 1.000 | 0.973 | 0.791 | 1.000 | 1.000 | 0.854 | 0.825 |
| 1621 | 0.822 | 1.000 | 0.840 | 1.000 | 0.984 | 1.000 | 0.867 | 1.000 | 0.980 | 1.000 | 0.962 | 1.000 | 0.973 | 0.794 | 1.000 | 1.000 | 0.856 | 0.827 |
| 1622 | 0.822 | 1.000 | 0.840 | 1.000 | 0.984 | 1.000 | 0.869 | 1.000 | 0.980 | 1.000 | 0.962 | 1.000 | 0.973 | 0.793 | 1.000 | 1.000 | 0.856 | 0.827 |
| 1623 | 0.823 | 1.000 | 0.840 | 1.000 | 0.984 | 1.000 | 0.869 | 1.000 | 0.980 | 1.000 | 0.962 | 1.000 | 0.973 | 0.794 | 1.000 | 1.000 | 0.857 | 0.827 |
| 1624 | 0.822 | 1.000 | 0.840 | 1.000 | 0.984 | 1.000 | 0.869 | 1.000 | 0.980 | 1.000 | 0.962 | 1.000 | 0.973 | 0.794 | 1.000 | 1.000 | 0.856 | 0.827 |
| 1625 | 0.823 | 1.000 | 0.840 | 1.000 | 0.984 | 1.000 | 0.869 | 1.000 | 0.980 | 1.000 | 0.962 | 1.000 | 0.973 | 0.791 | 1.000 | 1.000 | 0.856 | 0.827 |
| 1626 | 0.823 | 1.000 | 0.838 | 1.000 | 0.984 | 1.000 | 0.869 | 1.000 | 0.980 | 1.000 | 0.962 | 1.000 | 0.973 | 0.791 | 1.000 | 1.000 | 0.856 | 0.827 |
| 1627 | 0.824 | 1.000 | 0.836 | 1.000 | 0.984 | 1.000 | 0.869 | 1.000 | 0.980 | 1.000 | 0.962 | 1.000 | 0.973 | 0.791 | 1.000 | 1.000 | 0.856 | 0.827 |
| 1628 | 0.823 | 1.000 | 0.836 | 1.000 | 0.984 | 1.000 | 0.869 | 1.000 | 0.980 | 1.000 | 0.962 | 1.000 | 0.973 | 0.792 | 1.000 | 1.000 | 0.856 | 0.827 |
| 1629 | 0.820 | 1.000 | 0.838 | 1.000 | 0.979 | 1.000 | 0.871 | 1.000 | 0.980 | 1.000 | 0.965 | 1.000 | 0.973 | 0.793 | 1.000 | 1.000 | 0.856 | 0.826 |
| 1630 | 0.821 | 1.000 | 0.834 | 1.000 | 0.979 | 1.000 | 0.871 | 1.000 | 0.980 | 1.000 | 0.965 | 1.000 | 0.973 | 0.792 | 1.000 | 1.000 | 0.855 | 0.826 |
| 1631 | 0.821 | 1.000 | 0.830 | 1.000 | 0.984 | 1.000 | 0.872 | 1.000 | 0.980 | 1.000 | 0.965 | 1.000 | 0.973 | 0.791 | 1.000 | 1.000 | 0.855 | 0.825 |
| 1632 | 0.821 | 1.000 | 0.830 | 1.000 | 0.984 | 1.000 | 0.872 | 1.000 | 0.980 | 1.000 | 0.965 | 1.000 | 0.973 | 0.791 | 1.000 | 1.000 | 0.855 | 0.825 |
| 1633 | 0.820 | 1.000 | 0.832 | 1.000 | 0.984 | 1.000 | 0.872 | 1.000 | 0.980 | 1.000 | 0.965 | 1.000 | 0.973 | 0.791 | 1.000 | 1.000 | 0.855 | 0.826 |
| 1634 | 0.820 | 1.000 | 0.832 | 1.000 | 0.979 | 1.000 | 0.872 | 1.000 | 0.980 | 1.000 | 0.965 | 1.000 | 0.982 | 0.791 | 1.000 | 1.000 | 0.855 | 0.825 |
| 1635 | 0.820 | 1.000 | 0.832 | 1.000 | 0.979 | 1.000 | 0.872 | 1.000 | 0.980 | 1.000 | 0.965 | 1.000 | 0.982 | 0.791 | 1.000 | 1.000 | 0.855 | 0.825 |
| 1636 | 0.818 | 1.000 | 0.832 | 1.000 | 0.984 | 1.000 | 0.872 | 1.000 | 0.980 | 1.000 | 0.965 | 1.000 | 0.982 | 0.791 | 1.000 | 1.000 | 0.854 | 0.825 |
| 1637 | 0.818 | 1.000 | 0.832 | 1.000 | 0.984 | 1.000 | 0.871 | 1.000 | 0.980 | 1.000 | 0.965 | 1.000 | 0.982 | 0.791 | 1.000 | 1.000 | 0.854 | 0.825 |
| 1638 | 0.820 | 1.000 | 0.832 | 1.000 | 0.984 | 1.000 | 0.871 | 1.000 | 0.980 | 1.000 | 0.965 | 1.000 | 0.982 | 0.791 | 1.000 | 1.000 | 0.855 | 0.825 |
| 1639 | 0.820 | 1.000 | 0.834 | 1.000 | 0.984 | 1.000 | 0.871 | 1.000 | 0.980 | 1.000 | 0.962 | 1.000 | 0.982 | 0.791 | 1.000 | 1.000 | 0.855 | 0.825 |
| 1640 | 0.820 | 1.000 | 0.834 | 1.000 | 0.984 | 1.000 | 0.871 | 1.000 | 0.980 | 1.000 | 0.962 | 1.000 | 0.982 | 0.791 | 1.000 | 1.000 | 0.855 | 0.825 |
| 1641 | 0.816 | 1.000 | 0.838 | 1.000 | 0.984 | 1.000 | 0.869 | 1.000 | 0.980 | 1.000 | 0.965 | 1.000 | 0.982 | 0.792 | 1.000 | 1.000 | 0.854 | 0.825 |

| | | | | | | | | | | | | | | | | | | |
|---|---|---|---|---|---|---|---|---|---|---|---|---|---|---|---|---|---|---|
| 1642 | 0.815 | 1.000 | 0.838 | 1.000 | 0.979 | 1.000 | 0.869 | 1.000 | 0.969 | 1.000 | 0.965 | 1.000 | 0.982 | 0.791 | 1.000 | 1.000 | 0.853 | 0.824 |
| 1643 | 0.814 | 1.000 | 0.834 | 1.000 | 0.979 | 1.000 | 0.867 | 1.000 | 0.969 | 1.000 | 0.962 | 1.000 | 0.982 | 0.790 | 1.000 | 1.000 | 0.852 | 0.822 |
| 1644 | 0.814 | 1.000 | 0.834 | 1.000 | 0.979 | 1.000 | 0.867 | 1.000 | 0.969 | 1.000 | 0.962 | 1.000 | 0.973 | 0.789 | 1.000 | 1.000 | 0.851 | 0.821 |
| 1645 | 0.813 | 1.000 | 0.832 | 1.000 | 0.979 | 1.000 | 0.867 | 1.000 | 0.980 | 1.000 | 0.962 | 1.000 | 0.982 | 0.790 | 1.000 | 1.000 | 0.852 | 0.822 |
| 1646 | 0.815 | 1.000 | 0.838 | 1.000 | 0.979 | 1.000 | 0.871 | 1.000 | 0.980 | 1.000 | 0.962 | 1.000 | 0.982 | 0.789 | 1.000 | 1.000 | 0.853 | 0.823 |
| 1647 | 0.814 | 1.000 | 0.838 | 1.000 | 0.979 | 1.000 | 0.869 | 1.000 | 0.980 | 1.000 | 0.962 | 1.000 | 0.982 | 0.790 | 1.000 | 1.000 | 0.853 | 0.823 |
| 1648 | 0.813 | 1.000 | 0.844 | 1.000 | 0.979 | 1.000 | 0.867 | 1.000 | 0.969 | 1.000 | 0.962 | 1.000 | 0.982 | 0.789 | 1.000 | 1.000 | 0.852 | 0.823 |
| 1649 | 0.812 | 1.000 | 0.844 | 1.000 | 0.979 | 1.000 | 0.869 | 1.000 | 0.969 | 1.000 | 0.962 | 1.000 | 0.982 | 0.789 | 1.000 | 1.000 | 0.852 | 0.823 |
| 1650 | 0.812 | 1.000 | 0.844 | 1.000 | 0.979 | 1.000 | 0.869 | 1.000 | 0.969 | 1.000 | 0.962 | 1.000 | 0.982 | 0.789 | 1.000 | 1.000 | 0.852 | 0.822 |
| 1651 | 0.812 | 1.000 | 0.844 | 1.000 | 0.984 | 1.000 | 0.866 | 1.000 | 0.980 | 1.000 | 0.962 | 1.000 | 0.982 | 0.788 | 1.000 | 1.000 | 0.852 | 0.822 |
| 1652 | 0.812 | 1.000 | 0.844 | 1.000 | 0.984 | 1.000 | 0.866 | 1.000 | 0.980 | 1.000 | 0.965 | 1.000 | 0.982 | 0.787 | 1.000 | 1.000 | 0.852 | 0.822 |
| 1653 | 0.813 | 1.000 | 0.840 | 1.000 | 0.979 | 1.000 | 0.863 | 1.000 | 0.969 | 1.000 | 0.965 | 1.000 | 0.982 | 0.788 | 1.000 | 1.000 | 0.851 | 0.821 |
| 1654 | 0.813 | 1.000 | 0.840 | 1.000 | 0.979 | 1.000 | 0.863 | 1.000 | 0.969 | 1.000 | 0.965 | 1.000 | 0.982 | 0.788 | 1.000 | 1.000 | 0.851 | 0.821 |
| 1655 | 0.813 | 1.000 | 0.840 | 1.000 | 0.979 | 1.000 | 0.863 | 1.000 | 0.969 | 1.000 | 0.965 | 1.000 | 0.982 | 0.788 | 1.000 | 1.000 | 0.851 | 0.821 |
| 1656 | 0.813 | 1.000 | 0.840 | 1.000 | 0.979 | 1.000 | 0.863 | 1.000 | 0.969 | 1.000 | 0.965 | 1.000 | 0.982 | 0.788 | 1.000 | 1.000 | 0.851 | 0.821 |
| 1657 | 0.812 | 1.000 | 0.842 | 1.000 | 0.974 | 1.000 | 0.863 | 1.000 | 0.980 | 1.000 | 0.962 | 1.000 | 0.982 | 0.791 | 1.000 | 1.000 | 0.852 | 0.822 |
| 1658 | 0.813 | 1.000 | 0.842 | 1.000 | 0.974 | 1.000 | 0.866 | 1.000 | 0.980 | 1.000 | 0.962 | 1.000 | 0.982 | 0.791 | 1.000 | 1.000 | 0.852 | 0.823 |
| 1659 | 0.813 | 1.000 | 0.842 | 1.000 | 0.974 | 1.000 | 0.867 | 1.000 | 0.980 | 1.000 | 0.962 | 1.000 | 0.982 | 0.791 | 1.000 | 1.000 | 0.853 | 0.823 |
| 1660 | 0.816 | 1.000 | 0.840 | 1.000 | 0.974 | 1.000 | 0.864 | 1.000 | 0.980 | 1.000 | 0.962 | 1.000 | 0.982 | 0.791 | 1.000 | 1.000 | 0.853 | 0.823 |
| 1661 | 0.817 | 1.000 | 0.840 | 1.000 | 0.974 | 1.000 | 0.863 | 1.000 | 0.980 | 1.000 | 0.962 | 1.000 | 0.982 | 0.791 | 1.000 | 1.000 | 0.853 | 0.823 |
| 1662 | 0.817 | 1.000 | 0.840 | 1.000 | 0.974 | 1.000 | 0.861 | 1.000 | 0.980 | 1.000 | 0.962 | 1.000 | 0.982 | 0.791 | 1.000 | 1.000 | 0.853 | 0.823 |
| 1663 | 0.817 | 1.000 | 0.840 | 1.000 | 0.974 | 1.000 | 0.861 | 1.000 | 0.980 | 1.000 | 0.962 | 1.000 | 0.982 | 0.791 | 1.000 | 1.000 | 0.853 | 0.823 |
| 1664 | 0.817 | 1.000 | 0.840 | 1.000 | 0.974 | 1.000 | 0.861 | 1.000 | 0.980 | 1.000 | 0.962 | 1.000 | 0.982 | 0.791 | 1.000 | 1.000 | 0.853 | 0.823 |
| 1665 | 0.817 | 1.000 | 0.840 | 1.000 | 0.974 | 1.000 | 0.860 | 1.000 | 0.980 | 1.000 | 0.962 | 1.000 | 0.982 | 0.789 | 1.000 | 1.000 | 0.852 | 0.822 |
| 1666 | 0.817 | 1.000 | 0.840 | 1.000 | 0.974 | 1.000 | 0.860 | 1.000 | 0.980 | 1.000 | 0.962 | 1.000 | 0.982 | 0.789 | 1.000 | 1.000 | 0.852 | 0.822 |
| 1667 | 0.817 | 1.000 | 0.840 | 1.000 | 0.974 | 1.000 | 0.860 | 1.000 | 0.980 | 1.000 | 0.962 | 1.000 | 0.982 | 0.789 | 1.000 | 1.000 | 0.852 | 0.822 |
| 1668 | 0.818 | 1.000 | 0.840 | 1.000 | 0.974 | 1.000 | 0.861 | 1.000 | 0.980 | 1.000 | 0.962 | 1.000 | 0.982 | 0.788 | 1.000 | 1.000 | 0.852 | 0.822 |
| 1669 | 0.818 | 1.000 | 0.840 | 1.000 | 0.974 | 1.000 | 0.860 | 1.000 | 0.980 | 1.000 | 0.962 | 1.000 | 0.982 | 0.791 | 1.000 | 1.000 | 0.853 | 0.823 |
| 1670 | 0.818 | 1.000 | 0.840 | 1.000 | 0.974 | 1.000 | 0.860 | 1.000 | 0.980 | 1.000 | 0.962 | 1.000 | 0.982 | 0.791 | 1.000 | 1.000 | 0.853 | 0.823 |
| 1671 | 0.818 | 1.000 | 0.840 | 1.000 | 0.974 | 1.000 | 0.861 | 1.000 | 0.980 | 1.000 | 0.962 | 1.000 | 0.982 | 0.791 | 1.000 | 1.000 | 0.853 | 0.823 |
| 1672 | 0.818 | 1.000 | 0.840 | 1.000 | 0.974 | 1.000 | 0.861 | 1.000 | 0.980 | 1.000 | 0.962 | 1.000 | 0.982 | 0.788 | 1.000 | 1.000 | 0.853 | 0.823 |
| 1673 | 0.818 | 1.000 | 0.842 | 1.000 | 0.974 | 1.000 | 0.861 | 1.000 | 0.980 | 1.000 | 0.962 | 1.000 | 0.982 | 0.788 | 1.000 | 1.000 | 0.853 | 0.823 |
| 1674 | 0.818 | 1.000 | 0.842 | 1.000 | 0.974 | 1.000 | 0.861 | 1.000 | 0.980 | 1.000 | 0.962 | 1.000 | 0.982 | 0.788 | 1.000 | 1.000 | 0.853 | 0.823 |
| 1675 | 0.818 | 1.000 | 0.842 | 1.000 | 0.974 | 1.000 | 0.861 | 1.000 | 0.980 | 1.000 | 0.962 | 1.000 | 0.982 | 0.788 | 1.000 | 1.000 | 0.853 | 0.823 |
| 1676 | 0.818 | 1.000 | 0.842 | 1.000 | 0.974 | 1.000 | 0.861 | 1.000 | 0.980 | 1.000 | 0.962 | 1.000 | 0.982 | 0.788 | 1.000 | 1.000 | 0.853 | 0.823 |
| 1677 | 0.818 | 1.000 | 0.842 | 1.000 | 0.974 | 1.000 | 0.861 | 1.000 | 0.980 | 1.000 | 0.959 | 1.000 | 0.982 | 0.788 | 1.000 | 1.000 | 0.852 | 0.822 |
| 1678 | 0.818 | 1.000 | 0.842 | 1.000 | 0.974 | 1.000 | 0.861 | 1.000 | 0.980 | 1.000 | 0.959 | 1.000 | 0.982 | 0.788 | 1.000 | 1.000 | 0.852 | 0.822 |
| 1679 | 0.818 | 1.000 | 0.844 | 1.000 | 0.974 | 1.000 | 0.861 | 1.000 | 0.980 | 1.000 | 0.962 | 1.000 | 0.982 | 0.789 | 1.000 | 1.000 | 0.853 | 0.823 |
| 1680 | 0.818 | 1.000 | 0.844 | 1.000 | 0.974 | 1.000 | 0.861 | 1.000 | 0.980 | 1.000 | 0.962 | 1.000 | 0.982 | 0.788 | 1.000 | 1.000 | 0.853 | 0.823 |

| | | | | | | | | | | | | | | | | | | |
|---|---|---|---|---|---|---|---|---|---|---|---|---|---|---|---|---|---|---|
| 1681 | 0.818 | 1.000 | 0.842 | 1.000 | 0.974 | 1.000 | 0.863 | 1.000 | 0.980 | 1.000 | 0.962 | 1.000 | 0.982 | 0.790 | 1.000 | 1.000 | 0.853 | 0.823 |
| 1682 | 0.818 | 1.000 | 0.840 | 1.000 | 0.974 | 1.000 | 0.863 | 1.000 | 0.980 | 1.000 | 0.962 | 1.000 | 0.982 | 0.790 | 1.000 | 1.000 | 0.853 | 0.823 |
| 1683 | 0.816 | 1.000 | 0.840 | 1.000 | 0.974 | 1.000 | 0.863 | 1.000 | 0.980 | 1.000 | 0.962 | 1.000 | 0.982 | 0.790 | 1.000 | 1.000 | 0.853 | 0.823 |
| 1684 | 0.818 | 1.000 | 0.840 | 1.000 | 0.974 | 1.000 | 0.861 | 1.000 | 0.980 | 1.000 | 0.962 | 1.000 | 0.982 | 0.790 | 1.000 | 1.000 | 0.853 | 0.823 |
| 1685 | 0.818 | 1.000 | 0.842 | 1.000 | 0.974 | 1.000 | 0.863 | 1.000 | 0.980 | 1.000 | 0.962 | 1.000 | 0.982 | 0.790 | 1.000 | 1.000 | 0.853 | 0.824 |
| 1686 | 0.818 | 1.000 | 0.844 | 1.000 | 0.974 | 1.000 | 0.861 | 1.000 | 0.980 | 1.000 | 0.962 | 1.000 | 0.982 | 0.790 | 1.000 | 1.000 | 0.853 | 0.824 |
| 1687 | 0.818 | 1.000 | 0.844 | 1.000 | 0.974 | 1.000 | 0.861 | 1.000 | 0.980 | 1.000 | 0.962 | 1.000 | 0.982 | 0.791 | 1.000 | 1.000 | 0.853 | 0.824 |
| 1688 | 0.818 | 1.000 | 0.844 | 1.000 | 0.974 | 1.000 | 0.861 | 1.000 | 0.980 | 1.000 | 0.962 | 1.000 | 0.982 | 0.791 | 1.000 | 1.000 | 0.853 | 0.824 |
| 1689 | 0.818 | 1.000 | 0.844 | 1.000 | 0.974 | 1.000 | 0.860 | 1.000 | 0.980 | 1.000 | 0.962 | 1.000 | 0.982 | 0.790 | 1.000 | 1.000 | 0.853 | 0.823 |
| 1690 | 0.818 | 1.000 | 0.844 | 1.000 | 0.979 | 1.000 | 0.861 | 1.000 | 0.980 | 1.000 | 0.962 | 1.000 | 0.982 | 0.789 | 1.000 | 1.000 | 0.853 | 0.823 |
| 1691 | 0.818 | 1.000 | 0.844 | 1.000 | 0.979 | 1.000 | 0.863 | 1.000 | 0.980 | 1.000 | 0.962 | 1.000 | 0.982 | 0.788 | 1.000 | 1.000 | 0.853 | 0.823 |
| 1692 | 0.818 | 1.000 | 0.844 | 1.000 | 0.979 | 1.000 | 0.863 | 1.000 | 0.980 | 1.000 | 0.962 | 1.000 | 0.982 | 0.789 | 1.000 | 1.000 | 0.854 | 0.824 |
| 1693 | 0.819 | 1.000 | 0.844 | 1.000 | 0.979 | 1.000 | 0.863 | 1.000 | 0.980 | 1.000 | 0.962 | 1.000 | 0.982 | 0.788 | 1.000 | 1.000 | 0.854 | 0.824 |
| 1694 | 0.820 | 1.000 | 0.846 | 1.000 | 0.979 | 1.000 | 0.861 | 1.000 | 0.980 | 1.000 | 0.962 | 1.000 | 0.982 | 0.788 | 1.000 | 1.000 | 0.854 | 0.824 |
| 1695 | 0.821 | 1.000 | 0.842 | 1.000 | 0.979 | 1.000 | 0.864 | 1.000 | 0.980 | 1.000 | 0.965 | 1.000 | 0.982 | 0.791 | 1.000 | 1.000 | 0.855 | 0.826 |
| 1696 | 0.821 | 1.000 | 0.842 | 1.000 | 0.979 | 1.000 | 0.864 | 1.000 | 0.980 | 1.000 | 0.965 | 1.000 | 0.982 | 0.790 | 1.000 | 1.000 | 0.855 | 0.825 |
| 1697 | 0.821 | 1.000 | 0.838 | 1.000 | 0.979 | 1.000 | 0.864 | 1.000 | 0.980 | 1.000 | 0.965 | 1.000 | 0.982 | 0.791 | 1.000 | 1.000 | 0.855 | 0.825 |
| 1698 | 0.821 | 1.000 | 0.838 | 1.000 | 0.979 | 1.000 | 0.864 | 1.000 | 0.980 | 1.000 | 0.965 | 1.000 | 0.982 | 0.791 | 1.000 | 1.000 | 0.855 | 0.825 |
| 1699 | 0.821 | 1.000 | 0.838 | 1.000 | 0.979 | 1.000 | 0.864 | 1.000 | 0.980 | 1.000 | 0.965 | 1.000 | 0.982 | 0.791 | 1.000 | 1.000 | 0.855 | 0.825 |
| 1700 | 0.821 | 1.000 | 0.838 | 1.000 | 0.979 | 1.000 | 0.864 | 1.000 | 0.980 | 1.000 | 0.965 | 1.000 | 0.982 | 0.791 | 1.000 | 1.000 | 0.855 | 0.825 |
| 1701 | 0.821 | 1.000 | 0.838 | 1.000 | 0.979 | 1.000 | 0.864 | 1.000 | 0.980 | 1.000 | 0.965 | 1.000 | 0.982 | 0.790 | 1.000 | 1.000 | 0.854 | 0.825 |
| 1702 | 0.821 | 1.000 | 0.838 | 1.000 | 0.974 | 1.000 | 0.864 | 1.000 | 0.980 | 1.000 | 0.965 | 1.000 | 0.982 | 0.790 | 1.000 | 1.000 | 0.854 | 0.825 |
| 1703 | 0.820 | 1.000 | 0.840 | 1.000 | 0.974 | 1.000 | 0.864 | 1.000 | 0.980 | 1.000 | 0.965 | 1.000 | 0.982 | 0.790 | 1.000 | 1.000 | 0.854 | 0.825 |
| 1704 | 0.821 | 1.000 | 0.842 | 1.000 | 0.974 | 1.000 | 0.863 | 1.000 | 0.980 | 1.000 | 0.962 | 1.000 | 0.982 | 0.789 | 1.000 | 1.000 | 0.854 | 0.824 |
| 1705 | 0.820 | 1.000 | 0.840 | 1.000 | 0.974 | 1.000 | 0.863 | 1.000 | 0.980 | 1.000 | 0.965 | 1.000 | 0.982 | 0.791 | 1.000 | 1.000 | 0.854 | 0.824 |
| 1706 | 0.820 | 1.000 | 0.840 | 1.000 | 0.974 | 1.000 | 0.863 | 1.000 | 0.980 | 1.000 | 0.965 | 1.000 | 0.982 | 0.791 | 1.000 | 1.000 | 0.854 | 0.824 |
| 1707 | 0.820 | 1.000 | 0.844 | 1.000 | 0.979 | 1.000 | 0.861 | 1.000 | 0.980 | 1.000 | 0.957 | 1.000 | 0.982 | 0.789 | 1.000 | 1.000 | 0.854 | 0.824 |
| 1708 | 0.820 | 1.000 | 0.844 | 1.000 | 0.979 | 1.000 | 0.861 | 1.000 | 0.980 | 1.000 | 0.957 | 1.000 | 0.982 | 0.789 | 1.000 | 1.000 | 0.854 | 0.824 |
| 1709 | 0.820 | 1.000 | 0.844 | 1.000 | 0.979 | 1.000 | 0.861 | 1.000 | 0.980 | 1.000 | 0.957 | 1.000 | 0.982 | 0.789 | 1.000 | 1.000 | 0.854 | 0.824 |
| 1710 | 0.820 | 1.000 | 0.844 | 1.000 | 0.979 | 1.000 | 0.861 | 1.000 | 0.980 | 1.000 | 0.957 | 1.000 | 0.982 | 0.788 | 1.000 | 1.000 | 0.853 | 0.824 |
| 1711 | 0.820 | 1.000 | 0.842 | 1.000 | 0.979 | 1.000 | 0.861 | 1.000 | 0.980 | 1.000 | 0.957 | 1.000 | 0.982 | 0.788 | 1.000 | 1.000 | 0.853 | 0.823 |
| 1712 | 0.820 | 1.000 | 0.844 | 1.000 | 0.979 | 1.000 | 0.861 | 1.000 | 0.980 | 1.000 | 0.957 | 1.000 | 0.982 | 0.788 | 1.000 | 1.000 | 0.853 | 0.823 |
| 1713 | 0.819 | 1.000 | 0.844 | 1.000 | 0.979 | 1.000 | 0.863 | 1.000 | 0.980 | 1.000 | 0.957 | 1.000 | 0.982 | 0.788 | 1.000 | 1.000 | 0.853 | 0.823 |
| 1714 | 0.820 | 1.000 | 0.844 | 1.000 | 0.979 | 1.000 | 0.863 | 1.000 | 0.980 | 1.000 | 0.957 | 1.000 | 0.982 | 0.788 | 1.000 | 1.000 | 0.854 | 0.824 |
| 1715 | 0.820 | 1.000 | 0.846 | 1.000 | 0.979 | 1.000 | 0.863 | 1.000 | 0.980 | 1.000 | 0.957 | 1.000 | 0.982 | 0.788 | 1.000 | 1.000 | 0.854 | 0.824 |
| 1716 | 0.820 | 1.000 | 0.844 | 1.000 | 0.979 | 1.000 | 0.864 | 1.000 | 0.980 | 1.000 | 0.957 | 1.000 | 0.982 | 0.788 | 1.000 | 1.000 | 0.854 | 0.824 |
| 1717 | 0.821 | 1.000 | 0.844 | 1.000 | 0.979 | 1.000 | 0.864 | 1.000 | 0.980 | 1.000 | 0.957 | 1.000 | 0.982 | 0.788 | 1.000 | 1.000 | 0.854 | 0.824 |
| 1718 | 0.819 | 1.000 | 0.848 | 1.000 | 0.979 | 1.000 | 0.863 | 1.000 | 0.980 | 1.000 | 0.959 | 1.000 | 0.982 | 0.790 | 1.000 | 1.000 | 0.854 | 0.825 |
| 1719 | 0.820 | 1.000 | 0.848 | 1.000 | 0.979 | 1.000 | 0.863 | 1.000 | 0.980 | 1.000 | 0.959 | 1.000 | 0.982 | 0.791 | 1.000 | 1.000 | 0.855 | 0.825 |

| | | | | | | | | | | | | | | | | | | |
|---|---|---|---|---|---|---|---|---|---|---|---|---|---|---|---|---|---|---|
| 1720 | 0.821 | 1.000 | 0.844 | 1.000 | 0.979 | 1.000 | 0.863 | 1.000 | 0.980 | 1.000 | 0.962 | 1.000 | 0.982 | 0.789 | 1.000 | 1.000 | 0.854 | 0.825 |
| 1721 | 0.821 | 1.000 | 0.848 | 1.000 | 0.979 | 1.000 | 0.863 | 1.000 | 0.980 | 1.000 | 0.962 | 1.000 | 0.982 | 0.789 | 1.000 | 1.000 | 0.855 | 0.825 |
| 1722 | 0.822 | 1.000 | 0.844 | 1.000 | 0.979 | 1.000 | 0.863 | 1.000 | 0.980 | 1.000 | 0.962 | 1.000 | 0.982 | 0.789 | 1.000 | 1.000 | 0.855 | 0.825 |
| 1723 | 0.822 | 1.000 | 0.842 | 1.000 | 0.979 | 1.000 | 0.864 | 1.000 | 0.980 | 1.000 | 0.965 | 1.000 | 0.982 | 0.790 | 1.000 | 1.000 | 0.855 | 0.826 |
| 1724 | 0.824 | 1.000 | 0.842 | 1.000 | 0.979 | 1.000 | 0.864 | 1.000 | 0.980 | 1.000 | 0.965 | 1.000 | 0.982 | 0.792 | 1.000 | 1.000 | 0.856 | 0.827 |
| 1725 | 0.822 | 1.000 | 0.842 | 1.000 | 0.979 | 1.000 | 0.864 | 1.000 | 0.980 | 1.000 | 0.962 | 1.000 | 0.973 | 0.793 | 1.000 | 1.000 | 0.856 | 0.826 |
| 1726 | 0.822 | 1.000 | 0.844 | 1.000 | 0.979 | 1.000 | 0.864 | 1.000 | 0.980 | 1.000 | 0.962 | 1.000 | 0.973 | 0.792 | 1.000 | 1.000 | 0.856 | 0.826 |
| 1727 | 0.822 | 1.000 | 0.842 | 1.000 | 0.984 | 1.000 | 0.864 | 1.000 | 0.980 | 1.000 | 0.962 | 1.000 | 0.982 | 0.791 | 1.000 | 1.000 | 0.856 | 0.826 |
| 1728 | 0.822 | 1.000 | 0.838 | 1.000 | 0.984 | 1.000 | 0.864 | 1.000 | 0.980 | 1.000 | 0.962 | 1.000 | 0.982 | 0.792 | 1.000 | 1.000 | 0.855 | 0.826 |
| 1729 | 0.822 | 1.000 | 0.838 | 1.000 | 0.984 | 1.000 | 0.864 | 1.000 | 0.980 | 1.000 | 0.962 | 1.000 | 0.982 | 0.792 | 1.000 | 1.000 | 0.855 | 0.826 |
| 1730 | 0.822 | 1.000 | 0.838 | 1.000 | 0.984 | 1.000 | 0.864 | 1.000 | 0.980 | 1.000 | 0.962 | 1.000 | 0.982 | 0.792 | 1.000 | 1.000 | 0.855 | 0.826 |
| 1731 | 0.820 | 1.000 | 0.836 | 1.000 | 0.984 | 1.000 | 0.863 | 1.000 | 0.980 | 1.000 | 0.962 | 1.000 | 0.982 | 0.789 | 1.000 | 1.000 | 0.854 | 0.824 |
| 1732 | 0.820 | 1.000 | 0.836 | 1.000 | 0.984 | 1.000 | 0.863 | 1.000 | 0.980 | 1.000 | 0.962 | 1.000 | 0.982 | 0.789 | 1.000 | 1.000 | 0.854 | 0.824 |
| 1733 | 0.820 | 1.000 | 0.836 | 1.000 | 0.984 | 1.000 | 0.863 | 1.000 | 0.980 | 1.000 | 0.962 | 1.000 | 0.982 | 0.789 | 1.000 | 1.000 | 0.854 | 0.824 |
| 1734 | 0.822 | 1.000 | 0.836 | 1.000 | 0.984 | 1.000 | 0.863 | 1.000 | 0.980 | 1.000 | 0.965 | 1.000 | 0.982 | 0.790 | 1.000 | 1.000 | 0.855 | 0.825 |
| 1735 | 0.822 | 1.000 | 0.836 | 1.000 | 0.984 | 1.000 | 0.863 | 1.000 | 0.980 | 1.000 | 0.962 | 1.000 | 0.982 | 0.790 | 1.000 | 1.000 | 0.854 | 0.825 |
| 1736 | 0.822 | 1.000 | 0.836 | 1.000 | 0.984 | 1.000 | 0.863 | 1.000 | 0.980 | 1.000 | 0.962 | 1.000 | 0.982 | 0.791 | 1.000 | 1.000 | 0.855 | 0.825 |
| 1737 | 0.822 | 1.000 | 0.836 | 1.000 | 0.984 | 1.000 | 0.863 | 1.000 | 0.980 | 1.000 | 0.962 | 1.000 | 0.982 | 0.791 | 1.000 | 1.000 | 0.855 | 0.825 |
| 1738 | 0.822 | 1.000 | 0.836 | 1.000 | 0.984 | 1.000 | 0.863 | 1.000 | 0.980 | 1.000 | 0.962 | 1.000 | 0.982 | 0.791 | 1.000 | 1.000 | 0.854 | 0.825 |
| 1739 | 0.822 | 1.000 | 0.836 | 1.000 | 0.984 | 1.000 | 0.863 | 1.000 | 0.980 | 1.000 | 0.962 | 1.000 | 0.982 | 0.789 | 1.000 | 1.000 | 0.854 | 0.824 |
| 1740 | 0.822 | 1.000 | 0.834 | 1.000 | 0.984 | 1.000 | 0.863 | 1.000 | 0.980 | 1.000 | 0.962 | 1.000 | 0.973 | 0.790 | 1.000 | 1.000 | 0.854 | 0.824 |
| 1741 | 0.818 | 1.000 | 0.838 | 1.000 | 0.979 | 1.000 | 0.861 | 1.000 | 0.980 | 1.000 | 0.959 | 1.000 | 0.973 | 0.791 | 1.000 | 1.000 | 0.853 | 0.823 |
| 1742 | 0.818 | 1.000 | 0.838 | 1.000 | 0.979 | 1.000 | 0.861 | 1.000 | 0.980 | 1.000 | 0.959 | 1.000 | 0.973 | 0.791 | 1.000 | 1.000 | 0.853 | 0.823 |
| 1743 | 0.817 | 1.000 | 0.838 | 1.000 | 0.979 | 1.000 | 0.861 | 1.000 | 0.980 | 1.000 | 0.959 | 1.000 | 0.973 | 0.791 | 1.000 | 1.000 | 0.852 | 0.822 |
| 1744 | 0.818 | 1.000 | 0.838 | 1.000 | 0.979 | 1.000 | 0.864 | 1.000 | 0.980 | 1.000 | 0.962 | 1.000 | 0.973 | 0.791 | 1.000 | 1.000 | 0.853 | 0.823 |
| 1745 | 0.817 | 1.000 | 0.838 | 1.000 | 0.979 | 1.000 | 0.864 | 1.000 | 0.980 | 1.000 | 0.962 | 1.000 | 0.973 | 0.791 | 1.000 | 1.000 | 0.853 | 0.823 |
| 1746 | 0.817 | 1.000 | 0.838 | 1.000 | 0.979 | 1.000 | 0.866 | 1.000 | 0.980 | 1.000 | 0.962 | 1.000 | 0.973 | 0.791 | 1.000 | 1.000 | 0.853 | 0.823 |
| 1747 | 0.817 | 1.000 | 0.838 | 1.000 | 0.979 | 1.000 | 0.864 | 1.000 | 0.980 | 1.000 | 0.959 | 1.000 | 0.973 | 0.791 | 1.000 | 1.000 | 0.853 | 0.823 |
| 1748 | 0.817 | 1.000 | 0.838 | 1.000 | 0.979 | 1.000 | 0.863 | 1.000 | 0.980 | 1.000 | 0.959 | 1.000 | 0.973 | 0.790 | 1.000 | 1.000 | 0.852 | 0.822 |
| 1749 | 0.818 | 1.000 | 0.838 | 1.000 | 0.979 | 1.000 | 0.861 | 1.000 | 0.980 | 1.000 | 0.959 | 1.000 | 0.973 | 0.788 | 1.000 | 1.000 | 0.852 | 0.822 |
| 1750 | 0.818 | 1.000 | 0.838 | 1.000 | 0.979 | 1.000 | 0.861 | 1.000 | 0.980 | 1.000 | 0.959 | 1.000 | 0.973 | 0.788 | 1.000 | 1.000 | 0.852 | 0.822 |
| 1751 | 0.818 | 1.000 | 0.838 | 1.000 | 0.979 | 1.000 | 0.861 | 1.000 | 0.980 | 1.000 | 0.959 | 1.000 | 0.982 | 0.788 | 1.000 | 1.000 | 0.852 | 0.822 |
| 1752 | 0.818 | 1.000 | 0.840 | 1.000 | 0.979 | 1.000 | 0.861 | 1.000 | 0.980 | 1.000 | 0.962 | 1.000 | 0.973 | 0.788 | 1.000 | 1.000 | 0.852 | 0.822 |
| 1753 | 0.818 | 1.000 | 0.840 | 1.000 | 0.979 | 1.000 | 0.863 | 1.000 | 0.980 | 1.000 | 0.965 | 1.000 | 0.973 | 0.788 | 1.000 | 1.000 | 0.853 | 0.823 |
| 1754 | 0.818 | 1.000 | 0.840 | 1.000 | 0.979 | 1.000 | 0.863 | 1.000 | 0.980 | 1.000 | 0.965 | 1.000 | 0.973 | 0.788 | 1.000 | 1.000 | 0.853 | 0.823 |
| 1755 | 0.818 | 1.000 | 0.840 | 1.000 | 0.979 | 1.000 | 0.863 | 1.000 | 0.980 | 1.000 | 0.965 | 1.000 | 0.973 | 0.788 | 1.000 | 1.000 | 0.853 | 0.823 |
| 1756 | 0.818 | 1.000 | 0.842 | 1.000 | 0.979 | 1.000 | 0.863 | 1.000 | 0.980 | 1.000 | 0.965 | 1.000 | 0.973 | 0.789 | 1.000 | 1.000 | 0.853 | 0.823 |
| 1757 | 0.818 | 1.000 | 0.842 | 1.000 | 0.979 | 1.000 | 0.861 | 1.000 | 0.980 | 1.000 | 0.962 | 1.000 | 0.973 | 0.788 | 1.000 | 1.000 | 0.853 | 0.823 |
| 1758 | 0.818 | 1.000 | 0.842 | 1.000 | 0.979 | 1.000 | 0.861 | 1.000 | 0.980 | 1.000 | 0.962 | 1.000 | 0.973 | 0.788 | 1.000 | 1.000 | 0.853 | 0.823 |

| | | | | | | | | | | | | | | | | | | |
|---|---|---|---|---|---|---|---|---|---|---|---|---|---|---|---|---|---|---|
| 1759 | 0.818 | 1.000 | 0.840 | 1.000 | 0.979 | 1.000 | 0.861 | 1.000 | 0.980 | 1.000 | 0.962 | 1.000 | 0.973 | 0.788 | 1.000 | 1.000 | 0.852 | 0.822 |
| 1760 | 0.818 | 1.000 | 0.842 | 1.000 | 0.979 | 1.000 | 0.861 | 1.000 | 0.980 | 1.000 | 0.962 | 1.000 | 0.973 | 0.790 | 1.000 | 1.000 | 0.853 | 0.823 |
| 1761 | 0.818 | 1.000 | 0.840 | 1.000 | 0.979 | 1.000 | 0.861 | 1.000 | 0.980 | 1.000 | 0.962 | 1.000 | 0.973 | 0.788 | 1.000 | 1.000 | 0.852 | 0.822 |
| 1762 | 0.817 | 1.000 | 0.842 | 1.000 | 0.979 | 1.000 | 0.863 | 1.000 | 0.980 | 1.000 | 0.962 | 1.000 | 0.973 | 0.789 | 1.000 | 1.000 | 0.853 | 0.823 |
| 1763 | 0.817 | 1.000 | 0.842 | 1.000 | 0.979 | 1.000 | 0.863 | 1.000 | 0.980 | 1.000 | 0.962 | 1.000 | 0.982 | 0.790 | 1.000 | 1.000 | 0.853 | 0.823 |
| 1764 | 0.818 | 1.000 | 0.840 | 1.000 | 0.979 | 1.000 | 0.863 | 1.000 | 0.980 | 1.000 | 0.962 | 1.000 | 0.982 | 0.788 | 1.000 | 1.000 | 0.853 | 0.823 |
| 1765 | 0.818 | 1.000 | 0.840 | 1.000 | 0.979 | 1.000 | 0.863 | 1.000 | 0.980 | 1.000 | 0.962 | 1.000 | 0.973 | 0.789 | 1.000 | 1.000 | 0.853 | 0.823 |
| 1766 | 0.817 | 1.000 | 0.836 | 1.000 | 0.979 | 1.000 | 0.863 | 1.000 | 0.980 | 1.000 | 0.962 | 1.000 | 0.973 | 0.791 | 1.000 | 1.000 | 0.853 | 0.823 |
| 1767 | 0.817 | 1.000 | 0.836 | 1.000 | 0.979 | 1.000 | 0.861 | 1.000 | 0.980 | 1.000 | 0.962 | 1.000 | 0.973 | 0.791 | 1.000 | 1.000 | 0.852 | 0.822 |
| 1768 | 0.816 | 1.000 | 0.838 | 1.000 | 0.979 | 1.000 | 0.866 | 1.000 | 0.980 | 1.000 | 0.962 | 1.000 | 0.973 | 0.791 | 1.000 | 1.000 | 0.853 | 0.823 |
| 1769 | 0.818 | 1.000 | 0.838 | 1.000 | 0.984 | 1.000 | 0.864 | 1.000 | 0.980 | 1.000 | 0.962 | 1.000 | 0.973 | 0.790 | 1.000 | 1.000 | 0.853 | 0.823 |
| 1770 | 0.818 | 1.000 | 0.838 | 1.000 | 0.984 | 1.000 | 0.869 | 1.000 | 0.980 | 1.000 | 0.962 | 1.000 | 0.973 | 0.790 | 1.000 | 1.000 | 0.854 | 0.824 |
| 1771 | 0.818 | 1.000 | 0.836 | 1.000 | 0.984 | 1.000 | 0.867 | 1.000 | 0.980 | 1.000 | 0.962 | 1.000 | 0.973 | 0.789 | 1.000 | 1.000 | 0.853 | 0.823 |
| 1772 | 0.818 | 1.000 | 0.840 | 1.000 | 0.984 | 1.000 | 0.866 | 1.000 | 0.980 | 1.000 | 0.962 | 1.000 | 0.982 | 0.791 | 1.000 | 1.000 | 0.854 | 0.824 |
| 1773 | 0.818 | 1.000 | 0.840 | 1.000 | 0.984 | 1.000 | 0.866 | 1.000 | 0.980 | 1.000 | 0.962 | 1.000 | 0.982 | 0.791 | 1.000 | 1.000 | 0.854 | 0.824 |
| 1774 | 0.818 | 1.000 | 0.840 | 1.000 | 0.984 | 1.000 | 0.866 | 1.000 | 0.980 | 1.000 | 0.962 | 1.000 | 0.982 | 0.791 | 1.000 | 1.000 | 0.854 | 0.825 |
| 1775 | 0.818 | 1.000 | 0.840 | 1.000 | 0.984 | 1.000 | 0.866 | 1.000 | 0.980 | 1.000 | 0.962 | 1.000 | 0.982 | 0.791 | 1.000 | 1.000 | 0.854 | 0.825 |
| 1776 | 0.818 | 1.000 | 0.840 | 1.000 | 0.979 | 1.000 | 0.866 | 1.000 | 0.980 | 1.000 | 0.962 | 1.000 | 0.982 | 0.791 | 1.000 | 1.000 | 0.854 | 0.825 |
| 1777 | 0.818 | 1.000 | 0.840 | 1.000 | 0.984 | 1.000 | 0.866 | 1.000 | 0.980 | 1.000 | 0.962 | 1.000 | 0.982 | 0.791 | 1.000 | 1.000 | 0.854 | 0.825 |
| 1778 | 0.818 | 1.000 | 0.840 | 1.000 | 0.984 | 1.000 | 0.866 | 1.000 | 0.980 | 1.000 | 0.962 | 1.000 | 0.982 | 0.791 | 1.000 | 1.000 | 0.854 | 0.825 |
| 1779 | 0.818 | 1.000 | 0.842 | 1.000 | 0.979 | 1.000 | 0.867 | 1.000 | 0.980 | 1.000 | 0.962 | 1.000 | 0.982 | 0.791 | 1.000 | 1.000 | 0.854 | 0.825 |
| 1780 | 0.818 | 1.000 | 0.842 | 1.000 | 0.979 | 1.000 | 0.867 | 1.000 | 0.980 | 1.000 | 0.962 | 1.000 | 0.982 | 0.791 | 1.000 | 1.000 | 0.854 | 0.825 |
| 1781 | 0.816 | 1.000 | 0.840 | 1.000 | 0.979 | 1.000 | 0.866 | 1.000 | 0.980 | 1.000 | 0.962 | 1.000 | 0.982 | 0.792 | 1.000 | 1.000 | 0.854 | 0.824 |
| 1782 | 0.817 | 1.000 | 0.844 | 1.000 | 0.979 | 1.000 | 0.867 | 1.000 | 0.980 | 1.000 | 0.962 | 1.000 | 0.982 | 0.793 | 1.000 | 1.000 | 0.855 | 0.825 |
| 1783 | 0.814 | 1.000 | 0.842 | 1.000 | 0.979 | 1.000 | 0.861 | 1.000 | 0.980 | 1.000 | 0.962 | 1.000 | 0.982 | 0.793 | 1.000 | 1.000 | 0.853 | 0.823 |
| 1784 | 0.814 | 1.000 | 0.842 | 1.000 | 0.979 | 1.000 | 0.861 | 1.000 | 0.980 | 1.000 | 0.962 | 1.000 | 0.982 | 0.793 | 1.000 | 1.000 | 0.853 | 0.823 |
| 1785 | 0.815 | 1.000 | 0.842 | 1.000 | 0.979 | 1.000 | 0.861 | 1.000 | 0.980 | 1.000 | 0.962 | 1.000 | 1.000 | 0.793 | 1.000 | 1.000 | 0.854 | 0.824 |
| 1786 | 0.815 | 1.000 | 0.844 | 1.000 | 0.979 | 1.000 | 0.861 | 1.000 | 0.980 | 1.000 | 0.962 | 1.000 | 1.000 | 0.793 | 1.000 | 1.000 | 0.854 | 0.824 |
| 1787 | 0.815 | 1.000 | 0.844 | 1.000 | 0.984 | 1.000 | 0.861 | 1.000 | 0.980 | 1.000 | 0.962 | 1.000 | 1.000 | 0.793 | 1.000 | 1.000 | 0.854 | 0.824 |
| 1788 | 0.816 | 1.000 | 0.846 | 1.000 | 0.984 | 1.000 | 0.861 | 1.000 | 0.980 | 1.000 | 0.962 | 1.000 | 0.991 | 0.792 | 1.000 | 1.000 | 0.854 | 0.824 |
| 1789 | 0.815 | 1.000 | 0.844 | 1.000 | 0.984 | 1.000 | 0.861 | 1.000 | 0.980 | 1.000 | 0.962 | 1.000 | 0.991 | 0.792 | 1.000 | 1.000 | 0.854 | 0.824 |
| 1790 | 0.816 | 1.000 | 0.846 | 1.000 | 0.984 | 1.000 | 0.861 | 1.000 | 0.980 | 1.000 | 0.962 | 1.000 | 1.000 | 0.793 | 1.000 | 1.000 | 0.854 | 0.825 |
| 1791 | 0.816 | 1.000 | 0.846 | 1.000 | 0.984 | 1.000 | 0.861 | 1.000 | 0.980 | 1.000 | 0.962 | 1.000 | 0.991 | 0.792 | 1.000 | 1.000 | 0.854 | 0.824 |
| 1792 | 0.816 | 1.000 | 0.846 | 1.000 | 0.984 | 1.000 | 0.861 | 1.000 | 0.980 | 1.000 | 0.962 | 1.000 | 0.991 | 0.793 | 1.000 | 1.000 | 0.854 | 0.825 |
| 1793 | 0.815 | 1.000 | 0.844 | 1.000 | 0.979 | 1.000 | 0.863 | 1.000 | 0.980 | 1.000 | 0.962 | 1.000 | 0.991 | 0.791 | 1.000 | 1.000 | 0.853 | 0.824 |
| 1794 | 0.813 | 1.000 | 0.844 | 1.000 | 0.979 | 1.000 | 0.861 | 1.000 | 0.980 | 1.000 | 0.962 | 1.000 | 0.991 | 0.791 | 1.000 | 1.000 | 0.853 | 0.823 |
| 1795 | 0.813 | 1.000 | 0.846 | 1.000 | 0.979 | 1.000 | 0.861 | 1.000 | 0.980 | 1.000 | 0.962 | 1.000 | 0.991 | 0.791 | 1.000 | 1.000 | 0.853 | 0.823 |
| 1796 | 0.815 | 1.000 | 0.844 | 1.000 | 0.979 | 1.000 | 0.866 | 1.000 | 0.980 | 1.000 | 0.962 | 1.000 | 0.991 | 0.789 | 1.000 | 1.000 | 0.853 | 0.823 |
| 1797 | 0.816 | 1.000 | 0.840 | 1.000 | 0.974 | 1.000 | 0.867 | 1.000 | 0.980 | 1.000 | 0.962 | 1.000 | 0.991 | 0.789 | 1.000 | 1.000 | 0.853 | 0.823 |

| | | | | | | | | | | | | | | | | | | |
|---|---|---|---|---|---|---|---|---|---|---|---|---|---|---|---|---|---|---|
| 1798 | 0.816 | 1.000 | 0.840 | 1.000 | 0.979 | 1.000 | 0.867 | 1.000 | 0.980 | 1.000 | 0.962 | 1.000 | 0.991 | 0.789 | 1.000 | 1.000 | 0.853 | 0.823 |
| 1799 | 0.815 | 1.000 | 0.840 | 1.000 | 0.979 | 1.000 | 0.867 | 1.000 | 0.980 | 1.000 | 0.962 | 1.000 | 0.991 | 0.789 | 1.000 | 1.000 | 0.853 | 0.823 |
| 1800 | 0.815 | 1.000 | 0.842 | 1.000 | 0.979 | 1.000 | 0.867 | 1.000 | 0.980 | 1.000 | 0.962 | 1.000 | 0.991 | 0.789 | 1.000 | 1.000 | 0.853 | 0.823 |
| 1801 | 0.815 | 1.000 | 0.842 | 1.000 | 0.974 | 1.000 | 0.867 | 1.000 | 0.980 | 1.000 | 0.962 | 1.000 | 0.991 | 0.789 | 1.000 | 1.000 | 0.853 | 0.823 |
| 1802 | 0.815 | 1.000 | 0.846 | 1.000 | 0.974 | 1.000 | 0.864 | 1.000 | 0.990 | 1.000 | 0.962 | 1.000 | 0.991 | 0.790 | 1.000 | 1.000 | 0.853 | 0.824 |
| 1803 | 0.814 | 1.000 | 0.850 | 1.000 | 0.974 | 1.000 | 0.866 | 1.000 | 0.990 | 1.000 | 0.962 | 1.000 | 0.991 | 0.789 | 1.000 | 1.000 | 0.854 | 0.824 |
| 1804 | 0.815 | 1.000 | 0.850 | 1.000 | 0.974 | 1.000 | 0.866 | 1.000 | 0.990 | 1.000 | 0.962 | 1.000 | 0.991 | 0.790 | 1.000 | 1.000 | 0.854 | 0.825 |
| 1805 | 0.814 | 1.000 | 0.850 | 1.000 | 0.974 | 1.000 | 0.866 | 1.000 | 0.990 | 1.000 | 0.965 | 1.000 | 0.991 | 0.791 | 1.000 | 1.000 | 0.854 | 0.825 |
| 1806 | 0.813 | 1.000 | 0.850 | 1.000 | 0.979 | 1.000 | 0.866 | 1.000 | 0.990 | 1.000 | 0.962 | 1.000 | 0.991 | 0.792 | 1.000 | 1.000 | 0.854 | 0.825 |
| 1807 | 0.814 | 1.000 | 0.850 | 1.000 | 0.979 | 1.000 | 0.866 | 1.000 | 0.990 | 1.000 | 0.965 | 1.000 | 0.982 | 0.793 | 1.000 | 1.000 | 0.855 | 0.825 |
| 1808 | 0.813 | 1.000 | 0.850 | 1.000 | 0.979 | 1.000 | 0.866 | 1.000 | 0.990 | 1.000 | 0.965 | 1.000 | 0.982 | 0.793 | 1.000 | 1.000 | 0.854 | 0.825 |
| 1809 | 0.813 | 1.000 | 0.850 | 1.000 | 0.979 | 1.000 | 0.866 | 1.000 | 0.990 | 1.000 | 0.965 | 1.000 | 0.982 | 0.793 | 1.000 | 1.000 | 0.854 | 0.825 |
| 1810 | 0.814 | 1.000 | 0.852 | 1.000 | 0.979 | 1.000 | 0.864 | 1.000 | 0.990 | 1.000 | 0.965 | 1.000 | 0.982 | 0.792 | 1.000 | 1.000 | 0.855 | 0.825 |
| 1811 | 0.814 | 1.000 | 0.850 | 1.000 | 0.984 | 1.000 | 0.864 | 1.000 | 0.990 | 1.000 | 0.962 | 1.000 | 0.982 | 0.791 | 1.000 | 1.000 | 0.854 | 0.825 |
| 1812 | 0.814 | 1.000 | 0.850 | 1.000 | 0.984 | 1.000 | 0.864 | 1.000 | 0.990 | 1.000 | 0.962 | 1.000 | 0.982 | 0.791 | 1.000 | 1.000 | 0.854 | 0.825 |
| 1813 | 0.814 | 1.000 | 0.850 | 1.000 | 0.984 | 1.000 | 0.864 | 1.000 | 0.990 | 1.000 | 0.962 | 1.000 | 0.982 | 0.791 | 1.000 | 1.000 | 0.854 | 0.825 |
| 1814 | 0.815 | 1.000 | 0.850 | 1.000 | 0.984 | 1.000 | 0.866 | 1.000 | 0.990 | 1.000 | 0.962 | 1.000 | 0.982 | 0.791 | 1.000 | 1.000 | 0.855 | 0.825 |
| 1815 | 0.816 | 1.000 | 0.850 | 1.000 | 0.974 | 1.000 | 0.864 | 1.000 | 0.980 | 1.000 | 0.962 | 1.000 | 0.982 | 0.791 | 1.000 | 1.000 | 0.854 | 0.824 |
| 1816 | 0.816 | 1.000 | 0.850 | 1.000 | 0.974 | 1.000 | 0.863 | 1.000 | 0.980 | 1.000 | 0.962 | 1.000 | 0.982 | 0.791 | 1.000 | 1.000 | 0.854 | 0.824 |
| 1817 | 0.816 | 1.000 | 0.850 | 1.000 | 0.974 | 1.000 | 0.863 | 1.000 | 0.980 | 1.000 | 0.962 | 1.000 | 0.982 | 0.791 | 1.000 | 1.000 | 0.854 | 0.824 |
| 1818 | 0.815 | 1.000 | 0.854 | 1.000 | 0.974 | 1.000 | 0.864 | 1.000 | 0.980 | 1.000 | 0.962 | 1.000 | 1.000 | 0.791 | 1.000 | 1.000 | 0.854 | 0.825 |
| 1819 | 0.814 | 1.000 | 0.854 | 1.000 | 0.974 | 1.000 | 0.864 | 1.000 | 0.980 | 1.000 | 0.962 | 1.000 | 1.000 | 0.791 | 1.000 | 1.000 | 0.854 | 0.825 |
| 1820 | 0.813 | 1.000 | 0.854 | 1.000 | 0.974 | 1.000 | 0.863 | 1.000 | 0.980 | 1.000 | 0.962 | 1.000 | 1.000 | 0.791 | 1.000 | 1.000 | 0.854 | 0.824 |
| 1821 | 0.815 | 1.000 | 0.854 | 1.000 | 0.974 | 1.000 | 0.861 | 1.000 | 0.980 | 1.000 | 0.962 | 1.000 | 1.000 | 0.791 | 1.000 | 1.000 | 0.854 | 0.824 |
| 1822 | 0.816 | 1.000 | 0.850 | 1.000 | 0.974 | 1.000 | 0.861 | 1.000 | 0.980 | 1.000 | 0.962 | 1.000 | 1.000 | 0.791 | 1.000 | 1.000 | 0.854 | 0.824 |
| 1823 | 0.816 | 1.000 | 0.850 | 1.000 | 0.974 | 1.000 | 0.861 | 1.000 | 0.980 | 1.000 | 0.962 | 1.000 | 1.000 | 0.791 | 1.000 | 1.000 | 0.854 | 0.824 |
| 1824 | 0.816 | 1.000 | 0.850 | 1.000 | 0.974 | 1.000 | 0.864 | 1.000 | 0.980 | 1.000 | 0.962 | 1.000 | 1.000 | 0.790 | 1.000 | 1.000 | 0.854 | 0.825 |
| 1825 | 0.816 | 1.000 | 0.846 | 1.000 | 0.979 | 1.000 | 0.864 | 1.000 | 0.980 | 1.000 | 0.962 | 1.000 | 0.991 | 0.792 | 1.000 | 1.000 | 0.854 | 0.825 |
| 1826 | 0.816 | 1.000 | 0.846 | 1.000 | 0.979 | 1.000 | 0.864 | 1.000 | 0.980 | 1.000 | 0.962 | 1.000 | 0.991 | 0.791 | 1.000 | 1.000 | 0.854 | 0.825 |
| 1827 | 0.816 | 1.000 | 0.846 | 1.000 | 0.979 | 1.000 | 0.861 | 1.000 | 0.980 | 1.000 | 0.962 | 1.000 | 0.991 | 0.791 | 1.000 | 1.000 | 0.854 | 0.824 |
| 1828 | 0.816 | 1.000 | 0.846 | 1.000 | 0.979 | 1.000 | 0.866 | 1.000 | 0.980 | 1.000 | 0.962 | 1.000 | 0.991 | 0.791 | 1.000 | 1.000 | 0.854 | 0.825 |
| 1829 | 0.816 | 1.000 | 0.846 | 1.000 | 0.979 | 1.000 | 0.864 | 1.000 | 0.980 | 1.000 | 0.962 | 1.000 | 0.991 | 0.791 | 1.000 | 1.000 | 0.854 | 0.825 |
| 1830 | 0.816 | 1.000 | 0.850 | 1.000 | 0.979 | 1.000 | 0.864 | 1.000 | 0.980 | 1.000 | 0.962 | 1.000 | 0.991 | 0.791 | 1.000 | 1.000 | 0.854 | 0.825 |
| 1831 | 0.816 | 1.000 | 0.848 | 1.000 | 0.979 | 1.000 | 0.864 | 1.000 | 0.980 | 1.000 | 0.962 | 1.000 | 0.991 | 0.791 | 1.000 | 1.000 | 0.854 | 0.825 |
| 1832 | 0.815 | 1.000 | 0.848 | 1.000 | 0.979 | 1.000 | 0.864 | 1.000 | 0.980 | 1.000 | 0.962 | 1.000 | 0.991 | 0.791 | 1.000 | 1.000 | 0.854 | 0.824 |
| 1833 | 0.815 | 1.000 | 0.848 | 1.000 | 0.979 | 1.000 | 0.863 | 1.000 | 0.980 | 1.000 | 0.962 | 1.000 | 0.991 | 0.791 | 1.000 | 1.000 | 0.854 | 0.824 |
| 1834 | 0.815 | 1.000 | 0.848 | 1.000 | 0.979 | 1.000 | 0.863 | 1.000 | 0.980 | 1.000 | 0.962 | 1.000 | 1.000 | 0.791 | 1.000 | 1.000 | 0.854 | 0.824 |
| 1835 | 0.815 | 1.000 | 0.848 | 1.000 | 0.979 | 1.000 | 0.863 | 1.000 | 0.980 | 1.000 | 0.962 | 1.000 | 1.000 | 0.791 | 1.000 | 1.000 | 0.854 | 0.824 |
| 1836 | 0.816 | 1.000 | 0.848 | 1.000 | 0.979 | 1.000 | 0.861 | 1.000 | 0.980 | 1.000 | 0.962 | 1.000 | 1.000 | 0.791 | 1.000 | 1.000 | 0.854 | 0.824 |

| | | | | | | | | | | | | | | | | | | |
|---|---|---|---|---|---|---|---|---|---|---|---|---|---|---|---|---|---|---|
| 1837 | 0.816 | 1.000 | 0.848 | 1.000 | 0.979 | 1.000 | 0.861 | 1.000 | 0.980 | 1.000 | 0.962 | 1.000 | 1.000 | 0.791 | 1.000 | 1.000 | 0.854 | 0.824 |
| 1838 | 0.815 | 1.000 | 0.848 | 1.000 | 0.979 | 1.000 | 0.861 | 1.000 | 0.980 | 1.000 | 0.962 | 1.000 | 1.000 | 0.791 | 1.000 | 1.000 | 0.854 | 0.824 |
| 1839 | 0.815 | 1.000 | 0.850 | 1.000 | 0.979 | 1.000 | 0.863 | 1.000 | 0.980 | 1.000 | 0.962 | 1.000 | 1.000 | 0.791 | 1.000 | 1.000 | 0.854 | 0.824 |
| 1840 | 0.815 | 1.000 | 0.848 | 1.000 | 0.979 | 1.000 | 0.863 | 1.000 | 0.980 | 1.000 | 0.962 | 1.000 | 1.000 | 0.791 | 1.000 | 1.000 | 0.854 | 0.824 |
| 1841 | 0.813 | 1.000 | 0.852 | 1.000 | 0.979 | 1.000 | 0.863 | 1.000 | 0.980 | 1.000 | 0.962 | 1.000 | 1.000 | 0.790 | 1.000 | 1.000 | 0.853 | 0.824 |
| 1842 | 0.814 | 1.000 | 0.852 | 1.000 | 0.979 | 1.000 | 0.864 | 1.000 | 0.980 | 1.000 | 0.962 | 1.000 | 1.000 | 0.790 | 1.000 | 1.000 | 0.854 | 0.824 |
| 1843 | 0.815 | 1.000 | 0.852 | 1.000 | 0.974 | 1.000 | 0.863 | 1.000 | 0.980 | 1.000 | 0.962 | 1.000 | 1.000 | 0.791 | 1.000 | 1.000 | 0.854 | 0.825 |
| 1844 | 0.820 | 1.000 | 0.846 | 1.000 | 0.974 | 1.000 | 0.863 | 1.000 | 0.980 | 1.000 | 0.962 | 1.000 | 1.000 | 0.794 | 1.000 | 1.000 | 0.856 | 0.826 |
| 1845 | 0.820 | 1.000 | 0.844 | 1.000 | 0.979 | 1.000 | 0.860 | 1.000 | 0.980 | 1.000 | 0.962 | 1.000 | 0.991 | 0.792 | 1.000 | 1.000 | 0.855 | 0.825 |
| 1846 | 0.820 | 1.000 | 0.844 | 1.000 | 0.979 | 1.000 | 0.858 | 1.000 | 0.980 | 1.000 | 0.962 | 1.000 | 0.991 | 0.792 | 1.000 | 1.000 | 0.855 | 0.825 |
| 1847 | 0.820 | 1.000 | 0.844 | 1.000 | 0.979 | 1.000 | 0.858 | 1.000 | 0.980 | 1.000 | 0.962 | 1.000 | 0.991 | 0.791 | 1.000 | 1.000 | 0.854 | 0.825 |
| 1848 | 0.820 | 1.000 | 0.844 | 1.000 | 0.979 | 1.000 | 0.858 | 1.000 | 0.980 | 1.000 | 0.962 | 1.000 | 0.991 | 0.791 | 1.000 | 1.000 | 0.854 | 0.824 |
| 1849 | 0.820 | 1.000 | 0.844 | 1.000 | 0.979 | 1.000 | 0.858 | 1.000 | 0.980 | 1.000 | 0.962 | 1.000 | 0.991 | 0.791 | 1.000 | 1.000 | 0.854 | 0.824 |
| 1850 | 0.820 | 1.000 | 0.844 | 1.000 | 0.979 | 1.000 | 0.858 | 1.000 | 0.980 | 1.000 | 0.962 | 1.000 | 0.991 | 0.791 | 1.000 | 1.000 | 0.854 | 0.824 |
| 1851 | 0.820 | 1.000 | 0.844 | 1.000 | 0.979 | 1.000 | 0.858 | 1.000 | 0.980 | 1.000 | 0.962 | 1.000 | 0.991 | 0.791 | 1.000 | 1.000 | 0.854 | 0.824 |
| 1852 | 0.820 | 1.000 | 0.846 | 1.000 | 0.979 | 1.000 | 0.858 | 1.000 | 0.980 | 1.000 | 0.962 | 1.000 | 0.991 | 0.791 | 1.000 | 1.000 | 0.854 | 0.825 |
| 1853 | 0.820 | 1.000 | 0.844 | 1.000 | 0.979 | 1.000 | 0.860 | 1.000 | 0.980 | 1.000 | 0.962 | 1.000 | 0.991 | 0.791 | 1.000 | 1.000 | 0.854 | 0.825 |
| 1854 | 0.820 | 1.000 | 0.846 | 1.000 | 0.979 | 1.000 | 0.858 | 1.000 | 0.980 | 1.000 | 0.962 | 1.000 | 0.991 | 0.792 | 1.000 | 1.000 | 0.855 | 0.825 |
| 1855 | 0.819 | 1.000 | 0.846 | 1.000 | 0.979 | 1.000 | 0.860 | 1.000 | 0.980 | 1.000 | 0.962 | 1.000 | 0.991 | 0.792 | 1.000 | 1.000 | 0.855 | 0.825 |
| 1856 | 0.819 | 1.000 | 0.846 | 1.000 | 0.979 | 1.000 | 0.860 | 1.000 | 0.980 | 1.000 | 0.962 | 1.000 | 0.991 | 0.792 | 1.000 | 1.000 | 0.855 | 0.825 |
| 1857 | 0.819 | 1.000 | 0.846 | 1.000 | 0.979 | 1.000 | 0.860 | 1.000 | 0.980 | 1.000 | 0.962 | 1.000 | 0.991 | 0.792 | 1.000 | 1.000 | 0.855 | 0.825 |
| 1858 | 0.819 | 1.000 | 0.848 | 1.000 | 0.979 | 1.000 | 0.864 | 1.000 | 0.980 | 1.000 | 0.962 | 1.000 | 0.991 | 0.793 | 1.000 | 1.000 | 0.856 | 0.826 |
| 1859 | 0.819 | 1.000 | 0.848 | 1.000 | 0.979 | 1.000 | 0.864 | 1.000 | 0.980 | 1.000 | 0.962 | 1.000 | 0.991 | 0.793 | 1.000 | 1.000 | 0.856 | 0.826 |
| 1860 | 0.818 | 1.000 | 0.846 | 1.000 | 0.979 | 1.000 | 0.863 | 1.000 | 0.980 | 1.000 | 0.962 | 1.000 | 0.991 | 0.793 | 1.000 | 1.000 | 0.855 | 0.825 |
| 1861 | 0.818 | 1.000 | 0.848 | 1.000 | 0.979 | 1.000 | 0.864 | 1.000 | 0.980 | 1.000 | 0.962 | 1.000 | 0.991 | 0.792 | 1.000 | 1.000 | 0.855 | 0.826 |
| 1862 | 0.820 | 1.000 | 0.848 | 1.000 | 0.979 | 1.000 | 0.864 | 1.000 | 0.980 | 1.000 | 0.962 | 1.000 | 0.991 | 0.791 | 1.000 | 1.000 | 0.856 | 0.826 |
| 1863 | 0.820 | 1.000 | 0.846 | 1.000 | 0.979 | 1.000 | 0.864 | 1.000 | 0.980 | 1.000 | 0.962 | 1.000 | 0.991 | 0.792 | 1.000 | 1.000 | 0.855 | 0.826 |
| 1864 | 0.818 | 1.000 | 0.846 | 1.000 | 0.979 | 1.000 | 0.864 | 1.000 | 0.980 | 1.000 | 0.962 | 1.000 | 1.000 | 0.792 | 1.000 | 1.000 | 0.855 | 0.826 |
| 1865 | 0.821 | 1.000 | 0.844 | 1.000 | 0.979 | 1.000 | 0.863 | 1.000 | 0.980 | 1.000 | 0.962 | 1.000 | 0.982 | 0.793 | 1.000 | 1.000 | 0.855 | 0.826 |
| 1866 | 0.820 | 1.000 | 0.844 | 1.000 | 0.979 | 1.000 | 0.863 | 1.000 | 0.980 | 1.000 | 0.962 | 1.000 | 0.982 | 0.794 | 1.000 | 1.000 | 0.855 | 0.826 |
| 1867 | 0.820 | 1.000 | 0.844 | 1.000 | 0.979 | 1.000 | 0.863 | 1.000 | 0.980 | 1.000 | 0.962 | 1.000 | 0.982 | 0.794 | 1.000 | 1.000 | 0.855 | 0.826 |
| 1868 | 0.817 | 1.000 | 0.844 | 1.000 | 0.979 | 1.000 | 0.866 | 1.000 | 0.990 | 1.000 | 0.962 | 1.000 | 0.982 | 0.795 | 1.000 | 1.000 | 0.855 | 0.826 |
| 1869 | 0.816 | 1.000 | 0.844 | 1.000 | 0.979 | 1.000 | 0.864 | 1.000 | 0.990 | 1.000 | 0.962 | 1.000 | 0.982 | 0.795 | 1.000 | 1.000 | 0.855 | 0.825 |
| 1870 | 0.818 | 1.000 | 0.846 | 1.000 | 0.979 | 1.000 | 0.863 | 1.000 | 0.990 | 1.000 | 0.962 | 1.000 | 0.982 | 0.797 | 1.000 | 1.000 | 0.856 | 0.826 |
| 1871 | 0.819 | 1.000 | 0.852 | 1.000 | 0.979 | 1.000 | 0.863 | 1.000 | 0.990 | 1.000 | 0.962 | 1.000 | 0.982 | 0.797 | 1.000 | 1.000 | 0.857 | 0.828 |
| 1872 | 0.818 | 1.000 | 0.852 | 1.000 | 0.979 | 1.000 | 0.861 | 1.000 | 0.990 | 1.000 | 0.959 | 1.000 | 0.991 | 0.796 | 1.000 | 1.000 | 0.856 | 0.827 |
| 1873 | 0.818 | 1.000 | 0.854 | 1.000 | 0.979 | 1.000 | 0.863 | 1.000 | 0.990 | 1.000 | 0.959 | 1.000 | 1.000 | 0.797 | 1.000 | 1.000 | 0.857 | 0.828 |
| 1874 | 0.819 | 1.000 | 0.854 | 1.000 | 0.979 | 1.000 | 0.861 | 1.000 | 0.990 | 1.000 | 0.959 | 1.000 | 1.000 | 0.796 | 1.000 | 1.000 | 0.857 | 0.828 |
| 1875 | 0.819 | 1.000 | 0.852 | 1.000 | 0.979 | 1.000 | 0.860 | 1.000 | 0.990 | 1.000 | 0.959 | 1.000 | 1.000 | 0.795 | 1.000 | 1.000 | 0.856 | 0.827 |

| | | | | | | | | | | | | | | | | | | |
|---|---|---|---|---|---|---|---|---|---|---|---|---|---|---|---|---|---|---|
| 1876 | 0.819 | 1.000 | 0.852 | 1.000 | 0.979 | 1.000 | 0.860 | 1.000 | 0.990 | 1.000 | 0.959 | 1.000 | 1.000 | 0.795 | 1.000 | 1.000 | 0.856 | 0.827 |
| 1877 | 0.820 | 1.000 | 0.852 | 1.000 | 0.979 | 1.000 | 0.861 | 1.000 | 0.990 | 1.000 | 0.959 | 1.000 | 1.000 | 0.796 | 1.000 | 1.000 | 0.857 | 0.828 |
| 1878 | 0.820 | 1.000 | 0.852 | 1.000 | 0.979 | 1.000 | 0.866 | 1.000 | 0.990 | 1.000 | 0.959 | 1.000 | 1.000 | 0.795 | 1.000 | 1.000 | 0.857 | 0.828 |
| 1879 | 0.820 | 1.000 | 0.852 | 1.000 | 0.979 | 1.000 | 0.866 | 1.000 | 0.990 | 1.000 | 0.959 | 1.000 | 1.000 | 0.795 | 1.000 | 1.000 | 0.857 | 0.828 |
| 1880 | 0.820 | 1.000 | 0.852 | 1.000 | 0.979 | 1.000 | 0.866 | 1.000 | 0.990 | 1.000 | 0.959 | 1.000 | 1.000 | 0.795 | 1.000 | 1.000 | 0.857 | 0.828 |
| 1881 | 0.819 | 1.000 | 0.852 | 1.000 | 0.979 | 1.000 | 0.866 | 1.000 | 0.990 | 1.000 | 0.959 | 1.000 | 1.000 | 0.795 | 1.000 | 1.000 | 0.857 | 0.828 |
| 1882 | 0.818 | 1.000 | 0.852 | 1.000 | 0.984 | 1.000 | 0.866 | 1.000 | 0.990 | 1.000 | 0.959 | 1.000 | 1.000 | 0.795 | 1.000 | 1.000 | 0.857 | 0.828 |
| 1883 | 0.818 | 1.000 | 0.852 | 1.000 | 0.979 | 1.000 | 0.866 | 1.000 | 0.990 | 1.000 | 0.959 | 1.000 | 1.000 | 0.794 | 1.000 | 1.000 | 0.856 | 0.827 |
| 1884 | 0.818 | 1.000 | 0.852 | 1.000 | 0.979 | 1.000 | 0.866 | 1.000 | 0.990 | 1.000 | 0.959 | 1.000 | 1.000 | 0.794 | 1.000 | 1.000 | 0.856 | 0.827 |
| 1885 | 0.818 | 1.000 | 0.852 | 1.000 | 0.979 | 1.000 | 0.866 | 1.000 | 0.990 | 1.000 | 0.959 | 1.000 | 1.000 | 0.794 | 1.000 | 1.000 | 0.856 | 0.827 |
| 1886 | 0.818 | 1.000 | 0.852 | 1.000 | 0.979 | 1.000 | 0.866 | 1.000 | 0.990 | 1.000 | 0.959 | 1.000 | 1.000 | 0.794 | 1.000 | 1.000 | 0.856 | 0.827 |
| 1887 | 0.819 | 1.000 | 0.850 | 1.000 | 0.979 | 1.000 | 0.866 | 1.000 | 0.990 | 1.000 | 0.959 | 1.000 | 1.000 | 0.794 | 1.000 | 1.000 | 0.856 | 0.827 |
| 1888 | 0.818 | 1.000 | 0.852 | 1.000 | 0.979 | 1.000 | 0.866 | 1.000 | 0.990 | 1.000 | 0.959 | 1.000 | 1.000 | 0.794 | 1.000 | 1.000 | 0.856 | 0.827 |
| 1889 | 0.818 | 1.000 | 0.850 | 1.000 | 0.979 | 1.000 | 0.866 | 1.000 | 0.990 | 1.000 | 0.959 | 1.000 | 1.000 | 0.795 | 1.000 | 1.000 | 0.856 | 0.827 |
| 1890 | 0.820 | 1.000 | 0.852 | 1.000 | 0.979 | 1.000 | 0.867 | 1.000 | 0.990 | 1.000 | 0.959 | 1.000 | 0.991 | 0.794 | 1.000 | 1.000 | 0.857 | 0.828 |
| 1891 | 0.820 | 1.000 | 0.852 | 1.000 | 0.979 | 1.000 | 0.867 | 1.000 | 0.990 | 1.000 | 0.959 | 1.000 | 0.991 | 0.794 | 1.000 | 1.000 | 0.857 | 0.828 |
| 1892 | 0.820 | 1.000 | 0.854 | 1.000 | 0.984 | 1.000 | 0.867 | 1.000 | 0.990 | 1.000 | 0.959 | 1.000 | 0.991 | 0.794 | 1.000 | 1.000 | 0.857 | 0.828 |
| 1893 | 0.820 | 1.000 | 0.852 | 1.000 | 0.984 | 1.000 | 0.864 | 1.000 | 0.990 | 1.000 | 0.959 | 1.000 | 0.991 | 0.792 | 1.000 | 1.000 | 0.856 | 0.827 |
| 1894 | 0.821 | 1.000 | 0.852 | 1.000 | 0.984 | 1.000 | 0.864 | 1.000 | 0.990 | 1.000 | 0.959 | 1.000 | 0.991 | 0.793 | 1.000 | 1.000 | 0.857 | 0.828 |
| 1895 | 0.822 | 1.000 | 0.852 | 1.000 | 0.984 | 1.000 | 0.864 | 1.000 | 0.990 | 1.000 | 0.959 | 1.000 | 0.991 | 0.792 | 1.000 | 1.000 | 0.857 | 0.828 |
| 1896 | 0.821 | 1.000 | 0.852 | 1.000 | 0.984 | 1.000 | 0.863 | 1.000 | 0.990 | 1.000 | 0.959 | 1.000 | 0.991 | 0.794 | 1.000 | 1.000 | 0.857 | 0.828 |
| 1897 | 0.821 | 1.000 | 0.852 | 1.000 | 0.984 | 1.000 | 0.863 | 1.000 | 0.990 | 1.000 | 0.959 | 1.000 | 0.991 | 0.794 | 1.000 | 1.000 | 0.857 | 0.828 |
| 1898 | 0.820 | 1.000 | 0.852 | 1.000 | 0.984 | 1.000 | 0.864 | 1.000 | 0.990 | 1.000 | 0.959 | 1.000 | 0.991 | 0.794 | 1.000 | 1.000 | 0.857 | 0.827 |
| 1899 | 0.818 | 1.000 | 0.852 | 1.000 | 0.984 | 1.000 | 0.863 | 1.000 | 0.990 | 1.000 | 0.959 | 1.000 | 0.991 | 0.796 | 1.000 | 1.000 | 0.857 | 0.827 |
| 1900 | 0.818 | 1.000 | 0.852 | 1.000 | 0.984 | 1.000 | 0.863 | 1.000 | 0.990 | 1.000 | 0.962 | 1.000 | 0.991 | 0.795 | 1.000 | 1.000 | 0.856 | 0.827 |
| 1901 | 0.818 | 1.000 | 0.854 | 1.000 | 0.984 | 1.000 | 0.863 | 1.000 | 0.990 | 1.000 | 0.959 | 1.000 | 0.991 | 0.795 | 1.000 | 1.000 | 0.856 | 0.827 |
| 1902 | 0.818 | 1.000 | 0.854 | 1.000 | 0.984 | 1.000 | 0.864 | 1.000 | 0.990 | 1.000 | 0.959 | 1.000 | 0.991 | 0.795 | 1.000 | 1.000 | 0.857 | 0.827 |
| 1903 | 0.818 | 1.000 | 0.854 | 1.000 | 0.984 | 1.000 | 0.864 | 1.000 | 0.990 | 1.000 | 0.959 | 1.000 | 0.991 | 0.795 | 1.000 | 1.000 | 0.857 | 0.827 |
| 1904 | 0.818 | 1.000 | 0.850 | 1.000 | 0.984 | 1.000 | 0.863 | 1.000 | 0.990 | 1.000 | 0.957 | 1.000 | 0.991 | 0.795 | 1.000 | 1.000 | 0.856 | 0.826 |
| 1905 | 0.818 | 1.000 | 0.850 | 1.000 | 0.984 | 1.000 | 0.864 | 1.000 | 0.990 | 1.000 | 0.957 | 1.000 | 0.991 | 0.796 | 1.000 | 1.000 | 0.856 | 0.827 |
| 1906 | 0.818 | 1.000 | 0.854 | 1.000 | 0.979 | 1.000 | 0.864 | 1.000 | 0.990 | 1.000 | 0.959 | 1.000 | 0.991 | 0.796 | 1.000 | 1.000 | 0.857 | 0.828 |
| 1907 | 0.817 | 1.000 | 0.854 | 1.000 | 0.979 | 1.000 | 0.866 | 1.000 | 0.990 | 1.000 | 0.959 | 1.000 | 0.991 | 0.795 | 1.000 | 1.000 | 0.856 | 0.827 |
| 1908 | 0.818 | 1.000 | 0.852 | 1.000 | 0.979 | 1.000 | 0.866 | 1.000 | 0.990 | 1.000 | 0.959 | 1.000 | 0.991 | 0.793 | 1.000 | 1.000 | 0.856 | 0.826 |
| 1909 | 0.818 | 1.000 | 0.854 | 1.000 | 0.979 | 1.000 | 0.866 | 1.000 | 0.990 | 1.000 | 0.959 | 1.000 | 0.991 | 0.794 | 1.000 | 1.000 | 0.856 | 0.827 |
| 1910 | 0.818 | 1.000 | 0.856 | 1.000 | 0.979 | 1.000 | 0.866 | 1.000 | 0.990 | 1.000 | 0.959 | 1.000 | 0.991 | 0.792 | 1.000 | 1.000 | 0.856 | 0.827 |
| 1911 | 0.818 | 1.000 | 0.856 | 1.000 | 0.979 | 1.000 | 0.866 | 1.000 | 0.990 | 1.000 | 0.959 | 1.000 | 0.991 | 0.792 | 1.000 | 1.000 | 0.856 | 0.827 |
| 1912 | 0.819 | 1.000 | 0.854 | 1.000 | 0.979 | 1.000 | 0.866 | 1.000 | 0.990 | 1.000 | 0.959 | 1.000 | 0.991 | 0.793 | 1.000 | 1.000 | 0.856 | 0.827 |
| 1913 | 0.819 | 1.000 | 0.854 | 1.000 | 0.979 | 1.000 | 0.866 | 1.000 | 0.990 | 1.000 | 0.959 | 1.000 | 0.991 | 0.793 | 1.000 | 1.000 | 0.856 | 0.827 |
| 1914 | 0.819 | 1.000 | 0.854 | 1.000 | 0.979 | 1.000 | 0.866 | 1.000 | 0.990 | 1.000 | 0.959 | 1.000 | 0.991 | 0.793 | 1.000 | 1.000 | 0.856 | 0.827 |

| | | | | | | | | | | | | | | | | | |
|---|---|---|---|---|---|---|---|---|---|---|---|---|---|---|---|---|---|
| 1915 | 0.818 | 1.000 | 0.856 | 1.000 | 0.979 | 1.000 | 0.867 | 1.000 | 0.990 | 1.000 | 0.959 | 1.000 | 0.991 | 0.794 | 1.000 | 1.000 | 0.857 | 0.828 |
| 1916 | 0.819 | 1.000 | 0.856 | 1.000 | 0.979 | 1.000 | 0.866 | 1.000 | 0.990 | 1.000 | 0.959 | 1.000 | 0.991 | 0.793 | 1.000 | 1.000 | 0.857 | 0.827 |
| 1917 | 0.819 | 1.000 | 0.856 | 1.000 | 0.979 | 1.000 | 0.866 | 1.000 | 0.990 | 1.000 | 0.959 | 1.000 | 0.991 | 0.794 | 1.000 | 1.000 | 0.857 | 0.828 |
| 1918 | 0.819 | 1.000 | 0.854 | 1.000 | 0.979 | 1.000 | 0.866 | 1.000 | 0.990 | 1.000 | 0.959 | 1.000 | 0.991 | 0.794 | 1.000 | 1.000 | 0.857 | 0.827 |
| 1919 | 0.818 | 1.000 | 0.854 | 1.000 | 0.979 | 1.000 | 0.866 | 1.000 | 0.990 | 1.000 | 0.959 | 1.000 | 0.991 | 0.795 | 1.000 | 1.000 | 0.856 | 0.827 |
| 1920 | 0.818 | 1.000 | 0.854 | 1.000 | 0.979 | 1.000 | 0.867 | 1.000 | 0.990 | 1.000 | 0.959 | 1.000 | 0.991 | 0.793 | 1.000 | 1.000 | 0.856 | 0.827 |
| 1921 | 0.818 | 1.000 | 0.854 | 1.000 | 0.979 | 1.000 | 0.867 | 1.000 | 0.990 | 1.000 | 0.959 | 1.000 | 0.991 | 0.793 | 1.000 | 1.000 | 0.856 | 0.827 |
| 1922 | 0.818 | 1.000 | 0.854 | 1.000 | 0.979 | 1.000 | 0.867 | 1.000 | 0.990 | 1.000 | 0.959 | 1.000 | 0.991 | 0.793 | 1.000 | 1.000 | 0.856 | 0.827 |
| 1923 | 0.818 | 1.000 | 0.854 | 1.000 | 0.979 | 1.000 | 0.867 | 1.000 | 0.990 | 1.000 | 0.959 | 1.000 | 0.991 | 0.793 | 1.000 | 1.000 | 0.856 | 0.827 |
| 1924 | 0.817 | 1.000 | 0.854 | 1.000 | 0.979 | 1.000 | 0.867 | 1.000 | 0.990 | 1.000 | 0.959 | 1.000 | 0.991 | 0.794 | 1.000 | 1.000 | 0.856 | 0.827 |
| 1925 | 0.817 | 1.000 | 0.856 | 1.000 | 0.979 | 1.000 | 0.866 | 1.000 | 0.990 | 1.000 | 0.959 | 1.000 | 0.991 | 0.793 | 1.000 | 1.000 | 0.856 | 0.827 |
| 1926 | 0.818 | 1.000 | 0.858 | 1.000 | 0.979 | 1.000 | 0.866 | 1.000 | 0.990 | 1.000 | 0.959 | 1.000 | 0.991 | 0.794 | 1.000 | 1.000 | 0.857 | 0.827 |
| 1927 | 0.820 | 1.000 | 0.858 | 1.000 | 0.979 | 1.000 | 0.867 | 1.000 | 0.990 | 1.000 | 0.959 | 1.000 | 0.991 | 0.795 | 1.000 | 1.000 | 0.858 | 0.829 |
| 1928 | 0.820 | 1.000 | 0.860 | 1.000 | 0.979 | 1.000 | 0.867 | 1.000 | 0.990 | 1.000 | 0.959 | 1.000 | 0.991 | 0.795 | 1.000 | 1.000 | 0.858 | 0.829 |
| 1929 | 0.819 | 1.000 | 0.858 | 1.000 | 0.979 | 1.000 | 0.867 | 1.000 | 0.990 | 1.000 | 0.959 | 1.000 | 0.991 | 0.797 | 1.000 | 1.000 | 0.858 | 0.829 |
| 1930 | 0.819 | 1.000 | 0.858 | 1.000 | 0.979 | 1.000 | 0.867 | 1.000 | 0.990 | 1.000 | 0.959 | 1.000 | 0.991 | 0.797 | 1.000 | 1.000 | 0.858 | 0.829 |
| 1931 | 0.819 | 1.000 | 0.860 | 1.000 | 0.979 | 1.000 | 0.867 | 1.000 | 0.990 | 1.000 | 0.959 | 1.000 | 0.991 | 0.798 | 1.000 | 1.000 | 0.858 | 0.830 |
| 1932 | 0.818 | 1.000 | 0.858 | 1.000 | 0.979 | 1.000 | 0.867 | 1.000 | 0.990 | 1.000 | 0.959 | 1.000 | 0.991 | 0.798 | 1.000 | 1.000 | 0.858 | 0.829 |
| 1933 | 0.818 | 1.000 | 0.858 | 1.000 | 0.979 | 1.000 | 0.867 | 1.000 | 0.990 | 1.000 | 0.959 | 1.000 | 0.991 | 0.798 | 1.000 | 1.000 | 0.858 | 0.829 |
| 1934 | 0.818 | 1.000 | 0.858 | 1.000 | 0.979 | 1.000 | 0.867 | 1.000 | 0.990 | 1.000 | 0.959 | 1.000 | 0.991 | 0.798 | 1.000 | 1.000 | 0.858 | 0.829 |
| 1935 | 0.819 | 1.000 | 0.854 | 1.000 | 0.979 | 1.000 | 0.867 | 1.000 | 0.990 | 1.000 | 0.959 | 1.000 | 0.991 | 0.795 | 1.000 | 1.000 | 0.857 | 0.828 |
| 1936 | 0.819 | 1.000 | 0.854 | 1.000 | 0.979 | 1.000 | 0.867 | 1.000 | 0.990 | 1.000 | 0.959 | 1.000 | 0.991 | 0.795 | 1.000 | 1.000 | 0.857 | 0.828 |
| 1937 | 0.819 | 1.000 | 0.854 | 1.000 | 0.979 | 1.000 | 0.867 | 1.000 | 0.990 | 1.000 | 0.959 | 1.000 | 0.991 | 0.795 | 1.000 | 1.000 | 0.857 | 0.828 |
| 1938 | 0.819 | 1.000 | 0.854 | 1.000 | 0.979 | 1.000 | 0.867 | 1.000 | 0.990 | 1.000 | 0.959 | 1.000 | 0.991 | 0.795 | 1.000 | 1.000 | 0.857 | 0.828 |
| 1939 | 0.815 | 1.000 | 0.852 | 1.000 | 0.979 | 1.000 | 0.864 | 1.000 | 0.990 | 1.000 | 0.959 | 1.000 | 0.991 | 0.794 | 1.000 | 1.000 | 0.855 | 0.825 |
| 1940 | 0.816 | 1.000 | 0.852 | 1.000 | 0.979 | 1.000 | 0.864 | 1.000 | 0.990 | 1.000 | 0.959 | 1.000 | 0.991 | 0.794 | 1.000 | 1.000 | 0.855 | 0.826 |
| 1941 | 0.815 | 1.000 | 0.854 | 1.000 | 0.979 | 1.000 | 0.866 | 1.000 | 0.990 | 1.000 | 0.959 | 1.000 | 0.991 | 0.794 | 1.000 | 1.000 | 0.855 | 0.826 |
| 1942 | 0.815 | 1.000 | 0.854 | 1.000 | 0.979 | 1.000 | 0.867 | 1.000 | 0.990 | 1.000 | 0.959 | 1.000 | 0.991 | 0.794 | 1.000 | 1.000 | 0.856 | 0.826 |
| 1943 | 0.815 | 1.000 | 0.854 | 1.000 | 0.979 | 1.000 | 0.866 | 1.000 | 0.990 | 1.000 | 0.959 | 1.000 | 0.991 | 0.794 | 1.000 | 1.000 | 0.855 | 0.826 |
| 1944 | 0.815 | 1.000 | 0.854 | 1.000 | 0.979 | 1.000 | 0.866 | 1.000 | 0.990 | 1.000 | 0.959 | 1.000 | 0.991 | 0.794 | 1.000 | 1.000 | 0.855 | 0.826 |
| 1945 | 0.814 | 1.000 | 0.856 | 1.000 | 0.979 | 1.000 | 0.866 | 1.000 | 0.990 | 1.000 | 0.959 | 1.000 | 0.991 | 0.795 | 1.000 | 1.000 | 0.856 | 0.826 |
| 1946 | 0.814 | 1.000 | 0.854 | 1.000 | 0.979 | 1.000 | 0.866 | 1.000 | 0.990 | 1.000 | 0.959 | 1.000 | 0.991 | 0.797 | 1.000 | 1.000 | 0.856 | 0.827 |
| 1947 | 0.814 | 1.000 | 0.854 | 1.000 | 0.979 | 1.000 | 0.866 | 1.000 | 0.990 | 1.000 | 0.959 | 1.000 | 0.991 | 0.798 | 1.000 | 1.000 | 0.856 | 0.827 |
| 1948 | 0.815 | 1.000 | 0.854 | 1.000 | 0.979 | 1.000 | 0.866 | 1.000 | 0.990 | 1.000 | 0.959 | 1.000 | 0.991 | 0.798 | 1.000 | 1.000 | 0.856 | 0.827 |
| 1949 | 0.815 | 1.000 | 0.856 | 1.000 | 0.979 | 1.000 | 0.866 | 1.000 | 0.990 | 1.000 | 0.959 | 1.000 | 0.991 | 0.798 | 1.000 | 1.000 | 0.857 | 0.827 |
| 1950 | 0.815 | 1.000 | 0.856 | 1.000 | 0.979 | 1.000 | 0.866 | 1.000 | 0.990 | 1.000 | 0.959 | 1.000 | 0.991 | 0.798 | 1.000 | 1.000 | 0.857 | 0.827 |
| 1951 | 0.814 | 1.000 | 0.858 | 1.000 | 0.979 | 1.000 | 0.867 | 1.000 | 0.990 | 1.000 | 0.959 | 1.000 | 0.991 | 0.798 | 1.000 | 1.000 | 0.857 | 0.828 |
| 1952 | 0.815 | 1.000 | 0.858 | 1.000 | 0.979 | 1.000 | 0.867 | 1.000 | 0.990 | 1.000 | 0.959 | 1.000 | 0.991 | 0.798 | 1.000 | 1.000 | 0.857 | 0.828 |
| 1953 | 0.816 | 1.000 | 0.856 | 1.000 | 0.979 | 1.000 | 0.869 | 1.000 | 0.990 | 1.000 | 0.959 | 1.000 | 0.991 | 0.798 | 1.000 | 1.000 | 0.857 | 0.828 |

| | | | | | | | | | | | | | | | | | | |
|---|---|---|---|---|---|---|---|---|---|---|---|---|---|---|---|---|---|---|
| 1954 | 0.818 | 1.000 | 0.856 | 1.000 | 0.984 | 1.000 | 0.869 | 1.000 | 0.990 | 1.000 | 0.959 | 1.000 | 0.991 | 0.798 | 1.000 | 1.000 | 0.858 | 0.829 |
| 1955 | 0.818 | 1.000 | 0.856 | 1.000 | 0.979 | 1.000 | 0.867 | 1.000 | 0.990 | 1.000 | 0.959 | 1.000 | 0.991 | 0.797 | 1.000 | 1.000 | 0.858 | 0.829 |
| 1956 | 0.818 | 1.000 | 0.856 | 1.000 | 0.984 | 1.000 | 0.867 | 1.000 | 0.990 | 1.000 | 0.959 | 1.000 | 0.991 | 0.797 | 1.000 | 1.000 | 0.858 | 0.829 |
| 1957 | 0.819 | 1.000 | 0.856 | 1.000 | 0.984 | 1.000 | 0.867 | 1.000 | 0.990 | 1.000 | 0.959 | 1.000 | 0.991 | 0.798 | 1.000 | 1.000 | 0.858 | 0.829 |
| 1958 | 0.817 | 1.000 | 0.856 | 1.000 | 0.984 | 1.000 | 0.867 | 1.000 | 0.990 | 1.000 | 0.959 | 1.000 | 0.991 | 0.798 | 1.000 | 1.000 | 0.858 | 0.829 |
| 1959 | 0.817 | 1.000 | 0.856 | 1.000 | 0.984 | 1.000 | 0.867 | 1.000 | 0.990 | 1.000 | 0.959 | 1.000 | 0.991 | 0.798 | 1.000 | 1.000 | 0.858 | 0.829 |
| 1960 | 0.818 | 1.000 | 0.854 | 1.000 | 0.984 | 1.000 | 0.867 | 1.000 | 0.990 | 1.000 | 0.959 | 1.000 | 0.991 | 0.798 | 1.000 | 1.000 | 0.858 | 0.829 |
| 1961 | 0.818 | 1.000 | 0.856 | 1.000 | 0.984 | 1.000 | 0.867 | 1.000 | 0.990 | 1.000 | 0.959 | 1.000 | 0.991 | 0.798 | 1.000 | 1.000 | 0.858 | 0.829 |
| 1962 | 0.817 | 1.000 | 0.856 | 1.000 | 0.984 | 1.000 | 0.867 | 1.000 | 0.990 | 1.000 | 0.959 | 1.000 | 0.991 | 0.798 | 1.000 | 1.000 | 0.858 | 0.829 |
| 1963 | 0.818 | 1.000 | 0.856 | 1.000 | 0.984 | 1.000 | 0.867 | 1.000 | 0.990 | 1.000 | 0.959 | 1.000 | 0.991 | 0.798 | 1.000 | 1.000 | 0.858 | 0.829 |
| 1964 | 0.818 | 1.000 | 0.856 | 1.000 | 0.984 | 1.000 | 0.867 | 1.000 | 0.990 | 1.000 | 0.959 | 1.000 | 0.991 | 0.798 | 1.000 | 1.000 | 0.858 | 0.829 |
| 1965 | 0.818 | 1.000 | 0.858 | 1.000 | 0.984 | 1.000 | 0.869 | 1.000 | 0.990 | 1.000 | 0.959 | 1.000 | 0.991 | 0.798 | 1.000 | 1.000 | 0.858 | 0.830 |
| 1966 | 0.818 | 1.000 | 0.856 | 1.000 | 0.984 | 1.000 | 0.869 | 1.000 | 0.990 | 1.000 | 0.959 | 1.000 | 0.991 | 0.798 | 1.000 | 1.000 | 0.858 | 0.830 |
| 1967 | 0.818 | 1.000 | 0.856 | 1.000 | 0.984 | 1.000 | 0.869 | 1.000 | 0.990 | 1.000 | 0.959 | 1.000 | 0.991 | 0.798 | 1.000 | 1.000 | 0.858 | 0.829 |
| 1968 | 0.818 | 1.000 | 0.856 | 1.000 | 0.984 | 1.000 | 0.869 | 1.000 | 0.990 | 1.000 | 0.959 | 1.000 | 0.991 | 0.798 | 1.000 | 1.000 | 0.858 | 0.829 |
| 1969 | 0.818 | 1.000 | 0.856 | 1.000 | 0.984 | 1.000 | 0.869 | 1.000 | 0.990 | 1.000 | 0.959 | 1.000 | 0.991 | 0.798 | 1.000 | 1.000 | 0.858 | 0.830 |
| 1970 | 0.816 | 1.000 | 0.856 | 1.000 | 0.984 | 1.000 | 0.869 | 1.000 | 0.990 | 1.000 | 0.959 | 1.000 | 0.991 | 0.799 | 1.000 | 1.000 | 0.858 | 0.829 |
| 1971 | 0.820 | 1.000 | 0.858 | 1.000 | 0.979 | 1.000 | 0.871 | 1.000 | 0.990 | 1.000 | 0.959 | 1.000 | 0.991 | 0.798 | 1.000 | 1.000 | 0.859 | 0.830 |
| 1972 | 0.819 | 1.000 | 0.860 | 1.000 | 0.979 | 1.000 | 0.869 | 1.000 | 0.990 | 1.000 | 0.959 | 1.000 | 0.991 | 0.798 | 1.000 | 1.000 | 0.859 | 0.830 |
| 1973 | 0.818 | 1.000 | 0.860 | 1.000 | 0.979 | 1.000 | 0.871 | 1.000 | 0.990 | 1.000 | 0.959 | 1.000 | 0.991 | 0.797 | 1.000 | 1.000 | 0.858 | 0.830 |
| 1974 | 0.818 | 1.000 | 0.860 | 1.000 | 0.974 | 1.000 | 0.871 | 1.000 | 0.990 | 1.000 | 0.959 | 1.000 | 0.991 | 0.798 | 1.000 | 1.000 | 0.858 | 0.830 |
| 1975 | 0.820 | 1.000 | 0.856 | 1.000 | 0.974 | 1.000 | 0.872 | 1.000 | 0.990 | 1.000 | 0.959 | 1.000 | 0.991 | 0.798 | 1.000 | 1.000 | 0.859 | 0.830 |
| 1976 | 0.819 | 1.000 | 0.856 | 1.000 | 0.974 | 1.000 | 0.871 | 1.000 | 0.990 | 1.000 | 0.959 | 1.000 | 0.991 | 0.798 | 1.000 | 1.000 | 0.858 | 0.829 |
| 1977 | 0.820 | 1.000 | 0.858 | 1.000 | 0.979 | 1.000 | 0.871 | 1.000 | 0.990 | 1.000 | 0.959 | 1.000 | 0.991 | 0.798 | 1.000 | 1.000 | 0.859 | 0.830 |
| 1978 | 0.820 | 1.000 | 0.856 | 1.000 | 0.979 | 1.000 | 0.871 | 1.000 | 0.990 | 1.000 | 0.959 | 1.000 | 0.991 | 0.798 | 1.000 | 1.000 | 0.859 | 0.830 |
| 1979 | 0.820 | 1.000 | 0.856 | 1.000 | 0.979 | 1.000 | 0.871 | 1.000 | 0.990 | 1.000 | 0.959 | 1.000 | 0.991 | 0.798 | 1.000 | 1.000 | 0.859 | 0.830 |
| 1980 | 0.820 | 1.000 | 0.856 | 1.000 | 0.979 | 1.000 | 0.872 | 1.000 | 0.990 | 1.000 | 0.957 | 1.000 | 0.991 | 0.798 | 1.000 | 1.000 | 0.859 | 0.830 |
| 1981 | 0.820 | 1.000 | 0.854 | 1.000 | 0.979 | 1.000 | 0.871 | 1.000 | 0.990 | 1.000 | 0.959 | 1.000 | 0.991 | 0.798 | 1.000 | 1.000 | 0.858 | 0.830 |
| 1982 | 0.820 | 1.000 | 0.854 | 1.000 | 0.979 | 1.000 | 0.871 | 1.000 | 0.990 | 1.000 | 0.959 | 1.000 | 0.991 | 0.798 | 1.000 | 1.000 | 0.858 | 0.830 |
| 1983 | 0.820 | 1.000 | 0.854 | 1.000 | 0.979 | 1.000 | 0.871 | 1.000 | 0.990 | 1.000 | 0.959 | 1.000 | 0.991 | 0.798 | 1.000 | 1.000 | 0.859 | 0.830 |
| 1984 | 0.820 | 1.000 | 0.854 | 1.000 | 0.979 | 1.000 | 0.871 | 1.000 | 0.990 | 1.000 | 0.959 | 1.000 | 0.991 | 0.797 | 1.000 | 1.000 | 0.858 | 0.830 |
| 1985 | 0.819 | 1.000 | 0.854 | 1.000 | 0.979 | 1.000 | 0.871 | 1.000 | 0.990 | 1.000 | 0.959 | 1.000 | 0.991 | 0.797 | 1.000 | 1.000 | 0.858 | 0.829 |
| 1986 | 0.820 | 1.000 | 0.854 | 1.000 | 0.979 | 1.000 | 0.871 | 1.000 | 0.990 | 1.000 | 0.959 | 1.000 | 0.991 | 0.795 | 1.000 | 1.000 | 0.858 | 0.829 |
| 1987 | 0.820 | 1.000 | 0.852 | 1.000 | 0.979 | 1.000 | 0.874 | 1.000 | 0.990 | 1.000 | 0.957 | 1.000 | 0.991 | 0.795 | 1.000 | 1.000 | 0.858 | 0.829 |
| 1988 | 0.822 | 1.000 | 0.852 | 1.000 | 0.979 | 1.000 | 0.875 | 1.000 | 0.990 | 1.000 | 0.959 | 1.000 | 0.991 | 0.795 | 1.000 | 1.000 | 0.859 | 0.830 |
| 1989 | 0.821 | 1.000 | 0.852 | 1.000 | 0.979 | 1.000 | 0.875 | 1.000 | 0.990 | 1.000 | 0.959 | 1.000 | 0.991 | 0.794 | 1.000 | 1.000 | 0.858 | 0.829 |
| 1990 | 0.821 | 1.000 | 0.854 | 1.000 | 0.979 | 1.000 | 0.874 | 1.000 | 0.990 | 1.000 | 0.959 | 1.000 | 0.991 | 0.794 | 1.000 | 1.000 | 0.858 | 0.829 |
| 1991 | 0.821 | 1.000 | 0.852 | 1.000 | 0.979 | 1.000 | 0.872 | 1.000 | 0.990 | 1.000 | 0.959 | 1.000 | 0.991 | 0.793 | 1.000 | 1.000 | 0.858 | 0.829 |
| 1992 | 0.820 | 1.000 | 0.854 | 1.000 | 0.979 | 1.000 | 0.874 | 1.000 | 0.990 | 1.000 | 0.959 | 1.000 | 0.991 | 0.795 | 1.000 | 1.000 | 0.858 | 0.830 |

| | | | | | | | | | | | | | | | | | | |
|---|---|---|---|---|---|---|---|---|---|---|---|---|---|---|---|---|---|---|
| 1993 | 0.822 | 1.000 | 0.854 | 1.000 | 0.979 | 1.000 | 0.874 | 1.000 | 0.990 | 1.000 | 0.959 | 1.000 | 0.991 | 0.795 | 1.000 | 1.000 | 0.859 | 0.830 |
| 1994 | 0.821 | 1.000 | 0.854 | 1.000 | 0.979 | 1.000 | 0.874 | 1.000 | 0.990 | 1.000 | 0.959 | 1.000 | 0.991 | 0.795 | 1.000 | 1.000 | 0.858 | 0.830 |
| 1995 | 0.821 | 1.000 | 0.854 | 1.000 | 0.979 | 1.000 | 0.874 | 1.000 | 0.990 | 1.000 | 0.959 | 1.000 | 0.991 | 0.795 | 1.000 | 1.000 | 0.858 | 0.830 |
| 1996 | 0.821 | 1.000 | 0.854 | 1.000 | 0.979 | 1.000 | 0.874 | 1.000 | 0.990 | 1.000 | 0.959 | 1.000 | 0.991 | 0.795 | 1.000 | 1.000 | 0.859 | 0.830 |
| 1997 | 0.823 | 1.000 | 0.856 | 1.000 | 0.979 | 1.000 | 0.875 | 1.000 | 0.990 | 1.000 | 0.957 | 1.000 | 0.991 | 0.793 | 1.000 | 1.000 | 0.859 | 0.830 |
| 1998 | 0.823 | 1.000 | 0.856 | 1.000 | 0.979 | 1.000 | 0.875 | 1.000 | 0.990 | 1.000 | 0.957 | 1.000 | 0.991 | 0.794 | 1.000 | 1.000 | 0.859 | 0.830 |
| 1999 | 0.823 | 1.000 | 0.856 | 1.000 | 0.979 | 1.000 | 0.875 | 1.000 | 0.990 | 1.000 | 0.957 | 1.000 | 0.991 | 0.793 | 1.000 | 1.000 | 0.859 | 0.830 |
| 2000 | 0.824 | 1.000 | 0.856 | 1.000 | 0.979 | 1.000 | 0.875 | 1.000 | 0.990 | 1.000 | 0.957 | 1.000 | 0.991 | 0.794 | 1.000 | 1.000 | 0.859 | 0.831 |
| 2001 | 0.822 | 1.000 | 0.856 | 1.000 | 0.979 | 1.000 | 0.875 | 1.000 | 0.990 | 1.000 | 0.957 | 1.000 | 0.991 | 0.794 | 1.000 | 1.000 | 0.859 | 0.830 |
| 2002 | 0.823 | 1.000 | 0.856 | 1.000 | 0.979 | 1.000 | 0.875 | 1.000 | 0.990 | 1.000 | 0.957 | 1.000 | 0.991 | 0.794 | 1.000 | 1.000 | 0.859 | 0.830 |
| 2003 | 0.822 | 1.000 | 0.854 | 1.000 | 0.979 | 1.000 | 0.875 | 1.000 | 0.990 | 1.000 | 0.957 | 1.000 | 0.991 | 0.795 | 1.000 | 1.000 | 0.859 | 0.830 |
| 2004 | 0.822 | 1.000 | 0.854 | 1.000 | 0.979 | 1.000 | 0.875 | 1.000 | 0.990 | 1.000 | 0.957 | 1.000 | 0.991 | 0.795 | 1.000 | 1.000 | 0.859 | 0.830 |
| 2005 | 0.822 | 1.000 | 0.856 | 1.000 | 0.979 | 1.000 | 0.877 | 1.000 | 0.990 | 1.000 | 0.957 | 1.000 | 0.991 | 0.795 | 1.000 | 1.000 | 0.859 | 0.831 |
| 2006 | 0.822 | 1.000 | 0.856 | 1.000 | 0.979 | 1.000 | 0.878 | 1.000 | 0.990 | 1.000 | 0.957 | 1.000 | 0.991 | 0.794 | 1.000 | 1.000 | 0.859 | 0.831 |
| 2007 | 0.823 | 1.000 | 0.854 | 1.000 | 0.979 | 1.000 | 0.877 | 1.000 | 0.990 | 1.000 | 0.957 | 1.000 | 0.991 | 0.794 | 1.000 | 1.000 | 0.859 | 0.830 |
| 2008 | 0.823 | 1.000 | 0.854 | 1.000 | 0.979 | 1.000 | 0.877 | 1.000 | 0.990 | 1.000 | 0.957 | 1.000 | 0.991 | 0.794 | 1.000 | 1.000 | 0.859 | 0.830 |
| 2009 | 0.822 | 1.000 | 0.852 | 1.000 | 0.979 | 1.000 | 0.880 | 1.000 | 0.990 | 1.000 | 0.957 | 1.000 | 0.991 | 0.794 | 1.000 | 1.000 | 0.859 | 0.830 |
| 2010 | 0.822 | 1.000 | 0.852 | 1.000 | 0.979 | 1.000 | 0.880 | 1.000 | 0.990 | 1.000 | 0.957 | 1.000 | 0.991 | 0.794 | 1.000 | 1.000 | 0.859 | 0.830 |
| 2011 | 0.821 | 1.000 | 0.852 | 1.000 | 0.979 | 1.000 | 0.880 | 1.000 | 0.990 | 1.000 | 0.957 | 1.000 | 0.991 | 0.795 | 1.000 | 1.000 | 0.859 | 0.830 |
| 2012 | 0.824 | 1.000 | 0.856 | 1.000 | 0.979 | 1.000 | 0.880 | 1.000 | 0.990 | 1.000 | 0.957 | 1.000 | 0.991 | 0.797 | 1.000 | 1.000 | 0.861 | 0.832 |
| 2013 | 0.824 | 1.000 | 0.856 | 1.000 | 0.979 | 1.000 | 0.880 | 1.000 | 0.990 | 1.000 | 0.957 | 1.000 | 0.991 | 0.796 | 1.000 | 1.000 | 0.860 | 0.832 |
| 2014 | 0.824 | 1.000 | 0.858 | 1.000 | 0.979 | 1.000 | 0.880 | 1.000 | 0.990 | 1.000 | 0.957 | 1.000 | 0.991 | 0.798 | 1.000 | 1.000 | 0.861 | 0.833 |
| 2015 | 0.820 | 1.000 | 0.856 | 1.000 | 0.979 | 1.000 | 0.875 | 1.000 | 0.990 | 1.000 | 0.957 | 1.000 | 1.000 | 0.798 | 1.000 | 1.000 | 0.859 | 0.831 |
| 2016 | 0.821 | 1.000 | 0.856 | 1.000 | 0.979 | 1.000 | 0.877 | 1.000 | 0.990 | 1.000 | 0.957 | 1.000 | 1.000 | 0.795 | 1.000 | 1.000 | 0.859 | 0.831 |
| 2017 | 0.821 | 1.000 | 0.856 | 1.000 | 0.979 | 1.000 | 0.875 | 1.000 | 0.990 | 1.000 | 0.957 | 1.000 | 1.000 | 0.796 | 1.000 | 1.000 | 0.859 | 0.831 |
| 2018 | 0.822 | 1.000 | 0.856 | 1.000 | 0.979 | 1.000 | 0.877 | 1.000 | 0.990 | 1.000 | 0.957 | 1.000 | 1.000 | 0.798 | 1.000 | 1.000 | 0.860 | 0.832 |
| 2019 | 0.820 | 1.000 | 0.856 | 1.000 | 0.979 | 1.000 | 0.877 | 1.000 | 0.990 | 1.000 | 0.957 | 1.000 | 1.000 | 0.797 | 1.000 | 1.000 | 0.859 | 0.831 |
| 2020 | 0.820 | 1.000 | 0.856 | 1.000 | 0.979 | 1.000 | 0.877 | 1.000 | 0.990 | 1.000 | 0.957 | 1.000 | 1.000 | 0.797 | 1.000 | 1.000 | 0.859 | 0.831 |
| 2021 | 0.821 | 1.000 | 0.858 | 1.000 | 0.979 | 1.000 | 0.875 | 1.000 | 0.990 | 1.000 | 0.957 | 1.000 | 1.000 | 0.797 | 1.000 | 1.000 | 0.860 | 0.831 |
| 2022 | 0.821 | 1.000 | 0.858 | 1.000 | 0.979 | 1.000 | 0.877 | 1.000 | 0.990 | 1.000 | 0.957 | 1.000 | 1.000 | 0.798 | 1.000 | 1.000 | 0.860 | 0.832 |
| 2023 | 0.822 | 1.000 | 0.858 | 1.000 | 0.979 | 1.000 | 0.877 | 1.000 | 0.990 | 1.000 | 0.957 | 1.000 | 1.000 | 0.797 | 1.000 | 1.000 | 0.860 | 0.832 |
| 2024 | 0.822 | 1.000 | 0.858 | 1.000 | 0.979 | 1.000 | 0.877 | 1.000 | 0.990 | 1.000 | 0.957 | 1.000 | 1.000 | 0.797 | 1.000 | 1.000 | 0.860 | 0.832 |
| 2025 | 0.824 | 1.000 | 0.858 | 1.000 | 0.979 | 1.000 | 0.877 | 1.000 | 0.990 | 1.000 | 0.959 | 1.000 | 1.000 | 0.795 | 1.000 | 1.000 | 0.861 | 0.832 |
| 2026 | 0.823 | 1.000 | 0.858 | 1.000 | 0.979 | 1.000 | 0.878 | 1.000 | 0.990 | 1.000 | 0.959 | 1.000 | 1.000 | 0.796 | 1.000 | 1.000 | 0.861 | 0.832 |
| 2027 | 0.823 | 1.000 | 0.858 | 1.000 | 0.979 | 1.000 | 0.880 | 1.000 | 0.990 | 1.000 | 0.959 | 1.000 | 1.000 | 0.797 | 1.000 | 1.000 | 0.861 | 0.833 |
| 2028 | 0.823 | 1.000 | 0.860 | 1.000 | 0.979 | 1.000 | 0.878 | 1.000 | 0.990 | 1.000 | 0.959 | 1.000 | 1.000 | 0.795 | 1.000 | 1.000 | 0.861 | 0.832 |
| 2029 | 0.822 | 1.000 | 0.858 | 1.000 | 0.979 | 1.000 | 0.878 | 1.000 | 0.990 | 1.000 | 0.959 | 1.000 | 1.000 | 0.796 | 1.000 | 1.000 | 0.860 | 0.832 |
| 2030 | 0.821 | 1.000 | 0.860 | 1.000 | 0.979 | 1.000 | 0.878 | 1.000 | 0.990 | 1.000 | 0.959 | 1.000 | 1.000 | 0.795 | 1.000 | 1.000 | 0.860 | 0.832 |
| 2031 | 0.822 | 1.000 | 0.860 | 1.000 | 0.979 | 1.000 | 0.880 | 1.000 | 0.990 | 1.000 | 0.959 | 1.000 | 1.000 | 0.795 | 1.000 | 1.000 | 0.861 | 0.832 |

| | | | | | | | | | | | | | | | | | | |
|---|---|---|---|---|---|---|---|---|---|---|---|---|---|---|---|---|---|---|
| 2032 | 0.824 | 1.000 | 0.860 | 1.000 | 0.979 | 1.000 | 0.875 | 1.000 | 0.990 | 1.000 | 0.959 | 1.000 | 1.000 | 0.797 | 1.000 | 1.000 | 0.861 | 0.833 |
| 2033 | 0.825 | 1.000 | 0.858 | 1.000 | 0.979 | 1.000 | 0.875 | 1.000 | 0.990 | 1.000 | 0.959 | 1.000 | 1.000 | 0.797 | 1.000 | 1.000 | 0.861 | 0.833 |
| 2034 | 0.823 | 1.000 | 0.858 | 1.000 | 0.979 | 1.000 | 0.878 | 1.000 | 0.990 | 1.000 | 0.959 | 1.000 | 1.000 | 0.797 | 1.000 | 1.000 | 0.861 | 0.833 |
| 2035 | 0.822 | 1.000 | 0.858 | 1.000 | 0.979 | 1.000 | 0.878 | 1.000 | 0.990 | 1.000 | 0.959 | 1.000 | 1.000 | 0.795 | 1.000 | 1.000 | 0.860 | 0.832 |
| 2036 | 0.823 | 1.000 | 0.858 | 1.000 | 0.979 | 1.000 | 0.878 | 1.000 | 0.990 | 1.000 | 0.959 | 1.000 | 1.000 | 0.796 | 1.000 | 1.000 | 0.861 | 0.832 |
| 2037 | 0.824 | 1.000 | 0.858 | 1.000 | 0.979 | 1.000 | 0.878 | 1.000 | 0.990 | 1.000 | 0.959 | 1.000 | 1.000 | 0.796 | 1.000 | 1.000 | 0.861 | 0.833 |
| 2038 | 0.823 | 1.000 | 0.858 | 1.000 | 0.979 | 1.000 | 0.878 | 1.000 | 0.990 | 1.000 | 0.959 | 1.000 | 1.000 | 0.795 | 1.000 | 1.000 | 0.860 | 0.832 |
| 2039 | 0.825 | 1.000 | 0.860 | 1.000 | 0.979 | 1.000 | 0.880 | 1.000 | 0.990 | 1.000 | 0.959 | 1.000 | 1.000 | 0.795 | 1.000 | 1.000 | 0.861 | 0.833 |
| 2040 | 0.824 | 1.000 | 0.862 | 1.000 | 0.979 | 1.000 | 0.880 | 1.000 | 0.990 | 1.000 | 0.959 | 1.000 | 1.000 | 0.798 | 1.000 | 1.000 | 0.862 | 0.834 |
| 2041 | 0.824 | 1.000 | 0.860 | 1.000 | 0.979 | 1.000 | 0.883 | 1.000 | 0.990 | 1.000 | 0.959 | 1.000 | 1.000 | 0.798 | 1.000 | 1.000 | 0.862 | 0.834 |
| 2042 | 0.824 | 1.000 | 0.860 | 1.000 | 0.979 | 1.000 | 0.883 | 1.000 | 0.990 | 1.000 | 0.959 | 1.000 | 1.000 | 0.797 | 1.000 | 1.000 | 0.862 | 0.834 |
| 2043 | 0.823 | 1.000 | 0.860 | 1.000 | 0.979 | 1.000 | 0.883 | 1.000 | 0.990 | 1.000 | 0.959 | 1.000 | 1.000 | 0.797 | 1.000 | 1.000 | 0.862 | 0.834 |
| 2044 | 0.826 | 1.000 | 0.860 | 1.000 | 0.979 | 1.000 | 0.883 | 1.000 | 0.990 | 1.000 | 0.959 | 1.000 | 1.000 | 0.797 | 1.000 | 1.000 | 0.862 | 0.835 |
| 2045 | 0.824 | 1.000 | 0.860 | 1.000 | 0.979 | 1.000 | 0.881 | 1.000 | 0.990 | 1.000 | 0.959 | 1.000 | 1.000 | 0.797 | 1.000 | 1.000 | 0.862 | 0.834 |
| 2046 | 0.822 | 1.000 | 0.862 | 1.000 | 0.979 | 1.000 | 0.881 | 1.000 | 0.990 | 1.000 | 0.959 | 1.000 | 1.000 | 0.795 | 1.000 | 1.000 | 0.861 | 0.833 |
| 2047 | 0.824 | 1.000 | 0.864 | 1.000 | 0.979 | 1.000 | 0.881 | 1.000 | 0.990 | 1.000 | 0.959 | 1.000 | 1.000 | 0.798 | 1.000 | 1.000 | 0.863 | 0.835 |
| 2048 | 0.824 | 1.000 | 0.864 | 1.000 | 0.979 | 1.000 | 0.883 | 1.000 | 0.990 | 1.000 | 0.959 | 1.000 | 1.000 | 0.799 | 1.000 | 1.000 | 0.863 | 0.835 |
| 2049 | 0.822 | 1.000 | 0.862 | 1.000 | 0.979 | 1.000 | 0.881 | 1.000 | 0.990 | 1.000 | 0.959 | 1.000 | 1.000 | 0.798 | 1.000 | 1.000 | 0.862 | 0.834 |
| 2050 | 0.824 | 1.000 | 0.862 | 1.000 | 0.979 | 1.000 | 0.883 | 1.000 | 0.990 | 1.000 | 0.959 | 1.000 | 1.000 | 0.798 | 1.000 | 1.000 | 0.862 | 0.834 |
| 2051 | 0.823 | 1.000 | 0.862 | 1.000 | 0.979 | 1.000 | 0.881 | 1.000 | 0.990 | 1.000 | 0.959 | 1.000 | 1.000 | 0.798 | 1.000 | 1.000 | 0.862 | 0.834 |
| 2052 | 0.822 | 1.000 | 0.862 | 1.000 | 0.979 | 1.000 | 0.881 | 1.000 | 0.990 | 1.000 | 0.959 | 1.000 | 1.000 | 0.798 | 1.000 | 1.000 | 0.862 | 0.834 |
| 2053 | 0.822 | 1.000 | 0.862 | 1.000 | 0.979 | 1.000 | 0.881 | 1.000 | 0.990 | 1.000 | 0.959 | 1.000 | 1.000 | 0.798 | 1.000 | 1.000 | 0.862 | 0.834 |
| 2054 | 0.822 | 1.000 | 0.862 | 1.000 | 0.979 | 1.000 | 0.881 | 1.000 | 0.990 | 1.000 | 0.959 | 1.000 | 1.000 | 0.798 | 1.000 | 1.000 | 0.862 | 0.834 |
| 2055 | 0.822 | 1.000 | 0.862 | 1.000 | 0.979 | 1.000 | 0.881 | 1.000 | 0.990 | 1.000 | 0.959 | 1.000 | 1.000 | 0.798 | 1.000 | 1.000 | 0.862 | 0.834 |
| 2056 | 0.824 | 1.000 | 0.862 | 1.000 | 0.979 | 1.000 | 0.880 | 1.000 | 0.990 | 1.000 | 0.959 | 1.000 | 1.000 | 0.798 | 1.000 | 1.000 | 0.862 | 0.834 |
| 2057 | 0.822 | 1.000 | 0.866 | 1.000 | 0.979 | 1.000 | 0.880 | 1.000 | 0.990 | 1.000 | 0.959 | 1.000 | 1.000 | 0.798 | 1.000 | 1.000 | 0.862 | 0.834 |
| 2058 | 0.822 | 1.000 | 0.866 | 1.000 | 0.979 | 1.000 | 0.880 | 1.000 | 0.990 | 1.000 | 0.959 | 1.000 | 1.000 | 0.798 | 1.000 | 1.000 | 0.862 | 0.834 |
| 2059 | 0.822 | 1.000 | 0.866 | 1.000 | 0.979 | 1.000 | 0.880 | 1.000 | 0.990 | 1.000 | 0.959 | 1.000 | 1.000 | 0.798 | 1.000 | 1.000 | 0.862 | 0.834 |
| 2060 | 0.822 | 1.000 | 0.866 | 1.000 | 0.979 | 1.000 | 0.880 | 1.000 | 0.990 | 1.000 | 0.959 | 1.000 | 1.000 | 0.798 | 1.000 | 1.000 | 0.862 | 0.834 |
| 2061 | 0.823 | 1.000 | 0.866 | 1.000 | 0.979 | 1.000 | 0.880 | 1.000 | 0.990 | 1.000 | 0.959 | 1.000 | 1.000 | 0.795 | 1.000 | 1.000 | 0.861 | 0.833 |
| 2062 | 0.822 | 1.000 | 0.866 | 1.000 | 0.979 | 1.000 | 0.880 | 1.000 | 0.990 | 1.000 | 0.959 | 1.000 | 1.000 | 0.795 | 1.000 | 1.000 | 0.861 | 0.833 |
| 2063 | 0.822 | 1.000 | 0.864 | 1.000 | 0.979 | 1.000 | 0.880 | 1.000 | 0.990 | 1.000 | 0.959 | 1.000 | 1.000 | 0.796 | 1.000 | 1.000 | 0.861 | 0.833 |
| 2064 | 0.822 | 1.000 | 0.864 | 1.000 | 0.979 | 1.000 | 0.878 | 1.000 | 0.990 | 1.000 | 0.959 | 1.000 | 1.000 | 0.796 | 1.000 | 1.000 | 0.861 | 0.833 |
| 2065 | 0.822 | 1.000 | 0.864 | 1.000 | 0.979 | 1.000 | 0.878 | 1.000 | 0.990 | 1.000 | 0.959 | 1.000 | 1.000 | 0.796 | 1.000 | 1.000 | 0.861 | 0.833 |
| 2066 | 0.822 | 1.000 | 0.862 | 1.000 | 0.979 | 1.000 | 0.878 | 1.000 | 0.990 | 1.000 | 0.959 | 1.000 | 1.000 | 0.796 | 1.000 | 1.000 | 0.861 | 0.833 |
| 2067 | 0.824 | 1.000 | 0.862 | 1.000 | 0.979 | 1.000 | 0.878 | 1.000 | 0.990 | 1.000 | 0.959 | 1.000 | 1.000 | 0.795 | 1.000 | 1.000 | 0.861 | 0.833 |
| 2068 | 0.824 | 1.000 | 0.862 | 1.000 | 0.979 | 1.000 | 0.877 | 1.000 | 0.990 | 1.000 | 0.959 | 1.000 | 1.000 | 0.795 | 1.000 | 1.000 | 0.861 | 0.833 |
| 2069 | 0.824 | 1.000 | 0.862 | 1.000 | 0.979 | 1.000 | 0.877 | 1.000 | 0.990 | 1.000 | 0.959 | 1.000 | 1.000 | 0.795 | 1.000 | 1.000 | 0.861 | 0.833 |
| 2070 | 0.822 | 1.000 | 0.862 | 1.000 | 0.979 | 1.000 | 0.877 | 1.000 | 0.990 | 1.000 | 0.959 | 1.000 | 1.000 | 0.793 | 1.000 | 1.000 | 0.860 | 0.832 |

| | | | | | | | | | | | | | | | | | | |
|---|---|---|---|---|---|---|---|---|---|---|---|---|---|---|---|---|---|---|
| 2071 | 0.822 | 1.000 | 0.858 | 1.000 | 0.979 | 1.000 | 0.881 | 1.000 | 0.990 | 1.000 | 0.959 | 1.000 | 1.000 | 0.793 | 1.000 | 1.000 | 0.860 | 0.832 |
| 2072 | 0.822 | 1.000 | 0.858 | 1.000 | 0.979 | 1.000 | 0.881 | 1.000 | 0.990 | 1.000 | 0.959 | 1.000 | 1.000 | 0.794 | 1.000 | 1.000 | 0.860 | 0.832 |
| 2073 | 0.822 | 1.000 | 0.858 | 1.000 | 0.979 | 1.000 | 0.883 | 1.000 | 0.990 | 1.000 | 0.959 | 1.000 | 1.000 | 0.794 | 1.000 | 1.000 | 0.860 | 0.832 |
| 2074 | 0.822 | 1.000 | 0.862 | 1.000 | 0.979 | 1.000 | 0.883 | 1.000 | 0.990 | 1.000 | 0.959 | 1.000 | 1.000 | 0.795 | 1.000 | 1.000 | 0.861 | 0.833 |
| 2075 | 0.822 | 1.000 | 0.862 | 1.000 | 0.979 | 1.000 | 0.883 | 1.000 | 0.990 | 1.000 | 0.959 | 1.000 | 1.000 | 0.794 | 1.000 | 1.000 | 0.861 | 0.833 |
| 2076 | 0.822 | 1.000 | 0.862 | 1.000 | 0.979 | 1.000 | 0.883 | 1.000 | 0.990 | 1.000 | 0.959 | 1.000 | 1.000 | 0.795 | 1.000 | 1.000 | 0.861 | 0.833 |
| 2077 | 0.822 | 1.000 | 0.862 | 1.000 | 0.979 | 1.000 | 0.883 | 1.000 | 0.990 | 1.000 | 0.959 | 1.000 | 1.000 | 0.795 | 1.000 | 1.000 | 0.861 | 0.833 |
| 2078 | 0.822 | 1.000 | 0.862 | 1.000 | 0.979 | 1.000 | 0.883 | 1.000 | 0.990 | 1.000 | 0.959 | 1.000 | 1.000 | 0.795 | 1.000 | 1.000 | 0.861 | 0.833 |
| 2079 | 0.822 | 1.000 | 0.862 | 1.000 | 0.979 | 1.000 | 0.883 | 1.000 | 0.990 | 1.000 | 0.959 | 1.000 | 1.000 | 0.795 | 1.000 | 1.000 | 0.861 | 0.833 |
| 2080 | 0.822 | 1.000 | 0.860 | 1.000 | 0.979 | 1.000 | 0.883 | 1.000 | 0.990 | 1.000 | 0.959 | 1.000 | 1.000 | 0.795 | 1.000 | 1.000 | 0.861 | 0.833 |
| 2081 | 0.823 | 1.000 | 0.862 | 1.000 | 0.979 | 1.000 | 0.884 | 1.000 | 0.990 | 1.000 | 0.959 | 1.000 | 1.000 | 0.793 | 1.000 | 1.000 | 0.861 | 0.833 |
| 2082 | 0.824 | 1.000 | 0.862 | 1.000 | 0.979 | 1.000 | 0.883 | 1.000 | 0.990 | 1.000 | 0.959 | 1.000 | 1.000 | 0.793 | 1.000 | 1.000 | 0.861 | 0.833 |
| 2083 | 0.823 | 1.000 | 0.862 | 1.000 | 0.979 | 1.000 | 0.884 | 1.000 | 0.990 | 1.000 | 0.959 | 1.000 | 1.000 | 0.793 | 1.000 | 1.000 | 0.861 | 0.833 |
| 2084 | 0.824 | 1.000 | 0.860 | 1.000 | 0.979 | 1.000 | 0.881 | 1.000 | 0.990 | 1.000 | 0.965 | 1.000 | 1.000 | 0.791 | 1.000 | 1.000 | 0.861 | 0.833 |
| 2085 | 0.825 | 1.000 | 0.860 | 1.000 | 0.979 | 1.000 | 0.881 | 1.000 | 0.990 | 1.000 | 0.965 | 1.000 | 1.000 | 0.791 | 1.000 | 1.000 | 0.861 | 0.833 |
| 2086 | 0.826 | 1.000 | 0.860 | 1.000 | 0.979 | 1.000 | 0.881 | 1.000 | 0.990 | 1.000 | 0.965 | 1.000 | 1.000 | 0.791 | 1.000 | 1.000 | 0.861 | 0.833 |
| 2087 | 0.826 | 1.000 | 0.860 | 1.000 | 0.979 | 1.000 | 0.881 | 1.000 | 0.990 | 1.000 | 0.965 | 1.000 | 1.000 | 0.791 | 1.000 | 1.000 | 0.861 | 0.833 |
| 2088 | 0.824 | 1.000 | 0.858 | 1.000 | 0.979 | 1.000 | 0.881 | 1.000 | 0.990 | 1.000 | 0.962 | 1.000 | 1.000 | 0.792 | 1.000 | 1.000 | 0.860 | 0.832 |
| 2089 | 0.824 | 1.000 | 0.858 | 1.000 | 0.979 | 1.000 | 0.881 | 1.000 | 0.990 | 1.000 | 0.962 | 1.000 | 1.000 | 0.790 | 1.000 | 1.000 | 0.860 | 0.832 |
| 2090 | 0.824 | 1.000 | 0.858 | 1.000 | 0.979 | 1.000 | 0.883 | 1.000 | 0.990 | 1.000 | 0.962 | 1.000 | 1.000 | 0.792 | 1.000 | 1.000 | 0.861 | 0.833 |
| 2091 | 0.824 | 1.000 | 0.858 | 1.000 | 0.979 | 1.000 | 0.883 | 1.000 | 0.990 | 1.000 | 0.962 | 1.000 | 1.000 | 0.792 | 1.000 | 1.000 | 0.861 | 0.833 |
| 2092 | 0.824 | 1.000 | 0.858 | 1.000 | 0.979 | 1.000 | 0.883 | 1.000 | 0.990 | 1.000 | 0.962 | 1.000 | 1.000 | 0.793 | 1.000 | 1.000 | 0.861 | 0.833 |
| 2093 | 0.823 | 1.000 | 0.858 | 1.000 | 0.979 | 1.000 | 0.883 | 1.000 | 0.990 | 1.000 | 0.962 | 1.000 | 1.000 | 0.792 | 1.000 | 1.000 | 0.860 | 0.832 |
| 2094 | 0.822 | 1.000 | 0.858 | 1.000 | 0.979 | 1.000 | 0.881 | 1.000 | 0.990 | 1.000 | 0.962 | 1.000 | 1.000 | 0.792 | 1.000 | 1.000 | 0.860 | 0.831 |
| 2095 | 0.822 | 1.000 | 0.858 | 1.000 | 0.979 | 1.000 | 0.881 | 1.000 | 0.990 | 1.000 | 0.965 | 1.000 | 1.000 | 0.793 | 1.000 | 1.000 | 0.860 | 0.832 |
| 2096 | 0.820 | 1.000 | 0.860 | 1.000 | 0.979 | 1.000 | 0.878 | 1.000 | 0.990 | 1.000 | 0.965 | 1.000 | 1.000 | 0.794 | 1.000 | 1.000 | 0.860 | 0.831 |
| 2097 | 0.820 | 1.000 | 0.860 | 1.000 | 0.979 | 1.000 | 0.878 | 1.000 | 0.990 | 1.000 | 0.965 | 1.000 | 1.000 | 0.794 | 1.000 | 1.000 | 0.860 | 0.831 |
| 2098 | 0.821 | 1.000 | 0.860 | 1.000 | 0.979 | 1.000 | 0.878 | 1.000 | 0.990 | 1.000 | 0.965 | 1.000 | 1.000 | 0.794 | 1.000 | 1.000 | 0.860 | 0.832 |
| 2099 | 0.821 | 1.000 | 0.860 | 1.000 | 0.979 | 1.000 | 0.878 | 1.000 | 0.990 | 1.000 | 0.965 | 1.000 | 1.000 | 0.793 | 1.000 | 1.000 | 0.860 | 0.831 |
| 2100 | 0.822 | 1.000 | 0.860 | 1.000 | 0.979 | 1.000 | 0.878 | 1.000 | 0.990 | 1.000 | 0.965 | 1.000 | 1.000 | 0.793 | 1.000 | 1.000 | 0.860 | 0.832 |
| 2101 | 0.822 | 1.000 | 0.860 | 1.000 | 0.979 | 1.000 | 0.880 | 1.000 | 0.990 | 1.000 | 0.965 | 1.000 | 1.000 | 0.794 | 1.000 | 1.000 | 0.860 | 0.832 |
| 2102 | 0.823 | 1.000 | 0.860 | 1.000 | 0.979 | 1.000 | 0.880 | 1.000 | 0.990 | 1.000 | 0.965 | 1.000 | 1.000 | 0.792 | 1.000 | 1.000 | 0.860 | 0.832 |
| 2103 | 0.822 | 1.000 | 0.860 | 1.000 | 0.979 | 1.000 | 0.880 | 1.000 | 0.990 | 1.000 | 0.965 | 1.000 | 1.000 | 0.793 | 1.000 | 1.000 | 0.860 | 0.832 |
| 2104 | 0.824 | 1.000 | 0.860 | 1.000 | 0.979 | 1.000 | 0.880 | 1.000 | 0.990 | 1.000 | 0.965 | 1.000 | 1.000 | 0.792 | 1.000 | 1.000 | 0.861 | 0.832 |
| 2105 | 0.822 | 1.000 | 0.860 | 1.000 | 0.979 | 1.000 | 0.880 | 1.000 | 0.990 | 1.000 | 0.965 | 1.000 | 1.000 | 0.792 | 1.000 | 1.000 | 0.860 | 0.832 |
| 2106 | 0.823 | 1.000 | 0.860 | 1.000 | 0.979 | 1.000 | 0.880 | 1.000 | 0.990 | 1.000 | 0.965 | 1.000 | 1.000 | 0.794 | 1.000 | 1.000 | 0.861 | 0.833 |
| 2107 | 0.824 | 1.000 | 0.860 | 1.000 | 0.979 | 1.000 | 0.878 | 1.000 | 0.990 | 1.000 | 0.965 | 1.000 | 1.000 | 0.794 | 1.000 | 1.000 | 0.861 | 0.833 |
| 2108 | 0.824 | 1.000 | 0.860 | 1.000 | 0.979 | 1.000 | 0.880 | 1.000 | 0.990 | 1.000 | 0.965 | 1.000 | 1.000 | 0.793 | 1.000 | 1.000 | 0.861 | 0.833 |
| 2109 | 0.825 | 1.000 | 0.860 | 1.000 | 0.979 | 1.000 | 0.878 | 1.000 | 0.990 | 1.000 | 0.962 | 1.000 | 1.000 | 0.793 | 1.000 | 1.000 | 0.861 | 0.833 |

| | | | | | | | | | | | | | | | | | | |
|---|---|---|---|---|---|---|---|---|---|---|---|---|---|---|---|---|---|---|
| 2110 | 0.823 | 1.000 | 0.860 | 1.000 | 0.979 | 1.000 | 0.878 | 1.000 | 0.990 | 1.000 | 0.965 | 1.000 | 1.000 | 0.793 | 1.000 | 1.000 | 0.860 | 0.832 |
| 2111 | 0.824 | 1.000 | 0.860 | 1.000 | 0.979 | 1.000 | 0.878 | 1.000 | 0.990 | 1.000 | 0.965 | 1.000 | 1.000 | 0.792 | 1.000 | 1.000 | 0.861 | 0.832 |
| 2112 | 0.824 | 1.000 | 0.862 | 1.000 | 0.979 | 1.000 | 0.878 | 1.000 | 0.990 | 1.000 | 0.965 | 1.000 | 1.000 | 0.794 | 1.000 | 1.000 | 0.861 | 0.833 |
| 2113 | 0.824 | 1.000 | 0.862 | 1.000 | 0.979 | 1.000 | 0.878 | 1.000 | 0.990 | 1.000 | 0.965 | 1.000 | 1.000 | 0.793 | 1.000 | 1.000 | 0.861 | 0.833 |
| 2114 | 0.824 | 1.000 | 0.862 | 1.000 | 0.979 | 1.000 | 0.880 | 1.000 | 0.990 | 1.000 | 0.965 | 1.000 | 1.000 | 0.795 | 1.000 | 1.000 | 0.862 | 0.834 |
| 2115 | 0.824 | 1.000 | 0.862 | 1.000 | 0.979 | 1.000 | 0.880 | 1.000 | 0.990 | 1.000 | 0.965 | 1.000 | 1.000 | 0.796 | 1.000 | 1.000 | 0.862 | 0.834 |
| 2116 | 0.824 | 1.000 | 0.862 | 1.000 | 0.979 | 1.000 | 0.880 | 1.000 | 0.990 | 1.000 | 0.965 | 1.000 | 1.000 | 0.795 | 1.000 | 1.000 | 0.862 | 0.834 |
| 2117 | 0.824 | 1.000 | 0.862 | 1.000 | 0.979 | 1.000 | 0.880 | 1.000 | 0.990 | 1.000 | 0.965 | 1.000 | 1.000 | 0.796 | 1.000 | 1.000 | 0.862 | 0.834 |
| 2118 | 0.823 | 1.000 | 0.862 | 1.000 | 0.979 | 1.000 | 0.880 | 1.000 | 0.990 | 1.000 | 0.965 | 1.000 | 1.000 | 0.796 | 1.000 | 1.000 | 0.862 | 0.834 |
| 2119 | 0.823 | 1.000 | 0.862 | 1.000 | 0.979 | 1.000 | 0.881 | 1.000 | 0.990 | 1.000 | 0.965 | 1.000 | 1.000 | 0.796 | 1.000 | 1.000 | 0.862 | 0.834 |
| 2120 | 0.824 | 1.000 | 0.862 | 1.000 | 0.979 | 1.000 | 0.880 | 1.000 | 0.990 | 1.000 | 0.962 | 1.000 | 1.000 | 0.795 | 1.000 | 1.000 | 0.862 | 0.834 |
| 2121 | 0.824 | 1.000 | 0.864 | 1.000 | 0.979 | 1.000 | 0.880 | 1.000 | 0.990 | 1.000 | 0.962 | 1.000 | 1.000 | 0.795 | 1.000 | 1.000 | 0.862 | 0.834 |
| 2122 | 0.824 | 1.000 | 0.864 | 1.000 | 0.979 | 1.000 | 0.880 | 1.000 | 0.990 | 1.000 | 0.962 | 1.000 | 1.000 | 0.795 | 1.000 | 1.000 | 0.862 | 0.834 |
| 2123 | 0.820 | 1.000 | 0.864 | 1.000 | 0.979 | 1.000 | 0.878 | 1.000 | 0.990 | 1.000 | 0.962 | 1.000 | 1.000 | 0.795 | 1.000 | 1.000 | 0.860 | 0.832 |
| 2124 | 0.822 | 1.000 | 0.862 | 1.000 | 0.979 | 1.000 | 0.878 | 1.000 | 0.990 | 1.000 | 0.962 | 1.000 | 1.000 | 0.795 | 1.000 | 1.000 | 0.861 | 0.832 |
| 2125 | 0.822 | 1.000 | 0.864 | 1.000 | 0.979 | 1.000 | 0.878 | 1.000 | 0.990 | 1.000 | 0.962 | 1.000 | 1.000 | 0.795 | 1.000 | 1.000 | 0.861 | 0.832 |
| 2126 | 0.822 | 1.000 | 0.864 | 1.000 | 0.979 | 1.000 | 0.878 | 1.000 | 0.990 | 1.000 | 0.962 | 1.000 | 1.000 | 0.795 | 1.000 | 1.000 | 0.861 | 0.833 |
| 2127 | 0.822 | 1.000 | 0.862 | 1.000 | 0.979 | 1.000 | 0.877 | 1.000 | 0.990 | 1.000 | 0.962 | 1.000 | 1.000 | 0.794 | 1.000 | 1.000 | 0.860 | 0.832 |
| 2128 | 0.821 | 1.000 | 0.862 | 1.000 | 0.979 | 1.000 | 0.877 | 1.000 | 0.990 | 1.000 | 0.962 | 1.000 | 1.000 | 0.794 | 1.000 | 1.000 | 0.860 | 0.831 |
| 2129 | 0.821 | 1.000 | 0.862 | 1.000 | 0.979 | 1.000 | 0.877 | 1.000 | 0.990 | 1.000 | 0.962 | 1.000 | 1.000 | 0.794 | 1.000 | 1.000 | 0.860 | 0.831 |
| 2130 | 0.821 | 1.000 | 0.862 | 1.000 | 0.979 | 1.000 | 0.877 | 1.000 | 0.990 | 1.000 | 0.962 | 1.000 | 1.000 | 0.795 | 1.000 | 1.000 | 0.860 | 0.832 |
| 2131 | 0.823 | 1.000 | 0.862 | 1.000 | 0.979 | 1.000 | 0.875 | 1.000 | 0.990 | 1.000 | 0.962 | 1.000 | 1.000 | 0.795 | 1.000 | 1.000 | 0.861 | 0.832 |
| 2132 | 0.823 | 1.000 | 0.862 | 1.000 | 0.979 | 1.000 | 0.875 | 1.000 | 0.990 | 1.000 | 0.962 | 1.000 | 1.000 | 0.795 | 1.000 | 1.000 | 0.861 | 0.832 |
| 2133 | 0.824 | 1.000 | 0.862 | 1.000 | 0.979 | 1.000 | 0.877 | 1.000 | 0.990 | 1.000 | 0.962 | 1.000 | 1.000 | 0.795 | 1.000 | 1.000 | 0.861 | 0.833 |
| 2134 | 0.824 | 1.000 | 0.862 | 1.000 | 0.979 | 1.000 | 0.877 | 1.000 | 0.990 | 1.000 | 0.962 | 1.000 | 1.000 | 0.795 | 1.000 | 1.000 | 0.861 | 0.833 |
| 2135 | 0.824 | 1.000 | 0.862 | 1.000 | 0.979 | 1.000 | 0.877 | 1.000 | 0.990 | 1.000 | 0.962 | 1.000 | 1.000 | 0.795 | 1.000 | 1.000 | 0.861 | 0.833 |
| 2136 | 0.824 | 1.000 | 0.862 | 1.000 | 0.979 | 1.000 | 0.877 | 1.000 | 0.990 | 1.000 | 0.962 | 1.000 | 1.000 | 0.795 | 1.000 | 1.000 | 0.861 | 0.833 |
| 2137 | 0.822 | 1.000 | 0.862 | 1.000 | 0.979 | 1.000 | 0.877 | 1.000 | 0.990 | 1.000 | 0.962 | 1.000 | 1.000 | 0.794 | 1.000 | 1.000 | 0.860 | 0.832 |
| 2138 | 0.822 | 1.000 | 0.862 | 1.000 | 0.979 | 1.000 | 0.877 | 1.000 | 0.990 | 1.000 | 0.962 | 1.000 | 1.000 | 0.794 | 1.000 | 1.000 | 0.860 | 0.832 |
| 2139 | 0.823 | 1.000 | 0.862 | 1.000 | 0.979 | 1.000 | 0.877 | 1.000 | 0.990 | 1.000 | 0.962 | 1.000 | 1.000 | 0.794 | 1.000 | 1.000 | 0.860 | 0.832 |
| 2140 | 0.823 | 1.000 | 0.862 | 1.000 | 0.979 | 1.000 | 0.875 | 1.000 | 0.990 | 1.000 | 0.962 | 1.000 | 1.000 | 0.794 | 1.000 | 1.000 | 0.860 | 0.832 |
| 2141 | 0.823 | 1.000 | 0.862 | 1.000 | 0.979 | 1.000 | 0.875 | 1.000 | 0.990 | 1.000 | 0.965 | 1.000 | 1.000 | 0.794 | 1.000 | 1.000 | 0.860 | 0.832 |
| 2142 | 0.823 | 1.000 | 0.862 | 1.000 | 0.979 | 1.000 | 0.875 | 1.000 | 0.990 | 1.000 | 0.962 | 1.000 | 1.000 | 0.794 | 1.000 | 1.000 | 0.860 | 0.832 |
| 2143 | 0.823 | 1.000 | 0.862 | 1.000 | 0.979 | 1.000 | 0.875 | 1.000 | 0.990 | 1.000 | 0.962 | 1.000 | 1.000 | 0.794 | 1.000 | 1.000 | 0.860 | 0.832 |
| 2144 | 0.824 | 1.000 | 0.862 | 1.000 | 0.979 | 1.000 | 0.875 | 1.000 | 0.990 | 1.000 | 0.962 | 1.000 | 1.000 | 0.794 | 1.000 | 1.000 | 0.860 | 0.832 |
| 2145 | 0.824 | 1.000 | 0.862 | 1.000 | 0.979 | 1.000 | 0.875 | 1.000 | 0.990 | 1.000 | 0.962 | 1.000 | 1.000 | 0.794 | 1.000 | 1.000 | 0.860 | 0.832 |
| 2146 | 0.823 | 1.000 | 0.864 | 1.000 | 0.979 | 1.000 | 0.875 | 1.000 | 0.990 | 1.000 | 0.965 | 1.000 | 1.000 | 0.794 | 1.000 | 1.000 | 0.861 | 0.832 |
| 2147 | 0.823 | 1.000 | 0.864 | 1.000 | 0.979 | 1.000 | 0.875 | 1.000 | 0.990 | 1.000 | 0.965 | 1.000 | 1.000 | 0.793 | 1.000 | 1.000 | 0.860 | 0.832 |
| 2148 | 0.823 | 1.000 | 0.864 | 1.000 | 0.979 | 1.000 | 0.875 | 1.000 | 0.990 | 1.000 | 0.965 | 1.000 | 1.000 | 0.794 | 1.000 | 1.000 | 0.861 | 0.832 |

| | | | | | | | | | | | | | | | | | | |
|---|---|---|---|---|---|---|---|---|---|---|---|---|---|---|---|---|---|---|
| 2149 | 0.823 | 1.000 | 0.864 | 1.000 | 0.979 | 1.000 | 0.875 | 1.000 | 0.990 | 1.000 | 0.965 | 1.000 | 1.000 | 0.793 | 1.000 | 1.000 | 0.860 | 0.832 |
| 2150 | 0.823 | 1.000 | 0.862 | 1.000 | 0.979 | 1.000 | 0.875 | 1.000 | 0.990 | 1.000 | 0.965 | 1.000 | 1.000 | 0.794 | 1.000 | 1.000 | 0.860 | 0.832 |
| 2151 | 0.822 | 1.000 | 0.864 | 1.000 | 0.979 | 1.000 | 0.875 | 1.000 | 0.990 | 1.000 | 0.965 | 1.000 | 1.000 | 0.793 | 1.000 | 1.000 | 0.860 | 0.832 |
| 2152 | 0.823 | 1.000 | 0.864 | 1.000 | 0.979 | 1.000 | 0.875 | 1.000 | 0.990 | 1.000 | 0.965 | 1.000 | 1.000 | 0.793 | 1.000 | 1.000 | 0.860 | 0.832 |
| 2153 | 0.823 | 1.000 | 0.864 | 1.000 | 0.979 | 1.000 | 0.878 | 1.000 | 0.990 | 1.000 | 0.965 | 1.000 | 1.000 | 0.794 | 1.000 | 1.000 | 0.861 | 0.833 |
| 2154 | 0.824 | 1.000 | 0.864 | 1.000 | 0.979 | 1.000 | 0.878 | 1.000 | 0.990 | 1.000 | 0.965 | 1.000 | 1.000 | 0.794 | 1.000 | 1.000 | 0.861 | 0.833 |
| 2155 | 0.822 | 1.000 | 0.862 | 1.000 | 0.979 | 1.000 | 0.878 | 1.000 | 0.990 | 1.000 | 0.965 | 1.000 | 1.000 | 0.793 | 1.000 | 1.000 | 0.860 | 0.832 |
| 2156 | 0.823 | 1.000 | 0.862 | 1.000 | 0.979 | 1.000 | 0.878 | 1.000 | 0.990 | 1.000 | 0.965 | 1.000 | 1.000 | 0.794 | 1.000 | 1.000 | 0.861 | 0.833 |
| 2157 | 0.823 | 1.000 | 0.862 | 1.000 | 0.979 | 1.000 | 0.878 | 1.000 | 0.990 | 1.000 | 0.962 | 1.000 | 1.000 | 0.793 | 1.000 | 1.000 | 0.860 | 0.832 |
| 2158 | 0.824 | 1.000 | 0.862 | 1.000 | 0.979 | 1.000 | 0.878 | 1.000 | 0.990 | 1.000 | 0.962 | 1.000 | 1.000 | 0.794 | 1.000 | 1.000 | 0.861 | 0.833 |
| 2159 | 0.823 | 1.000 | 0.862 | 1.000 | 0.979 | 1.000 | 0.878 | 1.000 | 0.990 | 1.000 | 0.962 | 1.000 | 1.000 | 0.794 | 1.000 | 1.000 | 0.861 | 0.832 |
| 2160 | 0.824 | 1.000 | 0.862 | 1.000 | 0.979 | 1.000 | 0.878 | 1.000 | 0.990 | 1.000 | 0.962 | 1.000 | 1.000 | 0.793 | 1.000 | 1.000 | 0.861 | 0.832 |
| 2161 | 0.824 | 1.000 | 0.862 | 1.000 | 0.979 | 1.000 | 0.878 | 1.000 | 0.990 | 1.000 | 0.962 | 1.000 | 1.000 | 0.793 | 1.000 | 1.000 | 0.861 | 0.832 |
| 2162 | 0.824 | 1.000 | 0.862 | 1.000 | 0.979 | 1.000 | 0.880 | 1.000 | 0.990 | 1.000 | 0.959 | 1.000 | 1.000 | 0.795 | 1.000 | 1.000 | 0.861 | 0.833 |
| 2163 | 0.824 | 1.000 | 0.862 | 1.000 | 0.979 | 1.000 | 0.880 | 1.000 | 0.990 | 1.000 | 0.962 | 1.000 | 1.000 | 0.795 | 1.000 | 1.000 | 0.861 | 0.833 |
| 2164 | 0.824 | 1.000 | 0.864 | 1.000 | 0.979 | 1.000 | 0.880 | 1.000 | 0.990 | 1.000 | 0.959 | 1.000 | 1.000 | 0.795 | 1.000 | 1.000 | 0.861 | 0.833 |
| 2165 | 0.823 | 1.000 | 0.862 | 1.000 | 0.979 | 1.000 | 0.880 | 1.000 | 0.990 | 1.000 | 0.959 | 1.000 | 1.000 | 0.795 | 1.000 | 1.000 | 0.861 | 0.833 |
| 2166 | 0.822 | 1.000 | 0.862 | 1.000 | 0.979 | 1.000 | 0.880 | 1.000 | 0.990 | 1.000 | 0.959 | 1.000 | 1.000 | 0.795 | 1.000 | 1.000 | 0.861 | 0.833 |
| 2167 | 0.822 | 1.000 | 0.862 | 1.000 | 0.979 | 1.000 | 0.880 | 1.000 | 0.990 | 1.000 | 0.962 | 1.000 | 1.000 | 0.795 | 1.000 | 1.000 | 0.861 | 0.833 |
| 2168 | 0.822 | 1.000 | 0.862 | 1.000 | 0.979 | 1.000 | 0.880 | 1.000 | 0.990 | 1.000 | 0.962 | 1.000 | 1.000 | 0.796 | 1.000 | 1.000 | 0.861 | 0.833 |
| 2169 | 0.821 | 1.000 | 0.864 | 1.000 | 0.979 | 1.000 | 0.880 | 1.000 | 0.990 | 1.000 | 0.959 | 1.000 | 1.000 | 0.797 | 1.000 | 1.000 | 0.861 | 0.833 |
| 2170 | 0.824 | 1.000 | 0.864 | 1.000 | 0.979 | 1.000 | 0.878 | 1.000 | 0.990 | 1.000 | 0.965 | 1.000 | 1.000 | 0.795 | 1.000 | 1.000 | 0.862 | 0.834 |
| 2171 | 0.824 | 1.000 | 0.864 | 1.000 | 0.979 | 1.000 | 0.878 | 1.000 | 0.990 | 1.000 | 0.965 | 1.000 | 1.000 | 0.795 | 1.000 | 1.000 | 0.862 | 0.834 |
| 2172 | 0.824 | 1.000 | 0.864 | 1.000 | 0.979 | 1.000 | 0.878 | 1.000 | 0.990 | 1.000 | 0.965 | 1.000 | 1.000 | 0.795 | 1.000 | 1.000 | 0.861 | 0.833 |
| 2173 | 0.824 | 1.000 | 0.864 | 1.000 | 0.979 | 1.000 | 0.880 | 1.000 | 0.990 | 1.000 | 0.965 | 1.000 | 1.000 | 0.795 | 1.000 | 1.000 | 0.862 | 0.834 |
| 2174 | 0.822 | 1.000 | 0.864 | 1.000 | 0.979 | 1.000 | 0.878 | 1.000 | 0.990 | 1.000 | 0.965 | 1.000 | 1.000 | 0.796 | 1.000 | 1.000 | 0.861 | 0.833 |
| 2175 | 0.824 | 1.000 | 0.866 | 1.000 | 0.979 | 1.000 | 0.888 | 1.000 | 0.990 | 1.000 | 0.965 | 1.000 | 1.000 | 0.796 | 1.000 | 1.000 | 0.863 | 0.836 |
| 2176 | 0.825 | 1.000 | 0.866 | 1.000 | 0.979 | 1.000 | 0.886 | 1.000 | 0.990 | 1.000 | 0.962 | 1.000 | 1.000 | 0.796 | 1.000 | 1.000 | 0.863 | 0.836 |
| 2177 | 0.825 | 1.000 | 0.866 | 1.000 | 0.979 | 1.000 | 0.886 | 1.000 | 0.990 | 1.000 | 0.962 | 1.000 | 1.000 | 0.796 | 1.000 | 1.000 | 0.863 | 0.836 |
| 2178 | 0.825 | 1.000 | 0.866 | 1.000 | 0.979 | 1.000 | 0.886 | 1.000 | 0.990 | 1.000 | 0.962 | 1.000 | 1.000 | 0.796 | 1.000 | 1.000 | 0.863 | 0.836 |
| 2179 | 0.826 | 1.000 | 0.862 | 1.000 | 0.979 | 1.000 | 0.884 | 1.000 | 0.990 | 1.000 | 0.962 | 1.000 | 1.000 | 0.794 | 1.000 | 1.000 | 0.862 | 0.835 |
| 2180 | 0.826 | 1.000 | 0.858 | 1.000 | 0.979 | 1.000 | 0.884 | 1.000 | 0.990 | 1.000 | 0.962 | 1.000 | 1.000 | 0.791 | 1.000 | 1.000 | 0.861 | 0.833 |
| 2181 | 0.826 | 1.000 | 0.858 | 1.000 | 0.979 | 1.000 | 0.884 | 1.000 | 0.990 | 1.000 | 0.962 | 1.000 | 1.000 | 0.791 | 1.000 | 1.000 | 0.861 | 0.833 |
| 2182 | 0.826 | 1.000 | 0.858 | 1.000 | 0.979 | 1.000 | 0.884 | 1.000 | 0.990 | 1.000 | 0.962 | 1.000 | 1.000 | 0.790 | 1.000 | 1.000 | 0.861 | 0.833 |
| 2183 | 0.826 | 1.000 | 0.858 | 1.000 | 0.979 | 1.000 | 0.884 | 1.000 | 0.990 | 1.000 | 0.962 | 1.000 | 1.000 | 0.790 | 1.000 | 1.000 | 0.861 | 0.833 |
| 2184 | 0.826 | 1.000 | 0.862 | 1.000 | 0.979 | 1.000 | 0.886 | 1.000 | 0.990 | 1.000 | 0.962 | 1.000 | 1.000 | 0.791 | 1.000 | 1.000 | 0.862 | 0.834 |
| 2185 | 0.826 | 1.000 | 0.862 | 1.000 | 0.979 | 1.000 | 0.886 | 1.000 | 0.990 | 1.000 | 0.962 | 1.000 | 1.000 | 0.791 | 1.000 | 1.000 | 0.862 | 0.834 |
| 2186 | 0.826 | 1.000 | 0.864 | 1.000 | 0.979 | 1.000 | 0.884 | 1.000 | 0.990 | 1.000 | 0.962 | 1.000 | 1.000 | 0.791 | 1.000 | 1.000 | 0.862 | 0.834 |
| 2187 | 0.826 | 1.000 | 0.864 | 1.000 | 0.979 | 1.000 | 0.884 | 1.000 | 0.990 | 1.000 | 0.962 | 1.000 | 1.000 | 0.791 | 1.000 | 1.000 | 0.862 | 0.834 |

| | | | | | | | | | | | | | | | | | | |
|---|---|---|---|---|---|---|---|---|---|---|---|---|---|---|---|---|---|---|
| 2188 | 0.826 | 1.000 | 0.864 | 1.000 | 0.979 | 1.000 | 0.886 | 1.000 | 0.990 | 1.000 | 0.962 | 1.000 | 1.000 | 0.791 | 1.000 | 1.000 | 0.862 | 0.834 |
| 2189 | 0.826 | 1.000 | 0.864 | 1.000 | 0.979 | 1.000 | 0.886 | 1.000 | 0.990 | 1.000 | 0.962 | 1.000 | 1.000 | 0.791 | 1.000 | 1.000 | 0.862 | 0.834 |
| 2190 | 0.826 | 1.000 | 0.864 | 1.000 | 0.979 | 1.000 | 0.886 | 1.000 | 0.990 | 1.000 | 0.962 | 1.000 | 1.000 | 0.791 | 1.000 | 1.000 | 0.862 | 0.834 |
| 2191 | 0.826 | 1.000 | 0.862 | 1.000 | 0.979 | 1.000 | 0.886 | 1.000 | 0.990 | 1.000 | 0.962 | 1.000 | 1.000 | 0.791 | 1.000 | 1.000 | 0.862 | 0.834 |
| 2192 | 0.827 | 1.000 | 0.864 | 1.000 | 0.979 | 1.000 | 0.886 | 1.000 | 0.990 | 1.000 | 0.962 | 1.000 | 1.000 | 0.791 | 1.000 | 1.000 | 0.862 | 0.835 |
| 2193 | 0.827 | 1.000 | 0.864 | 1.000 | 0.979 | 1.000 | 0.886 | 1.000 | 0.990 | 1.000 | 0.962 | 1.000 | 1.000 | 0.791 | 1.000 | 1.000 | 0.862 | 0.835 |
| 2194 | 0.826 | 1.000 | 0.864 | 1.000 | 0.979 | 1.000 | 0.886 | 1.000 | 0.990 | 1.000 | 0.959 | 1.000 | 1.000 | 0.791 | 1.000 | 1.000 | 0.862 | 0.834 |
| 2195 | 0.826 | 1.000 | 0.864 | 1.000 | 0.979 | 1.000 | 0.886 | 1.000 | 0.990 | 1.000 | 0.959 | 1.000 | 1.000 | 0.791 | 1.000 | 1.000 | 0.862 | 0.834 |
| 2196 | 0.827 | 1.000 | 0.862 | 1.000 | 0.979 | 1.000 | 0.886 | 1.000 | 0.990 | 1.000 | 0.959 | 1.000 | 1.000 | 0.792 | 1.000 | 1.000 | 0.862 | 0.834 |
| 2197 | 0.825 | 1.000 | 0.862 | 1.000 | 0.979 | 1.000 | 0.878 | 1.000 | 0.990 | 1.000 | 0.959 | 1.000 | 1.000 | 0.791 | 1.000 | 1.000 | 0.860 | 0.832 |
| 2198 | 0.825 | 1.000 | 0.862 | 1.000 | 0.979 | 1.000 | 0.878 | 1.000 | 0.990 | 1.000 | 0.959 | 1.000 | 1.000 | 0.792 | 1.000 | 1.000 | 0.861 | 0.832 |
| 2199 | 0.825 | 1.000 | 0.862 | 1.000 | 0.979 | 1.000 | 0.877 | 1.000 | 0.990 | 1.000 | 0.959 | 1.000 | 1.000 | 0.793 | 1.000 | 1.000 | 0.861 | 0.832 |
| 2200 | 0.825 | 1.000 | 0.862 | 1.000 | 0.979 | 1.000 | 0.877 | 1.000 | 0.990 | 1.000 | 0.959 | 1.000 | 1.000 | 0.794 | 1.000 | 1.000 | 0.861 | 0.833 |
| 2201 | 0.825 | 1.000 | 0.862 | 1.000 | 0.979 | 1.000 | 0.877 | 1.000 | 0.990 | 1.000 | 0.959 | 1.000 | 1.000 | 0.794 | 1.000 | 1.000 | 0.861 | 0.833 |
| 2202 | 0.825 | 1.000 | 0.862 | 1.000 | 0.979 | 1.000 | 0.877 | 1.000 | 0.990 | 1.000 | 0.962 | 1.000 | 1.000 | 0.792 | 1.000 | 1.000 | 0.861 | 0.832 |
| 2203 | 0.825 | 1.000 | 0.862 | 1.000 | 0.979 | 1.000 | 0.877 | 1.000 | 0.990 | 1.000 | 0.962 | 1.000 | 1.000 | 0.794 | 1.000 | 1.000 | 0.861 | 0.833 |
| 2204 | 0.825 | 1.000 | 0.862 | 1.000 | 0.979 | 1.000 | 0.877 | 1.000 | 0.990 | 1.000 | 0.962 | 1.000 | 1.000 | 0.793 | 1.000 | 1.000 | 0.861 | 0.833 |
| 2205 | 0.826 | 1.000 | 0.862 | 1.000 | 0.979 | 1.000 | 0.877 | 1.000 | 0.990 | 1.000 | 0.962 | 1.000 | 1.000 | 0.795 | 1.000 | 1.000 | 0.862 | 0.833 |
| 2206 | 0.826 | 1.000 | 0.862 | 1.000 | 0.979 | 1.000 | 0.877 | 1.000 | 0.990 | 1.000 | 0.962 | 1.000 | 1.000 | 0.794 | 1.000 | 1.000 | 0.861 | 0.833 |
| 2207 | 0.826 | 1.000 | 0.864 | 1.000 | 0.979 | 1.000 | 0.877 | 1.000 | 0.990 | 1.000 | 0.962 | 1.000 | 1.000 | 0.795 | 1.000 | 1.000 | 0.862 | 0.834 |
| 2208 | 0.825 | 1.000 | 0.864 | 1.000 | 0.979 | 1.000 | 0.877 | 1.000 | 0.990 | 1.000 | 0.962 | 1.000 | 1.000 | 0.794 | 1.000 | 1.000 | 0.861 | 0.833 |
| 2209 | 0.825 | 1.000 | 0.864 | 1.000 | 0.979 | 1.000 | 0.877 | 1.000 | 0.990 | 1.000 | 0.962 | 1.000 | 1.000 | 0.792 | 1.000 | 1.000 | 0.861 | 0.833 |
| 2210 | 0.826 | 1.000 | 0.864 | 1.000 | 0.979 | 1.000 | 0.877 | 1.000 | 0.990 | 1.000 | 0.962 | 1.000 | 1.000 | 0.794 | 1.000 | 1.000 | 0.861 | 0.833 |
| 2211 | 0.825 | 1.000 | 0.864 | 1.000 | 0.979 | 1.000 | 0.877 | 1.000 | 0.990 | 1.000 | 0.962 | 1.000 | 1.000 | 0.793 | 1.000 | 1.000 | 0.861 | 0.833 |
| 2212 | 0.825 | 1.000 | 0.864 | 1.000 | 0.979 | 1.000 | 0.877 | 1.000 | 0.990 | 1.000 | 0.962 | 1.000 | 1.000 | 0.793 | 1.000 | 1.000 | 0.861 | 0.833 |
| 2213 | 0.825 | 1.000 | 0.864 | 1.000 | 0.979 | 1.000 | 0.880 | 1.000 | 0.990 | 1.000 | 0.962 | 1.000 | 1.000 | 0.793 | 1.000 | 1.000 | 0.861 | 0.833 |
| 2214 | 0.825 | 1.000 | 0.866 | 1.000 | 0.979 | 1.000 | 0.880 | 1.000 | 0.990 | 1.000 | 0.962 | 1.000 | 1.000 | 0.792 | 1.000 | 1.000 | 0.861 | 0.833 |
| 2215 | 0.829 | 1.000 | 0.864 | 1.000 | 0.979 | 1.000 | 0.874 | 1.000 | 0.990 | 1.000 | 0.962 | 1.000 | 1.000 | 0.794 | 1.000 | 1.000 | 0.862 | 0.834 |
| 2216 | 0.827 | 1.000 | 0.864 | 1.000 | 0.979 | 1.000 | 0.875 | 1.000 | 0.990 | 1.000 | 0.962 | 1.000 | 1.000 | 0.793 | 1.000 | 1.000 | 0.861 | 0.833 |
| 2217 | 0.826 | 1.000 | 0.864 | 1.000 | 0.979 | 1.000 | 0.875 | 1.000 | 0.990 | 1.000 | 0.962 | 1.000 | 1.000 | 0.794 | 1.000 | 1.000 | 0.861 | 0.833 |
| 2218 | 0.825 | 1.000 | 0.864 | 1.000 | 0.979 | 1.000 | 0.875 | 1.000 | 0.990 | 1.000 | 0.965 | 1.000 | 1.000 | 0.794 | 1.000 | 1.000 | 0.861 | 0.833 |
| 2219 | 0.826 | 1.000 | 0.864 | 1.000 | 0.979 | 1.000 | 0.875 | 1.000 | 0.990 | 1.000 | 0.962 | 1.000 | 1.000 | 0.794 | 1.000 | 1.000 | 0.861 | 0.833 |
| 2220 | 0.826 | 1.000 | 0.860 | 1.000 | 0.979 | 1.000 | 0.875 | 1.000 | 0.990 | 1.000 | 0.965 | 1.000 | 1.000 | 0.794 | 1.000 | 1.000 | 0.861 | 0.833 |
| 2221 | 0.826 | 1.000 | 0.860 | 1.000 | 0.979 | 1.000 | 0.875 | 1.000 | 0.990 | 1.000 | 0.965 | 1.000 | 1.000 | 0.794 | 1.000 | 1.000 | 0.861 | 0.833 |
| 2222 | 0.827 | 1.000 | 0.860 | 1.000 | 0.979 | 1.000 | 0.875 | 1.000 | 0.990 | 1.000 | 0.965 | 1.000 | 1.000 | 0.794 | 1.000 | 1.000 | 0.861 | 0.833 |
| 2223 | 0.829 | 1.000 | 0.860 | 1.000 | 0.979 | 1.000 | 0.877 | 1.000 | 0.990 | 1.000 | 0.962 | 1.000 | 1.000 | 0.797 | 1.000 | 1.000 | 0.863 | 0.835 |
| 2224 | 0.829 | 1.000 | 0.860 | 1.000 | 0.979 | 1.000 | 0.877 | 1.000 | 0.990 | 1.000 | 0.962 | 1.000 | 1.000 | 0.797 | 1.000 | 1.000 | 0.863 | 0.835 |
| 2225 | 0.827 | 1.000 | 0.860 | 1.000 | 0.979 | 1.000 | 0.877 | 1.000 | 0.990 | 1.000 | 0.962 | 1.000 | 1.000 | 0.797 | 1.000 | 1.000 | 0.862 | 0.834 |
| 2226 | 0.828 | 1.000 | 0.860 | 1.000 | 0.979 | 1.000 | 0.877 | 1.000 | 0.990 | 1.000 | 0.962 | 1.000 | 1.000 | 0.797 | 1.000 | 1.000 | 0.862 | 0.834 |

| | | | | | | | | | | | | | | | | | | |
|---|---|---|---|---|---|---|---|---|---|---|---|---|---|---|---|---|---|---|
| 2227 | 0.827 | 1.000 | 0.860 | 1.000 | 0.979 | 1.000 | 0.877 | 1.000 | 0.990 | 1.000 | 0.962 | 1.000 | 1.000 | 0.797 | 1.000 | 1.000 | 0.862 | 0.834 |
| 2228 | 0.829 | 1.000 | 0.860 | 1.000 | 0.979 | 1.000 | 0.877 | 1.000 | 0.990 | 1.000 | 0.965 | 1.000 | 1.000 | 0.797 | 1.000 | 1.000 | 0.863 | 0.835 |
| 2229 | 0.829 | 1.000 | 0.860 | 1.000 | 0.979 | 1.000 | 0.877 | 1.000 | 0.990 | 1.000 | 0.965 | 1.000 | 1.000 | 0.797 | 1.000 | 1.000 | 0.863 | 0.835 |
| 2230 | 0.826 | 1.000 | 0.858 | 1.000 | 0.979 | 1.000 | 0.877 | 1.000 | 0.990 | 1.000 | 0.965 | 1.000 | 1.000 | 0.797 | 1.000 | 1.000 | 0.862 | 0.834 |
| 2231 | 0.826 | 1.000 | 0.860 | 1.000 | 0.979 | 1.000 | 0.878 | 1.000 | 0.990 | 1.000 | 0.962 | 1.000 | 1.000 | 0.797 | 1.000 | 1.000 | 0.862 | 0.834 |
| 2232 | 0.826 | 1.000 | 0.860 | 1.000 | 0.979 | 1.000 | 0.878 | 1.000 | 0.990 | 1.000 | 0.962 | 1.000 | 1.000 | 0.797 | 1.000 | 1.000 | 0.862 | 0.834 |
| 2233 | 0.824 | 1.000 | 0.858 | 1.000 | 0.979 | 1.000 | 0.878 | 1.000 | 0.990 | 1.000 | 0.962 | 1.000 | 1.000 | 0.796 | 1.000 | 1.000 | 0.861 | 0.833 |
| 2234 | 0.824 | 1.000 | 0.860 | 1.000 | 0.979 | 1.000 | 0.878 | 1.000 | 0.990 | 1.000 | 0.962 | 1.000 | 1.000 | 0.797 | 1.000 | 1.000 | 0.862 | 0.834 |
| 2235 | 0.824 | 1.000 | 0.858 | 1.000 | 0.979 | 1.000 | 0.878 | 1.000 | 0.990 | 1.000 | 0.962 | 1.000 | 1.000 | 0.796 | 1.000 | 1.000 | 0.861 | 0.833 |
| 2236 | 0.825 | 1.000 | 0.858 | 1.000 | 0.979 | 1.000 | 0.878 | 1.000 | 0.990 | 1.000 | 0.962 | 1.000 | 1.000 | 0.798 | 1.000 | 1.000 | 0.862 | 0.834 |
| 2237 | 0.825 | 1.000 | 0.858 | 1.000 | 0.979 | 1.000 | 0.878 | 1.000 | 0.990 | 1.000 | 0.962 | 1.000 | 1.000 | 0.798 | 1.000 | 1.000 | 0.862 | 0.834 |
| 2238 | 0.824 | 1.000 | 0.858 | 1.000 | 0.979 | 1.000 | 0.877 | 1.000 | 0.990 | 1.000 | 0.962 | 1.000 | 1.000 | 0.798 | 1.000 | 1.000 | 0.861 | 0.833 |
| 2239 | 0.827 | 1.000 | 0.858 | 1.000 | 0.979 | 1.000 | 0.877 | 1.000 | 0.990 | 1.000 | 0.962 | 1.000 | 1.000 | 0.798 | 1.000 | 1.000 | 0.862 | 0.834 |
| 2240 | 0.827 | 1.000 | 0.858 | 1.000 | 0.979 | 1.000 | 0.877 | 1.000 | 0.990 | 1.000 | 0.962 | 1.000 | 1.000 | 0.798 | 1.000 | 1.000 | 0.862 | 0.834 |
| 2241 | 0.827 | 1.000 | 0.858 | 1.000 | 0.979 | 1.000 | 0.878 | 1.000 | 0.990 | 1.000 | 0.962 | 1.000 | 1.000 | 0.798 | 1.000 | 1.000 | 0.863 | 0.835 |
| 2242 | 0.827 | 1.000 | 0.858 | 1.000 | 0.979 | 1.000 | 0.878 | 1.000 | 0.990 | 1.000 | 0.962 | 1.000 | 1.000 | 0.798 | 1.000 | 1.000 | 0.863 | 0.835 |
| 2243 | 0.826 | 1.000 | 0.858 | 1.000 | 0.979 | 1.000 | 0.877 | 1.000 | 0.990 | 1.000 | 0.962 | 1.000 | 1.000 | 0.798 | 1.000 | 1.000 | 0.862 | 0.834 |
| 2244 | 0.826 | 1.000 | 0.858 | 1.000 | 0.979 | 1.000 | 0.877 | 1.000 | 0.990 | 1.000 | 0.962 | 1.000 | 1.000 | 0.798 | 1.000 | 1.000 | 0.862 | 0.834 |
| 2245 | 0.826 | 1.000 | 0.858 | 1.000 | 0.979 | 1.000 | 0.877 | 1.000 | 0.990 | 1.000 | 0.962 | 1.000 | 1.000 | 0.798 | 1.000 | 1.000 | 0.862 | 0.834 |
| 2246 | 0.826 | 1.000 | 0.858 | 1.000 | 0.979 | 1.000 | 0.877 | 1.000 | 0.990 | 1.000 | 0.962 | 1.000 | 1.000 | 0.798 | 1.000 | 1.000 | 0.862 | 0.834 |
| 2247 | 0.826 | 1.000 | 0.858 | 1.000 | 0.979 | 1.000 | 0.875 | 1.000 | 0.990 | 1.000 | 0.962 | 1.000 | 1.000 | 0.798 | 1.000 | 1.000 | 0.862 | 0.833 |
| 2248 | 0.826 | 1.000 | 0.858 | 1.000 | 0.979 | 1.000 | 0.875 | 1.000 | 0.990 | 1.000 | 0.962 | 1.000 | 1.000 | 0.798 | 1.000 | 1.000 | 0.862 | 0.833 |
| 2249 | 0.826 | 1.000 | 0.858 | 1.000 | 0.979 | 1.000 | 0.875 | 1.000 | 0.990 | 1.000 | 0.962 | 1.000 | 1.000 | 0.796 | 1.000 | 1.000 | 0.861 | 0.833 |
| 2250 | 0.826 | 1.000 | 0.856 | 1.000 | 0.979 | 1.000 | 0.875 | 1.000 | 0.990 | 1.000 | 0.962 | 1.000 | 1.000 | 0.798 | 1.000 | 1.000 | 0.862 | 0.833 |
| 2251 | 0.826 | 1.000 | 0.856 | 1.000 | 0.979 | 1.000 | 0.875 | 1.000 | 0.990 | 1.000 | 0.962 | 1.000 | 1.000 | 0.798 | 1.000 | 1.000 | 0.862 | 0.833 |
| 2252 | 0.827 | 1.000 | 0.858 | 1.000 | 0.979 | 1.000 | 0.875 | 1.000 | 0.990 | 1.000 | 0.962 | 1.000 | 1.000 | 0.797 | 1.000 | 1.000 | 0.862 | 0.834 |
| 2253 | 0.827 | 1.000 | 0.860 | 1.000 | 0.979 | 1.000 | 0.875 | 1.000 | 0.990 | 1.000 | 0.962 | 1.000 | 1.000 | 0.796 | 1.000 | 1.000 | 0.862 | 0.834 |
| 2254 | 0.826 | 1.000 | 0.860 | 1.000 | 0.979 | 1.000 | 0.878 | 1.000 | 0.990 | 1.000 | 0.965 | 1.000 | 1.000 | 0.798 | 1.000 | 1.000 | 0.863 | 0.835 |
| 2255 | 0.826 | 1.000 | 0.860 | 1.000 | 0.979 | 1.000 | 0.878 | 1.000 | 0.990 | 1.000 | 0.965 | 1.000 | 1.000 | 0.798 | 1.000 | 1.000 | 0.863 | 0.835 |
| 2256 | 0.826 | 1.000 | 0.860 | 1.000 | 0.979 | 1.000 | 0.875 | 1.000 | 0.990 | 1.000 | 0.962 | 1.000 | 1.000 | 0.798 | 1.000 | 1.000 | 0.862 | 0.834 |
| 2257 | 0.826 | 1.000 | 0.860 | 1.000 | 0.979 | 1.000 | 0.875 | 1.000 | 0.990 | 1.000 | 0.965 | 1.000 | 1.000 | 0.798 | 1.000 | 1.000 | 0.862 | 0.834 |
| 2258 | 0.826 | 1.000 | 0.860 | 1.000 | 0.979 | 1.000 | 0.875 | 1.000 | 0.990 | 1.000 | 0.965 | 1.000 | 1.000 | 0.800 | 1.000 | 1.000 | 0.863 | 0.835 |
| 2259 | 0.825 | 1.000 | 0.860 | 1.000 | 0.979 | 1.000 | 0.878 | 1.000 | 0.990 | 1.000 | 0.965 | 1.000 | 1.000 | 0.800 | 1.000 | 1.000 | 0.863 | 0.835 |
| 2260 | 0.825 | 1.000 | 0.860 | 1.000 | 0.979 | 1.000 | 0.880 | 1.000 | 0.990 | 1.000 | 0.962 | 1.000 | 1.000 | 0.800 | 1.000 | 1.000 | 0.863 | 0.835 |
| 2261 | 0.825 | 1.000 | 0.860 | 1.000 | 0.979 | 1.000 | 0.880 | 1.000 | 0.990 | 1.000 | 0.965 | 1.000 | 1.000 | 0.801 | 1.000 | 1.000 | 0.863 | 0.835 |
| 2262 | 0.824 | 1.000 | 0.860 | 1.000 | 0.979 | 1.000 | 0.880 | 1.000 | 0.990 | 1.000 | 0.965 | 1.000 | 1.000 | 0.801 | 1.000 | 1.000 | 0.863 | 0.835 |
| 2263 | 0.824 | 1.000 | 0.860 | 1.000 | 0.979 | 1.000 | 0.880 | 1.000 | 0.990 | 1.000 | 0.965 | 1.000 | 1.000 | 0.801 | 1.000 | 1.000 | 0.863 | 0.835 |
| 2264 | 0.826 | 1.000 | 0.858 | 1.000 | 0.979 | 1.000 | 0.880 | 1.000 | 0.990 | 1.000 | 0.962 | 1.000 | 1.000 | 0.802 | 1.000 | 1.000 | 0.863 | 0.835 |
| 2265 | 0.824 | 1.000 | 0.858 | 1.000 | 0.979 | 1.000 | 0.878 | 1.000 | 0.990 | 1.000 | 0.962 | 1.000 | 1.000 | 0.802 | 1.000 | 1.000 | 0.863 | 0.835 |

| | | | | | | | | | | | | | | | | | | |
|---|---|---|---|---|---|---|---|---|---|---|---|---|---|---|---|---|---|---|
| 2266 | 0.824 | 1.000 | 0.858 | 1.000 | 0.979 | 1.000 | 0.877 | 1.000 | 0.990 | 1.000 | 0.962 | 1.000 | 1.000 | 0.802 | 1.000 | 1.000 | 0.862 | 0.834 |
| 2267 | 0.824 | 1.000 | 0.856 | 1.000 | 0.979 | 1.000 | 0.878 | 1.000 | 0.990 | 1.000 | 0.962 | 1.000 | 1.000 | 0.800 | 1.000 | 1.000 | 0.862 | 0.834 |
| 2268 | 0.824 | 1.000 | 0.856 | 1.000 | 0.979 | 1.000 | 0.878 | 1.000 | 0.990 | 1.000 | 0.962 | 1.000 | 1.000 | 0.800 | 1.000 | 1.000 | 0.862 | 0.834 |
| 2269 | 0.825 | 1.000 | 0.858 | 1.000 | 0.979 | 1.000 | 0.878 | 1.000 | 0.990 | 1.000 | 0.962 | 1.000 | 1.000 | 0.799 | 1.000 | 1.000 | 0.862 | 0.834 |
| 2270 | 0.825 | 1.000 | 0.856 | 1.000 | 0.979 | 1.000 | 0.880 | 1.000 | 0.990 | 1.000 | 0.962 | 1.000 | 1.000 | 0.800 | 1.000 | 1.000 | 0.862 | 0.834 |
| 2271 | 0.826 | 1.000 | 0.858 | 1.000 | 0.979 | 1.000 | 0.880 | 1.000 | 0.990 | 1.000 | 0.962 | 1.000 | 1.000 | 0.800 | 1.000 | 1.000 | 0.863 | 0.835 |
| 2272 | 0.826 | 1.000 | 0.858 | 1.000 | 0.979 | 1.000 | 0.880 | 1.000 | 0.990 | 1.000 | 0.962 | 1.000 | 1.000 | 0.800 | 1.000 | 1.000 | 0.863 | 0.835 |
| 2273 | 0.827 | 1.000 | 0.856 | 1.000 | 0.979 | 1.000 | 0.878 | 1.000 | 0.990 | 1.000 | 0.962 | 1.000 | 1.000 | 0.801 | 1.000 | 1.000 | 0.863 | 0.835 |
| 2274 | 0.826 | 1.000 | 0.858 | 1.000 | 0.979 | 1.000 | 0.877 | 1.000 | 0.990 | 1.000 | 0.962 | 1.000 | 1.000 | 0.802 | 1.000 | 1.000 | 0.863 | 0.835 |
| 2275 | 0.826 | 1.000 | 0.858 | 1.000 | 0.979 | 1.000 | 0.877 | 1.000 | 0.990 | 1.000 | 0.962 | 1.000 | 1.000 | 0.802 | 1.000 | 1.000 | 0.863 | 0.835 |
| 2276 | 0.825 | 1.000 | 0.858 | 1.000 | 0.979 | 1.000 | 0.877 | 1.000 | 0.990 | 1.000 | 0.962 | 1.000 | 1.000 | 0.802 | 1.000 | 1.000 | 0.863 | 0.835 |
| 2277 | 0.824 | 1.000 | 0.858 | 1.000 | 0.979 | 1.000 | 0.877 | 1.000 | 0.990 | 1.000 | 0.962 | 1.000 | 1.000 | 0.802 | 1.000 | 1.000 | 0.862 | 0.834 |
| 2278 | 0.824 | 1.000 | 0.854 | 1.000 | 0.979 | 1.000 | 0.877 | 1.000 | 0.990 | 1.000 | 0.962 | 1.000 | 1.000 | 0.803 | 1.000 | 1.000 | 0.862 | 0.834 |
| 2279 | 0.824 | 1.000 | 0.854 | 1.000 | 0.979 | 1.000 | 0.875 | 1.000 | 0.990 | 1.000 | 0.962 | 1.000 | 1.000 | 0.803 | 1.000 | 1.000 | 0.862 | 0.834 |
| 2280 | 0.823 | 1.000 | 0.854 | 1.000 | 0.979 | 1.000 | 0.875 | 1.000 | 0.990 | 1.000 | 0.962 | 1.000 | 1.000 | 0.803 | 1.000 | 1.000 | 0.862 | 0.834 |
| 2281 | 0.824 | 1.000 | 0.852 | 1.000 | 0.979 | 1.000 | 0.875 | 1.000 | 0.990 | 1.000 | 0.962 | 1.000 | 1.000 | 0.803 | 1.000 | 1.000 | 0.862 | 0.834 |
| 2282 | 0.824 | 1.000 | 0.854 | 1.000 | 0.979 | 1.000 | 0.875 | 1.000 | 0.990 | 1.000 | 0.962 | 1.000 | 1.000 | 0.803 | 1.000 | 1.000 | 0.862 | 0.834 |
| 2283 | 0.824 | 1.000 | 0.852 | 1.000 | 0.979 | 1.000 | 0.875 | 1.000 | 0.990 | 1.000 | 0.962 | 1.000 | 1.000 | 0.803 | 1.000 | 1.000 | 0.862 | 0.834 |
| 2284 | 0.824 | 1.000 | 0.852 | 1.000 | 0.979 | 1.000 | 0.875 | 1.000 | 0.990 | 1.000 | 0.962 | 1.000 | 1.000 | 0.803 | 1.000 | 1.000 | 0.862 | 0.834 |
| 2285 | 0.823 | 1.000 | 0.856 | 1.000 | 0.979 | 1.000 | 0.875 | 1.000 | 0.990 | 1.000 | 0.962 | 1.000 | 1.000 | 0.803 | 1.000 | 1.000 | 0.862 | 0.834 |
| 2286 | 0.823 | 1.000 | 0.856 | 1.000 | 0.979 | 1.000 | 0.875 | 1.000 | 0.990 | 1.000 | 0.962 | 1.000 | 1.000 | 0.802 | 1.000 | 1.000 | 0.862 | 0.834 |
| 2287 | 0.822 | 1.000 | 0.856 | 1.000 | 0.979 | 1.000 | 0.875 | 1.000 | 0.990 | 1.000 | 0.962 | 1.000 | 1.000 | 0.803 | 1.000 | 1.000 | 0.862 | 0.834 |
| 2288 | 0.822 | 1.000 | 0.856 | 1.000 | 0.979 | 1.000 | 0.875 | 1.000 | 0.990 | 1.000 | 0.962 | 1.000 | 1.000 | 0.803 | 1.000 | 1.000 | 0.862 | 0.834 |
| 2289 | 0.821 | 1.000 | 0.852 | 1.000 | 0.979 | 1.000 | 0.875 | 1.000 | 0.990 | 1.000 | 0.962 | 1.000 | 1.000 | 0.802 | 1.000 | 1.000 | 0.861 | 0.832 |
| 2290 | 0.822 | 1.000 | 0.852 | 1.000 | 0.979 | 1.000 | 0.874 | 1.000 | 0.990 | 1.000 | 0.962 | 1.000 | 1.000 | 0.803 | 1.000 | 1.000 | 0.861 | 0.833 |
| 2291 | 0.823 | 1.000 | 0.854 | 1.000 | 0.979 | 1.000 | 0.874 | 1.000 | 0.990 | 1.000 | 0.962 | 1.000 | 1.000 | 0.802 | 1.000 | 1.000 | 0.861 | 0.833 |
| 2292 | 0.829 | 1.000 | 0.852 | 1.000 | 0.979 | 1.000 | 0.875 | 1.000 | 0.990 | 1.000 | 0.962 | 1.000 | 1.000 | 0.801 | 1.000 | 1.000 | 0.863 | 0.834 |
| 2293 | 0.829 | 1.000 | 0.852 | 1.000 | 0.979 | 1.000 | 0.875 | 1.000 | 0.990 | 1.000 | 0.962 | 1.000 | 1.000 | 0.801 | 1.000 | 1.000 | 0.863 | 0.834 |
| 2294 | 0.829 | 1.000 | 0.852 | 1.000 | 0.979 | 1.000 | 0.875 | 1.000 | 0.990 | 1.000 | 0.962 | 1.000 | 1.000 | 0.801 | 1.000 | 1.000 | 0.863 | 0.834 |
| 2295 | 0.829 | 1.000 | 0.852 | 1.000 | 0.979 | 1.000 | 0.875 | 1.000 | 0.990 | 1.000 | 0.962 | 1.000 | 1.000 | 0.801 | 1.000 | 1.000 | 0.863 | 0.834 |
| 2296 | 0.827 | 1.000 | 0.854 | 1.000 | 0.979 | 1.000 | 0.874 | 1.000 | 0.990 | 1.000 | 0.962 | 1.000 | 1.000 | 0.801 | 1.000 | 1.000 | 0.862 | 0.834 |
| 2297 | 0.829 | 1.000 | 0.854 | 1.000 | 0.979 | 1.000 | 0.875 | 1.000 | 0.990 | 1.000 | 0.959 | 1.000 | 1.000 | 0.802 | 1.000 | 1.000 | 0.863 | 0.835 |
| 2298 | 0.829 | 1.000 | 0.854 | 1.000 | 0.979 | 1.000 | 0.877 | 1.000 | 0.990 | 1.000 | 0.959 | 1.000 | 1.000 | 0.800 | 1.000 | 1.000 | 0.863 | 0.835 |
| 2299 | 0.829 | 1.000 | 0.854 | 1.000 | 0.979 | 1.000 | 0.875 | 1.000 | 0.990 | 1.000 | 0.959 | 1.000 | 1.000 | 0.801 | 1.000 | 1.000 | 0.863 | 0.835 |
| 2300 | 0.829 | 1.000 | 0.854 | 1.000 | 0.979 | 1.000 | 0.875 | 1.000 | 0.990 | 1.000 | 0.959 | 1.000 | 1.000 | 0.802 | 1.000 | 1.000 | 0.863 | 0.835 |
| 2301 | 0.829 | 1.000 | 0.854 | 1.000 | 0.979 | 1.000 | 0.875 | 1.000 | 0.990 | 1.000 | 0.959 | 1.000 | 1.000 | 0.802 | 1.000 | 1.000 | 0.863 | 0.835 |
| 2302 | 0.829 | 1.000 | 0.854 | 1.000 | 0.979 | 1.000 | 0.875 | 1.000 | 0.990 | 1.000 | 0.959 | 1.000 | 1.000 | 0.802 | 1.000 | 1.000 | 0.863 | 0.835 |
| 2303 | 0.829 | 1.000 | 0.854 | 1.000 | 0.979 | 1.000 | 0.874 | 1.000 | 0.990 | 1.000 | 0.959 | 1.000 | 1.000 | 0.801 | 1.000 | 1.000 | 0.863 | 0.834 |
| 2304 | 0.826 | 1.000 | 0.854 | 1.000 | 0.979 | 1.000 | 0.874 | 1.000 | 0.990 | 1.000 | 0.959 | 1.000 | 1.000 | 0.802 | 1.000 | 1.000 | 0.862 | 0.834 |

| | | | | | | | | | | | | | | | | | | |
|---|---|---|---|---|---|---|---|---|---|---|---|---|---|---|---|---|---|---|
| 2305 | 0.826 | 1.000 | 0.854 | 1.000 | 0.979 | 1.000 | 0.874 | 1.000 | 0.990 | 1.000 | 0.959 | 1.000 | 1.000 | 0.801 | 1.000 | 1.000 | 0.862 | 0.833 |
| 2306 | 0.826 | 1.000 | 0.854 | 1.000 | 0.979 | 1.000 | 0.874 | 1.000 | 0.990 | 1.000 | 0.959 | 1.000 | 1.000 | 0.801 | 1.000 | 1.000 | 0.862 | 0.833 |
| 2307 | 0.824 | 1.000 | 0.854 | 1.000 | 0.979 | 1.000 | 0.874 | 1.000 | 0.990 | 1.000 | 0.962 | 1.000 | 1.000 | 0.800 | 1.000 | 1.000 | 0.861 | 0.833 |
| 2308 | 0.826 | 1.000 | 0.856 | 1.000 | 0.979 | 1.000 | 0.874 | 1.000 | 0.990 | 1.000 | 0.962 | 1.000 | 1.000 | 0.799 | 1.000 | 1.000 | 0.862 | 0.833 |
| 2309 | 0.825 | 1.000 | 0.856 | 1.000 | 0.979 | 1.000 | 0.874 | 1.000 | 0.990 | 1.000 | 0.962 | 1.000 | 1.000 | 0.798 | 1.000 | 1.000 | 0.861 | 0.833 |
| 2310 | 0.824 | 1.000 | 0.854 | 1.000 | 0.979 | 1.000 | 0.874 | 1.000 | 0.990 | 1.000 | 0.962 | 1.000 | 1.000 | 0.798 | 1.000 | 1.000 | 0.861 | 0.832 |
| 2311 | 0.824 | 1.000 | 0.856 | 1.000 | 0.979 | 1.000 | 0.877 | 1.000 | 0.990 | 1.000 | 0.962 | 1.000 | 1.000 | 0.798 | 1.000 | 1.000 | 0.861 | 0.833 |
| 2312 | 0.825 | 1.000 | 0.856 | 1.000 | 0.979 | 1.000 | 0.875 | 1.000 | 0.990 | 1.000 | 0.959 | 1.000 | 1.000 | 0.798 | 1.000 | 1.000 | 0.861 | 0.833 |
| 2313 | 0.825 | 1.000 | 0.856 | 1.000 | 0.979 | 1.000 | 0.874 | 1.000 | 0.990 | 1.000 | 0.959 | 1.000 | 1.000 | 0.798 | 1.000 | 1.000 | 0.861 | 0.832 |
| 2314 | 0.824 | 1.000 | 0.856 | 1.000 | 0.979 | 1.000 | 0.874 | 1.000 | 0.990 | 1.000 | 0.959 | 1.000 | 1.000 | 0.798 | 1.000 | 1.000 | 0.860 | 0.832 |
| 2315 | 0.823 | 1.000 | 0.854 | 1.000 | 0.979 | 1.000 | 0.872 | 1.000 | 0.990 | 1.000 | 0.959 | 1.000 | 1.000 | 0.798 | 1.000 | 1.000 | 0.860 | 0.831 |
| 2316 | 0.824 | 1.000 | 0.856 | 1.000 | 0.979 | 1.000 | 0.874 | 1.000 | 0.990 | 1.000 | 0.959 | 1.000 | 1.000 | 0.798 | 1.000 | 1.000 | 0.861 | 0.832 |
| 2317 | 0.824 | 1.000 | 0.852 | 1.000 | 0.979 | 1.000 | 0.874 | 1.000 | 0.990 | 1.000 | 0.959 | 1.000 | 1.000 | 0.798 | 1.000 | 1.000 | 0.860 | 0.832 |
| 2318 | 0.825 | 1.000 | 0.854 | 1.000 | 0.979 | 1.000 | 0.874 | 1.000 | 0.990 | 1.000 | 0.959 | 1.000 | 1.000 | 0.798 | 1.000 | 1.000 | 0.861 | 0.832 |
| 2319 | 0.824 | 1.000 | 0.854 | 1.000 | 0.979 | 1.000 | 0.874 | 1.000 | 0.990 | 1.000 | 0.959 | 1.000 | 1.000 | 0.798 | 1.000 | 1.000 | 0.861 | 0.832 |
| 2320 | 0.825 | 1.000 | 0.854 | 1.000 | 0.979 | 1.000 | 0.874 | 1.000 | 0.990 | 1.000 | 0.959 | 1.000 | 1.000 | 0.798 | 1.000 | 1.000 | 0.861 | 0.832 |
| 2321 | 0.825 | 1.000 | 0.854 | 1.000 | 0.979 | 1.000 | 0.874 | 1.000 | 0.990 | 1.000 | 0.959 | 1.000 | 1.000 | 0.798 | 1.000 | 1.000 | 0.861 | 0.832 |
| 2322 | 0.825 | 1.000 | 0.850 | 1.000 | 0.979 | 1.000 | 0.874 | 1.000 | 0.990 | 1.000 | 0.959 | 1.000 | 1.000 | 0.798 | 1.000 | 1.000 | 0.860 | 0.832 |
| 2323 | 0.826 | 1.000 | 0.850 | 1.000 | 0.979 | 1.000 | 0.872 | 1.000 | 0.990 | 1.000 | 0.959 | 1.000 | 1.000 | 0.798 | 1.000 | 1.000 | 0.860 | 0.832 |
| 2324 | 0.824 | 1.000 | 0.852 | 1.000 | 0.979 | 1.000 | 0.874 | 1.000 | 0.990 | 1.000 | 0.959 | 1.000 | 1.000 | 0.798 | 1.000 | 1.000 | 0.860 | 0.831 |
| 2325 | 0.825 | 1.000 | 0.854 | 1.000 | 0.979 | 1.000 | 0.875 | 1.000 | 0.990 | 1.000 | 0.959 | 1.000 | 1.000 | 0.798 | 1.000 | 1.000 | 0.861 | 0.833 |
| 2326 | 0.826 | 1.000 | 0.854 | 1.000 | 0.979 | 1.000 | 0.874 | 1.000 | 0.990 | 1.000 | 0.959 | 1.000 | 1.000 | 0.799 | 1.000 | 1.000 | 0.861 | 0.833 |
| 2327 | 0.827 | 1.000 | 0.854 | 1.000 | 0.979 | 1.000 | 0.877 | 1.000 | 0.990 | 1.000 | 0.965 | 1.000 | 1.000 | 0.801 | 1.000 | 1.000 | 0.863 | 0.835 |
| 2328 | 0.827 | 1.000 | 0.854 | 1.000 | 0.979 | 1.000 | 0.877 | 1.000 | 0.990 | 1.000 | 0.965 | 1.000 | 1.000 | 0.801 | 1.000 | 1.000 | 0.863 | 0.835 |
| 2329 | 0.827 | 1.000 | 0.854 | 1.000 | 0.979 | 1.000 | 0.877 | 1.000 | 0.990 | 1.000 | 0.965 | 1.000 | 1.000 | 0.801 | 1.000 | 1.000 | 0.863 | 0.835 |
| 2330 | 0.828 | 1.000 | 0.852 | 1.000 | 0.979 | 1.000 | 0.878 | 1.000 | 0.990 | 1.000 | 0.965 | 1.000 | 1.000 | 0.801 | 1.000 | 1.000 | 0.863 | 0.835 |
| 2331 | 0.828 | 1.000 | 0.850 | 1.000 | 0.979 | 1.000 | 0.877 | 1.000 | 0.990 | 1.000 | 0.968 | 1.000 | 1.000 | 0.801 | 1.000 | 1.000 | 0.863 | 0.835 |
| 2332 | 0.828 | 1.000 | 0.852 | 1.000 | 0.979 | 1.000 | 0.877 | 1.000 | 0.990 | 1.000 | 0.968 | 1.000 | 1.000 | 0.801 | 1.000 | 1.000 | 0.863 | 0.835 |
| 2333 | 0.828 | 1.000 | 0.852 | 1.000 | 0.979 | 1.000 | 0.877 | 1.000 | 0.990 | 1.000 | 0.968 | 1.000 | 1.000 | 0.801 | 1.000 | 1.000 | 0.863 | 0.835 |
| 2334 | 0.829 | 1.000 | 0.852 | 1.000 | 0.979 | 1.000 | 0.877 | 1.000 | 0.990 | 1.000 | 0.968 | 1.000 | 1.000 | 0.800 | 1.000 | 1.000 | 0.863 | 0.835 |
| 2335 | 0.829 | 1.000 | 0.850 | 1.000 | 0.979 | 1.000 | 0.877 | 1.000 | 0.990 | 1.000 | 0.968 | 1.000 | 1.000 | 0.800 | 1.000 | 1.000 | 0.863 | 0.835 |
| 2336 | 0.828 | 1.000 | 0.858 | 1.000 | 0.979 | 1.000 | 0.877 | 1.000 | 0.990 | 1.000 | 0.965 | 1.000 | 1.000 | 0.799 | 1.000 | 1.000 | 0.863 | 0.835 |
| 2337 | 0.829 | 1.000 | 0.856 | 1.000 | 0.979 | 1.000 | 0.877 | 1.000 | 0.990 | 1.000 | 0.965 | 1.000 | 1.000 | 0.799 | 1.000 | 1.000 | 0.863 | 0.835 |
| 2338 | 0.829 | 1.000 | 0.856 | 1.000 | 0.979 | 1.000 | 0.877 | 1.000 | 0.990 | 1.000 | 0.965 | 1.000 | 1.000 | 0.799 | 1.000 | 1.000 | 0.863 | 0.835 |
| 2339 | 0.828 | 1.000 | 0.858 | 1.000 | 0.979 | 1.000 | 0.877 | 1.000 | 0.990 | 1.000 | 0.965 | 1.000 | 1.000 | 0.800 | 1.000 | 1.000 | 0.863 | 0.835 |
| 2340 | 0.829 | 1.000 | 0.856 | 1.000 | 0.979 | 1.000 | 0.875 | 1.000 | 0.990 | 1.000 | 0.968 | 1.000 | 1.000 | 0.802 | 1.000 | 1.000 | 0.864 | 0.836 |
| 2341 | 0.829 | 1.000 | 0.854 | 1.000 | 0.979 | 1.000 | 0.875 | 1.000 | 0.990 | 1.000 | 0.968 | 1.000 | 1.000 | 0.802 | 1.000 | 1.000 | 0.864 | 0.836 |
| 2342 | 0.829 | 1.000 | 0.854 | 1.000 | 0.979 | 1.000 | 0.875 | 1.000 | 0.990 | 1.000 | 0.968 | 1.000 | 1.000 | 0.802 | 1.000 | 1.000 | 0.864 | 0.836 |
| 2343 | 0.829 | 1.000 | 0.856 | 1.000 | 0.979 | 1.000 | 0.875 | 1.000 | 0.990 | 1.000 | 0.968 | 1.000 | 1.000 | 0.802 | 1.000 | 1.000 | 0.864 | 0.836 |

| | | | | | | | | | | | | | | | | | | |
|---|---|---|---|---|---|---|---|---|---|---|---|---|---|---|---|---|---|---|
| 2344 | 0.829 | 1.000 | 0.854 | 1.000 | 0.979 | 1.000 | 0.875 | 1.000 | 0.990 | 1.000 | 0.968 | 1.000 | 1.000 | 0.802 | 1.000 | 1.000 | 0.864 | 0.836 |
| 2345 | 0.828 | 1.000 | 0.856 | 1.000 | 0.979 | 1.000 | 0.875 | 1.000 | 0.990 | 1.000 | 0.968 | 1.000 | 1.000 | 0.802 | 1.000 | 1.000 | 0.864 | 0.836 |
| 2346 | 0.826 | 1.000 | 0.856 | 1.000 | 0.979 | 1.000 | 0.875 | 1.000 | 0.990 | 1.000 | 0.968 | 1.000 | 1.000 | 0.802 | 1.000 | 1.000 | 0.863 | 0.835 |
| 2347 | 0.827 | 1.000 | 0.856 | 1.000 | 0.979 | 1.000 | 0.875 | 1.000 | 0.990 | 1.000 | 0.965 | 1.000 | 1.000 | 0.802 | 1.000 | 1.000 | 0.863 | 0.835 |
| 2348 | 0.827 | 1.000 | 0.854 | 1.000 | 0.979 | 1.000 | 0.877 | 1.000 | 0.990 | 1.000 | 0.965 | 1.000 | 1.000 | 0.804 | 1.000 | 1.000 | 0.864 | 0.836 |
| 2349 | 0.827 | 1.000 | 0.854 | 1.000 | 0.979 | 1.000 | 0.877 | 1.000 | 0.990 | 1.000 | 0.965 | 1.000 | 1.000 | 0.804 | 1.000 | 1.000 | 0.864 | 0.836 |
| 2350 | 0.829 | 1.000 | 0.856 | 1.000 | 0.979 | 1.000 | 0.877 | 1.000 | 0.990 | 1.000 | 0.965 | 1.000 | 1.000 | 0.802 | 1.000 | 1.000 | 0.864 | 0.836 |
| 2351 | 0.829 | 1.000 | 0.854 | 1.000 | 0.979 | 1.000 | 0.877 | 1.000 | 0.990 | 1.000 | 0.968 | 1.000 | 1.000 | 0.802 | 1.000 | 1.000 | 0.864 | 0.836 |
| 2352 | 0.829 | 1.000 | 0.854 | 1.000 | 0.979 | 1.000 | 0.877 | 1.000 | 0.990 | 1.000 | 0.968 | 1.000 | 1.000 | 0.802 | 1.000 | 1.000 | 0.864 | 0.836 |
| 2353 | 0.829 | 1.000 | 0.854 | 1.000 | 0.979 | 1.000 | 0.877 | 1.000 | 0.990 | 1.000 | 0.968 | 1.000 | 1.000 | 0.802 | 1.000 | 1.000 | 0.864 | 0.836 |
| 2354 | 0.829 | 1.000 | 0.854 | 1.000 | 0.979 | 1.000 | 0.877 | 1.000 | 0.990 | 1.000 | 0.968 | 1.000 | 1.000 | 0.802 | 1.000 | 1.000 | 0.864 | 0.836 |
| 2355 | 0.828 | 1.000 | 0.856 | 1.000 | 0.979 | 1.000 | 0.877 | 1.000 | 0.990 | 1.000 | 0.968 | 1.000 | 1.000 | 0.803 | 1.000 | 1.000 | 0.864 | 0.836 |
| 2356 | 0.828 | 1.000 | 0.856 | 1.000 | 0.979 | 1.000 | 0.877 | 1.000 | 0.990 | 1.000 | 0.965 | 1.000 | 1.000 | 0.803 | 1.000 | 1.000 | 0.864 | 0.836 |
| 2357 | 0.829 | 1.000 | 0.860 | 1.000 | 0.979 | 1.000 | 0.877 | 1.000 | 0.990 | 1.000 | 0.965 | 1.000 | 1.000 | 0.805 | 1.000 | 1.000 | 0.865 | 0.837 |
| 2358 | 0.829 | 1.000 | 0.860 | 1.000 | 0.979 | 1.000 | 0.877 | 1.000 | 0.990 | 1.000 | 0.965 | 1.000 | 1.000 | 0.805 | 1.000 | 1.000 | 0.865 | 0.837 |
| 2359 | 0.829 | 1.000 | 0.860 | 1.000 | 0.979 | 1.000 | 0.877 | 1.000 | 0.990 | 1.000 | 0.965 | 1.000 | 1.000 | 0.805 | 1.000 | 1.000 | 0.865 | 0.837 |
| 2360 | 0.829 | 1.000 | 0.860 | 1.000 | 0.979 | 1.000 | 0.877 | 1.000 | 0.990 | 1.000 | 0.965 | 1.000 | 1.000 | 0.805 | 1.000 | 1.000 | 0.865 | 0.837 |
| 2361 | 0.828 | 1.000 | 0.860 | 1.000 | 0.979 | 1.000 | 0.878 | 1.000 | 0.990 | 1.000 | 0.965 | 1.000 | 1.000 | 0.805 | 1.000 | 1.000 | 0.865 | 0.837 |
| 2362 | 0.829 | 1.000 | 0.860 | 1.000 | 0.979 | 1.000 | 0.877 | 1.000 | 0.990 | 1.000 | 0.965 | 1.000 | 1.000 | 0.805 | 1.000 | 1.000 | 0.865 | 0.838 |
| 2363 | 0.829 | 1.000 | 0.860 | 1.000 | 0.979 | 1.000 | 0.877 | 1.000 | 0.990 | 1.000 | 0.965 | 1.000 | 1.000 | 0.805 | 1.000 | 1.000 | 0.865 | 0.838 |
| 2364 | 0.831 | 1.000 | 0.860 | 1.000 | 0.979 | 1.000 | 0.875 | 1.000 | 0.990 | 1.000 | 0.968 | 1.000 | 1.000 | 0.806 | 1.000 | 1.000 | 0.866 | 0.838 |
| 2365 | 0.829 | 1.000 | 0.858 | 1.000 | 0.979 | 1.000 | 0.877 | 1.000 | 0.990 | 1.000 | 0.968 | 1.000 | 1.000 | 0.804 | 1.000 | 1.000 | 0.865 | 0.837 |
| 2366 | 0.828 | 1.000 | 0.858 | 1.000 | 0.979 | 1.000 | 0.877 | 1.000 | 0.990 | 1.000 | 0.968 | 1.000 | 1.000 | 0.804 | 1.000 | 1.000 | 0.864 | 0.837 |
| 2367 | 0.826 | 1.000 | 0.858 | 1.000 | 0.979 | 1.000 | 0.877 | 1.000 | 0.990 | 1.000 | 0.968 | 1.000 | 1.000 | 0.804 | 1.000 | 1.000 | 0.864 | 0.836 |
| 2368 | 0.827 | 1.000 | 0.858 | 1.000 | 0.979 | 1.000 | 0.877 | 1.000 | 0.990 | 1.000 | 0.968 | 1.000 | 1.000 | 0.804 | 1.000 | 1.000 | 0.864 | 0.836 |
| 2369 | 0.826 | 1.000 | 0.858 | 1.000 | 0.979 | 1.000 | 0.878 | 1.000 | 0.990 | 1.000 | 0.968 | 1.000 | 1.000 | 0.803 | 1.000 | 1.000 | 0.864 | 0.836 |
| 2370 | 0.826 | 1.000 | 0.856 | 1.000 | 0.979 | 1.000 | 0.878 | 1.000 | 0.990 | 1.000 | 0.968 | 1.000 | 1.000 | 0.803 | 1.000 | 1.000 | 0.864 | 0.836 |
| 2371 | 0.828 | 1.000 | 0.852 | 1.000 | 0.979 | 1.000 | 0.877 | 1.000 | 0.990 | 1.000 | 0.971 | 1.000 | 1.000 | 0.800 | 1.000 | 1.000 | 0.863 | 0.835 |
| 2372 | 0.828 | 1.000 | 0.852 | 1.000 | 0.979 | 1.000 | 0.877 | 1.000 | 0.990 | 1.000 | 0.971 | 1.000 | 1.000 | 0.799 | 1.000 | 1.000 | 0.863 | 0.835 |
| 2373 | 0.827 | 1.000 | 0.852 | 1.000 | 0.979 | 1.000 | 0.877 | 1.000 | 0.990 | 1.000 | 0.971 | 1.000 | 1.000 | 0.799 | 1.000 | 1.000 | 0.863 | 0.834 |
| 2374 | 0.829 | 1.000 | 0.852 | 1.000 | 0.979 | 1.000 | 0.877 | 1.000 | 0.990 | 1.000 | 0.971 | 1.000 | 1.000 | 0.799 | 1.000 | 1.000 | 0.863 | 0.835 |
| 2375 | 0.829 | 1.000 | 0.852 | 1.000 | 0.979 | 1.000 | 0.877 | 1.000 | 0.990 | 1.000 | 0.971 | 1.000 | 1.000 | 0.799 | 1.000 | 1.000 | 0.863 | 0.835 |
| 2376 | 0.829 | 1.000 | 0.854 | 1.000 | 0.979 | 1.000 | 0.877 | 1.000 | 0.990 | 1.000 | 0.971 | 1.000 | 1.000 | 0.799 | 1.000 | 1.000 | 0.863 | 0.835 |
| 2377 | 0.829 | 1.000 | 0.854 | 1.000 | 0.979 | 1.000 | 0.877 | 1.000 | 0.990 | 1.000 | 0.971 | 1.000 | 1.000 | 0.799 | 1.000 | 1.000 | 0.863 | 0.835 |
| 2378 | 0.829 | 1.000 | 0.854 | 1.000 | 0.979 | 1.000 | 0.877 | 1.000 | 0.990 | 1.000 | 0.971 | 1.000 | 1.000 | 0.799 | 1.000 | 1.000 | 0.863 | 0.835 |
| 2379 | 0.829 | 1.000 | 0.854 | 1.000 | 0.979 | 1.000 | 0.877 | 1.000 | 0.990 | 1.000 | 0.971 | 1.000 | 1.000 | 0.800 | 1.000 | 1.000 | 0.864 | 0.836 |
| 2380 | 0.829 | 1.000 | 0.858 | 1.000 | 0.979 | 1.000 | 0.878 | 1.000 | 0.990 | 1.000 | 0.968 | 1.000 | 1.000 | 0.801 | 1.000 | 1.000 | 0.864 | 0.836 |
| 2381 | 0.828 | 1.000 | 0.858 | 1.000 | 0.979 | 1.000 | 0.880 | 1.000 | 0.990 | 1.000 | 0.971 | 1.000 | 1.000 | 0.802 | 1.000 | 1.000 | 0.864 | 0.837 |
| 2382 | 0.827 | 1.000 | 0.858 | 1.000 | 0.979 | 1.000 | 0.881 | 1.000 | 0.990 | 1.000 | 0.968 | 1.000 | 1.000 | 0.801 | 1.000 | 1.000 | 0.864 | 0.836 |

| | | | | | | | | | | | | | | | | | | |
|---|---|---|---|---|---|---|---|---|---|---|---|---|---|---|---|---|---|---|
| 2383 | 0.828 | 1.000 | 0.858 | 1.000 | 0.979 | 1.000 | 0.881 | 1.000 | 0.990 | 1.000 | 0.968 | 1.000 | 1.000 | 0.801 | 1.000 | 1.000 | 0.864 | 0.836 |
| 2384 | 0.828 | 1.000 | 0.858 | 1.000 | 0.979 | 1.000 | 0.881 | 1.000 | 0.990 | 1.000 | 0.968 | 1.000 | 1.000 | 0.802 | 1.000 | 1.000 | 0.864 | 0.837 |
| 2385 | 0.828 | 1.000 | 0.856 | 1.000 | 0.979 | 1.000 | 0.878 | 1.000 | 0.990 | 1.000 | 0.968 | 1.000 | 1.000 | 0.801 | 1.000 | 1.000 | 0.864 | 0.836 |
| 2386 | 0.828 | 1.000 | 0.856 | 1.000 | 0.979 | 1.000 | 0.881 | 1.000 | 0.990 | 1.000 | 0.968 | 1.000 | 1.000 | 0.802 | 1.000 | 1.000 | 0.864 | 0.836 |
| 2387 | 0.829 | 1.000 | 0.854 | 1.000 | 0.979 | 1.000 | 0.880 | 1.000 | 0.990 | 1.000 | 0.968 | 1.000 | 1.000 | 0.802 | 1.000 | 1.000 | 0.864 | 0.836 |
| 2388 | 0.829 | 1.000 | 0.854 | 1.000 | 0.979 | 1.000 | 0.883 | 1.000 | 0.990 | 1.000 | 0.968 | 1.000 | 1.000 | 0.801 | 1.000 | 1.000 | 0.864 | 0.836 |
| 2389 | 0.826 | 1.000 | 0.856 | 1.000 | 0.979 | 1.000 | 0.881 | 1.000 | 0.990 | 1.000 | 0.968 | 1.000 | 1.000 | 0.802 | 1.000 | 1.000 | 0.864 | 0.836 |
| 2390 | 0.827 | 1.000 | 0.856 | 1.000 | 0.979 | 1.000 | 0.881 | 1.000 | 0.990 | 1.000 | 0.968 | 1.000 | 1.000 | 0.802 | 1.000 | 1.000 | 0.864 | 0.836 |
| 2391 | 0.827 | 1.000 | 0.858 | 1.000 | 0.979 | 1.000 | 0.881 | 1.000 | 0.990 | 1.000 | 0.968 | 1.000 | 1.000 | 0.802 | 1.000 | 1.000 | 0.864 | 0.837 |
| 2392 | 0.828 | 1.000 | 0.858 | 1.000 | 0.979 | 1.000 | 0.881 | 1.000 | 0.990 | 1.000 | 0.968 | 1.000 | 1.000 | 0.802 | 1.000 | 1.000 | 0.865 | 0.837 |
| 2393 | 0.829 | 1.000 | 0.856 | 1.000 | 0.979 | 1.000 | 0.881 | 1.000 | 0.990 | 1.000 | 0.968 | 1.000 | 1.000 | 0.802 | 1.000 | 1.000 | 0.865 | 0.837 |
| 2394 | 0.829 | 1.000 | 0.856 | 1.000 | 0.979 | 1.000 | 0.881 | 1.000 | 0.990 | 1.000 | 0.968 | 1.000 | 1.000 | 0.803 | 1.000 | 1.000 | 0.865 | 0.837 |
| 2395 | 0.829 | 1.000 | 0.858 | 1.000 | 0.979 | 1.000 | 0.881 | 1.000 | 0.990 | 1.000 | 0.968 | 1.000 | 1.000 | 0.803 | 1.000 | 1.000 | 0.865 | 0.838 |
| 2396 | 0.831 | 1.000 | 0.860 | 1.000 | 0.979 | 1.000 | 0.881 | 1.000 | 0.990 | 1.000 | 0.965 | 1.000 | 1.000 | 0.803 | 1.000 | 1.000 | 0.866 | 0.838 |
| 2397 | 0.831 | 1.000 | 0.860 | 1.000 | 0.979 | 1.000 | 0.881 | 1.000 | 0.990 | 1.000 | 0.965 | 1.000 | 1.000 | 0.803 | 1.000 | 1.000 | 0.866 | 0.838 |
| 2398 | 0.831 | 1.000 | 0.858 | 1.000 | 0.979 | 1.000 | 0.881 | 1.000 | 0.990 | 1.000 | 0.965 | 1.000 | 1.000 | 0.803 | 1.000 | 1.000 | 0.866 | 0.838 |
| 2399 | 0.831 | 1.000 | 0.856 | 1.000 | 0.979 | 1.000 | 0.881 | 1.000 | 0.990 | 1.000 | 0.965 | 1.000 | 1.000 | 0.802 | 1.000 | 1.000 | 0.865 | 0.838 |
| 2400 | 0.831 | 1.000 | 0.858 | 1.000 | 0.979 | 1.000 | 0.881 | 1.000 | 0.990 | 1.000 | 0.965 | 1.000 | 1.000 | 0.804 | 1.000 | 1.000 | 0.866 | 0.838 |
| 2401 | 0.831 | 1.000 | 0.858 | 1.000 | 0.979 | 1.000 | 0.881 | 1.000 | 0.990 | 1.000 | 0.965 | 1.000 | 1.000 | 0.803 | 1.000 | 1.000 | 0.866 | 0.838 |
| 2402 | 0.831 | 1.000 | 0.856 | 1.000 | 0.979 | 1.000 | 0.881 | 1.000 | 0.990 | 1.000 | 0.965 | 1.000 | 1.000 | 0.802 | 1.000 | 1.000 | 0.865 | 0.837 |
| 2403 | 0.831 | 1.000 | 0.856 | 1.000 | 0.979 | 1.000 | 0.881 | 1.000 | 0.990 | 1.000 | 0.965 | 1.000 | 1.000 | 0.802 | 1.000 | 1.000 | 0.865 | 0.837 |
| 2404 | 0.831 | 1.000 | 0.856 | 1.000 | 0.979 | 1.000 | 0.881 | 1.000 | 0.990 | 1.000 | 0.965 | 1.000 | 1.000 | 0.802 | 1.000 | 1.000 | 0.865 | 0.838 |
| 2405 | 0.831 | 1.000 | 0.860 | 1.000 | 0.979 | 1.000 | 0.881 | 1.000 | 0.990 | 1.000 | 0.965 | 1.000 | 1.000 | 0.802 | 1.000 | 1.000 | 0.865 | 0.838 |
| 2406 | 0.831 | 1.000 | 0.860 | 1.000 | 0.979 | 1.000 | 0.881 | 1.000 | 0.990 | 1.000 | 0.965 | 1.000 | 1.000 | 0.803 | 1.000 | 1.000 | 0.866 | 0.838 |
| 2407 | 0.831 | 1.000 | 0.858 | 1.000 | 0.979 | 1.000 | 0.881 | 1.000 | 0.990 | 1.000 | 0.962 | 1.000 | 1.000 | 0.803 | 1.000 | 1.000 | 0.865 | 0.838 |
| 2408 | 0.831 | 1.000 | 0.856 | 1.000 | 0.979 | 1.000 | 0.881 | 1.000 | 0.990 | 1.000 | 0.962 | 1.000 | 1.000 | 0.803 | 1.000 | 1.000 | 0.865 | 0.837 |
| 2409 | 0.831 | 1.000 | 0.856 | 1.000 | 0.979 | 1.000 | 0.881 | 1.000 | 0.990 | 1.000 | 0.959 | 1.000 | 1.000 | 0.803 | 1.000 | 1.000 | 0.865 | 0.837 |
| 2410 | 0.830 | 1.000 | 0.856 | 1.000 | 0.979 | 1.000 | 0.881 | 1.000 | 0.990 | 1.000 | 0.959 | 1.000 | 1.000 | 0.803 | 1.000 | 1.000 | 0.865 | 0.837 |
| 2411 | 0.831 | 1.000 | 0.858 | 1.000 | 0.979 | 1.000 | 0.881 | 1.000 | 0.990 | 1.000 | 0.959 | 1.000 | 1.000 | 0.803 | 1.000 | 1.000 | 0.865 | 0.837 |
| 2412 | 0.831 | 1.000 | 0.858 | 1.000 | 0.979 | 1.000 | 0.881 | 1.000 | 0.990 | 1.000 | 0.959 | 1.000 | 1.000 | 0.802 | 1.000 | 1.000 | 0.865 | 0.837 |
| 2413 | 0.831 | 1.000 | 0.858 | 1.000 | 0.979 | 1.000 | 0.881 | 1.000 | 0.990 | 1.000 | 0.959 | 1.000 | 1.000 | 0.802 | 1.000 | 1.000 | 0.865 | 0.837 |
| 2414 | 0.831 | 1.000 | 0.858 | 1.000 | 0.979 | 1.000 | 0.881 | 1.000 | 0.990 | 1.000 | 0.959 | 1.000 | 1.000 | 0.802 | 1.000 | 1.000 | 0.865 | 0.837 |
| 2415 | 0.830 | 1.000 | 0.858 | 1.000 | 0.979 | 1.000 | 0.881 | 1.000 | 0.990 | 1.000 | 0.959 | 1.000 | 1.000 | 0.802 | 1.000 | 1.000 | 0.865 | 0.837 |
| 2416 | 0.830 | 1.000 | 0.858 | 1.000 | 0.979 | 1.000 | 0.881 | 1.000 | 0.990 | 1.000 | 0.959 | 1.000 | 1.000 | 0.802 | 1.000 | 1.000 | 0.864 | 0.837 |
| 2417 | 0.830 | 1.000 | 0.858 | 1.000 | 0.979 | 1.000 | 0.881 | 1.000 | 0.990 | 1.000 | 0.959 | 1.000 | 1.000 | 0.802 | 1.000 | 1.000 | 0.864 | 0.837 |
| 2418 | 0.829 | 1.000 | 0.858 | 1.000 | 0.979 | 1.000 | 0.881 | 1.000 | 0.990 | 1.000 | 0.959 | 1.000 | 1.000 | 0.802 | 1.000 | 1.000 | 0.864 | 0.836 |
| 2419 | 0.829 | 1.000 | 0.858 | 1.000 | 0.979 | 1.000 | 0.881 | 1.000 | 0.990 | 1.000 | 0.959 | 1.000 | 1.000 | 0.801 | 1.000 | 1.000 | 0.864 | 0.836 |
| 2420 | 0.829 | 1.000 | 0.856 | 1.000 | 0.979 | 1.000 | 0.881 | 1.000 | 0.990 | 1.000 | 0.965 | 1.000 | 1.000 | 0.800 | 1.000 | 1.000 | 0.864 | 0.836 |
| 2421 | 0.826 | 1.000 | 0.854 | 1.000 | 0.979 | 1.000 | 0.881 | 1.000 | 0.990 | 1.000 | 0.971 | 1.000 | 1.000 | 0.800 | 1.000 | 1.000 | 0.863 | 0.835 |

| | | | | | | | | | | | | | | | | | | |
|---|---|---|---|---|---|---|---|---|---|---|---|---|---|---|---|---|---|---|
| 2422 | 0.827 | 1.000 | 0.854 | 1.000 | 0.979 | 1.000 | 0.881 | 1.000 | 0.990 | 1.000 | 0.971 | 1.000 | 1.000 | 0.800 | 1.000 | 1.000 | 0.864 | 0.836 |
| 2423 | 0.826 | 1.000 | 0.858 | 1.000 | 0.979 | 1.000 | 0.881 | 1.000 | 0.990 | 1.000 | 0.968 | 1.000 | 1.000 | 0.802 | 1.000 | 1.000 | 0.864 | 0.836 |
| 2424 | 0.827 | 1.000 | 0.856 | 1.000 | 0.979 | 1.000 | 0.880 | 1.000 | 0.990 | 1.000 | 0.968 | 1.000 | 1.000 | 0.801 | 1.000 | 1.000 | 0.864 | 0.836 |
| 2425 | 0.827 | 1.000 | 0.856 | 1.000 | 0.979 | 1.000 | 0.880 | 1.000 | 0.990 | 1.000 | 0.968 | 1.000 | 1.000 | 0.801 | 1.000 | 1.000 | 0.864 | 0.836 |
| 2426 | 0.827 | 1.000 | 0.856 | 1.000 | 0.979 | 1.000 | 0.880 | 1.000 | 0.990 | 1.000 | 0.968 | 1.000 | 1.000 | 0.801 | 1.000 | 1.000 | 0.864 | 0.836 |
| 2427 | 0.828 | 1.000 | 0.860 | 1.000 | 0.979 | 1.000 | 0.880 | 1.000 | 0.990 | 1.000 | 0.968 | 1.000 | 1.000 | 0.802 | 1.000 | 1.000 | 0.864 | 0.837 |
| 2428 | 0.828 | 1.000 | 0.860 | 1.000 | 0.979 | 1.000 | 0.880 | 1.000 | 0.990 | 1.000 | 0.968 | 1.000 | 1.000 | 0.802 | 1.000 | 1.000 | 0.864 | 0.837 |
| 2429 | 0.828 | 1.000 | 0.860 | 1.000 | 0.979 | 1.000 | 0.880 | 1.000 | 0.990 | 1.000 | 0.968 | 1.000 | 1.000 | 0.802 | 1.000 | 1.000 | 0.864 | 0.837 |
| 2430 | 0.827 | 1.000 | 0.860 | 1.000 | 0.979 | 1.000 | 0.880 | 1.000 | 0.990 | 1.000 | 0.968 | 1.000 | 1.000 | 0.802 | 1.000 | 1.000 | 0.864 | 0.836 |
| 2431 | 0.828 | 1.000 | 0.860 | 1.000 | 0.979 | 1.000 | 0.881 | 1.000 | 0.990 | 1.000 | 0.968 | 1.000 | 1.000 | 0.802 | 1.000 | 1.000 | 0.865 | 0.837 |
| 2432 | 0.828 | 1.000 | 0.860 | 1.000 | 0.979 | 1.000 | 0.881 | 1.000 | 0.990 | 1.000 | 0.968 | 1.000 | 1.000 | 0.802 | 1.000 | 1.000 | 0.865 | 0.837 |
| 2433 | 0.827 | 1.000 | 0.860 | 1.000 | 0.979 | 1.000 | 0.880 | 1.000 | 1.000 | 1.000 | 0.965 | 1.000 | 1.000 | 0.800 | 1.000 | 1.000 | 0.864 | 0.836 |
| 2434 | 0.827 | 1.000 | 0.860 | 1.000 | 0.979 | 1.000 | 0.880 | 1.000 | 1.000 | 1.000 | 0.965 | 1.000 | 1.000 | 0.801 | 1.000 | 1.000 | 0.864 | 0.836 |
| 2435 | 0.827 | 1.000 | 0.860 | 1.000 | 0.979 | 1.000 | 0.880 | 1.000 | 1.000 | 1.000 | 0.965 | 1.000 | 1.000 | 0.800 | 1.000 | 1.000 | 0.864 | 0.836 |
| 2436 | 0.827 | 1.000 | 0.860 | 1.000 | 0.979 | 1.000 | 0.880 | 1.000 | 1.000 | 1.000 | 0.965 | 1.000 | 1.000 | 0.801 | 1.000 | 1.000 | 0.864 | 0.836 |
| 2437 | 0.827 | 1.000 | 0.860 | 1.000 | 0.979 | 1.000 | 0.880 | 1.000 | 1.000 | 1.000 | 0.965 | 1.000 | 1.000 | 0.801 | 1.000 | 1.000 | 0.864 | 0.836 |
| 2438 | 0.827 | 1.000 | 0.860 | 1.000 | 0.979 | 1.000 | 0.880 | 1.000 | 1.000 | 1.000 | 0.965 | 1.000 | 1.000 | 0.801 | 1.000 | 1.000 | 0.864 | 0.836 |
| 2439 | 0.827 | 1.000 | 0.860 | 1.000 | 0.979 | 1.000 | 0.881 | 1.000 | 1.000 | 1.000 | 0.962 | 1.000 | 1.000 | 0.800 | 1.000 | 1.000 | 0.864 | 0.836 |
| 2440 | 0.827 | 1.000 | 0.860 | 1.000 | 0.979 | 1.000 | 0.881 | 1.000 | 1.000 | 1.000 | 0.962 | 1.000 | 1.000 | 0.800 | 1.000 | 1.000 | 0.864 | 0.836 |
| 2441 | 0.827 | 1.000 | 0.860 | 1.000 | 0.979 | 1.000 | 0.881 | 1.000 | 1.000 | 1.000 | 0.962 | 1.000 | 1.000 | 0.801 | 1.000 | 1.000 | 0.864 | 0.836 |
| 2442 | 0.828 | 1.000 | 0.860 | 1.000 | 0.979 | 1.000 | 0.881 | 1.000 | 1.000 | 1.000 | 0.962 | 1.000 | 1.000 | 0.800 | 1.000 | 1.000 | 0.864 | 0.836 |
| 2443 | 0.829 | 1.000 | 0.858 | 1.000 | 0.979 | 1.000 | 0.880 | 1.000 | 1.000 | 1.000 | 0.962 | 1.000 | 1.000 | 0.800 | 1.000 | 1.000 | 0.864 | 0.836 |
| 2444 | 0.829 | 1.000 | 0.858 | 1.000 | 0.979 | 1.000 | 0.880 | 1.000 | 1.000 | 1.000 | 0.962 | 1.000 | 1.000 | 0.799 | 1.000 | 1.000 | 0.864 | 0.836 |
| 2445 | 0.826 | 1.000 | 0.858 | 1.000 | 0.979 | 1.000 | 0.881 | 1.000 | 1.000 | 1.000 | 0.965 | 1.000 | 1.000 | 0.802 | 1.000 | 1.000 | 0.864 | 0.836 |
| 2446 | 0.824 | 1.000 | 0.854 | 1.000 | 0.979 | 1.000 | 0.883 | 1.000 | 1.000 | 1.000 | 0.962 | 1.000 | 1.000 | 0.801 | 1.000 | 1.000 | 0.863 | 0.835 |
| 2447 | 0.824 | 1.000 | 0.854 | 1.000 | 0.979 | 1.000 | 0.881 | 1.000 | 1.000 | 1.000 | 0.962 | 1.000 | 1.000 | 0.802 | 1.000 | 1.000 | 0.863 | 0.835 |
| 2448 | 0.825 | 1.000 | 0.852 | 1.000 | 0.979 | 1.000 | 0.878 | 1.000 | 1.000 | 1.000 | 0.962 | 1.000 | 1.000 | 0.802 | 1.000 | 1.000 | 0.862 | 0.834 |
| 2449 | 0.826 | 1.000 | 0.854 | 1.000 | 0.979 | 1.000 | 0.877 | 1.000 | 1.000 | 1.000 | 0.962 | 1.000 | 1.000 | 0.802 | 1.000 | 1.000 | 0.863 | 0.835 |
| 2450 | 0.826 | 1.000 | 0.854 | 1.000 | 0.979 | 1.000 | 0.877 | 1.000 | 1.000 | 1.000 | 0.962 | 1.000 | 1.000 | 0.802 | 1.000 | 1.000 | 0.863 | 0.835 |
| 2451 | 0.827 | 1.000 | 0.854 | 1.000 | 0.979 | 1.000 | 0.877 | 1.000 | 1.000 | 1.000 | 0.962 | 1.000 | 1.000 | 0.802 | 1.000 | 1.000 | 0.863 | 0.835 |
| 2452 | 0.826 | 1.000 | 0.854 | 1.000 | 0.979 | 1.000 | 0.878 | 1.000 | 1.000 | 1.000 | 0.962 | 1.000 | 1.000 | 0.802 | 1.000 | 1.000 | 0.863 | 0.835 |
| 2453 | 0.826 | 1.000 | 0.852 | 1.000 | 0.979 | 1.000 | 0.877 | 1.000 | 1.000 | 1.000 | 0.962 | 1.000 | 1.000 | 0.803 | 1.000 | 1.000 | 0.863 | 0.835 |
| 2454 | 0.826 | 1.000 | 0.854 | 1.000 | 0.979 | 1.000 | 0.878 | 1.000 | 1.000 | 1.000 | 0.962 | 1.000 | 1.000 | 0.802 | 1.000 | 1.000 | 0.863 | 0.835 |
| 2455 | 0.824 | 1.000 | 0.854 | 1.000 | 0.979 | 1.000 | 0.874 | 1.000 | 1.000 | 1.000 | 0.962 | 1.000 | 1.000 | 0.802 | 1.000 | 1.000 | 0.862 | 0.834 |
| 2456 | 0.825 | 1.000 | 0.852 | 1.000 | 0.979 | 1.000 | 0.875 | 1.000 | 1.000 | 1.000 | 0.962 | 1.000 | 1.000 | 0.800 | 1.000 | 1.000 | 0.862 | 0.833 |
| 2457 | 0.826 | 1.000 | 0.852 | 1.000 | 0.979 | 1.000 | 0.874 | 1.000 | 1.000 | 1.000 | 0.962 | 1.000 | 1.000 | 0.800 | 1.000 | 1.000 | 0.862 | 0.833 |
| 2458 | 0.826 | 1.000 | 0.852 | 1.000 | 0.979 | 1.000 | 0.874 | 1.000 | 1.000 | 1.000 | 0.962 | 1.000 | 1.000 | 0.799 | 1.000 | 1.000 | 0.862 | 0.833 |
| 2459 | 0.824 | 1.000 | 0.852 | 1.000 | 0.979 | 1.000 | 0.874 | 1.000 | 1.000 | 1.000 | 0.962 | 1.000 | 1.000 | 0.802 | 1.000 | 1.000 | 0.861 | 0.833 |
| 2460 | 0.823 | 1.000 | 0.852 | 1.000 | 0.979 | 1.000 | 0.872 | 1.000 | 1.000 | 1.000 | 0.962 | 1.000 | 1.000 | 0.800 | 1.000 | 1.000 | 0.861 | 0.832 |

| | | | | | | | | | | | | | | | | | | |
|---|---|---|---|---|---|---|---|---|---|---|---|---|---|---|---|---|---|---|
| 2461 | 0.822 | 1.000 | 0.852 | 1.000 | 0.979 | 1.000 | 0.872 | 1.000 | 1.000 | 1.000 | 0.962 | 1.000 | 1.000 | 0.799 | 1.000 | 1.000 | 0.860 | 0.832 |
| 2462 | 0.823 | 1.000 | 0.852 | 1.000 | 0.979 | 1.000 | 0.874 | 1.000 | 1.000 | 1.000 | 0.962 | 1.000 | 1.000 | 0.799 | 1.000 | 1.000 | 0.861 | 0.832 |
| 2463 | 0.823 | 1.000 | 0.852 | 1.000 | 0.979 | 1.000 | 0.874 | 1.000 | 1.000 | 1.000 | 0.962 | 1.000 | 1.000 | 0.799 | 1.000 | 1.000 | 0.861 | 0.832 |
| 2464 | 0.824 | 1.000 | 0.852 | 1.000 | 0.979 | 1.000 | 0.874 | 1.000 | 1.000 | 1.000 | 0.962 | 1.000 | 1.000 | 0.798 | 1.000 | 1.000 | 0.861 | 0.832 |
| 2465 | 0.824 | 1.000 | 0.852 | 1.000 | 0.979 | 1.000 | 0.875 | 1.000 | 1.000 | 1.000 | 0.962 | 1.000 | 1.000 | 0.799 | 1.000 | 1.000 | 0.861 | 0.833 |
| 2466 | 0.824 | 1.000 | 0.852 | 1.000 | 0.979 | 1.000 | 0.875 | 1.000 | 1.000 | 1.000 | 0.962 | 1.000 | 1.000 | 0.799 | 1.000 | 1.000 | 0.861 | 0.833 |
| 2467 | 0.824 | 1.000 | 0.852 | 1.000 | 0.979 | 1.000 | 0.875 | 1.000 | 1.000 | 1.000 | 0.962 | 1.000 | 1.000 | 0.798 | 1.000 | 1.000 | 0.861 | 0.832 |
| 2468 | 0.824 | 1.000 | 0.852 | 1.000 | 0.979 | 1.000 | 0.875 | 1.000 | 1.000 | 1.000 | 0.962 | 1.000 | 1.000 | 0.800 | 1.000 | 1.000 | 0.861 | 0.833 |
| 2469 | 0.824 | 1.000 | 0.852 | 1.000 | 0.979 | 1.000 | 0.877 | 1.000 | 1.000 | 1.000 | 0.962 | 1.000 | 1.000 | 0.799 | 1.000 | 1.000 | 0.861 | 0.833 |
| 2470 | 0.824 | 1.000 | 0.852 | 1.000 | 0.979 | 1.000 | 0.874 | 1.000 | 1.000 | 1.000 | 0.962 | 1.000 | 1.000 | 0.799 | 1.000 | 1.000 | 0.861 | 0.833 |
| 2471 | 0.826 | 1.000 | 0.852 | 1.000 | 0.979 | 1.000 | 0.872 | 1.000 | 1.000 | 1.000 | 0.962 | 1.000 | 1.000 | 0.799 | 1.000 | 1.000 | 0.861 | 0.833 |
| 2472 | 0.828 | 1.000 | 0.852 | 1.000 | 0.979 | 1.000 | 0.875 | 1.000 | 1.000 | 1.000 | 0.962 | 1.000 | 1.000 | 0.798 | 1.000 | 1.000 | 0.862 | 0.834 |
| 2473 | 0.827 | 1.000 | 0.852 | 1.000 | 0.979 | 1.000 | 0.875 | 1.000 | 1.000 | 1.000 | 0.962 | 1.000 | 1.000 | 0.798 | 1.000 | 1.000 | 0.862 | 0.833 |
| 2474 | 0.828 | 1.000 | 0.852 | 1.000 | 0.979 | 1.000 | 0.875 | 1.000 | 1.000 | 1.000 | 0.962 | 1.000 | 1.000 | 0.800 | 1.000 | 1.000 | 0.862 | 0.834 |
| 2475 | 0.826 | 1.000 | 0.850 | 1.000 | 0.979 | 1.000 | 0.875 | 1.000 | 1.000 | 1.000 | 0.962 | 1.000 | 1.000 | 0.799 | 1.000 | 1.000 | 0.861 | 0.833 |
| 2476 | 0.824 | 1.000 | 0.850 | 1.000 | 0.979 | 1.000 | 0.874 | 1.000 | 1.000 | 1.000 | 0.962 | 1.000 | 1.000 | 0.798 | 1.000 | 1.000 | 0.860 | 0.832 |
| 2477 | 0.822 | 1.000 | 0.850 | 1.000 | 0.979 | 1.000 | 0.878 | 1.000 | 1.000 | 1.000 | 0.968 | 1.000 | 1.000 | 0.798 | 1.000 | 1.000 | 0.861 | 0.832 |
| 2478 | 0.823 | 1.000 | 0.852 | 1.000 | 0.979 | 1.000 | 0.877 | 1.000 | 1.000 | 1.000 | 0.968 | 1.000 | 1.000 | 0.798 | 1.000 | 1.000 | 0.861 | 0.833 |
| 2479 | 0.822 | 1.000 | 0.852 | 1.000 | 0.979 | 1.000 | 0.877 | 1.000 | 1.000 | 1.000 | 0.968 | 1.000 | 1.000 | 0.798 | 1.000 | 1.000 | 0.861 | 0.833 |
| 2480 | 0.823 | 1.000 | 0.852 | 1.000 | 0.979 | 1.000 | 0.877 | 1.000 | 1.000 | 1.000 | 0.968 | 1.000 | 1.000 | 0.799 | 1.000 | 1.000 | 0.861 | 0.833 |
| 2481 | 0.824 | 1.000 | 0.852 | 1.000 | 0.979 | 1.000 | 0.875 | 1.000 | 1.000 | 1.000 | 0.968 | 1.000 | 1.000 | 0.798 | 1.000 | 1.000 | 0.861 | 0.832 |
| 2482 | 0.823 | 1.000 | 0.850 | 1.000 | 0.979 | 1.000 | 0.875 | 1.000 | 1.000 | 1.000 | 0.968 | 1.000 | 1.000 | 0.798 | 1.000 | 1.000 | 0.861 | 0.832 |
| 2483 | 0.822 | 1.000 | 0.850 | 1.000 | 0.979 | 1.000 | 0.874 | 1.000 | 1.000 | 1.000 | 0.968 | 1.000 | 1.000 | 0.798 | 1.000 | 1.000 | 0.860 | 0.832 |
| 2484 | 0.823 | 1.000 | 0.850 | 1.000 | 0.979 | 1.000 | 0.874 | 1.000 | 1.000 | 1.000 | 0.968 | 1.000 | 1.000 | 0.799 | 1.000 | 1.000 | 0.861 | 0.832 |
| 2485 | 0.825 | 1.000 | 0.854 | 1.000 | 0.979 | 1.000 | 0.875 | 1.000 | 1.000 | 1.000 | 0.971 | 1.000 | 1.000 | 0.801 | 1.000 | 1.000 | 0.863 | 0.834 |
| 2486 | 0.826 | 1.000 | 0.852 | 1.000 | 0.979 | 1.000 | 0.875 | 1.000 | 1.000 | 1.000 | 0.971 | 1.000 | 1.000 | 0.802 | 1.000 | 1.000 | 0.863 | 0.835 |
| 2487 | 0.826 | 1.000 | 0.852 | 1.000 | 0.979 | 1.000 | 0.875 | 1.000 | 1.000 | 1.000 | 0.971 | 1.000 | 1.000 | 0.802 | 1.000 | 1.000 | 0.863 | 0.835 |
| 2488 | 0.825 | 1.000 | 0.852 | 1.000 | 0.979 | 1.000 | 0.877 | 1.000 | 1.000 | 1.000 | 0.971 | 1.000 | 1.000 | 0.800 | 1.000 | 1.000 | 0.862 | 0.834 |
| 2489 | 0.825 | 1.000 | 0.852 | 1.000 | 0.979 | 1.000 | 0.877 | 1.000 | 1.000 | 1.000 | 0.971 | 1.000 | 1.000 | 0.800 | 1.000 | 1.000 | 0.862 | 0.834 |
| 2490 | 0.826 | 1.000 | 0.850 | 1.000 | 0.979 | 1.000 | 0.875 | 1.000 | 1.000 | 1.000 | 0.971 | 1.000 | 1.000 | 0.802 | 1.000 | 1.000 | 0.863 | 0.834 |
| 2491 | 0.825 | 1.000 | 0.850 | 1.000 | 0.979 | 1.000 | 0.875 | 1.000 | 1.000 | 1.000 | 0.971 | 1.000 | 1.000 | 0.802 | 1.000 | 1.000 | 0.862 | 0.834 |
| 2492 | 0.825 | 1.000 | 0.850 | 1.000 | 0.979 | 1.000 | 0.872 | 1.000 | 1.000 | 1.000 | 0.971 | 1.000 | 1.000 | 0.802 | 1.000 | 1.000 | 0.862 | 0.834 |
| 2493 | 0.826 | 1.000 | 0.850 | 1.000 | 0.979 | 1.000 | 0.871 | 1.000 | 1.000 | 1.000 | 0.971 | 1.000 | 1.000 | 0.800 | 1.000 | 1.000 | 0.862 | 0.833 |
| 2494 | 0.829 | 1.000 | 0.850 | 1.000 | 0.979 | 1.000 | 0.877 | 1.000 | 1.000 | 1.000 | 0.971 | 1.000 | 1.000 | 0.798 | 1.000 | 1.000 | 0.863 | 0.835 |
| 2495 | 0.831 | 1.000 | 0.850 | 1.000 | 0.979 | 1.000 | 0.875 | 1.000 | 1.000 | 1.000 | 0.971 | 1.000 | 1.000 | 0.798 | 1.000 | 1.000 | 0.863 | 0.835 |
| 2496 | 0.831 | 1.000 | 0.850 | 1.000 | 0.979 | 1.000 | 0.875 | 1.000 | 1.000 | 1.000 | 0.971 | 1.000 | 1.000 | 0.799 | 1.000 | 1.000 | 0.864 | 0.835 |
| 2497 | 0.831 | 1.000 | 0.850 | 1.000 | 0.979 | 1.000 | 0.877 | 1.000 | 1.000 | 1.000 | 0.971 | 1.000 | 1.000 | 0.800 | 1.000 | 1.000 | 0.864 | 0.836 |
| 2498 | 0.831 | 1.000 | 0.850 | 1.000 | 0.979 | 1.000 | 0.877 | 1.000 | 1.000 | 1.000 | 0.971 | 1.000 | 1.000 | 0.800 | 1.000 | 1.000 | 0.864 | 0.836 |
| 2499 | 0.831 | 1.000 | 0.850 | 1.000 | 0.979 | 1.000 | 0.875 | 1.000 | 1.000 | 1.000 | 0.971 | 1.000 | 1.000 | 0.800 | 1.000 | 1.000 | 0.864 | 0.836 |

| | | | | | | | | | | | | | | | | | | |
|---|---|---|---|---|---|---|---|---|---|---|---|---|---|---|---|---|---|---|
| 2500 | 0.831 | 1.000 | 0.850 | 1.000 | 0.979 | 1.000 | 0.875 | 1.000 | 1.000 | 1.000 | 0.971 | 1.000 | 1.000 | 0.800 | 1.000 | 1.000 | 0.864 | 0.836 |
| 2501 | 0.832 | 1.000 | 0.850 | 1.000 | 0.979 | 1.000 | 0.877 | 1.000 | 1.000 | 1.000 | 0.971 | 1.000 | 1.000 | 0.799 | 1.000 | 1.000 | 0.864 | 0.836 |
| 2502 | 0.832 | 1.000 | 0.850 | 1.000 | 0.979 | 1.000 | 0.877 | 1.000 | 1.000 | 1.000 | 0.971 | 1.000 | 1.000 | 0.800 | 1.000 | 1.000 | 0.864 | 0.836 |
| 2503 | 0.832 | 1.000 | 0.850 | 1.000 | 0.979 | 1.000 | 0.877 | 1.000 | 1.000 | 1.000 | 0.971 | 1.000 | 1.000 | 0.799 | 1.000 | 1.000 | 0.864 | 0.836 |
| 2504 | 0.833 | 1.000 | 0.850 | 1.000 | 0.979 | 1.000 | 0.877 | 1.000 | 1.000 | 1.000 | 0.971 | 1.000 | 1.000 | 0.801 | 1.000 | 1.000 | 0.865 | 0.837 |
| 2505 | 0.833 | 1.000 | 0.850 | 1.000 | 0.979 | 1.000 | 0.878 | 1.000 | 1.000 | 1.000 | 0.971 | 1.000 | 1.000 | 0.801 | 1.000 | 1.000 | 0.865 | 0.837 |
| 2506 | 0.833 | 1.000 | 0.850 | 1.000 | 0.979 | 1.000 | 0.878 | 1.000 | 1.000 | 1.000 | 0.971 | 1.000 | 1.000 | 0.801 | 1.000 | 1.000 | 0.865 | 0.837 |
| 2507 | 0.833 | 1.000 | 0.850 | 1.000 | 0.979 | 1.000 | 0.878 | 1.000 | 1.000 | 1.000 | 0.971 | 1.000 | 1.000 | 0.801 | 1.000 | 1.000 | 0.865 | 0.837 |
| 2508 | 0.833 | 1.000 | 0.850 | 1.000 | 0.979 | 1.000 | 0.878 | 1.000 | 1.000 | 1.000 | 0.971 | 1.000 | 1.000 | 0.801 | 1.000 | 1.000 | 0.865 | 0.837 |
| 2509 | 0.833 | 1.000 | 0.850 | 1.000 | 0.979 | 1.000 | 0.880 | 1.000 | 1.000 | 1.000 | 0.971 | 1.000 | 1.000 | 0.801 | 1.000 | 1.000 | 0.865 | 0.837 |
| 2510 | 0.833 | 1.000 | 0.850 | 1.000 | 0.979 | 1.000 | 0.880 | 1.000 | 1.000 | 1.000 | 0.971 | 1.000 | 1.000 | 0.802 | 1.000 | 1.000 | 0.865 | 0.838 |
| 2511 | 0.833 | 1.000 | 0.850 | 1.000 | 0.979 | 1.000 | 0.878 | 1.000 | 1.000 | 1.000 | 0.971 | 1.000 | 1.000 | 0.802 | 1.000 | 1.000 | 0.865 | 0.837 |
| 2512 | 0.833 | 1.000 | 0.850 | 1.000 | 0.979 | 1.000 | 0.880 | 1.000 | 1.000 | 1.000 | 0.971 | 1.000 | 1.000 | 0.802 | 1.000 | 1.000 | 0.865 | 0.838 |
| 2513 | 0.834 | 1.000 | 0.850 | 1.000 | 0.979 | 1.000 | 0.878 | 1.000 | 1.000 | 1.000 | 0.971 | 1.000 | 1.000 | 0.802 | 1.000 | 1.000 | 0.865 | 0.838 |
| 2514 | 0.833 | 1.000 | 0.850 | 1.000 | 0.979 | 1.000 | 0.877 | 1.000 | 1.000 | 1.000 | 0.971 | 1.000 | 1.000 | 0.802 | 1.000 | 1.000 | 0.865 | 0.837 |
| 2515 | 0.833 | 1.000 | 0.850 | 1.000 | 0.979 | 1.000 | 0.877 | 1.000 | 1.000 | 1.000 | 0.971 | 1.000 | 1.000 | 0.801 | 1.000 | 1.000 | 0.865 | 0.837 |
| 2516 | 0.834 | 1.000 | 0.850 | 1.000 | 0.979 | 1.000 | 0.877 | 1.000 | 1.000 | 1.000 | 0.971 | 1.000 | 1.000 | 0.801 | 1.000 | 1.000 | 0.865 | 0.837 |
| 2517 | 0.835 | 1.000 | 0.850 | 1.000 | 0.979 | 1.000 | 0.877 | 1.000 | 1.000 | 1.000 | 0.971 | 1.000 | 1.000 | 0.801 | 1.000 | 1.000 | 0.865 | 0.838 |
| 2518 | 0.835 | 1.000 | 0.850 | 1.000 | 0.979 | 1.000 | 0.877 | 1.000 | 1.000 | 1.000 | 0.971 | 1.000 | 1.000 | 0.801 | 1.000 | 1.000 | 0.865 | 0.838 |
| 2519 | 0.835 | 1.000 | 0.850 | 1.000 | 0.979 | 1.000 | 0.877 | 1.000 | 1.000 | 1.000 | 0.971 | 1.000 | 1.000 | 0.801 | 1.000 | 1.000 | 0.865 | 0.837 |
| 2520 | 0.833 | 1.000 | 0.850 | 1.000 | 0.979 | 1.000 | 0.877 | 1.000 | 1.000 | 1.000 | 0.971 | 1.000 | 1.000 | 0.800 | 1.000 | 1.000 | 0.865 | 0.837 |
| 2521 | 0.832 | 1.000 | 0.848 | 1.000 | 0.979 | 1.000 | 0.878 | 1.000 | 1.000 | 1.000 | 0.971 | 1.000 | 1.000 | 0.800 | 1.000 | 1.000 | 0.864 | 0.836 |
| 2522 | 0.832 | 1.000 | 0.848 | 1.000 | 0.979 | 1.000 | 0.878 | 1.000 | 1.000 | 1.000 | 0.971 | 1.000 | 1.000 | 0.800 | 1.000 | 1.000 | 0.864 | 0.836 |
| 2523 | 0.833 | 1.000 | 0.848 | 1.000 | 0.979 | 1.000 | 0.878 | 1.000 | 1.000 | 1.000 | 0.971 | 1.000 | 1.000 | 0.800 | 1.000 | 1.000 | 0.864 | 0.836 |
| 2524 | 0.833 | 1.000 | 0.848 | 1.000 | 0.979 | 1.000 | 0.880 | 1.000 | 1.000 | 1.000 | 0.971 | 1.000 | 1.000 | 0.801 | 1.000 | 1.000 | 0.865 | 0.837 |
| 2525 | 0.834 | 1.000 | 0.850 | 1.000 | 0.979 | 1.000 | 0.880 | 1.000 | 1.000 | 1.000 | 0.974 | 1.000 | 1.000 | 0.800 | 1.000 | 1.000 | 0.865 | 0.838 |
| 2526 | 0.834 | 1.000 | 0.850 | 1.000 | 0.979 | 1.000 | 0.880 | 1.000 | 1.000 | 1.000 | 0.974 | 1.000 | 1.000 | 0.800 | 1.000 | 1.000 | 0.865 | 0.838 |
| 2527 | 0.835 | 1.000 | 0.852 | 1.000 | 0.979 | 1.000 | 0.880 | 1.000 | 1.000 | 1.000 | 0.974 | 1.000 | 1.000 | 0.800 | 1.000 | 1.000 | 0.866 | 0.838 |
| 2528 | 0.832 | 1.000 | 0.852 | 1.000 | 0.979 | 1.000 | 0.877 | 1.000 | 1.000 | 1.000 | 0.977 | 1.000 | 1.000 | 0.801 | 1.000 | 1.000 | 0.865 | 0.837 |
| 2529 | 0.832 | 1.000 | 0.852 | 1.000 | 0.979 | 1.000 | 0.877 | 1.000 | 1.000 | 1.000 | 0.977 | 1.000 | 1.000 | 0.802 | 1.000 | 1.000 | 0.865 | 0.838 |
| 2530 | 0.832 | 1.000 | 0.852 | 1.000 | 0.979 | 1.000 | 0.878 | 1.000 | 1.000 | 1.000 | 0.977 | 1.000 | 1.000 | 0.802 | 1.000 | 1.000 | 0.866 | 0.838 |
| 2531 | 0.832 | 1.000 | 0.852 | 1.000 | 0.979 | 1.000 | 0.878 | 1.000 | 1.000 | 1.000 | 0.977 | 1.000 | 1.000 | 0.802 | 1.000 | 1.000 | 0.866 | 0.838 |
| 2532 | 0.832 | 1.000 | 0.852 | 1.000 | 0.979 | 1.000 | 0.878 | 1.000 | 1.000 | 1.000 | 0.977 | 1.000 | 1.000 | 0.802 | 1.000 | 1.000 | 0.866 | 0.838 |
| 2533 | 0.832 | 1.000 | 0.850 | 1.000 | 0.979 | 1.000 | 0.883 | 1.000 | 1.000 | 1.000 | 0.977 | 1.000 | 1.000 | 0.802 | 1.000 | 1.000 | 0.866 | 0.838 |
| 2534 | 0.833 | 1.000 | 0.850 | 1.000 | 0.979 | 1.000 | 0.883 | 1.000 | 1.000 | 1.000 | 0.977 | 1.000 | 1.000 | 0.802 | 1.000 | 1.000 | 0.866 | 0.839 |
| 2535 | 0.833 | 1.000 | 0.852 | 1.000 | 0.979 | 1.000 | 0.881 | 1.000 | 1.000 | 1.000 | 0.977 | 1.000 | 1.000 | 0.803 | 1.000 | 1.000 | 0.866 | 0.839 |
| 2536 | 0.833 | 1.000 | 0.852 | 1.000 | 0.979 | 1.000 | 0.881 | 1.000 | 1.000 | 1.000 | 0.977 | 1.000 | 1.000 | 0.803 | 1.000 | 1.000 | 0.867 | 0.839 |
| 2537 | 0.833 | 1.000 | 0.852 | 1.000 | 0.979 | 1.000 | 0.881 | 1.000 | 1.000 | 1.000 | 0.977 | 1.000 | 1.000 | 0.803 | 1.000 | 1.000 | 0.867 | 0.839 |
| 2538 | 0.833 | 1.000 | 0.852 | 1.000 | 0.979 | 1.000 | 0.881 | 1.000 | 1.000 | 1.000 | 0.977 | 1.000 | 1.000 | 0.803 | 1.000 | 1.000 | 0.867 | 0.839 |

| | | | | | | | | | | | | | | | | | | |
|---|---|---|---|---|---|---|---|---|---|---|---|---|---|---|---|---|---|---|
| 2539 | 0.833 | 1.000 | 0.850 | 1.000 | 0.979 | 1.000 | 0.883 | 1.000 | 1.000 | 1.000 | 0.977 | 1.000 | 1.000 | 0.803 | 1.000 | 1.000 | 0.867 | 0.839 |
| 2540 | 0.833 | 1.000 | 0.850 | 1.000 | 0.979 | 1.000 | 0.883 | 1.000 | 1.000 | 1.000 | 0.977 | 1.000 | 1.000 | 0.803 | 1.000 | 1.000 | 0.867 | 0.839 |
| 2541 | 0.833 | 1.000 | 0.854 | 1.000 | 0.979 | 1.000 | 0.883 | 1.000 | 1.000 | 1.000 | 0.977 | 1.000 | 1.000 | 0.804 | 1.000 | 1.000 | 0.867 | 0.840 |
| 2542 | 0.833 | 1.000 | 0.852 | 1.000 | 0.979 | 1.000 | 0.883 | 1.000 | 1.000 | 1.000 | 0.977 | 1.000 | 1.000 | 0.804 | 1.000 | 1.000 | 0.867 | 0.839 |
| 2543 | 0.831 | 1.000 | 0.856 | 1.000 | 0.984 | 1.000 | 0.881 | 1.000 | 1.000 | 1.000 | 0.980 | 1.000 | 1.000 | 0.803 | 1.000 | 1.000 | 0.867 | 0.839 |
| 2544 | 0.830 | 1.000 | 0.858 | 1.000 | 0.984 | 1.000 | 0.884 | 1.000 | 1.000 | 1.000 | 0.980 | 1.000 | 1.000 | 0.803 | 1.000 | 1.000 | 0.867 | 0.840 |
| 2545 | 0.829 | 1.000 | 0.854 | 1.000 | 0.984 | 1.000 | 0.884 | 1.000 | 1.000 | 1.000 | 0.980 | 1.000 | 1.000 | 0.802 | 1.000 | 1.000 | 0.866 | 0.838 |
| 2546 | 0.829 | 1.000 | 0.854 | 1.000 | 0.984 | 1.000 | 0.884 | 1.000 | 1.000 | 1.000 | 0.980 | 1.000 | 1.000 | 0.801 | 1.000 | 1.000 | 0.866 | 0.838 |
| 2547 | 0.830 | 1.000 | 0.854 | 1.000 | 0.984 | 1.000 | 0.884 | 1.000 | 1.000 | 1.000 | 0.980 | 1.000 | 1.000 | 0.801 | 1.000 | 1.000 | 0.866 | 0.838 |
| 2548 | 0.831 | 1.000 | 0.854 | 1.000 | 0.984 | 1.000 | 0.884 | 1.000 | 1.000 | 1.000 | 0.977 | 1.000 | 1.000 | 0.801 | 1.000 | 1.000 | 0.866 | 0.838 |
| 2549 | 0.831 | 1.000 | 0.854 | 1.000 | 0.984 | 1.000 | 0.884 | 1.000 | 1.000 | 1.000 | 0.980 | 1.000 | 1.000 | 0.801 | 1.000 | 1.000 | 0.866 | 0.839 |
| 2550 | 0.831 | 1.000 | 0.854 | 1.000 | 0.984 | 1.000 | 0.884 | 1.000 | 1.000 | 1.000 | 0.977 | 1.000 | 1.000 | 0.801 | 1.000 | 1.000 | 0.866 | 0.839 |
| 2551 | 0.831 | 1.000 | 0.854 | 1.000 | 0.984 | 1.000 | 0.884 | 1.000 | 1.000 | 1.000 | 0.977 | 1.000 | 1.000 | 0.802 | 1.000 | 1.000 | 0.866 | 0.839 |
| 2552 | 0.830 | 1.000 | 0.854 | 1.000 | 0.984 | 1.000 | 0.884 | 1.000 | 1.000 | 1.000 | 0.977 | 1.000 | 1.000 | 0.801 | 1.000 | 1.000 | 0.866 | 0.838 |
| 2553 | 0.830 | 1.000 | 0.854 | 1.000 | 0.984 | 1.000 | 0.884 | 1.000 | 1.000 | 1.000 | 0.977 | 1.000 | 1.000 | 0.801 | 1.000 | 1.000 | 0.866 | 0.838 |
| 2554 | 0.829 | 1.000 | 0.856 | 1.000 | 0.984 | 1.000 | 0.884 | 1.000 | 1.000 | 1.000 | 0.977 | 1.000 | 1.000 | 0.801 | 1.000 | 1.000 | 0.866 | 0.838 |
| 2555 | 0.829 | 1.000 | 0.856 | 1.000 | 0.984 | 1.000 | 0.884 | 1.000 | 1.000 | 1.000 | 0.980 | 1.000 | 1.000 | 0.801 | 1.000 | 1.000 | 0.866 | 0.838 |
| 2556 | 0.829 | 1.000 | 0.852 | 1.000 | 0.984 | 1.000 | 0.888 | 1.000 | 1.000 | 1.000 | 0.980 | 1.000 | 1.000 | 0.800 | 1.000 | 1.000 | 0.866 | 0.838 |
| 2557 | 0.829 | 1.000 | 0.852 | 1.000 | 0.984 | 1.000 | 0.884 | 1.000 | 1.000 | 1.000 | 0.977 | 1.000 | 1.000 | 0.799 | 1.000 | 1.000 | 0.865 | 0.837 |
| 2558 | 0.829 | 1.000 | 0.852 | 1.000 | 0.984 | 1.000 | 0.888 | 1.000 | 1.000 | 1.000 | 0.977 | 1.000 | 1.000 | 0.800 | 1.000 | 1.000 | 0.866 | 0.838 |
| 2559 | 0.829 | 1.000 | 0.852 | 1.000 | 0.984 | 1.000 | 0.888 | 1.000 | 1.000 | 1.000 | 0.977 | 1.000 | 1.000 | 0.800 | 1.000 | 1.000 | 0.866 | 0.838 |
| 2560 | 0.829 | 1.000 | 0.852 | 1.000 | 0.984 | 1.000 | 0.886 | 1.000 | 1.000 | 1.000 | 0.977 | 1.000 | 1.000 | 0.800 | 1.000 | 1.000 | 0.865 | 0.837 |
| 2561 | 0.829 | 1.000 | 0.852 | 1.000 | 0.984 | 1.000 | 0.886 | 1.000 | 1.000 | 1.000 | 0.977 | 1.000 | 1.000 | 0.801 | 1.000 | 1.000 | 0.866 | 0.838 |
| 2562 | 0.830 | 1.000 | 0.852 | 1.000 | 0.984 | 1.000 | 0.884 | 1.000 | 1.000 | 1.000 | 0.977 | 1.000 | 1.000 | 0.800 | 1.000 | 1.000 | 0.865 | 0.838 |
| 2563 | 0.826 | 1.000 | 0.854 | 1.000 | 0.984 | 1.000 | 0.886 | 1.000 | 1.000 | 1.000 | 0.983 | 1.000 | 1.000 | 0.800 | 1.000 | 1.000 | 0.865 | 0.838 |
| 2564 | 0.828 | 1.000 | 0.854 | 1.000 | 0.984 | 1.000 | 0.884 | 1.000 | 1.000 | 1.000 | 0.980 | 1.000 | 1.000 | 0.800 | 1.000 | 1.000 | 0.865 | 0.837 |
| 2565 | 0.829 | 1.000 | 0.854 | 1.000 | 0.984 | 1.000 | 0.884 | 1.000 | 1.000 | 1.000 | 0.977 | 1.000 | 1.000 | 0.798 | 1.000 | 1.000 | 0.865 | 0.837 |
| 2566 | 0.828 | 1.000 | 0.854 | 1.000 | 0.984 | 1.000 | 0.884 | 1.000 | 1.000 | 1.000 | 0.977 | 1.000 | 1.000 | 0.798 | 1.000 | 1.000 | 0.864 | 0.837 |
| 2567 | 0.828 | 1.000 | 0.854 | 1.000 | 0.984 | 1.000 | 0.884 | 1.000 | 1.000 | 1.000 | 0.977 | 1.000 | 1.000 | 0.797 | 1.000 | 1.000 | 0.864 | 0.836 |
| 2568 | 0.828 | 1.000 | 0.854 | 1.000 | 0.984 | 1.000 | 0.883 | 1.000 | 1.000 | 1.000 | 0.977 | 1.000 | 1.000 | 0.798 | 1.000 | 1.000 | 0.864 | 0.836 |
| 2569 | 0.828 | 1.000 | 0.854 | 1.000 | 0.984 | 1.000 | 0.881 | 1.000 | 1.000 | 1.000 | 0.977 | 1.000 | 1.000 | 0.798 | 1.000 | 1.000 | 0.864 | 0.836 |
| 2570 | 0.828 | 1.000 | 0.854 | 1.000 | 0.984 | 1.000 | 0.881 | 1.000 | 1.000 | 1.000 | 0.977 | 1.000 | 1.000 | 0.798 | 1.000 | 1.000 | 0.864 | 0.836 |
| 2571 | 0.829 | 1.000 | 0.854 | 1.000 | 0.984 | 1.000 | 0.881 | 1.000 | 1.000 | 1.000 | 0.977 | 1.000 | 1.000 | 0.798 | 1.000 | 1.000 | 0.864 | 0.837 |
| 2572 | 0.829 | 1.000 | 0.854 | 1.000 | 0.984 | 1.000 | 0.881 | 1.000 | 1.000 | 1.000 | 0.977 | 1.000 | 1.000 | 0.796 | 1.000 | 1.000 | 0.864 | 0.836 |
| 2573 | 0.829 | 1.000 | 0.854 | 1.000 | 0.984 | 1.000 | 0.881 | 1.000 | 1.000 | 1.000 | 0.977 | 1.000 | 1.000 | 0.796 | 1.000 | 1.000 | 0.864 | 0.836 |
| 2574 | 0.831 | 1.000 | 0.854 | 1.000 | 0.984 | 1.000 | 0.883 | 1.000 | 1.000 | 1.000 | 0.977 | 1.000 | 1.000 | 0.798 | 1.000 | 1.000 | 0.865 | 0.838 |
| 2575 | 0.831 | 1.000 | 0.854 | 1.000 | 0.984 | 1.000 | 0.883 | 1.000 | 1.000 | 1.000 | 0.977 | 1.000 | 1.000 | 0.798 | 1.000 | 1.000 | 0.865 | 0.838 |
| 2576 | 0.831 | 1.000 | 0.854 | 1.000 | 0.984 | 1.000 | 0.883 | 1.000 | 1.000 | 1.000 | 0.977 | 1.000 | 1.000 | 0.798 | 1.000 | 1.000 | 0.865 | 0.838 |
| 2577 | 0.828 | 1.000 | 0.854 | 1.000 | 0.984 | 1.000 | 0.881 | 1.000 | 1.000 | 1.000 | 0.977 | 1.000 | 1.000 | 0.798 | 1.000 | 1.000 | 0.864 | 0.836 |

| | | | | | | | | | | | | | | | | | | |
|---|---|---|---|---|---|---|---|---|---|---|---|---|---|---|---|---|---|---|
| 2578 | 0.829 | 1.000 | 0.854 | 1.000 | 0.984 | 1.000 | 0.883 | 1.000 | 1.000 | 1.000 | 0.977 | 1.000 | 1.000 | 0.799 | 1.000 | 1.000 | 0.865 | 0.837 |
| 2579 | 0.830 | 1.000 | 0.854 | 1.000 | 0.984 | 1.000 | 0.878 | 1.000 | 1.000 | 1.000 | 0.977 | 1.000 | 1.000 | 0.799 | 1.000 | 1.000 | 0.865 | 0.837 |
| 2580 | 0.830 | 1.000 | 0.854 | 1.000 | 0.984 | 1.000 | 0.878 | 1.000 | 1.000 | 1.000 | 0.977 | 1.000 | 1.000 | 0.799 | 1.000 | 1.000 | 0.865 | 0.837 |
| 2581 | 0.831 | 1.000 | 0.856 | 1.000 | 0.984 | 1.000 | 0.880 | 1.000 | 1.000 | 1.000 | 0.977 | 1.000 | 1.000 | 0.799 | 1.000 | 1.000 | 0.865 | 0.837 |
| 2582 | 0.832 | 1.000 | 0.858 | 1.000 | 0.984 | 1.000 | 0.880 | 1.000 | 1.000 | 1.000 | 0.977 | 1.000 | 1.000 | 0.800 | 1.000 | 1.000 | 0.866 | 0.838 |
| 2583 | 0.832 | 1.000 | 0.858 | 1.000 | 0.984 | 1.000 | 0.880 | 1.000 | 1.000 | 1.000 | 0.977 | 1.000 | 1.000 | 0.801 | 1.000 | 1.000 | 0.866 | 0.839 |
| 2584 | 0.833 | 1.000 | 0.858 | 1.000 | 0.984 | 1.000 | 0.877 | 1.000 | 1.000 | 1.000 | 0.977 | 1.000 | 1.000 | 0.800 | 1.000 | 1.000 | 0.866 | 0.838 |
| 2585 | 0.834 | 1.000 | 0.858 | 1.000 | 0.984 | 1.000 | 0.877 | 1.000 | 1.000 | 1.000 | 0.977 | 1.000 | 1.000 | 0.800 | 1.000 | 1.000 | 0.866 | 0.839 |
| 2586 | 0.833 | 1.000 | 0.858 | 1.000 | 0.984 | 1.000 | 0.877 | 1.000 | 1.000 | 1.000 | 0.977 | 1.000 | 1.000 | 0.800 | 1.000 | 1.000 | 0.866 | 0.838 |
| 2587 | 0.834 | 1.000 | 0.858 | 1.000 | 0.984 | 1.000 | 0.877 | 1.000 | 1.000 | 1.000 | 0.977 | 1.000 | 1.000 | 0.801 | 1.000 | 1.000 | 0.866 | 0.839 |
| 2588 | 0.833 | 1.000 | 0.858 | 1.000 | 0.984 | 1.000 | 0.877 | 1.000 | 1.000 | 1.000 | 0.977 | 1.000 | 1.000 | 0.801 | 1.000 | 1.000 | 0.866 | 0.839 |
| 2589 | 0.833 | 1.000 | 0.858 | 1.000 | 0.984 | 1.000 | 0.877 | 1.000 | 1.000 | 1.000 | 0.977 | 1.000 | 1.000 | 0.801 | 1.000 | 1.000 | 0.866 | 0.839 |
| 2590 | 0.834 | 1.000 | 0.858 | 1.000 | 0.984 | 1.000 | 0.877 | 1.000 | 1.000 | 1.000 | 0.977 | 1.000 | 1.000 | 0.801 | 1.000 | 1.000 | 0.866 | 0.839 |
| 2591 | 0.830 | 1.000 | 0.858 | 1.000 | 0.984 | 1.000 | 0.878 | 1.000 | 1.000 | 1.000 | 0.977 | 1.000 | 1.000 | 0.802 | 1.000 | 1.000 | 0.866 | 0.838 |
| 2592 | 0.829 | 1.000 | 0.860 | 1.000 | 0.984 | 1.000 | 0.878 | 1.000 | 1.000 | 1.000 | 0.977 | 1.000 | 1.000 | 0.802 | 1.000 | 1.000 | 0.866 | 0.838 |
| 2593 | 0.831 | 1.000 | 0.858 | 1.000 | 0.984 | 1.000 | 0.877 | 1.000 | 1.000 | 1.000 | 0.977 | 1.000 | 1.000 | 0.802 | 1.000 | 1.000 | 0.866 | 0.838 |
| 2594 | 0.830 | 1.000 | 0.858 | 1.000 | 0.984 | 1.000 | 0.877 | 1.000 | 1.000 | 1.000 | 0.977 | 1.000 | 1.000 | 0.802 | 1.000 | 1.000 | 0.866 | 0.838 |
| 2595 | 0.831 | 1.000 | 0.860 | 1.000 | 0.984 | 1.000 | 0.875 | 1.000 | 1.000 | 1.000 | 0.977 | 1.000 | 1.000 | 0.802 | 1.000 | 1.000 | 0.866 | 0.838 |
| 2596 | 0.831 | 1.000 | 0.860 | 1.000 | 0.984 | 1.000 | 0.877 | 1.000 | 1.000 | 1.000 | 0.977 | 1.000 | 1.000 | 0.802 | 1.000 | 1.000 | 0.866 | 0.838 |
| 2597 | 0.831 | 1.000 | 0.860 | 1.000 | 0.984 | 1.000 | 0.875 | 1.000 | 1.000 | 1.000 | 0.977 | 1.000 | 1.000 | 0.802 | 1.000 | 1.000 | 0.866 | 0.838 |
| 2598 | 0.831 | 1.000 | 0.860 | 1.000 | 0.984 | 1.000 | 0.875 | 1.000 | 1.000 | 1.000 | 0.977 | 1.000 | 1.000 | 0.802 | 1.000 | 1.000 | 0.866 | 0.838 |
| 2599 | 0.833 | 1.000 | 0.860 | 1.000 | 0.984 | 1.000 | 0.877 | 1.000 | 1.000 | 1.000 | 0.977 | 1.000 | 1.000 | 0.802 | 1.000 | 1.000 | 0.867 | 0.839 |
| 2600 | 0.832 | 1.000 | 0.860 | 1.000 | 0.984 | 1.000 | 0.877 | 1.000 | 1.000 | 1.000 | 0.974 | 1.000 | 1.000 | 0.802 | 1.000 | 1.000 | 0.866 | 0.839 |
| 2601 | 0.832 | 1.000 | 0.860 | 1.000 | 0.984 | 1.000 | 0.877 | 1.000 | 1.000 | 1.000 | 0.974 | 1.000 | 1.000 | 0.802 | 1.000 | 1.000 | 0.866 | 0.839 |
| 2602 | 0.831 | 1.000 | 0.856 | 1.000 | 0.984 | 1.000 | 0.874 | 1.000 | 1.000 | 1.000 | 0.974 | 1.000 | 1.000 | 0.801 | 1.000 | 1.000 | 0.865 | 0.837 |
| 2603 | 0.831 | 1.000 | 0.860 | 1.000 | 0.984 | 1.000 | 0.877 | 1.000 | 1.000 | 1.000 | 0.974 | 1.000 | 1.000 | 0.801 | 1.000 | 1.000 | 0.866 | 0.838 |
| 2604 | 0.832 | 1.000 | 0.860 | 1.000 | 0.984 | 1.000 | 0.877 | 1.000 | 1.000 | 1.000 | 0.974 | 1.000 | 1.000 | 0.801 | 1.000 | 1.000 | 0.866 | 0.838 |
| 2605 | 0.831 | 1.000 | 0.858 | 1.000 | 0.984 | 1.000 | 0.877 | 1.000 | 1.000 | 1.000 | 0.974 | 1.000 | 1.000 | 0.801 | 1.000 | 1.000 | 0.865 | 0.838 |
| 2606 | 0.831 | 1.000 | 0.858 | 1.000 | 0.984 | 1.000 | 0.877 | 1.000 | 1.000 | 1.000 | 0.974 | 1.000 | 1.000 | 0.801 | 1.000 | 1.000 | 0.865 | 0.838 |
| 2607 | 0.831 | 1.000 | 0.858 | 1.000 | 0.984 | 1.000 | 0.877 | 1.000 | 1.000 | 1.000 | 0.974 | 1.000 | 1.000 | 0.802 | 1.000 | 1.000 | 0.866 | 0.838 |
| 2608 | 0.831 | 1.000 | 0.860 | 1.000 | 0.984 | 1.000 | 0.877 | 1.000 | 1.000 | 1.000 | 0.974 | 1.000 | 1.000 | 0.802 | 1.000 | 1.000 | 0.866 | 0.838 |
| 2609 | 0.831 | 1.000 | 0.860 | 1.000 | 0.984 | 1.000 | 0.877 | 1.000 | 1.000 | 1.000 | 0.974 | 1.000 | 1.000 | 0.802 | 1.000 | 1.000 | 0.866 | 0.838 |
| 2610 | 0.831 | 1.000 | 0.860 | 1.000 | 0.984 | 1.000 | 0.875 | 1.000 | 1.000 | 1.000 | 0.974 | 1.000 | 1.000 | 0.802 | 1.000 | 1.000 | 0.866 | 0.838 |
| 2611 | 0.832 | 1.000 | 0.858 | 1.000 | 0.984 | 1.000 | 0.877 | 1.000 | 1.000 | 1.000 | 0.974 | 1.000 | 1.000 | 0.802 | 1.000 | 1.000 | 0.866 | 0.838 |
| 2612 | 0.833 | 1.000 | 0.856 | 1.000 | 0.984 | 1.000 | 0.877 | 1.000 | 1.000 | 1.000 | 0.974 | 1.000 | 1.000 | 0.802 | 1.000 | 1.000 | 0.866 | 0.838 |
| 2613 | 0.833 | 1.000 | 0.856 | 1.000 | 0.984 | 1.000 | 0.877 | 1.000 | 1.000 | 1.000 | 0.974 | 1.000 | 1.000 | 0.802 | 1.000 | 1.000 | 0.866 | 0.839 |
| 2614 | 0.835 | 1.000 | 0.860 | 1.000 | 0.984 | 1.000 | 0.877 | 1.000 | 1.000 | 1.000 | 0.974 | 1.000 | 1.000 | 0.804 | 1.000 | 1.000 | 0.867 | 0.840 |
| 2615 | 0.835 | 1.000 | 0.858 | 1.000 | 0.984 | 1.000 | 0.877 | 1.000 | 1.000 | 1.000 | 0.974 | 1.000 | 1.000 | 0.802 | 1.000 | 1.000 | 0.867 | 0.839 |
| 2616 | 0.835 | 1.000 | 0.858 | 1.000 | 0.984 | 1.000 | 0.877 | 1.000 | 1.000 | 1.000 | 0.974 | 1.000 | 1.000 | 0.802 | 1.000 | 1.000 | 0.867 | 0.839 |

| | | | | | | | | | | | | | | | | | | |
|---|---|---|---|---|---|---|---|---|---|---|---|---|---|---|---|---|---|---|
| 2617 | 0.835 | 1.000 | 0.858 | 1.000 | 0.984 | 1.000 | 0.877 | 1.000 | 1.000 | 1.000 | 0.974 | 1.000 | 1.000 | 0.803 | 1.000 | 1.000 | 0.867 | 0.840 |
| 2618 | 0.835 | 1.000 | 0.858 | 1.000 | 0.984 | 1.000 | 0.877 | 1.000 | 1.000 | 1.000 | 0.974 | 1.000 | 1.000 | 0.802 | 1.000 | 1.000 | 0.867 | 0.840 |
| 2619 | 0.835 | 1.000 | 0.858 | 1.000 | 0.984 | 1.000 | 0.877 | 1.000 | 1.000 | 1.000 | 0.974 | 1.000 | 1.000 | 0.803 | 1.000 | 1.000 | 0.867 | 0.840 |
| 2620 | 0.835 | 1.000 | 0.858 | 1.000 | 0.984 | 1.000 | 0.877 | 1.000 | 1.000 | 1.000 | 0.974 | 1.000 | 1.000 | 0.802 | 1.000 | 1.000 | 0.867 | 0.839 |
| 2621 | 0.835 | 1.000 | 0.858 | 1.000 | 0.984 | 1.000 | 0.877 | 1.000 | 1.000 | 1.000 | 0.974 | 1.000 | 1.000 | 0.802 | 1.000 | 1.000 | 0.867 | 0.839 |
| 2622 | 0.835 | 1.000 | 0.858 | 1.000 | 0.984 | 1.000 | 0.877 | 1.000 | 1.000 | 1.000 | 0.974 | 1.000 | 1.000 | 0.802 | 1.000 | 1.000 | 0.867 | 0.840 |
| 2623 | 0.833 | 1.000 | 0.858 | 1.000 | 0.984 | 1.000 | 0.878 | 1.000 | 1.000 | 1.000 | 0.974 | 1.000 | 1.000 | 0.802 | 1.000 | 1.000 | 0.867 | 0.839 |
| 2624 | 0.833 | 1.000 | 0.858 | 1.000 | 0.984 | 1.000 | 0.878 | 1.000 | 1.000 | 1.000 | 0.974 | 1.000 | 1.000 | 0.803 | 1.000 | 1.000 | 0.867 | 0.839 |
| 2625 | 0.833 | 1.000 | 0.858 | 1.000 | 0.984 | 1.000 | 0.877 | 1.000 | 0.990 | 1.000 | 0.974 | 1.000 | 1.000 | 0.802 | 1.000 | 1.000 | 0.866 | 0.838 |
| 2626 | 0.833 | 1.000 | 0.858 | 1.000 | 0.984 | 1.000 | 0.875 | 1.000 | 0.990 | 1.000 | 0.974 | 1.000 | 1.000 | 0.805 | 1.000 | 1.000 | 0.866 | 0.839 |
| 2627 | 0.833 | 1.000 | 0.858 | 1.000 | 0.984 | 1.000 | 0.875 | 1.000 | 0.990 | 1.000 | 0.974 | 1.000 | 1.000 | 0.805 | 1.000 | 1.000 | 0.866 | 0.839 |
| 2628 | 0.833 | 1.000 | 0.858 | 1.000 | 0.984 | 1.000 | 0.875 | 1.000 | 0.990 | 1.000 | 0.974 | 1.000 | 1.000 | 0.805 | 1.000 | 1.000 | 0.867 | 0.839 |
| 2629 | 0.833 | 1.000 | 0.858 | 1.000 | 0.984 | 1.000 | 0.875 | 1.000 | 0.990 | 1.000 | 0.974 | 1.000 | 1.000 | 0.805 | 1.000 | 1.000 | 0.867 | 0.839 |
| 2630 | 0.832 | 1.000 | 0.858 | 1.000 | 0.984 | 1.000 | 0.875 | 1.000 | 0.990 | 1.000 | 0.974 | 1.000 | 1.000 | 0.805 | 1.000 | 1.000 | 0.866 | 0.839 |
| 2631 | 0.833 | 1.000 | 0.858 | 1.000 | 0.984 | 1.000 | 0.877 | 1.000 | 0.990 | 1.000 | 0.974 | 1.000 | 1.000 | 0.805 | 1.000 | 1.000 | 0.867 | 0.839 |
| 2632 | 0.831 | 1.000 | 0.856 | 1.000 | 0.984 | 1.000 | 0.878 | 1.000 | 0.990 | 1.000 | 0.974 | 1.000 | 1.000 | 0.804 | 1.000 | 1.000 | 0.866 | 0.838 |
| 2633 | 0.831 | 1.000 | 0.856 | 1.000 | 0.984 | 1.000 | 0.878 | 1.000 | 0.990 | 1.000 | 0.974 | 1.000 | 1.000 | 0.804 | 1.000 | 1.000 | 0.866 | 0.838 |
| 2634 | 0.831 | 1.000 | 0.856 | 1.000 | 0.984 | 1.000 | 0.877 | 1.000 | 0.990 | 1.000 | 0.974 | 1.000 | 1.000 | 0.804 | 1.000 | 1.000 | 0.866 | 0.838 |
| 2635 | 0.833 | 1.000 | 0.854 | 1.000 | 0.984 | 1.000 | 0.878 | 1.000 | 0.990 | 1.000 | 0.974 | 1.000 | 1.000 | 0.804 | 1.000 | 1.000 | 0.866 | 0.839 |
| 2636 | 0.833 | 1.000 | 0.854 | 1.000 | 0.984 | 1.000 | 0.877 | 1.000 | 0.990 | 1.000 | 0.974 | 1.000 | 1.000 | 0.804 | 1.000 | 1.000 | 0.866 | 0.838 |
| 2637 | 0.832 | 1.000 | 0.854 | 1.000 | 0.984 | 1.000 | 0.877 | 1.000 | 0.990 | 1.000 | 0.974 | 1.000 | 1.000 | 0.804 | 1.000 | 1.000 | 0.866 | 0.838 |
| 2638 | 0.833 | 1.000 | 0.854 | 1.000 | 0.984 | 1.000 | 0.877 | 1.000 | 0.990 | 1.000 | 0.974 | 1.000 | 1.000 | 0.804 | 1.000 | 1.000 | 0.866 | 0.838 |
| 2639 | 0.831 | 1.000 | 0.856 | 1.000 | 0.984 | 1.000 | 0.877 | 1.000 | 0.990 | 1.000 | 0.974 | 1.000 | 1.000 | 0.804 | 1.000 | 1.000 | 0.866 | 0.838 |
| 2640 | 0.831 | 1.000 | 0.856 | 1.000 | 0.984 | 1.000 | 0.877 | 1.000 | 0.990 | 1.000 | 0.974 | 1.000 | 1.000 | 0.804 | 1.000 | 1.000 | 0.866 | 0.838 |
| 2641 | 0.831 | 1.000 | 0.854 | 1.000 | 0.984 | 1.000 | 0.878 | 1.000 | 0.990 | 1.000 | 0.974 | 1.000 | 1.000 | 0.804 | 1.000 | 1.000 | 0.866 | 0.838 |
| 2642 | 0.831 | 1.000 | 0.854 | 1.000 | 0.984 | 1.000 | 0.877 | 1.000 | 0.990 | 1.000 | 0.974 | 1.000 | 1.000 | 0.804 | 1.000 | 1.000 | 0.866 | 0.838 |
| 2643 | 0.831 | 1.000 | 0.854 | 1.000 | 0.984 | 1.000 | 0.877 | 1.000 | 0.990 | 1.000 | 0.974 | 1.000 | 1.000 | 0.803 | 1.000 | 1.000 | 0.865 | 0.838 |
| 2644 | 0.831 | 1.000 | 0.854 | 1.000 | 0.984 | 1.000 | 0.877 | 1.000 | 0.990 | 1.000 | 0.974 | 1.000 | 1.000 | 0.803 | 1.000 | 1.000 | 0.865 | 0.838 |
| 2645 | 0.831 | 1.000 | 0.854 | 1.000 | 0.984 | 1.000 | 0.877 | 1.000 | 0.990 | 1.000 | 0.974 | 1.000 | 1.000 | 0.804 | 1.000 | 1.000 | 0.866 | 0.838 |
| 2646 | 0.831 | 1.000 | 0.854 | 1.000 | 0.984 | 1.000 | 0.878 | 1.000 | 0.990 | 1.000 | 0.974 | 1.000 | 1.000 | 0.803 | 1.000 | 1.000 | 0.866 | 0.838 |
| 2647 | 0.832 | 1.000 | 0.854 | 1.000 | 0.984 | 1.000 | 0.878 | 1.000 | 0.990 | 1.000 | 0.974 | 1.000 | 1.000 | 0.804 | 1.000 | 1.000 | 0.866 | 0.838 |
| 2648 | 0.831 | 1.000 | 0.856 | 1.000 | 0.984 | 1.000 | 0.878 | 1.000 | 0.990 | 1.000 | 0.974 | 1.000 | 1.000 | 0.805 | 1.000 | 1.000 | 0.866 | 0.839 |
| 2649 | 0.831 | 1.000 | 0.856 | 1.000 | 0.984 | 1.000 | 0.878 | 1.000 | 0.990 | 1.000 | 0.974 | 1.000 | 1.000 | 0.805 | 1.000 | 1.000 | 0.866 | 0.839 |
| 2650 | 0.831 | 1.000 | 0.858 | 1.000 | 0.984 | 1.000 | 0.877 | 1.000 | 0.990 | 1.000 | 0.974 | 1.000 | 1.000 | 0.805 | 1.000 | 1.000 | 0.866 | 0.838 |
| 2651 | 0.831 | 1.000 | 0.858 | 1.000 | 0.984 | 1.000 | 0.877 | 1.000 | 0.990 | 1.000 | 0.974 | 1.000 | 1.000 | 0.804 | 1.000 | 1.000 | 0.866 | 0.838 |
| 2652 | 0.831 | 1.000 | 0.858 | 1.000 | 0.984 | 1.000 | 0.877 | 1.000 | 0.990 | 1.000 | 0.974 | 1.000 | 1.000 | 0.805 | 1.000 | 1.000 | 0.866 | 0.839 |
| 2653 | 0.831 | 1.000 | 0.858 | 1.000 | 0.984 | 1.000 | 0.877 | 1.000 | 0.990 | 1.000 | 0.974 | 1.000 | 1.000 | 0.805 | 1.000 | 1.000 | 0.866 | 0.839 |
| 2654 | 0.831 | 1.000 | 0.858 | 1.000 | 0.984 | 1.000 | 0.877 | 1.000 | 0.990 | 1.000 | 0.974 | 1.000 | 1.000 | 0.805 | 1.000 | 1.000 | 0.866 | 0.839 |
| 2655 | 0.831 | 1.000 | 0.856 | 1.000 | 0.984 | 1.000 | 0.877 | 1.000 | 0.990 | 1.000 | 0.974 | 1.000 | 1.000 | 0.804 | 1.000 | 1.000 | 0.866 | 0.838 |

| | | | | | | | | | | | | | | | | | | |
|---|---|---|---|---|---|---|---|---|---|---|---|---|---|---|---|---|---|---|
| 2656 | 0.831 | 1.000 | 0.856 | 1.000 | 0.984 | 1.000 | 0.877 | 1.000 | 0.990 | 1.000 | 0.974 | 1.000 | 1.000 | 0.804 | 1.000 | 1.000 | 0.866 | 0.838 |
| 2657 | 0.829 | 1.000 | 0.856 | 1.000 | 0.984 | 1.000 | 0.877 | 1.000 | 0.990 | 1.000 | 0.977 | 1.000 | 1.000 | 0.805 | 1.000 | 1.000 | 0.866 | 0.838 |
| 2658 | 0.830 | 1.000 | 0.858 | 1.000 | 0.984 | 1.000 | 0.877 | 1.000 | 0.990 | 1.000 | 0.977 | 1.000 | 1.000 | 0.805 | 1.000 | 1.000 | 0.866 | 0.838 |
| 2659 | 0.829 | 1.000 | 0.858 | 1.000 | 0.984 | 1.000 | 0.877 | 1.000 | 0.990 | 1.000 | 0.977 | 1.000 | 1.000 | 0.805 | 1.000 | 1.000 | 0.866 | 0.838 |
| 2660 | 0.830 | 1.000 | 0.858 | 1.000 | 0.984 | 1.000 | 0.877 | 1.000 | 0.990 | 1.000 | 0.974 | 1.000 | 1.000 | 0.805 | 1.000 | 1.000 | 0.866 | 0.838 |
| 2661 | 0.833 | 1.000 | 0.854 | 1.000 | 0.984 | 1.000 | 0.877 | 1.000 | 0.990 | 1.000 | 0.974 | 1.000 | 1.000 | 0.804 | 1.000 | 1.000 | 0.866 | 0.838 |
| 2662 | 0.834 | 1.000 | 0.854 | 1.000 | 0.984 | 1.000 | 0.877 | 1.000 | 0.990 | 1.000 | 0.974 | 1.000 | 1.000 | 0.804 | 1.000 | 1.000 | 0.866 | 0.839 |
| 2663 | 0.833 | 1.000 | 0.854 | 1.000 | 0.984 | 1.000 | 0.877 | 1.000 | 0.990 | 1.000 | 0.974 | 1.000 | 1.000 | 0.804 | 1.000 | 1.000 | 0.866 | 0.838 |
| 2664 | 0.834 | 1.000 | 0.854 | 1.000 | 0.984 | 1.000 | 0.877 | 1.000 | 0.990 | 1.000 | 0.974 | 1.000 | 1.000 | 0.804 | 1.000 | 1.000 | 0.866 | 0.839 |
| 2665 | 0.835 | 1.000 | 0.854 | 1.000 | 0.984 | 1.000 | 0.877 | 1.000 | 0.990 | 1.000 | 0.977 | 1.000 | 1.000 | 0.805 | 1.000 | 1.000 | 0.867 | 0.840 |
| 2666 | 0.835 | 1.000 | 0.856 | 1.000 | 0.984 | 1.000 | 0.877 | 1.000 | 0.990 | 1.000 | 0.977 | 1.000 | 1.000 | 0.805 | 1.000 | 1.000 | 0.867 | 0.840 |
| 2667 | 0.835 | 1.000 | 0.856 | 1.000 | 0.984 | 1.000 | 0.877 | 1.000 | 0.990 | 1.000 | 0.977 | 1.000 | 1.000 | 0.805 | 1.000 | 1.000 | 0.867 | 0.840 |
| 2668 | 0.835 | 1.000 | 0.856 | 1.000 | 0.984 | 1.000 | 0.877 | 1.000 | 0.990 | 1.000 | 0.977 | 1.000 | 1.000 | 0.805 | 1.000 | 1.000 | 0.867 | 0.840 |
| 2669 | 0.835 | 1.000 | 0.856 | 1.000 | 0.984 | 1.000 | 0.877 | 1.000 | 0.990 | 1.000 | 0.977 | 1.000 | 1.000 | 0.805 | 1.000 | 1.000 | 0.867 | 0.840 |
| 2670 | 0.835 | 1.000 | 0.856 | 1.000 | 0.984 | 1.000 | 0.877 | 1.000 | 0.990 | 1.000 | 0.977 | 1.000 | 1.000 | 0.805 | 1.000 | 1.000 | 0.867 | 0.840 |
| 2671 | 0.836 | 1.000 | 0.856 | 1.000 | 0.984 | 1.000 | 0.877 | 1.000 | 0.990 | 1.000 | 0.977 | 1.000 | 1.000 | 0.805 | 1.000 | 1.000 | 0.868 | 0.840 |
| 2672 | 0.837 | 1.000 | 0.856 | 1.000 | 0.979 | 1.000 | 0.878 | 1.000 | 0.990 | 1.000 | 0.977 | 1.000 | 1.000 | 0.804 | 1.000 | 1.000 | 0.868 | 0.840 |
| 2673 | 0.835 | 1.000 | 0.856 | 1.000 | 0.979 | 1.000 | 0.878 | 1.000 | 0.990 | 1.000 | 0.977 | 1.000 | 1.000 | 0.804 | 1.000 | 1.000 | 0.867 | 0.840 |
| 2674 | 0.836 | 1.000 | 0.856 | 1.000 | 0.984 | 1.000 | 0.878 | 1.000 | 1.000 | 1.000 | 0.977 | 1.000 | 1.000 | 0.803 | 1.000 | 1.000 | 0.868 | 0.840 |
| 2675 | 0.836 | 1.000 | 0.856 | 1.000 | 0.984 | 1.000 | 0.878 | 1.000 | 1.000 | 1.000 | 0.977 | 1.000 | 1.000 | 0.803 | 1.000 | 1.000 | 0.868 | 0.840 |
| 2676 | 0.837 | 1.000 | 0.856 | 1.000 | 0.984 | 1.000 | 0.878 | 1.000 | 1.000 | 1.000 | 0.974 | 1.000 | 1.000 | 0.803 | 1.000 | 1.000 | 0.868 | 0.840 |
| 2677 | 0.837 | 1.000 | 0.856 | 1.000 | 0.984 | 1.000 | 0.878 | 1.000 | 1.000 | 1.000 | 0.974 | 1.000 | 1.000 | 0.803 | 1.000 | 1.000 | 0.868 | 0.840 |
| 2678 | 0.837 | 1.000 | 0.856 | 1.000 | 0.984 | 1.000 | 0.878 | 1.000 | 1.000 | 1.000 | 0.974 | 1.000 | 1.000 | 0.803 | 1.000 | 1.000 | 0.868 | 0.840 |
| 2679 | 0.837 | 1.000 | 0.856 | 1.000 | 0.984 | 1.000 | 0.878 | 1.000 | 1.000 | 1.000 | 0.974 | 1.000 | 1.000 | 0.805 | 1.000 | 1.000 | 0.868 | 0.841 |
| 2680 | 0.837 | 1.000 | 0.852 | 1.000 | 0.984 | 1.000 | 0.878 | 1.000 | 1.000 | 1.000 | 0.974 | 1.000 | 1.000 | 0.804 | 1.000 | 1.000 | 0.867 | 0.840 |
| 2681 | 0.835 | 1.000 | 0.852 | 1.000 | 0.984 | 1.000 | 0.878 | 1.000 | 1.000 | 1.000 | 0.974 | 1.000 | 1.000 | 0.805 | 1.000 | 1.000 | 0.867 | 0.839 |
| 2682 | 0.835 | 1.000 | 0.854 | 1.000 | 0.984 | 1.000 | 0.878 | 1.000 | 1.000 | 1.000 | 0.974 | 1.000 | 1.000 | 0.804 | 1.000 | 1.000 | 0.867 | 0.840 |
| 2683 | 0.835 | 1.000 | 0.852 | 1.000 | 0.984 | 1.000 | 0.878 | 1.000 | 1.000 | 1.000 | 0.974 | 1.000 | 1.000 | 0.804 | 1.000 | 1.000 | 0.867 | 0.839 |
| 2684 | 0.835 | 1.000 | 0.852 | 1.000 | 0.984 | 1.000 | 0.878 | 1.000 | 1.000 | 1.000 | 0.974 | 1.000 | 1.000 | 0.804 | 1.000 | 1.000 | 0.867 | 0.839 |
| 2685 | 0.835 | 1.000 | 0.852 | 1.000 | 0.984 | 1.000 | 0.877 | 1.000 | 1.000 | 1.000 | 0.974 | 1.000 | 1.000 | 0.804 | 1.000 | 1.000 | 0.867 | 0.839 |
| 2686 | 0.834 | 1.000 | 0.852 | 1.000 | 0.984 | 1.000 | 0.877 | 1.000 | 1.000 | 1.000 | 0.974 | 1.000 | 1.000 | 0.805 | 1.000 | 1.000 | 0.867 | 0.839 |
| 2687 | 0.834 | 1.000 | 0.854 | 1.000 | 0.984 | 1.000 | 0.880 | 1.000 | 1.000 | 1.000 | 0.974 | 1.000 | 1.000 | 0.804 | 1.000 | 1.000 | 0.867 | 0.839 |
| 2688 | 0.835 | 1.000 | 0.856 | 1.000 | 0.984 | 1.000 | 0.878 | 1.000 | 1.000 | 1.000 | 0.974 | 1.000 | 1.000 | 0.805 | 1.000 | 1.000 | 0.868 | 0.840 |
| 2689 | 0.835 | 1.000 | 0.856 | 1.000 | 0.984 | 1.000 | 0.878 | 1.000 | 1.000 | 1.000 | 0.974 | 1.000 | 1.000 | 0.804 | 1.000 | 1.000 | 0.867 | 0.840 |
| 2690 | 0.833 | 1.000 | 0.858 | 1.000 | 0.984 | 1.000 | 0.877 | 1.000 | 1.000 | 1.000 | 0.974 | 1.000 | 1.000 | 0.802 | 1.000 | 1.000 | 0.866 | 0.839 |
| 2691 | 0.833 | 1.000 | 0.856 | 1.000 | 0.984 | 1.000 | 0.877 | 1.000 | 1.000 | 1.000 | 0.971 | 1.000 | 1.000 | 0.803 | 1.000 | 1.000 | 0.866 | 0.838 |
| 2692 | 0.834 | 1.000 | 0.862 | 1.000 | 0.979 | 1.000 | 0.880 | 1.000 | 1.000 | 1.000 | 0.971 | 1.000 | 1.000 | 0.801 | 1.000 | 1.000 | 0.867 | 0.839 |
| 2693 | 0.834 | 1.000 | 0.862 | 1.000 | 0.979 | 1.000 | 0.880 | 1.000 | 1.000 | 1.000 | 0.971 | 1.000 | 1.000 | 0.801 | 1.000 | 1.000 | 0.867 | 0.839 |
| 2694 | 0.834 | 1.000 | 0.862 | 1.000 | 0.979 | 1.000 | 0.880 | 1.000 | 1.000 | 1.000 | 0.971 | 1.000 | 1.000 | 0.802 | 1.000 | 1.000 | 0.867 | 0.839 |

| | | | | | | | | | | | | | | | | | | |
|---|---|---|---|---|---|---|---|---|---|---|---|---|---|---|---|---|---|---|
| 2695 | 0.833 | 1.000 | 0.864 | 1.000 | 0.979 | 1.000 | 0.880 | 1.000 | 1.000 | 1.000 | 0.971 | 1.000 | 1.000 | 0.801 | 1.000 | 1.000 | 0.866 | 0.839 |
| 2696 | 0.831 | 1.000 | 0.862 | 1.000 | 0.979 | 1.000 | 0.881 | 1.000 | 1.000 | 1.000 | 0.971 | 1.000 | 1.000 | 0.800 | 1.000 | 1.000 | 0.866 | 0.838 |
| 2697 | 0.831 | 1.000 | 0.860 | 1.000 | 0.979 | 1.000 | 0.881 | 1.000 | 1.000 | 1.000 | 0.971 | 1.000 | 1.000 | 0.800 | 1.000 | 1.000 | 0.866 | 0.838 |
| 2698 | 0.831 | 1.000 | 0.862 | 1.000 | 0.979 | 1.000 | 0.881 | 1.000 | 1.000 | 1.000 | 0.971 | 1.000 | 1.000 | 0.800 | 1.000 | 1.000 | 0.866 | 0.838 |
| 2699 | 0.831 | 1.000 | 0.862 | 1.000 | 0.979 | 1.000 | 0.881 | 1.000 | 1.000 | 1.000 | 0.971 | 1.000 | 1.000 | 0.800 | 1.000 | 1.000 | 0.866 | 0.838 |
| 2700 | 0.831 | 1.000 | 0.864 | 1.000 | 0.979 | 1.000 | 0.881 | 1.000 | 1.000 | 1.000 | 0.971 | 1.000 | 1.000 | 0.800 | 1.000 | 1.000 | 0.866 | 0.838 |
| 2701 | 0.832 | 1.000 | 0.866 | 1.000 | 0.979 | 1.000 | 0.878 | 1.000 | 1.000 | 1.000 | 0.971 | 1.000 | 1.000 | 0.801 | 1.000 | 1.000 | 0.866 | 0.839 |
| 2702 | 0.831 | 1.000 | 0.868 | 1.000 | 0.979 | 1.000 | 0.881 | 1.000 | 1.000 | 1.000 | 0.971 | 1.000 | 1.000 | 0.802 | 1.000 | 1.000 | 0.867 | 0.839 |
| 2703 | 0.830 | 1.000 | 0.870 | 1.000 | 0.979 | 1.000 | 0.880 | 1.000 | 1.000 | 1.000 | 0.971 | 1.000 | 1.000 | 0.803 | 1.000 | 1.000 | 0.867 | 0.839 |
| 2704 | 0.830 | 1.000 | 0.866 | 1.000 | 0.979 | 1.000 | 0.884 | 1.000 | 1.000 | 1.000 | 0.974 | 1.000 | 1.000 | 0.805 | 1.000 | 1.000 | 0.868 | 0.840 |
| 2705 | 0.831 | 1.000 | 0.866 | 1.000 | 0.979 | 1.000 | 0.884 | 1.000 | 1.000 | 1.000 | 0.971 | 1.000 | 1.000 | 0.804 | 1.000 | 1.000 | 0.868 | 0.840 |
| 2706 | 0.831 | 1.000 | 0.866 | 1.000 | 0.979 | 1.000 | 0.884 | 1.000 | 1.000 | 1.000 | 0.971 | 1.000 | 1.000 | 0.804 | 1.000 | 1.000 | 0.868 | 0.840 |
| 2707 | 0.831 | 1.000 | 0.864 | 1.000 | 0.979 | 1.000 | 0.884 | 1.000 | 1.000 | 1.000 | 0.971 | 1.000 | 1.000 | 0.804 | 1.000 | 1.000 | 0.867 | 0.840 |
| 2708 | 0.831 | 1.000 | 0.862 | 1.000 | 0.979 | 1.000 | 0.884 | 1.000 | 1.000 | 1.000 | 0.971 | 1.000 | 1.000 | 0.804 | 1.000 | 1.000 | 0.867 | 0.840 |
| 2709 | 0.831 | 1.000 | 0.862 | 1.000 | 0.979 | 1.000 | 0.884 | 1.000 | 1.000 | 1.000 | 0.971 | 1.000 | 1.000 | 0.804 | 1.000 | 1.000 | 0.867 | 0.840 |
| 2710 | 0.831 | 1.000 | 0.862 | 1.000 | 0.979 | 1.000 | 0.884 | 1.000 | 1.000 | 1.000 | 0.971 | 1.000 | 1.000 | 0.804 | 1.000 | 1.000 | 0.867 | 0.840 |
| 2711 | 0.831 | 1.000 | 0.862 | 1.000 | 0.979 | 1.000 | 0.886 | 1.000 | 1.000 | 1.000 | 0.974 | 1.000 | 1.000 | 0.804 | 1.000 | 1.000 | 0.868 | 0.840 |
| 2712 | 0.831 | 1.000 | 0.862 | 1.000 | 0.979 | 1.000 | 0.886 | 1.000 | 1.000 | 1.000 | 0.971 | 1.000 | 1.000 | 0.805 | 1.000 | 1.000 | 0.868 | 0.841 |
| 2713 | 0.831 | 1.000 | 0.868 | 1.000 | 0.979 | 1.000 | 0.886 | 1.000 | 1.000 | 1.000 | 0.971 | 1.000 | 1.000 | 0.805 | 1.000 | 1.000 | 0.868 | 0.841 |
| 2714 | 0.831 | 1.000 | 0.868 | 1.000 | 0.979 | 1.000 | 0.886 | 1.000 | 1.000 | 1.000 | 0.971 | 1.000 | 1.000 | 0.806 | 1.000 | 1.000 | 0.869 | 0.842 |
| 2715 | 0.831 | 1.000 | 0.866 | 1.000 | 0.979 | 1.000 | 0.886 | 1.000 | 1.000 | 1.000 | 0.971 | 1.000 | 1.000 | 0.806 | 1.000 | 1.000 | 0.868 | 0.841 |
| 2716 | 0.832 | 1.000 | 0.866 | 1.000 | 0.979 | 1.000 | 0.886 | 1.000 | 1.000 | 1.000 | 0.971 | 1.000 | 1.000 | 0.805 | 1.000 | 1.000 | 0.868 | 0.841 |
| 2717 | 0.832 | 1.000 | 0.866 | 1.000 | 0.979 | 1.000 | 0.886 | 1.000 | 1.000 | 1.000 | 0.971 | 1.000 | 1.000 | 0.805 | 1.000 | 1.000 | 0.868 | 0.841 |
| 2718 | 0.831 | 1.000 | 0.864 | 1.000 | 0.979 | 1.000 | 0.886 | 1.000 | 1.000 | 1.000 | 0.971 | 1.000 | 1.000 | 0.805 | 1.000 | 1.000 | 0.868 | 0.841 |
| 2719 | 0.833 | 1.000 | 0.864 | 1.000 | 0.984 | 1.000 | 0.886 | 1.000 | 1.000 | 1.000 | 0.971 | 1.000 | 1.000 | 0.805 | 1.000 | 1.000 | 0.869 | 0.842 |
| 2720 | 0.831 | 1.000 | 0.864 | 1.000 | 0.984 | 1.000 | 0.886 | 1.000 | 1.000 | 1.000 | 0.971 | 1.000 | 1.000 | 0.807 | 1.000 | 1.000 | 0.869 | 0.842 |
| 2721 | 0.831 | 1.000 | 0.864 | 1.000 | 0.984 | 1.000 | 0.886 | 1.000 | 1.000 | 1.000 | 0.971 | 1.000 | 1.000 | 0.807 | 1.000 | 1.000 | 0.869 | 0.842 |
| 2722 | 0.829 | 1.000 | 0.864 | 1.000 | 0.979 | 1.000 | 0.886 | 1.000 | 1.000 | 1.000 | 0.974 | 1.000 | 1.000 | 0.806 | 1.000 | 1.000 | 0.868 | 0.841 |
| 2723 | 0.829 | 1.000 | 0.862 | 1.000 | 0.979 | 1.000 | 0.886 | 1.000 | 1.000 | 1.000 | 0.974 | 1.000 | 1.000 | 0.809 | 1.000 | 1.000 | 0.868 | 0.841 |
| 2724 | 0.829 | 1.000 | 0.862 | 1.000 | 0.979 | 1.000 | 0.886 | 1.000 | 1.000 | 1.000 | 0.974 | 1.000 | 1.000 | 0.808 | 1.000 | 1.000 | 0.868 | 0.841 |
| 2725 | 0.829 | 1.000 | 0.862 | 1.000 | 0.984 | 1.000 | 0.886 | 1.000 | 1.000 | 1.000 | 0.974 | 1.000 | 1.000 | 0.806 | 1.000 | 1.000 | 0.868 | 0.840 |
| 2726 | 0.828 | 1.000 | 0.862 | 1.000 | 0.984 | 1.000 | 0.884 | 1.000 | 1.000 | 1.000 | 0.974 | 1.000 | 1.000 | 0.807 | 1.000 | 1.000 | 0.867 | 0.840 |
| 2727 | 0.828 | 1.000 | 0.864 | 1.000 | 0.984 | 1.000 | 0.884 | 1.000 | 1.000 | 1.000 | 0.971 | 1.000 | 1.000 | 0.808 | 1.000 | 1.000 | 0.868 | 0.840 |
| 2728 | 0.830 | 1.000 | 0.860 | 1.000 | 0.984 | 1.000 | 0.884 | 1.000 | 1.000 | 1.000 | 0.971 | 1.000 | 1.000 | 0.809 | 1.000 | 1.000 | 0.868 | 0.841 |
| 2729 | 0.829 | 1.000 | 0.860 | 1.000 | 0.984 | 1.000 | 0.884 | 1.000 | 1.000 | 1.000 | 0.971 | 1.000 | 1.000 | 0.809 | 1.000 | 1.000 | 0.868 | 0.841 |
| 2730 | 0.829 | 1.000 | 0.858 | 1.000 | 0.984 | 1.000 | 0.883 | 1.000 | 1.000 | 1.000 | 0.974 | 1.000 | 1.000 | 0.809 | 1.000 | 1.000 | 0.868 | 0.841 |
| 2731 | 0.831 | 1.000 | 0.858 | 1.000 | 0.984 | 1.000 | 0.883 | 1.000 | 1.000 | 1.000 | 0.974 | 1.000 | 1.000 | 0.810 | 1.000 | 1.000 | 0.869 | 0.841 |
| 2732 | 0.829 | 1.000 | 0.862 | 1.000 | 0.984 | 1.000 | 0.883 | 1.000 | 1.000 | 1.000 | 0.974 | 1.000 | 1.000 | 0.808 | 1.000 | 1.000 | 0.868 | 0.840 |
| 2733 | 0.829 | 1.000 | 0.862 | 1.000 | 0.984 | 1.000 | 0.883 | 1.000 | 1.000 | 1.000 | 0.974 | 1.000 | 1.000 | 0.808 | 1.000 | 1.000 | 0.868 | 0.840 |

| | | | | | | | | | | | | | | | | | | |
|---|---|---|---|---|---|---|---|---|---|---|---|---|---|---|---|---|---|---|
| 2734 | 0.829 | 1.000 | 0.862 | 1.000 | 0.984 | 1.000 | 0.883 | 1.000 | 1.000 | 1.000 | 0.974 | 1.000 | 1.000 | 0.808 | 1.000 | 1.000 | 0.868 | 0.841 |
| 2735 | 0.829 | 1.000 | 0.860 | 1.000 | 0.984 | 1.000 | 0.883 | 1.000 | 1.000 | 1.000 | 0.974 | 1.000 | 1.000 | 0.809 | 1.000 | 1.000 | 0.868 | 0.841 |
| 2736 | 0.829 | 1.000 | 0.860 | 1.000 | 0.984 | 1.000 | 0.883 | 1.000 | 1.000 | 1.000 | 0.974 | 1.000 | 1.000 | 0.808 | 1.000 | 1.000 | 0.868 | 0.840 |
| 2737 | 0.832 | 1.000 | 0.860 | 1.000 | 0.984 | 1.000 | 0.880 | 1.000 | 1.000 | 1.000 | 0.974 | 1.000 | 1.000 | 0.809 | 1.000 | 1.000 | 0.868 | 0.841 |
| 2738 | 0.832 | 1.000 | 0.860 | 1.000 | 0.984 | 1.000 | 0.880 | 1.000 | 1.000 | 1.000 | 0.974 | 1.000 | 1.000 | 0.809 | 1.000 | 1.000 | 0.868 | 0.841 |
| 2739 | 0.831 | 1.000 | 0.858 | 1.000 | 0.984 | 1.000 | 0.880 | 1.000 | 1.000 | 1.000 | 0.974 | 1.000 | 1.000 | 0.809 | 1.000 | 1.000 | 0.868 | 0.841 |
| 2740 | 0.831 | 1.000 | 0.858 | 1.000 | 0.984 | 1.000 | 0.880 | 1.000 | 1.000 | 1.000 | 0.974 | 1.000 | 1.000 | 0.809 | 1.000 | 1.000 | 0.868 | 0.840 |
| 2741 | 0.831 | 1.000 | 0.860 | 1.000 | 0.984 | 1.000 | 0.883 | 1.000 | 1.000 | 1.000 | 0.974 | 1.000 | 1.000 | 0.811 | 1.000 | 1.000 | 0.869 | 0.842 |
| 2742 | 0.832 | 1.000 | 0.860 | 1.000 | 0.984 | 1.000 | 0.883 | 1.000 | 1.000 | 1.000 | 0.974 | 1.000 | 1.000 | 0.811 | 1.000 | 1.000 | 0.869 | 0.842 |
| 2743 | 0.832 | 1.000 | 0.860 | 1.000 | 0.984 | 1.000 | 0.881 | 1.000 | 1.000 | 1.000 | 0.974 | 1.000 | 1.000 | 0.812 | 1.000 | 1.000 | 0.869 | 0.842 |
| 2744 | 0.831 | 1.000 | 0.860 | 1.000 | 0.984 | 1.000 | 0.881 | 1.000 | 1.000 | 1.000 | 0.974 | 1.000 | 1.000 | 0.812 | 1.000 | 1.000 | 0.869 | 0.842 |
| 2745 | 0.831 | 1.000 | 0.862 | 1.000 | 0.984 | 1.000 | 0.881 | 1.000 | 1.000 | 1.000 | 0.974 | 1.000 | 1.000 | 0.810 | 1.000 | 1.000 | 0.869 | 0.842 |
| 2746 | 0.831 | 1.000 | 0.860 | 1.000 | 0.984 | 1.000 | 0.881 | 1.000 | 1.000 | 1.000 | 0.974 | 1.000 | 1.000 | 0.810 | 1.000 | 1.000 | 0.869 | 0.841 |
| 2747 | 0.831 | 1.000 | 0.860 | 1.000 | 0.984 | 1.000 | 0.881 | 1.000 | 1.000 | 1.000 | 0.974 | 1.000 | 1.000 | 0.810 | 1.000 | 1.000 | 0.868 | 0.841 |
| 2748 | 0.831 | 1.000 | 0.860 | 1.000 | 0.984 | 1.000 | 0.883 | 1.000 | 1.000 | 1.000 | 0.974 | 1.000 | 1.000 | 0.809 | 1.000 | 1.000 | 0.868 | 0.841 |
| 2749 | 0.830 | 1.000 | 0.862 | 1.000 | 0.984 | 1.000 | 0.883 | 1.000 | 1.000 | 1.000 | 0.974 | 1.000 | 1.000 | 0.809 | 1.000 | 1.000 | 0.868 | 0.841 |
| 2750 | 0.830 | 1.000 | 0.862 | 1.000 | 0.984 | 1.000 | 0.883 | 1.000 | 1.000 | 1.000 | 0.977 | 1.000 | 1.000 | 0.810 | 1.000 | 1.000 | 0.869 | 0.842 |
| 2751 | 0.831 | 1.000 | 0.862 | 1.000 | 0.984 | 1.000 | 0.883 | 1.000 | 1.000 | 1.000 | 0.977 | 1.000 | 1.000 | 0.811 | 1.000 | 1.000 | 0.869 | 0.842 |
| 2752 | 0.834 | 1.000 | 0.858 | 1.000 | 0.984 | 1.000 | 0.888 | 1.000 | 1.000 | 1.000 | 0.977 | 1.000 | 1.000 | 0.811 | 1.000 | 1.000 | 0.870 | 0.844 |
| 2753 | 0.835 | 1.000 | 0.858 | 1.000 | 0.984 | 1.000 | 0.888 | 1.000 | 1.000 | 1.000 | 0.977 | 1.000 | 1.000 | 0.810 | 1.000 | 1.000 | 0.870 | 0.844 |
| 2754 | 0.835 | 1.000 | 0.858 | 1.000 | 0.984 | 1.000 | 0.888 | 1.000 | 1.000 | 1.000 | 0.977 | 1.000 | 1.000 | 0.812 | 1.000 | 1.000 | 0.871 | 0.844 |
| 2755 | 0.835 | 1.000 | 0.858 | 1.000 | 0.984 | 1.000 | 0.888 | 1.000 | 1.000 | 1.000 | 0.977 | 1.000 | 1.000 | 0.809 | 1.000 | 1.000 | 0.870 | 0.843 |
| 2756 | 0.835 | 1.000 | 0.858 | 1.000 | 0.984 | 1.000 | 0.888 | 1.000 | 1.000 | 1.000 | 0.977 | 1.000 | 1.000 | 0.810 | 1.000 | 1.000 | 0.870 | 0.844 |
| 2757 | 0.835 | 1.000 | 0.858 | 1.000 | 0.984 | 1.000 | 0.888 | 1.000 | 1.000 | 1.000 | 0.977 | 1.000 | 1.000 | 0.809 | 1.000 | 1.000 | 0.870 | 0.843 |
| 2758 | 0.834 | 1.000 | 0.860 | 1.000 | 0.984 | 1.000 | 0.888 | 1.000 | 1.000 | 1.000 | 0.977 | 1.000 | 1.000 | 0.808 | 1.000 | 1.000 | 0.870 | 0.843 |
| 2759 | 0.834 | 1.000 | 0.860 | 1.000 | 0.984 | 1.000 | 0.888 | 1.000 | 1.000 | 1.000 | 0.977 | 1.000 | 1.000 | 0.808 | 1.000 | 1.000 | 0.870 | 0.843 |
| 2760 | 0.834 | 1.000 | 0.860 | 1.000 | 0.984 | 1.000 | 0.888 | 1.000 | 1.000 | 1.000 | 0.977 | 1.000 | 1.000 | 0.807 | 1.000 | 1.000 | 0.870 | 0.843 |
| 2761 | 0.833 | 1.000 | 0.858 | 1.000 | 0.984 | 1.000 | 0.884 | 1.000 | 1.000 | 1.000 | 0.971 | 1.000 | 1.000 | 0.809 | 1.000 | 1.000 | 0.869 | 0.842 |
| 2762 | 0.833 | 1.000 | 0.858 | 1.000 | 0.984 | 1.000 | 0.884 | 1.000 | 1.000 | 1.000 | 0.971 | 1.000 | 1.000 | 0.809 | 1.000 | 1.000 | 0.869 | 0.841 |
| 2763 | 0.833 | 1.000 | 0.858 | 1.000 | 0.984 | 1.000 | 0.884 | 1.000 | 1.000 | 1.000 | 0.971 | 1.000 | 1.000 | 0.809 | 1.000 | 1.000 | 0.869 | 0.841 |
| 2764 | 0.833 | 1.000 | 0.858 | 1.000 | 0.984 | 1.000 | 0.884 | 1.000 | 1.000 | 1.000 | 0.971 | 1.000 | 1.000 | 0.808 | 1.000 | 1.000 | 0.868 | 0.841 |
| 2765 | 0.833 | 1.000 | 0.858 | 1.000 | 0.984 | 1.000 | 0.884 | 1.000 | 1.000 | 1.000 | 0.971 | 1.000 | 1.000 | 0.808 | 1.000 | 1.000 | 0.868 | 0.841 |
| 2766 | 0.832 | 1.000 | 0.858 | 1.000 | 0.984 | 1.000 | 0.884 | 1.000 | 1.000 | 1.000 | 0.971 | 1.000 | 1.000 | 0.808 | 1.000 | 1.000 | 0.868 | 0.841 |
| 2767 | 0.832 | 1.000 | 0.858 | 1.000 | 0.984 | 1.000 | 0.884 | 1.000 | 1.000 | 1.000 | 0.971 | 1.000 | 1.000 | 0.809 | 1.000 | 1.000 | 0.868 | 0.841 |
| 2768 | 0.832 | 1.000 | 0.858 | 1.000 | 0.984 | 1.000 | 0.884 | 1.000 | 1.000 | 1.000 | 0.971 | 1.000 | 1.000 | 0.809 | 1.000 | 1.000 | 0.868 | 0.841 |
| 2769 | 0.832 | 1.000 | 0.858 | 1.000 | 0.984 | 1.000 | 0.884 | 1.000 | 1.000 | 1.000 | 0.971 | 1.000 | 1.000 | 0.809 | 1.000 | 1.000 | 0.868 | 0.841 |
| 2770 | 0.832 | 1.000 | 0.858 | 1.000 | 0.984 | 1.000 | 0.884 | 1.000 | 1.000 | 1.000 | 0.971 | 1.000 | 1.000 | 0.809 | 1.000 | 1.000 | 0.868 | 0.841 |
| 2771 | 0.832 | 1.000 | 0.858 | 1.000 | 0.984 | 1.000 | 0.884 | 1.000 | 1.000 | 1.000 | 0.971 | 1.000 | 1.000 | 0.809 | 1.000 | 1.000 | 0.868 | 0.841 |
| 2772 | 0.833 | 1.000 | 0.860 | 1.000 | 0.984 | 1.000 | 0.884 | 1.000 | 1.000 | 1.000 | 0.974 | 1.000 | 1.000 | 0.807 | 1.000 | 1.000 | 0.869 | 0.842 |

| | | | | | | | | | | | | | | | | | |
|---|---|---|---|---|---|---|---|---|---|---|---|---|---|---|---|---|---|
| 2773 | 0.835 | 1.000 | 0.858 | 1.000 | 0.984 | 1.000 | 0.884 | 1.000 | 1.000 | 1.000 | 0.971 | 1.000 | 1.000 | 0.808 | 1.000 | 1.000 | 0.869 | 0.842 |
| 2774 | 0.834 | 1.000 | 0.858 | 1.000 | 0.984 | 1.000 | 0.883 | 1.000 | 1.000 | 1.000 | 0.971 | 1.000 | 1.000 | 0.809 | 1.000 | 1.000 | 0.869 | 0.842 |
| 2775 | 0.835 | 1.000 | 0.858 | 1.000 | 0.984 | 1.000 | 0.883 | 1.000 | 1.000 | 1.000 | 0.968 | 1.000 | 1.000 | 0.809 | 1.000 | 1.000 | 0.869 | 0.842 |
| 2776 | 0.835 | 1.000 | 0.858 | 1.000 | 0.984 | 1.000 | 0.883 | 1.000 | 1.000 | 1.000 | 0.968 | 1.000 | 1.000 | 0.809 | 1.000 | 1.000 | 0.869 | 0.842 |
| 2777 | 0.834 | 1.000 | 0.858 | 1.000 | 0.984 | 1.000 | 0.883 | 1.000 | 1.000 | 1.000 | 0.968 | 1.000 | 1.000 | 0.809 | 1.000 | 1.000 | 0.869 | 0.841 |
| 2778 | 0.833 | 1.000 | 0.858 | 1.000 | 0.984 | 1.000 | 0.883 | 1.000 | 1.000 | 1.000 | 0.968 | 1.000 | 1.000 | 0.809 | 1.000 | 1.000 | 0.868 | 0.841 |
| 2779 | 0.834 | 1.000 | 0.858 | 1.000 | 0.984 | 1.000 | 0.883 | 1.000 | 1.000 | 1.000 | 0.968 | 1.000 | 1.000 | 0.809 | 1.000 | 1.000 | 0.869 | 0.841 |
| 2780 | 0.835 | 1.000 | 0.858 | 1.000 | 0.984 | 1.000 | 0.884 | 1.000 | 1.000 | 1.000 | 0.968 | 1.000 | 1.000 | 0.811 | 1.000 | 1.000 | 0.870 | 0.843 |
| 2781 | 0.835 | 1.000 | 0.858 | 1.000 | 0.984 | 1.000 | 0.884 | 1.000 | 1.000 | 1.000 | 0.968 | 1.000 | 1.000 | 0.811 | 1.000 | 1.000 | 0.870 | 0.843 |
| 2782 | 0.835 | 1.000 | 0.858 | 1.000 | 0.984 | 1.000 | 0.884 | 1.000 | 1.000 | 1.000 | 0.968 | 1.000 | 1.000 | 0.811 | 1.000 | 1.000 | 0.870 | 0.843 |
| 2783 | 0.831 | 1.000 | 0.860 | 1.000 | 0.984 | 1.000 | 0.884 | 1.000 | 1.000 | 1.000 | 0.968 | 1.000 | 1.000 | 0.809 | 1.000 | 1.000 | 0.868 | 0.841 |
| 2784 | 0.831 | 1.000 | 0.860 | 1.000 | 0.979 | 1.000 | 0.884 | 1.000 | 1.000 | 1.000 | 0.968 | 1.000 | 1.000 | 0.809 | 1.000 | 1.000 | 0.868 | 0.841 |
| 2785 | 0.833 | 1.000 | 0.860 | 1.000 | 0.979 | 1.000 | 0.884 | 1.000 | 1.000 | 1.000 | 0.968 | 1.000 | 1.000 | 0.809 | 1.000 | 1.000 | 0.868 | 0.841 |
| 2786 | 0.834 | 1.000 | 0.858 | 1.000 | 0.984 | 1.000 | 0.888 | 1.000 | 1.000 | 1.000 | 0.968 | 1.000 | 1.000 | 0.808 | 1.000 | 1.000 | 0.869 | 0.842 |
| 2787 | 0.835 | 1.000 | 0.856 | 1.000 | 0.984 | 1.000 | 0.889 | 1.000 | 1.000 | 1.000 | 0.968 | 1.000 | 1.000 | 0.807 | 1.000 | 1.000 | 0.869 | 0.842 |
| 2788 | 0.836 | 1.000 | 0.856 | 1.000 | 0.984 | 1.000 | 0.889 | 1.000 | 1.000 | 1.000 | 0.968 | 1.000 | 1.000 | 0.805 | 1.000 | 1.000 | 0.869 | 0.842 |
| 2789 | 0.837 | 1.000 | 0.856 | 1.000 | 0.984 | 1.000 | 0.889 | 1.000 | 1.000 | 1.000 | 0.968 | 1.000 | 1.000 | 0.805 | 1.000 | 1.000 | 0.869 | 0.842 |
| 2790 | 0.835 | 1.000 | 0.858 | 1.000 | 0.979 | 1.000 | 0.889 | 1.000 | 1.000 | 1.000 | 0.968 | 1.000 | 1.000 | 0.807 | 1.000 | 1.000 | 0.869 | 0.842 |
| 2791 | 0.835 | 1.000 | 0.856 | 1.000 | 0.984 | 1.000 | 0.888 | 1.000 | 1.000 | 1.000 | 0.968 | 1.000 | 1.000 | 0.808 | 1.000 | 1.000 | 0.869 | 0.842 |
| 2792 | 0.835 | 1.000 | 0.856 | 1.000 | 0.984 | 1.000 | 0.888 | 1.000 | 1.000 | 1.000 | 0.968 | 1.000 | 1.000 | 0.808 | 1.000 | 1.000 | 0.869 | 0.842 |
| 2793 | 0.835 | 1.000 | 0.856 | 1.000 | 0.984 | 1.000 | 0.886 | 1.000 | 1.000 | 1.000 | 0.968 | 1.000 | 1.000 | 0.808 | 1.000 | 1.000 | 0.869 | 0.842 |
| 2794 | 0.835 | 1.000 | 0.856 | 1.000 | 0.984 | 1.000 | 0.884 | 1.000 | 1.000 | 1.000 | 0.968 | 1.000 | 1.000 | 0.808 | 1.000 | 1.000 | 0.869 | 0.842 |
| 2795 | 0.835 | 1.000 | 0.858 | 1.000 | 0.984 | 1.000 | 0.884 | 1.000 | 1.000 | 1.000 | 0.968 | 1.000 | 1.000 | 0.808 | 1.000 | 1.000 | 0.869 | 0.842 |
| 2796 | 0.835 | 1.000 | 0.856 | 1.000 | 0.984 | 1.000 | 0.883 | 1.000 | 1.000 | 1.000 | 0.968 | 1.000 | 1.000 | 0.808 | 1.000 | 1.000 | 0.869 | 0.841 |
| 2797 | 0.835 | 1.000 | 0.856 | 1.000 | 0.984 | 1.000 | 0.883 | 1.000 | 1.000 | 1.000 | 0.968 | 1.000 | 1.000 | 0.808 | 1.000 | 1.000 | 0.869 | 0.841 |
| 2798 | 0.832 | 1.000 | 0.850 | 1.000 | 0.979 | 1.000 | 0.881 | 1.000 | 1.000 | 1.000 | 0.968 | 1.000 | 1.000 | 0.810 | 1.000 | 1.000 | 0.867 | 0.840 |
| 2799 | 0.832 | 1.000 | 0.850 | 1.000 | 0.979 | 1.000 | 0.881 | 1.000 | 1.000 | 1.000 | 0.968 | 1.000 | 1.000 | 0.810 | 1.000 | 1.000 | 0.867 | 0.840 |
| 2800 | 0.832 | 1.000 | 0.852 | 1.000 | 0.979 | 1.000 | 0.881 | 1.000 | 1.000 | 1.000 | 0.968 | 1.000 | 1.000 | 0.809 | 1.000 | 1.000 | 0.867 | 0.840 |
| 2801 | 0.832 | 1.000 | 0.852 | 1.000 | 0.979 | 1.000 | 0.881 | 1.000 | 1.000 | 1.000 | 0.968 | 1.000 | 1.000 | 0.809 | 1.000 | 1.000 | 0.867 | 0.840 |
| 2802 | 0.833 | 1.000 | 0.850 | 1.000 | 0.979 | 1.000 | 0.883 | 1.000 | 1.000 | 1.000 | 0.968 | 1.000 | 1.000 | 0.809 | 1.000 | 1.000 | 0.867 | 0.840 |
| 2803 | 0.833 | 1.000 | 0.852 | 1.000 | 0.979 | 1.000 | 0.881 | 1.000 | 1.000 | 1.000 | 0.968 | 1.000 | 1.000 | 0.809 | 1.000 | 1.000 | 0.867 | 0.840 |
| 2804 | 0.833 | 1.000 | 0.852 | 1.000 | 0.979 | 1.000 | 0.883 | 1.000 | 1.000 | 1.000 | 0.968 | 1.000 | 1.000 | 0.809 | 1.000 | 1.000 | 0.868 | 0.840 |
| 2805 | 0.833 | 1.000 | 0.852 | 1.000 | 0.979 | 1.000 | 0.881 | 1.000 | 1.000 | 1.000 | 0.968 | 1.000 | 1.000 | 0.810 | 1.000 | 1.000 | 0.868 | 0.840 |
| 2806 | 0.834 | 1.000 | 0.852 | 1.000 | 0.979 | 1.000 | 0.881 | 1.000 | 1.000 | 1.000 | 0.968 | 1.000 | 1.000 | 0.809 | 1.000 | 1.000 | 0.868 | 0.840 |
| 2807 | 0.833 | 1.000 | 0.852 | 1.000 | 0.979 | 1.000 | 0.880 | 1.000 | 1.000 | 1.000 | 0.968 | 1.000 | 1.000 | 0.810 | 1.000 | 1.000 | 0.868 | 0.840 |
| 2808 | 0.833 | 1.000 | 0.852 | 1.000 | 0.979 | 1.000 | 0.880 | 1.000 | 1.000 | 1.000 | 0.968 | 1.000 | 1.000 | 0.810 | 1.000 | 1.000 | 0.868 | 0.840 |
| 2809 | 0.833 | 1.000 | 0.854 | 1.000 | 0.979 | 1.000 | 0.881 | 1.000 | 1.000 | 1.000 | 0.968 | 1.000 | 1.000 | 0.809 | 1.000 | 1.000 | 0.868 | 0.840 |
| 2810 | 0.833 | 1.000 | 0.854 | 1.000 | 0.979 | 1.000 | 0.881 | 1.000 | 1.000 | 1.000 | 0.968 | 1.000 | 1.000 | 0.809 | 1.000 | 1.000 | 0.868 | 0.840 |
| 2811 | 0.833 | 1.000 | 0.854 | 1.000 | 0.979 | 1.000 | 0.881 | 1.000 | 1.000 | 1.000 | 0.968 | 1.000 | 1.000 | 0.809 | 1.000 | 1.000 | 0.868 | 0.840 |

| | | | | | | | | | | | | | | | | | | |
|---|---|---|---|---|---|---|---|---|---|---|---|---|---|---|---|---|---|---|
| 2812 | 0.832 | 1.000 | 0.856 | 1.000 | 0.979 | 1.000 | 0.880 | 1.000 | 1.000 | 1.000 | 0.968 | 1.000 | 1.000 | 0.809 | 1.000 | 1.000 | 0.867 | 0.840 |
| 2813 | 0.833 | 1.000 | 0.856 | 1.000 | 0.979 | 1.000 | 0.880 | 1.000 | 1.000 | 1.000 | 0.968 | 1.000 | 1.000 | 0.809 | 1.000 | 1.000 | 0.868 | 0.840 |
| 2814 | 0.834 | 1.000 | 0.854 | 1.000 | 0.979 | 1.000 | 0.880 | 1.000 | 1.000 | 1.000 | 0.968 | 1.000 | 1.000 | 0.809 | 1.000 | 1.000 | 0.868 | 0.840 |
| 2815 | 0.834 | 1.000 | 0.854 | 1.000 | 0.979 | 1.000 | 0.881 | 1.000 | 1.000 | 1.000 | 0.968 | 1.000 | 1.000 | 0.809 | 1.000 | 1.000 | 0.868 | 0.840 |
| 2816 | 0.834 | 1.000 | 0.856 | 1.000 | 0.979 | 1.000 | 0.881 | 1.000 | 1.000 | 1.000 | 0.968 | 1.000 | 1.000 | 0.809 | 1.000 | 1.000 | 0.868 | 0.841 |
| 2817 | 0.833 | 1.000 | 0.856 | 1.000 | 0.979 | 1.000 | 0.880 | 1.000 | 1.000 | 1.000 | 0.968 | 1.000 | 1.000 | 0.809 | 1.000 | 1.000 | 0.868 | 0.840 |
| 2818 | 0.833 | 1.000 | 0.856 | 1.000 | 0.979 | 1.000 | 0.881 | 1.000 | 1.000 | 1.000 | 0.968 | 1.000 | 1.000 | 0.809 | 1.000 | 1.000 | 0.868 | 0.840 |
| 2819 | 0.834 | 1.000 | 0.852 | 1.000 | 0.979 | 1.000 | 0.880 | 1.000 | 1.000 | 1.000 | 0.968 | 1.000 | 1.000 | 0.809 | 1.000 | 1.000 | 0.867 | 0.840 |
| 2820 | 0.833 | 1.000 | 0.852 | 1.000 | 0.979 | 1.000 | 0.880 | 1.000 | 1.000 | 1.000 | 0.968 | 1.000 | 1.000 | 0.809 | 1.000 | 1.000 | 0.867 | 0.839 |
| 2821 | 0.835 | 1.000 | 0.854 | 1.000 | 0.979 | 1.000 | 0.883 | 1.000 | 1.000 | 1.000 | 0.968 | 1.000 | 1.000 | 0.809 | 1.000 | 1.000 | 0.868 | 0.841 |
| 2822 | 0.836 | 1.000 | 0.854 | 1.000 | 0.979 | 1.000 | 0.883 | 1.000 | 1.000 | 1.000 | 0.968 | 1.000 | 1.000 | 0.809 | 1.000 | 1.000 | 0.869 | 0.842 |
| 2823 | 0.835 | 1.000 | 0.854 | 1.000 | 0.979 | 1.000 | 0.883 | 1.000 | 1.000 | 1.000 | 0.968 | 1.000 | 1.000 | 0.809 | 1.000 | 1.000 | 0.869 | 0.841 |
| 2824 | 0.836 | 1.000 | 0.854 | 1.000 | 0.979 | 1.000 | 0.881 | 1.000 | 1.000 | 1.000 | 0.968 | 1.000 | 1.000 | 0.809 | 1.000 | 1.000 | 0.869 | 0.841 |
| 2825 | 0.837 | 1.000 | 0.854 | 1.000 | 0.979 | 1.000 | 0.884 | 1.000 | 1.000 | 1.000 | 0.968 | 1.000 | 1.000 | 0.809 | 1.000 | 1.000 | 0.869 | 0.842 |
| 2826 | 0.836 | 1.000 | 0.854 | 1.000 | 0.979 | 1.000 | 0.883 | 1.000 | 1.000 | 1.000 | 0.968 | 1.000 | 1.000 | 0.809 | 1.000 | 1.000 | 0.869 | 0.841 |
| 2827 | 0.836 | 1.000 | 0.854 | 1.000 | 0.979 | 1.000 | 0.883 | 1.000 | 1.000 | 1.000 | 0.968 | 1.000 | 1.000 | 0.809 | 1.000 | 1.000 | 0.869 | 0.841 |
| 2828 | 0.837 | 1.000 | 0.854 | 1.000 | 0.979 | 1.000 | 0.883 | 1.000 | 1.000 | 1.000 | 0.968 | 1.000 | 1.000 | 0.809 | 1.000 | 1.000 | 0.869 | 0.842 |
| 2829 | 0.837 | 1.000 | 0.854 | 1.000 | 0.979 | 1.000 | 0.884 | 1.000 | 1.000 | 1.000 | 0.968 | 1.000 | 1.000 | 0.809 | 1.000 | 1.000 | 0.869 | 0.842 |
| 2830 | 0.837 | 1.000 | 0.856 | 1.000 | 0.979 | 1.000 | 0.883 | 1.000 | 1.000 | 1.000 | 0.974 | 1.000 | 1.000 | 0.805 | 1.000 | 1.000 | 0.869 | 0.841 |
| 2831 | 0.836 | 1.000 | 0.856 | 1.000 | 0.979 | 1.000 | 0.881 | 1.000 | 1.000 | 1.000 | 0.974 | 1.000 | 1.000 | 0.805 | 1.000 | 1.000 | 0.868 | 0.841 |
| 2832 | 0.836 | 1.000 | 0.856 | 1.000 | 0.979 | 1.000 | 0.881 | 1.000 | 1.000 | 1.000 | 0.974 | 1.000 | 1.000 | 0.805 | 1.000 | 1.000 | 0.868 | 0.841 |
| 2833 | 0.836 | 1.000 | 0.856 | 1.000 | 0.979 | 1.000 | 0.881 | 1.000 | 1.000 | 1.000 | 0.974 | 1.000 | 1.000 | 0.805 | 1.000 | 1.000 | 0.868 | 0.841 |
| 2834 | 0.836 | 1.000 | 0.856 | 1.000 | 0.979 | 1.000 | 0.881 | 1.000 | 1.000 | 1.000 | 0.974 | 1.000 | 1.000 | 0.805 | 1.000 | 1.000 | 0.868 | 0.841 |
| 2835 | 0.836 | 1.000 | 0.856 | 1.000 | 0.979 | 1.000 | 0.881 | 1.000 | 1.000 | 1.000 | 0.974 | 1.000 | 1.000 | 0.805 | 1.000 | 1.000 | 0.868 | 0.841 |
| 2836 | 0.837 | 1.000 | 0.856 | 1.000 | 0.979 | 1.000 | 0.883 | 1.000 | 1.000 | 1.000 | 0.974 | 1.000 | 1.000 | 0.805 | 1.000 | 1.000 | 0.869 | 0.841 |
| 2837 | 0.837 | 1.000 | 0.858 | 1.000 | 0.979 | 1.000 | 0.883 | 1.000 | 1.000 | 1.000 | 0.974 | 1.000 | 1.000 | 0.806 | 1.000 | 1.000 | 0.869 | 0.842 |
| 2838 | 0.836 | 1.000 | 0.858 | 1.000 | 0.979 | 1.000 | 0.881 | 1.000 | 1.000 | 1.000 | 0.974 | 1.000 | 1.000 | 0.805 | 1.000 | 1.000 | 0.868 | 0.841 |
| 2839 | 0.835 | 1.000 | 0.860 | 1.000 | 0.979 | 1.000 | 0.881 | 1.000 | 1.000 | 1.000 | 0.974 | 1.000 | 1.000 | 0.805 | 1.000 | 1.000 | 0.868 | 0.841 |
| 2840 | 0.835 | 1.000 | 0.860 | 1.000 | 0.979 | 1.000 | 0.881 | 1.000 | 1.000 | 1.000 | 0.974 | 1.000 | 1.000 | 0.805 | 1.000 | 1.000 | 0.868 | 0.841 |
| 2841 | 0.835 | 1.000 | 0.860 | 1.000 | 0.979 | 1.000 | 0.881 | 1.000 | 1.000 | 1.000 | 0.974 | 1.000 | 1.000 | 0.805 | 1.000 | 1.000 | 0.868 | 0.841 |
| 2842 | 0.835 | 1.000 | 0.860 | 1.000 | 0.979 | 1.000 | 0.881 | 1.000 | 1.000 | 1.000 | 0.974 | 1.000 | 1.000 | 0.805 | 1.000 | 1.000 | 0.868 | 0.841 |
| 2843 | 0.835 | 1.000 | 0.860 | 1.000 | 0.979 | 1.000 | 0.881 | 1.000 | 1.000 | 1.000 | 0.974 | 1.000 | 1.000 | 0.805 | 1.000 | 1.000 | 0.868 | 0.841 |
| 2844 | 0.835 | 1.000 | 0.858 | 1.000 | 0.979 | 1.000 | 0.881 | 1.000 | 1.000 | 1.000 | 0.974 | 1.000 | 1.000 | 0.805 | 1.000 | 1.000 | 0.868 | 0.841 |
| 2845 | 0.835 | 1.000 | 0.858 | 1.000 | 0.979 | 1.000 | 0.881 | 1.000 | 1.000 | 1.000 | 0.974 | 1.000 | 1.000 | 0.805 | 1.000 | 1.000 | 0.868 | 0.841 |
| 2846 | 0.835 | 1.000 | 0.858 | 1.000 | 0.979 | 1.000 | 0.881 | 1.000 | 1.000 | 1.000 | 0.971 | 1.000 | 1.000 | 0.805 | 1.000 | 1.000 | 0.868 | 0.841 |
| 2847 | 0.835 | 1.000 | 0.858 | 1.000 | 0.979 | 1.000 | 0.881 | 1.000 | 1.000 | 1.000 | 0.971 | 1.000 | 1.000 | 0.805 | 1.000 | 1.000 | 0.868 | 0.841 |
| 2848 | 0.836 | 1.000 | 0.858 | 1.000 | 0.979 | 1.000 | 0.884 | 1.000 | 1.000 | 1.000 | 0.971 | 1.000 | 1.000 | 0.805 | 1.000 | 1.000 | 0.868 | 0.841 |
| 2849 | 0.835 | 1.000 | 0.858 | 1.000 | 0.979 | 1.000 | 0.884 | 1.000 | 1.000 | 1.000 | 0.971 | 1.000 | 1.000 | 0.805 | 1.000 | 1.000 | 0.868 | 0.841 |
| 2850 | 0.835 | 1.000 | 0.858 | 1.000 | 0.979 | 1.000 | 0.884 | 1.000 | 1.000 | 1.000 | 0.971 | 1.000 | 1.000 | 0.805 | 1.000 | 1.000 | 0.868 | 0.841 |

| | | | | | | | | | | | | | | | | | | |
|---|---|---|---|---|---|---|---|---|---|---|---|---|---|---|---|---|---|---|
| 2851 | 0.835 | 1.000 | 0.858 | 1.000 | 0.979 | 1.000 | 0.884 | 1.000 | 1.000 | 1.000 | 0.971 | 1.000 | 1.000 | 0.805 | 1.000 | 1.000 | 0.868 | 0.841 |
| 2852 | 0.835 | 1.000 | 0.860 | 1.000 | 0.979 | 1.000 | 0.888 | 1.000 | 1.000 | 1.000 | 0.971 | 1.000 | 1.000 | 0.805 | 1.000 | 1.000 | 0.869 | 0.842 |
| 2853 | 0.835 | 1.000 | 0.860 | 1.000 | 0.979 | 1.000 | 0.888 | 1.000 | 1.000 | 1.000 | 0.971 | 1.000 | 1.000 | 0.805 | 1.000 | 1.000 | 0.869 | 0.842 |
| 2854 | 0.835 | 1.000 | 0.860 | 1.000 | 0.979 | 1.000 | 0.888 | 1.000 | 1.000 | 1.000 | 0.971 | 1.000 | 1.000 | 0.805 | 1.000 | 1.000 | 0.869 | 0.842 |
| 2855 | 0.835 | 1.000 | 0.860 | 1.000 | 0.979 | 1.000 | 0.888 | 1.000 | 1.000 | 1.000 | 0.971 | 1.000 | 1.000 | 0.805 | 1.000 | 1.000 | 0.869 | 0.842 |
| 2856 | 0.835 | 1.000 | 0.868 | 1.000 | 0.979 | 1.000 | 0.886 | 1.000 | 1.000 | 1.000 | 0.968 | 1.000 | 1.000 | 0.806 | 1.000 | 1.000 | 0.869 | 0.843 |
| 2857 | 0.834 | 1.000 | 0.868 | 1.000 | 0.979 | 1.000 | 0.886 | 1.000 | 1.000 | 1.000 | 0.968 | 1.000 | 1.000 | 0.805 | 1.000 | 1.000 | 0.869 | 0.842 |
| 2858 | 0.834 | 1.000 | 0.868 | 1.000 | 0.979 | 1.000 | 0.884 | 1.000 | 1.000 | 1.000 | 0.968 | 1.000 | 1.000 | 0.806 | 1.000 | 1.000 | 0.869 | 0.842 |
| 2859 | 0.834 | 1.000 | 0.868 | 1.000 | 0.979 | 1.000 | 0.886 | 1.000 | 1.000 | 1.000 | 0.968 | 1.000 | 1.000 | 0.805 | 1.000 | 1.000 | 0.869 | 0.842 |
| 2860 | 0.834 | 1.000 | 0.868 | 1.000 | 0.979 | 1.000 | 0.884 | 1.000 | 1.000 | 1.000 | 0.968 | 1.000 | 1.000 | 0.805 | 1.000 | 1.000 | 0.869 | 0.842 |
| 2861 | 0.834 | 1.000 | 0.868 | 1.000 | 0.979 | 1.000 | 0.886 | 1.000 | 1.000 | 1.000 | 0.968 | 1.000 | 1.000 | 0.805 | 1.000 | 1.000 | 0.869 | 0.842 |
| 2862 | 0.834 | 1.000 | 0.868 | 1.000 | 0.979 | 1.000 | 0.886 | 1.000 | 1.000 | 1.000 | 0.968 | 1.000 | 1.000 | 0.805 | 1.000 | 1.000 | 0.869 | 0.842 |
| 2863 | 0.834 | 1.000 | 0.868 | 1.000 | 0.979 | 1.000 | 0.886 | 1.000 | 1.000 | 1.000 | 0.968 | 1.000 | 1.000 | 0.805 | 1.000 | 1.000 | 0.869 | 0.842 |
| 2864 | 0.834 | 1.000 | 0.868 | 1.000 | 0.979 | 1.000 | 0.886 | 1.000 | 1.000 | 1.000 | 0.968 | 1.000 | 1.000 | 0.805 | 1.000 | 1.000 | 0.869 | 0.842 |
| 2865 | 0.834 | 1.000 | 0.866 | 1.000 | 0.979 | 1.000 | 0.886 | 1.000 | 1.000 | 1.000 | 0.968 | 1.000 | 1.000 | 0.805 | 1.000 | 1.000 | 0.869 | 0.842 |
| 2866 | 0.834 | 1.000 | 0.866 | 1.000 | 0.979 | 1.000 | 0.886 | 1.000 | 1.000 | 1.000 | 0.968 | 1.000 | 1.000 | 0.805 | 1.000 | 1.000 | 0.869 | 0.842 |
| 2867 | 0.834 | 1.000 | 0.866 | 1.000 | 0.979 | 1.000 | 0.886 | 1.000 | 1.000 | 1.000 | 0.968 | 1.000 | 1.000 | 0.806 | 1.000 | 1.000 | 0.869 | 0.842 |
| 2868 | 0.834 | 1.000 | 0.866 | 1.000 | 0.979 | 1.000 | 0.886 | 1.000 | 1.000 | 1.000 | 0.968 | 1.000 | 1.000 | 0.805 | 1.000 | 1.000 | 0.869 | 0.842 |
| 2869 | 0.834 | 1.000 | 0.866 | 1.000 | 0.979 | 1.000 | 0.886 | 1.000 | 1.000 | 1.000 | 0.968 | 1.000 | 1.000 | 0.805 | 1.000 | 1.000 | 0.869 | 0.842 |
| 2870 | 0.834 | 1.000 | 0.866 | 1.000 | 0.979 | 1.000 | 0.884 | 1.000 | 1.000 | 1.000 | 0.968 | 1.000 | 1.000 | 0.806 | 1.000 | 1.000 | 0.869 | 0.842 |
| 2871 | 0.834 | 1.000 | 0.866 | 1.000 | 0.979 | 1.000 | 0.884 | 1.000 | 1.000 | 1.000 | 0.968 | 1.000 | 1.000 | 0.806 | 1.000 | 1.000 | 0.869 | 0.842 |
| 2872 | 0.833 | 1.000 | 0.864 | 1.000 | 0.979 | 1.000 | 0.886 | 1.000 | 1.000 | 1.000 | 0.968 | 1.000 | 1.000 | 0.809 | 1.000 | 1.000 | 0.869 | 0.842 |
| 2873 | 0.833 | 1.000 | 0.864 | 1.000 | 0.979 | 1.000 | 0.886 | 1.000 | 1.000 | 1.000 | 0.968 | 1.000 | 1.000 | 0.809 | 1.000 | 1.000 | 0.869 | 0.842 |
| 2874 | 0.834 | 1.000 | 0.864 | 1.000 | 0.979 | 1.000 | 0.886 | 1.000 | 1.000 | 1.000 | 0.968 | 1.000 | 1.000 | 0.807 | 1.000 | 1.000 | 0.869 | 0.842 |
| 2875 | 0.833 | 1.000 | 0.864 | 1.000 | 0.979 | 1.000 | 0.886 | 1.000 | 1.000 | 1.000 | 0.968 | 1.000 | 1.000 | 0.807 | 1.000 | 1.000 | 0.869 | 0.842 |
| 2876 | 0.835 | 1.000 | 0.862 | 1.000 | 0.979 | 1.000 | 0.886 | 1.000 | 1.000 | 1.000 | 0.968 | 1.000 | 1.000 | 0.809 | 1.000 | 1.000 | 0.869 | 0.842 |
| 2877 | 0.835 | 1.000 | 0.862 | 1.000 | 0.979 | 1.000 | 0.886 | 1.000 | 1.000 | 1.000 | 0.968 | 1.000 | 1.000 | 0.808 | 1.000 | 1.000 | 0.869 | 0.842 |
| 2878 | 0.835 | 1.000 | 0.862 | 1.000 | 0.979 | 1.000 | 0.886 | 1.000 | 1.000 | 1.000 | 0.968 | 1.000 | 1.000 | 0.807 | 1.000 | 1.000 | 0.869 | 0.842 |
| 2879 | 0.835 | 1.000 | 0.862 | 1.000 | 0.979 | 1.000 | 0.886 | 1.000 | 1.000 | 1.000 | 0.968 | 1.000 | 1.000 | 0.808 | 1.000 | 1.000 | 0.869 | 0.842 |
| 2880 | 0.835 | 1.000 | 0.862 | 1.000 | 0.979 | 1.000 | 0.886 | 1.000 | 1.000 | 1.000 | 0.968 | 1.000 | 1.000 | 0.807 | 1.000 | 1.000 | 0.869 | 0.842 |
| 2881 | 0.835 | 1.000 | 0.862 | 1.000 | 0.979 | 1.000 | 0.886 | 1.000 | 1.000 | 1.000 | 0.968 | 1.000 | 1.000 | 0.808 | 1.000 | 1.000 | 0.869 | 0.842 |
| 2882 | 0.835 | 1.000 | 0.862 | 1.000 | 0.979 | 1.000 | 0.886 | 1.000 | 1.000 | 1.000 | 0.968 | 1.000 | 1.000 | 0.809 | 1.000 | 1.000 | 0.869 | 0.842 |
| 2883 | 0.834 | 1.000 | 0.868 | 1.000 | 0.979 | 1.000 | 0.886 | 1.000 | 1.000 | 1.000 | 0.968 | 1.000 | 1.000 | 0.809 | 1.000 | 1.000 | 0.870 | 0.843 |
| 2884 | 0.834 | 1.000 | 0.868 | 1.000 | 0.979 | 1.000 | 0.886 | 1.000 | 1.000 | 1.000 | 0.968 | 1.000 | 1.000 | 0.809 | 1.000 | 1.000 | 0.870 | 0.843 |
| 2885 | 0.833 | 1.000 | 0.868 | 1.000 | 0.979 | 1.000 | 0.886 | 1.000 | 1.000 | 1.000 | 0.968 | 1.000 | 1.000 | 0.809 | 1.000 | 1.000 | 0.870 | 0.843 |
| 2886 | 0.833 | 1.000 | 0.868 | 1.000 | 0.979 | 1.000 | 0.886 | 1.000 | 1.000 | 1.000 | 0.968 | 1.000 | 1.000 | 0.808 | 1.000 | 1.000 | 0.869 | 0.842 |
| 2887 | 0.835 | 1.000 | 0.870 | 1.000 | 0.979 | 1.000 | 0.886 | 1.000 | 1.000 | 1.000 | 0.968 | 1.000 | 1.000 | 0.809 | 1.000 | 1.000 | 0.870 | 0.844 |
| 2888 | 0.835 | 1.000 | 0.872 | 1.000 | 0.979 | 1.000 | 0.886 | 1.000 | 1.000 | 1.000 | 0.968 | 1.000 | 1.000 | 0.809 | 1.000 | 1.000 | 0.870 | 0.844 |
| 2889 | 0.833 | 1.000 | 0.866 | 1.000 | 0.979 | 1.000 | 0.886 | 1.000 | 1.000 | 1.000 | 0.968 | 1.000 | 1.000 | 0.807 | 1.000 | 1.000 | 0.869 | 0.842 |

| | | | | | | | | | | | | | | | | | | |
|---|---|---|---|---|---|---|---|---|---|---|---|---|---|---|---|---|---|---|
| 2890 | 0.832 | 1.000 | 0.870 | 1.000 | 0.979 | 1.000 | 0.883 | 1.000 | 1.000 | 1.000 | 0.968 | 1.000 | 1.000 | 0.807 | 1.000 | 1.000 | 0.869 | 0.842 |
| 2891 | 0.832 | 1.000 | 0.868 | 1.000 | 0.979 | 1.000 | 0.883 | 1.000 | 1.000 | 1.000 | 0.968 | 1.000 | 1.000 | 0.807 | 1.000 | 1.000 | 0.868 | 0.841 |
| 2892 | 0.833 | 1.000 | 0.870 | 1.000 | 0.979 | 1.000 | 0.883 | 1.000 | 1.000 | 1.000 | 0.968 | 1.000 | 1.000 | 0.806 | 1.000 | 1.000 | 0.869 | 0.842 |
| 2893 | 0.833 | 1.000 | 0.870 | 1.000 | 0.979 | 1.000 | 0.888 | 1.000 | 1.000 | 1.000 | 0.968 | 1.000 | 1.000 | 0.806 | 1.000 | 1.000 | 0.869 | 0.843 |
| 2894 | 0.833 | 1.000 | 0.870 | 1.000 | 0.979 | 1.000 | 0.888 | 1.000 | 1.000 | 1.000 | 0.968 | 1.000 | 1.000 | 0.806 | 1.000 | 1.000 | 0.869 | 0.843 |
| 2895 | 0.833 | 1.000 | 0.866 | 1.000 | 0.979 | 1.000 | 0.888 | 1.000 | 1.000 | 1.000 | 0.968 | 1.000 | 1.000 | 0.806 | 1.000 | 1.000 | 0.869 | 0.842 |
| 2896 | 0.833 | 1.000 | 0.868 | 1.000 | 0.979 | 1.000 | 0.888 | 1.000 | 1.000 | 1.000 | 0.968 | 1.000 | 1.000 | 0.806 | 1.000 | 1.000 | 0.869 | 0.842 |
| 2897 | 0.832 | 1.000 | 0.868 | 1.000 | 0.979 | 1.000 | 0.888 | 1.000 | 1.000 | 1.000 | 0.968 | 1.000 | 1.000 | 0.806 | 1.000 | 1.000 | 0.869 | 0.842 |
| 2898 | 0.831 | 1.000 | 0.866 | 1.000 | 0.979 | 1.000 | 0.888 | 1.000 | 1.000 | 1.000 | 0.968 | 1.000 | 1.000 | 0.807 | 1.000 | 1.000 | 0.869 | 0.842 |
| 2899 | 0.833 | 1.000 | 0.866 | 1.000 | 0.979 | 1.000 | 0.886 | 1.000 | 1.000 | 1.000 | 0.968 | 1.000 | 1.000 | 0.807 | 1.000 | 1.000 | 0.869 | 0.842 |
| 2900 | 0.833 | 1.000 | 0.866 | 1.000 | 0.979 | 1.000 | 0.886 | 1.000 | 1.000 | 1.000 | 0.968 | 1.000 | 1.000 | 0.807 | 1.000 | 1.000 | 0.869 | 0.842 |
| 2901 | 0.833 | 1.000 | 0.868 | 1.000 | 0.979 | 1.000 | 0.888 | 1.000 | 1.000 | 1.000 | 0.968 | 1.000 | 1.000 | 0.806 | 1.000 | 1.000 | 0.869 | 0.842 |
| 2902 | 0.833 | 1.000 | 0.868 | 1.000 | 0.979 | 1.000 | 0.888 | 1.000 | 1.000 | 1.000 | 0.968 | 1.000 | 1.000 | 0.807 | 1.000 | 1.000 | 0.869 | 0.842 |
| 2903 | 0.833 | 1.000 | 0.868 | 1.000 | 0.979 | 1.000 | 0.888 | 1.000 | 1.000 | 1.000 | 0.968 | 1.000 | 1.000 | 0.807 | 1.000 | 1.000 | 0.869 | 0.842 |
| 2904 | 0.833 | 1.000 | 0.868 | 1.000 | 0.979 | 1.000 | 0.888 | 1.000 | 1.000 | 1.000 | 0.968 | 1.000 | 1.000 | 0.807 | 1.000 | 1.000 | 0.869 | 0.842 |
| 2905 | 0.832 | 1.000 | 0.868 | 1.000 | 0.979 | 1.000 | 0.886 | 1.000 | 1.000 | 1.000 | 0.968 | 1.000 | 1.000 | 0.808 | 1.000 | 1.000 | 0.869 | 0.842 |
| 2906 | 0.834 | 1.000 | 0.866 | 1.000 | 0.979 | 1.000 | 0.884 | 1.000 | 1.000 | 1.000 | 0.968 | 1.000 | 1.000 | 0.808 | 1.000 | 1.000 | 0.869 | 0.842 |
| 2907 | 0.833 | 1.000 | 0.866 | 1.000 | 0.979 | 1.000 | 0.884 | 1.000 | 1.000 | 1.000 | 0.968 | 1.000 | 1.000 | 0.807 | 1.000 | 1.000 | 0.869 | 0.842 |
| 2908 | 0.833 | 1.000 | 0.868 | 1.000 | 0.979 | 1.000 | 0.884 | 1.000 | 1.000 | 1.000 | 0.968 | 1.000 | 1.000 | 0.807 | 1.000 | 1.000 | 0.869 | 0.842 |
| 2909 | 0.834 | 1.000 | 0.868 | 1.000 | 0.979 | 1.000 | 0.884 | 1.000 | 1.000 | 1.000 | 0.968 | 1.000 | 1.000 | 0.807 | 1.000 | 1.000 | 0.869 | 0.842 |
| 2910 | 0.834 | 1.000 | 0.866 | 1.000 | 0.979 | 1.000 | 0.888 | 1.000 | 1.000 | 1.000 | 0.968 | 1.000 | 1.000 | 0.807 | 1.000 | 1.000 | 0.869 | 0.843 |
| 2911 | 0.835 | 1.000 | 0.866 | 1.000 | 0.979 | 1.000 | 0.886 | 1.000 | 1.000 | 1.000 | 0.968 | 1.000 | 1.000 | 0.807 | 1.000 | 1.000 | 0.869 | 0.843 |
| 2912 | 0.834 | 1.000 | 0.866 | 1.000 | 0.979 | 1.000 | 0.886 | 1.000 | 1.000 | 1.000 | 0.968 | 1.000 | 1.000 | 0.807 | 1.000 | 1.000 | 0.869 | 0.842 |
| 2913 | 0.834 | 1.000 | 0.864 | 1.000 | 0.979 | 1.000 | 0.889 | 1.000 | 1.000 | 1.000 | 0.968 | 1.000 | 1.000 | 0.806 | 1.000 | 1.000 | 0.869 | 0.842 |
| 2914 | 0.834 | 1.000 | 0.864 | 1.000 | 0.979 | 1.000 | 0.889 | 1.000 | 1.000 | 1.000 | 0.968 | 1.000 | 1.000 | 0.807 | 1.000 | 1.000 | 0.869 | 0.843 |
| 2915 | 0.833 | 1.000 | 0.862 | 1.000 | 0.979 | 1.000 | 0.889 | 1.000 | 1.000 | 1.000 | 0.968 | 1.000 | 1.000 | 0.806 | 1.000 | 1.000 | 0.869 | 0.842 |
| 2916 | 0.834 | 1.000 | 0.862 | 1.000 | 0.979 | 1.000 | 0.889 | 1.000 | 1.000 | 1.000 | 0.968 | 1.000 | 1.000 | 0.806 | 1.000 | 1.000 | 0.869 | 0.842 |
| 2917 | 0.835 | 1.000 | 0.862 | 1.000 | 0.979 | 1.000 | 0.889 | 1.000 | 1.000 | 1.000 | 0.968 | 1.000 | 1.000 | 0.809 | 1.000 | 1.000 | 0.870 | 0.843 |
| 2918 | 0.835 | 1.000 | 0.862 | 1.000 | 0.979 | 1.000 | 0.889 | 1.000 | 1.000 | 1.000 | 0.968 | 1.000 | 1.000 | 0.809 | 1.000 | 1.000 | 0.870 | 0.843 |
| 2919 | 0.835 | 1.000 | 0.862 | 1.000 | 0.979 | 1.000 | 0.888 | 1.000 | 1.000 | 1.000 | 0.968 | 1.000 | 1.000 | 0.809 | 1.000 | 1.000 | 0.870 | 0.843 |
| 2920 | 0.835 | 1.000 | 0.862 | 1.000 | 0.979 | 1.000 | 0.889 | 1.000 | 1.000 | 1.000 | 0.968 | 1.000 | 1.000 | 0.809 | 1.000 | 1.000 | 0.870 | 0.843 |
| 2921 | 0.835 | 1.000 | 0.862 | 1.000 | 0.979 | 1.000 | 0.889 | 1.000 | 1.000 | 1.000 | 0.968 | 1.000 | 1.000 | 0.809 | 1.000 | 1.000 | 0.870 | 0.843 |
| 2922 | 0.835 | 1.000 | 0.862 | 1.000 | 0.979 | 1.000 | 0.888 | 1.000 | 1.000 | 1.000 | 0.968 | 1.000 | 1.000 | 0.809 | 1.000 | 1.000 | 0.870 | 0.843 |
| 2923 | 0.835 | 1.000 | 0.862 | 1.000 | 0.979 | 1.000 | 0.888 | 1.000 | 1.000 | 1.000 | 0.968 | 1.000 | 1.000 | 0.808 | 1.000 | 1.000 | 0.869 | 0.842 |
| 2924 | 0.834 | 1.000 | 0.862 | 1.000 | 0.979 | 1.000 | 0.888 | 1.000 | 1.000 | 1.000 | 0.968 | 1.000 | 1.000 | 0.808 | 1.000 | 1.000 | 0.869 | 0.842 |
| 2925 | 0.834 | 1.000 | 0.862 | 1.000 | 0.979 | 1.000 | 0.888 | 1.000 | 1.000 | 1.000 | 0.968 | 1.000 | 1.000 | 0.808 | 1.000 | 1.000 | 0.869 | 0.842 |
| 2926 | 0.834 | 1.000 | 0.862 | 1.000 | 0.979 | 1.000 | 0.888 | 1.000 | 1.000 | 1.000 | 0.968 | 1.000 | 1.000 | 0.807 | 1.000 | 1.000 | 0.869 | 0.842 |
| 2927 | 0.834 | 1.000 | 0.862 | 1.000 | 0.979 | 1.000 | 0.888 | 1.000 | 1.000 | 1.000 | 0.968 | 1.000 | 1.000 | 0.808 | 1.000 | 1.000 | 0.869 | 0.842 |
| 2928 | 0.834 | 1.000 | 0.862 | 1.000 | 0.979 | 1.000 | 0.888 | 1.000 | 1.000 | 1.000 | 0.968 | 1.000 | 1.000 | 0.808 | 1.000 | 1.000 | 0.869 | 0.842 |

| | | | | | | | | | | | | | | | | | | |
|---|---|---|---|---|---|---|---|---|---|---|---|---|---|---|---|---|---|---|
| 2929 | 0.834 | 1.000 | 0.862 | 1.000 | 0.979 | 1.000 | 0.888 | 1.000 | 1.000 | 1.000 | 0.968 | 1.000 | 1.000 | 0.808 | 1.000 | 1.000 | 0.869 | 0.842 |
| 2930 | 0.833 | 1.000 | 0.862 | 1.000 | 0.979 | 1.000 | 0.884 | 1.000 | 1.000 | 1.000 | 0.968 | 1.000 | 1.000 | 0.809 | 1.000 | 1.000 | 0.869 | 0.842 |
| 2931 | 0.833 | 1.000 | 0.864 | 1.000 | 0.979 | 1.000 | 0.884 | 1.000 | 1.000 | 1.000 | 0.968 | 1.000 | 1.000 | 0.808 | 1.000 | 1.000 | 0.869 | 0.842 |
| 2932 | 0.833 | 1.000 | 0.862 | 1.000 | 0.979 | 1.000 | 0.889 | 1.000 | 1.000 | 1.000 | 0.968 | 1.000 | 1.000 | 0.807 | 1.000 | 1.000 | 0.869 | 0.842 |
| 2933 | 0.833 | 1.000 | 0.862 | 1.000 | 0.979 | 1.000 | 0.889 | 1.000 | 1.000 | 1.000 | 0.968 | 1.000 | 1.000 | 0.807 | 1.000 | 1.000 | 0.869 | 0.842 |
| 2934 | 0.833 | 1.000 | 0.862 | 1.000 | 0.979 | 1.000 | 0.886 | 1.000 | 1.000 | 1.000 | 0.968 | 1.000 | 1.000 | 0.808 | 1.000 | 1.000 | 0.869 | 0.842 |
| 2935 | 0.833 | 1.000 | 0.862 | 1.000 | 0.979 | 1.000 | 0.886 | 1.000 | 1.000 | 1.000 | 0.968 | 1.000 | 1.000 | 0.809 | 1.000 | 1.000 | 0.869 | 0.842 |
| 2936 | 0.833 | 1.000 | 0.862 | 1.000 | 0.979 | 1.000 | 0.886 | 1.000 | 1.000 | 1.000 | 0.968 | 1.000 | 1.000 | 0.809 | 1.000 | 1.000 | 0.869 | 0.842 |
| 2937 | 0.833 | 1.000 | 0.862 | 1.000 | 0.979 | 1.000 | 0.886 | 1.000 | 1.000 | 1.000 | 0.968 | 1.000 | 1.000 | 0.809 | 1.000 | 1.000 | 0.869 | 0.842 |
| 2938 | 0.833 | 1.000 | 0.862 | 1.000 | 0.979 | 1.000 | 0.884 | 1.000 | 1.000 | 1.000 | 0.968 | 1.000 | 1.000 | 0.807 | 1.000 | 1.000 | 0.868 | 0.841 |
| 2939 | 0.831 | 1.000 | 0.862 | 1.000 | 0.979 | 1.000 | 0.888 | 1.000 | 1.000 | 1.000 | 0.968 | 1.000 | 1.000 | 0.809 | 1.000 | 1.000 | 0.869 | 0.842 |
| 2940 | 0.831 | 1.000 | 0.862 | 1.000 | 0.979 | 1.000 | 0.888 | 1.000 | 1.000 | 1.000 | 0.968 | 1.000 | 1.000 | 0.809 | 1.000 | 1.000 | 0.869 | 0.842 |
| 2941 | 0.831 | 1.000 | 0.862 | 1.000 | 0.979 | 1.000 | 0.888 | 1.000 | 1.000 | 1.000 | 0.968 | 1.000 | 1.000 | 0.809 | 1.000 | 1.000 | 0.869 | 0.842 |
| 2942 | 0.831 | 1.000 | 0.862 | 1.000 | 0.979 | 1.000 | 0.886 | 1.000 | 1.000 | 1.000 | 0.968 | 1.000 | 1.000 | 0.809 | 1.000 | 1.000 | 0.869 | 0.842 |
| 2943 | 0.831 | 1.000 | 0.862 | 1.000 | 0.979 | 1.000 | 0.888 | 1.000 | 1.000 | 1.000 | 0.968 | 1.000 | 1.000 | 0.809 | 1.000 | 1.000 | 0.869 | 0.842 |
| 2944 | 0.832 | 1.000 | 0.862 | 1.000 | 0.979 | 1.000 | 0.888 | 1.000 | 1.000 | 1.000 | 0.968 | 1.000 | 1.000 | 0.809 | 1.000 | 1.000 | 0.869 | 0.842 |
| 2945 | 0.832 | 1.000 | 0.862 | 1.000 | 0.979 | 1.000 | 0.888 | 1.000 | 1.000 | 1.000 | 0.968 | 1.000 | 1.000 | 0.809 | 1.000 | 1.000 | 0.869 | 0.842 |
| 2946 | 0.831 | 1.000 | 0.862 | 1.000 | 0.979 | 1.000 | 0.888 | 1.000 | 1.000 | 1.000 | 0.968 | 1.000 | 1.000 | 0.810 | 1.000 | 1.000 | 0.869 | 0.842 |
| 2947 | 0.831 | 1.000 | 0.862 | 1.000 | 0.979 | 1.000 | 0.888 | 1.000 | 1.000 | 1.000 | 0.968 | 1.000 | 1.000 | 0.809 | 1.000 | 1.000 | 0.869 | 0.842 |
| 2948 | 0.831 | 1.000 | 0.862 | 1.000 | 0.979 | 1.000 | 0.889 | 1.000 | 1.000 | 1.000 | 0.968 | 1.000 | 1.000 | 0.809 | 1.000 | 1.000 | 0.869 | 0.842 |
| 2949 | 0.831 | 1.000 | 0.862 | 1.000 | 0.979 | 1.000 | 0.889 | 1.000 | 1.000 | 1.000 | 0.968 | 1.000 | 1.000 | 0.810 | 1.000 | 1.000 | 0.869 | 0.842 |
| 2950 | 0.831 | 1.000 | 0.864 | 1.000 | 0.979 | 1.000 | 0.889 | 1.000 | 1.000 | 1.000 | 0.968 | 1.000 | 1.000 | 0.811 | 1.000 | 1.000 | 0.869 | 0.843 |
| 2951 | 0.828 | 1.000 | 0.862 | 1.000 | 0.979 | 1.000 | 0.884 | 1.000 | 1.000 | 1.000 | 0.968 | 1.000 | 1.000 | 0.810 | 1.000 | 1.000 | 0.868 | 0.840 |
| 2952 | 0.828 | 1.000 | 0.862 | 1.000 | 0.979 | 1.000 | 0.884 | 1.000 | 1.000 | 1.000 | 0.968 | 1.000 | 1.000 | 0.810 | 1.000 | 1.000 | 0.868 | 0.840 |
| 2953 | 0.828 | 1.000 | 0.862 | 1.000 | 0.979 | 1.000 | 0.884 | 1.000 | 1.000 | 1.000 | 0.968 | 1.000 | 1.000 | 0.811 | 1.000 | 1.000 | 0.868 | 0.841 |
| 2954 | 0.829 | 1.000 | 0.862 | 1.000 | 0.979 | 1.000 | 0.883 | 1.000 | 1.000 | 1.000 | 0.968 | 1.000 | 1.000 | 0.811 | 1.000 | 1.000 | 0.868 | 0.841 |
| 2955 | 0.829 | 1.000 | 0.864 | 1.000 | 0.979 | 1.000 | 0.884 | 1.000 | 1.000 | 1.000 | 0.968 | 1.000 | 1.000 | 0.810 | 1.000 | 1.000 | 0.868 | 0.841 |
| 2956 | 0.829 | 1.000 | 0.864 | 1.000 | 0.979 | 1.000 | 0.883 | 1.000 | 1.000 | 1.000 | 0.968 | 1.000 | 1.000 | 0.810 | 1.000 | 1.000 | 0.868 | 0.841 |
| 2957 | 0.829 | 1.000 | 0.864 | 1.000 | 0.979 | 1.000 | 0.883 | 1.000 | 1.000 | 1.000 | 0.968 | 1.000 | 1.000 | 0.811 | 1.000 | 1.000 | 0.868 | 0.841 |
| 2958 | 0.829 | 1.000 | 0.864 | 1.000 | 0.979 | 1.000 | 0.883 | 1.000 | 1.000 | 1.000 | 0.968 | 1.000 | 1.000 | 0.812 | 1.000 | 1.000 | 0.868 | 0.841 |
| 2959 | 0.829 | 1.000 | 0.864 | 1.000 | 0.979 | 1.000 | 0.883 | 1.000 | 1.000 | 1.000 | 0.968 | 1.000 | 1.000 | 0.811 | 1.000 | 1.000 | 0.868 | 0.841 |
| 2960 | 0.829 | 1.000 | 0.866 | 1.000 | 0.979 | 1.000 | 0.884 | 1.000 | 1.000 | 1.000 | 0.968 | 1.000 | 1.000 | 0.811 | 1.000 | 1.000 | 0.869 | 0.842 |
| 2961 | 0.829 | 1.000 | 0.866 | 1.000 | 0.979 | 1.000 | 0.884 | 1.000 | 1.000 | 1.000 | 0.968 | 1.000 | 1.000 | 0.809 | 1.000 | 1.000 | 0.868 | 0.841 |
| 2962 | 0.829 | 1.000 | 0.866 | 1.000 | 0.979 | 1.000 | 0.884 | 1.000 | 1.000 | 1.000 | 0.968 | 1.000 | 1.000 | 0.809 | 1.000 | 1.000 | 0.868 | 0.841 |
| 2963 | 0.829 | 1.000 | 0.866 | 1.000 | 0.979 | 1.000 | 0.884 | 1.000 | 1.000 | 1.000 | 0.968 | 1.000 | 1.000 | 0.810 | 1.000 | 1.000 | 0.868 | 0.841 |
| 2964 | 0.829 | 1.000 | 0.864 | 1.000 | 0.979 | 1.000 | 0.883 | 1.000 | 1.000 | 1.000 | 0.968 | 1.000 | 1.000 | 0.812 | 1.000 | 1.000 | 0.868 | 0.841 |
| 2965 | 0.829 | 1.000 | 0.864 | 1.000 | 0.979 | 1.000 | 0.884 | 1.000 | 1.000 | 1.000 | 0.968 | 1.000 | 1.000 | 0.812 | 1.000 | 1.000 | 0.868 | 0.841 |
| 2966 | 0.830 | 1.000 | 0.864 | 1.000 | 0.979 | 1.000 | 0.884 | 1.000 | 1.000 | 1.000 | 0.968 | 1.000 | 1.000 | 0.811 | 1.000 | 1.000 | 0.869 | 0.842 |
| 2967 | 0.831 | 1.000 | 0.866 | 1.000 | 0.979 | 1.000 | 0.884 | 1.000 | 1.000 | 1.000 | 0.968 | 1.000 | 1.000 | 0.811 | 1.000 | 1.000 | 0.869 | 0.842 |

| | | | | | | | | | | | | | | | | | | |
|---|---|---|---|---|---|---|---|---|---|---|---|---|---|---|---|---|---|---|
| 2968 | 0.830 | 1.000 | 0.864 | 1.000 | 0.979 | 1.000 | 0.884 | 1.000 | 1.000 | 1.000 | 0.968 | 1.000 | 1.000 | 0.811 | 1.000 | 1.000 | 0.869 | 0.842 |
| 2969 | 0.831 | 1.000 | 0.866 | 1.000 | 0.979 | 1.000 | 0.884 | 1.000 | 1.000 | 1.000 | 0.968 | 1.000 | 1.000 | 0.811 | 1.000 | 1.000 | 0.869 | 0.842 |
| 2970 | 0.830 | 1.000 | 0.864 | 1.000 | 0.979 | 1.000 | 0.884 | 1.000 | 1.000 | 1.000 | 0.968 | 1.000 | 1.000 | 0.811 | 1.000 | 1.000 | 0.869 | 0.842 |
| 2971 | 0.830 | 1.000 | 0.864 | 1.000 | 0.979 | 1.000 | 0.884 | 1.000 | 1.000 | 1.000 | 0.968 | 1.000 | 1.000 | 0.811 | 1.000 | 1.000 | 0.869 | 0.842 |
| 2972 | 0.830 | 1.000 | 0.864 | 1.000 | 0.979 | 1.000 | 0.884 | 1.000 | 1.000 | 1.000 | 0.968 | 1.000 | 1.000 | 0.810 | 1.000 | 1.000 | 0.868 | 0.841 |
| 2973 | 0.831 | 1.000 | 0.864 | 1.000 | 0.979 | 1.000 | 0.884 | 1.000 | 1.000 | 1.000 | 0.968 | 1.000 | 1.000 | 0.812 | 1.000 | 1.000 | 0.869 | 0.842 |
| 2974 | 0.830 | 1.000 | 0.864 | 1.000 | 0.979 | 1.000 | 0.884 | 1.000 | 1.000 | 1.000 | 0.968 | 1.000 | 1.000 | 0.811 | 1.000 | 1.000 | 0.869 | 0.842 |
| 2975 | 0.831 | 1.000 | 0.864 | 1.000 | 0.979 | 1.000 | 0.884 | 1.000 | 1.000 | 1.000 | 0.968 | 1.000 | 1.000 | 0.811 | 1.000 | 1.000 | 0.869 | 0.842 |
| 2976 | 0.835 | 1.000 | 0.862 | 1.000 | 0.979 | 1.000 | 0.884 | 1.000 | 1.000 | 1.000 | 0.974 | 1.000 | 1.000 | 0.810 | 1.000 | 1.000 | 0.870 | 0.843 |
| 2977 | 0.834 | 1.000 | 0.862 | 1.000 | 0.979 | 1.000 | 0.883 | 1.000 | 1.000 | 1.000 | 0.974 | 1.000 | 1.000 | 0.810 | 1.000 | 1.000 | 0.870 | 0.843 |
| 2978 | 0.834 | 1.000 | 0.862 | 1.000 | 0.979 | 1.000 | 0.884 | 1.000 | 1.000 | 1.000 | 0.974 | 1.000 | 1.000 | 0.810 | 1.000 | 1.000 | 0.870 | 0.843 |
| 2979 | 0.834 | 1.000 | 0.862 | 1.000 | 0.979 | 1.000 | 0.884 | 1.000 | 1.000 | 1.000 | 0.974 | 1.000 | 1.000 | 0.812 | 1.000 | 1.000 | 0.870 | 0.843 |
| 2980 | 0.833 | 1.000 | 0.862 | 1.000 | 0.979 | 1.000 | 0.881 | 1.000 | 1.000 | 1.000 | 0.974 | 1.000 | 1.000 | 0.811 | 1.000 | 1.000 | 0.869 | 0.842 |
| 2981 | 0.833 | 1.000 | 0.862 | 1.000 | 0.979 | 1.000 | 0.881 | 1.000 | 1.000 | 1.000 | 0.974 | 1.000 | 1.000 | 0.811 | 1.000 | 1.000 | 0.869 | 0.842 |
| 2982 | 0.833 | 1.000 | 0.860 | 1.000 | 0.979 | 1.000 | 0.881 | 1.000 | 1.000 | 1.000 | 0.974 | 1.000 | 1.000 | 0.811 | 1.000 | 1.000 | 0.869 | 0.842 |
| 2983 | 0.833 | 1.000 | 0.858 | 1.000 | 0.979 | 1.000 | 0.880 | 1.000 | 1.000 | 1.000 | 0.974 | 1.000 | 1.000 | 0.809 | 1.000 | 1.000 | 0.868 | 0.841 |
| 2984 | 0.833 | 1.000 | 0.858 | 1.000 | 0.979 | 1.000 | 0.880 | 1.000 | 1.000 | 1.000 | 0.974 | 1.000 | 1.000 | 0.809 | 1.000 | 1.000 | 0.868 | 0.841 |
| 2985 | 0.832 | 1.000 | 0.858 | 1.000 | 0.979 | 1.000 | 0.880 | 1.000 | 1.000 | 1.000 | 0.974 | 1.000 | 1.000 | 0.810 | 1.000 | 1.000 | 0.868 | 0.841 |
| 2986 | 0.833 | 1.000 | 0.858 | 1.000 | 0.979 | 1.000 | 0.880 | 1.000 | 1.000 | 1.000 | 0.974 | 1.000 | 1.000 | 0.810 | 1.000 | 1.000 | 0.869 | 0.841 |
| 2987 | 0.833 | 1.000 | 0.858 | 1.000 | 0.979 | 1.000 | 0.880 | 1.000 | 1.000 | 1.000 | 0.974 | 1.000 | 1.000 | 0.812 | 1.000 | 1.000 | 0.869 | 0.842 |
| 2988 | 0.833 | 1.000 | 0.858 | 1.000 | 0.979 | 1.000 | 0.880 | 1.000 | 1.000 | 1.000 | 0.974 | 1.000 | 1.000 | 0.812 | 1.000 | 1.000 | 0.869 | 0.842 |
| 2989 | 0.833 | 1.000 | 0.858 | 1.000 | 0.979 | 1.000 | 0.880 | 1.000 | 1.000 | 1.000 | 0.974 | 1.000 | 1.000 | 0.810 | 1.000 | 1.000 | 0.868 | 0.841 |
| 2990 | 0.833 | 1.000 | 0.858 | 1.000 | 0.979 | 1.000 | 0.880 | 1.000 | 1.000 | 1.000 | 0.974 | 1.000 | 1.000 | 0.810 | 1.000 | 1.000 | 0.868 | 0.841 |
| 2991 | 0.833 | 1.000 | 0.858 | 1.000 | 0.979 | 1.000 | 0.880 | 1.000 | 1.000 | 1.000 | 0.974 | 1.000 | 1.000 | 0.810 | 1.000 | 1.000 | 0.868 | 0.841 |
| 2992 | 0.832 | 1.000 | 0.858 | 1.000 | 0.979 | 1.000 | 0.880 | 1.000 | 1.000 | 1.000 | 0.974 | 1.000 | 1.000 | 0.810 | 1.000 | 1.000 | 0.868 | 0.841 |
| 2993 | 0.833 | 1.000 | 0.858 | 1.000 | 0.979 | 1.000 | 0.880 | 1.000 | 1.000 | 1.000 | 0.974 | 1.000 | 1.000 | 0.809 | 1.000 | 1.000 | 0.868 | 0.841 |
| 2994 | 0.833 | 1.000 | 0.858 | 1.000 | 0.979 | 1.000 | 0.880 | 1.000 | 1.000 | 1.000 | 0.974 | 1.000 | 1.000 | 0.810 | 1.000 | 1.000 | 0.868 | 0.841 |
| 2995 | 0.833 | 1.000 | 0.858 | 1.000 | 0.979 | 1.000 | 0.880 | 1.000 | 1.000 | 1.000 | 0.974 | 1.000 | 1.000 | 0.810 | 1.000 | 1.000 | 0.868 | 0.841 |
| 2996 | 0.833 | 1.000 | 0.858 | 1.000 | 0.979 | 1.000 | 0.880 | 1.000 | 1.000 | 1.000 | 0.974 | 1.000 | 1.000 | 0.810 | 1.000 | 1.000 | 0.868 | 0.841 |
| 2997 | 0.833 | 1.000 | 0.858 | 1.000 | 0.979 | 1.000 | 0.880 | 1.000 | 1.000 | 1.000 | 0.974 | 1.000 | 1.000 | 0.811 | 1.000 | 1.000 | 0.869 | 0.841 |
| 2998 | 0.833 | 1.000 | 0.858 | 1.000 | 0.979 | 1.000 | 0.880 | 1.000 | 1.000 | 1.000 | 0.974 | 1.000 | 1.000 | 0.811 | 1.000 | 1.000 | 0.869 | 0.841 |
| 2999 | 0.833 | 1.000 | 0.858 | 1.000 | 0.979 | 1.000 | 0.880 | 1.000 | 1.000 | 1.000 | 0.974 | 1.000 | 1.000 | 0.811 | 1.000 | 1.000 | 0.869 | 0.841 |
| 3000 | 0.833 | 1.000 | 0.858 | 1.000 | 0.979 | 1.000 | 0.880 | 1.000 | 1.000 | 1.000 | 0.974 | 1.000 | 1.000 | 0.811 | 1.000 | 1.000 | 0.869 | 0.841 |
| 3001 | 0.833 | 1.000 | 0.858 | 1.000 | 0.979 | 1.000 | 0.880 | 1.000 | 1.000 | 1.000 | 0.974 | 1.000 | 1.000 | 0.811 | 1.000 | 1.000 | 0.869 | 0.842 |
| 3002 | 0.833 | 1.000 | 0.858 | 1.000 | 0.979 | 1.000 | 0.881 | 1.000 | 1.000 | 1.000 | 0.974 | 1.000 | 1.000 | 0.811 | 1.000 | 1.000 | 0.869 | 0.842 |
| 3003 | 0.832 | 1.000 | 0.858 | 1.000 | 0.979 | 1.000 | 0.881 | 1.000 | 1.000 | 1.000 | 0.974 | 1.000 | 1.000 | 0.811 | 1.000 | 1.000 | 0.869 | 0.842 |
| 3004 | 0.833 | 1.000 | 0.858 | 1.000 | 0.979 | 1.000 | 0.881 | 1.000 | 1.000 | 1.000 | 0.974 | 1.000 | 1.000 | 0.812 | 1.000 | 1.000 | 0.869 | 0.842 |
| 3005 | 0.834 | 1.000 | 0.858 | 1.000 | 0.979 | 1.000 | 0.881 | 1.000 | 1.000 | 1.000 | 0.974 | 1.000 | 1.000 | 0.810 | 1.000 | 1.000 | 0.869 | 0.842 |
| 3006 | 0.834 | 1.000 | 0.858 | 1.000 | 0.979 | 1.000 | 0.881 | 1.000 | 1.000 | 1.000 | 0.974 | 1.000 | 1.000 | 0.810 | 1.000 | 1.000 | 0.869 | 0.842 |

| | | | | | | | | | | | | | | | | | |
|---|---|---|---|---|---|---|---|---|---|---|---|---|---|---|---|---|---|
| 3007 | 0.834 | 1.000 | 0.858 | 1.000 | 0.979 | 1.000 | 0.881 | 1.000 | 1.000 | 1.000 | 0.974 | 1.000 | 1.000 | 0.811 | 1.000 | 1.000 | 0.869 | 0.842 |
| 3008 | 0.835 | 1.000 | 0.858 | 1.000 | 0.979 | 1.000 | 0.881 | 1.000 | 1.000 | 1.000 | 0.974 | 1.000 | 1.000 | 0.810 | 1.000 | 1.000 | 0.869 | 0.842 |
| 3009 | 0.835 | 1.000 | 0.858 | 1.000 | 0.979 | 1.000 | 0.880 | 1.000 | 1.000 | 1.000 | 0.974 | 1.000 | 1.000 | 0.811 | 1.000 | 1.000 | 0.869 | 0.842 |
| 3010 | 0.833 | 1.000 | 0.858 | 1.000 | 0.979 | 1.000 | 0.880 | 1.000 | 1.000 | 1.000 | 0.974 | 1.000 | 1.000 | 0.811 | 1.000 | 1.000 | 0.869 | 0.842 |
| 3011 | 0.834 | 1.000 | 0.860 | 1.000 | 0.979 | 1.000 | 0.880 | 1.000 | 1.000 | 1.000 | 0.974 | 1.000 | 1.000 | 0.812 | 1.000 | 1.000 | 0.869 | 0.842 |
| 3012 | 0.835 | 1.000 | 0.856 | 1.000 | 0.979 | 1.000 | 0.880 | 1.000 | 1.000 | 1.000 | 0.974 | 1.000 | 1.000 | 0.812 | 1.000 | 1.000 | 0.869 | 0.842 |
| 3013 | 0.835 | 1.000 | 0.856 | 1.000 | 0.979 | 1.000 | 0.880 | 1.000 | 1.000 | 1.000 | 0.974 | 1.000 | 1.000 | 0.811 | 1.000 | 1.000 | 0.869 | 0.842 |
| 3014 | 0.835 | 1.000 | 0.858 | 1.000 | 0.979 | 1.000 | 0.880 | 1.000 | 1.000 | 1.000 | 0.974 | 1.000 | 1.000 | 0.811 | 1.000 | 1.000 | 0.869 | 0.842 |
| 3015 | 0.835 | 1.000 | 0.858 | 1.000 | 0.979 | 1.000 | 0.880 | 1.000 | 1.000 | 1.000 | 0.974 | 1.000 | 1.000 | 0.811 | 1.000 | 1.000 | 0.869 | 0.842 |
| 3016 | 0.835 | 1.000 | 0.858 | 1.000 | 0.979 | 1.000 | 0.880 | 1.000 | 1.000 | 1.000 | 0.974 | 1.000 | 1.000 | 0.811 | 1.000 | 1.000 | 0.869 | 0.842 |
| 3017 | 0.835 | 1.000 | 0.858 | 1.000 | 0.979 | 1.000 | 0.880 | 1.000 | 1.000 | 1.000 | 0.974 | 1.000 | 1.000 | 0.811 | 1.000 | 1.000 | 0.869 | 0.842 |
| 3018 | 0.835 | 1.000 | 0.858 | 1.000 | 0.979 | 1.000 | 0.880 | 1.000 | 1.000 | 1.000 | 0.974 | 1.000 | 1.000 | 0.811 | 1.000 | 1.000 | 0.869 | 0.842 |
| 3019 | 0.835 | 1.000 | 0.858 | 1.000 | 0.979 | 1.000 | 0.880 | 1.000 | 1.000 | 1.000 | 0.974 | 1.000 | 1.000 | 0.809 | 1.000 | 1.000 | 0.869 | 0.842 |
| 3020 | 0.835 | 1.000 | 0.858 | 1.000 | 0.979 | 1.000 | 0.880 | 1.000 | 1.000 | 1.000 | 0.974 | 1.000 | 1.000 | 0.810 | 1.000 | 1.000 | 0.869 | 0.842 |
| 3021 | 0.835 | 1.000 | 0.858 | 1.000 | 0.979 | 1.000 | 0.880 | 1.000 | 1.000 | 1.000 | 0.974 | 1.000 | 1.000 | 0.812 | 1.000 | 1.000 | 0.869 | 0.842 |
| 3022 | 0.835 | 1.000 | 0.858 | 1.000 | 0.979 | 1.000 | 0.880 | 1.000 | 1.000 | 1.000 | 0.974 | 1.000 | 1.000 | 0.812 | 1.000 | 1.000 | 0.869 | 0.842 |
| 3023 | 0.835 | 1.000 | 0.858 | 1.000 | 0.979 | 1.000 | 0.880 | 1.000 | 1.000 | 1.000 | 0.974 | 1.000 | 1.000 | 0.812 | 1.000 | 1.000 | 0.870 | 0.843 |
| 3024 | 0.834 | 1.000 | 0.856 | 1.000 | 0.979 | 1.000 | 0.884 | 1.000 | 1.000 | 1.000 | 0.974 | 1.000 | 1.000 | 0.810 | 1.000 | 1.000 | 0.869 | 0.842 |
| 3025 | 0.835 | 1.000 | 0.856 | 1.000 | 0.979 | 1.000 | 0.883 | 1.000 | 1.000 | 1.000 | 0.974 | 1.000 | 1.000 | 0.810 | 1.000 | 1.000 | 0.869 | 0.842 |
| 3026 | 0.835 | 1.000 | 0.856 | 1.000 | 0.979 | 1.000 | 0.880 | 1.000 | 1.000 | 1.000 | 0.974 | 1.000 | 1.000 | 0.812 | 1.000 | 1.000 | 0.869 | 0.842 |
| 3027 | 0.835 | 1.000 | 0.856 | 1.000 | 0.979 | 1.000 | 0.881 | 1.000 | 1.000 | 1.000 | 0.974 | 1.000 | 1.000 | 0.812 | 1.000 | 1.000 | 0.870 | 0.843 |
| 3028 | 0.834 | 1.000 | 0.856 | 1.000 | 0.979 | 1.000 | 0.878 | 1.000 | 1.000 | 1.000 | 0.977 | 1.000 | 1.000 | 0.809 | 1.000 | 1.000 | 0.868 | 0.841 |
| 3029 | 0.835 | 1.000 | 0.856 | 1.000 | 0.979 | 1.000 | 0.883 | 1.000 | 1.000 | 1.000 | 0.977 | 1.000 | 1.000 | 0.809 | 1.000 | 1.000 | 0.869 | 0.842 |
| 3030 | 0.835 | 1.000 | 0.856 | 1.000 | 0.979 | 1.000 | 0.884 | 1.000 | 1.000 | 1.000 | 0.977 | 1.000 | 1.000 | 0.808 | 1.000 | 1.000 | 0.869 | 0.842 |
| 3031 | 0.835 | 1.000 | 0.858 | 1.000 | 0.979 | 1.000 | 0.886 | 1.000 | 1.000 | 1.000 | 0.977 | 1.000 | 1.000 | 0.809 | 1.000 | 1.000 | 0.870 | 0.843 |
| 3032 | 0.834 | 1.000 | 0.858 | 1.000 | 0.979 | 1.000 | 0.886 | 1.000 | 1.000 | 1.000 | 0.977 | 1.000 | 1.000 | 0.809 | 1.000 | 1.000 | 0.870 | 0.843 |
| 3033 | 0.834 | 1.000 | 0.858 | 1.000 | 0.979 | 1.000 | 0.884 | 1.000 | 1.000 | 1.000 | 0.977 | 1.000 | 1.000 | 0.809 | 1.000 | 1.000 | 0.869 | 0.842 |
| 3034 | 0.835 | 1.000 | 0.858 | 1.000 | 0.979 | 1.000 | 0.886 | 1.000 | 1.000 | 1.000 | 0.977 | 1.000 | 1.000 | 0.810 | 1.000 | 1.000 | 0.870 | 0.843 |
| 3035 | 0.836 | 1.000 | 0.858 | 1.000 | 0.979 | 1.000 | 0.886 | 1.000 | 1.000 | 1.000 | 0.980 | 1.000 | 1.000 | 0.810 | 1.000 | 1.000 | 0.871 | 0.844 |
| 3036 | 0.835 | 1.000 | 0.858 | 1.000 | 0.979 | 1.000 | 0.886 | 1.000 | 1.000 | 1.000 | 0.980 | 1.000 | 1.000 | 0.810 | 1.000 | 1.000 | 0.870 | 0.844 |
| 3037 | 0.834 | 1.000 | 0.858 | 1.000 | 0.979 | 1.000 | 0.884 | 1.000 | 1.000 | 1.000 | 0.980 | 1.000 | 1.000 | 0.810 | 1.000 | 1.000 | 0.870 | 0.843 |
| 3038 | 0.835 | 1.000 | 0.858 | 1.000 | 0.979 | 1.000 | 0.884 | 1.000 | 1.000 | 1.000 | 0.980 | 1.000 | 1.000 | 0.810 | 1.000 | 1.000 | 0.870 | 0.843 |
| 3039 | 0.834 | 1.000 | 0.858 | 1.000 | 0.979 | 1.000 | 0.884 | 1.000 | 1.000 | 1.000 | 0.980 | 1.000 | 1.000 | 0.810 | 1.000 | 1.000 | 0.870 | 0.843 |
| 3040 | 0.833 | 1.000 | 0.858 | 1.000 | 0.979 | 1.000 | 0.886 | 1.000 | 1.000 | 1.000 | 0.980 | 1.000 | 1.000 | 0.810 | 1.000 | 1.000 | 0.870 | 0.843 |
| 3041 | 0.833 | 1.000 | 0.858 | 1.000 | 0.979 | 1.000 | 0.886 | 1.000 | 1.000 | 1.000 | 0.980 | 1.000 | 1.000 | 0.810 | 1.000 | 1.000 | 0.870 | 0.843 |
| 3042 | 0.833 | 1.000 | 0.858 | 1.000 | 0.979 | 1.000 | 0.886 | 1.000 | 1.000 | 1.000 | 0.980 | 1.000 | 1.000 | 0.809 | 1.000 | 1.000 | 0.869 | 0.842 |
| 3043 | 0.833 | 1.000 | 0.858 | 1.000 | 0.979 | 1.000 | 0.886 | 1.000 | 1.000 | 1.000 | 0.980 | 1.000 | 1.000 | 0.809 | 1.000 | 1.000 | 0.870 | 0.843 |
| 3044 | 0.833 | 1.000 | 0.856 | 1.000 | 0.979 | 1.000 | 0.886 | 1.000 | 1.000 | 1.000 | 0.980 | 1.000 | 1.000 | 0.810 | 1.000 | 1.000 | 0.870 | 0.843 |
| 3045 | 0.833 | 1.000 | 0.856 | 1.000 | 0.979 | 1.000 | 0.886 | 1.000 | 1.000 | 1.000 | 0.980 | 1.000 | 1.000 | 0.810 | 1.000 | 1.000 | 0.870 | 0.843 |

| | | | | | | | | | | | | | | | | | | |
|---|---|---|---|---|---|---|---|---|---|---|---|---|---|---|---|---|---|---|
| 3046 | 0.833 | 1.000 | 0.856 | 1.000 | 0.979 | 1.000 | 0.886 | 1.000 | 1.000 | 1.000 | 0.980 | 1.000 | 1.000 | 0.810 | 1.000 | 1.000 | 0.870 | 0.843 |
| 3047 | 0.833 | 1.000 | 0.856 | 1.000 | 0.979 | 1.000 | 0.888 | 1.000 | 1.000 | 1.000 | 0.980 | 1.000 | 1.000 | 0.810 | 1.000 | 1.000 | 0.870 | 0.843 |
| 3048 | 0.833 | 1.000 | 0.856 | 1.000 | 0.979 | 1.000 | 0.888 | 1.000 | 1.000 | 1.000 | 0.980 | 1.000 | 1.000 | 0.809 | 1.000 | 1.000 | 0.869 | 0.843 |
| 3049 | 0.833 | 1.000 | 0.856 | 1.000 | 0.979 | 1.000 | 0.886 | 1.000 | 1.000 | 1.000 | 0.980 | 1.000 | 1.000 | 0.809 | 1.000 | 1.000 | 0.869 | 0.842 |
| 3050 | 0.833 | 1.000 | 0.856 | 1.000 | 0.979 | 1.000 | 0.886 | 1.000 | 1.000 | 1.000 | 0.980 | 1.000 | 1.000 | 0.809 | 1.000 | 1.000 | 0.869 | 0.842 |
| 3051 | 0.833 | 1.000 | 0.856 | 1.000 | 0.979 | 1.000 | 0.886 | 1.000 | 1.000 | 1.000 | 0.980 | 1.000 | 1.000 | 0.810 | 1.000 | 1.000 | 0.870 | 0.843 |
| 3052 | 0.833 | 1.000 | 0.856 | 1.000 | 0.979 | 1.000 | 0.886 | 1.000 | 1.000 | 1.000 | 0.980 | 1.000 | 1.000 | 0.810 | 1.000 | 1.000 | 0.870 | 0.843 |
| 3053 | 0.836 | 1.000 | 0.856 | 1.000 | 0.979 | 1.000 | 0.891 | 1.000 | 1.000 | 1.000 | 0.980 | 1.000 | 1.000 | 0.812 | 1.000 | 1.000 | 0.871 | 0.845 |
| 3054 | 0.835 | 1.000 | 0.856 | 1.000 | 0.979 | 1.000 | 0.886 | 1.000 | 1.000 | 1.000 | 0.980 | 1.000 | 1.000 | 0.814 | 1.000 | 1.000 | 0.871 | 0.845 |
| 3055 | 0.835 | 1.000 | 0.856 | 1.000 | 0.979 | 1.000 | 0.886 | 1.000 | 1.000 | 1.000 | 0.980 | 1.000 | 1.000 | 0.814 | 1.000 | 1.000 | 0.871 | 0.845 |
| 3056 | 0.834 | 1.000 | 0.858 | 1.000 | 0.979 | 1.000 | 0.884 | 1.000 | 1.000 | 1.000 | 0.977 | 1.000 | 1.000 | 0.811 | 1.000 | 1.000 | 0.870 | 0.843 |
| 3057 | 0.836 | 1.000 | 0.860 | 1.000 | 0.979 | 1.000 | 0.884 | 1.000 | 1.000 | 1.000 | 0.977 | 1.000 | 1.000 | 0.811 | 1.000 | 1.000 | 0.871 | 0.844 |
| 3058 | 0.836 | 1.000 | 0.860 | 1.000 | 0.979 | 1.000 | 0.884 | 1.000 | 1.000 | 1.000 | 0.977 | 1.000 | 1.000 | 0.811 | 1.000 | 1.000 | 0.871 | 0.844 |
| 3059 | 0.835 | 1.000 | 0.860 | 1.000 | 0.979 | 1.000 | 0.884 | 1.000 | 1.000 | 1.000 | 0.980 | 1.000 | 1.000 | 0.810 | 1.000 | 1.000 | 0.870 | 0.843 |
| 3060 | 0.835 | 1.000 | 0.860 | 1.000 | 0.979 | 1.000 | 0.884 | 1.000 | 1.000 | 1.000 | 0.980 | 1.000 | 1.000 | 0.810 | 1.000 | 1.000 | 0.870 | 0.843 |
| 3061 | 0.835 | 1.000 | 0.860 | 1.000 | 0.979 | 1.000 | 0.886 | 1.000 | 1.000 | 1.000 | 0.980 | 1.000 | 1.000 | 0.810 | 1.000 | 1.000 | 0.870 | 0.844 |
| 3062 | 0.835 | 1.000 | 0.858 | 1.000 | 0.979 | 1.000 | 0.884 | 1.000 | 1.000 | 1.000 | 0.980 | 1.000 | 1.000 | 0.810 | 1.000 | 1.000 | 0.870 | 0.843 |
| 3063 | 0.835 | 1.000 | 0.858 | 1.000 | 0.979 | 1.000 | 0.884 | 1.000 | 1.000 | 1.000 | 0.980 | 1.000 | 1.000 | 0.810 | 1.000 | 1.000 | 0.870 | 0.843 |
| 3064 | 0.835 | 1.000 | 0.858 | 1.000 | 0.979 | 1.000 | 0.884 | 1.000 | 1.000 | 1.000 | 0.980 | 1.000 | 1.000 | 0.810 | 1.000 | 1.000 | 0.870 | 0.843 |
| 3065 | 0.833 | 1.000 | 0.858 | 1.000 | 0.979 | 1.000 | 0.886 | 1.000 | 1.000 | 1.000 | 0.980 | 1.000 | 1.000 | 0.809 | 1.000 | 1.000 | 0.869 | 0.843 |
| 3066 | 0.834 | 1.000 | 0.858 | 1.000 | 0.979 | 1.000 | 0.886 | 1.000 | 1.000 | 1.000 | 0.980 | 1.000 | 1.000 | 0.809 | 1.000 | 1.000 | 0.870 | 0.843 |
| 3067 | 0.834 | 1.000 | 0.858 | 1.000 | 0.979 | 1.000 | 0.886 | 1.000 | 1.000 | 1.000 | 0.980 | 1.000 | 1.000 | 0.809 | 1.000 | 1.000 | 0.870 | 0.843 |
| 3068 | 0.835 | 1.000 | 0.858 | 1.000 | 0.979 | 1.000 | 0.888 | 1.000 | 1.000 | 1.000 | 0.980 | 1.000 | 1.000 | 0.809 | 1.000 | 1.000 | 0.870 | 0.843 |
| 3069 | 0.833 | 1.000 | 0.858 | 1.000 | 0.979 | 1.000 | 0.886 | 1.000 | 1.000 | 1.000 | 0.980 | 1.000 | 1.000 | 0.810 | 1.000 | 1.000 | 0.870 | 0.843 |
| 3070 | 0.832 | 1.000 | 0.858 | 1.000 | 0.979 | 1.000 | 0.886 | 1.000 | 1.000 | 1.000 | 0.980 | 1.000 | 1.000 | 0.810 | 1.000 | 1.000 | 0.869 | 0.843 |
| 3071 | 0.832 | 1.000 | 0.858 | 1.000 | 0.979 | 1.000 | 0.886 | 1.000 | 1.000 | 1.000 | 0.980 | 1.000 | 1.000 | 0.811 | 1.000 | 1.000 | 0.870 | 0.843 |
| 3072 | 0.832 | 1.000 | 0.856 | 1.000 | 0.979 | 1.000 | 0.886 | 1.000 | 1.000 | 1.000 | 0.980 | 1.000 | 1.000 | 0.811 | 1.000 | 1.000 | 0.869 | 0.843 |
| 3073 | 0.832 | 1.000 | 0.856 | 1.000 | 0.979 | 1.000 | 0.886 | 1.000 | 1.000 | 1.000 | 0.980 | 1.000 | 1.000 | 0.811 | 1.000 | 1.000 | 0.869 | 0.843 |
| 3074 | 0.832 | 1.000 | 0.856 | 1.000 | 0.979 | 1.000 | 0.886 | 1.000 | 1.000 | 1.000 | 0.980 | 1.000 | 1.000 | 0.811 | 1.000 | 1.000 | 0.869 | 0.843 |
| 3075 | 0.831 | 1.000 | 0.856 | 1.000 | 0.979 | 1.000 | 0.886 | 1.000 | 1.000 | 1.000 | 0.980 | 1.000 | 1.000 | 0.811 | 1.000 | 1.000 | 0.869 | 0.842 |
| 3076 | 0.831 | 1.000 | 0.856 | 1.000 | 0.979 | 1.000 | 0.886 | 1.000 | 1.000 | 1.000 | 0.980 | 1.000 | 1.000 | 0.811 | 1.000 | 1.000 | 0.869 | 0.842 |
| 3077 | 0.831 | 1.000 | 0.856 | 1.000 | 0.979 | 1.000 | 0.886 | 1.000 | 1.000 | 1.000 | 0.980 | 1.000 | 1.000 | 0.811 | 1.000 | 1.000 | 0.869 | 0.842 |
| 3078 | 0.831 | 1.000 | 0.856 | 1.000 | 0.979 | 1.000 | 0.886 | 1.000 | 1.000 | 1.000 | 0.980 | 1.000 | 1.000 | 0.811 | 1.000 | 1.000 | 0.869 | 0.842 |
| 3079 | 0.831 | 1.000 | 0.856 | 1.000 | 0.979 | 1.000 | 0.886 | 1.000 | 1.000 | 1.000 | 0.980 | 1.000 | 1.000 | 0.811 | 1.000 | 1.000 | 0.869 | 0.842 |
| 3080 | 0.831 | 1.000 | 0.856 | 1.000 | 0.979 | 1.000 | 0.886 | 1.000 | 1.000 | 1.000 | 0.980 | 1.000 | 1.000 | 0.809 | 1.000 | 1.000 | 0.869 | 0.842 |
| 3081 | 0.833 | 1.000 | 0.856 | 1.000 | 0.979 | 1.000 | 0.884 | 1.000 | 1.000 | 1.000 | 0.980 | 1.000 | 1.000 | 0.809 | 1.000 | 1.000 | 0.869 | 0.842 |
| 3082 | 0.833 | 1.000 | 0.858 | 1.000 | 0.979 | 1.000 | 0.884 | 1.000 | 1.000 | 1.000 | 0.980 | 1.000 | 1.000 | 0.809 | 1.000 | 1.000 | 0.869 | 0.842 |
| 3083 | 0.831 | 1.000 | 0.858 | 1.000 | 0.979 | 1.000 | 0.884 | 1.000 | 1.000 | 1.000 | 0.980 | 1.000 | 1.000 | 0.809 | 1.000 | 1.000 | 0.869 | 0.842 |
| 3084 | 0.833 | 1.000 | 0.858 | 1.000 | 0.979 | 1.000 | 0.884 | 1.000 | 1.000 | 1.000 | 0.980 | 1.000 | 1.000 | 0.808 | 1.000 | 1.000 | 0.869 | 0.842 |

| | | | | | | | | | | | | | | | | | | |
|---|---|---|---|---|---|---|---|---|---|---|---|---|---|---|---|---|---|---|
| 3085 | 0.833 | 1.000 | 0.858 | 1.000 | 0.979 | 1.000 | 0.884 | 1.000 | 1.000 | 1.000 | 0.980 | 1.000 | 1.000 | 0.808 | 1.000 | 1.000 | 0.869 | 0.842 |
| 3086 | 0.833 | 1.000 | 0.858 | 1.000 | 0.979 | 1.000 | 0.884 | 1.000 | 1.000 | 1.000 | 0.980 | 1.000 | 1.000 | 0.808 | 1.000 | 1.000 | 0.869 | 0.842 |
| 3087 | 0.833 | 1.000 | 0.858 | 1.000 | 0.979 | 1.000 | 0.884 | 1.000 | 1.000 | 1.000 | 0.980 | 1.000 | 1.000 | 0.808 | 1.000 | 1.000 | 0.869 | 0.842 |
| 3088 | 0.833 | 1.000 | 0.858 | 1.000 | 0.979 | 1.000 | 0.884 | 1.000 | 1.000 | 1.000 | 0.980 | 1.000 | 1.000 | 0.808 | 1.000 | 1.000 | 0.869 | 0.842 |
| 3089 | 0.833 | 1.000 | 0.858 | 1.000 | 0.979 | 1.000 | 0.884 | 1.000 | 1.000 | 1.000 | 0.980 | 1.000 | 1.000 | 0.808 | 1.000 | 1.000 | 0.869 | 0.842 |
| 3090 | 0.833 | 1.000 | 0.858 | 1.000 | 0.979 | 1.000 | 0.884 | 1.000 | 1.000 | 1.000 | 0.980 | 1.000 | 1.000 | 0.809 | 1.000 | 1.000 | 0.869 | 0.842 |
| 3091 | 0.833 | 1.000 | 0.858 | 1.000 | 0.979 | 1.000 | 0.884 | 1.000 | 1.000 | 1.000 | 0.980 | 1.000 | 1.000 | 0.809 | 1.000 | 1.000 | 0.869 | 0.842 |
| 3092 | 0.833 | 1.000 | 0.858 | 1.000 | 0.979 | 1.000 | 0.884 | 1.000 | 1.000 | 1.000 | 0.980 | 1.000 | 1.000 | 0.807 | 1.000 | 1.000 | 0.869 | 0.842 |
| 3093 | 0.833 | 1.000 | 0.856 | 1.000 | 0.979 | 1.000 | 0.886 | 1.000 | 1.000 | 1.000 | 0.980 | 1.000 | 1.000 | 0.806 | 1.000 | 1.000 | 0.869 | 0.842 |
| 3094 | 0.833 | 1.000 | 0.856 | 1.000 | 0.979 | 1.000 | 0.886 | 1.000 | 1.000 | 1.000 | 0.980 | 1.000 | 1.000 | 0.806 | 1.000 | 1.000 | 0.869 | 0.842 |
| 3095 | 0.833 | 1.000 | 0.856 | 1.000 | 0.979 | 1.000 | 0.886 | 1.000 | 1.000 | 1.000 | 0.980 | 1.000 | 1.000 | 0.806 | 1.000 | 1.000 | 0.868 | 0.841 |
| 3096 | 0.829 | 1.000 | 0.854 | 1.000 | 0.979 | 1.000 | 0.883 | 1.000 | 1.000 | 1.000 | 0.980 | 1.000 | 1.000 | 0.805 | 1.000 | 1.000 | 0.867 | 0.839 |
| 3097 | 0.830 | 1.000 | 0.854 | 1.000 | 0.979 | 1.000 | 0.883 | 1.000 | 1.000 | 1.000 | 0.980 | 1.000 | 1.000 | 0.805 | 1.000 | 1.000 | 0.867 | 0.839 |
| 3098 | 0.830 | 1.000 | 0.854 | 1.000 | 0.979 | 1.000 | 0.883 | 1.000 | 1.000 | 1.000 | 0.980 | 1.000 | 1.000 | 0.806 | 1.000 | 1.000 | 0.867 | 0.840 |
| 3099 | 0.830 | 1.000 | 0.854 | 1.000 | 0.979 | 1.000 | 0.883 | 1.000 | 1.000 | 1.000 | 0.980 | 1.000 | 1.000 | 0.805 | 1.000 | 1.000 | 0.867 | 0.839 |
| 3100 | 0.830 | 1.000 | 0.856 | 1.000 | 0.979 | 1.000 | 0.881 | 1.000 | 1.000 | 1.000 | 0.980 | 1.000 | 1.000 | 0.805 | 1.000 | 1.000 | 0.867 | 0.839 |
| 3101 | 0.829 | 1.000 | 0.856 | 1.000 | 0.979 | 1.000 | 0.881 | 1.000 | 1.000 | 1.000 | 0.980 | 1.000 | 1.000 | 0.805 | 1.000 | 1.000 | 0.866 | 0.839 |
| 3102 | 0.829 | 1.000 | 0.856 | 1.000 | 0.979 | 1.000 | 0.881 | 1.000 | 1.000 | 1.000 | 0.980 | 1.000 | 1.000 | 0.805 | 1.000 | 1.000 | 0.866 | 0.839 |
| 3103 | 0.828 | 1.000 | 0.858 | 1.000 | 0.979 | 1.000 | 0.881 | 1.000 | 1.000 | 1.000 | 0.980 | 1.000 | 1.000 | 0.805 | 1.000 | 1.000 | 0.866 | 0.839 |
| 3104 | 0.829 | 1.000 | 0.858 | 1.000 | 0.979 | 1.000 | 0.881 | 1.000 | 1.000 | 1.000 | 0.980 | 1.000 | 1.000 | 0.805 | 1.000 | 1.000 | 0.866 | 0.839 |
| 3105 | 0.828 | 1.000 | 0.858 | 1.000 | 0.979 | 1.000 | 0.881 | 1.000 | 1.000 | 1.000 | 0.980 | 1.000 | 1.000 | 0.805 | 1.000 | 1.000 | 0.866 | 0.839 |
| 3106 | 0.831 | 1.000 | 0.860 | 1.000 | 0.979 | 1.000 | 0.880 | 1.000 | 1.000 | 1.000 | 0.980 | 1.000 | 1.000 | 0.804 | 1.000 | 1.000 | 0.867 | 0.839 |
| 3107 | 0.829 | 1.000 | 0.858 | 1.000 | 0.979 | 1.000 | 0.881 | 1.000 | 1.000 | 1.000 | 0.980 | 1.000 | 1.000 | 0.805 | 1.000 | 1.000 | 0.867 | 0.839 |
| 3108 | 0.830 | 1.000 | 0.860 | 1.000 | 0.979 | 1.000 | 0.881 | 1.000 | 1.000 | 1.000 | 0.980 | 1.000 | 1.000 | 0.805 | 1.000 | 1.000 | 0.867 | 0.840 |
| 3109 | 0.829 | 1.000 | 0.858 | 1.000 | 0.979 | 1.000 | 0.880 | 1.000 | 1.000 | 1.000 | 0.980 | 1.000 | 1.000 | 0.805 | 1.000 | 1.000 | 0.867 | 0.839 |
| 3110 | 0.827 | 1.000 | 0.858 | 1.000 | 0.979 | 1.000 | 0.881 | 1.000 | 1.000 | 1.000 | 0.980 | 1.000 | 1.000 | 0.805 | 1.000 | 1.000 | 0.866 | 0.839 |
| 3111 | 0.832 | 1.000 | 0.858 | 1.000 | 0.979 | 1.000 | 0.878 | 1.000 | 1.000 | 1.000 | 0.980 | 1.000 | 1.000 | 0.808 | 1.000 | 1.000 | 0.868 | 0.841 |
| 3112 | 0.832 | 1.000 | 0.860 | 1.000 | 0.979 | 1.000 | 0.878 | 1.000 | 1.000 | 1.000 | 0.980 | 1.000 | 1.000 | 0.808 | 1.000 | 1.000 | 0.868 | 0.841 |
| 3113 | 0.832 | 1.000 | 0.860 | 1.000 | 0.979 | 1.000 | 0.878 | 1.000 | 1.000 | 1.000 | 0.980 | 1.000 | 1.000 | 0.808 | 1.000 | 1.000 | 0.868 | 0.841 |
| 3114 | 0.832 | 1.000 | 0.860 | 1.000 | 0.979 | 1.000 | 0.880 | 1.000 | 1.000 | 1.000 | 0.980 | 1.000 | 1.000 | 0.807 | 1.000 | 1.000 | 0.868 | 0.841 |
| 3115 | 0.830 | 1.000 | 0.860 | 1.000 | 0.979 | 1.000 | 0.880 | 1.000 | 1.000 | 1.000 | 0.980 | 1.000 | 1.000 | 0.809 | 1.000 | 1.000 | 0.868 | 0.841 |
| 3116 | 0.831 | 1.000 | 0.860 | 1.000 | 0.979 | 1.000 | 0.880 | 1.000 | 1.000 | 1.000 | 0.980 | 1.000 | 1.000 | 0.809 | 1.000 | 1.000 | 0.868 | 0.841 |
| 3117 | 0.831 | 1.000 | 0.860 | 1.000 | 0.979 | 1.000 | 0.880 | 1.000 | 1.000 | 1.000 | 0.980 | 1.000 | 1.000 | 0.809 | 1.000 | 1.000 | 0.868 | 0.841 |
| 3118 | 0.832 | 1.000 | 0.858 | 1.000 | 0.979 | 1.000 | 0.881 | 1.000 | 1.000 | 1.000 | 0.980 | 1.000 | 1.000 | 0.809 | 1.000 | 1.000 | 0.868 | 0.841 |
| 3119 | 0.831 | 1.000 | 0.860 | 1.000 | 0.979 | 1.000 | 0.883 | 1.000 | 1.000 | 1.000 | 0.980 | 1.000 | 1.000 | 0.808 | 1.000 | 1.000 | 0.868 | 0.841 |
| 3120 | 0.829 | 1.000 | 0.860 | 1.000 | 0.979 | 1.000 | 0.886 | 1.000 | 1.000 | 1.000 | 0.980 | 1.000 | 1.000 | 0.807 | 1.000 | 1.000 | 0.868 | 0.841 |
| 3121 | 0.829 | 1.000 | 0.860 | 1.000 | 0.979 | 1.000 | 0.886 | 1.000 | 1.000 | 1.000 | 0.980 | 1.000 | 1.000 | 0.807 | 1.000 | 1.000 | 0.868 | 0.841 |
| 3122 | 0.829 | 1.000 | 0.860 | 1.000 | 0.979 | 1.000 | 0.884 | 1.000 | 1.000 | 1.000 | 0.980 | 1.000 | 1.000 | 0.807 | 1.000 | 1.000 | 0.868 | 0.841 |
| 3123 | 0.829 | 1.000 | 0.860 | 1.000 | 0.979 | 1.000 | 0.886 | 1.000 | 1.000 | 1.000 | 0.980 | 1.000 | 1.000 | 0.807 | 1.000 | 1.000 | 0.868 | 0.841 |

| | | | | | | | | | | | | | | | | | |
|---|---|---|---|---|---|---|---|---|---|---|---|---|---|---|---|---|---|
| 3124 | 0.830 | 1.000 | 0.860 | 1.000 | 0.979 | 1.000 | 0.886 | 1.000 | 1.000 | 1.000 | 0.980 | 1.000 | 1.000 | 0.807 | 1.000 | 1.000 | 0.868 | 0.841 |
| 3125 | 0.831 | 1.000 | 0.860 | 1.000 | 0.979 | 1.000 | 0.886 | 1.000 | 1.000 | 1.000 | 0.980 | 1.000 | 1.000 | 0.805 | 1.000 | 1.000 | 0.868 | 0.841 |
| 3126 | 0.831 | 1.000 | 0.860 | 1.000 | 0.979 | 1.000 | 0.886 | 1.000 | 1.000 | 1.000 | 0.980 | 1.000 | 1.000 | 0.805 | 1.000 | 1.000 | 0.868 | 0.841 |
| 3127 | 0.831 | 1.000 | 0.860 | 1.000 | 0.979 | 1.000 | 0.886 | 1.000 | 1.000 | 1.000 | 0.980 | 1.000 | 1.000 | 0.805 | 1.000 | 1.000 | 0.868 | 0.841 |
| 3128 | 0.831 | 1.000 | 0.860 | 1.000 | 0.979 | 1.000 | 0.886 | 1.000 | 1.000 | 1.000 | 0.980 | 1.000 | 1.000 | 0.805 | 1.000 | 1.000 | 0.868 | 0.841 |
| 3129 | 0.832 | 1.000 | 0.856 | 1.000 | 0.979 | 1.000 | 0.883 | 1.000 | 1.000 | 1.000 | 0.980 | 1.000 | 1.000 | 0.809 | 1.000 | 1.000 | 0.868 | 0.841 |
| 3130 | 0.833 | 1.000 | 0.856 | 1.000 | 0.979 | 1.000 | 0.883 | 1.000 | 1.000 | 1.000 | 0.980 | 1.000 | 1.000 | 0.809 | 1.000 | 1.000 | 0.869 | 0.842 |
| 3131 | 0.833 | 1.000 | 0.856 | 1.000 | 0.979 | 1.000 | 0.884 | 1.000 | 1.000 | 1.000 | 0.980 | 1.000 | 1.000 | 0.809 | 1.000 | 1.000 | 0.869 | 0.842 |
| 3132 | 0.833 | 1.000 | 0.860 | 1.000 | 0.979 | 1.000 | 0.884 | 1.000 | 1.000 | 1.000 | 0.980 | 1.000 | 1.000 | 0.808 | 1.000 | 1.000 | 0.869 | 0.842 |
| 3133 | 0.832 | 1.000 | 0.860 | 1.000 | 0.979 | 1.000 | 0.883 | 1.000 | 1.000 | 1.000 | 0.980 | 1.000 | 1.000 | 0.809 | 1.000 | 1.000 | 0.869 | 0.842 |
| 3134 | 0.832 | 1.000 | 0.860 | 1.000 | 0.979 | 1.000 | 0.883 | 1.000 | 1.000 | 1.000 | 0.980 | 1.000 | 1.000 | 0.809 | 1.000 | 1.000 | 0.869 | 0.842 |
| 3135 | 0.833 | 1.000 | 0.860 | 1.000 | 0.979 | 1.000 | 0.884 | 1.000 | 1.000 | 1.000 | 0.980 | 1.000 | 1.000 | 0.809 | 1.000 | 1.000 | 0.869 | 0.842 |
| 3136 | 0.833 | 1.000 | 0.860 | 1.000 | 0.979 | 1.000 | 0.883 | 1.000 | 1.000 | 1.000 | 0.980 | 1.000 | 1.000 | 0.809 | 1.000 | 1.000 | 0.869 | 0.842 |
| 3137 | 0.831 | 1.000 | 0.860 | 1.000 | 0.979 | 1.000 | 0.883 | 1.000 | 1.000 | 1.000 | 0.980 | 1.000 | 1.000 | 0.809 | 1.000 | 1.000 | 0.869 | 0.842 |
| 3138 | 0.832 | 1.000 | 0.860 | 1.000 | 0.979 | 1.000 | 0.883 | 1.000 | 1.000 | 1.000 | 0.980 | 1.000 | 1.000 | 0.809 | 1.000 | 1.000 | 0.869 | 0.842 |
| 3139 | 0.831 | 1.000 | 0.860 | 1.000 | 0.979 | 1.000 | 0.883 | 1.000 | 1.000 | 1.000 | 0.980 | 1.000 | 1.000 | 0.808 | 1.000 | 1.000 | 0.868 | 0.841 |
| 3140 | 0.831 | 1.000 | 0.860 | 1.000 | 0.979 | 1.000 | 0.883 | 1.000 | 1.000 | 1.000 | 0.980 | 1.000 | 1.000 | 0.807 | 1.000 | 1.000 | 0.868 | 0.841 |
| 3141 | 0.831 | 1.000 | 0.862 | 1.000 | 0.979 | 1.000 | 0.884 | 1.000 | 1.000 | 1.000 | 0.980 | 1.000 | 1.000 | 0.807 | 1.000 | 1.000 | 0.868 | 0.841 |
| 3142 | 0.831 | 1.000 | 0.864 | 1.000 | 0.979 | 1.000 | 0.884 | 1.000 | 1.000 | 1.000 | 0.980 | 1.000 | 1.000 | 0.805 | 1.000 | 1.000 | 0.868 | 0.841 |
| 3143 | 0.828 | 1.000 | 0.862 | 1.000 | 0.979 | 1.000 | 0.884 | 1.000 | 1.000 | 1.000 | 0.977 | 1.000 | 1.000 | 0.802 | 1.000 | 1.000 | 0.866 | 0.839 |
| 3144 | 0.829 | 1.000 | 0.862 | 1.000 | 0.979 | 1.000 | 0.884 | 1.000 | 1.000 | 1.000 | 0.977 | 1.000 | 1.000 | 0.801 | 1.000 | 1.000 | 0.866 | 0.839 |
| 3145 | 0.829 | 1.000 | 0.862 | 1.000 | 0.979 | 1.000 | 0.884 | 1.000 | 1.000 | 1.000 | 0.977 | 1.000 | 1.000 | 0.802 | 1.000 | 1.000 | 0.867 | 0.839 |
| 3146 | 0.830 | 1.000 | 0.862 | 1.000 | 0.979 | 1.000 | 0.884 | 1.000 | 1.000 | 1.000 | 0.977 | 1.000 | 1.000 | 0.802 | 1.000 | 1.000 | 0.867 | 0.839 |
| 3147 | 0.829 | 1.000 | 0.864 | 1.000 | 0.979 | 1.000 | 0.884 | 1.000 | 1.000 | 1.000 | 0.977 | 1.000 | 1.000 | 0.802 | 1.000 | 1.000 | 0.867 | 0.840 |
| 3148 | 0.830 | 1.000 | 0.864 | 1.000 | 0.979 | 1.000 | 0.884 | 1.000 | 1.000 | 1.000 | 0.977 | 1.000 | 1.000 | 0.802 | 1.000 | 1.000 | 0.867 | 0.840 |
| 3149 | 0.829 | 1.000 | 0.864 | 1.000 | 0.979 | 1.000 | 0.884 | 1.000 | 1.000 | 1.000 | 0.977 | 1.000 | 1.000 | 0.802 | 1.000 | 1.000 | 0.866 | 0.839 |
| 3150 | 0.830 | 1.000 | 0.864 | 1.000 | 0.979 | 1.000 | 0.884 | 1.000 | 1.000 | 1.000 | 0.977 | 1.000 | 1.000 | 0.802 | 1.000 | 1.000 | 0.867 | 0.840 |
| 3151 | 0.831 | 1.000 | 0.862 | 1.000 | 0.979 | 1.000 | 0.884 | 1.000 | 1.000 | 1.000 | 0.977 | 1.000 | 1.000 | 0.804 | 1.000 | 1.000 | 0.867 | 0.840 |
| 3152 | 0.831 | 1.000 | 0.862 | 1.000 | 0.979 | 1.000 | 0.884 | 1.000 | 1.000 | 1.000 | 0.977 | 1.000 | 1.000 | 0.804 | 1.000 | 1.000 | 0.867 | 0.840 |
| 3153 | 0.832 | 1.000 | 0.862 | 1.000 | 0.979 | 1.000 | 0.884 | 1.000 | 1.000 | 1.000 | 0.977 | 1.000 | 1.000 | 0.805 | 1.000 | 1.000 | 0.868 | 0.841 |
| 3154 | 0.831 | 1.000 | 0.862 | 1.000 | 0.979 | 1.000 | 0.884 | 1.000 | 1.000 | 1.000 | 0.977 | 1.000 | 1.000 | 0.804 | 1.000 | 1.000 | 0.868 | 0.840 |
| 3155 | 0.831 | 1.000 | 0.862 | 1.000 | 0.979 | 1.000 | 0.884 | 1.000 | 1.000 | 1.000 | 0.977 | 1.000 | 1.000 | 0.804 | 1.000 | 1.000 | 0.868 | 0.840 |
| 3156 | 0.831 | 1.000 | 0.862 | 1.000 | 0.979 | 1.000 | 0.884 | 1.000 | 1.000 | 1.000 | 0.977 | 1.000 | 1.000 | 0.805 | 1.000 | 1.000 | 0.868 | 0.841 |
| 3157 | 0.830 | 1.000 | 0.862 | 1.000 | 0.979 | 1.000 | 0.884 | 1.000 | 1.000 | 1.000 | 0.977 | 1.000 | 1.000 | 0.805 | 1.000 | 1.000 | 0.867 | 0.840 |
| 3158 | 0.830 | 1.000 | 0.862 | 1.000 | 0.979 | 1.000 | 0.884 | 1.000 | 1.000 | 1.000 | 0.974 | 1.000 | 1.000 | 0.804 | 1.000 | 1.000 | 0.867 | 0.840 |
| 3159 | 0.831 | 1.000 | 0.862 | 1.000 | 0.979 | 1.000 | 0.884 | 1.000 | 1.000 | 1.000 | 0.977 | 1.000 | 1.000 | 0.805 | 1.000 | 1.000 | 0.868 | 0.841 |
| 3160 | 0.831 | 1.000 | 0.862 | 1.000 | 0.979 | 1.000 | 0.884 | 1.000 | 1.000 | 1.000 | 0.974 | 1.000 | 1.000 | 0.806 | 1.000 | 1.000 | 0.868 | 0.841 |
| 3161 | 0.831 | 1.000 | 0.862 | 1.000 | 0.979 | 1.000 | 0.883 | 1.000 | 1.000 | 1.000 | 0.977 | 1.000 | 1.000 | 0.805 | 1.000 | 1.000 | 0.867 | 0.840 |
| 3162 | 0.831 | 1.000 | 0.862 | 1.000 | 0.979 | 1.000 | 0.883 | 1.000 | 1.000 | 1.000 | 0.974 | 1.000 | 1.000 | 0.804 | 1.000 | 1.000 | 0.867 | 0.840 |

| | | | | | | | | | | | | | | | | | | |
|---|---|---|---|---|---|---|---|---|---|---|---|---|---|---|---|---|---|---|
| 3163 | 0.832 | 1.000 | 0.864 | 1.000 | 0.979 | 1.000 | 0.883 | 1.000 | 1.000 | 1.000 | 0.974 | 1.000 | 1.000 | 0.805 | 1.000 | 1.000 | 0.868 | 0.841 |
| 3164 | 0.832 | 1.000 | 0.864 | 1.000 | 0.979 | 1.000 | 0.883 | 1.000 | 1.000 | 1.000 | 0.974 | 1.000 | 1.000 | 0.804 | 1.000 | 1.000 | 0.868 | 0.840 |
| 3165 | 0.832 | 1.000 | 0.864 | 1.000 | 0.979 | 1.000 | 0.884 | 1.000 | 1.000 | 1.000 | 0.974 | 1.000 | 1.000 | 0.804 | 1.000 | 1.000 | 0.868 | 0.841 |
| 3166 | 0.833 | 1.000 | 0.864 | 1.000 | 0.979 | 1.000 | 0.884 | 1.000 | 1.000 | 1.000 | 0.974 | 1.000 | 1.000 | 0.805 | 1.000 | 1.000 | 0.868 | 0.841 |
| 3167 | 0.831 | 1.000 | 0.864 | 1.000 | 0.979 | 1.000 | 0.884 | 1.000 | 1.000 | 1.000 | 0.974 | 1.000 | 1.000 | 0.805 | 1.000 | 1.000 | 0.868 | 0.841 |
| 3168 | 0.831 | 1.000 | 0.862 | 1.000 | 0.979 | 1.000 | 0.884 | 1.000 | 1.000 | 1.000 | 0.974 | 1.000 | 1.000 | 0.805 | 1.000 | 1.000 | 0.868 | 0.841 |
| 3169 | 0.831 | 1.000 | 0.862 | 1.000 | 0.979 | 1.000 | 0.883 | 1.000 | 1.000 | 1.000 | 0.974 | 1.000 | 1.000 | 0.805 | 1.000 | 1.000 | 0.867 | 0.840 |
| 3170 | 0.832 | 1.000 | 0.862 | 1.000 | 0.979 | 1.000 | 0.883 | 1.000 | 1.000 | 1.000 | 0.974 | 1.000 | 1.000 | 0.805 | 1.000 | 1.000 | 0.868 | 0.840 |
| 3171 | 0.831 | 1.000 | 0.862 | 1.000 | 0.979 | 1.000 | 0.883 | 1.000 | 1.000 | 1.000 | 0.974 | 1.000 | 1.000 | 0.805 | 1.000 | 1.000 | 0.867 | 0.840 |
| 3172 | 0.832 | 1.000 | 0.862 | 1.000 | 0.979 | 1.000 | 0.883 | 1.000 | 1.000 | 1.000 | 0.974 | 1.000 | 1.000 | 0.805 | 1.000 | 1.000 | 0.868 | 0.840 |
| 3173 | 0.831 | 1.000 | 0.862 | 1.000 | 0.979 | 1.000 | 0.883 | 1.000 | 1.000 | 1.000 | 0.974 | 1.000 | 1.000 | 0.805 | 1.000 | 1.000 | 0.868 | 0.840 |
| 3174 | 0.832 | 1.000 | 0.860 | 1.000 | 0.979 | 1.000 | 0.883 | 1.000 | 1.000 | 1.000 | 0.974 | 1.000 | 1.000 | 0.807 | 1.000 | 1.000 | 0.868 | 0.841 |
| 3175 | 0.832 | 1.000 | 0.860 | 1.000 | 0.979 | 1.000 | 0.883 | 1.000 | 1.000 | 1.000 | 0.974 | 1.000 | 1.000 | 0.809 | 1.000 | 1.000 | 0.868 | 0.841 |
| 3176 | 0.832 | 1.000 | 0.860 | 1.000 | 0.979 | 1.000 | 0.883 | 1.000 | 1.000 | 1.000 | 0.974 | 1.000 | 1.000 | 0.807 | 1.000 | 1.000 | 0.868 | 0.841 |
| 3177 | 0.832 | 1.000 | 0.860 | 1.000 | 0.979 | 1.000 | 0.883 | 1.000 | 1.000 | 1.000 | 0.974 | 1.000 | 1.000 | 0.808 | 1.000 | 1.000 | 0.868 | 0.841 |
| 3178 | 0.832 | 1.000 | 0.860 | 1.000 | 0.979 | 1.000 | 0.883 | 1.000 | 1.000 | 1.000 | 0.974 | 1.000 | 1.000 | 0.807 | 1.000 | 1.000 | 0.868 | 0.841 |
| 3179 | 0.829 | 1.000 | 0.858 | 1.000 | 0.979 | 1.000 | 0.883 | 1.000 | 1.000 | 1.000 | 0.974 | 1.000 | 1.000 | 0.809 | 1.000 | 1.000 | 0.867 | 0.840 |
| 3180 | 0.829 | 1.000 | 0.858 | 1.000 | 0.979 | 1.000 | 0.883 | 1.000 | 1.000 | 1.000 | 0.974 | 1.000 | 1.000 | 0.808 | 1.000 | 1.000 | 0.867 | 0.840 |
| 3181 | 0.833 | 1.000 | 0.860 | 1.000 | 0.979 | 1.000 | 0.881 | 1.000 | 1.000 | 1.000 | 0.974 | 1.000 | 1.000 | 0.809 | 1.000 | 1.000 | 0.869 | 0.841 |
| 3182 | 0.831 | 1.000 | 0.860 | 1.000 | 0.979 | 1.000 | 0.883 | 1.000 | 1.000 | 1.000 | 0.974 | 1.000 | 1.000 | 0.808 | 1.000 | 1.000 | 0.868 | 0.841 |
| 3183 | 0.831 | 1.000 | 0.860 | 1.000 | 0.979 | 1.000 | 0.883 | 1.000 | 1.000 | 1.000 | 0.974 | 1.000 | 1.000 | 0.807 | 1.000 | 1.000 | 0.868 | 0.841 |
| 3184 | 0.831 | 1.000 | 0.860 | 1.000 | 0.979 | 1.000 | 0.884 | 1.000 | 1.000 | 1.000 | 0.974 | 1.000 | 1.000 | 0.809 | 1.000 | 1.000 | 0.869 | 0.842 |
| 3185 | 0.830 | 1.000 | 0.860 | 1.000 | 0.979 | 1.000 | 0.883 | 1.000 | 1.000 | 1.000 | 0.974 | 1.000 | 1.000 | 0.809 | 1.000 | 1.000 | 0.868 | 0.841 |
| 3186 | 0.830 | 1.000 | 0.860 | 1.000 | 0.979 | 1.000 | 0.884 | 1.000 | 1.000 | 1.000 | 0.974 | 1.000 | 1.000 | 0.808 | 1.000 | 1.000 | 0.868 | 0.841 |
| 3187 | 0.830 | 1.000 | 0.860 | 1.000 | 0.979 | 1.000 | 0.884 | 1.000 | 1.000 | 1.000 | 0.974 | 1.000 | 1.000 | 0.808 | 1.000 | 1.000 | 0.868 | 0.841 |
| 3188 | 0.830 | 1.000 | 0.860 | 1.000 | 0.979 | 1.000 | 0.884 | 1.000 | 1.000 | 1.000 | 0.974 | 1.000 | 1.000 | 0.808 | 1.000 | 1.000 | 0.868 | 0.841 |
| 3189 | 0.829 | 1.000 | 0.860 | 1.000 | 0.979 | 1.000 | 0.884 | 1.000 | 1.000 | 1.000 | 0.974 | 1.000 | 1.000 | 0.809 | 1.000 | 1.000 | 0.868 | 0.841 |
| 3190 | 0.830 | 1.000 | 0.862 | 1.000 | 0.979 | 1.000 | 0.884 | 1.000 | 1.000 | 1.000 | 0.974 | 1.000 | 1.000 | 0.810 | 1.000 | 1.000 | 0.869 | 0.842 |
| 3191 | 0.829 | 1.000 | 0.864 | 1.000 | 0.979 | 1.000 | 0.884 | 1.000 | 1.000 | 1.000 | 0.974 | 1.000 | 1.000 | 0.810 | 1.000 | 1.000 | 0.868 | 0.841 |
| 3192 | 0.829 | 1.000 | 0.864 | 1.000 | 0.979 | 1.000 | 0.884 | 1.000 | 1.000 | 1.000 | 0.974 | 1.000 | 1.000 | 0.810 | 1.000 | 1.000 | 0.868 | 0.841 |
| 3193 | 0.827 | 1.000 | 0.864 | 1.000 | 0.979 | 1.000 | 0.886 | 1.000 | 1.000 | 1.000 | 0.974 | 1.000 | 1.000 | 0.809 | 1.000 | 1.000 | 0.868 | 0.841 |
| 3194 | 0.827 | 1.000 | 0.864 | 1.000 | 0.979 | 1.000 | 0.886 | 1.000 | 1.000 | 1.000 | 0.974 | 1.000 | 1.000 | 0.809 | 1.000 | 1.000 | 0.868 | 0.841 |
| 3195 | 0.828 | 1.000 | 0.868 | 1.000 | 0.979 | 1.000 | 0.886 | 1.000 | 1.000 | 1.000 | 0.974 | 1.000 | 1.000 | 0.811 | 1.000 | 1.000 | 0.869 | 0.842 |
| 3196 | 0.829 | 1.000 | 0.868 | 1.000 | 0.979 | 1.000 | 0.884 | 1.000 | 1.000 | 1.000 | 0.974 | 1.000 | 1.000 | 0.811 | 1.000 | 1.000 | 0.869 | 0.842 |
| 3197 | 0.831 | 1.000 | 0.868 | 1.000 | 0.979 | 1.000 | 0.883 | 1.000 | 1.000 | 1.000 | 0.974 | 1.000 | 1.000 | 0.810 | 1.000 | 1.000 | 0.869 | 0.842 |
| 3198 | 0.831 | 1.000 | 0.866 | 1.000 | 0.979 | 1.000 | 0.881 | 1.000 | 1.000 | 1.000 | 0.974 | 1.000 | 1.000 | 0.810 | 1.000 | 1.000 | 0.869 | 0.842 |
| 3199 | 0.831 | 1.000 | 0.866 | 1.000 | 0.979 | 1.000 | 0.881 | 1.000 | 1.000 | 1.000 | 0.974 | 1.000 | 1.000 | 0.811 | 1.000 | 1.000 | 0.869 | 0.842 |
| 3200 | 0.831 | 1.000 | 0.866 | 1.000 | 0.979 | 1.000 | 0.881 | 1.000 | 1.000 | 1.000 | 0.974 | 1.000 | 1.000 | 0.811 | 1.000 | 1.000 | 0.869 | 0.842 |
| 3201 | 0.830 | 1.000 | 0.866 | 1.000 | 0.979 | 1.000 | 0.881 | 1.000 | 1.000 | 1.000 | 0.974 | 1.000 | 1.000 | 0.810 | 1.000 | 1.000 | 0.869 | 0.842 |

| | | | | | | | | | | | | | | | | | | |
|---|---|---|---|---|---|---|---|---|---|---|---|---|---|---|---|---|---|---|
| 3202 | 0.829 | 1.000 | 0.866 | 1.000 | 0.979 | 1.000 | 0.883 | 1.000 | 1.000 | 1.000 | 0.974 | 1.000 | 1.000 | 0.810 | 1.000 | 1.000 | 0.868 | 0.841 |
| 3203 | 0.829 | 1.000 | 0.864 | 1.000 | 0.979 | 1.000 | 0.881 | 1.000 | 1.000 | 1.000 | 0.974 | 1.000 | 1.000 | 0.810 | 1.000 | 1.000 | 0.868 | 0.841 |
| 3204 | 0.829 | 1.000 | 0.864 | 1.000 | 0.979 | 1.000 | 0.881 | 1.000 | 1.000 | 1.000 | 0.974 | 1.000 | 1.000 | 0.809 | 1.000 | 1.000 | 0.868 | 0.841 |
| 3205 | 0.829 | 1.000 | 0.864 | 1.000 | 0.979 | 1.000 | 0.881 | 1.000 | 1.000 | 1.000 | 0.974 | 1.000 | 1.000 | 0.809 | 1.000 | 1.000 | 0.868 | 0.841 |
| 3206 | 0.829 | 1.000 | 0.864 | 1.000 | 0.979 | 1.000 | 0.883 | 1.000 | 1.000 | 1.000 | 0.974 | 1.000 | 1.000 | 0.809 | 1.000 | 1.000 | 0.868 | 0.841 |
| 3207 | 0.830 | 1.000 | 0.864 | 1.000 | 0.979 | 1.000 | 0.881 | 1.000 | 1.000 | 1.000 | 0.974 | 1.000 | 1.000 | 0.809 | 1.000 | 1.000 | 0.868 | 0.841 |
| 3208 | 0.829 | 1.000 | 0.864 | 1.000 | 0.979 | 1.000 | 0.881 | 1.000 | 1.000 | 1.000 | 0.974 | 1.000 | 1.000 | 0.809 | 1.000 | 1.000 | 0.868 | 0.841 |
| 3209 | 0.831 | 1.000 | 0.864 | 1.000 | 0.979 | 1.000 | 0.881 | 1.000 | 1.000 | 1.000 | 0.974 | 1.000 | 1.000 | 0.809 | 1.000 | 1.000 | 0.868 | 0.841 |
| 3210 | 0.831 | 1.000 | 0.864 | 1.000 | 0.979 | 1.000 | 0.883 | 1.000 | 1.000 | 1.000 | 0.974 | 1.000 | 1.000 | 0.809 | 1.000 | 1.000 | 0.869 | 0.842 |
| 3211 | 0.831 | 1.000 | 0.864 | 1.000 | 0.979 | 1.000 | 0.883 | 1.000 | 1.000 | 1.000 | 0.974 | 1.000 | 1.000 | 0.809 | 1.000 | 1.000 | 0.869 | 0.842 |
| 3212 | 0.831 | 1.000 | 0.864 | 1.000 | 0.979 | 1.000 | 0.883 | 1.000 | 1.000 | 1.000 | 0.974 | 1.000 | 1.000 | 0.809 | 1.000 | 1.000 | 0.869 | 0.842 |
| 3213 | 0.830 | 1.000 | 0.864 | 1.000 | 0.979 | 1.000 | 0.883 | 1.000 | 1.000 | 1.000 | 0.974 | 1.000 | 1.000 | 0.809 | 1.000 | 1.000 | 0.868 | 0.841 |
| 3214 | 0.830 | 1.000 | 0.864 | 1.000 | 0.979 | 1.000 | 0.883 | 1.000 | 1.000 | 1.000 | 0.974 | 1.000 | 1.000 | 0.809 | 1.000 | 1.000 | 0.868 | 0.841 |
| 3215 | 0.829 | 1.000 | 0.864 | 1.000 | 0.979 | 1.000 | 0.881 | 1.000 | 1.000 | 1.000 | 0.974 | 1.000 | 1.000 | 0.809 | 1.000 | 1.000 | 0.868 | 0.841 |
| 3216 | 0.829 | 1.000 | 0.864 | 1.000 | 0.979 | 1.000 | 0.881 | 1.000 | 1.000 | 1.000 | 0.974 | 1.000 | 1.000 | 0.809 | 1.000 | 1.000 | 0.868 | 0.841 |
| 3217 | 0.829 | 1.000 | 0.864 | 1.000 | 0.979 | 1.000 | 0.883 | 1.000 | 1.000 | 1.000 | 0.974 | 1.000 | 1.000 | 0.809 | 1.000 | 1.000 | 0.868 | 0.841 |
| 3218 | 0.829 | 1.000 | 0.864 | 1.000 | 0.979 | 1.000 | 0.883 | 1.000 | 1.000 | 1.000 | 0.974 | 1.000 | 1.000 | 0.809 | 1.000 | 1.000 | 0.868 | 0.841 |
| 3219 | 0.829 | 1.000 | 0.864 | 1.000 | 0.979 | 1.000 | 0.883 | 1.000 | 1.000 | 1.000 | 0.974 | 1.000 | 1.000 | 0.809 | 1.000 | 1.000 | 0.868 | 0.841 |
| 3220 | 0.829 | 1.000 | 0.866 | 1.000 | 0.979 | 1.000 | 0.881 | 1.000 | 1.000 | 1.000 | 0.974 | 1.000 | 1.000 | 0.809 | 1.000 | 1.000 | 0.868 | 0.841 |
| 3221 | 0.830 | 1.000 | 0.866 | 1.000 | 0.979 | 1.000 | 0.881 | 1.000 | 1.000 | 1.000 | 0.974 | 1.000 | 1.000 | 0.810 | 1.000 | 1.000 | 0.869 | 0.841 |
| 3222 | 0.830 | 1.000 | 0.866 | 1.000 | 0.979 | 1.000 | 0.881 | 1.000 | 1.000 | 1.000 | 0.974 | 1.000 | 1.000 | 0.811 | 1.000 | 1.000 | 0.869 | 0.842 |
| 3223 | 0.831 | 1.000 | 0.866 | 1.000 | 0.979 | 1.000 | 0.881 | 1.000 | 1.000 | 1.000 | 0.974 | 1.000 | 1.000 | 0.810 | 1.000 | 1.000 | 0.869 | 0.842 |
| 3224 | 0.829 | 1.000 | 0.866 | 1.000 | 0.979 | 1.000 | 0.883 | 1.000 | 1.000 | 1.000 | 0.974 | 1.000 | 1.000 | 0.811 | 1.000 | 1.000 | 0.869 | 0.842 |
| 3225 | 0.829 | 1.000 | 0.866 | 1.000 | 0.979 | 1.000 | 0.881 | 1.000 | 1.000 | 1.000 | 0.974 | 1.000 | 1.000 | 0.811 | 1.000 | 1.000 | 0.868 | 0.841 |
| 3226 | 0.829 | 1.000 | 0.866 | 1.000 | 0.979 | 1.000 | 0.883 | 1.000 | 1.000 | 1.000 | 0.974 | 1.000 | 1.000 | 0.811 | 1.000 | 1.000 | 0.869 | 0.842 |
| 3227 | 0.829 | 1.000 | 0.866 | 1.000 | 0.979 | 1.000 | 0.883 | 1.000 | 1.000 | 1.000 | 0.974 | 1.000 | 1.000 | 0.811 | 1.000 | 1.000 | 0.869 | 0.842 |
| 3228 | 0.829 | 1.000 | 0.862 | 1.000 | 0.979 | 1.000 | 0.883 | 1.000 | 1.000 | 1.000 | 0.974 | 1.000 | 1.000 | 0.811 | 1.000 | 1.000 | 0.868 | 0.841 |
| 3229 | 0.831 | 1.000 | 0.862 | 1.000 | 0.979 | 1.000 | 0.883 | 1.000 | 1.000 | 1.000 | 0.974 | 1.000 | 1.000 | 0.811 | 1.000 | 1.000 | 0.869 | 0.842 |
| 3230 | 0.831 | 1.000 | 0.864 | 1.000 | 0.979 | 1.000 | 0.883 | 1.000 | 1.000 | 1.000 | 0.974 | 1.000 | 1.000 | 0.811 | 1.000 | 1.000 | 0.869 | 0.842 |
| 3231 | 0.831 | 1.000 | 0.862 | 1.000 | 0.979 | 1.000 | 0.883 | 1.000 | 1.000 | 1.000 | 0.974 | 1.000 | 1.000 | 0.811 | 1.000 | 1.000 | 0.869 | 0.842 |
| 3232 | 0.831 | 1.000 | 0.862 | 1.000 | 0.979 | 1.000 | 0.883 | 1.000 | 1.000 | 1.000 | 0.974 | 1.000 | 1.000 | 0.811 | 1.000 | 1.000 | 0.869 | 0.842 |
| 3233 | 0.831 | 1.000 | 0.862 | 1.000 | 0.979 | 1.000 | 0.883 | 1.000 | 1.000 | 1.000 | 0.974 | 1.000 | 1.000 | 0.810 | 1.000 | 1.000 | 0.869 | 0.842 |
| 3234 | 0.831 | 1.000 | 0.862 | 1.000 | 0.979 | 1.000 | 0.883 | 1.000 | 1.000 | 1.000 | 0.974 | 1.000 | 1.000 | 0.810 | 1.000 | 1.000 | 0.869 | 0.842 |
| 3235 | 0.831 | 1.000 | 0.862 | 1.000 | 0.979 | 1.000 | 0.883 | 1.000 | 1.000 | 1.000 | 0.974 | 1.000 | 1.000 | 0.811 | 1.000 | 1.000 | 0.869 | 0.842 |
| 3236 | 0.831 | 1.000 | 0.862 | 1.000 | 0.979 | 1.000 | 0.883 | 1.000 | 1.000 | 1.000 | 0.974 | 1.000 | 1.000 | 0.810 | 1.000 | 1.000 | 0.869 | 0.841 |
| 3237 | 0.831 | 1.000 | 0.862 | 1.000 | 0.979 | 1.000 | 0.883 | 1.000 | 1.000 | 1.000 | 0.974 | 1.000 | 1.000 | 0.810 | 1.000 | 1.000 | 0.869 | 0.841 |
| 3238 | 0.831 | 1.000 | 0.862 | 1.000 | 0.979 | 1.000 | 0.883 | 1.000 | 1.000 | 1.000 | 0.974 | 1.000 | 1.000 | 0.810 | 1.000 | 1.000 | 0.869 | 0.841 |
| 3239 | 0.830 | 1.000 | 0.864 | 1.000 | 0.979 | 1.000 | 0.883 | 1.000 | 1.000 | 1.000 | 0.974 | 1.000 | 1.000 | 0.809 | 1.000 | 1.000 | 0.868 | 0.841 |
| 3240 | 0.830 | 1.000 | 0.864 | 1.000 | 0.979 | 1.000 | 0.883 | 1.000 | 1.000 | 1.000 | 0.974 | 1.000 | 1.000 | 0.810 | 1.000 | 1.000 | 0.869 | 0.841 |

| | | | | | | | | | | | | | | | | | | |
|---|---|---|---|---|---|---|---|---|---|---|---|---|---|---|---|---|---|---|
| 3241 | 0.830 | 1.000 | 0.864 | 1.000 | 0.979 | 1.000 | 0.883 | 1.000 | 1.000 | 1.000 | 0.974 | 1.000 | 1.000 | 0.809 | 1.000 | 1.000 | 0.868 | 0.841 |
| 3242 | 0.830 | 1.000 | 0.864 | 1.000 | 0.979 | 1.000 | 0.883 | 1.000 | 1.000 | 1.000 | 0.974 | 1.000 | 1.000 | 0.809 | 1.000 | 1.000 | 0.868 | 0.841 |
| 3243 | 0.831 | 1.000 | 0.864 | 1.000 | 0.979 | 1.000 | 0.883 | 1.000 | 1.000 | 1.000 | 0.974 | 1.000 | 1.000 | 0.809 | 1.000 | 1.000 | 0.869 | 0.841 |
| 3244 | 0.830 | 1.000 | 0.862 | 1.000 | 0.979 | 1.000 | 0.883 | 1.000 | 1.000 | 1.000 | 0.974 | 1.000 | 1.000 | 0.809 | 1.000 | 1.000 | 0.868 | 0.841 |
| 3245 | 0.830 | 1.000 | 0.864 | 1.000 | 0.979 | 1.000 | 0.883 | 1.000 | 1.000 | 1.000 | 0.974 | 1.000 | 1.000 | 0.809 | 1.000 | 1.000 | 0.868 | 0.841 |
| 3246 | 0.831 | 1.000 | 0.864 | 1.000 | 0.979 | 1.000 | 0.883 | 1.000 | 1.000 | 1.000 | 0.971 | 1.000 | 1.000 | 0.809 | 1.000 | 1.000 | 0.868 | 0.841 |
| 3247 | 0.831 | 1.000 | 0.864 | 1.000 | 0.979 | 1.000 | 0.883 | 1.000 | 1.000 | 1.000 | 0.971 | 1.000 | 1.000 | 0.808 | 1.000 | 1.000 | 0.868 | 0.841 |
| 3248 | 0.831 | 1.000 | 0.864 | 1.000 | 0.979 | 1.000 | 0.883 | 1.000 | 1.000 | 1.000 | 0.971 | 1.000 | 1.000 | 0.808 | 1.000 | 1.000 | 0.868 | 0.841 |
| 3249 | 0.830 | 1.000 | 0.864 | 1.000 | 0.979 | 1.000 | 0.883 | 1.000 | 1.000 | 1.000 | 0.971 | 1.000 | 1.000 | 0.808 | 1.000 | 1.000 | 0.868 | 0.841 |
| 3250 | 0.831 | 1.000 | 0.864 | 1.000 | 0.979 | 1.000 | 0.883 | 1.000 | 1.000 | 1.000 | 0.971 | 1.000 | 1.000 | 0.810 | 1.000 | 1.000 | 0.869 | 0.841 |
| 3251 | 0.831 | 1.000 | 0.864 | 1.000 | 0.979 | 1.000 | 0.883 | 1.000 | 1.000 | 1.000 | 0.971 | 1.000 | 1.000 | 0.810 | 1.000 | 1.000 | 0.869 | 0.841 |
| 3252 | 0.831 | 1.000 | 0.864 | 1.000 | 0.979 | 1.000 | 0.883 | 1.000 | 1.000 | 1.000 | 0.971 | 1.000 | 1.000 | 0.810 | 1.000 | 1.000 | 0.869 | 0.841 |
| 3253 | 0.831 | 1.000 | 0.864 | 1.000 | 0.979 | 1.000 | 0.883 | 1.000 | 1.000 | 1.000 | 0.971 | 1.000 | 1.000 | 0.810 | 1.000 | 1.000 | 0.869 | 0.841 |
| 3254 | 0.831 | 1.000 | 0.864 | 1.000 | 0.979 | 1.000 | 0.883 | 1.000 | 1.000 | 1.000 | 0.971 | 1.000 | 1.000 | 0.810 | 1.000 | 1.000 | 0.869 | 0.842 |
| 3255 | 0.829 | 1.000 | 0.864 | 1.000 | 0.979 | 1.000 | 0.884 | 1.000 | 1.000 | 1.000 | 0.971 | 1.000 | 1.000 | 0.811 | 1.000 | 1.000 | 0.869 | 0.841 |
| 3256 | 0.829 | 1.000 | 0.864 | 1.000 | 0.979 | 1.000 | 0.884 | 1.000 | 1.000 | 1.000 | 0.971 | 1.000 | 1.000 | 0.811 | 1.000 | 1.000 | 0.868 | 0.841 |
| 3257 | 0.826 | 1.000 | 0.862 | 1.000 | 0.979 | 1.000 | 0.884 | 1.000 | 1.000 | 1.000 | 0.971 | 1.000 | 1.000 | 0.809 | 1.000 | 1.000 | 0.867 | 0.840 |
| 3258 | 0.826 | 1.000 | 0.862 | 1.000 | 0.979 | 1.000 | 0.884 | 1.000 | 1.000 | 1.000 | 0.971 | 1.000 | 1.000 | 0.809 | 1.000 | 1.000 | 0.867 | 0.840 |
| 3259 | 0.826 | 1.000 | 0.864 | 1.000 | 0.979 | 1.000 | 0.884 | 1.000 | 1.000 | 1.000 | 0.971 | 1.000 | 1.000 | 0.808 | 1.000 | 1.000 | 0.867 | 0.840 |
| 3260 | 0.829 | 1.000 | 0.864 | 1.000 | 0.979 | 1.000 | 0.884 | 1.000 | 1.000 | 1.000 | 0.971 | 1.000 | 1.000 | 0.809 | 1.000 | 1.000 | 0.868 | 0.841 |
| 3261 | 0.828 | 1.000 | 0.862 | 1.000 | 0.979 | 1.000 | 0.884 | 1.000 | 1.000 | 1.000 | 0.971 | 1.000 | 1.000 | 0.809 | 1.000 | 1.000 | 0.868 | 0.840 |
| 3262 | 0.829 | 1.000 | 0.864 | 1.000 | 0.979 | 1.000 | 0.884 | 1.000 | 1.000 | 1.000 | 0.971 | 1.000 | 1.000 | 0.808 | 1.000 | 1.000 | 0.868 | 0.840 |
| 3263 | 0.830 | 1.000 | 0.862 | 1.000 | 0.979 | 1.000 | 0.884 | 1.000 | 1.000 | 1.000 | 0.971 | 1.000 | 1.000 | 0.809 | 1.000 | 1.000 | 0.868 | 0.841 |
| 3264 | 0.829 | 1.000 | 0.862 | 1.000 | 0.979 | 1.000 | 0.884 | 1.000 | 1.000 | 1.000 | 0.971 | 1.000 | 1.000 | 0.809 | 1.000 | 1.000 | 0.868 | 0.841 |
| 3265 | 0.828 | 1.000 | 0.862 | 1.000 | 0.979 | 1.000 | 0.884 | 1.000 | 1.000 | 1.000 | 0.971 | 1.000 | 1.000 | 0.808 | 1.000 | 1.000 | 0.867 | 0.840 |
| 3266 | 0.828 | 1.000 | 0.862 | 1.000 | 0.979 | 1.000 | 0.884 | 1.000 | 1.000 | 1.000 | 0.971 | 1.000 | 1.000 | 0.809 | 1.000 | 1.000 | 0.867 | 0.840 |
| 3267 | 0.829 | 1.000 | 0.862 | 1.000 | 0.979 | 1.000 | 0.884 | 1.000 | 1.000 | 1.000 | 0.974 | 1.000 | 1.000 | 0.809 | 1.000 | 1.000 | 0.868 | 0.841 |
| 3268 | 0.829 | 1.000 | 0.862 | 1.000 | 0.979 | 1.000 | 0.884 | 1.000 | 1.000 | 1.000 | 0.974 | 1.000 | 1.000 | 0.808 | 1.000 | 1.000 | 0.868 | 0.840 |
| 3269 | 0.830 | 1.000 | 0.862 | 1.000 | 0.979 | 1.000 | 0.884 | 1.000 | 1.000 | 1.000 | 0.974 | 1.000 | 1.000 | 0.808 | 1.000 | 1.000 | 0.868 | 0.841 |
| 3270 | 0.830 | 1.000 | 0.862 | 1.000 | 0.979 | 1.000 | 0.884 | 1.000 | 1.000 | 1.000 | 0.974 | 1.000 | 1.000 | 0.809 | 1.000 | 1.000 | 0.868 | 0.841 |
| 3271 | 0.829 | 1.000 | 0.862 | 1.000 | 0.979 | 1.000 | 0.884 | 1.000 | 1.000 | 1.000 | 0.974 | 1.000 | 1.000 | 0.809 | 1.000 | 1.000 | 0.868 | 0.841 |
| 3272 | 0.829 | 1.000 | 0.862 | 1.000 | 0.979 | 1.000 | 0.884 | 1.000 | 1.000 | 1.000 | 0.974 | 1.000 | 1.000 | 0.809 | 1.000 | 1.000 | 0.868 | 0.841 |
| 3273 | 0.828 | 1.000 | 0.862 | 1.000 | 0.979 | 1.000 | 0.884 | 1.000 | 1.000 | 1.000 | 0.974 | 1.000 | 1.000 | 0.809 | 1.000 | 1.000 | 0.868 | 0.840 |
| 3274 | 0.829 | 1.000 | 0.862 | 1.000 | 0.979 | 1.000 | 0.884 | 1.000 | 1.000 | 1.000 | 0.974 | 1.000 | 1.000 | 0.809 | 1.000 | 1.000 | 0.868 | 0.841 |
| 3275 | 0.829 | 1.000 | 0.860 | 1.000 | 0.979 | 1.000 | 0.884 | 1.000 | 1.000 | 1.000 | 0.974 | 1.000 | 1.000 | 0.809 | 1.000 | 1.000 | 0.868 | 0.840 |
| 3276 | 0.829 | 1.000 | 0.860 | 1.000 | 0.979 | 1.000 | 0.884 | 1.000 | 1.000 | 1.000 | 0.974 | 1.000 | 1.000 | 0.809 | 1.000 | 1.000 | 0.868 | 0.840 |
| 3277 | 0.828 | 1.000 | 0.860 | 1.000 | 0.979 | 1.000 | 0.884 | 1.000 | 1.000 | 1.000 | 0.974 | 1.000 | 1.000 | 0.808 | 1.000 | 1.000 | 0.867 | 0.840 |
| 3278 | 0.828 | 1.000 | 0.862 | 1.000 | 0.979 | 1.000 | 0.884 | 1.000 | 1.000 | 1.000 | 0.974 | 1.000 | 1.000 | 0.808 | 1.000 | 1.000 | 0.867 | 0.840 |
| 3279 | 0.829 | 1.000 | 0.862 | 1.000 | 0.979 | 1.000 | 0.884 | 1.000 | 1.000 | 1.000 | 0.977 | 1.000 | 1.000 | 0.808 | 1.000 | 1.000 | 0.868 | 0.841 |

| | | | | | | | | | | | | | | | | | | |
|---|---|---|---|---|---|---|---|---|---|---|---|---|---|---|---|---|---|---|
| 3280 | 0.828 | 1.000 | 0.862 | 1.000 | 0.979 | 1.000 | 0.884 | 1.000 | 1.000 | 1.000 | 0.971 | 1.000 | 1.000 | 0.808 | 1.000 | 1.000 | 0.867 | 0.840 |
| 3281 | 0.828 | 1.000 | 0.862 | 1.000 | 0.979 | 1.000 | 0.884 | 1.000 | 1.000 | 1.000 | 0.977 | 1.000 | 1.000 | 0.808 | 1.000 | 1.000 | 0.868 | 0.840 |
| 3282 | 0.829 | 1.000 | 0.862 | 1.000 | 0.979 | 1.000 | 0.884 | 1.000 | 1.000 | 1.000 | 0.977 | 1.000 | 1.000 | 0.808 | 1.000 | 1.000 | 0.868 | 0.841 |
| 3283 | 0.829 | 1.000 | 0.862 | 1.000 | 0.979 | 1.000 | 0.884 | 1.000 | 1.000 | 1.000 | 0.980 | 1.000 | 1.000 | 0.808 | 1.000 | 1.000 | 0.868 | 0.841 |
| 3284 | 0.828 | 1.000 | 0.862 | 1.000 | 0.979 | 1.000 | 0.884 | 1.000 | 1.000 | 1.000 | 0.974 | 1.000 | 1.000 | 0.808 | 1.000 | 1.000 | 0.867 | 0.840 |
| 3285 | 0.829 | 1.000 | 0.862 | 1.000 | 0.979 | 1.000 | 0.884 | 1.000 | 1.000 | 1.000 | 0.977 | 1.000 | 1.000 | 0.808 | 1.000 | 1.000 | 0.868 | 0.841 |
| 3286 | 0.829 | 1.000 | 0.862 | 1.000 | 0.979 | 1.000 | 0.884 | 1.000 | 1.000 | 1.000 | 0.977 | 1.000 | 1.000 | 0.808 | 1.000 | 1.000 | 0.868 | 0.841 |
| 3287 | 0.829 | 1.000 | 0.862 | 1.000 | 0.979 | 1.000 | 0.884 | 1.000 | 1.000 | 1.000 | 0.977 | 1.000 | 1.000 | 0.807 | 1.000 | 1.000 | 0.868 | 0.841 |
| 3288 | 0.829 | 1.000 | 0.860 | 1.000 | 0.979 | 1.000 | 0.884 | 1.000 | 1.000 | 1.000 | 0.977 | 1.000 | 1.000 | 0.806 | 1.000 | 1.000 | 0.867 | 0.840 |
| 3289 | 0.830 | 1.000 | 0.860 | 1.000 | 0.979 | 1.000 | 0.884 | 1.000 | 1.000 | 1.000 | 0.977 | 1.000 | 1.000 | 0.807 | 1.000 | 1.000 | 0.868 | 0.841 |
| 3290 | 0.830 | 1.000 | 0.860 | 1.000 | 0.979 | 1.000 | 0.884 | 1.000 | 1.000 | 1.000 | 0.977 | 1.000 | 1.000 | 0.807 | 1.000 | 1.000 | 0.868 | 0.841 |
| 3291 | 0.829 | 1.000 | 0.860 | 1.000 | 0.979 | 1.000 | 0.884 | 1.000 | 1.000 | 1.000 | 0.977 | 1.000 | 1.000 | 0.807 | 1.000 | 1.000 | 0.868 | 0.840 |
| 3292 | 0.828 | 1.000 | 0.860 | 1.000 | 0.979 | 1.000 | 0.884 | 1.000 | 1.000 | 1.000 | 0.977 | 1.000 | 1.000 | 0.809 | 1.000 | 1.000 | 0.868 | 0.840 |
| 3293 | 0.829 | 1.000 | 0.860 | 1.000 | 0.979 | 1.000 | 0.884 | 1.000 | 1.000 | 1.000 | 0.977 | 1.000 | 1.000 | 0.809 | 1.000 | 1.000 | 0.868 | 0.841 |
| 3294 | 0.829 | 1.000 | 0.862 | 1.000 | 0.979 | 1.000 | 0.884 | 1.000 | 1.000 | 1.000 | 0.977 | 1.000 | 1.000 | 0.809 | 1.000 | 1.000 | 0.868 | 0.841 |
| 3295 | 0.829 | 1.000 | 0.860 | 1.000 | 0.979 | 1.000 | 0.884 | 1.000 | 1.000 | 1.000 | 0.977 | 1.000 | 1.000 | 0.808 | 1.000 | 1.000 | 0.868 | 0.841 |
| 3296 | 0.829 | 1.000 | 0.860 | 1.000 | 0.979 | 1.000 | 0.884 | 1.000 | 1.000 | 1.000 | 0.977 | 1.000 | 1.000 | 0.808 | 1.000 | 1.000 | 0.868 | 0.840 |
| 3297 | 0.829 | 1.000 | 0.860 | 1.000 | 0.979 | 1.000 | 0.884 | 1.000 | 1.000 | 1.000 | 0.977 | 1.000 | 1.000 | 0.809 | 1.000 | 1.000 | 0.868 | 0.841 |
| 3298 | 0.829 | 1.000 | 0.860 | 1.000 | 0.979 | 1.000 | 0.884 | 1.000 | 1.000 | 1.000 | 0.977 | 1.000 | 1.000 | 0.809 | 1.000 | 1.000 | 0.868 | 0.841 |
| 3299 | 0.831 | 1.000 | 0.860 | 1.000 | 0.979 | 1.000 | 0.884 | 1.000 | 1.000 | 1.000 | 0.977 | 1.000 | 1.000 | 0.808 | 1.000 | 1.000 | 0.868 | 0.841 |
| 3300 | 0.828 | 1.000 | 0.864 | 1.000 | 0.979 | 1.000 | 0.883 | 1.000 | 1.000 | 1.000 | 0.977 | 1.000 | 1.000 | 0.809 | 1.000 | 1.000 | 0.868 | 0.841 |
| 3301 | 0.827 | 1.000 | 0.866 | 1.000 | 0.979 | 1.000 | 0.883 | 1.000 | 1.000 | 1.000 | 0.977 | 1.000 | 1.000 | 0.810 | 1.000 | 1.000 | 0.868 | 0.841 |
| 3302 | 0.827 | 1.000 | 0.866 | 1.000 | 0.979 | 1.000 | 0.883 | 1.000 | 1.000 | 1.000 | 0.977 | 1.000 | 1.000 | 0.810 | 1.000 | 1.000 | 0.868 | 0.841 |
| 3303 | 0.827 | 1.000 | 0.866 | 1.000 | 0.979 | 1.000 | 0.883 | 1.000 | 1.000 | 1.000 | 0.977 | 1.000 | 1.000 | 0.810 | 1.000 | 1.000 | 0.868 | 0.841 |
| 3304 | 0.827 | 1.000 | 0.864 | 1.000 | 0.979 | 1.000 | 0.883 | 1.000 | 1.000 | 1.000 | 0.977 | 1.000 | 1.000 | 0.810 | 1.000 | 1.000 | 0.868 | 0.841 |
| 3305 | 0.827 | 1.000 | 0.864 | 1.000 | 0.979 | 1.000 | 0.883 | 1.000 | 1.000 | 1.000 | 0.977 | 1.000 | 1.000 | 0.809 | 1.000 | 1.000 | 0.868 | 0.840 |
| 3306 | 0.826 | 1.000 | 0.864 | 1.000 | 0.979 | 1.000 | 0.883 | 1.000 | 1.000 | 1.000 | 0.977 | 1.000 | 1.000 | 0.809 | 1.000 | 1.000 | 0.867 | 0.840 |
| 3307 | 0.828 | 1.000 | 0.864 | 1.000 | 0.979 | 1.000 | 0.883 | 1.000 | 1.000 | 1.000 | 0.977 | 1.000 | 1.000 | 0.811 | 1.000 | 1.000 | 0.868 | 0.841 |
| 3308 | 0.828 | 1.000 | 0.864 | 1.000 | 0.979 | 1.000 | 0.883 | 1.000 | 1.000 | 1.000 | 0.977 | 1.000 | 1.000 | 0.811 | 1.000 | 1.000 | 0.868 | 0.841 |
| 3309 | 0.829 | 1.000 | 0.864 | 1.000 | 0.979 | 1.000 | 0.886 | 1.000 | 1.000 | 1.000 | 0.974 | 1.000 | 1.000 | 0.808 | 1.000 | 1.000 | 0.868 | 0.841 |
| 3310 | 0.827 | 1.000 | 0.864 | 1.000 | 0.979 | 1.000 | 0.889 | 1.000 | 1.000 | 1.000 | 0.977 | 1.000 | 1.000 | 0.807 | 1.000 | 1.000 | 0.868 | 0.841 |
| 3311 | 0.828 | 1.000 | 0.864 | 1.000 | 0.979 | 1.000 | 0.889 | 1.000 | 1.000 | 1.000 | 0.974 | 1.000 | 1.000 | 0.808 | 1.000 | 1.000 | 0.868 | 0.841 |
| 3312 | 0.828 | 1.000 | 0.864 | 1.000 | 0.979 | 1.000 | 0.889 | 1.000 | 1.000 | 1.000 | 0.974 | 1.000 | 1.000 | 0.808 | 1.000 | 1.000 | 0.868 | 0.841 |
| 3313 | 0.827 | 1.000 | 0.864 | 1.000 | 0.979 | 1.000 | 0.889 | 1.000 | 1.000 | 1.000 | 0.977 | 1.000 | 1.000 | 0.809 | 1.000 | 1.000 | 0.868 | 0.841 |
| 3314 | 0.828 | 1.000 | 0.864 | 1.000 | 0.979 | 1.000 | 0.888 | 1.000 | 1.000 | 1.000 | 0.977 | 1.000 | 1.000 | 0.809 | 1.000 | 1.000 | 0.868 | 0.841 |
| 3315 | 0.828 | 1.000 | 0.864 | 1.000 | 0.979 | 1.000 | 0.888 | 1.000 | 1.000 | 1.000 | 0.977 | 1.000 | 1.000 | 0.809 | 1.000 | 1.000 | 0.869 | 0.842 |
| 3316 | 0.827 | 1.000 | 0.864 | 1.000 | 0.979 | 1.000 | 0.888 | 1.000 | 1.000 | 1.000 | 0.977 | 1.000 | 1.000 | 0.809 | 1.000 | 1.000 | 0.868 | 0.841 |
| 3317 | 0.827 | 1.000 | 0.864 | 1.000 | 0.979 | 1.000 | 0.888 | 1.000 | 1.000 | 1.000 | 0.977 | 1.000 | 1.000 | 0.809 | 1.000 | 1.000 | 0.868 | 0.841 |
| 3318 | 0.827 | 1.000 | 0.864 | 1.000 | 0.979 | 1.000 | 0.888 | 1.000 | 1.000 | 1.000 | 0.977 | 1.000 | 1.000 | 0.809 | 1.000 | 1.000 | 0.868 | 0.841 |

| | | | | | | | | | | | | | | | | | | |
|---|---|---|---|---|---|---|---|---|---|---|---|---|---|---|---|---|---|---|
| 3319 | 0.827 | 1.000 | 0.864 | 1.000 | 0.979 | 1.000 | 0.889 | 1.000 | 1.000 | 1.000 | 0.977 | 1.000 | 1.000 | 0.809 | 1.000 | 1.000 | 0.869 | 0.842 |
| 3320 | 0.828 | 1.000 | 0.864 | 1.000 | 0.979 | 1.000 | 0.886 | 1.000 | 1.000 | 1.000 | 0.977 | 1.000 | 1.000 | 0.809 | 1.000 | 1.000 | 0.868 | 0.841 |
| 3321 | 0.828 | 1.000 | 0.864 | 1.000 | 0.979 | 1.000 | 0.888 | 1.000 | 1.000 | 1.000 | 0.977 | 1.000 | 1.000 | 0.809 | 1.000 | 1.000 | 0.869 | 0.842 |
| 3322 | 0.830 | 1.000 | 0.862 | 1.000 | 0.979 | 1.000 | 0.888 | 1.000 | 1.000 | 1.000 | 0.974 | 1.000 | 1.000 | 0.810 | 1.000 | 1.000 | 0.869 | 0.842 |
| 3323 | 0.830 | 1.000 | 0.862 | 1.000 | 0.979 | 1.000 | 0.888 | 1.000 | 1.000 | 1.000 | 0.974 | 1.000 | 1.000 | 0.810 | 1.000 | 1.000 | 0.869 | 0.842 |
| 3324 | 0.829 | 1.000 | 0.864 | 1.000 | 0.979 | 1.000 | 0.889 | 1.000 | 1.000 | 1.000 | 0.974 | 1.000 | 1.000 | 0.809 | 1.000 | 1.000 | 0.869 | 0.842 |
| 3325 | 0.829 | 1.000 | 0.864 | 1.000 | 0.979 | 1.000 | 0.889 | 1.000 | 1.000 | 1.000 | 0.974 | 1.000 | 1.000 | 0.809 | 1.000 | 1.000 | 0.869 | 0.842 |
| 3326 | 0.829 | 1.000 | 0.864 | 1.000 | 0.979 | 1.000 | 0.889 | 1.000 | 1.000 | 1.000 | 0.974 | 1.000 | 1.000 | 0.809 | 1.000 | 1.000 | 0.869 | 0.842 |
| 3327 | 0.829 | 1.000 | 0.864 | 1.000 | 0.979 | 1.000 | 0.888 | 1.000 | 1.000 | 1.000 | 0.974 | 1.000 | 1.000 | 0.809 | 1.000 | 1.000 | 0.869 | 0.842 |
| 3328 | 0.828 | 1.000 | 0.864 | 1.000 | 0.979 | 1.000 | 0.886 | 1.000 | 1.000 | 1.000 | 0.974 | 1.000 | 1.000 | 0.809 | 1.000 | 1.000 | 0.868 | 0.841 |
| 3329 | 0.831 | 1.000 | 0.864 | 1.000 | 0.979 | 1.000 | 0.881 | 1.000 | 1.000 | 1.000 | 0.974 | 1.000 | 1.000 | 0.809 | 1.000 | 1.000 | 0.868 | 0.841 |
| 3330 | 0.831 | 1.000 | 0.864 | 1.000 | 0.979 | 1.000 | 0.881 | 1.000 | 1.000 | 1.000 | 0.974 | 1.000 | 1.000 | 0.809 | 1.000 | 1.000 | 0.868 | 0.841 |
| 3331 | 0.832 | 1.000 | 0.864 | 1.000 | 0.979 | 1.000 | 0.883 | 1.000 | 1.000 | 1.000 | 0.974 | 1.000 | 1.000 | 0.809 | 1.000 | 1.000 | 0.869 | 0.842 |
| 3332 | 0.832 | 1.000 | 0.864 | 1.000 | 0.979 | 1.000 | 0.884 | 1.000 | 1.000 | 1.000 | 0.974 | 1.000 | 1.000 | 0.810 | 1.000 | 1.000 | 0.869 | 0.842 |
| 3333 | 0.832 | 1.000 | 0.864 | 1.000 | 0.979 | 1.000 | 0.884 | 1.000 | 1.000 | 1.000 | 0.974 | 1.000 | 1.000 | 0.810 | 1.000 | 1.000 | 0.869 | 0.842 |
| 3334 | 0.832 | 1.000 | 0.864 | 1.000 | 0.979 | 1.000 | 0.886 | 1.000 | 1.000 | 1.000 | 0.974 | 1.000 | 1.000 | 0.810 | 1.000 | 1.000 | 0.870 | 0.843 |
| 3335 | 0.832 | 1.000 | 0.864 | 1.000 | 0.979 | 1.000 | 0.884 | 1.000 | 1.000 | 1.000 | 0.974 | 1.000 | 1.000 | 0.809 | 1.000 | 1.000 | 0.869 | 0.842 |
| 3336 | 0.832 | 1.000 | 0.864 | 1.000 | 0.979 | 1.000 | 0.881 | 1.000 | 1.000 | 1.000 | 0.974 | 1.000 | 1.000 | 0.810 | 1.000 | 1.000 | 0.869 | 0.842 |
| 3337 | 0.833 | 1.000 | 0.864 | 1.000 | 0.979 | 1.000 | 0.883 | 1.000 | 1.000 | 1.000 | 0.977 | 1.000 | 1.000 | 0.812 | 1.000 | 1.000 | 0.870 | 0.843 |
| 3338 | 0.833 | 1.000 | 0.864 | 1.000 | 0.979 | 1.000 | 0.883 | 1.000 | 1.000 | 1.000 | 0.977 | 1.000 | 1.000 | 0.812 | 1.000 | 1.000 | 0.870 | 0.843 |
| 3339 | 0.832 | 1.000 | 0.864 | 1.000 | 0.979 | 1.000 | 0.883 | 1.000 | 1.000 | 1.000 | 0.977 | 1.000 | 1.000 | 0.812 | 1.000 | 1.000 | 0.870 | 0.843 |
| 3340 | 0.832 | 1.000 | 0.864 | 1.000 | 0.979 | 1.000 | 0.884 | 1.000 | 1.000 | 1.000 | 0.977 | 1.000 | 1.000 | 0.810 | 1.000 | 1.000 | 0.870 | 0.843 |
| 3341 | 0.832 | 1.000 | 0.864 | 1.000 | 0.979 | 1.000 | 0.884 | 1.000 | 1.000 | 1.000 | 0.977 | 1.000 | 1.000 | 0.810 | 1.000 | 1.000 | 0.870 | 0.843 |
| 3342 | 0.832 | 1.000 | 0.864 | 1.000 | 0.979 | 1.000 | 0.884 | 1.000 | 1.000 | 1.000 | 0.977 | 1.000 | 1.000 | 0.810 | 1.000 | 1.000 | 0.870 | 0.843 |
| 3343 | 0.832 | 1.000 | 0.864 | 1.000 | 0.979 | 1.000 | 0.884 | 1.000 | 1.000 | 1.000 | 0.977 | 1.000 | 1.000 | 0.810 | 1.000 | 1.000 | 0.870 | 0.843 |
| 3344 | 0.833 | 1.000 | 0.868 | 1.000 | 0.979 | 1.000 | 0.884 | 1.000 | 1.000 | 1.000 | 0.977 | 1.000 | 1.000 | 0.809 | 1.000 | 1.000 | 0.870 | 0.843 |
| 3345 | 0.834 | 1.000 | 0.868 | 1.000 | 0.979 | 1.000 | 0.883 | 1.000 | 1.000 | 1.000 | 0.977 | 1.000 | 1.000 | 0.809 | 1.000 | 1.000 | 0.870 | 0.843 |
| 3346 | 0.833 | 1.000 | 0.868 | 1.000 | 0.979 | 1.000 | 0.883 | 1.000 | 1.000 | 1.000 | 0.977 | 1.000 | 1.000 | 0.810 | 1.000 | 1.000 | 0.870 | 0.843 |
| 3347 | 0.833 | 1.000 | 0.868 | 1.000 | 0.979 | 1.000 | 0.884 | 1.000 | 1.000 | 1.000 | 0.977 | 1.000 | 1.000 | 0.811 | 1.000 | 1.000 | 0.871 | 0.844 |
| 3348 | 0.834 | 1.000 | 0.868 | 1.000 | 0.979 | 1.000 | 0.884 | 1.000 | 1.000 | 1.000 | 0.977 | 1.000 | 1.000 | 0.810 | 1.000 | 1.000 | 0.871 | 0.844 |
| 3349 | 0.834 | 1.000 | 0.868 | 1.000 | 0.979 | 1.000 | 0.884 | 1.000 | 1.000 | 1.000 | 0.977 | 1.000 | 1.000 | 0.811 | 1.000 | 1.000 | 0.871 | 0.844 |
| 3350 | 0.834 | 1.000 | 0.868 | 1.000 | 0.979 | 1.000 | 0.884 | 1.000 | 1.000 | 1.000 | 0.977 | 1.000 | 1.000 | 0.811 | 1.000 | 1.000 | 0.871 | 0.844 |
| 3351 | 0.835 | 1.000 | 0.866 | 1.000 | 0.979 | 1.000 | 0.883 | 1.000 | 1.000 | 1.000 | 0.977 | 1.000 | 1.000 | 0.811 | 1.000 | 1.000 | 0.871 | 0.844 |
| 3352 | 0.835 | 1.000 | 0.872 | 1.000 | 0.979 | 1.000 | 0.889 | 1.000 | 1.000 | 1.000 | 0.977 | 1.000 | 1.000 | 0.812 | 1.000 | 1.000 | 0.872 | 0.846 |
| 3353 | 0.835 | 1.000 | 0.872 | 1.000 | 0.979 | 1.000 | 0.886 | 1.000 | 1.000 | 1.000 | 0.977 | 1.000 | 1.000 | 0.812 | 1.000 | 1.000 | 0.872 | 0.846 |
| 3354 | 0.837 | 1.000 | 0.870 | 1.000 | 0.979 | 1.000 | 0.888 | 1.000 | 1.000 | 1.000 | 0.977 | 1.000 | 1.000 | 0.812 | 1.000 | 1.000 | 0.872 | 0.846 |
| 3355 | 0.836 | 1.000 | 0.872 | 1.000 | 0.979 | 1.000 | 0.889 | 1.000 | 1.000 | 1.000 | 0.977 | 1.000 | 1.000 | 0.812 | 1.000 | 1.000 | 0.873 | 0.846 |
| 3356 | 0.833 | 1.000 | 0.870 | 1.000 | 0.979 | 1.000 | 0.888 | 1.000 | 1.000 | 1.000 | 0.977 | 1.000 | 1.000 | 0.810 | 1.000 | 1.000 | 0.871 | 0.844 |
| 3357 | 0.833 | 1.000 | 0.870 | 1.000 | 0.979 | 1.000 | 0.891 | 1.000 | 1.000 | 1.000 | 0.977 | 1.000 | 1.000 | 0.812 | 1.000 | 1.000 | 0.872 | 0.845 |

| | | | | | | | | | | | | | | | | | | |
|---|---|---|---|---|---|---|---|---|---|---|---|---|---|---|---|---|---|---|
| 3358 | 0.835 | 1.000 | 0.868 | 1.000 | 0.979 | 1.000 | 0.891 | 1.000 | 1.000 | 1.000 | 0.977 | 1.000 | 1.000 | 0.813 | 1.000 | 1.000 | 0.872 | 0.846 |
| 3359 | 0.834 | 1.000 | 0.868 | 1.000 | 0.979 | 1.000 | 0.889 | 1.000 | 1.000 | 1.000 | 0.977 | 1.000 | 1.000 | 0.814 | 1.000 | 1.000 | 0.872 | 0.846 |
| 3360 | 0.834 | 1.000 | 0.868 | 1.000 | 0.979 | 1.000 | 0.889 | 1.000 | 1.000 | 1.000 | 0.977 | 1.000 | 1.000 | 0.813 | 1.000 | 1.000 | 0.872 | 0.846 |
| 3361 | 0.834 | 1.000 | 0.868 | 1.000 | 0.979 | 1.000 | 0.889 | 1.000 | 1.000 | 1.000 | 0.977 | 1.000 | 1.000 | 0.813 | 1.000 | 1.000 | 0.872 | 0.846 |
| 3362 | 0.835 | 1.000 | 0.868 | 1.000 | 0.979 | 1.000 | 0.889 | 1.000 | 1.000 | 1.000 | 0.977 | 1.000 | 1.000 | 0.813 | 1.000 | 1.000 | 0.872 | 0.846 |
| 3363 | 0.834 | 1.000 | 0.870 | 1.000 | 0.979 | 1.000 | 0.889 | 1.000 | 1.000 | 1.000 | 0.977 | 1.000 | 1.000 | 0.813 | 1.000 | 1.000 | 0.872 | 0.846 |
| 3364 | 0.833 | 1.000 | 0.868 | 1.000 | 0.979 | 1.000 | 0.889 | 1.000 | 1.000 | 1.000 | 0.977 | 1.000 | 1.000 | 0.813 | 1.000 | 1.000 | 0.872 | 0.845 |
| 3365 | 0.833 | 1.000 | 0.868 | 1.000 | 0.979 | 1.000 | 0.889 | 1.000 | 1.000 | 1.000 | 0.977 | 1.000 | 1.000 | 0.813 | 1.000 | 1.000 | 0.872 | 0.845 |
| 3366 | 0.833 | 1.000 | 0.868 | 1.000 | 0.979 | 1.000 | 0.889 | 1.000 | 1.000 | 1.000 | 0.977 | 1.000 | 1.000 | 0.814 | 1.000 | 1.000 | 0.872 | 0.846 |
| 3367 | 0.833 | 1.000 | 0.868 | 1.000 | 0.979 | 1.000 | 0.889 | 1.000 | 1.000 | 1.000 | 0.977 | 1.000 | 1.000 | 0.813 | 1.000 | 1.000 | 0.872 | 0.845 |
| 3368 | 0.833 | 1.000 | 0.868 | 1.000 | 0.979 | 1.000 | 0.889 | 1.000 | 1.000 | 1.000 | 0.977 | 1.000 | 1.000 | 0.813 | 1.000 | 1.000 | 0.872 | 0.845 |
| 3369 | 0.835 | 1.000 | 0.868 | 1.000 | 0.979 | 1.000 | 0.888 | 1.000 | 1.000 | 1.000 | 0.977 | 1.000 | 1.000 | 0.812 | 1.000 | 1.000 | 0.872 | 0.845 |
| 3370 | 0.835 | 1.000 | 0.868 | 1.000 | 0.979 | 1.000 | 0.886 | 1.000 | 1.000 | 1.000 | 0.977 | 1.000 | 1.000 | 0.812 | 1.000 | 1.000 | 0.872 | 0.845 |
| 3371 | 0.834 | 1.000 | 0.868 | 1.000 | 0.979 | 1.000 | 0.886 | 1.000 | 1.000 | 1.000 | 0.977 | 1.000 | 1.000 | 0.812 | 1.000 | 1.000 | 0.871 | 0.845 |
| 3372 | 0.835 | 1.000 | 0.868 | 1.000 | 0.979 | 1.000 | 0.886 | 1.000 | 1.000 | 1.000 | 0.977 | 1.000 | 1.000 | 0.812 | 1.000 | 1.000 | 0.871 | 0.845 |
| 3373 | 0.834 | 1.000 | 0.868 | 1.000 | 0.979 | 1.000 | 0.888 | 1.000 | 1.000 | 1.000 | 0.977 | 1.000 | 1.000 | 0.812 | 1.000 | 1.000 | 0.871 | 0.845 |
| 3374 | 0.835 | 1.000 | 0.866 | 1.000 | 0.979 | 1.000 | 0.888 | 1.000 | 1.000 | 1.000 | 0.977 | 1.000 | 1.000 | 0.813 | 1.000 | 1.000 | 0.872 | 0.845 |
| 3375 | 0.833 | 1.000 | 0.866 | 1.000 | 0.979 | 1.000 | 0.888 | 1.000 | 1.000 | 1.000 | 0.977 | 1.000 | 1.000 | 0.813 | 1.000 | 1.000 | 0.871 | 0.845 |
| 3376 | 0.833 | 1.000 | 0.866 | 1.000 | 0.979 | 1.000 | 0.888 | 1.000 | 1.000 | 1.000 | 0.977 | 1.000 | 1.000 | 0.813 | 1.000 | 1.000 | 0.871 | 0.845 |
| 3377 | 0.833 | 1.000 | 0.866 | 1.000 | 0.979 | 1.000 | 0.889 | 1.000 | 1.000 | 1.000 | 0.977 | 1.000 | 1.000 | 0.812 | 1.000 | 1.000 | 0.871 | 0.845 |
| 3378 | 0.833 | 1.000 | 0.866 | 1.000 | 0.979 | 1.000 | 0.889 | 1.000 | 1.000 | 1.000 | 0.977 | 1.000 | 1.000 | 0.812 | 1.000 | 1.000 | 0.871 | 0.845 |
| 3379 | 0.833 | 1.000 | 0.866 | 1.000 | 0.979 | 1.000 | 0.888 | 1.000 | 1.000 | 1.000 | 0.977 | 1.000 | 1.000 | 0.811 | 1.000 | 1.000 | 0.871 | 0.844 |
| 3380 | 0.834 | 1.000 | 0.868 | 1.000 | 0.979 | 1.000 | 0.888 | 1.000 | 1.000 | 1.000 | 0.977 | 1.000 | 1.000 | 0.812 | 1.000 | 1.000 | 0.871 | 0.845 |
| 3381 | 0.834 | 1.000 | 0.868 | 1.000 | 0.979 | 1.000 | 0.888 | 1.000 | 1.000 | 1.000 | 0.977 | 1.000 | 1.000 | 0.812 | 1.000 | 1.000 | 0.871 | 0.845 |
| 3382 | 0.833 | 1.000 | 0.868 | 1.000 | 0.979 | 1.000 | 0.888 | 1.000 | 1.000 | 1.000 | 0.977 | 1.000 | 1.000 | 0.812 | 1.000 | 1.000 | 0.871 | 0.845 |
| 3383 | 0.833 | 1.000 | 0.868 | 1.000 | 0.979 | 1.000 | 0.889 | 1.000 | 1.000 | 1.000 | 0.977 | 1.000 | 1.000 | 0.812 | 1.000 | 1.000 | 0.871 | 0.845 |
| 3384 | 0.833 | 1.000 | 0.868 | 1.000 | 0.979 | 1.000 | 0.889 | 1.000 | 1.000 | 1.000 | 0.977 | 1.000 | 1.000 | 0.812 | 1.000 | 1.000 | 0.871 | 0.845 |
| 3385 | 0.834 | 1.000 | 0.868 | 1.000 | 0.979 | 1.000 | 0.888 | 1.000 | 1.000 | 1.000 | 0.977 | 1.000 | 1.000 | 0.811 | 1.000 | 1.000 | 0.871 | 0.845 |
| 3386 | 0.834 | 1.000 | 0.868 | 1.000 | 0.979 | 1.000 | 0.886 | 1.000 | 1.000 | 1.000 | 0.977 | 1.000 | 1.000 | 0.811 | 1.000 | 1.000 | 0.871 | 0.844 |
| 3387 | 0.834 | 1.000 | 0.868 | 1.000 | 0.979 | 1.000 | 0.889 | 1.000 | 1.000 | 1.000 | 0.977 | 1.000 | 1.000 | 0.811 | 1.000 | 1.000 | 0.871 | 0.845 |
| 3388 | 0.834 | 1.000 | 0.868 | 1.000 | 0.979 | 1.000 | 0.889 | 1.000 | 1.000 | 1.000 | 0.977 | 1.000 | 1.000 | 0.812 | 1.000 | 1.000 | 0.872 | 0.845 |
| 3389 | 0.833 | 1.000 | 0.868 | 1.000 | 0.979 | 1.000 | 0.888 | 1.000 | 1.000 | 1.000 | 0.977 | 1.000 | 1.000 | 0.810 | 1.000 | 1.000 | 0.871 | 0.844 |
| 3390 | 0.832 | 1.000 | 0.868 | 1.000 | 0.979 | 1.000 | 0.888 | 1.000 | 1.000 | 1.000 | 0.977 | 1.000 | 1.000 | 0.812 | 1.000 | 1.000 | 0.871 | 0.845 |
| 3391 | 0.832 | 1.000 | 0.868 | 1.000 | 0.979 | 1.000 | 0.888 | 1.000 | 1.000 | 1.000 | 0.977 | 1.000 | 1.000 | 0.810 | 1.000 | 1.000 | 0.870 | 0.844 |
| 3392 | 0.833 | 1.000 | 0.868 | 1.000 | 0.979 | 1.000 | 0.888 | 1.000 | 1.000 | 1.000 | 0.977 | 1.000 | 1.000 | 0.812 | 1.000 | 1.000 | 0.871 | 0.845 |
| 3393 | 0.833 | 1.000 | 0.868 | 1.000 | 0.979 | 1.000 | 0.888 | 1.000 | 1.000 | 1.000 | 0.977 | 1.000 | 1.000 | 0.812 | 1.000 | 1.000 | 0.871 | 0.845 |
| 3394 | 0.833 | 1.000 | 0.868 | 1.000 | 0.979 | 1.000 | 0.888 | 1.000 | 1.000 | 1.000 | 0.977 | 1.000 | 1.000 | 0.811 | 1.000 | 1.000 | 0.871 | 0.844 |
| 3395 | 0.833 | 1.000 | 0.868 | 1.000 | 0.979 | 1.000 | 0.888 | 1.000 | 1.000 | 1.000 | 0.977 | 1.000 | 1.000 | 0.811 | 1.000 | 1.000 | 0.871 | 0.844 |
| 3396 | 0.833 | 1.000 | 0.868 | 1.000 | 0.979 | 1.000 | 0.888 | 1.000 | 1.000 | 1.000 | 0.977 | 1.000 | 1.000 | 0.812 | 1.000 | 1.000 | 0.871 | 0.845 |

| | | | | | | | | | | | | | | | | | | |
|---|---|---|---|---|---|---|---|---|---|---|---|---|---|---|---|---|---|---|
| 3397 | 0.833 | 1.000 | 0.868 | 1.000 | 0.979 | 1.000 | 0.888 | 1.000 | 1.000 | 1.000 | 0.977 | 1.000 | 1.000 | 0.811 | 1.000 | 1.000 | 0.871 | 0.844 |
| 3398 | 0.836 | 1.000 | 0.864 | 1.000 | 0.979 | 1.000 | 0.891 | 1.000 | 1.000 | 1.000 | 0.980 | 1.000 | 1.000 | 0.813 | 1.000 | 1.000 | 0.873 | 0.846 |
| 3399 | 0.832 | 1.000 | 0.864 | 1.000 | 0.979 | 1.000 | 0.891 | 1.000 | 1.000 | 1.000 | 0.980 | 1.000 | 1.000 | 0.812 | 1.000 | 1.000 | 0.871 | 0.845 |
| 3400 | 0.833 | 1.000 | 0.862 | 1.000 | 0.979 | 1.000 | 0.891 | 1.000 | 1.000 | 1.000 | 0.980 | 1.000 | 1.000 | 0.812 | 1.000 | 1.000 | 0.871 | 0.845 |
| 3401 | 0.832 | 1.000 | 0.862 | 1.000 | 0.979 | 1.000 | 0.891 | 1.000 | 1.000 | 1.000 | 0.980 | 1.000 | 1.000 | 0.812 | 1.000 | 1.000 | 0.871 | 0.844 |
| 3402 | 0.833 | 1.000 | 0.862 | 1.000 | 0.979 | 1.000 | 0.889 | 1.000 | 1.000 | 1.000 | 0.980 | 1.000 | 1.000 | 0.812 | 1.000 | 1.000 | 0.871 | 0.844 |
| 3403 | 0.833 | 1.000 | 0.862 | 1.000 | 0.979 | 1.000 | 0.891 | 1.000 | 1.000 | 1.000 | 0.980 | 1.000 | 1.000 | 0.812 | 1.000 | 1.000 | 0.871 | 0.845 |
| 3404 | 0.833 | 1.000 | 0.862 | 1.000 | 0.979 | 1.000 | 0.891 | 1.000 | 1.000 | 1.000 | 0.980 | 1.000 | 1.000 | 0.812 | 1.000 | 1.000 | 0.871 | 0.844 |
| 3405 | 0.833 | 1.000 | 0.864 | 1.000 | 0.979 | 1.000 | 0.889 | 1.000 | 1.000 | 1.000 | 0.980 | 1.000 | 1.000 | 0.812 | 1.000 | 1.000 | 0.871 | 0.845 |
| 3406 | 0.833 | 1.000 | 0.864 | 1.000 | 0.979 | 1.000 | 0.891 | 1.000 | 1.000 | 1.000 | 0.980 | 1.000 | 1.000 | 0.812 | 1.000 | 1.000 | 0.871 | 0.845 |
| 3407 | 0.833 | 1.000 | 0.866 | 1.000 | 0.979 | 1.000 | 0.892 | 1.000 | 1.000 | 1.000 | 0.977 | 1.000 | 1.000 | 0.809 | 1.000 | 1.000 | 0.871 | 0.844 |
| 3408 | 0.833 | 1.000 | 0.866 | 1.000 | 0.979 | 1.000 | 0.889 | 1.000 | 1.000 | 1.000 | 0.977 | 1.000 | 1.000 | 0.809 | 1.000 | 1.000 | 0.870 | 0.844 |
| 3409 | 0.832 | 1.000 | 0.864 | 1.000 | 0.979 | 1.000 | 0.889 | 1.000 | 1.000 | 1.000 | 0.977 | 1.000 | 1.000 | 0.809 | 1.000 | 1.000 | 0.870 | 0.843 |
| 3410 | 0.833 | 1.000 | 0.864 | 1.000 | 0.979 | 1.000 | 0.889 | 1.000 | 1.000 | 1.000 | 0.977 | 1.000 | 1.000 | 0.808 | 1.000 | 1.000 | 0.870 | 0.843 |
| 3411 | 0.833 | 1.000 | 0.864 | 1.000 | 0.979 | 1.000 | 0.889 | 1.000 | 1.000 | 1.000 | 0.977 | 1.000 | 1.000 | 0.809 | 1.000 | 1.000 | 0.870 | 0.844 |
| 3412 | 0.833 | 1.000 | 0.864 | 1.000 | 0.979 | 1.000 | 0.889 | 1.000 | 1.000 | 1.000 | 0.977 | 1.000 | 1.000 | 0.809 | 1.000 | 1.000 | 0.870 | 0.844 |
| 3413 | 0.833 | 1.000 | 0.864 | 1.000 | 0.979 | 1.000 | 0.889 | 1.000 | 1.000 | 1.000 | 0.977 | 1.000 | 1.000 | 0.810 | 1.000 | 1.000 | 0.870 | 0.844 |
| 3414 | 0.833 | 1.000 | 0.864 | 1.000 | 0.979 | 1.000 | 0.889 | 1.000 | 1.000 | 1.000 | 0.977 | 1.000 | 1.000 | 0.808 | 1.000 | 1.000 | 0.870 | 0.843 |
| 3415 | 0.833 | 1.000 | 0.864 | 1.000 | 0.979 | 1.000 | 0.889 | 1.000 | 1.000 | 1.000 | 0.977 | 1.000 | 1.000 | 0.809 | 1.000 | 1.000 | 0.870 | 0.843 |
| 3416 | 0.829 | 1.000 | 0.866 | 1.000 | 0.979 | 1.000 | 0.886 | 1.000 | 1.000 | 1.000 | 0.977 | 1.000 | 1.000 | 0.809 | 1.000 | 1.000 | 0.869 | 0.842 |
| 3417 | 0.829 | 1.000 | 0.866 | 1.000 | 0.979 | 1.000 | 0.889 | 1.000 | 1.000 | 1.000 | 0.977 | 1.000 | 1.000 | 0.809 | 1.000 | 1.000 | 0.869 | 0.843 |
| 3418 | 0.829 | 1.000 | 0.868 | 1.000 | 0.979 | 1.000 | 0.888 | 1.000 | 1.000 | 1.000 | 0.977 | 1.000 | 1.000 | 0.809 | 1.000 | 1.000 | 0.869 | 0.842 |
| 3419 | 0.829 | 1.000 | 0.868 | 1.000 | 0.979 | 1.000 | 0.889 | 1.000 | 1.000 | 1.000 | 0.977 | 1.000 | 1.000 | 0.809 | 1.000 | 1.000 | 0.870 | 0.843 |
| 3420 | 0.830 | 1.000 | 0.870 | 1.000 | 0.979 | 1.000 | 0.886 | 1.000 | 1.000 | 1.000 | 0.977 | 1.000 | 1.000 | 0.809 | 1.000 | 1.000 | 0.870 | 0.843 |
| 3421 | 0.830 | 1.000 | 0.870 | 1.000 | 0.979 | 1.000 | 0.888 | 1.000 | 1.000 | 1.000 | 0.977 | 1.000 | 1.000 | 0.809 | 1.000 | 1.000 | 0.870 | 0.843 |
| 3422 | 0.832 | 1.000 | 0.870 | 1.000 | 0.979 | 1.000 | 0.886 | 1.000 | 1.000 | 1.000 | 0.977 | 1.000 | 1.000 | 0.811 | 1.000 | 1.000 | 0.871 | 0.844 |
| 3423 | 0.831 | 1.000 | 0.870 | 1.000 | 0.979 | 1.000 | 0.886 | 1.000 | 1.000 | 1.000 | 0.977 | 1.000 | 1.000 | 0.810 | 1.000 | 1.000 | 0.870 | 0.844 |
| 3424 | 0.831 | 1.000 | 0.870 | 1.000 | 0.979 | 1.000 | 0.886 | 1.000 | 1.000 | 1.000 | 0.977 | 1.000 | 1.000 | 0.809 | 1.000 | 1.000 | 0.870 | 0.843 |
| 3425 | 0.832 | 1.000 | 0.870 | 1.000 | 0.979 | 1.000 | 0.886 | 1.000 | 1.000 | 1.000 | 0.977 | 1.000 | 1.000 | 0.809 | 1.000 | 1.000 | 0.870 | 0.844 |
| 3426 | 0.831 | 1.000 | 0.872 | 1.000 | 0.979 | 1.000 | 0.888 | 1.000 | 1.000 | 1.000 | 0.977 | 1.000 | 1.000 | 0.809 | 1.000 | 1.000 | 0.870 | 0.844 |
| 3427 | 0.831 | 1.000 | 0.872 | 1.000 | 0.979 | 1.000 | 0.888 | 1.000 | 1.000 | 1.000 | 0.977 | 1.000 | 1.000 | 0.809 | 1.000 | 1.000 | 0.870 | 0.843 |
| 3428 | 0.832 | 1.000 | 0.872 | 1.000 | 0.979 | 1.000 | 0.888 | 1.000 | 1.000 | 1.000 | 0.980 | 1.000 | 1.000 | 0.809 | 1.000 | 1.000 | 0.871 | 0.844 |
| 3429 | 0.832 | 1.000 | 0.872 | 1.000 | 0.979 | 1.000 | 0.888 | 1.000 | 1.000 | 1.000 | 0.980 | 1.000 | 1.000 | 0.809 | 1.000 | 1.000 | 0.871 | 0.844 |
| 3430 | 0.831 | 1.000 | 0.872 | 1.000 | 0.979 | 1.000 | 0.888 | 1.000 | 1.000 | 1.000 | 0.980 | 1.000 | 1.000 | 0.809 | 1.000 | 1.000 | 0.870 | 0.844 |
| 3431 | 0.831 | 1.000 | 0.872 | 1.000 | 0.979 | 1.000 | 0.888 | 1.000 | 1.000 | 1.000 | 0.980 | 1.000 | 1.000 | 0.809 | 1.000 | 1.000 | 0.870 | 0.844 |
| 3432 | 0.831 | 1.000 | 0.872 | 1.000 | 0.979 | 1.000 | 0.888 | 1.000 | 1.000 | 1.000 | 0.980 | 1.000 | 1.000 | 0.808 | 1.000 | 1.000 | 0.870 | 0.844 |
| 3433 | 0.831 | 1.000 | 0.872 | 1.000 | 0.979 | 1.000 | 0.886 | 1.000 | 1.000 | 1.000 | 0.980 | 1.000 | 1.000 | 0.808 | 1.000 | 1.000 | 0.870 | 0.843 |
| 3434 | 0.831 | 1.000 | 0.872 | 1.000 | 0.979 | 1.000 | 0.886 | 1.000 | 1.000 | 1.000 | 0.980 | 1.000 | 1.000 | 0.808 | 1.000 | 1.000 | 0.870 | 0.843 |
| 3435 | 0.832 | 1.000 | 0.872 | 1.000 | 0.979 | 1.000 | 0.888 | 1.000 | 1.000 | 1.000 | 0.980 | 1.000 | 1.000 | 0.808 | 1.000 | 1.000 | 0.870 | 0.844 |

| | | | | | | | | | | | | | | | | | | |
|---|---|---|---|---|---|---|---|---|---|---|---|---|---|---|---|---|---|---|
| 3436 | 0.832 | 1.000 | 0.872 | 1.000 | 0.979 | 1.000 | 0.888 | 1.000 | 1.000 | 1.000 | 0.980 | 1.000 | 1.000 | 0.808 | 1.000 | 1.000 | 0.870 | 0.844 |
| 3437 | 0.832 | 1.000 | 0.872 | 1.000 | 0.979 | 1.000 | 0.888 | 1.000 | 1.000 | 1.000 | 0.980 | 1.000 | 1.000 | 0.808 | 1.000 | 1.000 | 0.870 | 0.844 |
| 3438 | 0.831 | 1.000 | 0.872 | 1.000 | 0.979 | 1.000 | 0.888 | 1.000 | 1.000 | 1.000 | 0.980 | 1.000 | 1.000 | 0.809 | 1.000 | 1.000 | 0.870 | 0.844 |
| 3439 | 0.831 | 1.000 | 0.872 | 1.000 | 0.979 | 1.000 | 0.888 | 1.000 | 1.000 | 1.000 | 0.980 | 1.000 | 1.000 | 0.809 | 1.000 | 1.000 | 0.870 | 0.844 |
| 3440 | 0.832 | 1.000 | 0.872 | 1.000 | 0.979 | 1.000 | 0.888 | 1.000 | 1.000 | 1.000 | 0.980 | 1.000 | 1.000 | 0.808 | 1.000 | 1.000 | 0.870 | 0.844 |
| 3441 | 0.832 | 1.000 | 0.872 | 1.000 | 0.979 | 1.000 | 0.888 | 1.000 | 1.000 | 1.000 | 0.980 | 1.000 | 1.000 | 0.808 | 1.000 | 1.000 | 0.870 | 0.844 |
| 3442 | 0.833 | 1.000 | 0.872 | 1.000 | 0.979 | 1.000 | 0.888 | 1.000 | 1.000 | 1.000 | 0.980 | 1.000 | 1.000 | 0.808 | 1.000 | 1.000 | 0.871 | 0.844 |
| 3443 | 0.833 | 1.000 | 0.872 | 1.000 | 0.979 | 1.000 | 0.888 | 1.000 | 1.000 | 1.000 | 0.980 | 1.000 | 1.000 | 0.808 | 1.000 | 1.000 | 0.871 | 0.844 |
| 3444 | 0.833 | 1.000 | 0.874 | 1.000 | 0.979 | 1.000 | 0.888 | 1.000 | 1.000 | 1.000 | 0.980 | 1.000 | 1.000 | 0.808 | 1.000 | 1.000 | 0.871 | 0.845 |
| 3445 | 0.833 | 1.000 | 0.874 | 1.000 | 0.979 | 1.000 | 0.888 | 1.000 | 1.000 | 1.000 | 0.980 | 1.000 | 1.000 | 0.808 | 1.000 | 1.000 | 0.871 | 0.845 |
| 3446 | 0.833 | 1.000 | 0.874 | 1.000 | 0.979 | 1.000 | 0.889 | 1.000 | 1.000 | 1.000 | 0.980 | 1.000 | 1.000 | 0.807 | 1.000 | 1.000 | 0.871 | 0.844 |
| 3447 | 0.833 | 1.000 | 0.872 | 1.000 | 0.979 | 1.000 | 0.888 | 1.000 | 1.000 | 1.000 | 0.980 | 1.000 | 1.000 | 0.808 | 1.000 | 1.000 | 0.871 | 0.844 |
| 3448 | 0.833 | 1.000 | 0.872 | 1.000 | 0.979 | 1.000 | 0.888 | 1.000 | 1.000 | 1.000 | 0.980 | 1.000 | 1.000 | 0.808 | 1.000 | 1.000 | 0.871 | 0.844 |
| 3449 | 0.833 | 1.000 | 0.872 | 1.000 | 0.979 | 1.000 | 0.888 | 1.000 | 1.000 | 1.000 | 0.980 | 1.000 | 1.000 | 0.807 | 1.000 | 1.000 | 0.870 | 0.844 |
| 3450 | 0.833 | 1.000 | 0.872 | 1.000 | 0.979 | 1.000 | 0.888 | 1.000 | 1.000 | 1.000 | 0.980 | 1.000 | 1.000 | 0.809 | 1.000 | 1.000 | 0.871 | 0.845 |
| 3451 | 0.834 | 1.000 | 0.872 | 1.000 | 0.979 | 1.000 | 0.894 | 1.000 | 1.000 | 1.000 | 0.980 | 1.000 | 1.000 | 0.809 | 1.000 | 1.000 | 0.872 | 0.846 |
| 3452 | 0.835 | 1.000 | 0.872 | 1.000 | 0.979 | 1.000 | 0.894 | 1.000 | 1.000 | 1.000 | 0.980 | 1.000 | 1.000 | 0.809 | 1.000 | 1.000 | 0.872 | 0.846 |
| 3453 | 0.835 | 1.000 | 0.872 | 1.000 | 0.979 | 1.000 | 0.894 | 1.000 | 1.000 | 1.000 | 0.980 | 1.000 | 1.000 | 0.809 | 1.000 | 1.000 | 0.873 | 0.847 |
| 3454 | 0.835 | 1.000 | 0.872 | 1.000 | 0.979 | 1.000 | 0.894 | 1.000 | 1.000 | 1.000 | 0.980 | 1.000 | 1.000 | 0.809 | 1.000 | 1.000 | 0.873 | 0.847 |
| 3455 | 0.835 | 1.000 | 0.872 | 1.000 | 0.979 | 1.000 | 0.894 | 1.000 | 1.000 | 1.000 | 0.980 | 1.000 | 1.000 | 0.809 | 1.000 | 1.000 | 0.873 | 0.847 |
| 3456 | 0.835 | 1.000 | 0.872 | 1.000 | 0.979 | 1.000 | 0.894 | 1.000 | 1.000 | 1.000 | 0.980 | 1.000 | 1.000 | 0.809 | 1.000 | 1.000 | 0.873 | 0.847 |
| 3457 | 0.835 | 1.000 | 0.872 | 1.000 | 0.979 | 1.000 | 0.894 | 1.000 | 1.000 | 1.000 | 0.980 | 1.000 | 1.000 | 0.809 | 1.000 | 1.000 | 0.873 | 0.847 |
| 3458 | 0.836 | 1.000 | 0.872 | 1.000 | 0.979 | 1.000 | 0.895 | 1.000 | 1.000 | 1.000 | 0.980 | 1.000 | 1.000 | 0.809 | 1.000 | 1.000 | 0.873 | 0.847 |
| 3459 | 0.835 | 1.000 | 0.872 | 1.000 | 0.979 | 1.000 | 0.895 | 1.000 | 1.000 | 1.000 | 0.980 | 1.000 | 1.000 | 0.809 | 1.000 | 1.000 | 0.873 | 0.847 |
| 3460 | 0.835 | 1.000 | 0.872 | 1.000 | 0.979 | 1.000 | 0.895 | 1.000 | 1.000 | 1.000 | 0.980 | 1.000 | 1.000 | 0.809 | 1.000 | 1.000 | 0.873 | 0.846 |
| 3461 | 0.835 | 1.000 | 0.872 | 1.000 | 0.979 | 1.000 | 0.895 | 1.000 | 1.000 | 1.000 | 0.980 | 1.000 | 1.000 | 0.809 | 1.000 | 1.000 | 0.872 | 0.846 |
| 3462 | 0.835 | 1.000 | 0.872 | 1.000 | 0.979 | 1.000 | 0.895 | 1.000 | 1.000 | 1.000 | 0.980 | 1.000 | 1.000 | 0.809 | 1.000 | 1.000 | 0.873 | 0.847 |
| 3463 | 0.835 | 1.000 | 0.872 | 1.000 | 0.979 | 1.000 | 0.895 | 1.000 | 1.000 | 1.000 | 0.980 | 1.000 | 1.000 | 0.809 | 1.000 | 1.000 | 0.872 | 0.846 |
| 3464 | 0.835 | 1.000 | 0.872 | 1.000 | 0.979 | 1.000 | 0.895 | 1.000 | 1.000 | 1.000 | 0.980 | 1.000 | 1.000 | 0.809 | 1.000 | 1.000 | 0.873 | 0.846 |
| 3465 | 0.835 | 1.000 | 0.872 | 1.000 | 0.979 | 1.000 | 0.895 | 1.000 | 1.000 | 1.000 | 0.980 | 1.000 | 1.000 | 0.809 | 1.000 | 1.000 | 0.873 | 0.846 |
| 3466 | 0.836 | 1.000 | 0.872 | 1.000 | 0.979 | 1.000 | 0.895 | 1.000 | 1.000 | 1.000 | 0.980 | 1.000 | 1.000 | 0.809 | 1.000 | 1.000 | 0.873 | 0.847 |
| 3467 | 0.836 | 1.000 | 0.872 | 1.000 | 0.979 | 1.000 | 0.895 | 1.000 | 1.000 | 1.000 | 0.980 | 1.000 | 1.000 | 0.809 | 1.000 | 1.000 | 0.873 | 0.847 |
| 3468 | 0.835 | 1.000 | 0.872 | 1.000 | 0.979 | 1.000 | 0.895 | 1.000 | 1.000 | 1.000 | 0.980 | 1.000 | 1.000 | 0.809 | 1.000 | 1.000 | 0.873 | 0.847 |
| 3469 | 0.835 | 1.000 | 0.872 | 1.000 | 0.979 | 1.000 | 0.895 | 1.000 | 1.000 | 1.000 | 0.980 | 1.000 | 1.000 | 0.809 | 1.000 | 1.000 | 0.873 | 0.847 |
| 3470 | 0.835 | 1.000 | 0.872 | 1.000 | 0.979 | 1.000 | 0.895 | 1.000 | 1.000 | 1.000 | 0.980 | 1.000 | 1.000 | 0.809 | 1.000 | 1.000 | 0.873 | 0.847 |
| 3471 | 0.835 | 1.000 | 0.872 | 1.000 | 0.979 | 1.000 | 0.895 | 1.000 | 1.000 | 1.000 | 0.980 | 1.000 | 1.000 | 0.809 | 1.000 | 1.000 | 0.873 | 0.847 |
| 3472 | 0.834 | 1.000 | 0.872 | 1.000 | 0.979 | 1.000 | 0.895 | 1.000 | 1.000 | 1.000 | 0.980 | 1.000 | 1.000 | 0.809 | 1.000 | 1.000 | 0.872 | 0.846 |
| 3473 | 0.834 | 1.000 | 0.872 | 1.000 | 0.979 | 1.000 | 0.895 | 1.000 | 1.000 | 1.000 | 0.980 | 1.000 | 1.000 | 0.808 | 1.000 | 1.000 | 0.872 | 0.846 |
| 3474 | 0.834 | 1.000 | 0.872 | 1.000 | 0.979 | 1.000 | 0.895 | 1.000 | 1.000 | 1.000 | 0.980 | 1.000 | 1.000 | 0.808 | 1.000 | 1.000 | 0.872 | 0.846 |

| | | | | | | | | | | | | | | | | | | |
|---|---|---|---|---|---|---|---|---|---|---|---|---|---|---|---|---|---|---|
| 3475 | 0.835 | 1.000 | 0.872 | 1.000 | 0.979 | 1.000 | 0.895 | 1.000 | 1.000 | 1.000 | 0.980 | 1.000 | 1.000 | 0.808 | 1.000 | 1.000 | 0.872 | 0.846 |
| 3476 | 0.835 | 1.000 | 0.872 | 1.000 | 0.979 | 1.000 | 0.895 | 1.000 | 1.000 | 1.000 | 0.980 | 1.000 | 1.000 | 0.808 | 1.000 | 1.000 | 0.872 | 0.846 |
| 3477 | 0.835 | 1.000 | 0.872 | 1.000 | 0.979 | 1.000 | 0.895 | 1.000 | 1.000 | 1.000 | 0.980 | 1.000 | 1.000 | 0.808 | 1.000 | 1.000 | 0.872 | 0.846 |
| 3478 | 0.835 | 1.000 | 0.870 | 1.000 | 0.979 | 1.000 | 0.895 | 1.000 | 1.000 | 1.000 | 0.980 | 1.000 | 1.000 | 0.809 | 1.000 | 1.000 | 0.872 | 0.846 |
| 3479 | 0.837 | 1.000 | 0.870 | 1.000 | 0.979 | 1.000 | 0.895 | 1.000 | 1.000 | 1.000 | 0.980 | 1.000 | 1.000 | 0.807 | 1.000 | 1.000 | 0.872 | 0.846 |
| 3480 | 0.835 | 1.000 | 0.870 | 1.000 | 0.979 | 1.000 | 0.895 | 1.000 | 1.000 | 1.000 | 0.980 | 1.000 | 1.000 | 0.807 | 1.000 | 1.000 | 0.872 | 0.846 |
| 3481 | 0.836 | 1.000 | 0.870 | 1.000 | 0.979 | 1.000 | 0.894 | 1.000 | 1.000 | 1.000 | 0.980 | 1.000 | 1.000 | 0.807 | 1.000 | 1.000 | 0.872 | 0.846 |
| 3482 | 0.835 | 1.000 | 0.870 | 1.000 | 0.979 | 1.000 | 0.895 | 1.000 | 1.000 | 1.000 | 0.980 | 1.000 | 1.000 | 0.807 | 1.000 | 1.000 | 0.872 | 0.846 |
| 3483 | 0.835 | 1.000 | 0.868 | 1.000 | 0.979 | 1.000 | 0.892 | 1.000 | 1.000 | 1.000 | 0.980 | 1.000 | 1.000 | 0.809 | 1.000 | 1.000 | 0.872 | 0.845 |
| 3484 | 0.835 | 1.000 | 0.868 | 1.000 | 0.979 | 1.000 | 0.892 | 1.000 | 1.000 | 1.000 | 0.980 | 1.000 | 1.000 | 0.808 | 1.000 | 1.000 | 0.872 | 0.845 |
| 3485 | 0.835 | 1.000 | 0.868 | 1.000 | 0.979 | 1.000 | 0.892 | 1.000 | 1.000 | 1.000 | 0.980 | 1.000 | 1.000 | 0.809 | 1.000 | 1.000 | 0.872 | 0.845 |
| 3486 | 0.835 | 1.000 | 0.868 | 1.000 | 0.979 | 1.000 | 0.892 | 1.000 | 1.000 | 1.000 | 0.980 | 1.000 | 1.000 | 0.809 | 1.000 | 1.000 | 0.872 | 0.845 |
| 3487 | 0.835 | 1.000 | 0.868 | 1.000 | 0.979 | 1.000 | 0.892 | 1.000 | 1.000 | 1.000 | 0.980 | 1.000 | 1.000 | 0.809 | 1.000 | 1.000 | 0.872 | 0.845 |
| 3488 | 0.833 | 1.000 | 0.868 | 1.000 | 0.979 | 1.000 | 0.889 | 1.000 | 1.000 | 1.000 | 0.980 | 1.000 | 1.000 | 0.809 | 1.000 | 1.000 | 0.871 | 0.844 |
| 3489 | 0.833 | 1.000 | 0.868 | 1.000 | 0.979 | 1.000 | 0.888 | 1.000 | 1.000 | 1.000 | 0.980 | 1.000 | 1.000 | 0.809 | 1.000 | 1.000 | 0.871 | 0.844 |
| 3490 | 0.833 | 1.000 | 0.868 | 1.000 | 0.979 | 1.000 | 0.888 | 1.000 | 1.000 | 1.000 | 0.980 | 1.000 | 1.000 | 0.809 | 1.000 | 1.000 | 0.871 | 0.844 |
| 3491 | 0.833 | 1.000 | 0.868 | 1.000 | 0.979 | 1.000 | 0.888 | 1.000 | 1.000 | 1.000 | 0.980 | 1.000 | 1.000 | 0.809 | 1.000 | 1.000 | 0.871 | 0.844 |
| 3492 | 0.832 | 1.000 | 0.868 | 1.000 | 0.979 | 1.000 | 0.888 | 1.000 | 1.000 | 1.000 | 0.980 | 1.000 | 1.000 | 0.809 | 1.000 | 1.000 | 0.870 | 0.844 |
| 3493 | 0.832 | 1.000 | 0.868 | 1.000 | 0.979 | 1.000 | 0.888 | 1.000 | 1.000 | 1.000 | 0.980 | 1.000 | 1.000 | 0.809 | 1.000 | 1.000 | 0.870 | 0.844 |
| 3494 | 0.833 | 1.000 | 0.868 | 1.000 | 0.979 | 1.000 | 0.889 | 1.000 | 1.000 | 1.000 | 0.980 | 1.000 | 1.000 | 0.809 | 1.000 | 1.000 | 0.871 | 0.844 |
| 3495 | 0.833 | 1.000 | 0.868 | 1.000 | 0.979 | 1.000 | 0.889 | 1.000 | 1.000 | 1.000 | 0.980 | 1.000 | 1.000 | 0.809 | 1.000 | 1.000 | 0.871 | 0.844 |
| 3496 | 0.833 | 1.000 | 0.868 | 1.000 | 0.979 | 1.000 | 0.889 | 1.000 | 1.000 | 1.000 | 0.980 | 1.000 | 1.000 | 0.809 | 1.000 | 1.000 | 0.871 | 0.844 |
| 3497 | 0.833 | 1.000 | 0.868 | 1.000 | 0.979 | 1.000 | 0.889 | 1.000 | 1.000 | 1.000 | 0.980 | 1.000 | 1.000 | 0.809 | 1.000 | 1.000 | 0.871 | 0.844 |
| 3498 | 0.833 | 1.000 | 0.868 | 1.000 | 0.979 | 1.000 | 0.889 | 1.000 | 1.000 | 1.000 | 0.980 | 1.000 | 1.000 | 0.809 | 1.000 | 1.000 | 0.871 | 0.844 |
| 3499 | 0.833 | 1.000 | 0.868 | 1.000 | 0.979 | 1.000 | 0.889 | 1.000 | 1.000 | 1.000 | 0.980 | 1.000 | 1.000 | 0.809 | 1.000 | 1.000 | 0.871 | 0.844 |
| 3500 | 0.833 | 1.000 | 0.868 | 1.000 | 0.979 | 1.000 | 0.889 | 1.000 | 1.000 | 1.000 | 0.980 | 1.000 | 1.000 | 0.809 | 1.000 | 1.000 | 0.871 | 0.844 |
| 3501 | 0.833 | 1.000 | 0.868 | 1.000 | 0.979 | 1.000 | 0.889 | 1.000 | 1.000 | 1.000 | 0.980 | 1.000 | 1.000 | 0.809 | 1.000 | 1.000 | 0.871 | 0.844 |
| 3502 | 0.833 | 1.000 | 0.868 | 1.000 | 0.979 | 1.000 | 0.889 | 1.000 | 1.000 | 1.000 | 0.980 | 1.000 | 1.000 | 0.809 | 1.000 | 1.000 | 0.871 | 0.844 |
| 3503 | 0.832 | 1.000 | 0.864 | 1.000 | 0.979 | 1.000 | 0.888 | 1.000 | 1.000 | 1.000 | 0.980 | 1.000 | 1.000 | 0.810 | 1.000 | 1.000 | 0.870 | 0.843 |
| 3504 | 0.832 | 1.000 | 0.864 | 1.000 | 0.979 | 1.000 | 0.888 | 1.000 | 1.000 | 1.000 | 0.980 | 1.000 | 1.000 | 0.809 | 1.000 | 1.000 | 0.870 | 0.843 |
| 3505 | 0.833 | 1.000 | 0.864 | 1.000 | 0.979 | 1.000 | 0.886 | 1.000 | 1.000 | 1.000 | 0.980 | 1.000 | 1.000 | 0.809 | 1.000 | 1.000 | 0.870 | 0.843 |
| 3506 | 0.833 | 1.000 | 0.864 | 1.000 | 0.979 | 1.000 | 0.886 | 1.000 | 1.000 | 1.000 | 0.980 | 1.000 | 1.000 | 0.809 | 1.000 | 1.000 | 0.870 | 0.843 |
| 3507 | 0.833 | 1.000 | 0.864 | 1.000 | 0.979 | 1.000 | 0.886 | 1.000 | 1.000 | 1.000 | 0.980 | 1.000 | 1.000 | 0.809 | 1.000 | 1.000 | 0.870 | 0.843 |
| 3508 | 0.830 | 1.000 | 0.864 | 1.000 | 0.979 | 1.000 | 0.886 | 1.000 | 1.000 | 1.000 | 0.980 | 1.000 | 1.000 | 0.809 | 1.000 | 1.000 | 0.869 | 0.842 |
| 3509 | 0.831 | 1.000 | 0.864 | 1.000 | 0.979 | 1.000 | 0.886 | 1.000 | 1.000 | 1.000 | 0.980 | 1.000 | 1.000 | 0.809 | 1.000 | 1.000 | 0.869 | 0.842 |
| 3510 | 0.830 | 1.000 | 0.864 | 1.000 | 0.979 | 1.000 | 0.886 | 1.000 | 1.000 | 1.000 | 0.980 | 1.000 | 1.000 | 0.809 | 1.000 | 1.000 | 0.869 | 0.842 |
| 3511 | 0.830 | 1.000 | 0.864 | 1.000 | 0.979 | 1.000 | 0.886 | 1.000 | 1.000 | 1.000 | 0.980 | 1.000 | 1.000 | 0.809 | 1.000 | 1.000 | 0.869 | 0.842 |
| 3512 | 0.832 | 1.000 | 0.866 | 1.000 | 0.979 | 1.000 | 0.884 | 1.000 | 1.000 | 1.000 | 0.980 | 1.000 | 1.000 | 0.809 | 1.000 | 1.000 | 0.870 | 0.843 |
| 3513 | 0.833 | 1.000 | 0.866 | 1.000 | 0.979 | 1.000 | 0.884 | 1.000 | 1.000 | 1.000 | 0.980 | 1.000 | 1.000 | 0.809 | 1.000 | 1.000 | 0.870 | 0.843 |

| | | | | | | | | | | | | | | | | | | |
|---|---|---|---|---|---|---|---|---|---|---|---|---|---|---|---|---|---|---|
| 3514 | 0.833 | 1.000 | 0.868 | 1.000 | 0.979 | 1.000 | 0.886 | 1.000 | 1.000 | 1.000 | 0.980 | 1.000 | 1.000 | 0.809 | 1.000 | 1.000 | 0.870 | 0.843 |
| 3515 | 0.833 | 1.000 | 0.866 | 1.000 | 0.979 | 1.000 | 0.886 | 1.000 | 1.000 | 1.000 | 0.980 | 1.000 | 1.000 | 0.809 | 1.000 | 1.000 | 0.870 | 0.843 |
| 3516 | 0.833 | 1.000 | 0.866 | 1.000 | 0.979 | 1.000 | 0.886 | 1.000 | 1.000 | 1.000 | 0.980 | 1.000 | 1.000 | 0.809 | 1.000 | 1.000 | 0.870 | 0.844 |
| 3517 | 0.833 | 1.000 | 0.868 | 1.000 | 0.979 | 1.000 | 0.888 | 1.000 | 1.000 | 1.000 | 0.980 | 1.000 | 1.000 | 0.809 | 1.000 | 1.000 | 0.870 | 0.844 |
| 3518 | 0.833 | 1.000 | 0.868 | 1.000 | 0.979 | 1.000 | 0.888 | 1.000 | 1.000 | 1.000 | 0.980 | 1.000 | 1.000 | 0.809 | 1.000 | 1.000 | 0.871 | 0.844 |
| 3519 | 0.834 | 1.000 | 0.866 | 1.000 | 0.979 | 1.000 | 0.888 | 1.000 | 1.000 | 1.000 | 0.980 | 1.000 | 1.000 | 0.809 | 1.000 | 1.000 | 0.871 | 0.844 |
| 3520 | 0.834 | 1.000 | 0.868 | 1.000 | 0.979 | 1.000 | 0.888 | 1.000 | 1.000 | 1.000 | 0.980 | 1.000 | 1.000 | 0.809 | 1.000 | 1.000 | 0.871 | 0.844 |
| 3521 | 0.834 | 1.000 | 0.868 | 1.000 | 0.979 | 1.000 | 0.888 | 1.000 | 1.000 | 1.000 | 0.980 | 1.000 | 1.000 | 0.809 | 1.000 | 1.000 | 0.871 | 0.844 |
| 3522 | 0.834 | 1.000 | 0.868 | 1.000 | 0.979 | 1.000 | 0.888 | 1.000 | 1.000 | 1.000 | 0.980 | 1.000 | 1.000 | 0.809 | 1.000 | 1.000 | 0.871 | 0.844 |
| 3523 | 0.834 | 1.000 | 0.868 | 1.000 | 0.979 | 1.000 | 0.886 | 1.000 | 1.000 | 1.000 | 0.980 | 1.000 | 1.000 | 0.809 | 1.000 | 1.000 | 0.871 | 0.844 |
| 3524 | 0.834 | 1.000 | 0.870 | 1.000 | 0.979 | 1.000 | 0.886 | 1.000 | 1.000 | 1.000 | 0.980 | 1.000 | 1.000 | 0.809 | 1.000 | 1.000 | 0.871 | 0.844 |
| 3525 | 0.835 | 1.000 | 0.870 | 1.000 | 0.979 | 1.000 | 0.886 | 1.000 | 1.000 | 1.000 | 0.980 | 1.000 | 1.000 | 0.809 | 1.000 | 1.000 | 0.871 | 0.844 |
| 3526 | 0.833 | 1.000 | 0.870 | 1.000 | 0.979 | 1.000 | 0.888 | 1.000 | 1.000 | 1.000 | 0.980 | 1.000 | 1.000 | 0.808 | 1.000 | 1.000 | 0.871 | 0.844 |
| 3527 | 0.834 | 1.000 | 0.870 | 1.000 | 0.979 | 1.000 | 0.889 | 1.000 | 1.000 | 1.000 | 0.980 | 1.000 | 1.000 | 0.808 | 1.000 | 1.000 | 0.871 | 0.844 |
| 3528 | 0.832 | 1.000 | 0.870 | 1.000 | 0.979 | 1.000 | 0.889 | 1.000 | 1.000 | 1.000 | 0.980 | 1.000 | 1.000 | 0.809 | 1.000 | 1.000 | 0.871 | 0.844 |
| 3529 | 0.833 | 1.000 | 0.870 | 1.000 | 0.979 | 1.000 | 0.888 | 1.000 | 1.000 | 1.000 | 0.980 | 1.000 | 1.000 | 0.809 | 1.000 | 1.000 | 0.871 | 0.844 |
| 3530 | 0.833 | 1.000 | 0.870 | 1.000 | 0.979 | 1.000 | 0.888 | 1.000 | 1.000 | 1.000 | 0.980 | 1.000 | 1.000 | 0.809 | 1.000 | 1.000 | 0.871 | 0.844 |
| 3531 | 0.833 | 1.000 | 0.868 | 1.000 | 0.979 | 1.000 | 0.888 | 1.000 | 1.000 | 1.000 | 0.980 | 1.000 | 1.000 | 0.809 | 1.000 | 1.000 | 0.871 | 0.844 |
| 3532 | 0.834 | 1.000 | 0.868 | 1.000 | 0.979 | 1.000 | 0.889 | 1.000 | 1.000 | 1.000 | 0.980 | 1.000 | 1.000 | 0.809 | 1.000 | 1.000 | 0.871 | 0.845 |
| 3533 | 0.833 | 1.000 | 0.868 | 1.000 | 0.979 | 1.000 | 0.889 | 1.000 | 1.000 | 1.000 | 0.980 | 1.000 | 1.000 | 0.809 | 1.000 | 1.000 | 0.871 | 0.844 |
| 3534 | 0.833 | 1.000 | 0.868 | 1.000 | 0.979 | 1.000 | 0.889 | 1.000 | 1.000 | 1.000 | 0.980 | 1.000 | 1.000 | 0.809 | 1.000 | 1.000 | 0.871 | 0.844 |
| 3535 | 0.833 | 1.000 | 0.868 | 1.000 | 0.979 | 1.000 | 0.889 | 1.000 | 1.000 | 1.000 | 0.980 | 1.000 | 1.000 | 0.809 | 1.000 | 1.000 | 0.871 | 0.844 |
| 3536 | 0.833 | 1.000 | 0.868 | 1.000 | 0.979 | 1.000 | 0.889 | 1.000 | 1.000 | 1.000 | 0.980 | 1.000 | 1.000 | 0.809 | 1.000 | 1.000 | 0.871 | 0.844 |
| 3537 | 0.832 | 1.000 | 0.868 | 1.000 | 0.979 | 1.000 | 0.889 | 1.000 | 1.000 | 1.000 | 0.980 | 1.000 | 1.000 | 0.809 | 1.000 | 1.000 | 0.871 | 0.844 |
| 3538 | 0.833 | 1.000 | 0.868 | 1.000 | 0.979 | 1.000 | 0.889 | 1.000 | 1.000 | 1.000 | 0.980 | 1.000 | 1.000 | 0.809 | 1.000 | 1.000 | 0.871 | 0.844 |
| 3539 | 0.832 | 1.000 | 0.868 | 1.000 | 0.979 | 1.000 | 0.889 | 1.000 | 1.000 | 1.000 | 0.980 | 1.000 | 1.000 | 0.809 | 1.000 | 1.000 | 0.871 | 0.844 |
| 3540 | 0.831 | 1.000 | 0.868 | 1.000 | 0.979 | 1.000 | 0.889 | 1.000 | 1.000 | 1.000 | 0.980 | 1.000 | 1.000 | 0.810 | 1.000 | 1.000 | 0.870 | 0.844 |
| 3541 | 0.831 | 1.000 | 0.868 | 1.000 | 0.979 | 1.000 | 0.889 | 1.000 | 1.000 | 1.000 | 0.980 | 1.000 | 1.000 | 0.809 | 1.000 | 1.000 | 0.870 | 0.844 |
| 3542 | 0.831 | 1.000 | 0.868 | 1.000 | 0.979 | 1.000 | 0.889 | 1.000 | 1.000 | 1.000 | 0.980 | 1.000 | 1.000 | 0.809 | 1.000 | 1.000 | 0.870 | 0.844 |
| 3543 | 0.831 | 1.000 | 0.868 | 1.000 | 0.979 | 1.000 | 0.889 | 1.000 | 1.000 | 1.000 | 0.980 | 1.000 | 1.000 | 0.809 | 1.000 | 1.000 | 0.870 | 0.844 |
| 3544 | 0.832 | 1.000 | 0.868 | 1.000 | 0.979 | 1.000 | 0.889 | 1.000 | 1.000 | 1.000 | 0.980 | 1.000 | 1.000 | 0.812 | 1.000 | 1.000 | 0.871 | 0.845 |
| 3545 | 0.832 | 1.000 | 0.868 | 1.000 | 0.979 | 1.000 | 0.889 | 1.000 | 1.000 | 1.000 | 0.980 | 1.000 | 1.000 | 0.812 | 1.000 | 1.000 | 0.871 | 0.845 |
| 3546 | 0.832 | 1.000 | 0.868 | 1.000 | 0.979 | 1.000 | 0.889 | 1.000 | 1.000 | 1.000 | 0.980 | 1.000 | 1.000 | 0.812 | 1.000 | 1.000 | 0.871 | 0.845 |
| 3547 | 0.833 | 1.000 | 0.866 | 1.000 | 0.979 | 1.000 | 0.889 | 1.000 | 1.000 | 1.000 | 0.980 | 1.000 | 1.000 | 0.810 | 1.000 | 1.000 | 0.871 | 0.844 |
| 3548 | 0.833 | 1.000 | 0.866 | 1.000 | 0.979 | 1.000 | 0.889 | 1.000 | 1.000 | 1.000 | 0.980 | 1.000 | 1.000 | 0.810 | 1.000 | 1.000 | 0.871 | 0.844 |
| 3549 | 0.833 | 1.000 | 0.866 | 1.000 | 0.979 | 1.000 | 0.889 | 1.000 | 1.000 | 1.000 | 0.980 | 1.000 | 1.000 | 0.810 | 1.000 | 1.000 | 0.871 | 0.844 |
| 3550 | 0.832 | 1.000 | 0.866 | 1.000 | 0.979 | 1.000 | 0.891 | 1.000 | 1.000 | 1.000 | 0.980 | 1.000 | 1.000 | 0.809 | 1.000 | 1.000 | 0.870 | 0.844 |
| 3551 | 0.835 | 1.000 | 0.866 | 1.000 | 0.979 | 1.000 | 0.894 | 1.000 | 1.000 | 1.000 | 0.980 | 1.000 | 1.000 | 0.809 | 1.000 | 1.000 | 0.872 | 0.845 |
| 3552 | 0.834 | 1.000 | 0.866 | 1.000 | 0.979 | 1.000 | 0.894 | 1.000 | 1.000 | 1.000 | 0.980 | 1.000 | 1.000 | 0.809 | 1.000 | 1.000 | 0.871 | 0.845 |

| | | | | | | | | | | | | | | | | | | |
|---|---|---|---|---|---|---|---|---|---|---|---|---|---|---|---|---|---|---|
| 3553 | 0.834 | 1.000 | 0.866 | 1.000 | 0.979 | 1.000 | 0.894 | 1.000 | 1.000 | 1.000 | 0.980 | 1.000 | 1.000 | 0.808 | 1.000 | 1.000 | 0.871 | 0.845 |
| 3554 | 0.835 | 1.000 | 0.866 | 1.000 | 0.979 | 1.000 | 0.894 | 1.000 | 1.000 | 1.000 | 0.980 | 1.000 | 1.000 | 0.807 | 1.000 | 1.000 | 0.871 | 0.845 |
| 3555 | 0.835 | 1.000 | 0.866 | 1.000 | 0.979 | 1.000 | 0.894 | 1.000 | 1.000 | 1.000 | 0.980 | 1.000 | 1.000 | 0.806 | 1.000 | 1.000 | 0.871 | 0.845 |
| 3556 | 0.835 | 1.000 | 0.866 | 1.000 | 0.979 | 1.000 | 0.894 | 1.000 | 1.000 | 1.000 | 0.980 | 1.000 | 1.000 | 0.806 | 1.000 | 1.000 | 0.871 | 0.845 |
| 3557 | 0.835 | 1.000 | 0.864 | 1.000 | 0.979 | 1.000 | 0.894 | 1.000 | 1.000 | 1.000 | 0.980 | 1.000 | 1.000 | 0.805 | 1.000 | 1.000 | 0.871 | 0.844 |
| 3558 | 0.835 | 1.000 | 0.864 | 1.000 | 0.979 | 1.000 | 0.894 | 1.000 | 1.000 | 1.000 | 0.980 | 1.000 | 1.000 | 0.805 | 1.000 | 1.000 | 0.871 | 0.844 |
| 3559 | 0.835 | 1.000 | 0.864 | 1.000 | 0.979 | 1.000 | 0.894 | 1.000 | 1.000 | 1.000 | 0.980 | 1.000 | 1.000 | 0.806 | 1.000 | 1.000 | 0.871 | 0.844 |
| 3560 | 0.835 | 1.000 | 0.864 | 1.000 | 0.979 | 1.000 | 0.894 | 1.000 | 1.000 | 1.000 | 0.980 | 1.000 | 1.000 | 0.806 | 1.000 | 1.000 | 0.871 | 0.844 |
| 3561 | 0.835 | 1.000 | 0.864 | 1.000 | 0.979 | 1.000 | 0.894 | 1.000 | 1.000 | 1.000 | 0.980 | 1.000 | 1.000 | 0.806 | 1.000 | 1.000 | 0.871 | 0.844 |
| 3562 | 0.835 | 1.000 | 0.864 | 1.000 | 0.979 | 1.000 | 0.894 | 1.000 | 1.000 | 1.000 | 0.980 | 1.000 | 1.000 | 0.806 | 1.000 | 1.000 | 0.871 | 0.845 |
| 3563 | 0.835 | 1.000 | 0.864 | 1.000 | 0.979 | 1.000 | 0.894 | 1.000 | 1.000 | 1.000 | 0.980 | 1.000 | 1.000 | 0.805 | 1.000 | 1.000 | 0.871 | 0.844 |
| 3564 | 0.835 | 1.000 | 0.864 | 1.000 | 0.979 | 1.000 | 0.894 | 1.000 | 1.000 | 1.000 | 0.980 | 1.000 | 1.000 | 0.806 | 1.000 | 1.000 | 0.871 | 0.845 |
| 3565 | 0.834 | 1.000 | 0.864 | 1.000 | 0.979 | 1.000 | 0.894 | 1.000 | 1.000 | 1.000 | 0.980 | 1.000 | 1.000 | 0.806 | 1.000 | 1.000 | 0.871 | 0.844 |
| 3566 | 0.834 | 1.000 | 0.866 | 1.000 | 0.979 | 1.000 | 0.894 | 1.000 | 1.000 | 1.000 | 0.980 | 1.000 | 1.000 | 0.806 | 1.000 | 1.000 | 0.871 | 0.844 |
| 3567 | 0.833 | 1.000 | 0.864 | 1.000 | 0.979 | 1.000 | 0.894 | 1.000 | 1.000 | 1.000 | 0.980 | 1.000 | 1.000 | 0.808 | 1.000 | 1.000 | 0.871 | 0.844 |
| 3568 | 0.832 | 1.000 | 0.864 | 1.000 | 0.979 | 1.000 | 0.894 | 1.000 | 1.000 | 1.000 | 0.980 | 1.000 | 1.000 | 0.807 | 1.000 | 1.000 | 0.870 | 0.844 |
| 3569 | 0.833 | 1.000 | 0.864 | 1.000 | 0.979 | 1.000 | 0.894 | 1.000 | 1.000 | 1.000 | 0.980 | 1.000 | 1.000 | 0.806 | 1.000 | 1.000 | 0.870 | 0.844 |
| 3570 | 0.834 | 1.000 | 0.864 | 1.000 | 0.979 | 1.000 | 0.892 | 1.000 | 1.000 | 1.000 | 0.980 | 1.000 | 1.000 | 0.807 | 1.000 | 1.000 | 0.871 | 0.844 |
| 3571 | 0.834 | 1.000 | 0.864 | 1.000 | 0.979 | 1.000 | 0.892 | 1.000 | 1.000 | 1.000 | 0.980 | 1.000 | 1.000 | 0.807 | 1.000 | 1.000 | 0.871 | 0.844 |
| 3572 | 0.834 | 1.000 | 0.864 | 1.000 | 0.979 | 1.000 | 0.892 | 1.000 | 1.000 | 1.000 | 0.980 | 1.000 | 1.000 | 0.807 | 1.000 | 1.000 | 0.871 | 0.844 |
| 3573 | 0.834 | 1.000 | 0.864 | 1.000 | 0.979 | 1.000 | 0.894 | 1.000 | 1.000 | 1.000 | 0.980 | 1.000 | 1.000 | 0.807 | 1.000 | 1.000 | 0.871 | 0.844 |
| 3574 | 0.835 | 1.000 | 0.866 | 1.000 | 0.979 | 1.000 | 0.894 | 1.000 | 1.000 | 1.000 | 0.980 | 1.000 | 1.000 | 0.806 | 1.000 | 1.000 | 0.871 | 0.845 |
| 3575 | 0.835 | 1.000 | 0.864 | 1.000 | 0.979 | 1.000 | 0.894 | 1.000 | 1.000 | 1.000 | 0.980 | 1.000 | 1.000 | 0.806 | 1.000 | 1.000 | 0.871 | 0.844 |
| 3576 | 0.835 | 1.000 | 0.864 | 1.000 | 0.979 | 1.000 | 0.894 | 1.000 | 1.000 | 1.000 | 0.980 | 1.000 | 1.000 | 0.806 | 1.000 | 1.000 | 0.871 | 0.844 |
| 3577 | 0.835 | 1.000 | 0.866 | 1.000 | 0.979 | 1.000 | 0.894 | 1.000 | 1.000 | 1.000 | 0.980 | 1.000 | 1.000 | 0.806 | 1.000 | 1.000 | 0.871 | 0.845 |
| 3578 | 0.835 | 1.000 | 0.866 | 1.000 | 0.979 | 1.000 | 0.894 | 1.000 | 1.000 | 1.000 | 0.980 | 1.000 | 1.000 | 0.806 | 1.000 | 1.000 | 0.871 | 0.845 |
| 3579 | 0.833 | 1.000 | 0.866 | 1.000 | 0.979 | 1.000 | 0.892 | 1.000 | 1.000 | 1.000 | 0.980 | 1.000 | 1.000 | 0.807 | 1.000 | 1.000 | 0.871 | 0.844 |
| 3580 | 0.834 | 1.000 | 0.866 | 1.000 | 0.979 | 1.000 | 0.894 | 1.000 | 1.000 | 1.000 | 0.980 | 1.000 | 1.000 | 0.806 | 1.000 | 1.000 | 0.871 | 0.844 |
| 3581 | 0.834 | 1.000 | 0.866 | 1.000 | 0.979 | 1.000 | 0.894 | 1.000 | 1.000 | 1.000 | 0.980 | 1.000 | 1.000 | 0.806 | 1.000 | 1.000 | 0.871 | 0.844 |
| 3582 | 0.833 | 1.000 | 0.868 | 1.000 | 0.979 | 1.000 | 0.894 | 1.000 | 1.000 | 1.000 | 0.980 | 1.000 | 1.000 | 0.806 | 1.000 | 1.000 | 0.871 | 0.844 |
| 3583 | 0.833 | 1.000 | 0.868 | 1.000 | 0.979 | 1.000 | 0.894 | 1.000 | 1.000 | 1.000 | 0.980 | 1.000 | 1.000 | 0.806 | 1.000 | 1.000 | 0.871 | 0.844 |
| 3584 | 0.833 | 1.000 | 0.868 | 1.000 | 0.979 | 1.000 | 0.894 | 1.000 | 1.000 | 1.000 | 0.980 | 1.000 | 1.000 | 0.806 | 1.000 | 1.000 | 0.871 | 0.844 |
| 3585 | 0.833 | 1.000 | 0.868 | 1.000 | 0.979 | 1.000 | 0.894 | 1.000 | 1.000 | 1.000 | 0.980 | 1.000 | 1.000 | 0.806 | 1.000 | 1.000 | 0.871 | 0.844 |
| 3586 | 0.833 | 1.000 | 0.868 | 1.000 | 0.979 | 1.000 | 0.894 | 1.000 | 1.000 | 1.000 | 0.980 | 1.000 | 1.000 | 0.806 | 1.000 | 1.000 | 0.871 | 0.844 |
| 3587 | 0.833 | 1.000 | 0.868 | 1.000 | 0.979 | 1.000 | 0.894 | 1.000 | 1.000 | 1.000 | 0.980 | 1.000 | 1.000 | 0.806 | 1.000 | 1.000 | 0.871 | 0.844 |
| 3588 | 0.834 | 1.000 | 0.868 | 1.000 | 0.979 | 1.000 | 0.894 | 1.000 | 1.000 | 1.000 | 0.980 | 1.000 | 1.000 | 0.806 | 1.000 | 1.000 | 0.871 | 0.845 |
| 3589 | 0.835 | 1.000 | 0.866 | 1.000 | 0.979 | 1.000 | 0.894 | 1.000 | 1.000 | 1.000 | 0.980 | 1.000 | 1.000 | 0.807 | 1.000 | 1.000 | 0.871 | 0.845 |
| 3590 | 0.835 | 1.000 | 0.866 | 1.000 | 0.979 | 1.000 | 0.894 | 1.000 | 1.000 | 1.000 | 0.980 | 1.000 | 1.000 | 0.807 | 1.000 | 1.000 | 0.871 | 0.845 |
| 3591 | 0.835 | 1.000 | 0.868 | 1.000 | 0.979 | 1.000 | 0.894 | 1.000 | 1.000 | 1.000 | 0.980 | 1.000 | 1.000 | 0.806 | 1.000 | 1.000 | 0.871 | 0.845 |

| | | | | | | | | | | | | | | | | | | |
|---|---|---|---|---|---|---|---|---|---|---|---|---|---|---|---|---|---|---|
| 3592 | 0.835 | 1.000 | 0.868 | 1.000 | 0.979 | 1.000 | 0.894 | 1.000 | 1.000 | 1.000 | 0.980 | 1.000 | 1.000 | 0.806 | 1.000 | 1.000 | 0.871 | 0.845 |
| 3593 | 0.836 | 1.000 | 0.868 | 1.000 | 0.979 | 1.000 | 0.894 | 1.000 | 1.000 | 1.000 | 0.980 | 1.000 | 1.000 | 0.806 | 1.000 | 1.000 | 0.872 | 0.845 |
| 3594 | 0.836 | 1.000 | 0.868 | 1.000 | 0.979 | 1.000 | 0.894 | 1.000 | 1.000 | 1.000 | 0.980 | 1.000 | 1.000 | 0.806 | 1.000 | 1.000 | 0.872 | 0.845 |
| 3595 | 0.834 | 1.000 | 0.870 | 1.000 | 0.979 | 1.000 | 0.894 | 1.000 | 1.000 | 1.000 | 0.980 | 1.000 | 1.000 | 0.806 | 1.000 | 1.000 | 0.871 | 0.845 |
| 3596 | 0.834 | 1.000 | 0.868 | 1.000 | 0.979 | 1.000 | 0.894 | 1.000 | 1.000 | 1.000 | 0.980 | 1.000 | 1.000 | 0.805 | 1.000 | 1.000 | 0.871 | 0.844 |
| 3597 | 0.833 | 1.000 | 0.868 | 1.000 | 0.979 | 1.000 | 0.892 | 1.000 | 1.000 | 1.000 | 0.980 | 1.000 | 1.000 | 0.806 | 1.000 | 1.000 | 0.870 | 0.844 |
| 3598 | 0.833 | 1.000 | 0.868 | 1.000 | 0.979 | 1.000 | 0.892 | 1.000 | 1.000 | 1.000 | 0.980 | 1.000 | 1.000 | 0.806 | 1.000 | 1.000 | 0.870 | 0.844 |
| 3599 | 0.835 | 1.000 | 0.868 | 1.000 | 0.979 | 1.000 | 0.892 | 1.000 | 1.000 | 1.000 | 0.980 | 1.000 | 1.000 | 0.806 | 1.000 | 1.000 | 0.871 | 0.845 |
| 3600 | 0.834 | 1.000 | 0.870 | 1.000 | 0.979 | 1.000 | 0.892 | 1.000 | 1.000 | 1.000 | 0.980 | 1.000 | 1.000 | 0.806 | 1.000 | 1.000 | 0.871 | 0.845 |
| 3601 | 0.835 | 1.000 | 0.868 | 1.000 | 0.979 | 1.000 | 0.892 | 1.000 | 1.000 | 1.000 | 0.980 | 1.000 | 1.000 | 0.806 | 1.000 | 1.000 | 0.871 | 0.845 |
| 3602 | 0.835 | 1.000 | 0.870 | 1.000 | 0.979 | 1.000 | 0.894 | 1.000 | 1.000 | 1.000 | 0.980 | 1.000 | 1.000 | 0.807 | 1.000 | 1.000 | 0.872 | 0.845 |
| 3603 | 0.835 | 1.000 | 0.872 | 1.000 | 0.979 | 1.000 | 0.894 | 1.000 | 1.000 | 1.000 | 0.980 | 1.000 | 1.000 | 0.808 | 1.000 | 1.000 | 0.872 | 0.846 |
| 3604 | 0.834 | 1.000 | 0.872 | 1.000 | 0.979 | 1.000 | 0.894 | 1.000 | 1.000 | 1.000 | 0.980 | 1.000 | 1.000 | 0.808 | 1.000 | 1.000 | 0.872 | 0.846 |
| 3605 | 0.834 | 1.000 | 0.872 | 1.000 | 0.979 | 1.000 | 0.894 | 1.000 | 1.000 | 1.000 | 0.980 | 1.000 | 1.000 | 0.808 | 1.000 | 1.000 | 0.872 | 0.846 |
| 3606 | 0.833 | 1.000 | 0.870 | 1.000 | 0.979 | 1.000 | 0.894 | 1.000 | 1.000 | 1.000 | 0.980 | 1.000 | 1.000 | 0.808 | 1.000 | 1.000 | 0.871 | 0.845 |
| 3607 | 0.834 | 1.000 | 0.870 | 1.000 | 0.979 | 1.000 | 0.894 | 1.000 | 1.000 | 1.000 | 0.980 | 1.000 | 1.000 | 0.808 | 1.000 | 1.000 | 0.872 | 0.845 |
| 3608 | 0.833 | 1.000 | 0.870 | 1.000 | 0.979 | 1.000 | 0.894 | 1.000 | 1.000 | 1.000 | 0.980 | 1.000 | 1.000 | 0.808 | 1.000 | 1.000 | 0.871 | 0.845 |
| 3609 | 0.834 | 1.000 | 0.870 | 1.000 | 0.979 | 1.000 | 0.894 | 1.000 | 1.000 | 1.000 | 0.980 | 1.000 | 1.000 | 0.808 | 1.000 | 1.000 | 0.872 | 0.845 |
| 3610 | 0.833 | 1.000 | 0.870 | 1.000 | 0.979 | 1.000 | 0.894 | 1.000 | 1.000 | 1.000 | 0.980 | 1.000 | 1.000 | 0.808 | 1.000 | 1.000 | 0.871 | 0.845 |
| 3611 | 0.835 | 1.000 | 0.872 | 1.000 | 0.979 | 1.000 | 0.894 | 1.000 | 1.000 | 1.000 | 0.980 | 1.000 | 1.000 | 0.808 | 1.000 | 1.000 | 0.872 | 0.846 |
| 3612 | 0.835 | 1.000 | 0.872 | 1.000 | 0.979 | 1.000 | 0.894 | 1.000 | 1.000 | 1.000 | 0.980 | 1.000 | 1.000 | 0.809 | 1.000 | 1.000 | 0.872 | 0.846 |
| 3613 | 0.835 | 1.000 | 0.872 | 1.000 | 0.979 | 1.000 | 0.894 | 1.000 | 1.000 | 1.000 | 0.980 | 1.000 | 1.000 | 0.809 | 1.000 | 1.000 | 0.872 | 0.846 |
| 3614 | 0.835 | 1.000 | 0.872 | 1.000 | 0.979 | 1.000 | 0.894 | 1.000 | 1.000 | 1.000 | 0.980 | 1.000 | 1.000 | 0.809 | 1.000 | 1.000 | 0.872 | 0.846 |
| 3615 | 0.834 | 1.000 | 0.872 | 1.000 | 0.979 | 1.000 | 0.894 | 1.000 | 1.000 | 1.000 | 0.980 | 1.000 | 1.000 | 0.809 | 1.000 | 1.000 | 0.872 | 0.846 |
| 3616 | 0.835 | 1.000 | 0.872 | 1.000 | 0.979 | 1.000 | 0.894 | 1.000 | 1.000 | 1.000 | 0.980 | 1.000 | 1.000 | 0.809 | 1.000 | 1.000 | 0.872 | 0.846 |
| 3617 | 0.835 | 1.000 | 0.872 | 1.000 | 0.979 | 1.000 | 0.894 | 1.000 | 1.000 | 1.000 | 0.977 | 1.000 | 1.000 | 0.808 | 1.000 | 1.000 | 0.872 | 0.845 |
| 3618 | 0.833 | 1.000 | 0.870 | 1.000 | 0.979 | 1.000 | 0.894 | 1.000 | 1.000 | 1.000 | 0.977 | 1.000 | 1.000 | 0.805 | 1.000 | 1.000 | 0.871 | 0.844 |
| 3619 | 0.833 | 1.000 | 0.870 | 1.000 | 0.979 | 1.000 | 0.894 | 1.000 | 1.000 | 1.000 | 0.977 | 1.000 | 1.000 | 0.805 | 1.000 | 1.000 | 0.871 | 0.844 |
| 3620 | 0.833 | 1.000 | 0.870 | 1.000 | 0.979 | 1.000 | 0.894 | 1.000 | 1.000 | 1.000 | 0.977 | 1.000 | 1.000 | 0.805 | 1.000 | 1.000 | 0.871 | 0.844 |
| 3621 | 0.833 | 1.000 | 0.872 | 1.000 | 0.979 | 1.000 | 0.892 | 1.000 | 1.000 | 1.000 | 0.977 | 1.000 | 1.000 | 0.806 | 1.000 | 1.000 | 0.871 | 0.844 |
| 3622 | 0.833 | 1.000 | 0.872 | 1.000 | 0.979 | 1.000 | 0.892 | 1.000 | 1.000 | 1.000 | 0.977 | 1.000 | 1.000 | 0.806 | 1.000 | 1.000 | 0.871 | 0.844 |
| 3623 | 0.832 | 1.000 | 0.872 | 1.000 | 0.979 | 1.000 | 0.891 | 1.000 | 1.000 | 1.000 | 0.974 | 1.000 | 1.000 | 0.805 | 1.000 | 1.000 | 0.870 | 0.843 |
| 3624 | 0.832 | 1.000 | 0.872 | 1.000 | 0.979 | 1.000 | 0.891 | 1.000 | 1.000 | 1.000 | 0.974 | 1.000 | 1.000 | 0.805 | 1.000 | 1.000 | 0.870 | 0.843 |
| 3625 | 0.832 | 1.000 | 0.872 | 1.000 | 0.979 | 1.000 | 0.891 | 1.000 | 1.000 | 1.000 | 0.974 | 1.000 | 1.000 | 0.806 | 1.000 | 1.000 | 0.870 | 0.843 |
| 3626 | 0.832 | 1.000 | 0.870 | 1.000 | 0.979 | 1.000 | 0.891 | 1.000 | 1.000 | 1.000 | 0.974 | 1.000 | 1.000 | 0.806 | 1.000 | 1.000 | 0.870 | 0.843 |
| 3627 | 0.833 | 1.000 | 0.872 | 1.000 | 0.979 | 1.000 | 0.891 | 1.000 | 1.000 | 1.000 | 0.974 | 1.000 | 1.000 | 0.805 | 1.000 | 1.000 | 0.870 | 0.843 |
| 3628 | 0.833 | 1.000 | 0.872 | 1.000 | 0.979 | 1.000 | 0.891 | 1.000 | 1.000 | 1.000 | 0.974 | 1.000 | 1.000 | 0.805 | 1.000 | 1.000 | 0.870 | 0.843 |
| 3629 | 0.832 | 1.000 | 0.872 | 1.000 | 0.979 | 1.000 | 0.891 | 1.000 | 1.000 | 1.000 | 0.974 | 1.000 | 1.000 | 0.805 | 1.000 | 1.000 | 0.870 | 0.843 |
| 3630 | 0.832 | 1.000 | 0.874 | 1.000 | 0.979 | 1.000 | 0.895 | 1.000 | 1.000 | 1.000 | 0.974 | 1.000 | 1.000 | 0.806 | 1.000 | 1.000 | 0.871 | 0.844 |

| | | | | | | | | | | | | | | | | | | |
|---|---|---|---|---|---|---|---|---|---|---|---|---|---|---|---|---|---|---|
| 3631 | 0.831 | 1.000 | 0.872 | 1.000 | 0.979 | 1.000 | 0.895 | 1.000 | 1.000 | 1.000 | 0.974 | 1.000 | 1.000 | 0.806 | 1.000 | 1.000 | 0.870 | 0.844 |
| 3632 | 0.831 | 1.000 | 0.872 | 1.000 | 0.979 | 1.000 | 0.895 | 1.000 | 1.000 | 1.000 | 0.974 | 1.000 | 1.000 | 0.806 | 1.000 | 1.000 | 0.870 | 0.844 |
| 3633 | 0.831 | 1.000 | 0.872 | 1.000 | 0.979 | 1.000 | 0.895 | 1.000 | 1.000 | 1.000 | 0.974 | 1.000 | 1.000 | 0.806 | 1.000 | 1.000 | 0.870 | 0.844 |
| 3634 | 0.831 | 1.000 | 0.872 | 1.000 | 0.979 | 1.000 | 0.895 | 1.000 | 1.000 | 1.000 | 0.974 | 1.000 | 1.000 | 0.806 | 1.000 | 1.000 | 0.870 | 0.844 |
| 3635 | 0.831 | 1.000 | 0.872 | 1.000 | 0.979 | 1.000 | 0.895 | 1.000 | 1.000 | 1.000 | 0.974 | 1.000 | 1.000 | 0.806 | 1.000 | 1.000 | 0.870 | 0.844 |
| 3636 | 0.831 | 1.000 | 0.872 | 1.000 | 0.979 | 1.000 | 0.894 | 1.000 | 1.000 | 1.000 | 0.974 | 1.000 | 1.000 | 0.806 | 1.000 | 1.000 | 0.870 | 0.844 |
| 3637 | 0.831 | 1.000 | 0.872 | 1.000 | 0.979 | 1.000 | 0.894 | 1.000 | 1.000 | 1.000 | 0.974 | 1.000 | 1.000 | 0.806 | 1.000 | 1.000 | 0.870 | 0.843 |
| 3638 | 0.829 | 1.000 | 0.874 | 1.000 | 0.979 | 1.000 | 0.897 | 1.000 | 1.000 | 1.000 | 0.974 | 1.000 | 1.000 | 0.806 | 1.000 | 1.000 | 0.870 | 0.844 |
| 3639 | 0.829 | 1.000 | 0.874 | 1.000 | 0.979 | 1.000 | 0.895 | 1.000 | 1.000 | 1.000 | 0.974 | 1.000 | 1.000 | 0.806 | 1.000 | 1.000 | 0.870 | 0.843 |
| 3640 | 0.829 | 1.000 | 0.870 | 1.000 | 0.979 | 1.000 | 0.895 | 1.000 | 1.000 | 1.000 | 0.974 | 1.000 | 1.000 | 0.806 | 1.000 | 1.000 | 0.869 | 0.843 |
| 3641 | 0.829 | 1.000 | 0.870 | 1.000 | 0.979 | 1.000 | 0.895 | 1.000 | 1.000 | 1.000 | 0.974 | 1.000 | 1.000 | 0.806 | 1.000 | 1.000 | 0.869 | 0.843 |
| 3642 | 0.828 | 1.000 | 0.870 | 1.000 | 0.979 | 1.000 | 0.895 | 1.000 | 1.000 | 1.000 | 0.974 | 1.000 | 1.000 | 0.806 | 1.000 | 1.000 | 0.869 | 0.843 |
| 3643 | 0.828 | 1.000 | 0.872 | 1.000 | 0.979 | 1.000 | 0.897 | 1.000 | 1.000 | 1.000 | 0.974 | 1.000 | 1.000 | 0.807 | 1.000 | 1.000 | 0.870 | 0.843 |
| 3644 | 0.829 | 1.000 | 0.872 | 1.000 | 0.979 | 1.000 | 0.897 | 1.000 | 1.000 | 1.000 | 0.974 | 1.000 | 1.000 | 0.807 | 1.000 | 1.000 | 0.870 | 0.843 |
| 3645 | 0.828 | 1.000 | 0.872 | 1.000 | 0.979 | 1.000 | 0.897 | 1.000 | 1.000 | 1.000 | 0.974 | 1.000 | 1.000 | 0.807 | 1.000 | 1.000 | 0.870 | 0.843 |
| 3646 | 0.826 | 1.000 | 0.872 | 1.000 | 0.979 | 1.000 | 0.894 | 1.000 | 1.000 | 1.000 | 0.977 | 1.000 | 1.000 | 0.807 | 1.000 | 1.000 | 0.869 | 0.842 |
| 3647 | 0.824 | 1.000 | 0.872 | 1.000 | 0.979 | 1.000 | 0.894 | 1.000 | 1.000 | 1.000 | 0.977 | 1.000 | 1.000 | 0.807 | 1.000 | 1.000 | 0.868 | 0.842 |
| 3648 | 0.824 | 1.000 | 0.872 | 1.000 | 0.979 | 1.000 | 0.894 | 1.000 | 1.000 | 1.000 | 0.977 | 1.000 | 1.000 | 0.807 | 1.000 | 1.000 | 0.868 | 0.842 |
| 3649 | 0.824 | 1.000 | 0.872 | 1.000 | 0.979 | 1.000 | 0.894 | 1.000 | 1.000 | 1.000 | 0.977 | 1.000 | 1.000 | 0.807 | 1.000 | 1.000 | 0.868 | 0.842 |
| 3650 | 0.824 | 1.000 | 0.872 | 1.000 | 0.979 | 1.000 | 0.894 | 1.000 | 1.000 | 1.000 | 0.977 | 1.000 | 1.000 | 0.807 | 1.000 | 1.000 | 0.868 | 0.842 |
| 3651 | 0.824 | 1.000 | 0.872 | 1.000 | 0.979 | 1.000 | 0.894 | 1.000 | 1.000 | 1.000 | 0.977 | 1.000 | 1.000 | 0.809 | 1.000 | 1.000 | 0.869 | 0.842 |
| 3652 | 0.825 | 1.000 | 0.872 | 1.000 | 0.979 | 1.000 | 0.892 | 1.000 | 1.000 | 1.000 | 0.977 | 1.000 | 1.000 | 0.809 | 1.000 | 1.000 | 0.869 | 0.842 |
| 3653 | 0.824 | 1.000 | 0.872 | 1.000 | 0.979 | 1.000 | 0.892 | 1.000 | 1.000 | 1.000 | 0.977 | 1.000 | 1.000 | 0.808 | 1.000 | 1.000 | 0.869 | 0.842 |
| 3654 | 0.825 | 1.000 | 0.872 | 1.000 | 0.979 | 1.000 | 0.892 | 1.000 | 1.000 | 1.000 | 0.977 | 1.000 | 1.000 | 0.807 | 1.000 | 1.000 | 0.869 | 0.842 |
| 3655 | 0.825 | 1.000 | 0.872 | 1.000 | 0.979 | 1.000 | 0.892 | 1.000 | 1.000 | 1.000 | 0.977 | 1.000 | 1.000 | 0.807 | 1.000 | 1.000 | 0.869 | 0.842 |
| 3656 | 0.826 | 1.000 | 0.872 | 1.000 | 0.979 | 1.000 | 0.894 | 1.000 | 1.000 | 1.000 | 0.977 | 1.000 | 1.000 | 0.806 | 1.000 | 1.000 | 0.869 | 0.842 |
| 3657 | 0.826 | 1.000 | 0.874 | 1.000 | 0.979 | 1.000 | 0.894 | 1.000 | 1.000 | 1.000 | 0.977 | 1.000 | 1.000 | 0.805 | 1.000 | 1.000 | 0.869 | 0.842 |
| 3658 | 0.826 | 1.000 | 0.874 | 1.000 | 0.979 | 1.000 | 0.894 | 1.000 | 1.000 | 1.000 | 0.977 | 1.000 | 1.000 | 0.804 | 1.000 | 1.000 | 0.868 | 0.842 |
| 3659 | 0.825 | 1.000 | 0.874 | 1.000 | 0.979 | 1.000 | 0.895 | 1.000 | 1.000 | 1.000 | 0.977 | 1.000 | 1.000 | 0.804 | 1.000 | 1.000 | 0.868 | 0.842 |
| 3660 | 0.825 | 1.000 | 0.872 | 1.000 | 0.979 | 1.000 | 0.897 | 1.000 | 1.000 | 1.000 | 0.977 | 1.000 | 1.000 | 0.803 | 1.000 | 1.000 | 0.868 | 0.841 |
| 3661 | 0.826 | 1.000 | 0.874 | 1.000 | 0.979 | 1.000 | 0.894 | 1.000 | 1.000 | 1.000 | 0.977 | 1.000 | 1.000 | 0.804 | 1.000 | 1.000 | 0.868 | 0.842 |
| 3662 | 0.826 | 1.000 | 0.872 | 1.000 | 0.979 | 1.000 | 0.894 | 1.000 | 1.000 | 1.000 | 0.977 | 1.000 | 1.000 | 0.805 | 1.000 | 1.000 | 0.869 | 0.842 |
| 3663 | 0.826 | 1.000 | 0.872 | 1.000 | 0.979 | 1.000 | 0.894 | 1.000 | 1.000 | 1.000 | 0.977 | 1.000 | 1.000 | 0.805 | 1.000 | 1.000 | 0.868 | 0.842 |
| 3664 | 0.826 | 1.000 | 0.872 | 1.000 | 0.979 | 1.000 | 0.894 | 1.000 | 1.000 | 1.000 | 0.977 | 1.000 | 1.000 | 0.802 | 1.000 | 1.000 | 0.868 | 0.841 |
| 3665 | 0.826 | 1.000 | 0.872 | 1.000 | 0.979 | 1.000 | 0.894 | 1.000 | 1.000 | 1.000 | 0.977 | 1.000 | 1.000 | 0.802 | 1.000 | 1.000 | 0.868 | 0.841 |
| 3666 | 0.825 | 1.000 | 0.872 | 1.000 | 0.979 | 1.000 | 0.894 | 1.000 | 1.000 | 1.000 | 0.980 | 1.000 | 1.000 | 0.802 | 1.000 | 1.000 | 0.868 | 0.841 |
| 3667 | 0.826 | 1.000 | 0.872 | 1.000 | 0.979 | 1.000 | 0.894 | 1.000 | 1.000 | 1.000 | 0.980 | 1.000 | 1.000 | 0.803 | 1.000 | 1.000 | 0.868 | 0.841 |
| 3668 | 0.826 | 1.000 | 0.872 | 1.000 | 0.979 | 1.000 | 0.894 | 1.000 | 1.000 | 1.000 | 0.980 | 1.000 | 1.000 | 0.803 | 1.000 | 1.000 | 0.868 | 0.841 |
| 3669 | 0.826 | 1.000 | 0.872 | 1.000 | 0.979 | 1.000 | 0.894 | 1.000 | 1.000 | 1.000 | 0.980 | 1.000 | 1.000 | 0.803 | 1.000 | 1.000 | 0.868 | 0.842 |

| | | | | | | | | | | | | | | | | | | |
|---|---|---|---|---|---|---|---|---|---|---|---|---|---|---|---|---|---|---|
| 3670 | 0.826 | 1.000 | 0.872 | 1.000 | 0.979 | 1.000 | 0.894 | 1.000 | 1.000 | 1.000 | 0.980 | 1.000 | 1.000 | 0.803 | 1.000 | 1.000 | 0.868 | 0.841 |
| 3671 | 0.826 | 1.000 | 0.872 | 1.000 | 0.979 | 1.000 | 0.894 | 1.000 | 1.000 | 1.000 | 0.980 | 1.000 | 1.000 | 0.803 | 1.000 | 1.000 | 0.868 | 0.842 |
| 3672 | 0.826 | 1.000 | 0.872 | 1.000 | 0.979 | 1.000 | 0.894 | 1.000 | 1.000 | 1.000 | 0.980 | 1.000 | 1.000 | 0.804 | 1.000 | 1.000 | 0.869 | 0.842 |
| 3673 | 0.824 | 1.000 | 0.872 | 1.000 | 0.979 | 1.000 | 0.892 | 1.000 | 1.000 | 1.000 | 0.980 | 1.000 | 1.000 | 0.802 | 1.000 | 1.000 | 0.867 | 0.840 |
| 3674 | 0.826 | 1.000 | 0.872 | 1.000 | 0.979 | 1.000 | 0.894 | 1.000 | 1.000 | 1.000 | 0.980 | 1.000 | 1.000 | 0.801 | 1.000 | 1.000 | 0.868 | 0.841 |
| 3675 | 0.826 | 1.000 | 0.872 | 1.000 | 0.979 | 1.000 | 0.892 | 1.000 | 1.000 | 1.000 | 0.980 | 1.000 | 1.000 | 0.801 | 1.000 | 1.000 | 0.867 | 0.840 |
| 3676 | 0.826 | 1.000 | 0.872 | 1.000 | 0.979 | 1.000 | 0.892 | 1.000 | 1.000 | 1.000 | 0.980 | 1.000 | 1.000 | 0.801 | 1.000 | 1.000 | 0.867 | 0.840 |
| 3677 | 0.826 | 1.000 | 0.872 | 1.000 | 0.979 | 1.000 | 0.892 | 1.000 | 1.000 | 1.000 | 0.980 | 1.000 | 1.000 | 0.801 | 1.000 | 1.000 | 0.868 | 0.841 |
| 3678 | 0.826 | 1.000 | 0.872 | 1.000 | 0.979 | 1.000 | 0.892 | 1.000 | 1.000 | 1.000 | 0.980 | 1.000 | 1.000 | 0.801 | 1.000 | 1.000 | 0.868 | 0.841 |
| 3679 | 0.826 | 1.000 | 0.872 | 1.000 | 0.979 | 1.000 | 0.892 | 1.000 | 1.000 | 1.000 | 0.980 | 1.000 | 1.000 | 0.801 | 1.000 | 1.000 | 0.867 | 0.840 |
| 3680 | 0.826 | 1.000 | 0.872 | 1.000 | 0.979 | 1.000 | 0.892 | 1.000 | 1.000 | 1.000 | 0.980 | 1.000 | 1.000 | 0.802 | 1.000 | 1.000 | 0.868 | 0.841 |
| 3681 | 0.824 | 1.000 | 0.872 | 1.000 | 0.979 | 1.000 | 0.892 | 1.000 | 1.000 | 1.000 | 0.980 | 1.000 | 1.000 | 0.802 | 1.000 | 1.000 | 0.867 | 0.840 |
| 3682 | 0.825 | 1.000 | 0.872 | 1.000 | 0.979 | 1.000 | 0.892 | 1.000 | 1.000 | 1.000 | 0.980 | 1.000 | 1.000 | 0.802 | 1.000 | 1.000 | 0.868 | 0.841 |
| 3683 | 0.825 | 1.000 | 0.872 | 1.000 | 0.979 | 1.000 | 0.892 | 1.000 | 1.000 | 1.000 | 0.980 | 1.000 | 1.000 | 0.802 | 1.000 | 1.000 | 0.867 | 0.840 |
| 3684 | 0.825 | 1.000 | 0.872 | 1.000 | 0.979 | 1.000 | 0.892 | 1.000 | 1.000 | 1.000 | 0.980 | 1.000 | 1.000 | 0.801 | 1.000 | 1.000 | 0.867 | 0.840 |
| 3685 | 0.824 | 1.000 | 0.872 | 1.000 | 0.979 | 1.000 | 0.892 | 1.000 | 1.000 | 1.000 | 0.980 | 1.000 | 1.000 | 0.801 | 1.000 | 1.000 | 0.867 | 0.840 |
| 3686 | 0.824 | 1.000 | 0.872 | 1.000 | 0.979 | 1.000 | 0.892 | 1.000 | 1.000 | 1.000 | 0.980 | 1.000 | 1.000 | 0.802 | 1.000 | 1.000 | 0.867 | 0.840 |
| 3687 | 0.825 | 1.000 | 0.872 | 1.000 | 0.979 | 1.000 | 0.892 | 1.000 | 1.000 | 1.000 | 0.980 | 1.000 | 1.000 | 0.801 | 1.000 | 1.000 | 0.867 | 0.840 |
| 3688 | 0.824 | 1.000 | 0.872 | 1.000 | 0.979 | 1.000 | 0.892 | 1.000 | 1.000 | 1.000 | 0.980 | 1.000 | 1.000 | 0.801 | 1.000 | 1.000 | 0.867 | 0.840 |
| 3689 | 0.825 | 1.000 | 0.872 | 1.000 | 0.979 | 1.000 | 0.892 | 1.000 | 1.000 | 1.000 | 0.980 | 1.000 | 1.000 | 0.802 | 1.000 | 1.000 | 0.867 | 0.840 |
| 3690 | 0.825 | 1.000 | 0.872 | 1.000 | 0.979 | 1.000 | 0.892 | 1.000 | 1.000 | 1.000 | 0.980 | 1.000 | 1.000 | 0.802 | 1.000 | 1.000 | 0.867 | 0.840 |
| 3691 | 0.824 | 1.000 | 0.872 | 1.000 | 0.979 | 1.000 | 0.892 | 1.000 | 1.000 | 1.000 | 0.980 | 1.000 | 1.000 | 0.802 | 1.000 | 1.000 | 0.867 | 0.840 |
| 3692 | 0.824 | 1.000 | 0.872 | 1.000 | 0.979 | 1.000 | 0.892 | 1.000 | 1.000 | 1.000 | 0.980 | 1.000 | 1.000 | 0.802 | 1.000 | 1.000 | 0.867 | 0.840 |
| 3693 | 0.824 | 1.000 | 0.872 | 1.000 | 0.979 | 1.000 | 0.892 | 1.000 | 1.000 | 1.000 | 0.980 | 1.000 | 1.000 | 0.802 | 1.000 | 1.000 | 0.867 | 0.840 |
| 3694 | 0.825 | 1.000 | 0.870 | 1.000 | 0.979 | 1.000 | 0.892 | 1.000 | 1.000 | 1.000 | 0.980 | 1.000 | 1.000 | 0.801 | 1.000 | 1.000 | 0.867 | 0.840 |
| 3695 | 0.826 | 1.000 | 0.870 | 1.000 | 0.979 | 1.000 | 0.894 | 1.000 | 1.000 | 1.000 | 0.980 | 1.000 | 1.000 | 0.802 | 1.000 | 1.000 | 0.868 | 0.841 |
| 3696 | 0.826 | 1.000 | 0.870 | 1.000 | 0.979 | 1.000 | 0.894 | 1.000 | 1.000 | 1.000 | 0.980 | 1.000 | 1.000 | 0.802 | 1.000 | 1.000 | 0.868 | 0.841 |
| 3697 | 0.827 | 1.000 | 0.870 | 1.000 | 0.979 | 1.000 | 0.894 | 1.000 | 1.000 | 1.000 | 0.980 | 1.000 | 1.000 | 0.802 | 1.000 | 1.000 | 0.868 | 0.841 |
| 3698 | 0.826 | 1.000 | 0.872 | 1.000 | 0.979 | 1.000 | 0.894 | 1.000 | 1.000 | 1.000 | 0.980 | 1.000 | 1.000 | 0.801 | 1.000 | 1.000 | 0.868 | 0.841 |
| 3699 | 0.824 | 1.000 | 0.872 | 1.000 | 0.979 | 1.000 | 0.894 | 1.000 | 1.000 | 1.000 | 0.980 | 1.000 | 1.000 | 0.801 | 1.000 | 1.000 | 0.867 | 0.840 |
| 3700 | 0.824 | 1.000 | 0.872 | 1.000 | 0.979 | 1.000 | 0.894 | 1.000 | 1.000 | 1.000 | 0.980 | 1.000 | 1.000 | 0.802 | 1.000 | 1.000 | 0.867 | 0.840 |
| 3701 | 0.824 | 1.000 | 0.872 | 1.000 | 0.979 | 1.000 | 0.894 | 1.000 | 1.000 | 1.000 | 0.980 | 1.000 | 1.000 | 0.801 | 1.000 | 1.000 | 0.867 | 0.840 |
| 3702 | 0.824 | 1.000 | 0.872 | 1.000 | 0.979 | 1.000 | 0.892 | 1.000 | 1.000 | 1.000 | 0.980 | 1.000 | 1.000 | 0.801 | 1.000 | 1.000 | 0.867 | 0.840 |
| 3703 | 0.828 | 1.000 | 0.870 | 1.000 | 0.979 | 1.000 | 0.892 | 1.000 | 1.000 | 1.000 | 0.980 | 1.000 | 1.000 | 0.801 | 1.000 | 1.000 | 0.868 | 0.841 |
| 3704 | 0.828 | 1.000 | 0.870 | 1.000 | 0.979 | 1.000 | 0.892 | 1.000 | 1.000 | 1.000 | 0.980 | 1.000 | 1.000 | 0.801 | 1.000 | 1.000 | 0.868 | 0.841 |
| 3705 | 0.829 | 1.000 | 0.870 | 1.000 | 0.979 | 1.000 | 0.894 | 1.000 | 1.000 | 1.000 | 0.980 | 1.000 | 1.000 | 0.801 | 1.000 | 1.000 | 0.868 | 0.842 |
| 3706 | 0.829 | 1.000 | 0.870 | 1.000 | 0.979 | 1.000 | 0.894 | 1.000 | 1.000 | 1.000 | 0.980 | 1.000 | 1.000 | 0.801 | 1.000 | 1.000 | 0.868 | 0.842 |
| 3707 | 0.829 | 1.000 | 0.870 | 1.000 | 0.979 | 1.000 | 0.894 | 1.000 | 1.000 | 1.000 | 0.980 | 1.000 | 1.000 | 0.801 | 1.000 | 1.000 | 0.868 | 0.842 |
| 3708 | 0.829 | 1.000 | 0.870 | 1.000 | 0.979 | 1.000 | 0.894 | 1.000 | 1.000 | 1.000 | 0.980 | 1.000 | 1.000 | 0.801 | 1.000 | 1.000 | 0.868 | 0.842 |

| | | | | | | | | | | | | | | | | | | |
|---|---|---|---|---|---|---|---|---|---|---|---|---|---|---|---|---|---|---|
| 3709 | 0.829 | 1.000 | 0.870 | 1.000 | 0.979 | 1.000 | 0.894 | 1.000 | 1.000 | 1.000 | 0.980 | 1.000 | 1.000 | 0.802 | 1.000 | 1.000 | 0.869 | 0.842 |
| 3710 | 0.829 | 1.000 | 0.870 | 1.000 | 0.979 | 1.000 | 0.892 | 1.000 | 1.000 | 1.000 | 0.977 | 1.000 | 1.000 | 0.802 | 1.000 | 1.000 | 0.868 | 0.841 |
| 3711 | 0.829 | 1.000 | 0.870 | 1.000 | 0.979 | 1.000 | 0.895 | 1.000 | 1.000 | 1.000 | 0.977 | 1.000 | 1.000 | 0.801 | 1.000 | 1.000 | 0.868 | 0.841 |
| 3712 | 0.829 | 1.000 | 0.870 | 1.000 | 0.979 | 1.000 | 0.895 | 1.000 | 1.000 | 1.000 | 0.977 | 1.000 | 1.000 | 0.801 | 1.000 | 1.000 | 0.868 | 0.841 |
| 3713 | 0.829 | 1.000 | 0.870 | 1.000 | 0.979 | 1.000 | 0.895 | 1.000 | 1.000 | 1.000 | 0.977 | 1.000 | 1.000 | 0.801 | 1.000 | 1.000 | 0.868 | 0.842 |
| 3714 | 0.829 | 1.000 | 0.870 | 1.000 | 0.979 | 1.000 | 0.895 | 1.000 | 1.000 | 1.000 | 0.977 | 1.000 | 1.000 | 0.801 | 1.000 | 1.000 | 0.868 | 0.842 |
| 3715 | 0.829 | 1.000 | 0.870 | 1.000 | 0.979 | 1.000 | 0.895 | 1.000 | 1.000 | 1.000 | 0.977 | 1.000 | 1.000 | 0.801 | 1.000 | 1.000 | 0.868 | 0.841 |
| 3716 | 0.829 | 1.000 | 0.870 | 1.000 | 0.979 | 1.000 | 0.895 | 1.000 | 1.000 | 1.000 | 0.977 | 1.000 | 1.000 | 0.801 | 1.000 | 1.000 | 0.868 | 0.841 |
| 3717 | 0.829 | 1.000 | 0.870 | 1.000 | 0.979 | 1.000 | 0.895 | 1.000 | 1.000 | 1.000 | 0.977 | 1.000 | 1.000 | 0.801 | 1.000 | 1.000 | 0.868 | 0.841 |
| 3718 | 0.827 | 1.000 | 0.870 | 1.000 | 0.979 | 1.000 | 0.897 | 1.000 | 1.000 | 1.000 | 0.977 | 1.000 | 1.000 | 0.803 | 1.000 | 1.000 | 0.869 | 0.842 |
| 3719 | 0.827 | 1.000 | 0.870 | 1.000 | 0.979 | 1.000 | 0.897 | 1.000 | 1.000 | 1.000 | 0.977 | 1.000 | 1.000 | 0.803 | 1.000 | 1.000 | 0.869 | 0.842 |
| 3720 | 0.827 | 1.000 | 0.870 | 1.000 | 0.979 | 1.000 | 0.897 | 1.000 | 1.000 | 1.000 | 0.977 | 1.000 | 1.000 | 0.803 | 1.000 | 1.000 | 0.869 | 0.842 |
| 3721 | 0.827 | 1.000 | 0.870 | 1.000 | 0.979 | 1.000 | 0.897 | 1.000 | 1.000 | 1.000 | 0.977 | 1.000 | 1.000 | 0.803 | 1.000 | 1.000 | 0.869 | 0.842 |
| 3722 | 0.827 | 1.000 | 0.868 | 1.000 | 0.979 | 1.000 | 0.897 | 1.000 | 1.000 | 1.000 | 0.977 | 1.000 | 1.000 | 0.803 | 1.000 | 1.000 | 0.868 | 0.842 |
| 3723 | 0.827 | 1.000 | 0.868 | 1.000 | 0.979 | 1.000 | 0.897 | 1.000 | 1.000 | 1.000 | 0.977 | 1.000 | 1.000 | 0.804 | 1.000 | 1.000 | 0.869 | 0.842 |
| 3724 | 0.826 | 1.000 | 0.870 | 1.000 | 0.979 | 1.000 | 0.898 | 1.000 | 1.000 | 1.000 | 0.977 | 1.000 | 1.000 | 0.803 | 1.000 | 1.000 | 0.869 | 0.842 |
| 3725 | 0.828 | 1.000 | 0.870 | 1.000 | 0.979 | 1.000 | 0.898 | 1.000 | 1.000 | 1.000 | 0.977 | 1.000 | 1.000 | 0.803 | 1.000 | 1.000 | 0.869 | 0.842 |
| 3726 | 0.827 | 1.000 | 0.870 | 1.000 | 0.979 | 1.000 | 0.898 | 1.000 | 1.000 | 1.000 | 0.977 | 1.000 | 1.000 | 0.803 | 1.000 | 1.000 | 0.869 | 0.842 |
| 3727 | 0.829 | 1.000 | 0.870 | 1.000 | 0.979 | 1.000 | 0.898 | 1.000 | 1.000 | 1.000 | 0.977 | 1.000 | 1.000 | 0.803 | 1.000 | 1.000 | 0.869 | 0.843 |
| 3728 | 0.827 | 1.000 | 0.870 | 1.000 | 0.979 | 1.000 | 0.897 | 1.000 | 1.000 | 1.000 | 0.977 | 1.000 | 1.000 | 0.802 | 1.000 | 1.000 | 0.868 | 0.842 |
| 3729 | 0.826 | 1.000 | 0.870 | 1.000 | 0.979 | 1.000 | 0.894 | 1.000 | 1.000 | 1.000 | 0.977 | 1.000 | 1.000 | 0.802 | 1.000 | 1.000 | 0.868 | 0.841 |
| 3730 | 0.826 | 1.000 | 0.870 | 1.000 | 0.979 | 1.000 | 0.894 | 1.000 | 1.000 | 1.000 | 0.977 | 1.000 | 1.000 | 0.802 | 1.000 | 1.000 | 0.868 | 0.841 |
| 3731 | 0.826 | 1.000 | 0.870 | 1.000 | 0.979 | 1.000 | 0.894 | 1.000 | 1.000 | 1.000 | 0.977 | 1.000 | 1.000 | 0.802 | 1.000 | 1.000 | 0.868 | 0.841 |
| 3732 | 0.826 | 1.000 | 0.870 | 1.000 | 0.979 | 1.000 | 0.894 | 1.000 | 1.000 | 1.000 | 0.977 | 1.000 | 1.000 | 0.802 | 1.000 | 1.000 | 0.868 | 0.841 |
| 3733 | 0.826 | 1.000 | 0.870 | 1.000 | 0.979 | 1.000 | 0.894 | 1.000 | 1.000 | 1.000 | 0.977 | 1.000 | 1.000 | 0.802 | 1.000 | 1.000 | 0.868 | 0.841 |
| 3734 | 0.826 | 1.000 | 0.870 | 1.000 | 0.979 | 1.000 | 0.894 | 1.000 | 1.000 | 1.000 | 0.977 | 1.000 | 1.000 | 0.802 | 1.000 | 1.000 | 0.868 | 0.841 |
| 3735 | 0.826 | 1.000 | 0.870 | 1.000 | 0.979 | 1.000 | 0.895 | 1.000 | 1.000 | 1.000 | 0.977 | 1.000 | 1.000 | 0.802 | 1.000 | 1.000 | 0.868 | 0.841 |
| 3736 | 0.827 | 1.000 | 0.870 | 1.000 | 0.979 | 1.000 | 0.894 | 1.000 | 1.000 | 1.000 | 0.977 | 1.000 | 1.000 | 0.802 | 1.000 | 1.000 | 0.868 | 0.841 |
| 3737 | 0.825 | 1.000 | 0.870 | 1.000 | 0.979 | 1.000 | 0.895 | 1.000 | 1.000 | 1.000 | 0.977 | 1.000 | 1.000 | 0.802 | 1.000 | 1.000 | 0.868 | 0.841 |
| 3738 | 0.825 | 1.000 | 0.870 | 1.000 | 0.979 | 1.000 | 0.895 | 1.000 | 1.000 | 1.000 | 0.977 | 1.000 | 1.000 | 0.802 | 1.000 | 1.000 | 0.868 | 0.841 |
| 3739 | 0.825 | 1.000 | 0.870 | 1.000 | 0.979 | 1.000 | 0.895 | 1.000 | 1.000 | 1.000 | 0.977 | 1.000 | 1.000 | 0.802 | 1.000 | 1.000 | 0.868 | 0.841 |
| 3740 | 0.826 | 1.000 | 0.870 | 1.000 | 0.979 | 1.000 | 0.895 | 1.000 | 1.000 | 1.000 | 0.977 | 1.000 | 1.000 | 0.802 | 1.000 | 1.000 | 0.868 | 0.841 |
| 3741 | 0.826 | 1.000 | 0.870 | 1.000 | 0.979 | 1.000 | 0.895 | 1.000 | 1.000 | 1.000 | 0.977 | 1.000 | 1.000 | 0.802 | 1.000 | 1.000 | 0.868 | 0.841 |
| 3742 | 0.826 | 1.000 | 0.870 | 1.000 | 0.979 | 1.000 | 0.892 | 1.000 | 1.000 | 1.000 | 0.977 | 1.000 | 1.000 | 0.802 | 1.000 | 1.000 | 0.868 | 0.841 |
| 3743 | 0.826 | 1.000 | 0.870 | 1.000 | 0.979 | 1.000 | 0.892 | 1.000 | 1.000 | 1.000 | 0.977 | 1.000 | 1.000 | 0.803 | 1.000 | 1.000 | 0.868 | 0.841 |
| 3744 | 0.828 | 1.000 | 0.872 | 1.000 | 0.979 | 1.000 | 0.895 | 1.000 | 1.000 | 1.000 | 0.977 | 1.000 | 1.000 | 0.804 | 1.000 | 1.000 | 0.869 | 0.842 |
| 3745 | 0.828 | 1.000 | 0.872 | 1.000 | 0.979 | 1.000 | 0.895 | 1.000 | 1.000 | 1.000 | 0.977 | 1.000 | 1.000 | 0.804 | 1.000 | 1.000 | 0.869 | 0.842 |
| 3746 | 0.826 | 1.000 | 0.870 | 1.000 | 0.979 | 1.000 | 0.895 | 1.000 | 1.000 | 1.000 | 0.977 | 1.000 | 1.000 | 0.804 | 1.000 | 1.000 | 0.868 | 0.842 |
| 3747 | 0.826 | 1.000 | 0.870 | 1.000 | 0.979 | 1.000 | 0.895 | 1.000 | 1.000 | 1.000 | 0.977 | 1.000 | 1.000 | 0.804 | 1.000 | 1.000 | 0.868 | 0.842 |

| | | | | | | | | | | | | | | | | | | |
|---|---|---|---|---|---|---|---|---|---|---|---|---|---|---|---|---|---|---|
| 3748 | 0.826 | 1.000 | 0.872 | 1.000 | 0.979 | 1.000 | 0.895 | 1.000 | 1.000 | 1.000 | 0.977 | 1.000 | 1.000 | 0.804 | 1.000 | 1.000 | 0.869 | 0.842 |
| 3749 | 0.826 | 1.000 | 0.870 | 1.000 | 0.979 | 1.000 | 0.895 | 1.000 | 1.000 | 1.000 | 0.977 | 1.000 | 1.000 | 0.804 | 1.000 | 1.000 | 0.868 | 0.841 |
| 3750 | 0.826 | 1.000 | 0.870 | 1.000 | 0.979 | 1.000 | 0.895 | 1.000 | 1.000 | 1.000 | 0.977 | 1.000 | 1.000 | 0.804 | 1.000 | 1.000 | 0.868 | 0.842 |
| 3751 | 0.826 | 1.000 | 0.870 | 1.000 | 0.979 | 1.000 | 0.895 | 1.000 | 1.000 | 1.000 | 0.977 | 1.000 | 1.000 | 0.804 | 1.000 | 1.000 | 0.868 | 0.842 |
| 3752 | 0.826 | 1.000 | 0.870 | 1.000 | 0.979 | 1.000 | 0.895 | 1.000 | 1.000 | 1.000 | 0.977 | 1.000 | 1.000 | 0.804 | 1.000 | 1.000 | 0.868 | 0.841 |
| 3753 | 0.826 | 1.000 | 0.870 | 1.000 | 0.979 | 1.000 | 0.895 | 1.000 | 1.000 | 1.000 | 0.977 | 1.000 | 1.000 | 0.804 | 1.000 | 1.000 | 0.868 | 0.841 |
| 3754 | 0.826 | 1.000 | 0.870 | 1.000 | 0.979 | 1.000 | 0.895 | 1.000 | 1.000 | 1.000 | 0.977 | 1.000 | 1.000 | 0.804 | 1.000 | 1.000 | 0.868 | 0.841 |
| 3755 | 0.826 | 1.000 | 0.872 | 1.000 | 0.979 | 1.000 | 0.895 | 1.000 | 1.000 | 1.000 | 0.977 | 1.000 | 1.000 | 0.803 | 1.000 | 1.000 | 0.868 | 0.841 |
| 3756 | 0.826 | 1.000 | 0.870 | 1.000 | 0.979 | 1.000 | 0.895 | 1.000 | 1.000 | 1.000 | 0.977 | 1.000 | 1.000 | 0.803 | 1.000 | 1.000 | 0.868 | 0.841 |
| 3757 | 0.826 | 1.000 | 0.872 | 1.000 | 0.979 | 1.000 | 0.895 | 1.000 | 1.000 | 1.000 | 0.977 | 1.000 | 1.000 | 0.804 | 1.000 | 1.000 | 0.868 | 0.842 |
| 3758 | 0.826 | 1.000 | 0.872 | 1.000 | 0.979 | 1.000 | 0.895 | 1.000 | 1.000 | 1.000 | 0.977 | 1.000 | 1.000 | 0.803 | 1.000 | 1.000 | 0.868 | 0.841 |
| 3759 | 0.827 | 1.000 | 0.870 | 1.000 | 0.979 | 1.000 | 0.895 | 1.000 | 1.000 | 1.000 | 0.977 | 1.000 | 1.000 | 0.803 | 1.000 | 1.000 | 0.868 | 0.842 |
| 3760 | 0.827 | 1.000 | 0.870 | 1.000 | 0.979 | 1.000 | 0.894 | 1.000 | 1.000 | 1.000 | 0.977 | 1.000 | 1.000 | 0.803 | 1.000 | 1.000 | 0.868 | 0.841 |
| 3761 | 0.826 | 1.000 | 0.870 | 1.000 | 0.979 | 1.000 | 0.894 | 1.000 | 1.000 | 1.000 | 0.977 | 1.000 | 1.000 | 0.803 | 1.000 | 1.000 | 0.868 | 0.841 |
| 3762 | 0.826 | 1.000 | 0.870 | 1.000 | 0.979 | 1.000 | 0.894 | 1.000 | 1.000 | 1.000 | 0.977 | 1.000 | 1.000 | 0.803 | 1.000 | 1.000 | 0.868 | 0.841 |
| 3763 | 0.826 | 1.000 | 0.870 | 1.000 | 0.979 | 1.000 | 0.894 | 1.000 | 1.000 | 1.000 | 0.977 | 1.000 | 1.000 | 0.803 | 1.000 | 1.000 | 0.868 | 0.841 |
| 3764 | 0.826 | 1.000 | 0.872 | 1.000 | 0.979 | 1.000 | 0.894 | 1.000 | 1.000 | 1.000 | 0.977 | 1.000 | 1.000 | 0.804 | 1.000 | 1.000 | 0.868 | 0.842 |
| 3765 | 0.827 | 1.000 | 0.872 | 1.000 | 0.979 | 1.000 | 0.894 | 1.000 | 1.000 | 1.000 | 0.977 | 1.000 | 1.000 | 0.804 | 1.000 | 1.000 | 0.869 | 0.842 |
| 3766 | 0.828 | 1.000 | 0.872 | 1.000 | 0.979 | 1.000 | 0.894 | 1.000 | 1.000 | 1.000 | 0.977 | 1.000 | 1.000 | 0.804 | 1.000 | 1.000 | 0.869 | 0.842 |
| 3767 | 0.826 | 1.000 | 0.872 | 1.000 | 0.979 | 1.000 | 0.894 | 1.000 | 1.000 | 1.000 | 0.977 | 1.000 | 1.000 | 0.804 | 1.000 | 1.000 | 0.868 | 0.842 |
| 3768 | 0.828 | 1.000 | 0.872 | 1.000 | 0.979 | 1.000 | 0.894 | 1.000 | 1.000 | 1.000 | 0.977 | 1.000 | 1.000 | 0.804 | 1.000 | 1.000 | 0.869 | 0.842 |
| 3769 | 0.828 | 1.000 | 0.872 | 1.000 | 0.979 | 1.000 | 0.894 | 1.000 | 1.000 | 1.000 | 0.977 | 1.000 | 1.000 | 0.804 | 1.000 | 1.000 | 0.869 | 0.842 |
| 3770 | 0.828 | 1.000 | 0.874 | 1.000 | 0.979 | 1.000 | 0.894 | 1.000 | 1.000 | 1.000 | 0.977 | 1.000 | 1.000 | 0.804 | 1.000 | 1.000 | 0.869 | 0.842 |
| 3771 | 0.828 | 1.000 | 0.874 | 1.000 | 0.979 | 1.000 | 0.894 | 1.000 | 1.000 | 1.000 | 0.977 | 1.000 | 1.000 | 0.804 | 1.000 | 1.000 | 0.869 | 0.842 |
| 3772 | 0.829 | 1.000 | 0.874 | 1.000 | 0.979 | 1.000 | 0.894 | 1.000 | 1.000 | 1.000 | 0.977 | 1.000 | 1.000 | 0.804 | 1.000 | 1.000 | 0.869 | 0.843 |
| 3773 | 0.827 | 1.000 | 0.874 | 1.000 | 0.979 | 1.000 | 0.894 | 1.000 | 1.000 | 1.000 | 0.980 | 1.000 | 1.000 | 0.806 | 1.000 | 1.000 | 0.870 | 0.843 |
| 3774 | 0.826 | 1.000 | 0.872 | 1.000 | 0.979 | 1.000 | 0.894 | 1.000 | 1.000 | 1.000 | 0.980 | 1.000 | 1.000 | 0.805 | 1.000 | 1.000 | 0.869 | 0.842 |
| 3775 | 0.827 | 1.000 | 0.872 | 1.000 | 0.979 | 1.000 | 0.894 | 1.000 | 1.000 | 1.000 | 0.980 | 1.000 | 1.000 | 0.805 | 1.000 | 1.000 | 0.869 | 0.843 |
| 3776 | 0.827 | 1.000 | 0.872 | 1.000 | 0.979 | 1.000 | 0.894 | 1.000 | 1.000 | 1.000 | 0.980 | 1.000 | 1.000 | 0.805 | 1.000 | 1.000 | 0.869 | 0.843 |
| 3777 | 0.827 | 1.000 | 0.872 | 1.000 | 0.979 | 1.000 | 0.894 | 1.000 | 1.000 | 1.000 | 0.980 | 1.000 | 1.000 | 0.805 | 1.000 | 1.000 | 0.869 | 0.843 |
| 3778 | 0.827 | 1.000 | 0.872 | 1.000 | 0.979 | 1.000 | 0.894 | 1.000 | 1.000 | 1.000 | 0.980 | 1.000 | 1.000 | 0.805 | 1.000 | 1.000 | 0.869 | 0.843 |
| 3779 | 0.827 | 1.000 | 0.872 | 1.000 | 0.979 | 1.000 | 0.894 | 1.000 | 1.000 | 1.000 | 0.980 | 1.000 | 1.000 | 0.806 | 1.000 | 1.000 | 0.869 | 0.843 |
| 3780 | 0.826 | 1.000 | 0.872 | 1.000 | 0.979 | 1.000 | 0.894 | 1.000 | 1.000 | 1.000 | 0.980 | 1.000 | 1.000 | 0.805 | 1.000 | 1.000 | 0.869 | 0.842 |
| 3781 | 0.826 | 1.000 | 0.872 | 1.000 | 0.979 | 1.000 | 0.894 | 1.000 | 1.000 | 1.000 | 0.980 | 1.000 | 1.000 | 0.805 | 1.000 | 1.000 | 0.869 | 0.842 |
| 3782 | 0.826 | 1.000 | 0.872 | 1.000 | 0.979 | 1.000 | 0.894 | 1.000 | 1.000 | 1.000 | 0.980 | 1.000 | 1.000 | 0.805 | 1.000 | 1.000 | 0.869 | 0.842 |
| 3783 | 0.826 | 1.000 | 0.872 | 1.000 | 0.979 | 1.000 | 0.894 | 1.000 | 1.000 | 1.000 | 0.980 | 1.000 | 1.000 | 0.805 | 1.000 | 1.000 | 0.869 | 0.842 |
| 3784 | 0.829 | 1.000 | 0.872 | 1.000 | 0.979 | 1.000 | 0.894 | 1.000 | 1.000 | 1.000 | 0.980 | 1.000 | 1.000 | 0.805 | 1.000 | 1.000 | 0.870 | 0.843 |
| 3785 | 0.829 | 1.000 | 0.870 | 1.000 | 0.979 | 1.000 | 0.894 | 1.000 | 1.000 | 1.000 | 0.980 | 1.000 | 1.000 | 0.805 | 1.000 | 1.000 | 0.869 | 0.843 |
| 3786 | 0.829 | 1.000 | 0.872 | 1.000 | 0.979 | 1.000 | 0.894 | 1.000 | 1.000 | 1.000 | 0.980 | 1.000 | 1.000 | 0.805 | 1.000 | 1.000 | 0.870 | 0.843 |

| | | | | | | | | | | | | | | | | | | |
|---|---|---|---|---|---|---|---|---|---|---|---|---|---|---|---|---|---|---|
| 3787 | 0.827 | 1.000 | 0.870 | 1.000 | 0.979 | 1.000 | 0.894 | 1.000 | 1.000 | 1.000 | 0.980 | 1.000 | 1.000 | 0.805 | 1.000 | 1.000 | 0.869 | 0.842 |
| 3788 | 0.828 | 1.000 | 0.872 | 1.000 | 0.979 | 1.000 | 0.895 | 1.000 | 1.000 | 1.000 | 0.980 | 1.000 | 1.000 | 0.803 | 1.000 | 1.000 | 0.869 | 0.842 |
| 3789 | 0.829 | 1.000 | 0.872 | 1.000 | 0.979 | 1.000 | 0.895 | 1.000 | 1.000 | 1.000 | 0.980 | 1.000 | 1.000 | 0.804 | 1.000 | 1.000 | 0.869 | 0.843 |
| 3790 | 0.829 | 1.000 | 0.872 | 1.000 | 0.979 | 1.000 | 0.895 | 1.000 | 1.000 | 1.000 | 0.980 | 1.000 | 1.000 | 0.804 | 1.000 | 1.000 | 0.869 | 0.843 |
| 3791 | 0.829 | 1.000 | 0.872 | 1.000 | 0.979 | 1.000 | 0.895 | 1.000 | 1.000 | 1.000 | 0.980 | 1.000 | 1.000 | 0.804 | 1.000 | 1.000 | 0.869 | 0.843 |
| 3792 | 0.829 | 1.000 | 0.872 | 1.000 | 0.979 | 1.000 | 0.895 | 1.000 | 1.000 | 1.000 | 0.980 | 1.000 | 1.000 | 0.803 | 1.000 | 1.000 | 0.869 | 0.843 |
| 3793 | 0.828 | 1.000 | 0.872 | 1.000 | 0.979 | 1.000 | 0.895 | 1.000 | 1.000 | 1.000 | 0.980 | 1.000 | 1.000 | 0.803 | 1.000 | 1.000 | 0.869 | 0.842 |
| 3794 | 0.828 | 1.000 | 0.870 | 1.000 | 0.979 | 1.000 | 0.895 | 1.000 | 1.000 | 1.000 | 0.980 | 1.000 | 1.000 | 0.803 | 1.000 | 1.000 | 0.869 | 0.842 |
| 3795 | 0.828 | 1.000 | 0.870 | 1.000 | 0.979 | 1.000 | 0.895 | 1.000 | 1.000 | 1.000 | 0.980 | 1.000 | 1.000 | 0.803 | 1.000 | 1.000 | 0.869 | 0.842 |
| 3796 | 0.829 | 1.000 | 0.870 | 1.000 | 0.979 | 1.000 | 0.895 | 1.000 | 1.000 | 1.000 | 0.980 | 1.000 | 1.000 | 0.805 | 1.000 | 1.000 | 0.869 | 0.843 |
| 3797 | 0.829 | 1.000 | 0.870 | 1.000 | 0.979 | 1.000 | 0.895 | 1.000 | 1.000 | 1.000 | 0.980 | 1.000 | 1.000 | 0.805 | 1.000 | 1.000 | 0.870 | 0.843 |
| 3798 | 0.829 | 1.000 | 0.870 | 1.000 | 0.979 | 1.000 | 0.895 | 1.000 | 1.000 | 1.000 | 0.980 | 1.000 | 1.000 | 0.804 | 1.000 | 1.000 | 0.869 | 0.843 |
| 3799 | 0.829 | 1.000 | 0.870 | 1.000 | 0.979 | 1.000 | 0.895 | 1.000 | 1.000 | 1.000 | 0.980 | 1.000 | 1.000 | 0.804 | 1.000 | 1.000 | 0.869 | 0.843 |
| 3800 | 0.829 | 1.000 | 0.870 | 1.000 | 0.979 | 1.000 | 0.894 | 1.000 | 1.000 | 1.000 | 0.980 | 1.000 | 1.000 | 0.804 | 1.000 | 1.000 | 0.869 | 0.843 |
| 3801 | 0.829 | 1.000 | 0.870 | 1.000 | 0.979 | 1.000 | 0.894 | 1.000 | 1.000 | 1.000 | 0.980 | 1.000 | 1.000 | 0.804 | 1.000 | 1.000 | 0.869 | 0.842 |
| 3802 | 0.829 | 1.000 | 0.870 | 1.000 | 0.979 | 1.000 | 0.894 | 1.000 | 1.000 | 1.000 | 0.980 | 1.000 | 1.000 | 0.804 | 1.000 | 1.000 | 0.869 | 0.842 |
| 3803 | 0.828 | 1.000 | 0.870 | 1.000 | 0.979 | 1.000 | 0.894 | 1.000 | 1.000 | 1.000 | 0.980 | 1.000 | 1.000 | 0.805 | 1.000 | 1.000 | 0.869 | 0.842 |
| 3804 | 0.828 | 1.000 | 0.870 | 1.000 | 0.979 | 1.000 | 0.894 | 1.000 | 1.000 | 1.000 | 0.980 | 1.000 | 1.000 | 0.805 | 1.000 | 1.000 | 0.869 | 0.842 |
| 3805 | 0.829 | 1.000 | 0.870 | 1.000 | 0.979 | 1.000 | 0.894 | 1.000 | 1.000 | 1.000 | 0.980 | 1.000 | 1.000 | 0.805 | 1.000 | 1.000 | 0.869 | 0.843 |
| 3806 | 0.828 | 1.000 | 0.870 | 1.000 | 0.979 | 1.000 | 0.891 | 1.000 | 1.000 | 1.000 | 0.980 | 1.000 | 1.000 | 0.805 | 1.000 | 1.000 | 0.869 | 0.842 |
| 3807 | 0.826 | 1.000 | 0.868 | 1.000 | 0.979 | 1.000 | 0.891 | 1.000 | 1.000 | 1.000 | 0.980 | 1.000 | 1.000 | 0.808 | 1.000 | 1.000 | 0.869 | 0.842 |
| 3808 | 0.824 | 1.000 | 0.868 | 1.000 | 0.979 | 1.000 | 0.891 | 1.000 | 1.000 | 1.000 | 0.980 | 1.000 | 1.000 | 0.808 | 1.000 | 1.000 | 0.868 | 0.841 |
| 3809 | 0.825 | 1.000 | 0.868 | 1.000 | 0.979 | 1.000 | 0.891 | 1.000 | 1.000 | 1.000 | 0.980 | 1.000 | 1.000 | 0.808 | 1.000 | 1.000 | 0.868 | 0.842 |
| 3810 | 0.825 | 1.000 | 0.866 | 1.000 | 0.979 | 1.000 | 0.891 | 1.000 | 1.000 | 1.000 | 0.980 | 1.000 | 1.000 | 0.808 | 1.000 | 1.000 | 0.868 | 0.841 |
| 3811 | 0.826 | 1.000 | 0.870 | 1.000 | 0.979 | 1.000 | 0.891 | 1.000 | 1.000 | 1.000 | 0.980 | 1.000 | 1.000 | 0.809 | 1.000 | 1.000 | 0.869 | 0.843 |
| 3812 | 0.828 | 1.000 | 0.870 | 1.000 | 0.979 | 1.000 | 0.891 | 1.000 | 1.000 | 1.000 | 0.980 | 1.000 | 1.000 | 0.808 | 1.000 | 1.000 | 0.869 | 0.843 |
| 3813 | 0.826 | 1.000 | 0.870 | 1.000 | 0.979 | 1.000 | 0.892 | 1.000 | 1.000 | 1.000 | 0.980 | 1.000 | 1.000 | 0.808 | 1.000 | 1.000 | 0.869 | 0.842 |
| 3814 | 0.825 | 1.000 | 0.870 | 1.000 | 0.979 | 1.000 | 0.892 | 1.000 | 1.000 | 1.000 | 0.980 | 1.000 | 1.000 | 0.808 | 1.000 | 1.000 | 0.869 | 0.842 |
| 3815 | 0.829 | 1.000 | 0.868 | 1.000 | 0.979 | 1.000 | 0.892 | 1.000 | 1.000 | 1.000 | 0.980 | 1.000 | 1.000 | 0.808 | 1.000 | 1.000 | 0.870 | 0.843 |
| 3816 | 0.829 | 1.000 | 0.866 | 1.000 | 0.979 | 1.000 | 0.892 | 1.000 | 1.000 | 1.000 | 0.980 | 1.000 | 1.000 | 0.807 | 1.000 | 1.000 | 0.869 | 0.842 |
| 3817 | 0.828 | 1.000 | 0.866 | 1.000 | 0.979 | 1.000 | 0.892 | 1.000 | 1.000 | 1.000 | 0.980 | 1.000 | 1.000 | 0.807 | 1.000 | 1.000 | 0.869 | 0.842 |
| 3818 | 0.829 | 1.000 | 0.868 | 1.000 | 0.979 | 1.000 | 0.892 | 1.000 | 1.000 | 1.000 | 0.980 | 1.000 | 1.000 | 0.807 | 1.000 | 1.000 | 0.869 | 0.843 |
| 3819 | 0.828 | 1.000 | 0.866 | 1.000 | 0.979 | 1.000 | 0.891 | 1.000 | 1.000 | 1.000 | 0.980 | 1.000 | 1.000 | 0.807 | 1.000 | 1.000 | 0.869 | 0.842 |
| 3820 | 0.829 | 1.000 | 0.866 | 1.000 | 0.979 | 1.000 | 0.891 | 1.000 | 1.000 | 1.000 | 0.977 | 1.000 | 1.000 | 0.807 | 1.000 | 1.000 | 0.869 | 0.842 |
| 3821 | 0.829 | 1.000 | 0.866 | 1.000 | 0.979 | 1.000 | 0.889 | 1.000 | 1.000 | 1.000 | 0.977 | 1.000 | 1.000 | 0.807 | 1.000 | 1.000 | 0.869 | 0.842 |
| 3822 | 0.831 | 1.000 | 0.870 | 1.000 | 0.979 | 1.000 | 0.891 | 1.000 | 1.000 | 1.000 | 0.977 | 1.000 | 1.000 | 0.807 | 1.000 | 1.000 | 0.870 | 0.843 |
| 3823 | 0.831 | 1.000 | 0.870 | 1.000 | 0.979 | 1.000 | 0.891 | 1.000 | 1.000 | 1.000 | 0.977 | 1.000 | 1.000 | 0.807 | 1.000 | 1.000 | 0.870 | 0.843 |
| 3824 | 0.831 | 1.000 | 0.870 | 1.000 | 0.979 | 1.000 | 0.891 | 1.000 | 1.000 | 1.000 | 0.977 | 1.000 | 1.000 | 0.806 | 1.000 | 1.000 | 0.870 | 0.843 |
| 3825 | 0.830 | 1.000 | 0.870 | 1.000 | 0.979 | 1.000 | 0.889 | 1.000 | 1.000 | 1.000 | 0.977 | 1.000 | 1.000 | 0.808 | 1.000 | 1.000 | 0.870 | 0.843 |

| | | | | | | | | | | | | | | | | | | |
|---|---|---|---|---|---|---|---|---|---|---|---|---|---|---|---|---|---|---|
| 3826 | 0.829 | 1.000 | 0.870 | 1.000 | 0.979 | 1.000 | 0.889 | 1.000 | 1.000 | 1.000 | 0.977 | 1.000 | 1.000 | 0.808 | 1.000 | 1.000 | 0.869 | 0.843 |
| 3827 | 0.830 | 1.000 | 0.870 | 1.000 | 0.979 | 1.000 | 0.889 | 1.000 | 1.000 | 1.000 | 0.977 | 1.000 | 1.000 | 0.808 | 1.000 | 1.000 | 0.870 | 0.843 |
| 3828 | 0.830 | 1.000 | 0.870 | 1.000 | 0.979 | 1.000 | 0.891 | 1.000 | 1.000 | 1.000 | 0.977 | 1.000 | 1.000 | 0.808 | 1.000 | 1.000 | 0.870 | 0.843 |
| 3829 | 0.829 | 1.000 | 0.868 | 1.000 | 0.979 | 1.000 | 0.889 | 1.000 | 1.000 | 1.000 | 0.977 | 1.000 | 1.000 | 0.808 | 1.000 | 1.000 | 0.869 | 0.842 |
| 3830 | 0.829 | 1.000 | 0.868 | 1.000 | 0.979 | 1.000 | 0.889 | 1.000 | 1.000 | 1.000 | 0.977 | 1.000 | 1.000 | 0.808 | 1.000 | 1.000 | 0.869 | 0.842 |
| 3831 | 0.829 | 1.000 | 0.868 | 1.000 | 0.979 | 1.000 | 0.889 | 1.000 | 1.000 | 1.000 | 0.977 | 1.000 | 1.000 | 0.808 | 1.000 | 1.000 | 0.869 | 0.842 |
| 3832 | 0.829 | 1.000 | 0.868 | 1.000 | 0.979 | 1.000 | 0.889 | 1.000 | 1.000 | 1.000 | 0.977 | 1.000 | 1.000 | 0.808 | 1.000 | 1.000 | 0.869 | 0.842 |
| 3833 | 0.829 | 1.000 | 0.868 | 1.000 | 0.979 | 1.000 | 0.889 | 1.000 | 1.000 | 1.000 | 0.977 | 1.000 | 1.000 | 0.807 | 1.000 | 1.000 | 0.869 | 0.842 |
| 3834 | 0.829 | 1.000 | 0.868 | 1.000 | 0.979 | 1.000 | 0.889 | 1.000 | 1.000 | 1.000 | 0.977 | 1.000 | 1.000 | 0.807 | 1.000 | 1.000 | 0.869 | 0.842 |
| 3835 | 0.829 | 1.000 | 0.868 | 1.000 | 0.979 | 1.000 | 0.889 | 1.000 | 1.000 | 1.000 | 0.977 | 1.000 | 1.000 | 0.808 | 1.000 | 1.000 | 0.869 | 0.842 |
| 3836 | 0.829 | 1.000 | 0.868 | 1.000 | 0.979 | 1.000 | 0.889 | 1.000 | 1.000 | 1.000 | 0.977 | 1.000 | 1.000 | 0.807 | 1.000 | 1.000 | 0.869 | 0.842 |
| 3837 | 0.829 | 1.000 | 0.868 | 1.000 | 0.979 | 1.000 | 0.889 | 1.000 | 1.000 | 1.000 | 0.977 | 1.000 | 1.000 | 0.807 | 1.000 | 1.000 | 0.869 | 0.842 |
| 3838 | 0.829 | 1.000 | 0.870 | 1.000 | 0.979 | 1.000 | 0.889 | 1.000 | 1.000 | 1.000 | 0.977 | 1.000 | 1.000 | 0.809 | 1.000 | 1.000 | 0.870 | 0.843 |
| 3839 | 0.830 | 1.000 | 0.870 | 1.000 | 0.979 | 1.000 | 0.889 | 1.000 | 1.000 | 1.000 | 0.977 | 1.000 | 1.000 | 0.809 | 1.000 | 1.000 | 0.870 | 0.843 |
| 3840 | 0.830 | 1.000 | 0.870 | 1.000 | 0.979 | 1.000 | 0.889 | 1.000 | 1.000 | 1.000 | 0.977 | 1.000 | 1.000 | 0.809 | 1.000 | 1.000 | 0.870 | 0.843 |
| 3841 | 0.830 | 1.000 | 0.870 | 1.000 | 0.979 | 1.000 | 0.889 | 1.000 | 1.000 | 1.000 | 0.977 | 1.000 | 1.000 | 0.809 | 1.000 | 1.000 | 0.870 | 0.843 |
| 3842 | 0.830 | 1.000 | 0.872 | 1.000 | 0.979 | 1.000 | 0.889 | 1.000 | 1.000 | 1.000 | 0.977 | 1.000 | 1.000 | 0.809 | 1.000 | 1.000 | 0.870 | 0.843 |
| 3843 | 0.830 | 1.000 | 0.870 | 1.000 | 0.979 | 1.000 | 0.889 | 1.000 | 1.000 | 1.000 | 0.977 | 1.000 | 1.000 | 0.809 | 1.000 | 1.000 | 0.870 | 0.843 |
| 3844 | 0.831 | 1.000 | 0.872 | 1.000 | 0.979 | 1.000 | 0.889 | 1.000 | 1.000 | 1.000 | 0.977 | 1.000 | 1.000 | 0.809 | 1.000 | 1.000 | 0.870 | 0.844 |
| 3845 | 0.833 | 1.000 | 0.870 | 1.000 | 0.979 | 1.000 | 0.891 | 1.000 | 1.000 | 1.000 | 0.977 | 1.000 | 1.000 | 0.809 | 1.000 | 1.000 | 0.871 | 0.845 |
| 3846 | 0.833 | 1.000 | 0.870 | 1.000 | 0.979 | 1.000 | 0.891 | 1.000 | 1.000 | 1.000 | 0.977 | 1.000 | 1.000 | 0.809 | 1.000 | 1.000 | 0.871 | 0.845 |
| 3847 | 0.833 | 1.000 | 0.870 | 1.000 | 0.979 | 1.000 | 0.892 | 1.000 | 1.000 | 1.000 | 0.977 | 1.000 | 1.000 | 0.809 | 1.000 | 1.000 | 0.871 | 0.845 |
| 3848 | 0.833 | 1.000 | 0.870 | 1.000 | 0.979 | 1.000 | 0.892 | 1.000 | 1.000 | 1.000 | 0.977 | 1.000 | 1.000 | 0.809 | 1.000 | 1.000 | 0.871 | 0.845 |
| 3849 | 0.833 | 1.000 | 0.872 | 1.000 | 0.979 | 1.000 | 0.892 | 1.000 | 1.000 | 1.000 | 0.977 | 1.000 | 1.000 | 0.809 | 1.000 | 1.000 | 0.871 | 0.845 |
| 3850 | 0.833 | 1.000 | 0.870 | 1.000 | 0.979 | 1.000 | 0.892 | 1.000 | 1.000 | 1.000 | 0.977 | 1.000 | 1.000 | 0.809 | 1.000 | 1.000 | 0.871 | 0.845 |
| 3851 | 0.833 | 1.000 | 0.870 | 1.000 | 0.979 | 1.000 | 0.892 | 1.000 | 1.000 | 1.000 | 0.977 | 1.000 | 1.000 | 0.809 | 1.000 | 1.000 | 0.871 | 0.845 |
| 3852 | 0.833 | 1.000 | 0.870 | 1.000 | 0.979 | 1.000 | 0.892 | 1.000 | 1.000 | 1.000 | 0.977 | 1.000 | 1.000 | 0.809 | 1.000 | 1.000 | 0.871 | 0.845 |
| 3853 | 0.833 | 1.000 | 0.870 | 1.000 | 0.979 | 1.000 | 0.892 | 1.000 | 1.000 | 1.000 | 0.977 | 1.000 | 1.000 | 0.809 | 1.000 | 1.000 | 0.871 | 0.845 |
| 3854 | 0.833 | 1.000 | 0.870 | 1.000 | 0.979 | 1.000 | 0.894 | 1.000 | 1.000 | 1.000 | 0.977 | 1.000 | 1.000 | 0.809 | 1.000 | 1.000 | 0.871 | 0.845 |
| 3855 | 0.831 | 1.000 | 0.868 | 1.000 | 0.979 | 1.000 | 0.891 | 1.000 | 1.000 | 1.000 | 0.977 | 1.000 | 1.000 | 0.810 | 1.000 | 1.000 | 0.871 | 0.844 |
| 3856 | 0.831 | 1.000 | 0.868 | 1.000 | 0.979 | 1.000 | 0.891 | 1.000 | 1.000 | 1.000 | 0.977 | 1.000 | 1.000 | 0.810 | 1.000 | 1.000 | 0.871 | 0.844 |
| 3857 | 0.831 | 1.000 | 0.868 | 1.000 | 0.979 | 1.000 | 0.891 | 1.000 | 1.000 | 1.000 | 0.977 | 1.000 | 1.000 | 0.810 | 1.000 | 1.000 | 0.871 | 0.844 |
| 3858 | 0.832 | 1.000 | 0.870 | 1.000 | 0.979 | 1.000 | 0.891 | 1.000 | 1.000 | 1.000 | 0.977 | 1.000 | 1.000 | 0.810 | 1.000 | 1.000 | 0.871 | 0.845 |
| 3859 | 0.832 | 1.000 | 0.870 | 1.000 | 0.979 | 1.000 | 0.891 | 1.000 | 1.000 | 1.000 | 0.977 | 1.000 | 1.000 | 0.810 | 1.000 | 1.000 | 0.871 | 0.845 |
| 3860 | 0.831 | 1.000 | 0.870 | 1.000 | 0.979 | 1.000 | 0.892 | 1.000 | 1.000 | 1.000 | 0.977 | 1.000 | 1.000 | 0.809 | 1.000 | 1.000 | 0.871 | 0.844 |
| 3861 | 0.831 | 1.000 | 0.870 | 1.000 | 0.979 | 1.000 | 0.888 | 1.000 | 1.000 | 1.000 | 0.977 | 1.000 | 1.000 | 0.807 | 1.000 | 1.000 | 0.869 | 0.843 |
| 3862 | 0.830 | 1.000 | 0.870 | 1.000 | 0.979 | 1.000 | 0.888 | 1.000 | 1.000 | 1.000 | 0.977 | 1.000 | 1.000 | 0.807 | 1.000 | 1.000 | 0.869 | 0.842 |
| 3863 | 0.831 | 1.000 | 0.868 | 1.000 | 0.979 | 1.000 | 0.888 | 1.000 | 1.000 | 1.000 | 0.980 | 1.000 | 1.000 | 0.809 | 1.000 | 1.000 | 0.870 | 0.843 |
| 3864 | 0.831 | 1.000 | 0.868 | 1.000 | 0.979 | 1.000 | 0.888 | 1.000 | 1.000 | 1.000 | 0.980 | 1.000 | 1.000 | 0.809 | 1.000 | 1.000 | 0.870 | 0.843 |

| | | | | | | | | | | | | | | | | | | |
|---|---|---|---|---|---|---|---|---|---|---|---|---|---|---|---|---|---|---|
| 3865 | 0.831 | 1.000 | 0.868 | 1.000 | 0.979 | 1.000 | 0.888 | 1.000 | 1.000 | 1.000 | 0.980 | 1.000 | 1.000 | 0.809 | 1.000 | 1.000 | 0.870 | 0.843 |
| 3866 | 0.831 | 1.000 | 0.868 | 1.000 | 0.979 | 1.000 | 0.888 | 1.000 | 1.000 | 1.000 | 0.980 | 1.000 | 1.000 | 0.809 | 1.000 | 1.000 | 0.870 | 0.843 |
| 3867 | 0.831 | 1.000 | 0.868 | 1.000 | 0.979 | 1.000 | 0.888 | 1.000 | 1.000 | 1.000 | 0.980 | 1.000 | 1.000 | 0.809 | 1.000 | 1.000 | 0.870 | 0.843 |
| 3868 | 0.831 | 1.000 | 0.870 | 1.000 | 0.979 | 1.000 | 0.888 | 1.000 | 1.000 | 1.000 | 0.980 | 1.000 | 1.000 | 0.809 | 1.000 | 1.000 | 0.870 | 0.844 |
| 3869 | 0.830 | 1.000 | 0.870 | 1.000 | 0.979 | 1.000 | 0.888 | 1.000 | 1.000 | 1.000 | 0.980 | 1.000 | 1.000 | 0.809 | 1.000 | 1.000 | 0.870 | 0.843 |
| 3870 | 0.830 | 1.000 | 0.870 | 1.000 | 0.979 | 1.000 | 0.888 | 1.000 | 1.000 | 1.000 | 0.980 | 1.000 | 1.000 | 0.809 | 1.000 | 1.000 | 0.870 | 0.843 |
| 3871 | 0.831 | 1.000 | 0.870 | 1.000 | 0.979 | 1.000 | 0.888 | 1.000 | 1.000 | 1.000 | 0.980 | 1.000 | 1.000 | 0.809 | 1.000 | 1.000 | 0.870 | 0.844 |
| 3872 | 0.831 | 1.000 | 0.870 | 1.000 | 0.979 | 1.000 | 0.888 | 1.000 | 1.000 | 1.000 | 0.980 | 1.000 | 1.000 | 0.809 | 1.000 | 1.000 | 0.870 | 0.844 |
| 3873 | 0.831 | 1.000 | 0.870 | 1.000 | 0.979 | 1.000 | 0.889 | 1.000 | 1.000 | 1.000 | 0.980 | 1.000 | 1.000 | 0.809 | 1.000 | 1.000 | 0.871 | 0.844 |
| 3874 | 0.831 | 1.000 | 0.870 | 1.000 | 0.979 | 1.000 | 0.889 | 1.000 | 1.000 | 1.000 | 0.980 | 1.000 | 1.000 | 0.809 | 1.000 | 1.000 | 0.871 | 0.844 |
| 3875 | 0.831 | 1.000 | 0.870 | 1.000 | 0.979 | 1.000 | 0.889 | 1.000 | 1.000 | 1.000 | 0.980 | 1.000 | 1.000 | 0.809 | 1.000 | 1.000 | 0.871 | 0.844 |
| 3876 | 0.832 | 1.000 | 0.870 | 1.000 | 0.979 | 1.000 | 0.889 | 1.000 | 1.000 | 1.000 | 0.980 | 1.000 | 1.000 | 0.809 | 1.000 | 1.000 | 0.871 | 0.844 |
| 3877 | 0.831 | 1.000 | 0.870 | 1.000 | 0.979 | 1.000 | 0.889 | 1.000 | 1.000 | 1.000 | 0.980 | 1.000 | 1.000 | 0.809 | 1.000 | 1.000 | 0.871 | 0.844 |
| 3878 | 0.831 | 1.000 | 0.870 | 1.000 | 0.979 | 1.000 | 0.889 | 1.000 | 1.000 | 1.000 | 0.980 | 1.000 | 1.000 | 0.809 | 1.000 | 1.000 | 0.871 | 0.844 |
| 3879 | 0.831 | 1.000 | 0.872 | 1.000 | 0.979 | 1.000 | 0.888 | 1.000 | 1.000 | 1.000 | 0.980 | 1.000 | 1.000 | 0.809 | 1.000 | 1.000 | 0.871 | 0.844 |
| 3880 | 0.831 | 1.000 | 0.872 | 1.000 | 0.979 | 1.000 | 0.889 | 1.000 | 1.000 | 1.000 | 0.980 | 1.000 | 1.000 | 0.809 | 1.000 | 1.000 | 0.871 | 0.844 |
| 3881 | 0.831 | 1.000 | 0.872 | 1.000 | 0.979 | 1.000 | 0.889 | 1.000 | 1.000 | 1.000 | 0.980 | 1.000 | 1.000 | 0.809 | 1.000 | 1.000 | 0.871 | 0.844 |
| 3882 | 0.831 | 1.000 | 0.872 | 1.000 | 0.979 | 1.000 | 0.889 | 1.000 | 1.000 | 1.000 | 0.980 | 1.000 | 1.000 | 0.809 | 1.000 | 1.000 | 0.871 | 0.844 |
| 3883 | 0.831 | 1.000 | 0.872 | 1.000 | 0.979 | 1.000 | 0.889 | 1.000 | 1.000 | 1.000 | 0.980 | 1.000 | 1.000 | 0.809 | 1.000 | 1.000 | 0.871 | 0.844 |
| 3884 | 0.831 | 1.000 | 0.872 | 1.000 | 0.979 | 1.000 | 0.889 | 1.000 | 1.000 | 1.000 | 0.980 | 1.000 | 1.000 | 0.809 | 1.000 | 1.000 | 0.871 | 0.844 |
| 3885 | 0.831 | 1.000 | 0.872 | 1.000 | 0.979 | 1.000 | 0.889 | 1.000 | 1.000 | 1.000 | 0.980 | 1.000 | 1.000 | 0.809 | 1.000 | 1.000 | 0.871 | 0.844 |
| 3886 | 0.831 | 1.000 | 0.870 | 1.000 | 0.979 | 1.000 | 0.889 | 1.000 | 1.000 | 1.000 | 0.980 | 1.000 | 1.000 | 0.809 | 1.000 | 1.000 | 0.870 | 0.844 |
| 3887 | 0.831 | 1.000 | 0.872 | 1.000 | 0.979 | 1.000 | 0.888 | 1.000 | 1.000 | 1.000 | 0.980 | 1.000 | 1.000 | 0.812 | 1.000 | 1.000 | 0.871 | 0.845 |
| 3888 | 0.831 | 1.000 | 0.872 | 1.000 | 0.979 | 1.000 | 0.888 | 1.000 | 1.000 | 1.000 | 0.980 | 1.000 | 1.000 | 0.812 | 1.000 | 1.000 | 0.871 | 0.845 |
| 3889 | 0.833 | 1.000 | 0.870 | 1.000 | 0.979 | 1.000 | 0.888 | 1.000 | 1.000 | 1.000 | 0.980 | 1.000 | 1.000 | 0.812 | 1.000 | 1.000 | 0.871 | 0.845 |
| 3890 | 0.833 | 1.000 | 0.870 | 1.000 | 0.979 | 1.000 | 0.888 | 1.000 | 1.000 | 1.000 | 0.980 | 1.000 | 1.000 | 0.812 | 1.000 | 1.000 | 0.871 | 0.845 |
| 3891 | 0.832 | 1.000 | 0.870 | 1.000 | 0.979 | 1.000 | 0.888 | 1.000 | 1.000 | 1.000 | 0.980 | 1.000 | 1.000 | 0.812 | 1.000 | 1.000 | 0.871 | 0.845 |
| 3892 | 0.833 | 1.000 | 0.870 | 1.000 | 0.979 | 1.000 | 0.888 | 1.000 | 1.000 | 1.000 | 0.980 | 1.000 | 1.000 | 0.812 | 1.000 | 1.000 | 0.871 | 0.845 |
| 3893 | 0.831 | 1.000 | 0.870 | 1.000 | 0.979 | 1.000 | 0.888 | 1.000 | 1.000 | 1.000 | 0.980 | 1.000 | 1.000 | 0.812 | 1.000 | 1.000 | 0.871 | 0.844 |
| 3894 | 0.832 | 1.000 | 0.870 | 1.000 | 0.979 | 1.000 | 0.888 | 1.000 | 1.000 | 1.000 | 0.980 | 1.000 | 1.000 | 0.812 | 1.000 | 1.000 | 0.871 | 0.845 |
| 3895 | 0.832 | 1.000 | 0.870 | 1.000 | 0.979 | 1.000 | 0.888 | 1.000 | 1.000 | 1.000 | 0.980 | 1.000 | 1.000 | 0.812 | 1.000 | 1.000 | 0.871 | 0.845 |
| 3896 | 0.831 | 1.000 | 0.870 | 1.000 | 0.979 | 1.000 | 0.888 | 1.000 | 1.000 | 1.000 | 0.980 | 1.000 | 1.000 | 0.812 | 1.000 | 1.000 | 0.871 | 0.844 |
| 3897 | 0.831 | 1.000 | 0.870 | 1.000 | 0.979 | 1.000 | 0.888 | 1.000 | 1.000 | 1.000 | 0.980 | 1.000 | 1.000 | 0.812 | 1.000 | 1.000 | 0.871 | 0.844 |
| 3898 | 0.831 | 1.000 | 0.870 | 1.000 | 0.979 | 1.000 | 0.888 | 1.000 | 1.000 | 1.000 | 0.977 | 1.000 | 1.000 | 0.812 | 1.000 | 1.000 | 0.871 | 0.844 |
| 3899 | 0.831 | 1.000 | 0.870 | 1.000 | 0.979 | 1.000 | 0.888 | 1.000 | 1.000 | 1.000 | 0.977 | 1.000 | 1.000 | 0.811 | 1.000 | 1.000 | 0.870 | 0.844 |
| 3900 | 0.833 | 1.000 | 0.870 | 1.000 | 0.979 | 1.000 | 0.888 | 1.000 | 1.000 | 1.000 | 0.977 | 1.000 | 1.000 | 0.812 | 1.000 | 1.000 | 0.871 | 0.845 |
| 3901 | 0.833 | 1.000 | 0.870 | 1.000 | 0.979 | 1.000 | 0.891 | 1.000 | 1.000 | 1.000 | 0.977 | 1.000 | 1.000 | 0.811 | 1.000 | 1.000 | 0.871 | 0.845 |
| 3902 | 0.833 | 1.000 | 0.870 | 1.000 | 0.979 | 1.000 | 0.891 | 1.000 | 1.000 | 1.000 | 0.977 | 1.000 | 1.000 | 0.810 | 1.000 | 1.000 | 0.871 | 0.845 |
| 3903 | 0.832 | 1.000 | 0.870 | 1.000 | 0.979 | 1.000 | 0.891 | 1.000 | 1.000 | 1.000 | 0.977 | 1.000 | 1.000 | 0.811 | 1.000 | 1.000 | 0.871 | 0.845 |

| | | | | | | | | | | | | | | | | | | |
|---|---|---|---|---|---|---|---|---|---|---|---|---|---|---|---|---|---|---|
| 3904 | 0.832 | 1.000 | 0.868 | 1.000 | 0.979 | 1.000 | 0.892 | 1.000 | 1.000 | 1.000 | 0.977 | 1.000 | 1.000 | 0.812 | 1.000 | 1.000 | 0.871 | 0.845 |
| 3905 | 0.833 | 1.000 | 0.870 | 1.000 | 0.979 | 1.000 | 0.892 | 1.000 | 1.000 | 1.000 | 0.980 | 1.000 | 1.000 | 0.811 | 1.000 | 1.000 | 0.872 | 0.845 |
| 3906 | 0.832 | 1.000 | 0.870 | 1.000 | 0.979 | 1.000 | 0.892 | 1.000 | 1.000 | 1.000 | 0.980 | 1.000 | 1.000 | 0.811 | 1.000 | 1.000 | 0.872 | 0.845 |
| 3907 | 0.832 | 1.000 | 0.870 | 1.000 | 0.979 | 1.000 | 0.892 | 1.000 | 1.000 | 1.000 | 0.980 | 1.000 | 1.000 | 0.811 | 1.000 | 1.000 | 0.872 | 0.845 |
| 3908 | 0.833 | 1.000 | 0.870 | 1.000 | 0.979 | 1.000 | 0.892 | 1.000 | 1.000 | 1.000 | 0.980 | 1.000 | 1.000 | 0.810 | 1.000 | 1.000 | 0.872 | 0.845 |
| 3909 | 0.833 | 1.000 | 0.870 | 1.000 | 0.979 | 1.000 | 0.892 | 1.000 | 1.000 | 1.000 | 0.980 | 1.000 | 1.000 | 0.810 | 1.000 | 1.000 | 0.872 | 0.845 |
| 3910 | 0.833 | 1.000 | 0.870 | 1.000 | 0.979 | 1.000 | 0.892 | 1.000 | 1.000 | 1.000 | 0.980 | 1.000 | 1.000 | 0.810 | 1.000 | 1.000 | 0.872 | 0.845 |
| 3911 | 0.833 | 1.000 | 0.870 | 1.000 | 0.979 | 1.000 | 0.892 | 1.000 | 1.000 | 1.000 | 0.980 | 1.000 | 1.000 | 0.810 | 1.000 | 1.000 | 0.872 | 0.845 |
| 3912 | 0.833 | 1.000 | 0.870 | 1.000 | 0.979 | 1.000 | 0.892 | 1.000 | 1.000 | 1.000 | 0.980 | 1.000 | 1.000 | 0.810 | 1.000 | 1.000 | 0.872 | 0.845 |
| 3913 | 0.833 | 1.000 | 0.870 | 1.000 | 0.979 | 1.000 | 0.892 | 1.000 | 1.000 | 1.000 | 0.980 | 1.000 | 1.000 | 0.810 | 1.000 | 1.000 | 0.872 | 0.845 |
| 3914 | 0.833 | 1.000 | 0.870 | 1.000 | 0.979 | 1.000 | 0.892 | 1.000 | 1.000 | 1.000 | 0.980 | 1.000 | 1.000 | 0.810 | 1.000 | 1.000 | 0.872 | 0.845 |
| 3915 | 0.833 | 1.000 | 0.870 | 1.000 | 0.979 | 1.000 | 0.892 | 1.000 | 1.000 | 1.000 | 0.980 | 1.000 | 1.000 | 0.811 | 1.000 | 1.000 | 0.872 | 0.846 |
| 3916 | 0.833 | 1.000 | 0.870 | 1.000 | 0.979 | 1.000 | 0.892 | 1.000 | 1.000 | 1.000 | 0.980 | 1.000 | 1.000 | 0.811 | 1.000 | 1.000 | 0.872 | 0.846 |
| 3917 | 0.833 | 1.000 | 0.870 | 1.000 | 0.979 | 1.000 | 0.892 | 1.000 | 1.000 | 1.000 | 0.980 | 1.000 | 1.000 | 0.810 | 1.000 | 1.000 | 0.872 | 0.845 |
| 3918 | 0.833 | 1.000 | 0.870 | 1.000 | 0.979 | 1.000 | 0.892 | 1.000 | 1.000 | 1.000 | 0.980 | 1.000 | 1.000 | 0.811 | 1.000 | 1.000 | 0.872 | 0.846 |
| 3919 | 0.834 | 1.000 | 0.870 | 1.000 | 0.979 | 1.000 | 0.892 | 1.000 | 1.000 | 1.000 | 0.980 | 1.000 | 1.000 | 0.811 | 1.000 | 1.000 | 0.872 | 0.846 |
| 3920 | 0.834 | 1.000 | 0.874 | 1.000 | 0.979 | 1.000 | 0.892 | 1.000 | 1.000 | 1.000 | 0.980 | 1.000 | 1.000 | 0.810 | 1.000 | 1.000 | 0.872 | 0.846 |
| 3921 | 0.834 | 1.000 | 0.874 | 1.000 | 0.979 | 1.000 | 0.892 | 1.000 | 1.000 | 1.000 | 0.980 | 1.000 | 1.000 | 0.810 | 1.000 | 1.000 | 0.872 | 0.846 |
| 3922 | 0.833 | 1.000 | 0.874 | 1.000 | 0.979 | 1.000 | 0.892 | 1.000 | 1.000 | 1.000 | 0.980 | 1.000 | 1.000 | 0.810 | 1.000 | 1.000 | 0.872 | 0.846 |
| 3923 | 0.835 | 1.000 | 0.874 | 1.000 | 0.979 | 1.000 | 0.891 | 1.000 | 1.000 | 1.000 | 0.977 | 1.000 | 1.000 | 0.810 | 1.000 | 1.000 | 0.872 | 0.846 |
| 3924 | 0.834 | 1.000 | 0.874 | 1.000 | 0.979 | 1.000 | 0.891 | 1.000 | 1.000 | 1.000 | 0.977 | 1.000 | 1.000 | 0.810 | 1.000 | 1.000 | 0.872 | 0.846 |
| 3925 | 0.835 | 1.000 | 0.874 | 1.000 | 0.979 | 1.000 | 0.891 | 1.000 | 1.000 | 1.000 | 0.977 | 1.000 | 1.000 | 0.810 | 1.000 | 1.000 | 0.872 | 0.846 |
| 3926 | 0.835 | 1.000 | 0.874 | 1.000 | 0.979 | 1.000 | 0.891 | 1.000 | 1.000 | 1.000 | 0.977 | 1.000 | 1.000 | 0.810 | 1.000 | 1.000 | 0.872 | 0.846 |
| 3927 | 0.835 | 1.000 | 0.879 | 1.000 | 0.979 | 1.000 | 0.892 | 1.000 | 1.000 | 1.000 | 0.977 | 1.000 | 1.000 | 0.810 | 1.000 | 1.000 | 0.873 | 0.847 |
| 3928 | 0.835 | 1.000 | 0.879 | 1.000 | 0.979 | 1.000 | 0.892 | 1.000 | 1.000 | 1.000 | 0.977 | 1.000 | 1.000 | 0.811 | 1.000 | 1.000 | 0.873 | 0.847 |
| 3929 | 0.834 | 1.000 | 0.877 | 1.000 | 0.979 | 1.000 | 0.892 | 1.000 | 1.000 | 1.000 | 0.977 | 1.000 | 1.000 | 0.811 | 1.000 | 1.000 | 0.873 | 0.847 |
| 3930 | 0.835 | 1.000 | 0.877 | 1.000 | 0.979 | 1.000 | 0.892 | 1.000 | 1.000 | 1.000 | 0.977 | 1.000 | 1.000 | 0.811 | 1.000 | 1.000 | 0.873 | 0.847 |
| 3931 | 0.835 | 1.000 | 0.879 | 1.000 | 0.979 | 1.000 | 0.892 | 1.000 | 1.000 | 1.000 | 0.977 | 1.000 | 1.000 | 0.811 | 1.000 | 1.000 | 0.873 | 0.847 |
| 3932 | 0.835 | 1.000 | 0.879 | 1.000 | 0.979 | 1.000 | 0.892 | 1.000 | 1.000 | 1.000 | 0.977 | 1.000 | 1.000 | 0.811 | 1.000 | 1.000 | 0.873 | 0.847 |
| 3933 | 0.835 | 1.000 | 0.877 | 1.000 | 0.979 | 1.000 | 0.892 | 1.000 | 1.000 | 1.000 | 0.977 | 1.000 | 1.000 | 0.811 | 1.000 | 1.000 | 0.873 | 0.847 |
| 3934 | 0.835 | 1.000 | 0.877 | 1.000 | 0.979 | 1.000 | 0.891 | 1.000 | 1.000 | 1.000 | 0.977 | 1.000 | 1.000 | 0.812 | 1.000 | 1.000 | 0.873 | 0.847 |
| 3935 | 0.835 | 1.000 | 0.877 | 1.000 | 0.979 | 1.000 | 0.891 | 1.000 | 1.000 | 1.000 | 0.977 | 1.000 | 1.000 | 0.812 | 1.000 | 1.000 | 0.873 | 0.847 |
| 3936 | 0.834 | 1.000 | 0.872 | 1.000 | 0.979 | 1.000 | 0.894 | 1.000 | 1.000 | 1.000 | 0.977 | 1.000 | 1.000 | 0.812 | 1.000 | 1.000 | 0.873 | 0.846 |
| 3937 | 0.834 | 1.000 | 0.874 | 1.000 | 0.979 | 1.000 | 0.892 | 1.000 | 1.000 | 1.000 | 0.977 | 1.000 | 1.000 | 0.812 | 1.000 | 1.000 | 0.873 | 0.846 |
| 3938 | 0.834 | 1.000 | 0.872 | 1.000 | 0.979 | 1.000 | 0.892 | 1.000 | 1.000 | 1.000 | 0.977 | 1.000 | 1.000 | 0.812 | 1.000 | 1.000 | 0.872 | 0.846 |
| 3939 | 0.834 | 1.000 | 0.872 | 1.000 | 0.979 | 1.000 | 0.892 | 1.000 | 1.000 | 1.000 | 0.977 | 1.000 | 1.000 | 0.812 | 1.000 | 1.000 | 0.872 | 0.846 |
| 3940 | 0.834 | 1.000 | 0.874 | 1.000 | 0.979 | 1.000 | 0.894 | 1.000 | 1.000 | 1.000 | 0.977 | 1.000 | 1.000 | 0.812 | 1.000 | 1.000 | 0.873 | 0.847 |
| 3941 | 0.835 | 1.000 | 0.874 | 1.000 | 0.979 | 1.000 | 0.892 | 1.000 | 1.000 | 1.000 | 0.977 | 1.000 | 1.000 | 0.810 | 1.000 | 1.000 | 0.873 | 0.846 |
| 3942 | 0.835 | 1.000 | 0.874 | 1.000 | 0.979 | 1.000 | 0.892 | 1.000 | 1.000 | 1.000 | 0.977 | 1.000 | 1.000 | 0.810 | 1.000 | 1.000 | 0.873 | 0.846 |

| | | | | | | | | | | | | | | | | | | |
|---|---|---|---|---|---|---|---|---|---|---|---|---|---|---|---|---|---|---|
| 3943 | 0.834 | 1.000 | 0.874 | 1.000 | 0.979 | 1.000 | 0.892 | 1.000 | 1.000 | 1.000 | 0.977 | 1.000 | 1.000 | 0.810 | 1.000 | 1.000 | 0.872 | 0.846 |
| 3944 | 0.834 | 1.000 | 0.874 | 1.000 | 0.979 | 1.000 | 0.892 | 1.000 | 1.000 | 1.000 | 0.977 | 1.000 | 1.000 | 0.810 | 1.000 | 1.000 | 0.872 | 0.846 |
| 3945 | 0.835 | 1.000 | 0.874 | 1.000 | 0.979 | 1.000 | 0.892 | 1.000 | 1.000 | 1.000 | 0.977 | 1.000 | 1.000 | 0.811 | 1.000 | 1.000 | 0.873 | 0.846 |
| 3946 | 0.835 | 1.000 | 0.872 | 1.000 | 0.979 | 1.000 | 0.892 | 1.000 | 1.000 | 1.000 | 0.977 | 1.000 | 1.000 | 0.811 | 1.000 | 1.000 | 0.872 | 0.846 |
| 3947 | 0.834 | 1.000 | 0.874 | 1.000 | 0.979 | 1.000 | 0.892 | 1.000 | 1.000 | 1.000 | 0.977 | 1.000 | 1.000 | 0.811 | 1.000 | 1.000 | 0.872 | 0.846 |
| 3948 | 0.834 | 1.000 | 0.872 | 1.000 | 0.979 | 1.000 | 0.892 | 1.000 | 1.000 | 1.000 | 0.977 | 1.000 | 1.000 | 0.811 | 1.000 | 1.000 | 0.872 | 0.846 |
| 3949 | 0.834 | 1.000 | 0.872 | 1.000 | 0.979 | 1.000 | 0.892 | 1.000 | 1.000 | 1.000 | 0.977 | 1.000 | 1.000 | 0.811 | 1.000 | 1.000 | 0.872 | 0.846 |
| 3950 | 0.834 | 1.000 | 0.872 | 1.000 | 0.979 | 1.000 | 0.892 | 1.000 | 1.000 | 1.000 | 0.977 | 1.000 | 1.000 | 0.812 | 1.000 | 1.000 | 0.872 | 0.846 |
| 3951 | 0.835 | 1.000 | 0.872 | 1.000 | 0.979 | 1.000 | 0.892 | 1.000 | 1.000 | 1.000 | 0.977 | 1.000 | 1.000 | 0.812 | 1.000 | 1.000 | 0.873 | 0.847 |
| 3952 | 0.835 | 1.000 | 0.872 | 1.000 | 0.979 | 1.000 | 0.892 | 1.000 | 1.000 | 1.000 | 0.977 | 1.000 | 1.000 | 0.812 | 1.000 | 1.000 | 0.873 | 0.847 |
| 3953 | 0.835 | 1.000 | 0.874 | 1.000 | 0.979 | 1.000 | 0.892 | 1.000 | 1.000 | 1.000 | 0.980 | 1.000 | 1.000 | 0.812 | 1.000 | 1.000 | 0.873 | 0.847 |
| 3954 | 0.835 | 1.000 | 0.874 | 1.000 | 0.979 | 1.000 | 0.892 | 1.000 | 1.000 | 1.000 | 0.980 | 1.000 | 1.000 | 0.812 | 1.000 | 1.000 | 0.873 | 0.847 |
| 3955 | 0.835 | 1.000 | 0.872 | 1.000 | 0.979 | 1.000 | 0.892 | 1.000 | 1.000 | 1.000 | 0.980 | 1.000 | 1.000 | 0.812 | 1.000 | 1.000 | 0.873 | 0.847 |
| 3956 | 0.835 | 1.000 | 0.874 | 1.000 | 0.979 | 1.000 | 0.892 | 1.000 | 1.000 | 1.000 | 0.980 | 1.000 | 1.000 | 0.811 | 1.000 | 1.000 | 0.873 | 0.847 |
| 3957 | 0.834 | 1.000 | 0.872 | 1.000 | 0.979 | 1.000 | 0.889 | 1.000 | 1.000 | 1.000 | 0.980 | 1.000 | 1.000 | 0.813 | 1.000 | 1.000 | 0.873 | 0.846 |
| 3958 | 0.832 | 1.000 | 0.870 | 1.000 | 0.979 | 1.000 | 0.889 | 1.000 | 1.000 | 1.000 | 0.980 | 1.000 | 1.000 | 0.812 | 1.000 | 1.000 | 0.872 | 0.845 |
| 3959 | 0.833 | 1.000 | 0.870 | 1.000 | 0.979 | 1.000 | 0.889 | 1.000 | 1.000 | 1.000 | 0.980 | 1.000 | 1.000 | 0.815 | 1.000 | 1.000 | 0.872 | 0.846 |
| 3960 | 0.829 | 1.000 | 0.870 | 1.000 | 0.979 | 1.000 | 0.889 | 1.000 | 1.000 | 1.000 | 0.980 | 1.000 | 1.000 | 0.815 | 1.000 | 1.000 | 0.871 | 0.845 |
| 3961 | 0.829 | 1.000 | 0.870 | 1.000 | 0.979 | 1.000 | 0.889 | 1.000 | 1.000 | 1.000 | 0.980 | 1.000 | 1.000 | 0.812 | 1.000 | 1.000 | 0.871 | 0.844 |
| 3962 | 0.829 | 1.000 | 0.870 | 1.000 | 0.979 | 1.000 | 0.889 | 1.000 | 1.000 | 1.000 | 0.980 | 1.000 | 1.000 | 0.812 | 1.000 | 1.000 | 0.871 | 0.844 |
| 3963 | 0.829 | 1.000 | 0.870 | 1.000 | 0.979 | 1.000 | 0.889 | 1.000 | 1.000 | 1.000 | 0.980 | 1.000 | 1.000 | 0.813 | 1.000 | 1.000 | 0.871 | 0.844 |
| 3964 | 0.829 | 1.000 | 0.870 | 1.000 | 0.979 | 1.000 | 0.889 | 1.000 | 1.000 | 1.000 | 0.980 | 1.000 | 1.000 | 0.812 | 1.000 | 1.000 | 0.871 | 0.844 |
| 3965 | 0.831 | 1.000 | 0.872 | 1.000 | 0.979 | 1.000 | 0.889 | 1.000 | 1.000 | 1.000 | 0.980 | 1.000 | 1.000 | 0.811 | 1.000 | 1.000 | 0.871 | 0.844 |
| 3966 | 0.831 | 1.000 | 0.872 | 1.000 | 0.979 | 1.000 | 0.889 | 1.000 | 1.000 | 1.000 | 0.980 | 1.000 | 1.000 | 0.812 | 1.000 | 1.000 | 0.871 | 0.845 |
| 3967 | 0.830 | 1.000 | 0.872 | 1.000 | 0.979 | 1.000 | 0.889 | 1.000 | 1.000 | 1.000 | 0.980 | 1.000 | 1.000 | 0.813 | 1.000 | 1.000 | 0.871 | 0.845 |
| 3968 | 0.831 | 1.000 | 0.872 | 1.000 | 0.979 | 1.000 | 0.889 | 1.000 | 1.000 | 1.000 | 0.980 | 1.000 | 1.000 | 0.813 | 1.000 | 1.000 | 0.872 | 0.845 |
| 3969 | 0.830 | 1.000 | 0.872 | 1.000 | 0.979 | 1.000 | 0.889 | 1.000 | 1.000 | 1.000 | 0.980 | 1.000 | 1.000 | 0.815 | 1.000 | 1.000 | 0.872 | 0.845 |
| 3970 | 0.829 | 1.000 | 0.872 | 1.000 | 0.979 | 1.000 | 0.889 | 1.000 | 1.000 | 1.000 | 0.980 | 1.000 | 1.000 | 0.816 | 1.000 | 1.000 | 0.872 | 0.845 |
| 3971 | 0.830 | 1.000 | 0.872 | 1.000 | 0.979 | 1.000 | 0.892 | 1.000 | 1.000 | 1.000 | 0.980 | 1.000 | 1.000 | 0.818 | 1.000 | 1.000 | 0.873 | 0.847 |
| 3972 | 0.831 | 1.000 | 0.870 | 1.000 | 0.979 | 1.000 | 0.892 | 1.000 | 1.000 | 1.000 | 0.980 | 1.000 | 1.000 | 0.816 | 1.000 | 1.000 | 0.872 | 0.846 |
| 3973 | 0.831 | 1.000 | 0.872 | 1.000 | 0.979 | 1.000 | 0.892 | 1.000 | 1.000 | 1.000 | 0.980 | 1.000 | 1.000 | 0.816 | 1.000 | 1.000 | 0.873 | 0.846 |
| 3974 | 0.829 | 1.000 | 0.872 | 1.000 | 0.979 | 1.000 | 0.892 | 1.000 | 1.000 | 1.000 | 0.983 | 1.000 | 1.000 | 0.816 | 1.000 | 1.000 | 0.872 | 0.846 |
| 3975 | 0.829 | 1.000 | 0.872 | 1.000 | 0.979 | 1.000 | 0.892 | 1.000 | 1.000 | 1.000 | 0.983 | 1.000 | 1.000 | 0.816 | 1.000 | 1.000 | 0.872 | 0.846 |
| 3976 | 0.829 | 1.000 | 0.872 | 1.000 | 0.979 | 1.000 | 0.894 | 1.000 | 1.000 | 1.000 | 0.980 | 1.000 | 1.000 | 0.816 | 1.000 | 1.000 | 0.872 | 0.846 |
| 3977 | 0.831 | 1.000 | 0.870 | 1.000 | 0.979 | 1.000 | 0.897 | 1.000 | 1.000 | 1.000 | 0.980 | 1.000 | 1.000 | 0.815 | 1.000 | 1.000 | 0.873 | 0.847 |
| 3978 | 0.830 | 1.000 | 0.870 | 1.000 | 0.979 | 1.000 | 0.897 | 1.000 | 1.000 | 1.000 | 0.980 | 1.000 | 1.000 | 0.816 | 1.000 | 1.000 | 0.873 | 0.847 |
| 3979 | 0.829 | 1.000 | 0.874 | 1.000 | 0.979 | 1.000 | 0.897 | 1.000 | 1.000 | 1.000 | 0.980 | 1.000 | 1.000 | 0.818 | 1.000 | 1.000 | 0.874 | 0.848 |
| 3980 | 0.829 | 1.000 | 0.874 | 1.000 | 0.979 | 1.000 | 0.897 | 1.000 | 1.000 | 1.000 | 0.980 | 1.000 | 1.000 | 0.816 | 1.000 | 1.000 | 0.873 | 0.847 |
| 3981 | 0.829 | 1.000 | 0.874 | 1.000 | 0.979 | 1.000 | 0.897 | 1.000 | 1.000 | 1.000 | 0.980 | 1.000 | 1.000 | 0.816 | 1.000 | 1.000 | 0.873 | 0.847 |

| | | | | | | | | | | | | | | | | | | |
|---|---|---|---|---|---|---|---|---|---|---|---|---|---|---|---|---|---|---|
| 3982 | 0.829 | 1.000 | 0.874 | 1.000 | 0.979 | 1.000 | 0.897 | 1.000 | 1.000 | 1.000 | 0.980 | 1.000 | 1.000 | 0.816 | 1.000 | 1.000 | 0.873 | 0.847 |
| 3983 | 0.829 | 1.000 | 0.872 | 1.000 | 0.979 | 1.000 | 0.897 | 1.000 | 1.000 | 1.000 | 0.980 | 1.000 | 1.000 | 0.816 | 1.000 | 1.000 | 0.873 | 0.847 |
| 3984 | 0.829 | 1.000 | 0.872 | 1.000 | 0.979 | 1.000 | 0.897 | 1.000 | 1.000 | 1.000 | 0.980 | 1.000 | 1.000 | 0.816 | 1.000 | 1.000 | 0.873 | 0.847 |
| 3985 | 0.829 | 1.000 | 0.874 | 1.000 | 0.979 | 1.000 | 0.897 | 1.000 | 1.000 | 1.000 | 0.980 | 1.000 | 1.000 | 0.817 | 1.000 | 1.000 | 0.873 | 0.847 |
| 3986 | 0.829 | 1.000 | 0.875 | 1.000 | 0.979 | 1.000 | 0.897 | 1.000 | 1.000 | 1.000 | 0.980 | 1.000 | 1.000 | 0.819 | 1.000 | 1.000 | 0.874 | 0.848 |
| 3987 | 0.830 | 1.000 | 0.874 | 1.000 | 0.979 | 1.000 | 0.894 | 1.000 | 1.000 | 1.000 | 0.980 | 1.000 | 1.000 | 0.817 | 1.000 | 1.000 | 0.873 | 0.847 |
| 3988 | 0.830 | 1.000 | 0.874 | 1.000 | 0.979 | 1.000 | 0.894 | 1.000 | 1.000 | 1.000 | 0.980 | 1.000 | 1.000 | 0.818 | 1.000 | 1.000 | 0.873 | 0.847 |
| 3989 | 0.831 | 1.000 | 0.874 | 1.000 | 0.979 | 1.000 | 0.895 | 1.000 | 1.000 | 1.000 | 0.980 | 1.000 | 1.000 | 0.819 | 1.000 | 1.000 | 0.874 | 0.848 |
| 3990 | 0.831 | 1.000 | 0.874 | 1.000 | 0.979 | 1.000 | 0.895 | 1.000 | 1.000 | 1.000 | 0.980 | 1.000 | 1.000 | 0.819 | 1.000 | 1.000 | 0.874 | 0.848 |
| 3991 | 0.833 | 1.000 | 0.875 | 1.000 | 0.979 | 1.000 | 0.895 | 1.000 | 1.000 | 1.000 | 0.980 | 1.000 | 1.000 | 0.819 | 1.000 | 1.000 | 0.875 | 0.849 |
| 3992 | 0.832 | 1.000 | 0.874 | 1.000 | 0.979 | 1.000 | 0.895 | 1.000 | 1.000 | 1.000 | 0.980 | 1.000 | 1.000 | 0.818 | 1.000 | 1.000 | 0.874 | 0.848 |
| 3993 | 0.832 | 1.000 | 0.875 | 1.000 | 0.979 | 1.000 | 0.895 | 1.000 | 1.000 | 1.000 | 0.980 | 1.000 | 1.000 | 0.818 | 1.000 | 1.000 | 0.874 | 0.849 |
| 3994 | 0.832 | 1.000 | 0.875 | 1.000 | 0.979 | 1.000 | 0.895 | 1.000 | 1.000 | 1.000 | 0.980 | 1.000 | 1.000 | 0.818 | 1.000 | 1.000 | 0.874 | 0.849 |
| 3995 | 0.832 | 1.000 | 0.875 | 1.000 | 0.979 | 1.000 | 0.895 | 1.000 | 1.000 | 1.000 | 0.980 | 1.000 | 1.000 | 0.818 | 1.000 | 1.000 | 0.874 | 0.849 |
| 3996 | 0.832 | 1.000 | 0.875 | 1.000 | 0.979 | 1.000 | 0.895 | 1.000 | 1.000 | 1.000 | 0.983 | 1.000 | 1.000 | 0.816 | 1.000 | 1.000 | 0.874 | 0.848 |
| 3997 | 0.832 | 1.000 | 0.874 | 1.000 | 0.979 | 1.000 | 0.895 | 1.000 | 1.000 | 1.000 | 0.983 | 1.000 | 1.000 | 0.817 | 1.000 | 1.000 | 0.874 | 0.848 |
| 3998 | 0.832 | 1.000 | 0.874 | 1.000 | 0.979 | 1.000 | 0.895 | 1.000 | 1.000 | 1.000 | 0.983 | 1.000 | 1.000 | 0.816 | 1.000 | 1.000 | 0.874 | 0.848 |
| 3999 | 0.833 | 1.000 | 0.874 | 1.000 | 0.979 | 1.000 | 0.895 | 1.000 | 1.000 | 1.000 | 0.983 | 1.000 | 1.000 | 0.816 | 1.000 | 1.000 | 0.874 | 0.848 |
| 4000 | 0.833 | 1.000 | 0.874 | 1.000 | 0.979 | 1.000 | 0.897 | 1.000 | 1.000 | 1.000 | 0.983 | 1.000 | 1.000 | 0.816 | 1.000 | 1.000 | 0.874 | 0.849 |
| 4001 | 0.833 | 1.000 | 0.874 | 1.000 | 0.979 | 1.000 | 0.895 | 1.000 | 1.000 | 1.000 | 0.983 | 1.000 | 1.000 | 0.815 | 1.000 | 1.000 | 0.874 | 0.848 |
| 4002 | 0.832 | 1.000 | 0.874 | 1.000 | 0.979 | 1.000 | 0.895 | 1.000 | 1.000 | 1.000 | 0.983 | 1.000 | 1.000 | 0.815 | 1.000 | 1.000 | 0.874 | 0.848 |
| 4003 | 0.832 | 1.000 | 0.874 | 1.000 | 0.979 | 1.000 | 0.895 | 1.000 | 1.000 | 1.000 | 0.983 | 1.000 | 1.000 | 0.816 | 1.000 | 1.000 | 0.874 | 0.848 |
| 4004 | 0.832 | 1.000 | 0.874 | 1.000 | 0.979 | 1.000 | 0.894 | 1.000 | 1.000 | 1.000 | 0.983 | 1.000 | 1.000 | 0.816 | 1.000 | 1.000 | 0.874 | 0.848 |
| 4005 | 0.832 | 1.000 | 0.874 | 1.000 | 0.979 | 1.000 | 0.894 | 1.000 | 1.000 | 1.000 | 0.983 | 1.000 | 1.000 | 0.816 | 1.000 | 1.000 | 0.874 | 0.848 |
| 4006 | 0.832 | 1.000 | 0.875 | 1.000 | 0.979 | 1.000 | 0.894 | 1.000 | 1.000 | 1.000 | 0.983 | 1.000 | 1.000 | 0.816 | 1.000 | 1.000 | 0.874 | 0.848 |
| 4007 | 0.832 | 1.000 | 0.874 | 1.000 | 0.979 | 1.000 | 0.894 | 1.000 | 1.000 | 1.000 | 0.983 | 1.000 | 1.000 | 0.816 | 1.000 | 1.000 | 0.874 | 0.848 |
| 4008 | 0.832 | 1.000 | 0.874 | 1.000 | 0.979 | 1.000 | 0.894 | 1.000 | 1.000 | 1.000 | 0.983 | 1.000 | 1.000 | 0.816 | 1.000 | 1.000 | 0.874 | 0.848 |
| 4009 | 0.831 | 1.000 | 0.874 | 1.000 | 0.979 | 1.000 | 0.895 | 1.000 | 1.000 | 1.000 | 0.983 | 1.000 | 1.000 | 0.815 | 1.000 | 1.000 | 0.873 | 0.847 |
| 4010 | 0.831 | 1.000 | 0.874 | 1.000 | 0.979 | 1.000 | 0.895 | 1.000 | 1.000 | 1.000 | 0.983 | 1.000 | 1.000 | 0.815 | 1.000 | 1.000 | 0.873 | 0.847 |
| 4011 | 0.831 | 1.000 | 0.874 | 1.000 | 0.979 | 1.000 | 0.895 | 1.000 | 1.000 | 1.000 | 0.983 | 1.000 | 1.000 | 0.815 | 1.000 | 1.000 | 0.873 | 0.847 |
| 4012 | 0.831 | 1.000 | 0.874 | 1.000 | 0.979 | 1.000 | 0.895 | 1.000 | 1.000 | 1.000 | 0.983 | 1.000 | 1.000 | 0.816 | 1.000 | 1.000 | 0.873 | 0.847 |
| 4013 | 0.833 | 1.000 | 0.874 | 1.000 | 0.979 | 1.000 | 0.894 | 1.000 | 1.000 | 1.000 | 0.983 | 1.000 | 1.000 | 0.816 | 1.000 | 1.000 | 0.874 | 0.848 |
| 4014 | 0.833 | 1.000 | 0.874 | 1.000 | 0.979 | 1.000 | 0.894 | 1.000 | 1.000 | 1.000 | 0.980 | 1.000 | 1.000 | 0.816 | 1.000 | 1.000 | 0.874 | 0.848 |
| 4015 | 0.833 | 1.000 | 0.874 | 1.000 | 0.979 | 1.000 | 0.894 | 1.000 | 1.000 | 1.000 | 0.980 | 1.000 | 1.000 | 0.815 | 1.000 | 1.000 | 0.874 | 0.848 |
| 4016 | 0.832 | 1.000 | 0.872 | 1.000 | 0.979 | 1.000 | 0.894 | 1.000 | 1.000 | 1.000 | 0.980 | 1.000 | 1.000 | 0.816 | 1.000 | 1.000 | 0.873 | 0.847 |
| 4017 | 0.832 | 1.000 | 0.872 | 1.000 | 0.979 | 1.000 | 0.894 | 1.000 | 1.000 | 1.000 | 0.980 | 1.000 | 1.000 | 0.816 | 1.000 | 1.000 | 0.873 | 0.847 |
| 4018 | 0.832 | 1.000 | 0.872 | 1.000 | 0.979 | 1.000 | 0.894 | 1.000 | 1.000 | 1.000 | 0.983 | 1.000 | 1.000 | 0.817 | 1.000 | 1.000 | 0.874 | 0.848 |
| 4019 | 0.833 | 1.000 | 0.872 | 1.000 | 0.979 | 1.000 | 0.895 | 1.000 | 1.000 | 1.000 | 0.983 | 1.000 | 1.000 | 0.816 | 1.000 | 1.000 | 0.874 | 0.848 |
| 4020 | 0.833 | 1.000 | 0.872 | 1.000 | 0.979 | 1.000 | 0.895 | 1.000 | 1.000 | 1.000 | 0.980 | 1.000 | 1.000 | 0.816 | 1.000 | 1.000 | 0.874 | 0.848 |

| | | | | | | | | | | | | | | | | | | |
|---|---|---|---|---|---|---|---|---|---|---|---|---|---|---|---|---|---|---|
| 4021 | 0.832 | 1.000 | 0.872 | 1.000 | 0.979 | 1.000 | 0.895 | 1.000 | 1.000 | 1.000 | 0.980 | 1.000 | 1.000 | 0.816 | 1.000 | 1.000 | 0.873 | 0.847 |
| 4022 | 0.832 | 1.000 | 0.872 | 1.000 | 0.979 | 1.000 | 0.895 | 1.000 | 1.000 | 1.000 | 0.980 | 1.000 | 1.000 | 0.816 | 1.000 | 1.000 | 0.873 | 0.847 |
| 4023 | 0.831 | 1.000 | 0.872 | 1.000 | 0.979 | 1.000 | 0.895 | 1.000 | 1.000 | 1.000 | 0.980 | 1.000 | 1.000 | 0.819 | 1.000 | 1.000 | 0.874 | 0.848 |
| 4024 | 0.830 | 1.000 | 0.872 | 1.000 | 0.979 | 1.000 | 0.895 | 1.000 | 1.000 | 1.000 | 0.980 | 1.000 | 1.000 | 0.818 | 1.000 | 1.000 | 0.873 | 0.847 |
| 4025 | 0.830 | 1.000 | 0.872 | 1.000 | 0.979 | 1.000 | 0.895 | 1.000 | 1.000 | 1.000 | 0.980 | 1.000 | 1.000 | 0.819 | 1.000 | 1.000 | 0.874 | 0.848 |
| 4026 | 0.831 | 1.000 | 0.872 | 1.000 | 0.979 | 1.000 | 0.895 | 1.000 | 1.000 | 1.000 | 0.980 | 1.000 | 1.000 | 0.819 | 1.000 | 1.000 | 0.874 | 0.848 |
| 4027 | 0.830 | 1.000 | 0.872 | 1.000 | 0.979 | 1.000 | 0.895 | 1.000 | 1.000 | 1.000 | 0.980 | 1.000 | 1.000 | 0.820 | 1.000 | 1.000 | 0.874 | 0.848 |
| 4028 | 0.831 | 1.000 | 0.872 | 1.000 | 0.979 | 1.000 | 0.895 | 1.000 | 1.000 | 1.000 | 0.980 | 1.000 | 1.000 | 0.819 | 1.000 | 1.000 | 0.874 | 0.848 |
| 4029 | 0.831 | 1.000 | 0.872 | 1.000 | 0.979 | 1.000 | 0.895 | 1.000 | 1.000 | 1.000 | 0.980 | 1.000 | 1.000 | 0.819 | 1.000 | 1.000 | 0.874 | 0.848 |
| 4030 | 0.833 | 1.000 | 0.872 | 1.000 | 0.979 | 1.000 | 0.895 | 1.000 | 1.000 | 1.000 | 0.980 | 1.000 | 1.000 | 0.819 | 1.000 | 1.000 | 0.874 | 0.848 |
| 4031 | 0.833 | 1.000 | 0.872 | 1.000 | 0.979 | 1.000 | 0.895 | 1.000 | 1.000 | 1.000 | 0.980 | 1.000 | 1.000 | 0.819 | 1.000 | 1.000 | 0.874 | 0.848 |
| 4032 | 0.832 | 1.000 | 0.872 | 1.000 | 0.979 | 1.000 | 0.895 | 1.000 | 1.000 | 1.000 | 0.980 | 1.000 | 1.000 | 0.819 | 1.000 | 1.000 | 0.874 | 0.848 |
| 4033 | 0.832 | 1.000 | 0.872 | 1.000 | 0.979 | 1.000 | 0.891 | 1.000 | 1.000 | 1.000 | 0.980 | 1.000 | 1.000 | 0.819 | 1.000 | 1.000 | 0.874 | 0.848 |
| 4034 | 0.832 | 1.000 | 0.872 | 1.000 | 0.979 | 1.000 | 0.891 | 1.000 | 1.000 | 1.000 | 0.980 | 1.000 | 1.000 | 0.819 | 1.000 | 1.000 | 0.874 | 0.848 |
| 4035 | 0.832 | 1.000 | 0.872 | 1.000 | 0.979 | 1.000 | 0.891 | 1.000 | 1.000 | 1.000 | 0.980 | 1.000 | 1.000 | 0.819 | 1.000 | 1.000 | 0.874 | 0.847 |
| 4036 | 0.831 | 1.000 | 0.874 | 1.000 | 0.979 | 1.000 | 0.894 | 1.000 | 1.000 | 1.000 | 0.980 | 1.000 | 1.000 | 0.819 | 1.000 | 1.000 | 0.874 | 0.848 |
| 4037 | 0.831 | 1.000 | 0.874 | 1.000 | 0.979 | 1.000 | 0.895 | 1.000 | 1.000 | 1.000 | 0.980 | 1.000 | 1.000 | 0.818 | 1.000 | 1.000 | 0.874 | 0.848 |
| 4038 | 0.831 | 1.000 | 0.874 | 1.000 | 0.979 | 1.000 | 0.895 | 1.000 | 1.000 | 1.000 | 0.980 | 1.000 | 1.000 | 0.818 | 1.000 | 1.000 | 0.874 | 0.848 |
| 4039 | 0.831 | 1.000 | 0.874 | 1.000 | 0.979 | 1.000 | 0.895 | 1.000 | 1.000 | 1.000 | 0.980 | 1.000 | 1.000 | 0.817 | 1.000 | 1.000 | 0.874 | 0.848 |
| 4040 | 0.831 | 1.000 | 0.874 | 1.000 | 0.979 | 1.000 | 0.894 | 1.000 | 1.000 | 1.000 | 0.980 | 1.000 | 1.000 | 0.818 | 1.000 | 1.000 | 0.874 | 0.848 |
| 4041 | 0.832 | 1.000 | 0.874 | 1.000 | 0.979 | 1.000 | 0.892 | 1.000 | 1.000 | 1.000 | 0.980 | 1.000 | 1.000 | 0.818 | 1.000 | 1.000 | 0.874 | 0.848 |
| 4042 | 0.832 | 1.000 | 0.874 | 1.000 | 0.979 | 1.000 | 0.892 | 1.000 | 1.000 | 1.000 | 0.980 | 1.000 | 1.000 | 0.817 | 1.000 | 1.000 | 0.874 | 0.848 |
| 4043 | 0.832 | 1.000 | 0.874 | 1.000 | 0.979 | 1.000 | 0.894 | 1.000 | 1.000 | 1.000 | 0.980 | 1.000 | 1.000 | 0.817 | 1.000 | 1.000 | 0.874 | 0.848 |
| 4044 | 0.832 | 1.000 | 0.874 | 1.000 | 0.979 | 1.000 | 0.894 | 1.000 | 1.000 | 1.000 | 0.980 | 1.000 | 1.000 | 0.817 | 1.000 | 1.000 | 0.874 | 0.848 |
| 4045 | 0.831 | 1.000 | 0.874 | 1.000 | 0.979 | 1.000 | 0.894 | 1.000 | 1.000 | 1.000 | 0.980 | 1.000 | 1.000 | 0.817 | 1.000 | 1.000 | 0.874 | 0.848 |
| 4046 | 0.832 | 1.000 | 0.874 | 1.000 | 0.979 | 1.000 | 0.895 | 1.000 | 1.000 | 1.000 | 0.980 | 1.000 | 1.000 | 0.817 | 1.000 | 1.000 | 0.874 | 0.848 |
| 4047 | 0.831 | 1.000 | 0.874 | 1.000 | 0.979 | 1.000 | 0.892 | 1.000 | 1.000 | 1.000 | 0.980 | 1.000 | 1.000 | 0.816 | 1.000 | 1.000 | 0.873 | 0.847 |
| 4048 | 0.831 | 1.000 | 0.874 | 1.000 | 0.979 | 1.000 | 0.892 | 1.000 | 1.000 | 1.000 | 0.980 | 1.000 | 1.000 | 0.816 | 1.000 | 1.000 | 0.873 | 0.847 |
| 4049 | 0.831 | 1.000 | 0.874 | 1.000 | 0.979 | 1.000 | 0.892 | 1.000 | 1.000 | 1.000 | 0.980 | 1.000 | 1.000 | 0.816 | 1.000 | 1.000 | 0.873 | 0.847 |
| 4050 | 0.831 | 1.000 | 0.874 | 1.000 | 0.979 | 1.000 | 0.892 | 1.000 | 1.000 | 1.000 | 0.980 | 1.000 | 1.000 | 0.816 | 1.000 | 1.000 | 0.873 | 0.847 |
| 4051 | 0.831 | 1.000 | 0.874 | 1.000 | 0.979 | 1.000 | 0.892 | 1.000 | 1.000 | 1.000 | 0.980 | 1.000 | 1.000 | 0.816 | 1.000 | 1.000 | 0.873 | 0.847 |
| 4052 | 0.831 | 1.000 | 0.872 | 1.000 | 0.979 | 1.000 | 0.892 | 1.000 | 1.000 | 1.000 | 0.977 | 1.000 | 1.000 | 0.818 | 1.000 | 1.000 | 0.873 | 0.847 |
| 4053 | 0.831 | 1.000 | 0.872 | 1.000 | 0.979 | 1.000 | 0.892 | 1.000 | 1.000 | 1.000 | 0.977 | 1.000 | 1.000 | 0.818 | 1.000 | 1.000 | 0.873 | 0.847 |
| 4054 | 0.831 | 1.000 | 0.872 | 1.000 | 0.979 | 1.000 | 0.892 | 1.000 | 1.000 | 1.000 | 0.977 | 1.000 | 1.000 | 0.818 | 1.000 | 1.000 | 0.873 | 0.847 |
| 4055 | 0.832 | 1.000 | 0.872 | 1.000 | 0.979 | 1.000 | 0.892 | 1.000 | 1.000 | 1.000 | 0.977 | 1.000 | 1.000 | 0.817 | 1.000 | 1.000 | 0.873 | 0.847 |
| 4056 | 0.832 | 1.000 | 0.872 | 1.000 | 0.979 | 1.000 | 0.892 | 1.000 | 1.000 | 1.000 | 0.980 | 1.000 | 1.000 | 0.817 | 1.000 | 1.000 | 0.873 | 0.847 |
| 4057 | 0.832 | 1.000 | 0.872 | 1.000 | 0.979 | 1.000 | 0.892 | 1.000 | 1.000 | 1.000 | 0.977 | 1.000 | 1.000 | 0.817 | 1.000 | 1.000 | 0.873 | 0.847 |
| 4058 | 0.831 | 1.000 | 0.872 | 1.000 | 0.979 | 1.000 | 0.892 | 1.000 | 1.000 | 1.000 | 0.977 | 1.000 | 1.000 | 0.817 | 1.000 | 1.000 | 0.873 | 0.847 |
| 4059 | 0.830 | 1.000 | 0.874 | 1.000 | 0.979 | 1.000 | 0.892 | 1.000 | 1.000 | 1.000 | 0.977 | 1.000 | 1.000 | 0.816 | 1.000 | 1.000 | 0.873 | 0.846 |

| | | | | | | | | | | | | | | | | | | |
|---|---|---|---|---|---|---|---|---|---|---|---|---|---|---|---|---|---|---|
| 4060 | 0.830 | 1.000 | 0.872 | 1.000 | 0.979 | 1.000 | 0.892 | 1.000 | 1.000 | 1.000 | 0.977 | 1.000 | 1.000 | 0.816 | 1.000 | 1.000 | 0.872 | 0.846 |
| 4061 | 0.830 | 1.000 | 0.874 | 1.000 | 0.979 | 1.000 | 0.892 | 1.000 | 1.000 | 1.000 | 0.977 | 1.000 | 1.000 | 0.819 | 1.000 | 1.000 | 0.873 | 0.847 |
| 4062 | 0.830 | 1.000 | 0.874 | 1.000 | 0.979 | 1.000 | 0.892 | 1.000 | 1.000 | 1.000 | 0.977 | 1.000 | 1.000 | 0.819 | 1.000 | 1.000 | 0.873 | 0.847 |
| 4063 | 0.830 | 1.000 | 0.874 | 1.000 | 0.979 | 1.000 | 0.892 | 1.000 | 1.000 | 1.000 | 0.977 | 1.000 | 1.000 | 0.820 | 1.000 | 1.000 | 0.874 | 0.848 |
| 4064 | 0.829 | 1.000 | 0.874 | 1.000 | 0.979 | 1.000 | 0.892 | 1.000 | 1.000 | 1.000 | 0.977 | 1.000 | 1.000 | 0.818 | 1.000 | 1.000 | 0.873 | 0.847 |
| 4065 | 0.829 | 1.000 | 0.875 | 1.000 | 0.979 | 1.000 | 0.894 | 1.000 | 1.000 | 1.000 | 0.977 | 1.000 | 1.000 | 0.819 | 1.000 | 1.000 | 0.873 | 0.847 |
| 4066 | 0.829 | 1.000 | 0.875 | 1.000 | 0.979 | 1.000 | 0.894 | 1.000 | 1.000 | 1.000 | 0.977 | 1.000 | 1.000 | 0.819 | 1.000 | 1.000 | 0.873 | 0.847 |
| 4067 | 0.829 | 1.000 | 0.877 | 1.000 | 0.979 | 1.000 | 0.894 | 1.000 | 1.000 | 1.000 | 0.977 | 1.000 | 1.000 | 0.818 | 1.000 | 1.000 | 0.873 | 0.847 |
| 4068 | 0.830 | 1.000 | 0.877 | 1.000 | 0.979 | 1.000 | 0.894 | 1.000 | 1.000 | 1.000 | 0.980 | 1.000 | 1.000 | 0.818 | 1.000 | 1.000 | 0.874 | 0.848 |
| 4069 | 0.830 | 1.000 | 0.877 | 1.000 | 0.979 | 1.000 | 0.894 | 1.000 | 1.000 | 1.000 | 0.977 | 1.000 | 1.000 | 0.818 | 1.000 | 1.000 | 0.874 | 0.848 |
| 4070 | 0.829 | 1.000 | 0.877 | 1.000 | 0.979 | 1.000 | 0.894 | 1.000 | 1.000 | 1.000 | 0.977 | 1.000 | 1.000 | 0.818 | 1.000 | 1.000 | 0.873 | 0.847 |
| 4071 | 0.829 | 1.000 | 0.875 | 1.000 | 0.979 | 1.000 | 0.894 | 1.000 | 1.000 | 1.000 | 0.977 | 1.000 | 1.000 | 0.817 | 1.000 | 1.000 | 0.873 | 0.847 |
| 4072 | 0.829 | 1.000 | 0.877 | 1.000 | 0.979 | 1.000 | 0.894 | 1.000 | 1.000 | 1.000 | 0.977 | 1.000 | 1.000 | 0.817 | 1.000 | 1.000 | 0.873 | 0.847 |
| 4073 | 0.830 | 1.000 | 0.875 | 1.000 | 0.979 | 1.000 | 0.894 | 1.000 | 1.000 | 1.000 | 0.977 | 1.000 | 1.000 | 0.817 | 1.000 | 1.000 | 0.873 | 0.847 |
| 4074 | 0.830 | 1.000 | 0.875 | 1.000 | 0.979 | 1.000 | 0.894 | 1.000 | 1.000 | 1.000 | 0.977 | 1.000 | 1.000 | 0.819 | 1.000 | 1.000 | 0.874 | 0.848 |
| 4075 | 0.830 | 1.000 | 0.874 | 1.000 | 0.979 | 1.000 | 0.894 | 1.000 | 1.000 | 1.000 | 0.977 | 1.000 | 1.000 | 0.819 | 1.000 | 1.000 | 0.873 | 0.847 |
| 4076 | 0.830 | 1.000 | 0.875 | 1.000 | 0.979 | 1.000 | 0.894 | 1.000 | 1.000 | 1.000 | 0.977 | 1.000 | 1.000 | 0.819 | 1.000 | 1.000 | 0.874 | 0.848 |
| 4077 | 0.831 | 1.000 | 0.875 | 1.000 | 0.979 | 1.000 | 0.894 | 1.000 | 1.000 | 1.000 | 0.980 | 1.000 | 1.000 | 0.819 | 1.000 | 1.000 | 0.874 | 0.848 |
| 4078 | 0.831 | 1.000 | 0.875 | 1.000 | 0.979 | 1.000 | 0.894 | 1.000 | 1.000 | 1.000 | 0.977 | 1.000 | 1.000 | 0.819 | 1.000 | 1.000 | 0.874 | 0.848 |
| 4079 | 0.831 | 1.000 | 0.875 | 1.000 | 0.979 | 1.000 | 0.894 | 1.000 | 1.000 | 1.000 | 0.977 | 1.000 | 1.000 | 0.819 | 1.000 | 1.000 | 0.874 | 0.848 |
| 4080 | 0.830 | 1.000 | 0.875 | 1.000 | 0.979 | 1.000 | 0.894 | 1.000 | 1.000 | 1.000 | 0.980 | 1.000 | 1.000 | 0.819 | 1.000 | 1.000 | 0.874 | 0.848 |
| 4081 | 0.830 | 1.000 | 0.875 | 1.000 | 0.979 | 1.000 | 0.894 | 1.000 | 1.000 | 1.000 | 0.977 | 1.000 | 1.000 | 0.819 | 1.000 | 1.000 | 0.874 | 0.848 |
| 4082 | 0.830 | 1.000 | 0.874 | 1.000 | 0.979 | 1.000 | 0.894 | 1.000 | 1.000 | 1.000 | 0.977 | 1.000 | 1.000 | 0.819 | 1.000 | 1.000 | 0.873 | 0.847 |
| 4083 | 0.831 | 1.000 | 0.875 | 1.000 | 0.979 | 1.000 | 0.894 | 1.000 | 1.000 | 1.000 | 0.977 | 1.000 | 1.000 | 0.819 | 1.000 | 1.000 | 0.874 | 0.848 |
| 4084 | 0.831 | 1.000 | 0.874 | 1.000 | 0.979 | 1.000 | 0.894 | 1.000 | 1.000 | 1.000 | 0.977 | 1.000 | 1.000 | 0.819 | 1.000 | 1.000 | 0.874 | 0.848 |
| 4085 | 0.831 | 1.000 | 0.872 | 1.000 | 0.979 | 1.000 | 0.894 | 1.000 | 1.000 | 1.000 | 0.977 | 1.000 | 1.000 | 0.818 | 1.000 | 1.000 | 0.873 | 0.847 |
| 4086 | 0.831 | 1.000 | 0.872 | 1.000 | 0.979 | 1.000 | 0.894 | 1.000 | 1.000 | 1.000 | 0.980 | 1.000 | 1.000 | 0.818 | 1.000 | 1.000 | 0.874 | 0.848 |
| 4087 | 0.831 | 1.000 | 0.872 | 1.000 | 0.979 | 1.000 | 0.894 | 1.000 | 1.000 | 1.000 | 0.977 | 1.000 | 1.000 | 0.819 | 1.000 | 1.000 | 0.874 | 0.847 |
| 4088 | 0.832 | 1.000 | 0.872 | 1.000 | 0.979 | 1.000 | 0.894 | 1.000 | 1.000 | 1.000 | 0.977 | 1.000 | 1.000 | 0.818 | 1.000 | 1.000 | 0.874 | 0.847 |
| 4089 | 0.832 | 1.000 | 0.874 | 1.000 | 0.979 | 1.000 | 0.894 | 1.000 | 1.000 | 1.000 | 0.977 | 1.000 | 1.000 | 0.818 | 1.000 | 1.000 | 0.874 | 0.848 |
| 4090 | 0.832 | 1.000 | 0.872 | 1.000 | 0.979 | 1.000 | 0.894 | 1.000 | 1.000 | 1.000 | 0.977 | 1.000 | 1.000 | 0.818 | 1.000 | 1.000 | 0.874 | 0.847 |
| 4091 | 0.832 | 1.000 | 0.874 | 1.000 | 0.979 | 1.000 | 0.894 | 1.000 | 1.000 | 1.000 | 0.977 | 1.000 | 1.000 | 0.818 | 1.000 | 1.000 | 0.874 | 0.848 |
| 4092 | 0.832 | 1.000 | 0.874 | 1.000 | 0.979 | 1.000 | 0.894 | 1.000 | 1.000 | 1.000 | 0.977 | 1.000 | 1.000 | 0.818 | 1.000 | 1.000 | 0.874 | 0.848 |
| 4093 | 0.832 | 1.000 | 0.874 | 1.000 | 0.979 | 1.000 | 0.894 | 1.000 | 1.000 | 1.000 | 0.977 | 1.000 | 1.000 | 0.818 | 1.000 | 1.000 | 0.874 | 0.848 |
| 4094 | 0.834 | 1.000 | 0.872 | 1.000 | 0.979 | 1.000 | 0.894 | 1.000 | 1.000 | 1.000 | 0.977 | 1.000 | 1.000 | 0.819 | 1.000 | 1.000 | 0.874 | 0.848 |
| 4095 | 0.833 | 1.000 | 0.874 | 1.000 | 0.979 | 1.000 | 0.895 | 1.000 | 1.000 | 1.000 | 0.980 | 1.000 | 1.000 | 0.817 | 1.000 | 1.000 | 0.874 | 0.848 |
| 4096 | 0.833 | 1.000 | 0.870 | 1.000 | 0.979 | 1.000 | 0.897 | 1.000 | 1.000 | 1.000 | 0.980 | 1.000 | 1.000 | 0.818 | 1.000 | 1.000 | 0.874 | 0.848 |
| 4097 | 0.833 | 1.000 | 0.870 | 1.000 | 0.979 | 1.000 | 0.897 | 1.000 | 1.000 | 1.000 | 0.980 | 1.000 | 1.000 | 0.817 | 1.000 | 1.000 | 0.874 | 0.848 |
| 4098 | 0.833 | 1.000 | 0.870 | 1.000 | 0.979 | 1.000 | 0.897 | 1.000 | 1.000 | 1.000 | 0.980 | 1.000 | 1.000 | 0.818 | 1.000 | 1.000 | 0.874 | 0.848 |

| | | | | | | | | | | | | | | | | | | |
|---|---|---|---|---|---|---|---|---|---|---|---|---|---|---|---|---|---|---|
| 4099 | 0.833 | 1.000 | 0.870 | 1.000 | 0.979 | 1.000 | 0.897 | 1.000 | 1.000 | 1.000 | 0.980 | 1.000 | 1.000 | 0.817 | 1.000 | 1.000 | 0.874 | 0.848 |
| 4100 | 0.833 | 1.000 | 0.870 | 1.000 | 0.979 | 1.000 | 0.897 | 1.000 | 1.000 | 1.000 | 0.980 | 1.000 | 1.000 | 0.817 | 1.000 | 1.000 | 0.874 | 0.848 |
| 4101 | 0.833 | 1.000 | 0.870 | 1.000 | 0.979 | 1.000 | 0.897 | 1.000 | 1.000 | 1.000 | 0.980 | 1.000 | 1.000 | 0.817 | 1.000 | 1.000 | 0.874 | 0.848 |
| 4102 | 0.833 | 1.000 | 0.870 | 1.000 | 0.979 | 1.000 | 0.897 | 1.000 | 1.000 | 1.000 | 0.980 | 1.000 | 1.000 | 0.817 | 1.000 | 1.000 | 0.874 | 0.848 |
| 4103 | 0.833 | 1.000 | 0.870 | 1.000 | 0.979 | 1.000 | 0.897 | 1.000 | 1.000 | 1.000 | 0.980 | 1.000 | 1.000 | 0.817 | 1.000 | 1.000 | 0.874 | 0.848 |
| 4104 | 0.833 | 1.000 | 0.870 | 1.000 | 0.979 | 1.000 | 0.897 | 1.000 | 1.000 | 1.000 | 0.980 | 1.000 | 1.000 | 0.817 | 1.000 | 1.000 | 0.874 | 0.848 |
| 4105 | 0.833 | 1.000 | 0.870 | 1.000 | 0.979 | 1.000 | 0.897 | 1.000 | 1.000 | 1.000 | 0.980 | 1.000 | 1.000 | 0.818 | 1.000 | 1.000 | 0.874 | 0.848 |
| 4106 | 0.832 | 1.000 | 0.870 | 1.000 | 0.979 | 1.000 | 0.897 | 1.000 | 1.000 | 1.000 | 0.980 | 1.000 | 1.000 | 0.818 | 1.000 | 1.000 | 0.874 | 0.848 |
| 4107 | 0.833 | 1.000 | 0.872 | 1.000 | 0.979 | 1.000 | 0.897 | 1.000 | 1.000 | 1.000 | 0.980 | 1.000 | 1.000 | 0.818 | 1.000 | 1.000 | 0.874 | 0.848 |
| 4108 | 0.832 | 1.000 | 0.870 | 1.000 | 0.979 | 1.000 | 0.895 | 1.000 | 1.000 | 1.000 | 0.980 | 1.000 | 1.000 | 0.818 | 1.000 | 1.000 | 0.874 | 0.848 |
| 4109 | 0.833 | 1.000 | 0.870 | 1.000 | 0.979 | 1.000 | 0.895 | 1.000 | 1.000 | 1.000 | 0.980 | 1.000 | 1.000 | 0.818 | 1.000 | 1.000 | 0.874 | 0.848 |
| 4110 | 0.833 | 1.000 | 0.870 | 1.000 | 0.979 | 1.000 | 0.894 | 1.000 | 1.000 | 1.000 | 0.980 | 1.000 | 1.000 | 0.818 | 1.000 | 1.000 | 0.874 | 0.848 |
| 4111 | 0.833 | 1.000 | 0.870 | 1.000 | 0.979 | 1.000 | 0.895 | 1.000 | 1.000 | 1.000 | 0.980 | 1.000 | 1.000 | 0.818 | 1.000 | 1.000 | 0.874 | 0.848 |
| 4112 | 0.832 | 1.000 | 0.872 | 1.000 | 0.979 | 1.000 | 0.895 | 1.000 | 1.000 | 1.000 | 0.980 | 1.000 | 1.000 | 0.819 | 1.000 | 1.000 | 0.874 | 0.848 |
| 4113 | 0.832 | 1.000 | 0.872 | 1.000 | 0.979 | 1.000 | 0.895 | 1.000 | 1.000 | 1.000 | 0.980 | 1.000 | 1.000 | 0.819 | 1.000 | 1.000 | 0.874 | 0.848 |
| 4114 | 0.832 | 1.000 | 0.872 | 1.000 | 0.979 | 1.000 | 0.895 | 1.000 | 1.000 | 1.000 | 0.980 | 1.000 | 1.000 | 0.819 | 1.000 | 1.000 | 0.874 | 0.848 |
| 4115 | 0.832 | 1.000 | 0.870 | 1.000 | 0.979 | 1.000 | 0.895 | 1.000 | 1.000 | 1.000 | 0.980 | 1.000 | 1.000 | 0.819 | 1.000 | 1.000 | 0.874 | 0.848 |
| 4116 | 0.833 | 1.000 | 0.870 | 1.000 | 0.979 | 1.000 | 0.895 | 1.000 | 1.000 | 1.000 | 0.980 | 1.000 | 1.000 | 0.820 | 1.000 | 1.000 | 0.875 | 0.849 |
| 4117 | 0.833 | 1.000 | 0.870 | 1.000 | 0.979 | 1.000 | 0.895 | 1.000 | 1.000 | 1.000 | 0.980 | 1.000 | 1.000 | 0.819 | 1.000 | 1.000 | 0.874 | 0.848 |
| 4118 | 0.833 | 1.000 | 0.872 | 1.000 | 0.979 | 1.000 | 0.895 | 1.000 | 1.000 | 1.000 | 0.980 | 1.000 | 1.000 | 0.820 | 1.000 | 1.000 | 0.875 | 0.849 |
| 4119 | 0.833 | 1.000 | 0.872 | 1.000 | 0.979 | 1.000 | 0.895 | 1.000 | 1.000 | 1.000 | 0.980 | 1.000 | 1.000 | 0.821 | 1.000 | 1.000 | 0.875 | 0.849 |
| 4120 | 0.833 | 1.000 | 0.872 | 1.000 | 0.979 | 1.000 | 0.895 | 1.000 | 1.000 | 1.000 | 0.980 | 1.000 | 1.000 | 0.819 | 1.000 | 1.000 | 0.875 | 0.849 |
| 4121 | 0.833 | 1.000 | 0.874 | 1.000 | 0.979 | 1.000 | 0.895 | 1.000 | 1.000 | 1.000 | 0.980 | 1.000 | 1.000 | 0.819 | 1.000 | 1.000 | 0.875 | 0.849 |
| 4122 | 0.834 | 1.000 | 0.874 | 1.000 | 0.979 | 1.000 | 0.894 | 1.000 | 1.000 | 1.000 | 0.980 | 1.000 | 1.000 | 0.819 | 1.000 | 1.000 | 0.875 | 0.849 |
| 4123 | 0.833 | 1.000 | 0.874 | 1.000 | 0.979 | 1.000 | 0.894 | 1.000 | 1.000 | 1.000 | 0.980 | 1.000 | 1.000 | 0.819 | 1.000 | 1.000 | 0.875 | 0.849 |
| 4124 | 0.833 | 1.000 | 0.872 | 1.000 | 0.979 | 1.000 | 0.895 | 1.000 | 1.000 | 1.000 | 0.977 | 1.000 | 1.000 | 0.819 | 1.000 | 1.000 | 0.874 | 0.848 |
| 4125 | 0.832 | 1.000 | 0.870 | 1.000 | 0.979 | 1.000 | 0.895 | 1.000 | 1.000 | 1.000 | 0.977 | 1.000 | 1.000 | 0.819 | 1.000 | 1.000 | 0.874 | 0.848 |
| 4126 | 0.833 | 1.000 | 0.872 | 1.000 | 0.979 | 1.000 | 0.895 | 1.000 | 1.000 | 1.000 | 0.977 | 1.000 | 1.000 | 0.819 | 1.000 | 1.000 | 0.874 | 0.848 |
| 4127 | 0.833 | 1.000 | 0.870 | 1.000 | 0.979 | 1.000 | 0.895 | 1.000 | 1.000 | 1.000 | 0.977 | 1.000 | 1.000 | 0.819 | 1.000 | 1.000 | 0.874 | 0.848 |
| 4128 | 0.832 | 1.000 | 0.870 | 1.000 | 0.979 | 1.000 | 0.895 | 1.000 | 1.000 | 1.000 | 0.980 | 1.000 | 1.000 | 0.820 | 1.000 | 1.000 | 0.874 | 0.848 |
| 4129 | 0.833 | 1.000 | 0.868 | 1.000 | 0.979 | 1.000 | 0.895 | 1.000 | 1.000 | 1.000 | 0.977 | 1.000 | 1.000 | 0.820 | 1.000 | 1.000 | 0.874 | 0.848 |
| 4130 | 0.833 | 1.000 | 0.870 | 1.000 | 0.979 | 1.000 | 0.894 | 1.000 | 1.000 | 1.000 | 0.977 | 1.000 | 1.000 | 0.822 | 1.000 | 1.000 | 0.875 | 0.849 |
| 4131 | 0.833 | 1.000 | 0.870 | 1.000 | 0.979 | 1.000 | 0.894 | 1.000 | 1.000 | 1.000 | 0.977 | 1.000 | 1.000 | 0.821 | 1.000 | 1.000 | 0.875 | 0.849 |
| 4132 | 0.834 | 1.000 | 0.872 | 1.000 | 0.979 | 1.000 | 0.894 | 1.000 | 1.000 | 1.000 | 0.980 | 1.000 | 1.000 | 0.823 | 1.000 | 1.000 | 0.876 | 0.850 |
| 4133 | 0.834 | 1.000 | 0.872 | 1.000 | 0.979 | 1.000 | 0.894 | 1.000 | 1.000 | 1.000 | 0.980 | 1.000 | 1.000 | 0.823 | 1.000 | 1.000 | 0.876 | 0.850 |
| 4134 | 0.834 | 1.000 | 0.872 | 1.000 | 0.979 | 1.000 | 0.894 | 1.000 | 1.000 | 1.000 | 0.980 | 1.000 | 1.000 | 0.821 | 1.000 | 1.000 | 0.875 | 0.849 |
| 4135 | 0.834 | 1.000 | 0.872 | 1.000 | 0.979 | 1.000 | 0.894 | 1.000 | 1.000 | 1.000 | 0.980 | 1.000 | 1.000 | 0.823 | 1.000 | 1.000 | 0.876 | 0.850 |
| 4136 | 0.835 | 1.000 | 0.872 | 1.000 | 0.979 | 1.000 | 0.894 | 1.000 | 1.000 | 1.000 | 0.980 | 1.000 | 1.000 | 0.820 | 1.000 | 1.000 | 0.875 | 0.849 |
| 4137 | 0.835 | 1.000 | 0.872 | 1.000 | 0.979 | 1.000 | 0.894 | 1.000 | 1.000 | 1.000 | 0.980 | 1.000 | 1.000 | 0.820 | 1.000 | 1.000 | 0.875 | 0.849 |

| | | | | | | | | | | | | | | | | | | |
|---|---|---|---|---|---|---|---|---|---|---|---|---|---|---|---|---|---|---|
| 4138 | 0.835 | 1.000 | 0.872 | 1.000 | 0.979 | 1.000 | 0.894 | 1.000 | 1.000 | 1.000 | 0.980 | 1.000 | 1.000 | 0.820 | 1.000 | 1.000 | 0.875 | 0.849 |
| 4139 | 0.835 | 1.000 | 0.872 | 1.000 | 0.979 | 1.000 | 0.894 | 1.000 | 1.000 | 1.000 | 0.980 | 1.000 | 1.000 | 0.820 | 1.000 | 1.000 | 0.875 | 0.849 |
| 4140 | 0.835 | 1.000 | 0.870 | 1.000 | 0.979 | 1.000 | 0.894 | 1.000 | 1.000 | 1.000 | 0.980 | 1.000 | 1.000 | 0.819 | 1.000 | 1.000 | 0.875 | 0.849 |
| 4141 | 0.834 | 1.000 | 0.870 | 1.000 | 0.979 | 1.000 | 0.894 | 1.000 | 1.000 | 1.000 | 0.980 | 1.000 | 1.000 | 0.821 | 1.000 | 1.000 | 0.875 | 0.849 |
| 4142 | 0.834 | 1.000 | 0.870 | 1.000 | 0.979 | 1.000 | 0.894 | 1.000 | 1.000 | 1.000 | 0.980 | 1.000 | 1.000 | 0.821 | 1.000 | 1.000 | 0.875 | 0.849 |
| 4143 | 0.833 | 1.000 | 0.870 | 1.000 | 0.979 | 1.000 | 0.894 | 1.000 | 1.000 | 1.000 | 0.980 | 1.000 | 1.000 | 0.821 | 1.000 | 1.000 | 0.875 | 0.849 |
| 4144 | 0.834 | 1.000 | 0.870 | 1.000 | 0.979 | 1.000 | 0.894 | 1.000 | 1.000 | 1.000 | 0.980 | 1.000 | 1.000 | 0.821 | 1.000 | 1.000 | 0.875 | 0.849 |
| 4145 | 0.834 | 1.000 | 0.870 | 1.000 | 0.979 | 1.000 | 0.894 | 1.000 | 1.000 | 1.000 | 0.980 | 1.000 | 1.000 | 0.821 | 1.000 | 1.000 | 0.875 | 0.849 |
| 4146 | 0.833 | 1.000 | 0.870 | 1.000 | 0.979 | 1.000 | 0.894 | 1.000 | 1.000 | 1.000 | 0.980 | 1.000 | 1.000 | 0.820 | 1.000 | 1.000 | 0.875 | 0.849 |
| 4147 | 0.833 | 1.000 | 0.870 | 1.000 | 0.979 | 1.000 | 0.894 | 1.000 | 1.000 | 1.000 | 0.980 | 1.000 | 1.000 | 0.819 | 1.000 | 1.000 | 0.874 | 0.848 |
| 4148 | 0.833 | 1.000 | 0.870 | 1.000 | 0.979 | 1.000 | 0.894 | 1.000 | 1.000 | 1.000 | 0.980 | 1.000 | 1.000 | 0.819 | 1.000 | 1.000 | 0.874 | 0.848 |
| 4149 | 0.833 | 1.000 | 0.870 | 1.000 | 0.979 | 1.000 | 0.894 | 1.000 | 1.000 | 1.000 | 0.980 | 1.000 | 1.000 | 0.819 | 1.000 | 1.000 | 0.874 | 0.848 |
| 4150 | 0.833 | 1.000 | 0.870 | 1.000 | 0.979 | 1.000 | 0.892 | 1.000 | 1.000 | 1.000 | 0.980 | 1.000 | 1.000 | 0.819 | 1.000 | 1.000 | 0.874 | 0.848 |
| 4151 | 0.833 | 1.000 | 0.870 | 1.000 | 0.979 | 1.000 | 0.894 | 1.000 | 1.000 | 1.000 | 0.980 | 1.000 | 1.000 | 0.819 | 1.000 | 1.000 | 0.874 | 0.848 |
| 4152 | 0.833 | 1.000 | 0.870 | 1.000 | 0.979 | 1.000 | 0.892 | 1.000 | 1.000 | 1.000 | 0.980 | 1.000 | 1.000 | 0.819 | 1.000 | 1.000 | 0.874 | 0.848 |
| 4153 | 0.833 | 1.000 | 0.868 | 1.000 | 0.979 | 1.000 | 0.894 | 1.000 | 1.000 | 1.000 | 0.980 | 1.000 | 1.000 | 0.819 | 1.000 | 1.000 | 0.874 | 0.848 |
| 4154 | 0.833 | 1.000 | 0.868 | 1.000 | 0.979 | 1.000 | 0.894 | 1.000 | 1.000 | 1.000 | 0.980 | 1.000 | 1.000 | 0.819 | 1.000 | 1.000 | 0.874 | 0.848 |
| 4155 | 0.833 | 1.000 | 0.870 | 1.000 | 0.979 | 1.000 | 0.891 | 1.000 | 1.000 | 1.000 | 0.980 | 1.000 | 1.000 | 0.819 | 1.000 | 1.000 | 0.874 | 0.848 |
| 4156 | 0.833 | 1.000 | 0.870 | 1.000 | 0.979 | 1.000 | 0.891 | 1.000 | 1.000 | 1.000 | 0.980 | 1.000 | 1.000 | 0.819 | 1.000 | 1.000 | 0.874 | 0.848 |
| 4157 | 0.833 | 1.000 | 0.870 | 1.000 | 0.979 | 1.000 | 0.891 | 1.000 | 1.000 | 1.000 | 0.980 | 1.000 | 1.000 | 0.819 | 1.000 | 1.000 | 0.874 | 0.848 |
| 4158 | 0.832 | 1.000 | 0.870 | 1.000 | 0.979 | 1.000 | 0.891 | 1.000 | 1.000 | 1.000 | 0.980 | 1.000 | 1.000 | 0.820 | 1.000 | 1.000 | 0.874 | 0.848 |
| 4159 | 0.832 | 1.000 | 0.870 | 1.000 | 0.979 | 1.000 | 0.891 | 1.000 | 1.000 | 1.000 | 0.980 | 1.000 | 1.000 | 0.820 | 1.000 | 1.000 | 0.874 | 0.848 |
| 4160 | 0.832 | 1.000 | 0.870 | 1.000 | 0.979 | 1.000 | 0.891 | 1.000 | 1.000 | 1.000 | 0.980 | 1.000 | 1.000 | 0.820 | 1.000 | 1.000 | 0.874 | 0.848 |
| 4161 | 0.832 | 1.000 | 0.870 | 1.000 | 0.979 | 1.000 | 0.891 | 1.000 | 1.000 | 1.000 | 0.980 | 1.000 | 1.000 | 0.820 | 1.000 | 1.000 | 0.874 | 0.848 |
| 4162 | 0.831 | 1.000 | 0.870 | 1.000 | 0.979 | 1.000 | 0.894 | 1.000 | 1.000 | 1.000 | 0.983 | 1.000 | 1.000 | 0.819 | 1.000 | 1.000 | 0.874 | 0.847 |
| 4163 | 0.830 | 1.000 | 0.870 | 1.000 | 0.979 | 1.000 | 0.892 | 1.000 | 1.000 | 1.000 | 0.983 | 1.000 | 1.000 | 0.819 | 1.000 | 1.000 | 0.873 | 0.847 |
| 4164 | 0.830 | 1.000 | 0.870 | 1.000 | 0.979 | 1.000 | 0.894 | 1.000 | 1.000 | 1.000 | 0.983 | 1.000 | 1.000 | 0.818 | 1.000 | 1.000 | 0.873 | 0.847 |
| 4165 | 0.830 | 1.000 | 0.870 | 1.000 | 0.979 | 1.000 | 0.894 | 1.000 | 1.000 | 1.000 | 0.983 | 1.000 | 1.000 | 0.818 | 1.000 | 1.000 | 0.873 | 0.847 |
| 4166 | 0.830 | 1.000 | 0.870 | 1.000 | 0.979 | 1.000 | 0.894 | 1.000 | 1.000 | 1.000 | 0.983 | 1.000 | 1.000 | 0.818 | 1.000 | 1.000 | 0.873 | 0.847 |
| 4167 | 0.830 | 1.000 | 0.870 | 1.000 | 0.979 | 1.000 | 0.894 | 1.000 | 1.000 | 1.000 | 0.983 | 1.000 | 1.000 | 0.819 | 1.000 | 1.000 | 0.873 | 0.847 |
| 4168 | 0.830 | 1.000 | 0.870 | 1.000 | 0.979 | 1.000 | 0.894 | 1.000 | 1.000 | 1.000 | 0.983 | 1.000 | 1.000 | 0.819 | 1.000 | 1.000 | 0.874 | 0.847 |
| 4169 | 0.831 | 1.000 | 0.870 | 1.000 | 0.979 | 1.000 | 0.894 | 1.000 | 1.000 | 1.000 | 0.983 | 1.000 | 1.000 | 0.819 | 1.000 | 1.000 | 0.874 | 0.847 |
| 4170 | 0.831 | 1.000 | 0.870 | 1.000 | 0.979 | 1.000 | 0.894 | 1.000 | 1.000 | 1.000 | 0.983 | 1.000 | 1.000 | 0.819 | 1.000 | 1.000 | 0.874 | 0.848 |
| 4171 | 0.831 | 1.000 | 0.870 | 1.000 | 0.979 | 1.000 | 0.894 | 1.000 | 1.000 | 1.000 | 0.983 | 1.000 | 1.000 | 0.819 | 1.000 | 1.000 | 0.874 | 0.848 |
| 4172 | 0.831 | 1.000 | 0.870 | 1.000 | 0.979 | 1.000 | 0.894 | 1.000 | 1.000 | 1.000 | 0.983 | 1.000 | 1.000 | 0.820 | 1.000 | 1.000 | 0.874 | 0.848 |
| 4173 | 0.831 | 1.000 | 0.870 | 1.000 | 0.979 | 1.000 | 0.894 | 1.000 | 1.000 | 1.000 | 0.983 | 1.000 | 1.000 | 0.820 | 1.000 | 1.000 | 0.874 | 0.848 |
| 4174 | 0.831 | 1.000 | 0.870 | 1.000 | 0.979 | 1.000 | 0.894 | 1.000 | 1.000 | 1.000 | 0.983 | 1.000 | 1.000 | 0.820 | 1.000 | 1.000 | 0.874 | 0.848 |
| 4175 | 0.831 | 1.000 | 0.870 | 1.000 | 0.979 | 1.000 | 0.894 | 1.000 | 1.000 | 1.000 | 0.983 | 1.000 | 1.000 | 0.820 | 1.000 | 1.000 | 0.874 | 0.848 |
| 4176 | 0.831 | 1.000 | 0.870 | 1.000 | 0.979 | 1.000 | 0.894 | 1.000 | 1.000 | 1.000 | 0.980 | 1.000 | 1.000 | 0.819 | 1.000 | 1.000 | 0.874 | 0.847 |

| | | | | | | | | | | | | | | | | | | |
|---|---|---|---|---|---|---|---|---|---|---|---|---|---|---|---|---|---|---|
| 4177 | 0.831 | 1.000 | 0.870 | 1.000 | 0.979 | 1.000 | 0.894 | 1.000 | 1.000 | 1.000 | 0.980 | 1.000 | 1.000 | 0.820 | 1.000 | 1.000 | 0.874 | 0.848 |
| 4178 | 0.831 | 1.000 | 0.870 | 1.000 | 0.979 | 1.000 | 0.894 | 1.000 | 1.000 | 1.000 | 0.980 | 1.000 | 1.000 | 0.819 | 1.000 | 1.000 | 0.874 | 0.847 |
| 4179 | 0.831 | 1.000 | 0.870 | 1.000 | 0.979 | 1.000 | 0.894 | 1.000 | 1.000 | 1.000 | 0.977 | 1.000 | 1.000 | 0.820 | 1.000 | 1.000 | 0.874 | 0.848 |
| 4180 | 0.831 | 1.000 | 0.868 | 1.000 | 0.979 | 1.000 | 0.892 | 1.000 | 1.000 | 1.000 | 0.977 | 1.000 | 1.000 | 0.820 | 1.000 | 1.000 | 0.873 | 0.847 |
| 4181 | 0.831 | 1.000 | 0.868 | 1.000 | 0.979 | 1.000 | 0.892 | 1.000 | 1.000 | 1.000 | 0.977 | 1.000 | 1.000 | 0.820 | 1.000 | 1.000 | 0.873 | 0.847 |
| 4182 | 0.831 | 1.000 | 0.868 | 1.000 | 0.979 | 1.000 | 0.892 | 1.000 | 1.000 | 1.000 | 0.977 | 1.000 | 1.000 | 0.820 | 1.000 | 1.000 | 0.873 | 0.847 |
| 4183 | 0.831 | 1.000 | 0.868 | 1.000 | 0.979 | 1.000 | 0.892 | 1.000 | 1.000 | 1.000 | 0.977 | 1.000 | 1.000 | 0.820 | 1.000 | 1.000 | 0.873 | 0.847 |
| 4184 | 0.830 | 1.000 | 0.870 | 1.000 | 0.979 | 1.000 | 0.894 | 1.000 | 1.000 | 1.000 | 0.977 | 1.000 | 1.000 | 0.819 | 1.000 | 1.000 | 0.873 | 0.847 |
| 4185 | 0.831 | 1.000 | 0.870 | 1.000 | 0.979 | 1.000 | 0.891 | 1.000 | 1.000 | 1.000 | 0.977 | 1.000 | 1.000 | 0.817 | 1.000 | 1.000 | 0.872 | 0.846 |
| 4186 | 0.831 | 1.000 | 0.870 | 1.000 | 0.979 | 1.000 | 0.889 | 1.000 | 1.000 | 1.000 | 0.977 | 1.000 | 1.000 | 0.817 | 1.000 | 1.000 | 0.872 | 0.846 |
| 4187 | 0.832 | 1.000 | 0.868 | 1.000 | 0.979 | 1.000 | 0.894 | 1.000 | 1.000 | 1.000 | 0.977 | 1.000 | 1.000 | 0.815 | 1.000 | 1.000 | 0.872 | 0.846 |
| 4188 | 0.832 | 1.000 | 0.868 | 1.000 | 0.979 | 1.000 | 0.894 | 1.000 | 1.000 | 1.000 | 0.977 | 1.000 | 1.000 | 0.818 | 1.000 | 1.000 | 0.873 | 0.847 |
| 4189 | 0.831 | 1.000 | 0.868 | 1.000 | 0.979 | 1.000 | 0.892 | 1.000 | 1.000 | 1.000 | 0.977 | 1.000 | 1.000 | 0.819 | 1.000 | 1.000 | 0.873 | 0.847 |
| 4190 | 0.830 | 1.000 | 0.870 | 1.000 | 0.979 | 1.000 | 0.892 | 1.000 | 1.000 | 1.000 | 0.980 | 1.000 | 1.000 | 0.818 | 1.000 | 1.000 | 0.873 | 0.846 |
| 4191 | 0.830 | 1.000 | 0.870 | 1.000 | 0.979 | 1.000 | 0.892 | 1.000 | 1.000 | 1.000 | 0.977 | 1.000 | 1.000 | 0.819 | 1.000 | 1.000 | 0.873 | 0.846 |
| 4192 | 0.830 | 1.000 | 0.870 | 1.000 | 0.979 | 1.000 | 0.892 | 1.000 | 1.000 | 1.000 | 0.977 | 1.000 | 1.000 | 0.819 | 1.000 | 1.000 | 0.873 | 0.846 |
| 4193 | 0.833 | 1.000 | 0.870 | 1.000 | 0.979 | 1.000 | 0.891 | 1.000 | 1.000 | 1.000 | 0.977 | 1.000 | 1.000 | 0.819 | 1.000 | 1.000 | 0.874 | 0.847 |
| 4194 | 0.833 | 1.000 | 0.868 | 1.000 | 0.979 | 1.000 | 0.891 | 1.000 | 1.000 | 1.000 | 0.977 | 1.000 | 1.000 | 0.819 | 1.000 | 1.000 | 0.873 | 0.847 |
| 4195 | 0.833 | 1.000 | 0.868 | 1.000 | 0.979 | 1.000 | 0.891 | 1.000 | 1.000 | 1.000 | 0.977 | 1.000 | 1.000 | 0.819 | 1.000 | 1.000 | 0.873 | 0.847 |
| 4196 | 0.833 | 1.000 | 0.868 | 1.000 | 0.979 | 1.000 | 0.891 | 1.000 | 1.000 | 1.000 | 0.977 | 1.000 | 1.000 | 0.819 | 1.000 | 1.000 | 0.873 | 0.847 |
| 4197 | 0.834 | 1.000 | 0.870 | 1.000 | 0.979 | 1.000 | 0.889 | 1.000 | 1.000 | 1.000 | 0.983 | 1.000 | 1.000 | 0.818 | 1.000 | 1.000 | 0.874 | 0.848 |
| 4198 | 0.834 | 1.000 | 0.870 | 1.000 | 0.979 | 1.000 | 0.889 | 1.000 | 1.000 | 1.000 | 0.983 | 1.000 | 1.000 | 0.818 | 1.000 | 1.000 | 0.874 | 0.848 |
| 4199 | 0.834 | 1.000 | 0.870 | 1.000 | 0.979 | 1.000 | 0.889 | 1.000 | 1.000 | 1.000 | 0.980 | 1.000 | 1.000 | 0.818 | 1.000 | 1.000 | 0.874 | 0.847 |
| 4200 | 0.834 | 1.000 | 0.870 | 1.000 | 0.979 | 1.000 | 0.889 | 1.000 | 1.000 | 1.000 | 0.980 | 1.000 | 1.000 | 0.818 | 1.000 | 1.000 | 0.874 | 0.847 |
| 4201 | 0.834 | 1.000 | 0.870 | 1.000 | 0.979 | 1.000 | 0.891 | 1.000 | 1.000 | 1.000 | 0.980 | 1.000 | 1.000 | 0.818 | 1.000 | 1.000 | 0.874 | 0.848 |
| 4202 | 0.834 | 1.000 | 0.870 | 1.000 | 0.979 | 1.000 | 0.891 | 1.000 | 1.000 | 1.000 | 0.980 | 1.000 | 1.000 | 0.819 | 1.000 | 1.000 | 0.874 | 0.848 |
| 4203 | 0.834 | 1.000 | 0.870 | 1.000 | 0.979 | 1.000 | 0.891 | 1.000 | 1.000 | 1.000 | 0.983 | 1.000 | 1.000 | 0.819 | 1.000 | 1.000 | 0.874 | 0.848 |
| 4204 | 0.834 | 1.000 | 0.870 | 1.000 | 0.979 | 1.000 | 0.892 | 1.000 | 1.000 | 1.000 | 0.980 | 1.000 | 1.000 | 0.819 | 1.000 | 1.000 | 0.874 | 0.848 |
| 4205 | 0.834 | 1.000 | 0.870 | 1.000 | 0.979 | 1.000 | 0.892 | 1.000 | 1.000 | 1.000 | 0.980 | 1.000 | 1.000 | 0.819 | 1.000 | 1.000 | 0.874 | 0.848 |
| 4206 | 0.835 | 1.000 | 0.870 | 1.000 | 0.979 | 1.000 | 0.892 | 1.000 | 1.000 | 1.000 | 0.983 | 1.000 | 1.000 | 0.819 | 1.000 | 1.000 | 0.875 | 0.849 |
| 4207 | 0.835 | 1.000 | 0.870 | 1.000 | 0.979 | 1.000 | 0.892 | 1.000 | 1.000 | 1.000 | 0.980 | 1.000 | 1.000 | 0.819 | 1.000 | 1.000 | 0.875 | 0.849 |
| 4208 | 0.835 | 1.000 | 0.870 | 1.000 | 0.979 | 1.000 | 0.892 | 1.000 | 1.000 | 1.000 | 0.980 | 1.000 | 1.000 | 0.819 | 1.000 | 1.000 | 0.875 | 0.849 |
| 4209 | 0.835 | 1.000 | 0.870 | 1.000 | 0.979 | 1.000 | 0.892 | 1.000 | 1.000 | 1.000 | 0.980 | 1.000 | 1.000 | 0.819 | 1.000 | 1.000 | 0.875 | 0.849 |
| 4210 | 0.833 | 1.000 | 0.872 | 1.000 | 0.979 | 1.000 | 0.892 | 1.000 | 1.000 | 1.000 | 0.980 | 1.000 | 1.000 | 0.819 | 1.000 | 1.000 | 0.874 | 0.848 |
| 4211 | 0.833 | 1.000 | 0.872 | 1.000 | 0.979 | 1.000 | 0.892 | 1.000 | 1.000 | 1.000 | 0.980 | 1.000 | 1.000 | 0.818 | 1.000 | 1.000 | 0.874 | 0.848 |
| 4212 | 0.832 | 1.000 | 0.870 | 1.000 | 0.979 | 1.000 | 0.892 | 1.000 | 1.000 | 1.000 | 0.980 | 1.000 | 1.000 | 0.818 | 1.000 | 1.000 | 0.873 | 0.847 |
| 4213 | 0.833 | 1.000 | 0.870 | 1.000 | 0.979 | 1.000 | 0.892 | 1.000 | 1.000 | 1.000 | 0.980 | 1.000 | 1.000 | 0.818 | 1.000 | 1.000 | 0.874 | 0.847 |
| 4214 | 0.833 | 1.000 | 0.870 | 1.000 | 0.979 | 1.000 | 0.892 | 1.000 | 1.000 | 1.000 | 0.980 | 1.000 | 1.000 | 0.819 | 1.000 | 1.000 | 0.874 | 0.848 |
| 4215 | 0.833 | 1.000 | 0.870 | 1.000 | 0.979 | 1.000 | 0.892 | 1.000 | 1.000 | 1.000 | 0.980 | 1.000 | 1.000 | 0.819 | 1.000 | 1.000 | 0.874 | 0.848 |

| | | | | | | | | | | | | | | | | | | |
|---|---|---|---|---|---|---|---|---|---|---|---|---|---|---|---|---|---|---|
| 4216 | 0.833 | 1.000 | 0.870 | 1.000 | 0.979 | 1.000 | 0.892 | 1.000 | 1.000 | 1.000 | 0.980 | 1.000 | 1.000 | 0.819 | 1.000 | 1.000 | 0.874 | 0.848 |
| 4217 | 0.835 | 1.000 | 0.870 | 1.000 | 0.979 | 1.000 | 0.889 | 1.000 | 1.000 | 1.000 | 0.980 | 1.000 | 1.000 | 0.818 | 1.000 | 1.000 | 0.874 | 0.848 |
| 4218 | 0.835 | 1.000 | 0.870 | 1.000 | 0.979 | 1.000 | 0.889 | 1.000 | 1.000 | 1.000 | 0.980 | 1.000 | 1.000 | 0.818 | 1.000 | 1.000 | 0.874 | 0.848 |
| 4219 | 0.834 | 1.000 | 0.870 | 1.000 | 0.979 | 1.000 | 0.891 | 1.000 | 1.000 | 1.000 | 0.980 | 1.000 | 1.000 | 0.819 | 1.000 | 1.000 | 0.874 | 0.848 |
| 4220 | 0.833 | 1.000 | 0.870 | 1.000 | 0.979 | 1.000 | 0.891 | 1.000 | 1.000 | 1.000 | 0.980 | 1.000 | 1.000 | 0.818 | 1.000 | 1.000 | 0.874 | 0.847 |
| 4221 | 0.833 | 1.000 | 0.870 | 1.000 | 0.979 | 1.000 | 0.891 | 1.000 | 1.000 | 1.000 | 0.980 | 1.000 | 1.000 | 0.818 | 1.000 | 1.000 | 0.874 | 0.847 |
| 4222 | 0.834 | 1.000 | 0.870 | 1.000 | 0.979 | 1.000 | 0.891 | 1.000 | 1.000 | 1.000 | 0.980 | 1.000 | 1.000 | 0.818 | 1.000 | 1.000 | 0.874 | 0.848 |
| 4223 | 0.834 | 1.000 | 0.870 | 1.000 | 0.979 | 1.000 | 0.891 | 1.000 | 1.000 | 1.000 | 0.980 | 1.000 | 1.000 | 0.818 | 1.000 | 1.000 | 0.874 | 0.848 |
| 4224 | 0.833 | 1.000 | 0.870 | 1.000 | 0.979 | 1.000 | 0.891 | 1.000 | 1.000 | 1.000 | 0.980 | 1.000 | 1.000 | 0.818 | 1.000 | 1.000 | 0.874 | 0.847 |
| 4225 | 0.833 | 1.000 | 0.870 | 1.000 | 0.979 | 1.000 | 0.891 | 1.000 | 1.000 | 1.000 | 0.980 | 1.000 | 1.000 | 0.818 | 1.000 | 1.000 | 0.874 | 0.847 |
| 4226 | 0.833 | 1.000 | 0.870 | 1.000 | 0.979 | 1.000 | 0.891 | 1.000 | 1.000 | 1.000 | 0.980 | 1.000 | 1.000 | 0.818 | 1.000 | 1.000 | 0.874 | 0.847 |
| 4227 | 0.834 | 1.000 | 0.872 | 1.000 | 0.979 | 1.000 | 0.894 | 1.000 | 1.000 | 1.000 | 0.980 | 1.000 | 1.000 | 0.819 | 1.000 | 1.000 | 0.875 | 0.849 |
| 4228 | 0.833 | 1.000 | 0.872 | 1.000 | 0.979 | 1.000 | 0.894 | 1.000 | 1.000 | 1.000 | 0.980 | 1.000 | 1.000 | 0.819 | 1.000 | 1.000 | 0.874 | 0.848 |
| 4229 | 0.833 | 1.000 | 0.870 | 1.000 | 0.979 | 1.000 | 0.894 | 1.000 | 1.000 | 1.000 | 0.980 | 1.000 | 1.000 | 0.819 | 1.000 | 1.000 | 0.874 | 0.848 |
| 4230 | 0.833 | 1.000 | 0.872 | 1.000 | 0.979 | 1.000 | 0.894 | 1.000 | 1.000 | 1.000 | 0.980 | 1.000 | 1.000 | 0.819 | 1.000 | 1.000 | 0.874 | 0.848 |
| 4231 | 0.833 | 1.000 | 0.870 | 1.000 | 0.979 | 1.000 | 0.894 | 1.000 | 1.000 | 1.000 | 0.980 | 1.000 | 1.000 | 0.819 | 1.000 | 1.000 | 0.874 | 0.848 |
| 4232 | 0.833 | 1.000 | 0.870 | 1.000 | 0.979 | 1.000 | 0.894 | 1.000 | 1.000 | 1.000 | 0.980 | 1.000 | 1.000 | 0.819 | 1.000 | 1.000 | 0.874 | 0.848 |
| 4233 | 0.834 | 1.000 | 0.870 | 1.000 | 0.979 | 1.000 | 0.892 | 1.000 | 1.000 | 1.000 | 0.980 | 1.000 | 1.000 | 0.817 | 1.000 | 1.000 | 0.874 | 0.848 |
| 4234 | 0.833 | 1.000 | 0.870 | 1.000 | 0.979 | 1.000 | 0.892 | 1.000 | 1.000 | 1.000 | 0.980 | 1.000 | 1.000 | 0.817 | 1.000 | 1.000 | 0.874 | 0.847 |
| 4235 | 0.833 | 1.000 | 0.870 | 1.000 | 0.979 | 1.000 | 0.894 | 1.000 | 1.000 | 1.000 | 0.980 | 1.000 | 1.000 | 0.817 | 1.000 | 1.000 | 0.874 | 0.848 |
| 4236 | 0.833 | 1.000 | 0.870 | 1.000 | 0.979 | 1.000 | 0.894 | 1.000 | 1.000 | 1.000 | 0.980 | 1.000 | 1.000 | 0.817 | 1.000 | 1.000 | 0.874 | 0.848 |
| 4237 | 0.833 | 1.000 | 0.870 | 1.000 | 0.979 | 1.000 | 0.892 | 1.000 | 1.000 | 1.000 | 0.980 | 1.000 | 1.000 | 0.817 | 1.000 | 1.000 | 0.874 | 0.847 |
| 4238 | 0.833 | 1.000 | 0.870 | 1.000 | 0.979 | 1.000 | 0.894 | 1.000 | 1.000 | 1.000 | 0.980 | 1.000 | 1.000 | 0.817 | 1.000 | 1.000 | 0.874 | 0.848 |
| 4239 | 0.833 | 1.000 | 0.872 | 1.000 | 0.979 | 1.000 | 0.894 | 1.000 | 1.000 | 1.000 | 0.980 | 1.000 | 1.000 | 0.819 | 1.000 | 1.000 | 0.874 | 0.848 |
| 4240 | 0.834 | 1.000 | 0.872 | 1.000 | 0.979 | 1.000 | 0.894 | 1.000 | 1.000 | 1.000 | 0.980 | 1.000 | 1.000 | 0.819 | 1.000 | 1.000 | 0.875 | 0.849 |
| 4241 | 0.833 | 1.000 | 0.874 | 1.000 | 0.979 | 1.000 | 0.892 | 1.000 | 1.000 | 1.000 | 0.980 | 1.000 | 1.000 | 0.819 | 1.000 | 1.000 | 0.875 | 0.849 |
| 4242 | 0.833 | 1.000 | 0.874 | 1.000 | 0.979 | 1.000 | 0.894 | 1.000 | 1.000 | 1.000 | 0.980 | 1.000 | 1.000 | 0.819 | 1.000 | 1.000 | 0.875 | 0.849 |
| 4243 | 0.833 | 1.000 | 0.874 | 1.000 | 0.979 | 1.000 | 0.894 | 1.000 | 1.000 | 1.000 | 0.980 | 1.000 | 1.000 | 0.819 | 1.000 | 1.000 | 0.875 | 0.849 |
| 4244 | 0.832 | 1.000 | 0.874 | 1.000 | 0.979 | 1.000 | 0.894 | 1.000 | 1.000 | 1.000 | 0.980 | 1.000 | 1.000 | 0.819 | 1.000 | 1.000 | 0.874 | 0.848 |
| 4245 | 0.832 | 1.000 | 0.874 | 1.000 | 0.979 | 1.000 | 0.894 | 1.000 | 1.000 | 1.000 | 0.980 | 1.000 | 1.000 | 0.819 | 1.000 | 1.000 | 0.874 | 0.848 |
| 4246 | 0.832 | 1.000 | 0.874 | 1.000 | 0.979 | 1.000 | 0.894 | 1.000 | 1.000 | 1.000 | 0.980 | 1.000 | 1.000 | 0.819 | 1.000 | 1.000 | 0.874 | 0.848 |
| 4247 | 0.833 | 1.000 | 0.874 | 1.000 | 0.979 | 1.000 | 0.894 | 1.000 | 1.000 | 1.000 | 0.980 | 1.000 | 1.000 | 0.819 | 1.000 | 1.000 | 0.874 | 0.848 |
| 4248 | 0.833 | 1.000 | 0.874 | 1.000 | 0.979 | 1.000 | 0.891 | 1.000 | 1.000 | 1.000 | 0.980 | 1.000 | 1.000 | 0.819 | 1.000 | 1.000 | 0.874 | 0.848 |
| 4249 | 0.833 | 1.000 | 0.874 | 1.000 | 0.979 | 1.000 | 0.891 | 1.000 | 1.000 | 1.000 | 0.980 | 1.000 | 1.000 | 0.819 | 1.000 | 1.000 | 0.874 | 0.848 |
| 4250 | 0.833 | 1.000 | 0.874 | 1.000 | 0.979 | 1.000 | 0.891 | 1.000 | 1.000 | 1.000 | 0.980 | 1.000 | 1.000 | 0.819 | 1.000 | 1.000 | 0.874 | 0.848 |
| 4251 | 0.832 | 1.000 | 0.874 | 1.000 | 0.974 | 1.000 | 0.891 | 1.000 | 1.000 | 1.000 | 0.983 | 1.000 | 1.000 | 0.819 | 1.000 | 1.000 | 0.874 | 0.848 |
| 4252 | 0.832 | 1.000 | 0.874 | 1.000 | 0.979 | 1.000 | 0.891 | 1.000 | 1.000 | 1.000 | 0.983 | 1.000 | 1.000 | 0.819 | 1.000 | 1.000 | 0.874 | 0.848 |
| 4253 | 0.832 | 1.000 | 0.874 | 1.000 | 0.979 | 1.000 | 0.891 | 1.000 | 1.000 | 1.000 | 0.983 | 1.000 | 1.000 | 0.819 | 1.000 | 1.000 | 0.874 | 0.848 |
| 4254 | 0.832 | 1.000 | 0.874 | 1.000 | 0.974 | 1.000 | 0.891 | 1.000 | 1.000 | 1.000 | 0.983 | 1.000 | 1.000 | 0.820 | 1.000 | 1.000 | 0.874 | 0.848 |

| | | | | | | | | | | | | | | | | | | |
|---|---|---|---|---|---|---|---|---|---|---|---|---|---|---|---|---|---|---|
| 4255 | 0.833 | 1.000 | 0.874 | 1.000 | 0.974 | 1.000 | 0.891 | 1.000 | 1.000 | 1.000 | 0.983 | 1.000 | 1.000 | 0.820 | 1.000 | 1.000 | 0.874 | 0.848 |
| 4256 | 0.832 | 1.000 | 0.874 | 1.000 | 0.974 | 1.000 | 0.891 | 1.000 | 1.000 | 1.000 | 0.983 | 1.000 | 1.000 | 0.820 | 1.000 | 1.000 | 0.874 | 0.848 |
| 4257 | 0.833 | 1.000 | 0.874 | 1.000 | 0.974 | 1.000 | 0.891 | 1.000 | 1.000 | 1.000 | 0.983 | 1.000 | 1.000 | 0.820 | 1.000 | 1.000 | 0.874 | 0.848 |
| 4258 | 0.833 | 1.000 | 0.874 | 1.000 | 0.974 | 1.000 | 0.891 | 1.000 | 1.000 | 1.000 | 0.983 | 1.000 | 1.000 | 0.819 | 1.000 | 1.000 | 0.874 | 0.848 |
| 4259 | 0.833 | 1.000 | 0.874 | 1.000 | 0.974 | 1.000 | 0.891 | 1.000 | 1.000 | 1.000 | 0.983 | 1.000 | 1.000 | 0.819 | 1.000 | 1.000 | 0.874 | 0.848 |
| 4260 | 0.833 | 1.000 | 0.874 | 1.000 | 0.974 | 1.000 | 0.891 | 1.000 | 1.000 | 1.000 | 0.983 | 1.000 | 1.000 | 0.819 | 1.000 | 1.000 | 0.874 | 0.848 |
| 4261 | 0.833 | 1.000 | 0.874 | 1.000 | 0.974 | 1.000 | 0.892 | 1.000 | 1.000 | 1.000 | 0.983 | 1.000 | 1.000 | 0.819 | 1.000 | 1.000 | 0.875 | 0.849 |
| 4262 | 0.832 | 1.000 | 0.874 | 1.000 | 0.974 | 1.000 | 0.892 | 1.000 | 1.000 | 1.000 | 0.983 | 1.000 | 1.000 | 0.820 | 1.000 | 1.000 | 0.874 | 0.848 |
| 4263 | 0.832 | 1.000 | 0.872 | 1.000 | 0.974 | 1.000 | 0.892 | 1.000 | 1.000 | 1.000 | 0.983 | 1.000 | 1.000 | 0.819 | 1.000 | 1.000 | 0.874 | 0.848 |
| 4264 | 0.832 | 1.000 | 0.872 | 1.000 | 0.974 | 1.000 | 0.892 | 1.000 | 1.000 | 1.000 | 0.983 | 1.000 | 1.000 | 0.819 | 1.000 | 1.000 | 0.874 | 0.848 |
| 4265 | 0.831 | 1.000 | 0.872 | 1.000 | 0.974 | 1.000 | 0.892 | 1.000 | 1.000 | 1.000 | 0.983 | 1.000 | 1.000 | 0.819 | 1.000 | 1.000 | 0.874 | 0.848 |
| 4266 | 0.832 | 1.000 | 0.872 | 1.000 | 0.974 | 1.000 | 0.892 | 1.000 | 1.000 | 1.000 | 0.983 | 1.000 | 1.000 | 0.819 | 1.000 | 1.000 | 0.874 | 0.848 |
| 4267 | 0.833 | 1.000 | 0.870 | 1.000 | 0.974 | 1.000 | 0.892 | 1.000 | 1.000 | 1.000 | 0.983 | 1.000 | 1.000 | 0.819 | 1.000 | 1.000 | 0.874 | 0.848 |
| 4268 | 0.833 | 1.000 | 0.870 | 1.000 | 0.974 | 1.000 | 0.892 | 1.000 | 1.000 | 1.000 | 0.983 | 1.000 | 1.000 | 0.819 | 1.000 | 1.000 | 0.874 | 0.848 |
| 4269 | 0.833 | 1.000 | 0.870 | 1.000 | 0.974 | 1.000 | 0.892 | 1.000 | 1.000 | 1.000 | 0.983 | 1.000 | 1.000 | 0.818 | 1.000 | 1.000 | 0.874 | 0.848 |
| 4270 | 0.833 | 1.000 | 0.870 | 1.000 | 0.974 | 1.000 | 0.891 | 1.000 | 1.000 | 1.000 | 0.983 | 1.000 | 1.000 | 0.819 | 1.000 | 1.000 | 0.874 | 0.847 |
| 4271 | 0.833 | 1.000 | 0.870 | 1.000 | 0.974 | 1.000 | 0.891 | 1.000 | 1.000 | 1.000 | 0.983 | 1.000 | 1.000 | 0.819 | 1.000 | 1.000 | 0.874 | 0.847 |
| 4272 | 0.833 | 1.000 | 0.870 | 1.000 | 0.974 | 1.000 | 0.891 | 1.000 | 1.000 | 1.000 | 0.983 | 1.000 | 1.000 | 0.819 | 1.000 | 1.000 | 0.874 | 0.847 |
| 4273 | 0.833 | 1.000 | 0.870 | 1.000 | 0.974 | 1.000 | 0.891 | 1.000 | 1.000 | 1.000 | 0.983 | 1.000 | 1.000 | 0.819 | 1.000 | 1.000 | 0.874 | 0.848 |
| 4274 | 0.833 | 1.000 | 0.870 | 1.000 | 0.974 | 1.000 | 0.891 | 1.000 | 1.000 | 1.000 | 0.983 | 1.000 | 1.000 | 0.819 | 1.000 | 1.000 | 0.874 | 0.847 |
| 4275 | 0.833 | 1.000 | 0.870 | 1.000 | 0.974 | 1.000 | 0.888 | 1.000 | 1.000 | 1.000 | 0.983 | 1.000 | 1.000 | 0.819 | 1.000 | 1.000 | 0.873 | 0.847 |
| 4276 | 0.833 | 1.000 | 0.870 | 1.000 | 0.974 | 1.000 | 0.888 | 1.000 | 1.000 | 1.000 | 0.983 | 1.000 | 1.000 | 0.819 | 1.000 | 1.000 | 0.873 | 0.847 |
| 4277 | 0.833 | 1.000 | 0.870 | 1.000 | 0.974 | 1.000 | 0.888 | 1.000 | 1.000 | 1.000 | 0.983 | 1.000 | 1.000 | 0.819 | 1.000 | 1.000 | 0.873 | 0.847 |
| 4278 | 0.833 | 1.000 | 0.870 | 1.000 | 0.974 | 1.000 | 0.888 | 1.000 | 1.000 | 1.000 | 0.983 | 1.000 | 1.000 | 0.819 | 1.000 | 1.000 | 0.874 | 0.847 |
| 4279 | 0.833 | 1.000 | 0.870 | 1.000 | 0.974 | 1.000 | 0.888 | 1.000 | 1.000 | 1.000 | 0.983 | 1.000 | 1.000 | 0.819 | 1.000 | 1.000 | 0.874 | 0.847 |
| 4280 | 0.833 | 1.000 | 0.872 | 1.000 | 0.974 | 1.000 | 0.888 | 1.000 | 1.000 | 1.000 | 0.983 | 1.000 | 1.000 | 0.819 | 1.000 | 1.000 | 0.874 | 0.848 |
| 4281 | 0.832 | 1.000 | 0.870 | 1.000 | 0.974 | 1.000 | 0.888 | 1.000 | 1.000 | 1.000 | 0.983 | 1.000 | 1.000 | 0.819 | 1.000 | 1.000 | 0.873 | 0.847 |
| 4282 | 0.833 | 1.000 | 0.868 | 1.000 | 0.974 | 1.000 | 0.888 | 1.000 | 1.000 | 1.000 | 0.983 | 1.000 | 1.000 | 0.819 | 1.000 | 1.000 | 0.873 | 0.847 |
| 4283 | 0.833 | 1.000 | 0.870 | 1.000 | 0.974 | 1.000 | 0.888 | 1.000 | 1.000 | 1.000 | 0.983 | 1.000 | 1.000 | 0.819 | 1.000 | 1.000 | 0.873 | 0.847 |
| 4284 | 0.833 | 1.000 | 0.870 | 1.000 | 0.974 | 1.000 | 0.888 | 1.000 | 1.000 | 1.000 | 0.983 | 1.000 | 1.000 | 0.819 | 1.000 | 1.000 | 0.874 | 0.847 |
| 4285 | 0.833 | 1.000 | 0.870 | 1.000 | 0.974 | 1.000 | 0.888 | 1.000 | 1.000 | 1.000 | 0.983 | 1.000 | 1.000 | 0.819 | 1.000 | 1.000 | 0.874 | 0.847 |
| 4286 | 0.832 | 1.000 | 0.874 | 1.000 | 0.974 | 1.000 | 0.888 | 1.000 | 1.000 | 1.000 | 0.980 | 1.000 | 1.000 | 0.820 | 1.000 | 1.000 | 0.874 | 0.847 |
| 4287 | 0.833 | 1.000 | 0.872 | 1.000 | 0.974 | 1.000 | 0.888 | 1.000 | 1.000 | 1.000 | 0.983 | 1.000 | 1.000 | 0.820 | 1.000 | 1.000 | 0.874 | 0.848 |
| 4288 | 0.831 | 1.000 | 0.874 | 1.000 | 0.974 | 1.000 | 0.888 | 1.000 | 1.000 | 1.000 | 0.983 | 1.000 | 1.000 | 0.820 | 1.000 | 1.000 | 0.874 | 0.847 |
| 4289 | 0.831 | 1.000 | 0.874 | 1.000 | 0.974 | 1.000 | 0.888 | 1.000 | 1.000 | 1.000 | 0.983 | 1.000 | 1.000 | 0.820 | 1.000 | 1.000 | 0.874 | 0.847 |
| 4290 | 0.831 | 1.000 | 0.874 | 1.000 | 0.974 | 1.000 | 0.888 | 1.000 | 1.000 | 1.000 | 0.983 | 1.000 | 1.000 | 0.820 | 1.000 | 1.000 | 0.874 | 0.847 |
| 4291 | 0.832 | 1.000 | 0.874 | 1.000 | 0.974 | 1.000 | 0.888 | 1.000 | 1.000 | 1.000 | 0.983 | 1.000 | 1.000 | 0.820 | 1.000 | 1.000 | 0.874 | 0.848 |
| 4292 | 0.832 | 1.000 | 0.874 | 1.000 | 0.974 | 1.000 | 0.888 | 1.000 | 1.000 | 1.000 | 0.983 | 1.000 | 1.000 | 0.820 | 1.000 | 1.000 | 0.874 | 0.848 |
| 4293 | 0.832 | 1.000 | 0.872 | 1.000 | 0.974 | 1.000 | 0.888 | 1.000 | 1.000 | 1.000 | 0.983 | 1.000 | 1.000 | 0.820 | 1.000 | 1.000 | 0.874 | 0.847 |

| | | | | | | | | | | | | | | | | | | |
|---|---|---|---|---|---|---|---|---|---|---|---|---|---|---|---|---|---|---|
| 4294 | 0.832 | 1.000 | 0.870 | 1.000 | 0.974 | 1.000 | 0.888 | 1.000 | 1.000 | 1.000 | 0.983 | 1.000 | 1.000 | 0.821 | 1.000 | 1.000 | 0.874 | 0.847 |
| 4295 | 0.832 | 1.000 | 0.872 | 1.000 | 0.974 | 1.000 | 0.889 | 1.000 | 1.000 | 1.000 | 0.983 | 1.000 | 1.000 | 0.820 | 1.000 | 1.000 | 0.874 | 0.848 |
| 4296 | 0.833 | 1.000 | 0.872 | 1.000 | 0.974 | 1.000 | 0.889 | 1.000 | 1.000 | 1.000 | 0.983 | 1.000 | 1.000 | 0.820 | 1.000 | 1.000 | 0.874 | 0.848 |
| 4297 | 0.833 | 1.000 | 0.872 | 1.000 | 0.974 | 1.000 | 0.889 | 1.000 | 1.000 | 1.000 | 0.983 | 1.000 | 1.000 | 0.820 | 1.000 | 1.000 | 0.874 | 0.848 |
| 4298 | 0.832 | 1.000 | 0.870 | 1.000 | 0.974 | 1.000 | 0.889 | 1.000 | 1.000 | 1.000 | 0.983 | 1.000 | 1.000 | 0.820 | 1.000 | 1.000 | 0.874 | 0.847 |
| 4299 | 0.833 | 1.000 | 0.872 | 1.000 | 0.974 | 1.000 | 0.889 | 1.000 | 1.000 | 1.000 | 0.983 | 1.000 | 1.000 | 0.820 | 1.000 | 1.000 | 0.874 | 0.848 |
| 4300 | 0.832 | 1.000 | 0.872 | 1.000 | 0.974 | 1.000 | 0.889 | 1.000 | 1.000 | 1.000 | 0.983 | 1.000 | 1.000 | 0.821 | 1.000 | 1.000 | 0.874 | 0.848 |
| 4301 | 0.832 | 1.000 | 0.872 | 1.000 | 0.974 | 1.000 | 0.889 | 1.000 | 1.000 | 1.000 | 0.983 | 1.000 | 1.000 | 0.821 | 1.000 | 1.000 | 0.874 | 0.848 |
| 4302 | 0.832 | 1.000 | 0.872 | 1.000 | 0.974 | 1.000 | 0.889 | 1.000 | 1.000 | 1.000 | 0.983 | 1.000 | 1.000 | 0.819 | 1.000 | 1.000 | 0.873 | 0.847 |
| 4303 | 0.832 | 1.000 | 0.870 | 1.000 | 0.974 | 1.000 | 0.889 | 1.000 | 1.000 | 1.000 | 0.983 | 1.000 | 1.000 | 0.819 | 1.000 | 1.000 | 0.873 | 0.847 |
| 4304 | 0.833 | 1.000 | 0.870 | 1.000 | 0.974 | 1.000 | 0.889 | 1.000 | 1.000 | 1.000 | 0.983 | 1.000 | 1.000 | 0.819 | 1.000 | 1.000 | 0.873 | 0.847 |
| 4305 | 0.832 | 1.000 | 0.870 | 1.000 | 0.974 | 1.000 | 0.889 | 1.000 | 1.000 | 1.000 | 0.983 | 1.000 | 1.000 | 0.819 | 1.000 | 1.000 | 0.873 | 0.847 |
| 4306 | 0.831 | 1.000 | 0.872 | 1.000 | 0.974 | 1.000 | 0.889 | 1.000 | 1.000 | 1.000 | 0.983 | 1.000 | 1.000 | 0.819 | 1.000 | 1.000 | 0.873 | 0.847 |
| 4307 | 0.832 | 1.000 | 0.870 | 1.000 | 0.974 | 1.000 | 0.889 | 1.000 | 1.000 | 1.000 | 0.983 | 1.000 | 1.000 | 0.819 | 1.000 | 1.000 | 0.873 | 0.847 |
| 4308 | 0.832 | 1.000 | 0.868 | 1.000 | 0.974 | 1.000 | 0.889 | 1.000 | 1.000 | 1.000 | 0.983 | 1.000 | 1.000 | 0.819 | 1.000 | 1.000 | 0.873 | 0.847 |
| 4309 | 0.833 | 1.000 | 0.868 | 1.000 | 0.974 | 1.000 | 0.889 | 1.000 | 1.000 | 1.000 | 0.983 | 1.000 | 1.000 | 0.819 | 1.000 | 1.000 | 0.873 | 0.847 |
| 4310 | 0.832 | 1.000 | 0.868 | 1.000 | 0.974 | 1.000 | 0.889 | 1.000 | 1.000 | 1.000 | 0.983 | 1.000 | 1.000 | 0.819 | 1.000 | 1.000 | 0.873 | 0.847 |
| 4311 | 0.831 | 1.000 | 0.868 | 1.000 | 0.974 | 1.000 | 0.889 | 1.000 | 1.000 | 1.000 | 0.983 | 1.000 | 1.000 | 0.819 | 1.000 | 1.000 | 0.873 | 0.847 |
| 4312 | 0.831 | 1.000 | 0.868 | 1.000 | 0.974 | 1.000 | 0.888 | 1.000 | 1.000 | 1.000 | 0.983 | 1.000 | 1.000 | 0.819 | 1.000 | 1.000 | 0.873 | 0.846 |
| 4313 | 0.831 | 1.000 | 0.868 | 1.000 | 0.974 | 1.000 | 0.889 | 1.000 | 1.000 | 1.000 | 0.983 | 1.000 | 1.000 | 0.820 | 1.000 | 1.000 | 0.873 | 0.847 |
| 4314 | 0.831 | 1.000 | 0.868 | 1.000 | 0.974 | 1.000 | 0.889 | 1.000 | 1.000 | 1.000 | 0.983 | 1.000 | 1.000 | 0.820 | 1.000 | 1.000 | 0.873 | 0.847 |
| 4315 | 0.831 | 1.000 | 0.868 | 1.000 | 0.974 | 1.000 | 0.889 | 1.000 | 1.000 | 1.000 | 0.983 | 1.000 | 1.000 | 0.820 | 1.000 | 1.000 | 0.873 | 0.847 |
| 4316 | 0.831 | 1.000 | 0.868 | 1.000 | 0.974 | 1.000 | 0.889 | 1.000 | 1.000 | 1.000 | 0.983 | 1.000 | 1.000 | 0.820 | 1.000 | 1.000 | 0.873 | 0.847 |
| 4317 | 0.831 | 1.000 | 0.868 | 1.000 | 0.974 | 1.000 | 0.889 | 1.000 | 1.000 | 1.000 | 0.983 | 1.000 | 1.000 | 0.820 | 1.000 | 1.000 | 0.873 | 0.847 |
| 4318 | 0.833 | 1.000 | 0.868 | 1.000 | 0.974 | 1.000 | 0.889 | 1.000 | 1.000 | 1.000 | 0.983 | 1.000 | 1.000 | 0.820 | 1.000 | 1.000 | 0.874 | 0.847 |
| 4319 | 0.832 | 1.000 | 0.868 | 1.000 | 0.974 | 1.000 | 0.889 | 1.000 | 1.000 | 1.000 | 0.980 | 1.000 | 1.000 | 0.818 | 1.000 | 1.000 | 0.873 | 0.846 |
| 4320 | 0.833 | 1.000 | 0.868 | 1.000 | 0.974 | 1.000 | 0.889 | 1.000 | 1.000 | 1.000 | 0.980 | 1.000 | 1.000 | 0.818 | 1.000 | 1.000 | 0.873 | 0.846 |
| 4321 | 0.833 | 1.000 | 0.868 | 1.000 | 0.974 | 1.000 | 0.889 | 1.000 | 1.000 | 1.000 | 0.980 | 1.000 | 1.000 | 0.818 | 1.000 | 1.000 | 0.873 | 0.846 |
| 4322 | 0.833 | 1.000 | 0.868 | 1.000 | 0.974 | 1.000 | 0.889 | 1.000 | 1.000 | 1.000 | 0.980 | 1.000 | 1.000 | 0.818 | 1.000 | 1.000 | 0.873 | 0.846 |
| 4323 | 0.831 | 1.000 | 0.866 | 1.000 | 0.974 | 1.000 | 0.889 | 1.000 | 1.000 | 1.000 | 0.980 | 1.000 | 1.000 | 0.818 | 1.000 | 1.000 | 0.872 | 0.846 |
| 4324 | 0.831 | 1.000 | 0.866 | 1.000 | 0.974 | 1.000 | 0.889 | 1.000 | 1.000 | 1.000 | 0.980 | 1.000 | 1.000 | 0.818 | 1.000 | 1.000 | 0.872 | 0.846 |
| 4325 | 0.831 | 1.000 | 0.868 | 1.000 | 0.974 | 1.000 | 0.889 | 1.000 | 1.000 | 1.000 | 0.980 | 1.000 | 1.000 | 0.818 | 1.000 | 1.000 | 0.872 | 0.846 |
| 4326 | 0.831 | 1.000 | 0.866 | 1.000 | 0.974 | 1.000 | 0.891 | 1.000 | 1.000 | 1.000 | 0.980 | 1.000 | 1.000 | 0.817 | 1.000 | 1.000 | 0.872 | 0.846 |
| 4327 | 0.831 | 1.000 | 0.868 | 1.000 | 0.974 | 1.000 | 0.889 | 1.000 | 1.000 | 1.000 | 0.980 | 1.000 | 1.000 | 0.818 | 1.000 | 1.000 | 0.872 | 0.846 |
| 4328 | 0.831 | 1.000 | 0.870 | 1.000 | 0.974 | 1.000 | 0.889 | 1.000 | 1.000 | 1.000 | 0.980 | 1.000 | 1.000 | 0.818 | 1.000 | 1.000 | 0.872 | 0.846 |
| 4329 | 0.831 | 1.000 | 0.870 | 1.000 | 0.974 | 1.000 | 0.889 | 1.000 | 1.000 | 1.000 | 0.980 | 1.000 | 1.000 | 0.818 | 1.000 | 1.000 | 0.872 | 0.846 |
| 4330 | 0.831 | 1.000 | 0.870 | 1.000 | 0.974 | 1.000 | 0.891 | 1.000 | 1.000 | 1.000 | 0.980 | 1.000 | 1.000 | 0.818 | 1.000 | 1.000 | 0.873 | 0.846 |
| 4331 | 0.831 | 1.000 | 0.870 | 1.000 | 0.974 | 1.000 | 0.891 | 1.000 | 1.000 | 1.000 | 0.980 | 1.000 | 1.000 | 0.819 | 1.000 | 1.000 | 0.873 | 0.847 |
| 4332 | 0.831 | 1.000 | 0.870 | 1.000 | 0.974 | 1.000 | 0.891 | 1.000 | 1.000 | 1.000 | 0.980 | 1.000 | 1.000 | 0.819 | 1.000 | 1.000 | 0.873 | 0.847 |

| | | | | | | | | | | | | | | | | | | |
|---|---|---|---|---|---|---|---|---|---|---|---|---|---|---|---|---|---|---|
| 4333 | 0.831 | 1.000 | 0.872 | 1.000 | 0.974 | 1.000 | 0.889 | 1.000 | 1.000 | 1.000 | 0.980 | 1.000 | 1.000 | 0.819 | 1.000 | 1.000 | 0.873 | 0.847 |
| 4334 | 0.831 | 1.000 | 0.872 | 1.000 | 0.974 | 1.000 | 0.891 | 1.000 | 1.000 | 1.000 | 0.980 | 1.000 | 1.000 | 0.819 | 1.000 | 1.000 | 0.873 | 0.847 |
| 4335 | 0.831 | 1.000 | 0.874 | 1.000 | 0.974 | 1.000 | 0.889 | 1.000 | 1.000 | 1.000 | 0.980 | 1.000 | 1.000 | 0.819 | 1.000 | 1.000 | 0.873 | 0.847 |
| 4336 | 0.831 | 1.000 | 0.872 | 1.000 | 0.974 | 1.000 | 0.891 | 1.000 | 1.000 | 1.000 | 0.980 | 1.000 | 1.000 | 0.819 | 1.000 | 1.000 | 0.873 | 0.847 |
| 4337 | 0.831 | 1.000 | 0.870 | 1.000 | 0.974 | 1.000 | 0.891 | 1.000 | 1.000 | 1.000 | 0.980 | 1.000 | 1.000 | 0.819 | 1.000 | 1.000 | 0.873 | 0.847 |
| 4338 | 0.831 | 1.000 | 0.870 | 1.000 | 0.974 | 1.000 | 0.891 | 1.000 | 1.000 | 1.000 | 0.980 | 1.000 | 1.000 | 0.819 | 1.000 | 1.000 | 0.873 | 0.847 |
| 4339 | 0.831 | 1.000 | 0.870 | 1.000 | 0.974 | 1.000 | 0.891 | 1.000 | 1.000 | 1.000 | 0.980 | 1.000 | 1.000 | 0.819 | 1.000 | 1.000 | 0.873 | 0.846 |
| 4340 | 0.831 | 1.000 | 0.872 | 1.000 | 0.974 | 1.000 | 0.891 | 1.000 | 1.000 | 1.000 | 0.980 | 1.000 | 1.000 | 0.818 | 1.000 | 1.000 | 0.873 | 0.847 |
| 4341 | 0.831 | 1.000 | 0.870 | 1.000 | 0.974 | 1.000 | 0.891 | 1.000 | 1.000 | 1.000 | 0.980 | 1.000 | 1.000 | 0.817 | 1.000 | 1.000 | 0.873 | 0.846 |
| 4342 | 0.831 | 1.000 | 0.872 | 1.000 | 0.974 | 1.000 | 0.891 | 1.000 | 1.000 | 1.000 | 0.980 | 1.000 | 1.000 | 0.816 | 1.000 | 1.000 | 0.873 | 0.846 |
| 4343 | 0.831 | 1.000 | 0.875 | 1.000 | 0.974 | 1.000 | 0.891 | 1.000 | 1.000 | 1.000 | 0.980 | 1.000 | 1.000 | 0.819 | 1.000 | 1.000 | 0.873 | 0.847 |
| 4344 | 0.831 | 1.000 | 0.875 | 1.000 | 0.974 | 1.000 | 0.891 | 1.000 | 1.000 | 1.000 | 0.980 | 1.000 | 1.000 | 0.819 | 1.000 | 1.000 | 0.873 | 0.847 |
| 4345 | 0.830 | 1.000 | 0.875 | 1.000 | 0.974 | 1.000 | 0.891 | 1.000 | 1.000 | 1.000 | 0.980 | 1.000 | 1.000 | 0.819 | 1.000 | 1.000 | 0.873 | 0.847 |
| 4346 | 0.831 | 1.000 | 0.874 | 1.000 | 0.974 | 1.000 | 0.891 | 1.000 | 1.000 | 1.000 | 0.980 | 1.000 | 1.000 | 0.817 | 1.000 | 1.000 | 0.873 | 0.847 |
| 4347 | 0.831 | 1.000 | 0.874 | 1.000 | 0.974 | 1.000 | 0.891 | 1.000 | 1.000 | 1.000 | 0.980 | 1.000 | 1.000 | 0.816 | 1.000 | 1.000 | 0.873 | 0.846 |
| 4348 | 0.831 | 1.000 | 0.874 | 1.000 | 0.974 | 1.000 | 0.891 | 1.000 | 1.000 | 1.000 | 0.980 | 1.000 | 1.000 | 0.816 | 1.000 | 1.000 | 0.873 | 0.846 |
| 4349 | 0.831 | 1.000 | 0.874 | 1.000 | 0.974 | 1.000 | 0.892 | 1.000 | 1.000 | 1.000 | 0.980 | 1.000 | 1.000 | 0.815 | 1.000 | 1.000 | 0.873 | 0.846 |
| 4350 | 0.832 | 1.000 | 0.874 | 1.000 | 0.974 | 1.000 | 0.891 | 1.000 | 1.000 | 1.000 | 0.980 | 1.000 | 1.000 | 0.816 | 1.000 | 1.000 | 0.873 | 0.847 |
| 4351 | 0.832 | 1.000 | 0.874 | 1.000 | 0.974 | 1.000 | 0.891 | 1.000 | 1.000 | 1.000 | 0.980 | 1.000 | 1.000 | 0.816 | 1.000 | 1.000 | 0.873 | 0.847 |
| 4352 | 0.832 | 1.000 | 0.875 | 1.000 | 0.974 | 1.000 | 0.891 | 1.000 | 1.000 | 1.000 | 0.980 | 1.000 | 1.000 | 0.817 | 1.000 | 1.000 | 0.873 | 0.847 |
| 4353 | 0.832 | 1.000 | 0.875 | 1.000 | 0.974 | 1.000 | 0.892 | 1.000 | 1.000 | 1.000 | 0.980 | 1.000 | 1.000 | 0.816 | 1.000 | 1.000 | 0.873 | 0.847 |
| 4354 | 0.833 | 1.000 | 0.877 | 1.000 | 0.974 | 1.000 | 0.892 | 1.000 | 1.000 | 1.000 | 0.980 | 1.000 | 1.000 | 0.816 | 1.000 | 1.000 | 0.874 | 0.848 |
| 4355 | 0.831 | 1.000 | 0.877 | 1.000 | 0.974 | 1.000 | 0.892 | 1.000 | 1.000 | 1.000 | 0.980 | 1.000 | 1.000 | 0.817 | 1.000 | 1.000 | 0.874 | 0.848 |
| 4356 | 0.831 | 1.000 | 0.877 | 1.000 | 0.974 | 1.000 | 0.892 | 1.000 | 1.000 | 1.000 | 0.980 | 1.000 | 1.000 | 0.818 | 1.000 | 1.000 | 0.874 | 0.848 |
| 4357 | 0.831 | 1.000 | 0.877 | 1.000 | 0.974 | 1.000 | 0.892 | 1.000 | 1.000 | 1.000 | 0.980 | 1.000 | 1.000 | 0.818 | 1.000 | 1.000 | 0.874 | 0.848 |
| 4358 | 0.831 | 1.000 | 0.874 | 1.000 | 0.974 | 1.000 | 0.895 | 1.000 | 1.000 | 1.000 | 0.980 | 1.000 | 1.000 | 0.819 | 1.000 | 1.000 | 0.874 | 0.848 |
| 4359 | 0.831 | 1.000 | 0.872 | 1.000 | 0.974 | 1.000 | 0.894 | 1.000 | 1.000 | 1.000 | 0.980 | 1.000 | 1.000 | 0.820 | 1.000 | 1.000 | 0.874 | 0.848 |
| 4360 | 0.831 | 1.000 | 0.872 | 1.000 | 0.974 | 1.000 | 0.894 | 1.000 | 1.000 | 1.000 | 0.980 | 1.000 | 1.000 | 0.819 | 1.000 | 1.000 | 0.874 | 0.848 |
| 4361 | 0.831 | 1.000 | 0.872 | 1.000 | 0.974 | 1.000 | 0.891 | 1.000 | 1.000 | 1.000 | 0.980 | 1.000 | 1.000 | 0.819 | 1.000 | 1.000 | 0.873 | 0.847 |
| 4362 | 0.832 | 1.000 | 0.872 | 1.000 | 0.974 | 1.000 | 0.894 | 1.000 | 1.000 | 1.000 | 0.980 | 1.000 | 1.000 | 0.819 | 1.000 | 1.000 | 0.874 | 0.848 |
| 4363 | 0.831 | 1.000 | 0.872 | 1.000 | 0.974 | 1.000 | 0.892 | 1.000 | 1.000 | 1.000 | 0.980 | 1.000 | 1.000 | 0.819 | 1.000 | 1.000 | 0.874 | 0.847 |
| 4364 | 0.832 | 1.000 | 0.872 | 1.000 | 0.974 | 1.000 | 0.894 | 1.000 | 1.000 | 1.000 | 0.980 | 1.000 | 1.000 | 0.819 | 1.000 | 1.000 | 0.874 | 0.848 |
| 4365 | 0.832 | 1.000 | 0.872 | 1.000 | 0.974 | 1.000 | 0.894 | 1.000 | 1.000 | 1.000 | 0.980 | 1.000 | 1.000 | 0.819 | 1.000 | 1.000 | 0.874 | 0.848 |
| 4366 | 0.833 | 1.000 | 0.872 | 1.000 | 0.974 | 1.000 | 0.892 | 1.000 | 1.000 | 1.000 | 0.980 | 1.000 | 1.000 | 0.819 | 1.000 | 1.000 | 0.874 | 0.848 |
| 4367 | 0.833 | 1.000 | 0.872 | 1.000 | 0.974 | 1.000 | 0.894 | 1.000 | 1.000 | 1.000 | 0.980 | 1.000 | 1.000 | 0.819 | 1.000 | 1.000 | 0.874 | 0.848 |
| 4368 | 0.833 | 1.000 | 0.872 | 1.000 | 0.974 | 1.000 | 0.892 | 1.000 | 1.000 | 1.000 | 0.980 | 1.000 | 1.000 | 0.819 | 1.000 | 1.000 | 0.874 | 0.848 |
| 4369 | 0.833 | 1.000 | 0.872 | 1.000 | 0.974 | 1.000 | 0.892 | 1.000 | 1.000 | 1.000 | 0.980 | 1.000 | 1.000 | 0.819 | 1.000 | 1.000 | 0.874 | 0.848 |
| 4370 | 0.833 | 1.000 | 0.872 | 1.000 | 0.974 | 1.000 | 0.892 | 1.000 | 1.000 | 1.000 | 0.980 | 1.000 | 1.000 | 0.819 | 1.000 | 1.000 | 0.874 | 0.848 |
| 4371 | 0.833 | 1.000 | 0.872 | 1.000 | 0.974 | 1.000 | 0.892 | 1.000 | 1.000 | 1.000 | 0.980 | 1.000 | 1.000 | 0.819 | 1.000 | 1.000 | 0.874 | 0.848 |

| | | | | | | | | | | | | | | | | | | |
|---|---|---|---|---|---|---|---|---|---|---|---|---|---|---|---|---|---|---|
| 4372 | 0.833 | 1.000 | 0.872 | 1.000 | 0.974 | 1.000 | 0.892 | 1.000 | 1.000 | 1.000 | 0.980 | 1.000 | 1.000 | 0.819 | 1.000 | 1.000 | 0.874 | 0.848 |
| 4373 | 0.832 | 1.000 | 0.872 | 1.000 | 0.974 | 1.000 | 0.891 | 1.000 | 1.000 | 1.000 | 0.980 | 1.000 | 1.000 | 0.819 | 1.000 | 1.000 | 0.873 | 0.847 |
| 4374 | 0.832 | 1.000 | 0.872 | 1.000 | 0.974 | 1.000 | 0.891 | 1.000 | 1.000 | 1.000 | 0.980 | 1.000 | 1.000 | 0.819 | 1.000 | 1.000 | 0.874 | 0.847 |
| 4375 | 0.832 | 1.000 | 0.872 | 1.000 | 0.974 | 1.000 | 0.891 | 1.000 | 1.000 | 1.000 | 0.980 | 1.000 | 1.000 | 0.819 | 1.000 | 1.000 | 0.873 | 0.847 |
| 4376 | 0.832 | 1.000 | 0.872 | 1.000 | 0.974 | 1.000 | 0.891 | 1.000 | 1.000 | 1.000 | 0.980 | 1.000 | 1.000 | 0.819 | 1.000 | 1.000 | 0.874 | 0.847 |
| 4377 | 0.831 | 1.000 | 0.872 | 1.000 | 0.974 | 1.000 | 0.892 | 1.000 | 1.000 | 1.000 | 0.980 | 1.000 | 1.000 | 0.819 | 1.000 | 1.000 | 0.873 | 0.847 |
| 4378 | 0.831 | 1.000 | 0.872 | 1.000 | 0.974 | 1.000 | 0.891 | 1.000 | 1.000 | 1.000 | 0.980 | 1.000 | 1.000 | 0.819 | 1.000 | 1.000 | 0.873 | 0.847 |
| 4379 | 0.831 | 1.000 | 0.872 | 1.000 | 0.974 | 1.000 | 0.892 | 1.000 | 1.000 | 1.000 | 0.980 | 1.000 | 1.000 | 0.819 | 1.000 | 1.000 | 0.874 | 0.847 |
| 4380 | 0.831 | 1.000 | 0.872 | 1.000 | 0.974 | 1.000 | 0.892 | 1.000 | 1.000 | 1.000 | 0.980 | 1.000 | 1.000 | 0.819 | 1.000 | 1.000 | 0.874 | 0.847 |
| 4381 | 0.831 | 1.000 | 0.872 | 1.000 | 0.974 | 1.000 | 0.892 | 1.000 | 1.000 | 1.000 | 0.980 | 1.000 | 1.000 | 0.819 | 1.000 | 1.000 | 0.873 | 0.847 |
| 4382 | 0.831 | 1.000 | 0.872 | 1.000 | 0.974 | 1.000 | 0.892 | 1.000 | 1.000 | 1.000 | 0.980 | 1.000 | 1.000 | 0.819 | 1.000 | 1.000 | 0.873 | 0.847 |
| 4383 | 0.832 | 1.000 | 0.870 | 1.000 | 0.979 | 1.000 | 0.894 | 1.000 | 1.000 | 1.000 | 0.980 | 1.000 | 1.000 | 0.819 | 1.000 | 1.000 | 0.874 | 0.848 |
| 4384 | 0.832 | 1.000 | 0.872 | 1.000 | 0.979 | 1.000 | 0.895 | 1.000 | 1.000 | 1.000 | 0.980 | 1.000 | 1.000 | 0.819 | 1.000 | 1.000 | 0.874 | 0.848 |
| 4385 | 0.832 | 1.000 | 0.870 | 1.000 | 0.979 | 1.000 | 0.895 | 1.000 | 1.000 | 1.000 | 0.980 | 1.000 | 1.000 | 0.819 | 1.000 | 1.000 | 0.874 | 0.848 |
| 4386 | 0.833 | 1.000 | 0.870 | 1.000 | 0.979 | 1.000 | 0.897 | 1.000 | 1.000 | 1.000 | 0.980 | 1.000 | 1.000 | 0.819 | 1.000 | 1.000 | 0.875 | 0.849 |
| 4387 | 0.833 | 1.000 | 0.870 | 1.000 | 0.979 | 1.000 | 0.897 | 1.000 | 1.000 | 1.000 | 0.980 | 1.000 | 1.000 | 0.819 | 1.000 | 1.000 | 0.875 | 0.849 |
| 4388 | 0.833 | 1.000 | 0.870 | 1.000 | 0.979 | 1.000 | 0.897 | 1.000 | 1.000 | 1.000 | 0.980 | 1.000 | 1.000 | 0.818 | 1.000 | 1.000 | 0.874 | 0.848 |
| 4389 | 0.833 | 1.000 | 0.872 | 1.000 | 0.979 | 1.000 | 0.895 | 1.000 | 1.000 | 1.000 | 0.980 | 1.000 | 1.000 | 0.817 | 1.000 | 1.000 | 0.874 | 0.848 |
| 4390 | 0.833 | 1.000 | 0.872 | 1.000 | 0.979 | 1.000 | 0.895 | 1.000 | 1.000 | 1.000 | 0.980 | 1.000 | 1.000 | 0.817 | 1.000 | 1.000 | 0.874 | 0.848 |
| 4391 | 0.833 | 1.000 | 0.872 | 1.000 | 0.979 | 1.000 | 0.895 | 1.000 | 1.000 | 1.000 | 0.980 | 1.000 | 1.000 | 0.817 | 1.000 | 1.000 | 0.874 | 0.848 |
| 4392 | 0.833 | 1.000 | 0.870 | 1.000 | 0.979 | 1.000 | 0.895 | 1.000 | 1.000 | 1.000 | 0.980 | 1.000 | 1.000 | 0.817 | 1.000 | 1.000 | 0.874 | 0.848 |
| 4393 | 0.833 | 1.000 | 0.872 | 1.000 | 0.979 | 1.000 | 0.895 | 1.000 | 1.000 | 1.000 | 0.980 | 1.000 | 1.000 | 0.817 | 1.000 | 1.000 | 0.874 | 0.848 |
| 4394 | 0.833 | 1.000 | 0.870 | 1.000 | 0.979 | 1.000 | 0.897 | 1.000 | 1.000 | 1.000 | 0.980 | 1.000 | 1.000 | 0.817 | 1.000 | 1.000 | 0.874 | 0.848 |
| 4395 | 0.833 | 1.000 | 0.870 | 1.000 | 0.979 | 1.000 | 0.897 | 1.000 | 1.000 | 1.000 | 0.980 | 1.000 | 1.000 | 0.818 | 1.000 | 1.000 | 0.874 | 0.848 |
| 4396 | 0.833 | 1.000 | 0.872 | 1.000 | 0.979 | 1.000 | 0.897 | 1.000 | 1.000 | 1.000 | 0.980 | 1.000 | 1.000 | 0.818 | 1.000 | 1.000 | 0.875 | 0.849 |
| 4397 | 0.833 | 1.000 | 0.872 | 1.000 | 0.979 | 1.000 | 0.897 | 1.000 | 1.000 | 1.000 | 0.980 | 1.000 | 1.000 | 0.817 | 1.000 | 1.000 | 0.874 | 0.848 |
| 4398 | 0.833 | 1.000 | 0.872 | 1.000 | 0.979 | 1.000 | 0.897 | 1.000 | 1.000 | 1.000 | 0.980 | 1.000 | 1.000 | 0.819 | 1.000 | 1.000 | 0.875 | 0.849 |
| 4399 | 0.833 | 1.000 | 0.872 | 1.000 | 0.979 | 1.000 | 0.897 | 1.000 | 1.000 | 1.000 | 0.980 | 1.000 | 1.000 | 0.819 | 1.000 | 1.000 | 0.875 | 0.849 |
| 4400 | 0.833 | 1.000 | 0.872 | 1.000 | 0.979 | 1.000 | 0.895 | 1.000 | 1.000 | 1.000 | 0.980 | 1.000 | 1.000 | 0.819 | 1.000 | 1.000 | 0.875 | 0.849 |
| 4401 | 0.833 | 1.000 | 0.872 | 1.000 | 0.979 | 1.000 | 0.895 | 1.000 | 1.000 | 1.000 | 0.980 | 1.000 | 1.000 | 0.819 | 1.000 | 1.000 | 0.875 | 0.849 |
| 4402 | 0.833 | 1.000 | 0.872 | 1.000 | 0.979 | 1.000 | 0.895 | 1.000 | 1.000 | 1.000 | 0.980 | 1.000 | 1.000 | 0.819 | 1.000 | 1.000 | 0.875 | 0.849 |
| 4403 | 0.833 | 1.000 | 0.870 | 1.000 | 0.979 | 1.000 | 0.895 | 1.000 | 1.000 | 1.000 | 0.980 | 1.000 | 1.000 | 0.819 | 1.000 | 1.000 | 0.874 | 0.848 |
| 4404 | 0.834 | 1.000 | 0.872 | 1.000 | 0.979 | 1.000 | 0.895 | 1.000 | 1.000 | 1.000 | 0.980 | 1.000 | 1.000 | 0.819 | 1.000 | 1.000 | 0.875 | 0.849 |
| 4405 | 0.833 | 1.000 | 0.870 | 1.000 | 0.979 | 1.000 | 0.897 | 1.000 | 1.000 | 1.000 | 0.980 | 1.000 | 1.000 | 0.819 | 1.000 | 1.000 | 0.875 | 0.849 |
| 4406 | 0.833 | 1.000 | 0.870 | 1.000 | 0.979 | 1.000 | 0.897 | 1.000 | 1.000 | 1.000 | 0.980 | 1.000 | 1.000 | 0.819 | 1.000 | 1.000 | 0.875 | 0.849 |
| 4407 | 0.833 | 1.000 | 0.872 | 1.000 | 0.979 | 1.000 | 0.894 | 1.000 | 1.000 | 1.000 | 0.980 | 1.000 | 1.000 | 0.819 | 1.000 | 1.000 | 0.874 | 0.848 |
| 4408 | 0.833 | 1.000 | 0.872 | 1.000 | 0.979 | 1.000 | 0.894 | 1.000 | 1.000 | 1.000 | 0.980 | 1.000 | 1.000 | 0.819 | 1.000 | 1.000 | 0.874 | 0.848 |
| 4409 | 0.833 | 1.000 | 0.872 | 1.000 | 0.979 | 1.000 | 0.894 | 1.000 | 1.000 | 1.000 | 0.980 | 1.000 | 1.000 | 0.819 | 1.000 | 1.000 | 0.874 | 0.848 |
| 4410 | 0.833 | 1.000 | 0.870 | 1.000 | 0.979 | 1.000 | 0.895 | 1.000 | 1.000 | 1.000 | 0.980 | 1.000 | 1.000 | 0.819 | 1.000 | 1.000 | 0.875 | 0.849 |

| | | | | | | | | | | | | | | | | | | |
|---|---|---|---|---|---|---|---|---|---|---|---|---|---|---|---|---|---|---|
| 4411 | 0.833 | 1.000 | 0.868 | 1.000 | 0.979 | 1.000 | 0.895 | 1.000 | 1.000 | 1.000 | 0.980 | 1.000 | 1.000 | 0.819 | 1.000 | 1.000 | 0.874 | 0.848 |
| 4412 | 0.833 | 1.000 | 0.868 | 1.000 | 0.979 | 1.000 | 0.895 | 1.000 | 1.000 | 1.000 | 0.977 | 1.000 | 1.000 | 0.819 | 1.000 | 1.000 | 0.874 | 0.848 |
| 4413 | 0.833 | 1.000 | 0.868 | 1.000 | 0.979 | 1.000 | 0.895 | 1.000 | 1.000 | 1.000 | 0.977 | 1.000 | 1.000 | 0.819 | 1.000 | 1.000 | 0.874 | 0.848 |
| 4414 | 0.833 | 1.000 | 0.868 | 1.000 | 0.979 | 1.000 | 0.895 | 1.000 | 1.000 | 1.000 | 0.977 | 1.000 | 1.000 | 0.819 | 1.000 | 1.000 | 0.874 | 0.848 |
| 4415 | 0.835 | 1.000 | 0.868 | 1.000 | 0.979 | 1.000 | 0.895 | 1.000 | 1.000 | 1.000 | 0.977 | 1.000 | 1.000 | 0.819 | 1.000 | 1.000 | 0.875 | 0.849 |
| 4416 | 0.835 | 1.000 | 0.868 | 1.000 | 0.979 | 1.000 | 0.895 | 1.000 | 1.000 | 1.000 | 0.977 | 1.000 | 1.000 | 0.819 | 1.000 | 1.000 | 0.874 | 0.848 |
| 4417 | 0.835 | 1.000 | 0.868 | 1.000 | 0.979 | 1.000 | 0.895 | 1.000 | 1.000 | 1.000 | 0.977 | 1.000 | 1.000 | 0.819 | 1.000 | 1.000 | 0.875 | 0.849 |
| 4418 | 0.835 | 1.000 | 0.868 | 1.000 | 0.979 | 1.000 | 0.895 | 1.000 | 1.000 | 1.000 | 0.977 | 1.000 | 1.000 | 0.819 | 1.000 | 1.000 | 0.875 | 0.849 |
| 4419 | 0.835 | 1.000 | 0.868 | 1.000 | 0.979 | 1.000 | 0.897 | 1.000 | 1.000 | 1.000 | 0.977 | 1.000 | 1.000 | 0.819 | 1.000 | 1.000 | 0.875 | 0.849 |
| 4420 | 0.835 | 1.000 | 0.868 | 1.000 | 0.979 | 1.000 | 0.895 | 1.000 | 1.000 | 1.000 | 0.977 | 1.000 | 1.000 | 0.819 | 1.000 | 1.000 | 0.875 | 0.849 |
| 4421 | 0.835 | 1.000 | 0.868 | 1.000 | 0.979 | 1.000 | 0.895 | 1.000 | 1.000 | 1.000 | 0.977 | 1.000 | 1.000 | 0.819 | 1.000 | 1.000 | 0.875 | 0.849 |
| 4422 | 0.835 | 1.000 | 0.868 | 1.000 | 0.979 | 1.000 | 0.895 | 1.000 | 1.000 | 1.000 | 0.977 | 1.000 | 1.000 | 0.820 | 1.000 | 1.000 | 0.875 | 0.849 |
| 4423 | 0.836 | 1.000 | 0.868 | 1.000 | 0.979 | 1.000 | 0.895 | 1.000 | 1.000 | 1.000 | 0.977 | 1.000 | 1.000 | 0.821 | 1.000 | 1.000 | 0.875 | 0.850 |
| 4424 | 0.835 | 1.000 | 0.868 | 1.000 | 0.979 | 1.000 | 0.895 | 1.000 | 1.000 | 1.000 | 0.977 | 1.000 | 1.000 | 0.821 | 1.000 | 1.000 | 0.875 | 0.849 |
| 4425 | 0.835 | 1.000 | 0.868 | 1.000 | 0.979 | 1.000 | 0.895 | 1.000 | 1.000 | 1.000 | 0.977 | 1.000 | 1.000 | 0.820 | 1.000 | 1.000 | 0.875 | 0.849 |
| 4426 | 0.835 | 1.000 | 0.868 | 1.000 | 0.979 | 1.000 | 0.895 | 1.000 | 1.000 | 1.000 | 0.977 | 1.000 | 1.000 | 0.821 | 1.000 | 1.000 | 0.875 | 0.849 |
| 4427 | 0.835 | 1.000 | 0.868 | 1.000 | 0.979 | 1.000 | 0.895 | 1.000 | 1.000 | 1.000 | 0.977 | 1.000 | 1.000 | 0.821 | 1.000 | 1.000 | 0.875 | 0.849 |
| 4428 | 0.835 | 1.000 | 0.868 | 1.000 | 0.979 | 1.000 | 0.895 | 1.000 | 1.000 | 1.000 | 0.977 | 1.000 | 1.000 | 0.820 | 1.000 | 1.000 | 0.875 | 0.849 |
| 4429 | 0.835 | 1.000 | 0.868 | 1.000 | 0.979 | 1.000 | 0.895 | 1.000 | 1.000 | 1.000 | 0.977 | 1.000 | 1.000 | 0.820 | 1.000 | 1.000 | 0.875 | 0.849 |
| 4430 | 0.835 | 1.000 | 0.868 | 1.000 | 0.979 | 1.000 | 0.895 | 1.000 | 1.000 | 1.000 | 0.977 | 1.000 | 1.000 | 0.820 | 1.000 | 1.000 | 0.875 | 0.849 |
| 4431 | 0.834 | 1.000 | 0.868 | 1.000 | 0.979 | 1.000 | 0.895 | 1.000 | 1.000 | 1.000 | 0.977 | 1.000 | 1.000 | 0.819 | 1.000 | 1.000 | 0.874 | 0.848 |
| 4432 | 0.834 | 1.000 | 0.868 | 1.000 | 0.979 | 1.000 | 0.895 | 1.000 | 1.000 | 1.000 | 0.977 | 1.000 | 1.000 | 0.819 | 1.000 | 1.000 | 0.874 | 0.848 |
| 4433 | 0.835 | 1.000 | 0.868 | 1.000 | 0.979 | 1.000 | 0.895 | 1.000 | 1.000 | 1.000 | 0.977 | 1.000 | 1.000 | 0.820 | 1.000 | 1.000 | 0.875 | 0.849 |
| 4434 | 0.835 | 1.000 | 0.868 | 1.000 | 0.979 | 1.000 | 0.895 | 1.000 | 1.000 | 1.000 | 0.977 | 1.000 | 1.000 | 0.820 | 1.000 | 1.000 | 0.875 | 0.849 |
| 4435 | 0.835 | 1.000 | 0.868 | 1.000 | 0.979 | 1.000 | 0.895 | 1.000 | 1.000 | 1.000 | 0.977 | 1.000 | 1.000 | 0.821 | 1.000 | 1.000 | 0.875 | 0.849 |
| 4436 | 0.835 | 1.000 | 0.870 | 1.000 | 0.979 | 1.000 | 0.895 | 1.000 | 1.000 | 1.000 | 0.977 | 1.000 | 1.000 | 0.821 | 1.000 | 1.000 | 0.875 | 0.850 |
| 4437 | 0.835 | 1.000 | 0.868 | 1.000 | 0.979 | 1.000 | 0.895 | 1.000 | 1.000 | 1.000 | 0.977 | 1.000 | 1.000 | 0.819 | 1.000 | 1.000 | 0.875 | 0.849 |
| 4438 | 0.835 | 1.000 | 0.868 | 1.000 | 0.979 | 1.000 | 0.895 | 1.000 | 1.000 | 1.000 | 0.977 | 1.000 | 1.000 | 0.819 | 1.000 | 1.000 | 0.875 | 0.849 |
| 4439 | 0.835 | 1.000 | 0.868 | 1.000 | 0.979 | 1.000 | 0.895 | 1.000 | 1.000 | 1.000 | 0.977 | 1.000 | 1.000 | 0.819 | 1.000 | 1.000 | 0.875 | 0.849 |
| 4440 | 0.835 | 1.000 | 0.868 | 1.000 | 0.979 | 1.000 | 0.895 | 1.000 | 1.000 | 1.000 | 0.977 | 1.000 | 1.000 | 0.819 | 1.000 | 1.000 | 0.875 | 0.849 |
| 4441 | 0.835 | 1.000 | 0.868 | 1.000 | 0.979 | 1.000 | 0.895 | 1.000 | 1.000 | 1.000 | 0.977 | 1.000 | 1.000 | 0.819 | 1.000 | 1.000 | 0.874 | 0.848 |
| 4442 | 0.835 | 1.000 | 0.866 | 1.000 | 0.979 | 1.000 | 0.895 | 1.000 | 1.000 | 1.000 | 0.977 | 1.000 | 1.000 | 0.819 | 1.000 | 1.000 | 0.874 | 0.848 |
| 4443 | 0.835 | 1.000 | 0.866 | 1.000 | 0.979 | 1.000 | 0.895 | 1.000 | 1.000 | 1.000 | 0.977 | 1.000 | 1.000 | 0.819 | 1.000 | 1.000 | 0.875 | 0.849 |
| 4444 | 0.835 | 1.000 | 0.866 | 1.000 | 0.979 | 1.000 | 0.895 | 1.000 | 1.000 | 1.000 | 0.977 | 1.000 | 1.000 | 0.819 | 1.000 | 1.000 | 0.875 | 0.849 |
| 4445 | 0.836 | 1.000 | 0.866 | 1.000 | 0.979 | 1.000 | 0.895 | 1.000 | 1.000 | 1.000 | 0.977 | 1.000 | 1.000 | 0.819 | 1.000 | 1.000 | 0.875 | 0.849 |
| 4446 | 0.836 | 1.000 | 0.868 | 1.000 | 0.979 | 1.000 | 0.895 | 1.000 | 1.000 | 1.000 | 0.977 | 1.000 | 1.000 | 0.819 | 1.000 | 1.000 | 0.875 | 0.849 |
| 4447 | 0.833 | 1.000 | 0.870 | 1.000 | 0.979 | 1.000 | 0.894 | 1.000 | 1.000 | 1.000 | 0.977 | 1.000 | 1.000 | 0.819 | 1.000 | 1.000 | 0.874 | 0.848 |
| 4448 | 0.833 | 1.000 | 0.870 | 1.000 | 0.979 | 1.000 | 0.894 | 1.000 | 1.000 | 1.000 | 0.977 | 1.000 | 1.000 | 0.819 | 1.000 | 1.000 | 0.874 | 0.848 |
| 4449 | 0.833 | 1.000 | 0.870 | 1.000 | 0.979 | 1.000 | 0.894 | 1.000 | 1.000 | 1.000 | 0.977 | 1.000 | 1.000 | 0.819 | 1.000 | 1.000 | 0.874 | 0.848 |

| | | | | | | | | | | | | | | | | | | |
|---|---|---|---|---|---|---|---|---|---|---|---|---|---|---|---|---|---|---|
| 4450 | 0.832 | 1.000 | 0.870 | 1.000 | 0.979 | 1.000 | 0.894 | 1.000 | 1.000 | 1.000 | 0.980 | 1.000 | 1.000 | 0.820 | 1.000 | 1.000 | 0.874 | 0.848 |
| 4451 | 0.832 | 1.000 | 0.870 | 1.000 | 0.979 | 1.000 | 0.894 | 1.000 | 1.000 | 1.000 | 0.980 | 1.000 | 1.000 | 0.820 | 1.000 | 1.000 | 0.874 | 0.848 |
| 4452 | 0.832 | 1.000 | 0.870 | 1.000 | 0.979 | 1.000 | 0.894 | 1.000 | 1.000 | 1.000 | 0.980 | 1.000 | 1.000 | 0.819 | 1.000 | 1.000 | 0.874 | 0.848 |
| 4453 | 0.832 | 1.000 | 0.870 | 1.000 | 0.979 | 1.000 | 0.894 | 1.000 | 1.000 | 1.000 | 0.980 | 1.000 | 1.000 | 0.819 | 1.000 | 1.000 | 0.874 | 0.848 |
| 4454 | 0.832 | 1.000 | 0.870 | 1.000 | 0.979 | 1.000 | 0.894 | 1.000 | 1.000 | 1.000 | 0.980 | 1.000 | 1.000 | 0.819 | 1.000 | 1.000 | 0.874 | 0.848 |
| 4455 | 0.832 | 1.000 | 0.870 | 1.000 | 0.979 | 1.000 | 0.894 | 1.000 | 1.000 | 1.000 | 0.980 | 1.000 | 1.000 | 0.819 | 1.000 | 1.000 | 0.874 | 0.848 |
| 4456 | 0.833 | 1.000 | 0.870 | 1.000 | 0.979 | 1.000 | 0.894 | 1.000 | 1.000 | 1.000 | 0.980 | 1.000 | 1.000 | 0.819 | 1.000 | 1.000 | 0.874 | 0.848 |
| 4457 | 0.832 | 1.000 | 0.870 | 1.000 | 0.979 | 1.000 | 0.894 | 1.000 | 1.000 | 1.000 | 0.980 | 1.000 | 1.000 | 0.819 | 1.000 | 1.000 | 0.874 | 0.848 |
| 4458 | 0.833 | 1.000 | 0.870 | 1.000 | 0.979 | 1.000 | 0.892 | 1.000 | 1.000 | 1.000 | 0.980 | 1.000 | 1.000 | 0.819 | 1.000 | 1.000 | 0.874 | 0.848 |
| 4459 | 0.832 | 1.000 | 0.870 | 1.000 | 0.979 | 1.000 | 0.892 | 1.000 | 1.000 | 1.000 | 0.980 | 1.000 | 1.000 | 0.819 | 1.000 | 1.000 | 0.874 | 0.847 |
| 4460 | 0.834 | 1.000 | 0.870 | 1.000 | 0.979 | 1.000 | 0.892 | 1.000 | 1.000 | 1.000 | 0.980 | 1.000 | 1.000 | 0.819 | 1.000 | 1.000 | 0.874 | 0.848 |
| 4461 | 0.834 | 1.000 | 0.870 | 1.000 | 0.979 | 1.000 | 0.892 | 1.000 | 1.000 | 1.000 | 0.980 | 1.000 | 1.000 | 0.819 | 1.000 | 1.000 | 0.874 | 0.848 |
| 4462 | 0.834 | 1.000 | 0.870 | 1.000 | 0.979 | 1.000 | 0.892 | 1.000 | 1.000 | 1.000 | 0.980 | 1.000 | 1.000 | 0.819 | 1.000 | 1.000 | 0.874 | 0.848 |
| 4463 | 0.834 | 1.000 | 0.870 | 1.000 | 0.979 | 1.000 | 0.892 | 1.000 | 1.000 | 1.000 | 0.980 | 1.000 | 1.000 | 0.821 | 1.000 | 1.000 | 0.875 | 0.849 |
| 4464 | 0.834 | 1.000 | 0.870 | 1.000 | 0.979 | 1.000 | 0.892 | 1.000 | 1.000 | 1.000 | 0.980 | 1.000 | 1.000 | 0.818 | 1.000 | 1.000 | 0.874 | 0.848 |
| 4465 | 0.833 | 1.000 | 0.870 | 1.000 | 0.979 | 1.000 | 0.892 | 1.000 | 1.000 | 1.000 | 0.980 | 1.000 | 1.000 | 0.819 | 1.000 | 1.000 | 0.874 | 0.848 |
| 4466 | 0.833 | 1.000 | 0.870 | 1.000 | 0.979 | 1.000 | 0.892 | 1.000 | 1.000 | 1.000 | 0.980 | 1.000 | 1.000 | 0.818 | 1.000 | 1.000 | 0.874 | 0.848 |
| 4467 | 0.833 | 1.000 | 0.870 | 1.000 | 0.979 | 1.000 | 0.892 | 1.000 | 1.000 | 1.000 | 0.980 | 1.000 | 1.000 | 0.819 | 1.000 | 1.000 | 0.874 | 0.848 |
| 4468 | 0.832 | 1.000 | 0.870 | 1.000 | 0.979 | 1.000 | 0.892 | 1.000 | 1.000 | 1.000 | 0.980 | 1.000 | 1.000 | 0.819 | 1.000 | 1.000 | 0.874 | 0.848 |
| 4469 | 0.832 | 1.000 | 0.870 | 1.000 | 0.979 | 1.000 | 0.892 | 1.000 | 1.000 | 1.000 | 0.980 | 1.000 | 1.000 | 0.818 | 1.000 | 1.000 | 0.873 | 0.847 |
| 4470 | 0.832 | 1.000 | 0.870 | 1.000 | 0.979 | 1.000 | 0.892 | 1.000 | 1.000 | 1.000 | 0.980 | 1.000 | 1.000 | 0.818 | 1.000 | 1.000 | 0.873 | 0.847 |
| 4471 | 0.832 | 1.000 | 0.870 | 1.000 | 0.979 | 1.000 | 0.892 | 1.000 | 1.000 | 1.000 | 0.980 | 1.000 | 1.000 | 0.818 | 1.000 | 1.000 | 0.873 | 0.847 |
| 4472 | 0.831 | 1.000 | 0.870 | 1.000 | 0.979 | 1.000 | 0.892 | 1.000 | 1.000 | 1.000 | 0.980 | 1.000 | 1.000 | 0.819 | 1.000 | 1.000 | 0.873 | 0.847 |
| 4473 | 0.831 | 1.000 | 0.870 | 1.000 | 0.979 | 1.000 | 0.892 | 1.000 | 1.000 | 1.000 | 0.980 | 1.000 | 1.000 | 0.819 | 1.000 | 1.000 | 0.873 | 0.847 |
| 4474 | 0.832 | 1.000 | 0.870 | 1.000 | 0.979 | 1.000 | 0.892 | 1.000 | 1.000 | 1.000 | 0.980 | 1.000 | 1.000 | 0.819 | 1.000 | 1.000 | 0.874 | 0.848 |
| 4475 | 0.831 | 1.000 | 0.870 | 1.000 | 0.979 | 1.000 | 0.892 | 1.000 | 1.000 | 1.000 | 0.980 | 1.000 | 1.000 | 0.819 | 1.000 | 1.000 | 0.873 | 0.847 |
| 4476 | 0.831 | 1.000 | 0.870 | 1.000 | 0.979 | 1.000 | 0.892 | 1.000 | 1.000 | 1.000 | 0.980 | 1.000 | 1.000 | 0.819 | 1.000 | 1.000 | 0.874 | 0.847 |
| 4477 | 0.831 | 1.000 | 0.870 | 1.000 | 0.979 | 1.000 | 0.892 | 1.000 | 1.000 | 1.000 | 0.980 | 1.000 | 1.000 | 0.819 | 1.000 | 1.000 | 0.874 | 0.847 |
| 4478 | 0.830 | 1.000 | 0.870 | 1.000 | 0.979 | 1.000 | 0.892 | 1.000 | 1.000 | 1.000 | 0.980 | 1.000 | 1.000 | 0.819 | 1.000 | 1.000 | 0.873 | 0.847 |
| 4479 | 0.830 | 1.000 | 0.870 | 1.000 | 0.979 | 1.000 | 0.892 | 1.000 | 1.000 | 1.000 | 0.980 | 1.000 | 1.000 | 0.819 | 1.000 | 1.000 | 0.873 | 0.847 |
| 4480 | 0.831 | 1.000 | 0.870 | 1.000 | 0.979 | 1.000 | 0.891 | 1.000 | 1.000 | 1.000 | 0.980 | 1.000 | 1.000 | 0.819 | 1.000 | 1.000 | 0.873 | 0.847 |
| 4481 | 0.831 | 1.000 | 0.870 | 1.000 | 0.979 | 1.000 | 0.891 | 1.000 | 1.000 | 1.000 | 0.980 | 1.000 | 1.000 | 0.819 | 1.000 | 1.000 | 0.873 | 0.847 |
| 4482 | 0.831 | 1.000 | 0.870 | 1.000 | 0.979 | 1.000 | 0.891 | 1.000 | 1.000 | 1.000 | 0.980 | 1.000 | 1.000 | 0.820 | 1.000 | 1.000 | 0.873 | 0.847 |
| 4483 | 0.831 | 1.000 | 0.870 | 1.000 | 0.979 | 1.000 | 0.891 | 1.000 | 1.000 | 1.000 | 0.980 | 1.000 | 1.000 | 0.820 | 1.000 | 1.000 | 0.874 | 0.847 |
| 4484 | 0.831 | 1.000 | 0.870 | 1.000 | 0.979 | 1.000 | 0.891 | 1.000 | 1.000 | 1.000 | 0.980 | 1.000 | 1.000 | 0.819 | 1.000 | 1.000 | 0.873 | 0.847 |
| 4485 | 0.831 | 1.000 | 0.870 | 1.000 | 0.979 | 1.000 | 0.894 | 1.000 | 1.000 | 1.000 | 0.980 | 1.000 | 1.000 | 0.821 | 1.000 | 1.000 | 0.874 | 0.848 |
| 4486 | 0.831 | 1.000 | 0.870 | 1.000 | 0.979 | 1.000 | 0.894 | 1.000 | 1.000 | 1.000 | 0.983 | 1.000 | 1.000 | 0.821 | 1.000 | 1.000 | 0.874 | 0.848 |
| 4487 | 0.831 | 1.000 | 0.870 | 1.000 | 0.979 | 1.000 | 0.895 | 1.000 | 1.000 | 1.000 | 0.983 | 1.000 | 1.000 | 0.821 | 1.000 | 1.000 | 0.875 | 0.849 |
| 4488 | 0.832 | 1.000 | 0.870 | 1.000 | 0.979 | 1.000 | 0.894 | 1.000 | 1.000 | 1.000 | 0.983 | 1.000 | 1.000 | 0.820 | 1.000 | 1.000 | 0.874 | 0.848 |

| | | | | | | | | | | | | | | | | | | |
|---|---|---|---|---|---|---|---|---|---|---|---|---|---|---|---|---|---|---|
| 4489 | 0.831 | 1.000 | 0.870 | 1.000 | 0.979 | 1.000 | 0.894 | 1.000 | 1.000 | 1.000 | 0.983 | 1.000 | 1.000 | 0.820 | 1.000 | 1.000 | 0.874 | 0.848 |
| 4490 | 0.831 | 1.000 | 0.870 | 1.000 | 0.979 | 1.000 | 0.894 | 1.000 | 1.000 | 1.000 | 0.983 | 1.000 | 1.000 | 0.820 | 1.000 | 1.000 | 0.874 | 0.848 |
| 4491 | 0.831 | 1.000 | 0.870 | 1.000 | 0.979 | 1.000 | 0.894 | 1.000 | 1.000 | 1.000 | 0.983 | 1.000 | 1.000 | 0.819 | 1.000 | 1.000 | 0.874 | 0.847 |
| 4492 | 0.831 | 1.000 | 0.870 | 1.000 | 0.979 | 1.000 | 0.894 | 1.000 | 1.000 | 1.000 | 0.983 | 1.000 | 1.000 | 0.818 | 1.000 | 1.000 | 0.873 | 0.847 |
| 4493 | 0.831 | 1.000 | 0.870 | 1.000 | 0.979 | 1.000 | 0.894 | 1.000 | 1.000 | 1.000 | 0.983 | 1.000 | 1.000 | 0.818 | 1.000 | 1.000 | 0.873 | 0.847 |
| 4494 | 0.830 | 1.000 | 0.870 | 1.000 | 0.979 | 1.000 | 0.894 | 1.000 | 1.000 | 1.000 | 0.983 | 1.000 | 1.000 | 0.818 | 1.000 | 1.000 | 0.873 | 0.847 |
| 4495 | 0.830 | 1.000 | 0.870 | 1.000 | 0.979 | 1.000 | 0.894 | 1.000 | 1.000 | 1.000 | 0.983 | 1.000 | 1.000 | 0.818 | 1.000 | 1.000 | 0.873 | 0.847 |
| 4496 | 0.831 | 1.000 | 0.870 | 1.000 | 0.979 | 1.000 | 0.894 | 1.000 | 1.000 | 1.000 | 0.983 | 1.000 | 1.000 | 0.818 | 1.000 | 1.000 | 0.873 | 0.847 |
| 4497 | 0.831 | 1.000 | 0.870 | 1.000 | 0.979 | 1.000 | 0.894 | 1.000 | 1.000 | 1.000 | 0.983 | 1.000 | 1.000 | 0.819 | 1.000 | 1.000 | 0.874 | 0.847 |
| 4498 | 0.832 | 1.000 | 0.870 | 1.000 | 0.979 | 1.000 | 0.894 | 1.000 | 1.000 | 1.000 | 0.983 | 1.000 | 1.000 | 0.819 | 1.000 | 1.000 | 0.874 | 0.848 |
| 4499 | 0.831 | 1.000 | 0.870 | 1.000 | 0.979 | 1.000 | 0.894 | 1.000 | 1.000 | 1.000 | 0.983 | 1.000 | 1.000 | 0.819 | 1.000 | 1.000 | 0.874 | 0.848 |
| 4500 | 0.831 | 1.000 | 0.870 | 1.000 | 0.979 | 1.000 | 0.894 | 1.000 | 1.000 | 1.000 | 0.983 | 1.000 | 1.000 | 0.818 | 1.000 | 1.000 | 0.874 | 0.847 |
| 4501 | 0.831 | 1.000 | 0.870 | 1.000 | 0.979 | 1.000 | 0.894 | 1.000 | 1.000 | 1.000 | 0.983 | 1.000 | 1.000 | 0.819 | 1.000 | 1.000 | 0.874 | 0.848 |
| 4502 | 0.830 | 1.000 | 0.868 | 1.000 | 0.979 | 1.000 | 0.894 | 1.000 | 1.000 | 1.000 | 0.980 | 1.000 | 1.000 | 0.819 | 1.000 | 1.000 | 0.873 | 0.847 |
| 4503 | 0.831 | 1.000 | 0.870 | 1.000 | 0.979 | 1.000 | 0.894 | 1.000 | 1.000 | 1.000 | 0.980 | 1.000 | 1.000 | 0.819 | 1.000 | 1.000 | 0.873 | 0.847 |
| 4504 | 0.831 | 1.000 | 0.870 | 1.000 | 0.979 | 1.000 | 0.895 | 1.000 | 1.000 | 1.000 | 0.980 | 1.000 | 1.000 | 0.819 | 1.000 | 1.000 | 0.874 | 0.847 |
| 4505 | 0.832 | 1.000 | 0.870 | 1.000 | 0.979 | 1.000 | 0.895 | 1.000 | 1.000 | 1.000 | 0.980 | 1.000 | 1.000 | 0.819 | 1.000 | 1.000 | 0.874 | 0.848 |
| 4506 | 0.832 | 1.000 | 0.870 | 1.000 | 0.979 | 1.000 | 0.895 | 1.000 | 1.000 | 1.000 | 0.977 | 1.000 | 1.000 | 0.818 | 1.000 | 1.000 | 0.874 | 0.847 |
| 4507 | 0.831 | 1.000 | 0.868 | 1.000 | 0.979 | 1.000 | 0.892 | 1.000 | 1.000 | 1.000 | 0.977 | 1.000 | 1.000 | 0.819 | 1.000 | 1.000 | 0.873 | 0.847 |
| 4508 | 0.831 | 1.000 | 0.868 | 1.000 | 0.979 | 1.000 | 0.892 | 1.000 | 1.000 | 1.000 | 0.977 | 1.000 | 1.000 | 0.818 | 1.000 | 1.000 | 0.873 | 0.846 |
| 4509 | 0.831 | 1.000 | 0.868 | 1.000 | 0.979 | 1.000 | 0.892 | 1.000 | 1.000 | 1.000 | 0.977 | 1.000 | 1.000 | 0.818 | 1.000 | 1.000 | 0.873 | 0.846 |
| 4510 | 0.831 | 1.000 | 0.868 | 1.000 | 0.979 | 1.000 | 0.892 | 1.000 | 1.000 | 1.000 | 0.977 | 1.000 | 1.000 | 0.819 | 1.000 | 1.000 | 0.873 | 0.847 |
| 4511 | 0.832 | 1.000 | 0.868 | 1.000 | 0.979 | 1.000 | 0.892 | 1.000 | 1.000 | 1.000 | 0.977 | 1.000 | 1.000 | 0.819 | 1.000 | 1.000 | 0.873 | 0.847 |
| 4512 | 0.832 | 1.000 | 0.868 | 1.000 | 0.979 | 1.000 | 0.892 | 1.000 | 1.000 | 1.000 | 0.977 | 1.000 | 1.000 | 0.819 | 1.000 | 1.000 | 0.873 | 0.847 |
| 4513 | 0.832 | 1.000 | 0.868 | 1.000 | 0.979 | 1.000 | 0.892 | 1.000 | 1.000 | 1.000 | 0.977 | 1.000 | 1.000 | 0.819 | 1.000 | 1.000 | 0.873 | 0.847 |
| 4514 | 0.831 | 1.000 | 0.868 | 1.000 | 0.979 | 1.000 | 0.892 | 1.000 | 1.000 | 1.000 | 0.980 | 1.000 | 1.000 | 0.819 | 1.000 | 1.000 | 0.873 | 0.847 |
| 4515 | 0.831 | 1.000 | 0.868 | 1.000 | 0.979 | 1.000 | 0.892 | 1.000 | 1.000 | 1.000 | 0.980 | 1.000 | 1.000 | 0.819 | 1.000 | 1.000 | 0.873 | 0.847 |
| 4516 | 0.831 | 1.000 | 0.868 | 1.000 | 0.979 | 1.000 | 0.892 | 1.000 | 1.000 | 1.000 | 0.977 | 1.000 | 1.000 | 0.820 | 1.000 | 1.000 | 0.873 | 0.847 |
| 4517 | 0.831 | 1.000 | 0.868 | 1.000 | 0.979 | 1.000 | 0.892 | 1.000 | 1.000 | 1.000 | 0.980 | 1.000 | 1.000 | 0.819 | 1.000 | 1.000 | 0.873 | 0.847 |
| 4518 | 0.831 | 1.000 | 0.868 | 1.000 | 0.979 | 1.000 | 0.892 | 1.000 | 1.000 | 1.000 | 0.980 | 1.000 | 1.000 | 0.820 | 1.000 | 1.000 | 0.874 | 0.847 |
| 4519 | 0.831 | 1.000 | 0.868 | 1.000 | 0.979 | 1.000 | 0.892 | 1.000 | 1.000 | 1.000 | 0.980 | 1.000 | 1.000 | 0.820 | 1.000 | 1.000 | 0.874 | 0.847 |
| 4520 | 0.831 | 1.000 | 0.868 | 1.000 | 0.979 | 1.000 | 0.892 | 1.000 | 1.000 | 1.000 | 0.977 | 1.000 | 1.000 | 0.820 | 1.000 | 1.000 | 0.873 | 0.847 |
| 4521 | 0.831 | 1.000 | 0.868 | 1.000 | 0.979 | 1.000 | 0.892 | 1.000 | 1.000 | 1.000 | 0.980 | 1.000 | 1.000 | 0.820 | 1.000 | 1.000 | 0.874 | 0.847 |
| 4522 | 0.831 | 1.000 | 0.868 | 1.000 | 0.979 | 1.000 | 0.892 | 1.000 | 1.000 | 1.000 | 0.977 | 1.000 | 1.000 | 0.820 | 1.000 | 1.000 | 0.873 | 0.847 |
| 4523 | 0.831 | 1.000 | 0.868 | 1.000 | 0.979 | 1.000 | 0.894 | 1.000 | 1.000 | 1.000 | 0.977 | 1.000 | 1.000 | 0.820 | 1.000 | 1.000 | 0.874 | 0.847 |
| 4524 | 0.831 | 1.000 | 0.868 | 1.000 | 0.979 | 1.000 | 0.894 | 1.000 | 1.000 | 1.000 | 0.977 | 1.000 | 1.000 | 0.820 | 1.000 | 1.000 | 0.874 | 0.847 |
| 4525 | 0.832 | 1.000 | 0.868 | 1.000 | 0.979 | 1.000 | 0.894 | 1.000 | 1.000 | 1.000 | 0.977 | 1.000 | 1.000 | 0.819 | 1.000 | 1.000 | 0.874 | 0.847 |
| 4526 | 0.831 | 1.000 | 0.868 | 1.000 | 0.979 | 1.000 | 0.894 | 1.000 | 1.000 | 1.000 | 0.977 | 1.000 | 1.000 | 0.819 | 1.000 | 1.000 | 0.873 | 0.847 |
| 4527 | 0.831 | 1.000 | 0.868 | 1.000 | 0.979 | 1.000 | 0.894 | 1.000 | 1.000 | 1.000 | 0.977 | 1.000 | 1.000 | 0.819 | 1.000 | 1.000 | 0.873 | 0.847 |

| | | | | | | | | | | | | | | | | | | |
|---|---|---|---|---|---|---|---|---|---|---|---|---|---|---|---|---|---|---|
| 4528 | 0.831 | 1.000 | 0.868 | 1.000 | 0.979 | 1.000 | 0.894 | 1.000 | 1.000 | 1.000 | 0.977 | 1.000 | 1.000 | 0.819 | 1.000 | 1.000 | 0.873 | 0.847 |
| 4529 | 0.831 | 1.000 | 0.868 | 1.000 | 0.979 | 1.000 | 0.894 | 1.000 | 1.000 | 1.000 | 0.980 | 1.000 | 1.000 | 0.819 | 1.000 | 1.000 | 0.873 | 0.847 |
| 4530 | 0.831 | 1.000 | 0.868 | 1.000 | 0.979 | 1.000 | 0.894 | 1.000 | 1.000 | 1.000 | 0.977 | 1.000 | 1.000 | 0.818 | 1.000 | 1.000 | 0.873 | 0.846 |
| 4531 | 0.832 | 1.000 | 0.868 | 1.000 | 0.979 | 1.000 | 0.892 | 1.000 | 1.000 | 1.000 | 0.980 | 1.000 | 1.000 | 0.817 | 1.000 | 1.000 | 0.873 | 0.847 |
| 4532 | 0.832 | 1.000 | 0.868 | 1.000 | 0.979 | 1.000 | 0.898 | 1.000 | 1.000 | 1.000 | 0.980 | 1.000 | 1.000 | 0.817 | 1.000 | 1.000 | 0.874 | 0.848 |
| 4533 | 0.833 | 1.000 | 0.870 | 1.000 | 0.979 | 1.000 | 0.900 | 1.000 | 1.000 | 1.000 | 0.980 | 1.000 | 1.000 | 0.816 | 1.000 | 1.000 | 0.874 | 0.848 |
| 4534 | 0.833 | 1.000 | 0.872 | 1.000 | 0.979 | 1.000 | 0.900 | 1.000 | 1.000 | 1.000 | 0.980 | 1.000 | 1.000 | 0.816 | 1.000 | 1.000 | 0.874 | 0.849 |
| 4535 | 0.833 | 1.000 | 0.868 | 1.000 | 0.979 | 1.000 | 0.900 | 1.000 | 1.000 | 1.000 | 0.980 | 1.000 | 1.000 | 0.816 | 1.000 | 1.000 | 0.874 | 0.848 |
| 4536 | 0.833 | 1.000 | 0.870 | 1.000 | 0.979 | 1.000 | 0.900 | 1.000 | 1.000 | 1.000 | 0.980 | 1.000 | 1.000 | 0.816 | 1.000 | 1.000 | 0.874 | 0.848 |
| 4537 | 0.834 | 1.000 | 0.872 | 1.000 | 0.979 | 1.000 | 0.900 | 1.000 | 1.000 | 1.000 | 0.980 | 1.000 | 1.000 | 0.816 | 1.000 | 1.000 | 0.875 | 0.849 |
| 4538 | 0.834 | 1.000 | 0.872 | 1.000 | 0.979 | 1.000 | 0.900 | 1.000 | 1.000 | 1.000 | 0.980 | 1.000 | 1.000 | 0.816 | 1.000 | 1.000 | 0.875 | 0.849 |
| 4539 | 0.834 | 1.000 | 0.872 | 1.000 | 0.979 | 1.000 | 0.900 | 1.000 | 1.000 | 1.000 | 0.980 | 1.000 | 1.000 | 0.815 | 1.000 | 1.000 | 0.874 | 0.848 |
| 4540 | 0.837 | 1.000 | 0.872 | 1.000 | 0.979 | 1.000 | 0.901 | 1.000 | 1.000 | 1.000 | 0.980 | 1.000 | 1.000 | 0.814 | 1.000 | 1.000 | 0.875 | 0.849 |
| 4541 | 0.836 | 1.000 | 0.872 | 1.000 | 0.979 | 1.000 | 0.900 | 1.000 | 1.000 | 1.000 | 0.980 | 1.000 | 1.000 | 0.814 | 1.000 | 1.000 | 0.875 | 0.849 |
| 4542 | 0.836 | 1.000 | 0.872 | 1.000 | 0.979 | 1.000 | 0.900 | 1.000 | 1.000 | 1.000 | 0.980 | 1.000 | 1.000 | 0.814 | 1.000 | 1.000 | 0.875 | 0.849 |
| 4543 | 0.836 | 1.000 | 0.872 | 1.000 | 0.979 | 1.000 | 0.900 | 1.000 | 1.000 | 1.000 | 0.980 | 1.000 | 1.000 | 0.814 | 1.000 | 1.000 | 0.875 | 0.849 |
| 4544 | 0.836 | 1.000 | 0.872 | 1.000 | 0.979 | 1.000 | 0.900 | 1.000 | 1.000 | 1.000 | 0.980 | 1.000 | 1.000 | 0.814 | 1.000 | 1.000 | 0.875 | 0.849 |
| 4545 | 0.836 | 1.000 | 0.872 | 1.000 | 0.979 | 1.000 | 0.900 | 1.000 | 1.000 | 1.000 | 0.980 | 1.000 | 1.000 | 0.814 | 1.000 | 1.000 | 0.875 | 0.849 |
| 4546 | 0.836 | 1.000 | 0.872 | 1.000 | 0.979 | 1.000 | 0.900 | 1.000 | 1.000 | 1.000 | 0.980 | 1.000 | 1.000 | 0.814 | 1.000 | 1.000 | 0.875 | 0.849 |
| 4547 | 0.836 | 1.000 | 0.872 | 1.000 | 0.979 | 1.000 | 0.900 | 1.000 | 1.000 | 1.000 | 0.980 | 1.000 | 1.000 | 0.814 | 1.000 | 1.000 | 0.875 | 0.849 |
| 4548 | 0.836 | 1.000 | 0.872 | 1.000 | 0.979 | 1.000 | 0.900 | 1.000 | 1.000 | 1.000 | 0.980 | 1.000 | 1.000 | 0.814 | 1.000 | 1.000 | 0.875 | 0.849 |
| 4549 | 0.836 | 1.000 | 0.872 | 1.000 | 0.979 | 1.000 | 0.900 | 1.000 | 1.000 | 1.000 | 0.980 | 1.000 | 1.000 | 0.814 | 1.000 | 1.000 | 0.875 | 0.849 |
| 4550 | 0.836 | 1.000 | 0.872 | 1.000 | 0.979 | 1.000 | 0.900 | 1.000 | 1.000 | 1.000 | 0.980 | 1.000 | 1.000 | 0.814 | 1.000 | 1.000 | 0.875 | 0.849 |
| 4551 | 0.835 | 1.000 | 0.872 | 1.000 | 0.979 | 1.000 | 0.900 | 1.000 | 1.000 | 1.000 | 0.980 | 1.000 | 1.000 | 0.814 | 1.000 | 1.000 | 0.875 | 0.849 |
| 4552 | 0.835 | 1.000 | 0.872 | 1.000 | 0.979 | 1.000 | 0.900 | 1.000 | 1.000 | 1.000 | 0.983 | 1.000 | 1.000 | 0.814 | 1.000 | 1.000 | 0.875 | 0.849 |
| 4553 | 0.834 | 1.000 | 0.872 | 1.000 | 0.979 | 1.000 | 0.900 | 1.000 | 1.000 | 1.000 | 0.983 | 1.000 | 1.000 | 0.814 | 1.000 | 1.000 | 0.874 | 0.849 |
| 4554 | 0.834 | 1.000 | 0.872 | 1.000 | 0.979 | 1.000 | 0.900 | 1.000 | 1.000 | 1.000 | 0.983 | 1.000 | 1.000 | 0.814 | 1.000 | 1.000 | 0.874 | 0.849 |
| 4555 | 0.834 | 1.000 | 0.872 | 1.000 | 0.979 | 1.000 | 0.900 | 1.000 | 1.000 | 1.000 | 0.983 | 1.000 | 1.000 | 0.814 | 1.000 | 1.000 | 0.874 | 0.849 |
| 4556 | 0.834 | 1.000 | 0.872 | 1.000 | 0.979 | 1.000 | 0.900 | 1.000 | 1.000 | 1.000 | 0.983 | 1.000 | 1.000 | 0.814 | 1.000 | 1.000 | 0.874 | 0.849 |
| 4557 | 0.835 | 1.000 | 0.875 | 1.000 | 0.979 | 1.000 | 0.900 | 1.000 | 1.000 | 1.000 | 0.980 | 1.000 | 1.000 | 0.814 | 1.000 | 1.000 | 0.875 | 0.849 |
| 4558 | 0.835 | 1.000 | 0.874 | 1.000 | 0.979 | 1.000 | 0.900 | 1.000 | 1.000 | 1.000 | 0.980 | 1.000 | 1.000 | 0.814 | 1.000 | 1.000 | 0.875 | 0.849 |
| 4559 | 0.835 | 1.000 | 0.875 | 1.000 | 0.979 | 1.000 | 0.900 | 1.000 | 1.000 | 1.000 | 0.980 | 1.000 | 1.000 | 0.814 | 1.000 | 1.000 | 0.875 | 0.849 |
| 4560 | 0.834 | 1.000 | 0.875 | 1.000 | 0.979 | 1.000 | 0.900 | 1.000 | 1.000 | 1.000 | 0.980 | 1.000 | 1.000 | 0.814 | 1.000 | 1.000 | 0.875 | 0.849 |
| 4561 | 0.835 | 1.000 | 0.874 | 1.000 | 0.979 | 1.000 | 0.898 | 1.000 | 1.000 | 1.000 | 0.983 | 1.000 | 1.000 | 0.814 | 1.000 | 1.000 | 0.875 | 0.849 |
| 4562 | 0.835 | 1.000 | 0.874 | 1.000 | 0.979 | 1.000 | 0.898 | 1.000 | 1.000 | 1.000 | 0.983 | 1.000 | 1.000 | 0.813 | 1.000 | 1.000 | 0.875 | 0.849 |
| 4563 | 0.835 | 1.000 | 0.874 | 1.000 | 0.979 | 1.000 | 0.898 | 1.000 | 1.000 | 1.000 | 0.983 | 1.000 | 1.000 | 0.813 | 1.000 | 1.000 | 0.875 | 0.849 |
| 4564 | 0.835 | 1.000 | 0.874 | 1.000 | 0.979 | 1.000 | 0.898 | 1.000 | 1.000 | 1.000 | 0.983 | 1.000 | 1.000 | 0.813 | 1.000 | 1.000 | 0.875 | 0.849 |
| 4565 | 0.834 | 1.000 | 0.874 | 1.000 | 0.979 | 1.000 | 0.898 | 1.000 | 1.000 | 1.000 | 0.983 | 1.000 | 1.000 | 0.814 | 1.000 | 1.000 | 0.874 | 0.848 |
| 4566 | 0.834 | 1.000 | 0.874 | 1.000 | 0.979 | 1.000 | 0.898 | 1.000 | 1.000 | 1.000 | 0.983 | 1.000 | 1.000 | 0.815 | 1.000 | 1.000 | 0.875 | 0.849 |

| | | | | | | | | | | | | | | | | | | |
|---|---|---|---|---|---|---|---|---|---|---|---|---|---|---|---|---|---|---|
| 4567 | 0.835 | 1.000 | 0.874 | 1.000 | 0.979 | 1.000 | 0.898 | 1.000 | 1.000 | 1.000 | 0.983 | 1.000 | 1.000 | 0.815 | 1.000 | 1.000 | 0.875 | 0.849 |
| 4568 | 0.833 | 1.000 | 0.874 | 1.000 | 0.979 | 1.000 | 0.898 | 1.000 | 1.000 | 1.000 | 0.983 | 1.000 | 1.000 | 0.815 | 1.000 | 1.000 | 0.874 | 0.848 |
| 4569 | 0.833 | 1.000 | 0.874 | 1.000 | 0.979 | 1.000 | 0.898 | 1.000 | 1.000 | 1.000 | 0.983 | 1.000 | 1.000 | 0.815 | 1.000 | 1.000 | 0.874 | 0.848 |
| 4570 | 0.833 | 1.000 | 0.874 | 1.000 | 0.979 | 1.000 | 0.898 | 1.000 | 1.000 | 1.000 | 0.983 | 1.000 | 1.000 | 0.815 | 1.000 | 1.000 | 0.874 | 0.848 |
| 4571 | 0.835 | 1.000 | 0.872 | 1.000 | 0.979 | 1.000 | 0.898 | 1.000 | 1.000 | 1.000 | 0.983 | 1.000 | 1.000 | 0.815 | 1.000 | 1.000 | 0.875 | 0.849 |
| 4572 | 0.833 | 1.000 | 0.874 | 1.000 | 0.979 | 1.000 | 0.898 | 1.000 | 1.000 | 1.000 | 0.983 | 1.000 | 1.000 | 0.814 | 1.000 | 1.000 | 0.874 | 0.848 |
| 4573 | 0.833 | 1.000 | 0.874 | 1.000 | 0.979 | 1.000 | 0.898 | 1.000 | 1.000 | 1.000 | 0.983 | 1.000 | 1.000 | 0.814 | 1.000 | 1.000 | 0.874 | 0.848 |
| 4574 | 0.833 | 1.000 | 0.874 | 1.000 | 0.979 | 1.000 | 0.898 | 1.000 | 1.000 | 1.000 | 0.983 | 1.000 | 1.000 | 0.815 | 1.000 | 1.000 | 0.874 | 0.848 |
| 4575 | 0.833 | 1.000 | 0.874 | 1.000 | 0.979 | 1.000 | 0.898 | 1.000 | 1.000 | 1.000 | 0.983 | 1.000 | 1.000 | 0.815 | 1.000 | 1.000 | 0.874 | 0.848 |
| 4576 | 0.833 | 1.000 | 0.874 | 1.000 | 0.979 | 1.000 | 0.898 | 1.000 | 1.000 | 1.000 | 0.983 | 1.000 | 1.000 | 0.815 | 1.000 | 1.000 | 0.874 | 0.848 |
| 4577 | 0.833 | 1.000 | 0.874 | 1.000 | 0.979 | 1.000 | 0.898 | 1.000 | 1.000 | 1.000 | 0.983 | 1.000 | 1.000 | 0.815 | 1.000 | 1.000 | 0.874 | 0.848 |
| 4578 | 0.833 | 1.000 | 0.874 | 1.000 | 0.979 | 1.000 | 0.897 | 1.000 | 1.000 | 1.000 | 0.983 | 1.000 | 1.000 | 0.815 | 1.000 | 1.000 | 0.874 | 0.848 |
| 4579 | 0.833 | 1.000 | 0.874 | 1.000 | 0.979 | 1.000 | 0.898 | 1.000 | 1.000 | 1.000 | 0.983 | 1.000 | 1.000 | 0.816 | 1.000 | 1.000 | 0.875 | 0.849 |
| 4580 | 0.833 | 1.000 | 0.874 | 1.000 | 0.979 | 1.000 | 0.898 | 1.000 | 1.000 | 1.000 | 0.983 | 1.000 | 1.000 | 0.816 | 1.000 | 1.000 | 0.875 | 0.849 |
| 4581 | 0.835 | 1.000 | 0.875 | 1.000 | 0.979 | 1.000 | 0.898 | 1.000 | 1.000 | 1.000 | 0.983 | 1.000 | 1.000 | 0.816 | 1.000 | 1.000 | 0.875 | 0.849 |
| 4582 | 0.834 | 1.000 | 0.875 | 1.000 | 0.979 | 1.000 | 0.897 | 1.000 | 1.000 | 1.000 | 0.983 | 1.000 | 1.000 | 0.816 | 1.000 | 1.000 | 0.875 | 0.849 |
| 4583 | 0.833 | 1.000 | 0.875 | 1.000 | 0.979 | 1.000 | 0.898 | 1.000 | 1.000 | 1.000 | 0.983 | 1.000 | 1.000 | 0.816 | 1.000 | 1.000 | 0.875 | 0.849 |
| 4584 | 0.833 | 1.000 | 0.875 | 1.000 | 0.979 | 1.000 | 0.897 | 1.000 | 1.000 | 1.000 | 0.983 | 1.000 | 1.000 | 0.815 | 1.000 | 1.000 | 0.874 | 0.849 |
| 4585 | 0.834 | 1.000 | 0.875 | 1.000 | 0.979 | 1.000 | 0.898 | 1.000 | 1.000 | 1.000 | 0.983 | 1.000 | 1.000 | 0.813 | 1.000 | 1.000 | 0.874 | 0.849 |
| 4586 | 0.833 | 1.000 | 0.877 | 1.000 | 0.979 | 1.000 | 0.898 | 1.000 | 1.000 | 1.000 | 0.983 | 1.000 | 1.000 | 0.814 | 1.000 | 1.000 | 0.874 | 0.849 |
| 4587 | 0.833 | 1.000 | 0.879 | 1.000 | 0.979 | 1.000 | 0.898 | 1.000 | 1.000 | 1.000 | 0.983 | 1.000 | 1.000 | 0.814 | 1.000 | 1.000 | 0.875 | 0.849 |
| 4588 | 0.833 | 1.000 | 0.879 | 1.000 | 0.979 | 1.000 | 0.898 | 1.000 | 1.000 | 1.000 | 0.983 | 1.000 | 1.000 | 0.814 | 1.000 | 1.000 | 0.875 | 0.849 |
| 4589 | 0.834 | 1.000 | 0.879 | 1.000 | 0.979 | 1.000 | 0.900 | 1.000 | 1.000 | 1.000 | 0.983 | 1.000 | 1.000 | 0.816 | 1.000 | 1.000 | 0.876 | 0.850 |
| 4590 | 0.835 | 1.000 | 0.877 | 1.000 | 0.979 | 1.000 | 0.900 | 1.000 | 1.000 | 1.000 | 0.983 | 1.000 | 1.000 | 0.816 | 1.000 | 1.000 | 0.876 | 0.850 |
| 4591 | 0.834 | 1.000 | 0.877 | 1.000 | 0.979 | 1.000 | 0.901 | 1.000 | 1.000 | 1.000 | 0.983 | 1.000 | 1.000 | 0.815 | 1.000 | 1.000 | 0.875 | 0.850 |
| 4592 | 0.835 | 1.000 | 0.877 | 1.000 | 0.979 | 1.000 | 0.900 | 1.000 | 1.000 | 1.000 | 0.983 | 1.000 | 1.000 | 0.814 | 1.000 | 1.000 | 0.875 | 0.850 |
| 4593 | 0.834 | 1.000 | 0.877 | 1.000 | 0.979 | 1.000 | 0.901 | 1.000 | 1.000 | 1.000 | 0.983 | 1.000 | 1.000 | 0.815 | 1.000 | 1.000 | 0.875 | 0.850 |
| 4594 | 0.835 | 1.000 | 0.877 | 1.000 | 0.979 | 1.000 | 0.901 | 1.000 | 1.000 | 1.000 | 0.983 | 1.000 | 1.000 | 0.815 | 1.000 | 1.000 | 0.876 | 0.850 |
| 4595 | 0.834 | 1.000 | 0.877 | 1.000 | 0.979 | 1.000 | 0.900 | 1.000 | 1.000 | 1.000 | 0.983 | 1.000 | 1.000 | 0.815 | 1.000 | 1.000 | 0.875 | 0.850 |
| 4596 | 0.834 | 1.000 | 0.877 | 1.000 | 0.979 | 1.000 | 0.900 | 1.000 | 1.000 | 1.000 | 0.983 | 1.000 | 1.000 | 0.816 | 1.000 | 1.000 | 0.876 | 0.850 |
| 4597 | 0.834 | 1.000 | 0.877 | 1.000 | 0.979 | 1.000 | 0.900 | 1.000 | 1.000 | 1.000 | 0.983 | 1.000 | 1.000 | 0.816 | 1.000 | 1.000 | 0.876 | 0.850 |
| 4598 | 0.834 | 1.000 | 0.877 | 1.000 | 0.979 | 1.000 | 0.900 | 1.000 | 1.000 | 1.000 | 0.983 | 1.000 | 1.000 | 0.816 | 1.000 | 1.000 | 0.876 | 0.850 |
| 4599 | 0.834 | 1.000 | 0.877 | 1.000 | 0.979 | 1.000 | 0.901 | 1.000 | 1.000 | 1.000 | 0.983 | 1.000 | 1.000 | 0.816 | 1.000 | 1.000 | 0.876 | 0.850 |
| 4600 | 0.835 | 1.000 | 0.875 | 1.000 | 0.979 | 1.000 | 0.901 | 1.000 | 1.000 | 1.000 | 0.983 | 1.000 | 1.000 | 0.816 | 1.000 | 1.000 | 0.876 | 0.850 |
| 4601 | 0.835 | 1.000 | 0.875 | 1.000 | 0.979 | 1.000 | 0.901 | 1.000 | 1.000 | 1.000 | 0.983 | 1.000 | 1.000 | 0.816 | 1.000 | 1.000 | 0.876 | 0.850 |
| 4602 | 0.835 | 1.000 | 0.875 | 1.000 | 0.979 | 1.000 | 0.901 | 1.000 | 1.000 | 1.000 | 0.983 | 1.000 | 1.000 | 0.816 | 1.000 | 1.000 | 0.876 | 0.850 |
| 4603 | 0.835 | 1.000 | 0.875 | 1.000 | 0.979 | 1.000 | 0.901 | 1.000 | 1.000 | 1.000 | 0.983 | 1.000 | 1.000 | 0.816 | 1.000 | 1.000 | 0.876 | 0.850 |
| 4604 | 0.835 | 1.000 | 0.875 | 1.000 | 0.979 | 1.000 | 0.901 | 1.000 | 1.000 | 1.000 | 0.983 | 1.000 | 1.000 | 0.816 | 1.000 | 1.000 | 0.876 | 0.850 |
| 4605 | 0.835 | 1.000 | 0.875 | 1.000 | 0.979 | 1.000 | 0.901 | 1.000 | 1.000 | 1.000 | 0.983 | 1.000 | 1.000 | 0.816 | 1.000 | 1.000 | 0.876 | 0.850 |

| | | | | | | | | | | | | | | | | | | |
|---|---|---|---|---|---|---|---|---|---|---|---|---|---|---|---|---|---|---|
| 4606 | 0.833 | 1.000 | 0.875 | 1.000 | 0.979 | 1.000 | 0.901 | 1.000 | 1.000 | 1.000 | 0.983 | 1.000 | 1.000 | 0.816 | 1.000 | 1.000 | 0.875 | 0.849 |
| 4607 | 0.833 | 1.000 | 0.875 | 1.000 | 0.979 | 1.000 | 0.901 | 1.000 | 1.000 | 1.000 | 0.983 | 1.000 | 1.000 | 0.816 | 1.000 | 1.000 | 0.875 | 0.850 |
| 4608 | 0.833 | 1.000 | 0.875 | 1.000 | 0.979 | 1.000 | 0.901 | 1.000 | 1.000 | 1.000 | 0.983 | 1.000 | 1.000 | 0.817 | 1.000 | 1.000 | 0.876 | 0.850 |
| 4609 | 0.834 | 1.000 | 0.875 | 1.000 | 0.979 | 1.000 | 0.901 | 1.000 | 1.000 | 1.000 | 0.983 | 1.000 | 1.000 | 0.816 | 1.000 | 1.000 | 0.876 | 0.850 |
| 4610 | 0.833 | 1.000 | 0.875 | 1.000 | 0.979 | 1.000 | 0.901 | 1.000 | 1.000 | 1.000 | 0.983 | 1.000 | 1.000 | 0.817 | 1.000 | 1.000 | 0.876 | 0.850 |
| 4611 | 0.833 | 1.000 | 0.875 | 1.000 | 0.979 | 1.000 | 0.901 | 1.000 | 1.000 | 1.000 | 0.983 | 1.000 | 1.000 | 0.816 | 1.000 | 1.000 | 0.875 | 0.850 |
| 4612 | 0.834 | 1.000 | 0.875 | 1.000 | 0.979 | 1.000 | 0.901 | 1.000 | 1.000 | 1.000 | 0.983 | 1.000 | 1.000 | 0.816 | 1.000 | 1.000 | 0.876 | 0.850 |
| 4613 | 0.835 | 1.000 | 0.875 | 1.000 | 0.979 | 1.000 | 0.901 | 1.000 | 1.000 | 1.000 | 0.983 | 1.000 | 1.000 | 0.816 | 1.000 | 1.000 | 0.876 | 0.850 |
| 4614 | 0.834 | 1.000 | 0.875 | 1.000 | 0.979 | 1.000 | 0.901 | 1.000 | 1.000 | 1.000 | 0.983 | 1.000 | 1.000 | 0.816 | 1.000 | 1.000 | 0.876 | 0.850 |
| 4615 | 0.835 | 1.000 | 0.875 | 1.000 | 0.979 | 1.000 | 0.901 | 1.000 | 1.000 | 1.000 | 0.983 | 1.000 | 1.000 | 0.816 | 1.000 | 1.000 | 0.876 | 0.850 |
| 4616 | 0.834 | 1.000 | 0.875 | 1.000 | 0.979 | 1.000 | 0.901 | 1.000 | 1.000 | 1.000 | 0.983 | 1.000 | 1.000 | 0.816 | 1.000 | 1.000 | 0.876 | 0.850 |
| 4617 | 0.834 | 1.000 | 0.875 | 1.000 | 0.979 | 1.000 | 0.901 | 1.000 | 1.000 | 1.000 | 0.983 | 1.000 | 1.000 | 0.816 | 1.000 | 1.000 | 0.876 | 0.850 |
| 4618 | 0.834 | 1.000 | 0.875 | 1.000 | 0.979 | 1.000 | 0.901 | 1.000 | 1.000 | 1.000 | 0.983 | 1.000 | 1.000 | 0.816 | 1.000 | 1.000 | 0.876 | 0.850 |
| 4619 | 0.834 | 1.000 | 0.877 | 1.000 | 0.979 | 1.000 | 0.901 | 1.000 | 1.000 | 1.000 | 0.983 | 1.000 | 1.000 | 0.816 | 1.000 | 1.000 | 0.876 | 0.850 |
| 4620 | 0.834 | 1.000 | 0.879 | 1.000 | 0.979 | 1.000 | 0.901 | 1.000 | 1.000 | 1.000 | 0.983 | 1.000 | 1.000 | 0.817 | 1.000 | 1.000 | 0.876 | 0.851 |
| 4621 | 0.834 | 1.000 | 0.879 | 1.000 | 0.979 | 1.000 | 0.900 | 1.000 | 1.000 | 1.000 | 0.983 | 1.000 | 1.000 | 0.817 | 1.000 | 1.000 | 0.876 | 0.850 |
| 4622 | 0.833 | 1.000 | 0.879 | 1.000 | 0.979 | 1.000 | 0.900 | 1.000 | 1.000 | 1.000 | 0.983 | 1.000 | 1.000 | 0.816 | 1.000 | 1.000 | 0.876 | 0.850 |
| 4623 | 0.833 | 1.000 | 0.881 | 1.000 | 0.979 | 1.000 | 0.900 | 1.000 | 1.000 | 1.000 | 0.983 | 1.000 | 1.000 | 0.816 | 1.000 | 1.000 | 0.876 | 0.850 |
| 4624 | 0.833 | 1.000 | 0.881 | 1.000 | 0.979 | 1.000 | 0.900 | 1.000 | 1.000 | 1.000 | 0.983 | 1.000 | 1.000 | 0.816 | 1.000 | 1.000 | 0.876 | 0.850 |
| 4625 | 0.833 | 1.000 | 0.879 | 1.000 | 0.979 | 1.000 | 0.900 | 1.000 | 1.000 | 1.000 | 0.983 | 1.000 | 1.000 | 0.815 | 1.000 | 1.000 | 0.875 | 0.850 |
| 4626 | 0.834 | 1.000 | 0.879 | 1.000 | 0.979 | 1.000 | 0.900 | 1.000 | 1.000 | 1.000 | 0.983 | 1.000 | 1.000 | 0.816 | 1.000 | 1.000 | 0.876 | 0.850 |
| 4627 | 0.835 | 1.000 | 0.879 | 1.000 | 0.979 | 1.000 | 0.898 | 1.000 | 1.000 | 1.000 | 0.983 | 1.000 | 1.000 | 0.816 | 1.000 | 1.000 | 0.876 | 0.850 |
| 4628 | 0.835 | 1.000 | 0.879 | 1.000 | 0.979 | 1.000 | 0.898 | 1.000 | 1.000 | 1.000 | 0.983 | 1.000 | 1.000 | 0.816 | 1.000 | 1.000 | 0.876 | 0.850 |
| 4629 | 0.835 | 1.000 | 0.879 | 1.000 | 0.979 | 1.000 | 0.898 | 1.000 | 1.000 | 1.000 | 0.983 | 1.000 | 1.000 | 0.816 | 1.000 | 1.000 | 0.876 | 0.850 |
| 4630 | 0.835 | 1.000 | 0.877 | 1.000 | 0.979 | 1.000 | 0.900 | 1.000 | 1.000 | 1.000 | 0.983 | 1.000 | 1.000 | 0.816 | 1.000 | 1.000 | 0.876 | 0.850 |
| 4631 | 0.833 | 1.000 | 0.877 | 1.000 | 0.979 | 1.000 | 0.900 | 1.000 | 1.000 | 1.000 | 0.983 | 1.000 | 1.000 | 0.816 | 1.000 | 1.000 | 0.875 | 0.850 |
| 4632 | 0.835 | 1.000 | 0.877 | 1.000 | 0.979 | 1.000 | 0.898 | 1.000 | 1.000 | 1.000 | 0.983 | 1.000 | 1.000 | 0.816 | 1.000 | 1.000 | 0.876 | 0.850 |
| 4633 | 0.835 | 1.000 | 0.877 | 1.000 | 0.979 | 1.000 | 0.898 | 1.000 | 1.000 | 1.000 | 0.983 | 1.000 | 1.000 | 0.816 | 1.000 | 1.000 | 0.876 | 0.850 |
| 4634 | 0.835 | 1.000 | 0.875 | 1.000 | 0.979 | 1.000 | 0.898 | 1.000 | 1.000 | 1.000 | 0.983 | 1.000 | 1.000 | 0.816 | 1.000 | 1.000 | 0.875 | 0.850 |
| 4635 | 0.837 | 1.000 | 0.875 | 1.000 | 0.979 | 1.000 | 0.898 | 1.000 | 1.000 | 1.000 | 0.983 | 1.000 | 1.000 | 0.816 | 1.000 | 1.000 | 0.876 | 0.850 |
| 4636 | 0.837 | 1.000 | 0.875 | 1.000 | 0.979 | 1.000 | 0.898 | 1.000 | 1.000 | 1.000 | 0.983 | 1.000 | 1.000 | 0.816 | 1.000 | 1.000 | 0.876 | 0.850 |
| 4637 | 0.837 | 1.000 | 0.875 | 1.000 | 0.979 | 1.000 | 0.898 | 1.000 | 1.000 | 1.000 | 0.983 | 1.000 | 1.000 | 0.816 | 1.000 | 1.000 | 0.876 | 0.850 |
| 4638 | 0.837 | 1.000 | 0.877 | 1.000 | 0.979 | 1.000 | 0.898 | 1.000 | 1.000 | 1.000 | 0.983 | 1.000 | 1.000 | 0.816 | 1.000 | 1.000 | 0.876 | 0.850 |
| 4639 | 0.836 | 1.000 | 0.877 | 1.000 | 0.979 | 1.000 | 0.898 | 1.000 | 1.000 | 1.000 | 0.983 | 1.000 | 1.000 | 0.816 | 1.000 | 1.000 | 0.876 | 0.850 |
| 4640 | 0.836 | 1.000 | 0.877 | 1.000 | 0.979 | 1.000 | 0.900 | 1.000 | 1.000 | 1.000 | 0.983 | 1.000 | 1.000 | 0.816 | 1.000 | 1.000 | 0.876 | 0.850 |
| 4641 | 0.836 | 1.000 | 0.875 | 1.000 | 0.979 | 1.000 | 0.894 | 1.000 | 1.000 | 1.000 | 0.983 | 1.000 | 1.000 | 0.816 | 1.000 | 1.000 | 0.875 | 0.849 |
| 4642 | 0.837 | 1.000 | 0.875 | 1.000 | 0.979 | 1.000 | 0.894 | 1.000 | 1.000 | 1.000 | 0.983 | 1.000 | 1.000 | 0.816 | 1.000 | 1.000 | 0.875 | 0.849 |
| 4643 | 0.836 | 1.000 | 0.875 | 1.000 | 0.979 | 1.000 | 0.894 | 1.000 | 1.000 | 1.000 | 0.983 | 1.000 | 1.000 | 0.816 | 1.000 | 1.000 | 0.875 | 0.849 |
| 4644 | 0.837 | 1.000 | 0.875 | 1.000 | 0.979 | 1.000 | 0.894 | 1.000 | 1.000 | 1.000 | 0.983 | 1.000 | 1.000 | 0.816 | 1.000 | 1.000 | 0.875 | 0.849 |

| | | | | | | | | | | | | | | | | | | |
|---|---|---|---|---|---|---|---|---|---|---|---|---|---|---|---|---|---|---|
| 4645 | 0.836 | 1.000 | 0.875 | 1.000 | 0.979 | 1.000 | 0.894 | 1.000 | 1.000 | 1.000 | 0.983 | 1.000 | 1.000 | 0.816 | 1.000 | 1.000 | 0.875 | 0.849 |
| 4646 | 0.837 | 1.000 | 0.875 | 1.000 | 0.979 | 1.000 | 0.894 | 1.000 | 1.000 | 1.000 | 0.983 | 1.000 | 1.000 | 0.816 | 1.000 | 1.000 | 0.875 | 0.849 |
| 4647 | 0.837 | 1.000 | 0.875 | 1.000 | 0.979 | 1.000 | 0.894 | 1.000 | 1.000 | 1.000 | 0.983 | 1.000 | 1.000 | 0.818 | 1.000 | 1.000 | 0.876 | 0.850 |
| 4648 | 0.837 | 1.000 | 0.875 | 1.000 | 0.979 | 1.000 | 0.894 | 1.000 | 1.000 | 1.000 | 0.983 | 1.000 | 1.000 | 0.816 | 1.000 | 1.000 | 0.875 | 0.850 |
| 4649 | 0.836 | 1.000 | 0.874 | 1.000 | 0.979 | 1.000 | 0.894 | 1.000 | 1.000 | 1.000 | 0.983 | 1.000 | 1.000 | 0.816 | 1.000 | 1.000 | 0.875 | 0.849 |
| 4650 | 0.837 | 1.000 | 0.874 | 1.000 | 0.979 | 1.000 | 0.894 | 1.000 | 1.000 | 1.000 | 0.983 | 1.000 | 1.000 | 0.816 | 1.000 | 1.000 | 0.875 | 0.849 |
| 4651 | 0.837 | 1.000 | 0.874 | 1.000 | 0.979 | 1.000 | 0.894 | 1.000 | 1.000 | 1.000 | 0.983 | 1.000 | 1.000 | 0.816 | 1.000 | 1.000 | 0.875 | 0.849 |
| 4652 | 0.837 | 1.000 | 0.874 | 1.000 | 0.979 | 1.000 | 0.894 | 1.000 | 1.000 | 1.000 | 0.983 | 1.000 | 1.000 | 0.816 | 1.000 | 1.000 | 0.875 | 0.849 |
| 4653 | 0.836 | 1.000 | 0.874 | 1.000 | 0.979 | 1.000 | 0.894 | 1.000 | 1.000 | 1.000 | 0.983 | 1.000 | 1.000 | 0.816 | 1.000 | 1.000 | 0.875 | 0.849 |
| 4654 | 0.837 | 1.000 | 0.875 | 1.000 | 0.979 | 1.000 | 0.894 | 1.000 | 1.000 | 1.000 | 0.983 | 1.000 | 1.000 | 0.816 | 1.000 | 1.000 | 0.875 | 0.850 |
| 4655 | 0.837 | 1.000 | 0.875 | 1.000 | 0.979 | 1.000 | 0.894 | 1.000 | 1.000 | 1.000 | 0.983 | 1.000 | 1.000 | 0.816 | 1.000 | 1.000 | 0.875 | 0.849 |
| 4656 | 0.837 | 1.000 | 0.875 | 1.000 | 0.979 | 1.000 | 0.894 | 1.000 | 1.000 | 1.000 | 0.983 | 1.000 | 1.000 | 0.816 | 1.000 | 1.000 | 0.875 | 0.850 |
| 4657 | 0.837 | 1.000 | 0.875 | 1.000 | 0.979 | 1.000 | 0.894 | 1.000 | 1.000 | 1.000 | 0.983 | 1.000 | 1.000 | 0.816 | 1.000 | 1.000 | 0.875 | 0.850 |
| 4658 | 0.836 | 1.000 | 0.874 | 1.000 | 0.979 | 1.000 | 0.894 | 1.000 | 1.000 | 1.000 | 0.983 | 1.000 | 1.000 | 0.815 | 1.000 | 1.000 | 0.875 | 0.849 |
| 4659 | 0.836 | 1.000 | 0.874 | 1.000 | 0.979 | 1.000 | 0.894 | 1.000 | 1.000 | 1.000 | 0.983 | 1.000 | 1.000 | 0.815 | 1.000 | 1.000 | 0.875 | 0.849 |
| 4660 | 0.837 | 1.000 | 0.874 | 1.000 | 0.979 | 1.000 | 0.894 | 1.000 | 1.000 | 1.000 | 0.983 | 1.000 | 1.000 | 0.815 | 1.000 | 1.000 | 0.875 | 0.849 |
| 4661 | 0.837 | 1.000 | 0.874 | 1.000 | 0.979 | 1.000 | 0.894 | 1.000 | 1.000 | 1.000 | 0.983 | 1.000 | 1.000 | 0.815 | 1.000 | 1.000 | 0.875 | 0.849 |
| 4662 | 0.837 | 1.000 | 0.874 | 1.000 | 0.979 | 1.000 | 0.894 | 1.000 | 1.000 | 1.000 | 0.983 | 1.000 | 1.000 | 0.816 | 1.000 | 1.000 | 0.875 | 0.849 |
| 4663 | 0.837 | 1.000 | 0.874 | 1.000 | 0.979 | 1.000 | 0.894 | 1.000 | 1.000 | 1.000 | 0.983 | 1.000 | 1.000 | 0.816 | 1.000 | 1.000 | 0.875 | 0.849 |
| 4664 | 0.837 | 1.000 | 0.874 | 1.000 | 0.979 | 1.000 | 0.894 | 1.000 | 1.000 | 1.000 | 0.983 | 1.000 | 1.000 | 0.816 | 1.000 | 1.000 | 0.875 | 0.849 |
| 4665 | 0.837 | 1.000 | 0.875 | 1.000 | 0.979 | 1.000 | 0.894 | 1.000 | 1.000 | 1.000 | 0.983 | 1.000 | 1.000 | 0.816 | 1.000 | 1.000 | 0.875 | 0.849 |
| 4666 | 0.837 | 1.000 | 0.875 | 1.000 | 0.979 | 1.000 | 0.894 | 1.000 | 1.000 | 1.000 | 0.983 | 1.000 | 1.000 | 0.816 | 1.000 | 1.000 | 0.875 | 0.849 |
| 4667 | 0.837 | 1.000 | 0.875 | 1.000 | 0.979 | 1.000 | 0.894 | 1.000 | 1.000 | 1.000 | 0.983 | 1.000 | 1.000 | 0.816 | 1.000 | 1.000 | 0.875 | 0.849 |
| 4668 | 0.837 | 1.000 | 0.874 | 1.000 | 0.979 | 1.000 | 0.894 | 1.000 | 1.000 | 1.000 | 0.983 | 1.000 | 1.000 | 0.816 | 1.000 | 1.000 | 0.875 | 0.849 |
| 4669 | 0.837 | 1.000 | 0.874 | 1.000 | 0.979 | 1.000 | 0.894 | 1.000 | 1.000 | 1.000 | 0.983 | 1.000 | 1.000 | 0.815 | 1.000 | 1.000 | 0.875 | 0.849 |
| 4670 | 0.837 | 1.000 | 0.875 | 1.000 | 0.979 | 1.000 | 0.894 | 1.000 | 1.000 | 1.000 | 0.983 | 1.000 | 1.000 | 0.815 | 1.000 | 1.000 | 0.875 | 0.849 |
| 4671 | 0.836 | 1.000 | 0.872 | 1.000 | 0.979 | 1.000 | 0.894 | 1.000 | 1.000 | 1.000 | 0.983 | 1.000 | 1.000 | 0.816 | 1.000 | 1.000 | 0.875 | 0.849 |
| 4672 | 0.837 | 1.000 | 0.872 | 1.000 | 0.979 | 1.000 | 0.895 | 1.000 | 1.000 | 1.000 | 0.983 | 1.000 | 1.000 | 0.816 | 1.000 | 1.000 | 0.875 | 0.849 |
| 4673 | 0.837 | 1.000 | 0.872 | 1.000 | 0.979 | 1.000 | 0.895 | 1.000 | 1.000 | 1.000 | 0.983 | 1.000 | 1.000 | 0.816 | 1.000 | 1.000 | 0.875 | 0.849 |
| 4674 | 0.837 | 1.000 | 0.872 | 1.000 | 0.979 | 1.000 | 0.895 | 1.000 | 1.000 | 1.000 | 0.983 | 1.000 | 1.000 | 0.816 | 1.000 | 1.000 | 0.875 | 0.849 |
| 4675 | 0.837 | 1.000 | 0.872 | 1.000 | 0.979 | 1.000 | 0.895 | 1.000 | 1.000 | 1.000 | 0.983 | 1.000 | 1.000 | 0.816 | 1.000 | 1.000 | 0.875 | 0.849 |
| 4676 | 0.837 | 1.000 | 0.872 | 1.000 | 0.979 | 1.000 | 0.895 | 1.000 | 1.000 | 1.000 | 0.983 | 1.000 | 1.000 | 0.816 | 1.000 | 1.000 | 0.875 | 0.849 |
| 4677 | 0.836 | 1.000 | 0.872 | 1.000 | 0.979 | 1.000 | 0.895 | 1.000 | 1.000 | 1.000 | 0.983 | 1.000 | 1.000 | 0.816 | 1.000 | 1.000 | 0.875 | 0.849 |
| 4678 | 0.836 | 1.000 | 0.872 | 1.000 | 0.979 | 1.000 | 0.895 | 1.000 | 1.000 | 1.000 | 0.983 | 1.000 | 1.000 | 0.816 | 1.000 | 1.000 | 0.875 | 0.849 |
| 4679 | 0.837 | 1.000 | 0.872 | 1.000 | 0.979 | 1.000 | 0.895 | 1.000 | 1.000 | 1.000 | 0.983 | 1.000 | 1.000 | 0.816 | 1.000 | 1.000 | 0.875 | 0.849 |
| 4680 | 0.836 | 1.000 | 0.872 | 1.000 | 0.979 | 1.000 | 0.895 | 1.000 | 1.000 | 1.000 | 0.983 | 1.000 | 1.000 | 0.817 | 1.000 | 1.000 | 0.875 | 0.849 |
| 4681 | 0.836 | 1.000 | 0.872 | 1.000 | 0.979 | 1.000 | 0.895 | 1.000 | 1.000 | 1.000 | 0.983 | 1.000 | 1.000 | 0.817 | 1.000 | 1.000 | 0.875 | 0.849 |
| 4682 | 0.836 | 1.000 | 0.874 | 1.000 | 0.979 | 1.000 | 0.895 | 1.000 | 1.000 | 1.000 | 0.983 | 1.000 | 1.000 | 0.817 | 1.000 | 1.000 | 0.875 | 0.850 |
| 4683 | 0.835 | 1.000 | 0.874 | 1.000 | 0.979 | 1.000 | 0.895 | 1.000 | 1.000 | 1.000 | 0.983 | 1.000 | 1.000 | 0.817 | 1.000 | 1.000 | 0.875 | 0.849 |

| | | | | | | | | | | | | | | | | | | |
|---|---|---|---|---|---|---|---|---|---|---|---|---|---|---|---|---|---|---|
| 4684 | 0.835 | 1.000 | 0.874 | 1.000 | 0.979 | 1.000 | 0.895 | 1.000 | 1.000 | 1.000 | 0.983 | 1.000 | 1.000 | 0.817 | 1.000 | 1.000 | 0.875 | 0.849 |
| 4685 | 0.836 | 1.000 | 0.874 | 1.000 | 0.979 | 1.000 | 0.895 | 1.000 | 1.000 | 1.000 | 0.983 | 1.000 | 1.000 | 0.817 | 1.000 | 1.000 | 0.875 | 0.850 |
| 4686 | 0.835 | 1.000 | 0.874 | 1.000 | 0.979 | 1.000 | 0.895 | 1.000 | 1.000 | 1.000 | 0.983 | 1.000 | 1.000 | 0.817 | 1.000 | 1.000 | 0.875 | 0.849 |
| 4687 | 0.835 | 1.000 | 0.874 | 1.000 | 0.979 | 1.000 | 0.895 | 1.000 | 1.000 | 1.000 | 0.983 | 1.000 | 1.000 | 0.818 | 1.000 | 1.000 | 0.875 | 0.850 |
| 4688 | 0.836 | 1.000 | 0.874 | 1.000 | 0.979 | 1.000 | 0.895 | 1.000 | 1.000 | 1.000 | 0.983 | 1.000 | 1.000 | 0.818 | 1.000 | 1.000 | 0.876 | 0.850 |
| 4689 | 0.835 | 1.000 | 0.874 | 1.000 | 0.979 | 1.000 | 0.895 | 1.000 | 1.000 | 1.000 | 0.983 | 1.000 | 1.000 | 0.818 | 1.000 | 1.000 | 0.875 | 0.850 |
| 4690 | 0.835 | 1.000 | 0.874 | 1.000 | 0.979 | 1.000 | 0.895 | 1.000 | 1.000 | 1.000 | 0.983 | 1.000 | 1.000 | 0.818 | 1.000 | 1.000 | 0.875 | 0.850 |
| 4691 | 0.835 | 1.000 | 0.874 | 1.000 | 0.979 | 1.000 | 0.895 | 1.000 | 1.000 | 1.000 | 0.983 | 1.000 | 1.000 | 0.818 | 1.000 | 1.000 | 0.875 | 0.850 |
| 4692 | 0.835 | 1.000 | 0.874 | 1.000 | 0.979 | 1.000 | 0.895 | 1.000 | 1.000 | 1.000 | 0.983 | 1.000 | 1.000 | 0.818 | 1.000 | 1.000 | 0.875 | 0.850 |
| 4693 | 0.835 | 1.000 | 0.874 | 1.000 | 0.979 | 1.000 | 0.895 | 1.000 | 1.000 | 1.000 | 0.983 | 1.000 | 1.000 | 0.817 | 1.000 | 1.000 | 0.875 | 0.849 |
| 4694 | 0.835 | 1.000 | 0.874 | 1.000 | 0.979 | 1.000 | 0.895 | 1.000 | 1.000 | 1.000 | 0.983 | 1.000 | 1.000 | 0.818 | 1.000 | 1.000 | 0.875 | 0.849 |
| 4695 | 0.834 | 1.000 | 0.874 | 1.000 | 0.979 | 1.000 | 0.895 | 1.000 | 1.000 | 1.000 | 0.983 | 1.000 | 1.000 | 0.818 | 1.000 | 1.000 | 0.875 | 0.849 |
| 4696 | 0.834 | 1.000 | 0.874 | 1.000 | 0.979 | 1.000 | 0.895 | 1.000 | 1.000 | 1.000 | 0.983 | 1.000 | 1.000 | 0.818 | 1.000 | 1.000 | 0.875 | 0.849 |
| 4697 | 0.834 | 1.000 | 0.874 | 1.000 | 0.979 | 1.000 | 0.895 | 1.000 | 1.000 | 1.000 | 0.983 | 1.000 | 1.000 | 0.818 | 1.000 | 1.000 | 0.875 | 0.849 |
| 4698 | 0.835 | 1.000 | 0.874 | 1.000 | 0.979 | 1.000 | 0.895 | 1.000 | 1.000 | 1.000 | 0.983 | 1.000 | 1.000 | 0.818 | 1.000 | 1.000 | 0.875 | 0.849 |
| 4699 | 0.835 | 1.000 | 0.874 | 1.000 | 0.979 | 1.000 | 0.895 | 1.000 | 1.000 | 1.000 | 0.983 | 1.000 | 1.000 | 0.817 | 1.000 | 1.000 | 0.875 | 0.849 |
| 4700 | 0.835 | 1.000 | 0.875 | 1.000 | 0.979 | 1.000 | 0.895 | 1.000 | 1.000 | 1.000 | 0.983 | 1.000 | 1.000 | 0.817 | 1.000 | 1.000 | 0.875 | 0.850 |
| 4701 | 0.835 | 1.000 | 0.875 | 1.000 | 0.979 | 1.000 | 0.895 | 1.000 | 1.000 | 1.000 | 0.983 | 1.000 | 1.000 | 0.818 | 1.000 | 1.000 | 0.876 | 0.850 |
| 4702 | 0.835 | 1.000 | 0.875 | 1.000 | 0.979 | 1.000 | 0.895 | 1.000 | 1.000 | 1.000 | 0.983 | 1.000 | 1.000 | 0.818 | 1.000 | 1.000 | 0.876 | 0.850 |
| 4703 | 0.835 | 1.000 | 0.875 | 1.000 | 0.979 | 1.000 | 0.895 | 1.000 | 1.000 | 1.000 | 0.983 | 1.000 | 1.000 | 0.817 | 1.000 | 1.000 | 0.875 | 0.850 |
| 4704 | 0.835 | 1.000 | 0.875 | 1.000 | 0.979 | 1.000 | 0.895 | 1.000 | 1.000 | 1.000 | 0.983 | 1.000 | 1.000 | 0.818 | 1.000 | 1.000 | 0.876 | 0.850 |
| 4705 | 0.837 | 1.000 | 0.875 | 1.000 | 0.979 | 1.000 | 0.894 | 1.000 | 1.000 | 1.000 | 0.983 | 1.000 | 1.000 | 0.817 | 1.000 | 1.000 | 0.876 | 0.850 |
| 4706 | 0.837 | 1.000 | 0.875 | 1.000 | 0.979 | 1.000 | 0.895 | 1.000 | 1.000 | 1.000 | 0.983 | 1.000 | 1.000 | 0.817 | 1.000 | 1.000 | 0.876 | 0.850 |
| 4707 | 0.837 | 1.000 | 0.875 | 1.000 | 0.979 | 1.000 | 0.895 | 1.000 | 1.000 | 1.000 | 0.983 | 1.000 | 1.000 | 0.817 | 1.000 | 1.000 | 0.876 | 0.850 |
| 4708 | 0.836 | 1.000 | 0.877 | 1.000 | 0.979 | 1.000 | 0.895 | 1.000 | 1.000 | 1.000 | 0.983 | 1.000 | 1.000 | 0.817 | 1.000 | 1.000 | 0.876 | 0.850 |
| 4709 | 0.836 | 1.000 | 0.877 | 1.000 | 0.979 | 1.000 | 0.894 | 1.000 | 1.000 | 1.000 | 0.983 | 1.000 | 1.000 | 0.816 | 1.000 | 1.000 | 0.875 | 0.850 |
| 4710 | 0.837 | 1.000 | 0.877 | 1.000 | 0.979 | 1.000 | 0.894 | 1.000 | 1.000 | 1.000 | 0.983 | 1.000 | 1.000 | 0.817 | 1.000 | 1.000 | 0.876 | 0.850 |
| 4711 | 0.837 | 1.000 | 0.877 | 1.000 | 0.979 | 1.000 | 0.894 | 1.000 | 1.000 | 1.000 | 0.983 | 1.000 | 1.000 | 0.817 | 1.000 | 1.000 | 0.876 | 0.850 |
| 4712 | 0.837 | 1.000 | 0.877 | 1.000 | 0.979 | 1.000 | 0.894 | 1.000 | 1.000 | 1.000 | 0.983 | 1.000 | 1.000 | 0.817 | 1.000 | 1.000 | 0.876 | 0.850 |
| 4713 | 0.837 | 1.000 | 0.875 | 1.000 | 0.979 | 1.000 | 0.894 | 1.000 | 1.000 | 1.000 | 0.983 | 1.000 | 1.000 | 0.817 | 1.000 | 1.000 | 0.876 | 0.850 |
| 4714 | 0.836 | 1.000 | 0.877 | 1.000 | 0.979 | 1.000 | 0.894 | 1.000 | 1.000 | 1.000 | 0.983 | 1.000 | 1.000 | 0.817 | 1.000 | 1.000 | 0.876 | 0.850 |
| 4715 | 0.837 | 1.000 | 0.877 | 1.000 | 0.979 | 1.000 | 0.894 | 1.000 | 1.000 | 1.000 | 0.983 | 1.000 | 1.000 | 0.817 | 1.000 | 1.000 | 0.876 | 0.850 |
| 4716 | 0.836 | 1.000 | 0.877 | 1.000 | 0.979 | 1.000 | 0.894 | 1.000 | 1.000 | 1.000 | 0.983 | 1.000 | 1.000 | 0.817 | 1.000 | 1.000 | 0.876 | 0.850 |
| 4717 | 0.836 | 1.000 | 0.875 | 1.000 | 0.979 | 1.000 | 0.894 | 1.000 | 1.000 | 1.000 | 0.983 | 1.000 | 1.000 | 0.817 | 1.000 | 1.000 | 0.875 | 0.850 |
| 4718 | 0.835 | 1.000 | 0.875 | 1.000 | 0.979 | 1.000 | 0.894 | 1.000 | 1.000 | 1.000 | 0.983 | 1.000 | 1.000 | 0.816 | 1.000 | 1.000 | 0.875 | 0.849 |
| 4719 | 0.836 | 1.000 | 0.875 | 1.000 | 0.979 | 1.000 | 0.894 | 1.000 | 1.000 | 1.000 | 0.983 | 1.000 | 1.000 | 0.816 | 1.000 | 1.000 | 0.875 | 0.849 |
| 4720 | 0.835 | 1.000 | 0.875 | 1.000 | 0.979 | 1.000 | 0.894 | 1.000 | 1.000 | 1.000 | 0.983 | 1.000 | 1.000 | 0.816 | 1.000 | 1.000 | 0.875 | 0.849 |
| 4721 | 0.836 | 1.000 | 0.875 | 1.000 | 0.979 | 1.000 | 0.894 | 1.000 | 1.000 | 1.000 | 0.983 | 1.000 | 1.000 | 0.816 | 1.000 | 1.000 | 0.875 | 0.849 |
| 4722 | 0.836 | 1.000 | 0.875 | 1.000 | 0.979 | 1.000 | 0.894 | 1.000 | 1.000 | 1.000 | 0.983 | 1.000 | 1.000 | 0.816 | 1.000 | 1.000 | 0.875 | 0.849 |

| | | | | | | | | | | | | | | | | | | |
|---|---|---|---|---|---|---|---|---|---|---|---|---|---|---|---|---|---|---|
| 4723 | 0.836 | 1.000 | 0.875 | 1.000 | 0.979 | 1.000 | 0.894 | 1.000 | 1.000 | 1.000 | 0.983 | 1.000 | 1.000 | 0.816 | 1.000 | 1.000 | 0.875 | 0.849 |
| 4724 | 0.835 | 1.000 | 0.874 | 1.000 | 0.979 | 1.000 | 0.894 | 1.000 | 1.000 | 1.000 | 0.983 | 1.000 | 1.000 | 0.816 | 1.000 | 1.000 | 0.874 | 0.848 |
| 4725 | 0.834 | 1.000 | 0.874 | 1.000 | 0.979 | 1.000 | 0.894 | 1.000 | 1.000 | 1.000 | 0.983 | 1.000 | 1.000 | 0.816 | 1.000 | 1.000 | 0.874 | 0.848 |
| 4726 | 0.835 | 1.000 | 0.874 | 1.000 | 0.979 | 1.000 | 0.894 | 1.000 | 1.000 | 1.000 | 0.983 | 1.000 | 1.000 | 0.816 | 1.000 | 1.000 | 0.875 | 0.849 |
| 4727 | 0.835 | 1.000 | 0.874 | 1.000 | 0.979 | 1.000 | 0.894 | 1.000 | 1.000 | 1.000 | 0.983 | 1.000 | 1.000 | 0.816 | 1.000 | 1.000 | 0.875 | 0.849 |
| 4728 | 0.835 | 1.000 | 0.877 | 1.000 | 0.979 | 1.000 | 0.894 | 1.000 | 1.000 | 1.000 | 0.983 | 1.000 | 1.000 | 0.817 | 1.000 | 1.000 | 0.875 | 0.850 |
| 4729 | 0.832 | 1.000 | 0.879 | 1.000 | 0.979 | 1.000 | 0.897 | 1.000 | 1.000 | 1.000 | 0.983 | 1.000 | 1.000 | 0.818 | 1.000 | 1.000 | 0.875 | 0.849 |
| 4730 | 0.832 | 1.000 | 0.881 | 1.000 | 0.979 | 1.000 | 0.897 | 1.000 | 1.000 | 1.000 | 0.983 | 1.000 | 1.000 | 0.817 | 1.000 | 1.000 | 0.875 | 0.849 |
| 4731 | 0.833 | 1.000 | 0.885 | 1.000 | 0.979 | 1.000 | 0.894 | 1.000 | 1.000 | 1.000 | 0.983 | 1.000 | 1.000 | 0.817 | 1.000 | 1.000 | 0.876 | 0.850 |
| 4732 | 0.833 | 1.000 | 0.881 | 1.000 | 0.979 | 1.000 | 0.894 | 1.000 | 1.000 | 1.000 | 0.983 | 1.000 | 1.000 | 0.817 | 1.000 | 1.000 | 0.875 | 0.849 |
| 4733 | 0.833 | 1.000 | 0.885 | 1.000 | 0.979 | 1.000 | 0.894 | 1.000 | 1.000 | 1.000 | 0.983 | 1.000 | 1.000 | 0.817 | 1.000 | 1.000 | 0.876 | 0.850 |
| 4734 | 0.833 | 1.000 | 0.883 | 1.000 | 0.979 | 1.000 | 0.894 | 1.000 | 1.000 | 1.000 | 0.983 | 1.000 | 1.000 | 0.816 | 1.000 | 1.000 | 0.875 | 0.849 |
| 4735 | 0.833 | 1.000 | 0.883 | 1.000 | 0.979 | 1.000 | 0.894 | 1.000 | 1.000 | 1.000 | 0.983 | 1.000 | 1.000 | 0.816 | 1.000 | 1.000 | 0.875 | 0.849 |
| 4736 | 0.833 | 1.000 | 0.887 | 1.000 | 0.979 | 1.000 | 0.894 | 1.000 | 1.000 | 1.000 | 0.983 | 1.000 | 1.000 | 0.816 | 1.000 | 1.000 | 0.875 | 0.850 |
| 4737 | 0.832 | 1.000 | 0.885 | 1.000 | 0.979 | 1.000 | 0.894 | 1.000 | 1.000 | 1.000 | 0.983 | 1.000 | 1.000 | 0.818 | 1.000 | 1.000 | 0.875 | 0.850 |
| 4738 | 0.831 | 1.000 | 0.883 | 1.000 | 0.979 | 1.000 | 0.892 | 1.000 | 1.000 | 1.000 | 0.983 | 1.000 | 1.000 | 0.818 | 1.000 | 1.000 | 0.875 | 0.849 |
| 4739 | 0.831 | 1.000 | 0.885 | 1.000 | 0.979 | 1.000 | 0.891 | 1.000 | 1.000 | 1.000 | 0.983 | 1.000 | 1.000 | 0.819 | 1.000 | 1.000 | 0.875 | 0.849 |
| 4740 | 0.833 | 1.000 | 0.885 | 1.000 | 0.979 | 1.000 | 0.891 | 1.000 | 1.000 | 1.000 | 0.983 | 1.000 | 1.000 | 0.819 | 1.000 | 1.000 | 0.875 | 0.850 |
| 4741 | 0.831 | 1.000 | 0.885 | 1.000 | 0.979 | 1.000 | 0.891 | 1.000 | 1.000 | 1.000 | 0.983 | 1.000 | 1.000 | 0.820 | 1.000 | 1.000 | 0.875 | 0.850 |
| 4742 | 0.831 | 1.000 | 0.885 | 1.000 | 0.979 | 1.000 | 0.894 | 1.000 | 1.000 | 1.000 | 0.983 | 1.000 | 1.000 | 0.820 | 1.000 | 1.000 | 0.876 | 0.850 |
| 4743 | 0.831 | 1.000 | 0.883 | 1.000 | 0.979 | 1.000 | 0.894 | 1.000 | 1.000 | 1.000 | 0.983 | 1.000 | 1.000 | 0.820 | 1.000 | 1.000 | 0.875 | 0.850 |
| 4744 | 0.831 | 1.000 | 0.885 | 1.000 | 0.979 | 1.000 | 0.894 | 1.000 | 1.000 | 1.000 | 0.983 | 1.000 | 1.000 | 0.820 | 1.000 | 1.000 | 0.876 | 0.850 |
| 4745 | 0.831 | 1.000 | 0.885 | 1.000 | 0.979 | 1.000 | 0.892 | 1.000 | 1.000 | 1.000 | 0.983 | 1.000 | 1.000 | 0.819 | 1.000 | 1.000 | 0.875 | 0.849 |
| 4746 | 0.831 | 1.000 | 0.885 | 1.000 | 0.979 | 1.000 | 0.892 | 1.000 | 1.000 | 1.000 | 0.983 | 1.000 | 1.000 | 0.819 | 1.000 | 1.000 | 0.875 | 0.850 |
| 4747 | 0.832 | 1.000 | 0.885 | 1.000 | 0.979 | 1.000 | 0.892 | 1.000 | 1.000 | 1.000 | 0.983 | 1.000 | 1.000 | 0.819 | 1.000 | 1.000 | 0.876 | 0.850 |
| 4748 | 0.832 | 1.000 | 0.885 | 1.000 | 0.979 | 1.000 | 0.892 | 1.000 | 1.000 | 1.000 | 0.983 | 1.000 | 1.000 | 0.819 | 1.000 | 1.000 | 0.876 | 0.850 |
| 4749 | 0.832 | 1.000 | 0.885 | 1.000 | 0.979 | 1.000 | 0.892 | 1.000 | 1.000 | 1.000 | 0.983 | 1.000 | 1.000 | 0.819 | 1.000 | 1.000 | 0.875 | 0.850 |
| 4750 | 0.832 | 1.000 | 0.885 | 1.000 | 0.979 | 1.000 | 0.892 | 1.000 | 1.000 | 1.000 | 0.983 | 1.000 | 1.000 | 0.819 | 1.000 | 1.000 | 0.875 | 0.850 |
| 4751 | 0.832 | 1.000 | 0.885 | 1.000 | 0.979 | 1.000 | 0.892 | 1.000 | 1.000 | 1.000 | 0.983 | 1.000 | 1.000 | 0.819 | 1.000 | 1.000 | 0.876 | 0.850 |
| 4752 | 0.832 | 1.000 | 0.885 | 1.000 | 0.979 | 1.000 | 0.892 | 1.000 | 1.000 | 1.000 | 0.983 | 1.000 | 1.000 | 0.819 | 1.000 | 1.000 | 0.876 | 0.850 |
| 4753 | 0.833 | 1.000 | 0.885 | 1.000 | 0.979 | 1.000 | 0.892 | 1.000 | 1.000 | 1.000 | 0.983 | 1.000 | 1.000 | 0.820 | 1.000 | 1.000 | 0.876 | 0.850 |
| 4754 | 0.833 | 1.000 | 0.885 | 1.000 | 0.979 | 1.000 | 0.892 | 1.000 | 1.000 | 1.000 | 0.983 | 1.000 | 1.000 | 0.821 | 1.000 | 1.000 | 0.876 | 0.851 |
| 4755 | 0.831 | 1.000 | 0.885 | 1.000 | 0.979 | 1.000 | 0.892 | 1.000 | 1.000 | 1.000 | 0.983 | 1.000 | 1.000 | 0.823 | 1.000 | 1.000 | 0.876 | 0.851 |
| 4756 | 0.832 | 1.000 | 0.885 | 1.000 | 0.979 | 1.000 | 0.892 | 1.000 | 1.000 | 1.000 | 0.983 | 1.000 | 1.000 | 0.823 | 1.000 | 1.000 | 0.876 | 0.851 |
| 4757 | 0.832 | 1.000 | 0.885 | 1.000 | 0.979 | 1.000 | 0.892 | 1.000 | 1.000 | 1.000 | 0.983 | 1.000 | 1.000 | 0.822 | 1.000 | 1.000 | 0.876 | 0.851 |
| 4758 | 0.833 | 1.000 | 0.885 | 1.000 | 0.979 | 1.000 | 0.892 | 1.000 | 1.000 | 1.000 | 0.983 | 1.000 | 1.000 | 0.822 | 1.000 | 1.000 | 0.876 | 0.851 |
| 4759 | 0.833 | 1.000 | 0.885 | 1.000 | 0.979 | 1.000 | 0.892 | 1.000 | 1.000 | 1.000 | 0.983 | 1.000 | 1.000 | 0.822 | 1.000 | 1.000 | 0.876 | 0.851 |
| 4760 | 0.832 | 1.000 | 0.883 | 1.000 | 0.979 | 1.000 | 0.892 | 1.000 | 1.000 | 1.000 | 0.983 | 1.000 | 1.000 | 0.822 | 1.000 | 1.000 | 0.876 | 0.850 |
| 4761 | 0.834 | 1.000 | 0.885 | 1.000 | 0.979 | 1.000 | 0.892 | 1.000 | 1.000 | 1.000 | 0.983 | 1.000 | 1.000 | 0.820 | 1.000 | 1.000 | 0.876 | 0.851 |

| | | | | | | | | | | | | | | | | | |
|---|---|---|---|---|---|---|---|---|---|---|---|---|---|---|---|---|---|
| 4762 | 0.832 | 1.000 | 0.885 | 1.000 | 0.979 | 1.000 | 0.892 | 1.000 | 1.000 | 1.000 | 0.983 | 1.000 | 1.000 | 0.820 | 1.000 | 1.000 | 0.876 | 0.850 |
| 4763 | 0.833 | 1.000 | 0.885 | 1.000 | 0.979 | 1.000 | 0.892 | 1.000 | 1.000 | 1.000 | 0.983 | 1.000 | 1.000 | 0.821 | 1.000 | 1.000 | 0.876 | 0.851 |
| 4764 | 0.834 | 1.000 | 0.885 | 1.000 | 0.979 | 1.000 | 0.892 | 1.000 | 1.000 | 1.000 | 0.983 | 1.000 | 1.000 | 0.819 | 1.000 | 1.000 | 0.876 | 0.851 |
| 4765 | 0.833 | 1.000 | 0.885 | 1.000 | 0.979 | 1.000 | 0.892 | 1.000 | 1.000 | 1.000 | 0.983 | 1.000 | 1.000 | 0.821 | 1.000 | 1.000 | 0.876 | 0.851 |
| 4766 | 0.833 | 1.000 | 0.885 | 1.000 | 0.979 | 1.000 | 0.892 | 1.000 | 1.000 | 1.000 | 0.983 | 1.000 | 1.000 | 0.821 | 1.000 | 1.000 | 0.876 | 0.851 |
| 4767 | 0.833 | 1.000 | 0.887 | 1.000 | 0.979 | 1.000 | 0.892 | 1.000 | 1.000 | 1.000 | 0.983 | 1.000 | 1.000 | 0.820 | 1.000 | 1.000 | 0.876 | 0.851 |
| 4768 | 0.833 | 1.000 | 0.885 | 1.000 | 0.979 | 1.000 | 0.892 | 1.000 | 1.000 | 1.000 | 0.983 | 1.000 | 1.000 | 0.820 | 1.000 | 1.000 | 0.876 | 0.850 |
| 4769 | 0.833 | 1.000 | 0.883 | 1.000 | 0.979 | 1.000 | 0.892 | 1.000 | 1.000 | 1.000 | 0.983 | 1.000 | 1.000 | 0.820 | 1.000 | 1.000 | 0.876 | 0.850 |
| 4770 | 0.832 | 1.000 | 0.883 | 1.000 | 0.979 | 1.000 | 0.892 | 1.000 | 1.000 | 1.000 | 0.983 | 1.000 | 1.000 | 0.820 | 1.000 | 1.000 | 0.876 | 0.850 |
| 4771 | 0.832 | 1.000 | 0.883 | 1.000 | 0.979 | 1.000 | 0.892 | 1.000 | 1.000 | 1.000 | 0.983 | 1.000 | 1.000 | 0.822 | 1.000 | 1.000 | 0.876 | 0.850 |
| 4772 | 0.832 | 1.000 | 0.883 | 1.000 | 0.979 | 1.000 | 0.892 | 1.000 | 1.000 | 1.000 | 0.983 | 1.000 | 1.000 | 0.823 | 1.000 | 1.000 | 0.876 | 0.851 |
| 4773 | 0.833 | 1.000 | 0.883 | 1.000 | 0.979 | 1.000 | 0.892 | 1.000 | 1.000 | 1.000 | 0.983 | 1.000 | 1.000 | 0.823 | 1.000 | 1.000 | 0.876 | 0.851 |

**3. IFS results using RF and MCFS feature list**

| Number of features | Class 1 | Class 2 | Class 3 | Class 4 | Class 5 | Class 6 | Class 7 | Class 8 | Class 9 | Class 10 | Class 11 | Class 12 | Class 13 | Class 14 | Class 15 | Class 16 | ACC | MCC |
|---|---|---|---|---|---|---|---|---|---|---|---|---|---|---|---|---|---|---|
| 1 | 0.003 | 0.000 | 0.000 | 0.000 | 0.005 | 0.000 | 0.000 | 1.000 | 0.000 | 0.000 | 0.012 | 0.030 | 0.000 | 0.001 | 0.717 | 0.000 | 0.010 | 0.000 |
| 2 | 0.005 | 0.000 | 0.014 | 0.000 | 0.005 | 0.000 | 0.000 | 0.333 | 1.000 | 0.000 | 0.017 | 0.030 | 0.598 | 0.009 | 0.717 | 0.000 | 0.047 | 0.046 |
| 3 | 0.005 | 0.000 | 0.018 | 0.000 | 0.032 | 0.000 | 0.000 | 0.333 | 1.000 | 0.000 | 0.026 | 0.030 | 0.598 | 0.010 | 0.739 | 0.000 | 0.049 | 0.049 |
| 4 | 0.005 | 0.000 | 0.032 | 0.000 | 0.032 | 0.000 | 0.000 | 0.333 | 1.000 | 0.000 | 0.026 | 0.091 | 0.705 | 0.017 | 0.761 | 0.000 | 0.055 | 0.056 |
| 5 | 0.005 | 0.000 | 0.045 | 0.000 | 0.032 | 0.000 | 0.002 | 0.333 | 1.000 | 0.000 | 0.058 | 0.242 | 0.723 | 0.046 | 0.761 | 0.000 | 0.068 | 0.104 |
| 6 | 0.005 | 0.000 | 0.065 | 0.000 | 0.042 | 0.000 | 0.002 | 0.333 | 0.000 | 0.000 | 0.055 | 0.273 | 0.723 | 0.061 | 0.761 | 1.000 | 0.066 | 0.099 |
| 7 | 0.005 | 0.000 | 0.067 | 0.014 | 0.042 | 0.000 | 0.002 | 0.333 | 0.000 | 0.000 | 0.058 | 0.242 | 0.741 | 0.060 | 0.761 | 1.000 | 0.066 | 0.099 |
| 8 | 0.005 | 0.000 | 0.067 | 0.043 | 0.042 | 0.000 | 0.002 | 0.333 | 0.010 | 0.000 | 0.070 | 0.273 | 0.759 | 0.072 | 0.761 | 1.000 | 0.071 | 0.106 |
| 9 | 0.005 | 0.000 | 0.071 | 0.057 | 0.042 | 0.000 | 0.002 | 0.333 | 0.010 | 0.000 | 0.064 | 0.273 | 0.732 | 0.072 | 0.761 | 1.000 | 0.071 | 0.105 |
| 10 | 0.005 | 0.000 | 0.065 | 0.057 | 0.042 | 0.000 | 0.002 | 0.333 | 0.010 | 0.000 | 0.067 | 0.242 | 0.759 | 0.073 | 0.761 | 1.000 | 0.071 | 0.105 |
| 11 | 0.005 | 0.000 | 0.071 | 0.057 | 0.047 | 0.000 | 0.002 | 0.333 | 0.020 | 0.000 | 0.072 | 0.303 | 0.750 | 0.088 | 0.761 | 1.000 | 0.076 | 0.114 |
| 12 | 0.005 | 0.000 | 0.067 | 0.057 | 0.047 | 0.000 | 0.002 | 0.333 | 0.020 | 0.000 | 0.087 | 0.303 | 0.741 | 0.101 | 0.761 | 1.000 | 0.080 | 0.116 |
| 13 | 0.008 | 0.000 | 0.067 | 0.057 | 0.047 | 0.000 | 0.003 | 0.333 | 0.020 | 0.000 | 0.087 | 0.303 | 0.759 | 0.103 | 0.761 | 1.000 | 0.082 | 0.116 |
| 14 | 0.010 | 0.000 | 0.075 | 0.057 | 0.047 | 0.000 | 0.003 | 0.333 | 0.020 | 0.000 | 0.093 | 0.303 | 0.759 | 0.111 | 0.761 | 1.000 | 0.086 | 0.122 |
| 15 | 0.010 | 0.000 | 0.081 | 0.057 | 0.047 | 0.040 | 0.003 | 0.333 | 0.031 | 0.042 | 0.122 | 0.333 | 0.777 | 0.135 | 0.761 | 1.000 | 0.096 | 0.135 |
| 16 | 0.011 | 0.000 | 0.085 | 0.057 | 0.047 | 0.040 | 0.008 | 0.333 | 0.031 | 0.042 | 0.125 | 0.333 | 0.777 | 0.137 | 0.761 | 1.000 | 0.098 | 0.138 |
| 17 | 0.036 | 0.057 | 0.231 | 0.171 | 0.211 | 0.040 | 0.091 | 0.333 | 0.837 | 0.167 | 0.536 | 0.485 | 0.795 | 0.201 | 0.891 | 0.167 | 0.195 | 0.180 |
| 18 | 0.038 | 0.057 | 0.241 | 0.171 | 0.221 | 0.080 | 0.091 | 0.333 | 0.827 | 0.167 | 0.545 | 0.455 | 0.804 | 0.212 | 0.870 | 0.167 | 0.201 | 0.180 |
| 19 | 0.038 | 0.057 | 0.253 | 0.171 | 0.216 | 0.080 | 0.091 | 0.333 | 0.827 | 0.167 | 0.536 | 0.455 | 0.804 | 0.214 | 0.870 | 0.148 | 0.201 | 0.180 |
| 20 | 0.036 | 0.029 | 0.261 | 0.186 | 0.226 | 0.080 | 0.092 | 0.333 | 0.816 | 0.188 | 0.548 | 0.606 | 0.795 | 0.221 | 0.891 | 0.148 | 0.206 | 0.179 |
| 21 | 0.036 | 0.029 | 0.253 | 0.200 | 0.226 | 0.080 | 0.092 | 0.333 | 0.816 | 0.188 | 0.559 | 0.576 | 0.813 | 0.224 | 0.891 | 0.148 | 0.207 | 0.181 |
| 22 | 0.038 | 0.771 | 0.249 | 0.229 | 0.242 | 0.080 | 0.091 | 0.333 | 0.082 | 0.188 | 0.557 | 0.576 | 0.830 | 0.228 | 0.891 | 0.148 | 0.200 | 0.190 |
| 23 | 0.040 | 0.771 | 0.259 | 0.229 | 0.253 | 0.080 | 0.091 | 0.333 | 0.082 | 0.188 | 0.568 | 0.576 | 0.821 | 0.233 | 0.891 | 0.148 | 0.204 | 0.194 |
| 24 | 0.040 | 0.771 | 0.255 | 0.271 | 0.258 | 0.160 | 0.088 | 0.333 | 0.082 | 0.188 | 0.562 | 0.576 | 0.830 | 0.231 | 0.891 | 0.148 | 0.203 | 0.193 |
| 25 | 0.039 | 0.771 | 0.257 | 0.271 | 0.253 | 0.160 | 0.089 | 0.333 | 0.102 | 0.188 | 0.571 | 0.606 | 0.839 | 0.244 | 0.891 | 0.148 | 0.208 | 0.199 |
| 26 | 0.039 | 0.771 | 0.259 | 0.271 | 0.258 | 0.160 | 0.091 | 0.333 | 0.102 | 0.188 | 0.577 | 0.606 | 0.848 | 0.240 | 0.891 | 0.148 | 0.208 | 0.198 |
| 27 | 0.038 | 0.771 | 0.265 | 0.286 | 0.253 | 0.160 | 0.091 | 0.333 | 0.102 | 0.208 | 0.571 | 0.606 | 0.848 | 0.255 | 0.891 | 0.148 | 0.212 | 0.203 |
| 28 | 0.039 | 0.771 | 0.271 | 0.271 | 0.253 | 0.160 | 0.091 | 0.333 | 0.102 | 0.188 | 0.577 | 0.606 | 0.839 | 0.251 | 0.891 | 0.148 | 0.212 | 0.202 |
| 29 | 0.040 | 0.771 | 0.275 | 0.300 | 0.268 | 0.160 | 0.091 | 0.333 | 0.112 | 0.188 | 0.574 | 0.576 | 0.839 | 0.258 | 0.913 | 0.167 | 0.216 | 0.206 |
| 30 | 0.040 | 0.771 | 0.259 | 0.329 | 0.268 | 0.160 | 0.100 | 0.333 | 0.112 | 0.188 | 0.586 | 0.606 | 0.857 | 0.272 | 0.913 | 0.185 | 0.221 | 0.210 |
| 31 | 0.041 | 0.771 | 0.267 | 0.329 | 0.279 | 0.160 | 0.097 | 0.333 | 0.122 | 0.167 | 0.580 | 0.606 | 0.866 | 0.279 | 0.913 | 0.185 | 0.224 | 0.213 |
| 32 | 0.042 | 0.771 | 0.271 | 0.329 | 0.279 | 0.160 | 0.097 | 0.333 | 0.133 | 0.188 | 0.583 | 0.606 | 0.848 | 0.287 | 0.913 | 0.204 | 0.227 | 0.216 |
| 33 | 0.042 | 0.771 | 0.265 | 0.329 | 0.274 | 0.160 | 0.099 | 0.333 | 0.133 | 0.188 | 0.591 | 0.606 | 0.848 | 0.298 | 0.913 | 0.185 | 0.229 | 0.219 |
| 34 | 0.044 | 0.771 | 0.267 | 0.329 | 0.289 | 0.160 | 0.100 | 0.333 | 0.143 | 0.208 | 0.580 | 0.636 | 0.857 | 0.293 | 0.913 | 0.222 | 0.230 | 0.219 |
| 35 | 0.045 | 0.771 | 0.271 | 0.329 | 0.300 | 0.160 | 0.103 | 0.333 | 0.143 | 0.208 | 0.571 | 0.576 | 0.857 | 0.293 | 0.891 | 0.204 | 0.230 | 0.218 |
| 36 | 0.044 | 0.771 | 0.259 | 0.343 | 0.300 | 0.200 | 0.102 | 0.333 | 0.143 | 0.229 | 0.568 | 0.576 | 0.848 | 0.289 | 0.891 | 0.204 | 0.228 | 0.215 |
| 37 | 0.044 | 0.771 | 0.255 | 0.357 | 0.295 | 0.200 | 0.103 | 0.333 | 0.143 | 0.208 | 0.577 | 0.576 | 0.866 | 0.290 | 0.891 | 0.204 | 0.229 | 0.216 |

| | | | | | | | | | | | | | | | | | | |
|---|---|---|---|---|---|---|---|---|---|---|---|---|---|---|---|---|---|---|
| 38 | 0.393 | 0.143 | 0.296 | 0.471 | 0.511 | 0.280 | 0.133 | 0.333 | 0.194 | 0.229 | 0.620 | 0.606 | 0.839 | 0.317 | 0.913 | 0.370 | 0.359 | 0.251 |
| 39 | 0.396 | 0.143 | 0.302 | 0.471 | 0.516 | 0.280 | 0.137 | 0.333 | 0.184 | 0.208 | 0.629 | 0.606 | 0.866 | 0.317 | 0.913 | 0.389 | 0.362 | 0.255 |
| 40 | 0.395 | 0.143 | 0.304 | 0.471 | 0.521 | 0.280 | 0.140 | 0.333 | 0.194 | 0.208 | 0.641 | 0.636 | 0.866 | 0.320 | 0.913 | 0.389 | 0.365 | 0.258 |
| 41 | 0.395 | 0.143 | 0.314 | 0.471 | 0.532 | 0.280 | 0.139 | 0.333 | 0.194 | 0.208 | 0.643 | 0.606 | 0.866 | 0.325 | 0.913 | 0.389 | 0.368 | 0.262 |
| 42 | 0.395 | 0.143 | 0.322 | 0.471 | 0.532 | 0.280 | 0.137 | 0.333 | 0.194 | 0.229 | 0.672 | 0.606 | 0.866 | 0.328 | 0.913 | 0.389 | 0.371 | 0.266 |
| 43 | 0.395 | 0.143 | 0.320 | 0.486 | 0.537 | 0.280 | 0.142 | 0.333 | 0.194 | 0.208 | 0.678 | 0.636 | 0.875 | 0.325 | 0.913 | 0.389 | 0.372 | 0.267 |
| 44 | 0.684 | 0.171 | 0.318 | 0.471 | 0.532 | 0.280 | 0.136 | 0.333 | 0.194 | 0.208 | 0.690 | 0.636 | 0.866 | 0.332 | 0.913 | 0.389 | 0.459 | 0.345 |
| 45 | 0.683 | 0.171 | 0.326 | 0.471 | 0.532 | 0.280 | 0.142 | 0.333 | 0.214 | 0.208 | 0.678 | 0.636 | 0.866 | 0.339 | 0.935 | 0.389 | 0.462 | 0.348 |
| 46 | 0.683 | 0.171 | 0.328 | 0.471 | 0.542 | 0.280 | 0.142 | 0.333 | 0.214 | 0.208 | 0.675 | 0.667 | 0.875 | 0.339 | 0.935 | 0.389 | 0.463 | 0.350 |
| 47 | 0.685 | 0.143 | 0.330 | 0.486 | 0.532 | 0.280 | 0.145 | 0.333 | 0.214 | 0.208 | 0.675 | 0.636 | 0.875 | 0.341 | 0.913 | 0.389 | 0.464 | 0.350 |
| 48 | 0.687 | 0.143 | 0.334 | 0.486 | 0.542 | 0.280 | 0.148 | 0.333 | 0.224 | 0.188 | 0.670 | 0.667 | 0.884 | 0.342 | 0.935 | 0.389 | 0.466 | 0.353 |
| 49 | 0.684 | 0.143 | 0.326 | 0.486 | 0.537 | 0.280 | 0.143 | 0.333 | 0.224 | 0.208 | 0.672 | 0.606 | 0.875 | 0.332 | 0.935 | 0.389 | 0.460 | 0.347 |
| 50 | 0.684 | 0.171 | 0.326 | 0.500 | 0.532 | 0.280 | 0.143 | 0.333 | 0.224 | 0.208 | 0.687 | 0.636 | 0.875 | 0.346 | 0.913 | 0.389 | 0.466 | 0.353 |
| 51 | 0.104 | 0.429 | 0.413 | 0.671 | 0.658 | 0.480 | 0.245 | 0.333 | 0.337 | 0.813 | 0.765 | 0.727 | 0.902 | 0.358 | 0.957 | 0.648 | 0.345 | 0.306 |
| 52 | 0.102 | 0.429 | 0.413 | 0.686 | 0.663 | 0.480 | 0.230 | 0.333 | 0.347 | 0.813 | 0.745 | 0.697 | 0.902 | 0.368 | 0.957 | 0.667 | 0.345 | 0.304 |
| 53 | 0.104 | 0.429 | 0.425 | 0.700 | 0.647 | 0.480 | 0.240 | 0.333 | 0.357 | 0.813 | 0.759 | 0.697 | 0.902 | 0.374 | 0.978 | 0.648 | 0.350 | 0.310 |
| 54 | 0.102 | 0.429 | 0.419 | 0.700 | 0.647 | 0.480 | 0.237 | 0.333 | 0.357 | 0.813 | 0.759 | 0.697 | 0.902 | 0.361 | 0.957 | 0.667 | 0.345 | 0.306 |
| 55 | 0.098 | 0.429 | 0.415 | 0.700 | 0.663 | 0.480 | 0.231 | 0.333 | 0.357 | 0.854 | 0.757 | 0.697 | 0.902 | 0.370 | 0.978 | 0.667 | 0.346 | 0.307 |
| 56 | 0.098 | 0.429 | 0.427 | 0.686 | 0.663 | 0.520 | 0.231 | 0.333 | 0.357 | 0.854 | 0.762 | 0.727 | 0.902 | 0.375 | 0.978 | 0.685 | 0.349 | 0.310 |
| 57 | 0.096 | 0.429 | 0.421 | 0.714 | 0.674 | 0.520 | 0.236 | 0.333 | 0.347 | 0.833 | 0.765 | 0.727 | 0.902 | 0.366 | 0.957 | 0.667 | 0.347 | 0.308 |
| 58 | 0.102 | 0.429 | 0.417 | 0.700 | 0.668 | 0.520 | 0.242 | 0.333 | 0.347 | 0.854 | 0.768 | 0.727 | 0.902 | 0.369 | 0.957 | 0.685 | 0.350 | 0.311 |
| 59 | 0.102 | 0.429 | 0.417 | 0.714 | 0.674 | 0.520 | 0.239 | 0.333 | 0.337 | 0.833 | 0.762 | 0.727 | 0.902 | 0.370 | 0.978 | 0.667 | 0.349 | 0.309 |
| 60 | 0.103 | 0.429 | 0.419 | 0.714 | 0.679 | 0.520 | 0.240 | 0.333 | 0.357 | 0.854 | 0.768 | 0.727 | 0.893 | 0.378 | 0.978 | 0.667 | 0.353 | 0.314 |
| 61 | 0.101 | 0.429 | 0.409 | 0.700 | 0.663 | 0.520 | 0.245 | 0.333 | 0.347 | 0.854 | 0.768 | 0.727 | 0.902 | 0.384 | 0.978 | 0.667 | 0.353 | 0.313 |
| 62 | 0.103 | 0.429 | 0.423 | 0.714 | 0.674 | 0.520 | 0.245 | 0.333 | 0.357 | 0.854 | 0.765 | 0.727 | 0.902 | 0.381 | 0.978 | 0.667 | 0.355 | 0.315 |
| 63 | 0.108 | 0.429 | 0.419 | 0.714 | 0.689 | 0.520 | 0.250 | 0.333 | 0.357 | 0.854 | 0.765 | 0.727 | 0.902 | 0.381 | 0.978 | 0.667 | 0.357 | 0.317 |
| 64 | 0.106 | 0.429 | 0.421 | 0.714 | 0.679 | 0.520 | 0.248 | 0.333 | 0.347 | 0.854 | 0.771 | 0.697 | 0.902 | 0.382 | 0.978 | 0.667 | 0.356 | 0.317 |
| 65 | 0.108 | 0.457 | 0.419 | 0.729 | 0.679 | 0.520 | 0.245 | 0.333 | 0.337 | 0.833 | 0.759 | 0.697 | 0.902 | 0.382 | 0.978 | 0.685 | 0.356 | 0.316 |
| 66 | 0.106 | 0.457 | 0.427 | 0.729 | 0.679 | 0.520 | 0.251 | 0.333 | 0.347 | 0.854 | 0.754 | 0.727 | 0.902 | 0.397 | 0.978 | 0.667 | 0.361 | 0.321 |
| 67 | 0.110 | 0.457 | 0.431 | 0.714 | 0.679 | 0.520 | 0.250 | 0.333 | 0.347 | 0.833 | 0.777 | 0.758 | 0.902 | 0.384 | 0.978 | 0.667 | 0.360 | 0.321 |
| 68 | 0.108 | 0.457 | 0.425 | 0.729 | 0.668 | 0.520 | 0.253 | 0.333 | 0.347 | 0.854 | 0.777 | 0.758 | 0.902 | 0.382 | 0.957 | 0.667 | 0.358 | 0.320 |
| 69 | 0.149 | 0.971 | 0.464 | 0.757 | 0.689 | 0.480 | 0.274 | 0.333 | 0.418 | 0.500 | 0.791 | 0.788 | 0.920 | 0.419 | 0.957 | 0.667 | 0.391 | 0.347 |
| 70 | 0.147 | 0.971 | 0.458 | 0.757 | 0.684 | 0.480 | 0.276 | 0.333 | 0.418 | 0.500 | 0.803 | 0.788 | 0.920 | 0.428 | 0.978 | 0.667 | 0.393 | 0.349 |
| 71 | 0.146 | 0.971 | 0.472 | 0.729 | 0.705 | 0.520 | 0.282 | 0.333 | 0.439 | 0.479 | 0.800 | 0.788 | 0.911 | 0.428 | 0.957 | 0.667 | 0.396 | 0.352 |
| 72 | 0.144 | 0.971 | 0.462 | 0.771 | 0.684 | 0.480 | 0.285 | 0.333 | 0.429 | 0.479 | 0.817 | 0.788 | 0.911 | 0.430 | 0.978 | 0.667 | 0.396 | 0.353 |
| 73 | 0.159 | 0.971 | 0.480 | 0.757 | 0.689 | 0.480 | 0.288 | 0.333 | 0.429 | 0.500 | 0.812 | 0.788 | 0.911 | 0.426 | 0.978 | 0.667 | 0.401 | 0.357 |
| 74 | 0.153 | 0.971 | 0.466 | 0.757 | 0.689 | 0.560 | 0.287 | 0.333 | 0.429 | 0.521 | 0.820 | 0.788 | 0.911 | 0.411 | 0.957 | 0.667 | 0.395 | 0.351 |
| 75 | 0.484 | 0.543 | 0.480 | 0.786 | 0.732 | 0.680 | 0.299 | 0.667 | 0.490 | 0.479 | 0.855 | 0.788 | 0.911 | 0.430 | 0.978 | 0.704 | 0.505 | 0.413 |
| 76 | 0.482 | 0.543 | 0.480 | 0.786 | 0.721 | 0.680 | 0.300 | 0.667 | 0.490 | 0.500 | 0.858 | 0.788 | 0.911 | 0.433 | 0.978 | 0.704 | 0.505 | 0.414 |

| | | | | | | | | | | | | | | | | | | |
|---|---|---|---|---|---|---|---|---|---|---|---|---|---|---|---|---|---|---|
| 77 | 0.486 | 0.543 | 0.476 | 0.800 | 0.705 | 0.680 | 0.311 | 0.667 | 0.480 | 0.458 | 0.846 | 0.788 | 0.911 | 0.430 | 0.978 | 0.685 | 0.505 | 0.413 |
| 78 | 0.492 | 0.543 | 0.486 | 0.800 | 0.721 | 0.680 | 0.304 | 0.667 | 0.480 | 0.479 | 0.849 | 0.788 | 0.911 | 0.431 | 0.978 | 0.685 | 0.508 | 0.416 |
| 79 | 0.486 | 0.543 | 0.462 | 0.800 | 0.721 | 0.680 | 0.299 | 0.667 | 0.469 | 0.479 | 0.855 | 0.788 | 0.911 | 0.433 | 0.978 | 0.685 | 0.504 | 0.411 |
| 80 | 0.489 | 0.543 | 0.490 | 0.800 | 0.716 | 0.680 | 0.296 | 0.667 | 0.500 | 0.479 | 0.846 | 0.788 | 0.902 | 0.432 | 0.978 | 0.667 | 0.506 | 0.414 |
| 81 | 0.498 | 0.600 | 0.538 | 0.814 | 0.737 | 0.760 | 0.399 | 0.667 | 0.765 | 0.542 | 0.872 | 0.848 | 0.929 | 0.521 | 0.978 | 0.889 | 0.563 | 0.480 |
| 82 | 0.498 | 0.600 | 0.526 | 0.800 | 0.747 | 0.760 | 0.405 | 0.667 | 0.786 | 0.583 | 0.875 | 0.879 | 0.929 | 0.513 | 0.978 | 0.870 | 0.562 | 0.480 |
| 83 | 0.494 | 0.600 | 0.532 | 0.814 | 0.747 | 0.800 | 0.411 | 0.667 | 0.776 | 0.583 | 0.864 | 0.879 | 0.929 | 0.511 | 0.978 | 0.870 | 0.561 | 0.480 |
| 84 | 0.496 | 0.600 | 0.526 | 0.814 | 0.747 | 0.760 | 0.404 | 0.667 | 0.776 | 0.583 | 0.867 | 0.848 | 0.920 | 0.527 | 0.978 | 0.889 | 0.564 | 0.483 |
| 85 | 0.488 | 0.629 | 0.530 | 0.829 | 0.742 | 0.800 | 0.408 | 0.667 | 0.755 | 0.563 | 0.867 | 0.848 | 0.929 | 0.525 | 0.978 | 0.870 | 0.562 | 0.480 |
| 86 | 0.490 | 0.600 | 0.534 | 0.786 | 0.758 | 0.800 | 0.413 | 0.667 | 0.776 | 0.563 | 0.858 | 0.848 | 0.929 | 0.521 | 0.957 | 0.889 | 0.562 | 0.480 |
| 87 | 0.487 | 0.600 | 0.530 | 0.800 | 0.737 | 0.800 | 0.393 | 0.667 | 0.786 | 0.604 | 0.867 | 0.848 | 0.920 | 0.514 | 0.978 | 0.889 | 0.557 | 0.474 |
| 88 | 0.495 | 0.600 | 0.545 | 0.800 | 0.763 | 0.800 | 0.402 | 0.667 | 0.796 | 0.583 | 0.855 | 0.848 | 0.929 | 0.529 | 0.978 | 0.852 | 0.566 | 0.485 |
| 89 | 0.490 | 0.600 | 0.528 | 0.786 | 0.763 | 0.800 | 0.394 | 0.667 | 0.786 | 0.583 | 0.872 | 0.879 | 0.938 | 0.529 | 0.978 | 0.870 | 0.563 | 0.481 |
| 90 | 0.488 | 0.571 | 0.540 | 0.800 | 0.758 | 0.800 | 0.414 | 0.667 | 0.786 | 0.542 | 0.858 | 0.879 | 0.920 | 0.525 | 0.978 | 0.889 | 0.563 | 0.482 |
| 91 | 0.496 | 0.600 | 0.536 | 0.800 | 0.763 | 0.800 | 0.404 | 0.667 | 0.786 | 0.563 | 0.858 | 0.879 | 0.938 | 0.538 | 0.978 | 0.889 | 0.568 | 0.487 |
| 92 | 0.493 | 0.600 | 0.536 | 0.800 | 0.753 | 0.800 | 0.401 | 0.667 | 0.796 | 0.604 | 0.875 | 0.879 | 0.938 | 0.515 | 0.978 | 0.870 | 0.562 | 0.481 |
| 93 | 0.499 | 0.600 | 0.543 | 0.800 | 0.753 | 0.800 | 0.411 | 0.667 | 0.796 | 0.604 | 0.852 | 0.879 | 0.929 | 0.518 | 0.978 | 0.852 | 0.565 | 0.484 |
| 94 | 0.496 | 0.629 | 0.528 | 0.800 | 0.774 | 0.800 | 0.414 | 0.667 | 0.806 | 0.625 | 0.858 | 0.879 | 0.938 | 0.530 | 0.957 | 0.870 | 0.568 | 0.487 |
| 95 | 0.497 | 0.629 | 0.526 | 0.786 | 0.784 | 0.800 | 0.404 | 0.667 | 0.806 | 0.625 | 0.861 | 0.879 | 0.929 | 0.535 | 0.978 | 0.907 | 0.569 | 0.488 |
| 96 | 0.487 | 0.571 | 0.540 | 0.800 | 0.784 | 0.800 | 0.421 | 0.667 | 0.796 | 0.625 | 0.858 | 0.879 | 0.929 | 0.532 | 0.978 | 0.870 | 0.567 | 0.486 |
| 97 | 0.494 | 0.543 | 0.534 | 0.814 | 0.795 | 0.800 | 0.411 | 0.667 | 0.806 | 0.583 | 0.846 | 0.879 | 0.929 | 0.527 | 0.978 | 0.907 | 0.566 | 0.486 |
| 98 | 0.486 | 0.571 | 0.542 | 0.814 | 0.779 | 0.800 | 0.421 | 0.667 | 0.806 | 0.563 | 0.855 | 0.879 | 0.938 | 0.531 | 0.978 | 0.870 | 0.567 | 0.487 |
| 99 | 0.499 | 0.571 | 0.532 | 0.843 | 0.763 | 0.800 | 0.421 | 0.667 | 0.806 | 0.583 | 0.864 | 0.848 | 0.929 | 0.531 | 0.978 | 0.870 | 0.570 | 0.489 |
| 100 | 0.487 | 0.629 | 0.518 | 0.814 | 0.779 | 0.800 | 0.418 | 0.667 | 0.816 | 0.583 | 0.858 | 0.848 | 0.929 | 0.537 | 0.978 | 0.870 | 0.566 | 0.485 |
| 101 | 0.494 | 0.629 | 0.518 | 0.800 | 0.800 | 0.800 | 0.427 | 0.667 | 0.806 | 0.604 | 0.849 | 0.848 | 0.929 | 0.533 | 0.978 | 0.889 | 0.569 | 0.488 |
| 102 | 0.491 | 0.600 | 0.538 | 0.800 | 0.800 | 0.800 | 0.416 | 0.667 | 0.816 | 0.646 | 0.843 | 0.848 | 0.929 | 0.536 | 0.978 | 0.889 | 0.569 | 0.489 |
| 103 | 0.490 | 0.600 | 0.524 | 0.800 | 0.784 | 0.800 | 0.413 | 0.667 | 0.796 | 0.646 | 0.855 | 0.848 | 0.938 | 0.545 | 0.978 | 0.889 | 0.569 | 0.489 |
| 104 | 0.480 | 0.629 | 0.542 | 0.829 | 0.789 | 0.800 | 0.425 | 0.667 | 0.816 | 0.646 | 0.849 | 0.848 | 0.929 | 0.535 | 0.978 | 0.926 | 0.568 | 0.488 |
| 105 | 0.493 | 0.629 | 0.526 | 0.814 | 0.784 | 0.800 | 0.424 | 0.667 | 0.796 | 0.625 | 0.843 | 0.848 | 0.938 | 0.534 | 0.978 | 0.907 | 0.569 | 0.488 |
| 106 | 0.488 | 0.629 | 0.536 | 0.814 | 0.805 | 0.800 | 0.422 | 0.667 | 0.796 | 0.604 | 0.841 | 0.848 | 0.938 | 0.542 | 0.978 | 0.926 | 0.571 | 0.491 |
| 107 | 0.492 | 0.657 | 0.543 | 0.814 | 0.795 | 0.800 | 0.430 | 0.667 | 0.796 | 0.646 | 0.872 | 0.848 | 0.938 | 0.545 | 0.978 | 0.907 | 0.577 | 0.498 |
| 108 | 0.500 | 0.629 | 0.551 | 0.814 | 0.800 | 0.800 | 0.422 | 0.667 | 0.796 | 0.625 | 0.858 | 0.818 | 0.938 | 0.536 | 0.978 | 0.889 | 0.575 | 0.495 |
| 109 | 0.492 | 0.657 | 0.530 | 0.829 | 0.779 | 0.800 | 0.424 | 0.667 | 0.786 | 0.646 | 0.864 | 0.848 | 0.929 | 0.538 | 0.978 | 0.889 | 0.571 | 0.490 |
| 110 | 0.497 | 0.629 | 0.547 | 0.814 | 0.784 | 0.800 | 0.408 | 0.667 | 0.796 | 0.708 | 0.881 | 0.848 | 0.929 | 0.532 | 0.978 | 0.889 | 0.573 | 0.492 |
| 111 | 0.498 | 0.629 | 0.542 | 0.829 | 0.800 | 0.800 | 0.414 | 0.667 | 0.796 | 0.688 | 0.861 | 0.848 | 0.938 | 0.535 | 0.978 | 0.889 | 0.573 | 0.493 |
| 112 | 0.495 | 0.629 | 0.543 | 0.843 | 0.784 | 0.800 | 0.428 | 0.667 | 0.796 | 0.708 | 0.867 | 0.848 | 0.929 | 0.538 | 0.978 | 0.907 | 0.575 | 0.496 |
| 113 | 0.492 | 0.600 | 0.528 | 0.800 | 0.789 | 0.800 | 0.418 | 0.667 | 0.786 | 0.708 | 0.864 | 0.848 | 0.938 | 0.525 | 0.978 | 0.907 | 0.567 | 0.486 |
| 114 | 0.501 | 0.657 | 0.545 | 0.800 | 0.774 | 0.800 | 0.430 | 0.667 | 0.776 | 0.708 | 0.872 | 0.848 | 0.929 | 0.535 | 0.978 | 0.889 | 0.576 | 0.497 |
| 115 | 0.504 | 0.629 | 0.545 | 0.786 | 0.784 | 0.800 | 0.435 | 0.667 | 0.796 | 0.708 | 0.875 | 0.818 | 0.938 | 0.524 | 0.978 | 0.907 | 0.575 | 0.496 |

| | | | | | | | | | | | | | | | | | |
|---|---|---|---|---|---|---|---|---|---|---|---|---|---|---|---|---|---|
| 116 | 0.525 | 0.600 | 0.581 | 0.814 | 0.800 | 0.800 | 0.442 | 0.667 | 0.796 | 0.688 | 0.867 | 0.848 | 0.929 | 0.530 | 0.978 | 0.907 | 0.588 | 0.508 |
| 117 | 0.518 | 0.600 | 0.575 | 0.829 | 0.811 | 0.800 | 0.431 | 0.667 | 0.786 | 0.667 | 0.867 | 0.848 | 0.929 | 0.539 | 0.978 | 0.907 | 0.586 | 0.506 |
| 118 | 0.524 | 0.629 | 0.587 | 0.814 | 0.811 | 0.800 | 0.436 | 0.667 | 0.796 | 0.708 | 0.861 | 0.848 | 0.938 | 0.528 | 0.978 | 0.907 | 0.587 | 0.508 |
| 119 | 0.526 | 0.600 | 0.559 | 0.814 | 0.800 | 0.800 | 0.444 | 0.667 | 0.806 | 0.688 | 0.875 | 0.848 | 0.938 | 0.528 | 0.978 | 0.907 | 0.586 | 0.507 |
| 120 | 0.534 | 0.571 | 0.581 | 0.786 | 0.816 | 0.800 | 0.439 | 0.667 | 0.745 | 0.688 | 0.870 | 0.848 | 0.938 | 0.551 | 0.978 | 0.926 | 0.595 | 0.516 |
| 121 | 0.533 | 0.571 | 0.559 | 0.786 | 0.811 | 0.800 | 0.439 | 0.667 | 0.755 | 0.688 | 0.864 | 0.848 | 0.946 | 0.545 | 0.978 | 0.926 | 0.591 | 0.510 |
| 122 | 0.532 | 0.571 | 0.569 | 0.814 | 0.800 | 0.800 | 0.435 | 0.667 | 0.765 | 0.688 | 0.864 | 0.848 | 0.929 | 0.528 | 0.978 | 0.944 | 0.587 | 0.506 |
| 123 | 0.547 | 0.571 | 0.567 | 0.786 | 0.811 | 0.800 | 0.438 | 0.667 | 0.765 | 0.688 | 0.858 | 0.848 | 0.938 | 0.539 | 0.978 | 0.926 | 0.594 | 0.513 |
| 124 | 0.530 | 0.629 | 0.575 | 0.786 | 0.800 | 0.800 | 0.424 | 0.667 | 0.745 | 0.688 | 0.864 | 0.848 | 0.938 | 0.543 | 0.978 | 0.944 | 0.589 | 0.509 |
| 125 | 0.543 | 0.629 | 0.569 | 0.786 | 0.795 | 0.800 | 0.458 | 0.667 | 0.755 | 0.688 | 0.867 | 0.848 | 0.938 | 0.539 | 0.978 | 0.944 | 0.596 | 0.516 |
| 126 | 0.521 | 0.657 | 0.561 | 0.771 | 0.800 | 0.800 | 0.438 | 0.667 | 0.765 | 0.688 | 0.884 | 0.848 | 0.938 | 0.542 | 0.978 | 0.926 | 0.588 | 0.508 |
| 127 | 0.533 | 0.657 | 0.553 | 0.786 | 0.800 | 0.800 | 0.442 | 0.667 | 0.765 | 0.688 | 0.849 | 0.848 | 0.938 | 0.547 | 0.978 | 0.944 | 0.591 | 0.511 |
| 128 | 0.535 | 0.657 | 0.561 | 0.800 | 0.800 | 0.800 | 0.442 | 0.667 | 0.755 | 0.708 | 0.875 | 0.818 | 0.938 | 0.535 | 0.978 | 0.926 | 0.590 | 0.510 |
| 129 | 0.534 | 0.657 | 0.559 | 0.771 | 0.795 | 0.800 | 0.442 | 0.667 | 0.776 | 0.688 | 0.864 | 0.848 | 0.938 | 0.537 | 0.978 | 0.944 | 0.590 | 0.510 |
| 130 | 0.536 | 0.629 | 0.543 | 0.786 | 0.784 | 0.800 | 0.444 | 0.667 | 0.776 | 0.708 | 0.867 | 0.848 | 0.929 | 0.544 | 0.978 | 0.926 | 0.590 | 0.510 |
| 131 | 0.522 | 0.629 | 0.559 | 0.800 | 0.795 | 0.800 | 0.436 | 0.667 | 0.776 | 0.688 | 0.875 | 0.848 | 0.938 | 0.544 | 0.978 | 0.926 | 0.588 | 0.508 |
| 132 | 0.545 | 0.571 | 0.573 | 0.771 | 0.821 | 0.840 | 0.462 | 0.667 | 0.827 | 0.688 | 0.861 | 0.848 | 0.938 | 0.556 | 0.978 | 0.944 | 0.603 | 0.525 |
| 133 | 0.538 | 0.571 | 0.563 | 0.800 | 0.800 | 0.840 | 0.451 | 0.667 | 0.827 | 0.708 | 0.867 | 0.848 | 0.929 | 0.569 | 0.978 | 0.944 | 0.602 | 0.524 |
| 134 | 0.532 | 0.571 | 0.579 | 0.771 | 0.789 | 0.840 | 0.461 | 0.667 | 0.806 | 0.688 | 0.867 | 0.848 | 0.938 | 0.549 | 0.978 | 0.944 | 0.597 | 0.518 |
| 135 | 0.531 | 0.571 | 0.591 | 0.800 | 0.811 | 0.840 | 0.456 | 0.667 | 0.827 | 0.708 | 0.867 | 0.848 | 0.938 | 0.561 | 0.978 | 0.944 | 0.602 | 0.524 |
| 136 | 0.529 | 0.714 | 0.593 | 0.800 | 0.816 | 0.800 | 0.501 | 0.667 | 0.816 | 0.750 | 0.890 | 0.879 | 0.938 | 0.578 | 0.978 | 0.963 | 0.615 | 0.540 |
| 137 | 0.533 | 0.714 | 0.619 | 0.800 | 0.821 | 0.800 | 0.484 | 0.667 | 0.837 | 0.729 | 0.887 | 0.879 | 0.929 | 0.578 | 0.978 | 0.963 | 0.617 | 0.542 |
| 138 | 0.531 | 0.686 | 0.605 | 0.786 | 0.837 | 0.800 | 0.498 | 0.667 | 0.827 | 0.750 | 0.904 | 0.879 | 0.929 | 0.581 | 0.978 | 0.944 | 0.619 | 0.544 |
| 139 | 0.531 | 0.686 | 0.611 | 0.800 | 0.842 | 0.800 | 0.492 | 0.667 | 0.837 | 0.750 | 0.893 | 0.879 | 0.929 | 0.578 | 0.978 | 0.963 | 0.617 | 0.544 |
| 140 | 0.525 | 0.714 | 0.611 | 0.786 | 0.821 | 0.800 | 0.482 | 0.667 | 0.837 | 0.750 | 0.887 | 0.879 | 0.929 | 0.587 | 0.978 | 0.963 | 0.616 | 0.541 |
| 141 | 0.535 | 0.686 | 0.607 | 0.800 | 0.826 | 0.800 | 0.495 | 0.667 | 0.878 | 0.771 | 0.881 | 0.879 | 0.938 | 0.577 | 0.978 | 0.944 | 0.618 | 0.544 |
| 142 | 0.530 | 0.714 | 0.619 | 0.786 | 0.832 | 0.800 | 0.488 | 0.667 | 0.827 | 0.750 | 0.881 | 0.879 | 0.938 | 0.574 | 0.978 | 0.963 | 0.615 | 0.541 |
| 143 | 0.521 | 0.686 | 0.619 | 0.786 | 0.832 | 0.800 | 0.504 | 0.667 | 0.857 | 0.750 | 0.878 | 0.879 | 0.929 | 0.596 | 0.978 | 0.963 | 0.620 | 0.547 |
| 144 | 0.529 | 0.686 | 0.603 | 0.786 | 0.832 | 0.800 | 0.505 | 0.667 | 0.857 | 0.771 | 0.887 | 0.879 | 0.929 | 0.574 | 0.978 | 0.944 | 0.616 | 0.542 |
| 145 | 0.519 | 0.714 | 0.607 | 0.786 | 0.821 | 0.800 | 0.495 | 0.667 | 0.847 | 0.792 | 0.884 | 0.909 | 0.929 | 0.577 | 0.978 | 0.963 | 0.613 | 0.539 |
| 146 | 0.527 | 0.686 | 0.595 | 0.786 | 0.816 | 0.800 | 0.499 | 0.667 | 0.837 | 0.771 | 0.896 | 0.909 | 0.929 | 0.586 | 0.978 | 0.944 | 0.617 | 0.542 |
| 147 | 0.527 | 0.686 | 0.593 | 0.786 | 0.826 | 0.800 | 0.496 | 0.667 | 0.847 | 0.771 | 0.901 | 0.939 | 0.929 | 0.582 | 0.978 | 0.963 | 0.617 | 0.543 |
| 148 | 0.534 | 0.686 | 0.599 | 0.800 | 0.826 | 0.800 | 0.499 | 0.667 | 0.857 | 0.792 | 0.884 | 0.939 | 0.938 | 0.581 | 0.978 | 0.963 | 0.619 | 0.546 |
| 149 | 0.531 | 0.686 | 0.609 | 0.786 | 0.826 | 0.800 | 0.495 | 0.667 | 0.847 | 0.771 | 0.890 | 0.939 | 0.929 | 0.583 | 0.978 | 0.944 | 0.618 | 0.544 |
| 150 | 0.524 | 0.686 | 0.605 | 0.757 | 0.832 | 0.800 | 0.504 | 0.667 | 0.857 | 0.750 | 0.878 | 0.909 | 0.938 | 0.582 | 0.978 | 0.963 | 0.616 | 0.542 |
| 151 | 0.531 | 0.686 | 0.599 | 0.814 | 0.826 | 0.800 | 0.488 | 0.667 | 0.827 | 0.792 | 0.887 | 0.939 | 0.946 | 0.588 | 0.978 | 0.963 | 0.619 | 0.544 |
| 152 | 0.545 | 0.686 | 0.601 | 0.814 | 0.826 | 0.800 | 0.495 | 0.667 | 0.837 | 0.771 | 0.899 | 0.939 | 0.946 | 0.579 | 0.978 | 0.963 | 0.622 | 0.549 |
| 153 | 0.479 | 0.686 | 0.601 | 0.786 | 0.837 | 0.800 | 0.502 | 0.667 | 0.878 | 0.792 | 0.893 | 0.939 | 0.946 | 0.586 | 0.978 | 0.944 | 0.606 | 0.533 |
| 154 | 0.482 | 0.686 | 0.593 | 0.829 | 0.853 | 0.800 | 0.487 | 0.667 | 0.847 | 0.792 | 0.893 | 0.939 | 0.955 | 0.583 | 0.978 | 0.926 | 0.604 | 0.530 |

| | | | | | | | | | | | | | | | | | | |
|---|---|---|---|---|---|---|---|---|---|---|---|---|---|---|---|---|---|---|
| 155 | 0.492 | 0.686 | 0.611 | 0.786 | 0.832 | 0.800 | 0.492 | 0.667 | 0.847 | 0.792 | 0.893 | 0.939 | 0.946 | 0.578 | 0.978 | 0.944 | 0.606 | 0.533 |
| 156 | 0.497 | 0.686 | 0.593 | 0.800 | 0.842 | 0.800 | 0.487 | 0.667 | 0.837 | 0.792 | 0.890 | 0.970 | 0.955 | 0.582 | 0.978 | 0.944 | 0.607 | 0.534 |
| 157 | 0.496 | 0.686 | 0.611 | 0.786 | 0.837 | 0.800 | 0.490 | 0.667 | 0.837 | 0.792 | 0.890 | 0.939 | 0.955 | 0.586 | 0.978 | 0.944 | 0.609 | 0.537 |
| 158 | 0.577 | 0.743 | 0.585 | 0.800 | 0.847 | 0.920 | 0.541 | 0.667 | 0.847 | 0.833 | 0.887 | 0.909 | 0.955 | 0.653 | 0.978 | 0.963 | 0.657 | 0.590 |
| 159 | 0.572 | 0.743 | 0.605 | 0.786 | 0.863 | 0.880 | 0.538 | 0.667 | 0.857 | 0.813 | 0.893 | 0.939 | 0.946 | 0.656 | 0.978 | 0.963 | 0.658 | 0.592 |
| 160 | 0.575 | 0.771 | 0.601 | 0.814 | 0.858 | 0.880 | 0.553 | 0.667 | 0.867 | 0.833 | 0.904 | 0.909 | 0.955 | 0.656 | 0.978 | 0.944 | 0.662 | 0.596 |
| 161 | 0.570 | 0.743 | 0.617 | 0.829 | 0.837 | 0.920 | 0.538 | 0.667 | 0.857 | 0.813 | 0.904 | 0.909 | 0.946 | 0.645 | 0.978 | 0.944 | 0.656 | 0.590 |
| 162 | 0.576 | 0.771 | 0.599 | 0.786 | 0.853 | 0.920 | 0.539 | 0.667 | 0.857 | 0.813 | 0.901 | 0.909 | 0.946 | 0.651 | 0.978 | 0.963 | 0.658 | 0.592 |
| 163 | 0.575 | 0.800 | 0.625 | 0.829 | 0.858 | 0.920 | 0.541 | 0.667 | 0.867 | 0.833 | 0.904 | 0.909 | 0.955 | 0.643 | 0.978 | 0.963 | 0.660 | 0.595 |
| 164 | 0.570 | 0.800 | 0.609 | 0.857 | 0.874 | 0.920 | 0.538 | 0.667 | 0.827 | 0.833 | 0.910 | 0.909 | 0.955 | 0.653 | 0.978 | 0.963 | 0.660 | 0.594 |
| 165 | 0.576 | 0.800 | 0.611 | 0.814 | 0.842 | 0.920 | 0.542 | 0.667 | 0.878 | 0.833 | 0.904 | 0.909 | 0.955 | 0.653 | 0.957 | 0.944 | 0.661 | 0.595 |
| 166 | 0.579 | 0.800 | 0.593 | 0.843 | 0.837 | 0.920 | 0.550 | 0.667 | 0.827 | 0.833 | 0.904 | 0.909 | 0.964 | 0.658 | 0.978 | 0.963 | 0.662 | 0.596 |
| 167 | 0.555 | 0.971 | 0.611 | 0.900 | 0.900 | 0.880 | 0.607 | 0.667 | 0.878 | 0.875 | 0.907 | 0.909 | 0.964 | 0.661 | 0.978 | 0.963 | 0.671 | 0.611 |
| 168 | 0.545 | 0.971 | 0.650 | 0.886 | 0.879 | 0.880 | 0.604 | 0.667 | 0.878 | 0.896 | 0.910 | 0.909 | 0.964 | 0.670 | 0.978 | 0.944 | 0.673 | 0.614 |
| 169 | 0.547 | 0.971 | 0.630 | 0.871 | 0.879 | 0.880 | 0.616 | 0.667 | 0.888 | 0.875 | 0.896 | 0.909 | 0.946 | 0.659 | 0.978 | 0.944 | 0.669 | 0.608 |
| 170 | 0.543 | 0.971 | 0.617 | 0.886 | 0.889 | 0.920 | 0.609 | 0.667 | 0.867 | 0.875 | 0.901 | 0.939 | 0.955 | 0.665 | 0.978 | 0.944 | 0.668 | 0.607 |
| 171 | 0.546 | 0.971 | 0.623 | 0.900 | 0.874 | 0.880 | 0.609 | 0.667 | 0.878 | 0.896 | 0.899 | 0.939 | 0.955 | 0.669 | 0.978 | 0.944 | 0.670 | 0.609 |
| 172 | 0.548 | 0.971 | 0.626 | 0.857 | 0.879 | 0.920 | 0.615 | 0.667 | 0.867 | 0.896 | 0.896 | 0.939 | 0.955 | 0.668 | 0.978 | 0.944 | 0.671 | 0.610 |
| 173 | 0.559 | 0.914 | 0.642 | 0.871 | 0.916 | 0.880 | 0.609 | 0.667 | 0.918 | 0.917 | 0.887 | 0.939 | 0.938 | 0.667 | 0.978 | 0.926 | 0.676 | 0.616 |
| 174 | 0.566 | 0.943 | 0.640 | 0.900 | 0.905 | 0.880 | 0.606 | 0.667 | 0.918 | 0.917 | 0.881 | 0.909 | 0.938 | 0.665 | 0.978 | 0.926 | 0.676 | 0.616 |
| 175 | 0.566 | 0.914 | 0.634 | 0.929 | 0.911 | 0.840 | 0.606 | 0.667 | 0.918 | 0.917 | 0.893 | 0.939 | 0.973 | 0.668 | 0.957 | 0.926 | 0.678 | 0.618 |
| 176 | 0.568 | 0.943 | 0.656 | 0.886 | 0.900 | 0.880 | 0.610 | 0.667 | 0.929 | 0.917 | 0.899 | 0.939 | 0.964 | 0.661 | 0.978 | 0.944 | 0.680 | 0.621 |
| 177 | 0.562 | 0.943 | 0.654 | 0.900 | 0.874 | 0.880 | 0.613 | 0.667 | 0.898 | 0.875 | 0.901 | 0.939 | 0.964 | 0.664 | 0.978 | 0.944 | 0.678 | 0.618 |
| 178 | 0.556 | 0.943 | 0.650 | 0.900 | 0.889 | 0.840 | 0.624 | 0.667 | 0.898 | 0.917 | 0.896 | 0.939 | 0.964 | 0.669 | 0.978 | 0.963 | 0.679 | 0.620 |
| 179 | 0.554 | 0.943 | 0.664 | 0.886 | 0.889 | 0.920 | 0.599 | 0.667 | 0.918 | 0.917 | 0.890 | 0.939 | 0.929 | 0.668 | 0.978 | 0.944 | 0.675 | 0.615 |
| 180 | 0.569 | 0.943 | 0.670 | 0.871 | 0.889 | 0.880 | 0.599 | 0.667 | 0.929 | 0.917 | 0.893 | 0.939 | 0.946 | 0.657 | 0.957 | 0.944 | 0.678 | 0.617 |
| 181 | 0.568 | 0.943 | 0.642 | 0.886 | 0.905 | 0.880 | 0.601 | 0.667 | 0.908 | 0.917 | 0.899 | 0.939 | 0.946 | 0.668 | 0.978 | 0.944 | 0.679 | 0.619 |
| 182 | 0.553 | 0.943 | 0.623 | 0.929 | 0.889 | 0.920 | 0.586 | 0.667 | 0.918 | 0.917 | 0.884 | 0.939 | 0.964 | 0.676 | 0.978 | 0.944 | 0.672 | 0.612 |
| 183 | 0.554 | 0.943 | 0.636 | 0.900 | 0.884 | 0.880 | 0.587 | 0.667 | 0.918 | 0.917 | 0.890 | 0.939 | 0.946 | 0.666 | 0.978 | 0.944 | 0.671 | 0.610 |
| 184 | 0.572 | 0.971 | 0.642 | 0.857 | 0.874 | 0.880 | 0.604 | 0.667 | 0.918 | 0.896 | 0.899 | 0.909 | 0.955 | 0.666 | 0.978 | 0.944 | 0.679 | 0.617 |
| 185 | 0.572 | 0.971 | 0.640 | 0.871 | 0.879 | 0.920 | 0.607 | 0.667 | 0.939 | 0.896 | 0.913 | 0.909 | 0.955 | 0.674 | 0.957 | 0.944 | 0.683 | 0.623 |
| 186 | 0.560 | 0.971 | 0.654 | 0.871 | 0.868 | 0.840 | 0.606 | 0.667 | 0.918 | 0.896 | 0.893 | 0.909 | 0.946 | 0.675 | 0.957 | 0.944 | 0.678 | 0.617 |
| 187 | 0.574 | 0.971 | 0.632 | 0.871 | 0.884 | 0.840 | 0.619 | 0.667 | 0.918 | 0.896 | 0.907 | 0.909 | 0.964 | 0.668 | 0.957 | 0.963 | 0.682 | 0.621 |
| 188 | 0.574 | 0.971 | 0.640 | 0.871 | 0.868 | 0.880 | 0.601 | 0.667 | 0.918 | 0.896 | 0.904 | 0.909 | 0.946 | 0.672 | 0.957 | 0.944 | 0.680 | 0.619 |
| 189 | 0.578 | 0.971 | 0.638 | 0.886 | 0.895 | 0.840 | 0.607 | 0.667 | 0.908 | 0.896 | 0.907 | 0.939 | 0.955 | 0.661 | 0.957 | 0.944 | 0.681 | 0.620 |
| 190 | 0.566 | 0.971 | 0.646 | 0.900 | 0.858 | 0.920 | 0.601 | 0.667 | 0.918 | 0.896 | 0.916 | 0.909 | 0.955 | 0.672 | 0.957 | 0.944 | 0.679 | 0.618 |
| 191 | 0.564 | 0.971 | 0.642 | 0.886 | 0.879 | 0.920 | 0.607 | 0.667 | 0.908 | 0.896 | 0.910 | 0.909 | 0.964 | 0.675 | 0.957 | 0.926 | 0.680 | 0.620 |
| 192 | 0.560 | 0.971 | 0.630 | 0.886 | 0.884 | 0.920 | 0.610 | 0.667 | 0.908 | 0.917 | 0.904 | 0.909 | 0.938 | 0.672 | 0.957 | 0.944 | 0.677 | 0.616 |
| 193 | 0.584 | 0.971 | 0.628 | 0.857 | 0.884 | 0.880 | 0.610 | 0.667 | 0.929 | 0.896 | 0.896 | 0.909 | 0.955 | 0.666 | 0.957 | 0.926 | 0.682 | 0.621 |

| | | | | | | | | | | | | | | | | | | |
|---|---|---|---|---|---|---|---|---|---|---|---|---|---|---|---|---|---|---|
| 194 | 0.579 | 0.971 | 0.638 | 0.871 | 0.874 | 0.880 | 0.609 | 0.667 | 0.939 | 0.896 | 0.913 | 0.909 | 0.964 | 0.658 | 0.957 | 0.944 | 0.681 | 0.620 |
| 195 | 0.588 | 1.000 | 0.650 | 0.886 | 0.889 | 0.840 | 0.610 | 0.667 | 0.959 | 0.896 | 0.910 | 0.909 | 0.973 | 0.679 | 0.957 | 0.944 | 0.691 | 0.632 |
| 196 | 0.579 | 1.000 | 0.619 | 0.871 | 0.889 | 0.800 | 0.618 | 0.667 | 0.939 | 0.875 | 0.899 | 0.909 | 0.964 | 0.679 | 0.957 | 0.944 | 0.684 | 0.624 |
| 197 | 0.568 | 1.000 | 0.630 | 0.886 | 0.889 | 0.880 | 0.615 | 0.667 | 0.949 | 0.896 | 0.899 | 0.909 | 0.973 | 0.678 | 0.957 | 0.944 | 0.683 | 0.622 |
| 198 | 0.581 | 1.000 | 0.636 | 0.871 | 0.900 | 0.840 | 0.613 | 0.667 | 0.949 | 0.896 | 0.907 | 0.909 | 0.964 | 0.671 | 0.957 | 0.944 | 0.686 | 0.625 |
| 199 | 0.577 | 1.000 | 0.638 | 0.886 | 0.911 | 0.840 | 0.612 | 0.667 | 0.959 | 0.917 | 0.901 | 0.879 | 0.973 | 0.685 | 0.957 | 0.944 | 0.689 | 0.629 |
| 200 | 0.575 | 1.000 | 0.619 | 0.900 | 0.900 | 0.880 | 0.636 | 0.667 | 0.969 | 0.875 | 0.893 | 0.909 | 0.964 | 0.680 | 0.957 | 0.944 | 0.687 | 0.627 |
| 201 | 0.583 | 1.000 | 0.650 | 0.871 | 0.895 | 0.840 | 0.615 | 0.667 | 0.959 | 0.917 | 0.913 | 0.909 | 0.964 | 0.679 | 0.957 | 0.944 | 0.691 | 0.631 |
| 202 | 0.583 | 0.971 | 0.632 | 0.886 | 0.874 | 0.840 | 0.613 | 0.667 | 0.949 | 0.917 | 0.901 | 0.909 | 0.973 | 0.670 | 0.957 | 0.963 | 0.685 | 0.624 |
| 203 | 0.590 | 0.971 | 0.630 | 0.886 | 0.884 | 0.880 | 0.621 | 0.667 | 0.959 | 0.896 | 0.899 | 0.879 | 0.938 | 0.704 | 0.957 | 0.963 | 0.696 | 0.636 |
| 204 | 0.579 | 1.000 | 0.638 | 0.871 | 0.874 | 0.840 | 0.616 | 0.667 | 0.908 | 0.917 | 0.899 | 0.879 | 0.946 | 0.712 | 0.957 | 0.963 | 0.694 | 0.634 |
| 205 | 0.599 | 0.971 | 0.650 | 0.900 | 0.884 | 0.800 | 0.616 | 0.667 | 0.949 | 0.896 | 0.893 | 0.909 | 0.946 | 0.701 | 0.978 | 0.963 | 0.699 | 0.640 |
| 206 | 0.599 | 0.971 | 0.636 | 0.886 | 0.853 | 0.840 | 0.622 | 0.667 | 0.959 | 0.875 | 0.899 | 0.909 | 0.938 | 0.703 | 0.978 | 0.944 | 0.697 | 0.638 |
| 207 | 0.599 | 0.914 | 0.636 | 0.900 | 0.884 | 0.840 | 0.686 | 0.667 | 0.929 | 0.854 | 0.899 | 0.909 | 0.955 | 0.710 | 0.978 | 0.963 | 0.708 | 0.651 |
| 208 | 0.605 | 0.857 | 0.634 | 0.857 | 0.889 | 0.840 | 0.695 | 0.667 | 0.949 | 0.875 | 0.899 | 0.909 | 0.946 | 0.713 | 0.978 | 0.944 | 0.711 | 0.654 |
| 209 | 0.603 | 0.886 | 0.632 | 0.886 | 0.884 | 0.880 | 0.683 | 0.667 | 0.949 | 0.854 | 0.901 | 0.909 | 0.938 | 0.700 | 0.978 | 0.963 | 0.706 | 0.648 |
| 210 | 0.606 | 0.886 | 0.652 | 0.900 | 0.889 | 0.920 | 0.690 | 0.667 | 0.929 | 0.875 | 0.910 | 0.909 | 0.946 | 0.707 | 0.957 | 0.926 | 0.712 | 0.655 |
| 211 | 0.592 | 0.886 | 0.632 | 0.900 | 0.879 | 0.880 | 0.684 | 0.667 | 0.929 | 0.875 | 0.916 | 0.909 | 0.929 | 0.705 | 0.957 | 0.926 | 0.704 | 0.646 |
| 212 | 0.598 | 0.857 | 0.642 | 0.900 | 0.889 | 0.880 | 0.680 | 0.667 | 0.908 | 0.854 | 0.904 | 0.939 | 0.946 | 0.706 | 0.957 | 0.944 | 0.706 | 0.648 |
| 213 | 0.597 | 0.857 | 0.650 | 0.900 | 0.900 | 0.880 | 0.696 | 0.667 | 0.929 | 0.875 | 0.916 | 0.909 | 0.946 | 0.708 | 0.957 | 0.944 | 0.711 | 0.655 |
| 214 | 0.608 | 0.857 | 0.626 | 0.900 | 0.874 | 0.800 | 0.698 | 0.667 | 0.939 | 0.833 | 0.919 | 0.909 | 0.938 | 0.710 | 0.957 | 0.926 | 0.711 | 0.653 |
| 215 | 0.621 | 0.857 | 0.650 | 0.871 | 0.874 | 0.880 | 0.686 | 0.667 | 0.939 | 0.854 | 0.904 | 0.909 | 0.946 | 0.703 | 0.957 | 0.944 | 0.713 | 0.656 |
| 216 | 0.605 | 0.886 | 0.644 | 0.886 | 0.884 | 0.840 | 0.687 | 0.667 | 0.918 | 0.854 | 0.910 | 0.909 | 0.946 | 0.703 | 0.957 | 0.926 | 0.709 | 0.650 |
| 217 | 0.597 | 0.857 | 0.646 | 0.900 | 0.884 | 0.840 | 0.676 | 0.667 | 0.929 | 0.854 | 0.913 | 0.909 | 0.946 | 0.705 | 0.957 | 0.907 | 0.706 | 0.647 |
| 218 | 0.603 | 0.857 | 0.636 | 0.871 | 0.889 | 0.880 | 0.696 | 0.667 | 0.918 | 0.875 | 0.907 | 0.909 | 0.938 | 0.705 | 0.957 | 0.926 | 0.709 | 0.651 |
| 219 | 0.605 | 0.857 | 0.625 | 0.886 | 0.895 | 0.880 | 0.689 | 0.667 | 0.969 | 0.875 | 0.916 | 0.909 | 0.964 | 0.711 | 0.957 | 0.926 | 0.711 | 0.654 |
| 220 | 0.611 | 0.857 | 0.646 | 0.886 | 0.916 | 0.800 | 0.689 | 0.667 | 0.949 | 0.833 | 0.907 | 0.909 | 0.946 | 0.709 | 0.957 | 0.926 | 0.713 | 0.657 |
| 221 | 0.603 | 0.857 | 0.650 | 0.900 | 0.900 | 0.800 | 0.690 | 0.667 | 0.939 | 0.854 | 0.913 | 0.909 | 0.955 | 0.707 | 0.957 | 0.907 | 0.711 | 0.654 |
| 222 | 0.610 | 0.857 | 0.654 | 0.886 | 0.895 | 0.880 | 0.696 | 0.667 | 0.939 | 0.854 | 0.913 | 0.909 | 0.955 | 0.704 | 0.957 | 0.926 | 0.714 | 0.657 |
| 223 | 0.622 | 0.857 | 0.638 | 0.886 | 0.895 | 0.920 | 0.704 | 0.667 | 0.929 | 0.896 | 0.913 | 0.909 | 0.955 | 0.705 | 0.957 | 0.944 | 0.718 | 0.661 |
| 224 | 0.611 | 0.857 | 0.658 | 0.886 | 0.900 | 0.920 | 0.700 | 0.667 | 0.898 | 0.896 | 0.913 | 0.909 | 0.955 | 0.707 | 0.957 | 0.944 | 0.716 | 0.659 |
| 225 | 0.612 | 0.886 | 0.640 | 0.886 | 0.900 | 0.880 | 0.700 | 0.667 | 0.918 | 0.854 | 0.907 | 0.909 | 0.955 | 0.705 | 0.957 | 0.926 | 0.713 | 0.656 |
| 226 | 0.613 | 0.857 | 0.652 | 0.871 | 0.905 | 0.840 | 0.707 | 0.667 | 0.918 | 0.854 | 0.907 | 0.939 | 0.964 | 0.710 | 0.957 | 0.926 | 0.717 | 0.661 |
| 227 | 0.605 | 0.857 | 0.646 | 0.886 | 0.900 | 0.880 | 0.698 | 0.667 | 0.908 | 0.833 | 0.919 | 0.909 | 0.955 | 0.705 | 0.957 | 0.926 | 0.712 | 0.654 |
| 228 | 0.630 | 0.886 | 0.650 | 0.900 | 0.900 | 0.920 | 0.690 | 0.667 | 0.918 | 0.854 | 0.922 | 0.909 | 0.964 | 0.712 | 0.957 | 0.926 | 0.722 | 0.666 |
| 229 | 0.622 | 0.857 | 0.650 | 0.886 | 0.895 | 0.880 | 0.706 | 0.667 | 0.918 | 0.854 | 0.922 | 0.939 | 0.955 | 0.709 | 0.957 | 0.944 | 0.720 | 0.664 |
| 230 | 0.627 | 0.857 | 0.652 | 0.886 | 0.900 | 0.840 | 0.689 | 0.667 | 0.929 | 0.875 | 0.916 | 0.909 | 0.964 | 0.711 | 0.957 | 0.926 | 0.720 | 0.663 |
| 231 | 0.625 | 0.943 | 0.656 | 0.900 | 0.884 | 0.920 | 0.678 | 0.667 | 0.929 | 0.896 | 0.916 | 0.909 | 0.946 | 0.705 | 0.957 | 0.944 | 0.718 | 0.660 |
| 232 | 0.633 | 0.943 | 0.650 | 0.900 | 0.879 | 0.840 | 0.684 | 0.667 | 0.918 | 0.896 | 0.907 | 0.909 | 0.946 | 0.728 | 0.957 | 0.926 | 0.724 | 0.668 |

| | | | | | | | | | | | | | | | | | | |
|---|---|---|---|---|---|---|---|---|---|---|---|---|---|---|---|---|---|---|
| 233 | 0.639 | 0.943 | 0.650 | 0.914 | 0.916 | 0.840 | 0.698 | 0.667 | 0.929 | 0.875 | 0.922 | 0.879 | 0.946 | 0.734 | 0.957 | 0.944 | 0.732 | 0.677 |
| 234 | 0.638 | 0.914 | 0.652 | 0.900 | 0.874 | 0.880 | 0.693 | 0.667 | 0.929 | 0.875 | 0.913 | 0.879 | 0.946 | 0.728 | 0.957 | 0.963 | 0.728 | 0.672 |
| 235 | 0.629 | 0.943 | 0.660 | 0.914 | 0.889 | 0.880 | 0.684 | 0.667 | 0.939 | 0.896 | 0.916 | 0.909 | 0.938 | 0.731 | 0.957 | 0.963 | 0.727 | 0.672 |
| 236 | 0.640 | 0.943 | 0.642 | 0.914 | 0.895 | 0.880 | 0.689 | 0.667 | 0.939 | 0.896 | 0.922 | 0.879 | 0.946 | 0.738 | 0.957 | 0.926 | 0.731 | 0.675 |
| 237 | 0.638 | 0.943 | 0.654 | 0.914 | 0.905 | 0.920 | 0.689 | 0.667 | 0.929 | 0.854 | 0.919 | 0.879 | 0.946 | 0.723 | 0.978 | 0.963 | 0.728 | 0.673 |
| 238 | 0.638 | 0.943 | 0.638 | 0.914 | 0.900 | 0.880 | 0.700 | 0.667 | 0.918 | 0.896 | 0.913 | 0.909 | 0.955 | 0.732 | 0.978 | 0.926 | 0.729 | 0.674 |
| 239 | 0.633 | 0.943 | 0.680 | 0.929 | 0.916 | 0.880 | 0.672 | 0.667 | 0.929 | 0.917 | 0.913 | 0.909 | 0.946 | 0.729 | 0.957 | 0.944 | 0.729 | 0.674 |
| 240 | 0.635 | 0.943 | 0.638 | 0.914 | 0.889 | 0.880 | 0.655 | 0.667 | 0.918 | 0.896 | 0.907 | 0.909 | 0.938 | 0.725 | 0.978 | 0.944 | 0.720 | 0.662 |
| 241 | 0.660 | 0.943 | 0.654 | 0.943 | 0.926 | 0.920 | 0.669 | 0.667 | 0.929 | 0.896 | 0.925 | 0.939 | 0.946 | 0.722 | 0.978 | 0.944 | 0.734 | 0.680 |
| 242 | 0.641 | 0.914 | 0.650 | 0.929 | 0.900 | 0.920 | 0.655 | 0.667 | 0.918 | 0.917 | 0.913 | 0.909 | 0.955 | 0.726 | 0.957 | 0.926 | 0.725 | 0.668 |
| 243 | 0.647 | 0.943 | 0.648 | 0.914 | 0.889 | 0.880 | 0.673 | 0.667 | 0.918 | 0.917 | 0.913 | 0.909 | 0.955 | 0.716 | 0.978 | 0.926 | 0.726 | 0.669 |
| 244 | 0.644 | 0.943 | 0.658 | 0.929 | 0.900 | 0.920 | 0.672 | 0.667 | 0.918 | 0.896 | 0.916 | 0.909 | 0.946 | 0.735 | 0.957 | 0.944 | 0.731 | 0.676 |
| 245 | 0.650 | 0.943 | 0.638 | 0.900 | 0.895 | 0.880 | 0.667 | 0.667 | 0.939 | 0.896 | 0.910 | 0.909 | 0.938 | 0.724 | 0.978 | 0.944 | 0.726 | 0.670 |
| 246 | 0.640 | 0.914 | 0.664 | 0.914 | 0.895 | 0.880 | 0.676 | 0.667 | 0.939 | 0.896 | 0.916 | 0.909 | 0.938 | 0.719 | 0.957 | 0.926 | 0.726 | 0.670 |
| 247 | 0.639 | 0.943 | 0.664 | 0.914 | 0.884 | 0.880 | 0.673 | 0.667 | 0.929 | 0.896 | 0.919 | 0.909 | 0.964 | 0.734 | 0.978 | 0.944 | 0.730 | 0.675 |
| 248 | 0.640 | 0.914 | 0.648 | 0.929 | 0.895 | 0.880 | 0.686 | 0.667 | 0.929 | 0.917 | 0.899 | 0.909 | 0.946 | 0.734 | 0.978 | 1.000 | 0.730 | 0.674 |
| 249 | 0.653 | 0.943 | 0.650 | 0.914 | 0.900 | 0.960 | 0.689 | 0.667 | 0.939 | 0.917 | 0.904 | 0.909 | 0.938 | 0.719 | 0.978 | 0.963 | 0.731 | 0.675 |
| 250 | 0.645 | 0.943 | 0.650 | 0.914 | 0.863 | 0.840 | 0.686 | 0.667 | 0.929 | 0.896 | 0.901 | 0.909 | 0.938 | 0.727 | 0.957 | 0.963 | 0.727 | 0.671 |
| 251 | 0.646 | 0.943 | 0.644 | 0.929 | 0.911 | 0.800 | 0.681 | 0.667 | 0.939 | 0.917 | 0.904 | 0.909 | 0.946 | 0.727 | 0.978 | 0.981 | 0.729 | 0.674 |
| 252 | 0.644 | 0.943 | 0.658 | 0.900 | 0.895 | 0.880 | 0.673 | 0.667 | 0.908 | 0.896 | 0.904 | 0.879 | 0.955 | 0.732 | 0.978 | 0.963 | 0.729 | 0.673 |
| 253 | 0.636 | 1.000 | 0.668 | 0.929 | 0.895 | 0.960 | 0.681 | 0.667 | 0.949 | 0.958 | 0.919 | 0.909 | 0.955 | 0.733 | 0.957 | 0.963 | 0.732 | 0.678 |
| 254 | 0.641 | 1.000 | 0.644 | 0.914 | 0.895 | 0.960 | 0.684 | 0.667 | 0.929 | 0.938 | 0.922 | 0.879 | 0.955 | 0.723 | 0.978 | 0.981 | 0.729 | 0.674 |
| 255 | 0.666 | 1.000 | 0.664 | 0.886 | 0.900 | 0.960 | 0.664 | 0.667 | 0.949 | 0.938 | 0.907 | 0.909 | 0.946 | 0.739 | 0.957 | 0.944 | 0.739 | 0.684 |
| 256 | 0.645 | 1.000 | 0.648 | 0.929 | 0.916 | 0.920 | 0.673 | 0.667 | 0.949 | 0.938 | 0.916 | 0.909 | 0.946 | 0.728 | 0.978 | 0.944 | 0.731 | 0.675 |
| 257 | 0.644 | 1.000 | 0.664 | 0.914 | 0.916 | 0.960 | 0.687 | 0.667 | 0.908 | 0.896 | 0.919 | 0.909 | 0.946 | 0.745 | 0.978 | 0.981 | 0.738 | 0.684 |
| 258 | 0.646 | 0.971 | 0.658 | 0.929 | 0.921 | 0.960 | 0.701 | 0.667 | 0.908 | 0.917 | 0.919 | 0.939 | 0.946 | 0.732 | 0.957 | 0.963 | 0.736 | 0.683 |
| 259 | 0.658 | 1.000 | 0.662 | 0.914 | 0.921 | 0.960 | 0.686 | 0.667 | 0.929 | 0.896 | 0.907 | 0.939 | 0.964 | 0.739 | 0.957 | 0.963 | 0.740 | 0.686 |
| 260 | 0.645 | 0.971 | 0.644 | 0.914 | 0.921 | 0.920 | 0.700 | 0.667 | 0.929 | 0.917 | 0.919 | 0.909 | 0.955 | 0.754 | 0.957 | 0.981 | 0.740 | 0.687 |
| 261 | 0.640 | 1.000 | 0.664 | 0.914 | 0.900 | 0.960 | 0.696 | 0.667 | 0.929 | 0.896 | 0.933 | 0.909 | 0.964 | 0.745 | 0.957 | 0.944 | 0.738 | 0.685 |
| 262 | 0.642 | 0.943 | 0.664 | 0.914 | 0.916 | 0.920 | 0.690 | 0.667 | 0.918 | 0.896 | 0.919 | 0.879 | 0.946 | 0.739 | 0.957 | 0.981 | 0.735 | 0.681 |
| 263 | 0.644 | 1.000 | 0.656 | 0.914 | 0.900 | 0.920 | 0.695 | 0.667 | 0.918 | 0.938 | 0.916 | 0.939 | 0.955 | 0.753 | 0.957 | 0.981 | 0.740 | 0.686 |
| 264 | 0.641 | 1.000 | 0.652 | 0.914 | 0.911 | 0.960 | 0.715 | 0.667 | 0.908 | 0.896 | 0.913 | 0.879 | 0.946 | 0.741 | 0.978 | 0.944 | 0.737 | 0.683 |
| 265 | 0.649 | 1.000 | 0.676 | 0.914 | 0.911 | 0.960 | 0.713 | 0.667 | 0.929 | 0.896 | 0.925 | 0.939 | 0.973 | 0.759 | 0.978 | 0.981 | 0.749 | 0.697 |
| 266 | 0.646 | 0.943 | 0.664 | 0.929 | 0.926 | 0.960 | 0.721 | 0.667 | 0.898 | 0.958 | 0.928 | 0.909 | 0.964 | 0.753 | 0.978 | 0.981 | 0.747 | 0.695 |
| 267 | 0.635 | 1.000 | 0.672 | 0.929 | 0.911 | 0.960 | 0.707 | 0.667 | 0.939 | 0.938 | 0.919 | 0.909 | 0.946 | 0.747 | 0.978 | 0.981 | 0.740 | 0.688 |
| 268 | 0.647 | 1.000 | 0.670 | 0.929 | 0.932 | 0.920 | 0.703 | 0.667 | 0.939 | 0.917 | 0.913 | 0.909 | 0.964 | 0.748 | 0.978 | 0.963 | 0.743 | 0.691 |
| 269 | 0.672 | 0.971 | 0.686 | 0.929 | 0.905 | 0.920 | 0.696 | 0.667 | 0.908 | 0.958 | 0.919 | 0.909 | 0.946 | 0.755 | 0.978 | 0.944 | 0.752 | 0.700 |
| 270 | 0.648 | 1.000 | 0.650 | 0.914 | 0.900 | 0.960 | 0.701 | 0.667 | 0.929 | 0.917 | 0.933 | 0.879 | 0.955 | 0.755 | 0.978 | 0.926 | 0.742 | 0.690 |
| 271 | 0.660 | 1.000 | 0.696 | 0.914 | 0.916 | 0.960 | 0.730 | 0.667 | 0.908 | 0.958 | 0.925 | 0.909 | 0.955 | 0.746 | 0.978 | 0.981 | 0.753 | 0.702 |

| | | | | | | | | | | | | | | | | | | |
|---|---|---|---|---|---|---|---|---|---|---|---|---|---|---|---|---|---|---|
| 272 | 0.639 | 1.000 | 0.680 | 0.900 | 0.911 | 0.960 | 0.723 | 1.000 | 0.929 | 0.979 | 0.913 | 0.939 | 0.955 | 0.754 | 0.978 | 0.963 | 0.746 | 0.694 |
| 273 | 0.656 | 1.000 | 0.674 | 0.929 | 0.921 | 0.960 | 0.716 | 1.000 | 0.929 | 0.938 | 0.922 | 0.939 | 0.946 | 0.756 | 0.978 | 0.963 | 0.751 | 0.700 |
| 274 | 0.656 | 0.971 | 0.692 | 0.943 | 0.889 | 0.920 | 0.713 | 1.000 | 0.929 | 0.958 | 0.930 | 0.939 | 0.938 | 0.751 | 0.978 | 0.926 | 0.749 | 0.698 |
| 275 | 0.646 | 1.000 | 0.684 | 0.929 | 0.916 | 0.960 | 0.735 | 1.000 | 0.929 | 0.958 | 0.930 | 0.939 | 0.955 | 0.742 | 0.978 | 0.981 | 0.748 | 0.698 |
| 276 | 0.654 | 1.000 | 0.682 | 0.914 | 0.905 | 0.960 | 0.730 | 1.000 | 0.908 | 0.979 | 0.928 | 0.970 | 0.946 | 0.749 | 0.978 | 0.963 | 0.751 | 0.700 |
| 277 | 0.649 | 0.971 | 0.682 | 0.929 | 0.911 | 0.960 | 0.709 | 1.000 | 0.918 | 0.938 | 0.913 | 0.939 | 0.955 | 0.756 | 0.978 | 0.981 | 0.747 | 0.696 |
| 278 | 0.647 | 1.000 | 0.672 | 0.929 | 0.900 | 0.920 | 0.710 | 1.000 | 0.949 | 0.979 | 0.939 | 0.970 | 0.946 | 0.749 | 0.978 | 0.981 | 0.747 | 0.695 |
| 279 | 0.653 | 1.000 | 0.686 | 0.914 | 0.916 | 0.960 | 0.727 | 1.000 | 0.929 | 0.979 | 0.925 | 0.939 | 0.964 | 0.755 | 0.978 | 0.981 | 0.753 | 0.703 |
| 280 | 0.645 | 0.971 | 0.666 | 0.914 | 0.889 | 0.960 | 0.720 | 1.000 | 0.939 | 0.979 | 0.922 | 0.970 | 0.955 | 0.755 | 0.978 | 1.000 | 0.746 | 0.695 |
| 281 | 0.656 | 0.971 | 0.692 | 0.914 | 0.905 | 0.960 | 0.766 | 1.000 | 0.929 | 0.958 | 0.925 | 0.970 | 0.946 | 0.763 | 0.978 | 0.981 | 0.761 | 0.712 |
| 282 | 0.658 | 0.943 | 0.662 | 0.900 | 0.895 | 0.920 | 0.794 | 0.667 | 0.949 | 0.958 | 0.910 | 0.970 | 0.946 | 0.782 | 0.978 | 0.963 | 0.765 | 0.716 |
| 283 | 0.654 | 0.943 | 0.666 | 0.929 | 0.905 | 0.920 | 0.778 | 1.000 | 0.929 | 0.917 | 0.930 | 0.939 | 0.955 | 0.780 | 0.978 | 0.963 | 0.763 | 0.714 |
| 284 | 0.665 | 0.943 | 0.654 | 0.914 | 0.916 | 0.960 | 0.795 | 1.000 | 0.939 | 0.896 | 0.916 | 0.970 | 0.946 | 0.786 | 0.978 | 0.981 | 0.768 | 0.721 |
| 285 | 0.673 | 0.971 | 0.658 | 0.914 | 0.911 | 0.960 | 0.789 | 1.000 | 0.959 | 0.958 | 0.925 | 0.970 | 0.946 | 0.785 | 0.978 | 1.000 | 0.772 | 0.725 |
| 286 | 0.669 | 0.971 | 0.674 | 0.929 | 0.905 | 0.960 | 0.795 | 1.000 | 0.939 | 0.896 | 0.925 | 0.909 | 0.973 | 0.778 | 0.978 | 0.963 | 0.770 | 0.723 |
| 287 | 0.684 | 0.943 | 0.702 | 0.929 | 0.905 | 0.920 | 0.780 | 1.000 | 0.969 | 0.938 | 0.910 | 1.000 | 0.955 | 0.792 | 0.978 | 0.963 | 0.779 | 0.733 |
| 288 | 0.683 | 0.943 | 0.684 | 0.943 | 0.900 | 0.920 | 0.794 | 1.000 | 0.969 | 0.958 | 0.922 | 1.000 | 0.946 | 0.787 | 0.978 | 0.944 | 0.778 | 0.731 |
| 289 | 0.691 | 0.971 | 0.680 | 0.943 | 0.895 | 0.920 | 0.794 | 0.667 | 0.959 | 0.958 | 0.919 | 0.970 | 0.964 | 0.792 | 0.978 | 0.944 | 0.781 | 0.735 |
| 290 | 0.685 | 0.943 | 0.682 | 0.929 | 0.916 | 0.920 | 0.781 | 1.000 | 0.949 | 0.917 | 0.913 | 1.000 | 0.946 | 0.778 | 0.978 | 0.981 | 0.774 | 0.727 |
| 291 | 0.679 | 0.971 | 0.696 | 0.914 | 0.905 | 0.960 | 0.800 | 1.000 | 0.980 | 0.896 | 0.925 | 0.970 | 0.955 | 0.789 | 0.978 | 0.963 | 0.779 | 0.734 |
| 292 | 0.685 | 0.971 | 0.700 | 0.914 | 0.900 | 0.960 | 0.792 | 1.000 | 0.969 | 0.896 | 0.933 | 0.970 | 0.955 | 0.782 | 0.978 | 1.000 | 0.779 | 0.734 |
| 293 | 0.677 | 0.971 | 0.678 | 0.943 | 0.905 | 0.960 | 0.797 | 0.667 | 0.969 | 0.896 | 0.922 | 1.000 | 0.955 | 0.792 | 0.978 | 0.963 | 0.777 | 0.731 |
| 294 | 0.683 | 0.971 | 0.670 | 0.914 | 0.921 | 0.960 | 0.790 | 1.000 | 0.969 | 0.917 | 0.916 | 1.000 | 0.964 | 0.798 | 0.978 | 0.981 | 0.780 | 0.733 |
| 295 | 0.689 | 0.971 | 0.682 | 0.943 | 0.916 | 0.920 | 0.798 | 1.000 | 0.969 | 0.896 | 0.925 | 1.000 | 0.973 | 0.791 | 0.978 | 0.944 | 0.782 | 0.737 |
| 296 | 0.701 | 0.943 | 0.698 | 0.929 | 0.937 | 0.960 | 0.806 | 0.667 | 0.980 | 0.917 | 0.922 | 1.000 | 0.946 | 0.798 | 0.978 | 1.000 | 0.791 | 0.747 |
| 297 | 0.695 | 0.971 | 0.698 | 0.943 | 0.937 | 0.920 | 0.798 | 1.000 | 0.959 | 0.896 | 0.930 | 0.970 | 0.946 | 0.790 | 0.978 | 0.981 | 0.786 | 0.741 |
| 298 | 0.695 | 0.971 | 0.662 | 0.943 | 0.926 | 0.960 | 0.810 | 1.000 | 0.939 | 0.896 | 0.913 | 0.970 | 0.955 | 0.802 | 0.978 | 0.944 | 0.785 | 0.740 |
| 299 | 0.693 | 0.971 | 0.698 | 0.929 | 0.926 | 0.960 | 0.803 | 1.000 | 0.939 | 0.875 | 0.928 | 1.000 | 0.964 | 0.801 | 0.978 | 0.981 | 0.788 | 0.743 |
| 300 | 0.701 | 0.971 | 0.696 | 0.943 | 0.926 | 0.960 | 0.834 | 1.000 | 0.959 | 0.875 | 0.925 | 1.000 | 0.964 | 0.797 | 0.978 | 0.981 | 0.794 | 0.751 |
| 301 | 0.696 | 0.971 | 0.694 | 0.929 | 0.932 | 0.960 | 0.829 | 1.000 | 0.959 | 0.896 | 0.919 | 0.970 | 0.973 | 0.788 | 0.978 | 0.963 | 0.789 | 0.745 |
| 302 | 0.702 | 0.971 | 0.696 | 0.914 | 0.942 | 0.960 | 0.817 | 1.000 | 0.959 | 0.938 | 0.928 | 1.000 | 0.973 | 0.801 | 0.978 | 0.963 | 0.794 | 0.751 |
| 303 | 0.703 | 0.971 | 0.694 | 0.929 | 0.926 | 0.960 | 0.834 | 1.000 | 0.969 | 0.917 | 0.930 | 0.970 | 0.964 | 0.798 | 0.978 | 0.981 | 0.795 | 0.753 |
| 304 | 0.698 | 0.971 | 0.698 | 0.929 | 0.937 | 0.960 | 0.838 | 0.667 | 0.959 | 0.917 | 0.928 | 1.000 | 0.982 | 0.798 | 0.978 | 0.981 | 0.795 | 0.752 |
| 305 | 0.697 | 0.943 | 0.700 | 0.929 | 0.932 | 0.960 | 0.849 | 1.000 | 0.980 | 0.938 | 0.936 | 1.000 | 0.982 | 0.796 | 0.978 | 0.963 | 0.796 | 0.755 |
| 306 | 0.695 | 0.943 | 0.680 | 0.929 | 0.921 | 0.920 | 0.852 | 1.000 | 0.969 | 0.896 | 0.930 | 1.000 | 0.973 | 0.811 | 0.978 | 0.944 | 0.796 | 0.754 |
| 307 | 0.700 | 0.943 | 0.706 | 0.929 | 0.916 | 0.960 | 0.844 | 0.667 | 0.949 | 0.896 | 0.930 | 1.000 | 0.973 | 0.816 | 0.978 | 0.963 | 0.800 | 0.758 |
| 308 | 0.713 | 0.914 | 0.700 | 0.929 | 0.921 | 0.960 | 0.847 | 1.000 | 0.959 | 0.917 | 0.919 | 1.000 | 0.973 | 0.810 | 0.978 | 0.963 | 0.802 | 0.761 |
| 309 | 0.707 | 0.943 | 0.674 | 0.929 | 0.926 | 0.960 | 0.867 | 1.000 | 0.939 | 0.938 | 0.928 | 1.000 | 0.973 | 0.814 | 0.978 | 0.944 | 0.802 | 0.761 |
| 310 | 0.698 | 0.943 | 0.694 | 0.929 | 0.916 | 0.960 | 0.849 | 1.000 | 0.959 | 0.938 | 0.922 | 1.000 | 0.973 | 0.813 | 0.978 | 0.963 | 0.798 | 0.757 |

| | | | | | | | | | | | | | | | | | | |
|---|---|---|---|---|---|---|---|---|---|---|---|---|---|---|---|---|---|---|
| 311 | 0.715 | 0.971 | 0.682 | 0.943 | 0.921 | 0.960 | 0.847 | 1.000 | 0.959 | 0.917 | 0.907 | 1.000 | 0.973 | 0.809 | 1.000 | 0.963 | 0.800 | 0.759 |
| 312 | 0.704 | 0.971 | 0.678 | 0.929 | 0.921 | 0.960 | 0.852 | 1.000 | 0.949 | 0.938 | 0.933 | 0.939 | 0.973 | 0.827 | 0.978 | 0.944 | 0.803 | 0.762 |
| 313 | 0.713 | 0.971 | 0.686 | 0.929 | 0.916 | 0.960 | 0.851 | 1.000 | 0.969 | 0.938 | 0.930 | 0.939 | 0.991 | 0.812 | 0.978 | 0.963 | 0.803 | 0.762 |
| 314 | 0.709 | 0.971 | 0.688 | 0.914 | 0.926 | 0.960 | 0.835 | 1.000 | 0.959 | 0.938 | 0.930 | 0.970 | 0.973 | 0.810 | 0.978 | 0.944 | 0.799 | 0.757 |
| 315 | 0.711 | 0.971 | 0.729 | 0.914 | 0.916 | 0.960 | 0.855 | 1.000 | 0.990 | 0.938 | 0.919 | 0.939 | 0.973 | 0.821 | 0.978 | 1.000 | 0.809 | 0.769 |
| 316 | 0.701 | 0.971 | 0.686 | 0.914 | 0.921 | 0.960 | 0.854 | 0.667 | 0.969 | 0.938 | 0.930 | 0.970 | 0.982 | 0.821 | 0.978 | 1.000 | 0.802 | 0.761 |
| 317 | 0.691 | 0.971 | 0.733 | 0.900 | 0.921 | 0.960 | 0.854 | 1.000 | 0.969 | 0.938 | 0.916 | 0.970 | 0.982 | 0.812 | 0.978 | 1.000 | 0.801 | 0.760 |
| 318 | 0.720 | 0.971 | 0.713 | 0.929 | 0.926 | 0.960 | 0.858 | 0.667 | 0.949 | 0.938 | 0.928 | 0.970 | 0.973 | 0.813 | 0.978 | 0.981 | 0.808 | 0.769 |
| 319 | 0.712 | 0.971 | 0.672 | 0.943 | 0.921 | 0.960 | 0.851 | 1.000 | 0.980 | 0.938 | 0.928 | 0.970 | 0.982 | 0.816 | 0.978 | 0.981 | 0.803 | 0.762 |
| 320 | 0.718 | 0.971 | 0.684 | 0.943 | 0.926 | 0.960 | 0.846 | 0.667 | 0.959 | 0.938 | 0.930 | 0.970 | 0.991 | 0.807 | 0.978 | 1.000 | 0.803 | 0.762 |
| 321 | 0.714 | 0.971 | 0.680 | 0.929 | 0.921 | 0.960 | 0.860 | 0.667 | 0.969 | 0.938 | 0.919 | 0.970 | 0.973 | 0.816 | 1.000 | 0.981 | 0.804 | 0.763 |
| 322 | 0.704 | 0.971 | 0.700 | 0.914 | 0.921 | 0.960 | 0.852 | 0.667 | 0.990 | 0.958 | 0.916 | 0.970 | 0.982 | 0.820 | 0.978 | 1.000 | 0.804 | 0.763 |
| 323 | 0.719 | 0.971 | 0.690 | 0.929 | 0.926 | 0.960 | 0.861 | 0.667 | 0.980 | 0.938 | 0.925 | 0.970 | 0.973 | 0.822 | 0.978 | 0.963 | 0.809 | 0.769 |
| 324 | 0.705 | 0.971 | 0.715 | 0.929 | 0.921 | 0.960 | 0.860 | 0.667 | 0.969 | 0.938 | 0.922 | 0.970 | 0.973 | 0.826 | 0.978 | 0.981 | 0.808 | 0.768 |
| 325 | 0.706 | 0.971 | 0.702 | 0.943 | 0.926 | 0.960 | 0.869 | 1.000 | 0.969 | 0.938 | 0.928 | 0.970 | 0.982 | 0.820 | 0.978 | 0.963 | 0.807 | 0.768 |
| 326 | 0.704 | 0.971 | 0.700 | 0.914 | 0.900 | 0.960 | 0.863 | 0.667 | 0.949 | 0.938 | 0.901 | 0.909 | 0.964 | 0.830 | 0.978 | 0.981 | 0.804 | 0.763 |
| 327 | 0.699 | 0.971 | 0.709 | 0.929 | 0.926 | 1.000 | 0.861 | 0.667 | 0.990 | 0.938 | 0.907 | 0.970 | 0.964 | 0.825 | 0.978 | 1.000 | 0.805 | 0.765 |
| 328 | 0.699 | 0.971 | 0.702 | 0.900 | 0.921 | 0.960 | 0.854 | 1.000 | 0.969 | 0.958 | 0.919 | 0.939 | 0.973 | 0.824 | 0.978 | 1.000 | 0.803 | 0.763 |
| 329 | 0.702 | 0.971 | 0.711 | 0.900 | 0.932 | 0.960 | 0.871 | 1.000 | 0.959 | 0.958 | 0.933 | 0.939 | 0.964 | 0.825 | 0.978 | 1.000 | 0.808 | 0.769 |
| 330 | 0.716 | 0.971 | 0.719 | 0.900 | 0.926 | 1.000 | 0.866 | 0.667 | 0.969 | 0.958 | 0.919 | 0.909 | 0.964 | 0.835 | 0.978 | 0.981 | 0.814 | 0.776 |
| 331 | 0.718 | 1.000 | 0.737 | 0.900 | 0.921 | 0.960 | 0.866 | 0.667 | 0.959 | 0.938 | 0.930 | 0.909 | 0.973 | 0.832 | 0.978 | 1.000 | 0.816 | 0.778 |
| 332 | 0.701 | 0.971 | 0.725 | 0.943 | 0.921 | 1.000 | 0.857 | 1.000 | 0.959 | 0.979 | 0.922 | 0.879 | 0.973 | 0.842 | 1.000 | 0.981 | 0.811 | 0.773 |
| 333 | 0.712 | 1.000 | 0.711 | 0.929 | 0.926 | 0.960 | 0.852 | 1.000 | 0.959 | 0.958 | 0.925 | 0.909 | 0.973 | 0.830 | 0.978 | 1.000 | 0.810 | 0.771 |
| 334 | 0.719 | 0.971 | 0.717 | 0.914 | 0.926 | 0.920 | 0.846 | 1.000 | 0.980 | 0.958 | 0.922 | 0.939 | 0.973 | 0.830 | 0.978 | 0.981 | 0.811 | 0.772 |
| 335 | 0.710 | 0.971 | 0.715 | 0.900 | 0.932 | 0.960 | 0.864 | 1.000 | 0.949 | 0.938 | 0.919 | 0.939 | 0.973 | 0.834 | 0.978 | 0.981 | 0.811 | 0.772 |
| 336 | 0.714 | 0.971 | 0.727 | 0.929 | 0.921 | 0.960 | 0.863 | 1.000 | 0.959 | 0.938 | 0.925 | 0.939 | 0.982 | 0.836 | 0.978 | 0.981 | 0.814 | 0.776 |
| 337 | 0.728 | 1.000 | 0.706 | 0.929 | 0.911 | 0.960 | 0.878 | 0.667 | 0.980 | 0.938 | 0.928 | 0.909 | 0.964 | 0.829 | 0.978 | 0.981 | 0.816 | 0.778 |
| 338 | 0.723 | 0.971 | 0.723 | 0.929 | 0.926 | 0.960 | 0.858 | 0.667 | 0.980 | 0.958 | 0.925 | 0.879 | 0.982 | 0.823 | 0.978 | 1.000 | 0.813 | 0.775 |
| 339 | 0.724 | 0.971 | 0.719 | 0.943 | 0.921 | 0.960 | 0.864 | 0.667 | 0.990 | 0.938 | 0.925 | 0.970 | 0.973 | 0.837 | 0.978 | 0.981 | 0.818 | 0.780 |
| 340 | 0.707 | 0.971 | 0.702 | 0.943 | 0.937 | 0.960 | 0.886 | 0.667 | 0.980 | 0.958 | 0.925 | 0.939 | 0.973 | 0.827 | 0.978 | 1.000 | 0.812 | 0.774 |
| 341 | 0.708 | 1.000 | 0.713 | 0.943 | 0.921 | 0.960 | 0.858 | 0.667 | 0.969 | 0.958 | 0.928 | 0.909 | 0.973 | 0.831 | 0.978 | 1.000 | 0.810 | 0.771 |
| 342 | 0.722 | 1.000 | 0.702 | 0.914 | 0.926 | 0.960 | 0.880 | 1.000 | 0.980 | 0.958 | 0.916 | 0.909 | 0.964 | 0.827 | 0.978 | 1.000 | 0.814 | 0.776 |
| 343 | 0.711 | 1.000 | 0.713 | 0.957 | 0.926 | 0.960 | 0.866 | 1.000 | 0.990 | 0.958 | 0.930 | 0.939 | 0.982 | 0.836 | 0.978 | 1.000 | 0.815 | 0.777 |
| 344 | 0.712 | 1.000 | 0.727 | 0.943 | 0.937 | 1.000 | 0.866 | 0.667 | 0.969 | 0.958 | 0.919 | 0.939 | 0.982 | 0.838 | 1.000 | 1.000 | 0.816 | 0.779 |
| 345 | 0.724 | 0.971 | 0.731 | 0.943 | 0.921 | 0.960 | 0.867 | 0.667 | 0.959 | 0.979 | 0.922 | 0.909 | 0.973 | 0.826 | 0.978 | 0.981 | 0.816 | 0.778 |
| 346 | 0.710 | 0.971 | 0.698 | 0.957 | 0.921 | 0.960 | 0.864 | 0.667 | 0.949 | 0.938 | 0.933 | 0.909 | 0.982 | 0.834 | 0.978 | 1.000 | 0.811 | 0.772 |
| 347 | 0.721 | 1.000 | 0.698 | 0.957 | 0.911 | 0.960 | 0.861 | 0.667 | 0.969 | 0.958 | 0.925 | 0.939 | 0.991 | 0.834 | 0.978 | 1.000 | 0.814 | 0.775 |
| 348 | 0.722 | 0.971 | 0.719 | 0.957 | 0.905 | 0.960 | 0.861 | 0.667 | 0.959 | 0.979 | 0.925 | 0.939 | 0.973 | 0.826 | 0.978 | 1.000 | 0.813 | 0.774 |
| 349 | 0.724 | 1.000 | 0.698 | 0.943 | 0.926 | 0.960 | 0.858 | 0.667 | 0.959 | 0.958 | 0.928 | 0.909 | 0.991 | 0.839 | 0.978 | 1.000 | 0.816 | 0.777 |

| | | | | | | | | | | | | | | | | | | |
|---|---|---|---|---|---|---|---|---|---|---|---|---|---|---|---|---|---|---|
| 350 | 0.715 | 1.000 | 0.694 | 0.957 | 0.905 | 0.960 | 0.863 | 0.667 | 0.969 | 0.958 | 0.925 | 0.909 | 0.973 | 0.841 | 0.978 | 1.000 | 0.813 | 0.774 |
| 351 | 0.732 | 1.000 | 0.731 | 0.957 | 0.921 | 0.960 | 0.866 | 1.000 | 0.969 | 0.958 | 0.928 | 0.909 | 0.973 | 0.833 | 0.978 | 1.000 | 0.821 | 0.784 |
| 352 | 0.720 | 1.000 | 0.719 | 0.971 | 0.905 | 0.960 | 0.844 | 0.667 | 0.990 | 0.958 | 0.928 | 0.909 | 0.964 | 0.837 | 1.000 | 0.981 | 0.814 | 0.775 |
| 353 | 0.718 | 1.000 | 0.723 | 0.943 | 0.921 | 0.960 | 0.852 | 1.000 | 0.980 | 0.979 | 0.928 | 0.909 | 0.973 | 0.833 | 1.000 | 1.000 | 0.814 | 0.776 |
| 354 | 0.720 | 1.000 | 0.711 | 0.971 | 0.932 | 0.960 | 0.863 | 0.667 | 0.959 | 0.958 | 0.933 | 0.909 | 0.964 | 0.845 | 0.978 | 1.000 | 0.819 | 0.781 |
| 355 | 0.726 | 0.971 | 0.708 | 0.957 | 0.921 | 0.960 | 0.866 | 0.667 | 0.980 | 0.938 | 0.933 | 0.939 | 0.982 | 0.833 | 0.978 | 1.000 | 0.817 | 0.780 |
| 356 | 0.715 | 1.000 | 0.702 | 0.971 | 0.921 | 0.960 | 0.858 | 0.667 | 0.980 | 0.958 | 0.942 | 0.909 | 0.982 | 0.830 | 0.978 | 1.000 | 0.812 | 0.774 |
| 357 | 0.722 | 1.000 | 0.696 | 0.971 | 0.932 | 0.960 | 0.878 | 0.667 | 0.969 | 0.979 | 0.942 | 0.970 | 0.973 | 0.838 | 0.978 | 1.000 | 0.819 | 0.782 |
| 358 | 0.730 | 1.000 | 0.711 | 0.957 | 0.916 | 0.960 | 0.871 | 0.667 | 0.980 | 0.979 | 0.942 | 0.939 | 0.964 | 0.834 | 0.978 | 1.000 | 0.820 | 0.783 |
| 359 | 0.724 | 1.000 | 0.709 | 0.971 | 0.926 | 0.960 | 0.871 | 0.667 | 0.980 | 0.979 | 0.933 | 0.909 | 0.973 | 0.838 | 0.978 | 1.000 | 0.819 | 0.782 |
| 360 | 0.726 | 1.000 | 0.700 | 0.957 | 0.932 | 0.960 | 0.864 | 1.000 | 0.959 | 0.979 | 0.942 | 0.939 | 0.982 | 0.839 | 0.978 | 1.000 | 0.819 | 0.782 |
| 361 | 0.726 | 1.000 | 0.694 | 0.943 | 0.937 | 0.960 | 0.867 | 1.000 | 0.969 | 0.979 | 0.945 | 0.909 | 0.964 | 0.836 | 0.978 | 1.000 | 0.818 | 0.780 |
| 362 | 0.730 | 1.000 | 0.698 | 0.943 | 0.932 | 1.000 | 0.861 | 1.000 | 0.980 | 0.979 | 0.933 | 0.909 | 0.973 | 0.833 | 0.978 | 1.000 | 0.818 | 0.780 |
| 363 | 0.722 | 1.000 | 0.694 | 0.971 | 0.932 | 0.960 | 0.871 | 0.667 | 0.980 | 0.979 | 0.928 | 0.909 | 0.982 | 0.844 | 1.000 | 0.981 | 0.819 | 0.781 |
| 364 | 0.728 | 1.000 | 0.700 | 0.957 | 0.937 | 0.960 | 0.878 | 1.000 | 0.969 | 0.979 | 0.930 | 0.909 | 0.982 | 0.841 | 0.978 | 1.000 | 0.822 | 0.785 |
| 365 | 0.716 | 1.000 | 0.709 | 0.957 | 0.932 | 1.000 | 0.872 | 1.000 | 0.969 | 0.979 | 0.945 | 0.939 | 0.982 | 0.841 | 0.978 | 1.000 | 0.819 | 0.783 |
| 366 | 0.724 | 1.000 | 0.711 | 0.943 | 0.937 | 1.000 | 0.875 | 1.000 | 0.969 | 0.979 | 0.936 | 0.909 | 0.982 | 0.840 | 1.000 | 1.000 | 0.822 | 0.785 |
| 367 | 0.736 | 1.000 | 0.704 | 0.929 | 0.942 | 0.960 | 0.875 | 1.000 | 0.990 | 0.938 | 0.945 | 0.909 | 0.991 | 0.845 | 1.000 | 1.000 | 0.826 | 0.791 |
| 368 | 0.727 | 1.000 | 0.719 | 0.943 | 0.937 | 0.960 | 0.874 | 1.000 | 0.980 | 1.000 | 0.933 | 0.909 | 0.982 | 0.835 | 0.978 | 1.000 | 0.822 | 0.785 |
| 369 | 0.729 | 1.000 | 0.694 | 0.957 | 0.937 | 0.960 | 0.871 | 1.000 | 0.980 | 0.979 | 0.933 | 0.939 | 0.973 | 0.833 | 0.978 | 1.000 | 0.819 | 0.782 |
| 370 | 0.736 | 1.000 | 0.702 | 0.971 | 0.942 | 1.000 | 0.872 | 1.000 | 0.980 | 0.979 | 0.939 | 0.939 | 0.982 | 0.843 | 1.000 | 1.000 | 0.826 | 0.790 |
| 371 | 0.741 | 1.000 | 0.708 | 0.971 | 0.937 | 1.000 | 0.871 | 1.000 | 0.990 | 0.958 | 0.928 | 0.939 | 0.982 | 0.838 | 1.000 | 0.981 | 0.825 | 0.789 |
| 372 | 0.726 | 1.000 | 0.729 | 0.971 | 0.932 | 1.000 | 0.867 | 1.000 | 0.990 | 0.938 | 0.945 | 0.909 | 0.964 | 0.827 | 1.000 | 1.000 | 0.820 | 0.784 |
| 373 | 0.736 | 1.000 | 0.713 | 0.971 | 0.932 | 1.000 | 0.857 | 0.667 | 0.980 | 0.979 | 0.948 | 0.909 | 0.982 | 0.838 | 1.000 | 1.000 | 0.823 | 0.787 |
| 374 | 0.725 | 1.000 | 0.694 | 0.957 | 0.942 | 0.960 | 0.864 | 0.667 | 0.980 | 0.958 | 0.945 | 0.909 | 0.982 | 0.850 | 1.000 | 1.000 | 0.822 | 0.785 |
| 375 | 0.744 | 1.000 | 0.715 | 0.971 | 0.942 | 0.960 | 0.867 | 1.000 | 1.000 | 0.958 | 0.936 | 0.909 | 0.973 | 0.837 | 0.978 | 1.000 | 0.827 | 0.791 |
| 376 | 0.742 | 1.000 | 0.717 | 0.971 | 0.926 | 1.000 | 0.866 | 0.667 | 1.000 | 1.000 | 0.942 | 0.909 | 0.973 | 0.837 | 1.000 | 0.981 | 0.826 | 0.790 |
| 377 | 0.730 | 1.000 | 0.702 | 0.957 | 0.947 | 1.000 | 0.871 | 1.000 | 0.990 | 1.000 | 0.939 | 0.909 | 0.973 | 0.824 | 1.000 | 1.000 | 0.819 | 0.782 |
| 378 | 0.724 | 1.000 | 0.719 | 0.971 | 0.942 | 1.000 | 0.869 | 0.667 | 1.000 | 0.979 | 0.948 | 0.909 | 0.982 | 0.846 | 1.000 | 1.000 | 0.825 | 0.789 |
| 379 | 0.726 | 1.000 | 0.733 | 0.943 | 0.942 | 1.000 | 0.861 | 1.000 | 1.000 | 0.979 | 0.939 | 0.939 | 0.982 | 0.840 | 1.000 | 0.981 | 0.823 | 0.787 |
| 380 | 0.728 | 1.000 | 0.723 | 0.971 | 0.942 | 0.960 | 0.861 | 1.000 | 0.990 | 1.000 | 0.942 | 0.909 | 0.964 | 0.844 | 1.000 | 1.000 | 0.824 | 0.788 |
| 381 | 0.729 | 1.000 | 0.719 | 0.971 | 0.942 | 1.000 | 0.861 | 1.000 | 0.980 | 1.000 | 0.939 | 0.939 | 0.973 | 0.843 | 1.000 | 1.000 | 0.824 | 0.788 |
| 382 | 0.732 | 1.000 | 0.723 | 0.957 | 0.947 | 1.000 | 0.871 | 1.000 | 0.990 | 0.979 | 0.948 | 0.939 | 0.973 | 0.840 | 1.000 | 0.981 | 0.826 | 0.791 |
| 383 | 0.732 | 1.000 | 0.706 | 0.943 | 0.947 | 1.000 | 0.861 | 1.000 | 0.980 | 0.979 | 0.939 | 0.909 | 0.982 | 0.847 | 1.000 | 1.000 | 0.824 | 0.788 |
| 384 | 0.726 | 1.000 | 0.727 | 0.957 | 0.953 | 1.000 | 0.864 | 1.000 | 0.969 | 0.958 | 0.942 | 0.939 | 0.982 | 0.839 | 1.000 | 1.000 | 0.823 | 0.788 |
| 385 | 0.730 | 1.000 | 0.723 | 0.957 | 0.937 | 1.000 | 0.869 | 0.667 | 0.980 | 0.958 | 0.942 | 0.939 | 0.973 | 0.840 | 1.000 | 0.981 | 0.824 | 0.788 |
| 386 | 0.724 | 1.000 | 0.713 | 0.957 | 0.937 | 1.000 | 0.875 | 1.000 | 0.990 | 0.979 | 0.945 | 0.970 | 0.973 | 0.844 | 1.000 | 1.000 | 0.824 | 0.788 |
| 387 | 0.730 | 1.000 | 0.725 | 0.971 | 0.947 | 0.960 | 0.864 | 1.000 | 0.990 | 1.000 | 0.936 | 0.909 | 0.973 | 0.837 | 1.000 | 1.000 | 0.824 | 0.788 |
| 388 | 0.727 | 1.000 | 0.723 | 0.957 | 0.953 | 1.000 | 0.866 | 1.000 | 0.980 | 1.000 | 0.942 | 0.939 | 0.973 | 0.858 | 1.000 | 1.000 | 0.829 | 0.794 |

| | | | | | | | | | | | | | | | | | |
|---|---|---|---|---|---|---|---|---|---|---|---|---|---|---|---|---|---|
| 389 | 0.725 | 1.000 | 0.739 | 0.957 | 0.953 | 1.000 | 0.869 | 1.000 | 0.980 | 0.979 | 0.942 | 0.909 | 0.982 | 0.851 | 1.000 | 1.000 | 0.828 | 0.794 |
| 390 | 0.722 | 1.000 | 0.739 | 0.971 | 0.942 | 1.000 | 0.878 | 1.000 | 0.990 | 0.979 | 0.948 | 0.909 | 0.982 | 0.844 | 1.000 | 1.000 | 0.827 | 0.792 |
| 391 | 0.730 | 1.000 | 0.725 | 0.957 | 0.947 | 1.000 | 0.872 | 1.000 | 0.980 | 1.000 | 0.936 | 0.939 | 0.991 | 0.849 | 1.000 | 1.000 | 0.828 | 0.793 |
| 392 | 0.719 | 1.000 | 0.719 | 0.943 | 0.947 | 0.960 | 0.878 | 1.000 | 0.969 | 0.979 | 0.954 | 0.909 | 0.964 | 0.830 | 1.000 | 1.000 | 0.820 | 0.784 |
| 393 | 0.740 | 1.000 | 0.729 | 0.957 | 0.958 | 1.000 | 0.869 | 1.000 | 0.980 | 0.938 | 0.945 | 0.939 | 0.964 | 0.843 | 1.000 | 0.981 | 0.829 | 0.794 |
| 394 | 0.736 | 1.000 | 0.733 | 0.943 | 0.942 | 1.000 | 0.878 | 1.000 | 0.969 | 0.979 | 0.954 | 0.939 | 0.982 | 0.836 | 1.000 | 1.000 | 0.828 | 0.793 |
| 395 | 0.722 | 1.000 | 0.743 | 0.957 | 0.942 | 1.000 | 0.874 | 1.000 | 0.990 | 0.979 | 0.939 | 0.939 | 0.991 | 0.837 | 1.000 | 1.000 | 0.825 | 0.790 |
| 396 | 0.744 | 1.000 | 0.723 | 0.971 | 0.953 | 1.000 | 0.877 | 1.000 | 0.980 | 0.979 | 0.948 | 0.970 | 0.991 | 0.840 | 1.000 | 1.000 | 0.831 | 0.797 |
| 397 | 0.728 | 1.000 | 0.711 | 0.971 | 0.953 | 0.960 | 0.880 | 1.000 | 0.990 | 1.000 | 0.962 | 0.939 | 0.964 | 0.846 | 1.000 | 1.000 | 0.828 | 0.793 |
| 398 | 0.746 | 1.000 | 0.737 | 0.971 | 0.947 | 1.000 | 0.875 | 1.000 | 0.980 | 0.979 | 0.957 | 0.939 | 0.991 | 0.839 | 1.000 | 0.981 | 0.833 | 0.799 |
| 399 | 0.730 | 1.000 | 0.719 | 0.971 | 0.958 | 0.960 | 0.869 | 1.000 | 0.990 | 0.979 | 0.948 | 0.939 | 0.982 | 0.844 | 1.000 | 1.000 | 0.827 | 0.792 |
| 400 | 0.728 | 1.000 | 0.719 | 0.957 | 0.947 | 1.000 | 0.884 | 1.000 | 1.000 | 0.979 | 0.945 | 0.939 | 0.991 | 0.844 | 1.000 | 1.000 | 0.828 | 0.793 |
| 401 | 0.744 | 1.000 | 0.721 | 0.943 | 0.953 | 1.000 | 0.864 | 1.000 | 0.980 | 0.979 | 0.948 | 0.909 | 0.991 | 0.836 | 1.000 | 0.981 | 0.828 | 0.792 |
| 402 | 0.738 | 1.000 | 0.735 | 0.957 | 0.942 | 0.960 | 0.866 | 1.000 | 0.980 | 1.000 | 0.951 | 0.909 | 0.991 | 0.837 | 1.000 | 1.000 | 0.828 | 0.793 |
| 403 | 0.728 | 1.000 | 0.719 | 0.971 | 0.942 | 1.000 | 0.872 | 1.000 | 0.980 | 0.979 | 0.948 | 0.939 | 0.982 | 0.843 | 1.000 | 1.000 | 0.826 | 0.790 |
| 404 | 0.733 | 1.000 | 0.733 | 0.957 | 0.932 | 1.000 | 0.866 | 0.667 | 0.990 | 1.000 | 0.959 | 0.939 | 0.991 | 0.841 | 1.000 | 1.000 | 0.828 | 0.793 |
| 405 | 0.724 | 1.000 | 0.733 | 0.971 | 0.947 | 1.000 | 0.869 | 1.000 | 0.990 | 1.000 | 0.957 | 0.909 | 0.982 | 0.847 | 1.000 | 1.000 | 0.828 | 0.793 |
| 406 | 0.724 | 1.000 | 0.711 | 0.957 | 0.937 | 0.960 | 0.877 | 1.000 | 0.990 | 0.979 | 0.945 | 0.939 | 0.982 | 0.842 | 1.000 | 1.000 | 0.824 | 0.788 |
| 407 | 0.730 | 1.000 | 0.731 | 0.971 | 0.958 | 0.960 | 0.875 | 1.000 | 0.969 | 0.979 | 0.930 | 0.909 | 0.991 | 0.845 | 1.000 | 1.000 | 0.828 | 0.793 |
| 408 | 0.736 | 1.000 | 0.727 | 0.971 | 0.953 | 0.960 | 0.889 | 1.000 | 0.969 | 1.000 | 0.951 | 0.909 | 0.991 | 0.843 | 1.000 | 1.000 | 0.831 | 0.797 |
| 409 | 0.723 | 1.000 | 0.709 | 0.971 | 0.942 | 0.960 | 0.880 | 1.000 | 0.980 | 0.979 | 0.942 | 0.939 | 0.991 | 0.849 | 1.000 | 1.000 | 0.825 | 0.790 |
| 410 | 0.711 | 1.000 | 0.745 | 0.971 | 0.947 | 1.000 | 0.875 | 1.000 | 0.990 | 0.979 | 0.945 | 0.939 | 0.973 | 0.847 | 1.000 | 0.981 | 0.825 | 0.789 |
| 411 | 0.726 | 1.000 | 0.733 | 0.971 | 0.942 | 1.000 | 0.884 | 1.000 | 0.980 | 0.979 | 0.957 | 0.939 | 0.973 | 0.844 | 1.000 | 1.000 | 0.829 | 0.794 |
| 412 | 0.728 | 1.000 | 0.719 | 0.986 | 0.947 | 1.000 | 0.874 | 1.000 | 1.000 | 1.000 | 0.957 | 0.939 | 0.982 | 0.836 | 1.000 | 1.000 | 0.826 | 0.791 |
| 413 | 0.733 | 1.000 | 0.727 | 0.986 | 0.953 | 1.000 | 0.880 | 1.000 | 0.990 | 0.979 | 0.954 | 0.909 | 0.982 | 0.840 | 1.000 | 0.981 | 0.829 | 0.795 |
| 414 | 0.731 | 1.000 | 0.739 | 0.971 | 0.953 | 1.000 | 0.861 | 1.000 | 0.990 | 0.958 | 0.954 | 0.909 | 0.991 | 0.842 | 1.000 | 1.000 | 0.828 | 0.793 |
| 415 | 0.724 | 1.000 | 0.727 | 0.971 | 0.937 | 0.960 | 0.863 | 1.000 | 0.990 | 0.979 | 0.951 | 0.909 | 0.964 | 0.840 | 1.000 | 1.000 | 0.823 | 0.787 |
| 416 | 0.719 | 1.000 | 0.733 | 0.957 | 0.947 | 1.000 | 0.860 | 1.000 | 0.990 | 1.000 | 0.942 | 0.939 | 0.982 | 0.844 | 1.000 | 0.981 | 0.823 | 0.787 |
| 417 | 0.722 | 1.000 | 0.723 | 0.971 | 0.932 | 1.000 | 0.877 | 1.000 | 0.990 | 0.979 | 0.954 | 0.970 | 0.982 | 0.844 | 1.000 | 0.981 | 0.825 | 0.790 |
| 418 | 0.715 | 1.000 | 0.721 | 0.971 | 0.953 | 0.960 | 0.864 | 1.000 | 0.990 | 0.979 | 0.951 | 0.939 | 0.973 | 0.854 | 1.000 | 1.000 | 0.824 | 0.789 |
| 419 | 0.732 | 1.000 | 0.719 | 0.986 | 0.947 | 1.000 | 0.874 | 1.000 | 0.980 | 1.000 | 0.942 | 0.909 | 0.982 | 0.841 | 1.000 | 1.000 | 0.827 | 0.791 |
| 420 | 0.723 | 1.000 | 0.721 | 0.971 | 0.963 | 1.000 | 0.877 | 0.667 | 0.980 | 0.979 | 0.942 | 0.939 | 0.973 | 0.845 | 1.000 | 1.000 | 0.826 | 0.790 |
| 421 | 0.732 | 1.000 | 0.717 | 0.957 | 0.953 | 1.000 | 0.863 | 1.000 | 0.990 | 0.979 | 0.936 | 0.970 | 0.973 | 0.847 | 1.000 | 0.981 | 0.826 | 0.790 |
| 422 | 0.737 | 1.000 | 0.723 | 0.971 | 0.953 | 0.960 | 0.866 | 1.000 | 0.990 | 0.958 | 0.954 | 0.939 | 0.973 | 0.847 | 1.000 | 1.000 | 0.830 | 0.795 |
| 423 | 0.720 | 1.000 | 0.721 | 0.957 | 0.947 | 0.960 | 0.874 | 1.000 | 0.980 | 0.979 | 0.936 | 0.939 | 0.982 | 0.844 | 1.000 | 0.981 | 0.823 | 0.787 |
| 424 | 0.728 | 1.000 | 0.717 | 0.971 | 0.947 | 1.000 | 0.869 | 1.000 | 0.980 | 0.979 | 0.948 | 0.970 | 0.982 | 0.843 | 1.000 | 1.000 | 0.825 | 0.790 |
| 425 | 0.734 | 1.000 | 0.721 | 0.971 | 0.963 | 0.960 | 0.866 | 1.000 | 1.000 | 0.958 | 0.945 | 0.970 | 0.982 | 0.854 | 1.000 | 1.000 | 0.831 | 0.796 |
| 426 | 0.725 | 1.000 | 0.700 | 0.986 | 0.968 | 1.000 | 0.863 | 1.000 | 0.990 | 0.979 | 0.942 | 0.970 | 0.964 | 0.854 | 1.000 | 1.000 | 0.825 | 0.789 |
| 427 | 0.724 | 1.000 | 0.727 | 0.971 | 0.963 | 1.000 | 0.867 | 1.000 | 1.000 | 1.000 | 0.948 | 0.939 | 0.982 | 0.848 | 1.000 | 1.000 | 0.827 | 0.792 |

| | | | | | | | | | | | | | | | | | |
|---|---|---|---|---|---|---|---|---|---|---|---|---|---|---|---|---|---|
| 428 | 0.734 | 1.000 | 0.719 | 0.957 | 0.947 | 1.000 | 0.855 | 0.667 | 0.980 | 1.000 | 0.954 | 0.939 | 0.973 | 0.847 | 1.000 | 0.981 | 0.827 | 0.791 |
| 429 | 0.731 | 1.000 | 0.727 | 0.957 | 0.942 | 0.960 | 0.875 | 1.000 | 0.980 | 1.000 | 0.942 | 0.970 | 0.973 | 0.840 | 1.000 | 1.000 | 0.827 | 0.791 |
| 430 | 0.730 | 1.000 | 0.702 | 0.957 | 0.953 | 1.000 | 0.875 | 1.000 | 0.990 | 0.958 | 0.939 | 0.939 | 0.964 | 0.850 | 1.000 | 1.000 | 0.826 | 0.790 |
| 431 | 0.734 | 1.000 | 0.711 | 0.957 | 0.942 | 1.000 | 0.875 | 1.000 | 0.990 | 0.979 | 0.942 | 0.939 | 0.973 | 0.844 | 1.000 | 0.981 | 0.827 | 0.791 |
| 432 | 0.727 | 1.000 | 0.709 | 0.943 | 0.953 | 1.000 | 0.884 | 1.000 | 0.980 | 0.979 | 0.933 | 0.970 | 0.982 | 0.839 | 1.000 | 1.000 | 0.824 | 0.789 |
| 433 | 0.730 | 1.000 | 0.704 | 0.957 | 0.958 | 1.000 | 0.861 | 1.000 | 0.980 | 0.979 | 0.942 | 0.939 | 0.982 | 0.847 | 1.000 | 1.000 | 0.825 | 0.788 |
| 434 | 0.736 | 1.000 | 0.717 | 0.971 | 0.963 | 1.000 | 0.871 | 1.000 | 0.990 | 1.000 | 0.945 | 0.939 | 0.991 | 0.850 | 1.000 | 1.000 | 0.831 | 0.796 |
| 435 | 0.729 | 1.000 | 0.706 | 0.971 | 0.953 | 1.000 | 0.866 | 1.000 | 0.990 | 1.000 | 0.942 | 0.939 | 0.973 | 0.847 | 1.000 | 1.000 | 0.825 | 0.790 |
| 436 | 0.739 | 1.000 | 0.708 | 0.957 | 0.953 | 1.000 | 0.875 | 1.000 | 0.990 | 1.000 | 0.957 | 0.939 | 0.964 | 0.851 | 1.000 | 1.000 | 0.831 | 0.796 |
| 437 | 0.734 | 1.000 | 0.717 | 0.971 | 0.963 | 1.000 | 0.866 | 1.000 | 0.980 | 0.979 | 0.948 | 0.970 | 0.991 | 0.848 | 1.000 | 0.981 | 0.829 | 0.793 |
| 438 | 0.735 | 1.000 | 0.717 | 0.971 | 0.953 | 1.000 | 0.880 | 0.667 | 0.990 | 0.979 | 0.939 | 0.970 | 0.973 | 0.855 | 1.000 | 1.000 | 0.832 | 0.797 |
| 439 | 0.735 | 1.000 | 0.702 | 0.957 | 0.947 | 0.960 | 0.866 | 1.000 | 0.980 | 1.000 | 0.945 | 0.970 | 0.982 | 0.854 | 1.000 | 1.000 | 0.828 | 0.793 |
| 440 | 0.734 | 1.000 | 0.708 | 0.957 | 0.958 | 1.000 | 0.861 | 1.000 | 0.980 | 0.979 | 0.936 | 0.939 | 0.982 | 0.853 | 1.000 | 0.981 | 0.827 | 0.791 |
| 441 | 0.731 | 1.000 | 0.690 | 0.957 | 0.963 | 1.000 | 0.872 | 1.000 | 0.990 | 0.979 | 0.933 | 0.939 | 0.973 | 0.835 | 1.000 | 1.000 | 0.821 | 0.785 |
| 442 | 0.731 | 1.000 | 0.717 | 0.957 | 0.968 | 1.000 | 0.884 | 1.000 | 0.980 | 0.958 | 0.945 | 0.939 | 0.991 | 0.853 | 1.000 | 1.000 | 0.831 | 0.797 |
| 443 | 0.733 | 1.000 | 0.723 | 0.957 | 0.958 | 1.000 | 0.874 | 1.000 | 0.990 | 0.979 | 0.945 | 0.939 | 0.973 | 0.854 | 1.000 | 0.981 | 0.831 | 0.796 |
| 444 | 0.730 | 1.000 | 0.731 | 0.986 | 0.968 | 1.000 | 0.867 | 1.000 | 1.000 | 1.000 | 0.948 | 0.939 | 0.982 | 0.842 | 1.000 | 0.981 | 0.828 | 0.794 |
| 445 | 0.714 | 1.000 | 0.717 | 0.971 | 0.958 | 1.000 | 0.867 | 1.000 | 0.990 | 1.000 | 0.948 | 0.939 | 0.973 | 0.844 | 1.000 | 1.000 | 0.822 | 0.786 |
| 446 | 0.736 | 1.000 | 0.725 | 0.971 | 0.953 | 0.960 | 0.866 | 0.667 | 0.980 | 0.979 | 0.939 | 0.970 | 0.964 | 0.847 | 1.000 | 1.000 | 0.828 | 0.793 |
| 447 | 0.723 | 1.000 | 0.727 | 0.971 | 0.958 | 1.000 | 0.875 | 1.000 | 0.990 | 0.979 | 0.948 | 0.939 | 0.964 | 0.843 | 1.000 | 0.981 | 0.826 | 0.791 |
| 448 | 0.727 | 1.000 | 0.723 | 0.971 | 0.958 | 1.000 | 0.861 | 1.000 | 0.990 | 1.000 | 0.951 | 0.939 | 0.973 | 0.841 | 1.000 | 1.000 | 0.825 | 0.790 |
| 449 | 0.731 | 1.000 | 0.721 | 0.986 | 0.947 | 1.000 | 0.866 | 1.000 | 0.990 | 0.979 | 0.939 | 0.939 | 0.982 | 0.850 | 1.000 | 1.000 | 0.828 | 0.793 |
| 450 | 0.734 | 1.000 | 0.743 | 0.971 | 0.942 | 1.000 | 0.869 | 1.000 | 0.980 | 1.000 | 0.945 | 0.970 | 0.964 | 0.854 | 1.000 | 1.000 | 0.832 | 0.798 |
| 451 | 0.726 | 1.000 | 0.733 | 0.986 | 0.963 | 1.000 | 0.874 | 1.000 | 0.980 | 1.000 | 0.942 | 0.970 | 0.973 | 0.849 | 1.000 | 0.981 | 0.829 | 0.795 |
| 452 | 0.715 | 1.000 | 0.725 | 0.971 | 0.958 | 1.000 | 0.871 | 1.000 | 0.980 | 1.000 | 0.939 | 0.939 | 0.973 | 0.838 | 1.000 | 1.000 | 0.821 | 0.785 |
| 453 | 0.732 | 1.000 | 0.711 | 0.986 | 0.953 | 1.000 | 0.861 | 1.000 | 0.990 | 0.979 | 0.951 | 0.939 | 0.964 | 0.850 | 1.000 | 1.000 | 0.827 | 0.792 |
| 454 | 0.739 | 1.000 | 0.727 | 0.986 | 0.958 | 1.000 | 0.869 | 1.000 | 0.990 | 0.979 | 0.945 | 0.970 | 0.982 | 0.844 | 1.000 | 1.000 | 0.831 | 0.796 |
| 455 | 0.738 | 1.000 | 0.737 | 0.986 | 0.963 | 1.000 | 0.874 | 1.000 | 0.990 | 1.000 | 0.945 | 0.939 | 0.964 | 0.840 | 1.000 | 1.000 | 0.831 | 0.796 |
| 456 | 0.724 | 1.000 | 0.715 | 0.986 | 0.974 | 1.000 | 0.871 | 1.000 | 0.990 | 1.000 | 0.945 | 0.970 | 0.964 | 0.851 | 1.000 | 1.000 | 0.827 | 0.792 |
| 457 | 0.729 | 1.000 | 0.733 | 0.971 | 0.947 | 1.000 | 0.883 | 1.000 | 0.990 | 0.979 | 0.945 | 0.939 | 0.982 | 0.854 | 1.000 | 1.000 | 0.832 | 0.798 |
| 458 | 0.730 | 1.000 | 0.741 | 0.986 | 0.958 | 1.000 | 0.874 | 1.000 | 0.980 | 0.979 | 0.942 | 0.970 | 0.964 | 0.845 | 1.000 | 1.000 | 0.830 | 0.795 |
| 459 | 0.716 | 1.000 | 0.731 | 0.971 | 0.968 | 1.000 | 0.869 | 1.000 | 0.990 | 1.000 | 0.945 | 0.909 | 0.973 | 0.843 | 1.000 | 0.981 | 0.823 | 0.788 |
| 460 | 0.714 | 1.000 | 0.706 | 0.986 | 0.968 | 1.000 | 0.857 | 1.000 | 0.990 | 0.979 | 0.948 | 0.939 | 0.982 | 0.853 | 1.000 | 0.981 | 0.822 | 0.786 |
| 461 | 0.733 | 1.000 | 0.745 | 0.986 | 0.953 | 1.000 | 0.852 | 1.000 | 1.000 | 0.979 | 0.954 | 1.000 | 0.973 | 0.847 | 1.000 | 1.000 | 0.830 | 0.795 |
| 462 | 0.728 | 1.000 | 0.727 | 0.986 | 0.963 | 1.000 | 0.852 | 1.000 | 0.990 | 1.000 | 0.939 | 0.939 | 0.991 | 0.846 | 1.000 | 1.000 | 0.826 | 0.790 |
| 463 | 0.724 | 1.000 | 0.733 | 0.971 | 0.947 | 1.000 | 0.871 | 1.000 | 0.990 | 1.000 | 0.951 | 0.939 | 0.973 | 0.840 | 1.000 | 1.000 | 0.825 | 0.790 |
| 464 | 0.732 | 1.000 | 0.743 | 0.971 | 0.958 | 1.000 | 0.847 | 1.000 | 1.000 | 0.979 | 0.933 | 0.970 | 0.973 | 0.851 | 1.000 | 1.000 | 0.828 | 0.793 |
| 465 | 0.730 | 1.000 | 0.725 | 0.986 | 0.953 | 1.000 | 0.863 | 1.000 | 0.990 | 1.000 | 0.951 | 0.939 | 0.982 | 0.837 | 1.000 | 0.981 | 0.825 | 0.790 |
| 466 | 0.735 | 1.000 | 0.729 | 0.971 | 0.963 | 1.000 | 0.864 | 1.000 | 0.990 | 1.000 | 0.942 | 0.939 | 0.964 | 0.848 | 1.000 | 1.000 | 0.830 | 0.794 |

| | | | | | | | | | | | | | | | | | | |
|---|---|---|---|---|---|---|---|---|---|---|---|---|---|---|---|---|---|---|
| 467 | 0.727 | 1.000 | 0.735 | 0.971 | 0.953 | 1.000 | 0.863 | 1.000 | 0.990 | 0.979 | 0.942 | 0.970 | 0.964 | 0.848 | 1.000 | 0.981 | 0.827 | 0.792 |
| 468 | 0.739 | 1.000 | 0.733 | 0.971 | 0.953 | 1.000 | 0.866 | 1.000 | 0.980 | 0.979 | 0.948 | 0.939 | 0.982 | 0.856 | 1.000 | 0.981 | 0.833 | 0.799 |
| 469 | 0.723 | 1.000 | 0.727 | 0.971 | 0.963 | 1.000 | 0.864 | 1.000 | 0.990 | 0.979 | 0.948 | 0.970 | 0.973 | 0.848 | 1.000 | 1.000 | 0.826 | 0.791 |
| 470 | 0.730 | 1.000 | 0.739 | 0.986 | 0.968 | 1.000 | 0.857 | 1.000 | 0.980 | 1.000 | 0.942 | 1.000 | 0.973 | 0.836 | 1.000 | 1.000 | 0.826 | 0.790 |
| 471 | 0.742 | 1.000 | 0.745 | 0.971 | 0.947 | 0.960 | 0.858 | 1.000 | 0.990 | 0.979 | 0.942 | 0.970 | 0.991 | 0.842 | 1.000 | 1.000 | 0.831 | 0.796 |
| 472 | 0.738 | 1.000 | 0.727 | 0.986 | 0.947 | 1.000 | 0.860 | 1.000 | 0.980 | 0.979 | 0.945 | 0.970 | 0.982 | 0.846 | 1.000 | 1.000 | 0.829 | 0.794 |
| 473 | 0.723 | 1.000 | 0.747 | 1.000 | 0.942 | 1.000 | 0.875 | 1.000 | 0.990 | 1.000 | 0.948 | 0.970 | 0.973 | 0.847 | 1.000 | 1.000 | 0.829 | 0.795 |
| 474 | 0.734 | 1.000 | 0.715 | 0.986 | 0.958 | 1.000 | 0.863 | 1.000 | 0.980 | 0.979 | 0.948 | 0.970 | 0.991 | 0.848 | 1.000 | 0.981 | 0.828 | 0.793 |
| 475 | 0.740 | 1.000 | 0.741 | 0.971 | 0.947 | 1.000 | 0.866 | 1.000 | 0.990 | 0.958 | 0.945 | 0.970 | 0.982 | 0.846 | 1.000 | 1.000 | 0.832 | 0.797 |
| 476 | 0.735 | 1.000 | 0.749 | 1.000 | 0.947 | 0.960 | 0.866 | 1.000 | 0.990 | 0.979 | 0.936 | 0.970 | 0.973 | 0.857 | 1.000 | 1.000 | 0.833 | 0.799 |
| 477 | 0.739 | 1.000 | 0.737 | 0.986 | 0.953 | 1.000 | 0.861 | 1.000 | 0.980 | 0.979 | 0.942 | 1.000 | 1.000 | 0.852 | 1.000 | 1.000 | 0.833 | 0.798 |
| 478 | 0.737 | 1.000 | 0.737 | 0.986 | 0.974 | 1.000 | 0.861 | 1.000 | 0.980 | 1.000 | 0.939 | 1.000 | 0.991 | 0.841 | 1.000 | 1.000 | 0.830 | 0.795 |
| 479 | 0.732 | 1.000 | 0.743 | 0.986 | 0.953 | 1.000 | 0.866 | 1.000 | 0.980 | 0.979 | 0.951 | 0.939 | 0.991 | 0.847 | 1.000 | 1.000 | 0.831 | 0.796 |
| 480 | 0.738 | 1.000 | 0.743 | 0.971 | 0.968 | 1.000 | 0.861 | 0.667 | 0.980 | 1.000 | 0.939 | 0.970 | 0.973 | 0.833 | 1.000 | 1.000 | 0.828 | 0.792 |
| 481 | 0.734 | 1.000 | 0.747 | 0.986 | 0.963 | 1.000 | 0.858 | 1.000 | 0.980 | 1.000 | 0.925 | 0.970 | 0.982 | 0.844 | 1.000 | 1.000 | 0.828 | 0.793 |
| 482 | 0.736 | 1.000 | 0.731 | 1.000 | 0.947 | 1.000 | 0.863 | 1.000 | 0.980 | 1.000 | 0.951 | 0.939 | 0.973 | 0.847 | 1.000 | 1.000 | 0.830 | 0.795 |
| 483 | 0.739 | 1.000 | 0.731 | 0.986 | 0.963 | 1.000 | 0.861 | 1.000 | 0.990 | 1.000 | 0.939 | 0.970 | 0.973 | 0.848 | 1.000 | 1.000 | 0.831 | 0.796 |
| 484 | 0.728 | 1.000 | 0.735 | 0.986 | 0.963 | 1.000 | 0.874 | 1.000 | 0.990 | 0.979 | 0.959 | 0.970 | 0.982 | 0.859 | 1.000 | 1.000 | 0.834 | 0.801 |
| 485 | 0.738 | 1.000 | 0.741 | 1.000 | 0.942 | 1.000 | 0.863 | 1.000 | 0.980 | 0.979 | 0.939 | 0.970 | 0.982 | 0.858 | 1.000 | 1.000 | 0.833 | 0.799 |
| 486 | 0.738 | 1.000 | 0.729 | 1.000 | 0.958 | 1.000 | 0.857 | 0.667 | 0.990 | 0.958 | 0.933 | 0.939 | 0.982 | 0.856 | 1.000 | 0.981 | 0.831 | 0.796 |
| 487 | 0.738 | 1.000 | 0.723 | 1.000 | 0.963 | 1.000 | 0.869 | 1.000 | 1.000 | 1.000 | 0.951 | 0.970 | 0.973 | 0.851 | 1.000 | 0.981 | 0.833 | 0.798 |
| 488 | 0.740 | 1.000 | 0.721 | 1.000 | 0.958 | 1.000 | 0.869 | 1.000 | 0.990 | 1.000 | 0.945 | 0.970 | 0.982 | 0.856 | 1.000 | 0.981 | 0.834 | 0.800 |
| 489 | 0.731 | 1.000 | 0.733 | 0.986 | 0.963 | 1.000 | 0.863 | 1.000 | 0.980 | 0.979 | 0.936 | 0.970 | 0.973 | 0.847 | 1.000 | 1.000 | 0.828 | 0.793 |
| 490 | 0.731 | 1.000 | 0.717 | 0.986 | 0.947 | 1.000 | 0.867 | 0.667 | 0.980 | 1.000 | 0.939 | 0.970 | 0.982 | 0.868 | 1.000 | 1.000 | 0.832 | 0.798 |
| 491 | 0.752 | 1.000 | 0.719 | 1.000 | 0.968 | 1.000 | 0.867 | 1.000 | 0.980 | 1.000 | 0.948 | 0.970 | 0.982 | 0.856 | 1.000 | 0.981 | 0.837 | 0.803 |
| 492 | 0.732 | 1.000 | 0.733 | 1.000 | 0.953 | 1.000 | 0.861 | 1.000 | 0.990 | 0.979 | 0.945 | 0.970 | 0.991 | 0.855 | 1.000 | 1.000 | 0.831 | 0.797 |
| 493 | 0.741 | 1.000 | 0.727 | 0.986 | 0.963 | 1.000 | 0.854 | 1.000 | 0.980 | 1.000 | 0.948 | 0.970 | 1.000 | 0.856 | 1.000 | 0.981 | 0.833 | 0.798 |
| 494 | 0.741 | 1.000 | 0.741 | 1.000 | 0.968 | 1.000 | 0.869 | 1.000 | 0.990 | 1.000 | 0.951 | 0.970 | 0.982 | 0.854 | 1.000 | 0.981 | 0.836 | 0.803 |
| 495 | 0.745 | 1.000 | 0.737 | 1.000 | 0.963 | 1.000 | 0.855 | 1.000 | 0.990 | 1.000 | 0.945 | 0.970 | 0.973 | 0.862 | 1.000 | 0.981 | 0.837 | 0.803 |
| 496 | 0.728 | 1.000 | 0.743 | 1.000 | 0.963 | 1.000 | 0.857 | 1.000 | 0.990 | 1.000 | 0.942 | 1.000 | 0.982 | 0.862 | 1.000 | 0.981 | 0.832 | 0.798 |
| 497 | 0.731 | 1.000 | 0.729 | 1.000 | 0.963 | 1.000 | 0.867 | 1.000 | 0.980 | 0.979 | 0.951 | 0.970 | 1.000 | 0.868 | 1.000 | 1.000 | 0.835 | 0.802 |
| 498 | 0.741 | 1.000 | 0.729 | 1.000 | 0.953 | 1.000 | 0.854 | 1.000 | 0.990 | 0.979 | 0.951 | 0.970 | 0.991 | 0.867 | 1.000 | 1.000 | 0.836 | 0.802 |
| 499 | 0.740 | 1.000 | 0.739 | 0.986 | 0.947 | 1.000 | 0.858 | 1.000 | 0.990 | 0.979 | 0.954 | 0.970 | 0.991 | 0.864 | 1.000 | 1.000 | 0.836 | 0.802 |
| 500 | 0.741 | 1.000 | 0.725 | 1.000 | 0.942 | 1.000 | 0.861 | 1.000 | 0.980 | 0.979 | 0.945 | 0.970 | 0.973 | 0.865 | 1.000 | 0.981 | 0.834 | 0.800 |
| 501 | 0.734 | 1.000 | 0.727 | 0.986 | 0.942 | 1.000 | 0.878 | 1.000 | 0.980 | 0.979 | 0.948 | 0.970 | 0.991 | 0.860 | 1.000 | 0.981 | 0.834 | 0.800 |
| 502 | 0.743 | 1.000 | 0.741 | 1.000 | 0.953 | 1.000 | 0.858 | 1.000 | 0.990 | 0.979 | 0.951 | 0.970 | 0.964 | 0.860 | 1.000 | 0.981 | 0.836 | 0.802 |
| 503 | 0.742 | 1.000 | 0.743 | 1.000 | 0.953 | 1.000 | 0.867 | 1.000 | 0.980 | 0.979 | 0.945 | 0.970 | 0.982 | 0.868 | 1.000 | 1.000 | 0.839 | 0.806 |
| 504 | 0.738 | 1.000 | 0.733 | 0.971 | 0.953 | 1.000 | 0.846 | 1.000 | 0.969 | 1.000 | 0.951 | 0.970 | 0.982 | 0.866 | 1.000 | 0.981 | 0.833 | 0.799 |
| 505 | 0.737 | 1.000 | 0.741 | 0.986 | 0.942 | 1.000 | 0.851 | 1.000 | 0.990 | 0.979 | 0.939 | 0.970 | 0.991 | 0.865 | 1.000 | 1.000 | 0.834 | 0.799 |

| | | | | | | | | | | | | | | | | | | |
|---|---|---|---|---|---|---|---|---|---|---|---|---|---|---|---|---|---|---|
| 506 | 0.749 | 1.000 | 0.731 | 0.986 | 0.953 | 1.000 | 0.860 | 1.000 | 0.990 | 1.000 | 0.945 | 1.000 | 0.991 | 0.865 | 1.000 | 1.000 | 0.839 | 0.806 |
| 507 | 0.737 | 1.000 | 0.751 | 0.971 | 0.947 | 1.000 | 0.875 | 1.000 | 0.980 | 0.979 | 0.957 | 0.970 | 0.973 | 0.866 | 1.000 | 1.000 | 0.839 | 0.806 |
| 508 | 0.745 | 1.000 | 0.743 | 0.986 | 0.953 | 1.000 | 0.857 | 1.000 | 0.969 | 0.958 | 0.945 | 0.970 | 1.000 | 0.861 | 1.000 | 1.000 | 0.836 | 0.803 |
| 509 | 0.740 | 1.000 | 0.741 | 1.000 | 0.937 | 1.000 | 0.860 | 1.000 | 0.980 | 0.979 | 0.954 | 0.970 | 0.982 | 0.863 | 1.000 | 0.981 | 0.836 | 0.802 |
| 510 | 0.740 | 1.000 | 0.745 | 1.000 | 0.947 | 1.000 | 0.858 | 1.000 | 0.980 | 0.979 | 0.945 | 0.970 | 0.973 | 0.865 | 1.000 | 0.981 | 0.836 | 0.802 |
| 511 | 0.741 | 1.000 | 0.739 | 1.000 | 0.937 | 1.000 | 0.872 | 1.000 | 0.969 | 0.958 | 0.939 | 0.970 | 0.982 | 0.861 | 1.000 | 1.000 | 0.836 | 0.802 |
| 512 | 0.736 | 1.000 | 0.755 | 0.986 | 0.947 | 1.000 | 0.867 | 1.000 | 0.990 | 0.979 | 0.945 | 0.970 | 0.991 | 0.863 | 1.000 | 1.000 | 0.837 | 0.804 |
| 513 | 0.734 | 1.000 | 0.725 | 0.971 | 0.953 | 1.000 | 0.851 | 1.000 | 1.000 | 0.958 | 0.942 | 0.939 | 0.973 | 0.873 | 1.000 | 0.981 | 0.833 | 0.798 |
| 514 | 0.742 | 1.000 | 0.739 | 0.986 | 0.942 | 1.000 | 0.871 | 1.000 | 0.980 | 0.979 | 0.933 | 0.970 | 0.982 | 0.871 | 1.000 | 1.000 | 0.838 | 0.805 |
| 515 | 0.744 | 1.000 | 0.725 | 0.986 | 0.947 | 1.000 | 0.869 | 0.667 | 0.990 | 0.958 | 0.939 | 0.970 | 0.991 | 0.864 | 1.000 | 1.000 | 0.836 | 0.802 |
| 516 | 0.746 | 1.000 | 0.747 | 0.986 | 0.947 | 1.000 | 0.861 | 1.000 | 0.980 | 0.979 | 0.951 | 0.970 | 0.964 | 0.870 | 1.000 | 1.000 | 0.840 | 0.807 |
| 517 | 0.740 | 1.000 | 0.739 | 0.986 | 0.963 | 1.000 | 0.867 | 1.000 | 0.990 | 0.979 | 0.948 | 0.939 | 0.982 | 0.876 | 1.000 | 1.000 | 0.840 | 0.808 |
| 518 | 0.733 | 1.000 | 0.739 | 0.986 | 0.953 | 1.000 | 0.866 | 0.667 | 0.980 | 0.958 | 0.942 | 0.970 | 0.973 | 0.874 | 1.000 | 0.981 | 0.836 | 0.802 |
| 519 | 0.744 | 1.000 | 0.729 | 0.986 | 0.942 | 1.000 | 0.880 | 1.000 | 0.980 | 0.958 | 0.954 | 0.970 | 0.964 | 0.870 | 1.000 | 0.981 | 0.839 | 0.806 |
| 520 | 0.741 | 1.000 | 0.739 | 1.000 | 0.942 | 1.000 | 0.869 | 1.000 | 0.980 | 0.958 | 0.951 | 0.970 | 0.973 | 0.865 | 1.000 | 1.000 | 0.837 | 0.804 |
| 521 | 0.735 | 1.000 | 0.731 | 0.986 | 0.937 | 1.000 | 0.871 | 0.667 | 0.990 | 1.000 | 0.945 | 0.970 | 0.982 | 0.862 | 1.000 | 1.000 | 0.834 | 0.800 |
| 522 | 0.738 | 1.000 | 0.715 | 1.000 | 0.947 | 1.000 | 0.874 | 1.000 | 0.980 | 0.979 | 0.948 | 0.970 | 0.973 | 0.868 | 1.000 | 1.000 | 0.836 | 0.802 |
| 523 | 0.741 | 1.000 | 0.733 | 1.000 | 0.953 | 1.000 | 0.877 | 1.000 | 0.980 | 0.979 | 0.954 | 0.970 | 0.991 | 0.861 | 1.000 | 0.981 | 0.838 | 0.805 |
| 524 | 0.746 | 1.000 | 0.727 | 0.986 | 0.947 | 1.000 | 0.867 | 1.000 | 0.990 | 0.979 | 0.951 | 0.970 | 0.982 | 0.868 | 1.000 | 1.000 | 0.839 | 0.806 |
| 525 | 0.741 | 1.000 | 0.731 | 0.986 | 0.968 | 1.000 | 0.869 | 1.000 | 0.980 | 1.000 | 0.942 | 0.970 | 0.982 | 0.866 | 1.000 | 1.000 | 0.838 | 0.804 |
| 526 | 0.744 | 1.000 | 0.725 | 0.986 | 0.963 | 1.000 | 0.878 | 0.667 | 0.980 | 0.979 | 0.948 | 0.970 | 0.982 | 0.869 | 1.000 | 1.000 | 0.840 | 0.807 |
| 527 | 0.747 | 1.000 | 0.731 | 0.986 | 0.947 | 1.000 | 0.883 | 1.000 | 0.969 | 0.979 | 0.951 | 0.970 | 0.955 | 0.864 | 1.000 | 0.981 | 0.839 | 0.806 |
| 528 | 0.743 | 1.000 | 0.727 | 0.986 | 0.953 | 1.000 | 0.878 | 1.000 | 0.990 | 0.979 | 0.945 | 0.970 | 0.982 | 0.865 | 1.000 | 1.000 | 0.838 | 0.805 |
| 529 | 0.753 | 1.000 | 0.731 | 1.000 | 0.942 | 1.000 | 0.874 | 1.000 | 0.990 | 0.979 | 0.948 | 0.970 | 0.982 | 0.872 | 1.000 | 1.000 | 0.843 | 0.810 |
| 530 | 0.746 | 1.000 | 0.723 | 1.000 | 0.953 | 1.000 | 0.881 | 1.000 | 0.990 | 0.979 | 0.933 | 0.970 | 0.955 | 0.868 | 1.000 | 1.000 | 0.839 | 0.805 |
| 531 | 0.735 | 1.000 | 0.757 | 1.000 | 0.942 | 1.000 | 0.864 | 0.667 | 0.990 | 0.958 | 0.954 | 0.970 | 0.991 | 0.865 | 1.000 | 1.000 | 0.837 | 0.804 |
| 532 | 0.738 | 1.000 | 0.731 | 1.000 | 0.947 | 1.000 | 0.867 | 0.667 | 0.990 | 0.979 | 0.951 | 0.970 | 0.982 | 0.870 | 1.000 | 1.000 | 0.837 | 0.804 |
| 533 | 0.732 | 1.000 | 0.717 | 0.986 | 0.953 | 1.000 | 0.875 | 1.000 | 0.980 | 1.000 | 0.939 | 0.970 | 0.991 | 0.863 | 1.000 | 1.000 | 0.833 | 0.799 |
| 534 | 0.748 | 1.000 | 0.727 | 1.000 | 0.947 | 1.000 | 0.877 | 1.000 | 0.980 | 1.000 | 0.957 | 1.000 | 0.991 | 0.871 | 1.000 | 1.000 | 0.842 | 0.810 |
| 535 | 0.736 | 1.000 | 0.741 | 1.000 | 0.968 | 1.000 | 0.871 | 1.000 | 0.980 | 1.000 | 0.954 | 0.970 | 0.973 | 0.869 | 1.000 | 0.981 | 0.839 | 0.806 |
| 536 | 0.736 | 1.000 | 0.747 | 0.986 | 0.947 | 1.000 | 0.872 | 1.000 | 0.990 | 0.979 | 0.936 | 0.970 | 0.973 | 0.865 | 1.000 | 0.981 | 0.836 | 0.803 |
| 537 | 0.732 | 1.000 | 0.737 | 1.000 | 0.953 | 1.000 | 0.860 | 1.000 | 0.990 | 0.979 | 0.948 | 1.000 | 0.973 | 0.864 | 1.000 | 1.000 | 0.834 | 0.800 |
| 538 | 0.732 | 1.000 | 0.743 | 0.986 | 0.942 | 1.000 | 0.863 | 1.000 | 0.990 | 0.979 | 0.945 | 0.970 | 0.973 | 0.872 | 1.000 | 0.981 | 0.836 | 0.802 |
| 539 | 0.744 | 1.000 | 0.747 | 1.000 | 0.958 | 1.000 | 0.869 | 0.667 | 0.990 | 1.000 | 0.948 | 0.970 | 0.973 | 0.858 | 1.000 | 1.000 | 0.838 | 0.805 |
| 540 | 0.738 | 1.000 | 0.741 | 1.000 | 0.953 | 1.000 | 0.872 | 0.667 | 0.990 | 1.000 | 0.951 | 1.000 | 0.982 | 0.863 | 1.000 | 1.000 | 0.838 | 0.805 |
| 541 | 0.740 | 1.000 | 0.727 | 1.000 | 0.958 | 1.000 | 0.874 | 1.000 | 0.990 | 0.979 | 0.945 | 0.970 | 0.982 | 0.870 | 1.000 | 1.000 | 0.838 | 0.806 |
| 542 | 0.735 | 1.000 | 0.735 | 0.986 | 0.968 | 1.000 | 0.878 | 1.000 | 0.980 | 1.000 | 0.945 | 0.970 | 0.982 | 0.865 | 1.000 | 1.000 | 0.837 | 0.805 |
| 543 | 0.730 | 1.000 | 0.755 | 0.986 | 0.968 | 1.000 | 0.875 | 1.000 | 0.990 | 0.979 | 0.957 | 0.970 | 0.991 | 0.861 | 1.000 | 0.981 | 0.837 | 0.804 |
| 544 | 0.742 | 1.000 | 0.741 | 1.000 | 0.958 | 1.000 | 0.877 | 1.000 | 0.980 | 0.979 | 0.945 | 0.970 | 0.982 | 0.864 | 1.000 | 1.000 | 0.839 | 0.806 |

| | | | | | | | | | | | | | | | | | | |
|---|---|---|---|---|---|---|---|---|---|---|---|---|---|---|---|---|---|---|
| 545 | 0.753 | 1.000 | 0.745 | 1.000 | 0.953 | 1.000 | 0.864 | 1.000 | 0.990 | 1.000 | 0.954 | 0.970 | 0.973 | 0.860 | 1.000 | 1.000 | 0.841 | 0.808 |
| 546 | 0.744 | 1.000 | 0.733 | 0.986 | 0.968 | 1.000 | 0.878 | 1.000 | 0.990 | 1.000 | 0.951 | 0.970 | 0.991 | 0.858 | 1.000 | 0.981 | 0.838 | 0.806 |
| 547 | 0.740 | 1.000 | 0.739 | 1.000 | 0.968 | 1.000 | 0.872 | 1.000 | 0.990 | 0.979 | 0.957 | 1.000 | 0.982 | 0.860 | 1.000 | 1.000 | 0.838 | 0.805 |
| 548 | 0.756 | 1.000 | 0.743 | 0.986 | 0.958 | 1.000 | 0.877 | 1.000 | 1.000 | 1.000 | 0.948 | 1.000 | 0.955 | 0.867 | 1.000 | 0.981 | 0.844 | 0.812 |
| 549 | 0.755 | 1.000 | 0.755 | 1.000 | 0.963 | 1.000 | 0.877 | 1.000 | 0.990 | 1.000 | 0.951 | 0.970 | 0.973 | 0.861 | 1.000 | 0.981 | 0.844 | 0.812 |
| 550 | 0.734 | 1.000 | 0.737 | 0.986 | 0.958 | 1.000 | 0.867 | 1.000 | 1.000 | 0.979 | 0.948 | 0.970 | 0.991 | 0.874 | 1.000 | 1.000 | 0.838 | 0.806 |
| 551 | 0.741 | 1.000 | 0.739 | 0.986 | 0.958 | 1.000 | 0.871 | 1.000 | 0.980 | 1.000 | 0.948 | 0.970 | 0.991 | 0.870 | 1.000 | 0.981 | 0.840 | 0.807 |
| 552 | 0.734 | 1.000 | 0.737 | 1.000 | 0.963 | 1.000 | 0.872 | 1.000 | 0.980 | 1.000 | 0.957 | 0.970 | 0.982 | 0.864 | 1.000 | 0.981 | 0.837 | 0.804 |
| 553 | 0.736 | 1.000 | 0.719 | 1.000 | 0.953 | 1.000 | 0.872 | 1.000 | 0.990 | 1.000 | 0.942 | 0.970 | 0.991 | 0.868 | 1.000 | 1.000 | 0.836 | 0.803 |
| 554 | 0.730 | 1.000 | 0.723 | 0.986 | 0.937 | 1.000 | 0.880 | 1.000 | 0.990 | 1.000 | 0.942 | 0.970 | 0.982 | 0.865 | 1.000 | 0.981 | 0.834 | 0.800 |
| 555 | 0.734 | 1.000 | 0.741 | 0.986 | 0.958 | 1.000 | 0.878 | 1.000 | 0.990 | 1.000 | 0.951 | 0.970 | 0.982 | 0.866 | 1.000 | 1.000 | 0.838 | 0.805 |
| 556 | 0.740 | 1.000 | 0.727 | 0.986 | 0.947 | 1.000 | 0.884 | 1.000 | 0.990 | 1.000 | 0.959 | 0.970 | 0.973 | 0.862 | 1.000 | 0.981 | 0.838 | 0.805 |
| 557 | 0.740 | 1.000 | 0.745 | 1.000 | 0.958 | 1.000 | 0.875 | 1.000 | 0.990 | 1.000 | 0.942 | 1.000 | 0.991 | 0.868 | 1.000 | 1.000 | 0.840 | 0.808 |
| 558 | 0.739 | 1.000 | 0.739 | 1.000 | 0.947 | 1.000 | 0.877 | 1.000 | 0.980 | 1.000 | 0.939 | 0.970 | 0.973 | 0.870 | 1.000 | 0.981 | 0.839 | 0.806 |
| 559 | 0.750 | 1.000 | 0.735 | 1.000 | 0.953 | 1.000 | 0.874 | 1.000 | 0.990 | 1.000 | 0.945 | 0.970 | 0.982 | 0.860 | 1.000 | 1.000 | 0.840 | 0.807 |
| 560 | 0.739 | 1.000 | 0.731 | 0.986 | 0.953 | 1.000 | 0.878 | 1.000 | 0.990 | 1.000 | 0.951 | 0.970 | 0.982 | 0.870 | 1.000 | 1.000 | 0.839 | 0.807 |
| 561 | 0.744 | 1.000 | 0.749 | 1.000 | 0.958 | 1.000 | 0.880 | 1.000 | 0.980 | 1.000 | 0.954 | 1.000 | 0.973 | 0.868 | 1.000 | 1.000 | 0.843 | 0.811 |
| 562 | 0.744 | 1.000 | 0.735 | 1.000 | 0.958 | 1.000 | 0.883 | 1.000 | 0.980 | 0.979 | 0.948 | 0.970 | 0.973 | 0.860 | 1.000 | 1.000 | 0.839 | 0.806 |
| 563 | 0.740 | 1.000 | 0.729 | 1.000 | 0.953 | 1.000 | 0.889 | 1.000 | 0.980 | 1.000 | 0.959 | 0.970 | 0.991 | 0.859 | 1.000 | 1.000 | 0.839 | 0.807 |
| 564 | 0.736 | 1.000 | 0.749 | 1.000 | 0.963 | 1.000 | 0.880 | 1.000 | 0.990 | 1.000 | 0.957 | 0.970 | 0.973 | 0.861 | 1.000 | 0.981 | 0.839 | 0.807 |
| 565 | 0.738 | 1.000 | 0.751 | 1.000 | 0.953 | 1.000 | 0.883 | 1.000 | 0.980 | 1.000 | 0.939 | 0.970 | 0.973 | 0.864 | 1.000 | 0.981 | 0.839 | 0.806 |
| 566 | 0.737 | 1.000 | 0.735 | 1.000 | 0.958 | 1.000 | 0.878 | 1.000 | 0.980 | 0.979 | 0.948 | 1.000 | 0.991 | 0.858 | 1.000 | 0.981 | 0.836 | 0.803 |
| 567 | 0.743 | 1.000 | 0.735 | 1.000 | 0.947 | 1.000 | 0.881 | 1.000 | 0.990 | 1.000 | 0.939 | 0.970 | 0.973 | 0.866 | 1.000 | 0.981 | 0.839 | 0.806 |
| 568 | 0.740 | 1.000 | 0.741 | 1.000 | 0.942 | 1.000 | 0.875 | 1.000 | 0.980 | 0.979 | 0.948 | 1.000 | 0.982 | 0.862 | 1.000 | 1.000 | 0.838 | 0.805 |
| 569 | 0.733 | 1.000 | 0.743 | 0.986 | 0.953 | 1.000 | 0.897 | 1.000 | 1.000 | 0.979 | 0.939 | 0.970 | 0.964 | 0.861 | 1.000 | 1.000 | 0.838 | 0.805 |
| 570 | 0.741 | 1.000 | 0.745 | 1.000 | 0.958 | 1.000 | 0.889 | 1.000 | 0.990 | 1.000 | 0.951 | 0.970 | 0.982 | 0.865 | 1.000 | 1.000 | 0.842 | 0.810 |
| 571 | 0.736 | 1.000 | 0.771 | 1.000 | 0.963 | 1.000 | 0.881 | 1.000 | 0.980 | 1.000 | 0.954 | 1.000 | 0.991 | 0.871 | 1.000 | 0.963 | 0.844 | 0.813 |
| 572 | 0.741 | 1.000 | 0.737 | 1.000 | 0.942 | 1.000 | 0.871 | 1.000 | 0.990 | 1.000 | 0.948 | 0.970 | 0.982 | 0.865 | 1.000 | 1.000 | 0.838 | 0.805 |
| 573 | 0.736 | 1.000 | 0.727 | 1.000 | 0.953 | 1.000 | 0.874 | 1.000 | 0.990 | 1.000 | 0.945 | 0.970 | 0.982 | 0.865 | 1.000 | 0.981 | 0.836 | 0.802 |
| 574 | 0.738 | 1.000 | 0.737 | 0.986 | 0.932 | 1.000 | 0.880 | 1.000 | 0.990 | 0.979 | 0.951 | 0.970 | 0.964 | 0.865 | 1.000 | 1.000 | 0.837 | 0.804 |
| 575 | 0.753 | 1.000 | 0.735 | 1.000 | 0.953 | 1.000 | 0.867 | 1.000 | 1.000 | 0.979 | 0.948 | 0.970 | 0.973 | 0.868 | 1.000 | 1.000 | 0.842 | 0.810 |
| 576 | 0.743 | 1.000 | 0.741 | 0.986 | 0.958 | 1.000 | 0.878 | 1.000 | 0.990 | 1.000 | 0.948 | 1.000 | 0.982 | 0.865 | 1.000 | 0.981 | 0.840 | 0.808 |
| 577 | 0.725 | 1.000 | 0.745 | 0.986 | 0.953 | 1.000 | 0.867 | 1.000 | 0.990 | 1.000 | 0.954 | 0.970 | 0.973 | 0.870 | 1.000 | 0.981 | 0.835 | 0.802 |
| 578 | 0.742 | 1.000 | 0.745 | 1.000 | 0.947 | 1.000 | 0.881 | 1.000 | 1.000 | 1.000 | 0.945 | 0.970 | 0.991 | 0.862 | 1.000 | 1.000 | 0.840 | 0.808 |
| 579 | 0.744 | 1.000 | 0.749 | 1.000 | 0.958 | 1.000 | 0.884 | 1.000 | 0.980 | 0.979 | 0.951 | 0.970 | 0.973 | 0.872 | 1.000 | 1.000 | 0.844 | 0.812 |
| 580 | 0.729 | 1.000 | 0.745 | 1.000 | 0.947 | 1.000 | 0.881 | 1.000 | 0.980 | 0.979 | 0.957 | 0.939 | 0.991 | 0.861 | 1.000 | 1.000 | 0.836 | 0.803 |
| 581 | 0.746 | 1.000 | 0.735 | 1.000 | 0.953 | 1.000 | 0.878 | 1.000 | 1.000 | 1.000 | 0.954 | 0.970 | 0.991 | 0.858 | 1.000 | 1.000 | 0.839 | 0.807 |
| 582 | 0.734 | 1.000 | 0.759 | 1.000 | 0.947 | 1.000 | 0.878 | 1.000 | 1.000 | 1.000 | 0.942 | 1.000 | 0.973 | 0.862 | 1.000 | 1.000 | 0.838 | 0.806 |
| 583 | 0.741 | 1.000 | 0.741 | 1.000 | 0.963 | 1.000 | 0.880 | 1.000 | 0.990 | 1.000 | 0.951 | 0.970 | 0.982 | 0.860 | 1.000 | 1.000 | 0.839 | 0.807 |

| | | | | | | | | | | | | | | | | | | |
|---|---|---|---|---|---|---|---|---|---|---|---|---|---|---|---|---|---|---|
| 584 | 0.736 | 1.000 | 0.725 | 1.000 | 0.937 | 1.000 | 0.883 | 1.000 | 0.990 | 0.979 | 0.951 | 0.970 | 0.982 | 0.861 | 1.000 | 1.000 | 0.836 | 0.803 |
| 585 | 0.742 | 1.000 | 0.755 | 1.000 | 0.958 | 1.000 | 0.886 | 1.000 | 0.980 | 1.000 | 0.945 | 0.970 | 0.982 | 0.860 | 1.000 | 1.000 | 0.841 | 0.809 |
| 586 | 0.747 | 1.000 | 0.729 | 0.986 | 0.953 | 1.000 | 0.891 | 1.000 | 0.980 | 1.000 | 0.942 | 0.970 | 0.973 | 0.868 | 1.000 | 1.000 | 0.842 | 0.809 |
| 587 | 0.738 | 1.000 | 0.741 | 1.000 | 0.958 | 1.000 | 0.875 | 1.000 | 1.000 | 1.000 | 0.951 | 1.000 | 1.000 | 0.859 | 1.000 | 1.000 | 0.838 | 0.805 |
| 588 | 0.730 | 1.000 | 0.741 | 1.000 | 0.937 | 1.000 | 0.884 | 1.000 | 0.990 | 1.000 | 0.948 | 0.970 | 0.991 | 0.865 | 1.000 | 1.000 | 0.837 | 0.804 |
| 589 | 0.743 | 1.000 | 0.749 | 1.000 | 0.958 | 1.000 | 0.881 | 1.000 | 0.980 | 1.000 | 0.957 | 0.970 | 0.973 | 0.865 | 1.000 | 0.981 | 0.842 | 0.810 |
| 590 | 0.748 | 1.000 | 0.753 | 1.000 | 0.963 | 1.000 | 0.880 | 1.000 | 0.990 | 1.000 | 0.954 | 0.970 | 0.982 | 0.864 | 1.000 | 1.000 | 0.844 | 0.812 |
| 591 | 0.732 | 1.000 | 0.739 | 1.000 | 0.963 | 1.000 | 0.886 | 1.000 | 0.990 | 1.000 | 0.951 | 0.970 | 0.991 | 0.855 | 1.000 | 0.981 | 0.836 | 0.803 |
| 592 | 0.742 | 1.000 | 0.753 | 1.000 | 0.958 | 1.000 | 0.875 | 1.000 | 0.980 | 1.000 | 0.951 | 0.970 | 0.982 | 0.859 | 1.000 | 1.000 | 0.839 | 0.807 |
| 593 | 0.738 | 1.000 | 0.753 | 1.000 | 0.947 | 1.000 | 0.884 | 1.000 | 0.990 | 1.000 | 0.954 | 0.970 | 0.973 | 0.860 | 1.000 | 1.000 | 0.840 | 0.808 |
| 594 | 0.736 | 1.000 | 0.743 | 1.000 | 0.942 | 1.000 | 0.878 | 1.000 | 1.000 | 1.000 | 0.945 | 1.000 | 0.973 | 0.861 | 1.000 | 1.000 | 0.837 | 0.804 |
| 595 | 0.732 | 1.000 | 0.741 | 1.000 | 0.947 | 1.000 | 0.881 | 1.000 | 0.969 | 0.979 | 0.954 | 1.000 | 0.973 | 0.857 | 1.000 | 1.000 | 0.835 | 0.802 |
| 596 | 0.746 | 1.000 | 0.747 | 1.000 | 0.958 | 1.000 | 0.886 | 1.000 | 0.990 | 1.000 | 0.945 | 0.970 | 0.973 | 0.858 | 1.000 | 0.981 | 0.840 | 0.808 |
| 597 | 0.738 | 1.000 | 0.745 | 1.000 | 0.947 | 1.000 | 0.877 | 1.000 | 0.990 | 0.979 | 0.942 | 1.000 | 0.991 | 0.866 | 1.000 | 1.000 | 0.839 | 0.806 |
| 598 | 0.736 | 1.000 | 0.741 | 1.000 | 0.958 | 1.000 | 0.871 | 1.000 | 1.000 | 1.000 | 0.951 | 0.970 | 0.991 | 0.865 | 1.000 | 1.000 | 0.838 | 0.805 |
| 599 | 0.734 | 1.000 | 0.741 | 1.000 | 0.947 | 1.000 | 0.886 | 1.000 | 0.990 | 1.000 | 0.951 | 0.970 | 0.964 | 0.862 | 1.000 | 1.000 | 0.837 | 0.805 |
| 600 | 0.742 | 1.000 | 0.731 | 1.000 | 0.953 | 1.000 | 0.883 | 1.000 | 0.990 | 1.000 | 0.959 | 0.970 | 0.973 | 0.855 | 1.000 | 1.000 | 0.838 | 0.805 |
| 601 | 0.742 | 1.000 | 0.745 | 0.986 | 0.958 | 1.000 | 0.881 | 1.000 | 0.990 | 1.000 | 0.951 | 0.970 | 0.982 | 0.869 | 1.000 | 1.000 | 0.842 | 0.810 |
| 602 | 0.732 | 1.000 | 0.733 | 1.000 | 0.963 | 1.000 | 0.889 | 1.000 | 0.990 | 1.000 | 0.942 | 0.970 | 0.973 | 0.864 | 1.000 | 1.000 | 0.837 | 0.804 |
| 603 | 0.744 | 1.000 | 0.761 | 1.000 | 0.947 | 1.000 | 0.881 | 1.000 | 0.990 | 0.979 | 0.951 | 0.970 | 0.973 | 0.865 | 1.000 | 1.000 | 0.842 | 0.811 |
| 604 | 0.738 | 1.000 | 0.735 | 1.000 | 0.958 | 1.000 | 0.869 | 1.000 | 0.990 | 1.000 | 0.945 | 0.970 | 0.973 | 0.860 | 1.000 | 1.000 | 0.835 | 0.802 |
| 605 | 0.738 | 1.000 | 0.733 | 1.000 | 0.953 | 1.000 | 0.881 | 1.000 | 1.000 | 0.979 | 0.945 | 0.970 | 0.973 | 0.863 | 1.000 | 1.000 | 0.837 | 0.805 |
| 606 | 0.740 | 1.000 | 0.737 | 1.000 | 0.953 | 1.000 | 0.875 | 1.000 | 0.990 | 0.979 | 0.948 | 0.970 | 0.973 | 0.862 | 1.000 | 1.000 | 0.837 | 0.804 |
| 607 | 0.732 | 1.000 | 0.745 | 1.000 | 0.947 | 1.000 | 0.878 | 1.000 | 0.980 | 1.000 | 0.951 | 0.970 | 0.982 | 0.865 | 1.000 | 1.000 | 0.837 | 0.804 |
| 608 | 0.736 | 1.000 | 0.745 | 1.000 | 0.953 | 1.000 | 0.880 | 1.000 | 0.990 | 1.000 | 0.942 | 0.970 | 0.964 | 0.860 | 1.000 | 1.000 | 0.837 | 0.804 |
| 609 | 0.738 | 1.000 | 0.745 | 1.000 | 0.947 | 1.000 | 0.877 | 1.000 | 0.980 | 0.979 | 0.945 | 0.970 | 0.964 | 0.861 | 1.000 | 0.981 | 0.837 | 0.803 |
| 610 | 0.744 | 1.000 | 0.749 | 1.000 | 0.958 | 1.000 | 0.869 | 1.000 | 0.969 | 1.000 | 0.957 | 0.970 | 0.973 | 0.860 | 1.000 | 0.981 | 0.839 | 0.806 |
| 611 | 0.736 | 1.000 | 0.739 | 1.000 | 0.937 | 1.000 | 0.878 | 1.000 | 1.000 | 0.979 | 0.945 | 0.970 | 0.938 | 0.872 | 1.000 | 1.000 | 0.838 | 0.805 |
| 612 | 0.737 | 1.000 | 0.747 | 1.000 | 0.963 | 1.000 | 0.874 | 1.000 | 1.000 | 1.000 | 0.948 | 0.939 | 0.973 | 0.868 | 1.000 | 1.000 | 0.839 | 0.807 |
| 613 | 0.738 | 1.000 | 0.743 | 1.000 | 0.953 | 1.000 | 0.872 | 1.000 | 1.000 | 1.000 | 0.945 | 0.970 | 0.964 | 0.865 | 1.000 | 1.000 | 0.838 | 0.805 |
| 614 | 0.724 | 1.000 | 0.739 | 1.000 | 0.953 | 1.000 | 0.877 | 1.000 | 1.000 | 0.979 | 0.942 | 0.970 | 0.973 | 0.864 | 1.000 | 1.000 | 0.833 | 0.800 |
| 615 | 0.736 | 1.000 | 0.731 | 1.000 | 0.953 | 1.000 | 0.866 | 1.000 | 1.000 | 0.979 | 0.945 | 0.970 | 0.964 | 0.861 | 1.000 | 1.000 | 0.834 | 0.800 |
| 616 | 0.737 | 1.000 | 0.749 | 1.000 | 0.958 | 1.000 | 0.880 | 1.000 | 1.000 | 0.979 | 0.942 | 0.970 | 0.964 | 0.859 | 1.000 | 1.000 | 0.837 | 0.805 |
| 617 | 0.745 | 1.000 | 0.745 | 0.986 | 0.958 | 1.000 | 0.877 | 1.000 | 0.990 | 0.979 | 0.942 | 0.970 | 0.991 | 0.865 | 1.000 | 1.000 | 0.841 | 0.808 |
| 618 | 0.736 | 1.000 | 0.723 | 1.000 | 0.947 | 1.000 | 0.877 | 1.000 | 0.990 | 1.000 | 0.951 | 0.970 | 0.964 | 0.854 | 1.000 | 1.000 | 0.833 | 0.799 |
| 619 | 0.733 | 1.000 | 0.733 | 1.000 | 0.947 | 1.000 | 0.880 | 1.000 | 1.000 | 1.000 | 0.951 | 0.970 | 0.991 | 0.854 | 1.000 | 1.000 | 0.834 | 0.801 |
| 620 | 0.740 | 1.000 | 0.739 | 1.000 | 0.937 | 1.000 | 0.883 | 1.000 | 1.000 | 1.000 | 0.945 | 0.939 | 0.982 | 0.871 | 1.000 | 1.000 | 0.841 | 0.808 |
| 621 | 0.733 | 1.000 | 0.745 | 1.000 | 0.958 | 1.000 | 0.883 | 1.000 | 0.990 | 1.000 | 0.945 | 0.970 | 0.964 | 0.861 | 1.000 | 1.000 | 0.837 | 0.804 |
| 622 | 0.730 | 1.000 | 0.735 | 1.000 | 0.937 | 1.000 | 0.886 | 1.000 | 0.980 | 0.979 | 0.942 | 1.000 | 0.991 | 0.865 | 1.000 | 1.000 | 0.836 | 0.803 |

| | | | | | | | | | | | | | | | | | | |
|---|---|---|---|---|---|---|---|---|---|---|---|---|---|---|---|---|---|---|
| 623 | 0.739 | 1.000 | 0.739 | 1.000 | 0.937 | 1.000 | 0.881 | 1.000 | 0.990 | 1.000 | 0.954 | 0.970 | 0.973 | 0.862 | 1.000 | 1.000 | 0.838 | 0.806 |
| 624 | 0.736 | 1.000 | 0.729 | 1.000 | 0.942 | 1.000 | 0.874 | 1.000 | 0.990 | 1.000 | 0.957 | 0.970 | 0.991 | 0.862 | 1.000 | 1.000 | 0.836 | 0.803 |
| 625 | 0.742 | 1.000 | 0.739 | 1.000 | 0.942 | 1.000 | 0.889 | 1.000 | 0.980 | 0.979 | 0.945 | 0.970 | 0.991 | 0.863 | 1.000 | 0.981 | 0.840 | 0.807 |
| 626 | 0.724 | 1.000 | 0.743 | 1.000 | 0.942 | 1.000 | 0.875 | 1.000 | 0.980 | 1.000 | 0.948 | 0.970 | 0.964 | 0.857 | 1.000 | 1.000 | 0.831 | 0.798 |
| 627 | 0.733 | 1.000 | 0.725 | 1.000 | 0.958 | 1.000 | 0.871 | 1.000 | 0.990 | 0.979 | 0.936 | 0.970 | 0.964 | 0.861 | 1.000 | 1.000 | 0.833 | 0.798 |
| 628 | 0.738 | 1.000 | 0.725 | 1.000 | 0.942 | 1.000 | 0.886 | 1.000 | 0.990 | 0.979 | 0.948 | 0.970 | 0.955 | 0.871 | 1.000 | 1.000 | 0.839 | 0.806 |
| 629 | 0.733 | 1.000 | 0.751 | 1.000 | 0.958 | 1.000 | 0.874 | 1.000 | 0.990 | 0.979 | 0.942 | 0.970 | 0.973 | 0.865 | 1.000 | 1.000 | 0.837 | 0.804 |
| 630 | 0.740 | 1.000 | 0.725 | 1.000 | 0.947 | 1.000 | 0.880 | 1.000 | 1.000 | 1.000 | 0.942 | 0.970 | 0.982 | 0.862 | 1.000 | 1.000 | 0.837 | 0.804 |
| 631 | 0.740 | 1.000 | 0.737 | 1.000 | 0.947 | 1.000 | 0.869 | 1.000 | 0.990 | 1.000 | 0.939 | 1.000 | 0.991 | 0.857 | 1.000 | 1.000 | 0.835 | 0.802 |
| 632 | 0.736 | 1.000 | 0.729 | 1.000 | 0.942 | 1.000 | 0.875 | 1.000 | 0.990 | 1.000 | 0.936 | 0.970 | 0.955 | 0.861 | 1.000 | 1.000 | 0.834 | 0.800 |
| 633 | 0.739 | 1.000 | 0.741 | 0.986 | 0.942 | 1.000 | 0.874 | 1.000 | 1.000 | 1.000 | 0.942 | 0.970 | 0.973 | 0.854 | 1.000 | 1.000 | 0.835 | 0.801 |
| 634 | 0.728 | 1.000 | 0.727 | 1.000 | 0.947 | 1.000 | 0.883 | 1.000 | 0.990 | 1.000 | 0.942 | 1.000 | 0.964 | 0.861 | 1.000 | 1.000 | 0.833 | 0.800 |
| 635 | 0.732 | 1.000 | 0.733 | 0.986 | 0.953 | 1.000 | 0.881 | 1.000 | 0.990 | 1.000 | 0.936 | 0.970 | 0.973 | 0.864 | 1.000 | 1.000 | 0.835 | 0.802 |
| 636 | 0.735 | 1.000 | 0.733 | 0.986 | 0.947 | 1.000 | 0.874 | 1.000 | 0.990 | 1.000 | 0.948 | 0.970 | 0.964 | 0.872 | 1.000 | 1.000 | 0.838 | 0.805 |
| 637 | 0.724 | 1.000 | 0.741 | 0.986 | 0.942 | 1.000 | 0.867 | 1.000 | 0.990 | 1.000 | 0.954 | 0.970 | 0.973 | 0.867 | 1.000 | 1.000 | 0.833 | 0.799 |
| 638 | 0.730 | 1.000 | 0.743 | 0.986 | 0.953 | 1.000 | 0.891 | 1.000 | 0.990 | 1.000 | 0.945 | 0.970 | 0.955 | 0.854 | 1.000 | 1.000 | 0.834 | 0.801 |
| 639 | 0.734 | 1.000 | 0.721 | 1.000 | 0.942 | 1.000 | 0.877 | 1.000 | 0.990 | 1.000 | 0.939 | 0.970 | 0.973 | 0.863 | 1.000 | 1.000 | 0.834 | 0.800 |
| 640 | 0.738 | 1.000 | 0.729 | 1.000 | 0.958 | 1.000 | 0.888 | 1.000 | 0.980 | 1.000 | 0.933 | 0.970 | 0.973 | 0.865 | 1.000 | 1.000 | 0.838 | 0.805 |
| 641 | 0.736 | 1.000 | 0.733 | 0.986 | 0.942 | 1.000 | 0.874 | 1.000 | 0.990 | 1.000 | 0.942 | 0.970 | 0.964 | 0.868 | 1.000 | 0.981 | 0.836 | 0.802 |
| 642 | 0.738 | 1.000 | 0.723 | 0.971 | 0.953 | 1.000 | 0.872 | 1.000 | 0.980 | 1.000 | 0.957 | 0.970 | 0.955 | 0.861 | 1.000 | 1.000 | 0.834 | 0.801 |
| 643 | 0.736 | 1.000 | 0.735 | 0.986 | 0.926 | 1.000 | 0.880 | 1.000 | 0.980 | 1.000 | 0.942 | 0.970 | 0.955 | 0.862 | 1.000 | 1.000 | 0.835 | 0.801 |
| 644 | 0.728 | 1.000 | 0.731 | 0.986 | 0.932 | 1.000 | 0.875 | 1.000 | 0.980 | 1.000 | 0.948 | 0.970 | 0.982 | 0.854 | 1.000 | 1.000 | 0.831 | 0.797 |
| 645 | 0.735 | 1.000 | 0.747 | 0.986 | 0.926 | 1.000 | 0.880 | 1.000 | 0.990 | 1.000 | 0.948 | 0.970 | 0.973 | 0.866 | 1.000 | 1.000 | 0.838 | 0.804 |
| 646 | 0.724 | 1.000 | 0.739 | 0.986 | 0.947 | 1.000 | 0.867 | 1.000 | 0.980 | 0.979 | 0.942 | 0.970 | 0.973 | 0.862 | 1.000 | 1.000 | 0.831 | 0.797 |
| 647 | 0.727 | 1.000 | 0.735 | 0.986 | 0.926 | 1.000 | 0.858 | 1.000 | 0.980 | 1.000 | 0.936 | 0.970 | 0.973 | 0.861 | 1.000 | 1.000 | 0.829 | 0.794 |
| 648 | 0.726 | 1.000 | 0.735 | 1.000 | 0.947 | 1.000 | 0.878 | 1.000 | 0.980 | 1.000 | 0.948 | 0.970 | 0.982 | 0.862 | 1.000 | 1.000 | 0.833 | 0.800 |
| 649 | 0.733 | 1.000 | 0.737 | 0.986 | 0.942 | 1.000 | 0.875 | 1.000 | 0.980 | 1.000 | 0.939 | 0.970 | 0.964 | 0.865 | 1.000 | 0.981 | 0.834 | 0.801 |
| 650 | 0.723 | 1.000 | 0.753 | 1.000 | 0.942 | 1.000 | 0.883 | 1.000 | 0.990 | 1.000 | 0.945 | 0.970 | 0.973 | 0.863 | 1.000 | 1.000 | 0.835 | 0.802 |
| 651 | 0.734 | 1.000 | 0.729 | 0.971 | 0.942 | 1.000 | 0.886 | 1.000 | 0.990 | 1.000 | 0.945 | 0.970 | 0.973 | 0.861 | 1.000 | 0.981 | 0.835 | 0.802 |
| 652 | 0.733 | 1.000 | 0.725 | 1.000 | 0.953 | 1.000 | 0.872 | 1.000 | 0.990 | 1.000 | 0.951 | 0.970 | 0.973 | 0.859 | 1.000 | 0.981 | 0.833 | 0.800 |
| 653 | 0.736 | 1.000 | 0.737 | 0.986 | 0.947 | 1.000 | 0.875 | 1.000 | 0.980 | 0.979 | 0.939 | 0.939 | 0.982 | 0.859 | 1.000 | 1.000 | 0.834 | 0.801 |
| 654 | 0.742 | 1.000 | 0.735 | 0.986 | 0.947 | 1.000 | 0.875 | 1.000 | 0.980 | 1.000 | 0.933 | 1.000 | 0.973 | 0.859 | 1.000 | 1.000 | 0.836 | 0.803 |
| 655 | 0.725 | 1.000 | 0.747 | 0.986 | 0.947 | 1.000 | 0.866 | 1.000 | 0.990 | 1.000 | 0.945 | 0.970 | 0.964 | 0.860 | 1.000 | 1.000 | 0.832 | 0.798 |
| 656 | 0.736 | 1.000 | 0.739 | 1.000 | 0.942 | 1.000 | 0.874 | 1.000 | 0.980 | 1.000 | 0.951 | 0.970 | 0.973 | 0.865 | 1.000 | 1.000 | 0.837 | 0.804 |
| 657 | 0.733 | 1.000 | 0.759 | 0.971 | 0.937 | 1.000 | 0.875 | 1.000 | 0.990 | 1.000 | 0.939 | 0.970 | 0.964 | 0.859 | 1.000 | 1.000 | 0.835 | 0.802 |
| 658 | 0.732 | 1.000 | 0.743 | 0.971 | 0.932 | 1.000 | 0.866 | 1.000 | 0.990 | 1.000 | 0.939 | 0.970 | 0.973 | 0.857 | 1.000 | 1.000 | 0.832 | 0.797 |
| 659 | 0.732 | 1.000 | 0.723 | 0.986 | 0.947 | 1.000 | 0.877 | 1.000 | 0.990 | 1.000 | 0.945 | 1.000 | 0.964 | 0.860 | 1.000 | 1.000 | 0.833 | 0.799 |
| 660 | 0.730 | 1.000 | 0.729 | 1.000 | 0.947 | 1.000 | 0.875 | 1.000 | 0.990 | 1.000 | 0.933 | 1.000 | 0.973 | 0.859 | 1.000 | 1.000 | 0.832 | 0.798 |
| 661 | 0.732 | 1.000 | 0.725 | 1.000 | 0.953 | 1.000 | 0.880 | 1.000 | 0.980 | 1.000 | 0.951 | 0.970 | 0.973 | 0.864 | 1.000 | 1.000 | 0.835 | 0.802 |

| | | | | | | | | | | | | | | | | | | |
|---|---|---|---|---|---|---|---|---|---|---|---|---|---|---|---|---|---|---|
| 662 | 0.734 | 1.000 | 0.747 | 1.000 | 0.947 | 1.000 | 0.889 | 1.000 | 0.990 | 1.000 | 0.954 | 0.970 | 0.964 | 0.858 | 1.000 | 1.000 | 0.838 | 0.805 |
| 663 | 0.728 | 1.000 | 0.737 | 1.000 | 0.947 | 1.000 | 0.869 | 1.000 | 0.990 | 1.000 | 0.942 | 0.970 | 0.955 | 0.865 | 1.000 | 1.000 | 0.833 | 0.800 |
| 664 | 0.733 | 1.000 | 0.725 | 1.000 | 0.942 | 1.000 | 0.884 | 1.000 | 0.980 | 1.000 | 0.936 | 0.970 | 0.973 | 0.857 | 1.000 | 1.000 | 0.833 | 0.799 |
| 665 | 0.727 | 1.000 | 0.731 | 1.000 | 0.947 | 1.000 | 0.880 | 1.000 | 0.990 | 1.000 | 0.945 | 0.970 | 0.964 | 0.865 | 1.000 | 1.000 | 0.834 | 0.800 |
| 666 | 0.742 | 1.000 | 0.727 | 1.000 | 0.937 | 1.000 | 0.878 | 1.000 | 0.980 | 1.000 | 0.957 | 0.970 | 0.973 | 0.859 | 1.000 | 1.000 | 0.837 | 0.803 |
| 667 | 0.740 | 1.000 | 0.739 | 0.986 | 0.926 | 1.000 | 0.875 | 1.000 | 0.990 | 1.000 | 0.951 | 1.000 | 0.973 | 0.858 | 1.000 | 1.000 | 0.836 | 0.803 |
| 668 | 0.716 | 1.000 | 0.743 | 1.000 | 0.932 | 1.000 | 0.888 | 1.000 | 0.990 | 1.000 | 0.942 | 1.000 | 0.964 | 0.860 | 1.000 | 1.000 | 0.831 | 0.797 |
| 669 | 0.731 | 1.000 | 0.749 | 0.986 | 0.947 | 1.000 | 0.884 | 1.000 | 0.990 | 1.000 | 0.939 | 0.970 | 0.982 | 0.867 | 1.000 | 1.000 | 0.838 | 0.805 |
| 670 | 0.734 | 1.000 | 0.733 | 0.986 | 0.932 | 1.000 | 0.871 | 1.000 | 0.980 | 1.000 | 0.945 | 0.970 | 0.982 | 0.854 | 1.000 | 1.000 | 0.832 | 0.797 |
| 671 | 0.730 | 1.000 | 0.745 | 0.986 | 0.947 | 1.000 | 0.872 | 1.000 | 0.980 | 1.000 | 0.948 | 0.970 | 0.982 | 0.861 | 1.000 | 1.000 | 0.835 | 0.801 |
| 672 | 0.718 | 1.000 | 0.723 | 1.000 | 0.947 | 1.000 | 0.874 | 1.000 | 0.990 | 1.000 | 0.957 | 0.970 | 0.964 | 0.864 | 1.000 | 1.000 | 0.830 | 0.796 |
| 673 | 0.729 | 1.000 | 0.723 | 0.986 | 0.942 | 1.000 | 0.878 | 1.000 | 0.990 | 1.000 | 0.957 | 0.970 | 0.991 | 0.859 | 1.000 | 1.000 | 0.833 | 0.800 |
| 674 | 0.730 | 1.000 | 0.741 | 0.986 | 0.953 | 1.000 | 0.888 | 1.000 | 0.990 | 1.000 | 0.942 | 0.970 | 0.973 | 0.861 | 1.000 | 0.981 | 0.836 | 0.803 |
| 675 | 0.742 | 1.000 | 0.733 | 0.986 | 0.937 | 1.000 | 0.888 | 1.000 | 0.990 | 1.000 | 0.942 | 0.970 | 0.964 | 0.857 | 1.000 | 1.000 | 0.837 | 0.804 |
| 676 | 0.741 | 1.000 | 0.733 | 0.986 | 0.921 | 1.000 | 0.886 | 1.000 | 0.969 | 1.000 | 0.942 | 0.970 | 0.964 | 0.861 | 1.000 | 1.000 | 0.836 | 0.803 |
| 677 | 0.728 | 1.000 | 0.733 | 0.986 | 0.942 | 1.000 | 0.884 | 1.000 | 0.980 | 1.000 | 0.951 | 0.970 | 0.973 | 0.860 | 1.000 | 1.000 | 0.834 | 0.801 |
| 678 | 0.734 | 1.000 | 0.753 | 1.000 | 0.947 | 1.000 | 0.864 | 1.000 | 0.969 | 1.000 | 0.951 | 0.970 | 0.964 | 0.862 | 1.000 | 1.000 | 0.835 | 0.802 |
| 679 | 0.725 | 1.000 | 0.727 | 0.986 | 0.942 | 1.000 | 0.877 | 1.000 | 0.990 | 1.000 | 0.939 | 1.000 | 0.964 | 0.859 | 1.000 | 1.000 | 0.830 | 0.796 |
| 680 | 0.730 | 1.000 | 0.721 | 0.986 | 0.947 | 1.000 | 0.881 | 1.000 | 0.990 | 1.000 | 0.945 | 0.970 | 0.964 | 0.867 | 1.000 | 1.000 | 0.834 | 0.801 |
| 681 | 0.730 | 1.000 | 0.735 | 0.986 | 0.947 | 1.000 | 0.880 | 1.000 | 0.980 | 1.000 | 0.936 | 0.970 | 0.973 | 0.858 | 1.000 | 1.000 | 0.833 | 0.799 |
| 682 | 0.734 | 1.000 | 0.721 | 0.971 | 0.942 | 1.000 | 0.889 | 1.000 | 0.990 | 1.000 | 0.936 | 0.970 | 0.982 | 0.868 | 1.000 | 1.000 | 0.836 | 0.803 |
| 683 | 0.746 | 1.000 | 0.739 | 0.971 | 0.947 | 1.000 | 0.871 | 1.000 | 0.990 | 1.000 | 0.942 | 0.970 | 0.973 | 0.858 | 1.000 | 1.000 | 0.837 | 0.803 |
| 684 | 0.730 | 1.000 | 0.729 | 0.986 | 0.932 | 1.000 | 0.883 | 1.000 | 0.990 | 1.000 | 0.945 | 1.000 | 0.982 | 0.860 | 1.000 | 1.000 | 0.834 | 0.800 |
| 685 | 0.736 | 1.000 | 0.729 | 0.986 | 0.942 | 1.000 | 0.880 | 1.000 | 0.990 | 1.000 | 0.951 | 0.970 | 0.973 | 0.854 | 1.000 | 1.000 | 0.834 | 0.801 |
| 686 | 0.740 | 1.000 | 0.723 | 0.986 | 0.937 | 1.000 | 0.886 | 1.000 | 0.990 | 1.000 | 0.945 | 0.970 | 0.964 | 0.858 | 1.000 | 1.000 | 0.835 | 0.802 |
| 687 | 0.738 | 1.000 | 0.729 | 0.986 | 0.947 | 1.000 | 0.872 | 1.000 | 0.969 | 1.000 | 0.945 | 0.970 | 0.964 | 0.854 | 1.000 | 1.000 | 0.833 | 0.799 |
| 688 | 0.730 | 1.000 | 0.727 | 0.986 | 0.932 | 1.000 | 0.891 | 1.000 | 0.969 | 1.000 | 0.948 | 0.970 | 0.973 | 0.860 | 1.000 | 1.000 | 0.834 | 0.801 |
| 689 | 0.731 | 1.000 | 0.737 | 0.986 | 0.953 | 1.000 | 0.881 | 1.000 | 0.980 | 1.000 | 0.945 | 0.970 | 0.973 | 0.868 | 1.000 | 1.000 | 0.837 | 0.804 |
| 690 | 0.725 | 1.000 | 0.739 | 1.000 | 0.937 | 1.000 | 0.888 | 1.000 | 0.990 | 1.000 | 0.948 | 0.939 | 0.982 | 0.861 | 1.000 | 0.981 | 0.834 | 0.801 |
| 691 | 0.737 | 1.000 | 0.745 | 0.986 | 0.953 | 1.000 | 0.877 | 1.000 | 0.990 | 1.000 | 0.945 | 0.970 | 0.991 | 0.862 | 1.000 | 1.000 | 0.838 | 0.805 |
| 692 | 0.720 | 1.000 | 0.737 | 1.000 | 0.947 | 1.000 | 0.878 | 1.000 | 0.990 | 1.000 | 0.930 | 0.970 | 0.982 | 0.858 | 1.000 | 1.000 | 0.830 | 0.796 |
| 693 | 0.730 | 1.000 | 0.735 | 0.986 | 0.947 | 1.000 | 0.886 | 1.000 | 1.000 | 1.000 | 0.936 | 0.970 | 0.982 | 0.863 | 1.000 | 1.000 | 0.835 | 0.803 |
| 694 | 0.738 | 1.000 | 0.723 | 0.986 | 0.937 | 1.000 | 0.880 | 1.000 | 0.990 | 1.000 | 0.945 | 0.970 | 0.982 | 0.864 | 1.000 | 1.000 | 0.836 | 0.803 |
| 695 | 0.730 | 1.000 | 0.741 | 0.986 | 0.953 | 1.000 | 0.892 | 1.000 | 0.990 | 1.000 | 0.945 | 0.970 | 0.973 | 0.860 | 1.000 | 1.000 | 0.836 | 0.804 |
| 696 | 0.729 | 1.000 | 0.755 | 0.986 | 0.947 | 1.000 | 0.889 | 1.000 | 0.990 | 1.000 | 0.939 | 0.970 | 0.964 | 0.860 | 1.000 | 1.000 | 0.836 | 0.803 |
| 697 | 0.740 | 1.000 | 0.743 | 0.986 | 0.947 | 1.000 | 0.881 | 1.000 | 0.990 | 1.000 | 0.954 | 0.970 | 0.991 | 0.860 | 1.000 | 0.981 | 0.839 | 0.806 |
| 698 | 0.732 | 1.000 | 0.741 | 0.986 | 0.953 | 1.000 | 0.889 | 1.000 | 0.990 | 1.000 | 0.942 | 0.970 | 0.973 | 0.862 | 1.000 | 1.000 | 0.837 | 0.804 |
| 699 | 0.742 | 1.000 | 0.733 | 0.986 | 0.942 | 1.000 | 0.895 | 1.000 | 0.990 | 1.000 | 0.948 | 0.970 | 0.982 | 0.858 | 1.000 | 1.000 | 0.839 | 0.807 |
| 700 | 0.738 | 1.000 | 0.733 | 0.986 | 0.942 | 1.000 | 0.889 | 1.000 | 0.980 | 1.000 | 0.945 | 0.970 | 0.964 | 0.857 | 1.000 | 0.981 | 0.836 | 0.802 |

| | | | | | | | | | | | | | | | | | | |
|---|---|---|---|---|---|---|---|---|---|---|---|---|---|---|---|---|---|---|
| 701 | 0.729 | 1.000 | 0.733 | 1.000 | 0.953 | 1.000 | 0.881 | 1.000 | 0.990 | 1.000 | 0.942 | 0.970 | 0.973 | 0.866 | 1.000 | 1.000 | 0.835 | 0.803 |
| 702 | 0.727 | 1.000 | 0.745 | 0.986 | 0.942 | 1.000 | 0.895 | 1.000 | 0.990 | 1.000 | 0.942 | 0.970 | 0.973 | 0.861 | 1.000 | 1.000 | 0.836 | 0.803 |
| 703 | 0.738 | 1.000 | 0.723 | 0.986 | 0.953 | 1.000 | 0.884 | 1.000 | 0.990 | 1.000 | 0.948 | 0.970 | 0.964 | 0.861 | 1.000 | 1.000 | 0.836 | 0.803 |
| 704 | 0.732 | 1.000 | 0.743 | 0.986 | 0.947 | 1.000 | 0.886 | 1.000 | 0.990 | 1.000 | 0.948 | 0.970 | 0.991 | 0.855 | 1.000 | 1.000 | 0.836 | 0.803 |
| 705 | 0.732 | 1.000 | 0.733 | 1.000 | 0.958 | 1.000 | 0.877 | 1.000 | 0.990 | 1.000 | 0.948 | 1.000 | 0.964 | 0.863 | 1.000 | 1.000 | 0.836 | 0.803 |
| 706 | 0.734 | 1.000 | 0.737 | 1.000 | 0.942 | 1.000 | 0.888 | 1.000 | 0.990 | 1.000 | 0.948 | 0.970 | 0.982 | 0.859 | 1.000 | 1.000 | 0.837 | 0.804 |
| 707 | 0.731 | 1.000 | 0.743 | 1.000 | 0.958 | 1.000 | 0.880 | 1.000 | 0.980 | 1.000 | 0.957 | 0.970 | 0.973 | 0.858 | 1.000 | 1.000 | 0.836 | 0.803 |
| 708 | 0.727 | 1.000 | 0.733 | 0.986 | 0.947 | 1.000 | 0.881 | 1.000 | 0.969 | 1.000 | 0.959 | 0.970 | 0.964 | 0.862 | 1.000 | 1.000 | 0.834 | 0.801 |
| 709 | 0.724 | 1.000 | 0.723 | 0.986 | 0.947 | 1.000 | 0.886 | 1.000 | 0.980 | 1.000 | 0.945 | 0.970 | 0.982 | 0.862 | 1.000 | 1.000 | 0.832 | 0.799 |
| 710 | 0.734 | 1.000 | 0.727 | 1.000 | 0.942 | 0.960 | 0.892 | 1.000 | 0.990 | 1.000 | 0.942 | 0.970 | 0.964 | 0.862 | 1.000 | 1.000 | 0.836 | 0.803 |
| 711 | 0.732 | 1.000 | 0.743 | 0.986 | 0.953 | 1.000 | 0.863 | 1.000 | 0.990 | 1.000 | 0.942 | 0.970 | 0.982 | 0.855 | 1.000 | 0.981 | 0.832 | 0.798 |
| 712 | 0.736 | 1.000 | 0.747 | 0.986 | 0.947 | 1.000 | 0.877 | 1.000 | 0.990 | 1.000 | 0.945 | 1.000 | 0.982 | 0.858 | 1.000 | 1.000 | 0.836 | 0.804 |
| 713 | 0.732 | 1.000 | 0.743 | 0.986 | 0.937 | 1.000 | 0.880 | 1.000 | 0.980 | 1.000 | 0.933 | 0.970 | 0.973 | 0.861 | 1.000 | 1.000 | 0.834 | 0.801 |
| 714 | 0.735 | 1.000 | 0.737 | 1.000 | 0.947 | 1.000 | 0.883 | 1.000 | 0.990 | 1.000 | 0.945 | 1.000 | 0.973 | 0.855 | 1.000 | 1.000 | 0.835 | 0.802 |
| 715 | 0.736 | 1.000 | 0.747 | 1.000 | 0.953 | 1.000 | 0.892 | 1.000 | 0.990 | 1.000 | 0.942 | 0.970 | 0.973 | 0.868 | 1.000 | 1.000 | 0.841 | 0.809 |
| 716 | 0.734 | 1.000 | 0.745 | 0.986 | 0.942 | 1.000 | 0.881 | 1.000 | 0.980 | 1.000 | 0.939 | 1.000 | 0.982 | 0.865 | 1.000 | 1.000 | 0.837 | 0.805 |
| 717 | 0.736 | 1.000 | 0.743 | 1.000 | 0.942 | 1.000 | 0.895 | 1.000 | 0.980 | 1.000 | 0.954 | 1.000 | 0.964 | 0.854 | 1.000 | 1.000 | 0.837 | 0.805 |
| 718 | 0.728 | 1.000 | 0.733 | 0.986 | 0.958 | 1.000 | 0.877 | 1.000 | 0.980 | 1.000 | 0.936 | 0.970 | 0.982 | 0.865 | 1.000 | 1.000 | 0.834 | 0.801 |
| 719 | 0.724 | 1.000 | 0.743 | 0.986 | 0.953 | 1.000 | 0.880 | 1.000 | 0.990 | 1.000 | 0.954 | 0.970 | 0.973 | 0.858 | 1.000 | 1.000 | 0.833 | 0.801 |
| 720 | 0.724 | 1.000 | 0.729 | 1.000 | 0.953 | 1.000 | 0.884 | 1.000 | 0.980 | 1.000 | 0.945 | 1.000 | 0.982 | 0.866 | 1.000 | 1.000 | 0.834 | 0.801 |
| 721 | 0.732 | 1.000 | 0.729 | 0.986 | 0.947 | 1.000 | 0.878 | 1.000 | 0.990 | 1.000 | 0.942 | 1.000 | 0.973 | 0.858 | 1.000 | 1.000 | 0.833 | 0.800 |
| 722 | 0.730 | 1.000 | 0.733 | 0.986 | 0.937 | 1.000 | 0.892 | 1.000 | 0.990 | 1.000 | 0.948 | 0.970 | 0.982 | 0.860 | 1.000 | 0.981 | 0.835 | 0.803 |
| 723 | 0.735 | 1.000 | 0.731 | 0.986 | 0.953 | 1.000 | 0.884 | 1.000 | 0.990 | 1.000 | 0.957 | 0.970 | 0.982 | 0.856 | 1.000 | 1.000 | 0.836 | 0.803 |
| 724 | 0.738 | 1.000 | 0.747 | 0.986 | 0.937 | 1.000 | 0.878 | 1.000 | 1.000 | 1.000 | 0.957 | 0.970 | 0.991 | 0.865 | 1.000 | 1.000 | 0.840 | 0.807 |
| 725 | 0.733 | 1.000 | 0.751 | 1.000 | 0.947 | 1.000 | 0.889 | 1.000 | 1.000 | 1.000 | 0.965 | 0.970 | 0.964 | 0.865 | 1.000 | 1.000 | 0.841 | 0.809 |
| 726 | 0.736 | 1.000 | 0.751 | 0.986 | 0.947 | 1.000 | 0.880 | 1.000 | 0.980 | 1.000 | 0.942 | 1.000 | 0.964 | 0.854 | 1.000 | 1.000 | 0.835 | 0.802 |
| 727 | 0.730 | 1.000 | 0.739 | 1.000 | 0.958 | 1.000 | 0.886 | 1.000 | 0.990 | 1.000 | 0.948 | 0.970 | 0.973 | 0.861 | 1.000 | 1.000 | 0.836 | 0.804 |
| 728 | 0.731 | 1.000 | 0.735 | 0.986 | 0.937 | 1.000 | 0.898 | 1.000 | 1.000 | 1.000 | 0.951 | 0.970 | 0.982 | 0.860 | 1.000 | 1.000 | 0.837 | 0.805 |
| 729 | 0.728 | 1.000 | 0.755 | 1.000 | 0.953 | 1.000 | 0.881 | 1.000 | 0.990 | 1.000 | 0.948 | 0.970 | 0.982 | 0.859 | 1.000 | 1.000 | 0.836 | 0.804 |
| 730 | 0.726 | 1.000 | 0.753 | 1.000 | 0.942 | 1.000 | 0.871 | 1.000 | 0.980 | 1.000 | 0.945 | 0.970 | 0.982 | 0.858 | 1.000 | 1.000 | 0.833 | 0.800 |
| 731 | 0.730 | 1.000 | 0.757 | 0.986 | 0.953 | 1.000 | 0.892 | 1.000 | 0.969 | 1.000 | 0.951 | 1.000 | 0.973 | 0.864 | 1.000 | 1.000 | 0.839 | 0.807 |
| 732 | 0.736 | 1.000 | 0.739 | 0.986 | 0.947 | 1.000 | 0.897 | 1.000 | 0.990 | 1.000 | 0.948 | 0.970 | 0.973 | 0.854 | 1.000 | 1.000 | 0.837 | 0.805 |
| 733 | 0.736 | 1.000 | 0.739 | 0.986 | 0.942 | 1.000 | 0.881 | 1.000 | 0.980 | 1.000 | 0.945 | 0.970 | 0.973 | 0.854 | 1.000 | 1.000 | 0.835 | 0.801 |
| 734 | 0.734 | 1.000 | 0.755 | 0.986 | 0.942 | 1.000 | 0.891 | 1.000 | 0.990 | 1.000 | 0.936 | 0.970 | 0.964 | 0.868 | 1.000 | 1.000 | 0.839 | 0.808 |
| 735 | 0.739 | 1.000 | 0.761 | 1.000 | 0.932 | 1.000 | 0.883 | 1.000 | 0.990 | 1.000 | 0.945 | 1.000 | 0.991 | 0.865 | 1.000 | 1.000 | 0.841 | 0.810 |
| 736 | 0.740 | 1.000 | 0.743 | 1.000 | 0.937 | 1.000 | 0.889 | 1.000 | 0.990 | 1.000 | 0.948 | 0.970 | 0.982 | 0.859 | 1.000 | 0.981 | 0.839 | 0.806 |
| 737 | 0.734 | 1.000 | 0.741 | 0.986 | 0.942 | 1.000 | 0.886 | 1.000 | 1.000 | 1.000 | 0.951 | 0.970 | 0.991 | 0.853 | 1.000 | 1.000 | 0.835 | 0.803 |
| 738 | 0.737 | 1.000 | 0.745 | 0.986 | 0.947 | 1.000 | 0.891 | 1.000 | 0.990 | 1.000 | 0.945 | 0.970 | 0.964 | 0.858 | 1.000 | 0.981 | 0.837 | 0.805 |
| 739 | 0.740 | 1.000 | 0.743 | 0.986 | 0.958 | 1.000 | 0.892 | 1.000 | 1.000 | 1.000 | 0.951 | 0.970 | 0.982 | 0.862 | 1.000 | 1.000 | 0.841 | 0.809 |

| | | | | | | | | | | | | | | | | | | |
|---|---|---|---|---|---|---|---|---|---|---|---|---|---|---|---|---|---|---|
| 740 | 0.736 | 1.000 | 0.751 | 1.000 | 0.947 | 1.000 | 0.886 | 1.000 | 0.980 | 1.000 | 0.948 | 0.970 | 0.991 | 0.856 | 1.000 | 1.000 | 0.838 | 0.805 |
| 741 | 0.724 | 1.000 | 0.721 | 1.000 | 0.947 | 1.000 | 0.883 | 1.000 | 0.980 | 1.000 | 0.954 | 0.970 | 0.964 | 0.868 | 1.000 | 1.000 | 0.834 | 0.801 |
| 742 | 0.736 | 1.000 | 0.739 | 1.000 | 0.947 | 1.000 | 0.884 | 1.000 | 0.980 | 1.000 | 0.945 | 0.970 | 0.991 | 0.859 | 1.000 | 1.000 | 0.837 | 0.804 |
| 743 | 0.738 | 1.000 | 0.727 | 1.000 | 0.953 | 1.000 | 0.886 | 1.000 | 1.000 | 1.000 | 0.945 | 1.000 | 0.991 | 0.862 | 1.000 | 1.000 | 0.838 | 0.806 |
| 744 | 0.729 | 1.000 | 0.741 | 1.000 | 0.937 | 1.000 | 0.891 | 1.000 | 0.990 | 1.000 | 0.936 | 0.970 | 0.964 | 0.860 | 1.000 | 1.000 | 0.835 | 0.802 |
| 745 | 0.726 | 1.000 | 0.753 | 1.000 | 0.958 | 1.000 | 0.895 | 1.000 | 0.990 | 1.000 | 0.939 | 0.970 | 0.973 | 0.867 | 1.000 | 1.000 | 0.839 | 0.807 |
| 746 | 0.735 | 1.000 | 0.745 | 1.000 | 0.958 | 1.000 | 0.884 | 1.000 | 0.990 | 1.000 | 0.933 | 0.970 | 0.964 | 0.853 | 1.000 | 1.000 | 0.835 | 0.802 |
| 747 | 0.729 | 1.000 | 0.755 | 1.000 | 0.947 | 1.000 | 0.884 | 1.000 | 0.990 | 1.000 | 0.951 | 0.970 | 0.991 | 0.857 | 1.000 | 1.000 | 0.836 | 0.804 |
| 748 | 0.736 | 1.000 | 0.749 | 1.000 | 0.947 | 1.000 | 0.886 | 1.000 | 0.990 | 1.000 | 0.945 | 0.970 | 0.982 | 0.864 | 1.000 | 1.000 | 0.839 | 0.807 |
| 749 | 0.734 | 1.000 | 0.727 | 1.000 | 0.947 | 1.000 | 0.883 | 1.000 | 0.990 | 1.000 | 0.948 | 0.970 | 0.991 | 0.860 | 1.000 | 1.000 | 0.835 | 0.803 |
| 750 | 0.727 | 1.000 | 0.735 | 1.000 | 0.958 | 1.000 | 0.884 | 1.000 | 0.990 | 1.000 | 0.948 | 0.970 | 0.982 | 0.856 | 1.000 | 1.000 | 0.834 | 0.801 |
| 751 | 0.730 | 1.000 | 0.743 | 1.000 | 0.963 | 1.000 | 0.888 | 1.000 | 1.000 | 1.000 | 0.936 | 0.970 | 0.955 | 0.865 | 1.000 | 1.000 | 0.837 | 0.805 |
| 752 | 0.726 | 1.000 | 0.735 | 1.000 | 0.963 | 1.000 | 0.888 | 1.000 | 0.990 | 1.000 | 0.962 | 0.970 | 0.973 | 0.854 | 1.000 | 1.000 | 0.834 | 0.802 |
| 753 | 0.734 | 1.000 | 0.761 | 0.986 | 0.947 | 1.000 | 0.880 | 1.000 | 1.000 | 1.000 | 0.939 | 0.970 | 0.973 | 0.865 | 1.000 | 1.000 | 0.839 | 0.807 |
| 754 | 0.726 | 1.000 | 0.749 | 1.000 | 0.937 | 1.000 | 0.884 | 1.000 | 0.980 | 1.000 | 0.942 | 1.000 | 0.964 | 0.861 | 1.000 | 1.000 | 0.834 | 0.802 |
| 755 | 0.731 | 1.000 | 0.737 | 1.000 | 0.953 | 1.000 | 0.888 | 1.000 | 0.969 | 1.000 | 0.954 | 0.970 | 0.973 | 0.864 | 1.000 | 1.000 | 0.837 | 0.805 |
| 756 | 0.732 | 1.000 | 0.747 | 1.000 | 0.953 | 1.000 | 0.886 | 1.000 | 0.990 | 1.000 | 0.948 | 0.970 | 1.000 | 0.854 | 1.000 | 1.000 | 0.836 | 0.804 |
| 757 | 0.738 | 1.000 | 0.739 | 1.000 | 0.947 | 1.000 | 0.883 | 1.000 | 0.980 | 1.000 | 0.954 | 0.970 | 0.982 | 0.857 | 1.000 | 1.000 | 0.837 | 0.805 |
| 758 | 0.718 | 1.000 | 0.753 | 1.000 | 0.937 | 1.000 | 0.884 | 1.000 | 0.990 | 1.000 | 0.948 | 0.970 | 0.973 | 0.858 | 1.000 | 1.000 | 0.832 | 0.799 |
| 759 | 0.737 | 1.000 | 0.745 | 1.000 | 0.958 | 1.000 | 0.901 | 1.000 | 1.000 | 1.000 | 0.945 | 0.970 | 0.973 | 0.862 | 1.000 | 1.000 | 0.841 | 0.810 |
| 760 | 0.742 | 1.000 | 0.739 | 1.000 | 0.947 | 1.000 | 0.894 | 1.000 | 0.980 | 1.000 | 0.954 | 0.970 | 0.955 | 0.864 | 1.000 | 1.000 | 0.841 | 0.809 |
| 761 | 0.740 | 1.000 | 0.753 | 1.000 | 0.953 | 1.000 | 0.889 | 1.000 | 1.000 | 1.000 | 0.954 | 0.970 | 0.982 | 0.861 | 1.000 | 1.000 | 0.842 | 0.810 |
| 762 | 0.734 | 1.000 | 0.737 | 1.000 | 0.953 | 1.000 | 0.883 | 1.000 | 0.980 | 1.000 | 0.951 | 0.970 | 0.973 | 0.857 | 1.000 | 1.000 | 0.835 | 0.803 |
| 763 | 0.726 | 1.000 | 0.747 | 1.000 | 0.963 | 1.000 | 0.895 | 1.000 | 0.990 | 1.000 | 0.954 | 1.000 | 0.982 | 0.865 | 1.000 | 1.000 | 0.839 | 0.808 |
| 764 | 0.722 | 1.000 | 0.751 | 1.000 | 0.958 | 1.000 | 0.891 | 1.000 | 0.990 | 1.000 | 0.951 | 0.970 | 0.973 | 0.859 | 1.000 | 1.000 | 0.835 | 0.803 |
| 765 | 0.726 | 1.000 | 0.751 | 0.986 | 0.953 | 1.000 | 0.889 | 1.000 | 0.990 | 1.000 | 0.959 | 1.000 | 0.982 | 0.864 | 1.000 | 1.000 | 0.838 | 0.806 |
| 766 | 0.733 | 1.000 | 0.751 | 1.000 | 0.947 | 1.000 | 0.886 | 1.000 | 0.990 | 1.000 | 0.951 | 0.970 | 0.973 | 0.863 | 1.000 | 1.000 | 0.839 | 0.807 |
| 767 | 0.728 | 1.000 | 0.747 | 1.000 | 0.958 | 1.000 | 0.888 | 1.000 | 0.980 | 1.000 | 0.945 | 1.000 | 0.973 | 0.868 | 1.000 | 1.000 | 0.838 | 0.806 |
| 768 | 0.732 | 1.000 | 0.747 | 1.000 | 0.958 | 1.000 | 0.886 | 1.000 | 0.990 | 1.000 | 0.933 | 0.970 | 0.973 | 0.864 | 1.000 | 1.000 | 0.837 | 0.805 |
| 769 | 0.731 | 1.000 | 0.735 | 1.000 | 0.942 | 1.000 | 0.903 | 1.000 | 0.980 | 1.000 | 0.942 | 0.970 | 0.991 | 0.865 | 1.000 | 1.000 | 0.839 | 0.807 |
| 770 | 0.724 | 1.000 | 0.725 | 0.986 | 0.947 | 1.000 | 0.891 | 1.000 | 0.980 | 1.000 | 0.942 | 0.970 | 0.991 | 0.868 | 1.000 | 1.000 | 0.834 | 0.802 |
| 771 | 0.739 | 1.000 | 0.753 | 1.000 | 0.953 | 1.000 | 0.881 | 1.000 | 0.980 | 1.000 | 0.942 | 0.970 | 0.973 | 0.865 | 1.000 | 1.000 | 0.840 | 0.808 |
| 772 | 0.734 | 1.000 | 0.761 | 1.000 | 0.947 | 1.000 | 0.878 | 1.000 | 0.990 | 1.000 | 0.954 | 0.970 | 0.982 | 0.861 | 1.000 | 1.000 | 0.839 | 0.807 |
| 773 | 0.742 | 1.000 | 0.761 | 1.000 | 0.942 | 1.000 | 0.886 | 1.000 | 1.000 | 1.000 | 0.939 | 0.970 | 0.982 | 0.863 | 1.000 | 1.000 | 0.842 | 0.810 |
| 774 | 0.733 | 1.000 | 0.741 | 0.986 | 0.947 | 1.000 | 0.891 | 1.000 | 0.990 | 1.000 | 0.945 | 0.970 | 0.982 | 0.865 | 1.000 | 1.000 | 0.838 | 0.807 |
| 775 | 0.732 | 1.000 | 0.737 | 1.000 | 0.937 | 1.000 | 0.878 | 1.000 | 1.000 | 1.000 | 0.951 | 0.970 | 0.982 | 0.858 | 1.000 | 1.000 | 0.834 | 0.802 |
| 776 | 0.737 | 1.000 | 0.751 | 1.000 | 0.947 | 0.960 | 0.888 | 1.000 | 0.980 | 1.000 | 0.945 | 0.970 | 0.973 | 0.867 | 1.000 | 1.000 | 0.840 | 0.808 |
| 777 | 0.733 | 1.000 | 0.739 | 1.000 | 0.947 | 1.000 | 0.892 | 1.000 | 0.980 | 1.000 | 0.948 | 0.970 | 0.973 | 0.865 | 1.000 | 1.000 | 0.838 | 0.806 |
| 778 | 0.727 | 1.000 | 0.735 | 0.986 | 0.937 | 1.000 | 0.895 | 1.000 | 1.000 | 1.000 | 0.945 | 0.970 | 0.982 | 0.863 | 1.000 | 1.000 | 0.836 | 0.803 |

| | | | | | | | | | | | | | | | | | | |
|---|---|---|---|---|---|---|---|---|---|---|---|---|---|---|---|---|---|---|
| 779 | 0.732 | 1.000 | 0.723 | 0.986 | 0.947 | 1.000 | 0.894 | 1.000 | 0.990 | 1.000 | 0.948 | 0.970 | 0.973 | 0.857 | 1.000 | 1.000 | 0.835 | 0.802 |
| 780 | 0.730 | 1.000 | 0.727 | 1.000 | 0.947 | 1.000 | 0.886 | 1.000 | 1.000 | 1.000 | 0.945 | 0.970 | 0.973 | 0.865 | 1.000 | 1.000 | 0.836 | 0.803 |
| 781 | 0.728 | 1.000 | 0.733 | 1.000 | 0.947 | 1.000 | 0.886 | 1.000 | 0.980 | 1.000 | 0.942 | 0.970 | 0.973 | 0.856 | 1.000 | 1.000 | 0.833 | 0.800 |
| 782 | 0.727 | 1.000 | 0.717 | 1.000 | 0.958 | 1.000 | 0.892 | 1.000 | 0.990 | 1.000 | 0.942 | 0.970 | 0.982 | 0.863 | 1.000 | 1.000 | 0.834 | 0.801 |
| 783 | 0.739 | 1.000 | 0.755 | 0.986 | 0.958 | 1.000 | 0.883 | 1.000 | 0.990 | 1.000 | 0.939 | 0.970 | 0.982 | 0.856 | 1.000 | 1.000 | 0.838 | 0.806 |
| 784 | 0.727 | 1.000 | 0.739 | 1.000 | 0.953 | 1.000 | 0.894 | 1.000 | 1.000 | 1.000 | 0.942 | 0.970 | 0.982 | 0.857 | 1.000 | 1.000 | 0.835 | 0.803 |
| 785 | 0.738 | 1.000 | 0.719 | 0.986 | 0.968 | 1.000 | 0.888 | 1.000 | 1.000 | 1.000 | 0.951 | 0.970 | 0.973 | 0.861 | 1.000 | 1.000 | 0.837 | 0.805 |
| 786 | 0.733 | 1.000 | 0.733 | 1.000 | 0.932 | 1.000 | 0.875 | 1.000 | 0.990 | 1.000 | 0.954 | 0.970 | 0.973 | 0.857 | 1.000 | 1.000 | 0.833 | 0.800 |
| 787 | 0.729 | 1.000 | 0.715 | 0.986 | 0.942 | 1.000 | 0.875 | 1.000 | 0.980 | 1.000 | 0.951 | 1.000 | 0.982 | 0.865 | 1.000 | 1.000 | 0.833 | 0.799 |
| 788 | 0.738 | 1.000 | 0.733 | 0.986 | 0.932 | 1.000 | 0.895 | 1.000 | 1.000 | 1.000 | 0.957 | 0.970 | 0.982 | 0.866 | 1.000 | 1.000 | 0.840 | 0.809 |
| 789 | 0.737 | 1.000 | 0.757 | 1.000 | 0.947 | 1.000 | 0.881 | 1.000 | 1.000 | 1.000 | 0.939 | 0.970 | 0.973 | 0.866 | 1.000 | 1.000 | 0.840 | 0.808 |
| 790 | 0.725 | 1.000 | 0.733 | 1.000 | 0.942 | 1.000 | 0.881 | 1.000 | 1.000 | 1.000 | 0.945 | 0.970 | 0.991 | 0.854 | 1.000 | 1.000 | 0.832 | 0.799 |
| 791 | 0.738 | 1.000 | 0.743 | 0.986 | 0.942 | 1.000 | 0.880 | 1.000 | 1.000 | 1.000 | 0.959 | 0.970 | 0.964 | 0.865 | 1.000 | 1.000 | 0.839 | 0.807 |
| 792 | 0.739 | 1.000 | 0.737 | 0.986 | 0.932 | 1.000 | 0.892 | 1.000 | 1.000 | 1.000 | 0.945 | 1.000 | 0.973 | 0.858 | 1.000 | 1.000 | 0.838 | 0.806 |
| 793 | 0.737 | 1.000 | 0.741 | 1.000 | 0.947 | 1.000 | 0.897 | 1.000 | 1.000 | 1.000 | 0.957 | 0.970 | 0.982 | 0.864 | 1.000 | 0.981 | 0.841 | 0.810 |
| 794 | 0.732 | 1.000 | 0.737 | 1.000 | 0.947 | 1.000 | 0.886 | 1.000 | 1.000 | 1.000 | 0.945 | 1.000 | 0.973 | 0.861 | 1.000 | 0.981 | 0.836 | 0.804 |
| 795 | 0.734 | 1.000 | 0.729 | 1.000 | 0.942 | 1.000 | 0.894 | 1.000 | 0.990 | 1.000 | 0.945 | 1.000 | 0.973 | 0.868 | 1.000 | 1.000 | 0.838 | 0.806 |
| 796 | 0.740 | 1.000 | 0.737 | 1.000 | 0.942 | 1.000 | 0.891 | 1.000 | 1.000 | 1.000 | 0.942 | 0.970 | 0.973 | 0.863 | 1.000 | 0.981 | 0.839 | 0.807 |
| 797 | 0.724 | 1.000 | 0.729 | 1.000 | 0.953 | 1.000 | 0.892 | 1.000 | 1.000 | 1.000 | 0.951 | 0.970 | 0.982 | 0.862 | 1.000 | 1.000 | 0.835 | 0.802 |
| 798 | 0.735 | 1.000 | 0.719 | 1.000 | 0.953 | 1.000 | 0.886 | 1.000 | 0.990 | 1.000 | 0.948 | 0.970 | 0.982 | 0.862 | 1.000 | 1.000 | 0.836 | 0.803 |
| 799 | 0.721 | 1.000 | 0.737 | 1.000 | 0.942 | 1.000 | 0.892 | 1.000 | 1.000 | 1.000 | 0.948 | 0.970 | 0.982 | 0.868 | 1.000 | 1.000 | 0.836 | 0.804 |
| 800 | 0.732 | 1.000 | 0.735 | 1.000 | 0.947 | 1.000 | 0.883 | 1.000 | 1.000 | 1.000 | 0.948 | 0.970 | 0.964 | 0.861 | 1.000 | 0.981 | 0.835 | 0.803 |
| 801 | 0.730 | 1.000 | 0.745 | 1.000 | 0.947 | 1.000 | 0.888 | 1.000 | 1.000 | 1.000 | 0.945 | 0.970 | 0.982 | 0.856 | 1.000 | 0.981 | 0.835 | 0.803 |
| 802 | 0.734 | 1.000 | 0.729 | 0.986 | 0.958 | 1.000 | 0.897 | 1.000 | 0.990 | 1.000 | 0.948 | 1.000 | 0.964 | 0.857 | 1.000 | 1.000 | 0.836 | 0.804 |
| 803 | 0.726 | 1.000 | 0.741 | 1.000 | 0.947 | 1.000 | 0.906 | 1.000 | 1.000 | 1.000 | 0.951 | 0.970 | 0.973 | 0.857 | 1.000 | 1.000 | 0.837 | 0.805 |
| 804 | 0.732 | 1.000 | 0.735 | 1.000 | 0.947 | 1.000 | 0.895 | 1.000 | 1.000 | 1.000 | 0.954 | 0.970 | 0.973 | 0.857 | 1.000 | 1.000 | 0.837 | 0.805 |
| 805 | 0.727 | 1.000 | 0.733 | 1.000 | 0.937 | 1.000 | 0.884 | 1.000 | 1.000 | 1.000 | 0.951 | 0.970 | 0.973 | 0.860 | 1.000 | 1.000 | 0.834 | 0.801 |
| 806 | 0.736 | 1.000 | 0.727 | 0.986 | 0.942 | 1.000 | 0.892 | 1.000 | 1.000 | 1.000 | 0.954 | 0.970 | 0.982 | 0.853 | 1.000 | 1.000 | 0.836 | 0.803 |
| 807 | 0.728 | 1.000 | 0.743 | 0.986 | 0.953 | 1.000 | 0.886 | 1.000 | 1.000 | 1.000 | 0.948 | 1.000 | 0.982 | 0.865 | 1.000 | 1.000 | 0.837 | 0.805 |
| 808 | 0.729 | 1.000 | 0.743 | 1.000 | 0.947 | 1.000 | 0.886 | 1.000 | 1.000 | 1.000 | 0.954 | 0.970 | 0.982 | 0.856 | 1.000 | 1.000 | 0.835 | 0.803 |
| 809 | 0.737 | 1.000 | 0.745 | 1.000 | 0.947 | 1.000 | 0.894 | 1.000 | 1.000 | 1.000 | 0.951 | 0.970 | 0.973 | 0.854 | 1.000 | 1.000 | 0.838 | 0.806 |
| 810 | 0.732 | 1.000 | 0.741 | 1.000 | 0.953 | 1.000 | 0.892 | 1.000 | 0.990 | 1.000 | 0.951 | 0.970 | 0.982 | 0.862 | 1.000 | 1.000 | 0.838 | 0.806 |
| 811 | 0.722 | 1.000 | 0.731 | 1.000 | 0.947 | 1.000 | 0.894 | 1.000 | 1.000 | 1.000 | 0.948 | 1.000 | 0.982 | 0.861 | 1.000 | 0.981 | 0.834 | 0.802 |
| 812 | 0.736 | 1.000 | 0.715 | 1.000 | 0.937 | 1.000 | 0.903 | 1.000 | 0.990 | 1.000 | 0.951 | 0.970 | 0.991 | 0.866 | 1.000 | 1.000 | 0.839 | 0.807 |
| 813 | 0.743 | 1.000 | 0.757 | 1.000 | 0.942 | 1.000 | 0.892 | 1.000 | 1.000 | 1.000 | 0.948 | 0.970 | 0.973 | 0.851 | 1.000 | 1.000 | 0.840 | 0.808 |
| 814 | 0.728 | 1.000 | 0.729 | 1.000 | 0.947 | 1.000 | 0.884 | 1.000 | 1.000 | 1.000 | 0.948 | 0.970 | 0.964 | 0.861 | 1.000 | 0.981 | 0.834 | 0.801 |
| 815 | 0.734 | 1.000 | 0.739 | 1.000 | 0.932 | 1.000 | 0.888 | 1.000 | 1.000 | 1.000 | 0.948 | 1.000 | 0.991 | 0.854 | 1.000 | 1.000 | 0.836 | 0.804 |
| 816 | 0.736 | 1.000 | 0.731 | 1.000 | 0.953 | 1.000 | 0.892 | 1.000 | 1.000 | 1.000 | 0.951 | 0.970 | 0.982 | 0.857 | 1.000 | 1.000 | 0.837 | 0.805 |
| 817 | 0.733 | 1.000 | 0.719 | 1.000 | 0.958 | 1.000 | 0.889 | 1.000 | 1.000 | 1.000 | 0.957 | 0.970 | 0.973 | 0.854 | 1.000 | 1.000 | 0.835 | 0.802 |

| | | | | | | | | | | | | | | | | | | |
|---|---|---|---|---|---|---|---|---|---|---|---|---|---|---|---|---|---|---|
| 818 | 0.729 | 1.000 | 0.729 | 1.000 | 0.963 | 1.000 | 0.884 | 1.000 | 1.000 | 1.000 | 0.948 | 0.970 | 0.973 | 0.863 | 1.000 | 1.000 | 0.836 | 0.803 |
| 819 | 0.728 | 1.000 | 0.747 | 0.986 | 0.932 | 1.000 | 0.892 | 1.000 | 0.990 | 1.000 | 0.957 | 0.970 | 0.982 | 0.860 | 1.000 | 1.000 | 0.836 | 0.805 |
| 820 | 0.724 | 1.000 | 0.725 | 1.000 | 0.953 | 1.000 | 0.884 | 1.000 | 1.000 | 1.000 | 0.957 | 0.939 | 0.973 | 0.865 | 1.000 | 1.000 | 0.834 | 0.802 |
| 821 | 0.734 | 1.000 | 0.743 | 1.000 | 0.937 | 1.000 | 0.892 | 1.000 | 1.000 | 1.000 | 0.954 | 0.970 | 0.973 | 0.861 | 1.000 | 1.000 | 0.838 | 0.806 |
| 822 | 0.734 | 1.000 | 0.725 | 1.000 | 0.937 | 1.000 | 0.875 | 1.000 | 1.000 | 1.000 | 0.945 | 1.000 | 0.973 | 0.857 | 1.000 | 1.000 | 0.833 | 0.800 |
| 823 | 0.740 | 1.000 | 0.721 | 1.000 | 0.942 | 1.000 | 0.889 | 1.000 | 0.990 | 1.000 | 0.942 | 0.970 | 0.973 | 0.862 | 1.000 | 1.000 | 0.837 | 0.804 |
| 824 | 0.726 | 1.000 | 0.735 | 1.000 | 0.947 | 1.000 | 0.889 | 1.000 | 1.000 | 1.000 | 0.948 | 1.000 | 0.973 | 0.859 | 1.000 | 1.000 | 0.835 | 0.802 |
| 825 | 0.732 | 1.000 | 0.741 | 0.986 | 0.942 | 1.000 | 0.888 | 1.000 | 1.000 | 1.000 | 0.945 | 0.970 | 0.982 | 0.858 | 1.000 | 1.000 | 0.836 | 0.803 |
| 826 | 0.730 | 1.000 | 0.741 | 1.000 | 0.942 | 0.960 | 0.888 | 1.000 | 0.990 | 1.000 | 0.948 | 0.970 | 0.973 | 0.854 | 1.000 | 1.000 | 0.834 | 0.802 |
| 827 | 0.726 | 1.000 | 0.727 | 1.000 | 0.958 | 1.000 | 0.880 | 1.000 | 1.000 | 1.000 | 0.945 | 0.970 | 0.982 | 0.854 | 1.000 | 1.000 | 0.831 | 0.798 |
| 828 | 0.729 | 1.000 | 0.735 | 0.986 | 0.953 | 1.000 | 0.880 | 1.000 | 1.000 | 1.000 | 0.948 | 0.970 | 0.982 | 0.863 | 1.000 | 1.000 | 0.835 | 0.802 |
| 829 | 0.730 | 1.000 | 0.739 | 1.000 | 0.953 | 1.000 | 0.891 | 1.000 | 0.990 | 1.000 | 0.951 | 1.000 | 0.982 | 0.863 | 1.000 | 1.000 | 0.838 | 0.806 |
| 830 | 0.734 | 1.000 | 0.735 | 1.000 | 0.926 | 1.000 | 0.889 | 1.000 | 0.990 | 1.000 | 0.948 | 0.970 | 0.973 | 0.856 | 1.000 | 1.000 | 0.835 | 0.802 |
| 831 | 0.732 | 1.000 | 0.731 | 1.000 | 0.953 | 1.000 | 0.889 | 1.000 | 1.000 | 1.000 | 0.945 | 1.000 | 0.973 | 0.861 | 1.000 | 1.000 | 0.836 | 0.804 |
| 832 | 0.725 | 1.000 | 0.737 | 1.000 | 0.932 | 1.000 | 0.888 | 1.000 | 1.000 | 1.000 | 0.957 | 1.000 | 0.991 | 0.862 | 1.000 | 1.000 | 0.835 | 0.803 |
| 833 | 0.740 | 1.000 | 0.737 | 1.000 | 0.953 | 1.000 | 0.886 | 1.000 | 1.000 | 1.000 | 0.951 | 1.000 | 0.982 | 0.861 | 1.000 | 1.000 | 0.840 | 0.808 |
| 834 | 0.739 | 1.000 | 0.747 | 1.000 | 0.942 | 1.000 | 0.889 | 1.000 | 1.000 | 1.000 | 0.957 | 0.970 | 0.991 | 0.846 | 1.000 | 1.000 | 0.837 | 0.805 |
| 835 | 0.727 | 1.000 | 0.727 | 1.000 | 0.942 | 1.000 | 0.891 | 1.000 | 1.000 | 1.000 | 0.945 | 0.970 | 0.973 | 0.852 | 1.000 | 1.000 | 0.832 | 0.799 |
| 836 | 0.740 | 1.000 | 0.751 | 1.000 | 0.937 | 1.000 | 0.898 | 1.000 | 0.990 | 1.000 | 0.954 | 1.000 | 0.982 | 0.868 | 1.000 | 1.000 | 0.844 | 0.813 |
| 837 | 0.730 | 1.000 | 0.733 | 1.000 | 0.942 | 1.000 | 0.891 | 1.000 | 1.000 | 1.000 | 0.945 | 0.970 | 0.991 | 0.866 | 1.000 | 1.000 | 0.837 | 0.805 |
| 838 | 0.722 | 1.000 | 0.751 | 1.000 | 0.942 | 1.000 | 0.883 | 1.000 | 1.000 | 1.000 | 0.954 | 0.970 | 0.991 | 0.858 | 1.000 | 1.000 | 0.834 | 0.802 |
| 839 | 0.728 | 1.000 | 0.731 | 1.000 | 0.932 | 1.000 | 0.883 | 1.000 | 1.000 | 1.000 | 0.948 | 1.000 | 0.982 | 0.861 | 1.000 | 1.000 | 0.834 | 0.801 |
| 840 | 0.736 | 1.000 | 0.743 | 1.000 | 0.963 | 1.000 | 0.888 | 1.000 | 1.000 | 1.000 | 0.957 | 0.970 | 0.982 | 0.861 | 1.000 | 1.000 | 0.840 | 0.808 |
| 841 | 0.728 | 1.000 | 0.749 | 1.000 | 0.937 | 1.000 | 0.884 | 1.000 | 1.000 | 1.000 | 0.945 | 0.970 | 0.973 | 0.858 | 1.000 | 1.000 | 0.835 | 0.802 |
| 842 | 0.730 | 1.000 | 0.751 | 1.000 | 0.932 | 1.000 | 0.884 | 1.000 | 1.000 | 1.000 | 0.945 | 0.970 | 0.982 | 0.862 | 1.000 | 1.000 | 0.837 | 0.804 |
| 843 | 0.723 | 1.000 | 0.737 | 1.000 | 0.937 | 1.000 | 0.886 | 1.000 | 1.000 | 1.000 | 0.942 | 0.970 | 0.982 | 0.861 | 1.000 | 1.000 | 0.833 | 0.801 |
| 844 | 0.732 | 1.000 | 0.747 | 1.000 | 0.937 | 1.000 | 0.886 | 1.000 | 1.000 | 1.000 | 0.954 | 0.970 | 0.982 | 0.862 | 1.000 | 1.000 | 0.838 | 0.806 |
| 845 | 0.746 | 1.000 | 0.741 | 1.000 | 0.958 | 1.000 | 0.888 | 1.000 | 1.000 | 1.000 | 0.951 | 1.000 | 0.964 | 0.856 | 1.000 | 1.000 | 0.840 | 0.809 |
| 846 | 0.734 | 1.000 | 0.737 | 1.000 | 0.937 | 1.000 | 0.880 | 1.000 | 1.000 | 1.000 | 0.939 | 0.970 | 0.982 | 0.854 | 1.000 | 1.000 | 0.834 | 0.801 |
| 847 | 0.730 | 1.000 | 0.751 | 0.986 | 0.942 | 1.000 | 0.891 | 1.000 | 0.990 | 1.000 | 0.959 | 0.970 | 0.991 | 0.856 | 1.000 | 1.000 | 0.837 | 0.805 |
| 848 | 0.734 | 1.000 | 0.745 | 1.000 | 0.942 | 1.000 | 0.886 | 1.000 | 1.000 | 1.000 | 0.945 | 1.000 | 0.982 | 0.861 | 1.000 | 1.000 | 0.838 | 0.806 |
| 849 | 0.732 | 1.000 | 0.737 | 1.000 | 0.947 | 1.000 | 0.878 | 1.000 | 1.000 | 1.000 | 0.954 | 0.970 | 0.973 | 0.861 | 1.000 | 1.000 | 0.836 | 0.803 |
| 850 | 0.737 | 1.000 | 0.749 | 1.000 | 0.947 | 1.000 | 0.880 | 1.000 | 0.990 | 1.000 | 0.957 | 0.970 | 0.982 | 0.855 | 1.000 | 1.000 | 0.837 | 0.805 |
| 851 | 0.735 | 1.000 | 0.737 | 1.000 | 0.947 | 1.000 | 0.888 | 1.000 | 0.990 | 1.000 | 0.951 | 1.000 | 0.982 | 0.857 | 1.000 | 1.000 | 0.837 | 0.805 |
| 852 | 0.736 | 1.000 | 0.745 | 1.000 | 0.963 | 1.000 | 0.897 | 1.000 | 0.980 | 1.000 | 0.951 | 0.970 | 0.982 | 0.854 | 1.000 | 1.000 | 0.838 | 0.807 |
| 853 | 0.737 | 1.000 | 0.755 | 1.000 | 0.937 | 1.000 | 0.889 | 1.000 | 1.000 | 1.000 | 0.957 | 1.000 | 0.991 | 0.852 | 1.000 | 1.000 | 0.839 | 0.807 |
| 854 | 0.743 | 1.000 | 0.719 | 1.000 | 0.942 | 1.000 | 0.881 | 1.000 | 0.990 | 1.000 | 0.957 | 0.970 | 0.973 | 0.870 | 1.000 | 1.000 | 0.840 | 0.808 |
| 855 | 0.732 | 1.000 | 0.745 | 1.000 | 0.963 | 1.000 | 0.884 | 1.000 | 1.000 | 1.000 | 0.939 | 0.970 | 0.982 | 0.862 | 1.000 | 1.000 | 0.838 | 0.806 |
| 856 | 0.742 | 1.000 | 0.753 | 1.000 | 0.953 | 1.000 | 0.888 | 1.000 | 1.000 | 1.000 | 0.942 | 0.970 | 0.973 | 0.855 | 1.000 | 1.000 | 0.839 | 0.808 |

| | | | | | | | | | | | | | | | | | | |
|---|---|---|---|---|---|---|---|---|---|---|---|---|---|---|---|---|---|---|
| 857 | 0.734 | 1.000 | 0.727 | 1.000 | 0.942 | 1.000 | 0.894 | 1.000 | 1.000 | 1.000 | 0.954 | 1.000 | 0.973 | 0.853 | 1.000 | 0.981 | 0.835 | 0.803 |
| 858 | 0.735 | 1.000 | 0.739 | 1.000 | 0.937 | 1.000 | 0.895 | 1.000 | 0.990 | 1.000 | 0.945 | 0.970 | 1.000 | 0.859 | 1.000 | 1.000 | 0.838 | 0.806 |
| 859 | 0.740 | 1.000 | 0.735 | 1.000 | 0.953 | 1.000 | 0.888 | 1.000 | 0.990 | 1.000 | 0.954 | 0.970 | 0.982 | 0.860 | 1.000 | 1.000 | 0.839 | 0.807 |
| 860 | 0.726 | 1.000 | 0.739 | 1.000 | 0.953 | 1.000 | 0.891 | 1.000 | 0.990 | 1.000 | 0.954 | 0.970 | 0.991 | 0.846 | 1.000 | 1.000 | 0.832 | 0.799 |
| 861 | 0.739 | 1.000 | 0.747 | 1.000 | 0.937 | 1.000 | 0.888 | 1.000 | 0.990 | 1.000 | 0.954 | 0.970 | 0.973 | 0.862 | 1.000 | 1.000 | 0.840 | 0.808 |
| 862 | 0.735 | 1.000 | 0.741 | 1.000 | 0.942 | 1.000 | 0.891 | 1.000 | 0.990 | 1.000 | 0.954 | 1.000 | 0.982 | 0.861 | 1.000 | 1.000 | 0.839 | 0.807 |
| 863 | 0.730 | 1.000 | 0.761 | 1.000 | 0.953 | 1.000 | 0.888 | 1.000 | 1.000 | 1.000 | 0.954 | 0.970 | 0.964 | 0.857 | 1.000 | 1.000 | 0.838 | 0.806 |
| 864 | 0.724 | 1.000 | 0.747 | 1.000 | 0.942 | 1.000 | 0.897 | 1.000 | 1.000 | 1.000 | 0.948 | 0.970 | 0.991 | 0.861 | 1.000 | 1.000 | 0.837 | 0.805 |
| 865 | 0.724 | 1.000 | 0.743 | 1.000 | 0.958 | 1.000 | 0.886 | 1.000 | 1.000 | 1.000 | 0.974 | 0.970 | 0.991 | 0.858 | 1.000 | 0.981 | 0.836 | 0.804 |
| 866 | 0.729 | 1.000 | 0.741 | 1.000 | 0.947 | 1.000 | 0.886 | 1.000 | 1.000 | 1.000 | 0.957 | 0.970 | 0.982 | 0.857 | 1.000 | 0.981 | 0.835 | 0.803 |
| 867 | 0.742 | 1.000 | 0.741 | 1.000 | 0.947 | 1.000 | 0.900 | 1.000 | 1.000 | 1.000 | 0.951 | 1.000 | 0.982 | 0.852 | 1.000 | 1.000 | 0.840 | 0.808 |
| 868 | 0.734 | 1.000 | 0.743 | 1.000 | 0.947 | 1.000 | 0.889 | 1.000 | 1.000 | 1.000 | 0.954 | 0.970 | 0.982 | 0.862 | 1.000 | 0.981 | 0.839 | 0.807 |
| 869 | 0.736 | 1.000 | 0.749 | 1.000 | 0.926 | 1.000 | 0.884 | 1.000 | 1.000 | 1.000 | 0.954 | 0.970 | 0.973 | 0.859 | 1.000 | 1.000 | 0.838 | 0.806 |
| 870 | 0.746 | 1.000 | 0.741 | 1.000 | 0.953 | 1.000 | 0.892 | 1.000 | 0.980 | 1.000 | 0.942 | 0.970 | 0.982 | 0.861 | 1.000 | 1.000 | 0.841 | 0.810 |
| 871 | 0.729 | 1.000 | 0.739 | 1.000 | 0.937 | 1.000 | 0.884 | 1.000 | 0.990 | 1.000 | 0.948 | 0.970 | 0.973 | 0.860 | 1.000 | 1.000 | 0.835 | 0.802 |
| 872 | 0.742 | 1.000 | 0.735 | 1.000 | 0.958 | 1.000 | 0.892 | 1.000 | 1.000 | 1.000 | 0.945 | 0.970 | 0.964 | 0.867 | 1.000 | 1.000 | 0.841 | 0.810 |
| 873 | 0.732 | 1.000 | 0.745 | 1.000 | 0.958 | 1.000 | 0.881 | 1.000 | 0.990 | 1.000 | 0.948 | 0.970 | 0.973 | 0.854 | 1.000 | 1.000 | 0.835 | 0.802 |
| 874 | 0.736 | 1.000 | 0.749 | 1.000 | 0.953 | 1.000 | 0.884 | 1.000 | 0.980 | 1.000 | 0.959 | 0.970 | 0.973 | 0.865 | 1.000 | 0.981 | 0.840 | 0.808 |
| 875 | 0.736 | 1.000 | 0.723 | 1.000 | 0.947 | 1.000 | 0.886 | 1.000 | 0.980 | 1.000 | 0.945 | 0.970 | 0.982 | 0.851 | 1.000 | 1.000 | 0.833 | 0.800 |
| 876 | 0.740 | 1.000 | 0.725 | 1.000 | 0.937 | 1.000 | 0.886 | 1.000 | 0.990 | 1.000 | 0.954 | 1.000 | 0.982 | 0.860 | 1.000 | 1.000 | 0.838 | 0.805 |
| 877 | 0.735 | 1.000 | 0.747 | 1.000 | 0.937 | 1.000 | 0.901 | 1.000 | 0.990 | 1.000 | 0.951 | 1.000 | 0.991 | 0.857 | 1.000 | 1.000 | 0.839 | 0.808 |
| 878 | 0.736 | 1.000 | 0.723 | 1.000 | 0.947 | 1.000 | 0.883 | 1.000 | 0.980 | 1.000 | 0.951 | 0.970 | 0.982 | 0.863 | 1.000 | 0.981 | 0.836 | 0.803 |
| 879 | 0.732 | 1.000 | 0.747 | 1.000 | 0.963 | 1.000 | 0.897 | 1.000 | 0.990 | 1.000 | 0.962 | 0.970 | 0.982 | 0.860 | 1.000 | 1.000 | 0.840 | 0.809 |
| 880 | 0.732 | 1.000 | 0.723 | 1.000 | 0.953 | 1.000 | 0.892 | 1.000 | 0.980 | 1.000 | 0.948 | 1.000 | 0.973 | 0.861 | 1.000 | 1.000 | 0.836 | 0.803 |
| 881 | 0.738 | 1.000 | 0.741 | 1.000 | 0.958 | 1.000 | 0.884 | 1.000 | 0.990 | 1.000 | 0.939 | 0.970 | 0.982 | 0.861 | 1.000 | 1.000 | 0.838 | 0.806 |
| 882 | 0.744 | 1.000 | 0.747 | 0.986 | 0.942 | 1.000 | 0.886 | 1.000 | 0.969 | 1.000 | 0.954 | 0.970 | 0.991 | 0.872 | 1.000 | 1.000 | 0.844 | 0.813 |
| 883 | 0.729 | 1.000 | 0.743 | 1.000 | 0.947 | 1.000 | 0.889 | 1.000 | 0.990 | 1.000 | 0.968 | 0.970 | 0.982 | 0.847 | 1.000 | 1.000 | 0.834 | 0.802 |
| 884 | 0.740 | 1.000 | 0.749 | 1.000 | 0.963 | 1.000 | 0.891 | 1.000 | 0.990 | 1.000 | 0.948 | 0.970 | 0.973 | 0.862 | 1.000 | 0.981 | 0.841 | 0.810 |
| 885 | 0.742 | 1.000 | 0.739 | 1.000 | 0.926 | 1.000 | 0.891 | 1.000 | 0.990 | 1.000 | 0.951 | 1.000 | 0.991 | 0.859 | 1.000 | 1.000 | 0.839 | 0.807 |
| 886 | 0.734 | 1.000 | 0.741 | 1.000 | 0.947 | 1.000 | 0.877 | 1.000 | 0.980 | 1.000 | 0.948 | 0.970 | 0.982 | 0.861 | 1.000 | 1.000 | 0.836 | 0.803 |
| 887 | 0.724 | 1.000 | 0.735 | 1.000 | 0.942 | 1.000 | 0.878 | 1.000 | 0.990 | 1.000 | 0.962 | 1.000 | 0.991 | 0.861 | 1.000 | 0.981 | 0.834 | 0.802 |
| 888 | 0.735 | 1.000 | 0.751 | 1.000 | 0.953 | 1.000 | 0.883 | 1.000 | 0.980 | 1.000 | 0.957 | 0.970 | 0.982 | 0.860 | 1.000 | 1.000 | 0.839 | 0.806 |
| 889 | 0.738 | 1.000 | 0.743 | 0.986 | 0.942 | 1.000 | 0.883 | 1.000 | 0.990 | 1.000 | 0.959 | 1.000 | 0.964 | 0.857 | 1.000 | 0.981 | 0.837 | 0.805 |
| 890 | 0.737 | 1.000 | 0.763 | 0.986 | 0.958 | 1.000 | 0.886 | 1.000 | 0.980 | 1.000 | 0.962 | 0.970 | 1.000 | 0.856 | 1.000 | 0.981 | 0.840 | 0.809 |
| 891 | 0.736 | 1.000 | 0.751 | 1.000 | 0.953 | 1.000 | 0.889 | 1.000 | 0.980 | 1.000 | 0.951 | 0.970 | 0.964 | 0.862 | 1.000 | 1.000 | 0.840 | 0.808 |
| 892 | 0.722 | 1.000 | 0.727 | 1.000 | 0.953 | 1.000 | 0.894 | 1.000 | 0.990 | 1.000 | 0.939 | 1.000 | 0.991 | 0.857 | 1.000 | 1.000 | 0.832 | 0.799 |
| 893 | 0.744 | 1.000 | 0.747 | 1.000 | 0.953 | 1.000 | 0.886 | 1.000 | 0.990 | 1.000 | 0.951 | 1.000 | 0.991 | 0.858 | 1.000 | 1.000 | 0.841 | 0.809 |
| 894 | 0.734 | 1.000 | 0.729 | 1.000 | 0.937 | 1.000 | 0.889 | 1.000 | 0.990 | 1.000 | 0.959 | 0.970 | 0.973 | 0.858 | 1.000 | 1.000 | 0.836 | 0.804 |
| 895 | 0.736 | 1.000 | 0.737 | 1.000 | 0.958 | 1.000 | 0.886 | 1.000 | 0.990 | 1.000 | 0.959 | 0.970 | 0.982 | 0.854 | 1.000 | 1.000 | 0.837 | 0.805 |

| | | | | | | | | | | | | | | | | | | |
|---|---|---|---|---|---|---|---|---|---|---|---|---|---|---|---|---|---|---|
| 896 | 0.726 | 1.000 | 0.737 | 1.000 | 0.937 | 1.000 | 0.895 | 1.000 | 0.990 | 1.000 | 0.951 | 1.000 | 0.982 | 0.860 | 1.000 | 1.000 | 0.836 | 0.803 |
| 897 | 0.742 | 1.000 | 0.741 | 1.000 | 0.947 | 1.000 | 0.889 | 1.000 | 0.990 | 1.000 | 0.948 | 0.970 | 0.973 | 0.858 | 1.000 | 1.000 | 0.839 | 0.807 |
| 898 | 0.738 | 1.000 | 0.747 | 1.000 | 0.947 | 1.000 | 0.897 | 1.000 | 0.990 | 1.000 | 0.945 | 0.970 | 0.991 | 0.859 | 1.000 | 1.000 | 0.840 | 0.809 |
| 899 | 0.734 | 1.000 | 0.723 | 1.000 | 0.932 | 1.000 | 0.889 | 1.000 | 0.990 | 1.000 | 0.951 | 0.970 | 0.991 | 0.860 | 1.000 | 0.981 | 0.835 | 0.803 |
| 900 | 0.736 | 1.000 | 0.739 | 1.000 | 0.947 | 1.000 | 0.889 | 1.000 | 0.990 | 1.000 | 0.945 | 0.970 | 0.982 | 0.864 | 1.000 | 1.000 | 0.839 | 0.807 |
| 901 | 0.740 | 1.000 | 0.725 | 1.000 | 0.953 | 1.000 | 0.888 | 1.000 | 0.990 | 1.000 | 0.954 | 0.970 | 0.991 | 0.859 | 1.000 | 1.000 | 0.838 | 0.806 |
| 902 | 0.744 | 1.000 | 0.759 | 1.000 | 0.942 | 1.000 | 0.892 | 1.000 | 0.969 | 1.000 | 0.945 | 0.970 | 0.982 | 0.854 | 1.000 | 0.981 | 0.840 | 0.808 |
| 903 | 0.734 | 1.000 | 0.719 | 1.000 | 0.947 | 1.000 | 0.894 | 1.000 | 0.980 | 1.000 | 0.957 | 1.000 | 0.991 | 0.861 | 1.000 | 0.981 | 0.837 | 0.805 |
| 904 | 0.733 | 1.000 | 0.753 | 1.000 | 0.963 | 1.000 | 0.891 | 1.000 | 0.990 | 1.000 | 0.957 | 0.970 | 0.973 | 0.861 | 1.000 | 1.000 | 0.840 | 0.808 |
| 905 | 0.734 | 1.000 | 0.737 | 1.000 | 0.953 | 1.000 | 0.886 | 1.000 | 0.980 | 1.000 | 0.957 | 0.970 | 0.964 | 0.856 | 1.000 | 1.000 | 0.836 | 0.803 |
| 906 | 0.726 | 1.000 | 0.765 | 1.000 | 0.947 | 1.000 | 0.886 | 1.000 | 1.000 | 1.000 | 0.945 | 1.000 | 0.964 | 0.859 | 1.000 | 1.000 | 0.837 | 0.805 |
| 907 | 0.753 | 1.000 | 0.745 | 1.000 | 0.947 | 1.000 | 0.891 | 1.000 | 0.980 | 1.000 | 0.954 | 0.970 | 0.982 | 0.859 | 1.000 | 0.981 | 0.843 | 0.812 |
| 908 | 0.742 | 1.000 | 0.737 | 1.000 | 0.932 | 1.000 | 0.888 | 1.000 | 0.980 | 1.000 | 0.954 | 0.970 | 0.991 | 0.858 | 1.000 | 1.000 | 0.839 | 0.806 |
| 909 | 0.736 | 1.000 | 0.741 | 1.000 | 0.947 | 1.000 | 0.886 | 1.000 | 0.990 | 1.000 | 0.957 | 0.970 | 0.973 | 0.854 | 1.000 | 0.981 | 0.836 | 0.804 |
| 910 | 0.734 | 1.000 | 0.757 | 1.000 | 0.947 | 1.000 | 0.894 | 1.000 | 1.000 | 1.000 | 0.962 | 1.000 | 0.991 | 0.858 | 1.000 | 1.000 | 0.841 | 0.810 |
| 911 | 0.732 | 1.000 | 0.735 | 1.000 | 0.953 | 1.000 | 0.889 | 1.000 | 0.990 | 1.000 | 0.954 | 0.970 | 0.973 | 0.854 | 1.000 | 1.000 | 0.835 | 0.803 |
| 912 | 0.740 | 1.000 | 0.735 | 1.000 | 0.979 | 1.000 | 0.886 | 1.000 | 0.990 | 1.000 | 0.951 | 0.970 | 0.973 | 0.860 | 1.000 | 1.000 | 0.839 | 0.807 |
| 913 | 0.740 | 1.000 | 0.733 | 1.000 | 0.953 | 1.000 | 0.892 | 1.000 | 0.969 | 1.000 | 0.948 | 1.000 | 0.955 | 0.864 | 1.000 | 0.981 | 0.839 | 0.807 |
| 914 | 0.736 | 1.000 | 0.743 | 0.986 | 0.942 | 1.000 | 0.877 | 1.000 | 0.990 | 1.000 | 0.954 | 0.970 | 0.982 | 0.860 | 1.000 | 1.000 | 0.837 | 0.804 |
| 915 | 0.732 | 1.000 | 0.731 | 1.000 | 0.932 | 1.000 | 0.875 | 1.000 | 0.990 | 1.000 | 0.951 | 1.000 | 0.964 | 0.852 | 1.000 | 1.000 | 0.831 | 0.798 |
| 916 | 0.744 | 1.000 | 0.717 | 1.000 | 0.947 | 1.000 | 0.888 | 1.000 | 0.980 | 1.000 | 0.954 | 0.970 | 0.982 | 0.855 | 1.000 | 1.000 | 0.837 | 0.804 |
| 917 | 0.730 | 1.000 | 0.747 | 0.986 | 0.932 | 1.000 | 0.889 | 1.000 | 0.990 | 1.000 | 0.954 | 0.970 | 0.991 | 0.861 | 1.000 | 1.000 | 0.837 | 0.805 |
| 918 | 0.732 | 1.000 | 0.733 | 1.000 | 0.932 | 1.000 | 0.889 | 1.000 | 0.980 | 1.000 | 0.933 | 0.970 | 0.973 | 0.858 | 1.000 | 1.000 | 0.834 | 0.801 |
| 919 | 0.734 | 1.000 | 0.745 | 1.000 | 0.963 | 1.000 | 0.891 | 1.000 | 0.969 | 1.000 | 0.951 | 1.000 | 0.964 | 0.856 | 1.000 | 1.000 | 0.837 | 0.805 |
| 920 | 0.739 | 1.000 | 0.737 | 1.000 | 0.953 | 1.000 | 0.901 | 1.000 | 0.990 | 1.000 | 0.954 | 1.000 | 0.973 | 0.863 | 1.000 | 1.000 | 0.842 | 0.810 |
| 921 | 0.737 | 1.000 | 0.759 | 0.986 | 0.937 | 1.000 | 0.903 | 1.000 | 0.990 | 1.000 | 0.957 | 0.970 | 0.973 | 0.858 | 1.000 | 1.000 | 0.841 | 0.810 |
| 922 | 0.735 | 1.000 | 0.737 | 0.986 | 0.958 | 1.000 | 0.889 | 1.000 | 0.990 | 1.000 | 0.957 | 0.970 | 0.982 | 0.859 | 1.000 | 1.000 | 0.838 | 0.806 |
| 923 | 0.739 | 1.000 | 0.755 | 1.000 | 0.947 | 1.000 | 0.894 | 1.000 | 0.990 | 1.000 | 0.948 | 1.000 | 0.982 | 0.861 | 1.000 | 1.000 | 0.842 | 0.811 |
| 924 | 0.744 | 1.000 | 0.741 | 1.000 | 0.953 | 1.000 | 0.894 | 1.000 | 0.980 | 1.000 | 0.948 | 1.000 | 0.973 | 0.858 | 1.000 | 1.000 | 0.841 | 0.809 |
| 925 | 0.728 | 1.000 | 0.729 | 0.986 | 0.942 | 1.000 | 0.886 | 1.000 | 0.990 | 1.000 | 0.939 | 0.970 | 0.991 | 0.862 | 1.000 | 1.000 | 0.834 | 0.801 |
| 926 | 0.735 | 1.000 | 0.743 | 1.000 | 0.953 | 1.000 | 0.898 | 1.000 | 0.980 | 1.000 | 0.939 | 0.970 | 0.964 | 0.862 | 1.000 | 1.000 | 0.839 | 0.807 |
| 927 | 0.748 | 1.000 | 0.765 | 1.000 | 0.958 | 1.000 | 0.897 | 1.000 | 0.990 | 1.000 | 0.951 | 0.970 | 0.982 | 0.861 | 1.000 | 1.000 | 0.846 | 0.816 |
| 928 | 0.729 | 1.000 | 0.739 | 1.000 | 0.963 | 1.000 | 0.894 | 1.000 | 0.990 | 1.000 | 0.948 | 0.970 | 0.982 | 0.850 | 1.000 | 1.000 | 0.834 | 0.802 |
| 929 | 0.737 | 1.000 | 0.749 | 1.000 | 0.968 | 1.000 | 0.911 | 1.000 | 0.990 | 1.000 | 0.942 | 0.970 | 0.982 | 0.854 | 1.000 | 1.000 | 0.841 | 0.810 |
| 930 | 0.739 | 1.000 | 0.751 | 1.000 | 0.947 | 1.000 | 0.884 | 1.000 | 0.990 | 1.000 | 0.948 | 0.970 | 0.982 | 0.854 | 1.000 | 1.000 | 0.838 | 0.806 |
| 931 | 0.735 | 1.000 | 0.741 | 1.000 | 0.942 | 1.000 | 0.897 | 1.000 | 0.990 | 1.000 | 0.954 | 1.000 | 0.982 | 0.861 | 1.000 | 1.000 | 0.839 | 0.808 |
| 932 | 0.739 | 1.000 | 0.753 | 1.000 | 0.953 | 1.000 | 0.888 | 1.000 | 0.990 | 1.000 | 0.957 | 1.000 | 0.991 | 0.863 | 1.000 | 1.000 | 0.842 | 0.810 |
| 933 | 0.742 | 1.000 | 0.741 | 1.000 | 0.942 | 1.000 | 0.892 | 1.000 | 0.980 | 1.000 | 0.945 | 1.000 | 0.982 | 0.851 | 1.000 | 1.000 | 0.837 | 0.805 |
| 934 | 0.744 | 1.000 | 0.737 | 1.000 | 0.958 | 1.000 | 0.889 | 1.000 | 0.980 | 1.000 | 0.965 | 0.970 | 0.991 | 0.857 | 1.000 | 0.981 | 0.841 | 0.809 |

| | | | | | | | | | | | | | | | | | | |
|---|---|---|---|---|---|---|---|---|---|---|---|---|---|---|---|---|---|---|
| 935 | 0.731 | 1.000 | 0.755 | 1.000 | 0.953 | 1.000 | 0.891 | 1.000 | 0.980 | 1.000 | 0.962 | 0.970 | 0.991 | 0.859 | 1.000 | 1.000 | 0.839 | 0.808 |
| 936 | 0.734 | 1.000 | 0.749 | 1.000 | 0.937 | 1.000 | 0.897 | 1.000 | 0.990 | 1.000 | 0.951 | 0.970 | 0.991 | 0.854 | 1.000 | 1.000 | 0.838 | 0.806 |
| 937 | 0.743 | 1.000 | 0.753 | 1.000 | 0.947 | 1.000 | 0.886 | 1.000 | 0.990 | 1.000 | 0.951 | 0.970 | 0.991 | 0.861 | 1.000 | 1.000 | 0.842 | 0.810 |
| 938 | 0.728 | 1.000 | 0.749 | 1.000 | 0.942 | 1.000 | 0.888 | 1.000 | 0.990 | 1.000 | 0.959 | 0.970 | 0.991 | 0.856 | 1.000 | 1.000 | 0.836 | 0.804 |
| 939 | 0.740 | 1.000 | 0.745 | 1.000 | 0.958 | 1.000 | 0.895 | 1.000 | 0.990 | 1.000 | 0.951 | 0.970 | 0.991 | 0.859 | 1.000 | 1.000 | 0.841 | 0.809 |
| 940 | 0.739 | 1.000 | 0.753 | 1.000 | 0.953 | 1.000 | 0.891 | 1.000 | 0.990 | 1.000 | 0.962 | 0.970 | 0.973 | 0.856 | 1.000 | 1.000 | 0.840 | 0.809 |
| 941 | 0.729 | 1.000 | 0.753 | 1.000 | 0.947 | 1.000 | 0.892 | 1.000 | 0.990 | 1.000 | 0.939 | 1.000 | 0.973 | 0.855 | 1.000 | 1.000 | 0.836 | 0.804 |
| 942 | 0.730 | 1.000 | 0.753 | 1.000 | 0.968 | 1.000 | 0.888 | 1.000 | 1.000 | 1.000 | 0.957 | 0.970 | 0.982 | 0.851 | 1.000 | 1.000 | 0.837 | 0.805 |
| 943 | 0.735 | 1.000 | 0.765 | 1.000 | 0.953 | 1.000 | 0.898 | 1.000 | 0.980 | 1.000 | 0.945 | 1.000 | 0.982 | 0.864 | 1.000 | 1.000 | 0.842 | 0.811 |
| 944 | 0.744 | 1.000 | 0.737 | 1.000 | 0.953 | 1.000 | 0.875 | 1.000 | 0.990 | 1.000 | 0.957 | 1.000 | 0.973 | 0.863 | 1.000 | 1.000 | 0.840 | 0.808 |
| 945 | 0.736 | 1.000 | 0.749 | 1.000 | 0.958 | 1.000 | 0.903 | 1.000 | 0.969 | 1.000 | 0.957 | 1.000 | 0.982 | 0.854 | 1.000 | 1.000 | 0.840 | 0.809 |
| 946 | 0.747 | 1.000 | 0.741 | 1.000 | 0.942 | 1.000 | 0.888 | 1.000 | 1.000 | 1.000 | 0.957 | 0.970 | 0.991 | 0.862 | 1.000 | 1.000 | 0.843 | 0.811 |
| 947 | 0.730 | 1.000 | 0.741 | 1.000 | 0.958 | 1.000 | 0.897 | 1.000 | 0.969 | 1.000 | 0.959 | 0.970 | 0.973 | 0.852 | 1.000 | 1.000 | 0.836 | 0.804 |
| 948 | 0.734 | 1.000 | 0.743 | 1.000 | 0.932 | 1.000 | 0.891 | 1.000 | 0.980 | 1.000 | 0.951 | 0.970 | 0.982 | 0.852 | 1.000 | 1.000 | 0.835 | 0.803 |
| 949 | 0.734 | 1.000 | 0.759 | 1.000 | 0.942 | 1.000 | 0.892 | 1.000 | 0.980 | 1.000 | 0.945 | 0.970 | 0.982 | 0.854 | 1.000 | 1.000 | 0.837 | 0.805 |
| 950 | 0.734 | 1.000 | 0.747 | 1.000 | 0.942 | 1.000 | 0.888 | 1.000 | 0.980 | 1.000 | 0.945 | 0.970 | 0.991 | 0.856 | 1.000 | 1.000 | 0.836 | 0.804 |
| 951 | 0.740 | 1.000 | 0.743 | 1.000 | 0.953 | 1.000 | 0.888 | 1.000 | 0.980 | 1.000 | 0.948 | 0.970 | 0.991 | 0.861 | 1.000 | 1.000 | 0.840 | 0.808 |
| 952 | 0.727 | 1.000 | 0.751 | 1.000 | 0.958 | 1.000 | 0.888 | 1.000 | 0.990 | 1.000 | 0.948 | 0.970 | 0.973 | 0.858 | 1.000 | 1.000 | 0.836 | 0.804 |
| 953 | 0.728 | 1.000 | 0.747 | 0.986 | 0.958 | 1.000 | 0.883 | 1.000 | 0.990 | 1.000 | 0.954 | 1.000 | 0.982 | 0.864 | 1.000 | 1.000 | 0.837 | 0.805 |
| 954 | 0.744 | 1.000 | 0.763 | 1.000 | 0.958 | 1.000 | 0.880 | 1.000 | 0.990 | 1.000 | 0.959 | 0.970 | 0.973 | 0.848 | 1.000 | 1.000 | 0.839 | 0.808 |
| 955 | 0.747 | 1.000 | 0.751 | 1.000 | 0.953 | 1.000 | 0.888 | 1.000 | 0.990 | 1.000 | 0.957 | 0.970 | 0.973 | 0.856 | 1.000 | 1.000 | 0.842 | 0.810 |
| 956 | 0.743 | 1.000 | 0.745 | 1.000 | 0.953 | 1.000 | 0.891 | 1.000 | 0.980 | 1.000 | 0.951 | 0.970 | 0.982 | 0.858 | 1.000 | 1.000 | 0.840 | 0.808 |
| 957 | 0.726 | 1.000 | 0.749 | 1.000 | 0.937 | 1.000 | 0.888 | 1.000 | 0.980 | 1.000 | 0.951 | 0.970 | 0.973 | 0.863 | 1.000 | 1.000 | 0.836 | 0.804 |
| 958 | 0.731 | 1.000 | 0.759 | 1.000 | 0.947 | 1.000 | 0.889 | 1.000 | 0.990 | 1.000 | 0.954 | 0.970 | 0.991 | 0.852 | 1.000 | 1.000 | 0.837 | 0.805 |
| 959 | 0.748 | 1.000 | 0.763 | 1.000 | 0.942 | 1.000 | 0.886 | 1.000 | 0.980 | 1.000 | 0.942 | 0.970 | 0.973 | 0.851 | 1.000 | 1.000 | 0.840 | 0.808 |
| 960 | 0.736 | 1.000 | 0.739 | 1.000 | 0.953 | 1.000 | 0.889 | 1.000 | 0.990 | 1.000 | 0.954 | 1.000 | 0.982 | 0.852 | 1.000 | 1.000 | 0.837 | 0.804 |
| 961 | 0.727 | 1.000 | 0.749 | 1.000 | 0.947 | 1.000 | 0.884 | 1.000 | 0.969 | 1.000 | 0.948 | 0.970 | 0.991 | 0.853 | 1.000 | 1.000 | 0.834 | 0.801 |
| 962 | 0.744 | 1.000 | 0.753 | 1.000 | 0.953 | 1.000 | 0.897 | 1.000 | 0.980 | 1.000 | 0.962 | 0.970 | 0.982 | 0.857 | 1.000 | 1.000 | 0.843 | 0.812 |
| 963 | 0.736 | 1.000 | 0.745 | 1.000 | 0.942 | 1.000 | 0.906 | 1.000 | 0.980 | 1.000 | 0.948 | 0.970 | 0.973 | 0.861 | 1.000 | 1.000 | 0.840 | 0.809 |
| 964 | 0.735 | 1.000 | 0.757 | 1.000 | 0.942 | 1.000 | 0.880 | 1.000 | 0.980 | 1.000 | 0.948 | 0.970 | 0.982 | 0.854 | 1.000 | 1.000 | 0.836 | 0.804 |
| 965 | 0.737 | 1.000 | 0.757 | 1.000 | 0.958 | 1.000 | 0.883 | 1.000 | 0.969 | 1.000 | 0.948 | 1.000 | 0.982 | 0.854 | 1.000 | 1.000 | 0.838 | 0.806 |
| 966 | 0.738 | 1.000 | 0.753 | 1.000 | 0.947 | 1.000 | 0.891 | 1.000 | 0.990 | 1.000 | 0.948 | 0.970 | 0.982 | 0.848 | 1.000 | 1.000 | 0.837 | 0.805 |
| 967 | 0.742 | 1.000 | 0.749 | 1.000 | 0.953 | 1.000 | 0.892 | 1.000 | 0.980 | 1.000 | 0.957 | 0.970 | 0.991 | 0.860 | 1.000 | 1.000 | 0.842 | 0.810 |
| 968 | 0.733 | 1.000 | 0.751 | 1.000 | 0.947 | 1.000 | 0.892 | 1.000 | 0.980 | 1.000 | 0.945 | 1.000 | 0.982 | 0.861 | 1.000 | 1.000 | 0.839 | 0.807 |
| 969 | 0.742 | 1.000 | 0.753 | 1.000 | 0.942 | 1.000 | 0.892 | 1.000 | 0.980 | 1.000 | 0.957 | 0.970 | 0.982 | 0.859 | 1.000 | 1.000 | 0.842 | 0.810 |
| 970 | 0.736 | 1.000 | 0.759 | 1.000 | 0.937 | 1.000 | 0.888 | 1.000 | 0.980 | 1.000 | 0.945 | 1.000 | 0.991 | 0.854 | 1.000 | 1.000 | 0.838 | 0.806 |
| 971 | 0.732 | 1.000 | 0.759 | 0.986 | 0.953 | 1.000 | 0.894 | 1.000 | 0.980 | 1.000 | 0.957 | 0.970 | 0.982 | 0.858 | 1.000 | 1.000 | 0.839 | 0.808 |
| 972 | 0.732 | 1.000 | 0.757 | 0.986 | 0.942 | 1.000 | 0.886 | 1.000 | 0.980 | 1.000 | 0.951 | 0.970 | 0.982 | 0.850 | 1.000 | 1.000 | 0.835 | 0.803 |
| 973 | 0.749 | 1.000 | 0.747 | 1.000 | 0.958 | 1.000 | 0.884 | 1.000 | 0.980 | 1.000 | 0.957 | 1.000 | 0.973 | 0.860 | 1.000 | 1.000 | 0.843 | 0.811 |

| | | | | | | | | | | | | | | | | | | |
|---|---|---|---|---|---|---|---|---|---|---|---|---|---|---|---|---|---|---|
| 974 | 0.744 | 1.000 | 0.749 | 1.000 | 0.937 | 1.000 | 0.889 | 1.000 | 1.000 | 1.000 | 0.959 | 1.000 | 0.982 | 0.852 | 1.000 | 1.000 | 0.840 | 0.808 |
| 975 | 0.728 | 1.000 | 0.769 | 1.000 | 0.942 | 1.000 | 0.897 | 1.000 | 0.990 | 1.000 | 0.954 | 0.970 | 0.982 | 0.858 | 1.000 | 1.000 | 0.839 | 0.808 |
| 976 | 0.742 | 1.000 | 0.757 | 1.000 | 0.958 | 1.000 | 0.891 | 1.000 | 0.959 | 1.000 | 0.957 | 0.970 | 0.991 | 0.858 | 1.000 | 1.000 | 0.842 | 0.810 |
| 977 | 0.741 | 1.000 | 0.751 | 1.000 | 0.947 | 1.000 | 0.884 | 1.000 | 0.990 | 1.000 | 0.959 | 0.970 | 0.991 | 0.860 | 1.000 | 1.000 | 0.841 | 0.809 |
| 978 | 0.738 | 1.000 | 0.761 | 1.000 | 0.947 | 1.000 | 0.894 | 1.000 | 0.969 | 1.000 | 0.957 | 0.970 | 0.982 | 0.858 | 1.000 | 1.000 | 0.841 | 0.810 |
| 979 | 0.736 | 1.000 | 0.749 | 1.000 | 0.958 | 1.000 | 0.895 | 1.000 | 1.000 | 1.000 | 0.951 | 1.000 | 0.991 | 0.853 | 1.000 | 1.000 | 0.839 | 0.807 |
| 980 | 0.738 | 1.000 | 0.775 | 1.000 | 0.947 | 1.000 | 0.889 | 1.000 | 0.980 | 1.000 | 0.954 | 1.000 | 0.973 | 0.854 | 1.000 | 1.000 | 0.841 | 0.809 |
| 981 | 0.738 | 1.000 | 0.757 | 0.986 | 0.953 | 1.000 | 0.884 | 1.000 | 0.969 | 1.000 | 0.954 | 0.970 | 1.000 | 0.848 | 1.000 | 1.000 | 0.837 | 0.805 |
| 982 | 0.734 | 1.000 | 0.761 | 1.000 | 0.947 | 1.000 | 0.883 | 1.000 | 0.980 | 1.000 | 0.951 | 0.970 | 0.973 | 0.852 | 1.000 | 1.000 | 0.836 | 0.804 |
| 983 | 0.734 | 1.000 | 0.771 | 1.000 | 0.947 | 1.000 | 0.880 | 1.000 | 0.980 | 1.000 | 0.951 | 0.970 | 0.991 | 0.858 | 1.000 | 1.000 | 0.839 | 0.807 |
| 984 | 0.734 | 1.000 | 0.775 | 1.000 | 0.953 | 1.000 | 0.892 | 1.000 | 0.990 | 1.000 | 0.951 | 1.000 | 1.000 | 0.852 | 1.000 | 1.000 | 0.840 | 0.809 |
| 985 | 0.726 | 1.000 | 0.763 | 1.000 | 0.937 | 1.000 | 0.894 | 1.000 | 0.990 | 1.000 | 0.962 | 1.000 | 0.991 | 0.852 | 1.000 | 1.000 | 0.837 | 0.805 |
| 986 | 0.745 | 1.000 | 0.769 | 1.000 | 0.947 | 1.000 | 0.892 | 1.000 | 0.980 | 1.000 | 0.954 | 1.000 | 0.982 | 0.851 | 1.000 | 1.000 | 0.842 | 0.811 |
| 987 | 0.748 | 1.000 | 0.763 | 1.000 | 0.953 | 1.000 | 0.888 | 1.000 | 0.990 | 1.000 | 0.951 | 0.970 | 0.982 | 0.851 | 1.000 | 1.000 | 0.842 | 0.810 |
| 988 | 0.730 | 1.000 | 0.777 | 0.986 | 0.963 | 1.000 | 0.889 | 1.000 | 0.990 | 1.000 | 0.945 | 1.000 | 0.982 | 0.854 | 1.000 | 1.000 | 0.839 | 0.807 |
| 989 | 0.738 | 1.000 | 0.769 | 1.000 | 0.947 | 1.000 | 0.895 | 1.000 | 0.990 | 1.000 | 0.951 | 0.970 | 0.991 | 0.857 | 1.000 | 1.000 | 0.842 | 0.811 |
| 990 | 0.738 | 1.000 | 0.777 | 1.000 | 0.958 | 1.000 | 0.884 | 1.000 | 0.980 | 1.000 | 0.959 | 0.970 | 0.982 | 0.854 | 1.000 | 1.000 | 0.841 | 0.810 |
| 991 | 0.743 | 1.000 | 0.769 | 1.000 | 0.947 | 1.000 | 0.888 | 1.000 | 0.980 | 1.000 | 0.951 | 0.970 | 0.982 | 0.854 | 1.000 | 1.000 | 0.841 | 0.810 |
| 992 | 0.736 | 1.000 | 0.755 | 1.000 | 0.963 | 1.000 | 0.883 | 1.000 | 0.980 | 1.000 | 0.945 | 0.970 | 0.991 | 0.847 | 1.000 | 1.000 | 0.836 | 0.804 |
| 993 | 0.736 | 1.000 | 0.767 | 1.000 | 0.947 | 1.000 | 0.886 | 1.000 | 0.980 | 1.000 | 0.939 | 0.970 | 0.991 | 0.865 | 1.000 | 1.000 | 0.841 | 0.809 |
| 994 | 0.736 | 1.000 | 0.759 | 1.000 | 0.942 | 0.960 | 0.884 | 1.000 | 0.990 | 1.000 | 0.959 | 0.970 | 0.982 | 0.857 | 1.000 | 1.000 | 0.839 | 0.807 |
| 995 | 0.736 | 1.000 | 0.765 | 1.000 | 0.958 | 0.960 | 0.886 | 1.000 | 0.990 | 1.000 | 0.957 | 1.000 | 0.982 | 0.852 | 1.000 | 1.000 | 0.839 | 0.807 |
| 996 | 0.738 | 1.000 | 0.765 | 1.000 | 0.953 | 0.960 | 0.888 | 1.000 | 0.990 | 1.000 | 0.957 | 0.970 | 0.982 | 0.860 | 1.000 | 1.000 | 0.841 | 0.810 |
| 997 | 0.734 | 1.000 | 0.759 | 1.000 | 0.953 | 0.960 | 0.889 | 1.000 | 0.980 | 1.000 | 0.948 | 0.970 | 0.991 | 0.859 | 1.000 | 1.000 | 0.839 | 0.807 |
| 998 | 0.729 | 1.000 | 0.757 | 1.000 | 0.942 | 0.960 | 0.880 | 1.000 | 0.969 | 1.000 | 0.954 | 0.970 | 0.973 | 0.850 | 1.000 | 1.000 | 0.833 | 0.800 |
| 999 | 0.726 | 1.000 | 0.751 | 1.000 | 0.968 | 0.960 | 0.878 | 1.000 | 0.990 | 1.000 | 0.957 | 0.970 | 0.991 | 0.848 | 1.000 | 1.000 | 0.833 | 0.800 |
| 1000 | 0.746 | 1.000 | 0.757 | 1.000 | 0.942 | 0.960 | 0.895 | 1.000 | 0.969 | 1.000 | 0.948 | 0.970 | 1.000 | 0.856 | 1.000 | 1.000 | 0.842 | 0.811 |
| 1001 | 0.740 | 1.000 | 0.767 | 1.000 | 0.942 | 0.960 | 0.881 | 1.000 | 0.990 | 1.000 | 0.957 | 0.970 | 0.991 | 0.847 | 1.000 | 1.000 | 0.838 | 0.806 |
| 1002 | 0.740 | 1.000 | 0.761 | 1.000 | 0.953 | 0.960 | 0.897 | 1.000 | 0.990 | 1.000 | 0.957 | 1.000 | 0.982 | 0.861 | 1.000 | 1.000 | 0.843 | 0.813 |
| 1003 | 0.739 | 1.000 | 0.773 | 0.986 | 0.942 | 0.960 | 0.889 | 1.000 | 0.980 | 1.000 | 0.965 | 0.970 | 0.982 | 0.849 | 1.000 | 1.000 | 0.840 | 0.808 |
| 1004 | 0.754 | 1.000 | 0.765 | 1.000 | 0.953 | 0.960 | 0.892 | 1.000 | 0.990 | 1.000 | 0.965 | 0.970 | 1.000 | 0.854 | 1.000 | 1.000 | 0.846 | 0.816 |
| 1005 | 0.744 | 1.000 | 0.763 | 1.000 | 0.937 | 0.960 | 0.888 | 1.000 | 0.969 | 1.000 | 0.954 | 0.970 | 0.982 | 0.851 | 1.000 | 1.000 | 0.840 | 0.808 |
| 1006 | 0.734 | 1.000 | 0.775 | 1.000 | 0.953 | 0.960 | 0.886 | 1.000 | 0.969 | 1.000 | 0.948 | 0.970 | 0.964 | 0.862 | 1.000 | 1.000 | 0.840 | 0.809 |
| 1007 | 0.736 | 1.000 | 0.755 | 1.000 | 0.953 | 0.960 | 0.889 | 1.000 | 0.990 | 1.000 | 0.957 | 1.000 | 0.991 | 0.854 | 1.000 | 1.000 | 0.839 | 0.808 |
| 1008 | 0.736 | 1.000 | 0.751 | 1.000 | 0.937 | 0.960 | 0.888 | 1.000 | 0.969 | 1.000 | 0.957 | 0.970 | 0.982 | 0.854 | 1.000 | 1.000 | 0.837 | 0.805 |
| 1009 | 0.737 | 1.000 | 0.765 | 1.000 | 0.947 | 0.960 | 0.891 | 1.000 | 0.980 | 1.000 | 0.959 | 0.970 | 0.991 | 0.851 | 1.000 | 1.000 | 0.839 | 0.808 |
| 1010 | 0.734 | 1.000 | 0.757 | 1.000 | 0.947 | 0.960 | 0.889 | 1.000 | 0.980 | 1.000 | 0.951 | 0.970 | 0.991 | 0.845 | 1.000 | 1.000 | 0.835 | 0.803 |
| 1011 | 0.739 | 1.000 | 0.767 | 1.000 | 0.958 | 0.960 | 0.886 | 1.000 | 0.980 | 1.000 | 0.959 | 0.970 | 0.991 | 0.856 | 1.000 | 1.000 | 0.841 | 0.810 |
| 1012 | 0.740 | 1.000 | 0.763 | 1.000 | 0.947 | 0.960 | 0.883 | 1.000 | 0.980 | 1.000 | 0.954 | 0.970 | 0.991 | 0.854 | 1.000 | 1.000 | 0.840 | 0.808 |

| | | | | | | | | | | | | | | | | | | |
|---|---|---|---|---|---|---|---|---|---|---|---|---|---|---|---|---|---|---|
| 1013 | 0.736 | 1.000 | 0.761 | 1.000 | 0.947 | 0.960 | 0.895 | 1.000 | 0.990 | 1.000 | 0.959 | 1.000 | 0.991 | 0.852 | 1.000 | 1.000 | 0.840 | 0.809 |
| 1014 | 0.742 | 1.000 | 0.753 | 1.000 | 0.953 | 0.960 | 0.888 | 1.000 | 0.980 | 1.000 | 0.959 | 1.000 | 0.982 | 0.852 | 1.000 | 1.000 | 0.840 | 0.808 |
| 1015 | 0.735 | 1.000 | 0.765 | 0.986 | 0.947 | 0.960 | 0.883 | 1.000 | 1.000 | 1.000 | 0.959 | 1.000 | 0.991 | 0.850 | 1.000 | 1.000 | 0.838 | 0.806 |
| 1016 | 0.728 | 1.000 | 0.767 | 1.000 | 0.947 | 0.960 | 0.891 | 1.000 | 0.980 | 1.000 | 0.959 | 0.970 | 0.991 | 0.854 | 1.000 | 1.000 | 0.837 | 0.806 |
| 1017 | 0.740 | 1.000 | 0.765 | 1.000 | 0.937 | 0.960 | 0.892 | 1.000 | 0.969 | 1.000 | 0.951 | 0.970 | 0.991 | 0.860 | 1.000 | 1.000 | 0.841 | 0.810 |
| 1018 | 0.732 | 1.000 | 0.749 | 1.000 | 0.947 | 0.960 | 0.881 | 1.000 | 0.990 | 1.000 | 0.954 | 1.000 | 0.991 | 0.851 | 1.000 | 1.000 | 0.835 | 0.803 |
| 1019 | 0.734 | 1.000 | 0.769 | 1.000 | 0.942 | 0.960 | 0.898 | 1.000 | 0.990 | 1.000 | 0.936 | 0.970 | 0.991 | 0.852 | 1.000 | 1.000 | 0.839 | 0.807 |
| 1020 | 0.740 | 1.000 | 0.749 | 0.986 | 0.942 | 0.960 | 0.880 | 1.000 | 0.969 | 1.000 | 0.954 | 0.970 | 0.991 | 0.853 | 1.000 | 1.000 | 0.837 | 0.804 |
| 1021 | 0.740 | 1.000 | 0.743 | 0.986 | 0.953 | 0.960 | 0.888 | 1.000 | 0.990 | 1.000 | 0.948 | 1.000 | 0.982 | 0.848 | 1.000 | 1.000 | 0.836 | 0.804 |
| 1022 | 0.742 | 1.000 | 0.773 | 0.986 | 0.942 | 0.960 | 0.888 | 1.000 | 0.980 | 1.000 | 0.945 | 0.970 | 0.973 | 0.856 | 1.000 | 1.000 | 0.841 | 0.809 |
| 1023 | 0.742 | 1.000 | 0.753 | 0.986 | 0.953 | 0.960 | 0.881 | 1.000 | 0.980 | 1.000 | 0.951 | 0.970 | 1.000 | 0.850 | 1.000 | 1.000 | 0.838 | 0.805 |
| 1024 | 0.744 | 1.000 | 0.759 | 1.000 | 0.958 | 0.960 | 0.884 | 1.000 | 0.980 | 1.000 | 0.936 | 0.970 | 0.991 | 0.850 | 1.000 | 1.000 | 0.838 | 0.806 |
| 1025 | 0.729 | 1.000 | 0.763 | 1.000 | 0.947 | 0.960 | 0.891 | 1.000 | 0.969 | 1.000 | 0.954 | 1.000 | 0.982 | 0.854 | 1.000 | 1.000 | 0.837 | 0.805 |
| 1026 | 0.744 | 1.000 | 0.733 | 1.000 | 0.947 | 0.960 | 0.884 | 1.000 | 0.980 | 1.000 | 0.957 | 0.970 | 0.991 | 0.854 | 1.000 | 1.000 | 0.838 | 0.805 |
| 1027 | 0.745 | 1.000 | 0.753 | 0.986 | 0.947 | 0.960 | 0.894 | 1.000 | 0.980 | 1.000 | 0.954 | 0.970 | 0.973 | 0.857 | 1.000 | 1.000 | 0.841 | 0.810 |
| 1028 | 0.742 | 1.000 | 0.759 | 1.000 | 0.932 | 0.960 | 0.883 | 1.000 | 0.969 | 1.000 | 0.939 | 0.939 | 0.991 | 0.846 | 1.000 | 1.000 | 0.835 | 0.802 |
| 1029 | 0.747 | 1.000 | 0.757 | 1.000 | 0.937 | 0.960 | 0.891 | 1.000 | 0.980 | 1.000 | 0.951 | 0.970 | 0.982 | 0.852 | 1.000 | 1.000 | 0.841 | 0.809 |
| 1030 | 0.746 | 1.000 | 0.761 | 0.986 | 0.958 | 0.960 | 0.892 | 1.000 | 1.000 | 1.000 | 0.942 | 0.970 | 0.991 | 0.852 | 1.000 | 1.000 | 0.842 | 0.810 |
| 1031 | 0.744 | 1.000 | 0.749 | 1.000 | 0.947 | 0.960 | 0.883 | 1.000 | 0.969 | 1.000 | 0.957 | 0.970 | 0.964 | 0.852 | 1.000 | 1.000 | 0.838 | 0.805 |
| 1032 | 0.747 | 1.000 | 0.731 | 1.000 | 0.942 | 0.960 | 0.888 | 1.000 | 0.980 | 1.000 | 0.951 | 0.970 | 0.991 | 0.853 | 1.000 | 1.000 | 0.838 | 0.806 |
| 1033 | 0.743 | 1.000 | 0.767 | 1.000 | 0.947 | 0.960 | 0.886 | 1.000 | 0.990 | 1.000 | 0.945 | 0.970 | 1.000 | 0.850 | 1.000 | 1.000 | 0.840 | 0.808 |
| 1034 | 0.740 | 1.000 | 0.743 | 1.000 | 0.963 | 0.960 | 0.894 | 1.000 | 1.000 | 1.000 | 0.954 | 0.970 | 0.973 | 0.856 | 1.000 | 1.000 | 0.840 | 0.808 |
| 1035 | 0.754 | 1.000 | 0.769 | 1.000 | 0.942 | 0.960 | 0.909 | 1.000 | 0.980 | 1.000 | 0.945 | 1.000 | 0.991 | 0.845 | 1.000 | 1.000 | 0.845 | 0.814 |
| 1036 | 0.736 | 1.000 | 0.777 | 1.000 | 0.942 | 0.960 | 0.897 | 1.000 | 0.990 | 1.000 | 0.942 | 0.970 | 0.991 | 0.849 | 1.000 | 1.000 | 0.839 | 0.808 |
| 1037 | 0.748 | 1.000 | 0.749 | 1.000 | 0.947 | 0.960 | 0.877 | 1.000 | 0.959 | 1.000 | 0.951 | 0.970 | 1.000 | 0.850 | 1.000 | 1.000 | 0.838 | 0.806 |
| 1038 | 0.738 | 1.000 | 0.765 | 1.000 | 0.947 | 0.960 | 0.891 | 1.000 | 0.980 | 1.000 | 0.959 | 1.000 | 1.000 | 0.848 | 1.000 | 1.000 | 0.839 | 0.808 |
| 1039 | 0.741 | 1.000 | 0.761 | 0.986 | 0.942 | 0.960 | 0.877 | 1.000 | 0.969 | 1.000 | 0.936 | 1.000 | 1.000 | 0.847 | 1.000 | 1.000 | 0.835 | 0.803 |
| 1040 | 0.748 | 1.000 | 0.761 | 1.000 | 0.953 | 0.960 | 0.889 | 1.000 | 0.969 | 1.000 | 0.954 | 0.970 | 0.982 | 0.852 | 1.000 | 1.000 | 0.842 | 0.810 |
| 1041 | 0.743 | 1.000 | 0.755 | 1.000 | 0.942 | 0.960 | 0.881 | 1.000 | 0.969 | 1.000 | 0.957 | 0.970 | 0.982 | 0.850 | 1.000 | 1.000 | 0.838 | 0.805 |
| 1042 | 0.741 | 1.000 | 0.751 | 1.000 | 0.947 | 0.960 | 0.886 | 1.000 | 0.969 | 1.000 | 0.948 | 1.000 | 0.991 | 0.854 | 1.000 | 1.000 | 0.838 | 0.806 |
| 1043 | 0.739 | 1.000 | 0.767 | 1.000 | 0.947 | 0.960 | 0.886 | 1.000 | 0.969 | 1.000 | 0.942 | 0.970 | 0.982 | 0.853 | 1.000 | 1.000 | 0.838 | 0.806 |
| 1044 | 0.740 | 1.000 | 0.763 | 1.000 | 0.942 | 0.960 | 0.897 | 1.000 | 0.969 | 1.000 | 0.945 | 1.000 | 0.973 | 0.851 | 1.000 | 1.000 | 0.839 | 0.807 |
| 1045 | 0.747 | 1.000 | 0.765 | 0.986 | 0.942 | 0.960 | 0.889 | 1.000 | 0.969 | 1.000 | 0.951 | 0.970 | 0.982 | 0.845 | 1.000 | 1.000 | 0.839 | 0.807 |
| 1046 | 0.742 | 1.000 | 0.753 | 1.000 | 0.963 | 0.960 | 0.891 | 1.000 | 0.980 | 1.000 | 0.957 | 0.970 | 0.982 | 0.854 | 1.000 | 1.000 | 0.841 | 0.809 |
| 1047 | 0.752 | 1.000 | 0.757 | 1.000 | 0.958 | 0.960 | 0.889 | 1.000 | 0.969 | 1.000 | 0.945 | 0.970 | 0.991 | 0.847 | 1.000 | 1.000 | 0.841 | 0.809 |
| 1048 | 0.753 | 1.000 | 0.753 | 1.000 | 0.942 | 0.960 | 0.894 | 1.000 | 0.980 | 1.000 | 0.942 | 0.970 | 0.991 | 0.854 | 1.000 | 1.000 | 0.843 | 0.811 |
| 1049 | 0.742 | 1.000 | 0.743 | 1.000 | 0.947 | 0.960 | 0.875 | 1.000 | 0.980 | 1.000 | 0.954 | 0.970 | 0.991 | 0.856 | 1.000 | 1.000 | 0.838 | 0.805 |
| 1050 | 0.735 | 1.000 | 0.745 | 1.000 | 0.942 | 0.960 | 0.894 | 1.000 | 0.980 | 1.000 | 0.951 | 0.970 | 0.991 | 0.854 | 1.000 | 1.000 | 0.837 | 0.805 |
| 1051 | 0.734 | 1.000 | 0.747 | 0.986 | 0.947 | 0.960 | 0.880 | 1.000 | 0.969 | 1.000 | 0.959 | 0.970 | 0.982 | 0.854 | 1.000 | 1.000 | 0.835 | 0.803 |

| | | | | | | | | | | | | | | | | | |
|---|---|---|---|---|---|---|---|---|---|---|---|---|---|---|---|---|---|
| 1052 | 0.741 | 1.000 | 0.749 | 0.986 | 0.942 | 0.960 | 0.884 | 1.000 | 0.980 | 1.000 | 0.948 | 0.970 | 0.991 | 0.848 | 1.000 | 1.000 | 0.836 | 0.804 |
| 1053 | 0.738 | 1.000 | 0.751 | 1.000 | 0.942 | 0.960 | 0.889 | 1.000 | 0.959 | 1.000 | 0.957 | 0.970 | 0.991 | 0.855 | 1.000 | 1.000 | 0.838 | 0.806 |
| 1054 | 0.751 | 1.000 | 0.765 | 1.000 | 0.947 | 0.960 | 0.878 | 1.000 | 0.969 | 1.000 | 0.948 | 0.970 | 0.982 | 0.857 | 1.000 | 1.000 | 0.842 | 0.810 |
| 1055 | 0.737 | 1.000 | 0.763 | 1.000 | 0.958 | 0.960 | 0.888 | 1.000 | 0.980 | 1.000 | 0.951 | 0.970 | 0.973 | 0.858 | 1.000 | 1.000 | 0.840 | 0.808 |
| 1056 | 0.736 | 1.000 | 0.753 | 0.986 | 0.926 | 0.960 | 0.880 | 1.000 | 0.959 | 1.000 | 0.936 | 0.970 | 1.000 | 0.859 | 1.000 | 1.000 | 0.836 | 0.803 |
| 1057 | 0.741 | 1.000 | 0.765 | 1.000 | 0.947 | 0.960 | 0.891 | 1.000 | 0.959 | 1.000 | 0.954 | 0.970 | 0.982 | 0.851 | 1.000 | 1.000 | 0.840 | 0.808 |
| 1058 | 0.750 | 1.000 | 0.757 | 0.986 | 0.947 | 0.960 | 0.883 | 1.000 | 0.980 | 1.000 | 0.936 | 1.000 | 0.982 | 0.853 | 1.000 | 1.000 | 0.840 | 0.808 |
| 1059 | 0.740 | 1.000 | 0.755 | 1.000 | 0.947 | 0.960 | 0.891 | 1.000 | 0.959 | 1.000 | 0.945 | 1.000 | 0.982 | 0.851 | 1.000 | 1.000 | 0.838 | 0.805 |
| 1060 | 0.747 | 1.000 | 0.773 | 1.000 | 0.947 | 0.960 | 0.875 | 1.000 | 0.980 | 1.000 | 0.954 | 0.970 | 0.982 | 0.851 | 1.000 | 1.000 | 0.840 | 0.809 |
| 1061 | 0.749 | 1.000 | 0.775 | 0.986 | 0.947 | 0.960 | 0.886 | 1.000 | 0.969 | 1.000 | 0.954 | 0.970 | 0.982 | 0.847 | 1.000 | 1.000 | 0.841 | 0.809 |
| 1062 | 0.747 | 1.000 | 0.761 | 1.000 | 0.942 | 0.960 | 0.892 | 1.000 | 0.969 | 1.000 | 0.954 | 0.939 | 1.000 | 0.857 | 1.000 | 1.000 | 0.843 | 0.811 |
| 1063 | 0.745 | 1.000 | 0.759 | 0.986 | 0.958 | 0.960 | 0.888 | 1.000 | 0.949 | 1.000 | 0.951 | 0.970 | 0.991 | 0.847 | 1.000 | 1.000 | 0.839 | 0.806 |
| 1064 | 0.748 | 1.000 | 0.753 | 0.971 | 0.942 | 0.960 | 0.883 | 1.000 | 0.980 | 1.000 | 0.945 | 1.000 | 0.973 | 0.854 | 1.000 | 1.000 | 0.840 | 0.807 |
| 1065 | 0.743 | 1.000 | 0.775 | 0.986 | 0.958 | 0.960 | 0.884 | 1.000 | 0.959 | 1.000 | 0.957 | 0.970 | 0.973 | 0.844 | 1.000 | 1.000 | 0.839 | 0.807 |
| 1066 | 0.740 | 1.000 | 0.767 | 1.000 | 0.953 | 0.960 | 0.884 | 1.000 | 0.980 | 1.000 | 0.948 | 0.970 | 0.991 | 0.847 | 1.000 | 1.000 | 0.838 | 0.806 |
| 1067 | 0.749 | 1.000 | 0.753 | 1.000 | 0.953 | 0.960 | 0.891 | 1.000 | 0.969 | 1.000 | 0.962 | 0.970 | 0.982 | 0.853 | 1.000 | 1.000 | 0.842 | 0.811 |
| 1068 | 0.738 | 1.000 | 0.749 | 1.000 | 0.937 | 0.960 | 0.877 | 1.000 | 0.980 | 1.000 | 0.957 | 0.970 | 0.964 | 0.843 | 1.000 | 1.000 | 0.833 | 0.800 |
| 1069 | 0.748 | 1.000 | 0.743 | 1.000 | 0.932 | 0.960 | 0.892 | 1.000 | 0.969 | 1.000 | 0.948 | 1.000 | 0.991 | 0.852 | 1.000 | 1.000 | 0.840 | 0.808 |
| 1070 | 0.748 | 1.000 | 0.745 | 0.986 | 0.958 | 0.960 | 0.889 | 1.000 | 0.969 | 1.000 | 0.951 | 0.970 | 0.973 | 0.863 | 1.000 | 1.000 | 0.843 | 0.811 |
| 1071 | 0.760 | 1.000 | 0.741 | 1.000 | 0.947 | 0.960 | 0.891 | 1.000 | 0.959 | 1.000 | 0.962 | 0.970 | 0.991 | 0.855 | 1.000 | 1.000 | 0.845 | 0.813 |
| 1072 | 0.749 | 1.000 | 0.749 | 1.000 | 0.947 | 0.960 | 0.892 | 1.000 | 0.969 | 1.000 | 0.948 | 1.000 | 0.991 | 0.857 | 1.000 | 1.000 | 0.842 | 0.811 |
| 1073 | 0.747 | 1.000 | 0.759 | 1.000 | 0.947 | 0.960 | 0.889 | 1.000 | 0.969 | 1.000 | 0.954 | 0.970 | 0.982 | 0.844 | 1.000 | 1.000 | 0.839 | 0.807 |
| 1074 | 0.744 | 1.000 | 0.739 | 1.000 | 0.953 | 0.960 | 0.892 | 1.000 | 0.969 | 1.000 | 0.951 | 0.970 | 0.973 | 0.855 | 1.000 | 1.000 | 0.839 | 0.807 |
| 1075 | 0.750 | 1.000 | 0.761 | 0.986 | 0.963 | 0.960 | 0.888 | 1.000 | 0.980 | 1.000 | 0.945 | 0.970 | 0.991 | 0.851 | 1.000 | 1.000 | 0.842 | 0.810 |
| 1076 | 0.744 | 1.000 | 0.751 | 1.000 | 0.953 | 0.960 | 0.888 | 1.000 | 0.980 | 1.000 | 0.954 | 0.970 | 0.991 | 0.855 | 1.000 | 1.000 | 0.841 | 0.809 |
| 1077 | 0.740 | 1.000 | 0.755 | 1.000 | 0.974 | 0.960 | 0.888 | 1.000 | 0.959 | 1.000 | 0.948 | 1.000 | 0.973 | 0.859 | 1.000 | 1.000 | 0.840 | 0.809 |
| 1078 | 0.748 | 1.000 | 0.759 | 1.000 | 0.968 | 0.960 | 0.895 | 1.000 | 0.990 | 1.000 | 0.948 | 0.970 | 0.991 | 0.850 | 1.000 | 1.000 | 0.843 | 0.811 |
| 1079 | 0.751 | 1.000 | 0.775 | 0.986 | 0.947 | 0.960 | 0.888 | 1.000 | 0.969 | 1.000 | 0.945 | 1.000 | 0.982 | 0.853 | 1.000 | 1.000 | 0.843 | 0.812 |
| 1080 | 0.736 | 1.000 | 0.779 | 0.971 | 0.953 | 0.960 | 0.881 | 1.000 | 0.969 | 1.000 | 0.951 | 0.970 | 0.982 | 0.857 | 1.000 | 1.000 | 0.840 | 0.808 |
| 1081 | 0.741 | 1.000 | 0.769 | 0.986 | 0.953 | 0.960 | 0.883 | 1.000 | 0.969 | 1.000 | 0.954 | 0.970 | 0.991 | 0.856 | 1.000 | 1.000 | 0.841 | 0.809 |
| 1082 | 0.749 | 1.000 | 0.789 | 0.986 | 0.968 | 0.960 | 0.875 | 1.000 | 0.980 | 1.000 | 0.954 | 0.970 | 0.982 | 0.849 | 1.000 | 1.000 | 0.843 | 0.811 |
| 1083 | 0.744 | 1.000 | 0.755 | 1.000 | 0.953 | 0.960 | 0.900 | 1.000 | 0.980 | 1.000 | 0.945 | 0.970 | 0.982 | 0.850 | 1.000 | 1.000 | 0.840 | 0.809 |
| 1084 | 0.746 | 1.000 | 0.763 | 0.986 | 0.953 | 0.960 | 0.883 | 1.000 | 0.980 | 1.000 | 0.951 | 0.970 | 0.955 | 0.850 | 1.000 | 1.000 | 0.839 | 0.807 |
| 1085 | 0.752 | 1.000 | 0.763 | 0.986 | 0.958 | 0.960 | 0.883 | 1.000 | 0.969 | 1.000 | 0.951 | 0.939 | 0.991 | 0.851 | 1.000 | 1.000 | 0.842 | 0.810 |
| 1086 | 0.748 | 1.000 | 0.769 | 1.000 | 0.947 | 0.960 | 0.886 | 1.000 | 0.959 | 1.000 | 0.951 | 0.970 | 0.982 | 0.846 | 1.000 | 1.000 | 0.840 | 0.808 |
| 1087 | 0.748 | 1.000 | 0.753 | 1.000 | 0.953 | 0.960 | 0.878 | 1.000 | 0.959 | 1.000 | 0.951 | 0.970 | 0.991 | 0.851 | 1.000 | 1.000 | 0.839 | 0.807 |
| 1088 | 0.742 | 1.000 | 0.753 | 0.986 | 0.947 | 0.960 | 0.884 | 1.000 | 0.990 | 1.000 | 0.957 | 0.970 | 0.982 | 0.855 | 1.000 | 1.000 | 0.840 | 0.807 |
| 1089 | 0.747 | 1.000 | 0.751 | 1.000 | 0.958 | 0.960 | 0.881 | 1.000 | 0.969 | 1.000 | 0.948 | 1.000 | 0.991 | 0.857 | 1.000 | 1.000 | 0.841 | 0.809 |
| 1090 | 0.744 | 1.000 | 0.759 | 0.986 | 0.963 | 0.960 | 0.875 | 1.000 | 0.969 | 1.000 | 0.948 | 0.970 | 0.982 | 0.855 | 1.000 | 1.000 | 0.839 | 0.807 |

| | | | | | | | | | | | | | | | | | | |
|---|---|---|---|---|---|---|---|---|---|---|---|---|---|---|---|---|---|---|
| 1091 | 0.747 | 1.000 | 0.765 | 1.000 | 0.947 | 0.960 | 0.877 | 1.000 | 0.980 | 1.000 | 0.962 | 0.970 | 0.991 | 0.851 | 1.000 | 1.000 | 0.841 | 0.809 |
| 1092 | 0.748 | 1.000 | 0.775 | 1.000 | 0.953 | 0.960 | 0.883 | 1.000 | 0.990 | 1.000 | 0.957 | 0.970 | 1.000 | 0.853 | 1.000 | 1.000 | 0.843 | 0.812 |
| 1093 | 0.744 | 1.000 | 0.775 | 1.000 | 0.953 | 0.960 | 0.895 | 1.000 | 0.969 | 1.000 | 0.959 | 0.970 | 0.991 | 0.857 | 1.000 | 1.000 | 0.844 | 0.814 |
| 1094 | 0.740 | 1.000 | 0.759 | 1.000 | 0.958 | 0.960 | 0.884 | 1.000 | 0.969 | 1.000 | 0.954 | 1.000 | 0.991 | 0.848 | 1.000 | 1.000 | 0.838 | 0.806 |
| 1095 | 0.755 | 1.000 | 0.759 | 0.986 | 0.937 | 0.960 | 0.883 | 1.000 | 0.969 | 1.000 | 0.959 | 0.970 | 0.982 | 0.851 | 1.000 | 1.000 | 0.842 | 0.810 |
| 1096 | 0.760 | 1.000 | 0.765 | 0.986 | 0.963 | 0.960 | 0.880 | 1.000 | 0.980 | 1.000 | 0.951 | 0.970 | 0.982 | 0.844 | 1.000 | 1.000 | 0.843 | 0.811 |
| 1097 | 0.740 | 1.000 | 0.745 | 1.000 | 0.953 | 0.960 | 0.884 | 1.000 | 0.980 | 1.000 | 0.957 | 0.970 | 0.991 | 0.849 | 1.000 | 1.000 | 0.837 | 0.804 |
| 1098 | 0.748 | 1.000 | 0.777 | 0.986 | 0.947 | 0.960 | 0.875 | 1.000 | 0.969 | 1.000 | 0.962 | 0.970 | 0.991 | 0.850 | 1.000 | 1.000 | 0.841 | 0.810 |
| 1099 | 0.745 | 1.000 | 0.757 | 1.000 | 0.947 | 0.960 | 0.883 | 1.000 | 0.969 | 1.000 | 0.959 | 0.970 | 1.000 | 0.848 | 1.000 | 1.000 | 0.839 | 0.807 |
| 1100 | 0.748 | 1.000 | 0.763 | 0.986 | 0.942 | 0.960 | 0.883 | 1.000 | 0.969 | 1.000 | 0.962 | 0.970 | 0.982 | 0.853 | 1.000 | 1.000 | 0.841 | 0.809 |
| 1101 | 0.749 | 1.000 | 0.763 | 0.986 | 0.958 | 0.960 | 0.884 | 1.000 | 0.980 | 1.000 | 0.951 | 0.970 | 0.982 | 0.846 | 1.000 | 1.000 | 0.840 | 0.808 |
| 1102 | 0.759 | 1.000 | 0.773 | 1.000 | 0.937 | 0.960 | 0.886 | 1.000 | 0.980 | 1.000 | 0.951 | 0.970 | 0.991 | 0.851 | 1.000 | 1.000 | 0.845 | 0.814 |
| 1103 | 0.749 | 1.000 | 0.759 | 1.000 | 0.947 | 0.960 | 0.878 | 1.000 | 0.959 | 1.000 | 0.948 | 0.970 | 0.973 | 0.854 | 1.000 | 1.000 | 0.840 | 0.808 |
| 1104 | 0.750 | 1.000 | 0.757 | 0.986 | 0.947 | 0.960 | 0.881 | 1.000 | 0.990 | 1.000 | 0.945 | 0.970 | 0.973 | 0.851 | 1.000 | 1.000 | 0.840 | 0.807 |
| 1105 | 0.744 | 1.000 | 0.757 | 0.986 | 0.953 | 0.960 | 0.874 | 1.000 | 0.980 | 1.000 | 0.954 | 0.970 | 0.973 | 0.862 | 1.000 | 1.000 | 0.840 | 0.808 |
| 1106 | 0.748 | 1.000 | 0.769 | 0.971 | 0.963 | 0.960 | 0.884 | 1.000 | 0.990 | 1.000 | 0.951 | 0.970 | 0.973 | 0.853 | 1.000 | 1.000 | 0.842 | 0.811 |
| 1107 | 0.743 | 1.000 | 0.759 | 0.986 | 0.958 | 0.960 | 0.884 | 1.000 | 0.980 | 1.000 | 0.957 | 0.970 | 0.973 | 0.862 | 1.000 | 1.000 | 0.842 | 0.810 |
| 1108 | 0.752 | 1.000 | 0.759 | 1.000 | 0.953 | 0.960 | 0.883 | 1.000 | 0.990 | 1.000 | 0.951 | 0.939 | 0.973 | 0.854 | 1.000 | 1.000 | 0.842 | 0.810 |
| 1109 | 0.751 | 1.000 | 0.765 | 1.000 | 0.963 | 0.960 | 0.881 | 1.000 | 0.969 | 1.000 | 0.948 | 0.970 | 0.955 | 0.861 | 1.000 | 1.000 | 0.843 | 0.812 |
| 1110 | 0.753 | 1.000 | 0.765 | 1.000 | 0.958 | 0.960 | 0.881 | 1.000 | 0.980 | 1.000 | 0.954 | 0.970 | 0.964 | 0.851 | 1.000 | 1.000 | 0.842 | 0.810 |
| 1111 | 0.744 | 1.000 | 0.755 | 1.000 | 0.947 | 0.960 | 0.892 | 1.000 | 0.980 | 1.000 | 0.948 | 1.000 | 0.973 | 0.848 | 1.000 | 1.000 | 0.839 | 0.807 |
| 1112 | 0.742 | 1.000 | 0.767 | 0.986 | 0.953 | 0.960 | 0.878 | 1.000 | 1.000 | 1.000 | 0.954 | 1.000 | 0.964 | 0.854 | 1.000 | 1.000 | 0.840 | 0.808 |
| 1113 | 0.753 | 1.000 | 0.759 | 0.986 | 0.968 | 0.960 | 0.880 | 1.000 | 0.980 | 1.000 | 0.948 | 0.970 | 0.973 | 0.858 | 1.000 | 1.000 | 0.843 | 0.812 |
| 1114 | 0.742 | 1.000 | 0.779 | 1.000 | 0.963 | 0.960 | 0.889 | 1.000 | 0.969 | 1.000 | 0.957 | 0.939 | 0.964 | 0.854 | 1.000 | 1.000 | 0.842 | 0.811 |
| 1115 | 0.755 | 1.000 | 0.755 | 0.986 | 0.947 | 0.960 | 0.884 | 1.000 | 0.980 | 1.000 | 0.951 | 0.970 | 0.964 | 0.857 | 1.000 | 1.000 | 0.843 | 0.811 |
| 1116 | 0.748 | 1.000 | 0.757 | 1.000 | 0.963 | 0.960 | 0.871 | 1.000 | 0.980 | 1.000 | 0.962 | 1.000 | 0.964 | 0.861 | 1.000 | 1.000 | 0.842 | 0.810 |
| 1117 | 0.746 | 1.000 | 0.751 | 1.000 | 0.937 | 0.960 | 0.883 | 1.000 | 0.980 | 1.000 | 0.948 | 1.000 | 0.964 | 0.854 | 1.000 | 1.000 | 0.839 | 0.806 |
| 1118 | 0.748 | 1.000 | 0.757 | 0.986 | 0.953 | 0.960 | 0.881 | 1.000 | 0.990 | 1.000 | 0.959 | 1.000 | 0.982 | 0.856 | 1.000 | 1.000 | 0.842 | 0.810 |
| 1119 | 0.747 | 1.000 | 0.773 | 1.000 | 0.968 | 0.960 | 0.875 | 1.000 | 0.980 | 1.000 | 0.954 | 0.970 | 0.991 | 0.858 | 1.000 | 1.000 | 0.843 | 0.812 |
| 1120 | 0.742 | 1.000 | 0.757 | 0.986 | 0.963 | 0.960 | 0.871 | 1.000 | 0.969 | 1.000 | 0.948 | 0.939 | 0.973 | 0.850 | 1.000 | 1.000 | 0.836 | 0.803 |
| 1121 | 0.742 | 1.000 | 0.779 | 1.000 | 0.942 | 0.960 | 0.881 | 1.000 | 0.980 | 1.000 | 0.954 | 0.970 | 0.982 | 0.853 | 1.000 | 1.000 | 0.841 | 0.809 |
| 1122 | 0.756 | 1.000 | 0.767 | 1.000 | 0.968 | 0.960 | 0.886 | 1.000 | 0.980 | 1.000 | 0.959 | 0.970 | 0.982 | 0.856 | 1.000 | 1.000 | 0.846 | 0.816 |
| 1123 | 0.754 | 1.000 | 0.765 | 0.971 | 0.958 | 0.960 | 0.875 | 1.000 | 0.980 | 1.000 | 0.945 | 0.970 | 0.964 | 0.855 | 1.000 | 1.000 | 0.842 | 0.810 |
| 1124 | 0.757 | 1.000 | 0.785 | 1.000 | 0.963 | 0.960 | 0.883 | 1.000 | 0.990 | 1.000 | 0.957 | 1.000 | 0.964 | 0.854 | 1.000 | 1.000 | 0.847 | 0.817 |
| 1125 | 0.742 | 1.000 | 0.745 | 1.000 | 0.942 | 0.960 | 0.875 | 1.000 | 0.959 | 1.000 | 0.954 | 1.000 | 0.964 | 0.853 | 1.000 | 1.000 | 0.836 | 0.803 |
| 1126 | 0.748 | 1.000 | 0.761 | 1.000 | 0.958 | 0.960 | 0.877 | 1.000 | 0.959 | 1.000 | 0.959 | 0.970 | 0.964 | 0.858 | 1.000 | 1.000 | 0.841 | 0.809 |
| 1127 | 0.755 | 1.000 | 0.763 | 1.000 | 0.963 | 0.960 | 0.889 | 1.000 | 0.959 | 1.000 | 0.948 | 0.970 | 0.982 | 0.850 | 1.000 | 1.000 | 0.843 | 0.812 |
| 1128 | 0.743 | 1.000 | 0.763 | 0.986 | 0.958 | 0.960 | 0.880 | 1.000 | 0.990 | 1.000 | 0.959 | 1.000 | 0.964 | 0.863 | 1.000 | 1.000 | 0.843 | 0.811 |
| 1129 | 0.747 | 1.000 | 0.777 | 0.986 | 0.968 | 0.960 | 0.875 | 1.000 | 0.969 | 1.000 | 0.957 | 1.000 | 0.964 | 0.842 | 1.000 | 1.000 | 0.839 | 0.807 |

| | | | | | | | | | | | | | | | | | | |
|---|---|---|---|---|---|---|---|---|---|---|---|---|---|---|---|---|---|---|
| 1130 | 0.743 | 1.000 | 0.769 | 0.986 | 0.963 | 0.960 | 0.872 | 1.000 | 0.969 | 1.000 | 0.957 | 0.970 | 0.973 | 0.853 | 1.000 | 1.000 | 0.839 | 0.807 |
| 1131 | 0.752 | 1.000 | 0.759 | 0.986 | 0.963 | 0.960 | 0.878 | 1.000 | 0.969 | 1.000 | 0.954 | 0.970 | 0.964 | 0.854 | 1.000 | 1.000 | 0.841 | 0.809 |
| 1132 | 0.747 | 1.000 | 0.757 | 1.000 | 0.947 | 0.960 | 0.889 | 1.000 | 0.969 | 1.000 | 0.959 | 0.970 | 0.973 | 0.861 | 1.000 | 1.000 | 0.843 | 0.812 |
| 1133 | 0.738 | 1.000 | 0.779 | 1.000 | 0.958 | 0.960 | 0.888 | 1.000 | 0.959 | 1.000 | 0.954 | 0.939 | 0.973 | 0.844 | 1.000 | 1.000 | 0.838 | 0.806 |
| 1134 | 0.756 | 1.000 | 0.767 | 1.000 | 0.958 | 0.960 | 0.883 | 1.000 | 0.980 | 1.000 | 0.962 | 0.970 | 0.982 | 0.855 | 1.000 | 1.000 | 0.846 | 0.815 |
| 1135 | 0.748 | 1.000 | 0.773 | 1.000 | 0.963 | 0.960 | 0.886 | 1.000 | 0.969 | 1.000 | 0.959 | 0.970 | 0.973 | 0.854 | 1.000 | 1.000 | 0.843 | 0.812 |
| 1136 | 0.748 | 1.000 | 0.759 | 1.000 | 0.953 | 0.960 | 0.894 | 1.000 | 0.980 | 1.000 | 0.951 | 0.970 | 0.982 | 0.861 | 1.000 | 1.000 | 0.844 | 0.813 |
| 1137 | 0.747 | 1.000 | 0.759 | 1.000 | 0.947 | 0.960 | 0.883 | 1.000 | 0.980 | 1.000 | 0.959 | 0.970 | 0.973 | 0.854 | 1.000 | 1.000 | 0.841 | 0.809 |
| 1138 | 0.748 | 1.000 | 0.775 | 1.000 | 0.958 | 0.960 | 0.883 | 1.000 | 0.969 | 1.000 | 0.957 | 0.970 | 0.964 | 0.850 | 1.000 | 1.000 | 0.842 | 0.810 |
| 1139 | 0.755 | 1.000 | 0.763 | 0.986 | 0.963 | 0.960 | 0.884 | 1.000 | 0.969 | 1.000 | 0.957 | 1.000 | 0.973 | 0.862 | 1.000 | 1.000 | 0.846 | 0.815 |
| 1140 | 0.739 | 1.000 | 0.777 | 0.986 | 0.947 | 0.960 | 0.877 | 1.000 | 0.980 | 1.000 | 0.951 | 1.000 | 0.964 | 0.854 | 1.000 | 1.000 | 0.839 | 0.807 |
| 1141 | 0.753 | 1.000 | 0.787 | 0.986 | 0.958 | 0.960 | 0.875 | 1.000 | 0.980 | 1.000 | 0.951 | 1.000 | 0.991 | 0.856 | 1.000 | 1.000 | 0.845 | 0.814 |
| 1142 | 0.743 | 1.000 | 0.751 | 1.000 | 0.974 | 0.960 | 0.892 | 1.000 | 0.969 | 1.000 | 0.945 | 0.970 | 0.973 | 0.859 | 1.000 | 1.000 | 0.841 | 0.810 |
| 1143 | 0.755 | 1.000 | 0.755 | 1.000 | 0.968 | 0.960 | 0.871 | 1.000 | 0.969 | 1.000 | 0.957 | 0.970 | 0.973 | 0.856 | 1.000 | 1.000 | 0.842 | 0.810 |
| 1144 | 0.748 | 1.000 | 0.751 | 1.000 | 0.953 | 0.960 | 0.880 | 1.000 | 0.990 | 1.000 | 0.965 | 0.970 | 0.982 | 0.847 | 1.000 | 1.000 | 0.839 | 0.807 |
| 1145 | 0.746 | 1.000 | 0.769 | 1.000 | 0.947 | 0.960 | 0.891 | 1.000 | 0.969 | 1.000 | 0.959 | 0.970 | 0.955 | 0.865 | 1.000 | 1.000 | 0.845 | 0.814 |
| 1146 | 0.758 | 1.000 | 0.761 | 1.000 | 0.953 | 0.960 | 0.888 | 1.000 | 0.980 | 1.000 | 0.959 | 1.000 | 0.973 | 0.851 | 1.000 | 1.000 | 0.845 | 0.813 |
| 1147 | 0.759 | 1.000 | 0.745 | 1.000 | 0.958 | 0.960 | 0.889 | 1.000 | 0.969 | 1.000 | 0.951 | 0.970 | 0.955 | 0.851 | 1.000 | 1.000 | 0.842 | 0.810 |
| 1148 | 0.741 | 1.000 | 0.763 | 0.986 | 0.947 | 0.960 | 0.892 | 1.000 | 0.959 | 1.000 | 0.948 | 0.970 | 0.964 | 0.860 | 1.000 | 1.000 | 0.841 | 0.809 |
| 1149 | 0.740 | 1.000 | 0.749 | 1.000 | 0.953 | 0.960 | 0.883 | 1.000 | 0.980 | 1.000 | 0.951 | 0.939 | 0.955 | 0.856 | 1.000 | 1.000 | 0.838 | 0.805 |
| 1150 | 0.742 | 1.000 | 0.755 | 1.000 | 0.947 | 0.960 | 0.895 | 1.000 | 0.969 | 1.000 | 0.957 | 1.000 | 0.964 | 0.861 | 1.000 | 1.000 | 0.842 | 0.811 |
| 1151 | 0.758 | 1.000 | 0.753 | 1.000 | 0.947 | 0.960 | 0.889 | 1.000 | 0.969 | 1.000 | 0.962 | 0.970 | 0.973 | 0.853 | 1.000 | 1.000 | 0.844 | 0.813 |
| 1152 | 0.755 | 1.000 | 0.781 | 1.000 | 0.937 | 0.960 | 0.889 | 1.000 | 0.990 | 1.000 | 0.962 | 0.970 | 0.973 | 0.860 | 1.000 | 1.000 | 0.848 | 0.818 |
| 1153 | 0.751 | 1.000 | 0.769 | 1.000 | 0.953 | 0.960 | 0.884 | 1.000 | 0.990 | 1.000 | 0.954 | 0.970 | 0.955 | 0.858 | 1.000 | 1.000 | 0.844 | 0.813 |
| 1154 | 0.747 | 1.000 | 0.763 | 0.986 | 0.974 | 0.960 | 0.884 | 1.000 | 0.969 | 1.000 | 0.957 | 0.970 | 0.973 | 0.858 | 1.000 | 1.000 | 0.843 | 0.812 |
| 1155 | 0.738 | 1.000 | 0.761 | 0.986 | 0.968 | 0.960 | 0.892 | 1.000 | 0.969 | 1.000 | 0.954 | 0.970 | 0.973 | 0.854 | 1.000 | 1.000 | 0.840 | 0.808 |
| 1156 | 0.749 | 1.000 | 0.777 | 0.986 | 0.942 | 0.960 | 0.881 | 1.000 | 0.969 | 1.000 | 0.965 | 0.970 | 0.973 | 0.857 | 1.000 | 1.000 | 0.844 | 0.813 |
| 1157 | 0.749 | 1.000 | 0.773 | 1.000 | 0.968 | 0.960 | 0.884 | 1.000 | 0.980 | 1.000 | 0.959 | 1.000 | 0.964 | 0.851 | 1.000 | 1.000 | 0.843 | 0.812 |
| 1158 | 0.756 | 1.000 | 0.761 | 0.986 | 0.958 | 0.960 | 0.891 | 1.000 | 0.969 | 1.000 | 0.957 | 1.000 | 0.973 | 0.861 | 1.000 | 1.000 | 0.847 | 0.816 |
| 1159 | 0.755 | 1.000 | 0.751 | 0.986 | 0.968 | 0.960 | 0.884 | 1.000 | 0.990 | 1.000 | 0.957 | 0.970 | 0.991 | 0.849 | 1.000 | 1.000 | 0.843 | 0.811 |
| 1160 | 0.748 | 1.000 | 0.783 | 1.000 | 0.963 | 0.960 | 0.888 | 1.000 | 0.969 | 1.000 | 0.959 | 0.970 | 0.982 | 0.854 | 1.000 | 1.000 | 0.845 | 0.814 |
| 1161 | 0.748 | 1.000 | 0.767 | 1.000 | 0.958 | 0.960 | 0.894 | 1.000 | 0.969 | 1.000 | 0.954 | 0.970 | 0.964 | 0.858 | 1.000 | 1.000 | 0.844 | 0.813 |
| 1162 | 0.758 | 1.000 | 0.757 | 0.971 | 0.953 | 0.960 | 0.889 | 1.000 | 0.959 | 1.000 | 0.957 | 0.970 | 0.973 | 0.855 | 1.000 | 1.000 | 0.844 | 0.813 |
| 1163 | 0.746 | 1.000 | 0.761 | 0.986 | 0.958 | 0.960 | 0.886 | 1.000 | 0.980 | 1.000 | 0.954 | 0.970 | 0.973 | 0.862 | 1.000 | 1.000 | 0.843 | 0.812 |
| 1164 | 0.750 | 1.000 | 0.779 | 1.000 | 0.963 | 0.960 | 0.883 | 1.000 | 0.969 | 1.000 | 0.951 | 0.970 | 0.955 | 0.867 | 1.000 | 1.000 | 0.847 | 0.816 |
| 1165 | 0.753 | 1.000 | 0.761 | 1.000 | 0.947 | 0.960 | 0.874 | 1.000 | 0.980 | 1.000 | 0.957 | 0.970 | 0.955 | 0.849 | 1.000 | 1.000 | 0.840 | 0.807 |
| 1166 | 0.745 | 1.000 | 0.759 | 1.000 | 0.947 | 0.960 | 0.886 | 1.000 | 0.969 | 1.000 | 0.959 | 0.970 | 0.973 | 0.858 | 1.000 | 1.000 | 0.842 | 0.810 |
| 1167 | 0.752 | 1.000 | 0.771 | 0.986 | 0.963 | 0.960 | 0.884 | 1.000 | 0.980 | 1.000 | 0.954 | 0.970 | 0.964 | 0.857 | 1.000 | 1.000 | 0.844 | 0.813 |
| 1168 | 0.755 | 1.000 | 0.755 | 0.986 | 0.968 | 0.960 | 0.888 | 1.000 | 0.969 | 1.000 | 0.957 | 1.000 | 0.964 | 0.854 | 1.000 | 1.000 | 0.844 | 0.813 |

| | | | | | | | | | | | | | | | | | | |
|---|---|---|---|---|---|---|---|---|---|---|---|---|---|---|---|---|---|---|
| 1169 | 0.747 | 1.000 | 0.757 | 1.000 | 0.963 | 0.960 | 0.886 | 1.000 | 0.980 | 1.000 | 0.957 | 0.939 | 0.964 | 0.863 | 1.000 | 1.000 | 0.844 | 0.812 |
| 1170 | 0.742 | 1.000 | 0.763 | 0.986 | 0.947 | 0.960 | 0.888 | 1.000 | 0.980 | 1.000 | 0.962 | 0.970 | 0.982 | 0.854 | 1.000 | 1.000 | 0.841 | 0.809 |
| 1171 | 0.751 | 1.000 | 0.763 | 1.000 | 0.963 | 0.960 | 0.883 | 1.000 | 0.980 | 1.000 | 0.942 | 0.970 | 0.982 | 0.853 | 1.000 | 1.000 | 0.842 | 0.810 |
| 1172 | 0.745 | 1.000 | 0.763 | 1.000 | 0.963 | 0.960 | 0.892 | 1.000 | 0.969 | 1.000 | 0.957 | 0.970 | 0.964 | 0.852 | 1.000 | 1.000 | 0.842 | 0.810 |
| 1173 | 0.755 | 1.000 | 0.777 | 1.000 | 0.947 | 0.960 | 0.888 | 1.000 | 0.980 | 1.000 | 0.954 | 0.970 | 0.964 | 0.851 | 1.000 | 1.000 | 0.844 | 0.813 |
| 1174 | 0.766 | 1.000 | 0.771 | 0.986 | 0.963 | 0.960 | 0.892 | 1.000 | 0.990 | 1.000 | 0.957 | 0.970 | 0.964 | 0.860 | 1.000 | 1.000 | 0.851 | 0.821 |
| 1175 | 0.744 | 1.000 | 0.759 | 0.986 | 0.963 | 0.960 | 0.888 | 1.000 | 0.990 | 1.000 | 0.959 | 1.000 | 0.973 | 0.853 | 1.000 | 1.000 | 0.841 | 0.810 |
| 1176 | 0.746 | 1.000 | 0.765 | 0.986 | 0.958 | 0.960 | 0.888 | 1.000 | 0.969 | 1.000 | 0.951 | 0.970 | 0.973 | 0.857 | 1.000 | 1.000 | 0.842 | 0.811 |
| 1177 | 0.746 | 1.000 | 0.763 | 0.986 | 0.968 | 0.960 | 0.877 | 1.000 | 0.969 | 1.000 | 0.957 | 0.970 | 0.991 | 0.857 | 1.000 | 1.000 | 0.842 | 0.810 |
| 1178 | 0.755 | 1.000 | 0.761 | 0.986 | 0.963 | 0.960 | 0.888 | 1.000 | 0.969 | 1.000 | 0.957 | 0.970 | 0.964 | 0.860 | 1.000 | 1.000 | 0.845 | 0.814 |
| 1179 | 0.755 | 1.000 | 0.765 | 1.000 | 0.953 | 0.960 | 0.883 | 1.000 | 0.969 | 1.000 | 0.954 | 0.970 | 0.973 | 0.862 | 1.000 | 1.000 | 0.846 | 0.815 |
| 1180 | 0.756 | 1.000 | 0.753 | 1.000 | 0.963 | 0.960 | 0.875 | 1.000 | 0.980 | 1.000 | 0.948 | 0.970 | 0.973 | 0.858 | 1.000 | 1.000 | 0.843 | 0.811 |
| 1181 | 0.751 | 1.000 | 0.763 | 1.000 | 0.963 | 0.960 | 0.883 | 1.000 | 0.959 | 1.000 | 0.957 | 1.000 | 0.955 | 0.851 | 1.000 | 1.000 | 0.842 | 0.810 |
| 1182 | 0.746 | 1.000 | 0.751 | 1.000 | 0.958 | 0.960 | 0.888 | 1.000 | 0.980 | 1.000 | 0.959 | 0.970 | 0.964 | 0.858 | 1.000 | 1.000 | 0.842 | 0.810 |
| 1183 | 0.747 | 1.000 | 0.761 | 0.986 | 0.953 | 0.960 | 0.884 | 1.000 | 0.969 | 1.000 | 0.954 | 0.970 | 0.973 | 0.854 | 1.000 | 1.000 | 0.841 | 0.809 |
| 1184 | 0.744 | 1.000 | 0.771 | 0.986 | 0.958 | 0.960 | 0.891 | 1.000 | 0.969 | 1.000 | 0.959 | 0.970 | 0.964 | 0.859 | 1.000 | 1.000 | 0.843 | 0.812 |
| 1185 | 0.752 | 1.000 | 0.769 | 0.986 | 0.974 | 0.960 | 0.881 | 1.000 | 0.969 | 1.000 | 0.951 | 0.970 | 0.973 | 0.857 | 1.000 | 1.000 | 0.844 | 0.813 |
| 1186 | 0.750 | 1.000 | 0.765 | 1.000 | 0.958 | 0.960 | 0.889 | 1.000 | 0.980 | 1.000 | 0.954 | 0.970 | 0.973 | 0.860 | 1.000 | 1.000 | 0.845 | 0.814 |
| 1187 | 0.746 | 1.000 | 0.767 | 1.000 | 0.947 | 0.960 | 0.878 | 1.000 | 0.980 | 1.000 | 0.954 | 1.000 | 0.964 | 0.855 | 1.000 | 1.000 | 0.841 | 0.809 |
| 1188 | 0.756 | 1.000 | 0.765 | 1.000 | 0.968 | 0.960 | 0.877 | 1.000 | 0.969 | 1.000 | 0.954 | 0.970 | 0.973 | 0.863 | 1.000 | 1.000 | 0.846 | 0.815 |
| 1189 | 0.737 | 1.000 | 0.755 | 1.000 | 0.942 | 0.960 | 0.880 | 1.000 | 0.969 | 1.000 | 0.948 | 0.970 | 0.964 | 0.854 | 1.000 | 1.000 | 0.836 | 0.803 |
| 1190 | 0.755 | 1.000 | 0.759 | 1.000 | 0.958 | 0.960 | 0.891 | 1.000 | 0.959 | 1.000 | 0.951 | 0.970 | 0.964 | 0.857 | 1.000 | 1.000 | 0.844 | 0.813 |
| 1191 | 0.753 | 1.000 | 0.759 | 0.986 | 0.963 | 0.960 | 0.891 | 1.000 | 0.969 | 1.000 | 0.954 | 0.970 | 0.964 | 0.850 | 1.000 | 1.000 | 0.842 | 0.811 |
| 1192 | 0.758 | 1.000 | 0.761 | 1.000 | 0.947 | 0.960 | 0.886 | 1.000 | 0.980 | 1.000 | 0.959 | 0.970 | 0.973 | 0.854 | 1.000 | 1.000 | 0.845 | 0.814 |
| 1193 | 0.753 | 1.000 | 0.771 | 1.000 | 0.963 | 0.960 | 0.881 | 1.000 | 0.980 | 1.000 | 0.945 | 0.970 | 0.964 | 0.858 | 1.000 | 1.000 | 0.844 | 0.813 |
| 1194 | 0.740 | 1.000 | 0.761 | 1.000 | 0.974 | 0.960 | 0.892 | 1.000 | 0.969 | 1.000 | 0.957 | 0.970 | 0.964 | 0.854 | 1.000 | 1.000 | 0.841 | 0.809 |
| 1195 | 0.750 | 1.000 | 0.755 | 0.986 | 0.963 | 0.960 | 0.886 | 1.000 | 0.969 | 1.000 | 0.957 | 0.970 | 0.973 | 0.856 | 1.000 | 1.000 | 0.842 | 0.811 |
| 1196 | 0.753 | 1.000 | 0.765 | 1.000 | 0.963 | 0.960 | 0.888 | 1.000 | 0.969 | 1.000 | 0.954 | 0.970 | 0.973 | 0.847 | 1.000 | 1.000 | 0.842 | 0.811 |
| 1197 | 0.746 | 1.000 | 0.775 | 0.986 | 0.958 | 0.960 | 0.888 | 1.000 | 0.980 | 1.000 | 0.954 | 0.970 | 0.964 | 0.862 | 1.000 | 1.000 | 0.845 | 0.814 |
| 1198 | 0.761 | 1.000 | 0.757 | 1.000 | 0.968 | 0.960 | 0.891 | 1.000 | 0.969 | 1.000 | 0.954 | 0.970 | 0.964 | 0.858 | 1.000 | 1.000 | 0.847 | 0.816 |
| 1199 | 0.751 | 1.000 | 0.769 | 0.986 | 0.963 | 0.960 | 0.888 | 1.000 | 0.969 | 1.000 | 0.951 | 0.970 | 0.964 | 0.856 | 1.000 | 1.000 | 0.844 | 0.812 |
| 1200 | 0.749 | 1.000 | 0.767 | 0.986 | 0.963 | 0.960 | 0.894 | 1.000 | 0.980 | 1.000 | 0.954 | 0.970 | 0.973 | 0.857 | 1.000 | 1.000 | 0.845 | 0.814 |
| 1201 | 0.751 | 1.000 | 0.773 | 1.000 | 0.974 | 0.960 | 0.883 | 1.000 | 0.980 | 1.000 | 0.962 | 0.970 | 0.973 | 0.858 | 1.000 | 1.000 | 0.846 | 0.815 |
| 1202 | 0.757 | 1.000 | 0.763 | 1.000 | 0.963 | 0.960 | 0.880 | 1.000 | 0.980 | 1.000 | 0.954 | 0.939 | 0.964 | 0.856 | 1.000 | 1.000 | 0.844 | 0.813 |
| 1203 | 0.755 | 1.000 | 0.741 | 1.000 | 0.963 | 0.960 | 0.881 | 1.000 | 0.969 | 1.000 | 0.951 | 0.970 | 0.964 | 0.849 | 1.000 | 1.000 | 0.840 | 0.807 |
| 1204 | 0.738 | 1.000 | 0.765 | 1.000 | 0.968 | 0.960 | 0.891 | 1.000 | 0.969 | 1.000 | 0.957 | 0.970 | 0.964 | 0.860 | 1.000 | 1.000 | 0.842 | 0.811 |
| 1205 | 0.758 | 1.000 | 0.757 | 1.000 | 0.958 | 0.960 | 0.883 | 1.000 | 0.969 | 1.000 | 0.957 | 0.970 | 0.964 | 0.856 | 1.000 | 1.000 | 0.844 | 0.813 |
| 1206 | 0.749 | 1.000 | 0.773 | 0.986 | 0.974 | 0.960 | 0.892 | 1.000 | 0.980 | 1.000 | 0.951 | 0.970 | 0.955 | 0.851 | 1.000 | 1.000 | 0.843 | 0.812 |
| 1207 | 0.755 | 1.000 | 0.767 | 1.000 | 0.953 | 0.960 | 0.881 | 1.000 | 0.969 | 1.000 | 0.957 | 0.970 | 0.964 | 0.853 | 1.000 | 1.000 | 0.843 | 0.812 |

| | | | | | | | | | | | | | | | | | |
|---|---|---|---|---|---|---|---|---|---|---|---|---|---|---|---|---|---|
| 1208 | 0.755 | 1.000 | 0.759 | 1.000 | 0.958 | 0.960 | 0.886 | 1.000 | 0.969 | 1.000 | 0.957 | 0.970 | 0.964 | 0.849 | 1.000 | 1.000 | 0.842 | 0.810 |
| 1209 | 0.755 | 1.000 | 0.755 | 1.000 | 0.968 | 0.960 | 0.891 | 1.000 | 0.969 | 1.000 | 0.965 | 0.970 | 0.955 | 0.859 | 1.000 | 1.000 | 0.846 | 0.815 |
| 1210 | 0.743 | 1.000 | 0.767 | 0.986 | 0.958 | 0.960 | 0.892 | 1.000 | 0.969 | 1.000 | 0.951 | 0.970 | 0.964 | 0.860 | 1.000 | 1.000 | 0.843 | 0.811 |
| 1211 | 0.754 | 1.000 | 0.767 | 1.000 | 0.958 | 0.960 | 0.880 | 1.000 | 0.969 | 1.000 | 0.957 | 0.970 | 0.955 | 0.860 | 1.000 | 1.000 | 0.845 | 0.813 |
| 1212 | 0.749 | 1.000 | 0.777 | 1.000 | 0.974 | 0.960 | 0.884 | 1.000 | 0.959 | 1.000 | 0.957 | 1.000 | 0.973 | 0.854 | 1.000 | 1.000 | 0.844 | 0.813 |
| 1213 | 0.753 | 1.000 | 0.761 | 0.986 | 0.963 | 0.960 | 0.898 | 1.000 | 0.969 | 1.000 | 0.965 | 0.970 | 0.964 | 0.858 | 1.000 | 1.000 | 0.846 | 0.816 |
| 1214 | 0.752 | 1.000 | 0.753 | 1.000 | 0.974 | 0.960 | 0.895 | 1.000 | 0.969 | 1.000 | 0.954 | 0.970 | 0.973 | 0.861 | 1.000 | 1.000 | 0.846 | 0.815 |
| 1215 | 0.753 | 1.000 | 0.759 | 1.000 | 0.968 | 0.960 | 0.881 | 1.000 | 0.969 | 1.000 | 0.968 | 0.970 | 0.973 | 0.857 | 1.000 | 1.000 | 0.845 | 0.813 |
| 1216 | 0.753 | 1.000 | 0.759 | 1.000 | 0.974 | 0.960 | 0.888 | 1.000 | 0.969 | 1.000 | 0.965 | 0.970 | 0.973 | 0.856 | 1.000 | 1.000 | 0.845 | 0.814 |
| 1217 | 0.752 | 1.000 | 0.759 | 0.986 | 0.974 | 0.960 | 0.894 | 1.000 | 0.980 | 1.000 | 0.962 | 0.970 | 0.973 | 0.854 | 1.000 | 1.000 | 0.845 | 0.814 |
| 1218 | 0.749 | 1.000 | 0.751 | 0.986 | 0.963 | 0.960 | 0.888 | 1.000 | 0.969 | 1.000 | 0.957 | 0.970 | 0.964 | 0.867 | 1.000 | 1.000 | 0.845 | 0.813 |
| 1219 | 0.759 | 1.000 | 0.763 | 1.000 | 0.958 | 0.960 | 0.898 | 1.000 | 0.959 | 1.000 | 0.957 | 0.970 | 0.973 | 0.849 | 1.000 | 1.000 | 0.845 | 0.815 |
| 1220 | 0.758 | 1.000 | 0.773 | 0.986 | 0.968 | 0.960 | 0.892 | 1.000 | 0.969 | 1.000 | 0.945 | 1.000 | 0.982 | 0.849 | 1.000 | 1.000 | 0.845 | 0.814 |
| 1221 | 0.748 | 1.000 | 0.761 | 0.986 | 0.963 | 0.960 | 0.889 | 1.000 | 0.969 | 1.000 | 0.959 | 0.970 | 0.973 | 0.861 | 1.000 | 1.000 | 0.844 | 0.813 |
| 1222 | 0.751 | 1.000 | 0.749 | 1.000 | 0.963 | 0.960 | 0.884 | 1.000 | 0.969 | 1.000 | 0.959 | 0.970 | 0.955 | 0.854 | 1.000 | 1.000 | 0.841 | 0.809 |
| 1223 | 0.745 | 1.000 | 0.759 | 1.000 | 0.963 | 0.960 | 0.883 | 1.000 | 0.969 | 1.000 | 0.954 | 0.970 | 0.964 | 0.855 | 1.000 | 1.000 | 0.841 | 0.809 |
| 1224 | 0.752 | 1.000 | 0.771 | 1.000 | 0.963 | 0.960 | 0.894 | 1.000 | 0.969 | 1.000 | 0.945 | 0.970 | 0.964 | 0.858 | 1.000 | 1.000 | 0.845 | 0.814 |
| 1225 | 0.755 | 1.000 | 0.769 | 1.000 | 0.958 | 0.960 | 0.888 | 1.000 | 0.980 | 1.000 | 0.962 | 1.000 | 0.964 | 0.853 | 1.000 | 1.000 | 0.845 | 0.814 |
| 1226 | 0.749 | 1.000 | 0.769 | 0.986 | 0.963 | 0.960 | 0.900 | 1.000 | 0.949 | 1.000 | 0.959 | 0.970 | 0.982 | 0.858 | 1.000 | 1.000 | 0.846 | 0.816 |
| 1227 | 0.757 | 1.000 | 0.767 | 1.000 | 0.963 | 0.960 | 0.892 | 1.000 | 0.980 | 1.000 | 0.948 | 0.970 | 0.973 | 0.858 | 1.000 | 1.000 | 0.847 | 0.816 |
| 1228 | 0.755 | 1.000 | 0.755 | 1.000 | 0.953 | 0.960 | 0.880 | 1.000 | 0.969 | 1.000 | 0.959 | 0.970 | 0.973 | 0.858 | 1.000 | 1.000 | 0.843 | 0.812 |
| 1229 | 0.758 | 1.000 | 0.767 | 0.986 | 0.958 | 0.960 | 0.891 | 1.000 | 0.980 | 1.000 | 0.945 | 0.970 | 0.955 | 0.855 | 1.000 | 1.000 | 0.845 | 0.814 |
| 1230 | 0.761 | 1.000 | 0.777 | 1.000 | 0.942 | 0.960 | 0.901 | 1.000 | 0.969 | 1.000 | 0.957 | 1.000 | 0.955 | 0.847 | 1.000 | 1.000 | 0.847 | 0.816 |
| 1231 | 0.759 | 1.000 | 0.763 | 1.000 | 0.963 | 0.960 | 0.886 | 1.000 | 0.980 | 1.000 | 0.954 | 0.970 | 0.982 | 0.855 | 1.000 | 1.000 | 0.846 | 0.815 |
| 1232 | 0.757 | 1.000 | 0.761 | 0.986 | 0.963 | 0.960 | 0.875 | 1.000 | 0.980 | 1.000 | 0.954 | 0.970 | 0.964 | 0.854 | 1.000 | 1.000 | 0.843 | 0.811 |
| 1233 | 0.751 | 1.000 | 0.773 | 1.000 | 0.958 | 0.960 | 0.898 | 1.000 | 0.969 | 1.000 | 0.965 | 0.970 | 0.982 | 0.861 | 1.000 | 1.000 | 0.848 | 0.818 |
| 1234 | 0.751 | 1.000 | 0.773 | 0.986 | 0.963 | 0.960 | 0.883 | 1.000 | 0.980 | 1.000 | 0.942 | 1.000 | 0.955 | 0.866 | 1.000 | 1.000 | 0.846 | 0.815 |
| 1235 | 0.755 | 1.000 | 0.747 | 1.000 | 0.953 | 0.960 | 0.894 | 1.000 | 0.990 | 1.000 | 0.954 | 0.970 | 0.964 | 0.856 | 1.000 | 1.000 | 0.844 | 0.813 |
| 1236 | 0.752 | 1.000 | 0.763 | 1.000 | 0.963 | 0.960 | 0.888 | 1.000 | 0.980 | 1.000 | 0.962 | 0.970 | 0.955 | 0.858 | 1.000 | 1.000 | 0.845 | 0.814 |
| 1237 | 0.753 | 1.000 | 0.751 | 1.000 | 0.974 | 0.960 | 0.886 | 1.000 | 0.980 | 1.000 | 0.951 | 0.970 | 0.964 | 0.851 | 1.000 | 1.000 | 0.842 | 0.810 |
| 1238 | 0.756 | 1.000 | 0.763 | 1.000 | 0.953 | 0.960 | 0.881 | 1.000 | 0.990 | 1.000 | 0.951 | 0.970 | 0.973 | 0.861 | 1.000 | 1.000 | 0.845 | 0.814 |
| 1239 | 0.759 | 1.000 | 0.767 | 1.000 | 0.968 | 0.960 | 0.883 | 1.000 | 0.980 | 1.000 | 0.954 | 0.970 | 0.964 | 0.854 | 1.000 | 1.000 | 0.846 | 0.814 |
| 1240 | 0.751 | 1.000 | 0.747 | 0.986 | 0.963 | 0.960 | 0.881 | 1.000 | 0.990 | 1.000 | 0.954 | 0.970 | 0.973 | 0.854 | 1.000 | 1.000 | 0.841 | 0.809 |
| 1241 | 0.759 | 1.000 | 0.767 | 0.986 | 0.958 | 0.960 | 0.888 | 1.000 | 0.969 | 1.000 | 0.954 | 0.970 | 0.964 | 0.862 | 1.000 | 1.000 | 0.848 | 0.817 |
| 1242 | 0.748 | 1.000 | 0.767 | 1.000 | 0.958 | 0.960 | 0.875 | 1.000 | 0.980 | 1.000 | 0.957 | 0.970 | 0.973 | 0.851 | 1.000 | 1.000 | 0.841 | 0.809 |
| 1243 | 0.748 | 1.000 | 0.781 | 1.000 | 0.968 | 0.960 | 0.888 | 1.000 | 0.980 | 1.000 | 0.951 | 1.000 | 0.964 | 0.858 | 1.000 | 1.000 | 0.845 | 0.815 |
| 1244 | 0.758 | 1.000 | 0.775 | 1.000 | 0.963 | 0.960 | 0.888 | 1.000 | 0.969 | 1.000 | 0.957 | 0.970 | 0.964 | 0.854 | 1.000 | 1.000 | 0.846 | 0.816 |
| 1245 | 0.759 | 1.000 | 0.767 | 0.986 | 0.968 | 0.960 | 0.900 | 1.000 | 0.969 | 1.000 | 0.954 | 0.970 | 0.964 | 0.853 | 1.000 | 1.000 | 0.847 | 0.817 |
| 1246 | 0.760 | 1.000 | 0.759 | 1.000 | 0.963 | 0.960 | 0.888 | 1.000 | 0.990 | 1.000 | 0.954 | 0.970 | 0.964 | 0.858 | 1.000 | 1.000 | 0.847 | 0.816 |

| | | | | | | | | | | | | | | | | | | |
|---|---|---|---|---|---|---|---|---|---|---|---|---|---|---|---|---|---|---|
| 1247 | 0.758 | 1.000 | 0.763 | 1.000 | 0.963 | 0.960 | 0.884 | 1.000 | 0.980 | 1.000 | 0.951 | 0.939 | 0.982 | 0.855 | 1.000 | 1.000 | 0.845 | 0.814 |
| 1248 | 0.749 | 1.000 | 0.781 | 1.000 | 0.958 | 0.960 | 0.892 | 1.000 | 0.969 | 1.000 | 0.962 | 0.939 | 0.973 | 0.857 | 1.000 | 1.000 | 0.846 | 0.815 |
| 1249 | 0.750 | 1.000 | 0.767 | 1.000 | 0.963 | 0.960 | 0.895 | 1.000 | 0.980 | 1.000 | 0.957 | 0.970 | 0.982 | 0.853 | 1.000 | 1.000 | 0.845 | 0.814 |
| 1250 | 0.754 | 1.000 | 0.759 | 1.000 | 0.974 | 0.960 | 0.892 | 1.000 | 0.969 | 1.000 | 0.962 | 0.939 | 0.964 | 0.855 | 1.000 | 1.000 | 0.845 | 0.814 |
| 1251 | 0.744 | 1.000 | 0.765 | 0.986 | 0.958 | 0.960 | 0.892 | 1.000 | 0.959 | 1.000 | 0.959 | 1.000 | 0.973 | 0.853 | 1.000 | 1.000 | 0.842 | 0.811 |
| 1252 | 0.756 | 1.000 | 0.759 | 1.000 | 0.968 | 0.960 | 0.897 | 1.000 | 0.980 | 1.000 | 0.951 | 0.970 | 0.964 | 0.868 | 1.000 | 1.000 | 0.849 | 0.819 |
| 1253 | 0.753 | 1.000 | 0.777 | 0.986 | 0.974 | 0.960 | 0.884 | 1.000 | 0.969 | 1.000 | 0.954 | 0.939 | 0.964 | 0.853 | 1.000 | 1.000 | 0.844 | 0.813 |
| 1254 | 0.753 | 1.000 | 0.761 | 0.986 | 0.958 | 0.960 | 0.891 | 1.000 | 0.969 | 1.000 | 0.962 | 0.970 | 0.964 | 0.852 | 1.000 | 1.000 | 0.843 | 0.812 |
| 1255 | 0.771 | 1.000 | 0.785 | 0.971 | 0.974 | 0.960 | 0.883 | 1.000 | 0.969 | 1.000 | 0.948 | 0.970 | 0.982 | 0.854 | 1.000 | 1.000 | 0.851 | 0.820 |
| 1256 | 0.751 | 1.000 | 0.771 | 1.000 | 0.968 | 0.960 | 0.894 | 1.000 | 0.969 | 1.000 | 0.951 | 0.939 | 0.973 | 0.852 | 1.000 | 1.000 | 0.844 | 0.813 |
| 1257 | 0.757 | 1.000 | 0.767 | 0.986 | 0.958 | 0.960 | 0.884 | 1.000 | 0.980 | 1.000 | 0.951 | 0.970 | 0.964 | 0.862 | 1.000 | 1.000 | 0.847 | 0.816 |
| 1258 | 0.751 | 1.000 | 0.755 | 1.000 | 0.968 | 0.960 | 0.897 | 1.000 | 0.959 | 1.000 | 0.957 | 1.000 | 0.964 | 0.856 | 1.000 | 1.000 | 0.844 | 0.813 |
| 1259 | 0.761 | 1.000 | 0.771 | 0.986 | 0.979 | 0.960 | 0.884 | 1.000 | 0.969 | 1.000 | 0.954 | 0.970 | 0.955 | 0.858 | 1.000 | 1.000 | 0.847 | 0.817 |
| 1260 | 0.743 | 1.000 | 0.773 | 0.986 | 0.963 | 0.960 | 0.892 | 1.000 | 0.969 | 1.000 | 0.957 | 0.970 | 0.964 | 0.858 | 1.000 | 1.000 | 0.843 | 0.812 |
| 1261 | 0.749 | 1.000 | 0.761 | 1.000 | 0.958 | 0.960 | 0.894 | 1.000 | 0.969 | 1.000 | 0.948 | 0.970 | 0.973 | 0.851 | 1.000 | 1.000 | 0.842 | 0.810 |
| 1262 | 0.745 | 1.000 | 0.781 | 0.986 | 0.968 | 0.960 | 0.895 | 1.000 | 0.980 | 1.000 | 0.957 | 0.970 | 0.964 | 0.851 | 1.000 | 1.000 | 0.844 | 0.813 |
| 1263 | 0.753 | 1.000 | 0.771 | 1.000 | 0.968 | 0.960 | 0.883 | 1.000 | 0.969 | 1.000 | 0.959 | 0.970 | 0.964 | 0.865 | 1.000 | 1.000 | 0.847 | 0.817 |
| 1264 | 0.748 | 1.000 | 0.769 | 0.986 | 0.974 | 0.960 | 0.894 | 1.000 | 0.969 | 1.000 | 0.951 | 0.970 | 0.964 | 0.865 | 1.000 | 1.000 | 0.846 | 0.815 |
| 1265 | 0.748 | 1.000 | 0.769 | 0.986 | 0.968 | 0.960 | 0.888 | 1.000 | 0.980 | 1.000 | 0.957 | 0.970 | 0.973 | 0.854 | 1.000 | 1.000 | 0.843 | 0.812 |
| 1266 | 0.757 | 1.000 | 0.767 | 0.986 | 0.963 | 0.960 | 0.897 | 1.000 | 0.980 | 1.000 | 0.951 | 0.970 | 0.973 | 0.865 | 1.000 | 1.000 | 0.849 | 0.819 |
| 1267 | 0.761 | 1.000 | 0.763 | 0.986 | 0.963 | 0.960 | 0.897 | 1.000 | 0.980 | 1.000 | 0.954 | 0.970 | 0.973 | 0.856 | 1.000 | 1.000 | 0.848 | 0.817 |
| 1268 | 0.748 | 1.000 | 0.751 | 1.000 | 0.958 | 0.960 | 0.894 | 1.000 | 0.980 | 1.000 | 0.948 | 0.970 | 0.964 | 0.857 | 1.000 | 1.000 | 0.842 | 0.811 |
| 1269 | 0.753 | 1.000 | 0.765 | 1.000 | 0.968 | 0.960 | 0.894 | 1.000 | 0.990 | 1.000 | 0.959 | 1.000 | 0.973 | 0.855 | 1.000 | 1.000 | 0.846 | 0.816 |
| 1270 | 0.753 | 1.000 | 0.763 | 0.986 | 0.953 | 0.960 | 0.886 | 1.000 | 0.980 | 1.000 | 0.951 | 0.970 | 0.973 | 0.858 | 1.000 | 1.000 | 0.844 | 0.813 |
| 1271 | 0.748 | 1.000 | 0.777 | 0.986 | 0.979 | 0.960 | 0.891 | 1.000 | 0.990 | 1.000 | 0.957 | 1.000 | 0.964 | 0.852 | 1.000 | 1.000 | 0.845 | 0.814 |
| 1272 | 0.753 | 1.000 | 0.763 | 1.000 | 0.968 | 0.960 | 0.888 | 1.000 | 0.980 | 1.000 | 0.951 | 0.970 | 0.982 | 0.851 | 1.000 | 1.000 | 0.843 | 0.812 |
| 1273 | 0.757 | 1.000 | 0.771 | 0.986 | 0.968 | 0.960 | 0.886 | 1.000 | 0.969 | 1.000 | 0.954 | 0.970 | 0.973 | 0.854 | 1.000 | 1.000 | 0.846 | 0.814 |
| 1274 | 0.758 | 1.000 | 0.773 | 0.986 | 0.968 | 0.960 | 0.880 | 1.000 | 0.980 | 1.000 | 0.959 | 0.970 | 0.982 | 0.852 | 1.000 | 1.000 | 0.846 | 0.815 |
| 1275 | 0.751 | 1.000 | 0.769 | 0.986 | 0.968 | 0.960 | 0.897 | 1.000 | 0.980 | 1.000 | 0.954 | 1.000 | 0.964 | 0.852 | 1.000 | 1.000 | 0.845 | 0.814 |
| 1276 | 0.746 | 1.000 | 0.771 | 0.986 | 0.963 | 0.960 | 0.883 | 1.000 | 0.990 | 1.000 | 0.948 | 0.939 | 0.964 | 0.849 | 1.000 | 1.000 | 0.840 | 0.808 |
| 1277 | 0.751 | 1.000 | 0.781 | 0.986 | 0.968 | 0.960 | 0.883 | 1.000 | 0.980 | 1.000 | 0.951 | 0.970 | 0.964 | 0.854 | 1.000 | 1.000 | 0.844 | 0.813 |
| 1278 | 0.750 | 1.000 | 0.759 | 1.000 | 0.968 | 0.960 | 0.889 | 1.000 | 0.980 | 1.000 | 0.948 | 0.970 | 0.973 | 0.864 | 1.000 | 1.000 | 0.845 | 0.814 |
| 1279 | 0.749 | 1.000 | 0.783 | 1.000 | 0.974 | 0.960 | 0.895 | 1.000 | 0.980 | 1.000 | 0.951 | 1.000 | 0.973 | 0.844 | 1.000 | 1.000 | 0.844 | 0.813 |
| 1280 | 0.759 | 1.000 | 0.769 | 0.986 | 0.963 | 0.960 | 0.880 | 1.000 | 0.990 | 1.000 | 0.959 | 1.000 | 0.964 | 0.864 | 1.000 | 1.000 | 0.848 | 0.818 |
| 1281 | 0.753 | 1.000 | 0.765 | 0.986 | 0.968 | 0.960 | 0.897 | 1.000 | 0.980 | 1.000 | 0.954 | 0.970 | 0.973 | 0.847 | 1.000 | 1.000 | 0.844 | 0.813 |
| 1282 | 0.755 | 1.000 | 0.773 | 1.000 | 0.974 | 0.960 | 0.891 | 1.000 | 0.980 | 1.000 | 0.948 | 0.970 | 0.982 | 0.861 | 1.000 | 1.000 | 0.848 | 0.818 |
| 1283 | 0.750 | 1.000 | 0.785 | 1.000 | 0.968 | 0.960 | 0.891 | 1.000 | 0.969 | 1.000 | 0.951 | 0.970 | 0.973 | 0.852 | 1.000 | 1.000 | 0.845 | 0.814 |
| 1284 | 0.746 | 1.000 | 0.769 | 1.000 | 0.958 | 0.960 | 0.880 | 1.000 | 0.980 | 1.000 | 0.957 | 0.970 | 0.955 | 0.850 | 1.000 | 1.000 | 0.840 | 0.808 |
| 1285 | 0.757 | 1.000 | 0.755 | 1.000 | 0.968 | 0.960 | 0.897 | 1.000 | 0.969 | 1.000 | 0.954 | 1.000 | 0.964 | 0.854 | 1.000 | 1.000 | 0.845 | 0.814 |

| | | | | | | | | | | | | | | | | | | |
|---|---|---|---|---|---|---|---|---|---|---|---|---|---|---|---|---|---|---|
| 1286 | 0.759 | 1.000 | 0.759 | 0.986 | 0.968 | 1.000 | 0.892 | 1.000 | 0.969 | 1.000 | 0.954 | 0.970 | 0.982 | 0.861 | 1.000 | 1.000 | 0.848 | 0.817 |
| 1287 | 0.752 | 1.000 | 0.757 | 1.000 | 0.963 | 0.960 | 0.889 | 1.000 | 0.990 | 1.000 | 0.951 | 0.970 | 0.973 | 0.854 | 1.000 | 1.000 | 0.843 | 0.812 |
| 1288 | 0.752 | 1.000 | 0.779 | 1.000 | 0.974 | 0.960 | 0.891 | 1.000 | 0.969 | 1.000 | 0.951 | 0.970 | 0.973 | 0.858 | 1.000 | 1.000 | 0.847 | 0.816 |
| 1289 | 0.761 | 1.000 | 0.775 | 1.000 | 0.963 | 0.960 | 0.897 | 1.000 | 0.969 | 1.000 | 0.954 | 0.970 | 0.955 | 0.858 | 1.000 | 1.000 | 0.849 | 0.819 |
| 1290 | 0.755 | 1.000 | 0.785 | 1.000 | 0.958 | 0.960 | 0.894 | 1.000 | 0.969 | 1.000 | 0.959 | 0.970 | 0.973 | 0.854 | 1.000 | 1.000 | 0.848 | 0.817 |
| 1291 | 0.753 | 1.000 | 0.765 | 0.986 | 0.963 | 0.960 | 0.886 | 1.000 | 0.980 | 1.000 | 0.954 | 0.970 | 0.955 | 0.858 | 1.000 | 1.000 | 0.844 | 0.813 |
| 1292 | 0.751 | 1.000 | 0.765 | 0.986 | 0.968 | 0.960 | 0.891 | 1.000 | 0.990 | 1.000 | 0.957 | 0.939 | 0.955 | 0.856 | 1.000 | 1.000 | 0.844 | 0.813 |
| 1293 | 0.763 | 1.000 | 0.779 | 1.000 | 0.979 | 0.960 | 0.889 | 1.000 | 0.980 | 1.000 | 0.951 | 0.970 | 0.973 | 0.857 | 1.000 | 1.000 | 0.850 | 0.820 |
| 1294 | 0.761 | 1.000 | 0.765 | 0.986 | 0.958 | 0.960 | 0.880 | 1.000 | 0.980 | 1.000 | 0.965 | 0.970 | 0.973 | 0.858 | 1.000 | 1.000 | 0.847 | 0.816 |
| 1295 | 0.755 | 1.000 | 0.775 | 1.000 | 0.974 | 0.960 | 0.889 | 1.000 | 0.969 | 1.000 | 0.962 | 1.000 | 0.964 | 0.851 | 1.000 | 1.000 | 0.846 | 0.816 |
| 1296 | 0.758 | 1.000 | 0.775 | 1.000 | 0.968 | 0.960 | 0.880 | 1.000 | 0.990 | 1.000 | 0.951 | 0.939 | 0.973 | 0.861 | 1.000 | 1.000 | 0.847 | 0.816 |
| 1297 | 0.751 | 1.000 | 0.761 | 1.000 | 0.968 | 0.960 | 0.895 | 1.000 | 0.980 | 1.000 | 0.965 | 1.000 | 0.973 | 0.860 | 1.000 | 1.000 | 0.847 | 0.817 |
| 1298 | 0.744 | 1.000 | 0.767 | 1.000 | 0.974 | 0.960 | 0.898 | 1.000 | 0.969 | 1.000 | 0.951 | 0.970 | 0.973 | 0.853 | 1.000 | 1.000 | 0.843 | 0.812 |
| 1299 | 0.762 | 1.000 | 0.777 | 1.000 | 0.974 | 0.960 | 0.886 | 1.000 | 0.969 | 1.000 | 0.954 | 1.000 | 0.964 | 0.855 | 1.000 | 1.000 | 0.848 | 0.818 |
| 1300 | 0.752 | 1.000 | 0.783 | 1.000 | 0.968 | 0.960 | 0.892 | 1.000 | 0.969 | 1.000 | 0.959 | 0.970 | 0.964 | 0.863 | 1.000 | 1.000 | 0.849 | 0.818 |
| 1301 | 0.767 | 1.000 | 0.753 | 0.986 | 0.963 | 0.960 | 0.900 | 1.000 | 0.980 | 1.000 | 0.959 | 0.939 | 0.955 | 0.859 | 1.000 | 1.000 | 0.850 | 0.819 |
| 1302 | 0.757 | 1.000 | 0.777 | 1.000 | 0.958 | 0.960 | 0.886 | 1.000 | 0.969 | 1.000 | 0.951 | 1.000 | 0.964 | 0.852 | 1.000 | 1.000 | 0.845 | 0.814 |
| 1303 | 0.755 | 1.000 | 0.761 | 1.000 | 0.968 | 0.960 | 0.892 | 1.000 | 0.980 | 1.000 | 0.951 | 0.970 | 0.964 | 0.862 | 1.000 | 1.000 | 0.847 | 0.816 |
| 1304 | 0.751 | 1.000 | 0.763 | 1.000 | 0.968 | 0.960 | 0.892 | 1.000 | 0.969 | 1.000 | 0.965 | 0.970 | 0.973 | 0.855 | 1.000 | 1.000 | 0.845 | 0.814 |
| 1305 | 0.754 | 1.000 | 0.781 | 1.000 | 0.963 | 0.960 | 0.881 | 1.000 | 0.980 | 1.000 | 0.957 | 0.970 | 0.964 | 0.861 | 1.000 | 1.000 | 0.847 | 0.817 |
| 1306 | 0.760 | 1.000 | 0.777 | 0.986 | 0.968 | 0.960 | 0.886 | 1.000 | 0.980 | 1.000 | 0.959 | 0.970 | 0.973 | 0.856 | 1.000 | 1.000 | 0.848 | 0.818 |
| 1307 | 0.754 | 1.000 | 0.763 | 1.000 | 0.968 | 0.960 | 0.883 | 1.000 | 0.980 | 1.000 | 0.951 | 0.970 | 0.964 | 0.857 | 1.000 | 1.000 | 0.844 | 0.813 |
| 1308 | 0.755 | 1.000 | 0.767 | 0.986 | 0.979 | 0.960 | 0.888 | 1.000 | 0.969 | 1.000 | 0.959 | 0.970 | 0.964 | 0.858 | 1.000 | 1.000 | 0.846 | 0.816 |
| 1309 | 0.759 | 1.000 | 0.771 | 1.000 | 0.968 | 0.960 | 0.878 | 1.000 | 0.980 | 1.000 | 0.954 | 0.970 | 0.982 | 0.856 | 1.000 | 1.000 | 0.846 | 0.815 |
| 1310 | 0.758 | 1.000 | 0.763 | 0.986 | 0.958 | 0.960 | 0.900 | 1.000 | 0.980 | 1.000 | 0.957 | 1.000 | 0.973 | 0.855 | 1.000 | 1.000 | 0.847 | 0.817 |
| 1311 | 0.755 | 1.000 | 0.775 | 0.986 | 0.968 | 0.960 | 0.898 | 1.000 | 0.969 | 1.000 | 0.957 | 0.970 | 0.973 | 0.855 | 1.000 | 1.000 | 0.847 | 0.817 |
| 1312 | 0.765 | 1.000 | 0.755 | 1.000 | 0.963 | 0.960 | 0.891 | 1.000 | 0.980 | 1.000 | 0.954 | 0.970 | 0.964 | 0.860 | 1.000 | 1.000 | 0.848 | 0.818 |
| 1313 | 0.762 | 1.000 | 0.763 | 1.000 | 0.947 | 0.960 | 0.900 | 1.000 | 0.969 | 1.000 | 0.957 | 0.970 | 0.964 | 0.862 | 1.000 | 1.000 | 0.850 | 0.819 |
| 1314 | 0.752 | 1.000 | 0.755 | 1.000 | 0.968 | 0.960 | 0.912 | 1.000 | 0.969 | 1.000 | 0.957 | 0.970 | 0.973 | 0.860 | 1.000 | 1.000 | 0.848 | 0.818 |
| 1315 | 0.766 | 1.000 | 0.765 | 1.000 | 0.974 | 0.960 | 0.886 | 1.000 | 0.980 | 1.000 | 0.954 | 0.939 | 0.982 | 0.860 | 1.000 | 1.000 | 0.850 | 0.819 |
| 1316 | 0.751 | 1.000 | 0.755 | 1.000 | 0.968 | 0.960 | 0.889 | 1.000 | 0.969 | 1.000 | 0.959 | 1.000 | 0.964 | 0.861 | 1.000 | 1.000 | 0.845 | 0.814 |
| 1317 | 0.741 | 1.000 | 0.775 | 1.000 | 0.968 | 0.960 | 0.897 | 1.000 | 0.990 | 1.000 | 0.948 | 0.970 | 0.964 | 0.854 | 1.000 | 1.000 | 0.843 | 0.812 |
| 1318 | 0.751 | 1.000 | 0.763 | 0.986 | 0.974 | 0.960 | 0.892 | 1.000 | 0.969 | 1.000 | 0.962 | 0.939 | 0.982 | 0.858 | 1.000 | 1.000 | 0.846 | 0.815 |
| 1319 | 0.759 | 1.000 | 0.781 | 1.000 | 0.953 | 0.960 | 0.900 | 1.000 | 0.969 | 1.000 | 0.954 | 0.970 | 0.964 | 0.861 | 1.000 | 1.000 | 0.850 | 0.820 |
| 1320 | 0.757 | 1.000 | 0.761 | 0.986 | 0.968 | 0.960 | 0.908 | 1.000 | 0.969 | 1.000 | 0.954 | 0.970 | 0.955 | 0.857 | 1.000 | 1.000 | 0.848 | 0.817 |
| 1321 | 0.763 | 1.000 | 0.771 | 1.000 | 0.968 | 0.960 | 0.894 | 1.000 | 0.969 | 1.000 | 0.951 | 0.970 | 0.973 | 0.857 | 1.000 | 1.000 | 0.849 | 0.819 |
| 1322 | 0.761 | 1.000 | 0.765 | 1.000 | 0.963 | 0.960 | 0.894 | 1.000 | 0.980 | 1.000 | 0.954 | 0.970 | 0.973 | 0.854 | 1.000 | 1.000 | 0.847 | 0.817 |
| 1323 | 0.751 | 1.000 | 0.771 | 1.000 | 0.968 | 0.960 | 0.886 | 1.000 | 0.980 | 1.000 | 0.945 | 0.970 | 0.964 | 0.863 | 1.000 | 1.000 | 0.846 | 0.815 |
| 1324 | 0.753 | 1.000 | 0.763 | 0.986 | 0.968 | 0.960 | 0.901 | 1.000 | 0.969 | 1.000 | 0.954 | 0.970 | 0.973 | 0.852 | 1.000 | 1.000 | 0.845 | 0.814 |

| | | | | | | | | | | | | | | | | | | |
|---|---|---|---|---|---|---|---|---|---|---|---|---|---|---|---|---|---|---|
| 1325 | 0.754 | 1.000 | 0.773 | 1.000 | 0.958 | 0.960 | 0.889 | 1.000 | 0.980 | 1.000 | 0.954 | 1.000 | 0.964 | 0.854 | 1.000 | 1.000 | 0.845 | 0.814 |
| 1326 | 0.762 | 1.000 | 0.787 | 0.986 | 0.963 | 0.960 | 0.894 | 1.000 | 0.969 | 1.000 | 0.962 | 0.970 | 0.964 | 0.855 | 1.000 | 1.000 | 0.850 | 0.820 |
| 1327 | 0.748 | 1.000 | 0.763 | 0.986 | 0.974 | 0.960 | 0.888 | 1.000 | 0.969 | 1.000 | 0.959 | 0.970 | 0.964 | 0.860 | 1.000 | 1.000 | 0.844 | 0.813 |
| 1328 | 0.760 | 1.000 | 0.779 | 0.986 | 0.963 | 0.960 | 0.891 | 1.000 | 0.980 | 1.000 | 0.959 | 0.970 | 0.964 | 0.859 | 1.000 | 1.000 | 0.849 | 0.819 |
| 1329 | 0.755 | 1.000 | 0.775 | 1.000 | 0.963 | 0.960 | 0.898 | 1.000 | 0.980 | 1.000 | 0.942 | 0.939 | 0.964 | 0.857 | 1.000 | 1.000 | 0.847 | 0.816 |
| 1330 | 0.763 | 1.000 | 0.781 | 0.986 | 0.953 | 0.960 | 0.884 | 1.000 | 0.990 | 1.000 | 0.957 | 0.970 | 0.973 | 0.855 | 1.000 | 1.000 | 0.848 | 0.818 |
| 1331 | 0.749 | 1.000 | 0.775 | 0.986 | 0.974 | 0.960 | 0.894 | 1.000 | 0.969 | 1.000 | 0.957 | 0.970 | 0.973 | 0.858 | 1.000 | 1.000 | 0.846 | 0.816 |
| 1332 | 0.752 | 1.000 | 0.767 | 1.000 | 0.968 | 0.960 | 0.878 | 1.000 | 0.969 | 1.000 | 0.965 | 0.970 | 0.964 | 0.854 | 1.000 | 1.000 | 0.844 | 0.812 |
| 1333 | 0.759 | 1.000 | 0.771 | 0.986 | 0.974 | 0.960 | 0.897 | 1.000 | 1.000 | 1.000 | 0.951 | 0.970 | 0.973 | 0.860 | 1.000 | 1.000 | 0.850 | 0.820 |
| 1334 | 0.757 | 1.000 | 0.775 | 1.000 | 0.974 | 0.960 | 0.891 | 1.000 | 0.980 | 1.000 | 0.957 | 0.939 | 0.973 | 0.861 | 1.000 | 1.000 | 0.849 | 0.819 |
| 1335 | 0.763 | 1.000 | 0.783 | 1.000 | 0.968 | 0.960 | 0.903 | 1.000 | 0.969 | 1.000 | 0.959 | 0.970 | 0.982 | 0.863 | 1.000 | 1.000 | 0.854 | 0.824 |
| 1336 | 0.759 | 1.000 | 0.775 | 1.000 | 0.963 | 0.960 | 0.886 | 1.000 | 0.969 | 1.000 | 0.957 | 0.970 | 0.982 | 0.857 | 1.000 | 1.000 | 0.848 | 0.817 |
| 1337 | 0.757 | 1.000 | 0.775 | 0.986 | 0.958 | 0.960 | 0.888 | 1.000 | 0.980 | 1.000 | 0.959 | 0.970 | 0.964 | 0.851 | 1.000 | 1.000 | 0.845 | 0.814 |
| 1338 | 0.758 | 1.000 | 0.783 | 0.986 | 0.974 | 0.960 | 0.895 | 1.000 | 0.969 | 1.000 | 0.962 | 0.970 | 0.964 | 0.866 | 1.000 | 1.000 | 0.852 | 0.822 |
| 1339 | 0.755 | 1.000 | 0.769 | 0.986 | 0.958 | 0.960 | 0.898 | 1.000 | 0.959 | 1.000 | 0.957 | 0.970 | 0.955 | 0.856 | 1.000 | 1.000 | 0.846 | 0.815 |
| 1340 | 0.760 | 1.000 | 0.759 | 0.986 | 0.979 | 0.960 | 0.897 | 1.000 | 0.969 | 1.000 | 0.954 | 0.939 | 0.973 | 0.856 | 1.000 | 1.000 | 0.847 | 0.817 |
| 1341 | 0.756 | 1.000 | 0.763 | 0.986 | 0.953 | 0.960 | 0.880 | 1.000 | 0.969 | 1.000 | 0.948 | 1.000 | 0.982 | 0.861 | 1.000 | 1.000 | 0.845 | 0.814 |
| 1342 | 0.756 | 1.000 | 0.765 | 1.000 | 0.947 | 0.960 | 0.895 | 1.000 | 0.990 | 1.000 | 0.959 | 0.970 | 0.964 | 0.864 | 1.000 | 1.000 | 0.848 | 0.818 |
| 1343 | 0.755 | 1.000 | 0.759 | 1.000 | 0.953 | 0.960 | 0.901 | 1.000 | 0.990 | 1.000 | 0.959 | 0.970 | 0.982 | 0.859 | 1.000 | 1.000 | 0.848 | 0.818 |
| 1344 | 0.756 | 1.000 | 0.777 | 0.986 | 0.963 | 0.960 | 0.881 | 1.000 | 0.990 | 1.000 | 0.948 | 0.970 | 0.982 | 0.865 | 1.000 | 1.000 | 0.848 | 0.817 |
| 1345 | 0.755 | 1.000 | 0.779 | 1.000 | 0.958 | 0.960 | 0.888 | 1.000 | 0.990 | 1.000 | 0.951 | 0.970 | 0.955 | 0.864 | 1.000 | 1.000 | 0.848 | 0.818 |
| 1346 | 0.755 | 1.000 | 0.759 | 0.971 | 0.953 | 0.960 | 0.892 | 1.000 | 0.990 | 1.000 | 0.968 | 0.970 | 0.964 | 0.867 | 1.000 | 1.000 | 0.848 | 0.818 |
| 1347 | 0.761 | 1.000 | 0.773 | 1.000 | 0.963 | 0.960 | 0.891 | 1.000 | 0.980 | 1.000 | 0.942 | 1.000 | 0.973 | 0.867 | 1.000 | 1.000 | 0.851 | 0.821 |
| 1348 | 0.755 | 1.000 | 0.769 | 0.986 | 0.958 | 0.960 | 0.892 | 1.000 | 0.980 | 1.000 | 0.957 | 0.939 | 0.964 | 0.863 | 1.000 | 1.000 | 0.847 | 0.817 |
| 1349 | 0.767 | 1.000 | 0.769 | 1.000 | 0.979 | 0.960 | 0.884 | 1.000 | 0.980 | 1.000 | 0.951 | 0.970 | 0.973 | 0.856 | 1.000 | 1.000 | 0.849 | 0.819 |
| 1350 | 0.760 | 1.000 | 0.771 | 0.986 | 0.963 | 0.960 | 0.897 | 1.000 | 0.980 | 1.000 | 0.945 | 0.939 | 0.973 | 0.858 | 1.000 | 1.000 | 0.848 | 0.818 |
| 1351 | 0.759 | 1.000 | 0.761 | 0.986 | 0.963 | 0.960 | 0.895 | 1.000 | 0.980 | 1.000 | 0.957 | 0.970 | 0.982 | 0.863 | 1.000 | 1.000 | 0.849 | 0.818 |
| 1352 | 0.757 | 1.000 | 0.773 | 0.986 | 0.974 | 0.960 | 0.892 | 1.000 | 0.990 | 1.000 | 0.959 | 0.970 | 0.964 | 0.857 | 1.000 | 1.000 | 0.848 | 0.818 |
| 1353 | 0.758 | 1.000 | 0.769 | 0.986 | 0.953 | 0.960 | 0.886 | 1.000 | 0.980 | 1.000 | 0.959 | 0.939 | 0.973 | 0.856 | 1.000 | 1.000 | 0.846 | 0.815 |
| 1354 | 0.765 | 1.000 | 0.759 | 0.986 | 0.974 | 0.960 | 0.886 | 1.000 | 0.990 | 1.000 | 0.957 | 0.970 | 0.964 | 0.867 | 1.000 | 1.000 | 0.851 | 0.820 |
| 1355 | 0.746 | 1.000 | 0.769 | 1.000 | 0.974 | 0.960 | 0.892 | 1.000 | 0.980 | 1.000 | 0.959 | 0.970 | 0.964 | 0.861 | 1.000 | 1.000 | 0.846 | 0.815 |
| 1356 | 0.751 | 1.000 | 0.767 | 0.986 | 0.958 | 0.960 | 0.880 | 1.000 | 0.980 | 1.000 | 0.957 | 0.970 | 0.964 | 0.861 | 1.000 | 1.000 | 0.844 | 0.813 |
| 1357 | 0.755 | 1.000 | 0.769 | 0.986 | 0.968 | 0.960 | 0.884 | 1.000 | 0.990 | 1.000 | 0.942 | 0.970 | 0.955 | 0.858 | 1.000 | 1.000 | 0.845 | 0.813 |
| 1358 | 0.760 | 1.000 | 0.767 | 1.000 | 0.968 | 0.960 | 0.895 | 1.000 | 0.990 | 1.000 | 0.951 | 0.970 | 0.973 | 0.852 | 1.000 | 1.000 | 0.847 | 0.817 |
| 1359 | 0.755 | 1.000 | 0.761 | 0.986 | 0.968 | 0.960 | 0.888 | 1.000 | 0.980 | 1.000 | 0.962 | 0.939 | 0.964 | 0.864 | 1.000 | 1.000 | 0.847 | 0.816 |
| 1360 | 0.749 | 1.000 | 0.773 | 0.986 | 0.963 | 0.960 | 0.900 | 1.000 | 0.990 | 1.000 | 0.959 | 0.970 | 0.964 | 0.865 | 1.000 | 1.000 | 0.848 | 0.818 |
| 1361 | 0.755 | 1.000 | 0.773 | 1.000 | 0.958 | 0.960 | 0.895 | 1.000 | 0.990 | 1.000 | 0.954 | 0.970 | 0.982 | 0.859 | 1.000 | 1.000 | 0.848 | 0.818 |
| 1362 | 0.763 | 1.000 | 0.783 | 1.000 | 0.958 | 0.960 | 0.901 | 1.000 | 0.990 | 1.000 | 0.959 | 0.970 | 0.973 | 0.856 | 1.000 | 1.000 | 0.852 | 0.822 |
| 1363 | 0.752 | 1.000 | 0.765 | 0.986 | 0.963 | 0.960 | 0.892 | 1.000 | 0.969 | 1.000 | 0.959 | 0.970 | 0.964 | 0.861 | 1.000 | 1.000 | 0.846 | 0.815 |

| | | | | | | | | | | | | | | | | | | |
|---|---|---|---|---|---|---|---|---|---|---|---|---|---|---|---|---|---|---|
| 1364 | 0.759 | 1.000 | 0.777 | 0.986 | 0.974 | 0.960 | 0.895 | 1.000 | 0.990 | 1.000 | 0.957 | 0.970 | 0.964 | 0.854 | 1.000 | 1.000 | 0.849 | 0.818 |
| 1365 | 0.757 | 1.000 | 0.753 | 0.971 | 0.963 | 0.960 | 0.900 | 1.000 | 0.990 | 1.000 | 0.965 | 0.970 | 0.973 | 0.852 | 1.000 | 1.000 | 0.846 | 0.815 |
| 1366 | 0.764 | 1.000 | 0.771 | 1.000 | 0.953 | 0.960 | 0.889 | 1.000 | 0.980 | 1.000 | 0.965 | 0.970 | 0.973 | 0.856 | 1.000 | 1.000 | 0.849 | 0.819 |
| 1367 | 0.755 | 1.000 | 0.783 | 1.000 | 0.974 | 0.960 | 0.895 | 1.000 | 0.990 | 1.000 | 0.948 | 0.939 | 0.973 | 0.863 | 1.000 | 1.000 | 0.850 | 0.820 |
| 1368 | 0.763 | 1.000 | 0.783 | 1.000 | 0.968 | 0.960 | 0.888 | 1.000 | 0.969 | 1.000 | 0.965 | 1.000 | 0.964 | 0.863 | 1.000 | 1.000 | 0.852 | 0.823 |
| 1369 | 0.767 | 1.000 | 0.765 | 0.986 | 0.963 | 0.960 | 0.881 | 1.000 | 0.990 | 1.000 | 0.957 | 0.970 | 0.964 | 0.868 | 1.000 | 1.000 | 0.851 | 0.821 |
| 1370 | 0.763 | 1.000 | 0.755 | 1.000 | 0.968 | 0.960 | 0.897 | 1.000 | 0.980 | 1.000 | 0.959 | 0.939 | 0.955 | 0.852 | 1.000 | 1.000 | 0.847 | 0.816 |
| 1371 | 0.761 | 1.000 | 0.789 | 1.000 | 0.958 | 0.960 | 0.891 | 1.000 | 0.980 | 1.000 | 0.959 | 0.939 | 0.964 | 0.856 | 1.000 | 1.000 | 0.850 | 0.820 |
| 1372 | 0.759 | 1.000 | 0.773 | 1.000 | 0.953 | 0.960 | 0.891 | 1.000 | 0.990 | 1.000 | 0.951 | 0.970 | 0.982 | 0.860 | 1.000 | 1.000 | 0.848 | 0.818 |
| 1373 | 0.761 | 1.000 | 0.753 | 0.986 | 0.947 | 0.960 | 0.895 | 1.000 | 0.990 | 1.000 | 0.954 | 0.939 | 0.982 | 0.861 | 1.000 | 1.000 | 0.847 | 0.816 |
| 1374 | 0.755 | 1.000 | 0.789 | 1.000 | 0.968 | 0.960 | 0.900 | 1.000 | 0.990 | 1.000 | 0.948 | 0.939 | 0.955 | 0.864 | 1.000 | 1.000 | 0.851 | 0.821 |
| 1375 | 0.760 | 1.000 | 0.783 | 1.000 | 0.947 | 0.960 | 0.884 | 1.000 | 0.980 | 1.000 | 0.948 | 0.970 | 0.973 | 0.858 | 1.000 | 1.000 | 0.848 | 0.817 |
| 1376 | 0.759 | 1.000 | 0.771 | 1.000 | 0.953 | 0.960 | 0.894 | 1.000 | 0.980 | 1.000 | 0.954 | 0.970 | 0.964 | 0.860 | 1.000 | 1.000 | 0.848 | 0.818 |
| 1377 | 0.755 | 1.000 | 0.771 | 0.986 | 0.974 | 0.960 | 0.897 | 1.000 | 0.990 | 1.000 | 0.948 | 0.970 | 0.973 | 0.865 | 1.000 | 1.000 | 0.849 | 0.819 |
| 1378 | 0.753 | 1.000 | 0.777 | 1.000 | 0.963 | 0.960 | 0.901 | 1.000 | 0.969 | 1.000 | 0.959 | 0.970 | 0.982 | 0.861 | 1.000 | 1.000 | 0.849 | 0.820 |
| 1379 | 0.750 | 1.000 | 0.781 | 1.000 | 0.974 | 0.960 | 0.897 | 1.000 | 0.980 | 1.000 | 0.957 | 0.939 | 0.964 | 0.861 | 1.000 | 1.000 | 0.848 | 0.818 |
| 1380 | 0.758 | 1.000 | 0.773 | 0.986 | 0.968 | 0.960 | 0.894 | 1.000 | 1.000 | 1.000 | 0.954 | 1.000 | 0.982 | 0.856 | 1.000 | 1.000 | 0.849 | 0.818 |
| 1381 | 0.761 | 1.000 | 0.767 | 1.000 | 0.968 | 0.960 | 0.901 | 1.000 | 0.990 | 1.000 | 0.962 | 0.970 | 0.964 | 0.858 | 1.000 | 1.000 | 0.850 | 0.820 |
| 1382 | 0.747 | 1.000 | 0.775 | 0.986 | 0.974 | 0.960 | 0.903 | 1.000 | 0.990 | 1.000 | 0.957 | 0.939 | 0.973 | 0.854 | 1.000 | 1.000 | 0.846 | 0.815 |
| 1383 | 0.765 | 1.000 | 0.777 | 1.000 | 0.968 | 0.960 | 0.881 | 1.000 | 0.969 | 1.000 | 0.948 | 0.970 | 0.964 | 0.862 | 1.000 | 1.000 | 0.850 | 0.819 |
| 1384 | 0.759 | 1.000 | 0.775 | 0.986 | 0.963 | 0.960 | 0.892 | 1.000 | 0.980 | 1.000 | 0.965 | 0.939 | 0.964 | 0.868 | 1.000 | 1.000 | 0.851 | 0.822 |
| 1385 | 0.746 | 1.000 | 0.767 | 0.986 | 0.953 | 0.960 | 0.894 | 1.000 | 0.980 | 1.000 | 0.951 | 1.000 | 0.973 | 0.860 | 1.000 | 1.000 | 0.844 | 0.813 |
| 1386 | 0.753 | 1.000 | 0.763 | 0.986 | 0.963 | 0.960 | 0.900 | 1.000 | 0.969 | 1.000 | 0.951 | 0.970 | 0.973 | 0.857 | 1.000 | 1.000 | 0.846 | 0.815 |
| 1387 | 0.761 | 1.000 | 0.767 | 1.000 | 0.984 | 0.960 | 0.888 | 1.000 | 0.980 | 1.000 | 0.951 | 0.970 | 0.955 | 0.860 | 1.000 | 1.000 | 0.848 | 0.818 |
| 1388 | 0.756 | 1.000 | 0.763 | 1.000 | 0.947 | 0.960 | 0.889 | 1.000 | 1.000 | 1.000 | 0.957 | 0.970 | 0.973 | 0.857 | 1.000 | 1.000 | 0.846 | 0.815 |
| 1389 | 0.757 | 1.000 | 0.785 | 0.986 | 0.963 | 0.960 | 0.903 | 1.000 | 0.980 | 1.000 | 0.951 | 0.970 | 0.982 | 0.853 | 1.000 | 1.000 | 0.849 | 0.819 |
| 1390 | 0.755 | 1.000 | 0.773 | 0.986 | 0.968 | 0.960 | 0.894 | 1.000 | 0.990 | 1.000 | 0.945 | 0.970 | 0.973 | 0.865 | 1.000 | 1.000 | 0.849 | 0.819 |
| 1391 | 0.759 | 1.000 | 0.755 | 0.986 | 0.963 | 0.960 | 0.895 | 1.000 | 0.980 | 1.000 | 0.951 | 0.970 | 0.964 | 0.855 | 1.000 | 1.000 | 0.846 | 0.814 |
| 1392 | 0.752 | 1.000 | 0.775 | 1.000 | 0.968 | 0.960 | 0.897 | 1.000 | 0.980 | 1.000 | 0.957 | 0.939 | 0.973 | 0.859 | 1.000 | 1.000 | 0.848 | 0.817 |
| 1393 | 0.758 | 1.000 | 0.765 | 1.000 | 0.968 | 0.960 | 0.891 | 1.000 | 0.990 | 1.000 | 0.939 | 0.970 | 0.982 | 0.862 | 1.000 | 1.000 | 0.848 | 0.817 |
| 1394 | 0.755 | 1.000 | 0.765 | 0.986 | 0.953 | 0.960 | 0.901 | 1.000 | 0.980 | 1.000 | 0.959 | 0.970 | 0.982 | 0.861 | 1.000 | 1.000 | 0.849 | 0.818 |
| 1395 | 0.761 | 1.000 | 0.765 | 0.986 | 0.963 | 0.960 | 0.883 | 1.000 | 0.980 | 1.000 | 0.951 | 0.970 | 0.964 | 0.856 | 1.000 | 1.000 | 0.846 | 0.815 |
| 1396 | 0.760 | 1.000 | 0.777 | 0.986 | 0.968 | 0.960 | 0.901 | 1.000 | 1.000 | 1.000 | 0.951 | 0.970 | 0.973 | 0.862 | 1.000 | 1.000 | 0.852 | 0.822 |
| 1397 | 0.749 | 1.000 | 0.767 | 1.000 | 0.968 | 0.960 | 0.886 | 1.000 | 0.990 | 1.000 | 0.959 | 0.970 | 0.973 | 0.859 | 1.000 | 1.000 | 0.845 | 0.815 |
| 1398 | 0.754 | 1.000 | 0.773 | 1.000 | 0.968 | 0.960 | 0.895 | 1.000 | 0.980 | 1.000 | 0.945 | 0.970 | 0.964 | 0.858 | 1.000 | 1.000 | 0.847 | 0.816 |
| 1399 | 0.757 | 1.000 | 0.792 | 0.986 | 0.974 | 0.960 | 0.908 | 1.000 | 0.980 | 1.000 | 0.959 | 0.970 | 0.973 | 0.854 | 1.000 | 1.000 | 0.851 | 0.822 |
| 1400 | 0.759 | 1.000 | 0.767 | 0.986 | 0.958 | 0.960 | 0.891 | 1.000 | 1.000 | 1.000 | 0.957 | 0.970 | 0.964 | 0.858 | 1.000 | 1.000 | 0.848 | 0.817 |
| 1401 | 0.752 | 1.000 | 0.761 | 1.000 | 0.968 | 0.960 | 0.895 | 1.000 | 0.980 | 1.000 | 0.954 | 0.970 | 0.982 | 0.858 | 1.000 | 1.000 | 0.846 | 0.815 |
| 1402 | 0.769 | 1.000 | 0.773 | 0.986 | 0.974 | 0.960 | 0.903 | 1.000 | 0.990 | 1.000 | 0.962 | 0.970 | 0.973 | 0.858 | 1.000 | 1.000 | 0.854 | 0.824 |

| | | | | | | | | | | | | | | | | | | |
|---|---|---|---|---|---|---|---|---|---|---|---|---|---|---|---|---|---|---|
| 1403 | 0.751 | 1.000 | 0.773 | 0.986 | 0.953 | 0.960 | 0.886 | 1.000 | 0.980 | 1.000 | 0.951 | 0.970 | 0.982 | 0.858 | 1.000 | 1.000 | 0.845 | 0.813 |
| 1404 | 0.759 | 1.000 | 0.769 | 1.000 | 0.963 | 0.960 | 0.904 | 1.000 | 0.980 | 1.000 | 0.957 | 0.970 | 0.973 | 0.858 | 1.000 | 1.000 | 0.850 | 0.820 |
| 1405 | 0.755 | 1.000 | 0.767 | 0.986 | 0.968 | 0.960 | 0.884 | 1.000 | 0.959 | 1.000 | 0.948 | 0.970 | 0.955 | 0.860 | 1.000 | 1.000 | 0.845 | 0.814 |
| 1406 | 0.759 | 1.000 | 0.769 | 0.986 | 0.953 | 0.960 | 0.898 | 1.000 | 0.980 | 1.000 | 0.962 | 0.970 | 0.964 | 0.861 | 1.000 | 1.000 | 0.849 | 0.819 |
| 1407 | 0.755 | 1.000 | 0.775 | 1.000 | 0.958 | 0.960 | 0.891 | 1.000 | 0.990 | 1.000 | 0.962 | 0.970 | 0.973 | 0.862 | 1.000 | 1.000 | 0.849 | 0.818 |
| 1408 | 0.755 | 1.000 | 0.785 | 1.000 | 0.963 | 0.960 | 0.892 | 1.000 | 0.990 | 1.000 | 0.948 | 0.970 | 0.973 | 0.867 | 1.000 | 1.000 | 0.850 | 0.820 |
| 1409 | 0.763 | 1.000 | 0.777 | 1.000 | 0.968 | 0.960 | 0.895 | 1.000 | 0.990 | 1.000 | 0.962 | 0.970 | 0.991 | 0.858 | 1.000 | 1.000 | 0.852 | 0.822 |
| 1410 | 0.756 | 1.000 | 0.761 | 1.000 | 0.974 | 0.960 | 0.900 | 1.000 | 0.980 | 1.000 | 0.951 | 0.939 | 0.964 | 0.859 | 1.000 | 1.000 | 0.847 | 0.817 |
| 1411 | 0.763 | 1.000 | 0.775 | 0.986 | 0.963 | 0.960 | 0.892 | 1.000 | 0.980 | 1.000 | 0.948 | 0.970 | 0.973 | 0.856 | 1.000 | 1.000 | 0.849 | 0.818 |
| 1412 | 0.760 | 1.000 | 0.751 | 0.986 | 0.963 | 0.960 | 0.895 | 1.000 | 0.980 | 1.000 | 0.968 | 0.970 | 0.964 | 0.861 | 1.000 | 1.000 | 0.848 | 0.818 |
| 1413 | 0.759 | 1.000 | 0.771 | 0.971 | 0.963 | 0.960 | 0.897 | 1.000 | 1.000 | 1.000 | 0.948 | 1.000 | 0.982 | 0.853 | 1.000 | 1.000 | 0.847 | 0.817 |
| 1414 | 0.765 | 1.000 | 0.771 | 1.000 | 0.963 | 0.960 | 0.901 | 1.000 | 0.990 | 1.000 | 0.951 | 0.970 | 0.964 | 0.856 | 1.000 | 1.000 | 0.851 | 0.821 |
| 1415 | 0.750 | 1.000 | 0.757 | 1.000 | 0.974 | 0.960 | 0.889 | 1.000 | 0.990 | 1.000 | 0.957 | 0.970 | 0.973 | 0.858 | 1.000 | 1.000 | 0.844 | 0.813 |
| 1416 | 0.768 | 1.000 | 0.781 | 0.986 | 0.963 | 0.960 | 0.891 | 1.000 | 0.980 | 1.000 | 0.939 | 0.970 | 0.964 | 0.861 | 1.000 | 1.000 | 0.851 | 0.821 |
| 1417 | 0.760 | 1.000 | 0.781 | 0.986 | 0.963 | 0.960 | 0.888 | 1.000 | 0.980 | 1.000 | 0.951 | 0.939 | 0.973 | 0.865 | 1.000 | 1.000 | 0.850 | 0.820 |
| 1418 | 0.765 | 1.000 | 0.775 | 1.000 | 0.963 | 0.960 | 0.895 | 1.000 | 0.990 | 1.000 | 0.962 | 1.000 | 0.973 | 0.858 | 1.000 | 1.000 | 0.852 | 0.822 |
| 1419 | 0.766 | 1.000 | 0.771 | 0.986 | 0.974 | 0.960 | 0.898 | 1.000 | 0.990 | 1.000 | 0.965 | 0.970 | 0.973 | 0.855 | 1.000 | 1.000 | 0.852 | 0.822 |
| 1420 | 0.759 | 1.000 | 0.775 | 1.000 | 0.963 | 0.960 | 0.898 | 1.000 | 0.990 | 1.000 | 0.959 | 1.000 | 0.964 | 0.864 | 1.000 | 1.000 | 0.852 | 0.822 |
| 1421 | 0.767 | 1.000 | 0.759 | 1.000 | 0.958 | 0.960 | 0.898 | 1.000 | 0.990 | 1.000 | 0.962 | 0.939 | 0.973 | 0.854 | 1.000 | 1.000 | 0.849 | 0.819 |
| 1422 | 0.755 | 1.000 | 0.771 | 0.986 | 0.974 | 0.960 | 0.894 | 1.000 | 0.980 | 1.000 | 0.948 | 0.970 | 0.991 | 0.851 | 1.000 | 1.000 | 0.846 | 0.815 |
| 1423 | 0.768 | 1.000 | 0.777 | 1.000 | 0.958 | 0.960 | 0.898 | 1.000 | 0.980 | 1.000 | 0.962 | 0.970 | 0.991 | 0.857 | 1.000 | 1.000 | 0.853 | 0.823 |
| 1424 | 0.766 | 1.000 | 0.785 | 0.986 | 0.963 | 0.960 | 0.897 | 1.000 | 0.990 | 1.000 | 0.954 | 0.939 | 0.982 | 0.854 | 1.000 | 1.000 | 0.851 | 0.821 |
| 1425 | 0.765 | 1.000 | 0.773 | 0.986 | 0.958 | 0.960 | 0.914 | 1.000 | 0.990 | 1.000 | 0.954 | 0.970 | 0.982 | 0.856 | 1.000 | 1.000 | 0.853 | 0.823 |
| 1426 | 0.758 | 1.000 | 0.769 | 1.000 | 0.963 | 0.960 | 0.898 | 1.000 | 1.000 | 1.000 | 0.957 | 0.970 | 0.973 | 0.859 | 1.000 | 1.000 | 0.849 | 0.820 |
| 1427 | 0.755 | 1.000 | 0.771 | 0.986 | 0.963 | 0.960 | 0.895 | 1.000 | 0.990 | 1.000 | 0.959 | 0.970 | 0.964 | 0.853 | 1.000 | 1.000 | 0.846 | 0.816 |
| 1428 | 0.757 | 1.000 | 0.781 | 0.986 | 0.953 | 0.960 | 0.891 | 1.000 | 0.990 | 1.000 | 0.951 | 0.970 | 0.964 | 0.854 | 1.000 | 1.000 | 0.846 | 0.816 |
| 1429 | 0.770 | 1.000 | 0.781 | 1.000 | 0.958 | 0.960 | 0.901 | 1.000 | 0.969 | 1.000 | 0.951 | 0.970 | 0.973 | 0.865 | 1.000 | 1.000 | 0.855 | 0.825 |
| 1430 | 0.759 | 1.000 | 0.767 | 0.986 | 0.958 | 0.960 | 0.895 | 1.000 | 0.980 | 1.000 | 0.959 | 0.970 | 0.973 | 0.854 | 1.000 | 1.000 | 0.847 | 0.817 |
| 1431 | 0.757 | 1.000 | 0.775 | 1.000 | 0.968 | 0.960 | 0.891 | 1.000 | 0.990 | 1.000 | 0.957 | 0.970 | 0.964 | 0.853 | 1.000 | 1.000 | 0.847 | 0.816 |
| 1432 | 0.746 | 1.000 | 0.787 | 0.986 | 0.968 | 0.960 | 0.895 | 1.000 | 0.980 | 1.000 | 0.959 | 0.970 | 0.964 | 0.858 | 1.000 | 1.000 | 0.847 | 0.817 |
| 1433 | 0.759 | 1.000 | 0.777 | 0.986 | 0.974 | 0.960 | 0.903 | 1.000 | 0.980 | 1.000 | 0.957 | 0.970 | 0.982 | 0.856 | 1.000 | 1.000 | 0.850 | 0.820 |
| 1434 | 0.759 | 1.000 | 0.763 | 0.986 | 0.963 | 0.960 | 0.898 | 1.000 | 0.959 | 1.000 | 0.957 | 0.970 | 0.964 | 0.862 | 1.000 | 1.000 | 0.849 | 0.818 |
| 1435 | 0.756 | 1.000 | 0.779 | 1.000 | 0.958 | 0.960 | 0.897 | 1.000 | 0.969 | 1.000 | 0.954 | 0.970 | 0.973 | 0.861 | 1.000 | 1.000 | 0.849 | 0.819 |
| 1436 | 0.750 | 1.000 | 0.771 | 0.986 | 0.963 | 0.960 | 0.892 | 1.000 | 0.980 | 1.000 | 0.951 | 0.939 | 0.964 | 0.852 | 1.000 | 1.000 | 0.843 | 0.812 |
| 1437 | 0.748 | 1.000 | 0.773 | 1.000 | 0.958 | 0.960 | 0.884 | 1.000 | 0.980 | 1.000 | 0.957 | 0.970 | 0.964 | 0.858 | 1.000 | 1.000 | 0.844 | 0.813 |
| 1438 | 0.761 | 1.000 | 0.763 | 0.986 | 0.963 | 0.960 | 0.897 | 1.000 | 0.990 | 1.000 | 0.954 | 0.970 | 0.964 | 0.854 | 1.000 | 1.000 | 0.847 | 0.817 |
| 1439 | 0.764 | 1.000 | 0.763 | 0.986 | 0.963 | 0.960 | 0.895 | 1.000 | 0.969 | 1.000 | 0.948 | 0.970 | 0.973 | 0.854 | 1.000 | 1.000 | 0.848 | 0.817 |
| 1440 | 0.758 | 1.000 | 0.783 | 1.000 | 0.968 | 0.960 | 0.895 | 1.000 | 0.980 | 1.000 | 0.959 | 0.970 | 0.964 | 0.854 | 1.000 | 1.000 | 0.849 | 0.819 |
| 1441 | 0.756 | 1.000 | 0.783 | 1.000 | 0.963 | 0.960 | 0.894 | 1.000 | 0.980 | 1.000 | 0.951 | 0.970 | 0.973 | 0.861 | 1.000 | 1.000 | 0.849 | 0.819 |

| | | | | | | | | | | | | | | | | | |
|---|---|---|---|---|---|---|---|---|---|---|---|---|---|---|---|---|---|
| 1442 | 0.759 | 1.000 | 0.777 | 0.986 | 0.958 | 0.960 | 0.894 | 1.000 | 0.990 | 1.000 | 0.962 | 0.970 | 0.964 | 0.854 | 1.000 | 1.000 | 0.848 | 0.818 |
| 1443 | 0.759 | 1.000 | 0.775 | 0.986 | 0.963 | 0.960 | 0.900 | 1.000 | 0.980 | 1.000 | 0.951 | 0.970 | 0.973 | 0.852 | 1.000 | 1.000 | 0.848 | 0.817 |
| 1444 | 0.758 | 1.000 | 0.781 | 1.000 | 0.958 | 0.960 | 0.903 | 1.000 | 0.969 | 1.000 | 0.954 | 0.970 | 0.982 | 0.861 | 1.000 | 1.000 | 0.851 | 0.821 |
| 1445 | 0.759 | 1.000 | 0.787 | 1.000 | 0.968 | 0.960 | 0.892 | 1.000 | 0.969 | 1.000 | 0.954 | 0.939 | 0.982 | 0.861 | 1.000 | 1.000 | 0.851 | 0.821 |
| 1446 | 0.761 | 1.000 | 0.771 | 1.000 | 0.958 | 0.960 | 0.901 | 1.000 | 0.969 | 1.000 | 0.957 | 0.939 | 0.973 | 0.860 | 1.000 | 1.000 | 0.850 | 0.820 |
| 1447 | 0.764 | 1.000 | 0.779 | 1.000 | 0.968 | 0.960 | 0.877 | 1.000 | 0.969 | 1.000 | 0.954 | 0.970 | 0.973 | 0.861 | 1.000 | 1.000 | 0.849 | 0.819 |
| 1448 | 0.755 | 1.000 | 0.773 | 0.986 | 0.958 | 0.960 | 0.894 | 1.000 | 0.980 | 1.000 | 0.954 | 0.970 | 0.973 | 0.859 | 1.000 | 1.000 | 0.847 | 0.816 |
| 1449 | 0.759 | 1.000 | 0.789 | 0.986 | 0.963 | 0.960 | 0.892 | 1.000 | 0.980 | 1.000 | 0.948 | 0.970 | 0.982 | 0.858 | 1.000 | 1.000 | 0.850 | 0.819 |
| 1450 | 0.757 | 1.000 | 0.771 | 1.000 | 0.968 | 0.960 | 0.894 | 1.000 | 0.969 | 1.000 | 0.951 | 0.970 | 0.964 | 0.859 | 1.000 | 1.000 | 0.848 | 0.817 |
| 1451 | 0.758 | 1.000 | 0.771 | 0.986 | 0.953 | 0.960 | 0.895 | 1.000 | 0.980 | 1.000 | 0.945 | 0.970 | 0.982 | 0.860 | 1.000 | 1.000 | 0.848 | 0.817 |
| 1452 | 0.749 | 1.000 | 0.783 | 1.000 | 0.963 | 0.960 | 0.898 | 1.000 | 0.969 | 1.000 | 0.951 | 0.970 | 0.973 | 0.860 | 1.000 | 1.000 | 0.847 | 0.817 |
| 1453 | 0.754 | 1.000 | 0.791 | 0.986 | 0.958 | 0.960 | 0.900 | 1.000 | 0.980 | 1.000 | 0.959 | 0.970 | 0.955 | 0.861 | 1.000 | 1.000 | 0.850 | 0.820 |
| 1454 | 0.757 | 1.000 | 0.781 | 0.986 | 0.968 | 0.960 | 0.892 | 1.000 | 0.980 | 1.000 | 0.965 | 0.970 | 0.982 | 0.861 | 1.000 | 1.000 | 0.850 | 0.821 |
| 1455 | 0.752 | 1.000 | 0.777 | 1.000 | 0.958 | 0.960 | 0.897 | 1.000 | 0.980 | 1.000 | 0.959 | 0.939 | 0.973 | 0.856 | 1.000 | 1.000 | 0.847 | 0.816 |
| 1456 | 0.757 | 1.000 | 0.783 | 0.986 | 0.968 | 0.960 | 0.900 | 1.000 | 0.980 | 1.000 | 0.959 | 0.970 | 0.973 | 0.854 | 1.000 | 1.000 | 0.849 | 0.819 |
| 1457 | 0.759 | 1.000 | 0.773 | 0.986 | 0.968 | 0.960 | 0.894 | 1.000 | 0.969 | 1.000 | 0.959 | 0.970 | 0.973 | 0.858 | 1.000 | 1.000 | 0.849 | 0.818 |
| 1458 | 0.759 | 1.000 | 0.767 | 1.000 | 0.947 | 0.960 | 0.898 | 1.000 | 0.990 | 1.000 | 0.954 | 0.939 | 0.973 | 0.854 | 1.000 | 1.000 | 0.847 | 0.816 |
| 1459 | 0.752 | 1.000 | 0.773 | 0.986 | 0.968 | 0.960 | 0.895 | 1.000 | 0.980 | 1.000 | 0.948 | 0.970 | 0.955 | 0.859 | 1.000 | 1.000 | 0.846 | 0.815 |
| 1460 | 0.761 | 1.000 | 0.763 | 1.000 | 0.963 | 0.960 | 0.898 | 1.000 | 0.990 | 1.000 | 0.951 | 0.970 | 0.964 | 0.850 | 1.000 | 1.000 | 0.846 | 0.816 |
| 1461 | 0.768 | 1.000 | 0.769 | 1.000 | 0.968 | 0.960 | 0.900 | 1.000 | 0.980 | 1.000 | 0.959 | 0.970 | 0.973 | 0.858 | 1.000 | 1.000 | 0.852 | 0.822 |
| 1462 | 0.763 | 1.000 | 0.777 | 0.986 | 0.958 | 0.960 | 0.883 | 1.000 | 0.980 | 1.000 | 0.957 | 0.970 | 0.964 | 0.859 | 1.000 | 1.000 | 0.849 | 0.818 |
| 1463 | 0.760 | 1.000 | 0.781 | 1.000 | 0.963 | 0.960 | 0.904 | 1.000 | 0.980 | 1.000 | 0.948 | 0.970 | 0.973 | 0.861 | 1.000 | 1.000 | 0.852 | 0.822 |
| 1464 | 0.765 | 1.000 | 0.773 | 1.000 | 0.963 | 0.960 | 0.897 | 1.000 | 0.990 | 1.000 | 0.954 | 0.970 | 0.973 | 0.855 | 1.000 | 1.000 | 0.850 | 0.820 |
| 1465 | 0.764 | 1.000 | 0.775 | 0.986 | 0.963 | 0.960 | 0.894 | 1.000 | 0.990 | 1.000 | 0.957 | 0.970 | 0.982 | 0.856 | 1.000 | 1.000 | 0.850 | 0.820 |
| 1466 | 0.760 | 1.000 | 0.779 | 1.000 | 0.958 | 0.960 | 0.891 | 1.000 | 0.980 | 1.000 | 0.965 | 0.939 | 0.955 | 0.857 | 1.000 | 1.000 | 0.849 | 0.818 |
| 1467 | 0.766 | 1.000 | 0.785 | 0.986 | 0.968 | 0.960 | 0.894 | 1.000 | 0.990 | 1.000 | 0.957 | 0.970 | 0.973 | 0.851 | 1.000 | 1.000 | 0.851 | 0.821 |
| 1468 | 0.760 | 1.000 | 0.779 | 1.000 | 0.963 | 0.960 | 0.906 | 1.000 | 0.980 | 1.000 | 0.957 | 0.970 | 0.964 | 0.856 | 1.000 | 1.000 | 0.851 | 0.821 |
| 1469 | 0.759 | 1.000 | 0.757 | 1.000 | 0.963 | 0.960 | 0.889 | 1.000 | 0.980 | 1.000 | 0.957 | 0.939 | 0.973 | 0.853 | 1.000 | 1.000 | 0.845 | 0.814 |
| 1470 | 0.756 | 1.000 | 0.789 | 0.986 | 0.974 | 0.960 | 0.892 | 1.000 | 0.980 | 1.000 | 0.968 | 0.970 | 0.973 | 0.862 | 1.000 | 1.000 | 0.851 | 0.821 |
| 1471 | 0.751 | 1.000 | 0.757 | 0.986 | 0.953 | 0.960 | 0.898 | 1.000 | 0.980 | 1.000 | 0.957 | 0.970 | 0.973 | 0.851 | 1.000 | 1.000 | 0.843 | 0.812 |
| 1472 | 0.762 | 1.000 | 0.763 | 1.000 | 0.958 | 0.960 | 0.892 | 1.000 | 0.990 | 1.000 | 0.959 | 0.970 | 0.973 | 0.860 | 1.000 | 1.000 | 0.849 | 0.819 |
| 1473 | 0.761 | 1.000 | 0.763 | 1.000 | 0.968 | 0.960 | 0.889 | 1.000 | 0.990 | 1.000 | 0.951 | 0.939 | 0.973 | 0.847 | 1.000 | 1.000 | 0.845 | 0.813 |
| 1474 | 0.767 | 1.000 | 0.775 | 0.986 | 0.968 | 0.960 | 0.897 | 1.000 | 0.980 | 1.000 | 0.951 | 0.970 | 0.973 | 0.859 | 1.000 | 1.000 | 0.852 | 0.822 |
| 1475 | 0.755 | 1.000 | 0.759 | 0.986 | 0.963 | 0.960 | 0.889 | 1.000 | 0.969 | 1.000 | 0.948 | 0.970 | 0.973 | 0.847 | 1.000 | 1.000 | 0.842 | 0.810 |
| 1476 | 0.756 | 1.000 | 0.769 | 1.000 | 0.968 | 0.960 | 0.900 | 1.000 | 0.980 | 1.000 | 0.951 | 0.970 | 0.964 | 0.856 | 1.000 | 1.000 | 0.847 | 0.817 |
| 1477 | 0.756 | 1.000 | 0.787 | 0.986 | 0.958 | 0.960 | 0.884 | 1.000 | 0.980 | 1.000 | 0.962 | 0.939 | 0.973 | 0.851 | 1.000 | 1.000 | 0.846 | 0.815 |
| 1478 | 0.752 | 1.000 | 0.771 | 0.986 | 0.963 | 0.960 | 0.892 | 1.000 | 0.980 | 1.000 | 0.951 | 0.970 | 0.973 | 0.853 | 1.000 | 1.000 | 0.844 | 0.813 |
| 1479 | 0.768 | 1.000 | 0.781 | 1.000 | 0.968 | 0.960 | 0.884 | 1.000 | 0.980 | 1.000 | 0.959 | 0.970 | 0.973 | 0.863 | 1.000 | 1.000 | 0.853 | 0.823 |
| 1480 | 0.764 | 1.000 | 0.767 | 1.000 | 0.958 | 0.960 | 0.894 | 1.000 | 0.980 | 1.000 | 0.954 | 0.970 | 0.973 | 0.854 | 1.000 | 1.000 | 0.848 | 0.818 |

| | | | | | | | | | | | | | | | | | | |
|---|---|---|---|---|---|---|---|---|---|---|---|---|---|---|---|---|---|---|
| 1481 | 0.759 | 1.000 | 0.783 | 0.986 | 0.974 | 0.960 | 0.898 | 1.000 | 0.969 | 1.000 | 0.962 | 0.970 | 0.973 | 0.866 | 1.000 | 1.000 | 0.853 | 0.823 |
| 1482 | 0.754 | 1.000 | 0.763 | 0.986 | 0.963 | 0.960 | 0.891 | 1.000 | 0.980 | 1.000 | 0.957 | 0.970 | 0.973 | 0.858 | 1.000 | 1.000 | 0.846 | 0.815 |
| 1483 | 0.753 | 1.000 | 0.761 | 1.000 | 0.958 | 0.960 | 0.898 | 1.000 | 0.980 | 1.000 | 0.951 | 0.970 | 0.973 | 0.868 | 1.000 | 1.000 | 0.848 | 0.818 |
| 1484 | 0.761 | 1.000 | 0.755 | 0.986 | 0.958 | 0.960 | 0.901 | 1.000 | 0.990 | 1.000 | 0.954 | 0.970 | 0.973 | 0.867 | 1.000 | 1.000 | 0.851 | 0.820 |
| 1485 | 0.757 | 1.000 | 0.771 | 1.000 | 0.963 | 0.960 | 0.894 | 1.000 | 0.980 | 1.000 | 0.954 | 0.939 | 0.973 | 0.861 | 1.000 | 1.000 | 0.849 | 0.818 |
| 1486 | 0.759 | 1.000 | 0.765 | 0.986 | 0.974 | 0.960 | 0.897 | 1.000 | 0.990 | 1.000 | 0.948 | 0.939 | 0.964 | 0.858 | 1.000 | 1.000 | 0.848 | 0.817 |
| 1487 | 0.755 | 1.000 | 0.763 | 1.000 | 0.963 | 0.960 | 0.892 | 1.000 | 0.980 | 1.000 | 0.957 | 0.970 | 0.973 | 0.854 | 1.000 | 1.000 | 0.845 | 0.814 |
| 1488 | 0.757 | 1.000 | 0.761 | 1.000 | 0.958 | 0.960 | 0.898 | 1.000 | 0.980 | 1.000 | 0.957 | 0.970 | 0.964 | 0.862 | 1.000 | 1.000 | 0.848 | 0.818 |
| 1489 | 0.753 | 1.000 | 0.787 | 1.000 | 0.958 | 0.960 | 0.901 | 1.000 | 0.959 | 1.000 | 0.962 | 0.970 | 0.964 | 0.854 | 1.000 | 1.000 | 0.848 | 0.818 |
| 1490 | 0.759 | 1.000 | 0.779 | 0.986 | 0.963 | 0.960 | 0.894 | 1.000 | 0.980 | 1.000 | 0.959 | 0.970 | 0.964 | 0.860 | 1.000 | 1.000 | 0.850 | 0.820 |
| 1491 | 0.759 | 1.000 | 0.773 | 1.000 | 0.963 | 0.960 | 0.892 | 1.000 | 0.980 | 1.000 | 0.951 | 0.970 | 0.973 | 0.864 | 1.000 | 1.000 | 0.850 | 0.819 |
| 1492 | 0.756 | 1.000 | 0.763 | 0.986 | 0.974 | 0.960 | 0.891 | 1.000 | 0.980 | 1.000 | 0.951 | 0.970 | 0.973 | 0.856 | 1.000 | 1.000 | 0.846 | 0.815 |
| 1493 | 0.765 | 1.000 | 0.787 | 1.000 | 0.953 | 0.960 | 0.895 | 1.000 | 0.980 | 1.000 | 0.959 | 0.970 | 0.973 | 0.863 | 1.000 | 1.000 | 0.853 | 0.824 |
| 1494 | 0.762 | 1.000 | 0.757 | 0.986 | 0.968 | 0.960 | 0.894 | 1.000 | 0.980 | 1.000 | 0.957 | 0.970 | 0.973 | 0.861 | 1.000 | 1.000 | 0.849 | 0.818 |
| 1495 | 0.762 | 1.000 | 0.771 | 0.986 | 0.963 | 0.960 | 0.895 | 1.000 | 0.990 | 1.000 | 0.957 | 0.970 | 0.964 | 0.859 | 1.000 | 1.000 | 0.850 | 0.820 |
| 1496 | 0.762 | 1.000 | 0.767 | 1.000 | 0.968 | 0.960 | 0.901 | 1.000 | 0.969 | 1.000 | 0.962 | 0.939 | 0.964 | 0.860 | 1.000 | 1.000 | 0.851 | 0.820 |
| 1497 | 0.761 | 1.000 | 0.773 | 1.000 | 0.963 | 0.960 | 0.891 | 1.000 | 0.990 | 1.000 | 0.951 | 0.970 | 0.955 | 0.852 | 1.000 | 1.000 | 0.847 | 0.816 |
| 1498 | 0.748 | 1.000 | 0.767 | 0.986 | 0.968 | 0.960 | 0.891 | 1.000 | 0.969 | 1.000 | 0.957 | 0.970 | 0.964 | 0.858 | 1.000 | 1.000 | 0.844 | 0.813 |
| 1499 | 0.757 | 1.000 | 0.777 | 0.971 | 0.963 | 0.960 | 0.912 | 1.000 | 0.990 | 1.000 | 0.959 | 0.970 | 0.973 | 0.854 | 1.000 | 1.000 | 0.850 | 0.820 |
| 1500 | 0.761 | 1.000 | 0.769 | 0.986 | 0.958 | 0.960 | 0.900 | 1.000 | 0.990 | 1.000 | 0.957 | 0.970 | 0.964 | 0.859 | 1.000 | 1.000 | 0.850 | 0.819 |
| 1501 | 0.760 | 1.000 | 0.769 | 0.986 | 0.968 | 0.960 | 0.901 | 1.000 | 0.980 | 1.000 | 0.962 | 0.970 | 0.964 | 0.855 | 1.000 | 1.000 | 0.849 | 0.819 |
| 1502 | 0.762 | 1.000 | 0.781 | 1.000 | 0.963 | 0.960 | 0.895 | 1.000 | 0.969 | 1.000 | 0.945 | 0.970 | 0.964 | 0.859 | 1.000 | 1.000 | 0.850 | 0.819 |
| 1503 | 0.759 | 1.000 | 0.783 | 0.986 | 0.963 | 0.960 | 0.894 | 1.000 | 0.990 | 1.000 | 0.951 | 0.970 | 0.973 | 0.857 | 1.000 | 1.000 | 0.849 | 0.819 |
| 1504 | 0.758 | 1.000 | 0.783 | 1.000 | 0.968 | 0.960 | 0.903 | 1.000 | 0.969 | 1.000 | 0.954 | 0.970 | 0.964 | 0.853 | 1.000 | 1.000 | 0.849 | 0.819 |
| 1505 | 0.757 | 1.000 | 0.785 | 1.000 | 0.974 | 0.960 | 0.900 | 1.000 | 0.990 | 1.000 | 0.965 | 0.970 | 0.955 | 0.858 | 1.000 | 1.000 | 0.851 | 0.821 |
| 1506 | 0.753 | 1.000 | 0.763 | 1.000 | 0.963 | 0.960 | 0.897 | 1.000 | 0.980 | 1.000 | 0.971 | 1.000 | 0.964 | 0.855 | 1.000 | 1.000 | 0.847 | 0.816 |
| 1507 | 0.760 | 1.000 | 0.785 | 0.986 | 0.974 | 0.960 | 0.889 | 1.000 | 0.980 | 1.000 | 0.951 | 1.000 | 0.982 | 0.852 | 1.000 | 1.000 | 0.848 | 0.818 |
| 1508 | 0.772 | 1.000 | 0.775 | 0.986 | 0.974 | 0.960 | 0.892 | 1.000 | 0.990 | 1.000 | 0.962 | 1.000 | 0.973 | 0.854 | 1.000 | 1.000 | 0.853 | 0.823 |
| 1509 | 0.765 | 1.000 | 0.769 | 0.986 | 0.963 | 0.960 | 0.897 | 1.000 | 0.990 | 1.000 | 0.951 | 0.970 | 0.964 | 0.858 | 1.000 | 1.000 | 0.850 | 0.820 |
| 1510 | 0.757 | 1.000 | 0.769 | 0.986 | 0.963 | 0.960 | 0.908 | 1.000 | 0.969 | 1.000 | 0.954 | 1.000 | 0.973 | 0.856 | 1.000 | 1.000 | 0.849 | 0.819 |
| 1511 | 0.755 | 1.000 | 0.783 | 0.986 | 0.963 | 0.960 | 0.900 | 1.000 | 0.980 | 1.000 | 0.957 | 1.000 | 0.973 | 0.858 | 1.000 | 1.000 | 0.850 | 0.820 |
| 1512 | 0.759 | 1.000 | 0.765 | 0.986 | 0.974 | 0.960 | 0.880 | 1.000 | 0.990 | 1.000 | 0.954 | 1.000 | 0.973 | 0.851 | 1.000 | 1.000 | 0.845 | 0.813 |
| 1513 | 0.761 | 1.000 | 0.763 | 0.986 | 0.974 | 0.960 | 0.895 | 1.000 | 0.980 | 1.000 | 0.957 | 1.000 | 0.964 | 0.861 | 1.000 | 1.000 | 0.850 | 0.819 |
| 1514 | 0.768 | 1.000 | 0.789 | 1.000 | 0.963 | 0.960 | 0.898 | 1.000 | 0.969 | 1.000 | 0.948 | 1.000 | 0.973 | 0.853 | 1.000 | 1.000 | 0.852 | 0.822 |
| 1515 | 0.756 | 1.000 | 0.787 | 1.000 | 0.974 | 0.960 | 0.904 | 1.000 | 0.980 | 1.000 | 0.951 | 0.970 | 0.964 | 0.861 | 1.000 | 1.000 | 0.851 | 0.822 |
| 1516 | 0.758 | 1.000 | 0.781 | 0.986 | 0.979 | 0.960 | 0.898 | 1.000 | 0.980 | 1.000 | 0.965 | 0.970 | 0.973 | 0.851 | 1.000 | 1.000 | 0.849 | 0.819 |
| 1517 | 0.755 | 1.000 | 0.775 | 0.986 | 0.968 | 0.960 | 0.894 | 1.000 | 0.969 | 1.000 | 0.951 | 1.000 | 0.964 | 0.854 | 1.000 | 1.000 | 0.846 | 0.815 |
| 1518 | 0.758 | 1.000 | 0.779 | 1.000 | 0.968 | 0.960 | 0.891 | 1.000 | 0.990 | 1.000 | 0.951 | 1.000 | 0.964 | 0.854 | 1.000 | 1.000 | 0.848 | 0.817 |
| 1519 | 0.767 | 1.000 | 0.755 | 1.000 | 0.963 | 0.960 | 0.884 | 1.000 | 0.990 | 1.000 | 0.951 | 0.970 | 0.973 | 0.861 | 1.000 | 1.000 | 0.849 | 0.818 |

| | | | | | | | | | | | | | | | | | | |
|---|---|---|---|---|---|---|---|---|---|---|---|---|---|---|---|---|---|---|
| 1520 | 0.753 | 1.000 | 0.775 | 0.986 | 0.974 | 0.960 | 0.897 | 1.000 | 0.990 | 1.000 | 0.948 | 1.000 | 0.973 | 0.856 | 1.000 | 1.000 | 0.847 | 0.817 |
| 1521 | 0.765 | 1.000 | 0.769 | 0.986 | 0.958 | 0.960 | 0.888 | 1.000 | 0.990 | 1.000 | 0.948 | 1.000 | 0.973 | 0.858 | 1.000 | 1.000 | 0.849 | 0.818 |
| 1522 | 0.756 | 1.000 | 0.767 | 1.000 | 0.968 | 1.000 | 0.901 | 1.000 | 0.980 | 1.000 | 0.957 | 1.000 | 0.964 | 0.858 | 1.000 | 1.000 | 0.849 | 0.818 |
| 1523 | 0.761 | 1.000 | 0.755 | 0.986 | 0.953 | 0.960 | 0.892 | 1.000 | 0.980 | 1.000 | 0.957 | 0.970 | 0.973 | 0.861 | 1.000 | 1.000 | 0.848 | 0.817 |
| 1524 | 0.757 | 1.000 | 0.785 | 1.000 | 0.963 | 0.960 | 0.897 | 1.000 | 0.980 | 1.000 | 0.954 | 0.970 | 0.964 | 0.857 | 1.000 | 1.000 | 0.849 | 0.819 |
| 1525 | 0.769 | 1.000 | 0.773 | 0.986 | 0.958 | 0.960 | 0.894 | 1.000 | 0.980 | 1.000 | 0.954 | 0.970 | 0.964 | 0.845 | 1.000 | 1.000 | 0.848 | 0.817 |
| 1526 | 0.760 | 1.000 | 0.777 | 1.000 | 0.968 | 0.960 | 0.904 | 1.000 | 0.980 | 1.000 | 0.959 | 0.970 | 0.964 | 0.853 | 1.000 | 1.000 | 0.850 | 0.820 |
| 1527 | 0.755 | 1.000 | 0.775 | 0.986 | 0.963 | 0.960 | 0.895 | 1.000 | 0.990 | 1.000 | 0.954 | 1.000 | 0.973 | 0.856 | 1.000 | 1.000 | 0.848 | 0.817 |
| 1528 | 0.761 | 1.000 | 0.767 | 1.000 | 0.963 | 0.960 | 0.897 | 1.000 | 0.959 | 1.000 | 0.962 | 0.970 | 0.973 | 0.858 | 1.000 | 1.000 | 0.849 | 0.819 |
| 1529 | 0.760 | 1.000 | 0.775 | 1.000 | 0.968 | 0.960 | 0.900 | 1.000 | 0.990 | 1.000 | 0.951 | 1.000 | 0.964 | 0.854 | 1.000 | 1.000 | 0.849 | 0.819 |
| 1530 | 0.763 | 1.000 | 0.771 | 1.000 | 0.968 | 0.960 | 0.892 | 1.000 | 0.980 | 1.000 | 0.954 | 0.939 | 0.964 | 0.862 | 1.000 | 1.000 | 0.850 | 0.820 |
| 1531 | 0.757 | 1.000 | 0.775 | 0.986 | 0.974 | 0.960 | 0.894 | 1.000 | 0.980 | 1.000 | 0.962 | 1.000 | 0.964 | 0.858 | 1.000 | 1.000 | 0.849 | 0.819 |
| 1532 | 0.755 | 1.000 | 0.771 | 0.986 | 0.968 | 0.960 | 0.888 | 1.000 | 0.980 | 1.000 | 0.957 | 1.000 | 0.955 | 0.858 | 1.000 | 1.000 | 0.846 | 0.815 |
| 1533 | 0.771 | 1.000 | 0.777 | 0.986 | 0.963 | 0.960 | 0.906 | 1.000 | 0.990 | 1.000 | 0.968 | 0.939 | 0.955 | 0.854 | 1.000 | 1.000 | 0.854 | 0.824 |
| 1534 | 0.769 | 1.000 | 0.777 | 0.986 | 0.963 | 0.960 | 0.891 | 1.000 | 0.980 | 1.000 | 0.957 | 0.970 | 0.973 | 0.859 | 1.000 | 1.000 | 0.852 | 0.822 |
| 1535 | 0.755 | 1.000 | 0.757 | 0.986 | 0.953 | 0.960 | 0.895 | 1.000 | 0.990 | 1.000 | 0.957 | 0.939 | 0.982 | 0.858 | 1.000 | 1.000 | 0.846 | 0.815 |
| 1536 | 0.757 | 1.000 | 0.773 | 0.986 | 0.953 | 0.960 | 0.901 | 1.000 | 0.969 | 1.000 | 0.957 | 1.000 | 0.982 | 0.852 | 1.000 | 1.000 | 0.847 | 0.817 |
| 1537 | 0.766 | 1.000 | 0.757 | 1.000 | 0.958 | 0.960 | 0.891 | 1.000 | 0.990 | 1.000 | 0.957 | 1.000 | 0.973 | 0.850 | 1.000 | 1.000 | 0.847 | 0.816 |
| 1538 | 0.753 | 1.000 | 0.767 | 1.000 | 0.968 | 0.960 | 0.898 | 1.000 | 0.980 | 1.000 | 0.968 | 0.970 | 0.964 | 0.858 | 1.000 | 1.000 | 0.848 | 0.818 |
| 1539 | 0.762 | 1.000 | 0.785 | 1.000 | 0.963 | 0.960 | 0.895 | 1.000 | 0.969 | 1.000 | 0.957 | 1.000 | 0.973 | 0.861 | 1.000 | 1.000 | 0.852 | 0.822 |
| 1540 | 0.760 | 1.000 | 0.785 | 1.000 | 0.963 | 0.960 | 0.892 | 1.000 | 0.969 | 1.000 | 0.962 | 1.000 | 0.973 | 0.857 | 1.000 | 1.000 | 0.850 | 0.820 |
| 1541 | 0.762 | 1.000 | 0.777 | 1.000 | 0.979 | 0.960 | 0.891 | 1.000 | 0.990 | 1.000 | 0.954 | 0.970 | 0.973 | 0.857 | 1.000 | 1.000 | 0.850 | 0.820 |
| 1542 | 0.756 | 1.000 | 0.771 | 0.986 | 0.974 | 0.960 | 0.898 | 1.000 | 0.990 | 1.000 | 0.962 | 1.000 | 0.982 | 0.862 | 1.000 | 1.000 | 0.851 | 0.821 |
| 1543 | 0.761 | 1.000 | 0.757 | 1.000 | 0.963 | 0.960 | 0.886 | 1.000 | 0.980 | 1.000 | 0.954 | 0.970 | 0.973 | 0.848 | 1.000 | 1.000 | 0.844 | 0.813 |
| 1544 | 0.756 | 1.000 | 0.775 | 0.986 | 0.953 | 0.960 | 0.894 | 1.000 | 0.980 | 1.000 | 0.962 | 0.970 | 0.964 | 0.859 | 1.000 | 1.000 | 0.848 | 0.817 |
| 1545 | 0.760 | 1.000 | 0.767 | 0.986 | 0.968 | 0.960 | 0.891 | 1.000 | 0.980 | 1.000 | 0.954 | 1.000 | 0.973 | 0.853 | 1.000 | 1.000 | 0.847 | 0.816 |
| 1546 | 0.751 | 1.000 | 0.749 | 1.000 | 0.968 | 0.960 | 0.901 | 1.000 | 0.969 | 1.000 | 0.951 | 0.970 | 0.973 | 0.861 | 1.000 | 1.000 | 0.845 | 0.814 |
| 1547 | 0.757 | 1.000 | 0.777 | 1.000 | 0.958 | 0.960 | 0.888 | 1.000 | 0.980 | 1.000 | 0.954 | 0.970 | 0.964 | 0.859 | 1.000 | 1.000 | 0.847 | 0.817 |
| 1548 | 0.762 | 1.000 | 0.779 | 0.986 | 0.968 | 0.960 | 0.895 | 1.000 | 0.990 | 1.000 | 0.954 | 1.000 | 0.973 | 0.851 | 1.000 | 1.000 | 0.849 | 0.819 |
| 1549 | 0.763 | 1.000 | 0.771 | 0.986 | 0.963 | 0.960 | 0.889 | 1.000 | 0.969 | 1.000 | 0.962 | 0.970 | 0.973 | 0.855 | 1.000 | 1.000 | 0.848 | 0.818 |
| 1550 | 0.755 | 1.000 | 0.757 | 1.000 | 0.979 | 0.960 | 0.891 | 1.000 | 0.980 | 1.000 | 0.951 | 1.000 | 0.973 | 0.858 | 1.000 | 1.000 | 0.846 | 0.815 |
| 1551 | 0.760 | 1.000 | 0.771 | 0.986 | 0.968 | 0.960 | 0.894 | 1.000 | 0.969 | 1.000 | 0.957 | 0.970 | 0.973 | 0.856 | 1.000 | 1.000 | 0.848 | 0.818 |
| 1552 | 0.763 | 1.000 | 0.773 | 1.000 | 0.963 | 0.960 | 0.888 | 1.000 | 0.969 | 1.000 | 0.954 | 1.000 | 0.955 | 0.853 | 1.000 | 1.000 | 0.847 | 0.816 |
| 1553 | 0.758 | 1.000 | 0.763 | 0.986 | 0.958 | 0.960 | 0.889 | 1.000 | 0.980 | 1.000 | 0.965 | 1.000 | 0.973 | 0.858 | 1.000 | 1.000 | 0.847 | 0.817 |
| 1554 | 0.759 | 1.000 | 0.777 | 1.000 | 0.968 | 0.960 | 0.897 | 1.000 | 0.980 | 1.000 | 0.962 | 0.939 | 0.973 | 0.858 | 1.000 | 1.000 | 0.850 | 0.820 |
| 1555 | 0.757 | 1.000 | 0.767 | 0.986 | 0.958 | 0.960 | 0.891 | 1.000 | 0.980 | 1.000 | 0.957 | 0.970 | 0.964 | 0.861 | 1.000 | 1.000 | 0.847 | 0.817 |
| 1556 | 0.761 | 1.000 | 0.765 | 1.000 | 0.953 | 0.960 | 0.895 | 1.000 | 0.980 | 1.000 | 0.962 | 0.970 | 0.964 | 0.854 | 1.000 | 1.000 | 0.848 | 0.817 |
| 1557 | 0.761 | 1.000 | 0.777 | 0.986 | 0.963 | 0.960 | 0.897 | 1.000 | 0.969 | 1.000 | 0.957 | 1.000 | 0.964 | 0.858 | 1.000 | 1.000 | 0.850 | 0.819 |
| 1558 | 0.761 | 1.000 | 0.769 | 0.986 | 0.958 | 0.960 | 0.891 | 1.000 | 0.980 | 1.000 | 0.965 | 1.000 | 0.964 | 0.857 | 1.000 | 1.000 | 0.848 | 0.818 |

| | | | | | | | | | | | | | | | | | | |
|---|---|---|---|---|---|---|---|---|---|---|---|---|---|---|---|---|---|---|
| 1559 | 0.759 | 1.000 | 0.767 | 1.000 | 0.968 | 0.960 | 0.898 | 1.000 | 0.980 | 1.000 | 0.948 | 0.970 | 0.964 | 0.865 | 1.000 | 1.000 | 0.850 | 0.819 |
| 1560 | 0.762 | 1.000 | 0.761 | 0.986 | 0.963 | 0.960 | 0.894 | 1.000 | 0.980 | 1.000 | 0.957 | 0.970 | 0.982 | 0.851 | 1.000 | 1.000 | 0.847 | 0.816 |
| 1561 | 0.759 | 1.000 | 0.775 | 0.986 | 0.968 | 0.960 | 0.903 | 1.000 | 0.990 | 1.000 | 0.959 | 0.970 | 0.964 | 0.860 | 1.000 | 1.000 | 0.851 | 0.821 |
| 1562 | 0.765 | 1.000 | 0.792 | 1.000 | 0.979 | 0.960 | 0.901 | 1.000 | 0.990 | 1.000 | 0.957 | 1.000 | 0.982 | 0.859 | 1.000 | 1.000 | 0.855 | 0.826 |
| 1563 | 0.760 | 1.000 | 0.765 | 1.000 | 0.968 | 0.960 | 0.895 | 1.000 | 0.990 | 1.000 | 0.962 | 1.000 | 0.973 | 0.858 | 1.000 | 1.000 | 0.850 | 0.819 |
| 1564 | 0.768 | 1.000 | 0.781 | 0.986 | 0.953 | 0.960 | 0.901 | 1.000 | 0.969 | 1.000 | 0.954 | 0.970 | 0.964 | 0.858 | 1.000 | 1.000 | 0.852 | 0.822 |
| 1565 | 0.759 | 1.000 | 0.777 | 1.000 | 0.953 | 0.960 | 0.906 | 1.000 | 0.980 | 1.000 | 0.957 | 1.000 | 0.982 | 0.851 | 1.000 | 1.000 | 0.849 | 0.819 |
| 1566 | 0.763 | 1.000 | 0.769 | 0.971 | 0.958 | 0.960 | 0.900 | 1.000 | 0.980 | 1.000 | 0.951 | 0.970 | 0.964 | 0.859 | 1.000 | 1.000 | 0.849 | 0.819 |
| 1567 | 0.757 | 1.000 | 0.775 | 1.000 | 0.979 | 0.960 | 0.892 | 1.000 | 0.980 | 1.000 | 0.965 | 0.970 | 0.973 | 0.852 | 1.000 | 1.000 | 0.848 | 0.817 |
| 1568 | 0.763 | 1.000 | 0.777 | 1.000 | 0.974 | 1.000 | 0.883 | 1.000 | 0.969 | 1.000 | 0.951 | 0.970 | 0.973 | 0.857 | 1.000 | 1.000 | 0.849 | 0.818 |
| 1569 | 0.756 | 1.000 | 0.781 | 1.000 | 0.968 | 0.960 | 0.888 | 1.000 | 0.990 | 1.000 | 0.954 | 0.970 | 0.964 | 0.856 | 1.000 | 1.000 | 0.847 | 0.817 |
| 1570 | 0.759 | 1.000 | 0.765 | 0.986 | 0.963 | 0.960 | 0.909 | 1.000 | 0.980 | 1.000 | 0.954 | 1.000 | 0.964 | 0.854 | 1.000 | 1.000 | 0.849 | 0.819 |
| 1571 | 0.757 | 1.000 | 0.775 | 0.986 | 0.974 | 0.960 | 0.892 | 1.000 | 0.980 | 1.000 | 0.948 | 1.000 | 0.964 | 0.858 | 1.000 | 1.000 | 0.848 | 0.818 |
| 1572 | 0.761 | 1.000 | 0.777 | 0.986 | 0.974 | 0.960 | 0.895 | 1.000 | 0.990 | 1.000 | 0.954 | 0.939 | 0.964 | 0.855 | 1.000 | 1.000 | 0.849 | 0.819 |
| 1573 | 0.758 | 1.000 | 0.777 | 0.986 | 0.979 | 0.960 | 0.895 | 1.000 | 0.969 | 1.000 | 0.957 | 1.000 | 0.964 | 0.861 | 1.000 | 1.000 | 0.850 | 0.820 |
| 1574 | 0.760 | 1.000 | 0.771 | 0.986 | 0.968 | 0.960 | 0.898 | 1.000 | 0.980 | 1.000 | 0.954 | 0.970 | 0.955 | 0.852 | 1.000 | 1.000 | 0.847 | 0.817 |
| 1575 | 0.761 | 1.000 | 0.773 | 1.000 | 0.974 | 0.960 | 0.903 | 1.000 | 0.969 | 1.000 | 0.962 | 1.000 | 0.964 | 0.856 | 1.000 | 1.000 | 0.851 | 0.821 |
| 1576 | 0.752 | 1.000 | 0.765 | 1.000 | 0.968 | 0.960 | 0.897 | 1.000 | 0.980 | 1.000 | 0.959 | 1.000 | 0.964 | 0.856 | 1.000 | 1.000 | 0.846 | 0.816 |
| 1577 | 0.754 | 1.000 | 0.785 | 1.000 | 0.958 | 0.960 | 0.900 | 1.000 | 0.959 | 1.000 | 0.959 | 1.000 | 0.964 | 0.856 | 1.000 | 1.000 | 0.848 | 0.818 |
| 1578 | 0.740 | 1.000 | 0.771 | 1.000 | 0.963 | 0.960 | 0.881 | 1.000 | 0.980 | 1.000 | 0.962 | 1.000 | 0.982 | 0.858 | 1.000 | 1.000 | 0.842 | 0.811 |
| 1579 | 0.757 | 1.000 | 0.757 | 1.000 | 0.963 | 0.960 | 0.898 | 1.000 | 0.980 | 1.000 | 0.957 | 0.970 | 0.964 | 0.855 | 1.000 | 1.000 | 0.846 | 0.815 |
| 1580 | 0.760 | 1.000 | 0.775 | 0.986 | 0.968 | 0.960 | 0.895 | 1.000 | 0.980 | 1.000 | 0.951 | 0.939 | 0.973 | 0.851 | 1.000 | 1.000 | 0.847 | 0.816 |
| 1581 | 0.761 | 1.000 | 0.763 | 0.986 | 0.963 | 0.960 | 0.891 | 1.000 | 0.969 | 1.000 | 0.965 | 0.970 | 0.973 | 0.855 | 1.000 | 1.000 | 0.848 | 0.817 |
| 1582 | 0.757 | 1.000 | 0.767 | 1.000 | 0.958 | 0.960 | 0.898 | 1.000 | 0.990 | 1.000 | 0.951 | 1.000 | 0.964 | 0.848 | 1.000 | 1.000 | 0.845 | 0.815 |
| 1583 | 0.775 | 1.000 | 0.769 | 0.986 | 0.968 | 0.960 | 0.898 | 1.000 | 0.969 | 1.000 | 0.957 | 1.000 | 0.973 | 0.857 | 1.000 | 1.000 | 0.853 | 0.824 |
| 1584 | 0.764 | 1.000 | 0.771 | 0.986 | 0.958 | 0.960 | 0.886 | 1.000 | 0.980 | 1.000 | 0.954 | 1.000 | 0.982 | 0.853 | 1.000 | 1.000 | 0.848 | 0.817 |
| 1585 | 0.759 | 1.000 | 0.771 | 0.986 | 0.979 | 0.960 | 0.898 | 1.000 | 0.990 | 1.000 | 0.962 | 1.000 | 0.973 | 0.854 | 1.000 | 1.000 | 0.850 | 0.819 |
| 1586 | 0.763 | 1.000 | 0.791 | 1.000 | 0.974 | 0.960 | 0.898 | 1.000 | 0.980 | 1.000 | 0.959 | 1.000 | 0.955 | 0.859 | 1.000 | 1.000 | 0.853 | 0.823 |
| 1587 | 0.762 | 1.000 | 0.779 | 0.986 | 0.958 | 0.960 | 0.903 | 1.000 | 0.980 | 1.000 | 0.948 | 0.970 | 0.964 | 0.860 | 1.000 | 1.000 | 0.851 | 0.821 |
| 1588 | 0.765 | 1.000 | 0.769 | 0.986 | 0.968 | 0.960 | 0.891 | 1.000 | 0.990 | 1.000 | 0.957 | 0.970 | 0.982 | 0.858 | 1.000 | 1.000 | 0.850 | 0.820 |
| 1589 | 0.762 | 1.000 | 0.773 | 0.986 | 0.968 | 0.960 | 0.886 | 1.000 | 0.969 | 1.000 | 0.959 | 1.000 | 0.955 | 0.853 | 1.000 | 1.000 | 0.847 | 0.816 |
| 1590 | 0.757 | 1.000 | 0.773 | 0.971 | 0.974 | 0.960 | 0.903 | 1.000 | 0.980 | 1.000 | 0.959 | 1.000 | 0.973 | 0.854 | 1.000 | 1.000 | 0.849 | 0.819 |
| 1591 | 0.765 | 1.000 | 0.763 | 0.986 | 0.968 | 0.960 | 0.889 | 1.000 | 0.990 | 1.000 | 0.959 | 1.000 | 0.982 | 0.857 | 1.000 | 1.000 | 0.850 | 0.819 |
| 1592 | 0.759 | 1.000 | 0.771 | 0.986 | 0.974 | 0.960 | 0.895 | 1.000 | 0.980 | 1.000 | 0.962 | 0.970 | 0.964 | 0.859 | 1.000 | 1.000 | 0.850 | 0.819 |
| 1593 | 0.757 | 1.000 | 0.767 | 0.986 | 0.968 | 0.960 | 0.897 | 1.000 | 0.980 | 1.000 | 0.954 | 1.000 | 0.964 | 0.852 | 1.000 | 1.000 | 0.846 | 0.815 |
| 1594 | 0.763 | 1.000 | 0.773 | 1.000 | 0.974 | 0.960 | 0.897 | 1.000 | 0.969 | 1.000 | 0.957 | 1.000 | 0.964 | 0.861 | 1.000 | 1.000 | 0.851 | 0.821 |
| 1595 | 0.765 | 1.000 | 0.767 | 1.000 | 0.968 | 0.960 | 0.895 | 1.000 | 0.990 | 1.000 | 0.971 | 1.000 | 0.964 | 0.848 | 1.000 | 1.000 | 0.849 | 0.819 |
| 1596 | 0.763 | 1.000 | 0.771 | 0.986 | 0.968 | 0.960 | 0.903 | 1.000 | 0.969 | 1.000 | 0.954 | 0.970 | 0.973 | 0.856 | 1.000 | 1.000 | 0.850 | 0.820 |
| 1597 | 0.762 | 1.000 | 0.777 | 0.986 | 0.968 | 0.960 | 0.901 | 1.000 | 0.980 | 1.000 | 0.965 | 0.970 | 0.955 | 0.861 | 1.000 | 1.000 | 0.852 | 0.822 |

| | | | | | | | | | | | | | | | | | | |
|---|---|---|---|---|---|---|---|---|---|---|---|---|---|---|---|---|---|---|
| 1598 | 0.769 | 1.000 | 0.767 | 1.000 | 0.963 | 0.960 | 0.901 | 1.000 | 0.969 | 1.000 | 0.948 | 1.000 | 0.973 | 0.851 | 1.000 | 1.000 | 0.850 | 0.819 |
| 1599 | 0.758 | 1.000 | 0.765 | 0.986 | 0.984 | 0.960 | 0.897 | 1.000 | 0.990 | 1.000 | 0.965 | 0.939 | 0.973 | 0.854 | 1.000 | 1.000 | 0.848 | 0.818 |
| 1600 | 0.761 | 1.000 | 0.775 | 0.986 | 0.968 | 0.960 | 0.898 | 1.000 | 1.000 | 1.000 | 0.962 | 1.000 | 0.964 | 0.857 | 1.000 | 1.000 | 0.851 | 0.821 |
| 1601 | 0.763 | 1.000 | 0.759 | 1.000 | 0.968 | 0.960 | 0.897 | 1.000 | 1.000 | 1.000 | 0.959 | 0.939 | 0.955 | 0.855 | 1.000 | 1.000 | 0.849 | 0.818 |
| 1602 | 0.767 | 1.000 | 0.769 | 1.000 | 0.963 | 0.960 | 0.886 | 1.000 | 0.980 | 1.000 | 0.965 | 0.970 | 0.973 | 0.847 | 1.000 | 1.000 | 0.848 | 0.817 |
| 1603 | 0.760 | 1.000 | 0.767 | 0.986 | 0.974 | 0.960 | 0.892 | 1.000 | 0.990 | 1.000 | 0.945 | 1.000 | 0.964 | 0.860 | 1.000 | 1.000 | 0.848 | 0.818 |
| 1604 | 0.767 | 1.000 | 0.765 | 1.000 | 0.968 | 0.960 | 0.895 | 1.000 | 0.980 | 1.000 | 0.959 | 1.000 | 0.982 | 0.858 | 1.000 | 1.000 | 0.852 | 0.822 |
| 1605 | 0.761 | 1.000 | 0.783 | 0.986 | 0.963 | 0.960 | 0.898 | 1.000 | 0.969 | 1.000 | 0.959 | 0.970 | 0.964 | 0.860 | 1.000 | 1.000 | 0.851 | 0.821 |
| 1606 | 0.759 | 1.000 | 0.773 | 0.986 | 0.974 | 0.960 | 0.900 | 1.000 | 0.980 | 1.000 | 0.962 | 0.939 | 0.964 | 0.854 | 1.000 | 1.000 | 0.849 | 0.819 |
| 1607 | 0.762 | 1.000 | 0.771 | 1.000 | 0.963 | 0.960 | 0.892 | 1.000 | 1.000 | 1.000 | 0.954 | 0.939 | 0.964 | 0.860 | 1.000 | 1.000 | 0.850 | 0.819 |
| 1608 | 0.765 | 1.000 | 0.775 | 0.986 | 0.963 | 0.960 | 0.906 | 1.000 | 0.980 | 1.000 | 0.959 | 1.000 | 0.955 | 0.857 | 1.000 | 1.000 | 0.852 | 0.822 |
| 1609 | 0.764 | 1.000 | 0.767 | 0.986 | 0.974 | 0.960 | 0.898 | 1.000 | 0.980 | 1.000 | 0.962 | 0.970 | 0.973 | 0.858 | 1.000 | 1.000 | 0.851 | 0.821 |
| 1610 | 0.759 | 1.000 | 0.773 | 1.000 | 0.963 | 0.960 | 0.886 | 1.000 | 0.990 | 1.000 | 0.965 | 0.970 | 0.982 | 0.854 | 1.000 | 1.000 | 0.848 | 0.817 |
| 1611 | 0.770 | 1.000 | 0.767 | 0.986 | 0.974 | 0.960 | 0.903 | 1.000 | 0.980 | 1.000 | 0.959 | 0.970 | 0.964 | 0.849 | 1.000 | 1.000 | 0.851 | 0.820 |
| 1612 | 0.752 | 1.000 | 0.775 | 0.986 | 0.974 | 0.960 | 0.897 | 1.000 | 0.980 | 1.000 | 0.957 | 1.000 | 0.964 | 0.856 | 1.000 | 1.000 | 0.847 | 0.817 |
| 1613 | 0.778 | 1.000 | 0.777 | 0.986 | 0.963 | 0.960 | 0.904 | 1.000 | 0.980 | 1.000 | 0.965 | 1.000 | 0.964 | 0.850 | 1.000 | 1.000 | 0.855 | 0.825 |
| 1614 | 0.755 | 1.000 | 0.767 | 1.000 | 0.968 | 0.960 | 0.889 | 1.000 | 0.980 | 1.000 | 0.959 | 0.970 | 0.964 | 0.861 | 1.000 | 1.000 | 0.848 | 0.817 |
| 1615 | 0.756 | 1.000 | 0.771 | 1.000 | 0.963 | 0.960 | 0.908 | 1.000 | 0.990 | 1.000 | 0.957 | 0.970 | 0.964 | 0.853 | 1.000 | 1.000 | 0.848 | 0.818 |
| 1616 | 0.766 | 1.000 | 0.773 | 0.971 | 0.974 | 0.960 | 0.901 | 1.000 | 0.980 | 1.000 | 0.962 | 1.000 | 0.964 | 0.857 | 1.000 | 1.000 | 0.852 | 0.822 |
| 1617 | 0.763 | 1.000 | 0.777 | 0.986 | 0.968 | 0.960 | 0.895 | 1.000 | 0.980 | 1.000 | 0.962 | 0.970 | 0.973 | 0.858 | 1.000 | 1.000 | 0.851 | 0.821 |
| 1618 | 0.763 | 1.000 | 0.763 | 0.986 | 0.974 | 0.960 | 0.891 | 1.000 | 0.969 | 1.000 | 0.954 | 0.970 | 0.964 | 0.858 | 1.000 | 1.000 | 0.848 | 0.817 |
| 1619 | 0.765 | 1.000 | 0.767 | 0.986 | 0.963 | 0.960 | 0.903 | 1.000 | 0.990 | 1.000 | 0.951 | 0.970 | 0.973 | 0.854 | 1.000 | 1.000 | 0.850 | 0.820 |
| 1620 | 0.766 | 1.000 | 0.783 | 0.986 | 0.968 | 0.960 | 0.900 | 1.000 | 0.969 | 1.000 | 0.951 | 1.000 | 0.973 | 0.858 | 1.000 | 1.000 | 0.852 | 0.823 |
| 1621 | 0.757 | 1.000 | 0.773 | 1.000 | 0.968 | 0.960 | 0.901 | 1.000 | 1.000 | 1.000 | 0.962 | 1.000 | 0.955 | 0.854 | 1.000 | 1.000 | 0.849 | 0.819 |
| 1622 | 0.768 | 1.000 | 0.763 | 1.000 | 0.963 | 0.960 | 0.891 | 1.000 | 0.990 | 1.000 | 0.962 | 1.000 | 0.982 | 0.854 | 1.000 | 1.000 | 0.850 | 0.820 |
| 1623 | 0.763 | 1.000 | 0.777 | 1.000 | 0.958 | 0.960 | 0.895 | 1.000 | 0.980 | 1.000 | 0.954 | 1.000 | 0.973 | 0.847 | 1.000 | 1.000 | 0.847 | 0.817 |
| 1624 | 0.759 | 1.000 | 0.773 | 1.000 | 0.963 | 0.960 | 0.904 | 1.000 | 0.980 | 1.000 | 0.965 | 0.970 | 0.955 | 0.858 | 1.000 | 1.000 | 0.850 | 0.821 |
| 1625 | 0.769 | 1.000 | 0.757 | 0.986 | 0.974 | 0.960 | 0.908 | 1.000 | 0.990 | 1.000 | 0.959 | 0.970 | 0.973 | 0.858 | 1.000 | 1.000 | 0.853 | 0.823 |
| 1626 | 0.763 | 1.000 | 0.769 | 0.986 | 0.958 | 0.960 | 0.903 | 1.000 | 0.990 | 1.000 | 0.951 | 1.000 | 0.964 | 0.861 | 1.000 | 1.000 | 0.851 | 0.821 |
| 1627 | 0.771 | 1.000 | 0.763 | 1.000 | 0.963 | 0.960 | 0.903 | 1.000 | 0.980 | 1.000 | 0.968 | 0.970 | 0.973 | 0.856 | 1.000 | 1.000 | 0.853 | 0.823 |
| 1628 | 0.767 | 1.000 | 0.765 | 1.000 | 0.968 | 0.960 | 0.897 | 1.000 | 0.980 | 1.000 | 0.957 | 1.000 | 0.973 | 0.854 | 1.000 | 1.000 | 0.850 | 0.820 |
| 1629 | 0.753 | 1.000 | 0.755 | 0.986 | 0.968 | 0.960 | 0.891 | 1.000 | 0.980 | 1.000 | 0.954 | 0.970 | 0.964 | 0.850 | 1.000 | 1.000 | 0.842 | 0.810 |
| 1630 | 0.756 | 1.000 | 0.775 | 0.986 | 0.958 | 0.960 | 0.897 | 1.000 | 0.990 | 1.000 | 0.951 | 0.970 | 0.964 | 0.864 | 1.000 | 1.000 | 0.849 | 0.819 |
| 1631 | 0.755 | 1.000 | 0.777 | 0.986 | 0.953 | 0.960 | 0.898 | 1.000 | 0.990 | 1.000 | 0.959 | 0.970 | 0.946 | 0.861 | 1.000 | 1.000 | 0.849 | 0.818 |
| 1632 | 0.762 | 1.000 | 0.779 | 0.986 | 0.974 | 0.960 | 0.892 | 1.000 | 0.980 | 1.000 | 0.959 | 0.970 | 0.973 | 0.863 | 1.000 | 1.000 | 0.852 | 0.822 |
| 1633 | 0.755 | 1.000 | 0.763 | 0.986 | 0.968 | 0.960 | 0.897 | 1.000 | 0.980 | 1.000 | 0.954 | 0.939 | 0.982 | 0.858 | 1.000 | 1.000 | 0.847 | 0.816 |
| 1634 | 0.754 | 1.000 | 0.773 | 0.986 | 0.963 | 0.960 | 0.897 | 1.000 | 1.000 | 1.000 | 0.957 | 1.000 | 0.973 | 0.856 | 1.000 | 1.000 | 0.848 | 0.817 |
| 1635 | 0.767 | 1.000 | 0.783 | 0.986 | 0.963 | 0.960 | 0.891 | 1.000 | 0.990 | 1.000 | 0.945 | 0.970 | 0.982 | 0.862 | 1.000 | 1.000 | 0.852 | 0.823 |
| 1636 | 0.754 | 1.000 | 0.783 | 1.000 | 0.968 | 0.960 | 0.895 | 1.000 | 0.980 | 1.000 | 0.962 | 0.970 | 0.973 | 0.860 | 1.000 | 1.000 | 0.850 | 0.820 |

| | | | | | | | | | | | | | | | | | |
|---|---|---|---|---|---|---|---|---|---|---|---|---|---|---|---|---|---|
| 1637 | 0.763 | 1.000 | 0.781 | 1.000 | 0.963 | 0.960 | 0.903 | 1.000 | 0.980 | 1.000 | 0.959 | 0.970 | 0.973 | 0.865 | 1.000 | 1.000 | 0.854 | 0.825 |
| 1638 | 0.761 | 1.000 | 0.779 | 1.000 | 0.968 | 0.960 | 0.908 | 1.000 | 0.980 | 1.000 | 0.965 | 1.000 | 0.955 | 0.858 | 1.000 | 1.000 | 0.852 | 0.823 |
| 1639 | 0.762 | 1.000 | 0.791 | 0.986 | 0.958 | 0.960 | 0.894 | 1.000 | 0.980 | 1.000 | 0.954 | 0.970 | 0.973 | 0.858 | 1.000 | 1.000 | 0.851 | 0.821 |
| 1640 | 0.757 | 1.000 | 0.769 | 0.986 | 0.958 | 0.960 | 0.897 | 1.000 | 0.990 | 1.000 | 0.957 | 0.970 | 0.964 | 0.853 | 1.000 | 0.981 | 0.846 | 0.816 |
| 1641 | 0.762 | 1.000 | 0.763 | 1.000 | 0.968 | 0.960 | 0.900 | 1.000 | 0.980 | 1.000 | 0.962 | 0.970 | 0.973 | 0.860 | 1.000 | 1.000 | 0.851 | 0.820 |
| 1642 | 0.765 | 1.000 | 0.769 | 0.986 | 0.963 | 0.960 | 0.888 | 1.000 | 0.980 | 1.000 | 0.951 | 1.000 | 0.973 | 0.856 | 1.000 | 1.000 | 0.848 | 0.818 |
| 1643 | 0.754 | 1.000 | 0.775 | 0.986 | 0.963 | 0.960 | 0.906 | 1.000 | 0.980 | 1.000 | 0.962 | 0.970 | 0.964 | 0.864 | 1.000 | 1.000 | 0.851 | 0.821 |
| 1644 | 0.767 | 1.000 | 0.781 | 0.986 | 0.958 | 0.960 | 0.906 | 1.000 | 0.980 | 1.000 | 0.959 | 0.970 | 0.955 | 0.868 | 1.000 | 1.000 | 0.856 | 0.826 |
| 1645 | 0.768 | 1.000 | 0.771 | 0.986 | 0.974 | 0.960 | 0.888 | 1.000 | 0.990 | 1.000 | 0.957 | 1.000 | 0.964 | 0.860 | 1.000 | 1.000 | 0.851 | 0.821 |
| 1646 | 0.761 | 1.000 | 0.785 | 1.000 | 0.974 | 0.960 | 0.895 | 1.000 | 0.990 | 1.000 | 0.957 | 1.000 | 0.973 | 0.856 | 1.000 | 1.000 | 0.851 | 0.821 |
| 1647 | 0.761 | 1.000 | 0.773 | 1.000 | 0.963 | 0.960 | 0.895 | 1.000 | 0.969 | 1.000 | 0.945 | 0.970 | 0.964 | 0.863 | 1.000 | 1.000 | 0.850 | 0.819 |
| 1648 | 0.759 | 1.000 | 0.779 | 0.986 | 0.958 | 0.960 | 0.901 | 1.000 | 0.990 | 1.000 | 0.962 | 0.970 | 0.982 | 0.854 | 1.000 | 1.000 | 0.850 | 0.820 |
| 1649 | 0.757 | 1.000 | 0.759 | 0.986 | 0.974 | 0.960 | 0.897 | 1.000 | 0.980 | 1.000 | 0.962 | 0.970 | 0.973 | 0.862 | 1.000 | 1.000 | 0.849 | 0.819 |
| 1650 | 0.757 | 1.000 | 0.775 | 0.971 | 0.953 | 0.960 | 0.904 | 1.000 | 1.000 | 1.000 | 0.968 | 1.000 | 0.955 | 0.839 | 1.000 | 1.000 | 0.845 | 0.814 |
| 1651 | 0.757 | 1.000 | 0.765 | 0.986 | 0.953 | 0.960 | 0.897 | 1.000 | 0.980 | 1.000 | 0.959 | 0.970 | 0.964 | 0.856 | 1.000 | 1.000 | 0.847 | 0.816 |
| 1652 | 0.761 | 1.000 | 0.771 | 0.986 | 0.963 | 0.960 | 0.898 | 1.000 | 0.980 | 1.000 | 0.954 | 1.000 | 0.964 | 0.868 | 1.000 | 1.000 | 0.852 | 0.822 |
| 1653 | 0.762 | 1.000 | 0.759 | 1.000 | 0.963 | 0.960 | 0.891 | 1.000 | 0.990 | 1.000 | 0.962 | 0.970 | 0.964 | 0.859 | 1.000 | 1.000 | 0.849 | 0.818 |
| 1654 | 0.761 | 1.000 | 0.775 | 1.000 | 0.963 | 0.960 | 0.901 | 1.000 | 0.980 | 1.000 | 0.959 | 0.970 | 0.964 | 0.862 | 1.000 | 1.000 | 0.852 | 0.822 |
| 1655 | 0.761 | 1.000 | 0.763 | 0.986 | 0.963 | 0.960 | 0.897 | 1.000 | 0.990 | 1.000 | 0.954 | 1.000 | 0.973 | 0.857 | 1.000 | 1.000 | 0.848 | 0.818 |
| 1656 | 0.756 | 1.000 | 0.765 | 1.000 | 0.963 | 0.960 | 0.901 | 1.000 | 1.000 | 1.000 | 0.954 | 1.000 | 0.964 | 0.860 | 1.000 | 1.000 | 0.849 | 0.819 |
| 1657 | 0.766 | 1.000 | 0.763 | 0.986 | 0.968 | 0.960 | 0.903 | 1.000 | 0.969 | 1.000 | 0.959 | 1.000 | 0.964 | 0.859 | 1.000 | 1.000 | 0.851 | 0.821 |
| 1658 | 0.755 | 1.000 | 0.769 | 1.000 | 0.968 | 0.960 | 0.900 | 1.000 | 0.990 | 1.000 | 0.962 | 1.000 | 0.955 | 0.857 | 1.000 | 1.000 | 0.848 | 0.818 |
| 1659 | 0.762 | 1.000 | 0.775 | 0.986 | 0.963 | 0.960 | 0.903 | 1.000 | 0.980 | 1.000 | 0.959 | 1.000 | 0.964 | 0.858 | 1.000 | 1.000 | 0.851 | 0.821 |
| 1660 | 0.763 | 1.000 | 0.771 | 0.986 | 0.968 | 0.960 | 0.906 | 1.000 | 0.990 | 1.000 | 0.951 | 0.970 | 0.955 | 0.863 | 1.000 | 1.000 | 0.852 | 0.822 |
| 1661 | 0.753 | 1.000 | 0.759 | 1.000 | 0.958 | 0.960 | 0.901 | 1.000 | 1.000 | 1.000 | 0.945 | 1.000 | 0.973 | 0.861 | 1.000 | 1.000 | 0.847 | 0.816 |
| 1662 | 0.754 | 1.000 | 0.767 | 0.986 | 0.968 | 0.960 | 0.891 | 1.000 | 0.990 | 1.000 | 0.954 | 1.000 | 0.964 | 0.858 | 1.000 | 1.000 | 0.846 | 0.815 |
| 1663 | 0.757 | 1.000 | 0.765 | 1.000 | 0.974 | 0.960 | 0.895 | 1.000 | 1.000 | 1.000 | 0.959 | 0.970 | 0.973 | 0.854 | 1.000 | 1.000 | 0.848 | 0.817 |
| 1664 | 0.761 | 1.000 | 0.765 | 0.986 | 0.947 | 0.960 | 0.894 | 1.000 | 0.990 | 1.000 | 0.948 | 1.000 | 0.973 | 0.854 | 1.000 | 1.000 | 0.847 | 0.816 |
| 1665 | 0.753 | 1.000 | 0.767 | 1.000 | 0.968 | 0.960 | 0.888 | 1.000 | 0.959 | 1.000 | 0.959 | 1.000 | 0.955 | 0.858 | 1.000 | 1.000 | 0.845 | 0.814 |
| 1666 | 0.759 | 1.000 | 0.769 | 0.986 | 0.979 | 0.960 | 0.900 | 1.000 | 0.980 | 1.000 | 0.962 | 1.000 | 0.973 | 0.861 | 1.000 | 1.000 | 0.851 | 0.821 |
| 1667 | 0.755 | 1.000 | 0.771 | 0.986 | 0.958 | 0.960 | 0.892 | 1.000 | 1.000 | 1.000 | 0.957 | 1.000 | 0.973 | 0.854 | 1.000 | 1.000 | 0.846 | 0.815 |
| 1668 | 0.767 | 1.000 | 0.769 | 0.986 | 0.958 | 0.960 | 0.898 | 1.000 | 0.980 | 1.000 | 0.948 | 0.970 | 0.964 | 0.858 | 1.000 | 1.000 | 0.850 | 0.820 |
| 1669 | 0.763 | 1.000 | 0.781 | 0.986 | 0.963 | 0.960 | 0.906 | 1.000 | 0.990 | 1.000 | 0.954 | 0.970 | 0.973 | 0.867 | 1.000 | 1.000 | 0.855 | 0.825 |
| 1670 | 0.765 | 1.000 | 0.779 | 1.000 | 0.958 | 0.960 | 0.901 | 1.000 | 0.990 | 1.000 | 0.957 | 1.000 | 0.964 | 0.857 | 1.000 | 1.000 | 0.852 | 0.822 |
| 1671 | 0.756 | 1.000 | 0.789 | 1.000 | 0.958 | 0.960 | 0.895 | 1.000 | 0.990 | 1.000 | 0.954 | 1.000 | 0.964 | 0.857 | 1.000 | 1.000 | 0.849 | 0.819 |
| 1672 | 0.766 | 1.000 | 0.765 | 0.986 | 0.958 | 0.960 | 0.904 | 1.000 | 1.000 | 1.000 | 0.957 | 0.970 | 0.964 | 0.854 | 1.000 | 1.000 | 0.850 | 0.820 |
| 1673 | 0.766 | 1.000 | 0.767 | 1.000 | 0.953 | 0.960 | 0.895 | 1.000 | 0.969 | 1.000 | 0.954 | 0.939 | 0.964 | 0.859 | 1.000 | 1.000 | 0.850 | 0.819 |
| 1674 | 0.768 | 1.000 | 0.773 | 0.986 | 0.968 | 0.960 | 0.898 | 1.000 | 0.990 | 1.000 | 0.954 | 0.970 | 0.946 | 0.851 | 1.000 | 1.000 | 0.850 | 0.819 |
| 1675 | 0.771 | 1.000 | 0.777 | 1.000 | 0.974 | 0.960 | 0.898 | 1.000 | 0.980 | 1.000 | 0.954 | 0.970 | 0.982 | 0.858 | 1.000 | 1.000 | 0.854 | 0.824 |

| | | | | | | | | | | | | | | | | | |
|---|---|---|---|---|---|---|---|---|---|---|---|---|---|---|---|---|---|
| 1676 | 0.754 | 1.000 | 0.775 | 0.986 | 0.958 | 0.960 | 0.898 | 1.000 | 0.980 | 1.000 | 0.965 | 0.970 | 0.955 | 0.863 | 1.000 | 1.000 | 0.849 | 0.819 |
| 1677 | 0.763 | 1.000 | 0.779 | 0.986 | 0.958 | 0.960 | 0.901 | 1.000 | 0.980 | 1.000 | 0.959 | 1.000 | 0.973 | 0.859 | 1.000 | 1.000 | 0.852 | 0.822 |
| 1678 | 0.748 | 1.000 | 0.763 | 1.000 | 0.968 | 0.960 | 0.884 | 1.000 | 0.980 | 1.000 | 0.959 | 0.970 | 0.973 | 0.863 | 1.000 | 1.000 | 0.845 | 0.814 |
| 1679 | 0.755 | 1.000 | 0.783 | 0.986 | 0.968 | 0.960 | 0.901 | 1.000 | 0.990 | 1.000 | 0.954 | 1.000 | 0.973 | 0.861 | 1.000 | 1.000 | 0.851 | 0.821 |
| 1680 | 0.761 | 1.000 | 0.771 | 0.986 | 0.958 | 0.960 | 0.898 | 1.000 | 0.990 | 1.000 | 0.962 | 0.970 | 0.973 | 0.854 | 1.000 | 1.000 | 0.849 | 0.819 |
| 1681 | 0.761 | 1.000 | 0.769 | 0.971 | 0.958 | 0.960 | 0.897 | 1.000 | 1.000 | 1.000 | 0.959 | 0.970 | 0.964 | 0.858 | 1.000 | 1.000 | 0.849 | 0.819 |
| 1682 | 0.751 | 1.000 | 0.783 | 0.986 | 0.963 | 0.960 | 0.897 | 1.000 | 0.969 | 1.000 | 0.971 | 1.000 | 0.973 | 0.863 | 1.000 | 1.000 | 0.850 | 0.820 |
| 1683 | 0.756 | 1.000 | 0.779 | 1.000 | 0.963 | 0.960 | 0.904 | 1.000 | 0.969 | 1.000 | 0.954 | 1.000 | 0.964 | 0.853 | 1.000 | 1.000 | 0.848 | 0.818 |
| 1684 | 0.761 | 1.000 | 0.773 | 0.986 | 0.968 | 0.960 | 0.894 | 1.000 | 0.990 | 1.000 | 0.954 | 0.970 | 0.982 | 0.861 | 1.000 | 1.000 | 0.850 | 0.820 |
| 1685 | 0.757 | 1.000 | 0.771 | 1.000 | 0.963 | 0.960 | 0.895 | 1.000 | 0.980 | 1.000 | 0.962 | 1.000 | 0.964 | 0.858 | 1.000 | 1.000 | 0.849 | 0.818 |
| 1686 | 0.765 | 1.000 | 0.765 | 0.986 | 0.968 | 0.960 | 0.889 | 1.000 | 1.000 | 1.000 | 0.965 | 0.970 | 0.973 | 0.854 | 1.000 | 1.000 | 0.849 | 0.819 |
| 1687 | 0.759 | 1.000 | 0.779 | 1.000 | 0.963 | 1.000 | 0.898 | 1.000 | 0.969 | 1.000 | 0.959 | 1.000 | 0.973 | 0.854 | 1.000 | 1.000 | 0.849 | 0.819 |
| 1688 | 0.756 | 1.000 | 0.771 | 1.000 | 0.968 | 0.960 | 0.906 | 1.000 | 0.980 | 1.000 | 0.957 | 1.000 | 0.955 | 0.863 | 1.000 | 1.000 | 0.851 | 0.821 |
| 1689 | 0.770 | 1.000 | 0.773 | 0.986 | 0.974 | 0.960 | 0.894 | 1.000 | 0.990 | 1.000 | 0.957 | 1.000 | 0.973 | 0.858 | 1.000 | 1.000 | 0.853 | 0.823 |
| 1690 | 0.762 | 1.000 | 0.767 | 0.986 | 0.958 | 0.960 | 0.895 | 1.000 | 0.980 | 1.000 | 0.959 | 0.970 | 0.964 | 0.861 | 1.000 | 1.000 | 0.850 | 0.819 |
| 1691 | 0.759 | 1.000 | 0.761 | 1.000 | 0.958 | 0.960 | 0.898 | 1.000 | 0.990 | 1.000 | 0.971 | 1.000 | 0.955 | 0.851 | 1.000 | 1.000 | 0.847 | 0.816 |
| 1692 | 0.759 | 1.000 | 0.767 | 0.986 | 0.963 | 0.960 | 0.897 | 1.000 | 0.980 | 1.000 | 0.959 | 1.000 | 0.973 | 0.865 | 1.000 | 1.000 | 0.851 | 0.821 |
| 1693 | 0.763 | 1.000 | 0.769 | 0.986 | 0.968 | 0.960 | 0.906 | 1.000 | 0.980 | 1.000 | 0.951 | 0.939 | 0.964 | 0.854 | 1.000 | 1.000 | 0.849 | 0.819 |
| 1694 | 0.759 | 1.000 | 0.751 | 0.986 | 0.968 | 0.960 | 0.891 | 1.000 | 0.969 | 1.000 | 0.965 | 0.970 | 0.973 | 0.860 | 1.000 | 1.000 | 0.847 | 0.816 |
| 1695 | 0.751 | 1.000 | 0.783 | 0.986 | 0.963 | 0.960 | 0.889 | 1.000 | 0.990 | 1.000 | 0.965 | 0.970 | 0.964 | 0.858 | 1.000 | 1.000 | 0.847 | 0.817 |
| 1696 | 0.767 | 1.000 | 0.779 | 1.000 | 0.958 | 0.960 | 0.892 | 1.000 | 0.980 | 1.000 | 0.965 | 1.000 | 0.955 | 0.854 | 1.000 | 1.000 | 0.851 | 0.821 |
| 1697 | 0.761 | 1.000 | 0.775 | 1.000 | 0.958 | 0.960 | 0.898 | 1.000 | 0.980 | 1.000 | 0.959 | 1.000 | 0.973 | 0.852 | 1.000 | 1.000 | 0.849 | 0.819 |
| 1698 | 0.754 | 1.000 | 0.785 | 1.000 | 0.974 | 1.000 | 0.900 | 1.000 | 0.980 | 1.000 | 0.948 | 1.000 | 0.964 | 0.855 | 1.000 | 1.000 | 0.849 | 0.819 |
| 1699 | 0.766 | 1.000 | 0.769 | 0.986 | 0.963 | 0.960 | 0.898 | 1.000 | 0.969 | 1.000 | 0.957 | 1.000 | 0.955 | 0.858 | 1.000 | 1.000 | 0.851 | 0.821 |
| 1700 | 0.763 | 1.000 | 0.775 | 1.000 | 0.963 | 0.960 | 0.900 | 1.000 | 0.980 | 1.000 | 0.957 | 1.000 | 0.973 | 0.856 | 1.000 | 1.000 | 0.851 | 0.820 |
| 1701 | 0.761 | 1.000 | 0.775 | 0.986 | 0.974 | 0.960 | 0.897 | 1.000 | 0.980 | 1.000 | 0.968 | 1.000 | 0.964 | 0.848 | 1.000 | 1.000 | 0.848 | 0.818 |
| 1702 | 0.758 | 1.000 | 0.767 | 0.986 | 0.963 | 0.960 | 0.886 | 1.000 | 0.980 | 1.000 | 0.957 | 0.970 | 0.982 | 0.858 | 1.000 | 1.000 | 0.847 | 0.816 |
| 1703 | 0.761 | 1.000 | 0.767 | 1.000 | 0.958 | 0.960 | 0.889 | 1.000 | 0.990 | 1.000 | 0.957 | 1.000 | 0.964 | 0.853 | 1.000 | 1.000 | 0.847 | 0.816 |
| 1704 | 0.763 | 1.000 | 0.773 | 0.986 | 0.974 | 0.960 | 0.906 | 1.000 | 1.000 | 1.000 | 0.954 | 0.970 | 0.955 | 0.855 | 1.000 | 1.000 | 0.851 | 0.821 |
| 1705 | 0.763 | 1.000 | 0.773 | 0.986 | 0.968 | 0.960 | 0.908 | 1.000 | 0.980 | 1.000 | 0.965 | 1.000 | 0.973 | 0.859 | 1.000 | 1.000 | 0.853 | 0.823 |
| 1706 | 0.763 | 1.000 | 0.773 | 0.971 | 0.968 | 0.960 | 0.903 | 1.000 | 1.000 | 1.000 | 0.954 | 0.970 | 0.964 | 0.861 | 1.000 | 1.000 | 0.852 | 0.822 |
| 1707 | 0.759 | 1.000 | 0.794 | 1.000 | 0.968 | 0.960 | 0.908 | 1.000 | 0.980 | 1.000 | 0.954 | 0.939 | 0.973 | 0.860 | 1.000 | 1.000 | 0.853 | 0.824 |
| 1708 | 0.767 | 1.000 | 0.763 | 0.986 | 0.958 | 0.960 | 0.891 | 1.000 | 0.990 | 1.000 | 0.957 | 0.970 | 0.955 | 0.859 | 1.000 | 1.000 | 0.849 | 0.819 |
| 1709 | 0.768 | 1.000 | 0.773 | 0.986 | 0.968 | 0.960 | 0.892 | 1.000 | 0.980 | 1.000 | 0.959 | 0.970 | 0.973 | 0.860 | 1.000 | 1.000 | 0.852 | 0.822 |
| 1710 | 0.756 | 1.000 | 0.781 | 0.986 | 0.953 | 0.960 | 0.894 | 1.000 | 0.980 | 1.000 | 0.957 | 1.000 | 0.964 | 0.858 | 1.000 | 1.000 | 0.848 | 0.818 |
| 1711 | 0.765 | 1.000 | 0.773 | 1.000 | 0.979 | 0.960 | 0.897 | 1.000 | 1.000 | 1.000 | 0.951 | 1.000 | 0.973 | 0.858 | 1.000 | 1.000 | 0.852 | 0.822 |
| 1712 | 0.753 | 1.000 | 0.761 | 0.986 | 0.979 | 0.960 | 0.892 | 1.000 | 0.969 | 1.000 | 0.957 | 1.000 | 0.964 | 0.854 | 1.000 | 1.000 | 0.845 | 0.814 |
| 1713 | 0.774 | 1.000 | 0.777 | 0.986 | 0.974 | 0.960 | 0.901 | 1.000 | 0.969 | 1.000 | 0.954 | 0.970 | 0.964 | 0.864 | 1.000 | 1.000 | 0.856 | 0.827 |
| 1714 | 0.762 | 1.000 | 0.767 | 0.986 | 0.958 | 0.960 | 0.904 | 1.000 | 0.980 | 1.000 | 0.962 | 0.970 | 0.964 | 0.861 | 1.000 | 1.000 | 0.851 | 0.821 |

| | | | | | | | | | | | | | | | | | | |
|---|---|---|---|---|---|---|---|---|---|---|---|---|---|---|---|---|---|---|
| 1715 | 0.754 | 1.000 | 0.769 | 1.000 | 0.968 | 0.960 | 0.900 | 1.000 | 0.990 | 1.000 | 0.959 | 0.970 | 0.964 | 0.859 | 1.000 | 1.000 | 0.848 | 0.818 |
| 1716 | 0.767 | 1.000 | 0.781 | 0.986 | 0.958 | 0.960 | 0.888 | 1.000 | 0.980 | 1.000 | 0.954 | 0.970 | 0.964 | 0.855 | 1.000 | 1.000 | 0.850 | 0.819 |
| 1717 | 0.767 | 1.000 | 0.791 | 1.000 | 0.963 | 0.960 | 0.901 | 1.000 | 0.990 | 1.000 | 0.957 | 0.970 | 0.973 | 0.862 | 1.000 | 1.000 | 0.855 | 0.826 |
| 1718 | 0.765 | 1.000 | 0.781 | 0.986 | 0.963 | 0.960 | 0.889 | 1.000 | 0.969 | 1.000 | 0.962 | 0.970 | 0.964 | 0.862 | 1.000 | 1.000 | 0.852 | 0.822 |
| 1719 | 0.757 | 1.000 | 0.769 | 0.986 | 0.974 | 0.960 | 0.897 | 1.000 | 0.969 | 1.000 | 0.957 | 0.939 | 0.973 | 0.861 | 1.000 | 1.000 | 0.849 | 0.818 |
| 1720 | 0.765 | 1.000 | 0.785 | 1.000 | 0.968 | 0.960 | 0.898 | 1.000 | 0.990 | 1.000 | 0.962 | 0.970 | 0.964 | 0.858 | 1.000 | 1.000 | 0.853 | 0.824 |
| 1721 | 0.765 | 1.000 | 0.777 | 0.986 | 0.963 | 0.960 | 0.903 | 1.000 | 0.980 | 1.000 | 0.954 | 1.000 | 0.964 | 0.855 | 1.000 | 1.000 | 0.851 | 0.821 |
| 1722 | 0.759 | 1.000 | 0.781 | 0.986 | 0.958 | 0.960 | 0.892 | 1.000 | 1.000 | 1.000 | 0.954 | 1.000 | 0.964 | 0.850 | 1.000 | 1.000 | 0.847 | 0.817 |
| 1723 | 0.761 | 1.000 | 0.781 | 0.986 | 0.968 | 0.960 | 0.894 | 1.000 | 0.980 | 1.000 | 0.962 | 1.000 | 0.973 | 0.858 | 1.000 | 1.000 | 0.851 | 0.821 |
| 1724 | 0.763 | 1.000 | 0.771 | 1.000 | 0.979 | 0.960 | 0.898 | 1.000 | 0.969 | 1.000 | 0.942 | 1.000 | 0.964 | 0.856 | 1.000 | 1.000 | 0.849 | 0.819 |
| 1725 | 0.765 | 1.000 | 0.771 | 0.986 | 0.958 | 0.960 | 0.895 | 1.000 | 0.980 | 1.000 | 0.951 | 1.000 | 0.973 | 0.861 | 1.000 | 1.000 | 0.851 | 0.820 |
| 1726 | 0.761 | 1.000 | 0.779 | 1.000 | 0.963 | 0.960 | 0.904 | 1.000 | 0.980 | 1.000 | 0.957 | 0.970 | 0.964 | 0.851 | 1.000 | 1.000 | 0.850 | 0.819 |
| 1727 | 0.759 | 1.000 | 0.771 | 0.986 | 0.963 | 0.960 | 0.912 | 1.000 | 0.990 | 1.000 | 0.959 | 0.939 | 0.955 | 0.858 | 1.000 | 1.000 | 0.851 | 0.821 |
| 1728 | 0.765 | 1.000 | 0.792 | 1.000 | 0.963 | 0.960 | 0.892 | 1.000 | 0.980 | 1.000 | 0.957 | 0.970 | 0.982 | 0.860 | 1.000 | 1.000 | 0.853 | 0.823 |
| 1729 | 0.764 | 1.000 | 0.773 | 0.986 | 0.968 | 0.960 | 0.895 | 1.000 | 0.980 | 1.000 | 0.959 | 1.000 | 0.955 | 0.861 | 1.000 | 1.000 | 0.851 | 0.821 |
| 1730 | 0.762 | 1.000 | 0.769 | 0.986 | 0.974 | 0.960 | 0.891 | 1.000 | 0.969 | 1.000 | 0.948 | 0.970 | 0.973 | 0.861 | 1.000 | 1.000 | 0.849 | 0.819 |
| 1731 | 0.752 | 1.000 | 0.765 | 1.000 | 0.958 | 1.000 | 0.901 | 1.000 | 0.980 | 1.000 | 0.957 | 0.939 | 0.964 | 0.854 | 1.000 | 1.000 | 0.845 | 0.815 |
| 1732 | 0.760 | 1.000 | 0.761 | 0.986 | 0.979 | 0.960 | 0.900 | 1.000 | 0.990 | 1.000 | 0.965 | 0.970 | 0.964 | 0.854 | 1.000 | 1.000 | 0.849 | 0.818 |
| 1733 | 0.767 | 1.000 | 0.765 | 1.000 | 0.963 | 0.960 | 0.889 | 1.000 | 0.980 | 1.000 | 0.957 | 0.970 | 0.973 | 0.857 | 1.000 | 1.000 | 0.850 | 0.819 |
| 1734 | 0.752 | 1.000 | 0.779 | 1.000 | 0.968 | 0.960 | 0.900 | 1.000 | 1.000 | 1.000 | 0.951 | 0.939 | 0.973 | 0.862 | 1.000 | 1.000 | 0.849 | 0.819 |
| 1735 | 0.767 | 1.000 | 0.794 | 1.000 | 0.968 | 0.960 | 0.894 | 1.000 | 0.980 | 1.000 | 0.954 | 1.000 | 0.973 | 0.862 | 1.000 | 1.000 | 0.855 | 0.825 |
| 1736 | 0.753 | 1.000 | 0.765 | 0.986 | 0.968 | 0.960 | 0.898 | 1.000 | 0.969 | 1.000 | 0.959 | 0.970 | 0.973 | 0.862 | 1.000 | 1.000 | 0.848 | 0.817 |
| 1737 | 0.764 | 1.000 | 0.773 | 0.986 | 0.963 | 0.960 | 0.900 | 1.000 | 0.969 | 1.000 | 0.954 | 1.000 | 0.955 | 0.865 | 1.000 | 1.000 | 0.852 | 0.822 |
| 1738 | 0.758 | 1.000 | 0.789 | 1.000 | 0.963 | 0.960 | 0.894 | 1.000 | 0.980 | 1.000 | 0.957 | 0.970 | 0.973 | 0.858 | 1.000 | 1.000 | 0.850 | 0.820 |
| 1739 | 0.761 | 1.000 | 0.769 | 1.000 | 0.968 | 0.960 | 0.901 | 1.000 | 0.980 | 1.000 | 0.959 | 1.000 | 0.973 | 0.865 | 1.000 | 1.000 | 0.852 | 0.823 |
| 1740 | 0.775 | 1.000 | 0.777 | 1.000 | 0.947 | 0.960 | 0.903 | 1.000 | 0.980 | 1.000 | 0.959 | 0.970 | 0.964 | 0.858 | 1.000 | 1.000 | 0.855 | 0.825 |
| 1741 | 0.769 | 1.000 | 0.771 | 1.000 | 0.968 | 0.960 | 0.895 | 1.000 | 0.980 | 1.000 | 0.959 | 0.970 | 0.973 | 0.860 | 1.000 | 1.000 | 0.853 | 0.823 |
| 1742 | 0.763 | 1.000 | 0.779 | 1.000 | 0.968 | 0.960 | 0.915 | 1.000 | 0.990 | 1.000 | 0.948 | 0.970 | 0.964 | 0.861 | 1.000 | 1.000 | 0.854 | 0.825 |
| 1743 | 0.754 | 1.000 | 0.785 | 1.000 | 0.963 | 0.960 | 0.900 | 1.000 | 0.980 | 1.000 | 0.965 | 0.970 | 0.982 | 0.864 | 1.000 | 1.000 | 0.852 | 0.822 |
| 1744 | 0.756 | 1.000 | 0.787 | 0.986 | 0.958 | 0.960 | 0.901 | 1.000 | 0.990 | 1.000 | 0.954 | 1.000 | 0.973 | 0.859 | 1.000 | 1.000 | 0.850 | 0.821 |
| 1745 | 0.759 | 1.000 | 0.773 | 0.986 | 0.974 | 0.960 | 0.904 | 1.000 | 0.990 | 1.000 | 0.962 | 1.000 | 0.982 | 0.856 | 1.000 | 1.000 | 0.851 | 0.821 |
| 1746 | 0.765 | 1.000 | 0.779 | 1.000 | 0.968 | 0.960 | 0.889 | 1.000 | 0.969 | 1.000 | 0.951 | 0.970 | 0.973 | 0.861 | 1.000 | 1.000 | 0.851 | 0.821 |
| 1747 | 0.756 | 1.000 | 0.771 | 1.000 | 0.974 | 0.960 | 0.892 | 1.000 | 0.980 | 1.000 | 0.954 | 1.000 | 0.973 | 0.859 | 1.000 | 1.000 | 0.848 | 0.818 |
| 1748 | 0.756 | 1.000 | 0.779 | 1.000 | 0.968 | 0.960 | 0.889 | 1.000 | 0.990 | 1.000 | 0.954 | 0.970 | 0.973 | 0.860 | 1.000 | 1.000 | 0.849 | 0.818 |
| 1749 | 0.762 | 1.000 | 0.783 | 0.986 | 0.974 | 0.960 | 0.900 | 1.000 | 0.980 | 1.000 | 0.959 | 1.000 | 0.982 | 0.861 | 1.000 | 1.000 | 0.853 | 0.824 |
| 1750 | 0.761 | 1.000 | 0.777 | 1.000 | 0.968 | 0.960 | 0.898 | 1.000 | 0.980 | 1.000 | 0.954 | 0.970 | 0.973 | 0.868 | 1.000 | 1.000 | 0.853 | 0.823 |
| 1751 | 0.759 | 1.000 | 0.767 | 0.986 | 0.963 | 0.960 | 0.894 | 1.000 | 0.980 | 1.000 | 0.954 | 1.000 | 0.964 | 0.858 | 1.000 | 1.000 | 0.848 | 0.817 |
| 1752 | 0.759 | 1.000 | 0.792 | 0.986 | 0.958 | 0.960 | 0.894 | 1.000 | 1.000 | 1.000 | 0.948 | 1.000 | 0.991 | 0.861 | 1.000 | 1.000 | 0.852 | 0.822 |
| 1753 | 0.765 | 1.000 | 0.753 | 1.000 | 0.974 | 0.960 | 0.892 | 1.000 | 0.990 | 1.000 | 0.959 | 1.000 | 0.982 | 0.858 | 1.000 | 1.000 | 0.850 | 0.819 |

| | | | | | | | | | | | | | | | | | | |
|---|---|---|---|---|---|---|---|---|---|---|---|---|---|---|---|---|---|---|
| 1754 | 0.776 | 1.000 | 0.769 | 1.000 | 0.958 | 0.960 | 0.891 | 1.000 | 0.980 | 1.000 | 0.948 | 1.000 | 0.991 | 0.858 | 1.000 | 1.000 | 0.853 | 0.823 |
| 1755 | 0.757 | 1.000 | 0.779 | 0.986 | 0.979 | 0.960 | 0.889 | 1.000 | 0.990 | 1.000 | 0.954 | 1.000 | 0.982 | 0.854 | 1.000 | 1.000 | 0.848 | 0.818 |
| 1756 | 0.757 | 1.000 | 0.781 | 0.986 | 0.958 | 0.960 | 0.901 | 1.000 | 0.980 | 1.000 | 0.954 | 1.000 | 1.000 | 0.855 | 1.000 | 1.000 | 0.849 | 0.819 |
| 1757 | 0.755 | 1.000 | 0.777 | 1.000 | 0.974 | 0.960 | 0.900 | 1.000 | 0.980 | 1.000 | 0.957 | 1.000 | 0.982 | 0.866 | 1.000 | 1.000 | 0.852 | 0.822 |
| 1758 | 0.760 | 1.000 | 0.765 | 0.986 | 0.974 | 0.960 | 0.900 | 1.000 | 0.980 | 1.000 | 0.957 | 0.970 | 0.973 | 0.858 | 1.000 | 1.000 | 0.849 | 0.819 |
| 1759 | 0.773 | 1.000 | 0.775 | 0.986 | 0.968 | 0.960 | 0.897 | 1.000 | 0.980 | 1.000 | 0.948 | 1.000 | 0.991 | 0.859 | 1.000 | 1.000 | 0.854 | 0.824 |
| 1760 | 0.763 | 1.000 | 0.763 | 0.986 | 0.968 | 0.960 | 0.911 | 1.000 | 0.980 | 1.000 | 0.957 | 1.000 | 1.000 | 0.858 | 1.000 | 1.000 | 0.852 | 0.822 |
| 1761 | 0.768 | 1.000 | 0.767 | 0.986 | 0.968 | 0.960 | 0.888 | 1.000 | 0.969 | 1.000 | 0.951 | 1.000 | 0.991 | 0.854 | 1.000 | 1.000 | 0.849 | 0.818 |
| 1762 | 0.757 | 1.000 | 0.775 | 1.000 | 0.968 | 0.960 | 0.891 | 1.000 | 0.990 | 1.000 | 0.959 | 1.000 | 0.982 | 0.859 | 1.000 | 1.000 | 0.849 | 0.819 |
| 1763 | 0.759 | 1.000 | 0.777 | 0.986 | 0.958 | 0.960 | 0.888 | 1.000 | 0.980 | 1.000 | 0.954 | 1.000 | 0.982 | 0.859 | 1.000 | 1.000 | 0.849 | 0.818 |
| 1764 | 0.755 | 1.000 | 0.785 | 1.000 | 0.958 | 0.960 | 0.898 | 1.000 | 0.990 | 1.000 | 0.948 | 1.000 | 0.991 | 0.859 | 1.000 | 1.000 | 0.850 | 0.820 |
| 1765 | 0.759 | 1.000 | 0.777 | 1.000 | 0.963 | 0.960 | 0.897 | 1.000 | 1.000 | 1.000 | 0.962 | 1.000 | 0.982 | 0.857 | 1.000 | 1.000 | 0.850 | 0.820 |
| 1766 | 0.759 | 1.000 | 0.777 | 0.986 | 0.968 | 0.960 | 0.903 | 1.000 | 0.980 | 1.000 | 0.957 | 1.000 | 0.982 | 0.858 | 1.000 | 1.000 | 0.851 | 0.821 |
| 1767 | 0.764 | 1.000 | 0.779 | 0.986 | 0.968 | 0.960 | 0.901 | 1.000 | 0.990 | 1.000 | 0.959 | 1.000 | 0.973 | 0.852 | 1.000 | 1.000 | 0.851 | 0.821 |
| 1768 | 0.757 | 1.000 | 0.767 | 0.986 | 0.968 | 0.960 | 0.895 | 1.000 | 0.990 | 1.000 | 0.962 | 0.970 | 0.982 | 0.857 | 1.000 | 1.000 | 0.848 | 0.818 |
| 1769 | 0.762 | 1.000 | 0.785 | 0.986 | 0.958 | 0.960 | 0.898 | 1.000 | 0.980 | 1.000 | 0.957 | 1.000 | 0.973 | 0.859 | 1.000 | 1.000 | 0.852 | 0.822 |
| 1770 | 0.767 | 1.000 | 0.775 | 0.986 | 0.958 | 0.960 | 0.892 | 1.000 | 0.980 | 1.000 | 0.945 | 1.000 | 0.973 | 0.856 | 1.000 | 1.000 | 0.850 | 0.819 |
| 1771 | 0.760 | 1.000 | 0.787 | 0.986 | 0.958 | 0.960 | 0.903 | 1.000 | 0.990 | 1.000 | 0.959 | 1.000 | 0.973 | 0.859 | 1.000 | 1.000 | 0.852 | 0.823 |
| 1772 | 0.759 | 1.000 | 0.769 | 1.000 | 0.974 | 0.960 | 0.903 | 1.000 | 0.990 | 1.000 | 0.965 | 1.000 | 0.982 | 0.867 | 1.000 | 1.000 | 0.854 | 0.824 |
| 1773 | 0.755 | 1.000 | 0.783 | 0.986 | 0.968 | 0.960 | 0.903 | 1.000 | 0.969 | 1.000 | 0.954 | 1.000 | 0.982 | 0.864 | 1.000 | 1.000 | 0.851 | 0.822 |
| 1774 | 0.752 | 1.000 | 0.787 | 0.986 | 0.968 | 0.960 | 0.900 | 1.000 | 0.980 | 1.000 | 0.957 | 1.000 | 0.991 | 0.854 | 1.000 | 1.000 | 0.848 | 0.818 |
| 1775 | 0.755 | 1.000 | 0.769 | 1.000 | 0.963 | 0.960 | 0.894 | 1.000 | 0.980 | 1.000 | 0.951 | 0.970 | 0.982 | 0.861 | 1.000 | 1.000 | 0.848 | 0.817 |
| 1776 | 0.755 | 1.000 | 0.789 | 1.000 | 0.968 | 0.960 | 0.898 | 1.000 | 0.980 | 1.000 | 0.954 | 0.970 | 0.991 | 0.855 | 1.000 | 1.000 | 0.849 | 0.820 |
| 1777 | 0.765 | 1.000 | 0.767 | 0.971 | 0.968 | 0.960 | 0.909 | 1.000 | 0.990 | 1.000 | 0.954 | 0.970 | 0.991 | 0.858 | 1.000 | 1.000 | 0.852 | 0.823 |
| 1778 | 0.766 | 1.000 | 0.767 | 1.000 | 0.974 | 0.960 | 0.901 | 1.000 | 0.990 | 1.000 | 0.957 | 0.970 | 0.991 | 0.861 | 1.000 | 1.000 | 0.853 | 0.823 |
| 1779 | 0.765 | 1.000 | 0.767 | 0.986 | 0.968 | 0.960 | 0.898 | 1.000 | 1.000 | 1.000 | 0.957 | 0.970 | 0.982 | 0.862 | 1.000 | 1.000 | 0.852 | 0.823 |
| 1780 | 0.763 | 1.000 | 0.769 | 1.000 | 0.963 | 0.960 | 0.895 | 1.000 | 0.980 | 1.000 | 0.959 | 1.000 | 0.982 | 0.865 | 1.000 | 1.000 | 0.852 | 0.822 |
| 1781 | 0.760 | 1.000 | 0.792 | 1.000 | 0.968 | 0.960 | 0.897 | 1.000 | 0.980 | 1.000 | 0.954 | 1.000 | 0.973 | 0.859 | 1.000 | 1.000 | 0.852 | 0.823 |
| 1782 | 0.767 | 1.000 | 0.771 | 1.000 | 0.958 | 0.960 | 0.892 | 1.000 | 0.980 | 1.000 | 0.962 | 1.000 | 0.991 | 0.853 | 1.000 | 1.000 | 0.850 | 0.820 |
| 1783 | 0.763 | 1.000 | 0.792 | 1.000 | 0.963 | 0.960 | 0.903 | 1.000 | 0.990 | 1.000 | 0.951 | 1.000 | 0.982 | 0.859 | 1.000 | 1.000 | 0.854 | 0.824 |
| 1784 | 0.765 | 1.000 | 0.781 | 0.986 | 0.963 | 0.960 | 0.898 | 1.000 | 0.980 | 1.000 | 0.951 | 1.000 | 0.982 | 0.857 | 1.000 | 1.000 | 0.851 | 0.822 |
| 1785 | 0.761 | 1.000 | 0.783 | 0.986 | 0.963 | 0.960 | 0.897 | 1.000 | 0.990 | 1.000 | 0.954 | 1.000 | 0.991 | 0.858 | 1.000 | 1.000 | 0.851 | 0.822 |
| 1786 | 0.754 | 1.000 | 0.783 | 0.986 | 0.979 | 0.960 | 0.901 | 1.000 | 0.990 | 1.000 | 0.954 | 0.970 | 0.991 | 0.859 | 1.000 | 1.000 | 0.850 | 0.821 |
| 1787 | 0.769 | 1.000 | 0.783 | 1.000 | 0.979 | 0.960 | 0.901 | 1.000 | 0.969 | 1.000 | 0.957 | 1.000 | 0.982 | 0.850 | 1.000 | 1.000 | 0.852 | 0.823 |
| 1788 | 0.761 | 1.000 | 0.785 | 1.000 | 0.963 | 0.960 | 0.891 | 1.000 | 0.990 | 1.000 | 0.951 | 1.000 | 1.000 | 0.853 | 1.000 | 1.000 | 0.849 | 0.819 |
| 1789 | 0.765 | 1.000 | 0.783 | 1.000 | 0.968 | 0.960 | 0.895 | 1.000 | 0.980 | 1.000 | 0.957 | 1.000 | 0.982 | 0.860 | 1.000 | 1.000 | 0.853 | 0.823 |
| 1790 | 0.768 | 1.000 | 0.785 | 0.986 | 0.963 | 0.960 | 0.898 | 1.000 | 0.980 | 1.000 | 0.957 | 1.000 | 0.991 | 0.856 | 1.000 | 1.000 | 0.853 | 0.824 |
| 1791 | 0.758 | 1.000 | 0.791 | 0.986 | 0.968 | 0.960 | 0.908 | 1.000 | 0.980 | 1.000 | 0.959 | 1.000 | 0.982 | 0.856 | 1.000 | 1.000 | 0.852 | 0.823 |
| 1792 | 0.759 | 1.000 | 0.769 | 1.000 | 0.974 | 0.960 | 0.901 | 1.000 | 0.969 | 1.000 | 0.957 | 0.970 | 0.982 | 0.856 | 1.000 | 1.000 | 0.849 | 0.819 |

| | | | | | | | | | | | | | | | | | | |
|---|---|---|---|---|---|---|---|---|---|---|---|---|---|---|---|---|---|---|
| 1793 | 0.761 | 1.000 | 0.773 | 1.000 | 0.968 | 0.960 | 0.903 | 1.000 | 1.000 | 1.000 | 0.954 | 1.000 | 1.000 | 0.854 | 1.000 | 1.000 | 0.851 | 0.821 |
| 1794 | 0.762 | 1.000 | 0.771 | 0.986 | 0.963 | 0.960 | 0.903 | 1.000 | 0.990 | 1.000 | 0.957 | 1.000 | 0.991 | 0.863 | 1.000 | 1.000 | 0.853 | 0.823 |
| 1795 | 0.761 | 1.000 | 0.783 | 0.986 | 0.968 | 0.960 | 0.881 | 1.000 | 0.990 | 1.000 | 0.948 | 1.000 | 0.991 | 0.863 | 1.000 | 1.000 | 0.850 | 0.820 |
| 1796 | 0.761 | 1.000 | 0.769 | 0.986 | 0.958 | 0.960 | 0.903 | 1.000 | 0.980 | 1.000 | 0.962 | 0.970 | 0.982 | 0.859 | 1.000 | 1.000 | 0.851 | 0.821 |
| 1797 | 0.769 | 1.000 | 0.785 | 0.986 | 0.979 | 0.960 | 0.903 | 1.000 | 0.990 | 1.000 | 0.954 | 1.000 | 0.982 | 0.868 | 1.000 | 1.000 | 0.857 | 0.829 |
| 1798 | 0.767 | 1.000 | 0.785 | 1.000 | 0.968 | 0.960 | 0.891 | 1.000 | 1.000 | 1.000 | 0.957 | 1.000 | 0.991 | 0.861 | 1.000 | 1.000 | 0.854 | 0.825 |
| 1799 | 0.753 | 1.000 | 0.773 | 1.000 | 0.963 | 0.960 | 0.895 | 1.000 | 0.980 | 1.000 | 0.959 | 1.000 | 0.973 | 0.854 | 1.000 | 1.000 | 0.847 | 0.816 |
| 1800 | 0.759 | 1.000 | 0.796 | 0.986 | 0.968 | 0.960 | 0.891 | 1.000 | 0.969 | 1.000 | 0.951 | 1.000 | 0.991 | 0.851 | 1.000 | 1.000 | 0.849 | 0.819 |
| 1801 | 0.760 | 1.000 | 0.771 | 1.000 | 0.963 | 0.960 | 0.895 | 1.000 | 0.990 | 1.000 | 0.954 | 1.000 | 0.991 | 0.866 | 1.000 | 1.000 | 0.852 | 0.822 |
| 1802 | 0.763 | 1.000 | 0.783 | 0.986 | 0.963 | 0.960 | 0.901 | 1.000 | 0.980 | 1.000 | 0.959 | 1.000 | 0.991 | 0.855 | 1.000 | 1.000 | 0.852 | 0.822 |
| 1803 | 0.760 | 1.000 | 0.789 | 1.000 | 0.963 | 0.960 | 0.888 | 1.000 | 0.990 | 1.000 | 0.962 | 1.000 | 0.991 | 0.857 | 1.000 | 1.000 | 0.851 | 0.821 |
| 1804 | 0.761 | 1.000 | 0.783 | 0.986 | 0.974 | 0.960 | 0.889 | 1.000 | 0.990 | 1.000 | 0.959 | 0.970 | 0.973 | 0.855 | 1.000 | 1.000 | 0.850 | 0.819 |
| 1805 | 0.756 | 1.000 | 0.779 | 0.986 | 0.963 | 0.960 | 0.908 | 1.000 | 0.980 | 1.000 | 0.954 | 1.000 | 0.982 | 0.861 | 1.000 | 1.000 | 0.851 | 0.822 |
| 1806 | 0.760 | 1.000 | 0.773 | 0.986 | 0.963 | 0.960 | 0.897 | 1.000 | 0.990 | 1.000 | 0.959 | 0.970 | 0.982 | 0.860 | 1.000 | 1.000 | 0.850 | 0.821 |
| 1807 | 0.766 | 1.000 | 0.792 | 1.000 | 0.963 | 0.960 | 0.903 | 1.000 | 0.990 | 1.000 | 0.957 | 1.000 | 0.973 | 0.861 | 1.000 | 1.000 | 0.855 | 0.826 |
| 1808 | 0.763 | 1.000 | 0.777 | 0.986 | 0.963 | 0.960 | 0.895 | 1.000 | 0.990 | 1.000 | 0.954 | 1.000 | 0.982 | 0.853 | 1.000 | 1.000 | 0.849 | 0.819 |
| 1809 | 0.765 | 1.000 | 0.781 | 1.000 | 0.979 | 0.960 | 0.891 | 1.000 | 0.990 | 1.000 | 0.959 | 1.000 | 0.982 | 0.856 | 1.000 | 1.000 | 0.852 | 0.822 |
| 1810 | 0.764 | 1.000 | 0.781 | 0.986 | 0.968 | 0.960 | 0.903 | 1.000 | 0.990 | 1.000 | 0.954 | 0.970 | 0.991 | 0.857 | 1.000 | 1.000 | 0.852 | 0.823 |
| 1811 | 0.763 | 1.000 | 0.787 | 0.986 | 0.958 | 0.960 | 0.895 | 1.000 | 0.990 | 1.000 | 0.951 | 1.000 | 0.991 | 0.854 | 1.000 | 1.000 | 0.851 | 0.821 |
| 1812 | 0.758 | 1.000 | 0.779 | 1.000 | 0.968 | 0.960 | 0.903 | 1.000 | 1.000 | 1.000 | 0.957 | 0.970 | 0.982 | 0.863 | 1.000 | 1.000 | 0.852 | 0.823 |
| 1813 | 0.755 | 1.000 | 0.791 | 0.986 | 0.953 | 0.960 | 0.895 | 1.000 | 0.990 | 1.000 | 0.957 | 1.000 | 0.991 | 0.861 | 1.000 | 1.000 | 0.851 | 0.821 |
| 1814 | 0.753 | 1.000 | 0.789 | 0.986 | 0.963 | 0.960 | 0.894 | 1.000 | 0.980 | 1.000 | 0.957 | 1.000 | 0.982 | 0.858 | 1.000 | 1.000 | 0.849 | 0.819 |
| 1815 | 0.757 | 1.000 | 0.787 | 0.986 | 0.958 | 0.960 | 0.904 | 1.000 | 0.969 | 1.000 | 0.959 | 0.970 | 0.982 | 0.862 | 1.000 | 1.000 | 0.852 | 0.822 |
| 1816 | 0.756 | 1.000 | 0.789 | 0.986 | 0.979 | 0.960 | 0.911 | 1.000 | 0.980 | 1.000 | 0.951 | 1.000 | 0.982 | 0.865 | 1.000 | 1.000 | 0.854 | 0.825 |
| 1817 | 0.765 | 1.000 | 0.773 | 0.986 | 0.979 | 0.960 | 0.898 | 1.000 | 0.990 | 1.000 | 0.957 | 0.970 | 0.982 | 0.865 | 1.000 | 1.000 | 0.854 | 0.824 |
| 1818 | 0.758 | 1.000 | 0.785 | 0.986 | 0.963 | 0.960 | 0.909 | 1.000 | 0.969 | 1.000 | 0.957 | 1.000 | 0.991 | 0.864 | 1.000 | 1.000 | 0.853 | 0.824 |
| 1819 | 0.755 | 1.000 | 0.785 | 0.986 | 0.953 | 0.960 | 0.906 | 1.000 | 0.980 | 1.000 | 0.957 | 1.000 | 0.991 | 0.846 | 1.000 | 1.000 | 0.847 | 0.817 |
| 1820 | 0.763 | 1.000 | 0.769 | 1.000 | 0.953 | 0.960 | 0.900 | 1.000 | 0.980 | 1.000 | 0.954 | 0.970 | 0.991 | 0.858 | 1.000 | 1.000 | 0.850 | 0.820 |
| 1821 | 0.766 | 1.000 | 0.777 | 1.000 | 0.958 | 0.960 | 0.901 | 1.000 | 0.980 | 1.000 | 0.957 | 1.000 | 0.982 | 0.858 | 1.000 | 1.000 | 0.853 | 0.823 |
| 1822 | 0.762 | 1.000 | 0.779 | 0.986 | 0.963 | 0.960 | 0.897 | 1.000 | 0.969 | 1.000 | 0.954 | 1.000 | 0.982 | 0.858 | 1.000 | 1.000 | 0.851 | 0.821 |
| 1823 | 0.760 | 1.000 | 0.783 | 1.000 | 0.974 | 0.960 | 0.906 | 1.000 | 0.980 | 1.000 | 0.954 | 1.000 | 0.982 | 0.864 | 1.000 | 1.000 | 0.854 | 0.825 |
| 1824 | 0.758 | 1.000 | 0.773 | 0.986 | 0.968 | 0.960 | 0.900 | 1.000 | 0.980 | 1.000 | 0.942 | 1.000 | 0.991 | 0.853 | 1.000 | 1.000 | 0.848 | 0.817 |
| 1825 | 0.751 | 1.000 | 0.769 | 0.986 | 0.974 | 0.960 | 0.900 | 1.000 | 0.980 | 1.000 | 0.957 | 1.000 | 0.982 | 0.858 | 1.000 | 1.000 | 0.847 | 0.817 |
| 1826 | 0.759 | 1.000 | 0.791 | 0.986 | 0.963 | 0.960 | 0.908 | 1.000 | 0.990 | 1.000 | 0.954 | 1.000 | 0.982 | 0.859 | 1.000 | 1.000 | 0.853 | 0.824 |
| 1827 | 0.759 | 1.000 | 0.779 | 0.986 | 0.963 | 0.960 | 0.904 | 1.000 | 1.000 | 1.000 | 0.959 | 1.000 | 1.000 | 0.855 | 1.000 | 1.000 | 0.851 | 0.822 |
| 1828 | 0.751 | 1.000 | 0.777 | 1.000 | 0.968 | 0.960 | 0.897 | 1.000 | 0.990 | 1.000 | 0.951 | 1.000 | 0.991 | 0.863 | 1.000 | 1.000 | 0.849 | 0.819 |
| 1829 | 0.762 | 1.000 | 0.789 | 0.986 | 0.958 | 0.960 | 0.903 | 1.000 | 0.969 | 1.000 | 0.951 | 1.000 | 0.991 | 0.858 | 1.000 | 1.000 | 0.852 | 0.823 |
| 1830 | 0.771 | 1.000 | 0.781 | 0.986 | 0.963 | 0.960 | 0.897 | 1.000 | 0.990 | 1.000 | 0.959 | 1.000 | 0.991 | 0.859 | 1.000 | 1.000 | 0.855 | 0.825 |
| 1831 | 0.767 | 1.000 | 0.773 | 0.986 | 0.963 | 0.960 | 0.900 | 1.000 | 0.990 | 1.000 | 0.962 | 0.970 | 0.991 | 0.854 | 1.000 | 1.000 | 0.852 | 0.822 |

| | | | | | | | | | | | | | | | | | |
|---|---|---|---|---|---|---|---|---|---|---|---|---|---|---|---|---|---|
| 1832 | 0.761 | 1.000 | 0.802 | 1.000 | 0.979 | 0.960 | 0.894 | 1.000 | 1.000 | 1.000 | 0.954 | 0.970 | 0.982 | 0.861 | 1.000 | 1.000 | 0.854 | 0.825 |
| 1833 | 0.773 | 1.000 | 0.781 | 0.986 | 0.974 | 0.960 | 0.897 | 1.000 | 1.000 | 1.000 | 0.954 | 0.970 | 0.991 | 0.851 | 1.000 | 1.000 | 0.853 | 0.824 |
| 1834 | 0.766 | 1.000 | 0.765 | 0.986 | 0.958 | 0.960 | 0.900 | 1.000 | 0.990 | 1.000 | 0.959 | 1.000 | 0.982 | 0.863 | 1.000 | 1.000 | 0.853 | 0.823 |
| 1835 | 0.763 | 1.000 | 0.787 | 0.986 | 0.968 | 0.960 | 0.894 | 1.000 | 0.980 | 1.000 | 0.962 | 1.000 | 0.991 | 0.863 | 1.000 | 1.000 | 0.854 | 0.824 |
| 1836 | 0.765 | 1.000 | 0.771 | 0.986 | 0.958 | 0.960 | 0.891 | 1.000 | 0.980 | 1.000 | 0.959 | 1.000 | 0.982 | 0.856 | 1.000 | 1.000 | 0.850 | 0.820 |
| 1837 | 0.765 | 1.000 | 0.785 | 1.000 | 0.963 | 0.960 | 0.908 | 1.000 | 0.990 | 1.000 | 0.945 | 1.000 | 0.991 | 0.859 | 1.000 | 1.000 | 0.854 | 0.825 |
| 1838 | 0.767 | 1.000 | 0.787 | 1.000 | 0.968 | 0.960 | 0.884 | 1.000 | 0.980 | 1.000 | 0.965 | 1.000 | 0.964 | 0.855 | 1.000 | 1.000 | 0.851 | 0.821 |
| 1839 | 0.771 | 1.000 | 0.787 | 0.986 | 0.984 | 0.960 | 0.897 | 1.000 | 0.990 | 1.000 | 0.957 | 1.000 | 0.991 | 0.857 | 1.000 | 1.000 | 0.855 | 0.826 |
| 1840 | 0.757 | 1.000 | 0.773 | 0.986 | 0.958 | 0.960 | 0.895 | 1.000 | 0.980 | 1.000 | 0.965 | 1.000 | 0.982 | 0.861 | 1.000 | 1.000 | 0.850 | 0.820 |
| 1841 | 0.755 | 1.000 | 0.792 | 0.986 | 0.968 | 0.960 | 0.904 | 1.000 | 0.980 | 1.000 | 0.962 | 1.000 | 1.000 | 0.862 | 1.000 | 1.000 | 0.853 | 0.824 |
| 1842 | 0.757 | 1.000 | 0.783 | 0.986 | 0.953 | 0.960 | 0.900 | 1.000 | 0.990 | 1.000 | 0.942 | 1.000 | 0.991 | 0.855 | 1.000 | 1.000 | 0.848 | 0.818 |
| 1843 | 0.762 | 1.000 | 0.800 | 0.986 | 0.974 | 0.960 | 0.897 | 1.000 | 0.980 | 1.000 | 0.948 | 1.000 | 1.000 | 0.858 | 1.000 | 1.000 | 0.853 | 0.824 |
| 1844 | 0.765 | 1.000 | 0.779 | 1.000 | 0.958 | 0.960 | 0.898 | 1.000 | 0.990 | 1.000 | 0.954 | 0.970 | 0.991 | 0.862 | 1.000 | 1.000 | 0.853 | 0.824 |
| 1845 | 0.768 | 1.000 | 0.775 | 0.986 | 0.974 | 0.960 | 0.908 | 1.000 | 1.000 | 1.000 | 0.948 | 1.000 | 0.982 | 0.857 | 1.000 | 1.000 | 0.854 | 0.824 |
| 1846 | 0.771 | 1.000 | 0.777 | 0.986 | 0.963 | 0.960 | 0.900 | 1.000 | 0.990 | 1.000 | 0.951 | 1.000 | 0.982 | 0.862 | 1.000 | 1.000 | 0.855 | 0.825 |
| 1847 | 0.762 | 1.000 | 0.775 | 0.986 | 0.968 | 0.960 | 0.908 | 1.000 | 0.990 | 1.000 | 0.959 | 1.000 | 0.964 | 0.851 | 1.000 | 1.000 | 0.850 | 0.821 |
| 1848 | 0.756 | 1.000 | 0.787 | 0.986 | 0.974 | 0.960 | 0.904 | 1.000 | 0.990 | 1.000 | 0.948 | 1.000 | 0.973 | 0.854 | 1.000 | 1.000 | 0.850 | 0.820 |
| 1849 | 0.756 | 1.000 | 0.783 | 1.000 | 0.968 | 0.960 | 0.898 | 1.000 | 0.980 | 1.000 | 0.962 | 1.000 | 1.000 | 0.854 | 1.000 | 1.000 | 0.850 | 0.820 |
| 1850 | 0.759 | 1.000 | 0.796 | 0.986 | 0.963 | 0.960 | 0.898 | 1.000 | 0.980 | 1.000 | 0.954 | 1.000 | 0.973 | 0.864 | 1.000 | 1.000 | 0.853 | 0.823 |
| 1851 | 0.761 | 1.000 | 0.802 | 0.986 | 0.958 | 0.960 | 0.901 | 1.000 | 0.969 | 1.000 | 0.959 | 0.970 | 0.973 | 0.857 | 1.000 | 1.000 | 0.853 | 0.824 |
| 1852 | 0.766 | 1.000 | 0.777 | 0.986 | 0.963 | 0.960 | 0.900 | 1.000 | 0.980 | 1.000 | 0.959 | 1.000 | 0.991 | 0.859 | 1.000 | 1.000 | 0.853 | 0.823 |
| 1853 | 0.764 | 1.000 | 0.779 | 0.986 | 0.968 | 0.960 | 0.906 | 1.000 | 0.990 | 1.000 | 0.957 | 1.000 | 0.982 | 0.860 | 1.000 | 1.000 | 0.854 | 0.825 |
| 1854 | 0.775 | 1.000 | 0.785 | 1.000 | 0.968 | 0.960 | 0.894 | 1.000 | 0.969 | 1.000 | 0.962 | 0.970 | 1.000 | 0.853 | 1.000 | 1.000 | 0.855 | 0.825 |
| 1855 | 0.769 | 1.000 | 0.794 | 1.000 | 0.974 | 0.960 | 0.909 | 1.000 | 0.980 | 1.000 | 0.954 | 1.000 | 0.982 | 0.858 | 1.000 | 1.000 | 0.857 | 0.828 |
| 1856 | 0.751 | 1.000 | 0.783 | 1.000 | 0.974 | 0.960 | 0.914 | 1.000 | 0.969 | 1.000 | 0.957 | 0.970 | 1.000 | 0.859 | 1.000 | 1.000 | 0.851 | 0.822 |
| 1857 | 0.752 | 1.000 | 0.773 | 1.000 | 0.979 | 0.960 | 0.895 | 1.000 | 0.980 | 1.000 | 0.957 | 1.000 | 0.982 | 0.861 | 1.000 | 1.000 | 0.849 | 0.818 |
| 1858 | 0.763 | 1.000 | 0.789 | 1.000 | 0.968 | 0.960 | 0.892 | 1.000 | 0.980 | 1.000 | 0.957 | 1.000 | 0.973 | 0.863 | 1.000 | 1.000 | 0.853 | 0.824 |
| 1859 | 0.764 | 1.000 | 0.771 | 0.986 | 0.968 | 0.960 | 0.900 | 1.000 | 0.990 | 1.000 | 0.954 | 1.000 | 0.982 | 0.857 | 1.000 | 1.000 | 0.851 | 0.821 |
| 1860 | 0.762 | 1.000 | 0.779 | 0.986 | 0.974 | 0.960 | 0.900 | 1.000 | 0.980 | 1.000 | 0.959 | 0.970 | 0.991 | 0.857 | 1.000 | 1.000 | 0.852 | 0.822 |
| 1861 | 0.749 | 1.000 | 0.806 | 0.986 | 0.958 | 0.960 | 0.903 | 1.000 | 0.990 | 1.000 | 0.954 | 1.000 | 0.973 | 0.854 | 1.000 | 1.000 | 0.849 | 0.820 |
| 1862 | 0.764 | 1.000 | 0.783 | 0.986 | 0.968 | 0.960 | 0.894 | 1.000 | 0.980 | 1.000 | 0.957 | 1.000 | 0.991 | 0.866 | 1.000 | 1.000 | 0.854 | 0.825 |
| 1863 | 0.761 | 1.000 | 0.785 | 0.986 | 0.963 | 0.960 | 0.914 | 1.000 | 0.980 | 1.000 | 0.951 | 0.970 | 0.973 | 0.861 | 1.000 | 1.000 | 0.853 | 0.824 |
| 1864 | 0.763 | 1.000 | 0.781 | 1.000 | 0.958 | 0.960 | 0.894 | 1.000 | 0.969 | 1.000 | 0.957 | 1.000 | 1.000 | 0.857 | 1.000 | 1.000 | 0.851 | 0.821 |
| 1865 | 0.760 | 1.000 | 0.781 | 1.000 | 0.963 | 0.960 | 0.903 | 1.000 | 0.990 | 1.000 | 0.962 | 1.000 | 0.982 | 0.864 | 1.000 | 1.000 | 0.854 | 0.824 |
| 1866 | 0.763 | 1.000 | 0.791 | 0.986 | 0.963 | 0.960 | 0.901 | 1.000 | 0.969 | 1.000 | 0.951 | 1.000 | 0.982 | 0.865 | 1.000 | 1.000 | 0.854 | 0.825 |
| 1867 | 0.770 | 1.000 | 0.789 | 1.000 | 0.974 | 0.960 | 0.901 | 1.000 | 0.980 | 1.000 | 0.954 | 0.970 | 0.982 | 0.854 | 1.000 | 1.000 | 0.854 | 0.825 |
| 1868 | 0.764 | 1.000 | 0.787 | 0.986 | 0.968 | 0.960 | 0.889 | 1.000 | 0.980 | 1.000 | 0.957 | 1.000 | 0.973 | 0.852 | 1.000 | 1.000 | 0.850 | 0.820 |
| 1869 | 0.765 | 1.000 | 0.783 | 0.986 | 0.974 | 0.960 | 0.897 | 1.000 | 0.990 | 1.000 | 0.954 | 0.970 | 1.000 | 0.856 | 1.000 | 1.000 | 0.852 | 0.823 |
| 1870 | 0.777 | 1.000 | 0.781 | 1.000 | 0.963 | 0.960 | 0.898 | 1.000 | 0.959 | 1.000 | 0.962 | 1.000 | 0.991 | 0.859 | 1.000 | 1.000 | 0.856 | 0.827 |

| | | | | | | | | | | | | | | | | | | |
|---|---|---|---|---|---|---|---|---|---|---|---|---|---|---|---|---|---|---|
| 1871 | 0.763 | 1.000 | 0.792 | 0.986 | 0.963 | 0.960 | 0.897 | 1.000 | 1.000 | 1.000 | 0.948 | 0.970 | 0.973 | 0.855 | 1.000 | 1.000 | 0.851 | 0.822 |
| 1872 | 0.762 | 1.000 | 0.785 | 0.986 | 0.974 | 0.960 | 0.906 | 1.000 | 0.990 | 1.000 | 0.954 | 1.000 | 0.982 | 0.855 | 1.000 | 1.000 | 0.852 | 0.823 |
| 1873 | 0.765 | 1.000 | 0.785 | 1.000 | 0.968 | 0.960 | 0.895 | 1.000 | 0.980 | 1.000 | 0.962 | 0.970 | 0.991 | 0.858 | 1.000 | 1.000 | 0.853 | 0.823 |
| 1874 | 0.765 | 1.000 | 0.804 | 1.000 | 0.963 | 0.960 | 0.895 | 1.000 | 0.990 | 1.000 | 0.957 | 1.000 | 0.982 | 0.862 | 1.000 | 1.000 | 0.856 | 0.827 |
| 1875 | 0.769 | 1.000 | 0.789 | 0.986 | 0.968 | 0.960 | 0.903 | 1.000 | 0.980 | 1.000 | 0.957 | 1.000 | 0.991 | 0.859 | 1.000 | 1.000 | 0.856 | 0.826 |
| 1876 | 0.755 | 1.000 | 0.789 | 1.000 | 0.974 | 0.960 | 0.900 | 1.000 | 0.990 | 1.000 | 0.957 | 0.970 | 0.991 | 0.854 | 1.000 | 1.000 | 0.850 | 0.821 |
| 1877 | 0.771 | 1.000 | 0.785 | 1.000 | 0.958 | 0.960 | 0.898 | 1.000 | 0.980 | 1.000 | 0.959 | 1.000 | 0.973 | 0.860 | 1.000 | 1.000 | 0.855 | 0.825 |
| 1878 | 0.775 | 1.000 | 0.791 | 1.000 | 0.963 | 0.960 | 0.903 | 1.000 | 0.980 | 1.000 | 0.948 | 1.000 | 0.982 | 0.852 | 1.000 | 1.000 | 0.855 | 0.826 |
| 1879 | 0.761 | 1.000 | 0.781 | 0.986 | 0.963 | 0.960 | 0.903 | 1.000 | 0.980 | 1.000 | 0.957 | 1.000 | 0.964 | 0.861 | 1.000 | 1.000 | 0.852 | 0.823 |
| 1880 | 0.757 | 1.000 | 0.792 | 0.986 | 0.963 | 0.960 | 0.903 | 1.000 | 1.000 | 1.000 | 0.968 | 1.000 | 0.982 | 0.858 | 1.000 | 1.000 | 0.853 | 0.824 |
| 1881 | 0.768 | 1.000 | 0.787 | 1.000 | 0.963 | 0.960 | 0.897 | 1.000 | 0.980 | 1.000 | 0.959 | 0.970 | 0.991 | 0.860 | 1.000 | 1.000 | 0.854 | 0.825 |
| 1882 | 0.750 | 1.000 | 0.781 | 1.000 | 0.958 | 0.960 | 0.895 | 1.000 | 0.980 | 1.000 | 0.962 | 1.000 | 0.991 | 0.857 | 1.000 | 1.000 | 0.848 | 0.818 |
| 1883 | 0.773 | 1.000 | 0.775 | 0.986 | 0.984 | 0.960 | 0.894 | 1.000 | 0.969 | 1.000 | 0.954 | 1.000 | 0.982 | 0.858 | 1.000 | 1.000 | 0.854 | 0.824 |
| 1884 | 0.769 | 1.000 | 0.791 | 0.986 | 0.968 | 0.960 | 0.903 | 1.000 | 0.980 | 1.000 | 0.957 | 1.000 | 0.973 | 0.854 | 1.000 | 1.000 | 0.854 | 0.824 |
| 1885 | 0.758 | 1.000 | 0.792 | 0.986 | 0.963 | 0.960 | 0.897 | 1.000 | 0.980 | 1.000 | 0.959 | 1.000 | 0.991 | 0.863 | 1.000 | 1.000 | 0.853 | 0.823 |
| 1886 | 0.767 | 1.000 | 0.785 | 1.000 | 0.963 | 0.960 | 0.901 | 1.000 | 0.969 | 1.000 | 0.959 | 1.000 | 0.973 | 0.854 | 1.000 | 1.000 | 0.853 | 0.823 |
| 1887 | 0.757 | 1.000 | 0.791 | 1.000 | 0.979 | 0.960 | 0.898 | 1.000 | 1.000 | 1.000 | 0.957 | 1.000 | 0.982 | 0.858 | 1.000 | 1.000 | 0.852 | 0.823 |
| 1888 | 0.771 | 1.000 | 0.789 | 0.986 | 0.963 | 0.960 | 0.895 | 1.000 | 0.980 | 1.000 | 0.959 | 0.970 | 0.982 | 0.858 | 1.000 | 1.000 | 0.854 | 0.825 |
| 1889 | 0.767 | 1.000 | 0.777 | 0.986 | 0.974 | 0.960 | 0.897 | 1.000 | 0.980 | 1.000 | 0.957 | 1.000 | 0.973 | 0.854 | 1.000 | 1.000 | 0.851 | 0.821 |
| 1890 | 0.761 | 1.000 | 0.779 | 0.986 | 0.958 | 0.960 | 0.900 | 1.000 | 0.969 | 1.000 | 0.954 | 1.000 | 0.991 | 0.857 | 1.000 | 1.000 | 0.850 | 0.820 |
| 1891 | 0.770 | 1.000 | 0.787 | 1.000 | 0.968 | 0.960 | 0.892 | 1.000 | 0.980 | 1.000 | 0.957 | 1.000 | 1.000 | 0.861 | 1.000 | 1.000 | 0.855 | 0.826 |
| 1892 | 0.767 | 1.000 | 0.806 | 0.986 | 0.968 | 0.960 | 0.897 | 1.000 | 0.990 | 1.000 | 0.957 | 0.970 | 0.982 | 0.851 | 1.000 | 1.000 | 0.853 | 0.824 |
| 1893 | 0.760 | 1.000 | 0.800 | 1.000 | 0.958 | 0.960 | 0.895 | 1.000 | 0.969 | 1.000 | 0.954 | 1.000 | 0.991 | 0.859 | 1.000 | 1.000 | 0.852 | 0.823 |
| 1894 | 0.761 | 1.000 | 0.789 | 1.000 | 0.974 | 0.960 | 0.894 | 1.000 | 1.000 | 1.000 | 0.959 | 1.000 | 0.982 | 0.863 | 1.000 | 1.000 | 0.854 | 0.825 |
| 1895 | 0.753 | 1.000 | 0.789 | 1.000 | 0.963 | 0.960 | 0.900 | 1.000 | 0.990 | 1.000 | 0.957 | 1.000 | 0.973 | 0.864 | 1.000 | 1.000 | 0.851 | 0.822 |
| 1896 | 0.767 | 1.000 | 0.775 | 0.986 | 0.963 | 0.960 | 0.912 | 1.000 | 0.980 | 1.000 | 0.957 | 0.970 | 0.982 | 0.862 | 1.000 | 1.000 | 0.855 | 0.826 |
| 1897 | 0.759 | 1.000 | 0.791 | 1.000 | 0.963 | 0.960 | 0.901 | 1.000 | 0.980 | 1.000 | 0.957 | 1.000 | 0.982 | 0.856 | 1.000 | 1.000 | 0.851 | 0.822 |
| 1898 | 0.765 | 1.000 | 0.779 | 0.986 | 0.968 | 0.960 | 0.901 | 1.000 | 0.969 | 1.000 | 0.957 | 1.000 | 0.973 | 0.865 | 1.000 | 1.000 | 0.854 | 0.825 |
| 1899 | 0.765 | 1.000 | 0.775 | 1.000 | 0.963 | 0.960 | 0.909 | 1.000 | 0.980 | 1.000 | 0.954 | 1.000 | 0.982 | 0.861 | 1.000 | 1.000 | 0.854 | 0.824 |
| 1900 | 0.755 | 1.000 | 0.783 | 0.986 | 0.968 | 0.960 | 0.888 | 1.000 | 0.980 | 1.000 | 0.951 | 1.000 | 0.973 | 0.858 | 1.000 | 1.000 | 0.848 | 0.817 |
| 1901 | 0.768 | 1.000 | 0.789 | 1.000 | 0.963 | 0.960 | 0.908 | 1.000 | 0.980 | 1.000 | 0.948 | 1.000 | 0.982 | 0.863 | 1.000 | 1.000 | 0.856 | 0.827 |
| 1902 | 0.763 | 1.000 | 0.802 | 0.986 | 0.968 | 0.960 | 0.909 | 1.000 | 0.990 | 1.000 | 0.962 | 1.000 | 0.973 | 0.853 | 1.000 | 1.000 | 0.855 | 0.826 |
| 1903 | 0.760 | 1.000 | 0.787 | 0.986 | 0.968 | 0.960 | 0.908 | 1.000 | 0.969 | 1.000 | 0.951 | 1.000 | 0.991 | 0.861 | 1.000 | 1.000 | 0.853 | 0.824 |
| 1904 | 0.763 | 1.000 | 0.781 | 1.000 | 0.958 | 0.960 | 0.901 | 1.000 | 0.969 | 1.000 | 0.959 | 1.000 | 0.964 | 0.856 | 1.000 | 1.000 | 0.851 | 0.822 |
| 1905 | 0.775 | 1.000 | 0.791 | 1.000 | 0.963 | 0.960 | 0.904 | 1.000 | 1.000 | 1.000 | 0.959 | 1.000 | 0.973 | 0.861 | 1.000 | 1.000 | 0.859 | 0.830 |
| 1906 | 0.770 | 1.000 | 0.779 | 0.986 | 0.963 | 0.960 | 0.897 | 1.000 | 0.990 | 1.000 | 0.951 | 1.000 | 0.973 | 0.858 | 1.000 | 1.000 | 0.853 | 0.823 |
| 1907 | 0.766 | 1.000 | 0.787 | 1.000 | 0.974 | 0.960 | 0.895 | 1.000 | 0.990 | 1.000 | 0.951 | 1.000 | 0.982 | 0.856 | 1.000 | 1.000 | 0.853 | 0.823 |
| 1908 | 0.765 | 1.000 | 0.783 | 0.986 | 0.958 | 0.960 | 0.895 | 1.000 | 0.990 | 1.000 | 0.951 | 1.000 | 0.982 | 0.857 | 1.000 | 1.000 | 0.851 | 0.822 |
| 1909 | 0.769 | 1.000 | 0.787 | 1.000 | 0.968 | 0.960 | 0.898 | 1.000 | 1.000 | 1.000 | 0.954 | 1.000 | 0.982 | 0.860 | 1.000 | 1.000 | 0.855 | 0.826 |

| | | | | | | | | | | | | | | | | | | |
|---|---|---|---|---|---|---|---|---|---|---|---|---|---|---|---|---|---|---|
| 1910 | 0.761 | 1.000 | 0.802 | 0.986 | 0.974 | 0.960 | 0.891 | 1.000 | 0.980 | 1.000 | 0.959 | 0.970 | 0.991 | 0.853 | 1.000 | 1.000 | 0.851 | 0.822 |
| 1911 | 0.765 | 1.000 | 0.783 | 0.986 | 0.963 | 0.960 | 0.898 | 1.000 | 0.980 | 1.000 | 0.954 | 1.000 | 0.973 | 0.864 | 1.000 | 1.000 | 0.853 | 0.824 |
| 1912 | 0.767 | 1.000 | 0.798 | 0.986 | 0.963 | 0.960 | 0.909 | 1.000 | 0.990 | 1.000 | 0.959 | 1.000 | 0.991 | 0.862 | 1.000 | 1.000 | 0.858 | 0.830 |
| 1913 | 0.765 | 1.000 | 0.781 | 1.000 | 0.963 | 0.960 | 0.903 | 1.000 | 0.990 | 1.000 | 0.957 | 0.970 | 0.982 | 0.852 | 1.000 | 1.000 | 0.851 | 0.822 |
| 1914 | 0.766 | 1.000 | 0.802 | 0.986 | 0.968 | 0.960 | 0.889 | 1.000 | 0.969 | 1.000 | 0.954 | 1.000 | 0.973 | 0.859 | 1.000 | 1.000 | 0.853 | 0.824 |
| 1915 | 0.776 | 1.000 | 0.791 | 0.986 | 0.968 | 0.960 | 0.898 | 1.000 | 0.980 | 1.000 | 0.954 | 0.970 | 0.991 | 0.854 | 1.000 | 1.000 | 0.856 | 0.826 |
| 1916 | 0.772 | 1.000 | 0.777 | 0.986 | 0.963 | 0.960 | 0.904 | 1.000 | 0.990 | 1.000 | 0.954 | 1.000 | 0.982 | 0.857 | 1.000 | 1.000 | 0.854 | 0.825 |
| 1917 | 0.759 | 1.000 | 0.785 | 0.986 | 0.974 | 0.960 | 0.894 | 1.000 | 0.969 | 1.000 | 0.965 | 1.000 | 0.973 | 0.858 | 1.000 | 1.000 | 0.851 | 0.821 |
| 1918 | 0.771 | 1.000 | 0.779 | 0.986 | 0.968 | 0.960 | 0.898 | 1.000 | 0.980 | 1.000 | 0.959 | 1.000 | 0.991 | 0.857 | 1.000 | 1.000 | 0.854 | 0.825 |
| 1919 | 0.763 | 1.000 | 0.791 | 0.986 | 0.968 | 0.960 | 0.909 | 1.000 | 0.980 | 1.000 | 0.957 | 1.000 | 0.964 | 0.856 | 1.000 | 1.000 | 0.853 | 0.824 |
| 1920 | 0.768 | 1.000 | 0.773 | 0.986 | 0.968 | 0.960 | 0.912 | 1.000 | 0.990 | 1.000 | 0.965 | 0.970 | 1.000 | 0.852 | 1.000 | 1.000 | 0.854 | 0.825 |
| 1921 | 0.773 | 1.000 | 0.787 | 1.000 | 0.963 | 0.960 | 0.895 | 1.000 | 0.990 | 1.000 | 0.959 | 1.000 | 0.973 | 0.858 | 1.000 | 1.000 | 0.855 | 0.826 |
| 1922 | 0.762 | 1.000 | 0.785 | 1.000 | 0.968 | 0.960 | 0.895 | 1.000 | 0.990 | 1.000 | 0.948 | 1.000 | 0.982 | 0.858 | 1.000 | 1.000 | 0.851 | 0.822 |
| 1923 | 0.769 | 1.000 | 0.791 | 1.000 | 0.974 | 0.960 | 0.898 | 1.000 | 0.990 | 1.000 | 0.959 | 1.000 | 0.982 | 0.854 | 1.000 | 1.000 | 0.854 | 0.825 |
| 1924 | 0.765 | 1.000 | 0.777 | 1.000 | 0.963 | 0.960 | 0.897 | 1.000 | 0.980 | 1.000 | 0.968 | 1.000 | 1.000 | 0.859 | 1.000 | 1.000 | 0.853 | 0.824 |
| 1925 | 0.767 | 1.000 | 0.800 | 0.986 | 0.974 | 0.960 | 0.897 | 1.000 | 0.980 | 1.000 | 0.957 | 1.000 | 0.973 | 0.858 | 1.000 | 1.000 | 0.855 | 0.826 |
| 1926 | 0.775 | 1.000 | 0.777 | 0.986 | 0.958 | 0.960 | 0.898 | 1.000 | 0.980 | 1.000 | 0.954 | 1.000 | 0.982 | 0.861 | 1.000 | 1.000 | 0.855 | 0.826 |
| 1927 | 0.763 | 1.000 | 0.787 | 1.000 | 0.968 | 0.960 | 0.903 | 1.000 | 0.969 | 1.000 | 0.951 | 1.000 | 0.991 | 0.865 | 1.000 | 1.000 | 0.854 | 0.825 |
| 1928 | 0.773 | 1.000 | 0.783 | 0.986 | 0.958 | 0.960 | 0.898 | 1.000 | 0.969 | 1.000 | 0.948 | 1.000 | 0.982 | 0.855 | 1.000 | 1.000 | 0.853 | 0.824 |
| 1929 | 0.771 | 1.000 | 0.787 | 1.000 | 0.958 | 0.960 | 0.892 | 1.000 | 0.969 | 1.000 | 0.948 | 1.000 | 0.973 | 0.858 | 1.000 | 1.000 | 0.853 | 0.823 |
| 1930 | 0.768 | 1.000 | 0.791 | 1.000 | 0.968 | 0.960 | 0.895 | 1.000 | 0.980 | 1.000 | 0.959 | 1.000 | 0.982 | 0.861 | 1.000 | 1.000 | 0.855 | 0.826 |
| 1931 | 0.772 | 1.000 | 0.779 | 0.986 | 0.974 | 0.960 | 0.901 | 1.000 | 0.990 | 1.000 | 0.951 | 0.970 | 0.982 | 0.857 | 1.000 | 1.000 | 0.854 | 0.825 |
| 1932 | 0.771 | 1.000 | 0.773 | 0.986 | 0.968 | 0.960 | 0.895 | 1.000 | 0.969 | 1.000 | 0.962 | 1.000 | 0.973 | 0.854 | 1.000 | 1.000 | 0.852 | 0.822 |
| 1933 | 0.761 | 1.000 | 0.783 | 1.000 | 0.963 | 0.960 | 0.901 | 1.000 | 1.000 | 1.000 | 0.951 | 1.000 | 0.973 | 0.858 | 1.000 | 1.000 | 0.852 | 0.822 |
| 1934 | 0.777 | 1.000 | 0.791 | 1.000 | 0.974 | 0.960 | 0.912 | 1.000 | 0.969 | 1.000 | 0.951 | 0.970 | 0.973 | 0.864 | 1.000 | 1.000 | 0.860 | 0.832 |
| 1935 | 0.769 | 1.000 | 0.792 | 1.000 | 0.968 | 0.960 | 0.912 | 1.000 | 0.990 | 1.000 | 0.959 | 1.000 | 1.000 | 0.855 | 1.000 | 1.000 | 0.857 | 0.828 |
| 1936 | 0.775 | 1.000 | 0.779 | 1.000 | 0.968 | 0.960 | 0.894 | 1.000 | 0.959 | 1.000 | 0.957 | 1.000 | 0.964 | 0.855 | 1.000 | 1.000 | 0.853 | 0.824 |
| 1937 | 0.764 | 1.000 | 0.792 | 1.000 | 0.974 | 0.960 | 0.900 | 1.000 | 0.990 | 1.000 | 0.951 | 1.000 | 0.973 | 0.858 | 1.000 | 1.000 | 0.854 | 0.824 |
| 1938 | 0.761 | 1.000 | 0.769 | 1.000 | 0.958 | 0.960 | 0.901 | 1.000 | 1.000 | 1.000 | 0.957 | 1.000 | 0.982 | 0.867 | 1.000 | 1.000 | 0.853 | 0.823 |
| 1939 | 0.773 | 1.000 | 0.785 | 0.986 | 0.963 | 0.960 | 0.901 | 1.000 | 0.980 | 1.000 | 0.962 | 1.000 | 0.982 | 0.865 | 1.000 | 1.000 | 0.858 | 0.829 |
| 1940 | 0.775 | 1.000 | 0.779 | 0.986 | 0.974 | 0.960 | 0.906 | 1.000 | 0.980 | 1.000 | 0.957 | 1.000 | 0.982 | 0.863 | 1.000 | 1.000 | 0.858 | 0.829 |
| 1941 | 0.757 | 1.000 | 0.781 | 0.986 | 0.963 | 0.960 | 0.901 | 1.000 | 0.949 | 1.000 | 0.962 | 1.000 | 1.000 | 0.860 | 1.000 | 1.000 | 0.851 | 0.821 |
| 1942 | 0.765 | 1.000 | 0.785 | 0.986 | 0.958 | 0.960 | 0.897 | 1.000 | 0.990 | 1.000 | 0.957 | 1.000 | 0.973 | 0.851 | 1.000 | 1.000 | 0.850 | 0.820 |
| 1943 | 0.772 | 1.000 | 0.785 | 1.000 | 0.963 | 0.960 | 0.906 | 1.000 | 0.969 | 1.000 | 0.959 | 0.939 | 0.991 | 0.854 | 1.000 | 1.000 | 0.855 | 0.826 |
| 1944 | 0.767 | 1.000 | 0.800 | 1.000 | 0.953 | 0.960 | 0.889 | 1.000 | 1.000 | 1.000 | 0.954 | 1.000 | 0.973 | 0.865 | 1.000 | 1.000 | 0.855 | 0.826 |
| 1945 | 0.770 | 1.000 | 0.765 | 0.986 | 0.968 | 0.960 | 0.918 | 1.000 | 0.990 | 1.000 | 0.959 | 1.000 | 0.982 | 0.860 | 1.000 | 1.000 | 0.856 | 0.827 |
| 1946 | 0.774 | 1.000 | 0.787 | 1.000 | 0.963 | 0.960 | 0.901 | 1.000 | 1.000 | 1.000 | 0.959 | 1.000 | 0.982 | 0.852 | 1.000 | 1.000 | 0.855 | 0.826 |
| 1947 | 0.771 | 1.000 | 0.787 | 1.000 | 0.963 | 0.960 | 0.906 | 1.000 | 0.980 | 1.000 | 0.954 | 0.970 | 0.991 | 0.856 | 1.000 | 1.000 | 0.855 | 0.826 |
| 1948 | 0.773 | 1.000 | 0.792 | 1.000 | 0.953 | 0.960 | 0.914 | 1.000 | 0.990 | 1.000 | 0.954 | 1.000 | 0.982 | 0.854 | 1.000 | 1.000 | 0.857 | 0.828 |

| | | | | | | | | | | | | | | | | | | |
|---|---|---|---|---|---|---|---|---|---|---|---|---|---|---|---|---|---|---|
| 1949 | 0.781 | 1.000 | 0.794 | 1.000 | 0.984 | 0.960 | 0.895 | 1.000 | 0.969 | 1.000 | 0.962 | 1.000 | 0.973 | 0.854 | 1.000 | 1.000 | 0.858 | 0.829 |
| 1950 | 0.769 | 1.000 | 0.779 | 1.000 | 0.958 | 0.960 | 0.901 | 1.000 | 0.990 | 1.000 | 0.968 | 1.000 | 0.991 | 0.866 | 1.000 | 1.000 | 0.857 | 0.828 |
| 1951 | 0.772 | 1.000 | 0.785 | 1.000 | 0.974 | 0.960 | 0.904 | 1.000 | 0.980 | 1.000 | 0.957 | 1.000 | 0.973 | 0.858 | 1.000 | 1.000 | 0.856 | 0.827 |
| 1952 | 0.764 | 1.000 | 0.798 | 0.986 | 0.974 | 0.960 | 0.900 | 1.000 | 0.990 | 1.000 | 0.957 | 1.000 | 0.973 | 0.862 | 1.000 | 1.000 | 0.855 | 0.826 |
| 1953 | 0.763 | 1.000 | 0.773 | 0.986 | 0.963 | 0.960 | 0.892 | 1.000 | 0.969 | 1.000 | 0.954 | 1.000 | 0.982 | 0.855 | 1.000 | 1.000 | 0.849 | 0.818 |
| 1954 | 0.765 | 1.000 | 0.781 | 1.000 | 0.963 | 0.960 | 0.900 | 1.000 | 0.980 | 1.000 | 0.959 | 1.000 | 0.973 | 0.865 | 1.000 | 1.000 | 0.855 | 0.825 |
| 1955 | 0.767 | 1.000 | 0.794 | 0.971 | 0.963 | 0.960 | 0.881 | 1.000 | 0.980 | 1.000 | 0.959 | 0.970 | 0.973 | 0.858 | 1.000 | 1.000 | 0.851 | 0.822 |
| 1956 | 0.777 | 1.000 | 0.791 | 0.986 | 0.963 | 0.960 | 0.891 | 1.000 | 0.980 | 1.000 | 0.951 | 1.000 | 0.982 | 0.858 | 1.000 | 1.000 | 0.855 | 0.826 |
| 1957 | 0.771 | 1.000 | 0.791 | 0.986 | 0.958 | 0.960 | 0.895 | 1.000 | 0.969 | 1.000 | 0.962 | 1.000 | 0.964 | 0.846 | 1.000 | 1.000 | 0.851 | 0.821 |
| 1958 | 0.773 | 1.000 | 0.787 | 1.000 | 0.974 | 0.960 | 0.903 | 1.000 | 0.969 | 1.000 | 0.962 | 0.970 | 0.973 | 0.864 | 1.000 | 1.000 | 0.858 | 0.829 |
| 1959 | 0.769 | 1.000 | 0.789 | 0.986 | 0.963 | 0.960 | 0.895 | 1.000 | 0.980 | 1.000 | 0.957 | 1.000 | 0.991 | 0.862 | 1.000 | 1.000 | 0.855 | 0.826 |
| 1960 | 0.772 | 1.000 | 0.792 | 1.000 | 0.968 | 0.960 | 0.901 | 1.000 | 0.990 | 1.000 | 0.954 | 0.970 | 0.982 | 0.857 | 1.000 | 1.000 | 0.856 | 0.827 |
| 1961 | 0.761 | 1.000 | 0.789 | 1.000 | 0.963 | 0.960 | 0.895 | 1.000 | 0.980 | 1.000 | 0.957 | 1.000 | 0.982 | 0.847 | 1.000 | 1.000 | 0.849 | 0.819 |
| 1962 | 0.765 | 1.000 | 0.779 | 0.986 | 0.968 | 0.960 | 0.909 | 1.000 | 0.969 | 1.000 | 0.948 | 1.000 | 0.991 | 0.861 | 1.000 | 1.000 | 0.854 | 0.824 |
| 1963 | 0.769 | 1.000 | 0.781 | 1.000 | 0.963 | 0.960 | 0.903 | 1.000 | 1.000 | 1.000 | 0.957 | 1.000 | 0.964 | 0.861 | 1.000 | 1.000 | 0.855 | 0.826 |
| 1964 | 0.761 | 1.000 | 0.791 | 1.000 | 0.963 | 0.960 | 0.911 | 1.000 | 0.990 | 1.000 | 0.957 | 0.970 | 0.982 | 0.860 | 1.000 | 1.000 | 0.854 | 0.825 |
| 1965 | 0.779 | 1.000 | 0.789 | 0.986 | 0.963 | 0.960 | 0.904 | 1.000 | 0.990 | 1.000 | 0.948 | 1.000 | 0.973 | 0.866 | 1.000 | 1.000 | 0.860 | 0.831 |
| 1966 | 0.761 | 1.000 | 0.769 | 1.000 | 0.968 | 0.960 | 0.903 | 1.000 | 0.990 | 1.000 | 0.957 | 1.000 | 0.973 | 0.865 | 1.000 | 1.000 | 0.853 | 0.823 |
| 1967 | 0.769 | 1.000 | 0.802 | 1.000 | 0.968 | 0.960 | 0.891 | 1.000 | 0.980 | 1.000 | 0.959 | 1.000 | 0.982 | 0.865 | 1.000 | 1.000 | 0.857 | 0.829 |
| 1968 | 0.765 | 1.000 | 0.781 | 0.986 | 0.974 | 0.960 | 0.906 | 1.000 | 0.959 | 1.000 | 0.951 | 1.000 | 0.973 | 0.847 | 1.000 | 1.000 | 0.850 | 0.820 |
| 1969 | 0.764 | 1.000 | 0.794 | 0.986 | 0.974 | 0.960 | 0.897 | 1.000 | 0.990 | 1.000 | 0.957 | 1.000 | 0.973 | 0.865 | 1.000 | 1.000 | 0.855 | 0.826 |
| 1970 | 0.761 | 1.000 | 0.800 | 1.000 | 0.974 | 0.960 | 0.909 | 1.000 | 0.969 | 1.000 | 0.959 | 1.000 | 0.973 | 0.858 | 1.000 | 1.000 | 0.855 | 0.826 |
| 1971 | 0.762 | 1.000 | 0.794 | 0.986 | 0.968 | 0.960 | 0.895 | 1.000 | 0.990 | 1.000 | 0.954 | 0.939 | 0.991 | 0.865 | 1.000 | 1.000 | 0.854 | 0.825 |
| 1972 | 0.781 | 1.000 | 0.775 | 1.000 | 0.963 | 0.960 | 0.906 | 1.000 | 0.990 | 1.000 | 0.959 | 1.000 | 1.000 | 0.859 | 1.000 | 1.000 | 0.859 | 0.830 |
| 1973 | 0.765 | 1.000 | 0.785 | 0.986 | 0.974 | 0.960 | 0.889 | 1.000 | 0.990 | 1.000 | 0.965 | 1.000 | 0.973 | 0.863 | 1.000 | 1.000 | 0.854 | 0.824 |
| 1974 | 0.765 | 1.000 | 0.775 | 1.000 | 0.974 | 0.960 | 0.886 | 1.000 | 0.990 | 1.000 | 0.954 | 0.970 | 0.982 | 0.859 | 1.000 | 1.000 | 0.851 | 0.820 |
| 1975 | 0.767 | 1.000 | 0.796 | 1.000 | 0.968 | 0.960 | 0.900 | 1.000 | 0.980 | 1.000 | 0.959 | 1.000 | 0.991 | 0.862 | 1.000 | 1.000 | 0.856 | 0.828 |
| 1976 | 0.777 | 1.000 | 0.785 | 1.000 | 0.963 | 0.960 | 0.892 | 1.000 | 0.980 | 1.000 | 0.954 | 1.000 | 0.973 | 0.861 | 1.000 | 1.000 | 0.856 | 0.827 |
| 1977 | 0.755 | 1.000 | 0.798 | 1.000 | 0.979 | 0.960 | 0.904 | 1.000 | 0.980 | 1.000 | 0.962 | 0.970 | 0.982 | 0.851 | 1.000 | 1.000 | 0.851 | 0.822 |
| 1978 | 0.766 | 1.000 | 0.794 | 0.971 | 0.963 | 0.960 | 0.895 | 1.000 | 0.980 | 1.000 | 0.959 | 1.000 | 0.973 | 0.857 | 1.000 | 1.000 | 0.853 | 0.824 |
| 1979 | 0.768 | 1.000 | 0.804 | 1.000 | 0.968 | 0.960 | 0.908 | 1.000 | 0.980 | 1.000 | 0.951 | 1.000 | 0.973 | 0.853 | 1.000 | 1.000 | 0.855 | 0.826 |
| 1980 | 0.774 | 1.000 | 0.789 | 0.986 | 0.974 | 0.960 | 0.903 | 1.000 | 0.969 | 1.000 | 0.962 | 1.000 | 0.973 | 0.853 | 1.000 | 1.000 | 0.855 | 0.826 |
| 1981 | 0.763 | 1.000 | 0.787 | 0.986 | 0.974 | 0.960 | 0.898 | 1.000 | 0.990 | 1.000 | 0.959 | 1.000 | 0.973 | 0.851 | 1.000 | 1.000 | 0.851 | 0.821 |
| 1982 | 0.768 | 1.000 | 0.783 | 0.986 | 0.968 | 0.960 | 0.895 | 1.000 | 0.980 | 1.000 | 0.957 | 0.939 | 0.982 | 0.858 | 1.000 | 1.000 | 0.853 | 0.823 |
| 1983 | 0.763 | 1.000 | 0.798 | 1.000 | 0.958 | 0.960 | 0.898 | 1.000 | 0.980 | 1.000 | 0.962 | 1.000 | 0.973 | 0.858 | 1.000 | 1.000 | 0.853 | 0.824 |
| 1984 | 0.763 | 1.000 | 0.787 | 0.986 | 0.974 | 0.960 | 0.898 | 1.000 | 0.990 | 1.000 | 0.959 | 1.000 | 0.973 | 0.847 | 1.000 | 1.000 | 0.850 | 0.820 |
| 1985 | 0.761 | 1.000 | 0.783 | 1.000 | 0.968 | 0.960 | 0.901 | 1.000 | 0.969 | 1.000 | 0.957 | 1.000 | 0.973 | 0.861 | 1.000 | 1.000 | 0.852 | 0.823 |
| 1986 | 0.767 | 1.000 | 0.789 | 0.986 | 0.979 | 0.960 | 0.900 | 1.000 | 0.980 | 1.000 | 0.957 | 1.000 | 0.973 | 0.860 | 1.000 | 1.000 | 0.855 | 0.825 |
| 1987 | 0.767 | 1.000 | 0.779 | 1.000 | 0.979 | 0.960 | 0.903 | 1.000 | 0.969 | 1.000 | 0.954 | 1.000 | 0.982 | 0.858 | 1.000 | 1.000 | 0.854 | 0.825 |

| | | | | | | | | | | | | | | | | | | |
|---|---|---|---|---|---|---|---|---|---|---|---|---|---|---|---|---|---|---|
| 1988 | 0.769 | 1.000 | 0.787 | 1.000 | 0.968 | 0.960 | 0.892 | 1.000 | 0.990 | 1.000 | 0.959 | 1.000 | 0.982 | 0.855 | 1.000 | 1.000 | 0.853 | 0.824 |
| 1989 | 0.772 | 1.000 | 0.796 | 1.000 | 0.974 | 0.960 | 0.898 | 1.000 | 0.969 | 1.000 | 0.957 | 1.000 | 0.982 | 0.865 | 1.000 | 1.000 | 0.858 | 0.829 |
| 1990 | 0.777 | 1.000 | 0.791 | 1.000 | 0.968 | 0.960 | 0.904 | 1.000 | 0.990 | 1.000 | 0.954 | 1.000 | 0.982 | 0.851 | 1.000 | 1.000 | 0.856 | 0.827 |
| 1991 | 0.773 | 1.000 | 0.781 | 0.986 | 0.963 | 0.960 | 0.889 | 1.000 | 0.980 | 1.000 | 0.959 | 1.000 | 0.982 | 0.859 | 1.000 | 1.000 | 0.854 | 0.824 |
| 1992 | 0.761 | 1.000 | 0.785 | 0.986 | 0.968 | 0.960 | 0.901 | 1.000 | 0.980 | 1.000 | 0.957 | 0.970 | 0.982 | 0.854 | 1.000 | 1.000 | 0.851 | 0.821 |
| 1993 | 0.770 | 1.000 | 0.771 | 1.000 | 0.963 | 0.960 | 0.904 | 1.000 | 0.980 | 1.000 | 0.957 | 1.000 | 0.982 | 0.854 | 1.000 | 1.000 | 0.853 | 0.823 |
| 1994 | 0.769 | 1.000 | 0.779 | 1.000 | 0.968 | 0.960 | 0.911 | 1.000 | 0.990 | 1.000 | 0.954 | 0.970 | 0.991 | 0.855 | 1.000 | 1.000 | 0.854 | 0.825 |
| 1995 | 0.769 | 1.000 | 0.791 | 1.000 | 0.968 | 0.960 | 0.892 | 1.000 | 0.990 | 1.000 | 0.965 | 1.000 | 0.982 | 0.858 | 1.000 | 1.000 | 0.855 | 0.826 |
| 1996 | 0.763 | 1.000 | 0.798 | 1.000 | 0.963 | 0.960 | 0.904 | 1.000 | 0.990 | 1.000 | 0.954 | 1.000 | 0.982 | 0.858 | 1.000 | 1.000 | 0.854 | 0.825 |
| 1997 | 0.773 | 1.000 | 0.791 | 1.000 | 0.958 | 0.960 | 0.903 | 1.000 | 0.980 | 1.000 | 0.951 | 1.000 | 0.991 | 0.848 | 1.000 | 1.000 | 0.853 | 0.824 |
| 1998 | 0.774 | 1.000 | 0.779 | 0.986 | 0.968 | 0.960 | 0.906 | 1.000 | 0.969 | 1.000 | 0.954 | 1.000 | 0.982 | 0.858 | 1.000 | 1.000 | 0.856 | 0.827 |
| 1999 | 0.767 | 1.000 | 0.783 | 1.000 | 0.958 | 0.960 | 0.898 | 1.000 | 0.990 | 1.000 | 0.962 | 1.000 | 0.973 | 0.857 | 1.000 | 1.000 | 0.853 | 0.824 |
| 2000 | 0.773 | 1.000 | 0.796 | 1.000 | 0.968 | 0.960 | 0.903 | 1.000 | 1.000 | 1.000 | 0.957 | 1.000 | 0.973 | 0.864 | 1.000 | 1.000 | 0.859 | 0.831 |
| 2001 | 0.760 | 1.000 | 0.789 | 1.000 | 0.958 | 0.960 | 0.900 | 1.000 | 0.969 | 1.000 | 0.959 | 1.000 | 0.982 | 0.857 | 1.000 | 1.000 | 0.851 | 0.822 |
| 2002 | 0.763 | 1.000 | 0.781 | 0.986 | 0.963 | 0.960 | 0.911 | 1.000 | 0.980 | 1.000 | 0.959 | 1.000 | 0.982 | 0.859 | 1.000 | 1.000 | 0.854 | 0.825 |
| 2003 | 0.775 | 1.000 | 0.789 | 0.986 | 0.963 | 0.960 | 0.892 | 1.000 | 0.980 | 1.000 | 0.954 | 1.000 | 0.973 | 0.861 | 1.000 | 1.000 | 0.856 | 0.826 |
| 2004 | 0.765 | 1.000 | 0.781 | 0.971 | 0.968 | 0.960 | 0.918 | 1.000 | 0.969 | 1.000 | 0.959 | 1.000 | 0.973 | 0.860 | 1.000 | 1.000 | 0.855 | 0.826 |
| 2005 | 0.764 | 1.000 | 0.779 | 1.000 | 0.968 | 0.960 | 0.900 | 1.000 | 0.969 | 1.000 | 0.968 | 1.000 | 0.964 | 0.854 | 1.000 | 1.000 | 0.852 | 0.822 |
| 2006 | 0.757 | 1.000 | 0.785 | 0.986 | 0.958 | 0.960 | 0.898 | 1.000 | 1.000 | 1.000 | 0.962 | 1.000 | 0.982 | 0.861 | 1.000 | 1.000 | 0.852 | 0.822 |
| 2007 | 0.759 | 1.000 | 0.806 | 1.000 | 0.963 | 0.960 | 0.900 | 1.000 | 0.969 | 1.000 | 0.959 | 1.000 | 0.973 | 0.854 | 1.000 | 1.000 | 0.852 | 0.823 |
| 2008 | 0.772 | 1.000 | 0.796 | 1.000 | 0.968 | 0.960 | 0.904 | 1.000 | 0.980 | 1.000 | 0.951 | 1.000 | 1.000 | 0.862 | 1.000 | 1.000 | 0.858 | 0.830 |
| 2009 | 0.762 | 1.000 | 0.787 | 0.986 | 0.963 | 0.960 | 0.903 | 1.000 | 0.990 | 1.000 | 0.959 | 0.970 | 0.964 | 0.859 | 1.000 | 0.981 | 0.852 | 0.823 |
| 2010 | 0.767 | 1.000 | 0.779 | 0.986 | 0.963 | 0.960 | 0.895 | 1.000 | 0.990 | 1.000 | 0.954 | 1.000 | 0.973 | 0.858 | 1.000 | 1.000 | 0.852 | 0.822 |
| 2011 | 0.766 | 1.000 | 0.787 | 1.000 | 0.963 | 0.960 | 0.901 | 1.000 | 0.990 | 1.000 | 0.948 | 1.000 | 0.982 | 0.861 | 1.000 | 1.000 | 0.854 | 0.825 |
| 2012 | 0.771 | 1.000 | 0.781 | 1.000 | 0.958 | 0.960 | 0.903 | 1.000 | 0.990 | 1.000 | 0.962 | 0.970 | 0.964 | 0.861 | 1.000 | 1.000 | 0.855 | 0.826 |
| 2013 | 0.769 | 1.000 | 0.785 | 1.000 | 0.963 | 0.960 | 0.904 | 1.000 | 0.969 | 1.000 | 0.954 | 0.970 | 0.973 | 0.858 | 1.000 | 1.000 | 0.854 | 0.825 |
| 2014 | 0.777 | 1.000 | 0.791 | 1.000 | 0.963 | 0.960 | 0.901 | 1.000 | 0.969 | 1.000 | 0.962 | 1.000 | 0.964 | 0.857 | 1.000 | 1.000 | 0.857 | 0.828 |
| 2015 | 0.772 | 1.000 | 0.777 | 1.000 | 0.963 | 0.960 | 0.903 | 1.000 | 0.980 | 1.000 | 0.954 | 1.000 | 0.982 | 0.852 | 1.000 | 1.000 | 0.853 | 0.824 |
| 2016 | 0.767 | 1.000 | 0.787 | 1.000 | 0.963 | 0.960 | 0.911 | 1.000 | 0.990 | 1.000 | 0.957 | 1.000 | 0.964 | 0.856 | 1.000 | 1.000 | 0.854 | 0.826 |
| 2017 | 0.783 | 1.000 | 0.771 | 1.000 | 0.968 | 0.960 | 0.909 | 1.000 | 0.969 | 1.000 | 0.959 | 0.970 | 0.982 | 0.854 | 1.000 | 1.000 | 0.857 | 0.829 |
| 2018 | 0.762 | 1.000 | 0.798 | 0.986 | 0.968 | 0.960 | 0.901 | 1.000 | 0.980 | 1.000 | 0.951 | 1.000 | 0.982 | 0.857 | 1.000 | 1.000 | 0.853 | 0.824 |
| 2019 | 0.770 | 1.000 | 0.781 | 0.986 | 0.974 | 0.960 | 0.900 | 1.000 | 1.000 | 1.000 | 0.959 | 1.000 | 0.982 | 0.866 | 1.000 | 1.000 | 0.857 | 0.828 |
| 2020 | 0.761 | 1.000 | 0.796 | 1.000 | 0.958 | 0.960 | 0.894 | 1.000 | 0.980 | 1.000 | 0.959 | 0.970 | 0.991 | 0.847 | 1.000 | 1.000 | 0.849 | 0.820 |
| 2021 | 0.771 | 1.000 | 0.798 | 0.986 | 0.963 | 0.960 | 0.898 | 1.000 | 0.980 | 1.000 | 0.957 | 1.000 | 0.982 | 0.863 | 1.000 | 1.000 | 0.857 | 0.829 |
| 2022 | 0.771 | 1.000 | 0.800 | 1.000 | 0.963 | 0.960 | 0.904 | 1.000 | 0.990 | 1.000 | 0.954 | 1.000 | 0.964 | 0.856 | 1.000 | 1.000 | 0.856 | 0.827 |
| 2023 | 0.767 | 1.000 | 0.792 | 0.986 | 0.963 | 0.960 | 0.891 | 1.000 | 0.990 | 1.000 | 0.965 | 1.000 | 0.964 | 0.855 | 1.000 | 1.000 | 0.853 | 0.823 |
| 2024 | 0.768 | 1.000 | 0.783 | 1.000 | 0.958 | 0.960 | 0.897 | 1.000 | 1.000 | 1.000 | 0.945 | 0.970 | 0.982 | 0.854 | 1.000 | 1.000 | 0.851 | 0.822 |
| 2025 | 0.771 | 1.000 | 0.804 | 0.986 | 0.968 | 0.960 | 0.903 | 1.000 | 0.980 | 1.000 | 0.962 | 1.000 | 0.973 | 0.854 | 1.000 | 1.000 | 0.856 | 0.828 |
| 2026 | 0.768 | 1.000 | 0.781 | 1.000 | 0.958 | 0.960 | 0.908 | 1.000 | 0.990 | 1.000 | 0.957 | 1.000 | 1.000 | 0.861 | 1.000 | 1.000 | 0.856 | 0.827 |

| | | | | | | | | | | | | | | | | | | |
|---|---|---|---|---|---|---|---|---|---|---|---|---|---|---|---|---|---|---|
| 2027 | 0.761 | 1.000 | 0.785 | 1.000 | 0.958 | 0.960 | 0.895 | 1.000 | 1.000 | 1.000 | 0.965 | 1.000 | 0.973 | 0.863 | 1.000 | 1.000 | 0.853 | 0.824 |
| 2028 | 0.766 | 1.000 | 0.802 | 1.000 | 0.968 | 0.960 | 0.895 | 1.000 | 0.980 | 1.000 | 0.954 | 1.000 | 0.991 | 0.864 | 1.000 | 1.000 | 0.856 | 0.828 |
| 2029 | 0.749 | 1.000 | 0.785 | 0.986 | 0.953 | 0.960 | 0.904 | 1.000 | 0.990 | 1.000 | 0.954 | 1.000 | 0.982 | 0.857 | 1.000 | 1.000 | 0.848 | 0.818 |
| 2030 | 0.763 | 1.000 | 0.796 | 1.000 | 0.968 | 0.960 | 0.898 | 1.000 | 0.969 | 1.000 | 0.965 | 0.970 | 0.982 | 0.864 | 1.000 | 1.000 | 0.855 | 0.826 |
| 2031 | 0.760 | 1.000 | 0.785 | 0.986 | 0.958 | 0.960 | 0.904 | 1.000 | 0.969 | 1.000 | 0.959 | 1.000 | 0.973 | 0.852 | 1.000 | 1.000 | 0.850 | 0.820 |
| 2032 | 0.773 | 1.000 | 0.781 | 1.000 | 0.963 | 0.960 | 0.891 | 1.000 | 0.980 | 1.000 | 0.954 | 1.000 | 0.955 | 0.863 | 1.000 | 1.000 | 0.854 | 0.825 |
| 2033 | 0.769 | 1.000 | 0.792 | 1.000 | 0.963 | 0.960 | 0.900 | 1.000 | 0.980 | 1.000 | 0.959 | 1.000 | 0.991 | 0.856 | 1.000 | 1.000 | 0.855 | 0.826 |
| 2034 | 0.759 | 1.000 | 0.773 | 1.000 | 0.979 | 0.960 | 0.892 | 1.000 | 0.969 | 1.000 | 0.959 | 1.000 | 0.973 | 0.852 | 1.000 | 1.000 | 0.848 | 0.817 |
| 2035 | 0.773 | 1.000 | 0.771 | 1.000 | 0.958 | 0.960 | 0.901 | 1.000 | 1.000 | 1.000 | 0.954 | 1.000 | 0.991 | 0.860 | 1.000 | 1.000 | 0.855 | 0.826 |
| 2036 | 0.768 | 1.000 | 0.785 | 1.000 | 0.958 | 0.960 | 0.898 | 1.000 | 0.990 | 1.000 | 0.962 | 1.000 | 0.982 | 0.859 | 1.000 | 1.000 | 0.854 | 0.825 |
| 2037 | 0.759 | 1.000 | 0.785 | 1.000 | 0.958 | 0.960 | 0.895 | 1.000 | 0.959 | 1.000 | 0.965 | 1.000 | 0.973 | 0.858 | 1.000 | 1.000 | 0.850 | 0.821 |
| 2038 | 0.751 | 1.000 | 0.789 | 1.000 | 0.958 | 0.960 | 0.898 | 1.000 | 0.990 | 1.000 | 0.954 | 1.000 | 0.982 | 0.854 | 1.000 | 1.000 | 0.848 | 0.818 |
| 2039 | 0.779 | 1.000 | 0.787 | 1.000 | 0.974 | 0.960 | 0.906 | 1.000 | 1.000 | 1.000 | 0.954 | 1.000 | 0.982 | 0.858 | 1.000 | 1.000 | 0.859 | 0.830 |
| 2040 | 0.757 | 1.000 | 0.781 | 1.000 | 0.974 | 0.960 | 0.901 | 1.000 | 0.980 | 1.000 | 0.965 | 1.000 | 0.991 | 0.847 | 1.000 | 1.000 | 0.849 | 0.819 |
| 2041 | 0.769 | 1.000 | 0.787 | 0.986 | 0.953 | 0.960 | 0.903 | 1.000 | 1.000 | 1.000 | 0.965 | 1.000 | 0.973 | 0.857 | 1.000 | 1.000 | 0.855 | 0.826 |
| 2042 | 0.767 | 1.000 | 0.791 | 1.000 | 0.953 | 0.960 | 0.901 | 1.000 | 0.980 | 1.000 | 0.957 | 1.000 | 0.982 | 0.855 | 1.000 | 1.000 | 0.853 | 0.824 |
| 2043 | 0.769 | 1.000 | 0.781 | 1.000 | 0.963 | 0.960 | 0.897 | 1.000 | 0.990 | 1.000 | 0.962 | 1.000 | 0.982 | 0.858 | 1.000 | 1.000 | 0.854 | 0.825 |
| 2044 | 0.769 | 1.000 | 0.787 | 0.986 | 0.963 | 0.960 | 0.895 | 1.000 | 0.980 | 1.000 | 0.957 | 1.000 | 1.000 | 0.864 | 1.000 | 1.000 | 0.855 | 0.826 |
| 2045 | 0.767 | 1.000 | 0.794 | 1.000 | 0.968 | 0.960 | 0.900 | 1.000 | 0.969 | 1.000 | 0.965 | 1.000 | 0.973 | 0.855 | 1.000 | 1.000 | 0.854 | 0.825 |
| 2046 | 0.770 | 1.000 | 0.802 | 1.000 | 0.968 | 0.960 | 0.895 | 1.000 | 0.969 | 1.000 | 0.965 | 1.000 | 0.991 | 0.849 | 1.000 | 1.000 | 0.854 | 0.825 |
| 2047 | 0.767 | 1.000 | 0.783 | 0.986 | 0.953 | 0.960 | 0.900 | 1.000 | 0.980 | 1.000 | 0.957 | 1.000 | 0.982 | 0.865 | 1.000 | 1.000 | 0.854 | 0.825 |
| 2048 | 0.767 | 1.000 | 0.804 | 1.000 | 0.968 | 0.960 | 0.901 | 1.000 | 0.980 | 1.000 | 0.957 | 1.000 | 0.973 | 0.862 | 1.000 | 1.000 | 0.857 | 0.829 |
| 2049 | 0.766 | 1.000 | 0.777 | 0.986 | 0.958 | 0.960 | 0.904 | 1.000 | 0.969 | 1.000 | 0.965 | 1.000 | 0.991 | 0.855 | 1.000 | 0.981 | 0.852 | 0.823 |
| 2050 | 0.767 | 1.000 | 0.781 | 0.986 | 0.963 | 0.960 | 0.903 | 1.000 | 0.949 | 1.000 | 0.962 | 1.000 | 0.982 | 0.861 | 1.000 | 1.000 | 0.854 | 0.825 |
| 2051 | 0.766 | 1.000 | 0.789 | 0.971 | 0.953 | 0.960 | 0.897 | 1.000 | 0.980 | 1.000 | 0.959 | 1.000 | 0.991 | 0.860 | 1.000 | 1.000 | 0.853 | 0.824 |
| 2052 | 0.773 | 1.000 | 0.791 | 1.000 | 0.968 | 0.960 | 0.901 | 1.000 | 0.980 | 1.000 | 0.954 | 1.000 | 0.982 | 0.860 | 1.000 | 1.000 | 0.857 | 0.828 |
| 2053 | 0.765 | 1.000 | 0.802 | 1.000 | 0.963 | 0.960 | 0.906 | 1.000 | 0.969 | 1.000 | 0.965 | 1.000 | 0.982 | 0.853 | 1.000 | 1.000 | 0.855 | 0.826 |
| 2054 | 0.767 | 1.000 | 0.792 | 0.986 | 0.963 | 0.960 | 0.904 | 1.000 | 0.980 | 1.000 | 0.965 | 0.970 | 0.991 | 0.858 | 1.000 | 1.000 | 0.855 | 0.826 |
| 2055 | 0.761 | 1.000 | 0.789 | 1.000 | 0.968 | 0.960 | 0.898 | 1.000 | 0.980 | 1.000 | 0.965 | 1.000 | 0.991 | 0.859 | 1.000 | 1.000 | 0.853 | 0.824 |
| 2056 | 0.767 | 1.000 | 0.796 | 1.000 | 0.968 | 0.960 | 0.900 | 1.000 | 0.990 | 1.000 | 0.962 | 1.000 | 0.964 | 0.861 | 1.000 | 1.000 | 0.856 | 0.828 |
| 2057 | 0.770 | 1.000 | 0.783 | 1.000 | 0.968 | 0.960 | 0.895 | 1.000 | 0.959 | 1.000 | 0.965 | 1.000 | 0.982 | 0.854 | 1.000 | 1.000 | 0.853 | 0.824 |
| 2058 | 0.767 | 1.000 | 0.787 | 0.986 | 0.963 | 0.960 | 0.904 | 1.000 | 0.990 | 1.000 | 0.962 | 1.000 | 0.964 | 0.853 | 1.000 | 1.000 | 0.853 | 0.824 |
| 2059 | 0.765 | 1.000 | 0.794 | 0.986 | 0.963 | 0.960 | 0.892 | 1.000 | 0.980 | 1.000 | 0.968 | 1.000 | 0.973 | 0.860 | 1.000 | 1.000 | 0.854 | 0.825 |
| 2060 | 0.765 | 1.000 | 0.792 | 1.000 | 0.963 | 0.960 | 0.903 | 1.000 | 0.959 | 1.000 | 0.954 | 1.000 | 0.964 | 0.856 | 1.000 | 1.000 | 0.853 | 0.823 |
| 2061 | 0.761 | 1.000 | 0.800 | 1.000 | 0.968 | 0.960 | 0.900 | 1.000 | 0.980 | 1.000 | 0.971 | 0.970 | 0.991 | 0.859 | 1.000 | 1.000 | 0.855 | 0.826 |
| 2062 | 0.769 | 1.000 | 0.781 | 0.986 | 0.958 | 0.960 | 0.895 | 1.000 | 0.990 | 1.000 | 0.959 | 1.000 | 0.982 | 0.861 | 1.000 | 1.000 | 0.854 | 0.825 |
| 2063 | 0.775 | 1.000 | 0.783 | 1.000 | 0.963 | 0.960 | 0.900 | 1.000 | 0.980 | 1.000 | 0.965 | 1.000 | 0.982 | 0.855 | 1.000 | 1.000 | 0.856 | 0.827 |
| 2064 | 0.771 | 1.000 | 0.792 | 0.986 | 0.968 | 0.960 | 0.898 | 1.000 | 0.969 | 1.000 | 0.959 | 1.000 | 0.991 | 0.857 | 1.000 | 1.000 | 0.855 | 0.826 |
| 2065 | 0.767 | 1.000 | 0.779 | 1.000 | 0.963 | 0.960 | 0.900 | 1.000 | 0.990 | 1.000 | 0.971 | 1.000 | 0.991 | 0.857 | 1.000 | 1.000 | 0.854 | 0.825 |

| | | | | | | | | | | | | | | | | | |
|---|---|---|---|---|---|---|---|---|---|---|---|---|---|---|---|---|---|
| 2066 | 0.764 | 1.000 | 0.779 | 1.000 | 0.958 | 0.960 | 0.900 | 1.000 | 0.980 | 1.000 | 0.959 | 1.000 | 0.973 | 0.854 | 1.000 | 1.000 | 0.851 | 0.821 |
| 2067 | 0.761 | 1.000 | 0.783 | 0.986 | 0.968 | 0.960 | 0.889 | 1.000 | 0.980 | 1.000 | 0.962 | 1.000 | 0.982 | 0.859 | 1.000 | 1.000 | 0.851 | 0.821 |
| 2068 | 0.762 | 1.000 | 0.794 | 1.000 | 0.963 | 0.960 | 0.898 | 1.000 | 0.969 | 1.000 | 0.962 | 0.970 | 0.973 | 0.860 | 1.000 | 1.000 | 0.853 | 0.824 |
| 2069 | 0.765 | 1.000 | 0.777 | 1.000 | 0.963 | 0.960 | 0.901 | 1.000 | 0.980 | 1.000 | 0.957 | 1.000 | 0.991 | 0.854 | 1.000 | 1.000 | 0.852 | 0.822 |
| 2070 | 0.761 | 1.000 | 0.806 | 0.986 | 0.958 | 0.960 | 0.895 | 1.000 | 0.980 | 1.000 | 0.957 | 1.000 | 0.973 | 0.855 | 1.000 | 1.000 | 0.852 | 0.822 |
| 2071 | 0.761 | 1.000 | 0.777 | 0.986 | 0.963 | 0.960 | 0.897 | 1.000 | 0.990 | 1.000 | 0.959 | 1.000 | 0.973 | 0.864 | 1.000 | 1.000 | 0.852 | 0.823 |
| 2072 | 0.759 | 1.000 | 0.804 | 1.000 | 0.963 | 0.960 | 0.901 | 1.000 | 0.980 | 1.000 | 0.962 | 1.000 | 1.000 | 0.862 | 1.000 | 1.000 | 0.855 | 0.827 |
| 2073 | 0.768 | 1.000 | 0.789 | 1.000 | 0.968 | 0.960 | 0.904 | 1.000 | 0.990 | 1.000 | 0.957 | 1.000 | 0.982 | 0.851 | 1.000 | 1.000 | 0.853 | 0.824 |
| 2074 | 0.769 | 1.000 | 0.798 | 0.986 | 0.963 | 0.960 | 0.898 | 1.000 | 0.980 | 1.000 | 0.965 | 1.000 | 0.973 | 0.861 | 1.000 | 1.000 | 0.856 | 0.828 |
| 2075 | 0.775 | 1.000 | 0.800 | 1.000 | 0.963 | 0.960 | 0.897 | 1.000 | 0.980 | 1.000 | 0.962 | 1.000 | 0.982 | 0.861 | 1.000 | 1.000 | 0.859 | 0.830 |
| 2076 | 0.763 | 1.000 | 0.777 | 1.000 | 0.968 | 0.960 | 0.892 | 1.000 | 0.980 | 1.000 | 0.965 | 1.000 | 0.973 | 0.851 | 1.000 | 1.000 | 0.850 | 0.820 |
| 2077 | 0.763 | 1.000 | 0.789 | 1.000 | 0.963 | 0.960 | 0.901 | 1.000 | 0.969 | 1.000 | 0.968 | 1.000 | 0.964 | 0.864 | 1.000 | 1.000 | 0.855 | 0.826 |
| 2078 | 0.769 | 1.000 | 0.808 | 0.986 | 0.958 | 0.960 | 0.900 | 1.000 | 0.969 | 1.000 | 0.951 | 1.000 | 0.964 | 0.865 | 1.000 | 1.000 | 0.857 | 0.828 |
| 2079 | 0.765 | 1.000 | 0.792 | 1.000 | 0.958 | 0.960 | 0.897 | 1.000 | 0.990 | 1.000 | 0.954 | 1.000 | 0.973 | 0.861 | 1.000 | 1.000 | 0.854 | 0.825 |
| 2080 | 0.767 | 1.000 | 0.787 | 0.986 | 0.963 | 0.960 | 0.892 | 1.000 | 0.980 | 1.000 | 0.968 | 1.000 | 0.973 | 0.861 | 1.000 | 1.000 | 0.854 | 0.825 |
| 2081 | 0.776 | 1.000 | 0.785 | 1.000 | 0.958 | 0.960 | 0.900 | 1.000 | 0.990 | 1.000 | 0.962 | 0.970 | 0.964 | 0.858 | 1.000 | 0.981 | 0.856 | 0.827 |
| 2082 | 0.755 | 1.000 | 0.785 | 1.000 | 0.968 | 0.960 | 0.898 | 1.000 | 0.990 | 1.000 | 0.948 | 1.000 | 0.964 | 0.863 | 1.000 | 1.000 | 0.851 | 0.821 |
| 2083 | 0.769 | 1.000 | 0.781 | 1.000 | 0.958 | 0.960 | 0.898 | 1.000 | 0.990 | 1.000 | 0.957 | 1.000 | 0.973 | 0.857 | 1.000 | 1.000 | 0.853 | 0.824 |
| 2084 | 0.766 | 1.000 | 0.804 | 1.000 | 0.963 | 0.960 | 0.900 | 1.000 | 0.969 | 1.000 | 0.957 | 1.000 | 0.955 | 0.854 | 1.000 | 1.000 | 0.854 | 0.824 |
| 2085 | 0.769 | 1.000 | 0.804 | 1.000 | 0.968 | 0.960 | 0.906 | 1.000 | 0.969 | 1.000 | 0.962 | 1.000 | 0.973 | 0.860 | 1.000 | 1.000 | 0.858 | 0.829 |
| 2086 | 0.763 | 1.000 | 0.796 | 1.000 | 0.968 | 0.960 | 0.901 | 1.000 | 0.990 | 1.000 | 0.962 | 0.970 | 0.955 | 0.854 | 1.000 | 1.000 | 0.853 | 0.823 |
| 2087 | 0.762 | 1.000 | 0.791 | 0.986 | 0.953 | 0.960 | 0.894 | 1.000 | 0.969 | 1.000 | 0.957 | 1.000 | 0.973 | 0.861 | 1.000 | 1.000 | 0.852 | 0.822 |
| 2088 | 0.767 | 1.000 | 0.802 | 1.000 | 0.963 | 0.960 | 0.894 | 1.000 | 0.990 | 1.000 | 0.957 | 1.000 | 0.964 | 0.851 | 1.000 | 1.000 | 0.853 | 0.824 |
| 2089 | 0.776 | 1.000 | 0.802 | 1.000 | 0.968 | 0.960 | 0.894 | 1.000 | 0.980 | 1.000 | 0.968 | 1.000 | 0.955 | 0.860 | 1.000 | 1.000 | 0.858 | 0.830 |
| 2090 | 0.770 | 1.000 | 0.808 | 1.000 | 0.968 | 0.960 | 0.909 | 1.000 | 0.980 | 1.000 | 0.959 | 0.970 | 0.973 | 0.867 | 1.000 | 1.000 | 0.860 | 0.833 |
| 2091 | 0.761 | 1.000 | 0.779 | 1.000 | 0.974 | 0.960 | 0.908 | 1.000 | 0.969 | 1.000 | 0.951 | 1.000 | 0.955 | 0.856 | 1.000 | 1.000 | 0.851 | 0.821 |
| 2092 | 0.765 | 1.000 | 0.796 | 1.000 | 0.958 | 0.960 | 0.906 | 1.000 | 0.980 | 1.000 | 0.968 | 1.000 | 0.982 | 0.859 | 1.000 | 1.000 | 0.856 | 0.827 |
| 2093 | 0.761 | 1.000 | 0.806 | 1.000 | 0.968 | 0.960 | 0.901 | 1.000 | 1.000 | 1.000 | 0.959 | 1.000 | 0.973 | 0.858 | 1.000 | 1.000 | 0.855 | 0.826 |
| 2094 | 0.769 | 1.000 | 0.804 | 1.000 | 0.958 | 0.960 | 0.904 | 1.000 | 0.990 | 1.000 | 0.959 | 1.000 | 0.982 | 0.857 | 1.000 | 1.000 | 0.857 | 0.828 |
| 2095 | 0.761 | 1.000 | 0.783 | 1.000 | 0.963 | 0.960 | 0.898 | 1.000 | 0.980 | 1.000 | 0.957 | 1.000 | 0.982 | 0.861 | 1.000 | 1.000 | 0.852 | 0.823 |
| 2096 | 0.761 | 1.000 | 0.777 | 1.000 | 0.974 | 0.960 | 0.897 | 1.000 | 0.990 | 1.000 | 0.962 | 1.000 | 0.982 | 0.857 | 1.000 | 1.000 | 0.851 | 0.822 |
| 2097 | 0.757 | 1.000 | 0.777 | 1.000 | 0.968 | 0.960 | 0.900 | 1.000 | 0.990 | 1.000 | 0.959 | 0.970 | 0.946 | 0.850 | 1.000 | 1.000 | 0.847 | 0.817 |
| 2098 | 0.767 | 1.000 | 0.792 | 1.000 | 0.963 | 0.960 | 0.889 | 1.000 | 0.980 | 1.000 | 0.962 | 0.970 | 0.991 | 0.861 | 1.000 | 1.000 | 0.854 | 0.825 |
| 2099 | 0.761 | 1.000 | 0.792 | 1.000 | 0.968 | 0.960 | 0.901 | 1.000 | 1.000 | 1.000 | 0.971 | 1.000 | 0.955 | 0.853 | 1.000 | 1.000 | 0.852 | 0.823 |
| 2100 | 0.767 | 1.000 | 0.783 | 1.000 | 0.963 | 0.960 | 0.897 | 1.000 | 0.990 | 1.000 | 0.959 | 1.000 | 0.991 | 0.853 | 1.000 | 1.000 | 0.852 | 0.823 |
| 2101 | 0.769 | 1.000 | 0.800 | 1.000 | 0.963 | 0.960 | 0.901 | 1.000 | 1.000 | 1.000 | 0.962 | 1.000 | 0.982 | 0.854 | 1.000 | 1.000 | 0.856 | 0.827 |
| 2102 | 0.768 | 1.000 | 0.794 | 0.986 | 0.968 | 0.960 | 0.897 | 1.000 | 0.990 | 1.000 | 0.971 | 1.000 | 0.964 | 0.861 | 1.000 | 1.000 | 0.856 | 0.827 |
| 2103 | 0.766 | 1.000 | 0.791 | 1.000 | 0.968 | 0.960 | 0.903 | 1.000 | 0.980 | 1.000 | 0.962 | 1.000 | 0.973 | 0.862 | 1.000 | 1.000 | 0.856 | 0.827 |
| 2104 | 0.759 | 1.000 | 0.769 | 0.986 | 0.968 | 0.960 | 0.908 | 1.000 | 0.969 | 1.000 | 0.965 | 1.000 | 0.964 | 0.858 | 1.000 | 1.000 | 0.851 | 0.821 |

| | | | | | | | | | | | | | | | | | | |
|---|---|---|---|---|---|---|---|---|---|---|---|---|---|---|---|---|---|---|
| 2105 | 0.763 | 1.000 | 0.789 | 1.000 | 0.963 | 0.960 | 0.894 | 1.000 | 0.990 | 1.000 | 0.959 | 1.000 | 0.991 | 0.860 | 1.000 | 1.000 | 0.853 | 0.824 |
| 2106 | 0.767 | 1.000 | 0.800 | 0.986 | 0.963 | 0.960 | 0.891 | 1.000 | 1.000 | 1.000 | 0.959 | 0.939 | 0.991 | 0.861 | 1.000 | 1.000 | 0.855 | 0.826 |
| 2107 | 0.772 | 1.000 | 0.794 | 1.000 | 0.974 | 0.960 | 0.898 | 1.000 | 0.959 | 1.000 | 0.968 | 1.000 | 0.973 | 0.860 | 1.000 | 1.000 | 0.857 | 0.828 |
| 2108 | 0.765 | 1.000 | 0.789 | 1.000 | 0.963 | 0.960 | 0.895 | 1.000 | 0.990 | 1.000 | 0.974 | 1.000 | 0.964 | 0.856 | 1.000 | 1.000 | 0.853 | 0.824 |
| 2109 | 0.758 | 1.000 | 0.796 | 0.986 | 0.958 | 0.960 | 0.898 | 1.000 | 0.980 | 1.000 | 0.959 | 1.000 | 0.964 | 0.864 | 1.000 | 1.000 | 0.853 | 0.824 |
| 2110 | 0.758 | 1.000 | 0.791 | 0.986 | 0.968 | 0.960 | 0.906 | 1.000 | 0.990 | 1.000 | 0.962 | 1.000 | 0.982 | 0.860 | 1.000 | 1.000 | 0.853 | 0.825 |
| 2111 | 0.763 | 1.000 | 0.791 | 1.000 | 0.958 | 0.960 | 0.892 | 1.000 | 0.980 | 1.000 | 0.957 | 1.000 | 0.982 | 0.861 | 1.000 | 1.000 | 0.852 | 0.823 |
| 2112 | 0.761 | 1.000 | 0.779 | 1.000 | 0.963 | 0.960 | 0.892 | 1.000 | 1.000 | 1.000 | 0.962 | 1.000 | 0.955 | 0.850 | 1.000 | 1.000 | 0.848 | 0.818 |
| 2113 | 0.773 | 1.000 | 0.794 | 1.000 | 0.974 | 0.960 | 0.898 | 1.000 | 0.990 | 1.000 | 0.957 | 1.000 | 0.964 | 0.848 | 1.000 | 1.000 | 0.854 | 0.825 |
| 2114 | 0.756 | 1.000 | 0.798 | 1.000 | 0.974 | 0.960 | 0.898 | 1.000 | 0.980 | 1.000 | 0.959 | 1.000 | 0.973 | 0.861 | 1.000 | 1.000 | 0.853 | 0.823 |
| 2115 | 0.773 | 1.000 | 0.775 | 1.000 | 0.968 | 0.960 | 0.903 | 1.000 | 0.990 | 1.000 | 0.957 | 0.970 | 0.964 | 0.864 | 1.000 | 1.000 | 0.856 | 0.827 |
| 2116 | 0.772 | 1.000 | 0.808 | 0.986 | 0.968 | 0.960 | 0.897 | 1.000 | 0.990 | 1.000 | 0.965 | 1.000 | 0.964 | 0.851 | 1.000 | 1.000 | 0.856 | 0.827 |
| 2117 | 0.769 | 1.000 | 0.791 | 1.000 | 0.963 | 0.960 | 0.901 | 1.000 | 1.000 | 1.000 | 0.959 | 1.000 | 0.973 | 0.859 | 1.000 | 1.000 | 0.856 | 0.827 |
| 2118 | 0.776 | 1.000 | 0.796 | 1.000 | 0.968 | 0.960 | 0.901 | 1.000 | 1.000 | 1.000 | 0.959 | 1.000 | 1.000 | 0.854 | 1.000 | 1.000 | 0.858 | 0.829 |
| 2119 | 0.772 | 1.000 | 0.791 | 1.000 | 0.963 | 0.960 | 0.892 | 1.000 | 1.000 | 1.000 | 0.965 | 0.970 | 0.964 | 0.851 | 1.000 | 1.000 | 0.853 | 0.824 |
| 2120 | 0.765 | 1.000 | 0.798 | 1.000 | 0.963 | 0.960 | 0.895 | 1.000 | 0.980 | 1.000 | 0.962 | 1.000 | 0.973 | 0.854 | 1.000 | 1.000 | 0.853 | 0.824 |
| 2121 | 0.761 | 1.000 | 0.787 | 1.000 | 0.958 | 0.960 | 0.904 | 1.000 | 0.969 | 1.000 | 0.962 | 1.000 | 0.973 | 0.855 | 1.000 | 1.000 | 0.852 | 0.822 |
| 2122 | 0.777 | 1.000 | 0.791 | 1.000 | 0.963 | 0.960 | 0.906 | 1.000 | 0.990 | 1.000 | 0.965 | 1.000 | 0.955 | 0.854 | 1.000 | 1.000 | 0.857 | 0.829 |
| 2123 | 0.760 | 1.000 | 0.794 | 1.000 | 0.963 | 0.960 | 0.897 | 1.000 | 0.969 | 1.000 | 0.954 | 1.000 | 0.982 | 0.851 | 1.000 | 1.000 | 0.850 | 0.820 |
| 2124 | 0.762 | 1.000 | 0.787 | 0.986 | 0.958 | 0.960 | 0.901 | 1.000 | 0.990 | 1.000 | 0.968 | 1.000 | 0.964 | 0.861 | 1.000 | 1.000 | 0.854 | 0.825 |
| 2125 | 0.767 | 1.000 | 0.792 | 1.000 | 0.958 | 0.960 | 0.897 | 1.000 | 0.980 | 1.000 | 0.965 | 1.000 | 0.973 | 0.857 | 1.000 | 1.000 | 0.854 | 0.825 |
| 2126 | 0.767 | 1.000 | 0.806 | 0.986 | 0.963 | 0.960 | 0.895 | 1.000 | 0.990 | 1.000 | 0.954 | 1.000 | 0.991 | 0.854 | 1.000 | 1.000 | 0.854 | 0.825 |
| 2127 | 0.763 | 1.000 | 0.792 | 1.000 | 0.974 | 0.960 | 0.892 | 1.000 | 1.000 | 1.000 | 0.959 | 1.000 | 0.955 | 0.861 | 1.000 | 1.000 | 0.853 | 0.824 |
| 2128 | 0.763 | 1.000 | 0.781 | 1.000 | 0.953 | 0.960 | 0.894 | 1.000 | 0.990 | 1.000 | 0.962 | 1.000 | 0.955 | 0.861 | 1.000 | 1.000 | 0.852 | 0.822 |
| 2129 | 0.771 | 1.000 | 0.800 | 0.971 | 0.963 | 0.960 | 0.894 | 1.000 | 0.990 | 1.000 | 0.959 | 1.000 | 0.991 | 0.857 | 1.000 | 1.000 | 0.856 | 0.827 |
| 2130 | 0.767 | 1.000 | 0.787 | 0.986 | 0.968 | 0.960 | 0.895 | 1.000 | 0.980 | 1.000 | 0.959 | 1.000 | 0.955 | 0.858 | 1.000 | 1.000 | 0.853 | 0.823 |
| 2131 | 0.754 | 1.000 | 0.787 | 1.000 | 0.963 | 0.960 | 0.901 | 1.000 | 1.000 | 1.000 | 0.959 | 1.000 | 0.973 | 0.861 | 1.000 | 1.000 | 0.851 | 0.822 |
| 2132 | 0.773 | 1.000 | 0.787 | 0.971 | 0.963 | 0.960 | 0.903 | 1.000 | 0.990 | 1.000 | 0.962 | 1.000 | 0.991 | 0.854 | 1.000 | 1.000 | 0.855 | 0.826 |
| 2133 | 0.766 | 1.000 | 0.779 | 1.000 | 0.974 | 0.960 | 0.903 | 1.000 | 0.980 | 1.000 | 0.965 | 1.000 | 0.964 | 0.858 | 1.000 | 1.000 | 0.854 | 0.825 |
| 2134 | 0.768 | 1.000 | 0.806 | 1.000 | 0.968 | 0.960 | 0.901 | 1.000 | 0.980 | 1.000 | 0.962 | 1.000 | 0.964 | 0.855 | 1.000 | 1.000 | 0.856 | 0.827 |
| 2135 | 0.764 | 1.000 | 0.773 | 0.986 | 0.958 | 0.960 | 0.903 | 1.000 | 1.000 | 1.000 | 0.959 | 1.000 | 0.964 | 0.862 | 1.000 | 1.000 | 0.853 | 0.823 |
| 2136 | 0.757 | 1.000 | 0.792 | 1.000 | 0.963 | 0.960 | 0.908 | 1.000 | 1.000 | 1.000 | 0.954 | 1.000 | 0.973 | 0.848 | 1.000 | 1.000 | 0.850 | 0.821 |
| 2137 | 0.759 | 1.000 | 0.792 | 0.986 | 0.963 | 0.960 | 0.892 | 1.000 | 0.990 | 1.000 | 0.962 | 1.000 | 0.973 | 0.860 | 1.000 | 1.000 | 0.852 | 0.822 |
| 2138 | 0.755 | 1.000 | 0.792 | 0.986 | 0.968 | 0.960 | 0.897 | 1.000 | 0.980 | 1.000 | 0.957 | 1.000 | 0.964 | 0.858 | 1.000 | 1.000 | 0.850 | 0.820 |
| 2139 | 0.763 | 1.000 | 0.789 | 1.000 | 0.968 | 0.960 | 0.895 | 1.000 | 0.990 | 1.000 | 0.962 | 1.000 | 0.991 | 0.851 | 1.000 | 1.000 | 0.852 | 0.822 |
| 2140 | 0.767 | 1.000 | 0.792 | 0.986 | 0.974 | 0.960 | 0.900 | 1.000 | 0.990 | 1.000 | 0.948 | 0.970 | 0.946 | 0.852 | 1.000 | 1.000 | 0.852 | 0.822 |
| 2141 | 0.773 | 1.000 | 0.798 | 1.000 | 0.963 | 0.960 | 0.897 | 1.000 | 0.990 | 1.000 | 0.965 | 1.000 | 0.955 | 0.856 | 1.000 | 1.000 | 0.856 | 0.827 |
| 2142 | 0.761 | 1.000 | 0.800 | 0.986 | 0.958 | 0.960 | 0.886 | 1.000 | 0.980 | 1.000 | 0.959 | 1.000 | 0.973 | 0.854 | 1.000 | 0.981 | 0.850 | 0.820 |
| 2143 | 0.765 | 1.000 | 0.800 | 0.986 | 0.958 | 0.960 | 0.904 | 1.000 | 0.969 | 1.000 | 0.959 | 1.000 | 0.982 | 0.865 | 1.000 | 1.000 | 0.856 | 0.828 |

| | | | | | | | | | | | | | | | | | |
|---|---|---|---|---|---|---|---|---|---|---|---|---|---|---|---|---|---|
| 2144 | 0.770 | 1.000 | 0.794 | 1.000 | 0.963 | 0.960 | 0.900 | 1.000 | 0.980 | 1.000 | 0.962 | 1.000 | 0.973 | 0.857 | 1.000 | 1.000 | 0.855 | 0.827 |
| 2145 | 0.762 | 1.000 | 0.804 | 1.000 | 0.963 | 0.960 | 0.892 | 1.000 | 0.969 | 1.000 | 0.957 | 1.000 | 0.973 | 0.857 | 1.000 | 1.000 | 0.852 | 0.823 |
| 2146 | 0.775 | 1.000 | 0.787 | 0.986 | 0.963 | 0.960 | 0.897 | 1.000 | 0.990 | 1.000 | 0.951 | 1.000 | 0.964 | 0.856 | 1.000 | 1.000 | 0.854 | 0.825 |
| 2147 | 0.767 | 1.000 | 0.798 | 1.000 | 0.953 | 0.960 | 0.897 | 1.000 | 0.990 | 1.000 | 0.957 | 1.000 | 0.964 | 0.851 | 1.000 | 1.000 | 0.852 | 0.823 |
| 2148 | 0.773 | 1.000 | 0.808 | 0.986 | 0.963 | 0.960 | 0.901 | 1.000 | 1.000 | 1.000 | 0.951 | 1.000 | 0.973 | 0.852 | 1.000 | 1.000 | 0.856 | 0.827 |
| 2149 | 0.767 | 1.000 | 0.787 | 0.986 | 0.968 | 0.960 | 0.894 | 1.000 | 0.980 | 1.000 | 0.962 | 1.000 | 0.946 | 0.858 | 1.000 | 1.000 | 0.853 | 0.823 |
| 2150 | 0.774 | 1.000 | 0.783 | 1.000 | 0.963 | 0.960 | 0.895 | 1.000 | 0.980 | 1.000 | 0.959 | 1.000 | 0.964 | 0.860 | 1.000 | 1.000 | 0.855 | 0.826 |
| 2151 | 0.762 | 1.000 | 0.798 | 1.000 | 0.968 | 0.960 | 0.904 | 1.000 | 0.969 | 1.000 | 0.962 | 0.970 | 0.991 | 0.855 | 1.000 | 1.000 | 0.854 | 0.825 |
| 2152 | 0.767 | 1.000 | 0.775 | 1.000 | 0.963 | 0.960 | 0.904 | 1.000 | 0.980 | 1.000 | 0.959 | 1.000 | 0.973 | 0.852 | 1.000 | 1.000 | 0.852 | 0.822 |
| 2153 | 0.769 | 1.000 | 0.791 | 1.000 | 0.968 | 0.960 | 0.894 | 1.000 | 0.990 | 1.000 | 0.957 | 1.000 | 0.955 | 0.850 | 1.000 | 1.000 | 0.852 | 0.822 |
| 2154 | 0.775 | 1.000 | 0.789 | 0.986 | 0.963 | 0.960 | 0.897 | 1.000 | 0.990 | 1.000 | 0.954 | 1.000 | 0.955 | 0.856 | 1.000 | 1.000 | 0.855 | 0.825 |
| 2155 | 0.770 | 1.000 | 0.773 | 1.000 | 0.958 | 0.960 | 0.909 | 1.000 | 0.959 | 1.000 | 0.959 | 1.000 | 0.982 | 0.844 | 1.000 | 1.000 | 0.850 | 0.821 |
| 2156 | 0.769 | 1.000 | 0.792 | 0.986 | 0.958 | 0.960 | 0.888 | 1.000 | 0.980 | 1.000 | 0.948 | 1.000 | 0.964 | 0.858 | 1.000 | 1.000 | 0.852 | 0.822 |
| 2157 | 0.774 | 1.000 | 0.798 | 1.000 | 0.963 | 0.960 | 0.908 | 1.000 | 0.980 | 1.000 | 0.951 | 1.000 | 0.964 | 0.856 | 1.000 | 1.000 | 0.857 | 0.828 |
| 2158 | 0.767 | 1.000 | 0.796 | 1.000 | 0.968 | 0.960 | 0.901 | 1.000 | 0.980 | 1.000 | 0.951 | 0.970 | 0.946 | 0.861 | 1.000 | 1.000 | 0.854 | 0.825 |
| 2159 | 0.781 | 1.000 | 0.810 | 1.000 | 0.963 | 0.960 | 0.892 | 1.000 | 0.980 | 1.000 | 0.959 | 1.000 | 0.964 | 0.864 | 1.000 | 1.000 | 0.861 | 0.832 |
| 2160 | 0.767 | 1.000 | 0.792 | 1.000 | 0.963 | 0.960 | 0.895 | 1.000 | 0.990 | 1.000 | 0.959 | 1.000 | 0.982 | 0.861 | 1.000 | 1.000 | 0.855 | 0.826 |
| 2161 | 0.769 | 1.000 | 0.810 | 1.000 | 0.958 | 0.960 | 0.888 | 1.000 | 0.990 | 1.000 | 0.959 | 1.000 | 0.973 | 0.860 | 1.000 | 1.000 | 0.856 | 0.827 |
| 2162 | 0.760 | 1.000 | 0.791 | 0.986 | 0.963 | 0.960 | 0.906 | 1.000 | 0.980 | 1.000 | 0.951 | 0.970 | 0.973 | 0.861 | 1.000 | 1.000 | 0.853 | 0.823 |
| 2163 | 0.752 | 1.000 | 0.789 | 0.986 | 0.958 | 0.960 | 0.906 | 1.000 | 1.000 | 1.000 | 0.968 | 1.000 | 0.973 | 0.849 | 1.000 | 1.000 | 0.849 | 0.819 |
| 2164 | 0.768 | 1.000 | 0.808 | 1.000 | 0.968 | 0.960 | 0.894 | 1.000 | 0.969 | 1.000 | 0.965 | 1.000 | 0.955 | 0.860 | 1.000 | 1.000 | 0.856 | 0.827 |
| 2165 | 0.770 | 1.000 | 0.800 | 1.000 | 0.963 | 0.960 | 0.901 | 1.000 | 0.980 | 1.000 | 0.957 | 1.000 | 0.964 | 0.865 | 1.000 | 1.000 | 0.858 | 0.829 |
| 2166 | 0.772 | 1.000 | 0.792 | 1.000 | 0.958 | 0.960 | 0.898 | 1.000 | 0.980 | 1.000 | 0.962 | 1.000 | 0.982 | 0.854 | 1.000 | 1.000 | 0.855 | 0.826 |
| 2167 | 0.762 | 1.000 | 0.796 | 0.971 | 0.968 | 0.960 | 0.901 | 1.000 | 0.969 | 1.000 | 0.965 | 1.000 | 0.973 | 0.858 | 1.000 | 1.000 | 0.853 | 0.824 |
| 2168 | 0.759 | 1.000 | 0.773 | 0.986 | 0.963 | 0.960 | 0.904 | 1.000 | 1.000 | 1.000 | 0.957 | 1.000 | 0.973 | 0.848 | 1.000 | 1.000 | 0.848 | 0.818 |
| 2169 | 0.765 | 1.000 | 0.775 | 1.000 | 0.963 | 0.960 | 0.900 | 1.000 | 0.980 | 1.000 | 0.954 | 1.000 | 0.964 | 0.861 | 1.000 | 1.000 | 0.852 | 0.822 |
| 2170 | 0.767 | 1.000 | 0.792 | 1.000 | 0.963 | 0.960 | 0.881 | 1.000 | 0.980 | 1.000 | 0.962 | 1.000 | 0.964 | 0.850 | 1.000 | 1.000 | 0.850 | 0.820 |
| 2171 | 0.765 | 1.000 | 0.781 | 1.000 | 0.963 | 0.960 | 0.897 | 1.000 | 0.969 | 1.000 | 0.957 | 1.000 | 0.964 | 0.855 | 1.000 | 1.000 | 0.851 | 0.821 |
| 2172 | 0.772 | 1.000 | 0.796 | 1.000 | 0.958 | 0.960 | 0.892 | 1.000 | 0.969 | 1.000 | 0.959 | 1.000 | 0.964 | 0.862 | 1.000 | 1.000 | 0.856 | 0.827 |
| 2173 | 0.771 | 1.000 | 0.789 | 1.000 | 0.958 | 0.960 | 0.900 | 1.000 | 0.980 | 1.000 | 0.968 | 1.000 | 0.973 | 0.856 | 1.000 | 0.981 | 0.855 | 0.826 |
| 2174 | 0.775 | 1.000 | 0.789 | 1.000 | 0.963 | 0.960 | 0.892 | 1.000 | 0.980 | 1.000 | 0.954 | 1.000 | 0.973 | 0.862 | 1.000 | 1.000 | 0.856 | 0.827 |
| 2175 | 0.768 | 1.000 | 0.800 | 1.000 | 0.963 | 0.960 | 0.903 | 1.000 | 0.980 | 1.000 | 0.948 | 1.000 | 0.955 | 0.857 | 1.000 | 1.000 | 0.854 | 0.825 |
| 2176 | 0.771 | 1.000 | 0.791 | 1.000 | 0.968 | 0.960 | 0.898 | 1.000 | 0.980 | 1.000 | 0.959 | 1.000 | 0.982 | 0.861 | 1.000 | 1.000 | 0.856 | 0.827 |
| 2177 | 0.753 | 1.000 | 0.806 | 1.000 | 0.958 | 0.960 | 0.877 | 1.000 | 0.969 | 1.000 | 0.951 | 1.000 | 0.982 | 0.858 | 1.000 | 1.000 | 0.848 | 0.817 |
| 2178 | 0.763 | 1.000 | 0.792 | 1.000 | 0.968 | 0.960 | 0.897 | 1.000 | 0.980 | 1.000 | 0.957 | 1.000 | 0.964 | 0.864 | 1.000 | 1.000 | 0.854 | 0.825 |
| 2179 | 0.764 | 1.000 | 0.791 | 1.000 | 0.963 | 0.960 | 0.900 | 1.000 | 0.990 | 1.000 | 0.954 | 1.000 | 0.964 | 0.859 | 1.000 | 1.000 | 0.853 | 0.824 |
| 2180 | 0.771 | 1.000 | 0.789 | 1.000 | 0.968 | 0.960 | 0.903 | 1.000 | 0.980 | 1.000 | 0.962 | 1.000 | 0.973 | 0.852 | 1.000 | 1.000 | 0.854 | 0.825 |
| 2181 | 0.777 | 1.000 | 0.789 | 0.986 | 0.958 | 0.960 | 0.901 | 1.000 | 0.990 | 1.000 | 0.957 | 1.000 | 0.964 | 0.852 | 1.000 | 1.000 | 0.855 | 0.826 |
| 2182 | 0.764 | 1.000 | 0.785 | 1.000 | 0.963 | 0.960 | 0.911 | 1.000 | 0.969 | 1.000 | 0.957 | 1.000 | 0.964 | 0.851 | 1.000 | 1.000 | 0.852 | 0.822 |

| | | | | | | | | | | | | | | | | | |
|---|---|---|---|---|---|---|---|---|---|---|---|---|---|---|---|---|---|
| 2183 | 0.765 | 1.000 | 0.792 | 0.986 | 0.958 | 0.960 | 0.894 | 1.000 | 0.980 | 1.000 | 0.959 | 1.000 | 0.955 | 0.854 | 1.000 | 1.000 | 0.851 | 0.821 |
| 2184 | 0.758 | 1.000 | 0.783 | 0.986 | 0.968 | 0.960 | 0.894 | 1.000 | 0.969 | 1.000 | 0.965 | 1.000 | 0.991 | 0.857 | 1.000 | 1.000 | 0.850 | 0.820 |
| 2185 | 0.764 | 1.000 | 0.783 | 1.000 | 0.963 | 0.960 | 0.901 | 1.000 | 0.980 | 1.000 | 0.954 | 1.000 | 0.964 | 0.851 | 1.000 | 1.000 | 0.850 | 0.821 |
| 2186 | 0.768 | 1.000 | 0.775 | 1.000 | 0.968 | 0.960 | 0.898 | 1.000 | 1.000 | 1.000 | 0.951 | 1.000 | 0.955 | 0.858 | 1.000 | 1.000 | 0.852 | 0.822 |
| 2187 | 0.765 | 1.000 | 0.791 | 1.000 | 0.958 | 0.960 | 0.901 | 1.000 | 1.000 | 1.000 | 0.957 | 1.000 | 0.955 | 0.855 | 1.000 | 1.000 | 0.853 | 0.823 |
| 2188 | 0.768 | 1.000 | 0.771 | 0.986 | 0.963 | 0.960 | 0.888 | 1.000 | 0.969 | 1.000 | 0.951 | 0.970 | 0.973 | 0.854 | 1.000 | 1.000 | 0.849 | 0.818 |
| 2189 | 0.765 | 1.000 | 0.796 | 0.986 | 0.968 | 0.960 | 0.901 | 1.000 | 0.980 | 1.000 | 0.954 | 0.970 | 0.973 | 0.865 | 1.000 | 1.000 | 0.855 | 0.827 |
| 2190 | 0.775 | 1.000 | 0.796 | 0.986 | 0.963 | 0.960 | 0.903 | 1.000 | 0.969 | 1.000 | 0.959 | 1.000 | 0.982 | 0.865 | 1.000 | 1.000 | 0.859 | 0.831 |
| 2191 | 0.769 | 1.000 | 0.794 | 0.986 | 0.963 | 0.960 | 0.898 | 1.000 | 0.980 | 1.000 | 0.951 | 1.000 | 0.955 | 0.857 | 1.000 | 1.000 | 0.853 | 0.824 |
| 2192 | 0.771 | 1.000 | 0.789 | 0.986 | 0.963 | 0.960 | 0.900 | 1.000 | 0.990 | 1.000 | 0.962 | 1.000 | 0.955 | 0.851 | 1.000 | 1.000 | 0.853 | 0.824 |
| 2193 | 0.763 | 1.000 | 0.812 | 1.000 | 0.963 | 0.960 | 0.900 | 1.000 | 0.980 | 1.000 | 0.957 | 1.000 | 0.982 | 0.849 | 1.000 | 1.000 | 0.853 | 0.824 |
| 2194 | 0.773 | 1.000 | 0.792 | 1.000 | 0.968 | 0.960 | 0.898 | 1.000 | 0.969 | 1.000 | 0.962 | 1.000 | 0.982 | 0.854 | 1.000 | 1.000 | 0.855 | 0.827 |
| 2195 | 0.757 | 1.000 | 0.798 | 0.971 | 0.968 | 0.960 | 0.898 | 1.000 | 0.980 | 1.000 | 0.954 | 1.000 | 0.964 | 0.854 | 1.000 | 1.000 | 0.850 | 0.820 |
| 2196 | 0.758 | 1.000 | 0.787 | 1.000 | 0.963 | 0.960 | 0.895 | 1.000 | 0.980 | 1.000 | 0.959 | 1.000 | 0.955 | 0.856 | 1.000 | 1.000 | 0.850 | 0.820 |
| 2197 | 0.773 | 1.000 | 0.792 | 1.000 | 0.963 | 0.960 | 0.895 | 1.000 | 0.990 | 1.000 | 0.962 | 1.000 | 0.982 | 0.853 | 1.000 | 1.000 | 0.855 | 0.826 |
| 2198 | 0.771 | 1.000 | 0.791 | 0.986 | 0.958 | 0.960 | 0.900 | 1.000 | 0.969 | 1.000 | 0.959 | 1.000 | 0.973 | 0.860 | 1.000 | 1.000 | 0.855 | 0.826 |
| 2199 | 0.779 | 1.000 | 0.796 | 1.000 | 0.963 | 0.960 | 0.900 | 1.000 | 0.969 | 1.000 | 0.957 | 1.000 | 0.982 | 0.858 | 1.000 | 1.000 | 0.858 | 0.830 |
| 2200 | 0.763 | 1.000 | 0.789 | 1.000 | 0.974 | 0.960 | 0.903 | 1.000 | 0.990 | 1.000 | 0.965 | 1.000 | 0.982 | 0.860 | 1.000 | 1.000 | 0.855 | 0.826 |
| 2201 | 0.763 | 1.000 | 0.781 | 1.000 | 0.963 | 0.960 | 0.900 | 1.000 | 0.980 | 1.000 | 0.959 | 1.000 | 0.982 | 0.851 | 1.000 | 1.000 | 0.851 | 0.821 |
| 2202 | 0.776 | 1.000 | 0.773 | 1.000 | 0.974 | 0.960 | 0.897 | 1.000 | 0.990 | 1.000 | 0.951 | 1.000 | 0.955 | 0.865 | 1.000 | 1.000 | 0.856 | 0.827 |
| 2203 | 0.769 | 1.000 | 0.798 | 1.000 | 0.968 | 0.960 | 0.892 | 1.000 | 0.980 | 1.000 | 0.951 | 1.000 | 0.964 | 0.857 | 1.000 | 1.000 | 0.854 | 0.825 |
| 2204 | 0.770 | 1.000 | 0.796 | 0.986 | 0.974 | 0.960 | 0.901 | 1.000 | 0.969 | 1.000 | 0.962 | 1.000 | 0.982 | 0.862 | 1.000 | 1.000 | 0.857 | 0.829 |
| 2205 | 0.760 | 1.000 | 0.777 | 0.986 | 0.963 | 0.960 | 0.891 | 1.000 | 0.990 | 1.000 | 0.945 | 1.000 | 0.955 | 0.858 | 1.000 | 0.981 | 0.848 | 0.817 |
| 2206 | 0.765 | 1.000 | 0.791 | 0.986 | 0.958 | 0.960 | 0.906 | 1.000 | 0.990 | 1.000 | 0.962 | 1.000 | 0.964 | 0.864 | 1.000 | 1.000 | 0.856 | 0.827 |
| 2207 | 0.757 | 1.000 | 0.796 | 0.986 | 0.963 | 0.960 | 0.901 | 1.000 | 0.980 | 1.000 | 0.957 | 1.000 | 0.964 | 0.856 | 1.000 | 1.000 | 0.851 | 0.822 |
| 2208 | 0.763 | 1.000 | 0.791 | 0.971 | 0.958 | 0.960 | 0.895 | 1.000 | 0.980 | 1.000 | 0.957 | 0.970 | 0.991 | 0.853 | 1.000 | 1.000 | 0.851 | 0.821 |
| 2209 | 0.766 | 1.000 | 0.791 | 1.000 | 0.963 | 0.960 | 0.891 | 1.000 | 0.969 | 1.000 | 0.957 | 1.000 | 0.964 | 0.857 | 1.000 | 0.981 | 0.852 | 0.822 |
| 2210 | 0.771 | 1.000 | 0.789 | 0.986 | 0.963 | 0.960 | 0.897 | 1.000 | 0.969 | 1.000 | 0.968 | 1.000 | 0.946 | 0.854 | 1.000 | 1.000 | 0.853 | 0.824 |
| 2211 | 0.767 | 1.000 | 0.785 | 1.000 | 0.963 | 0.960 | 0.909 | 1.000 | 0.969 | 1.000 | 0.957 | 1.000 | 0.964 | 0.858 | 1.000 | 1.000 | 0.854 | 0.826 |
| 2212 | 0.761 | 1.000 | 0.800 | 0.986 | 0.958 | 0.960 | 0.906 | 1.000 | 0.990 | 1.000 | 0.962 | 1.000 | 0.946 | 0.860 | 1.000 | 1.000 | 0.854 | 0.825 |
| 2213 | 0.771 | 1.000 | 0.802 | 1.000 | 0.958 | 0.960 | 0.898 | 1.000 | 0.969 | 1.000 | 0.954 | 1.000 | 0.964 | 0.862 | 1.000 | 1.000 | 0.856 | 0.828 |
| 2214 | 0.763 | 1.000 | 0.791 | 0.986 | 0.958 | 0.960 | 0.903 | 1.000 | 0.980 | 1.000 | 0.968 | 1.000 | 0.955 | 0.854 | 1.000 | 1.000 | 0.852 | 0.823 |
| 2215 | 0.769 | 1.000 | 0.812 | 1.000 | 0.968 | 0.960 | 0.894 | 1.000 | 1.000 | 1.000 | 0.965 | 1.000 | 0.973 | 0.865 | 1.000 | 1.000 | 0.859 | 0.831 |
| 2216 | 0.765 | 1.000 | 0.785 | 1.000 | 0.968 | 0.960 | 0.903 | 1.000 | 0.980 | 1.000 | 0.957 | 0.970 | 0.964 | 0.853 | 1.000 | 1.000 | 0.852 | 0.822 |
| 2217 | 0.766 | 1.000 | 0.789 | 0.986 | 0.958 | 0.960 | 0.892 | 1.000 | 0.980 | 1.000 | 0.948 | 1.000 | 0.973 | 0.858 | 1.000 | 1.000 | 0.852 | 0.822 |
| 2218 | 0.776 | 1.000 | 0.792 | 1.000 | 0.963 | 0.960 | 0.898 | 1.000 | 0.969 | 1.000 | 0.957 | 1.000 | 0.973 | 0.855 | 1.000 | 1.000 | 0.856 | 0.827 |
| 2219 | 0.772 | 1.000 | 0.800 | 0.986 | 0.963 | 0.960 | 0.891 | 1.000 | 0.980 | 1.000 | 0.954 | 1.000 | 0.982 | 0.854 | 1.000 | 1.000 | 0.854 | 0.825 |
| 2220 | 0.774 | 1.000 | 0.791 | 1.000 | 0.968 | 0.960 | 0.908 | 1.000 | 0.990 | 1.000 | 0.957 | 1.000 | 0.955 | 0.865 | 1.000 | 1.000 | 0.859 | 0.831 |
| 2221 | 0.765 | 1.000 | 0.789 | 1.000 | 0.953 | 0.960 | 0.906 | 1.000 | 0.990 | 1.000 | 0.951 | 1.000 | 0.964 | 0.858 | 1.000 | 1.000 | 0.853 | 0.824 |

| | | | | | | | | | | | | | | | | | |
|---|---|---|---|---|---|---|---|---|---|---|---|---|---|---|---|---|---|
| 2222 | 0.763 | 1.000 | 0.791 | 1.000 | 0.953 | 0.960 | 0.900 | 1.000 | 0.980 | 1.000 | 0.954 | 1.000 | 0.973 | 0.862 | 1.000 | 1.000 | 0.853 | 0.824 |
| 2223 | 0.763 | 1.000 | 0.792 | 0.986 | 0.958 | 0.960 | 0.891 | 1.000 | 0.969 | 1.000 | 0.954 | 0.970 | 0.973 | 0.853 | 1.000 | 0.981 | 0.849 | 0.819 |
| 2224 | 0.764 | 1.000 | 0.792 | 1.000 | 0.963 | 0.960 | 0.897 | 1.000 | 0.969 | 1.000 | 0.962 | 1.000 | 0.982 | 0.861 | 1.000 | 1.000 | 0.854 | 0.825 |
| 2225 | 0.762 | 1.000 | 0.787 | 1.000 | 0.953 | 0.960 | 0.903 | 1.000 | 0.969 | 1.000 | 0.962 | 1.000 | 0.982 | 0.854 | 1.000 | 0.981 | 0.851 | 0.822 |
| 2226 | 0.778 | 1.000 | 0.779 | 1.000 | 0.963 | 0.960 | 0.904 | 1.000 | 0.969 | 1.000 | 0.954 | 1.000 | 0.964 | 0.854 | 1.000 | 1.000 | 0.855 | 0.826 |
| 2227 | 0.773 | 1.000 | 0.794 | 1.000 | 0.958 | 0.960 | 0.901 | 1.000 | 0.990 | 1.000 | 0.957 | 1.000 | 0.982 | 0.854 | 1.000 | 1.000 | 0.856 | 0.827 |
| 2228 | 0.775 | 1.000 | 0.794 | 0.986 | 0.953 | 0.960 | 0.909 | 1.000 | 0.990 | 1.000 | 0.951 | 1.000 | 0.964 | 0.861 | 1.000 | 1.000 | 0.858 | 0.829 |
| 2229 | 0.765 | 1.000 | 0.777 | 0.986 | 0.953 | 0.960 | 0.906 | 1.000 | 0.990 | 1.000 | 0.951 | 1.000 | 0.982 | 0.860 | 1.000 | 1.000 | 0.853 | 0.823 |
| 2230 | 0.766 | 1.000 | 0.773 | 0.986 | 0.958 | 0.960 | 0.892 | 1.000 | 0.980 | 1.000 | 0.957 | 1.000 | 0.955 | 0.858 | 1.000 | 1.000 | 0.850 | 0.820 |
| 2231 | 0.773 | 1.000 | 0.804 | 0.986 | 0.958 | 0.960 | 0.903 | 1.000 | 0.969 | 1.000 | 0.959 | 1.000 | 0.973 | 0.860 | 1.000 | 1.000 | 0.858 | 0.829 |
| 2232 | 0.761 | 1.000 | 0.792 | 0.986 | 0.963 | 0.960 | 0.903 | 1.000 | 0.959 | 1.000 | 0.959 | 1.000 | 0.973 | 0.858 | 1.000 | 1.000 | 0.852 | 0.823 |
| 2233 | 0.766 | 1.000 | 0.781 | 0.986 | 0.963 | 0.960 | 0.889 | 1.000 | 0.969 | 1.000 | 0.962 | 1.000 | 0.991 | 0.856 | 1.000 | 1.000 | 0.851 | 0.821 |
| 2234 | 0.764 | 1.000 | 0.789 | 1.000 | 0.963 | 0.960 | 0.897 | 1.000 | 0.980 | 1.000 | 0.959 | 1.000 | 0.955 | 0.855 | 1.000 | 0.981 | 0.851 | 0.822 |
| 2235 | 0.760 | 1.000 | 0.789 | 1.000 | 0.958 | 0.960 | 0.906 | 1.000 | 1.000 | 1.000 | 0.957 | 1.000 | 0.982 | 0.856 | 1.000 | 1.000 | 0.852 | 0.823 |
| 2236 | 0.770 | 1.000 | 0.804 | 0.986 | 0.958 | 0.960 | 0.891 | 1.000 | 0.959 | 1.000 | 0.951 | 1.000 | 0.973 | 0.857 | 1.000 | 1.000 | 0.854 | 0.824 |
| 2237 | 0.766 | 1.000 | 0.792 | 0.986 | 0.968 | 0.960 | 0.894 | 1.000 | 0.959 | 1.000 | 0.959 | 1.000 | 0.982 | 0.856 | 1.000 | 1.000 | 0.853 | 0.823 |
| 2238 | 0.763 | 1.000 | 0.775 | 1.000 | 0.953 | 0.960 | 0.889 | 1.000 | 0.980 | 1.000 | 0.954 | 1.000 | 0.964 | 0.861 | 1.000 | 1.000 | 0.850 | 0.820 |
| 2239 | 0.763 | 1.000 | 0.783 | 0.986 | 0.963 | 0.960 | 0.900 | 1.000 | 0.980 | 1.000 | 0.959 | 1.000 | 0.946 | 0.851 | 1.000 | 0.981 | 0.849 | 0.819 |
| 2240 | 0.768 | 1.000 | 0.785 | 1.000 | 0.958 | 0.960 | 0.901 | 1.000 | 0.969 | 1.000 | 0.957 | 1.000 | 0.964 | 0.852 | 1.000 | 1.000 | 0.852 | 0.822 |
| 2241 | 0.767 | 1.000 | 0.775 | 0.986 | 0.968 | 0.960 | 0.901 | 1.000 | 0.969 | 1.000 | 0.962 | 1.000 | 0.955 | 0.855 | 1.000 | 1.000 | 0.852 | 0.822 |
| 2242 | 0.771 | 1.000 | 0.787 | 1.000 | 0.963 | 0.960 | 0.897 | 1.000 | 0.980 | 1.000 | 0.951 | 1.000 | 0.973 | 0.862 | 1.000 | 1.000 | 0.855 | 0.826 |
| 2243 | 0.769 | 1.000 | 0.787 | 1.000 | 0.953 | 0.960 | 0.901 | 1.000 | 0.969 | 1.000 | 0.951 | 1.000 | 0.964 | 0.856 | 1.000 | 1.000 | 0.853 | 0.823 |
| 2244 | 0.765 | 1.000 | 0.785 | 0.986 | 0.958 | 0.960 | 0.903 | 1.000 | 0.990 | 1.000 | 0.954 | 1.000 | 0.964 | 0.856 | 1.000 | 1.000 | 0.852 | 0.823 |
| 2245 | 0.771 | 1.000 | 0.789 | 1.000 | 0.953 | 0.960 | 0.911 | 1.000 | 0.990 | 1.000 | 0.962 | 0.970 | 0.946 | 0.847 | 1.000 | 1.000 | 0.853 | 0.824 |
| 2246 | 0.761 | 1.000 | 0.808 | 1.000 | 0.963 | 0.960 | 0.900 | 1.000 | 0.980 | 1.000 | 0.962 | 1.000 | 0.973 | 0.857 | 1.000 | 1.000 | 0.854 | 0.825 |
| 2247 | 0.781 | 1.000 | 0.804 | 0.986 | 0.968 | 0.960 | 0.904 | 1.000 | 0.969 | 1.000 | 0.962 | 1.000 | 0.955 | 0.858 | 1.000 | 1.000 | 0.860 | 0.832 |
| 2248 | 0.763 | 1.000 | 0.798 | 0.986 | 0.963 | 0.960 | 0.906 | 1.000 | 0.969 | 1.000 | 0.959 | 0.970 | 0.982 | 0.859 | 1.000 | 1.000 | 0.855 | 0.826 |
| 2249 | 0.772 | 1.000 | 0.791 | 1.000 | 0.947 | 0.960 | 0.900 | 1.000 | 0.980 | 1.000 | 0.951 | 1.000 | 0.964 | 0.852 | 1.000 | 0.981 | 0.853 | 0.823 |
| 2250 | 0.772 | 1.000 | 0.785 | 0.986 | 0.968 | 0.960 | 0.897 | 1.000 | 0.990 | 1.000 | 0.954 | 1.000 | 0.964 | 0.852 | 1.000 | 1.000 | 0.853 | 0.823 |
| 2251 | 0.767 | 1.000 | 0.785 | 0.986 | 0.968 | 0.960 | 0.898 | 1.000 | 0.980 | 1.000 | 0.957 | 1.000 | 0.982 | 0.851 | 1.000 | 1.000 | 0.852 | 0.822 |
| 2252 | 0.761 | 1.000 | 0.791 | 1.000 | 0.968 | 0.960 | 0.901 | 1.000 | 0.969 | 1.000 | 0.951 | 1.000 | 0.964 | 0.855 | 1.000 | 1.000 | 0.851 | 0.822 |
| 2253 | 0.766 | 1.000 | 0.806 | 1.000 | 0.963 | 0.960 | 0.900 | 1.000 | 0.969 | 1.000 | 0.954 | 1.000 | 0.973 | 0.855 | 1.000 | 1.000 | 0.854 | 0.825 |
| 2254 | 0.771 | 1.000 | 0.792 | 1.000 | 0.968 | 0.960 | 0.912 | 1.000 | 0.980 | 1.000 | 0.962 | 1.000 | 0.955 | 0.861 | 1.000 | 0.981 | 0.858 | 0.830 |
| 2255 | 0.767 | 1.000 | 0.789 | 0.986 | 0.968 | 0.960 | 0.912 | 1.000 | 0.980 | 1.000 | 0.959 | 1.000 | 0.982 | 0.858 | 1.000 | 1.000 | 0.856 | 0.828 |
| 2256 | 0.776 | 1.000 | 0.816 | 0.986 | 0.963 | 0.960 | 0.901 | 1.000 | 0.990 | 1.000 | 0.968 | 1.000 | 0.973 | 0.852 | 1.000 | 0.981 | 0.859 | 0.830 |
| 2257 | 0.773 | 1.000 | 0.777 | 1.000 | 0.963 | 0.960 | 0.891 | 1.000 | 0.969 | 1.000 | 0.959 | 1.000 | 0.982 | 0.854 | 1.000 | 1.000 | 0.853 | 0.823 |
| 2258 | 0.757 | 1.000 | 0.794 | 1.000 | 0.958 | 0.960 | 0.897 | 1.000 | 0.980 | 1.000 | 0.959 | 1.000 | 0.982 | 0.858 | 1.000 | 1.000 | 0.851 | 0.822 |
| 2259 | 0.761 | 1.000 | 0.791 | 1.000 | 0.974 | 0.960 | 0.897 | 1.000 | 0.990 | 1.000 | 0.959 | 1.000 | 0.964 | 0.854 | 1.000 | 1.000 | 0.852 | 0.822 |
| 2260 | 0.763 | 1.000 | 0.802 | 1.000 | 0.963 | 0.960 | 0.894 | 1.000 | 0.969 | 1.000 | 0.954 | 1.000 | 0.964 | 0.854 | 1.000 | 0.981 | 0.851 | 0.822 |

| | | | | | | | | | | | | | | | | | |
|---|---|---|---|---|---|---|---|---|---|---|---|---|---|---|---|---|---|
| 2261 | 0.767 | 1.000 | 0.792 | 1.000 | 0.963 | 0.960 | 0.906 | 1.000 | 0.980 | 1.000 | 0.954 | 1.000 | 0.973 | 0.854 | 1.000 | 1.000 | 0.854 | 0.825 |
| 2262 | 0.769 | 1.000 | 0.796 | 0.986 | 0.963 | 0.960 | 0.895 | 1.000 | 0.990 | 1.000 | 0.962 | 1.000 | 0.982 | 0.855 | 1.000 | 1.000 | 0.855 | 0.825 |
| 2263 | 0.767 | 1.000 | 0.779 | 0.986 | 0.958 | 0.960 | 0.895 | 1.000 | 0.980 | 1.000 | 0.954 | 1.000 | 0.991 | 0.855 | 1.000 | 1.000 | 0.851 | 0.822 |
| 2264 | 0.775 | 1.000 | 0.789 | 0.986 | 0.963 | 0.960 | 0.903 | 1.000 | 0.980 | 1.000 | 0.954 | 1.000 | 0.964 | 0.858 | 1.000 | 1.000 | 0.856 | 0.827 |
| 2265 | 0.768 | 1.000 | 0.781 | 1.000 | 0.953 | 0.960 | 0.903 | 1.000 | 0.980 | 1.000 | 0.957 | 1.000 | 0.973 | 0.852 | 1.000 | 1.000 | 0.852 | 0.822 |
| 2266 | 0.767 | 1.000 | 0.792 | 1.000 | 0.963 | 0.960 | 0.906 | 1.000 | 0.959 | 1.000 | 0.954 | 1.000 | 0.964 | 0.861 | 1.000 | 1.000 | 0.855 | 0.826 |
| 2267 | 0.771 | 1.000 | 0.794 | 0.986 | 0.968 | 0.960 | 0.895 | 1.000 | 0.990 | 1.000 | 0.959 | 1.000 | 0.955 | 0.855 | 1.000 | 1.000 | 0.854 | 0.825 |
| 2268 | 0.772 | 1.000 | 0.789 | 1.000 | 0.963 | 0.960 | 0.898 | 1.000 | 0.969 | 1.000 | 0.962 | 1.000 | 0.964 | 0.858 | 1.000 | 1.000 | 0.855 | 0.826 |
| 2269 | 0.771 | 1.000 | 0.789 | 0.986 | 0.958 | 0.960 | 0.894 | 1.000 | 0.990 | 1.000 | 0.951 | 1.000 | 0.964 | 0.861 | 1.000 | 1.000 | 0.854 | 0.825 |
| 2270 | 0.760 | 1.000 | 0.781 | 0.986 | 0.963 | 0.960 | 0.900 | 1.000 | 0.969 | 1.000 | 0.954 | 1.000 | 0.946 | 0.852 | 1.000 | 1.000 | 0.848 | 0.818 |
| 2271 | 0.769 | 1.000 | 0.789 | 1.000 | 0.963 | 0.960 | 0.900 | 1.000 | 0.980 | 1.000 | 0.962 | 1.000 | 0.955 | 0.857 | 1.000 | 1.000 | 0.854 | 0.825 |
| 2272 | 0.769 | 1.000 | 0.775 | 0.986 | 0.958 | 0.960 | 0.891 | 1.000 | 0.969 | 1.000 | 0.951 | 1.000 | 0.964 | 0.853 | 1.000 | 0.981 | 0.849 | 0.819 |
| 2273 | 0.779 | 1.000 | 0.785 | 0.986 | 0.968 | 0.960 | 0.895 | 1.000 | 0.990 | 1.000 | 0.959 | 1.000 | 0.973 | 0.858 | 1.000 | 1.000 | 0.857 | 0.828 |
| 2274 | 0.769 | 1.000 | 0.796 | 1.000 | 0.968 | 0.960 | 0.904 | 1.000 | 0.980 | 1.000 | 0.962 | 1.000 | 0.973 | 0.852 | 1.000 | 0.981 | 0.855 | 0.826 |
| 2275 | 0.762 | 1.000 | 0.791 | 1.000 | 0.958 | 0.960 | 0.886 | 1.000 | 0.969 | 1.000 | 0.954 | 1.000 | 0.964 | 0.858 | 1.000 | 1.000 | 0.850 | 0.820 |
| 2276 | 0.767 | 1.000 | 0.794 | 1.000 | 0.968 | 0.960 | 0.891 | 1.000 | 0.980 | 1.000 | 0.959 | 1.000 | 0.964 | 0.858 | 1.000 | 1.000 | 0.854 | 0.824 |
| 2277 | 0.766 | 1.000 | 0.791 | 0.986 | 0.958 | 0.960 | 0.900 | 1.000 | 0.980 | 1.000 | 0.965 | 1.000 | 0.955 | 0.851 | 1.000 | 1.000 | 0.852 | 0.823 |
| 2278 | 0.769 | 1.000 | 0.775 | 1.000 | 0.963 | 0.960 | 0.892 | 1.000 | 0.990 | 1.000 | 0.962 | 1.000 | 0.964 | 0.856 | 1.000 | 1.000 | 0.852 | 0.822 |
| 2279 | 0.773 | 1.000 | 0.796 | 0.986 | 0.963 | 0.960 | 0.898 | 1.000 | 0.980 | 1.000 | 0.951 | 1.000 | 0.964 | 0.863 | 1.000 | 1.000 | 0.857 | 0.828 |
| 2280 | 0.771 | 1.000 | 0.785 | 0.986 | 0.963 | 0.960 | 0.903 | 1.000 | 0.980 | 1.000 | 0.962 | 1.000 | 0.973 | 0.857 | 1.000 | 1.000 | 0.855 | 0.826 |
| 2281 | 0.767 | 1.000 | 0.779 | 1.000 | 0.963 | 0.960 | 0.906 | 1.000 | 0.990 | 1.000 | 0.959 | 1.000 | 0.973 | 0.861 | 1.000 | 1.000 | 0.855 | 0.826 |
| 2282 | 0.767 | 1.000 | 0.794 | 1.000 | 0.953 | 0.960 | 0.897 | 1.000 | 0.969 | 1.000 | 0.971 | 1.000 | 0.955 | 0.858 | 1.000 | 1.000 | 0.854 | 0.825 |
| 2283 | 0.763 | 1.000 | 0.794 | 0.986 | 0.963 | 0.960 | 0.898 | 1.000 | 0.980 | 1.000 | 0.968 | 1.000 | 0.973 | 0.854 | 1.000 | 1.000 | 0.853 | 0.824 |
| 2284 | 0.763 | 1.000 | 0.800 | 1.000 | 0.958 | 0.960 | 0.894 | 1.000 | 0.969 | 1.000 | 0.962 | 1.000 | 0.964 | 0.858 | 1.000 | 1.000 | 0.853 | 0.823 |
| 2285 | 0.774 | 1.000 | 0.789 | 1.000 | 0.963 | 0.960 | 0.897 | 1.000 | 0.969 | 1.000 | 0.959 | 1.000 | 0.973 | 0.845 | 1.000 | 1.000 | 0.852 | 0.823 |
| 2286 | 0.775 | 1.000 | 0.777 | 1.000 | 0.963 | 0.960 | 0.894 | 1.000 | 1.000 | 1.000 | 0.962 | 1.000 | 0.973 | 0.852 | 1.000 | 1.000 | 0.853 | 0.824 |
| 2287 | 0.772 | 1.000 | 0.789 | 1.000 | 0.953 | 0.960 | 0.894 | 1.000 | 0.980 | 1.000 | 0.954 | 1.000 | 0.982 | 0.860 | 1.000 | 1.000 | 0.855 | 0.825 |
| 2288 | 0.763 | 1.000 | 0.792 | 0.986 | 0.958 | 0.960 | 0.895 | 1.000 | 0.969 | 1.000 | 0.959 | 1.000 | 0.982 | 0.855 | 1.000 | 0.981 | 0.851 | 0.822 |
| 2289 | 0.761 | 1.000 | 0.792 | 0.986 | 0.963 | 1.000 | 0.901 | 1.000 | 0.980 | 1.000 | 0.957 | 1.000 | 0.964 | 0.856 | 1.000 | 0.981 | 0.852 | 0.822 |
| 2290 | 0.769 | 1.000 | 0.792 | 0.986 | 0.963 | 0.960 | 0.901 | 1.000 | 0.980 | 1.000 | 0.959 | 1.000 | 0.964 | 0.858 | 1.000 | 1.000 | 0.855 | 0.826 |
| 2291 | 0.779 | 1.000 | 0.794 | 1.000 | 0.963 | 0.960 | 0.894 | 1.000 | 0.990 | 1.000 | 0.959 | 1.000 | 0.964 | 0.852 | 1.000 | 1.000 | 0.856 | 0.827 |
| 2292 | 0.766 | 1.000 | 0.800 | 1.000 | 0.974 | 0.960 | 0.898 | 1.000 | 0.980 | 1.000 | 0.959 | 1.000 | 0.946 | 0.854 | 1.000 | 1.000 | 0.854 | 0.825 |
| 2293 | 0.763 | 1.000 | 0.794 | 0.986 | 0.958 | 0.960 | 0.908 | 1.000 | 0.969 | 1.000 | 0.965 | 1.000 | 0.955 | 0.850 | 1.000 | 0.981 | 0.852 | 0.822 |
| 2294 | 0.768 | 1.000 | 0.794 | 0.986 | 0.953 | 0.960 | 0.900 | 1.000 | 0.980 | 1.000 | 0.954 | 1.000 | 0.982 | 0.850 | 1.000 | 1.000 | 0.852 | 0.823 |
| 2295 | 0.759 | 1.000 | 0.785 | 0.986 | 0.953 | 0.960 | 0.894 | 1.000 | 0.980 | 1.000 | 0.954 | 1.000 | 0.982 | 0.857 | 1.000 | 1.000 | 0.849 | 0.819 |
| 2296 | 0.769 | 1.000 | 0.785 | 0.986 | 0.968 | 0.960 | 0.898 | 1.000 | 0.980 | 1.000 | 0.965 | 1.000 | 0.973 | 0.857 | 1.000 | 1.000 | 0.854 | 0.825 |
| 2297 | 0.767 | 1.000 | 0.785 | 0.986 | 0.963 | 0.960 | 0.884 | 1.000 | 0.959 | 1.000 | 0.951 | 1.000 | 0.982 | 0.852 | 1.000 | 1.000 | 0.849 | 0.819 |
| 2298 | 0.773 | 1.000 | 0.796 | 1.000 | 0.963 | 0.960 | 0.901 | 1.000 | 0.990 | 1.000 | 0.962 | 1.000 | 0.973 | 0.858 | 1.000 | 1.000 | 0.857 | 0.829 |
| 2299 | 0.777 | 1.000 | 0.794 | 1.000 | 0.958 | 0.960 | 0.908 | 1.000 | 0.990 | 1.000 | 0.965 | 1.000 | 0.973 | 0.857 | 1.000 | 1.000 | 0.859 | 0.830 |

| | | | | | | | | | | | | | | | | | |
|---|---|---|---|---|---|---|---|---|---|---|---|---|---|---|---|---|---|
| 2300 | 0.769 | 1.000 | 0.794 | 0.986 | 0.963 | 0.960 | 0.903 | 1.000 | 0.969 | 1.000 | 0.948 | 1.000 | 0.982 | 0.857 | 1.000 | 1.000 | 0.854 | 0.825 |
| 2301 | 0.769 | 1.000 | 0.773 | 1.000 | 0.963 | 0.960 | 0.892 | 1.000 | 1.000 | 1.000 | 0.959 | 1.000 | 0.964 | 0.851 | 1.000 | 1.000 | 0.850 | 0.820 |
| 2302 | 0.771 | 1.000 | 0.769 | 1.000 | 0.958 | 0.960 | 0.894 | 1.000 | 0.980 | 1.000 | 0.965 | 1.000 | 0.955 | 0.858 | 1.000 | 1.000 | 0.852 | 0.822 |
| 2303 | 0.769 | 1.000 | 0.785 | 1.000 | 0.958 | 0.960 | 0.895 | 1.000 | 0.969 | 1.000 | 0.954 | 1.000 | 0.955 | 0.855 | 1.000 | 1.000 | 0.852 | 0.822 |
| 2304 | 0.769 | 1.000 | 0.798 | 0.986 | 0.963 | 0.960 | 0.903 | 1.000 | 0.990 | 1.000 | 0.959 | 1.000 | 0.955 | 0.856 | 1.000 | 0.981 | 0.855 | 0.826 |
| 2305 | 0.775 | 1.000 | 0.791 | 1.000 | 0.968 | 0.960 | 0.903 | 1.000 | 0.990 | 1.000 | 0.951 | 1.000 | 0.982 | 0.856 | 1.000 | 1.000 | 0.857 | 0.828 |
| 2306 | 0.784 | 1.000 | 0.798 | 0.986 | 0.963 | 0.960 | 0.911 | 1.000 | 0.980 | 1.000 | 0.962 | 1.000 | 0.955 | 0.854 | 1.000 | 1.000 | 0.860 | 0.832 |
| 2307 | 0.775 | 1.000 | 0.785 | 1.000 | 0.968 | 0.960 | 0.906 | 1.000 | 0.969 | 1.000 | 0.971 | 1.000 | 0.982 | 0.852 | 1.000 | 0.981 | 0.856 | 0.827 |
| 2308 | 0.777 | 1.000 | 0.789 | 1.000 | 0.958 | 0.960 | 0.897 | 1.000 | 0.980 | 1.000 | 0.962 | 1.000 | 0.973 | 0.845 | 1.000 | 1.000 | 0.853 | 0.824 |
| 2309 | 0.776 | 1.000 | 0.789 | 0.986 | 0.963 | 0.960 | 0.903 | 1.000 | 0.969 | 1.000 | 0.954 | 1.000 | 0.973 | 0.858 | 1.000 | 1.000 | 0.856 | 0.827 |
| 2310 | 0.773 | 1.000 | 0.798 | 0.986 | 0.974 | 0.960 | 0.900 | 1.000 | 0.980 | 1.000 | 0.965 | 1.000 | 0.973 | 0.853 | 1.000 | 1.000 | 0.856 | 0.827 |
| 2311 | 0.767 | 1.000 | 0.771 | 1.000 | 0.963 | 0.960 | 0.904 | 1.000 | 0.969 | 1.000 | 0.962 | 1.000 | 0.964 | 0.846 | 1.000 | 1.000 | 0.849 | 0.819 |
| 2312 | 0.764 | 1.000 | 0.789 | 1.000 | 0.958 | 0.960 | 0.900 | 1.000 | 0.980 | 1.000 | 0.959 | 1.000 | 0.964 | 0.854 | 1.000 | 1.000 | 0.852 | 0.822 |
| 2313 | 0.769 | 1.000 | 0.783 | 0.986 | 0.958 | 0.960 | 0.898 | 1.000 | 0.990 | 1.000 | 0.957 | 1.000 | 0.973 | 0.854 | 1.000 | 1.000 | 0.852 | 0.823 |
| 2314 | 0.767 | 1.000 | 0.800 | 1.000 | 0.958 | 0.960 | 0.900 | 1.000 | 0.990 | 1.000 | 0.959 | 1.000 | 0.973 | 0.847 | 1.000 | 1.000 | 0.852 | 0.823 |
| 2315 | 0.769 | 1.000 | 0.810 | 0.986 | 0.963 | 0.960 | 0.898 | 1.000 | 0.969 | 1.000 | 0.959 | 1.000 | 0.973 | 0.852 | 1.000 | 1.000 | 0.855 | 0.826 |
| 2316 | 0.765 | 1.000 | 0.785 | 1.000 | 0.963 | 0.960 | 0.900 | 1.000 | 0.980 | 1.000 | 0.965 | 0.970 | 0.955 | 0.857 | 1.000 | 1.000 | 0.853 | 0.823 |
| 2317 | 0.760 | 1.000 | 0.781 | 1.000 | 0.958 | 0.960 | 0.888 | 1.000 | 0.980 | 1.000 | 0.965 | 1.000 | 0.964 | 0.849 | 1.000 | 1.000 | 0.847 | 0.817 |
| 2318 | 0.775 | 1.000 | 0.791 | 1.000 | 0.963 | 0.960 | 0.897 | 1.000 | 0.990 | 1.000 | 0.957 | 1.000 | 0.955 | 0.854 | 1.000 | 1.000 | 0.855 | 0.826 |
| 2319 | 0.761 | 1.000 | 0.800 | 0.986 | 0.963 | 0.960 | 0.904 | 1.000 | 0.969 | 1.000 | 0.959 | 1.000 | 0.982 | 0.857 | 1.000 | 1.000 | 0.854 | 0.825 |
| 2320 | 0.769 | 1.000 | 0.777 | 1.000 | 0.963 | 0.960 | 0.894 | 1.000 | 0.990 | 1.000 | 0.954 | 0.970 | 0.973 | 0.855 | 1.000 | 0.981 | 0.851 | 0.821 |
| 2321 | 0.762 | 1.000 | 0.800 | 0.986 | 0.958 | 0.960 | 0.898 | 1.000 | 0.990 | 1.000 | 0.957 | 1.000 | 0.973 | 0.854 | 1.000 | 1.000 | 0.852 | 0.822 |
| 2322 | 0.769 | 1.000 | 0.789 | 1.000 | 0.963 | 0.960 | 0.898 | 1.000 | 0.980 | 1.000 | 0.962 | 1.000 | 0.991 | 0.861 | 1.000 | 0.981 | 0.855 | 0.827 |
| 2323 | 0.772 | 1.000 | 0.783 | 1.000 | 0.968 | 0.960 | 0.901 | 1.000 | 0.969 | 1.000 | 0.957 | 1.000 | 0.964 | 0.850 | 1.000 | 1.000 | 0.853 | 0.823 |
| 2324 | 0.768 | 1.000 | 0.785 | 1.000 | 0.968 | 0.960 | 0.895 | 1.000 | 0.980 | 1.000 | 0.959 | 0.970 | 0.973 | 0.851 | 1.000 | 1.000 | 0.851 | 0.822 |
| 2325 | 0.769 | 1.000 | 0.794 | 0.986 | 0.958 | 0.960 | 0.901 | 1.000 | 0.990 | 1.000 | 0.962 | 1.000 | 0.982 | 0.855 | 1.000 | 1.000 | 0.855 | 0.826 |
| 2326 | 0.772 | 1.000 | 0.800 | 0.986 | 0.974 | 0.960 | 0.898 | 1.000 | 0.990 | 1.000 | 0.957 | 1.000 | 0.955 | 0.855 | 1.000 | 1.000 | 0.856 | 0.827 |
| 2327 | 0.768 | 1.000 | 0.796 | 1.000 | 0.963 | 0.960 | 0.895 | 1.000 | 0.969 | 1.000 | 0.954 | 1.000 | 0.973 | 0.850 | 1.000 | 1.000 | 0.852 | 0.822 |
| 2328 | 0.765 | 1.000 | 0.804 | 1.000 | 0.958 | 0.960 | 0.897 | 1.000 | 1.000 | 1.000 | 0.957 | 1.000 | 0.973 | 0.852 | 1.000 | 1.000 | 0.853 | 0.824 |
| 2329 | 0.782 | 1.000 | 0.796 | 1.000 | 0.958 | 0.960 | 0.897 | 1.000 | 0.969 | 1.000 | 0.965 | 1.000 | 0.973 | 0.854 | 1.000 | 1.000 | 0.858 | 0.829 |
| 2330 | 0.774 | 1.000 | 0.775 | 1.000 | 0.963 | 0.960 | 0.908 | 1.000 | 0.969 | 1.000 | 0.965 | 0.970 | 0.982 | 0.851 | 1.000 | 0.981 | 0.854 | 0.825 |
| 2331 | 0.765 | 1.000 | 0.804 | 0.971 | 0.974 | 1.000 | 0.895 | 1.000 | 0.980 | 1.000 | 0.965 | 1.000 | 0.982 | 0.852 | 1.000 | 1.000 | 0.854 | 0.825 |
| 2332 | 0.767 | 1.000 | 0.789 | 1.000 | 0.958 | 0.960 | 0.903 | 1.000 | 0.980 | 1.000 | 0.951 | 1.000 | 0.982 | 0.855 | 1.000 | 1.000 | 0.853 | 0.824 |
| 2333 | 0.763 | 1.000 | 0.791 | 0.986 | 0.963 | 0.960 | 0.903 | 1.000 | 0.990 | 1.000 | 0.959 | 1.000 | 0.964 | 0.853 | 1.000 | 1.000 | 0.852 | 0.823 |
| 2334 | 0.774 | 1.000 | 0.791 | 1.000 | 0.958 | 0.960 | 0.895 | 1.000 | 0.969 | 1.000 | 0.968 | 1.000 | 0.973 | 0.857 | 1.000 | 1.000 | 0.856 | 0.827 |
| 2335 | 0.765 | 1.000 | 0.798 | 1.000 | 0.974 | 0.960 | 0.903 | 1.000 | 0.990 | 1.000 | 0.957 | 1.000 | 0.964 | 0.852 | 1.000 | 1.000 | 0.853 | 0.825 |
| 2336 | 0.773 | 1.000 | 0.798 | 0.986 | 0.974 | 0.960 | 0.911 | 1.000 | 0.959 | 1.000 | 0.962 | 1.000 | 0.973 | 0.863 | 1.000 | 1.000 | 0.860 | 0.832 |
| 2337 | 0.770 | 1.000 | 0.791 | 1.000 | 0.958 | 0.960 | 0.904 | 1.000 | 0.980 | 1.000 | 0.965 | 1.000 | 0.982 | 0.847 | 1.000 | 0.981 | 0.853 | 0.824 |
| 2338 | 0.782 | 1.000 | 0.796 | 0.986 | 0.958 | 0.960 | 0.894 | 1.000 | 0.949 | 1.000 | 0.965 | 1.000 | 0.982 | 0.847 | 1.000 | 1.000 | 0.855 | 0.826 |

| | | | | | | | | | | | | | | | | | | |
|---|---|---|---|---|---|---|---|---|---|---|---|---|---|---|---|---|---|---|
| 2339 | 0.776 | 1.000 | 0.812 | 1.000 | 0.968 | 0.960 | 0.897 | 1.000 | 0.990 | 1.000 | 0.971 | 1.000 | 0.973 | 0.847 | 1.000 | 1.000 | 0.857 | 0.829 |
| 2340 | 0.769 | 1.000 | 0.791 | 1.000 | 0.963 | 0.960 | 0.903 | 1.000 | 0.980 | 1.000 | 0.951 | 1.000 | 0.982 | 0.849 | 1.000 | 1.000 | 0.853 | 0.823 |
| 2341 | 0.762 | 1.000 | 0.791 | 1.000 | 0.963 | 0.960 | 0.901 | 1.000 | 0.949 | 1.000 | 0.954 | 1.000 | 0.973 | 0.858 | 1.000 | 1.000 | 0.852 | 0.823 |
| 2342 | 0.769 | 1.000 | 0.796 | 0.986 | 0.963 | 0.960 | 0.898 | 1.000 | 0.980 | 1.000 | 0.951 | 1.000 | 0.964 | 0.846 | 1.000 | 1.000 | 0.851 | 0.822 |
| 2343 | 0.764 | 1.000 | 0.800 | 1.000 | 0.958 | 0.960 | 0.908 | 1.000 | 0.959 | 1.000 | 0.957 | 1.000 | 0.973 | 0.857 | 1.000 | 1.000 | 0.854 | 0.825 |
| 2344 | 0.773 | 1.000 | 0.806 | 0.986 | 0.958 | 0.960 | 0.897 | 1.000 | 0.990 | 1.000 | 0.954 | 1.000 | 0.964 | 0.851 | 1.000 | 1.000 | 0.855 | 0.826 |
| 2345 | 0.765 | 1.000 | 0.810 | 0.986 | 0.953 | 0.960 | 0.892 | 1.000 | 0.980 | 1.000 | 0.962 | 1.000 | 0.982 | 0.859 | 1.000 | 0.981 | 0.855 | 0.826 |
| 2346 | 0.759 | 1.000 | 0.781 | 0.986 | 0.958 | 0.960 | 0.903 | 1.000 | 0.969 | 1.000 | 0.968 | 1.000 | 0.982 | 0.854 | 1.000 | 0.981 | 0.850 | 0.821 |
| 2347 | 0.779 | 1.000 | 0.789 | 1.000 | 0.974 | 0.960 | 0.906 | 1.000 | 0.990 | 1.000 | 0.959 | 1.000 | 0.973 | 0.857 | 1.000 | 1.000 | 0.859 | 0.831 |
| 2348 | 0.769 | 1.000 | 0.787 | 0.986 | 0.958 | 0.960 | 0.911 | 1.000 | 0.990 | 1.000 | 0.951 | 1.000 | 0.946 | 0.853 | 1.000 | 1.000 | 0.853 | 0.824 |
| 2349 | 0.771 | 1.000 | 0.802 | 0.986 | 0.968 | 0.960 | 0.900 | 1.000 | 0.969 | 1.000 | 0.959 | 1.000 | 0.982 | 0.850 | 1.000 | 1.000 | 0.855 | 0.826 |
| 2350 | 0.769 | 1.000 | 0.787 | 0.986 | 0.963 | 0.960 | 0.914 | 1.000 | 0.969 | 1.000 | 0.962 | 1.000 | 0.955 | 0.856 | 1.000 | 1.000 | 0.855 | 0.826 |
| 2351 | 0.772 | 1.000 | 0.789 | 1.000 | 0.963 | 0.960 | 0.897 | 1.000 | 0.980 | 1.000 | 0.957 | 1.000 | 0.964 | 0.854 | 1.000 | 0.981 | 0.853 | 0.824 |
| 2352 | 0.779 | 1.000 | 0.783 | 1.000 | 0.958 | 0.960 | 0.891 | 1.000 | 0.980 | 1.000 | 0.959 | 1.000 | 0.973 | 0.853 | 1.000 | 1.000 | 0.854 | 0.825 |
| 2353 | 0.772 | 1.000 | 0.802 | 1.000 | 0.968 | 0.960 | 0.892 | 1.000 | 0.969 | 1.000 | 0.957 | 1.000 | 0.955 | 0.855 | 1.000 | 0.981 | 0.854 | 0.825 |
| 2354 | 0.770 | 1.000 | 0.781 | 1.000 | 0.963 | 0.960 | 0.888 | 1.000 | 0.990 | 1.000 | 0.962 | 1.000 | 0.982 | 0.847 | 1.000 | 1.000 | 0.850 | 0.820 |
| 2355 | 0.767 | 1.000 | 0.798 | 1.000 | 0.963 | 0.960 | 0.903 | 1.000 | 0.980 | 1.000 | 0.965 | 1.000 | 0.955 | 0.852 | 1.000 | 1.000 | 0.854 | 0.825 |
| 2356 | 0.759 | 1.000 | 0.791 | 0.986 | 0.958 | 0.960 | 0.894 | 1.000 | 0.990 | 1.000 | 0.962 | 1.000 | 0.964 | 0.858 | 1.000 | 1.000 | 0.851 | 0.821 |
| 2357 | 0.769 | 1.000 | 0.794 | 1.000 | 0.958 | 0.960 | 0.900 | 1.000 | 0.990 | 1.000 | 0.954 | 1.000 | 0.955 | 0.858 | 1.000 | 1.000 | 0.855 | 0.826 |
| 2358 | 0.759 | 1.000 | 0.796 | 1.000 | 0.968 | 0.960 | 0.895 | 1.000 | 0.959 | 1.000 | 0.959 | 1.000 | 0.964 | 0.855 | 1.000 | 1.000 | 0.851 | 0.821 |
| 2359 | 0.769 | 1.000 | 0.789 | 0.986 | 0.963 | 0.960 | 0.894 | 1.000 | 0.980 | 1.000 | 0.957 | 1.000 | 0.973 | 0.855 | 1.000 | 1.000 | 0.853 | 0.823 |
| 2360 | 0.769 | 1.000 | 0.796 | 1.000 | 0.968 | 0.960 | 0.904 | 1.000 | 0.980 | 1.000 | 0.962 | 1.000 | 0.964 | 0.851 | 1.000 | 1.000 | 0.854 | 0.825 |
| 2361 | 0.777 | 1.000 | 0.779 | 1.000 | 0.968 | 0.960 | 0.912 | 1.000 | 0.980 | 1.000 | 0.962 | 1.000 | 0.964 | 0.847 | 1.000 | 1.000 | 0.855 | 0.826 |
| 2362 | 0.772 | 1.000 | 0.794 | 1.000 | 0.963 | 0.960 | 0.895 | 1.000 | 0.969 | 1.000 | 0.957 | 1.000 | 0.955 | 0.852 | 1.000 | 1.000 | 0.853 | 0.824 |
| 2363 | 0.775 | 1.000 | 0.802 | 0.986 | 0.963 | 0.960 | 0.898 | 1.000 | 0.969 | 1.000 | 0.954 | 1.000 | 0.964 | 0.853 | 1.000 | 1.000 | 0.855 | 0.826 |
| 2364 | 0.772 | 1.000 | 0.808 | 1.000 | 0.968 | 0.960 | 0.897 | 1.000 | 0.980 | 1.000 | 0.959 | 1.000 | 0.955 | 0.851 | 1.000 | 1.000 | 0.855 | 0.826 |
| 2365 | 0.772 | 1.000 | 0.802 | 1.000 | 0.958 | 0.960 | 0.894 | 1.000 | 0.980 | 1.000 | 0.962 | 1.000 | 0.964 | 0.855 | 1.000 | 1.000 | 0.855 | 0.826 |
| 2366 | 0.767 | 1.000 | 0.796 | 0.986 | 0.968 | 0.960 | 0.900 | 1.000 | 0.969 | 1.000 | 0.959 | 1.000 | 0.964 | 0.850 | 1.000 | 0.981 | 0.852 | 0.823 |
| 2367 | 0.773 | 1.000 | 0.792 | 0.986 | 0.963 | 0.960 | 0.900 | 1.000 | 0.969 | 1.000 | 0.968 | 1.000 | 0.973 | 0.858 | 1.000 | 1.000 | 0.856 | 0.827 |
| 2368 | 0.769 | 1.000 | 0.791 | 0.986 | 0.963 | 0.960 | 0.900 | 1.000 | 1.000 | 1.000 | 0.962 | 1.000 | 0.964 | 0.854 | 1.000 | 1.000 | 0.854 | 0.825 |
| 2369 | 0.768 | 1.000 | 0.789 | 0.986 | 0.958 | 0.960 | 0.894 | 1.000 | 1.000 | 1.000 | 0.957 | 1.000 | 0.982 | 0.858 | 1.000 | 1.000 | 0.853 | 0.824 |
| 2370 | 0.766 | 1.000 | 0.791 | 1.000 | 0.953 | 0.960 | 0.906 | 1.000 | 0.980 | 1.000 | 0.957 | 1.000 | 0.955 | 0.854 | 1.000 | 1.000 | 0.853 | 0.823 |
| 2371 | 0.774 | 1.000 | 0.792 | 1.000 | 0.958 | 0.960 | 0.904 | 1.000 | 0.969 | 1.000 | 0.959 | 1.000 | 0.964 | 0.851 | 1.000 | 1.000 | 0.855 | 0.826 |
| 2372 | 0.769 | 1.000 | 0.791 | 1.000 | 0.963 | 0.960 | 0.898 | 1.000 | 0.980 | 1.000 | 0.957 | 1.000 | 0.982 | 0.854 | 1.000 | 1.000 | 0.853 | 0.824 |
| 2373 | 0.770 | 1.000 | 0.804 | 1.000 | 0.953 | 0.960 | 0.895 | 1.000 | 0.990 | 1.000 | 0.962 | 1.000 | 0.973 | 0.858 | 1.000 | 1.000 | 0.856 | 0.827 |
| 2374 | 0.771 | 1.000 | 0.796 | 0.986 | 0.958 | 0.960 | 0.909 | 1.000 | 0.959 | 1.000 | 0.957 | 1.000 | 0.955 | 0.857 | 1.000 | 1.000 | 0.855 | 0.827 |
| 2375 | 0.767 | 1.000 | 0.796 | 1.000 | 0.963 | 0.960 | 0.904 | 1.000 | 0.980 | 1.000 | 0.957 | 0.970 | 0.982 | 0.852 | 1.000 | 1.000 | 0.854 | 0.825 |
| 2376 | 0.774 | 1.000 | 0.791 | 0.986 | 0.953 | 0.960 | 0.898 | 1.000 | 0.969 | 1.000 | 0.965 | 1.000 | 0.964 | 0.856 | 1.000 | 0.981 | 0.855 | 0.826 |
| 2377 | 0.777 | 1.000 | 0.783 | 1.000 | 0.968 | 0.960 | 0.901 | 1.000 | 0.969 | 1.000 | 0.971 | 1.000 | 0.964 | 0.857 | 1.000 | 1.000 | 0.857 | 0.828 |

| | | | | | | | | | | | | | | | | | |
|---|---|---|---|---|---|---|---|---|---|---|---|---|---|---|---|---|---|
| 2378 | 0.763 | 1.000 | 0.783 | 1.000 | 0.963 | 0.960 | 0.892 | 1.000 | 0.969 | 1.000 | 0.959 | 1.000 | 0.991 | 0.847 | 1.000 | 0.981 | 0.848 | 0.818 |
| 2379 | 0.769 | 1.000 | 0.802 | 1.000 | 0.968 | 0.960 | 0.895 | 1.000 | 0.980 | 1.000 | 0.954 | 1.000 | 0.964 | 0.855 | 1.000 | 1.000 | 0.854 | 0.825 |
| 2380 | 0.768 | 1.000 | 0.804 | 1.000 | 0.958 | 0.960 | 0.894 | 1.000 | 0.990 | 1.000 | 0.954 | 1.000 | 0.964 | 0.861 | 1.000 | 1.000 | 0.855 | 0.827 |
| 2381 | 0.774 | 1.000 | 0.802 | 1.000 | 0.968 | 0.960 | 0.886 | 1.000 | 0.980 | 1.000 | 0.962 | 1.000 | 0.955 | 0.853 | 1.000 | 1.000 | 0.854 | 0.825 |
| 2382 | 0.766 | 1.000 | 0.800 | 1.000 | 0.958 | 0.960 | 0.904 | 1.000 | 0.980 | 1.000 | 0.968 | 1.000 | 0.973 | 0.858 | 1.000 | 1.000 | 0.856 | 0.828 |
| 2383 | 0.763 | 1.000 | 0.808 | 1.000 | 0.968 | 0.960 | 0.900 | 1.000 | 0.990 | 1.000 | 0.968 | 1.000 | 0.973 | 0.851 | 1.000 | 1.000 | 0.854 | 0.826 |
| 2384 | 0.767 | 1.000 | 0.783 | 1.000 | 0.968 | 0.960 | 0.894 | 1.000 | 0.969 | 1.000 | 0.959 | 1.000 | 0.964 | 0.853 | 1.000 | 1.000 | 0.851 | 0.821 |
| 2385 | 0.777 | 1.000 | 0.794 | 0.986 | 0.963 | 0.960 | 0.901 | 1.000 | 0.980 | 1.000 | 0.951 | 1.000 | 0.982 | 0.856 | 1.000 | 1.000 | 0.857 | 0.828 |
| 2386 | 0.767 | 1.000 | 0.794 | 0.986 | 0.963 | 0.960 | 0.886 | 1.000 | 0.980 | 1.000 | 0.954 | 1.000 | 0.955 | 0.859 | 1.000 | 0.981 | 0.852 | 0.822 |
| 2387 | 0.769 | 1.000 | 0.800 | 1.000 | 0.968 | 0.960 | 0.897 | 1.000 | 0.990 | 1.000 | 0.954 | 1.000 | 0.973 | 0.849 | 1.000 | 1.000 | 0.853 | 0.824 |
| 2388 | 0.777 | 1.000 | 0.785 | 1.000 | 0.963 | 0.960 | 0.894 | 1.000 | 0.980 | 1.000 | 0.959 | 1.000 | 0.964 | 0.851 | 1.000 | 1.000 | 0.854 | 0.824 |
| 2389 | 0.769 | 1.000 | 0.800 | 1.000 | 0.947 | 0.960 | 0.898 | 1.000 | 0.990 | 1.000 | 0.951 | 1.000 | 0.973 | 0.859 | 1.000 | 1.000 | 0.855 | 0.826 |
| 2390 | 0.774 | 1.000 | 0.794 | 1.000 | 0.968 | 0.960 | 0.903 | 1.000 | 0.959 | 1.000 | 0.957 | 1.000 | 0.991 | 0.857 | 1.000 | 0.981 | 0.857 | 0.828 |
| 2391 | 0.773 | 1.000 | 0.794 | 1.000 | 0.953 | 0.960 | 0.912 | 1.000 | 0.969 | 1.000 | 0.954 | 1.000 | 0.964 | 0.851 | 1.000 | 1.000 | 0.855 | 0.826 |
| 2392 | 0.767 | 1.000 | 0.796 | 1.000 | 0.947 | 0.960 | 0.904 | 1.000 | 0.980 | 1.000 | 0.957 | 1.000 | 0.973 | 0.848 | 1.000 | 1.000 | 0.852 | 0.823 |
| 2393 | 0.768 | 1.000 | 0.796 | 1.000 | 0.958 | 0.960 | 0.895 | 1.000 | 0.980 | 1.000 | 0.959 | 1.000 | 0.964 | 0.858 | 1.000 | 1.000 | 0.854 | 0.825 |
| 2394 | 0.772 | 1.000 | 0.792 | 0.986 | 0.958 | 0.960 | 0.897 | 1.000 | 1.000 | 1.000 | 0.962 | 1.000 | 0.955 | 0.856 | 1.000 | 1.000 | 0.855 | 0.825 |
| 2395 | 0.765 | 1.000 | 0.791 | 1.000 | 0.958 | 0.960 | 0.894 | 1.000 | 0.969 | 1.000 | 0.962 | 1.000 | 0.964 | 0.861 | 1.000 | 0.981 | 0.853 | 0.824 |
| 2396 | 0.764 | 1.000 | 0.796 | 0.986 | 0.963 | 0.960 | 0.889 | 1.000 | 0.980 | 1.000 | 0.965 | 1.000 | 0.982 | 0.857 | 1.000 | 1.000 | 0.853 | 0.823 |
| 2397 | 0.767 | 1.000 | 0.791 | 1.000 | 0.963 | 0.960 | 0.898 | 1.000 | 0.969 | 1.000 | 0.951 | 1.000 | 0.964 | 0.854 | 1.000 | 0.981 | 0.852 | 0.822 |
| 2398 | 0.768 | 1.000 | 0.785 | 1.000 | 0.968 | 0.960 | 0.900 | 1.000 | 0.969 | 1.000 | 0.954 | 1.000 | 0.964 | 0.861 | 1.000 | 0.981 | 0.854 | 0.825 |
| 2399 | 0.767 | 1.000 | 0.800 | 0.986 | 0.968 | 0.960 | 0.904 | 1.000 | 0.990 | 1.000 | 0.962 | 1.000 | 0.991 | 0.857 | 1.000 | 1.000 | 0.856 | 0.828 |
| 2400 | 0.767 | 1.000 | 0.798 | 1.000 | 0.963 | 0.960 | 0.897 | 1.000 | 0.969 | 1.000 | 0.959 | 1.000 | 0.973 | 0.849 | 1.000 | 0.981 | 0.852 | 0.823 |
| 2401 | 0.769 | 1.000 | 0.792 | 1.000 | 0.958 | 0.960 | 0.895 | 1.000 | 0.969 | 1.000 | 0.962 | 1.000 | 0.973 | 0.865 | 1.000 | 1.000 | 0.856 | 0.827 |
| 2402 | 0.769 | 1.000 | 0.792 | 1.000 | 0.958 | 0.960 | 0.900 | 1.000 | 0.990 | 1.000 | 0.965 | 1.000 | 0.982 | 0.852 | 1.000 | 1.000 | 0.854 | 0.825 |
| 2403 | 0.774 | 1.000 | 0.796 | 0.986 | 0.963 | 0.960 | 0.897 | 1.000 | 0.980 | 1.000 | 0.965 | 1.000 | 0.973 | 0.856 | 1.000 | 1.000 | 0.856 | 0.827 |
| 2404 | 0.774 | 1.000 | 0.787 | 0.986 | 0.968 | 0.960 | 0.889 | 1.000 | 0.969 | 1.000 | 0.962 | 1.000 | 0.973 | 0.862 | 1.000 | 0.981 | 0.855 | 0.826 |
| 2405 | 0.766 | 1.000 | 0.796 | 1.000 | 0.963 | 0.960 | 0.895 | 1.000 | 0.980 | 1.000 | 0.962 | 1.000 | 0.973 | 0.857 | 1.000 | 1.000 | 0.854 | 0.825 |
| 2406 | 0.773 | 1.000 | 0.806 | 0.986 | 0.953 | 0.960 | 0.891 | 1.000 | 0.969 | 1.000 | 0.957 | 1.000 | 0.973 | 0.855 | 1.000 | 1.000 | 0.855 | 0.826 |
| 2407 | 0.759 | 1.000 | 0.794 | 1.000 | 0.963 | 0.960 | 0.904 | 1.000 | 0.980 | 1.000 | 0.959 | 1.000 | 0.964 | 0.848 | 1.000 | 1.000 | 0.850 | 0.821 |
| 2408 | 0.769 | 1.000 | 0.796 | 1.000 | 0.963 | 0.960 | 0.898 | 1.000 | 0.969 | 1.000 | 0.954 | 1.000 | 0.964 | 0.849 | 1.000 | 0.981 | 0.852 | 0.823 |
| 2409 | 0.769 | 1.000 | 0.787 | 1.000 | 0.968 | 0.960 | 0.897 | 1.000 | 0.969 | 1.000 | 0.962 | 1.000 | 0.964 | 0.861 | 1.000 | 1.000 | 0.855 | 0.825 |
| 2410 | 0.768 | 1.000 | 0.777 | 0.986 | 0.968 | 0.960 | 0.906 | 1.000 | 0.969 | 1.000 | 0.959 | 1.000 | 0.973 | 0.858 | 1.000 | 1.000 | 0.854 | 0.825 |
| 2411 | 0.775 | 1.000 | 0.812 | 0.986 | 0.963 | 0.960 | 0.903 | 1.000 | 0.990 | 1.000 | 0.959 | 1.000 | 0.991 | 0.862 | 1.000 | 1.000 | 0.861 | 0.833 |
| 2412 | 0.771 | 1.000 | 0.791 | 0.986 | 0.963 | 0.960 | 0.903 | 1.000 | 0.990 | 1.000 | 0.959 | 0.970 | 0.973 | 0.861 | 1.000 | 1.000 | 0.856 | 0.827 |
| 2413 | 0.764 | 1.000 | 0.785 | 0.971 | 0.963 | 0.960 | 0.911 | 1.000 | 0.980 | 1.000 | 0.965 | 1.000 | 0.964 | 0.852 | 1.000 | 1.000 | 0.852 | 0.823 |
| 2414 | 0.773 | 1.000 | 0.792 | 1.000 | 0.958 | 0.960 | 0.909 | 1.000 | 0.980 | 1.000 | 0.954 | 1.000 | 0.955 | 0.865 | 1.000 | 1.000 | 0.858 | 0.830 |
| 2415 | 0.775 | 1.000 | 0.781 | 1.000 | 0.963 | 0.960 | 0.891 | 1.000 | 0.980 | 1.000 | 0.957 | 1.000 | 0.973 | 0.849 | 1.000 | 1.000 | 0.852 | 0.822 |
| 2416 | 0.773 | 1.000 | 0.798 | 1.000 | 0.958 | 0.960 | 0.889 | 1.000 | 0.969 | 1.000 | 0.954 | 1.000 | 0.955 | 0.854 | 1.000 | 1.000 | 0.853 | 0.824 |

| | | | | | | | | | | | | | | | | | |
|---|---|---|---|---|---|---|---|---|---|---|---|---|---|---|---|---|---|
| 2417 | 0.769 | 1.000 | 0.777 | 1.000 | 0.958 | 0.960 | 0.906 | 1.000 | 0.969 | 1.000 | 0.962 | 1.000 | 0.982 | 0.859 | 1.000 | 1.000 | 0.855 | 0.826 |
| 2418 | 0.773 | 1.000 | 0.785 | 1.000 | 0.963 | 0.960 | 0.903 | 1.000 | 0.980 | 1.000 | 0.962 | 1.000 | 0.964 | 0.851 | 1.000 | 1.000 | 0.854 | 0.825 |
| 2419 | 0.773 | 1.000 | 0.806 | 1.000 | 0.958 | 0.960 | 0.903 | 1.000 | 0.969 | 1.000 | 0.957 | 1.000 | 0.964 | 0.854 | 1.000 | 1.000 | 0.856 | 0.828 |
| 2420 | 0.775 | 1.000 | 0.798 | 1.000 | 0.963 | 0.960 | 0.906 | 1.000 | 0.990 | 1.000 | 0.962 | 1.000 | 0.973 | 0.858 | 1.000 | 1.000 | 0.859 | 0.830 |
| 2421 | 0.769 | 1.000 | 0.785 | 1.000 | 0.963 | 0.960 | 0.897 | 1.000 | 0.969 | 1.000 | 0.959 | 1.000 | 0.982 | 0.853 | 1.000 | 1.000 | 0.853 | 0.823 |
| 2422 | 0.773 | 1.000 | 0.791 | 0.986 | 0.968 | 0.960 | 0.901 | 1.000 | 0.990 | 1.000 | 0.962 | 1.000 | 0.982 | 0.852 | 1.000 | 1.000 | 0.855 | 0.826 |
| 2423 | 0.769 | 1.000 | 0.789 | 1.000 | 0.963 | 0.960 | 0.901 | 1.000 | 0.980 | 1.000 | 0.959 | 1.000 | 0.973 | 0.851 | 1.000 | 1.000 | 0.853 | 0.824 |
| 2424 | 0.773 | 1.000 | 0.785 | 1.000 | 0.958 | 0.960 | 0.903 | 1.000 | 0.990 | 1.000 | 0.971 | 1.000 | 0.964 | 0.853 | 1.000 | 1.000 | 0.855 | 0.826 |
| 2425 | 0.767 | 1.000 | 0.787 | 1.000 | 0.963 | 0.960 | 0.898 | 1.000 | 0.969 | 1.000 | 0.951 | 1.000 | 0.991 | 0.851 | 1.000 | 1.000 | 0.851 | 0.822 |
| 2426 | 0.771 | 1.000 | 0.792 | 0.971 | 0.958 | 0.960 | 0.904 | 1.000 | 0.980 | 1.000 | 0.962 | 1.000 | 0.973 | 0.855 | 1.000 | 0.981 | 0.855 | 0.826 |
| 2427 | 0.774 | 1.000 | 0.787 | 0.986 | 0.968 | 0.960 | 0.909 | 1.000 | 0.980 | 1.000 | 0.957 | 1.000 | 0.973 | 0.859 | 1.000 | 1.000 | 0.857 | 0.829 |
| 2428 | 0.767 | 1.000 | 0.802 | 0.971 | 0.958 | 0.960 | 0.906 | 1.000 | 0.969 | 1.000 | 0.957 | 1.000 | 0.973 | 0.847 | 1.000 | 1.000 | 0.852 | 0.823 |
| 2429 | 0.765 | 1.000 | 0.775 | 1.000 | 0.963 | 0.960 | 0.900 | 1.000 | 0.959 | 1.000 | 0.954 | 1.000 | 0.973 | 0.856 | 1.000 | 1.000 | 0.851 | 0.821 |
| 2430 | 0.762 | 1.000 | 0.798 | 0.971 | 0.947 | 0.960 | 0.904 | 1.000 | 0.969 | 1.000 | 0.951 | 1.000 | 0.955 | 0.861 | 1.000 | 1.000 | 0.853 | 0.823 |
| 2431 | 0.776 | 1.000 | 0.796 | 1.000 | 0.968 | 0.960 | 0.906 | 1.000 | 0.980 | 1.000 | 0.962 | 1.000 | 0.973 | 0.857 | 1.000 | 0.981 | 0.858 | 0.830 |
| 2432 | 0.766 | 1.000 | 0.794 | 0.986 | 0.963 | 0.960 | 0.903 | 1.000 | 0.980 | 1.000 | 0.957 | 1.000 | 0.982 | 0.850 | 1.000 | 1.000 | 0.852 | 0.823 |
| 2433 | 0.782 | 1.000 | 0.785 | 1.000 | 0.968 | 0.960 | 0.906 | 1.000 | 0.980 | 1.000 | 0.954 | 1.000 | 0.964 | 0.858 | 1.000 | 1.000 | 0.858 | 0.830 |
| 2434 | 0.770 | 1.000 | 0.787 | 1.000 | 0.963 | 0.960 | 0.901 | 1.000 | 0.980 | 1.000 | 0.957 | 1.000 | 0.955 | 0.863 | 1.000 | 1.000 | 0.856 | 0.827 |
| 2435 | 0.769 | 1.000 | 0.804 | 1.000 | 0.963 | 0.960 | 0.898 | 1.000 | 0.959 | 1.000 | 0.965 | 1.000 | 0.982 | 0.860 | 1.000 | 1.000 | 0.857 | 0.828 |
| 2436 | 0.766 | 1.000 | 0.779 | 1.000 | 0.968 | 0.960 | 0.889 | 1.000 | 0.980 | 1.000 | 0.954 | 1.000 | 0.964 | 0.855 | 1.000 | 1.000 | 0.850 | 0.820 |
| 2437 | 0.754 | 1.000 | 0.791 | 1.000 | 0.963 | 0.960 | 0.895 | 1.000 | 1.000 | 1.000 | 0.954 | 1.000 | 0.982 | 0.848 | 1.000 | 1.000 | 0.847 | 0.818 |
| 2438 | 0.776 | 1.000 | 0.798 | 0.986 | 0.958 | 0.960 | 0.906 | 1.000 | 0.980 | 1.000 | 0.957 | 1.000 | 0.973 | 0.855 | 1.000 | 1.000 | 0.857 | 0.829 |
| 2439 | 0.778 | 1.000 | 0.796 | 1.000 | 0.963 | 0.960 | 0.897 | 1.000 | 0.980 | 1.000 | 0.957 | 1.000 | 0.955 | 0.852 | 1.000 | 1.000 | 0.856 | 0.827 |
| 2440 | 0.775 | 1.000 | 0.816 | 1.000 | 0.963 | 0.960 | 0.903 | 1.000 | 0.990 | 1.000 | 0.962 | 1.000 | 0.955 | 0.848 | 1.000 | 1.000 | 0.857 | 0.829 |
| 2441 | 0.770 | 1.000 | 0.802 | 1.000 | 0.958 | 0.960 | 0.904 | 1.000 | 0.969 | 1.000 | 0.945 | 1.000 | 0.982 | 0.858 | 1.000 | 1.000 | 0.856 | 0.827 |
| 2442 | 0.777 | 1.000 | 0.792 | 1.000 | 0.963 | 0.960 | 0.895 | 1.000 | 0.980 | 1.000 | 0.962 | 1.000 | 0.991 | 0.851 | 1.000 | 0.981 | 0.855 | 0.827 |
| 2443 | 0.771 | 1.000 | 0.789 | 1.000 | 0.963 | 0.960 | 0.894 | 1.000 | 0.980 | 1.000 | 0.959 | 1.000 | 0.955 | 0.860 | 1.000 | 1.000 | 0.854 | 0.825 |
| 2444 | 0.772 | 1.000 | 0.789 | 0.986 | 0.963 | 0.960 | 0.886 | 1.000 | 0.969 | 1.000 | 0.965 | 1.000 | 0.964 | 0.854 | 1.000 | 1.000 | 0.852 | 0.823 |
| 2445 | 0.769 | 1.000 | 0.796 | 1.000 | 0.963 | 0.960 | 0.901 | 1.000 | 0.990 | 1.000 | 0.957 | 1.000 | 0.964 | 0.853 | 1.000 | 1.000 | 0.854 | 0.825 |
| 2446 | 0.771 | 1.000 | 0.798 | 1.000 | 0.968 | 0.960 | 0.898 | 1.000 | 0.980 | 1.000 | 0.962 | 1.000 | 0.982 | 0.863 | 1.000 | 1.000 | 0.858 | 0.829 |
| 2447 | 0.763 | 1.000 | 0.785 | 1.000 | 0.958 | 0.960 | 0.900 | 1.000 | 0.980 | 1.000 | 0.957 | 1.000 | 0.973 | 0.847 | 1.000 | 1.000 | 0.849 | 0.819 |
| 2448 | 0.775 | 1.000 | 0.791 | 1.000 | 0.958 | 0.960 | 0.897 | 1.000 | 0.980 | 1.000 | 0.971 | 1.000 | 0.964 | 0.863 | 1.000 | 1.000 | 0.858 | 0.829 |
| 2449 | 0.764 | 1.000 | 0.783 | 0.986 | 0.953 | 0.960 | 0.900 | 1.000 | 0.969 | 1.000 | 0.959 | 1.000 | 0.973 | 0.857 | 1.000 | 1.000 | 0.851 | 0.822 |
| 2450 | 0.775 | 1.000 | 0.783 | 1.000 | 0.953 | 0.960 | 0.903 | 1.000 | 0.980 | 1.000 | 0.962 | 1.000 | 0.991 | 0.854 | 1.000 | 1.000 | 0.855 | 0.826 |
| 2451 | 0.774 | 1.000 | 0.806 | 0.986 | 0.968 | 0.960 | 0.906 | 1.000 | 0.969 | 1.000 | 0.962 | 1.000 | 0.982 | 0.856 | 1.000 | 1.000 | 0.858 | 0.830 |
| 2452 | 0.779 | 1.000 | 0.779 | 1.000 | 0.958 | 0.960 | 0.892 | 1.000 | 0.980 | 1.000 | 0.962 | 1.000 | 0.964 | 0.859 | 1.000 | 1.000 | 0.856 | 0.827 |
| 2453 | 0.765 | 1.000 | 0.796 | 1.000 | 0.953 | 0.960 | 0.895 | 1.000 | 0.990 | 1.000 | 0.962 | 1.000 | 0.955 | 0.863 | 1.000 | 0.981 | 0.854 | 0.825 |
| 2454 | 0.769 | 1.000 | 0.802 | 1.000 | 0.958 | 0.960 | 0.894 | 1.000 | 0.990 | 1.000 | 0.965 | 1.000 | 0.991 | 0.850 | 1.000 | 1.000 | 0.854 | 0.825 |
| 2455 | 0.769 | 1.000 | 0.787 | 1.000 | 0.963 | 0.960 | 0.901 | 1.000 | 0.990 | 1.000 | 0.959 | 1.000 | 0.973 | 0.862 | 1.000 | 1.000 | 0.856 | 0.827 |

| | | | | | | | | | | | | | | | | | | |
|---|---|---|---|---|---|---|---|---|---|---|---|---|---|---|---|---|---|---|
| 2456 | 0.763 | 1.000 | 0.785 | 1.000 | 0.963 | 0.960 | 0.894 | 1.000 | 0.969 | 1.000 | 0.957 | 1.000 | 0.973 | 0.855 | 1.000 | 1.000 | 0.851 | 0.821 |
| 2457 | 0.763 | 1.000 | 0.798 | 1.000 | 0.963 | 0.960 | 0.901 | 1.000 | 0.969 | 1.000 | 0.968 | 1.000 | 0.982 | 0.858 | 1.000 | 1.000 | 0.854 | 0.826 |
| 2458 | 0.762 | 1.000 | 0.796 | 1.000 | 0.958 | 0.960 | 0.903 | 1.000 | 0.980 | 1.000 | 0.962 | 1.000 | 0.964 | 0.850 | 1.000 | 1.000 | 0.851 | 0.822 |
| 2459 | 0.775 | 1.000 | 0.796 | 1.000 | 0.968 | 0.960 | 0.898 | 1.000 | 0.969 | 1.000 | 0.957 | 1.000 | 0.955 | 0.858 | 1.000 | 1.000 | 0.856 | 0.827 |
| 2460 | 0.759 | 1.000 | 0.796 | 1.000 | 0.968 | 0.960 | 0.900 | 1.000 | 0.980 | 1.000 | 0.971 | 1.000 | 0.973 | 0.858 | 1.000 | 0.981 | 0.853 | 0.825 |
| 2461 | 0.774 | 1.000 | 0.792 | 1.000 | 0.963 | 0.960 | 0.894 | 1.000 | 0.969 | 1.000 | 0.959 | 1.000 | 0.973 | 0.848 | 1.000 | 1.000 | 0.853 | 0.824 |
| 2462 | 0.767 | 1.000 | 0.820 | 1.000 | 0.968 | 0.960 | 0.903 | 1.000 | 0.969 | 1.000 | 0.965 | 1.000 | 0.955 | 0.861 | 1.000 | 1.000 | 0.858 | 0.830 |
| 2463 | 0.767 | 1.000 | 0.783 | 1.000 | 0.958 | 0.960 | 0.900 | 1.000 | 0.969 | 1.000 | 0.957 | 1.000 | 0.991 | 0.858 | 1.000 | 1.000 | 0.853 | 0.824 |
| 2464 | 0.770 | 1.000 | 0.791 | 1.000 | 0.968 | 0.960 | 0.895 | 1.000 | 0.980 | 1.000 | 0.971 | 1.000 | 0.982 | 0.860 | 1.000 | 1.000 | 0.856 | 0.828 |
| 2465 | 0.775 | 1.000 | 0.800 | 0.986 | 0.963 | 0.960 | 0.889 | 1.000 | 0.980 | 1.000 | 0.954 | 1.000 | 0.982 | 0.855 | 1.000 | 0.981 | 0.855 | 0.826 |
| 2466 | 0.773 | 1.000 | 0.804 | 1.000 | 0.968 | 0.960 | 0.894 | 1.000 | 0.980 | 1.000 | 0.965 | 1.000 | 0.973 | 0.859 | 1.000 | 1.000 | 0.857 | 0.829 |
| 2467 | 0.773 | 1.000 | 0.798 | 1.000 | 0.958 | 0.960 | 0.901 | 1.000 | 0.959 | 1.000 | 0.959 | 1.000 | 0.973 | 0.855 | 1.000 | 1.000 | 0.856 | 0.827 |
| 2468 | 0.769 | 1.000 | 0.781 | 1.000 | 0.968 | 0.960 | 0.903 | 1.000 | 0.969 | 1.000 | 0.957 | 1.000 | 0.955 | 0.857 | 1.000 | 1.000 | 0.853 | 0.824 |
| 2469 | 0.773 | 1.000 | 0.794 | 1.000 | 0.968 | 0.960 | 0.908 | 1.000 | 0.969 | 1.000 | 0.959 | 1.000 | 0.973 | 0.847 | 1.000 | 1.000 | 0.855 | 0.826 |
| 2470 | 0.767 | 1.000 | 0.791 | 1.000 | 0.974 | 0.960 | 0.894 | 1.000 | 0.980 | 1.000 | 0.962 | 1.000 | 0.973 | 0.858 | 1.000 | 1.000 | 0.854 | 0.825 |
| 2471 | 0.771 | 1.000 | 0.791 | 0.986 | 0.968 | 0.960 | 0.903 | 1.000 | 0.969 | 1.000 | 0.957 | 1.000 | 0.964 | 0.852 | 1.000 | 0.981 | 0.854 | 0.824 |
| 2472 | 0.776 | 1.000 | 0.787 | 0.986 | 0.963 | 0.960 | 0.895 | 1.000 | 0.990 | 1.000 | 0.957 | 1.000 | 0.946 | 0.858 | 1.000 | 1.000 | 0.855 | 0.826 |
| 2473 | 0.769 | 1.000 | 0.802 | 0.986 | 0.963 | 0.960 | 0.904 | 1.000 | 0.969 | 1.000 | 0.965 | 1.000 | 0.982 | 0.861 | 1.000 | 1.000 | 0.858 | 0.830 |
| 2474 | 0.765 | 1.000 | 0.789 | 1.000 | 0.963 | 0.960 | 0.898 | 1.000 | 0.980 | 1.000 | 0.951 | 1.000 | 0.973 | 0.851 | 1.000 | 1.000 | 0.851 | 0.821 |
| 2475 | 0.767 | 1.000 | 0.802 | 1.000 | 0.953 | 0.960 | 0.904 | 1.000 | 0.969 | 1.000 | 0.968 | 1.000 | 0.991 | 0.855 | 1.000 | 1.000 | 0.856 | 0.827 |
| 2476 | 0.771 | 1.000 | 0.798 | 1.000 | 0.968 | 0.960 | 0.891 | 1.000 | 0.980 | 1.000 | 0.962 | 1.000 | 0.946 | 0.857 | 1.000 | 1.000 | 0.855 | 0.825 |
| 2477 | 0.770 | 1.000 | 0.785 | 1.000 | 0.958 | 0.960 | 0.900 | 1.000 | 0.980 | 1.000 | 0.959 | 1.000 | 0.973 | 0.852 | 1.000 | 1.000 | 0.853 | 0.823 |
| 2478 | 0.767 | 1.000 | 0.785 | 1.000 | 0.963 | 0.960 | 0.894 | 1.000 | 0.980 | 1.000 | 0.959 | 1.000 | 0.955 | 0.856 | 1.000 | 1.000 | 0.852 | 0.822 |
| 2479 | 0.772 | 1.000 | 0.798 | 1.000 | 0.968 | 1.000 | 0.901 | 1.000 | 0.969 | 1.000 | 0.959 | 1.000 | 0.955 | 0.864 | 1.000 | 1.000 | 0.858 | 0.830 |
| 2480 | 0.769 | 1.000 | 0.802 | 1.000 | 0.958 | 0.960 | 0.897 | 1.000 | 0.990 | 1.000 | 0.962 | 1.000 | 0.964 | 0.860 | 1.000 | 0.981 | 0.856 | 0.827 |
| 2481 | 0.765 | 1.000 | 0.789 | 0.986 | 0.963 | 0.960 | 0.906 | 1.000 | 0.980 | 1.000 | 0.959 | 0.970 | 0.991 | 0.858 | 1.000 | 1.000 | 0.854 | 0.825 |
| 2482 | 0.766 | 1.000 | 0.802 | 1.000 | 0.968 | 0.960 | 0.904 | 1.000 | 0.990 | 1.000 | 0.954 | 1.000 | 0.964 | 0.858 | 1.000 | 1.000 | 0.856 | 0.827 |
| 2483 | 0.764 | 1.000 | 0.796 | 1.000 | 0.963 | 0.960 | 0.904 | 1.000 | 0.980 | 1.000 | 0.954 | 1.000 | 0.964 | 0.851 | 1.000 | 1.000 | 0.852 | 0.823 |
| 2484 | 0.770 | 1.000 | 0.787 | 1.000 | 0.958 | 0.960 | 0.897 | 1.000 | 1.000 | 1.000 | 0.959 | 1.000 | 0.973 | 0.861 | 1.000 | 1.000 | 0.855 | 0.826 |
| 2485 | 0.768 | 1.000 | 0.796 | 1.000 | 0.974 | 0.960 | 0.906 | 1.000 | 1.000 | 1.000 | 0.957 | 1.000 | 0.964 | 0.855 | 1.000 | 1.000 | 0.856 | 0.827 |
| 2486 | 0.775 | 1.000 | 0.787 | 0.986 | 0.963 | 0.960 | 0.901 | 1.000 | 0.990 | 1.000 | 0.962 | 1.000 | 0.973 | 0.857 | 1.000 | 1.000 | 0.856 | 0.828 |
| 2487 | 0.768 | 1.000 | 0.794 | 0.971 | 0.958 | 0.960 | 0.904 | 1.000 | 0.990 | 1.000 | 0.957 | 1.000 | 0.982 | 0.859 | 1.000 | 1.000 | 0.855 | 0.827 |
| 2488 | 0.768 | 1.000 | 0.794 | 0.986 | 0.963 | 0.960 | 0.900 | 1.000 | 0.969 | 1.000 | 0.959 | 1.000 | 0.964 | 0.858 | 1.000 | 1.000 | 0.854 | 0.825 |
| 2489 | 0.773 | 1.000 | 0.798 | 1.000 | 0.953 | 0.960 | 0.891 | 1.000 | 0.990 | 1.000 | 0.959 | 1.000 | 0.946 | 0.851 | 1.000 | 1.000 | 0.853 | 0.823 |
| 2490 | 0.769 | 1.000 | 0.804 | 1.000 | 0.963 | 0.960 | 0.900 | 1.000 | 0.980 | 1.000 | 0.965 | 1.000 | 0.973 | 0.864 | 1.000 | 1.000 | 0.858 | 0.829 |
| 2491 | 0.767 | 1.000 | 0.785 | 1.000 | 0.958 | 0.960 | 0.909 | 1.000 | 0.980 | 1.000 | 0.957 | 1.000 | 0.982 | 0.854 | 1.000 | 1.000 | 0.853 | 0.824 |
| 2492 | 0.777 | 1.000 | 0.783 | 1.000 | 0.968 | 0.960 | 0.901 | 1.000 | 0.990 | 1.000 | 0.951 | 1.000 | 0.973 | 0.858 | 1.000 | 1.000 | 0.856 | 0.827 |
| 2493 | 0.767 | 1.000 | 0.798 | 0.986 | 0.968 | 0.960 | 0.900 | 1.000 | 0.990 | 1.000 | 0.957 | 1.000 | 0.982 | 0.851 | 1.000 | 1.000 | 0.853 | 0.824 |
| 2494 | 0.765 | 1.000 | 0.808 | 0.986 | 0.958 | 0.960 | 0.897 | 1.000 | 0.990 | 1.000 | 0.954 | 1.000 | 0.955 | 0.861 | 1.000 | 0.981 | 0.855 | 0.826 |

| | | | | | | | | | | | | | | | | | | |
|---|---|---|---|---|---|---|---|---|---|---|---|---|---|---|---|---|---|---|
| 2495 | 0.773 | 1.000 | 0.792 | 1.000 | 0.958 | 0.960 | 0.895 | 1.000 | 0.990 | 1.000 | 0.965 | 1.000 | 0.982 | 0.853 | 1.000 | 1.000 | 0.855 | 0.826 |
| 2496 | 0.769 | 1.000 | 0.796 | 1.000 | 0.963 | 0.960 | 0.904 | 1.000 | 0.990 | 1.000 | 0.965 | 1.000 | 0.973 | 0.852 | 1.000 | 1.000 | 0.855 | 0.826 |
| 2497 | 0.771 | 1.000 | 0.808 | 0.986 | 0.968 | 0.960 | 0.897 | 1.000 | 0.990 | 1.000 | 0.965 | 1.000 | 0.973 | 0.852 | 1.000 | 1.000 | 0.856 | 0.827 |
| 2498 | 0.768 | 1.000 | 0.796 | 1.000 | 0.958 | 0.960 | 0.900 | 1.000 | 0.990 | 1.000 | 0.965 | 1.000 | 0.982 | 0.854 | 1.000 | 1.000 | 0.855 | 0.826 |
| 2499 | 0.778 | 1.000 | 0.796 | 0.986 | 0.958 | 0.960 | 0.892 | 1.000 | 0.990 | 1.000 | 0.965 | 1.000 | 0.964 | 0.854 | 1.000 | 1.000 | 0.856 | 0.827 |
| 2500 | 0.767 | 1.000 | 0.789 | 1.000 | 0.968 | 0.960 | 0.900 | 1.000 | 0.990 | 1.000 | 0.957 | 1.000 | 0.964 | 0.852 | 1.000 | 1.000 | 0.852 | 0.823 |
| 2501 | 0.762 | 1.000 | 0.806 | 0.986 | 0.963 | 0.960 | 0.895 | 1.000 | 0.990 | 1.000 | 0.957 | 1.000 | 0.973 | 0.864 | 1.000 | 1.000 | 0.855 | 0.826 |
| 2502 | 0.773 | 1.000 | 0.794 | 0.986 | 0.968 | 1.000 | 0.897 | 1.000 | 0.990 | 1.000 | 0.962 | 1.000 | 0.991 | 0.859 | 1.000 | 1.000 | 0.857 | 0.829 |
| 2503 | 0.771 | 1.000 | 0.806 | 1.000 | 0.953 | 0.960 | 0.895 | 1.000 | 0.980 | 1.000 | 0.959 | 1.000 | 0.991 | 0.860 | 1.000 | 1.000 | 0.857 | 0.828 |
| 2504 | 0.761 | 1.000 | 0.804 | 0.986 | 0.968 | 0.960 | 0.898 | 1.000 | 0.980 | 1.000 | 0.965 | 1.000 | 0.964 | 0.853 | 1.000 | 0.981 | 0.852 | 0.823 |
| 2505 | 0.769 | 1.000 | 0.806 | 0.986 | 0.958 | 0.960 | 0.897 | 1.000 | 1.000 | 1.000 | 0.951 | 1.000 | 0.973 | 0.846 | 1.000 | 1.000 | 0.852 | 0.823 |
| 2506 | 0.773 | 1.000 | 0.796 | 1.000 | 0.958 | 0.960 | 0.897 | 1.000 | 1.000 | 1.000 | 0.965 | 1.000 | 0.955 | 0.858 | 1.000 | 1.000 | 0.856 | 0.827 |
| 2507 | 0.763 | 1.000 | 0.785 | 1.000 | 0.963 | 0.960 | 0.895 | 1.000 | 0.990 | 1.000 | 0.957 | 1.000 | 0.982 | 0.858 | 1.000 | 1.000 | 0.852 | 0.822 |
| 2508 | 0.770 | 1.000 | 0.808 | 0.986 | 0.953 | 0.960 | 0.904 | 1.000 | 0.990 | 1.000 | 0.959 | 1.000 | 0.955 | 0.859 | 1.000 | 0.981 | 0.857 | 0.828 |
| 2509 | 0.775 | 1.000 | 0.794 | 0.986 | 0.963 | 0.960 | 0.895 | 1.000 | 0.990 | 1.000 | 0.968 | 1.000 | 0.973 | 0.854 | 1.000 | 1.000 | 0.856 | 0.827 |
| 2510 | 0.768 | 1.000 | 0.802 | 1.000 | 0.958 | 0.960 | 0.903 | 1.000 | 1.000 | 1.000 | 0.968 | 1.000 | 0.955 | 0.856 | 1.000 | 1.000 | 0.856 | 0.828 |
| 2511 | 0.769 | 1.000 | 0.791 | 1.000 | 0.968 | 0.960 | 0.888 | 1.000 | 0.990 | 1.000 | 0.957 | 1.000 | 1.000 | 0.857 | 1.000 | 1.000 | 0.854 | 0.825 |
| 2512 | 0.767 | 1.000 | 0.816 | 0.986 | 0.958 | 0.960 | 0.894 | 1.000 | 0.990 | 1.000 | 0.962 | 1.000 | 0.964 | 0.854 | 1.000 | 1.000 | 0.855 | 0.826 |
| 2513 | 0.769 | 1.000 | 0.798 | 1.000 | 0.963 | 0.960 | 0.904 | 1.000 | 1.000 | 1.000 | 0.951 | 1.000 | 0.973 | 0.855 | 1.000 | 1.000 | 0.855 | 0.826 |
| 2514 | 0.764 | 1.000 | 0.802 | 1.000 | 0.968 | 0.960 | 0.895 | 1.000 | 0.980 | 1.000 | 0.954 | 1.000 | 0.982 | 0.852 | 1.000 | 1.000 | 0.852 | 0.823 |
| 2515 | 0.770 | 1.000 | 0.789 | 0.986 | 0.963 | 0.960 | 0.904 | 1.000 | 0.990 | 1.000 | 0.965 | 0.970 | 0.946 | 0.847 | 1.000 | 0.981 | 0.852 | 0.823 |
| 2516 | 0.775 | 1.000 | 0.808 | 1.000 | 0.968 | 0.960 | 0.898 | 1.000 | 0.990 | 1.000 | 0.965 | 1.000 | 0.973 | 0.851 | 1.000 | 0.981 | 0.857 | 0.828 |
| 2517 | 0.765 | 1.000 | 0.802 | 1.000 | 0.963 | 0.960 | 0.906 | 1.000 | 1.000 | 1.000 | 0.957 | 1.000 | 0.946 | 0.854 | 1.000 | 1.000 | 0.854 | 0.826 |
| 2518 | 0.763 | 1.000 | 0.798 | 1.000 | 0.963 | 0.960 | 0.906 | 1.000 | 1.000 | 1.000 | 0.962 | 1.000 | 0.964 | 0.866 | 1.000 | 1.000 | 0.857 | 0.829 |
| 2519 | 0.770 | 1.000 | 0.804 | 1.000 | 0.963 | 0.960 | 0.908 | 1.000 | 0.980 | 1.000 | 0.965 | 1.000 | 0.982 | 0.851 | 1.000 | 1.000 | 0.856 | 0.828 |
| 2520 | 0.774 | 1.000 | 0.804 | 0.986 | 0.963 | 0.960 | 0.903 | 1.000 | 0.980 | 1.000 | 0.959 | 1.000 | 0.982 | 0.859 | 1.000 | 1.000 | 0.858 | 0.830 |
| 2521 | 0.773 | 1.000 | 0.794 | 1.000 | 0.963 | 0.960 | 0.900 | 1.000 | 0.990 | 1.000 | 0.968 | 1.000 | 0.991 | 0.856 | 1.000 | 1.000 | 0.857 | 0.828 |
| 2522 | 0.769 | 1.000 | 0.785 | 1.000 | 0.968 | 0.960 | 0.908 | 1.000 | 0.980 | 1.000 | 0.957 | 1.000 | 0.991 | 0.857 | 1.000 | 1.000 | 0.855 | 0.827 |
| 2523 | 0.767 | 1.000 | 0.802 | 0.971 | 0.968 | 0.960 | 0.900 | 1.000 | 0.980 | 1.000 | 0.959 | 1.000 | 0.955 | 0.858 | 1.000 | 1.000 | 0.855 | 0.826 |
| 2524 | 0.778 | 1.000 | 0.787 | 1.000 | 0.958 | 1.000 | 0.898 | 1.000 | 0.990 | 1.000 | 0.959 | 1.000 | 0.991 | 0.851 | 1.000 | 1.000 | 0.856 | 0.827 |
| 2525 | 0.767 | 1.000 | 0.806 | 0.986 | 0.968 | 0.960 | 0.894 | 1.000 | 0.990 | 1.000 | 0.951 | 1.000 | 0.955 | 0.847 | 1.000 | 1.000 | 0.851 | 0.822 |
| 2526 | 0.777 | 1.000 | 0.792 | 1.000 | 0.958 | 0.960 | 0.909 | 1.000 | 0.990 | 1.000 | 0.962 | 1.000 | 0.955 | 0.849 | 1.000 | 1.000 | 0.856 | 0.827 |
| 2527 | 0.761 | 1.000 | 0.796 | 1.000 | 0.942 | 0.960 | 0.903 | 1.000 | 1.000 | 1.000 | 0.954 | 1.000 | 0.973 | 0.858 | 1.000 | 1.000 | 0.853 | 0.823 |
| 2528 | 0.771 | 1.000 | 0.792 | 0.986 | 0.963 | 0.960 | 0.897 | 1.000 | 0.990 | 1.000 | 0.951 | 1.000 | 0.982 | 0.861 | 1.000 | 1.000 | 0.856 | 0.827 |
| 2529 | 0.775 | 1.000 | 0.808 | 1.000 | 0.958 | 0.960 | 0.901 | 1.000 | 1.000 | 1.000 | 0.957 | 1.000 | 0.964 | 0.856 | 1.000 | 1.000 | 0.858 | 0.830 |
| 2530 | 0.768 | 1.000 | 0.810 | 1.000 | 0.963 | 0.960 | 0.898 | 1.000 | 0.980 | 1.000 | 0.948 | 1.000 | 0.955 | 0.855 | 1.000 | 1.000 | 0.854 | 0.825 |
| 2531 | 0.776 | 1.000 | 0.791 | 1.000 | 0.958 | 0.960 | 0.895 | 1.000 | 0.990 | 1.000 | 0.962 | 1.000 | 0.964 | 0.854 | 1.000 | 1.000 | 0.855 | 0.826 |
| 2532 | 0.771 | 1.000 | 0.781 | 0.986 | 0.963 | 0.960 | 0.903 | 1.000 | 0.990 | 1.000 | 0.959 | 1.000 | 0.964 | 0.856 | 1.000 | 0.981 | 0.854 | 0.825 |
| 2533 | 0.769 | 1.000 | 0.800 | 0.986 | 0.958 | 0.960 | 0.895 | 1.000 | 0.990 | 1.000 | 0.957 | 1.000 | 0.973 | 0.857 | 1.000 | 1.000 | 0.854 | 0.825 |

| | | | | | | | | | | | | | | | | | |
|---|---|---|---|---|---|---|---|---|---|---|---|---|---|---|---|---|---|
| 2534 | 0.772 | 1.000 | 0.804 | 1.000 | 0.968 | 0.960 | 0.904 | 1.000 | 0.990 | 1.000 | 0.954 | 1.000 | 0.955 | 0.846 | 1.000 | 1.000 | 0.854 | 0.825 |
| 2535 | 0.771 | 1.000 | 0.796 | 1.000 | 0.958 | 0.960 | 0.904 | 1.000 | 0.990 | 1.000 | 0.962 | 1.000 | 0.973 | 0.858 | 1.000 | 1.000 | 0.857 | 0.828 |
| 2536 | 0.765 | 1.000 | 0.791 | 1.000 | 0.968 | 0.960 | 0.904 | 1.000 | 0.990 | 1.000 | 0.965 | 1.000 | 0.991 | 0.871 | 1.000 | 1.000 | 0.859 | 0.831 |
| 2537 | 0.777 | 1.000 | 0.791 | 1.000 | 0.963 | 0.960 | 0.901 | 1.000 | 0.980 | 1.000 | 0.959 | 1.000 | 0.982 | 0.858 | 1.000 | 1.000 | 0.858 | 0.829 |
| 2538 | 0.767 | 1.000 | 0.802 | 1.000 | 0.958 | 0.960 | 0.894 | 1.000 | 0.990 | 1.000 | 0.962 | 1.000 | 0.955 | 0.852 | 1.000 | 1.000 | 0.853 | 0.824 |
| 2539 | 0.765 | 1.000 | 0.798 | 1.000 | 0.953 | 0.960 | 0.904 | 1.000 | 0.980 | 1.000 | 0.959 | 1.000 | 0.991 | 0.858 | 1.000 | 0.981 | 0.855 | 0.826 |
| 2540 | 0.773 | 1.000 | 0.791 | 1.000 | 0.968 | 0.960 | 0.895 | 1.000 | 1.000 | 1.000 | 0.948 | 1.000 | 0.982 | 0.855 | 1.000 | 1.000 | 0.855 | 0.826 |
| 2541 | 0.767 | 1.000 | 0.791 | 1.000 | 0.968 | 0.960 | 0.906 | 1.000 | 0.980 | 1.000 | 0.954 | 1.000 | 0.964 | 0.861 | 1.000 | 1.000 | 0.855 | 0.827 |
| 2542 | 0.761 | 1.000 | 0.785 | 0.986 | 0.963 | 0.960 | 0.901 | 1.000 | 1.000 | 1.000 | 0.959 | 1.000 | 0.964 | 0.857 | 1.000 | 1.000 | 0.852 | 0.822 |
| 2543 | 0.776 | 1.000 | 0.791 | 1.000 | 0.963 | 0.960 | 0.894 | 1.000 | 0.980 | 1.000 | 0.965 | 1.000 | 0.973 | 0.853 | 1.000 | 1.000 | 0.855 | 0.826 |
| 2544 | 0.773 | 1.000 | 0.798 | 1.000 | 0.963 | 0.960 | 0.886 | 1.000 | 0.990 | 1.000 | 0.962 | 1.000 | 0.964 | 0.861 | 1.000 | 1.000 | 0.856 | 0.827 |
| 2545 | 0.762 | 1.000 | 0.791 | 1.000 | 0.963 | 0.960 | 0.897 | 1.000 | 0.980 | 1.000 | 0.959 | 1.000 | 0.955 | 0.861 | 1.000 | 1.000 | 0.853 | 0.824 |
| 2546 | 0.771 | 1.000 | 0.806 | 0.971 | 0.963 | 0.960 | 0.898 | 1.000 | 0.990 | 1.000 | 0.962 | 1.000 | 0.964 | 0.858 | 1.000 | 1.000 | 0.856 | 0.828 |
| 2547 | 0.779 | 1.000 | 0.816 | 1.000 | 0.958 | 0.960 | 0.900 | 1.000 | 0.980 | 1.000 | 0.959 | 1.000 | 0.964 | 0.861 | 1.000 | 1.000 | 0.861 | 0.833 |
| 2548 | 0.764 | 1.000 | 0.796 | 1.000 | 0.963 | 0.960 | 0.894 | 1.000 | 0.990 | 1.000 | 0.957 | 1.000 | 0.973 | 0.859 | 1.000 | 1.000 | 0.853 | 0.824 |
| 2549 | 0.775 | 1.000 | 0.796 | 0.986 | 0.963 | 0.960 | 0.895 | 1.000 | 0.990 | 1.000 | 0.962 | 1.000 | 0.973 | 0.857 | 1.000 | 1.000 | 0.856 | 0.828 |
| 2550 | 0.767 | 1.000 | 0.802 | 1.000 | 0.958 | 0.960 | 0.892 | 1.000 | 0.990 | 1.000 | 0.962 | 1.000 | 0.973 | 0.859 | 1.000 | 1.000 | 0.855 | 0.826 |
| 2551 | 0.763 | 1.000 | 0.792 | 1.000 | 0.958 | 0.960 | 0.894 | 1.000 | 0.980 | 1.000 | 0.957 | 1.000 | 0.964 | 0.856 | 1.000 | 1.000 | 0.851 | 0.822 |
| 2552 | 0.776 | 1.000 | 0.798 | 0.986 | 0.963 | 0.960 | 0.901 | 1.000 | 1.000 | 1.000 | 0.968 | 1.000 | 0.973 | 0.864 | 1.000 | 1.000 | 0.860 | 0.832 |
| 2553 | 0.770 | 1.000 | 0.808 | 1.000 | 0.953 | 0.960 | 0.904 | 1.000 | 0.990 | 1.000 | 0.968 | 1.000 | 0.938 | 0.858 | 1.000 | 0.981 | 0.857 | 0.829 |
| 2554 | 0.765 | 1.000 | 0.804 | 1.000 | 0.958 | 0.960 | 0.897 | 1.000 | 0.980 | 1.000 | 0.959 | 1.000 | 0.964 | 0.852 | 1.000 | 1.000 | 0.853 | 0.823 |
| 2555 | 0.767 | 1.000 | 0.789 | 0.986 | 0.963 | 0.960 | 0.901 | 1.000 | 0.990 | 1.000 | 0.962 | 1.000 | 0.964 | 0.863 | 1.000 | 0.981 | 0.855 | 0.827 |
| 2556 | 0.754 | 1.000 | 0.812 | 1.000 | 0.968 | 0.960 | 0.894 | 1.000 | 0.990 | 1.000 | 0.962 | 1.000 | 0.973 | 0.850 | 1.000 | 1.000 | 0.850 | 0.821 |
| 2557 | 0.768 | 1.000 | 0.791 | 0.986 | 0.968 | 0.960 | 0.895 | 1.000 | 0.990 | 1.000 | 0.965 | 1.000 | 0.955 | 0.854 | 1.000 | 1.000 | 0.853 | 0.824 |
| 2558 | 0.773 | 1.000 | 0.802 | 0.986 | 0.963 | 0.960 | 0.900 | 1.000 | 0.990 | 1.000 | 0.957 | 1.000 | 0.955 | 0.858 | 1.000 | 1.000 | 0.857 | 0.828 |
| 2559 | 0.775 | 1.000 | 0.798 | 1.000 | 0.963 | 0.960 | 0.895 | 1.000 | 0.990 | 1.000 | 0.971 | 1.000 | 0.955 | 0.863 | 1.000 | 1.000 | 0.859 | 0.831 |
| 2560 | 0.777 | 1.000 | 0.800 | 1.000 | 0.958 | 0.960 | 0.906 | 1.000 | 0.990 | 1.000 | 0.959 | 1.000 | 0.955 | 0.858 | 1.000 | 1.000 | 0.859 | 0.830 |
| 2561 | 0.763 | 1.000 | 0.779 | 1.000 | 0.968 | 0.960 | 0.895 | 1.000 | 1.000 | 1.000 | 0.962 | 1.000 | 0.955 | 0.860 | 1.000 | 1.000 | 0.852 | 0.823 |
| 2562 | 0.771 | 1.000 | 0.810 | 1.000 | 0.968 | 0.960 | 0.897 | 1.000 | 0.990 | 1.000 | 0.957 | 0.970 | 0.964 | 0.855 | 1.000 | 1.000 | 0.856 | 0.828 |
| 2563 | 0.771 | 1.000 | 0.800 | 1.000 | 0.958 | 0.960 | 0.897 | 1.000 | 0.980 | 1.000 | 0.957 | 0.970 | 0.964 | 0.856 | 1.000 | 1.000 | 0.855 | 0.825 |
| 2564 | 0.773 | 1.000 | 0.802 | 1.000 | 0.963 | 0.960 | 0.898 | 1.000 | 0.980 | 1.000 | 0.962 | 1.000 | 0.964 | 0.852 | 1.000 | 1.000 | 0.856 | 0.827 |
| 2565 | 0.765 | 1.000 | 0.806 | 1.000 | 0.974 | 0.960 | 0.904 | 1.000 | 0.990 | 1.000 | 0.962 | 1.000 | 0.964 | 0.854 | 1.000 | 1.000 | 0.855 | 0.827 |
| 2566 | 0.773 | 1.000 | 0.789 | 0.986 | 0.958 | 0.960 | 0.888 | 1.000 | 0.990 | 1.000 | 0.954 | 1.000 | 0.955 | 0.854 | 1.000 | 1.000 | 0.852 | 0.822 |
| 2567 | 0.767 | 1.000 | 0.787 | 0.986 | 0.963 | 0.960 | 0.911 | 1.000 | 0.980 | 1.000 | 0.962 | 1.000 | 0.955 | 0.858 | 1.000 | 1.000 | 0.855 | 0.826 |
| 2568 | 0.762 | 1.000 | 0.802 | 1.000 | 0.963 | 0.960 | 0.889 | 1.000 | 1.000 | 1.000 | 0.965 | 1.000 | 0.964 | 0.854 | 1.000 | 1.000 | 0.852 | 0.823 |
| 2569 | 0.763 | 1.000 | 0.783 | 1.000 | 0.958 | 0.960 | 0.903 | 1.000 | 0.990 | 1.000 | 0.965 | 1.000 | 0.982 | 0.856 | 1.000 | 1.000 | 0.853 | 0.824 |
| 2570 | 0.776 | 1.000 | 0.798 | 1.000 | 0.963 | 0.960 | 0.909 | 1.000 | 0.980 | 1.000 | 0.965 | 1.000 | 0.964 | 0.859 | 1.000 | 1.000 | 0.859 | 0.831 |
| 2571 | 0.773 | 1.000 | 0.808 | 1.000 | 0.968 | 0.960 | 0.903 | 1.000 | 0.990 | 1.000 | 0.948 | 1.000 | 0.964 | 0.860 | 1.000 | 1.000 | 0.858 | 0.830 |
| 2572 | 0.766 | 1.000 | 0.794 | 0.986 | 0.963 | 0.960 | 0.898 | 1.000 | 0.990 | 1.000 | 0.965 | 1.000 | 0.982 | 0.854 | 1.000 | 1.000 | 0.854 | 0.824 |

| | | | | | | | | | | | | | | | | | | |
|---|---|---|---|---|---|---|---|---|---|---|---|---|---|---|---|---|---|---|
| 2573 | 0.776 | 1.000 | 0.792 | 1.000 | 0.974 | 0.960 | 0.897 | 1.000 | 1.000 | 1.000 | 0.954 | 1.000 | 0.964 | 0.850 | 1.000 | 1.000 | 0.855 | 0.826 |
| 2574 | 0.769 | 1.000 | 0.785 | 1.000 | 0.963 | 0.960 | 0.897 | 1.000 | 0.990 | 1.000 | 0.962 | 1.000 | 0.973 | 0.858 | 1.000 | 1.000 | 0.854 | 0.825 |
| 2575 | 0.771 | 1.000 | 0.798 | 1.000 | 0.953 | 0.960 | 0.891 | 1.000 | 0.990 | 1.000 | 0.959 | 1.000 | 0.973 | 0.863 | 1.000 | 1.000 | 0.856 | 0.827 |
| 2576 | 0.770 | 1.000 | 0.800 | 1.000 | 0.963 | 0.960 | 0.901 | 1.000 | 0.990 | 1.000 | 0.962 | 1.000 | 0.955 | 0.847 | 1.000 | 0.981 | 0.853 | 0.824 |
| 2577 | 0.771 | 1.000 | 0.798 | 0.986 | 0.963 | 0.960 | 0.894 | 1.000 | 0.980 | 1.000 | 0.962 | 1.000 | 0.955 | 0.865 | 1.000 | 1.000 | 0.857 | 0.828 |
| 2578 | 0.777 | 1.000 | 0.800 | 1.000 | 0.958 | 0.960 | 0.906 | 1.000 | 0.980 | 1.000 | 0.959 | 1.000 | 0.964 | 0.858 | 1.000 | 1.000 | 0.859 | 0.830 |
| 2579 | 0.769 | 1.000 | 0.794 | 0.986 | 0.963 | 0.960 | 0.892 | 1.000 | 0.990 | 1.000 | 0.959 | 1.000 | 0.973 | 0.860 | 1.000 | 1.000 | 0.855 | 0.826 |
| 2580 | 0.774 | 1.000 | 0.789 | 0.986 | 0.963 | 0.960 | 0.898 | 1.000 | 0.990 | 1.000 | 0.957 | 1.000 | 0.964 | 0.859 | 1.000 | 0.981 | 0.856 | 0.826 |
| 2581 | 0.779 | 1.000 | 0.789 | 0.986 | 0.968 | 0.960 | 0.901 | 1.000 | 0.990 | 1.000 | 0.968 | 1.000 | 0.964 | 0.860 | 1.000 | 1.000 | 0.859 | 0.831 |
| 2582 | 0.772 | 1.000 | 0.792 | 1.000 | 0.958 | 0.960 | 0.914 | 1.000 | 0.980 | 1.000 | 0.965 | 1.000 | 0.964 | 0.855 | 1.000 | 1.000 | 0.857 | 0.828 |
| 2583 | 0.770 | 1.000 | 0.800 | 1.000 | 0.963 | 0.960 | 0.903 | 1.000 | 0.980 | 1.000 | 0.962 | 1.000 | 0.973 | 0.855 | 1.000 | 1.000 | 0.856 | 0.827 |
| 2584 | 0.767 | 1.000 | 0.794 | 1.000 | 0.958 | 0.960 | 0.911 | 1.000 | 0.980 | 1.000 | 0.951 | 1.000 | 0.982 | 0.857 | 1.000 | 1.000 | 0.855 | 0.826 |
| 2585 | 0.775 | 1.000 | 0.787 | 1.000 | 0.968 | 0.960 | 0.894 | 1.000 | 0.980 | 1.000 | 0.954 | 1.000 | 0.964 | 0.857 | 1.000 | 1.000 | 0.855 | 0.825 |
| 2586 | 0.761 | 1.000 | 0.810 | 1.000 | 0.963 | 0.960 | 0.894 | 1.000 | 0.990 | 1.000 | 0.962 | 1.000 | 0.973 | 0.851 | 1.000 | 1.000 | 0.852 | 0.823 |
| 2587 | 0.769 | 1.000 | 0.804 | 1.000 | 0.963 | 0.960 | 0.901 | 1.000 | 0.980 | 1.000 | 0.957 | 1.000 | 0.964 | 0.856 | 1.000 | 1.000 | 0.856 | 0.827 |
| 2588 | 0.763 | 1.000 | 0.775 | 1.000 | 0.963 | 0.960 | 0.900 | 1.000 | 0.980 | 1.000 | 0.954 | 0.970 | 0.955 | 0.850 | 1.000 | 1.000 | 0.848 | 0.818 |
| 2589 | 0.773 | 1.000 | 0.794 | 0.986 | 0.958 | 0.960 | 0.901 | 1.000 | 1.000 | 1.000 | 0.957 | 1.000 | 0.955 | 0.855 | 1.000 | 1.000 | 0.855 | 0.826 |
| 2590 | 0.769 | 1.000 | 0.785 | 1.000 | 0.963 | 0.960 | 0.906 | 1.000 | 0.980 | 1.000 | 0.957 | 1.000 | 0.982 | 0.859 | 1.000 | 1.000 | 0.855 | 0.826 |
| 2591 | 0.769 | 1.000 | 0.808 | 0.986 | 0.968 | 0.960 | 0.901 | 1.000 | 0.990 | 1.000 | 0.957 | 1.000 | 0.982 | 0.862 | 1.000 | 1.000 | 0.858 | 0.830 |
| 2592 | 0.772 | 1.000 | 0.789 | 0.986 | 0.963 | 0.960 | 0.891 | 1.000 | 0.990 | 1.000 | 0.959 | 1.000 | 0.964 | 0.860 | 1.000 | 1.000 | 0.855 | 0.825 |
| 2593 | 0.775 | 1.000 | 0.808 | 1.000 | 0.963 | 0.960 | 0.908 | 1.000 | 0.990 | 1.000 | 0.965 | 1.000 | 0.946 | 0.858 | 1.000 | 1.000 | 0.859 | 0.831 |
| 2594 | 0.780 | 1.000 | 0.779 | 1.000 | 0.958 | 0.960 | 0.901 | 1.000 | 0.980 | 1.000 | 0.965 | 1.000 | 0.973 | 0.854 | 1.000 | 1.000 | 0.856 | 0.828 |
| 2595 | 0.770 | 1.000 | 0.806 | 1.000 | 0.963 | 0.960 | 0.912 | 1.000 | 1.000 | 1.000 | 0.959 | 1.000 | 0.973 | 0.868 | 1.000 | 1.000 | 0.861 | 0.834 |
| 2596 | 0.778 | 1.000 | 0.789 | 1.000 | 0.968 | 0.960 | 0.898 | 1.000 | 0.990 | 1.000 | 0.962 | 1.000 | 0.964 | 0.854 | 1.000 | 1.000 | 0.856 | 0.827 |
| 2597 | 0.765 | 1.000 | 0.804 | 0.986 | 0.963 | 0.960 | 0.894 | 1.000 | 0.980 | 1.000 | 0.965 | 1.000 | 0.982 | 0.851 | 1.000 | 1.000 | 0.853 | 0.823 |
| 2598 | 0.765 | 1.000 | 0.806 | 1.000 | 0.963 | 0.960 | 0.906 | 1.000 | 0.980 | 1.000 | 0.962 | 1.000 | 0.964 | 0.862 | 1.000 | 1.000 | 0.857 | 0.829 |
| 2599 | 0.767 | 1.000 | 0.785 | 1.000 | 0.968 | 0.960 | 0.909 | 1.000 | 1.000 | 1.000 | 0.965 | 1.000 | 0.973 | 0.851 | 1.000 | 1.000 | 0.854 | 0.825 |
| 2600 | 0.769 | 1.000 | 0.802 | 0.986 | 0.963 | 0.960 | 0.889 | 1.000 | 0.980 | 1.000 | 0.959 | 1.000 | 0.964 | 0.851 | 1.000 | 1.000 | 0.853 | 0.823 |
| 2601 | 0.771 | 1.000 | 0.818 | 1.000 | 0.968 | 0.960 | 0.895 | 1.000 | 0.990 | 1.000 | 0.971 | 0.970 | 0.964 | 0.861 | 1.000 | 1.000 | 0.859 | 0.831 |
| 2602 | 0.775 | 1.000 | 0.812 | 1.000 | 0.974 | 0.960 | 0.898 | 1.000 | 0.990 | 1.000 | 0.965 | 1.000 | 0.964 | 0.856 | 1.000 | 1.000 | 0.859 | 0.831 |
| 2603 | 0.771 | 1.000 | 0.794 | 0.986 | 0.963 | 0.960 | 0.891 | 1.000 | 0.990 | 1.000 | 0.962 | 1.000 | 0.973 | 0.859 | 1.000 | 1.000 | 0.855 | 0.826 |
| 2604 | 0.775 | 1.000 | 0.783 | 1.000 | 0.963 | 0.960 | 0.903 | 1.000 | 0.980 | 1.000 | 0.962 | 1.000 | 0.955 | 0.858 | 1.000 | 1.000 | 0.856 | 0.827 |
| 2605 | 0.765 | 1.000 | 0.802 | 1.000 | 0.968 | 0.960 | 0.900 | 1.000 | 0.990 | 1.000 | 0.962 | 1.000 | 0.973 | 0.857 | 1.000 | 1.000 | 0.855 | 0.827 |
| 2606 | 0.764 | 1.000 | 0.804 | 1.000 | 0.974 | 0.960 | 0.904 | 1.000 | 0.980 | 1.000 | 0.959 | 1.000 | 0.964 | 0.865 | 1.000 | 1.000 | 0.857 | 0.829 |
| 2607 | 0.765 | 1.000 | 0.792 | 1.000 | 0.974 | 0.960 | 0.900 | 1.000 | 0.980 | 1.000 | 0.957 | 1.000 | 0.964 | 0.858 | 1.000 | 1.000 | 0.854 | 0.825 |
| 2608 | 0.778 | 1.000 | 0.794 | 0.986 | 0.963 | 0.960 | 0.895 | 1.000 | 0.980 | 1.000 | 0.957 | 1.000 | 0.982 | 0.856 | 1.000 | 1.000 | 0.857 | 0.828 |
| 2609 | 0.763 | 1.000 | 0.806 | 1.000 | 0.968 | 0.960 | 0.901 | 1.000 | 1.000 | 1.000 | 0.962 | 1.000 | 0.973 | 0.857 | 1.000 | 1.000 | 0.855 | 0.827 |
| 2610 | 0.769 | 1.000 | 0.791 | 1.000 | 0.963 | 0.960 | 0.901 | 1.000 | 0.990 | 1.000 | 0.957 | 1.000 | 0.964 | 0.851 | 1.000 | 1.000 | 0.853 | 0.824 |
| 2611 | 0.761 | 1.000 | 0.791 | 1.000 | 0.958 | 0.960 | 0.888 | 1.000 | 0.990 | 1.000 | 0.965 | 1.000 | 0.973 | 0.858 | 1.000 | 1.000 | 0.851 | 0.821 |

| | | | | | | | | | | | | | | | | | |
|---|---|---|---|---|---|---|---|---|---|---|---|---|---|---|---|---|---|
| 2612 | 0.769 | 1.000 | 0.791 | 1.000 | 0.958 | 0.960 | 0.901 | 1.000 | 0.980 | 1.000 | 0.962 | 1.000 | 0.973 | 0.857 | 1.000 | 1.000 | 0.855 | 0.826 |
| 2613 | 0.771 | 1.000 | 0.802 | 1.000 | 0.963 | 0.960 | 0.898 | 1.000 | 0.990 | 1.000 | 0.965 | 1.000 | 0.964 | 0.860 | 1.000 | 1.000 | 0.857 | 0.829 |
| 2614 | 0.773 | 1.000 | 0.785 | 0.986 | 0.958 | 0.960 | 0.906 | 1.000 | 0.969 | 1.000 | 0.957 | 1.000 | 0.982 | 0.861 | 1.000 | 1.000 | 0.856 | 0.827 |
| 2615 | 0.780 | 1.000 | 0.798 | 1.000 | 0.958 | 0.960 | 0.900 | 1.000 | 0.980 | 1.000 | 0.971 | 1.000 | 0.964 | 0.856 | 1.000 | 1.000 | 0.859 | 0.830 |
| 2616 | 0.771 | 1.000 | 0.800 | 0.986 | 0.958 | 0.960 | 0.908 | 1.000 | 1.000 | 1.000 | 0.962 | 1.000 | 0.973 | 0.854 | 1.000 | 1.000 | 0.857 | 0.828 |
| 2617 | 0.765 | 1.000 | 0.800 | 0.986 | 0.968 | 0.960 | 0.914 | 1.000 | 0.980 | 1.000 | 0.962 | 1.000 | 0.973 | 0.858 | 1.000 | 1.000 | 0.857 | 0.828 |
| 2618 | 0.772 | 1.000 | 0.789 | 1.000 | 0.958 | 0.960 | 0.891 | 1.000 | 0.990 | 1.000 | 0.959 | 1.000 | 0.973 | 0.859 | 1.000 | 1.000 | 0.855 | 0.825 |
| 2619 | 0.776 | 1.000 | 0.794 | 1.000 | 0.968 | 0.960 | 0.897 | 1.000 | 0.980 | 1.000 | 0.951 | 1.000 | 0.973 | 0.857 | 1.000 | 0.981 | 0.856 | 0.827 |
| 2620 | 0.770 | 1.000 | 0.798 | 1.000 | 0.963 | 0.960 | 0.901 | 1.000 | 0.980 | 1.000 | 0.959 | 1.000 | 0.955 | 0.847 | 1.000 | 1.000 | 0.853 | 0.824 |
| 2621 | 0.775 | 1.000 | 0.785 | 1.000 | 0.974 | 0.960 | 0.892 | 1.000 | 0.990 | 1.000 | 0.968 | 1.000 | 0.955 | 0.857 | 1.000 | 1.000 | 0.856 | 0.826 |
| 2622 | 0.767 | 1.000 | 0.804 | 0.986 | 0.958 | 0.960 | 0.898 | 1.000 | 1.000 | 1.000 | 0.962 | 1.000 | 0.982 | 0.857 | 1.000 | 0.981 | 0.855 | 0.827 |
| 2623 | 0.770 | 1.000 | 0.806 | 1.000 | 0.963 | 0.960 | 0.895 | 1.000 | 0.990 | 1.000 | 0.959 | 1.000 | 0.973 | 0.857 | 1.000 | 1.000 | 0.856 | 0.827 |
| 2624 | 0.763 | 1.000 | 0.791 | 1.000 | 0.963 | 0.960 | 0.903 | 1.000 | 0.980 | 1.000 | 0.971 | 1.000 | 0.991 | 0.856 | 1.000 | 1.000 | 0.854 | 0.825 |
| 2625 | 0.772 | 1.000 | 0.785 | 0.986 | 0.953 | 0.960 | 0.894 | 1.000 | 0.980 | 1.000 | 0.962 | 1.000 | 0.964 | 0.858 | 1.000 | 1.000 | 0.854 | 0.824 |
| 2626 | 0.777 | 1.000 | 0.804 | 0.986 | 0.958 | 0.960 | 0.906 | 1.000 | 0.990 | 1.000 | 0.971 | 1.000 | 0.973 | 0.854 | 1.000 | 1.000 | 0.859 | 0.831 |
| 2627 | 0.771 | 1.000 | 0.802 | 0.986 | 0.968 | 0.960 | 0.908 | 1.000 | 0.980 | 1.000 | 0.957 | 1.000 | 0.973 | 0.854 | 1.000 | 0.981 | 0.856 | 0.828 |
| 2628 | 0.781 | 1.000 | 0.808 | 1.000 | 0.963 | 0.960 | 0.906 | 1.000 | 0.990 | 1.000 | 0.965 | 1.000 | 0.955 | 0.865 | 1.000 | 1.000 | 0.863 | 0.835 |
| 2629 | 0.763 | 1.000 | 0.794 | 1.000 | 0.968 | 0.960 | 0.906 | 1.000 | 0.990 | 1.000 | 0.962 | 0.970 | 0.982 | 0.858 | 1.000 | 1.000 | 0.855 | 0.826 |
| 2630 | 0.767 | 1.000 | 0.792 | 1.000 | 0.974 | 0.960 | 0.903 | 1.000 | 0.990 | 1.000 | 0.962 | 1.000 | 0.982 | 0.850 | 1.000 | 1.000 | 0.854 | 0.825 |
| 2631 | 0.766 | 1.000 | 0.794 | 1.000 | 0.968 | 0.960 | 0.904 | 1.000 | 0.980 | 1.000 | 0.959 | 1.000 | 0.964 | 0.860 | 1.000 | 1.000 | 0.855 | 0.827 |
| 2632 | 0.773 | 1.000 | 0.794 | 0.986 | 0.963 | 0.960 | 0.903 | 1.000 | 0.990 | 1.000 | 0.962 | 1.000 | 0.973 | 0.858 | 1.000 | 1.000 | 0.857 | 0.829 |
| 2633 | 0.765 | 1.000 | 0.792 | 0.986 | 0.958 | 0.960 | 0.895 | 1.000 | 1.000 | 1.000 | 0.957 | 1.000 | 0.973 | 0.852 | 1.000 | 1.000 | 0.852 | 0.822 |
| 2634 | 0.773 | 1.000 | 0.796 | 1.000 | 0.963 | 0.960 | 0.894 | 1.000 | 0.990 | 1.000 | 0.951 | 1.000 | 0.955 | 0.858 | 1.000 | 1.000 | 0.855 | 0.826 |
| 2635 | 0.775 | 1.000 | 0.781 | 0.986 | 0.968 | 0.960 | 0.891 | 1.000 | 0.980 | 1.000 | 0.959 | 1.000 | 0.964 | 0.856 | 1.000 | 1.000 | 0.854 | 0.824 |
| 2636 | 0.764 | 1.000 | 0.794 | 1.000 | 0.968 | 0.960 | 0.900 | 1.000 | 0.980 | 1.000 | 0.959 | 1.000 | 0.964 | 0.850 | 1.000 | 0.981 | 0.851 | 0.822 |
| 2637 | 0.777 | 1.000 | 0.792 | 1.000 | 0.979 | 0.960 | 0.911 | 1.000 | 0.990 | 1.000 | 0.962 | 1.000 | 0.955 | 0.860 | 1.000 | 1.000 | 0.860 | 0.832 |
| 2638 | 0.768 | 1.000 | 0.792 | 1.000 | 0.958 | 0.960 | 0.884 | 1.000 | 0.990 | 1.000 | 0.951 | 1.000 | 0.982 | 0.858 | 1.000 | 1.000 | 0.852 | 0.822 |
| 2639 | 0.769 | 1.000 | 0.796 | 1.000 | 0.958 | 0.960 | 0.900 | 1.000 | 0.990 | 1.000 | 0.954 | 1.000 | 0.973 | 0.861 | 1.000 | 0.981 | 0.856 | 0.827 |
| 2640 | 0.777 | 1.000 | 0.779 | 1.000 | 0.963 | 0.960 | 0.895 | 1.000 | 0.990 | 1.000 | 0.968 | 1.000 | 0.982 | 0.854 | 1.000 | 1.000 | 0.855 | 0.826 |
| 2641 | 0.765 | 1.000 | 0.791 | 0.986 | 0.963 | 0.960 | 0.908 | 1.000 | 1.000 | 1.000 | 0.957 | 1.000 | 0.955 | 0.860 | 1.000 | 1.000 | 0.855 | 0.826 |
| 2642 | 0.769 | 1.000 | 0.792 | 0.986 | 0.958 | 0.960 | 0.895 | 1.000 | 0.980 | 1.000 | 0.959 | 1.000 | 0.982 | 0.854 | 1.000 | 0.981 | 0.853 | 0.824 |
| 2643 | 0.775 | 1.000 | 0.783 | 1.000 | 0.968 | 0.960 | 0.897 | 1.000 | 0.990 | 1.000 | 0.962 | 1.000 | 0.982 | 0.860 | 1.000 | 1.000 | 0.857 | 0.828 |
| 2644 | 0.763 | 1.000 | 0.791 | 1.000 | 0.958 | 0.960 | 0.900 | 1.000 | 1.000 | 1.000 | 0.959 | 1.000 | 0.982 | 0.853 | 1.000 | 1.000 | 0.852 | 0.823 |
| 2645 | 0.767 | 1.000 | 0.781 | 1.000 | 0.963 | 0.960 | 0.911 | 1.000 | 0.980 | 1.000 | 0.959 | 1.000 | 0.982 | 0.863 | 1.000 | 1.000 | 0.856 | 0.827 |
| 2646 | 0.767 | 1.000 | 0.802 | 0.986 | 0.968 | 0.960 | 0.900 | 1.000 | 0.990 | 1.000 | 0.957 | 1.000 | 0.973 | 0.863 | 1.000 | 1.000 | 0.857 | 0.828 |
| 2647 | 0.769 | 1.000 | 0.804 | 0.986 | 0.963 | 0.960 | 0.892 | 1.000 | 0.990 | 1.000 | 0.965 | 1.000 | 0.955 | 0.857 | 1.000 | 1.000 | 0.855 | 0.826 |
| 2648 | 0.770 | 1.000 | 0.789 | 1.000 | 0.968 | 0.960 | 0.906 | 1.000 | 1.000 | 1.000 | 0.957 | 1.000 | 0.964 | 0.858 | 1.000 | 1.000 | 0.856 | 0.827 |
| 2649 | 0.764 | 1.000 | 0.796 | 1.000 | 0.963 | 0.960 | 0.897 | 1.000 | 0.990 | 1.000 | 0.962 | 1.000 | 0.973 | 0.861 | 1.000 | 1.000 | 0.855 | 0.826 |
| 2650 | 0.771 | 1.000 | 0.794 | 0.986 | 0.963 | 0.960 | 0.901 | 1.000 | 0.990 | 1.000 | 0.965 | 1.000 | 0.973 | 0.856 | 1.000 | 1.000 | 0.856 | 0.827 |

| | | | | | | | | | | | | | | | | | | |
|---|---|---|---|---|---|---|---|---|---|---|---|---|---|---|---|---|---|---|
| 2651 | 0.771 | 1.000 | 0.806 | 1.000 | 0.968 | 0.960 | 0.892 | 1.000 | 0.980 | 1.000 | 0.965 | 1.000 | 0.964 | 0.860 | 1.000 | 1.000 | 0.857 | 0.828 |
| 2652 | 0.771 | 1.000 | 0.794 | 1.000 | 0.958 | 0.960 | 0.908 | 1.000 | 0.990 | 1.000 | 0.965 | 1.000 | 0.964 | 0.863 | 1.000 | 1.000 | 0.858 | 0.830 |
| 2653 | 0.763 | 1.000 | 0.777 | 0.986 | 0.963 | 0.960 | 0.903 | 1.000 | 0.990 | 1.000 | 0.939 | 1.000 | 0.973 | 0.851 | 1.000 | 1.000 | 0.849 | 0.819 |
| 2654 | 0.780 | 1.000 | 0.789 | 0.986 | 0.958 | 0.960 | 0.903 | 1.000 | 0.990 | 1.000 | 0.954 | 1.000 | 0.955 | 0.851 | 1.000 | 1.000 | 0.856 | 0.827 |
| 2655 | 0.765 | 1.000 | 0.798 | 1.000 | 0.963 | 0.960 | 0.903 | 1.000 | 0.990 | 1.000 | 0.959 | 1.000 | 0.973 | 0.859 | 1.000 | 1.000 | 0.855 | 0.827 |
| 2656 | 0.767 | 1.000 | 0.798 | 1.000 | 0.974 | 0.960 | 0.914 | 1.000 | 0.980 | 1.000 | 0.959 | 1.000 | 0.973 | 0.854 | 1.000 | 1.000 | 0.856 | 0.828 |
| 2657 | 0.759 | 1.000 | 0.798 | 1.000 | 0.958 | 0.960 | 0.904 | 1.000 | 0.990 | 1.000 | 0.954 | 1.000 | 0.973 | 0.850 | 1.000 | 1.000 | 0.851 | 0.821 |
| 2658 | 0.771 | 1.000 | 0.800 | 1.000 | 0.963 | 0.960 | 0.895 | 1.000 | 0.980 | 1.000 | 0.962 | 1.000 | 0.973 | 0.857 | 1.000 | 1.000 | 0.856 | 0.827 |
| 2659 | 0.768 | 1.000 | 0.791 | 1.000 | 0.963 | 0.960 | 0.904 | 1.000 | 0.990 | 1.000 | 0.959 | 1.000 | 0.973 | 0.853 | 1.000 | 0.981 | 0.854 | 0.825 |
| 2660 | 0.767 | 1.000 | 0.771 | 1.000 | 0.958 | 0.960 | 0.906 | 1.000 | 1.000 | 1.000 | 0.959 | 1.000 | 0.955 | 0.856 | 1.000 | 1.000 | 0.852 | 0.823 |
| 2661 | 0.769 | 1.000 | 0.785 | 1.000 | 0.963 | 0.960 | 0.908 | 1.000 | 0.990 | 1.000 | 0.951 | 1.000 | 0.973 | 0.858 | 1.000 | 1.000 | 0.855 | 0.826 |
| 2662 | 0.773 | 1.000 | 0.812 | 0.986 | 0.963 | 0.960 | 0.904 | 1.000 | 0.969 | 1.000 | 0.954 | 1.000 | 0.982 | 0.859 | 1.000 | 1.000 | 0.858 | 0.830 |
| 2663 | 0.773 | 1.000 | 0.794 | 1.000 | 0.963 | 0.960 | 0.908 | 1.000 | 1.000 | 1.000 | 0.951 | 1.000 | 0.973 | 0.853 | 1.000 | 1.000 | 0.856 | 0.827 |
| 2664 | 0.770 | 1.000 | 0.789 | 1.000 | 0.968 | 0.960 | 0.897 | 1.000 | 0.990 | 1.000 | 0.957 | 1.000 | 0.973 | 0.857 | 1.000 | 0.981 | 0.854 | 0.825 |
| 2665 | 0.769 | 1.000 | 0.804 | 0.986 | 0.974 | 0.960 | 0.903 | 1.000 | 0.990 | 1.000 | 0.965 | 1.000 | 0.955 | 0.863 | 1.000 | 1.000 | 0.858 | 0.830 |
| 2666 | 0.770 | 1.000 | 0.791 | 1.000 | 0.963 | 0.960 | 0.898 | 1.000 | 1.000 | 1.000 | 0.954 | 1.000 | 0.973 | 0.850 | 1.000 | 1.000 | 0.853 | 0.823 |
| 2667 | 0.771 | 1.000 | 0.785 | 1.000 | 0.968 | 0.960 | 0.911 | 1.000 | 0.990 | 1.000 | 0.954 | 1.000 | 0.982 | 0.860 | 1.000 | 1.000 | 0.857 | 0.828 |
| 2668 | 0.765 | 1.000 | 0.800 | 1.000 | 0.968 | 0.960 | 0.898 | 1.000 | 0.990 | 1.000 | 0.965 | 1.000 | 0.964 | 0.854 | 1.000 | 1.000 | 0.854 | 0.825 |
| 2669 | 0.783 | 1.000 | 0.794 | 1.000 | 0.963 | 0.960 | 0.911 | 1.000 | 0.980 | 1.000 | 0.968 | 1.000 | 0.955 | 0.851 | 1.000 | 1.000 | 0.859 | 0.831 |
| 2670 | 0.780 | 1.000 | 0.779 | 1.000 | 0.968 | 0.960 | 0.898 | 1.000 | 0.990 | 1.000 | 0.959 | 1.000 | 0.973 | 0.854 | 1.000 | 1.000 | 0.856 | 0.827 |
| 2671 | 0.765 | 1.000 | 0.783 | 0.986 | 0.963 | 0.960 | 0.897 | 1.000 | 0.990 | 1.000 | 0.959 | 0.970 | 0.955 | 0.857 | 1.000 | 1.000 | 0.852 | 0.822 |
| 2672 | 0.775 | 1.000 | 0.808 | 1.000 | 0.958 | 0.960 | 0.906 | 1.000 | 0.980 | 1.000 | 0.951 | 1.000 | 0.982 | 0.850 | 1.000 | 0.981 | 0.856 | 0.828 |
| 2673 | 0.775 | 1.000 | 0.792 | 0.986 | 0.963 | 0.960 | 0.903 | 1.000 | 0.990 | 1.000 | 0.959 | 1.000 | 0.973 | 0.858 | 1.000 | 1.000 | 0.857 | 0.829 |
| 2674 | 0.768 | 1.000 | 0.808 | 0.986 | 0.958 | 0.960 | 0.908 | 1.000 | 1.000 | 1.000 | 0.959 | 1.000 | 0.982 | 0.855 | 1.000 | 1.000 | 0.857 | 0.829 |
| 2675 | 0.769 | 1.000 | 0.787 | 1.000 | 0.974 | 0.960 | 0.888 | 1.000 | 0.990 | 1.000 | 0.957 | 1.000 | 0.964 | 0.858 | 1.000 | 1.000 | 0.853 | 0.823 |
| 2676 | 0.771 | 1.000 | 0.792 | 0.971 | 0.968 | 1.000 | 0.897 | 1.000 | 0.990 | 1.000 | 0.962 | 1.000 | 0.982 | 0.856 | 1.000 | 1.000 | 0.855 | 0.826 |
| 2677 | 0.769 | 1.000 | 0.783 | 1.000 | 0.958 | 0.960 | 0.909 | 1.000 | 0.980 | 1.000 | 0.965 | 1.000 | 0.973 | 0.851 | 1.000 | 0.981 | 0.854 | 0.825 |
| 2678 | 0.770 | 1.000 | 0.781 | 1.000 | 0.974 | 0.960 | 0.894 | 1.000 | 0.990 | 1.000 | 0.968 | 1.000 | 0.955 | 0.851 | 1.000 | 1.000 | 0.852 | 0.823 |
| 2679 | 0.769 | 1.000 | 0.792 | 1.000 | 0.963 | 0.960 | 0.897 | 1.000 | 1.000 | 1.000 | 0.971 | 1.000 | 0.964 | 0.856 | 1.000 | 1.000 | 0.855 | 0.826 |
| 2680 | 0.775 | 1.000 | 0.800 | 1.000 | 0.958 | 0.960 | 0.903 | 1.000 | 0.980 | 1.000 | 0.951 | 1.000 | 0.982 | 0.860 | 1.000 | 1.000 | 0.858 | 0.829 |
| 2681 | 0.770 | 1.000 | 0.794 | 1.000 | 0.963 | 0.960 | 0.903 | 1.000 | 0.990 | 1.000 | 0.954 | 1.000 | 0.982 | 0.858 | 1.000 | 1.000 | 0.856 | 0.827 |
| 2682 | 0.773 | 1.000 | 0.792 | 0.986 | 0.958 | 0.960 | 0.900 | 1.000 | 0.990 | 1.000 | 0.959 | 1.000 | 0.964 | 0.858 | 1.000 | 0.981 | 0.855 | 0.826 |
| 2683 | 0.765 | 1.000 | 0.791 | 1.000 | 0.963 | 0.960 | 0.892 | 1.000 | 0.990 | 1.000 | 0.957 | 1.000 | 0.964 | 0.853 | 1.000 | 1.000 | 0.851 | 0.822 |
| 2684 | 0.760 | 1.000 | 0.804 | 1.000 | 0.958 | 0.960 | 0.895 | 1.000 | 1.000 | 1.000 | 0.965 | 1.000 | 0.982 | 0.854 | 1.000 | 1.000 | 0.853 | 0.823 |
| 2685 | 0.770 | 1.000 | 0.791 | 0.986 | 0.968 | 0.960 | 0.889 | 1.000 | 0.990 | 1.000 | 0.951 | 1.000 | 0.982 | 0.861 | 1.000 | 1.000 | 0.854 | 0.825 |
| 2686 | 0.778 | 1.000 | 0.800 | 1.000 | 0.958 | 0.960 | 0.895 | 1.000 | 0.990 | 1.000 | 0.948 | 1.000 | 0.982 | 0.854 | 1.000 | 0.981 | 0.856 | 0.827 |
| 2687 | 0.764 | 1.000 | 0.785 | 1.000 | 0.974 | 0.960 | 0.892 | 1.000 | 0.990 | 1.000 | 0.954 | 1.000 | 0.973 | 0.858 | 1.000 | 1.000 | 0.852 | 0.822 |
| 2688 | 0.771 | 1.000 | 0.800 | 0.986 | 0.963 | 0.960 | 0.909 | 1.000 | 0.990 | 1.000 | 0.968 | 1.000 | 0.964 | 0.858 | 1.000 | 1.000 | 0.858 | 0.830 |
| 2689 | 0.775 | 1.000 | 0.798 | 0.971 | 0.958 | 0.960 | 0.891 | 1.000 | 1.000 | 1.000 | 0.962 | 1.000 | 0.973 | 0.851 | 1.000 | 1.000 | 0.854 | 0.825 |

| | | | | | | | | | | | | | | | | | |
|---|---|---|---|---|---|---|---|---|---|---|---|---|---|---|---|---|---|
| 2690 | 0.766 | 1.000 | 0.794 | 1.000 | 0.963 | 0.960 | 0.898 | 1.000 | 0.990 | 1.000 | 0.959 | 1.000 | 0.982 | 0.858 | 1.000 | 1.000 | 0.854 | 0.826 |
| 2691 | 0.779 | 1.000 | 0.787 | 1.000 | 0.968 | 0.960 | 0.891 | 1.000 | 0.980 | 1.000 | 0.959 | 1.000 | 0.982 | 0.847 | 1.000 | 1.000 | 0.854 | 0.824 |
| 2692 | 0.768 | 1.000 | 0.792 | 1.000 | 0.963 | 0.960 | 0.886 | 1.000 | 0.990 | 1.000 | 0.962 | 1.000 | 0.964 | 0.854 | 1.000 | 1.000 | 0.852 | 0.822 |
| 2693 | 0.771 | 1.000 | 0.800 | 1.000 | 0.963 | 0.960 | 0.894 | 1.000 | 0.980 | 1.000 | 0.951 | 1.000 | 0.964 | 0.852 | 1.000 | 1.000 | 0.853 | 0.824 |
| 2694 | 0.767 | 1.000 | 0.787 | 0.986 | 0.958 | 0.960 | 0.911 | 1.000 | 0.980 | 1.000 | 0.957 | 1.000 | 0.982 | 0.861 | 1.000 | 1.000 | 0.856 | 0.827 |
| 2695 | 0.775 | 1.000 | 0.783 | 0.986 | 0.963 | 0.960 | 0.892 | 1.000 | 1.000 | 1.000 | 0.957 | 1.000 | 0.973 | 0.851 | 1.000 | 1.000 | 0.853 | 0.823 |
| 2696 | 0.775 | 1.000 | 0.779 | 0.971 | 0.963 | 0.960 | 0.894 | 1.000 | 0.980 | 1.000 | 0.957 | 1.000 | 0.964 | 0.857 | 1.000 | 1.000 | 0.854 | 0.824 |
| 2697 | 0.769 | 1.000 | 0.796 | 0.986 | 0.963 | 0.960 | 0.911 | 1.000 | 0.990 | 1.000 | 0.959 | 1.000 | 0.964 | 0.861 | 1.000 | 1.000 | 0.857 | 0.829 |
| 2698 | 0.767 | 1.000 | 0.800 | 1.000 | 0.958 | 0.960 | 0.895 | 1.000 | 0.980 | 1.000 | 0.968 | 1.000 | 0.955 | 0.852 | 1.000 | 1.000 | 0.853 | 0.824 |
| 2699 | 0.759 | 1.000 | 0.792 | 1.000 | 0.968 | 0.960 | 0.901 | 1.000 | 0.990 | 1.000 | 0.954 | 1.000 | 0.973 | 0.854 | 1.000 | 1.000 | 0.851 | 0.822 |
| 2700 | 0.769 | 1.000 | 0.802 | 0.986 | 0.963 | 0.960 | 0.895 | 1.000 | 0.980 | 1.000 | 0.954 | 1.000 | 0.973 | 0.863 | 1.000 | 1.000 | 0.856 | 0.827 |
| 2701 | 0.769 | 1.000 | 0.789 | 1.000 | 0.968 | 0.960 | 0.908 | 1.000 | 0.990 | 1.000 | 0.959 | 1.000 | 0.955 | 0.855 | 1.000 | 1.000 | 0.855 | 0.826 |
| 2702 | 0.772 | 1.000 | 0.802 | 1.000 | 0.963 | 0.960 | 0.903 | 1.000 | 0.990 | 1.000 | 0.954 | 1.000 | 0.973 | 0.861 | 1.000 | 1.000 | 0.858 | 0.830 |
| 2703 | 0.767 | 1.000 | 0.792 | 0.986 | 0.968 | 0.960 | 0.892 | 1.000 | 0.980 | 1.000 | 0.965 | 1.000 | 0.982 | 0.855 | 1.000 | 1.000 | 0.853 | 0.824 |
| 2704 | 0.775 | 1.000 | 0.792 | 1.000 | 0.958 | 0.960 | 0.900 | 1.000 | 0.990 | 1.000 | 0.962 | 1.000 | 0.964 | 0.851 | 1.000 | 1.000 | 0.855 | 0.826 |
| 2705 | 0.768 | 1.000 | 0.812 | 0.986 | 0.968 | 0.960 | 0.901 | 1.000 | 0.969 | 1.000 | 0.965 | 1.000 | 0.973 | 0.862 | 1.000 | 1.000 | 0.858 | 0.830 |
| 2706 | 0.779 | 1.000 | 0.796 | 1.000 | 0.968 | 0.960 | 0.901 | 1.000 | 0.990 | 1.000 | 0.959 | 1.000 | 0.973 | 0.858 | 1.000 | 1.000 | 0.859 | 0.831 |
| 2707 | 0.766 | 1.000 | 0.794 | 0.986 | 0.958 | 0.960 | 0.906 | 1.000 | 0.990 | 1.000 | 0.957 | 1.000 | 0.982 | 0.853 | 1.000 | 1.000 | 0.854 | 0.824 |
| 2708 | 0.779 | 1.000 | 0.787 | 1.000 | 0.968 | 0.960 | 0.909 | 1.000 | 0.990 | 1.000 | 0.965 | 1.000 | 0.955 | 0.861 | 1.000 | 0.981 | 0.860 | 0.831 |
| 2709 | 0.773 | 1.000 | 0.791 | 0.986 | 0.958 | 0.960 | 0.901 | 1.000 | 1.000 | 1.000 | 0.957 | 1.000 | 0.964 | 0.855 | 1.000 | 1.000 | 0.855 | 0.826 |
| 2710 | 0.777 | 1.000 | 0.802 | 1.000 | 0.968 | 0.960 | 0.901 | 1.000 | 0.990 | 1.000 | 0.971 | 1.000 | 0.964 | 0.858 | 1.000 | 1.000 | 0.860 | 0.831 |
| 2711 | 0.769 | 1.000 | 0.796 | 0.986 | 0.953 | 0.960 | 0.897 | 1.000 | 0.990 | 1.000 | 0.954 | 1.000 | 0.964 | 0.857 | 1.000 | 1.000 | 0.854 | 0.825 |
| 2712 | 0.765 | 1.000 | 0.806 | 1.000 | 0.963 | 0.960 | 0.908 | 1.000 | 0.990 | 1.000 | 0.962 | 1.000 | 0.973 | 0.850 | 1.000 | 1.000 | 0.855 | 0.826 |
| 2713 | 0.772 | 1.000 | 0.808 | 0.986 | 0.963 | 0.960 | 0.883 | 1.000 | 0.980 | 1.000 | 0.959 | 1.000 | 0.982 | 0.851 | 1.000 | 1.000 | 0.854 | 0.824 |
| 2714 | 0.771 | 1.000 | 0.791 | 1.000 | 0.963 | 0.960 | 0.888 | 1.000 | 0.990 | 1.000 | 0.959 | 1.000 | 0.955 | 0.848 | 1.000 | 0.981 | 0.851 | 0.821 |
| 2715 | 0.767 | 1.000 | 0.789 | 1.000 | 0.963 | 0.960 | 0.900 | 1.000 | 0.980 | 1.000 | 0.957 | 1.000 | 0.964 | 0.856 | 1.000 | 1.000 | 0.853 | 0.824 |
| 2716 | 0.765 | 1.000 | 0.789 | 1.000 | 0.958 | 0.960 | 0.892 | 1.000 | 0.990 | 1.000 | 0.962 | 0.970 | 0.973 | 0.861 | 1.000 | 1.000 | 0.853 | 0.824 |
| 2717 | 0.765 | 1.000 | 0.789 | 0.986 | 0.963 | 0.960 | 0.892 | 1.000 | 0.990 | 1.000 | 0.957 | 1.000 | 0.973 | 0.851 | 1.000 | 1.000 | 0.850 | 0.820 |
| 2718 | 0.777 | 1.000 | 0.779 | 1.000 | 0.963 | 0.960 | 0.904 | 1.000 | 0.990 | 1.000 | 0.951 | 1.000 | 0.955 | 0.854 | 1.000 | 1.000 | 0.855 | 0.825 |
| 2719 | 0.769 | 1.000 | 0.810 | 1.000 | 0.963 | 0.960 | 0.895 | 1.000 | 0.990 | 1.000 | 0.962 | 1.000 | 0.964 | 0.853 | 1.000 | 0.981 | 0.855 | 0.826 |
| 2720 | 0.778 | 1.000 | 0.802 | 1.000 | 0.958 | 0.960 | 0.897 | 1.000 | 0.980 | 1.000 | 0.962 | 1.000 | 0.982 | 0.863 | 1.000 | 1.000 | 0.860 | 0.832 |
| 2721 | 0.768 | 1.000 | 0.798 | 1.000 | 0.963 | 0.960 | 0.906 | 1.000 | 0.969 | 1.000 | 0.962 | 1.000 | 0.955 | 0.861 | 1.000 | 1.000 | 0.857 | 0.828 |
| 2722 | 0.765 | 1.000 | 0.806 | 0.986 | 0.974 | 0.960 | 0.900 | 1.000 | 0.980 | 1.000 | 0.954 | 1.000 | 0.991 | 0.849 | 1.000 | 1.000 | 0.853 | 0.824 |
| 2723 | 0.767 | 1.000 | 0.789 | 1.000 | 0.963 | 0.960 | 0.906 | 1.000 | 0.990 | 1.000 | 0.962 | 1.000 | 0.982 | 0.856 | 1.000 | 1.000 | 0.855 | 0.826 |
| 2724 | 0.767 | 1.000 | 0.800 | 1.000 | 0.974 | 0.960 | 0.900 | 1.000 | 1.000 | 1.000 | 0.962 | 1.000 | 0.964 | 0.859 | 1.000 | 1.000 | 0.856 | 0.828 |
| 2725 | 0.775 | 1.000 | 0.806 | 1.000 | 0.963 | 0.960 | 0.894 | 1.000 | 0.980 | 1.000 | 0.962 | 1.000 | 0.982 | 0.861 | 1.000 | 1.000 | 0.859 | 0.830 |
| 2726 | 0.771 | 1.000 | 0.791 | 1.000 | 0.963 | 0.960 | 0.895 | 1.000 | 0.990 | 1.000 | 0.951 | 1.000 | 0.973 | 0.858 | 1.000 | 1.000 | 0.854 | 0.825 |
| 2727 | 0.763 | 1.000 | 0.779 | 1.000 | 0.963 | 0.960 | 0.898 | 1.000 | 0.990 | 1.000 | 0.974 | 1.000 | 0.973 | 0.857 | 1.000 | 1.000 | 0.852 | 0.823 |
| 2728 | 0.775 | 1.000 | 0.775 | 1.000 | 0.963 | 0.960 | 0.901 | 1.000 | 0.990 | 1.000 | 0.957 | 1.000 | 0.973 | 0.856 | 1.000 | 1.000 | 0.855 | 0.825 |

| | | | | | | | | | | | | | | | | | |
|---|---|---|---|---|---|---|---|---|---|---|---|---|---|---|---|---|---|
| 2729 | 0.767 | 1.000 | 0.798 | 0.986 | 0.968 | 0.960 | 0.892 | 1.000 | 1.000 | 1.000 | 0.965 | 1.000 | 0.955 | 0.853 | 1.000 | 1.000 | 0.853 | 0.824 |
| 2730 | 0.769 | 1.000 | 0.794 | 0.986 | 0.963 | 0.960 | 0.895 | 1.000 | 1.000 | 1.000 | 0.957 | 1.000 | 0.982 | 0.856 | 1.000 | 1.000 | 0.854 | 0.825 |
| 2731 | 0.766 | 1.000 | 0.794 | 0.986 | 0.963 | 0.960 | 0.912 | 1.000 | 0.990 | 1.000 | 0.962 | 1.000 | 0.955 | 0.854 | 1.000 | 1.000 | 0.855 | 0.826 |
| 2732 | 0.765 | 1.000 | 0.789 | 1.000 | 0.953 | 0.960 | 0.898 | 1.000 | 0.980 | 1.000 | 0.959 | 1.000 | 0.964 | 0.857 | 1.000 | 1.000 | 0.852 | 0.823 |
| 2733 | 0.777 | 1.000 | 0.804 | 1.000 | 0.968 | 0.960 | 0.900 | 1.000 | 0.980 | 1.000 | 0.962 | 1.000 | 0.973 | 0.853 | 1.000 | 1.000 | 0.858 | 0.829 |
| 2734 | 0.774 | 1.000 | 0.802 | 0.986 | 0.963 | 0.960 | 0.906 | 1.000 | 0.980 | 1.000 | 0.957 | 1.000 | 0.973 | 0.847 | 1.000 | 1.000 | 0.855 | 0.827 |
| 2735 | 0.766 | 1.000 | 0.787 | 1.000 | 0.968 | 0.960 | 0.897 | 1.000 | 0.980 | 1.000 | 0.962 | 1.000 | 0.964 | 0.858 | 1.000 | 1.000 | 0.853 | 0.824 |
| 2736 | 0.774 | 1.000 | 0.792 | 0.986 | 0.974 | 0.960 | 0.894 | 1.000 | 0.980 | 1.000 | 0.962 | 1.000 | 0.973 | 0.860 | 1.000 | 1.000 | 0.857 | 0.828 |
| 2737 | 0.759 | 1.000 | 0.792 | 1.000 | 0.968 | 0.960 | 0.889 | 1.000 | 0.990 | 1.000 | 0.962 | 1.000 | 0.964 | 0.858 | 1.000 | 1.000 | 0.851 | 0.821 |
| 2738 | 0.773 | 1.000 | 0.794 | 1.000 | 0.963 | 0.960 | 0.891 | 1.000 | 0.990 | 1.000 | 0.951 | 1.000 | 0.955 | 0.855 | 1.000 | 1.000 | 0.854 | 0.825 |
| 2739 | 0.765 | 1.000 | 0.800 | 0.986 | 0.963 | 0.960 | 0.898 | 1.000 | 0.990 | 1.000 | 0.968 | 1.000 | 0.964 | 0.852 | 1.000 | 1.000 | 0.853 | 0.824 |
| 2740 | 0.767 | 1.000 | 0.785 | 1.000 | 0.963 | 0.960 | 0.901 | 1.000 | 1.000 | 1.000 | 0.962 | 1.000 | 0.973 | 0.863 | 1.000 | 1.000 | 0.856 | 0.827 |
| 2741 | 0.781 | 1.000 | 0.796 | 1.000 | 0.968 | 0.960 | 0.912 | 1.000 | 0.990 | 1.000 | 0.968 | 1.000 | 0.973 | 0.859 | 1.000 | 1.000 | 0.862 | 0.834 |
| 2742 | 0.773 | 1.000 | 0.792 | 0.986 | 0.958 | 0.960 | 0.900 | 1.000 | 0.990 | 1.000 | 0.957 | 1.000 | 0.955 | 0.854 | 1.000 | 1.000 | 0.854 | 0.825 |
| 2743 | 0.778 | 1.000 | 0.781 | 1.000 | 0.963 | 0.960 | 0.903 | 1.000 | 0.969 | 1.000 | 0.959 | 1.000 | 0.964 | 0.862 | 1.000 | 1.000 | 0.858 | 0.829 |
| 2744 | 0.773 | 1.000 | 0.787 | 0.986 | 0.963 | 0.960 | 0.911 | 1.000 | 0.980 | 1.000 | 0.948 | 1.000 | 0.982 | 0.864 | 1.000 | 1.000 | 0.858 | 0.829 |
| 2745 | 0.778 | 1.000 | 0.800 | 1.000 | 0.958 | 0.960 | 0.901 | 1.000 | 0.980 | 1.000 | 0.959 | 1.000 | 0.982 | 0.858 | 1.000 | 1.000 | 0.859 | 0.830 |
| 2746 | 0.773 | 1.000 | 0.783 | 1.000 | 0.963 | 0.960 | 0.898 | 1.000 | 0.980 | 1.000 | 0.957 | 1.000 | 0.982 | 0.857 | 1.000 | 1.000 | 0.855 | 0.825 |
| 2747 | 0.777 | 1.000 | 0.794 | 0.986 | 0.963 | 0.960 | 0.900 | 1.000 | 0.980 | 1.000 | 0.948 | 1.000 | 0.973 | 0.854 | 1.000 | 1.000 | 0.856 | 0.827 |
| 2748 | 0.773 | 1.000 | 0.806 | 1.000 | 0.963 | 0.960 | 0.895 | 1.000 | 0.990 | 1.000 | 0.968 | 1.000 | 0.973 | 0.851 | 1.000 | 1.000 | 0.856 | 0.827 |
| 2749 | 0.771 | 1.000 | 0.796 | 1.000 | 0.968 | 0.960 | 0.898 | 1.000 | 0.990 | 1.000 | 0.968 | 1.000 | 0.964 | 0.862 | 1.000 | 1.000 | 0.858 | 0.829 |
| 2750 | 0.773 | 1.000 | 0.802 | 1.000 | 0.958 | 0.960 | 0.908 | 1.000 | 0.980 | 1.000 | 0.971 | 1.000 | 0.973 | 0.852 | 1.000 | 1.000 | 0.857 | 0.829 |
| 2751 | 0.765 | 1.000 | 0.800 | 0.986 | 0.953 | 0.960 | 0.911 | 1.000 | 0.990 | 1.000 | 0.959 | 1.000 | 0.991 | 0.858 | 1.000 | 1.000 | 0.856 | 0.828 |
| 2752 | 0.770 | 1.000 | 0.787 | 1.000 | 0.963 | 0.960 | 0.904 | 1.000 | 0.990 | 1.000 | 0.962 | 1.000 | 0.982 | 0.851 | 1.000 | 1.000 | 0.854 | 0.825 |
| 2753 | 0.771 | 1.000 | 0.808 | 1.000 | 0.963 | 0.960 | 0.888 | 1.000 | 1.000 | 1.000 | 0.959 | 1.000 | 0.982 | 0.851 | 1.000 | 1.000 | 0.855 | 0.826 |
| 2754 | 0.765 | 1.000 | 0.794 | 1.000 | 0.974 | 0.960 | 0.891 | 1.000 | 0.990 | 1.000 | 0.962 | 1.000 | 0.946 | 0.860 | 1.000 | 1.000 | 0.853 | 0.824 |
| 2755 | 0.771 | 1.000 | 0.789 | 1.000 | 0.974 | 0.960 | 0.888 | 1.000 | 0.969 | 1.000 | 0.959 | 1.000 | 0.973 | 0.847 | 1.000 | 1.000 | 0.851 | 0.821 |
| 2756 | 0.770 | 1.000 | 0.789 | 0.986 | 0.963 | 0.960 | 0.900 | 1.000 | 1.000 | 1.000 | 0.959 | 1.000 | 0.973 | 0.858 | 1.000 | 1.000 | 0.855 | 0.826 |
| 2757 | 0.770 | 1.000 | 0.796 | 1.000 | 0.963 | 0.960 | 0.895 | 1.000 | 0.990 | 1.000 | 0.951 | 1.000 | 0.964 | 0.851 | 1.000 | 0.981 | 0.852 | 0.823 |
| 2758 | 0.761 | 1.000 | 0.796 | 1.000 | 0.968 | 0.960 | 0.906 | 1.000 | 0.980 | 1.000 | 0.962 | 1.000 | 0.973 | 0.860 | 1.000 | 1.000 | 0.855 | 0.826 |
| 2759 | 0.761 | 1.000 | 0.798 | 1.000 | 0.974 | 0.960 | 0.901 | 1.000 | 1.000 | 1.000 | 0.959 | 1.000 | 0.991 | 0.855 | 1.000 | 1.000 | 0.854 | 0.825 |
| 2760 | 0.764 | 1.000 | 0.792 | 0.986 | 0.958 | 0.960 | 0.898 | 1.000 | 0.990 | 1.000 | 0.959 | 1.000 | 0.973 | 0.854 | 1.000 | 0.981 | 0.852 | 0.822 |
| 2761 | 0.775 | 1.000 | 0.796 | 1.000 | 0.979 | 0.960 | 0.906 | 1.000 | 1.000 | 1.000 | 0.957 | 1.000 | 0.955 | 0.851 | 1.000 | 1.000 | 0.857 | 0.828 |
| 2762 | 0.771 | 1.000 | 0.800 | 1.000 | 0.958 | 0.960 | 0.889 | 1.000 | 0.980 | 1.000 | 0.965 | 1.000 | 0.973 | 0.854 | 1.000 | 1.000 | 0.854 | 0.825 |
| 2763 | 0.773 | 1.000 | 0.802 | 1.000 | 0.963 | 0.960 | 0.908 | 1.000 | 0.980 | 1.000 | 0.968 | 0.970 | 0.982 | 0.861 | 1.000 | 1.000 | 0.860 | 0.832 |
| 2764 | 0.773 | 1.000 | 0.787 | 1.000 | 0.968 | 0.960 | 0.900 | 1.000 | 1.000 | 1.000 | 0.951 | 1.000 | 0.964 | 0.855 | 1.000 | 1.000 | 0.855 | 0.826 |
| 2765 | 0.767 | 1.000 | 0.785 | 1.000 | 0.974 | 0.960 | 0.906 | 1.000 | 1.000 | 1.000 | 0.959 | 1.000 | 0.964 | 0.854 | 1.000 | 1.000 | 0.854 | 0.826 |
| 2766 | 0.772 | 1.000 | 0.798 | 1.000 | 0.963 | 0.960 | 0.909 | 1.000 | 0.990 | 1.000 | 0.959 | 1.000 | 0.982 | 0.854 | 1.000 | 1.000 | 0.857 | 0.829 |
| 2767 | 0.779 | 1.000 | 0.798 | 0.986 | 0.958 | 0.960 | 0.900 | 1.000 | 0.980 | 1.000 | 0.959 | 1.000 | 0.955 | 0.858 | 1.000 | 1.000 | 0.858 | 0.829 |

| | | | | | | | | | | | | | | | | | | |
|---|---|---|---|---|---|---|---|---|---|---|---|---|---|---|---|---|---|---|
| 2768 | 0.769 | 1.000 | 0.787 | 0.986 | 0.963 | 0.960 | 0.904 | 1.000 | 1.000 | 1.000 | 0.965 | 1.000 | 0.964 | 0.855 | 1.000 | 1.000 | 0.855 | 0.826 |
| 2769 | 0.771 | 1.000 | 0.789 | 0.986 | 0.958 | 0.960 | 0.898 | 1.000 | 1.000 | 1.000 | 0.957 | 1.000 | 0.955 | 0.854 | 1.000 | 1.000 | 0.854 | 0.824 |
| 2770 | 0.768 | 1.000 | 0.789 | 1.000 | 0.963 | 0.960 | 0.889 | 1.000 | 0.990 | 1.000 | 0.962 | 1.000 | 0.991 | 0.858 | 1.000 | 1.000 | 0.854 | 0.824 |
| 2771 | 0.771 | 1.000 | 0.789 | 1.000 | 0.958 | 0.960 | 0.897 | 1.000 | 1.000 | 1.000 | 0.962 | 1.000 | 0.946 | 0.861 | 1.000 | 1.000 | 0.855 | 0.826 |
| 2772 | 0.769 | 1.000 | 0.792 | 1.000 | 0.963 | 0.960 | 0.894 | 1.000 | 1.000 | 1.000 | 0.951 | 1.000 | 0.964 | 0.855 | 1.000 | 1.000 | 0.853 | 0.824 |
| 2773 | 0.771 | 1.000 | 0.789 | 1.000 | 0.963 | 0.960 | 0.889 | 1.000 | 0.980 | 1.000 | 0.971 | 1.000 | 0.982 | 0.857 | 1.000 | 1.000 | 0.855 | 0.825 |
| 2774 | 0.776 | 1.000 | 0.789 | 0.971 | 0.968 | 0.960 | 0.897 | 1.000 | 0.980 | 1.000 | 0.962 | 1.000 | 0.964 | 0.857 | 1.000 | 1.000 | 0.856 | 0.827 |
| 2775 | 0.763 | 1.000 | 0.798 | 1.000 | 0.963 | 0.960 | 0.900 | 1.000 | 0.990 | 1.000 | 0.957 | 1.000 | 0.946 | 0.854 | 1.000 | 1.000 | 0.852 | 0.823 |
| 2776 | 0.765 | 1.000 | 0.802 | 1.000 | 0.963 | 0.960 | 0.897 | 1.000 | 1.000 | 1.000 | 0.962 | 1.000 | 0.964 | 0.860 | 1.000 | 1.000 | 0.855 | 0.827 |
| 2777 | 0.769 | 1.000 | 0.791 | 0.986 | 0.963 | 0.960 | 0.895 | 1.000 | 0.980 | 1.000 | 0.965 | 1.000 | 0.982 | 0.851 | 1.000 | 0.981 | 0.852 | 0.823 |
| 2778 | 0.773 | 1.000 | 0.783 | 1.000 | 0.968 | 0.960 | 0.901 | 1.000 | 0.980 | 1.000 | 0.948 | 1.000 | 0.982 | 0.853 | 1.000 | 1.000 | 0.854 | 0.824 |
| 2779 | 0.771 | 1.000 | 0.783 | 0.986 | 0.963 | 0.960 | 0.901 | 1.000 | 0.990 | 1.000 | 0.948 | 1.000 | 0.973 | 0.864 | 1.000 | 1.000 | 0.856 | 0.827 |
| 2780 | 0.779 | 1.000 | 0.794 | 1.000 | 0.958 | 0.960 | 0.900 | 1.000 | 0.990 | 1.000 | 0.959 | 1.000 | 0.973 | 0.863 | 1.000 | 1.000 | 0.860 | 0.831 |
| 2781 | 0.776 | 1.000 | 0.785 | 0.986 | 0.968 | 0.960 | 0.895 | 1.000 | 0.990 | 1.000 | 0.959 | 1.000 | 0.982 | 0.856 | 1.000 | 1.000 | 0.856 | 0.827 |
| 2782 | 0.763 | 1.000 | 0.791 | 1.000 | 0.968 | 0.960 | 0.898 | 1.000 | 1.000 | 1.000 | 0.962 | 1.000 | 0.964 | 0.860 | 1.000 | 1.000 | 0.854 | 0.825 |
| 2783 | 0.769 | 1.000 | 0.791 | 0.971 | 0.963 | 0.960 | 0.897 | 1.000 | 0.990 | 1.000 | 0.959 | 1.000 | 0.973 | 0.851 | 1.000 | 1.000 | 0.852 | 0.823 |
| 2784 | 0.769 | 1.000 | 0.789 | 1.000 | 0.968 | 0.960 | 0.898 | 1.000 | 0.990 | 1.000 | 0.948 | 1.000 | 0.973 | 0.844 | 1.000 | 1.000 | 0.850 | 0.820 |
| 2785 | 0.773 | 1.000 | 0.792 | 0.986 | 0.963 | 0.960 | 0.903 | 1.000 | 0.980 | 1.000 | 0.957 | 1.000 | 0.964 | 0.855 | 1.000 | 1.000 | 0.855 | 0.827 |
| 2786 | 0.761 | 1.000 | 0.800 | 1.000 | 0.968 | 0.960 | 0.892 | 1.000 | 0.980 | 1.000 | 0.962 | 1.000 | 0.982 | 0.859 | 1.000 | 1.000 | 0.853 | 0.824 |
| 2787 | 0.772 | 1.000 | 0.796 | 1.000 | 0.958 | 0.960 | 0.911 | 1.000 | 0.980 | 1.000 | 0.954 | 1.000 | 0.964 | 0.851 | 1.000 | 0.981 | 0.855 | 0.826 |
| 2788 | 0.767 | 1.000 | 0.789 | 0.971 | 0.963 | 0.960 | 0.898 | 1.000 | 0.990 | 1.000 | 0.951 | 1.000 | 0.964 | 0.862 | 1.000 | 1.000 | 0.854 | 0.825 |
| 2789 | 0.772 | 1.000 | 0.789 | 0.986 | 0.958 | 0.960 | 0.908 | 1.000 | 0.990 | 1.000 | 0.951 | 1.000 | 0.955 | 0.859 | 1.000 | 1.000 | 0.856 | 0.827 |
| 2790 | 0.769 | 1.000 | 0.794 | 1.000 | 0.963 | 0.960 | 0.895 | 1.000 | 0.990 | 1.000 | 0.957 | 1.000 | 0.955 | 0.867 | 1.000 | 1.000 | 0.856 | 0.828 |
| 2791 | 0.774 | 1.000 | 0.779 | 0.986 | 0.963 | 0.960 | 0.889 | 1.000 | 0.980 | 1.000 | 0.959 | 1.000 | 0.973 | 0.851 | 1.000 | 1.000 | 0.852 | 0.822 |
| 2792 | 0.764 | 1.000 | 0.792 | 0.986 | 0.968 | 0.960 | 0.898 | 1.000 | 1.000 | 1.000 | 0.962 | 1.000 | 0.964 | 0.864 | 1.000 | 1.000 | 0.855 | 0.826 |
| 2793 | 0.770 | 1.000 | 0.794 | 1.000 | 0.958 | 0.960 | 0.898 | 1.000 | 0.990 | 1.000 | 0.962 | 1.000 | 0.973 | 0.849 | 1.000 | 1.000 | 0.853 | 0.824 |
| 2794 | 0.773 | 1.000 | 0.804 | 1.000 | 0.963 | 0.960 | 0.906 | 1.000 | 0.990 | 1.000 | 0.957 | 1.000 | 0.982 | 0.857 | 1.000 | 1.000 | 0.858 | 0.830 |
| 2795 | 0.768 | 1.000 | 0.791 | 1.000 | 0.968 | 0.960 | 0.898 | 1.000 | 0.990 | 1.000 | 0.968 | 1.000 | 0.982 | 0.856 | 1.000 | 1.000 | 0.855 | 0.826 |
| 2796 | 0.771 | 1.000 | 0.789 | 0.986 | 0.963 | 0.960 | 0.900 | 1.000 | 0.980 | 1.000 | 0.965 | 1.000 | 0.982 | 0.851 | 1.000 | 1.000 | 0.854 | 0.825 |
| 2797 | 0.772 | 1.000 | 0.794 | 1.000 | 0.968 | 0.960 | 0.897 | 1.000 | 0.990 | 1.000 | 0.948 | 1.000 | 0.955 | 0.851 | 1.000 | 1.000 | 0.853 | 0.824 |
| 2798 | 0.768 | 1.000 | 0.794 | 0.986 | 0.963 | 0.960 | 0.892 | 1.000 | 0.990 | 1.000 | 0.957 | 1.000 | 0.982 | 0.852 | 1.000 | 1.000 | 0.852 | 0.823 |
| 2799 | 0.775 | 1.000 | 0.789 | 0.986 | 0.963 | 0.960 | 0.888 | 1.000 | 0.990 | 1.000 | 0.962 | 1.000 | 0.964 | 0.856 | 1.000 | 1.000 | 0.854 | 0.825 |
| 2800 | 0.768 | 1.000 | 0.791 | 1.000 | 0.958 | 0.960 | 0.889 | 1.000 | 1.000 | 1.000 | 0.959 | 1.000 | 0.964 | 0.863 | 1.000 | 1.000 | 0.854 | 0.825 |
| 2801 | 0.777 | 1.000 | 0.800 | 1.000 | 0.963 | 0.960 | 0.904 | 1.000 | 0.980 | 1.000 | 0.968 | 1.000 | 0.973 | 0.863 | 1.000 | 1.000 | 0.861 | 0.833 |
| 2802 | 0.768 | 1.000 | 0.800 | 1.000 | 0.968 | 0.960 | 0.903 | 1.000 | 0.980 | 1.000 | 0.962 | 1.000 | 0.964 | 0.859 | 1.000 | 1.000 | 0.856 | 0.828 |
| 2803 | 0.773 | 1.000 | 0.792 | 0.971 | 0.968 | 0.960 | 0.889 | 1.000 | 0.969 | 1.000 | 0.962 | 1.000 | 0.964 | 0.850 | 1.000 | 1.000 | 0.852 | 0.823 |
| 2804 | 0.767 | 1.000 | 0.787 | 0.986 | 0.968 | 0.960 | 0.898 | 1.000 | 0.980 | 1.000 | 0.962 | 1.000 | 0.982 | 0.862 | 1.000 | 1.000 | 0.855 | 0.826 |
| 2805 | 0.766 | 1.000 | 0.796 | 1.000 | 0.968 | 0.960 | 0.903 | 1.000 | 0.990 | 1.000 | 0.957 | 1.000 | 0.964 | 0.858 | 1.000 | 1.000 | 0.855 | 0.826 |
| 2806 | 0.776 | 1.000 | 0.804 | 0.986 | 0.963 | 0.960 | 0.886 | 1.000 | 0.990 | 1.000 | 0.957 | 1.000 | 0.982 | 0.857 | 1.000 | 1.000 | 0.856 | 0.827 |

| | | | | | | | | | | | | | | | | | | |
|---|---|---|---|---|---|---|---|---|---|---|---|---|---|---|---|---|---|---|
| 2807 | 0.776 | 1.000 | 0.792 | 1.000 | 0.968 | 0.960 | 0.900 | 1.000 | 1.000 | 1.000 | 0.974 | 1.000 | 0.982 | 0.857 | 1.000 | 1.000 | 0.859 | 0.830 |
| 2808 | 0.779 | 1.000 | 0.800 | 1.000 | 0.968 | 0.960 | 0.897 | 1.000 | 0.980 | 1.000 | 0.948 | 1.000 | 0.973 | 0.858 | 1.000 | 1.000 | 0.858 | 0.829 |
| 2809 | 0.773 | 1.000 | 0.787 | 1.000 | 0.968 | 0.960 | 0.908 | 1.000 | 1.000 | 1.000 | 0.962 | 1.000 | 0.964 | 0.853 | 1.000 | 1.000 | 0.856 | 0.827 |
| 2810 | 0.773 | 1.000 | 0.794 | 0.986 | 0.958 | 0.960 | 0.895 | 1.000 | 0.980 | 1.000 | 0.959 | 1.000 | 0.973 | 0.852 | 1.000 | 1.000 | 0.854 | 0.825 |
| 2811 | 0.761 | 1.000 | 0.808 | 1.000 | 0.963 | 0.960 | 0.906 | 1.000 | 0.980 | 1.000 | 0.962 | 1.000 | 0.964 | 0.854 | 1.000 | 1.000 | 0.854 | 0.825 |
| 2812 | 0.769 | 1.000 | 0.800 | 0.986 | 0.963 | 0.960 | 0.898 | 1.000 | 0.980 | 1.000 | 0.962 | 1.000 | 0.982 | 0.852 | 1.000 | 1.000 | 0.854 | 0.825 |
| 2813 | 0.767 | 1.000 | 0.791 | 1.000 | 0.963 | 0.960 | 0.883 | 1.000 | 0.990 | 1.000 | 0.957 | 1.000 | 0.955 | 0.861 | 1.000 | 1.000 | 0.852 | 0.823 |
| 2814 | 0.769 | 1.000 | 0.800 | 0.986 | 0.963 | 0.960 | 0.891 | 1.000 | 0.980 | 1.000 | 0.954 | 1.000 | 0.973 | 0.858 | 1.000 | 1.000 | 0.854 | 0.825 |
| 2815 | 0.765 | 1.000 | 0.789 | 1.000 | 0.958 | 0.960 | 0.901 | 1.000 | 1.000 | 1.000 | 0.959 | 1.000 | 0.982 | 0.854 | 1.000 | 1.000 | 0.853 | 0.824 |
| 2816 | 0.765 | 1.000 | 0.798 | 0.971 | 0.968 | 0.960 | 0.895 | 1.000 | 0.990 | 1.000 | 0.968 | 1.000 | 0.973 | 0.860 | 1.000 | 1.000 | 0.855 | 0.826 |
| 2817 | 0.773 | 1.000 | 0.787 | 1.000 | 0.958 | 0.960 | 0.903 | 1.000 | 0.980 | 1.000 | 0.957 | 1.000 | 0.991 | 0.857 | 1.000 | 0.981 | 0.856 | 0.827 |
| 2818 | 0.767 | 1.000 | 0.785 | 0.986 | 0.968 | 0.960 | 0.909 | 1.000 | 1.000 | 1.000 | 0.954 | 1.000 | 0.982 | 0.855 | 1.000 | 1.000 | 0.854 | 0.825 |
| 2819 | 0.769 | 1.000 | 0.787 | 1.000 | 0.963 | 0.960 | 0.903 | 1.000 | 0.990 | 1.000 | 0.959 | 1.000 | 0.973 | 0.857 | 1.000 | 1.000 | 0.855 | 0.826 |
| 2820 | 0.771 | 1.000 | 0.804 | 1.000 | 0.958 | 0.960 | 0.901 | 1.000 | 1.000 | 1.000 | 0.968 | 1.000 | 0.973 | 0.852 | 1.000 | 1.000 | 0.856 | 0.828 |
| 2821 | 0.767 | 1.000 | 0.792 | 1.000 | 0.963 | 0.960 | 0.903 | 1.000 | 0.980 | 1.000 | 0.957 | 1.000 | 0.982 | 0.857 | 1.000 | 1.000 | 0.855 | 0.826 |
| 2822 | 0.767 | 1.000 | 0.787 | 1.000 | 0.963 | 0.960 | 0.897 | 1.000 | 0.990 | 1.000 | 0.959 | 1.000 | 0.991 | 0.861 | 1.000 | 1.000 | 0.855 | 0.826 |
| 2823 | 0.769 | 1.000 | 0.791 | 0.986 | 0.963 | 0.960 | 0.891 | 1.000 | 0.980 | 1.000 | 0.957 | 1.000 | 0.964 | 0.858 | 1.000 | 1.000 | 0.853 | 0.824 |
| 2824 | 0.763 | 1.000 | 0.800 | 1.000 | 0.963 | 0.960 | 0.904 | 1.000 | 0.980 | 1.000 | 0.959 | 1.000 | 0.991 | 0.854 | 1.000 | 0.981 | 0.854 | 0.825 |
| 2825 | 0.772 | 1.000 | 0.783 | 0.986 | 0.968 | 0.960 | 0.895 | 1.000 | 0.990 | 1.000 | 0.959 | 1.000 | 0.982 | 0.861 | 1.000 | 1.000 | 0.856 | 0.826 |
| 2826 | 0.765 | 1.000 | 0.804 | 0.986 | 0.958 | 0.960 | 0.897 | 1.000 | 0.990 | 1.000 | 0.965 | 0.970 | 0.964 | 0.861 | 1.000 | 1.000 | 0.855 | 0.826 |
| 2827 | 0.772 | 1.000 | 0.810 | 1.000 | 0.963 | 0.960 | 0.904 | 1.000 | 0.990 | 1.000 | 0.948 | 1.000 | 0.955 | 0.853 | 1.000 | 1.000 | 0.856 | 0.827 |
| 2828 | 0.779 | 1.000 | 0.775 | 1.000 | 0.963 | 0.960 | 0.898 | 1.000 | 0.990 | 1.000 | 0.962 | 1.000 | 0.955 | 0.854 | 1.000 | 1.000 | 0.855 | 0.825 |
| 2829 | 0.767 | 1.000 | 0.810 | 0.986 | 0.968 | 0.960 | 0.900 | 1.000 | 0.990 | 1.000 | 0.962 | 1.000 | 0.964 | 0.847 | 1.000 | 1.000 | 0.854 | 0.825 |
| 2830 | 0.775 | 1.000 | 0.789 | 0.986 | 0.953 | 0.960 | 0.901 | 1.000 | 0.990 | 1.000 | 0.948 | 1.000 | 0.973 | 0.856 | 1.000 | 1.000 | 0.855 | 0.826 |
| 2831 | 0.775 | 1.000 | 0.789 | 0.986 | 0.958 | 0.960 | 0.900 | 1.000 | 0.980 | 1.000 | 0.954 | 1.000 | 0.973 | 0.847 | 1.000 | 1.000 | 0.853 | 0.823 |
| 2832 | 0.761 | 1.000 | 0.783 | 1.000 | 0.963 | 0.960 | 0.897 | 1.000 | 0.990 | 1.000 | 0.957 | 1.000 | 0.964 | 0.865 | 1.000 | 1.000 | 0.853 | 0.824 |
| 2833 | 0.773 | 1.000 | 0.794 | 0.986 | 0.963 | 0.960 | 0.897 | 1.000 | 0.990 | 1.000 | 0.957 | 1.000 | 0.964 | 0.855 | 1.000 | 1.000 | 0.855 | 0.825 |
| 2834 | 0.768 | 1.000 | 0.794 | 1.000 | 0.963 | 0.960 | 0.906 | 1.000 | 0.990 | 1.000 | 0.957 | 1.000 | 0.973 | 0.865 | 1.000 | 0.981 | 0.857 | 0.829 |
| 2835 | 0.764 | 1.000 | 0.800 | 1.000 | 0.958 | 0.960 | 0.903 | 1.000 | 1.000 | 1.000 | 0.948 | 1.000 | 0.964 | 0.858 | 1.000 | 1.000 | 0.854 | 0.825 |
| 2836 | 0.775 | 1.000 | 0.787 | 0.986 | 0.963 | 0.960 | 0.895 | 1.000 | 0.990 | 1.000 | 0.959 | 1.000 | 0.991 | 0.856 | 1.000 | 1.000 | 0.855 | 0.826 |
| 2837 | 0.772 | 1.000 | 0.796 | 0.986 | 0.963 | 0.960 | 0.895 | 1.000 | 0.980 | 1.000 | 0.965 | 1.000 | 0.964 | 0.856 | 1.000 | 1.000 | 0.855 | 0.826 |
| 2838 | 0.769 | 1.000 | 0.802 | 0.986 | 0.958 | 0.960 | 0.904 | 1.000 | 1.000 | 1.000 | 0.962 | 1.000 | 0.964 | 0.860 | 1.000 | 1.000 | 0.857 | 0.828 |
| 2839 | 0.769 | 1.000 | 0.781 | 1.000 | 0.963 | 0.960 | 0.894 | 1.000 | 0.990 | 1.000 | 0.954 | 0.970 | 0.964 | 0.849 | 1.000 | 1.000 | 0.850 | 0.820 |
| 2840 | 0.767 | 1.000 | 0.787 | 1.000 | 0.958 | 0.960 | 0.898 | 1.000 | 0.980 | 1.000 | 0.954 | 1.000 | 0.964 | 0.855 | 1.000 | 1.000 | 0.852 | 0.823 |
| 2841 | 0.772 | 1.000 | 0.789 | 1.000 | 0.958 | 0.960 | 0.903 | 1.000 | 0.990 | 1.000 | 0.965 | 1.000 | 0.964 | 0.854 | 1.000 | 1.000 | 0.855 | 0.826 |
| 2842 | 0.771 | 1.000 | 0.783 | 0.986 | 0.968 | 0.960 | 0.892 | 1.000 | 1.000 | 1.000 | 0.962 | 1.000 | 0.973 | 0.858 | 1.000 | 1.000 | 0.854 | 0.825 |
| 2843 | 0.767 | 1.000 | 0.794 | 1.000 | 0.968 | 0.960 | 0.901 | 1.000 | 0.980 | 1.000 | 0.965 | 1.000 | 0.955 | 0.856 | 1.000 | 1.000 | 0.855 | 0.826 |
| 2844 | 0.765 | 1.000 | 0.787 | 1.000 | 0.968 | 0.960 | 0.904 | 1.000 | 0.990 | 1.000 | 0.965 | 1.000 | 0.946 | 0.860 | 1.000 | 1.000 | 0.854 | 0.825 |
| 2845 | 0.767 | 1.000 | 0.791 | 1.000 | 0.963 | 0.960 | 0.897 | 1.000 | 0.980 | 1.000 | 0.951 | 1.000 | 0.973 | 0.854 | 1.000 | 1.000 | 0.852 | 0.823 |

| | | | | | | | | | | | | | | | | | | |
|---|---|---|---|---|---|---|---|---|---|---|---|---|---|---|---|---|---|---|
| 2846 | 0.775 | 1.000 | 0.804 | 0.986 | 0.963 | 0.960 | 0.908 | 1.000 | 0.990 | 1.000 | 0.962 | 1.000 | 0.973 | 0.867 | 1.000 | 1.000 | 0.862 | 0.834 |
| 2847 | 0.780 | 1.000 | 0.808 | 1.000 | 0.968 | 0.960 | 0.897 | 1.000 | 0.990 | 1.000 | 0.962 | 1.000 | 0.964 | 0.851 | 1.000 | 1.000 | 0.858 | 0.829 |
| 2848 | 0.777 | 1.000 | 0.800 | 1.000 | 0.963 | 0.960 | 0.911 | 1.000 | 0.980 | 1.000 | 0.954 | 1.000 | 0.973 | 0.859 | 1.000 | 1.000 | 0.860 | 0.832 |
| 2849 | 0.770 | 1.000 | 0.791 | 1.000 | 0.968 | 0.960 | 0.903 | 1.000 | 0.990 | 1.000 | 0.959 | 1.000 | 0.991 | 0.854 | 1.000 | 1.000 | 0.855 | 0.827 |
| 2850 | 0.771 | 1.000 | 0.808 | 1.000 | 0.968 | 0.960 | 0.903 | 1.000 | 0.990 | 1.000 | 0.971 | 1.000 | 0.964 | 0.855 | 1.000 | 1.000 | 0.858 | 0.830 |
| 2851 | 0.770 | 1.000 | 0.787 | 0.986 | 0.958 | 0.960 | 0.908 | 1.000 | 0.980 | 1.000 | 0.939 | 1.000 | 0.964 | 0.857 | 1.000 | 1.000 | 0.853 | 0.824 |
| 2852 | 0.763 | 1.000 | 0.794 | 0.986 | 0.974 | 0.960 | 0.898 | 1.000 | 0.980 | 1.000 | 0.962 | 1.000 | 0.964 | 0.863 | 1.000 | 1.000 | 0.855 | 0.826 |
| 2853 | 0.767 | 1.000 | 0.781 | 0.986 | 0.968 | 0.960 | 0.897 | 1.000 | 1.000 | 1.000 | 0.959 | 1.000 | 0.964 | 0.862 | 1.000 | 1.000 | 0.854 | 0.825 |
| 2854 | 0.766 | 1.000 | 0.810 | 0.986 | 0.958 | 0.960 | 0.898 | 1.000 | 0.990 | 1.000 | 0.957 | 1.000 | 0.955 | 0.862 | 1.000 | 1.000 | 0.856 | 0.827 |
| 2855 | 0.772 | 1.000 | 0.794 | 1.000 | 0.968 | 0.960 | 0.897 | 1.000 | 0.990 | 1.000 | 0.965 | 1.000 | 0.964 | 0.858 | 1.000 | 1.000 | 0.856 | 0.827 |
| 2856 | 0.764 | 1.000 | 0.804 | 0.986 | 0.963 | 0.960 | 0.903 | 1.000 | 0.980 | 1.000 | 0.962 | 1.000 | 0.982 | 0.851 | 1.000 | 1.000 | 0.853 | 0.824 |
| 2857 | 0.761 | 1.000 | 0.802 | 0.986 | 0.968 | 0.960 | 0.894 | 1.000 | 0.990 | 1.000 | 0.962 | 1.000 | 0.964 | 0.852 | 1.000 | 1.000 | 0.852 | 0.822 |
| 2858 | 0.774 | 1.000 | 0.775 | 0.986 | 0.953 | 0.960 | 0.901 | 1.000 | 0.980 | 1.000 | 0.962 | 1.000 | 0.964 | 0.854 | 1.000 | 1.000 | 0.853 | 0.824 |
| 2859 | 0.763 | 1.000 | 0.800 | 1.000 | 0.963 | 0.960 | 0.904 | 1.000 | 0.980 | 1.000 | 0.951 | 1.000 | 0.973 | 0.859 | 1.000 | 1.000 | 0.854 | 0.826 |
| 2860 | 0.767 | 1.000 | 0.802 | 0.986 | 0.963 | 0.960 | 0.900 | 1.000 | 0.990 | 1.000 | 0.962 | 1.000 | 0.982 | 0.852 | 1.000 | 1.000 | 0.854 | 0.826 |
| 2861 | 0.771 | 1.000 | 0.798 | 0.971 | 0.958 | 0.960 | 0.904 | 1.000 | 0.980 | 1.000 | 0.965 | 1.000 | 0.955 | 0.858 | 1.000 | 1.000 | 0.856 | 0.827 |
| 2862 | 0.775 | 1.000 | 0.792 | 1.000 | 0.963 | 0.960 | 0.904 | 1.000 | 0.990 | 1.000 | 0.948 | 1.000 | 0.964 | 0.857 | 1.000 | 1.000 | 0.856 | 0.827 |
| 2863 | 0.779 | 1.000 | 0.785 | 1.000 | 0.958 | 0.960 | 0.898 | 1.000 | 1.000 | 1.000 | 0.962 | 1.000 | 0.982 | 0.861 | 1.000 | 1.000 | 0.858 | 0.830 |
| 2864 | 0.775 | 1.000 | 0.804 | 0.986 | 0.968 | 0.960 | 0.900 | 1.000 | 0.980 | 1.000 | 0.962 | 1.000 | 0.973 | 0.857 | 1.000 | 1.000 | 0.858 | 0.829 |
| 2865 | 0.771 | 1.000 | 0.796 | 1.000 | 0.963 | 0.960 | 0.904 | 1.000 | 1.000 | 1.000 | 0.959 | 1.000 | 0.973 | 0.860 | 1.000 | 1.000 | 0.857 | 0.829 |
| 2866 | 0.772 | 1.000 | 0.800 | 1.000 | 0.963 | 0.960 | 0.903 | 1.000 | 0.980 | 1.000 | 0.957 | 1.000 | 0.964 | 0.858 | 1.000 | 1.000 | 0.857 | 0.828 |
| 2867 | 0.773 | 1.000 | 0.787 | 0.986 | 0.963 | 0.960 | 0.904 | 1.000 | 1.000 | 1.000 | 0.977 | 1.000 | 0.964 | 0.856 | 1.000 | 1.000 | 0.857 | 0.828 |
| 2868 | 0.771 | 1.000 | 0.796 | 0.986 | 0.968 | 0.960 | 0.900 | 1.000 | 0.990 | 1.000 | 0.957 | 1.000 | 0.982 | 0.849 | 1.000 | 1.000 | 0.854 | 0.825 |
| 2869 | 0.778 | 1.000 | 0.792 | 1.000 | 0.958 | 0.960 | 0.898 | 1.000 | 0.980 | 1.000 | 0.951 | 1.000 | 0.982 | 0.859 | 1.000 | 1.000 | 0.857 | 0.828 |
| 2870 | 0.760 | 1.000 | 0.794 | 1.000 | 0.963 | 0.960 | 0.906 | 1.000 | 0.980 | 1.000 | 0.954 | 1.000 | 0.955 | 0.851 | 1.000 | 1.000 | 0.851 | 0.821 |
| 2871 | 0.775 | 1.000 | 0.794 | 1.000 | 0.958 | 0.960 | 0.900 | 1.000 | 0.980 | 1.000 | 0.959 | 1.000 | 0.982 | 0.862 | 1.000 | 1.000 | 0.858 | 0.829 |
| 2872 | 0.776 | 1.000 | 0.791 | 1.000 | 0.968 | 0.960 | 0.901 | 1.000 | 1.000 | 1.000 | 0.962 | 0.970 | 0.955 | 0.858 | 1.000 | 1.000 | 0.857 | 0.829 |
| 2873 | 0.771 | 1.000 | 0.791 | 1.000 | 0.958 | 0.960 | 0.900 | 1.000 | 0.980 | 1.000 | 0.962 | 0.970 | 0.982 | 0.850 | 1.000 | 1.000 | 0.853 | 0.824 |
| 2874 | 0.776 | 1.000 | 0.787 | 1.000 | 0.963 | 0.960 | 0.898 | 1.000 | 0.990 | 1.000 | 0.959 | 1.000 | 0.964 | 0.861 | 1.000 | 0.981 | 0.857 | 0.828 |
| 2875 | 0.761 | 1.000 | 0.789 | 0.986 | 0.963 | 0.960 | 0.895 | 1.000 | 0.990 | 1.000 | 0.968 | 1.000 | 0.955 | 0.851 | 1.000 | 1.000 | 0.850 | 0.820 |
| 2876 | 0.768 | 1.000 | 0.789 | 1.000 | 0.953 | 0.960 | 0.903 | 1.000 | 0.990 | 1.000 | 0.957 | 1.000 | 0.982 | 0.866 | 1.000 | 1.000 | 0.857 | 0.828 |
| 2877 | 0.766 | 1.000 | 0.783 | 0.986 | 0.963 | 0.960 | 0.904 | 1.000 | 0.990 | 1.000 | 0.954 | 1.000 | 0.982 | 0.863 | 1.000 | 1.000 | 0.855 | 0.826 |
| 2878 | 0.769 | 1.000 | 0.792 | 0.986 | 0.947 | 0.960 | 0.904 | 1.000 | 0.990 | 1.000 | 0.951 | 1.000 | 0.955 | 0.860 | 1.000 | 1.000 | 0.854 | 0.825 |
| 2879 | 0.767 | 1.000 | 0.789 | 1.000 | 0.958 | 0.960 | 0.901 | 1.000 | 0.990 | 1.000 | 0.962 | 1.000 | 0.973 | 0.861 | 1.000 | 1.000 | 0.855 | 0.826 |
| 2880 | 0.768 | 1.000 | 0.812 | 0.986 | 0.963 | 0.960 | 0.900 | 1.000 | 0.969 | 1.000 | 0.962 | 1.000 | 0.982 | 0.858 | 1.000 | 1.000 | 0.857 | 0.828 |
| 2881 | 0.777 | 1.000 | 0.791 | 1.000 | 0.963 | 0.960 | 0.914 | 1.000 | 0.990 | 1.000 | 0.965 | 1.000 | 0.973 | 0.861 | 1.000 | 1.000 | 0.861 | 0.833 |
| 2882 | 0.771 | 1.000 | 0.785 | 0.986 | 0.968 | 1.000 | 0.909 | 1.000 | 0.980 | 1.000 | 0.959 | 1.000 | 0.946 | 0.848 | 1.000 | 1.000 | 0.853 | 0.824 |
| 2883 | 0.774 | 1.000 | 0.785 | 1.000 | 0.963 | 0.960 | 0.900 | 1.000 | 0.980 | 1.000 | 0.957 | 1.000 | 0.964 | 0.859 | 1.000 | 1.000 | 0.856 | 0.827 |
| 2884 | 0.779 | 1.000 | 0.785 | 1.000 | 0.963 | 0.960 | 0.895 | 1.000 | 0.990 | 1.000 | 0.957 | 1.000 | 0.982 | 0.858 | 1.000 | 1.000 | 0.857 | 0.828 |

| | | | | | | | | | | | | | | | | | |
|---|---|---|---|---|---|---|---|---|---|---|---|---|---|---|---|---|---|
| 2885 | 0.765 | 1.000 | 0.808 | 1.000 | 0.953 | 0.960 | 0.904 | 1.000 | 0.990 | 1.000 | 0.951 | 1.000 | 0.982 | 0.861 | 1.000 | 1.000 | 0.856 | 0.828 |
| 2886 | 0.771 | 1.000 | 0.804 | 1.000 | 0.953 | 0.960 | 0.895 | 1.000 | 0.990 | 1.000 | 0.954 | 1.000 | 0.964 | 0.854 | 1.000 | 0.981 | 0.854 | 0.825 |
| 2887 | 0.770 | 1.000 | 0.779 | 1.000 | 0.963 | 0.960 | 0.903 | 1.000 | 0.980 | 1.000 | 0.968 | 1.000 | 0.982 | 0.858 | 1.000 | 1.000 | 0.855 | 0.826 |
| 2888 | 0.770 | 1.000 | 0.783 | 1.000 | 0.958 | 0.960 | 0.901 | 1.000 | 0.990 | 1.000 | 0.962 | 1.000 | 0.982 | 0.854 | 1.000 | 1.000 | 0.854 | 0.825 |
| 2889 | 0.774 | 1.000 | 0.781 | 0.971 | 0.958 | 0.960 | 0.901 | 1.000 | 0.990 | 1.000 | 0.959 | 1.000 | 0.955 | 0.856 | 1.000 | 1.000 | 0.854 | 0.825 |
| 2890 | 0.773 | 1.000 | 0.796 | 1.000 | 0.958 | 0.960 | 0.898 | 1.000 | 0.990 | 1.000 | 0.951 | 1.000 | 0.973 | 0.851 | 1.000 | 1.000 | 0.854 | 0.825 |
| 2891 | 0.767 | 1.000 | 0.804 | 0.986 | 0.968 | 0.960 | 0.901 | 1.000 | 0.990 | 1.000 | 0.959 | 1.000 | 0.964 | 0.859 | 1.000 | 1.000 | 0.856 | 0.828 |
| 2892 | 0.769 | 1.000 | 0.792 | 0.986 | 0.968 | 0.960 | 0.901 | 1.000 | 0.990 | 1.000 | 0.962 | 1.000 | 0.982 | 0.858 | 1.000 | 1.000 | 0.856 | 0.827 |
| 2893 | 0.776 | 1.000 | 0.781 | 0.986 | 0.968 | 0.960 | 0.901 | 1.000 | 0.980 | 1.000 | 0.962 | 1.000 | 0.973 | 0.855 | 1.000 | 1.000 | 0.856 | 0.826 |
| 2894 | 0.769 | 1.000 | 0.796 | 1.000 | 0.963 | 0.960 | 0.895 | 1.000 | 0.990 | 1.000 | 0.954 | 1.000 | 0.973 | 0.853 | 1.000 | 1.000 | 0.853 | 0.824 |
| 2895 | 0.777 | 1.000 | 0.792 | 0.986 | 0.968 | 0.960 | 0.909 | 1.000 | 0.990 | 1.000 | 0.954 | 1.000 | 0.973 | 0.861 | 1.000 | 1.000 | 0.859 | 0.831 |
| 2896 | 0.767 | 1.000 | 0.792 | 0.971 | 0.974 | 0.960 | 0.908 | 1.000 | 0.980 | 1.000 | 0.954 | 1.000 | 0.982 | 0.852 | 1.000 | 1.000 | 0.854 | 0.825 |
| 2897 | 0.769 | 1.000 | 0.787 | 0.986 | 0.958 | 0.960 | 0.889 | 1.000 | 0.990 | 1.000 | 0.962 | 1.000 | 0.991 | 0.854 | 1.000 | 1.000 | 0.852 | 0.823 |
| 2898 | 0.779 | 1.000 | 0.787 | 0.986 | 0.968 | 0.960 | 0.906 | 1.000 | 0.969 | 1.000 | 0.954 | 1.000 | 0.973 | 0.854 | 1.000 | 1.000 | 0.856 | 0.827 |
| 2899 | 0.763 | 1.000 | 0.798 | 0.986 | 0.968 | 0.960 | 0.903 | 1.000 | 1.000 | 1.000 | 0.957 | 1.000 | 0.964 | 0.852 | 1.000 | 1.000 | 0.853 | 0.823 |
| 2900 | 0.771 | 1.000 | 0.794 | 1.000 | 0.968 | 0.960 | 0.898 | 1.000 | 0.980 | 1.000 | 0.954 | 1.000 | 0.973 | 0.858 | 1.000 | 0.981 | 0.855 | 0.826 |
| 2901 | 0.757 | 1.000 | 0.773 | 1.000 | 0.963 | 0.960 | 0.901 | 1.000 | 0.980 | 1.000 | 0.954 | 1.000 | 0.973 | 0.858 | 1.000 | 1.000 | 0.849 | 0.819 |
| 2902 | 0.765 | 1.000 | 0.785 | 1.000 | 0.963 | 0.960 | 0.900 | 1.000 | 0.990 | 1.000 | 0.959 | 1.000 | 0.955 | 0.855 | 1.000 | 1.000 | 0.852 | 0.822 |
| 2903 | 0.765 | 1.000 | 0.791 | 1.000 | 0.968 | 0.960 | 0.900 | 1.000 | 0.980 | 1.000 | 0.959 | 1.000 | 0.973 | 0.864 | 1.000 | 1.000 | 0.855 | 0.826 |
| 2904 | 0.770 | 1.000 | 0.792 | 0.986 | 0.963 | 0.960 | 0.898 | 1.000 | 0.990 | 1.000 | 0.959 | 1.000 | 0.955 | 0.856 | 1.000 | 0.981 | 0.854 | 0.825 |
| 2905 | 0.762 | 1.000 | 0.783 | 1.000 | 0.968 | 0.960 | 0.903 | 1.000 | 0.990 | 1.000 | 0.974 | 1.000 | 0.964 | 0.858 | 1.000 | 1.000 | 0.854 | 0.824 |
| 2906 | 0.763 | 1.000 | 0.820 | 0.986 | 0.968 | 0.960 | 0.897 | 1.000 | 0.980 | 1.000 | 0.959 | 1.000 | 0.982 | 0.853 | 1.000 | 1.000 | 0.855 | 0.826 |
| 2907 | 0.763 | 1.000 | 0.789 | 1.000 | 0.968 | 0.960 | 0.900 | 1.000 | 0.980 | 1.000 | 0.959 | 1.000 | 0.964 | 0.854 | 1.000 | 1.000 | 0.852 | 0.822 |
| 2908 | 0.767 | 1.000 | 0.789 | 0.986 | 0.968 | 0.960 | 0.901 | 1.000 | 0.980 | 1.000 | 0.968 | 1.000 | 0.982 | 0.853 | 1.000 | 1.000 | 0.854 | 0.825 |
| 2909 | 0.771 | 1.000 | 0.763 | 0.986 | 0.958 | 0.960 | 0.898 | 1.000 | 0.980 | 1.000 | 0.951 | 1.000 | 0.991 | 0.851 | 1.000 | 1.000 | 0.850 | 0.820 |
| 2910 | 0.768 | 1.000 | 0.791 | 1.000 | 0.968 | 0.960 | 0.904 | 1.000 | 1.000 | 1.000 | 0.968 | 1.000 | 0.964 | 0.853 | 1.000 | 1.000 | 0.855 | 0.826 |
| 2911 | 0.761 | 1.000 | 0.789 | 0.986 | 0.958 | 0.960 | 0.892 | 1.000 | 0.990 | 1.000 | 0.957 | 1.000 | 0.973 | 0.857 | 1.000 | 1.000 | 0.851 | 0.821 |
| 2912 | 0.767 | 1.000 | 0.794 | 0.986 | 0.953 | 0.960 | 0.908 | 1.000 | 1.000 | 1.000 | 0.962 | 1.000 | 0.964 | 0.859 | 1.000 | 1.000 | 0.856 | 0.827 |
| 2913 | 0.771 | 1.000 | 0.789 | 0.986 | 0.968 | 0.960 | 0.901 | 1.000 | 0.990 | 1.000 | 0.954 | 1.000 | 0.973 | 0.854 | 1.000 | 1.000 | 0.854 | 0.825 |
| 2914 | 0.776 | 1.000 | 0.791 | 1.000 | 0.963 | 0.960 | 0.909 | 1.000 | 0.980 | 1.000 | 0.954 | 1.000 | 0.982 | 0.851 | 1.000 | 1.000 | 0.856 | 0.827 |
| 2915 | 0.777 | 1.000 | 0.789 | 1.000 | 0.963 | 0.960 | 0.894 | 1.000 | 0.980 | 1.000 | 0.957 | 1.000 | 0.991 | 0.863 | 1.000 | 1.000 | 0.858 | 0.829 |
| 2916 | 0.767 | 1.000 | 0.789 | 1.000 | 0.963 | 0.960 | 0.900 | 1.000 | 0.980 | 1.000 | 0.948 | 1.000 | 0.973 | 0.854 | 1.000 | 1.000 | 0.852 | 0.823 |
| 2917 | 0.771 | 1.000 | 0.791 | 0.986 | 0.968 | 0.960 | 0.906 | 1.000 | 1.000 | 1.000 | 0.962 | 1.000 | 0.982 | 0.857 | 1.000 | 1.000 | 0.857 | 0.828 |
| 2918 | 0.763 | 1.000 | 0.783 | 0.986 | 0.968 | 0.960 | 0.894 | 1.000 | 0.990 | 1.000 | 0.959 | 1.000 | 0.973 | 0.854 | 1.000 | 1.000 | 0.851 | 0.821 |
| 2919 | 0.776 | 1.000 | 0.779 | 1.000 | 0.968 | 0.960 | 0.908 | 1.000 | 1.000 | 1.000 | 0.968 | 1.000 | 0.973 | 0.862 | 1.000 | 1.000 | 0.859 | 0.831 |
| 2920 | 0.765 | 1.000 | 0.798 | 0.986 | 0.968 | 0.960 | 0.897 | 1.000 | 0.969 | 1.000 | 0.959 | 1.000 | 0.973 | 0.856 | 1.000 | 1.000 | 0.853 | 0.824 |
| 2921 | 0.776 | 1.000 | 0.802 | 0.986 | 0.963 | 0.960 | 0.891 | 1.000 | 0.990 | 1.000 | 0.959 | 1.000 | 0.955 | 0.861 | 1.000 | 1.000 | 0.857 | 0.829 |
| 2922 | 0.778 | 1.000 | 0.787 | 1.000 | 0.958 | 0.960 | 0.911 | 1.000 | 0.990 | 1.000 | 0.968 | 1.000 | 0.973 | 0.858 | 1.000 | 1.000 | 0.859 | 0.831 |
| 2923 | 0.765 | 1.000 | 0.796 | 0.986 | 0.968 | 0.960 | 0.901 | 1.000 | 0.990 | 1.000 | 0.957 | 1.000 | 0.973 | 0.854 | 1.000 | 1.000 | 0.853 | 0.824 |

| | | | | | | | | | | | | | | | | | | |
|---|---|---|---|---|---|---|---|---|---|---|---|---|---|---|---|---|---|---|
| 2924 | 0.761 | 1.000 | 0.791 | 1.000 | 0.963 | 0.960 | 0.903 | 1.000 | 0.990 | 1.000 | 0.971 | 1.000 | 0.982 | 0.862 | 1.000 | 1.000 | 0.855 | 0.827 |
| 2925 | 0.781 | 1.000 | 0.792 | 1.000 | 0.963 | 0.960 | 0.900 | 1.000 | 0.990 | 1.000 | 0.962 | 1.000 | 0.991 | 0.856 | 1.000 | 1.000 | 0.859 | 0.830 |
| 2926 | 0.770 | 1.000 | 0.806 | 1.000 | 0.968 | 0.960 | 0.895 | 1.000 | 0.990 | 1.000 | 0.968 | 1.000 | 0.973 | 0.855 | 1.000 | 1.000 | 0.856 | 0.828 |
| 2927 | 0.776 | 1.000 | 0.787 | 0.986 | 0.958 | 0.960 | 0.904 | 1.000 | 0.980 | 1.000 | 0.965 | 1.000 | 0.973 | 0.860 | 1.000 | 1.000 | 0.858 | 0.829 |
| 2928 | 0.762 | 1.000 | 0.798 | 0.986 | 0.963 | 0.960 | 0.892 | 1.000 | 0.990 | 1.000 | 0.957 | 1.000 | 0.982 | 0.855 | 1.000 | 1.000 | 0.852 | 0.822 |
| 2929 | 0.771 | 1.000 | 0.814 | 1.000 | 0.968 | 0.960 | 0.904 | 1.000 | 0.990 | 1.000 | 0.951 | 1.000 | 0.973 | 0.862 | 1.000 | 1.000 | 0.859 | 0.831 |
| 2930 | 0.773 | 1.000 | 0.789 | 0.986 | 0.958 | 0.960 | 0.900 | 1.000 | 0.990 | 1.000 | 0.957 | 1.000 | 0.964 | 0.851 | 1.000 | 1.000 | 0.853 | 0.824 |
| 2931 | 0.771 | 1.000 | 0.787 | 1.000 | 0.974 | 0.960 | 0.897 | 1.000 | 1.000 | 1.000 | 0.957 | 1.000 | 0.955 | 0.862 | 1.000 | 1.000 | 0.856 | 0.827 |
| 2932 | 0.757 | 1.000 | 0.775 | 0.986 | 0.958 | 0.960 | 0.908 | 1.000 | 0.980 | 1.000 | 0.948 | 1.000 | 0.991 | 0.864 | 1.000 | 0.981 | 0.851 | 0.822 |
| 2933 | 0.780 | 1.000 | 0.783 | 1.000 | 0.974 | 0.960 | 0.898 | 1.000 | 1.000 | 1.000 | 0.957 | 1.000 | 0.973 | 0.851 | 1.000 | 1.000 | 0.856 | 0.827 |
| 2934 | 0.775 | 1.000 | 0.800 | 1.000 | 0.968 | 0.960 | 0.900 | 1.000 | 0.990 | 1.000 | 0.968 | 1.000 | 0.955 | 0.862 | 1.000 | 1.000 | 0.859 | 0.831 |
| 2935 | 0.771 | 1.000 | 0.792 | 1.000 | 0.963 | 0.960 | 0.897 | 1.000 | 0.990 | 1.000 | 0.959 | 1.000 | 0.982 | 0.866 | 1.000 | 1.000 | 0.858 | 0.829 |
| 2936 | 0.763 | 1.000 | 0.789 | 1.000 | 0.968 | 0.960 | 0.906 | 1.000 | 1.000 | 1.000 | 0.957 | 0.970 | 0.982 | 0.853 | 1.000 | 1.000 | 0.853 | 0.824 |
| 2937 | 0.774 | 1.000 | 0.789 | 1.000 | 0.968 | 0.960 | 0.901 | 1.000 | 0.990 | 1.000 | 0.962 | 1.000 | 0.982 | 0.858 | 1.000 | 1.000 | 0.857 | 0.829 |
| 2938 | 0.761 | 1.000 | 0.789 | 0.986 | 0.974 | 0.960 | 0.900 | 1.000 | 0.980 | 1.000 | 0.954 | 1.000 | 0.964 | 0.859 | 1.000 | 1.000 | 0.852 | 0.823 |
| 2939 | 0.764 | 1.000 | 0.802 | 0.986 | 0.974 | 0.960 | 0.914 | 1.000 | 0.980 | 1.000 | 0.957 | 1.000 | 0.982 | 0.857 | 1.000 | 1.000 | 0.856 | 0.828 |
| 2940 | 0.772 | 1.000 | 0.798 | 1.000 | 0.963 | 0.960 | 0.901 | 1.000 | 0.990 | 1.000 | 0.954 | 1.000 | 0.973 | 0.857 | 1.000 | 1.000 | 0.856 | 0.827 |
| 2941 | 0.771 | 1.000 | 0.791 | 0.986 | 0.968 | 0.960 | 0.900 | 1.000 | 1.000 | 1.000 | 0.965 | 1.000 | 0.955 | 0.854 | 1.000 | 0.981 | 0.855 | 0.825 |
| 2942 | 0.767 | 1.000 | 0.777 | 1.000 | 0.968 | 0.960 | 0.897 | 1.000 | 1.000 | 1.000 | 0.957 | 1.000 | 0.964 | 0.857 | 1.000 | 1.000 | 0.852 | 0.823 |
| 2943 | 0.765 | 1.000 | 0.781 | 0.986 | 0.963 | 0.960 | 0.898 | 1.000 | 0.980 | 1.000 | 0.962 | 1.000 | 0.955 | 0.860 | 1.000 | 1.000 | 0.853 | 0.823 |
| 2944 | 0.769 | 1.000 | 0.792 | 1.000 | 0.968 | 0.960 | 0.911 | 1.000 | 0.990 | 1.000 | 0.957 | 1.000 | 0.982 | 0.850 | 1.000 | 1.000 | 0.855 | 0.826 |
| 2945 | 0.771 | 1.000 | 0.796 | 1.000 | 0.974 | 0.960 | 0.900 | 1.000 | 1.000 | 1.000 | 0.959 | 1.000 | 0.964 | 0.850 | 1.000 | 1.000 | 0.854 | 0.825 |
| 2946 | 0.769 | 1.000 | 0.789 | 1.000 | 0.968 | 0.960 | 0.906 | 1.000 | 0.990 | 1.000 | 0.959 | 1.000 | 0.991 | 0.856 | 1.000 | 1.000 | 0.856 | 0.827 |
| 2947 | 0.767 | 1.000 | 0.787 | 1.000 | 0.974 | 0.960 | 0.904 | 1.000 | 0.980 | 1.000 | 0.962 | 1.000 | 0.964 | 0.850 | 1.000 | 1.000 | 0.853 | 0.824 |
| 2948 | 0.764 | 1.000 | 0.792 | 0.986 | 0.968 | 0.960 | 0.912 | 1.000 | 1.000 | 1.000 | 0.957 | 1.000 | 0.982 | 0.858 | 1.000 | 1.000 | 0.855 | 0.827 |
| 2949 | 0.769 | 1.000 | 0.785 | 1.000 | 0.963 | 0.960 | 0.904 | 1.000 | 0.990 | 1.000 | 0.959 | 1.000 | 0.964 | 0.859 | 1.000 | 1.000 | 0.855 | 0.826 |
| 2950 | 0.759 | 1.000 | 0.804 | 1.000 | 0.963 | 0.960 | 0.903 | 1.000 | 0.980 | 1.000 | 0.962 | 1.000 | 0.973 | 0.852 | 1.000 | 1.000 | 0.852 | 0.823 |
| 2951 | 0.770 | 1.000 | 0.791 | 1.000 | 0.974 | 0.960 | 0.901 | 1.000 | 0.990 | 1.000 | 0.954 | 1.000 | 0.964 | 0.855 | 1.000 | 1.000 | 0.855 | 0.826 |
| 2952 | 0.769 | 1.000 | 0.787 | 1.000 | 0.968 | 0.960 | 0.894 | 1.000 | 1.000 | 1.000 | 0.951 | 1.000 | 0.973 | 0.861 | 1.000 | 1.000 | 0.854 | 0.825 |
| 2953 | 0.765 | 1.000 | 0.785 | 1.000 | 0.968 | 0.960 | 0.909 | 1.000 | 1.000 | 1.000 | 0.954 | 1.000 | 0.973 | 0.853 | 1.000 | 1.000 | 0.853 | 0.824 |
| 2954 | 0.769 | 1.000 | 0.787 | 1.000 | 0.968 | 0.960 | 0.894 | 1.000 | 0.990 | 1.000 | 0.965 | 1.000 | 0.973 | 0.861 | 1.000 | 1.000 | 0.855 | 0.826 |
| 2955 | 0.767 | 1.000 | 0.796 | 1.000 | 0.968 | 0.960 | 0.906 | 1.000 | 0.990 | 1.000 | 0.954 | 1.000 | 0.955 | 0.854 | 1.000 | 1.000 | 0.854 | 0.825 |
| 2956 | 0.773 | 1.000 | 0.794 | 1.000 | 0.968 | 0.960 | 0.906 | 1.000 | 0.990 | 1.000 | 0.962 | 1.000 | 0.973 | 0.852 | 1.000 | 1.000 | 0.856 | 0.828 |
| 2957 | 0.771 | 1.000 | 0.794 | 0.986 | 0.968 | 0.960 | 0.886 | 1.000 | 0.990 | 1.000 | 0.957 | 1.000 | 0.973 | 0.849 | 1.000 | 1.000 | 0.852 | 0.822 |
| 2958 | 0.768 | 1.000 | 0.792 | 0.986 | 0.963 | 0.960 | 0.900 | 1.000 | 0.980 | 1.000 | 0.959 | 1.000 | 0.955 | 0.849 | 1.000 | 1.000 | 0.852 | 0.822 |
| 2959 | 0.773 | 1.000 | 0.798 | 0.986 | 0.963 | 0.960 | 0.901 | 1.000 | 0.980 | 1.000 | 0.954 | 1.000 | 0.973 | 0.852 | 1.000 | 1.000 | 0.855 | 0.826 |
| 2960 | 0.761 | 1.000 | 0.794 | 1.000 | 0.963 | 0.960 | 0.911 | 1.000 | 0.990 | 1.000 | 0.957 | 1.000 | 0.982 | 0.869 | 1.000 | 1.000 | 0.857 | 0.829 |
| 2961 | 0.773 | 1.000 | 0.785 | 1.000 | 0.974 | 0.960 | 0.901 | 1.000 | 0.969 | 1.000 | 0.959 | 1.000 | 0.973 | 0.857 | 1.000 | 1.000 | 0.855 | 0.827 |
| 2962 | 0.764 | 1.000 | 0.791 | 1.000 | 0.968 | 0.960 | 0.903 | 1.000 | 0.990 | 1.000 | 0.962 | 1.000 | 0.964 | 0.856 | 1.000 | 1.000 | 0.854 | 0.825 |

| | | | | | | | | | | | | | | | | | |
|---|---|---|---|---|---|---|---|---|---|---|---|---|---|---|---|---|---|
| 2963 | 0.771 | 1.000 | 0.796 | 1.000 | 0.968 | 0.960 | 0.900 | 1.000 | 1.000 | 1.000 | 0.957 | 1.000 | 0.964 | 0.861 | 1.000 | 0.981 | 0.857 | 0.828 |
| 2964 | 0.772 | 1.000 | 0.783 | 0.986 | 0.968 | 0.960 | 0.900 | 1.000 | 0.990 | 1.000 | 0.962 | 1.000 | 0.955 | 0.854 | 1.000 | 1.000 | 0.854 | 0.824 |
| 2965 | 0.765 | 1.000 | 0.792 | 0.986 | 0.974 | 0.960 | 0.903 | 1.000 | 1.000 | 1.000 | 0.962 | 1.000 | 0.964 | 0.854 | 1.000 | 1.000 | 0.854 | 0.825 |
| 2966 | 0.771 | 1.000 | 0.775 | 1.000 | 0.979 | 0.960 | 0.897 | 1.000 | 1.000 | 1.000 | 0.957 | 1.000 | 0.973 | 0.854 | 1.000 | 1.000 | 0.853 | 0.824 |
| 2967 | 0.776 | 1.000 | 0.796 | 0.986 | 0.968 | 0.960 | 0.898 | 1.000 | 0.990 | 1.000 | 0.965 | 1.000 | 0.973 | 0.852 | 1.000 | 1.000 | 0.856 | 0.828 |
| 2968 | 0.775 | 1.000 | 0.804 | 1.000 | 0.968 | 0.960 | 0.900 | 1.000 | 1.000 | 1.000 | 0.959 | 1.000 | 0.973 | 0.853 | 1.000 | 1.000 | 0.857 | 0.829 |
| 2969 | 0.764 | 1.000 | 0.785 | 1.000 | 0.968 | 0.960 | 0.906 | 1.000 | 0.980 | 1.000 | 0.957 | 1.000 | 0.964 | 0.853 | 1.000 | 1.000 | 0.852 | 0.823 |
| 2970 | 0.758 | 1.000 | 0.796 | 1.000 | 0.968 | 0.960 | 0.903 | 1.000 | 0.990 | 1.000 | 0.962 | 1.000 | 0.973 | 0.851 | 1.000 | 1.000 | 0.851 | 0.822 |
| 2971 | 0.772 | 1.000 | 0.787 | 1.000 | 0.963 | 0.960 | 0.898 | 1.000 | 0.980 | 1.000 | 0.954 | 1.000 | 0.982 | 0.856 | 1.000 | 1.000 | 0.854 | 0.825 |
| 2972 | 0.769 | 1.000 | 0.785 | 1.000 | 0.963 | 0.960 | 0.903 | 1.000 | 0.980 | 1.000 | 0.959 | 1.000 | 0.973 | 0.855 | 1.000 | 1.000 | 0.854 | 0.825 |
| 2973 | 0.780 | 1.000 | 0.794 | 1.000 | 0.968 | 0.960 | 0.906 | 1.000 | 0.990 | 1.000 | 0.965 | 1.000 | 0.964 | 0.859 | 1.000 | 0.981 | 0.860 | 0.832 |
| 2974 | 0.773 | 1.000 | 0.771 | 0.986 | 0.958 | 0.960 | 0.898 | 1.000 | 0.990 | 1.000 | 0.965 | 1.000 | 0.973 | 0.854 | 1.000 | 1.000 | 0.853 | 0.823 |
| 2975 | 0.768 | 1.000 | 0.789 | 0.986 | 0.963 | 0.960 | 0.906 | 1.000 | 0.980 | 1.000 | 0.957 | 1.000 | 0.973 | 0.861 | 1.000 | 1.000 | 0.855 | 0.827 |
| 2976 | 0.777 | 1.000 | 0.787 | 1.000 | 0.963 | 0.960 | 0.894 | 1.000 | 0.990 | 1.000 | 0.954 | 0.970 | 0.973 | 0.856 | 1.000 | 1.000 | 0.855 | 0.826 |
| 2977 | 0.769 | 1.000 | 0.792 | 0.986 | 0.963 | 0.960 | 0.904 | 1.000 | 0.990 | 1.000 | 0.954 | 1.000 | 0.955 | 0.858 | 1.000 | 1.000 | 0.855 | 0.826 |
| 2978 | 0.771 | 1.000 | 0.798 | 1.000 | 0.968 | 0.960 | 0.903 | 1.000 | 0.990 | 1.000 | 0.948 | 1.000 | 0.964 | 0.847 | 1.000 | 1.000 | 0.853 | 0.824 |
| 2979 | 0.768 | 1.000 | 0.798 | 1.000 | 0.958 | 0.960 | 0.895 | 1.000 | 1.000 | 1.000 | 0.965 | 1.000 | 0.982 | 0.853 | 1.000 | 1.000 | 0.854 | 0.825 |
| 2980 | 0.770 | 1.000 | 0.781 | 1.000 | 0.963 | 0.960 | 0.898 | 1.000 | 0.980 | 1.000 | 0.954 | 1.000 | 0.982 | 0.857 | 1.000 | 1.000 | 0.853 | 0.824 |
| 2981 | 0.765 | 1.000 | 0.800 | 1.000 | 0.963 | 0.960 | 0.898 | 1.000 | 0.980 | 1.000 | 0.957 | 1.000 | 0.973 | 0.856 | 1.000 | 1.000 | 0.854 | 0.825 |
| 2982 | 0.765 | 1.000 | 0.810 | 1.000 | 0.963 | 0.960 | 0.900 | 1.000 | 0.990 | 1.000 | 0.959 | 1.000 | 0.982 | 0.854 | 1.000 | 1.000 | 0.855 | 0.827 |
| 2983 | 0.769 | 1.000 | 0.789 | 0.971 | 0.963 | 0.960 | 0.894 | 1.000 | 0.990 | 1.000 | 0.962 | 1.000 | 0.982 | 0.854 | 1.000 | 1.000 | 0.853 | 0.824 |
| 2984 | 0.772 | 1.000 | 0.783 | 0.986 | 0.968 | 0.960 | 0.903 | 1.000 | 0.980 | 1.000 | 0.962 | 1.000 | 0.982 | 0.855 | 1.000 | 1.000 | 0.855 | 0.826 |
| 2985 | 0.767 | 1.000 | 0.791 | 1.000 | 0.968 | 0.960 | 0.900 | 1.000 | 0.990 | 1.000 | 0.962 | 1.000 | 0.964 | 0.850 | 1.000 | 1.000 | 0.853 | 0.823 |
| 2986 | 0.767 | 1.000 | 0.800 | 1.000 | 0.958 | 0.960 | 0.900 | 1.000 | 0.980 | 1.000 | 0.954 | 1.000 | 0.964 | 0.854 | 1.000 | 1.000 | 0.854 | 0.825 |
| 2987 | 0.768 | 1.000 | 0.787 | 0.971 | 0.963 | 0.960 | 0.906 | 1.000 | 0.980 | 1.000 | 0.959 | 1.000 | 0.973 | 0.858 | 1.000 | 0.981 | 0.854 | 0.825 |
| 2988 | 0.765 | 1.000 | 0.785 | 1.000 | 0.963 | 0.960 | 0.897 | 1.000 | 0.990 | 1.000 | 0.954 | 1.000 | 0.982 | 0.852 | 1.000 | 1.000 | 0.851 | 0.821 |
| 2989 | 0.767 | 1.000 | 0.791 | 1.000 | 0.963 | 0.960 | 0.898 | 1.000 | 0.980 | 1.000 | 0.957 | 1.000 | 0.982 | 0.858 | 1.000 | 1.000 | 0.854 | 0.825 |
| 2990 | 0.768 | 1.000 | 0.794 | 1.000 | 0.968 | 0.960 | 0.884 | 1.000 | 0.990 | 1.000 | 0.959 | 1.000 | 0.991 | 0.861 | 1.000 | 1.000 | 0.854 | 0.825 |
| 2991 | 0.769 | 1.000 | 0.800 | 0.986 | 0.974 | 0.960 | 0.906 | 1.000 | 1.000 | 1.000 | 0.962 | 1.000 | 0.955 | 0.851 | 1.000 | 1.000 | 0.855 | 0.827 |
| 2992 | 0.769 | 1.000 | 0.794 | 0.986 | 0.963 | 0.960 | 0.900 | 1.000 | 0.990 | 1.000 | 0.971 | 1.000 | 0.982 | 0.860 | 1.000 | 1.000 | 0.857 | 0.828 |
| 2993 | 0.769 | 1.000 | 0.787 | 1.000 | 0.974 | 0.960 | 0.906 | 1.000 | 0.980 | 1.000 | 0.962 | 1.000 | 0.955 | 0.854 | 1.000 | 1.000 | 0.854 | 0.825 |
| 2994 | 0.773 | 1.000 | 0.789 | 1.000 | 0.968 | 0.960 | 0.912 | 1.000 | 0.980 | 1.000 | 0.957 | 1.000 | 0.964 | 0.860 | 1.000 | 1.000 | 0.858 | 0.829 |
| 2995 | 0.772 | 1.000 | 0.792 | 0.971 | 0.974 | 0.960 | 0.901 | 1.000 | 0.980 | 1.000 | 0.954 | 1.000 | 0.982 | 0.864 | 1.000 | 1.000 | 0.857 | 0.829 |
| 2996 | 0.765 | 1.000 | 0.794 | 1.000 | 0.963 | 0.960 | 0.909 | 1.000 | 0.980 | 1.000 | 0.954 | 1.000 | 0.991 | 0.854 | 1.000 | 1.000 | 0.854 | 0.825 |
| 2997 | 0.767 | 1.000 | 0.794 | 1.000 | 0.963 | 0.960 | 0.901 | 1.000 | 0.990 | 1.000 | 0.954 | 1.000 | 0.955 | 0.858 | 1.000 | 1.000 | 0.854 | 0.825 |
| 2998 | 0.761 | 1.000 | 0.816 | 1.000 | 0.968 | 0.960 | 0.895 | 1.000 | 0.980 | 1.000 | 0.957 | 1.000 | 0.973 | 0.856 | 1.000 | 1.000 | 0.854 | 0.825 |
| 2999 | 0.768 | 1.000 | 0.791 | 1.000 | 0.968 | 0.960 | 0.900 | 1.000 | 0.980 | 1.000 | 0.957 | 1.000 | 0.982 | 0.850 | 1.000 | 1.000 | 0.853 | 0.823 |
| 3000 | 0.773 | 1.000 | 0.796 | 1.000 | 0.963 | 0.960 | 0.903 | 1.000 | 0.980 | 1.000 | 0.951 | 1.000 | 0.982 | 0.851 | 1.000 | 1.000 | 0.855 | 0.826 |
| 3001 | 0.767 | 1.000 | 0.798 | 0.971 | 0.963 | 0.960 | 0.906 | 1.000 | 1.000 | 1.000 | 0.957 | 1.000 | 0.964 | 0.853 | 1.000 | 1.000 | 0.854 | 0.825 |

| | | | | | | | | | | | | | | | | | | |
|---|---|---|---|---|---|---|---|---|---|---|---|---|---|---|---|---|---|---|
| 3002 | 0.767 | 1.000 | 0.806 | 1.000 | 0.974 | 0.960 | 0.900 | 1.000 | 0.990 | 1.000 | 0.957 | 1.000 | 0.982 | 0.855 | 1.000 | 1.000 | 0.856 | 0.827 |
| 3003 | 0.771 | 1.000 | 0.791 | 1.000 | 0.968 | 0.960 | 0.909 | 1.000 | 0.990 | 1.000 | 0.965 | 1.000 | 0.955 | 0.845 | 1.000 | 1.000 | 0.854 | 0.825 |
| 3004 | 0.763 | 1.000 | 0.791 | 1.000 | 0.958 | 0.960 | 0.904 | 1.000 | 0.990 | 1.000 | 0.954 | 1.000 | 0.982 | 0.859 | 1.000 | 1.000 | 0.854 | 0.825 |
| 3005 | 0.767 | 1.000 | 0.802 | 0.986 | 0.958 | 0.960 | 0.898 | 1.000 | 0.990 | 1.000 | 0.959 | 1.000 | 0.982 | 0.846 | 1.000 | 1.000 | 0.852 | 0.823 |
| 3006 | 0.775 | 1.000 | 0.800 | 1.000 | 0.958 | 0.960 | 0.906 | 1.000 | 1.000 | 1.000 | 0.965 | 1.000 | 0.973 | 0.851 | 1.000 | 1.000 | 0.857 | 0.829 |
| 3007 | 0.770 | 1.000 | 0.787 | 1.000 | 0.963 | 1.000 | 0.904 | 1.000 | 0.980 | 1.000 | 0.957 | 1.000 | 0.955 | 0.854 | 1.000 | 1.000 | 0.854 | 0.825 |
| 3008 | 0.769 | 1.000 | 0.779 | 1.000 | 0.968 | 0.960 | 0.897 | 1.000 | 0.990 | 1.000 | 0.957 | 1.000 | 0.973 | 0.854 | 1.000 | 1.000 | 0.852 | 0.823 |
| 3009 | 0.764 | 1.000 | 0.806 | 1.000 | 0.968 | 0.960 | 0.897 | 1.000 | 1.000 | 1.000 | 0.962 | 1.000 | 0.955 | 0.856 | 1.000 | 1.000 | 0.854 | 0.826 |
| 3010 | 0.772 | 1.000 | 0.781 | 0.986 | 0.958 | 0.960 | 0.901 | 1.000 | 0.990 | 1.000 | 0.951 | 1.000 | 0.973 | 0.857 | 1.000 | 1.000 | 0.854 | 0.825 |
| 3011 | 0.771 | 1.000 | 0.798 | 0.986 | 0.974 | 0.960 | 0.898 | 1.000 | 0.980 | 1.000 | 0.957 | 1.000 | 0.955 | 0.849 | 1.000 | 1.000 | 0.853 | 0.824 |
| 3012 | 0.772 | 1.000 | 0.787 | 1.000 | 0.968 | 0.960 | 0.915 | 1.000 | 0.990 | 1.000 | 0.959 | 1.000 | 0.973 | 0.861 | 1.000 | 1.000 | 0.858 | 0.830 |
| 3013 | 0.769 | 1.000 | 0.796 | 0.986 | 0.974 | 0.960 | 0.901 | 1.000 | 0.990 | 1.000 | 0.951 | 1.000 | 0.991 | 0.856 | 1.000 | 1.000 | 0.855 | 0.826 |
| 3014 | 0.764 | 1.000 | 0.785 | 1.000 | 0.968 | 0.960 | 0.901 | 1.000 | 0.990 | 1.000 | 0.957 | 1.000 | 0.964 | 0.855 | 1.000 | 1.000 | 0.852 | 0.823 |
| 3015 | 0.765 | 1.000 | 0.794 | 1.000 | 0.963 | 0.960 | 0.898 | 1.000 | 0.990 | 1.000 | 0.954 | 1.000 | 0.973 | 0.854 | 1.000 | 1.000 | 0.853 | 0.824 |
| 3016 | 0.774 | 1.000 | 0.783 | 1.000 | 0.953 | 0.960 | 0.906 | 1.000 | 0.980 | 1.000 | 0.945 | 1.000 | 0.991 | 0.865 | 1.000 | 1.000 | 0.857 | 0.828 |
| 3017 | 0.771 | 1.000 | 0.777 | 1.000 | 0.968 | 0.960 | 0.909 | 1.000 | 0.969 | 1.000 | 0.968 | 1.000 | 0.973 | 0.854 | 1.000 | 1.000 | 0.855 | 0.826 |
| 3018 | 0.768 | 1.000 | 0.794 | 1.000 | 0.963 | 0.960 | 0.906 | 1.000 | 0.990 | 1.000 | 0.954 | 1.000 | 0.964 | 0.862 | 1.000 | 1.000 | 0.856 | 0.828 |
| 3019 | 0.763 | 1.000 | 0.794 | 1.000 | 0.968 | 0.960 | 0.903 | 1.000 | 1.000 | 1.000 | 0.957 | 1.000 | 0.973 | 0.857 | 1.000 | 1.000 | 0.854 | 0.825 |
| 3020 | 0.763 | 1.000 | 0.785 | 0.986 | 0.968 | 0.960 | 0.909 | 1.000 | 0.990 | 1.000 | 0.959 | 1.000 | 0.964 | 0.863 | 1.000 | 1.000 | 0.855 | 0.826 |
| 3021 | 0.765 | 1.000 | 0.783 | 0.971 | 0.963 | 0.960 | 0.917 | 1.000 | 0.990 | 1.000 | 0.957 | 1.000 | 0.964 | 0.846 | 1.000 | 1.000 | 0.851 | 0.822 |
| 3022 | 0.760 | 1.000 | 0.800 | 1.000 | 0.968 | 0.960 | 0.900 | 1.000 | 0.969 | 1.000 | 0.965 | 1.000 | 0.991 | 0.855 | 1.000 | 1.000 | 0.853 | 0.824 |
| 3023 | 0.759 | 1.000 | 0.802 | 0.986 | 0.968 | 0.960 | 0.894 | 1.000 | 0.980 | 1.000 | 0.962 | 1.000 | 0.964 | 0.865 | 1.000 | 1.000 | 0.854 | 0.825 |
| 3024 | 0.767 | 1.000 | 0.771 | 0.986 | 0.963 | 0.960 | 0.898 | 1.000 | 0.990 | 1.000 | 0.959 | 1.000 | 0.964 | 0.852 | 1.000 | 1.000 | 0.850 | 0.820 |
| 3025 | 0.770 | 1.000 | 0.800 | 0.971 | 0.963 | 0.960 | 0.906 | 1.000 | 0.990 | 1.000 | 0.959 | 1.000 | 1.000 | 0.853 | 1.000 | 1.000 | 0.856 | 0.827 |
| 3026 | 0.761 | 1.000 | 0.804 | 1.000 | 0.963 | 0.960 | 0.901 | 1.000 | 0.990 | 1.000 | 0.959 | 1.000 | 0.964 | 0.849 | 1.000 | 1.000 | 0.852 | 0.823 |
| 3027 | 0.766 | 1.000 | 0.789 | 0.986 | 0.963 | 0.960 | 0.895 | 1.000 | 0.980 | 1.000 | 0.957 | 1.000 | 0.964 | 0.858 | 1.000 | 1.000 | 0.852 | 0.823 |
| 3028 | 0.763 | 1.000 | 0.783 | 0.986 | 0.968 | 0.960 | 0.904 | 1.000 | 0.990 | 1.000 | 0.957 | 1.000 | 0.973 | 0.853 | 1.000 | 1.000 | 0.851 | 0.822 |
| 3029 | 0.772 | 1.000 | 0.804 | 1.000 | 0.968 | 0.960 | 0.900 | 1.000 | 0.980 | 1.000 | 0.962 | 1.000 | 0.964 | 0.859 | 1.000 | 1.000 | 0.858 | 0.829 |
| 3030 | 0.770 | 1.000 | 0.781 | 1.000 | 0.974 | 0.960 | 0.908 | 1.000 | 1.000 | 1.000 | 0.962 | 1.000 | 0.964 | 0.852 | 1.000 | 1.000 | 0.854 | 0.826 |
| 3031 | 0.775 | 1.000 | 0.796 | 1.000 | 0.968 | 0.960 | 0.900 | 1.000 | 1.000 | 1.000 | 0.965 | 1.000 | 0.982 | 0.858 | 1.000 | 1.000 | 0.858 | 0.830 |
| 3032 | 0.757 | 1.000 | 0.779 | 1.000 | 0.974 | 0.960 | 0.906 | 1.000 | 0.980 | 1.000 | 0.962 | 1.000 | 0.964 | 0.858 | 1.000 | 1.000 | 0.851 | 0.822 |
| 3033 | 0.773 | 1.000 | 0.779 | 0.986 | 0.974 | 0.960 | 0.908 | 1.000 | 0.990 | 1.000 | 0.965 | 1.000 | 0.955 | 0.859 | 1.000 | 1.000 | 0.857 | 0.828 |
| 3034 | 0.769 | 1.000 | 0.798 | 1.000 | 0.968 | 0.960 | 0.912 | 1.000 | 0.990 | 1.000 | 0.957 | 1.000 | 0.955 | 0.861 | 1.000 | 1.000 | 0.858 | 0.830 |
| 3035 | 0.769 | 1.000 | 0.787 | 1.000 | 0.968 | 0.960 | 0.901 | 1.000 | 1.000 | 1.000 | 0.957 | 1.000 | 0.973 | 0.863 | 1.000 | 1.000 | 0.856 | 0.828 |
| 3036 | 0.771 | 1.000 | 0.794 | 1.000 | 0.968 | 0.960 | 0.901 | 1.000 | 0.980 | 1.000 | 0.948 | 1.000 | 0.955 | 0.856 | 1.000 | 1.000 | 0.854 | 0.825 |
| 3037 | 0.777 | 1.000 | 0.798 | 0.986 | 0.968 | 0.960 | 0.903 | 1.000 | 0.980 | 1.000 | 0.957 | 1.000 | 0.955 | 0.854 | 1.000 | 1.000 | 0.857 | 0.828 |
| 3038 | 0.768 | 1.000 | 0.792 | 1.000 | 0.974 | 0.960 | 0.898 | 1.000 | 1.000 | 1.000 | 0.954 | 1.000 | 0.982 | 0.847 | 1.000 | 1.000 | 0.852 | 0.823 |
| 3039 | 0.767 | 1.000 | 0.792 | 1.000 | 0.963 | 0.960 | 0.898 | 1.000 | 1.000 | 1.000 | 0.959 | 1.000 | 0.991 | 0.859 | 1.000 | 1.000 | 0.855 | 0.827 |
| 3040 | 0.767 | 1.000 | 0.796 | 1.000 | 0.974 | 0.960 | 0.897 | 1.000 | 0.969 | 1.000 | 0.954 | 1.000 | 0.955 | 0.854 | 1.000 | 1.000 | 0.853 | 0.824 |

| | | | | | | | | | | | | | | | | | |
|---|---|---|---|---|---|---|---|---|---|---|---|---|---|---|---|---|---|
| 3041 | 0.754 | 1.000 | 0.796 | 1.000 | 0.974 | 0.960 | 0.898 | 1.000 | 0.990 | 1.000 | 0.954 | 1.000 | 0.991 | 0.858 | 1.000 | 1.000 | 0.851 | 0.822 |
| 3042 | 0.766 | 1.000 | 0.785 | 1.000 | 0.963 | 0.960 | 0.898 | 1.000 | 0.980 | 1.000 | 0.959 | 1.000 | 0.964 | 0.858 | 1.000 | 1.000 | 0.853 | 0.823 |
| 3043 | 0.773 | 1.000 | 0.787 | 1.000 | 0.979 | 0.960 | 0.900 | 1.000 | 0.980 | 1.000 | 0.965 | 1.000 | 0.973 | 0.858 | 1.000 | 1.000 | 0.857 | 0.828 |
| 3044 | 0.773 | 1.000 | 0.796 | 1.000 | 0.963 | 0.960 | 0.903 | 1.000 | 0.980 | 1.000 | 0.951 | 1.000 | 0.973 | 0.851 | 1.000 | 1.000 | 0.855 | 0.826 |
| 3045 | 0.776 | 1.000 | 0.808 | 1.000 | 0.968 | 0.960 | 0.915 | 1.000 | 0.980 | 1.000 | 0.954 | 1.000 | 0.982 | 0.851 | 1.000 | 1.000 | 0.859 | 0.831 |
| 3046 | 0.773 | 1.000 | 0.783 | 0.971 | 0.958 | 0.960 | 0.906 | 1.000 | 1.000 | 1.000 | 0.957 | 1.000 | 0.991 | 0.865 | 1.000 | 1.000 | 0.858 | 0.829 |
| 3047 | 0.770 | 1.000 | 0.785 | 1.000 | 0.963 | 0.960 | 0.892 | 1.000 | 0.980 | 1.000 | 0.959 | 1.000 | 0.964 | 0.848 | 1.000 | 1.000 | 0.851 | 0.821 |
| 3048 | 0.766 | 1.000 | 0.775 | 0.986 | 0.968 | 1.000 | 0.909 | 1.000 | 1.000 | 1.000 | 0.954 | 1.000 | 0.973 | 0.855 | 1.000 | 1.000 | 0.853 | 0.824 |
| 3049 | 0.781 | 1.000 | 0.781 | 0.986 | 0.963 | 0.960 | 0.897 | 1.000 | 0.980 | 1.000 | 0.968 | 1.000 | 0.973 | 0.860 | 1.000 | 1.000 | 0.858 | 0.829 |
| 3050 | 0.768 | 1.000 | 0.800 | 1.000 | 0.963 | 0.960 | 0.908 | 1.000 | 0.980 | 1.000 | 0.965 | 1.000 | 0.982 | 0.846 | 1.000 | 1.000 | 0.854 | 0.825 |
| 3051 | 0.772 | 1.000 | 0.791 | 1.000 | 0.963 | 0.960 | 0.898 | 1.000 | 0.980 | 1.000 | 0.962 | 1.000 | 0.973 | 0.855 | 1.000 | 1.000 | 0.855 | 0.826 |
| 3052 | 0.767 | 1.000 | 0.792 | 1.000 | 0.963 | 0.960 | 0.909 | 1.000 | 0.980 | 1.000 | 0.968 | 1.000 | 0.973 | 0.852 | 1.000 | 1.000 | 0.855 | 0.826 |
| 3053 | 0.765 | 1.000 | 0.789 | 1.000 | 0.968 | 0.960 | 0.904 | 1.000 | 0.990 | 1.000 | 0.959 | 1.000 | 0.955 | 0.854 | 1.000 | 1.000 | 0.853 | 0.824 |
| 3054 | 0.758 | 1.000 | 0.806 | 0.986 | 0.968 | 0.960 | 0.906 | 1.000 | 0.980 | 1.000 | 0.948 | 1.000 | 0.973 | 0.861 | 1.000 | 1.000 | 0.854 | 0.825 |
| 3055 | 0.776 | 1.000 | 0.785 | 1.000 | 0.963 | 0.960 | 0.903 | 1.000 | 0.980 | 1.000 | 0.951 | 1.000 | 0.973 | 0.858 | 1.000 | 1.000 | 0.856 | 0.827 |
| 3056 | 0.778 | 1.000 | 0.789 | 0.986 | 0.963 | 0.960 | 0.911 | 1.000 | 0.990 | 1.000 | 0.962 | 1.000 | 0.982 | 0.854 | 1.000 | 1.000 | 0.858 | 0.830 |
| 3057 | 0.773 | 1.000 | 0.796 | 0.971 | 0.968 | 0.960 | 0.903 | 1.000 | 0.980 | 1.000 | 0.957 | 1.000 | 0.973 | 0.851 | 1.000 | 1.000 | 0.855 | 0.826 |
| 3058 | 0.767 | 1.000 | 0.810 | 1.000 | 0.974 | 0.960 | 0.892 | 1.000 | 1.000 | 1.000 | 0.968 | 1.000 | 0.973 | 0.858 | 1.000 | 1.000 | 0.857 | 0.828 |
| 3059 | 0.760 | 1.000 | 0.787 | 1.000 | 0.968 | 0.960 | 0.898 | 1.000 | 0.980 | 1.000 | 0.959 | 1.000 | 0.973 | 0.857 | 1.000 | 1.000 | 0.851 | 0.822 |
| 3060 | 0.771 | 1.000 | 0.791 | 1.000 | 0.968 | 0.960 | 0.904 | 1.000 | 0.980 | 1.000 | 0.957 | 1.000 | 0.982 | 0.858 | 1.000 | 1.000 | 0.856 | 0.828 |
| 3061 | 0.771 | 1.000 | 0.792 | 1.000 | 0.968 | 0.960 | 0.903 | 1.000 | 0.990 | 1.000 | 0.957 | 1.000 | 0.973 | 0.857 | 1.000 | 1.000 | 0.856 | 0.827 |
| 3062 | 0.761 | 1.000 | 0.787 | 1.000 | 0.968 | 0.960 | 0.904 | 1.000 | 1.000 | 1.000 | 0.965 | 1.000 | 0.973 | 0.854 | 1.000 | 1.000 | 0.853 | 0.823 |
| 3063 | 0.767 | 1.000 | 0.787 | 1.000 | 0.968 | 0.960 | 0.903 | 1.000 | 0.990 | 1.000 | 0.968 | 0.970 | 0.964 | 0.852 | 1.000 | 1.000 | 0.853 | 0.824 |
| 3064 | 0.768 | 1.000 | 0.806 | 1.000 | 0.963 | 0.960 | 0.903 | 1.000 | 0.990 | 1.000 | 0.959 | 1.000 | 0.973 | 0.864 | 1.000 | 1.000 | 0.858 | 0.830 |
| 3065 | 0.773 | 1.000 | 0.794 | 1.000 | 0.974 | 0.960 | 0.912 | 1.000 | 0.990 | 1.000 | 0.954 | 1.000 | 0.991 | 0.860 | 1.000 | 1.000 | 0.859 | 0.831 |
| 3066 | 0.764 | 1.000 | 0.804 | 1.000 | 0.963 | 0.960 | 0.900 | 1.000 | 0.980 | 1.000 | 0.965 | 1.000 | 0.973 | 0.859 | 1.000 | 1.000 | 0.855 | 0.827 |
| 3067 | 0.759 | 1.000 | 0.806 | 1.000 | 0.968 | 0.960 | 0.901 | 1.000 | 1.000 | 1.000 | 0.965 | 1.000 | 0.964 | 0.864 | 1.000 | 1.000 | 0.856 | 0.828 |
| 3068 | 0.770 | 1.000 | 0.792 | 0.986 | 0.963 | 0.960 | 0.900 | 1.000 | 1.000 | 1.000 | 0.959 | 1.000 | 0.964 | 0.856 | 1.000 | 1.000 | 0.855 | 0.826 |
| 3069 | 0.773 | 1.000 | 0.798 | 0.986 | 0.968 | 0.960 | 0.908 | 1.000 | 0.990 | 1.000 | 0.957 | 1.000 | 0.982 | 0.851 | 1.000 | 1.000 | 0.856 | 0.828 |
| 3070 | 0.771 | 1.000 | 0.792 | 0.986 | 0.968 | 0.960 | 0.903 | 1.000 | 0.990 | 1.000 | 0.962 | 1.000 | 0.973 | 0.860 | 1.000 | 1.000 | 0.857 | 0.828 |
| 3071 | 0.775 | 1.000 | 0.804 | 1.000 | 0.968 | 0.960 | 0.901 | 1.000 | 0.990 | 1.000 | 0.959 | 1.000 | 0.973 | 0.853 | 1.000 | 1.000 | 0.857 | 0.829 |
| 3072 | 0.773 | 1.000 | 0.798 | 0.986 | 0.963 | 0.960 | 0.904 | 1.000 | 0.990 | 1.000 | 0.954 | 1.000 | 0.964 | 0.854 | 1.000 | 1.000 | 0.856 | 0.827 |
| 3073 | 0.773 | 1.000 | 0.800 | 0.971 | 0.968 | 0.960 | 0.901 | 1.000 | 0.990 | 1.000 | 0.965 | 1.000 | 0.964 | 0.860 | 1.000 | 1.000 | 0.858 | 0.830 |
| 3074 | 0.771 | 1.000 | 0.796 | 0.986 | 0.963 | 0.960 | 0.906 | 1.000 | 0.990 | 1.000 | 0.957 | 1.000 | 0.982 | 0.856 | 1.000 | 1.000 | 0.856 | 0.827 |
| 3075 | 0.777 | 1.000 | 0.794 | 1.000 | 0.963 | 0.960 | 0.900 | 1.000 | 0.990 | 1.000 | 0.965 | 1.000 | 0.964 | 0.858 | 1.000 | 1.000 | 0.858 | 0.830 |
| 3076 | 0.761 | 1.000 | 0.785 | 1.000 | 0.968 | 0.960 | 0.903 | 1.000 | 1.000 | 1.000 | 0.959 | 1.000 | 0.964 | 0.855 | 1.000 | 1.000 | 0.852 | 0.823 |
| 3077 | 0.769 | 1.000 | 0.785 | 1.000 | 0.968 | 0.960 | 0.900 | 1.000 | 0.990 | 1.000 | 0.951 | 1.000 | 0.973 | 0.859 | 1.000 | 1.000 | 0.854 | 0.825 |
| 3078 | 0.773 | 1.000 | 0.792 | 1.000 | 0.963 | 0.960 | 0.901 | 1.000 | 0.990 | 1.000 | 0.959 | 1.000 | 0.973 | 0.859 | 1.000 | 1.000 | 0.857 | 0.828 |
| 3079 | 0.763 | 1.000 | 0.791 | 1.000 | 0.963 | 0.960 | 0.904 | 1.000 | 0.990 | 1.000 | 0.957 | 1.000 | 0.955 | 0.862 | 1.000 | 1.000 | 0.854 | 0.826 |

| | | | | | | | | | | | | | | | | | |
|---|---|---|---|---|---|---|---|---|---|---|---|---|---|---|---|---|---|
| 3080 | 0.774 | 1.000 | 0.785 | 1.000 | 0.968 | 0.960 | 0.908 | 1.000 | 0.980 | 1.000 | 0.962 | 1.000 | 0.973 | 0.858 | 1.000 | 1.000 | 0.857 | 0.829 |
| 3081 | 0.775 | 1.000 | 0.814 | 0.986 | 0.968 | 0.960 | 0.903 | 1.000 | 0.980 | 1.000 | 0.957 | 1.000 | 0.973 | 0.855 | 1.000 | 1.000 | 0.859 | 0.830 |
| 3082 | 0.763 | 1.000 | 0.794 | 1.000 | 0.963 | 0.960 | 0.908 | 1.000 | 1.000 | 1.000 | 0.959 | 1.000 | 0.964 | 0.852 | 1.000 | 1.000 | 0.853 | 0.824 |
| 3083 | 0.771 | 1.000 | 0.779 | 1.000 | 0.963 | 0.960 | 0.892 | 1.000 | 0.990 | 1.000 | 0.954 | 1.000 | 0.955 | 0.858 | 1.000 | 1.000 | 0.853 | 0.823 |
| 3084 | 0.765 | 1.000 | 0.806 | 1.000 | 0.968 | 0.960 | 0.900 | 1.000 | 0.990 | 1.000 | 0.957 | 1.000 | 0.973 | 0.854 | 1.000 | 1.000 | 0.854 | 0.825 |
| 3085 | 0.755 | 1.000 | 0.800 | 1.000 | 0.963 | 0.960 | 0.895 | 1.000 | 0.980 | 1.000 | 0.959 | 1.000 | 0.982 | 0.861 | 1.000 | 1.000 | 0.852 | 0.823 |
| 3086 | 0.757 | 1.000 | 0.794 | 1.000 | 0.974 | 0.960 | 0.900 | 1.000 | 0.990 | 1.000 | 0.959 | 1.000 | 0.955 | 0.854 | 1.000 | 1.000 | 0.851 | 0.821 |
| 3087 | 0.761 | 1.000 | 0.810 | 1.000 | 0.963 | 0.960 | 0.894 | 1.000 | 0.990 | 1.000 | 0.959 | 1.000 | 0.964 | 0.856 | 1.000 | 1.000 | 0.853 | 0.824 |
| 3088 | 0.769 | 1.000 | 0.796 | 0.986 | 0.968 | 0.960 | 0.901 | 1.000 | 0.990 | 1.000 | 0.965 | 1.000 | 0.973 | 0.854 | 1.000 | 1.000 | 0.855 | 0.826 |
| 3089 | 0.769 | 1.000 | 0.785 | 0.971 | 0.968 | 0.960 | 0.909 | 1.000 | 0.990 | 1.000 | 0.951 | 1.000 | 0.973 | 0.845 | 1.000 | 1.000 | 0.852 | 0.822 |
| 3090 | 0.769 | 1.000 | 0.798 | 1.000 | 0.968 | 0.960 | 0.903 | 1.000 | 0.990 | 1.000 | 0.962 | 1.000 | 0.964 | 0.858 | 1.000 | 1.000 | 0.856 | 0.828 |
| 3091 | 0.768 | 1.000 | 0.791 | 1.000 | 0.968 | 0.960 | 0.895 | 1.000 | 0.990 | 1.000 | 0.959 | 1.000 | 0.964 | 0.851 | 1.000 | 1.000 | 0.852 | 0.823 |
| 3092 | 0.769 | 1.000 | 0.792 | 1.000 | 0.974 | 0.960 | 0.909 | 1.000 | 0.980 | 1.000 | 0.957 | 1.000 | 0.982 | 0.853 | 1.000 | 1.000 | 0.855 | 0.826 |
| 3093 | 0.769 | 1.000 | 0.794 | 1.000 | 0.963 | 0.960 | 0.906 | 1.000 | 0.980 | 1.000 | 0.965 | 1.000 | 0.973 | 0.853 | 1.000 | 1.000 | 0.855 | 0.827 |
| 3094 | 0.769 | 1.000 | 0.785 | 1.000 | 0.968 | 0.960 | 0.908 | 1.000 | 1.000 | 1.000 | 0.965 | 1.000 | 0.973 | 0.857 | 1.000 | 1.000 | 0.856 | 0.827 |
| 3095 | 0.777 | 1.000 | 0.787 | 1.000 | 0.968 | 0.960 | 0.894 | 1.000 | 0.990 | 1.000 | 0.968 | 1.000 | 0.982 | 0.854 | 1.000 | 1.000 | 0.856 | 0.827 |
| 3096 | 0.783 | 1.000 | 0.791 | 1.000 | 0.974 | 0.960 | 0.900 | 1.000 | 0.990 | 1.000 | 0.957 | 1.000 | 0.955 | 0.859 | 1.000 | 1.000 | 0.859 | 0.831 |
| 3097 | 0.775 | 1.000 | 0.789 | 1.000 | 0.968 | 0.960 | 0.908 | 1.000 | 0.990 | 1.000 | 0.959 | 1.000 | 0.982 | 0.858 | 1.000 | 1.000 | 0.858 | 0.830 |
| 3098 | 0.771 | 1.000 | 0.808 | 1.000 | 0.968 | 0.960 | 0.904 | 1.000 | 0.990 | 1.000 | 0.954 | 1.000 | 0.982 | 0.857 | 1.000 | 1.000 | 0.858 | 0.830 |
| 3099 | 0.771 | 1.000 | 0.787 | 1.000 | 0.968 | 0.960 | 0.901 | 1.000 | 0.980 | 1.000 | 0.962 | 1.000 | 0.991 | 0.855 | 1.000 | 1.000 | 0.855 | 0.827 |
| 3100 | 0.763 | 1.000 | 0.796 | 1.000 | 0.968 | 0.960 | 0.906 | 1.000 | 0.969 | 1.000 | 0.954 | 1.000 | 0.955 | 0.851 | 1.000 | 1.000 | 0.852 | 0.823 |
| 3101 | 0.758 | 1.000 | 0.794 | 0.986 | 0.968 | 0.960 | 0.906 | 1.000 | 0.990 | 1.000 | 0.965 | 1.000 | 0.964 | 0.855 | 1.000 | 1.000 | 0.852 | 0.824 |
| 3102 | 0.764 | 1.000 | 0.779 | 1.000 | 0.968 | 0.960 | 0.904 | 1.000 | 0.990 | 1.000 | 0.945 | 1.000 | 0.982 | 0.865 | 1.000 | 1.000 | 0.854 | 0.825 |
| 3103 | 0.770 | 1.000 | 0.787 | 0.986 | 0.968 | 0.960 | 0.900 | 1.000 | 0.980 | 1.000 | 0.951 | 1.000 | 0.973 | 0.854 | 1.000 | 1.000 | 0.853 | 0.824 |
| 3104 | 0.778 | 1.000 | 0.794 | 0.986 | 0.968 | 0.960 | 0.912 | 1.000 | 1.000 | 1.000 | 0.965 | 1.000 | 0.955 | 0.863 | 1.000 | 1.000 | 0.861 | 0.833 |
| 3105 | 0.761 | 1.000 | 0.800 | 0.986 | 0.963 | 1.000 | 0.909 | 1.000 | 0.990 | 1.000 | 0.957 | 1.000 | 0.982 | 0.855 | 1.000 | 1.000 | 0.854 | 0.825 |
| 3106 | 0.769 | 1.000 | 0.781 | 0.986 | 0.958 | 0.960 | 0.897 | 1.000 | 0.990 | 1.000 | 0.962 | 1.000 | 0.991 | 0.854 | 1.000 | 1.000 | 0.853 | 0.824 |
| 3107 | 0.771 | 1.000 | 0.779 | 0.986 | 0.968 | 0.960 | 0.898 | 1.000 | 0.990 | 1.000 | 0.959 | 1.000 | 0.973 | 0.849 | 1.000 | 1.000 | 0.852 | 0.823 |
| 3108 | 0.773 | 1.000 | 0.802 | 1.000 | 0.968 | 0.960 | 0.911 | 1.000 | 0.980 | 1.000 | 0.965 | 1.000 | 0.982 | 0.859 | 1.000 | 1.000 | 0.860 | 0.832 |
| 3109 | 0.764 | 1.000 | 0.802 | 1.000 | 0.968 | 0.960 | 0.900 | 1.000 | 1.000 | 1.000 | 0.965 | 1.000 | 0.973 | 0.854 | 1.000 | 1.000 | 0.855 | 0.826 |
| 3110 | 0.774 | 1.000 | 0.794 | 1.000 | 0.963 | 0.960 | 0.898 | 1.000 | 1.000 | 1.000 | 0.968 | 1.000 | 0.982 | 0.860 | 1.000 | 1.000 | 0.858 | 0.830 |
| 3111 | 0.782 | 1.000 | 0.796 | 1.000 | 0.963 | 0.960 | 0.900 | 1.000 | 0.990 | 1.000 | 0.962 | 1.000 | 0.982 | 0.864 | 1.000 | 1.000 | 0.861 | 0.833 |
| 3112 | 0.759 | 1.000 | 0.792 | 0.986 | 0.968 | 0.960 | 0.906 | 1.000 | 1.000 | 1.000 | 0.959 | 1.000 | 0.964 | 0.857 | 1.000 | 1.000 | 0.853 | 0.824 |
| 3113 | 0.775 | 1.000 | 0.796 | 1.000 | 0.968 | 0.960 | 0.891 | 1.000 | 0.980 | 1.000 | 0.954 | 1.000 | 0.964 | 0.854 | 1.000 | 1.000 | 0.855 | 0.826 |
| 3114 | 0.772 | 1.000 | 0.796 | 1.000 | 0.968 | 0.960 | 0.897 | 1.000 | 0.990 | 1.000 | 0.951 | 1.000 | 0.973 | 0.855 | 1.000 | 1.000 | 0.855 | 0.826 |
| 3115 | 0.767 | 1.000 | 0.785 | 1.000 | 0.974 | 0.960 | 0.892 | 1.000 | 0.980 | 1.000 | 0.962 | 1.000 | 0.964 | 0.858 | 1.000 | 1.000 | 0.853 | 0.824 |
| 3116 | 0.781 | 1.000 | 0.800 | 1.000 | 0.968 | 0.960 | 0.892 | 1.000 | 0.990 | 1.000 | 0.957 | 1.000 | 0.946 | 0.861 | 1.000 | 1.000 | 0.859 | 0.830 |
| 3117 | 0.773 | 1.000 | 0.800 | 1.000 | 0.963 | 0.960 | 0.906 | 1.000 | 0.990 | 1.000 | 0.951 | 1.000 | 0.973 | 0.861 | 1.000 | 1.000 | 0.858 | 0.830 |
| 3118 | 0.768 | 1.000 | 0.775 | 1.000 | 0.963 | 0.960 | 0.904 | 1.000 | 0.990 | 1.000 | 0.965 | 1.000 | 0.973 | 0.858 | 1.000 | 1.000 | 0.854 | 0.825 |

| | | | | | | | | | | | | | | | | | |
|---|---|---|---|---|---|---|---|---|---|---|---|---|---|---|---|---|---|
| 3119 | 0.765 | 1.000 | 0.800 | 1.000 | 0.974 | 0.960 | 0.908 | 1.000 | 0.990 | 1.000 | 0.959 | 1.000 | 0.973 | 0.859 | 1.000 | 1.000 | 0.856 | 0.828 |
| 3120 | 0.773 | 1.000 | 0.781 | 1.000 | 0.968 | 0.960 | 0.898 | 1.000 | 0.990 | 1.000 | 0.957 | 1.000 | 0.964 | 0.853 | 1.000 | 1.000 | 0.853 | 0.824 |
| 3121 | 0.766 | 1.000 | 0.794 | 1.000 | 0.968 | 0.960 | 0.904 | 1.000 | 0.990 | 1.000 | 0.951 | 1.000 | 0.973 | 0.856 | 1.000 | 1.000 | 0.854 | 0.825 |
| 3122 | 0.774 | 1.000 | 0.794 | 1.000 | 0.968 | 0.960 | 0.895 | 1.000 | 0.990 | 1.000 | 0.951 | 1.000 | 0.973 | 0.851 | 1.000 | 1.000 | 0.854 | 0.825 |
| 3123 | 0.772 | 1.000 | 0.775 | 1.000 | 0.968 | 0.960 | 0.911 | 1.000 | 1.000 | 1.000 | 0.957 | 1.000 | 0.964 | 0.854 | 1.000 | 1.000 | 0.855 | 0.826 |
| 3124 | 0.777 | 1.000 | 0.781 | 0.971 | 0.963 | 0.960 | 0.900 | 1.000 | 0.990 | 1.000 | 0.957 | 1.000 | 0.973 | 0.857 | 1.000 | 1.000 | 0.855 | 0.826 |
| 3125 | 0.768 | 1.000 | 0.794 | 1.000 | 0.963 | 0.960 | 0.900 | 1.000 | 0.990 | 1.000 | 0.954 | 1.000 | 0.982 | 0.857 | 1.000 | 1.000 | 0.855 | 0.826 |
| 3126 | 0.769 | 1.000 | 0.785 | 0.971 | 0.968 | 0.960 | 0.906 | 1.000 | 0.990 | 1.000 | 0.948 | 1.000 | 0.955 | 0.862 | 1.000 | 1.000 | 0.855 | 0.826 |
| 3127 | 0.765 | 1.000 | 0.794 | 1.000 | 0.963 | 0.960 | 0.900 | 1.000 | 0.990 | 1.000 | 0.957 | 1.000 | 0.964 | 0.860 | 1.000 | 1.000 | 0.854 | 0.826 |
| 3128 | 0.774 | 1.000 | 0.789 | 0.986 | 0.974 | 0.960 | 0.895 | 1.000 | 0.980 | 1.000 | 0.957 | 1.000 | 0.955 | 0.861 | 1.000 | 1.000 | 0.856 | 0.826 |
| 3129 | 0.775 | 1.000 | 0.796 | 1.000 | 0.963 | 0.960 | 0.904 | 1.000 | 0.990 | 1.000 | 0.959 | 1.000 | 0.973 | 0.858 | 1.000 | 1.000 | 0.858 | 0.830 |
| 3130 | 0.776 | 1.000 | 0.802 | 1.000 | 0.963 | 0.960 | 0.895 | 1.000 | 0.990 | 1.000 | 0.965 | 1.000 | 0.973 | 0.861 | 1.000 | 1.000 | 0.859 | 0.831 |
| 3131 | 0.765 | 1.000 | 0.800 | 1.000 | 0.974 | 0.960 | 0.895 | 1.000 | 0.990 | 1.000 | 0.959 | 1.000 | 0.946 | 0.851 | 1.000 | 1.000 | 0.852 | 0.823 |
| 3132 | 0.769 | 1.000 | 0.789 | 1.000 | 0.968 | 0.960 | 0.911 | 1.000 | 0.990 | 1.000 | 0.959 | 1.000 | 0.973 | 0.860 | 1.000 | 1.000 | 0.857 | 0.829 |
| 3133 | 0.772 | 1.000 | 0.791 | 1.000 | 0.963 | 0.960 | 0.898 | 1.000 | 0.990 | 1.000 | 0.959 | 1.000 | 0.991 | 0.854 | 1.000 | 1.000 | 0.855 | 0.826 |
| 3134 | 0.773 | 1.000 | 0.791 | 1.000 | 0.963 | 0.960 | 0.894 | 1.000 | 0.990 | 1.000 | 0.957 | 1.000 | 0.973 | 0.852 | 1.000 | 1.000 | 0.854 | 0.825 |
| 3135 | 0.766 | 1.000 | 0.779 | 0.986 | 0.968 | 0.960 | 0.898 | 1.000 | 0.990 | 1.000 | 0.962 | 1.000 | 0.973 | 0.853 | 1.000 | 1.000 | 0.852 | 0.822 |
| 3136 | 0.767 | 1.000 | 0.792 | 0.986 | 0.968 | 0.960 | 0.900 | 1.000 | 0.980 | 1.000 | 0.968 | 1.000 | 0.973 | 0.854 | 1.000 | 1.000 | 0.854 | 0.825 |
| 3137 | 0.761 | 1.000 | 0.794 | 0.986 | 0.958 | 0.960 | 0.898 | 1.000 | 0.980 | 1.000 | 0.954 | 1.000 | 0.955 | 0.860 | 1.000 | 1.000 | 0.852 | 0.823 |
| 3138 | 0.775 | 1.000 | 0.794 | 1.000 | 0.968 | 0.960 | 0.898 | 1.000 | 0.990 | 1.000 | 0.962 | 1.000 | 0.955 | 0.858 | 1.000 | 1.000 | 0.857 | 0.829 |
| 3139 | 0.765 | 1.000 | 0.787 | 1.000 | 0.974 | 0.960 | 0.903 | 1.000 | 0.990 | 1.000 | 0.954 | 0.970 | 0.964 | 0.860 | 1.000 | 1.000 | 0.854 | 0.825 |
| 3140 | 0.778 | 1.000 | 0.781 | 1.000 | 0.968 | 0.960 | 0.906 | 1.000 | 0.990 | 1.000 | 0.957 | 1.000 | 0.982 | 0.863 | 1.000 | 1.000 | 0.859 | 0.831 |
| 3141 | 0.771 | 1.000 | 0.781 | 1.000 | 0.963 | 0.960 | 0.903 | 1.000 | 0.990 | 1.000 | 0.959 | 1.000 | 0.982 | 0.863 | 1.000 | 1.000 | 0.857 | 0.828 |
| 3142 | 0.763 | 1.000 | 0.791 | 0.986 | 0.968 | 0.960 | 0.897 | 1.000 | 0.990 | 1.000 | 0.962 | 1.000 | 0.964 | 0.862 | 1.000 | 1.000 | 0.854 | 0.825 |
| 3143 | 0.775 | 1.000 | 0.804 | 1.000 | 0.968 | 0.960 | 0.901 | 1.000 | 0.990 | 1.000 | 0.968 | 1.000 | 0.973 | 0.853 | 1.000 | 1.000 | 0.858 | 0.829 |
| 3144 | 0.764 | 1.000 | 0.794 | 0.986 | 0.963 | 0.960 | 0.892 | 1.000 | 0.980 | 1.000 | 0.965 | 1.000 | 0.973 | 0.856 | 1.000 | 1.000 | 0.852 | 0.823 |
| 3145 | 0.755 | 1.000 | 0.796 | 1.000 | 0.974 | 0.960 | 0.901 | 1.000 | 0.990 | 1.000 | 0.959 | 1.000 | 0.973 | 0.852 | 1.000 | 1.000 | 0.850 | 0.821 |
| 3146 | 0.767 | 1.000 | 0.789 | 0.986 | 0.968 | 0.960 | 0.898 | 1.000 | 0.990 | 1.000 | 0.957 | 1.000 | 0.964 | 0.862 | 1.000 | 1.000 | 0.855 | 0.826 |
| 3147 | 0.771 | 1.000 | 0.783 | 1.000 | 0.974 | 0.960 | 0.900 | 1.000 | 1.000 | 1.000 | 0.959 | 1.000 | 0.973 | 0.861 | 1.000 | 0.981 | 0.856 | 0.827 |
| 3148 | 0.767 | 1.000 | 0.798 | 1.000 | 0.968 | 0.960 | 0.901 | 1.000 | 1.000 | 1.000 | 0.962 | 1.000 | 0.964 | 0.857 | 1.000 | 1.000 | 0.855 | 0.827 |
| 3149 | 0.759 | 1.000 | 0.791 | 1.000 | 0.968 | 0.960 | 0.908 | 1.000 | 0.980 | 1.000 | 0.954 | 1.000 | 0.955 | 0.851 | 1.000 | 1.000 | 0.850 | 0.821 |
| 3150 | 0.777 | 1.000 | 0.787 | 1.000 | 0.968 | 0.960 | 0.903 | 1.000 | 0.980 | 1.000 | 0.954 | 1.000 | 0.973 | 0.859 | 1.000 | 1.000 | 0.857 | 0.829 |
| 3151 | 0.767 | 1.000 | 0.794 | 1.000 | 0.968 | 0.960 | 0.906 | 1.000 | 0.990 | 1.000 | 0.957 | 1.000 | 0.964 | 0.851 | 1.000 | 1.000 | 0.853 | 0.825 |
| 3152 | 0.769 | 1.000 | 0.781 | 1.000 | 0.968 | 0.960 | 0.901 | 1.000 | 0.969 | 1.000 | 0.962 | 1.000 | 0.982 | 0.859 | 1.000 | 1.000 | 0.855 | 0.825 |
| 3153 | 0.776 | 1.000 | 0.789 | 0.971 | 0.963 | 0.960 | 0.897 | 1.000 | 0.990 | 1.000 | 0.962 | 1.000 | 0.973 | 0.859 | 1.000 | 1.000 | 0.857 | 0.828 |
| 3154 | 0.767 | 1.000 | 0.792 | 1.000 | 0.968 | 0.960 | 0.904 | 1.000 | 0.980 | 1.000 | 0.957 | 1.000 | 0.982 | 0.861 | 1.000 | 1.000 | 0.856 | 0.828 |
| 3155 | 0.768 | 1.000 | 0.792 | 1.000 | 0.968 | 0.960 | 0.908 | 1.000 | 0.990 | 1.000 | 0.962 | 1.000 | 0.964 | 0.862 | 1.000 | 1.000 | 0.857 | 0.829 |
| 3156 | 0.765 | 1.000 | 0.794 | 1.000 | 0.968 | 0.960 | 0.906 | 1.000 | 1.000 | 1.000 | 0.965 | 1.000 | 0.982 | 0.848 | 1.000 | 1.000 | 0.854 | 0.825 |
| 3157 | 0.769 | 1.000 | 0.783 | 0.986 | 0.963 | 0.960 | 0.892 | 1.000 | 0.980 | 1.000 | 0.957 | 1.000 | 0.973 | 0.864 | 1.000 | 1.000 | 0.854 | 0.825 |

| | | | | | | | | | | | | | | | | | | |
|---|---|---|---|---|---|---|---|---|---|---|---|---|---|---|---|---|---|---|
| 3158 | 0.771 | 1.000 | 0.783 | 1.000 | 0.968 | 0.960 | 0.908 | 1.000 | 1.000 | 1.000 | 0.968 | 1.000 | 0.964 | 0.858 | 1.000 | 1.000 | 0.857 | 0.828 |
| 3159 | 0.775 | 1.000 | 0.783 | 1.000 | 0.963 | 1.000 | 0.895 | 1.000 | 0.990 | 1.000 | 0.957 | 1.000 | 0.973 | 0.850 | 1.000 | 1.000 | 0.853 | 0.824 |
| 3160 | 0.765 | 1.000 | 0.789 | 1.000 | 0.968 | 0.960 | 0.903 | 1.000 | 0.990 | 1.000 | 0.957 | 1.000 | 0.973 | 0.856 | 1.000 | 1.000 | 0.854 | 0.825 |
| 3161 | 0.772 | 1.000 | 0.779 | 0.986 | 0.968 | 0.960 | 0.906 | 1.000 | 0.990 | 1.000 | 0.965 | 1.000 | 0.973 | 0.858 | 1.000 | 1.000 | 0.856 | 0.827 |
| 3162 | 0.774 | 1.000 | 0.802 | 0.986 | 0.968 | 0.960 | 0.901 | 1.000 | 0.990 | 1.000 | 0.971 | 1.000 | 0.973 | 0.852 | 1.000 | 1.000 | 0.857 | 0.829 |
| 3163 | 0.764 | 1.000 | 0.783 | 0.986 | 0.963 | 0.960 | 0.898 | 1.000 | 0.980 | 1.000 | 0.971 | 1.000 | 0.982 | 0.864 | 1.000 | 1.000 | 0.855 | 0.826 |
| 3164 | 0.761 | 1.000 | 0.789 | 1.000 | 0.968 | 0.960 | 0.906 | 1.000 | 0.990 | 1.000 | 0.948 | 1.000 | 0.973 | 0.858 | 1.000 | 1.000 | 0.853 | 0.824 |
| 3165 | 0.770 | 1.000 | 0.806 | 1.000 | 0.968 | 0.960 | 0.906 | 1.000 | 0.980 | 1.000 | 0.959 | 1.000 | 0.973 | 0.861 | 1.000 | 1.000 | 0.858 | 0.830 |
| 3166 | 0.764 | 1.000 | 0.792 | 0.971 | 0.968 | 0.960 | 0.895 | 1.000 | 0.990 | 1.000 | 0.948 | 1.000 | 0.964 | 0.861 | 1.000 | 1.000 | 0.853 | 0.823 |
| 3167 | 0.772 | 1.000 | 0.777 | 1.000 | 0.974 | 0.960 | 0.901 | 1.000 | 0.990 | 1.000 | 0.959 | 1.000 | 0.982 | 0.861 | 1.000 | 1.000 | 0.856 | 0.827 |
| 3168 | 0.775 | 1.000 | 0.796 | 0.986 | 0.974 | 0.960 | 0.898 | 1.000 | 0.990 | 1.000 | 0.959 | 1.000 | 0.964 | 0.857 | 1.000 | 1.000 | 0.857 | 0.828 |
| 3169 | 0.771 | 1.000 | 0.792 | 0.986 | 0.958 | 0.960 | 0.908 | 1.000 | 0.990 | 1.000 | 0.974 | 1.000 | 0.982 | 0.855 | 1.000 | 1.000 | 0.857 | 0.829 |
| 3170 | 0.768 | 1.000 | 0.792 | 1.000 | 0.958 | 0.960 | 0.906 | 1.000 | 0.969 | 1.000 | 0.965 | 1.000 | 0.973 | 0.853 | 1.000 | 1.000 | 0.854 | 0.825 |
| 3171 | 0.766 | 1.000 | 0.779 | 1.000 | 0.968 | 0.960 | 0.903 | 1.000 | 0.990 | 1.000 | 0.962 | 1.000 | 0.964 | 0.854 | 1.000 | 1.000 | 0.853 | 0.823 |
| 3172 | 0.768 | 1.000 | 0.814 | 0.986 | 0.963 | 0.960 | 0.900 | 1.000 | 0.990 | 1.000 | 0.968 | 1.000 | 0.946 | 0.856 | 1.000 | 1.000 | 0.856 | 0.828 |
| 3173 | 0.777 | 1.000 | 0.789 | 1.000 | 0.963 | 0.960 | 0.900 | 1.000 | 0.980 | 1.000 | 0.965 | 1.000 | 0.991 | 0.858 | 1.000 | 1.000 | 0.858 | 0.829 |
| 3174 | 0.769 | 1.000 | 0.787 | 0.971 | 0.968 | 0.960 | 0.904 | 1.000 | 0.980 | 1.000 | 0.962 | 1.000 | 0.955 | 0.857 | 1.000 | 1.000 | 0.854 | 0.825 |
| 3175 | 0.767 | 1.000 | 0.804 | 1.000 | 0.968 | 0.960 | 0.906 | 1.000 | 0.990 | 1.000 | 0.968 | 0.970 | 0.991 | 0.858 | 1.000 | 1.000 | 0.858 | 0.830 |
| 3176 | 0.759 | 1.000 | 0.787 | 1.000 | 0.968 | 0.960 | 0.901 | 1.000 | 0.990 | 1.000 | 0.962 | 1.000 | 0.973 | 0.858 | 1.000 | 1.000 | 0.852 | 0.823 |
| 3177 | 0.767 | 1.000 | 0.787 | 0.986 | 0.963 | 0.960 | 0.892 | 1.000 | 0.990 | 1.000 | 0.965 | 1.000 | 0.982 | 0.862 | 1.000 | 1.000 | 0.854 | 0.825 |
| 3178 | 0.771 | 1.000 | 0.789 | 1.000 | 0.963 | 0.960 | 0.906 | 1.000 | 0.990 | 1.000 | 0.971 | 1.000 | 0.982 | 0.854 | 1.000 | 1.000 | 0.856 | 0.828 |
| 3179 | 0.772 | 1.000 | 0.802 | 0.986 | 0.968 | 0.960 | 0.906 | 1.000 | 0.980 | 1.000 | 0.959 | 1.000 | 0.973 | 0.861 | 1.000 | 1.000 | 0.858 | 0.830 |
| 3180 | 0.771 | 1.000 | 0.806 | 1.000 | 0.968 | 0.960 | 0.898 | 1.000 | 0.980 | 1.000 | 0.954 | 1.000 | 0.973 | 0.856 | 1.000 | 1.000 | 0.856 | 0.827 |
| 3181 | 0.766 | 1.000 | 0.798 | 1.000 | 0.968 | 0.960 | 0.894 | 1.000 | 0.980 | 1.000 | 0.962 | 1.000 | 0.964 | 0.854 | 1.000 | 1.000 | 0.853 | 0.824 |
| 3182 | 0.771 | 1.000 | 0.787 | 1.000 | 0.968 | 0.960 | 0.903 | 1.000 | 0.980 | 1.000 | 0.957 | 1.000 | 0.973 | 0.858 | 1.000 | 1.000 | 0.856 | 0.827 |
| 3183 | 0.772 | 1.000 | 0.802 | 1.000 | 0.968 | 0.960 | 0.892 | 1.000 | 0.990 | 1.000 | 0.962 | 1.000 | 0.973 | 0.857 | 1.000 | 1.000 | 0.856 | 0.827 |
| 3184 | 0.765 | 1.000 | 0.785 | 0.971 | 0.974 | 0.960 | 0.912 | 1.000 | 0.969 | 1.000 | 0.957 | 1.000 | 0.991 | 0.861 | 1.000 | 1.000 | 0.855 | 0.827 |
| 3185 | 0.767 | 1.000 | 0.785 | 1.000 | 0.974 | 0.960 | 0.906 | 1.000 | 0.990 | 1.000 | 0.959 | 1.000 | 0.964 | 0.853 | 1.000 | 1.000 | 0.853 | 0.824 |
| 3186 | 0.770 | 1.000 | 0.798 | 1.000 | 0.968 | 0.960 | 0.898 | 1.000 | 0.980 | 1.000 | 0.962 | 1.000 | 0.982 | 0.858 | 1.000 | 1.000 | 0.856 | 0.828 |
| 3187 | 0.767 | 1.000 | 0.794 | 0.986 | 0.963 | 0.960 | 0.903 | 1.000 | 0.980 | 1.000 | 0.968 | 1.000 | 0.982 | 0.859 | 1.000 | 1.000 | 0.856 | 0.827 |
| 3188 | 0.770 | 1.000 | 0.804 | 0.986 | 0.968 | 0.960 | 0.903 | 1.000 | 0.990 | 1.000 | 0.959 | 1.000 | 0.964 | 0.855 | 1.000 | 1.000 | 0.856 | 0.828 |
| 3189 | 0.767 | 1.000 | 0.779 | 0.986 | 0.968 | 0.960 | 0.909 | 1.000 | 0.980 | 1.000 | 0.962 | 1.000 | 0.982 | 0.859 | 1.000 | 1.000 | 0.855 | 0.826 |
| 3190 | 0.771 | 1.000 | 0.779 | 1.000 | 0.968 | 0.960 | 0.903 | 1.000 | 1.000 | 1.000 | 0.959 | 1.000 | 0.973 | 0.859 | 1.000 | 1.000 | 0.855 | 0.827 |
| 3191 | 0.773 | 1.000 | 0.789 | 1.000 | 0.968 | 0.960 | 0.901 | 1.000 | 0.980 | 1.000 | 0.957 | 1.000 | 0.982 | 0.865 | 1.000 | 1.000 | 0.858 | 0.829 |
| 3192 | 0.776 | 1.000 | 0.791 | 0.986 | 0.974 | 0.960 | 0.900 | 1.000 | 0.990 | 1.000 | 0.968 | 1.000 | 0.973 | 0.858 | 1.000 | 1.000 | 0.858 | 0.829 |
| 3193 | 0.766 | 1.000 | 0.802 | 0.986 | 0.974 | 0.960 | 0.892 | 1.000 | 0.990 | 1.000 | 0.968 | 1.000 | 0.982 | 0.857 | 1.000 | 1.000 | 0.855 | 0.826 |
| 3194 | 0.773 | 1.000 | 0.796 | 0.986 | 0.963 | 0.960 | 0.898 | 1.000 | 0.990 | 1.000 | 0.974 | 1.000 | 0.946 | 0.852 | 1.000 | 1.000 | 0.855 | 0.826 |
| 3195 | 0.772 | 1.000 | 0.802 | 0.986 | 0.958 | 0.960 | 0.904 | 1.000 | 0.990 | 1.000 | 0.962 | 1.000 | 0.973 | 0.856 | 1.000 | 1.000 | 0.857 | 0.829 |
| 3196 | 0.766 | 1.000 | 0.810 | 0.986 | 0.968 | 0.960 | 0.908 | 1.000 | 0.990 | 1.000 | 0.954 | 1.000 | 0.955 | 0.853 | 1.000 | 1.000 | 0.855 | 0.827 |

| | | | | | | | | | | | | | | | | | |
|---|---|---|---|---|---|---|---|---|---|---|---|---|---|---|---|---|---|
| 3197 | 0.765 | 1.000 | 0.789 | 1.000 | 0.968 | 0.960 | 0.903 | 1.000 | 1.000 | 1.000 | 0.954 | 1.000 | 0.973 | 0.858 | 1.000 | 1.000 | 0.854 | 0.825 |
| 3198 | 0.767 | 1.000 | 0.791 | 0.986 | 0.968 | 0.960 | 0.901 | 1.000 | 1.000 | 1.000 | 0.957 | 1.000 | 0.973 | 0.855 | 1.000 | 1.000 | 0.854 | 0.825 |
| 3199 | 0.765 | 1.000 | 0.781 | 1.000 | 0.974 | 0.960 | 0.911 | 1.000 | 0.990 | 1.000 | 0.959 | 1.000 | 0.964 | 0.854 | 1.000 | 1.000 | 0.854 | 0.825 |
| 3200 | 0.774 | 1.000 | 0.810 | 1.000 | 0.968 | 0.960 | 0.900 | 1.000 | 0.980 | 1.000 | 0.962 | 1.000 | 0.973 | 0.854 | 1.000 | 1.000 | 0.858 | 0.829 |
| 3201 | 0.769 | 1.000 | 0.781 | 1.000 | 0.968 | 0.960 | 0.908 | 1.000 | 0.990 | 1.000 | 0.971 | 1.000 | 0.973 | 0.855 | 1.000 | 1.000 | 0.855 | 0.827 |
| 3202 | 0.764 | 1.000 | 0.798 | 0.986 | 0.963 | 0.960 | 0.914 | 1.000 | 0.980 | 1.000 | 0.959 | 1.000 | 0.964 | 0.858 | 1.000 | 1.000 | 0.856 | 0.827 |
| 3203 | 0.773 | 1.000 | 0.798 | 1.000 | 0.963 | 0.960 | 0.892 | 1.000 | 0.990 | 1.000 | 0.971 | 1.000 | 0.955 | 0.861 | 1.000 | 1.000 | 0.857 | 0.829 |
| 3204 | 0.765 | 1.000 | 0.792 | 1.000 | 0.968 | 0.960 | 0.900 | 1.000 | 0.990 | 1.000 | 0.965 | 1.000 | 0.964 | 0.854 | 1.000 | 1.000 | 0.854 | 0.825 |
| 3205 | 0.774 | 1.000 | 0.792 | 0.986 | 0.958 | 0.960 | 0.906 | 1.000 | 1.000 | 1.000 | 0.959 | 1.000 | 0.964 | 0.867 | 1.000 | 1.000 | 0.859 | 0.831 |
| 3206 | 0.776 | 1.000 | 0.794 | 0.986 | 0.974 | 0.960 | 0.901 | 1.000 | 0.980 | 1.000 | 0.957 | 1.000 | 0.973 | 0.865 | 1.000 | 1.000 | 0.859 | 0.831 |
| 3207 | 0.773 | 1.000 | 0.800 | 0.986 | 0.963 | 0.960 | 0.909 | 1.000 | 0.980 | 1.000 | 0.962 | 1.000 | 0.982 | 0.859 | 1.000 | 1.000 | 0.859 | 0.831 |
| 3208 | 0.763 | 1.000 | 0.789 | 1.000 | 0.979 | 0.960 | 0.897 | 1.000 | 0.990 | 1.000 | 0.957 | 1.000 | 0.964 | 0.854 | 1.000 | 1.000 | 0.852 | 0.823 |
| 3209 | 0.777 | 1.000 | 0.800 | 0.986 | 0.968 | 0.960 | 0.904 | 1.000 | 0.980 | 1.000 | 0.959 | 1.000 | 0.964 | 0.854 | 1.000 | 1.000 | 0.857 | 0.829 |
| 3210 | 0.769 | 1.000 | 0.794 | 1.000 | 0.963 | 0.960 | 0.900 | 1.000 | 0.990 | 1.000 | 0.959 | 1.000 | 0.964 | 0.858 | 1.000 | 1.000 | 0.855 | 0.827 |
| 3211 | 0.764 | 1.000 | 0.785 | 0.986 | 0.958 | 1.000 | 0.900 | 1.000 | 0.980 | 1.000 | 0.957 | 1.000 | 0.973 | 0.855 | 1.000 | 1.000 | 0.852 | 0.822 |
| 3212 | 0.771 | 1.000 | 0.781 | 1.000 | 0.963 | 0.960 | 0.901 | 1.000 | 0.980 | 1.000 | 0.962 | 1.000 | 0.973 | 0.858 | 1.000 | 1.000 | 0.855 | 0.826 |
| 3213 | 0.773 | 1.000 | 0.796 | 1.000 | 0.963 | 0.960 | 0.908 | 1.000 | 0.980 | 1.000 | 0.959 | 1.000 | 0.964 | 0.854 | 1.000 | 1.000 | 0.856 | 0.828 |
| 3214 | 0.767 | 1.000 | 0.789 | 1.000 | 0.953 | 0.960 | 0.900 | 1.000 | 0.990 | 1.000 | 0.965 | 1.000 | 0.973 | 0.861 | 1.000 | 1.000 | 0.855 | 0.826 |
| 3215 | 0.771 | 1.000 | 0.802 | 1.000 | 0.968 | 0.960 | 0.909 | 1.000 | 0.990 | 1.000 | 0.957 | 1.000 | 0.973 | 0.863 | 1.000 | 1.000 | 0.859 | 0.832 |
| 3216 | 0.769 | 1.000 | 0.785 | 1.000 | 0.958 | 1.000 | 0.901 | 1.000 | 0.980 | 1.000 | 0.959 | 1.000 | 0.973 | 0.862 | 1.000 | 1.000 | 0.856 | 0.827 |
| 3217 | 0.765 | 1.000 | 0.789 | 1.000 | 0.953 | 0.960 | 0.911 | 1.000 | 0.980 | 1.000 | 0.959 | 1.000 | 0.982 | 0.857 | 1.000 | 1.000 | 0.854 | 0.825 |
| 3218 | 0.760 | 1.000 | 0.792 | 0.986 | 0.963 | 0.960 | 0.900 | 1.000 | 0.990 | 1.000 | 0.957 | 1.000 | 0.982 | 0.854 | 1.000 | 1.000 | 0.851 | 0.822 |
| 3219 | 0.776 | 1.000 | 0.789 | 0.957 | 0.963 | 0.960 | 0.891 | 1.000 | 0.990 | 1.000 | 0.965 | 1.000 | 0.982 | 0.852 | 1.000 | 1.000 | 0.854 | 0.825 |
| 3220 | 0.767 | 1.000 | 0.787 | 1.000 | 0.963 | 0.960 | 0.900 | 1.000 | 0.980 | 1.000 | 0.951 | 1.000 | 0.982 | 0.850 | 1.000 | 1.000 | 0.851 | 0.822 |
| 3221 | 0.764 | 1.000 | 0.789 | 1.000 | 0.958 | 0.960 | 0.895 | 1.000 | 0.980 | 1.000 | 0.959 | 1.000 | 0.973 | 0.854 | 1.000 | 1.000 | 0.851 | 0.822 |
| 3222 | 0.773 | 1.000 | 0.806 | 1.000 | 0.963 | 0.960 | 0.904 | 1.000 | 0.990 | 1.000 | 0.951 | 1.000 | 0.982 | 0.855 | 1.000 | 1.000 | 0.857 | 0.829 |
| 3223 | 0.768 | 1.000 | 0.794 | 0.986 | 0.974 | 0.960 | 0.901 | 1.000 | 0.990 | 1.000 | 0.957 | 1.000 | 0.982 | 0.858 | 1.000 | 1.000 | 0.856 | 0.827 |
| 3224 | 0.764 | 1.000 | 0.800 | 0.971 | 0.968 | 0.960 | 0.903 | 1.000 | 0.980 | 1.000 | 0.974 | 1.000 | 0.973 | 0.861 | 1.000 | 1.000 | 0.856 | 0.828 |
| 3225 | 0.767 | 1.000 | 0.792 | 0.971 | 0.963 | 0.960 | 0.898 | 1.000 | 0.990 | 1.000 | 0.959 | 1.000 | 0.982 | 0.851 | 1.000 | 1.000 | 0.852 | 0.823 |
| 3226 | 0.768 | 1.000 | 0.794 | 1.000 | 0.963 | 0.960 | 0.895 | 1.000 | 0.990 | 1.000 | 0.959 | 1.000 | 0.973 | 0.861 | 1.000 | 1.000 | 0.855 | 0.826 |
| 3227 | 0.763 | 1.000 | 0.789 | 0.986 | 0.968 | 0.960 | 0.901 | 1.000 | 1.000 | 1.000 | 0.951 | 1.000 | 0.991 | 0.858 | 1.000 | 1.000 | 0.853 | 0.824 |
| 3228 | 0.763 | 1.000 | 0.808 | 1.000 | 0.968 | 0.960 | 0.911 | 1.000 | 0.990 | 1.000 | 0.965 | 1.000 | 0.982 | 0.861 | 1.000 | 1.000 | 0.858 | 0.830 |
| 3229 | 0.772 | 1.000 | 0.789 | 0.986 | 0.958 | 0.960 | 0.892 | 1.000 | 0.990 | 1.000 | 0.962 | 1.000 | 0.955 | 0.854 | 1.000 | 1.000 | 0.853 | 0.824 |
| 3230 | 0.768 | 1.000 | 0.798 | 1.000 | 0.963 | 0.960 | 0.901 | 1.000 | 0.990 | 1.000 | 0.962 | 1.000 | 1.000 | 0.861 | 1.000 | 1.000 | 0.857 | 0.829 |
| 3231 | 0.773 | 1.000 | 0.802 | 1.000 | 0.974 | 0.960 | 0.895 | 1.000 | 0.980 | 1.000 | 0.957 | 1.000 | 0.982 | 0.858 | 1.000 | 1.000 | 0.857 | 0.828 |
| 3232 | 0.772 | 1.000 | 0.796 | 0.986 | 0.963 | 0.960 | 0.895 | 1.000 | 0.980 | 1.000 | 0.962 | 1.000 | 0.973 | 0.858 | 1.000 | 1.000 | 0.856 | 0.827 |
| 3233 | 0.772 | 1.000 | 0.800 | 1.000 | 0.958 | 0.960 | 0.911 | 1.000 | 0.980 | 1.000 | 0.962 | 1.000 | 0.991 | 0.858 | 1.000 | 1.000 | 0.858 | 0.830 |
| 3234 | 0.775 | 1.000 | 0.798 | 0.986 | 0.968 | 0.960 | 0.891 | 1.000 | 0.980 | 1.000 | 0.957 | 1.000 | 0.964 | 0.859 | 1.000 | 1.000 | 0.856 | 0.827 |
| 3235 | 0.753 | 1.000 | 0.792 | 1.000 | 0.953 | 0.960 | 0.903 | 1.000 | 0.980 | 1.000 | 0.968 | 1.000 | 0.955 | 0.857 | 1.000 | 1.000 | 0.850 | 0.821 |

| | | | | | | | | | | | | | | | | | |
|---|---|---|---|---|---|---|---|---|---|---|---|---|---|---|---|---|---|
| 3236 | 0.771 | 1.000 | 0.779 | 1.000 | 0.963 | 0.960 | 0.901 | 1.000 | 0.980 | 1.000 | 0.945 | 1.000 | 0.964 | 0.858 | 1.000 | 1.000 | 0.853 | 0.824 |
| 3237 | 0.772 | 1.000 | 0.783 | 1.000 | 0.968 | 0.960 | 0.904 | 1.000 | 0.990 | 1.000 | 0.962 | 1.000 | 0.964 | 0.856 | 1.000 | 1.000 | 0.855 | 0.826 |
| 3238 | 0.761 | 1.000 | 0.798 | 1.000 | 0.968 | 0.960 | 0.906 | 1.000 | 0.980 | 1.000 | 0.954 | 1.000 | 0.973 | 0.860 | 1.000 | 1.000 | 0.854 | 0.825 |
| 3239 | 0.771 | 1.000 | 0.802 | 1.000 | 0.958 | 0.960 | 0.904 | 1.000 | 0.990 | 1.000 | 0.962 | 1.000 | 0.982 | 0.851 | 1.000 | 1.000 | 0.856 | 0.827 |
| 3240 | 0.765 | 1.000 | 0.794 | 0.986 | 0.963 | 0.960 | 0.901 | 1.000 | 0.990 | 1.000 | 0.957 | 1.000 | 0.982 | 0.844 | 1.000 | 1.000 | 0.851 | 0.821 |
| 3241 | 0.771 | 1.000 | 0.791 | 1.000 | 0.968 | 0.960 | 0.900 | 1.000 | 1.000 | 1.000 | 0.959 | 1.000 | 1.000 | 0.850 | 1.000 | 1.000 | 0.855 | 0.826 |
| 3242 | 0.760 | 1.000 | 0.789 | 1.000 | 0.968 | 0.960 | 0.909 | 1.000 | 1.000 | 1.000 | 0.962 | 1.000 | 0.955 | 0.860 | 1.000 | 1.000 | 0.854 | 0.825 |
| 3243 | 0.776 | 1.000 | 0.800 | 0.986 | 0.968 | 0.960 | 0.903 | 1.000 | 0.980 | 1.000 | 0.962 | 1.000 | 0.973 | 0.861 | 1.000 | 1.000 | 0.859 | 0.831 |
| 3244 | 0.752 | 1.000 | 0.808 | 1.000 | 0.963 | 0.960 | 0.911 | 1.000 | 0.980 | 1.000 | 0.954 | 1.000 | 0.982 | 0.856 | 1.000 | 1.000 | 0.852 | 0.824 |
| 3245 | 0.773 | 1.000 | 0.789 | 1.000 | 0.968 | 0.960 | 0.906 | 1.000 | 0.980 | 1.000 | 0.965 | 1.000 | 0.982 | 0.857 | 1.000 | 1.000 | 0.857 | 0.829 |
| 3246 | 0.768 | 1.000 | 0.787 | 0.986 | 0.963 | 0.960 | 0.903 | 1.000 | 1.000 | 1.000 | 0.959 | 1.000 | 0.982 | 0.858 | 1.000 | 1.000 | 0.855 | 0.826 |
| 3247 | 0.767 | 1.000 | 0.773 | 1.000 | 0.963 | 0.960 | 0.901 | 1.000 | 1.000 | 1.000 | 0.965 | 1.000 | 0.964 | 0.853 | 1.000 | 1.000 | 0.852 | 0.822 |
| 3248 | 0.763 | 1.000 | 0.800 | 1.000 | 0.968 | 0.960 | 0.908 | 1.000 | 0.990 | 1.000 | 0.965 | 1.000 | 0.964 | 0.857 | 1.000 | 1.000 | 0.855 | 0.827 |
| 3249 | 0.767 | 1.000 | 0.802 | 1.000 | 0.974 | 0.960 | 0.909 | 1.000 | 0.969 | 1.000 | 0.959 | 1.000 | 0.955 | 0.851 | 1.000 | 1.000 | 0.854 | 0.826 |
| 3250 | 0.762 | 1.000 | 0.791 | 1.000 | 0.963 | 0.960 | 0.897 | 1.000 | 0.980 | 1.000 | 0.959 | 1.000 | 0.946 | 0.849 | 1.000 | 1.000 | 0.849 | 0.819 |
| 3251 | 0.771 | 1.000 | 0.798 | 1.000 | 0.968 | 0.960 | 0.906 | 1.000 | 0.990 | 1.000 | 0.957 | 1.000 | 0.964 | 0.858 | 1.000 | 1.000 | 0.857 | 0.829 |
| 3252 | 0.766 | 1.000 | 0.806 | 0.986 | 0.958 | 0.960 | 0.900 | 1.000 | 0.980 | 1.000 | 0.965 | 1.000 | 0.973 | 0.857 | 1.000 | 1.000 | 0.855 | 0.826 |
| 3253 | 0.773 | 1.000 | 0.806 | 0.986 | 0.968 | 0.960 | 0.898 | 1.000 | 1.000 | 1.000 | 0.965 | 1.000 | 0.973 | 0.858 | 1.000 | 1.000 | 0.858 | 0.830 |
| 3254 | 0.765 | 1.000 | 0.802 | 1.000 | 0.963 | 0.960 | 0.892 | 1.000 | 0.990 | 1.000 | 0.965 | 1.000 | 0.973 | 0.864 | 1.000 | 1.000 | 0.856 | 0.827 |
| 3255 | 0.776 | 1.000 | 0.791 | 1.000 | 0.963 | 0.960 | 0.903 | 1.000 | 1.000 | 1.000 | 0.959 | 1.000 | 0.982 | 0.861 | 1.000 | 1.000 | 0.859 | 0.830 |
| 3256 | 0.766 | 1.000 | 0.806 | 1.000 | 0.958 | 0.960 | 0.901 | 1.000 | 0.990 | 1.000 | 0.959 | 1.000 | 0.982 | 0.860 | 1.000 | 1.000 | 0.856 | 0.828 |
| 3257 | 0.763 | 1.000 | 0.806 | 0.986 | 0.974 | 0.960 | 0.903 | 1.000 | 0.990 | 1.000 | 0.954 | 1.000 | 0.964 | 0.861 | 1.000 | 1.000 | 0.856 | 0.827 |
| 3258 | 0.767 | 1.000 | 0.806 | 1.000 | 0.963 | 0.960 | 0.895 | 1.000 | 0.980 | 1.000 | 0.965 | 1.000 | 0.982 | 0.859 | 1.000 | 1.000 | 0.856 | 0.828 |
| 3259 | 0.758 | 1.000 | 0.810 | 1.000 | 0.974 | 0.960 | 0.906 | 1.000 | 0.980 | 1.000 | 0.957 | 1.000 | 0.973 | 0.862 | 1.000 | 1.000 | 0.856 | 0.827 |
| 3260 | 0.771 | 1.000 | 0.785 | 1.000 | 0.974 | 0.960 | 0.906 | 1.000 | 0.980 | 1.000 | 0.965 | 1.000 | 0.973 | 0.858 | 1.000 | 1.000 | 0.856 | 0.828 |
| 3261 | 0.768 | 1.000 | 0.791 | 0.986 | 0.968 | 0.960 | 0.908 | 1.000 | 1.000 | 1.000 | 0.959 | 1.000 | 0.964 | 0.861 | 1.000 | 1.000 | 0.856 | 0.828 |
| 3262 | 0.771 | 1.000 | 0.789 | 1.000 | 0.963 | 0.960 | 0.904 | 1.000 | 0.980 | 1.000 | 0.957 | 1.000 | 0.964 | 0.860 | 1.000 | 1.000 | 0.856 | 0.827 |
| 3263 | 0.769 | 1.000 | 0.794 | 1.000 | 0.968 | 0.960 | 0.900 | 1.000 | 0.980 | 1.000 | 0.971 | 1.000 | 0.982 | 0.861 | 1.000 | 1.000 | 0.857 | 0.829 |
| 3264 | 0.765 | 1.000 | 0.798 | 1.000 | 0.968 | 0.960 | 0.909 | 1.000 | 0.990 | 1.000 | 0.951 | 1.000 | 0.973 | 0.854 | 1.000 | 1.000 | 0.854 | 0.826 |
| 3265 | 0.756 | 1.000 | 0.789 | 0.986 | 0.963 | 0.960 | 0.894 | 1.000 | 0.980 | 1.000 | 0.954 | 1.000 | 0.973 | 0.854 | 1.000 | 1.000 | 0.848 | 0.818 |
| 3266 | 0.775 | 1.000 | 0.785 | 0.986 | 0.963 | 0.960 | 0.909 | 1.000 | 0.969 | 1.000 | 0.957 | 1.000 | 0.973 | 0.861 | 1.000 | 1.000 | 0.857 | 0.829 |
| 3267 | 0.773 | 1.000 | 0.792 | 1.000 | 0.963 | 0.960 | 0.904 | 1.000 | 0.990 | 1.000 | 0.957 | 1.000 | 0.982 | 0.854 | 1.000 | 1.000 | 0.856 | 0.827 |
| 3268 | 0.772 | 1.000 | 0.785 | 0.986 | 0.963 | 0.960 | 0.898 | 1.000 | 0.990 | 1.000 | 0.962 | 1.000 | 0.973 | 0.855 | 1.000 | 1.000 | 0.854 | 0.825 |
| 3269 | 0.777 | 1.000 | 0.771 | 0.971 | 0.963 | 0.960 | 0.906 | 1.000 | 1.000 | 1.000 | 0.954 | 1.000 | 0.973 | 0.857 | 1.000 | 1.000 | 0.855 | 0.826 |
| 3270 | 0.773 | 1.000 | 0.798 | 1.000 | 0.963 | 0.960 | 0.895 | 1.000 | 0.990 | 1.000 | 0.968 | 1.000 | 0.964 | 0.857 | 1.000 | 1.000 | 0.857 | 0.828 |
| 3271 | 0.771 | 1.000 | 0.781 | 0.986 | 0.968 | 0.960 | 0.909 | 1.000 | 0.980 | 1.000 | 0.959 | 1.000 | 0.964 | 0.855 | 1.000 | 1.000 | 0.855 | 0.826 |
| 3272 | 0.761 | 1.000 | 0.791 | 1.000 | 0.963 | 0.960 | 0.915 | 1.000 | 0.980 | 1.000 | 0.957 | 1.000 | 0.964 | 0.854 | 1.000 | 1.000 | 0.853 | 0.824 |
| 3273 | 0.773 | 1.000 | 0.794 | 1.000 | 0.963 | 0.960 | 0.895 | 1.000 | 1.000 | 1.000 | 0.965 | 1.000 | 0.982 | 0.857 | 1.000 | 1.000 | 0.857 | 0.828 |
| 3274 | 0.767 | 1.000 | 0.783 | 0.986 | 0.958 | 0.960 | 0.911 | 1.000 | 0.990 | 1.000 | 0.965 | 1.000 | 0.982 | 0.857 | 1.000 | 1.000 | 0.855 | 0.826 |

| | | | | | | | | | | | | | | | | | | |
|---|---|---|---|---|---|---|---|---|---|---|---|---|---|---|---|---|---|---|
| 3275 | 0.779 | 1.000 | 0.794 | 0.986 | 0.963 | 0.960 | 0.901 | 1.000 | 0.990 | 1.000 | 0.959 | 1.000 | 0.991 | 0.857 | 1.000 | 1.000 | 0.858 | 0.830 |
| 3276 | 0.761 | 1.000 | 0.785 | 0.986 | 0.968 | 0.960 | 0.900 | 1.000 | 0.990 | 1.000 | 0.962 | 1.000 | 0.964 | 0.851 | 1.000 | 1.000 | 0.850 | 0.821 |
| 3277 | 0.769 | 1.000 | 0.779 | 0.986 | 0.968 | 0.960 | 0.894 | 1.000 | 1.000 | 1.000 | 0.959 | 1.000 | 0.973 | 0.854 | 1.000 | 1.000 | 0.852 | 0.823 |
| 3278 | 0.771 | 1.000 | 0.800 | 0.986 | 0.968 | 0.960 | 0.894 | 1.000 | 0.990 | 1.000 | 0.962 | 1.000 | 0.973 | 0.866 | 1.000 | 1.000 | 0.858 | 0.830 |
| 3279 | 0.772 | 1.000 | 0.800 | 1.000 | 0.968 | 0.960 | 0.886 | 1.000 | 1.000 | 1.000 | 0.962 | 1.000 | 0.982 | 0.858 | 1.000 | 1.000 | 0.856 | 0.827 |
| 3280 | 0.772 | 1.000 | 0.798 | 0.971 | 0.968 | 0.960 | 0.906 | 1.000 | 1.000 | 1.000 | 0.959 | 1.000 | 0.973 | 0.849 | 1.000 | 1.000 | 0.855 | 0.826 |
| 3281 | 0.773 | 1.000 | 0.806 | 1.000 | 0.968 | 0.960 | 0.904 | 1.000 | 0.990 | 1.000 | 0.968 | 1.000 | 0.991 | 0.859 | 1.000 | 1.000 | 0.860 | 0.832 |
| 3282 | 0.771 | 1.000 | 0.796 | 1.000 | 0.968 | 0.960 | 0.901 | 1.000 | 1.000 | 1.000 | 0.971 | 1.000 | 0.973 | 0.854 | 1.000 | 1.000 | 0.857 | 0.828 |
| 3283 | 0.766 | 1.000 | 0.794 | 1.000 | 0.968 | 0.960 | 0.894 | 1.000 | 1.000 | 1.000 | 0.959 | 1.000 | 0.973 | 0.859 | 1.000 | 1.000 | 0.854 | 0.825 |
| 3284 | 0.771 | 1.000 | 0.792 | 1.000 | 0.968 | 0.960 | 0.898 | 1.000 | 0.990 | 1.000 | 0.962 | 1.000 | 0.964 | 0.862 | 1.000 | 1.000 | 0.857 | 0.828 |
| 3285 | 0.775 | 1.000 | 0.794 | 1.000 | 0.963 | 0.960 | 0.901 | 1.000 | 1.000 | 1.000 | 0.957 | 1.000 | 0.955 | 0.862 | 1.000 | 1.000 | 0.858 | 0.830 |
| 3286 | 0.769 | 1.000 | 0.794 | 0.986 | 0.963 | 0.960 | 0.897 | 1.000 | 0.980 | 1.000 | 0.957 | 1.000 | 0.964 | 0.855 | 1.000 | 1.000 | 0.853 | 0.824 |
| 3287 | 0.768 | 1.000 | 0.785 | 1.000 | 0.963 | 0.960 | 0.897 | 1.000 | 0.990 | 1.000 | 0.948 | 1.000 | 0.964 | 0.858 | 1.000 | 1.000 | 0.853 | 0.823 |
| 3288 | 0.768 | 1.000 | 0.787 | 0.986 | 0.958 | 0.960 | 0.904 | 1.000 | 0.990 | 1.000 | 0.959 | 1.000 | 0.964 | 0.854 | 1.000 | 1.000 | 0.853 | 0.824 |
| 3289 | 0.763 | 1.000 | 0.802 | 1.000 | 0.958 | 0.960 | 0.903 | 1.000 | 0.990 | 1.000 | 0.965 | 1.000 | 0.982 | 0.868 | 1.000 | 1.000 | 0.858 | 0.830 |
| 3290 | 0.767 | 1.000 | 0.792 | 1.000 | 0.963 | 0.960 | 0.901 | 1.000 | 0.990 | 1.000 | 0.957 | 1.000 | 0.973 | 0.855 | 1.000 | 1.000 | 0.854 | 0.825 |
| 3291 | 0.773 | 1.000 | 0.802 | 1.000 | 0.974 | 0.960 | 0.906 | 1.000 | 0.990 | 1.000 | 0.962 | 1.000 | 0.973 | 0.849 | 1.000 | 1.000 | 0.856 | 0.828 |
[truncated: 2,461,872 more chars]
